# Transcriptome Analysis and Comparison of *Marmota monax* and *Marmota himalayana*

**DOI:** 10.1371/journal.pone.0165875

**Published:** 2016-11-02

**Authors:** Yanan Liu, Baoju Wang, Lu Wang, Vikash Vikash, Qin Wang, Michael Roggendorf, Mengji Lu, Dongliang Yang, Jia Liu

**Affiliations:** 1 Department of Infectious Diseases, Institute of Infection and Immunology, Union Hospital, Tongji Medical College, Huazhong University of Science and Technology, Wuhan, China; 2 Institute for Virology, University Hospital of Essen, University of Duisburg-Essen, Essen, Germany; Centre de Recherche en Cancerologie de Lyon, FRANCE

## Abstract

The Eastern woodchuck (*Marmota monax*) is a classical animal model for studying hepatitis B virus (HBV) infection and hepatocellular carcinoma (HCC) in humans. Recently, we found that *Marmota himalayana*, an Asian animal species closely related to *Marmota monax*, is susceptible to woodchuck hepatitis virus (WHV) infection and can be used as a new mammalian model for HBV infection. However, the lack of genomic sequence information of both *Marmota* models strongly limited their application breadth and depth. To address this major obstacle of the *Marmota* models, we utilized Illumina RNA-Seq technology to sequence the cDNA libraries of liver and spleen samples of two *Marmota monax* and four *Marmota himalayana*. In total, over 13 billion nucleotide bases were sequenced and approximately 1.5 billion clean reads were obtained. Following assembly, 106,496 consensus sequences of *Marmota monax* and 78,483 consensus sequences of *Marmota himalayana* were detected. For functional annotation, in total 73,603 Unigenes of *Marmota monax* and 78,483 Unigenes of *Marmota himalayana* were identified using different databases (NR, NT, Swiss-Prot, KEGG, COG, GO). The Unigenes were aligned by blastx to protein databases to decide the coding DNA sequences (CDS) and in total 41,247 CDS of *Marmota monax* and 34,033 CDS of *Marmota himalayana* were predicted. The single nucleotide polymorphisms (SNPs) and the simple sequence repeats (SSRs) were also analyzed for all Unigenes obtained. Moreover, a large-scale transcriptome comparison was performed and revealed a high similarity in transcriptome sequences between the two *marmota* species. Our study provides an extensive amount of novel sequence information for *Marmota monax* and *Marmota himalayana*. This information may serve as a valuable genomics resource for further molecular, developmental and comparative evolutionary studies, as well as for the identification and characterization of functional genes that are involved in WHV infection and HCC development in the woodchuck model.

## Introduction

It is estimated that more than 248 million people are chronically infected with Hepatitis B virus (HBV) worldwide [1]. Every year, over 500,000 people die due to HBV-associated liver diseases, such as cirrhosis and hepatocellular carcinoma (HCC) [2, 3]. Pegylated interferon-α (IFN-α) and various nucleos(t)ides are currently licensed for the treatment of chronic hepatitis B (CHB), but they rarely lead to cure [4]. Research on the development of new treatment options for CHB is urgently needed.

The progress in HBV research is strongly dependent on the availability of suitable animal models. Except humans, HBV can infect only apes, tree shrews and macaques [5]. However, due to various restraints encountered using these primate animal models for HBV infection, animal models based on a series of HBV-related hepadnaviruses were discovered in the past decades (ducks, geese, herons, woodchucks, squirrels and woolly monkeys) [6]. Among them, the Eastern woodchuck (*Marmota monax*, *M*. *monax*) represents a well established animal model for investigating HBV-related disease and served in preclinical efficacy studies to investigate therapeutic strategies for CHB [7–9]. *M*. *monax* is naturally infected with woodchuck hepatitis virus (WHV), which is genetically closely related to human HBV, and has a disease course similar to that in HBV-infected patients. Recently, we found that *Marmota himalayana* (*M*. *himalayana*), an Asian animal species closely related to *M*. *monax*, is susceptible to WHV infection and can be used as a new mammalian model for HBV infection [10]. It is proposed that *M*. *himalayana* and *M*. *monax* are derived from a common ancestor, and belong to the same subgenera *Marmota* [11, 12]. Our previous genetic and immunogenetic analysis also indicated that the *M*. *himalayana* are closely related to *M*. *monax*, as the genes of the immune system of both species are nearly identical in their nucleotide sequences, as analyzed so far [13–15]. However, the transcriptional comparison analysis between the two species was restricted to only a few sequenced gene segments due to the lack of accessible genomic resources for both species. The lack of transcriptional sequence information has also hampered the application of the woodchuck model for functional genomics analysis and immunological mechanism studies in HBV and HCC.

In recent years, the use of deep-sequencing technologies has offered a powerful tool for a global and rapid transcriptome analysis via a high-throughput approach [16]. For example, RNA-Seq can generate millions of short cDNA reads from the transcriptome of an organism. The resulting reads are either aligned to a reference genome or reference transcripts, or assembled de novo (without the genomic sequence) to produce a genome-scale transcription map that consists of gene expression profiling, genomics function annotation, single-nucleotide polymorphism (SNP) and simple sequence repeats (SSR) marker identification, as well as protein coding sequence (CDS) prediction [17–19].

The first woodchuck transcriptome analysis was carried out by S.P.Fletcher et al using deep-sequencing technology (the Roche 454 Genome Sequencer FLX). In the study, the authors briefly described the sequencing, assembly and annotation of the woodchuck transcriptome, and focused on the characterization of the transcriptional response to persistent WHV infection and WHV-induced HCC (S.P.Fletcher et al., Hepatology 2012) [20]. By taking the power of deep-sequencing technologies, the authors were able to establish the translational utility of the woodchuck model and provide new insights into various immune pathways which may play a role in HBV persistence or associated liver diseases. However, the data of basic woodchuck transcriptome analysis of this report, such as CDS information, were still inaccessible. Besides, to our knowledge, there was still no transcriptome information of *M*. *himalayana* available so far.

In this study, we report and focus on the sequencing, assembly, annotation, and comparison of the transcriptomes of *M*. *monax* and *M*. *himalayana* by using Illumina RNA-Seq technology. This transcriptomic resource may serve as a valuable foundation for further molecular, developmental and comparative evolutionary studies, as well as for the identification and characterization of functional genes that are involved in WHV infection and HCC development in the woodchuck model.

## Materials and Methods

### Animals

*M*. *monax* were purchased from North Eastern Wildlife (Harrison, ID, USA) and *M*. *himalayana* were captured in the area of Tongren county (N35°31′25.42″, E102°01′33.42), Qinghai province, China. The capture of *M*. *himalayana* for this study was performed in 2009 and 2010, and no specific permissions for the capture activities were needed in Qinghai province at that time. Each animal was housed in a single cage with two separate chambers. One chamber was for food and drink, and the other one was for living with its floor covered by sterilized hay. The conditions of experimental facility were kept as follows, the room temperature: 25 ± 1°C, the air humidity: 60% ± 10%, the light cycle: 12:12 hours. The animals were euthanized by intracardiac injection of 1ml T-61. Abdomen incision was made to sufficiently expose the abdominal organs of the sacrificed animal, and the sections of spleen and liver were quickly collected and frozen in liquid nitrogen. This study was carried out in strict accordance with the recommendations in the Guide for the Care and Use of Laboratory Animals of the National Institutes of Health. The protocol was approved by the Committee on the Ethics of Animal Experiments of the University of Tongji Medical College, Huazhong University of Science & Technology, China (Permit Number: 491). All surgery was performed under sodium pentobarbital anesthesia, and all efforts were made to minimize suffering. The information on age, gender, body weight, duration in the laboratory environment and the date of euthanasia of used animals were provided in S1 Table.

### RNA Extraction, cDNA Library Synthesis and RNA Sequencing

Total RNA was extracted from the liver and spleen samples of two *M*. *monax* and four *M*. *himalayana* (Table 1). RNA was extracted from frozen tissue samples using Trizol total RNA extraction reagent according to the manufacturer's instructions (TaKaRa, Dalian, China). Briefly, 60mg tissue sample was ground with liquid nitrogen into powder and transferred the powder sample into the 2 ml tube containing of 1ml Trizol reagent, and then the total RNA was extracted by protein depletion processing with Chloroform/isoamyl (24:1) alcohol and washing with isopropyl alcohol. At last, the dry RNA was dissolved in 25μL RNase-free water and measuring concentration with NanoDrop Spectrophotometer (Thermo Scientific Inc). RNA-seq library preparation and sequencing was carried out by Beijing Genomics Institute BGI (Hong-Kong, China). In brief, DNase I digestion was performed to remove any traces of genomic DAN and magnetic beads with oligo (dT) were used to isolate mRNA. Following purification, the mRNA was fragmented using divalent cations at elevated temperature. Taking these short fragments as templates, the first-strand cDNA was synthesized using random hexamer primers and Superscript TM III (Invitrogen^™^, Carlsbad, CA, USA). The second strand cDNA was synthesized using buffer, dNTPs, RNaseH and DNA polymerase I. Short fragments were purified with a QiaQuick PCR extraction kit (Qiagen) and resolved with EB buffer for end reparation and poly(A) addition. The short fragments were then connected using sequencing adapters. The suitable fragments were selected as templates for the PCR amplification. For the quality control steps, Agilent 2100 Bioanaylzer and ABI StepOnePlus Real-Time PCR System were used in quantification and qualification of the sample library. The libraries were sequenced using Illumina HiSeq^™^ 2000 (Illumina Inc., San Diego, CA, USA).

**Table 1:** Summary of transcriptome Illumina sequencing of *M*. *monax* and *M*. *himalayana*.

Samples	Tissue	Total Raw Reads	Total Clean Reads	Total Clean Nucleotides (nt)	Q20 percentage	N percentage	GC percentage
**Mm1**	Liver	27,087,676	25,307,006	2,277,630,540	97.55%	0.00%	48.01%
**Mm2**	Spleen	25,820,696	23,795,870	2,141,628,300	97.49%	0.00%	47.36%
**Mh1**	Spleen	25,994,178	23,958,494	2,156,264,460	97.50%	0.00%	49.98%
**Mh2**	Spleen	30,093,280	25,783,508	2,320,515,720	97.36%	0.00%	49.33%
**Mh3**	Liver	29,709,094	26,970,972	2,427,387,480	97.61%	0.00%	47.78%
**Mh4**	Liver	27,671,692	24,055,738	2,165,016,420	96.35%	0.01%	49.29%

Total numbers of raw reads, clean reads and clean nucleotides generated from Illumina sequencing are listed. Q20 percentage is proportion of nucleotides with quality value larger than 20 in reads. N percentage is proportion of unknown nucleotides in clean reads. GC percentage is proportion of guanidine and cytosine nucleotides among total nucleotides.

### Filter RNA-Seq raw reads and sequence assembly

Raw reads generated from the RNA-seq were filtered by removing reads with adaptors, reads with unknown nucleotides larger than 5% and low-quality reads which the percentage of low-quality bases (base quality≤10) was more than 20%. The filtered reads were assembled into transcripts using Trinity (version: release-20121005) [21], a reference genome-independent assembler which combines three independent software modules: Inchworm, Chrysalis, and Butterfly. In brief, Inchworm assembled the RNA-seq reads into the unique sequences of transcripts (contigs). Chrysalis clustered the Inchworm contigs and constructed complete de Bruijn graphs for each cluster. Each cluster represented the full transcriptional complexity for a given gene (or sets of genes that share sequences in common). Chrysalis then partitioned the full read set among these disjoint graphs. Butterfly processed the individual graphs in parallel, including tracing the paths that reads and pairs of reads take within the graph, ultimately reporting full-length transcripts for alternatively spliced isoforms, and teasing apart transcripts that corresponds to paralogous genes. The ultimate result sequences of Trinity were called Unigenes. When multiple samples from the same species were sequenced, Unigenes from each sample's assembly were taken into the further process of sequence splicing and redundancy removing with sequence clustering software [22] to acquire non-redundant Unigenes as long as possible.

### Annotation of Unigenes

Unigenes were used for BLAST searches and annotation against an NCBI Nr protein database [23] (NCBI non-redundant sequence database) using an E-value cut-off of 0.00001 (E-value≤ 0.00001). Consensus sequences were further aligned by BLASTX to protein databases such as Swiss Institute of Bioinformatics databases (Swiss-Prot) [24], Kyoto Encyclopedia of Genes and Genomes (KEGG) [25] and Clusters of Orthologous Groups of proteins (COG) [26], retrieving proteins with the highest sequence similarity with the given sequences along with the functional annotations for their proteins. If results of different databases conflicted, a priority order of Nr, Swiss-Prot, KEGG and COG was followed. The Blast2GO program [27] (version: release 2012-10-01) was used to obtain Gene Ontology (GO) [28] annotations for the sequences, as well as for KEGG and COG analysis. The WEGO software [29] was then used to perform GO functional classification of all sequences to view the distribution of gene functions of the species at the macro level. The analysis mapped all of the annotated sequences to GO terms in the database and calculated the number of sequences associated with every term.

### CDS prediction of Unigenes

Unigenes were firstly aligned to protein databases and then the sequences with the highest rank in blast results were selected to decide the CDS of Unigenes. For Unigenes that could not be aligned to any database were scanned by ESTScan [30] with software ESTScan (version: v3.0.2) producing nucleotide sequence in (5' − 3') direction and amino sequence of the predicted coding region. The numbers of CDS were summarized, and the length distribution of Unigenes blast and ESTScan CDS were added up.

### Unigene SNP and SSR analysis

The SNPs were identified on the consensus sequence through the comparison with the Unigenes using software SOAPsnp. The SSR detection was done with software MicroSAtellite (MISA) using Unigenes as reference.

### Similarity Analysis of GO Classification

A total number of *M*. *himalayana* Unigenes annotated by GO classification were aligned in consistency with *M*. *monax* in four range values, compartmentalized by genetic similarity.

## Results and Discussion

### Sample preparation and Illumina sequencing

To globally characterize the transcriptome features of the two species of woodchuck, total RNA was isolated from different tissues of five animals: liver samples of *M*. *monax* (Mm1) and *M*. *himalayana* (Mh3 and Mh4); spleen samples of *M*. *monax* (Mm2) and *M*. *himalayana* (Mh1 and Mh2) (Table 1). After cleaning and quality checks, six independent rounds of Illumina sequencing for each sample were performed and generated 1,663,766,16 raw reads in total. The raw reads were preprocessed to eliminate primer/adaptor contamination and low quality sections of reads because these sequences can substantially compromise de novo assembly. At last, in total 1,498,715,88 clean reads were generated, encompassing 13,488,422,920 total nucleotides (nt) (Table 1). These data were comprised of high quality reads with the Q20 percentages greater than 96%, hardly any N percentages and nearly half GC percentages for each of the six libraries (Table 1). The raw sequence data sets are available in the Sequence Read Archive (SRA) database (accession number: SRP074653 and SRP075170).

### De novo assembly of sequence reads without a reference genome

**Fig 1:**
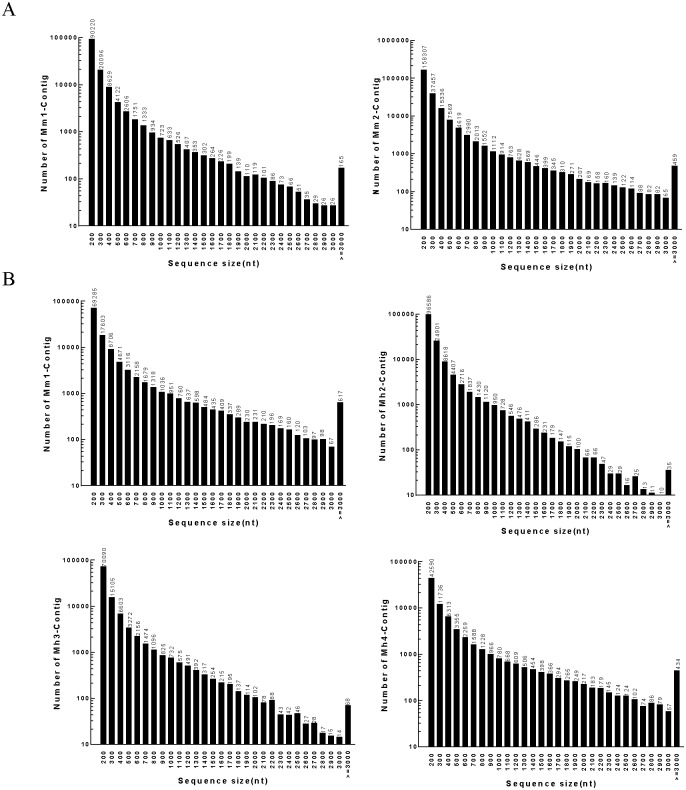
The length distribution of Contigs of *M*. *monax and M*. *himalayana*. (A) The length distribution of Contigs of *M*. *monax*. (B) The length distribution of Contigs of *M*. *himalayana*.

**Fig 2:**
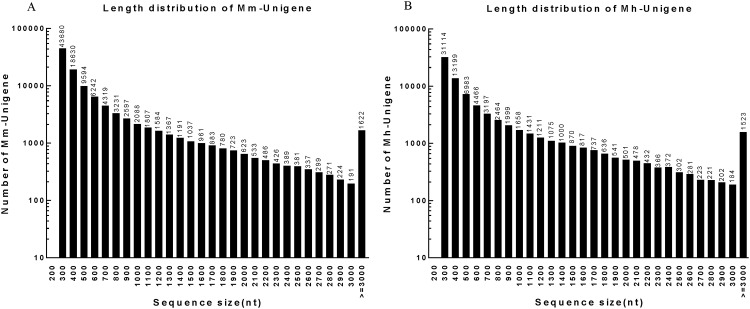
The length distribution of Unigenes of *M*. *monax and M*. *himalayana*. (A) The length distribution of Unigenes of *M*. *monax*. (B) The length distribution of Unigenes of *M*. *himalayana*.

Transcriptome de novo assembly was carried out with short reads assembling program-Trinity [31], which combines three independent software modules: Inchworm, Chrysalis, and Butterfly, applied sequentially to process large volumes of RNA-seq reads. Firstly, the RNA-seq data were assembled into the unique sequences of transcripts by Inchworm to generate Contigs for each of the six libraries. Table 2 shows the general information of Contigs, including total number, total length, mean length and N50 Contig length (50% of the total assembled sequence was contained in Contigs of this length or longer). Fig 1 shows the length distribution of Contigs ranging from 200 bp to over 3,000 bp. In all samples, the most abundant Contigs were 200 bp and the least abundant were 3000 bp. Next, we pooled the Contigs of *M*. *monax* and *M*. *himalayana* respectively. The pooled Contigs were clustered into sets of connected components by Chrysalis. Each cluster represents the full transcriptional complexity for a given gene (or sets of genes that share sequences in common). Complete de Bruijn graphs for each cluster were constructed by Chrysalis. The individual de Bruijn graphs were processed in parallel by Butterfly to reconstruct plausible full-length, linear transcripts. The ultimate result sequences of Trinity were called Unigenes. These Unigenes were then clustered using the TIGR Gene Indices clustering tools (TGICL) to join further sequences and remove any redundant sequences [22]. Finally, 106,496 Unigenes of *M*. *monax* and 78,483 Unigenes of *M*. *himalayana* were generated respectively (Table 2). Fig 2 shows the length distribution of Unigenes ranging from 200 bp to over 3,000 bp. The most abundant Unigenes of both species were 200 bp (43,680 for *M*. *monax* and 31,114 for *M*. *himalayana*) and the least abundant were 3000 bp (191 for *M*. *monax* and 184 for *M*. *himalayana*).

**Table 2:** Summary of de novo assembly of transcriptome sequence reads.

	Sample	Total Number	Total Length(nt)	Mean Length(nt)	N50	Total Consensus Sequences	Distinct Clusters	Distinct Singletons
**Contig**	Mm1	134,350	32,464,306	242	298	-	-	-
	Mm2	237,435	57,536,327	242	292	-	-	-
	Mh1	116,770	37,553,862	322	532	-	-	-
	Mh2	146,131	34,301,191	235	266	-	-	-
	Mh3	104,611	25,665,536	245	305	-	-	-
	Mh4	76,428	26,802,677	351	609	-	-	-
**Unigene**	Mm	106,496	64,429,411	605	898	106,496	20,897	85,599
	Mh	78,483	50,577,229	644	1030	78,483	15,125	63,358

The RNA-seq data were assembled into the unique sequences of transcripts by Inchworm to generate Contigs for each of the six libraries. The total number, total length, mean length and N50 of Contigs are listed. N50 means the N50 Contig length (i.e., 50% of the total assembled sequence was contained in Contigs of this length or longer). The Contigs of M. monax and M. himalayana were pooled respectively, and the numbers of the consensus sequences as well as the Clusters and the Singletons are listed.

Moreover, gene family clustering divided the Unigenes into two classes: One class comprised the Clusters, for which the prefix CL followed by the cluster ID and the number of contigs in each cluster was given (S2 and S3 Tables). In any one cluster, there were several Unigenes for which similarity between the consensus sequences was more than 70%. The other class comprised the Singletons, for which the prefix Unigene was given. For *M*. *monax*, 20,897 Unigenes were grouped into 6,383 different clusters, and 85,599 Unigenes were Singletons. For *M*. *himalayana*, 15,125 Unigenes were grouped into 6,882 different clusters, and 63,358 Unigenes were Singletons (Table 2).

### Annotation and classification of *Marmota* Unigenes

For annotation, the Unigenes were aligned by Blastx (e-value < 0.00001) with protein databases such as NR, Swiss-Prot, KEGG, COG and GO. The KEGG pathway database records networks of molecular interactions in cells, and variants of them, specific to particular organisms. Pathway-based analysis helped to understand further the biological functions of genes. Pathway information for all annotated sequences was obtained from KEGG pathway annotations. COG is a database where orthologous gene products are classified. The whole database is built on genes encoding proteins from species with complete genome sequences as well as the evolutionary relationships between bacteria, algae and eukaryotes. All Unigenes were aligned to the COG database to predict and classify possible functions. GO offers three ontologies of genes: molecular function, cellular component and biological process. The basic unit of GO is the GO-term which belongs to a type of ontology. Based on the NR annotation, the Blast2GO program was used to get the GO annotation of all Unigenes. WEGO software was then used for GO functional classification and to understand the distribution of gene functions of the species at a macro level. In each database, two criteria were used, the score and the e-value. Each gene was analyzed independently, and the annotation was made according to these criteria. As a result, 41,301, 72,951, 39,499, 29,156, 11,894, 31,314 Unigenes for *M*. *monax* and 33,957, 58,403, 32,318, 24,258, 10,040, 26,163 Unigenes for *M*. *himalayana* were annotated using the NR, NT, Swiss-Prot, KEGG, COG, GO databases, respectively. In total, 73,603 and 58,893 annotated sequences were identified *for M*. *monax* and *M*. *himalayana*, respectively (Table 3).

**Table 3:** The statistics of Unigenes annotated by different databases.

Sequence File	NR	NT	Swiss-Prot	KEGG	COG	GO	ALL
**Mm-Unigene. fa**	41,301	72,951	39,499	29,156	11,894	31,314	73,603
**Mh-Unigene. fa**	33,957	58,403	32,318	24,258	10,040	26,163	58,893

The Unigenes were aligned by Blastx (e-value < 0.00001) with protein databases such as the NCBI ‘non-redundant’ database (NR), Swiss Institute of Bioinformatics databases (Swiss-Prot), Kyoto Encyclopedia of Genes and Genomes (KEGG), Clusters of Orthologous Groups of proteins (COG), Gene Ontology (GO). The numbers of annotated Unigenes are listed.

**Fig 3:**
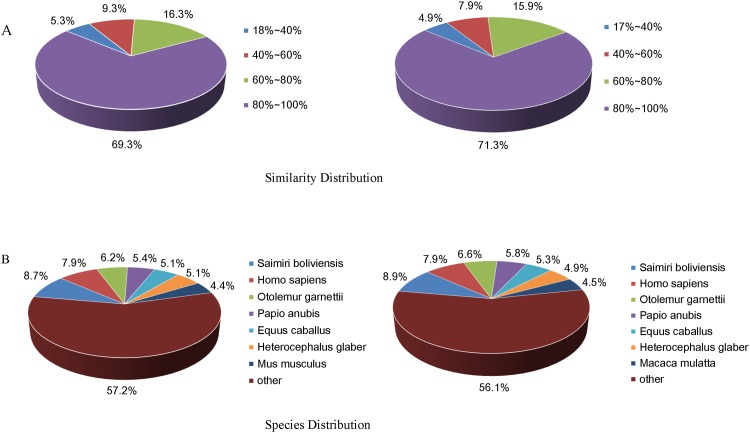
The identity and species distribution of Unigenes of *M*. *monax and M*. *himalayana* to the NR database. All Unigene sequences of *M*. *monax and M*. *himalayana* were aligned to the NR database by blastx. (A) The distribution of Unigenes with different sequence identities to the NR database is shown. (B) Species distribution is shown as the percentage of total homologous sequences of Unigenes with an e-value of at least 1e-05.

The identity distribution of top hits in the NR database indicated that 69.3% of the *M*. *monax* Unigenes and 71.3% of the *M*. *himalayana* Unigenes have sequence identity higher than 80% (Fig 3A). Among the annotated sequences, the species with the highest number of best hits was *Saimiri boliviensis* (8.7% matched genes of *M*. *monax* and 8.9% of *M*. *himalayana*), followed by *Homo sapiens* (7.9% of *M*. *monax* and *M*. *himalayana)* and *Otolemur garnettii* (6.2% of *M*. *monax* and 6.6% of *M*. *himalayana*) (Fig 3B).

**Fig 4:**
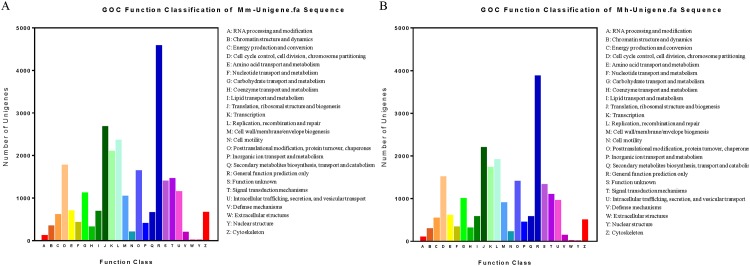
Histogram of GO classification of *M*. *monax and M*. *himalayana* transcripts. The Unigenes of *M*. *monax (A) and M*. *himalayana* (B) were annotated by GO classification analysis and the results are summarized for the three main GO categories: biological process, cellular component and molecular function. The left axis indicate the percent of sequences of each category, and right axis shows the total number of genes in each category.

Based on sequence homology, Unigenes of both species were categorized into 40 functional groups, belonging to three main GO ontologies: molecular function, cellular component and biological process. Similar category distribution of Unigenes was observed between *M*. *monax* and *M*. *himalayana*. Results showed a high proportion of genes from the categories of: “biologic regulation”, “cellular process”, “metabolic process”, “cell”, “cell part”, “organelle”, “binding” and “catalytic” with only a few genes related to “cell killing”, “extracellular matrix part”, “nucleoid”, “channel regulator activity”, “antioxidant activity”, “receptor regulator activity” and “translation regulator activity”. No Unigenes were clustered as “carbon utilization”, “virion”, “virion part”, “chemoattractant activity”, “chemorepellent activity”, “merallochapter activity” or “protein tag”(Fig 4).

**Fig 5:**
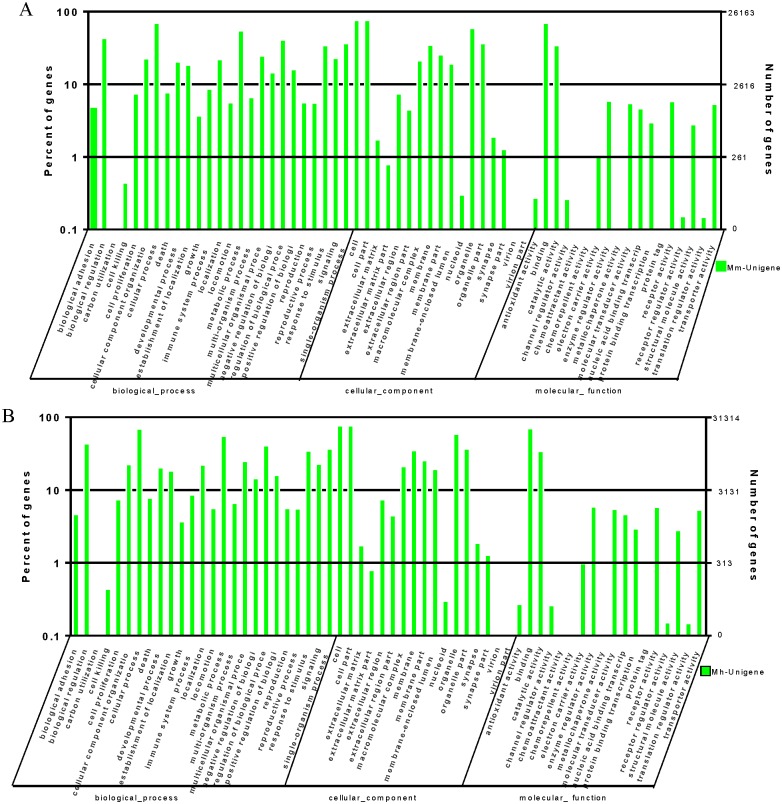
Histogram of COG classification of *M*. *monax and M*. *himalayana* transcripts. The Unigenes of *M*. *monax* (A) and *M*. *himalayana* (B) were annotated by COG classification analysis and the results are summarized into 25 clusters with indicated functions.

To further differentiate the annotated sequences at the protein level, all Unigenes were aligned to COG database and were grouped into 25 categories based on their putative functions. A high similarity of the distribution pattern of GOC function classification between *M*. *monax* and *M*. *himalayana* was observed. The category with the most number of Unigenes classified is “general function prediction” (4576 for *M*. *monax* and 3870 for *M*. *himalayana*), followed by “translation, ribosomal structure” (2671 for *M*. *monax* and 2190 for *M*. *himalayana*) and “replication, combination and repair” (2351 for *M*. *monax* and 1904 for *M*. *himalayana*). “Nuclear structure” represents the smallest category for both species (4 for *M*. *monax* and 5 for *M*. *himalayana*) (Fig 5).

To further identify active biochemical pathways and to know about biological complex behaviors in *Marmota*, the transcript sequences were mapped to the Kyoto Encyclopedia of Genes and Genomes (KEGG) database. A Total of 29,156 and 24,258 transcripts were mapped in *M*. *monax* and *M*. *himalayana* respectively, and 258 pathways were predicted for both species. The data from KEGG annotation demonstrated that the largest predicted category was metabolic pathway (11.5% for *M*. *monax* and 10.7% for *M*.*himalayana*), which contained 3,347 and 2,591 transcripts for *M*. *monax* and *M*. *himalayana* respectively, and the second largest category was focal adhesion (4.2% and 4.3%). Other pathways predicted in Marmota included the following: pathway in cancer (3.7% and 3.9%); regulation of action cytoskeleton (4.0% and 3.9%); amoebiasis (3.4% and 3.0%); endocytosis (2.9% and 2.8%); MAPK signal pathway (2.8% and 2.7%); and HTLV-I infection (3.3% and 2.9%), et al (S4 Table).

The results of annotation of transcriptome provide a comprehensive fundamental functional classification and pathway identification for the two Marmota breeds, and will be a valuable resource for future researches involving metabolic processes and pathway function in the *Marmota* animal models.

### Protein Coding Region Prediction

**Fig 6:**
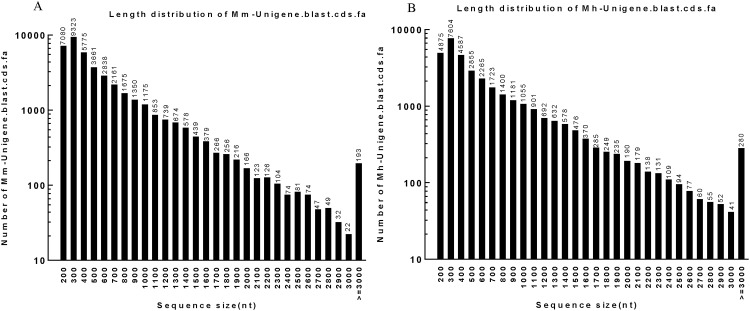
The length distribution of blast CDS of *M*. *monax and M*. *himalayana*. (A) The length distribution of blast CDS of *M*. *monax*. (B) The length distribution of blast CDS of *M*. *himalayana*.

**Fig 7:**
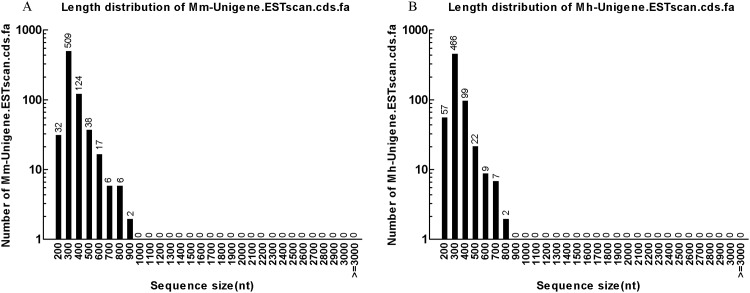
The length distribution of ESTscan CDS of *M*. *monax and M*. *himalayana*. (A) The length distribution of ESTscan CDS of *M*. *monax*. (B) The length distribution of ESTscan CDS of *M*. *himalayana*.

Next, the Unigenes were aligned by blastx (evalue<0.00001) to protein databases to predict their coding DNA sequences (CDS) in the priority order of NR, Swiss-Prot, KEGG and COG. Proteins with highest ranks in blast results were taken to decide the CDS of Unigenes, which were then translated into amino acid sequences with the standard codon table. The orientation and CDS of Unigenes that had no hit in blast were predicted using ESTScan. In total, 41,247 CDS (S1 and S2 Files) of *M*. *monax* and 34,033 CDS (S3 and S4 Files) of *M*. *himalayana* were obtained by blastx and ESTScan (Table 4). Fig 6 shows the length distribution of CDS by blastx of both woodchuck species ranging from 200 bp to over 3,000 bp, and the most abundant CDS were 300 bp and the least abundant were 3000 bp. The most abundant CDS predicted by ESTscan were also 300 bp in length for both species (Fig 7). Detailed CDS information is available in S1, S2, S3 and S4 Files.

**Table 4:** The statistics of CDS prediction of the Unigenes.

Sequence File	Sequence Number
**Mm-Unigene. blast. cds. fa**	40,511
**Mm-Unigene. ESTscan. cds. fa**	736
**Total**	41,247
**Mh-Unigene. blast. cds. fa**	33,369
**Mh-Unigene. ESTscan. cds. fa**	664
**Total**	34,033

The Unigenes were firstly aligned to protein databases and proteins with highest ranks in blast results were taken to decide the coding region sequences of Unigenes. The Unigenes that could not be aligned to any database were scanned by ESTScan. Numbers of the CDS of Unigenes are listed.

### Unigene SSR Analysis

**Fig 8:**
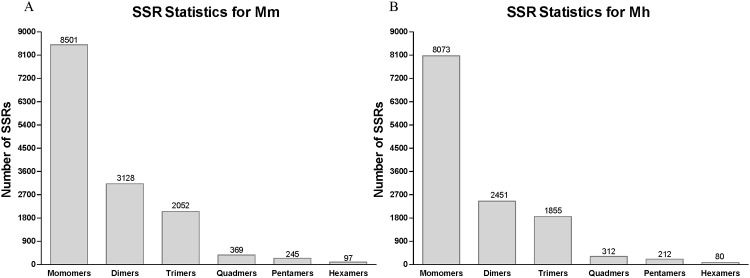
Unigene SSR Analysis. SSR analysis of all Unigenes was done by software MicroSAtellite. The number statistics of monomers to hexamers of SSR is shown. (A) SSR of *M*. *monax*. (B) SSR of *M*. *himalayana*.

Simple Sequence Repeats (SSRs), also known as microsatellites, are repeating sequences of 1–6 base pairs of DNA with conservative flanking sequence,which can generate SSR markers used for genomic mapping, DNA fingerprinting, and marker-assisted selection in many species [32, 33]. To identify the SSR for *M*. *monax* and *M*. *himalayana*, software MicroSAtellite (MISA) was utilized by using Unigenes as reference. In total, 14,392 SSRs for *M*. *monax* and 12,983 for *M*. *himalayana* have been identified. The mononucleotide SSRs accounted for the largest amounts of identified SSRs (8,501 for *M*. *monax* and 8,073 for *M*. *himalayana*), followed by dinucleotide (3128 and 2451), tetra-nucleotide (2,052 and 1,855), quad-nucleotide (369 and 312), penta-nucleotide (245 and 212) and the smallest amounts of hexanucleotide (97 and 80) (Fig 8). These SSRs will be useful as molecular markers for assaying the functional diversity in natural populations or germplasm collections.

### Heterozygous SNP Analysis

A single nucleotide polymorphism (SNP) is a variation in a single nucleotide that occurs at a specific position in the genome. SNP can be used to follow the inheritance patterns of chromosomal regions from generation to generation and are powerful tools in the study of genetic factors associated with diseases [34]. Here, the software SOAPsnp was utilized to identify the SNPs of all individual animals. In brief, the consensus sequences for the genome of each individual animal were assembled based on the alignment of the raw sequencing reads with the Unigenes of *M*. *monax* or *M*. *himalayana*. The SNPs were then identified on the consensus sequences by using the corresponding species Unigenes as references. Table 5 shows the numbers of different types of SNPs identified for all six animals used in this study. In all animals, the most frequent SNPs are A-G transition and the least are A-T transversion.

**Table 5:** The statistics of SNP Analysis.

SNP Type	Mm1	Mm2	Mh1	Mh2	Mh3	Mh4
**Transition**	7,688	10,910	7,918	5,428	4,196	4,720
**A-G**	3,849	5,521	3,989	2,659	2,044	2,309
**C-T**	3,839	5,389	3,929	2,769	2,152	2,411
**Transversion**	3,454	5,126	3,411	2,317	1,874	1,934
**A-C**	878	1,348	882	639	508	523
**A-T**	656	985	682	513	441	383
**C-G**	1,000	1,436	979	601	479	579
**G-T**	920	1,357	868	564	446	449
**Total**	11,142	16,036	11,329	7,745	6,070	6,654

The numbers of different types of SNP in all six transcriptome libraries are shown.

### Transcriptome sequences homology analysis

**Fig 9:**
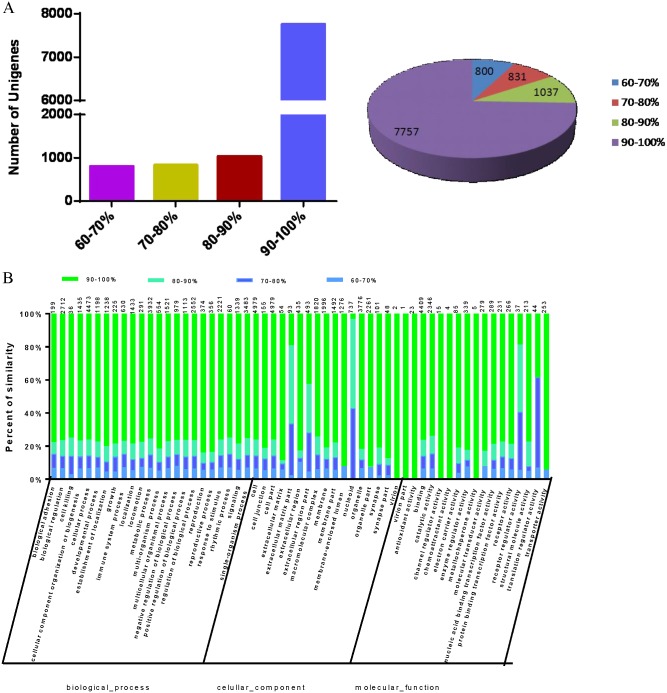
Transcriptome sequences homology analysis between *M*. *himalayana* and *M*. *monax*. A large-scale sequence comparison of the Unigenes with identical annotation between *M*. *monax* and *M*. *himalayana* was performed. (A) The distribution of *M*. *himalayana* Unigenes with different sequence identities to the *M*. *monax* Unigenes is shown. (B) GO functional classification was performed to analyze the homology distribution of the Unigenes in different functional categories.

Next, to characterize the transcriptome sequences homology between the two *marmota* species, we performed a large-scale sequence comparison of the Unigenes with identical annotation between *M*. *monax* and *M*. *himalayana*. When using the Unigenes of *M*. *monax* as the reference sequences, more than 74% (7,757) *M*. *himalayana* Unigenes with identical annotation showed a homology of 90–100%. This is followed by 9.9% (1,037) *M*. *himalayana* Unigenes with a homology of 80–90% to corresponding *M*. *monax* Unigenes. The *M*. *himalayana* Unigenes with a homology of 60–70% is about 7.67% (800) (Fig 9A). GO functional classification was also performed to further analyze the homology distribution of the Unigenes in different functional categories. The Unigenes in category of biological process showed the highest similarity in sequences between the two *Marmota* species. In all 57 functional classifications, only the Unigenes of extracellular matrix part, nucleoid, receptor regulator activity and translation regulator activity showed relatively lower homology (Fig 9B). Thus, these results indicated that *M*. *himalayana* shares a high similarity in transcriptome sequences with *M*. *monax*.

## Conclusions

Although both *M*. *monax* and *M*. *himalayana* have been proven to be useful animal models for studying HBV infection and HCC, the lack of genomic sequence information of them strongly limited their application breadth and depth. In this study, we successfully performed Illumina RNA-Seq technology to sequence the transcriptome of liver and spleen samples of *M*. *monax and M*. *himalayana*. Consensus sequences and Unigenes were generated by de novo assembly and functional annotation for both species. The Unigenes were aligned by blastx to protein databases to obtain the CDS information. The SNPs and SSRs were also analyzed for all Unigenes obtained. Moreover, a large-scale transcriptome comparison was performed and revealed a high similarity in transcriptome sequences between the two *marmota* species. This is the first time that the transcriptome of *M*. *himalayana* was fully sequenced and analyzed. Our study provides an extensive amount of novel sequence information for *M*. *monax* and *M*. *himalayana*. This information may serve as a valuable genomics resource for further molecular, developmental and comparative evolutionary studies, as well as for the identification and characterization of functional genes that are involved in WHV infection and HCC development in the woodchuck model.

## Supporting Information

S1 File Predicted CDS by blastx for *M*. *monax*. (DOC)

S2 File Predicted CDS by ESTScan for *M*.*monax*. (DOC)

S3 File Predicted CDS by blastx for *M*. *himalayana*. (DOC)

S4 File Predicted CDS by ESTScan for *M*.*himalayana*. (DOC)

S1 Table The basic information of *M*. *monax* and *M*. *himalayana* used in the study. (DOC)

S2 Table The information of Clusters for contigs for *M*. *monax*. (XLS)

S3 Table The information of Clusters for contigs for *M*. *himalayana*. (XLS)

S4 Table KEGG analysis of transcripts. (XLS)
