# Supplementary material for: Transcriptome Analysis and Comparison of Marmota monax and Marmota himalayana
Source: PLoS One. 2016 Nov 2;11(11):e0165875. doi: 10.1371/journal.pone.0165875 (PMC5091844; doi:10.1371/journal.pone.0165875)
Supplement: S2 File — (DOC) [file pone.0165875.s002.doc]

>CL66.Contig2_A-W 1 203 LEN=203

TGCAAGCATAATCAGAGAAGCTCCATAAATAACGAATCTAACCCCTTGTCTGGAACTXAG

CGAGACGAGAAGGCGGCAAAGATGTCGGAGCGCAAAGTTTTAAACAAATACTACCCTCCA

GACTTCGACCCTTCAAAGATCCCCAAACTCAAGCTCCCCAAGGACCGGCAGCGCGTGCAC

TTGATATAGAACCTGAAGATGGGC

>CL95.Contig2_A-W 1 235 LEN=235

XXTTCCAAATATATCTTCTCAGAAAGCAAGCACATTCCTACTGATGAGCATGCTAAAACA

CCCGATTTCCAGAGGTGGAAAGGCATTGCTATTGAAAAATACGAGCTAGAAGCAGCCAGG

ACTCAGGCAGCAGGACAAAATTCATCTGATTCAAAATATTTCAGCTGTCTATCCACTACC

ACTAAACTCCTTCCTACTGAGGAGCATGGGTTCCAGATATTCCAACAGGTTGCTCCT

>CL174.Contig1_A-W 1 399 LEN=427; minus strand

CCGACCATCAAAAGCAAAGATACAGATCCCCAAAAACCACAATGGAAGAATGGAATAAAA

ATACTGGAATCAGATTCACCTTGGGATAGTTCATCCGAAGAAGAGAAGGAGTCGCCTGAT

GGCACAGTAAATAGGCCTCAACAGAGTGTCTGTGAGAAGCCTCCAGAGGTTTGTGCTTCT

AGCATATCTGGTGAGTCCCATTCAGGAGCAAAGAATCGAATAAATGGACATAGAGAAGAT

ACAGGACATGTATCTTTGCACAAGGGTGAAAAAGAAAACATTCTATTGCCTAAAACAAAG

GAAGCAGGATTAGAAGATGTGCCAGGGTCCCAGACGGGTATGAAAAAAACACACATATAT

GTATTACCTGTTATTCTAAATGCATATATCTGGTCCAAT

>CL174.Contig2_A-W 1 326 LEN=354; minus strand

XAGTCACATGAGAATATGTCATCATCACGAGAGAAGGAAAGGCTTGATGGCATGGAAAAG

AGCAATCAGCAGAGTGTCTGTGAGAAGCCTCCAGAGGTTTGTGCTTCTAGCATATCTGGT

GAGTCCCATTCAGGAGCAAAGAATCGAATAAATGGACATAGAGAAGATACAGGACATGTA

TCTTTGCACAAGGGTGAAAAAGAAAACATTCTATTGCCTAAAACAAAGGAAGCAGGATTA

GAAGATGTGCCAGGGTCCCAGACGGGTATGAAAAAAACACACATATATGTATTACCTGTT

ATTCTAAATGCATATATCTGGTCCAAT

>CL174.Contig3_A-W 1 200 LEN=200; minus strand

CCGACCATCAAAAGCAAAGATACAGATCCCCAAAAACCACAATGGAAGAATGGAATAAAA

ATACTGGAATCAGATTCACCTTGGGATAGTTCATCCGAAGAAGAGAAGGAGTCGCCTGAT

GGCACAGTAAATAGGCCTCAACAGGTATGTAAATGTGTCAACTGTTTGTCCCTTCTTTGC

TTTGTGAAGGGACATTATGGX

>CL242.Contig5_A-W 1 261 LEN=261

XXCAGAATATTTGTTAAAATGATTTACAATTTCAAATGGTGGATGAGAGAACAGAGGTAT

GTAGTATGTGATATTCATGAAGGAGGAAGAGAGAATCTAGAAGTAGAAATTCACAGGAAG

AGTGGAAGAGTTGCTGACAAAGATTGGCTGTTGGTTGGAGGAAAATTGGAAAGAAAATCC

AAGAAAATAGACAAGAGAGAGGAAGAATACAAAGAAAGAGAAAATATTAAAGACAGAAGA

ATACAACAAAAATGCAAAACACCX

>CL242.Contig6_A-W 1 257 LEN=257

GAAAATTTGTTAAAACGGTTTACAATTTCAAATAGACAAGAGAACAGAGGTAGTATAGTA

TGTGATGTTCTTGAGGGAAAAAGAGAGAAGCTAGAATTAAAAATTCACAGGAAAAGTGGA

AGAGGAGTCAACAGAGATTGGCTGTTGGTTGGAGAAAAGTTGGAAAGAAAATCCAAGAAA

ATAGACAAGAGAGAGGAAGAATACAAAGAAAGAGAAAATATTAAAGACAGAAGAATACAA

CAAAAATGCAAAACACCX

>CL327.Contig2_A-W 1 351 LEN=859; minus strand

CACCAGCAAAAGAGTGATTCTGAGAAACATCAAAAGGTATGTGCTGCTTGTGGGTCCAGG

ACGTTTAAACCAAGCACAAAGACAATAAATGGACATGGACAAGGTAAATCTGAAATAAGG

AAAACCACAGAAGAATGGGGAGTGAAGACTGAGAAAGGCAATGTTATATCACTTTCTTCA

TACAAGAGAGGAAATTTACAACTGATGTTGGTTAAACGAGTGGATAAAGGAAAAGATGAC

ACACCAGTTTCCCACTTGGAGCCACATTTGGACAGATCATCCAAGGCAGAACAGAAGAGT

TTTCATGATATTGATGATACTGCACATCGCCAGCAAAAGGTAGCACATGTG

>CL327.Contig3_A-W 553 1250 LEN=2171; minus strand

GACGTACATGCTTCCTACAAGCCGACCACCAAAAGCAAAGATACAGTTCCCCCAAAACGA

AAACGGAACAATGGCCCAAAAACACTGGAATCAGAGTCACATTGGGACAAGTCATCATCT

GAACAGAAGGAATGGCTTGATGTCACAGAAAATCXGTCATCAACAGATATGGAACGACTA

TCTTCACACAAGACAGAAAAAAGCAATTTGATGTTGTTTAAACCAGAGGATACAGGAAAA

GGAAATGCACCATTTTTCCACATGGCAGAGTCACATTTGGACATATCATCAGAGGCAAAA

CAGAAGAGGCCTGATGATACTGGACATAGCCAGCAAGAGAGTGATTCTGAGCAAGATAAT

GAGTGTACTTCTTGTCTATTTGGTGTATTCCGTTCACCCACAAAAAAAGGAATAAAAGGA

TATGTAAAAGGACAAAAACACAGTGAAGAAACAGCAAATGGAAAAGGCAAAAGAAGTACA

AATAGTTGTGGAGCATTCCTGAAACCCACAGCAGAGTTAAATTTGGACAGATCATCTGAG

GCAGAACAGAAGAGGCTTCATGATATGGATGATACTGCACATCACCAGCAAAAGAGTGAT

TCTGAGAAACATCAAAAGGTATGTGCTGCTTGTGGGTCCAGGACGTTTAAACCAAGCACA

AAGACAATAAATGGACATGGACAAGGTAAGAACCATATA

>CL327.Contig4_A-W 471 1427 LEN=2394; minus strand

AAGGGTGAAAAAGAAAACATTCTATTGCCTAAAACAAAGGAAGCAGGATTAGAAGATGTG

CCAGGGTCCCAGACGGGCTCTAAGGACGTACATGCTTCCTACAAGCCGACCACCAAAAGC

AAAGATACAGTTCCCCCAAAACGAAAACGGAACAATGGCCCAAAAACACTGGAATCAGAG

TCACATTGGGACAAGTCATCATCTGAACAGAAGGAATGGCTTGATGTCACAGAAAATCGT

CATCAACAGAGTCACTCTAAGCAACATCCAGAGGTGTCTGCTTCTTGTGTATCTGGTGAG

TTTCCTCAAAGAGCAAAGAAAGAAGATATGGAACGACTATCTTCACACAAGACAGAAAAA

AGCAATTTGATGTTGTTTAAACCAGAGGATACAGGAAAAGGAAATGCACCATTTTTCCAC

ATGGCAGAGTCACATTTGGACATATCATCAGAGGCAAAACAGAAGAGGCCTGATGATACT

GGACATAGCCAGCAAGAGAGTGATTCTGAGCAAGATAATGAGTGTACTTCTTGTCTATTT

GGTGTATTCCGTTCACCCACAAAAAAAGGAATAAAAGGATATGTAAAAGGACAAAAACAC

AGTGAAGAAACAGCAAATGGAAAAGGCAAAAGAAGTACAAATAGTTGTGGAGCATTCCTG

AAACCCACAGATGTTGGATCACTTTCTTCACACAACAGAGGAAAAATAAAATTGGTGTTG

GTTGAGCCACCTTATACAGGAAAGGAAGCTATATCAGTTTCCCACATGGCAGAGTTAAAT

TTGGACAGATCATCTGAGGCAGAACAGAAGAGGCTTCATGATATGGATGATACTGCACAT

CACCAGCAAAAGAGTGATTCTGAGAAACATCAAAAGGTATGTGCTGCTTGTGGGTCCAGG

ACGTTTAAACCAAGCACAAAGACAATAAATGGACATGGACAAGGTAAGAACCATATA

>CL327.Contig5_A-W 553 1395 LEN=2316; minus strand

GACGTACATGCTTCCTACAAGCCGACCACCAAAAGCAAAGATACAGTTCCCCCAAAACGA

AAACGGAACAATGGCCCAAAAACACTGGAATCAGAGTCACATTGGGACAAGTCATCATCT

GAACAGAAGGAATGGCTTGATGTCACAGAAAATCGTCATCAACAGAGTCACTCTAAGCAA

CATCCAGAGGTGTCTGCTTCTTGTGTATCTGATATGGAACGACTATCTTCACACAAGACA

GAAAAAAGCAATTTGATGTTGTTTAAACCAGAGGATACAGGAAAAGGAAATGCACCATTT

TTCCACATGGCAGAGTCACATTTGGACATATCATCAGAGGCAAAACAGAAGAGGCCTGAT

GATACTGGACATAGCCAGCAAGAGAGTGATTCTGAGCAAGATAATGAGTGTACTTCTTGT

CTATTTGGTGTATTCCGTTCACCCACAAAAAAAGGAATAAAAGGATATGTAAAAGGACAA

AAACACAGTGAAGAAACAGCAAATGGAAAAGGCAAAAGAAGTACAAATAGTTGTGGAGCA

TTCCTGAAACCCACAGATGTTGGATCACTTTCTTCACACAACAGAGGAAAAATAAAATTG

GTGTTGGTTGAGCCACCTTATACAGGAAAGGAAGCTATATCAGTTTCCCACATGGCAGAG

TTAAATTTGGACAGATCATCTGAGGCAGAACAGAAGAGGCTTCATGATATGGATGATACT

GCACATCACCAGCAAAAGAGTGATTCTGAGAAACATCAAAAGGTATGTGCTGCTTGTGGG

TCCAGGACGTTTAAACCAAGCACAAAGACAATAAATGGACATGGACAAGGTAAGAACCAT

ATA

>CL327.Contig6_A-W 553 1349 LEN=2270; minus strand

GACGTACATGCTTCCTACAAGCCGACCACCAAAAGCAAAGATACAGTTCCCCCAAAACGA

AAACGGAACAATGGCCCAAAAACACTGGAATCAGAGTCACATTGGGACAAGTCATCATCT

GAACAGAAGGAATGGCTTGATGTCACAGAAAATCXGTCATCAACAGATATGGAACGACTA

TCTTCACACAAGACAGAAAAAAGCAATTTGATGTTGTTTAAACCAGAGGATACAGGAAAA

GGAAATGCACCATTTTTCCACATGGCAGAGTCACATTTGGACATATCATCAGAGGCAAAA

CAGAAGAGGCCTGATGATACTGGACATAGCCAGCAAGAGAGTGATTCTGAGCAAGATAAT

GAGTGTACTTCTTGTCTATTTGGTGTATTCCGTTCACCCACAAAAAAAGGAATAAAAGGA

TATGTAAAAGGACAAAAACACAGTGAAGAAACAGCAAATGGAAAAGGCAAAAGAAGTACA

AATAGTTGTGGAGCATTCCTGAAACCCACAGATGTTGGATCACTTTCTTCACACAACAGA

GGAAAAATAAAATTGGTGTTGGTTGAGCCACCTTATACAGGAAAGGAAGCTATATCAGTT

TCCCACATGGCAGAGTTAAATTTGGACAGATCATCTGAGGCAGAACAGAAGAGGCTTCAT

GATATGGATGATACTGCACATCACCAGCAAAAGAGTGATTCTGAGAAACATCAAAAGGTA

TGTGCTGCTTGTGGGTCCAGGACGTTTAAACCAAGCACAAAGACAATAAATGGACATGGA

CAAGGTAAGAACCATATA

>CL327.Contig7_A-W 471 1328 LEN=2249; minus strand

AAGGGTGAAAAAGAAAACATTCTATTGCCTAAAACAAAGGAAGCAGGATTAGAAGATGTG

CCAGGGTCCCAGACGGGCTCTAAGGACGTACATGCTTCCTACAAGCCGACCACCAAAAGC

AAAGATACAGTTCCCCCAAAACGAAAACGGAACAATGGCCCAAAAACACTGGAATCAGAG

TCACATTGGGACAAGTCATCATCTGAACAGAAGGAATGGCTTGATGTCACAGAAAATCGT

CATCAACAGAGTCACTCTAAGCAACATCCAGAGGTGTCTGCTTCTTGTGTATCTGGTGAG

TTTCCTCAAAGAGCAAAGAAAGAAGATATGGAACGACTATCTTCACACAAGACAGAAAAA

AGCAATTTGATGTTGTTTAAACCAGAGGATACAGGAAAAGGAAATGCACCATTTTTCCAC

ATGGCAGAGTCACATTTGGACATATCATCAGAGGCAAAACAGAAGAGGCCTGATGATACT

GGACATAGCCAGCAAGAGAGTGATTCTGAGCAAGATAATGAGTGTACTTCTTGTCTATTT

GGTGTATTCCGTTCACCCACAAAAAAAGGAATAAAAGGATATGTAAAAGGACAAAAACAC

AGTGAAGAAACAGCAAATGGAAAAGGCAAAAGAAGTACAAATAGTTGTGGAGCATTCCTG

AAACCCACAGCAGAGTTAAATTTGGACAGATCATCTGAGGCAGAACAGAAGAGGCTTCAT

GATATGGATGATACTGCACATCACCAGCAAAAGAGTGATTCTGAGAAACATCAAAAGGTA

TGTGCTGCTTGTGGGTCCAGGACGTTTAAACCAAGCACAAAGACAATAAATGGACATGGA

CAAGGTAAGAACCATATA

>CL327.Contig8_A-W 553 1296 LEN=2217; minus strand

GACGTACATGCTTCCTACAAGCCGACCACCAAAAGCAAAGATACAGTTCCCCCAAAACGA

AAACGGAACAATGGCCCAAAAACACTGGAATCAGAGTCACATTGGGACAAGTCATCATCT

GAACAGAAGGAATGGCTTGATGTCACAGAAAATCGTCATCAACAGAGTCACTCTAAGCAA

CATCCAGAGGTGTCTGCTTCTTGTGTATCTGATATGGAACGACTATCTTCACACAAGACA

GAAAAAAGCAATTTGATGTTGTTTAAACCAGAGGATACAGGAAAAGGAAATGCACCATTT

TTCCACATGGCAGAGTCACATTTGGACATATCATCAGAGGCAAAACAGAAGAGGCCTGAT

GATACTGGACATAGCCAGCAAGAGAGTGATTCTGAGCAAGATAATGAGTGTACTTCTTGT

CTATTTGGTGTATTCCGTTCACCCACAAAAAAAGGAATAAAAGGATATGTAAAAGGACAA

AAACACAGTGAAGAAACAGCAAATGGAAAAGGCAAAAGAAGTACAAATAGTTGTGGAGCA

TTCCTGAAACCCACAGCAGAGTTAAATTTGGACAGATCATCTGAGGCAGAACAGAAGAGG

CTTCATGATATGGATGATACTGCACATCACCAGCAAAAGAGTGATTCTGAGAAACATCAA

AAGGTATGTGCTGCTTGTGGGTCCAGGACGTTTAAACCAAGCACAAAGACAATAAATGGA

CATGGACAAGGTAAGAACCATATA

>CL343.Contig1_A-W 1 413 LEN=413; minus strand

CAGGAAGCCCGGCCCAACGAGGCCCATCCTGCACGAGGCCCCGCCCCGGAGGAAGCCCGG

CCCACCGTGACCCCGCCCCTCGCCGTGGTTTCCACGCCGGCGGCCGCCACAGCTCTTCTC

TTGGCCGTTGTCCCTCGCCGCCTGTCCACTCGCCTCACGCTGGCCGCTGACAGCTCCTTC

CCAGTACCTGAGGAAGGATGGTGAGGGGAGTCCATGACGCCCTACCTGCTGGCCCTCTCC

TCCCTGCCGCCTTATCAGTGGTCCAGGACCTCCAGCTGTCCCCTGGCGCCCGCGCGCTCC

CGAAGCACCTTTCTGCGGCGCGTCGAGATGACGGCCCAGCGTTTCGGGTCTTCGCCGCCC

CAGAGCCTGCTGAGGGCCGCCATGTTCGTGTGGAGGGGCGGGGTCACGGGGCGX

>CL584.Contig2_A-W 1 205 LEN=205

XACAAAAACAGCATGGTATTGGCAGCAAAACAGACTGGTAGACCAATGGAACAGAATAGA

GCATCTGGAAATAAGTCCACATGTACAACAGAACTGACTTTCAACAAAGATACCAAGAAT

ACATAGGCAAATGATGTTGGAACACTGAATATCCACAAGCAGAAAATGAAATTAGACCTT

TTTTTTGGGCTGGGGATGTGGCTCAAX

>CL809.Contig2_A-W 1 547 LEN=547

XATCTTGAGTTACCTCCGCTATTGCCACCACCACCTTTAGTTGCAGTAGCATCCATCGTG

GACATTGGCTGGGGAATTGAAGCTCAATACCAAAGTGAGGTACAGGTAATCTTTAGGGGC

ACTATAGGGTTATAGGGTAAAAACTGTTAATACCTGAGATCTGCATTGCAAGACAGGAAG

ACACATAAACAAAATGGAAAACAAGGGAAGAAAGTGCTCCAAACAAATCAAGATACAACA

ATAATAGAATTCATGGACAGCGCAGTTGATGAAATATCAGAAAAAGAGTTCAGAATGTAC

ATAATTAAAATGATCTATGAATTAAAGAATGACTTAAGTGAGCAAATACAGGCAAAAATT

GATCACTCCAACAAAGAGATAAGGGAGCAAATACAGATAGCAAAAGACTACTACAAAAAA

GACAGAGATTCTGAAAAAAAAATCAGAAATCTTTGAAATGAAGGAAACAATAAATTAAAT

TTAAAAAATTCAACAGAAAGCATCACCAACAGACTAGATCACTTGGAAGACAGAAGATCA

GATAAGGXX

>CL1306.Contig1_A-W 1 494 LEN=779; minus strand

GAATCAGGGAACATTTAGGTGGTGTTCATGTTACACATTATTAGCATTCGTGTCAGTCAA

TGGAGAAGGAATGGATTAAAGCTGTCCAGCTGCATTGGAGGGATTCCACACCCTGGAGGG

ACATCCAAAGATGCAGAAGAGCXGTACTCCCTGGGGGACCTGGAGGAGACCCTGGAGCCT

GCCTTCTTCTATGACTGTTTCTCTTCGGCCTCAAACATCTCGAGTTCCTCCACCGAACAA

GAGATGGGCCAAATGTTGGGAAGCCAGGTCACTGTCACAGGCCCCGAGGAGGAGGACCAA

GGACACCAGGTCCCAGCCTTGACAGAAAACCCTGAGCCCATGGAGGAACAGGCCAAAGCA

GCTCTGGTGCTTGTGGAAGCTCCCGTTCGAGGTCCAACATCTCCACCATCTGATAGAGAA

CTGGAGCTGGACGCACTTGCCTTGGACCATCAAGGCCAACGTATCCTGCTGGCCCTTCAG

TCAGCACTGCATCTA

>CL1403.Contig3_A-W 1 274 LEN=274; minus strand

XXTTGGAAGGCAGCTGATGTGGAACAAGCAAATAAGTTCTCCAGGGGGAGCCAGCGCCAT

GCTGTAAGAGAGGACCCCATGGCTATCCGGGAAGTGGATCTTCTAGCCCCAGTCAAGCCT

TAGCTGACTGCAGCCCTGGCTGACATCTTCACTGCAAACTCCTGAGACAGCTTGATCCAG

AAGCATCCAGCTAAGCTGCCCAAATTCCTCACCAACACAAACTGTGAGACAATAAAAGCT

TGTTGTTTAAACACACCCTCAATAACCATAGGTCCC

>CL1599.Contig3_A-W 1 95 LEN=560

XCCCCCATGCTGACTGTCCCTCTGTCTCGGCTCCAGCCTCATCCCAACGTCAAGCCCATG

GCCTACGCCAACACGCTGCTGCAGCTAGGAATGATG

>CL1691.Contig2_A-W 1 322 LEN=322; minus strand

CCTTATCTCCGATGTCGGTGGAGCAAAGGAGTCCTGGTGAAAGAAGAGCGGTTATGGGAA

CAAGAAAAGGGCCCCATGCACAACACTCAGATAAACACAAGACAGACGGCCGGCGAACAG

CAAGAAGACGCTGGAGAAACGACAAACAGCAGAACAGACACAGGACAAGCAACAATCGTT

GAGATCACACCCAGAGACCTCTAATGGGGCAGAGACCCCGCAAACAGAACAAACGGCTGG

CGAACTGACACTGAACAAATGACCAACAGCAGAAGACCCTGGAGAAATGACAAACAGCAG

AACAGACACTGGACAAGCAACAXX

>CL1714.Contig2_A-W 1 203 LEN=203; minus strand

XTTCGAATCATGATCCTAAATGACCTAGTGTCCTCGTGGCTGCAGGAAGAGGAAGCCAAT

GGGGAGCAGGATGCCTTGGACCTGTACCAACACAGCCTGGGGGAGCTGCTGCTGCTGCTG

GCAGGTGAGATGCCAGCACCACCCACACCAGACCCCTGGCAGCTTTGTCCTCACCACCTG

CCCTCTCTTTACAGCGGAACCCCC

>CL1904.Contig3_A-W 1 304 LEN=304

XACTCAATCTTTCTTTCCTTCCATAAAAGGTATTTTACAGCTTTCACCACAGCTGCATCA

GCTTACAGTTATTATCATTATGGTCGGAAAACTGTTCAGGTGATAAAAGGCAGATGAGAG

TCACCCATCACCATTAGCAAGGAAGCAATACACATCATCCGTGGCAATGCCAGCGAAAAT

CTACAGGAGTTTCCCTATTTTGGTGTTCAGCTTGTAAAAGGACTTGTCAGAACAAACCAT

GTCATCAAAATTGAAGTAATGTGCATTGAAAATAAGCTTGATCATGGGCATATGCAGAAT

TTCCAX

>CL2160.Contig1_A-W 1 508 LEN=508; minus strand

CTGTGGTTTAAAATGGCTGGCAGGGAAGATCAGGAAGGTCTGTACCAGGGCATGGTGGCT

TTCCAATCACAGGGCAAGAACAAAGGCAAGATCATGGAACACACTAGAATGAAGCTGATC

AAAGACCTTTATTCTGGTCCTCATCATTCATTAAGCCACCTATGCAAACAATTTCTTCAT

CTCTGCCCCAAGCAGCAACAGCTGCATCCAGGGCTAAAATGGAAGACAGACATAAGTAGT

GGAAGACCTTATAAAGAAAAGTTATCAGGTAAGAGCTCTGCTTATCCCGGCTTAAGTAAA

GGTAGAGCAGGAAGATGTGCATATGTGCAGAGATTCTGTTCTTCAAAGCTGAGGCCTGCA

GAGAGGCAAGCTGAAGAATGCAGTGAAGGCTCAAAAACTGTCCAGTTCAGCAATCAATCC

AAAGTACCTTCCAGAAAAGCAAATTCATCCACAGCTTCTATTGCATTATCTGAAACACAA

GGAAAAGGGACACTTGACTGTAGTCATTXX

>CL2371.Contig2_A-W 1 214 LEN=214; minus strand

CCTAACCCAAAGCTCTTCAAGGAGACGGAGCCCGAGGACCTGCCCGTGGCCCCGCGTTCT

GTGGACGGACGTGAGCATCAGGTGCTGXATGTACAGATGGAGACTGGCCCTTCTCCTGGT

CCTCTCGTGGTCCTGAGCTCTCAGCCGAAGGCCCCCGCCCCGACAGACAGCCGACTGCAA

GCTGGACTGAGCACGGCCCTCCTGGACACGGGCACX

>CL2475.Contig1_A-W 1 543 LEN=543; minus strand

XXTTGGACAGATAATGAAGATAAGCAAGTCTTTGGTGACTGTCAGTCTTGGGCTGGTGTT

TATTTGAACGTGGCCGTGGGTGGAAACAGAACAGGGGCTGTCCAGATTTTTGTGGGTGGC

ACATATTCAACAATCCTTTTTGAATGGCCCCTCAGTTTGTCACTCACAGTCATCACCCTC

AGTAACACCGTTCTATTGAGAATAACAAGTACATCGTGGGAGGATAACGAGTGGACTCCA

TGTAGACTCCAGGGTCTTGGTGTCCAAGCATCATTTGGTAGAAATAGTCACTGGTAGAGC

CACTCAAAGACTCAGGTCCTAGACTTCACAATTGCTGTTTTACTGGCAAAGAGATAGAGG

TCTAGCGATGTGCCCATGGGCCCCCATCTAGTGAATTATGGAGGTAAGACTGGAGTCCAG

CAGTCTCAGGATGGAGTCTGGGGTGCTAGTGGCCGAGTTCCAAATCCCCTTTTGCTGTAC

CTGGAACATAAXTTAAAATCCAATAAAAAGGCGGCAGATGTCACTGCTGTTGATGTTATT

ATGAGG

>CL2475.Contig3_A-W 1 796 LEN=796; minus strand

XXTTGGACAGATAATGAAGATAAGCAAGTCTTTGGTGACTGTCAGTCTTGGGCTGGTGTT

TATTTGAACGTGGCCGTGGGTGGAAACAGAACAGGGGCTGTCCAGATTTTTGTGGGTGGC

ACATATTCAACAATCCTTTTTGAATGGCCCCTCAGTTTGTCACTCACAGTCATCACCCTC

AGTAACACCGTTCTATTGAGAATAACAAGTACATCGTGGGAGGATAACGAGTGGACTCCA

TGTAGACTCCAGGGTCTTGGTGTCCAAGCATCATTTGGTAGAAATAGTCACTGGTAGAGC

CACTCAAAGACTCAGGTCCTAGACTTCACAATTGCTGTTTTACTGGCAAAGAGATAGAGG

TCTAGCGATGTGCCCATGGGCCCCCATCTAGTGAATTATGGAGGTAAGACTGGAGTCCAG

CAGTCTCAGGATGGAGTCTGGGGTGCTAGTGGCCGAGTTCCAAATCCCCTTTTGCTGTAC

CTGCAAGGGAGTGAGGAATTCTGGGCCCTGTGTCCTGGGGTAGCTGCGCTGATGCTGACC

GCCAATCACAGTCGTGGGGAGAAGGTATCTGGAGGGACAGCTTCTTCCAAGTTGAAGCTT

CCAGCTTCCTCGAAGACCATTGTTCCTTCCCTTTCGCCTTCTGTGACTGCACAGGTCCCT

CAGAGACATCCATTACTTCTCCTGCATTCAGGTAGAACTCCGTGGGTCTCATTTAGAGGA

CACATTACACAGAATTACAGCCATTGGTTTATCTTCATCTCTCCACTAGACTGTGAACTC

AGCAAAGTGCACAGCTCT

>CL2475.Contig4_A-W 1 639 LEN=639; minus strand

XXTTGGACAGATAATGAAGATAAGCAAGTCTTTGGTGACTGTCAGTCTTGGGCTGGTGTT

TATTTGAACGTGGCCGTGGGTGGAAACAGAACAGGGGCTGTCCAGATTTTTGTGGGTGGC

ACATATTCAACAATCCTTTTTGAATGGCCCCTCAGTTTGTCACTCACAGTCATCACCCTC

AGTAACACCGTTCTATTGAGAATAACAAGTACATCGTGGGAGGATAACGAGTGGACTCCA

TGTAGACTCCAGGGTCTTGGTGTCCAAGCATCATTTGGTAGAAATAGTCACTGGTAGAGC

CACTCAAAGACTCAGGTCCTAGACTTCACAATTGCTGTTTTACTGGCAAAGAGATAGAGG

TCTAGCGATGTGCCCATGGGCCCCCATCTAGTGAATTATGGAGGTAAGACTGGAGTCCAG

CAGTCTCAGGATGGAGTCTGGGGTGCTAGTGGCCGAGTTCCAAATCCCCTTTTGCTGTAC

CTTCTGTGACTGCACAGGTCCCTCAGAGACATCCATTACTTCTCCTGCATTCAGGTAGAA

CTCCGTGGGTCTCATTTAGAGGACACATTACACAGAATTACAGCCATTGGTTTATCTTCA

TCTCTCCACTAGACTGTGAACTCAGCAAAGTGCACAGCTCTX

>CL2715.Contig2_A-W 1 205 LEN=205

XXGTTGAAGTCATCTGATTCAAGGCCAGTTATTGCCCTTTGTAACTCACACTTTGACCTA

GAAAATCTTGGTCACTTGTACAAGTTCTACAGTCAGTCATCTATATACCAGAACCTAGTG

AGCAAAACCTTTAAGTTGGCTGGGTTACTGATCTTGGGATGTAAGAGATCTGGTTTTGAA

TGTAAAATTCATATTGCACAATCACCA

>CL3151.Contig1_A-W 1 215 LEN=215; minus strand

XXCCTTTTCTTTGAGAAGCAAGGACCTAAATTCAGCAAGCCAGTCTTTTTGGGAAACAAC

AAGGAAAACTGTGACAGATTCTGCTCAAAATGTAGAAATAACATCAATAAAGTGAGATTT

CAGACAGCAACAGTGTGCAGGCAAAAGATACTTCCACTGCCACAAGAGCAGCTTTTGAAA

AGCAAGGAGACCTATGCTGACCACACCCAAAACACAGXX

>CL3304.Contig1_A-W 1 268 LEN=268; minus strand

XGCCACTGTGGACATCCAGCTTCAGGTCAAGTAGGCCAATCTAATAAGTCCCCTTTTATA

ATCACACTTCGTGTTGATGCTGTTCCTCTAGAAATCATTGATTAACAAACTATAAGTCTA

AAATTGCTCATTTTTGGCTTCTCTGGTGACCTGACTCCTCTGGAGAAACCTCCGGGCACC

CGAGATCCCCGGGAACACGTGCAGCGCAGAGTCACCTGGGTCCCTGGAGCAGAGAACACG

GGGCCTGAAGAAGCAGTAGCCCAGTCGGXX

>CL3384.Contig1_A-W 1 316 LEN=316

TGAAGATCAGTGTACTTGAGTCAGCGATGGAGTCCTCTAAGTCATACCTTTGTTGGAACA

GGAAAGTTGACTGTGGAAAAGAACATAGGTCAGTATTGGGATGGGGTCTTGGTGGGTAGG

GGTGCTCTGGTCTCCCCACCTTCTCACATTATGCCAACAAGGACACAGAGACATTTAGAC

ACTTTGCAGGAAGAGAATCCAGGAGTTCTTAGAGTCACAAAGAGGAGGCTTGAACAAATC

TTCTTTCAGTCCCAAACAAAGCAGCTGTCTCAGACTACAGAACAAAATAATCAAGAACAA

ATTCAGGTCAAGCGCTXX

>CL3446.Contig5_A-W 1 400 LEN=815; minus strand

XXAGGAGCCAATCAGCTAGATGTTGCTGGGGCCGCTGTGAGCCAATCATCAGCCAGCAGC

TGGAAGTTTGCTGGCAGCTGGAAGTTTGCTGGGGCCCCTTCGGCTGTGGCTCTCAACATC

TCCCCCTCTCTGTTTAAACAACAAGCATGTGGCTTAGGGACCGTGCCTGTTAGGCAGTCC

AATTCAACATATGGTCCTTACCCGTCATCGGATGAACTGACCTCTAGGCGTCAGCCTCCT

GTCTTAGGTTGGTACCACTGCAATTGGATCATACCCGTCACTGACTACCGGTCCAGCATA

AGCCATTGGCCCCCAAATTTTAGACCATCACTAGTAGAGGAGGATGCAAAATGCCATGAC

ATTAAGCCAATTGAGGGCTCCTTTGAAAAATTGTACCACCGG

>CL3446.Contig7_A-W 1 214 LEN=214; minus strand

XXTCATCCCAGTGCCGGTGAAGACAAAGATAAAACCAAACTACAGCTTCTTCGATAGTTA

TCACAACAAACTGTAAAAACTGATGCATCAATGAATAATAACTCAACAAGTAATGCTCAA

TTAAGGAGTCGATTTACTTTGGGAGGAAATGGACATATTGACAGATTCCTCTGCTTTGAA

TTGCTTGCAGACCTTGCCTGGACTATGTATCACTTG

>CL3543.Contig2_A-W 1 224 LEN=224; minus strand

TATGGGGTCTGGATTAAGAAAATTCACCATAACATGAATGTAAGCAATGGTGCTTGCAAG

TGGGCCTTCCGTGAGAAGGGGAAGTCATGCTCCATCTCCAGGAAAATTTAAACTACTAAA

GATCCCTTTATAGGGACCAACGGTCCCCGGGTTGACCAGAACGTCACTGGATCTCTAATG

ATGTCCCCAACTGACCAATGCAATCACGGTGATGGGGACACCATX

>CL3592.Contig3_A-W 349 865 LEN=865; minus strand

AAGACAGAACAGCCTATCTGCAGCCCAGAGAAGGCTCTTGGAACTCAACAAGAAAATTAT

GACTGTGCCCAAGCAGTTGCTGGTGAATTCAGTGCTCTCCCAATGAAGAGAGAGGAAGGG

TCGGAAGAGAAGCCCAGCCCGCTGCAGCGAGGTGGACAAGGGGCAAGGCGATGCAGATAC

GAGGATCTGGTGCCCACTTCCCTTGTGAGTCCGGAGGACCAGCAGAAGGCAAGGACCATG

CAGAGCCAAGTGGACGAAATCATAGTTATCAGCAGTGAGGACTCGCAATCCAGTGACGAG

GAAGAGCCCCAGGTGTCTCCTGCTCAGCACCAAGGTGCAGGATTCAGGCGGGTGAGGAGG

GCGCGGACCTCTGCCCCAGACTCCAGGATCTTAGAGCTCCTGCTTGCTGGTGCACATGGC

TCGGAGGGCACTACCAGCCAGGGCCCTGCTAACCCTGGGCGCCCATTCTTCACACTTGCC

TCGGCTGGCCCTTTGGGAAACATGGGAAGGCAGGGCGXX

>CL3675.Contig1_A-W 1 204 LEN=204

XGTGTCCATCTAAAACTAAAAATAATAATAATAATAAAAATAAAGGGCTGGGGAGGCCTT

TTAACGTATTCCAAGGGCCGGACTGTGGGTCTCTGCGGCGGTGGCATGAGAGCAGAGTCA

GAGCTGCAGTGGCATGAGTCACGGCCAGACAAGCACTTAGAAAGTTTCTTCCACTTTTTG

GCCGAGTATTGGTCGAAAGGAGTGCXX

>CL3783.Contig2_A-W 1 342 LEN=342

XXATAAAAACAAAATAAATCAAAACAGGAACACATTGTTTTTTGAATTGATCACCCATTC

TCTCGCCTTCCCTCAGGGGTGAGCAATTTCACTTACCTCCAAGCTTTAGAAGTTCCACGC

ACGGGCCACCAAATGCCACAGTCTGGCTGGGCACACAATAACCGGGAGGTCACGAGCCAC

TTTTAGGTTCAAAACGGGAACTACTTTATTGCCAGAACCCCACGGGAACTCTCCAGAACT

CCACGGAAACTCCGCAAGAACCAACGGGAACTCCGTGAGAACTCAAAAGTAGAGGGCACC

CGAGGTAGCAGGAGCCGCCTTATTGCTGGACAGCAGAGGTTTATX

>CL4142.Contig1_A-W 1 372 LEN=372

XXCCGGGAACGGAGCGGAGCCTCCAGGAAGCGGATCTGTGCTGCCGCCTCGCCCGCCGGC

CGTGATGCTGCTCGCTCGCGGACCCCGGTCCCGGGAACCCCGGCCTGCCGAGCAGATGGT

GATGTCTTGGTGCTGAAGATCTATATCGCAGCTCAGAAGAGCCGGTGGAGGAAGCTTTAC

GTCATCAGAAGGGCGCTACTAGATGAGGACCTCAGCACTGTCTGTGTGAGCAGCACCAAC

TCCCACAGCGTTGCCTCAGAGCTGGCCTTTCGGCATGGGGAGGTGAGTGATGGGCTAATG

GCCGTCTTGAAACTGAGATTTGGGTGCCAAAAAACCAAGAGGGAGAGGTTTGGGGCTGTA

GCTCAGTGGTAGAXX

>CL4346.Contig1_A-W 1 488 LEN=488

CGCAGCTCCGCCCCTCGGTCTTTCCGAAGCTGCTTGGCCCAGGGTGTCATCAACGTCATC

GGGATCGCTGTGGTGGAGGAACTGGCCCTCATTTCGCTTTCTGCGATGGTGCGTAGCGGG

CGCCCAGGGACTCTGGTTGCCTCCCAGCCACTGGCTTCCGACCTCCTCCTCTCATTCTCG

CGCGGCCCTGGCTATTACCCGGAGAAGGTGGACCTAAAGCAAAGGATCCTGGATTCATCA

TTTGTTTGGTAGCACTTTGATGGGAAGAAGGTGGCTTGGGCTGTGAGAGGACATGATGAT

GTGGCCTGTGAAAAAGAAGATGATGATGAAGATAATTATGATTGGCCACTGGCGACCCCT

ATGATGATCATGGAAACAGATCGGTTACGTGGAGACTGCTCGGTAACTGGGACTCTTGAT

GTTGTTGCTAAAACAAATAAAAGAGCAAAATCGCTGTCAGATTTGAAAATATCCATTCTT

CAGCAC

>CL4346.Contig2_A-W 1 371 LEN=371

CGCAGCTCCGCCCCTCGGTCTTTCCGAAGCTGCTTGGCCCAGGGTGTCATCAACGTCATC

GGGATCGCTGTGGTGGTAGGAACTGGCCCTCATTTCGCTTTCTGCGATGATCCTGGATTC

ATCATTTGTTTGGAGCACTTTGATGGGAAGAAGGTGGCTTGGGCTGTGAGAGGACATGAT

GATGTGGCCTGTGAAAAAGAAGATGATGATGAAGATAATTATGATTGGCCACTGGCGACC

CCTATGATGATCATGGAAACAGATCGGTTACGTGGAGACTGCTCGGTAACTGGGACTCTT

GATGTTGTTGCTAAAACAAATAAAAGAGCAAAATCGCTGTCAGATTTGAAAATATCCATT

CTTCAGCAC

>CL4414.Contig3_A-W 1 267 LEN=267; minus strand

XXCCACCACCTCTCACTTTGCACCCTGTGGCCTCCCGAGAGCCCAGCGCAGGGTGCGATC

CCGGCTCCCGACCGGCCAGCAGGGAGCAGGGGCCCCCCAGGCCAGCGGGGACTGAGCTCC

ATGGAGACGCGGAGTCCAGTGGAGGAGGTGGCCCTGCAGCCCCTGCTGACAAAGGCCGCC

GGGGCCGTGGATGGGGAGGGCGCGCCGCCCGGCGGTCCCAGCGAACAGGCGGCCACGATG

AGGGTCAACGAGAAGTACTCGACGCTCCCX

>CL4494.Contig3_A-W 1 269 LEN=269

AGATCTGGAGCTGGTGGTGAAGGAGTTTCTGGCAACGGCTCCTCTGGTTCTCAGGATGGT

GATGGAGCTGGAGCTAGAGCAAGCTTCTTTACATTCTTCAGGGAACATGTCCTTGTTTCT

TCAGAATGGAAAATTTAACCATGGAAGAGTCAGGACCTTATTTCTCACCTTCAAGAAGAT

CAGGATTTTGATACACCTCAGCAAAAGGAGAGAATCAACCAAGCATGCTCATTTTTTCAG

CAATTTTCTTTGAAAGCTCCATACATGATX

>CL4544.Contig1_A-W 1 201 LEN=201

CTGGAGGCTCGTGGCGCTGGTTGTTCCGGCGAGCAGTTCGAGGAGGTTGACTATGAACTC

CTCAAGGAGCAAGAACTATATCTTAGTCACCTATGTAAACCCAGGTCAAATTGCATGTGC

TCAATAAAACCTGTTGAAGGAATGAATACACTGACGAATATGAACAATCTGTTCCAGCCA

AACTGGTCCATTTGGAGTCTC

>CL4549.Contig1_A-W 1 270 LEN=270; minus strand

XXTGCTTTTCGGCTGTCCAAATACAGGATGCTTATAGATTTTACCAACATCCCACCAATT

CAAGTCCTTCTTCCAGAAGCAGGAAATAAATTGATCAGAAAAGTGAAAGGTGCTGCTGGG

CATTATTTGGAAGGAATCCTTTATGGGGTGCTATGGAGAAAGCAGGCTCAATGAGATTAT

ACCCATGCCTACATAGTTAGTATTACATGGCAGCAGTACCTGTCCAAAGGTCACTCTAGT

CCCACCTGGTTCAACGAGCAGCACAACCACACX

>CL4549.Contig2_A-W 1 213 LEN=213; minus strand

XXTGCTTTTCGGCTGTCCAAATACAGGATGCTTATAGATTTTACCAACATCCCACCAATT

CAAGTCCTTCTTCCAGAAGCAGGAAATAAATTGATCAGAAAAGTGAAAGGTGCTGCTGGG

CATTATTTGGAAGGAATCCTTTATGGGGTGCTGCAGCAGTACCTGTCCAAAGGTCACTCT

AGTCCCACCTGGTTCAACGAGCAGCACAACCACACX

>CL4584.Contig2_A-W 1 795 LEN=795

XXTACAAACTGGATCATAGACCTAAGCAGAAGAGCTACAACTATAGAAGTTTTAAAAGAA

AACATAGGAACAACTCTTCATAACATTGGAGTAGTACAGATCTTCTTACATACAGCTCCA

AACGCACAAGGGAATTTTAAAAGTAGATTAGACTTCATCAAAATTAAAGACTTGTGGAGG

GGAAGATGGCGGCGAGGGGAGTGCATTACCCCCGTGTGCTGCTTCACTGTGTGGGAGTAT

GACAAGTCAGGACGGCTAAAGGATATCTTGTTAGGAATTTCCAGCAATAGTGGGGTGCGC

CGGAACCTGGAGGAAGAGGAAGGATTTCCATCGCACGAGGATCGGCTACGGGGACTCAAA

CGCGAGAGGTTTGTCGCACGGAGGGTAACACTGCTTATTCAGCAAATCACCCCGCGGCTA

GAGTCTGCAAGGCGCGCTGGGATAGAGACGCAGGGTTACAGCGCTGCAGTTTCTGCCAGA

CGCGCAAGCACCGCACTCAGGGCTGAATTCTGGGTTCGAGACGGGGGAAGGAAGCGGTCC

ATCTCGGTTCTCCACACCGGTCAGACCACAGAGGAGGCCAGCAGCCACCATGTTGGTAAA

CTGACGTCACCACACCTGTTTTTGACTGACCGCAGCTCATTCAGCCATAGAACAGGTAAT

TTCAGGCTGCAATTCGCCTGCGGCTTGCAGACAGATTGCTAGGGTTCAGCGCCTGGGGGC

TTCTTAAAATCTGATCTTATCGGAGTGAACACGGAGCGCGGGGCGGCCGAGTCCCGGCTC

CCGGAACCGTCCCGGAAX

>CL4857.Contig1_A-W 1 112 LEN=213

XXTGATCATGTTGATGGTCGTGGTGGTGTTGGTGATGATGATCATGTTGATGGTCGTGGT

GGTGTTGGTGATGATGATCATGGTACTGTTTGGCTCCCATCCTTATTTCTTAAA

>CL4876.Contig2_A-W 1 253 LEN=253; minus strand

TAAAAAAGAGAAAAGAGTAGAATTCAAAAAGATGATGATGATGATGATGATGATAAAGCA

XCAAAGGAATTAATGAACTGGAAGATAGACCAGAAGAAAACACCAAAAGTAAAGCAGGGA

TCTGAGAGTGTAGCTCAGGGAGACATTGGATTCAATCCCCAACATGATAATGATGATGAT

GATGATGATGATGTCATTTTCTGCTCTGAGATTTTGCCTGCAAAGGAGACTTTGCCAAAT

AACAATGCAGTGACX

>CL4996.Contig2_A-W 1 579 LEN=579

XCTGCTTCGGTTTCCGGGGGGGTTGACACCCCGGATTACATCCTCCCCCCGCACCAAGCC

GAGGGCAACATTGGAGCCCCCCCGGAAGGCTCCACCATCCAGATTCTTCACTGCAGCGGC

CTCCCTGCCAAGAACAGCATTCCGCCCTGCGTCCTGCAGCCACACCCCTGGCGAGCCGCG

CCTGGCTACGCCCACGCGACCCCTCCCGTTCGGCCAATGGAGAACTACGAATGGCACTAC

CAGCCCAAGCTTGGCAGTCGGGAGTTACTTCGTGCTTGGGAGAACTTAGTTGGAAGAGGA

GGCGGGGAGAAGGTGCTGGAGGCCGTGGGGAGATGGCCCTTGCACCTATTCCAACTTCCC

TGTTTCCCTGGAGTCGCCGAACCACAGCGCAACCAAGCGGCTCAGATATGGCGGGTTGCC

TATTCCCTGTGAGTCTATGCGGCACTCTTCTGCCTGGTGACTGACGACTTGGAATTGAAG

TTTGTGACGTCATCGCTTCGACAGACCAATAGAAGAAGCTCCCGCGGAGATGGGATCCAC

CTCCTTCGACTTTGCTACTTCACGCGCGTGAGCGAGCGAGXX

>CL5265.Contig2_A-W 1 225 LEN=225

XXCCCAGGTCCCAACCCTAGAGGTTCTGATTTAATTTACAAGGTGGACACAGGGAATCAA

ATGACTTCACCAGGTGATTTCATAGGACTCAGGTCTGGGGAGCACTGCACTACATGGGAA

CCTCTGCAGATGCGACCAACCAATGGGCAGGATCAAGACTGGAAACCGTTGCACCCTGGG

GACCCCACCTTTGTGACTCTTGACGGAAAAACTATCCTGTTGGGTGGX

>CL5275.Contig3_A-W 1 325 LEN=567

CTAAACACTGCAAACACACTTAATCATGAAAGGCTCATGACAGACATTCCTTTTGCGTGC

TAAGTTCAAGTGTCAGATCAAAAGTCTCAAGCCTTAGGCCGCAAGTCCAGCAGATGCACA

CTCACTAAGACCGCGGTGATCACAGATCAGGAACCAAGGCAAGCTTCTCCTGGGGTGGAG

CTGACGGGTCGTAGGGAGAAGCTGTGGCTCTCCCAGGGTCAAGATGTGGCTGTAGAGTTT

GAGAGTGATGAGGTAGATGCAGAGGATCAGCAAATGATGAACAGTGTTGGGATGTCAGTC

CAGGTCCTCATCAGGTTCTCAGCG

>CL5445.Contig1_A-W 1 287 LEN=656

XAGAAGAAGAAGAAGAAGAAGAAGACGGAGATGGAGATGGAGAGAGGGTTGGAGGTTTCC

CTGGCACAGAATCAAGCTCCAGTCTGTCAAGACTCAGATGCGGGATTGCCAGAGGACCCC

AGGGCCAAGGACAATGCAGTCTATGCACAGCTGACGGCACAGGATTCTCTGAAGGGCAAG

CGGAAGGTGAAAACTGATATTGATGACAAACATCTGGAAGGAAAGGGGCCCTGCACCAGT

GTTTACAGCCTGGTCCAGAGACCAGGCCAGGCCTTGAAGATGACTTAT

>CL5445.Contig2_A-W 1 347 LEN=716

XAGAAGAAGAAGAAGAAGAAGAAGACGGAGATGGAGATGGAGAGAGGGTTGGAGGTTTCC

CTGGCACAGAATCAAGCTCCAGTCTGTCAAGACTCAGATGCGGGATTGCCAGAGGACCCC

AGGGCCAAGGACAATGCAGTCTATGCACAGCTGACGGCACAGGATTCTCTGAAGGGCAAG

CGGAAGGTGAAAACTGTGAGTGGTGACCAGCCAGGTCTCATGGCACCCAGCCCTGGGCTG

CCTGCCCAAAGCCAGGATATTGATGACAAACATCTGGAAGGAAAGGGGCCCTGCACCAGT

GTTTACAGCCTGGTCCAGAGACCAGGCCAGACCTTGAAGATGACTTAT

>CL5503.Contig2_A-W 1 224 LEN=396

XGCCTAACTGGGCCCACCTGGTTCCACCTGGTTGAGCAGCTGTGCCTTTTCCAGAGCGAG

AGGCTCCTGCTCTATGACACGCTGCAGGGGGAGCTGCAGGAGCGGATCCAGAGGCTGGAG

GAGGACCGCCAGAGTCTGGACATCAGTTCTGGTGAGGGCGGGGCCCAGCTCTGCCAGTGC

CCACCTCTCCGGACCTCAGCTTTCCTGGCTGCACAAGGGAGGGTC

>CL5528.Contig1_A-W 1 290 LEN=290; minus strand

XXTGGTGCAAATAAAGAACTCAAGGGTACCTGTGCCACCCAACGACTGTCCATGCACGTG

TTTTGTGAGGAGGAGTGGTGTGAACTGGCTCATCCCCACAGGATGCCAGGATACTGGACG

CAGTATGCAGACATGGTCCACATGGAGCAGGCTTTCAGTTACGAACAGGTGATCCTGGAG

ACTCTTCCTGCAGGCAGGTGTGCAGGCATGGGGTATCAGGCCCTGCTCTTCTGTGGCCCA

CTTCTTCCTTTAAACACATAATACTTGAACCCTGAAGCAGGAAGCCAGGACTXX

>CL5608.Contig2_A-W 1 407 LEN=407

XATTTTGTTTTTGTTTCAAGCTGCCTATCCCTAGAAATTTCTCAGGCAAACTGGAAAAAA

TGGTTGACTAAAGGTTTGAAGTTTGTCGGCCCTGAGGAAGAGAAAATTGATACAACGATT

TTTTATTCCGTTTTTGTTTCATTCAGTTTCGGTTTTGTTTTGTGTTATCTTATTGGGTTG

CGTTATCTTTATAGTAGATTAGAAATTAGTAAAAAACAAACCGAAAAGATGTTAAGTAAA

TTGTTAGAGGTTCAGACCATGGAGAAAGACATTTTAGATCAAGCAAAAGAGAAGGTCTCT

CGAGCTAGTCAGACAGAGGAAGAAAATTTAAAGGAAAAGGGGTTGTTAGGAAAAAGGCCA

CAACAGGAGGCTGTTACTAACTCCGTTTTATCACAAGAGGGCGTAATT

>CL5688.Contig1_A-W 1 350 LEN=350; minus strand

CTTTGCCCCTTGTTGGCAGCGGATGAAAGGTGCCTGAGGAAATACCCCGCACCGTCCACC

CACTGCCAGATCTACTCCTTAAATTCCGAGCCCAGAGAGATCCGCGCACAGCGGATCGGG

CCTGCACAACCATCCAGCGTGCAACAGCAGAGAGGTGTTCCGAGCGCAGCTCCACCGATC

GCCCCGCTCAAGCGGGCCCCTGAACCGAAGTCCACCCTCAGTTTCAGCTGTGCCAAATTC

CTCCGCAGCCTCAGCCCCGGGAGCGTGATCCCAGCACCCGAGAGCCAGATGCCCGCCCAC

ATGCTGCAGGAGATATCCAGCTCCTACACCACCACCACCACCACCACCAXX

>CL5804.Contig1_A-W 1 300 LEN=300

XGGGGTTTATGGAGGCTTCTACCAAGATTTCGAAGGAAAGCCAGGCAGGGTTGTAGTCCC

TGCAGGCACCCCCTGAAAAGTCAGTGTGGAGATGTGAGGAGGAAGCCAAAGGTGCAGTGG

AGACCCTCGAGATTAAGAAATGCCAGTAATGTGGAACATCATCCTAGGAAAGCTGCAGGA

ATTGAGCAGAGACAAGCCAACATAAAGGCCACTTGGGCTTCAACAAACAAGGCCATAGGG

ATAGAGCTACACATACCCTTTGGAGAGAACTTCAAGACACCATGTGCCCCAGATGCCGGA

CXX

>CL5924.Contig2_A-W 1 395 LEN=703; minus strand

XCACATCGGTTACACATCCACTGATCCAAATTTGATCAATTTCATCTGATCAACATAAAA

ATCGATAATAGTTTACATTCTTCTTTTCATACCGTCTCGGCAATTCAGTACGTGATTTCC

ACGAACCCCTCGTCTCCATCGGGGCCGGCTGCATTTCGGGTGCTCCGGGCACGTGTGGCC

AGTGCTGTGCCCGGCAGGGAGCTCGGGGCGCAGGCTGCCCTTCACTCGGCCTCAGCTGAC

CCGACGCCACAGGCCCAGGCGCGGCCGGAGGGAGCGGGGCTGGGACTCCGGGGCTCCCTG

TGGAAGACGCTGCGCTACCCGGGCCGGACCTTCTCCACCGTCAGCCCGGCTCGCTACGCC

CGTCGCCTCTGCCAGTGGGTAGAGGAGCACACCGAG

>CL6361.Contig2_A-W 1 332 LEN=332

XXCTATTCCATGATGGGTCCATCTGGGCTTTCTCAGGCCCTGGATGAGGAAGGGGTCGTG

CCAAGTGGGGCCCAGGGACATCGTTCAACCAATATTCAGACAGAGCTGTATCCCCACCCT

ACAGCTAATGGGAGGCGCGCGCAAATCAACACCAGAGATTTAAACCCGGACCCAGTTCCT

CGGGCCTGTGAGCCTACTTTCAGACGGGAAATGGTCCAGTTCTTCGTGGCCAGGTCTCTG

ACTGTCCCCTTTATGGACCTTGCCCTACCTAGAGGGTCCAAGGACGTGGAAGAGAGGAGT

GATAGGCCGGGCCCCAGGTTCAGTCTAGAGAAAGXX

>CL6757.Contig1_A-W 1 256 LEN=256

XXCAAATCCATAACAACTGATATCCAGAGCTCCATCTTGGCTATTCTTACATCTGCCACA

GTGGTTCTCCCACACCTGGAGGCTAATGAATAATGAACACCATTAAAGCCTATTTCAAAT

GTGAAAAACCTTGGGGCTTGCTTGTGGGAATACCTTCAGACCCATTATGCAAGTTGCAGG

AAAAAGGATGGGATATCCAAAGAAGAGTCAAACCCAGGTGGTAACCGGGATGTCTTTTTC

AATATCTATTTTATTATT

>CL6829.Contig1_A-W 1 229 LEN=229; minus strand

XTCAAGGCATACCTAACTCAGATGGAACGGAATATTAGAGAAGACATGAGACAGCAAGTC

CAAGCAATGAAAGTATATTTTGAAAACGAATTAAACAAACAAATTCAAACTGCAAAGAAC

GAGCTTTACCAGGAGATAGAGATCTTAAAAAAAAAACAAACAGTGATTTTAGAAATGCAA

GAAACTATAAACCAGATTAAAAATGCTAACGAGAATGTTACAAATAGACCX

>CL6845.Contig3_A-W 1 222 LEN=222

XGGCCCCTTTGCGTGCATGGCATGGCCAGAAGAATGGTGGCCGCTGGAATGCCGAATGCC

TTGGAGGCCAGCACGGGCTTCAAGATCCCAAGAAGGACAGCTGACGCCTGCAAGCTCGGC

CACGTGTTGGACAGCGGAGCTGCACAGACTAGGGCCCTCGTGAGCGCTCAGCGCCGCCTG

CTAGACCTCACCTTGTCTCGCCAACAAAGACGGAACCGTTCTGXX

>Unigene797_A-W 1 249 LEN=249

XXGTGGTTATATGATTTCTCTCCAGTTAGTGCCTATCAGCTCCCTGTTAGTGCAAAGAGG

CAGGXAGATAGTGGGACAGATTCAAGGGTGGGGTTTGAAAGCAGAATTGTGAAAGGAGAA

GAAATTGAGTATGCTGATAAGATAGTGGTTGATCTAATGATCTTGAATGTAGGCTGGAAA

GAAGAGGAAGTGTCGAGTTGTGAGAAAGCAGAAGGAACCATGAGCTTTCGTAATAAAAAA

CTAAAAGAGCCA

>Unigene1033_A-W 1 291 LEN=291; minus strand

TCTTATTGGTGGACTCCAAACAGAATTCTGAAAGGGGAATCTGAAAAATACAATCTTTTA

TTTATTTCTGGCTCCTGGTCATGTGAGACCTTAGAAGGACTGGGAGAGTTCTGGGAGCTA

ACAGATAGTGTCTACCACAAAATGCATTCGGAAATTAAAAAACAAAAAGAAAGAAAGAAA

GAAAAAGTCATGCTAAAGCAGCAGGCTAATGGGCAGCTGAGAGGCAAGAGAGATGAAGTC

TAAGCTAAACTGAGGCAGCAGGGACTGCAGGAGGGAAAGACACCAGGACCC

>Unigene1503_A-W 1 124 LEN=468

XXGTCAGAACAGCCCCAGGTTGAGGTTGATGGCTCCAACCTCAACCCTCCCAAGACTGTC

ATGTTCTCCGACCCACAGTGTGGTGAGGTTCTGCCGCACCACATCCGCCTTTTACCTCCT

ACTCCG

>Unigene1526_A-W 368 616 LEN=840

GTAGCCTCTGCAGCAAATGAGAAATTTCCCCCAAATGACATGGTGGAGTTCATTTATGGA

GAAAGCATGGGTCAGGAGGCCCCATCAAAAGCCATGGAGGATTCAAGAGGATCCATAATT

GGTTGGACATGGAACAAAGATTATGATTCAAGCTGTGATGAGGAAGAGCCTGCAGGCCAC

ATCATCACCGTCATGGCCATGGTCCACAGAGAAGACATAGAAACTGATGCAGAGGAGGAG

GAGGAGGAA

>Unigene2686_A-W 1 314 LEN=314

GTTGCAAGAAGAAAGGAAGGTGGGTGGGTGGGGGGAGATTTTAAAAGAAAGGAGATTTTA

AGAGAAAAAGAAGAAAGACACTTTGTACACGGAGAGGGAATAATCCCCAACGCCTCAGGT

GAGTGTGACAAGAAAGGAAGCGACATGGCAATGGCTGGAGCCCTGTCCTCGCTGCAGCCC

CACAGCTGTCCTGATGTTTGCTCAGAGAGGCCTGAGGCACGGGTGGGGCTGCTGGGAACA

CAGTGGTTTCCAGGCAAGAAACAAGGAAATTGCCCAACTGGACGTGTGTCCGAGGGCGGG

CAGGCTGCAGTCTTX

>Unigene2726_A-W 1 201 LEN=201; minus strand

XGCTCTTTCATGCATAATAGTCTTCATAAAATGCTTACTGGCTCAGAATATCAGGTTTTA

GAAGAGACAAGGTAAGACCGAGAAAGCTGGTTTGAGGCTCGGGCAGGAGCTCAACGGCAG

AGCACCTACCCAGCATACACAGGTCCTGAGTGTGGTTGCCAGTATCACAAAAAGAGCAAA

ACAAACAAACAACAACAACAAAXX

>Unigene3294_A-W 1 318 LEN=318

XGGTCCACTAAAGTATATCAGTATTCCTCCTATAACAAAAGTAAATTAACCCTCACAGTT

GAAAAAACAGAAATATTTGGCATXAGGCTTCTGAGTGAGCTTGAAAAACAAGTTGTGTTT

CAACTCTGTAGAGGAACATACAAAATCCACCTAGAAACATCTGACAGAAAAACTGGTCTG

TTTGCTATTGGGATTCTTCTGCTTCAGAAGATGAGTGAAGGTATACTAGAGGGTGATGAA

GACCCACAACAGCCACAGAATCACTTACTGAGAATTAATCAAAATTTAATACAGAATAAC

ACAGGACAACGGTACAATCTX

>Unigene3451_A-W 1 624 LEN=624; minus strand

XATCCAGAGAACTCTATAGATTATGATAGTATTGAAGGCTTACCCTGATTCTGCAGAACA

GCTTTTGAAGGTACTGTGAGGATCTCAGAGGGGCATGTTTTGGATGGAAAATCTCCACAG

GTTGAAATGCCAAAAACGTATTCTGTTGACTCACATAATTGAGTCCACTTCAGCAAAGCT

TTTGTTATTAAACCACAGATGAAGAGTGTTGTGTATGTGCCTCTCCCAGGAGGGCTGTTC

CCCCCACCACCACCACCACCAAATGATTCTGGGCTCTTTCTAGGCTGGGCCAGAGCAACC

AACACCTACCTGTCCACATTCTTATTGGACAGGCTTGTTCACGGTCGTAGATCAATCTGT

CTTTCAGAAATGGTACAGGAGGAGGAAATAGAATCACGTATTCCTCTGTGTAGAAGTGGG

GAGGGGGAGAAACAGATTGCAGACCAGAATGTGAATCCGTATCATGTCGCCTGGGCCTTC

GTCGGGAACAAACCAGCATCTCTTAATGGTCAGCACTGTCCACCGGACACCCCTGAGCCT

GTGGCCTATCCCATCTCTGTGGGTTGGGATGCATTGAGAGGTGACCTACCCCTGATATAC

TCCCGCGAAATCAACTGCCAGCAX

>Unigene3524_A-W 1 585 LEN=585

XXCCACTATTTTATTCTGCACACAGATTCTGTAACTCAGGAATCTGAACAGGCACAGCAC

AGCGAGGCGGCTTCTCTCTCTCTGGATGTGAGACGGAAAGCTGAAGGTGAACTGATGCCA

GGGGCACAATCACCTGTGACTGAGGCTGGGCTGGCAGGATTTGAAGGCCTGGCCCTCCTG

GGTCTGCAAGTGAGGGCACCTGCCCACAGCCTTGGTGTCACCTGGGCTTCCTCACAGAAT

GGTGGCTTCTGCTTCAGGGTAGCTGGATTTCCTATGGACAGTCTTGGGGACCCAAAAGCA

AGTGTCCTAGCCACAGGGCAGGACTGTGGGTATCATGTTGGGGGAGTCATTAGCCGGCCT

AGATTCTAGGGGAACACCCCACCTTTTGATTCAAGGAAAAACAACCTCACCTCATTTCTA

CGATCACCACACCTGAATTCTCTCCAAACCCTATGGTCACTACCAACTCCTGTGAAGCTG

TCATTAATACCATCTGGCCTGCCACAAGTTTTCAGAGAAAGGGAAAGCAGGTCCCCAGAA

AACACTGTCCCAAAGAGCAGTGAAGAGCCTGAGGCAGTGCCTGGCATX

>Unigene4615_A-W 1 471 LEN=1297

ATTGGAACTCATCTGAACATCAGATATACCCGGTGTTGGTTAGCAAGAACCGTTTGTACA

TTGGATATTGATGGTGCCAAAACCAAAACAGTGACTGGACATGATGGGGAGAGAAGATGT

TAAGAACAAAAACCTGATTTCAGAGAGGAAAATAAAAGCCAGTCAGATTCAGTGAAGATG

CAAAACCTGGTACAACAAAATCCTTTTACAAAATATAAATTTATTACGAAAACCTGGAAG

GATAATCTGAGAAAGGTAAAGAAAGAGAAAAGGAGGCCAGCAGGAAGGAGACAGGAAGAG

GGGGAGAAGAAAAAAGAGAAGATGAGAGAAGAAAATATTGTTGAGCACAGCGACAAGGTG

CTGAAGCTTACCTGCCCAAATGGCATTAAAGCCTTGCTATGTAATCATATCAGAAAACAA

AGCATGCTGAAATACAGTGCTCTACCGCCCATCCATTCGAGTTCTAAGAGG

>Unigene4792_A-W 1 261 LEN=261; minus strand

CCTCATACCTGGAACTTGGCCCCTGCCTCCTCCTGGTGTCCTCCTCCTGAACACCAGGTA

CGTGTTCAGCTCATGGAGGACAGGCAGGCCGTCCTCATCTTGGCTGCACCCCAGGACTTG

TGCTTGTCTGTGGTGCAGCTCCAGCTGATGCCAAAGTGGCACAGGGAGAACCTCTGGGAC

CAGCAAGGGTCCCTGGGCTTCTGTAAGCAGCACCCATGCCAGGCCCTGTGCAGCCTCACC

TCAGCCACCTGTGGGCTCATC

>Unigene5059_A-W 1 270 LEN=270

GAAAGCGAAGTTGCAGAATTTCTGAAAAATATTAGTAATGAAGAAAGATATAAGAATCTA

AGGGATATAATTAAAACAGTGATTAGAAGATTCATAGCCTATGTACTTACAGTAATAAAG

AAGAAGAAATTACATTCTCAAAAAAAGGAAAAGAATAAAACAAGTCAAATCAGAATAAAG

AACAAGGAAGAAAACAAGAGGTCAAAGCAAAAATGAAGTAGAAAACAGAAAGACAATAGA

GTTAAAAACAAAAAGAGAAAAATGCTGGTT

>Unigene5225_A-W 1 210 LEN=210

XAAAAAGAAAGAAAAGAAAAAGAAATGGAAATTGCCTGTTTGTATGCCCCCTCATTGTTG

GAGGATCACTTGTTCATCTTTCAAGGCTCTCTCCTGGGAACAAATTACTCATGTAAGCGG

CCAATTAGCCCTTCTCACACGAGTAGGGATGGCAATACCGTTGAGACACACCAGAAGCTA

ATATTAATGAATGCCTTACACTATAGTCCAGXX

>Unigene5620_A-W 1 103 LEN=939

XXCCCAGATGGCTACCTGTATGAGCGGGAGGCGATCCTGGAGTACATTCTGCACCAGAAG

AAGGAGATCGCCCGGCAAATGAAGGTGTTGGGGATGGGGATAAAG

>Unigene6013_A-W 1 393 LEN=393; minus strand

XXCTTCCACGGAGCTACATCTTCTGCTCCTTTGGAAATTTTTCTTTTGGATAGCAGGTCG

CTAAATCATCCAGGCCTCCTGCTTCAGCCTCCAGGGAGGTGGGAACACAGGCAAGTGCAG

AAGCACTTGATGAGGATTAATGTCTTAAAGTTTCCTGTGGTCACATTCATTGTTATCTCC

TCAGGATCCAAXTCATCTGAACATCCATCCAACCACCAGAAAAAGAAGATGATGATGAAG

ATAATTATGATTAGGCCACTGGCGACCCCTATGATGATCATGGAAACAGATCGGTTACGT

GGAGACTGCTCGGTAACTGGGACTCTTGATGTTGTTGCTAAAACAAATAAAAGAGCAAAA

TCGCTGTCAGATTTGAAAATATCCATTCTTCAGCAC

>Unigene6691_A-W 1 218 LEN=218

GTTTCTGAAAGGCTTTCAATTCAGGAAAAAAAGAAAAATACCATAAAAAACAATATGTGC

TAAATATTTGGAGCAACTAAGATGCCCTTCGATGAGGAAGTGGATAGAGACCTCCATACA

ATGGGGTGTTATTTAACCATAAACAGAAATGGACTGTCGAGCCACACTGGACACAGAGGA

ACCTTAAATGCAGGTTATGCAATGAAAGACGCCAATCTX

>Unigene6719_A-W 1 296 LEN=296

GTCTTAGCAAAGGATAAAGTCTACAATGGCAGCGCCAAACACTTTAATAGACCTGGGGGG

GCGGGGGAATCTGCACGGTGGCCTCCCCATCCTGCTTGCCTCTCCCGATGCCCAAGGGCA

CCAGGAGACAGTAGGGCGACCTTCCTGCTGAACCCCAGGACTTTAGGACTTGAGGCTTGT

CCTCTCAGTCGCTTCAAGGCCAAGATGCGGTATGAGAGTTATAATGGCAAGGGACTGAGA

AAAGTTCCACCTAAACCGAAATTTCCAACTTTCTGGGAAGAGAAGAGAAAACCAAGX

>Unigene6791_A-W 1 245 LEN=245; minus strand

XTGCTGTATACCTAAACCATCTTCCTCATCTTTAAGTATGGATACGTCCTCTTTCACTAA

AACATAAACTTTGAAGATGAGGTGAAGTACCATGCAGCTGCTGCTGATCCCATTAGATCA

TCTGCAGAAAAACTGAAGTTCCCAACGGCCACACTGCTGGTGTCTCTTTTTAGAACATGA

CACATACAGCTTATTGGCACGCTGAATGAATCAAGACCTGGCTCCAAGGTGAACCTAATC

TCCTGC

>Unigene7154_A-W 1 217 LEN=217

XXTATAAAGCATTCGGGGTGATTAACACACATTCGATTAAAACACGTCTGGGTACTTTAT

ATGGCCGATAAGCACCAAACTCCACCAGGAACCCTCCTACCTAAAGCAGGAACAGAGCCT

AGGGCTCATCTCACTAAGGAACTACCACCACATTCTCAGCTCAGAAACATGGAGTCGGCG

TCCGGAGACGCGTACCTTAATGAAGCCTATATCCTTCGC

>Unigene7756_A-W 1 126 LEN=371; minus strand

XACGGCCGTGTGGAGCCCTACGTGGACTTCGCCGAATTTTATCGCCTTTGGAGCGTGGAG

CATGGCGAGCAGAGCGTGATGCGGCGCCGTAGCCTGGCCGGCGAGTGTAACCCAGTGGAC

CTCGAG

>Unigene7812_A-W 1 294 LEN=294; minus strand

CCTACTTCTGTAATTACAGTAACCAAGAACAGTGTGGTAATGGCAGAGGAACAAATAATT

GGAACTGAATTAAAAAATCCAGAAATAGACCCATACAATATGCCCAACTGATATTTGAAA

AAATCTGAAAGATCCCTCAACAGAGGAAGGTTATCATTTTCAACAAATAATGCTGGTATA

TTAGACCAAAAGAAAAATAACGTAAGTTTTGGAAGTAATGCTAAGCAAAGAGTTCATAGA

CTTGACCCCAAAAGCAAGATCACTAAAAGAAAAACTGATATATTAGACCTGGTG

>Unigene7948_A-W 1 234 LEN=398

XXCTCAGATCTAAACCATAACAAGCACAAAGATAAAGAGATGGAAAATATGAAAAAGAAT

GAGAAAGAAAGGATAGATTTAGAAGGTGTACCATACATCTATTTGGAGTTCCTXACAGAA

AAGGATAGAAATGGAGAGAAGGCAGTGTTTGAAGAGATATTGATGGAGAATATTCCAGAA

TTGAAGGCAGACATAAATTCTTTATTGAAAACCATATTGATTGCTCAGGAGAACGAA

>Unigene9288_A-W 1 421 LEN=421

XGGTTTTTATGATCTTCCCACATGCTTCAAAGAAACACACATTCTACAACACAGCGCTAT

ATCAACATCACTACGGCCTCAGCGTCTGGACAGACTAGAACCACAAACCTCCCTGTAAGT

CCCTTGGCTCTCAGGATCCCAGCAGCGCGTCTCTTGATGGAGTACCTGACGAGCAGGGGA

ATGAAACCTCGTGCCAATTCAGCTGCCAGGATGCGAATCCAAGAAGGAATGGAGCTCAAG

GGCAAAGGAGTGAGCTCAGAAAAACACCCATTTCACGGCCACAACGCAGCTAACGCGGTG

GACAGGGCCTCTGGAGTCACAGCCAGGAAGCATGGTGCTGCTAGAGGTAAAGCCACAGAG

GGCTACTAAGGCCGAGGAAATGACCACGTCCTAGGGTCGGCCTCCCAACACCCACTGGTA

CAX

>Unigene9776_A-W 1 290 LEN=290; minus strand

XXGGCTCCTGAGTTTCTGGCATTACAGGCCTGCACAGTGTGCCTGGCACACTTTTGTGTC

TTGCATGACGACAGTATAGCAGTGAACCAGGAATGTGACGTGGGTACGGTGCTCCAAGTC

ATTGCAGGGTGCCTTCGAGGTGCAGGACTGTCCCCATCACAGACCTCCCCTTCTTATTCA

CACCCCTACTACGAGCCCAGCCCACTCTCAAGAGGCGATCAGCTCTTGAAAGACTGCCCC

AACTTCACTCCACAGATGGGCACTTGCAATAAACAAGGTGCTGAGATTGGTCXX

>Unigene9778_A-W 1 496 LEN=496

GGTCCTGTTGAAGGTTTGGTGGCAGTGTGAGCAAAATGGTATTTTGGGACAACTGATATG

TCTATGGGACAGAGAAGGACAGGCTAGGGAGTGAGGGCCAATTTAGAATTGAGGAATGAG

GTGGACAATTTACAATCTGGCAGGACAGGTGGGAAGCCAAGACATTTAGGAAAGGGCCTG

CTAGAAGACTGGACTAGAAAAGGGGCCAGTTCTCAAAGTAAGTGCTGGGAGTCTTCAACA

GAAATGGATAGGATGAATTCCACCCCAGATGCACAGAGCTGTTGGGGACTTCCAGATGGA

ACCATTCAGCAGGCAGTTGGCAGTGTGGAGCTGCAGCTTGAGAAAGGGGTCAGAAGAGAA

GATAAAGAATTGGAAGGCATCCACAAAGAAGTGTTAGAGAAGCCAAAGAGTGGATGAGGT

CACTGTGAAGTGAAAGGAATAGAAAAGAGAACTGAGATGGGAAGAGAAAGAGGACCTGGG

AAAGGATTAAAAGAAGXX

>Unigene9843_A-W 1 408 LEN=408; minus strand

CGGGCGAAGAAGGAAAGGGAAGGAGAGCAAACACAGCACACATTACAGTGTCCAGGTACA

CGGGGAGCCCAGCACGAGGAAAGGGCTCGGCACACATTGAGTAACAGTCCTGGTGATATT

TGGTCATATATTCATCAACTACTGACTCTGGAGCGGCTGTTATTGATTGAAGCAGTGACA

AATCAAATGGAAAAGGAGGCCGTGGTAGCAAGGGAAGGAGCAGGCTTCAAGTTCTGTAAG

CCTTTTGCACTCCACTGTCCAGCTCCCCAGAAACATTGAACCGGGTGGGCCCTGCTCCAT

CCGGGCAGGGCTAGAGGTCACCGGGTGAGGCTAACTCAGGCGCTGATTTCACATCAGTCA

CAAAGCACACACGTGGACACCCACCTGTCAGTGGCCAATCAGCGGCAA

>Unigene9857_A-W 1 257 LEN=257

AAAAAAAAAAAAAGACAAGAATATGCTGAAAACCGTGAAAATTCCAAAGCACATAAAATT

GAGATCATGGAGATACACGAAGAAATGGAGGCTGAGAGAAGGAAGTCAGAAGTGGAAACA

GCGGAGCCAGAGAGCATCGCAGACCAACAGGCAGGCTCCTTGAAGGGCCTCACCAGGGAA

AGTGGGGAGAGAGCTGGGAGGATGGCCCTCAGCCCAGGCCGGCAGGGGCCCCGGGGAGCA

GACCAGGCAAAACGGAGX

>Unigene10667_A-W 1 211 LEN=211

XXGACAATGAAAGATCACTTCAACAACAAATTATATAAACAAATACAAGAAGCAGAAGAA

CAACTCTACAGGGTACAAAAAAATCAAACAGAAATCCTGGAAATGAAGGAAATTATAAAC

CAAATTAAAAATTCAAATGAGAGTATCACCAGTAGAGTAGACCAATTAGAAGTCAGAACA

TCAGACAACGAAGACAAAATATATCATCTTGAA

>Unigene10682_A-W 1 317 LEN=317

AGGGATTATAAGGTCCTTGATCATTTTCAGTGGGAAATGACTCCAAACCACACCTGTATC

TTTGATCTGGAATGGGTATGGAAAAATATCATATGGTAGCACTGGTCCCAAGCTCTGGGA

ATGGGGACATTGGCAGGACATGGCTATGGGAAGAAAACATGTTACAGAAAAGAGGGAGCT

CAAACAAATGGAATCCCTGAGCCCCAGATGTTTTTTAGAATAAAGCAGATACAGGCCAGT

CTTGGAACTGAAGCTTCTGTGAAAGAGAAGCAATTATATTTGGTCAAGGGCCAGTCTGTT

CTCAGACTAAAGCCATCX

>Unigene10947_A-W 1 440 LEN=440

XXGAGCAGAGAGGGACCCATCTATAGATACCAGATGGAACTTGAGGATCAAGGCCATGTG

CGGGTACGCATGCGGGAAGAGATGTGCAATAGAGACATGCAGATCTTGAGTGACCAGAGC

AGCAATGAAATTGGAACTAGAAAGTCAGAGGCAGGTGTCACAGTCTTTAGGAACATTTTC

AAGTCATGGCGGATATGCTTTCTCAGCTATAAGGGCTTAGAGAAAGGATTGGCGGATTTC

AATCTGTGGGCTGAATCTGGCCTGCCATTTGCTTTTATAAATAAAGCTTTATTGGAACAC

AGTCAAGCTCACTTATTTACATGTCGTCTGACACTTTCACTCCGGGAAAGTATTAAGTAC

TTAAGTGCAGCATTGAGTAGTTACCGAATGGGCTGGCCCTTACAGAATGTTTGCCAACCT

CTGCTTTAGAGGAGTCAAGGCAXX

>Unigene10956_A-W 1 354 LEN=354

AAGAAGCTCATAAGAGGAAACTCCCTAACTCAGCCTGATGAAGTCAGAAAAGATTTGACA

GAGAAAATGCTTAAGCTAAATATAAAAACTAGTCAGCAGGAACATGGGGGAGTAAACATT

CTGGCCCAAGGAATTAAGGCCCACAATTTTTGGAGAATACAAGGAGATGATATGTTTTTG

AGTAATAGGGCAGGAAGAAACTCCACAGGGCTAGGGGGAGTGTTGACAGAGAGTAACCAG

ACAGTAGCTGCAACAGXTCTAGACATGACAATGAGACAGACCTATGGTCTTGGCAAAGGG

AGAGTTGAGAGGCAGATTGATTTGCTATTCCAGATGCACAAGGATAAGGCACAAAXX

>Unigene11217_A-W 1 270 LEN=270

XGGGACCCCAAATGCACCTCAGAGTCAGCAACAGTCCTGGATCCTGCCCTTCCTTACAGG

AGACTCACAAATCCTGAATTTTCAATTAAGCAACCGCTGTGGCTTTGTCCCTGCACAGAG

TTTGTGATAAAACTTGTCAGCATTGCCAAACTGACGCTCACTGCATCAGCTTGGCAGAAG

CCACCGCCTCAAGAAGAGTCAGCAACCCAGGGAGAGAGGCATATGGGGACTACAGAAACA

CCAAGGCCTCTGACAGCTCTTAGCCAAGGAAXX

>Unigene11702_A-W 1 259 LEN=259

GGGACTGAAACACTGGCATTGTACTGCATTGGTCATTGGCGTTCGTGGATGTTGGCATTG

GGCATGGGCATTCAATCAGAAGTTGTCAAGTATGGTACCCGTTTGGCAACGAGCACATTT

TTGGGCATAGCCAACCAGGGTTTTCACATGGGCGTTCAGGGGCGGATGGGCCAACGTCAT

GGTGGGCAACGCAGGGGTAAGTCGATCCAGTACATGGGCAACGGAGATATATGGCCAAAA

AAACAAGGAGAGGGAAAAGXX

>Unigene11723_A-W 1 220 LEN=284

XXGGAGTTCAGGGTCAAGGGTCAGAGGCAAGGGATGAGGATCCCCAGTCGAGGGTATGGG

TCGGGGTTCAGAGAGAACAAGGCTCCCCGCGCGAAGCCGTACGCCCCCGGCTCCGCGAGG

TCAGCGACGTCTTCTGGGCCGCAGCACCGGAAGCCTCGCGGCTGGACCTTCGGGATCCGG

CATTCGGACTACCTGGCCCCAATGGTGACCAACGTGGACGAC

>Unigene12106_A-W 1 1584 LEN=1584

XXCGTGGACAAATACATTGTTAACCAGACAGTGTGGAAAAACTACACACAATATTATAAT

ATATTTAATTGGACATGGTGTTATAATTTTAGCAGACAAAATTGTGTACAGAATCCTCTT

CATGCTGTATATTTGAATGCCTCTCATATGCATCTTGAGGTGAATAATACGTGGCATAGG

TTTGATAATCAGTCCTGGCAACCTGTATTGGTGGTCACACCTGGAAATGCTGATGACTAC

TGGTCATTGAAGACTGGGTGCCCTAGAGCACAGCTCTGGGATACTATAATTAGATACCAT

GTGGATGTAGCAGATAGAAGAACTCTGAAATGGTGGTTGACACAAGGAATGGATTTCTCT

ACTTGGCCTAGTAAAATATATCCAGGAAAATTACCTTATTACTGGTATATGTTATTACCA

GGAAGTGAAAATGAATGGAATCTTTATGATATTGTAACACATGATGTTTTACATAATAAT

ACTGATGTGTTTGATGCACATGCAGGTAATCATATTATATATAGTACAAAGATGGATATG

CAGAAATGCTACATGGAACCGACCTCTTTGAATCCTTGCTTAATTGATAGAGGAGGATTT

AATGAAACCAAGGGATGGGTTACTGCCCATTTCCCAAGAGCGGTCCTCTTCCCACGCAAG

GAAAAGGACGGTAATTTTATGAAGACTTTAGAGGCTCCAGGATTTTTACCTTTACTACAT

CAGCCAGTTAGATTTTTGATACAACCACAAGTACAGCCAATTGTGTTACGATTGTCTGTT

AGTGATTTGTTGAAAGGTGAAACTGTATGTCCTTCTTTTGAGTCTACTGTGTTGCCAGAT

TTGTTTTATTCAGTTCAAAGAAATGCAGCAGGACGGAGACCTTTGCAGTTCGCAGCGATT

GGCGCTTTCCTGGCCGGAGCAGCTTTGGGGGCAGCAATCGCTGGAGCAATGACGGAGGAA

CTTCGAGTGGAAATTTCAGCTCTTCGAGGACTTCAGAATAAACAGAACTTTGTTATCTCA

CAATTATCTAAAAATCTGCACTCCGCATCTTTACTTATGGAAAGACTTTCTGCAGAACAG

GAACTTTTGAAACATCATGTTGATTCATTAAGTAAGGTGATTGAGACCATGGATGCATCA

TGGAATGTACGTATGAAACAATTGGAGGCAACTCAGGAGTGTGAACATCTGCAGGCAATG

ATCACCGCGGGATTAGAAGAAATAAGACTGTTATTTAGAACTGGCATAGGCATGGAAACT

GCATCAGCTATAACCATATTAGATCCTACAAAGGACTCTTGGTGTGAAACTGGTGTTTGC

ATGATTAATTTATGGCAAATGTCGACATCTAAGGTTGTAATAGGATATTCAACTAAGGCT

ATACCGAAGAAAATTGGTAAAGATTGGATAATACCATTTGATAAGTTTTATTGGTTGAAA

TGGAACAATATGTCATATTATGTGCCTAGTGAAGCCACTTATGCGATAGGTAAAACAACT

GTTATAGCCTATGATCTTTTCTCAGATGAACCTAGGACTTCTGATATAACTATATCTATG

CCTCAGGCTACTTTGTTAGATCTTGXX

>Unigene12506_A-W 1 452 LEN=843; minus strand

XCTTTCCAGATGCACACTAACGTGGCTATTAGAGATGATGTGCCAATGTTACCTGGAGGC

TCTAGTGTCTTCAGTGGAGAGTTCCAATGTTTCATCTTTGCATCCACTGCAGGAATCTGG

GGAGCTAAGGTTTGTGCTGTCCTAAAGAAGTTCCGTATCCTATGGTGGTTTATCCCCAGG

TGTGACCAAAAAGGACAGAGGGAGATTAGAAATCAGAGCAGGACATTGTGCACTATTCAG

AAGCATAACTTGTGGGTGAATCACAAAGATACTGGGGCCTCCTGCGAAGGTCCTCCTGGA

GCACATTTAAGAAACCTTGAAAATCTTCTTTTTGAACTGACTGACCAGGTGTGCTTTTCA

CTCAAGATAAATGTGCTCAAGGGCATCAAGGAATTTGCTTCAAGCAAAGAGCTCCCTTGG

AGGAACCACATGAAGCCAGTTTCCAATCTCCTA

>Unigene13078_A-W 1 318 LEN=318

AGACTGTATAAAAAAACCAAGTGGATGGGATCAATAGAGTATATGCCATTAAAGAGAGAA

TTAATGAACTGGAAGATAGACCAGAAGAAAACACCAAAAGTAAAGCAGGGATCTGAGAGT

GTAGCTCAGGGAGACATTGGATTCAATCCCCAACATGATAATGATGATGATGATGATGAT

GATGGAGATGATCCTGATGAAAGAGAAGAAAAACAGAAAGATGTAGAGGATAGCCGAGAT

GGTATTCCACAGACATGTGTACAATATGTTAGAAGTAAGTGAAACACGAAGTTGATGATT

TCTTACAAAATCAGCTTT

>Unigene13175_A-W 1 262 LEN=262; minus strand

XXCTTTGGATCTCTGGGTTTGTTCATTGAGATGGCAGTAGCAGAGAAATGCACAGGGTGA

CAAGAGACTCCTGATGTTTTCACAAAACACAGCCCTAATAATTCTGATGATGTGAGACCT

GGTGGTCATGGAAATGATACTAGTCTTGGAGATCAGGAAGAGATTCTCGTTCCCCTGGTG

CTGAGAATTAAACACCCGAGAAACATTGAAGCAAGCATAGAAAGCAAGTTTCATTTAAGA

AAAAGATAGATACAGGGCTGGGGA

>Unigene13179_A-W 1 241 LEN=241

XXCCAAATCACAATTTTTGAAAAAATTGATTAAAAATGGACAGTAAGATCTAAAGAGACA

TTTCTCAAACAAATTCATATAGATCGTCAACAGGTACGTGTAAAAATGTTCAACATCACT

AATCAGGAATTTGCAAATTACATCACAATGAAATAGCACCTCACCCTAGTAAGAATGGCT

AATATCAACAACAACAACAACAAAATAGACATAACACATGATGGTGAGGATGTGGAGAAA

AGG

>Unigene13250_A-W 1 374 LEN=374; minus strand

TTCCCAACACCTACCACATTCCTACCACAGGCAGCGCAAATAGACCAGTGGGGGAAGAAT

GAAGTAAGTGAAATAATCATGCCAATAAATGTGAAACTGAATAACAAGTGTTTGGGAGAA

CGTGAATATGGTAATATGGTACCAAGACCAGTTATAAAAGGAATCTGCCAGGAGCTTGAG

CCTAGATCTGAAGAGCAGGCATTATTCGGAGGAACACTGGAGAAGCATAGAACAGCATAT

GCAAAGGTCCTGTGGCAAGAGCCACCCAACAGTGAGAGAATTAAGCCTTACAGAGCAACG

TCACTTCTCAATTCCTTCAGGGATGGTCCAAGGCTGCAACCCCATTATATGCAAAGGAGA

GGCTTTTGCACAGAX

>Unigene13670_A-W 1 284 LEN=284

XGTCCTGAAGTAGTAGCATCCAACCCAGGATACAGTCATTATTCCTTTTCGGTCACTGTA

GGGACAGAACAGACTTCAGTCTACATAGCAGCCTGTGTCATTGATAGCATTGACAATATA

TCTCGACATCGCAGAACAACAGGAAGGAATTAAATTCCAATATTGGAGGAGAGATTTAAC

TTGGCTCCCAAAGACACCGCAAAGATGTGTCCTTGTGATAGAACCCACCTGATCAAATTT

GAAAACGTGTAGGATATTCGATTACACAGCAACCAGATAAAAGAG

>Unigene13938_A-W 1 308 LEN=308; minus strand

XTACTTCTTCACATATGCAGACATCGGCATAAAGCAACAGAAAATGTGAAGAACCAAGTC

AACAAGAAGTATGGGAAATCAGCTAAAGAACACAATAATCTCGCAGTTACTGACACCAAA

GAAAGAGAGATATATGAACTCCCTGACAAAGAATTGTTTTCAGGTGGCTTAGTGAACTTC

AGGAAAATAACAGAGAAATAATTCAGTGAAATTCAGAAAACAATAAGAGACCAAAATTTG

AGAAAATCAACAGAGATTGAAATTACATATGTCAAAAAAAAAATCAGCCAGGCATGGTGG

TGCACACCT

>Unigene14060_A-W 1 420 LEN=420; minus strand

XTGCCTGAGCCCCTCACCCTGAGATTCGATCCACTTTCTCAGCCCAACGAACACACCAGT

GAGAAATCTGAAGCTGTAGTCACTGTAACTTTCATTGCTATGATACCTCTGCTCATCGTG

GGAATTGXTGTACTGGAGAAGACGTCAAGTGATGACGAGGTTCATGCTGCATCAGTCTAC

CATAAGAATCTGAAGAGAGTTTTCATGGATATCAATGCAGAATGGGACAACAATGACAGA

TTGAGGGTCTCTGATGACTTCATGACTGTGGCATATCTGATGCCTGGGAAAGTTTCTATG

CAAGGCATAGGATGCTTAGCTGCTATGGAAACACCTTGCTTGAGGAGGCTCTGTGCAAGA

GTCTTATCAGCTCTGGGCTGGGGATGTGGCTCAAGCGGCAGCGCGCTCGCCTGGCATGCG

TGX

>Unigene14612_A-W 711 1373 LEN=1373

GATGCGCTGTATAAAGACAAACCCCCACGACGGCGAGATGGCACCAGAGGATCACATCAA

GCCTCTCACGGTGAGGACACACGGAGCCTCGGCGACTCCACAGCCTTCCACGGTGTTTCT

GTTATTGGGAAAAAGGCGATAAACTCACATGCTCAAAGCAAGTCAGGTACTTCACAGGCA

GCTCAGCTCCTAGCTAATCAGGTGCGTCTGCTGCCTGAAGCAAGTGGTGAAGCTGTCTCG

GCCATGTCGGGCGTCACCGCCATTCCCGATATCACAGATGCTAAACTGACTTCACAGATT

AGGTGTTTTTAAAATGATGCACTGATTAAAAGAGAAAAACAACCTAAAACXGTCCAGGCT

TTGCAGATAGTGCTCCTCCTGGCTTCAAACTGTTGTAGTGTTGGGAGAGCTCGAGGAGGT

GTCTGCACTCACAAGTGCGACCTGCACGAGGTGCACCGACTCCCAGACTCACCAAGGTGC

TGCAAAGCAGGTTACATGAGGGGCCACATGGGCATGTGGGAAAACTACTCTACCCACCCA

ACCCACCGCACACACAACTCATCTAGAACCTTCTCTCCAACACAAGATGATATAAGAAGC

TACATTACATCACAGCCACAAGAGCAGCTAGCTGCAGCAGCCAAAGATTTCTTTGGGGTG

ATAAXX

>Unigene15379_A-W 1 327 LEN=327

XCTGGTAGAGGAAATTCAAAGCAATATAGCATTCAGGCACTGGCATATGTATTGCTAGAA

GCTTTTAGCCAGGTTTACTATGATAATCAGGAGCATAAAGCAGAGAAGAAATATTTTAAA

AATTTACAGGTTGGCCAAAAAAGTCCTTATAAACTTGAGGTCAAGGAAGAGGTGGTTGCT

AAAGATATCACTCTCATTGAAAAGAAACTAAGTACTTTGCACAGAGCAATAGAAAAGATG

CCTTGATGGCATCTCAGGAATCAGGACCATACCTATGGCAGCTTCAAGGGTATAAAATCA

GAAATGTAATTTAAGAGACCAAGGGGAGXX

>Unigene15469_A-W 1 290 LEN=320; minus strand

XTGGGCTGTTTCAGAGGGTTTGGTCTGCAGACAGCTGATTCTGTTGCTCTTGGCCCAAGT

GTAGCAGAATATCATGGCAGAAGGGTGTGGAGGAGGAAATTGGTTCAGGACACCGGCCAC

CAGAAAGCAGAGGTGGAGAGTTCTCCTCTTACCCAAGTCATCATGGAGAACTTCAGAGCT

CTGGGACAGAGTCTGAAAACTCCACAGCTCTTTGAGATGTGCTCAGCTGGTTTTTCTAAT

GAGCTGGATGGCTACTGCATCTATATCGTTTATGAATCCTCAGACGATGTC

>Unigene15481_A-W 1 402 LEN=402; minus strand

GCAGGTCTTCCAAAGATTCATTCAGCATTTATTTTCAAAGTAGAAATAATATTTTTAAAA

GGTGGCATAAAGAAATGCCCTCCTTGTGATTCCTTCCATATGGTGAAATTCTAGGTGGTG

ATGGAGGTTATTCCTTATTCTAGAAACATTTCGGGAAGCATTAAGACAGAGCATGACAGA

AAGTATAAAAAAGTTGTGTTCTGTTCTGAAGATGAATCATATAAATCTGCTTTGGAAGGA

CAGTTTCTTGAAGCTGCAGACCCAGGATTCCTGGCTAGAAAGGATGAGAGTGCCCTGGTC

CTCATTATTGTTAGGACCTGGGTAGCATTTCTTCCATGTCTGCAACAACATAAGAGACAA

CAGTCCAAACGGCAGCAGCAATGTTCATCTGCTTTGGATCCX

>Unigene15931_A-W 1 286 LEN=286

XGCGCCGTGCAGCAGCAGTCCCACATAGGCAGGGTAGCACCTGCAGCCTGGTCACGGCGT

GGAGAACTGGTGCAGATGGGATGGGAACTAGATGCCACCATCCCTGAGACCTGCCAGGGC

AAACCTGGCCGACACTGCCTGGACCAGTGGAGAGACATTTCAGAGAGACACAGGCACCTC

AGGGGTGCCACTGTCAATGCCTAGATCTCTCCAAGCCCTAAAGCCTATGGGAGACCTGGA

GGCAGGCAGGCAAGGCTCACGGCAGCACTCAGCCGCATCGACAATGCX

>Unigene15983_A-W 1 415 LEN=415; minus strand

XCTTATATTGATTGCTGGCAAGAGATAATACAAAAACAGCCCCCTTGGTTGAGAGCCTGT

CTAGATGGACAGTCCAAGCTGAGGTTAGCCAGGAGCATAGTGGTCTCTAAGACCCAGCGG

AAAATAGCTAAACAGAAAACCTGACTAGGAAAGACAGGAAAGAAAACAGTTTTCCCCATG

AGCCAGAAGAGGACCCAAGACCAATTTTGCCAAGATCACTGGGACAGCAGACTGTCCCCT

CAGCCCCCTTGTCCTCAGAGGTAACAAATGATGGCCCCCAATACTGGCTATTCTTTCCTC

TGTGATGGCCTCAAAGCCAAAGGCTTCCTAGAACCAAAGATTCTTTCACCTCCAAAGATG

AAGGTGGGAGATTTGTCCCAAGATCAGAACCACAACCCAGATGAAAACTCTCCAGAX

>Unigene16405_A-W 1 707 LEN=707; minus strand

XXTGCTGAAGTGACATTTTATTTATCTTTTGGGGAAACAGTTTCCAATTTTTTTTAAATC

ACAAGACCCCACAATTTAGAAGATCTGACCCATATATTGATCCTACCATCTCTCTTCTTG

GAGCTCTGGAGGGAAAGCCCGGCCACCATGGCTGAAAACCTGTGCAGCGATGAAGGCCAC

AGTATCTGGAGACACGGGAGCACCAGCTGCAACCCAGAAGCCACATCCCCAGATGGACTG

GATCTTCCTTCTCTCAGTCCCAGGACAACAGCTCAAGGAGGCTCAGAATCAAAAACCTGG

AGATCACGGACTGCCAGCGTTCCTGGCCACAGAGAACAGCCTGCAATTCCTGATGCGTCT

GGAATCCTCCCAGGAAACACAGTTGCTGGAATACTGCGGACGTCTCCCTACGAGTGCCTC

AGGCCCTCCACTTTCCTAGACCCCGTTTATATTACTTCACTCAGTATTTCTGCACTCACT

CACCTTACCCAACTGGTTTCATCTGGAACTTACCTACATATCCTAGATTTTAGAGATCTG

GCTTCTGAGCTGATTCTGCAAAGGCTTCCGAACACAGCTGGAAGATATGGACAAATCCCT

GGCAACAAGAGACCTTTTGCTGTCCTTAGACACACCGAGGCCCTCATACCCACCACTGCC

TTCTTCAGGCTGAGCTCTGGACCCACAAATAAGCCAGACAGACTACAAGXX

>Unigene16672_A-W 1 226 LEN=226; minus strand

GCAGGGGGTTCCAAAAGGGCGCAGCAGACCATCAAGGACAAGATGTCCCTTGTGTTTCCA

CCACAGGGGGCCAAGCTGCAGCCCAGGGTGCTCCCCCTTGTGGAGATTCAGGCCAAAACT

AGCTGGGCTTTAGGCAAGCCCATGCAGGTGGCGCAGGCACTGTCTTGGCTTCACAAGTTC

AGGAAGTTCCTCGATCTTTCCCACTCCACAGCTGCTCGGCCGACTTXX

>Unigene17029_A-W 1 359 LEN=359

GCTAGTGTCAGGCATGTTGATCTTCAGGTAGAAAACTTGGCTCCCTTTGATGTGTTCTCA

TTTCTTAATCCTGATGATGATCCAGCTCTCTGTACTGCTTTTCGGAAGGATAATGGTTTG

TCAGATATTATCAATCGAGAGCTAGAGAGTGTGGGTCCTGGTTCTGTAGATGCTGCCCAA

TGTGAGATAACCTATACCATTTTTGGTCAGGTTTCCAGTTGTACTTATGAGTCTTCTGCC

ATCCGCATGCATAGCATACAGAGATACAGTTCTCTTTTATCTTCTCAAACCGCTAATTGT

ATGTTACATCCCATGGATATCCCAAGGCATCTTGAAGTCTGTGAATGCCTGGCACAG

>Unigene17209_A-W 1 225 LEN=225; minus strand

CCAAGCATGGGACGTGTGGATGAGTCAGCTCCTTCTTACTCCCAGAACCCCGAGAAGGGT

GGGCGGACTGGAAAGCTAAAGAACTTGCCCAGAGGTGCAGGGCTAATGGGCATCAGAGCA

GATATGTAACCCCAGGTTACATTTCCATCTTCCCTGACAGAGCTTCAGAGGACACCTCCT

TCGGATTCACTGGAGGTTGTAATGGTAAATGGGTCTGGAAAACTA

>Unigene18877_A-W 1 220 LEN=1032

XXTGGAGAAGAAGTGACCCTGGGCCTGGCAGAGGAGGAGGAGGAAGGGGAGGAGGAGGAG

GAGAGACGGAGGAGGAGGGGGCTCCCAGGCGGCCTTTCCTTGGTGCATGACAAACTCTAC

ACTGAGGCTCAGCACGGCGGCCTCGGGCGGGCTCTGGCGTGGAACTCGGATCAGCCACAC

GCGAGGAAGATCGGGCTGAGGGAACCCAGAGGGACCAAGCGC

>Unigene19053_A-W 1 314 LEN=314; minus strand

XACAAGTTCTGCGAGCCTCCAGGCGATCCGCAGGACAACTGGTGCCGCGCCCGGGGTGAC

AGGCGGCCCGGACTCCCCAGCGACTTCGGCCTCCCCAGCCGCGCCGTCTGCCACGTGCAT

CCCGGGCCCTCGCAATTTGCGCCCGGCCACCCGGCAGTCACCTTTTCGGGCTCCGGCTCC

AGTTCGCGCTCTGGCTCGGGCTCCGGACGACGCTTTCCGGGCTGCACTCTCAGCTGCTCG

CGCCTGGAACCCCTCTCCCCAGCCATGCCGAGCGGCTCCGGGCTCCCGACGGACCGCCGC

TTCCGGCAGCGAGCG

>Unigene19231_A-W 1 502 LEN=502; minus strand

XCCTCAATGGACCAGAACACTCATGATTTCCTGTAGGGAACAGGAGAGCAAGAAGAGATT

GCCTTCCTTGGGCAGAATGGTGGGATCATCCATAGACTTGGTGGCAGTAAGATGGCTTCT

GCCACAGGTACTGGCAGTGATGTTACCTCTGGAGTCACGAACAGTCTAGAAACTCCTATA

CACCTCAGGAAAAGATCATGCATATGTGTGCCTACTGGAAATCTCACCAGAGTAGATGTA

ACAATGTACAAGGTTCATATGCAGGCTGATGATTCTCTCACGAAAGGAACTCAGAGATTT

TATTTGATGTGTGCACTATTACTTGAAAAAGGAAATAACAAATTTTCTGCATTTGTAAGA

CCTCCCTCTAATGTGACATTTTTGCTGCTAAACAAATTTAAGAAATACAGCTAAACAGTC

ACCAAAACTGTTGCACACAGGGCCTTTCCCCAATGTGAACTTTCTGGTGTCTCACAAGAT

GGGAGCTTCTGCTATACGCTTTCX

>Unigene19423_A-W 1 138 LEN=356

CAGGTTTACATGGGAATTCGTCCCAGTTATTTTACAGGTCATACTTACAAACCACACCCC

AAGTCTTCTGTGCAGAGCTGCACTGGATGTGACTATTGGCTGGAAATGCCTGATGACAGG

CGATTTACACGTACACAG

>Unigene19673_A-W 1 409 LEN=409; minus strand

XGTTATCTATCAGTTATCACAAACAGCAACAAGGACCTTAGAGCCATGTTAGAAAACCGT

AAAGCTGGAATTTAATTCTAACAATCTCACTTGCTCTCAAGAGAACACATCCATTTCAAA

TCAGTGTCACAAACTCCAAAAGAGCAGGGGGGTGGGAGTGAGGAGGGCCAGGGTGGTAAA

CACGGGGGCCAGTACACAGGTGACAGACAGAAAAGAAACACAGCTCTCGAGGAAGCCAAA

ACCAATGCGATATTACAGCTAAGGACAAGGAAAAGCCTATGGCTTTTAAAAAACGACTTT

TCTGCCAGTACCATACAGTCTTACAAAGAGATGTGCGACAAGTCAACTGACTTAAAAGGA

CAGAGGCTACATTTTCAGATCATTTCAACTGCAAATGGTCGACCCTTTCCX

>Unigene20130_A-W 1 280 LEN=280; minus strand

XATGCATTTAACAAATCTGACAAAAAGGACAGTATCCAGTTTATTTGCGTTATTAACTAT

GAGGTTTTAACTCGACAGCCAATTAACAGATATTTAAAATAATTAGGTACAGGTAACAAT

GGAAAAACTTCAATGCATTAATCAGATGGCACCCTATCACTATGCATATGTTCATATAAC

ACTACTAGTAATGACCCTGAAGCACAGGCAAATGGAGATGGAGGACTCTCCCTAAAAAGC

AAGTTTAAGGGATTAAATATCATCATCATCATCATCATCATX

>Unigene20165_A-W 1 318 LEN=550

TTCGTCCTATTTGGGTGCCTGAACACACAATCAGACATGGAAGAGATAGAACCCAGATTC

AAGCGACTGAAGATTGACCCAGAGGAGCCCCTATCCAAAAGGAAGAGATATCGGAAAAAG

ATGAAGAAAGACCAGATTGACCCAGCCGAAAAGCTGATTTCATGGGAAGATGTGAAGAAG

CTAATAACCCAGGCTTCCCGAATACTTCAACACCTAGGAAAGAACAGGACCCCAGTGATG

ATGGTAGCAACAGTAATTGCCCTGTTGGGCTGTCAGGATGTCGAGTCTTTGGAACAAGAA

TACAGAAACTCCAAAGAG

>Unigene20362_A-W 1 325 LEN=325

XXGGAGAAGGAATGGATTAAAGCTGTCCAGCTGCATTGGAGGGATTCCACACCCTGGAGG

GACATCCAAAGATGCAGAAGAGCGTACTCCCTGGGGGACCTGGAGGAGACCCTGGAGCCT

GCCTTCTTCTATGACTGTTTCTCTTCGGCCTCAAACATCTCGAGTTCCTCCACCGAACAA

GAGATGGGCCAAATGTTGGGAAGCCAGGTCACTGTCACAGGCCCCGAGGAGGAGGACCAA

GGACACCAGGTCCCAGCCTTGACAGAAAACCCTGAGCCCATGGAGGAACAGGCCAAAGGC

GTACTCCCTGGGGAACCTGGAGGAGAC

>Unigene20363_A-W 1 276 LEN=276

GATGACACCAGCATTGGATATGTGTTCCAGGCGTACTCCCTGGGGGACCTGGAGGAGACC

CTGGAGCCTGCCTTCTTCTATGACTGTTTCTCTTCGGCCTCAAACATCTCGAGTTCCTCC

ACCGAACAAGAGATGGGCCAAATGTTGGGAAGCCAGGTCACTGTCACAGGCCCCGAGGAG

GAGGACCAAGGACACCAGGTCCCAGCCTTGACAGAAAACCCTGAGCCCATGGAGGAACAG

GCCAAAGGCGTACTCCCTGGGGAACCTGGAGGAGAC

>Unigene20503_A-W 1 515 LEN=515; minus strand

XTCTGTGTTATTTATTCCATCTTTGTAAGGCCAGGAGGTGACGGTAGCATACCTGTTTTA

CTGGCAAAGAGATAGAGGTCTAGCGATGTGCCCATGGGCCCCCATCTAGTGAATTATGGA

GGTAAGACTGGAGTCCAGCAGTCTCAGGATGGAGTCTGGGGTGCTAGTGGCCGAGTTCCA

AATCCCCTTTTGCTGTACCTGCAAGGGAGTGAGGAATTCTGGGCCCTGTGTCCTGGGGTA

GCTGCGCTGATGCTGACCGCCAATCACAGTCGTGGGGAGAAGGTATCTGGAGGGACAGCT

TCTTCCAAGTTGAAGCTTCCAGCTTCCTCGAAGACCATTGTTCCTTCCCTTTCGCCTTCT

GTGACTGCACAGGTCCCTCAGAGACATCCATTACTTCTCCTGCATTCAGGTAGAACTCCG

TGGGTCTCATTTAGAGGACACATTACACAGAATTACAGCCATTGGTTTATCTTCATCTCT

CCACTAGACTGTGAACTCAGCAAAGTGCACAGCTCT

>Unigene20600_A-W 1 227 LEN=227; minus strand

CAGGGAACAGGGGACATCTCCAAAGAGCTTTGCAACCCTGGGCCACATAACCTCCCAATC

AGGGAGATTAATGAGACCACTAGAGGACAGAAAGCAAATCAGTGCATTTGCTATGAAGAG

GAGGGTCATCAGAAAAAGGAGATATCCCTGATGCTTCCAAGGGAAGCCACATGGTTAAGC

AAGGCCTGGACCCATCCTAGTGCAAATGGCACTAGCAGAACTGAGAAX

>Unigene20796_A-W 1 657 LEN=657

TGGCACTCATCAAAGTCAGCAAAGATGTTTAAACTGATAAAACCCCATGCTGGTGAGGAT

GTGGTGAAACGGGCATTTTCAAAACTGCTTAGAGGTAAGTGTGGGCATGGGCTGGCGAAG

CCGTTTGACATGCGTGGCACAGGGAAGGCAGGCCCAGACGGCACTGTCCAGCTCCCAAGA

GTCTTCCCTAAACTTGGAAAAGGCAAGGATGCCCTGAAGATGTTCATTGTCATAGACTCA

GAAATTGAAATGAATCCGTGTTCTCTGTTTGAAAACGGTCCCTCTGCGCTGTTCAGTCAC

CTGACCACGCCTTATGAAGACCTGGGCCGTGGACACAGTGTTCCTGTTGACACTCCATGT

CCCTCTGTGTTGTGTGCGTTAAGAGGGACTTTTTTTAAAAACCTCCAAGAGTCAGTTTTG

ACCCTGTTAAGAGAGCTCTGAGCTGTGGTAACGGGGTGGTGCCTGGGGGTCGTCACAGCC

TTAAAAACCCGCGCAGGTCCAATAAGGAGAAGGCAGAAAACATCAAAATGTGAGAAGGCT

CATCACAAAAGGCAGAGCGCAGGCGGTCAGCAGGAACGTGAGAGGCCCAGCCTCACCAGA

GGCCGAGAAGATGTGCGACGAGATCCCAGCGGGGCCTCCGCTTACTCCCCTCTCACX

>Unigene20891_A-W 1 458 LEN=458; minus strand

XXTTCTCCACCAGAGAAACTGTTCTATGAGGTAGCTTCCAGAGTCAAGACCAGACCCAGG

CCTGTGCCACCAGGCCCCGTCCCATCCCAGGCCCTGGAGACTCCTGAGGCCACAGGCCAC

AGCAGAGTGCAGAGGAGTGCAACCTTGGACTCCGGCAGCAGCTTGACTCTCAGCAGCAGC

CCCAGCTGCAGCCCTAGCCCCAGAGCCAGGGGCTGCCTGCACTGCTGTCTGGAACCTAGC

TCCATAGGCCCTGCCCTGCCCTCCAGCTTCCAGGAATCGGCCTCCCTGCTGGGGACCCAG

AGCTCTGAGGAGCAGTTTGAGAGGAAGTTCTCAGTCAAGATTCAAGCCCTGCAGGGGCTG

CTGCAGCACCCACACAGGGTTGCCCCGCTGGACCCCCGGCCCTGCCCGCTGGAGCTCCGT

GACCCCCAGGGGCAGCTCAAGCCTTTTTCCCCTGCAGGCAXX

>Unigene20896_A-W 1 323 LEN=323; minus strand

XXAATACAGGCAGTGAAAGATCACTTCGACAAGGAGCTACATAAGCAAATACAGGAAGCA

AAAGATTACTTCAATAGGGAGAAAGAGGTTCTGAAGAAAAACCAAACAGAAATCCTTGAA

ATGAAAGAAACAATAAACCAAATTAAAAGCTCAAATGAAAGCATCACCAACAGAGTAGAC

TACTTGGAAGATAGGACATCAGACGAGGAAGATAAAATATACAATCTTGAAAAGAACATA

GACCCACATAGTGATAATGGTAAGAAACCTGAGCAGAACATTCAAGAAACATGGGATAGC

ATAAAAAGACCAAATTTAAGAGTT

>Unigene20900_A-W 1 583 LEN=583; minus strand

CCCAGAATTGACCCTTTACGAGAGCCTTCTCCTCTGAAATACTGCACGACTATGCGATTG

CAGGACAAAGACTTGGAAATGTATGTAAAGTCTGCTTCTGAACAGATTCTAGTAAACTAC

GCCAGGAACAGACAGGATTATGAACAGATGGTTGGATCTTTGGATGTGGAATCTGTGAAT

ATGAAGGGCCAAGTGGACAGTCCTCTGGATTCTCACCTGAGCATGGAGATTCCTAGTTCC

ATCAACGATGGAGATACGATGTGTGGGACTCCTGTGGAAATCCTGGCTGGGCACCGGAGG

GGAGAAACCAAACACTGGCTGCTGGATCTGACTTCACGCAAACCTGAAAAACTGCCCAAA

CTCGGTTTCAGCACGGGGTGTCCAGACAGCCCTCTGTGGACTGTGGCCGTGAAAAACCAT

CAGCCCAGACGAGCAAATCCAGAGTCTACTAAATACCAACAATTGTCCTGCCCAGAGCCA

TCCTGGGTTCTGTGGGGATGGGTGATTCTAGCCCAGGAAATTATAGGCTGTCCAGCCACC

AGGCCTTCTAAAAAGATTATGGGGTGACTTACTGATCTTTAX

>Unigene20959_A-W 1 202 LEN=202; minus strand

XCGCCACCCGAGTCGGCCGCGAGAGCGAGGGGCGCACTGGGTCAGAGGCGGCTGCTGGCC

AGCGACTACCCGCGCGCGCTCCTCCGCGAAGTGAAGTTGCTGACACACAGGAAACCCTCC

GGACCGCCGAGCCCCGCGCCGCTGGCGCCCGAGCTGGCGTCCTGGAGCACAAGTGTCCGG

GGCCCAGCCCAGCAAATAAACCGX

>Unigene20987_A-W 1 287 LEN=287

GGGAAAGCCACATCAACCCCAGGGTTTTAGAACACAGTAATGAAAATGTGTGAGATACCA

CTACTGATTATGCCAAAACCCAGAAAGTGCACCTCTACCCAGGGGACAACGGAAACCAAC

ACTGAGGTGACACAGATGTTGGAGCTACATGACAAGGATTCAGAACAGCCATCTGGAAAT

GCTTCAGCGCAAATCACAAACAACACCAAACAGCAAAAAACTGGAAAGTCTCGGCAAAGA

AATAGAAGATATCAGGAAGAATCAAATGGAAATTTAGAACTGGAACXX

>Unigene21062_A-W 1 283 LEN=283

GCAAGGTCATCCAGGGGTGCTGACGGTCTACACCAGGGGCTGGGGACAGCGTCCATCCCG

TCAGAAGGCAGAGTAAGAGCAGTCTCCCAGGCTGAGGAGCACACCCAGGCCACACCAGCA

CTGTTGTCACTCCTGCTGGGATCTCCGGACAGGCAAGTCACCAACATGACTAACAGTGAC

TTTGTCAAGCCAGGGGCCATCATGGAGCAGGGGCACACGGTGGCTGGCAGGATGGTGACC

ACTGACAGTACGGGCCACAGCCTGGATGGGGATGTGGCGGGGGXX

>Unigene21126_A-W 1 265 LEN=279; minus strand

XXCATCCCGGAGGGCAGGGCTCACCAGCTGGACTCTGATGAAAAGGCTTTCAGAAATTTG

GACCTCACATTTTTGCTGCACCCGCATGCCCCAAGGTTGGAGGAGAACATTCCACAAGGC

TGGGAAGAGAAAACTTCAATGATGCCAGCTGGTCCCCTGAAGTCACTTCGTCGTGGCCTC

GCTGACCCTGCGAAGGACAGTGACCCAAGTTTGACCGCAGAGACAGAACGCAGTGTTTGT

TTCGCAGGTTGCTGCAGTTCCAATCCG

>Unigene21227_A-W 1 257 LEN=257

XGGGGGAGGTGACACGCCGAGGAGAGGGCTAACTATCCTCACGATTTCTGGGGACACCAA

AGGGCAGGAGCCAAGCCAGAAAGGGAAAGAAGTGATGATGGCATCACAAGACAAAAACTG

GCACAGCGATTCAGAAAGGGAGCGGAAGACATCCACATCCATGTCCACTTACTGATACGT

AACGACACTCACGTTTATCTAGAACTTAACAATGTGCCAGGAATTGATCTAAGTCTGCAG

CTAGATCACGTCACTGAG

>Unigene21344_A-W 1 517 LEN=517; minus strand

XXCTTTGGGCAGGCAGAAGGTGCTGAGACACTGACTTGGGGACCTGAGAAAGCGGTGTGT

CATGATGGATCCATTAGTGGGACTCAAACCCCAGTTGAAACGATTGCAGGTCAATCCACA

AGTGGTGGTGCTGCACAAATCACTGTCTGCCATTTCCACAAGAGAAAAAGGTATAGGAAA

AGGAAGCGAATTCCATATCTCCCAACTTGGACTCAGATGGAGGGGCTGATAAGGGAATCT

CGGAATACAATTGAGAGTCAAGGAAACTCTGTGACGCCAGCTACCATGTTTATGACAATG

CTGGCCATTGTAAGTTGCCAGTCAGCTGCAAAGTCCTTGGATCCCAGTTCCAATTCAGAA

GGTATACATCGGTTTTGGTTGAAAAATAAAGAGGAAGATGAGGAGAGTGAATATGCCCCA

GATGATCCTGCTGTGTTGGCAAAAGCCAAGCCTGGGGAACATTCCTTGCCAGAAGATGAG

AATATATGAACAGCCTTGACACTCAGCTCTGATGCCAAC

>Unigene21421_A-W 1 239 LEN=239

XXGCTTTCCTTTAATTTTAACGCAGCATCCCTCCCCAGACGCCCTTGTGTGAATCCTGTC

ATCGTGGTTCACAGACCCACTGAATGTACACGTGAATTTGCTCAGACTCAGCAAAGGAGA

GCCATCATCCCCGCAGAACCCGCCGAAGCATCGAGTCCGAGAGCTGGACACAGGTGTCCC

ATCACCGTCATGGGACCATCTCCTTGTGTTGTAGTTGCTCTGCAGGTACTTTATGGTCAA

GXX

>Unigene21477_A-W 1 218 LEN=218; minus strand

XTGGGAATTGGAGTTTTGGTTTTGGGGAGTAATGCTGTTTTCCCAATTCTCAGTCCAAAT

GCTCACACACTGGAAAATTCCAAATTAAAAGCCACAGAGGGGAAAGGGGTTTTGAACACA

TTATCTCTTTGCTCGTACAAAGTACAAGGCGTTTGTTTTTGGGTAGTACTTCACATTCTG

CTTCTTGTCCATGAGTCCTCCGAATATGATGAGTTCGCC

>Unigene21543_A-W 1 320 LEN=320; minus strand

ATAGAATACAAAATAAGCATGTTCGACTTGTTTTAAGAACCTATTAAAAGGATGGTAAAA

GAAGAAGAAACTATCAAAACTGAACAGGTACATGTGAAAATGAATATAATTTCTAGAAAT

GGAAAAACACGTAATGATATTAGAAACCTCAGTGGATTAGACACAGCCGAAGAAAGCATT

AATGAGCTGGAAGAAATCACCCGGAATGCAGTATATAGCAAGCTACAGAGTAAAAAATGA

GAAAGAAAAATTAAAACAGACATCATTCTAACAATATCCAACCTATTACCAATTGGAATT

CTACAAGGAAAAGAAAAGACX

>Unigene21596_A-W 1 262 LEN=262

XXAGAAAATATAGAGTAGAAAACATTATATATGAAGAAACAAGGTGTTACAGGAATCATG

CATTTTCTTTCAGTATTTGCTAAAATGAGGAGAGGCGCCATGAGAGATTCAGGAAAGGAG

CTACAGAAGGTCAGTGCTAAAAAGAAAAAGAAAAAGAAAAAGGCAATGTCTCCTACAGAG

AGCAGAGGACATGAACAGCAGAGGAAAATGTGGCCTGTAGAGGGATTGTGTAAGGGTGAA

AGCAACAGGACCAAACATCAGGAG

>Unigene21668_A-W 1 93 LEN=333; minus strand

AAGAAGAAGGACGAAGGCAGCTACACGCTGGAGGAGCCCAAGCAGGCGAGCGTCACCTAC

CAGAAGCCTGACAAGCAGGAGGAATTCTATGCC

>Unigene21896_A-W 1 387 LEN=387; minus strand

XXCAATGTGCCTGGATTTTTCTCTCCCTCTAATGGTTTTGGGATTCGCCCACGTGGCAGT

GAGTCAGGTACAACTTCTCAAACAGATCTATGCACCACCACCAGCCAGGAAGAGCCAGCA

ATCTAAGACACCGGGACCAAACCCAATAGAAGGTTCTTCCAGTTCCATCGCCCTGAGCTG

GTGAGCAGGAAAGTGGAGAGCCATTTCAACCAGCGACATTCCCGTCAGCTCCAGCTCACA

GAGAGGTGCAAATTACAAAAGGAGTTGGGTTCAATATCCCCCAGCCCTAGCTGGACCTCA

GAGGCACATAACCCAAGAAGCAAGGGACAGAACATGACCTGGAAGGGTACAAATACTCAA

TTTAGCTCCCTCTGTGGAAAAATTCAAGCX

>Unigene21947_A-W 1 250 LEN=250; minus strand

XXGTGGAAACGGTCTATGCCTGGTGATTTTCACAAACAGATGAATATACTCCTATTTCCA

AGCATGCGATTCAGCCACTTGGACTCCAGCAATAACTGCCACCCGTTTCAAAGGAACTGG

TGTAATGCAGTCCTTATATCTGAGCTCAGATCTATTACGAAAATCAAGCTGCATGTTTTT

CCAGGTGCTACCATCTTGGAAACAAGGCACAAGGGGACCAAAAAATGTGTTTCAGAACCT

AATGTGAAAGCC

>Unigene22052_A-W 1 440 LEN=440

ATCCTATTTCAGTCCGGAAGCAGGCATCATCCCTGGTTTCATGAAGAACCACACAACCCA

GAAGCAGGAGTCCCCATTTCCCCCAGGAGTGTTCCGAGAACCCTGAAGCATCCCCAAGGA

TCTAGTGCTGTCGACTCCACTCCAAAGCCCAACTTGTGCCTTCCAAAGATCACACGGGAA

AGAAAAAGAAAGAGAGGCAATTTCTTTGAGGAGGAAAAGAAGTTGAGTTTGGCAATAAGA

AAAAGCAGAATCCAGCCTGTTGAGAATCCAGGAAGCCTGCTCATGGGTCTTGCAGCGTCT

TACCGCGAGCATTCACCACCAGATGGCAGCATCGGCCCACCATGCTACAACCAGCCACGT

CGGGACAAACAGGTTGAGGATAAGGGGCTAATTCCAAGAGCATCTCCCGATGAGCGCCAG

CCCTTTGCACCCTCCAGAAXX

>Unigene22199_A-W 1 343 LEN=343

XTAACTTCCCTAAGTGTGTATATGAATGCATGATCAAGTGAGACTCCTCCTCATGTACAA

CTGAAGAATAGGATCCTAATTAGAATAAGTTATATTCCATGTATGTATAATATGTCAAAT

ATGCTCTATGTCATGAACATCTAAAAAGAATAAATGAAGAAAAAGAAGGAGAAGGAGAAG

AGGAGGGAGAAGGAGGAGGAGGAGAAGAAGAAAAAGAGGAGGAAGCAGCAGCAGCAGCAG

CAGCAAGGTGTGCCATTCCAGCATTACAACGGTGACATTGGAAGTGTGCCTCCGCCAAAG

AGTTGGGCTGGATACAGGGTTGGTGGCCCAAAAAGAGTGCTGAAX

>Unigene22581_A-W 1 259 LEN=259

XGCCTGGAGCCCTATGAGGCCTGCGACCTGGCCTCCCCGACCCTCCAGAGCATCACTGTG

GGTCCAGGCTCTCAGCTGACGTTCCCAACTAGCCAGAGCATCCCTGCGGGTTCCAGCTCT

GCTGCAGCCTCATTGTCGCTGACGCTGCTGCTTGGATCGCTGCTGCTGCTGCTGGGGGCA

GTGCCCAGCCCTGACCACAGGATCTCAGGGTGCTCCACTGTGCTCCTGATTGTCCCTGGT

TTCACCTCAGGTCCCCACGXX

>Unigene22637_A-W 1 207 LEN=207

GTTGGGGTGTCGGCCTTCATAAACCCACCCTCAAGGTGGGGCAGGCAAGCAGCCACAGAA

AAACTAGGCAAAGTGTCCTGGATCAACCCCAGTCCCAGGTCTAAGAGAAGTGGGAACCAG

CTGGCACTTTTCAAGATGAGACGTAATCCAGCCAAGCAAGCTGCTGGTGACAGCAAGCAA

CCAGCCAGAAGCAGCTACAGAAATGTG

>Unigene22642_A-W 1 214 LEN=214

TGGGGGATGTGGGATCCACTACAGAGACAGGCCAACATAACTTTATCACAAAGCAAATTG

GGGCAAGTGTTACTGCAGATTAACCCAGCTGGAGGTCCAGAGCAGGTTGGCTAGGAGGAT

CTGGGCTGCTCCTTGAAAGAGGAGCAGGACTTGCAGAAGATGATAGAAAGAGCATTCGAG

GTAGAAGGAGTAGATGAGTGGAGGTATACAGGTGXX

>Unigene22674_A-W 1 397 LEN=397

XXGGGACCTCACTCTTCTGGAATAGTTCAGGCCGACCACCAGAAAACCACGGGCAACGTG

GATGGCTGACCACCACCACACACCCCAGGTAGTAGTGGGGAGAAGGCCATGCAAACTGGT

CACACTAAGACCAGGAAGAAAACCCGAGGGCTGCTGTCATGGGTTCAATCTCATCCCTGC

CTCATTCCAAAAATACTCTCCTGTTGCAGTGCTAAGCCAGGACCTTGGAACCTGAGAGAT

TTGGAGACGGAGTCTTTACTGAGGTCAAGTGAAGAGGACATTGTTTGGTGTCAGGCCTTA

GTCCTGCACAGCTGGTACCCTTATAAAGGAGGGAAATTGGACAAAGACACAGGTGACGCA

GGGAGGTAAGGTGAAGAAGCTACCACCTGCAAGCCAAGC

>Unigene22731_A-W 1 265 LEN=265; minus strand

XXCAAGCTGCAATCACAGAGAGGCCTAGGCTGTGGTCATCTACCACCTACCTGGGGAAAC

ATTGATGTCAAGCTCACGTGGTTACCAGAAAGACTGAGCTCTCTGAAGTTTGCAGGACTG

AGGTCTGCAGTTCCTTCTTGGCCATTAGCTGGAGGTGACATTCAGTTCCTTGCTACTCAG

GTCTCTCCTACATGGCCAAGTGATTCATCAAAGTACACAGGCCAAGAAGACAACGGTGAT

GGTGCAGGCCAGGTGGATGTCACCACC

>Unigene22803_A-W 1 212 LEN=212; minus strand

XCGCGGCGGGCGGAGGGCTCAGCGTGCAGCGCCCGGACTCAGCAGGCGGCGCGCATGCTG

GGCGCGGGCTGGGCAGGGCTGGGACGCAGGGTCGGGCTATTGGGTTATGAGGCTTCGCTG

CTCTTCTTGCTCAGCTCAAACGCCTGCTTTCGCCGCCACCGCCGCCTCTGCTGCCGCCAG

TGCCGCCGCCTCTACATCCCCGCTCAGGCTTCG

>Unigene22900_A-W 1 248 LEN=248

XGTACACAGCATTATAGAGCAGTGAAATGACGATTCCAGAAACAGACCTGTCCATATCTG

GCTACTCAGTGTTCACAGAAGCAGCAGAGCAATCCCGGGGGAGGAAAGCCTTTTCACTAT

GGCTGGACATGCACACAAGAGAAAACCAGCCTTGATCCCCACTCCACACCATGCCCCCAA

AAACACCTGGAGGGGACGGTGGACCTAGATGCAAAGGCCACACTACAGACATCCAAAGGC

AACAAAGGC

>Unigene22941_A-W 1 215 LEN=215

XGGAGCAGTGACAGTGACGATGACGATGATGATGATGGTGATGATGATTTTCCTTTGAGA

ACCACATTGACTCATGCCTAAGGAGATCCTTCAGTGGAACAAAAATTCAGCATTTGGGTC

AAGGGGCTTGGTGAAGGTTGGGTATCTGTGAGGGCGAAGGATAGGAGCTGAGACTGTGCT

GGATCAGATACCTACCAGCCTCTTCCCCCTGCTTCT

>Unigene23059_A-W 1 253 LEN=253; minus strand

ATTGGTTCTCAAGGACATATAATTGGTTCTCAAGGACATATAATTGGTTCTCAAGGACAT

ATATATGGTGTGGTGATGTGCTGGATGTCTTTTGGAATAACTGTGATAGTTATGGATGTT

GCACAACTTGGTGTCTTCAAAAACACCAACCAAGCTGGGTGTGGTGGTGGCATGCCTGTA

ATCCCAGCAACTGGGGAGGCCGAGGTAGGAAGATCACAAGTTCGAGGCCAGCCTCGGCAA

CTTAGTGGGGCCCXX

>Unigene23061_A-W 1 233 LEN=233; minus strand

XXCAAAAAGATAATACAAGGTGTGGCTTTGGATGTGGACACATTGGAACCCTCATAGGCT

GTGGTGAGAATGTAAAACAGTGCAGCCACTTTGGAAAACAGTCTGGCAATTCCTCAAAAG

GTTAAACACATGGAGTTACCATATGACCCAACAATTCCAAATTTTACTCCTAGATATATA

CTCAAAGAAAGGAAAACCATGTTCACACAGAATCTTACATATGAACATTCACAGCXX

>Unigene23074_A-W 1 294 LEN=294; minus strand

TGTGGGCTATCTCACCCCCCAAGCAACCCTTTCCACAGAGCAGTGACCACATATAATCCC

TTCAACAAACAGGGGAAACCAAAGAATTACAAGCTCACCTGACACCCCAGAACTCCTCTT

GGTGACATCAGAAGACGCCAGCAGCAGCTCCCTGGACTGGCAGGGGCCATGGAAACAGGT

GTTGAGTGCAGGCCACAGGGAAGGGGACACTTGGATGGCTGCTGTACAGACACTTACGGT

GAGAGACTGACACTCAAGAAGAAACGTACACATATGGCTGTTCGTTTACAGGCT

>Unigene23151_A-W 1 227 LEN=227; minus strand

XXCCAGAGCACCAGCACCTGGAATCCTGCTGCCCTTCGCTAGACTGAACAACTCATAAAT

GAGACTGCCACAATCCTACTTTGTTCCTTCCAACCCAAAACCAGAGCCTTAAGCATCATG

GCACCCCCTGAACTTTTTACAGTAAGTGCCAACCTACTGGATAAGTGGCCTTCAAATAGT

GCTCTAGGAACCCTACAGGCCCCAAACAACCTACATCCATCAGTGGCCCXX

>Unigene23164_A-W 1 249 LEN=249

XATTTTGTATGGGAAACTCTATTTGTGGCCCAACTCCAGGAGTCATCTCTCTATTTGGGA

ATCTATTCATGGAGCACCCACCATATGCCACCATATGCCATGTGTTCTTCCAGGTGCTAC

AGACACAACCACTCTGAACAAACTAGAGAAGGTTTCTGCATTCATGCTGTGGAGGGAAGG

GGTGACCAAGATGAAACAAGCAAAAAGGGCATTTCAGATAGTGATAAATGCAGTGATAAA

AATAAAGCAGXX

>Unigene23166_A-W 1 183 LEN=268; minus strand

AACAGAGGCACGTTCTCTGACTTTGATAGCTCCCTGGGTTCCCGTGAAAAACAACCGCCG

CGTTGCCAGAGCGTGTTTCTCAAATACCAGCATCAGGGGATCGCAGAATTACAGCAACAA

ATGTTACGAGGTTCTGGACTTCTTCACAGCAAAGAAGAGAAAGAAGTTAGCAGCTACCAA

GCA

>Unigene23186_A-W 1 266 LEN=266; minus strand

XXCGAGGTGAATGCCCCAGAACAGACCCAAACCACTGACTTGCACCAAATCAGAGCTCCT

GGTGGAGGTAAGAGAGGCAGTGAAAGAGGAGGCACTATTAAAGGATGTGATGACTTAACA

TCTTCCAGAGTTGTCAAAAGAAATCAATCCTTAGATGCAGAAGTCCCAGGAAACCCCAGC

AGGAATAGTAAAGAAAACAATCCTTCCGTGCTCACATCAGAGGAAACTGGGAAGAACATC

ACACAGACAGACAAACACCAAATACAGAXX

>Unigene23323_A-W 1 244 LEN=244

AGAACAGCCTCTATAGGAACAGGGATGACAAGACTGATGGGGAAGGTGATGCAGAAAGTG

CAGAATAGAGAGGCGACAGCTATAAGGGGGTCTATCCTGAAGGAGAGCTTGCTTATTGCT

AAGATGGGTGAAGAAGCCTACTGCATGCCCATGGGAATGGGCCAGTGGAAGGGGAGAAAT

TCACGACAGTAAAAACAAGGGATAGCTGTAGGAATGTGCAAGAAGCAGGAAGACCCAAAA

TACAXX

>Unigene23679_A-W 1 226 LEN=226

XGCACAAACACCAAGGATACCCAGCCGCCTTTTGGCAATCCTACTGTTCCTCCTGGCGAT

GCTTTTGACATTATAGCACAGTCTCCTCCCGTCACCTGTCACAGAAAACATCAGGGTCTT

GGAACACCAGAGATCCACCTAACTGCTCATCCTAAGAAGGGACTTGTTATTGGTTTTTGG

CAGATGTCAGATTTTTGTTTAGGATTCAGACTGAAGGAAAGAAAACAX

>Unigene23710_A-W 1 296 LEN=296

XXCGTCCTCTGTGCAAAACCAAGTGCTTATGACCATAGTGGGTTGAGTGGTGGCCCCCAA

AACTTACATCTACCTCCTACCCCCAGCATCTGTGATCGTGACCTGCTGGGGAAAAGTGTC

TCTGCAGATGTCATCAAGGATCTTGGAGGTGAGCTGATCCTGGTTTATCCAGTGACAAAC

GTCTTCATGAGTGACTGGGGAGAACATGCAAGAGAAGGTCATGTGAAGACCAAGATGGGA

GCCATACAGCCCCGAGCTGAGGGATACCAAGAGTTACCAGGATCTACCAGAAGCTGG

>Unigene23764_A-W 1 240 LEN=240; minus strand

CTTGTTACTGGCTTTGCAGTGGCATTATATGACATCTCCATTCGGTATGTTTGGGCCAAC

AACAGCCCAACTTGTGAAATTGAAGTCTCTGGCCTGAGAGATCCCCTTTCTGTAAATGGA

GTCCTGAGCCCAGTTCTCTGTGGCTCTGGGTCCAGTGGCTGCTGGGCTTGGGAACACAAT

CAGTCTGCCCCCATGGCCCGGGTGACTGAGTTCCTCCCAGAGATGCTCGAGGAGTACCCX

>Unigene23941_A-W 1 202 LEN=202; minus strand

XXCGCCATCCCTGACCTCATCTGCAAAAAGCACGTACATGTGGCTGGTGGTGACTTCTCC

AGAGCACCCCTTAGCCTGCAGACCCCCGAGACCCGCCCAGTACCCAGTGAGCGGACTCCA

TCCCCGTGGTGCGCGGTCTGGCCAGCTGCAGGGTTGCTCCAGCGTCCTCCTCCCTCGACG

GGCAGCACCATGGACCACGCCGCC

>Unigene24304_A-W 1 245 LEN=245

XXGGCCCATGCCCCAGGAGCTCTGCCCCAGAAGGACACAGACCACCAGCCCCAGGGTCTC

CAGCAAGCAAACCTCATGCCACGCTTCCCACGCTGGGCTCCTTACGAAAGGCCGGTTGCC

CACACTGGGATCCACAGGCAGACGCGCATTTAATAGACTGCCCTTCTGAAAACTCATTTC

AAAATAGAGAGCTCTCCCCGAATTGTAAAAGCCATAAAAATGCAAAATACTTCTTTAGGA

AAAATTGXX

>Unigene24305_A-W 1 336 LEN=336

GACCCCGGTGCCCGGAAACGCAACGGCCCGGACGGCGCGGGCCACGGCGCCCCCTAGCGG

ACGGCGCCCGCGCAGGCCAACCCCGACTTCCGCTGGGCCCGAGTTGACGTTCTCCCGTCT

GCGGATTGGGCGCCTCGGCACCGGGTGAAXAACGCACCCAGTCGTGGTCCCTGCATGGGA

TTAGCCTTGCTCTGCGAAATGAACGCCTACTCACGCAACCCCCAGCAGAAATCATCATGT

CTTCGCAACTTCGACCACCACCCTGTACAATTAATTGCCCAAACACATCCCCCTAAGACT

GTGTGGACCCACGGAATGGAATGCAACCACCCAAACAXX

>Unigene24382_A-W 1 227 LEN=227

XAAAGAAAGAAAGAAAAAAAGAAACCAGGGCATCATGAGCCTGGAGTAGAGGAGGCTAGG

CGAGATGGGGTGATCAGAAAGGGCTTCTTGGAAGAGGTGACATTAAAGCTGAAAGATGAG

AAGGAGAAGCCCTATGAAGATTCCCAGGGAAGCATCCCAGGCAGCCAGAAAGGCACATAT

AAAGCCATTCTGCCTGAAGGTGGCCATAAGGTAAGAGGAAGGCCACTG

>Unigene24407_A-W 1 228 LEN=228

XGGAAGGCCTGGTCTGAGCTTGGGAAGCTATATTCTACCCGACACAGCTCTTCTCGAGAT

GTAAGAAGCAATGATGACCAACAGAAACACGCAGGAAAAGAGAAGAAGGAAAATTCCTGA

CTAGACACAGGTAAAGACCCTTGTCTTTGGAAGCCAAGACTTTCCAGCAAACAGTGTACC

AGACTTTCCAAGGAAACCATCAAACCGCAAAGTACCCGCTGTGCAGGAGXX

>Unigene24551_A-W 1 242 LEN=242; minus strand

TTTTTACGTCACAGCTTCTGGAAGGAAATACAGTTTCCCAAATACTATTTACATTCCTAG

ATAACTGAGAACGAATCGTTTTTCGTGGGCTTCCCTTACTCAGAGGCCCTTTGCTCTGTC

CTGAACCCAGGTGTGGTTGAAATTAGAAATGACTCATATCAAAAATCAGCTCCTGACATA

GCCAAGTTTTCTTTTATAACTGCAGCAAAAGAGATCAACCCCCAGGTCACTGCTACGTGT

GCX

>Unigene24722_A-W 1 290 LEN=290; minus strand

GCCGCTGCCCTGCGCGGGCCAGGTGAGATCCTGGATCCCTTTATTGTTTGGACTGAGGTA

TCTCCTGAAATCTTATGGGTGAGACAAGGCAGGAATATTCAGAGGCCTTTTGGTCATAGC

AGTGAAGAGGTGACCAACAGTACTCATGGAGACTATGGCAGACATCATAAACTTGACTTT

CCTGCATCTGGTCTTCCCAGTCCAATAGTGATTATACCCGTGACTTTTGAACTACTCTGG

AGCTGTGGGCCGGGGGCTGGCTGCCTGGGGCTCCGGGTTCTTATTCCTCCX

>Unigene24844_A-W 1 215 LEN=215

CAGGACTCCCTCAGCCATAAACTTGTAGCCTCTCGGAAGACACAAAGCCACGGGGCTGGT

GCCTATGGCCCCCTGCAAGATTTGCATCACATTAGAGATGCTGCTCCAGGTCATATGGGT

GGTGTGGTTAACGGGCTTAGTAAAGCTGTTTTGAAGAGGTTAACCCAGCTTGTAGTAAGG

GTAAATAAACATAACAACCAGCCATTGTTCCCTATX

>Unigene24943_A-W 1 258 LEN=258

TCCAGGGAAGACAATAGGATTGCATATCATCATCACCATGCCATGTGAGAGTCAGCTTTC

CGTTGCTGTGATAAAATGTTTGAGAAAATCAACTTTCAGGAAGAGAGATTTCTTTTGGCT

CATGGTTTCAGAGGCTTCAGTCCATGGTATCTTACCTCCATCGTTCCTGGGCCTGTAGTG

AGGCAGGACATCACGGAAGAAGGGCATGGTGGAGGAAAGCTGTTTACTTACTACATGGCA

GTCAGGAAGCAGAGTGGG

>Unigene24979_A-W 1 184 LEN=391

XXGGGCCGCACCCTGCAGGGTCCTGGCAGCGGGAGGGCGGCTGCGATTAGAGACAGTGTG

GCAGGACTGCCCGGGTGGCCAGCCCCTGACTGCCTGCCCCTCTCCGCACAGCGCTGCAAC

TCCAAGTGCGTGTCGGAGCCGACCTTCTACCGGCTGGTGCAGTCCTTCGCGGTGGTGCTG

TTCGAG

>Unigene25020_A-W 1 320 LEN=320; minus strand

ACATTGAAGCCACAGTGCAGGGGCCAGAGGCTGGTGAAACCAATTCCTGGCAAACCTGAG

TTTGGCCTGAAATACAGGCTTGGCAAACCCAAACTGCATGGCATGGAGTGCGCCTAAGTG

ATCAGTAGAAAATCAAACATGGATATAGGTTTACACAGGGGAATGCTGGTTATGGAAGCC

CATCTGGTTCTCCTTCATCCCCTCCAGTCTGGTGGCCTTCAGGACCAGTTTGGAGAGATG

CACCTGGTGGGGAATTTGAAAGGGCACAGGCACAAGAGACTGAGTTTGGAGACTAAACCT

GAGACTAGGAAATGCGGGCTX

>Unigene25204_A-W 1 215 LEN=215; minus strand

XCTCTGCAGTTGGTGGAGGGAGGGGACCCTGGTATGCCAGACTTGGCCAGAGGACCCAGG

CCCAAGACCCCCATCTCCCCTCTCTGGGAGCTGGGAGCAGCTGCCCGTGAAGGGAAGGTG

GCTGTGCCAGGGACTCTCAGAGCCCAGTGGGGCCAGGAGCACAGTGAGGACAGGGCACAT

GCTAATGAGCTAAAGAGGGCCGACTGGGCCAAGGCT

>Unigene25309_A-W 1 340 LEN=340

XXACCAAGTATGCAGTAGTGCTTTGCCACGGTGGAGACTCTGAGGCCATTATTAGCAATG

CTAGAGGCAGTTAGAGTAACTGATGGTATCGGAGGCCTTCTTTTAAACCTCAAACAGGTA

ACATATTATCACTATGGCTCTTCACGCCTGTTCAATGATTTGGACATAAAAGAAATGCTA

ATTATGGAAATGAGCCATGAATTCAGAGTGACCGCAGGAACAAAATGTTGATCTGACTGG

TGTTATGTGGCTGGAAATTTATTGGAGGTTTTAAGATCATGTGAACTGATTTCCACCTTA

AGTGCTCTACCTGAAGGAGCATTAAAGGCTCCTGATAAGGAA

>Unigene25436_A-W 1 261 LEN=261; minus strand

TTTTTGCAGGAAAATATAATTTTTTTTACAAGAAAATTATAAAAGAGCAGTTTCACAGTG

ATGGCCAAAGATGATGAAGCTCTAAGACAGCAACCATTGCCACTATGGAAGGGGAGTATA

CCTCCAGCCTCCATACCCTTGCCAAGTGTCGGCATGGGAGAGAAGAGACGTGGGCAGGAA

CTGGACATTGGCAGCTTCAGAATGAGCAGCAGCAGCAGGACCCTTGGTGGCATTCCAACA

CAACTGGCTTCAGCAGGCACC

>Unigene25476_A-W 1 234 LEN=234

XXGCCAGGTGTGGAATTGTCTACTTGTGACATCATGTCTGTGCTCAAAGTTTCAGGTTTT

GAAATATTTCAGAGTTCAGGTTTCCGTATTACAAATGCTCAACCCGTTCGGGAGACCCTG

AATAAGCCCTTTGCCTGCAAGCACCCTGGGTTCCTCTCTGGAGTTGCTGAGGGGATGAAA

AAGACTCCACAGGGCCAATCACAGTGCCTGACAAACCTGTGTGCCCACGAACCCAAX

>Unigene25593_A-W 1 207 LEN=207; minus strand

XCAAAAAAGGTACAGAACATAAAAGACATGGGATATAATGAAATGATCTAACATCATGTA

CTTTTAAGTCTCAGAGAAGAGAATGGAACAGAAGTAACATTTAAAGAAGTAATGAAACAA

ACAGCCATATTTGAAAAGATAATGGCTAAGAATTTCCTTAAACTGGTAAAAGATACCAAG

TACAGATTCCACCCCACCCCACCTCCAAXX

>Unigene25640_A-W 1 207 LEN=207

XXTATGGGTTCTGATTTGGGTGATGCTTTCTTTTTCTGCATAGTTGACTTTGGCTAAAAG

ATGATGCTTCAGAAAGAAAATAAGCAAACAGATTTAGGGTTAAGTTGGAGTCAACCTGTT

CAGGAACTAGATCGACTGAGAGATTATTTGGCTTTTTACCTCAAGATTGCCAGGCCTACA

AACACGGATAATAAAGGGGGAAACCTGGGX

>Unigene25738_A-W 1 263 LEN=263; minus strand

GAAAGAAAAGCTTTACATGACAGGGATAAGCTAAAAGAATTTATGATCACCAAAGAAGCA

CTACAAAGAATACTCAAAGATAAACTGCACAAAGATGTACCCAAAAATAACCCCCAAAGC

TCCTAAATAGATGATGACCATGAGAAGAACAACCAACTAAATGAAAATTAAGACAGAATT

AAATATAGTAAACAAGCCAAAATATCAGAAATCTACAAACACATATCCATAATAACCCTG

AATGCATATGGTCTCAACTCCCCX

>Unigene25798_A-W 1 238 LEN=238

XXGATGCCTGATTCCATCCACAAAGGCTCCACTCTTCTGCCTAATCACCTTCCAAAGATC

CCACATCCTGATACGATCACATTGGTATTTAAGATGTCAACACACAAATTTCGAGGGGAC

ACAGACTTCCAGTTTGTAGCAACAGTTAAGCACACAAGCTCAGAGACTCATATGCACCTG

TATTTGAATCCGCTGCCACCACTTGTTTCAACTATGCGACCTGACACTTTGCTTTACCTG

>Unigene25949_A-W 1 295 LEN=295; minus strand

XTGTCAAACTTCTCCAAGATGGATCTTATCCAGAAAGTATCAGGTGTGCAGAAGCCACCA

GGAAGCTCTTCACAGCAGCAGCAGAGAGCCCAGCAGCCCCTTCTGACGAACACCTACGTC

CCCACAACCTCACCTGTCATGACTTTCAGGTCTGGAGGCACTACGTCTCCGTCAGCCATG

CCTTTCCACACAACACACCGGTTCATGGCCATTCCTACAGAGCTGTTTGTGCTGGGGGAC

ATTCGCAGAGCAGGCAGCAACCTTGTGCCTGAGGCTCCGAGCCCCTTGGATAGAACX

>Unigene25979_A-W 1 220 LEN=220

AGGGGGAGCAGATATTTAGGGGAGGAGACAGCTGGAAAAAAGGACAGAAGACAAGCCAAT

GTGGAACCAGGGCCTGCCAAAACATCCAGAAGAGAGTTAGAGGCCAGGAAACCAGTGGTG

TACCTGGGGGTTGTCTTATTCCTGTCCCCAATCCCTGATGCTCAGGAGATTCAGACACCC

CCTGTGAGGTCCCAGTACTGGGGTCTATCTTCAGTCCTGGXX

>Unigene26217_A-W 1 349 LEN=349

XTTGAGCCACATCCCCAGCCCCACAGTCATATTTCAACAATAATACAAAAACAAATGAAA

GGGAAAAATCACCAAGCTAATGACCAGGACCTGGAAAAAAAAATATATCTAGAGGGCTCT

CAGGTCTCACTCATGAATATATTCATACAGTACAAAAAAGAGAAAATTAACACAAAAATT

GTGGAGACAACATTATTGAGGTGTCTCAATATAGACCACATCCACATTCCTTTAGAATAT

AGAGAGATTTTAAGTGAAGAAGAGCTTTCACTAAGGGTATACCACAAGTATTCTGCAGTG

TACAACACAATTGCCAGGTCTCTAAAGAGGATCCCACTTTTTGACAGGGAX

>Unigene26229_A-W 1 269 LEN=269

XXGCTTGAGATTCAGGGCAAGGAGCACAGGAAGTGGGACTTTGGGCAAATGCTTCCCTTC

TGTGACTCTCAGTTTTCCATCTGCATAGTGAAGGGGTCAGACGGTGTTGCACTTCAGAGT

GGTGCCACTCTACCACTCTCATCCTCTAGTTCCATGGTTTCAAAAACCAATATAGAGCTG

CAGTGCAAGGCTGCCACCCTGTCCCTGGTGGAGCCTGAGGACCATGGCCATGAACAATGC

AGGGAGGATAGCTCTTCAAAGAAAGGAAAAGXX

>Unigene26666_A-W 1 311 LEN=311; minus strand

XXCTGCACTCAAGTGCCAAGCCTTGAGGAAAAGGAGACTAATCTGTTCCATGGTCCTGTT

CATTTCTTGTTGGAGATCTTAAAGACCACGAAGATTGAGCTCCTGTGTAAGAACCCAAGA

AGCCCAGCTCCAAGCCCTTTAGCACAGATCAGTGCAACTGGTACCCAGAGATTGCTGGGG

ACCTTTGTAGCCCAACATTATGACATGCCGCTGCACCAGGTGCTGCAGGGAATAGAGGGC

CTGGGCACTGCTGGTCGCTGTGTTTCTGTTTCCAGACAAGACCAACAAGAGTGGCTCCCA

CCTTGCAACCCCCXX

>Unigene26878_A-W 1 247 LEN=247; minus strand

TGTGGTGCTGAGGACCGAACCCAGCGCCCTGCGCATGCCAGGCGAGCTCGTTACCGCTXT

GAGCCACCTCCCCAGCCCCTAGAGTTTCTCTTGATCCAACGGAACCGGAACCTTTTCGAC

AATGAAAAGCGGAGTGGGAGGAGGTCTTTGCAATGGCTACGACACAAAGCATTCAAATCC

AGAACTGTGAAGAGTCTAGAACTGGAGAAGAAAAGGCAGAGACCCCCAAAGAAACATCAG

CAAAATCGX

>Unigene26997_A-W 1 419 LEN=419

XXAAGTGGCTTCCAGGACCACCATGGTGGTGGATTCAAATTTCAACTTGCCTTGGACAGC

GCAAATGTCCCTTCTCCCTCGGTCATCCAGGCTCCCAGTCCTGCTATCAAAAAGAACCAG

AAGATGCTGAGATCTCAGGTGCTGTGGCTATGGAGAATTTGTTTCAGTAGTTCAGGAGAC

AGATCCCAAGTCTGTGCCCATTTCCATTATTTCCGGCCAGATCTTCTTTTCCATGCGGTT

CAGAGGGCAGGGCAGCACCCATCTCACGATGCTTTATTTTTAGCAAACTCTCGATATTGT

GGTTGGGATGGCTTTGCTCCAAATCTCTAGGGATGCCCATTTGTGAGGGCTCCACAGGAG

CTCTTCATTACTTACAAATGGCATCTTGTCTACCTGACATCTCCAAACACACAGGCTCTG

CXX

>Unigene27153_A-W 1 301 LEN=301

XGGACTGCTACTCTTGCGCCCTTGCATCTGCATCCTTCTGGGCCTGTGGAGGAAGAAGAG

GGAGAGGAGGTGTGCATCACTGAGTCGGGGCAGGAGGTGGTCGAGGAGAGCAAGGCAGAC

GTGAGCAAGAGGTCAGACAGCCTCTAATCATGGGACCTTCTGCTTTCCACCCAAGAAGGC

TCATGGGCTCCTGTTGCCTTCAGAAGAAACGCCTTAACTGGAAGAGTTAATGATAACGTC

AATCACCATAAGCTAATTGTCTACAGTGTGACAAGCACTACACTAGGAGCCTTCCAGGCA

CTX

>Unigene27282_A-W 1 224 LEN=505; minus strand

XGTCTTCTGCCTGAGAAGTATTTCAACAGGCGCTTTCGGCAACGGGTGCAGATAAAGAAG

GATAAGAACGAGGTTTCTAACTTGACAACCTAAACTGGAGGCGACCCCGATGGTACCCCT

GAAATTCATCCTGGCCGAATACTTCTCAGGAAATGGTGGAAAAGTTCTGAAACCAAACCG

CTGGGACAGCCCACCGTCCGCCACTCCCGCCCAGGACCAACCCCG

>Unigene27345_A-W 1 347 LEN=347

XXTGAACTCCGGACCGAGGCTGTACTCCCTTCTAAGGGCTCAAAGTCTCCTACCGGTTGC

TGGCTCCACACGGAGTCAGAACCCCGCTCACAGCTTAGTTCAGGGCCATATCAATTATTT

CCTGTTAATATTAATCCAGGTGGTAATCGCCCTGCAGCCTGGTATCCCTGGGATCCAGGT

GATCTCAAGGAGTTACTTCAGGGCTTTGCCCATCAACCATGCACCTTGGCAGATTGGAGG

GCTTTGGTCCTGGCGATTCTTCCTACCCCAACAATGGGCAGCCAACTCCTTGTTGTTTGC

CTCACTATGGTAACCATGGGGTGCCTGGCGGAAAGAGCAAGTTTCCTGGXX

>Unigene27473_A-W 1 205 LEN=205

XGACAAGGACAGCAAGAAGAAGACTTCGAGCTAGGATCCCCAGGGAACCCAGGTGCAGAA

ATCCTTAAGTAAATGCTGGAAGCTGGATTCAAGAGTACAGTTAAAAGACCATTCAACTGT

GATGAAGTGGGATTCATTCCAGGGAGGCAAAGATGGTGCAGTGGTGTTACCCGGATATCT

CATATCGACAGGCTGAAGGATGATAGX

>Unigene27519_A-W 1 470 LEN=470

XXAGCAGCTAAAGCCGTTCTTAGAGTAAAATTAATAGCATTAATATCTTACCTCAAAGAG

AATAAAGATATAAGCCAATCTCTAATTTCCGCCTTCAAATCCAGACTGAGGAAAAGGAGA

GAAATAATAGATGGGCAGAAATTATTAAAGCTGAAACCACAAAAATAGGAAAAATGTATA

CAAAACAGAAAAAAAGAAAGAAGACCAGGGATGATCAGGACTGAAACAGGGGCCAACACT

ACAGACCTCACCAACGTGAAAAAACCCGTGGGCAGACCTGCAAATGCCCTCACCAGCGGT

GACAATCAGGTGAATAGACCACTTCCTGGGAAGTCACAAGCCCCCAGCACAGACAACCCA

CAGGGACAGAAGACCACAAACCATGCTGTGGTGGATGAGGCCATTGCCTTCCCATCCACT

GCTTTAGGAGGCCAGTGTCCTGGTGGAACAGCTTTAGGGACAGATTCTCTG

>Unigene27608_A-W 1 70 LEN=722

XXGGTGCAGATGCAGCGCAAGGGTGCGCCCCGCGTGCTGCTCTACTGCAAGCGGTCCCTG

CAGGAGTGGGTG

>Unigene27919_A-W 1 269 LEN=269; minus strand

CCCAGAATTGACCCTTTACGAGAGCCTTCTCCTCTGAAATACTGCACGACTATGCGATTG

CAGGACAAAGACTTGGAAATGTATGTAAAGTCTGCTTCTGAACAGATTCTAGTAAACTAC

GCCAGGAACAGACAGGATTATGAACAGGTGCACAGAGAAAAGGCCATGTGCAAACACCAT

GAACAGGAGGCCATCTGAAAGCCAGCAAGACAGACCTCATCAGAGACCAACCCTGTTGGC

AACTTGATCTTGGACCTCCAGCCCCGAAXX

>Unigene28185_A-W 1 338 LEN=338; minus strand

XXAGAAGATACAGTTCCTATGTGTTCTCAAATGAATGAATCAAGAAACTTTGAGTGGGTC

AGACCAGAGGATATATTAAAAGAAGATACACCACTGTCCTCCATGGTATTTCAGAATTTC

ACACATCCTGTTGTGAAGCCTATCTTAGACAGGAGGTATTTTCTTCAAAAGAATACAATG

GGGACAACAGAAGAAAGGATATGCAAATCAGCTGAACCACTTTGGTTTTCATCATCTGAA

GAAGAGAAGGAAGGTCTTGATGACACCAAAAATAAGCAGCCACAGGTATTTTCTGAGGGT

GGTCCACAGATGTGCATTTCTCACTCATCTGGAGAGAAACXX

>Unigene28187_A-W 1 205 LEN=205; minus strand

TCTCACTCATCTGGAGAGAAACATTATATACCAAAAGAGAAAGCAGATGGACATGTCCAT

GCCTCTAGGGATTCATCTGATTTCAGAAAGCACACCGATCCCATCACAATAAAAGAGGCC

CTTCTATGGAAAGGATTAGGCAAAGTGAAAGAAAAGACAGTCAAATTGGCAGATACCCAT

TTTGACATGACTTCAGAGGAAGAGCXX

>Unigene28401_A-W 1 242 LEN=242; minus strand

CCCCCGCCCCGACTTCTCCGCGTACACTGTCACACGCGCGAAAGCGGCCAAGTCTGCCAG

CCCAAGCGGCACCTGGCTCTCCGCTTCCTCGCGGGGTTCTCCTTAGCCCCCGAAGGGCAG

CACCTGGTGGCGGCCAGCTCCTGCCGGCCTGTCCTGCTTTGCCTCGGCCGCTGCGTCCGC

CCGGCACCGCCTCCTCTCTCCAAACGGTGGCGGCCCGCCCTCAGCGCCTGGACGCGCAAC

GGX

>Unigene28542_A-W 1 207 LEN=207

TCTGGGCCTGAGTCAGAGATGGCAAGAGGAACACTTCAGCAGCACCTCGCACGACTGGCA

GAAAGTGGTGAAATCTTCCAGTCAGCTCAAGCTGAGGACTGCCAAGGCTCCTTCTCTGTT

TTCAAATTCCTGATTCATCCCACAGAGCCAGTGTCCCACTTTTTTTTCTTCCAGGAAAAA

GTGACACATCAGGAAAATAGAATAAAG

>Unigene28905_A-W 1 269 LEN=269; minus strand

XGCCTGAACAGAAATTTTCGATATGCTACTTTCAAGCTTATGGCTGACCACCCAAATTTT

CCATTTGCTGATGAAATTAATCTGGACACCTTTGTCCTGACTTCTACTGGTATCCCTGAC

TTCAGTTGCGATTCCGGAGGTGGCTCAGATGCTGATGATTGGACACATCGCTTCTGTTGT

TAATCCATGCTTCTTAACAACTTACATTCTGCTACAGCAAAGTTCACCAGTCCAGTAATT

ACATTTTTGTCAATTTCTGAAAACCGAACX

>Unigene29181_A-W 1 344 LEN=344

GAATTTCATATCAGGAAATACCTTTTTATGTCTGTTAAATATTCAAACAATTTGCTACAG

TGTTAATTTGCATGGTCCAAGCCTGCTGTAGTTGCAATGCAGAGAGTGCATGAAGAAGTA

CAGGGCTGGGAAACCTGCGAAGGCACCAAGCCTCAGAAGGGTGCACGGAGACCTGGCATC

CTTGAACTAATGAGATCGCTGATTCTAGGACACAAAACGCCAATGGAATTGTATATGCTT

GTACAGATTGATCCTGTGTGCTCCTCTGAACAAACCAGAGAAAATGATGATATCATCAAT

ACTGAAATTCAACTTCCAACTTCTCTAATAAAAAATGAAAACACX

>Unigene29573_A-W 1 211 LEN=211; minus strand

TTGAGCCACATCCCCAGCCCCATGCTATTTCTTAATGATACTCCATTTGAAATTCCACTT

GGCATGCCTGCAATACCAACAACTCAGAAGGCTGAGGAAGGAGGATCCAAGTTCAATGCC

TGCCTCAGGTACTTAAAGCAAGTTAGTAAGACCCTGTCTCAAATTTTTTTTGAAAGGGAT

AAGGCGGGCTCGGTGACGCAAGCCTGTAATTXX

>Unigene29711_A-W 208 288 LEN=288; minus strand

GTGCCGGTCATGGAGGTGCTGCGGCGCTCCTCGGTCTTCGCCGCCGAGATCATGGACGCC

TTTGACCGCTCGCCCACCGAC

>Unigene30380_A-W 1 200 LEN=200; minus strand

XXCACATGCTCTGGAAATGATTCAGATCCCCAGCAGTTTCAAGCTAGGCTTTCTCAATAG

ATTATAGCAGGAGAAAAGGTCAATGCACTGGTTCCTCCAAGCCTGAGTGAAGTCACAGAT

GAAGAACACGGTTTATTCCCTGAAACTGGAAATGCTTTGGAATGTGATTTCAGGAAGAAA

AGAAAGTTCTTCCCAACTGGCCXX

>Unigene30388_A-W 1 318 LEN=318; minus strand

CTCAGTTCCCACCCTGATCCTCTCACGCTTCGGCGACGGGTATCGGGGGACGGCGCGGTG

TCGGAGGCTCCGAACGGCAGTCTGCCCTTAGGCGAGGATACCCTCGTGGCCGAATGGGGG

CGACGCGGTTCCGATGGCGCGGGAAGGTTCCGCAGCGACCCCCGGCCTCCGAGGCGGCGC

TTCCGGTGCACCGTGCXGAACCCGCCCTTCTCGCCTCCTGAGGCGCTGACGCGTGAACCA

TGTGCTGCTCGGGGGCGCGGGTGGGTCGCAGCCGTGGGTTATGACAACTTGCTCAACCGC

ACCGTAGTTTACCGAAGGAXX

>Unigene30405_A-W 1 286 LEN=286

XAGAGAGAGAGAGAGAGAGAAGCAGTTAAAGCAGGTAGCAGGAACAATGGAGTGTGGGGT

AAGTAGGAATCTCTAAGTCTCTCAAGGGATGTGAGTCAAGAAGATACATGGTCTATGCGT

TATTTCAAAGAAGGGACTCAGGGCTCAGTTAGACAAGAAGAAACACTTCCAGTCTGAGAG

TTAGGCCTGGAGCAGGCTACTAGGCATCTGGGGATTGTGAGGAAAAAGAGGCTTCGAGAA

CTAGAGTCTAGGACCAAGCCTCCTCAACCAGACCCACTCAACAGGGGX

>Unigene30553_A-W 1 249 LEN=249

GCAGGAATTCTCAGAGCCTTTTGTATACCTTATTTACACATTGCAAATAGTTTTGAGGGT

GGGACATTATGTGTGTTGGACTCTTGTTCAGAGAAGCAGTCTCACCGAACTGGACTTCCT

CAGATCACTTGTAGGGAAGACCAGAGCACTGAACCAGGGGTCCGTTGACTTTTTCAGCAG

CCTCGGTCACACAGAGAAGAAGGTGCCGTCACAGAAAGATCTATCACGCACTTCAACCAA

GTAGACACA

>Unigene30664_A-W 1 95 LEN=247

XGCTCTGGCTTCAATCCCAACAGTGAAATAAATAATGATGATGCTGCTGACAATGATGGT

GATGATGATGATGATGATGTGGAGGAGGAGGAGTAC

>Unigene30700_A-W 1 301 LEN=301

XXAAATTGCTCCTTTCCCATTGTGAACATATCAGTGGCTTCTAAGAAAATTCCAGATCCC

AACCTCATCTTACACCTTCACAGTTCTCTGAGTCCTTGAGTGATGACTCTGAGTTCGTCA

TTCTTTTTTCTGCCTGTTCCTGTATCAAAAGGCCATGTCCGAGACTCTCATTTGTACAGT

GTTGCTTGGACTGAGGACAGAGATGAAGACAAACTAAACAGAAAACCTACACTGGCAGTT

CTGAGTCCAGAGTCTAAAAACTTTGGGACAAATAATTCCATTGTTATGGCCACCAGTCCC

AGG

>Unigene30786_A-W 1 235 LEN=235

XGTTCTTCTGAGGACAACTGTGAGTTTTCCAGAGGAGAAAATGGTGTGCGACATGTGAAG

TCTGTTGGCTGGGAAAGCTGATTCCTGTCTCAGAAGAAAAGGGATCTACACAGGCTCTCC

CCTGCTGCTGCCAGAGGAAACTTTCTGAAGGTCAACACTGATCATGTCGATCCTGTTAAA

AGGAATTATAGAAGAAGAAGAAAAAAGGCTGAGAGTTTTCAGCGGACACAACATCTX

>Unigene30815_A-W 1 293 LEN=293

XGGTCTTCTCACTTAGATCCTGATCCTCTCACGGAAACAGGAATGAACAGTGCAGAGAGC

CTGGAGATCTAGTGGTGCTGCCTAACCTCAAGCAGAGTGCCTGCTTCCAGCTAAGAAATT

GCCTCCATGATGCCAAAGGAGATGAAGGCTGGAGACAGCAAAACAGGCAGTGACTGGTTA

GTGGAGGCCCAGGCCCGGGACACAGCCTTCTCTGCACTAGACTATTACACGGTCCCTTCA

GCTCAAGCACGGCAACTTGCCCGGGACAGAAAATGCCGTGTCAAAAGCAAGAGG

>Unigene31059_A-W 1 262 LEN=262

XXGGGTAAAAATAAGGCACGCTTTTACAAAGAAGACCAAGAGTGCAAGGAAGAGGTCCTC

CGGAGGCCTGCAGAGCGCGGGGATGCCGTCCCAGCGGAACCCAGGTCAGGGGAAGTGAGA

TCCCCGCGGAGCCATGGCAAAGGCATTACCAAGAAACCCCGCGATTCAGAGGAGCTAGAC

CTCGCTTTCCCGGGCAGCGAACACCAAGAACAAGCTCGAGAAGCGAAGCTAGCCGCAGTC

TTCACACCACCTCGTCCGCCACCG

>Unigene31063_A-W 1 252 LEN=252; minus strand

TGGGCTGTGTGTTTCTGTCAGGCAGCAGCATTACTGGGAATTGGGCAGTGCAAGCAGTTT

CATAAACACTTTCTTGGGCTCTTAAAAAGAGCCAATTATGCAAAGCAAAATAAGCATATT

GAGAAGTCTTCATCTCATCTACACAAACACCATGAACATCTTGGTTTTGTGGTAGGAACG

GGTTTGTCTGCAGAGCTGTTTCCTCCATCAGCCAGAATTCAAGTATTCCAGGGCCACCTC

GTAGCAGAACTT

>Unigene31066_A-W 1 255 LEN=255

GGGGAAACCCAGGTGCTGTCCATCATCTTAGAATCCCCGTGAAGTGAGGAGGCTCTCTTT

CTCCAAACAGAGGCTGAAATTGCTACCCAGGCCCCCCAGCATAGACATGAACAGAGTTTT

GCTGCCTACTGTGGGGAATTTCTCCTTGCTGCTAGCTTGTTTGGCCATCGGGAGGAAAAA

CCGCCCCTCCCTCCCCAGCTGGCTGTTGATAAACTGTCCTCTCGGACACTCCAGGACTTG

GCTGTGAAAATAAAC

>Unigene31216_A-W 1 225 LEN=225; minus strand

XXAGGGAACCCAGGGCAGGAGCAGCAGCAGCAATCACTCCATGGGGACCTCGGAACTCTG

AGAAAAAAGCTTCATCACCACCCTGGCATAGCCATGCAGGGTGGAGGTGGCTGCAGTTTG

CTCATCAATGAAATGCTCATGAACACTGAAGACCTCAAGAAGXTGAGACATGGACTAGAG

AAAGAAGAAAATGAAAAGGAGGAACGGAAGGATACTTTAGTAGACGAC

>Unigene31358_A-W 1 323 LEN=323

XTAATCTGGGGACAAAGGGAGACTGATGGAGAGAAGATCATGGTGGAGAGGCTGGGAGTT

GGAGCTGCAGATCAGAGGACACAGGAGGGGTACCGGAAAGACCAGCTGAAATATCCGGAG

AGCTGTTGCTTGCCCTTGGAAATCCAGATCGCATTTGAGGACCAAATAGGAAAGAAGCGT

GTTTCATCCCGGGGAACTAAGAAAGTGAAGGTCATCATCTTCTGGACAATCTCAAGGGAG

GCAAAGGAGCAAGCTCTGGCTACAGGGAGACCATGCTTTAGGGAGAGAAGTCCGCCTCCG

TGTGGTTTTGGTCGTGGCAGCAGX

>Unigene31439_A-W 1 242 LEN=242

XXGCTGTATTATATAAATTAAAAAAGAAAAAATAGCCACAACATGCCACCTGATAGTAGT

GAAAAAGAATCTGGCTTTGCTGTGACTCTTGGAGATGATGCCCAGGCATCCCTGCTGTGA

TTAGCTGCTAATACTAAAAATGGGCTTTCTCAGCTGCAGCGGCCTGCAACTTACACAGCT

CCCACCCACCAGCAGAAGCAGAGAATCGAGTTTCATCTCAAACTCTCATTTCAAAACCGT

AAGCXX

>Unigene31528_A-W 1 287 LEN=287; minus strand

CTGTTGGATAAGAGGAATGGTCGAGTAGAAAAGGAGAAATTGAAAGTATGCCCAGAGAAG

CCATGGTGATGGAAAAAGATGTTTGAGGGAATGGAATTTACCATACAGGCTAGATGGGTT

AACTTTGGAAAAATGCTAGAGCATTTCTCATACAAGAAAATATTTCAGAATACTAAAGCA

CAAGAACGGTTTATCAGTATCTTTGTAGACCAAGAACAGGACAAAGCCCAAGGTACATTT

TGTTGGCAACTACAGGTTGGAATAGATTGCTCCTTATTTCAGCCCAAX

>Unigene31587_A-W 1 389 LEN=389; minus strand

XTCTGTGAAAGCACATCCCACTCACAGGATTTTTATGGGGATCCAGCAAGGTCACAGATG

AGAAAAAGTACACAGTACCGCAGAAAAGTCAGGCCTATTACAGCAGCTGAAGAATCACAG

AAACGGGTCAAATCATCTACCCACCACCCTACTGGCAAAGTCTATATCCTGATGGGAAGG

TGAGAGGACAAAACTGAGGACCAGTGGCAAAGTGTCTCTGCTTCTTTCTGAACCTCAGGT

GTTGTTTATGCAAAGCAGGAATTGATTATCTTGGGTCATTCCTTGGACAGGAGGCAGATG

GGTGGGCCACAGGTGCAATCTCTTCGGACCAAGTCATCAACAGACATTGTCCTCTCAGGC

CACTGTGCTGGCAAGGGGAAGATTAGCATA

>Unigene31699_A-W 1 284 LEN=284

XGCTGTTTTCCAAAGTGGCTGCACCATTTCATGTCCCCAACAACGGTGCACGAGTTTCCA

ATTTCTCCACATTCCTGCCAATACCAATTCTGTGGTCTTGTTGGTGTTTGGAGCAGATAC

TCTGACTGGTGGGAGGCAACAACTCTTTGTGCTTTCGACCTGCATTTCTCTACCAATGAC

ACCATCACTTGTCCAGACAGGGGTCCTCCACCCTCACAGCTCTTCTACCCCATCTCTAAT

GGCTTTGGACACCTTCAGTCTGGGGTAGCCATTCCTCAGGGCTCT

>Unigene32223_A-W 1 258 LEN=258

XXGAAGAGATTTATAGTTCAGGTTTTATAAAAGACTAGTGCTATTCACATCAGTCTTTAT

AGCATTTTCAAAGTGGAACTAGAGACACACAGACAGATGGCCAAAGAGATATCGGCAAGT

AGATATAGAGATAGGAATGGGATAGATAAAGACATGGATGTGCAGAAAGGGAAGGATAGA

CCTATGAGAGACAGAAGCATACAGATGGGAAAAAAAAAAACAGGAAAGCACAGAGGGATA

AAGGCCAAGATCAACAGAGGX

>Unigene32224_A-W 1 223 LEN=223; minus strand

XCTTGGGAACTGAGGAGCAAAGAGGGTCAAAGAGTTGGGAGGAAGGAAAGTCCAGGTTTA

ACAGGATCTCTCTATCACTTTAAAATGCCTCTTAGAGGAAAGCTTAAAACAGGGACCAGA

CCACAGCACCTCAGGGCTGGAAGAGACCATGAAGAGGGTCTGCAACAACCACCCAATATG

AAAACAGATGCTCAGAGAGGTGCCATGACTTGCAAGAGTCACATX

>Unigene32250_A-W 1 262 LEN=262; minus strand

XCTGGATTAGCGACCTGGGAGCCCGAGCCCCGCGGCCTGGCGGTCACACTTTCCTGGGAG

CGGCGGCCACCGCGGCAATATGAAGAAGTCGTACTCAGGTGGTGTCCTTCATCTCCAGAA

TCTCCTGCCAACCCTGAAGCCTTCTGTGTTCCTCACTTAACCTATTTGGACATGGAGCTT

TCTGCCAAGCACCCACGAATTAACTTTTATTGATTAAGTCAGAATCATGGGTCTTGGTTG

GAACTAAAGAGGTCATTTGGGCCX

>Unigene32361_A-W 1 308 LEN=308

XCAAAGGTGCACATCCTTAGTCCTGCAGAGTTAAGTGTCAATAATTCTGTGATTATGATA

GCAACAGCCATGGTCCTGGTGGCATGGGATATGGAGATAACTTTGTCTCACAGGGCAACT

ATTGTTGATGCTGGGTGTTGTGGCTCCTTCGTTTGTGGTCCTAGAGCCACAGAATATAGA

GACGAACATTCCCACATCCAGCAGGATGAACATAGCTAATCCTGGTATGTGTTTTTGGCA

CAGGCAACCGTGGTCTTGGAATTGTGGAACACAAAGAGGACTTCTCCATATTATATGGGA

CAGGCAAAG

>Unigene32426_A-W 1 257 LEN=257

XGAAGTGTTCCAATCAGAATCAGAAAGTTACCAGCATCCACAAAGGCCTACAAACACCAG

TGAAGTGGTGATGGGGTAACAGAGATAGAAGAAGACATTTCCAAAACTGCCCAAAGCACA

GCTAGCACAGAGGATCCTGTGGGGCTTGGGCCATGAGAGGCTGGTGTGGAAAGTGAGAGA

GAGAGGCCAGGCAGACACACACAGCCTGTTCATGAAGGTCTTCCTGTGTTACGAGAATGT

GGACTTTCCTCTGAGGAC

>Unigene32603_A-W 1 62 LEN=218; minus strand

XGCCTGGCGCAGGAGCACGCGGAGCGCAGGAAGCGGAGGCGCGAAAGCGAGTGCAAGGCC

GAC

>Unigene32637_A-W 1 345 LEN=345

XXTTCAGCTCAGCTGGTTCTGGCTGAGGCCCCGAGGCAAGCTCGGCTTTCCAGTGGGTTC

TGCCGCACTTCCCGGATGCTGTCCGAAGCCCATCGCTGCTGCTCCAGCTCCCTTTTCTAA

GAAAGATTACACAGAGTTCTGCTTTTCATGGAAGGGTCTTTGGTTTTCAGGGTCAACACT

GGGAACTGGGGTTCTGCTCAGCATCTAGACGACAGTGTCACCCTCCCACCAACTTCTGAG

CTACAGTCGCTTCGTCAGAGGCAGGTTGCCCAGAGCAGCAGAGCTGGTGTGTTCCTGGAC

TCTAGTTCTCAGTGGATTCAGAAAAAGAGCAGTTTGGCATTTTGTGAX

>Unigene32725_A-W 1 264 LEN=264; minus strand

CATAAGGAAAATCACAACTGGGCAACTCACCATGAAATGACTAAACTCAAGACAGAGAAG

ATCTCCAAACCAGTCAGAGGGAAAAAGACACATGACCGGCAGCTGCTTCTTCCTXAGGAA

CAACAGAAGCCAGGAGACAACAGGGATGGAAACCAACAGCCAACCTGTGGAAAATCACCT

TCCAAAATGAAGGCCAAACGAAAACATTTCAAAGGAACAACAATGAAAGGAGCAAAAAGC

AAAGGCAAAGGCAAACCTGAGACAAXX

>Unigene32743_A-W 1 284 LEN=284

ATAGAAATCCCAAAGCTGAACCTGAACTACCCTGAAAAAAAGCTGGAGAACAGGAGATTC

TAGCAGAGAAGTCTCTCAGGAGGACAACAGGCCAAGTCAGCAGATGGGGAAGCTTGTTTT

CAAGATCAAGGATCTGGTGCTGCAGCTGCAAGCTGCTCTACAGATGAACCCAAGAATGCG

GCAGTTCTGAAGTGCCGGGAATCTGCAGAGGCACTAAAAGTTCACAGGGTAGAGACACTG

TTTGCTGAATTAAACCCAGCCAAGAGACAGTGCAACTTGCCCCAX

>Unigene32748_A-W 1 232 LEN=232; minus strand

XXATCCAATGTTCACTATAAAGTGGCAGCCAGATGGGGCAGGGAAGAACAAGCAATCAGA

GTCTTCCAGGCCAAGATTCAGATTTTAGTTCAGTCATTAATCTGTGTGATCCAGAGAAAT

CTTTGAATTTCCCGAGCCTTAGTATCCTCGTCTATCAAGTTAACAGTCATTTTTCTTTCA

GAGTTGTATGACGAGCATAGTGACAAATGTGTCTCAGGGACTCAAAAGTGGAAC

>Unigene32916_A-W 1 211 LEN=211; minus strand

GCATTAGTGCAGGCCTCTGGACAAATGACCCTGACCACATATGATGGCTATGTTCTGGCT

GGTGAATGTCTTTTCTTGTCTTCTGATGAGGGAAGTGAGGACTGTGCTCTCTGTCCCTCA

GATGGACCAGAATCCTTCCCTACAAAAAGACCAGTTGCATTAGAAAAAGACCATCCTAGC

TCAGATCCACCAAGCCCAGCAGTTTTGCTTCXX

>Unigene32941_A-W 1 326 LEN=326; minus strand

XXGCAGCGCAAATCTAATAGACTAAATACGTTCATCTCCTCCCAAACAGGCATTGGATTT

CAAAACAAACCAGAACAGAGCGTACGCCACAAGGAAACATGGTACTTACACATATTGTCC

AATAACTCTGCAGAGCTCTTTAACAAAGAAGAGAAAGGTTCTXCAGAGACTGAGAAAGCA

GTAGAAGCACTCAAGCACTCGCCGGCAGGTAACCGACCAGGTGTTTTGCACATCACGGGC

TCCGGACCACCTGTGACTTCAGAAAGGCTGCCGGCGGGGAAGAAGGAAACAGGTGCCGAC

CCTAACTGCAGACGACTTCACCACAAGCCX

>Unigene32956_A-W 1 266 LEN=266; minus strand

XCGCAGCAGCCGGAGCCGCAGCCTCAGCGCCTCCCTCCCGGGCCGCCGCTCCGCCGCCGT

CCGGCCTCCTCCAGCCGGAGCGCACATCGGGCTCCCGCCACGCGGGTCAAAAGCCTCGCG

CTCGTCCCTGCGGCCTCGGGAACCGCGTCCTACGAGATAGCCGCCTTCCCGCCCGCCCCG

TCACCTCCTGGCTCCTCGGGCAGCCGCTTCCATGGGCACCGACAGCCGCGCGGCCGGGGC

GCTCCTGGCGCGGGCCCGCACCCTGCA

>Unigene32991_A-W 1 224 LEN=224

TGTCCTGTAGTGCCAGGAGGAGGCGTTCTGGGTGCCACTCAGAGCGCTATCTACTACGAA

GCCCGGGACCTGCAGCGGGAGACCAGCCCGGAGAAAGACAGCTCACAGCCAAAGCGGAAG

TAAAACGCTACCCAGCAGGCTCTGCGCGCCTACAACTCCCATCATGCTTCATCTGTGATT

GCGAAGCTCCTCGCAGAAATCGGCGGCTCGACTTTTCACCTGTGX

>Unigene32996_A-W 1 230 LEN=230

XGGGGTCTTCACATGCATCCAGCCATGGTGGGTGGTGGCAATCCCTGGGAAACAGCACAA

TCCACAAACATGGATAAGGACAATACAAGGGCTACCTCTGGTGGCAACATGGTAGCCACC

AAAACAGCCCAATGGGCAGAATGCTGGAGGCCCCCCTATGACCACATCTTTTCCAGATTT

GCAGCAAAGGCACAATTGGGGTTTCCCACATGGCCTGTCCCAAATGACATG

>Unigene33080_A-W 1 250 LEN=250

GGCTGGCGATTTCCCTGTGGCCACACACATAGTCAGGACCTGCTGGGACTCGGTTCAAAT

CCAGCTCTGTGCCATACAGAACCTGGGGTCCCTCTGGCAACTTGTACCATAGCTGGAGAT

CAGTGGCTCATCGACAGAATGGGCACCATCATACCCCTCACTCAGAGTTGCTGGAAAGCC

CTGACTAAGAGGTTCTATGATGGTGCCTGGCACAGAGATGAAGGAAAGCCAGAAAATGAC

ACTCCCTCTGXX

>Unigene33102_A-W 1 225 LEN=225

TTTGGTGCTGGGAGTGGGGTGGGTCAGAACCAGGGCATTCGGCTTGCTAAGCAAGTGTTC

TACCACTCAGTTGTACTCCCAGCCTTGCAGTTGCTCTTTTCAACTACAGGTCTAGGTGGC

AGCAGGCAGCTATTGTACACTTATTTCTTGGGAGACAATGGTGCTGACACTTTCTCAGAT

TCATGGATGGTTCTAATTTCTCCTTCACTAATGAAGATGCTGGTG

>Unigene33115_A-W 1 210 LEN=210; minus strand

XCTGGTGGCTGGCATGTCTGGCAATGGCCTCTTAAGACCAGTGTGCCAATCCTGCTAGAT

CAGCTAGGATACAGGGGGGCGAAAGGCCTAGAACCATTTTTCAGTCTTGTTCACGAGGTA

CCAGGCAAGCACGACCACATGAAAATACGGTGTCTTGCAAAAGCAGAATCAAACCTTGTT

GAGGACACTGAATTCAAGATTTTGAGCTTCAXX

>Unigene33207_A-W 1 314 LEN=314

AAGAATCCAGCAGATAATTTCAAAAGAGATGAAGAAACTGAAGTCACCTATTTTGTTAAC

GATGCGAGGACTTAAAACATTATATGGAACGGTGTCTACCCCTTAAGACGCTTCTCTAAG

CAAGTGTCAGGCTCGATAACCTGGATGAAGTCAGGCATGCCATATGCCATAATGTTCAAC

CATAAATGTTTTTCAGATTACAAACTTGACATTGATGGCCTCAGGGTCCGAGGAGGAAAA

GGCACACCTGACCCAGAGATCTCTCAGGCTAGTGGGTCAAAGGCACATTTTTCAGTGTGT

ATGACAAAACAAGGX

>Unigene33359_A-W 1 253 LEN=253

GCGGGCTCCAGGATCAGCATCGCTGCCATGCTGGACTTTGAGCAGAGACTCTTGGAAGTG

GTGGCTGGTACCGTGATTGAGTCCCAGTGCGGGTTTGGAGAACCGAGGCGTCAGGAGCGC

TGGGAGGCTGAACGGATCACGGTGTATCTGGGACCTTATACCTGTGGACTCCACGCCAAG

AACTGGTCTGAGATCCAGGAACTAGCCCCTGGGCGGGCTGAGGGGACGTCAGAAGTTTTT

GCAGTAGGGCTG

>Unigene33455_A-W 1 290 LEN=290

XAGGGAAAATCAGCAAACATTCAGAAGACCCCAAAGATTCCGTCATTCAGTGGATTGTGG

TTGAAATGTTTGAAATGCTCCTCTCAAGCAGAAGGTGACCTTCTCAAGCGCTTTCATGGA

ACATTCATTAAGACAGACCACAACCAGGGTCACAGAACAGCCACACTGAAATTAATGAAA

TATGTTATCTTTACAATGATGGAATTATGCTGGAAACCAGAAACAAAAAGAAAGGAGAGG

AATTCACGAACACCTGAACACAAAACAGGAGACAAAAATAACCCACTCTCA

>Unigene33537_A-W 1 222 LEN=222

XCAGAAAATGACAATTTCATTGTAAAGGGAGAAGAACTTTTGGCTTATGGATCAATTCTA

GTGAGGGCCCTGTCTGAGAAGGGGGTCGATATGATAGAATTTGTGGAGCAAAAAGACTAT

GGAAGGCTGCTGGTGTTTGCAGGTAGAGGGGATATGTAGGAGAAAATGAAAAACATAGGA

GATGATGACAGAGAATCAGAAGAAGAAGCAGAAAGCATGGAG

>Unigene33561_A-W 1 274 LEN=274; minus strand

CTGACAATGATTTTGCGTTCCTTTCAGGAAAAGGATCAGAAAATCATAGAAAGATCTCCA

TATATGTCAAAATGGAAAGACGTTGCAAGAATCATAGAAAACAGCAGCTACAGATCTGTT

ATTAAATTCATAATAATGAGAAACATATGGATTGATTCCCATAAGTACTGAAGTTCCACA

ATTCAGGGCAAGGAGGTGAAGATGGACATCTGGGGCCTTGTTGCTGACTCCATCACTTTT

TGGAAACTAAGACAGGTTGCCAAGACAATCAAACXX

>Unigene33655_A-W 1 200 LEN=200; minus strand

CTTCAGACATATCAATTTGATCATAAAAATATAATGAAAATAACCAACAGCAGCAAGGAT

GTGGAGAGATTGGACCCTCATATGTTGCTGGTGGAAGTGTGAAAAATAGTTCCTCAAAAA

GCTAAACACAGAATGACCAAGCAATTCTACCTCCTTAGTCATGTATCCAAAATAATTGAA

AAGAGGGCCTCAAACAGCAGX

>Unigene33733_A-W 1 267 LEN=267; minus strand

XCTCACAAGAACTTTATCAAGTATATCATGGGGACCTGGAAGAAATGAAATGGAGGCCCT

GTTTTCTGTGAACTTAAAGTCAATTTGGGAGCAAGATCCACTTACCTGGTATGCATAAGA

GGTGACAAAAGTACACCAAAGAAGCTTTTAGAAGTCAGGGTCATCAGTGTAACAACAGGA

GCAATGTCACAGGCAATATTAAGCATTCTCTATGTAGATTGTTTAGAAAAGTCTAATAAT

GTAATGATCTCAGGAGAGGAGGAAGTAGXX

>Unigene33804_A-W 1 242 LEN=242

XXGAGGATCAAACCCAGTGCTTCACGAATGCAAGGCAAGTGCTCTGCCACTTTCATTCTA

TTCCTTCTTGAGGAACAAACATTTCCACCTAAAAATAATAGGACAGCTGCCCAATTAAAA

ATAATACGGCATCTGTACAGCAAGCAGTCTTCTACACCTGGTGAAATAAATGGGCTGTTG

AGTGAAATGGTCTCTACTGGTAATTGGAATGTCCAGAAATAGCACCTGAAAATAATCCCC

TTGAXX

>Unigene33934_A-W 1 291 LEN=291; minus strand

ATACTAACTAAAACAGAAATTGGTACCAAGAGTGAGGTCGTTGCTGTGACTATTACGTTC

AAAAGACTTTGGAATTTTTTGGAATTTGTTGGAGAAATTTTGAGATGTGTAGCAGTACAA

GCTGGAAATGCTATAGATTGTTGTAAGCAGAGCTTAAGGGCCAACTGTGGTTTGATCCCA

GAAGACCAGAATTCCAATAGGACTGTGGATACTGAAGACATTATTCAAAAAGTTTTAGAG

GAAAAGGAGGACACTATTTGTATTGGACTAGAAGCCATTCGTGTTATGTTC

>Unigene33960_A-W 1 217 LEN=217; minus strand

TGGGGATCTTGGGGAAGTTCCTCAGAAGTTCTTCAGTGGCTACACCCAAGCTTTGGGGCA

CAAGTCCAGACTGGGAGTGAGAGAGGGATCCTAAGGTCTCTGGTACCAAAGATTCAAGAT

GAGTCACCTCTAGGTTATCTCTTTGAAGAAAGCAACAAAGCTCTTAAGCTATTTCTTAAC

AATGTCCACATCACAGACCCCTTCAAATGTGGAATGCXX

>Unigene34070_A-W 1 321 LEN=321

XXGCTTTCTTCATTTCACCCAGGTGATTTGCAAACCTACAGAACAGGGAATGTGTGTTCC

AAAGACATGGTCAGAGTAGCAGTGGCAGCTGCAGAGACACTGGTGGTGGTGACCATGAGG

AAGAGGAGGAAGGAGAGGACCTTTTTCTTTACCAAAGATCAGCAGTGTGACACATTGCTT

GAAATGAGAACCCACACATTCTACAAAAGTCCCCAGCATATGTCTCTCTTTACTTCCCCC

TCTAGGGCTCCCAGAGAGGACAGAGCCCTGGATGAGACCCTGCTCTGTCATGACCATGTG

TATGGCCACACTTGCAAGTTTCTX

>Unigene34219_A-W 1 266 LEN=266; minus strand

XCATCTCTAGAGTCCTTGTTCTTGCCTGGGGAGTGGAGAACATGAGCCCTTTTGGTTGGG

GGTGGAGAACCTGGCCCTCAGCCAGAGCAGGAGAATCGGTATCTAGAACTGCAGTCATCG

AAAGCCTTGGTGGTGGCCAAAAAGACAACCAAAGGTGACCCGTCCTGGAGTTAGCACAAG

CAGGAGGTCAGTGACCAAGTAAGTAAGTCCCTGGGAGCGTACAGAGAGGCCCAGCTCTTA

GATCAGACAGGTCAAGGTGAACAGTTC

>Unigene34223_A-W 1 290 LEN=290

GTCCAAGGTCATACAGCTGTTAAAGAGCAAAGCAGACAGGTTCCAGGGCCCACAGTCTTA

ACAGGGGGCTCTTCTCGGGTTCAGCACCTAAGGCAGGGTTTATCAGGCCCAACACCGTTG

ACATTTGGGTTGGATGGCCTCTGTCCACTATCTGCTAGCCGTACCACCCCACCCCAGGCG

AATGACAACCAAAAATGTCTCCAGGCACTGTCAGCTGTCCTCTCGGGGCAGAATCACTGT

CATTCAGAAACTGCAGACATCAAGTATAAAACCATAAGCTTCACTAGGACX

>Unigene34316_A-W 1 225 LEN=225

XGCTTCCCGCTGGACCAGCAGCTCTTCTTTGGTACTGCTGGGTACTCTCCAGGTGCTGAG

CTCCTCACAGAGCATGTGGTAGGAGCTCAGTCCTTGTGTGGAATGGGTGGGACCCCAGGC

TCCTGGGTCCTTTCTCGACTTTCACCTCCAACTCTCATTTCACCTGGCTTTCAAGCACCA

TGGAATTTGCCAATTGGCTGTACCCCATCCAAAGACAATAATGGATXX

>Unigene34360_A-W 1 248 LEN=248

XTCACCCCCTCAGCCCATCAGGTGGCCGCTATAAAGAAAAAGAAGATAGGCATTGGTGGG

ACCAGGGAGAAACTGAAGACCTGGGCCCCCGTGGTGGGACGTAAAACAGGCCAGTTGTCT

GAAGACAGGACGGAGGTCACCCGAAAACTAAAAAATAGGATTACCATGGATCCGCAGCCG

GCTTTCATCAAAAGACCTGACAAGGTCAACTTGGAGGAGGGAAAGTTTACTGGGCTCACA

GTTTCAGAX

>Unigene34406_A-W 1 202 LEN=202

TTACTGAGAGAAATGAAACAATACCTAAATAAAAGATAGTCCATGTTAAAGGATAAAAAG

ACAATTGTTAAGATGGTATTACTCAGATTAAAAGCAATTCCTGTTGAAATCCTAATGATG

GTTTTTGCAGAAATAGAAAAACCTTTTCTGAGTTCATATGGAATCTCAGGGGACCCAGAT

AGCCAAAACAATCTTGAAAAAGXX

>Unigene34420_A-W 1 251 LEN=251; minus strand

CAGGAGAGCACCTCTTCTCTACAGTTCCAGTTTGCAGATCTATCTCTGAACAGCATGCCA

TCTCTGGGTGCAGTTCATGATGACACACGAGTGACTCTTTCCTTGGAAGGTAACAAGAAA

AAGAGCTCATGGATAGTGAGAGGATTTGTCATAAGATCATCAATTATTACAACTGTATTT

GAGAAGAAAAGAAAAATGTTAGACAACTCGAGATGTGAAAATGTTCAAATTTTCAGAAAG

TATAAAAAGGCX

>Unigene34427_A-W 1 296 LEN=296

AGAGATTATGCAAACCAAAGAGAGAAAACCATAAAGAAATGAACAGAGACTTGGAGAAAT

GTGGGACATCATTCAGGGCACCAACGGAAGTATAATGGGAATACCAGAAAGAAAGGAGAA

GAAAAAGATATTTAAAAAGTAATGGCTAAAAACTTCCAAGATGTATTGAAAAACAATAAT

GTACACATCCAGGAAGCTAAAGAATTCCCAAGATAAATACAGAGCTCACAGACAGACATG

GTAAAAGTGCTGAAAGAAAAGGGGAAAAATCTTGAAAGTTGCAAAGAGGAGCAACGX

>Unigene34468_A-W 1 253 LEN=253

XXGGTGTTCACTTCAGGGTTCTCTGAAAAGGGGAAAGTCAGGGACCCAAGGGGGCGACCT

CCACCCTTACTGGAGCAGCCCTGGGCCGCTTCCCCAGGCCTCATCGCCACCCAGGGGCCG

CCCTTGGAGCTGCAGGGCCCTGCGCTCGCGGCACCGGGACCCGGCTGCCTGCTGTCTGGA

CGCAGGGACCACGAGCTGGAGCACGGGAGAGGGGCCCGGCGCAGGATTCTGAGCTCCCTT

CTGAGGCGGTTTCTG

>Unigene34499_A-W 1 242 LEN=242

XXTTGGGGGAACTTGGTGGCAACGGTGATGATGATGGAGGGTGATCCTAAGGCTTCCTTT

CTTCTCCGTCTGGTGGTGGTGGTGGATTCAGCAGCTGCTAAAGAACCACGGATATTCCTT

GAATTTACACAGTACTCAGATCTCAAGCTATTTTTGCGGGATACATGGAACCTACAGATT

ACCACCAGCAGAACAGGGCAGATTTATCATGGAGCCAGGTCTGAGTCCTGGCCACACTTT

CTGAXX

>Unigene34508_A-W 1 251 LEN=251

XXTAAATCCTGTTTACATGCTCAATATGATGATCTGTTTGGAAACAAAATTGGAAGTATT

CAAATGCCCAAGATCAAGAAGTCTCTAAGTGATGACAAATGTTCCAAAATGAATTTTAAA

CCTTCAGGAAAAGGGCTGGCCTTCTTATCCCAGAATGGGCCTGGAATACATATAGAAGCA

GTGATAGATGTCAAAGAAGATAATGGATTGCCACACATTGATGAAGAAGATGATGCTCTT

AGTAAAGGACCAGXX

>Unigene34512_A-W 1 213 LEN=213; minus strand

XXCAAAGTTCCACATGGAGTATTAGGAATTCAGGAACTCAGGTTATTATATGGTGAATGG

TTACCTTTGAAGAGATTGAAGTGTTTTGAAACAAAAATTTTAAGTCAAGTAAAGATTGAT

CCAGTAAAATTGCCCCATACTCCACCAGTATTTACTAAACAGTATCCTATTAAAGGAGGA

CATGAAGAGATTAGCACCACAATAAAGGAAATGTTX

>Unigene34529_A-W 1 218 LEN=218; minus strand

XACTGTCCCTCCTCTGGGCGCCAAATAGGAACATTCCCAATGAACATGTTGTATTCTAAA

ACGTCTAATGGCAGTATTAGAAGCCTGCCATTCATCTTAATCATGTTTACCAGCTCAAAC

GAAGACTTTGGAATTGTGTCTGACTATGAGGCAATCATTTTAATCAACTCCAGGCTTGCC

TTTATACGAGCCATTTCTTTTCGAGATTTGAATACCAGC

>Unigene34534_A-W 1 204 LEN=204

XATGTCGATGTCCGGCTTACCCTGCCCCATCAAGAAGCTCTGTCACTTGTGGATCCCAAA

GAGCATCAGAAAGTGGGGGTGGGGATGGGGCAGGTGTCAGTAAATTCAAAGACGGATGTA

CAAAGGGCTTTATGGAGTTTAGCAGCACCTGGACTAGAGTCAGCAGTCCTCCATTCCAGT

GTGGCTCTGTACTGTGACTTCCAGGXX

>Unigene34549_A-W 1 202 LEN=202; minus strand

CGGGGGAAAGACTGTTAAGTGGGAAAAGAACCAGTACTTGATGAGTTAAGAGATTCCAAG

CCTATTCAGAATGTAAAAGAGGCTAAAGTTAGGAGATTCAATATTAGGAAAGTGTACTCT

GAAGATAGGGCCAAGGCTAAGGATGTGGCTGGATATTCTTTGCCTAGTGCTAAAGAGATC

AGGCATTGAGTCATGGATTTACXX

>Unigene34588_A-W 1 251 LEN=251; minus strand

XXTGGACAGAACAATCAAATGGGATCATATACATGATACAGTAACCAAAGATATTATGAG

ATGGGAATAATAGTAATCAGTAAGACCATGTTTTCAAAAGAGGTTTTAAAACAACAGCAC

CTAAACTCAAAAGTGGTTATCATGGTGGAATTTCAAACTTGCTACGATGTGACTGATCCT

GAAGAAGAAATGACAGATGAGTACTTACTACCCCACCCAATATTGTACGCAGAAGTACTT

GGTTTAGCCAGCCXX

>Unigene34818_A-W 1 240 LEN=240; minus strand

XXTGAAGATTCCACCATGATGCCTCCTTGGCCCATTAGCAGAAGGGGCTTTGGAGGAAAA

AGACATTAGAAAGCAAGTTCATATTAGTCCCCAGTACCTTCTCCACGGACAGAGAATGTG

GGCCAGAAGTTGGAACTCAAATCTTGTGTGCAGTTTGAACAGAATCGGTTATTTCGGGCC

TTTGCCTTAGCTACCAACAGCGAAGCCCTGTCCTATCTTCTTGCCTTCAGCCCTGTTCCT

CCX

>Unigene34919_A-W 1 237 LEN=237; minus strand

XXGGAAGTCAGGCCAAATACAGAGAAACTGATGTTCTCCGTCAAGAGGAAAAAGACAAAA

CTCCAGCAGAGCAAGCTGAAATCCCAGCTGTGCAGGGGACGGGCCTCCCTGAGCCTGCAG

ATGTTTGGGTCACACTCAAAACATGAGAACTGCCTGTTCCCTCCACCCACAGACACACCT

CACCCCGTGAACTTGGCTCCTCGTCTGAGCTGCAAGACGGAGCACAGGGAAAGTACCCCX

>Unigene34921_A-W 1 216 LEN=216; minus strand

XCTGAGTGGAGATATAGATATATTAATTTGGAGCTTATAGGAGAAGGCGAGATTAAAATC

TGGGACGTGCAGAGGTATACAAAGAAGCAGGAGAGCTCTGCCAAGAGAGAGGAACTGAGA

AAAAGAGAAAGGTAAGAAGTGAGCCTTGGGGGTCAGGAGAAGGAGGACCAGCCTGCAAAA

GCAATGGAGGAGAGACCCAGGAGACAGCAGGAAAATGXX

>Unigene34952_A-W 1 286 LEN=286

TCCCTTCTCAATCCTGCTTCATTAGGGTATAAGATTTCTCACTTCCACTGCAAAGTTGTC

AAGCCTCCAGTTGCAGCCACTGGTGCTCTGGTAGAGGTGAAGGTGAGACGCTGGTCATCT

CCCAGAGCCAAGGCCACTGATACCATTTATACAAAAAAGGAGGTGAAAAGTGTGAGGAGC

CTACCATCCAACCAAGCACCCGTGCTTCAAGGCAAGGGTCGGGTCCTGAGCCCTTGCCCA

CCAATTCAATCTGTGATGATCACCCTCTCTCTGCACTCTTCCTGTGXX

>Unigene34961_A-W 1 244 LEN=244; minus strand

XXTAAGCCTGAAAACCACAAAAGGGATGTTCCGGGGGTCTAGCCAATGCAACTAAAAGAG

ATCCTAGAACTGAAAAATTTACAAGTGGGACTCAACAGCTGGTCTGAGCTGGCAGAAGAA

TCAATCAGTGACTTCAGAGACAAGTCAAATGAGATTATCCTTCTGAGAACAAATGAAAGA

GTAAAGATTTTTCAAAAAAACAACATCATCTCAGAGCCCAGTGGGACACTATCAAGTGTA

ACAAAC

>Unigene35183_A-W 1 231 LEN=231

XXATTTTTACTTACAAAAAACCCAAATAGGCACATCCTAGCAGAATGTAAAGGCAAGCCT

CCATAAATTCAGTTGTAAGTAGTAGTTGAACTGCCTGCTGTAGTGATACTTTCAGGGAAA

GGCAGCGTGTTAGAGCACTACATCAGCACCACAAGCCAGTGTTCGAGCAAAATCCACTTG

GCAAACAATTCAAGAGCAAAGGTGCCCTATGCTGAAGAGAAAAGTAGTAGTAGX

>Unigene35206_A-W 1 250 LEN=250; minus strand

XCTTGCACTAATTTGGAGATTGGGCCAAGTGCTGCCAGTTTGGCTTGAACCACGTTCAAG

GGTAAGTTTCAAGATTCTCCTGAGTCCCTAGAAATTGCCATGGAGCTGATCATGTACAGG

TTCATCTTTGTCTTGGAACGTAACAAATGGAAAAGGTTTGGCAGTCAAGAACTGTTGATT

GAAAGACTGTTGGGTTGTTCCCTGGGCATCAAGCTCACCCAAATCCTTCCAAGAGGACTC

ATCACCAGTCCX

>Unigene35676_A-W 1 244 LEN=244

GCTATTAGGTTTAAGGCTACATATTATATGCTGAGTAATATTGATATTGGTCTCACCATT

AAGGGTGAGGCTCTGGAAACCTTCAGACACCTAGACACTATAAGTCTACAGGGTAGATTT

TATGCTGCCTTAGATCCATACTTCTAGATGGGAAAACAGACGAACAACATGAATAAACAA

GGGAATAAAGCGCCCCAAACAAACCAAGATGCTTCAACAACAGAAGCTACTGATAGCACA

GGAGXX

>Unigene35772_A-W 1 293 LEN=438; minus strand

XACTCCTCCTCCCCTGTCACCTCGCTCCCGCGCATCCTCCAGCTCCCGCGGCTCCCACCA

CAGGCGCAGAGTCCAGAACCTGCCGCGGGGACGTGGACCGGCGGGACAGAGCCCGCAGGG

AAAGTCTGGGGACTGAGGAAGTTCGATATGCAACTCACCTGCCCGCGACGCCAGAAGAAA

GTTGGGCAGGAACTGAGCGAGGACGCCGAGAACGAGCGCCGCGCGGACCGCACCCTCCAT

GGCCTGGAGCTCTGGGCCCTGGAGACCCGCCTCCGGGCGCCAAGACTTAAAGCC

>Unigene35962_A-W 1 203 LEN=203

XXTGGTAATTGTGCCCAGAACATTCACAAAGATGACAGATGTAAGAAAGTCCCTCCTCAT

TGCTCAAAGCACCATCGAAGTACTCAGAGCCAAGAGATGCCTTGCAAATATGAGGAATGG

GAGAGAGCTTCTTATCCAACTGCAAGCATTCCTCAATACAGTGGAACTCATCCTGAAAGG

GAGCCCCACATATTTAAGGAATATGXX

>Unigene36211_A-W 1 257 LEN=257

XGGAAAAGTGAAGAAAGATTTACTAAATATTAGGCTTCCTGCTCATGGGAACATTCAGAC

AAGGAGCAGTGGTAACAAGAGGTTAAGGGAATAATTCTACAAAATGGGCCAGTGTTCATG

ATTTCTCCTTTAAAAACCAATTTGCCTGCACTGCCTGGCTTGAGTCAACAGGATATGTTA

CATCCCTCAGCAAACAACCTGTTATCTGCAGGAGAGATGCAGGCCCTAAATGCTGATAAA

AAGAACACAGGTCAAGAA

>Unigene36315_A-W 1 232 LEN=232

XXCTTGGTAGTGAATTCTGTCACTACCTTCATGGTGGCTGCTGCTGAACTGGAAGTCTTC

TCTCAATCAACTGCCACCTGCACCACTACTGCCATTGCTACTGTCTCCGATTTCCCCGTT

GCCAGCCTGGATAGCCTGGAGGTCTTCTTTCTGGGTGCCGCTGCCCCAAAAAGAACTGCT

GCCCTGGATGCAACTGAAGCCGACACTGCTGCTGACTCCCAGAGATCGTATGGX

>Unigene36330_A-W 1 282 LEN=282; minus strand

CCTGACATCCCATCACTTGTCATTATTCCAGAGCCTCTGCGTGATCCTCACCCCTCTCAA

ACTCCACCGCCTCCTCTGGACCTTGATGAGAGCTGCAACTTCTCCCTATGCAACTGGTCT

TCCATTCCACGCCAGGAAAAGCCTGCCTTGGTTCCGGACCTGATCACTGTTACCGCTGTT

TACCGGTGATGAGTCTTTGCTTCCCGAATGCCAAAGTATAACACCAAGAAGCACGCCGAG

GCAAGCCAAGAGTGGAAAGTGGAAGGCTTTATTAAAGGACAG

>Unigene36678_A-W 1 101 LEN=261; minus strand

XAGGTGATCGGAGGTGCTGGGTTGGACGTGGATTTCACGCTGGAGAGCCCTCAGGGTGTG

CTGTTGGTCAGCGAGTCCCGAAAGGCTGACGGGGTGCACACG

>Unigene36694_A-W 1 203 LEN=203

GTCTGCCAAAAACATATGAATCCATTTGTACAAAATGTTCAGAAGAGACCAATCCACAAA

ATCCAGACATGGGTGAGAAGCTGGCAGGGCTGGGAACGGAGAATCAGAAACGAGTTCTCA

CTGTGTGTGGTTTCTCCCTGGGACGATGAAATCCTCTGTGGCCAGCACATCCGAGACGAT

GGCATTCGACGGTACTTGGAAAGX

>Unigene36871_A-W 1 403 LEN=403; minus strand

XCCCCTACAACCTTTGGCCGCCCTTCACACAGGCTATTTAGAGAATCAAAAGCGAGAACC

CGTGTGCAAACCTTTCGTGAACTAGAGGTTGCCGAGCCTGATGAAGCATTACGAAGCCAG

TCTGCCCTCAATACAATGACTGTGTTCCCTTCCTCTGACAAAAATTCTTTTGACTCCTCA

GGCAATCCAAAAGTGACCCTGCTTGTCTTGACGTTACTTCTAGTCCATGTTGGTAATTTA

CAGCTTTCTTCTGTAGTTCAGACATCAAGTGCTTCATTAATACAAGGCTTAGCTACCAAG

GGTCTCTTTGTGTCCTTCCCCAAATCAACACAGTTAAACCAGGTGTACATTCACCCGCAA

AGAGGTTGACATTATGTAACTGCATTCAGGCCTGAAACCCAGACX

>Unigene36875_A-W 1 152 LEN=437

XCCTCCGGGAACCCGTGGGATGGCAGAGGCGACATCACAGGGAGCAGCAGGCAGGTGGTC

GCTGATTGGACAAGAGGTCTCCGCTCATCAGCCCCAGTTACCGCGGAGGTAGCCCGAGGT

TTTGGTGGCCGGGAGACCTCTGGGCACCCGCAA

>Unigene36896_A-W 1 92 LEN=226; minus strand

XCCGAGGAGCGGCCCACCTTTGAGTACCTGCAGGCCTTCCTGGAGGACTACTTCACGTCC

ACGGAGCCCCAGTACCAGCCCGGGGAGAACCTA

>Unigene36971_A-W 1 207 LEN=207

GTCCGGATAGTTACTGTGTCATTCTTTTTTGGTGGCATTCCTTTCCAATACCTCAGATTC

CCTATTAAGAATCAGGAGGATCCATTTGGCCGACAAAGTTTTCTATTTTACACGTAGAAA

CGATTTCAACTCGCCCAGACGCCGGCAGCACCTTCTTCACGTTGCATGGAGAAACCACCT

GTAAGCATGATGGCGGTAGACGTTCAG

>Unigene37132_A-W 1 399 LEN=399

XXCTCTGAAGCTGGGATCCCCTTAAATAAAACTTTTCCTCTTCTACAATTATGCTGGTTG

GGTCCTTTAGTCACAGCAACAAAAAAGCTCACTAAAACAATTATCTATTGCACCAAATTT

TTACAGGAAAGGGTAGCAGAAGGCTACGTGGCTGCTGATGATTTCTTGGAAATTTCACCA

AAACACAGACAGCAACAACATCAAACACAGACAGACCACACAGACTGGAAACTTGAGCAC

TGCAAAAGAAACAAAGCACAGAGTAAAGGGACACCTGGAGGAGGGGAGGAAGTCATTGCC

CACCTCCTGCTTGATAAAGGCTTAACCTACAAAATACATAAGGAGCCGCCCCCAGCTCAA

CAGCCACAAGCACTAAAAACCCAATTAAAAACAGCCGAAGGX

>Unigene37284_A-W 1 373 LEN=373

AGTGAATTCATACCTGTATTATCAACTCCATCAAACTCTGCTTTTTGTTTTGCCTTTTCG

TTTCCCTGCCTTGATGTGATTCCTGTGGXTTACCAGCGACTCAAGATCAAGAAGTCTGTA

CCGTTGGTGAATAAGCAAGAAAAAATTTCTTCAAGTTTTCAGGAGGGGCAGGAAATGGAA

GCCACGAATAGCAAAACAAATAACGTGAAGGCCCAGGAATCTCAGGGACAGCGTCAACTT

CCACAACTGTCAGAAATTGGCGTAAAACCACCTCAGTGTATTATCCACACTCATCAGATG

CCTCCAACAGCACCAACCAGCAGTGTCCACACTCTTCAGAAGCCTCCAACAGCACCAACC

AGCATTGTCCACACX

>Unigene37396_A-W 1 278 LEN=278; minus strand

AAAGAAATGATGTATCATTGCAAACACAAAACTGCTATGGCTATGTTAACAGATGATGTA

AATTTTAGAAGGAGTATTTCAAGGGATAAAGAAGGACCTTTCAACATGATAAAGCAGTTT

GTTCATTATGAATCCATTAAGCACCCAACAGTAGAACTTCAGAAGTACAGGAAGCCAAAA

TCGATAGAACTGAAAGGAGAAAAAGAAGAAXTCTACAATTTAGCTGGAGATTTCAATACT

CATCCCATAGTAACTAATGGACCATTAGAGCATGTGAAC

>Unigene37424_A-W 1 328 LEN=328; minus strand

XXCTCTCCTGAAGGGCCAAGGAAGGGAGAAGCTACTAGCATAGGAGCCATGGACTCTGAC

ACAGTGAATAACCAGCCACCCAAGGAGAGCCAGAGGAATAAGTTCCTACTGCTTTTGCTG

TCCGCACTCCTCCCAGGGCCTCGAGTTGGTGGGTCCCTCTTGGAGATGGACAACTCACAG

AGGTCAGCCCTAGGGTACAGAGCAGGATGGAGAGCAGAGAGTGGACCTGTAGGAGCAAAT

GGAAACTGCCTAGCACAAATTCTTTTCAAAATCTTCACACTCATCTTGAGAAAATCAGCT

CTTTTTGTAGAGAAGAACCAATGGCACCCT

>Unigene37458_A-W 1 337 LEN=337

GAAGTCCAAGCGCAGTCCCTGGGTGCCAAGATGGTCAATAAGGCCTGCTGGGGCAGGGCC

ATTTTTGGAGAGCGTCTGGGGTGACCCCGAAAGCGACCTCGAGAGGCGCCGCGACCCAGA

GCTCTGGAACGGCAGGGCCAGATCTCGGGTCGCCTGGGCGAACGGGTCCCAGGCCTACTG

TCCCCCTTCCCGGACCAGTCCAGGGCCCGCGGGCGCTTCCCGGCGGTGAGTCACCGCGGG

CACGGCCGGCTGCAGCGCATTCCTCCGCGGGGCCGGCGCCCGGGTGGGGTGGAGATCGCG

AGTCACCCTACCGGGCCGGGCAGCTGGGACGTCAGGAXX

>Unigene37493_A-W 1 177 LEN=287; minus strand

CAGTGTGAAGCTCATTTGGTCATGGGTCAATGTCTCTGTCAGTCTCTCTGTGACAGTCAA

GACCAACAACACAAATCAACTGCAAAGTCTTCTATGGTGGTAGATGCAGGATTCAGTGAA

ACAGGAACTATGGAACTCCTGAATCCAATGCAAACAAAGTCACCAACAGGTAACAAC

>Unigene37519_A-W 1 230 LEN=230; minus strand

GCCAGCTCTATGACCTGGCTGCTGCTGTTCAGACTTCACTTGAGTAGCAAGGGACCAGCT

AAAGAGTCTAACAACGTGCCTGCCTGCCCACCTGGTACATATGACTGGCAACAACCCAAA

GATCAGCACAAGTTTCCTGGGGCCTATCTCATAGGTGTGCAGTTTCCTACTGACCTACCT

GAAAAGTATTGCTGCAACATATTTAAGAGCAGGAGAGAAGATCATGGGACX

>Unigene37570_A-W 1 261 LEN=261

XGTCTGGTGGGATATCCATATTCTTCAGATGAGGAGCCAGCAGCAGAGAAGCGAGGGGAC

AATGACAAGTGGTAGAAGTGGGGCTGCCTAATGCCAGAGCCTTTTCTTTTTTCTTCTGAT

TTTAGGGATGGAACCCAGGGCCTAATCTGTGCTAATAACATACTCTCCCTCTGTCATACC

CGCTCCCCTGAGCCCTTGCTTTTAACCAGCACCACAGTCACCTGGGTCACAGTTGCAGTG

GTTAAGATCAAGGATTCTGCTGXX

>Unigene37631_A-W 1 285 LEN=285

XGAAAAGTAAATCAGTCTATCCCTCATGTGACATTGCTTACAAATATCATGAATCTTGGC

ATGGCCAGTTTCAGCTATATTGATGGAGAGCCAAATACTTCCATAAGCTCTTGGGTTGAA

CTGGAACAGAGAGGGCAGGAGCTTCTTTTCATTGCTTTAAACAGTCACAGGTTATAGATA

TGGAAGAAAATCTCCATGTATGAAATGCTCAGGCTGGATAATAACCAAAAGGTGATGTAT

CAGAATCCACGAATACCTCCAATTCACATATTTTTAAATTTTGTGAXX

>Unigene37698_A-W 1 300 LEN=300; minus strand

XTGGGTGTTAAAATTTTTGTTCTGGATATGTTAGAATTTGAGGTGCCTGTGACACTGCTG

TACCAGCAGTACAGCACTCAGGAAATCAGTCTACACTGCAATTTAGATTTGAAAATCGTC

GTCATTGCTGGAATGATGCCTAAGCACAAGAATTCAGAGAAAGGTTTGTTAGAAACAGAT

AGAAAAGATGGATCCTGGGAAAAGCAATATCGACAAGATGTGGAGTGGAGATGGAGCTTG

GGGAAACTTACACCGAGGTCTGATCACTGTGCTCATGTTGTACTTATCAATGTGCTGACC

>Unigene37718_A-W 1 205 LEN=205

XXAAGTCACACACTGCTCAGACTCAAGCGTTGGCACCTAGGAGGTCCTCAAGTACTTTCT

GAGAGCAACTGGATCCTCTGGGACAAGCACACATCAGTCATCTTTGAACTGTGACACCCA

GTAACACCTGAGAACTGTGAAATTGCCAGATCAGACAGCTCTCGACTGTATCGTGATGAC

AGTCCCATGTGGGTCCCTGACTGCATG

>Unigene37732_A-W 1 267 LEN=267; minus strand

XXTCAGACTCAGGCAGCTGGTGTCCAGATGGCTTATGTTAAACCGAAGCTGCTTTCCAGA

GGGAAGTGGATGGGGAACGGGACCCCACCACAGACAGACAGGTACAACCAGGTCTGGGGC

CCAGTCCTTGCCTTCTGTGGCCTCAAGCTTCACTATGAACAACAAGAAAACAGTTTTTAC

CCGCCCTGCCTTTTCCTGAAGCTTCCAGTGGTGTCTGGAAGACAATTTTCCACCCACAGA

GCTGCCCTGCTTCCCTCTACCACTCTGGCX

>Unigene37786_A-W 1 237 LEN=237; minus strand

XCTTTTCTGTCATCTTCATGTCCTCGACCTGTGGTTTCCTCTGTCCTGTCTCTGCCCGCT

GTTTTGATCTGTGGGAACTTTCTGAAAGTGACAGGCTTTCTTCGAGGCTGGCTTGTTCGT

GACCTCGCCAGGTTGCCTGCGGTTTTTGCTGCGCTGTTTGTCTTTGGGCTCTTTTTTGAG

GCTTTCCCCATCTCTGACCCCTGGGTACTGGTTTCTATTTCAACAGTGGAGTCAAAGCXX

>Unigene37842_A-W 1 247 LEN=247; minus strand

XCGCAGCCAGGCGCCGAGGCTCGGCGCGCGCTCCGGGAGAGTTCTGGCGACGGGCAGGGG

CAGGGCCAGGGCCGGGCGCCTGCGGCCGCAGGTTACGCGGACTTCAGCTCACGTGCCCCA

CTGGTCTCCTTGGGCCGGAGGCCTCAGTTCGGAGGCTACCGAGACGCGGCCCAGGAGGAC

CAGACCCGTGAAGGTTCCGGGGATGGCCCTCCTAGGGAGCCXGTAGCCAAGACGGTCGCG

GTCTCCCTG

>Unigene37852_A-W 1 171 LEN=216

GGAGAAGGAGAAGGGGGCGGATACTGCACCTTCCCCCCCGGCCCCGCCCGGACCGTCCTG

ACGCGCGCGCGCTACGGAGCGGGCCTGCGCCGCTTCATGCCGCGCCGCCTCCACTCCGGG

CTGGGCTACAGCAGCTTCCTCGGCGGCGGCGCCGGTTGCAACCCCGACGGC

>Unigene37853_A-W 1 284 LEN=284; minus strand

XTTAGTGCTTTGCTGGACTCGTATGAGATTGGGAAAATCTCCAAAGTCATCTTTCCCTGC

AAAAATAAAAATGAGAGAACAAATGTCAGGCAGGAATCCAGGCAGGATGAGGAGGTAAGC

AGGAAGCAAGAGCGAGATCAGGCCTGTCCTCAGAGCGGGCGTGGATAGTATAGATTGGAC

CTGCCCATGCTCAGTGTTTCCCACTCCCAAAAGCAATGGCTTCACACGTATCCCTCTAAT

AGCAGATATTTTCAGGATAGCGATGCTGAGTCTCGGCCTAAAGGC

>Unigene37916_A-W 1 200 LEN=200

XXCTTTATGACAAAAGGTAAACAAAAGGACCTGGAGACTATCCTCTACGTGAAGTAGGCC

AGTTCCAAAAAACCCAAGGTTGAATTTGCTGAGATGTGGAAGCTAACCCATAATAAGCGG

GGAGAGGGGAAGAATAGATATTCAGTAGATCAAACAAAGGGGAAGGAAAGGGGCATAGGG

AAAAAGAAAGACAGTAGAATGAXX

>Unigene37955_A-W 1 324 LEN=324; minus strand

XTAAATGGCAGTAATGAGCCCAATGGAATAACCCAGCCTGTCTTTTTCAAAATAAGATCT

GCTGAAGTCAAGATTAGTACAGAGAGAAGCCCGCTTCCTGTCCTGCTAGTGTGCTTAATA

TTGGGATTTTATTTTAAGCCTTTGAATTCAACGTTGTGCATTGAAAAATCAAAGTGCCAA

ATAATCCATGACAAAGGCATGTATAAGGATGCAGGTTACCCTTACCCTGACCTGGAGGCT

GAAGGGCTAGAACCCACCCTGGGCAAGACAGCAGGCCAAGTTTTGCTCCAGCAAGGAGAC

ACGGATGACTTCAGGCAAGCCCCTCXX

>Unigene38090_A-W 1 248 LEN=248

XXGCGACTGGCTGACCCCGACCTGGAGCAGGCTGCTCATCTCATCCAGGCTCTTGGCAAC

TATGAGGATGTGATTCATGTCTATGACAACATTGATGACCAGGACATATGTGCTTGTGGG

CTCCTTCTTAGGCAAGGGACAGCTCATTCTATAGCACAGGCATCTGCAGCAATCTTTGAG

ACTGAAGCAGGTGGGAAGCCTAACAGGTTAGGAGGTCAAAGGCCATTTCTTGTGGCTTTG

AGGTCAGGG

>Unigene38394_A-W 1 272 LEN=272

XXGATGGACTGTAAGAAAGGATGCTCTAACACTGCTGTGTTTGGTCAGGAGGAAAAGGCA

GATGGAAGAAAGTTTGGGGAAAGGGAACAAGAAGATATTTACATCTCACATGGAGATGAA

CTACAAAACAATTTTCACATCGTGGCTTCATTTCTTCGTCATGGGAGTTTTTATTTTTTT

ACGGGCCAGCAGCAATTGAGGCATCTCAGTTTAGTTCATCTCCCGTCAGATGCACTTGCC

AATGTTAAAGCAGTGTTACGTTTTACAAAACCAGXX

>Unigene38525_A-W 1 302 LEN=302

GTCGAGGCGCTGGGCGTGGTGAGCACATGTCGCCAGGCCCGGGCCCACCCCCTGCCGGCT

CCAGCCTGGTCCTGGCAGCTGTGGGGCGTGAGTCAGCAGGCCGAGCTGCTCACCTCCACA

CTCCAGCCCACGCCTCCCTTGAGGTACAGCCGGCTGCGGGAGGGTGGCCAACGCCATGAA

GCAGGGCGCCTGGGCCAACTGGCTCAGGGGACTGGTGGGTGTCCGGCCTGCCCACTGAAA

GCTTATCAGAGCCCTGGTCCTCCTGTGAGCCACGGGGCCTTTGCTCTCTGGCTCAAGGTC

AAX

>Unigene38564_A-W 1 243 LEN=243; minus strand

XTTTGGGTGCCCATAAGTGCCAAGGGTATAGCCTATGGGGGACAGAGAGAGAAGCAAAAA

TGAGACACCACCACTAAAAGGGCTGCAGGTGGATACCCCCTCCTGACCCTGGAGCAAGAT

AAAGACGACTTGGAAATAGGAAGTCCTTCCTCATCCCACAATCAGGAAAATGAGGAAGGA

GATGAGGAGGATCACAGGTGGGGAGGAGAAGAGGACCATGCTGGATCCTCTTTGATAGTA

AGCCXX

>Unigene38742_A-W 1 318 LEN=318

XXTCTCAGCTTAAACTGTCTCTTCATCAGTGCTGCCATGGAGCTTGGTGAAGTCAGTGAT

GTGGAAGGCTGGCAGAGTATGACACCCAAAATACACCCCTATGGCCTGAAGCTTATTTTG

AAGTAAAGGCATTTGAAAAAGGGTCCATGAACAAAGGGCACTCTGATTTTCCCTTTTCTT

CCTCAAGTAGGAGATAACTACTCGCATGTGAAAGATGTCCTCTCCAAAACCAGGAAGAAA

CAAACATTTTTATCACCAGGGTGTCGAGGTAGCAGGAAATCTGCACAAACCAACCTGGTT

CCACTCATCTTCATCTTCTGX

>Unigene39123_A-W 1 266 LEN=266; minus strand

AGGCCTCAGGAGCTGATCAGCACTGGCCTAAGCTCTTTGGTGCCAGCTGGGCTTGGAGAG

CCCCCAGGGATGAGGATGAAGGCTCAGGTGGATCCCACAGAGGCTTCCAGCCTCTTCTCC

AGCCCTTCTCTGCCCATGAGCCATTCCAGGGCTAACTCAGAAGGACCCCCAGCAGAAGAT

GAGGATTCCCTGGGACTGGCCTTACAGCTGTTGAGGTCTGAAACTGTAAAAGTTTATGTA

AACAATGAGATAAATATATTAGCATCX

>Unigene39153_A-W 1 316 LEN=316; minus strand

CTCACACCACCTTCTGAGGTAGGTATCATTGTAACCTGTTTCATAGATGAAGAAACTGAG

GTTGAGGGCAGTGCCAGGTTTTCTCCCGAGGTCACACAGCTGCGCAGCTGGCTGGCCACG

ATAGACCTGGGTCTCTCCAGCTGCCATTCTGTCCTGTTCGCCTTGTTCACTGGGAGACAG

GTGACCACCAGGAAATCTGAAGAGGACAAGTACACAGCCATGTTTCCATCTCAGTCTCAG

ATGAGAAATGAGTCTGTGACCACAGCCGTGGCTTTCAATATTGCCTTCGGACAGAGTCTC

CTAGTGACAGTGTCA

>Unigene39239_A-W 1 266 LEN=266; minus strand

CTGTGGCTTGGTCCTACGTCTGGACATATTTATAGTGATCAGCTTTCAGATCAGTTGAGG

GGGAACTAGTATCTGACATTCCAAAAGGAAAAGGCAATTGAAAGTGATGAAAATTCAAGA

ATAGTTTCCTTAAAGACATTTCTAACTTTAGAACAGGGAGAGAGGAGTGGGAAGTCTTAT

CGGAATGTGTACATTTGTAGAAGGGAAACACTTCAAGTGAGTCTTGCCCCAACAGTTTCC

TGCGACCCAATCACTGCATGCAAAGCX

>Unigene39291_A-W 1 240 LEN=240

AAAAAGGATATGGGAGATGAATTCAAGTATCATTGTAAGTGGGATTCACAAGCCATTGAA

TGGAAAGGCAACCTGACAATTCTAAGTATGACTGTCCCATCAAAGTTGGATCTAGTTATA

GCTCCCTCTCATTCTCATGTCAGACCTGGATTTTTGTTGGTCCTGAGTGCACAACACATC

TCCATTCAGACACTCCCTCTGCTCCAGACACAATCTGACTCCTATCACTTTTCCCCAGAG

>Unigene39501_A-W 1 255 LEN=255; minus strand

XXCCTCAGCCCTGAGATCGAAGCTTCGCCCGTCCCTGCCCCCCTCCCGCCTCGAGGACTG

GTTGGGGGTTCGATCCCTCTGCCGCCGCCGCGGGGCCTCCTCTTCCTCTCTTGGGTTCTA

ATGGCCCGGGGGGAACCCGATGGGGCCAAGACCAAAATCTACAACCTGCAGTCTCTTCTG

CCTCCCACGCTTCGGAAGCAGGAGCCTCAGCGGGGCCAGTTCTTCCGCGTCCGGGAACCC

AACGGGTCCTGCGCACGX

>Unigene39512_A-W 1 313 LEN=313

AGCCAGTTGACTGTTTTCCTTAAAGGCCTCCGGGTGCTCAGAGCTTCTTTAGACGTTCAG

CCAAAATTTTTAGAGTTCGGTTAGCGGGAGAAGGAACTCACCCTGAATTCATTTAGCGCC

ATCATTGAACCTCGGGTTCCTAGACCAGGGCACAAAAACAGATCCAAAATGGTTTCAGGA

TATTGGAAAACAGCTAATTTTGCAGGTGCTTCGACATCAATGTGGCAAGGAGTAACATCC

ATGAGGGGCGGGGGCGGGGGATTGATTTTAGATGAAATGGGTAATACCTTGATTGAAATG

CAATGTGAAAATTXX

>Unigene39537_A-W 1 303 LEN=303; minus strand

XXCAGGAAGAATCCAAATCCGTTAACAGCTCATTCATTTCCCTGCCAGACAGACCACAGA

AGCAAAAGCGAATCCAACTCGGCAGCTACAGGCAGTGTGGGGCAGCAGTGGGGACAGCAT

ATCACCAAAGATGAGACCATTAGAACCAAGACCAATAAAAACGACAATGTATACAAAGCT

TTATATTAACACGACCATCCAAAGCTCCCAGGATACAGAAAAAGCACATATGATCCTAAA

GCTAAAACAAAAAGATCTTTGGAGTTTACTGATGGCAGCAGTAAACAAGCATTCAACAAG

CATTTX

>Unigene39929_A-W 1 310 LEN=310; minus strand

XTTTCCATGAAAAAAGATGGTGACACATATCAAAGGATTCTCATCTGGGATCTTCCTCTT

GCCCTCTACCATTATCCTGGCTAACAACCCAACCTAAATATGAACACACAGACTTACCAG

CTTTGCCACAGTGTCTGTGACAAACTAGACAAAGGAAGGCATAGGCTGAAAAAACCTAAG

TTACTTGTCAAACACTATGAAGATACTAAATCTATTTTAGGCTTTAAGTATTATATTAAA

CCAAAATACCAGAGTTTCTTACAAAAAGATTCAAATTATATCAGCATCAAGTATGCTAAG

AACTACACAGCX

>Unigene39968_A-W 1 255 LEN=255; minus strand

XCTAGTGAGCGAGTCCTGGGTGGAGCTGTTTGTAGAAGGAGACTAAGAGGCATTAGAGAA

GGTGTCGATGCCACATTAGCACCCCGTCAAGGATTCCTCTCTTTGCAGAAATTAAAGGGA

GCTGAAGAAAATGGAGAGATGTCACACCGGCCGTCAGCAGCACTTATGCCAGGGACGAAC

ATTCTCAGATTTGCCTGGACCCCGGGATCTGCAGAAACCGCTTCCAGGGCTGGGTCCCAG

ACACTAGCCACAGCCCXX

>Unigene40053_A-W 1 252 LEN=252

XGTGTGCTTATTTCAGGCAGAGCCACCTTTGCCGTCCTTGGGCCTCATCTTACTGTGCCA

GGAGGGTTCAAATACAGAAATGGTCACAGTGCTGTATCTCTGCTGTCCCTGGCTGGGCAG

CCTGGCTCTCTGCTGTTGAATGGAAGCTCTCTCCTCTTAGGCAAGGCACTTAAGAATAAG

GAAATTTTCAATGACTTCACAATTGACGAATACTTCAACAGCAGCTTCTCAAAAACCACC

CCCAGAGAAGAAGXX

>Unigene40147_A-W 1 225 LEN=225; minus strand

CACGAACCGGCTGGGGGTCAACTGCTCAAAGCTGGCTTATCAGCACTTGGACCCTGCAAA

CAGAGGCTCAAGGCAGATGACGATGCAGATGGAACTGAGATCTCCATGGCTGCTGTTTCC

CAGACGAGCCCAACAGATCCAAGCTGTGACXGTAACATGTACAACACCAAGGACCTAAGA

ACCACCAGAGAGCAACCTCCAAATGACAACGATTTCAAAGCAGCCAXX

>Unigene40253_A-W 1 523 LEN=523; minus strand

TGGGTTCAATCTCCAGTACCCAAAGTAAGAAAAAATAAAAAGAAGAAGAAGAAAAAGCTT

AGCCCTTTCACGCATCCTCATAAACCCATGATGACAGACACCCTCCACTACACACATCCC

CGTGGTGGACACTTCTCCCAACACCTACACTCGAGGACTCATGTACGTCTTTTAGAACAA

GCAGCCTATAAATGCAGTTTCCTAGACAAGCTCACCCGAGCATGTCGTCACACACAGGTC

ATACTTGTGAACACTCAGCCCACAGGCCCGCCACATGCACAACATGCCAGAAGTACATAT

GCACCAGAGATATCTCAAAATGTTCATACAAACAGACAAACGCACTGGTTCCTTTGCATA

CACCCAGCCATCCAGGACGGCTGCACGGCCAACAGCAGCAGGAACAGGCACAGGCACACG

TGTGTACAGGGGGAGCAGTCACAGTGCGGACACCCCAACTCACACCCGCCCACAGCCATG

CTGGCGTGCAAAGCGGACAGCATGGCCCCACCCTCTCCAAAC

>Unigene40454_A-W 1 450 LEN=450

XXGCAGAAGTTGCATTTTAGAAAAGACCAGCAGCGTAACCCTCAGGTGGGCAGGAAACTG

GGCCTGTTCCTCAGGTTGGCTGCCTCTGGCGCTCAGAACCAGCACCCATCTACAGTTGGA

GTCAGATAACTATGTTTGCTGAACAACAGACAGTTCCCAAGGGGAAAAAATTAAAAAGAG

GAAGGCAAAACTACAGATAATGCAGAGATCATAAAAACCAAAGCAGCAAGAAAACAAGAA

GTAGCCAAGGACTGGGAGGGAATAACTGCAAAAGGCTTGAGCAATAGAGAACCGCTACGT

AAACTATACCAAGAACGTTCAATCCCAGCAGTAAGAAACGAGAAGCCCCATTTAAAAGTG

GGCATGAGACCCGAACAGACACCTCATGGAAGTGGTTGTAGGGATGGGGAAGGAGCATAT

CAAGAGCTGCTCGGTGACAGGTGTCTTTGGAGX

>Unigene40834_A-W 1 251 LEN=251; minus strand

XXCCCCTCACAAGGTACTCGCCAGCCAATGAACTCTTTTACATGCAACCACCTATTCATC

GGTAGTGGGCAGCAGATGCACAGTGCACAGCACACTTAAGGGAACTCTTCACTAACCCAC

AATGGGAGCAGCTCCCAGACCCAGAAGTTCAAGGGTAAGCAACTCCCTGACTATCCAGAC

CCTCAACAGAGGCCTGGCAGAGCCAATGGGGAACTGAAATCAGGAGGCCCTCTGAGAGCA

GAAAGGATCGTGTXX

>Unigene40922_A-W 1 337 LEN=337

TAACATAGCAGATTTCATCTTAAAAATAAGAAAGTGAGCCCAGCACAGTCTCCATACCAG

AGGCCTGTGCTCCAGGACCCCCTCCCCCCAGAAGATAAAAATCCGTCAGAGCTTGTCTCA

CCAAGGGTTAGGACAAATCTGCAGTGGAACAGTGGTGGATTGCCTCTGAAGGAGCTTCGA

AGACGGACTGGATTGAAGAAGTTCTGAGGGAAACCCAAAGACAACAGAGATAAAACCAAG

GACGTTAGAGGAATTAAAAACTTCTGGCAGTTACAGTAGACAGACATTAATCTTCATCCA

ACTCCTAAACAGATTAACATTGAACCTCACACTAGAGXX

>Unigene40939_A-W 1 354 LEN=354; minus strand

CCCACGGCCACTCTGGATGCACTGTGAAGAGCTGGTGGAGCTCCCTGTTCAAATACTGTC

TCTGATGTGATCACTGGGAGCATCTGTCTTCCTGGGAGGGCCTGGTGTGAAGCCGAAGCC

CTCCGAGACAGAGCCCAGTGCAGCAGTTTGCGGAATCAGGTGAAGGAGAAGCTGGAGAAG

GAGCTGGAGGGAGCGCCCGCTGACCTGGAAGATGGCGCTCAGCGCGTGTGCCCTGTGTGC

ATGTGTGTGCGGGAGTACGTGAAGGGACGCACCTGGTGCCTAAACAGAGCCTTCTCCTTC

AAGGCATTCAGTGAGCTGCTCATCTGGGAGGAGGCGGAGCTGTCCTTTCAGGTX

>Unigene40962_A-W 1 302 LEN=439; minus strand

XTCATCCTGGCCGCCCGTCAGAATGAGCAACTGCGTACAAGCAAAATCGGTGGCCTCTGG

ATTCCGCTCCCTGGTGTCGCACTGGGAGACAGTGCAGGGAGATCTGCAGGTGCCAAAGGC

AAAAGACACGGTGGCATGGACTCTACCCGGCTGCTTTTGAAATATCCCAGCAACAATCTA

CAGATGAGAGAACTGATGGAATACAGCTCCCACGGCCCGTCAAAGGTAGAAAGACTACAT

CATGATAAACGTTTGCCAAGAAAACGATACATTAGGGATGAAAAAAGAGAGAGTGGTGAC

CAA

>Unigene41119_A-W 1 218 LEN=218

CAGAAAGAGGCTAAACAAATTCAATCAAAAGCCACAGGTCTTTCTTTATGGGTTATTCCT

CCCTCCTTATAGAGAGGGGTGATTGACAGGATGCAAGCACTTTCGGAACGTCCAACACCC

ATTCCTACCTCTGTTGGTGTTTCTATTAATAGAGTTACCAGCAATAGCAATAACGAAGAA

CATCATAACTCCACCTTTCACCCAATTCAGCGGAGCCAX

>Unigene41571_A-W 1 241 LEN=241; minus strand

CTGGGTGCTTCTCGCCGCAGCAGTCCCGAGGCCCGCGGGGAGGACGGCAGAAAAGGAGGA

AGCCGAGGGCCGACGCTGCTGGAGACCAAAGAAGAGGCAGAAGCGACGACTCCGCGGCCG

GGTCCCCGGAACGCCAGAGTCGTGGTCCTCGTAGTCGCCGCAACTGGCCGTGCGACCCCG

AACACCGCACCCAGCGGCCGCACCACCCTTGCCAGGCTCCGCCGCTACGGGTAGCCCGGC

CXX

>Unigene41582_A-W 1 228 LEN=228; minus strand

XTAAGTGAGAATTATTCAGTTCGAAGGGGCATTTACTGAACATCCTACCATATGATGGTC

ATTATAAGTGAAATGATCAGCAAGGCCTGCGTCTTACCCTCCAGTTCACAGCGTGGTTGG

AACCACCACCGTAAACTTACACAGTGTGGAAAGTGCAGCAGTAGACGTCAGTGCAAGGCT

CCAGGTTCTCACAGGTCTGGAGATAGTGGGAAGTCATTCATTGAGCCCAXX

>Unigene41583_A-W 1 361 LEN=361

XXTGGGGAAGGACAGAAAGATCAAGAGGGTATTGAGGAGGGAAGGAGAACAGAGGATAGG

GAGGCCAGAGAGCTCAGCAAGGTGTTTTGTGTTTGTGTATCCCGATGTGTGACCGCGCAA

TCTTTTCAAGTTCCCTGAGGCTTTGGAGGTGAAATGGGGTCTGAAAAGGAGTGGGTTCAA

GCATATCATCATTCAGTTTCATTACCTGCTCCTCCTGCTGGCGCTGGCAACAGAGAATCA

TCTGCAAATATGGTAGCTGAGGCAGAACCAGGATATATGGAAAATAAAGGGACAAACTTT

GACAATAGAACTCCACCATTAGAAGGGAAAGCTATAATGTCAGGAAAGGGGAAAGGCTGG

GAG

>Unigene41627_A-W 1 505 LEN=505; minus strand

CGCGTGCGCGCCGAGAAGCCCGCTAAGGAGCGGCGCTTGCGGACGTCTGGCCGGCGGCCC

GTGACGTCTGAGGAGAGCTTTAAAGTGCGGGCCGGGCCGGGCGTCTGAGGGTCTGGCTGG

GAGTCGGGCCGAGCCTACGCGGCAGCCCTCTGCGGCATGAGGCGCTTCTGGGCCCTTGCC

CCCCGGGACGTGGAGAAGGTGGAGGAGGAGGAAGCCCCGTTGTCGCCGCCGCTGCATGAC

CTGCCXGCTACTGAGTTCCGACCCCGCTCCTGGCCCCTAGACGCCCCCCGCTGGGTCCTG

AGGCAACGCATGGGGGTCCGGCACTGAGGACCCCGTTCCTTCCGGGGGCACCGGGGCGCG

TCCCCCAAGAGCCATGCCTGGCCGCCCCGCCCGCCCCGGAGGACCCTAGAGAGCGTCGCG

GGGGCCATGGCGGCCGCCAGCGGCTACACAGACCTGCGTGAGAAGCTCAAGTCCATGACG

TCCCGGGACAACTATAAGGCGGGC

>Unigene41673_A-W 1 229 LEN=229

ACACAGAACTTCAAACATTCTTGGAATTTCCTGACAGGAGTGCCTTTTGTTGCTCAAAGT

AAGCCTCTTGGGACCCTACCTGGATTAATGCTAACAAAGGGACCCATTGTCAGTCTTGAG

AGCCCTGAGATAGCTTCACCTGAAAAACTTATCATCTCTGGCTGGGGTTGTGGCTCAGCC

ATAGAGCACTCGTATAGCACATGCGTGGCCCTGGGTTTGATCCTCAACAXX

>Unigene41714_A-W 1 205 LEN=205

GGGAGCAGGCCTTGTCTTCTTGCTCCAGATGCCAAACTGGTGAGGATTAACTTGGAGCCA

CAGGTGGCTATCTTTGCCACCAACAACAAGATGTGCCTAATAATAAAGCCTGCACAGGGG

AAAGCAAAGCTGAAGGATGAACACAGATGGATTTCTGGCAAATGTTTTGAGCACCTGGAT

CCAGCTATGCCTGAAACAATTGCCTXX

>Unigene41723_A-W 1 262 LEN=396

XXGGCTCCAGAGGCTTCTGCTGCAAATGAAGCTCTCGCCAGTCACTGGCTGAGTGATTTC

ACACATGATTGGGACAATGGGTTTTGCCTCATAGATCCATTTTGGTGCAAATATATTGTA

GATGTGATTGCAACTATCTGCCAAGTGCCTAAATCTCAGGTAGGGGCAAAAGGGTTTGGT

GTCAATCACTTCCAGGGTTCTGTTGCCGGGAAAGTAGACATGGAGGAAATACAGAACATT

CTGAGATTAAAAACAGGAAATGGG

>Unigene41964_A-W 1 228 LEN=228

XGGCGCCTGCTGAAAGGCGCCTGGTGGCAGGCGCCGGGCCTGGAGCCTGCTCTGATGAAG

TTTAATGCCCGGCAGATGCTCAAGGAGAGCGGGGCAGAGCAGCCCACACCGGGGGCCCTC

TCGGGCGGCTGGATCACAGCCCGGCAGATCCCTTGCGGGGGCCTGGACTTTCTGCAGCGA

GTACAAGTCGAGGGGAAGGCAGCCCACGAACAAATACCCAATCAGACA

>Unigene42013_A-W 1 254 LEN=254; minus strand

XGTGGGAAAAATAATGAACCTCAGAGTGAAAGGGAACACAGGAGGCTTCAGAGAGAAGGT

GGCATTTTTAAACTGGCACTCAAAAAAATAGATATACGTAGGCGTTCCCCAGGCAGAGAA

GGTCAAGGAGGGGCATCTAGGGCAAAGGGCATGGCAGATACAAAGCACAAAGGCATGACA

CAGCATGATTCTGTTTCTCAACAGGGTGGCTACCCACGATTCCAGGTAGGACACATCCAT

GTCCAGAAGACTTTC

>Unigene42036_A-W 1 215 LEN=215; minus strand

XXTGGAGCCAATGAACACCCTGGACTTCAGTTATCTCGACATGGAAGGAATCAACCCCAT

GGTTCCAGGCTTTCAGCTGAGGTTCAGTTTCAGACTCCATGGCTTTTTCTAGACACAGCT

ATGTCCTGCCTCCGAGGCCTGTGGCACAAGTTGTGCTGCTGCAGGCAGGCTGCTTTAGAG

CTTCCAGTGAACTGGAAAGCTCATCCTGTGACCCCCAXX

>Unigene42281_A-W 1 248 LEN=248

XCTGTTGAATGGGACTGGGAAAGAGGTGGGGTCGAAGGTCAGCTGACGGGGCAAGACAAC

TGCAAAGACGGTGGAGAATTATAAAAATGTAGAGGGAACCTACTGCAACTCAAACCAAAG

CTGCAGCTGCGCCTCCCGTGCACAGACAAGCTCCAAGTTAAGAGAAAAGGCTTAGATTCG

AGGTGTAGCCGTTTCTGGATGCTCCGTCTAGACCCAAGCTGGAGTTACCCAGCCTACGAG

TCACCTAAG

>Unigene42324_A-W 1 275 LEN=275

XAGGATGTGCTCCACATCACCAACGGAGCCTGCCTGCAGGCAGGCCTGCCCTGGGCCAAG

GCATGTGGAGGAGGTGGGGAGGGGCATGTGCAGGACAAGGTGGGCAGCAACATCCCAGGT

AAAGATGAATCCACAAAGTCCTGGACACAGAAGAAACAGTCTTCCAATTTAAAAAAGGAC

AGGAAGAGAAAGGAGAAAAGACTCTTCTTAAGAATGCCAACCAGCATGTGCAGAAGGGAC

GACTTAGAAACACTACCTTCAGCCGCAGGCAAGAAA

>Unigene42331_A-W 1 242 LEN=242; minus strand

ACAGGAGATACAGTGGACTTCATTCAGATCTTAAAAATTGGGCTCTGTCATAAATATAAA

TTGAATTGTCCTCCTCAGAGCAGTGATGATAGTAAGTGGTGGGGGACTGATAAGCTTCTT

CATGAGCTGGCAAGGCCGGTAATTGTAAAGGCCACAAAATAAAAACAACACAAAATCCTG

AAAGCCAGAGCTCTGGATACTACTGACAGCCCTCTTATCATGACTTCAAGTCCAACACAA

TCX

>Unigene42434_A-W 1 267 LEN=267; minus strand

CTTCCCTACTGGGGCTATCAGCAAGCCTCCAGTCTCACCCACACCTGCACACACTTCATG

GCCCATTTCATTAACATACAACTGCAAAAATCCAAAACAGAAGTATTTAGGAAGAACAAA

ATTAAGTTTTGTATGAGCCTCTCGGCTGACAATGACCCAAGGACTGTTTGTTCCGTGGCC

CCAGCACCTGAGATGGAATTACAGTCCATGAGAACAGGCCGTCTCTTCACCAGCGATCGG

CAGCACAGGATGGTTCACAAGATGCAG

>Unigene42477_A-W 1 205 LEN=205; minus strand

TCAGAAAATCAAGTGTTGAATGCTTTCTCACATATGTGTAAAGTAAAGCGAGATAAGGAG

AAAATGGGGTCATTTCATGAAAATATATGGGAAATCAGTGAAGTAGAAGAAGGAGATAAA

TGGGAAGAGAGGAGGGAGGTGAAAAGGGAGGAACTGCAGAATGAAATTGACCCCGTTATG

CCATGGGCATGTATGAATATACCACXX

>Unigene42553_A-W 1 219 LEN=219; minus strand

XXTGTGTTGGAGTACCCAGGCAATTTAACACCTGAGGACACTGAAACTGAACTCCTTGAC

TCCACCGGGCCTAGCCTGCAATTTCAAAAAGCCCTACACTATGTTAAAGATGTGCATCAA

CTCACTCACCTTGGTTCAAAAAAACTACAGGCATTCCTGAAGGACCAAGAGCAGAAATTT

CCGCTGACCAGCACACAACGAAAAGAAATAGCCAAACGGATX

>Unigene42720_A-W 1 114 LEN=413; minus strand

GCCCAGGCCCAGGCCCAGGCCCAGTCCCAGCAGCCTGACACAGGGACCCTGGACAGCAAG

GACATGGAGAGCAAAGGCCTGCTTTCTGGCATCAGCGCCTCCACAGAGGACCCC

>Unigene42725_A-W 1 218 LEN=218

XXAAATAAAGTTTTATTGGAACACAGTGAAGCCTACTTCATTCCAAGTGGCTCACGGCTC

TGTGTTCACGCTACAGTGGTAACGTTGAGCAGTTACTACCGCAAGCCCCCCAAACCTGAA

CTATTTACCACCTGCCCTGAAAGAAAAGGATTGCTGACCTCTAATCTAGACAGACAATTT

TTCTTCTTATGGAACAATTCTGGGATTTTGGTTTTCTTGGXX

>Unigene42976_A-W 1 265 LEN=265; minus strand

XXCGAGGGGGAGGTGGCCGGCTGCTTCTCCCGCGTCCGCCATTTTGTTGCTGTGGCTCTT

GGGAACAGGCTGGGCAAAGCCGTCCCGGAGGCGCCACGCTCCCTCGAGTCCGGTGCTTCG

TGGTCGGCGGGTGTCAGTGCAGACCCGTACGGCTGCGGAGCCGGAGCTTTCCAGGTCGCG

ATCCACTGGAACCACCCCCGGTACGTCAGACAGCCCAAGAACCCCAGGGCGGCCTCCGCT

GCGGCTCGACTTCGCCCACCCGACCCC

>Unigene43058_A-W 1 317 LEN=317; minus strand

XCCGCGCAGACTAACAAGTTTCGAGGGTTTCGGGTCAGCTTTCCCGGAGCCCCTCGGCCT

CCGCTCCGGGGTGCCCGGCACGCTCCCCGCGGGCAGCACGTCTACCCGCGCTGCTTCCCG

CCCCCTCCCCCGCACACAAAGAGTAGGCTGCCTGGGGGCACCGCGCTGCCGGCGGGCTCC

CCGCGGGAGGTAGCAACTGAGTGCACCGAGCAGGTCAGCCTCCXGCGCGCCAAACACCGC

ACGGGCGACGGCGGCGGCGCGCGGACCCCGAGGGGACGGGCGGCGTCCAGTCTTACCTTG

GTGGTGATTTCAGAGCTAGXX

>Unigene43289_A-W 1 300 LEN=300

XXGATGTTCCTTGTTTTACCTACATGTATACCATTTCTGAAGTTCCTGTATGAGTCTTCT

AAGAGTAAGAAGTTTTGTGTGGAAAAAAATGTGTTCCCTCTTTTTCGTGTCAAAATTGAG

TTTTCAAGTGATAACCGTTACTCTGGTAAGTTAGCAAACGAAAATGCTTACTCTGAGAGG

AATACAGCCCCAGGAAGTTTTAACACAAGTGTGGAGAAAGTTAAGGAAGCAACCTGGGAA

CTCATGGGGAAACACTACACATTCATCAGTAATTACAGAGCACTCACAGAATGTAATACA

TTX

>Unigene43354_A-W 1 202 LEN=202

XGTAAACCTGGAGGGTTTGCTCAGCCTCATTGTGAAGAGAGATGCAGACCTGTTGAATGA

ATACAGATTCCTGTCATCATCTCCTTAGAAACACTTCTTGGAGTTCATCATCAGCATCAG

GTTTGGGTGACTTCTGATTCCTCTCTCACCTTGCTACAGGAGGGCATTAACCCTCTCACC

AAAAACAAAACAAAACAACAACAX

>Unigene43394_A-W 1 428 LEN=428; minus strand

XXAGCTTTAATATGTACAGAATATGAGAATTCTCCTTTGTCGCTTTTTGCCTACTGTGCT

CACAGGGCTGGCGTGAGTAATGAGCTCATCTCTATGAAGCATTTGGAAGGATGTCTGGGC

TTTGGTAAGAATGGTAATGACTACGATGACGATGATGGTGAATCTTGAAGTAAGGACCCT

AATATGTCAGTACAAAATGTGGACATCAGTTTCCACACCAAGGGCTGGACTGCTGAGCAA

TCGATTTGCTGGGAGTGGTATAATATGACAAGCAAGCAGCCCTGGTATTTGCAGGGAACT

GGTTCCAGGACCTTCACCCCAACCCCTACTCAAATATACCAAAACCCAGAGATGCTCAAG

TCCCTTATAAAAGGTAGTATAACATCTGCATATAACATGTACAACCCCCCCCCTTATACT

CTGATCATCTXX

>Unigene43498_A-W 1 404 LEN=404

XAGGAAGTCCATCATGGTGGCTGTATTAAGAAACTGGAAGTGGTTACTTTGGCTCTTCGC

AGACAGGATTCTGGACCGGGATTGAAACTTGAGATAATCAAACTAGAAATTGTTCATGAT

ATGACAAGGAACGAAAAGCAGCAATTCGCACTCATGAAGTAATTCAAAGTCTCCCGCCTA

CATATTTCTGTTGGTTGTATGCTTTATCAGCTGGTGGCTCGGGCATTCCTCTTTCCAAAC

ATCCACGGAGACTTAAGGTGGCAGATCCGCTTCGCAAGTGGTGTGCCCCTGGTACAACAG

GCAGCATCCAAAACTGCAGGAGCCGTGGTTCCTTCAGGAGGTCGGATCGGACCCCAGCCT

CCACCACAAAGCCGGGACCAAGCCGGCGCCCTCAGGAGACTTCCG

>Unigene43648_A-W 1 312 LEN=312

GGTTTGCTGTTTTACTTTCTAGAAAGTCGAAATTATTCCAATTTTGAACAGTTTTGTCTG

AACCAAGGTATTAATATTAATATGTATGATACTTGTACCAAGGTTAATAGTTTGTATGAT

TACTCCTTTTATCAATTGGCACCATCAGCTAATACAAAGGTGACAAGCTGGCTGACAGGA

AAACATCTGAATACAATGAACTATGCTCAACATGGGGCTAGTTGTACATCCATCTGGATT

TCCTTATTCACAGCCCAAAGTAGTATTCCTTTAGAGGAATATTTTAAATCAAAAGATAAA

ATAATCTTAAAG

>Unigene43702_A-W 1 243 LEN=243

GGGGGGTGGGGAGGAATGTCTGATAAAGATGATGTCTCTTCTCAGATGAAAAGTGAATCA

GGGCATAATTATCAGATGTCCATACAAACACTGAACGGCTTTCAATTCAAGAAAGTGGAC

ACTTGAACCAAAAGCCTTTTAAGAATGACAGGTTGGGGAGAAGCCTGTGAAGATGTTTAT

AAGTTCTTCTCATTTCTTTCTCGAATACACACTGAAAACTGGCATGCAGAGAGCATTCCA

GAA

>Unigene43713_A-W 1 211 LEN=211; minus strand

XXCAGTGCTGAAATATTTGCTATGAATGATGCTCAGAAGGAAGGAAGGGAATATCAACCA

GGGCCAGGAATTGAAAGCACAGCCCCTCCAGTGCCCTCAGCCATCTTGGCTCAGATGGTC

AAGATGAAAAACACCAGAGGGCCAGGGGGCAGCTCATGGAAAAGCACTTGCCTAGCACAG

GTGAGGTACAGGGTTCCATCCTCAGCACCACAT

>Unigene43844_A-W 1 239 LEN=239

XGGAGACTGAACCCAAGACCAGGAAAGGTGGAGTCTGCAGATGACGTAGGGGACAAAGAA

GTGGCTCCCCCACCACACGAGTGGAACCTAGGGGAGCTCTCTGGAGTAAAGTCTTCCAGC

GTGGACCAGGAAATACTGGGTGATGACGGAGGTACGCTGATTTATAATCTCCAGACCAAT

TAGCATTCAGCAAAGAGGCTGAGTCCACCTAACCAAACCCATCTATGGGATTACTTCTAT

>Unigene43964_A-W 1 260 LEN=260

GGACTAGCAGTGGCCCCTACCCTCCACCCGCCAGGACTGCTTCATCATCTTGCAAAAACA

GAAAAAGACACATGGACAGATGGTCAGGAGGCCCAGAGCCATCCCACCTGCCCTCCATCA

GCATTTTTCAGAGTTGTGAAGTTGAATCCTCAAGGCACAAAAAGCCCTCTGCAAGTGCTT

GCAGAAGCTCAGGGAGAATTCACGTATTCAGAAAGTATCAGTAGTTCATGGTTGTCCCCA

CCTACCTGGCACCTTTCTATX

>Unigene44035_A-W 1 209 LEN=209

XGTAGTAAAATTCGGAAACAGTCATTCATAGACTATCTTTCAAAGGTATTGTTGGATTAT

TAAATTCCTATTACCATTTATTCTGCTAAGTGCTTCTATTCAAATTTTAAATTCCTGGTG

GGCGGGAATATGGTTAACCTAAAATCAGGGTCAGAGTTAGTAATAGAAAAGAATGAAGAA

GGACAAGAGTATGGAAGCGATGGGCGTGCA

>Unigene44489_A-W 1 240 LEN=240

XXGGGTGTGCCACCATTCCTGGCTTTAAGGGAATGTTTTGGTACAACTGACAGAGAAGGT

AACTCCAACATGCTTGCATTACCTTTGAGACCAGGGAAGTCACCCCATGGCCATGGAAAA

GTCAGCCTAAAGCTTATCACTTATCACTGTGTGCTGGTCAAAGAGGTCTTCTGCCATGAC

AGATTCAGGGGCTCCTATTCTGCAGAAGTTGATTACAAAGTTACCCAGTTCTTTGAGGAA

TTX

>Unigene44537_A-W 1 216 LEN=216

XTGCACTGTCAGTGTCCCTCTAATGTTGGTCTTCTGGGATCCTGGTTGAATCCTGGGAAG

GGAGGAAGAGTAAAATGTGCCCACCCACGGCTATCTTTATACCTCCGATTGCTGGGCCAT

GGACGTTATTTGGTGTGTGACTTTATTATACAGAATGCCTTAGGTGTCCTTGTTGTAAAA

CATGGCACATCCCATCAGACCATCGTGGGACTGATAAXX

>Unigene44654_A-W 1 242 LEN=242; minus strand

XGTCCCAAAGGCAAGAAGGGGAAGACTTTGAAAATACTAAAACATGAGCTCCAGGTAGTA

TGATTATTAATTACGATAATGAAGAAAACAGAAAAACTTAAAGAGATCCATGTGGCTAAA

GAGGGAGATGCTCAGATGAAAGTGCTCGTGCATCAACAGCAGATAGAAAGAGGAACACAG

CGACAGAAATGAGAAGCAGGAGCTTCTACAACACCCTCCGACAAGCACAATAAATGAACA

ACC

>Unigene45038_A-W 1 276 LEN=276; minus strand

TTACAGGCCTGCACCACTGTGCCTGGCCCTTCTCCAGTATTGGTAGCTTTCTGAAACAGG

AAGAAAAGCAAACATGGCTCCATCGAGCTCTGTGCCCCCACAGTTGACCAGTGGACCCAA

GGAAAGCAGAATAAACCTCAAGTTAAAAGGGACAAAAATCTATTTCAAATGAGATTGCCT

CTAGCCCAGAGCTTCTCGACTGCAGAAGAGCTGGCATTGGGTCAGGTGGTTCTTGGTCAT

CGGCCTCCTGCACGCTGGAGGCTGTACAGCAGCACC

>Unigene45061_A-W 1 118 LEN=239; minus strand

XXCCATTTTGCCACCATCACCATGATAGCTGATTCAGACAAGGATCATGAATGGATGCTG

AAACTCCTGGGTTCAAGCCTCAAAGTACCTCCTTACCAACTACTTATCAATGACAAACGC

>Unigene45237_A-W 1 205 LEN=205

GGAACCAACCCTGTGACATCCTTGGACCTGCAGAACCCAGCCACGGGGTTCTGCAGCCAC

CTGATCCTTGGTACGTCCTTACAGCAGCCCCAGGAGTTCAACAGACCGTGCAACAAGGTG

AGTCAACCAGCCACCAACCCAAGTGCTCTGCAGACCTGCCTGGGCCTGGCACCACAGACA

CAGTCCCGAGGAGCCACCAACTCACXX

>Unigene45306_A-W 1 258 LEN=258

XGCGGCCAGGCCCACCTAAGTCCTGACTCTCTGCCTGAGGGGCAGCCCCTAGATCCCTTC

CCTTCCATCACTGTGGCTGCTCTTGAATTCAGAGGCAGCAGCAGGGTTGAACTTCATTCT

GGGGCTTTTCAACAATATGGAGACAAGACATGTGACAAAAAGTTTCTAAAGACATCTGAG

TGCTGAAGAAAGGAGAATCGCAGAGAGAACCCAGCTTTGTGTCAGGTGGACATGGCTCGT

GAAGTCCCACAGTGGCCCAXX

>Unigene45646_A-W 1 302 LEN=302; minus strand

AAAATACACAGGTAAGAGCATCTCAGTGCTCGTGGGGCTGCAGGAGGCAAAGCCGGCACC

CTGCAGGTAGCAGCACCCACACTACAACCCTGCAGGAAAGCCACTGGCGATAGGCATCAG

AAGCCACAGACGTTTTGGTTCACTCATAAGATCCTGTTCCAAAGAAATAATTTAGCCCAA

GGAAATCATCTAGCAGAAGTAAAAAGCAAAACACCTCAAAAGCAGGGGCCACAGGGAGCT

CAACCCACTCGCCTAGCACAGGAGTCAGCCAGTCCCACGAAGGACCAAGTGGGAGATAAA

GCX

>Unigene45748_A-W 1 215 LEN=215; minus strand

XXCATTCTACCACCGAACTATATCTCTAGCCCTAATGGTAAATCTTTGGCATTCATCTCT

GTCATCATTTTCTTCTTCTACGTCAAGTGCATCCCTATGCATCATGCCCTACAAGACCTA

GTCCATTACTCTACTCAAAATATTCCCTCTGCCTGGACCATCCTTTCCTTGCCATCATTC

TTCATCCAGCTAACTCCTATGTTCGGCCCAAATGTCAXX

>Unigene45791_A-W 1 252 LEN=252; minus strand

CCAAAGACCCACAAGGACTATGGGGACACTGGTACAATGGTGACCAGTCAGATGGGACTT

CTTCGACTCCCCCTTCGCTTCCTACCGTCTGTCCTAAAGTCCAGGGAATCAGAATACAAG

GGAGTTTCCCATGGAGTAGTTCCAGGGTAGAGAGACAGACTGGCTGGAAACAGGAACTTT

GAAAGACAGTAATAAATACAGCAGGCAAAAAGTCAAAAAGAAAAAGAAACAAAGAAAAAG

AAATTAAAGGCA

>Unigene45991_A-W 1 437 LEN=437

AAATAGATTCCAAATTAGAGAAGCATAAATTCAAAAATTGCTGACACTGTTTCTCACATG

GGACTGGCGAAAAAATTTTTTAGAAGAATGGCACCTCCTATTAGGAACATTATGGAGATA

CAGGTTCCTGAAACACTGCTGTGGCAGTGTAGGTTGGTACAGTTTCTGAGGGAAATGGGA

AAATGTATGACTTTTTATCCAGAAACTGCCCTGGAGGAATTAAACCGAAAATATAACTCA

AAACTACCCAGATACGTAACCACGGATTTGGAACAACTGAATGTGCACACAGAAGAGAGT

CCTGAGTACGCTTTGCAGCCCTCACACAAAGACTACTCTGGAGTGATTTTTAATAATGAG

GAAGATGTTGTGATCTGACAGGAAGAACTTTTCCAGGGCATGCTGTTCACTGAGAAAAGC

AAAGCCGAAGATACCCGX

>Unigene46054_A-W 1 211 LEN=211

XTGCTGTTCCTCCGTACCTCAGCTGTTCCTCCGTACCTCAGCAGCGATGATGACCTAACC

TCTCTGAGTGATGATGACCAAGTCTCTTATGAGCCTCCTCCAAAAGTAGTCCAAACAGCC

AGCAAGTTAAACCCAGAAGAGGAGCACCGGACCAGCTGCACACTCAGGAAGTCCCTGGCT

ATGGAAAAACGATACTTCCTGGACCTTGTAGXX

>Unigene46092_A-W 1 200 LEN=200

XXGTGGGCAGCAGGGGAGGAGCTGGCAGGAGCCACAGGTGTGCGGGGAGGGGGCAGGGAG

GTGGCACCCTCCACTGAACAGCAAATGCACTTCCAGGTGGAGGTGGACATGTGGCGTGAT

GCAGTCGGCCAGCAGCGGACGCAGAAGGGCCCTATGTGTCCCACACACCCTGAGGTTCCC

AGTGACACGGGTACCATGTCGGXX

>Unigene46140_A-W 1 215 LEN=215; minus strand

XCACAAAATCGTATGAGTCACGGACGGAGCCAGAAAGCTCCCTGCCCGGACGAGGCACCT

GTCTTCATCTTCCACACAGTGCTCCCTCAGCCTCCACAGCCAGAGTCCACGAGTTCGCCA

CACACCGAGAATGCTAGCCATGGTCCCCGGACCCACGCTGCCCTGGTCCTTCCTCATGTG

ACAGAGACCAGTCTGGAGCTGGGCCCAGAGCACCCA

>Unigene46200_A-W 1 289 LEN=289

GTAGAACTGGCGGCTAGATACACCAGCTGCATACACCAGCTGCGGTCAGGTTACAGTCGT

AGCGAGCTGTCAGCGTTCGCTGGAGTCGGGTTTCGGTCCTGGGGTACTCGTAAAACACGA

CTTTGTTTTAGGTCAATAAAACACCCAACAAAGCCACTAGAACACAACGGTCTGGGGCTT

GGAGGAGAAAGGACAGACCCCTAGCATCCCAGTTTCAAGAAAGCTAAGCAGCGAAATAGG

AGGAAAAAGCAAGTCCAGTCACACACCCGGAACCGCAAGTTACAAATGTXX

>Unigene46367_A-W 1 154 LEN=302; minus strand

XXTGCCGTTTTTAATAAGTTTTTGTCCTTTGGAGATGTTGAAACTATGGGCAATGTTAAT

GATGATTCTAAAACAGCTGTTCATTTAGAGAAATTTCGAGGAACCTCGTCTGGTACCCCT

GACACAGGTGCCATCACAGATTCAATTAGAGGGTCA

>Unigene46413_A-W 1 242 LEN=242; minus strand

GGCTGGGGGAATAATTCTGCACCACAAAACACCCATGGAAAACCCACAACCAGAGTTCCT

GCACTCACAGACAGTGACCAGGACCATTACCACTACAAAAAGGATCCTGCAGAGGGGAGC

AGGTACAGGATGATCCACAGCAATCACAGGAACATTCCAGAGCCTGCATCCTGAAATCTA

GCACCCAATAGAAATAACCAAGCTGCAATACTACAACCCTCAGAGCAGTCAGCTGCAGGC

CCX

>Unigene46445_A-W 1 296 LEN=296; minus strand

TTCCAGGTCTTGACCCTGCCACCTACAGAGCAAAGAGAGAGGAGCTTGGACAGGAGACCC

AGCGCCCGGTCCACAACACGCGGCACTGTCTTCTGGGGAGGGGCGGGGCAGGGTGTCGGG

GAACATTTAGACCATGGCAAAGCTGACACCAGTGACAAGACACAGCCAAGACAAAGCATC

TTTCACGACGTGAAAGTCTATGGGTACGAGCAGAGCAAGCGTGTCCAGATGACTCCCGAG

ATGGGTCAGACAGCAGATGAGAGAGAGCATGGCTGGGGAGGACCCCAGGGGCGACAX

>Unigene46451_A-W 1 268 LEN=268; minus strand

TTGTGGGATTCTCATAGAACCCCTGGGAAACCAGTGGAGCATGTCAAAAGGAAAGGTGAC

TTGCCCAAGGTCATGCACCACAAAAACCCCAAAGCAACACTTGACCTGGGGGGCTTCTGC

CAGTGGGGACTGTTCCTGTTATACCAGAGTGCACCCAAAAAAGCCATCAGGGGTTTCCAT

ACCTGGGCCCATCCTGCCAAGCCTCTACTCATATGTAAGAAAACATTACAGATAGACCTG

CTCTGCCTTCCAAGCCTCCCTAGAAAGCXX

>Unigene46456_A-W 1 220 LEN=220; minus strand

XXAAAGCTGAACTCTTCTTGGACTCTTCACACAGACAAGATCAACAGTCTGCAGCTGGAA

GCAAGAGATGATCATGGGGCAGCAGCAGCAGCTTCAGTTAGATCTAAAACAGGAGAGAGG

TTTACAAAACCACAGGAGCATTCCAGGGAAGGGTTCTCAGAGCCAAAACCCAAGGATGGG

CAGTTCGAAATTCAAGAGGAAAAGGTTAAACTAAAAAGAGCC

>Unigene46825_A-W 1 243 LEN=243; minus strand

XCAGAAACTCACTGGGGGCTGACTGCAGCTTCTAGAACATCCAGGTGTTCTGCAGATGAG

AGAATTTGTCCTGCAGTCACCAGATGGAGTCCCAGACAGATGCCAAGCTTGGGCTGCGGC

TCTAACATCCCTGAATACAGAGGGGTCACTTGCAGAGTTGCCAGAAGGGCCCTCAATGAC

ACCGACACGTCTAGTCATCTTTATGAAGCTGCCGTGGGAAACATCTCCCCTCGTGAGTCA

AGCCXX

>Unigene46938_A-W 1 252 LEN=252

XAAGATTCCTGGAGTGCAGTATCCCAGCACAGATGGGCAGCAGCTGGATGGGTCCTGGAG

ATGCGCCCACTTAAAAACTTCAGACCATTCAATGAAGAGCCTGCACCTATGGAGACCCAA

ACCCTGCCTTCGGAAGCTCACCTCTGGAACTCTACTCACAGAAAACTACAGCAACCCCAC

CCTGAGGGTGGAGCAGACATCACACTCCAGCCCACTCCACCTAACACAGGAGAAGGGAAG

CTGAGAACTTCTGXX

>Unigene47134_A-W 1 345 LEN=345; minus strand

XAGATCATGTCAAAAAAAATTGACAATGGGCCTCATATTGAGGATGATTCAATAAGAATT

ACACCTTTCCATGTCCTGATTTTCATTCGAGCCACAGGCAACACAAGAATGGGCTTTTTC

ATCCCCCTCGTCTTATCCTTCTACACTGTGGCCAAAGCAAGGAAACTACACTGCAGTGCT

GTTGGGGCAGGCCGAGGGAAAGACCACGAGCAGGTACCACTGGGAAGGGAAGAGCAGGAG

GCTGTGCAGAAAGGAAGGGAGACAGCATCTGGGCTAGATCTGAAGGTGGGGGATATGATG

TTAGAATGCTGGGCAAAGATAGGAATGTGGTTTCAAATGCAGGTGCXX

>Unigene47160_A-W 1 202 LEN=262; minus strand

XCGTCCCAACACCTACACCCGCCAGAGCTGATGCCCCCGCACTCTGGCCCCACCTCCAGC

CCCGACATTGGCCCGCAGTCCGGGAAACTGACCCGACCCCXGATCCGCCGTCCCGGACAG

ACCCCCTCCCGGCGGACGCCCCCTACCCCGGCTGACGACGACCACCCGACGCTCCGGCCC

GCGGCCGTGATCCCGCGCGAACTC

>Unigene47369_A-W 1 227 LEN=227; minus strand

XGTTTCTCTAGCTTAGCACTTCTCAAAACGTTACCAGAAATCACAAGTCACCGGAAACCA

CAGCTTTCAAATGCTCAGAAAGACAAATGGCAGTGGAGAGTGTACAAGTCTGAGCTGCTC

TCACTGGGGAGCTCCTCCCCAGAGCCCACTGGGACTCAGCTGCAGACTGTTGGAGATAGT

CTCTGCTGCACATCTACCCAGGAGGCAAAGCCGACCACCTGTTCACAC

>Unigene47496_A-W 1 219 LEN=219; minus strand

XXTCGGCTGTGGCTCTCAACACCCTCAGCCAAAAGAAAAAGAAAGAAAACCCATATTAAC

AAAATTAAAGATGAAAAAGGAGAAATTACCACAGACTTTGCAGAAATCCAGCXGGATTAT

TGGGGCTCTTTTGAAAAGTTATACTCCAATAAGTTGGAAAATCTAGAAGAAATGGCTATG

TTTCTAGACAAATATGACTGGCTAAAACTGAATCAAAAGGAC

>Unigene47572_A-W 1 370 LEN=370

GCTGTCATGCAGTACCCCAGCACAGATGCACAGGATGGGAGGCAGCCAGAGACAGAGGCA

GTCATGGAAAGAGGTGTCAAGCATTCTAGCACTGTGGGGAACTGTGATCAGGGTCCCATC

GTGGAGGGACAGTTAAGTTCTACTTAACTTAACCATTGCAAGGTGAACATGTATGTTCAT

AAGGAGGACTCTGAATCATCAACAAAGGCTAAATGTGGTCACTATACTATCAAACACTGT

ACTTTTATAGGGCAGGGAAGATTACAACACAGCTTAGAGAGACACATTCATTCAAAAGAA

ATCCAGCAGAGTGAGGTGTGGTGTTGCACACCTGCCCAGCTCCTGAGGAGGCTGAGACAG

GAGGGTCACAXX

>Unigene47580_A-W 1 220 LEN=220

GAAAGTTTGGTGATGCAGGTGAGGGAAGCCATAGAATGCTGTAAGCACACCTTAACGGGC

AATTTTGGAGTGAGCTTTAAAGACCAGAATAGACATGCTCTTGAGGTTTCAGATGCAATA

ATTGTTAAATTTGGTGGAGGAAATTTCAAAGCAGCATAGCATGCAGGCAGTGGCATAGAT

ACTGCTGAAGCTTTTAGCCATCTTCACTGTGAGAATTGGGXX

>Unigene47839_A-W 1 233 LEN=233

GGGGAGTGACTGGCCATGCATACTGGTACATTTGGTAGACATCATCGATATTCAGCAAGG

GGTGTTAACAGTGGCATGATATCCTTGATACTTGCTGATCGTGAGAACCCCCAAACATCT

CAGGCTGTAAGTGAGCTGTTGATCCGGTACTACTTCAATCCTACAACCCTCTATAAGTCT

ATAGTCCCAGGACCCCTAAGAAATCTGATGAGACCAAACAGAAAGCCAGCAGCX

>Unigene47847_A-W 1 261 LEN=261

XXGGTGCTCATCCTTGTGTACAGTTCTGAAAACAACGTTATGAGAGAAGCAACCCTATTG

CCACTTACATATGATAAATACGGAAAAGATATCCGGCCACTTGTCCTCGCTGTGGAAAAT

AGTGTGGAGCTGGAATCTGAACTCAAGCTCTCAGGACACTTGAAAACAAGAAGAGGTGCT

ATTCAGGACACCTTGAGATGACAGATGCAAACAGGCAGGGAATCAGGCAAACTAAGGCGA

GTGGCCATAGAGCCCTGCAGTAGX

>Unigene47862_A-W 1 201 LEN=201; minus strand

CTTCCTCTTCAAACTCCTGGTTTGTGTGTTTATGATTTCAGCTTTGAAAATTCCGAAACA

ACAGGTTCCAGGGACAACAATTCTTCTTACAAGACATCCTATCCAACCACCTGTGCAAAA

CAGATTCCTCATTTGACAAATGTCCATGATAATCTGATGTTCTGTGACTTACCTTATGGG

ACCAGTTTTGCCATTTCTCTT

>Unigene48201_A-W 1 209 LEN=209

CAGAGGCTCAAGGTGTGGCAGAAATCATGGTGGAAGGATGTAGCAGAGGAAAGAAGTTCA

GCATATCACATCCGGAAGCAGAGAGGGAAGCAGAGAGAGAGCACGCAGCTCAGCAAGGAC

AAAATGTATATCCCAAAGGCATACCTCCAGGGAGCCACCTCTCCAGCCACACCCTACCTG

CCTATAATTATTGCCCAGTTAATCCATATX

>Unigene48389_A-W 1 292 LEN=292

XTTTTATAATGATAAAGGTCAGTTCTCCAAGAAGACACAACAATCCTTAATGTGCATGTG

ACCAACAACAGAATATCTCTCAAGGCAAAACTGATGAACCGCAAAGAGAAATCAATCCAC

TTTCACAACTGGAAACCCCAACACCCTGTATCAGATGTGGGCGGATCCAGAAGGCTGACA

GTCAGTAGGGACATAGATGAACTCAACACCACCATTTATCCACCCGATGTGATTGACATC

TATGGACCACTTCACTCAGTGAGAGCAGAGTGCACATCCTCCTCAAGTTCACGX

>Unigene48533_A-W 1 202 LEN=202; minus strand

XCTGCTACTTCTATGTCTTTTCTTCTACCTCCAAAGCAGGCAGGTGTGGCCATGGAGCAG

TGCTCCAGAACACCTGCACATAACCAAACAGCAACAGCAGCTTCCACCAACCCAATACCT

ACCACACACCAGATCTTCAGGACTGCAGAGGCTCTGTGGACTCTGCCTTTGCATTTGTAT

CTTGTGGACACTACTGAAAAAGCX

>Unigene48562_A-W 1 219 LEN=219

CTAATAGAAGAAAAAGACCCAAATCTTCATCGTGTCAGATTAGGTCCTGATTTCCTTAAC

AAAACTCCTAAAGTGCAAGAAATAAAAAAAAAAAATAAGTATGATGGCATCAAATTAGAA

AGCTTCTCTATAGCAAAGAAAACAATTAAAAACATGAATAGAGCGCCTATCTAAGGAGCA

GGTGGAAAAGATCTTCTCCCATTCTATAGGCGCTCTATT

>Unigene48604_A-W 1 244 LEN=244

XGTGGTGTTGAGGATCGAACCCAGCTCCCAGCACTACTGCTTGAGCATCACATCCCCATC

CCTGGAATTCTTAAGTGTCTTTCCAGCAAGATTGTCAGCTTTTTGAGTAATCCAAATGAT

GCGTGCCTTCATTTTGAGGAAATGGAGCACTTTCTAAAAGAACAAGAACCACATAAAAAA

CTGAGCAGCAACTATACACGAGGCCCTTTCCCAAAATTAACCTTAGTATTGTTTAAAGAA

CTCAAX

>Unigene48793_A-W 1 202 LEN=202

GGGGTCCCATTTTACAGGAGAAACCAGAAGTCCCAGAAAGCCTCAAGTGACTGTGGTCAT

ATGTCAAATCTAGTCGAGCAGGCAGGACTAAACCAAGAACCTTCTGACTCTCCTCTCTGT

GCTCGAAAAAAGCCCAAACTGTACTTTCTTGGCAAAGAAATGCTTCATCTTCTATTCATC

ATAATCAATCCTAGGACTTTGGXX

>Unigene48827_A-W 1 260 LEN=260; minus strand

XCATTACTGTGGCTGTCATCTATTCATTCTCACAGCTGTATAGGAATGCACTGGGTGAAT

GTTCCACAGTTTATTGTTATTTCTATTGCTGATGCACGTGAGTGTGACTTTCCGTTTGGA

TCCAGTTTGGACATTATTGCCATGCACATTTCTTATAACATGGATGCATTTGAAAAACCC

AATGATGAAAATAGTAATAAATGCTGCAGAGGATATGGAGAAAAAGGAACATTTTTCCAC

TGTTGGTGGGATTGTAAATTA

>Unigene48960_A-W 1 207 LEN=207

XGTCAGGAACTGCATGGGGGCAAACCAAGCAGTGACAAAAACAGCCTAAGGAAATAAAAA

CCTTTGATCTTTGTTCTTCAAGTTGCAGAACTGAAGAAACACCTAGTGATTAGGATCAGG

ATTGTGTCTTTTGGCCACAAACCTCCACCTGTCAGAAGGCCTACCATCCTCCATCTCCTA

TATAAGTTGCCCCTAACTCAGCCTCACAXX

>Unigene49132_A-W 1 292 LEN=292

ACACACAACCAGTTATATGCCAGTTCTATGACCATTTTTGTCATAGAAAATACTAAAAGT

AAAACTTGTGTTTACAGAGTAAGTGAAGCACACATTTATTTCTCATCATACTACAAAAGG

CGAAAAAAACAGCAATGAGAACAAAAGCAAATGAGAGAGAGATACTACAATGATTTTGAA

AATTTGAGGAATTCTGATACCAAAAGGCACATGAATGACACAGGAGAGGCAATTCACTGT

GTAAAACCCAAGTTCCAGCTGGGCATGATGGCACACACCTGTAATTCCAGCAXX

>Unigene49421_A-W 1 201 LEN=201

XXACATCAACAAATTTCTAGAGACATCCTTCCAAACTAAATCATGAGGACATACACAAAT

AGATCAATTTTGAGGAATGAAATAGTAGATGCCATCAAAATCTACCAACAAAGCCCAGAA

CCAGATGAATTCTTATCTGAGTTTTGCAAGACCTGCAAAGAAGAATTAATACCAATCCAC

CTCAAATTATTCCATGAAATAGAX

>Unigene49652_A-W 1 470 LEN=470

XGCTTGAATCATGTGCGATTTATGATATATGGCAAAGATCTTCTGAGAAGAGGTTTGGGC

ATGTGTGTTCAGGGAGCAGAAGCTGCCTGGGCCCAGGGACAGCTGCACACACCCAGCAGG

AATGCTGGCCAGCCGTGTGGGAAGCCTGCAGCTGGGGTCGAGGGTCCTTCTCATGGAGAT

GTTTACCAGAACCAGTTTCCTGAACGTAGTTCACCTTTGGTGGAGGCTGGAAAGGAGCGT

TCCAACTACGATGTTACAAAGAAATACAGGAGTTGTTCTGACAGCATGGCAACAGAACTC

TCCCCCAAATTGTCAAAATCTACTTTTTACAGAACTCTGAAAATTAATCCAAAGTTTGCC

AACAATCCAAGGGGCATTTATTCAAGAAAACAAGCTAAACAGAGGTAAGAACAGAAAGTT

CTAAGGCACTCAACTGGTGCTATTCCCATTACCACCACCCAAGTCCACGGX

>Unigene49653_A-W 1 204 LEN=204; minus strand

XXCGACTCCGAGGATGACGATGTTGAAGAGCTTTTTGGACTCCGGGGACATTTTGGCTCC

GCCCTGCCCGAGCTCGACAGCGGCCCAGGGCAGTCAGCGCTCCCGGGGCGCAGCGGCGAC

GACCCTGGGGACGAGACGCGGCGGTGCGGAGCCGGCAGCGCCAGGAGCCGAGCGGCCGAA

CTGGCAGCTTGCAGTCGAGGGCCGGCX

>Unigene49744_A-W 1 323 LEN=323; minus strand

XXCCCTTTGCCCTTGGTGGTCATGCGGCTGCAAAAGGCAGTGGGTGTCAATCATGCTGGG

CGTGTGCAGGCTGAGCCACCCCATCCAGAACTCTCAAATCCAAAAATGCTCCCAAATCTG

AAATTTTTGAGTGCTGACATGATGCCAAAAGTGGACAGTTTCACCCCTGACCTCATGCGA

GAGGTGGCAATCAAGACGCAGGGTGCACTAAAAGTAGTGAACAAAATCACCTTCAGGTCA

GACACGGACACATGTGGAACAAAGGCAGCTCACATGCAGACTTGGGTCCATCCCGGGATG

TCTATGAATATGCAAATACACCAA

>Unigene49905_A-W 1 239 LEN=239; minus strand

XGTCTGCTTTACCTCAATTTGGGGGGAGACGGGTCAGAAATTGGGAGGTTAAGGTCATCA

GATGCTTCTGCTGTGGGCATCCCGGCGAGTACCCAGGCAGGGCGCCTGGATGACGGGTTG

CCCTCTGTGCACACCATGCCTGCTGGGAGCCCCACGTGTCGGCTGGCCAGTGCGGGCCAC

AACGTGGCCATCACTTTGCTGCAGCTCCTTGGCAGGGACCACTACGCCGGCTTCCGGTCC

>Unigene50252_A-W 1 388 LEN=388; minus strand

XCAAAGACCTCTGTCTCCCTCCTGTTGGGACCATCACAATTCCAGTGGCTAATGGGCCAA

CCTGTTAGGGGGAGATTTGCTGCAGTTAGGGCTTTCTGGTGCACTCGAGACAGTGATGAA

ATGCACCTGCAGGCGCTTCTTCCAGGAAAGGAATCACCACAGTACAGGTGCAAGCCTGAG

CTAAAGATGAAGAAGAAATAGTTCAGCTCATGTGGGAAGTGTCAGGTTTATCTTCACAGA

GGAGACCGCCTCTGGCTGAACTTTGAAGCAGGAATAGAXAAGATGGCAGCTCACCAGACA

GAGAATGAAAGTTCTGATGACCTGGAAGTCCAGTTCCAGAAAGTGGAACTTGGAGAGATG

GCAGGGGCTAGAGCAGTGCCTGACAGGGCC

>Unigene50369_A-W 1 221 LEN=221

XXAAGATTCCAAGACAAGGGCAAGCAGGACCTCAGTCTAGGAGACACTTTCCCGGTGCTT

ATCAGAGGCCAGAGTGGCAGCAGGCAGCGTCTGCCCAGCAGGGGCGCTGTGAGCCCTGGA

GAGGAACTGCACAGGCTGCTCAGCAGAGCACCAAGAGCACCAGGGGGCCTGGCGTGCTGG

CACCAGGGTGATGGCATGAAGAGGCAGAGCCTGGTCAGACACGXX

>Unigene50845_A-W 1 235 LEN=235

AGAAATTTCCTTTTAGCAGGCTCTCATTCAAAAGCCAGCGAGGACCGTTCTTCCCGTGCC

CTGCAGTTCCACCGGGAAAGCTTCCTGCAGGGGGGAGGAGCCCTGAACTGTGCAGCAAGC

TGTGGGTTCCCAAGGGCCGCGGTTCTGTCTTGGCAGGATGGCTACGAGCAGCTCCGGCAG

CTCTCCCAGCACGCCATGAAGGGCGTGATCCGTGTGAAGTTCGTCAACGACCTCGXX

>Unigene50948_A-W 1 248 LEN=248

XXCAGAAAAATAATCAAGGAAATGATAATGAAAAATTGCCAAAACTTGGTGAAAGACATA

AAGATTCATGAAGTTAACTCTGATAAGATAAATATGAAGAAAACTCCACCTAAAAATGCC

ACAATTGGACTGCTAAAAACCAGAGATGAAGAGAAAATCTTGAGAGCAGACAGAGAAAAA

GACATGATCTAGTATAGTAATGGATATAAGTGACAGCATGCTACTCTAAAAAACAGTAAA

AACAAGCAGGXX

>Unigene51026_A-W 1 233 LEN=233; minus strand

XGCCGTGTGCACCAGCTTTCCGGCAACGCAGTGGCCGTCACAGTGCAGCAATTTCGCTTA

CTGGATCCTTCTGACTGGTCCCTATTTTACTTTCCTTGTGCATCTTGGCGGCTGGAGGCT

CGGGAAGAATGCGGAGCCACTTGCGAGAGATTTGGGGTGGAGCAAGTAAAACCCTCGTAC

AACCAGGAAGTGCTGCTGCCCCGCTTTGTGACCCTAAAGAAACCCTTGCGCGCC

>Unigene51441_A-W 1 251 LEN=251; minus strand

XCAAGAAAAAAATACAAACTAGGGTAGACATTAAGAGTCTTCAGTGTAAGTCCCGTTTAT

AGTTTAGATTATTGTGAGGATGACTTCCCCAGGGAGAGGGATGGTGAAATGAGAAGCCAA

GATGGAAACTGGAAAATCTTGACAATTAAGGAGAGAGATGAGGCAGAAGAGCCTACAGTG

GAACTCAAGGAGGGGCTAGGGAGACTGGAGAAAACCAGGGAAAAAGTGGTATTATGGAAA

CCAAGGGAGGAT

>Unigene51529_A-W 1 323 LEN=323; minus strand

XXCATACGAACTTTATGGGGACACCAACTTTCAGGCCACAGCGATGGCCAACTGAAGATC

CCGGAGGATCAGATTAGACTAAAAGTCTAGGCCCAACTATGTGTTACCTGTAAGAAATGC

CCCTTCAATGTAAAGGCACAGAGAAGTGGGAAGAAAAAGGTCAGAAAAGTCACACCAGGT

AGCAGTGGTCGAAGGAAAGCTAGGAGAAATATCACAGAGGAAAAGAGGATCACTGCTGAT

CATGAAAGTGTCCCATGATCAAGAAGACATGCAATCTGTAATATCAATCCACATGACCTC

AGAGCCTCAAAGTTCATGAAGCAGCXX

>Unigene51538_A-W 1 334 LEN=334

AGGAGATATAGATGCCCCCGAGAGCATAGAGTCTCGCCCTCCTCTCCGCCAGAGAAACGA

AGGACAATAGACAGAGCACAGCAAACCGACCTGAATTTGAGTGCCTGCTCCGTCTCAGAC

AAGCTGTGGTGCGGTGGGCAGATTTCTTCACTCTCCAAGTCTCGGTTTCCTCATCTGGAA

CATGGGAATAAGATGGATTGCTTAATACAGTTGTTCAATACAATTCCCATTATACTAACA

CACTCCAAATTACACATGAAACAACAGTCACACTTCAGAATTAAATATGGGTATATGGGT

CTTAATTTGCGTTTCCCCAGAAGCAGACCCCAGAXX

>Unigene51743_A-W 1 207 LEN=207

CTTAAAGCAGGACATGGAACCATCTTTATTCAGCAGCCTGTGGTTGGCATCCACCAGCTG

GCGGGTCAAGGGGCAGGCTGCAAGTCTACACCCTTCGACTCCCTTCTGGGGTTAGAGCAC

ACCTTTACAGTCCAAAACCACATTAATCATAAGTGGCTGTTGCTGATTCAAGACCATAAG

CAAACATCAGGAGATCATAACGGGAAG

>Unigene51796_A-W 1 215 LEN=215

XXAGGAACAGGGGTAGAAGACATGGTGGCAGCAGCCAGCCCTGCAGGATTCGGTGGAGGA

GTCCCAGGTGGTGTTGTCTCTATTCCACTTTCTTCCATCATTTTTCTTCAACAATGGTAT

GCTGGGGTCCCGAGCGAGCGCGTGTGTGACAGGCGCAAAGTGGGGAATAGGGAGGTCACG

GTGTAGAACTATGGCTGGAGAACACAAGGAAGATGCAXX

>Unigene51918_A-W 1 427 LEN=664

XXGGTAGAAGCCAATGAATGCACATCGTCTAGCTTGGTCCACCATGTTGGAGCTGTTCAT

GCTTCTACAGGGTCATTATGTCCACATCAATGGAACCTACCCACAGCAATGACTCATGGA

AGTGGGAACCAAACACAAGATGCAGCTTCCTCAGCACTACCATCTTATAAACAATTAAAT

AGCACTTGCAGAACCATTGGCCTCAACTTCTTAGCTGCCAGGAAACATGGAGACCATGAG

CCAGTCTCCAATGCTATCAATGACTGACCTTCTAAGGGAGAAATGGTAGCTTGGCTTGGA

GTGAAAGAAAGTGCTCTTGACAGCCATGGGCTGGGCCTGTGGCTGTGCAGATCTCCAAAT

GAAGGAGCCAACAAAGGATTAAATGAGAAAGCAGATGGGGTGCCTTGTGAAGAACAACAC

AAACGTAAG

>Unigene52144_A-W 1 400 LEN=400; minus strand

GCTCCAAGAACCTCACTGAGGCGTCAGATGTCAGCCTTCGAAGGGCTCGTCTGCCACCCT

CTGTACCGCAGGGAGGTCTTACAAGGGACCCGGGAGACGTCCCACTTCCTGCTCATCAGG

TGTGATGAGCACAAAATCTTCAATGTCCTGTGTCAGTCTGCTGCTCTACGGAGTGTCCGT

GATGAAGGTCCCTGCAAATGCAAACTGCTGTCTATCTTGGTAGCTGCAGATCACTTCCGA

TACGTCTTCCTCATGGGGCCTGCAAAGGGCATGCTCACCCAGTCTACAGCTTCATTCCAA

ATCCCAGAGGCTGTGTTGATCTGCTCCACTGCAGAGAGAATGCATGTCACGACGGCAGCT

TGGGGCTTCCATTGGGTCACGTTCACCGTGATCCATCCC

>Unigene52236_A-W 1 366 LEN=366; minus strand

GAAAAGAGCAAATTTTAAAAATATTCTGAGATACCATTGTATGCATATATTGGCAAGACT

CTAGGATTTGATCACACTGTTGAAAAAACTGTGGAAAGTAAAAAATGTCAAAAACCTTAT

GGGAAAGGACTTATCAAAATTAGAGGCTTTATCCTCTGGGAATTTAGGGTCAAGGTACAA

ATTGCTGGAACTAAAGCTCTGGAATTAGAAGGGCTCCATAAGGAAATCAACTGGTTTGCC

ATACCCCGAATCAGTGCAGCTCTCCTGAGCAGGTATCACCAGTACTAGATAACATCACCA

TCCCTACCTTCCCTTCATTGCTGTGTTGTTCTGCAGGTCCTTAGGTCCCTAGCTAGTGTG

TCTTCC

>Unigene52284_A-W 1 124 LEN=271

XXTGCACGGATCATTGACACGGCACTACATAACGGCTCCTTAGCAGCCATTATTGGGTAT

CAAGTCAATGCATACTCAGGGAGACTTTATCGCACAGGCCTGCACAAGCAGACACATGCC

GAATAC

>Unigene52297_A-W 1 215 LEN=215

TGTGCACTGAGAGGTGACAGGGACTTCCAGCCATGGGAGCCTGGGAGTGCAAAAGGAGAC

TGCTCTCTGGATAGACCAGGAGCCACAGGAACAGGATGTGTGCGAGCACATCAAACCCAG

TCTCAGGAAGAAAAAGCAGCAGCTCCACTGCCCACAGGAAAGCCTCCAAAGGTCATCACA

CTGTTTTTGGAACCAGAATACGATCAAGACTTTGXX

>Unigene52474_A-W 1 200 LEN=200

XXAGATATAGGATCTCACAGAGTGCAATTTATTATGAGACATAATGATGAAAGTGTGGTC

CTTGGCAGCAGCAAGACCAAGTGGATCCCACAGTTTGAGGAAGAAGGAGGGGCATATATC

CAAGTTAAGACAAAAGTGACTTCATTCAAGCCAGAATACCTGGTTTGTTTTAAGGTAATA

TTAGGAACATTCTCATCACCAGXX

>Unigene52475_A-W 1 201 LEN=201

GCTCTCACCCCCACGCAGCCGCCCAGCCATCACTGGCGCTCGGCTCGAGGCCCACGGCTG

CGTCCCCAGATCCGCTGCCCCCGGGTGCGGAAGAACGCGTCTCGTAGAGCCGTCAAGTCG

CGAGCCTTTAACTCAGCCCGAGGCTGCGAGGCGGGCACAGGGACTCCCAGTCCTCCTGGA

GTTTCCGGGGCCGCCCTCGAG

>Unigene52504_A-W 1 212 LEN=212; minus strand

TGTGGGGAAGGGGTTGAGGACAAGGGTGGGGTTGGGAGAGAAAAGGTTTATGTGATCAAT

CCACTTAAAAATGCTAATGCCTTCACTCTCTCTCCCAAGATGCAAAACTCCAAAGTCAAT

CAGGAGGCCGAAGAAATTCTTATGAACATTCAGAAAAACTCGATTTTAATCCGGTTAAAA

ATCATCAGTGTCATTATCATCATCATCATCACX

>Unigene52531_A-W 1 206 LEN=206

XTCCACAGGGAGGGTGTGGAGAAGATCCAGGCAGAAGATGCCTGCCAGCACTGGCTGCTC

CTCACCACCCTCATCCCGTGGGTGGTCTGCGGCAGGTACGAAGAGCAAGCCTCCACACGA

AGCCAGTGCTTCCTGAATTTGCTCTCCGTAGCAGCCAGTGCCCCACAGAAGCCCACCCAG

CCTGTCTTCCCTCACTTATTCTCCAGG

>Unigene52577_A-W 1 224 LEN=224; minus strand

XCCAGCCGGTACCGTCCAGAAAAACGGGATGGAATTTAGGAAGAAACCCATTCTCCCTCC

CCATCGTCTACAAATCAAACATTTGCCCTTGCTCCCCGCGTCTTGCGGAAAGATGTCCCG

TTGTTGGAACACGGTCCCTTAGTTGCTAATAGACGAGTCCCAGAAGTTTCTCTAGGAGTA

AAAGCGAGGCTCTTCTCTTTTCCACTTCGAAATCCCTCCACCATX

>Unigene52698_A-W 1 342 LEN=342; minus strand

XAAAGACACATGCTCTTTGAACCATTAGCTCTTACAGTTAATATGATTTCTGTCAAAACA

ACTCCTCACAGGAGTTGCATACCGAAGAATGCTACCTGCATCTTCGTTTCAAGGGCACTT

TACCTTAAAAGGGTGTTAGAAAAAATGGAAGGAAAAATGAAAAAGAAAGCAGCAACACGA

AGGTGCCTTCAGAAGCTAACAAGGGGCTTAGAATCACACCCCAGCAAGAGCCAGTTGATG

CAGAGAGGATGGTGTGTAAACAGGAAAGCAAAATCACTCAGGACTTTTAAGAGCTGGGTT

TATGATGATGATAGGGAGTATTTTCCTAAATTCTATGCCCAAGXX

>Unigene52749_A-W 1 228 LEN=228

CATGGTCAAGGGTATAACAAAAGTGAAGAGAAGCCCACCAGGCTCAGTGCTGGAGACCAC

ATTTGGAATAATTGGTATTTGAAGCATTTTAGGAGGTATAGTGATAATCTACAGCTCAAA

GACTATCTGAAGGCTTGGGGACAAGGAATCTTTGTGAGAAAGGACTATGTTCAAGCACTG

GTAGAAGAATCATAGGCAAGTGATACAGGTGTTAATGTGGCTATGGAG

>Unigene53129_A-W 1 245 LEN=245

GGGCAATTCACCAGAAATGTTTACACAGAAATGCTTCCTGATTGCAGTCTGGCTTCTCCT

CTCCTGATAATACCTAGCATCTACCAGCAGCTTGTTGTTCCCGTGGGGGCCCATGCTGAT

GAAAAGCCTAAAGCTGAAGCTCTGTTACTTTCAAAGAGAGGGAAAGTTGTGGTTGGAGGC

AAAGGACTCCTGGACCTTGGAGCAGAGAAGAGACTACTGGGTGGGGTAGCCGAGATCACT

GGCTCX

>Unigene53219_A-W 1 211 LEN=211

XTAAATGAAGTTGCATCTTCCCTTTGTCCCACTCCACTGAGTAAGAGTCCAAGGATTCTC

TCCACCAGCAGCATCTGGGGACTGGCCCAGCATTCACCCACAACTTTTTCCCCCCTCAAA

CAGGCCATTGAAGAAAAGAATGTTCATTCTGAGGGTGGTGAGAGAAAATTTAATCTTTTC

TTCCTGTGGTCTGAATGGGAAGCTCCTCTAAAX

>Unigene53222_A-W 1 277 LEN=277; minus strand

CTCGGCAAGAACATGAATAGGTCAGAACAGACAGACGGCTCATTCCTCAGGGGACTCAAG

AAGTTGGCTGGCGGATCCACACAAACGGTTTTCCTCTCCACGGAGGAGGACACGAAAGTA

GGAACACTGGGAGTGAAGAGTGTAAATACAATTACTCTGGAGAAAAGCAGCGAAGGTGCA

AACAGATGAAGACAGAAAAGGAGAAAAGATGCTGACCACATGACACATGCCGAGGTGAGG

AAAATGTGGAAACTACTTTATAATGACCAACGCCCCCXX

>Unigene53233_A-W 1 264 LEN=264

XGGCTGACCTGTCCTGGCTTGCTTGCGTGGTCTTTCTCAGTTAAGCACATAAAGTCCTGG

GAAAAGTATGATGCATGGTCACCATGGTGGCATCAACTCAGCAGCCAGCTGCACCAGAAG

CTGATACTGTACCTGGGACAAGAGGGTGACCTGTGTCTACAGCACATGATAGATGGCCTG

ACAGTGCAGGAGGAGGACCGCACAGGGGGCGAGGTGAAATCGCTGGTGAACGCACCTGCC

GAGAAGCCCGCACAAGGCTCTCAACXX

>Unigene53270_A-W 1 211 LEN=211; minus strand

XXCTCTGCAGGTCTGCGCCCGCGAGGGCCCGCAGAGGAGCCGCTGCTCCCCGAGCTCACC

CCGTGTCGTGAGCGTCCTTTCGGGTCCGGGAACCCTGCTCCCCGCGTCTGCTCCGTGCCC

GAGTCTCTGGGATTCCGGGGCGAACCAGGGTCTAGCGGCAGGGGCTTCTCCGGAGGCGGG

GCGCTGGATGGTGCAGAGCTAGCGTCGCGATCG

>Unigene53798_A-W 1 256 LEN=256

XXCAAGAGGCCAAAGAAAGAAATTGCCAGAAAATGAATTCAACATAATGATTTTTGGAAA

CTTATTGAGATGCAAGAGAATAGAGGCAGGCGGTTCGACAAAATTGGGGAGAAAACCCAT

GATCTTGTTGAGAATGTCATCAAAAAAAGAGACCATTTGCAGAAGAAACAAATAGAAATC

TTAGAATCAAATAACTCAATAAATGAAGTAAAAATAAAAACAAAAATGAGAAGCCCAACA

GTAGACTAAGTCAAACAG

>Unigene53804_A-W 1 283 LEN=500; minus strand

XXTTTTGGGCCCCGGGAACAAATCCCAGTAGCTGATCAGTCTGCTAAGACAACACTTGGT

CTTCAGATGGAGATGAAAGAAAGCAACAAGAAACTCCCCTGGCCTACCAAAGGGGAAAGT

CCCAAAAGGTTGATGGCAGAATCTGAGCAAATCCAGTTTTCAAGGAGGTTTGGGCAGATC

AACATGTCAACATTATTGGATTTCTATAGACAAACTGAGTGTCCACTGTACCAGGGTTTC

CCTAGTGAAGTCAGCTACAGATACTGGCTGGGAAAGTATCCTAAG

>Unigene53877_A-W 1 217 LEN=217

GGTGGAGGCGGGGCTGCCGCTGTCATGGACGCCTGGGTCCGCTTCAGTGCTCAGAGCCAG

GCCCGGGAGCGGCTGTGTAGGAGACTGGCTGTCGGGTTTCCGCCCCTCTGCGTCGAGTGT

GCACGCCGGGCGGACGCGCTCGCCGGGACCCTGCTCCTCCGGCGCCTCCGGGCCGTCAGG

GCCCGAGACCCCACGCTCCAGATTACCCTTCCCAACCXX

>Unigene54413_A-W 1 298 LEN=298

ATCTCCAGCCCTTTACTAACTCTTAAAACTGAAAAGTCTAAGGATTGAGGTGGCTTGGAA

ATGGCCATGGGTTCAAATAAGTCATCAACAAATCATATTGCAAGAGTGATGACAGAAAGG

AGGAGAAAACAGGAGGCATATGCAGCAGTCCAAGCAAGAGATGACAGTAGTAATGTAGAT

AAGTATTCAGGGGCTGATCACTTGTTAGTGGGGAAAGAAGAGGAAGGAATGAAGGATGAC

CTTGATTTAGGGGTTGAGCAGCTGGTGATGCAACAACTGGGAAGACAGGAGGAGGAG

>Unigene54528_A-W 1 294 LEN=294; minus strand

CTGTGCTCCCAGGGTCTAGTTTTGATAGCCAGTATCACCAAATCTATCAGTTAAACTGCT

GGCCAACGAGGATGTCTGCAGGTCTTTCTGGAGTTAGCAAATGAATTCATCTGGAGAAGA

CAGTCCAAGAAGTCCTGGCAAGAAAACATGAAGTACCACATACAAGACAGGGATGTGAGG

GATGCAAGCAGTAGAGGGGAACTTATGGTCAATGAAGAGGTCATCTTTGGATCTCAACTC

TTAAAAGAGAATTCTGGAACCATTACTAAAATGAACAAGGCCAGCAGATTTCAG

>Unigene54590_A-W 1 239 LEN=239

XXGCTCTCAGCTCAGGTAAGATTCACAAGACACGGGACACTGCATATAGCTTCTAATGTG

GAGACTTTCACACGGCCTTCACCCCCAGGAAGTGGGGGTAGGAAACTGTGGCTTGGAACC

ACAGTAATGAAAACCAAAGAAAGCAAAGACCTAAAATGAATCATGCATCACCAGGAACCT

TCACCCAGAGCCCAAGTGAAGCACCCAGCACATCAGCGAAGACTGAACCAGTCTAGCTCC

TXX

>Unigene54681_A-W 1 237 LEN=237; minus strand

XCTTGCATCTACAAACATTTTCAAAACCCAAGGAGCAATGTGGTACAGAAAGATAACAAC

TTTAATTAGAAAGCTACCTCCAGAGTCAGACAGGATGGCTCTACGGAAATGTTAGATGAT

CCGTGCATCCAGAGTGCATCTGGAATCACTAAGGAGGATTCAAAGGGCATTAGGAAATCA

GAGCATGTAGACAGAATAGATGAAAGGAGATTGAGGAAGGCTAGATTTTATTCAAAACXX

>Unigene54710_A-W 1 309 LEN=309

XCGAGGAAGGAGCTTCTAAAAAGGAAGACTCTTCAGGACTTCTTGGGAGCCAGAACAAAA

GTGAAAGTGAAACGGGTGCTGGGCAGTACTTTCCCAGAAGGAGTTCACTGCATCCTGCCC

TTCTCAGAACCACTCCTGACAGTGGAGAGAGCATCACCTTCCTTTGTGACTAGAGAGGAA

GAAGTCACAGATATATAGAACCTGAGCTTGCCAGCTCCTCATGTACATCACGACCATGGG

ACAGAACTGGATACCAGACTCCAGTTCTTTGTGGGTACTGCTTCTGTATCTCCACATCGT

CACTCTTTTGXX

>Unigene54900_A-W 1 220 LEN=220

GTATACTTTGGGATTGTGCAAATCGGGATTCTGAATATATGGCATAAGCCCAAGGGAAAC

GTTATCGGAAATCATTTCAAAGTGATGTTCTCCAAACCTAAAGTCCCACATAAATCCTTT

AGAAGTAAGTTCTACCAAAAATTAGTTTTAGAAAAAAACACAAAAATAAATAAGGCAACA

ATCCACTATAAGCTGGAGACGGGAGATGCAACAGGAGGACXX

>Unigene55080_A-W 1 236 LEN=236

XXGGTTCCATCCCCAGCACCACAGAACAAACTGAAAAAGCCCTCAATCCAAGAGAAGGCA

GGAAAAGAGGAGAAGGGGAACAAGGAACCGGCGGCCCCAAAGAAACGAAGGCACAGCCTG

GGAAGTTCAAACCAGCTGTTCCTCAACCGCACTAAGTGCAAGCCGTCTGAACACGTGTGC

ACATCCTGTCAGCGCACTGTGCTGTGTCCTGTTGTGCTTAACTTTCTCCTGGTGTTCCXX

>Unigene55213_A-W 1 583 LEN=583

XCGGGCACAGAAAGAATACAGACTGGTGGTGCAGGAAGCCATCAAACAATCGGTGTGAAA

GCACAAGGTTCATACAAACGCTGGTGCTGGGAGGAAAAAGACCTGGGAAATGAACTATTC

AGGTGCTCTTTGGCAGAGAAGCCACCCCAGGTGTCTGGTGGCCATGGTGTACCATGGCAT

CCTCCATGACAACACCTGTCCTCTATTCTTATCACGGATGATTGGCAGCACACGGACCTC

TGGTCTTGGCTGATGTTAGTGTGTGTCATGTGCAAAGACCAGCTAATGTCCTTAATGGAA

CACTCAGGTGGTCAGTGTGCAGATGAAGAAATTAAATTTAATGGGAGGTGAAAGAAGAGA

GGGGCAATGGGCACGGGTGCTGGACAGGGACTGGAAGGGGATCTGTGCTTTGGTGTTCAT

GGGGCTTTCACTCTGCCCCCCACCACTTCACAAATGACCATAACTGTCACATGGGAAGGA

GCTTTTCCCATGGGTTGGTTTTCAGAATGGAAAGATGCCAACGTGTTTGGGATAGGTTCT

CTTTTTTTGCTGACTCAGTTGGTTGTTCAACATGAGGGTATGGAX

>Unigene55214_A-W 1 349 LEN=349; minus strand

XXTCATAATAATTATTGTCACTTTGATCATGATGATGACAGCCACAAATATGACTGGCCT

GGGAACTCGAGTCAGACCTCAATAAAAAGGACAGCAGGGAGGCATCATTCTGATCATAAC

AACATTTTGCCTGGGTCTGTCACACTCACCAGATTCTGCAAGTGGGAAAGTGGCCAACCT

TACAAAGTTAAAGATGTCTCTTTTCCTAATGAGGCTTCCATAGATATTCAGCGAAAAGCA

CCCAAGAACAAGGTCATTTTTCAGCCTTATCTGTGTTCTCCTCCATCGTCCCTTGCACCA

TGTGAGAATGGATTTGTTCTTTGTACCCTGCCTGGAACACGCCTCGTCTCC

>Unigene55237_A-W 1 269 LEN=269; minus strand

XCAAAGAAGAAAGAGGCTGGAGGCCGGCCGAGGGGTTCGGTGTCAGTGTCAGCCACTCCT

GCAGAGAACAAGCACCCTTCTGGGAGAGAGTGGGGTTCTGGGGTAGGAGAGGTGGCTGCC

TTGCTGCTAAGAGCTGTCAGCTGGGCTTTCGAGGCCATGATTCCTGGGGAAATCCCAATC

CCAGTAAATAAGGATATTACAAATGTTATAATTGGCGAGAAGCATGAACCAGAACTCAGA

GACTTTGCCACACCTTTACCTACATACCAC

>Unigene55314_A-W 1 256 LEN=256; minus strand

AGAGAGAGAGAGAGAGAAAAGGAATGGGAGCATGAGACAGAAGTCAGTGAAGAAGACAGA

TGGGAAAATGAACAAAGGATGCCAAAGGGAAAGGAGAACCAGAACAAACAGAGCACAGGA

CTCAGGCAGAGACACGGCCCAGAGTTGCTGAGCCAGCCCCAAAAATGGACCTCTGAAGGC

ACCACCTCCTTCCAATACTCCTATCCACAAGGTTTTTGCCACATGTCTCTGCAACAATAT

GGTAACCATATGTCCTXX

>Unigene55372_A-W 1 220 LEN=220

XXCAGGCGCCCGCGGTGTTGCGGGGAGTCCCGGAACCCGCGAGCCGGAGCCACCGGGCTC

GAGTGGGACTGGCGCGCGGCCGGCGCTTCCCGTGTGGCGACACGACTGCTGAGCGCTTGG

GGGAGGCTGCGCAGTGGCCGCCGCGGAAGCCGTGCGGGGAGCCGAGCCTCCATGTTCAAC

CAGCAGCAGCAGCAGTTCCAGCAGCAGCAGCTGCAGCAGCAG

>Unigene55513_A-W 1 349 LEN=406; minus strand

XXAATCACATCAGTTACACATCCATGTCGAGTCCAGACAGACACAGAGTCCAAGGCCAAG

GCTTTATTAGCCCCGCAAAGAATGACTCTTACACACACACACCCAGAGGACTCAGCTACT

GAAACGTCCTCCTCCCACTCCCAGAAGGACCGGCTCAGGGCAGTCACCCCTATAAACACT

GCAGCTCACAATATGTTGATCACGGTTGCTGAGTGGCTCTCGTCACAGAAACGAGACAGC

AAAGGCTGTTGTAATATGCTGACCCCAAAACCTTCCATGGGTTCTGTGATCAAACAAAAA

TATCAACATTGTCTCAAGTTTCCCAAATTGAGTGACATTCACACCAAACGG

>Unigene55688_A-W 1 426 LEN=426

GAGCATTTCATGAGGATGGGTAAGAAGAGTGCTCTGAGAGATGCAGGAGAGTGGAATGCT

TGGGTCACCGAGACCCAGGAGAGTGAGAACACCCAGGAAGACATTGTCACCAAATGACAA

CATCAACCAAGTCCAGTGAACACAGGATTTAAAGGTCCTGAGGGATTGAGCAATAAGAAG

GTCATTGGTGACCTTTATGACAACGAGTGAAATGGTGGGGGCAGAAGCCAGCTTGTACTG

TACCAGAGAAGAATTCCCAGATGTTTGGCTGTGAGCGGAAGGAAAATAATGGATCAGAAA

GGGACCCAGGGAAGGGAGAAGCCTTTGCAGGACATCCTGTCTCAATGGGAAACGGGCTCA

CGTTCTAATGCATCTCCAGTATTCCAAACAACAATGACAGATAAAATATCAAAAGAGGAA

GCCAGA

>Unigene55714_A-W 1 284 LEN=284

GTGCATAAGGAACACCTGCTTGAATTCTGGCAACACTGTGGGATAGGTGTGGATTATGCT

GGAATTTGTCACCGGGCTTTTAACGAAGGAGTAGCCAATCCCCAGCCCCGAAAATCCAAT

GGATGGAACAACCACACGGTTGAGGCCCTGCCCTTCAGAGATGCCTCTTGGAGGCTACAG

GGATTGGAAGAGGGTGGGATTTCAGGTGGACAGAGGCGGGATGAAGATTCACAACTTGGA

CAACTGGATACTGACTCTGTAAGAGTGGACACAACGTCCTAGAXX

>Unigene55853_A-W 1 344 LEN=344; minus strand

XXCCCGCCTAAGACGCAGGATCCAGACTCTTCCTGGCATTTGTCCCTAGATTTCCGTCTG

ATTCCTAGGAAGGCTCCAAAGATACTGTAGGGGGAGAGTTCGAAAATCCGAGAGTCAGCA

CGTGGTGTTGATACTGGATTTAAGACTCAGTTTTCTTATCTGGAAAATGGCAATGACTGC

ACAGCCACTGGAGCTGAGGTGCGCATAGAACGAAAAGATGCCCCGATTAACGCCAGTACC

CTATGTCCTCACGAGGACAGAGCTTGGCCGCAGTCCCCAGATGTTCTATTGCATAGTAAA

AGGCTGGCTCGACTGTGCCCTAGGCTCTGCGAGGCAAACCGAGGCAXX

>Unigene55929_A-W 1 313 LEN=313; minus strand

TATCATCTTAGATTCTTGGCTGACTTTTAAAGCTTGAGACATAGGAGGGCCCTGAGCAAG

AGAGAAAATAAAACTTTATCAATTAGACCAGGAATGGTAATTAGGTTCTGTTTCATGGAG

CTGTTAGATCACAAAGCTGAAAGAGAAGGACTGACTGAAGTAGACCCAGCAGGTCAAAAT

ACTTGGTCAGCTGGTGATGTTGCCATTGGTGACAGAGGGGCAGCACCTGGCTATGCAGTT

GCCACCTTAGGCTCAAGTGAAGAAAGATGTGGGGATAAAAGAAGAGCAACAATTTTTACT

TATGCACATTTATXX

>Unigene55933_A-W 1 217 LEN=217

XGTGGGCAGGGGGTTGGGGACTCTGACCCATGGTTCTCAGAATGGGGTGTCTGCACCCAC

AGCAGCATAACTTGGGAGCTGGTAGAAATGCAAGTTCCAGAGTCCACTGAGACCTGTGGA

ATCAGACGCTCTGGGCCAGGCACAGATACTAGATGCAATGGGCCCTTCAGCTACTCTAAC

AATCACAGAGTTCTGAGCCACTGCTCCAACTCTATCCAX

>Unigene55939_A-W 1 342 LEN=342; minus strand

XCAGATGCTTGTCAGGAGACAGGCAGTCAGTCTACCGTTGGAGGTAAACAAATTAGGGTC

CAGCTTAGGCTAAAAGAAAAACGTCTTGCTAAACTACGGACAACGGAATGTGCCTTTTGA

GATCATGTGTTGGATACACGTGTACTTAAAACAGCCCCGTCGCTGTGTAGAGCTCTTGAA

GTACGCCAGAGCTTTATCAGAGCTATTGCACTTAAGCCTCGGGACAGCCTTACGAGGTAG

GAACTGATATTATTCTCGGCTAAGACCAGGAAACCAATGATTGGGAACGTTAAATAATCT

GCCCGAGGTAGCACAGGTACCAAACACGTGGCTGAGTCCGCGTXX

>Unigene56093_A-W 1 254 LEN=254

CAGCACATGCTCTCGCTGCGTCGTCACGAGAGCACAGGGTGCACAGGCTGGAAGCCAAGA

GCCTCTAACGCTGGGGAGCGTGGGCTTCCGAACCCCAACCAACCCCCCACTGGACAACAC

CAGAAAATCACCCTGGGCACGTGGGAGCAGAGAGCACAGAGACAGCCCTCAGGCAGCCAG

AGAGACCTGCACGCCTCCACGGCTGAAGAAGATCGGCCAGTCCCGGATTCGAAGGCTCAA

AATAAGGGCATTAGX

>Unigene56266_A-W 1 203 LEN=203; minus strand

CTGGATGCGACACAAAAGGGTCAGCCCCATGTTAGGACCGCTCTGCTGAGCTGCAGCATG

CTCTTGGCCAAGTCCCTGGTGCAAGGGGAGGTCTTCGAGGCTGTGCATGTGCATGTCCTC

AAGTTCACAAGTGTGCACAACTTCCTTCAGCTCCCTCTGGTGGCCAGAGAGGAGGGAGAG

TCTCCGCCCAGCCCAGCCCACACX

>Unigene56551_A-W 1 209 LEN=209; minus strand

XXTGGGAATGGTGGTTCAGGGAGGTGGTGGGAGCTGGGGTCACATTGCAAAGGTGATGAG

AAAATAGCCAAGAAGCACTTGAAAAGATGGACAATTCAAGTCAAAACCACCATGAGATAC

CACTTCACGCCCACGAGCATGGGTAGGATCCAGAAAGGGGAAATCAGCAAGTGCTTGCTG

GCCAAGATGTGGAGAAGTCACGCCCCTCTTTXX

>Unigene56779_A-W 1 265 LEN=265; minus strand

XXCATACACACACACACACACACACCGGTTGTCAATCTGTAGTTGGTTGAGCCTGTAGAT

GCAGAACCCATGTACACAGAGGCTGACGGTTTTGACTGTGATGTAGACCTGACGTGTGGA

GTCTTGGTTCTCCCTGCAGAATCCTCAGTTTTGAGCGGAAAGGGGATCAGGCCAGGTGCT

GAATATGGTGACCACTTAGTTGTGCAAGAATGTGGGCAAGAGTGTGGGTGCTCTGTGGGT

GAGCGCATAGGCACCCTCAAGGAAGCC

>Unigene57160_A-W 1 217 LEN=217

XXGAGAGGTTTGCGAGAGTCCCCGCAAGCCTGTAGCCAAGTGGCACAGTCTGAGCGAGTA

CTAATCCTGTTCTTCTCGCCTCTAACCCTGGGGCCAGGCTGGGGAGGGACATGGTGGGGA

GAGATGTCAGAGAAGCGGACTCAAAACCTCATTTTAGTTGTAGAAGAAAATTTCTTACAA

AACAAACCCGACAAGGAACATCCCACATCACCACAGCCG

>Unigene57188_A-W 1 254 LEN=254; minus strand

XCTCCAACCCACCGAGAGCCTCGGGCCATTGAAAAAGAAAAATGGGAGAAAGGTGAGGTA

GTGATAGTCCCTTGAAAAAGCTGCAAAATATGTCGGAAAGGCTCAGCAGTCAGCAAGGCA

GTTCAAACAATTCAGGAATGGAAAGAACGTCTCATTGTGGGGAAAGGAGGAGAAGAGAAG

CAGGAAAGGAAGAGAGGGAAAGTAAAGGAGGGGGCCAGAAAACACAGATGTGGTCCCCAA

CTGCAGAGCACTTCT

>Unigene57713_A-W 1 402 LEN=487; minus strand

ACTCAGACCCTGTTTCTAAGTAAAATACAAAATGGGTTGGGGATGTGGCTCAGTGGTCAA

GCATCCATGAGATCAAACACTGGTACCTCCCCCCCACCACCACCAAAAAAAAAAAAAGAT

GATGATGATGATGAAAATGATGACGACAACGAGGACGACCAGTCAGAATGTCTTAACTGA

GCCACAGTGATGCATGCTTTTCGGGAAAACCTTCATTTACAAGTATTCCAGACACCACCA

GGCTTCAGGCATGGCCATGAGCAAGACCAGCAAATAACAGCTTTTTGCCTTGCAGCTCTG

ACCCCAATGTTGTCTGCTGTCTCCAACATGGCAACTCTGAACATGGCCTACAGCAGGATG

CTGTTGAATAACACCATGGCTTCATCAGAGGATGTGGGGTTG

>Unigene57818_A-W 1 241 LEN=241

XXGCCGGACTTTCGCCGGACTTTCGCGGGACTTTCGCGGGACTTTCGCCGGACCTTCGCC

GGACCTTCGCCGGACTTTCGCCGGAGTTCCATGGAAGTTTCACCCTGGTTTGGTTCCCAG

AGGTGATTCTTCGACTTCCAAGAGACTGCTGAAAGAAAGAAGACATGGTTGAGGATCTGC

TGGGGTCTGCTGCAGCGCTGGATGGAATGGCCCGCCATAAGGAGGAATGTGTCCTGGGAT

GAG

>Unigene57931_A-W 1 277 LEN=277; minus strand

CAGCAGTACGGGGGCTACCGAGCATCGCAGACGGGACCCTCTGCCCAGCAGCAGCGGCCT

TATGGCTATGAGCAGGGCCAGTACGGAAACTACCAGAATGAAGGACCCGTGTTCTCGTTG

GAGCCTGGTGGGAGTGTGTCTGGGCGCTGGACAGCTGTGGTGGTTCTGATGAACTGTGCC

ATGTTGCTGGCCCCTCACCCAGCGCCGTGCCTGCATGTGAACGTGCCCACCTTATGTTGG

GTACGAAGCCACCTGCACCTCTGGTGCAAGACAGCA

>Unigene57951_A-W 1 363 LEN=363

XGCCTATCCACCGGAAATTTTGGAGCGCTACAGCCAAGGGAGGTTACGGTTCGCTCTGTA

GAGATTCTGAAAATTGAAATTGCAAGAAAATTCACCCATTCCCGTTTCTTTCAGCGTCCT

TCAACTGTGAAGGAGATGAAGGATCAGAACCTGGAACCCAGAGCTTTGGGAGTATTAAAA

GACCGAGCCCTTTTCTCTGTGCATCATAACAGTCTTGGGCTGTAGCTGTATACTACCCTC

ATTTTACAGAAGGAGAAATTTGAGGCTCAGTGACTTGCTGGACATCACATAGCGGAGGAA

TGGCAGAAACTTGAATTAGAGCCCAAGGCTTCTAACTCGCAACTCGGATTTGCCTCTCAT

CCTGXX

>Unigene58019_A-W 1 237 LEN=237; minus strand

XCTCTGAATTTACACCCCACTCATCTCCTGAAGTCCCAGTTAGTTCTGCAGGAAGTGAAT

GGTGTCTTAAGGAGGTTTATGAGTTCAAGTAAGGGAACAAGAGGAAAAAACCAAGGTAGC

ATATACCACCGAGCTCTGCAGCCTGAGATTTTTGTGGAGAAAGTGGCTGATGCCTCATCA

CTTCACGATACATCACCTCGTCTCTTCTCTTCAGTTCTACTTTTCTCCACTGCCCAAGXX

>Unigene58085_A-W 1 278 LEN=278; minus strand

GCTCCAACGGCCAGTGTAAGTTAGCTGGAGGGTCTACTTCATTTGTCCAAAGCTAATGGA

GAGTCTTTAGCATCTGGAATATTGGTATATATCCCCACATTGGTGAGGGAGGAGGGTGTG

GCAAATGGAGGACTGGCTTTTGAGGCTCCTGACTGGAACCAATACCAGGACTTGGTTGAA

AGAAGTGACATGGTCACACCCAATTCAGGAGCATGGCAGTACTCTCCTACCACATGTCAA

AAGGAGACCCTGAAATGTTTGACGAATAATGCTGCCACX

>Unigene58297_A-W 1 265 LEN=265; minus strand

XCCTACTTCATCCTGGATGAGTTTTTGATGGGAGGGGACGTCCAGGACACCTCTAAGAAG

AGTGTGCTGAAGGCTATTGAGCAGGAGCTCCACATACCGATGGATCAGCTCCAGCGTGAT

GAGCTCATTGTCTTGGCCCTCAATGGCGCAGCAGAAATAGAGGCTGGCATATCATCCTGC

AGCCTCCGGCTCGGCGCGTCCCTCTTCGGCCACCGTAGGTGCCAGCTCCACTCCCTTTCT

ATCCTTTCTCTTCCTTCCCTCCCTCCX

>Unigene58338_A-W 1 212 LEN=212

GAGAGAGAGAGAGAGAGAGAGCGCTCAGCCGGAATCTGCAATTTGGAAGTCATCATAATG

GAGGACACGAAGCTCATGGACAACGGTGAGACCATTCATAAGTATTTAGAGGGAGATGAG

AGCAGGAATGGGGAAAGAACCCTGAGGAAGACCAGCATGGAAGAGAAGGAGCAATTATGA

AAGTTACAGGTAGGTCAGGCGTGGTTGCGCATX

>Unigene58352_A-W 1 96 LEN=282

CTGACAGAGCTGGAGGCCATCCGAGAGGACTTCCTGCGTGAGCGGGACACCAGCCCTGAC

AAAGGCGAGCTGGTCAGCGATGAGGAGGAGGACACC

>Unigene58474_A-W 1 237 LEN=237

GAGAACCTGTTGACAGGTCAAAAGCTCTCTCATCGCACTGTGTTCAAGAAGGCCTCCACG

GCCCACATTGGAGGGCTCATCTTTCTACACTACAACCCTAAAGTGACTTGGCAACTGAAT

GGCCTTAAGGATGTGATGCTAAGTGAAACAAGCCAGTCACAAAAAGACAAACACCACAGG

AATCCTCTTACTCGAGGTACCAGGTGACTCAAAGTCATGGGTGGAGAGTCGTCTGGG

>Unigene58491_A-W 1 327 LEN=327

XCTGCTACTTGTCAGGGATTCTTTAAAGTACTGGAGATAGCCAGAGAGATAGTGAAGCAG

AAAATGACTGGTGTGGTGGTGGCTGGAGGTGATTTTATTTAGGTCTCTGAGAGGAAAGCA

AGATTTGTGCACAGACCTGAAGGAAAGGAAGGAGCAAGTCATACAGACATGTGGACGGAA

GAGATTTCCAGACTGGATGACAGCAAGGGCATAGACCCCGAGTCAGGTGGAGTGGTGGAT

GTGGATAGGGAGGTACAAAAGATATGGCAGATTACCTCTATGGCCTTATCAGCCCTGGGA

GGAAGATAATGTTGTGGAGATGATGGGGXX

>Unigene58569_A-W 1 277 LEN=277; minus strand

TCAAGCCTGGTGAGGGTGCCTCTGTACAGCATGTGGGACTTACTTCTAATGGACCTGCAT

GGCCAGGCCAGCAAGGCTCCCCTGGAGGCCGACATAGAGGGGCACTTTTCAGAGCTGTTG

CTGCTTTCTGTGATCGTTGCTGGGGGCAGCAGTGGCTATGTCCTTGAGCCACCCCTGTGC

AGTGGTGTGGGTGCCCCACCTTGAACAGCACCAGCACATGGAGTGCCAGCAGCAGCCATT

GGGAGCCCCACTATCCTCCGCGAGCCCTACATGTCCCXX

>Unigene58626_A-W 1 301 LEN=301; minus strand

AACATGGGGACCCGGAAAGAAAAGAGAAGGTGGTAAGATCTTACACGGGAGGTAAAACAG

GATAAAGAGAGGAACCCTGATGAGAAGGGGTCAGGGGACCCATGGAAGAACACTGCAGCA

AGAACCAGGCCCAAGGGAGAGAGGCCACACCTCTGCTTTAACAGACCAGGTGACCGAGTC

AGGAAGAAGGCACAGGTGGATGGGATAAGGTCAGAAGAGAGGAAGACTCTGCTCATTCAG

ATAGAGGGTTCAGGGTCTCGTGGGCGACCAGCTGGATCTCAATGGAGAGCAATACGTCGT

CXX

>Unigene58669_A-W 1 262 LEN=262; minus strand

XXTGTTGGGAGAGACACATCTGGCCAGGAATTCAGAGAGGCAGAAGCAGGAACAATTAAG

TTTGGAGACTGTACCAAAGACCAGGGGTCTGCAGGTGATGTCATGAACTAAGAATTGGCT

CCTGCACCACATGAGTGGAACCCAGGGTGGATGCCTGGGGTACATACAGTCTTCCAGTGT

GGACCAGCAATTGCTAGGCCCTGGAGGTACACTGATTTAAAATATCCAGAACAACTGGCA

TTCAGCAAAGAGCCCATCAAAGCC

>Unigene58789_A-W 1 211 LEN=211

XXACTTGATTTACAGTCTCCCTATGGCAAAACTGGAAAAGGAAAAACACTCATAGAACAT

GACAGAGAAATGGTCATGGTCTTAATAGAAAAGGCACTCACAAATCAATAACAAGAAGTT

CAAATACCTAAGAGAAAACTGGGAAAACTGACATCAACAGGTATTTCACAGAAAAAGAAA

CACCTACGACTTAAAAGCCCATTAAAATATGCA

>Unigene58847_A-W 1 385 LEN=385; minus strand

XXCGCGCACGATCCCACCCGTCACGAAACGGACCTTTGGCTTGCCACTGCACGACAAATC

CAGAAGATCTCCTCTTACCCCTGGCCGGGGTACTCCTCCAGCGACATCAAAGTCTCCCGG

GACCTGCCTCCAACCCTGATCGCCTCCCACGTCCAAGTTTTCGTCAAGCACAGCAGCCCC

CCACCCGCAACCCGCAAAGCGTTGGCGGTGCGCCGCCTGCTCTGCGGCTCAGCACTACCC

AGCAAGCCCCAAGAAAAGCAAGTCCCCGGGCTTCTTGACCACCCGGAGCTAGTTGTCTAC

TGCCCGCAGCACGCGCTGGCCGCCGAGCCCCAATTCCCAAGCACCGGTGACCTGCCGCCG

CTGCAGCTCCCGGGTAGGTTCCGCCAX

>Unigene59032_A-W 1 286 LEN=286; minus strand

XXCTGTTTGGACCTGAAAGTGGGGTCTGATATCAGTCGTGCTGAAGATTCAATGAATGGA

ACCATAAACAAAACAAAAACCTGTGGGAGAAGAGGTGGGTGGAGGAGAAACTCAGAGGGA

AAAGCAGCCAATAAGGAAAATGCCCAAAAGCAGTTCATTAAGACAGAGGGGAAGAGAGGT

TGCCAGCTCTGCCTCTTGCTCATCCCTTCTAGAGCTCCGGATTCCCTGCTCCCCATGTGT

GGACTCTCACCACTTTTACTCAGTGGGTGGAACAGAGCTTGTGCTCCA

>Unigene59160_A-W 1 255 LEN=255; minus strand

AAGAGGACCAGCATTTCTGCTGCCTGCTACTTGGGGAAAGTAGAAGGAACCAACAGCTTA

CAGCAGGTTAAGAAGGCCACAGGACAAAGGGAAAGATTACAAAGTGGAGATTCTGAATTT

TCAGGCTTCCGAGTAGTTGATATCGAAGTAGGACTAAGTTTGGAGGCACTGTCCATGCAC

AAACACAATGCCATGACCAAGGTTCAGCAGCAGCAGAGCAGCAGCAATGCAGAAATTGCT

GATCTTCTGGGCTXX

>Unigene59257_A-W 1 339 LEN=546

ACAGAGAAAACTAGGGAACTCAGATACCAAAACCAAACTTGCACTCTCGGACTAACCCGG

GCCTCTGTCACTTGTGCAATTGCACTTCATCCCCTCCTCTCCAGAGCCCCAGAATCTCTT

CGACACAACTACATGTGCAACACTGATGTTAACGAAAGCTCAACAGCTGCCCCAGGGCTT

CCAGAGAACTCTGCTGCCCCAAAGACCGGGCTGACCGATGACCGTCTTTCTGTGTTCACC

ACAATGGGTGGCCTCTACCTCAGAGAGACGGTCTCTGAACTCACCAAGGCACATTCAGAG

GTACTTCACTTGAGTGCTTTCTGGGACAAAACTAAAAGG

>Unigene59696_A-W 1 247 LEN=570

XXATGAGTTGAAGGAGGAGACAATGAACAAGAAGGTCAAGATGACTATAAGAAGAAGCAT

GAGGAGAAAATGAAGAAGGACAAGAACAAGAAGGAGAAACACAAGCCAAACAACTAATAT

AACAATAAATCGATTAAAACTGTGAATGAGAATTATACTGATGAGGAGGAAATGCAGAAG

TGGGGAACTCAAGAGAAACATATTTCTCTGTATGGCACAGCCCTGCTGTGGACATCCTCC

ACCCACGCC

>Unigene59784_A-W 1 262 LEN=262; minus strand

GTTTTAGAGGAGGAAAAATTTACTTGGGGCTCAGGATTTCAGGGGTCTCAGTCCACAGAT

GGCCGGCTCCATTGCTGTGGGTCAGAGGTCAGGAAGAACATCATGGCAGAAGGGGGTGGG

GGAGAAAGGGACTCAGGAGATGACACCAAGAAGCAGAGGGAGACAGACAGCTCCACTTGC

CAGGGACAAAATATAAACCCTAAAGGCACACCCCCAGTGGTCTGCTTCCTCCAACCACAT

CCTGTCTGCCTACAGTTACCACXX

>Unigene59817_A-W 1 578 LEN=578

XAGAAAGAAAGAAAAAAAAAAAAGAGTCAACAGTACTTTCCTGATCAAAACCAAGAAATC

ACTCTGGTTCCTAGGCACAGGGGCTTCATATTAGTTTCAGCCTACCCCAGATCTGGAAAG

ACAAGTCCAAGCCATCTTACAGTGAGCACCCCGAGAAGAAAGAGCCCTTGTGGGACCATG

GGGACAGAGCCCAAACACATCCTTCAGAATAAGCAGCTGCTTGGGTTGGCTCCTGGCTTG

ACGTCTGATCATTCAGAACAGAATGCCTCAGAACTTTGCCTTCTAGTCAGGAGAGACTGG

CAGCACCTGCAGAAGGGACTCGGCAGCATGCAGTTTACAGCGACAGAGGAGGAGGAAATT

GGGACAGCTTCCAGGGGAGGAGGATGGAATCAAGAAGAGTCAAATGAGTTTTATGAGGAA

GAATGTCCAAGAGATATCAGAGAAAACGACAGCAGCTGGTGGCCCCGATGCTTCCCGAGC

CAGGAGTTCTGCTAAGTCATTTGCAACGTCACCAGCCAGCGAGCCAGGGTCCCACCTGTG

CTCCACTCAATCCCACGATCTCGACTTTCCTGCTTTACA

>Unigene59953_A-W 1 348 LEN=348

XGCTTGACATGTTACTCTGAGATACAGAATTTTTACAATATCGAAAGATCTCCACTTGAA

GTCAGTATTTATTACTCATGGTGTTTTCCCATCTTCGTCCCAAATCGACATTCCAATAAG

TGGGTGGGGACAGTTTCTAGTAGGCTGCCAAAGAAGATAGCTTTTAGTGGGAAACGGAGA

CAGAAAACCACAGCAACAAAACATAAACAGTACCATGGAGAAATCCCTTTAGAAATAGGT

GGGTTAAGTGAGTCCTGCTCTTCAATAGCTCCTGCCAACACTTCCACTGAGTTTCAAGAC

GGCGGAATGGAAGAGGCCTGTCTGCTTTCTTGGTTTTACACCACAGAGCXX

>Unigene60016_A-W 1 299 LEN=1085

XAGAAAGAAACAAATATATTAAGTCAACCAATTAATTTTTCAAAAGTGCAATTCACCTTT

AAAATAACAAATAAACAACAACGACGACAAACCAAAACCCCGACCATAACCCCGAAGGCA

AAACGTGTGGATAGACAAGCGTCAAGATGTGATGGTCTACGTTTTGATGTTGCACTTGAC

AAAAGCAGATTTGGTTTCATAGAAGCCTGCAGACCTGACATTGCTTACAAGGACCTGGAA

ACAGGTTTGGAAAGAAATGCGACACAAGTCAGCAGGGACAGAAAGCAGCCAGCTAGCGTA

>Unigene60545_A-W 1 220 LEN=220

GGGATTCCACCACCCTACGACAAGGTGAGCCACACCAAGCCTCCAGCACAGTGTCCTGCA

TCCAGCGTCCATGATGTTACACAATTACCTACATTGTTTGAGCCTCATGAAAACATAACT

GGCTGAGACGCCTACTGCAGCCAGCCTCCTTTTCCTAGTGTCTTTGCACTAGGTCTTTAA

GCCCCTCTCCTCCAACAGAAAAAGCGGATGGTGGTTCCTGXX

>Unigene60591_A-W 1 273 LEN=273; minus strand

XGCATCCTCAATGCTATCATGACCTACTCCAAACAAAAAGCAGAGGGTGCATATCCTAAT

AATGGCAGTGAAGGTAACAACATCAACAACAACAACGTAACAAACAGCACTTACAATGTG

CCAGGAACTGTTCTGAGCCCTTTGCATATAACAACTCATTTAATCATCACAAAACCCTAC

AAAGAAGATAACATTATTATCCATTTTCCAGAAAAGGAAAGTGAGGTACAGAGATTGACT

TCCTTCCCAGGGTCACACTACTACAAAAATGAC

>Unigene60736_A-W 1 97 LEN=375; minus strand

XXCTCGGTGCATGACTTCCAGGAAGAGTTTGGCCTGGACCAGGCCCAGGCTCTTGCCATC

AGTGACCATTTTCCAGTGGAGGTGACCCTCAAGTCCCAC

>Unigene60894_A-W 1 240 LEN=240

XGGAATCTCAACAACTCCCCTGCCAGACAACTCACTGAGAAGAGAAGTGAGAAGATAATC

ATCTGTGAACAAGGAAGTGAGCCCTCAGCAGACATCAGATCTGCTGGCACCTTGATCTTG

GACTTCAAAGTCTCCAGAACTGTGAGCAATAATTAATTTAAAAGCTTCTTGACAGCAAAA

GAGATAATCAAGAACATGAACAGAGAGCCTACAGAAAGAGAGAAAATCTTTGCCACCTGC

AXX

>Unigene60905_A-W 1 260 LEN=260; minus strand

XCTGCCCCCATCCCCTTCTGGCATGGCCAACCTATCTCCTCCCAGTTACCTCTATTCATT

CATTCCCCAAGAGCAAGACTGTGCTTCTCAGCCATCCTCCCACCTAAAACCAAACAGGAA

GTAGAAACATTCCATAAGAGTGGGACTGGCAAATACAGAGCTCAGATTGATGCTGGTACT

AAAGAGACCTTTAAGGAAGGGCACCTAGTGAAAAATTTAGCCATTAGATATTTATCTATT

GAAGACACTGACAAAGGATTC

>Unigene61025_A-W 1 81 LEN=966; minus strand

TTCCTGCCCTACGAGATGCTGCTCATGTGGGACGCGCTGAGCCAGCCTGACTTCACCTGT

GGCAAGCGGGTGCAGGTCTTC

>Unigene61127_A-W 1 327 LEN=327; minus strand

XCCGGGGACTCGGCTTCGGCGGGCTCCGGAGGCTGGAACCGCGGCGAGGCAGGACTTCAC

TCCTTCATCCTGGGACCAGGCAGCACATTACAGACAGCCGTGCACTTCCTTGAGAGGCAG

CCCAGGCAGCTCGGCCGCCGAGCCACGGCTTATGCTGAGACGGGAGGTGGCGGCATCACC

TGCGGCGGGCGGCGGCTGCACCGGGACGCGCCGAAGCAGCGAAGGCGCGCGGCGCCCTGG

ACCCTGCTCCCGCGGCCCCTCCCGCGGCTGCCACCTCGAAGCGCGGCGGAGGAGCCGGGG

CACCACGGGCGGCGGCAGCGGCGGCGACXX

>Unigene61150_A-W 1 266 LEN=266

XGGTCTGATGATGTTGCCTTCCCATGGTCCACAGAATTCATAGTTGATGATGAAACAGCT

TCTTTAATTGTGTCTTTTTTTTTCCTCCAGAAAAAGAAGATGATGATGAAGATAATTATG

ATTAGGCCACTGGCGACCCCTATGATGATCATGGAAACAGATCGGTTACGTGGAGACTGC

TCGGTAACTGGGACTCTTGATGTTGTTGCTAAAACAAATAAAAGAGCAAAATCGCTGTCA

GATTTGAAAATATCCATTCTTCAGCAC

>Unigene61204_A-W 1 221 LEN=221

ATTGGATTGTTCCCATTGCCCACCATTTACATGCTAGACTCTAGTAACATCTTTAAAGGG

ATGAAAATCTTCCATAGTTCAATTAATACTAGCAAATGTCATGCCAGGTCTACAGATAAC

CAAGCCAGGTTAGAAACTCACCAGCTTGCAACAAAGTTGGAAGATGCTGTGGATGTACCC

CCTGGGCCAGCATCCTCTGAACCAATCCTGGGTGAAGTTGAX

>Unigene61262_A-W 1 206 LEN=206; minus strand

XXTTCTGCAGAAGAATGGATTCTTGGTATCAGCTGCAGAGCAGGTTTCTCTGGTACTGAA

TTGACAGCTGAGGATCTGGATAATGCCXCCATGCAGCCCCTGCCCAGCTCTGCTGTGGTG

ATGATCATCACTGAAGACACTGTTGCTGTAAAGAAAGAAGATGGTGAACGGAAAACAAGT

GGCTCAGCACAGCTATATCATGGCCCCCAX

>Unigene61283_A-W 1 239 LEN=239

GGGGTCAAAGAGTCCAAAGTTGGGTCATAACCAGACATGGAGGAGAGAAAGTGAAAAGAG

ATCACCATCCAGGGAGTAGCTCCAAATCACAAACAGCAATGCAATCAGCTACAGAGAAAT

TTGAGTGAGCTCTCTGTGGTCGCACCTTCGGTACAACCTGGTCCAAAAGGCAGCAGTGCA

AGTGACAGTCCAGGCATTCTTACAAATCAAGGAAAAATTCACAATGTCCTCAGGACGGAX

>Unigene61652_A-W 1 310 LEN=310; minus strand

XXTGATTGTGGACACTTTTCTCTACCTGTCCTCATCTGTAAAGTGGGCACAATAATAGTG

TCAACGCGATACGTGATCTTTCACATTAGCACACACAGTCGGCGTTCAATAGATGCTTGC

GTTTATTTCATTCCTTGTCCTGGCGTCCGGGAACGGAGGGGAGTGAAACCAATGTTAGTG

GGTGACACAGGTCTATCAAGGATCTTATATCCTCGAAAAGAAGCATTTTCGAAGTCCAGC

GACCCCTGAAGTTACGCCTCTAACTACAGCGACCCAGAGACTCAGCCTCAGGAACTTCTA

GCCACACCGACT

>Unigene61959_A-W 1 63 LEN=213

GACTGGAGGAAGAACATCGAGGAGAAGTCTGGCATGGAGGGCCGGAAGAAGATGTTCGAG

TCG

>Unigene62027_A-W 1 274 LEN=274

XCCAGGGGGAACGTGTTGCAGCGTATGCGCATGTGCAGGGACACCGCCTCAGGACTGGAC

GTGGTTGTGGAAGTGCGCATGCGCCTGGTTCGGAACTGCCTCTGTGGCATCCTTGGGCGG

TCCGCCAAACCTACGCGCAAAATTAGCAGTTCTGCTGCCGGAAAACCGGCTATTAGAACT

ATTACTGAGCCCAGTGCCTGTGCTGTCCGCGAACGTCTGCGCTGTGGCCTGCCAGTCCGC

GCGTCTCCCCTACCCATACCCTGGATGACTCGGGGX

>Unigene62033_A-W 1 238 LEN=238; minus strand

CCTGTCTACACAGATAGGCTGGGCCCCACCTCAATATTCCTGTCCAGTGGCAACTTATAT

CCACCAAAGGAAACTGTCAAGCTGGATCAGCCATCAGCTGAGAAAGGTGGATATGTGAGG

CCAGCTCCCTGTTCCACCTGCATCCCAGCTTCTGACGTTACTTCCACAGGACGGCTAGGC

AAGAAATTTGGACAGCCTCTTTCCAAGCATCTACTTGGCCATAGCCAGTCTCCCATCCXX

>Unigene62128_A-W 1 235 LEN=235; minus strand

XXACCCCTACTGTCTGCTATGCCTCTAAGTTCCAGGCCCTCACCCAAGGGATCCCTACTG

AGCCCTGGGGCTGAGAGCCCCGAAGAGGGAAGCTCCCTGTCGGAGCCACGTGCAGGCAAA

CATGTGCTGTGCTACAGCTCGGATGTCCGGGACCTCCGGACCTCACAGGCCCCAAAGAGG

CACGAGGTCGTCACCATTCCTGGCTACAACTTGGGACTGCTGCAGGATCAGTCTTCT

>Unigene62187_A-W 1 295 LEN=295

XXCCCTTGTATTTGCCAAGTCGGCAGCCAACAGTGTTATCTTCCCCATCCACCCCAACGC

CAACCAGAAACTCACACTTATTACGATACTCAGTCTGTGGGGAGAGAAGGCAGAAAGAAG

CAGCCAGACCTTACCCTGGCTATCCAGCACTTGTGTAACACAGCAGCAAGAGCAATACCC

CAACTTCCCCACAGAGGTTGGCAGTTGAAAAACAAGACAACTACTGGTCACATGATGAAA

CCAAAGAAACCAGGAAGAAACAAAACAGGAAGAGAGAGAAGAGCAAAAAGAAAGTTT

>Unigene62214_A-W 1 264 LEN=264; minus strand

XCAATTAGCATATTAATAAAGCCCGGCCCAGCCCGGCTGGGGCTGGAGAGCCGGCGCCTG

CGAGAGGCAGAGCGCGAGCGCGAGCGCGAGCGCGAGGGCGAGGGGGGCGAACGGCGGCAG

CGGCGGCGGGAGCGCACAGACACGCACACGCACACGCCACACACGGACACACACTCCGGG

GACGCACACCCGCGCGCACACACGGAGAGGCGGCTCGCCTTCCTGCCTGGCTTCCTTCCT

GTCCGCTCGGCCCCTGACAGGCCC

>Unigene62547_A-W 1 230 LEN=230

TGCATCACTGAGCATGAACCTCAGATTAACAGGATGGTGACTATGAGGAGAACATACCAC

TGGGTGGATGGCAATGTGTCTCAGGCAACTTGCTCTGTTTTACATTTAGGAACAAGAGTC

TGACAGGAGGAGGCTGCAAAGTTGAAGATCAAGACACTGGAAGATCTGTTATCTGCTAAA

ATCATACAGAAAGAAAATGAAGATAATGATAAACAAAATGAAAAGGAGAAX

>Unigene62559_A-W 1 245 LEN=245

XXGTTGAGTACTGAGACGGCCGCCGCCCTTCCCGAGCAGCAGCAGCAACAGGTCCTACCC

GGGATACTGGGCGCCCGGAAGCCCACTTTCTGCACCTCCAATCGGGGTCACACCTGTGAT

AAGATCAGCTCTAGGCGCCTCAGTCTCCCTGCGGACACTGAGACAAGAACGCGAACTGTG

CGGAACGGACCCCGACCTACGGTCACGAATACCACAGGAAGAACTACGCACCTGCGTAGG

TGCCGGGXX

>Unigene62892_A-W 1 254 LEN=254; minus strand

XXCCCAGGTCCCAGCGCCCGGCCGCTTGCCGCCGCGTGGGAGCAGCGGGCTCGACGGCGC

GGCCGCCCAGCCGCCATGGCGGCGCACGGGAAGCTTCGGCGGGAGCGGGGGCTGCAGGCC

GAGTACGAGGCGCAAGTGAAAGGTGAGAGGGGCGGAGACCCCCCAGTTCGTGGTGGAGCC

CGAAGCGTCCTCAGGAGAGAGGCGGAGGGAGCCCGAGCTCCTGCCCGCGTAATGGGTCCC

TTCGCCCCGAGCGCCCXX

>Unigene62928_A-W 1 203 LEN=203; minus strand

XCCTCCCCTAACCCTTCATCCCTACCAGGATCCAGAGTAACCACCTGCTGCTACCAGGAG

CTGAAGTCCCCAAAACCCCGACTGGGCTTTTTCTTGGATATGGATGCAGCTGTACTGGTT

GATGGTTCTTCTTCAGCTGCTCGTTTCCTGAGAAAACAAACAGGCTATAGCAGCCACACA

TGTGCCAGGAGCCCATGCCCTCCC

>Unigene62938_A-W 1 228 LEN=228; minus strand

XTGCCTGAGTTCCAGCTCTACCATTTATTCCGCTTTGGTTTTATCGTCCCACAAGATTAT

TTTGGGAGATCAGTGAAATAACGCATGTGGAAAGGCTCTGGTGGTTGTGAGGATGAAGCT

GTGGTGTCTACACTAGGCCCTTGGACTGTAGAAGAATTTGTGCTCCCTGTAGCAAAGCCG

GTCTTCATGAGGCTATTTCTTGTTGCAGGTCCAGGTGGCCCAGGAGGTCXX

>Unigene62968_A-W 1 284 LEN=284

GGGGTGTACTCTCAAGACTACTCAGTGAATGCTATGGGAAATTACATTGAATACCTTCTA

ATTTTTCAGGGACATTTTGCGGTGACTGAGTTTGAGACTGAATTTGTGAAACAGATGAAG

CGTCATCTGAGAGTAATTCCTGCTCAGCATCTGTTGGATGAGAGTTTAGTTAAAAGCCAT

GCAAACAAAATAAAGAAGCATAAGAGTTCAGGGAGGTTAGTGCATACATCAAATATCTGC

ATACACAGAGATGAGAAATTCTTCAGCTCTGAGCTGAGTCTGCAX

>Unigene63505_A-W 1 297 LEN=297; minus strand

XTGGTGGCAGAATTATCAGGGCCTGTGAAATAAATGAGAATGCCACAAGTGAGTGACCAA

ACTATGTCAGGCCTCCCTTCCCCACCCACTTATGTCATCTGCAGTGTCCACACCACCTTC

TCTGAATTCCATCCAGAGCAGCCTGGACTCTCAGGTGTTCCTCAGACCAGGCTAGATCTA

GGAGCCTCACCCAGTGATGGCTCTGGGCCTGAGATGCTGATGGCTGGGAGCCTTAGAGTT

GATCCATCTTATTCGAGAACGATGGTCCCTGAGGAGTTACCCCTAACCCAGGTGCTATXX

>Unigene63562_A-W 1 214 LEN=214

CGGAAGGTGCGGAAGGTGCGTTGGAGAGGGGCATTTACAGAGTGCATTGTCATGACAAGG

CTGCACTGAGAGAGAAATGCTCAGGAAGAAAGGCTGCGTGGAAAACAGAAGGACACAATC

ATCAACAGTGTGGTCCCGACCTGGTGGGAAGAAAATACACGCACAGTAAATAGACACAAC

AAGATGAACAGTCCCAATGTAAGGAGGACTACTGXX

>Unigene63589_A-W 1 210 LEN=210; minus strand

XATGACCTAAATGAGCAAATACAGGAACAAATTGATCACTCCAATGATGAAATAAGAGCA

AATACAGGTAGCAAAAGATTACTTCAAGAGAGAGATACTCTGGGGAAAAAAAAAAAAAAA

GAACCAGTCAGAAATCCTCGAAATGAAGGACACAATAAATCAAATAAAAAACTCAATATA

AAGCATTACCAACAGACTAGATCACTTGGAAXX

>Unigene63744_A-W 1 754 LEN=754

XXCGTGGACAAATACATTGTTAACCAGACAGTGTGGAAAAACTACACACAATATTATAAT

ATATTTAATTGGACATGGTGTTATAATTTTAGCAGACAAAATTGTGTACAGAATCCTCTT

CATGCTGTATATTTGAATGCCTCTCATATGCATCTTGAGGTGAATAATACGTGGCATAGG

TTTGATAATCAGTCCTGGCAACCTGTATTGGTGGTCACACCTAAGGATATTTTGGAAAAA

CACTTTTATAATGTTACATTTCTGCAGGTAGATTATACTTATAATGGATCCAAAGGAGAA

TCTTCAAAGTGGCCTTATTATAAATGCGAAGACTGTACATTTAAGTTTTATGATAATTTC

TTTGGGACTTTGATTCCCGTGTTTAATTTGAGTGATGACAAAATCAGAGTGAATATCCAT

AATATGTCAATCCCGATGGGAGTTGATTCTAATTATGTGGACACTAAAAATGTTAGTTTA

GCTATGGAAAGATCTATTTGCAGATGGTTTCCACATTCATGGCAATATGTGATTGGTATT

CATAGGTCTATTATGCATAATGATTCCACGTACTATGATATTCCTCTTGATGTAAATATA

ACTGGAAATGTTTCCAAACATTATGAAGGTGGTAATGTTGTATTAGGCAATGAGGAAAAA

CAAACGAGGGATTATACTTTACATCCAACAGTATATTCTCTTGAATTTGTACCACCAGAT

TTGGATGAAGTTACTTTAGTGAATTACAAAACAGAC

>Unigene63746_A-W 1 543 LEN=543; minus strand

XXGTCCTTTCGCTTCATGGTCCCTGCTCCGGAGCTCTTCTTCCGTATCCTGCCGACTACG

CCAACTGTCACGAGCAGCTTCTCAGCCTTGTCTGGTTACCAGGAGTTCCCTTCCGGATAT

CTGCACTGGACCCCGGACCGCGACGTACGCGAACACCAAGCACGAACTCGATTGCCTTTT

GAAATGCTGGTTTATTCGCACACCACACAAAGCAATACACGAGGTGGCTGGCACACAACT

GCACGGTCTTACTCTCAGGCAACGAGAGTTAGTTGTGAGCAAATACATGGAGTTTCCCTC

GCGCTTATATACTCTATATGTTTTACATTACATCATTCACTACTAACTTATCTAACATTG

GCATACTACCTCTACCTTACATCATTTACTTATCATAGGCATACTGCCTACATTACATCA

TCTTCTTCAACTTATCTAACACGTACACACTATCTAGTCATCCTCCAGCCAGAGCATGTT

TGTAACTAAAAATCACTCAGTCACTGCAAAGCCACTACAAAGTCCAAAACAAGTTCAAAC

AAGTTX

>Unigene63750_A-W 1 401 LEN=401

XCTTTGTTAGATCTTGGGAATTTTAATATGTTGTCTTGTTATCCTGAACCGTACATGGTT

CAACGATGGCAAAACATTTCTTATAGTGATTCTTTGAGTCTGAATGCTGGTTGCCATATA

CATACTAATGTCATGATGATGGAACATATGAATCTAACTGTTAAGAAACATCTATTTAGG

TCTAAGTATTTTACTGCTACGCAAAACTTACGAGTCCTGACTCAGGTGGTTTATATGTCT

AATCATACTTTTGGGTTACATTCTTTTGTACCCCAGCCACCTCTGTCAGAAGAATATAAA

AATATGATGAAAGCAACAGAGGCAGCCAAATCCGTTTATGCTTCATTAGGAAAGGAAATT

GAGACAGTACATAACCAAACTACCGAACTTCTCCAAAGGTTT

>Unigene64120_A-W 1 413 LEN=413

GAGGCTGGCTTTGAACTTGTGATGTTCCTGCCATAGCCTCCCAAGTCACTGGGATTGCAG

GCATATGTCGCCACACCCGGCCACACAATTTTTTTCTATCTCCAGTATGTTGCAGTATCT

TCTGGGGACATGGAAGCAGGTGCTGCTGAAACATCAAACCATCCTTGTGATGGAGACCTT

GTGATGGAACAGGAAAGCATTCTTCCCTTAGGTATAGTAGGTGAACAGAGGGTGATTGGG

AAGCCAGACCTATCGCAAGAGATGCCTCTTTACATCATGACCAACATCATAGACAGGACA

ATGTGCACATTGGGAATGTGCTGAGCCATGGATGGAACAAGACTAGCAGCTTGTTGCCCT

CTTGGTGCTTCTAACCTGACACAGAGTCTCAGCAACCTCACAGATCCCCACACX

>Unigene64172_A-W 1 213 LEN=213

XXGGGTTCGATTCTCAGCACCATATACAAATAAATAAATAAAAATAAAGAAACCCAACCT

GCTGAGGAGGTGACTGTCATCTGGGCTGATTTGATGACAGCCACATACAGACAGGTCAAT

AAAGCAGATTCCGCCTGTGGCACTATCCTGGGCCAGGCTGAGCTGGCTGCATCAAGACAG

GAACAGGTCTGGCAGGGGGCATCTGGACAACTGGCX

>Unigene64460_A-W 1 241 LEN=241

XXCAGAATGAATTACAGAGTAGATAAACTGAAGAATGAATTAATGAACTGGATGACTGTA

GAGAGAAATTTTCCCAGAATGCAATAAGAAAGGGAAATAGATGGAAATATGAAAGAGGAT

TTAGACAACATGGTAGATGTACGTGTGCTAATTCCAGTCAAATGGGAGTGTCAAGAGAAA

TTGGAGAAACTTGGGAAGATCCTGTCTCAAACAAATACAACAACAACAACAACAAACCAG

GCA

>Unigene64566_A-W 1 290 LEN=290

XXTATATACGAAGGTGTGAAGAAACAGCAGGACACATGCAAAACTTGTTTTTGTCTGGGC

ATAATCTTAGTAGGAAAGAAAAAATTTCAAAACAAAAAACAATACAAAAACAAAACAAAA

CAAAAAGCATCACATTTTGCTGTGGAGACTGTAGGAACAAGTATGGAGGAGAGCCCTGTG

GGGCTGAGGGAAGGGGCAAGAGGGAGCCAAAGCTCCCTGCAGGGTAGCTCCAGGGAACAA

CCACAAAAGAAGAAAAAGAAAAAAGTTCTTTGGGAATATCACTCGCTAGATGXX

>Unigene64663_A-W 1 437 LEN=437

XXACCTTTCCTGGCAACAGGGTCCATTTTGTTCACTGGTTACCATAGGAGCCTGCTTGCT

CTCAAGAGCAGTAAGTGTTCCCGGGAAGTCAGCAAACTACAGGAAAGGACGGATGAACAG

TCTCTTGCCCAGATTATGAGTCAACAGATCATGAGCATTTTTGCTGGCTTTTCAAAAGXT

TTCATGCCTATACAGTCCAATGAATTAAAATCCCCAATGATTCAGCTCGTGCTCAACATT

CTGAATAAGTGCCACGCTTTCTCAGAACTGGGCCATATAGTAAAGGCACAGTCAGTGACA

ACTCATCCAGATACCCTAAGGGCCAGCTACATTCCTGCACCAAACTACTCTGGAGACTCC

AAAGTCAGTGCAGATTGGATTCCATTTAATACCAACAATCCTGACCTTTCAGATGATCAA

ATTAAGGAGAAGAAACACAXX

>Unigene64743_A-W 1 364 LEN=702

XXGGCCGTAGAGATCCAGTTTAATTTGTGTCGTATGTACATCGTCTCCCTCATACAGCCG

TCATCTGTGAAGCGGAACCCGGGAGGGCACGGATTGACGGTCGCCACACTCACTCCTATA

CAAAGACAAACTATGTACACACACCCAGGAAGCAACTGGAGAAAGCAACCGGTTTGGGGG

TTAACAGACCAACGGGAAGGAAAAGTCATGGCAGGTGACCTTTACCCACTGGCCGTGCAC

GGGGGAGCCTGCAGCTCCCTTCATGGAAGCCACACACCACGATGTTCATTCGGCAAATAT

AAATACATGTTCATAAAGCGGGAGAAAAATAAACCCAAAACTTTCCTCACGTTCACGCCA

CGTCGT

>Unigene66121_A-W 1 213 LEN=213

AATATCACAAAGGAACAGGTGAGCGTAGCTGGACCCATGGGGCTGCAGGAAGGCGTGCCT

ACGCATGAAGACACATTTGGCGACATTTTTAAGACCCTCAAGATCGGGGGCACAGTCTAC

AGCCACTTTGATGTAGCCAGATCACGCAAAGTGACTGACAGGGCCTCACCAATCAGGAAA

ATGGTGGCCACAAGGCGTGGCAGCCTCCTACAT

>Unigene66295_A-W 1 466 LEN=466

XXGGAATCCATAGCAAACGCCGTAACTCCTCAGCCAGTCTTCCAGAGGGACAGAAGGAAC

CGCCAAGAGCAAACCAGAAGAGAATCACACAGTGATATATTTCCAAGAAAAGTTATTAAA

ATATTGACATTTCATCTGCACAGGCTTATCCCTCAGACGTCCTCCAGACGGCCCTCAGAC

AGGGCTAATTTAATTATGTTCCACCTGCACCTGCTCGACATCAATCGGATCCTTCCAGAA

AGGGCGGCACTTATGTACTTTCAGTTTTGGAGGCTTTCCTAGAACTTATCTCAATTTCCA

GTGGGCTTTAAGGAATTCTCATTCCTGAAACCGATGAGGGTGGACTTCTCAGGTGATGAC

GCCGCCGCGGTGGCAGCCACGCAGGTACCTCCTCTGTGGATGGACCCTGCTAGGAAAGGG

AAACGGCCTGGAACCAGACGTGAGCCCACGGCCACTCTGGATGCACTX

>Unigene66532_A-W 1 278 LEN=278; minus strand

XCCACGCTGGATTCCAACATGCTGGTCCAGAACGCGGCTTGGCTAGAAGCTTCTCCAACA

GGCTTTCCCTTCCCCGCCATGTACATCATTGACTCTGATCCTACTCCTGTCCCAAATCCA

GAGGTATTGAATAAAGACACTCTTGATGCAAACAGTGAGTGGTTACAACACACGGATGGG

GGTGGCACGGTTTCCACGACACAGAGTGGAATCCAGGAAAATGACGCGCAGAGGCAAGAC

GCCCCCTTCATTGCTAATGGTATCATGGAGCTCAAAACX

>Unigene66862_A-W 1 320 LEN=320; minus strand

XXAAAAAATGATGGTAACAATATGGTTGTTTCTATGCTGAAAGGAATAAAGTGGCTCTGT

AAAGTTCTACAGCCCCCTGAGTTACAGGGAGGAGCACACTTCTGGAATGGGGGGAAAGGA

GACCTTACTATACCTGCTAAGCCATCCTTCAAATCTCAAACAGCAAACACAGGCCTGCAT

GATAATCACACAAGGGATAGATCTGGGATCCAAGCTAGTTCTCTGGAAACCACCAGTTTG

GAAACCAGATTTCCAATCTTAACCTTCTACTTCCTGTGTTACCTGGGCAAGCCAGGACCT

CTGAATCTGTCGGACAAAGAGCXX

>Unigene66877_A-W 1 284 LEN=284

XCACCCACATCTAAGCTGGGCTCACACTCAGGCTCCATCACACGCACGCCCTGTCACCCC

GTCACACACTCCTCCAGGAGAATTCGCGTTCGGGACTCGCTCGGGGACCAGACCCAGGCC

TCGGGCTCGGCCGCCAAGAGCCAGGGTCAGGAACACGGTCGACAGATGGACCTGGAGACG

GACGGAGAGACCGATGAACTGTCGCGTGGACGGGCGAAAGAGCAGACCGGCCTCCTACAA

AAGGACAGAGACAGCCACAAAGAGCAGACCAGCGACCACCGAGGX

>Unigene67087_A-W 1 252 LEN=252

XGGTTGGCTTACATGACCAGAAGCTGGATATTTCACAATGGCTGTCTGCAGGCACCAGTA

GCTGTGCAGTCCGAGAAGCTGAAGCCCCAGAACAAGAGGGTCAAGGGTGCTACCCCAGAC

CCAGACCAAAGGCTTCTGGAAACTCCCTGGAGAATCACTGGCAGAGTCTGCTTTGGAAGA

GTGAGGAATCGAGAGTCCGATATCTTCGGGCGTTTGCAGCAGCAATCAAGAACTTATTCA

AGAAGAATGGAGCXX

>Unigene67127_A-W 1 687 LEN=687

XGAACTTGCGATCCTCCTGTCTCAGACTCCCAGGCCGCTGGAATTAACAGACATGCGCCA

CCACGCCTGGCCCTTTATGACTGTCTTAATCCTTCTGATCTTGCAATCTTCAAGGGAAAC

CTCAAATAAAAGACAGTGGGACACGAAGACAGAAATAAAGGGAAAAACAAAAACTTTTGG

AGGAAATAAATTATACAGATTTAAAAGAATTTAGAGTGCTATCTAAAAAAGGGAACAGGA

GGAGAACAATATAAAAATGAAAGAGGAGAAGAGAGAGTGGAAGAAGGACAACTCTGGGAA

AGGAAAAACTATAAAAATGTAGAAAATAAAAACTTGATGGAAGGGTGGGAGGTAAAAAGA

AGGGAATTGCCTGAAAGCAGAACAGTGGGGCAGAGCATTGGAAGACAGCAGAGGTGAAAT

GCTTACGGAGGCCCAAGGCCACACGAGGAAGCCCTGGAGGAAGAGCCCCGGGAAAGATCA

AGTGCAAACCAGACTGTTGGGAGTCCTCAGATCACTCGCAGGTTCCGTGATGCGTGAGGA

GAAACCAGGAGGCCACTGGGCGTGGGAGCGCATTCACAGCTGGGCCTTAGTGCCTGGAAA

GGACTCCGCAGAATCAACAGAAGAGGCACCGGGCAAGAGCTGAAGGGAACCAGGACACGG

TTCCTGCCCATCAGCCAAGAGAGCACACXX

>Unigene67137_A-W 1 321 LEN=321

XAAGAGAAGCTGAGGTTTAACCACTCTCCTCTCTTATTTCCAAGTCCTGAGGACCCGTAT

CCCCTGGAAGTAGGAAATCCCTACAGCGCCCAAGATATTTCTGCTAACAGAGAGGCAGAA

ATCCCCAAGAGGTCAAAAACAATGCAGAAAAATGAACTAGTCGTCCTCAGCCTCTCTCCT

TACAGTTCAAGGGAATCGAGCCCAAGAACGGAGAAAATCTTTACGGGTGAAGGCTGCTGA

ATTCCGTCTAGACAAGAAACGGCGAAACAGAAAACTAGTACTGTGATTTGGGACACGTCA

ATCATTCCCAATCCACCAGACAXX

>Unigene67210_A-W 1 240 LEN=240

XAGGGGATGAAGATACTTAGGACAAAAATTTTCATTCAATTTGGGGAGACAAAAATGTCT

TCTGTTCTAGTCAGGTTCCCAAAGAAACAAACCAGCAGAATACTTGTAGATATACCTAAG

AGGAGATACATCACAGGAATTGCTCAAGAGATTACAGGAGCAAAGAAGTCCTTCGATGTG

TCATCTGCAAACTGGAAAACCACTAAAATTTATGGTATAACTCAGTCTAAGTCCAGAGAA

GXX

>Unigene67245_A-W 1 382 LEN=382; minus strand

XTTTGCCAAATGAAAGGTCTGCCATTGACCTCACCAAAACATGTAAAGAGGGCAGAACAT

TTGGAAAAACAGTTGGATTCTCCAGATGGGCTGTTAGAAAACAACTTGATTCTGCAGCTA

ATTATTGGATTTTATAGACCTGGCTCTAGGAAGCCCTGTTTTGATAGTTTTGAGATGATA

ACGATGTCTCAAATTGCATTTCTCCTCTCTGACAATTTACACCCACTGTTGAAAATTCCT

GTTTTTGGATCCAAGTCAGAGGGCCATCTCTACACCAGATCACCCAGAGTTGCCCTCTTT

CAAAGGTGTTATGGGAAAGTGGTAGGATGTTGGGGAGTAGAGAATCTCCTGATGGAAAAC

AAAACAAAAAACAAACAAACAAAX

>Unigene67580_A-W 1 407 LEN=604

XGTGGTGGTCAAGGCCCTGAGGGAGAGCAGACAGACAGCGCTCCTGGAAGCCAGAGTGCC

CACAACACGTGAGTAAGCCCAGTACCACCTCACAGTGCCTCAGAATGTGACAACGGCTAT

CAAGACCGCTTTAGGGGTGGTCGGGACAAACTGGGGCCTGGAGGAGTAAGTCCTACAGAT

ACTGGAAAAGACACATTGCAAGACGTGGGGCCACAGACAGCAGACCTGGACAACAGATAC

CACATCACTGCGCCTCCTGACCAACGCAGCTGCGGCCTCCTCTTCCAAGACTGGGCTAGT

TCTGCCCACCAATGCCCTCCTGAGCCCCAGCCCTATGTGCGGGTCCATCTCTGCACCCCA

CAGGGTCTCCTGCCTAGTGCTTCCCCTGCTGTCCTGCAATTACCGATT

>Unigene67767_A-W 1 585 LEN=585; minus strand

XXCTCCCTGTGGTTCCTTGTTACGGGCACTGGGGACATTGGCCTGAATGAAGGTCTTCTT

AAGCCTATTGAGTTCAAGTGCAAAGACATAACCACAAAGACCTTGAGACCACAGAGTCCA

GCAGGTCTGCAGTTCCTCATCTCACCCAAGGTCCATGAGCCAGAGAGGAAGAGGAATATG

GCTGAAGAACACAGAGCTTTGGGCTGCCACGCAAAGGTGCAGGACATAGAGAAGCCGCTG

CCTGGAGTTCAGACCCATGTTAAGTGGCCAAGAACAACTTACGTGGAAAAGGAGGACACC

TTCGAACCCAACTCCCAGGGTGGGTCTAATTGCTACCTCAGGGAAAGCTGTCTTCAGATC

CATGGTTATAACAGGGATGGGGAAGGAGAACCAGGCATGCATGATAAGCTAAAGAGACCC

AAGGAGGTCAGCACCCCATTGGTGGCATCTTTACTTCTAAATGCATATCCTGGGCTACCA

GGCCATGCTATCAGAGAAGCGAAAGGAGTGTTTGGGGGACTTGGTCCACTCCTACACCAT

ATGCATCTCTAGTGCAATTATAAGGGAGAGAGAGAGAGAGAGAGAGXX

>Unigene67838_A-W 1 250 LEN=250; minus strand

CTGCTGCAGGTCTATCAGACGGAGGTAAGGTCTGCCCTGCCCATTCCATCTCCTTACCCA

CACTCCCTGGCAGCCAGGGTCCGCATCGCCAAGTCCCAGGCTATGTCGTGCTGGGCCTAT

CGGCCCCCACCCACTCCCACCCGCTGACATCAGCTAGACTGGAGGAGCCGGGTGGGACCT

CCCCGCGCAGAGGTCCACACTAATGCCCGTGCTCCTGCCCTCTCCAAGCTGACCTTCGAC

ACTGGGGAGAXX

>Unigene67900_A-W 1 340 LEN=340; minus strand

CGCGGCCTCAGGGAGCCGGGCGTGGGGCGGAGGGCTGGGCCAGGCGGTGGGACGGTGGTG

GGTGTGCGCTCCTGTCTGCTAGAGGGCGCGCCCCGGGAGTGCAGGACGAGAGGTCCCAGT

CCTGGAGGTCGCAGGGACGCGGCCATGCTCCTGTCAGAGTTGGAGCAGAAGCAAACCGTG

GTCTTGGGAGAGGAAATGAAGTGGCCGCGTCTCAGCCGGCGGGAACTCCTGGCCCAGCGC

CAGAGGGAGGCTGCCGGGCGGCCCGGGGAGGGGCGCAGAGGAGGCGGACCGGCCGGCAAG

AAGCGCGTCCCGGCAGGGGTCGGCTCGGGGAGCACGGACAXX

>Unigene67974_A-W 1 152 LEN=501; minus strand

XGTGGGTCCTACGTGTGCTGGGTGAACATGGGGAGGGGACATGTCTGGACAGTGGATCTA

CAGATGTCACAGTTCATCTCTAAGTTCATTATGCTGAAGGACCAAGTCTCCAATGTGGAG

CAGACTGAGGTGACCCAGGTGTGTGGCGTAGTG

>Unigene68171_A-W 1 261 LEN=261; minus strand

XCTCCCCCGGACACTAAATGCCTGTCTCTCTCTGTGGCTTGTAGCTCTAATTCTCCATAT

GGAACTGGATGTAGGAACTTTTCTGGCACTTACCCTCCAAATTTCACAGGCTGTTCTCCA

AATCTAGCTCTACAGAAAAGGTCTGAAAAAGTAGTCTTTGATAAGATGACAATGATGAAG

TTCTCTCTGGCACTGCTAGCAAGGCAGAAGCATCCCCAAATGAGGAGGTTACCAGTTGCC

TGTGAAGCAGCTGGACCATTACXX

>Unigene68514_A-W 1 431 LEN=431; minus strand

AGAGGTGCCGTTTCTCTGAATCTTGAGCTGATTGACTTGTAGACTAGGATCCCAGTCAGA

GTCACAGCAGGCTTTTTGGTAGAAATGGACAAGCCGATTCTAAAATATGTCTGGAAACTC

ACAGGACTTGGGAGAGCCACAACACGTGAGGAAGAGGACAAAGTGGAGAGCCTGCATGTC

CTGCAGGATTCGCAGTCTTTAAAACGACAGGACTCCGATCCAGGAGGACACAGACAGGTG

GACGGAGACTCATGGAGGGTGCGGGAACAGCCTCACAGGTGCACAGACACAGATTTTCGG

CAAGGAACCAGGCGGTTCAGCCACAGGGCACTGCCCCTGAGCACTGGGGCTGGAGGAGTA

GACGTTTATATGTAAACCCACACCCATGCAGAAATGACTCGGACTGATCAGGGGCCCGAG

TGTAAAACCTXX

>Unigene68893_A-W 1 309 LEN=309; minus strand

XXTCGAGAGCTGGGCACGTCCACCCGCCCGAGGCCGCCGCCGCTGCTGGGGCCGGTCAGC

GCGTCCCCGCTGTATTTCTCCTCCATCCCGGGCTCAGGAGCCGCCACCGCTACCCCTGCC

GCGGGGTCTCCTCATGTCTTGTCGCTGCCGCGGAACAACGGGCGCAGGCGCGTCACTCCC

CCATGGCAGCAGCAACCCTTGTCGGGTTCCTGCAGGCCGGACCGCCACCGCTATTCCCAG

CTTCGGTTTACAAGCGCACCCGAGTTGGGACTACGCCGCGAGCAGTTTCGATTCGCCGGT

TCCCGGGAGGCX

>Unigene68943_A-W 1 202 LEN=202

XXGGTACAGCACTTGCCTACCATGCAGAAGGCCCTGTGTTAAATCACCAACACCACAAAA

GGAGGAAAAAAGAATACATATTCTGATCTCTATAATCATTAAAAAATAATACAAAGGCAG

GAGGGTTACAAGTTCAATAGCAGCCTGGTCAATATAGTGATACCTGTCAATAACAATAAC

AATGATGATGATGATGATGATGAT

>Unigene69143_A-W 1 289 LEN=289

XGAATGAAAGGAACAGAGAGGGCTTCCAGTGTGGAGAGTGTTACCCGTAGAGTCAGCTTT

ACTCAAATACTCGATGGTGGTGGAGAGGCGGAAAATGCGGCAGAAGCCCCAAGGGAACAC

CAGGTGAACTTCGAAGGAGCCCTGGACAGAATGACCACCTGGATTTCTAGGGGACCTGAC

TTCCTGGTGACAGCTCCCATTTCCAAATTGACATTGAAGTGATCAAAAGACTATCTCAGT

AACAATAAAGGGAAATCTGAAATTAGTGAAATGAAAGCTGAAATGATAGCX

>Unigene69197_A-W 1 241 LEN=241; minus strand

GCTCCACTGTGCCCAGTGGAAACATTTGAGAAGAATTATATCTACAAAAGCCCACTGAGT

GGTGGAAGACTAAAACCTTTCCACTATGGCCAAGAAAGACAAGGATGTTAGAGCTCTCCA

CTTCTAGTCAACATTATAAGGGAAGATCTAACCAGGGCAATTAAACAAGAAAAAGAAAGT

GGAGGCAGGCAAATAAGAAAGAAACCTATGTTTGCAGATATTACTCTATATACAAAATCC

CXX

>Unigene69344_A-W 1 243 LEN=243; minus strand

XXCATCTACAACTATGAAAAATATTTTTTTAAAAAAAGAAGTAAACAATTATTCCATGTT

GCTGATGAAATCAAGTCAGAAGATGCGCTGGCTGGAGGTACCATCACTGAGCAGGAACGC

AGGGTAACTGAGTGCTCCCCATGGAAGGAGCCCAGCCAGTCATCCCGCCCTTTGACCAGG

GAAATCACACTGAGAGCGAAGGCTGCCCAGCCAGGCCTGGCCAGAAGCAGCACCCTCCAC

CAACCX

>Unigene69354_A-W 1 249 LEN=249; minus strand

TGGGCCTTGGGTGCCGATCTAACAGTGGAACAACAGTGTTGGTTGCAGCATAAGGTTGTG

GTGTACAATCGAGGTGCAAAGAAAAAGGCATGGCATGTGGTCAGTGGTCAAGCACAACCC

AGGAAAAAATGTGGCCAGACACTGATGCCCTCAACCATCACAATATCACACAGCTCCTAT

GACTATGGCAACTGAATCATGAAGCCAAATTATGATTTACTTATTTCCAAGAAGAAAGAG

CAGAACTTX

>Unigene69391_A-W 1 238 LEN=238

XGTTGCCATGGCTTCATGAATAGAGAGTATGAACTAATAGTAAGCTTCCTCAATTCTGTA

TATATCTATGCCAATAGACTGGAGATTAAGCATCACAGCACATCATTATTTTAGGTTCAT

GTCTATAAAATGCTGGTAGCATCACTGATTGCTTCATTTCCAATTTTTGAGGAGTTAGAT

GAATTGAATATAAGTAATATGTCAGACAGGCAAGATGCTATATTTAAAATAAGCAACAAX

>Unigene69486_A-W 1 228 LEN=228; minus strand

XXCCGCTTGAGCCACATCCCCGGCCCGTATGTTATAATTTTTAAGTCACTAAGTTTTAGA

GTAACTGGTTACTTGGCAATGGATAATGGAAAACTAACAGGAAGCCATGCAGCAGATATT

TTATTTGATGCAAGCTGTACGGTTTCTATGGCAGTTATGCAACACTACTACTGTAGCACA

AAAGCTGCCAGAGCAAACACACAAATGAACGAACATGGCTCTGTTCTAATX

>Unigene69578_A-W 1 241 LEN=241; minus strand

CCAATGTTGGAGAAAAGCCCCATCATGAAAAATAAAGAAAAAAAGGCAACACATGGAAAA

AATAACTTGGAGGAAACAGACTCTGCATCCAGGAAGATGAAATTGATAAATTTCATGATA

AAATCTCAATATCTTAAAATGCAGATGGCAGTCAGACATAGATTCTGTGGTAGAGTGAAA

TACTATAGAGCAAACAAAGCAAACTATAAAGCCCCAGGAAAACAAGAAAATTTGCCAGAA

AXX

>Unigene69606_A-W 1 336 LEN=336; minus strand

XCAATAGATGAAATAGTCAGCAACTTAAGGTATAAAGTATTTCCAGAATTGATTAAAGAC

GTGGGTTACATGTATAAATTAATGTCCAGGGAAAAATGCCAATACATGTACTTAGAACAT

TTTCAAACTTCAGAGAGGAAGAATCCTGAAACATCTGGAAAGAAAGGCCAGATGACATTC

CAAATACTGATTTCAGCTGCAGCCACTATTAAGCCCAGGAACCCCCCAAGGAAATGAAAT

AACATCTGCAACTGTATTTATGGAAAAAGTAATCCAGGCAAGAGTTCCATTCATTTGGGA

AAGCTGAAGAAGGATTTTTCACATATCCCACCAACCCXX

>Unigene69622_A-W 1 261 LEN=261; minus strand

XCGGGGGTTATAGGTTGCTATGTCATGGGTATTATAGGAAATAAGGTGATTGTGTTCCAG

GATTTCCTGGGGGTATGTTCACGCTGTGGTCTATATAAACTGAGCTTGCTGGAGTTGTGC

AGTGCACAACCAATGCAGCAAATCTGGTTACATTATTCCATGAAGTTTACAGAAATATCA

GCAAACAGCACAGATAACCATCTATATATGGGACTGCTTATAGAGTGTGCATCTTTCTAT

AAGATGATGAGTGAGGTAGTACXX

>Unigene69651_A-W 1 569 LEN=569; minus strand

XXCTCACCTTTGTGCTGGAAGGAGATGGATGGAAAATGTGCCTTGTATTTCTATCTCTCC

GACACACACAGCACCAAGGACCTACCTTTGATTGAAGCCAAGCACTGTGTCGCACGGTGG

GGACGTGGGGCTGAACAAGACATGGTAAGGGGCTCACAGGCTAATAGAGAAGCTCCATAT

GCAAACAGATCATCACAATCGGACATTCAACAAGCTAGTGAAGACACGCCTGAAGGATTC

TGGGAGCATGGACAGACGCACTAACAACTCCCTGGAGAAAGACAAGGCCTCACAGAGCGC

ACCTCCCGCAGACTCTTCAAGGATGXCTTAGAAAATAGAAGCTCTCAGGTGGAGAAAGTC

TTTTGAGAAACAGAGAAAACCATGTTCAAAGGCCCAGAGGCATCTGATGTGAGGCCAGAT

GAGAGAAGGAAGCAAAGCACTCGGGAAAGAACCAGGAGAGGACGATGGACAGAATCCAGA

GCCCAAGGACCTGGTTATGCAGGGGAGATGGGTGCTGATCCTGTGAGGCAGGAGGAAGCC

CTGAGAGGTGATAGGCGAGGAGGTGACACAGCX

>Unigene69685_A-W 1 251 LEN=251; minus strand

CAGGCTGTGAAGCTGGTGAAGCTGGGGGGTGGAGGCTGGTGTGGGTATGAAGGGGTGCCT

GAGGGAGATGTGAAGCATTTTGATCCTGTGGCTACACGGATTGACATGTGTGACACAGTG

TCATAGAACTCTGCACCTGTAGCACCACCACTTTCTATTTGCTCTGCTCTTTCGCCATGT

AGTACACTCCACGGCTTCCACCTCCACAACGGCCTTGAGAAGGATGCTTCTCCTCTCCAG

GCACATTTTTTX

>Unigene69917_A-W 1 256 LEN=256; minus strand

XAAGGCGGGGGTGCCCCTCAGCAGACACACCAGGAGCTGATTTGTGCAGTGTTGTGAAAT

ACTGGTTTCCAAAGAGGACCAGGCCATGGCAGAATCAAAAGGGACACAAGTTTTGTCCCT

TCTCAGTCTGCTGGGAACCAGAGGTTAGATGGACAGCTTTTGCCTCTCACTGAGGAATTA

GCCAAAAACAAACAAAACAACAACAACGAAAAGCAAACTAGTGCTTCAAATGCACCTCCA

GCCCAACTCTATAAACCX

>Unigene69955_A-W 1 223 LEN=223

XXGGCTGGAGGTATGGCTCGGTGGTCCAGTGCCCGGGAGTTCAATCTCTGGTACACCCCA

AAAATCAATTTTATTGAGGTAAAACTTACATATAACAGACTGGACCCATTTAAAGGGTGT

TGTTCCATGAGTTTGACAAATGAACAGTGGGAACCAACAGGGCCAAGAGAAGATGGCTCT

AGCGATTCCCAGGTCAAGCTGACCAGTCCCTCCTCACCCAGCCAG

>Unigene70025_A-W 1 224 LEN=224

TGTTGCAGCAGCTGGAGGGAACTCATGACTTTGGTGAATGGGAAAGACACTCTGGCAAGT

GACGGGAGCCTGTGGTCCAAGGGGCTTCAGGTGGAGACAGCTCCGTCTTCCAGGGGCCAC

CTCAGGCCCCCAGGGCCATCATTAGGCAGGATTAACATGCAGCAAAGGCAAGAAGAGAAT

GATGGTAAACTAACAAGGCAGAATAATAAAGAGGAAAAGCCAAGX

>Unigene70048_A-W 1 233 LEN=233

XXAGCCTGCATAGCCCTGGAGCCAGAATCTATCATCTGCAAGTCTTTGAAGCATAAAGAT

CTCAGTAAACCCTTACAGGATGGCCTTTGGCTCAATGGATACTACTTAGAGGGTTTAATT

CTAAAGTCCAAGCTTAAACTTAAAGCTCTGAGTGAGGTTGGGGATGTAGCTCATGGTACC

CACCATGCACCTCAGCAAGTACCTAGCACACAGAGGAACCAATTCCCAGCACCAAXX

>Unigene70078_A-W 1 409 LEN=409; minus strand

XCCTGTGGGTCAGATGGTCAGCACCAGTCCAGAGTCCCCTGCCCATCATCAGCCCCACCA

CCAGGAATAGCATGGCACCACTGCAGTCACAACATGACCTGGGAGAAACTGGACAGACAG

CAGTGATCACCAGTGGACAAGGAAATAGGAGGAAAGTTAAACCATCGGTGGCCAGTATGG

CACCAACCAGACGACGCCATGAGAAAGGAGATGGGGCTACTGGTCAGGCACAACGGTCGT

AAAAACGCTGTTAACCTCGTAAGATGCACTGCTCACACTGACCTCCTAGGCAGGTATCCA

CCTAATCCAGCTGCAGAACGAGGGATGCATGGGACACGAGCCTCGAACTTGCTCAGAAAG

CTTCAGCCACAAAGGGGAGGAGCAAATGTAGAGAAAAGATTGAAAATGAGX

>Unigene70130_A-W 1 214 LEN=214

XXGTCCTGCTACCAGTTAAAAGTTTGTGGTTCAGATGGAAGAGAATCAGGATGGATGAAG

AAGGAAGACCCAGTGCTTTCTAATGTCACTGTGACTGCACTGACCTTTCTCAACTTCCTT

CTTTACCAGGGCCAGAAAGAGGAATCTCTGCTAACAATGCAACATGGGTCCCTTCCTTCC

TCACCCTCTCCTCTCCTGCGGAAATTTGGGAGAATT

>Unigene70133_A-W 1 318 LEN=318

GGAAAAGACCTCATAGTGAACAGTATGAGGTACAATATTCAGAAGGATACTGCCTCAGTG

GAGAAAGGTAGCCCTAAAGACCGTGTGGCCCACCTAACAAAGCTTATAAAAGCAAGCCTT

GAAATGATGAAGCTGTTTCCAATAACTGCTTGTGAAAATATAAAACAATATGAAAACTAT

GAACCCAAAGATTCAGTAAGTCCAACAAACCTAAACAAAAATCATGAAGAAAACTATACC

AGATACACATTACAATTAAATTGTACAAATTCAATAGTAAAAAAATTAAAATCTGGGGTT

GGGGATGTGGCTCAAGTG

>Unigene70189_A-W 1 225 LEN=225; minus strand

XXTTCTCCCCTTCCATGTCATGAGCCCTTTGCAGGTTGTGACTACGCTGGTATCTGTCCC

AGTGATCTGGCAAACCCTGTGGAACACATTCTCCTACCAGTATCAACTGGTAAAAAGCTT

CAGTCTTCAGCTTTCATTATACCTCTCACTGATTTAAAGGCCCTTTACAAACTTATTTCA

ATAATCCCAAGCAGTCAGGAGAAACTAGAGACCAGTAAGCTACATTCX

>Unigene70273_A-W 1 238 LEN=238; minus strand

XTGCAGGTACCAACCATTTCTCTTTGGGTCCTAAATGTGAATCCGATTCTGGGGACAGGG

CCCAGCAAACTGTATGTCGATTCCCTGGGAATCTCTATGGCCGAGTTTAGAAACTACCAA

AACAAGACTGATGCAATTGAGCAGGCTCACACTTCACCAGTTCCGATCACGTTCCACAGG

GGCTCTCAGGAGACAGCACCATGTCGCCTAACAGAACATGGGTGTTCCCAAGCCTGCCCX

>Unigene70390_A-W 1 245 LEN=245

XXTTTCAGTTTCCCAGTGTTAGCAATTACTCAACTATAAATAAATCCTTCTAATGAAGAA

TCAAGAGTTACTGGATCTGTATTAGGTGAAGTAGCCTTGAAAATAAGACTATTCAACAGA

GTCTCTGCTTATAATAAGCCAAAGAAAACCAACTTCACCTTCTACTTCATAGTACCTCAT

CAGGACCTCCAAATTCTACCACCAAATGTTTCTCTAGTCAGCAAACATCTTTCTCTGCCA

CCACCAGXX

>Unigene70418_A-W 1 233 LEN=233; minus strand

XCTGTCACAGCTATTCAGCTCTGCCCTTATGGCATGACCACAATCACTGACAGCACAATA

CTTAAACAGAAGTATGAAGAGTACATAATATTTCCAAGAATTCCTAAAATTCAGAAATCT

TCCTTTATATTAAACATTCAGCTTTTCTTAAATTTCTGCCACCGAATCCTATATTTCTCC

CTAGCAATTGTTAACACAGAACCAGTGATCCTAGGAGCACATAAGTTGAAAAAT

>Unigene70525_A-W 1 215 LEN=215

AGGAAACAACAGAGTGAAGACACAACACACAGATTAGAAGAAAATGTTTGTGAGTCGCAA

ATTAGATTAGGGGCTAATACCCCAAATATACATGGAACTCACACTACTCGGCAACAAGAA

AACAACAACTGTTTCTCAATAGAAGAAACACAAATGGCCACAAAAACGAAAACGTTCAAC

ATCACTCACTAATCATCAGCAAGGTGCAAATTAAGX

>Unigene70541_A-W 1 222 LEN=222

AATAAAGGTCCATCAACAACTAACAACAACAACAAAACTGAAATGGAATGAGCTACAGTG

CACAGAACACCTCCAATAATCTATCGGCATAATGTTGAACAAAGGGAACAAGAATGCCAA

CCGCGTAATCCCACTTCTACAGTTCAGAACCAGGAAGAGCTACACATCATGTTCTATGAG

GATGGTGGAACTAAAGAGAAACTAAGGAAAAAATCACTGCAG

>Unigene70543_A-W 1 253 LEN=516

XXCACAGTCACTGAGCTGGCCCAGGTAGCAGCCTTATTCAGACTGGTTCTAGATCATCCT

TTCAGTGGGTGCTCAAATCCCAGGTTGGCCATGCACGAGTATAAGCAGTTTGGTTACAAA

GCCAAAGAGAAGAAGAGACACCAGTACCTTGGAACTAGCCTGTCTTCACTCTCCACCATC

AAAGCCTCAGCTAGTCTGAACAGCCATGAGACATTGCTGCCGGCTGAACGGTCCATGGTT

GCCAGTCCTACAGTG

>Unigene70654_A-W 1 307 LEN=307; minus strand

CCTCTTTTCCCCAAAGATGAACCCACCTGCGGCCTTCTTGTCAGGATCCACTTGCAACCT

CCACAGTCTGCCATAAAGAGGCCTGACAAGAACACAGAACTGTTAAAAGGCACCGGGCAA

CTTGGGCTACCAGAACCTGCTGCGGCTCCGCAGCCTCCCACATACCCCATATCACAGGGC

ATACGCATGGCATGTCTGATGTCCACCCATGGGGTGTTTAACAAATCCTTTGGTAAACAT

GTCAGCTTGCTGAGTGCTTACAGTGCACATGTTTATAAAACATACCTTACAGTCAACTTG

GATGAGCXX

>Unigene70706_A-W 1 246 LEN=246

GCTCTGTTTTGCACCTTACGGGGAAAGAACATGTTTACAATTAAGTTGGTCACTGACTTG

GGGTGATTACCAAGCAAAGCCTTGAAGTACATGCAGGCTAGTGGTTTCTTCTTGCTATCA

GTACAAGGAAAGATAAAATCAAGGGAAGAAGTTAATAAAATGGAACAAGGACCTGATGAC

ATGAGAAATTCTCAGTCTATCTGGATGGCAAAAGATGATAAAGAGATTCACTGTCAGAAA

ATCATG

>Unigene70753_A-W 1 246 LEN=246

TGCGGGCAGCTGTCCACAGCTCCCAGAGATCACTTTCACCAAGCTGGGGCTTCCAGGAAG

CGCAATGGACGCCTGGACACTGCACACCTCGCCAGTGCCAGTTACAAAGACCCTTCCTCC

TCTCAATTCTCTTTTTGGGACAATGCTCTGGCTAACGTCCCAGGCAAAGGGACCTTGCGA

GGTCCTAGATCAGCCCTGGATTCAAGAAAAGCAGGGAGAAAAAGAGAAGTACAGAGGGTA

TACAAG

>Unigene70764_A-W 1 259 LEN=259

XGCCTTGAGTATAGTCCAGATGGAATAGCTACTAAGATTAAGGATTGTGGGCAAGACCAG

CAGGTGGATAAGGCTGGGGAGAAAATGGTGACAGGAGAGACCATGAGAACTGACTCTGGA

GAACAACGCAGCAGTTTTCATACTTTGCCAGAACCAGTAGAGGGAAGGCTATTGGAGGCA

AAAACAGATGCCTTACCACCCATATATGCATTCCTAAGAAACATATTGTTTAGTTTTCTC

AATCTTAAACTTGATATGTAX

>Unigene70829_A-W 1 227 LEN=227

XXTCTGGGCCTCAGTTTCCCCATCTGCTGTATGAGTTTACCAAACCCAGGGCTCTTCGTG

TTTTCTGGATGTGGGGCCCTTGAGGAAATGAAGTCTCTGGGAACCCCAGATGTCATCATG

ACCTGTGGCCAGGTAGAACTGGGAATCACAGGAAGACAGTGTCTTCCCAAGAGGGACCAT

AAAGCCTCGACTGCTCTCATGGCTAAACCAAGGCATCACGTGACCCCCGXX

>Unigene70981_A-W 1 269 LEN=269

XXCGGCTGCTGCTCTGCTCCGGGTTCTGTCACCGTGTCGGCGGTGCCCAGCTCACTGGCC

CCCTCCCCCTCCCGGCGAGCGTGTTCGCCAGTGGCTCCTTCAGCCCTGCTCCGCGGGGTC

CACAGCGGGGCGCGGGTGTCCGGCGGCCGCGGCGAGCCTGTGGGCAGTGGGGGTTGGTCC

CGTGGCTCCGGCCCCCGGTGCAGAATGGCGGCGGCGGTTCGGATGAACATCCAGATGCTG

CTGGAGGCGGCCGACTATCTGGAGCGGCGGGXX

>Unigene71133_A-W 1 244 LEN=244; minus strand

XXCACAGACCTTGCGCCTGTAGCCTTCCTGCTTAACTGTCCCACGCTGGGCAGTACTGCC

CTTGGTGGGTGGCTGCAGGGGCTAGGGCTGGGAGCCTCAGTGGTCCTGGTGCTGCATTTC

TGTAGCTTGGTCATCATGGGGCTTAAGGCTTGGCTCCTGGCCAGACAGGCTCCTGTGACC

CACAGCCCCTATGAGAACAAGGCAAGACCCATTCCTGCTGCAGTCCTGGGCCTCTCCAAA

GCAGCC

>Unigene71158_A-W 1 246 LEN=246; minus strand

XXTTATTTTGAGACAGGGCCTCACCAGGTTGCTGAGGCTGGCCTCAGTCTCCCAAGCCAT

GGGGTTGAAGGTGTGCGCCATCACACGGAGCTGGACGGTCTAAGTTTAGCAAAATATGAG

ATATGTTGCCTCTTTCCACTCTACACTCTCCAGAGCTCTCGGCTTCAAACAAGAGCTGGT

AGTGGACACACAGAACTGGCTGAGGCCCCTTTTACACTGGCAAATATCACCGCTCAGGGT

ATTGAGCCX

>Unigene71226_A-W 1 337 LEN=337; minus strand

XXTTGCTACACCAAAGGTGTGCCCTGCCTTCCTGACACTGAACTGCAGGCTCCCCAGAAG

ACGCTACGCGGACCAAACAGACTCATACTACTACCAACCCGTCCACATCACAAAGAGCCT

CTGCAGGTTTTGCATGAACGTGTGGCAGCCAGGCTGACTGATGGATTACAGGGGGCATTT

AGCCTTGAACACCCTCCGTGCTGCCCTAGAGAAATGGATGGGAGGCTCTCAGCACCCATC

CTGGAGCTGAGAGGAGAGAAGCCATACAACCCTAAGACTTCTTACCCTACAAGGGAACAA

GCAATGGATCAGCTGCACCCATAGGAAAGACATCCCATC

>Unigene71456_A-W 1 220 LEN=220

XGCGTGGCATTTGATGACAGCTATGAGGAACCCTTTCCCCCAAAAATGACCACGCTGACC

CTTGCCGAACACCTCCCTGCGCGGGCCCCGGTCACGTTCTCGGCTCCCTCTCTGCCTCTC

GTCTCATCCCATGGATGCGAAGTGGAGGCTGTGTCCTGGTCACTGGCCGGGTCCCAGCAG

GGTGCGTCTCCTCCACAGCTCTTCGCCTTCCTGGGCTTCCTX

>Unigene71571_A-W 1 282 LEN=282; minus strand

XXTCCTGAAATAGCAAATACAGAAAGCAACTACTTCCTGTATAGACTGAGAGTGAAGGAA

GAAATTAAGAACTTAGAAGAGGCCCCTCCGCCCAAGGATCTCTTTGAAGAGGGTAAAGTT

GCCATAGGAACCCACAGCTTAAGGATGGTGAAGAGATATGCCAAAAGGTGTGGGAAGAAT

TGGCCCCTCAAAGAGGTCTGCTGGATTGCAGAGAAACTTGCCAGAGAGTTTGAATTAACC

AAAACCAGGCCTTCTGTGTTTTAAAACAAAACAAAACAACAACAX

>Unigene71706_A-W 1 157 LEN=250; minus strand

XXCCGGGAATTTGGAGAGTTAAAAGAAGGTTGCTTGGCAGTAGTGATGCTTAGGCTAAAA

CCTGCTGATGAAGAAGATTTATCTGGGCAGCAATTATTGGTGGCAGAGGACTTAGAGAAA

ACAGCCTGTACCAAAGGCCTAAGAGTGAGAATGACACAG

>Unigene71782_A-W 1 207 LEN=207

TGGTGGATCAACTAGAAAGCCATGCAGAAAAAGATGTACCTGCAGCTCTCCAAAGAACAA

ATTCAAGGTAGAAAGAGGCCCTCAGCTGACACTCTGCGAGTGCTGAAGACTAGAACACAG

GGGAGGTGGGAAGAGGCCCTCAGCCCACACCCTGTTATTGCTGAATGGCAGTTTACAGTG

GCTGATGTTTCTTTATCTTGCTTCACA

>Unigene71802_A-W 1 256 LEN=256

CGAGGGCGGAAGGGGAAAAACTTTATTTTTTCTCTTCAGGCGCTGAAGCGAAAGGTTGGC

GACTGCGCCATCGTGGGCTTCCCTGGGAGAGCCCGGCGCGACCTCAGCCTGCTGTGGCXG

CAGAGGATCCGGGCCGCGAGGCCCGACGGGGCGCCCAGCATGTGGGACCCGCAGGAGTTT

GAACGTCACTGGAAGGCCGAATTCCCCGGGGAGGATGCACCGGTCATGAGGCTGGAGTCG

GTGCTGGACATGGAACGX

>Unigene71811_A-W 1 250 LEN=250; minus strand

XXAAGAGGGAGAAGTTGGATCCGTCAGGTTCCCATCAAAGATGGAAAGTTTAGAAATTCT

TGGATGCCACGGAGTTCGGGGTTTGGAAGTCCTCCCGCTTCATCAGGCTTGGTCATGAAG

AAGCCGGCCTGTGTCCAGCGGCTCCCAGCTCCAAGCCGACTGAAGCAGTTAACAGACGTG

GCCTCCCTGTTCTGCTGCCTTCGTATGAGTGATGAGAATCGATTATTTTGAATTTTTCTT

ATTGGGCGGGGC

>Unigene71893_A-W 1 241 LEN=241; minus strand

XAGAGCCGGGACCTTCGGAAGCGGTCGGTAGTGCAGCGGTGGCGGCTGCGGCGGTGGCTG

CCACGGAGGGGCGCCCCGGCAGCTGGGGCTCGGGATCGCTCCGAGCGGCTGAACCCCGTC

CGCACCGCCTCCTCCTCCGTCGCTGCGGACGGCCGCATGAGTCGAGGGACCATGCCCCAG

CCCGGAGCGTGGTCGGGCGCGAGCTGCGGCGAGACGCCGGCGCGGGAGGGCGGGAAGGCG

GCX

>Unigene71960_A-W 1 226 LEN=226; minus strand

XTAAGAATACAACAAATCATCCAAATCCTTCACTCCTGTGTATATACAATTGCCATCTGG

CTTAAGAAAAATAAAACACTTAAAAATGAACAGAAGGTGGCAGACAACATCCATATAAAG

TTACTTCAAGTAGAAAACAAAATGAGAGTCAAAACATTTCAGTTGATGAAAAGCTTCCCC

AAAACATTCAAGTATAGAGCTGAAGAAAACTACAGCACAATGCCCCAX

>Unigene71994_A-W 1 255 LEN=255; minus strand

XXAGAGGAGGAGCCTCCCACTGCAGCCAAGGGCGACCCCTAGGCCACCGAGGGAAGGCTG

GCTCTCTGCGGCAGCGTGGGGCTTCTTACGGGAGCAGAGCAGAGCAAGGCTTTCTCGGGA

GAAGCTGCTCCCCGGGGCAGGGCACAACCCAGTAGTCCCCGGAGTCCTGCTCACCTGCAC

CCCATCCTGGGCGAAGAAGGCACGGGCATCGGCCTGCATGGCAAGCTGGAGGCAGGTGGC

GTCCCCCCACAGCGGGCX

>Unigene72015_A-W 1 302 LEN=302; minus strand

CGCTGCTTCCCTGCAGGAGGGTCACAGTCAAAGAAGGTGCTTGCAGAAGAAGGTGCCCGA

TTTCGTCTGCGTGGTGCTTGGTGCTCACCTGCTTTAAGTCAGGATCCCAATACAGACCAG

GGCCTGCCTTCAGCTCACCCCCAGTCCACATTACCAGTTGGAATCACGATGCCCCCCAGG

GAAACACTGGCCACCATGTTGAAAGAGTACATCAACTAAGAAACACAGCTGAGATTGACT

TTCCGGAACAGATGGGATCTGCATTTAAATGCTTTCAATTTATTTAAGGACGCAAGAGAA

GAX

>Unigene72099_A-W 1 399 LEN=399; minus strand

XXAAAAAAAAAAAGAAAAGAAAAAGCTCATCTGATTGCAGGGCTGTTACCCAAAACACAC

ACACAACTCCCAACTCAACTAAAAGAAAGCAAGACCAGCCTGATGAACAACAGGCTAAAG

ACCTTAACAGACACTTCATAGACACCTCATGAAAAAGAGACACAGATAACAAACAGCATG

TGCAACGGTGTTCTGCATCATGTCATTGAGGAAAAACAAATTAAAACAACACCACTGTAC

ACCTCTCAGAAAGGCXCCAATCAAGCCCTGACCATACCTGCTGCTGGTGAGGCTGTGCAG

CAGCGGGAACTACTGACTGTTGCTATGGGAATGCAAAATGGTGCATACACACTTGTGACC

CGAGATCAGCAGGCCAAGGCAAGAGGAACCCACAGTCAAGGC

>Unigene72289_A-W 1 314 LEN=314

AGGGCAGGCATGACAACTGTGAAACTACTTCCTGTTGTGATATTTTTAAAAGGTTACTTT

GAAAGTTATTGTATTCTTTTTATAGCAGAGGGTGATTTTCAGGCATATTTATTAAATAAT

GCAATCAACTTACATACTCCAGTGGTCTCTCAGCCTTTTCTGTTTACTTCTGTGTTGGCC

CTCAGAATTGATGGGAGACAAGAACATCAGGACATCAGTGAAGGACAACAGGAGTCTCTA

GCAGCATACAGGCTGCCCATAACAGATAGCAGTGAACAGAGGGAAAGCCAGCAAAATATT

CAAACCCATGGAGGX

>Unigene72311_A-W 1 218 LEN=218

XGTAGGGCTGAGGACACCATCGCTTATAAAGACCGAGAATCCAGTGGAACCTGAGAGATC

CTTCAGTTTCCTGGGAAGCTGGTAGAATGGCTTCTATAGCTGTCCCCCTCCTCCAGTGGT

GTTCACACCTCAGAGATCCTTGAAGTTTTTTTGGGTCACTTTAAGAACCTTCTGGAGAAG

GAGGATGTTGTCTGCTTCAAGGACCCCAACCACTTTGAC

>Unigene72436_A-W 1 213 LEN=213

XTGCAGCTCAGTGGTAAAGCACCACAAAATAATAATGACGATGATGATGGTGAAGGAGAG

CCATCAAGACTGATGGACAAGGAAAGGTGGAGAAACCACGGCAGGCCGGAGGAGACTGAA

GAGACGTGGACACTGTGGCACCCTGGACTGCATCCCAGAACAGACAGAGACACCGTGAGG

GAAACTGGAAAAATAGGAACAGAGGGTTCAGTTAXX

>Unigene72529_A-W 1 328 LEN=328

XGCCCTGTCAGCTCCACCACCAATGACTTCTGTACCCGTCCACTTTTCTTCACTTCCCCT

TCCACAACCAGTCTCAGCCACGTTATCTTCCGTTATCGCGGAGCTTCCTCTTTTGTCCCC

ACAGCCTGGAGTCCACGAAGCAGCCAAAGGGGTGGCCTACACACACAGAAAGCAGACCAC

GCCCTCCGAGGGCGGATCCAGTTCCTCGGCTCGGCCCGAGAGGAGGCCGCCTACCACCTG

GCCTCCGCCGACCTCTACACCTCCTCCTACCGCTGTCCCCGGCCCCCGCCGCCCGGCTTC

CAGCGGCTGGTCAGGCACATCAAGTCCGXX

>Unigene72653_A-W 1 216 LEN=216

GTCATTGATGATGCAATTGAACAGTCACTATTAAAACCAACCTTCACCCCTAGTGGGCGG

TTGTTAGAGCCACAGGCCTTTAAGCTGTGGCAGCTGTGCCTGCCATTTCAGACGTTACAA

TCAGACAGTATTGGGTATCCGAAATGTGAAAATCTCACTCAAAGAAGGAAAGTGTTCATA

GAAAATGACCCACAAAGCAATCCAGCAATAAGAAGC

>Unigene72733_A-W 1 309 LEN=309; minus strand

XXCCTCTACCTCATTTTCCTGGCATTCCTTATATGCACATACCTATTCTCTAACAATCCA

GCATCCATGCTGCCCTCATCAGATCCCTATACACCTGACAGCCTCTGGCTTGAAATTACC

CCTGCTAGACTACTGACCTCTTACAAGAGTCAGGGTATTCACCAGTTAAAACTGACATAC

GATCGGACCACATGCCTCCCACAAGAGACCTCAGAACTTACTGACATTGACTTTGGCAAA

TTAAATAACATGGCATAAAACTGCTCATTTTCCCTATCTGCCCAGTTTTCCATTGCAGTG

GGAAATACCACX

>Unigene72957_A-W 1 203 LEN=250; minus strand

XCTACGAACTTTGCCACGTGGGAGCCCTCCTACTTGTCTACAGAAGAGCTTGCTGAAAAT

TTGGGAGAGGAAGGACTAGAGCAACAGCCAATACAGGATGATTTATACCCAGATCAAAGT

CTCCTGAGCTATGTTGATGAGAACTTTGTCAACAAGGTGAGCTGGGCCTACAATCTTGGT

GTATACAAGGTTTTGGCACTCATG

>Unigene72987_A-W 1 221 LEN=221

TCTAGAAAACGAGAAGGGAGAGGAGAGTGTCGATTGAAAATTTTTGTAGTGGTAAAGAAG

ATTCCAGGGAATCTAAAGGTAGTCCGAGGAAAAATAATTGGACCCACTGACCGAGATGAC

GGGAGGCTATCAACATCCTATCATTATTACAATATCTCTTAAAAAGGAGCGCTGTCCAAC

GTTGGAAGGGACCGCTTCCCAATCACCATTCCTGCGGCGGGX

>Unigene72988_A-W 1 294 LEN=294; minus strand

ATTCTAGAAAATAAATCTCGGACAGGAGAGGCGTATGAACGAGCTGTCTTGTTTGCCCTC

TTCCGTGCTCTGCAACCAATAATGACGACAAAGACCACGGTGGACTCGGAAATTCTTTGT

GGCCCACAGAAAAATAAGCAGAGGGAAATGACGGGGAGGGTTGCGGGCACCCGCGTGGCA

GAAACCTGCCCGACACCTATTGTGTGTGCTGGAGAGCCCCGTGGTGGCTXTGGCAGTGAC

AAGGACAATGTACAAACCCACTTGAAACCAGCTGGATTGAAACCCCATGCAGAACXX

>Unigene72991_A-W 1 280 LEN=280; minus strand

XTCTCCGGAGCCGTGTTCGGTTTCTGCCCCAGGCCAGGAGCCTCCCCACCGTGTGTCTGA

GGTGGCGCGGTGCCTGCTGGTCTGCTGCTGATGGTGAGAGCCTTGGTCGACGCCATTGTT

TCCATCGCTCAATCTGGCGAAAGTCGAGCTTTTGGCTTTGATCATCTTACCTGTTTGGAT

TTCATTTTTTCTCGAGTGTTTCTTCCTTCCAGTGACTTTGGCTTTGACTTGCCCTTCTTC

TTCCAGCTGCTCCTGGAGGAAGCCTGCATCTTTCATCTTACX

>Unigene73013_A-W 1 283 LEN=283; minus strand

CTAATGTCTGATGTATTCATCTCCTATTGCTGTAACAAATTACTACAAACTCGGTTACTT

GGAACAACACAAGTTTATTTTCTTGCAGGAGGTGAGAAACTCAACATTAGCTTCAGTACA

CTAAACCCAAAGCGTCCAAAGGGCTGGTTCTTTCTGGGAGATTATTGTGACCCCAACCCC

TCTTCTGTTCCTGCCCCCTTTGTTCTTCTTGGCCATCATGAAGTGAGCAGTTTTGCTCCA

CCACACACTCCCTACCATGATGTTCTAGCTCACAACAAGCCCAXX

>Unigene73102_A-W 1 228 LEN=228

GTGTGGGGTGGCTCTGAACAGGAGCTGAGTACGCACCCCTCAGCTTTGAGTAATGGGGGT

GTGGAACTGGCATCCCACGCAGCACGAAGCAAGCGGGACTTACCTTCGCAGGTAAGGAAG

TACAGCTCTGGGATTGGGCATCACCCAATGAGACTGGGCTACAGCCCCCTGTTTGTTGCC

TTATTTGGGTGGAATATTCTATCAAATGTCTTTCCCCTAATAAAAACA

>Unigene73171_A-W 1 141 LEN=214; minus strand

CCCTGGATGAGCAACCTGGCCACCTCCGTCTGTGGCCCGACCTTCAATGCTAACTACAGC

AGTAATGGTAGTGCTATTGGAAAGCATCCAGAGCTGGAGGTAAAACCAAAGCAAGCTCCA

AATGCCATGATGATGAACATG

>Unigene73243_A-W 1 215 LEN=215; minus strand

XXAGCCATTCCTCACACAGGGCTGTCAGGAACCCAGAGCAAGAACCACACTCCCTGCCAT

GGGAGCTTACGGCCTGAGGGTCCACTGGAAAGTCAGGCCTGGCAGTGTGCCTGGTGGGCC

GTGGAAGGTGAGAAACTGTACTTTCTTACTTCCTTATTTGCAAAGTCCACAGTGGCGATG

GCTTGCTGCAGGGGTACATTTGACAAGATTGACAAACXX

>Unigene73417_A-W 1 225 LEN=225; minus strand

XXTGTATCTTGTGTAGCGGATTTTCCTAATTTACCTATATATGGAAAGTTCGAGATTCTA

CAGAATCCTGATAGATCCTTAGAAATACGGAACCAATCTTATCTTACTATAGATAATTGG

TGGAAAGTCAATACCTTAGGTACCGCCACTTGGCGATCACCTGTTGTGTTGACCGACCCT

TTACCACAGCCATTTACCCATAACTTGGTTAAACCAGTGGCCACGCCX

>Unigene73664_A-W 1 238 LEN=238

XGGCTGGAGGAAGGCTTTGAAGAGAGAGACGTGTGGAGTTAGTGCTCAGAAGTGGAGGAG

CACAGTAAGAGTGGGGAAGACTTGGAGCCCACCAGGAAAGCGTGTGAACCAAAGGACAGG

GCTCAGAGAGACAAGGACAAGGACACACAGAGAAAGTAGACCAGCAATTCTAAAGAGCAG

CAAGTGGCTGCTCTACTCAATCGAAGGCATCTGGAGGGCAGAGGTGCATTCTATTCAGGX

>Unigene73740_A-W 1 256 LEN=256; minus strand

XCGCCCGGCTCCCCAGCCGCCTCCGCCCGCCGCAACAGCCGCGGCCGACAACTCCTTGCG

CCGGGTCAGCAAGTTTCTCCGCGAGGCCGGATTCCGCCGCCCGCCCCTCACCGAGACCCG

GGACGCCGCGAGCGCCCTCGAAACCCTTTCCTGACAGAAATGTCCCCCGACTTCTACGTC

GCGAGCCTAGCGCAGACTAGTCGCCTCGCGACCGACATTCAAAAACTACTGGAACCGGGC

CCGGGGGAGATTCCCGCX

>Unigene73749_A-W 1 227 LEN=227; minus strand

XTGGATAAAATCCCTGATGGTGGTTTTGCAGATGTATTGGCCTTGTACGTTCATCAGACA

GACAGCCCAGAAAGAAGGGAGAGCAGTCCTGTCATCGTCTGCCTGAGTGAAACTTCCATC

TCAGAAGAGGCACTATTTCTCTGCCAAAAAAAGAAACAGGATAAAAATTCTAAATCCTTC

ATCCACAACTGCCTACATGAGCCCAGTAAAGCCTTCATCTCCCATTTC

>Unigene73750_A-W 1 243 LEN=243; minus strand

XCAGAGGGGAAACTGAAGCTCAGGGAGGCGACCTGTCACGCCCAGTGCATCTGCCACGTG

GAGATGGCAGGACCCACACAGCGGACAGGGCTGATTCCTAACGCCCCCAGCTGTGACCTG

GGCAGCCACAGGGCTGGACCTCGAGCAGGGGCGCCCTACCACAGCTGTTCCCTGACAGGC

GGTGCCAAGGACCACTGCCAGGAGAAGGCCCTGGCAGGTGGCAGGGTCAGTGGCAACACT

CGCAXX

>Unigene73766_A-W 1 252 LEN=252

GGGCCAGGCTTGGAGAGCCAGTGTGAGGTTGGTTTAGTGGAGATTTGGTTGGTGAACATT

GGGAGCATGTTGAAGAACTTTAATCAGACCAATATATATTGTGCATTTCCCCCCAACATT

GAACATTATAGTGGGACCATGGATCTGTTTGGAGATATAGATGACATTTCTTCAGAGAGT

GATGAGGACAATCTACTACCCATTCCAAGACAGCCTGTTGTAAGTACCATCATGAATCTC

AATTGATCTCAT

>Unigene73868_A-W 1 351 LEN=351; minus strand

CCCATTACCAAAAAAGAAAAGAAAAAGAAAAAAGGCTGCAGTTCTTGACAATTTTCAGGT

GACTGGCCTCTGAGCAGTGACCCAACAGATGACCACAACCTGAGAACAGGGATCAAACTG

GATGGAAAGAATGCTGTTCAGCGTGGGGCTGTGAAGGATGTGCTTGCGGGGGAAACACTT

GGAATTCACGAGTCAATACGAGCAGACTGGGATATGTCCCCATACCAGCTACTTTTGTCA

TCTCCCTTAGAAGCCACAGTTTTTAGACTACCGAAGCGGTCACTCCTACAAGTCTCTGTT

GAATTTGAGCACTTTCCCAAGATAGCAGAAAGTAAGCTGAAAGGTCACATX

>Unigene73883_A-W 1 223 LEN=223; minus strand

CGCCCCGTGCGTCCTTCCCCGCGGGTTCAACCCGGAGTGCGGCGCTCTCCGGGTCCCTTC

TACCCCGGTGCGCTGTGGCCCCGCATCGGGCTGGAGCGGACCCCGGGCTCTCAGACGCAG

GTCCTGATGGCCGCGCCCGTTACTCAGCAGGTCAGCGGCGGCGCCGCCCCGTCCCCGAGC

CCGGCGCTGGCCGGCCCTGAGAGCGGGCAGCCCCTGGCGCCCTXX

>Unigene73997_A-W 1 264 LEN=264

XGTTGGAAGAAGGGTGATGGGAAACCTGGGAAGAGTGGGGAACCTATAGCCTTCATCATC

TCACCACCCACCTCTCTGTTGGGTCAGGAGGAGCCCACTGCCCTCAGCCAGCTTTCCTGG

AGCATGTGGACAGTGGGCCCAAGATATGGGATCAAGTGCCAGGGAATGAATAATTCCTGC

CCTCGTCCTTCTAGGTCACTGGAGCTGGCATATCTAGTGACAGGCATCAACTACACTGTC

ATGATCGAGCAGGATAGAACCTAGGXX

>Unigene74217_A-W 1 246 LEN=246; minus strand

GCGAGTCAAACACTTTTAGTCCCTGCAGCAAATCAGAAAGATTCCAACCAAGTTTCTACC

CTGGCTGGTGACCCTGCCACGGATGCAGGAGAAATATTCAATACTAAGGGAATAAGTGAT

AACAAAATACTGAGCCACAGCTACAGTGGGACAATCCAGAAGGAAGAGGGAAAGTACCAG

AATTTTGCTACTTGTGTCACACAAGCTAGAAAAACCCTACCTCTCCCACTGGAGAAGCAA

AACTAC

>Unigene74594_A-W 1 234 LEN=234

ACACCGCAGGACCTGGCTGAGGATGTACGAGGCATAAACTTCTCCATTCTGGTTTCAAGG

CCTCTCACACCCATAGCAGAGATAGGGACTGTCAATAGTTGGAAGGACCAGAGAGTTAGG

AGTCTTGGGGTACAACTGGGACTCTGCCACTTACCAACCAATCGTGCAACCATGGGCCAA

CCTTTATCCCTCTCCAAACCGGTTTCCTTAGTTGATCAGGACCAAGAGCCCCAA

>Unigene74618_A-W 1 244 LEN=244

XGTCTTGTGATCATCGGTCTTCATGATGTGGTGTGTAACAGGCCACTGGCTGCAACTTGT

TGCGTTTTCTTTCAGAAAATGATGGTTTTTCTCCTTGGGACTTGGTCAGAGCCTTGGAGT

GTACGTTGGGATTACCTGGCTGCAGCCAGCAGTATCAAGAGGAACAATACTGTCAGCCTT

CTGACATCCCACCTGGACACTCTTCCACGCTCAGGATCCTCGCGGTCCTTCATTCAGCAG

CAACGX

>Unigene74690_A-W 1 286 LEN=286

XCACCCAAACACCCATCAGTAGCAAAGAGAGCCAGTGAATTGAGCATCAATGGACAAGAG

TGCGCCGCGTCCCGTGGCACTAGCCGCGATATTGTGTACTAATACTGTAATGACGAAGGG

TTCTCACCAACAGAACGACGAGTAAAAGACTGTAGGTGGTCTAATTCCTTTTGTAAAATC

TTTAACAACTGGGCTGGATTCTTAAGCACACTTTTGGAGGAGATCAAATCCCTAAAAAGT

ACAGGAATGATCACAAATGTCACTTTAGTTTTGGCCGCAGAGGAGAGX

>Unigene74699_A-W 1 218 LEN=218; minus strand

XXTATACAAGAACTCTGGGACAACATAAAAAAACCAAACTTAAGATCCATTGACACTGAG

AAAGTAGACATACTGGCCAATGACACTGATAACATATTCAGTGAAGTAACAGAAAATGTT

CCAAATCTTGGGATTAAGCACATAGGACATATCCAGAACTTCAAATGGACAGGGCCAAAA

AAGAATCTCTCCACAACACATTATAATTAAAATACTAAACXX

>Unigene74711_A-W 1 270 LEN=270; minus strand

XCTTTGATGATTGTGAAAGGGGAGATGAGAAATAAGATTAAGCATGTGGGTTTATGCACA

GGCCCATTTTATCAGGGAATTCAGTGTCAGCAAAGACCAAATAAACATGGAAGTTTAGGA

TCTGGAAATTGATTACCCTTAACCTTGCTTACCTCCATTCCCCTCTTCAGATATCTTGAA

GGTTTTGTGAACCCCGCTATAAATATTGCTGATCTATACAATAAATCACCCAAGTTCTTA

GAGATGTTAATGGACATTTCTGAGATTTCATXX

>Unigene74736_A-W 1 111 LEN=285

AACACGCAGCAATCCATCCTGCGCCTGCGTTTCATTGAAACCACGGATGCCAAAGGCGGA

GGAGGGATGAAGGCCGTCTCCGAGAAACGACAGGTTGTCTGCGACGGGGAC

>Unigene74759_A-W 1 289 LEN=289; minus strand

TGTTTTGAGGTAAGAACAATGGAGGCTGGGAGGCAGGAATCAAACAGGGGAGGAAGCTTC

CGGGTACAGCGGTACAGCTCCCGGGGCTCGAGGGTCAGAAGGGGAGGCTGGAATGGACCC

AACGAGCCAGGTGTGCTCTGGGAGGAGGACCAGCATGCACCCCCGCCTGGAGGCAAACGG

GAGGCGGAGGATCACCGCACTCGAGTGAGGACAGAGGGGTGGCCAGAGGCCGGCCAGACT

GCGGAGCCTCTGCTCTTCCAGTGGGTGCTGGGGAACCACGGGAGGAGCCXX

>Unigene74827_A-W 1 211 LEN=211; minus strand

XXCATAAAACTACCAAGCTTCTTCACAGCCAAGGAAACAATCAAGAATGTTGAAAAGATC

CTACAGACTGGGAGAAGATTTTTGGCACCTACACCTTGGATAGAGCATTAATCTCCAAGA

TATACAGAGATAAATCAAGATAATCAAGATAAAATTATCCAAGATATACAGAGAACTCAA

AAACACTCCAAAACAAATAACACAATCAATAAA

>Unigene74948_A-W 1 231 LEN=231; minus strand

XCTGGGGGCCATTTTCTTTCTAAATAATTAACTCTGAAAGGCATTTTAAAGGGGTCTCTT

CAGGAAGTCAGACAAGAGAGGGAGAGAGTTGGCATCCAAGAGAACATTACTGTGAACCCT

GAGTGGGATGTCAAGTCAGCTGCTGCTGCTTTCTACGTGATAAGAAGACATCAGCGCAGT

TCCAGGACGGGAATGATGAGAAGTCTGTCCGCGGTGGCTGCTTGCAACATACXX

>Unigene74951_A-W 1 258 LEN=258; minus strand

XXCACAGGTGTGCGCCACTGTGCCCGATGCACACAGGTAGCTCCTGGAGCAAGACACATC

ACGGGAAGTGAGGACATTCCTGGACCTGCTACCCCCATTTGTGGAGACACCATTGACTGG

AGACTTAAGATTTCTAACCAGAAATTTTGGAGCAGTTCAAGAAGGAACTGAAGGCACAGT

CCCCAGGTTATTGGCCATCTCCCAATAGCAGAAGAACATGATCAACCCCCAGGAGTTGCT

GACACTGGAGGACGTGGCCGX

>Unigene75050_A-W 1 266 LEN=266; minus strand

XXCTCGGGGACCGAAGGGAGGGTGCAAGACCCAAGCTCACAAGGGCGACACAGGGCACGG

GGCATGGCACCTGGGCGGGCAGGGAAGGAAACGCAGGAGAAACTGGAAAATCACCATGTG

GACAGGCGACCTAAAGGAAACAGCAGCCACATGAACGAGACGACCCTCCACATGCAGCTG

GTGCTCTGGAGGCAGTCTGGCAAACGCACCCATGCCTCTGATGTGGCAGCTCCATTTCCA

GATGGAATAAGGGTGCTGCACTCCCTCGXX

>Unigene75162_A-W 1 331 LEN=331; minus strand

XGTGGACAATGGAAAGGATCTGCTTGGAGATCTGAAATACCAGCAGCATGTTTAAAAACA

GGAGGGAATGCCAGGGGCCAAGACCACGGAACCGCTTTTCTCAGGAAGAAGTATTGATTG

ACTGATTTGTTCGCTGATGAACAAGGGAAGAACTTACTCACGTTAGAAAATACCCAAAGA

AAATCCTCAACACAGGAGCAGAGTCTGGAATTAACGCACACAGGTGGCAGATCTGTGTCC

CGATGTTCTGGGGACGAGGAAGTGCAGGCACAGCGCGAGAGAAGCTGTCTGTCCACAGAG

GTGCGTCATGTGAGCCACCTGGACCTGTCCACX

>Unigene75198_A-W 1 271 LEN=271

XAGGGGTTAATAGATTTTAGAGATAAGAATAGGTGACTTGCTGACATAGAAGTGGATGTG

ACAAAAAGAGTTGTGGCCAAAGACAAXTTCCGAGGTTTGGAGCTTAAATGGAAAAAGGAT

AAAACTGCCACTGACTGCAATGGTGAGGAAAAACAAGAACAAATTTGGGGTGAAGGTCAA

GGGCTCAATATGGGACATTATGGCTTTGAGTTGCCACTTAGAAAACTCCCCCTGCAATTT

GCTGGTGGAAATAGCAAGGAAAGGAGGCAGCAG

>Unigene75230_A-W 1 203 LEN=203; minus strand

CTGGTGGAAGAGGGGGATACTCTCTTAGAAGGATACACAGAGCTAATGCTGCCAGTTTCT

TTTGAGTGCAGCAATGTGTGTCAAGAGGGGGAAGGAGTCCTAATTTTAGCCAAATATTCT

ACAGGTTCATTCTTCCAGGTTGCAGATGAGTGCATAAATTGACAAGTCCACACAGCTATA

TCCAGCAACAAACAAGCAGCTATX

>Unigene75446_A-W 1 301 LEN=301; minus strand

XXAGGCCGGTGGGATGGAGGCAGCGGGATCGGCTCACGGCCACGGATCCCGGTGAAGCGG

GGCGGGAGCCGGAACCGGAACCGGAGCCGGAACCGGAGCCGGGCGGAGCCAGAGGCGCAG

ATCGGCGCGGGCCGATCGGTCCAGGAAACCCGCGGTGTGGCCCTAGGCCCGGTGCCCGCC

TGCCCGCCCCTCGCCATCACAGGCGGCGCGACCATGGCCACGATGGTGCTTCCGCGGGAG

GAGAAGCTGAGCCAGGATGAGATCGTTCTGGGCACCAAGGCCGTCATCCAGGGATTAGAG

ACC

>Unigene75648_A-W 1 219 LEN=219; minus strand

XXAAAAAACGCCATTGATGTGACCTACTTGCTGGAGGAAGGATCAGGCAGGAGACTGCGA

AGGCGTACTTTGTTTATCCCAGAAAACAGTTTTCGGAAATGACCCTCAAAGAATGAGAAC

TTCAAGCATCTGGGATCCAGTGGAGCTAATCAGTTCCGCTTCCTGCTCTCTGGGTATAGA

CAGGGACAGGAAGGGTCCGTCCAGGCAAGGGAAATGGGGTCX

>Unigene75651_A-W 1 243 LEN=243

XGGGGAACTGAGAAGAGAGTGGGGCTGTTCCCAGTCCTGACCTGCAGCGAGTCGAAGGGC

AATCTCCTGAAGCTATGTCTACTCAAGTCCCCGGAACTGAAGATGTTATCTGGAAAAAAG

ATCCTTGCAGATGTAGGGGAACATCTTGAGAGGAGTTCACCTGGATTATCTGGGTGGGCC

CTACATCCAGTGACCATGCAGGCAGAGCTGGAGGGACCCAGCCACAGATCAAAGATGCTG

GGAGXX

>Unigene75684_A-W 1 277 LEN=277; minus strand

GAAAGAAGTGGCAGGGTCAAAGTCAAGAAAGAAGTGGCAGGGTCAAAGTCAATCAAGGAG

GCTGACAACACTATACTGCTGGCATTCAAGACGGAGGAAGAGGCCACAAACAGGAATACA

GGTGGCAAAGGCAAGGAAACAGATTCTTACCGAGAATCTCCAGAAAGAATCAACTCTGCT

GACACCCTAACGTTAGCCCAGAAGCCTGATTTTTGGATACCTCTAGAACTACAAGTAATA

CATGGACATTTCTGGTTTTGCTTTGCAGTACTAGAC

>Unigene75850_A-W 1 227 LEN=227

XXGTCCACTGATCGCCCCGCGGGAAACGCGTTTGAAGATGACCGCCCCACCACGTCCTTC

CCACCCTGGCTCTTTTCTTGGGGGCATGAGTTTCTGAAGCCAGTGTCTGTTTCAGAACAC

AGCTCCTGGGGTGCCATGGATGGCAGTGGAGGAGTGAGGGCGACCCTCGACCTCCTGCTC

CGCACAACCAACGAGAACACAGCAAACGTGGCCCGTCAGCATTCTCGGGXX

>Unigene76203_A-W 1 403 LEN=403; minus strand

XXCTACCGAGAAACTGGAAAGAAGCAGTTCTGTCTCCCCGTCTACTGGGGAGCCCGAACT

GTCTGTTGTGTTTTAGATCAGGTGCAGAAAACTGCCTGTCGAAGCCGGACTAGGCAGGTG

AAGCTAACACACTGCGTCAACAACATTGAACTCTGGGACAGGTCTTTGAACTTTCAGCAC

AATAGCAAAGCTCTTGAAGACCTTAAAACATATGCAATACGGGCAGTGTGGTGCTTACAC

CCAAGGTCATTACCAGAGGCCAAGGGCTGGACAGTTTACCCACTGAAAACCAAAAAAACT

ACAGCTTTGCTTGATTCTCTGAAGACTTACCTAAGTTTGGATATTAGATAATATACACAG

CCACAAACAGGTAAAACTTCTTATATTTTAAAACGTAACTTCAGC

>Unigene76239_A-W 1 235 LEN=235; minus strand

XCGGGCATGAGTAAAGAGATCAGCGTTCAAATCTCAGTTTCTTTACCTTATAAAATGGAA

CTAATACCCACTTACTTTAGAATTGTTATAAATGGCAAATATAATAAGAAAGGATTAGCT

AACACAGGCTCTGCTGTCAAATGCCTGCAACAGAGCTTTAGAACGTATGGTTTCTTTGCT

GCTTATTTCCAGGCATATCCACTAGACTTGGTTTCTAAGCTAGCCAATTCTTACAAX

>Unigene76256_A-W 1 264 LEN=264

XAGAGAAAGAAAACTGTTATGAGGGAGCAAAAAACAGGTATAAAGCATGAGGAAGATCAT

TCCAAAGAGAACACAGAACAGTTAGAAATATAGGACAAGGTAAAGAAAAGAGGCTTCCTA

AAAGTGAGTGATGAGTGCCCTGATCCATCCTTCAAGTCAGGTTTGGTCTTGAAGGATGGA

TCTGGGTACTGGCAGCCAAACAGGACACTGCTGGGAGAAGCTTCAGGAAAGGTGCAGCAT

GGGAAGAGGCAGGGCAGGAAGTATGXX

>Unigene76490_A-W 1 242 LEN=242

GCTAAAGAAGGAGGAGCTGGAGGTGACCTGGGTGTTTGGAGTGGTGACAATGGCCTGGTG

TGCAGTTGGTTACTTATTGCTTGGGGAGAGATTTCAGAGTTTAAGTGTGCATGGAAAGGA

GTCAACTTGGAGAAAGTTAAGGATGGCCAAGCGGAAGGAGTTCTTCCTGCAGGGGCCTTG

AAAAGGTCCAATATTGGGAGCACGGAGAAGGCTCCCAAGCTGCAGGGTCTGGTGGGTTTG

GGX

>Unigene76527_A-W 1 307 LEN=307; minus strand

CAGAGCTCACTCAGTTCTCAGATACCTTGCTCCAGGGCAGAGGGGCTCCTGGAAGGGCAG

GAGCAGGAAACTGACAGGAATCAAATCCTCTATTATGTGCCAGGCAGTATGGTGGGTGCT

ACACATACTTGATCCCTCTCAATCCACATTATTAGCATCATCCACATTGTGCAGAGGAAG

AAACTGAGAATCCGAGAAGTCATAGAGCTTGCCCAGGGTCATATAGCTGGAAATGGTGGA

GCCAACATTCTGGAAGCAGGTCTTCCTGATTCCAGAGCTTGGGCTTTCCGGGCTTCCTTA

TACCCCCXX

>Unigene76608_A-W 1 222 LEN=222

GTCTTTGGAGGATCTGTTTTTCTTTCTTCCTTTATCATGGCTTCAATGCATGATAAGGAA

ATTGATTTGGCTTCAGCTATTCAAGGACCAAGGAGACTCTCAGTTTGTCCCAATCTTGAA

TTTCCAAGGGAGGAGGATCATTGGCCCAGCTTGGATCCATTGCTGGCCAAAATTGTACCT

CACAAGTATAACCAGGGAGATGAACCTACAAGAACAGAGCAT

>Unigene76680_A-W 1 276 LEN=276; minus strand

XXCCAGGAGAGCCATGGGCCAGGGCAGAGCACCTGCTGCTCATTCTTCTGCAGCCTGGCC

CCTGAAAATGGACTGATCATTGCAGCAGAACCAGCTCCAGAGCAGATGTTGAGATACAGT

GGTAAGTTGGAGCTCATACAGTGCTTCAGAAACTCGGGTGTTTCCAAGAGAACATGTCCT

GAGACACATGGCAGAGGTGGCACCCGCATTCCTGCCCTCAGGCTGCTCCTGGAAAAATCC

ACAGTGGAGACATGGCTGGCCAGCACGTCCCACATGTGX

>Unigene76737_A-W 1 93 LEN=279

CGCGGCCCCACCTACGTCTGGACCTTTAACCTCAAGACTAAGGAGAAGGCAGCCAAGTGG

ATCCTGGCGGCGGTGGCGCTGCTCCTGCAGGTG

>Unigene76771_A-W 1 291 LEN=291; minus strand

XXCAGGACTGTTGTTATGAGGGCAGAGAGGAATATGGCTGGCATCCAGGTCACATTTGCA

AGTCATATTGTATGCCTGCCTAATAATGAGAGTGAAAAGAAATGTGCAGCAGCCATGCCA

GAGTTCACCAGAGCCAGAGGCTCAGAACCCTCAAGGATGAGTCTGAATCACGAATCAGGT

AAACTATCCAAGCCAGGTGCCAGTCATTATTGTAAAGGAGTATATGCACACTTTCTTCCC

TTGATGAAGCATAGGAAAGGTGAACCCCATCGTGAGATGGCTCTTAGTCCCAAX

>Unigene76833_A-W 1 237 LEN=237

XXGCCCCACAGCCCAGCTCTAGATCCTGTCAAATCCACCATGATATTCACACCACGGACT

GCACCTGGGCGCTGCCAGCATCGAGCTCAAAATCCTTCAAAGATTAGACCATCATACACG

TTTAAGATACATAGAAAAATAATATAATTAGGATTTATACAAAGTAGATTTTTACAAAAC

AACTTCACTACAAGAAATACATCTCAGATCCAAGCACAAAAACGTGAAGTCTACGTGGAX

>Unigene77100_A-W 1 200 LEN=200; minus strand

CTGAGAAGTAGATATGAACCGAAGATGCTCATTGTGCTTCAGATAGAATTTCAGTGTTTT

CTCGTTGAACTTGATATTTTTAAAAATACCAACAAACGTTTTTATGGAGTTAGGAAAGTT

GATGATGAAGTTTGTATGGAAAAACAAACAAGTAGGAATGCTGAAAAAAATGTTGAGAAA

GAAGTTGCGCAGGACGGCTGX

>Unigene77117_A-W 1 256 LEN=256; minus strand

XGTCCTCCCACAAATGGCCACTCTGAGGTACAACGGCTGTCACAGCATCACACAGAAGGG

GACCCAGAAATTCACCTACATGAAGAAACTTCAGAGACATTTGTAACATGTGTGTCACCA

GTTAGTGACATGCTGACTGTCCCCACACAGAGAGGGACTGAGGGGAAGGGACTGCAGGAG

GTAACAGGCACCAACGGACCACTCCATCGCTGGGGACTCTGGTCCACGAGCCTGTCACTA

GGTCATGGAGCATCTCCX

>Unigene77222_A-W 1 384 LEN=384; minus strand

XXCCTCAGTAAGCTCTGGTCCATCCATTCTGGAGAGAAACCTGTGAGCACCGTCTCTGTG

GTAAAGCTTTCAGCCCACACTCACTCCGACGTGCCCGAAAAACCTGAGGAGTGGAATGAT

GATAGGAAACCCTTCAGTGATAGCTCCCACTCAGGGGAAAGCCCTCTGAAGGTCAAGAGT

GTGTGTCTGACACACGTGAGATCACACAACAGAGGAAGACTCCAGAAATGTGTAAAACCC

TGTGGCCCAAATGTCCACATTACTGACCACCAGACATTGAGGTATGCCCACAGTGACTAT

GGGAAAGACTTGAATGTTCTGGAGCTTGAACACAGGGCCTTGCGCATGAGTGCTCTACCG

CTAGCTACACCTCCAGCCCTTTTTATX

>Unigene77226_A-W 1 266 LEN=266; minus strand

XGCCACCCACAACAAAGAAGTTATATTCTCCACTCTCCACCCCTCAAAGCTTGCAGACAC

ATCTTGTTTCCTGTGGTTGTTATGGGCCAAGCAGAACAATTAGAAAAAGGAAAATCACAA

CTGAACAATAAAGAAAAGAAGTCAGGCCTGGATGCTTATACCAGGCTTGGCAAAAGATCA

CAGTTCATTGAGGAGGAAGCAAACTGAATGAAACTTATTTCCTCCTATGTAAGTTTCCCC

CGAGTTTCTCTTGGGACAAACCTCTTC

>Unigene77359_A-W 1 242 LEN=242; minus strand

XTGGAGAGTCCAAGGTCAGTCAGCAGAACCAATGAGGTGTTCCACCTTGTGGCAGAAGAC

CTAAAATTAAGATCTCATGTTACACAGTGCCTCAGCACCCAGGAGAACCAGGAGGCCCTA

ACAAGCCTTCACACAACTCCTCTCCCCAGTCCTCCCTCGGAAAAGGTCACCTTCTCAGCA

GCCTGCCTCATCAAACGGACCAGATGCAGCTCCTGCTTCTCCCATGGAACTGTCCCAGCG

AAC

>Unigene77395_A-W 1 238 LEN=238

XXTTGCGAACAGTGCGTCACAAAGACTTCATACTCTTGTTATGTTCCTTCCAGGACTATC

GCTATTTCTGCTGTACACACAATTAACCGAGCTGGACTCTCAGAGCTCTGTTCTCCCCAC

AGAGTGAGACGGCAGAACTCCGTGCAGTTCTACCAGCTGCAGCTACGCAGCCTGAGATCT

GGCTATGAATGTGCAAAGGCCATTCAGGAAGCAAACAGAGCCACGAGCAGTGTACACGCG

>Unigene77838_A-W 1 282 LEN=282

XGAAAGAGGAAGTCAGAGGACAGCCACGGGGAGGAGGAGGACGTGGGCCTGGGAAAGAGC

AAGAGGCTCAAACTGGAGGACAGGAAAAGACCTGGAGAGAAACGGGGGCGAGTAGCAGAA

GTGGAAGGAGAAGTGGGAAACCCTGGAGCGCAGGAGGAGAGAGCAGCGGTCACTTCAGGA

CATGCCCCTGGACACGAGGAGACCCAGGAGGAACCCGAGGGAAGAGGCAGGGCCTTCCTC

AGGACCAAGATCAAGAGTGTGCAGACTGGATTCCAGCACGAG

>Unigene77850_A-W 1 381 LEN=381; minus strand

XCGTTGGACGTGGCCACCAGGAAGGACCCATGCTCTGTGGAACCCCGTGGGGAAGCTGGA

GTAGGAGGGACCAGCTCACATGGTGAGGAGCATGGGTTTGTCATCACAGTGAGAGGAAAC

CATGGTGCTCCTCTGGTGGATCTGTGACACATCACACACTGCGATTTTTCAGCAAGAACA

GGAAAGAAGCTGCTGTATCAGGGCAAATGGACACTGAAGCAGGGGTGGAGGGGTCACGTC

CTCAGACGTTTTAGAGAAAAGTCATTAGAATTTGTTCAACAGTTGAATGGTAAAGAAAGA

GGGAAGGAAAATAATTTTGAGTCTCCAGTGCTTGAGGCCAGTGGGATAGTGGACGCGGAT

ACTGAATAAAGACTGCACAGACXX

>Unigene77892_A-W 1 353 LEN=353; minus strand

XCTGTGTCAGACAAAAACCCTATGAAGAAGATGCCATTAGGTACCCTGTTATACAGAGAA

GGAGACAAAGATACAGAGCGGTGAAGTCATCTGTCCAATGTCACACGATCAGCGAGCAGT

GGAGCCTCGGCGGGGACCCAGGAGGCCACAGCTCTTCCCACCAGCTCTGCCAGGGCTCGA

GGAAGGCAGTGGCATTGCTGAGAGCAGTCTCCTCACTGTGACAGGAAAATCAGACGACCT

CAGGGGTTGCTGAGAAGATCACGTGAGTCGGCCCAGGACTCCAAGAGCATGACTGTTGTC

ACGATCAGTGCTATGATCATCACCATCACCTTAAACACGACTCTGGAAAGTGAC

>Unigene77895_A-W 1 343 LEN=343

XXATTGATTCCCAGGGAGCTACAGAGGAGTGAGCCCCAGAAACAGAGTCACAGGGCCAGA

GGGACAAATAGAGGATCAGAGGGAAAGGGACAGAGAAGAGAAAATGGAGAGACGCTGAAT

CACAAAGAGATACGGGGAGGAGAGACAGAGGCAAATATTCAGAGCTTTTCCCTAGCGACA

GAGAGATTGACAGAACCAGAGAAGGACAGGCAAAGAGAAACATTAAACCACAGATTGAGG

AGAAAGAGGCAAGCCCAACGGATTAGCCATAAAGAGAAACAGACAGGCAGGTATAAAGGC

AGGGATGTGGGAGAGAGAAGAGAGGCAAGAACAGTCAGAGAGAGG

>Unigene77968_A-W 1 252 LEN=252

XXGAGCCACATCCCCAGCCCCTTTCTGAGGAATTTAACCATAATGGAGAGGAAGGAGAGC

TTGAGAGTTAGAGGAATATGTGGAGTGGTTTTTATGAAGGCAAAGGCTTCAGCAGATTTG

ATTGCCAACCGGAAGGACATTGACTTTTGGGTCATGTCTAAATCCTGTAACGGAAGCAGA

AGATACAAAGGGGAGGAAATGGCAGTGGAACCCTCCTTACAGCAAGGAATGGGAGGCTCG

ACCCAGAGCACAGGX

>Unigene78053_A-W 1 263 LEN=263; minus strand

CAGGTGGGAGACAATAAGAGATCCAGGCAGACAGAGGAGTGTGTCCTGGAGGCTGCAGAG

GCTGGCAGTGGACCAGTGGGAAAGACTCTCATCCAAGCCTTTCCTAGGGGACATTCTCAC

CACGATGGACACATCTCCTTGGAGGTGGCTGATCCAACTAGGAGAAATAACAGAGGCCTC

CACACCCAGGCCTTCCTACGACCCTTCAAATCAAACAGCACCCAGCTCTTCAGGAAGATT

CTGGAGCTGCCTGTACCTCTGAAX

>Unigene78521_A-W 1 230 LEN=230; minus strand

ATGTCGGGGGTCGGGGTGCGGGAGCGGAATTCCGGGGTGGAGCTCACCTCTAGCTGGGAT

CACAGTAGGATCTACAGGATGCGCGGACTGACCCAGATCGTCCACTTGTCCTCCAGCCTG

ACGCCCTACAATTGGGTCCCCCGGAAGCAGGAGGGCTTCAGGGTCTGGTCTGGAAAGCTG

CCCATCACCCCTTCTCCTGTGCCTGGTTTTCCATATCCCACCGTCGCCGCX

>Unigene78695_A-W 1 231 LEN=231; minus strand

CTGGAACTTCAGTTCACTTTCTATGAACTGGAATGTTGGCCCAAGGCTAAATACTTTGTC

TTATCTCATCAAATTTTACAAGGTGAGGTAAGGAGACATGAGCAAGCAGATGAAGATTCC

TTGAATTTGAAAAGGGTCTAGGCAATGCACACTCATGAAGATTCAACTGCACTTGTCCCT

GAGAGCAACCCAAGCTCTGTGGTGTTAACTCCCAGGGACACAGGAAAGGAT

>Unigene78854_A-W 1 270 LEN=270; minus strand

XTCACTACAAAAGCAATACACACACTACCCTACAACAAAGAACCCCCACTTCCACAAGAG

GAGACAGCTGTATCCTCTGTGCACAATTCGGTTCTAGATATGAGTAGATATGCTTCTAAC

CTCCTGGATAGGTCCAGAATAAAACATGAGAGAAGACAGACAAAGATTGACATCCTGTTG

GTGCAAAAATCTCAGGCAGAGAGAAGTAAGTGGAAATACAACAGCTTTGAAAATTAGAAA

GAAATTGGAATCTGGCTCTTTTCTGGTCTCCXX

>Unigene78950_A-W 1 214 LEN=214

XATCAAAACCAGGAGCCCAGCCGCAAGGTTGCTGTGGGTCCAGGAAACTCCACCCAGGCC

GCAGAGCTGAGCTGGGTGGCAGGAGGGCTGCGGAGGGCCGCTGGTGCCGCCGGGGCCTGT

GCCAAGCACCACACGCACAGCGCCCTTTCCCGGGGGCTCTGGCTGCTGCCATGGGACCCT

GCAGTGCCCAGAGTGCCCCAGAGTGCCCATGATGTX

>Unigene79060_A-W 1 200 LEN=200

XXGTTGTCACCCAAGCCTGGGGCTAATCTGAACTCTCAAGGAGCCCAGGATCAAGGGACT

TTTGGCTTAGACCTAGAAGGATCTGCTGAGGCTGCCGACTGGATGCTGGCAAAACAGACA

CTGGGAACAGCAGGGAGACCCAGGTTAAAGCACCGTGACAAGGAGGGAACCTGGGGTCTC

TTCTGTCACTTACGGAGAACATXX

>Unigene79265_A-W 1 229 LEN=229

XXGACCCAGAATAGCCAAGCAATTCTGGAGAAAAAGAGCAATGGAGGCAACACAGTACCC

CATTTCAAATCATACTATATTGCCACGTATCCGAGCTGGCGACCTATGCTGGGTTCACAA

ACTCTGACGACTTCAACAACTAAGAAAACGGTGAAAGAAGCACGCACCACACAGAAGTAT

CTTTTCTAAATCCGGGATGGCTCTCCAACCTTAGGGGTTCATGGAAAGAGT

>Unigene79348_A-W 1 224 LEN=224

XGAGCATATGAGGGAGGCCCAAGTGATGCACAGATGCCCTGCCTGGGAAATCAGGAGAAC

ACAGGAGCTGGGTTTTCACTAAGAAAGCGGGTTGCTTTCAAGTCTACCCAGAAGGCTCCA

CGCCCATCTGCACAACCAGAGCAGCTTTATCAAAACAGAGCCTGCCTTCCAGTAGACAGA

GAGCTCCTAAATCTAATGTTTAATGTTCATCTCTTTGACCCAAGA

>Unigene79362_A-W 1 261 LEN=261

XXGGGGTCATGTCCTCCGCTCTGGCTCAGCAGCTCATCTGGACATCCTGGAGAGACCAGG

CGAGGGAGCCAGAGAAGCGCAGAGAGGGTATGCCAGGAAGATGCCAACTACACAGATCAT

GCCAGCCCGGTGAGTAACACCAAGAGGGCAGCCCTTGTCTACCTGCTCAATGAGTCCCAC

TTGGGCCTGGAAGTCCAGGACACTATCTGGAGAACCCCAGAGACCAATGAAATTGTTGTC

GCAGACTTTGAAGTTCTGGGAAAX

>Unigene79389_A-W 1 210 LEN=210; minus strand

XXCTGGCAGACAGGAAGAGAGAGCAGGGAGTCACGGGGACACAGAAGCAGCCACTCAGAA

CAGGAAAGCCCCTCCAGTGCACGCAAGAGCAGCAGACCAGGCTTTCTGGCCTTTTCAAAG

ATGTTCTTGAAACACCGAGACAAGGAAACAGCCCATATCCCTGAGGCAGGTCAGAATCAA

AAGCTGGTACCACAGGGAAGAGAAGATGCTCTX

>Unigene79396_A-W 1 209 LEN=209

XXGGTGGGACCATGGGTGAGAGACAGTGGGAGGTCTAGTGTGTGTGTCTGGGGACAGCAG

TTTGAGCTGGAACTTGAAGATTTGGGTGGAATGCTTGAAAAATGGAAACCAGACCCAGTG

CTTTGTCTAGAGATTAGTCAGAAAGGAGTCAGAACACAGGAGAAATGGTTGAATTTGAGC

AAGACAAATACAGATACTAAAAATGGCAATCXX

>Unigene79406_A-W 1 237 LEN=237; minus strand

XXCAAAATTAACCTGTTTATTAAAGAGAAGACAGAAAAAGAGGAAAAAGGGGGGGAAAAA

AAAACAGAAGAGACAAGTGGAACAGGACTCCATCTGACGCGCACCCACCATGATGCTCTA

ACCCTCCGCCAGAACAGGCGGACTGACGAGAGGAAGACTGACAGCCGCTGCCAGGAACCC

CAGGGGACTCGTGCAAACACTAGGTTAGAACTGAAGAGTGGAGACAGAGGGAAGAGGACX

>Unigene79491_A-W 1 382 LEN=382; minus strand

XXGTGTGAATGGACAGGTGTGAGAACCATCGTGGAGCAGGTGGTGATCTTGAGGACACAG

TGCATGTCTTTAGGAACTGATTTAGAAGTTGATCCAACACTATGTTCCAGGCACTGTGCT

TGTACTGCTGATGGAGGAAAGTTAAGGCAAAATCCTTGCTCTAAAAGAAAAACAACTGAA

AATGTGTGAGCCATGATAAGTGCTATAGTACAGCCAGGTGGGAGGCACCAGGGAGACTGG

GTAGGAGGAGAGCCATTCTGCTTTCAGTTCAGAGGCACGAAGGTCCAGGGAGAACTGGAG

CTGCAGGTGAGGGAGGCCAACACCAGCGGCCATGGAGGTGCGGACATCCTTACTAGGTGG

GAGAGGTCATCTAGGGGTGCCCAC

>Unigene79678_A-W 1 284 LEN=284; minus strand

CCGCTCCATTGGGTCCGCGTCTCCTGCTACTGCGCCCAGCGCATGGCCGGGCGCCGCGGC

TCCCTCCAGCCGGGCTCCTCCGACCCTCGGGATAGCGCCGCGCCGCTGTTCCCGCGCTGG

CTCCGAGCGGACGCCTCCTCGGCTGTCGGCGTCCTCTAXAGGACCCAGGGCCAGGTGGGC

TCAGTCCGGAGCTCGCGGGATGTACCTCCAGTGGGCGCGTCCTTCTCCGGGCTCACCTCG

GACCCGAGCCGGCCAGGGCGCGCCCTGGGATTCCGGCCCCCTGGC

>Unigene79754_A-W 1 205 LEN=205; minus strand

XCCAATGCAGAAAATTAAAATAACAGGGGATTTAACAAGCTAGAAGGTAAATCAAGTTTG

AAGGCAGTATACTGATATGGCAGCAAAGTCACCCAGGCTTTCTCCTTCTATCTCAACGCT

TTGCCATCCTCAACAGGGACCAAGATTGCTTTGCAGCGACAGCCATCATATCCACTTTCA

GACACCAAGGACAAAGGAGTGCAAAGX

>Unigene79970_A-W 1 206 LEN=206

TCCAAAGCCGGCTCTGACTTCAGATCTCATCCTCTATTTCCCAAAGTGCCTCGCCCCTGC

CTACCTCAGGCCCACGGTGACCCTGGGATTCTGGACTTCAGCCAGCGGCCCAAGGCCAAG

GGCACCGTCGAGCTGCTAGGAATTCAGTATGTGGTTCCAGCAGGTGGGTGGAACACGGGC

CTCTCAGAAGCCAACACTCTGGCCCGX

>Unigene80024_A-W 1 305 LEN=305

GAAAGAGGAAAAGAAAAGAACATGGAAGAGTTGCACAATCGCAATAAAAATGAAACAGGA

GTATCCAAAGAGAAGAGGAGAGAACAGAAGAAATATTTGGAGTAATAATAGCCAAGACCT

TTCCAAAATGGACAGACACCAAACCAAAGGGCTCAGAAAACACCTATCAGGAAAGGAGCA

ACCAAAAACCGTGCTTATGCATATCATTTTCAGACCAAAAAGGAGGAAAAAAAGTCTTGA

AAAAACACGAGAAAACCATATCTGCTGAAAAATACTCAACTAGCATTCCTGAAAGCTGTC

AAGATX

>Unigene80121_A-W 1 249 LEN=249; minus strand

XXCACTTCTAGAGAACCCGGGATGAGTACCGCCTCAGGACCACAGAGTCCAGACCCAAGT

CACATCCCTTCACAGGTGTCCTTGAGTTGGGTGAGCCATGTCAACGTCATTTTCCTTGCG

GGACCCACTTGCAGTACGGATTTCAATGGTGCTGGCATCAGAACCTGGAGAGGAAAAGAG

AAGACTCTGATGGCACCTGCATTCTTGAAGGTTTTTCCTCCAGCTGCAGAAAATGCCCTG

AGGACTCGGCAX

>Unigene80180_A-W 1 255 LEN=255; minus strand

TCCCGACTCTGAAGACCAGGGAGCCTTCATGGAGATGAGCTCTGTGAATCACCCAGTAAA

TTCCCTAAATCCAAAGGAAATCCAGCAGAGGACTTTCTTAGGAGGAGGAGTGTTATGTGT

TTAAACAAGCAAGTGGTTGATTCATACCTATACACGGATGGTATAGATATACGAGGCTAT

AATGCACAGAAGCTGACCATTGATCACCAGAGACCTCACCAATACAGAGACAGTCACAAT

TCAGAACTTACCAGC

>Unigene80344_A-W 1 211 LEN=211

XGGCTTCGTTGGGAAGAGCCCTTTGGCCCTTCTTACTGCCTGGAACTAGAATTCAGAGAT

GATTCCTGGAGCTTCAGCTACCAAATTAGACCAGGAAGTGGCCTTGAAGGTGACAGGATA

AACAACACAGAAATCTGGGTCCTAGATGCCTCCGAGGAGCCTCCACGGACTGCCTATCTC

TAGACTTTTTATATGAGACAGAAATATGCATCX

>Unigene80438_A-W 1 254 LEN=254; minus strand

CTCACATGTGAGTTCCAGCAGGGCAAAGATCCTTCTTATGATTCCTCCTGCTATTTCCAG

ATCCTTAGGCTGACACCTGGGACATGGAAGGCACATGGGTCGGCAGAAAAGCAGTCTTGT

TTCTGCCAAGAGAATCAGGATGTTAGAAATGGACATGAATATGTCTGTCCTCTGGAACAG

TTATCCTACCTGGAGTTGGAAAGCACAAGGATTCTGGAGGAAAGGGACATGGAGCAGATG

AGGAGGTATAGCTGX

>Unigene80471_A-W 1 272 LEN=272

XGTCATCGGAAGCGCGGTCTAGGCGAATGTCCTCAGAGGCGCTGTCCGAGGGTGTGGGGG

TTTGCTGAAGAGGAGTTTTGGAGCATGGTCTCAGATGAACTCCTCACCAGGTGTTCATTA

ATTCCAAATAGAGAAGCAGTGACTGTGAAACTGGGGAGGATGGGCCTCATGTGCCTCCAG

GTGGACACTCAAGACCAGCACAACCCTTACAAAATACACAAGCTGAAAATCTGTAACCTG

ATCACAGCTGTGGAGAAGCAGTAGAGAGACCCA

>Unigene80492_A-W 1 200 LEN=200

AAAAACAAGCAAGCCAATGAAAATATCGGCAAAAGATTTGAAAAATACTTCACCAAAGAT

GAAAAACTGAGGACAACTGAGCACAAGAGAAGATGATCACTATGGCGAGTCATTAGAGAA

AAACCACTGAAAACCACAGGACACTGCTGTATGTCTAGTAGAATGGCTAAAATTAAAAAG

ACGACCATACTGAGCACTAGX

>Unigene80501_A-W 1 235 LEN=235

XXGAGGCTGGCTGGAGCCAATGAGGCACCCAGACCAGATCCACATCTCCAAGACTCCAGT

GGATTACTCCCTACCAATGGCCTTCTCTCACACTCCTCCTTCACCCTCCCAGTCCCTTAC

GACTCAGTCTACAAGTCTGCTGTGCACCAGGCAAGCACCATGCTCACACTCAGAATGCAG

TCACAAACAGGAGATACTGTGTTTACCTTCATGGAGCTAATGGTCTCAGCAGAAGAG

>Unigene80522_A-W 1 253 LEN=253

CTCTCAGTTGGCCGTAACCAGATTAACTTTGTCAAGATCAAAGCTCCTGATGGGGGTGGG

AGGTGCCTAACCTTGACACTAGTCTTTCTCCATGACACAGTCTTGATAGCCACAATGACT

TTGAAAACCTTCAACTTTGTATTGTTTCTGTTAAAAGATACAAGTGTAGTGTATAAAGCC

TCTTATTTAGAACAGAGGATGTTCAAGTCTTCACAGGTTAGACATTGGAAAGAGAAGCTC

CAGGTAGAAAAGGXX

>Unigene80562_A-W 1 200 LEN=200

XGGGGGAGAAAATGTCTTACGTTCATAAGCATCTTCCGAAGTCAGATGCTGGGGCTGGAG

CAAGACAAGAAATGTCAGCCCCGGAAGTCCAGCGTAGCCAAGGTCAGGATGAAAAAGACG

TCGTTCCACCATCAAAATGTTGCAGCCTCCCCGTCGGGGAATCGAACCCCGGTCTCCCGC

GTGACAGGCGGGGATACTTAC

>Unigene80574_A-W 1 273 LEN=273; minus strand

XTTCGACTAATACAGTTTGGACGAGAGGTTAGTAAAGAAGTAAATGGCATCTTTAACTTC

TGGATTCAAAAAATAATCAGAAACTTGAAAAAGGAGGAACTACATATTCTTCACCTTATG

AAGAAGTGTGCCTCGTGTTTGAGGTTTGTGGTTCCCTATTAGGCCTTCTATCAGTTACCG

GGCAGCAGCGAGAGTGTGAAAACAGAGTCCAAACACCAATGTGACCTCCCGGTTTTAGGG

GAACCACCACCTACTTATTTCTGTGAGTGCTGCCXX

>Unigene80579_A-W 1 221 LEN=221

GGGAGGCAGCATTCTGTGTCGCACCATGACCTAGGATTCAAGTACTTAGAATACAGCTTC

TCTGTGAACAATATTCCTGCCCCCTGCACAGGAAGCACCATGCCTGTCCAGGGCTCCAGG

CCTCAGCCTCAGAGTAGGTCTCCCAACTATTCACTCACGCAGGACTACCGCCCAAGCGAG

ACCATCAGCAGATGCAGCAGCACCCTCAAAGAACTTTTGGCX

>Unigene80609_A-W 1 206 LEN=206; minus strand

XXCTCTGAGTCACCAGTGCCCATGATCGTCAACATGATTAACGAGATTTGAGAACAGGAC

AATCTGGAAGAAGTGGCATCTCCTTATACAGAACTTTCTCTTTTTTTCACACCATGGCCC

TGCCATTTGTTTGTGGCCATGGCTATATTACTTAACCCCTTCAAGTCCCATTATTCATCA

TCAAATGAAAATGGTATGGCTGGCCCCAXX

>Unigene80698_A-W 1 212 LEN=212

ATCCCACTCTTTGGAATTTATCCAGAGGTGCCAAAAACATGTTTGAAGAAAGATATCCTG

CTTGAACACAATGGTGAAGGGAAAACTGGATATCCATGTGGAAGACTGAAACTAGATCCC

TACTTCTCACACTACACAAAAATCATATCAAAGTGTATCAAAGATCTAGGAATTATACCA

GAAACACTGAAACTAAAAACATCATCCAACAGX

>Unigene80713_A-W 1 245 LEN=245; minus strand

GAAAGTCTTATTGTCATTGTGCCTGGGAGAAGGCACAGTCAGGCCAAGGCTGGTAGTCTC

AAGACAACCAGAAAAAGCTTCCTGGCGTCCTCAGACAAAAAGAACTTCAAAGTCTTTAGG

TGCTCTTTCCAGCTATGCCGGGGAATGTGGGCTGCCCAGACCCCAGCCCCAACAACACTA

GAGGATGGCAGGGCCACAGGACATCTCACAGAGTGTGCCCCAACCTGTCAGTCAAGTGCT

GATCCX

>Unigene81022_A-W 1 233 LEN=233

XXTTGCTGGCTCTTTAAGGCGGAGGCGAACCCCGGCAAGATGGCGGTGGAGAACCGCTGT

GTGTCTTTTCAGGCACCCGTGACGCAGCGTTTGGAAATTCTTGCTGGAGAGATCGGAGGT

GTCCTGCGCAATTTCAGACATCAGCGGAGCTGTCAGGACTGGATTGTCCCAGACAACAGG

ATTCCTTTACTTGTTTCAAAACTCAAACGCCACAGTCGGTACATCTTTCCAAAAGXX

>Unigene81036_A-W 1 260 LEN=260

XTGGGAGTCATGAGAATTTTTGTTAAAGGATGGTATCCACATATTTGCTCAGGTTTGAGA

AATCTTGGCATATGGTGTGAAGCTGAAAATGTAGAAGGGATGTTCACCGTCACAGACTTT

AAGATCACAGTCTGCTCTGGGACCTGGAATGAAGGAACAAATGTGACCAGTGAGGACCTG

GGAATGATGGAGGAGAAATATTTAAAAAGAGAGAGAGCCTACAATTGAGAGAGGGTTCTA

GGCTACTACATTCCAGTTTCA

>Unigene81216_A-W 1 210 LEN=210; minus strand

XXCCACTGTGCTGGTTCTACCGTGGGTCTGGACCGGTCCCCATGTCTTGTTGTGGGGCTT

CTGCAGCCTTTGGATTGTGAAGAXCTGTGGAAAAAGAGACTTGAAATCCAGTCACATAAT

TATAGTAAAGAACTATTAGCCCTCATGGAAGAAGAGCAAGATTTACCAGACCAACCAGTG

AAAAAAGCCAAGATGCAGGAACCAGGAGAGCAA

>Unigene81227_A-W 1 265 LEN=265

GTGCTAATGGTCACCTGCACCTCTCATGAGAAACCTCATAACGATTGGAATCAACACAGA

AGACAATGTGGTGGTAAGGAAGAGAGAGAAATAGGGGCAGAAAGAGAACGGACTGTGTTG

ACATGGACTAAGCCACTTGATCAACCCATGCCTGCAGCTGAGAGGCCTAGGAATGTTTCA

GTTTTACCAATCCATAAAACTCCACTTTATTTTGAAGCCAATTTGAATGCTATTTCCATA

ATATCTAACAGAACCTTGGCTAACGXX

>Unigene81341_A-W 1 323 LEN=323; minus strand

XXCTGTGGCATGAAACTAAGAAAGCGGATAGGCAGCATCTCAGCAAGGTGGGTGGAAAAA

AAAGGGACTTTTCTGGGATTAATACTGGATGCTCAAGGCAAATCAGGGACTGAGGAGTTT

GGATATAATAAGAAATATCCAGCACCACCACTGCTGCCCGAGCCAGAAGGGTCCCTCCAC

AAGCAAACTGGAGGAATAAGGAAATTAAAGAAACCCACATTTTTGAGAGGCCTGAGTTGT

GATTTAGAAACCAAAGACACTGCTGGGCCACCCTGAAAGGCAGGCTGCACGGTATCCTTG

TGTGGGAAAGAAGCCGCACTTCGCCXX

>Unigene81372_A-W 1 209 LEN=209; minus strand

CGTGCGGCTCCCGGGGCTCCCCCGCGGGCCGAGCCGGAGCAGCTCTGCACCTTCGCCGCG

GAGAAAGACAACAGACGGCCGCTCTGCGGCGACGGCTACTAGGCGAGGAGCCGACTCGGC

GGTTTCGACCGAGCCCAGGCGCTTCACCACGGCCGCTTCGGCTCCGCGCTGGCCCAGCAC

ACAACCAGCTACCCACGCATGCGCCGCAAX

>Unigene81588_A-W 1 208 LEN=208

TCTGGCTTGAAACTTAAAATCGCTGTTGCAGAGCATGCGGACAGTGCTCACTCTCTCCCC

AGAGGTTGTCGATGGCAACGCCAAGTGAGCACCTATGCAGTGGTTACTGGATGTCAGGGG

CAGGGCTCCTCGCCTCCTATGGACTCTCATTTAACCCACACAGCAGTCCAACAAGGTCGC

TGTCACCATGGTGACCATGTTACAGTTGXX

>Unigene81643_A-W 1 263 LEN=263

GACACTCAACACCCACTGGTAGAGGAAATGCAGTGCAAAGAGGCACTATACCGGTGTCCC

GATTATCCCACTGCTGCACTGGCAGGAAAGAGGAATAATAAAGCTCACTACACAGGGCTG

AGGACTAATCCACACAAAGCAACTGACATGGTGCCCACCCAGCATAGTCAGTATTCAGAA

AACACTGTAGCCACACACTTGGAGCCCTGCTTAGCTCTGACTTCCATGCAACAGGATGAG

CCTCTTTCCTCATCTGCTTCCTGX

>Unigene81646_A-W 1 226 LEN=226; minus strand

TGCACTCTTGACAGGAGTGCAAAAAACAGCCACCGTGGAAAAACATGTTACATGAGTGTT

CCTCAAAAAATGAGAATCAGCAAATCACCCAACAGTTCCGCTGTGGGTATGTACCCAAAA

GAATGGAAAGCAGGGTCTCGGAGGTTTGTGACTCATGTCCACAGCAGCAGCATGCACAGC

GGCCAGCAGGTGGGAGCAGCTCAGGTGTCCCTCACAGATGAGCAACXX

>Unigene81666_A-W 1 283 LEN=283; minus strand

CCTCAGTGTAGAAGTAAATCTTGTAGCCAGAGGCTGTGTGGCAGAGTGGGAGATGAGCTA

GATGATGGGAGCACTGGCATGAGAGAAGCCACAGACAGTGCTATGCAGGCTAAGGTGAGA

CCAGGCATGGCCCAGAACACTGGATGCCAAAGGCAGGAGCAAGCTGAGAGGATGTTTGTG

GGGAAAGATTGTGCAAAGGGTATGGAGCAAAAAGACTTCTGTAAAATTGAAAAAGGTAGG

AAAGAACAAGAAAGAATCTTTAACCCAGGGAACATTTTTTTTTXX

>Unigene81676_A-W 1 266 LEN=266; minus strand

XXTGGGAAGATTCACTCAGGTTATGGGACAAAAATGTTCTGTCACAGTGCCAAGCTCATT

AGAGCAGTGCAAGAACAGAAAGAGGGCCGGGGTCTAAGTAAGCAAGAGGGGACAGCAATG

GCACTACCCGACTCCAGTTCTGGCTACTCCAGCATGGATGAAGGGAACATGGCCACCAGC

CTAGGTAGGATAGGGGTGGTAGAGAACTTTTCATTTTTCATAAATAAACCATGCCCTAAG

AAAAAGGTACACAGGTTTCACATACTTCXX

>Unigene81771_A-W 1 153 LEN=212; minus strand

CTGCGGCAGGTCCAGGTGGCAGCTCTGGCCGATGGAGCTGAAGGCGTCCTGCAGCTTGTT

GACCAGCGGAATCAGCTCTTCCATTCCGCGGTTGCCCATGGCGCCGGCGGCCCCGGCCCG

AGCGCCCGGCGCTCCGCGCCCACCCCCGACCCT

>Unigene81826_A-W 1 239 LEN=239; minus strand

XGCCTGGTATGAGACCAACACTGCCCACTTCAGTCTGTCTCCTTAGCAATGATGGCAGTG

GTGGTGGTGGGAGATGGCATTAACTTTGTTACTTTCACTAATACCAATCTTTTAACACTG

CTCAACGTCAAAACACAGGATCCAGAAGGCTTTAAATATCCAGGTTCTCAGATCTTTGGT

GATCCCTGCATCCTGGATGTGTGTCCTCCTCTCCCACAAAGGCAGCATGGCGGCTGCCTC

>Unigene81832_A-W 1 236 LEN=236

XXTGGAGTCATCGGGCCTCCACGGTTGGAGACTGCACCTCAGTTCTATCAGGTACTGGCT

GTGCAGTCTGTGGCAAGTTACTTACACTTTGATTCAATTTTCTTACCCAAAACCAGAGGT

AATGCCATCTAGTTAGATGAAGGGTCATGGAGGTTAAATCCAGATTATACATTTAAACTG

TTTGGAAAAACTGTAGATGGTGATTGGCTACAATTTCAGATTAAAGACATATTTACCAXX

>Unigene81882_A-W 1 240 LEN=240

XGGGGAGTCCTGGACAGCCCGTTAGTGCAGCCTGGTTGTGGTTGGAGAGATGGTACTAAA

AGACATCAGAGCCAAGCTCCAGTCTGTGACAXACCCAAGGACATCCAGGAGCCACTGGAA

GATGATGAGATCAGATTTGCCTTTAGCACCATCACTCTGATCATGGTGTGCAATACAGAT

GGGGGTGGGGACTATGAGAAGACCTTAGATGCAGTTAAAATTATTCAAGCAAGAGAGACT

GGX

>Unigene81993_A-W 1 212 LEN=212

XXGGGGGAGAGTCAGGTGATAAGCATGTGTCTTCACGGTGTGTTCAAGACGAAACAGGAC

ACATGGGAAGGCAAGGATGGCAGTTTATATACTGTGCAGGGAAGACCACCACCTCTTATA

AGAGGGCATTTGAGCAGAGACCTGGAGCAACTGGGGAAACAATACCTTGAAGAAGCACAG

CAAGAACAGAGGAATGACAAGAGCAAAGGTCTCTXX

>Unigene82217_A-W 1 252 LEN=252; minus strand

CCCCTCTTTGATCCAGAAAGTAAAACTCCACAAGGGAAGAACTACGTCTGTCTTTTACAT

AGTGCCTAATACAGTCCTAGCATAAAGCAGGTACTTAGTAAAGAAAAAGAACAAGTAATC

CCCTTCATGATCCAGCCCCTGCTCATCAACTTACCTTTCTTTCTGTTACCCATCCTGTCC

AACAATGCTCTAACCATATCAAACTATTTCAAGTCTCCAAATGCTCCACCTTCTCTCCTG

TCTCTGGGCTTT

>Unigene82242_A-W 1 260 LEN=260

TTCAGAAGCTTAAATAGCGATCTCCGGCAGCTCAAGAGAGTAGCCATCATACAGCAAAAT

AGATGGTCATGGAAAGAACTCAGAATCTCCCCAGAATTTAGGGAAGATCAAGTCAAGGAA

ATCCCAGGAAGTAGGAAAAGCAAAAAGCCAAACAAAAAATCAGACAAGGAATTGGGAAGT

TCAGAGACCAGATACAGGAAACAGAGTCAGTCCAGAAAGTTGAATATGAAAATGTTTGGA

GTCAAGAAAGAGGAAACAGGX

>Unigene82317_A-W 1 205 LEN=205; minus strand

XCTCCATTCCGGAAAGAGGAGCTCAAAGCTAAGCACCGGGCTGGCTCCCAAGAGCTGAGA

CTGCGAAGTGGCCGGGGCCGGAGAGCCGGTGGTTCTCAAGTGGCACGCACCGAGGAGCCC

ACAGGCAAAGGAAGCTTCCGACTCGCCTACGCCCGCTCTGGTCCCCGGGTTTATACCACT

TACTGCGCTCTCAATCCCTCGCAGCXX

>Unigene82322_A-W 1 233 LEN=233; minus strand

XXCATAAACATCACTGCTAAATATCAAGATCACAACCTTCTAGAACGAATTGCTGAAATA

ATTGTTCATTGCTTCTATCACAACGGTGAATTCCAGTCTAAGTTGGTTTTATTACCCTTA

GCAAAGTGCACTTGGAGCTCTTTCCAAGTGAACCTACAGAGATTTCTTTATCCTTCTAAA

CAGCAGCAAAAGCCCGGCATGGTGGCACATGCCTATAGTTCCAAGTACTCAGGAAXX

>Unigene82419_A-W 1 338 LEN=338; minus strand

CAGGCCCTTAATTCAGCAGATTCCAGCATTCCAGGGTTCACCAAGAGAGGACTTCTGTGT

AGATTATGTCCCAGAAAGCTAGACCGCTTTGCTTGTGCAGCATCTGTAGCTCCCAGAGTG

GATACAGACCATGATGCTGAGCCCCATGGGAATCTGAGATCCCATGTAAAGAGGTGTTAT

GAAGCTACCCTCCAGCCTCTGGGAAGGGGTGGATTCCTGAACCAGGAAGAGGCCAGAGGT

GGAGTGCAGGGCAGCCTTCCAGCCTGGGCCCAGACAGAGGAGACCAGGAGTCAAGATGGA

CGTGAGGAGATGGCATTCATGGTTGCTGACAATGAGCAX

>Unigene82428_A-W 1 282 LEN=282

GGCGGATTTGCGCCCGCAGAGCCAACCATCCTGGAGCTCTAGGCGCTTTCGTCTGCCTTC

ACCAATATGGCGGACCCCCCTCCCCGGTTCCCTGAAGGGAGTATCTTTGGCAGTGGTCCT

CGGAGCAATTGAAAGACAGAGGTGTTTTCCCCTCGGATTACCACGAAACGTTCTGACATC

TGCAATAAGCAGGTCCGCCCACCCCGGCACGCACCCAGTTGTCCCAATTCCGAGCCTGGG

GTGGGTGTGAGCCGGAGACCCGAAGGGCTAACCCGATGGCAG

>Unigene82521_A-W 1 219 LEN=219; minus strand

GTGCCTCACAGAGTGGTTGCAGGAATGGATTCAGATAACATGCTGGGCATCCACAGGTGC

TTGGTGCAAGGTGCCTGGTTATTTTGCTCATCTCCTGGGCTTGCTAGAGTAGGTCTGCCC

AGGACTCCTCTTTCATTTCCCTATTTTCTGGTAGGTTTCTGCCAGCTCTACTTGAATGAG

AAGGACTACCCACCTGGCTATGCCTTCGACGTGGAGGCC

>Unigene82530_A-W 1 310 LEN=310

GGTAGTGAAAGTCTGAAGGCAGGACATCCTCAGGGAGAAGTCAGGGGTGGACAGAGGTGG

ACAGACCTTGAGGATGTACCATGCTCAGCAGATTTGCAAGGAATAGATTCTTCCAGAATG

CCCATGTATAGTTGGAAATGTAAGCACACAGAGGACTGGAGTGCTCAGGTTGGCTGGTCA

GCCAAACACACAGGGATGCCAACACAGTCAGGAGATTGTGGTGAGGGAGAAGGAACAAGG

GACAATCAGGAGATTTATCTAAAGGCATTAAACTACGACGGTCTGATGCAGCCATTGCCT

TTACGTGAACXX

>Unigene82550_A-W 1 230 LEN=230; minus strand

CCAATCCCAGCCCTTCATGTCAGCAGCAGAGGAAGGACCAGTGGTTTTCTCTGTGGTCCA

GTTCTTCTATATTTGCCACTCTTTGAGGCCATTCATCCCAAGAGCATCCCTGACATTCAG

CATGAGCACAGGCCTGTAACCACTGCATACGTTCATCATTGGATGATGACAGATACCAGC

AAGGCGGTGGCCATCAATTTGAGGGCAGCTGATGGAGCTGTCTCCGAGCGX

>Unigene82727_A-W 1 321 LEN=321

XAGCCTCAACAATTTAGCAAGGCTCAAATAAGGGCTGAGAATGTACTCATGCTAGGTGGA

GCACTTGGTAGCAGGTGGGAGGCCCTGGGCTCCATCCGATCCAGCACCACAAAACAAAGA

CCCTGTCAAGAGTCAAAAGGCAACTTGAGAATGGGAGGTAAGGGGTTAATATCCAGAGCA

TACAGAGACTCCCTAAAGCTCGGTAACCAAGCAGACAATCCCATCTGGAAATGGGCAAGA

ATCCTGAGTCGACATTCCTCCAAGAATCACAAGATGCCAAGACGCACGGTGGACGATGCC

AGCCGCCACATTCGGAAACGCAXX

>Unigene82738_A-W 1 241 LEN=241; minus strand

XCAATTTTACGTGTGACCCATCGCATCAGGGTGTGTGAAGGACTGTGTTTAGTTCTCGTT

AACCTTGTTGGAGTGGCACACCTGAAATGCTTCCTAGAGCTTTCACAGCTGCAGGCATGT

GTCCTACAGAAAGGCCCATTCCCTTCATGGCCAGGATTAAGAGGTCCCAATATTCCCCTC

AAGGGCACATCCAATGACCTAAAAACCTTCTGTGAGGTCCCACCTCTTAATGTTTCCACC

CCX

>Unigene82745_A-W 1 239 LEN=239; minus strand

TTGAGGCTTATCCTACTAGCAAAAGAAAATCTAAGGAAATTACAAAAGCCATCTTAAATG

AGCCAGCCCCAAGACATGGATTCCTCTCCTGGGTACAGACTGACAAATGACTATCTTCCA

TTACAAAGTATCACAATAAGTGAGCAAAACATAGGATATCCAATAGAAGCTGCTTGTATC

CTGAAGACCCCAATCTACAGGAAAAATGGAAAAGATGAATCAGACCTTAAAGAAATGGTX

>Unigene82769_A-W 1 231 LEN=231; minus strand

XXCAAGGCGGTTCTGCTGATGGCCACAGCGCACAGGACCCTGGTCCCAGCGCGGGTGAGA

GCAGCAGGGCTCTCCTGTGCTGCCGCTGGAAATGCCACTGGGAACCAGCGCGTGGCAGAG

GCCGTGGCGTCTCAGTTCCCTCAGGAGCAGGTCAACTCTCGGCCACCCTCCCACCCCCCG

GGCGAAATGGCTCAGCCACAGGACGGGATGTGGTCGGCCCGGGAAGCACAGACX

>Unigene82780_A-W 1 232 LEN=232; minus strand

XXGCCCTACACTGAACATTGCCCTGGACAAGTCTTCAAAAAATCACAGATTATGACCCCA

AAAGATCAAACTCTTTCCAAAGAACTTAATGTATTACAGAACAAAGCTCTAGAATATTTA

TGGAAATACAAAAGTATTCATCCTGAACAATGTGAAATCCATTCAAATATTAAAAGTGCA

AAGAAGCAGGGAAACATGATCCAAAATGCCAAGGATAGTCAATCAAAACCAACC

>Unigene82896_A-W 1 253 LEN=253

GGACTCATGCTTTCTGAAATCCTCTTCAGATGGAAAACTCCTGAATTCAAAGATGATGTG

TGAGAGAAGGAAAAGGAGCATTCCAAACAGCTGTCAGACTATAGGGAATGGATGGAGTCA

GCAGGGGAGGAGGAGAGAGAGCGAAAGAGAAGCTTTCAGCCTTCACTCCTGGTCTTATAT

GAATATCAAAATCTTCCCAGAGATCCACATGTCATGGCTCAGGACTATAATCCTAGCTGC

TCAAGAAACTGAGXX

>Unigene82952_A-W 1 223 LEN=223; minus strand

ATAGTACTCTGCTTCTTCTTCTTTGGATTCTTGAAAATGAGGTTAAGGTGTACTGCACTT

GATGAGGAAGAATATTTTTGCATAGGTGATCGGAGGAAACAGACAAATCTGGCAGCAAAT

GAGCTCTGCACCCGTGTATCTGCTTTTCTCCGGAGGTTCCAGGAGGCAGCTGAAGATCCT

TATGAAATGGCCACATTACAATTAAATGATCTTCACACCAAGCXX

>Unigene83019_A-W 1 248 LEN=248

XGTATAGCCCTTTCAAATACAGCCAATGCTGGGAAATGTGATTGCAGTGAACTCCATGCC

TCCATCTCTCTGGGGAAAGAGGAGTGCAGCAAGAGAAAGGCAGAGGATAAATTTGGCCAT

AATCGCACTTGGAAAGAGCAGATTATTTTCATTCTCCTCAACAGGTTTGCTGCATTCCTG

GCTCATCCCTCAAAGAGGTGGATGAGATGCAGCAGGGCATCAGGTGCCACCTGGTTGGTC

CTCGCAGGG

>Unigene83036_A-W 1 248 LEN=248

XXATACAAGGAAGGAAACAAGATGAAAGCTATATGAATGGAACTAGTGTCAATACAGCTG

GGGGAAATGACTGAAGCTTTTGTGGCTCTTTTAATCAGCACTGTTCCAGGAAAGACTCAA

GAGTACTGCACAAGTCCCAAGTCTGGAGTCACTCGAATTCTATATTCCAACCAGAGTCCA

ATGGAGCCATCCTTTTCTAAACATCCAAGACTGTTGGCAGATGCCAAGCTTTCCTTAGTT

CATTTTTATGXX

>Unigene83045_A-W 1 295 LEN=295; minus strand

ACCAGGCAGAATGAACCCAAAGAAATCTGTGTTAAGATACATCATTGGGAGACTGTTGAA

AGCCGAAGTAAAGAGCAATGAGAGAAGGACGCATTTCAAACTAAGGAGCCAGCTAATCGC

TGACTCTACCTCAGGCTCCATGGAGGCCCAGAGCAGATGAACACGGGTCAAGTCCTAGAA

GAAAAGAAAAAGCCTGTTAAACCAGAATTGTCTATCAAAAATACACCTTCAAGAACAGCA

GTGATTCTGGGATTAGCAGTGATGATAGCACAACCTTGTGAATATATTAAACACCXX

>Unigene83148_A-W 1 252 LEN=252; minus strand

XXTGCTGATGTGCGGCTCCAGAACGCTATTCCTCTGTATCCTGATCAACCTGGTGATGGA

CACGGGGCAGCTGTGTGCATAGTGACCCGAGACCGCCCTACTTATTACAGTTATCACACA

GTGGAAACTATGGCAACCCTAGTTAGGGCCTTGGTGTTGATCCATTCAGACCTCCTAAAT

CAGTCCAAAGAAAAGGATTTTTTAAAAATTCTGCTTTCATTCTCTAATCTGGTTAATAGA

AGAATCTTTGCCACX

>Unigene83422_A-W 1 216 LEN=216

XTCATGGAAACCATGAGAACACTCAGGACCCCAGTTCCCATAGGACATGCTGAGGACTCC

CCTTACTGCTCTGTGCTGGGCACTGACTCCGCTATCCTCTCATTGCAAACAAATATTACC

TGCTTCCCAGGGCAAACGGTGACCCACAGACGGCAGGCAGACAGACTCTGGGGCCACTTA

GACCTACACCCAAGGGATTTCCGAGGGGATAAATGTGXX

>Unigene83892_A-W 1 206 LEN=206

XGGGATGACCATAAGCATATTCACTGTGTGGCAGGAGAAGAGGCGCTGCCCCCTGCCATC

AATTCTCAGGGCAAGTCTGAGATGCCCCTGCTGCCCTCTCAGGTCTTGGGAGGTGAAGGG

ACAGGCAAAATGACCAGAGAGGGCCCACAAGCCCACAGAGTCCCTTACCAGCACTAAGTC

TTAACTGTCATGTTAAATAAAAATGAC

>Unigene83893_A-W 1 248 LEN=248; minus strand

XXCGACCAAAAACTAAGGTGCTCTCTGACAAACGAAGCAAAGAAAGAGCAGTGACTTTGT

AAAGTAAGAGCTAATGACTACCTGAATCAGCTACTTACCGCCCTTCAGCATCTGTCTTTT

ACTCTTGGCTTCTTCATTGTGGATGCAACATTGAAATCTCCTCCTATTCATTGTGGATAC

AACATGAAATCTCCTCATCAGTCCTTAGGTCATATTGGCATTTACATACTTAAACATGGG

AAAGAGCCACXX

>Unigene83935_A-W 1 210 LEN=210; minus strand

XXCCAAGGGATTAAAATAGACACTTACTTGCCTAGCATATACCATATTCCAGAATTAGAA

GGAAATCAGGTTTTTGGCAAAAATCGTAGTTTACATAAAGAGTTTGGGCACAGTGAACCA

TCAGTTATGGAATGGGAAGAATCATCCCCCAATCCAAATCTTTATTTTGAGCTAATGGGG

ATTGACAAGAGCATCCCACCACCACCTCCCCCX

>Unigene83963_A-W 1 218 LEN=218; minus strand

XCTGGGCAGGAAGTAATGGGGATGTTAGAACAGTCGGGTCAACAAATGTTCAAATGTAAG

TCCTTTAAAAAGGAGCACTCCAACGTTAAATATAAAAGGGTGATCTGTGGTGGCAGCTGG

CTAACAGGGGCTTCCCCCTCTTTACTTGGAGAAGAGATGTGTAGAATTGAGTGTGCTCGG

CATGAGCTGCAGATTTTAGGAAAGAATGGTCCCTGGTTC

>Unigene84120_A-W 1 234 LEN=234

ATCAAAACAACAGATAGAAAACGTTCTTTTGTCACCAGAGACCTAGGCCCAGGAATTCTG

AAGATCCCAACCAATCAGGTCCAGCCACCTACCCCAAAAAACAAAAGGAAAAGTAATGGT

GAAAAACCGTATATATGTCTATGTATGTCAGAAAAACAAAGGGACACGCGTTTGCAGCCT

GTCTTTGGGGATGCTAATGTGAGCTTCCTGGTCTATATAGACCAAGGGGACCAG

>Unigene84217_A-W 1 252 LEN=252; minus strand

XTGGCGCTGCAGGACAAAGCAAGATGGGTGGGAGCTGCCCAGCTTGGCCTGGTGGTGACA

GGAAGGGGCTGACTGGGGGAAGGGGGACTGAGGATTCCAGCACAAGGCTATCCAGGTTTA

GGCAAGATTCGACTCTGGAACCAAAATGCTCTCAATAGAGTTCACTGCCGGCATGAGGAA

GTTCACTCAGCCATTGTAAGTAATCCTGACCAATGTGAGATTAACAAGAATCCTAAAGGC

TCACCTAGGATTTXX

>Unigene84222_A-W 1 305 LEN=305

XAGCAGCAGCAGCAGCAGCAGCTTAATGTGCCTGAACAAATTCAGATTTCGCTGGCTGGA

ACGAGTGAGTCCCCTGGAATGGAGTGGACACGACCCAAAGCTGGGGAGGGTTATAACGGG

AGAAGTCCCAGAGTCATTAACGGGTCCAGAGCGTCCTGGCAGCGTCTCAGCGTCTTGAAG

ACGGTTTCGCTGGAAATGGTGGCGTACACCTGTACGCCAGAGGCTGAGGCAGAGAAGGAA

CATGAGTTCAGTATAAACAGCCTAGGCAACAATAACGATACCGTGCTTTAAAAAAAAGAA

GAAAAG

>Unigene84227_A-W 1 255 LEN=255

GGGCCTTATGTTGGAGGAACCACCCTTACTAGGAAACACAGGCAGTACCTGGGGAACCGG

AAGGAGGGTGGAGGCAGGGTGCAACCCCAACAAGCAAAAGTCACAGAAGGCAGAGCAGGG

CTGGAGCTCTCTAGCCCGTGTGGACCAAAGAAAGCCCACAGGCTCTTTGCAGAACACACA

GATACTGATAACATTCAACTTCAGTATGCACGGTCTTATGTGGAGCTCAACCTCTCGGTA

CAGCTTAGTCCCTGX

>Unigene84272_A-W 1 230 LEN=230

XXGGGGTTTCTGACAGTGACTATCCCTCTGGTGCTCAATGTGGTGATGGGTCAGCAGCTG

AAGCTGCAAGAAGTGCTAGAGGTAGCCCTGCAACTGGAAGAGGAGAAAAGATCACCGAAT

ATTCACATTCCTGCCAGGATACTGTCCACCTTCTCTCAGAGAATGGGCCACACAAAAACC

CAGGCTGGTAAAGAGTTCCCAGACTGCTTCCAACCTATGATTGCTCTGTTCCXX

>Unigene84435_A-W 1 232 LEN=232; minus strand

XXCCCCTACGTTATTCCAAACATCCAGTATAAAACCAAGAAGATGCAACAAAGTAATGGG

CACTATACTTCACAAGGATTTGCACCATTCTCTTTTGGACTGAAGCCCAAATTTCAAGTA

TCAGTTGAGATTGATCCTTCAGAAGTTTCTAGACTCTCAGGAGTTGTAGAGGTCTATGTA

TGAAGCATCACCATAGTCCCTCTGGCTGATGTAGTTGTCCTCACTGGATATAAA

>Unigene84498_A-W 1 246 LEN=246; minus strand

TGCCCAGAAGACAACACTAGCAAGCTGAAGTCACAGTCTTTTGTGACAATCACAGAAGTG

ACATCCAGTCGCTTTTTACTCAGTAGGCCAGCCTGTACTCAAGGAACATAGATTAGACAG

GGTATGACTGTCAGAAGGCAGAAATCATTGAGAGCCATGTTAGAAGTTGTCTGCCACAGG

CAGTGTTTCTGTGGACCACCAGCATCAAAATCATTATGGGAGCTTTTGAAGTTAAGCCTT

GCAGCC

>Unigene84628_A-W 1 218 LEN=218

GATGGAGACAAAGACAGCGAGAGCATCAGGGCTTCTAGGATGCAGCGACTGAGGAATGGA

CTCTTGAAGGTCATTCAAGGAATTGAAGATTCACAGAGGCACAAGAAGGGGCCTTCCAGC

CAACAAAGAGTGGCCCGAACAAAGCACTGGGGGTGGGACTGTGCCATACAGACCGGTGGC

AGCCGAGTCCGGAACTGGATCCCTTCGCGCCTGCGCACX

>Unigene84631_A-W 1 214 LEN=214; minus strand

XXCAGAAAGAACGAAAAACACAGACAAATGGTGGGGAATAACAGGCAGATCCGAAAGTTC

AATCCAGGACAAACGCTGGACTTCACCTGGTCAAAACCAGAGCAGCAGATGGAGGAAAAT

CACCGACTATCCCAGGGAAGGGGAGAAGACCAGGCAGCAGCGCCCACACCCCGACTGCAG

AGCCACAGGGGCTCAGTGTTCAAAGGGAAACACGGC

>Unigene84751_A-W 1 214 LEN=214

XGTGGGAACGGGGGGCCTGTCTTAGCAGGGAGAGTTTGGGGTATTGTGGTGTCTAGCTGG

ACCTTCTGGCTGCATGCCCTCAAATCATCATCTATGCTCATTTACATTATCTGGAAAGCT

GTCAACGAGCTGTCCAGACACAAAGTACTAGCTTGTGCAAGATTACTGGCAGCCTGTGCA

TCTATCGTGATAACTTGTGAAGGCACCTTTGGAAGX

>Unigene84809_A-W 1 220 LEN=220

GGCCTGGATATCAAACACCAGAGTATTAAGGGCAATTCTGGTGGGGTTAAGAAGACAAGA

TGCATAGAGAAACCTGGACTACTTAAAGAGTACTTAAGTAATTGTGTCCAGAATACTGGT

AGAAATATAGACAGCAAAGCCCATTCTGATACAGTCCCAGAGAGAAATGAGGAATATCCT

ACTAGGAACTGGAATAAGGGCCAACCTTATTATAAATTAGXX

>Unigene84928_A-W 1 222 LEN=222; minus strand

XXAAATTTTTATAAATCGAAACAAGACAATGGGTATGGTTATGGAAAGAAAACAGTGAAA

AAGGCTGCCAACATCCTTGCCTCTGATTCTGTTTTGACCATGAAACAGAACTGTTCACTG

CATACCTTCTAGAAAGCCTGCAACTCCAATACGGTAAAAACTGAGCAAATGAACAGAAAC

AACAAACAGACATGGAACGACTTTGGATTGCTCTCTACTCAAACX

>Unigene84974_A-W 1 201 LEN=201

TGGCCAGAGATGGTGCAGTATAAGTGTGAGGAAAATTCGGCATGGGATGGAGAAGGTGAG

GGACTGGCTCAGAGACAGGCTAAGGTGAGGAGCAGGGTCCCTGGAATCTCCAGTGGATTT

TCACAAGTCACTGAATGCATCCTTTTGGTGAACGGATCAGAAATGACTGAGAACAGAATG

CTCATTCATGAGGCTACCAAG

>Unigene85021_A-W 1 236 LEN=236; minus strand

XTGTTCCTCAAACTCTTCTTTCAGGGATCTGAAGTCTTTGTCTACAGTGTTCCAGGAAAC

ATGCAGCTGTCTCACTGCACAGCTGCCTTCAAGGATGAGCCATTCCCATCAGGCTCAGAG

GCCTACATTCTAGTAGAGAGCTCCAACTCTTGCCATAGCACTCTCTGCTACAACCAGAAT

CCTAATCCACCTGGAACATATGCAAGTTTTGAGGCAGGACTTTTCTCTGTATACAGC

>Unigene85133_A-W 1 236 LEN=236

XAAAAGCAGTCCAAGTGGGAACACTCTATTCCTTGCATGTTCCAGAAGCTTCTTTCTGTG

AAAATGAAGGCATTTCTCAGAGCAGAGAAGACCGGATCTCCTGTAGGTCCTGGGTATGGT

GATGATGGTGGAGGAGAAATGAGAGTCACTTCCCAGCAGTCACTTCAGCCATTAAGGAGA

ACAGGAAAGCTCACAGGCAGTATGGGATTGAGAGAAGAGGAACAGAGAAGTAAAAGG

>Unigene85207_A-W 1 204 LEN=204

TTTGGTCTGGATTCTCACCCTCCTATCTATGTGACCCCAGGAAATGTCCTAACCCATGTG

GACCTCTGGTTCCTCCTCATCTATAGAAAGGGGACAGTGATGACGACGATGACGGTGATG

GTGACGACGGTGACAATGACAGTGGTGATGATGGTAAACACCAGGACTGTGCTCAGAGTG

AACCGGGCTGGCCTGTGTAAGGAG

>Unigene85398_A-W 1 240 LEN=240

XGGGCATATGGAATACCTAAAACAGGTAAATCCAGAGACAGATTAGTGGTTGTCAGCGAT

TGGGAGGAGCTGGGAGTCCGACTGTTTAATGGCTACAGGTCTCCTTGGGGACATCATGGA

AGTGGTTGGGAACTAGATGGCTGCAAAACACTGTTGATTGTATCAAGTAGTACTACTGAA

TCGTTCCGCTTCAAAATAAATCTTATGTAACTTTCACCTCAAGAAAATTGTTACAGCCAC

AXX

>Unigene85567_A-W 1 200 LEN=200

GATGATCCTGGAGGAATTCGGAAGATGTTTGTCGCAGATTCTCCGCATCGAGTTTAAGTC

CAAGGGGTTGAAAATCGAGTCAAGTACCATGTAGTATAGCAAGCCCTGAGTGCATGCAAA

CACGTACCTCGGCGTTCTGAGGAAGCTCTGGTTCAGAAAGGGGGTTGGAGGAGCAAGAGG

CCAGCCTGCGGCCACACGAGX
