# Supplementary material for: Transcriptome Analysis and Comparison of Marmota monax and Marmota himalayana
Source: PLoS One. 2016 Nov 2;11(11):e0165875. doi: 10.1371/journal.pone.0165875 (PMC5091844; doi:10.1371/journal.pone.0165875)
Supplement: S3 File — (DOC) [file pone.0165875.s003.doc]

>CL1.Contig1_All 2 1294 minus strand PREDICTED: LOW QUALITY PROTEIN: perilipin-2-like [Equus caballus]

GCTCTTCCTATCATCCAGAAGCTAGAGCCACAAATTGCAGTTGCTAATACCTATGCCTGC

AAGGGGCTGGACAGGATTGAAGAGAAATTGCCTATTCTGAATCAGCCAACATCACAGGTT

GTTGCCAGTGCCAAAGGGGCTGTGACTGGGGCGAGAGATGCTGTGACGACTACTGTGACT

GGGGCCAAGGATTCTGTAGCCAGCACGGTCACAGGGGTGATGGACAAGACCAAAGGAGCG

GTGACTGGCGGGGTGGAGAAGACCAAGTCTGTGGTCAATGGCGGCATTAACACAGTTTTG

GAAAGTCGGATGGTGCAGTTTGTGAGCAGTGGAGTAGAAAATGCAATCAGCAAATCCGAG

ATGCTGGTAGATCAGTACCTTCCTCTCACTGAAGAAGAAATAGAAAAAGAAGCAAAAAAA

GTTGAAGGATTTGATGAAGTTCAGAAACCAAGTTATTATGTGAGACTGGGGTCTCTGTCT

ACCAAGCTCCGCGCTCGGGCCTACCAGCAGGCACTCAACAGGGTTAAAGAAGCTAAGCAA

ATGAGCCAAGAGACTATTTCTCAGCTTCATTCTACTGTTCACCTGATTGAATTTGCCAGA

AAGAATGTGCATAGTGCCAACCAGAAAATTCAGGATGCTCAGGATAAACTCTATCTCTCA

TGGGTGGACTGGAAGAGGAGCATCGGCCATGATGATACAGATGAATCCCACTGTGCTGAG

CACATCGAGTCACGTACTCTTGCTGTTGCCCGAAACCTGACTCAGCAGCTCCAGACCACA

TGCCACACCCTCCTGTCCAGCATTCAAGGGTTACCACAGAACATCCAAGATCAGGCCAAG

CACTTGGGGGTGATGGCAGGCGACATCTACTCAGTATTCCGCAATGCTGCCTCCTTTAAA

GAAGTGTCTGATGGCTTCCTCACTTCTAGCAAGGGGCAGCTACAGAAAATGAAGGAATCT

TTAGATGATGTGGTGGATTATCTTGTTAACAACACCCCCCTCAACTGGCTGGTAGGTCCC

TTTTATCCTCAGCTGACCGAGTCTCAGAATGCTCAGGACCAAAGTGCAAAAATGGAGAAG

ACCAGCCAGAAGGTCCAGCAGTCTCAGCACAAAACTCATTAAACCTTTCTTGTCACCAGT

GCATGTTGTAGCTATATAGATGACATCATTTGTCATGTTAAAATTAACCTGCTAGATAAC

TCTAAACTGGAAAAGCAGCCAATTAGGAAAAAGGCCTTCAGTTGTAGTCATTTACAGCTG

GTAAGAGCCTTAAAGTTTCTGGCATTAGGAGAC

>CL1.Contig2_All 3 1136 minus strand adipophilin [Pan troglodytes]

CTTCCTATCATCCAGAAGCTAGAGCCACAAATTGCAGTTGCTAATACCTATGCCTGCAAG

GGGCTGGACAGGATTGAAGAGAAATTGCCTATTCTGAATCAGCCAACATCACAGGTTGTT

GCCAGTGCCAAAGGGGCTGTGACTGGGGCGAGAGATGCTGTGACGACTACTGTGACTGGG

GCCAAGGATTCTGTAGCCAGCACGGTCACAGGGGTGATGGACAAGACCAAAGGAGCGGTG

ACTGGCGGGGTGGAGAAGACCAAGTCTGTGGTCAATGGCGGCATTAACACAGTTTTGGAA

AGTCGGATGGTGCAGTTTGTGAGCAGTGGAGTAGAAAATGCAATCAGCAAATCCGAGATG

CTGGTAGATCAGTACCTTCCTCTCACTGAAGAAGAAATAGAAAAAGAAGCAAAAAAAGTT

GAAGGATTTGATGAAGTTCAGAAACCAAGTTATTATGTGAGACTGGGGTCTCTGTCTACC

AAGCTCCGCGCTCGGGCCTACCAGCAGGCACTCAACAGGGTTAAAGAAGCTAAGCAAATG

AGCCAAGAGACTATTTCTCAGCTTCATTCTACTGTTCACCTGATTGAATTTGCCAGAAAG

AATGTGCATAGTGCCAACCAGAAAATTCAGGATGCTCAGGATAAACTCTATCTCTCATGG

GTGGACTGGAAGAGGAGCATCGGCCATGATGATACAGATGAATCCCACTGTGCTGAGCAC

ATCGAGTCACGTACTCTTGCTGTTGCCCGAAACCTGACTCAGCAGCTCCAGACCACATGC

CACACCCTCCTGTCCAGCATTCAAGGGTTACCACAGAACATCCAAGATCAGGCCAAGCAC

TTGGGGGTGATGGCAGGCGACATCTACTCAGTATTCCGCAATGCTGCCTCCTTTAAAGAA

GTGTCTGATGGCTTCCTCACTTCTAGCAAGGGGCAGCTACAGAAAATGAAGGAATCTTTA

GATGATGTGGTGGATTATCTTGTTAACAACACCCCCCTCAACTGGCTGGTGGTTGATTTC

ACTATCATAGACTTGACATCAGAGACTGATGAAATTCCAGATATTATAGCTTTGGAAGAG

GAGGATGGAGCAAATCATTCACACGCTAATGGTCCTGAACTCTCAGGGAATAAT

>CL3.Contig1_All 2 571 PREDICTED: transcription factor SOX-13 [Saimiri boliviensis boliviensis]

ATCAAGAGACCCATGAATGCCTTCATGGTGTGGGCCAAGGATGAGAGGAGAAAGATCCTC

CAAGCCTTCCCGGATATGCACAACTCCAGCATCAGCAAGATCCTTGGCTCCCGCTGGAAG

TCCATGACCAACCAGGAGAAGCAGCCCTATTATGAGGAGCAGGCACGGTTGAGCCGGCAG

CACCTGGAGAAGTACCCCGACTACAAGTACAAGCCTCGGCCCAAGCGCACCTGCATCGTG

GAGGGCAAGCGGCTGCGCGTGGGCGAGTACAAGGCCCTGATGAGGACCCGGCGCCAGGAT

GCCCGTCAGAGCTACGTGATCCCCCCACAGGCCGGCCAGGTGCAGATGAGCTCCTCAGAC

GTCCTATACCCTCGGGCAGCGGGCATGCCCCTGGCACAGCCCCTGGTGGAGCACTGTGTC

CCCCACAGCCTGGACCCCAACATGCCTGTCATCGTCAATACCTGCAGCCTCAGGGAGGAG

GGTGAGAGCACAGATGACAGGCACTCGGTGGCTGATGGCGAGATGTACCGGTACAGCGAG

GACGAGGACTCGGAGGGTGAAGAGAAGAGT

>CL3.Contig2_All 2 394 minus strand PREDICTED: transcription factor SOX-5 isoform 3 [Saimiri boliviensis boliviensis]

TTCATGGTATGGGCTAAAGATGAGCGGAGGAAAATTCTTCAAGCCTTTCCTGACATGCAC

AACTCCAACATCAGCAAGATACTGGGATCTCGCTGGAAAGCTATGACAAACCTAGAGAAA

CAGCCATATTATGAGGAGCAAGCCCGTCTCAGCAAGCAGCACCTGGAGAAGTACCCTGAC

TACAAGTACAAGCCCAGGCCAAAGCGCACCTGCCTTGTGGATGGCAAAAAGCTGCGCATT

GGAGAATACAAGGCGATCATGAGGAACCGGCGACAGGAAATGCGGCAATACTTCAATGTT

GGGCAACAAGCACAGATCCCCATCGCCACCGCGGGTGTTGTGTACCCTGGAGCCATCGCC

ATGGCTGGGATGCCCTCCCCTCACCTGCCCTCG

>CL4.Contig1_All 624 1280 mCG1026173 [Mus musculus]

ACATCTGAGTTAAATATAACCACTTTGGCCATTACTTCCCAGGTGTTAATTCCGTTGTCT

CCTTCTAATCAGACCTCTAATGCCACTCATTTTAAGGCTTCTCCTTATTGCACCCCTGAG

TATGGCTTCCCCCCAACATTCACGCCTTGCCAAGATCATTCTTGGGAGAAACATCAGGTC

GCTAAAGGTTTCTCCCTTTCCCCCTCTCTTAAAAGATACTTGTATAATTCTTCCAACAAT

AATTCCTCTATAGGAGCAGGTTGGTCTTGGTTTCAATGGTTAATTTCCAATGAAAAGGGA

GCCACAGCTGATATTTCCGCTTTGGCTCAATTACGAGGAGTCCAAAGTTGGCTGTCTAAT

GTTTCTGGCAGTACCCAAGACTCGGAGCGAGGTAGGAGGCTTAAAATTTCTGCAAATAAT

TCACAATTATCTAATGCTACATTACCCTCCACAGCAGTCTGTCTCCAATCTCCGTTCCTG

TTTATTTTGGCTAATGATACATCACAGGGGATGCTTAATTGTTCTAATATTACCTGCTAT

TTGTCTGAGTGTTGGAATGGCTCCTGGACTATGGCAGTGGTTATGAAAGTTCCGACCTTT

GTCCCGATTCCGGTTACTGCAGATCCAGAGTCTTTTCCTATTGTTGAACTGATTAGA

>CL4.Contig2_All 579 2285 mCG1029965 [Mus musculus]

CTGGTTCTGGTGCTGGTGCCGATGGCACAGACGACACCAATGCAGTGGTGGGCAGTGGCA

CGGGCTTGGCCAATGCCGATGCCGGTTCATAGTAATTCTAGTGTCCTCCCCACTCTTTTC

TCTACCTCATGTGAGATGTCTGCTCCCTGTGCCTCTCCTAGAGGGGACATGGAGAGAATT

TTTAATCAATCTCAAGTTAATCTCACAGGAGTTTTTTGTTTCAGCCTTGGAAACGCTAAT

TGCATCAACCTTAAGACCAAAAATTTGACTAACTGGGAGGACCCATTGCGGTCCAGTCAG

GTCTCAGGGAGCATTATCAGTGCCGTACTGAGCCAAGTAGTTTCGGGGGAGCAATCAGGT

TCAGGAACCCCCGCGAATAGCTCTGCTTTAAACATAACTACCTTGGCTATTATCTCCAGG

GTACTAATCCCAGTGCCTCCTTCTTCTAATAGTACTTACAATGCCACTCATTTCAAGGCC

TCCCCTTACTGTACCCCTAATCTTGGTTTCCCCCCAACATTTACGCCTTGTCAGGATCAT

TCTTGGGAGAAACATCAAGTTGCTAAAGGTTTCTCCTTTTCCCCCTCTCTTAAGAGGTAT

GTTTATAACTTTAACAACAGTGCCAACTCCTCTACAGGAGTAGGTTGGTCCTGGTTTCAA

TGGCTGGTTTCCAATGAGAAGGGGGCTTCAGCTGATATTTCTGCATTGGCTCAATTGCTG

GGAGTCAAAAGCTGGCTGGCTAATGTTTCTGGCACTGTTAGGGAGAGTGTACGAGGTAAT

AGACTTACATCTGTTTCATTCAACTCTTTGCTATTTAATGCCACATTGCCCCCTGCAATG

GTCTGCGTCCAATCCCCGTTCTTACTCCTTTTGGCTAATGACACCTCGTCGGGGATGCTG

GATTGTTCTAATATTACCTGCTATTTGTCTGAGTGTTGGAATGGCTCCTGGACCACGGCA

GTGGTTATGAAAATTCCAACTTTTGTCCCAATCCCAGTCACTGCGGACCCAGATAAATTT

CCCATTGTTGAACTACTTAGAGTCCGCAGGGATTTTGGAATTACGGCAGCTATAGTGACA

GCTGTGGCGGTATCTGCTGCTGCAGCAGTTACAGCTGGAGTAGCCATGGCCAGCCAGGTA

CAAACTGCTGCCACTATTAATCAGGTTATTCAACAAACATCCACCATACTTATATCCCAA

AATACAATTAATCAACATATTTTGTCAGGGATTTTAGCTGCCAACCAACGAATAGATTTA

CTCCAAGCTCAGGTAGAAGAATTGGCTGACTTGGTGCTTTTGGGTTGTGTTGACCAACGT

GCACATTTATGCATAACCTCTGTCAGATTTAATGATTCCAGGAATGCCTCCCGCATCATT

GGCGAATATTTGGCCGGAAATTGGTCCATGGCAGCGGAAGACATGATCCAGTCTCAACTA

ACCCAGATAGCTGTCTTGAACAGTACCCATGTCGATCCCGTGACTTTGGGACAATTCACC

GATTGGATATCTTCTGCTTTTTCCTTCTTCAAAGAGTGGGTGGGGGTAGGCATCTTTGGT

GCAATGTGTTGCTTTGGTATGTTCCTCTGCTTGTGGTTTCTCTGTCGCCTTAAGGCCCGT

AGTGCTCATGATAAGGCTATGATCATACAAGCTCTTGCAGCTTTAGAAAATGGCAACTCG

CCTCAAGTCTGGCTTGCGCATCTTAAA

>CL4.Contig3_All 419 676 PREDICTED: rap guanine nucleotide exchange factor 5-like [Nomascus leucogenys]

ATGAGAATGGCGGTGGGCTCAGTCAAGATGCAGCCGCCGTGCGAGAACCCGGCCCTGGCC

GCGGTGGTGGCGGTGGCGGTGGCGGACAGCGCTCTGCGCCGCAGTCCCAGCGCCCGCGAG

CCGGAGCGCGAGCAGCCGCTGGCGTCGCTGCGGCCGCGGCTGAAAGACCTGCCTGCACTG

CTGCGGAGCGGGCTCACGCTGCGGAGAAAACGGAGCACCGCCGGGGGCCAAAACCGGACG

GAGCCGGTGTGGGTTCCA

>CL4.Contig4_All 456 1484 mCG1029965 [Mus musculus]

CTGGCTCTAATGCTGATATTGATGGTACAGGGGACATCAATGCAGTGGTGGGTGGTAGCT

CGAACGTGGCCAATGCCGGTTCATAGCAATTCTAGTGTTCTCCCCACTCTTTTTTCTACT

TCATGTGAGATGTCTGCTCCTTGTGTTTCCCCTAGAGGGGACCAAGATCGGTCCTTCAAT

CTCTCCAGCATCAATCTCACAGGAGTTTTTTGCTTCAGTTTTTCCAAGGGTGATTGTATC

CATTTAAAACTTAAGAACTTGACTAACTGGGAGGACCCATTGCAGTACAGTCGGATCTCG

CAGGGAGTTTTGGTTGCAGCGCTCCTACAAGTTAGCTCAGGGAAAAATGAGGGCTCAGGA

ACCCCGACAAATAATTCTGAACTTAATATTACCACTCTGGCCATTACTTCCCAGGTGTTA

ATTCCGTTGTCTCCTTCTAATCAGACCTTTAATGCCACTCATTTTAAGGCTTCTCCTTAT

TGCACTCCTGAGTGTGGCTTCCCCCCAACATTCACGCCTTGCCAAGATCATTCTTGGGAG

AAACATCAGGTCGCTAAAGGTTTCTCCCTTTCCCCCTCTCTTAAAAGATACTTGTATAAT

TCTTCCAACAATAATTCCTCTATAGGAGCAAGTTGGTCTTGGTTTCAATGGTTGATTTCC

AATGAAAAGGGAGCCACAGCTGATATTTCCGCTTTGGCTCAATTACGAGGAGTCCAAAGT

TGGCTGTCTAATGTTTCTGGCAGTACCCAAGATTCGGAGCAAGGTAGGAGGCTTAAAATT

TTTGCAAATAATTCACAATTATCTAATGCTACATTACCCTCCACAGCAGTCTGTCTCCAA

TCTCCGTTCCTGTTTATTTTGGCTAATGATACATCACAGGGGATGCTTAATTGTTCTAAT

ATTACCTGCTATTTGTCTGAGTGTTGGAATGGCTCCTGGACTATGGCAGTGGTTATGAAA

GTTCCGACCTTTGTCCCGATTCCGGTTACTGCAGATCCAGAGTCTTTTCCTATTGTTGAA

CTGATTAGA

>CL4.Contig5_All 166 1194 mCG1029965 [Mus musculus]

CTGGCTCTAATGCTGATATTGATGGTACAGGGGACATCAATGCAGTGGTGGGTGGTAGCT

CGAACGTGGCCAATGCCGGTTCATAGCAATTCTAGTGTTCTCCCCACTCTTTTTTCTACT

TCATGTGAGATGTCTGCTCCTTGTGTTTCCCCTAGAGGGGACCAAGATCGGTCCTTCAAT

CTCTCCAGCATCAATCTCACAGGAGTTTTTTGCTTCAGTTTTTCCAAGGGTGATTGTATC

CATTTAAAACTTAAGAACTTGACTAACTGGGAGGACCCATTGCAGTACAGTCGGATCTCG

CAGGGAGTTTTGGTTGCAGCGCTCCTACAAGTTAGCTCAGGGAAAAATGAGGGCTCAGGA

ACCCCGACAAATAATTCTGAACTTAATATTACCACTCTGGCCATTACTTCCCAGGTGTTA

ATTCCGTTGTCTCCTTCTAATCAGACCTTTAATGCCACTCATTTTAAGGCTTCTCCTTAT

TGCACTCCTGAGTGTGGCTTCCCCCCAACATTCACGCCTTGCCAAGATCATTCTTGGGAG

AAACATCAGGTCGCTAAAGGTTTCTCCCTTTCCCCCTCTCTTAAAAGATACTTGTATAAT

TCTTCCAACAATAATTCCTCTATAGGAGCAGGTTGGTCTTGGTTTCAATGGTTAATTTCC

AATGAAAAGGGAGCCACAGCTGATATTTCCGCTTTGGCTCAATTACGAGGAGTCCAAAGT

TGGCTGTCTAATGTTTCTGGCAGTACCCAAGACTCGGAGCGAGGTAGGAGGCTTAAAATT

TCTGCAAATAATTCACAATTATCTAATGCTACATTACCCTCCACAGCAGTCTGTCTCCAA

TCTCCGTTCCTGTTTCTTTTGGCTAATGATACATCACAGGGGATGCTTAATTGTTCTAAT

ATTACCTGCTATTTGTCTGAGTGTTGGAATGGCTCCTGGACTATGGCAGTGGTTATGAAA

GTTCCGACCTTTGTCCCGATTCCGGTTACTGCAGATCCAGAGTCTTTTCCTATTGTTGAA

CTGATTAGA

>CL4.Contig6_All 315 1343 mCG1029965 [Mus musculus]

CTGGCTCTAATGCTGATATTGATGGTACAGGGGACATCAATGCAGTGGTGGGTGGTAGCT

CGAACGTGGCCAATGCCGGTTCATAGCAATTCTAGTGTTCTCCCCACTCTTTTTTCTACT

TCATGTGAGATGTCTGCTCCTTGTGTTTCCCCTAGAGGGGACCAAGATCGGTCCTTCAAT

CTCTCCAGCATCAATCTCACAGGAGTTTTTTGCTTCAGTTTTTCCAAGGGTGATTGTATC

CATTTAAAACTTAAGAACTTGACTAACTGGGAGGACCCATTGCAGTACAGTCGGATCTCG

CAGGGAGTTTTGGTTGCAGCGCTCCTACAAGTTAGCTCAGGGAAAAATGAGGGCTCAGGA

ACCCCGACAAATAATTCTGAACTTAATATTACCACTCTGGCCATTACTTCCCAGGTGTTA

ATTCCGTTGTCTCCTTCTAATCAGACCTTTAATGCCACTCATTTTAAGGCTTCTCCTTAT

TGCACTCCTGAGTGTGGCTTCCCCCCAACATTCACGCCTTGCCAAGATCATTCTTGGGAG

AAACATCAGGTCGCTAAAGGTTTCTCCCTTTCCCCCTCTCTTAAAAGATACTTGTATAAT

TCTTCCAACAATAATTCCTCTATAGGAGCAAGTTGGTCTTGGTTTCAATGGTTGATTTCC

AATGAAAAGGGAGCCACAGCTGATATTTCCGCTTTGGCTCAATTACGAGGAGTCCAAAGT

TGGCTGTCTAATGTTTCTGGCAGTACCCAAGATTCGGAGCAAGGTAGGAGGCTTAAAATT

TTTGCAAATAATTCACAATTATCTAATGCTACATTACCCTCCACAGCAGTCTGTCTCCAA

TCTCCGTTCCTGTTTCTTTTGGCTAATGATACATCACAGGGGATGCTTAATTGTTCTAAT

ATTACCTGCTATTTGTCTGAGTGTTGGAATGGCTCCTGGACTATGGCAGTGGTTATGAAA

GTTCCGACCTTTGTCCCGATTCCGGTTACTGCAGATCCAGAGTCTTTTCCTATTGTTGAA

CTGATTAGA

>CL4.Contig7_All 2 730 minus strand PREDICTED: pol-like [Oryctolagus cuniculus]

AAGCTTAAATATGTCCATGTGTTTGTAGATACTTGTTCTGGCATTATCCATGCTTCTGCT

TTGTCTGGTGAAAGGGCTGGAAATGTTATCACGCATTGCTTGGAAGCCTGGGCAGCATGG

GGCTTACCACAGACCATTAAAATGGACAATGGTCCAGCGTATACTGGATGACAATTTTCT

TCCTTTTGCAGCCAGATGGGTATCCAGCTTGTCCATGGCTTACCCTACAATCCACAAGGT

CAGGGTATTGTAGAGCGTGCTCATAAGACTCTAAAAGAGATGTTAATAAAACAAAAAGGG

GAAATTGGTTTAGGCCACACCCCAAAAGAGCGCCTCTCCCTGGCTCTATTTACTATTAAT

TTCTTGAATTTGGACATCCAAGGGCGCTCTGCTGCGACTAGGCACTGTTCGCCCTCTGCC

CCGAACTTTGGGCACGTTAAGTGGAAAGATGTACTTTCTGGACAATGGTATGGGCCTGAT

CCCGTGCTGGCATGGGCACGAGGTTCCGTCTGTGTTTTTCCACAGGACCGGACGGAGCCG

GTGTGGGTTCCAGAACGCCTGACAAGAAGAGTCCCGACATCAATCAACCAACAACAGGAG

GCGCTACAACAATCTCATGATGAGGACCATCCTTCTACTGAGTGTCCTGGTTCTAATGCT

GATATCGACGGTACAGGGGACATCAATGCAGTGGTGGGCGGTAGCTCGAACGTGGCCAAT

GCCAATGCC

>CL4.Contig8_All 18 233 PREDICTED: Pro-Pol-like [Oryctolagus cuniculus]

TGGCCAGTGCAGCTCTCAAATCAATCACTAAGAGGTTTGGGATACGCTCAAGCTCCACAA

GTCAGCTGCAGGCATTTATCATGGAAAGATTCTGAAGGACGTTCTGGCACTTTTCAACCA

TATGTGCTCGATCTCCCAATCTCTCTATGGGGACGTGATCTTATGAAGGACATGGGGTTT

CAACTTAGTAATGAATATTCACCAGTTGCCCAAAAG

>CL4.Contig9_All 166 483 minus strand mCG1029965 [Mus musculus]

CTGGTTCTAATGCTGATATCGACGGTACAGGGGACATCAATGCAGTGGTGGGCGGTAGCT

CGAACGTGGCCAATGCCAATGCCGGTTCACAGCAATTCTAGTGTCCTCCCCACTCTTTTT

TCTACTTCATGTGAGATGTCTGCTCCTTGTGTTTCCCCTAGAGGGGACCAAGACTGGTCC

TTCAATCTCTCCAGTATCAATCTCACAGGAGTTTTTTGCTTCAGTTTTTCCAAGGGAGTT

TTGGGTGCAATGCTCCTACAAGTTAGCTCAGGGAAAACCCAAGGCTCAGGAAACTTGACA

AATACTTCTGAGCTTAAT

>CL4.Contig10_All 251 907 mCG1026173 [Mus musculus]

ACATCTGAGTTAAATATAACCACTTTGGCCATTACTTCCCAGGTGTTAATTCCGTTGTCT

CCTTCTAATCAGACCTCTAATGCCACTCATTTTAAGGCTTCTCCTTATTGCACCCCTGAG

TATGGCTTCCCCCCAACATTCACGCCTTGCCAAGATCATTCTTGGGAGAAACATCAGGTC

GCTAAAGGTTTCTCCCTTTCCCCCTCTCTTAAAAGATACTTGTATAATTCTTCCAACAAT

AATTCCTCTATAGGAGCAGGTTGGTCTTGGTTTCAATGGTTAATTTCCAATGAAAAGGGA

GCCACAGCTGATATTTCCGCTTTGGCTCAATTACGAGGAGTCCAAAGTTGGCTGTCTAAT

GTTTCTGGCAGTACCCAAGACTCGGAGCGAGGTAGGAGGCTTAAAATTTCTGCAAATAAT

TCACAATTATCTAATGCTACATTACCCTCCACAGCAGTCTGTCTCCAATCTCCGTTCCTG

TTTATTTTGGCTAATGATACATCACAGGGGATGCTTAATTGTTCTAATATTACCTGCTAT

TTGTCTGAGTGTTGGAATGGCTCCTGGACTATGGCAGTGGTTATGAAAGTTCCGACCTTT

GTCCCGATTCCGGTTACTGCAGATCCAGAGTCTTTTCCTATTGTTGAACTGATTAGA

>CL5.Contig1_All 48 500 PREDICTED: THAP domain-containing protein 3 [Otolemur garnettii]

CAGCCTGTGAGGGAGAACACAAACCGAGCCAGGGAGAGAGGAGATGCCGGCTCTCAGGGA

GAGAAGGTCTGCCCTGAGGCAGGGGCCGAGGAGGACGGCCCAAGGAAGAGCATGGACATT

GCGCTGGAAGAGCTGCAGCTGCCCCCAAACACTGAAGGCCCCCTGCAGCAGGTTTTGCCA

CAGAGATCAGAAGCAGCAGAGGCTCCTGGCTGGCCAGCCAGCCCCGTTGGGCTGAAGAGG

GCCCTCCCCATGCAGGCTTCTGACCACAGCTATGCCCTTTTGGACTTAGATGCCCTGAAA

AAAAAACTCTTCTTCACTTTGAAAGAAAACGAAAAGCTCAGAAAGCGCTTGAAGGCCCAG

AGGCTGGTGATGCAGAGGATGTCCAGCCGCCTCCGCACACACAGAGGGGGTCAGCAGGGA

CCCCAGGCCAGGCCACGGCCGGAGCAGCGGAGC

>CL5.Contig2_All 3 254 PREDICTED: THAP domain-containing protein 3 [Papio anubis]

AACCGCAAAAACCTGAAGCACAATGCTGTGCCTACAGTGTTTGCCTTTCAGGACTCCACG

CAGCCTGTGAGGGAGAACACAAACCGAGCCAGGGAGAGAGGAGATGCCGGCTCTCAGGGA

GAGAAGGTCTGCCCTGAGGCAGGGGCCGAGGAGGACGGCCCAAGGAAGAGCATGGACATT

GCGCTGGAAGAGCTGCAGCTGCCCCCAAACACTGAAGGCCCCCTGCAGCAGGTAAGACAT

GGGTCCCCACAG

>CL5.Contig3_All 48 239 PREDICTED: THAP domain-containing protein 3 [Papio anubis]

CAGCCTGTGAGGGAGAACACAAACCGAGCCAGGGAGAGAGGAGATGCCGGCTCTCAGGGA

GAGAAGGTCTGCCCTGAGGCAGGGGCCGAGGAGGACGGCCCAAGGAAGAGCATGGACATT

GCGCTGGAAGAGCTGCAGCTGCCCCCAAACACTGAAGGCCCCCTGCAGCAGGTAAGACAT

GGGTCCCCACAG

>CL6.Contig1_All 55 918 PREDICTED: uridine-cytidine kinase-like 1 isoform 2 [Cavia porcellus]

GGATGTGGGAACGCGGTGGCCATTGACCTGATCGTGCAGCATGTGCACAGCCAGCTGGAG

GAGCGTGAACTCAGCGTCAGGGCGGCCCTGGCCTCAGCGCACCAGTGCCACCCACTTCCC

CAAACGCTGAGCGTCCTCAAGAGCACCCCGCAAGTGCGCGGTATGCACACCATCATCAGG

GACAGGGAGACTAGTCGGGACGAGTTCATCTTCTACTCCAAGAGACTGATGCGGCTGCTC

ATCGAGCATGCGCTTTCCTTCCTGCCCTTCCAGGACTGTGTGGTGCAGACCCCACAGGGG

CAGGACTACGCAGGCAAGTGCTATGCGGGAAAGCAGATCACTGGAGTATCCATCCTGCGT

GCTGGGGAGACCATGGAGCCTGCTCTGCGTGCTGTGTGCAAAGACGTGCGCATTGGCACC

ATCCTCATCCAGACCAACCAGCTCACGGGGGAGCCCGAGCTCCACTATCTTCGGCTGCCC

AAGGACATCAGTGACGACCACGTGATCCTGATGGACTGTACGGTGTCTACTGGCGCTGCG

GCCATGATGGCCGTCCGTGTCCTCCTGGACCACGATGTGCCCGAGGACAAGATCTTCTTG

CTGTCACTGCTGATGGCGGAGATGGGTGTCCACTCCGTGGCCTATGCTTTCCCACGAGTG

AGAATCATCACCACAGCTGTGGACAAGCGGGTCAATGACCTTTTCCGCATCATCCCAGGC

ATAGGTGAGCCGCCAGCCTATTCAGTGGTGTGTGTGAGCAAGGCTGGTCTCCCCAGGAGG

CTCCATACTGGGCTGCTTCTTTCTCCAGGGAACTTTGGGGATCGCTACTTTGGGACAGAT

GCGGTCCCTGATGGCAGCGACGAG

>CL6.Contig2_All 1471 2193 PREDICTED: uridine-cytidine kinase-like 1 [Cricetulus griseus]

AGGGCGGCCCTGGCCTCAGCGCACCAGTGCCACCCACTTCCCCAAACGCTGAGCGTCCTC

AAGAGCACCCCGCAAGTGCGCGGTATGCACACCATCATCAGGGACAGGGAGACTAGTCGG

GACGAGTTCATCTTCTACTCCAAGAGACTGATGCGGCTGCTCATCGAGCATGCGCTTTCC

TTCCTGCCCTTCCAGGACTGTGTGGTGCAGACCCCACAGGGGCAGGACTACGCAGGCAAG

TGCTATGCGGGAAAGCAGATCACTGGAGTATCCATCCTGCGTGCTGGGGAGACCATGGAG

CCTGCTCTGCGTGCTGTGTGCAAAGACGTGCGCATTGGCACCATCCTCATCCAGACCAAC

CAGCTCACGGGGGAGCCCGAGCTCCACTATCTTCGGCTGCCCAAGGACATCAGTGACGAC

CACGTGATCCTGATGGACTGTACGGTGTCTACTGGCGCTGCGGCCATGATGGCCGTCCGT

GTCCTCCTGGACCACGATGTGCCCGAGGACAAGATCTTCTTGCTGTCACTGCTGATGGCG

GAGATGGGTGTCCACTCCGTGGCCTATGCTTTCCCACGAGTGAGAATCATCACCACAGCT

GTGGACAAGCGGGTCAATGACCTTTTCCGCATCATCCCAGGCATAGGGAACTTTGGGGAT

CGCTACTTTGGGACAGATGCGGTCCCTGATGGCAGCGACGAGGAAGAGGTGGCTTCCACT

GGT

>CL6.Contig3_All 27 611 PREDICTED: uridine-cytidine kinase-like 1 isoform 1 [Cavia porcellus]

ATGGCTGCGCCACCGGCCTCCGCGGACGCCGCCCCCTCGTCCCCGCCGCCTCCTGCGGCC

CCGGACGCGCCCGGAGCCTGCGCGGGAGGACAGAGCGAGACCGCGTGCGAGGACCGCAGC

AATGCAGGGTCCCTGGACAGGCTTCTCCCCACCGTGGGCACCAGGCGCTCACCCCGGAAG

CGCACCACCAGCCAGTGCAAGTCGGAGCCACCCCTGCTGCGCACCAGCAAGCGCACCATC

TACACGGCAGGGCGGCCACCCTGGTACAATGAGCATGGCACACAGTCCAAGGAGGCCTTT

GCCATCGGCCTGGGAGGTGGCAGTGCCTCGGGGAAGACCACCGTGGCCAGGATGATCATT

GAGGCTCTAGATGTGCCCTGGGTGGTCTTGCTGTCCATGGACTCCTTCTACAAGGTGCTG

ACCCAGCAGCAGCAGGAGCAGGCCGCCCACAACGACTTCAACTTTGACCACCCCGACGCC

TTCGACTTCGACCTCATCATCTCCACCCTCAAGAAGCTGAAGCAGGGCAGGAGCGTCCAA

GTGCCCATCTATGACTTCACCACCCACAGCCGGAAGAAGGACTGG

>CL6.Contig4_All 974 1693 PREDICTED: uridine-cytidine kinase 1-like 1 isoform 1 [Pongo abelii]

GCGGCCCTGGCCTCAGCGCACCAGTGCCACCCACTTCCCCAAACGCTGAGCGTCCTCAAG

AGCACCCCGCAAGTGCGCGGTATGCACACCATCATCAGGGACAGGGAGACTAGTCGGGAC

GAGTTCATCTTCTACTCCAAGAGACTGATGCGGCTGCTCATCGAGCATGCGCTTTCCTTC

CTGCCCTTCCAGGACTGTGTGGTGCAGACCCCACAGGGGCAGGACTACGCAGGCAAGTGC

TATGCGGGAAAGCAGATCACTGGAGTATCCATCCTGCGTGCTGGGGAGACCATGGAGCCT

GCTCTGCGTGCTGTGTGCAAAGACGTGCGCATTGGCACCATCCTCATCCAGACCAACCAG

CTCACGGGGGAGCCCGAGCTCCACTATCTTCGGCTGCCCAAGGACATCAGTGACGACCAC

GTGATCCTGATGGACTGTACGGTGTCTACTGGCGCTGCGGCCATGATGGCCGTCCGTGTC

CTCCTGGACCACGATGTGCCCGAGGACAAGATCTTCTTGCTGTCACTGCTGATGGCGGAG

ATGGGTGTCCACTCCGTGGCCTATGCTTTCCCACGAGTGAGAATCATCACCACAGCTGTG

GACAAGCGGGTCAATGACCTTTTCCGCATCATCCCAGGCATAGGGAACTTTGGGGATCGC

TACTTTGGGACAGATGCGGTCCCTGATGGCAGCGACGAGGAAGAGGTGGCTTCCACTGGT

>CL6.Contig5_All 622 1428 PREDICTED: uridine-cytidine kinase-like 1 isoform 2 [Cavia porcellus]

AGGGCGGCCCTGGCCTCAGCGCACCAGTGCCACCCACTTCCCCAAACGCTGAGCGTCCTC

AAGAGCACCCCGCAAGTGCGCGGTATGCACACCATCATCAGGGACAGGGAGACTAGTCGG

GACGAGTTCATCTTCTACTCCAAGAGACTGATGCGGCTGCTCATCGAGCATGCGCTTTCC

TTCCTGCCCTTCCAGGACTGTGTGGTGCAGACCCCACAGGGGCAGGACTACGCAGGCAAG

TGCTATGCGGGAAAGCAGATCACTGGAGTATCCATCCTGCGTGCTGGGGAGACCATGGAG

CCTGCTCTGCGTGCTGTGTGCAAAGACGTGCGCATTGGCACCATCCTCATCCAGACCAAC

CAGCTCACGGGGGAGCCCGAGCTCCACTATCTTCGGCTGCCCAAGGACATCAGTGACGAC

CACGTGATCCTGATGGACTGTACGGTGTCTACTGGCGCTGCGGCCATGATGGCCGTCCGT

GTCCTCCTGGACCACGATGTGCCCGAGGACAAGATCTTCTTGCTGTCACTGCTGATGGCG

GAGATGGGTGTCCACTCCGTGGCCTATGCTTTCCCACGAGTGAGAATCATCACCACAGCT

GTGGACAAGCGGGTCAATGACCTTTTCCGCATCATCCCAGGCATAGGTGAGCCGCCAGCC

TATTCAGTGGTGTGTGTGAGCAAGGCTGGTCTCCCCAGGAGGCTCCATACTGGGCTGCTT

CTTTCTCCAGGGAACTTTGGGGATCGCTACTTTGGGACAGATGCGGTCCCTGATGGCAGC

GACGAGGAAGAGGTGGCTTCCACTGGT

>CL6.Contig6_All 55 834 PREDICTED: uridine-cytidine kinase-like 1 isoform 2 [Cavia porcellus]

GGATGTGGGAACGCGGTGGCCATTGACCTGATCGTGCAGCATGTGCACAGCCAGCTGGAG

GAGCGTGAACTCAGCGTCAGGGCGGCCCTGGCCTCAGCGCACCAGTGCCACCCACTTCCC

CAAACGCTGAGCGTCCTCAAGAGCACCCCGCAAGTGCGCGGTATGCACACCATCATCAGG

GACAGGGAGACTAGTCGGGACGAGTTCATCTTCTACTCCAAGAGACTGATGCGGCTGCTC

ATCGAGCATGCGCTTTCCTTCCTGCCCTTCCAGGACTGTGTGGTGCAGACCCCACAGGGG

CAGGACTACGCAGGCAAGTGCTATGCGGGAAAGCAGATCACTGGAGTATCCATCCTGCGT

GCTGGGGAGACCATGGAGCCTGCTCTGCGTGCTGTGTGCAAAGACGTGCGCATTGGCACC

ATCCTCATCCAGACCAACCAGCTCACGGGGGAGCCCGAGCTCCACTATCTTCGGCTGCCC

AAGGACATCAGTGACGACCACGTGATCCTGATGGACTGTACGGTGTCTACTGGCGCTGCG

GCCATGATGGCCGTCCGTGTCCTCCTGGACCACGATGTGCCCGAGGACAAGATCTTCTTG

CTGTCACTGCTGATGGCGGAGATGGGTGTCCACTCCGTGGCCTATGCTTTCCCACGAGTG

AGAATCATCACCACAGCTGTGGACAAGCGGGTCAATGACCTTTTCCGCATCATCCCAGGC

ATAGGGAACTTTGGGGATCGCTACTTTGGGACAGATGCGGTCCCTGATGGCAGCGACGAG

>CL6.Contig7_All 55 714 Uridine-cytidine kinase-like 1 [Heterocephalus glaber]

GGATGTGGGAACGCGGTGGCCATTGACCTGATCGTGCAGCATGTGCACAGCCAGCTGGAG

GAGCGTGAACTCAGCGTCAGGGCGGCCCTGGCCTCAGCGCACCAGTGCCACCCACTTCCC

CAAACGCTGAGCGTCCTCAAGAGCACCCCGCAAGTGCGCGGTATGCACACCATCATCAGG

GACAGGGAGACTAGTCGGGACGAGTTCATCTTCTACTCCAAGAGACTGATGCGGCTGCTC

ATCGAGCATGCGCTTTCCTTCCTGCCCTTCCAGGACTGTGTGGTGCAGACCCCACAGGGG

CAGGACTACGCAGGCAAGTGCTATGCGGGAAAGCAGATCACTGGAGTATCCATCCTGCGT

GCTGGGGAGACCATGGAGCCTGCTCTGCGTGCTGTGTGCAAAGACGTGCGCATTGGCACC

ATCCTCATCCAGACCAACCAGCTCACGGGGGAGCCCGAGCTCCACTATCTTCGGCTGCCC

AAGGACATCAGTGACGACCACGTGATCCTGATGGACTGTACGGTGTCTACTGGCGCTGCG

GCCATGATGGCCGTCCGTGTCCTCCTGGTACGTGGGTCCTGAATGGGCTGCAGGCAGGCC

CCTGTGCAGTTGGTATTTACCTGGCCCTTGGCTTGGCAGGACCACGATGTGCCCGAGGAC

>CL6.Contig8_All 116 697 Uridine-cytidine kinase-like 1 OS=Homo sapiens GN=UCKL1 PE=1 SV=2

AGGGCGGCCCTGGCCTCAGCGCACCAGTGCCACCCACTTCCCCAAACGCTGAGCGTCCTC

AAGAGCACCCCGCAAGTGCGCGGTATGCACACCATCATCAGGGACAGGGAGACTAGTCGG

GACGAGTTCATCTTCTACTCCAAGAGACTGATGCGGCTGCTCATCGAGCATGCGCTTTCC

TTCCTGCCCTTCCAGGACTGTGTGGTGCAGACCCCACAGGGGCAGGACTACGCAGGCAAG

TGCTATGCGGGAAAGCAGATCACTGGAGTATCCATCCTGCGTGCTGGGGAGACCATGGAG

CCTGCTCTGCGTGCTGTGTGCAAAGACGTGCGCATTGGCACCATCCTCATCCAGACCAAC

CAGCTCACGGGGGAGCCCGAGCTCCACTATCTTCGGCTGCCCAAGGACATCAGTGACGAC

CACGTGATCCTGATGGACTGTACGGTGTCTACTGGCGCTGCGGCCATGATGGCCGTCCGT

GTCCTCCTGGTACGTGGGTCCTGAATGGGCTGCAGGCAGGCCCCTGTGCAGTTGGTATTT

ACCTGGCCCTTGGCTTGGCAGGACCACGATGTGCCCGAGGAC

>CL6.Contig9_All 116 817 Uridine-cytidine kinase-like 1 OS=Homo sapiens GN=UCKL1 PE=1 SV=2

AGGGCGGCCCTGGCCTCAGCGCACCAGTGCCACCCACTTCCCCAAACGCTGAGCGTCCTC

AAGAGCACCCCGCAAGTGCGCGGTATGCACACCATCATCAGGGACAGGGAGACTAGTCGG

GACGAGTTCATCTTCTACTCCAAGAGACTGATGCGGCTGCTCATCGAGCATGCGCTTTCC

TTCCTGCCCTTCCAGGACTGTGTGGTGCAGACCCCACAGGGGCAGGACTACGCAGGCAAG

TGCTATGCGGGAAAGCAGATCACTGGAGTATCCATCCTGCGTGCTGGGGAGACCATGGAG

CCTGCTCTGCGTGCTGTGTGCAAAGACGTGCGCATTGGCACCATCCTCATCCAGACCAAC

CAGCTCACGGGGGAGCCCGAGCTCCACTATCTTCGGCTGCCCAAGGACATCAGTGACGAC

CACGTGATCCTGATGGACTGTACGGTGTCTACTGGCGCTGCGGCCATGATGGCCGTCCGT

GTCCTCCTGGACCACGATGTGCCCGAGGACAAGATCTTCTTGCTGTCACTGCTGATGGCG

GAGATGGGTGTCCACTCCGTGGCCTATGCTTTCCCACGAGTGAGAATCATCACCACAGCT

GTGGACAAGCGGGTCAATGACCTTTTCCGCATCATCCCAGGCATAGGGAACTTTGGGGAT

CGCTACTTTGGGACAGATGCGGTCCCTGATGGCAGCGACGAG

>CL6.Contig10_All 224 925 PREDICTED: uridine-cytidine kinase-like 1 isoform 2 [Cavia porcellus]

AGGGCGGCCCTGGCCTCAGCGCACCAGTGCCACCCACTTCCCCAAACGCTGAGCGTCCTC

AAGAGCACCCCGCAAGTGCGCGGTATGCACACCATCATCAGGGACAGGGAGACTAGTCGG

GACGAGTTCATCTTCTACTCCAAGAGACTGATGCGGCTGCTCATCGAGCATGCGCTTTCC

TTCCTGCCCTTCCAGGACTGTGTGGTGCAGACCCCACAGGGGCAGGACTACGCAGGCAAG

TGCTATGCGGGAAAGCAGATCACTGGAGTATCCATCCTGCGTGCTGGGGAGACCATGGAG

CCTGCTCTGCGTGCTGTGTGCAAAGACGTGCGCATTGGCACCATCCTCATCCAGACCAAC

CAGCTCACGGGGGAGCCCGAGCTCCACTATCTTCGGCTGCCCAAGGACATCAGTGACGAC

CACGTGATCCTGATGGACTGTACGGTGTCTACTGGCGCTGCGGCCATGATGGCCGTCCGT

GTCCTCCTGGACCACGATGTGCCCGAGGACAAGATCTTCTTGCTGTCACTGCTGATGGCG

GAGATGGGTGTCCACTCCGTGGCCTATGCTTTCCCACGAGTGAGAATCATCACCACAGCT

GTGGACAAGCGGGTCAATGACCTTTTCCGCATCATCCCAGGCATAGGGAACTTTGGGGAT

CGCTACTTTGGGACAGATGCGGTCCCTGATGGCAGCGACGAG

>CL6.Contig11_All 56 337 PREDICTED: uridine-cytidine kinase 1-like 1 isoform 1 [Pongo abelii]

CTCCTGGACATGAGAATCTTTGTGGACACGGACTCCGACATCCGCTTAGTGCGGCGGCTG

CGTCGGGACATCAGTGAACGAGGCCGGGACATCGAGGGTGTCATCAAGCAGTACCACAAG

TTTGTCAAGCCTGCCTTTGACCAATACATCCAGCCCACCATGCGCCTGGCAGACATTGTG

GTGCCCAGAGGGAGTGGGAACGCGGTGGCCATTGACCTGATCGTGCAGCATGTGCACAGC

CAGCTGGAGGAGCGTGAACTCAGCGTCAGAGGGAGCTGCGCT

>CL6.Contig12_All 56 307 PREDICTED: uridine-cytidine kinase-like 1 isoform 1 [Cavia porcellus]

CTCCTGGACATGAGAATCTTTGTGGACACGGACTCCGACATCCGCTTAGTGCGGCGGCTG

CGTCGGGACATCAGTGAACGAGGCCGGGACATCGAGGGTGTCATCAAGCAGTACCACAAG

TTTGTCAAGCCTGCCTTTGACCAATACATCCAGCCCACCATGCGCCTGGCAGACATTGTG

GTGCCCAGAGGGAGTGGGAACGCGGTGGCCATTGACCTGATCGTGCAGCATGTGCACAGC

CAGCTGGAGGAG

>CL7.Contig1_All 147 548 H2afvl protein [Danio rerio]

GCTTCAGCAGAATTTGAGATGGCTGGTGGTAAGGCTGGGAAGGACTCCGGAAAGGCCAAG

ACAAAGGCGGTTTCCCGCTCGCAGAGAGCCGGCTTGCAGTTCCCTGTGGGCCGTATTCAT

CGACACCTGAAATCCAGGACAACCAGCCATGGACGTGTGGGCGCGACTGCCGCTGTGTAT

AGCGCAGCCATCCTGGAGTACCTCACCGCAGAGGTACTTGAATTGGCAGGAAATGCATCA

AAAGACTTAAAGGTAAAGCGTATCACCCCTCGTCACTTGCAACTTGCTATTCGTGGAGAT

GAAGAATTGGACTCTCTGATCAAGGCTACAATTGCTGGTGGTGGTGTCATCCCTCATATC

CACAAATCTCTGATTGGGAAGAAAGGACAACAGAAGACTGTT

>CL7.Contig2_All 59 451 PREDICTED: uncharacterized protein LOC101021735 [Papio anubis]

TGCGACGAAGGAGCTGGTGGTAAGGCTGGGAAGGACTCCGGAAAGGCCAAGACAAAGGCG

GTTTCCCGCTCGCAGAGAGCCGGCTTGCAGTTCCCTGTGGGCCGTATTCATCGACACCTG

AAATCCAGGACAACCAGCCATGGACGTGTGGGCGCGACTGCCGCTGTGTATAGCGCAGCC

ATCCTGGAGTACCTCACCGCAGAGGTACTTGAATTGGCAGGAAATGCATCAAAAGACTTA

AAGGTAAAGCGTATCACCCCTCGTCACTTGCAACTTGCTATTCGTGGAGATGAAGAATTG

GACTCTCTGATCAAGGCTACAATTGCTGGTGGTGGTGTCATCCCTCATATCCACAAATCT

CTGATTGGGAAGAAAGGACAACAGAAGACTGTT

>CL8.Contig1_All 3 563 phosphatidylinositol-4-phosphate 5-kinase, type I, gamma, isoform CRA_b [Homo sapiens]

CGGCGCACGCAGTCCTCTGGACAGGATGGCCGGCCCCAGCAGGAGCCGCACATGGAGGAG

GACCTGCAGCAGATCACGGTGCAGGTGGAGCCTGCGTGCGGCGTGGAGATGGCGGTCCCC

AAGGAGGAGGACGCAAGGGTGGAGGCCTCCTTGGCCGGTGCATCTGCCACCACCGTGGAG

GTGGAGACCGCCAGCCAGGCCTCGGAACCCGCCAGCCAGGCCTCGGACGAGGAGGACACG

CCTGCTGCGGACATCTACTTCCCCACCGACGAGAGGAGCTGGGTGTACTCCCCGCTTCAC

TATAGCGCGCAGGCCCAGCCCGCCCCCGATGGCGGCGGCGGCGGCGGCGGCAGCGGCAAC

AACACATAATCTCTATGCAGCCCCCAACCTGGAGCCCAGCGCCCAGAGCGCCCACGCCGC

GTGCTGCTCAGAGGCGTCACCTACCCCTGAGGCCTGGAGCCCGGAGACCTGGCCCAACCT

CATCCTCCTCCGTCCTCCGGGGACACTGATGGCCGAGCCCCTCCCCACCCCGACAGCTCT

GGGCCACATGGCGACCTGGGG

>CL8.Contig2_All 230 766 PREDICTED: phosphatidylinositol-4-phosphate 5-kinase type-1 gamma-like [Oreochromis niloticus]

GGCCACGGGAAGAAGCTGGGTCATCGAGGCGTAGACGCATCTGGAGAGACCACCTACAAG

AAGACCACCTCCTCCACCCTGAAGGGGGCCATCCAGCTGGGCATCGGCTACACCGTGGGC

AACCTGAGCTCCAAGCCCGAGCGGGATGTGCTCATGCAGGACTTCTACGTGGTGGAGAGC

ATCTTCTTCCCCAGTGAAGGCAGCAACCTCACCCCCGCCCACCACTTCCAGGACTTCCGG

TTCAAGACCTACGCGCCCGTGGCCTTCCGCTACTTCCGGGAGCTCTTTGGGATCCGGCCT

GACGACTACTTGTACTCCCTGTGCAACGAGCCACTCATTGAGCTGTCCAACCCGGGCGCC

AGCGGCTCCCTGTTCTACGTCACCAGTGACGACGAGTTCATCATCAAGACTGTCATGCAC

AAGGAGGCCGAGTTCCTGCAGAAGCTGCTGCCCGGCTACTACATGAACCTGAACCAGAAC

CCGCGGACGCTGCTGCCCAAGTTCTACGGGCTGTACTGCGTGCAGTCGGGGGGGCAA

>CL9.Contig1_All 660 920 PREDICTED: ribosome biogenesis protein NEP1-like [Oryctolagus cuniculus]

GTAATTAAGAATCCAGTGTCAGATCACTTCCCAGTTGGTTGTATGAAAATTGGCACTTCA

TTTTCTGTTCCAGTCATCAGTGATGTGCGACAACTGGTGCCTAGGAGTGACCCTATTGTT

TTTGTGGTGGGGGCCTTTGCCCATGGCAAGGTCAATGTGGAATATACAGAGAAGACGGTG

TCCATGAGCAACTACCCTCTTTCTGCTGCCCTCACCTGTGCTAAACTTACCACAGCCTTT

GAAGAAGTATGGGGGGTTATT

>CL9.Contig2_All 111 281 PREDICTED: ribosomal RNA small subunit methyltransferase NEP1-like [Equus caballus]

ATGGCCGCGCCCAGTGATGAGTTCCAACCTCGTGAGCTGCGCTTTGGCGAGCAGGAGCAG

GACTGGGATGCTGTGGCTCCTAAGAGGCCTCGACTCGGGGCAGGAAGCAAGAGTGGAGGT

CGTAGGCTCATTGTGGTGTTGGAAGGGGCCAATCTGGAAACCGTCAAGGTA

>CL9.Contig3_All 95 265 PREDICTED: ribosomal RNA small subunit methyltransferase NEP1-like [Equus caballus]

ATGGCCGCGCCCAGTGATGAGTTCCAACCTCGTGAGCTGCGCTTTGGCGAGCAGGAGCAG

GACTGGGATGCTGTGGCTCCTAAGAGGCCTCGACTCGGGGCAGGAAGCAAGAGTGGAGGT

CGTAGGCTCATTGTGGTGTTGGAAGGGGCCAATCTGGAAACCGTCAAGGTA

>CL9.Contig4_All 660 812 PREDICTED: ribosome biogenesis protein NEP1-like [Oryctolagus cuniculus]

GTAATTAAGAATCCAGTGTCAGATCACTTCCCAGTTGGTTGTATGAAAATTGGCACTTCA

TTTTCTGTTCCAGTCATCAGTGATGTGCGACAACTGGTGCCTAGGAGTGACCCTATTGTT

TTTGTGGTGGGGGCCTTTGCCCATGGCAAGGTA

>CL9.Contig5_All 420 572 Ribosomal RNA small subunit methyltransferase NEP1 OS=Homo sapiens GN=EMG1 PE=1 SV=4

GTAATTAAGAATCCAGTGTCAGATCACTTCCCAGTTGGTTGTATGAAAATTGGCACTTCA

TTTTCTGTTCCAGTCATCAGTGATGTGCGACAACTGGTGCCTAGGAGTGACCCTATTGTT

TTTGTGGTGGGGGCCTTTGCCCATGGCAAGGTA

>CL9.Contig6_All 296 790 minus strand PREDICTED: ribosome biogenesis protein NEP1-like [Oryctolagus cuniculus]

ACGTTCTCTGTTCTTCAGGTAGGAAAGACATATGAGCTACTCAACTGCGACAAGCACAAG

TCCATGTTGTTGAAGAATGGACGGGATCCTGGGGAAGTGAGACCAGACATAACCCACCAG

AGCTTATTGATGCTGATGGACAGTCCCCTAAATAGAGCTGGCTTGCTGCAGGTTTATATC

CACACACAGAAGAATGTGCTGATTGAAGTGAACCCACAGACTCGAATTCCTAGAACCTTT

GACCGCTTCTGTGGCCTAATGGTTCAGCTTTTACACAAGCTCAGTGTACGAGCAGCTGAT

GGTCCCCAGAAGCTTTTGAAGGTAATTAAGAATCCAGTGTCAGATCACTTCCCAGTTGGT

TGTATGAAAATTGGCACTTCATTTTCTGTTCCAGTCATCAGTGATGTGCGACAACTGGTG

CCTAGGAGTGACCCTATTGTTTTTGTGGTGGGGGCCTTTGCCCATGGCAAGGTCAATGTG

GAATATACAGAGAAG

>CL9.Contig7_All 1055 1294 PREDICTED: ribosome biogenesis protein NEP1-like [Oryctolagus cuniculus]

CTTCTAGTTCAGCTTTTACACAAGCTCAGTGTACGAGCAGCTGATGGTCCCCAGAAGCTT

TTGAAGGTAATTAAGAATCCAGTGTCAGATCACTTCCCAGTTGGTTGTATGAAAATTGGC

ACTTCATTTTCTGTTCCAGTCATCAGTGATGTGCGACAACTGGTGCCTAGGAGTGACCCT

ATTGTTTTTGTGGTGGGGGCCTTTGCCCATGGCAAGGTCAATGTGGAATATACAGAGAAG

>CL9.Contig8_All 296 568 minus strand EMG1 nucleolar protein homolog (S. cerevisiae), isoform CRA_a [Homo sapiens]

ACGTTCTCTGTTCTTCAGGTAGGAAAGACATATGAGCTACTCAACTGCGACAAGCACAAG

TCCATGTTGTTGAAGAATGGACGGGATCCTGGGGAAGTGAGACCAGACATAACCCACCAG

AGCTTATTGATGCTGATGGACAGTCCCCTAAATAGAGCTGGCTTGCTGCAGGTTTATATC

CACACACAGAAGAATGTGCTGATTGAAGTGAACCCACAGACTCGAATTCCTAGAACCTTT

GACCGCTTCTGTGGCCTAATGGGTGAGAAGCCT

>CL9.Contig9_All 1053 1292 minus strand PREDICTED: ribosome biogenesis protein NEP1-like [Oryctolagus cuniculus]

CTTCTAGTTCAGCTTTTACACAAGCTCAGTGTACGAGCAGCTGATGGTCCCCAGAAGCTT

TTGAAGGTAATTAAGAATCCAGTGTCAGATCACTTCCCAGTTGGTTGTATGAAAATTGGC

ACTTCATTTTCTGTTCCAGTCATCAGTGATGTGCGACAACTGGTGCCTAGGAGTGACCCT

ATTGTTTTTGTGGTGGGGGCCTTTGCCCATGGCAAGGTCAATGTGGAATATACAGAGAAG

>CL10.Contig1_All 79 459 PREDICTED: phosphopantothenoylcysteine decarboxylase-like [Equus caballus]

ATGAAGTCCAGGCTTTGCAGGCTCCCAGAATTTTAAGCCATTAGACCCCACATGGAATCA

TGTCCAGGTGCTGCATCCTTGGTGGAGAGAAAATTCCATGTTCTTGTGGGTGTCACCGGA

AGTGTTGCTGCTCTGAAGTTGCCTTTTTTGGTGTCAAAGCTTTTGGACATTCCTGGGCTG

GAAGTCACAGTGGTCACAACTGAAAGAGCCAAACATTTCTACAACCCCCAGGACATTCCT

GTCACCCTCTACAGTGACTCTGATGAATGGGAGATGTGGAAGCACCGCTCTGACCCAGTT

CTCCACGTTGATCTGCGGAGGTGGGCTGACCTGATGCTAGTGGCTCCTCTAGATGCCACT

GGCCACCTTCCCCAGGGTGTT

>CL10.Contig2_All 48 428 PREDICTED: phosphopantothenoylcysteine decarboxylase-like [Equus caballus]

ATGAAGTCCAGGCTTTGCAGGCTCCCAGAATTTTAAGCCATTAGACCCCACATGGAATCA

TGTCCAGGTGCTGCATCCTTGGTGGAGAGAAAATTCCATGTTCTTGTGGGTGTCACCGGA

AGTGTTGCTGCTCTGAAGTTGCCTTTTTTGGTGTCAAAGCTTTTGGACATTCCTGGGCTG

GAAGTCACAGTGGTCACAACTGAAAGAGCCAAACATTTCTACAACCCCCAGGACATTCCT

GTCACCCTCTACAGTGACTCTGATGAATGGGAGATGTGGAAGCACCGCTCTGACCCAGTT

CTCCACGTTGATCTGCGGAGGTGGGCTGACCTGATGCTAGTGGCTCCTCTAGATGCCACT

GGCCACCTTCCCCAGGGTGTT

>CL10.Contig3_All 77 685 minus strand PREDICTED: peroxiredoxin-1 isoform 6 [Papio anubis]

GCTGATAGCAGGATGTCTTCAGGAAATGCCAAAATTGGGCACCCTGCCCCCAACTTCAAA

GCTACAGCTGTGATGCCAGATGGCCAGTTCAAAGATATTAGTCTATCTGACTACAGAGGA

AAATACATTGTGTTCTTCTTTTACCCTCTTGACTTCACCTTTGTGTGCCCTACGGAGATC

ATTGCTTTTAGTGACAGGGCAGAAGAATTTAAAAAACTTAACTGCCAAGTGATTGGTGCT

TCTGTGGATTCTCACTTCTGTCATCTGGCATGGATCAATACACCCAAGAAACAAGGAGGA

CTGGGACCCATGAATATTCCCTTGATATCAGATCCTAAGCGCACCATTGCTCAGGATTAT

GGAGTCTTAAAGGCTGATGAAGGCATTTCCTTCAGGGGCCTCTTTATCATTGATGATAAG

GGTATCCTTCGCCAGATCACTGTGAATGACCTTCCTGTTGGCCGCTCTGTGGATGAAACT

CTGAGACTAGTTCAGGCCTTCCAGTTCACTGACAAACATGGGGAAGTGTGCCCTGCTGGC

TGGAAGCCTGGCAGTGATACCATCAAGCCTGATGTCCAAAGAAGCAAAGAATATTTCTCC

AAGCAGAAG

>CL11.Contig1_All 198 1604 minus strand unnamed protein product [Homo sapiens]

ATGAGGCTGCTGGGTCTTGTCCTGTGCCTGGTCACAGCTCCCCAAGGTGTCCTGTGCCAG

GTGCAGCTGCAGGAGTCAGGACCTGGCCTGGTGAAGCCCTCGCAGACCCTGTCCCTCACC

TGTGCTGTCTCTGGATTCTCCATCACAACCAGTGGTTACTGCTGGAGCTGGATCCGTCAG

CCCCCAGGGAAGGGGCTGGAGTACATAGGGTACATAAGTTATGATGGTAGCACTTACTAT

AGCCCATCCCTCAAGAGCCGAGTGTCCATCTCCAGAGACACGTCCAAGAACCAGTTCTCC

CTGCAGCTGAACTCCCTGACCACTGAGGACACAGCCACCTATTACTGTGCCAGAAGGGGT

ATCCCGAGGGCGGCATATTACTTTGATTACTGGGGCCCTGGCACCCTGGTCACCGTCTCC

TCAGCTTCGACCACGGCCCCATCAGTCTTCCCCCTGGCCCCCACCTCTAGGGACACATCT

GGCTCCTCAGTGAACCTGGGCTGCCTGGTCAAGGGCTACTTCCCTGAGCCGGTGACCGTG

ACATGGAACTCAGGATCCCTGTCCAGTGGAGTCCGCACCTTCCCAGCTGTCCTGCAGTCA

GGGCTCTACTCTGTCACCAGCATGGTGACTGTGCCCACCAGCACCTGGCCCAGCCAGACC

GTCACCTGCAATGTAGCCCACCCGGCCAGCAGCACCAAGGTGGACAAGACCATTGCTCCC

TGTGGTCCTGTACCTACCCCATGTCCACCATGTCCACCTCCTGAAATCCTGGGGGGACCA

TCCGTCTTCATCTTCCCCCCAAAGCCCAAGGACACGCTCATGATCTCCCTGACCCCCAAG

GTCACATGCGTGGTGGTAGATGTGAGCCAGGATGACCCCGAGGTCCAGTTCAGCTGGTAT

GTAGACAACGTGGAGGTGAGCACAGCTCAGACAAAGCCACAGGAGAAGCAACTCAACAGC

ACATTCCGCGTGGTCAGTGTCCTCCCCATCAAGCACCAGGACTGGCTGAATGGCAAGAAG

TTCAAGTGCAAGGTCAACAACAAAGCCCTGCCCGCCCCCATCGAGAAGACCATCTCCAAA

GCCGCAGGGCAACCCCGGGCGCCAGAGGTGTACGTCTTGCCCCCGGCCCAGGAGGAGCTG

ACCAAGAACACCGTCAGTCTGACCTGCTTGATCCAAGGCTTCTACCCTGCCGACATCTCT

GTGGAGTGGGAGAAGCAGGGGCAGCCGGAGCAGGACTCCAAGACCACCGAGCCCGTCCTG

GACTCCGACGGGACCTACTTCCTCTACAGCAAGCTCAGGGTGGACAGGAACAGATGGCAG

CGAGGAGACCTCTACACATGCTCAGTGATGCACGAGGCTCTGCATAACCACTTCACCCAG

AAGACCCTGTCCCCGTCCCCGGGTAAA

>CL11.Contig2_All 2 289 immunoglobulin heavy chain variable region [Mus musculus]

CTCACCTGTACTGTCTCTGGATTTTCCATCACAACTAAGAATTACTGGTGGAGCTGGATC

CGACAGACCCCAGGGAAGGGGCTGGAATATATAGGCTACATTAGTTATACTGGTGTCAGT

TACTCTAGTCCTTCCCTCGAGAGCCGAGTGTCCATCTCCAGAGACACGTCCAAGAATCAG

TTCTCCCTGCAGCTGAGTTCCCTGACCACTGAGGACACAGCCACCTATTACTGTGCCAGA

GACCCGACTTATATAGCTGGGGCTACATCTTACTGGGGCCCTGGCACC

>CL11.Contig3_All 3 278 immunoglobulin heavy chain variable region 194-103, partial [Homo sapiens]

TCTGGATTCTCCATCACAACCACTGGTCATGACTGGAGCTGGATTCGTCAGTCCCCAGGG

AAGGGCCTGGAGTGGATAGGATATATAACTTATAGTGGTCATACTGACTACAGTCCATCC

CTCAAGAGCCGAGTGTCCATCTCCAGAGACACGTCCAAGAATCAGTTCTCCCTGCAGCTG

ACTTCCCTGACCACAGAAGACACGGCCACCTATTACTGTGCCAGAGACCCGACTTATATA

GCTGGGGCTACATCTTACTGGGGCCCTGGCACCCTG

>CL11.Contig4_All 1 234 minus strand immunoglobulin heavy chain variable region [Homo sapiens]

TCACAGACCCTGTCCCTCACCTGTCCTGTCTCTGGTTACTACATCACCAGAGGTTATTAC

TGGAGCTGGATCCGTCGGCCCCCTGGGAAGGGACTGGAATGGATAGGAGACATAAGTTTT

GATGGTAGCACTTACTATGACCCGTCCATGAAGAGCCGAGTGTCCATTTCCAGAGACACG

TCCAAGAACCAGATCTCCCTGCAGCTGAGCTCTCTGACCACTGAGGACACAGCC

>CL11.Contig5_All 2 220 minus strand immunoglobulin mu heavy chain [Homo sapiens]

CTCACCTGTAGTGTCTCTGGTTACTCCATTAGCAGTGGTTACCTCTGGAGCTGGATCCGT

CAGCCCCCCGGGAAGGGGCTGGAATACATAGGATATATATGTTATAATGGTAACTTTAAT

TACAATCCATCCCTCGAGAGCCGAGCGTCAATCTCCAGAGACACGTTGAAGGACCAGTTC

TCCCTGCAACTCAGCTCACTGACCACTGAGGACACAGCC

>CL12.Contig1_All 151 1197 minus strand CD1d [Macaca mulatta]

ATGGGATGCGCGTGGTTCCTGCTGTTGTGGGTGCTTTCCCAGCTTTCGGGAAATTATGAG

GGCCACTCGCTGTACACTTCCAAGCTTCACTCATTCTCAGTCCCGCAAAAGAATTTCACC

TTCCTCTGGCTCCAGACCGCGTCCTTTCCCAGCAACACCTCCACACGCGCGGAGGGCGTG

GCGTGGCTGGGGGAGCTGCAGACGCACAGCTGGAGCAACACCGCAGACACTATTCGTTTT

GTGAAGCCCTGGGCTCGCGGCACGTTCAGCAGCCAACAATGGGACAAAATGCAGCACCTG

TATCTGGGCTTCCGAAGCAGTTTCACAAAAGACATTCGGAAGTTTGCTCAAATGCTGCGA

TTCAAGTATCCCATGGAGCTGCAGCTGTCTGTTGGCTGTGAGGTGCACCCTGGGAACGTC

TCAGAACACTTTCTCCATGTAGCATATCAAGGAGAATATATCCTGAGTTTCCAGGGAACT

GCTTGGGAGGCTGCCCCACAGGCCCCCCAGAGGGTAGATTTGGTCATCAAAGTGCTCAAC

CAGGACGAAGGGACAAAGGAAACAGTGCAGTGGCTCCTCAATGACATCTGCCCCCAATAT

GTGAATGGCCTCCTAGAGACAGGGAAGTCAGAACTGGAGAAACAAGTGAAGCCTGAGGCC

TGGCTGTCCAGTGGCCCCAGTCCTGGGCCTGGGCATCTGCTGCTGGTGTGCCACGTGTCT

GGCTTCTACCCAAAGCCCGTGTGGGTGATGTGGATGCGAGGGGAACAGGAGCAGCAGGGC

ACTCAGAGAGGTGACTTCCTGCCCAACGCAGATGGGACCTGGAACCTCCGAGCAACCCTG

GATGTGGCAGCTGGGGAGGCGGCTGGCCTGGCCTGCAGGGTGAAGCACAGCAGCCTAGGA

GGGCAGGACCTCGTCCTCTACTGGGGTGGGAGCCACACCTCTGTGGGCTTGGTCATCCTG

GTGGTACTGGCTTGCCTGGTGCTGCTCTTTGTGCTCATTCTAGGCTTCTATTGGATCAGG

AGACACCGCTCCTATGAAGACATCTTG

>CL13.Contig1_All 3 233 immunoglobulin heavy chain variable region [Homo sapiens]

AAGGGGTTGGAGTGGGTTGCTCATATAAGAAACAAAGCTAATAGTTACAGCACAGTATAC

GCTGCATCTGTAAAAGACCGATTCACCATCTCCAGAGAGGATGCCAAGAACACACTGTAC

CTGCAAATGAGCAGTCTGAGAGCAGAGGACACTGCCACCTATTACTGTGCTTTATTGAGT

TCTGAGTACTACGATGAAACTCCTAGAAGTTACTATAGCTACGAATTAGAT

>CL14.Contig1_All 436 3528 minus strand platelet endothelial aggregation receptor 1 precursor [Homo sapiens] >gi|74757035|sp|Q5VY43.1|PEAR1_HUMAN RecName: Full=Platelet endothelial aggregation receptor 1; Short=hPEAR1; AltName: Full=Multiple epidermal growth factor-like domains protein 12; Short=Multiple EGF-like domains protein 12; Flags: Precursor >gi|55662090|emb|CAH70011.1| platelet endothelial aggregation receptor 1 [Homo sapiens] >gi|55662302|emb|CAH72941.1| platelet endothelial aggregation receptor 1 [Homo sapiens]

ATGTTGCCACCTCTGCGGACCCTCCTTCTCCTGGCCCTAGGCCTGGGACTGGCCAGGACG

CTCAGCTCCAGTGATCCTAATGCCTGCAGCTTCTGGGAAAGCTTCACTACGACCACCAAG

GAGTCCCACTCCCGCCCGTTCAGCCTCCACCCCTCGGAGCCCTGCGACAGGCCCTGGGAG

GGCCCCCAAACCTGCTCCCAGCCCACGGTCGTCTACCGGACTGTGTATCGGCAGGTGGTA

AAGACGGGCCACCGCCCGCGCTTACAGTGCTGCCAGGGCTTCTACGAGAGCAGCGGGACC

TGTGTCCCGCTCTGTGCCCAGGAATGTGTCCATGGCCGTTGTGTGGCGCCCAACCAGTGC

CAGTGTGCACCAGGCTGGAAGGGTGACGATTGCTCTAGCGAGTGTGGTCCCGGGGTGTGG

GGGCCACGGTGTGACAAGCCCTGCGGCTGTGGCAACAGCAGCTCCTGTGACCCCAAGAGC

GGGGTATGTTCTTGCCCTCCTGGCCTGCAGCCTCCCAACTGCCTTCGGCCTTGTTCCCCT

GGCTACTATGGCCCTGCCTGCCGGTTCAGCTGCCAGTGTCACGGGGCACCCTGCGATCCC

CAGACCGGAGCCTGCTTCTGCCCTCCAGGGAGAACGGGGCCCAGCTGTGATGTGTCCTGT

CCCCAGGGCACGGCTGGCTTCTTCTGTCCCAGCACCTCTCCTTGCCAAAATGGGGGTATC

TTCCAGGACTCCCAGGGCTCCTGCAGCTGCCCACCTGGCTGGATGGGCATCATCTGCTCC

CTGCCCTGCCCAGAGGGCTTTCATGGACCCAACTGCTCCCAGGAATGTCTCTGCCACAAT

GGTGGCCTCTGTGACCGATTCACTGGACAGTGCCGCTGCGCTCCAGGCTACACGGGGGAT

CGGTGCCACGAGGAGTGCCCAGTGGGCCGCTTTGGCCAGGACTGTGCTGAAACGTGCGAC

TGCGCCTCAGGCGCCCGCTGCTTCCCGGCCAACGGCGCGTGCCTGTGCGAGCACGGCTTC

ACCGGGGACCGCTGTGCGGAGCGCCTCTGCCCCGACGGCCGGTACGGCCTCGGATGTCAG

TCGTCCTGCACCTGCGACCCGGAGCACAGTCTCAGCTGCCACCCGATGCACGGGGAGTGC

TCCTGCCGCCCGGGCTGGGCCGGCCTCCACTGCAACGAGAGCTGCCCGCAGGACACGCAC

GGGCCGGGTTGCCAGGAGCACTGCCTCTGCCTGCACGGCGGCGTCTGCCTGGCAGACAGC

GGCCTCTGCCGGTGCGCTCCCGGCTACACGGGGCCTCACTGCGCCAGCCTCTGTCCTCCC

GACACTTATGGGTTCAACTGCTCCTCGCGCTGCTCCTGCGAAAATGCCATCGCCTGCTCA

CCCCTCGACGGCTCCTGCATCTGCAAGGAAGGTTGGCAGCGTGGGAACTGTTCTGTGCCC

TGCCCGCCTGGATCCTGGGGCTTCAACTGCAATGCCAGCTGCCAGTGTGCCCATGACGGA

GTCTGTAGCCCCCAAACTGGTGCCTGTACCTGCACCCCAGGGTGGCACGGGGCCCGCTGC

CAGCTGCCCTGCCCGAAGGGGCAGTTTGGCAAAGGTTGTGCCAGTCACTGTGACTGTGAC

CATTCTGATGGCTGTGACCCGGTCCATGGACACTGCCAATGCCAGGCTGGCTGGATGGGT

AACCGCTGCCACCTGCCCTGCCCCGAGGGCTTCTGGGGAGCCAACTGTAGCAATACCTGC

ACCTGCAAGAATGGAGGCACCTGTGTCCCTGAAAGTGGCAGTTGTGTGTGTGCACCTGGA

TTTCGAGGCCCCTCCTGCCAGAGACCCTGCCCACCTGGCCGCTATGGCAAACGCTGCGTA

CCCTGCAAGTGTAATAACCACTCCTCCTGCCACCCCTCGGATGGGACCTGCTACTGCCTG

GCTGGCTGGACGGGCCCTGACTGCTCCCAACTATGCCCCCTAGGCCACTGGGGAACCAAC

TGTGCCCAGGCCTGCCAGTGTCACCACGGTGGGACCTGCCACCCCCAGAATGGAAGCTGC

ATCTGTGCCCCAGGCTGGACCGGACTCCATTGCTTGGAAGGCTGCCCCCCAGGGGTGTTT

GGTGCCAACTGCTCCCAGCCATGCCAGTGTGGTCCAGGAGAGCGGTGCCACCCAGAGACT

GGCGCCTGTGTGTGTCCCCCAGGGCACAGTGGTGCACCTTGCAGGATTGGGAGCCAGGAG

CCCTTCACCATGATGCCTACCTCCCCTGTGGCCTATAACTCACTGGGCGCAGTGATCGGC

GTTGCAGTGCTGGGATCCCTGGTAGTGGCCCTGGTGGCACTGTTCATTGGCTACCGCCAT

TGGCAAAAGGGCAAGGAGCACCAGCACCTGGCGGTGGCCTACAGCACTGGACGGCTGGAT

GGCTCCGAGTATGTCATGCCAGATGTCCCTCCAAGCTACAGTCACTACTACTCCAACCCC

AGCTACCACACCCTGTCCCAGTGCTCTCCGAATCCCCCGCCCCCTAACAAGGTTCCAGGC

AGTCAGCTCTTCGCCAGCCTCCAGGGACCCGAGCGGCCAGGTGGAGCCCACGGGCATGAC

AACCACGCCACCCTGCCTGCTGACTGGAAGCACCGGCCTCTGGACAGGGGTGGCAGTCGC

CTGGATCGAAGTTACAGCTACAGCCACAGTAATGGCACAGGCCCCTTCTATAATAAAGGG

CCCATCTCTGAAGAACAGCTGGGGGCCAGCATGGCTTCTCTGAGCAGTGAGAACCCCTAT

GCCACCATCCGGGATCTGCCCGGCCTGCCAGGGGGCCCCCGGGAGAGCAGCTATGTGGAG

ATGAAAGGCCCTCCCTCAGGGTCTCCCCCCAGGCCTCTCCAATTCCGGGACAGCCAGAGG

CGGCGGCACCCACAGCCACAGAGAGACAGTGGCACCTATGAACAGCCCAGCCCCCTGACC

CACGATCAAGACTCTGTGGGCTCCCTGCCCCCACTTCCTCCAGGTCTGCCCCCCGGCCAC

TACGACTCACCCAAGAACAGCCACATCCCCGGACACTATGACTTGCCTCCAGTACGACAC

CCCCCATCACCCCCACTCTGGCGCCAGGATCGC

>CL14.Contig2_All 493 3585 minus strand platelet endothelial aggregation receptor 1 precursor [Homo sapiens] >gi|74757035|sp|Q5VY43.1|PEAR1_HUMAN RecName: Full=Platelet endothelial aggregation receptor 1; Short=hPEAR1; AltName: Full=Multiple epidermal growth factor-like domains protein 12; Short=Multiple EGF-like domains protein 12; Flags: Precursor >gi|55662090|emb|CAH70011.1| platelet endothelial aggregation receptor 1 [Homo sapiens] >gi|55662302|emb|CAH72941.1| platelet endothelial aggregation receptor 1 [Homo sapiens]

ATGTTGCCACCTCTGCGGACCCTCCTTCTCCTGGCCCTAGGCCTGGGACTGGCCAGGACG

CTCAGCTCCAGTGATCCTAATGCCTGCAGCTTCTGGGAAAGCTTCACTACGACCACCAAG

GAGTCCCACTCCCGCCCGTTCAGCCTCCACCCCTCGGAGCCCTGCGACAGGCCCTGGGAG

GGCCCCCAAACCTGCTCCCAGCCCACGGTCGTCTACCGGACTGTGTATCGGCAGGTGGTA

AAGACGGGCCACCGCCCGCGCTTACAGTGCTGCCAGGGCTTCTACGAGAGCAGCGGGACC

TGTGTCCCGCTCTGTGCCCAGGAATGTGTCCATGGCCGTTGTGTGGCGCCCAACCAGTGC

CAGTGTGCACCAGGCTGGAAGGGTGACGATTGCTCTAGCGAGTGTGGTCCCGGGGTGTGG

GGGCCACGGTGTGACAAGCCCTGCGGCTGTGGCAACAGCAGCTCCTGTGACCCCAAGAGC

GGGGTATGTTCTTGCCCTCCTGGCCTGCAGCCTCCCAACTGCCTTCGGCCTTGTTCCCCT

GGCTACTATGGCCCTGCCTGCCGGTTCAGCTGCCAGTGTCACGGGGCACCCTGCGATCCC

CAGACCGGAGCCTGCTTCTGCCCTCCAGGGAGAACGGGGCCCAGCTGTGATGTGTCCTGT

CCCCAGGGCACGGCTGGCTTCTTCTGTCCCAGCACCTCTCCTTGCCAAAATGGGGGTATC

TTCCAGGACTCCCAGGGCTCCTGCAGCTGCCCACCTGGCTGGATGGGCATCATCTGCTCC

CTGCCCTGCCCAGAGGGCTTTCATGGACCCAACTGCTCCCAGGAATGTCTCTGCCACAAT

GGTGGCCTCTGTGACCGATTCACTGGACAGTGCCGCTGCGCTCCAGGCTACACGGGGGAT

CGGTGCCACGAGGAGTGCCCAGTGGGCCGCTTTGGCCAGGACTGTGCTGAAACGTGCGAC

TGCGCCTCAGGCGCCCGCTGCTTCCCGGCCAACGGCGCGTGCCTGTGCGAGCACGGCTTC

ACCGGGGACCGCTGTGCGGAGCGCCTCTGCCCCGACGGCCGGTACGGCCTCGGATGTCAG

TCGTCCTGCACCTGCGACCCGGAGCACAGTCTCAGCTGCCACCCGATGCACGGGGAGTGC

TCCTGCCGCCCGGGCTGGGCCGGCCTCCACTGCAACGAGAGCTGCCCGCAGGACACGCAC

GGGCCGGGTTGCCAGGAGCACTGCCTCTGCCTGCACGGCGGCGTCTGCCTGGCAGACAGC

GGCCTCTGCCGGTGCGCTCCCGGCTACACGGGGCCTCACTGCGCCAGCCTCTGTCCTCCC

GACACTTATGGGTTCAACTGCTCCTCGCGCTGCTCCTGCGAAAATGCCATCGCCTGCTCA

CCCCTCGACGGCTCCTGCATCTGCAAGGAAGGTTGGCAGCGTGGGAACTGTTCTGTGCCC

TGCCCGCCTGGATCCTGGGGCTTCAACTGCAATGCCAGCTGCCAGTGTGCCCATGACGGA

GTCTGTAGCCCCCAAACTGGTGCCTGTACCTGCACCCCAGGGTGGCACGGGGCCCGCTGC

CAGCTGCCCTGCCCGAAGGGGCAGTTTGGCAAAGGTTGTGCCAGTCACTGTGACTGTGAC

CATTCTGATGGCTGTGACCCGGTCCATGGACACTGCCAATGCCAGGCTGGCTGGATGGGT

AACCGCTGCCACCTGCCCTGCCCCGAGGGCTTCTGGGGAGCCAACTGTAGCAATACCTGC

ACCTGCAAGAATGGAGGCACCTGTGTCCCTGAAAGTGGCAGTTGTGTGTGTGCACCTGGA

TTTCGAGGCCCCTCCTGCCAGAGACCCTGCCCACCTGGCCGCTATGGCAAACGCTGCGTA

CCCTGCAAGTGTAATAACCACTCCTCCTGCCACCCCTCGGATGGGACCTGCTACTGCCTG

GCTGGCTGGACGGGCCCTGACTGCTCCCAACTATGCCCCCTAGGCCACTGGGGAACCAAC

TGTGCCCAGGCCTGCCAGTGTCACCACGGTGGGACCTGCCACCCCCAGAATGGAAGCTGC

ATCTGTGCCCCAGGCTGGACCGGACTCCATTGCTTGGAAGGCTGCCCCCCAGGGGTGTTT

GGTGCCAACTGCTCCCAGCCATGCCAGTGTGGTCCAGGAGAGCGGTGCCACCCAGAGACT

GGCGCCTGTGTGTGTCCCCCAGGGCACAGTGGTGCACCTTGCAGGATTGGGAGCCAGGAG

CCCTTCACCATGATGCCTACCTCCCCTGTGGCCTATAACTCACTGGGCGCAGTGATCGGC

GTTGCAGTGCTGGGATCCCTGGTAGTGGCCCTGGTGGCACTGTTCATTGGCTACCGCCAT

TGGCAAAAGGGCAAGGAGCACCAGCACCTGGCGGTGGCCTACAGCACTGGACGGCTGGAT

GGCTCCGAGTATGTCATGCCAGATGTCCCTCCAAGCTACAGTCACTACTACTCCAACCCC

AGCTACCACACCCTGTCCCAGTGCTCTCCGAATCCCCCGCCCCCTAACAAGGTTCCAGGC

AGTCAGCTCTTCGCCAGCCTCCAGGGACCCGAGCGGCCAGGTGGAGCCCACGGGCATGAC

AACCACGCCACCCTGCCTGCTGACTGGAAGCACCGGCCTCTGGACAGGGGTGGCAGTCGC

CTGGATCGAAGTTACAGCTACAGCCACAGTAATGGCACAGGCCCCTTCTATAATAAAGGG

CCCATCTCTGAAGAACAGCTGGGGGCCAGCATGGCTTCTCTGAGCAGTGAGAACCCCTAT

GCCACCATCCGGGATCTGCCCGGCCTGCCAGGGGGCCCCCGGGAGAGCAGCTATGTGGAG

ATGAAAGGCCCTCCCTCAGGGTCTCCCCCCAGGCCTCTCCAATTCCGGGACAGCCAGAGG

CGGCGGCACCCACAGCCACAGAGAGACAGTGGCACCTATGAACAGCCCAGCCCCCTGACC

CACGATCAAGACTCTGTGGGCTCCCTGCCCCCACTTCCTCCAGGTCTGCCCCCCGGCCAC

TACGACTCACCCAAGAACAGCCACATCCCCGGACACTATGACTTGCCTCCAGTACGACAC

CCCCCATCACCCCCACTCTGGCGCCAGGATCGC

>CL15.Contig1_All 11 304 CD55 antigen [Mus musculus]

AAGCCCACCACAGTCAGTGTACCAGGTACAAGAGCCCCACCAACACCGCAGAAACCCACC

ACAGTAAATATTCCAGCTACAAGAGCCCCACCAACACCCGAGAAGCCCACCACAACCAGT

GTACCAGCAACACAGCGCCCACCTGTTCCCAAAGCAACCACGAGTTTTTATACAACAAGA

GTAACGGAAGGAAAAGAAACTTCTCCTTCAGGTGCTGTCAGTCTTATATATGGGCACACA

TGTATAACTTTGACAGTTTTGCTTGTGACGCTAGGAACCATTGGCTATCTGACT

>CL15.Contig2_All 135 320 minus strand RecName: Full=Complement decay-accelerating factor; AltName: CD_antigen=CD55; Flags: Precursor

GTTGCTGCTGCTATAATAATTGGTATCCTAATTCTAGTCAAAATTTTCTGGGACTATGGA

AAATCAGGCTCTTATAACACACATGAGAACAACAAAGCAGTCAATGTTATGTTTCATAAT

TTAAGTGAGACTGATGATACCTCAGAAGTCAGACATTCTGATAAATTATAAAAGGGCACA

CATGTA

>CL15.Contig3_All 2 1123 decay-accelerating factor splicing variant 1 [Homo sapiens]

TGCAACGAAGGCTTTGTCAAAATTCCTGGGAAGCCAGACTCAGTGGTCTGTCTTTCCAAC

AATCAATGGTCAGAGTTTATAGAATTCTGTAATCGTAGCTGTGATGTTCCAACTAGGCTA

CTTTTTGCATCCCTCAAAAAGGATTATAATATGCAGAATTATTTCCCAGTTGGTTTCACT

GTGGAATATGAGTGCCGACCTGGATACGCAAGGGACCCTTCTCAGTCTGTAAAACTAACT

TGCCTTCAGAATTTAACATGGTCCAAAGCAGCTGAATTTTGTAAAAAAAAATCATGTCCT

AATCCTGGAGAACTACGAAATGGTGATATCAATATCACAACTGGCCTATTTTATGGTTCA

CCCATCTTCTTCTCATGTAACACAGGGTATAGATTAGTTGGCGCATCTTCTAGTTACTGT

ACTCTTTTTGACAGAACTCTTAAATGGAGTGAACCATTGCCAGAGTGCGTAGAAATTTAC

TGTCCAGAACCAACCCAAATTGACAATGGCTATATTCAAGAGGAACACGAAGTTTATGTA

TATAGACAGTCTATAACATATGGATGTAATAAAGGATTCAACCTGGTTGGAAAGAGCTCC

ATTTATTGTACTGTCAAAGAAAACGAAGGAGAATGGAGTGACCTGCCACCTCAGTGCATA

GGAAAATCCTCAGGTTCCAAGCCTTCACCGACAGCTCAGAAGCCCACCACAGTCAGTGTA

CCAGGTACAAGAGCCCCACCAACACCGCAGAAACCCACCACAGTAAATATTCCAGCTACA

AGAGCCCCACCAACACCCGAGAAGCCCACCACAACCAGTGTACCAGCAACACAGCGCCCA

CCTGTTCCCAAAGCAACCACGAGTTTTTATACAACAAGAGTAACGGAAGGAAAAGAAACT

TCTCCTTCAGGTGCTGTCAGTCTTATATATGGATTTGTTGCTGCTGCTATAATAATTGGT

ATCCTAATTCTAGTCAAAATTTTCTGGGACTATGGAAAATCAGGGGCACACATGTATAAC

TTTGACAGTTTTGCTTGTGACGCTAGGAACCATTGGCTATCTGACTTAGCCAAAGAAGAG

TTAAGAAGAAAGTGTACTCATGTACACAGAATACTTGTAGTT

>CL15.Contig4_All 205 327 minus strand RecName: Full=Complement decay-accelerating factor; AltName: CD_antigen=CD55; Flags: Precursor

TCCAGCTCTTATAACACACATGAGAACAACAAAGCAGTCAATGTTATGTTTCATAATTTA

AGTGAGACTGATGATACCTCAGAAGTCAGACATTCTGATAAATTATAAAAGGGCACACAT

GTA

>CL15.Contig5_All 2 1204 decay-accelerating factor splicing variant 4 [Homo sapiens]

TGCAACGAAGGCTTTGTCAAAATTCCTGGGAAGCCAGACTCAGTGGTCTGTCTTTCCAAC

AATCAATGGTCAGAGTTTATAGAATTCTGTAATCGTAGCTGTGATGTTCCAACTAGGCTA

CTTTTTGCATCCCTCAAAAAGGATTATAATATGCAGAATTATTTCCCAGTTGGTTTCACT

GTGGAATATGAGTGCCGACCTGGATACGCAAGGGACCCTTCTCAGTCTGTAAAACTAACT

TGCCTTCAGAATTTAACATGGTCCAAAGCAGCTGAATTTTGTAAAAAAAAATCATGTCCT

AATCCTGGAGAACTACGAAATGGTGATATCAATATCACAACTGGCCTATTTTATGGTTCA

CCCATCTTCTTCTCATGTAACACAGGGTATAGATTAGTTGGCGCATCTTCTAGTTACTGT

ACTCTTTTTGACAGAACTCTTAAATGGAGTGAACCATTGCCAGAGTGCGTAGAAATTTAC

TGTCCAGAACCAACCCAAATTGACAATGGCTATATTCAAGAGGAACACGAAGTTTATGTA

TATAGACAGTCTATAACATATGGATGTAATAAAGGATTCAACCTGGTTGGAAAGAGCTCC

ATTTATTGTACTGTCAAAGAAAACGAAGGAGAATGGAGTGACCTGCCACCTCAGTGCATA

GGAAAATCCTCAGGTTCCAAGCCTTCACCGACAGCTCAGAAGCCCACCACAGTCAGTGTA

CCAGGTACAAGAGCCCCACCAACACCGCAGAAACCCACCACAGTAAATATTCCAGCTACA

AGAGCCCCACCAACACCCGAGAAGCCCACCACAACCAGTGTACCAGCAACACAGCGCCCA

CCTGTTCCCAAAGCAACCACGAGTTTTTATACAACAAGAGTAACGGAAGGAAAAGAAACT

TCTCCTTCAGGTGCTGTCAGTCTTATATATGGATTTGTTGCTGCTGCTATAATAATTGGT

ATCCTAATTCTAGTCAAAATTTTCTGGGACTATGGAAAATCAGGCTCTTATAACACACAT

GAGAACAACAAAGCAGTCAATGTTATGTTTCATAATTTAAGTGAGACTGATGATACCTCA

GAAGTCAGACATTCTGATAAATTATAAAAGGTTGTCTGTAGGAGATAATCATTGATTTTT

GGGCACACATGTATAACTTTGACAGTTTTGCTTGTGACGCTAGGAACCATTGGCTATCTG

ACT

>CL15.Contig6_All 164 658 decay-accelerating factor [Gorilla gorilla]

TTCCCAGTTGGTTTCACTGTGGAATATGAGTGCCGACCTGGATACGCAAGGGACCCTTCT

CAGTCTGTAAAACTAACTTGCCTTCAGAATTTAACATGGTCCAAAGCAGCTGAATTTTGT

AAAAAAAAATCATGTCCTAATCCTGGAGAACTACGAAATGGTGATATCAATATCACAACT

GGCCTATTTTATGGTTCACCCATCTTCTTCTCATGTAACACAGGGTATAGATTAGTTGGC

GCATCTTCTAGTTACTGTACTCTTTTTGACAGAACTCTTAAATGGAGTGAACCATTGCCA

GAGTGCGTAGAAATTTACTGTCCAGAACCAACCCAAATTGACAATGGCTATATTCAAGAG

GAACACGAAGTTTATGTATATAGACAGTCTATAACATATGGATGTAATAAAGGATTCAAC

CTGGTTGGAAAGAGCTCCATTTATTGTACTGTCAAAGAAAACGAAGGAGAATGGAGTGAC

CTGCCACCTCAGTGC

>CL15.Contig7_All 1359 1475 complement decay-accelerating factor isoform 2 precursor [Homo sapiens] >gi|119613891|gb|EAW93485.1| CD55 antigen, decay accelerating factor for complement (Cromer blood group), isoform CRA_a [Homo sapiens] >gi|119613893|gb|EAW93487.1| CD55 antigen, decay accelerating factor for complement (Cromer blood group), isoform CRA_a [Homo sapiens] >gi|225451|prf||1303335A decay accelerating factor long

GCACACATGTATAACTTTGACAGTTTTGCTTGTGACGCTAGGAACCATTGGCTATCTGAC

TTAGCCAAAGAAGAGTTAAGAAGAAAGTGTACTCATGTACACAGAATACTTGTAGTT

>CL15.Contig8_All 137 1183 minus strand decay-accelerating factor splicing variant 4 [Homo sapiens]

ATGAGCCCCGCGCGGCCGAGCGTGCTGCCCGCAGTGCTGCGCCTGCTGTCCCTGCTGCTG

CTGCTGCAGCTGCCGACTCCACCGGGTGTCCGGGGTGACTGCGGCCTTCCGCCAGAAGTG

CCTAATGCCCAGCCAACTCTTGAAGGGAGTACCAGTTTTCCTGAGAAAACCACAATAACA

TATAAATGCAACGAAGGCTTTGTCAAAATTCCTGGGAAGCCAGACTCAGTGGTCTGTCTT

TCCAACAATCAATGGTCAGAGTTTATAGAATTCTGTAATCGTAGCTGTGATGTTCCAACT

AGGCTACTTTTTGCATCCCTCAAAAAGGATTATAATATGCAGAATTATTTCCCAGTTGGT

TTCACTGTGGAATATGAGTGCCGACCTGGATACGCAAGGGACCCTTCTCAGTCTGTAAAA

CTAACTTGCCTTCAGAATTTAACATGGTCCAAAGCAGCTGAATTTTGTAAAAAAAAATCA

TGTCCTAATCCTGGAGAACTACGAAATGGTGATATCAATATCACAACTGGCCTATTTTAT

GGTTCACCCATCTTCTTCTCATGTAACACAGGGTATAGATTAGTTGGCGCATCTTCTAGT

TACTGTACTCTTTTTGACAGAACTCTTAAATGGAGTGAACCATTGCCAGAGTGCGTAGAA

ATTTACTGTCCAGAACCAACCCAAATTGACAATGGCTATATTCAAGAGGAACACGAAGTT

TATGTATATAGACAGTCTATAACATATGGATGTAATAAAGGATTCAACCTGGTTGGAAAG

AGCTCCATTTATTGTACTGTCAAAGAAAACGAAGGAGAATGGAGTGACCTGCCACCTCAG

TGCATAGGAAAATCCTCAGGTTCCAAGCCTTCACCGACAGCTCAGAAGCCCACCACAGTC

AGTGTACCAGGTACAAGAGCCCCACCAACACCGCAGAAACCCACCACAGTAAATATTCCA

GCTACAAGAGCCCCACCAACACCCGAGAAGCCCACCACAACCAGTGTACCAGCAACACAG

CGCCCACCTGTTCCCAAAGCAACCACG

>CL15.Contig9_All 1257 1373 PREDICTED: complement decay-accelerating factor isoform 3 [Nomascus leucogenys]

GCACACATGTATAACTTTGACAGTTTTGCTTGTGACGCTAGGAACCATTGGCTATCTGAC

TTAGCCAAAGAAGAGTTAAGAAGAAAGTGTACTCATGTACACAGAATACTTGTAGTT

>CL15.Contig10_All 137 1120 decay-accelerating factor splicing variant 4 [Homo sapiens]

ATGAGCCCCGCGCGGCCGAGCGTGCTGCCCGCAGTGCTGCGCCTGCTGTCCCTGCTGCTG

CTGCTGCAGCTGCCGACTCCACCGGGTGTCCGGGGTGACTGCGGCCTTCCGCCAGAAGTG

CCTAATGCCCAGCCAACTCTTGAAGGGAGTACCAGTTTTCCTGAGAAAACCACAATAACA

TATAAATGCAACGAAGGCTTTGTCAAAATTCCTGGGAAGCCAGACTCAGTGGTCTGTCTT

TCCAACAATCAATGGTCAGAGTTTATAGAATTCTGTAATCGTAGCTGTGATGTTCCAACT

AGGCTACTTTTTGCATCCCTCAAAAAGGATTATAATATGCAGAATTATTTCCCAGTTGGT

TTCACTGTGGAATATGAGTGCCGACCTGGATACGCAAGGGACCCTTCTCAGTCTGTAAAA

CTAACTTGCCTTCAGAATTTAACATGGTCCAAAGCAGCTGAATTTTGTAAAAAAAAATCA

TGTCCTAATCCTGGAGAACTACGAAATGGTGATATCAATATCACAACTGGCCTATTTTAT

GGTTCACCCATCTTCTTCTCATGTAACACAGGGTATAGATTAGTTGGCGCATCTTCTAGT

TACTGTACTCTTTTTGACAGAACTCTTAAATGGAGTGAACCATTGCCAGAGTGCGTAGAA

ATTTACTGTCCAGAACCAACCCAAATTGACAATGGCTATATTCAAGAGGAACACGAAGTT

TATGTATATAGACAGTCTATAACATATGGATGTAATAAAGGATTCAACCTGGTTGGAAAG

AGCTCCATTTATTGTACTGTCAAAGAAAACGAAGGAGAATGGAGTGACCTGCCACCTCAG

TGCATAGGAAAATCCTCAGGTTCCAAGCCTTCACCGACAGCTCAGAAGCCCACCACAGTC

AGTGTACCAGCAACACAGCGCCCACCTGTTCCCAAAGCAACCACGAGTTTTTATACAACA

AGAGTAACGGAAGGAAAAGAAACT

>CL16.Contig1_All 34 255 PREDICTED: death-associated protein kinase 3 [Bos taurus]

AGAAGAGTGGTGCTGGGCGCCGGGAACCCCGCCGCGCCCCGTCCCGTCGTGCTCGCTGCG

GCCGCCGCTTCGCAGCCTCCAGCCGGTTGTATGGTCCAGGCCTCCATGAGGAGCCCAAAC

ATGGAGACGTTCAAGCAGCAGAAGGTGGAGGACTTTTATGACATCGGAGAGGAGCTGGGG

AGTGGCCAGTTTGCTATCGTGAAGAAGTGCCGGGAGAAGAGC

>CL17.Contig1_All 1373 1582 minus strand PREDICTED: amyloid beta A4 precursor protein-binding family A member 3 [Canis lupus familiaris]

ATCTGCAGCCTCCTCCGCGGCGGCATCGCGGAGCGCGGGGGTGTGCGTGTGGGGCACCGC

ATCATTGAGATCAACGGGCAGAGCGTGGTGGCCACGCGGCACGAGCGCATCATCGAGCTG

CTCACCGAGGCCCACAGTGAGGTGCACATCAAGACCATGCCGGCCGCCACCTACCGCCTA

CTGACGGGCCAGGAGCAGCCCGTGTACCTG

>CL17.Contig2_All 84 821 PREDICTED: amyloid beta A4 precursor protein-binding family A member 3 [Sus scrofa]

TGGCACCTCCCGCAGGCCCAGCTCATTGCACGAGCCATAGGCCAGGCCTTCACCGTGGCC

TACAGCCAGTTCCTGCGCGAGAGCGGCATTGACCCCAGCCAGGTGGGCACCCAGCCCAGC

CAGGGGGCCGCCAGCCCTGGCCACCTCCACAACGGGGACCTGGACCACTTCTCCAACAGT

GACAACTGCAGGGAGGTGTGCATCGAGAAGCGCAGGGGTGAAGGCCTGGGTGTGGCCCTG

GTGGAGTCGGGCTGGGGCTCCCTGCTGCCCACAGCGGTCATCGCCAACCTGCTGCACGGG

GGCCCCGCCGAGCGCTCGGGCGCCCTCAGCATCGGCGACCGCCTCACTGCCATCAACGGG

ACCAGCCTGGTGGGGCTGCCCCTGGCCGCGTGCCAGGCAGCTGTGCGTGAGGTGAAGCCA

CAGACCTCAGTGAGGCTCAGCATCGTCCACTGCCCACCCGTCACCACTGCCATCATCCGG

AAACCCCATGCGCGTGAGCAGCTGGGCTTCTGTGTGGAGGACGGCATTATCTGCAGCCTC

CTCCGCGGCGGCATCGCGGAGCGCGGGGGTGTGCGTGTGGGGCACCGCATCATTGAGATC

AACGGGCAGAGCGTGGTGGCCACGCGGCACGAGCGCATCATCGAGCTGCTCACCGAGGCC

CACAGTGAGGTGCACATCAAGACCATGCCGGCCGCCACCTACCGCCTACTGACGGGCCAG

GAGCAGCCCGTGTACCTG

>CL17.Contig3_All 1676 1885 PREDICTED: amyloid beta A4 precursor protein-binding family A member 3 [Canis lupus familiaris]

ATCTGCAGCCTCCTCCGCGGCGGCATCGCGGAGCGCGGGGGTGTGCGTGTGGGGCACCGC

ATCATTGAGATCAACGGGCAGAGCGTGGTGGCCACGCGGCACGAGCGCATCATCGAGCTG

CTCACCGAGGCCCACAGTGAGGTGCACATCAAGACCATGCCGGCCGCCACCTACCGCCTA

CTGACGGGCCAGGAGCAGCCCGTGTACCTG

>CL17.Contig4_All 1559 1768 minus strand PREDICTED: amyloid beta A4 precursor protein-binding family A member 3 [Canis lupus familiaris]

ATCTGCAGCCTCCTCCGCGGCGGCATCGCGGAGCGCGGGGGTGTGCGTGTGGGGCACCGC

ATCATTGAGATCAACGGGCAGAGCGTGGTGGCCACGCGGCACGAGCGCATCATCGAGCTG

CTCACCGAGGCCCACAGTGAGGTGCACATCAAGACCATGCCGGCCGCCACCTACCGCCTA

CTGACGGGCCAGGAGCAGCCCGTGTACCTG

>CL17.Contig5_All 205 1920 minus strand PREDICTED: amyloid beta A4 precursor protein-binding family A member 3 [Canis lupus familiaris]

GCCCCCCGGCCGCGCTCAGAGCCTCGCGCCATGGACTTGGAGAAGCCCAAGGACCCGCTG

GTGCCCTCGGAGGACCTCAGCACTGAGAGTCAGTGGGACGAGGTGCCTGAGGCCCCCGGC

AGCCTGGGTCCGGTGGACCTCGAGGGGTCAAGCGTTCCGGGCCTGGTGCAGCAGTTTGAG

GCTCTGCCCGGAGACCTGATGGGCCTGCCCCCAGACAGCGCACCCTGCCCTCTGCACATT

GCCACTGGCCAGGGCCTGGACCCCCACGACACGACTGACGCCCACGGCCTCTTGTCTGCT

GAGGCTGGTCGGGATGATCTTCTGAGGCTTCTGCAGGGTGAGGGGCACTCGCCTTCTAAG

CCTGGTGCCCGGGAGCCTCCAGAGCCTGCCCATCCCCTGTTGCAGCCCCCTGAGGATGCA

GATGGAGAGTCCGAGCCCCCGGGATGGGCAGAGGGAGCCTCTGCCGAGCAGGGTGGTAGC

AGGAGCTCCAGCAGCTCCCCTGAGCCCTGGCTGGAGACCTCTCCTTTGGTCACACCCGAA

GAGCCACCTGACGGTGTCCAGGGCCCCAAGAGCCTGGCTTCATACCCTGCCCTCCAGGAG

GTACCCGGTCCCTGTGACCATGAGGACCTCATGGATGGTGTCATATTTGGGGCCAAATAC

TTGGGCTCCACCCAGCTGGTGTCGGAGCGGAACCCGCCCCCCAGCACGCGCATGGCCCAG

GCCCAGGAGGCGATGGACCGTGTCAAGGCCCCCGACGGGGAGACCCAGCCCATGACGGAG

GTGGACCTCTTCGTCTCCACCAAGAGGATCAAGGTCCTCACGGCCGATTCCCAGGAGGCC

ATGATGGACCACGCCCTGCAGACCATCTCCTACATCGCTGACATCGGCCAGGTCCTGGTG

CTCATGGCGCGGCGGCGGCTGGCACGGAGGTCACCCCAGGACCGAGGGCGCCGCCTGTGC

AAGATGATGTGCCACGTCTTCCACTCAGAGGACGCCCAGCTCATTGCACGAGCCATAGGC

CAGGCCTTCACCGTGGCCTACAGCCAGTTCCTGCGCGAGAGCGGCATTGACCCCAGCCAG

GTGGGCACCCAGCCCAGCCAGGGGGCCGCCAGCCCTGGCCACCTCCACAACGGGGACCTG

GACCACTTCTCCAACAGTGACAACTGCAGGGAGGTGTGCATCAAGAAGCGCAGGGGTGAA

GGCCTGGGTGTGGCCCTGGTGGAGTCGGGCTGGGGCTCCCTGCTGCCCACAGCGGTCATC

GCCAACCTGCTGCACGGGGGCCCCGCCGAGCGCTCGGGCGCCCTCAGCATCGGCGACCGC

CTCACTGCCATCAACGGGACCAGCCTGGTGGGGCTGCCCCTGGCCGCGTGCCAGGCAGCT

GTGCGTGAGGTGAAGCCACAGACCTCAGTGAGGCTCAGCATCGTCCACTGCCCACCCGTC

ACCACTGCCATCATCCGGAAACCCCATGCGCGTGAGCAGCTGGGCTTCTGTGTGGAGGAC

GGCATTATCTGCAGCCTCCTCCGCGGCGGCATCGCGGAGCGCGGGGGTGTGCGTGTGGGG

CACCGCATCATTGAGATCAACGGGCAGAGCGTGGTGGCCACGCGGCACGAGCGCATCATC

GAGCTGCTCACCGAGGCCCACAGTGAGGTGCACATCAAGACCATGCCGGCCGCCACCTAC

CGCCTACTGACGGGCCAGGAGCAGCCCGTGTACCTG

>CL17.Contig6_All 250 1617 minus strand PREDICTED: amyloid beta A4 precursor protein-binding family A member 3 [Canis lupus familiaris]

CCCAAGGACCCGCTGGTGCCCTCGGAGGACCTCAGCACTGAGAGTCAGTGGGACGAGCCC

CCTGAGGATGCAGATGGAGAGTCCGAGCCCCCGGGATGGGCAGAGGGAGCCTCTGCCGAG

CAGGGTGGTAGCAGGAGCTCCAGCAGCTCCCCTGAGCCCTGGCTGGAGACCTCTCCTTTG

GTCACACCCGAAGAGCCACCTGACGGTGTCCAGGGCCCCAAGAGCCTGGCTTCATACCCT

GCCCTCCAGGAGGTACCCGGTCCCTGTGACCATGAGGACCTCATGGATGGTGTCATATTT

GGGGCCAAATACTTGGGCTCCACCCAGCTGGTGTCGGAGCGGAACCCGCCCCCCAGCACG

CGCATGGCCCAGGCCCAGGAGGCGATGGACCGTGTCAAGGCCCCCGACGGGGAGACCCAG

CCCATGACGGAGGTGGACCTCTTCGTCTCCACCAAGAGGATCAAGGTCCTCACGGCCGAT

TCCCAGGAGGCCATGATGGACCACGCCCTGCAGACCATCTCCTACATCGCTGACATCGGC

CAGGTCCTGGTGCTCATGGCGCGGCGGCGGCTGGCACGGAGGTCACCCCAGGACCGAGGG

CGCCGCCTGTGCAAGATGATGTGCCACGTCTTCCACTCAGAGGACGCCCAGCTCATTGCA

CGAGCCATAGGCCAGGCCTTCACCGTGGCCTACAGCCAGTTCCTGCGCGAGAGCGGCATT

GACCCCAGCCAGGTGGGCACCCAGCCCAGCCAGGGGGCCGCCAGCCCTGGCCACCTCCAC

AACGGGGACCTGGACCACTTCTCCAACAGTGACAACTGCAGGGAGGTGTGCATCAAGAAG

CGCAGGGGTGAAGGCCTGGGTGTGGCCCTGGTGGAGTCGGGCTGGGGCTCCCTGCTGCCC

ACAGCGGTCATCGCCAACCTGCTGCACGGGGGCCCCGCCGAGCGCTCGGGCGCCCTCAGC

ATCGGCGACCGCCTCACTGCCATCAACGGGACCAGCCTGGTGGGGCTGCCCCTGGCCGCG

TGCCAGGCAGCTGTGCGTGAGGTGAAGCCACAGACCTCAGTGAGGCTCAGCATCGTCCAC

TGCCCACCCGTCACCACTGCCATCATCCGGAAACCCCATGCGCGTGAGCAGCTGGGCTTC

TGTGTGGAGGACGGCATTATCTGCAGCCTCCTCCGCGGCGGCATCGCGGAGCGCGGGGGT

GTGCGTGTGGGGCACCGCATCATTGAGATCAACGGGCAGAGCGTGGTGGCCACGCGGCAC

GAGCGCATCATCGAGCTGCTCACCGAGGCCCACAGTGAGGTGCACATCAAGACCATGCCG

GCCGCCACCTACCGCCTACTGACGGGCCAGGAGCAGCCCGTGTACCTG

>CL17.Contig7_All 16 426 minus strand PREDICTED: amyloid beta A4 precursor protein-binding family A member 3 [Canis lupus familiaris]

CCCAAGGACCCGCTGGTGCCCTCGGAGGACCTCAGCACTGAGAGTCAGTGGGACGAGCCC

CCTGAGGATGCAGATGGAGAGTCCGAGCCCCCGGGATGGGCAGAGGGAGCCTCTGCCGAG

CAGGGTGGTAGCAGGAGCTCCAGCAGCTCCCCTGAGCCCTGGCTGGAGACCTCTCCTTTG

GTCACACCCGAAGAGCCACCTGACGGTGTCCAGGGCCCCAAGAGCCTGGCTTCATACCCT

GCCCTCCAGGAGGTACCCGGTCCCTGTGACCATGAGGACCTCATGGATGGTGTCATATTT

GGGGCCAAATACTTGGGCTCCACCCAGCTGGTGTCGGAGCGGAACCCGCCCCCCAGCACG

CGCATGGCCCAGGCCCAGGAGGCGATGGACCGTGTCAAGGTGAGGCCAGGC

>CL18.Contig1_All 33 1082 PREDICTED: HEAT repeat-containing protein 7A-like [Ailuropoda melanoleuca]

AGCACGTCGCTGGAGGCCCTCAAGAGCCTCCTGTCCACCACGGGGCACTGGCATGACTTT

GCCCACCTTGAGCTGCAGGGCGCCTGGGAGCACTTCACCTCCATCAGCACCTACGCACAG

GGCGTGGGCCTCCTGGCCAGGGCTATGGTGCAGAACCACTGCCGGCAGATCAAGGCCGTG

CTCAGCTGGCTGCTGGGCCGCATGCAGAGCCAGGAGGAGCGGGAGAGGAAGGCGGCCGTC

CTCGTGCTCACTGAGGGCCTCCGGGATGAGAGCCCCGACATCCGCGTATGGAGCCTGCAG

GGCCTCGGGAACATCCTCTTCCACCCAGAGAAGGAAAGGCTGCTCCGAGGGCAGCTGCCG

CCCTTCCTCAACGGCTTCTTCCAGAACAGCGAGCCCGTGGTGGTGGACATCATGGGCACC

GTGTCCGACGTGCTGCACCGTTTGGGCACGCACGGCGCAGGGGCCCAGAGCCTCAGCGTG

GCCATCAATGCCCGCTCCTTCTTTGATGACGAGCGGGACAGGATTCGAGCAGCAGCCATG

GCACTGTTTGGGGATTTGGTGGCAACCATGGAGGGCAAGGAGCTAAGCGGCCTTCGAACC

CAGGTGTACCAGAGCATGGTACCTCTGCTGCTGCACCTGAAGGACAAGTGCCCCAAGGTT

GCCATGCAGGCCAAGTTCGCCTTCTACCGCTGTGCTCTGCTGCTGGGGTGGCGGCCGCGC

CACACCCTCTTCTGCACGCTGGCCTGGGAGCAAAGCCTCAGCGCCCGCCACTTCCTCTGG

ACCTGCCTGATGCGCGCCAGCTCTGAGGAGTTCAGCATCCACCTGGCCCAGGCCCTCAGC

TACCTGCACAGCCACCACCAGCACATGAAGACCTGGGCCGCGCTCTTCATAGGTTACACC

ATCTGCTACCACCCCCAGGCCGTGTCCCGGATGGTGAGCGAGGTGGACACCAACCTGCTG

TTCTGCACTTTTGAAGACCTCAAAAAGGACCCAGAGCCTGGCGTCCGGGAATTTGCCACC

AGGCAGCTCTCCTTCCTTCGGGAGGTGTCA

>CL18.Contig2_All 925 1056 PREDICTED: HEAT repeat-containing protein 7A-like [Ailuropoda melanoleuca]

GCCGTGTCCCGGATGGTGAGCGAGGTGGACACCAACCTGCTGTTCTGCACTTTTGAAGAC

CTCAAAAAGGACCCAGAGCCTGGCGTCCGGGAATTTGCCACCAGGCAGCTCTCCTTCCTT

CGGGAGGTGTCA

>CL18.Contig3_All 33 959 PREDICTED: HEAT repeat-containing protein 7A-like [Ailuropoda melanoleuca]

AGCACGTCGCTGGAGGCCCTCAAGAGCCTCCTGTCCACCACGGGGCACTGGCATGACTTT

GCCCACCTTGAGCTGCAGGGCGCCTGGGAGCACTTCACCTCCATCAGCACCTACGCACAG

GGCGTGGGCCTCCTGGCCAGGGCTATGGTGCAGAACCACTGCCGGCAGATCAAGGCCGTG

CTCAGCTGGCTGCTGGGCCGCATGCAGAGCCAGGAGGAGCGGGAGAGGAAGGCGGCCGTC

CTCGTGCTCACTGAGGGCCTCCGGGATGAGAGCCCCGACATCCGCGTATGGAGCCTGCAG

GGCCTCGGGAACATCCTCTTCCACCCAGAGAAGGAAAGGCTGCTCCGAGGGCAGCTGCCG

CCCTTCCTCAACGGCTTCTTCCAGAACAGCGAGCCCGTGGTGGTGGACATCATGGGCACC

GTGTCCGACGTGCTGCACCGTTTGGGCACGCACGGCGCAGGGGCCCAGAGCCTCAGCGTG

GCCATCAATGCCCGCTCCTTCTTTGATGACGAGCGGGACAGGATTCGAGCAGCAGCCATG

GCACTGTTTGGGGATTTGGTGGCAACCATGGAGGGCAAGGAGCTAAGCGGCCTTCGAACC

CAGGTGTACCAGAGCATGGTACCTCTGCTGCTGCACCTGAAGGACAAGTGCCCCAAGGTT

GCCATGATGCGCGCCAGCTCTGAGGAGTTCAGCATCCACCTGGCCCAGGCCCTCAGCTAC

CTGCACAGCCACCACCAGCACATGAAGACCTGGGCCGCGCTCTTCATAGGTTACACCATC

TGCTACCACCCCCAGGCCGTGTCCCGGATGGTGAGCGAGGTGGACACCAACCTGCTGTTC

TGCACTTTTGAAGACCTCAAAAAGGACCCAGAGCCTGGCGTCCGGGAATTTGCCACCAGG

CAGCTCTCCTTCCTTCGGGAGGTGTCA

>CL18.Contig4_All 802 933 PREDICTED: HEAT repeat-containing protein 7A-like [Ailuropoda melanoleuca]

GCCGTGTCCCGGATGGTGAGCGAGGTGGACACCAACCTGCTGTTCTGCACTTTTGAAGAC

CTCAAAAAGGACCCAGAGCCTGGCGTCCGGGAATTTGCCACCAGGCAGCTCTCCTTCCTT

CGGGAGGTGTCA

>CL18.Contig5_All 991 1122 PREDICTED: HEAT repeat-containing protein 7A-like [Ailuropoda melanoleuca]

GCCGTGTCCCGGATGGTGAGCGAGGTGGACACCAACCTGCTGTTCTGCACTTTTGAAGAC

CTCAAAAAGGACCCAGAGCCTGGCGTCCGGGAATTTGCCACCAGGCAGCTCTCCTTCCTT

CGGGAGGTGTCA

>CL18.Contig6_All 33 1148 PREDICTED: HEAT repeat-containing protein 7A-like [Ailuropoda melanoleuca]

AGCACGTCGCTGGAGGCCCTCAAGAGCCTCCTGTCCACCACGGGGCACTGGCATGACTTT

GCCCACCTTGAGCTGCAGGGCGCCTGGGAGCACTTCACCTCCATCAGCACCTACGCACAG

GGCGTGGGCCTCCTGGCCAGGGCTATGGTGCAGAACCACTGCCGGCAGATCAAGGCCGTG

CTCAGCTGGCTGCTGGGCCGCATGCAGAGCCAGGAGGAGCGGGAGAGGAAGGCGGCCGTC

CTCGTGCTCACTGAGTTCCTCTACAGCCCTGTCCTGCTGGAAGTGCTTCCCAAGCAGTCT

GCCCTGACCCTCCTGGCACAGGGCCTCCGGGATGAGAGCCCCGACATCCGCGTATGGAGC

CTGCAGGGCCTCGGGAACATCCTCTTCCACCCAGAGAAGGAAAGGCTGCTCCGAGGGCAG

CTGCCGCCCTTCCTCAACGGCTTCTTCCAGAACAGCGAGCCCGTGGTGGTGGACATCATG

GGCACCGTGTCCGACGTGCTGCACCGTTTGGGCACGCACGGCGCAGGGGCCCAGAGCCTC

AGCGTGGCCATCAATGCCCGCTCCTTCTTTGATGACGAGCGGGACAGGATTCGAGCAGCA

GCCATGGCACTGTTTGGGGATTTGGTGGCAACCATGGAGGGCAAGGAGCTAAGCGGCCTT

CGAACCCAGGTGTACCAGAGCATGGTACCTCTGCTGCTGCACCTGAAGGACAAGTGCCCC

AAGGTTGCCATGCAGGCCAAGTTCGCCTTCTACCGCTGTGCTCTGCTGCTGGGGTGGCGG

CCGCGCCACACCCTCTTCTGCACGCTGGCCTGGGAGCAAAGCCTCAGCGCCCGCCACTTC

CTCTGGACCTGCCTGATGCGCGCCAGCTCTGAGGAGTTCAGCATCCACCTGGCCCAGGCC

CTCAGCTACCTGCACAGCCACCACCAGCACATGAAGACCTGGGCCGCGCTCTTCATAGGT

TACACCATCTGCTACCACCCCCAGGCCGTGTCCCGGATGGTGAGCGAGGTGGACACCAAC

CTGCTGTTCTGCACTTTTGAAGACCTCAAAAAGGACCCAGAGCCTGGCGTCCGGGAATTT

GCCACCAGGCAGCTCTCCTTCCTTCGGGAGGTGTCA

>CL18.Contig7_All 33 1025 PREDICTED: HEAT repeat-containing protein 7A-like [Ailuropoda melanoleuca]

AGCACGTCGCTGGAGGCCCTCAAGAGCCTCCTGTCCACCACGGGGCACTGGCATGACTTT

GCCCACCTTGAGCTGCAGGGCGCCTGGGAGCACTTCACCTCCATCAGCACCTACGCACAG

GGCGTGGGCCTCCTGGCCAGGGCTATGGTGCAGAACCACTGCCGGCAGATCAAGGCCGTG

CTCAGCTGGCTGCTGGGCCGCATGCAGAGCCAGGAGGAGCGGGAGAGGAAGGCGGCCGTC

CTCGTGCTCACTGAGTTCCTCTACAGCCCTGTCCTGCTGGAAGTGCTTCCCAAGCAGTCT

GCCCTGACCCTCCTGGCACAGGGCCTCCGGGATGAGAGCCCCGACATCCGCGTATGGAGC

CTGCAGGGCCTCGGGAACATCCTCTTCCACCCAGAGAAGGAAAGGCTGCTCCGAGGGCAG

CTGCCGCCCTTCCTCAACGGCTTCTTCCAGAACAGCGAGCCCGTGGTGGTGGACATCATG

GGCACCGTGTCCGACGTGCTGCACCGTTTGGGCACGCACGGCGCAGGGGCCCAGAGCCTC

AGCGTGGCCATCAATGCCCGCTCCTTCTTTGATGACGAGCGGGACAGGATTCGAGCAGCA

GCCATGGCACTGTTTGGGGATTTGGTGGCAACCATGGAGGGCAAGGAGCTAAGCGGCCTT

CGAACCCAGGTGTACCAGAGCATGGTACCTCTGCTGCTGCACCTGAAGGACAAGTGCCCC

AAGGTTGCCATGATGCGCGCCAGCTCTGAGGAGTTCAGCATCCACCTGGCCCAGGCCCTC

AGCTACCTGCACAGCCACCACCAGCACATGAAGACCTGGGCCGCGCTCTTCATAGGTTAC

ACCATCTGCTACCACCCCCAGGCCGTGTCCCGGATGGTGAGCGAGGTGGACACCAACCTG

CTGTTCTGCACTTTTGAAGACCTCAAAAAGGACCCAGAGCCTGGCGTCCGGGAATTTGCC

ACCAGGCAGCTCTCCTTCCTTCGGGAGGTGTCA

>CL18.Contig8_All 868 999 PREDICTED: HEAT repeat-containing protein 7A-like [Ailuropoda melanoleuca]

GCCGTGTCCCGGATGGTGAGCGAGGTGGACACCAACCTGCTGTTCTGCACTTTTGAAGAC

CTCAAAAAGGACCCAGAGCCTGGCGTCCGGGAATTTGCCACCAGGCAGCTCTCCTTCCTT

CGGGAGGTGTCA

>CL18.Contig9_All 33 1082 PREDICTED: HEAT repeat-containing protein 7A-like [Ailuropoda melanoleuca]

AGCACGTCGCTGGAGGCCCTCAAGAGCCTCCTGTCCACCACGGGGCACTGGCATGACTTT

GCCCACCTTGAGCTGCAGGGCGCCTGGGAGCACTTCACCTCCATCAGCACCTACGCACAG

GGCGTGGGCCTCCTGGCCAGGGCTATGGTGCAGAACCACTGCCGGCAGATCAAGGCCGTG

CTCAGCTGGCTGCTGGGCCGCATGCAGAGCCAGGAGGAGCGGGAGAGGAAGGCGGCCGTC

CTCGTGCTCACTGAGTTCCTCTACAGCCCTGTCCTGCTGGAAGTGCTTCCCAAGCAGTCT

GCCCTGACCCTCCTGGCACAGGGCCTCCGGGATGAGAGCCCCGACATCCGCGTATGGAGC

CTGCAGGGCCTCGGGAACATCCTCTTCCACCCAGAGAAGGAAAGGCTGCTCCGAGGGCAG

CTGCCGCCCTTCCTCAACGGCTTCTTCCAGAACAGCGAGCCCGTGGTGGTGGACATCATG

GGCACCGTGTCCGACGTGCTGCACCGTTTGGGCACGCACGGCGCAGGGGCCCAGAGCCTC

AGCGTGGCCATCAATGCCCGCTCCTTCTTTGATGACGAGCGGGACAGGATTCGAGCAGCA

GCCATGGCACTGTTTGGGGATTTGGTGGCAACCATGGAGGGCAAGGAGCTAAGCGGCCTT

CGAACCCAGGTGTACCAGAGCATGGTACCTCTGCTGCTGCACCTGAAGGACAAGTGCCCC

AAGGTTGCCATGCAGGCCAAGTTCGCCTTCTACCGCTGTGCTCTGCTGCTGGGGTGGCGG

CCGCGCCACACCCTCTTCTGCACGCTGGCCTGGGAGCAAAGCCTCAGCGCCCGCCACTTC

CTCTGGACCTGCCTGATGCGCGCCAGCTCTGAGGAGTTCAGCATCCACCTGGCCCAGGCC

CTCAGCTACCTGCACAGCCACCACCAGCACATGAAGACCTGGGCCGCGCTCTTCATAGGT

TACACCATCTGCTACCACCCCCAGGCCGTGTCCCGGATGGTGAGCGAGGTGGACACCAAC

CTGCTGTTCTGCAGTAAGCAGCCCCAGCCC

>CL19.Contig1_All 1 1254 PREDICTED: ubiquitin carboxyl-terminal hydrolase 14 isoform 3 [Canis lupus familiaris]

GCTCTTCCGGAGGAACCCTCATCTAAAACTGTCTTCGTAGAAGACATGACAGAAGAGCAG

TTAGCATCTGCTATGGAATTACCATGTGGGTTGACAAATCTTGGTAACACTTGTTACATG

AATGCTACAGTTCAATGTATTCGATCTGTGCCTGAACTCAAAGATGCCCTTAAAAGGTAT

GCAGGTGCCTTGAGAGCTTCAGGGGAAATGGCTTCAGCACAGTATATTACTGCAGCCCTT

AGAGATTTGTTTGATTCCATGGATAAAACTTCTTCTAGTATTCCACCTATTATTCTACTG

CAGTTTTTGCACATGGCTTTCCCACAGTTTGCGGAGAAGGGTGAACAAGGACAATATCTT

CAACAGGATGCTAATGAATGTTGGATACAAATGATGCGAGTATTACAACAAAAATTGGAA

GCCATAGATGATGATTCTGTTAAAGAGTCAGATTCTTCATCTGCATCAGCAGTGACACCT

TCTAAAAAGAAAAGTTTAATTGATCAGTTCTTCGGTGTTGAATTTGAAACAACCATGAAA

TGTACAGAATCTGACGAAGAAGAAGTTACCAAAGGAAAGGAAAATCAACTTCAGCTTAGC

TGTTTTATCAATCAGGAAGTCAAGTATCTTTTTACAGGACTTAAATTGCGACTTCAGGAA

GAAATCACCAAACAGTCTCCGACATTGCAAAGAAATGCTTTATATATCAAATCTTCCAAG

ATCAGCCGGCTACCTGCTTACTTAACTATTCAGATGGTTCGATTTTTTTATAAAGAGAAG

GAATCTGTGAATGCCAAAGTTCTTAAGGATGTTAAATTTCCTCTTATGTTGGATGTATAT

GAACTGTGTACACCAGAACTTCAAGAGAAAATGATCTCTTTTCGATCAAAATTCAAGGAT

CTAGAAGATAAAAAAGTGAATCAACAGCCAAATACAGGTGACAAGAAGAGTAGTCCTCAT

AAAGAAGTTAAGTATGAACCCTTTTCATTTGCAGATGATATTGGCTCCAATAATTGTGGA

TACTATGACTTACAAGCAGTGCTAACGCACCAGGGAAGGTCTAGTTCTTCAGGTCATTAT

GTGTCATGGGTGAAAAGGAAACAAGATGAATGGATTAAATTTGATGATGATAAAGTCAGC

ATTGTAACACCAGAAGATATCTTGCGGCTTTCTGGTGGTGGAGACTGGCATATTGCTTAT

GTTTTACTCTACGGGCCTCGCAGAGTTGAAATAATGGAAGAGGAAAGTGAACAG

>CL20.Contig1_All 2 832 nuclear factor 1 B-type [Bos taurus] >gi|122145582|sp|Q0VCL6.1|NFIB_BOVIN RecName: Full=Nuclear factor 1 B-type; Short=NF1-B; Short=Nuclear factor 1/B; AltName: Full=Nuclear factor I/B; Short=NF-I/B; Short=NFI-B >gi|51972047|dbj|BAA25292.2| NF1-B3 [Rattus norvegicus] >gi|111304940|gb|AAI20108.1| Nuclear factor I/B [Bos taurus] >gi|149059532|gb|EDM10470.1| nuclear factor I/B, isoform CRA_b [Rattus norvegicus] >gi|378792882|gb|AFC41215.1| nuclear factor 1 B-type isoform 1 [Ovis aries]

CGGCTGGACCTGGTCATGGTGATTTTGTTTAAGGGGATCCCCCTGGAAAGTACTGATGGG

GAGCGGCTCTACAAGTCGCCCCAGTGCTCGAACCCCGGCCTGTGCGTCCAGCCACATCAT

ATTGGCGTCACAATCAAAGAACTGGATCTTTATCTGGCTTACTTTGTCCACACTCCGGAA

TCCGGACAATCAGATAGTTCAAACCAGCAAGGAGATGCGGACATCAAACCACTGCCCAAC

GGGCACTTAAGTTTCCAGGACTGTTTTGTGACTTCTGGGGTCTGGAATGTGACGGAGCTG

GTGAGAGTATCACAGACTCCTGTGGCAACAGCGTCAGGGCCCAACTTCTCGCTGGCCGAC

TTGGAGAGTCCCAGCTACTACAACATAAACCAGGTGACCCTGGGGCGGCGGTCCATCACC

TCCCCACCTTCCACCAGCACCACCAAGCGCCCCAAGTCCATTGATGACAGTGAGATGGAG

AGCCCTGTCGACGACGTGTTCTATCCCGGGACAGGCCGCTCCCCAGCAGCTGGCAGCAGC

CAGTCCAGTGGGTGGCCCAACGATGTGGATGCAGGCCCGGCTTCTCTAAAGAAGTCAGGA

AAGCTGGACTTCTGCAGTGCCCTCTCCTCTCAGGGCAGCTCCCCACGCATGGCTTTCACC

CACCACCCGCTGCCTGTGCTTGCTGGAGTCAGACCAGGGAGCCCCCGGGCCACAGCGTCA

GCCCTGCACTTCCCCTCCACGTCCATCATCCAGCAGTCGAGCCCATACTTCACACACCCG

ACCATCCGCTACCACCACCACCATGGGCAGGACTCGCTGAAGGAGTTTGTG

>CL20.Contig2_All 119 808 nuclear factor I/B, isoform CRA_f [Mus musculus]

GGCGAAGTCATGATGTATTCTCCCATCTGTCTCACTCAGGATGAATTTCACCCATTCATC

GAGGCACTTCTTCCACATGTCCGTGCAATCGCCTATACTTGGTTCAACCTGCAGGCTCGA

AAACGCAAGTACTTTAAAAAGCATGAGAAGCGAATGTCAAAGGATGAAGAAAGAGCAGTC

AAAGATGAGCTTCTCAGTGAAAAGCCTGAAATCAAACAGAAGTGGGCATCCAGGCTCCTG

GCCAAACTACGCAAAGATATCCGCCAGGAGTACCGAGAGGACTTTGTGCTCACCGTGACT

GGCAAGAAGCACCCGTGCTGTGTCTTATCCAATCCTGACCAGAAGGGTAAGATTAGGAGA

ATCGACTGCCTGCGACAGGCAGACAAAGTCTGGCGTCTGGATCTAGTCATGGTGATCCTG

TTCAAAGGCATCCCTTTGGAAAGTACCGATGGAGAGCGGCTCATGAAATCCCCACATTGC

ACAAACCCAGCACTTTGTGTCCAGCCACATCATATCACAGTATCAGTTAAGGAGCTTGAT

TTGTTTTTGGCATACTACGTGCAGGAGCAAGATTCTGGACAATCAGGAAGTCCAAGCCAC

AATGATCCTGCCAAGAATCCTCCAGGATACCTTGAGGATAGTTTTGTAAAATCTGGAGTC

TTCAATGTATCAGAACTTGTGAGGGTATCC

>CL22.Contig1_All 22 1284 PREDICTED: caiB/baiF CoA-transferase family protein C7orf10-like [Ailuropoda melanoleuca]

ATGCAGGCGACGCTTGCGAGGGTGGCGGCTCTGCGGAGAATCGGTCTCCCCTCCGGCTGG

GTCGGCGGGAGGGGGCTGTGGACAGGCCGCCCGCAGTCAGATATTGACAACGTGAAGCCA

TTGGAGGGTGTAAAAATTCTAGATCTAACAAGAGTGCTGGCAGGACCTTTTGCTACTATG

AATTTAGGAGATCTTGGAGCAGAAGTTATAAAAGTGGAAAGACCAGGAGCCGGTGATGAT

ACACGAACTTGGGGGCCACCTTTTGTGGGTACAGAAAGTACATATTTTCTCAGTGTTAAC

CGAAATAAAAAACTTGCAGCTGTTTGTGATGTATTTGTGGAAAACTATGTCCCTGGAAAA

CTGTCCACAATGGGCCTGGGGTATGAAGATATAGACAAGATTGCTCCTCACATCATCTAT

TGCTCCATCACAGGTTATGGTCAGACAGGCCCCATGTCTCAAAGAGCTGGTTATGATGCC

ATTGCCTCTGCTATTTCTGGTCTGATGCACATCACAGGGCCTGAGGATGGTGATCCAGTT

CGCCCAGGAGTGGCCATGACTGATCTTGCCACTGGCCTGTATGCATATGGAGCCATTATG

GCTGGATTGATACAAAGATATAAAACTGGAAGAGGACTGTTCATTGATTGTAACCTACTG

TCATCTCAGGTGGCATGTTTGACCCAGGTAGCTGTTAATTATCTCATTGGCCAAAAGGAA

GCAAAACGTTGGGGCACTGCTCATGGCAGTATTGTTCCTTACCAGGCTTTTAAAACCAAG

GATGGCTATTTTGTAGTTGGAGCAGGAAATGACCAACAGTTTGCTACTGTGTGCAAGATC

TTGAATTTGCCTGAGTTGATTGATGATTCCAAGTATAAAACCAACCATCTTCGAGTGGAG

AATAGAAAAGAGCTTATTAAAATACTATCTGCACGGTTTGAAGAAGAAATGACCACTAAG

TGGTTATATTTCTTTGAAGGCAGCGGGGTCCCATATGGCCCAATCAACAATATGAAGGGT

GTATTTACAGAACCTCAGGTGCTACACAATGGCCTCATTATGGAAATGAAGCATCCGACT

GTGGGGAAGATTTCAGTCCCAGGCCCAGCTGTGAGATACAGTAAGTTCAAGATGTCAGAG

GCCAGGCCACCTCCCCTGCTTGGGCAGCACACAACGTGCATCCTGAAGGAGGTCCTTGGA

TACGATGACAGAGCTGTTGGGGAGCTGCTCAGCGCTGGAGTCGTGACCCAACATAAAACC

AAG

>CL22.Contig2_All 22 1335 PREDICTED: caiB/baiF CoA-transferase family protein C7orf10-like [Ailuropoda melanoleuca]

ATGCAGGCGACGCTTGCGAGGGTGGCGGCTCTGCGGAGAATCGGTCTCCCCTCCGGCTGG

GTCGGCGGGAGGGGGCTGTGGACAGGCCGCCCGCAGTCAGATATTGACAACGTGAAGCCA

TTGGAGGGTGTAAAAATTCTAGATCTAACAAGAGTGCTGGCAGGACCTTTTGCTACTATG

AATTTAGGAGATCTTGGAGCAGAAGTTATAAAAGTGGAAAGACCAGGAGCCGGTGATGAT

ACACGAACTTGGGGGCCACCTTTTGTGGGTACAGAAAGTACATATTTTCTCAGTGTTAAC

CGAAATAAAAAAAGCATAGCTGTCAATATCAAGGATCCAAAAGGGGTGAAAATCATCAAA

GAGCTTGCAGCTGTTTGTGATGTATTTGTGGAAAACTATGTCCCTGGAAAACTGTCCACA

ATGGGCCTGGGGTATGAAGATATAGACAAGATTGCTCCTCACATCATCTATTGCTCCATC

ACAGGTTATGGTCAGACAGGCCCCATGTCTCAAAGAGCTGGTTATGATGCCATTGCCTCT

GCTATTTCTGGTCTGATGCACATCACAGGGCCTGAGGATGGTGATCCAGTTCGCCCAGGA

GTGGCCATGACTGATCTTGCCACTGGCCTGTATGCATATGGAGCCATTATGGCTGGATTG

ATACAAAGATATAAAACTGGAAGAGGACTGTTCATTGATTGTAACCTACTGTCATCTCAG

GTGGCATGTTTGACCCAGGTAGCTGTTAATTATCTCATTGGCCAAAAGGAAGCAAAACGT

TGGGGCACTGCTCATGGCAGTATTGTTCCTTACCAGGCTTTTAAAACCAAGGATGGCTAT

TTTGTAGTTGGAGCAGGAAATGACCAACAGTTTGCTACTGTGTGCAAGATCTTGAATTTG

CCTGAGTTGATTGATGATTCCAAGTATAAAACCAACCATCTTCGAGTGGAGAATAGAAAA

GAGCTTATTAAAATACTATCTGCACGGTTTGAAGAAGAAATGACCACTAAGTGGTTATAT

TTCTTTGAAGGCAGCGGGGTCCCATATGGCCCAATCAACAATATGAAGGGTGTATTTACA

GAACCTCAGGTGCTACACAATGGCCTCATTATGGAAATGAAGCATCCGACTGTGGGGAAG

ATTTCAGTCCCAGGCCCAGCTGTGAGATACAGTAAGTTCAAGATGTCAGAGGCCAGGCCA

CCTCCCCTGCTTGGGCAGCACACAACGTGCATCCTGAAGGAGGTCCTTGGATACGATGAC

AGAGCTGTTGGGGAGCTGCTCAGCGCTGGAGTCGTGACCCAACATAAAACCAAG

>CL23.Contig1_All 22 192 LINE [Rattus norvegicus]

CATATATACACAATGAAAGTTTACTGAGCAATAAAGACGAATGGAATAATGGCATTTGCC

AGTAAATGGATAGAACTGGAGAATATCATGCTAAGGGAAATAAGCCAATCCCAAAAACCA

AAAGCCAAATGTTCTCTCTGATATGTTGATGCTAACCCACAAAAACAGATT

>CL25.Contig1_All 2 283 minus strand gag-myb protein, partial [Mus musculus]

CAACTACAATATTGGCCCTTTTCTGCTTCTGATCTATATAACTGGAAAAACAATAACCCT

CCTTTCTCTAGAGACCCCTCTATCTTAACCTCTCTCATTGAGTCTATCCTAGTTACTCAC

CAGCCCACTTGGGATGACTGTCAACAGCTTCTCCAGGCATTGTTGACTACCGAGGAAAAA

CAACGGGTCATCCTAGAAGCCCGCAAGAATGTTCCAGGAGAGAATGGCCGTCCCACCCAG

TTGCCTAATGAGATTGATGCTGCATTTCCCCTCGAGCATCCC

>CL27.Contig1_All 2 199 PREDICTED: ribosomal RNA processing protein 1 homolog B [Saimiri boliviensis boliviensis]

TTCAACCGGAAACGTCTCTACAAACTCATCAAAAAGTTCCAAGGCCTTTCTGAAGGTGGT

ATATCTCAACTCAGTTTTCCTGAGGACATTGCTGCTGATGAAGGTGATCAAGCTCTCAGT

CAAAGAAGACATAAGAAAAAAAGACGTAAGCTCTTAGAGAAAGCTGACTTGGAGAAGGAA

ACAGGAAACAGAGTTTTT

>CL27.Contig2_All 33 2063 PREDICTED: ribosomal RNA processing protein 1 homolog B [Papio anubis]

ATGGCCCCCGCCGTGCAGCCCGCCGAGGTCCAGTTCGCTCAGCGGCTGGCGTCCAGCGAG

AAGGGCGTCCGCGACCGCGCGGTGAGGAAGCTGCGCCAGTACCTCAGCGTGAAGACGCAG

AGCGAGACAGGAGGTTTCAGCCAAGAAGAACTCCTAAAAATATGGAAAGGACTCTTCTAC

TGCATGTGGGTGCAGGACGAACCCCTTCTGCAGGAGGAGCTAGCGAACTCTATTTCCCAA

CTCATCCATGTTGTTAACAGCTCAGAGGCTCAGCACCTGTTCATTCAGACCTTCTGGCAA

ACCATGAATCGAGAATGGAAGGGAATAGACAAGCTGCGCCTGGACAAATACTACATGCTG

ATTCGCCTGGTCCTGAGGCAGTCTTTTGAAGTTCTGAAACGGAATGGCTGGGAGGAAAGC

CGAATCAAGCTTTTTTTGGATGTTCTGATGAAGGAGATCCTGTGTCCTGAGAGCCAGTCT

CCTGATGGAGTGAGGTTCCATTTCATCGATATTTATCTGGACGAACTATCCAAAGTGGGA

GGAAAAGAGCTTTTGGCAGATCAGAACCTCAAGTTTATTGACCCATTCTGCAAAATTGCT

GCCAAGACCAAGGACCACACACTGGTACAGACTATAGCTAGGGGTGTTTTTGAAGTCATT

GTAGATCAGTCTCCTCTTGTACCTGAAGACACAGTGGAGGAACAGAAAACCAAAGTGGGT

GATGGTGACCTCTCTGAAGAGGAGACTTTTGAAAACGAGGCAACTTGGAAAAAAGTAGTT

AGCAGAAAGAAGACAGCACTGAGCAAATGCCCCTCCAGGAGAGATGGAATCAGTGATAAA

AGAGGAAGAAAGGACTGTGGTACCTTGGAGGACATGGGGCTACTTCTCCAGTTTGACTAT

AAGGCTGTTGCGGACCGACTCTTGGAAATAACTCATAAGAAAAACATTCCTCCCTTCAAC

CGGAAACGTCTCTACAAACTCATCAAAAAGTTCCAAGGCCTTTCTGAAGGTGGTATATCT

CAACTCAGTTTTCCTGAGGACATTGCTGCTGATGAAGGTGATCAAGCTCTCAGTCAAAGA

AGACATAAGAAAAAAAGACGTAAGCTCTTAGAGAAAGCTGACTTGGAGAAGGAAACAGGA

AACAGAGTCTTTCTTGCTGAGGAAAAGGACAGTGAAGGCCGCATTCAGAAAAGAAAAAGG

AAAAAAAAGAAGAGGAGCCACCCCCAGCCTGAGACTCTGGACCTGAGGGACACAGCCTCA

CCCTCAGCACAGAGCAGGGGCGGGGAGCCTGAGCCTGCTCAGAGGCAGGTCCCCCAAGTG

CGAGCGGCCGAGTCCAGCACCAGGGAGGAGAGCAGCTCGGAGCACCCCGCCTCCGTCCCC

ACACGTGGCAGGAAGAATCAGCTGAAGAGAAAGAGCCTGGGCGCACAGGGAGAGATCCGG

GACCCCACAGAGCTGCCTCTGGAGGACAAGGCCCAGAGTGGCCCTGGCAGCAGCCATCCT

CATGGACCTGCTGCCAGAGGCTCCCCAACAGATGGAACCCAAGTCCCAAAAAGGAAGAGG

AAGCTTGGAGTGCACCCTGTCAATGGCAGTGGTCAGACCACCTTGCCCCAGTGCAGGAGG

CCACAGAAAAAGAAGGCAGAGCCCAGCAGCCTTGACCTCTATAGTCTGTCCAGTCAGAAG

ACAATTTTGAAAAAGAGGAAGAAGATGAAAGAAATGTCAAGCTTGATAGAATACAACGGA

ATACTGGGATCCAGAGTTAGACAGCTCCAAGCTCTGCTGGACAGAACAACCAGCAGCGCC

AAGAAAGTCACCTTTGGGTTGAACAGAAATACCACCGCGGAATTCAAGAAGACGGACAAG

AGTATCCTGGTCAGCCCCACAGGCCTCTCCCGAGTGGCCTTCAACCCTGAGCAGAGACCC

CTCCATGGAGTGCTGAAGACCCCCACCAGCTCTCCCGCCAGCACTCCCCTGGAAACCAAG

AAGCCGCTGGCCGCCACTCAAAAGAGAAGGCCAACGGCTGCGGACTTCTTC

>CL27.Contig3_All 33 2216 PREDICTED: ribosomal RNA processing protein 1 homolog B [Papio anubis]

ATGGCCCCCGCCGTGCAGCCCGCCGAGGTCCAGTTCGCTCAGCGGCTGGCGTCCAGCGAG

AAGGGCGTCCGCGACCGCGCGGTGAGGAAGCTGCGCCAGTACCTCAGCGTGAAGACGCAG

AGCGAGACAGGAGGTTTCAGCCAAGAAGAACTCCTAAAAATATGGAAAGGACTCTTCTAC

TGCATGTGGGTGCAGGACGAACCCCTTCTGCAGGAGGAGCTAGCGAACTCTATTTCCCAA

CTCATCCATGTTGTTAACAGCTCAGAGGCTCAGCACCTGTTCATTCAGACCTTCTGGCAA

ACCATGAATCGAGAATGGAAGGGAATAGACAAGCTGCGCCTGGACAAATACTACATGCTG

ATTCGCCTGGTCCTGAGGCAGTCTTTTGAAGTTCTGAAACGGAATGGCTGGGAGGAAAGC

CGAATCAAGCTTTTTTTGGATGTTCTGATGAAGGAGATCCTGTGTCCTGAGAGCCAGTCT

CCTGATGGAGTGAGGTTCCATTTCATCGATATTTATCTGGACGAACTATCCAAAGTGGGA

GGAAAAGAGCTTTTGGCAGATCAGAACCTCAAGTTTATTGACCCATTCTGCAAAATTGCT

GCCAAGACCAAGGACCACACACTGGTACAGACTATAGCTAGGGGTGTTTTTGAAGTCATT

GTAGATCAGTCTCCTCTTGTACCTGAAGACACAGTGGAGGAACAGAAAACCAAAGTGGGT

GATGGTGACCTCTCTGAAGAGGAGACTTTTGAAAACGAGGCAACTTGGAAAAAAGTAGTT

AGCAGAAAGAAGACAGCACTGAGCAAATGCCCCTCCAGGAGAGATGGAATCAGTGATAAA

AGAGGAAGAAAGGACTGTGGTACCTTGGAGGACATGGGGCTACTTCTCCAGTTTGACTAT

AAGGCTGTTGCGGACCGACTCTTGGAAATAACTCATAAGAAAAACATTCCTCCCTTCAAC

CGGAAACGTCTCTACAAACTCATCAAAAAGTTCCAAGGCCTTTCTGAAGGTGGTATATCT

CAACTCAGTTTTCCTGAGGACATTGCTGCTGATGAAGGTGATCAAGCTCTCAGTCAAAGA

AGACATAAGAAAAAAAGACGTAAGCTCTTAGAGAAAGCTGACTTGGAGAAGGAAACAGGA

AACAGAGTCTTTCTTGCTGAGGAAAAGGACAGTGAAGGCCGCATTCAGAAAAGAAAAAGG

AAAAAAAAGAAGAGGAGCCACCCCCAGCCTGAGACTCTGGACCTGAGGGACACAGCCTCA

CCCTCAGCACAGAGCAGGGGCGGGGAGCCTGAGCCTGCTCAGAGGCAGGTCCCCCAAGTG

CGAGCGGCCGAGTCCAGCACCAGGGAGGAGAGCAGCTCGGAGCACCCCGCCTCCGTCCCC

ACACGTGGCAGGAAGAATCAGCTGAAGAGAAAGAGCCTGGGCGCACAGGGAGAGATCCGG

GACCCCACAGAGCTGCCTCTGGAGGACAAGGCCCAGAGTGGCCCTGGCAGCAGCCATCCT

CATGGACCTGCTGCCAGAGGCTCCCCAACAGATGGAACCCAAGTCCCAAAAAGGAAGAGG

AAGCTTGGAGTGCACCCTGTCAATGGCAGTGGTCAGACCACCTTGCCCCAGTGCAGGAGG

CCACAGAAAAAGAAGGCAGAGCCCAGCAGCCTTGACCTCTATAGTCTGTCCAGTCAGAAG

ACAATTTTGAAAAAGAGGAAGAAGATGAAAGAAATGTCAAGCTTGATAGAATACAACGGA

ATACTGGGATCCAGAGTTAGACAGCTCCAAGCTCTGGGAAGCAGCAGGACTGTTGCCCCT

TTGAAGAAGCAGCCACTGAGGACAGAAAAGGATTTTGTGAAGTTTGACACCCACTTCTTA

CCAAAGCCCCTCTTCTTCAGAAAAGCCAAGCTCAGTGCTGCCCCTCGCCCTGCAGAGCGA

GCCGTCCAGCTGGACAGAACAACCAGCAGCGCCAAGAAAGTCACCTTTGGGTTGAACAGA

AATACCACCGCGGAATTCAAGAAGACGGACAAGAGTATCCTGGTCAGCCCCACAGGCCTC

TCCCGAGTGGCCTTCAACCCTGAGCAGAGACCCCTCCATGGAGTGCTGAAGACCCCCACC

AGCTCTCCCGCCAGCACTCCCCTGGAAACCAAGAAGCCGCTGGCCGCCACTCAAAAGAGA

AGGCCAACGGCTGCGGACTTCTTC

>CL28.Contig1_All 93 923 PREDICTED: solute carrier family 22 member 9-like [Equus caballus]

ATGGCCTTTCAGGACCTCCTGCATCAAGTTGGTGGTCTGGGGAGGTTCCAGATAATTCAG

ATGGCTTTTCTTCTTATCTACAATGCCATGGCACAGACTCATACTTTATTGGAGAATTTC

ACTGCAGTCATCCTTGGTCATCGCTGCTGGGTCCACATACTCGATAATGCCACTGTCTCT

GACAATGACACTGGGACCCTCAGCCAAGAAGCCCTCCTGAGGATCTCCATCCCACTAGAC

TCAAGCCTGAGGCCAGACAAATGTCATCGCTTCATCCACCCACAATGGCAGCTCCTTCAT

ATGAATGGAACCTTCTCAAACATGAGTGAGGTGGACACGGAGCCCTGCATGGATGGCTGG

GTATATGACCGGAGCTCGTTCCTCTCCACCACCGTGACTGAGTGGGATCTGGTATGTGAA

TCTCAGTCACTGAATTCAGTATCTAAATTCTTCTTCATGGCTGGAATGTTGATTGGAAAC

ATTGTATATGGCACTTTGTCAGACAGATTTGGGAGGAGGTTACTTCTGACATGGTGTCTT

CTCCAGCTGGCCGTTGCTGACACCTGTGCTACCTTTGCTCCCACCTTCCTGGTGTACTGT

TCACTACGCTTCCTGGCTGGCATGTCCACCTCGACCATCCTGACAAATGCTATTCTTCTC

ATTGTAGAATGGACAAGTCCCAAATACCAAGCTATGGGAACAACAATGGCAGTGTGTGCT

ACTAGTTTCGGGAATATCCTACTGGGAAGCCTGGCTTTTGCTATTCGAAACTGGCATACT

CTTCAGCTGGTTCTGTCTATACCAATGTTCTTCTTTTTTATTTCCTCAAGA

>CL29.Contig1_All 2807 3454 minus strand microtubule-associated protein 4 [Felis catus] >gi|269819863|gb|ACK86564.2| microtubule-associated protein 4 [Felis catus]

ACAAGAAGCCCACCTTAGCCAAGCCCAGCTCCTCTGCCCCCAGGCTGAATCGCCTGGCCA

CCAATGCCTCTATCCCTGATCTGAAGAATGTCCGCTCCAAGGTCGGCTCCACAGAAAACA

TCAAGCATCAGCCTGGAGGTGGCCGGGCCAAAATAGAGAAAAAAAACAGAGGCAGTGGCT

ACAACTCGAAAGCCTGAACCTAACGCAGTCACTAAAGCATCCAGCCCCATTGCAAGTGTG

CAGAAACCGCCTGCTGGGAAAGTTCAGATTCAGAACAAGAAAGTGGACATCTCTAAGGTC

TCTTCCAAGTGTGGGTCCAAGGCTAACATCAAACACAAGCCTGGTGGGGGAGATGTCAAG

ATTGAAAGTCAGAAGTTGAACTTCAAGGAGAAGGCCCAGGCCAAGGTGGGATCCCTGGAT

AACGTGGGCCACCTGCCTGCGGGAGGTGCTGTGAAGACTGAGGGCGGTGGCAGCGAGGCC

CCTCCGTGTGCGGGCCCCCCCGCTGGGGAGGAGCCGGCCATCTCTGAGGCAGCACCTGGA

GCTGGCGCCCCCACTTCAGCCAGTGGCCTCAGTGGCCACACCACCCTGTCGGGGGGTGGT

GACCAAAGGGAGGCCCAGACCTTGGACTGCCAGATCCAGGAGACAAAT

>CL29.Contig2_All 136 3339 minus strand microtubule-associated protein 4 [Felis catus] >gi|269819863|gb|ACK86564.2| microtubule-associated protein 4 [Felis catus]

ATGGCTGACCTCAGTCTTGCAGATGCATTAACAGAGCCACCTCCAGAAATTGAGGAAGAG

ATAAAGCGGGACTTCATTGCCACACTGGAGTCAGAGGCCTTTGAGGATGTTGTGGGGGAA

ACTGTTGGAAAAACAGACTATATTCCTCTCCTGGATGTGGACGAGAAAACTGAGAACTCT

GAGTCAAAGAAGAAACCATTCTCAGATACCAGCCAGATTGAAGGTACTCCATCTTCTAAA

CCAACAGTGTTAGCCAATGGTGACCATGGAATAGAAGGGAATGACACTATAGGGTCTCCA

ACTGAATTCCTCGAAGAGAAAATGGCCTACCAGGAATATCAGAACAGCCAGAACTGGCCA

GAAAATACAAACTTTTGCTTCCAGCCTGAGCAAGTGGTGAATCCTATCCAGACTGATCCC

TTTAAGATGCACCATGATGACGGACTAGCAGATTTGCTCTTTCTCTCCAGTGGAACAACA

AATGATTTGACATTTACAGGGCATAATAATCCTTTGGAAGACAGTTACGGTATGCCTCCC

TGCGACACATTTGCTCCTGCAACTGTTGTACCTCAGGGGTGGTCTATGGAAGCCCCAAAC

CCTCCATACTCGGAGTCCTTTGTTTCTCTAGAGGCCACTGCAAATCTTCCACAGATGACA

GAGTTACCCAAGGAAGGAGAAATGGCATCAGTAGAAGAACAGCCACCAACTAAAGCATTG

GAAATAATAATAGGACAGAAGACTACAGAAATGGCACCGTCTGGAGAAAAAGTGGTAGCC

CTGGTCAAGGAAATGGCACCCGCCACAGAAACAAAGGTGGAATTGACTAAAGATATGGAA

CAACCCACCAAATCAGATGTGATACTGCCCAAGGACTTGCAGTCATCCATTGAATCAGAT

ATGTCTCTGGTCAAGGACATGGTCCTACCTATAGAAAAAGAAGTGGTCCCAGTTAAGGAT

GTCATATCACCCGCAAAAACAGATGTGTCTTTGACTAAAGATATGGTGCTGCCCACAGAA

ATAGAGGTGGCTGCAGGCAAGGATGTGACACTATTTGAAGAAACAGAGAGAGTACCACCT

GTAAAAATGGATTTGTTTTCACATGAGGGCATGGCATCACCCAAAGAAACAGAGTCAATC

TCAGGCAAGGGTATGGTATCATTTTCAGAAATGGAGGTGGCACTAGCTGTGGACACTATA

TCATCCACAGAAAAATCCTCAGCTGAGGAGATGGTCTTGTTATCAGAAAAAGAGGTGGCT

GTGCCAAGGGACATGACACTGCCTTCAGAAGCAACAGTAGTCTTCACGAAGGATGCAGTA

GAAACAGAAGGGCCCTCAGACAAGGACATTACTCCACCTCCAGTAACAGAAGTGACCATG

GGTAAGGAAGTGGCTCTGTCCCCAGAATCAAAGGTGGGCCTTGTCAAGGATGTGGCTCCA

CTCCCAGAAACAGAAGTAACCCTGGTCAAGAATATGGGTACACCCTCTGAAACAAAGTTG

GCCCTGGGTAAGGATTTGGCTGTGTCTCCAGAAGTAGAGGTGGCTCAGGTCAAGGATATA

ACTCCACTCCCAGAAAAAGAAGTAACCTTGATCAAGGATATGCTGCTGCCTCCAGAAGCA

GATGTGGCCCTGGCTAAGGATGTGGCTCTGTCCCTTGGAACAGAGATGACCCTGGCCAAC

AGTGTGACTCCAGCCAAGGATGTTGCACCACCCTCAGGAACAGAGGTGGCATCAGTTCTA

GTTAAAGACATGGAAATTGCCCAGACTCAGGAAGGAATAAGTGAAAATTCCCAGATACAG

TCTCTCCAAAAAGATGGGCAGTTAGCTGCACCTACTTTCATGATTTCACCAGAACCAGTC

ACAGCCACGGGCCAAAAGGACAACTTGCCAGATTCTGTGTTAGAGAAATTAGAACAGAAG

AAACCATTCAGCAGTCAGCCTTCTGAACTTTCTTCAGAGACCTCAGGAATAGCCAGGCCA

GAAGAAGGAAAGGCTGCTGTGAGTGTGACCGGAAATGACATCACTACCCCACCAAACAAG

GAGCTCCCACCAAGCCCAGAGAAGAAAACAAAGCCTTTGGCCACCACTCAACCTGCAAAG

ACTTCAACATCGAAAGCCAAAACACAGCCCACTTCTCTCCCTAAGCAGCCAGCAGCTCCC

ACCACCTCTGGTGGGTCGAATAAAAAACCTATGAGCCTTTCTTCAGGCTTAGTACCAGCT

GCCCCACCCAAACGCCCTGCTGCTGCCACTGCCAGGCCTTCCATCTTACCTGCAAAAGAC

GTGAAGCCAAAGTCCGTTGCAGAGGCAAAGACTCCTGAGAAGCGGGCCTTACCATCCAAG

CCCACCTCTGCCCCAGCCCTCAGACCTGGACCCAAGAGCACCCCAGCTGCTCCGAAAGCT

ACTGCAGCTGCCTCAGCTGCGCCAAGCAATAGAAGTCCCCCCATGCCACTGCCCAAACGG

CCCACTACCATTAAGACTGAGGGGAAACCTGCAGAAGTGAAGAAGATGACTGCAAAGTCT

GCACCAGCTGACTTGAATCGTTCAAAGACCACCTCCACCAGTTCTGTGAAGAAAAATACC

ACTCCCACTGGGGCAGCACCTCCAACAGGGGTGGCTTCCACTCGAGTCAAGTCCACTCCC

ACACCTCCCCGGCCTTCTACAACTCCCATGGACAAGAAGCCCACCTTAGCCAAGCCCAGC

TCCTCTGCCCCCAGGCTGAATCGCCTGGCCACCAATGCCTCTATCCCTGATCTGAAGAAT

GTCCGCTCCAAGGTCGGCTCCACAGAAAACATCAAGCATCAGCCTGGAGGTGGCCGGGTT

CAGATTCAGAACAAGAAAGTGGACATCTCTAAGGTCTCTTCCAAGTGTGGGTCCAAGGCT

AACATCAAACACAAGCCTGGTGGGGGAGATGTCAAGATTGAAAGTCAGAAGTTGAACTTC

AAGGAGAAGGCCCAGGCCAAGGTGGGATCCCTGGATAACGTGGGCCACCTGCCTGCGGGA

GGTGCTGTGAAGACTGAGGGCGGTGGCAGCGAGGCCCCTCCGTGTGCGGGCCCCCCCGCT

GGGGAGGAGCCGGCCATCTCTGAGGCAGCACCTGGAGCTGGCGCCCCCACTTCAGCCAGT

GGCCTCAGTGGCCACACCACCCTGTCGGGGGGTGGTGACCAAAGGGAGGCCCAGACCTTG

GACTGCCAGATCCAGGAGACAAAT

>CL29.Contig3_All 2807 3457 minus strand microtubule-associated protein 4 [Felis catus] >gi|269819863|gb|ACK86564.2| microtubule-associated protein 4 [Felis catus]

ACAAGAAGCCCACCTTAGCCAAGCCCAGCTCCTCTGCCCCCAGGCTGAATCGCCTGGCCA

CCAATGCCTCTATCCCTGATCTGAAGAATGTCCGCTCCAAGGTCGGCTCCACAGAAAACA

TCAAGCATCAGCCTGGAGGTGGCCGGGCCAAAATAGAGAAAAAAAACAGAGGCAGTGGCT

ACAACTCGAAAGCCTGAACCTAACGCAGTCACTAAAGCATCCAGCCCCATTGCAAGTGTG

CAGAAACCGCCTGCTGGGAAAGTTCAGATTCAGAACAAGAAAGTGGACATCTCTAAGGTC

TCTTCCAAGTGTGGGTCCAAGGCTAACATCAAACACAAGCCTGGTGGGGGAGATGTCAAG

ATTGAAAGTCAGAAGTTGAACTTCAAGGAGAAGGCCCAGGCCAAGGTGGGATCCCTGGAT

AACGTGGGCCACCTGCCTGCGGGAGGTGCTGTGAAGACTGAGGGCGGTGGCAGCGAGGCC

CCTCCGTGTGCGGGCCCCCCCGCTGGGGAGGAGCCGGCCATCTCTGAGGCAGCACCTGGA

GCTGGCGCCCCCACTTCAGCCAGTGGCCTCAGTGGCCACACCACCCTGTCGGGGGGTGGT

GACCAAAGGGAGGCCCAGACCTTGGACTGCCAGATCCAGGAGACAAGCATC

>CL29.Contig4_All 2807 3547 minus strand microtubule-associated protein 4 [Felis catus] >gi|269819863|gb|ACK86564.2| microtubule-associated protein 4 [Felis catus]

ACAAGAAGCCCACCTTAGCCAAGCCCAGCTCCTCTGCCCCCAGGCTGAATCGCCTGGCCA

CCAATGCCTCTATCCCTGATCTGAAGAATGTCCGCTCCAAGGTCGGCTCCACAGAAAACA

TCAAGCATCAGCCTGGAGGTGGCCGGGCCAAAATAGAGAAAAAAAACAGAGGCAGTGGCT

ACAACTCGAAAGCCTGAACCTAACGCAGTCACTAAAGCATCCAGCCCCATTGCAAGTGTG

CAGAAACCGCCTGCTGGGAAAGTCCAGATAGTCTCCAAAAAAGTGAGCTACAGCCATATT

CAGTCCAAGTGTGGTTCCAAGGACAATATTAAGCATGTCCCTGGAGGTGGTAATGTTCAG

ATTCAGAACAAGAAAGTGGACATCTCTAAGGTCTCTTCCAAGTGTGGGTCCAAGGCTAAC

ATCAAACACAAGCCTGGTGGGGGAGATGTCAAGATTGAAAGTCAGAAGTTGAACTTCAAG

GAGAAGGCCCAGGCCAAGGTGGGATCCCTGGATAACGTGGGCCACCTGCCTGCGGGAGGT

GCTGTGAAGACTGAGGGCGGTGGCAGCGAGGCCCCTCCGTGTGCGGGCCCCCCCGCTGGG

GAGGAGCCGGCCATCTCTGAGGCAGCACCTGGAGCTGGCGCCCCCACTTCAGCCAGTGGC

CTCAGTGGCCACACCACCCTGTCGGGGGGTGGTGACCAAAGGGAGGCCCAGACCTTGGAC

TGCCAGATCCAGGAGACAAAT

>CL29.Contig5_All 3647 3784 minus strand PREDICTED: microtubule-associated protein 4 [Equus caballus]

ATTGAGACCTACAGGCTGACGTTCCGGGCAAACGCCAGGGCCCGCACCGACCATGGGGCC

GACATTGTCTCCCGGCCCCCCCACTTCCCTGGCGGCCCCAGCTTAGGCTCCCGGGCCCTT

GGCTCCCTTTCCCGGGCT

>CL29.Contig6_All 136 3342 minus strand microtubule-associated protein 4 [Felis catus] >gi|269819863|gb|ACK86564.2| microtubule-associated protein 4 [Felis catus]

ATGGCTGACCTCAGTCTTGCAGATGCATTAACAGAGCCACCTCCAGAAATTGAGGAAGAG

ATAAAGCGGGACTTCATTGCCACACTGGAGTCAGAGGCCTTTGAGGATGTTGTGGGGGAA

ACTGTTGGAAAAACAGACTATATTCCTCTCCTGGATGTGGACGAGAAAACTGAGAACTCT

GAGTCAAAGAAGAAACCATTCTCAGATACCAGCCAGATTGAAGGTACTCCATCTTCTAAA

CCAACAGTGTTAGCCAATGGTGACCATGGAATAGAAGGGAATGACACTATAGGGTCTCCA

ACTGAATTCCTCGAAGAGAAAATGGCCTACCAGGAATATCAGAACAGCCAGAACTGGCCA

GAAAATACAAACTTTTGCTTCCAGCCTGAGCAAGTGGTGAATCCTATCCAGACTGATCCC

TTTAAGATGCACCATGATGACGGACTAGCAGATTTGCTCTTTCTCTCCAGTGGAACAACA

AATGATTTGACATTTACAGGGCATAATAATCCTTTGGAAGACAGTTACGGTATGCCTCCC

TGCGACACATTTGCTCCTGCAACTGTTGTACCTCAGGGGTGGTCTATGGAAGCCCCAAAC

CCTCCATACTCGGAGTCCTTTGTTTCTCTAGAGGCCACTGCAAATCTTCCACAGATGACA

GAGTTACCCAAGGAAGGAGAAATGGCATCAGTAGAAGAACAGCCACCAACTAAAGCATTG

GAAATAATAATAGGACAGAAGACTACAGAAATGGCACCGTCTGGAGAAAAAGTGGTAGCC

CTGGTCAAGGAAATGGCACCCGCCACAGAAACAAAGGTGGAATTGACTAAAGATATGGAA

CAACCCACCAAATCAGATGTGATACTGCCCAAGGACTTGCAGTCATCCATTGAATCAGAT

ATGTCTCTGGTCAAGGACATGGTCCTACCTATAGAAAAAGAAGTGGTCCCAGTTAAGGAT

GTCATATCACCCGCAAAAACAGATGTGTCTTTGACTAAAGATATGGTGCTGCCCACAGAA

ATAGAGGTGGCTGCAGGCAAGGATGTGACACTATTTGAAGAAACAGAGAGAGTACCACCT

GTAAAAATGGATTTGTTTTCACATGAGGGCATGGCATCACACAAAGAAACAGAGTCAATC

TCAGGCAAGGGTATGGTATCATTTTCAGAAATGGAGGTGGCACTAGCTGTGGACACTATA

TCATCCACAGAAAAATCCTCAGCTGAGGAGATGGTCTTGTTATCAGAAAAAGAGGTGGCT

GTGCCAAGGGACATGACACTGCCTTCAGAAGCAACAGTAGTCTTCACGAAGGATGCAGTA

GAAACAGAAGGGCCCTCAGACAAGGACATTACTCCACCTCCAGTAACAGAAGTGACCATG

GGTAAGGAAGTGGCTCTGTCCCCAGAATCAAAGGTGGGCCTTGTCAAGGATGTGGCTCCA

CTCCCAGAAACAGAAGTAACCCTGGTCAAGAATATGGGTACACCCTCTGAAACAAAGTTG

GCCCTGGGTAAGGATTTGGCTGTGTCTCCAGAAGTAGAGGTGGCTCAGGTCAAGGATATA

ACTCCACTCCCAGAAAAAGAAGTAACCTTGATCAAGGATATGCTGCTGCCTCCAGAAGCA

GATGTGGCCCTGGCTAAGGATGTGGCTCTGTCCCTTGGAACAGAGATGACCCTGGCCAAC

AGTGTGACTCCAGCCAAGGATGTTGCACCACCCTCAGGAACAGAGGTGGCATCAGTTCTA

GTTAAAGACATGGAAATTGCCCAGACTCAGGAAGGAATAAGTGAAAATTCCCAGATACAG

TCTCTCCAAAAAGATGGGCAGTTAGCTGCACCTACTTTCATGATTTCACCAGAACCAGTC

ACAGCCACGGGCCAAAAGGACAACTTGCCAGATTCTGTGTTAGAGAAATTAGAACAGAAG

AAACCATTCAGCAGTCAGCCTTCTGAACTTTCTTCAGAGACCTCAGGAATAGCCAGGCCA

GAAGAAGGAAAGGCTGCTGTGAGTGTGACCGGAAATGACATCACTACCCCACCAAACAAG

GAGCTCCCACCAAGCCCAGAGAAGAAAACAAAGCCTTTGGCCACCACTCAACCTGCAAAG

ACTTCAACATCGAAAGCCAAAACACAGCCCACTTCTCTCCCTAAGCAGCCAGCAGCTCCC

ACCACCTCTGGTGGGTCGAATAAAAAACCTATGAGCCTTTCTTCAGGCTTAGTACCAGCT

GCCCCACCCAAACGCCCTGCTGCTGCCACTGCCAGGCCTTCCATCTTACCTGCAAAAGAC

GTGAAGCCAAAGTCCGTTGCAGAGGCAAAGACTCCTGAGAAGCGGGCCTTACCATCCAAG

CCCACCTCTGCCCCAGCCCTCAGACCTGGACCCAAGAGCACCCCAGCTGCTCCGAAAGCT

ACTGCAGCTGCCTCAGCTGCGCCAAGCAATAGAAGTCCCCCCATGCCACTGCCCAAACGG

CCCACTACCATTAAGACTGAGGGGAAACCTGCAGAAGTGAAGAAGATGACTGCAAAGTCT

GCACCAGCTGACTTGAATCGTTCAAAGACCACCTCCACCAGTTCTGTGAAGAAAAATACC

ACTCCCACTGGGGCAGCACCTCCAACAGGGGTGGCTTCCACTCGAGTCAAGTCCACTCCC

ACACCTCCCCGGCCTTCTACAACTCCCATGGACAAGAAGCCCACCTTAGCCAAGCCCAGC

TCCTCTGCCCCCAGGCTGAATCGCCTGGCCACCAATGCCTCTATCCCTGATCTGAAGAAT

GTCCGCTCCAAGGTCGGCTCCACAGAAAACATCAAGCATCAGCCTGGAGGTGGCCGGGTT

CAGATTCAGAACAAGAAAGTGGACATCTCTAAGGTCTCTTCCAAGTGTGGGTCCAAGGCT

AACATCAAACACAAGCCTGGTGGGGGAGATGTCAAGATTGAAAGTCAGAAGTTGAACTTC

AAGGAGAAGGCCCAGGCCAAGGTGGGATCCCTGGATAACGTGGGCCACCTGCCTGCGGGA

GGTGCTGTGAAGACTGAGGGCGGTGGCAGCGAGGCCCCTCCGTGTGCGGGCCCCCCCGCT

GGGGAGGAGCCGGCCATCTCTGAGGCAGCACCTGGAGCTGGCGCCCCCACTTCAGCCAGT

GGCCTCAGTGGCCACACCACCCTGTCGGGGGGTGGTGACCAAAGGGAGGCCCAGACCTTG

GACTGCCAGATCCAGGAGACAAGCATC

>CL32.Contig1_All 292 1050 PREDICTED: PAXIP1-associated protein 1-like [Nomascus leucogenys]

ATGTCCCTTGCCCGGGGTCATGGAGACATTGCGTCCACCACGGCGGCGCCTCTGTCTGAA

GAAGGGGAAGTGACTTCCGGCCTCCAGGCTCTGGCCGTGGAGGATACCGGAGGTCCCTCT

GTCTTGGCCAATAAGGCCGAGGAAGAGGGGGAAGGAGGACAGGAGGAGGCCGAGCATGCC

GGGCCCGGGACTGAGGAGGCAACTGAAGTACCCAGCGCCGAGGGGGAGGAGCGTGTCGAG

GGAGAATCCGAGGACTGGTGCGTGCCCTGCAGCGATGAGGAGGTGGAGCTGCCCGAGGAC

GGACAGCCCTGGATGCCCCCGCCCTCTGAAATCCAGCGGCTCTATGAGTTGCTGGCTGTC

CAAGGTACCTTGGAGCTTCAAGCGGAGATCCTGCCCCGACGGCCCCCTACACCCGAAGCC

CAGAGTGAAGAGGAGAGATCTGATGAGGAGCCGGAGGCCAAGGAAGAAGAAGAGGAAAAA

CCTCACATGCCTACGGAATTTGACTTCGATGATGAGCCAATGACACCAAAGGACTCCTTG

ATTGACCGGAGACGCACCCCAGGAAGTTCAGCCCGGAGCCAAAAAAGGGAGGCCCGCCTG

GACAAGGTCCTCTCAGACATGAAGCGACACAAGAAACTGGAGGAGCAGATTCTTCGTACT

GGGAGGGACCTCTTCAGCTTGGACTCAGAGGACCCCAGCCCAGCCAGCCCCCCACTCCGG

TCATCGGGGAGTAGTCTCTTCCCTCGACAGCGGAAATAC

>CL33.Contig1_All 2 742 Lysine-specific demethylase 2A [Heterocephalus glaber]

CGCAGAGAACTTTGTGAATGTATGCGAGTGTGCAAGACGTGGTATAAATGGTGCTGCGAC

AAGAGACTTTGGACAAAAATTGACTTGAGTAGGTGTAAGGCCATTGTACCCCAGGCTCTC

AGTGGCATCATCAAGAGGCAACCAGTTAGCCTTGACCTCAGTTGGACCAATATCTCCAAA

AAGCAGCTGACATGGCTGGTCAATAGGCTGCCAGGACTGAAAGACCTCCTCCTAGCAGGC

TGCTCGTGGTCTGCAGTCTCTGCCCTCAGCACCTCCAGCTGCCCCCTTCTCAGGACCCTT

GATCTTCGGTGGGCAGTAGGAATCAAGGACCCTCAAATTCGGGACTTGCTGACTCCATCG

GCCGATAAGCCGGGTCAGGACAATCGCAGCAAGCTCCGGAACATGACTGACTTCCGGCTG

GCAGGCCTTGACATCACGGATGCCACGCTTCGCCTCATCATTCGCCACATGCCCCTCTTG

TCTCGACTCGACCTTAGTCACTGCAGCCACCTTACAGATCAGTCCTCCAACTTACTCACT

GCCGTCGGGTCTTCCACTCGCTATTCCCTCACAGAGCTCAATATGGCAGGTTGCAATAAA

TTGACAGACCAGACGCTGATCTACCTACGGCGCATCGCCAATGTCACCTTGATCGACCTT

CGAGGATGCAAGCAGATCACTCGAAAAGCCTGTGAGCACTTCATCTCAGACTTGTCCATC

AACAGCCTCTACTGCATGTCT

>CL33.Contig2_All 3 263 PREDICTED: lysine-specific demethylase 2A [Saimiri boliviensis boliviensis]

CGAGTGTGCAAGACGTGGTATAAATGGTGCTGCGACAAGAGACTTTGGACAAAAATTGAC

TTGAGTAGGTGTAAGGCCATTGTACCCCAGGCTCTCAGTGGCATCATCAAGAGGCAACCA

GTTAGCCTTGACCTCAGTTGGACCAATATCTCCAAAAAGCAGCTGACATGGCTGGTCAAT

AGGCTGCCAGGACTGAAAGACCTCCTCCTAGCAGGCTGCTCGTGGTCTGCAGTCTCTGCC

CTCAGCACCTCCAGCTGCCCC

>CL34.Contig1_All 132 500 minus strand Casein kinase II subunit beta OS=Xenopus laevis GN=csnk2b PE=1 SV=1

ATGAGTAGCTCAGAGGAGGTGTCTTGGATTTCCTGGTTCTGTGGGCTCCGTGGCAATGAA

TTCTTCTGTGAAGTGGATGAAGACTATATCCAGGACAAATTTAATCTTACTGGACTCAAT

GAGCAGGTGCCTCACTATCGACAAGCTCTAGACATGATCTTGGACCTGGAGCCTGATGAA

GAGCTGGAAGACAACCCCAACCAGAGTGACCTGATTGAGCAGGCAGCTGAGATGCTGTAT

GGATTGATCCACGCCCGCTACATCCTCACCAACCGTGGCATCGCCCAGATGTTGGAAAAG

TACCAGCAGGGGGATTTTGGCTACTGTCCCCGTGTGTACTGTGAGAACCAGCCAATGCTT

CCCATCGGC

>CL34.Contig2_All 120 776 minus strand PREDICTED: casein kinase II subunit beta-like, partial [Sarcophilus harrisii]

GCCGACGTGAAGATGAGTAGCTCAGAGGAGGTGTCTTGGATTTCCTGGTTCTGTGGGCTC

CGTGGCAATGAATTCTTCTGTGAAGTGGATGAAGACTATATCCAGGACAAATTTAATCTT

ACTGGACTCAATGAGCAGGTGCCTCACTATCGACAAGCTCTAGACATGATCTTGGACCTG

GAGCCTGATGAAGAGCTGGAAGACAACCCCAACCAGAGTGACCTGATTGAGCAGGCAGCT

GAGATGCTGTATGGATTGATCCACGCCCGCTACATCCTCACCAACCGTGGCATCGCCCAG

ATGTTGGAAAAGTACCAGCAGGGGGATTTTGGCTACTGTCCCCGTGTGTACTGTGAGAAC

CAGCCAATGCTTCCCATCGGCCTTTCAGACATCCCAGGCGAGGCTATGGTGAAGCTCTAC

TGTCCCAAGTGCATGGATGTGTACACACCCAAGTCATCGAGGCACCATCACACGGACGGC

GCCTACTTCGGCACCGGTTTCCCTCACATGCTCTTCATGGTGCATCCAGAATACCGGCCC

AAGCGACCTGCCAACCAGTTTGTGCCCAGGCTCTACGGTTTCAAGATCCATCCGATGGCT

TACCAGCTGCAACTCCAAGCCGCCAGCAACTTCAAGAGCCCAGTCAAGACGATTCGC

>CL36.Contig1_All 752 916 minus strand B-cell receptor-associated protein 29 isoform a [Homo sapiens] >gi|119603814|gb|EAW83408.1| B-cell receptor-associated protein 29, isoform CRA_c [Homo sapiens]

TCGTTTAGAAAGAGACAATAAGAAAGGTCTGTGAACTTCATGGAAGAAGATTCTGATACA

CCGTGTCAAGATAATAAATTTGTTATCATGGTAGCCACTAGAAAATTTAAAATTCAATTT

AGAAAAATGTACTATGACTGGCTAATAGTGTTTTTTAAATGCCAC

>CL36.Contig2_All 3 416 minus strand PREDICTED: B-cell receptor-associated protein 29 [Papio anubis]

TTTCTAGATGCTGTGAGAGAAGTAAGGAAATATGCCTCTATTCATACCATTGACAAGAGC

TCAACTAGCAAACTTGGTGCCTATGAACATACACAGATGAAACTTTTTAGGTCTCAAAGA

AATCTTTACATTTCTGGATTTTCATTATTTTTTTGGCTAGTGTTGAGACGTCTGGTTACA

CTTATAACTCAGCTGGCAAAAGAACTCTCAAACAAAGGAATGCTTAAAAGTCAAGCAGAA

AATACTAATAAGGCTGCCAAAAGATTCATGGAAGAGAATGAAAAACTAAAAAGGATTTTG

AAAACCACAGATGAAGAACACACTTTGGAAACAGAAAATAAAAAACTAGTAGAAGACCAG

GAAAAACTGAAAGCTGAATTAAAGAAGACTTTAGATGGTAACTTTGTGTGCATG

>CL37.Contig1_All 3 200 minus strand sulfatase modifying factor 2 [Mustela putorius furo]

CACGTGAGCTGGAACGACGCCCGGGCCTACTGTGCCTGGCGGGGGAAGCGGCTGCCCACC

GAGGAAGAGTGGGAGTTTGCTGCCCGAGGGGGCTTGAAGGGTCAGGTCTACCCATGGGGG

AACCGGTTCCAGCCAAACCGTACCAACCTGTGGCAGGGATCTATGACCTCATGGGCAATG

TGTGGGAGTGGACCGCAT

>CL37.Contig2_All 3 1313 minus strand GTP-binding protein era-like protein [Heterocephalus glaber]

ATGGCTGCCCCTGGCAGGTGTGGGGTTGAGCTTGTTCGCGCAGGGTTGAAAGTCTGGCAG

CTGGGTCCCCACGCGGTGAGGGAATGGGTGGCCCCGTTTCGTTCACTTTTAGGCTGTCAA

AGGAGGTGCGTGTCCAGTGTGGGGGCTGCCACTTTCTCTAATCCCCGCCTGGCCTCAGCC

TCTCGCCATTATGGCCAGGGCTCAGCTATGGACCACTTCGTTGGAGTCTCTCAGCCCGAG

AATTCGCTGACTGCATGTGCGCCGGCGGTGTCCATGTATAGAGATGAGCAGAGTCTCCTC

TTGGTTCAACCCCCTGACATGCCTGAGAATCCCCGAGTGCTACGAGTGGTCCTCCTGGGA

GCTCCCAATGCAGGGAAGTCAACGCTTTCTAACCAGCTGCTAGGCCGAAACGTGTTCCCT

GTCTCCAAGAAGGTGCACACTACACGTTGCCAGGCTCTGGGGGTCATCACAGAGAAGGAA

TCTCAGGTGATTCTGCTTGACACACCTGGAATCATCAGTCCTGTTAAACAGAAGAGGCAC

CATCTGGAGCTCTCTTTGTTGGAAGATCCATGGAAGAGCATGGAATCTGCTGACCTTGTT

TTAGTTCTTGTGGATGTCTCAGACAAGTGGACTCGAAATCAGCTCAGCCCTCAGGTGCTC

CAGTGCTTGACCCAGTTCTCTCAGGTCCCCAGCATTCTTGTCTTGAACAAGGTAGATTGC

CTGAAGCAGAAGTCAATTCTCCTGGAGCTCACAGCAGCTCTCACTGAAGGTGTGGTTAAT

GGAAAGAAGCTTAATATAAGACAGGCCTTCCGCCCATGTCCTGACACCCATTGTCCTAGA

CCAGCAGCCAAGGACTTAAACACACAGTCTGAGAAAAATGCTGGGAAGATTGGCTGGCCC

AACTTCCAGGAGATCTTCATGTTGTCAGCTCTAAGCCAAGAGGATGTGAAGACACTAAAG

CAATACCTCTTGGCACAGGCCCAACCAGGACCCTGGGAGTTCCACAGTGGAGTCCTCACT

AGCCAGACACCTGAAGAGATCTGTATCAACAAAATCCGAGAGAAACTCCTAGAGTACCTC

CCCCAGGAGGTGCCCTACAATGTGCAGCAGAAGACAGTGGTGTGGGAGGAAGGACCAAGT

GGGGAGCTGATAATCCATCAGAACCTTCTGGTACCCAAAGAATCTCATATGAGGATCCTG

ATTGGTGAGAAGGGCCATCTTATCTCCCAGATTGCCCAGGAGGTGGGCCGCGACCTAATG

GACATCTTCCTCTGTGACATTCAGATACGCCTTTCTGTGAAGCTACTCAAG

>CL38.Contig1_All 272 1555 Spliceosome RNA helicase DDX39B OS=Pongo abelii GN=DDX39B PE=2 SV=1

ATGGCAGAGAATGATGTGGACAATGAGCTCTTGGACTATGAAGATGATGAGGTGGAAACG

GCAGCTGGGGGAGATGGAGCTGAGGCTCCTGCCAAGAAGGATGTCAAGGGCTCCTATGTC

TCCATCCATAGCTCTGGCTTTCGGGACTTCCTGCTCAAGCCAGAGTTACTCCGGGCTATT

GTTGACTGTGGCTTTGAGCATCCATCAGAAGTCCAGCATGAATGTATCCCTCAGGCCATT

CTGGGAATGGATGTCCTGTGCCAGGCCAAATCAGGCATGGGAAAGACAGCAGTGTTTGTG

TTGGCCACACTGCAACAGCTGGAGCCAGTTACTGGACAGGTGTCCGTGCTGGTGATGTGT

CACACTAGGGAGTTGGCTTTTCAGATCAGCAAGGAATATGAGCGCTTTTCTAAATACATG

CCCAATGTCAAGGTTGCAGTTTTTTTTGGTGGTCTGTCTATCAAGAAGGATGAAGAGGTG

CTGAAGAAGAACTGCCCGCATATCGTCGTGGGGACTCCTGGCCGCATCCTAGCCCTGGCT

CGAAATAAGAGCCTCAACCTCAAACATATTAAACACTTTATCTTGGATGAATGTGATAAG

ATGCTTGACCAGCTCGACATGCGTCGGGATGTCCAGGAAATTTTTCGCATGACCCCCCAT

GAGAAGCAGGTCATGATGTTCAGTGCTACCTTGGGCAAAGAGATCCGTCCAGTCTGCCGC

AAGTTCATGCAAGATCCAATGGAGATCTTCGTGGATGATGAGACGAAGTTGACGCTGCAC

GGGTTGCAGCAGTACTACGTGAAACTGAAGGACAACGAGAAGAACCGGAAACTCTTTGAC

CTTCTGGATGTCCTTGAGTTCAACCAGGTGGTGATCTTTGTGAAGTCTGTGCAGCGGTGC

ATCGCCCTGGCCCAGCTCCTAGTGGAGCAGAACTTTCCAGCCATTGCCATCCACCGTGGG

ATGCCTCAGGAGGAGAGGCTTTCTCGGTATCAGCAGTTTAAAGATTTTCAACGACGGATT

CTTGTGGCCACCAACCTATTTGGCCGAGGCATGGACATTGAGCGGGTGAACATTGCCTTT

AACTATGACATGCCTGAGGATTCTGACACCTACCTACATCGGGTGGCCAGAGCAGGTCGC

TTTGGCACCAAGGGCTTGGCTATCACATTTGTGTCAGATGAGAATGACGCCAAGATTCTT

AATGATGTGCAGGACCGCTTTGAGGTCAACATCAGTGAGCTGCCTGATGAGATAGACATC

TCCTCCTATATTGAACAGACGCGG

>CL38.Contig2_All 272 1189 unnamed protein product [Mus musculus]

ATGGCAGAGAATGATGTGGACAATGAGCTCTTGGACTATGAAGATGATGAGGTGGAAACG

GCAGCTGGGGGAGATGGAGCTGAGGCTCCTGCCAAGAAGGATGTCAAGGGCTCCTATGTC

TCCATCCATAGCTCTGGCTTTCGGGACTTCCTGCTCAAGCCAGAGTTACTCCGGGCTATT

GTTGACTGTGGCTTTGAGCATCCATCAGAAGTCCAGCATGAATGTATCCCTCAGGCCATT

CTGGGAATGGATGTCCTGTGCCAGGCCAAATCAGGCATGGGAAAGACAGCAGTGTTTGTG

TTGGCCACACTGCAACAGCTGGAGCCAGTTACTGGACAGGTGTCCGTGCTGGTGATGTGT

CACACTAGGGAGTTGGCTTTTCAGATCAGCAAGGAATATGAGCGCTTTTCTAAATACATG

CCCAATGTCAAGGTTGCAGTTTTTTTTGGTGGTCTGTCTATCAAGAAGGATGAAGAGGTG

CTGAAGAAGAACTGCCCGCATATCGTCGTGGGGACTCCTGGCCGCATCCTAGCCCTGGCT

CGAAATAAGAGCCTCAACCTCAAACATATTAAACACTTTATCTTGGATGAATGTGATAAG

ATGCTTGACCAGCTCGACATGCGTCGGGATGTCCAGGAAATTTTTCGCATGACCCCCCAT

GAGAAGCAGGTCATGATGTTCAGTGCTACCTTGGGCAAAGAGATCCGTCCAGTCTGCCGC

AAGTTCATGCAAGATGTAAATACCCTTCTACCTTCTCTCCCTCCACTCCCCGCCCACTGC

CTCCTCCCCTTCCTCGCCCTCTTCCTCCAGACTCCCTTGTCCTTCAAGTGCCAAGAAGGG

GGCTTTTGCCCGTCTGGGAGCAACAACTCCTTGAAGAGACACACAGAGGCAGAGACAGCT

AGTGTTAGGGTCTGCGCG

>CL38.Contig3_All 674 1222 Spliceosome RNA helicase Ddx39b OS=Rattus norvegicus GN=Ddx39b PE=1 SV=3

CCAATGGAGATCTTCGTGGATGATGAGACGAAGTTGACGCTGCACGGGTTGCAGCAGTAC

TACGTGAAACTGAAGGACAACGAGAAGAACCGGAAACTCTTTGACCTTCTGGATGTCCTT

GAGTTCAACCAGGTGGTGATCTTTGTGAAGTCTGTGCAGCGGTGCATCGCCCTGGCCCAG

CTCCTAGTGGAGCAGAACTTTCCAGCCATTGCCATCCACCGTGGGATGCCTCAGGAGGAG

AGGCTTTCTCGGTATCAGCAGTTTAAAGATTTTCAACGACGGATTCTTGTGGCCACCAAC

CTATTTGGCCGAGGCATGGACATTGAGCGGGTGAACATTGCCTTTAACTATGACATGCCT

GAGGATTCTGACACCTACCTACATCGGGTGGCCAGAGCAGGTCGCTTTGGCACTAAGGGC

TTGGCTATCACATTTGTGTCAGATGAGAATGACGCCAAGATTCTTAATGATGTGCAGGAC

CGCTTTGAGGTCAACATCAGTGAGCTGCCTGATGAGATAGACATCTCCTCCTATATTGAA

CAGACGCGG

>CL38.Contig4_All 272 1189 minus strand unnamed protein product [Mus musculus]

ATGGCAGAGAATGATGTGGACAATGAGCTCTTGGACTATGAAGATGATGAGGTGGAAACG

GCAGCTGGGGGAGATGGAGCTGAGGCTCCTGCCAAGAAGGATGTCAAGGGCTCCTATGTC

TCCATCCATAGCTCTGGCTTTCGGGACTTCCTGCTCAAGCCAGAGTTACTCCGGGCTATT

GTTGACTGTGGCTTTGAGCATCCATCAGAAGTCCAGCATGAATGTATCCCTCAGGCCATT

CTGGGAATGGATGTCCTGTGCCAGGCCAAATCAGGCATGGGAAAGACAGCAGTGTTTGTG

TTGGCCACACTGCAACAGCTGGAGCCAGTTACTGGACAGGTGTCCGTGCTGGTGATGTGT

CACACTAGGGAGTTGGCTTTTCAGATCAGCAAGGAATATGAGCGCTTTTCTAAATACATG

CCCAATGTCAAGGTTGCAGTTTTTTTTGGTGGTCTGTCTATCAAGAAGGATGAAGAGGTG

CTGAAGAAGAACTGCCCGCATATCGTCGTGGGGACTCCTGGCCGCATCCTAGCCCTGGCT

CGAAATAAGAGCCTCAACCTCAAACATATTAAACACTTTATCTTGGATGAATGTGATAAG

ATGCTTGACCAGCTCGACATGCGTCGGGATGTCCAGGAAATTTTTCGCATGACCCCCCAT

GAGAAGCAGGTCATGATGTTCAGTGCTACCTTGGGCAAAGAGATCCGTCCAGTCTGCCGC

AAGTTCATGCAAGATGTAAATACCCTTCTACCTTCTCTCCCTCCACTCCCCGCCCACTGC

CTCCTCCCCTTCCTCGCCCTCTTCCTCCAGACTCCCTTGTCCTTCAAGTGCCAAGAAGGG

GGCTTTTGCCCGTCTGGGAGCAACAACTCCTTGAAGAGACACACAGAGGCAGAGACAGCT

AGTGTTAGGGTCTGCGCG

>CL38.Contig5_All 1269 1817 ATP-dependent RNA helicase DDX39 [Macaca mulatta]

CCCATGGAGGTGTTTGTGGACGACGAGACCAAGCTCACGCTGCATGGGCTGCAGCAGTAC

TATGTCAAGCTCAAGGACAGCGAGAAGAACCGAAAGCTCTTTGACCTCCTGGATGTGCTG

GAGTTTAACCAGGTGGTGATCTTCGTCAAATCCGTGCAGCGCTGCATGGCCCTGGCCCAG

CTCCTTGTGGAGCAGAACTTCCCAGCCATCGCCATTCACCGGGGCATGGCCCAGGAGGAG

CGTCTGTCACGCTACCAGCAGTTCAAGGACTTCCAGCGGCGGATCTTGGTGGCCACCAAT

CTCTTTGGCCGAGGGATGGATATTGAGCGCGTCAACATCGTCTTCAACTATGACATGCCC

GAGGACTCGGACACCTACCTCCACCGGGTGGCCCGCGCAGGTCGCTTTGGTACCAAAGGC

CTGGCCATCACTTTTGTGTCTGATGAAAATGATGCCAAAATTCTCAATGATGTCCAGGAC

CGGTTTGAAGTGAATGTGGCTGAGCTTCCGGAAGAAATCGATATCTCCACATACATCGAG

CAGAGCCGG

>CL38.Contig6_All 7 720 ATP-dependent RNA helicase DDX39 [Macaca mulatta]

AGGAGGCAGGAAGCGCCGCAGCTCGTGTCGCGGCGCGTGCTGGGTAACCGGAGTCAGGCG

GCTCTCCTCGTAGCACGCGGAGGCGAACGGAGACCCAGTACCATGGCAGAGCAGGATGTG

GAGAACGAGCTCTTGGATTATGAAGAAGATGAAGAGCCCCAGGCTCCTCCAGAGAGTACT

CCAGCTCCACCTAAGAAAGACGTCAAGGGGTCCTACGTCTCCATCCATAGCTCTGGCTTC

CGAGACTTTTTGCTGAAGCCAGAGCTCCTGAGGGCCATAGTGGACTGTGGTTTTGAACAC

CCATCCGAGGTCCAGCATGAGTGTATCCCCCAGGCCATCCTGGGCATGGACGTCCTGTGC

CAGGCCAAGTCTGGGATGGGCAAGACGGCTGTCTTCGTGCTGGCTACTCTGCAGCAGATC

GAGCCAGTTAATGGACAGGTGACAGTCCTGGTCATGTGCCATACGAGGGAGTTGGCCTTC

CAAATCAGCAAGGAGTATGAGCGCTTCTCCAAATACATGCCCAGTGTCAAGGTGTCTGTG

TTCTTTGGGGGCCTCTCCATCAAGAAAGATGAAGAAGTGTTGAAGAAGAATTGTCCCCAC

GTAGTGGTGGGGACACCAGGCAGGATCCTGGCCCTCGTGCGGAACAGGAGCCTCAACTTA

AAAAACGTGAAGCACTTTGTGTTGGACGAGTGTGACAAGATGCTGGAGCAGCTG

>CL38.Contig7_All 7 1389 ATP-dependent RNA helicase DDX39 [Macaca mulatta]

AGGAGGCAGGAAGCGCCGCAGCTCGTGTCGCGGCGCGTGCTGGGTAACCGGAGTCAGGCG

GCTCTCCTCGTAGCACGCGGAGGCGAACGGAGACCCAGTACCATGGCAGAGCAGGATGTG

GAGAACGAGCTCTTGGATTATGAAGAAGATGAAGAGCCCCAGGCTCCTCCAGAGAGTACT

CCAGCTCCACCTAAGAAAGACGTCAAGGGGTCCTACGTCTCCATCCATAGCTCTGGCTTC

CGAGACTTTTTGCTGAAGCCAGAGCTCCTGAGGGCCATAGTGGACTGTGGTTTTGAACAC

CCATCCGAGGTCCAGCATGAGTGTATCCCCCAGGCCATCCTGGGCATGGACGTCCTGTGC

CAGGCCAAGTCTGGGATGGGCAAGACGGCTGTCTTCGTGCTGGCTACTCTGCAGCAGATC

GAGCCAGTTAATGGACAGGTGACAGTCCTGGTCATGTGCCATACGAGGGAGTTGGCCTTC

CAAATCAGCAAGGAGTATGAGCGCTTCTCCAAATACATGCCCAGTGTCAAGGTGTCTGTG

TTCTTTGGGGGCCTCTCCATCAAGAAAGATGAAGAAGTGTTGAAGAAGAATTGTCCCCAC

GTAGTGGTGGGGACACCAGGCAGGATCCTGGCCCTCGTGCGGAACAGGAGCCTCAACTTA

AAAAACGTGAAGCACTTTGTGTTGGACGAGTGTGACAAGATGCTGGAGCAGCTGGACATG

CGGCGGGATGTGCAGGAGATCTTCCGCCTAACGCCTCATGAGAAGCAGTGCATGATGTTC

AGTGCCACCCTGAGCAAGGAGATCCGGCCAGTCTGCAGGAAGTTCATGCAGGATCCCATG

GAGGTGTTTGTGGACGACGAGACCAAGCTCACGCTGCATGGGCTGCAGCAGTACTATGTC

AAGCTCAAGGACAGCGAGAAGAACCGAAAGCTCTTTGACCTCCTGGATGTGCTGGAGTTT

AACCAGGTGGTGATCTTCGTCAAATCCGTGCAGCGCTGCATGGCCCTGGCCCAGCTCCTT

GTGGAGCAGAACTTCCCAGCCATCGCCATTCACCGGGGCATGGCCCAGGAGGAGCGTCTG

TCACGCTACCAGCAGTTCAAGGACTTCCAGCGGCGGATCTTGGTGGCCACCAATCTCTTT

GGCCGAGGGATGGATATTGAGCGCGTCAACATCGTCTTCAACTATGACATGCCCGAGGAC

TCGGACACCTACCTCCACCGGGTGGCCCGCGCAGGTCGCTTTGGTACCAAAGGCCTGGCC

ATCACTTTTGTGTCTGATGAAAATGATGCCAAAATTCTCAATGATGTCCAGGACCGGTTT

GAAGTGAATGTGGCTGAGCTTCCGGAAGAAATCGATATCTCCACATACATCGAGCAGAGC

CGG

>CL38.Contig8_All 1208 1756 minus strand ATP-dependent RNA helicase DDX39 [Macaca mulatta]

CCCATGGAGGTGTTTGTGGACGACGAGACCAAGCTCACGCTGCATGGGCTGCAGCAGTAC

TATGTCAAGCTCAAGGACAGCGAGAAGAACCGAAAGCTCTTTGACCTCCTGGATGTGCTG

GAGTTTAACCAGGTGGTGATCTTCGTCAAATCCGTGCAGCGCTGCATGGCCCTGGCCCAG

CTCCTTGTGGAGCAGAACTTCCCAGCCATCGCCATTCACCGGGGCATGGCCCAGGAGGAG

CGTCTGTCACGCTACCAGCAGTTCAAGGACTTCCAGCGGCGGATCTTGGTGGCCACCAAT

CTCTTTGGCCGAGGGATGGATATTGAGCGCGTCAACATCGTCTTCAACTATGACATGCCC

GAGGACTCGGACACCTACCTCCACCGGGTGGCCCGCGCAGGTCGCTTTGGTACCAAAGGC

CTGGCCATCACTTTTGTGTCTGATGAAAATGATGCCAAAATTCTCAATGATGTCCAGGAC

CGGTTTGAAGTGAATGTGGCTGAGCTTCCGGAAGAAATCGATATCTCCACATACATCGAG

CAGAGCCGG

>CL40.Contig1_All 247 504 coiled-coil and C2 domain-containing protein 1A [Bos taurus] >gi|148743922|gb|AAI42502.1| CC2D1A protein [Bos taurus] >gi|296485971|gb|DAA28086.1| coiled-coil and C2 domain containing 1A [Bos taurus]

CCCCCAAAGGCCCCCCCGTCAGGATCAGCGCCAACAGCCAAAGCAGCCCCCAAAGCCCCG

TCCACCAGAGCCCAGCAGCAGCTGGCCTTCCTGGAGGGCCGCAAGAAGCAACTCCTGCAG

GCCGCTCTGCGCGCCAAGCAGAAGAACGACGTGGAGGGTGCCAAGATGCACCTGCGCCAG

GCCAAGGGGCTGGAGCCCATGCTGGAGGCCTCCCGCAACGGACTGCCTGTGGACATCACC

AAGGTGCCGCCTGCTCCT

>CL40.Contig2_All 19 246 PREDICTED: coiled-coil and C2 domain-containing protein 1A [Pan paniscus]

AGGATCTTTTCCGACCTAAGCAGCAGCGACATGCTCCTCTTCATCGTGAAGGGCGTCAAC

CTGCCCACGCCCCCAGGGCTGTCCCCTGGCGACCTGGACATCTTTGTGCGGTTCGACTTC

CCCTATCCCAATGTGGAGGAAGCTCAGAAAGACAAGACCAGCGTGGTCAGGAACACGGAC

TCGCCCGAATTCAAGGAGCAGTTCAAGCTGGGCATCAACCGCGACCAC

>CL41.Contig1_All 147 455 protein max isoform c [Homo sapiens] >gi|34471|emb|CAA47338.1| max [Homo sapiens] >gi|599793|emb|CAA42827.1| max [Homo sapiens] >gi|119601307|gb|EAW80901.1| MYC associated factor X, isoform CRA_d [Homo sapiens] >gi|119601312|gb|EAW80906.1| MYC associated factor X, isoform CRA_d [Homo sapiens]

ATGAGCGATAACGATGACATCGAGGTGGAGAGCGACGAAGAGCAACCGAGGTTTCAATCT

GCGGCTGACAAACGGGCTCATCATAATGCACTGGAACGAAAACGCAGGGACCACATCAAA

GACAGCTTTCACAGTTTGCGGGACTCAGTCCCGTCACTCCAAGGAGAGAAGGCATCCCGG

GCCCAAATCCTAGACAAAGCCACAGAGTATATCCAGTATATGCGAAGGAAAAACCACACG

CACCAGCAAGATATCGATGACCTCAAGCGGCAGAATGCTCTTCTGGAGCAGCAAGGGGAA

AGCCAGAGC

>CL41.Contig2_All 147 428 MAX protein [Homo sapiens] >gi|49457378|emb|CAG46988.1| MAX [Homo sapiens]

ATGAGCGATAACGATGACATCGAGGTGGAGAGCGACGCTGACAAACGGGCTCATCATAAT

GCACTGGAACGAAAACGCAGGGACCACATCAAAGACAGCTTTCACAGTTTGCGGGACTCA

GTCCCGTCACTCCAAGGAGAGAAGGCATCCCGGGCCCAAATCCTAGACAAAGCCACAGAG

TATATCCAGTATATGCGAAGGAAAAACCACACGCACCAGCAAGATATCGATGACCTCAAG

CGGCAGAATGCTCTTCTGGAGCAGCAAGGGGAAAGCCAGAGC

>CL41.Contig3_All 149 424 protein max isoform c [Homo sapiens] >gi|34471|emb|CAA47338.1| max [Homo sapiens] >gi|599793|emb|CAA42827.1| max [Homo sapiens] >gi|119601307|gb|EAW80901.1| MYC associated factor X, isoform CRA_d [Homo sapiens] >gi|119601312|gb|EAW80906.1| MYC associated factor X, isoform CRA_d [Homo sapiens]

GAGGAAGAGCAACCGAGGTTTCAATCTGCGGCTGACAAACGGGCTCATCATAATGCACTG

GAACGAAAACGCAGGGACCACATCAAAGACAGCTTTCACAGTTTGCGGGACTCAGTCCCG

TCACTCCAAGGAGAGAAGGCATCCCGGGCCCAAATCCTAGACAAAGCCACAGAGTATATC

CAGTATATGCGAAGGAAAAACCACACGCACCAGCAAGATATCGATGACCTCAAGCGGCAG

AATGCTCTTCTGGAGCAGCAAGGGGAAAGCCAGAGC

>CL41.Contig4_All 149 397 MAX protein [Homo sapiens] >gi|49457378|emb|CAG46988.1| MAX [Homo sapiens]

GAGGCTGACAAACGGGCTCATCATAATGCACTGGAACGAAAACGCAGGGACCACATCAAA

GACAGCTTTCACAGTTTGCGGGACTCAGTCCCGTCACTCCAAGGAGAGAAGGCATCCCGG

GCCCAAATCCTAGACAAAGCCACAGAGTATATCCAGTATATGCGAAGGAAAAACCACACG

CACCAGCAAGATATCGATGACCTCAAGCGGCAGAATGCTCTTCTGGAGCAGCAAGGGGAA

AGCCAGAGC

>CL41.Contig5_All 8 286 PREDICTED: protein L-Myc-1-like [Cricetulus griseus]

CGGGGCTCCCAGCGGGGCGACGGCGGGAGGGGGCCGGGCTGCCGCCGCCTTCTTCCCCTT

CCCCGGGGGCTGGGATCGCCTGCGTCGCGCTCCCGGCTCGCTCCCCTCCGGGCTCCTGGC

CGGAGCGGCCTCCTGGGACTGGAGGAAGAGCAACCGAGGTTTCAATCTGCGGCTGACAAA

CGGGCTCATCATAATGCACTGGAACGAAAACGCAGGGACCACATCAAAGACAGCTTTCAC

AGTTTGCGGGACTCAGTCCCGTCACTCCAAGGAGAGAAG

>CL41.Contig6_All 47 259 PREDICTED: protein max-like [Meleagris gallopavo]

TGCCGCCGCCTTCTTCCCCTTCCCCGGGGGCTGGGATCGCCTGCGTCGCGCTCCCGGCTC

GCTCCCCTCCGGGCTCCTGGCCGGAGCGGCCTCCTGGGACTGGAGGCTGACAAACGGGCT

CATCATAATGCACTGGAACGAAAACGCAGGGACCACATCAAAGACAGCTTTCACAGTTTG

CGGGACTCAGTCCCGTCACTCCAAGGAGAGAAG

>CL41.Contig7_All 8 595 PREDICTED: protein L-Myc-1-like [Cricetulus griseus]

CGGGGCTCCCAGCGGGGCGACGGCGGGAGGGGGCCGGGCTGCCGCCGCCTTCTTCCCCTT

CCCCGGGGGCTGGGATCGCCTGCGTCGCGCTCCCGGCTCGCTCCCCTCCGGGCTCCTGGC

CGGAGCGGCCTCCTGGGACTGGAGGAAGAGCAACCGAGGTTTCAATCTGCGGCTGACAAA

CGGGCTCATCATAATGCACTGGAACGAAAACGCAGGGACCACATCAAAGACAGCTTTCAC

AGTTTGCGGGACTCAGTCCCGTCACTCCAAGGAGAGAAGGCATCCCGGGCCCAAATCCTA

GACAAAGCCACAGAGTATATCCAGTATATGCGAAGGAAAAACCACACGCACCAGCAAGAT

ATCGATGACCTCAAGCGGCAGAATGCTCTTCTGGAGCAGCAAGTCCGTGCACTGGAGAAG

GCGAGGTCGAGTGCCCAACTGCAGACCAACTACCCCTCCTCAGACAACAGCCTCTACACC

AACGCCAAGGGCAGCACCATCTCTGCCTTCGATGGGGGCTCTGACTCCAGCTCAGAGTCA

GAGCCTGAAGAGCCCCAAAGCAGGAAGAAACTCCGGATGGAGGCCAGC

>CL41.Contig8_All 8 289 PREDICTED: protein L-Myc-1-like [Cricetulus griseus]

CGGGGCTCCCAGCGGGGCGACGGCGGGAGGGGGCCGGGCTGCCGCCGCCTTCTTCCCCTT

CCCCGGGGGCTGGGATCGCCTGCGTCGCGCTCCCGGCTCGCTCCCCTCCGGGCTCCTGGC

CGGAGCGGCCTCCTGGGACTGGAGGAAGAGCAACCGAGGTTTCAATCTGCGGCTGACAAA

CGGGCTCATCATAATGCACTGGAACGAAAACGCAGGGACCACATCAAAGACAGCTTTCAC

AGTTTGCGGGACTCAGTCCCGTCACTCCAAGGAGAGAAGTCC

>CL43.Contig1_All 1 300 minus strand PREDICTED: UDP-glucuronosyltransferase 2B31-like isoform 2 [Cavia porcellus]

ATAACTCATGGTGGAACCAATGGCATCTATGAGGCCATCTATCATGGGATCCCTCTTGTG

GGCATTCCCTTGTTTTCGGATCAACCTGATAATGTCATGCACATGAAAGCCAAAGGAGCA

GCTCTTAGACTGAATTTTATTACAATGTCAAGTATGGATTTACTCAGTGCGTTGAATACA

GTCATTAATGAACCTTCATATAAAGAGAATGTTATGAGATTATCAAGAATTCACCATGAA

CAGCCAGTGAAGCCTCTGGACCAAGCTGTCTTCTGGATAGAGTTTGTCATGCGCCACAAA

>CL43.Contig2_All 45 1463 UDP glucuronosyltransferase 2 family, polypeptide A3 [Bos taurus]

ATGATGTTTGAGAAATGGGTTTCGACAATTCTTTTCTTGCAGTTCTGCTGGGCCGGCTGT

GGATTCTGTGGGAAGGTCCTAGTGTGGCCCTGTGATATGAGCCATTGGCTCAACCAAAAG

ATCATTCTTGAGGAACTCCTGCAGAGAGGACATGAAGTGACAGTTTTGACTTCTTCACAG

AATCTCTTGGTTGACTATGAAAACTCTTCTGGATTGAATTTCGAGGTGATCTTTGTGTCA

CAGGACAGAGAAAGTACAGAGAAAAATCTAGAGGAGTTCTTATATTTGGCTGTGAACATC

ATGCCATCATTGTCACTCTGGCAATCAGCAAAATTATTTCAAAACTTCTTTCTTCAAGCA

ACTGAATATTTTGAAGAACTCTGTCGGAAAGCAGTTTATAATCAGTCCCTCATGGAGAAG

CTCCGGGAAACCAAGTATGATGTCATGTTTATAGACCCTGTGATTCCCTGTGGGGAGTTG

GTGGCTGAGTTGCTTGGGATCCCTTTTGTGCACACGATAAGGCTCTCCATAGGCAACACT

GTGGAGAAATACTGTGGGCATCTTCCAGTTCCACCTTCCTATGTGCCTGCTGCCATGGGA

GGACTAATAGACAGAATGACCTTTTTGGAAAGGGTGAAAAATATGATGTTTATATTCTTC

TTTGACTTTGTGCTCCAGCAATATGACTTTAAATTTTGGAATCAATTTTACAGTGAAGCA

TTAGGAAGACCCACTACATTATGTGAGACTATGGGCAAAGCTGAAATTTGGCTAATCCGG

ACATATTGGGATTTTGAATTTCCTCGTCCATACTTACCTAATTTTGAGTTTGTTGGAGGA

TTGCACTGTAAACCTGCCAAACCTTTACCTAAGGAAATGGAAGACTTTGTCCAGAGCTCA

GGGGAAGATGGTGTTGTTGCATTTTCTCTGGGGTCAATGGTCAAAAACCTCACAGAAGAA

AAGGCCAATCTCATTGCCTCGGCCCTTGCCCAGATTCCACAAAAGGTCTTATGGAGATAC

CAAGGAAAGAAACCAGCCACATTAGGAGCCAATACTCGAATTTTTGATTGGCTACCTCAG

AATGACCTTCTTGGTCATCCTAAAACCAAAGCTTTTATCACTCATGGTGGAACTAATGGG

ATCTATGAAGCTATTTATCATGGGATCCCTATGGTGGGAATCCCCATGTTTGCTGATCAA

CCTGATAACATTGCTCACATGAGGGCCAAAGGAGCAGCTGTGGAGGTGAACTTCAACACA

ATGACAAGTGCGGATTTGCTCAGTGCTTTGAGAACAGTCATTAATGACCCTTCTTATAAA

GAAAATGCCATGAGGTTATCAAGAATTCATCATGATCAGCCTGTAAAGCCCCTGGACCGA

GCTGTCTTCTGGATCGAGTTTGTCATGCGCCACAAAGGA

>CL43.Contig3_All 3 227 minus strand PREDICTED: UDP-glucuronosyltransferase 2B31-like isoform 1 [Cavia porcellus]

TTTTGGTTCCAAACATTTGATGAGAAGAAGTGGAATCAGTTTTACAGTGAAATTTTAGGA

CGACCCACTACATTACTAGAGACAATGTCAAAAGCAGATATCTGGCTCATTAGAACCTAT

TGGGATTTGGAATTTCCTCGCCCAACCCTACCAAATGTTGATTTCGTTGGAGGACTCCAC

TGCAGACCTGCCAAACCTCTGCCTAAGGAAATGGAAGACTTTGTC

>CL45.Contig1_All 95 613 minus strand PREDICTED: transmembrane protein 208-like [Loxodonta africana]

ATGGCGCCTAAGGGCAAAGTGGGCACCAGAGGGAAGAAGCAGATATTTGAAGAGAACAGA

GAGACCCTAAAGTTCTATCTGAGGATCATACTGGGGGCCAACGCCATTTACTGCCTTGTG

ACCTTGGTTGTCTTCTACTCATCTGCCTCATTTTGGGCCTGGATGGCCCTTGGCTTTAGT

TTGGCAGTATATGGGGCCAGCTACCACTCTATGAGCTCGATGGCTCGGGCAGCCTTCTCT

GAAGATGGGGCCTTGATGGATGGTGGCATGGACCTCAACATGGAACAGGGCATGGCAGAG

CACCTTAAGGATGTGATCCTGCTGACAGCTATTGTGCAGGTTCTGAGCTGCTTCTCCCTC

TATATCTGGTCCTTTTGGCTTCTGGCTCCAGGCCGGGCCCTTTATCTCTTATGGGTGAAT

GTGCTGGGACCCTGGTTCACAGCAGACAGTGGCACCCCAGCACCGGAGCACAACGAGAAA

CGGCAGCGCCGACAGGAGCGGCGGCAGATGAAGCGGCTA

>CL45.Contig2_All 417 635 minus strand PREDICTED: transmembrane protein 208-like [Loxodonta africana]

CACCTTAAGGATGTGATCCTGCTGACAGCTATTGTGCAGGTTCTGAGCTGCTTCTCCCTC

TATATCTGGTCCTTTTGGCTTCTGGCTCCAGGCCGGGCCCTTTATCTCTTATGGGTGAAT

GTGCTGGGACCCTGGTTCACAGCAGACAGTGGCACCCCAGCACCGGAGCACAACGAGAAA

CGGCAGCGCCGACAGGAGCGGCGGCAGATGAAGCGGCTA

>CL45.Contig3_All 95 487 minus strand PREDICTED: transmembrane protein 208-like [Loxodonta africana]

ATGGCGCCTAAGGGCAAAGTGGGCACCAGAGGGAAGAAGCAGATATTTGAAGAGAACAGA

GAGACCCTAAAGTTCTATCTGAGGATCATACTGGGGGCCAACGCCATTTACTGCCTTGTG

ACCTTGGTTGTCTTCTACTCATCTGCCTCATTTTGGGCCTGGATGGCCCTTGGCTTTAGT

TTGGCAGTATATGGGGCCAGCTACCACTCTATGAGCTCGATGGCTCGGGCAGCCTTCTCT

GAAGATGGGGCCTTGATGGATGGTGGCATGGACCTCAACATGGAACAGGGCATGGCAGAG

CACCTTAAGGATGTGATCCTGCTGACAGCTATTGTGCAGGTTCTGAGCTGCTTCTCCCTC

TATATCTGGTCCTTTTGGCTTCTGGTATGTGGG

>CL45.Contig4_All 89 388 minus strand Transmembrane protein 208 OS=Homo sapiens GN=TMEM208 PE=2 SV=1

ATGGCGCCTAAGGGCAAAGTGGGCACCAGAGGGAAGAAGCAGATATTTGAAGAGAACAGA

GAGACCCTAAAGTTCTATCTGAGGATCATACTGGGGGCCAACGCCATTTACTGCCTTGTG

ACCTTGGTTGTCTTCTACTCATCTGCCTCATTTTGGGCCTGGATGGCCCTTGGCTTTAGT

TTGGCAGTATATGGGGCCAGCTACCACTCTATGAGCTCGATGGCTCGGGCAGCCTTCTCT

GAAGATGGGGCCTTGATGGATGGTGGCATGGACCTCAACATGGAACAGGGCATGGCAGAG

>CL46.Contig1_All 454 807 minus strand PREDICTED: multiple C2 and transmembrane domain-containing protein 2 [Saimiri boliviensis boliviensis]

ATTTTACAAGTGAAGGTTTTGAAGGCAGTAGATCTCCTAGCTGCAGATTTCTCGGGGAAG

AGTGATCCTTTTTGCTTGTTGGAGGTAGGCAATGACCGGCTTCAGACACATACCATTTAC

AAAAACCTCAACCCGGAGTGGAACAAAGTTTTTACATTTCCCATTAAAGACATCCATGAT

GTTTTGGAAGTGACAGTGTTTGATGAAGATGGAGATAAACCCCCTGATTTTCTTGGAAAA

GTTGCCATTCCCTTGCTGTCTATTAAAGATGGGCAACCAAATTGTTATGTACTGAAGAAC

AAAGACTTAGAACAAGCTTTTAAAGGAGTTATTTACTTAGAGATGGACCTCATA

>CL46.Contig2_All 334 687 minus strand PREDICTED: multiple C2 and transmembrane domain-containing protein 2 [Pan paniscus]

ATTTTACAAGTGAAGGTTTTGAAGGCAGTAGATCTCCTAGCTGCAGATTTCTCGGGGAAG

AGTGATCCTTTTTGCTTGTTGGAGGTAGGCAATGACCGGCTTCAGACACATACCATTTAC

AAAAACCTCAACCCGGAGTGGAACAAAGTTTTTACATTTCCCATTAAAGACATCCATGAT

GTTTTGGAAGTGACAGTGTTTGATGAAGATGGAGATAAACCCCCTGATTTTCTTGGAAAA

GTTGCCATTCCCTTGCTGTCTATTAAAGATGGGCAACCAAATTGTTATGTACTGAAGAAC

AAAGACTTAGAACAAGCTTTTAAAGGAGTTATTTACTTAGAGATGGACCTCATA

>CL47.Contig1_All 45 1016 minus strand SAM pointed domain containing ets transcription factor [Mustela putorius furo]

GAGGCCCGGGGCCTACCTCAGAGGAGCGGGGTCGCGGCGCCGGCTAGCAACGGGCCCCCT

CCCGGTTCCCGGGCCTGGCGGCGCGGCGGCGGCTCGGTAGTGAGGAGGCGGGGAAGCGGG

TGTCCGGCCCCCGCCATGGAGGGCATGGACGTGGATCTGGACCCGGAGCTGATGCAGAAG

TTCAGCTGTTTGGGCACCACCGACAAGGACGTGCTCATCTCCGAGTTCCAGCGGCTGCTT

GGCTTCCAGCTCAACCCGGCCGGCTGCGCCTTCTTCCTGGACATGACCAACTGGAACTTA

CAGGCAGCAATTGGAGCCTATTATGACTTTGAGAGCCCAAACATCAGTGTGCCCTCCATG

TCCTTTGTTAAAGATGTCACCATAGGAGAAGGGGAGTCAATACCTCCTGATACTCAGTTT

ATAAAAACATGGCGGATCCAGAATTCTGGGGCAGAGGCCTGGCCTCCAGGGGTTTGTCTT

AAGTATGTCGGGGGAGACCAGTTTGGACATGTGAACATGGTGATGGTGAGATCATTAGAG

CCACAAGAAATTGCAGATGTCAGCGTCCAGATGTGCAGCCCCAGCAGAGCAGGAATGTAT

CAGGGACAGTGGCGGATGTGCACTGCTACAGGACTCTATTATGGAGATGTCATCTGGGTG

ATTCTCAGTGTGGAGGTGGGTGGACTGTTAGGAGTAACGCAGCAGCTGTCATCTTTTGAA

ACGGAGTTCAACACACAGCCTCATCGCAAGGTAGAAGGAAACTTCAACCCGTTTGCCTCT

CCCCAAAAGAACCGACAATCAGATGAAAACAACTTAAAAGACCCTGGGGGTTCCGGGTTC

GACTCGATCAGCAAAAACACATGGGCTCCTGCTCCTGACCAAACCGAGCAAGATCAGAAT

AGACTCTCACAGAACTCTGTAAATCTGTCCCCCAGCAGTCACGCAAACAACTTATCAGTA

GTGACTTACAGT

>CL47.Contig2_All 45 1016 minus strand SAM pointed domain containing ets transcription factor [Mustela putorius furo]

GAGGCCCGGGGCCTACCTCAGAGGAGCGGGGTCGCGGCGCCGGCTAGCAACGGGCCCCCT

CCCGGTTCCCGGGCCTGGCGGCGCGGCGGCGGCTCGGTAGTGAGGAGGCGGGGAAGCGGG

TGTCCGGCCCCCGCCATGGAGGGCATGGACGTGGATCTGGACCCGGAGCTGATGCAGAAG

TTCAGCTGTTTGGGCACCACCGACAAGGACGTGCTCATCTCCGAGTTCCAGCGGCTGCTT

GGCTTCCAGCTCAACCCGGCCGGCTGCGCCTTCTTCCTGGACATGACCAACTGGAACTTA

CAGGCAGCAATTGGAGCCTATTATGACTTTGAGAGCCCAAACATCAGTGTGCCCTCCATG

TCCTTTGTTAAAGATGTCACCATAGGAGAAGGGGAGTCAATACCTCCTGATACTCAGTTT

ATAAAAACATGGCGGATCCAGAATTCTGGGGCAGAGGCCTGGCCTCCAGGGGTTTGTCTT

AAGTATGTCGGGGGAGACCAGTTTGGACATGTGAACATGGTGATGGTGAGATCATTAGAG

CCACAAGAAATTGCAGATGTCAGCGTCCAGATGTGCAGCCCCAGCAGAGCAGGAATGTAT

CAGGGACAGTGGCGGATGTGCACTGCTACAGGACTCTATTATGGAGATGTCATCTGGGTG

ATTCTCAGTGTGGAGGTGGGTGGACTGTTAGGAGTAACGCAGCAGCTGTCATCTTTTGAA

ACGGAGTTCAACACACAGCCTCATCGCAAGGTAGAAGGAAACTTCAACCCGTTTGCCTCT

CCCCAAAAGAACCGACAATCAGATGAAAACAACTTAAAAGACCCTGGGGGTTCCGGGTTC

GACTCGATCAGCAAAAACACATGGGCTCCTGCTCCTGACCAAACCGAGCAAGATCAGAAT

AGACTCTCACAGAACTCTGTAAATCTGTCCCCCAGCAGTCACGCAAACAACTTATCAGTA

GTGACTTACAGT

>CL48.Contig1_All 36 794 PREDICTED: transmembrane emp24 domain-containing protein 4 isoform 4 [Pan troglodytes] >gi|397467116|ref|XP_003805273.1| PREDICTED: transmembrane emp24 domain-containing protein 4 [Pan paniscus]

ATGGCAGGTGTCGTGGGTAGGGGCCTGCCGGCTATGGGGCGACCGGCGTTGCTGTTGCTC

GTGGTGTGTGCTGCCGGCGCCCAGGGGCTCTACTTCCACATCGGCGAGACGGAGAAGCGT

TGCTTCATCGAGGAAATCCCCGACGAGACCATGGTCATCGGTCAGGCGGGCTGAGGGTGG

AGAGGCCCTTTGTTTTCGCCCGGCCCTCGGAAGTGCCCCTCCTCCCGAGCTTGGCCAGGC

AACTATCGAACCCAGATGTGGGACAAGCAGAAGGAGGTTTTCCTTCCCTCGACCCCTGGC

CTGGGCATGCATGTGGAGGTGAAGGACCCCGACGGTAAGGTGGTACTGTCCCGACAGTAT

GGCTCCGAGGGCCGTTTCACGTTCACCTCCCACACCCCCGGTGAACATCAGATTTGTCTG

CACTCCAATTCCACTAGAATGGCCCTCTTCGCCGGTGGCAAACTGCGTGTGCACCTAGAC

ATCCAGGTCGGGGAGCATGCCAACAACTACCCTGAGATTGCAGCTAAGGATAAGCTGACG

GAACTGCAGCTCCGTGCTCGCCAGTTGCTTGATCAAGTGGAACAGATCCAGAAGGAGCAG

GATTACCAGAGGTATCGTGAAGAACGCTTCCGTCTGACCAGTGAGAGTACCAACCAGAGG

GTCCTGTGGTGGTCCATCGCTCAGACAGTCATCCTTATCCTTACTGGCATTTGGCAGATG

CGTCATCTCAAGAGCTTCTTTGAGGCCAAGAAGCTGGTG

>CL48.Contig2_All 36 716 PREDICTED: transmembrane emp24 domain-containing protein 4 isoform 4 [Pan troglodytes] >gi|397467116|ref|XP_003805273.1| PREDICTED: transmembrane emp24 domain-containing protein 4 [Pan paniscus]

ATGGCAGGTGTCGTGGGTAGGGGCCTGCCGGCTATGGGGCGACCGGCGTTGCTGTTGCTC

GTGGTGTGTGCTGCCGGCGCCCAGGGGCTCTACTTCCACATCGGCGAGACGGAGAAGCGT

TGCTTCATCGAGGAAATCCCCGACGAGACCATGGTCATCGGCAACTATCGAACCCAGATG

TGGGACAAGCAGAAGGAGGTTTTCCTTCCCTCGACCCCTGGCCTGGGCATGCATGTGGAG

GTGAAGGACCCCGACGGTAAGGTGGTACTGTCCCGACAGTATGGCTCCGAGGGCCGTTTC

ACGTTCACCTCCCACACCCCCGGTGAACATCAGATTTGTCTGCACTCCAATTCCACTAGA

ATGGCCCTCTTCGCCGGTGGCAAACTGCGTGTGCACCTAGACATCCAGGTCGGGGAGCAT

GCCAACAACTACCCTGAGATTGCAGCTAAGGATAAGCTGACGGAACTGCAGCTCCGTGCT

CGCCAGTTGCTTGATCAAGTGGAACAGATCCAGAAGGAGCAGGATTACCAGAGGTATCGT

GAAGAACGCTTCCGTCTGACCAGTGAGAGTACCAACCAGAGGGTCCTGTGGTGGTCCATC

GCTCAGACAGTCATCCTTATCCTTACTGGCATTTGGCAGATGCGTCATCTCAAGAGCTTC

TTTGAGGCCAAGAAGCTGGTG

>CL49.Contig1_All 194 343 cell division cycle and apoptosis regulator 1 [Mustela putorius furo]

AGAAATCTGAAAAAGAAGAGGAAGAAGAGGATGATAGGAAATCTGAAGATGATAAGGAGG

AGGAAAAAAGAAGAACAGAAGGAATTAGAGAAATCTGAAAAAGAAGAGGAAGAAGAGGAT

GATAGGAAATCTGAAGATGATAAGGAGGAG

>CL49.Contig2_All 2 862 PREDICTED: cell division cycle and apoptosis regulator protein 1 [Saimiri boliviensis boliviensis]

AGACGTAGATCCAGAGAAAGATCACCTCAAAGGAAACGTTCCAGGGAGAGGTCTCCCAGA

AGAGAGAGAGAAAGATCGCCTCGGAGAGTTCGGCGTGTTGTTCCACGTTACACAGTGCAG

TTTTCAAAGTTTTCTTTAGATTGTCCTAGTTGTGACATGATGGAACTAAGGCGCCGTTAT

CAGAATTTATATATACCTAGTGACTTTTTTGATGCTCAGTTTACATGGGTGGATGCTTTC

CCTTTGTCAAGACCATTTCAGCTTGGAAATTACTGCAATTTCTATGTTATGCACAGAGAA

GTAGAGTCATTAGAAAAAAATATGGCCATTCTTGATCCTCCAGATGCTGACCATTTATAC

AGTGCAAAGGTAATGCTGATGGCTAGTCCTAGTATGGAAGATTTGTATCATAAGTCTTGT

GCTCTTGCTGAAGACCCACAAGAACTTCGAGATGGATTTCAACATCCTGCTAGACTTGTT

AAGTTTTTAGTGGGCATGAAAGGCAAGGATGAAGCCATGGCCATTGGTGGGCACTGGTCT

CCTTCGTTGGATGGACCAGACCCTGAAAAAGATCCCTCCGTGTTGATTAAGACTGCTATT

CGTTGTTGTAAGGCTCTGACAGGCATTGATCTAAGTGTTTGCACACAATGGTACCGTTTT

GCAGAGATTCGCTACCATCGCCCTGAGGAGACCCACAAGGGGCGTACAGTTCCAGCTCAT

GTGGAGACAGTGGTTTTATTTTTCCCGGATGTTTGGCATTGCCTTCCCACCCGCTCAGAG

TGGGAAACCCTCTCCCGAGGATACAAGCAGCAGCTGGTCGAGAAGCTTCAGGGTGAACGC

AAGGAGGCTGATGGAGAACAG

>CL49.Contig3_All 2 2476 cell division cycle and apoptosis regulator protein 1 [Macaca mulatta] >gi|383408293|gb|AFH27360.1| cell division cycle and apoptosis regulator protein 1 [Macaca mulatta] >gi|384943438|gb|AFI35324.1| cell division cycle and apoptosis regulator protein 1 [Macaca mulatta]

AGACGTAGATCCAGAGAAAGATCACCTCAAAGGAAACGTTCCAGGGAGAGGTCTCCCAGA

AGAGAGAGAGAAAGATCGCCTCGGAGAGTTCGGCGTGTTGTTCCACGTTACACAGTGCAG

TTTTCAAAGTTTTCTTTAGATTGTCCTAGTTGTGACATGATGGAACTAAGGCGCCGTTAT

CAGAATTTATATATACCTAGTGACTTTTTTGATGCTCAGTTTACATGGGTGGATGCTTTC

CCTTTGTCAAGACCATTTCAGCTTGGAAATTACTGCAATTTCTATGTTATGCACAGAGAA

GTAGAGTCATTAGAAAAAAATATGGCCATTCTTGATCCTCCAGATGCTGACCATTTATAC

AGTGCAAAGGTAATGCTGATGGCTAGTCCTAGTATGGAAGATTTGTATCATAAGTCTTGT

GCTCTTGCTGAAGACCCACAAGAACTTCGAGATGGATTTCAACATCCTGCTAGACTTGTT

AAGTTTTTAGTGGGCATGAAAGGCAAGGATGAAGCCATGGCCATTGGTGGGCACTGGTCT

CCTTCGTTGGATGGACCAGACCCTGAAAAAGATCCCTCCGTGTTGATTAAGACTGCTATT

CGTTGTTGTAAGGCTCTGACAGGCATTGATCTAAGTGTTTGCACACAATGGTACCGTTTT

GCAGAGATTCGCTACCATCGCCCTGAGGAGACCCACAAGGGGCGTACAGTTCCAGCTCAT

GTGGAGACAGTGGTTTTATTTTTCCCGGATGTTTGGCATTGCCTTCCCACCCGCTCAGAG

TGGGAAACCCTCTCCCGAGGATACAAGCAGCAGCTGGTCGAGAAGCTTCAGGGTGAACGC

AAGGAGGCTGATGGAGAACAGGATGAAGAAGAAAAGGATGATGGTGAAGCTAAAGAAATT

TCCACTCCTACCCATTGGTCTAAACTTGATCCAAAGACAATGAAGGTAAATGATCTCCGA

AAAGAATTAGAAAGTCGAGCCCTTAGTTCCAAAGGATTAAAATCCCAGTTAATAGCTCGA

TTGACAAAACAACTTAAAGTAGAAGAACAAAAAGAAGAACAGAAGGAATTAGAGAAATCT

GAAAAAGAAGAGGAAGAAGAGGATGATAGGAAATCTGAAGATGATAAGGAGGAGGAAGAA

AGAAAACGTCAAGAGGAAATGGAACGCCAGCGTCGGGAAAGAAGATACATTTTGCCTGAT

GAACCAGCCATCATTGTGCATCCAAATTGGGCTGCAAAAAGTGGCAAGTTTGATTGTAGC

ATCATGTCTTTGAGTGTCCTTTTGGACTACAGATTAGAAGATAATAAAGAACATTCATTT

GAGGTTTCATTGTTTGCAGAACTTTTCAATGAAATGCTTCAGAGAGATTTTGGTGTCAGA

ATATACAAATCATTACTCTCTCTTCCTGAGAAAGAGGACAAAAAAGAAAAGGATAAGAAA

AGCAAAAAAGATGAGAGAAAAGATAAAAAAGAAGAAAGAGATGACGAAAATGATGAGCCA

AAACCCAAACGGAGAAAATCAGGAGATGATAAAGATAAAAAAGAAGATAGAGATGAAAGG

AAGAAAGAAGATAAAAGGAAAGATGATTCTAAAGATGATGATGAAACTGAAGAAGATAAT

AATCAAGATGAATATGACCCAATGGAAGCAGAAGAAGCTGAGGATGAAGAAGATGATCGG

GATGAGGAAGAAATGAACAAACGGGATGACAAAAGAGAGATCAACAGATACTGCAAGGAA

AGGCCTTCTAAAGATAAGGAAAAAGAAAAAACTCAGATGATCACAATTAACAGGGATCTA

TTAATGGCCTTTGTTTATTTTGATCAAAGCCATTGTGGTTACCTTCTTGAAAAGGATTTG

GAAGAGATACTCTATACTCTTGGATTACATCTTTCTCGGGCTCAGGTAAAGAAGCTTCTT

AATAAAGTAGTTCTCCGTGAATCTTGCTTTTATCGGAAATTAACAGACACTTCAAAAGAT

GAAGAAAACCATGAAGAGTCTGAATCATTGCAGGAAGATATGTTAGGAAATAGGTTGTTA

CTGCCAACACCAACAGTAAAGCAGGAATCAAAGGATGTGGAAGAAAATGTAGGACTCATT

GTGTACAATGGTGCAATGGTTGATGTAGGAAGCCTTTTGCAAAAATTGGAAAAGAGTGAA

AAAGTAAGGGCTGAAGTAGAACAGAAGCTGCAGTTACTAGAAGAAAAAACAGATGAAGAT

GAAAAAACCATATTAAATTTGGAGAATTCCAACAAAAGCCTCTCTGGTGAACTCAAAGAA

GTTAAAAAGGACCTTAGTCAATTACAGGAAAACTTAAAGGTTTCAGAAAACATGAATTTG

CAATTTGAAAACCAACTGAATAAGACAATCAGAAACTTGTCTACAGTAATGGATGAAATC

CACACTGTTCTCAGAAAGGATAATGTAAAGAATGAAGACAAAGATCAGAAATCTAAGGAG

AATGGTGCAAATGTA

>CL50.Contig1_All 3 1850 minus strand stromal interaction molecule 1 (predicted), isoform CRA_b [Rattus norvegicus]

CCAACAGTGAAGCACAGCACCTTCCATGGTGAGGACAAGCTCATCAGCGTGGAGGACCTG

TGGAAAGCATGGAAGTCCTCAGAAGTATACAATTGGACCGTGGATGAGGTGGTACAGTGG

CTGATTACATATGTGGAACTGCCTCAGTATGAGGAGACCTTCCGGAAGCTGCAGCTCAGT

GGCCATGCTATGCCAAGGCTTGCTGTAACCAATACCACCATGACAGGAACTGTGCTGAAG

ATGACAGATCGGAGCCATCGGCAGAAGCTGCAGCTGAAGGCCCTGGACACAGTGCTCTTT

GGGCCTCCTCTCTTGACCCGCCACAATCACCTCAAGGACTTCATGCTGGTGGTATCTATT

GTTATTGGTGTGGGCGGCTGCTGGTTTGCCTATATCCAGAACCGTTACTCCAAGGAGCAC

ATGAAGAAGATGATGAAGGATTTGGAGGGTTTACACAGAGCTGAGCAGAGTTTGCATGAC

CTTCAGGAAAGGCTACACAAGGCCCAGGAGGAACACCGCACAGTGGAGGTGGAGAAAGTC

CATCTGGAGAAGAAGCTACGTGATGAGATCAACCTGGCCAAGCAGGAAGCCCAGCGGCTG

AAGGAACTGCGGGAGGGTACTGAGAATGAGCGGAGCCGCCAAAAATATGCTGAGGAAGAG

CTGGAGCAGGTTCGGGAGGCCTTGAGAAAAGCAGAGAAGGAGCTGGAATCACACAGCTCA

TGGTATGCTCCAGAGGCCCTTCAGAAGTGGCTACAGCTGACACATGAGGTGGAGGTGCAG

TACTACAACATCAAGAAGCAAAATGCTGAGAAGCAGCTGCTGGTGGCTAAGGAGGGGGCT

GAGAAGATAAAAAAGAAGAGAAACACACTCTTTGGTACCTTCCATGTGGCCCACAGCTCT

TCCCTGGATGATGTGGATCATAAAATCCTAACAGCTAAGCAAGCCCTGAGCGAGGTGACA

GCAGCACTGAGGGAGCGCCTGCACCGCTGGCAACAGATCGAGATCCTCTGTGGTTTCCAG

ATTGTTAATAACCCTGGCATGCACTCACTGGTAGCTGCCCTCAATATAGATCCCAGCTGG

ATGGGCAGTACACGCCCAAACCCCACCCACTTCATCATGACCGACGACGTGGATGACATG

GATGAGGAAATTGTGTCACCGTTGTCCATGCAGTATGCTGCCTGGCTGATGGGGCGTAGG

TTCAGTGACCGCTCTCTCTGCTCTACATCCGCCGGCTCGGATGATCAGTCCCTCTGGAAA

TACCCGGCCCCCAGCCTGCAGAGCAGTGTCCGGCAGCGCCTGACGGAACCACAGCATGGC

CTGGGATCTCAGAGGGATTTGACCCATTCCGATTCGGAGTCCTCCCTCCACACGAGTGAC

CGCCAGCGTATGGCCCCCAAGCCTCCTCAAATGGGCCGTGCTGCAGATGAGGCTGTCAAT

GTGATGACTTCCAATGGTAGCCACCGGCTGATTGAGGGGGTTCACCCAGGATGCCTGGTG

GAGAAACTGCCTGACAGCCCTGCCCTGGCCAAGAAGGCATTACTGGCGCTGAACCATGGG

CTGGACAAGGCCCATAGCCTGATGGAGCTGAGCCCCTCAGCCCCATCTGGTGGCTCCCCA

CCTTTGGATTCTTCCAGTTCTCATGGCCCGAGTTCCCCAGACCCAGACACACCATCTCCA

GTTGGGGATAGCCGAGTCCTGCAGGCCAGCCGAAACACACGCATTCCCCACTTGGCTGGC

AAGAAGGCTGTGGCTGAGGAGGATAATGGTTCTATTGGTGAGGAGACAGACTCCAGTCCA

GGCAGGAAGAAGTTTCCCCTAAAAATTTTTAAGAAGCCTCTTAAGAAA

>CL50.Contig2_All 160 2214 stromal interaction molecule 1 precursor [Homo sapiens] >gi|209572721|sp|Q13586.3|STIM1_HUMAN RecName: Full=Stromal interaction molecule 1; Flags: Precursor >gi|20987365|gb|AAH21300.1| Stromal interaction molecule 1 [Homo sapiens] >gi|119622986|gb|EAX02581.1| stromal interaction molecule 1, isoform CRA_b [Homo sapiens]

ATGGATGTTTGCGCTCGTCTTGCCCTGTGGCTCCTCTGGGGGCTTCTCCTGCATCAGGGC

CAGAGCCTCAGTCATAGTCATAGCGAGAAGGCCACAGGAACCGGCTCAGGGGCCACTTCT

GAGGAGTCCACTGCAGCAGAGTTCTGCCGGATTGACAAGCCCCTGTGTCACAGTGAGGAT

GAGAAGCTCAGCTTTGAGGCAGTCCGCAATATCCACAAGCTGATGGATGATGATGCCAAT

GGGGATGTGGATGTGGAAGAAAGTGATGAGTTCCTGAGGGAAGACCTCAATTACCATGAC

CCAACAGTGAAGCACAGCACCTTCCATGGTGAGGACAAGCTCATCAGCGTGGAGGACCTG

TGGAAAGCATGGAAGTCCTCAGAAGTATACAATTGGACCGTGGATGAGGTGGTACAGTGG

CTGATTACATATGTGGAACTGCCTCAGTATGAGGAGACCTTCCGGAAGCTGCAGCTCAGT

GGCCATGCTATGCCAAGGCTTGCTGTAACCAATACCACCATGACAGGAACTGTGCTGAAG

ATGACAGATCGGAGCCATCGGCAGAAGCTGCAGCTGAAGGCCCTGGACACAGTGCTCTTT

GGGCCTCCTCTCTTGACCCGCCACAATCACCTCAAGGACTTCATGCTGGTGGTATCTATT

GTTATTGGTGTGGGCGGCTGCTGGTTTGCCTATATCCAGAACCGTTACTCCAAGGAGCAC

ATGAAGAAGATGATGAAGGATTTGGAGGGTTTACACAGAGCTGAGCAGAGTTTGCATGAC

CTTCAGGAAAGGCTACACAAGGCCCAGGAGGAACACCGCACAGTGGAGGTGGAGAAAGTC

CATCTGGAGAAGAAGCTACGTGATGAGATCAACCTGGCCAAGCAGGAAGCCCAGCGGCTG

AAGGAACTGCGGGAGGGTACTGAGAATGAGCGGAGCCGCCAAAAATATGCTGAGGAAGAG

CTGGAGCAGGTTCGGGAGGCCTTGAGAAAAGCAGAGAAGGAGCTGGAATCACACAGCTCA

TGGTATGCTCCAGAGGCCCTTCAGAAGTGGCTACAGCTGACACATGAGGTGGAGGTGCAG

TACTACAACATCAAGAAGCAAAATGCTGAGAAGCAGCTGCTGGTGGCTAAGGAGGGGGCT

GAGAAGATAAAAAAGAAGAGAAACACACTCTTTGGTACCTTCCATGTGGCCCACAGCTCT

TCCCTGGATGATGTGGATCATAAAATCCTAACAGCTAAGCAAGCCCTGAGCGAGGTGACA

GCAGCACTGAGGGAGCGCCTGCACCGCTGGCAACAGATCGAGATCCTCTGTGGTTTCCAG

ATTGTTAATAACCCTGGCATGCACTCACTGGTAGCTGCCCTCAATATAGATCCCAGCTGG

ATGGGCAGTACACGCCCAAACCCCACCCACTTCATCATGACCGACGACGTGGATGACATG

GATGAGGAAATTGTGTCACCGTTGTCCATGCAGTCCCCCAGCCTGCAGAGCAGTGTCCGG

CAGCGCCTGACGGAACCACAGCATGGCCTGGGATCTCAGAGGGATTTGACCCATTCCGAT

TCGGAGTCCTCCCTCCACACGAGTGACCGCCAGCGTATGGCCCCCAAGCCTCCTCAAATG

GGCCGTGCTGCAGATGAGGCTGTCAATGTGATGACTTCCAATGGTAGCCACCGGCTGATT

GAGGGGGTTCACCCAGGATGCCTGGTGGAGAAACTGCCTGACAGCCCTGCCCTGGCCAAG

AAGGCATTACTGGCGCTGAACCATGGGCTGGACAAGGCCCATAGCCTGATGGAGCTGAGC

CCCTCAGCCCCATCTGGTGGCTCCCCACCTTTGGATTCTTCCAGTTCTCATGGCCCGAGT

TCCCCAGACCCAGACACACCATCTCCAGTTGGGGATAGCCGAGTCCTGCAGGCCAGCCGA

AACACACGCATTCCCCACTTGGCTGGCAAGAAGGCTGTGGCTGAGGAGGATAATGGTTCT

ATTGGTGAGGAGACAGACTCCAGTCCAGGCAGGAAGAAGTTTCCCCTAAAAATTTTTAAG

AAGCCTCTTAAGAAA

>CL50.Contig3_All 44 208 PREDICTED: stromal interaction molecule 1 isoform 2 [Saimiri boliviensis boliviensis]

GTTCGGGAGGCCTTGAGAAAAGCAGAGAAGGAGCTGGAATCACACAGCTCATGGTATGCT

CCAGAGGCCCTTCAGAAGTGGCTACAGCTGACACATGAGGTGGAGGTGCAGTACTACAAC

ATCAAGAAGCAAAATGCTGAGAAGCAGCTGCTGGTGGCTAAGGAG

>CL51.Contig1_All 33 1670 PREDICTED: LOW QUALITY PROTEIN: growth factor receptor-bound protein 7 [Equus caballus]

GGACAAGGCTTGCGAGCAGCTGGAATCAGTCCACCTCCCTTAGATGTCATGGAGCTGGAT

CTCTCCCCACCTCAGCTCAGCAGCTCCCCAGAAGACCTGTGCCCAACCCCTGGCACCCCG

CCTGGAACTCCCCTGCCCCAGGACGCCCCTCTGTCTGGGGAGGTGAAGAGGTCCCAGCCT

CTGCCCATCCCGACCAGCAGGAGACTCCCAGAGGAGGAGCGGCAGGCAACCTCTCTCCCC

TCCATCCCGAACCCCTTCCCTGAGCTCTGCAGTCCTCCCTCAAAGACCCCCATTCTCGGG

GGGCCCTCCAGTGCAAAGGGACTGCTACCACGAGATGCTAGCTGCCCTCATGTGGTGAAG

GTGTACAGTGAGGACGGGGCCTGTCGCTCGGTGGAGGTGGCGGCAGGTGCCACGGCTCGC

CACGTGTGTGAAATGCTGGTGCAGCGAGCGCATGCCCTCAGCGACGAGCACTGGGGGCTG

GTGGAGTGCCACCCCTGTCTAGCCCTGGAGCGGGGTCTGGAGGACCATGAGTCTGTGGTG

GAAGTGCAGGCGACCTGGCCCGTCGGTGGAGACAGCCGCTTCATCTTCCGGAAAAACTTC

GCCAAGTACGAACTGTTCAAGAGCCCCCCACACTCCCTGTTCCCAGAAAAGATGGTCTCC

AGCTGTCTCGATGCCCACACCGGTGCATCCCATGAAGACCTCATCCAGAACTTCCTGAAT

GCAGGCAGCTTCCCCGAGATCCAGGGCTTCCTGCAGCTGAGGGGCTCAGGTCGCAAGCTG

TGGAAACGCTTCTTCTGCTTCCTGCGCCGGTCTGGCCTCTATTACTCCACCAAGGGCACC

TCCAAGGATCCAAGGCACCTACAATATGTGGCAGATGTGAATGAGTCCAATGTCTATGTG

GTGACCCAGGGCCGAAAACTCTACGGGATGCCCACGGACTTTGGCTTCTGTGTCAAGCCC

AACAAGCTTCGGAATGGCCACAAGGGGCTTCGCATCTTCTGCAGTGAGGATGAACAGAGC

CGCACCTGCTGGCTGTCTGCCTTCCGTCTCTTCAAGTATGGGATGCAGCTGTATAAGAAT

TATCAGCAGGCCCAGTCTCGCCACCTGCGTCCATCCTATTTGGGTTCCCCGCCCTTGAGG

AGTGTCTCGGATAACACCCTGGTGGCTATGGACTTCTCCGGCCATGCTGGGCGAGTCATT

GAGAACCCCCGGGAAGCTCTGAGTGCAGCCCTGGAGGAGGCCCAGGCCTGGAGGAAGAAG

ACAAACCACCGCCTCAGCCTGCCCACCCCATGCTCAGGCACGAGCCTCAGTGCAGCTATC

CACCGCACCCAGCCCTGGTTCCATGGACGCATTTCCCGTGAGGAGAGCCAGCGGCTCATT

GGGCAGCAGGGCCTGGTGGATGGCCTGTTCCTGGTCCGGGAGAGTCAGCGAAACCCACAG

GGCTTTGTCCTGTCCTTGTGCCACCTAAAGAAAGTCAAGCACTATCTCATCCTGCCGAGC

GAGGACGAGGGCTGCCTCTACTTCAGCATGGACGATGGCCAGACCCGCTTCACGGACCTG

CTGCAGCTCGTGGAGTTCCACCAGCTGAACCGCGGCATCCTGCCATGCCTGCTGCGCCTC

TGCTGCACCCGCGTGGCC

>CL51.Contig2_All 25 354 PREDICTED: growth factor receptor-bound protein 7-like [Ailuropoda melanoleuca]

ACTCCAGCTATCCACCGCACCCAGCCCTGGTTCCATGGACGCATTTCCCGTGAGGAGAGC

CAGCGGCTCATTGGGCAGCAGGGCCTGGTGGATGGCCTGTTCCTGGTCCGGGAGAGTCAG

CGAAACCCACAGGGCTTTGTCCTGTCCTTGTGCCACCTAAAGAAAGTCAAGCACTATCTC

ATCCTGCCGAGCGAGGACGAGGGCTGCCTCTACTTCAGCATGGACGATGGCCAGACCCGC

TTCACGGACCTGCTGCAGCTCGTGGAGTTCCACCAGCTGAACCGCGGCATCCTGCCATGC

CTGCTGCGCCTCTGCTGCACCCGCGTGGCC

>CL51.Contig3_All 1 189 minus strand PREDICTED: growth factor receptor-bound protein 7 isoform 1 [Papio anubis] >gi|402900044|ref|XP_003912990.1| PREDICTED: growth factor receptor-bound protein 7 isoform 2 [Papio anubis] >gi|402900046|ref|XP_003912991.1| PREDICTED: growth factor receptor-bound protein 7 isoform 3 [Papio anubis]

TCCAGCTGTCTCGATGCCCACACCGGTGCATCCCATGAAGACCTCATCCAGAACTTCCTG

AATGCAGGCAGCTTCCCCGAGATCCAGGGCTTCCTGCAGCTGAGGGGCTCAGGTCGCAAG

CTGTGGAAACGCTTCTTCTGCTTCCTGCGCCGGTCTGGCCTCTATTACTCCACCAAGGGC

ACCTCCAAG

>CL52.Contig2_All 5 2296 minus strand inverted formin-2 isoform 2 [Homo sapiens]

CCTCCCCCAGTACTGCCCCCACCCCCACCTCCCTTGCCAGGCCTGGGGGCCATGTCTCCC

CCAGCACCCCCTCTGCCACCTCCCCTGCCTGGCTCCTGCGGGATCTTGCCCCCACCACCT

CCTCCACTCCCCCCCCCACCGCCTGGAGGGGGCTGGGGCCCCCCTCCACCTCCAACCCTG

CCTGCGGGTGGCGTGGAAGAGCTCATTGTGGCCCACGTGGACCACAGCCTGGGCTCGGCC

TGGGTCCCCAGCCACCGCAGGGTGAACCCACCCACACTGCGCATGAAGAAACTGAACTGG

CAGAAACTTCCGTCCAACGTGGCCCGAGAGCGCAACTCCATGTGGGCGACACTCAGCAGC

CCTGGCACAGACGCGGTGGAGCCTGACTTCTCCAGCATTGAGCGGCTCTTCTCCTTCCCC

GTGGCCAAGCCCAAGGAGCCCACAGCTGCCCCAGCCAGGAAGGAGCCCAAGGAGGTCACT

TTTCTTGACTCCAAGAAGAGCCTGAACCTCAACATCTTCCTGAAGCAGTTTAAGTGCTCC

AACGAGGAGGTCACCGCCATGATCCGGGCTGGGGACACCACCAAGTTTGACGTGGAGGTT

CTCAAACAGCTCCTGAAACTACTTCCAGAGAAGCACGAGATCGAAAACCTGAGGACATTC

ACAGAGGAGCGAGCCAAGCTGGCCAACGCCGACCAGTTCTACGTCCTCCTGCTGGACATT

CCTTGCTACCAGCTGCGGTTGGAGTGCATGCTGCTGTGTGAGGGCACGGCCATTGTGCTG

GACATGGTGCGACCCAAGGCCCAGCTGGTGCTCGCCGCCTGTGAGAGCCTGCTCACCAGT

CACCGGCTGCCTGTCTTCTGCCAATTGATCCTGAAAATCGGGAACTTCCTCAACTATGGC

AGCCACACCGGGGACGCTGATGGCTTCAAGATGAGCACCTTGCTGAAGCTCACAGAGACC

AAGTCGCAGCAGAACCGGGTGACGCTATTGCACCACGTGCTAGAGGAAGTGGAAAAGAGC

CACCCTGACCTCCTGCAGCTGCCCCAGGACCTGGCACAGCCCTCCCAGGCCGCAGGAATC

AACCTGGAGGTTATCCGCTCCGAAGCCAGCACCAACCTGAAGAAGCTTCTGGGGACGGAG

CGGAAGGTGTCCACCTCTGACCCTGAGGTGCAGGAGCAGTATGCCCAGCGCCTCCAGGAC

AGCATCGCGGCCTCCCAGGCTTTGGACGAGGTGTTTGACACCATCGAGCAGAAGAAGCTG

GAGCTGGCCGACTACCTGTGTGAGGACCCCCAACAGCTGTCCCTGGAGGACACCTTCAGC

ACCATGAAGATCTTCCGGGACCTCTTCACCCGTGCCCTGAAGGAGAACAAGGACAGGAAG

GAGCAGGTGGCAAAGGCAGAGAGGAGGAAGCAGCAGCTGGCAGAGGAGGAGGCACGGAGG

CCGCGGGGCGAGGATGGGAAGCCTGTCAGGAAGGGCCCTGGGAGGCAGGAGGAGGTGTGT

GTCATCGATGCCCTGCTGGCCGACATCAGGAAGGGCTTCCAGCTGCGCAAGACGGCCCGA

GGCCGGGGGGACAGCGAGGTGGGCAGCAAGGCCTCAGCAGAACTCCCGCGGGCCACAGTG

CCAGCAGCCGCCAGTGACCCTGCACAGAGCCCCCGTGAGGGTGCCAGCCACCCTGCCCCT

GAGCTGGGCCTTGATGCTGCTGCAGCTGGGAGGCCACAGGGCTGGGACCTTGTGGATGCT

GTGACTCCCAGCCCAGGGCCCCCTGGGGAGGAGGGCAGTCCGCAGCCCTTGGAGAGGCGC

TCCTCCTGGTACGTGGATGCCAGTGACTTCCTGGCCCCCGAGGACACGCTGAATCCCCAG

CCTCCCGAGGGGGCCTGGCCAGTGACTCTGGGAGACACCCGGGCCCTGAAGCCCCTCCAG

TTCTCTGGAGACAAGCCCCCCGGGGCCGAGAGTTCAAGCCAGGACCCGGAGGAGCTTGGA

GGCTTGCAGGGCGTCCACCAGGCCAAGGCACACGGCACAGGCCAAGGACCAGAGGACACA

GCTATCCAGGGCCAAAGTGCTGGCCTCCCTGCCACAGGCCCTGGTGGGGAGGAGGATGAG

GAAGACACAGCCCCTGAGTCTGCACTGGACACATCCTTGGACAAGTCCTTCTCCGAGGAC

GCAGTGACAGACTCCTCGGGGTCTGGCACCCTCCCCAGGTCCCGAGGCCGGGCCTCAAAG

GGGACAAGCAAGCGAAGGAAGAAGCGTCCCTCAAGGAGCCAGGAAGGCCTCAGACCCAAG

CCCAAAGCCAAG

>CL52.Contig3_All 5 2323 minus strand inverted formin-2 isoform 1 [Homo sapiens] >gi|166215588|sp|Q27J81.2|INF2_HUMAN RecName: Full=Inverted formin-2; AltName: Full=HBEBP2-binding protein C

CCTCCCCCAGTACTGCCCCCACCCCCACCTCCCTTGCCAGGCCTGGGGGCCATGTCTCCC

CCAGCACCCCCTCTGCCACCTCCCCTGCCTGGCTCCTGCGGGATCTTGCCCCCACCACCT

CCTCCACTCCCCCCCCCACCGCCTGGAGGGGGCTGGGGCCCCCCTCCACCTCCAACCCTG

CCTGCGGGTGGCGTGGAAGAGCTCATTGTGGCCCACGTGGACCACAGCCTGGGCTCGGCC

TGGGTCCCCAGCCACCGCAGGGTGAACCCACCCACACTGCGCATGAAGAAACTGAACTGG

CAGAAACTTCCGTCCAACGTGGCCCGAGAGCGCAACTCCATGTGGGCGACACTCAGCAGC

CCTGGCACAGACGCGGTGGAGCCTGACTTCTCCAGCATTGAGCGGCTCTTCTCCTTCCCC

GTGGCCAAGCCCAAGGAGCCCACAGCTGCCCCAGCCAGGAAGGAGCCCAAGGAGGTCACT

TTTCTTGACTCCAAGAAGAGCCTGAACCTCAACATCTTCCTGAAGCAGTTTAAGTGCTCC

AACGAGGAGGTCACCGCCATGATCCGGGCTGGGGACACCACCAAGTTTGACGTGGAGGTT

CTCAAACAGCTCCTGAAACTACTTCCAGAGAAGCACGAGATCGAAAACCTGAGGACATTC

ACAGAGGAGCGAGCCAAGCTGGCCAACGCCGACCAGTTCTACGTCCTCCTGCTGGACATT

CCTTGCTACCAGCTGCGGTTGGAGTGCATGCTGCTGTGTGAGGGCACGGCCATTGTGCTG

GACATGGTGCGACCCAAGGCCCAGCTGGTGCTCGCCGCCTGTGAGAGCCTGCTCACCAGT

CACCGGCTGCCTGTCTTCTGCCAACTGATCCTGAAAATCGGGAACTTCCTCAACTATGGC

AGCCACACCGGGGACGCTGATGGCTTCAAGATGAGCACCTTGCTGAAGCTCACAGAGACC

AAGTCGCAGCAGAACCGGGTGACGCTATTGCACCACGTGCTAGAGGAAGTGGAAAAGAGC

CACCCTGACCTCCTGCAGCTGCCCCAGGACCTGGCACAGCCCTCCCAGGCCGCAGGAATC

AACCTGGAGGTTATCCGCTCCGAAGCCAGCACCAACCTGAAGAAGCTTCTGGGGACGGAG

CGGAAGGTGTCCACCTCTGACCCTGAGGTGCAGGAGCAGTATGCCCAGCGCCTCCAGGAC

AGCATCGCGGCCTCCCAGGCTTTGGACGAGGTGTTTGACACCATCGAGCAGAAGAAGCTG

GAGCTGGCCGACTACCTGTGTGAGGACCCCCAGCAGCTGTCCCTGGAGGACACCTTCAGC

ACCATGAAGATCTTCCGGGACCTCTTCACCCGTGCCCTGAAGGAGAACAAGGACAGGAAG

GAGCAGGTGGCAAAGGCAGAGAGGAGGAAGCAGCAGCTGGCAGAGGAGGAGGCACGGAGG

CCGCGGGGCGAGGATGGGAAGCCTGTCAGGAAGGGCCCTGGGAGGCAGGAGGAGGTGTGT

GTCATCGATGCCCTGCTGGCCGACATCAGGAAGGGCTTCCAGCTGCGCAAGACGGCCCGA

GGCCGGGGGGACAGCGAGGTGGGCAGCAAGGCCTCAGCAGAACTCCCGCGGGCCACAGTG

CCAGCAGCCGCCAGTGACCCTGCACAGAGCCCCCGTGAGGGTGCCAGCCACCCTGCCCCT

GAGCTGGGCCTTGATGCTGCTGCAGCTGGGAGGCCACAGGGCTGGGACCTTGTGGATGCT

GTGACTCCCAGCCCAGGGCCCCCTGGGGAGGAGGGCAGTCCGCAGCCCTTGGAGAGGCGC

TCCTCCTGGTACGTGGATGCCAGTGACTTCCTGGCCCCCGAGGACACGCTGAATCCCCAG

CCTCCCGAGGGGGCCTGGCCAGTGACTCTGGGAGACACCCGGGCCCTGAAGCCCCTCCAG

TTCTCTGGAGACAAGCCCCCCGGGGCCGAGAGTTCAAGCCAGGACCCGGAGGAGCTTGGA

GGCTTGCAGGGCGTCCACCAGGCCAAGGCACACGGCACAGGCCAAGGACCAGAGGACACA

GCTATCCAGGGCCAAAGTGCTGGCCTCCCTGCCACAGGCCCTGGTGGGGAGGAGGATGAG

GAAGACACAGCCCCTGAGTCTGCACTGGACACATCCTTGGACAAGTCCTTCTCCGAGGAC

GCAGTGACAGACTCCTCGGGGTCTGGCACCCTCCCCAGGTCCCGAGGCCGGGCCTCAAAG

GGGACAAGCAAGCGAAGGAAGAAGCGTCCCTCAAGGAGCCAGGAAGAAGTTGCCCCTGGT

TCTGACGCTAATGTAACAAAAAGACTGTGTGCCGTCCAG

>CL53.Contig1_All 107 331 minus strand PREDICTED: active breakpoint cluster region-related protein [Papio anubis]

CTCCCCCTGGCCGACTTAGTGTTCCCATCCCCCGAGGAGTCTGAGGCCAGCCCCCACGTG

CACCCCTTCCCAGACCATGAGCTAGAGGACATGAAAATGAAGATCTCTGCCCTCAAGAGT

GAAATCCAGAAGGAGAAAGCCAACAAAGGGCAGAGCCGGGCCATCGAGCGCCTCAAAAAG

AAGATGTTTGAGAATGAGTTCTTACTGCTGCTCAACTCCCCCACA

>CL53.Contig2_All 1 297 minus strand active breakpoint cluster region-related protein isoform b [Macaca mulatta]

GATGTCCTGCTGTGCGCCAAGCTGAAGAAGACCTCTGCAGGGAAGCACCAGCAGTATGAC

TGTAAGTGGTACATCCCCCTGGCCGACTTAGTGTTCCCATCCCCCGAGGAGTCTGAGGCC

AGCCCCCACGTGCACCCCTTCCCAGACCATGAGCTAGAGGACATGAAAATGAAGATCTCT

GCCCTCAAGAGTGAAATCCAGAAGGAGAAAGCCAACAAAGGGCAGAGCCGGGCCATCGAG

CGCCTCAAAAAGAAGATGTTTGAGAATGAGTTCTTACTGCTGCTCAACTCCCCCACA

>CL55.Contig1_All 208 1746 PREDICTED: neutrophil cytosol factor 2 isoform 1 [Saimiri boliviensis boliviensis] >gi|403266341|ref|XP_003925347.1| PREDICTED: neutrophil cytosol factor 2 isoform 2 [Saimiri boliviensis boliviensis]

ATGTCCCTGGCTGAGGCCATCAGCCTGTGGAATGAAGGGGTGCTGGCAGCAGACAAGAAG

GACTGGAAGAGAGCCCTGGACGCCTTCAGTGCCGTCCAGGACCCTCACTCCAGGATCTGT

TTCAACATCGGTTGCATGCACACCATCCTGGGAAACATGCCCGAGGCCGAGAAGGCCTTT

ACCAGAAGCATCAACCGGGACAAGCATTTGGCGGTGGCCTACTTCCAACGCGGGATGCTC

TACTACAGGACAGAGAAATACGACTTAGCTATTAAAGACCTTAAGGAGGCCTTGATTCAG

CTTCGAGGGAACCAGCTGATAGACTACAAGATCCTGGGGCTGCAGTTCAAGCTATTTGCC

TGTGAGGAGGAATGGAAGAAGGCCGAAGAACAACTCGCACTGGCCACCAACATGAAGTCT

GAGCCCAGGCACTCCAAGATCGACAAGGCCATGGAAAGCATCTGGAAACAGAAGCTGTAT

GAGCCTGTGGTGATCCCCGAGGGAAGATTGTTTCGTCCAAATGAGAGACAAGTGGCTCAG

CTGGCCAAGAAGGATTACCTGGGCAAGGCCACGGTTGTGGCGTCTGTGGTGGATCAAGAC

AGCTTCTCCGGGTTTGCCCCTCTGCAACCACAGGCAGCTGAGCCTCCACCCAGACCCAAA

ACCCCAGAGATCTTCAGGGCTCTGGAAGGGGAAGCTCACCGGGTGCTGTTTGGGTTTGTG

CCTGAGACACCAGAGGAGCTCCAGGTCATGCCAGGGAACATCGTCTTCGTCTTGAAGAAG

GGCAATGATAACTGGGCCACGGTCATGTTCAATGGGCAGAAGGGGCTTGTTCCCTGCAAC

TACCTGGAGCCTGTCGAGCTTCGAATCCACCCTCAGCAGCAGCCCCAGGAGGAAACTCCC

CCGGAGTCTGAAATCCCACCTCCTCCTAGTTCCAATGCCCCAGGGAGACCCCAGTTGTCT

CCAGGTCAGAAACAAAAAGAAGAGCTCAAGGAAGTGAAGCTCAGCGTCCCCATGCCCTAC

ACACTCAAGGTGCACTACAAGTACACGGTGGTCATGGAGACACAGCCTGGGCTCCCATAC

AGCCAGGTCCGGGACATGGTGTCTCAGAAACTGGACTTGCTGCCGGAACACACTAAGCTG

AGCTATCGGCCCCGGGACAGCAATGAGCTGGTGCGCCTTTTGGAAGAAAACATGAAGGAT

GCCTGGAGCCAAGTGAAAAACTACTGCCTGACTCTTTGGTGTGAGAACACAGTGGGTGAC

CAAGGCTTTCTCGACAAATCCAAGGAACATGAAAAATGTGACTCTAATCAGAAAACAGAA

CCTCAGTTTAAGGAAGGCAGCCAAGTGGTAGCACTCTTCAGCTACGAGGCTACCCAACCG

GAGGACCTGGAATTCCAGGAAGGGGACGTAATCCTGGTTCTATCAACAGTGAATGAAGAA

TGGTTGGAAGGGGAGTGTAAAGGGAAAGTTGGCATTTTCCCTAAAGCCTTTGTTGAAGAG

TGTGCAACTACAGACTTGGAGAACACTCCTAGAGGAGTC

>CL55.Contig2_All 208 1782 PREDICTED: neutrophil cytosol factor 2 isoform 1 [Saimiri boliviensis boliviensis] >gi|403266341|ref|XP_003925347.1| PREDICTED: neutrophil cytosol factor 2 isoform 2 [Saimiri boliviensis boliviensis]

ATGTCCCTGGCTGAGGCCATCAGCCTGTGGAATGAAGGGGTGCTGGCAGCAGACAAGAAG

GACTGGAAGAGAGCCCTGGACGCCTTCAGTGCCGTCCAGGACCCTCACTCCAGGATCTGT

TTCAACATCGGTTGCATGCACACCATCCTGGGAAACATGCCCGAGGCCGAGAAGGCCTTT

ACCAGAAGCATCAACCGGGACAAGCATTTGGCGGTGGCCTACTTCCAACGCGGGATGCTC

TACTACAGGACAGAGAAATACGACTTAGCTATTAAAGACCTTAAGGAGGCCTTGATTCAG

CTTCGAGGGAACCAGCTGATAGACTACAAGATCCTGGGGCTGCAGTTCAAGCTATTTGCC

TGTGAGGTGTTGTATAACATCGCTTTCCTCTATGCCAAGAAGGAGGAATGGAAGAAGGCC

GAAGAACAACTCGCACTGGCCACCAACATGAAGTCTGAGCCCAGGCACTCCAAGATCGAC

AAGGCCATGGAAAGCATCTGGAAACAGAAGCTGTATGAGCCTGTGGTGATCCCCGAGGGA

AGATTGTTTCGTCCAAATGAGAGACAAGTGGCTCAGCTGGCCAAGAAGGATTACCTGGGC

AAGGCCACGGTTGTGGCGTCTGTGGTGGATCAAGACAGCTTCTCCGGGTTTGCCCCTCTG

CAACCACAGGCAGCTGAGCCTCCACCCAGACCCAAAACCCCAGAGATCTTCAGGGCTCTG

GAAGGGGAAGCTCACCGGGTGCTGTTTGGGTTTGTGCCTGAGACACCAGAGGAGCTCCAG

GTCATGCCAGGGAACATCGTCTTCGTCTTGAAGAAGGGCAATGATAACTGGGCCACGGTC

ATGTTCAATGGGCAGAAGGGGCTTGTTCCCTGCAACTACCTGGAGCCTGTCGAGCTTCGA

ATCCACCCTCAGCAGCAGCCCCAGGAGGAAACTCCCCCGGAGTCTGAAATCCCACCTCCT

CCTAGTTCCAATGCCCCAGGGAGACCCCAGTTGTCTCCAGGTCAGAAACAAAAAGAAGAG

CTCAAGGAAGTGAAGCTCAGCGTCCCCATGCCCTACACACTCAAGGTGCACTACAAGTAC

ACGGTGGTCATGGAGACACAGCCTGGGCTCCCATACAGCCAGGTCCGGGACATGGTGTCT

CAGAAACTGGACTTGCTGCCGGAACACACTAAGCTGAGCTATCGGCCCCGGGACAGCAAT

GAGCTGGTGCGCCTTTTGGAAGAAAACATGAAGGATGCCTGGAGCCAAGTGAAAAACTAC

TGCCTGACTCTTTGGTGTGAGAACACAGTGGGTGACCAAGGCTTTCTCGACAAATCCAAG

GAACATGAAAAATGTGACTCTAATCAGAAAACAGAACCTCAGTTTAAGGAAGGCAGCCAA

GTGGTAGCACTCTTCAGCTACGAGGCTACCCAACCGGAGGACCTGGAATTCCAGGAAGGG

GACGTAATCCTGGTTCTATCAACAGTGAATGAAGAATGGTTGGAAGGGGAGTGTAAAGGG

AAAGTTGGCATTTTCCCTAAAGCCTTTGTTGAAGAGTGTGCAACTACAGACTTGGAGAAC

ACTCCTAGAGGAGTC

>CL56.Contig1_All 112 930 PREDICTED: hydroxysteroid (17-beta) dehydrogenase 13 [Oryctolagus cuniculus]

ATGAACATTGTCTTGGATATCCTCCTGCTTCTGGTCACCATCATCTACTCCTACTTGGAG

TCATTGGTGAAGTTTTTCCTTCCCCGAAGGAGAAAATCTGTGGCTGGAGAGATTGTTCTC

ATTACCGGAGCTGGGCATGGAATAGGCAGGCTGACTGCCTATGAATTTGCAAAGCGGAAA

AGCAGACTGGTTCTTTGGGATATTAATAAGCATGGTGTTGAGGAAACTGCAACTGAATGT

CAAAAATTAGGGGCTACTGTGCATGCATTTGTGGTCGACTGCAGTAGCCGAGAGGAGATT

TACAGCTCCGTGAATCAGATAAAGAAAGAAGTGGGTGATGTAACCATCGTGGTGAATAAT

GCTGGAGCAATATATCCAGCTGATCTTCTTAGTACCAAGGATGAGGAAATTACCAAAACA

TTTGAAGTCAACATCCTGGGACATTTTTGGATCATAAAAGCACTTCTTCCTTCAATGATA

AAGAGAAATTACGGCCACATTGTCACAGTGGCTTCAGTGTGTGGCCATGGAGTAATTCCC

TATCTTATCCCATATTGTTCCAGCAAATTTGCGGCTGTTGGCTTTCACAGGGCTCTGACA

GCAGAACTTGAAACCTTGGGGAAAACTGGTATCAAAACCTCATGTCTCTGCCCAGTTTTT

GTGGATACTGGGTTCACCAAAAACCCAAGCACAAGATTATGGCCTGTATTAGAGACAGAT

GAAGTTGCAAGAAGTCTGATAGATGGAATACTTACTAATAAGAAAATGATTTTTGTTCCA

TCATATATTGGTATTTCTCTAATACTGGAAAAGGTAATA

>CL56.Contig2_All 122 1021 PREDICTED: hydroxysteroid (17-beta) dehydrogenase 13 [Oryctolagus cuniculus]

ATGAACATTGTCTTGGATATCCTCCTGCTTCTGGTCACCATCATCTACTCCTACTTGGAG

TCATTGGTGAAGTTTTTCCTTCCCCGAAGGAGAAAATCTGTGGCTGGAGAGATTGTTCTC

ATTACCGGAGCTGGGCATGGAATAGGCAGGCTGACTGCCTATGAATTTGCAAAGCGGAAA

AGCAGACTGGTTCTTTGGGATATTAATAAGCATGGTGTTGAGGAAACTGCAACTGAATGT

CAAAAATTAGGGGCTACTGTGCATGCATTTGTGGTCGACTGCAGTAGCCGAGAGGAGATT

TACAGCTCCGTGAATCAGATAAAGAAAGAAGTGGGTGATGTAACCATCGTGGTGAATAAT

GCTGGAGCAATATATCCAGCTGATCTTCTTAGTACCAAGGATGAGGAAATTACCAAAACA

TTTGAAGTCAACATCCTGGGACATTTTTGGATCATAAAAGCACTTCTTCCTTCAATGATA

AAGAGAAATTACGGCCACATTGTCACAGTGGCTTCAGTGTGTGGCCATGGAGTAATTCCC

TATCTTATCCCATATTGTTCCAGCAAATTTGCGGCTGTTGGCTTTCACAGGGCTCTGACA

GCAGAACTTGAAACCTTGGGGAAAACTGGTATCAAAACCTCATGTCTCTGCCCAGTTTTT

GTGGATACTGGGTTCACCAAAAACCCAAGCACAAGATTATGGCCTGTATTAGAGACAGAT

GAAGTTGCAAGAAGTCTGATAGATGGAATACTTACTAATAAGAAAATGATTTTTGTTCCA

TCATATATTGGTATTTCTCTAATACTGGAAAAGTTTCTTCCTGAACGTGCCTTGGCAGCT

TTAAGTCGTATACAGAATATACAATTTGAAGCAGTGGTTGGTCACAAAACCAGAATGAAA

>CL59.Contig1_All 1 879 minus strand proline synthetase co-transcribed homolog (bacterial), isoform CRA_b [Homo sapiens] >gi|119583768|gb|EAW63364.1| proline synthetase co-transcribed homolog (bacterial), isoform CRA_b [Homo sapiens]

TGGTCCCAGCCCCTGGGTTCCGAGGGTCCGGGGCTCTGCGTCTGTCCCCGGGGAATGTGG

AGAGCTGGCAGCATGTCCGCAGAGCTGGGAGTCGGGTTCGCATTGCGGGCAGTGAACGAG

CGCGTGCAGCAGGCTGTGGCGAGGCGGCCGAGGGATCTCCCAGCCATCCAGCCCCGGCTA

GTAGCAGTCAGCAAAACCAAACCAGCAGATATGGTGATTGAGGCCTATGGTCACGGCCAG

CGCACTTTTGGAGAGAACTATGTTCAGGAACTTCTAGAAAAAGCATCAAATCCTAAAATT

CTGTCTTCATGTCCTGAGATCAAATGGCATTTCATTGGCCACCTACAGAAACAAAACGTC

AACAAATTGATGGCTGTTCCCAACCTCTTCATGCTGGAAACAGTGGATTCTATGAAGTTA

GCAGACAAAGTGAACAGTTCCTGGCAGAAGAAAGGTTCTGCTGAAAGGCTGAAGGTTATG

GTCCAGATTAACACCAGTGGAGAGGAGAGTAAACATGGCCTTCCACCTTCAGAGACAATA

CCCATGGTGGAACACATAAATACCAAGTGTCCCAGCCTGGAATTCGTGGGACTGATGACC

ATAGGAAGCTTTGGGCATGATCTTAGCCAAGGACCAAACCCCGACTTCCAGGTGTTACTG

TCCCTGCGGGAGGAGCTGTGTAGAAAGCTGAGCATCCCCGCTGACCAAGTTGAGCTGAGC

ATGGGCATGTCCATGGACTTCCAGCATGCAATTGAAGTAGGCTCTACAAACGTCCGGATA

GGAAGCACCATTTTTGGAGCACGGGATTTCTCAAAGAAACCTGCCCTGGATAAGAGTACA

ACAGACCTGAAGGCCCCAGTGGGGGTGGCACAGGAACAC

>CL60.Contig1_All 87 254 minus strand PREDICTED: valine--tRNA ligase-like, partial [Papio anubis]

ATGTCCATCCTCTACGTCTCCCCTCACCCAGATGCCTTCCCCAGCCTTCGAGCCCTCATA

GCCGCTCGCTATGGGGAGGCTGGGGAGGGTCCTGGATGGGGAGGAGCCCACCCTCGCATC

TGTCTCCAGCCGCCTCCCACCAGCCGCACTCCTTTTCCCCCACCCCGC

>CL60.Contig2_All 216 383 minus strand PREDICTED: valine--tRNA ligase-like, partial [Papio anubis]

ATGTCCATCCTCTACGTCTCCCCTCACCCAGATGCCTTCCCCAGCCTTCGAGCCCTCATA

GCCGCTCGCTATGGGGAGGCTGGGGAGGGTCCTGGATGGGGAGGAGCCCACCCTCGCATC

TGTCTCCAGCCGCCTCCCACCAGCCGCACTCCTTTTCCCCCACCCCGC

>CL61.Contig1_All 122 685 PREDICTED: solute carrier family 35, member B3 [Oryctolagus cuniculus]

CAGCAAACAGAAGAATTAAACGAAATACAGCAAACCAACAGAGATAACTCTGGAAGCCCT

GAATGCAGCAAAATAAGAACAATGGACCTCAAGTTTAACAACTCCAGAAAATACATCTCT

ATCACTGTGCCATCCAAAACCCAAACAATGTCACCACACATTAAGTCAATTGATGACATT

ATAGTACTTGGCATAAATCTCAGCAAATTTAACAAACTTACTCAGTTTTTCATATGTGTT

GCTGGAGTTTTTGTTTTTTACCTAATTTATGGATATTTGCAGGAATTAATATTTTCAGTG

GAGGGTTTTAAGTCCTATGGCTGGTACCTTACTTTAGTGCAATTTGCATTTTACTCCATA

TTTGGCCTAATAGAACTTCAGCTTATTCAGGATAAAAGGAGAAGAATACCAGGAAAAACC

TACATGATAATAGCTTTTCTAACAGTGGGTACTATGGGGTTATCAAACACTTCCTTGGGC

TACCTGAATTATCCCACCCAAGTCATCTTCAAGTGCTGCAAATTGATTCCTGTTATGCTA

GGAGGAGTTTTTATTCAAGGAAAG

>CL61.Contig2_All 1 153 minus strand PREDICTED: adenosine 3&apos;-phospho 5&apos;-phosphosulfate transporter 2-like [Equus caballus]

GCCGGTGATGAGAGCGCCTTCCGGGGAGGAGGCAGCGGCTGTGGAAAGTCCAGAAGTAGG

AGAGGAAGCGGAGGAAGAGGAGGTGATGGCGACCGACTTGGGGAGGGGGCGACGTTTCGT

CTGGCCGAGGCGCCGTCCGGGCTAAGGTCACCC

>CL62.Contig1_All 145 1581 minus strand zinc finger protein 207 isoform a [Homo sapiens] >gi|6137311|sp|O43670.1|ZN207_HUMAN RecName: Full=Zinc finger protein 207 >gi|2895870|gb|AAC78561.1| zinc finger transcription factor [Homo sapiens] >gi|119600639|gb|EAW80233.1| zinc finger protein 207, isoform CRA_c [Homo sapiens] >gi|119600643|gb|EAW80237.1| zinc finger protein 207, isoform CRA_c [Homo sapiens] >gi|380816382|gb|AFE80065.1| zinc finger protein 207 isoform a [Macaca mulatta] >gi|384939358|gb|AFI33284.1| zinc finger protein 207 isoform a [Macaca mulatta]

ATGGGTCGCAAGAAGAAGAAGCAGCTGAAACCGTGGTGCTGGTATTGTAATAGAGATTTT

GATGATGAGAAGATCCTTATACAGCACCAAAAGGCAAAGCATTTTAAATGCCATATATGT

CATAAGAAATTGTATACAGGACCTGGCTTAGCTATTCATTGCATGCAGGTGCATAAAGAG

ACAATAGATGCTGTACCAAATGCAATACCTGGGAGAACAGACATAGAGTTGGAAATATAC

GGTATGGAAGGTATTCCAGAAAAAGACATGGATGAAAGACGACGACTTCTTGAACAGAAA

ACACAGGCAGAGAGTCAAAAAAAGAAGCAACAGGATGATTCTGATGAGTATGATGATGAT

GACTCTGCAGCCTCAACTTCATTTCAACCACAGCCTGTTCAACCTCAGCAAGGTTATATC

CCTCCAATGGCTCAGCCAGGACTGCCACCAGTTCCAGGAGCACCAGGAATGCCTCCAGGC

ATACCTCCACTAATGCCAGGTGTTCCTCCTCTGATGCCAGGAATGCCACCAGTTATGCCA

GGCATGCCACCTGGAATGATGCCAATGGGTGGAATGATGCCACCTGGACCAGGAATACCA

CCTCTTATGCCTGGTATGCCACCAGGTATGCCCCCCCCTGTTCCCCGTCCTGGAATTCCT

CCAATGACTCAAGCACAGGCTGTGTCAGCACCAGGTATTCTTAATAGACCACCTGCACCA

ACAGCAACAGTACCTGCTCCACAGCCTCCAGTTACTAAGCCTCTTTTCCCCAGTGCTGGA

CAGATGGGGACACCTGTAACAAGCTCAAGTACAGCTTCATCCAATTCAGAAAGTCTGTCT

GCATCTTCTAAAGCTCTGTTTCCTAGCACAGCACAAGCTCAGGCAGCTGTGCAAGGACCT

GTTGGTACAGATTTCAAGCCCTTAAATAGTACCCCTGCAACAACTACAGAACCCCCAAAG

CCTACATTCCCTGCTTATACACAGTCTACAGCTTCAACCACTAGTACAACAAATAGTACT

GCAGCAAAACCAGCAGCTTCAATAACAAGTAAGCCTGCTACACTTACAACCACCAGTGCA

ACCAGTAAGTTGATCCATCCAGATGAGGATATATCACTGGAAGAGAGAAGGGCACAGTTA

CCTAAATATCAGCGTAATCTTCCTCGACCAGGACAGGCCCCAATCGGTAATCCACCAGTT

GGACCAATTGGAGGTATGATGCCACCACAGCCAGGCATCCCACAGCAGCAAGGAATGAGA

CCCCCAATGCCACCTCATGGTCAGTATGGTGGTCATCATCAAGGCATGCCAGGTTACCTT

CCTGGTGCTATGCCACCGTATGGGCAGGGACCGCCAATGGTACCCCCTTACCAAGGTGGG

CCTCCTCGACCTCCAATGGGAATGAGACCTCCTGTAATGTCGCAAGGTGGCCGTTAC

>CL62.Contig2_All 145 1488 minus strand zinc finger protein 207 isoform b [Macaca mulatta]

ATGGGTCGCAAGAAGAAGAAGCAGCTGAAACCGTGGTGCTGGTATTGTAATAGAGATTTT

GATGATGAGAAGATCCTTATACAGCACCAAAAGGCAAAGCATTTTAAATGCCATATATGT

CATAAGAAATTGTATACAGGACCTGGCTTAGCTATTCATTGCATGCAGGTGCATAAAGAG

ACAATAGATGCTGTACCAAATGCAATACCTGGGAGAACAGACATAGAGTTGGAAATATAC

GGTATGGAAGGTATTCCAGAAAAAGACATGGATGAAAGACGACGACTTCTTGAACAGAAA

ACACAGGCAGAGAGTCAAAAAAAGAAGCAACAGGATGATTCTGATGAGTATGATGATGAT

GACTCTGCAGCCTCAACTTCATTTCAACCACAGCCTGTTCAACCTCAGCAAGGTTATATC

CCTCCAATGGCTCAGCCAGGACTGCCACCAGTTCCAGGAGCACCAGGAATGCCTCCAGGC

ATACCTCCACTAATGCCAGGTGTTCCTCCTCTGATGCCAGGAATGCCACCAGTTATGCCA

GGCATGCCACCTGGAATGATGCCAATGGGTGGAATGATGCCACCTGGACCAGGAATACCA

CCTCTTATGCCTGGTATGCCACCAGGTATGCCCCCCCCTGTTCCCCGTCCTGGAATTCCT

CCAATGACTCAAGCACAGGCTGTGTCAGCACCAGGTATTCTTAATAGACCACCTGCACCA

ACAGCAACAGTACCTGCTCCACAGCCTCCAGTTACTAAGCCTCTTTTCCCCAGTGCTGGA

CAGGCTCAGGCAGCTGTGCAAGGACCTGTTGGTACAGATTTCAAGCCCTTAAATAGTACC

CCTGCAACAACTACAGAACCCCCAAAGCCTACATTCCCTGCTTATACACAGTCTACAGCT

TCAACCACTAGTACAACAAATAGTACTGCAGCAAAACCAGCAGCTTCAATAACAAGTAAG

CCTGCTACACTTACAACCACCAGTGCAACCAGTAAGTTGATCCATCCAGATGAGGATATA

TCACTGGAAGAGAGAAGGGCACAGTTACCTAAATATCAGCGTAATCTTCCTCGACCAGGA

CAGGCCCCAATCGGTAATCCACCAGTTGGACCAATTGGAGGTATGATGCCACCACAGCCA

GGCATCCCACAGCAGCAAGGAATGAGACCCCCAATGCCACCTCATGGTCAGTATGGTGGT

CATCATCAAGGCATGCCAGGTTACCTTCCTGGTGCTATGCCACCGTATGGGCAGGGACCG

CCAATGGTACCCCCTTACCAAGGTGGGCCTCCTCGACCTCCAATGGGAATGAGACCTCCT

GTAATGTCGCAAGGTGGCCGTTAC

>CL62.Contig3_All 145 1533 zinc finger protein 207 isoform b [Homo sapiens] >gi|347543797|ref|NP_001231559.1| zinc finger protein 207 [Macaca mulatta] >gi|12803137|gb|AAH02372.1| Zinc finger protein 207 [Homo sapiens] >gi|119600641|gb|EAW80235.1| zinc finger protein 207, isoform CRA_d [Homo sapiens] >gi|119600642|gb|EAW80236.1| zinc finger protein 207, isoform CRA_d [Homo sapiens] >gi|380816384|gb|AFE80066.1| zinc finger protein 207 isoform b [Macaca mulatta] >gi|383408769|gb|AFH27598.1| zinc finger protein 207 isoform b [Macaca mulatta] >gi|384939360|gb|AFI33285.1| zinc finger protein 207 isoform b [Macaca mulatta]

ATGGGTCGCAAGAAGAAGAAGCAGCTGAAACCGTGGTGCTGGTATTGTAATAGAGATTTT

GATGATGAGAAGATCCTTATACAGCACCAAAAGGCAAAGCATTTTAAATGCCATATATGT

CATAAGAAATTGTATACAGGACCTGGCTTAGCTATTCATTGCATGCAGGTGCATAAAGAG

ACAATAGATGCTGTACCAAATGCAATACCTGGGAGAACAGACATAGAGTTGGAAATATAC

GGTATGGAAGGTATTCCAGAAAAAGACATGGATGAAAGACGACGACTTCTTGAACAGAAA

ACACAGGAGAGTCAAAAAAAGAAGCAACAGGATGATTCTGATGAGTATGATGATGATGAC

TCTGCAGCCTCAACTTCATTTCAACCACAGCCTGTTCAACCTCAGCAAGGTTATATCCCT

CCAATGGCTCAGCCAGGACTGCCACCAGTTCCAGGAGCACCAGGAATGCCTCCAGGCATA

CCTCCACTAATGCCAGGTGTTCCTCCTCTGATGCCAGGAATGCCACCAGTTATGCCAGGC

ATGCCACCTGGATTGCATCATCAGAGAAAATACACCCAGTCATTTTGCGGTGAAAACATA

ATGATGCCAATGGGTGGAATGATGCCACCTGGACCAGGAATACCACCTCTTATGCCTGGT

ATGCCACCAGGTATGCCCCCCCCTGTTCCCCGTCCTGGAATTCCTCCAATGACTCAAGCA

CAGGCTGTGTCAGCACCAGGTATTCTTAATAGACCACCTGCACCAACAGCAACAGTACCT

GCTCCACAGCCTCCAGTTACTAAGCCTCTTTTCCCCAGTGCTGGACAGGCTCAGGCAGCT

GTGCAAGGACCTGTTGGTACAGATTTCAAGCCCTTAAATAGTACCCCTGCAACAACTACA

GAACCCCCAAAGCCTACATTCCCTGCTTATACACAGTCTACAGCTTCAACCACTAGTACA

ACAAATAGTACTGCAGCAAAACCAGCAGCTTCAATAACAAGTAAGCCTGCTACACTTACA

ACCACCAGTGCAACCAGTAAGTTGATCCATCCAGATGAGGATATATCACTGGAAGAGAGA

AGGGCACAGTTACCTAAATATCAGCGTAATCTTCCTCGACCAGGACAGGCCCCAATCGGT

AATCCACCAGTTGGACCAATTGGAGGTATGATGCCACCACAGCCAGGCATCCCACAGCAG

CAAGGAATGAGACCCCCAATGCCACCTCATGGTCAGTATGGTGGTCATCATCAAGGCATG

CCAGGTTACCTTCCTGGTGCTATGCCACCGTATGGGCAGGGACCGCCAATGGTACCCCCT

TACCAAGGTGGGCCTCCTCGACCTCCAATGGGAATGAGACCTCCTGTAATGTCGCAAGGT

GGCCGTTAC

>CL62.Contig4_All 145 1626 zinc finger protein 207 isoform c [Homo sapiens] >gi|119600637|gb|EAW80231.1| zinc finger protein 207, isoform CRA_a [Homo sapiens] >gi|119600640|gb|EAW80234.1| zinc finger protein 207, isoform CRA_a [Homo sapiens] >gi|380816380|gb|AFE80064.1| zinc finger protein 207 isoform c [Macaca mulatta] >gi|383408767|gb|AFH27597.1| zinc finger protein 207 isoform c [Macaca mulatta] >gi|384939362|gb|AFI33286.1| zinc finger protein 207 isoform c [Macaca mulatta]

ATGGGTCGCAAGAAGAAGAAGCAGCTGAAACCGTGGTGCTGGTATTGTAATAGAGATTTT

GATGATGAGAAGATCCTTATACAGCACCAAAAGGCAAAGCATTTTAAATGCCATATATGT

CATAAGAAATTGTATACAGGACCTGGCTTAGCTATTCATTGCATGCAGGTGCATAAAGAG

ACAATAGATGCTGTACCAAATGCAATACCTGGGAGAACAGACATAGAGTTGGAAATATAC

GGTATGGAAGGTATTCCAGAAAAAGACATGGATGAAAGACGACGACTTCTTGAACAGAAA

ACACAGGAGAGTCAAAAAAAGAAGCAACAGGATGATTCTGATGAGTATGATGATGATGAC

TCTGCAGCCTCAACTTCATTTCAACCACAGCCTGTTCAACCTCAGCAAGGTTATATCCCT

CCAATGGCTCAGCCAGGACTGCCACCAGTTCCAGGAGCACCAGGAATGCCTCCAGGCATA

CCTCCACTAATGCCAGGTGTTCCTCCTCTGATGCCAGGAATGCCACCAGTTATGCCAGGC

ATGCCACCTGGATTGCATCATCAGAGAAAATACACCCAGTCATTTTGCGGTGAAAACATA

ATGATGCCAATGGGTGGAATGATGCCACCTGGACCAGGAATACCACCTCTTATGCCTGGT

ATGCCACCAGGTATGCCCCCCCCTGTTCCCCGTCCTGGAATTCCTCCAATGACTCAAGCA

CAGGCTGTGTCAGCACCAGGTATTCTTAATAGACCACCTGCACCAACAGCAACAGTACCT

GCTCCACAGCCTCCAGTTACTAAGCCTCTTTTCCCCAGTGCTGGACAGATGGGGACACCT

GTAACAAGCTCAAGTACAGCTTCATCCAATTCAGAAAGTCTGTCTGCATCTTCTAAAGCT

CTGTTTCCTAGCACAGCACAAGCTCAGGCAGCTGTGCAAGGACCTGTTGGTACAGATTTC

AAGCCCTTAAATAGTACCCCTGCAACAACTACAGAACCCCCAAAGCCTACATTCCCTGCT

TATACACAGTCTACAGCTTCAACCACTAGTACAACAAATAGTACTGCAGCAAAACCAGCA

GCTTCAATAACAAGTAAGCCTGCTACACTTACAACCACCAGTGCAACCAGTAAGTTGATC

CATCCAGATGAGGATATATCACTGGAAGAGAGAAGGGCACAGTTACCTAAATATCAGCGT

AATCTTCCTCGACCAGGACAGGCCCCAATCGGTAATCCACCAGTTGGACCAATTGGAGGT

ATGATGCCACCACAGCCAGGCATCCCACAGCAGCAAGGAATGAGACCCCCAATGCCACCT

CATGGTCAGTATGGTGGTCATCATCAAGGCATGCCAGGTTACCTTCCTGGTGCTATGCCA

CCGTATGGGCAGGGACCGCCAATGGTACCCCCTTACCAAGGTGGGCCTCCTCGACCTCCA

ATGGGAATGAGACCTCCTGTAATGTCGCAAGGTGGCCGTTAC

>CL62.Contig5_All 3 1025 minus strand zinc finger protein 207 [Pan troglodytes]

CCAGGAATGCCTCCAGGCATACCTCCACTAATGCCAGGTGTTCCTCCTCTGATGCCAGGA

ATGCCACCAGTTATGCCAGGCATGCCACCTGGATTGCATCATCAGAGAAAATACACCCAG

TCATTTTGCGGTGAAAACATAATGATGCCAATGGGTGGAATGATGCCACCTGGACCAGGA

ATACCACCTCTTATGCCTGGTATGCCACCAGGTATGCCCCCCCCTGTTCCCCGTCCTGGA

ATTCCTCCAATGACTCAAGCACAGGCTGTGTCAGCACCAGGTATTCTTAATAGACCACCT

GCACCAACAGCAACAGTACCTGCTCCACAGCCTCCAGTTACTAAGCCTCTTTTCCCCAGT

GCTGGACAGATGGGGACACCTGTAACAAGCTCAAGTACAGCTTCATCCAATTCAGAAAGT

CTGTCTGCATCTTCTAAAGCTCTGTTTCCTAGCACAGCACAAGCTCAGGCAGCTGTGCAA

GGACCTGTTGGTACAGATTTCAAGCCCTTAAATAGTACCCCTGCAACAACTACAGAACCC

CCAAAGCCTACATTCCCTGCTTATACACAGTCTACAGCTTCAACCACTAGTACAACAAAT

AGTACTGCAGCAAAACCAGCAGCTTCAATAACAAGTAAGCCTGCTACACTTACAACCACC

AGTGCAACCAGTAAGTTGATCCATCCAGATGAGGATATATCACTGGAAGAGAGAAGGGCA

CAGTTACCTAAATATCAGCGTAATCTTCCTCGACCAGGACAGGCCCCAATCGGTAATCCA

CCAGTTGGACCAATTGGAGGTATGATGCCACCACAGCCAGGCATCCCACAGCAGCAAGGA

ATGAGACCCCCAATGCCACCTCATGGTCAGTATGGTGGTCATCATCAAGGCATGCCAGGT

TACCTTCCTGGTGCTATGCCACCGTATGGGCAGGGACCGCCAATGGTACCCCCTTACCAA

GGTGGGCCTCCTCGACCTCCAATGGGAATGAGACCTCCTGTAATGTCGCAAGGTGGCCGT

TAC

>CL62.Contig6_All 3 932 minus strand zinc finger protein 207 isoform b [Homo sapiens] >gi|347543797|ref|NP_001231559.1| zinc finger protein 207 [Macaca mulatta] >gi|12803137|gb|AAH02372.1| Zinc finger protein 207 [Homo sapiens] >gi|119600641|gb|EAW80235.1| zinc finger protein 207, isoform CRA_d [Homo sapiens] >gi|119600642|gb|EAW80236.1| zinc finger protein 207, isoform CRA_d [Homo sapiens] >gi|380816384|gb|AFE80066.1| zinc finger protein 207 isoform b [Macaca mulatta] >gi|383408769|gb|AFH27598.1| zinc finger protein 207 isoform b [Macaca mulatta] >gi|384939360|gb|AFI33285.1| zinc finger protein 207 isoform b [Macaca mulatta]

CCAGGAATGCCTCCAGGCATACCTCCACTAATGCCAGGTGTTCCTCCTCTGATGCCAGGA

ATGCCACCAGTTATGCCAGGCATGCCACCTGGATTGCATCATCAGAGAAAATACACCCAG

TCATTTTGCGGTGAAAACATAATGATGCCAATGGGTGGAATGATGCCACCTGGACCAGGA

ATACCACCTCTTATGCCTGGTATGCCACCAGGTATGCCCCCCCCTGTTCCCCGTCCTGGA

ATTCCTCCAATGACTCAAGCACAGGCTGTGTCAGCACCAGGTATTCTTAATAGACCACCT

GCACCAACAGCAACAGTACCTGCTCCACAGCCTCCAGTTACTAAGCCTCTTTTCCCCAGT

GCTGGACAGGCTCAGGCAGCTGTGCAAGGACCTGTTGGTACAGATTTCAAGCCCTTAAAT

AGTACCCCTGCAACAACTACAGAACCCCCAAAGCCTACATTCCCTGCTTATACACAGTCT

ACAGCTTCAACCACTAGTACAACAAATAGTACTGCAGCAAAACCAGCAGCTTCAATAACA

AGTAAGCCTGCTACACTTACAACCACCAGTGCAACCAGTAAGTTGATCCATCCAGATGAG

GATATATCACTGGAAGAGAGAAGGGCACAGTTACCTAAATATCAGCGTAATCTTCCTCGA

CCAGGACAGGCCCCAATCGGTAATCCACCAGTTGGACCAATTGGAGGTATGATGCCACCA

CAGCCAGGCATCCCACAGCAGCAAGGAATGAGACCCCCAATGCCACCTCATGGTCAGTAT

GGTGGTCATCATCAAGGCATGCCAGGTTACCTTCCTGGTGCTATGCCACCGTATGGGCAG

GGACCGCCAATGGTACCCCCTTACCAAGGTGGGCCTCCTCGACCTCCAATGGGAATGAGA

CCTCCTGTAATGTCGCAAGGTGGCCGTTAC

>CL63.Contig1_All 182 601 Acyl-coenzyme A thioesterase 13 [Cricetulus griseus]

ATGAGCGGCCTGATGCAGAACGCGCGGGAAGTGATGAAGGCCCTGTTCAGGTCGCCCGGC

TTCGATAGAGTTTTGGAAAAGGTGACTCTCGTCTCTGCTGCTCCTGAGAAAGTGATTTGT

GAAATGAAAGTAGAAGAGCAGCATGCTAATAAAATGGGCACACTCCATGGTGGTTTGACA

GCCACCTTAATAGACAGCATATCAACTGTGGCTCTGCTGTGCTCAGAAAGGGGTCTGCCT

GGAGTCAGCGTTGACATGAACGTAACGTACATGTCACCTGCTAAAATAGGAGAAGAAATA

GTGATTACAGCACAAATTCTGAAGCAAGGAAGAACACTTGCATTTGCTTCTGTGGATCTG

ACCAACAAGGCCACAGGAAAATTAATAGCACAAGGCAGGCATACGAAACACCTGGGAAAC

>CL63.Contig2_All 46 198 acyl-coenzyme A thioesterase 13 [Rattus norvegicus]

TACATGTCACCTGCTAAAATAGGAGAAGAAATAGTGATTACAGCACAAATTCTGAAGCAA

GGAAGAACACTTGCATTTGCTTCTGTGGATCTGACCAACAAGGCCACAGGAAAATTAATA

GCACAAGGCAGGCATACGAAACACCTGGGAAAC

>CL64.Contig1_All 98 712 PREDICTED: V-type proton ATPase 21 kDa proteolipid subunit [Otolemur garnettii]

ATGACGGGGCTAGCGCTGCTCTACTCCGGGGTCTTCGTGGCCTTCTGGGCCTGCACGCTC

GTCGTGGGGATCTGCTACACCATTTTTGACTTGGGCTTCCGCTTTGATGTGGCATGGTTC

CTGACTGAGACTTCCCCCTTCATGTGGTCGAACCTGGGCATTGGCCTAGCAATCTCCCTG

TCTGTGGTTGGGGCAGCCTGGGGCATCTATATTACAGGCTCCTCCATCATTGGGGGAGGG

GTGAAGGCCCCCAGAATCAAGACCAAGAACCTGGTCAGCATCATCTTCTGTGAGGCTGTG

GCTATCTACGGCATCATCATGGCAATTGTCATTAGCAACATGGCTGAGCCTTTCAGTGCC

ACTGACCCTCAGGCCATTGGTCATCGAAACTACCATGCAGGCTATTCCATGTTTGGGGCT

GGTCTCACAGTGGGTCTGTCTAACCTCTTCTGTGGAGTCTGCGTGGGCATCGTGGGCAGT

GGGGCCGCCCTGGCTGATGCTCAGAACCCCAGTCTCTTTGTAAAGATTCTCATCGTGGAA

ATATTTGGAAGTGCCATCGGCCTCTTTGGGGTCATCGTCGCAATCCTTCAGACCTCCAGA

GTGAAGATGGGTGAC

>CL64.Contig2_All 162 659 PREDICTED: V-type proton ATPase 21 kDa proteolipid subunit [Otolemur garnettii]

TTCCTGACTGAGACTTCCCCCTTCATGTGGTCGAACCTGGGCATTGGCCTAGCAATCTCC

CTGTCTGTGGTTGGGGCAGCCTGGGGCATCTATATTACAGGCTCCTCCATCATTGGGGGA

GGGGTGAAGGCCCCCAGAATCAAGACCAAGAACCTGGTCAGCATCATCTTCTGTGAGGCT

GTGGCTATCTACGGCATCATCATGGCAATTGTCATTAGCAACATGGCTGAGCCTTTCAGT

GCCACTGACCCTCAGGCCATTGGTCATCGAAACTACCATGCAGGCTATTCCATGTTTGGG

GCTGGTCTCACAGTGGGTCTGTCTAACCTCTTCTGTGGAGTCTGCGTGGGCATCGTGGGC

AGTGGGGCCGCCCTGGCTGATGCTCAGAACCCCAGTCTCTTTGTAAAGATTCTCATCGTG

GAAATATTTGGAAGTGCCATCGGCCTCTTTGGGGTCATCGTCGCAATCCTTCAGACCTCC

AGAGTGAAGATGGGTGAC

>CL66.Contig1_All 4 171 likely orthologue of Mus musculus enhancer trap locus 4 [Homo sapiens] >gi|150172273|emb|CAI12211.2| KIAA1217 [Homo sapiens]

TTCTCTTCCCCCTCTCGCTGGTCCTTCCCACAGGGCTCCAGTGGGACCCCACAGACAAGC

AGGATGCCCGTCCCTATGAGTTCCAAGAATAGACCCGGAAGCCTGGACAAACCTGGCAAG

CAATCCAAACTGCAGGATCCTCGCCAATATCGTCAGGTAGTTTTACCT

>CL66.Contig2_All 3 2150 PREDICTED: sickle tail protein homolog isoform 1 [Equus caballus]

AGTATTTCTGATGCATCCAGAACATCAGAATATAAAACTGACATCTTAATGAAGGAAAAT

TCCATTTCTAATAAGAATTTATATAGAGACAGTAGAAACTATTCCCAGAAAAATATGCCA

AAGGTCAGTTTCAGCTTCTCTGGCACGAACTCATTAGAAGATGAAATAAACCAAGGGCCC

AAAGACTCAGGACTGCAGTACCCTGAGAATCAGAAGTTGAATTACGGAAAGACAAAGGAG

ATGGGTGAACGAGGACCGGAAAATACAGATAAGTGTCACGTTCCCTCCTCTGCCAGATCA

ACAGAATTGAATAGTCATGACATCAGAACACAAGATCAAGATGGGCTCATGACAAATATT

AGCCAAGTTGTTCTAAGACCCAGAGGGACGAGGCATGGAAACCTGAATCCTCCTGATGAT

GGAGACTCAATTCCAGGTTCTCCCACTGAAGAAAATTCAGCCACTGACAACATCGCCTTC

ATGATCACCAAAACCGCTGTCCAGGTGCTGTCCAGTGGGGAGGTCCATGATATAGTGAGC

CAAAAGGGACAAGATGTGCAGACAGTCAACATCGATACCAAGAAAGAGACGACCTCCCAC

CCAGAGGTGACTGAAGGCGAAGAGCCGGTCGTGTGCCTAGACAAGAAACCAGTTATCATC

ATTTTTGATGAACCCATGGACATCCGGTCTGCATATAAGAGACTTTCCACCATCTTTGAG

GAATGCGATGAGGAATTGGAGAGAATGATGACGGAAGAAAAGATAGAGGAGGAGGAAGAA

GAGGAAAATGGAGACTCCGTAGTCAAGAATGATGCTTCCGACATGTTTCCTACGCAGGTT

GCCTCAGGAAGTCTGATTGTGGGACAGCAGACAGAGATGAAATTGAAGCCACTCTCCCTA

TCAGCAGAGACCAAGCCCCCGGCAGGACAGGCCATGAACCAAACGGAGCCAAGCAAGTTG

AGCCTGGCAGATTCTCCAGATTCCGAAAGCAAAGGGGACGTGGCCGAGGACCAATTTGAA

AGCCCCAAGAAAAAATTTAAATTCAAGTTCCCTAAAAAGCAACTTGCTGCTCTCACTCAG

GCAATCCGCACGGGAACCAAGACAGGCAAGAAGACCCTGCAGGTGGTGGTCTACGAGGAA

GAGGAAGAGGATGGCACTTTGAAACAGCACAAAGAAGCCAAGCGATTCGAAATCACCAGG

TCTCAACCTGAAGACACCCCGAAAAATATGGCCAGGAGACAGGAGCAGCCCAGTTTAGAG

AGCACAGTTCAGGTTTCAAGAACTGATGAAATTAGGAAAAATACCTACAGAACATTGGAC

AGTCTGGAGAAGACCATCAAACAGCTTGAAAGTACAATCAGTGAAATGAGTCCCAAAGCC

TTAGTCGAGACCTCGTGTTCCTCCAACAGAGAATCTGTTGCAAGCTCATCCCACACAGCC

CACGAGGCCCCTCCCCGAAACTTGCTAGTTCTGGATGAAGCCCCCCCTGCCCTAGAGCCC

CCCACGTCAGTATCTTCAGCTTCACGTAAGGGCTCCAGTGGGACCCCACAGACAAGCAGG

ATGCCCGTCCCTATGAGTTCCAAGAATAGACCCGGAAGCCTGGACAAACCTGGCAAGCAA

TCCAAACTGCAGGATCCTCGCCAATATCGTCAGGCTAATGGAAGTGCTAAGAAAGCTGTT

GGGGACTGTAAGCCTACTTCCCCCTCCTTACCTGCTTCTAAGATTCCAGCCCTTTCCCCC

AGCTCTGGGAAAAGCAGTTCTCTGCCCTCTTCTAGTGGTGACAGCTCTAACCTTCCTAAT

CCGCCTGCTACTAAACCATCGATTACTTCTAACCCTCTCAGCCCTCAAACAGGACGATCC

ACTCACTCCGCCTCCCTCATCCCTTCTGTCTCTAATGGCTCCTTAAAATTTCAGAGCCCC

CCTCACACAGGTAAAGGTCACCACCTTTCATTCTCACTGCAGACTCAAAATGGCCGAGCA

GTCCCTCCTTCCTCTTCCTCCTCCTCCCCTCCCTCCCCTGCCTCCCCGACCTCCCTCAGC

CAAGGTGCCAAGGGCATCAGGACCATCCATACTCCCAGCCTCACCAGCTACAAGGCACAG

AATGGAAGTTCAAGCAAAGCCACCCCTTCCACAGCCAAGGAGACCTCT

>CL66.Contig3_All 3 1655 PREDICTED: sickle tail protein homolog isoform 1 [Equus caballus]

AGTATTTCTGATGCATCCAGAACATCAGAATATAAAACTGACATCTTAATGAAGGAAAAT

TCCATTTCTAATAAGAATTTATATAGAGACAGTAGAAACTATTCCCAGAAAAATATGCCA

AAGGTCAGTTTCAGCTTCTCTGGCACGAACTCATTAGAAGATGAAATAAACCAAGGGCCC

AAAGACTCAGGACTGCAGTACCCTGAGAATCAGAAGTTGAATTACGGAAAGACAAAGGAG

ATGGGTGAACGAGGACCGGAAAATACAGATAAGTGTCACGTTCCCTCCTCTGCCAGATCA

ACAGAATTGAATAGTCATGACATCAGAACACAAGATCAAGATGGGCTCATGACAAATATT

AGCCAAGTTGTTCTAAGACCCAGAGGGACGAGGCATGGAAACCTGAATCCTCCTGATGAT

GGAGACTCAATTCCAGGTTCTCCCACTGAAGAAAATTCAGCCACTGACAACATCGCCTTC

ATGATCACCAAAACCGCTGTCCAGGTGCTGTCCAGTGGGGAGGTCCATGATATAGTGAGC

CAAAAGGGACAAGATGTGCAGACAGTCAACATCGATACCAAGAAAGAGACGACCTCCCAC

CCAGAGGTGACTGAAGGCGAAGAGCCGGTCGTGTGCCTAGACAAGAAACCAGTTATCATC

ATTTTTGATGAACCCATGGACATCCGGTCTGCATATAAGAGACTTTCCACCATCTTTGAG

GAATGCGATGAGGAATTGGAGAGAATGATGACGGAAGAAAAGATAGAGGAGGAGGAAGAA

GAGGAAAATGGAGACTCCGTAGTCAAGAATGATGCTTCCGACATGTTTCCTACGCAGGTT

GCCTCAGGAAGTCTGATTGTGGGACAGCAGACAGAGATGAAATTGAAGCCACTCTCCCTA

TCAGCAGAGACCAAGCCCCCGGCAGGACAGGCCATGAACCAAACGGAGCCAAGCAAGTTG

AGCCTGGCAGATTCTCCAGATTCCGAAAGCAAAGGGGACGTGGCCGAGGACCAATTTGAA

AGCCCCAAGAAAAAATTTAAATTCAAGTTCCCTAAAAAGCAACTTGCTGCTCTCACTCAG

GCAATCCGCACGGGAACCAAGACAGGCAAGAAGACCCTGCAGGTGGTGGTCTACGAGGAA

GAGGAAGAGGATGGCACTTTGAAACAGCACAAAGAAGCCAAGCGATTCGAAATCACCAGG

TCTCAACCTGAAGACACCCCGAAAAATATGGCCAGGAGACAGGAGCAGCCCAGTTTAGAG

AGCACAGTTCAGGTTTCAAGAACTGATGAAATTAGGAAAAATACCTACAGAACATTGGAC

AGTCTGGAGAAGACCATCAAACAGCTTGAAAGTACAATCAGTGAAATGAGTCCCAAAGCC

TTAGTCGAGACCTCGTGTTCCTCCAACAGAGAATCTGTTGCAAGCTCATCCCACACAGCC

CACGAGGCCCCTCCCCGAAACTTGCTAGTTCTGGATGAAGCCCCCCCTGCCCTAGAGCCC

CCCACGTCAGTATCTTCAGCTTCACGTAAGGGCTCCAGTGGGACCCCACAGACAAGCAGG

ATGCCCGTCCCTATGAGTTCCAAGAATAGACCCGGAAGCCTGGACAAACCTGGCAAGCAA

TCCAAACTGCAGGATCCTCGCCAATATCGTCAG

>CL68.Contig1_All 70 1023 60S acidic ribosomal protein P0 [Sus scrofa] >gi|182705234|sp|Q29214.2|RLA0_PIG RecName: Full=60S acidic ribosomal protein P0; AltName: Full=60S ribosomal protein L10E >gi|83854099|gb|ABC47963.1| ribosomal phosphoprotein large PO subunit [Sus scrofa]

ATGCCCAGGGAAGACAGGGCGACCTGGAAGTCCAACTACTTCCTTAAGATCATCCAACTT

TTGGATGACTATCCAAAATGCTTCATTGTGGGAGCAGACAATGTGGGCTCCAAGCAGATG

CAGCAGATCCGAATGTCCCTCCGGGGGAAGGCTGTGGTGCTGATGGGCAAAAACACTATG

ATGCGCAAGGCCATTCGAGGGCATCTGGAAAACAACCCAGCTCTAGAGAAACTGTTGCCT

CATATCAGGGGGAATGTGGGCTTTGTGTTCACCAAGGAGGACCTCACTGAGATCAGGGAC

ATGCTGCTGGCCAATAAGGTTCCAGCTGCTGCCCGTGCTGGTGCCATTGCACCATGTGAA

GTCACCGTACCAGCCCAGAACACTGGTCTGGGGCCCGAGAAGACTTCCTTCTTCCAGGCT

TTGGGCATTACCACTAAAATCTCTAGGGGCACCATTGAAATCCTGAGTGATGTGCAGCTG

ATAAAGACTGGAGACAAAGTGGGAGCCAGCGAAGCTACCCTGCTGAACATGCTGAACATA

TCCCCCTTCTCCTTTGGGCTGATCATTCAGCAAGTGTTTGACAATGGCAGCATCTACAAC

CCTGAAGTGCTTGACATCACAGAGGAGACTCTGCATTCACGCTTCCTGGAAGGTGTCCGC

AATGTTGCTAGTGTTTGTCTGCAGATTGGTTATCCAACTGTTGCATCAGTACCCCATTCT

ATCATCAATGGGTACAAGAGGGTCCTGGCTTTGTCTGTGGAGACTGAATACACCTTCCCA

CTTGCTGAGAAGGTCAAGGCCTTCTTGGCTGATCCATCTGCATTTGTGGCTGCTGCCCCA

GTGGCTGCTGCTACTACTGCTGCTCCTGCTGCTGCTGCTGCGGCCCCAGCCAAGGTTGAA

GCCAAGGAAGAGTCGGAGGAGTCGGATGAGGATATGGGATTTGGTCTCTTTGAC

>CL68.Contig2_All 101 265 60S acidic ribosomal protein P0 [Macaca mulatta]

CAGGTCAAGGCCTTCTTGGCTGATCCATCTGCATTTGTGGCTGCTGCCCCAGTGGCTGCT

GCTACTACTGCTGCTCCTGCTGCTGCTGCTGCGGCCCCAGCCAAGGTTGAAGCCAAGGAA

GAGTCGGAGGAGTCGGATGAGGATATGGGATTTGGTCTCTTTGAC

>CL69.Contig1_All 151 1383 minus strand PREDICTED: lysosome-associated membrane glycoprotein 2 isoform 1 [Papio anubis]

ATGGTGTACTTCCGTCCCTCCCGGCTTTCAGGCTCCGGGCTTCTGCTGCTCTGTCTCATC

CTGGGAGCTGACCAGTCTCATGCCTTGGAACTTAATTTGACGGATTCATCAAATGCTACT

TGCCTTTATGCAAAGTGGCAGATGAATTTCACAGTAACGTATGAAACTACAAACAAAACT

TATAAAACGGTAAACATTTCAGACCTTAGCAATGTGACATATGAGGGAAGCACTTGTGGA

GATGACCAGAATGGTCCCAGAATAGCAGTGCAATTTGGATCTGGTTTTTCCTGGATTGTA

AACTTCACCAAACATGCTCCTGATTATTTGATTGATACCATCTCGTTTTCCTACAATATT

CATAATCACACAACATTTCCTGATGCTGAACATAAAGGAATTATTACCATTGACAACCGT

GTGGCATTAAATGTTCCATTGAATGACATCTTTAGATGCAATAGTTTGTTAACTATAGAA

AATAATAATGTCACTCAGCACATTTGGAATGTTCTTGTACAAGCTTTTGTCCAAAATGGC

ACGGTGAGCACAAAAGAATATGTATGTGATGAAGACAAACATGCCACAACAGTGGCACCC

ATCATTCACACCACTGTGCCATCTCCTACTCCAACACCCACTCCAAAGGAAAATCCCTAT

GTGGGAAACTATTCGGTTCGTGATGGTAATAGCACTTGTCTACTGGCTACCATGGGGCTG

CAGCTGAACATCACTCAAGATAAGGTTTCTTCAGTCATTAACATTAACCCCAATTCAACT

GGTGTCACTGGCAGCTGCCATCCTCAGACTGCTCAGCTTAGGCTAAACAGCAGCAACATT

AAGTATCTTGACTTCGTCTTTGCTGTCAAAAATGAAAACCGATTCTATCTGAAGGAAGTG

AATGTCAGCTTGAATTTGGCTAATGGCTCTGTTTTCAGCATTGCAAATAACAACCTTAGC

TACTGGGATGCTCCTCTGGGAAGTTCTTATATGTGCCACAAAGAGCAGACTGTTTCAGTA

TCTAGAACATTTCAGATAAATACCTTTGAATTAAGAGTTCAGCCTTTCAATGTGATGGAA

GGAAAGTATTCTACAGCTCAAGACTGCAGTGCAGATGACGACAACTTCCTTGTGCCCATA

GCGGTGGGAGCAGCCTTGGCAGGAGTACTTATTCTAGTGTTGCTGGCTTATTTTATTGGT

CTCAAGCGTCATCATGCTGGATATGAGCAATTT

>CL69.Contig2_All 1 306 minus strand PREDICTED: lysosome-associated membrane glycoprotein 2 isoform 4 [Canis lupus familiaris]

TCTGTTTTCAGCATTGCAAATAACAACCTTAGCTACTGGGATGCTCCTCTGGGAAGTTCT

TATATGTGCCACAAAGAGCAGACTGTTTCAGTATCTAGAACATTTCAGATAAATACCTTT

GAATTAAGAGTTCAGCCTTTCAATGTGATGGAAGGAAAGTATTCTACAGCTCAAGACTGC

AGTGCAGATGACGACAACTTCCTTGTGCCCATAGCGGTGGGAGCAGCCTTGGCAGGAGTA

CTTATTCTAGTGTTGCTGGCTTATTTTATTGGTCTCAAGCGTCATCATGCTGGATATGAG

CAATTT

>CL70.Contig1_All 1 504 minus strand PREDICTED: glutamate carboxypeptidase 2-like isoform 1 [Ailuropoda melanoleuca]

ATGGTGTTTGAATTGGCCAATTCCATAGTGCTTCCTTTTGACTGTCGAGATTATGCTGTC

GTTTTAAAAAAGTATGCTGACAAAATCTACGATATTTCAATGAAACATCCACAGGAAATG

AAAACATACAGTGTATCATTTGATGCACTGTTTTCTGCAGTAAAGAATTTTACAGAAATT

GCTTCTAAGTTCAGCAAGAGACTTCAAGATTTAGACAAAAGCAACCTAATATTATTGAGG

ATTATGAATGATCAGCTGATGTTTCTGGAACGGGCATTTATTGATCCTTTAGGGTTACCA

GACAGGCCTTTCTATAGGCATGTCATCTATGCTCCAAGCAGCCACAACAAGTATGCAGGG

GAATCATTCCCAGGAATTTATGATGCTTTGTTTGATATTGAAAGCAAAGTGGACCCTTCC

AAGGCCTGGGAAGAAGTGAAGAGACAGATTTCGATTGCAGCCTTCACCGTGCAGGCTGCA

GCAGGGACTTTGAGAGAAGTAGCC

>CL70.Contig2_All 1 411 minus strand PREDICTED: glutamate carboxypeptidase 2-like isoform 2 [Ailuropoda melanoleuca]

ATGGTGTTTGAATTGGCCAATTCCATAGTGCTTCCTTTTGACTGTCGAGATTATGCTGTC

GTTTTAAAAAAGTATGCTGACAAAATCTACGATATTTCAATGAAACATCCACAGGAAATG

AAAACATACAGTGTATCATTTGATGCACTGTTTTCTGCAGTAAAGAATTTTACAGAAATT

GCTTCTAAGTTCAGCAAGAGACTTCAAGATTTAGACAAAAGCAAGCATGTCATCTATGCT

CCAAGCAGCCACAACAAGTATGCAGGGGAATCATTCCCAGGAATTTATGATGCTTTGTTT

GATATTGAAAGCAAAGTGGACCCTTCCAAGGCCTGGGAAGAAGTGAAGAGACAGATTTCG

ATTGCAGCCTTCACCGTGCAGGCTGCAGCAGGGACTTTGAGAGAAGTAGCC

>CL71.Contig1_All 96 746 minus strand KIAA0677 protein [Homo sapiens]

CAAAAAGGAGGGAAAATGGCTTCTGAATCTGAAACCCTGAATCCAAGTGCTCGGATAATG

ACCTTTTATCCAACCATGGAAGAATTCCGGAACTTCAGTCGATACATTGCCTACATTGAA

TCCCAAGGAGCTCATCGTGCTGGACTAGCTAAGGTTGTTCCTCCAAAAGAGTGGAAGCCA

CGAGCATCCTATGATGACATTGATGATCTGGTCATCCCTGCACCCATCCAGCAACTGGTA

ACAGGGCAGTCTGGCCTCTTTACTCAGTACAACATTCAGAAGAAAGCCATGACTGTTCGA

GAGTTCCGCAAGATTGCTAATAGTGATAAGTACTGTACCCCACGGTATAGTGAGTTTGAA

GAGCTTGAGCGGAAATACTGGAAAAATCTCACATTCAACCCTCCAATTTATGGCGCAGAT

GTGAATGGTACTCTCTATGAAAAGCATATTGATGAGTGGAACATTGGCCGGCTGAAGACC

ATTTTGGATTTGGTTGAAAAGGAGAGTGGGATCACCATTGAGGGTGTGAACACCCCATAC

CTGTACTTTGGCATGTGGAAGACTTCCTTTGCCTGGCATACAGAAGACATGGACCTGTAC

AGCATCAATTACCTGCACTTCGGAGAGCCAAAGTCCTGGTACTCTGTTCCA

>CL72.Contig1_All 2 496 minus strand PREDICTED: guanine nucleotide-binding protein subunit alpha-11 [Saimiri boliviensis boliviensis]

CTCTTCCTCAACAAGAAGGACCTGCTGGAGGACAAGATCCTCTGCTCCCACCTGGTGGAC

TACTTCCCCGAGTTCGACGGGCCGCAGCGGGACGCGCAGGCCGCCCGGGAGTTCATCCTG

AAGATGTTCGTGGACCTGAACCCCGACAGCGACAAGATCATCTACTCGCACTTCACCTGC

GCCACGGACACGGAGAACATCCGCTTCGTGTTCGCGGCCGTCAAGGACACCATCCTGCAG

CTGAACCTCAAGGAGTACAACCTGGTGTGAGCCCGGCCGCCCGGCCACGGATGCCCCACG

CCTCCCACTCCCTCGCGCCCTCCAGGGCCACGGCAGCCTTTTTCTGCCTTGATCTGTGGC

TCGGTTTTTTTCTAAAAAAAAGAAAAGAGAAGAATAAACAAAAAACAGATCGGAGGCAGG

TGGCCGAGAGCCGGCGCCCCAGGCCCTGAGCGCCCGCGACCCTGGGCTCCCACAGGGAGG

TGTGGGGTCCTGGGC

>CL72.Contig2_All 2 265 Guanine nucleotide-binding protein subunit alpha-11 [Heterocephalus glaber]

CTCTTCCTCAACAAGAAGGACCTGCTGGAGGACAAGATCCTCTGCTCCCACCTGGTGGAC

TACTTCCCCGAGTTCGACGGGCCGCAGCGGGACGCGCAGGCCGCCCGGGAGTTCATCCTG

AAGATGTTCGTGGACCTGAACCCCGACAGCGACAAGATCATCTACTCGCACTTCACCTGC

GCCACGGACACGGAGAACATCCGCTTCGTGTTCGCGGCCGTCAAGGACACCATCCTGCAG

CTGAACCTCAAGGAGTACAACCTG

>CL73.Contig1_All 87 785 PREDICTED: COMM domain-containing protein 5 isoform 3 [Papio anubis]

TGGCTGATCAAGGAGGAGGAAGCAGCGATGTCTGCCTTAGGGACTGCAGCTCCATACCTG

CATCATCCTGCTGACAGTCACAGTGGCCGGGTCAGTTTTCTGGGGTCCCAAGTCCCTCCA

GAGGTGGCAGCAATGGCCCGGTTTCTGGGAGACCTGGACAGGAGCACATTCAGAAAGTTG

CTGAAGCTTGTGGTTAGCAGCTTGCAGGGAGAGGACTGCCGAGAGGCCATGCAGCACCTG

GGTGCCAGTTCCAGCCTGCCAGAGGAGCAGCTAGGTGCCCTACTTGCTGGCACACACACA

CTACTCCAGCAGGCTCTCCGGCTTCCCTCTCCCAGCCTGAAGCCTGATGCCTTCAAGGAC

CAACTCCAGGAGCTCGGCATCCCCCAAGACCTGGTTGGGGACTTGACCAGTGTGGTTTTT

GGTGGTCAGCGCCCCCTCCTGGACTCTGTGGCCCAGCAGCAGGGTTCCTGGCTTCCCCAC

ATTGCTGACTTTAAGTGGAGGGTGGATGTGGCAATTTCCACCAGTGCCCTGGCCCGCTCC

CTGAAGCCAAGCATCTTGATGCAGCTGAAGCTTTCAGATGGGTCAGCATATCGCTTCGAG

GTTCCCACAGCCAAGTTCCAGGAACTGCGGTACAGTGTGGCGCTGGTCCTGAAGGAGATG

ACAGATCTGGAGAGGAGGTGCGATCGCAGACTGCAGGAC

>CL73.Contig2_All 132 830 minus strand PREDICTED: COMM domain-containing protein 5 isoform 3 [Papio anubis]

TGGCTGATCAAGGAGGAGGAAGCAGCGATGTCTGCCTTAGGGACTGCAGCTCCATACCTG

CATCATCCTGCTGACAGTCACAGTGGCCGGGTCAGTTTTCTGGGGTCCCAAGTCCCTCCA

GAGGTGGCAGCAATGGCCCGGTTTCTGGGAGACCTGGACAGGAGCACATTCAGAAAGTTG

CTGAAGCTTGTGGTTAGCAGCTTGCAGGGAGAGGACTGCCGAGAGGCCATGCAGCACCTG

GGTGCCAGTTCCAGCCTGCCAGAGGAGCAGCTAGGTGCCCTACTTGCTGGCACACACACA

CTACTCCAGCAGGCTCTCCGGCTTCCCTCTCCCAGCCTGAAGCCTGATGCCTTCAAGGAC

CAACTCCAGGAGCTCGGCATCCCCCAAGACCTGGTTGGGGACTTGACCAGTGTGGTTTTT

GGTGGTCAGCGCCCCCTCCTGGACTCTGTGGCCCAGCAGCAGGGTTCCTGGCTTCCCCAC

ATTGCTGACTTTAAGTGGAGGGTGGATGTGGCAATTTCCACCAGTGCCCTGGCCCGCTCC

CTGAAGCCAAGCATCTTGATGCAGCTGAAGCTTTCAGATGGGTCAGCATATCGCTTCGAG

GTTCCCACAGCCAAGTTCCAGGAACTGCGGTACAGTGTGGCGCTGGTCCTGAAGGAGATG

ACAGATCTGGAGAGGAGGTGCGATCGCAGACTGCAGGAC

>CL73.Contig3_All 57 425 PREDICTED: COMM domain-containing protein 5 isoform 4 [Pan troglodytes] >gi|114622261|ref|XP_001160190.1| PREDICTED: COMM domain-containing protein 5 isoform 1 [Pan troglodytes] >gi|114622263|ref|XP_001160239.1| PREDICTED: COMM domain-containing protein 5 isoform 2 [Pan troglodytes] >gi|114622265|ref|XP_001160283.1| PREDICTED: COMM domain-containing protein 5 isoform 3 [Pan troglodytes]

TCCAGCCTGAAGCCTGATGCCTTCAAGGACCAACTCCAGGAGCTCGGCATCCCCCAAGAC

CTGGTTGGGGACTTGACCAGTGTGGTTTTTGGTGGTCAGCGCCCCCTCCTGGACTCTGTG

GCCCAGCAGCAGGGTTCCTGGCTTCCCCACATTGCTGACTTTAAGTGGAGGGTGGATGTG

GCAATTTCCACCAGTGCCCTGGCCCGCTCCCTGAAGCCAAGCATCTTGATGCAGCTGAAG

CTTTCAGATGGGTCAGCATATCGCTTCGAGGTTCCCACAGCCAAGTTCCAGGAACTGCGG

TACAGTGTGGCGCTGGTCCTGAAGGAGATGACAGATCTGGAGAGGAGGTGCGATCGCAGA

CTGCAGGAC

>CL74.Contig1_All 3 425 PREDICTED: transcription initiation factor TFIID subunit 1 [Papio anubis]

CAGGCCCGCTCTGGAGAGGGGCCCATGAGTAAATTTGCGCGTGGATCAAGGTTTTCTGTG

GCTGAGCATCAAGAACGTTACAAAGAGGAATGTCAGCGCATCTTTGACCTACAAAATAAA

GTTTTGTCATCAACTGAAGTCTTATCAACTGACACGGACAGTAGCTCAGCTGAAGACAGT

GACTTTGAAGAAATGGGAAAGAATATTGAGAACATGTTACAGAACAAGAAAACCAGCTCT

CAACTGTCACGTGAGCGGGAGGAGCAGGAGCGGAAGGAACTACAGCGGATGCTACTGGCA

GCAGGCTCAGCAGCATCAGGAAACAATCACAGAGATGATGACACTGCTTCTGTGACTAGC

CTTAACTCTTCTGCCACTGGCCGCTGTCTCAAGATTTATCGTACATTTCGAGATGAAGAG

GGG

>CL74.Contig2_All 3 320 PREDICTED: LOW QUALITY PROTEIN: transcription initiation factor TFIID subunit 1-like [Macaca mulatta]

CAGGCCCGCTCTGGAGAGGGGCCCATGAGTAAATTTGCGCGTGGATCAAGGTTTTCTGTG

GCTGAGCATCAAGAACGTTACAAAGAGGAATGTCAGCGCATCTTTGACCTACAAAATAAA

GTTTTGTCATCAACTGAAGTCTTATCAACTGACACGGACAGTAGCTCAGCTGAAGACAGT

GACTTTGAAGAAATGGGAAAGAATATTGAGAACATGTTACAGAACAAGAAAACCAGCTCT

CAACTGTCACGTGAGCGGGAGGAGCAGGAGCGGAAGGAACTACAGCGGATGCTACTGGGT

GAGGGTAGTGGCTTCACA

>CL75.Contig1_All 714 1106 interferon alpha responsive protein isoform a [Homo sapiens] >gi|197099768|ref|NP_001125531.1| interferon responsive gene 15 [Pongo abelii] >gi|387763497|ref|NP_001248560.1| interferon alpha responsive protein isoform a [Macaca mulatta] >gi|10862828|emb|CAC13974.1| IFRG15 protein [Homo sapiens] >gi|68532429|gb|AAH98348.1| Interferon responsive gene 15 [Homo sapiens] >gi|68532431|gb|AAH96704.1| Interferon responsive gene 15 [Homo sapiens] >gi|68532563|gb|AAH98170.1| Interferon responsive gene 15 [Homo sapiens] >gi|68532577|gb|AAH98309.1| Interferon responsive gene 15 [Homo sapiens] >gi|119611471|gb|EAW91065.1| interferon responsive gene 15 [Homo sapiens] >gi|380785693|gb|AFE64722.1| interferon alpha responsive protein isoform a [Macaca mulatta] >gi|383409841|gb|AFH28134.1| interferon alpha responsive protein isoform a [Macaca mulatta] >gi|384943124|gb|AFI35167.1| interferon alpha responsive protein isoform a [Macaca mulatta] >gi|384943126|gb|AFI35168.1| interferon alpha responsive protein isoform a [Macaca mulatta]

ATGTTCTCCGATAATTCACATTGCCCTGATTGTGGGCAGCAGTGGTTCCCTAGTTTAGAA

CTAGGCCATTGGTTGTACCAAACCGAACTTGTTGAAAATGAATGCTACCAAGTATTCTTA

GACCGTATTAACAGAGCTGATTATTGCCCAGAATGCTATCCTGATAATCCTGCTAATAGA

AGCCTTGTTCTTCCTTGGTCTTTCCCACTTGAGTGGGCTCCCCAAAATCTTACCAGGTGG

ACCTTTGAAAAAGCCTGCCATCCATTTCTTCTGGGTCCTCCACTGGTTAGAAAAAGGATA

CATGACTCCAGAGTAGCTGGTTTTAACCCTGCATTACAGTTAATCTTGACCAGAACAGAT

AAAACCTTAAACAAAAAACTTGGCCAAAATAAA

>CL75.Contig2_All 872 1264 interferon alpha responsive protein isoform a [Homo sapiens] >gi|197099768|ref|NP_001125531.1| interferon responsive gene 15 [Pongo abelii] >gi|387763497|ref|NP_001248560.1| interferon alpha responsive protein isoform a [Macaca mulatta] >gi|10862828|emb|CAC13974.1| IFRG15 protein [Homo sapiens] >gi|68532429|gb|AAH98348.1| Interferon responsive gene 15 [Homo sapiens] >gi|68532431|gb|AAH96704.1| Interferon responsive gene 15 [Homo sapiens] >gi|68532563|gb|AAH98170.1| Interferon responsive gene 15 [Homo sapiens] >gi|68532577|gb|AAH98309.1| Interferon responsive gene 15 [Homo sapiens] >gi|119611471|gb|EAW91065.1| interferon responsive gene 15 [Homo sapiens] >gi|380785693|gb|AFE64722.1| interferon alpha responsive protein isoform a [Macaca mulatta] >gi|383409841|gb|AFH28134.1| interferon alpha responsive protein isoform a [Macaca mulatta] >gi|384943124|gb|AFI35167.1| interferon alpha responsive protein isoform a [Macaca mulatta] >gi|384943126|gb|AFI35168.1| interferon alpha responsive protein isoform a [Macaca mulatta]

ATGTTCTCCGATAATTCACATTGCCCTGATTGTGGGCAGCAGTGGTTCCCTAGTTTAGAA

CTAGGCCATTGGTTGTACCAAACCGAACTTGTTGAAAATGAATGCTACCAAGTATTCTTA

GACCGTATTAACAGAGCTGATTATTGCCCAGAATGCTATCCTGATAATCCTGCTAATAGA

AGCCTTGTTCTTCCTTGGTCTTTCCCACTTGAGTGGGCTCCCCAAAATCTTACCAGGTGG

ACCTTTGAAAAAGCCTGCCATCCATTTCTTCTGGGTCCTCCACTGGTTAGAAAAAGGATA

CATGACTCCAGAGTAGCTGGTTTTAACCCTGCATTACAGTTAATCTTGACCAGAACAGAT

AAAACCTTAAACAAAAAACTTGGCCAAAATAAA

>CL76.Contig1_All 664 780 minus strand PREDICTED: CST complex subunit TEN1-like [Equus caballus]

GGCGGTTACGTGGTGAAGGCCCGGCTGCTGACCTGTGTGGAAGGGGTAAATCTGCCTTCC

TTAGAACAAGCCATCCTGGAGCAGCGGCTGTACCAGCAAGAGAGAGGCAGCAGCCAA

>CL76.Contig2_All 222 587 minus strand PREDICTED: CST complex subunit TEN1-like [Equus caballus]

ATGCTGCCCAAACCTGGGATCTATTACTTCCCCTGGGAGGTCAGTGCTGGCCAGGTTCCT

GATGGGGGCACCCTGAGAACATTTGGCAGGTTGTGCCTCTATGACGTGACCCAGTCCAGG

GTGACCCTGAGGGCTCAGCACGGATCTGATCAGCACCAGGTTCTTGTCTGTACCAAGTTG

GTGGAGCCGTTCCAGGCCCAGGTGGGCTCCTTGTACGTGGTCCTTGGGGAGCTCGAGCAA

GAGGACGATGGCGGTTACGTGGTGAAGGCCCGGCTGCTGACCTGTGTGGAAGGGGTAAAT

CTGCCTTCCTTAGAACAAGCCATCCTGGAGCAGCGGCTGTACCAGCAAGAGAGAGGCAGC

AGCCAA

>CL76.Contig3_All 450 815 minus strand PREDICTED: CST complex subunit TEN1-like [Equus caballus]

ATGCTGCCCAAACCTGGGATCTATTACTTCCCCTGGGAGGTCAGTGCTGGCCAGGTTCCT

GATGGGGGCACCCTGAGAACATTTGGCAGGTTGTGCCTCTATGACGTGACCCAGTCCAGG

GTGACCCTGAGGGCTCAGCACGGATCTGATCAGCACCAGGTTCTTGTCTGTACCAAGTTG

GTGGAGCCGTTCCAGGCCCAGGTGGGCTCCTTGTACGTGGTCCTTGGGGAGCTCGAGCAA

GAGGACGATGGCGGTTACGTGGTGAAGGCCCGGCTGCTGACCTGTGTGGAAGGGGTAAAT

CTGCCTTCCTTAGAACAAGCCATCCTGGAGCAGCGGCTGTACCAGCAAGAGAGAGGCAGC

AGCCAA

>CL78.Contig1_All 177 3242 minus strand PREDICTED: LOW QUALITY PROTEIN: uncharacterized protein C15orf39 homolog [Equus caballus]

ATGGCAGAGAAGCGCCCACTGGGGACTCTGGGGCCTGTGATATATGGCAAACTGCCCCGC

CTGGAAGCAGACTCAGGGCCTGGACATAGCCTGCCCCCCTCTGCTGGTAACCAGGACCCC

TGCAACTACAAGGGCGCTTACTTCTCCTGTCCCATGGGGGGCACTTCCAAGGCAGGATCT

GAGCGGTTGGCTTCCTGGACCCCATACTCACCCTTGTACTCCACAAGCGTAGCAGGACCT

CCACTTCGGCCAGACAGCCTGTTTACCAACTGCCTGCTGTACCGCCCACCAGCAGAAGGT

TCTGAGAAGATGCAGGACTCCAGCCCTGTTGAGCTCCTGCCGTTCAACCCCCAGTCTCAC

ACATACCCAGGCCCACCGCTGGCTGCACCCAAACCCGTCTACCGCAGCCCCCTGTGTTAT

GGGCTCTCAACTTGCCTGGGGGAGGGGCCAGCTAAGAGGCCACTGGATGTTGACTGGACC

CTGGTGACTGGGCCCCTGTTGTCCTCTGCTGACCCACCCTGTTCTCTGGGCCCAGCTCCT

GGGAAGGGCCAGGCCCTGGACAGCACCTTTTTACGTGGAGTGCCAGCTGGGGGATCTGGC

AAAGACCCCTCAGTTAGTTTCTCCTCATGCCAGGCGTTCCTGGAAAAGTATCGGACCATC

CACAGCACAGGCTTTCTGGCCTCTAAGTACACAGGTCCTTACTCTGGGGACTCCAAACAA

GCAATGGTAGAGGGGCCTTCAAGTCCTTGGACACAGCTGGCTCAGCCCCTGGGGCCATCG

TGCCAGGAGGTATTGCCTACCCACTACCCACTCCCTCCCCCTCCACAGGCCCTGCCTTGC

CCCCCAGCTTGTCGTCACCCAGAGAAGCAGGGCAGCTACAGCTCTGTGATCCCACTGCAG

CCTCTGGGAGTCCATAAGGGGACTGGATACCAGGTTGGTGGGCTGGGCAGCCCCTACCTG

AGGCAGCAGGCAGCCCAGACTCCATACATACCCCCAGTGGGACTGGACACTTATTCCTAC

CCCTCTGCCCCACTCCCGGCACCCTCACCAGGCCTCAAGCTGGAGCCTCCTCTTGCTCCA

CGGTGCCCACTGGACTTTGCCCCGCAGTCTCTGGGGTTTCCGTATGCCCGGGATGACCTG

TCTCTCTATGGGGCATCCCCTGGGCTTGGAGGGACACCACCTTCCCAGAACAGTGTGCAG

TCTGTGCCACAGCCCAGTGCCTTCCAGCGGGCATGCCAGCCTTTGCCAGCCAGTCAGCCA

TGCTCTGAGCCTACAAGGCCTGCTGAAAAGCCAGCACAGGAAACCGAAGAAAGGACATGG

CTGCCCAGCTGCAGGAAAGAGCAGCTCCAGACCCCGCTCAGCGAACACTCTGGAGCTCCC

ATTGTCATCCGAGATAGTCCAGTGCCCCGCACCTCACCAGCATTGCACCCCTGTGCCCAA

GAGCACCAGTCTGTCCCACAGAAGGAGGGTGCTAGACCGCCTGGCTCTCCACCAATGCCT

GTCATCGACAATGTCTTCAGCCTGGCCCCCTACCGTGACTATCTCGATGTGCAGACGCCA

GAGGCCACAGCTGAGCCAGATTCAGCCCCAGCTACCAGTGAAAGCCAAGACAAAGACTGC

AGTGGGACTTTAACTACCCAGGAGGGATCCTCAGAGCCTTGCTGCTCGCTTCGGGAGGAG

GTGGCCTTGGACTTGAGTGTGAAGAAGCCCACAGCAGAGGCTGCTCCCATCCAAGTCCCT

GGTCCTACAGTGCAAACCAAGCCCACTGCAACCGCGGACACGCCAGACACAGGGAACATA

GGTTCAGGTCTGTCAGGGCTGAAAAAGATGGTCACAGAAGCACCTGGGTTGCCAGTGGCC

ACAGAGGCCACACCAAGGACCAACTTTCACAGCTCTGTGGCCTTCATGTTCCGAAAATTC

AAGATTCTCCGTCCATCACCCTTACCTGCAGCTTCAGACCCAGCCACACCCACCTCAGCT

CCTGCCTCTGTACAGCCTGCACTCACCCCACCATCTGTACCCATGGGACTGCAGATTCTC

ACTCAGCCCTTGCCAGTGGCATGCTTCAACCTGGCCCTGCCTAGCCCTCCAGCCATAGCT

GTGGCGGCCCCAGCCTCGACTCTAGCCCCTGCCCCTGCCCCTGCTCCTGCTCCTGCCCCA

GCTCCTGCTCTAGTTCCAGCTCCAGCTCCTACTGTAGCCCCAGAAGACACCCCAGAGCAG

CGCTTTACAGGGCTGCATGCATCCCTGTGTGATGCCATCTCAGGCTCTGTGGCCCACTCC

CCACCAGAGAAGCTGCGGGAGTGGCTTGAGATGGCCGGACCGTGGGGCCGGGCTGCATGG

CAGGACTGCCAGGGAGTGCAGGGACTGCTCAGCAAGCTGCTGTCCCAGCTGCAGAGGTTT

GTATGCACGCAACAGTGCCCCTTTCCCCACGTAGTGCGAGCAGGTGCCATCTTCGTGCCC

ATCCACCTGGTGAAGGAGCGGCTCTTCCCACGGCTGCCCCCTGCCTCTGTGGACCATGTG

CTGCAGGAGCACCGTGTGGAGCTGCGGCCCACTACACTGTCAGAGGAGCGTGCGCTGCGG

GAGCGTGCCCTGCATGGCTGCACCTCACGCATGCTGAAGCTGCTGGCGCTGCGCCAGCTG

CCTGACATCTACCCTGATCTGCTGGGCCTACAGTGGCGAGACTGCGTGCGCCGCCAGCTG

GGTGACTTTGACACTGAGGCTGGAGCTATTCCCACCTCAGAACCCACTGTGGCCAGAGAT

GAGCCAGAGAGTATAGCCCAGGCTCGGAAGTCACCAGCCCCCAAGGTCAGGAAACCAGGG

CGGAAGCCACCAACCCCTGGCCTGGAGAAAGCAGAGGCAACTGCTGGGGTTGGCTCACGT

GGTCCCTCACCTACTCCTGCCAACAGCACCGGCCCACCTGGCCCCACGCTGAAGGCCCGC

TTCCGAAGCCTTCTGGAAACTGCCTGGCTCAATGGCCTAGCATTGCCCACGTGGGGCCAC

AAGGCCTCAGGACCTGACCGGCCCCCACCCTGCCCACAGCTACTGGGCAGCCAGAGCCAT

CACCTG

>CL78.Contig2_All 1315 1689 minus strand PREDICTED: LOW QUALITY PROTEIN: uncharacterized protein C15orf39 homolog [Equus caballus]

CGAGTGACAGGTGACTTTGACACTGAGGCTGGAGCTATTCCCACCTCAGAACCCACTGTG

GCCAGAGATGAGCCAGAGAGTATAGCCCAGGCTCGGAAGTCACCAGCCCCCAAGGTCAGG

AAACCAGGGCGGAAGCCACCAACCCCTGGCCTGGAGAAAGCAGAGGCAACTGCTGGGGTT

GGCTCACGTGGTCCCTCACCTACTCCTGCCAACAGCACCGGCCCACCTGGCCCCACGCTG

AAGGCCCGCTTCCGAAGCCTTCTGGAAACTGCCTGGCTCAATGGCCTAGCATTGCCCACG

TGGGGCCACAAGGCCTCAGGACCTGACCGGCCCCCACCCTGCCCACAGCTACTGGGCAGC

CAGAGCCATCACCTG

>CL78.Contig3_All 209 3280 minus strand PREDICTED: LOW QUALITY PROTEIN: uncharacterized protein C15orf39 homolog [Equus caballus]

ATGGCAGAGAAGCGCCCACTGGGGACTCTGGGGCCTGTGATATATGGCAAACTGCCCCGC

CTGGAAGCAGACTCAGGGCCTGGACATAGCCTGCCCCCCTCTGCTGGTAACCAGGACCCC

TGCAACTACAAGGGCGCTTACTTCTCCTGTCCCATGGGGGGCACTTCCAAGGCAGGATCT

GAGCGGTTGGCTTCCTGGACCCCATACTCACCCTTGTACTCCACAAGCGTAGCAGGACCT

CCACTTCGGCCAGACAGCCTGTTTACCAACTGCCTGCTGTACCGCCCACCAGCAGAAGGT

TCTGAGAAGATGCAGGACTCCAGCCCTGTTGAGCTCCTGCCGTTCAACCCCCAGTCTCAC

ACATACCCAGGCCCACCGCTGGCTGCACCCAAACCCGTCTACCGCAGCCCCCTGTGTTAT

GGGCTCTCAACTTGCCTGGGGGAGGGGCCAGCTAAGAGGCCACTGGATGTTGACTGGACC

CTGGTGACTGGGCCCCTGTTGTCCTCTGCTGACCCACCCTGTTCTCTGGGCCCAGCTCCT

GGGAAGGGCCAGGCCCTGGACAGCACCTTTTTACGTGGAGTGCCAGCTGGGGGATCTGGC

AAAGACCCCTCAGTTAGTTTCTCCTCATGCCAGGCGTTCCTGGAAAAGTATCGGACCATC

CACAGCACAGGCTTTCTGGCCTCTAAGTACACAGGTCCTTACTCTGGGGACTCCAAACAA

GCAATGGTAGAGGGGCCTTCAAGTCCTTGGACACAGCTGGCTCAGCCCCTGGGGCCATCG

TGCCAGGAGGTATTGCCTACCCACTACCCACTCCCTCCCCCTCCACAGGCCCTGCCTTGC

CCCCCAGCTTGTCGTCACCCAGAGAAGCAGGGCAGCTACAGCTCTGTGATCCCACTGCAG

CCTCTGGGAGTCCATAAGGGGACTGGATACCAGGTTGGTGGGCTGGGCAGCCCCTACCTG

AGGCAGCAGGCAGCCCAGACTCCATACATACCCCCAGTGGGACTGGACACTTATTCCTAC

CCCTCTGCCCCACTCCCGGCACCCTCACCAGGCCTCAAGCTGGAGCCTCCTCTTGCTCCA

CGGTGCCCACTGGACTTTGCCCCGCAGTCTCTGGGGTTTCCGTATGCCCGGGATGACCTG

TCTCTCTATGGGGCATCCCCTGGGCTTGGAGGGACACCACCTTCCCAGAACAGTGTGCAG

TCTGTGCCACAGCCCAGTGCCTTCCAGCGGGCATGCCAGCCTTTGCCAGCCAGTCAGCCA

TGCTCTGAGCCTACAAGGCCTGCTGAAAAGCCAGCACAGGAAACCGAAGAAAGGACATGG

CTGCCCAGCTGCAGGAAAGAGCAGCTCCAGACCCCGCTCAGCGAACACTCTGGAGCTCCC

ATTGTCATCCGAGATAGTCCAGTGCCCCGCACCTCACCAGCATTGCACCCCTGTGCCCAA

GAGCACCAGTCTGTCCCACAGAAGGAGGGTGCTAGACCGCCTGGCTCTCCACCAATGCCT

GTCATCGACAATGTCTTCAGCCTGGCCCCCTACCGTGACTATCTCGATGTGCAGACGCCA

GAGGCCACAGCTGAGCCAGATTCAGCCCCAGCTACCAGTGAAAGCCAAGACAAAGACTGC

AGTGGGACTTTAACTACCCAGGAGGGATCCTCAGAGCCTTGCTGCTCGCTTCGGGAGGAG

GTGGCCTTGGACTTGAGTGTGAAGAAGCCCACAGCAGAGGCTGCTCCCATCCAAGTCCCT

GGTCCTACAGTGCAAACCAAGCCCACTGCAACCGCGGACACGCCAGACACAGGGAACATA

GGTTCAGGTCTGTCAGGGCTGAAAAAGATGGTCACAGAAGCACCTGGGTTGCCAGTGGCC

ACAGAGGCCACACCAAGGACCAACTTTCACAGCTCTGTGGCCTTCATGTTCCGAAAATTC

AAGATTCTCCGTCCATCACCCTTACCTGCAGCTTCAGACCCAGCCACACCCACCTCAGCT

CCTGCCTCTGTACAGCCTGCACTCACCCCACCATCTGTACCCATGGGACTGCAGATTCTC

ACTCAGCCCTTGCCAGTGGCATGCTTCAACCTGGCCCTGCCTAGCCCTCCAGCCATAGCT

GTGGCGGCCCCAGCCTCGACTCTAGCCCCTGCCCCTGCCCCTGCTCCTGCTCCTGCCCCA

GCTCCTGCTCTAGTTCCAGCTCCAGCTCCAGCTCCTACTGTAGCCCCAGAAGACACCCCA

GAGCAGCGCTTTACAGGGCTGCATGCATCCCTGTGTGATGCCATCTCAGGCTCTGTGGCC

CACTCCCCACCAGAGAAGCTGCGGGAGTGGCTTGAGATGGCCGGACCGTGGGGCCGGGCT

GCATGGCAGGACTGCCAGGGAGTGCAGGGACTGCTCAGCAAGCTGCTGTCCCAGCTGCAG

AGGTTTGTATGCACGCAACAGTGCCCCTTTCCCCACGTAGTGCGAGCAGGTGCCATCTTC

GTGCCCATCCACCTGGTGAAGGAGCGGCTCTTCCCACGGCTGCCCCCTGCCTCTGTGGAC

CATGTGCTGCAGGAGCACCGTGTGGAGCTGCGGCCCACTACACTGTCAGAGGAGCGTGCG

CTGCGGGAGCGTGCCCTGCATGGCTGCACCTCACGCATGCTGAAGCTGCTGGCGCTGCGC

CAGCTGCCTGACATCTACCCTGATCTGCTGGGCCTACAGTGGCGAGACTGCGTGCGCCGC

CAGCTGGGTGACTTTGACACTGAGGCTGGAGCTATTCCCACCTCAGAACCCACTGTGGCC

AGAGATGAGCCAGAGAGTATAGCCCAGGCTCGGAAGTCACCAGCCCCCAAGGTCAGGAAA

CCAGGGCGGAAGCCACCAACCCCTGGCCTGGAGAAAGCAGAGGCAACTGCTGGGGTTGGC

TCACGTGGTCCCTCACCTACTCCTGCCAACAGCACCGGCCCACCTGGCCCCACGCTGAAG

GCCCGCTTCCGAAGCCTTCTGGAAACTGCCTGGCTCAATGGCCTAGCATTGCCCACGTGG

GGCCACAAGGCCTCAGGACCTGACCGGCCCCCACCCTGCCCACAGCTACTGGGCAGCCAG

AGCCATCACCTG

>CL81.Contig1_All 3 2837 minus strand PREDICTED: proteasome (prosome, macropain) activator subunit 4 [Oryctolagus cuniculus]

GACCTTTTACAATTCCAAGGATCTCACAAGCATGAATTTGACTCTAGATGGAAAAGTTTT

AACTTAGTAAAGAAATCAATGGAAAATCGGCTTCATGGGAAAAAACAACATATCAGAGCA

CTATTGATTGACAGAGTAATGTTGCAGCATGAGCTACGAACACTAACTGTTGAGGGTTGT

GAGTACAAAAAGATACATCAAGATATGATCAGAGATCTTCTCCGATTATCTACAAGTTCA

TATAGTCAGGTCAGAAATAAGGCTCAGCAAACATTTTTTGCTGCTTTAGGAGCATATAAC

TTCTGTTGCAGAGATATTATTCCCTTGGTTTTGGAGTTTTTACGTCCCGACAGACAAGAT

GTCACACAACAGCAATTTAAGGGTGCTTTGTATTGTCTCCTTGGAAATCACAGTGGTGTG

TGTTTGGCAAACCTTCATGATTGGGACTGTATTGTGCAGACATGGCCAGCAATTGTTTCT

TCAGGGCTTAGCCAAGCAATGTCACTAGAAAAGCCATCAATTGTGAGACTGTTTGATGAT

CTTGCAGAAAAGATTCACAGGCAATATGAAACAATTGGCTTGGACTTCACAATTCCAAAG

TCATGTGTTGCAATAGCAGAATTACTTCAACAGTCACCGAATCCTTCTATCAACCAGTTA

TCACTTAGCCCAGAAAAAATTAAGGAAGGGCTTAAACGCCAACAAGAAAAGAATGCTGAT

GCTCTAAGGAACTATGAGAATTTGGTAAACACTTTACTGGATGGTGTGGAACAAAGAAAC

CTGCCCTGGAAATTTGAACATATAGGCATTGGACTTCTGTCTCTACTGTTGAGAGATGAT

CGAGTATTGCCTCTTCGTGCCATACGGTTTTTTGTTGAGAATCTCAACCATGATGCAATT

GTAGTTCGAAAGATGGCTATCTCAGCTGTTACTGGAATTCTCAAGCAGCTAAAGAGAACA

CACAAAAAATTAACTATCAACCCTTATGAAATCAGTGGATATCCTAAACCCACCCAAATT

CTTGCTGGTGATAGGCCTGATAATCATTGGCTGCATTATAATAGCAAAAGTATACCAAGA

ACTAAAAAAGAATGGGAGTCAAGTTGCTTTGTTGAAAAAACTCACTGGGGATACTATACT

TGGCCAAAGAATATGGTTGTTTATGCTGGTGTGGAAGAGCAGCCTAAGCTTGGCAGAAGC

AGGGAGGATATGACAGAGGCAGAACAGATTATATTTGATCATTTTTCTGATCCTAAATTT

GTTGAACAGTTAATTACTTTTCTATCTTTAGAAGACAGAAAAGGAAAAGATAAGTTTAAT

CCCCGACGTTTTTGCCTCTTTAAGGGTATATTTAGAAATTTTGATGATGCCTTTCTGCCA

GTTCTGAAGCCCCATTTAGAACGTTTGGTTGCAGATTCTCATGAAAGTACCCAGCGATGT

GTTGCAGAAATTATAGCTGGTTTAATCCGAGGTTCTAAGCATTGGACATTTGAAAAGGTG

GAGAAGCTTTGGGAGCTTCTGTGCCCTCTGCTTAGAACAGCTTTGTCCAACATTACAGTA

GAAACTTATAATGACTGGGGAACTTGCATAGCAACATCCTGTGAAAGCAGAGATCCTCGG

AAACTTCATTGGCTTTTTGAGCTCTTATTGGAATCACCATTGAGTGGTGAAGGAGGATCC

TTTGTAGATGCATGTCGACTTTATGTACTACAAGGTGGCCTTGCCCAGCAAGAGTGGAGA

GTGCCTGAATTATTGCACAGACTACTGAAGTACTTGGAACCCAAACTCACCCAGGTTTAC

AAAAATGTCAGGGAAAGAATAGGGAGTGTGCTGACCTACATATTCATGATAGATGTTTCT

TTGCCAAATACTGCGCCAACAACATCCCCTTGTATTCCTGAGTTTACTGCTCGAATTTTA

GAGAAATTAAAACCCCTTATGGATGTGGATGAAGAAATTCAGAACCATGTTATGGAAGAA

AATGGAATTGGTGAAGAAGATGAACGAACTCAGGGCATTAAACTCTTAAAAACTATATTG

AAGTGGCTGATGGCAAGTGCAGGCAGATCCTTCTCTACAGCAGTCACAGAACAACTTCAG

CTTCTGCCATTGTTTTTTAAGATTGCCCCGGTGGAAAATGACAATAGCTATGATGAACTG

AAAAGAGATGCAAAACTTTGTTTATCACTAATGTCTCAGGGGTTGCTTTACCCTCATCAA

GTGCCTTTGGTACTTCAGGTGCTAAACCAAACAGCAAGAAGCAGTTCTTGGCATGCGAGA

TATACAGTACTGACCTACCTCCAGACCATGGTGTTTTATAACCTCTTTATTTTCCTAAAC

AATGAAGATGCAGTTAAAGACATCAGGTGGCTTGTTATAAACCTTTTGGAGGATGAGCAA

TTAGAGGTTCGAGAAATGGCTGCTACCACCTTAAGTGGTCTGTTACAGTGTAACTTCCTT

ACCATGGACAGTCCTATGCAGATTCATTTTGAACAACTTTGCAAAACAAAACTACCTAAG

AAAAGAAAGCGAGACCCTGGTTCTGTAGGAGATACCATTCCTTCTGCAGAGTTGGTGAAA

CGCCATGCTGGGGTGCTAGGACTTGGTGCTTGTGTTCTTTCAAGCCCTTACAATGTTCCC

ACCTGGATGCCCCAGCTACTTATGAATCTCAGTGCACATTTGAATGATCCTCAGCCTATT

GAGATGACTGTAAAAAAGACCTTATCCAATTTTCGAAGGACACACCATGACAACTGGCAA

GAACACAAACAGCAATTCACTGATGACCAACTGCTTGTTCTCACGGATCTCCTTGTGTCA

CCATGCTATTATGCA

>CL81.Contig3_All 2 391 PREDICTED: proteasome activator complex subunit 4 [Saimiri boliviensis boliviensis]

GATCATTTTTCTGATCCTAAATTTGTTGAACAGTTAATTACTTTTCTATCTTTAGAAGAC

AGAAAAGGAAAAGATAAGTTTAATCCCCGACGTTTTTGCCTCTTTAAGGGTATATTTAGA

AATTTTGATGATGCCTTTCTGCCAGTTCTGAAGCCCCATTTAGAACGTTTGGTTGCAGAT

TCTCATGAAAGTACCCAGCGATGTGTTGCAGAAATTATAGCTGGTTTAATCCGAGGTTCT

AAGCATTGGACATTTGAAAAGGTGGAGAAGCTTTGGGAGCTTCTGTGCCCTCTGCTTAGA

ACAGCTTTGTCCAACATTACAGTAGAAACTTATAATGACTGGGGATCTCTGCTTTCACAG

GATGTTGCTATGCAAGTTCCCCAGTCATTA

>CL82.Contig1_All 1617 2111 PREDICTED: SAFB-like transcription modulator [Papio anubis]

AAGGGAAAAAAAGCACGACCAACTGCACGAAGAGAAGATCCAGGCTTTGAAAGGTATCCC

AAAAATTTCAGTGATTCCAGAAGAAATGAGCCTCCACCACCAAGAAACGAACTTAGAGAA

ACAGACAGACGAGAGGTACGAGGGGAACGAGATGAGAGGAGAACAGTGATCATTCATGAC

AGGCCTGATATTGCTCACACTAGACACCCTCGAGAAGCAGGGCCCAATCCTTCTAGACCA

ACCTCCTGGAAAAGTGAAGGAAGCATGTCTGCAGATAAACGTGAGGCAAGAGTTGAAAGG

CCGGAACGGTCCGGTAGAGAAGTATCAGGACACAGTGTGAGAGGCGCTCCCCCTGGAAAT

CGCAGTAGTGCCACAGGTTATGGAAGCAGAGAAGGAGACAGAGGAATCATCTCAGATCGA

GGGAGTGGAGCCCAGCACTATCCTGAAGAACGACATGTGGTTGAACGCCATGGACGGGAC

ACAAGTGGACCAAGG

>CL82.Contig2_All 62 1267 minus strand PREDICTED: SAFB-like transcription modulator [Pongo abelii]

ATGGCTGCCGCTACCGGTGCTGTGGCAGCCTCGGCTGCCCCGGGTCAGGCGGAAGGTAAA

AAGATCACCGATCTGCGGGTCATCGATCTCAAGTCCGAGCTGAAGCGGCGGAACTTAGAC

ATCACCGGAGTCAAGACTGTGCTGGTCTCCCGACTCAAGCAGGCTATTGAAGAGGAAGGA

GGCGATCCAGATAATATTGAATTAACTGTTTCAACTGATACTCCAAACAAGAAACCAACC

AAAGGCAAAGGTAAAAAACAAGAAGCAGATGAGTTGAGTGGAGATGCTTCTGTGGAAGAT

GATGCTTTTGTCAAGGAAATTGAAGCTCAAGAAGGTGAAGATGATACCTTTCTAACAGCC

CAAGATGGTGAGGAAGAAGAAAATGAGAAAGAAGGGAACCTAGCTGAGGCTGATCACACA

GCTCATGAAGAGATGGAAGCTAATGCGACTGTGAAAGAAGCTGAGGATGACAACATCTCG

GTCACAATCCAGGCTGAAGATGCCATCACTCTGGATTTTGATGGTGATGACCTCCTAGAA

ACAGGTAAAAATGTGAAAATTACAGATTCTGAAGCAAGTAAGCCAAAAGATGGGCAGGAC

GCCATTGCACAGAGCCCGGAGAAGGAAACCAAGGATTATGAGATGAATGCGAACCATAAA

GATGGTAAGAAGGAAGACTGCGTGAAGGGTGATCCTGTCGAGAAGGAAGCCAGAGAAAGT

TCTAAGAAAGCAGAATCTGGAGAAAAAGAAAAGGATACTTTGAAGAAAGGGCCCTCGTCT

ACTGGGGCCTCTGGTCAAGCAAAGAGCTCTTCAAAGGAATCTAAAGACAGCAAGACATCA

TCTAAAGATGACAAAGGAAGTACAAGTAGTACTAGTGGTAGCAGTGGAAGCTCAACTAAA

AATATCTGGGTTAGTGGACTTTCTTCTAATACCAAAGCTGCTGATTTGAAGAACCTCTTT

GGCAAATATGGAAAGGTTCTTAGTGCAAAGGTAGTTACAAATGCTCGAAGTCCTGGGGCA

AAATGCTATGGCATTGTAACTATGTCATCAAGCACAGAGGTGGCCAGATGTATTGCACAT

CTTCATCGCACTGAGCTGCATGGTCAGCTGATTTCTGTTGAAAAAGTAAAAGGTGATCCT

TCTAAGAAAGAAATGAAGAAAGAAAATGATGAAAAGAGTAGTTCAAGAAGTTCTGGAGAT

AAAAAA

>CL82.Contig3_All 2503 3003 SAFB-like, transcription modulator, isoform CRA_b [Homo sapiens] >gi|119597959|gb|EAW77553.1| SAFB-like, transcription modulator, isoform CRA_b [Homo sapiens]

AAGGGAAAAAAAGCACGACCAACTGCACGAAGAGAAGATCCAGGCTTTGAAAGGTATCCC

AAAAATTTCAGTGATTCCAGAAGAAATGAGCCTCCACCACCAAGAAACGAACTTAGAGAA

ACAGACAGACGAGAGGTACGAGGGGAACGAGATGAGAGGAGAACAGTGATCATTCATGAC

AGGCCTGATATTGCTCACACTAGACACCCTCGAGAAGCAGGGCCCAATCCTTCTAGACCA

ACCTCCTGGAAAAGTGAAGGAAGCATGTCTGCAGATAAACGTGAGGCAAGAGTTGAAAGG

CCGGAACGGTCCGGTAGAGAAGTATCAGGACACAGTGTGAGAGGCGCTCCCCCTGGAAAT

CGCAGTAGTGCCACAGGTTATGGAAGCAGAGAAGGAGACAGAGGAATCATCTCAGATCGA

GGGAGTGGAGCCCAGCACTATCCTGAAGAACGACATGTGGTTGAACGCCATGGACGGGAC

ACAAGTGGACCAAGGAAAGAG

>CL82.Contig4_All 3 2948 SAFB-like, transcription modulator, isoform CRA_b [Homo sapiens] >gi|119597959|gb|EAW77553.1| SAFB-like, transcription modulator, isoform CRA_b [Homo sapiens]

GCGCGGCGCCGCCGGCCGCCGAGGCCTGGGTGGAAGTTGGCGGTGTTGCAGCCGCCCTGC

AGCCCACTCGCTGCCTCGGCAGCGCGCTGCTCTTCTAAGATGGCTGCCGCTACCGGTGCT

GTGGCAGCCTCGGCTGCCCCGGGTCAGGCGGAAGGTAAAAAGATCACCGATCTGCGGGTC

ATCGATCTCAAGTCCGAGCTGAAGCGGCGGAACTTAGACATCACCGGAGTCAAGACTGTG

CTGGTCTCCCGACTCAAGCAGGCTATTGAAGAGGAAGGAGGCGATCCAGATAATATTGAA

TTAACTGTTTCAACTGATACTCCAAACAAGAAACCAACCAAAGGCAAAGGTAAAAAACAA

GAAGCAGATGAGTTGAGTGGAGATGCTTCTGTGGAAGATGATGCTTTTGTCAAGGACTGT

GAATTGGAGAATCAAGAGGCACATGAACAAGACGGAAATGATGAACTAAAGGACTCTGAA

GAATTTGGTGAAAATGAAGAAGACTGTGTGCATTCCAAGGAGTTACTTTCTGCAGAAGAA

AACAAGAGAGCTCATGAATTAATAGAGGCAGAAGCAATAGAAGATATAGAAAAAGAGGAC

ATCGAAAGTCAGGAAATTGAAGCTCAAGAAGGTGAAGATGATACCTTTCTAACAGCCCAA

GATGGTGAGGAAGAAGAAAATGAGAAAGAAGGGAACCTAGCTGAGGCTGATCACACAGCT

CATGAAGAGATGGAAGCTAATGCGACTGTGAAAGAAGCTGAGGATGACAACATCTCGGTC

ACAATCCAGGCTGAAGATGCCATCACTCTGGATTTTGATGGTGATGACCTCCTAGAAACA

GGTAAAAATGTGAAAATTACAGATTCTGAAGCAAGTAAGCCAAAAGATGGGCAGGACGCC

ATTGCACAGAGCCCGGAGAAGGAAACCAAGGATTATGAGATGAATGCGAACCATAAAGAT

GGTAAGAAGGAAGACTGCGTGAAGGGTGATCCTGTCGAGAAGGAAGCCAGAGAAAGTTCT

AAGAAAGCAGAATCTGGAGAAAAAGAAAAGGATACTTTGAAGAAAGGGCCCTCGTCTACT

GGGGCCTCTGGTCAAGCAAAGAGCTCTTCAAAGGAATCTAAAGACAGCAAGACATCATCT

AAAGATGACAAAGGAAGTACAAGTAGTACTAGTGGTAGCAGTGGAAGCTCAACTAAAAAT

ATCTGGGTTAGTGGACTTTCTTCTAATACCAAAGCTGCTGATTTGAAGAACCTCTTTGGC

AAATATGGAAAGGTTCTTAGTGCAAAGGTAGTTACAAATGCTCGAAGTCCTGGGGCAAAA

TGCTATGGCATTGTAACTATGTCATCAAGCACAGAGGTGGCCAGATGTATTGCACATCTT

CATCGCACTGAGCTGCATGGTCAGCTGATTTCTGTTGAAAAAGTAAAAGGTGATCCTTCT

AAGAAAGAAATGAAGAAAGAAAATGATGAAAAGAGTAGTTCAAGAAGTTCTGGAGATAAA

AAAAATATGAGTGATAGAAGTAGCAAGATACAAGCCTCTGTCAAAAAAGAAGAGAAAAGG

TCATCTGAAAAATCTGAAAAAAAAGAAAGCAAGGATGCTAAGAAAATAGAAGGTAAAGAT

GAGAAGAATGATAATGGAGGAAGTGGCCAAACTTCAGAATCGATTAAAAAAAGTGAAGAA

AAGAAGCGAATAAGTTCAAAGAGTCCAGGACACATGGTAATATTAGACAAAACTAAGGGA

GATCATTGTAGGCCATCAAGAAGAGGAAGATATGAAAAAATTCATGGAAGAAGCAAGGAA

AAGGAGAGAGCTAGCCTAGATAAAAAAAGGGACAAAGACTACAGAAGGAAAGAGATTTTG

CCTTTTGAAAAGATGAAGGAACAACGATTGAGAGAACATTTAGTTCGTTTTGAAAGACTG

CGACGAGCAATTTCCCTCCGAAGACGAAGAGAGATTGCAGAAAGAGAGCGTCGAGAGCGA

GAACGCATTAGAATAATTCGTGAACGGGAAGAACGGGAACGCTTACAGAGAGAGAGAGAG

CGCCTAGAAATTGAAAGGCAAAAACTAGAGAGAGAGAGAATGGAACGCGAACGCTTGGAA

AGGGAACGCATTCGTATTGAACAGGAACGTCGTAAGGAAGCTGAACGGATTGCTCGAGAA

CGAGAGGAACTCAGAAGGCAGCAGCAGCAGCTACGTTATGAACAAGAAAAAAGGAATTCT

TTGAAACGCCCACATGATGTAGATCATAGGCGAGATGACCCTTACTGGAGCGAGAATAAA

AAGTTGTCTCTAGATACAGATGCACGATTCAGTCATGGATCTGACTACTCTCGGCAACAG

AACAGATTTAATGACTTTGATCACCGGGAAAGGGGCAGGTTTCCCGAGAGTTCAGCTGTA

CAGTCTTCATCTTTTGAAAGGCGAGAACGCTTTGTAGGTCAAAGTGAAGGGAAAAAAGCA

CGACCAACTGCACGAAGAGAAGATCCAGGCTTTGAAAGGTATCCCAAAAATTTCAGTGAT

TCCAGAAGAAATGAGCCTCCACCACCAAGAAACGAACTTAGAGAAACAGACAGACGAGAG

GTACGAGGGGAACGAGATGAGAGGAGAACAGTGATCATTCATGACAGGCCTGATATTGCT

CACACTAGACACCCTCGAGAAGCAGGGCCCAATCCTTCTAGACCAACCTCCTGGAAAAGT

GAAGGAAGCATGTCTGCAGATAAACGTGAGGCAAGAGTTGAAAGGCCGGAACGGTCCGGT

AGAGAAGTATCAGGACACAGTGTGAGAGGCGCTCCCCCTGGAAATCGCAGTAGTGCCACA

GGTTATGGAAGCAGAGAAGGAGACAGAGGAATCATCTCAGATCGAGGGAGTGGAGCCCAG

CACTATCCTGAAGAACGACATGTGGTTGAACGCCATGGACGGGACACAAGTGGACCAAGG

AAAGAG

>CL82.Contig5_All 2 1873 minus strand PREDICTED: SAFB-like transcription modulator isoform 2 [Saimiri boliviensis boliviensis]

CGACTCAAGCAGGCTATTGAAGAGGAAGGAGGCGATCCAGATAATATTGAATTAACTGTT

TCAACTGATACTCCAAACAAGAAACCAACCAAAGGCAAAGGTAAAAAACAAGAAGCAGAT

GAGTTGAGTGGAGATGCTTCTGTGGAAGATGATGCTTTTGTCAAGGACTGTGAATTGGAG

AATCAAGAGGCACATGAACAAGACGGAAATGATGAACTAAAGGACTCTGAAGAATTTGGT

GAAAATGAAGAAGACTGTGTGCATTCCAAGGAGTTACTTTCTGCAGAAGAAAACAAGAGA

GCTCATGAATTAATAGAGGCAGAAGCAATAGAAGATATAGAAAAAGAGGACATCGAAAGT

CAGGAAATTGAAGCTCAAGAAGGTGAAGATGATACCTTTCTAACAGCCCAAGATGGTGAG

GAAGAAGAAAATGAGAAAGAAGGGAACCTAGCTGAGGCTGATCACACAGCTCATGAAGAG

ATGGAAGCTAATGCGACTGTGAAAGAAGCTGAGGATGACAACATCTCGGTCACAATCCAG

GCTGAAGATGCCATCACTCTGGATTTTGATGGTGATGACCTCCTAGAAACAGGTAAAAAT

GTGAAAATTACAGATTCTGAAGCAAGTAAGCCAAAAGATGGGCAGGACGCCATTGCACAG

AGCCCGGAGAAGGAAACCAAGGATTATGAGATGAATGCGAACCATAAAGATGGTAAGAAG

GAAGACTGCGTGAAGGGTGATCCTGTCGAGAAGGAAGCCAGAGAAAGTTCTAAGAAAGCA

GAATCTGGAGAAAAAGAAAAGGATACTTTGAAGAAAGGGCCCTCGTCTACTGGGGCCTCT

GGTCAAGCAAAGAGCTCTTCAAAGGAATCTAAAGACAGCAAGACATCATCTAAAGATGAC

AAAGGAAGTACAAGTAGTACTAGTGGTAGCAGTGGAAGCTCAACTAAAAATATCTGGGTT

AGTGGACTTTCTTCTAATACCAAAGCTGCTGATTTGAAGAACCTCTTTGGCAAATATGGA

AAGGTTCTTAGTGCAAAGGTAGTTACAAATGCTCGAAGTCCTGGGGCAAAATGCTATGGC

ATTGTAACTATGTCATCAAGCACAGAGGTGGCCAGATGTATTGCACATCTTCATCGCACT

GAGCTGCATGGTCAGCTGATTTCTGTTGAAAAAGTAAAAGGTGATCCTTCTAAGAAAGAA

ATGAAGAAAGAAAATGATGAAAAGAGTAGTTCAAGAAGTTCTGGAGATAAAAAAAATATG

AGTGATAGAAGTAGCAAGATACAAGCCTCTGTCAAAAAAAGAAGAGAAAAGGTCATCTGA

AAAATCTGAAAAAAAAGAAAGCAAGGATGCTAAGAAAATAGAAGGTAAAGATGAGAAGAA

TGATAATGGAGGAAGTGGCCAAACTTCAGAATCGATTAAAAAAAGTGAAGAAAAGAAGCG

AATAAGTTCAAAGAGTCCAGGACACATGGTAATATTAGACAAAACTAAGGGAGATCATTG

TAGGCCATCAAGAAGAGGAAGATATGAAAAAATTCATGGAAGAAGCAAGGAAAAGGAGAG

AGCTAGCCTAGATAAAAAAAGGGACAAAGACTACAGAAGGAAAGAGATTTTGCCTTTTGA

AAAGATGAAGGAACAACGATTGAGAGAACATTTAGTTCGTTTTGAAAGACTGCGACGAGC

AATTTCCCTCCGAAGGAACGTCGTAAGGAAGCTGAACGGATTGCTCGAGAACGAGAGGAA

CTCAGAAGGCAGCAGCAGCAGCTACGTTATGAACAAGAAAAAAGGAATTCTTTGAAACGC

CCACATGATGTAGATCATAGGCGAGATGACCCTTACTGGAGCGAGAATAAAAAGTTGTCT

CTAGATACAGAT

>CL82.Contig6_All 61 837 minus strand PREDICTED: SAFB-like transcription modulator [Papio anubis]

AGGATACAAGCCTCTGTCAAAAAAGAAGAGAAAAGGTCATCTGAAAAATCTGAAAAAAAA

GAAAGCAAGGATGCTAAGAAAATAGAAGGTAAAGATGAGAAGAATGATAATGGAGGAAGT

GGCCAAACTTCAGAATCGATTAAAAAAAGTGAAGAAAAGAAGCGAATAAGTTCAAAGAGT

CCAGGACACATGGTAATATTAGACAAAACTAAGGGAGATCATTGTAGGCCATCAAGAAGA

GGAAGATATGAAAAAATTCATGGAAGAAGCAAGGAAAAGGAGAGAGCTAGCCTAGATAAA

AAAAGGGACAAAGACTACAGAAGGAAAGAGATTTTGCCTTTTGAAAAGATGAAGGAACAA

CGATTGAGAGAACATTTAGTTCGTTTTGAAAGACTGCGACGAGCAATTTCCCTCCGAAGA

CGAAGAGAGATTGCAGAAAGAGAGCGTCGAGAGCGAGAACGCATTAGAATAATTCGTGAA

CGGGAAGAACGGGAACGCTTACAGAGAGAGAGAGAGCGCCTAGAAATTGAAAGGCAAAAA

CTAGAGAGAGAGAGAATGGAACGCGAACGCTTGGAAAGGGAACGCATTCGTATTGAACAG

GAACGTCGTAAGGAAGCTGAACGGATTGCTCGAGAACGAGAGGAACTCAGAAGGCAGCAG

CAGCAGCTACGTTATGAACAAGAAAAAAGGAATTCTTTGAAACGCCCACATGATGTAGAT

CATAGGCGAGATGACCCTTACTGGAGCGAGAATAAAAAGTTGTCTCTAGATACAGAT

>CL82.Contig7_All 477 656 minus strand PREDICTED: SAFB-like transcription modulator [Papio anubis]

AAGGAACGTCGTAAGGAAGCTGAACGGATTGCTCGAGAACGAGAGGAACTCAGAAGGCAG

CAGCAGCAGCTACGTTATGAACAAGAAAAAAGGAATTCTTTGAAACGCCCACATGATGTA

GATCATAGGCGAGATGACCCTTACTGGAGCGAGAATAAAAAGTTGTCTCTAGATACAGAT

>CL84.Contig1_All 295 1236 Mitochondrial fission factor OS=Homo sapiens GN=MFF PE=1 SV=1

GTAAGTGGGGCAGCATTTCCTTCACCCACTGCTGCTGAGATGGCAGAAATTAGTCGAATT

CAGTATGAAATGGAATATACCGAAGGTATTAGTCAGAGAATGAGGGTCCCTGAAAAACTA

AAAGTAGCACCACCAAATGCTGACCTGGAACAAGGATTTCAAGAAGGAGTTCCAAATGCT

AGTGTGATAATGCAAGTTCCAGAGAGGATTGTTGTAGCAGGAAATAATGAAGACATTTCA

TTTTCAAGACCAGCAGATCTTGACCTTATTCAGTCAACTCCCTTTAAGCCTCTGGCACTA

ACAACACCACCTCGTGTACTTACACTAAGTGAAAGACCACTAGATTTTCTGGATTTAGAA

AGATCTCCAACACCTCAAAATGAAGAAATCCGTGCAGTTGGCAGGCTAAAAAGAGAGCGC

TCTATGAGTGAAAATGCTGTTCGCCAAAATGGACAGCTGGTCAGAAACGATTCCATTGTG

ACACCATCGCCACAACAGGCTCGGGTCTGTCCTTCCCATATGTTACCTGAAGATGGAGCT

AATCTTTCCTCTGCTCGTGGCATTTTGTCGCTTATCCAGTCTTCTACTCGTAGGGCTTAC

CAGCAGATCTTGGATGTGCTGGATGAAAATCGCAGTGTGAGAAGACAAAATGAAATACGT

TGTGAAAGACCTGTGTTGCGCGGTGGGTCTGCTGCCGCCACTTCTAATCCTCATCATGAC

AACGTCAGGTATGGCATTTCAAATATAGAAACAACAATTGAAGGCTCATCAGATGACATG

ACTGTTGTAGATGCAGCTTCATTAAGACGCCAGATAATCAAACTAAATAGACGTCTACAA

CTTCTAGAAGAGGAGAACAAAGAGCGTGCTAAAAGAGAAATGGTCATGTATTCAATTACT

GTAGCATTCTGGCTGCTTAATAGCTGGCTCTGGTTTCGCCGC

>CL84.Contig2_All 2 349 PREDICTED: mitochondrial fission factor-like [Otolemur garnettii]

TGGCACAGATCAGATTCTGCCCCGAGAAATAAAATTTCAAGGTTCCAGGCATCGATTTCT

GCACCGGAATACACACCTGTGTTGCGCGGTGGGTCTGCTGCCGCCACTTCTAATCCTCAT

CATGACAACGTCAGGTATGGCATTTCAAATATAGAAACAACAATTGAAGGCTCATCAGAT

GACATGACTGTTGTAGATGCAGCTTCATTAAGACGCCAGATAATCAAACTAAATAGACGT

CTACAACTTCTAGAAGAGGAGAACAAAGAGCGTGCTAAAAGAGAAATGGTCATGTATTCA

ATTACTGTAGCATTCTGGCTGCTTAATAGCTGGCTCTGGTTTCGCCGC

>CL84.Contig3_All 1 390 RIKEN cDNA 5230400G24, isoform CRA_i [Mus musculus]

GTCAGAAACGATTCCATTGTGACACCATCGCCACAACAGGCTCGGGTCTGTCCTTCCCAT

ATGTTACCTGAAGATGGAGCTAATCTTTCCTCTGCTCGTGGCATTTTGTCGCTTATCCAG

TCTTCTACTCGTAGGGCTTACCAGCAGATCTTGGATGTGCTGGATGAAAATCGCAGGTAT

GGCATTTCAAATATAGAAACAACAATTGAAGGCTCATCAGATGACATGACTGTTGTAGAT

GCAGCTTCATTAAGACGCCAGATAATCAAACTAAATAGACGTCTACAACTTCTAGAAGAG

GAGAACAAAGAGCGTGCTAAAAGAGAAATGGTCATGTATTCAATTACTGTAGCATTCTGG

CTGCTTAATAGCTGGCTCTGGTTTCGCCGC

>CL84.Contig4_All 37 252 PREDICTED: mitochondrial fission factor isoform 2 [Papio anubis]

AGGTATGGCATTTCAAATATAGAAACAACAATTGAAGGCTCATCAGATGACATGACTGTT

GTAGATGCAGCTTCATTAAGACGCCAGATAATCAAACTAAATAGACGTCTACAACTTCTA

GAAGAGGAGAACAAAGAGCGTGCTAAAAGAGAAATGGTCATGTATTCAATTACTGTAGCA

TTCTGGCTGCTTAATAGCTGGCTCTGGTTTCGCCGC

>CL84.Contig5_All 1 228 PREDICTED: mitochondrial fission factor-like isoform 11 [Macaca mulatta] >gi|402889547|ref|XP_003908074.1| PREDICTED: mitochondrial fission factor isoform 1 [Papio anubis]

GTCAGAAACGATTCCATTGTGACACCATCGCCACAACAGGCTCGGGTCTGTCCTTCCCAT

ATGTTACCTGAAGATGGAGCTAATCTTTCCTCTGCTCGTGGCATTTTGTCGCTTATCCAG

TCTTCTACTCGTAGGGCTTACCAGCAGATCTTGGATGTGCTGGATGAAAATAGCAGACCC

ACCGCGCAACACAGGTCTACGGCAGGTTTTTGCGGGAGAAGGCACAAG

>CL85.Contig1_All 26 142 minus strand PREDICTED: tetratricopeptide repeat protein 8 isoform 6 [Otolemur garnettii]

CAGATTGGAGATCTACAGAGAAGTTATATTGCTGCTCAGAAGTCTGAAGCAGCATTTCCT

GATCATGTGGATACACAACATTTAATTAAACAATTAAAGCAGCATTTTGCTATGCTC

>CL85.Contig2_All 2 874 PREDICTED: LOW QUALITY PROTEIN: tetratricopeptide repeat protein 8 isoform 1 [Equus caballus]

TGGTGGTGGAAGGTGCAGATTGGGAAATGTTACTGCAGGTTAGGAATGTATCGTGAAGCA

GAAAAACAGTTTAAATCCTCTCTGAAGCAGCAGGAAATGGTAGATACATTTCTCTATTTG

GCAAAAGTTTACATCTCATTGGATCAACCTGTGACTGCTTTAAATCTTTTCAAACAAGGC

TTAGATAAGTTTCCAGGAGAAGTAAGCCTGCTTTGTGGAATTGCCAGGATCTATGAGGAA

ATGAACAATGGTTCATCAGCAGCTGAATACTACAAAGAGGTTTTAAAACAAGATAATACT

CACATGGAAGCCATTGCATGCATTGGAAGCAACCACTTTTATTCTGATCAACCAGAAATA

GCTCTTCGGTTTTACAGGCGACTCCTGCAGATGGGCGTTTACAATTGCCAGCTTTTTAAC

AATCTGGGGCTGTGCTGCTTCTATGCCCAGCAGTATGATATGACTCTGACCTCATTTGAA

CGTGCCCTTTCTTTGGCTGAAAATGAAGAAGAGACAGCTGACGTCTGGTACAACTTGGGA

CATATAGCTGTGGGAATAGGTGATACGAATTTGGCCCATCAGTGCTTCAGGCTGGCTCTG

GTCAACAACAACAACCATGCTGAGGCCTACAACAACCTGGCTGTGCTGGAGATGCGGAAG

GGCCATGTTGAACAGGCAAGGGCACTTTTGCAAACTGCGTCAACTTTAGCACCTCATATG

TATGAACCCCATTTTAATTTTGCAACAATATCTGATAAGATTGGAGATCTACAGAGAAGT

TATATTGCTGCTCAGAAGTCTGAAGCAGCATTTCCTGATCATGTGGATACACAACATTTA

ATTAAACAATTAAAGCAGCATTTTGCTATGCTC

>CL86.Contig1_All 15 215 minus strand hypothetical protein NEMVEDRAFT_v1g221633 [Nematostella vectensis] >gi|156207949|gb|EDO29629.1| predicted protein [Nematostella vectensis]

AACCCTAACCCTATCACTAAACCTAACCCTAACCCTAATCCTAACACTAACCCTAACCCT

AACTCTAACACTAACCCTAACCCTAACCATAACACTAACCCTAACCCTAACACAAACCCT

AACCCTAACCCTAACACTTACCCTAAACCCTTTCCTGAACCTAACACTAACCCTAACCGT

AACCATAACCATAACACTAAC

>CL87.Contig1_All 117 1121 minus strand PREDICTED: transmembrane protein 59-like isoform 1 [Ailuropoda melanoleuca] >gi|281353576|gb|EFB29160.1| hypothetical protein PANDA_003815 [Ailuropoda melanoleuca]

ATGGCGGCGCCGAAGGCGAGCCTCTGGGTTCGGGCTCAACTGGGGCTCCCGCCGCTGCTG

CTGCTGACCATGGCCCTGGCCGGAGGTTCGGGGACCGCTGCGGCTGAAGCGTTTGACTCG

GTCTTGGGTGATACGGCGTCCTGCCACCGGGCCTGCCAGTTGACCTACCCCTTGCACACC

TACCCCAAGGTGCCTGCCAGTTTGTACACAGTGGACCACACAGAAGAGGAGTTGTATGCT

TGTCAAAGAGGTTGCAGGCTGTTTTCAATTTGTCAGTTTGTGGATGATGGAATTGATTTA

AATCGGACCAAATTGGAATGTGAATCTGCATGTACAGAAGCATATTCCCAATCTGATGAG

CAATATGCTTGCCATCTTGGTTGCCAGAATCAGCTGCCATTTGCTGAATTGAGACAAGAA

CAACTCATGTCCCTGATGCCAAAAATGCATCTCTTGTTCCCTCTAACTCTCGTGAGGTCA

TTCTGGAGTGACGTGATGGACTCTGCACAGAGCTTCATAACCTCTTCATGGACTTTTTAT

CTTCAAGCTGATGATGGAAAAATAGTTATATTCCAGTCTAAGCCAGAAATTCAGTATGCA

CCACAGTTGGAGCAGGAACCTACAAATTTGAGAGAATCATCTCTAAGCAAAATGTCCTCA

GATCTACAAATGAGAAGTGCTCAAGCACACAGGAACTATCTTGAGGATGGAGAAAGCGAT

GGCTTTTTAAGATGCCTCTCTCTTAACTCTGGGTGGATTTTAACCATGACTCTTGTCCTT

TCGGTGATGGTGTTGCTCTGGATTTGTTGTGCAGCTGTTGCTACAGCTGTAGAGCAGTAT

GTTCCCTCTGAGAAGCTGAGTATCTATGGTGACTTGGAATTTGTGAATGAACAAAAGCTA

AGCAGATATCCAGCTTCTTCTCTCGTGGTTGTTAGATCTAAAACTGAAGATCATGAAGAA

GCAGGGCCTCTACCTACAAAAGTGAATCTTGCTCATTCAGAAATT

>CL87.Contig2_All 117 1088 minus strand Transmembrane protein 59 [Homo sapiens] >gi|119627114|gb|EAX06709.1| transmembrane protein 59, isoform CRA_e [Homo sapiens] >gi|119627115|gb|EAX06710.1| transmembrane protein 59, isoform CRA_e [Homo sapiens]

ATGGCGGCGCCGAAGGCGAGCCTCTGGGTTCGGGCTCAACTGGGGCTCCCGCCGCTGCTG

CTGCTGACCATGGCCCTGGCCGGAGGTTCGGGGACCGCTGCGGCTGAAGCGTTTGACTCG

GTCTTGGGTGATACGGCGTCCTGCCACCGGGCCTGCCAGTTGACCTACCCCTTGCACACC

TACCCCAAGGAAGAGGAGTTGTATGCTTGTCAAAGAGGTTGCAGGCTGTTTTCAATTTGT

CAGTTTGTGGATGATGGAATTGATTTAAATCGGACCAAATTGGAATGTGAATCTGCATGT

ACAGAAGCATATTCCCAATCTGATGAGCAATATGCTTGCCATCTTGGTTGCCAGAATCAG

CTGCCATTTGCTGAATTGAGACAAGAACAACTCATGTCCCTGATGCCAAAAATGCATCTC

TTGTTCCCTCTAACTCTCGTGAGGTCATTCTGGAGTGACGTGATGGACTCTGCACAGAGC

TTCATAACCTCTTCATGGACTTTTTATCTTCAAGCTGATGATGGAAAAATAGTTATATTC

CAGTCTAAGCCAGAAATTCAGTATGCACCACAGTTGGAGCAGGAACCTACAAATTTGAGA

GAATCATCTCTAAGCAAAATGTCCTCAGATCTACAAATGAGAAGTGCTCAAGCACACAGG

AACTATCTTGAGGATGGAGAAAGCGATGGCTTTTTAAGATGCCTCTCTCTTAACTCTGGG

TGGATTTTAACCATGACTCTTGTCCTTTCGGTGATGGTGTTGCTCTGGATTTGTTGTGCA

GCTGTTGCTACAGCTGTAGAGCAGTATGTTCCCTCTGAGAAGCTGAGTATCTATGGTGAC

TTGGAATTTGTGAATGAACAAAAGCTAAGCAGATATCCAGCTTCTTCTCTCGTGGTTGTT

AGATCTAAAACTGAAGATCATGAAGAAGCAGGGCCTCTACCTACAAAAGTGAATCTTGCT

CATTCAGAAATT

>CL87.Contig3_All 213 1184 Transmembrane protein 59 [Homo sapiens] >gi|119627114|gb|EAX06709.1| transmembrane protein 59, isoform CRA_e [Homo sapiens] >gi|119627115|gb|EAX06710.1| transmembrane protein 59, isoform CRA_e [Homo sapiens]

ATGGCGGCGCCGAAGGCGAGCCTCTGGGTTCGGGCTCAACTGGGGCTCCCGCCGCTGCTG

CTGCTGACCATGGCCCTGGCCGGAGGTTCGGGGACCGCTGCGGCTGAAGCGTTTGACTCG

GTCTTGGGTGATACGGCGTCCTGCCACCGGGCCTGCCAGTTGACCTACCCCTTGCACACC

TACCCCAAGGAAGAGGAGTTGTATGCTTGTCAAAGAGGTTGCAGGCTGTTTTCAATTTGT

CAGTTTGTGGATGATGGAATTGATTTAAATCGGACCAAATTGGAATGTGAATCTGCATGT

ACAGAAGCATATTCCCAATCTGATGAGCAATATGCTTGCCATCTTGGTTGCCAGAATCAG

CTGCCATTTGCTGAATTGAGACAAGAACAACTCATGTCCCTGATGCCAAAAATGCATCTC

TTGTTCCCTCTAACTCTCGTGAGGTCATTCTGGAGTGACGTGATGGACTCTGCACAGAGC

TTCATAACCTCTTCATGGACTTTTTATCTTCAAGCTGATGATGGAAAAATAGTTATATTC

CAGTCTAAGCCAGAAATTCAGTATGCACCACAGTTGGAGCAGGAACCTACAAATTTGAGA

GAATCATCTCTAAGCAAAATGTCCTCAGATCTACAAATGAGAAGTGCTCAAGCACACAGG

AACTATCTTGAGGATGGAGAAAGCGATGGCTTTTTAAGATGCCTCTCTCTTAACTCTGGG

TGGATTTTAACCATGACTCTTGTCCTTTCGGTGATGGTGTTGCTCTGGATTTGTTGTGCA

GCTGTTGCTACAGCTGTAGAGCAGTATGTTCCCTCTGAGAAGCTGAGTATCTATGGTGAC

TTGGAATTTGTGAATGAACAAAAGCTAAGCAGATATCCAGCTTCTTCTCTCGTGGTTGTT

AGATCTAAAACTGAAGATCATGAAGAAGCAGGGCCTCTACCTACAAAAGTGAATCTTGCT

CATTCAGAAATT

>CL88.Contig1_All 119 1198 minus strand unnamed protein product [Mus musculus]

CACATAAAGAAGACCCCTATCAATCATTTCTCAACAATTATATAGTCAACTAATGTAACA

ATGGTACTAATATTGGGACGCAGACTAAACAGAGAGGATCTTGGGGTGCGTGATTCCCCA

GCAACTAAGCGTAAAGTTTTTGAAATGGACCCTAAATCTCTGACAGGTCATGAGTTTTTT

GACTTCTCTTCAGGATCATCCCATGCTGAAAACATACTCCAGATATTTAATGAGTTCCGA

GACAGCCGCTTATTCACAGATGTTATCATCTGTGTGGAAGGAAAGGAGTTTCCTTGCCAT

AGAGCTGTTCTTTCAGCCTGTAGCAGCTACTTCAGAGCTATGTTTTGTAATGACCACAGG

GAAAGCCGAGAAATGTTGGTTGAGATCAATGGTATTTTAGCTGAAGCCATGGAATGTTTT

TTGCAGTATGTTTATACTGGAAAGGTGAAGATCACTACAGAGAACGTTCAATATCTCTTT

GAAACCTCAAGCCTCTTTCAGATTAGTGTTCTCCGTGATGCATGTGCCAAGTTCTTGGAG

GAGCAACTGGATCCTTGTAATTGCTTAGGAATTCAGCGCTTTGCTGACACCCATTCACTC

AAAACACTCTTCACAAAATGTAAAACCTTTGCATTACAGACTTTTGAGGATGTGTCCCAG

CATGAAGAATTTCTTGAGCTTGACAAAGATGAACTTATTGATTATATTTGTAGTGATGAA

CTTGTTATTGGTAAAGAGGAGATGGTTTTTGAAGCAGTCATGCGTTGGGTCTATCGTGCT

GTTGATCTGAGAAGACCATTGTTACATGAGCTCCTGACACATGTGAGACTCCCTCTTTTG

CATCCCAACTACTTTGTTCAAACAGTCGAGGTGGACCAATTGATCCAGAATTCTCCTGAG

TGTTACCAGTTGTTGCATGAAGCAAGACGGTACCACATACTTGGGAATGAAATGATGTCC

CCAAGGACTAGGCCACGCAGGTCCACTGGCTATTCAGAGGTGATAGTTGTTGTTGGAGGC

TGTGAACGAGTTGGAGGATTTAACCTTCCATATACTGAGTGTTATGATCCTGTAACAGGA

>CL89.Contig1_All 171 713 minus strand granzyme B [Marmota monax]

GGCAGGCCAATGAATGTCACCCTGGGGGCACACAACATTAAGAAGCAGGAGAAGACCCAG

CAGGTCATCCCAGTGAAAAGAACCATCCCCCATCCAGACTATGACGATCATTACTTCTAC

AATGACATCATGTTATTGGAGCTAGAGAAAAAAGCCAACCTTAATCCAGCTGTGCAGCCT

ATCAAGCTGCCCAGGGGCAAGGACAAGGTGAAGCCTGGGAAGGTGTGTCTTGTGGCTGGC

TGGGGCAGAATGGCCCGAAATGGCAAATACCCCAACACACTGCAGGAGGTAAAGCTGAAA

GTGCAGAAGCACCAGGTGTGCGAGCGTGAGGAATTATTAAAAGAGTACTACAAGAGTAGC

ATCCAGATATGTGTGGGGGATCCAAAGGAAAACAAAGCTTCCTTTCAGGGGGACTCCGGA

GGCCCTCTTGTGTGTAACCATGTGGCCCAAGGAATTGTCTCTTATGGAAATAAAAATGGG

AAACCTCCCCGTGTCTACACCAAAGTCTCCAGATTCCTACAATGGATAAAGGAAACCATG

AAA

>CL89.Contig3_All 171 704 minus strand granzyme B [Marmota monax]

GGCAGGCCAATGAATGTCACCCTGGGGGCACACAACATTAAGAAGCAGGAGAAGACCCAG

CAGGTCATCCCAGTGAAAAGAACCATCCCCCATCCAGACTATGACGATCATTACTTCTAC

AATGACATCATGTTATTGCAGCTAGAGAAAAAAGCCAAGTTGAATCCAGCTGTGAAGCTT

ATCAAGCTGCCCAGGGGCAAGGACAAGGTGAAGCCTGGGAAGGTGTGTCTTGTGGCTGGC

TGGGGCAGAATGGCCCGAAATGGCAAATACCCCAACACACTGCAGGAAGTGGAACTGACA

TTGCAGGAGGACCAGGTGTGCAAGAACCACTTCAGCAATTACAATAGTAACATAGAGACA

TGTGTGGGGGATCCAAAGAAGAAAAATGCTTCTTTTCAGGGGGACTCTGGAGGCCCTCTT

GTTTGTAACCGTGTGGCCCAAGGAATTGTCTCTTACGGAAAAAAAAATGGGACACCTCCA

GGCGTCTACACCAAAGTCTCCAGATTCCTACAATGGATAAAGGAAACCATGAAA

>CL89.Contig4_All 114 869 Granzyme B [Heterocephalus glaber]

ATGCATTTGCTCCTGCTCTTGTTGGCCTTCTTTCTGCCTCCCAAGGCACAGGCAGGGGAG

ATCATCGGGGGACATGAATCCAAGCCCCACTCCCGGCCCTACATGGCCTATCTTCAGTTT

GTTGCTCAGGGTGTACAGAAGAAGTGTGGTGGGTTCCTGATAAGAGAAGACTTTGTGCTG

ACGGCTGCTCACTGCTTAGGAAGGCCAATGAACATCACTCTGGGGGCACACAACATTGAG

AATCTGGAGAAGACCCAGCAGGTCATCTCAGTGAAAAGAACCATCCCCCACCCAGACTAT

GATGCTCATTATTTGTACAATGACATCATGTTATTGGAGCTAGAGAAAAAAGCCAACCTT

AATCCAGCTGTGCAGCCTATCAAGCTGCCCAGGGGCAAGGACAAGGTGAAGCCTGGGAAG

GTGTGTCTTGTGGCTGGCTGGGGCAGAATGGCCCGAAATGGCAAATACCCCAACACACTG

CAGGAAGTGGAACTGACATTGCAGGAGGACCAGGTGTGCAAGAACCACTTCAGCAATTAC

AATAGTAACATAGAGACATGTGTGGGGGATCCAAAGAAGAAAAATGCTTCTTTTCAGGGG

GACTCTGGAGGCCCTCTTGTTTGTAACCGTGTGGCCCAAGGAATTGTCTCTTACGGAAAA

AAAAATGGGACACCTCCAGGCGTCTACACCAAAGTCTCCAGATTCCTACAGTGGATAAAG

GAAACCATGAAAAGCCACCAAGAACAGAACACAGAC

>CL89.Contig5_All 199 798 minus strand granzyme B [Marmota monax]

GCCAAGCCCCACTCCCGCCCCTACATGGCTTATCTTCAGTTCTTGAGTCAGAATTCATTG

CGCACTTGTGGTGGCTTCCTGATACGAGAAGACTTTGTGCTGACGGCCGCTCACTGCTTA

GGGAGGCCAATGAATGTCACCCTGGGGGCACACAACATTAAGAAGCAGGAGAAGACCCAG

CAGGTCATCCCAGTGAAAAGAACCATCCCCCATCCAGACTATGACGATCATTACTTCTAC

AATGACATCATGTTATTGGAGCTAGAGAAAAAAGCCAACCTTAATCCAGCTGTGCAGCCT

ATCAAGCTGCCCAGGGGCAAGGACAAGGTGAAGCCTGGGAAGGTGTGTCTTGTGGCTGGC

TGGGGCAGAATGGCCCGAAATGGCAAATACCCCAACACACTGCAGGAGGTAAAGCTGAAA

GTGCAGAAGCACCAGGTGTGCGAGCGTGAGGAATTATTAAAAGAGTACTACAAGAGTAGC

ATCCAGATATGTGTGGGGGATCCAAAGGAAAACAAAGCTTCCTTTCAGGGGGACTCCGGA

GGCCCTCTTGTGTGTAACCATGTGGCCCAAGGAATTGTCTCTTATGGAAATAAAAATGGG

>CL90.Contig1_All 55 276 minus strand unnamed protein product [Homo sapiens]

CCCACCGTCCCAAGCAGGCCTGGGATGGGTAATGGTGTGAAGGAAGGCTCGGTGCGCCTG

CGTGAGGATGCCGAAGCCGTCCTGCCCTCGCCGGTCTCTTCAAAGAGAGACCACAGGCAA

GTGCTCAGCTCCCTGCTGTCCGGCGCCCTGGCTGGTGCTCTCGCCAAGACGGCAGTAGCT

CCCCTGGACCGAACCAAAATCATCTTCCAAGGTAATGCTCGT

>CL90.Contig2_All 56 577 unnamed protein product [Homo sapiens]

CCCACCGTCCCAAGCAGGCCTGGGATGGGTAATGGTGTGAAGGAAGGCTCGGTGCGCCTG

CGTGAGGATGCCGAAGCCGTCCTGCCCTCGCCGGTCTCTTCAAAGAGAGACCACAGGCAA

GTGCTCAGCTCCCTGCTGTCCGGCGCCCTGGCTGGTGCTCTCGCCAAGACGGCAGTAGCT

CCCCTGGACCGAACCAAAATCATCTTCCAAGTGTCTTCAAAAAGATTTTCTGCCAAGGAG

GCCTTCCGGCTCCTCTACTTCACCTACCTCAACGAGGGCTTTCTCAGCCTGTGGCGCGGG

AACTCGGCCACCATGGTCCGCGTGGTGCCCTACGCTGCCATCCAGTTCAGCGCGCACGAG

GAGTACAAGCGCATCCTGGGCCGCTACTATGGTTTCCGCGGAGAGGCCCTGCCCCCTTGG

CCTCGCCTCCTTGCTGGCGCTCTGGCTGGAACCACCGCTGCTTCCCTCACCTACCCCCTG

GACCTGGTCCGAGCAAGGATGGCCGTGACTCCCAAGGAAATG

>CL90.Contig3_All 56 577 unnamed protein product [Homo sapiens]

CCCACCGTCCCAAGCAGGCCTGGGATGGGTAATGGTGTGAAGGAAGGCTCGGTGCGCCTG

CGTGAGGATGCCGAAGCCGTCCTGCCCTCGCCGGTCTCTTCAAAGAGAGACCACAGGCAA

GTGCTCAGCTCCCTGCTGTCCGGCGCCCTGGCTGGTGCTCTCGCCAAGACGGCAGTAGCT

CCCCTGGACCGAACCAAAATCATCTTCCAAGTGTCTTCAAAAAGATTTTCTGCCAAGGAG

GCCTTCCGGCTCCTCTACTTCACCTACCTCAACGAGGGCTTTCTCAGCCTGTGGCGCGGG

AACTCGGCCACCATGGTCCGCGTGGTGCCCTACGCTGCCATCCAGTTCAGCGCGCACGAG

GAGTACAAGCGCATCCTGGGCCGCTACTATGGTTTCCGCGGAGAGGCCCTGCCCCCTTGG

CCTCGCCTCCTTGCTGGCGCTCTGGCTGGAACCACCGCTGCTTCCCTCACCTACCCCCTG

GACCTGGTCCGAGCAAGGATGGCCGTGACTCCCAAGGAAATC

>CL90.Contig4_All 55 576 minus strand unnamed protein product [Homo sapiens]

CCCACCGTCCCAAGCAGGCCTGGGATGGGTAATGGTGTGAAGGAAGGCTCGGTGCGCCTG

CGTGAGGATGCCGAAGCCGTCCTGCCCTCGCCGGTCTCTTCAAAGAGAGACCACAGGCAA

GTGCTCAGCTCCCTGCTGTCCGGCGCCCTGGCTGGTGCTCTCGCCAAGACGGCAGTAGCT

CCCCTGGACCGAACCAAAATCATCTTCCAAGTGTCTTCAAAAAGATTTTCTGCCAAGGAG

GCCTTCCGGCTCCTCTACTTCACCTACCTCAACGAGGGCTTTCTCAGCCTGTGGCGCGGG

AACTCGGCCACCATGGTCCGCGTGGTGCCCTACGCTGCCATCCAGTTCAGCGCGCACGAG

GAGTACAAGCGCATCCTGGGCCGCTACTATGGTTTCCGCGGAGAGGCCCTGCCCCCTTGG

CCTCGCCTCCTTGCTGGCGCTCTGGCTGGAACCACCGCTGCTTCCCTCACCTACCCCCTG

GACCTGGTCCGAGCAAGGATGGCCGTGACTCCCAAGGAAATG

>CL91.Contig2_All 360 497 minus strand hypothetical protein (L1H 3&apos; region) - human

AATAAGTCTCCATCACAGGAAAGTCCAGGACCTGATTACTTTACTGCTGAATTCTATAAA

ATATTCAAATTTCAATTCTTATTAAACTCCTCCAAAAAAGTAGGGGGAGAAAACACTTTC

AAAACATTTTATGAAGCT

>CL94.Contig1_All 2 556 PREDICTED: transportin-3 isoform 4 [Otolemur garnettii]

TCATCTAACCTACATGAAGCTGCTTCAGACTGTGTGTGCTCAGCTCTTTATGCCATTGAG

AATGTGGAAACTAACTTGCCATTAGCAATGCAACTTTTTCAGGGAGTCCTGACATTGGAG

ACTGCCTATCATATGGCTGTGGCACGTGAAGATTTAGACAAAGTTCTGAATTACTGCCGT

ATTTTCACTGAACTATGTGAAACTTTTCTTGAAAAAATTGTTTGTACTCCAGGCCAAGGT

CTTGGGGACCTGCGAACTCTGGAATTGCTTCTTATTTGTGCAGGGCATCCTCAGTATGAG

GTAGTAGAAATTTCCTTTAACTTTTGGTACCGACTAGGGGAGCATTTGTACAAAACTAAC

GATGAAGTTATTCATGGCATCTTCAAAGCTTACATTCAGAGGCTGCTTCATGCCTTGGCT

CGACACTGCCAGCTAGAACCGGACCATGAGGGGGTTCCTGAGGAAACTGATGACTTTGGC

GAGTTTCGGATGCGGGTATCAGACCTGGTGAAAGATTTGATTTTCTTGATTGGGTCTATG

GAGTGTTTTGCTCAG

>CL94.Contig2_All 267 1856 Transportin-3 [Heterocephalus glaber]

ATGGAAGGAGCAAAACCGACATTGCAGCTTGTGTACCAGGCAGTGCAGGCGCTTTACCAC

GACCCGGATCCCAGCGGAAAGGAGCGCGCCTCGTTTTGGCTTGGGGAGCTGCAGCGTTCG

GTTCATGCCTGGGAGATTTCTGATCAGTTGTTACAGATCCGGCAGGATGTGGAATCATGC

TATTTTGCTGCACAGACCATGAAGATGAAGATTCAGACCTCATTTTATGAGCTCCCTACA

GACTCTCATGCCTCCTTAAGGGACTCATTGCTAACCCATATCCAGAACTTGAAAGATTTA

TCACCTGTCATTGTAACGCAGCTGGCTTTAGCAATAGCAGATCTCGCTCTACAGATGCCT

TCTTGGAAGGGTTGTGTACAAACATTGGTGGAAAAATATAGCAATGATGTAACTTCTCTG

CCATTTTTGCTGGAGATTCTTACGGTGTTACCTGAAGAAGTACATAGTCGTTCTTTACGA

ATTGGAGCTAACAGACGCACAGAAATTATAGAAGATTTGGCCTTCTACTCCAGTACAGTA

GTATCTCTGTTGATGACTTGTGTAGAAAAAGCAGGAACAGATGAGAAAATGCTTATGAAG

GTCTTTCGCTGTTTGGGAAGTTGGTTTAACTTGGGAGTTTTGGACAGTAACTTCATGGCT

AACAATAAGTTACTAGCCCTTCTTTTTGAGGTTTTGCAACAGGATAAGACCTCATCTAAC

CTACACGAAGCTGCTTCAGACTGTGTGTGCTCAGCTCTTTATGCCATTGAGAATGTGGAA

ACTAACTTGCCATTAGCAATGCAACTTTTTCAGGGAGTCCTGACATTGGAGACTGCCTAT

CATATGGCTGTGGCACGTGAAGATTTAGACAAAGTTCTGAATTACTGCCGTATTTTCACT

GAACTATGTGAAACTTTTCTTGAAAAAATTGTTTGTACTCCAGGCCAAGGTCTTGGGGAC

CTGCGAACTCTGGAATTGCTTCTTATTTGTGCAGGGCATCCTCAGTATGAGGTAGTAGAA

ATTTCCTTTAACTTTTGGTACCGACTAGGGGAGCATTTGTACAAAACTAACGATGAAGTT

ATTCATGGCATCTTCAAAGCTTACATTCAGAGGCTGCTTCATGCCTTGGCTCGACACTGC

CAGCTAGAACCGGACCATGAGGGGGTTCCTGAGGAAACTGATGACTTTGGCGAGTTTCGG

ATGCGGGTATCAGACCTGGTGAAAGATTTGATTTTCTTGATTGGGTCTATGGAGTGTTTT

GCTCAGTTATATTCTACTCTGAAAGAAGGCAACCCACCCTGGGAGGTGACAGAAGCGGTT

CTCTTTATCATGGCTGCTATAGCAAAGAGTGTTGATCCGGAGAACAATCCAACATTGGTG

GAGGTCCTAGAAGGAGTCGTTCGTCTCCCAGAGACTGTCCATACAGCCGTGCGATACACC

AGCATTGAGTTGGTTGGAGAAATGAGCGAAGTGGTTGATCGAAATCCTCAGTTCCTTGAC

CCTGTGTTGGGCTATTTGATGAAAGGCCTATGTGAAAAGCCTCTGGCTTCTGCTGCAGCC

AAAGCCATTCATAATATTTGCTCTGTCTGT

>CL94.Contig3_All 1452 3422 A-kinase anchor protein 9 isoform 2 [Macaca mulatta]

TTACAGGCTAGTGAAACTCTACGAAACAGCACTCACAGTAACACAGCTGCAGATTTACTG

CAAGCCAAACAACAGATTCTCACTCATCAACAACAGCTTGAAGAACAAGACCATCTATTA

GAAGATTATCAAAAAAAGAAAGAAGACTTCAAAATGCAAATTAATTTCTTACAGGAGAAA

ATTAAAGTATATGAAATGGAACAAGATGAAGTAGAAAAGTCAAATAAAAAAGAATTACAG

GAAAAGGAGGCAATCATCCAGGCGTTAAATATAAGAATAATAGAAGAAGAAAAAAAGAAT

CTTGAGCTAAAAGATCAAGTCACAGCCACTGAAAAATTAATGAGAGAATTACAAGAACAA

ATTGCAAGTATGAAATTAGAGCTGGCTAATTCTAAGCAAAAAGAAAAACAATGTTCTGAA

GAAATAAAACAGTTTATGGGTACAGTTGAAGACCTTCAGAAAAGACATCATTACACAGAT

AGCCAGTTTGAATCTGATGCGGTACAGAGAATGGTGGAACAAGAAATGCAGAAAAGATTG

GAACAACTCCAAGCAGAGATGGATGAAATGTATGGGCAACAGATAGTACAGATGAAACAA

GAGTTAATAAAGCAACACATGTCACAGATAGAGGAACTTAAAGCACAACATAAGGAGGAA

ATTGAGAGTGTTTTAAAATCACATTTAAATATTACAATTAATGAAGATCAAATAAAGTTA

ATGAACGTGGCAATAAATGAACTGAATATGAAATTACAAGATACTAACGCTCAAAAGGAA

AAACTCCAAGAAGAACTAGGAATAGTTTCAGGAGAAAAATCTACTCTGCAGAGACGACTT

GATGACCTTTTTGAAGAATTGAACTTTTTAAGGGACCAAATTCAGAGAGCTAGATTGATA

ATAGCTGAACAAGAAAGTCAACTGAATGAAGCACATAAGTCCCTTAGTACAGTGGAAGCT

TTAAAAGCTGAGATTGTTCTGGCATCTGAATCTAGGAAAGAACTAGAGTTAAAACATGAA

GCAGAAGTTACAAATTACAAGATAAAACTTGAAATGTTGGAAAAAGAAAAAAATGCTGTA

TTAGACAGAATGGCTGAATCACAAGAAGCTGAATTAGAGAGGCTGAGAACACAGCTTCTA

TTTAGTCATGAAGAAGAACTTTCGAAACTAAAAGAAGATTTAGAAGTTGAACATCGAATA

AATATTGAAAAACTTAAAGATAGCTTAGGCATTCACTATAAACAGCAGATATATGGCTTA

CAAAATGAAATGAGTCAAAAGATAGAAACAATGCAATTTGAAAAAGACAATTTGATAACT

AAGCAGAATCAGTTGGTTTTGGAAATTTCAAAACTAAAAGATTTACAGAAGTCCATCATG

GATTCAAAATCAGAAGAAATGACCCTTCAGATGCATGAACTCCAAGAGGAAATCCAAATA

TTAAGACAAGAAAAAAAAGAAAAAGGTACGCTTGAACAAGAAGTTCAGGAATTACAACTT

AAAATTGAATTATTGGAGAAAGAAATAAAGGAAAAAGAGGATGACCTTAAAGAAAAGTTT

TCACAACTTGAAGCAGAAAATAACATTCTTAAAAATGAGAAAAAAGCTATTGAGGACATG

TTGAAAATTTATGTTCCTGCTGAAGAAGAAGAAAAAATGATTTTCATTGACTCCAGCAAG

TCCATATCCAAAGATTGTAGCTGGCAAAAAGAAATGGAAATGCTTACAAAAGAGAATGAG

GCCCTCAAGCAACAGTGTATTCAGCTAAATGAAGAAATTGAAAAGCAAAGAAGTACATTT

TCATTTGCTGAAAAAAATTTTGAAGTTAACTATCAAGAGTTACAGGAGGAGTATACTTGC

CTTCTCAAGGTAAAAGATGATTTAGAAGACAGTAAAAACAAACAAGAATTAGAGTATAAA

AGTAAACTTAAAGCACTTAATGAAGAGCTTCATTTACAAAGAATAAATCCA

>CL94.Contig4_All 1157 2863 PREDICTED: A-kinase anchor protein 9 [Otolemur garnettii]

CAACAACAGCTTGAAGAACAAGACCATCTATTAGAAGATTATCAAAAAAAGAAAGAAGAC

TTCAAAATGCAAATTAATTTCTTACAGGAGAAAATTAAAGTATATGAAATGGAACAAGAT

GAAGTAGAAAAGTCAAATAAAAAAGAATTACAGGAAAAGGAGGCAATCATCCAGGCGTTA

AATATAAGAATAATAGAAGAAGAAAAAAAGAATCTTGAGCTAAAAGATCAAGTCACAGCC

ACTGAAAAATTAATGAGAGAATTACAAGAACAAATTGCAAGTATGAAATTAGAGCTGGCT

AATTCTAAGCAAAAAGAAAAACAATGTTCTGAAGAAATAAAACAGTTTATGGGTACAGTT

GAAGACCTTCAGAAAAGACATCATTACACAGATAGCCAGTTTGAATCTGATGTACAGAGA

ATGGTGGAACAAGAAATGCAGAAAAGATTAGAACAACTCCAAGCAGAGATGGATGAAATG

TATGGGCAACAGATAGTACAGATGAAACAAGAGTTAATAAAGCAACACATGTCACAGATA

GAGGAACTTAAAGCACAACATAAGGAGGAAATTGAGAGTGTTTTAAAATCACATTTAAAT

ATTACAATTAATGAAGATCAAATAAAGTTAATGAACGTGGCAATAAATGAACTGAATATG

AAATTACAAGATACTAACGCTCAAAAGGAAAAACTCCAAGAAGAACTAGGAATAGTTTCA

GGAGAAAAATCTACTCTGCAGAGACGACTTGATGACCTTTTTGAAGAATTGAACTTTTTA

AGGGACCAAATTCAGAGAGCTAGATTGATAATAGCTGAACAAGAAAGTCAACTGAATGAA

GCACATAAGTCCCTTAGTACAGTGGAAGCTTTAAAAGCTGAGATTGTTCTGGCATCTGAA

TCTAGGAAAGAACTAGAGTTAAAACATGAAGCAGAAGTTACAAATTACAAGATAAAACTT

GAAATGTTGGAAAAAGAAAAAAATGCTGTATTAGACAGAATGGCTGAATCACAAGAAGCT

GAATTAGAGAGGCTGAGAACACAGCTTCTATTTAGTCATGAAGAAGAACTTTCGAAACTA

AAAGAAGATTTAGAAGTTGAACATCGAATAAATATTGAAAAACTTAAAGATAGCTTAGGC

ATTCACTATAAACAGCAGATATATGGCTTACAAAATGAAATGAGTCAAAAGATAGAAACA

ATGCAATTTGAAAAAGACAATTTGATAACTAAGCAGAATCAGTTGGTTTTGGAAATTTCA

AAACTAAAAGATTTACAGAAGTCCATCATGGATTCAAAATCAGAAGAAATGACCCTTCAG

ATGCATGAACTCCAAGAGGAAATCCAAATATTAAGACAAGAAAAAAAAGAAAAAGGTACG

CTTGAACAAGAAGTTCAGGAATTACAACTTAAAATTGAATTATTGGAGAAAGAAATAAAG

GAAAAAGAGGATGACCTTAAAGAAAAGTTTTCACAACTTGAAGCAGAAAATAACATTCTT

AAAAATGAGAAAAAAGCTATTGAGGACATGTTGAAAATTTATGTTCCTGCTGAAGAAGAA

GAAAAAATGATTTTCATTGACTCCAGCAAGTCCATATCCAAAGATTGTAGCTGGCAAAAA

GAAATGGAAATGCTTACAAAAGAGAATGAGGCCCTCAAGCAACAGTGTATTCAGCTAAAT

GAAGAAATTGAAAAGCAAAGAAGTACA

>CL94.Contig5_All 722 3283 A-kinase anchor protein 9 isoform 2 [Macaca mulatta]

TTAGAAGCCATGGAGGACGAAGAAAGAAGGAGAAAGATCGAAATTGGCAGAGCGAAGCTT

GCCCAGTTTCGACAAAGAAGAGCTCGATTGGATGGACAAAACCCTCATAAGAAGCCAAAA

AAGAAGAAAACCTCAAGCAGTAAAAATGATGTTCATGGCTTGAATATTGATCAACCAAAG

AGTGAGGAGATGTTGATAAATAGTTCTCAGGGTGTTGGATCAGCCGTGATGCCTGAATCC

ACAATAATGAGAACTCTAAACAATGAAGAAATGCTTAAGCATGAGCAGGTCTTTTCTTTT

GATCCGGAAAGTGAGATTTCAACCACAGCAGATGATTACACTTCGGAGGTAAATGACTGC

TGTAATGTGGTGAAAACAGGAAAGCCTACCAATTTATTAATGGAAGAAGAATTTGGTGTC

GTTGATTCTTATTCTGAACAAGGCGCACACTATAGTGAGACTCGCCTAGAGTTGATAGAA

AACGAATCAGTTGGGAAACCTGAACATGAGCCCGAAGAACTGAACAGAGAACTGGAAGAA

ATGAGGGCCACTTATGGGACTGAAGGATTGCATCAGTTACAGGCTAGTGAAACTCTACGA

AACAGCACTCACAGTAACACAGCTGCAGATTTACTGCAAGCCAAACAACAGATTCTCACT

CATCAACAACAGCTTGAAGAACAAGACCATCTATTAGAAGATTATCAAAAAAAGAAAGAA

GACTTCAAAATGCAAATTAATTTCTTACAGGAGAAAATTAAAGTATATGAAATGGAACAA

GATGAAGTAGAAAAGTCAAATAAAAAAGAATTACAGGAAAAGGAGGCAATCATCCAGGCG

TTAAATATAAGAATAATAGAAGAAGAAAAAAAGAATCTTGAGCTAAAAGATCAAGTCACA

GCCACTGAAAAATTAATGAGAGAATTACAAGAACAAATTGCAAGTATGAAATTAGAGCTG

GCTAATTCTAAGCAAAAAGAAAAACAATGTTCTGAAGAAATAAAACAGTTTATGGGTACA

GTTGAAGACCTTCAGAAAAGACATCATTACACAGATAGCCAGTTTGAATCTGATGTACAG

AGAATGGTGGAACAAGAAATGCAGAAAAGATTAGAACAACTCCAAGCAAAGATGGATGAA

ATGTATGGGCAACAGATAGTACAGATGAAACAAGAGTTAATAAAGCAACACATGTCACAG

ATAGAGGAACTTAAAGCACAACATAAGGAGGAAATTGAGAGTGTTTTAAAATCACATTTA

AATATTACAATTAATGAAGATCAAATAAAGTTAATGAACGTGGCAATAAATGAACTGAAT

ATGAAATTACAAGATACTAACGCTCAAAAGGAAAAACTCCAAGAAGAACTAGGAATAGTT

TCAGGAGAAAAATCTACTCTGCAGAGACGACTTGATGACCTTTTTGAAGAATTGAACTTT

TTAAGGGACCAAATTCAGAGAGCTAGAATGATAATAGCTGAACAAGAAAGTCAACTGAAT

GAAGCACATAAGTCCCTTAGTACAGTGGAAGCTTTAAAAGCTGAGATTGTTCTGGCATCT

GAATCTAGGAAAGAACTAGAGTTAAAACATGAAGCAGAAGTTACAAATTACAAGATAAAA

CTTGAAATGTTGGAAAAAGAAAAAAATGCTGTATTAGACAGAATGGCTGAATCACAAGAA

GCTGAATTAGAGAGGCTGAGAACACAGCTTCTATTTAGTCATGAAGAAGAACTTTCGAAA

CTAAAAGAAGATTTAGAAGTTGAACATCGAATAAATATTGAAAAACTTAAAGATAGCTTA

GGCATTCACTATAAACAGCAGATATATGGCTTACAAAATGAAATGAGTCAAAAGATAGAA

ACAATGCAATTTGAAAAAGACAATTTGATAACTAAGCAGAATCAGTTGGTTTTGGAAATT

TCAAAACTAAAAGATTTACAGAAGTCCATCATGGATTCAAAATCAGAAGAAATGACCCTT

CAGATGCATGAACTCCAAGAGGAAATCCAAATATTAAGACAAGAAAAAAAAGAAAAAGGT

ACGCTTGAACAAGAAGTTCAGGAATTACAACTTAAAATTGAATTATTGGAGAAAGAAATA

AAGGAAAAAGAGGATGACCTTAAAGAAAAGTTTTCACAACTTGAAGCAGAAAATAACATT

CTTAAAAATGAGAAAAAAGCTATTGAGGACATGTTGAAAATTTATGTTCCTGCTGAAGAA

GAAGAAAAAATGATTTTCATTGACTCCAGCAAGTCCATATCCAAAGATTGTAGCTGGCAA

AAAGAAATGGAAATGCTTACAAAAGAGAATGAGGCCCTCAAGCAACAGTGTATTCAGCTA

AATGAAGAAATTGAAAAGCAAAGAAGTACATTTTCATTTGCTGAAAAAAATTTTGAAGTT

AACTATCAAGAGTTACAGGAGGAGTATACTTGCCTTCTCAAGGTAAAAGATGATTTAGAA

GACAGTAAAAACAAACAAGAATTAGAGTATAAAAGTAAACTTAAAGCACTTAATGAAGAG

CTTCATTTACAAAGAATAAATCCAGCTACAATCAAAGTGAAA

>CL94.Contig6_All 7 327 PREDICTED: transportin-3 [Saimiri boliviensis boliviensis]

CATCCCCAGCCCCGAGAGGTAGTAGAAATTTCCTTTAACTTTTGGTACCGACTAGGGGAG

CATTTGTACAAAACTAACGATGAAGTTATTCATGGCATCTTCAAAGCTTACATTCAGAGG

CTGCTTCATGCCTTGGCTCGACACTGCCAGCTAGAACCGGACCATGAGGGGGTTCCTGAG

GAAACTGATGACTTTGGCGAGTTTTGGATGCGGGTATCAGACCTGGTGAAAGATTTGATT

TTCTTGATTGGGTCTATGGAGTGTTTTGCTCAGTTATATTCTACTCTGAAAGAAGGCAAC

CCACCCTGGGAGGTGACAGAA

>CL95.Contig1_All 20 385 PREDICTED: prefoldin subunit 1-like [Equus caballus] >gi|291387451|ref|XP_002710297.1| PREDICTED: prefoldin subunit 1 [Oryctolagus cuniculus] >gi|348582858|ref|XP_003477193.1| PREDICTED: prefoldin subunit 1-like [Cavia porcellus]

ATGGCGGCCCCCGTGGATCTCGAGCTGAAGAAGGCCTTCACAGAGCTTCAAGCCAAAGTT

ATTGACACTCAACAGAAGGTGAAGCTTGCAGACATACAGATTGAACAGCTAAACAGAACG

AAAAAGCATGCGCATCTTACAGATACAGAGATTATGACTTTGGTAGATGAGACTAACATG

TATGAAGGTGTAGGAAGAATGTTTATTCTTCAATCCAAGGAAGTAATTCATAATCAGCTA

TTAGAGAAACAGAAAATAGCAGAAGAAAAAATTAAAGAATTAGAGCAGAAAAAGTCCTAC

CTGGAGCGGAGTGTTAAGGAAGCTGAGGACAACATCCGGGAGATGCTGATGGCGCGAAGG

GCACAG

>CL95.Contig2_All 47 217 minus strand PREDICTED: prefoldin subunit 1-like [Ailuropoda melanoleuca]

AGAAGGTTTATTCTTCAATCCAAGGAAGTAATTCATAATCAGCTATTAGAGAAACAGAAA

ATAGCAGAAGAAAAAATTAAAGAATTAGAGCAGAAAAAGTCCTACCTGGAGCGGAGTGTT

AAGGAAGCTGAGGACAACATCCGGGAGATGCTGATGGCGCGAAGGGCACAG

>CL97.Contig1_All 2 2221 oxysterol binding protein-like 9, isoform CRA_c [Homo sapiens]

GCGGCTCCCAAGATGGCGTCCATCGTGGAAGGGCCGCTGAGCAAATGGACTAACGTGATG

AAGGGTTGGCAGTACCGTTGGTTTGTGCTGGACTACAATGCGGGGCTGTTGTCCTACTAC

ACGTCCAAAGACAAAATGATGAGAGGCTCTCGCAGAGGATGCGTTAGACTAAGAGGAGCT

GTGATTGGTATAGACGATGAGGACGACAGCACCTTCACAATAACTGTTGATCAGAAAACC

TTCCATTTCCAGGCTCGTGATGCTGATGAGCGAGAGAAGTGGATCCATGCTTTAGAAGAA

ACAATTCTTCGACATACTCTTCAGCTTCAAGGTTTGGATTCAGGATTTGTTCCTAGTGTC

CAAGACTTTGACAAGAAACTAACAGAGGCAGATGCTTACCTACAAATCTTGATAGAACAA

CTAAAGCTTTTTGATGACAAGCTTCAAAACTGTAAAGATGATGAACAGAGAAAGAAAATT

GAAACTCTCAAAGAAACAACAAATAGCATGGTAGAATCTATTAAACACTGCATTGTGTTG

CTGCAGATTGCTAAAGACCAGAGTAATGCGGAGAAGCACGCAGATGGAATTATAAGTACT

ATTAATCCTGTAGATGCAATATATCAACCCAGTCCCTTGGAACCTGTAATCAGCACAATG

CCTTCCCAGACTGTCTTACCTCCAGAACCAGCTCAGTTGTGTAAGTCAGAGCAGCGTCCG

TCTTCCTTACCCGTTGGACCTGTATTAGCTACCTTGGGACATCATCAGACTCCAACACCA

AATAGTACAGGCAGTGGGCATTCACCACCTAGTAGCAGTCTAACTTCTCCAAGTCATGTC

AACTTGTCTCCAAATACAGTCCCAGAGTTTTCTTACTCTAGCAGTGAAGATGAATTCTAT

GATGCTGATGAATTCCATCAGAGTGGCTCATCCCCAAAGCGCTTAATAGATTCTTCTGGT

TCTGCCTCAGTCTTGACACACAGCAGTTCGGGAAATAGCCTAAAACGCCCAGATACCACA

GAATCACTGAATTCTTCCATGTCCAACGGTACAAGTGATGCTGACCTTTTTGATTCACAT

GACGATAGAGACGATGACGGAGAGGCAGGGTCAGTGGAGGAGCACAAGAGTGTTATCATG

CACCTCTTGTCACAGGTTAGGCTCGGAATGGATCTTACTAAGGTAGTTCTTCCAACATTT

ATTCTTGAAAGAAGATCTCTTTTAGAAATGTATGCAGACTTTTTTGCACATCCGGACCTG

TTTGTGAGCATTAGCGACCAGAAAGATCCTAGGGATCGAATGGTTCAGGTTGTGAAATGG

TACCTCTCAGCCTTTCATGCAGGAAGGAAAGGATCGGTTGCTAAAAAGCCATACAATCCC

ATTTTGGGTGAGATCTTTCAGTGTCACTGGACGTTACCAAATGACACTGAAGAGAACACA

GAGCTAGTTTCAGAAGGACCAGTTCCCTGGGTTTCCAAAAACAGTGTAACATTCGTGGCT

GAGCAGGTTTCCCATCATCCACCCATTTCAGCCTTTTATGCTGAATGCTTTAACAAGAAA

ATCCAATTCAATGCTCATATCTGGACTAAATCAAAATTCCTTGGGATGTCAATTGGGGTG

CACAACATAGGGCAAGGCTGTGTCTCATGTCTAGACTATGATGAGCATTACATTCTCACA

TTCCCCAATGGTTATGGAAGGTCTATCCTCACAGTGCCCTGGGTGGAACTAGGAGGAGAA

TGCAATATTAATTGTTCCAAAACTGGTTATAGTGCAAATATCGTCTTCCACACTAAACCT

TTCTATGGAGGCAAGAAACACAGAATTACTGCTGAGATTTTTTCTCCAAATGACAAGAAG

TCTTTTTGCTCAATTGAAGGGGAATGGAATGGTGTAATGTATGCAAAATATGCAACAGGG

GAAAATACGGTCTTTGTAGATACCAAGAAGTTGCCTATAATCAAGAAGAAAGTGAGGAAG

TTAGAAGATCAGAATGAATATGAGTCCCGCTGCCTTTGGAAGGATGTCACTTTCAATTTA

AAAATCAGAGACATTGATGCAGCAACTGAAGCAAAGCACAGACTTGAAGAAAGACAAAGA

GCAGAAGCCCGAGAAAGAAAGGAGAAGGAAATTCAGTGGGAGACAAGGTTATTCCATGAA

GATGGAGAATGCTGGGTTTATGATGAACCATTACTGAAACGTCTTGGTGCTGTGAAGCAT

>CL97.Contig2_All 22 456 PREDICTED: oxysterol-binding protein-related protein 9-like isoform 4 [Cavia porcellus]

AGCATTAGCGACCAGAAAGATCCTAGGGATCGAATGGTTCAGGTTGTGAAATGGTACCTC

TCAGCCTTTCATGCAGGAAGGAAAGGATCGGTTGCTAAAAAGCCATACAATCCCATTTTG

GGTGAGATCTTTCAGTGTCACTGGACGTTACCAAATGACACTGAAGAGAACACAGAGCTA

GTTTCAGAAGGACCAGTTCCCTGGGTTTCCAAAAACAGTGTAACATTCGTGGCTGAGCAG

GTTTCCCATCATCCACCCATTTCAGCCTTTTATGCTGAATGCTTTAACAAGAAAATCCAA

TTCAATGCTCATATCTGGACTAAATCAAAATTCCTTGGGATGTCAATTGGGGTGCACAAC

ATAGGGCAAGGCTGTGTCTCATGTCTAGACTATGATGAGCATTACATTCTCACATTCCCC

AATGGTTATGGAAGG

>CL99.Contig1_All 99 1016 minus strand PREDICTED: 39S ribosomal protein L2, mitochondrial [Papio anubis]

ATGGCCCTATGGACACTGACTCGTGCTCTGGGCTCTCTCAGTCTGGCGCCTCCGGCCGTT

ACCGCCCCCGGCACGAGTCTACTCCCTGCTGCGCAGGTGGTGAGCAATGCCCTCCTCCAA

CTGCCCTCTGCGTTGATGTTGCTCCCCTGCCGCCCTATACTTACTTCTGGGGCCCTTCAT

GCCAAGTTTATATCCTGGAAGAGTCGTACTAAGTACACCATCGTACCAGTGAAAAAGAGG

AAATCTGGGGGTCGAGACCACACAGGCCGAATCCGAGTACATGGTATTGGTGGGGGCCAC

AAGCAACTTTATCGCATGATTGACTTTCTGCGGTTCCGGCCAGAGCAGGAGACTAAGCCA

GGACCCTTTGAGGAAAAGGTTATCAGAGTCCGCTATGATCCTTGTAGGTCAGCAGACATA

GCTCTGGTTGCTGGGGGCAGTCGGAAACGTTGGATCATTGCCACAGAGAACATGCAGGCT

GGAGATATAATCCTGAACTCTAACCACATAGGCCGAATGGCAGTTGCTGCTCGGGAAGGG

GATGCACATCCTCTTGGGGCCCTGCCTGTGGGGACTCTCATTAACAACGTGGAAAGTGAG

CCAGGCCGAGGGGCCCAGTATATTCGAGCTGCAGGGACTTGTGGTGTGCTGCTGCGGAAG

GTGAATGGAACAGCCATTATCCAGTTGCCCTCTAAGAGGCAGATGCAGGTACTAGAAACG

TGCACAGCAACAGTCGGCAGAGTATCCAATGTTGATCATAACAAACGGGTCATCGGCAAA

GCCGGTCGGAACCGCTGGCTGGGCAAGAGGCCTTCCAGTGGGCTGTGGCACCGCAAGGGG

GGGTGGGCTGGCCGAAAGATTCGGCCACTGCCCCCCATGAAGAGTTATGTGAAGCTGCCC

TCCGCTGCTGCCCAAAGT

>CL99.Contig2_All 44 868 minus strand PREDICTED: 39S ribosomal protein L2, mitochondrial-like [Equus caballus]

AAGGTGGTGAGCAATGCCCTCCTCCAACTGCCCTCTGCGTTGATGTTGCTCCCCTGCCGC

CCTATACTTACTTCTGGGGCCCTTCATGCCAAGTTTATATCCTGGAAGAGTCGTACTAAG

TACACCATCGTACCAGTGAAAAAGAGGAAATCTGGGGGTCGAGACCACACAGGCCGAATC

CGAGTACATGGTATTGGTGGGGGCCACAAGCAACTTTATCGCATGATTGACTTTCTGCGG

TTCCGGCCAGAGCAGGAGACTAAGCCAGGACCCTTTGAGGAAAAGGTTATCAGAGTCCGC

TATGATCCTTGTAGGTCAGCAGACATAGCTCTGGTTGCTGGGGGCAGTCGGAAACGTTGG

ATCATTGCCACAGAGAACATGCAGGCTGGAGATATAATCCTGAACTCTAACCACATAGGC

CGAATGGCAGTTGCTGCTCGGGAAGGGGATGCACATCCTCTTGGGGCCCTGCCTGTGGGG

ACTCTCATTAACAACGTGGAAAGTGAGCCAGGCCGAGGGGCCCAGTATATTCGAGCTGCA

GGGACTTGTGGTGTGCTGCTGCGGAAGGTGAATGGAACAGCCATTATCCAGTTGCCCTCT

AAGAGGCAGATGCAGGTACTAGAAACGTGCACAGCAACAGTCGGCAGAGTATCCAATGTT

GATCATAACAAACGGGTCATCGGCAAAGCCGGTCGGAACCGCTGGCTGGGCAAGAGGCCT

TCCAGTGGGCTGTGGCACCGCAAGGGGGGGTGGGCTGGCCGAAAGATTCGGCCACTGCCC

CCCATGAAGAGTTATGTGAAGCTGCCCTCCGCTGCTGCCCAAAGT

>CL99.Contig3_All 646 747 minus strand hypothetical protein [Pongo abelii]

AGGGTGGCAAAGCTGCTTCTCCCTTCCCCAGGGACTTGTGGTGTGCTGCTGCGGAAGGTG

AATGGAACAGCCATTATCCAGTTGCCCTCTAAGAGGCAGATG

>CL99.Contig4_All 106 207 minus strand hypothetical protein [Pongo abelii]

AGGGTGGCAAAGCTGCTTCTCCCTTCCCCAGGGACTTGTGGTGTGCTGCTGCGGAAGGTG

AATGGAACAGCCATTATCCAGTTGCCCTCTAAGAGGCAGATG

>CL99.Contig5_All 824 1108 PREDICTED: 39S ribosomal protein L2, mitochondrial [Papio anubis]

GGGACTTGTGGTGTGCTGCTGCGGAAGGTGAATGGAACAGCCATTATCCAGTTGCCCTCT

AAGAGGCAGATGCAGGTACTAGAAACGTGCACAGCAACAGTCGGCAGAGTATCCAATGTT

GATCATAACAAACGGGTCATCGGCAAAGCCGGTCGGAACCGCTGGCTGGGCAAGAGGCCT

TCCAGTGGGCTGTGGCACCGCAAGGGGGGGTGGGCTGGCCGAAAGATTCGGCCACTGCCC

CCCATGAAGAGTTATGTGAAGCTGCCCTCCGCTGCTGCCCAAAGT

>CL101.Contig1_All 101 676 minus strand apoptosis regulator BAX [Bos taurus] >gi|5915766|sp|O02703.1|BAX_BOVIN RecName: Full=Apoptosis regulator BAX >gi|1938371|gb|AAC48806.1| bax-alpha [Bos taurus] >gi|296477397|gb|DAA19512.1| apoptosis regulator BAX [Bos taurus]

ATGGACGGGTCCGGGGAGCAGCCCAGAGGAGGCGGGCCCACCAGCTCTGAGCAGATCATG

AAGACAGGGGCCCTTTTGCTTCAGGGTTTCATCCAGGATCGAGCAGGACGGATGGGGGGG

GACACATCAGAGCTGGCCTTGGAGCAGGTGCCCCAGGATGCTTCCACCAAGAAGCTGAGC

GAGTGTCTGAAGCGCATCGGTGATGAACTAGACAGTAACATGGAGCTTCAGAGGATGATT

GCAGCTGTGGACACAGACTCCCCCCGAGAGGTCTTTTTCCGAGTGGCAGCTGATATGTTT

GCTGACGGCAACTTCAACTGGGGCCGGGTTGTCGCCCTTTTCTACTTTGCCAGCAAACTG

GTGCTCAAGGCCCTGTGTACCAAGGTGCCAGAGTTGATCAGAACCATCATGGGCTGGACG

CTGGACTTCCTCCGAGAGCGGCTACTGGGCTGGATTCAAGATCAGGGTGGTTGGGATGGC

CTCCTCTCCTACTTTGGGACCCCCACGTGGCAGACAGTGACCATCTTTGTGGCCGGAGTG

CTCACCGCCTCACTCACCATCTGGAAGAAGATGGGT

>CL101.Contig2_All 41 532 minus strand Apoptosis regulator BAX OS=Bos taurus GN=BAX PE=2 SV=1

GGTTTCATCCAGGATCGAGCAGGACGGATGGGGGGGGACACATCAGAGCTGGCCTTGGAG

CAGGTGCCCCAGGATGCTTCCACCAAGAAGCTGAGCGAGTGTCTGAAGCGCATCGGTGAT

GAACTAGACAGTAACATGGAGCTTCAGAGGATGATTGCAGCTGTGGACACAGACTCCCCC

CGAGAGGTCTTTTTCCGAGTGGCAGCTGATATGTTTGCTGACGGCAACTTCAACTGGGGC

CGGGTTGTCGCCCTTTTCTACTTTGCCAGCAAACTGGTGCTCAAGGCCCTGTGTACCAAG

GTGCCAGAGTTGATCAGAACCATCATGGGCTGGACGCTGGACTTCCTCCGAGAGCGGCTA

CTGGGCTGGATTCAAGATCAGGGTGGTTGGGATGGCCTCCTCTCCTACTTTGGGACCCCC

ACGTGGCAGACAGTGACCATCTTTGTGGCCGGAGTGCTCACCGCCTCACTCACCATCTGG

AAGAAGATGGGT

>CL102.Contig1_All 2 523 PREDICTED: RNA 3&apos;-terminal phosphate cyclase [Papio anubis]

AGGGGCTATTATCCAAAAGGTGGTGGTGAAGTAATTGTTCGGATGTCACCAGTTACACAG

TTGAACCCAATAAATTTGACTGATCGTGGCTCTGTGACTAAGATATATGGAAGAGCTTTT

GTTGCTGGTGTTCTGCCATTTAAAGTAGCAAAAGATATGGCAGCAGCGGCTGTGAGATGC

ATCAGAAAGGAGATCAGAGATCTATATGTTAACATCCAGCCTGTTCAGGAACCCAAAGAC

CAAGCATTTGGCAATGGAAATGGAATAATAATTATTGCTGAGACATCCACAGGCTGTTTG

TTTGCTGGATCATCGCTTGGGAAACGAGGTGTAAATGCAGACAAGGTTGGAATTGATGCT

GCTGAAATGCTGTTAGCTAATCTTAGACATGGTGGAACTGTGGATGAGTTTCTGCAAGAC

CAGGCTAAATTTACTGTGAAGAAATCAGAAGATGAAGAAGATTCCTCTAAAGACACTTAT

ATTATTGAATGCCAAGGAATTGGGATGAGAAATCCAAATCTG

>CL102.Contig2_All 140 1252 minus strand PREDICTED: RNA terminal phosphate cyclase domain 1 isoform 2 [Oryctolagus cuniculus]

ATGGCGGGGCAGCGGGTGGAGGTCGACGGCGGGATCTTGGAAGGGGGCGGCCAGATCCTC

AGGGTCTCCACCGCCCTCAGCTGTGTCCTGGGCCTCCCCTTGCGGGTGCAAAAGATCCGG

GCGGGCCGCAGCACGCCAGGCCTGAGTATTATGACCTGAAGGCCTCAACATTTATCTGGA

CTGGAAATGATTCGAGATTTGTGTGATGGGCAGCTGGAGGGGGCAGCAATTGGTTCAACT

GAAGTAACCTTCACACCAGAGAAGATCAAAGGTGGAATCCACACAGCAGATACCAAGACA

GCAGGGAGTGTGTGCCTCTTGATGCAGGTTTCAATGCCATGTGTTCTGTTTGCTGCTTCT

CCATCAGAACTTCATTTGAAAGGTGGAACTAATGCTGAAATGGCACCACAAATCGACTAC

ACAGTGATGGTCTTCAAGCCAATTGTTGAAAAATTTGGTTTTAATTTTAATTGTGACATC

AAAATGAGGGGCTATTATCCAAAAGGTGGTGGTGAAGTAATTGTTCGGATGTCACCAGTT

ACACAGTTGAACCCAATAAATTTGACTGATCGTGGCTCTGTGACTAAGATATATGGAAGA

GCTTTTGTTGCTGGTGTTCTGCCATTTAAAGTAGCAAAAGATATGGCAGCAGCGGCTGTG

AGATGCATCAGAAAGGAGATCAGAGATCTATATGTTAACATCCAGCCTGTTCAGGAACCC

AAAGACCAAGCATTTGGCAATGGAAATGGAATAATAATTATTGCTGAGACATCCACAGGC

TGTTTGTTTGCTGGATCATCGCTTGGGAAACGAGGTGTAAATGCAGACAAGGTTGGAATT

GATGCTGCTGAAATGCTGTTAGCTAATCTTAGACATGGTGGAACTGTGGATGAGTTTCTG

CAAGACCAGCTGATTATTTTCATGGCATTAGCCAATGGAGTTTCCAGAATAAAAACAGGA

CCAGTTACACTCCATACACAAACTGCTATACATTTTGCTGAACAACTAGCAAAGGCTAAA

TTTACTGTGAAGAAATCAGAAGATGAAGAAGATTCCTCTAAAGACACTTATATTATTGAA

TGCCAAGGAATTGGGATGAGAAATCCAAATCTG

>CL105.Contig1_All 1184 2275 PREDICTED: neurexin I-like isoform 3 [Oryctolagus cuniculus]

TTCCAGTTCAAGACTACATCCCTTGATGGGCTAATTCTATATAACAGTGGGGATGGAAAT

GACTTTATCGTAGTTGAATTAGTTAAAGGGTACTTACATTATGTGTTTGATTTGGGAAAT

GGTGCTAACCTCATCAAAGGGAGTTCTAATAAACCACTCAATGACAATCAGTGGCACAAC

GTGATGATATCAAGGGACACCAGCAATCTCCACACTGTAAAGATTGACACAAAAATCACA

ACACAGATCACCGCAGGAGCCAGGAATTTAGACCTCAAGAGTGACTTGTATATAGGAGGA

GTGGCTAAAGAAACATACAAATCCTTGCCAAAACTCGTCCATGCCAAGGAAGGCTTTCAA

GGCTGCCTGGCATCGGTTGATTTAAATGGACGGCTTCCGGACCTCATCTCAGATGCTCTT

TTCTGCAATGGACAGATTGAGAGAGGATGTGAAGGGCCCAGCACAACCTGCCAAGAGGAC

TCGTGTTCCAATCAAGGTGTCTGCTTACAGCAGTGGGATGGCTTCAGCTGTGACTGTAGC

ATGACTTCCTTCAGTGGGCCACTCTGCAATGACCCTGGGACAACGTATATCTTTAGCAAA

GGTGGAGGACAAATCACGTATAAGTGGCCTCCTAATGACCGGCCAAGTACAAGAGCAGAC

AGACTGGCCATAGGGTTTAGTACTGTTCAGAAAGAAGCAGTCCTGGTACGAGTGGACAGT

TCTTCAGGCTTGGGGGACTACCTAGAACTGCATATACACCAAGGAAAAATTGGAGTTAAG

TTTAATGTCGGGACGGATGACATCGCTATTGAAGAGTCCAATGCAATCATTAATGATGGG

AAGTACCATGTGGTCCGATTCACGAGGAGTGGTGGCAATGCCACGTTACAGGTGGACAGC

TGGCCAGTGATCGAGCGCTACCCAGCAGGGCGTCAGCTCACAATCTTCAATAGCCAAGCA

ACCATAATAATTGGCGGGAAAGAGCAGGGCCAGCCCTTCCAGGGCCAGCTCTCTGGGCTT

TACTACAATGGCTTGAAAGTTCTGAATATGGCAGCCGAAAACGATGCCAACATCGCCATA

GTGGGAAATGTG

>CL105.Contig2_All 1184 2080 neurexin-1-alpha isoform 2 precursor [Mus musculus]

TTCCAGTTCAAGACTACATCCCTTGATGGGCTAATTCTATATAACAGTGGGGATGGAAAT

GACTTTATCGTAGTTGAATTAGTTAAAGGGTACTTACATTATGTGTTTGATTTGGGAAAT

GGTGCTAACCTCATCAAAGGGAGTTCTAATAAACCACTCAATGACAATCAGTGGCACAAC

GTGATGATATCAAGGGACACCAGCAATCTCCACACTGTAAAGATTGACACAAAAATCACA

ACACAGATCACCGCAGGAGCCAGGAATTTAGACCTCAAGAGTGACTTGTATATAGGAGGA

GTGGCTAAAGAAACATACAAATCCTTGCCAAAACTCGTCCATGCCAAGGAAGGCTTTCAA

GGCTGCCTGGCATCGGTTGATTTAAATGGACGGCTTCCGGACCTCATCTCAGATGCTCTT

TTCTGCAATGGACAGATTGAGAGAGGATGTGAAGGGCCCAGCACAACCTGCCAAGAGGAC

TCGTGTTCCAATCAAGGTGTCTGCTTACAGCAGTGGGATGGCTTCAGCTGTGACTGTAGC

ATGACTTCCTTCAGTGGGCCACTCTGCAATGACCCTGGGACAACGTATATCTTTAGCAAA

GGTGGAGGACAAATCACGTATAAGTGGCCTCCTAATGACCGGCCAAGTACAAGAGCAGAC

AGACTGGCCATAGGGTTTAGTACTGTTCAGAAAGAAGCAGTCCTGGTACGAGTGGACAGT

TCTTCAGGCTTGGGGGACTACCTAGAACTGCATATACACCAAGGAAAAATTGGAGTTAAG

TTTAATGTCGGGACGGATGACATCGCTATTGAAGAGTCCAATGCAATCATTAATGATGGG

AAGTACCATGTGGTCCGATTCACGAGGAGTGGTGGCAATGCCACGTTACAGGTGGAC

>CL106.Contig1_All 443 553 minus strand Replication factor C subunit 2 [Heterocephalus glaber]

CAGGAAATTGGATACACGCACATGAAAGTCACCGAAGGCGTGAACTCCCTCCTGCAGATG

GCGGGGCTCCTGGCCAGGCTGTGTCAGAAGACAATAGCCCCTGTGGCCAGT

>CL106.Contig2_All 1288 1398 minus strand replication factor C subunit 2 [Bos taurus] >gi|122131746|sp|Q05B83.1|RFC2_BOVIN RecName: Full=Replication factor C subunit 2; AltName: Full=Activator 1 subunit 2 >gi|115545402|gb|AAI22636.1| Replication factor C (activator 1) 2, 40kDa [Bos taurus] >gi|296472929|gb|DAA15044.1| replication factor C subunit 2 [Bos taurus]

CAGGAAATTGGATACACGCACATGAAAGTCACCGAAGGCGTGAACTCCCTCCTGCAGATG

GCGGGGCTCCTGGCCAGGCTGTGTCAGAAGACAATAGCCCCTGTGGCCAGT

>CL106.Contig3_All 121 1176 minus strand replication factor C subunit 2 [Bos taurus] >gi|122131746|sp|Q05B83.1|RFC2_BOVIN RecName: Full=Replication factor C subunit 2; AltName: Full=Activator 1 subunit 2 >gi|115545402|gb|AAI22636.1| Replication factor C (activator 1) 2, 40kDa [Bos taurus] >gi|296472929|gb|DAA15044.1| replication factor C subunit 2 [Bos taurus]

ATGGAGGCGCAGCCTGGCGGCAGTGGCGCAGCAGACCGCGGGGCCGAGGACTCTGCGCCT

GGCCCCAGCAAGGCCTCTGGCGGCTCCGGCCACTACGAGCTGCCGTGGGTTGAAAAATAC

AGGCCAATAAAGCTGAATGAAATTGTTGGGAATGAAGACACTGTGAGCCGGCTAGAGGTC

TTTGCGAGAGAAGGGAATCTGCCCAATATCATCATTGCGGGTCCCCCGGGAACTGGCAAA

ACTACCAGCATCCTGTGTCTGGCCCGCGCTCTGCTGGGCCCTGCGCTGAAGGACGCTGTC

CTGGAACTCAACGCCTCCAACGACAGGGGCATCGACGTTGTGAGGAATAAAATCAAGATG

TTTGCTCAACAGAAGGTCACCCTTCCCAAAGGCCGGCACAAGATCATCATCCTGGACGAG

GCAGACAGCATGACGGATGGAGCCCAGCAGGCCTTGAGGAGGACCATGGAGATCTACTCC

AAGACCACGCGCTTCGCTCTGGCCTGTAACGCCTCCGACAAGATCATAGAGCCCATCCAG

TCGCGCTGTGCGGTGCTCCGCTACACCAAGCTCACGGATGCCCAGGTGCTGGCCAGGCTC

ATGAGTGTCCTGGAGAAGGAGGAGGTGCGGTACACGGACGACGGCCTGGAGGCCATCATC

TTCACTGCCCAGGGGGACATGCGGCAGGCTCTGAACAACCTGCAGTCCACCTTCTCGGGG

TTTGGCTTCATCAATAGTGAGAACGTGTTTAAGGTCTGCGACGAGCCGCATCCGCTGCTG

GTGAAGGAGATGATCCAGCACTGCGTGCACGGCGACATCGACGAGGCCTACAAGATCCTG

GCCCACCTGTGGCATCTGGGCTATTCGCCAGAGGATGTCATCGGCAACATCTTTCGAGTG

TGTAAAACTTTCCAAATGGCCGAGTACTTGAAGCTGGAGTTTATCAAGGAAATTGGATAC

ACGCACATGAAAGTCACCGAAGGCGTGAACTCCCTCCTGCAGATGGCGGGGCTCCTGGCC

AGGCTGTGTCAGAAGACAATAGCCCCTGTGGCCAGT

>CL106.Contig4_All 110 331 minus strand PREDICTED: replication factor C subunit 2 [Otolemur garnettii]

ATCCTGGCCCACCTGTGGCATCTGGGCTATTCGCCAGAGGATGTCATCGGCAACATCTTT

CGAGTGTGTAAAACTTTCCAAATGGCCGAGTACTTGAAGCTGGAGTTTATCAAGGAAATT

GGATACACGCACATGAAAGTCACCGAAGGCGTGAACTCCCTCCTGCAGATGGCGGGGCTC

CTGGCCAGGCTGTGTCAGAAGACAATAGCCCCTGTGGCCAGT

>CL106.Contig5_All 1101 1211 minus strand replication factor C subunit 2 [Bos taurus] >gi|122131746|sp|Q05B83.1|RFC2_BOVIN RecName: Full=Replication factor C subunit 2; AltName: Full=Activator 1 subunit 2 >gi|115545402|gb|AAI22636.1| Replication factor C (activator 1) 2, 40kDa [Bos taurus] >gi|296472929|gb|DAA15044.1| replication factor C subunit 2 [Bos taurus]

CAGGAAATTGGATACACGCACATGAAAGTCACCGAAGGCGTGAACTCCCTCCTGCAGATG

GCGGGGCTCCTGGCCAGGCTGTGTCAGAAGACAATAGCCCCTGTGGCCAGT

>CL106.Contig6_All 38 985 minus strand replication factor C subunit 2 [Bos taurus] >gi|122131746|sp|Q05B83.1|RFC2_BOVIN RecName: Full=Replication factor C subunit 2; AltName: Full=Activator 1 subunit 2 >gi|115545402|gb|AAI22636.1| Replication factor C (activator 1) 2, 40kDa [Bos taurus] >gi|296472929|gb|DAA15044.1| replication factor C subunit 2 [Bos taurus]

ATGGAGGCGCAGCCTGGCGGCAGTGGCGCAGCAGACCGCGGGGCCGAGGACTCTGCGCCT

GGCCCCAGCAAGGCCTCTGGCGGCTCCGGCCACTACGAGCTGCCGTGGGTTGAAAAATAC

AGGCCAATAAAGCTGAATGAAATTGTTGGGAATGAAGACACTGTGAGCCGGCTAGAGGTC

TTTGCGAGAGAAGGGAATCTGCCCAATATCATCATTGCGGGTCCCCCGGGAACTGGCAAA

ACTACCAGCATCCTGTGTCTGGCCCGCGCTCTGCTGGGCCCTGCGCTGAAGGACGCTGTC

CTGGAACTCAACGCCTCCAACGACAGGGGCATCGACGTTGTGAGGAATAAAATCAAGATG

TTTGCTCAACAGAAGGTCACCCTTCCCAAAGGCCGGCACAAGATCATCATCCTGGACGAG

GCAGACAGCATGACGGATGGAGCCCAGCAGGCCTTGAGGAGGACCATGGAGATCTACTCC

AAGACCACGCGCTTCGCTCTGGCCTGTAACGCCTCCGACAAGATCATAGAGCCCATCCAG

TCGCGCTGTGCGGTGCTCCGCTACACCAAGCTCACGGATGCCCAGGTGCTGGCCAGGCTC

ATGAGTGTCCTGGAGAAGGAGGAGGTGCGGTACACGGACGACGGCCTGGAGGCCATCATC

TTCACTGCCCAGGGGGACATGCGGCAGGCTCTGAACAACCTGCAGTCCACCTTCTCGGGG

TTTGGCTTCATCAATAGTGAGAACGTGTTTAAGGTCTGCGACGAGCCGCATCCGCTGCTG

GTGAAGGAGATGATCCAGCACTGCGTGCACGGCGACATCGACGAGGCCTACAAGATCCTG

GCCCACCTGTGGCATCTGGGCTATTCGCCAGAGGATGTCATCGGCAACATCTTTCGAGTG

TGTAAAACTTTCCAAATGGCCGAGTACTTGAAGCTGGAGTTTATCAAG

>CL106.Contig7_All 312 422 minus strand PREDICTED: replication factor C subunit 2 [Otolemur garnettii]

CAGGAAATTGGATACACGCACATGAAAGTCACCGAAGGCGTGAACTCCCTCCTGCAGATG

GCGGGGCTCCTGGCCAGGCTGTGTCAGAAGACAATAGCCCCTGTGGCCAGT

>CL106.Contig8_All 306 416 minus strand PREDICTED: replication factor C subunit 2 [Otolemur garnettii]

CAGGAAATTGGATACACGCACATGAAAGTCACCGAAGGCGTGAACTCCCTCCTGCAGATG

GCGGGGCTCCTGGCCAGGCTGTGTCAGAAGACAATAGCCCCTGTGGCCAGT

>CL106.Contig9_All 83 196 minus strand PREDICTED: replication factor C subunit 2 [Otolemur garnettii]

ATCCTGGCCCACCTGTGGCATCTGGGCTATTCGCCAGAGGATGTCATCGGCAACATCTTT

CGAGTGTGTAAAACTTTCCAAATGGCCGAGTACTTGAAGCTGGAGTTTATCAAG

>CL107.Contig1_All 688 843 ERV-F(c)1 provirus ancestral Env polyprotein OS=Pan troglodytes PE=3 SV=1

CTTCCCCAGTGGGCACTTCCTTTCCTGACCTCTCTCCTTGTCATAGGCCTTTTGCTAATG

CTTGCCCCCTGCATCATTAGATTTATTCAAGACCAGATTCAAAGGGTCTCTAACCAAATC

ATGAATCAGCTTCTGTTACAGGACTTCCAACCCCTC

>CL107.Contig10_All 5 235 minus strand unnamed protein product [Trypanosoma congolense IL3000]

CGTCAGAGAGAGAGAGAGGCCCAAAAAGGGTGGTATGAGTCTTGGTTCACACAGTCTCCC

TGGTTAACTACGCTTTTATCAACCCTGGCCGATCCTTTAATAATTCTTATATTGTTGCTA

ACTCTAGGACCCTGTTTGCTCAACCGTGTAGTTTCCTTTCTCCAAGCCCAAATTGGACAA

GTCGAACTTATGGTAATCAGACAACAATATACCCCACTAGCAGACACAGAA

>CL107.Contig11_All 1781 2218 minus strand env protein [Sus scrofa]

CCACCCCAATACCAAGTGGGAGACTTCATCTATATAAGACGACATCAGGTGTCATCCCTG

GAGCCTTGCTGGAAAGGAACCTGGTGGGCATGTGACTCAGGGTTGACTCCTTGCATTTCA

GCCCAGATATTTAACAACTCTGTAAATTATTGTGTAATGGTACAAATATTTCCCAAAGTG

CTCTATCATGACGCAAATACATTTGAAGATCAAATAAGGGGATATACCACCCGATTCCGC

TGGGAACCTGTGTCCCTCACTTTAGCCATATTATTAGAAATAGGAGTTGCTACAGGAGTG

GGTACAGGAACCGCTGCCCTGGTCCATGGGTCTCAACAAATGGCCCAATTACAAGCAGCA

ATAGATCAGGACTTAAAAACATTAGAAACCTCCATCACAGCCTTACAAGAGTCCCTAACC

TCTCTCTCTGAAGTTGTT

>CL108.Contig1_All 96 1055 minus strand PREDICTED: Down syndrome critical region protein 3-like [Loxodonta africana]

ATGGGGACTTCCCTGGACATCAAGATTAAAAGAGCGAATAAAGTTTATCACGCCGGGGTG

GACACAATGTCTTTATTTTACTTTATGTGGAGCTGAGGATCGAACCCAGTGCCGCCTCAC

GCATGCTAGGAAATGCTCTCAGGCGTGGTGGTCGTCTCTAGTAAGGATTCCGTCCAGCAC

CAGGGAGTGTCTTTGACCATGGAAGGAACCGTGAACCTCCAGCTCAGTGCCAAAAGTGTG

GGAGTGTTCGAAGCATTTTACAATTCTGTTAAGCCGATCCAGATTATCAACAGCACCATA

GAAATGGTGAAGCCAGGAAAGTTTCCCAGTGGCAAAACAGAAATTCCTTTTGAATTTCCT

CTCTACGTGAAGGGTAACAAAGTCCTGCACGAGACCTACCATGGTGTGTTTGTCAACATT

CAGTACACGCTGCGCTGTGACATGAGGCGGTCTCTGCTGGCCAAAGACTTGACAAAGACC

TGTGAATTCATTGTTCACTCTGCTCCTCAGAAGGGGACATTGACTCCGAGCCCTGTGGAC

TTCACCATCACCCCAGAAACTTTACAGAACGTCAAAGAGAGAGCTTTGCTCCCCAAATTC

CTCATTAGAGGACATCTCAACTCAACCAACTGTGTTATCACACAGCCACTGACAGGAGAG

CTGGTAGTGGAGCACTCGGACGCCGCCATCCGCAGCATAGAGCTGCAGCTGGTCCGCGTG

GAGACCTGTGGGTGTGCAGAAGGCTATGCACGTGATGCCACAGAGATCCAGAACATTCAG

ATTGCCGATGGGGACGTCTGCAGGGGCCTCTCTGTCCCTATACACATGGTATTTCCCAGG

CTCTTCACCTGCCCCACGCTGGAGACCACCAACTTCAAAGTGGAGTTCGAGGTGAACATC

GTGGTGCTGCTTCACGCCGACCACCTCATCACAGAGAACTTCCCGCTGAAGCTCTGCCGG

>CL108.Contig2_All 138 1025 minus strand PREDICTED: Down syndrome critical region protein 3-like [Loxodonta africana]

ATGGGGACTTCCCTGGACATCAAGATTAAAAGAGCGAATAAAGTTTATCACGCCGGGGAA

ATGCTCTCAGGCGTGGTGGTCGTCTCTAGTAAGGATTCCGTCCAGCACCAGGGAGTGTCT

TTGACCATGGAAGGAACCGTGAACCTCCAGCTCAGTGCCAAAAGTGTGGGAGTGTTCGAA

GCATTTTACAATTCTGTTAAGCCGATCCAGATTATCAACAGCACCATAGAAATGGTGAAG

CCAGGAAAGTTTCCCAGTGGCAAAACAGAAATTCCTTTTGAATTTCCTCTCTACGTGAAG

GGTAACAAAGTCCTGCACGAGACCTACCATGGTGTGTTTGTCAACATTCAGTACACGCTG

CGCTGTGACATGAGGCGGTCTCTGCTGGCCAAAGACTTGACAAAGACCTGTGAATTCATT

GTTCACTCTGCTCCTCAGAAGGGGACATTGACTCCGAGCCCTGTGGACTTCACCATCACC

CCAGAAACTTTACAGAACGTCAAAGAGAGAGCTTTGCTCCCCAAATTCCTCATTAGAGGA

CATCTCAACTCAACCAACTGTGTTATCACACAGCCACTGACAGGAGAGCTGGTAGTGGAG

CACTCGGACGCCGCCATCCGCAGCATAGAGCTGCAGCTGGTCCGCGTGGAGACCTGTGGG

TGTGCAGAAGGCTATGCACGTGATGCCACAGAGATCCAGAACATTCAGATTGCCGATGGG

GACGTCTGCAGGGGCCTCTCTGTCCCTATACACATGGTATTTCCCAGGCTCTTCACCTGC

CCCACGCTGGAGACCACCAACTTCAAAGTGGAGTTCGAGGTGAACATCGTGGTGCTGCTT

CACGCCGACCACCTCATCACAGAGAACTTCCCGCTGAAGCTCTGCCGG

>CL108.Contig3_All 3 368 PREDICTED: Down syndrome critical region protein 3-like [Cavia porcellus]

TTCCTCATTAGAGGACATCTCAACTCAACCAACTGTGTTATCACACAGCCACTGACAGGA

GAGCTGGTAGTGGAGCACTCGGACGCCGCCATCCGCAGCATAGAGCTGCAGCTGGTCCGC

GTGGAGACCTGTGGGTGTGCAGAAGGCTATGCACGTGATGCCACAGAGATCCAGAACATT

CAGATTGCCGATGGGGACGTCTGCAGGGGCCTCTCTGTCCCTATACACATGGTATTTCCC

AGGCTCTTCACCTGCCCCACGCTGGAGACCACCAACTTCAAAGTGGAGTTCGAGGTGAAC

ATCGTGGTGCTGCTTCACGCCGACCACCTCATCACAGAGAACTTCCCGCTGAAGCTCTGC

CGGACA

>CL109.Contig1_All 412 3360 PREDICTED: ring finger protein 20-like [Oryctolagus cuniculus]

AAACGGCTATCATCTTATGCCAAGATGTCAGGAATCGGAAATAAAAGAGCAGCTGGTGAG

CCAGGCACATCCATGCCTCCAGAGAAGAAGGCAGCTGTTGAAGATTCAGGGACCACCGTG

GAAACAATTAAGCTAGGGGGTGTCTCTTCAACGGAGGAGTTAGACATTCGAACACTACAA

ACAAAAAATCGCAAGCTAGCAGAAATGCTCGATCAGCGTCAGGCCATCGAAGATGAACTT

CGGGAGCACATTGAAAAACTAGAACGACGACAGGCCACTGATGATGCTTCACTGTTGATT

GTCAATCGGTACTGGAGTCAGTTCGATGAAAACATCCGTATCATCCTTAAACGTTATGAT

CTGGAGCAGGGTTTGGGAGACCTACTCACAGAAAGGAAAGCCCTTGTTGTGCCGGAACCA

GAACCTGACTCTGATAGCAATCAGGAACGCAAAGATGACCGAGAGAGAGGGGAAGGGCAA

GAGCCAGCTTTCTCTTTCCTTGCTACTTTGGCCAGCAGTTCCAGTGAAGAGATGGAGTCT

CAGCTGCAGGAGCGTGTGGAGTCCTCCCGGCGAGCTGTGTCCCAGATTGTGACTGTTTAT

GATAAATTGCAAGAAAAAGTGGAGCTCTTATCCCGGAAACTAAACAGTGGAGATAATCTG

ATAGTAGACGAAGCAGTACAGGAACTGAATTCCTTCCTCGTGCAAGAGAATATGAGGCTA

CAGGAACTGACAGACCTCCTTCAGGAGAAGCATTGCACCATGTCTCAGGAGTTTTCTAAG

TTGCAGAGTAAAGTGGAGACCGCGGAGTCACGGGTATCTGTCCTGGAGTCTATGATTGAT

GATCTGCAGTGGGATATTGACAAAATTCGTAAGAGGGAACAGCGACTCAACCGACACTTG

GCAGAAGTCCTAGAACGGGTGAATTCAAAAGGCTATAAGGTATATGGAGCAGGAAGCAGT

CTCTATGGTGGCACAATCACTATCAATGCCCGGAAGTTTGAGGAAATGAATGCAGAGCTT

GAAGAGAACAAAGAGTTGGCCCAGAACCGTCACTGTGAGCTGGAGAAACTTCGGCAAGAC

TTTGAGGAGGTCACTACCCAGAATGAGAAGTTGAAGGTGGAATTGCGGAGTGCAGTGGAG

GAGGTGGTTAAGGAAACTCCAGAATATCGTTGCATGCAGTCACAGTTCTCTGTTCTGTAC

AATGAGAGCCTACAGTTGAAAGCTCACTTGGATGAGGCTCGGACCCTGCTTCATGGCACC

AGGGGAACCCACCAGCGCCAGGTTGAACTCATTGAGCGAGATGAGGTTAGTCTTCATAAG

AAGCTGAGAACTGAAGTGATCCAGCTAGAAGATACACTGGCTCAGGTCCGCAAGGAATAT

GAAATGCTGAGGATAGAATTTGAGCAGACCCTTGCTGCCAATGAACAAGCAGGTCCTATA

AACCGGGAAATGCGCCACCTCATCAGTAGCCTCCAGAATCACAATCACCAGCTGAAAGGG

GAGGTCCTAAGGTATAAACGGAAGTTGAGAGAAGCCCAATCTGATCTGAACAAGACACGT

CTGCGCAGTGGCAGTGCCCTCCTGCAGTCTCAGTCTAGTACAGAGGATGCCAAGGATGAA

CCTGCAGAGTTAAAACAAGATTCTGAGGACTTACCCACCCAGTCCTCAGCACTGAAAACC

TCTCAGGAGGATGTCAATGAAATTAAATCCAAACGGGATGAGGAAGAGCGAGAACGAGAA

AGGAGGGAGAAAGAAAGGGAGCGAGAAAGAGAACGAGAGAAGGAAAAGGAGAGAGAGCGA

GAGAAGCAGAAACTGAAAGAATCAGAAAAAGAAAGAGATTCTGCTAAGGATAAGGAAAAA

GGAAAACATGATGATGGAAGGAAAAAGGAAGCAGAAGTTATCAAACAATTGAAGATTGAA

CTCAAGAAGGCACAAGAGAGCCAAAAGGAGATGAAATTGTTGCTAGATATGTACCGCTCT

GCCCCGAAGGAACAGAGAGACAAAGTTCAACTAATGGCAGCAGAGAAGAAGTCTAAGGCA

GAGTTGGAAGATCTAAGGCAAAGACTCAAGGATCTAGAGGATAAGGAGAAGAAAGAAAAC

AAGAAAATGGCTGATGAGGATGCCTTGAGGAAGATTCGGGCAGTAGAAGAGCAGATTGAA

TACCTACAGAAAAAACTGGCCATGGCCAAGCAGGAAGAAGAAGCTCTGCTCTCTGAGATG

GATGTCACAGGCCAAGCCTTTGAAGACATGCAGGAACAAAATATCCGTTTGATGCAGCAG

TTGCGAGAAAAGGATGATGCAAATTTTAAACTCATGTCAGAGCGTATCAAGTCTAATCAG

ATCCATAAATTACTCAAAGAAGAGAAGGAGGAACTGGCAGACCAGGTTTTGACTCTGAAG

ACTCAGGTTGATGCCCAGTTACAGGTAGTAAGGAAACTGGAAGAGAAGGAGCATTTATTG

CAAAGCAACATTGGCACAGGGGAGAAGGAGCTGGGTCTTAGGACCCAAGCCTTGGAGATG

AATAAACGTAAGGCAATGGAGGCAGCCCAACTTGCAGATGATCTCAAAGCACAACTTGAG

TTGGCGCAGAAGAAGCTCCATGATTTTCAGGATGAGATTGTGGAGAACAGTGTCACCAAA

GAAAAAGATATGTTCAATTTCAAACGAGCCCAGGAGGACATCTCAAGACTTCGAAGGAAG

CTGGAGACCACAAAGAAACCAGATAATGTGCCCAAGTGTGATGAGATTCTGATGGAGGAG

ATTAAGGATTATAAGGCTCGTCTGACCTGTCCATGCTGTAATATGCGCAAAAAGGATGCT

GTACTTACCAAGTGTTTTCATGTTTTCTGCTTTGAGTGTGTGAAAACACGCTATGACACC

CGTCAGCGCAAATGTCCCAAGTGTAATGCTGCTTTTGGTGCCAATGATTTTCATCGCATC

TACATTGGT

>CL109.Contig2_All 28 441 minus strand ring finger protein 20 [Mustela putorius furo]

TCTGCTAAGGATAAGGAAAAAGGAAAACATGATGATGGAAGGAAAAAGGAAGCAGAAGTT

ATCAAACAATTGAAGATTGAACTCAAGAAGGCACAAGAGAGCCAAAAGGAGATGAAATTG

TTGCTAGATATGTACCGCTCTGCCCCGAAGGAACAGAGAGACAAAGTTCAACTAATGGCA

GCAGAGAAGAAGTCTAAGGCAGAGTTGGAAGATCTAAGGCAAAGACTCAAGGATCTAGAG

GATAAGGAGAAGAAAGAAAACAAGAAAATGGCTGATGAGGATGCCTTGAGGAAGATTCGG

GCAGTAGAAGAGCAGATTGAATACCTACAGAAAAAACTGGCCATGGCCAAGCAGGAAGAA

GAAGCTCTGCTCTCTGAGATGGATGTCACAGGCCAAGCCTTTGAAGACATGCAG

>CL111.Contig1_All 381 554 minus strand thioredoxin-like protein 4A [Homo sapiens] >gi|13384656|ref|NP_079575.1| thioredoxin-like protein 4A isoform a [Mus musculus] >gi|386780898|ref|NP_001247795.1| thioredoxin-like protein 4A [Macaca mulatta] >gi|46577662|sp|P83876.1|TXN4A_HUMAN RecName: Full=Thioredoxin-like protein 4A; AltName: Full=DIM1 protein homolog; AltName: Full=Spliceosomal U5 snRNP-specific 15 kDa protein; AltName: Full=Thioredoxin-like U5 snRNP protein U5-15kD >gi|46577663|sp|P83877.1|TXN4A_MOUSE RecName: Full=Thioredoxin-like protein 4A; AltName: Full=DIM1 protein homolog; AltName: Full=Spliceosomal U5 snRNP-specific 15 kDa protein; AltName: Full=Thioredoxin-like U5 snRNP protein U5-15kD >gi|6730460|pdb|1QGV|A Chain A, Human Spliceosomal Protein U5-15kd >gi|82407298|pdb|1SYX|A Chain A, The Crystal Structure Of A Binary U5 Snrnp Complex >gi|82407300|pdb|1SYX|C Chain C, The Crystal Structure Of A Binary U5 Snrnp Complex >gi|82407302|pdb|1SYX|E Chain E, The Crystal Structure Of A Binary U5 Snrnp Complex >gi|6572636|gb|AAF17332.1|AF146373_1 thioredoxin-like U5 snRNP protein U5-15kD [Homo sapiens] >gi|2565275|gb|AAB81950.1| Dim1p homolog [Homo sapiens] >gi|12654441|gb|AAH01046.1| Thioredoxin-like 4A [Homo sapiens] >gi|17939455|gb|AAH19272.1| Thioredoxin-like 4A [Homo sapiens] >gi|21595023|gb|AAH31634.1| Txnl4a protein [Mus musculus] >gi|119587044|gb|EAW66640.1| thioredoxin-like 4A, isoform CRA_b [Homo sapiens] >gi|148677469|gb|EDL09416.1| mCG18172, isoform CRA_c [Mus musculus] >gi|380783349|gb|AFE63550.1| thioredoxin-like protein 4A [Macaca mulatta] >gi|383414455|gb|AFH30441.1| thioredoxin-like protein 4A [Macaca mulatta] >gi|384944932|gb|AFI36071.1| thioredoxin-like protein 4A [Macaca mulatta]

TTCAGGAACAAGCACATCATGATTGACTTGGGCACAGGCAACAACAACAAGATCAACTGG

GCCATGGAAGACAAGCAGGAGATGGTTGACATAATTGAGACTGTATACCGTGGTGCCCGT

AAGGGCCGGGGCCTGGTGGTGTCCCCCAAGGACTACTCCACCAAGTACAGATAC

>CL111.Contig2_All 85 510 minus strand thioredoxin-like protein 4A [Homo sapiens] >gi|13384656|ref|NP_079575.1| thioredoxin-like protein 4A isoform a [Mus musculus] >gi|386780898|ref|NP_001247795.1| thioredoxin-like protein 4A [Macaca mulatta] >gi|46577662|sp|P83876.1|TXN4A_HUMAN RecName: Full=Thioredoxin-like protein 4A; AltName: Full=DIM1 protein homolog; AltName: Full=Spliceosomal U5 snRNP-specific 15 kDa protein; AltName: Full=Thioredoxin-like U5 snRNP protein U5-15kD >gi|46577663|sp|P83877.1|TXN4A_MOUSE RecName: Full=Thioredoxin-like protein 4A; AltName: Full=DIM1 protein homolog; AltName: Full=Spliceosomal U5 snRNP-specific 15 kDa protein; AltName: Full=Thioredoxin-like U5 snRNP protein U5-15kD >gi|6730460|pdb|1QGV|A Chain A, Human Spliceosomal Protein U5-15kd >gi|82407298|pdb|1SYX|A Chain A, The Crystal Structure Of A Binary U5 Snrnp Complex >gi|82407300|pdb|1SYX|C Chain C, The Crystal Structure Of A Binary U5 Snrnp Complex >gi|82407302|pdb|1SYX|E Chain E, The Crystal Structure Of A Binary U5 Snrnp Complex >gi|6572636|gb|AAF17332.1|AF146373_1 thioredoxin-like U5 snRNP protein U5-15kD [Homo sapiens] >gi|2565275|gb|AAB81950.1| Dim1p homolog [Homo sapiens] >gi|12654441|gb|AAH01046.1| Thioredoxin-like 4A [Homo sapiens] >gi|17939455|gb|AAH19272.1| Thioredoxin-like 4A [Homo sapiens] >gi|21595023|gb|AAH31634.1| Txnl4a protein [Mus musculus] >gi|119587044|gb|EAW66640.1| thioredoxin-like 4A, isoform CRA_b [Homo sapiens] >gi|148677469|gb|EDL09416.1| mCG18172, isoform CRA_c [Mus musculus] >gi|380783349|gb|AFE63550.1| thioredoxin-like protein 4A [Macaca mulatta] >gi|383414455|gb|AFH30441.1| thioredoxin-like protein 4A [Macaca mulatta] >gi|384944932|gb|AFI36071.1| thioredoxin-like protein 4A [Macaca mulatta]

ATGTCGTACATGCTCCCCCACCTGCACAACGGCTGGCAGGTGGACCAGGCCATCCTCTCG

GAGGAGGACCGCGTCGTGGTCATTCGTTTCGGGCACGACTGGGACCCCACCTGCATGAAG

ATGGACGAGGTTCTGTACAGCATAGCGGAGAAGGTAAAAAATTTTGCAGTTATTTATCTT

GTGGATATTACAGAAGTACCTGACTTCAACAAAATGTATGAATTATACGATCCATGTACT

GTCATGTTTTTCTTCAGGAACAAGCACATCATGATTGACTTGGGCACAGGCAACAACAAC

AAGATCAACTGGGCCATGGAAGACAAGCAGGAGATGGTTGACATAATTGAGACTGTATAC

CGTGGTGCCCGTAAGGGCCGGGGCCTGGTGGTGTCCCCCAAGGACTACTCCACCAAGTAC

AGATAC

>CL111.Contig3_All 122 547 minus strand thioredoxin-like protein 4A [Homo sapiens] >gi|13384656|ref|NP_079575.1| thioredoxin-like protein 4A isoform a [Mus musculus] >gi|386780898|ref|NP_001247795.1| thioredoxin-like protein 4A [Macaca mulatta] >gi|46577662|sp|P83876.1|TXN4A_HUMAN RecName: Full=Thioredoxin-like protein 4A; AltName: Full=DIM1 protein homolog; AltName: Full=Spliceosomal U5 snRNP-specific 15 kDa protein; AltName: Full=Thioredoxin-like U5 snRNP protein U5-15kD >gi|46577663|sp|P83877.1|TXN4A_MOUSE RecName: Full=Thioredoxin-like protein 4A; AltName: Full=DIM1 protein homolog; AltName: Full=Spliceosomal U5 snRNP-specific 15 kDa protein; AltName: Full=Thioredoxin-like U5 snRNP protein U5-15kD >gi|6730460|pdb|1QGV|A Chain A, Human Spliceosomal Protein U5-15kd >gi|82407298|pdb|1SYX|A Chain A, The Crystal Structure Of A Binary U5 Snrnp Complex >gi|82407300|pdb|1SYX|C Chain C, The Crystal Structure Of A Binary U5 Snrnp Complex >gi|82407302|pdb|1SYX|E Chain E, The Crystal Structure Of A Binary U5 Snrnp Complex >gi|6572636|gb|AAF17332.1|AF146373_1 thioredoxin-like U5 snRNP protein U5-15kD [Homo sapiens] >gi|2565275|gb|AAB81950.1| Dim1p homolog [Homo sapiens] >gi|12654441|gb|AAH01046.1| Thioredoxin-like 4A [Homo sapiens] >gi|17939455|gb|AAH19272.1| Thioredoxin-like 4A [Homo sapiens] >gi|21595023|gb|AAH31634.1| Txnl4a protein [Mus musculus] >gi|119587044|gb|EAW66640.1| thioredoxin-like 4A, isoform CRA_b [Homo sapiens] >gi|148677469|gb|EDL09416.1| mCG18172, isoform CRA_c [Mus musculus] >gi|380783349|gb|AFE63550.1| thioredoxin-like protein 4A [Macaca mulatta] >gi|383414455|gb|AFH30441.1| thioredoxin-like protein 4A [Macaca mulatta] >gi|384944932|gb|AFI36071.1| thioredoxin-like protein 4A [Macaca mulatta]

ATGTCGTACATGCTCCCCCACCTGCACAACGGCTGGCAGGTGGACCAGGCCATCCTCTCG

GAGGAGGACCGCGTCGTGGTCATTCGTTTCGGGCACGACTGGGACCCCACCTGCATGAAG

ATGGACGAGGTTCTGTACAGCATAGCGGAGAAGGTAAAAAATTTTGCAGTTATTTATCTT

GTGGATATTACAGAAGTACCTGACTTCAACAAAATGTATGAATTATACGATCCATGTACT

GTCATGTTTTTCTTCAGGAACAAGCACATCATGATTGACTTGGGCACAGGCAACAACAAC

AAGATCAACTGGGCCATGGAAGACAAGCAGGAGATGGTTGACATAATTGAGACTGTATAC

CGTGGTGCCCGTAAGGGCCGGGGCCTGGTGGTGTCCCCCAAGGACTACTCCACCAAGTAC

AGATAC

>CL111.Contig4_All 418 591 minus strand thioredoxin-like protein 4A [Homo sapiens] >gi|13384656|ref|NP_079575.1| thioredoxin-like protein 4A isoform a [Mus musculus] >gi|386780898|ref|NP_001247795.1| thioredoxin-like protein 4A [Macaca mulatta] >gi|46577662|sp|P83876.1|TXN4A_HUMAN RecName: Full=Thioredoxin-like protein 4A; AltName: Full=DIM1 protein homolog; AltName: Full=Spliceosomal U5 snRNP-specific 15 kDa protein; AltName: Full=Thioredoxin-like U5 snRNP protein U5-15kD >gi|46577663|sp|P83877.1|TXN4A_MOUSE RecName: Full=Thioredoxin-like protein 4A; AltName: Full=DIM1 protein homolog; AltName: Full=Spliceosomal U5 snRNP-specific 15 kDa protein; AltName: Full=Thioredoxin-like U5 snRNP protein U5-15kD >gi|6730460|pdb|1QGV|A Chain A, Human Spliceosomal Protein U5-15kd >gi|82407298|pdb|1SYX|A Chain A, The Crystal Structure Of A Binary U5 Snrnp Complex >gi|82407300|pdb|1SYX|C Chain C, The Crystal Structure Of A Binary U5 Snrnp Complex >gi|82407302|pdb|1SYX|E Chain E, The Crystal Structure Of A Binary U5 Snrnp Complex >gi|6572636|gb|AAF17332.1|AF146373_1 thioredoxin-like U5 snRNP protein U5-15kD [Homo sapiens] >gi|2565275|gb|AAB81950.1| Dim1p homolog [Homo sapiens] >gi|12654441|gb|AAH01046.1| Thioredoxin-like 4A [Homo sapiens] >gi|17939455|gb|AAH19272.1| Thioredoxin-like 4A [Homo sapiens] >gi|21595023|gb|AAH31634.1| Txnl4a protein [Mus musculus] >gi|119587044|gb|EAW66640.1| thioredoxin-like 4A, isoform CRA_b [Homo sapiens] >gi|148677469|gb|EDL09416.1| mCG18172, isoform CRA_c [Mus musculus] >gi|380783349|gb|AFE63550.1| thioredoxin-like protein 4A [Macaca mulatta] >gi|383414455|gb|AFH30441.1| thioredoxin-like protein 4A [Macaca mulatta] >gi|384944932|gb|AFI36071.1| thioredoxin-like protein 4A [Macaca mulatta]

TTCAGGAACAAGCACATCATGATTGACTTGGGCACAGGCAACAACAACAAGATCAACTGG

GCCATGGAAGACAAGCAGGAGATGGTTGACATAATTGAGACTGTATACCGTGGTGCCCGT

AAGGGCCGGGGCCTGGTGGTGTCCCCCAAGGACTACTCCACCAAGTACAGATAC

>CL111.Contig5_All 113 280 minus strand Thioredoxin-like protein 4A [Heterocephalus glaber]

ATGTCGTACATGCTCCCCCACCTGCACAACGGCTGGCAGGTGGACCAGGCCATCCTCTCG

GAGGAGGACCGCGTCGTGGTCATTCGTTTCGGGCACGACTGGGACCCCACCTGCATGAAG

ATGGACGAGGTTCTGTACAGCATAGCGGAGAAGGAACAAGCACATCAT

>CL112.Contig2_All 1 1464 Cytochrome P450 51A1 [Mustela putorius furo]

GGACAGGCGATGGAGCGGGTGACTGGCGGCAACCTTCTGTCCATGCTGCTGATCGCCTGC

GCCTTCACACTCAGCCTGGTTTATCTGTTCCGCCTCGCTGTCAGTCACCTGGTCCCACTG

CCAGCCGGGGCGAAAAGTCCACCATACATTTTCTCTCCAATTCCATTCCTTGGGCATGCT

ATAGCATTTGGGAAAAGTCCAATTGAATTCCTAGAAAATGCATATGAGAAGTATGGACCT

GTATTTAGTTTTACCATGGTGGGCAAGACGTTTACTTACCTTTTGGGGAGTGATGCTGCT

GCACTGTTTTTTAATAGTAAAAATGAAGACCTGAATGCTGAGGATGTTTATAGCCGTCTG

ACAACACCTGTGTTTGGGAAGGGAGTTGGATATGATGTGCCTAATGCAGTTTTCTTGGAG

CAGAAGAAAATGTTAAAGAGTGGCCTTAACATAGCCCACTTTAAACAGTGTGTTTCTATA

ATTGAAAAAGAAACAAAGGAATACTTTCAGAGTTGGGGAGAAAGTGGAGAAAAAAATGTG

TTTGAAGCTCTTTCTGAGCTCATAATTTTAACAGCCAGTCATTGTTTACACGGAAAGGAA

ATCAGAAGTCAACTGAATGAGAAGGTGGCACAGCTGTATGCAGATTTGGATGGAGGTTTT

AGTCATGCAGCCTGGCTGTTGCCAGGTTGGCTGCCCCTGCCTAGTTTCAGACGCAGAGAC

AGAGCTCATCAAGAGATCAAGAATATTTTCTATAAGGCAATCCAAAAACGCAGGCAGTCA

GAGGAAAAAAACGATGACATTCTCCAAACTCTACTAGATTCTTCATACAAGGATGGGCGT

CCTTTGACAGATGAAGAAATAGCAGGGATGCTGATTGGACTCCTCTTGGCAGGACAGCAT

ACGTCTTCAACTACCAGTGCCTGGATGGGCTTCTTTTTGGCCAGAGATAAAACACTTCAA

GAAAAATGTTATTTAGAACAGCGTACAGTTTGCGGGGAGGATCTTCCTCCTTTAACTTAT

GATCAGCTCAAGGATTTAAATTTACTTGATCGCTGCATAAAAGAAACATTAAGACTTAGA

CCTCCTATAATGACCATGATGAGAATGGCCAAAACTCCTCAGACTGTGGCAGGGTATACC

ATTCCTCCAGGACATCAGGTGTGTGTTTCTCCTACCGTCAATCAAAGACTTAAAGACTCA

TGGGTAGAACGTCTGGACTTCAATCCTGATCGCTATTTACAGGATAACCCAGCATCAGGA

GAGAAGTTTGCCTATGTGCCATTTGGAGCTGGACGCCATCGTTGTATTGGGGAAAATTTC

GCTTATGTTCAAATCAAGACGATTTGGTCCACTATGCTTCGTCTATATGAATTTGATCTC

ATTAATGGATATTTTCCTACTGTGAATTATACAACTATGATTCATACGCCTGAAAACCCA

GTTATTCGTTACAAACGAAGATCA

>CL113.Contig1_All 269 2410 PREDICTED: sialic acid-binding Ig-like lectin 11 [Papio anubis]

CCTCCCAAGGCCGAGATGCTACTCCTGCCGCCACTGCTGTCCTTGTTGTTGGGTGGTGAG

TGGCAGGGGACTGGAGGGACCCGGCCCGGCCGGGACTGGGATTGCAGGTTGAGCCTCTGT

CTCCCCTCAGGGTCCCAGGCTCAGCTAGAGAAGTACTGGCTGCAGGTGCAGGAGTCGGTG

TCGGTGCAGGAGGGCCTGTGTGTGCTGGTGTCCTGCTCCTTCTCCTACCCCAGGGACGAC

CCCAGAACTGCTTCTAACCCGGCTTATGGATACTGGTTCGAAGAAGGGACCAACACGAAC

AAGGGGGATCCAGTGGCCACGAACAACCCACATCGAAGGGTGCAATCTAGGACCCAAGGC

CGATTCCAGCTCATGGGGGACCCCAACAGAGATGACTGCTCCCTGATGATCAAAGACGCA

TACAGATGGGACAGGGCGGCCTACTTCTTCCGCGTGGAGAGAGGACCCATAGTGAAATTT

AATTTCCAGAAAAAGTTCTCTCTGGAAGTGACAGACCTAACTCAGAAGCCAGACGTCTTC

ATTCCCGAGATCCTGGAGCCCGGGCAGCCTGTGAGCGTTCTCTGTGTGTTTAACCAAGCG

TTTGAGCAATGCCCGGCCCCTTCTTTCTCCTGGACCACGGCTGCCATCCCCTCCCAAAGA

ACAAGGCCACCAACCTCCCACTCCTCAGTGCTCAGCCTCACCCCACAGCCCCAGGACCAC

GGCACTACGCTTACCTGTCGAGTGGACTTCGGCAGACAAGGCGTCAGCACACACAGGACC

GTCCAACTGAGTGTGGCCTATGCCCCTCAAGAACCTGTCATCCGAGTTCTCCACCACAAT

GCATCCGCCCCAGTGGCCCAGGGAAACTTCTCACATCTGGAAGTCCAGAAAGGCCAGTTC

CTGCGGCTCCTCTGTGCTGCTGACAGCCATCCCCCGGCCACTCTGAGCTGGGTCCTGGAG

GACAGAGTCCTCTCCTGGTCCTGCCCCTTGGGCCCCAGAACCCTGGAGCTGCAGCTGCCC

GTGGTGAAGCCTGGAGATTCGGGGCTCTACACCTGCCGAGCAGAGAACAGGCTGGGCTCC

CAACAGGGCACCCTGGACCTCTCTGTGCAGTATCCTCCAGAGGACCTGAGGGTGACGGTT

TCCCAAGAAAATAGGACAGTCACGGAAGTCATCAGGAATGGCACGTCCTTCCCAGTCCTG

GAAGGCCAAAGCCTGCGCCTGGTCTGTGTCACCCACAGCAACCCCCCAGCCAGGCTGAGC

TGGACCTGGGGGACGCAGACCCTGAGCCCTGAGTGGCTTTCCGACGCTGGGCTCCTGGAG

CTGCCCCGGGTGCAGACAGAGCATGGAGGAGAATTCACCTGCCATGCCCGCAGCCCCCTG

GGCTCCCAGCACCTCTCCCTGAGCCTCTCTGTGCACTACCCCCCAAGGCTGCTGGGACCC

TCCTGCTCCTGGGAGGCCCAGGCTCTGCTCTGCAGCTGCTCCTCCCGCGCCTGGCCGGCC

CCCTCCCTGCACTGGCGGCTGGGGGAGGGGCTGCTGGAGGGGAACAGCAGCAATGCCTCC

CTCAGGGTCACCTCCAGCTCCGAGGGGCCCTGGGCCAACAGCTCCCTGAGCCTCCACGGG

GCGCTCAGCTCCGACCTCAGGCTGAGCTGTGAGGCCCAGAACGCCCAGGGCACCCAGAGC

GCCACCGTCCTGCTGCTGCCAGGGAAGCCAGAGCTCAGGGGAGGAGCTCTTCTGGGGACA

CTGGGGGCTGCTGGTGGGGCCACTCTACTCTCCCTCTGTGCCTGCCTCGTCTTCTCCTGG

TTAAAGACTCGCAGGAAGAAAGCTGCCAGCCCAACAGGAACTGCACAACAGGACACCCCC

GTTGACCTGGGCCCTGTCTGTGGGGGTCCCAAGGATGCATCCTGGTTGGACAGCCCCTCA

TATGCTCCCACCCCCACTGAGGCCACGCTGGCCGTGGGAGAGGAGCCAGAGCCCCAGGAG

CTGCACTACGCCTCCCTCAGCTTCCTGGAGTTGGAGTCTCGAGAGCCCAAGGTGCAGGAG

GCCACCAGCAGGACAGAATACTCAGAGATCAAGGTCTGCAATGGCAGCCTTCAGGAGCAG

CCGGATGTCCTGGGGAGAGAGACATCAGGGACAGTTCCTAGA

>CL113.Contig2_All 269 2377 PREDICTED: sialic acid-binding Ig-like lectin 11 [Papio anubis]

CCTCCCAAGGCCGAGATGCTACTCCTGCCGCCACTGCTGTCCTTGTTGTTGGGTGGGTCC

CAGGCTCAGCTAGAGAAGTACTGGCTGCAGGTGCAGGAGTCGGTGTCGGTGCAGGAGGGC

CTGTGTGTGCTGGTGTCCTGCTCCTTCTCCTACCCCAGGGACGACCCCAGAACTGCTTCT

AACCCGGCTTATGGATACTGGTTCGAAGAAGGGACCAACACGAACAAGGGGGATCCAGTG

GCCACGAACAACCCACATCGAAGGGTGCAATCTAGGACCCAAGGCCGATTCCAGCTCATG

GGGGACCCCAACAGAGATGACTGCTCCCTGATGATCAAAGACGCATACAGATGGGACAGG

GCGGCCTACTTCTTCCGCGTGGAGAGAGGACCCATAGTGAAATTTAATTTCCAGAAAAAG

TTCTCTCTGGAAGTGACAGACCTAACTCAGAAGCCAGACGTCTTCATTCCCGAGATCCTG

GAGCCCGGGCAGCCTGTGAGCGTTCTCTGTGTGTTTAACCAAGCGTTTGAGCAATGCCCG

GCCCCTTCTTTCTCCTGGACCACGGCTGCCATCCCCTCCCAAAGAACAAGGCCACCAACC

TCCCACTCCTCAGTGCTCAGCCTCACCCCACAGCCCCAGGACCACGGCACTACGCTTACC

TGTCGAGTGGACTTCGGCAGACAAGGCGTCAGCACACACAGGACCGTCCAACTGAGTGTG

GCCTATGCCCCTCAAGAACCTGTCATCCGAGTTCTCCACCACAATGCATCCGCCCCAGTG

GCCCAGGGAAACTTCTCACATCTGGAAGTCCAGAAAGGCCAGTTCCTGCGGCTCCTCTGT

GCTGCTGACAGCCATCCCCCGGCCACTCTGAGCTGGGTCCTGGAGGACAGAGTCCTCTCC

TGGTCCTGCCCCTTGGGCCCCAGAACCCTGGAGCTGCAGCTGCCCGTGGTGAAGCCTGGA

GATTCGGGGCTCTACACCTGCCGAGCAGAGAACAGGCTGGGCTCCCAACAGGGCACCCTG

GACCTCTCTGTGCAGTATCCTCCAGAGGACCTGAGGGTGACGGTTTCCCAAGAAAATAGG

ACAGTCACGGAAGTCATCAGGAATGGCACGTCCTTCCCAGTCCTGGAAGGCCAAAGCCTG

CGCCTGGTCTGTGTCACCCACAGCAACCCCCCAGCCAGGCTGAGCTGGACCTGGGGGACG

CAGACCCTGAGCCCTGAGTGGCTTTCCGACGCTGGGCTCCTGGAGCTGCCCCGGGTGCAG

ACAGAGCATGGAGGAGAATTCACCTGCCATGCCCGCAGCCCCCTGGGCTCCCAGCACCTC

TCCCTGAGCCTCTCTGTGCACTACCCCCCAAGGCTGCTGGGACCCTCCTGCTCCTGGGAG

GCCCAGGCTCTGCTCTGCAGCTGCTCCTCCCGCGCCTGGCCGGCCCCCTCCCTGCACTGG

CGGCTGGGGGAGGGGCTGCTGGAGGGGAACAGCAGCAATGCCTCCCTCAGGGTCACCTCC

AGCTCCGAGGGGCCCTGGGCCAACAGCTCCCTGAGCCTCCACGGGGCGCTCAGCTCCGAC

CTCAGGCTGAGCTGTGAGGCCCAGAACGCCCAGGGCACCCAGAGCGCCACCGTCCTGCTG

CTGCCAGGGAAGCCAGAGCTCAGGGGAGGAGCTCTTCTGGGGACACTGGGGGCTGCTGGT

GGGGCCACTCTACTCTCCCTCTGTGCCTGCCTCGTCTTCTCCTGGTTAAAGACTCGCAGG

AAGAAAGCTGCCAGCCCAACAGGAACTGCACAACAGGACACCCCCATTGACCTGGGCCCT

GTCTGTGGGCCCCGTCATCCTCATTGGATCCCGACTCCTGCCCCACTTCAGGGTCCCAAG

GATGCATCCTGGTTGGACAGCCCCTCATATGCTCCCACCCCCACTGAGGCCACGCTGGCC

GTGGGAGAGGAGCCAGAGCCCCAGGAGCTGCACTACGCCTCCCTCAGCTTCCTGGAGTTG

GAGTCTCGAGAGCCCAAGGTGCAGGAGGCCACCAGCAGGACAGAATACTCAGAGATCAAG

GTCTGCAATGGCAGCCTTCAGGAGCAGCCGGATGTCCTGGGGAGAGAGACATCAGGGACA

GTTCCTAGA

>CL113.Contig3_All 18 2276 PREDICTED: sialic acid binding Ig-like lectin 11 isoform 1 [Pan troglodytes]

CCTCCCCCTGGCCTCCTGAGGATGTCCTCTCAGAGAGGCAGAGCCAGAGGCCAGAGGATC

CCAGGACAGGACAGTCCCCTGAGCCCCAAGATGCTGCTCCTGGCCCTGCTGCTGCTTCAG

TTGGGGACGGGTGAGTGGGCTGAGGACCAGGGATCACAAGTGTGGGCTGGGACTGCAGCT

GAGCCTCTGTGTCCCCCAGGGTCCCTGCAGAAGAATCAGTCTTTCTGGCTGCAGATGCAG

AAGGCGGTGACAGTGCAGGAGGGTCTGTGTGTGCTTGTGTCCTGCTCCTTCTCCTACCCC

GAGGCTGGCTGGAAGCACTCCACACCTACCTACGGCTCCTGGTACAAGACAAAGAAGAAT

TCCAAACCTCATGCAAAAGACAATGACCTCGTGGCCACAAACAACCCAGACAAGGAAGCA

AAAGAGAAGTACAAACTTCATTTCCGGCTCCTCGGAGACCCCCAATCTAACAACTGCTCC

CTGAGCATCTCCGAGGTCCAGAAGGACGACAGCGGAACATACTATTTTCATCTAGAGCAA

GGAGCTGTGAATCACACTTACGAAAGTAACCTGCTCACCCTGACTGTCACAGAGTTGACC

CAAACCCCAGACATCCACATCCAGGAGCCCCTACAGTCGGGCCACCTTAGCCATGTGACA

TGCTCCATGCCTGGGGCCTGTGACTGGCCCACGGCCCCCCGTATCTCCTGGGCTGGGGCT

GCCCTGAGAGCAGCGGGGTCAGGCCTGGAGCCCTCCACCTCAGAGATCCTGCTCACGCCC

CACCCCGAGGACCATGGCACCCACCTCTCCTGCCGAGCGACCTTCCCCAGGGCTGGCGTG

TCTTCAAACAGAACTGTGCAGCTCAACGTGTCCTATGCTCCTCAGAACCTGACCATCAGC

ATCTCCGGAGCAGGTGACCCAGCCCCAGTGGCCCAGGGAAACTTCTCACATCTGGAAGTC

CAGAAAGGCCAGTTCCTGCGGCTCCTCTGTGCTGCTGACAGCCATCCCCCGGCCACTCTG

AGCTGGGTCCTGGAGGACAGAGTCCTCTCCTGGTCCTGCCCCTTGGGCCCCAGAACCCTG

GAGCTGCAGCTGCCCGTGGTGAAGCCTGGAGATTCGGGGCTCTACACCTGCCGAGCAGAG

AACAGGCTGGGCTCCCAACAGGGCACCCTGGACCTCTCTGTGCAGTATCCTCCAGAGGAC

CTGAGGGTGACGGTTTCCCAAGAAAATAGGACAGTCACGGAAGTCATCAGGAATGGCACG

TCCTTCCCAGTCCTGGAAGGCCAAAGCCTGCGCCTGGTCTGTGTCACCCACAGCAACCCC

CCAGCCAGGCTGAGCTGGACCTGGGGGACGCAGACCCTGAGCCCTGAGTGGCTTTCCGAC

GCTGGGCTCCTGGAGCTGCCCCGGGTGCAGACAGAGCATGGAGGAGAATTCACCTGCCAT

GCCCGCAGCCCCCTGGGCTCCCAGCACCTCTCCCTGAGCCTCTCTGTGCACTACCCCCCA

AGGCTGCTGGGACCCTCCTGCTCCTGGGAGGCCCAGGCTCTGCTCTGCAGCTGCTCCTCC

CGCGCCTGGCCGGCCCCCTCCCTGCACTGGCGGCTGGGGGAGGGGCTGCTGGAGGGGAAC

AGCAGCAATGCCTCCCTCAGGGTCACCTCCAGCTCCGAGGGGCCCTGGGCCAACAGCTCC

CTGAGCCTCCACGGGGCGCTCAGCTCCGACCTCAGGCTGAGCTGTGAGGCCCAGAACGCC

CAGGGCACCCAGAGCGCCACCGTCCTGCTGCTGCCAGGGAAGCCAGAGCTCAGGGGAGGA

GCTCTTCTGGGGACACTGGGGGCTGCTGGTGGGGCCACTCTACTCTCCCTCTGTGCCTGC

CTCGTCTTCTCCTGGTTAAAGACTCGCAGGAAGAAAGCTGCCAGCCCAACAGGAACTGCA

CAACAGGACACCCCCATTGACCTGGGCCCTGTCTGTGGGCCCCGTCATCCTCATTGGATC

CCGACTCCTGCCCCACTTCAGGGTCCCAAGGATGCATCCTGGTTGGACAGCCCCTCATAT

GCTCCCACCCCCACTGAGGCCACGCTGGCCGTGGGAGAGGAGCCAGAGCCCCAGGAGCTG

CACTACGCCTCCCTCAGCTTCCTGGAGTTGGAGTCTCGAGAGCCCAAGGTGCAGGAGGCC

ACCAGCAGGACAGAATACTCAGAGATCAAGGTCTGCAATGGCAGCCTTCAGGAGCAGCCG

GATGTCCTGGGGAGAGAGACATCAGGGACAGTTCCTAGA

>CL113.Contig4_All 18 2234 PREDICTED: sialic acid binding Ig-like lectin 11 isoform 1 [Pongo abelii]

CCTCCCCCTGGCCTCCTGAGGATGTCCTCTCAGAGAGGCAGAGCCAGAGGCCAGAGGATC

CCAGGACAGGACAGTCCCCTGAGCCCCAAGATGCTGCTCCTGGCCCTGCTGCTGCTTCAG

TTGGGGACGGGTGAGTGGGCTGAGGACCAGGGATCACAAGTGTGGGCTGGGACTGCAGCT

GAGCCTCTGTGTCCCCCAGGGTCCCTGCAGAAGAATCAGTCTTTCTGGCTGCAGATGCAG

AAGGCGGTGACAGTGCAGGAGGGTCTGTGTGTGCTTGTGTCCTGCTCCTTCTCCTACCCC

GAGGCTGGCTGGAAGCACTCCACACCTACCTACGGCTCCTGGTACAAGACAAAGAAGAAT

TCCAAACCTCATGCAAAAGACAATGACCTCGTGGCCACAAACAACCCAGACAAGGAAGCA

AAAGAGAAGTACAAACTTCATTTCCGGCTCCTCGGAGACCCCCAATCTAACAACTGCTCC

CTGAGCATCTCCGAGGTCCAGAAGGACGACAGCGGAACATACTATTTTCATCTAGAGCAA

GGAGCTGTGAATCACACTTACGAAAGTAACCTGCTCACCCTGACTGTCACAGAGTTGACC

CAAACCCCAGACATCCACATCCAGGAGCCCCTACAGTCGGGCCACCTTAGCCATGTGACA

TGCTCCATGCCTGGGGCCTGTGACTGGCCCACGGCCCCCCGTATCTCCTGGGCTGGGGCT

GCCCTGAGAGCAGCGGGGTCAGGCCTGGAGCCCTCCACCTCAGAGATCCTGCTCACGCCC

CACCCCGAGGACCATGGCACCCACCTCTCCTGCCGAGCGACCTTCCCCAGGGCTGGCGTG

TCTTCAAACAGAACTGTGCAGCTCAACGTGTCCTATGCTCCTCAGAACCTGACCATCAGC

ATCTCCGGAGCAGGTGACCCAGCCCCAGTGGCCCAGGGAAACTTCTCACATCTGGAAGTC

CAGAAAGGCCAGTTCCTGCGGCTCCTCTGTGCTGCTGACAGCCATCCCCCGGCCACTCTG

AGCTGGGTCCTGGAGGACAGAGTCCTCTCCTGGTCCTGCCCCTTGGGCCCCAGAACCCTG

GAGCTGCAGCTGCCCGTGGTGAAGCCTGGAGATTCGGGGCTCTACACCTGCCGAGCAGAG

AACAGGCTGGGCTCCCAACAGGGCACCCTGGACCTCTCTGTGCAGTATCCTCCAGAGGAC

CTGAGGGTGACGGTTTCCCAAGAAAATAGGACAGTCACGGAAGTCATCAGGAATGGCACG

TCCTTCCCAGTCCTGGAAGGCCAAAGCCTGCGCCTGGTCTGTGTCACCCACAGCAACCCC

CCAGCCAGGCTGAGCTGGACCTGGGGGACGCAGACCCTGAGCCCTGAGTGGCTTTCCGAC

GCTGGGCTCCTGGAGCTGCCCCGGGTGCAGACAGAGCATGGAGGAGAATTCACCTGCCAT

GCCCGCAGCCCCCTGGGCTCCCAGCACCTCTCCCTGAGCCTCTCTGTGCACTACCCCCCA

AGGCTGCTGGGACCCTCCTGCTCCTGGGAGGCCCAGGCTCTGCTCTGCAGCTGCTCCTCC

CGCGCCTGGCCGGCCCCCTCCCTGCACTGGCGGCTGGGGGAGGGGCTGCTGGAGGGGAAC

AGCAGCAATGCCTCCCTCAGGGTCACCTCCAGCTCCGAGGGGCCCTGGGCCAACAGCTCC

CTGAGCCTCCACGGGGCGCTCAGCTCCGACCTCAGGCTGAGCTGTGAGGCCCAGAACGCC

CAGGGCACCCAGAGCGCCACCGTCCTGCTGCTGCCAGGGAAGCCAGAGCTCAGGGGAGGA

GCTCTTCTGGGGACACTGGGGGCTGCTGGTGGGGCCACTCTACTCTCCCTCTGTGCCTGC

CTCGTCTTCTCCTGGTTAAAGACTCGCAGGAAGAAAGCTGCCAGCCCAACAGGAACTGCA

CAACAGGACACCCCCGTTGACCTGGGCCCTGTCTGTGGGGGTCCCAAGGATGCATCCTGG

TTGGACAGCCCCTCATATGCTCCCACCCCCACTGAGGCCACGCTGGCCGTGGGAGAGGAG

CCAGAGCCCCAGGAGCTGCACTACGCCTCCCTCAGCTTCCTGGAGTTGGAGTCTCGAGAG

CCCAAGGTGCAGGAGGCCACCAGCAGGACAGAATACTCAGAGATCAAGGTCTGCAATGGC

AGCCTTCAGGAGCAGCCGGATGTCCTGGGGAGAGAGACATCAGGGACAGTTCCTAGA

>CL113.Contig5_All 18 2207 PREDICTED: sialic acid binding Ig-like lectin 11 isoform 1 [Pongo abelii]

CCTCCCCCTGGCCTCCTGAGGATGTCCTCTCAGAGAGGCAGAGCCAGAGGCCAGAGGATC

CCAGGACAGGACAGTCCCCTGAGCCCCAAGATGCTGCTCCTGGCCCTGCTGCTGCTTCAG

TTGGGGACGGGGTCCCTGCAGAAGAATCAGTCTTTCTGGCTGCAGATGCAGAAGGCGGTG

ACAGTGCAGGAGGGTCTGTGTGTGCTTGTGTCCTGCTCCTTCTCCTACCCCGAGGCTGGC

TGGAAGCACTCCACACCTACCTACGGCTCCTGGTACAAGACAAAGAAGAATTCCAAACCT

CATGCAAAAGACAATGACCTCGTGGCCACAAACAACCCAGACAAGGAAGCAAAAGAGAAG

TACAAACTTCATTTCCGGCTCCTCGGAGACCCCCAATCTAACAACTGCTCCCTGAGCATC

TCCGAGGTCCAGAAGGACGACAGCGGAACATACTATTTTCATCTAGAGCAAGGAGCTGTG

AATCACACTTACGAAAGTAACCTGCTCACCCTGACTGTCACAGAGTTGACCCAAACCCCA

GACATCCACATCCAGGAGCCCCTACAGTCGGGCCACCTTAGCCATGTGACATGCTCCATG

CCTGGGGCCTGTGACTGGCCCACGGCCCCCCGTATCTCCTGGGCTGGGGCTGCCCTGAGA

GCAGCGGGGTCAGGCCTGGAGCCCTCCACCTCAGAGATCCTGCTCACGCCCCACCCCGAG

GACCATGGCACCCACCTCTCCTGCCGAGCGACCTTCCCCAGGGCTGGCGTGTCTTCAAAC

AGAACTGTGCAGCTCAACGTGTCCTATGCTCCTCAGAACCTGACCATCAGCATCTCCGGA

GCAGGTGACCCAGCCCCAGTGGCCCAGGGAAACTTCTCACATCTGGAAGTCCAGAAAGGC

CAGTTCCTGCGGCTCCTCTGTGCTGCTGACAGCCATCCCCCGGCCACTCTGAGCTGGGTC

CTGGAGGACAGAGTCCTCTCCTGGTCCTGCCCCTTGGGCCCCAGAACCCTGGAGCTGCAG

CTGCCCGTGGTGAAGCCTGGAGATTCGGGGCTCTACACCTGCCGAGCAGAGAACAGGCTG

GGCTCCCAACAGGGCACCCTGGACCTCTCTGTGCAGTATCCTCCAGAGGACCTGAGGGTG

ACGGTTTCCCAAGAAAATAGGACAGTCACGGAAGTCATCAGGAATGGCACGTCCTTCCCA

GTCCTGGAAGGCCAAAGCCTGCGCCTGGTCTGTGTCACCCACAGCAACCCCCCAGCCAGG

CTGAGCTGGACCTGGGGGACGCAGACCCTGAGCCCTGAGTGGCTTTCCGACGCTGGGCTC

CTGGAGCTGCCCCGGGTGCAGACAGAGCATGGAGGAGAATTCACCTGCCATGCCCGCAGC

CCCCTGGGCTCCCAGCACCTCTCCCTGAGCCTCTCTGTGCACTACCCCCCAAGGCTGCTG

GGACCCTCCTGCTCCTGGGAGGCCCAGGCTCTGCTCTGCAGCTGCTCCTCCCGCGCCTGG

CCGGCCCCCTCCCTGCACTGGCGGCTGGGGGAGGGGCTGCTGGAGGGGAACAGCAGCAAT

GCCTCCCTCAGGGTCACCTCCAGCTCCGAGGGGCCCTGGGCCAACAGCTCCCTGAGCCTC

CACGGGGCGCTCAGCTCCGACCTCAGGCTGAGCTGTGAGGCCCAGAACGCCCAGGGCACC

CAGAGCGCCACCGTCCTGCTGCTGCCAGGGAAGCCAGAGCTCAGGGGAGGAGCTCTTCTG

GGGACACTGGGGGCTGCTGGTGGGGCCACTCTACTCTCCCTCTGTGCCTGCCTCGTCTTC

TCCTGGTTAAAGACTCGCAGGAAGAAAGCTGCCAGCCCAACAGGAACTGCACAACAGGAC

ACCCCCATTGACCTGGGCCCTGTCTGTGGGCCCCGTCATCCTCATTGGATCCCGACTCCT

GCCCCACTTCAGGGTCCCAAGGATGCATCCTGGTTGGACAGCCCCTCATATGCTCCCACC

CCCACTGAGGCCACGCTGGCCGTGGGAGAGGAGCCAGAGCCCCAGGAGCTGCACTACGCC

TCCCTCAGCTTCCTGGAGTTGGAGTCTCGAGAGCCCAAGGTGCAGGAGGCCACCAGCAGG

ACAGAATACTCAGAGATCAAGGTCTGCAATGGCAGCCTTCAGGAGCAGCCGGATGTCCTG

GGGAGAGAGACATCAGGGACAGTTCCTAGA

>CL113.Contig6_All 18 2165 PREDICTED: sialic acid binding Ig-like lectin 11 isoform 1 [Pongo abelii]

CCTCCCCCTGGCCTCCTGAGGATGTCCTCTCAGAGAGGCAGAGCCAGAGGCCAGAGGATC

CCAGGACAGGACAGTCCCCTGAGCCCCAAGATGCTGCTCCTGGCCCTGCTGCTGCTTCAG

TTGGGGACGGGGTCCCTGCAGAAGAATCAGTCTTTCTGGCTGCAGATGCAGAAGGCGGTG

ACAGTGCAGGAGGGTCTGTGTGTGCTTGTGTCCTGCTCCTTCTCCTACCCCGAGGCTGGC

TGGAAGCACTCCACACCTACCTACGGCTCCTGGTACAAGACAAAGAAGAATTCCAAACCT

CATGCAAAAGACAATGACCTCGTGGCCACAAACAACCCAGACAAGGAAGCAAAAGAGAAG

TACAAACTTCATTTCCGGCTCCTCGGAGACCCCCAATCTAACAACTGCTCCCTGAGCATC

TCCGAGGTCCAGAAGGACGACAGCGGAACATACTATTTTCATCTAGAGCAAGGAGCTGTG

AATCACACTTACGAAAGTAACCTGCTCACCCTGACTGTCACAGAGTTGACCCAAACCCCA

GACATCCACATCCAGGAGCCCCTACAGTCGGGCCACCTTAGCCATGTGACATGCTCCATG

CCTGGGGCCTGTGACTGGCCCACGGCCCCCCGTATCTCCTGGGCTGGGGCTGCCCTGAGA

GCAGCGGGGTCAGGCCTGGAGCCCTCCACCTCAGAGATCCTGCTCACGCCCCACCCCGAG

GACCATGGCACCCACCTCTCCTGCCGAGCGACCTTCCCCAGGGCTGGCGTGTCTTCAAAC

AGAACTGTGCAGCTCAACGTGTCCTATGCTCCTCAGAACCTGACCATCAGCATCTCCGGA

GCAGGTGACCCAGCCCCAGTGGCCCAGGGAAACTTCTCACATCTGGAAGTCCAGAAAGGC

CAGTTCCTGCGGCTCCTCTGTGCTGCTGACAGCCATCCCCCGGCCACTCTGAGCTGGGTC

CTGGAGGACAGAGTCCTCTCCTGGTCCTGCCCCTTGGGCCCCAGAACCCTGGAGCTGCAG

CTGCCCGTGGTGAAGCCTGGAGATTCGGGGCTCTACACCTGCCGAGCAGAGAACAGGCTG

GGCTCCCAACAGGGCACCCTGGACCTCTCTGTGCAGTATCCTCCAGAGGACCTGAGGGTG

ACGGTTTCCCAAGAAAATAGGACAGTCACGGAAGTCATCAGGAATGGCACGTCCTTCCCA

GTCCTGGAAGGCCAAAGCCTGCGCCTGGTCTGTGTCACCCACAGCAACCCCCCAGCCAGG

CTGAGCTGGACCTGGGGGACGCAGACCCTGAGCCCTGAGTGGCTTTCCGACGCTGGGCTC

CTGGAGCTGCCCCGGGTGCAGACAGAGCATGGAGGAGAATTCACCTGCCATGCCCGCAGC

CCCCTGGGCTCCCAGCACCTCTCCCTGAGCCTCTCTGTGCACTACCCCCCAAGGCTGCTG

GGACCCTCCTGCTCCTGGGAGGCCCAGGCTCTGCTCTGCAGCTGCTCCTCCCGCGCCTGG

CCGGCCCCCTCCCTGCACTGGCGGCTGGGGGAGGGGCTGCTGGAGGGGAACAGCAGCAAT

GCCTCCCTCAGGGTCACCTCCAGCTCCGAGGGGCCCTGGGCCAACAGCTCCCTGAGCCTC

CACGGGGCGCTCAGCTCCGACCTCAGGCTGAGCTGTGAGGCCCAGAACGCCCAGGGCACC

CAGAGCGCCACCGTCCTGCTGCTGCCAGGGAAGCCAGAGCTCAGGGGAGGAGCTCTTCTG

GGGACACTGGGGGCTGCTGGTGGGGCCACTCTACTCTCCCTCTGTGCCTGCCTCGTCTTC

TCCTGGTTAAAGACTCGCAGGAAGAAAGCTGCCAGCCCAACAGGAACTGCACAACAGGAC

ACCCCCGTTGACCTGGGCCCTGTCTGTGGGGGTCCCAAGGATGCATCCTGGTTGGACAGC

CCCTCATATGCTCCCACCCCCACTGAGGCCACGCTGGCCGTGGGAGAGGAGCCAGAGCCC

CAGGAGCTGCACTACGCCTCCCTCAGCTTCCTGGAGTTGGAGTCTCGAGAGCCCAAGGTG

CAGGAGGCCACCAGCAGGACAGAATACTCAGAGATCAAGGTCTGCAATGGCAGCCTTCAG

GAGCAGCCGGATGTCCTGGGGAGAGAGACATCAGGGACAGTTCCTAGA

>CL113.Contig7_All 287 2356 PREDICTED: sialic acid binding Ig-like lectin 10 isoform 6 [Pan troglodytes]

CTACTCCTGCCGCCACTGCTGTCCTTGTTGTTGGGTGGGTCCCAGGCTCAGCTAGAGAAG

TACTGGCTGCAGGTGCAGGAGTCGGTGTCGGTGCAGGAGGGCCTGTGTGTGCTGGTGTCC

TGCTCCTTCTCCTACCCCAGGGACGACCCCAGAACTGCTTCTAACCCGGCTTATGGATAC

TGGTTCGAAGAAGGGACCAACACGAACAAGGGGGATCCAGTGGCCACGAACAACCCACAT

CGAAGGGTGCAATCTAGGACCCAAGGCCGATTCCAGCTCATGGGGGACCCCAACAGAGAT

GACTGCTCCCTGATGATCAAAGACGCATACAGATGGGACAGGGCGGCCTACTTCTTCCGC

GTGGAGAGAGGACCCATAGTGAAATTTAATTTCCAGAAAAAGTTCTCTCTGGAAGTGACA

GACCTAACTCAGAAGCCAGACGTCTTCATTCCCGAGATCCTGGAGCCCGGGCAGCCTGTG

AGCGTTCTCTGTGTGTTTAACCAAGCGTTTGAGCAATGCCCGGCCCCTTCTTTCTCCTGG

ACCACGGCTGCCATCCCCTCCCAAAGAACAAGGCCACCAACCTCCCACTCCTCAGTGCTC

AGCCTCACCCCACAGCCCCAGGACCACGGCACTACGCTTACCTGTCGAGTGGACTTCGGC

AGACAAGGCGTCAGCACACACAGGACCGTCCAACTGAGTGTGGCCTATGCCCCTCAAGAA

CCTGTCATCCGAGTTCTCCACCACAATGCATCCGCCCCAGTGGCCCAGGGAAACTTCTCA

CATCTGGAAGTCCAGAAAGGCCAGTTCCTGCGGCTCCTCTGTGCTGCTGACAGCCATCCC

CCGGCCACTCTGAGCTGGGTCCTGGAGGACAGAGTCCTCTCCTGGTCCTGCCCCTTGGGC

CCCAGAACCCTGGAGCTGCAGCTGCCCGTGGTGAAGCCTGGAGATTCGGGGCTCTACACC

TGCCGAGCAGAGAACAGGCTGGGCTCCCAACAGGGCACCCTGGACCTCTCTGTGCAGTAT

CCTCCAGAGGACCTGAGGGTGACGGTTTCCCAAGAAAATAGGACAGTCACGGAAGTCATC

AGGAATGGCACGTCCTTCCCAGTCCTGGAAGGCCAAAGCCTGCGCCTGGTCTGTGTCACC

CACAGCAACCCCCCAGCCAGGCTGAGCTGGACCTGGGGGACGCAGACCCTGAGCCCTGAG

TGGCTTTCCGACGCTGGGCTCCTGGAGCTGCCCCGGGTGCAGACAGAGCATGGAGGAGAA

TTCACCTGCCATGCCCGCAGCCCCCTGGGCTCCCAGCACCTCTCCCTGAGCCTCTCTGTG

CACTACCCCCCAAGGCTGCTGGGACCCTCCTGCTCCTGGGAGGCCCAGGCTCTGCTCTGC

AGCTGCTCCTCCCGCGCCTGGCCGGCCCCCTCCCTGCACTGGCGGCTGGGGGAGGGGCTG

CTGGAGGGGAACAGCAGCAATGCCTCCCTCAGGGTCACCTCCAGCTCCGAGGGGCCCTGG

GCCAACAGCTCCCTGAGCCTCCACGGGGCGCTCAGCTCCGACCTCAGGCTGAGCTGTGAG

GCCCAGAACGCCCAGGGCACCCAGAGCGCCACCGTCCTGCTGCTGCCAGGCGAGGCGTCT

GTCCCCACTGCGTTCTCCCGCGGAGTGTTTCTGGGAATCGGCGTCACCATGGTCCTCTCC

CTCTGCATCATCCTGGCCATTGTGAAGACTCTGAGGAAGGCGCCGCCCCAGACGGGGGCC

AGGCCCAAGGTGTCCCGCGGCAGCACCATCCTGGAGTACATCAACGTGGTCCCCAAGGCT

GCCCGCAGGGCTCAGGATCAGAAGGCCACGCCAAGCAGCCCTTCTCGGACCCCCTCTCCA

GGCACTCACTCCCCAGAACCAAAGAAGAATCCAGAGGAGCTGTATTCTGTTCCCCTTGGT

TGCTCAAAACCCAGATCGTCCACCCAAGCTCTGGACCCCAAGAATGACCCCGAGGAGCTC

CACTATGCTACTCTCACCTTCCCGGGCCACAGACCGTGGGCCACCCAGATGCCCAAAGAC

ACCCACACAGATTACGAGGAAATCAAGTTC

>CL113.Contig8_All 111 2186 PREDICTED: sialic acid binding Ig-like lectin 10 isoform 6 [Pan troglodytes]

CTGCTCCTGGCCCTGCTGCTGCTTCAGTTGGGGACGGGGTCCCTGCAGAAGAATCAGTCT

TTCTGGCTGCAGATGCAGAAGGCGGTGACAGTGCAGGAGGGTCTGTGTGTGCTTGTGTCC

TGCTCCTTCTCCTACCCCGAGGCTGGCTGGAAGCACTCCACACCTACCTATGGCTCCTGG

TACAAGACAAAGAAGAATTCCAAACCTCATGCAAAAGACAATGACCTCGTGGCCACAAAC

AACCCAGACAAGGAAGCAAAAGAGAAGTACAAACTTCATTTCCGGCTCCTCGGAGACCCC

CAATCTAACAACTGCTCCCTGAGCATCTCCGAGGTCCAGAAGGACGACAGCGGAACATAC

TATTTTCATCTAGAGCAAGGAGATGAGAATCACACTTACGAAAGTAACCTGCTCACCCTG

ACTGTCACAGAGTTGACCCAAACCCCAGACATCCACATCCAGGAGCCCCTACAGTCGGGC

CACCTTAGCCATGTGACATGCTCCATGCCTGGGGCCTGTGACTGGCCCACGGCCCCCCGT

ATCTCCTGGGCTGGGGCTGCCCTGAGAGCAGCGGGGTCAGGCCTGGAGCCCTCCACCTCA

GAGATCCTGCTCACGCCCCACCCCGAGGACCATGGCACCCACCTCTCCTGCCGAGCGACC

TTCCCCAGGGCTGGCGTGTCTTCAAACAGAACTGTGCAGCTCAACGTGTCCTATGCTCCT

CAGAACCTGACCATCAGCATCTCCGGAGCAGGTGACCCAGCCCCAGTGGCCCAGGGAAAC

TTCTCACATCTGGAAGTCCAGAAAGGCCAGTTCCTGCGGCTCCTCTGTGCTGCTGACAGC

CATCCCCCGGCCACTCTGAGCTGGGTCCTGGAGGACAGAGTCCTCTCCTGGTCCTGCCCC

TTGGGCCCCAGAACCCTGGAGCTGCAGCTGCCCGTGGTGAAGCCTGGAGATTCGGGGCTC

TACACCTGCCGAGCAGAGAACAGGCTGGGCTCCCAACAGGGCACCCTGGACCTCTCTGTG

CAGTATCCTCCAGAGGACCTGAGGGTGACGGTTTCCCAAGAAAATAGGACAGTCACGGAA

GTCATCAGGAATGGCACGTCCTTCCCAGTCCTGGAAGGCCAAAGCCTGCGCCTGGTCTGT

GTCACCCACAGCAACCCCCCAGCCAGGCTGAGCTGGACCTGGGGGACGCAGACCCTGAGC

CCTGAGTGGCTTTCCGACGCTGGGCTCCTGGAGCTGCCCCGGGTGCAGACAGAGCATGGA

GGAGAATTCACCTGCCATGCCCGCAGCCCCCTGGGCTCCCAGCACCTCTCCCTGAGCCTC

TCTGTGCACTACCCCCCAAGGCTGCTGGGACCCTCCTGCTCCTGGGAGGCCCAGGCTCTG

CTCTGCAGCTGCTCCTCCCGCGCCTGGCCGGCCCCCTCCCTGCACTGGCGGCTGGGGGAG

GGGCTGCTGGAGGGGAACAGCAGCAATGCCTCCCTCAGGGTCACCTCCAGCTCCGAGGGG

CCCTGGGCCAACAGCTCCCTGAGCCTCCACGGGGCGCTCAGCTCCGACCTCAGGCTGAGC

TGTGAGGCCCAGAACGCCCAGGGCACCCAGAGCGCCACCGTCCTGCTGCTGCCAGGCGAG

GCGTCTGTCCCCACTGCGTTCTCCCGCGGAGTGTTTCTGGGAATCGGCGTCACCATGGTC

CTCTCCCTCTGCATCATCCTGGCCATTGTGAAGACTCTGAGGAAGGCGCCGCCCCAGACG

GGGGCCAGGCCCAAGGTGTCCCGCGGCAGCACCATCCTGGAGTACATCAACGTGGTCCCC

AAGGCTGCCCGCAGGGCTCAGGATCAGAAGGCCACGCCAAGCAGCCCTTCTCGGACCCCC

TCTCCAGGCACTCACTCCCCAGAACCAAAGAAGAATCCAGAGGAGCTGTATTCTGTTCCC

CTTGGTTGCTCAAAACCCAGATCGTCCACCCAAGCTCTGGACCCCAAGAATGACCCCGAG

GAGCTCCACTATGCTACTCTCACCTTCCCGGGCCACAGACCGTGGGCCACCCAGATGCCC

AAAGACACCCACACAGATTACGAGGAAATCAAGTTC

>CL113.Contig9_All 2038 2364 PREDICTED: sialic acid-binding Ig-like lectin 16 isoform 1 [Saimiri boliviensis boliviensis]

AGAAAGCTGCCAGCCCAACAGGAACTGCACAACAGGACACCCCCCGTTGACCTGGGCCCT

GTCTGTGGGGGTCCCAAGGATGCATCCTGGTTGGACAGCCCCTCATATGCTCCCACCCCC

ACTGAGGCCACGCTGGCCGTGGGAGAGGAGCCAGAGCCCCAGGAGCTGCACTACGCCTCC

CTCAGCTTCCTGGAGTTGGAGTCTCGAGAGCCCAAGGTGCAGGAGGCCACCAGCAGGACA

GAATACTCAGAGATCAAGGTCTGCAATGGCAGCCTTCAGGAGCAGCCGGATGTCCTGGGG

AGAGAGACATCAGGGACAGTTCCTAGA

>CL113.Contig10_All 2010 2336 PREDICTED: sialic acid-binding Ig-like lectin 11 [Papio anubis]

AGAAAGCTGCCAGCCCAACAGGAACTGCACAACAGGACACCCCCCGTTGACCTGGGCCCT

GTCTGTGGGGGTCCCAAGGATGCATCCTGGTTGGACAGCCCCTCATATGCTCCCACCCCC

ACTGAGGCCACGCTGGCCGTGGGAGAGGAGCCAGAGCCCCAGGAGCTGCACTACGCCTCC

CTCAGCTTCCTGGAGTTGGAGTCTCGAGAGCCCAAGGTGCAGGAGGCCACCAGCAGGACA

GAATACTCAGAGATCAAGGTCTGCAATGGCAGCCTTCAGGAGCAGCCGGATGTCCTGGGG

AGAGAGACATCAGGGACAGTTCCTAGA

>CL113.Contig11_All 287 2431 PREDICTED: sialic acid binding Ig-like lectin 10 isoform 6 [Pan troglodytes]

CTACTCCTGCCGCCACTGCTGTCCTTGTTGTTGGGTGGTGAGTGGCAGGGGACTGGAGGG

ACCCGGCCCGGCCGGGACTGGGATTGCAGGTTGAGCCTCTGTCTCCCCTCAGGGTCCCAG

GCTCAGCTAGAGAAGTACTGGCTGCAGGTGCAGGAGTCGGTGTCGGTGCAGGAGGGCCTG

TGTGTGCTGGTGTCCTGCTCCTTCTCCTACCCCAGGGACGACCCCAGAACTGCTTCTAAC

CCGGCTTATGGATACTGGTTCGAAGAAGGGACCAACACGAACAAGGGGGATCCAGTGGCC

ACGAACAACCCACATCGAAGGGTGCAATCTAGGACCCAAGGCCGATTCCAGCTCATGGGG

GACCCCAACAGAGATGACTGCTCCCTGATGATCAAAGACGCATACAGATGGGACAGGGCG

GCCTACTTCTTCCGCGTGGAGAGAGGACCCATAGTGAAATTTAATTTCCAGAAAAAGTTC

TCTCTGGAAGTGACAGACCTAACTCAGAAGCCAGACGTCTTCATTCCCGAGATCCTGGAG

CCCGGGCAGCCTGTGAGCGTTCTCTGTGTGTTTAACCAAGCGTTTGAGCAATGCCCGGCC

CCTTCTTTCTCCTGGACCACGGCTGCCATCCCCTCCCAAAGAACAAGGCCACCAACCTCC

CACTCCTCAGTGCTCAGCCTCACCCCACAGCCCCAGGACCACGGCACTACGCTTACCTGT

CGAGTGGACTTCGGCAGACAAGGCGTCAGCACACACAGGACCGTCCAACTGAGTGTGGCC

TATGCCCCTCAAGAACCTGTCATCCGAGTTCTCCACCACAATGCATCCGCCCCAGTGGCC

CAGGGAAACTTCTCACATCTGGAAGTCCAGAAAGGCCAGTTCCTGCGGCTCCTCTGTGCT

GCTGACAGCCATCCCCCGGCCACTCTGAGCTGGGTCCTGGAGGACAGAGTCCTCTCCTGG

TCCTGCCCCTTGGGCCCCAGAACCCTGGAGCTGCAGCTGCCCGTGGTGAAGCCTGGAGAT

TCGGGGCTCTACACCTGCCGAGCAGAGAACAGGCTGGGCTCCCAACAGGGCACCCTGGAC

CTCTCTGTGCAGTATCCTCCAGAGGACCTGAGGGTGACGGTTTCCCAAGAAAATAGGACA

GTCACGGAAGTCATCAGGAATGGCACGTCCTTCCCAGTCCTGGAAGGCCAAAGCCTGCGC

CTGGTCTGTGTCACCCACAGCAACCCCCCAGCCAGGCTGAGCTGGACCTGGGGGACGCAG

ACCCTGAGCCCTGAGTGGCTTTCCGACGCTGGGCTCCTGGAGCTGCCCCGGGTGCAGACA

GAGCATGGAGGAGAATTCACCTGCCATGCCCGCAGCCCCCTGGGCTCCCAGCACCTCTCC

CTGAGCCTCTCTGTGCACTACCCCCCAAGGCTGCTGGGACCCTCCTGCTCCTGGGAGGCC

CAGGCTCTGCTCTGCAGCTGCTCCTCCCGCGCCTGGCCGGCCCCCTCCCTGCACTGGCGG

CTGGGGGAGGGGCTGCTGGAGGGGAACAGCAGCAATGCCTCCCTCAGGGTCACCTCCAGC

TCCGAGGGGCCCTGGGCCAACAGCTCCCTGAGCCTCCACGGGGCGCTCAGCTCCGACCTC

AGGCTGAGCTGTGAGGCCCAGAACGCCCAGGGCACCCAGAGCGCCACCGTCCTGCTGCTG

CCAGGCGAGGCGTCTGTCCCCACTGCGTTCTCCCGCGGAGTGTTTCTGGGAATCGGCGTC

ACCATGGTCCTCTCCCTCTGCATCATCCTGGCCATTGTGAAGACTCTGAGGAAGGCGCCG

CCCCAGACGGGGGCCAGGCCCAAGGTGTCCCGCGGCAGCACCATCCTGGAGTACATCAAC

GTGGTCCCCAAGGCTGCCCGCAGGGCTCAGGATCAGAAGGCCACGCCAAGCAGCCCTTCT

CGGACCCCCTCTCCAGGCACTCACTCCCCAGAACCAAAGAAGAATCCAGAGGAGCTGTAT

TCTGTTCCCCTTGGTTGCTCAAAACCCAGATCGTCCACCCAAGCTCTGGACCCCAAGAAT

GACCCCGAGGAGCTCCACTATGCTACTCTCACCTTCCCGGGCCACAGACCGTGGGCCACC

CAGATGCCCAAAGACACCCACACAGATTACGAGGAAATCAAGTTC

>CL113.Contig12_All 228 896 PREDICTED: LOW QUALITY PROTEIN: sialic acid binding Ig-like lectin 10 [Equus caballus]

AAGAATCAGTCTTTCTGGCTGCAGATGCAGAAGGCGGTGACAGTGCAGGAGGGTCTGTGT

GTGCTTGTGTCCTGCTCCTTCTCCTACCCCGAGGCTGGCTGGAAGCACTCCACACCTACC

TACGGCTCCTGGTACAAGACAAAGAAGAATTCCAAACCTCATGCAAAAGACAATGACCTC

GTGGCCACAAACAACCCAGACAAGGAAGCAAAAGAGAAGTACAAACTTCATTTCCGGCTC

CTCGGAGACCCCCAATCTAACAACTGCTCCCTGAGCATCTCCGAGGTCCAGAAGGACGAC

AGCGGAACATACTATTTTCATCTAGAGCAAGGAGCTGTGAATCACACTTACGAAAGTAAC

CTGCTCACCCTGACTGTCACAGAGTTGACCCAAACCCCAGACATCCACATCCAGGAGCCC

CTACAGTCGGGCCACCTTAGCCATGTGACATGCTCCATGCCTGGGGCCTGTGACTGGCCC

ACGGCCCCCCGTATCTCCTGGGCTGGGGCTGCCCTGAGAGCAGCGGGGTCAGGCCTGGAG

CCCTCCACCTCAGAGATCCTGCTCACGCCCCACCCCGAGGACCATGGCACCCACCTCTCC

TGCCGAGCGACCTTCCCCAGGGCTGGCGTGTCTTCAAACAGAACTGTGCAGCTCAACGTG

TCCTATGCT

>CL113.Contig13_All 1110 1436 PREDICTED: sialic acid-binding Ig-like lectin 16 isoform 1 [Saimiri boliviensis boliviensis]

AGAAAGCTGCCAGCCCAACAGGAACTGCACAACAGGACACCCCCCGTTGACCTGGGCCCT

GTCTGTGGGGGTCCCAAGGATGCATCCTGGTTGGACAGCCCCTCATATGCTCCCACCCCC

ACTGAGGCCACGCTGGCCGTGGGAGAGGAGCCAGAGCCCCAGGAGCTGCACTACGCCTCC

CTCAGCTTCCTGGAGTTGGAGTCTCGAGAGCCCAAGGTGCAGGAGGCCACCAGCAGGACA

GAATACTCAGAGATCAAGGTCTGCAATGGCAGCCTTCAGGAGCAGCCGGATGTCCTGGGG

AGAGAGACATCAGGGACAGTTCCTAGA

>CL113.Contig14_All 66 1967 PREDICTED: sialic acid binding Ig-like lectin 10 isoform 7 [Pan troglodytes]

CTGCTCCTGGCCCTGCTGCTGCTTCAGTTGGGGACGGGGTCCCTGCAGAAGAATCAGTCT

TTCTGGCTGCAGATGCAGAAGGCGGTGACAGTGCAGGAGGGTCTGTGTGTGCTTGTGTCC

TGCTCCTTCTCCTACCCCGAGGCTGGCTGGAAGCACTCCACACCTACCTATGGCTCCTGG

TACAAGACAAAGAAGAATTCCAAACCTCATGCAAAAGACAATGACCTCGTGGCCACAAAC

AACCCAGACAAGGAAGCAAAAGAGAAGTACAAACTTCATTTCCGGCTCCTCGGAGACCCC

CAATCTAACAACTGCTCCCTGAGCATCTCCGAGGTCCAGAAGGACGACAGCGGAACATAC

TATTTTCATCTAGAGCAAGGAGATGAGAATCACAGTTACAAGAAATTGCTCACCCTGACT

GTCACAGAGATCCTGCTCACGCCCCACCCCGAGGACCATGGCACCCACCTCTCCTGCCGA

GCGACCTTCCCCAGGGCTGGCGTGTCTTCAAACAGAACTGTGCAGCTCAACGTGTCCTAT

GCTCCTCAGAACCTGACCATCAGCATCTCCGGAGCAGGTGACCCAGCCCCAGTGGCCCAG

GGAAACTTCTCACATCTGGAAGTCCAGAAAGGCCAGTTCCTGCGGCTCCTCTGTGCTGCT

GACAGCCATCCCCCGGCCACTCTGAGCTGGGTCCTGGAGGACAGAGTCCTCTCCTGGTCC

TGCCCCTTGGGCCCCAGAACCCTGGAGCTGCAGCTGCCCGTGGTGAAGCCTGGAGATTCG

GGGCTCTACACCTGCCGAGCAGAGAACAGGCTGGGCTCCCAACAGGGCACCCTGGACCTC

TCTGTGCAGTATCCTCCAGAGGACCTGAGGGTGACGGTTTCCCAAGAAAATAGGACAGTC

ACGGAAGTCATCAGGAATGGCACGTCCTTCCCAGTCCTGGAGGGCCAAAGCCTGCGCCTG

GTCTGTGTCACCCACAGCAACCCCCCAGCCAGGCTGAGCTGGACCTGGGGGACGCAGACC

CTGAGCCCTGAGTGGCTTTCCGACGCTGGGCTCCTGGAGCTGCCCCGGGTGCAGACAGAG

CATGGAGGAGAATTCACCTGCCATGCCCGCAGCCCCCTGGGCTCCCAGCACCTCTCCCTG

AGCCTCTCTGTGCACTACCCCCCAAGGCTGCTGGGACCCTCCTGCTCCTGGGAGGCCCAG

GCTCTGCTCTGCAGCTGCTCCTCCCGCGCCTGGCCGGCCCCCTCCCTGCACTGGCGGCTG

GGGGAGGGGCTGCTGGAGGGGAACAGCAGCAATGCCTCCCTCAGGGTCACCTCCAGCTCC

GAGGGGCCCTGGGCCAACAGCTCCCTGAGCCTCCACGGGGCGCTCAGCTCCGACCTCAGG

CTGAGCTGTGAGGCCCAGAACGCCCAGGGCACCCAGAGCGCCACCGTCCTGCTGCTGCCA

GGCGAGGCGTCTGTCCCCACTGCGTTCTCCCGCGGAGTGTTTCTGGGAATCGGCGTCACC

ATGGTCCTCTCCCTCTGCATCATCCTGGCCATTGTGAAGACTCTGAGGAAGGCGCCGCCC

CAGACGGGGGCCAGGCCCAAGGTGTCCCGCGGCAGCACCATCCTGGAGTACATCAACGTG

GTCCCCAAGGCTGCCCGCAGGGCTCAGGATCAGAAGGCCACGCCAAGCAGCCCTTCTCGG

ACCCCCTCTCCAGGCACTCACTCCCCAGAACCAAAGAAGAATCCAGAGGAGCTGTATTCT

GTTCCCCTTGGTTGCTCAAAACCCAGATCGTCCACCCAAGCTCTGGACCCCAAGAATGAC

CCCGAGGAGCTCCACTATGCTACTCTCACCTTCCCGGGCCACAGACCGTGGGCCACCCAG

ATGCCCAAAGACACCCACACAGATTACGAGGAAATCAAGTTC

>CL113.Contig15_All 1621 1947 PREDICTED: sialic acid-binding Ig-like lectin 11 isoform 1 [Callithrix jacchus]

AGAAAGCTGCCAGCCCAACAGGAACTGCACAACAGGACACCCCCCGTTGACCTGGGCCCT

GTCTGTGGGGGTCCCAAGGATGCATCCTGGTTGGACAGCCCCTCATATGCTCCCACCCCC

ACTGAGGCCACGCTGGCCGTGGGAGAGGAGCCAGAGCCCCAGGAGCTGCACTACGCCTCC

CTCAGCTTCCTGGAGTTGGAGTCTCGAGAGCCCAAGGTGCAGGAGGCCACCAGCAGGACA

GAATACTCAGAGATCAAGGTCTGCAATGGCAGCCTTCAGGAGCAGCCGGATGTCCTGGGG

AGAGAGACATCAGGGACAGTTCCTAGA

>CL113.Contig16_All 2010 2336 PREDICTED: sialic acid-binding Ig-like lectin 11 [Papio anubis]

AGAAAGCTGCCAGCCCAACAGGAACTGCACAACAGGACACCCCCCGTTGACCTGGGCCCT

GTCTGTGGGGGTCCCAAGGATGCATCCTGGTTGGACAGCCCCTCATATGCTCCCACCCCC

ACTGAGGCCACGCTGGCCGTGGGAGAGGAGCCAGAGCCCCAGGAGCTGCACTACGCCTCC

CTCAGCTTCCTGGAGTTGGAGTCTCGAGAGCCCAAGGTGCAGGAGGCCACCAGCAGGACA

GAATACTCAGAGATCAAGGTCTGCAATGGCAGCCTTCAGGAGCAGCCGGATGTCCTGGGG

AGAGAGACATCAGGGACAGTTCCTAGA

>CL113.Contig17_All 269 2452 PREDICTED: sialic acid-binding Ig-like lectin 11 [Papio anubis]

CCTCCCAAGGCCGAGATGCTACTCCTGCCGCCACTGCTGTCCTTGTTGTTGGGTGGTGAG

TGGCAGGGGACTGGAGGGACCCGGCCCGGCCGGGACTGGGATTGCAGGTTGAGCCTCTGT

CTCCCCTCAGGGTCCCAGGCTCAGCTAGAGAAGTACTGGCTGCAGGTGCAGGAGTCGGTG

TCGGTGCAGGAGGGCCTGTGTGTGCTGGTGTCCTGCTCCTTCTCCTACCCCAGGGACGAC

CCCAGAACTGCTTCTAACCCGGCTTATGGATACTGGTTCGAAGAAGGGACCAACACGAAC

AAGGGGGATCCAGTGGCCACGAACAACCCACATCGAAGGGTGCAATCTAGGACCCAAGGC

CGATTCCAGCTCATGGGGGACCCCAACAGAGATGACTGCTCCCTGATGATCAAAGACGCA

TACAGATGGGACAGGGCGGCCTACTTCTTCCGCGTGGAGAGAGGACCCATAGTGAAATTT

AATTTCCAGAAAAAGTTCTCTCTGGAAGTGACAGACCTAACTCAGAAGCCAGACGTCTTC

ATTCCCGAGATCCTGGAGCCCGGGCAGCCTGTGAGCGTTCTCTGTGTGTTTAACCAAGCG

TTTGAGCAATGCCCGGCCCCTTCTTTCTCCTGGACCACGGCTGCCATCCCCTCCCAAAGA

ACAAGGCCACCAACCTCCCACTCCTCAGTGCTCAGCCTCACCCCACAGCCCCAGGACCAC

GGCACTACGCTTACCTGTCGAGTGGACTTCGGCAGACAAGGCGTCAGCACACACAGGACC

GTCCAACTGAGTGTGGCCTATGCCCCTCAAGAACCTGTCATCCGAGTTCTCCACCACAAT

GCATCCGCCCCAGTGGCCCAGGGAAACTTCTCACATCTGGAAGTCCAGAAAGGCCAGTTC

CTGCGGCTCCTCTGTGCTGCTGACAGCCATCCCCCGGCCACTCTGAGCTGGGTCCTGGAG

GACAGAGTCCTCTCCTGGTCCTGCCCCTTGGGCCCCAGAACCCTGGAGCTGCAGCTGCCC

GTGGTGAAGCCTGGAGATTCGGGGCTCTACACCTGCCGAGCAGAGAACAGGCTGGGCTCC

CAACAGGGCACCCTGGACCTCTCTGTGCAGTATCCTCCAGAGGACCTGAGGGTGACGGTT

TCCCAAGAAAATAGGACAGTCACGGAAGTCATCAGGAATGGCACGTCCTTCCCAGTCCTG

GAAGGCCAAAGCCTGCGCCTGGTCTGTGTCACCCACAGCAACCCCCCAGCCAGGCTGAGC

TGGACCTGGGGGACGCAGACCCTGAGCCCTGAGTGGCTTTCCGACGCTGGGCTCCTGGAG

CTGCCCCGGGTGCAGACAGAGCATGGAGGAGAATTCACCTGCCATGCCCGCAGCCCCCTG

GGCTCCCAGCACCTCTCCCTGAGCCTCTCTGTGCACTACCCCCCAAGGCTGCTGGGACCC

TCCTGCTCCTGGGAGGCCCAGGCTCTGCTCTGCAGCTGCTCCTCCCGCGCCTGGCCGGCC

CCCTCCCTGCACTGGCGGCTGGGGGAGGGGCTGCTGGAGGGGAACAGCAGCAATGCCTCC

CTCAGGGTCACCTCCAGCTCCGAGGGGCCCTGGGCCAACAGCTCCCTGAGCCTCCACGGG

GCGCTCAGCTCCGACCTCAGGCTGAGCTGTGAGGCCCAGAACGCCCAGGGCACCCAGAGC

GCCACCGTCCTGCTGCTGCCAGGGAAGCCAGAGCTCAGGGGAGGAGCTCTTCTGGGGACA

CTGGGGGCTGCTGGTGGGGCCACTCTACTCTCCCTCTGTGCCTGCCTCGTCTTCTCCTGG

TTAAAGACTCGCAGGAAGAAAGCTGCCAGCCCAACAGGAACTGCACAACAGGACACCCCC

ATTGACCTGGGCCCTGTCTGTGGGCCCCGTCATCCTCATTGGATCCCGACTCCTGCCCCA

CTTCAGGGTCCCAAGGATGCATCCTGGTTGGACAGCCCCTCATATGCTCCCACCCCCACT

GAGGCCACGCTGGCCGTGGGAGAGGAGCCAGAGCCCCAGGAGCTGCACTACGCCTCCCTC

AGCTTCCTGGAGTTGGAGTCTCGAGAGCCCAAGGTGCAGGAGGCCACCAGCAGGACAGAA

TACTCAGAGATCAAGGTCTGCAATGGCAGCCTTCAGGAGCAGCCGGATGTCCTGGGGAGA

GAGACATCAGGGACAGTTCCTAGA

>CL114.Contig1_All 2 820 minus strand PREDICTED: REST corepressor 3 [Pongo abelii]

TTTAGTTTTCATGGGAAGAGCTTTCACAGGATTCAACAAATGCTTCCAGATAAGACAATT

GCAAGCCTCGTAAAATATTACTATTCTTGGAAAAAAACCCGTTCTAGAACAAGTTTGATG

GATCGTCAAGCTCGTAAACTAGCCAATAGACATAATCAGGGTGACAGTGATGATGATGTG

GAAGAAACACACCCAATGGATGGGAATGATAGTGATTACGATCCTAAAAAAGAAGCCAAA

AAAGAGGGAAACACTGAACAACCTGTCCAAACTAGCAAGATTGGACTTGGAAGGAGAGAA

TATCAGAGTTTACAACATCGCCATCATTCTCAGCGCTCTAAGTGCCGTCCACCTAAGGGC

ATGTATTTAACCCAGGAAGATGTGGTAGCTGTTTCTTGTAGTCCCAATGCAGCCAACACC

ATTCTGAGGCAACTGGACATGGAGTTGATCTCTCTGAAACGTCAGGTTCAGAATGCTAAG

CAAGTAAACAGTGCACTTAAACAGAAAATGGAAGGTGGAATTGAAGAATTCAAACCTCCT

GAGTCAAATCAGAAAATTAATGCCCGTTGGACCACAGAGGAGCAGCTTCTAGCAGTGCAA

GGTGTCCGCAAATATGGTAAAGATTTTCAAGCTATTGCAGATGTAATTGGCAACAAGACT

GTTGGCCAAGTGAAGAACTTCTTTGTAAACTACAGGCGTCGGTTTAACTTAGAGGAGGTA

TTGCAGGAGTGGGAAGCAGAACAAGGAACCCAGGCTTCTAATGGTGATGCTTCTACTTTA

GGGGAGGAGACAAAAAGTGCTTCTAATGTGCCATCAGGG

>CL114.Contig2_All 600 944 minus strand RecName: Full=REST corepressor 3

AAGGCACAGACCCCACAGGCTCCTCGGACTCTGGGTCCATCACCTCCTGCCCCATCATCC

ACTCCAACACCAACAGCCCCCATTGCCACTCTGAACCAGCCTCCACCACTTCTTCGTCCA

ACACTGCCTGCTGCCCCTGCTCTTCATCGGCAGCCTCCTCCACTCCAGCAGCAGGCTCGG

TTCATCCAGCCCCGGCCAACTCTAAATCAGCCTCCACCACCTCTCATTCGCCCTGCTAAT

TCTATGCCACCCCGTCTAAACCCAAGACCAGTGTTGTCCACGGTTGGTGGTCAACAGCCA

CCATCACTTATTGGAATTCAGACAGATTCACAGTCCTCACTGCAC

>CL115.Contig1_All 237 818 PREDICTED: YTH domain-containing protein 1 isoform 2 [Saimiri boliviensis boliviensis]

CGGCGTCGACCAGAAGATTATGATATTCATAACAGCAGAAAGAAACCAAGGATTGACTAT

CCCCCTGAGTTTCACCAGAGACCAGGGTATTTAAAGGATCCACGATACCAGGAAGTAGAC

AGACGATTTTCAGGAGTTCGCCGAGATGTGTTTTTAAATGGGTCCTACAATGATTATGTG

AGGGAATTTCATAACATGGGACCACCACCACCATGGCAAGGAATGCCCCCCTATCCGGGA

ATGGAACAGCCTCCACACCATCCTTACTATCAGCACCATGCTCCACCTCCTCAAGCCCAT

CCCCCTTACTCAGGACATCATCCAGTACCACATGAAGCAAGATACAGAGATAAACGAGTA

CATGATTATGATATGAGAGTGGATGATTTCCTTCGTCGCACACAAGCTGTTGTCAGTGGT

CGGAGAAGTAGACCCCGTGAAAGAGACCGGGAGCGAGAACGAGATCGCCCTAGAGATAAC

AGAAGAGACAGAGAGCGAGATAGAGGACGTGATCGAGAAAGAGAAAGAGAGCGATTATGT

GATCGGGACAGAGACCGAGGGGAGAGAGGTCGATATCGAAGA

>CL115.Contig2_All 237 842 splicing factor YT521-B, isoform CRA_c [Rattus norvegicus]

CGGCGTCGACCAGAAGATTATGATATTCATAACAGCAGAAAGAAACCAAGGATTGACTAT

CCCCCTGAGTTTCACCAGAGACCAGGGTATTTAAAGGATCCACGATACCAGGAAGTAGAC

AGTTTCACAAATCTTATTCCCAACAGACGATTTTCAGGAGTTCGCCGAGATGTGTTTTTA

AATGGGTCCTACAATGATTATGTGAGGGAATTTCATAACATGGGACCACCACCACCATGG

CAAGGAATGCCCCCCTATCCGGGAATGGAACAGCCTCCACACCATCCTTACTATCAGCAC

CATGCTCCACCTCCTCAAGCCCATCCCCCTTACTCAGGACATCATCCAGTACCACATGAA

GCAAGATACAGAGATAAACGAGTACATGATTATGATATGAGAGTGGATGATTTCCTTCGT

CGCACACAAGCTGTTGTCAGTGGTCGGAGAAGTAGACCCCGTGAAAGAGACCGGGAGCGA

GAACGAGATCGCCCTAGAGATAACAGAAGAGACAGAGAGCGAGATAGAGGACGTGATCGA

GAAAGAGAAAGAGAGCGATTATGTGATCGGGACAGAGACCGAGGGGAGAGAGGTCGATAT

CGAAGA

>CL115.Contig3_All 2 1483 splicing factor YT521-B, isoform CRA_c [Rattus norvegicus]

GAGGAAGAGGAGGAAGAGGAAGAATATGAACAGGATGAGAGAGACCAGAAGGAAGAGGGA

AATGATTATGATACTCGAAGTGAGGCCAGTGACTCTGGTTCTGAATCTGTTTCCTTTACA

GATGGTTCTGTCAGATCTGGCTCAGGCACAGATGGATCAGATGAGAAAAAGAAGGAAAGA

AAGAGAGCTAGAGGCATATCACCAATTGTTTTTGATAGAAGTGGAAGCTCTGCATCAGAG

TCATATGCAGGTTCAGAAAAGAAGCATGAGAAATTATCATCTTCCGTTCGTGCTGTCCGA

AAAGATCAAACCAGTAAACTCAAATATGTACTTCAGGATGCAAGATTTTTCCTCATAAAG

AGTAACAACCATGAGAATGTGTCTCTTGCGAAAGCTAAGGGTGTATGGTCTACACTCCCT

GTAAATGAGAAAAAATTAAATCTTGCATTCAGATCTGCGAGGAGTGTTATTTTAATATTT

TCTGTCAGAGAAAGTGGAAAGTTTCAAGGATTTGCAAGACTTTCTTCAGAATCACATCAT

GGAGGATCTCCTATACACTGGGTACTTCCAGCAGGAATGAGTGCTAAGATGCTGGGAGGT

GTCTTTAAAATTGACTGGATTTGCAGGCGTGAATTACCCTTTACTAAGTCGGCTCATCTC

ACCAATCCTTGGAATGAACATAAGCCAGTAAAGATAGGACGCGATGGACAGGAAATTGAA

CTTGAATGTGGAACCCAGCTTTGTCTTCTATTTCCCCCCGATGAAAGTATTGACTTGTAT

CAGGTCATTCATAAAATGCGTCACAAGAGAAGAATGCATTCTCAGCCCAGATCAAGAGGA

CGTCCATCCCGTCGAGAACCAGTCCGGGATGTGGGAAGGCGTCGACCAGAAGATTATGAT

ATTCATAACAGCAGAAAGAAACCAAGGATTGACTATCCCCCTGAGTTTCACCAGAGACCA

GGGTATTTAAAGGATCCACGATACCAGGAAGTAGACAGTTTCACAAATCTTATTCCCAAC

AGACGATTTTCAGGAGTTCGCCGAGATGTGTTTTTAAATGGGTCCTACAATGATTATGTG

AGGGAATTTCATAACATGGGACCACCACCACCATGGCAAGGAATGCCCCCCTATCCGGGA

ATGGAACAGCCTCCACACCATCCTTACTATCAGCACCATGCTCCACCTCCTCAAGCCCAT

CCCCCTTACTCAGGACATCATCCAGTACCACATGAAGCAAGATACAGAGATAAACGAGTA

CATGATTATGATATGAGAGTGGATGATTTCCTTCGTCGCACACAAGCTGTTGTCAGTGGT

CGGAGAAGTAGACCCCGTGAAAGAGACCGGGAGCGAGAACGAGATCGCCCTAGAGATAAC

AGAAGAGACAGAGAGCGAGATAGAGGACGTGATCGAGAAAGAGAAAGAGAGCGATTATGT

GATCGGGACAGAGACCGAGGGGAGAGAGGTCGATATCGAAGA

>CL116.Contig1_All 36 1556 minus strand cytochrome P450 4A6 [Oryctolagus cuniculus] >gi|117168|sp|P14580.1|CP4A6_RABIT RecName: Full=Cytochrome P450 4A6; AltName: Full=CYPIVA6; AltName: Full=Cytochrome P450-KA-1; AltName: Full=Lauric acid omega-hydroxylase; Flags: Precursor >gi|164977|gb|AAA31230.1| lauric acid omega-hydroxylase [Oryctolagus cuniculus]

ATGAATGTCTCTGCACTGAGCCTCACCAGACTCCCAGGGAGCATCTCTGGGTTCCTCCAG

CTGGCCTCCCTGCTCGGCTTGCTTCTGTTCCTGTTCAAGGCAGCCCAGCTCTACCTGCGC

AGGCAGTGGCTGCTCAAAGCCTTCCAGCAGTTTCCATCCGCACCCTCCCATTGGCTCTTT

GGCCACAAGATCCAAAAGGATCAGGAGCTGCAACAGATTCTGTCATGGGTAGAGAAGTTC

CCCAGTGCCTTTCCCCGCTGGCTCTGGGGCAGCAAAGCTCACATGGTTATCTATGACCCT

GACTACATGAAGGTGATTCTGGGGAGATCAGATCCAAAGGCCTCTGGTACCTACAGATTC

CTGGCTCCCTGGATTGGATATGGTTTGCTCCTGCTAAACGAACAGACGTGGTTCCAGCAC

CGGCGGATGTTGACCCCAGCCTTCCACTACGACATCCTGAAGCCCTACGTGGGAATCGTG

GCTGACTCTGTCCGAGTGATGTTGGACAAATGGGAAAAACTCATTGACAAGGACTCCTCT

GTGGAGGTCTTCCAGCACATCTCCATGATGACCCTGGACTCCATCATGAAGTGTGCCTTC

AGTTACCAAGGCAATGTCCAGTTGGACAGGCATTCCCAGTCCTACATCAAAGCCATTGGG

GACCTGAACAACCTGTTTTTTGACCGTGTAAGAAATGCCTTTCACCAGAACGACACCATA

TATGGTCTGTCCTCTAATGGCCGTTTGTTCCACCGTGCCTGCCAACTTGCCCATGAGCAC

ACAGACCAAGTGATCAAAGAAAGGAAGGCTCAGCTGCAGAAAGAGAGAGAGTTGGAGAAG

ATCAGGTGGAAGAGGCGCTTGGATTTCCTGGACATCCTCTTATTTGCCAAAATGGAGAAT

GGGAGCAGTCTGTCTGATGAGGACCTGCGTGCCGAGGTGGACACCTTCATGTTTGAGGGC

CATGACACCACAGCCAGTGCTATCTCCTGGATCTTCTATGCTCTGGCTGCACACCCTGAG

CATCAGAAGAAGTGCAGGGAGGAGGTCCAGAGCCTCCTGGGGGATGGAGCCTCCATCACC

TGGGACCACATGGACCAGATGCCCTACACCACCATGTGCATCAAGGAGGCCCTGAGGATC

TACCCACCAGTACCAATTGTTGTCAGAGAGCTCAAAACACCTGTCACCTTCCCTGATGGA

CGCTCTTTACCCAAAGGCATCATAGTCACACTGTCCTTTTATGGCCTTCACCACAACTCA

AAAGCGTGGCCAAACCCAGAGGTATATGATCCTTTCCGGTTTGCACCAGATTCTTCCCGA

CACAGTCACTCATTCCTGCCTTTCTCAGGAGGAGCCAGGAACTGCATCGGGAAACAGTTT

GCCATGAATGAGCTGAAGGTGGCTGTGGCCCTGACCCTGCTCCGCTTTGAGCTGCTGCCA

GATCCCACCAGGATCCCCTTTCCCATTCCGCGACTTGTATTAAAGTCCAAGAATGGGATC

CACCTGCGTCTCAGAAAACTC

>CL116.Contig2_All 54 1409 minus strand cytochrome P450 4A6 [Oryctolagus cuniculus] >gi|117168|sp|P14580.1|CP4A6_RABIT RecName: Full=Cytochrome P450 4A6; AltName: Full=CYPIVA6; AltName: Full=Cytochrome P450-KA-1; AltName: Full=Lauric acid omega-hydroxylase; Flags: Precursor >gi|164977|gb|AAA31230.1| lauric acid omega-hydroxylase [Oryctolagus cuniculus]

ATGAGCATGTCTTCACTGAGCCTCACCAGATTCCCGGGCAGCATCTCTGGGCTCCTCCAA

GTGGCCTCCCTGCTCAGCTTGCTTCTGCTGCTGTTCAAGGCAGCCCAGTTCCTCCTGCGC

AGGCAATGGCTGCTCAAAGCCCTTCAGCAGTTTCCATCTCCACCATCCCACTGGTTCTTT

GGCCACAAGATCCCAAAGGACCAGGAGCTACAATACATTCTGAAGTGGGTTAACGAGTTC

CCGTGCGCCTTCCCACAATGGCTGTGGGGCAGCAAAGTGCGCGTCCTCCTCTATGATCCT

GAATACATGAAGGCGATTCTGGGGAGATCAGATCCAAAAGCTCAGAGTATATACAGACTT

GTGGCTCCCTGGATTGGGTACGGTTTGCTCCTGTTGAATGGGGAGACATGGTTCCAGCAC

CGCCGGATGTTGACCCCAGCCTTCCACTACGACATCCTGAAGCCCTACGTGGGAATCACA

GCTGACTCTGTCAGAGTGATGCTGGACAAATGGGAGCAGCTTATTGGACAGGACTCCCTA

GAGATTTTTCAGCATGTCTCCCTGATGACCCTGGACACCATCATGAAGTGTGCCTTCAGC

CATCAGGGCAGTATCCAGCTGGAGAGGAATTCCAAGTCCTACATTCAGGCTGTTGGGGAT

CTGAACTACCTGTTTTTTTCTCGTATGAGGAATGTCTTTCACCAGAACGACACCATCTAC

AGACAGACCTCTGATGGCCGCTTGTTCCACCATGCCTGCCAGCTTGCCCATGAGCACACA

GATCAAGTGATCGGGCAAAGGAAGGCTCAGCTGCAGAATGAGGGAGAGCTGGAGAAGATC

AGGCAGAAGAGGCACTTGGATTTCTTGGACATCCTTCTGTGTGCCAAAATGGAGAATGGG

AGCAGTCTGTCTGATGAGGACCTGCGTGCCGAGGTGGACACCTTCATGTTTGAGGGCCAT

GACACCACAGCCAGTGCTATCTCCTGGATCTTCTATGCTCTGGCTGCACACCCTGAGCAT

CAGAAGAAGTGCAGGGAGGAGGTCCAGAGCCTCCTGGGGGATGGAGCCTCCATCACCTGG

GACCACATGGACCAGATGCCCTACACCACCATGTGCATCAAGGAGGCCCTGAGGCTCTAT

CCACCAGTACCAAGTGTGAGCAGAGATCTCAGTAAACCTATCACTTTCCCTGATGGACGC

TCCCTACCCAAAGGTTTCATGGTCACACTCTCCATTTATGGCCTTCATCACAATCCGAAG

GTGTGGCCAAACCCAGAGGTGTTTGATCCTTCTCGATTTGCACCTGGATCTTCTCGACAC

AGCCACTCCTTCCTGCCCTTCTCAGGAGGATCAAGA

>CL116.Contig3_All 82 1437 minus strand cytochrome P450 4A6 [Oryctolagus cuniculus] >gi|117168|sp|P14580.1|CP4A6_RABIT RecName: Full=Cytochrome P450 4A6; AltName: Full=CYPIVA6; AltName: Full=Cytochrome P450-KA-1; AltName: Full=Lauric acid omega-hydroxylase; Flags: Precursor >gi|164977|gb|AAA31230.1| lauric acid omega-hydroxylase [Oryctolagus cuniculus]

ATGAGCATGTCTTCACTGAGCCTCACCAGATTCCCGGGCAGCATCTCTGGGCTCCTCCAA

GTGGCCTCCCTGCTCAGCTTGCTTCTGCTGCTGTTCAAGGCAGCCCAGTTCCTCCTGCGC

AGGCAATGGCTGCTCAAAGCCCTTCAGCAGTTTCCATCTCCACCATCCCACTGGTTCTTT

GGCCACAAGATCCCAAAGGACCAGGAGCTACAATACATTCTGAAGTGGGTTAACGAGTTC

CCGTGCGCCTTCCCACAATGGCTGTGGGGCAGCAAAGTGCGCGTCCTCCTCTATGATCCT

GAATACATGAAGGCGATTCTGGGGAGATCAGATCCAAAAGCTCAGAGTATATACAGACTT

GTGGCTCCCTGGATTGGGTACGGTTTGCTCCTGTTGAATGGGGAGACATGGTTCCAGCAC

CGCCGGATGTTGACCCCAGCCTTCCACTACGACATCCTGAAGCCCTACGTGGGAATCACA

GCTGACTCTGTCAGAGTGATGCTGGACAAATGGGAGCAGCTTATTGGACAGGACTCCCTA

GAGATTTTTCAGCATGTCTCCCTGATGACCCTGGACACCATCATGAAGTGTGCCTTCAGC

CATCAGGGCAGTATCCAGCTGGAGAGGAATTCCAAGTCCTACATTCAGGCTGTTGGGGAT

CTGAACTACCTGTTTTTTTCTCGTATGAGGAATGTCTTTCACCAGAACGACACCATCTAC

AGACAGACCTCTGATGGCCGCTTGTTCCACCATGCCTGCCAGCTTGCCCATGAGCACACA

GATCAAGTGATCGGGCAAAGGAAGGCTCAGCTGCAGAATGAGGGAGAGCTGGAGAAGATC

AGGCAGAAGAGGCACTTGGATTTCTTGGACATCCTTCTGTGTGCCAAAATGGAGAATGGG

AGCAGTCTGTCTGATGAGGACCTGCGTGCCGAGGTGGACACCTTCATGTTTGAGGGCCAT

GACACCACAGCCAGTGCTATCTCCTGGATCTTCTATGCTCTGGCTGCACACCCTGAGCAT

CAGAAGAAGTGCAGGGAGGAGGTCCAGAGCCTCCTGGGGGATGGAGCCTCCATCACCTGG

GACCACATGGACCAGATGCCCTACACCACCATGTGCATCAAGGAGGCCCTGAGGCTCTAT

CCACCAGTACCAAGTGTGAGCAGAGATCTCAGTAAACCTATCACTTTCCCTGATGGACGC

TCCCTACCCAAAGGTTTCATGGTCACACTCTCCATTTATGGCCTTCATCACAATCCGAAG

GTGTGGCCAAACCCAGAGGTGTTTGATCCTTCTCGATTTGCACCTGGATCTTCTCGACAC

AGCCACTCCTTCCTGCCCTTCTCAGGAGGATCAAGG

>CL116.Contig4_All 33 1391 minus strand cytochrome P450 4A6 [Oryctolagus cuniculus] >gi|117168|sp|P14580.1|CP4A6_RABIT RecName: Full=Cytochrome P450 4A6; AltName: Full=CYPIVA6; AltName: Full=Cytochrome P450-KA-1; AltName: Full=Lauric acid omega-hydroxylase; Flags: Precursor >gi|164977|gb|AAA31230.1| lauric acid omega-hydroxylase [Oryctolagus cuniculus]

ATGAATGTCTCTGCACTGAGCCTCACCAGACTCCCAGGGAGCATCTCTGGGTTCCTCCAG

CTGGCCTCCCTGCTCGGCTTGCTTCTGTTCCTGTTCAAGGCAGCCCAGCTCTACCTGCGC

AGGCAGTGGCTGCTCAAAGCCTTCCAGCAGTTTCCATCCGCACCCTCCCATTGGCTCTTT

GGCCACAAGATCCAAAAGGATCAGGAGCTGCAACAGATTCTGTCATGGGTAGAGAAGTTC

CCCAGTGCCTTTCCCCGCTGGCTCTGGGGCAGCAAAGCTCACATGGTTATCTATGACCCT

GACTACATGAAGGTGATTCTGGGGAGATCAGATCCAAAGGCCTCTGGTACCTACAGATTC

CTGGCTCCCTGGATTGGATATGGTTTGCTCCTGCTAAACGAACAGACGTGGTTCCAGCAC

CGGCGGATGTTGACCCCAGCCTTCCACTACGACATCCTGAAGCCCTACGTGGGAATCGTG

GCTGACTCTGTCCGAGTGATGTTGGACAAATGGGAAAAACTCATTGACAAGGACTCCTCT

GTGGAGGTCTTCCAGCACATCTCCATGATGACCCTGGACTCCATCATGAAGTGTGCCTTC

AGTTACCAAGGCAATGTCCAGTTGGACAGGCATTCCCAGTCCTACATCAAAGCCATTGGG

GACCTGAACAACCTGTTTTTTGACCGTGTAAGAAATGCCTTTCACCAGAACGACACCATA

TATGGTCTGTCCTCTAATGGCCGTTTGTTCCACCGTGCCTGCCAACTTGCCCATGAGCAC

ACAGACCAAGTGATCAAAGAAAGGAAGGCTCAGCTGCAGAAAGAGAGAGAGTTGGAGAAG

ATCAGGTGGAAGAGGCGCTTGGATTTCCTGGACATCCTCTTATTTGCCAAAATGGAGAAT

GGGAGCAGTCTGTCTGATGAGGACCTGCGTGCCGAGGTGGACACCTTCATGTTTGAGGGC

CATGACACCACAGCCAGTGCTATCTCCTGGATCTTCTATGCTCTGGCTGCACACCCTGAG

CATCAGAAGAAGTGCAGGGAGGAGGTCCAGAGCCTCCTGGGGGATGGAGCCTCCATCACC

TGGGACCACATGGACCAGATGCCCTACACCACCATGTGCATCAAGGAGGCCCTGAGGCTC

TATCCACCAGTACCAAGTGTGAGCAGAGATCTCAGTAAACCTATCACTTTCCCTGATGGA

CGCTCCCTACCCAAAGGTTTCATGGTCACACTCTCCATTTATGGCCTTCATCACAATCCG

AAGGTGTGGCCAAACCCAGAGGTGTTTGATCCTTCTCGATTTGCACCTGGATCTTCTCGA

CACAGCCACTCCTTCCTGCCCTTCTCAGGAGGATCAAGG

>CL116.Contig5_All 33 1391 minus strand cytochrome P450 4A6 [Oryctolagus cuniculus] >gi|117168|sp|P14580.1|CP4A6_RABIT RecName: Full=Cytochrome P450 4A6; AltName: Full=CYPIVA6; AltName: Full=Cytochrome P450-KA-1; AltName: Full=Lauric acid omega-hydroxylase; Flags: Precursor >gi|164977|gb|AAA31230.1| lauric acid omega-hydroxylase [Oryctolagus cuniculus]

ATGAATGTCTCTGCACTGAGCCTCACCAGACTCCCAGGGAGCATCTCTGGGTTCCTCCAG

CTGGCCTCCCTGCTCGGCTTGCTTCTGTTCCTGTTCAAGGCAGCCCAGCTCTACCTGCGC

AGGCAGTGGCTGCTCAAAGCCTTCCAGCAGTTTCCATCCGCACCCTCCCATTGGCTCTTT

GGCCACAAGATCCAAAAGGATCAGGAGCTGCAACAGATTCTGTCATGGGTAGAGAAGTTC

CCCAGTGCCTTTCCCCGCTGGCTCTGGGGCAGCAAAGCTCACATGGTTATCTATGACCCT

GACTACATGAAGGTGATTCTGGGGAGATCAGATCCAAAGGCCTCTGGTACCTACAGATTC

CTGGCTCCCTGGATTGGATATGGTTTGCTCCTGCTAAACGAACAGACGTGGTTCCAGCAC

CGGCGGATGTTGACCCCAGCCTTCCACTACGACATCCTGAAGCCCTACGTGGGAATCGTG

GCTGACTCTGTCCGAGTGATGTTGGACAAATGGGAAAAACTCATTGACAAGGACTCCTCT

GTGGAGGTCTTCCAGCACATCTCCATGATGACCCTGGACTCCATCATGAAGTGTGCCTTC

AGTTACCAAGGCAATGTCCAGTTGGACAGGCATTCCCAGTCCTACATCAAAGCCATTGGG

GACCTGAACAACCTGTTTTTTGACCGTGTAAGAAATGCCTTTCACCAGAACGACACCATA

TATGGTCTGTCCTCTAATGGCCGTTTGTTCCACCGTGCCTGCCAACTTGCCCATGAGCAC

ACAGACCAAGTGATCAAAGAAAGGAAGGCTCAGCTGCAGAAAGAGAGAGAGTTGGAGAAG

ATCAGGTGGAAGAGGCGCTTGGATTTCCTGGACATCCTCTTATTTGCCAAAATGGAGAAT

GGGAGCAGTCTGTCTGATGAGGACCTGCGTGCCGAGGTGGACACCTTCATGTTTGAGGGC

CATGACACCACAGCCAGTGCTATCTCCTGGATCTTCTATGCTCTGGCTGCACACCCTGAG

CATCAGAAGAAGTGCAGGGAGGAGGTCCAGAGCCTCCTGGGGGATGGAGCCTCCATCACC

TGGGACCACATGGACCAGATGCCCTACACCACCATGTGCATCAAGGAGGCCCTGAGGCTC

TATCCACCAGTACCAAGTGTGAGCAGAGATCTCAGTAAACCTATCACTTTCCCTGATGGA

CGCTCCCTACCCAAAGGTTTCATGGTCACACTCTCCATTTATGGCCTTCATCACAATCCG

AAGGTGTGGCCAAACCCAGAGGTGTTTGATCCTTCTCGATTTGCACCTGGATCTTCTCGA

CACAGCCACTCCTTCCTGCCCTTCTCAGGAGGATCAAGA

>CL116.Contig6_All 36 1559 minus strand cytochrome P450 4A6 [Oryctolagus cuniculus] >gi|117168|sp|P14580.1|CP4A6_RABIT RecName: Full=Cytochrome P450 4A6; AltName: Full=CYPIVA6; AltName: Full=Cytochrome P450-KA-1; AltName: Full=Lauric acid omega-hydroxylase; Flags: Precursor >gi|164977|gb|AAA31230.1| lauric acid omega-hydroxylase [Oryctolagus cuniculus]

ATGAATGTCTCTGCACTGAGCCTCACCAGACTCCCAGGGAGCATCTCTGGGTTCCTCCAG

CTGGCCTCCCTGCTCGGCTTGCTTCTGTTCCTGTTCAAGGCAGCCCAGCTCTACCTGCGC

AGGCAGTGGCTGCTCAAAGCCTTCCAGCAGTTTCCATCCGCACCCTCCCATTGGCTCTTT

GGCCACAAGATCCAAAAGGATCAGGAGCTGCAACAGATTCTGTCATGGGTAGAGAAGTTC

CCCAGTGCCTTTCCCCGCTGGCTCTGGGGCAGCAAAGCTCACATGGTTATCTATGACCCT

GACTACATGAAGGTGATTCTGGGGAGATCAGATCCAAAGGCCTCTGGTACCTACAGATTC

CTGGCTCCCTGGATTGGATATGGTTTGCTCCTGCTAAACGAACAGACGTGGTTCCAGCAC

CGGCGGATGTTGACCCCAGCCTTCCACTACGACATCCTGAAGCCCTACGTGGGAATCGTG

GCTGACTCTGTCCGAGTGATGTTGGACAAATGGGAAAAACTCATTGACAAGGACTCCTCT

GTGGAGGTCTTCCAGCACATCTCCATGATGACCCTGGACTCCATCATGAAGTGTGCCTTC

AGTTACCAAGGCAATGTCCAGTTGGACAGGCATTCCCAGTCCTACATCAAAGCCATTGGG

GACCTGAACAACCTGTTTTTTGACCGTGTAAGAAATGCCTTTCACCAGAACGACACCATA

TATGGTCTGTCCTCTAATGGCCGTTTGTTCCACCGTGCCTGCCAACTTGCCCATGAGCAC

ACAGACCAAGTGATCAAAGAAAGGAAGGCTCAGCTGCAGAAAGAGAGAGAGTTGGAGAAG

ATCAGGTGGAAGAGGCGCTTGGATTTCCTGGACATCCTCTTATTTGCCAAAATGGAGAAT

GGGAGCAGTCTGTCTGATGAGGACCTGCGTGCCGAGGTGGACACCTTCATGTTTGAGGGC

CATGACACCACAGCCAGTGCTATCTCCTGGATCTTCTATGCTCTGGCTGCACACCCTGAG

CATCAGAAGAAGTGCAGGGAGGAGGTCCAGAGCCTCCTGGGGGATGGAGCCTCCATCACC

TGGGACCACATGGACCAGATGCCCTACACCACCATGTGCATCAAGGAGGCCCTGAGGCTC

TATCCACCAGTACCAAGTGTGAGCAGAGATCTCAGTAAACCTATCACTTTCCCTGATGGA

CGCTCCCTACCCAAAGGTTTCATGGTCACACTCTCCATTTATGGCCTTCATCACAATCCG

AAGGTGTGGCCAAACCCAGAGGTGTTTGATCCTTCTCGATTTGCACCTGGATCTTCTCGA

CACAGCCACTCCTTCCTGCCCTTCTCAGGAGGATCAAGGAACTGCATTGGGAAACAATTT

GCCATGAATGAGCTGAAAGTGGCCGTGGCCCTTACTCTACTTCGTTTTGAGCTACTACCA

GATCCCACCAGGGTCCCTGTACCCATGTTACTCCCTGTGATGAAGTCCAAGAATGGAATC

CACCTGCATCTCAGGAAGCTCCAA

>CL116.Contig7_All 1 396 minus strand PREDICTED: cytochrome P450 4A11 [Pongo abelii]

CACTATGACATCCTGAAGCCCTACCTGGGACTCATGGCTGACTCTGTCAGAGTGATGCTG

AACAAGTGGGAGAAGCTTACCAACCAGGACTCATTTGAGATCTCTCAAGACATCTCTTTG

ATGACTCTGGACTCCATCATGAAGTGCGCCTTCACTTATCAAGACAGTGTTCAGCTGGAC

AGGAATTCCCAGTCCTACATCAAGGCTATTAGTGACCTGAGCTACTTGATACCTTCTCGT

GTGCGGAATGCCTTTCATCAGAATGACATCATCTATAGACTGACCTCTGCTGGTCGCTGG

ACCAACCATGCCTGCCAGATTGCCCATAAGCACACAGACCAAGTGATCAGGCAGAGGAAA

GCTCAATTGAAGGATGAGGGAGAGCTGGAGAAGATC

>CL116.Contig8_All 4 291 minus strand PREDICTED: cytochrome P450 4A11-like [Cavia porcellus]

AGGCAATGGCTGCTCAAAGCCATCCAGCAGTTCCCATCCCCGCCCTCCCACTGGCTCTTT

GGGCACAAGATCCAAATGGACCAGGAACTGCAACTGCTGCTGAAATGGGTAGAGAAATTC

CCAAGTGCCTGTCCTCGCTGGGTATGGGGAAGTAGGATCTTCTTCATGGTTTACGATCCT

GACTACATGAAGATGATTCTGGGGAGATCAGACCCCAAAGCTAAAACTACTTACAGATTC

CTGTCTCCCTGGATTGGGTATGGCTTGCTCCTGTTGAATGGGGAGACA

>CL117.Contig1_All 112 2547 minus strand PREDICTED: transferrin receptor protein 2 [Saimiri boliviensis boliviensis]

AGCCTGCAGGGCTCAGGAGGGGGCACAAGCATGGAGCGGCTTCGGGGTCTACTCCAGAGA

GCGCAACAGTTGTCCCCACAAGGCTCTCAGAACATCTACAAACGTGTGGAAGGTCCCCAT

CAGGGGCGACTGGAGGAGGAAGAGGAAGACAGGGAGGAGGGGGCCGAGCTGCCTGTCCAC

TTCTGCCCCATGGAGCTGAAGGGCCCTGATTCCCCAGGCTCTAGAGCTGGGCTGCCAAAC

CTCATACCGTGGGCAGCAGCAGGACGAAGGGCTGCCCCCTACCTGGTCTTGACTGCCCTC

CTGATCTTCACTGGGGCCTTCCTTCTGGGCTACGTGGCCTTCCGAAGGTCCTGTCAAGCA

TGTGGGGACTCCTTGTTGGTGGTCAGTGAGGACGTCAACTATGAGCTGGGCCTGGACTCC

CGTCAGGGCACTTTGTACTGGAGCGACCTCCAGGCCATGTTCCTGCGGCTCTTGGGGGAG

GGGCGCCTGGAAGACACCATCAGGGAAACCAGCCTTCGCCAACGGGTGGCCGGCTCCCCC

GGGATGGCTGCCCTGGCTCAGGACGTCGTTGCAGCGCTCTCCCGCCAGAAGCTGGACCAC

GTGTGGACAGACACTCACTACGTGGGGCTGCAGTTCCCCGACCTGGCTCATCCCAACACT

CTGCGCTGGCTCGACGAGACCGGGAGGGCCGGGGAGCAGCTGCCGCTGGAGGACCCCGAC

GCCTACTGTCCCTACAGCGCCACCGGCAACGCCACGGGAAAGCTGGTGTACGCCCACTAC

GGGCGACCGGAAGACCTGCAGGGCCTGCGGGCCAGGGGCGTGGAGCTAACCGACAGCCTC

CTGCTGGTGCGTGTCGGGGTGACCAGCTTTGCCCAGAAGGTGGCCAGTGCCCAGGACTTT

GGGGCCAAAGGAGTGCTCATATACCCTGACCCAGCAGATTTCTCCCAGGACCCACACAAG

CCAAGCCTGTCCAGCCACCGGGCTGTATATGGACATGTGCATCTGGGAACTGGTGACCCT

TATACGCCTGGCTTCCCTTCCTTCAACCAAACCCAGTTCCCTCCAGTGGCATCCTCTGGC

CTCCCCAGCATCCCTGCCCAGCCCATCAGTGCAGACATTGCCTTCCGCCTGCTAAGGAAG

CTTAAAGGCCCTGTGGTCCCTCAGGAATGGCAAGGGCGCCTCCCAGGCTCCCCTTATCGC

CTGGGCCCCGGGCCAGGCCTGCACCTAGAGGTCAACAACCATAGAACCTCCACTCCCATC

AGCAACATCTTTGGCTGCATTGAGGGCCTGGCAGAGCCAGATCACTATGTTGTCATCGGG

GCCCAGAGAGATGCATGGGGCCCAGGAGCAGCCAAGTCGGCTGTGGGGACAGGCATACTA

CTAGAGCTGGTGCGGACCTTTTCCTCCATGGTGAGCAATGGCTTCCGACCCCGCAGAAGT

CTTCTCTTCATCAGCTGGGATGGAGGCGACTTTGGGAGTGTGGGCTCCACAGAGTGGCTG

GAGGGCTACCTCAGTGTGCTGCACCTCAAAGCTGTAGCCTACGTGAGCCTGGATAACGCA

GTGCTGGGGGACGACAAGTTCCATGCCAAGACCAGCCCACTGCTGATCAGCCTCATTGAG

AACATCCTGAAGCAGGTGGACTCTCCTAACCACAGTGGGCAGACTCTCTATGAGCAGGTG

GTATTCACCAATCCCAGCTGGGATGCCGAGGTGATCCGGCCGCTGCCCATGGACAGCAGC

GCCTATTCCTTCACGGCCTTTGCCGGGGTCCCTGCGGTGGAGTTCTCCTTCTTGGAGGAT

GATCCGGCGTACCCATTCCTGCACACGAAGGAAGACACATACGAAAACCTGCACAAGATG

CTGCAAGGCCGACTGCCTGCCGTGGCCCAGGCTGTGGCCCAGCTCGCTGGGCAGCTCCTC

ATCCGGCTGAGCCACGACCACCTGCTGCCCTTCGACTTCGGCCGCTATGGGGACGTGGTC

CTCCGGCACATCGGCAGCCTCAACGAATTCTCTGGGGACCTCAAGGCCCGCGGGCTGACC

CTGCAGTGGGTGTACTCGGCGCGGGGAGACTACATCCGGGCGGCCGAGAAGCTGCGGAAG

GAGATCTACAGCTCAGAGGAGAGGGACGAGAGGCTGATGCGCATGTACAACGTGCGCATC

ATGCGGGTGGAGTATTACTTGCTGTCCCAGTACGTGTCGCCGGCCGACTTCCCGTTCCGA

CACATCTTTCTGGGCCATGGAGACCACACGCTGGGCGCCCTGCTCGACCACCTGCAGCTG

CTGCGCTCCAACAGTTCCGGGGCCTCCCCCAAGTTGTCCCAGGCTCTGGGCTTCCAGGAG

AGCCGCTTCCGGCGCCAGCTGGCCCTGCTCACCTGGACGTTACAGGGGGCAGCCAACGCG

CTTGGAGGAGACGTCTGGAACATTGATAACAACTTC

>CL117.Contig2_All 58 162 minus strand transferrin receptor 2, isoform CRA_e [Homo sapiens]

CCCAACCTACCTGCCCCCAGGATCCGGCCGCTGCCCATGGACAGCAGCGCCTATTCCTTC

ACGGCCTTTGCCGGGGTCCCTGCGGTGGAGTTCTCCTTCTTGGAG

>CL117.Contig3_All 2046 2435 minus strand PREDICTED: transferrin receptor protein 2 [Saimiri boliviensis boliviensis]

CAGGATGATCCGGCGTACCCATTCCTGCACACGAAGGAAGACACATACGAAAACCTGCAC

AAGATGCTGCAAGGCCGACTGCCTGCCGTGGCCCAGGCTGTGGCCCAGCTCGCTGGGCAG

CTCCTCATCCGGCTGAGCCACGACCACCTGCTGCCCTTCGACTTCGGCCGCTATGGGGAC

GTGGTCCTCCGGCACATCGGCAGCCTCAACGAATTCTCTGGGGACCTCAAGGCCCGCGGG

CTGACCCTGCAGTGGGTGTACTCGGCGCGGGGAGACTACATCCGGGCGGCCGAGAAGCTG

CGGAAGGAGATCTACAGCTCAGAGGAGAGGGACGAGAGGCTGATGCGCATGTACAACGTG

CGCATCATGCGGGTCTTGGTTTAATTTCTG

>CL117.Contig4_All 2485 2898 minus strand PREDICTED: transferrin receptor protein 2 [Saimiri boliviensis boliviensis]

CAGGCCCGCGGGCTGACCCTGCAGTGGGTGTACTCGGCGCGGGGAGACTACATCCGGGCG

GCCGAGAAGCTGCGGAAGGAGATCTACAGCTCAGAGGAGAGGGACGAGAGGCTGATGCGC

ATGTACAACGTGCGCATCATGCGGGTGGAGTATTACTTGCTGTCCCAGTACGTGTCGCCG

GCCGACTTCCCGTTCCGACACATCTTTCTGGGCCATGGAGACCACACGCTGGGCGCCCTG

CTCGACCACCTGCAGCTGCTGCGCTCCAACAGTTCCGGGGCCTCCCCCAAGTTGTCCCAG

GCTCTGGGCTTCCAGGAGAGCCGCTTCCGGCGCCAGCTGGCCCTGCTCACCTGGACGTTA

CAGGGGGCAGCCAACGCGCTTGGAGGAGACGTCTGGAACATTGATAACAACTTC

>CL117.Contig5_All 2485 2646 minus strand PREDICTED: transferrin receptor protein 2 [Saimiri boliviensis boliviensis]

CAGGCCCGCGGGCTGACCCTGCAGTGGGTGTACTCGGCGCGGGGAGACTACATCCGGGCG

GCCGAGAAGCTGCGGAAGGAGATCTACAGCTCAGAGGAGAGGGACGAGAGGCTGATGCGC

ATGTACAACGTGCGCATCATGCGGGTCTTGGTTTAATTTCTG

>CL120.Contig1_All 102 1943 SAM domain and HD domain-containing protein 1 [Heterocephalus glaber]

ATGCGGAGATCCGATCCTGAGCAGCTCTCAAAGCGGCCCAGATGCGATGGCAGCCCGAGA

ACCCCACCAAACACTCCTTCCGCAGCGGCGGACAGGACCCCGGAACTCCACCCGGACCAC

CAGACCTGGGGCCCGGAGCAGGTGTGCGCCTTCCTAGCGCGCAGTGGCTTCCAGGAGCCC

AAACTGCTGGAAAACTTCCGAGACAATAAAATCACAGGTTCATTGCTGCCCTTTCTTGAT

GAATCACTTCTTGAAAATCTTGGAGTTAGTTCCTTCAGGGAGAGGAATAAGCTGCTTCAT

TGTATCCAACGATTGAGTAAAACTCAGGTTGAGACAACTAAGGTGATCAATGATCCTGTC

CATGGCCACATTGAGTTCCACCCTCTTCTTATCCGGATCATTGACACTCCTCAGTTTCAG

CGTCTTCGATACATCAAACAGTTGGGAGGCGGTTACTATGTTTTTCCAGGAGCTTCACAT

AATCGATTTGAGCATAGTCTAGGGGTGGCGTACTTAGGAGGATGTCTGGTCCGCTCACTG

CGTGAAAAACAGCCAGAGCTGCAGATCAGTAATCGAGATGTACTCTGTGTTCAGATTGCT

GGGCTTTGTCATGATCTCGGTCATGGGCCATTTTCTCATATGTTTGATGGGAGATTTATT

CCACTTGCTCGCCCAGGAGTGAAATGGACGCATGAACAGGGCTCAGTTCAAATGTTTGAG

CATCTAGTAAATTCTAATGGACTCAGACCTATCATGGAACAGTATGGTCTTATCCCTGAG

GAAGATATTTGCTTTATCAAGGAACAAATTATAGGACCACTTGAAACACCAGTAAAAGAT

TCTGTGTGGCCATATAAAGGACGTCCTAAAGAGAAAAGCTTTCTTTATGAGATAGTGTCT

AATAAAAGAAATGGCATTGATGTGGATAAATGGGATTATTTTGCCAGGGACTGCCATCAT

CTTGGAATCCCAAATAATTTTGATTACAACCGCTTTATTATGTTTGCCCGTGTCTGTGAA

GAAGGTAACGAGAAGTGTATTTGTATTAGAGATAAGGAGGCTGGGAATCTATATGATATG

TTCCACACACGGAATCGGTTACACCACAAAGCCTATCAACACAAAGTTAGCAACATCATT

GATACAATGATTACAGATGCTCTCCTCAAAGCTGACCCCTACATAGAGATTACAGGTGCT

CAAGGAAAAAAATACCACATTTCTACAGCAATTGATGACATGGAAGCCTTCACTAAGCTA

ACAGATAACGTTTTTCTGGAGATTTTATACTCTTCTGATCCCAAATTGGATGCAGCACAA

AAGGTTTTAAAAAATATTGAATACCGTAATCTATACAAGTATGTGGGTGAGACCCAGCCA

GGAGAAGCAAGGAAGATTGAAAGGAAAGACTATGAACACCTTCCAAAAAAGGTTGCTGAT

GCTAAACCTGATGGTATATTCCTGGAGGCTGAAATGAAGGCTGAAGATTTCATAGTGGAT

GTTATCAACATGGATTATGGGATGAAAGACAAGAATCCAATTGATCATGTTCGCTTCTAT

TGTAAGAGTGACCTCAGCAGAACCGTCAGTATAGATAAAAGTCAGGTTTCACAGCTTCTA

CCAGAGAAATTTGCAGAGCAGGTGATTCGGGTGTATTGTAAGAAGAAAGATGAAAAGAGT

TTGTTTGCTGCACGACAACTTTTCGTTCATTGGTGTTTAATCAACAACTTCAGCAAGCCA

AAGGATGGTGATGTTGTAGCCCCACTTATTACACCTCGAAAACCGGAGTGGAATTACAGG

GATTCAGCCCACAGTCCAGCCCACTTCCCAGGAGCAGCCAAA

>CL120.Contig2_All 13 294 minus strand PREDICTED: SAM domain and HD domain-containing protein 1 [Equus caballus]

CATAAAGCAAGTATTCAAAGCTTTCTAAGTAATTTTCACACAATCTACTTTAGGATTACA

GATGCTCTCCTCAAAGCTGACCCCTACATAGAGATTACAGGTGCTCAAGGAAAAAAATAC

CACATTTCTACAGCAATTGATGACATGGAAGCCTTCACTAAGCTAACAGATAACGTTTTT

CTGGAGATTTTATACTCTTCTGATCCCAAATTGGATGCAGCACAAAAGGTTTTAAAAAAT

ATTGAATACCGTAATCTATACAAGTATGTGGGTGAGACCCAG

>CL121.Contig1_All 19 132 PREDICTED: 28S ribosomal protein S35, mitochondrial isoform 2 [Saimiri boliviensis boliviensis]

ATGGCGGCCCCCGTGGTCCGGCTGTGGCTCGGCCTTCAGCCGGGGGTTAGGACTCTGAGG

ACCCTCTCCACCGCCGTGTCCCCGGCCACTCACGCTCGGAGACCTGGCCTGCGC

>CL121.Contig2_All 12 983 minus strand mitochondrial ribosomal protein S35 [Sus scrofa]

ATGGCGGCCCCCGTGGTCCGGCTGTGGCTCGGCCTTCAGCCGGGGGTTAGGACTCTGAGG

ACCCTCTCCACCGCCGTGTCCCCGGCCACTCACGCTCGGAGACCTGGCCTGCGTACAATA

GAAAGAACATCCAAAAGTGAAAGGCAGCTGAGAAGAAAGGCACTGCCTCCTAGGACAGAG

AAAATGGCTGTTGACCAGGACTGGCCTAGTGTTTACCCTGTTGCAGCACCATTTAAACCC

TCCACAGTACCTCTACCTATTCGAATGGGTTATCCAGTAAACAAAGGAGTGCCTATGGCA

AAGGAGGGAAATCTGGAACTTTTAAAGATTCCCAATTTTCTGCATTTGACTCCTGTAGCA

ATTAAAAAACACTGTGAAGCTCTTAAAGATTTCTGTACTGAATGGCCAGCTGCACTAGAC

AGTGATGAGAAATGTGAGAAGCATTTTCCGATTGAAATTGACACAGCTGATTATGTTTCA

TCAGGACCATCTATTCGAAACCCTAAAGCTCGAGTAGTAACCTTAAGGGTGAAACTTTCC

AGTTTGAATTTAGATGATCATGCAAAGAAGAAACTTATTAAACTTGTAGGAGAACGATAC

TGCAAGACCACAGATGTACTTACCATCAAAACAGATAGGTGCCCTTTAAAAAGACAGAAT

TATGATTATGCAATGTATCTACTAACAGTTTTATACCATGAATCTTGGAAAACTGAAGAA

TGGGAAAAAAATAAGACTGAAGCAGACATGGAAGAATATATATGGAAAAATAGTGCCTCA

GAAAAAAATATCCTGGAAACACTTCTTCAAATTAAAGCTGCTGAGAACACAGAAGTAAAT

AAAGAAGAACTTCTTGGTACTAAAGAAGTTGAAGATTACCAAAAATCTGTTGTTACTCTT

AAGAATGAGGGGGAAAATGAAATTAGCCTTTCTCAGTACAAAGAATCAGTGAAAAGAATA

TTAAATTTGGCA

>CL122.Contig1_All 109 2016 PREDICTED: kinesin light chain 1 isoform 7 [Pan troglodytes]

ATGTATGACAACATGTCCACAATGGTGTACATAAAGGAAGACAAGTTGGAGAAGCTTACA

CAGGATGAGATCATCTCCAAGACCAAGCAAGTAATTCAGGGCCTGGAGGCTTTGAAGAAT

GAGCACAACTCCATTTTGCAGAGTTTGCTGGAGACACTGAAGTGTCTGAAGAAAGATGAT

GAGAGCAACCTGGTGGAGGAGAAATCCAACATGATACGGAAATCACTGGAGATGCTAGAG

CTCGGCCTGAGTGAGGCGCAGGTTATGATGGCCTTGTCAAATCACCTAAATGCTGTTGAG

TCAGAGAAACAGAAACTACGCGCGCAGGTTCGTCGCCTGTGCCAGGAGAATCAGTGGCTG

CGGGATGAGCTGGCCAACACACAACAGAAGTTACAGAAGAGTGAGCAGTCCGTGGCTCAG

CTGGAGGAAGAAAAGAAACACCTGGAATTTATGAACCAGCTCAAAAAGTATGACGATGAC

ATTTCCCCCTCGGAGGACAAGGACACTGATTCTGCCAAAGAGCCCCTGGATGATCTCTTC

CCAAATGACGAGGACGAGCCAGGCCAAGGAATCCAGCAGCAGCATAGCAGTGCTGCCGCT

GCTGCCCAGCAGGGAGGCTACGAGATCCCGGCGCGGCTACGCACACTCCACAACCTGGTG

ATCCAGTATGCCTCTCAGGGGCGCTACGAGGTGGCTGTGCCGCTCTGCAAACAGGCCCTG

GAGGATCTGGAGAAGACTTCAGGCCATGACCACCCTGATGTGGCCACCATGCTCAACATC

CTGGCCCTGGTGTACAGGGATCAGAACAAATACAAAGATGCAGCGAATCTGCTGAATGAT

GCCTTGGCCATCCGTGAGAAGACACTGGGCAAGGATCACCCTGCGGTGGCTGCGACCCTG

AATAACCTCGCTGTCCTGTATGGTAAACGAGGGAAGTACAAAGAGGCCGAGCCATTGTGT

AAGAGGGCCCTGGAGATCAGAGAAAAGGTTCTAGGAAAGGATCACCCTGATGTTGCTAAG

CAGTTAAATAACCTGGCCTTACTGTGCCAGAACCAGGGCAAATATGAAGAAGTAGAATAT

TATTACCAGAGAGCCCTGGAGATCTACCAGACAAAATTGGGACCCGACGACCCCAATGTG

GCCAAGACGAAAAACAACCTGGCATCCTGCTACCTGAAACAAGGAAAGTTCAAGCAAGCA

GAAACATTGTACAAAGAGATTCTCACTCGTGCACATGAGCGGGAGTTTGGTTCTGTGGAT

GATGAAAACAAGCCCATCTGGATGCACGCTGAGGAAAGGGAAGAGTGCAAAGGAAAGCAG

AAGGACGGGTCGTCTTTTGGAGAGTACGGAGGCTGGTACAAAGCCTGCAAAGTTGACAGC

CCAACTGTTACAACTACTTTGAAAAACCTTGGAGCACTTTACCGACGTCAAGGGAAATTT

GAAGCTGCAGAGACATTAGAAGAAGCTGCCATGAGGTCTCGTAAACAGGGTCTTGACACT

GTTCACAAACAGAGAGTGGCTGAAGTGCTAAATGACCCTGAGAACATGGAGAAGCGGAGG

AGCCGGGAGAGTCTCAATGTGGACGTGGTCAAGTACGAGAGTGGCCCTGACGGAGGGGAG

GAAGTGAGTATGAGCGTAGAGTGGAACGGGGATGGCACTGGATCTTTAAAACGCAGTGGT

TCCTTTAGCAAACTCCGGGCCTCCATTAGACGCAGCAGTGAGAAGCTGGTTAGGAAGCTG

AAGGGAGGAAGTTCACGAGAGAGTGAGCCAAGGAACCCCGGCATGAAGCATGCCAGTTCT

CTGAATGCTCTTAACGTGGGTGGCGAGGCTGCAGAAGATCATTCCCAAGAACGAAGTAAC

TGTTTGGCAGACTCGAGAGCTCTGAGTGCCAGCCACACTGACCTGTCC

>CL122.Contig2_All 109 2001 PREDICTED: kinesin light chain 1 [Pan troglodytes]

ATGTATGACAACATGTCCACAATGGTGTACATAAAGGAAGACAAGTTGGAGAAGCTTACA

CAGGATGAGATCATCTCCAAGACCAAGCAAGTAATTCAGGGCCTGGAGGCTTTGAAGAAT

GAGCACAACTCCATTTTGCAGAGTTTGCTGGAGACACTGAAGTGTCTGAAGAAAGATGAT

GAGAGCAACCTGGTGGAGGAGAAATCCAACATGATACGGAAATCACTGGAGATGCTAGAG

CTCGGCCTGAGTGAGGCGCAGGTTATGATGGCCTTGTCAAATCACCTAAATGCTGTTGAG

TCAGAGAAACAGAAACTACGCGCGCAGGTTCGTCGCCTGTGCCAGGAGAATCAGTGGCTG

CGGGATGAGCTGGCCAACACACAACAGAAGTTACAGAAGAGTGAGCAGTCCGTGGCTCAG

CTGGAGGAAGAAAAGAAACACCTGGAATTTATGAACCAGCTCAAAAAGTATGACGATGAC

ATTTCCCCCTCGGAGGACAAGGACACTGATTCTGCCAAAGAGCCCCTGGATGATCTCTTC

CCAAATGACGAGGACGAGCCAGGCCAAGGAATCCAGCAGCAGCATAGCAGTGCTGCCGCT

GCTGCCCAGCAGGGAGGCTACGAGATCCCGGCGCGGCTACGCACACTCCACAACCTGGTG

ATCCAGTATGCCTCTCAGGGGCGCTACGAGGTGGCTGTGCCGCTCTGCAAACAGGCCCTG

GAGGATCTGGAGAAGACTTCAGGCCATGACCACCCTGATGTGGCCACCATGCTCAACATC

CTGGCCCTGGTGTACAGGGATCAGAACAAATACAAAGATGCAGCGAATCTGCTGAATGAT

GCCTTGGCCATCCGTGAGAAGACACTGGGCAAGGATCACCCTGCGGTGGCTGCGACCCTG

AATAACCTCGCTGTCCTGTATGGTAAACGAGGGAAGTACAAAGAGGCCGAGCCATTGTGT

AAGAGGGCCCTGGAGATCAGAGAAAAGGTTCTAGGAAAGGATCACCCTGATGTTGCTAAG

CAGTTAAATAACCTGGCCTTACTGTGCCAGAACCAGGGCAAATATGAAGAAGTAGAATAT

TATTACCAGAGAGCCCTGGAGATCTACCAGACAAAATTGGGACCCGACGACCCCAATGTG

GCCAAGACGAAAAACAACCTGGCATCCTGCTACCTGAAACAAGGAAAGTTCAAGCAAGCA

GAAACATTGTACAAAGAGATTCTCACTCGTGCACATGAGCGGGAGTTTGGTTCTGTGGAT

GATGAAAACAAGCCCATCTGGATGCACGCTGAGGAAAGGGAAGAGTGCAAAGGAAAGCAG

AAGGACGGGTCGTCTTTTGGAGAGTACGGAGGCTGGTACAAAGCCTGCAAAGTTGACAGC

CCAACTGTTACAACTACTTTGAAAAACCTTGGAGCACTTTACCGACGTCAAGGGAAATTT

GAAGCTGCAGAGACATTAGAAGAAGCTGCCATGAGGTCTCGTAAACAGGGTCTTGACACT

GTTCACAAACAGAGAGTGGCTGAAGTGCTAAATGACCCTGAGAACATGGAGAAGCGGAGG

AGCCGGGAGAGTCTCAATGTGGACGTGGTCAAGTACGAGAGTGGCCCTGACGGAGGGGAG

GAAGTGAGTATGAGCGTAGAGTGGAACGGGGATGGCACTGGATCTTTAAAACGCAGTGGT

TCCTTTAGCAAACTCCGGGCCTCCATTAGACGCAGCAGTGAGAAGCTGGTTAGGAAGCTG

AAGGGAGGAAGTTCACGAGAGAGTGAGCCAAGGAACCCCGGCATGAAGCATGCCAGTTCT

CTGAATGCTCTTAACGTGGGTGGCGAGGCTGCAGAAGATCATTCCCAAGGCATCCCCAGC

GGAGCCTCTTTGTGTGGAGAATGACAGCAGCAG

>CL122.Contig3_All 109 1956 kinesin light chain 1 isoform 1D [Mus musculus]

ATGTATGACAACATGTCCACAATGGTGTACATAAAGGAAGACAAGTTGGAGAAGCTTACA

CAGGATGAGATCATCTCCAAGACCAAGCAAGTAATTCAGGGCCTGGAGGCTTTGAAGAAT

GAGCACAACTCCATTTTGCAGAGTTTGCTGGAGACACTGAAGTGTCTGAAGAAAGATGAT

GAGAGCAACCTGGTGGAGGAGAAATCCAACATGATACGGAAATCACTGGAGATGCTAGAG

CTCGGCCTGAGTGAGGCGCAGGTTATGATGGCCTTGTCAAATCACCTAAATGCTGTTGAG

TCAGAGAAACAGAAACTACGCGCGCAGGTTCGTCGCCTGTGCCAGGAGAATCAGTGGCTG

CGGGATGAGCTGGCCAACACACAACAGAAGTTACAGAAGAGTGAGCAGTCCGTGGCTCAG

CTGGAGGAAGAAAAGAAACACCTGGAATTTATGAACCAGCTCAAAAAGTATGACGATGAC

ATTTCCCCCTCGGAGGACAAGGACACTGATTCTGCCAAAGAGCCCCTGGATGATCTCTTC

CCAAATGACGAGGACGAGCCAGGCCAAGGAATCCAGCAGCAGCATAGCAGTGCTGCCGCT

GCTGCCCAGCAGGGAGGCTACGAGATCCCGGCGCGGCTACGCACACTCCACAACCTGGTG

ATCCAGTATGCCTCTCAGGGGCGCTACGAGGTGGCTGTGCCGCTCTGCAAACAGGCCCTG

GAGGATCTGGAGAAGACTTCAGGCCATGACCACCCTGATGTGGCCACCATGCTCAACATC

CTGGCCCTGGTGTACAGGGATCAGAACAAATACAAAGATGCAGCGAATCTGCTGAATGAT

GCCTTGGCCATCCGTGAGAAGACACTGGGCAAGGATCACCCTGCGGTGGCTGCGACCCTG

AATAACCTCGCTGTCCTGTATGGTAAACGAGGGAAGTACAAAGAGGCCGAGCCATTGTGT

AAGAGGGCCCTGGAGATCAGAGAAAAGGTTCTAGGAAAGGATCACCCTGATGTTGCTAAG

CAGTTAAATAACCTGGCCTTACTGTGCCAGAACCAGGGCAAATATGAAGAAGTAGAATAT

TATTACCAGAGAGCCCTGGAGATCTACCAGACAAAATTGGGACCCGACGACCCCAATGTG

GCCAAGACGAAAAACAACCTGGCATCCTGCTACCTGAAACAAGGAAAGTTCAAGCAAGCA

GAAACATTGTACAAAGAGATTCTCACTCGTGCACATGAGCGGGAGTTTGGTTCTGTGGAT

GATGAAAACAAGCCCATCTGGATGCACGCTGAGGAAAGGGAAGAGTGCAAAGGAAAGCAG

AAGGACGGGTCGTCTTTTGGAGAGTACGGAGGCTGGTACAAAGCCTGCAAAGTTGACAGC

CCAACTGTTACAACTACTTTGAAAAACCTTGGAGCACTTTACCGACGTCAAGGGAAATTT

GAAGCTGCAGAGACATTAGAAGAAGCTGCCATGAGGTCTCGTAAACAGGGTCTTGACACT

GTTCACAAACAGAGAGTGGCTGAAGTGCTAAATGACCCTGAGAACATGGAGAAGCGGAGG

AGCCGGGAGAGTCTCAATGTGGACGTGGTCAAGTACGAGAGTGGCCCTGACGGAGGGGAG

GAAGTGAGTATGAGCGTAGAGTGGAACGGGGATGGCACTGGATCTTTAAAACGCAGTGGT

TCCTTTAGCAAACTCCGGGCCTCCATTAGACGCAGCAGTGAGAAGCTGGTTAGGAAGCTG

AAGGGAGGAAGTTCACGAGAGAGTGAGCCAAGGAACCCCGGGGCATCCCCAGCGGAGCCT

CTTTGTGTGGAGAATGACAGCAGCAGCCTGGAGGATGCCAACTCTAAC

>CL122.Contig4_All 3 761 minus strand PREDICTED: kinesin light chain 4 [Equus caballus]

CGCACGTCTGGCCGTGGCCACCCTGATGTGGCTACCATGCTCAACATCCTTGCTTTGGTA

TATCGGGATCAGAATAAGTATAAGGAAGCTGCCCATCTGCTGAATGATGCTCTCAGCATC

CGGGAGAGCACCCTGGGCCGGGACCATCCTGCTGTGGCTGCCACACTCAATAATCTGGCT

GTGCTGTATGGCAAAAGGGGAAAATACAAGGAGGCAGAGCCACTGTGCCAGCGAGCACTG

GAGATTCGAGAAAAGGTCCTGGGCACTGACCACCCAGATGTGGCAAAGCAGCTGAACAAC

TTGGCCCTATTGTGCCAAAACCAGGGCAAGTATGAGGCTGTGGAACGCTATTACCAGCGG

GCACTGGCTATCTACGAGGGGCAGCTGGGGCCAGATAACCCTAATGTAGCTCGGACCAAG

AACAACCTGGCTTCCTGTTACCTGAAGCAGGGCAAATACGCTGAGGCTGAGACCCTGTAC

AAGGAGATCCTGACTCGTGCCCATGTGCAGGAGTTTGGGTCTGTGGATGATCATCACAAG

CCCATCTGGATGCATGCAGAGGAACGGGAAGAAATGAGCAAAAGCCGGCACCGTGAGGGA

GGCACACCCTATGCTGAATATGGAGGCTGGTACAAGGCCTGCAAAGTGAGCAGCCCCACA

GTGAACACTACTCTGAGAAACCTAGGGGCTCTATACAGGCGCCAAGGAAAACTGGAGGCA

GCTGAGACCCTGGAGGAATGTGCTCTGCGTTCCCGGAAA

>CL123.Contig1_All 24 266 thrombopoietin-like; K06854 thrombopoietin

CTGCAGTGGAGGGGCTGTGCCCCAACGCCACATGTCTTTCTACTCATCTGCTCCCCAGAG

TGCTGCCTGCTGTGCACCTGGGTCCTTAAGCCCTTCTCTACCCGGATAGTTTCCAGACTG

TCTTCCTTGGCCCACCTGTGCCCCACCCCACTCTGCCCAGAAGTGCAAAGAGCCCAAGCC

GCCTCCATGGCCCCAGGAAGGATTCAGGGGAGAGGCCCCATACAGGGAGCCACTCCAGCC

AGA

>CL123.Contig2_All 24 266 thrombopoietin (myeloproliferative leukemia virus oncogene ligand, megakaryocyte growth and development factor), isoform CRA_c [Homo sapiens]

CTGCAGTGGAGGGGCTGTGCCCCAACGCCACATGTCTTTCTACTCATCTGCTCCCCAGAG

TGCTGCCTGCTGTGCACCTGGGTCCTTAAGCCCTTCTCTACCCGGATAGTTTCCAGACTG

TCTTCCTTGGCCCACCTGTGCCCCACCCCACTCTGCCCAGAAGTGCAAAGAGCCCAAGCC

GCCTCCATGGCCCCAGGAAGGATTCAGGGGAGAGGCCCCATACAGGGAGCCACTCCAGCC

AGA

>CL127.Contig1_All 2 316 minus strand PREDICTED: disco-interacting protein 2 homolog B [Otolemur garnettii]

CAGTACAAAATAAGGGACACTTTCTGCTCCTACTCAGTAATGGAGCTTTGCACTAAAGGA

TTGGGAAACCAAGTGGAGGTGCTAAAGACAAGGGGGATTAACCTCTCCTGCATCCGGACC

TGTGTGGTGGTGGCAGAGGAGCGGCCCCGCATTGCCCTCCAGCAGTCCTTCTCCAAGCTC

TTTAAAGACATTGGTCTGTCCCCTCGAGCCGTCAGTACCACTTTTGGATCAAGAGTCAAC

GTAGCAATATGTTTACAGGGAACCTCAGGGCCTGATCCAACTACTGTATATGTAGATCTG

AAATCATTAAGACAT

>CL128.Contig1_All 3 242 YPEL3 protein [Bos taurus]

GCCGCGGCAGCAGCTGGTCTCGGTGTAAACAAGTCGAGGCGGCTGCGAACCCGGGCCGGG

GGGGACGGCGCCCACCAGGAGCGCCCCCCACTCCCAGGCCAGGCCACCCCGCGGACCGGG

GCCCCGCGCGCCCAGGCGAGGCACTGGGCTCCCTCTGCTCCCCGTGGGCCGCTCCCCGCG

TGGGGCCACTGCCCCTGGCCCCCGCCATGGTGCGGATTTCAAAGCCCAAGACGTTTCAGG

>CL128.Contig2_All 2 214 PREDICTED: protein yippee-like 1 [Papio anubis]

TACCTCTTCAATTCTGTGGTGAACGTGGGCTGCGGTCCCGCTGAGGAGAGGGTCCTTCTC

ACTGGGCTGCATGCAGTTGCAGACATCTACTGTGAGAACTGCAAGACCACACTCGGGTGG

AAATACGAACATGCCTTTGAGAGCAGTCAGAAATATAAGGAAGGAAAATTTATTATTGAG

CTTGCCCACATGATCAAAGACAATGGTTGGGAG

>CL128.Contig3_All 19 297 PREDICTED: protein yippee-like 3 [Canis lupus familiaris]

GACTCACCTTGAGCTCCTCCTAGCCTCAGTTTCCCTGCTGATGCAACCTGAGCCTCCTCA

CTGAGTCAAAACTGAAACGACTCTGGTCCCTGGCGAGGCTGCTGGCTCTGGGGAAAGAAG

AGAGCAGCCAAGTGTGACAGAGAGTCCCCATGGCTCTGGGCGTGCCTGCATGTGTGTGGC

CTGGGTCCTGACAGCCCACCTATTCCCTCCCTGGCAGGCACTGGGCTCCCTCTGCTCCCC

GTGGGCCGCTCCCCGCGTGGGGCCACTGCCCCTGGCCCC

>CL129.Contig1_All 618 842 minus strand PREDICTED: zinc finger protein 354B [Equus caballus]

TCAGTAGTTGTTAGGCAACAGATAGTTCCCAGAGAGAAACCACCACCAAAATGTGAAAAA

AAAGGAAACACTTTGAAACATAATTCAAATTTACCTAATCAGCCAAAAATGACAGCAGAT

AAACGCTATCAATGTAGTGTGTGTGAGAAGACCTTCATTAATACTTCATCCCTTCGTAAA

CATGAGAAAAATCATAGTGGAGAGAAATTATTTAAATGTAAAGAA

>CL129.Contig2_All 1132 2073 zinc finger protein 354A [Bos taurus] >gi|296485537|gb|DAA27652.1| zinc finger protein 354A isoform 1 [Bos taurus] >gi|296485538|gb|DAA27653.1| zinc finger protein 354A isoform 2 [Bos taurus]

ACCCACACTGTGGAGAAATCCTATAGATGTAAGGAATGTGGTAAGTCCTTCAGCCGAAGG

TCAGGGCTTTTTATACATCAGAAAATCCATGCTGGAGAAAACCCTTATAAATATAATCCA

GGTAGGAAAGCATCTGGTTGCAACACATCCCTTCCTGGTCAAAGAGTTCATCCCAGAAAG

AAGTCCTATTTGTGTAACGAATGTGGCAACACCTTTAAATCTAGTTCATCCCTTCGTTAT

CATCAGAGAATTCACACCGGAGAGAAACCTTTCAAATGTGGTGAATGTGGGAGAGCCTTT

AGTCAGAGTGCATCACTTATTCAACATGAACGAATTCACACTGGTGAAAAGCCCTATCGA

TGTAATGAATGTGGAAAAGGCTTCACTTCTATTTCACGACTCAATAGACATCGAATAATT

CATACTGGTGAGAAGTTTTATAATTGTAATGAATGTGGCAAGGCCTTAAGTTCCCATTCA

ACTCTTATCATTCATGAGAGGATTCACACCGGAGAGAAACCATGTAAATGTAAAGTGTGT

GGGAAAGCCTTCAGGCAGAGTTCATCTCTCATTCAGCATCAGAGAATGCACACTGGAGAA

AGACCCTACAAATGCAATGAGTGTGGGAAAACATTCAGGTGTAACTCATCCCTTAGTAAT

CATCAGAACATCCATACTGGAGAGAAACCTTATCAATGTGGGGAATGTGGGATGTCATTT

GGCCAAAGTTCAGCCCTTATTCAACATCGAAGGATTCATACAGGAGAGAAACCCTTTAAA

TGTAATACTTGTGGGAAAACTTTTAGACAAAGCTCCTCACGTATTGCACACCAGAGAATT

CATACAGGGGAGAAACCCTATGAGTGTGACACATGTGGGAAATGTTTCAACCATAGGTCA

TCCCTTACTAATCACTATAAAATCCATATGGAAGAAGAACCC

>CL129.Contig3_All 708 1706 minus strand zinc finger protein 354A [Mus musculus]

AAATGTGAAAAAAAAGGAAACACTTTGAAACATAATTCAAATTTACCTAATCAGCCAAAA

ATGACAGCAGATAAACGCTATCAATGTAGTGTGTGTGAGAAGACCTTCATTAATACTTCA

TCCCTTCGTAAACATGAGAAAAATCATAGTGGAGAGAAATTATTTAAATGTAAAGACTGT

TCCAAAGCCTTTAGCCAACGTTCAGCTCTTATTCAACCTCAAATAACTCATACCGGAGAG

AAACCTTACATATGTAAAGAATGTGGGAAAGCCTTCACTCTCAGTACATCCCTGTATAAG

CATCTAAGAACCCACACTGTGGAGAAATCCTATAGATGTAAGGAATGTGGTAAGTCCTTC

AGCCGAAGGTCAGGGCTTTTTATACATCAGAAAATCCATGCTGGAGAAAACCCTTATAAA

TATAATCCAGGTAGGAAAGCATCTGGTTGCAACACATCCCTTCCTGGTCAAAGAGTTCAT

CCCAGAAAGAAGTCCTATTTGTGTAACGAATGTGGCAACACCTTTAAATCTAGTTCATCC

CTTCGTTATCATCAGAGAATTCACACCGGAGAGAAACCTTTCAAATGTGGTGAATGTGGG

AGAGCCTTTAGTCAGAGTGCATCACTTATTCAACATGAACGAATTCACACTGGTGAAAAG

CCCTATCGATGTAATGAATGTGGAAAAGGCTTCACTTCTATTTCACGACTCAATAGACAT

CGAATAATTCATACTGGTGAGAAGTTTTATAATTGTAATGAATGTGGCAAGGCCTTAAGT

TCCCATTCAACTCTTATCATTCATGAGAGGATTCACACCGGAGAGAAACCATGTAAATGT

AAAGTGTGTGGGAAAGCCTTCAGGCAGAGTTCAGCTCTCATTCAGCATCAGAGAATGCAC

ACTGGAGAAAGACCCTACAAATGCAATGAGTGTGAGAAAACATTCAGGTGTAACTCATCC

CTTAGTAATCATCAGAAAATCCATACTGGAGAGAAACCT

>CL129.Contig4_All 108 413 minus strand PREDICTED: zinc finger protein 354B [Oryctolagus cuniculus]

GCCTGCTGGCGGGCGGGGCGGTTCCGGACCTTCCTGCCTGAGGAGCGCGGCCCAGCGAGG

CATCGCCCCAGAAGCCCCGAGCCTGGGGAGATCTGCCTTCTGGAAACTGCACCCACCCTG

GGGAGAAGAGCCCAGAAAAAGGACATGGCTCCTGAGCAAAGGGAAGCAAGGTCCCAGGTA

TCAGTGACGTTCGAGGATGTGGCTGTGCTCTTTACTCGGGATGAGTGGAAAAAGCTGGAT

CCTTCCCAGAGAAACTTGTATCGGGAGGTGATGCTGGAGAACTATAGTAACCTGGCCTCA

CTGGGT

>CL129.Contig5_All 605 829 PREDICTED: zinc finger protein 354A [Canis lupus familiaris]

TCAGTAGTTGTTAGGCAACAGATAGTTCCCAGAGAGAAACCACCACCAAAATGTGAAAAA

AAAGGAAACACTTTGAAACATAATTCAAATTTACCTAATCAGCCAAAAATGACAGCAGAT

AAACGCTATCAATGTAGTGTGTGTGAGAAGACCTTCATTAATACTTCATCCCTTCGTAAA

CATGAGAAAAATCATAGTGGAGAGAAATTATTTAAATGTAAAGAA

>CL130.Contig1_All 80 880 PREDICTED: lys-63-specific deubiquitinase BRCC36-like isoform 2 [Ailuropoda melanoleuca]

ATGGCGGTGCAGGTGGTACAGGCGGTGCAGGCGGTTCATCTCGAGTCTGATGCTTTCCTA

GTTTGTCTCAACCACGCTCTGAGCACAGAAAAGGAGGAGGTGATGGGACTGTGTATAGGG

GAGGTTGACACCGTCAGAATTGTTCATATTCATTCTGTCATCATCTTGCGACGTTCTGAT

AAGAGGAAGGACCGAGTAGAAATTTCTCCAGAGCAGCTATCTGCGGCCTCAACAGAGGCA

GAGAGGTTGGCTGAATTAACAGGTCGCCCCATGAGAGTTGTGGGCTGGTATCATTCCCAT

CCTCATATAACTGTCTGGCCTTCACATGTTGATGTTCGCACTCAAGCCATGTACCAGATG

ATGGATCAAGGTTTTGTAGGACTTATTTTTTCCTGTTTCATAGAGGATAAAAACACAAAG

ACTGGTCGGGTACTCTACACTTGCTTCCAATCCATACAGGCCCAAAAGAGCTCAGAGTAT

GAGAGAATTGAAATTCCAATCCATATTGTACCCCATGTCACCATTGGAAAAGTATGCCTT

GAATCAGCAGTGGAGCTGCCCAAGATCCTTTGTCAGGAGGAACAGGATGCATATAGGAGG

ATTCACAGCCTTACACATCTGGACTCAGTAACCAAGATCCATAATGGTTCAGTGTTCACC

AAGAATCTGTGCAGTCAGATGTCAGCAGTCAGCGGGCCTCTCCTACAGTGGTTGGAGGAT

AGATTGGAGCAAAACCAACAGCATTTGCAGGAGTTACAACAAGAAAAAGAAGAACTTATG

CAAGAACTTTACTCCCTAGAA

>CL130.Contig2_All 80 955 lys-63-specific deubiquitinase BRCC36 isoform 3 [Homo sapiens] >gi|150171080|emb|CAO03601.1| BRCA1/BRCA2-containing complex, subunit 3 [Homo sapiens] >gi|150171084|emb|CAO03575.1| BRCA1/BRCA2-containing complex, subunit 3 [Homo sapiens]

ATGGCGGTGCAGGTGGTACAGGCGGTGCAGGCGGTTCATCTCGAGTCTGATGCTTTCCTA

GTTTGTCTCAACCACGCTCTGAGCACAGAAAAGGAGGAGGTGATGGGACTGTGTATAGGG

GAGTTGAATGATGACACAAGTAGGAGTGACTCCAAATTTGCATATACTGGAACTGAAATG

CGCACAGTTGCTGAAAAGGTTGACACCGTCAGAATTGTTCATATTCATTCTGTCATCATC

TTGCGACGTTCTGATAAGAGGAAGGACCGAGTAGAAATTTCTCCAGAGCAGCTATCTGCG

GCCTCAACAGAGGCAGAGAGGTTGGCTGAATTAACAGGTCGCCCCATGAGAGTTGTGGGC

TGGTATCATTCCCATCCTCATATAACTGTCTGGCCTTCACATGTTGATGTTCGCACTCAA

GCCATGTACCAGATGATGGATCAAGGTTTTGTAGGACTTATTTTTTCCTGTTTCATAGAG

GATAAAAACACAAAGACTGGTCGGGTACTCTACACTTGCTTCCAATCCATACAGGCCCAA

AAGAGCTCAGAGTATGAGAGAATTGAAATTCCAATCCATATTGTACCCCATGTCACCATT

GGAAAAGTATGCCTTGAATCAGCAGTGGAGCTGCCCAAGATCCTTTGTCAGGAGGAACAG

GATGCATATAGGAGGATTCACAGCCTTACACATCTGGACTCAGTAACCAAGATCCATAAT

GGTTCAGTGTTCACCAAGAATCTGTGCAGTCAGATGTCAGCAGTCAGCGGGCCTCTCCTA

CAGTGGTTGGAGGATAGATTGGAGCAAAACCAACAGCATTTGCAGGAGTTACAACAAGAA

AAAGAAGAACTTATGCAAGAACTTTACTCCCTAGAA

>CL133.Contig1_All 7 1356 minus strand PREDICTED: fas-activated serine/threonine kinase isoform 1 [Otolemur garnettii]

TTGGCGTGCAGGCTGCGATTCGGGGGCTGCTGGGAAGATGGCGGACTCGGTGGGCCGCCG

ATGAGGAGGCCGCGGGGGGAGCCCGGCTCTCGGGCCCCGAGACCGACTGAGGGAGCGACC

TGCGCGGGGCCCGGGGAGTCATGGTCTCCATCACCCAACTCCATGCTTCGAGTCCTGCTC

TCTGCTCAGGCCTCCCCTGCTCGGCTGTCTGGCCTGCTGCTGATCCCTCCAGTACAGCCC

TGCTGTTTGGGGCCCAGCAAGTGGGGGGACCGGCCTCTTGGAGGAGGGCCCCGTGCAGGC

CATGTGCAAGGACTGCAGCGGCTTCTGGAACAGGCGAAGAGTCCTGGGGAGCTACTGCGC

TGGCTGGGCCAGAACCCCACCAAGGTGCGCGCCCACCACTATCCTGTGGCACTTCGTCGC

CTGGGCCAGCTCTTGGGGTCTCAGCCTCGGCCCCCTCCTGTGGAGCAGGCCACACTGCAG

GACTTGAGTCAGCTCATCATCCGAAACTGCCCCTCCTTTGACATTCATACCATCCACGTG

TGTCTGCACCTTGCAGTCTTACTTGGCTTTCCATCAGATGGACCTCTGGTGTGTGCCCTG

GAGCAGGAGCGAAGGTTTCGTCTCCCTCCAAAGCCACCTCCCCCTCTGCAGCCTGTCCTC

CGTGGTGGACAAAGGCTAGAAGCTGCTCTGAGCTGCCCCCGTTTCCTGCGGTATCCGAGG

CAGAATCTGATCAGCAGCCTGGCAGAGGCAAGGCCAGAAGAACTGACTCCTCATGTGATG

GTCCTTCTGGCCCAGCATCTCGCTCGGCATCGGTTGCGGGAGCCCCAGCTTCTGGAAGCC

ATTGCCCACTTCCTGGTGGTCCAGGAAGCCCAGCTCAGCAGCAAGGTGGTACAGAAGCTG

GTCTTGCCCTTTGGGCGGCTGAACTACCTACCCCTGGAGCAGCAGTTTATGCCCTGCCTT

GAGAGGATCCTGGCTCGGGAGGCAGGGGTGTCACCCCTGGCCACAGTCAACATTTTGATG

TCACTGTGCCAGTTACGGTGCCTGCCCTTCAGAGCCCTGCACTTTGTCTTCTCCCCGGGT

TTTATCAACCACATCAGTGGCACCCCTCACGCTCTGATTGTTCGACGCTACCTCTCCCTG

CTTGACGCGGCTGTGGAGCTGGAGCTCCCAGGATACCGGGGCCCCCGCCTTCCCCGAAGG

CAGCAAGTGCCCATCTTTCCGCAGCCACTCATCACCGACCGTGCCCGCTGCAAGTACAGT

CACAAGGACATAGTAGCTGAGGGGCTGCGCCAGCTGCTGGGGGAGGAGAAATACCGCCAG

GACCTGACTGTGCCTCCCGGCTACTGCACA

>CL133.Contig2_All 1683 1787 minus strand PREDICTED: fas-activated serine/threonine kinase isoform 1 [Otolemur garnettii]

CTGCCCTTTGAGGAACTGGAGTCACAGAGAGGCCTGCCCCAGCTCAAGAGCTACCTTAGG

CAGAAGCTCCAGGCCTTAGGCCTCCGCTGGGGGCCTGAAGGAGGG

>CL133.Contig3_All 36 1742 minus strand PREDICTED: fas-activated serine/threonine kinase isoform 1 [Otolemur garnettii]

TTGGCGTGCAGGCTGCGATTCGGGGGCTGCTGGGAAGATGGCGGACTCGGTGGGCCGCCG

ATGAGGAGGCCGCGGGGGGAGCCCGGCTCTCGGGCCCCGAGACCGACTGAGGGAGCGACC

TGCGCGGGGCCCGGGGAGTCATGGTCTCCATCACCCAACTCCATGCTTCGAGTCCTGCTC

TCTGCTCAGGCCTCCCCTGCTCGGCTGTCTGGCCTGCTGCTGATCCCTCCAGTACAGCCC

TGCTGTTTGGGGCCCAGCAAGTGGGGGGACCGGCCTCTTGGAGGAGGGCCCCGTGCAGGC

CATGTGCAAGGACTGCAGCGGCTTCTGGAACAGGCGAAGAGTCCTGGGGAGCTACTGCGC

TGGCTGGGCCAGAACCCCACCAAGGTGCGCGCCCACCACTATCCTGTGGCACTTCGTCGC

CTGGGCCAGCTCTTGGGGTCTCAGCCTCGGCCCCCTCCTGTGGAGCAGGCCACACTGCAG

GACTTGAGTCAGCTCATCATCCGAAACTGCCCCTCCTTTGACATTCATACCATCCACGTG

TGTCTGCACCTTGCAGTCTTACTTGGCTTTCCATCAGATGGACCTCTGGTGTGTGCCCTG

GAGCAGGAGCGAAGGTTTCGTCTCCCTCCAAAGCCACCTCCCCCTCTGCAGCCTGTCCTC

CGTGGTGGACAAAGGCTAGAAGCTGCTCTGAGCTGCCCCCGTTTCCTGCGGTATCCGAGG

CAGAATCTGATCAGCAGCCTGGCAGAGGCAAGGCCAGAAGAACTGACTCCTCATGTGATG

GTCCTTCTGGCCCAGCATCTCGCTCGGCATCGGTTGCGGGAGCCCCAGCTTCTGGAAGCC

ATTGCCCACTTCCTGGTGGTCCAGGAAGCCCAGCTCAGCAGCAAGGTGGTACAGAAGCTG

GTCTTGCCCTTTGGGCGGCTGAACTACCTACCCCTGGAGCAGCAGTTTATGCCCTGCCTT

GAGAGGATCCTGGCTCGGGAGGCAGGGGTGTCACCCCTGGCCACAGTCAACATTTTGATG

TCACTGTGCCAGTTACGGTGCCTGCCCTTCAGAGCCCTGCACTTTGTCTTCTCCCCGGGT

TTTATCAACCACATCAGTGGCACCCCTCACGCTCTGATTGTTCGACGCTACCTCTCCCTG

CTTGACGCGGCTGTGGAGCTGGAGCTCCCAGGATACCGGGGCCCCCGCCTTCCCCGAAGG

CAGCAAGTGCCCATCTTTCCGCAGCCACTCATCACCGACCGTGCCCGCTGCAAGTACAGT

CACAAGGACATAGTAGCTGAGGGGCTGCGCCAGCTGCTGGGGGAGGAGAAATACCGCCAG

GACCTGACTGTGCCTCCCGGCTACTGCACAGACTTCCTGCTGTGTGTCAGCAGCTCTGGT

GCTGTGCTTCCTGTGAGGACCCAAGACCCCTTTCTACCTTACCCACCAAGGTCCTGTCCA

CAGGGACAGGCTGCCTCTAATCCTACGACCCGTGACCCTGCCCAAAGGGTGGTGCTGATG

CTGCGTGAACGCTGGCATTTCTGCCGTGATGGCAGGGTGCTGCTGGGCTCCCGTGCCCTG

AGGGAGCGGCACCTGGGCCTAATGGGCTACCAGCTCCTGCCGCTGCCCTTTGAGGAACTG

GAGTCACAGAGAGGCCTGCCCCAGCTCAAGAGCTACCTTAGGCAGAAGCTCCAGGCCTTA

GGCCTCCGCTGGGGGCCTGAAGGAGGG

>CL133.Contig4_All 31 210 PREDICTED: fas-activated serine/threonine kinase isoform 1 [Nomascus leucogenys]

TCTGGGCCACCCTCAGGGTCTCCATCACCCAACTCCATGCTTCGAGTCCTGCTCTCTGCT

CAGGCCTCCCCTGCTCGGCTGTCTGGCCTGCTGCTGATCCCTCCAGTACAGCCCTGCTGT

TTGGGGCCCAGCAAGTGGGGGGACCGGCCTCTTGGAGGAGGGCCCCGTGCAGGCCATGTG

>CL134.Contig1_All 191 628 Retrovirus-related Pol polyprotein LINE-1 OS=Mus musculus GN=Pol PE=1 SV=2

AAAACTACAGAACACTCCAGAAAGAAATTGAAGAAGACCTTAGAAAATGCAAAATCCCCC

ATGTTCATGGATAGGCAAATTAATTTTATCAAAATGGTCATGCTCTCCAAAGCACGAAAC

AGATTTAATGTAATTCCCATTAAAATTCCAATGATGTTCTTCATAGAAATGGAAAAAGCA

GTTATGAAATTTATTTGGAAAAACAAGAAGCCCAGAATAGCCAAAGCAATCCTTAGTGAG

AAAAGTGATGCAGGAGGCATCACAACACCAGATTTTACATGATACTGCAGAACCATAGTA

ACAAAAATGCCATGGTACTGGCACCGAAACAGACATGAAGCCCAATGGTACAGAATAGAA

GACATAGAGACAAACCCACATAAATACGGTTGTGTCATACTAGACAAAGGTGCTAAAAAC

ATTCATTGGAGTAAAGAG

>CL134.Contig2_All 5 217 minus strand endonuclease/reverse transcriptase [Sus scrofa]

AGAAGATGGAAAGACCTCCCATGTTCATGGATAGGCAGAATTAATATTGTTAAAATGGCC

ATACTACCAAAAGCAATATACAGATTCAATGCAATCCCCATCAAAATTCTAATGACATTC

TTCTCAGAACTAGAAAAAGCATTTAGGAAATTCATTTGGAAAAATAAGAGGCCCAGAATA

GCCAAAGCAATCCTCAGCAAGAAGAGTGAAGCA

>CL134.Contig3_All 10 309 minus strand Retrovirus-related Pol polyprotein LINE-1 OS=Mus musculus GN=Pol PE=1 SV=2

ATAGGTAGCATTAATATTGTCAAAATGGCCATAGTATCAAAAGTGTTATACAGATTTAAT

GCACTTCCTATTAAAATCCTAATGACATTCTTCAAAGAAATAGAAAAAGCAACCAATAAA

CTTACTTGGAAAAATAAGATACCCAGAATAGCTAAAACAATCCTTAGCAAGAAAAGTGAA

GCAGGAGGCATCACAATACCAGACCTTAAATTATATTATACTACCAATGTAAATACAGTT

ATCTCAAATAAAGGCACTGTAAACATACATTGGAGAAAAGATAGGTTCTTCAACAAATGG

>CL134.Contig4_All 2 433 minus strand Retrovirus-related Pol polyprotein LINE-1 OS=Mus musculus GN=Pol PE=1 SV=2

AAAATGACCATTCTACCAAAAGCACTATACAGGTTCAATACAATTCCGATCAAAATCCCA

ATGGCATTCCTCATAGAAATAGAAAATGCAATCCTGAAATTCATCTGGAAAAATAAGAGA

CCCAAATAGCTAAAGCAATCCTAAGCAAGAAGAGTGAAGCAGGTGGTATTGCTATACCAG

ACCTTAAATTATACTACCAAGCAATAGTAACAAATAGCATAGTACTGGCACCAAAACAGG

CCTGTAGAGCAATGGTACACAATAGAGGACACAGAGACTAACTCACAAAATTACAATTAT

CTTATATTAGACAAAGGTGCCAAAAACATGCATTAGAGAAAAGATAGCCTCTTCAACAAC

TGGTGCTGTGAAAACTGGAAATCCACATGTAACAGAATGAAAGTTAGCCCCTATCTCTCA

CCTGTACAAAAG

>CL134.Contig5_All 221 445 unnamed protein product [Rattus norvegicus]

AAATTGGTCCTTGAATTTATTTGGAAGAATAAGACCCAGAATAGCCAAGCAATTCTTAAG

TCAAAGAGCAATGCTGGAACCATCACAATACCTAACTTCAAATTAGTAAAGATCTATAGT

AACAAAAACTTCAAAGTACTTGTATAAAACAAATGGTAGACCAATGATACAGAGTAGAAG

ACACAAAGGCACTATAGTCATCTGATCTTTGACAAAGTTGCCAAA

>CL134.Contig6_All 87 368 LRRG00130 [Rattus norvegicus]

AGATAGAAAGACCTCCCTAGTTCATGAATAGGAAAAATTAATACTGTTAAAATGGCCACA

GTACCAAAAGCAATATACAGATTCACTGCAGTCCCCATCAAAATACCAGTGACATTCTTC

ACAGAACTAGAAAAAAATTGGTCCTTGAATTTATTTGGAAGAATAAGACCCAGAATAGCC

AAGCAATTCTTAAGTCAAAGAGCAATGCTGGAACCATCACAATACCTAACTTCAAATTAG

TAAAGATCTATAGTAACAAAAACTTCAAAGTACTTGTATAAA

>CL134.Contig7_All 391 618 minus strand unknown [Homo sapiens]

AAAAATGTATATTGGAGAAAAGATAGCCTTTTAAACAAATAGTGCATGGAAAACCAGAAA

GCCATATGTAGAAGAATGAAACTAGATCCCTATCTATCACCGTGCACAAAGGGCAAATAA

AAATGGATCAAAGACATAGGAATTAGACCCCAAACCTTGCAACTGCTAGTAAAAAGCATA

GGGTCAACATTTCATTATACATGTGGTGGCACCAACTTCCTTAGCAAA

>CL134.Contig8_All 167 292 LRRGT00057 [Rattus norvegicus]

TTTGAAGAAAAAAAGCCCAGAATAGCCAAAGCAGTCTTAAGCAAGAAGAGTGATGCTAGA

GAGCTCATAATGCCTAATCTCATATTACAGAGCTATAGTAACAATAGACACAAAGAACAG

TGGAAT

>CL134.Contig10_All 145 294 L1 repetitive element ORF

AAGAAACCCAGAATAGCCAAAGCAATCCTCAGCAAGAAGAGTGAAGCAAGAGGCATCACA

ATACCAGATCTTAAATTATACTACAGAGTTATAGTAATGAAAACAGCAGGGTATTGGCAC

CAAAACAGACATGTAGACCAATGGTACAGA

>CL134.Contig11_All 3 335 minus strand Retrovirus-related Pol polyprotein LINE-1 OS=Mus musculus GN=Pol PE=1 SV=2

GAAGACCTTAGAAGATGGAAATATCTCCCTTGTTTTTGGATAGGCAGAATTAATATTGTA

AAGTGACCATAGTACCAAAAACACTATGCAGATTTAATGCAATTTCAATCAAAATCTCAA

TGATCATTCCTCAAAGAAATAGAAAAAGAAGTCATGAAATTCATCTTGGAAAATAAGAGA

CCCAGAATAATTAAAGCAGTCCTTAGCAAGAAGAGTAAAGCAGGTGACATCACTATACCA

GACCTTAAGCTATACTACAGAGCAATAGTAACAGAAATGACATGCTTTTGGCACCGAAAT

AGACATGTAGACTAATGGTACAAAATAGAAGAA

>CL134.Contig12_All 29 175 minus strand Retrovirus-related Pol polyprotein LINE-1 OS=Mus musculus GN=Pol PE=1 SV=2

CCATATATTTAGAGCCATCTGATACTTGACAAAGGTCTCAAAAATGTGTCTTGGAGAAAA

GACAATCTTTTTAACAGGTGGTGCTGGGAAAACTGGACAGCTATATGTATAAAAATGAAA

TTGGATCCCTATCTCTCACCCTGCACA

>CL134.Contig13_All 17 157 LRRGT00085 [Rattus norvegicus]

AAAAAAAAGAAAGACCTTTACATTGAAAACTACAGAACATACAAGAAGGAAATTGAAGAA

AACCTCAGAAGATGGAAAGATCTCCCATGCTCCTGGATAGGCAGAATTAATATTGTCAAA

ATGGCCATACTACCAAAAGCA

>CL134.Contig14_All 27 428 putative p150 [Homo sapiens]

AAAAGAAAACAAAACCTAGGAATAAATCTAACCAAGACAGTGAAAGACATGTACAGTGAA

AATTATAGAATATTAAAAAAGAAACTGAAGAGGCATAAGAGGGAAAGACCTCCCATGTTC

ATTGGTAGACATGATTAATATTGATAAAATGGCCATACTCCCAAAGCAATATACAGATTC

AATGCCATCCCCATCAAAATACCAATGATATTCTTCACAGAACTAGAAAAAACAGTCCTA

AAATTCATTTGGAGAAATAAAAGACTCAGAATAGTCAAAGCAATTCTAAGCCAAAAAAGC

AGCTGGAGGCATCACAATGCCTGACTTCAGCTATCTAACTACAGAGCTATAGTAACAAAA

ATGGCATGATATTGGCACCAAAATAGACTTGAAGACCAATGG

>CL134.Contig15_All 3 293 LRRGT00194 [Rattus norvegicus]

AACTACAGAACACTAAAGAAAGGAATTGAAGAAGACCTTAGAAGATGAAAAGATCTCTCA

TGCTCCTGGATAGGCAAAATTAATATTGTCAAAATGGCCATGCTACCAAAAGCTTTATAC

ACATTTAATGCAATTTGAATTATAATCCCAGTGACATTCTTCATAGAATTAGAAAAAGCA

ATCATGAAATTTAGTTGGAAAAATAAGAAACCCAGACTAGCCAATGCAATCCTTAGCAAG

AAGAGTGAAACAGGAGACATCACGATATCAGACCTTAAACTATACTACAGA

>CL134.Contig16_All 269 433 endonuclease/reverse transcriptase [Sus scrofa]

TACTACAGAGCTATCATAACAAAAACACTGTGGTTTTGGCATCAAAATAGACACAAAGAC

CAATGGAACAGAATAAAAGACACAGAGACAAATCCACTGACCTACGGCCATCTAATACTT

GATAAAGGTGCAAAAAAAAAAAAAAGATTTTGGAGAAAAGACACA

>CL134.Contig17_All 1 189 minus strand reverse transcriptase, partial [Peromyscus maniculatus]

AGAGAAATAGAAGAAGATCTTAGATGGAAAGATCTACCTTGCTCATGGATAGGCAGAATT

AATATTATCAAAATGACCATTCTACCAAAAGCACTATACAGGTTCAATACAATTCCGATC

AAAATCCCAATGGCATTCCTCATAGAAATAGAAAATGCAATCCTGAAATTCATCTGGAAA

AATAAGAAG

>CL134.Contig18_All 48 365 minus strand Retrovirus-related Pol polyprotein LINE-1 OS=Mus musculus GN=Pol PE=1 SV=2

AAAACTACAGAACACTCCAGAAAGAAATTGAAGAAGACCTTAGAAAATGCAAAATCCCCC

ATGTTCATGGATAGGCAAATTAATTTTATCAAAATGGTCATGCTCTCCAAAGCACGAAAC

AGATTTAATGTAATTCCCATTAAAATTCCAATGATGTTCTTCATAGAAATGGAAAAAGCA

GTTATGAAATTTATTTGGAAAAACAAGAAGCCCAGAATAGCCAAAGCAATCCTTAGTGAG

AAAAGTGATGCAGGAGGCATCACAACACCAGATTTTACATGATACTGCAGTACCATGGCA

TTTTTGTTACTATGGTTC

>CL134.Contig19_All 230 331 minus strand endonuclease/reverse transcriptase [Sus scrofa]

AAAAAAGCAATCATGAAATTCATCTAGAAAAATAAGAGACCCAGAATAGTCAAAGCAATC

CTTAGCAGGAAGAATGAAGCAGGAGGCACCACAATACCAGAC

>CL134.Contig20_All 2 214 hypothetical protein EGM_18659, partial [Macaca fascicularis]

AAAATGGCCATACTACCCAAAGCCCTATACAGATTCAATGCAATTCCAATTAAAATACCA

GCATCATTCCTTATAGAAGTAGAAAACGCAATCATGAAATTCATTTGGAAAGATAAGAGA

CCCAGAATAGCCAAAGTAGTCCTTAGCAAGAAGAGTAAAGCAGAAGGTATCACAATACCA

GAATTTTAACTATACTACAGAGCCATAGTAACA

>CL134.Contig21_All 19 291 reverse transcriptase, partial [Peromyscus maniculatus]

AGAAAAGAAAAAAAATACATAGGAATCAATCTATTTAAAGAAGTGAAAGACCTCTACAAT

GAAAACAGAACACTAAAGAAAGAAGTTGAAGAGGACCTTAGAAAATGGAAATACCTCCCA

ATCTCCTGGATAGGCAGAATTGATATTGTTAAAATAATAATACTACCCAAAATGTTGTAT

GAATTTAATGCAAATCCTATTAAAATTCCAATGACATTCTTCACAGAAATGGAAAAAGCA

ATCATGAAATTCATTGGAAAAATAAGAGACCCA

>CL134.Contig22_All 3 200 minus strand LINE-1 element ORF2 (predicted) [Sorex araneus]

GAAATTGAAGAAAACCTCAGAAAATGGAAATATTTCCCATGCTCCTGGATAGACAGAATT

AATGTAGTCAAAATGGCTGTACTACCAAAAGCATTATATAGGTTTAATGCACTTCCTATT

ATAATCCCAATGACCTTCTTTATAGAACTAGAAAAAGCAGTCATAAAATTCATTTGAAAA

AATAAGAGACCCAGAATA

>CL135.Contig1_All 42 1115 minus strand Synembryn-A [Heterocephalus glaber]

GCACTCCGCACTGATGTGCGCCAGCAGCTGTTTCAGGAGCTGCATGGTGTGCACCTGCTG

ACCCACACACTGGAGCTGACACTGGGGATGACCCCAGAAGAGAGCCCACCTGATCTCCTT

CCTCCCCAAGAGACTGAGCGGGCTATGGAGATCCTCAAGGTGCTATTTAATATCACCTTT

GACTCCATCAAGAGGGAAGTGGACGAGGAAGACACTGCCCTTTACCAGTACCTGGGAACC

CTTCTGCGGCACTGTGTGATGGTTGCTGCTGCTGGAGACCGCACGGAGGAGTTCCACGGC

CACACAGTGAATCTCCTGGGGAACTTGCCTCTCAAGTGTTTGGACGTCCTCCTCGCCCTA

GAGCTGCATGAAGGCTCCTTGGAGTTCATGGGAGTGAACATGGATGTGATTGGTGTCCTC

CTCGCCTTCCTAGAGAAGCGTTTGCACCAGACCCACAGGCTAAAGGAGAGTGTGGCTCCT

GTGCTGAGTGTGCTGACAGAGTGTGCCCGCATGCACCGTCCAGTCAGGAAATTCCTGAAG

GCCCAGGTGCTGCCACCTCTGAGGGACGTGAGGACCCGGCCTGAGGTTGGGGAGCTACTG

CGGAACAAGCTTGTCCGCCTCATGACACACCTGGACACGGATGTGAAGAGGGTAGCTGCC

GAATTCCTCTTTGTGCTGTGTTCTGAGAGTGTGCCCCGATTCATCAAGTATACAGGCTAC

GGGAACGCCGCAGGCCTCTTGGCTGCCAGGGGCCTCATGGCAGGGGGGCGGCCTGAGGGC

CAGTATTCAGAGGACGAGGATACGGACACTGATGAGTACAAGGAAGCCAAGGCCAGCATC

AACCCTGTGACAGGGAGGGTGGAGGAGAAGCCACCCAATCCTATGGAGGGCATGACAGAG

GAGCAGAAGGAGCATGAGGCCATGAAGCTGGTGAACATGTTTGACAAGCTCTCCAGGCAC

AGAGTCATACAACCCATGGGGATGAGTCCCCGGGGTCACCTCACATCTCTGCAAGACGCC

ATGTGTGAGACCATGGAGGGACAGCTCTCCTCAGACCCCGACTCGGACCCTGAC

>CL135.Contig2_All 2976 4055 minus strand Synembryn-A [Heterocephalus glaber]

CTCACAGCACTCCGCACTGATGTGCGCCAGCAGCTGTTTCAGGAGCTGCATGGTGTGCAC

CTGCTGACCCACACACTGGAGCTGACACTGGGGATGACCCCAGAAGAGAGCCCACCTGAT

CTCCTTCCTCCCCAAGAGACTGAGCGGGCTATGGAGATCCTCAAGGTGCTATTTAATATC

ACCTTTGACTCCATCAAGAGGGAAGTGGACGAGGAAGACACTGCCCTTTACCAGTACCTG

GGAACCCTTCTGCGGCACTGTGTGATGGTTGCTGCTGCTGGAGACCGCACGGAGGAGTTC

CACGGCCACACAGTGAATCTCCTGGGGAACTTGCCTCTCAAGTGTTTGGACGTCCTCCTC

GCCCTAGAGCTGCATGAAGGCTCCTTGGAGTTCATGGGAGTGAACATGGATGTGATTGGT

GTCCTCCTCGCCTTCCTAGAGAAGCGTTTGCACCAGACCCACAGGCTAAAGGAGAGTGTG

GCTCCTGTGCTGAGTGTGCTGACAGAGTGTGCCCGCATGCACCGTCCAGTCAGGAAATTC

CTGAAGGCCCAGGTGCTGCCACCTCTGAGGGACGTGAGGACCCGGCCTGAGGTTGGGGAG

CTACTGCGGAACAAGCTTGTCCGCCTCATGACACACCTGGACACGGATGTGAAGAGGGTA

GCTGCCGAATTCCTCTTTGTGCTGTGTTCTGAGAGTGTGCCCCGATTCATCAAGTATACA

GGCTACGGGAACGCCGCAGGCCTCTTGGCTGCCAGGGGCCTCATGGCAGGGGGGCGGCCT

GAGGGCCAGTATTCAGAGGACGAGGATACGGACACTGATGAGTACAAGGAAGCCAAGGCC

AGCATCAACCCTGTGACAGGGAGGGTGGAGGAGAAGCCACCCAATCCTATGGAGGGCATG

ACAGAGGAGCAGAAGGAGCATGAGGCCATGAAGCTGGTGAACATGTTTGACAAGCTCTCC

AGGCACAGAGTCATACAACCCATGGGGATGAGTCCCCGGGGTCACCTCACATCTCTGCAA

GACGCCATGTGTGAGACCATGGAGGGACAGCTCTCCTCAGACCCCGACTCGGACCCTGAC

>CL135.Contig3_All 254 1843 minus strand Synembryn-A [Heterocephalus glaber]

ATGGAGCCCCGGGCGGTTGCGGATGCCCTAGAGACGGGAAAGGAAGACGTGATGACGGAA

GCTCTACGAACGTACAACCGGGAGCACTCCCAGAGCTTCACCTTTGACGAGGCCCAACAG

GAGGACAGGAAGAGACTTGCAGAGTTGTTGGTCACTGTCTTGGAGCAGGGGTTGCCACCC

TCGCACCGTGTCACCTGGCTACAGACTGTCCGTATCCTGTCCCGGGACCGCAGCTGCCTG

GACCCATTCACCAGTCGCCAGAGCTTACATGCACTAGCTTGCTATGCTGGCATCTCTGCC

TCTGAGGGGTCTGTCCCAGAATCCCCAGATATGGATGTCATACTAGAGTCCCTGAAGTGC

CTCTGCAACCTCGTGCTCAGCAGTCCAGTGGCACAGACACTGGCAGCAGAGGCCCATCTG

GTGGTGCGGCTAGCAGAGCGTGTGGGGATGTACAGCAAGAAGAACTTCCCACATGATGTC

CAGTTCTTTGATTTAAGGCTCCTCTTCCTACTAACAGCACTCCGCACTGATGTGCGCCAG

CAGCTGTTTCAGGAGCTGCATGGTGTGCACCTGCTGACCCACACACTGGAGCTGACACTG

GGGATGACCCCAGAAGAGAGCCCACCTGATCTCCTTCCTCCCCAAGAGACTGAGCGGGCT

ATGGAGATCCTCAAGGTGCTATTTAATATCACCTTTGACTCCATCAAGAGGGAAGTGGAC

GAGGAAGACACTGCCCTTTACCAGTACCTGGGAACCCTTCTGCGGCACTGTGTGATGGTT

GCTGCTGCTGGAGACCGCACGGAGGAGTTCCACGGCCACACAGTGAATCTCCTGGGGAAC

TTGCCTCTCAAGTGTTTGGACGTCCTCCTCGCCCTAGAGCTGCATGAAGGCTCCTTGGAG

TTCATGGGAGTGAACATGGATGTGATTGGTGTCCTCCTCGCCTTCCTAGAGAAGCGTTTG

CACCAGACCCACAGGCTAAAGGAGAGTGTGGCTCCTGTGCTGAGTGTGCTGACAGAGTGT

GCCCGCATGCACCGTCCAGTCAGGAAATTCCTGAAGGCCCAGGTGCTGCCACCTCTGAGG

GACGTGAGGACCCGGCCTGAGGTTGGGGAGCTACTGCGGAACAAGCTTGTCCGCCTCATG

ACACACCTGGACACGGATGTGAAGAGGGTAGCTGCCGAATTCCTCTTTGTGCTGTGTTCT

GAGAGTGTGCCCCGATTCATCAAGTATACAGGCTACGGGAACGCCGCAGGCCTCTTGGCT

GCCAGGGGCCTCATGGCAGGGGGGCGGCCTGAGGGCCAGTATTCAGAGGACGAGGATACG

GACACTGATGAGTACAAGGAAGCCAAGGCCAGCATCAACCCTGTGACAGGGAGGGTGGAG

GAGAAGCCACCCAATCCTATGGAGGGCATGACAGAGGAGCAGAAGGAGCATGAGGCCATG

AAGCTGGTGAACATGTTTGACAAGCTCTCCAGGCACAGAGTCATACAACCCATGGGGATG

AGTCCCCGGGGTCACCTCACATCTCTGCAAGACGCCATGTGTGAGACCATGGAGGGACAG

CTCTCCTCAGACCCCGACTCGGACCCTGAC

>CL135.Contig4_All 42 887 minus strand Synembryn-A [Heterocephalus glaber]

GCACTCCGCACTGATGTGCGCCAGCAGCTGTTTCAGGAGCTGCATGGTGTGCACCTGCTG

ACCCACACACTGGAGCTGACACTGGGGATGACCCCAGAAGAGAGCCCACCTGATCTCCTT

CCTCCCCAAGAGACTGAGCGGGCTATGGAGATCCTCAAGGTGCTATTTAATATCACCTTT

GACTCCATCAAGAGGGAAGTGGACGAGGAAGACACTGCCCTTTACCAGTACCTGGGAACC

CTTCTGCGGCACTGTGTGATGGTTGCTGCTGCTGGAGACCGCACGGAGGAGTTCCACGGC

CACACAGTGAATCTCCTGGGGAACTTGCCTCTCAAGTGTTTGGACGTCCTCCTCGCCCTA

GAGCTGCATGAAGGCTCCTTGGAGTTCATGGGAGTGAACATGGATGTGATTGGTGTCCTC

CTCGCCTTCCTAGAGAAGCGTTTGCACCAGACCCACAGGCTAAAGGAGAGTGTGGCTCCT

GTGCTGAGTGTGCTGACAGAGTGTGCCCGCATGCACCGTCCAGTCAGGAAATTCCTGAAG

GCCCAGGTGCTGCCACCTCTGAGGGACGTGAGGACCCGGCCTGAGGTTGGGGAGCTACTG

CGGAACAAGCTTGTCCGCCTCATGACACACCTGGACACGGATGTGAAGAGGGTAGCTGCC

GAATTCCTCTTTGTGCTGTGTTCTGAGAGTGTGCCCCGATTCATCAAGTATACAGGCTAC

GGGAACGCCGCAGGCCTCTTGGCTGCCAGGGGCCTCATGGCAGGGGGGCGGCCTGAGGGC

CAGTATTCAGAGGACGAGGATACGGACACTGATGAGTACAAGGAAGCCAAGGCCAGGTGT

GTACCC

>CL135.Contig5_All 42 731 minus strand Synembryn-A [Heterocephalus glaber]

GCACTCCGCACTGATGTGCGCCAGCAGCTGTTTCAGGAGCTGCATGGTGTGCACCTGCTG

ACCCACACACTGGAGCTGACACTGGGGATGACCCCAGAAGAGAGCCCACCTGATCTCCTT

CCTCCCCAAGAGACTGAGCGGGCTATGGAGATCCTCAAGGTGCTATTTAATATCACCTTT

GACTCCATCAAGAGGGAAGTGGACGAGGAAGACACTGCCCTTTACCAGTACCTGGGAACC

CTTCTGCGGCACTGTGTGATGGTTGCTGCTGCTGGAGACCGCACGGAGGAGTTCCACGGC

CACACAGTGAATCTCCTGGGGAACTTGCCTCTCAAGTGTTTGGACGTCCTCCTCGCCCTA

GAGCTGCATGAAGGCTCCTTGGAGTTCATGGGAGTGAACATGGATGTGATTGGTGTCCTC

CTCGCCTTCCTAGAGAAGCGTTTGCACCAGACCCACAGGCTAAAGGAGAGTGTGGCTCCT

GTGCTGAGTGTGCTGACAGAGTGTGCCCGCATGCACCGTCCAGTCAGGAAATTCCTGAAG

GCCCAGGTGCTGCCACCTCTGAGGGACGTGAGGACCCGGCCTGAGGTTGGGGAGCTACTG

CGGAACAAGCTTGTCCGCCTCATGACACACCTGGACACGGATGTGAAGAGGGTAGCTGCC

GAATTCCTCTTTGTGCTGTGTTCTGAGAGT

>CL136.Contig1_All 85 231 minus strand CTD nuclear envelope phosphatase 1 [Homo sapiens] >gi|219555649|ref|NP_001137247.1| CTD nuclear envelope phosphatase 1 [Homo sapiens] >gi|262527541|sp|O95476.2|CNEP1_HUMAN RecName: Full=CTD nuclear envelope phosphatase 1; AltName: Full=Serine/threonine-protein phosphatase dullard >gi|14424544|gb|AAH09295.1| Dullard homolog (Xenopus laevis) [Homo sapiens] >gi|119610639|gb|EAW90233.1| hCG1987397, isoform CRA_a [Homo sapiens] >gi|119610641|gb|EAW90235.1| hCG1987397, isoform CRA_a [Homo sapiens] >gi|119610642|gb|EAW90236.1| hCG1987397, isoform CRA_a [Homo sapiens] >gi|351701548|gb|EHB04467.1| Serine/threonine-protein phosphatase dullard [Heterocephalus glaber] >gi|380785173|gb|AFE64462.1| CTD nuclear envelope phosphatase 1 [Macaca mulatta] >gi|384940074|gb|AFI33642.1| CTD nuclear envelope phosphatase 1 [Macaca mulatta]

CCAGACAACGCCATCCCCATCAAATCCTGGTTCAGTGACCCCAGTGACACGGCCCTTCTC

AATCTGCTTCCAATGCTGGATGCCCTCAGGTTCACCGCTGATGTTCGATCCGTGTTGAGC

CGAAATCTTCACCAACATAGGCTCTGG

>CL136.Contig2_All 85 231 PREDICTED: CTD nuclear envelope phosphatase 1 [Otolemur garnettii]

CCAGACAACGCCATCCCCATCAAATCCTGGTTCAGTGACCCCAGTGACACGGCCCTTCTC

AATCTGCTTCCAATGCTGGATGCCCTCAGGTTCACCGCTGATGTTCGATCCGTGTTGAGC

CGAAATCGTCACCAGAGCCTATGTTGG

>CL137.Contig1_All 186 359 minus strand HVEM [Cavia porcellus]

ATGGAGCCTCTGCCAGGCTGGGGGCTTCCCTGGTGGCGCCAGGAGCCTACAGCCAATGCC

CTGAAGCTGGCCCTGTGTCTCCTCCTCCTCCTGGGATCCCCCCCGTGTGCCCTTGCCCAG

TTCCTGTGCAGAGAGGAGGAGTACCCTGTGGGCACCGAATGCTGCCCCAAGTGC

>CL137.Contig2_All 186 1031 HVEM [Cavia porcellus]

ATGGAGCCTCTGCCAGGCTGGGGGCTTCCCTGGTGGCGCCAGGAGCCTACAGCCAATGCC

CTGAAGCTGGCCCTGTGTCTCCTCCTCCTCCTGGGATCCCCCCCGTGTGCCCTTGCCCAG

TTCCTGTGCAGAGAGGAGGAGTACCCTGTGGGCACCGAATGCTGCCCCAAGTGCACCCAG

GTTACCACGTGAAGCAAGCCTGCAGTGAGCTGACAGGCACAGTGTGCATACCCTGCTCCC

CCCGAGACCCACACCGCCCACCCCAACGGCCTAAGCCGGTGTCTGCCTTGCCGAGTCTGT

GACCCAGCCTTGGGCTTGGACACCAGGCAGAAGTGCTCCAGCATGCAGGACACGGTGTGC

GGCTGTCGCCAAGGCTACTTCTGCGAGGCCGAGGACAGGGAGCACTGCATCATGTGCTTG

CCCCACACCATCTGCCACCCTGGCCAGAGGGTACAGAGGAGAGGTACAGATAGCCAGGAC

ACAGTGTGTGCCAACTGCCCACTGGGGACCTTCTCTCCCAACGGGACCCTGGACCAATGT

CTTCCCTGGACCACGGGACTTTTTGCCATGGAAGCCGAGCCGGCGACCAGCAGCAGGGAC

GTCTCCTGCTCCCTGTCCCGGGGGTTCTATGTCTTTGTGTCCGTCCTCCCCCTCTCCGTG

GTGATCGTCCTGATGGTGTGGGTGAGGAGAAAAAGACCGTGTGGCTCTGAGCGCAGTGAG

ACGGTTGTGCTGCAGCGGCAAGTAGAGGAGGAAGCCGGTGAGTCTGCAGTCACCCAGGCT

CCAAAAGTCACCACGGTGGCGGAGGAGGAGACAGTACCTGTGTTGAGGGCAATGGGTCCC

CATCGA

>CL138.Contig1_All 22 1536 minus strand PREDICTED: C-type lectin domain family 4 member F-like [Cavia porcellus]

CTGAAGGACGCAGAGATGGAAAGTGACACGGTCCACTTCTGCACAGATAACCAGCGTGTC

TCCCTGCATCCCCGAGAGGTGGATTCTGAGACAGTGGCTCTGGCAGCCCCCAGGATGTCA

AGGCTTGTTGTGGCCACCCTGGCTGTGATCCTGGTCTCCTCTCTTGTGGCTCTCTTTGTT

GTGGCTCTCCAGTCCCGAAGCCCTGCACCGGGGGATCATCCTTCCTTCCAAGAGTTTAAA

GCCATGTTTCCGGGAGACAACACTACTGGCCAGTCACCTGTGGAGCCCCACAATCATCAT

TATTTTGACAGCATAGCAGGAATGCAAGAGGTGATCCAGATGTTCAGAGGGCATATGGAA

AATTCCTACACTTGGCATGAAGAGATCCAGATGTTGAAGTACAGAGTGGACAATGTCAGT

TCTCAGATCCAGACACTTGGCAGTCATCTGGGAGATGCCAGGGCTGACATCCAGATGGTA

AGAGGTGCTCTTAAGGCCGCCAATACCTTGAGCTCGCAGACCCAGACATTAAACAGCTCC

ATGGAGAGGGCCAATAGCGAGATTGAGAGGCTGAAGGGAAGTCTACAAGATGCAAATGCT

CTAAACACCCAGGCCCAGATCTTTCTGCAGGACAGTTTAGACAACACCAGTGCTGAGATA

CAGGTGCTGAGAGGTCATTTGAAAAGGGCAAATGATGACAGTTCCGTGTTAAAAAGAGAG

TTGGAAACTGCCACTGCCCAGATCCAAATAGCAAGTGGCCATCTGGAACAGACGGATGCT

CAGATCCAAGAGCTAAAATCAGAGCTGGAAAATGCCAGTACTTTAAATTTCCAGATTCAG

GTGTTAAATGGTCAGTTGAAAAATGCCAGCAGAGAGATACAGACCCTAAAAGAAGGAATG

AAGGGTGCTGCAGCCTTAAGTTCCCAGACTCAGATGTTAGAGAGCAATTTGCAGAAGGCC

AAGGCTGAGATGCAGAGGTTAAAAGAGGATTTGGAGAACACCAAGAATCTAACTGCAAAA

ATCCAGGAGGAGCAGAGTTGCCTGGGGAACCTCTCTGCAACCACTGCTTCACAGGAGCAG

CTACAGAGCACCCAAAATCAACTACTCCAGCTGATCCTGCAAGGCTGGAAAGCCTATGAG

GAAAACTTGTATTACTTTTCTCACGTCAAGAAGTCTTGGCATGAGGCTGAGCGGTCCTGT

GTGTCCAAGGGAGCCCACCTGGCATCGGTGACCTCTCAGGAAGAGCAGGCATTCCTGGTA

CAGTTCACAAGTAACTCCTACCACTGGATTGGCCTCACTGACATGGGCATGGAGGGCACC

TGGCGCTGGGCAGATGGGACACAGTTCAATAATGCTGGGAGCAAGGCGTTTTGGGACAGA

AATCAGCCAGACAACTGGAGGCACAAGAATGGGCAGACTGAAGACTGCGTCCATCTTCGC

AGCTTGTGGAATGACATGAACTGCAATAGCCCCTATCACTGGGTCTGCAAGAAGCCCATG

GGCCAGCAGGTGGCA

>CL138.Contig2_All 3 287 C-type lectin domain family 4 member F [Heterocephalus glaber]

GCTCTCCAGTCCCGAAGCCCTGCACCGGGGGATCATCCTTCCTTCCAAGAGTTTAAAGCC

ATGTTTCCGGGAGACAACACTACTGGCCAGTCACCTGTGGAGCCCCACAATCATCATTAT

TTTGACAGCATAGCAGGAATGCAAGAGGTGATCCAGATGTTCAGAGGGCATATGGAAAAT

TCCTACACTTGGCATGAAGAGATCCAGATGTTGAAGTACAGAGTGGACAATGTCAGCCCT

GGCATCTCCCAGATGACTGCCAAGTGTCTGGATCTGAGAACTGAC

>CL139.Contig1_All 237 353 mCG4061 [Mus musculus]

ACCATGCCTATGATACTGGGTTATTGGGACGTCCGTGGGCTTACTAACCCCATCCGCCTG

CTCCTGGAATACACAGACTCAAACTATGAGGAGAAGAGATACACCATGGGGGACGCT

>CL139.Contig2_All 237 893 mCG4061 [Mus musculus]

ACCATGCCTATGATACTGGGTTATTGGGACGTCCGTGGGCTTACTAACCCCATCCGCCTG

CTCCTGGAATACACAGACTCAAACTATGAGGAGAAGAGATACACCATGGGGGACGCTCCC

AATTATGACAGAAGCCAGTGGCTGGATGAGAAATTCAAGCTGGGCCTGGACTTTCCCAAT

CTGCCCTACTTGATTGACGGGCCTCACAGGATCACCCAGAGCAACGCCATCCTGCGCTAC

CTTGCCCGCAAGCACAACCTGTGTGGGGAGACAGAAGAGGAGATGATTCGTGTGGACATT

TTGGAGAACCAGGCTATGGACACTCGTGTACAACTTGCCGTGATCTGCTACAGCCCTGAC

TTTGAGAAAAAGAAGCCTGAGTTCTTGGAGACCCTGCCTGAGAAGATGAAGCTCTACTCA

CAGTTCCTGGGGAAGCGGCCCTGGTTTGCAGGGGACAAGATCACCTACGTGGATTTCATC

ACTTATGATGTCCTGGACCAGCACCTTCTATTTGAACCCAAGTGCCTGGAGGCATTCCCA

AACCTGAAGGACTTCATGTCCCGCTTTGAGGGCCTGAGGAAGATCTCTGACTACATGAAG

TCTAGCCGCTTCCTCCCCAGGCCTATTTTTACAAAGATGGCCCATTGGTATAAAAAA

>CL139.Contig3_All 1 267 minus strand mCG4061 [Mus musculus]

CGTGTGGACATTTTGGAGAACCAGGCTTTGGACACTCGCATGCAGCTTGCCATGGTCTGC

TACAGCCCTGACTTTGAGAAAAGGAAGCCTGAGTACTTGGAGACCCTGCCTGAGAAGATG

AAGCTCTACTCACAGTTCCTGGGGAAGCACCCATGGTTTGCAGGGGAGAAGATCACCTTT

GTGGATTTCATCGCTTATGATGTCCTTGACCTACACCGTCTGTTTGAACCCAAGTGCCTT

GATGCATTCCCAAACCTGAAGGACTTC

>CL139.Contig4_All 240 893 PREDICTED: glutathione S-transferase Y1-like [Cricetulus griseus]

ATGCCTATGATACTGGGTTATTGGGACGTCCGTGGGCTTACTAACCCCATCCGCCTGCTC

CTGGAATACACAGACTCAAACTATGAGGAGAAGAGATACACCATGGGGGACGCTCCCAAT

TATGACAGAAGCCAGTGGCTGGATGAGAAATTCAAGCTGGGCCTGGACTTTCCCAATCTG

CCCTACTTGATTGACGGGCCTCACAGGATCACCCAGAGCAACGCCATCCTGCGCTACCTT

GCCCGCAAGCACAACCTGTGTGGGGAGACAGAAGAGGAGATGATTCGTGTGGACATTTTG

GAGAACCAGGCTATGGACACTCGTGTACAACTTGCCGTGATCTGCTACAGCCCTGACTTT

GAGAAAAAGAAGCCTGAGTTCTTGGAGACCCTGCCTGAGAAGATGAAGCTCTACTCACAG

TTCCTGGGGAAGCGGCCCTGGTTTGCAGGGGACAAGATCACCTACGTGGATTTCATCACT

TATGATGTCCTGGACCAGCACCTTCTATTTGAACCCAAGTGCCTGGAGGCATTCCCAAAC

CTGAAGGACTTCATGTCCCGCTTTGAGGGCCTGAAGAAGATCTCTGCCTACATGAAGTCC

AGCCGCTTCCTCCCAACACCTATGTATTCCAAGATGGCCACATGGGGCAACAAG

>CL139.Contig5_All 3 299 PREDICTED: glutathione S-transferase Mu 4 isoform 9 [Pan troglodytes] >gi|397466370|ref|XP_003804935.1| PREDICTED: glutathione S-transferase Mu 4 isoform 1 [Pan paniscus]

TTGGAGAACCAGGCTATGGACATTACCAATGAGCTGGCTAGAGTCTGCTATAGCCCAGAC

TTTGAGAAACTGAAGCCAGAGTACTTGGAGCGCATCCCAGAGGCACTGAAGCTCTTTTCA

CAGTTCCTGGGGAAGAAGCCATGGTTTGTTGGTGATAAGATCACCTTTGTGGATTTCCTT

GCTTATGATTTACTTGACCTGCACCGAATATTTCACCCTAAGTGCCTGGATGAATTTTCG

AACCTGAAGGACTTCGTATCCCGCTTTGAGGGCTTGAAGAAGATCTCTGCCTACATG

>CL139.Contig6_All 240 893 PREDICTED: glutathione S-transferase Y1-like [Cricetulus griseus]

ATGCCTATGATACTGGGTTATTGGGACGTCCGTGGGCTTACTAACCCCATCCGCCTGCTC

CTGGAATACACAGACTCAAACTATGAGGAGAAGAGATACACCATGGGGGACGCTCCCAAT

TATGACAGAAGCCAGTGGCTGGATGAGAAATTCAAGCTGGGCCTGGACTTTCCCAATCTG

CCCTACTTGATTGACGGGCCTCACAGGATCACCCAGAGCAACGCCATCCTGCGCTACCTT

GCCCGCAAGCACAACCTGTGTGGGGAGACAGAAGAGGAGATGATTCGTGTGGACATTTTG

GAGAACCAGGCTATGGACACTCGTGTACAACTTGCCGTGATCTGCTACAGCCCTGACTTT

GAGAAAAAGAAGCCTGAGTTCTTGGAGACCCTGCCTGAGAAGATGAAGCTCTACTCACAG

TTCCTGGGGAAGCGGCCCTGGTTTGCAGGGGACAAGATCACCTACGTGGATTTCATCACT

TATGATGTCCTGGACCAGCACCTTCTATTTGAACCCAAGTGCCTGGAGGCATTCCCAAAC

CTGAAGGACTTCATGTCCCGCTTTGAGGGCCTGAAGAAGATCTCTGCCTACATGAAGTCC

AGCCGCTTCCTCCCAACACCTATGTATTCCAAGATGGCTGCATGGGGCAACAAG

>CL139.Contig7_All 240 893 minus strand PREDICTED: glutathione S-transferase Y1-like [Cricetulus griseus]

ATGCCTATGATACTGGGTTATTGGGACGTCCGTGGGCTTACTAACCCCATCCGCCTGCTC

CTGGAATACACAGACTCAAACTATGAGGAGAAGAGATACACCATGGGGGACGCTCCCAAT

TATGACAGAAGCCAGTGGCTGGATGAGAAATTCAAGCTGGGCCTGGACTTTCCCAATCTG

CCCTACTTGATTGACGGGCCTCACAGGATCACCCAGAGCAACGCCATCCTGCGCTACCTT

GCCCGCAAGCACAACCTGTGTGGGGAGACAGAAGAGGAGATGATTCGTGTGGACATTTTG

GAGAACCAGGCTATGGACACTCGTGTACAACTTGCCGTGATCTGCTACAGCCCTGACTTT

GAGAAAAAGAAGCCTGAGTTCTTGGAGACCCTGCCTGAGAAGATGAAGCTCTACTCACAG

TTCCTGGGGAAGCGGCCCTGGTTTGCAGGGGACAAGATCACCTACGTGGATTTCATCACT

TATGATGTCCTGGACCAGCACCTTCTATTTGAACCCAAGTGCCTGGAGGCATTCCCAAAC

CTGAAGGACTTCATGTCCCGCTTTGAGGGCCTGAAGAAGATCTCTGCCTACATGAAGTCC

AGCCGCTTCCTCCCCAAACCTCTGTACACAAAGGTGGCTACATGGGGCAACAAG

>CL139.Contig8_All 240 365 PREDICTED: glutathione S-transferase Y1-like [Cricetulus griseus]

ATGCCTATGATACTGGGTTATTGGGACGTCCGTGGGCTTACTAACCCCATCCGCCTGCTC

CTGGAATACACAGACTCAAACTATGAGGAGAAGAGATACACCATGGGGGACGCTGCCCTA

CTTGAT

>CL139.Contig9_All 240 893 PREDICTED: glutathione S-transferase Y1-like [Cricetulus griseus]

ATGCCTATGATACTGGGTTATTGGGACGTCCGTGGGCTTACTAACCCCATCCGCCTGCTC

CTGGAATACACAGACTCAAACTATGAGGAGAAGAGATACACCATGGGGGACGCTCCCAAT

TATGACAGAAGCCAGTGGCTGGATGAGAAATTCAAGCTGGGCCTGGACTTTCCCAATCTG

CCCTACTTGATTGACGGGCCTCACAGGATCACCCAGAGCAACGCCATCCTGCGCTACCTT

GCCCGCAAGCACAACCTGTGTGGGGAGACAGAAGAGGAGATGATTCGTGTGGACATTTTG

GAGAACCAGGCTATGGACACTCGTGTACAACTTGCCGTGATCTGCTACAGCCCTGACTTT

GAGAAAAAGAAGCCTGAGTTCTTGGAGACCCTGCCTGAGAAGATGAAGCTCTACTCACAG

TTCCTGGGGAAGCGGCCCTGGTTTGCAGGGGACAAGATCACCTACGTGGATTTCATCACT

TATGATGTCCTGGACCAGCACCTTCTATTTGAACCCAAGTGCCTGGAGGCATTCCCAAAC

CTGAAGGACTTCATGTCCCGCTTTGAGGGACTGAAGAAGATATCTACCTACATGAAGTCC

AGCCAATTCCTTCGCAGTCCTCTGTATTTAAAACTGGCCCAGTGGAGCAATAAA

>CL140.Contig1_All 200 517 Protein LBH [Heterocephalus glaber]

ATGTCTGTATATTTCCCCATTCACTGCCCTGACTATCTGAGATCGGCCGAGATGACTGAG

GTAATGATGAACACCCCATCCATGGAGGAGATTGGCCTCAACCCCCGGAAGGATGGCCTT

TCCTATCAGATTTTCCCCGACCCGTCAGACTTTGACCGCTGCTGCAAACTGAAGGACCGC

CTGCCCTCCATAGTGGTGGAACCCACCGAGGGGGAGGTGGAGAGCGGGGAGCTCCGGTGG

CCCCCCGAGGAGTTCTTGGTTCAGGAGGATGAGCCGGACGACAACTGCGAAGAGACAGCG

AAAGAAAACAAGGAGCAG

>CL140.Contig2_All 98 394 Protein LBH [Heterocephalus glaber]

TACAGCCCTGACTATCTGAGATCGGCCGAGATGACTGAGGTAATGATGAACACCCCATCC

ATGGAGGAGATTGGCCTCAACCCCCGGAAGGATGGCCTTTCCTATCAGATTTTCCCCGAC

CCGTCAGACTTTGACCGCTGCTGCAAACTGAAGGACCGCCTGCCCTCCATAGTGGTGGAA

CCCACCGAGGGGGAGGTGGAGAGCGGGGAGCTCCGGTGGCCCCCCGAGGAGTTCTTGGTT

CAGGAGGATGAGCCGGACGACAACTGCGAAGAGACAGCGAAAGAAAACAAGGAGCAG

>CL140.Contig3_All 26 157 Protein LBH [Heterocephalus glaber]

ATGTCTGTATATTTCCCCATTCACTGCCCTGACTATCTGAGATCGGCCGAGATGACTGAG

GTAATGATGAACACCCCATCCATGGAGGAGATTGGCCTCAACCCCCGGAAGGATGGCCTT

TCCTTCAGTTTG

>CL141.Contig1_All 2 313 minus strand PREDICTED: coiled-coil domain-containing protein 167 [Otolemur garnettii]

TGGGGGATCCCTGCAAGCAACATGACTAAAAAGAAGCGGGAGAATCTGGGCGTCGCTCTC

GAGATTGATGGGCTGGAGGAAAAGTTGTCCCAGTGTCGGAGAGACCTGGAGGCTGTGGAC

TGCAGGCTCCACAGGGCAGAGCTGAGCCCAGAGGACAGGAGGTCTCTGGAGAAGGAGAAA

AACAGCCTAATGAGCAGAGCCTCCAACTACGAGAAGGAACTAAAATTGCTTCGGCAAGAG

AACCGGAAGAACATGCTGCTCTCGGTGGCCATCTTCATCCTCCTGGCCCTGGTCTATGCC

CACTGGACCACG

>CL141.Contig2_All 21 380 minus strand hypothetical protein EGK_14857 [Macaca mulatta] >gi|355748522|gb|EHH53005.1| hypothetical protein EGM_13557 [Macaca fascicularis]

ATGACTAAAAAGAAGCGGGAGAATCTGGGCGTCGCTCTCGAGATTGATGGGCTGGAGGAA

AAGTTGTCCCAGTGTCGGAGAGACCTGGAGGCTGTGGACTGCAGGCTCCACAGGGCAGAG

CTGAGCCCAGAGGACAGAGAAGGAACTAAAATTGCTTCGGCAAGAGAACCGGAAGAACAT

GCTGCTCTCGGTGGCCATCTTCATCCTCCTGGCCCTGGTCTATGCCCACTGGACCACGTG

AGTCTGCAATGCCCCGCAGCCACTGCAGGCTTCCCCTCGGCTCCTTAATTGGGACCAAGC

AGGCCCTTCAAGTCTCAAGAGGACTACAGTGCCGGGGTGCTTCCCCCACCTAGGCCTTGG

>CL142.Contig2_All 1 1080 minus strand PREDICTED: plakophilin-2 [Saimiri boliviensis boliviensis]

GAAGCCTTACTCACCTTGACTGAGAATATCATCATCCCCTTCTCGGGGTGGCCTGAAGGA

GACTACCCAAAAGCAAATGGTTTGCTGGATTTTGATATATTCTACAACGTCACCGGATGC

CTAAGAAACATGAGCTCAGCTGGCCCTGATGGGAGGAAAGTGATGAGAAGGTGTGATGGA

CTCATTGACTCTCTGGTCCATTATGTCAGAGGAACCATTGCAGATTACCAGCCAGATGAC

AAGGCAACAGAGAATTGTGTGTGCATTCTTCATAACCTCTCCTACCAGCTGGAGGCAGAG

CTCCCAGAGAAGTATTCCCAGAGTATCTATATTCAAAACCGGAATATCCAGACTGACAAC

AACAAAAGTATTGGGTGTTTTGGCAGTCGAAGCAGGAAAGTAAAAGAGCAATACCAGGAT

GTGCCAATGCCAGAGGAAAAGAGCAACCCCAAGGGTGTGGAGTGGCTGTGGCATTCCATT

GTGATAAGGATGTATTTGTCCTTGATTGCCAAGAGCATCCGAAATTACACACAGGAAGCA

TCCTTAGGAGCTCTCCAGAATCTCACAGCAGGGAGTGGCCCGATGCCAACATCAGTAGCC

CAGACGGTTGTCCAGAAGGAAAATGGCCTCCAGCACACCCGAAAGATGCTGCACATTGGT

GATCCAAGCGTGAAAAAGACTGCAGTCTCCCTCCTGAGAAATCTGTCTCGGAATCTTTCT

CTGCAGAATGAAATTGCCAAAGAAACCTTACCCGATTTGGTGTCTATAATTCCCGACACA

GTCCCAAGTAATGACCTTCTCATTGAAACTACAGCCTCCGCCTGTTACACCCTCAACAAT

ATAATCCAAAACAGTTACCAGAATGCACGAGACCTTCTGAACACAGGGGGCCTGCAGAAA

ATTATGACCATCAGCACAGGCGACGCCTATGCCCCCAGCAAAGCCAGCAAGGCTGCCTCT

GTGCTGCTGTATTCTCTGTGGGCACACACGGAGCTCCACAACGCCTACAAGAAGGCTCAG

TTTAAGAAGACAGATTTTGTCAACAGCCGGACTGCCAAAGCCTACCACTCCCTTAAAGAC

>CL143.Contig1_All 70 2604 vav 1 guanine nucleotide exchange factor [Sus scrofa]

ATGGAGCTGTGGCGCCAGTGCACCCACTGGCTCATCCAGTGCCGGGTGCTGCCACCCAGC

CACCGCGTGACCTGGGAGGGGGCCCAGGTGTGCGAGCTGGCGCAGGCCCTCCGGGATGGC

GTCCTCCTGTGCCAGCTGCTCAACAACCTGCTGCCCCAGGCCATCAACCTGCGAGAGGTC

AACCTGCGCCCCCAGATGTCTCAGTTCCTGTGCCTTAAGAACATCCGGACCTTCCTGTCC

ACCTGTGGAGAGAAGTTCGGCCTCAAGCGGAGTGAGCTCTTTGAAGCCTTCGACCTCTTT

GATGTGCAGGATTTTGGAAAGGTCATCTGCACCCTCTCTGCTCTGTCTTGGACCCCGATC

GCCCAGAACAAGGGGATCATGCCCTTCCCCACTGAGGAGGACAGTTTGGGTGACGAAGAC

ATCTACAGTGGCCTGTCTGACCAGATTGACGACACTGCGGAGGAGGATGAGGACCTGTAC

GACTGCGTGGAGAACGAGGAGGCCGAGGGCGACGAGATCTACGAGGACCTCATGCGCTCG

GAGCCCGTGCCCATGCCGCCCAAGATGACGGAGTACGACAAGCGGTGCTGCTGCCTGCGG

GAGATCCAGCAGACGGAGGAGAAGTACACGGACACCCTGGGCTCCATCCTGCAGCACTTC

ATGAAACCCTTGCAGCGGTTCCTCACGCCCCAGGACATGGAGAACATCTTTGTCAACATC

GAGGACCTGCTTCGTGTGCACACCCTCTTCCTAAAGGAGATGAAGGAGGCCCTGGCCACC

CCTGGATCACCCACCCTGTACCAAGTCTTCATCAAATACAAGGAGAGGTTCCTTGTCTAT

GGCCGCTACTGCAGCCAGGTGGAGTCTGCCAGTAAGCACCTGGACCAAGTGGCCACGGCC

CGGGAGGACGTGCAGATGAAGCTGGAGGAATGTTCTCAGCGCGCCAACAACGGCAGGTTC

ACCCTGCGGGACCTGCTCATGGTGCCCATGCAGCGGGTGCTCAAGTACCACCTCCTTCTC

CAGGAGCTGGTGAAGCATACGCAGGACACAGTGGACAAGGAGAACCTGCGGCTGGCGCTG

GACGCCATGAGGGACCTGGCGCAGTGCGTGAATGAGGTCAAGAGGGACAACGAGACCCTG

CGGCAGATCACCCACTTCCAGCTGTCCATTGAGAACCTGGGCCAGTCTCTGGCCAACTAT

GGCCGGCCCAAGATCGACGGGGAGCTCAAGATCACTTCCGTGGAAAGGCGCACCAAGACG

GACAGGTACGCGTTCCTGCTGGACAAAGCTCTGCTCATCTGCAAGCGCCGGGGGGACTCC

TACGACCTCAAGGTCTTTGTGAACCTGCACAGCTTCCAGATCCGGGATGACTCCTCCGGA

GACCGAGAGAACAAGAAGTGGAGCCACATGTTTCTCCTGATCGAGGACCAAGGTGCCCAG

GGCTATGAGCTGTTCTTCAAGACTCGAGAGCTGAAGAAGAAGTGGATGGAGCAGTTTGAG

ATGGCCATCTCCAACATCTACCCAGAGAACGCCACCGCCAATGGGCACGACTTTCAGATG

TTCTCCTTTGAGGACACCACCTCCTGCAAAGCCTGCCAGATGCTGCTCAGAGGCACCTTC

TACCAGGGCTACCGCTGCAATCGGTGCCGGGCACCTGCACACAAGGAGTGTCTGGGGAGG

GTCCCTCCGTGTGGCCGACACGGGCAAGACTGTGCCGGAACTATGAGGAAGGATAAGCTG

CATCGAAGGGCTCAGGACAGAAAAAGGAGTGAACTCGGCCTGCCTAAGATGGAGGTGTTT

CAGGAGTACTACGGGCTCCCCCCGCCTCCCGGAGCCTTCGGGCCTTTTCTCCGCCTCAGC

CCCGGTGACATCGTGGAGCTGACGAAGGCCGAGGCTGAGCAGAACTGGTGGGAGGGCAGG

AATACAGCTACCAACGAAGTCGGCTGGTTTCCCTGCAACAGGGTGAAGCCCTATGTCCAC

GGCCCTCCTCAGGACCTGTCTGTCCATCTCTGGTACGCTGGCCCCATGGAGCGCGCGGGC

GCAGAGAACATCCTCACCAACCGCTCAGACGGGACCTTCTTGGTGCGGCAGAGGGTGAAA

GACTCAGCGGAATTTGCCATCAGCATTAAGTATAATGTCGAGGTCAAGCACATCAAAATC

ATGACGTCAGAAGGACTGTACCGGATCACCGAGAAGAAGGCTTTCCGGGGCCTTGTGGAG

CTGGTGGAATTTTACCAGCAGAACTCCCTGAAGGATTGCTTCAAGTCCCTGGACACCACC

TTGCAGTTCCCCTACAAGGAGCCCGAGAGGAGAGCCATCAGCAAGCCAGCAGCAGGGAGC

GCCAAGTACTTTGGCACAGCCAAAGCCCGCTATGACTTCTGTGCCCGGGACCGATCGGAG

CTGTCCCTTAAGGAGGGCGACATCATCAAGATCCTCAACAAAAGGGGGCAGCAAGGCTGG

TGGAGAGGGGAGATCTACGGCCGGGTCGGCTGGTTCCCTTCCAACTATGTAGAAGAAGAT

TACTCTGAATACTGC

>CL143.Contig2_All 1 183 PREDICTED: guanine nucleotide exchange factor VAV2 isoform 1 [Papio anubis]

CAGAATTACCTCGGCAACCCTGCCCCTCCCGGGAAGCCCGTGTTAACCTTCCAGACGGGC

GACGTGATCGAGCTGCTGCGGGGCGACCCTGAGTCCCAGTGGTGGGAGGGCCGGCTGGTG

CACAGCAGGAAGTCAGGGTGCTTCCCCAGCTCATCTGTGAAGCCCTGCCCTGTGGACGGA

AGG

>CL143.Contig3_All 3 2261 Vav 2 guanine nucleotide exchange factor [Homo sapiens]

CTGAGGAGCAGCGAGCTGTTTGACCCCTTTGACCTCTTTGACGTCCGAGACTTCGGGAAG

GTCATTTCCGCGGTGTCCCGACTGTCCCTGCACAGCATCGCACAGACCAAAGGCATCAGG

CCTTTCCCCTCCGAGGAGACTGCAGAGAGTGACGATGACGTCTACCGCAGCCTGGAGGAG

CTGGCTGATGAGCATGACCTGGGTGAGGACATCTACGACTGTGTCCCGCGGGAGGACGAA

GGTGACGACATCTACGAGGACATCATCAAGGTGGAGGTGCGGCAGCCTATGAAAATGGGC

ATGACAGAGGATGACAAGAGGAGCTGTTGCCTGCTGGAGATCCACGAGACCGAGGCCAAG

TACTGCCGGACCCTGGAGGACATCGAGAAGAACTATATGGGCCCGCTGCGGCTGGTGCTG

AGCCCTGTGGACATGGCAGCCGTCTTCATCAACCTGGAGGACCTGATGAAGGTGCACCAC

AGCCTCCTGAGGGCTATCGACGTGTCCATGATGGCCGGGGGCAGCACACTGGCCAAGGTC

TTCCTCGAGTTCAAGGAAAGGCTCCTCATCTACGGGGATTACTGCAGCCACCTGGAGCGT

GCCCAGACCACACTCAGCCAGCTCCTCACCAGCCGTGACGACTTCAGGCAGAAAGTAGAG

GAGTGCACGCTGAAGGTCCAGGACGGCAAGTTCAAACTGCAGGACCTGCTGGTGGTGCCC

ATGCAGCGGGTGCTCAAGTACCACCTTCTGCTCAAGGAGCTCCTGAGCCATTCTGCTGAC

CGGCCCGAGAGGCAGCAGCTCAAAGAAGCCCTGGAAGCCATGCAGGACTTGGCCATGTAC

ATAAACGAGGTGAAGCGGGACAAGGAGACCTTGAAGAAGATCCGCGAGTTTCAGAGCTCC

ATAGAGAACCTGCAGGTGAAGCTGGAGGAGTACGGGAGGCCAAAGATCGACGGGGAGCTG

AAAGTCCGGTCCATAGTCAACCACACCAAGCAAGACAGGTACTTGTTCCTGTTTGACAAG

GTGGTCATCGTCTGCAAGAGGAAGGGCTACAGCTACGAGCTGAAGGAGGTCATCGAGCTG

CTGTCCCACAAGATGACCGACGACCCCATGCACAACAAGGACATCAAGAAGTGGTCCTAC

GGCTTCTACCTGATTCACCTCCAAGGAAAGCAGGGCTTCCAGTTCTTCTGCAAGACGGAG

GACATGAAGCGGAAGTGGATGGAGCAGTTTGAGATGGCCATGTCCAACATCAAGCCAGAC

AAAGCTAACGCCAACCACCACAGCTTCCAGATGTACACGTTCGACAGGACCACCAGCTGC

AGAGCCTGCGGGATGTTCCTCAGGGGCACCTTCTACCAGGGTTACCTGTGCACCAGGTGT

GGCGTGGGGGCACACAAGGAGTGCCTGGAGGTGATTCCCCCCTGCAGGATCAGTTCCCCG

GCCGACCTGGATGCCTCCCCGGGAGCAGGACCTGGTCCCAAGATGGTGGCTGTGCAGAAT

TACCTCGGCAACCCTGCCCCTCCCGGGAAGCCCGTGTTAACCTTCCAGACGGGCGACGTG

ATCGAGCTGCTGCGGGGCGACCCTGAGTCCCAGTGGTGGGAGGGCCGGCTGGTGCACAGC

AGGAAGTCAGGGTGCTTCCCCAGCTCATCTGTGAAGCCCTGCCCTGTGGACGGAAGGCCG

CCCGTTAGCCGGCCGCCTTCCCGGGAGATCGACTACACCGTGTACCCCTGGTTTGCTGGC

AACATGGAGAGGCAGCAGACGGACAGCCTGCTCAAGGCCCACGCCAGCGGCACCTACCTC

ATCAGGGAGCGGCCTGCCGAGGCCGAACGCTTCGCCATAAGCATCAAGTTCAACGATGAG

GTGAAGCACATCAAAGTGGTGGAGAAAGACAACTGGATTCACATCACAGAAGCTAAGAAG

TTCGAAAGCCTCTTGGAGCTGGTGGAGTACTACCAGGGCCACTCTCTCAAGGAGAGCTTC

AAGCAGCTGGACACCACGCTCAAATACCCCTACAAGTCCCGGGAGCGCCCTGCCTCCAGG

ACCTCCAGCCGGTCCCCAGTGTTTACTCCCCGGGTCATCGGCACGGCTGTGGCCAGGTAC

AACTTCGCTGCGCGGGACATGCGGGAGCTCTCTCTGCGGGAGGGCGACGTGGTGAAGATC

TACAGCCGCATAGGTGGGGACCAGGGCTGGTGGAAAGGCGAGACAAACGGACGGATCGGC

TGGTTCCCTTCGACATACGTGGAAGAGGAGGGTGTCCAG

>CL144.Contig1_All 168 314 hypothetical protein PANDA_001527 [Ailuropoda melanoleuca]

AGCCAGGGCATCCAGGGCTGGAGTGGAGCAGACACTGTCCATGGAGCTGGTGGACAAGGT

GGACAGGGGGCTGCGGTGATGGCGCAGTTTGACACTGAATACCAGCGCCTGGAGGCATCC

TATAGTGATTCCCCTCCAGGGGAGGAG

>CL144.Contig2_All 150 296 hypothetical protein PANDA_001527 [Ailuropoda melanoleuca]

AGCCAGGGCATCCAGGGCTGGAGTGGAGCAGACACTGTCCATGGAGCTGGTGGACAAGGT

GGACAGGGGGCTGCGGTGATGGCGCAGTTTGACACTGAATACCAGCGCCTGGAGGCATCC

TATAGTGATTCCCCTCCAGGGGAGGAG

>CL147.Contig1_All 2 2278 minus strand PREDICTED: polyribonucleotide nucleotidyltransferase 1 [Oryctolagus cuniculus]

CGGAATCCGGCGCTCACGTATTTGCAGATGCGAGCACTATCGGGTAGCGTGGGGTCCCGA

GCTGTGGCTGTGGACCTAGGCAACAGAAAAATAGAAATATCCTCTGGGAAACTGGCCAGA

TTTGCAGATGGCTCTGCTGTAGTACAGTCAGGTGACACTGCAGTGATGGTCACAGCTGTC

AGTAAAACAAAACCTTCACCTTCCCAGTTCATGCCTTTGGTGGTTGACTACAGACAGAAG

GCTGCTGCAGCAGGTAGAATTCCCACAAACTATCTTAGAAGAGAGATTGGTTCTTCTGAT

AAAGAAATTCTTACAAGTCGAATAATAGATCGTTCAATTAGACCTCTCTTTCCCGCTGGC

TACTTTTATGATACACAAGTCCTTTGTAATCTGTTAGCAGTAGATGGTATTAATGAACCT

GATGTCCTAGCAATTAATGGTGCTTCTGTAGCCCTCTCATTATCAGATATTCCTTGGAAT

GGACCTGTTGGGGCAGTACGAATAGGAATGATTGATGGGGAATGTATTGTTAATCCAACA

AGAAAAGAAATGTCTTCAAGTACTTTAAATTTAGTGGTTGCTGGAGCACCTAAAAGTCAG

ATTGTCATGTTGGAAGCCTCTGCAGAGAACATTTTACAGCAAGACTTTTGCCATGCTATC

AAAGTGGGAGTGAAACACACCCAACAAATAATTCAGGGCATCCAGCAGTTGGTGAAAGAA

ATTGGTGTTACCAAGAGGACACCTCAGAAGATATTTACCCCTTCACAAGAGATTGTGAAA

TATGTTCATAAACTTGCCATGGAGAAACTCTATGCAGTTTTTACAAATTATGAACATGAT

AAAATTTCCAGAGATGAAGCTGTTAACAAGATAAGATTAGATACAGAGGAACAACTAAAG

GAAACATTTCCAGATGTCGATCCATATGAAATAATAGAATCCTTCAATGTTGTTGCAAAG

GAGGTTTTCAGAAGTATTATTTTGAATGAATACAAAAGGTGTGATGGACGCGATTTGACT

TCACTTAGGAATATAAGTTGTGAGGTAGATATGTTTAAAACTCTTCATGGATCAGCATTA

TTTCAAAGGGGACAAACACAGGTGCTTTGTACTGTTACGTTTGATTCATTAGAATCTAGT

ATTAAATCAGACCGAATTATAACAGCAATAAATGGGATAAAAGACAAAAATTTCATGCTA

CACTATGAGTTTCCTCCTTATGCAACTAATGAAATTGGCAAAGTCACTGGCATAAATAGA

AGAGAGCTTGGGCATGGTGCTCTTGCTGAGAAAGCTTTGTATCCTGTTATTCCCAAAGAT

TTTCCTTTTACCATAAGAGTGACATCTGAAGTCTTAGAGTCGAACGGGTCATCTTCTATG

GCATCTGCATGTGGTGGAAGTTTGGCATTAATGGATGCAGGGGTTCCAATTTCATCTGCT

GTTGCAGGTGTAGCAATAGGATTGGTCACCAAAAGCAATCCTGAGACGGGTGAAATAGAA

GATTATCGTTTGCTGACAGATATTCTGGGAATTGAAGATTACAATGGAGACATGGATTTC

AAAATAGCTGGTACTAATAAGGGAATAACTGCATTACAGGCTGATATTAAATTACCTGGA

ATACCAATGAAAATTGTAATGGAAGCCATTCAGCAAGCATCAGTGGCAAAGAAAGAGATA

CTACAGATTATGAACAAAACTATTTCAAAACCTCGAGCATCTAGAAAAGAAAATGGACCT

GTTGTAGAAACTGTTCAGGTTCCATTATCAAAACGAGCAAGATTTGTTGGACCAGGTGGA

TATCATTTAAAAAAACTTCAAGCTGAAACAGGTGTAACTATTAATCAGTTGGATGAAGAA

ACATTTTCTGTATTTGCACCAACACCCAGTGCAATGCATGAAGCAAAAGAATTCATTAGT

GAAATCTGCAAAGATGATCAAGAGCAACAATTAGAGTTTGGAGCAGTTTATACTGCCACA

ATAACTGAAATCAGAGACACTGGAGTAATGGTAAAACTATATCCAAATATGACTGCTGTG

CTTCTTCATAACACACAACTTGATCAACGAAAGATTAAACATCCCACTGCCCTAGGATTA

GAAGTTGGCCAAGAAATTCAGGTGAAATACTTCGGTCGTGATCCAGCTGATGGAAGAATG

AGGCTTTCTCGTAAAGTGCTTCAGTCTCCAGCTACAAATGTTGTCAAAACTCTAAATGAC

AGAAGCAGTATTGTAATGGGAGAACCTATTTCACAAACATCATCTAATTCTTCCCAG

>CL147.Contig2_All 23 271 minus strand PREDICTED: polyribonucleotide nucleotidyltransferase 1, mitochondrial [Saimiri boliviensis boliviensis]

ATTACTCCAGTGCTTCTTCATAACACACAACTTGATCAACGAAAGATTAAACATCCCACT

GCCCTAGGATTAGAAGTTGGCCAAGAAATTCAGGTGAAATACTTCGGTCGTGATCCAGCT

GATGGAAGAATGAGGCTTTCTCGTAAAGTGCTTCAGTCTCCAGCTACAAATGTTGTCAAA

ACTCTAAATGACAGAAGCAGTATTGTAATGGGAGAACCTATTTCACAAACATCATCTAAT

TCTTCCCAG

>CL148.Contig1_All 353 1003 protein CDV3 homolog isoform a [Macaca mulatta]

ATGGCTGAGACGGAGGAGCGGAGCCTAGACAACTTCTTTGCTAAGAGGGATAAGAAGAAG

AAGAAGGAGCGGAGCAGTCGGGCGGCGAGCGCCGCAGGCGCGGCGGGCAACGCGGGCGGG

AGCAGCGGAACGGCGGGCGGCGGGCCGGGCGCGGGGACCAGGCCGGGCGACGGCGGGACG

GCAGGCACAGGGTCCCGGCCGGGCGATGGAGGGACCGCAGGCACGGGGGCCACGGGCCCC

GGGGCCGCCACCAAGGCCGTGACGAAGGATGAAGATGAATGGAAAGAATTTGAGCAGAAA

GAGGTTGATTACAGCGGACTCAGAGTTCAGGCAATGCAGATAAGTGAAAAAGAAGAAGAT

GACAATGAAAAGAGAGAAGATCCAGGTGATAACTGGGAAGAAGGAGGAGGTGGTGGAGGT

ATAGAAAAATCTTCAGGTCCCTGGAATAAAACAGCTCCGGTACAAGCGCCTCCTACTCCA

GTAGTTGTTACAGAAACCCCAGAACCAGCAATGACTAGTGGTGTGTATAGGCCTCCTGGG

GCCAGGCTAACCACAACAAGGAAAACGCCACAAGGACCACCAGAAATATACAGCGACACA

CAGTTCCCATCCCTGCAGTCCACTGCCAAGCATGTAGAAAGCCGAAAATAC

>CL148.Contig2_All 353 1012 minus strand protein CDV3 homolog isoform a [Macaca mulatta]

ATGGCTGAGACGGAGGAGCGGAGCCTAGACAACTTCTTTGCTAAGAGGGATAAGAAGAAG

AAGAAGGAGCGGAGCAGTCGGGCGGCGAGCGCCGCAGGCGCGGCGGGCAACGCGGGCGGG

AGCAGCGGAACGGCGGGCGGCGGGCCGGGCGCGGGGACCAGGCCGGGCGACGGCGGGACG

GCAGGCACAGGGTCCCGGCCGGGCGATGGAGGGACCGCAGGCACGGGGGCCACGGGCCCC

GGGGCCGCCACCAAGGCCGTGACGAAGGATGAAGATGAATGGAAAGAATTTGAGCAGAAA

GAGGTTGATTACAGCGGACTCAGAGTTCAGGCAATGCAGATAAGTGAAAAAGAAGAAGAT

GACAATGAAAAGAGAGAAGATCCAGGTGATAACTGGGAAGAAGGAGGAGGTGGTGGAGGT

ATAGAAAAATCTTCAGGTCCCTGGAATAAAACAGCTCCGGTACAAGCGCCTCCTACTCCA

GTAGTTGTTACAGAAACCCCAGAACCAGCAATGACTAGTGGTGTGTATAGGCCTCCTGGG

GCCAGGCTAACCACAACAAGGAAAACGCCACAAGGACCACCAGAAATATACAGCGACACA

CAGTTCCCATCCCTGCAGTCCACTGCCAAGCATGTAGAAAGCCGAAACAGGTACTTAAAA

>CL148.Contig3_All 353 1147 protein CDV3 homolog isoform b [Macaca mulatta]

ATGGCTGAGACGGAGGAGCGGAGCCTAGACAACTTCTTTGCTAAGAGGGATAAGAAGAAG

AAGAAGGAGCGGAGCAGTCGGGCGGCGAGCGCCGCAGGCGCGGCGGGCAACGCGGGCGGG

AGCAGCGGAACGGCGGGCGGCGGGCCGGGCGCGGGGACCAGGCCGGGCGACGGCGGGACG

GCAGGCACAGGGTCCCGGCCGGGCGATGGAGGGACCGCAGGCACGGGGGCCACGGGCCCC

GGGGCCGCCACCAAGGCCGTGACGAAGGATGAAGATGAATGGAAAGAATTTGAGCAGAAA

GAGGTTGATTACAGCGGACTCAGAGTTCAGGCAATGCAGATAAGTGAAAAAGAAGAAGAT

GACAATGAAAAGAGAGAAGATCCAGGTGATAACTGGGAAGAAGGAGGAGGTGGTGGAGGT

ATAGAAAAATCTTCAGGTCCCTGGAATAAAACAGCTCCGGTACAAGCGCCTCCTACTCCA

GTAGTTGTTACAGAAACCCCAGAACCAGCAATGACTAGTGGTGTGTATAGGCCTCCTGGG

GCCAGGCTAACCACAACAAGGAAAACGCCACAAGGACCACCAGAAATATACAGCGACACA

CAGTTCCCATCCCTGCAGTCCACTGCCAAGCATGTAGAAAGCCGAAAGGATAAAGAAATG

GAGAAGAACTTTGAAGTAGTAAGACACAAAAATAGAGGTAGGGATGAGGTTTCAAAAAAC

CAGGCCCTTAAACTTCAGCTAGACAACCAGTATGCTGTGCTTGAAAATCAGAAAAGCAGC

CACACACAGTACAAT

>CL149.Contig1_All 1 2073 minus strand PREDICTED: DDB1- and CUL4-associated factor 8 [Otolemur garnettii]

CTCGAGCACATCGTCGCAAAGGAGGCCGGAAAGCGTGGCGGCGCAGGCGCAAGCGCAGAG

AGCGGAGGCGGTGGTGGTGGCGGCCGCTGGCCAGTGTAAGATGGCGGCGGCGTTGGTGGC

GGCAGTGGACGGGACGGCTGGGCCTGGAACAGTCGGAGCAGCCGCTGGCAGAAGGATGAC

CCAAGACGGGAAGCGGGCTGCTGAGCCTCGCCGGCTCCCGTGCTTGTAACTGCCCCAGCC

GGACACCCCCCCCTTGACCTACCCGGCTGGCTATAGCAAACATCATTCAAGATGTCCAGC

AAAGGAAGCAGCACAGATGGCAAAACAGACTTAGCTAATGGAAGCCTGTCTAGCAGTCCA

GAGGAGATGTCTGGTGCTGAAGAGGGAAGAGAGACATCCTCAGGCATTGAAGTAGAGGCC

TCAGACCTGAGTTTAAGCTTGACTGGGGATGATGGTGGCCCCAATCGCACCAGTACAGAA

AGTCGAGGCACAGACACAGAAAGCTCAGGTGAAGAAAAGGACTCTGACAGCATGGAGGAC

ACTGGCCATTACTCCATCAATGATGAAAACCGAGTTCGTGACCGCTCAGAGGAAGAGGAA

GAAGAGGAGGAAGAACAACAACCTCGGCGCCGTGTACAACGCAAGCGAGCTAACCGTGAC

CAGGACTCATCAGACGATGAGCAGGCCTTGGAGGATTGGGTGTCCTCAGAGACAACAGCC

CTGCCCCGACCTCGATGGCAGGCCCTCCCTGCCCTTCGGGAACGGGAGCTGGGTTCAAGT

GCCCGCTTTGTGTATGAAGCCTGTGGGGCAAGAGTCTTTGTACAGAGGTTCCGCCTGCAG

CATGGGCTTGAGGGCCATACTGGTTGTGTCAATACCCTGCACTTTAACCAGCGCGGCACC

TGGCTGGCCAGTGGCAGCGATGACCTGAAGGTGGTAGTGTGGGATTGGGTACGGCGGCAG

CCAGTACTGGACTTCGAGAGTGGCCACAAAAGTAATGTCTTTCAGGCCAAATTCCTTCCC

AATAGTGGTGATTCCACCCTGGCGATGTGTGCCCGGGATGGGCAGGTTCGGGTAGCAGAA

CTGTCTGCCACACAGTGCTGCAAGAATACAAAGCGTGTGGCCCAGCACAAGGGAGCATCC

CATAAGTTGGCCCTGGAACCAGACTCTCCCTGTACGTTCCTGTCTGCAGGTGAAGATGCA

GTTGTCTTCACTATTGACCTGAGACAAGATCGGCCAGCTTCGAAACTGGTGGTGACAAAA

GAGAAAGAGAAGAAAGTTGGGCTATATACAATCTATGTGAATCCTGCCAATACCCACCAG

TTTGCAGTAGGTGGACGAGATCAATTTGTAAGGATTTATGACCAGAGGAAAATTGATGAG

AACGAGAACAATGGTGTACTCAAGAAATTCTGTCCTCATCACCTGGTGAACAGTGAGTCC

AAAGCAAACATCACCTGTCTTGTGTACAGCCACGACGGCACAGAGCTCCTGGCCAGTTAT

AATGATGAAGACATTTACCTCTTCAACTCCTCTCACAGTGATGGGGCCCAGTATGTTAAG

AGATATAAGGGCCACAGAAATAATGCCACAGTAAAAGGCGTCAATTTCTATGGCCCCAAG

AGTGAATTTGTGGTGAGCGGTAGTGACTGCGGGCACATCTTCCTCTGGGAAAAATCATCC

TGCCAGATCATTCAGTTCATGGAGGGGGACAAGGGAGGTGTGGTCAACTGTCTTGAGCCC

CACCCTCATCTGCCAGTGTTGGCAACCAGTGGCCTAGACCATGATGTGAAGATCTGGGCA

CCCACAGCTGAAGCTTCTACTGAGCTGACTGGGTTAAAGGATGTGATTAAGAAGAACAAG

CGAGAGCGGGATGAAGATAGCTTACACCACACTGACCTGTTTGATAGCCACATGCTCTGG

TTTCTTATGCATCACCTGAGACAGAGACGCCATCACCGGCGCTGGCGAGAACCTGGGGTT

GGGGCCACAGACGCGGACTCTGACGAGTCTCCCAGCTCCTCAGATACATCGGACGAGGAG

GAGGGCCCCGACCGGGTGCAATGCATGCCATCC

>CL149.Contig2_All 267 2147 minus strand PREDICTED: DDB1- and CUL4-associated factor 8 [Otolemur garnettii]

CCTTCAGTGAATCTGCAGACCTATTTTCTCAGGAGTTCAGCCTGGCCTTACTTCAGTGAT

AAAAGGAGGAAAGGCTGGCTATAGCAAACATCATTCAAGATGTCCAGCAAAGGAAGCAGC

ACAGATGGCAAAACAGACTTAGCTAATGGAAGCCTGTCTAGCAGTCCAGAGGAGATGTCT

GGTGCTGAAGAGGGAAGAGAGACATCCTCAGGCATTGAAGTAGAGGCCTCAGACCTGAGT

TTAAGCTTGACTGGGGATGATGGTGGCCCCAATCGCACCAGTACAGAAAGTCGAGGCACA

GACACAGAAAGCTCAGGTGAAGAAAAGGACTCTGACAGCATGGAGGACACTGGCCATTAC

TCCATCAATGATGAAAACCGAGTTCGTGACCGCTCAGAGGAAGAGGAAGAAGAGGAGGAA

GAACAACAACCTCGGCGCCGTGTACAACGCAAGCGAGCTAACCGTGACCAGGACTCATCA

GACGATGAGCAGGCCTTGGAGGATTGGGTGTCCTCAGAGACAACAGCCCTGCCCCGACCT

CGATGGCAGGCCCTCCCTGCCCTTCGGGAACGGGAGCTGGGTTCAAGTGCCCGCTTTGTG

TATGAAGCCTGTGGGGCAAGAGTCTTTGTACAGAGGTTCCGCCTGCAGCATGGGCTTGAG

GGCCATACTGGTTGTGTCAATACCCTGCACTTTAACCAGCGCGGCACCTGGCTGGCCAGT

GGCAGCGATGACCTGAAGGTGGTAGTGTGGGATTGGGTACGGCGGCAGCCAGTACTGGAC

TTCGAGAGTGGCCACAAAAGTAATGTCTTTCAGGCCAAATTCCTTCCCAATAGTGGTGAT

TCCACCCTGGCGATGTGTGCCCGGGATGGGCAGGTTCGGGTAGCAGAACTGTCTGCCACA

CAGTGCTGCAAGAATACAAAGCGTGTGGCCCAGCACAAGGGAGCATCCCATAAGTTGGCC

CTGGAACCAGACTCTCCCTGTACGTTCCTGTCTGCAGGTGAAGATGCAGTTGTCTTCACT

ATTGACCTGAGACAAGATCGGCCAGCTTCGAAACTGGTGGTGACAAAAGAGAAAGAGAAG

AAAGTTGGGCTATATACAATCTATGTGAATCCTGCCAATACCCACCAGTTTGCAGTAGGT

GGACGAGATCAATTTGTAAGGATTTATGACCAGAGGAAAATTGATGAGAACGAGAACAAT

GGTGTACTCAAGAAATTCTGTCCTCATCACCTGGTGAACAGTGAGTCCAAAGCAAACATC

ACCTGTCTTGTGTACAGCCACGACGGCACAGAGCTCCTGGCCAGTTATAATGATGAAGAC

ATTTACCTCTTCAACTCCTCTCACAGTGATGGGGCCCAGTATGTTAAGAGATATAAGGGC

CACAGAAATAATGCCACAGTAAAAGGCGTCAATTTCTATGGCCCCAAGAGTGAATTTGTG

GTGAGCGGTAGTGACTGCGGGCACATCTTCCTCTGGGAAAAATCATCCTGCCAGATCATT

CAGTTCATGGAGGGGGACAAGGGAGGTGTGGTCAACTGTCTTGAGCCCCACCCTCATCTG

CCAGTGTTGGCAACCAGTGGCCTAGACCATGATGTGAAGATCTGGGCACCCACAGCTGAA

GCTTCTACTGAGCTGACTGGGTTAAAGGATGTGATTAAGAAGAACAAGCGAGAGCGGGAT

GAAGATAGCTTACACCACACTGACCTGTTTGATAGCCACATGCTCTGGTTTCTTATGCAT

CACCTGAGACAGAGACGCCATCACCGGCGCTGGCGAGAACCTGGGGTTGGGGCCACAGAC

GCGGACTCTGACGAGTCTCCCAGCTCCTCAGATACATCGGACGAGGAGGAGGGCCCCGAC

CGGGTGCAATGCATGCCATCC

>CL150.Contig1_All 9 437 PREDICTED: F-box only protein 4 [Pongo abelii]

ATGGCAGGGAGCGAGCCGCGCAGCGGAACCAGCTCCTCCCCGCCGCCCCCGAGCGACTGG

GGCCGCCTGGAGGCTGCCATCCTCAGCGGCTGGAGGACGTTCTGGCAGTCGGTGGGCAAG

GAGAGGACTGTGCGGACGGCCTCCCGAGACGAGGCGGACGAGGACACCAGCACCTTGACG

CAGCTGCCGATTGATGTCCAGCTATATATTTTGTCATTTCTTTCACCCCATGATCTGTGT

CAGCTGGGAAGTACAAATCATTATTGGAACGAAACTGTACGAGATCCAATTCTGTGGAGA

TACTTTCTGTTGCGAGATCTTCCTTCTTGGTCTTCTGTTGACTGGAAGTCTCTTCCAGAT

GTAGAAATCTTAAAAAAGCCTATATCTGAGGTCACTGACAATGCATCTTTTGATTACATG

GCAGTGTAT

>CL150.Contig2_All 3 245 minus strand PREDICTED: F-box only protein 4-like [Cricetulus griseus]

GGTTTGGAAGAACTGAATACATCTTTGGTGTTGAGCTTGATGTCTTCTGAGGAACTTAGT

CCAACTGCTGGTTTGCCTCACAGGCAGATTGATGGTATTGGATCAGGAGTCAGTTTTCAG

TTGAGCAACCAACATAAATTCAACATCCTGATATTGTATTCAACTACCAGAAAGGAAAGA

GACAGAGCAAGGGAAGAACATACAAGTGCAGTCAACAAGATGTTCAGCTTACAACGTACC

GGT

>CL153.Contig1_All 3 383 Pericentrin [Heterocephalus glaber]

GCTGAGGGCCTCCTGGAAGGGTTCAAAGTGGAGAAAGCAGATCTGCAGGAGGCTCTGGGC

CGCAAGGAGGAGTCGGAGCAGCAGCTGGTCCTGGAGCTGGAGAGCCTGGGGCGGCAGCTG

GAGCAGGCCGCCCAGGAGAAGGCAGCCCTGCAGGAGGAGCACTCCATCTTGCGGAGCCAG

AGGGAAGCTTGGGCTGCTGAGGCGGAAACAAGAGAAGCAGCACTGAAGAAGGAAGTGGAA

TCTCTAACCAGGGAGCAGGTGGAGGCCAGGGAGCAGGCAGAGAAGGACCGCGCAGCCTTG

CTCTCCCAGGTGCGGCTGTTAGAGTTGGAGCTGGAGGAGCAGCTCTCGCAACACCGTGGC

TGTGCCCGGCAGGCCGAGGAG

>CL153.Contig2_All 41 202 Pericentrin [Heterocephalus glaber]

GCACTGAAGAAGGAAGTGGAATCTCTAACCAGGGAGCAGGTGGAGGCCAGGGAGCAGGCA

GAGAAGGACCGCGCAGCCTTGCTCTCCCAGGTGCGGCTGTTAGAGTTGGAGCTGGAGGAG

CAGCTCTCGCAACACCGTGGCTGTGCCCGGCAGGCCGAGGAG

>CL154.Contig1_All 562 852 minus strand Envelope glycoprotein [Cricetulus griseus]

GAGAAAGGACCCCAACCCCAAACTAATGGTCGGAACCTGACCTCCCCACTTCCTCCGGAT

ACTCCTAACTTAACCCCTAGCCCCGACCAAACTCCCCAAGGGATTTCCCATATCTCAGGG

ATCCCTACTCTGGATACCCCGATCCCCACAACTAGGCCGGGCCAATCTCCTCAAAGACCA

GGTACTGGAGACCGGCTACTAAGCCTAATCCAAGGGGCTTTCACGGCCCTCAATCATTCA

GACCCAAACAAAACCCAAGAATGCTGGTTATGCTTGCTTTCACGACCCCCC

>CL154.Contig2_All 2 352 minus strand envelope polyprotein precursor [Felis catus]

TTATACTTTGACTTATGTGACTTAGTAGGAAACACCTGGAACCCTTCTGACCAGGAGCCT

TTCCCAGGTTATGGTTGTCATCACCCCGGGGGACGCATAGGGACTCAAAGTAAGAAATTT

TATGTTTGTCCATCGCACAAACGAGGGAAAAATTGTGGGGGACCCGCGGATGGTTTTTGT

GCCAGATGGGGTTGTGAGACCACTGGAACTGTCTATTGGAACCCCTCCTCCGATTGGGAC

TACATTACCCTCCGACGCGGTAAGACTCCCCATGGGTCTGCTTGCTATGATTCCAGCAAA

GTCACCCGCACTGGCTCCACTCCCGGGGGCAGATGCAACCCTCTAGTTCTA

>CL155.Contig1_All 1 612 PREDICTED: polypeptide N-acetylgalactosaminyltransferase 11-like [Cavia porcellus]

GTCTGGCTGGACGAGTACAAGGAGCAGTATTTCTCCTTAAGGCCTGATCTGAAGACCAAA

AGCTATGGAAATATCAGTGAGCGGGTTGAGCTGAGGAAGAGGCTGGGCTGTCAGTCGTTC

AGGTGGTACTTGGATCATGTCTACCCAGAGATGCAGATACCTGGGCCCAACGCCAGGCCC

CAGCAGCCCATTTTCATCAACAGAGGACCCAAGCGGCCCCGGGTCCTGCAGCGTGGCAGG

CTCTACCACTTCCAGACCAATAAATGTCTGGTGGCCCAGGGCCGCCCCAGCCAGAAGGGT

AGCCTGGTCGTGCTAAAGGCCTGCGACTACCGGGACCCGACGCAGATCTGGATTTACAAT

GAAGAGCACGAGTTGGTTTTAAATAACCTCCTTTGTCTAGACATGTCCGAGACCCGCTCC

TCAGACCCGCCTCGGCTCATGAAGTGCCACGGTTCGGGTGGATCCCAGCAGTGGACCTTT

GGGAAAAATAACTGGCTGTACCAGGTGTCCGTCGGACAGTGCCTGAGTGTGGTGGACACG

CTGGGTCCCAAGGGCTCCGTAGCCATGGCCATCTGTGATGGCTCCTCCTCACAGCAGTGG

CACCTGGAAGGG

>CL155.Contig2_All 1 285 PREDICTED: polypeptide N-acetylgalactosaminyltransferase 11-like [Cavia porcellus]

TACCGGGACCCGACGCAGATCTGGATTTACAATGAAGAGCACGAGTTGGTTTTAAATAAC

CTCCTTTGTCTAGACATGTCCGAGACCCGCTCCTCAGACCCGCCTCGGCTCATGAAGTGC

CACGGTTCGGGTGGATCCCAGCAGTGGACCTTTGGGAAAAATAACTGGCTGTACCAGGTG

TCCGTCGGACAGTGCCTGAGTGTGGTGGACACGCTGGGTCCCAAGGGCTCCGTAGCCATG

GCCATCTGTGATGGCTCCTCCTCACAGCAGTGGCACCTGGAAGGG

>CL156.Contig1_All 107 2176 PREDICTED: rho GTPase-activating protein 27-like isoform 1 [Papio anubis]

ATGGTGGACATGATTGCCAAACTGACCAGGAGGCAGAGTCGAGCCCTACGGGCACAGGCG

GACGACCCCCAAGAGCCCGTGTATGCGAACGTAGAGCGGCAACCTCGCGCCACCTCGCCG

CGCGCTGTCGCAGCCCCCGGCCCCAGCCCGGTGTGGGAGACGCACACGGACGCGGGCACC

GGACGCCTCTACTACTACAACCCCGACACGGGCGTGACCACTTGGGAGTCGCCCTTCGAG

GCTGCGGAGGGCCCCGCCAGCCCGGCCACCTCCCCGGCTTCAGTGGGCAGCCCCGAGAGC

CTCGAGGGCGAGTGGGGCCAGTACTGGGACGAGGAGAGCCGCAGGGTGTTCTTCTACAAC

GCGCTGACGGGCGAGACCGCCTGGGAGGACGACCCCGAAGACCAGCTGGAGATGCAGCCC

GGCCTGAGCCCCGGCAGCCCAATGGACCAGAGACCCCCCACCCCTGAGACAGACTACCCC

GAATTGCTGACTAGTTACCCTGAAGAGGACTATTCCCCCGTGGGTTCCTTCAGTGAGCCC

AGCCCCACCTGTCCTTTGGCCGCACCCCCAGGCTGGTCCTGTCACATCAGCCCGGACAAG

CAGATGATCTATACCAACCACTTCACCCAAGAGCAGTGGATGAAGCTGGAGGACCAGCAG

GGGAAGTCATACTTCTACAACCCAGAGGACTCCTCTGTTCGGTGGGAGCTGCCCCAGGTA

ACCAACTGGTGAATGGAGCGCACTGGGGAGAGTGGAGGGGATTATATACGACAGCCTCTC

TCTCCCCCCCAGGTCCCCATCCCTGCCCCTCGAAGCATCTGCAAATCCAGCCAAGACAGT

GACACCCCAGCCCAGCCCAGCCCTCCAGAGGAAAAAATCAAGACTCTGGACAAGGCAGGA

GTACTCTATCGCACCAAAACTGTGGACAAGGGGAAGCGGCTCCGGAAGAAGCACTGGAGT

GCCTCCTGGACAGTGCTGGAGGGTGGCGTCCTCACCTTCTTCAAGGACTCAAAGACCTCA

GCTGCTGGCGGCCTGAGGCAGCCTTACAAGCTCTCCACTCCTGAGTACACCGTGGAGCTG

AAGGGGGCCACGCTCTCCTGGGCCCCCAAAGACAAATCCAGCAAGAAGAATGTGCTGGAG

CTGCAGAGCCGTGATGGCTCCGAGTACCTGATCCAGCATGACTCAGAGGCCATCATCAGC

ACCTGGCACAAAGCCATTGCCCAGGGCATCCAGGAGCTGTCTGCAGACCTGCCCGCTGAG

GAGGAAAGTGAGACCAGCAGCGCAGATTTCGGGTCCAGTGAGCGCCTGGGAAGCTGGCGG

GAGAGAGAGGATGACACTCGGTCCAGTGCAGCGGCCGGACCTGTTCTGAGCCCTGGAGGC

CAGGAGAGCGACTTGAGCAAGGTCCGGCACAAGCTCCGCAGGTTCCTACAGCGGCGGCCC

ACGCTACAATCGCTGCGGGAGAAGGGTTACATCAGAGATCAGGTGTTCGGCTGCGCGCTG

GCGGTGCTGTGTGAGCGCGAGAGGAGCTCGGTGCCGCGCTTCGTGCAGCAGTGTATCCGG

ACCGTCGAGGCCCGGGGGCTGGACATCGACGGGCTGTACCGCATCAGCGGAAACCTGGCC

ACCATCCAGAAGCTGCGCTATAGGGTGGACCACGATGAGCGTCTGGACCTGGACGATGGG

CGCTGGGAGGACGTTCATGTCATCACTGGCGCTCTGAAGCTCTTCTTCCGGGAGCTGCCC

GAGCCCCTCTTCCCCTTCTCGCATTTCAGCCAGTTCATCGCAGCCATCAAGCTACAGGAC

CAGGCACAGCGCAGCCGCTGTGTGCGAGACCTGGTGCGCTCACTGCCAACCCCCAACCAT

GACACGCTGCGGCTACTCTTCCAGCACCTGTGCAGGGTTATCCAGCACGGCGAGCAGAAC

CGCATGTCGGTGCAGAGCGTGGCCATAGTGTTTGGGCCTACGCTGCTGCGGCCTGAGACA

GAGGAGAACACCATGACCATGACCATGGTGTTCCAGAACCAAGTGGTGGAGCTCATCCTG

CAGCAGTGCTCAGATATCTTCCCGCCCCAC

>CL156.Contig2_All 278 2236 PREDICTED: rho GTPase-activating protein 27 isoform 2 [Otolemur garnettii]

CCTCCGCCAACTCCAGGAATTGCGGACGACCCCCAAGAGCCCGTGTATGCGAACGTAGAG

CGGCAACCTCGCGCCACCTCGCCGCGCGCTGTCGCAGCCCCCGGCCCCAGCCCGGTGTGG

GAGACGCACACGGACGCGGGCACCGGACGCCTCTACTACTACAACCCCGACACGGGCGTG

ACCACTTGGGAGTCGCCCTTCGAGGCTGCGGAGGGCCCCGCCAGCCCGGCCACCTCCCCG

GCTTCAGTGGGCAGCCCCGAGAGCCTCGAGGGCGAGTGGGGCCAGTACTGGGACGAGGAG

AGCCGCAGGGTGTTCTTCTACAACGCGCTGACGGGCGAGACCGCCTGGGAGGACGACCCC

GAAGACCAGCTGGAGATGCAGCCCGGCCTGAGCCCCGGCAGCCCAATGGACCAGAGACCC

CCCACCCCTGAGACAGACTACCCCGAATTGCTGACTAGTTACCCTGAAGAGGACTATTCC

CCCGTGGGTTCCTTCAGTGAGCCCAGCCCCACCTGTCCTTTGGCCGCACCCCCAGGCTGG

TCCTGTCACATCAGCCCGGACAAGCAGATGATCTATACCAACCACTTCACCCAAGAGCAG

TGGATGAAGCTGGAGGACCAGCAGGGGAAGTCATACTTCTACAACCCAGAGGACTCCTCT

GTTCGGTGGGAGCTGCCCCAGGTCCCCATCCCTGCCCCTCGAAGCATCTGCAAATCCAGC

CAAGACAGTGACACCCCAGCCCAGCCCAGCCCTCCAGAGGAAAAAATCAAGACTCTGGAC

AAGGCAGGAGTACTCTATCGCACCAAAACTGTGGACAAGGGGAAGCGGCTCCGGAAGAAG

CACTGGAGTGCCTCCTGGACAGTGCTGGAGGGTGGCGTCCTCACCTTCTTCAAGGACTCA

AAGACCTCAGCTGCTGGCGGCCTGAGGCAGCCTTACAAGCTCTCCACTCCTGAGTACACC

GTGGAGCTGAAGGGGGCCACGCTCTCCTGGGCCCCCAAAGACAAATCCAGCAAGAAGAAT

GTGCTGGAGCTGCAGAGCCGTGATGGCTCCGAGTACCTGATCCAGCATGACTCAGAGGCC

ATCATCAGCACCTGGCACAAAGCCATTGCCCAGGGCATCCAGGAGCTGTCTGCAGACCTG

CCCGCTGAGGAGGAAAGTGAGACCAGCAGCGCAGATTTCGGGTCCAGTGAGCGCCTGGGA

AGCTGGCGGGAGAGAGAGGATGACACTCGGTCCAGTGCAGCGGCCGGACCTGTTCTGAGC

CCTGGAGGCCAGGAGAGCGACTTGAGCAAGGTCCGGCACAAGCTCCGCAGGTTCCTACAG

CGGCGGCCCACGCTACAATCGCTGCGGGAGAAGGGTTACATCAGAGATCAGGTGTTCGGC

TGCGCGCTGGCGGTGCTGTGTGAGCGCGAGAGGAGCTCGGTGCCGCGCTTCGTGCAGCAG

TGTATCCGGACCGTCGAGGCCCGGGGGCTGGACATCGACGGGCTGTACCGCATCAGCGGA

AACCTGGCCACCATCCAGAAGCTGCGCTATAGGGTGGACCACGATGAGCGTCTGGACCTG

GACGATGGGCGCTGGGAGGACGTTCATGTCATCACTGGCGCTCTGAAGCTCTTCTTCCGG

GAGCTGCCCGAGCCCCTCTTCCCCTTCTCGCATTTCAGCCAGTTCATCGCAGCCATCAAG

CTACAGGACCAGGCACAGCGCAGCCGCTGTGTGCGAGACCTGGTGCGCTCACTGCCAACC

CCCAACCATGACACGCTGCGGCTACTCTTCCAGCACCTGTGCAGGGTTATCCAGCACGGC

GAGCAGAACCGCATGTCGGTGCAGAGCGTGGCCATAGTGTTTGGGCCTACGCTGCTGCGG

CCTGAGACAGAGGAGAACACCATGACCATGACCATGGTGTTCCAGAACCAAGTGGTGGAG

CTCATCCTGCAGCAGTGCTCAGATATCTTCCCGCCCCAC

>CL156.Contig3_All 278 2311 PREDICTED: rho GTPase-activating protein 27 isoform 2 [Otolemur garnettii]

CCTCCGCCAACTCCAGGAATTGCGGACGACCCCCAAGAGCCCGTGTATGCGAACGTAGAG

CGGCAACCTCGCGCCACCTCGCCGCGCGCTGTCGCAGCCCCCGGCCCCAGCCCGGTGTGG

GAGACGCACACGGACGCGGGCACCGGACGCCTCTACTACTACAACCCCGACACGGGCGTG

ACCACTTGGGAGTCGCCCTTCGAGGCTGCGGAGGGCCCCGCCAGCCCGGCCACCTCCCCG

GCTTCAGTGGGCAGCCCCGAGAGCCTCGAGGGCGAGTGGGGCCAGTACTGGGACGAGGAG

AGCCGCAGGGTGTTCTTCTACAACGCGCTGACGGGCGAGACCGCCTGGGAGGACGACCCC

GAAGACCAGCTGGAGATGCAGCCCGGCCTGAGCCCCGGCAGCCCAATGGACCAGAGACCC

CCCACCCCTGAGACAGACTACCCCGAATTGCTGACTAGTTACCCTGAAGAGGACTATTCC

CCCGTGGGTTCCTTCAGTGAGCCCAGCCCCACCTGTCCTTTGGCCGCACCCCCAGGCTGG

TCCTGTCACATCAGCCCGGACAAGCAGATGATCTATACCAACCACTTCACCCAAGAGCAG

TGGATGAAGCTGGAGGACCAGCAGGGGAAGTCATACTTCTACAACCCAGAGGACTCCTCT

GTTCGGTGGGAGCTGCCCCAGGTAACCAACTGGTGAATGGAGCGCACTGGGGAGAGTGGA

GGGGATTATATACGACAGCCTCTCTCTCCCCCCCAGGTCCCCATCCCTGCCCCTCGAAGC

ATCTGCAAATCCAGCCAAGACAGTGACACCCCAGCCCAGCCCAGCCCTCCAGAGGAAAAA

ATCAAGACTCTGGACAAGGCAGGAGTACTCTATCGCACCAAAACTGTGGACAAGGGGAAG

CGGCTCCGGAAGAAGCACTGGAGTGCCTCCTGGACAGTGCTGGAGGGTGGCGTCCTCACC

TTCTTCAAGGACTCAAAGACCTCAGCTGCTGGCGGCCTGAGGCAGCCTTACAAGCTCTCC

ACTCCTGAGTACACCGTGGAGCTGAAGGGGGCCACGCTCTCCTGGGCCCCCAAAGACAAA

TCCAGCAAGAAGAATGTGCTGGAGCTGCAGAGCCGTGATGGCTCCGAGTACCTGATCCAG

CATGACTCAGAGGCCATCATCAGCACCTGGCACAAAGCCATTGCCCAGGGCATCCAGGAG

CTGTCTGCAGACCTGCCCGCTGAGGAGGAAAGTGAGACCAGCAGCGCAGATTTCGGGTCC

AGTGAGCGCCTGGGAAGCTGGCGGGAGAGAGAGGATGACACTCGGTCCAGTGCAGCGGCC

GGACCTGTTCTGAGCCCTGGAGGCCAGGAGAGCGACTTGAGCAAGGTCCGGCACAAGCTC

CGCAGGTTCCTACAGCGGCGGCCCACGCTACAATCGCTGCGGGAGAAGGGTTACATCAGA

GATCAGGTGTTCGGCTGCGCGCTGGCGGTGCTGTGTGAGCGCGAGAGGAGCTCGGTGCCG

CGCTTCGTGCAGCAGTGTATCCGGACCGTCGAGGCCCGGGGGCTGGACATCGACGGGCTG

TACCGCATCAGCGGAAACCTGGCCACCATCCAGAAGCTGCGCTATAGGGTGGACCACGAT

GAGCGTCTGGACCTGGACGATGGGCGCTGGGAGGACGTTCATGTCATCACTGGCGCTCTG

AAGCTCTTCTTCCGGGAGCTGCCCGAGCCCCTCTTCCCCTTCTCGCATTTCAGCCAGTTC

ATCGCAGCCATCAAGCTACAGGACCAGGCACAGCGCAGCCGCTGTGTGCGAGACCTGGTG

CGCTCACTGCCAACCCCCAACCATGACACGCTGCGGCTACTCTTCCAGCACCTGTGCAGG

GTTATCCAGCACGGCGAGCAGAACCGCATGTCGGTGCAGAGCGTGGCCATAGTGTTTGGG

CCTACGCTGCTGCGGCCTGAGACAGAGGAGAACACCATGACCATGACCATGGTGTTCCAG

AACCAAGTGGTGGAGCTCATCCTGCAGCAGTGCTCAGATATCTTCCCGCCCCAC

>CL157.Contig1_All 252 374 minus strand PREDICTED: HRAS-like suppressor 2 [Oryctolagus cuniculus]

ATGACTTTGGATGGAAAACAACCAAAACTTGGAGACCTAATTGAAATTTCTCGCTACTGC

TATCAGCACTGGGCCATCTATGTGGGAAATGGCTATGTGGTCCATCTGGCTCCACCAAGT

GAC

>CL157.Contig2_All 252 737 minus strand PREDICTED: HRAS-like suppressor 2 [Oryctolagus cuniculus]

ATGACTTTGGATGGAAAACAACCAAAACTTGGAGACCTAATTGAAATTTCTCGCTACTGC

TATCAGCACTGGGCCATCTATGTGGGAAATGGCTATGTGGTCCATCTGGCTCCACCAAGT

GAAGTGGCAGGAGCTGGCATGAGCAGCATCATATCCATCGTGGCTGACAGAGCCATTGTG

AAGAAGGAGCTGCTGTCCGTGGTGGCCGGAGGAGACAAGTACTGGGTCAATAACAAGCAC

GATGACAAGTATGACCCACTGCCATCCAACAAAATTGTCAAGCAGGCAGAGAAAATGGTG

GGGAAGGAAGTCCCCTATTCAGTGACAAGTGACAACTGTGAGCACTTTGTGAACACGTTG

CGCTATGGAGTTTCCCGCAGTGACCAGGTGACTGACACCTTCACTGTGATTGGTACTGCA

TCAGTTGTCCTGGGTGTTGCTGGCCTCATTGGGATGATGCTGTACAGAAGCAAGCGGGAA

AAGCAG

>CL157.Contig3_All 296 544 PREDICTED: HRAS-like suppressor 2 [Oryctolagus cuniculus]

ATGACTTTGGATGGAAAACAACCAAAACTTGGAGACCTAATTGAAATTTCTCGCTACTGC

TATCAGCACTGGGCCATCTATGTGGGAAATGGCTATGTGGTCCATCTGGCTCCACCAAGT

GAAGTGGCAGGAGCTGGCATGAGCAGCATCATATCCATCGTGGCTGACAGAGCCATTGTG

AAGAAGGAGCTGCTGTCCGTGGTGGCCGGAGGAGACAAGTACTGGGTCAATAACAAGCAC

GATGACAAG

>CL157.Contig4_All 345 467 PREDICTED: HRAS-like suppressor 2 [Oryctolagus cuniculus]

ATGACTTTGGATGGAAAACAACCAAAACTTGGAGACCTAATTGAAATTTCTCGCTACTGC

TATCAGCACTGGGCCATCTATGTGGGAAATGGCTATGTGGTCCATCTGGCTCCACCAAGT

GAC

>CL157.Contig5_All 203 451 PREDICTED: HRAS-like suppressor 2 [Oryctolagus cuniculus]

ATGACTTTGGATGGAAAACAACCAAAACTTGGAGACCTAATTGAAATTTCTCGCTACTGC

TATCAGCACTGGGCCATCTATGTGGGAAATGGCTATGTGGTCCATCTGGCTCCACCAAGT

GAAGTGGCAGGAGCTGGCATGAGCAGCATCATATCCATCGTGGCTGACAGAGCCATTGTG

AAGAAGGAGCTGCTGTCCGTGGTGGCCGGAGGAGACAAGTACTGGGTCAATAACAAGCAC

GATGACAAG

>CL157.Contig6_All 345 830 PREDICTED: HRAS-like suppressor 2 [Oryctolagus cuniculus]

ATGACTTTGGATGGAAAACAACCAAAACTTGGAGACCTAATTGAAATTTCTCGCTACTGC

TATCAGCACTGGGCCATCTATGTGGGAAATGGCTATGTGGTCCATCTGGCTCCACCAAGT

GAAGTGGCAGGAGCTGGCATGAGCAGCATCATATCCATCGTGGCTGACAGAGCCATTGTG

AAGAAGGAGCTGCTGTCCGTGGTGGCCGGAGGAGACAAGTACTGGGTCAATAACAAGCAC

GATGACAAGTATGACCCACTGCCATCCAACAAAATTGTCAAGCAGGCAGAGAAAATGGTG

GGGAAGGAAGTCCCCTATTCAGTGACAAGTGACAACTGTGAGCACTTTGTGAACACGTTG

CGCTATGGAGTTTCCCGCAGTGACCAGGTGACTGACACCTTCACTGTGATTGGTACTGCA

TCAGTTGTCCTGGGTGTTGCTGGCCTCATTGGGATGATGCTGTACAGAAGCAAGCGGGAA

AAGCAG

>CL157.Contig7_All 362 610 PREDICTED: HRAS-like suppressor 2 [Oryctolagus cuniculus]

ATGACTTTGGATGGAAAACAACCAAAACTTGGAGACCTAATTGAAATTTCTCGCTACTGC

TATCAGCACTGGGCCATCTATGTGGGAAATGGCTATGTGGTCCATCTGGCTCCACCAAGT

GAAGTGGCAGGAGCTGGCATGAGCAGCATCATATCCATCGTGGCTGACAGAGCCATTGTG

AAGAAGGAGCTGCTGTCCGTGGTGGCCGGAGGAGACAAGTACTGGGTCAATAACAAGCAC

GATGACAAG

>CL157.Contig8_All 362 484 PREDICTED: HRAS-like suppressor 2 [Oryctolagus cuniculus]

ATGACTTTGGATGGAAAACAACCAAAACTTGGAGACCTAATTGAAATTTCTCGCTACTGC

TATCAGCACTGGGCCATCTATGTGGGAAATGGCTATGTGGTCCATCTGGCTCCACCAAGT

GAC

>CL157.Contig9_All 381 866 minus strand PREDICTED: HRAS-like suppressor 2 [Oryctolagus cuniculus]

ATGACTTTGGATGGAAAACAACCAAAACTTGGAGACCTAATTGAAATTTCTCGCTACTGC

TATCAGCACTGGGCCATCTATGTGGGAAATGGCTATGTGGTCCATCTGGCTCCACCAAGT

GAAGTGGCAGGAGCTGGCATGAGCAGCATCATATCCATCGTGGCTGACAGAGCCATTGTG

AAGAAGGAGCTGCTGTCCGTGGTGGCCGGAGGAGACAAGTACTGGGTCAATAACAAGCAC

GATGACAAGTATGACCCACTGCCATCCAACAAAATTGTCAAGCAGGCAGAGAAAATGGTG

GGGAAGGAAGTCCCCTATTCAGTGACAAGTGACAACTGTGAGCACTTTGTGAACACGTTG

CGCTATGGAGTTTCCCGCAGTGACCAGGTGACTGACACCTTCACTGTGATTGGTACTGCA

TCAGTTGTCCTGGGTGTTGCTGGCCTCATTGGGATGATGCTGTACAGAAGCAAGCGGGAA

AAGCAG

>CL158.Contig1_All 126 677 ras-related protein Rap-1A [Homo sapiens] >gi|21704066|ref|NP_663516.1| ras-related protein Rap-1A precursor [Mus musculus] >gi|27806115|ref|NP_776873.1| ras-related protein Rap-1A precursor [Bos taurus] >gi|54114993|ref|NP_001005765.1| ras-related protein Rap-1A precursor [Rattus norvegicus] >gi|58331202|ref|NP_001010935.1| ras-related protein Rap-1A [Homo sapiens] >gi|387763528|ref|NP_001248567.1| ras-related protein Rap-1A [Macaca mulatta] >gi|51338596|sp|P62836.1|RAP1A_RAT RecName: Full=Ras-related protein Rap-1A; AltName: Full=Ras-related protein Krev-1; Flags: Precursor >gi|51338606|sp|P62833.1|RAP1A_BOVIN RecName: Full=Ras-related protein Rap-1A; AltName: Full=GTP-binding protein smg p21A; Flags: Precursor >gi|51338607|sp|P62834.1|RAP1A_HUMAN RecName: Full=Ras-related protein Rap-1A; AltName: Full=C21KG; AltName: Full=G-22K; AltName: Full=GTP-binding protein smg p21A; AltName: Full=Ras-related protein Krev-1; Flags: Precursor >gi|51338608|sp|P62835.1|RAP1A_MOUSE RecName: Full=Ras-related protein Rap-1A; AltName: Full=Ras-related protein Krev-1; Flags: Precursor >gi|20147717|gb|AAM12626.1|AF493912_1 Ras family small GTP binding protein RAP1A [Homo sapiens] >gi|162758|gb|AAA30415.1| GTP-binding protein [Bos taurus] >gi|511856|gb|AAA36150.1| ras-related protein [Homo sapiens] >gi|15559438|gb|AAH14086.1| RAP1A, member of RAS oncogene family [Homo sapiens] >gi|37572303|gb|AAH51419.1| RAS-related protein-1a [Mus musculus] >gi|53236951|gb|AAH83128.1| RAS-related protein-1a [Mus musculus] >gi|53733573|gb|AAH83813.1| RAP1A, member of RAS oncogene family [Rattus norvegicus] >gi|56203144|emb|CAI22712.1| RAP1A, member of RAS oncogene family [Homo sapiens] >gi|119576907|gb|EAW56503.1| RAP1A, member of RAS oncogene family [Homo sapiens] >gi|134024651|gb|AAI34567.1| RAP1A, member of RAS oncogene family [Bos taurus] >gi|148675597|gb|EDL07544.1| mCG10748, isoform CRA_a [Mus musculus] >gi|149030393|gb|EDL85430.1| rCG51808, isoform CRA_a [Rattus norvegicus] >gi|197692171|dbj|BAG70049.1| Ras-related protein Rap-1A precursor [Homo sapiens] >gi|197692421|dbj|BAG70174.1| Ras-related protein Rap-1A precursor [Homo sapiens] >gi|296489325|gb|DAA31438.1| ras-related protein Rap-1A precursor [Bos taurus] >gi|344254631|gb|EGW10735.1| Ras-related protein Rap-1A [Cricetulus griseus] >gi|351708065|gb|EHB10984.1| Ras-related protein Rap-1A [Heterocephalus glaber] >gi|380815262|gb|AFE79505.1| ras-related protein Rap-1A precursor [Macaca mulatta] >gi|383410959|gb|AFH28693.1| ras-related protein Rap-1A precursor [Macaca mulatta] >gi|384944334|gb|AFI35772.1| ras-related protein Rap-1A precursor [Macaca mulatta] >gi|387017998|gb|AFJ51117.1| ras-related protein Rap-1A [Crotalus adamanteus]

GGTGGAGGAGGCGCCGGACCCGGGGGATTGTCAGTATTTAAACAGACCACATCATGCACA

GTTCAGTTTGTTCAGGGAATTTTTGTTGAAAAATATGACCCGACGATAGAGGATTCCTAC

AGAAAGCAAGTTGAAGTAGATTGCCAACAGTGTATGCTGGAAATCCTGGACACAGCAGGA

ACAGAGCAGTTTACAGCGATGAGGGATTTATATATGAAGAATGGCCAAGGATTCGCACTA

GTATATTCTATTACAGCTCAGTCCACGTTTAATGACTTACAGGACTTGAGGGAACAGATT

TTACGGGTTAAGGATACAGAAGATGTTCCAATGATTTTGGTTGGCAATAAATGTGACCTG

GAAGATGAGCGAGTAGTTGGCAAAGAACAGGGCCAGAATTTAGCAAGACAGTGGTGTAAC

TGTGCCTTTTTAGAATCTTCTGCAAAGTCAAAGATCAACGTTAATGAGATATTTTATGAC

CTGGTCAGACAGATAAATAGAAAAACACCAGTGGAAAAGAAGAAGCCTAAAAAGAAATCA

TGCCTGCTGCTC

>CL158.Contig2_All 219 770 minus strand ras-related protein Rap-1A [Homo sapiens] >gi|21704066|ref|NP_663516.1| ras-related protein Rap-1A precursor [Mus musculus] >gi|27806115|ref|NP_776873.1| ras-related protein Rap-1A precursor [Bos taurus] >gi|54114993|ref|NP_001005765.1| ras-related protein Rap-1A precursor [Rattus norvegicus] >gi|58331202|ref|NP_001010935.1| ras-related protein Rap-1A [Homo sapiens] >gi|387763528|ref|NP_001248567.1| ras-related protein Rap-1A [Macaca mulatta] >gi|51338596|sp|P62836.1|RAP1A_RAT RecName: Full=Ras-related protein Rap-1A; AltName: Full=Ras-related protein Krev-1; Flags: Precursor >gi|51338606|sp|P62833.1|RAP1A_BOVIN RecName: Full=Ras-related protein Rap-1A; AltName: Full=GTP-binding protein smg p21A; Flags: Precursor >gi|51338607|sp|P62834.1|RAP1A_HUMAN RecName: Full=Ras-related protein Rap-1A; AltName: Full=C21KG; AltName: Full=G-22K; AltName: Full=GTP-binding protein smg p21A; AltName: Full=Ras-related protein Krev-1; Flags: Precursor >gi|51338608|sp|P62835.1|RAP1A_MOUSE RecName: Full=Ras-related protein Rap-1A; AltName: Full=Ras-related protein Krev-1; Flags: Precursor >gi|20147717|gb|AAM12626.1|AF493912_1 Ras family small GTP binding protein RAP1A [Homo sapiens] >gi|162758|gb|AAA30415.1| GTP-binding protein [Bos taurus] >gi|511856|gb|AAA36150.1| ras-related protein [Homo sapiens] >gi|15559438|gb|AAH14086.1| RAP1A, member of RAS oncogene family [Homo sapiens] >gi|37572303|gb|AAH51419.1| RAS-related protein-1a [Mus musculus] >gi|53236951|gb|AAH83128.1| RAS-related protein-1a [Mus musculus] >gi|53733573|gb|AAH83813.1| RAP1A, member of RAS oncogene family [Rattus norvegicus] >gi|56203144|emb|CAI22712.1| RAP1A, member of RAS oncogene family [Homo sapiens] >gi|119576907|gb|EAW56503.1| RAP1A, member of RAS oncogene family [Homo sapiens] >gi|134024651|gb|AAI34567.1| RAP1A, member of RAS oncogene family [Bos taurus] >gi|148675597|gb|EDL07544.1| mCG10748, isoform CRA_a [Mus musculus] >gi|149030393|gb|EDL85430.1| rCG51808, isoform CRA_a [Rattus norvegicus] >gi|197692171|dbj|BAG70049.1| Ras-related protein Rap-1A precursor [Homo sapiens] >gi|197692421|dbj|BAG70174.1| Ras-related protein Rap-1A precursor [Homo sapiens] >gi|296489325|gb|DAA31438.1| ras-related protein Rap-1A precursor [Bos taurus] >gi|344254631|gb|EGW10735.1| Ras-related protein Rap-1A [Cricetulus griseus] >gi|351708065|gb|EHB10984.1| Ras-related protein Rap-1A [Heterocephalus glaber] >gi|380815262|gb|AFE79505.1| ras-related protein Rap-1A precursor [Macaca mulatta] >gi|383410959|gb|AFH28693.1| ras-related protein Rap-1A precursor [Macaca mulatta] >gi|384944334|gb|AFI35772.1| ras-related protein Rap-1A precursor [Macaca mulatta] >gi|387017998|gb|AFJ51117.1| ras-related protein Rap-1A [Crotalus adamanteus]

ATGCGTGAGTACAAGCTAGTGGTCCTTGGTTCAGGAGGCGTGGGGAAGTCTGCTCTGACA

GTTCAGTTTGTTCAGGGAATTTTTGTTGAAAAATATGACCCGACGATAGAGGATTCCTAC

AGAAAGCAAGTTGAAGTAGATTGCCAACAGTGTATGCTGGAAATCCTGGACACAGCAGGA

ACAGAGCAGTTTACAGCGATGAGGGATTTATATATGAAGAATGGCCAAGGATTCGCACTA

GTATATTCTATTACAGCTCAGTCCACGTTTAATGACTTACAGGACTTGAGGGAACAGATT

TTACGGGTTAAGGATACAGAAGATGTTCCAATGATTTTGGTTGGCAATAAATGTGACCTG

GAAGATGAGCGAGTAGTTGGCAAAGAACAGGGCCAGAATTTAGCAAGACAGTGGTGTAAC

TGTGCCTTTTTAGAATCTTCTGCAAAGTCAAAGATCAACGTTAATGAGATATTTTATGAC

CTGGTCAGACAGATAAATAGAAAAACACCAGTGGAAAAGAAGAAGCCTAAAAAGAAATCA

TGCCTGCTGCTC

>CL158.Contig3_All 179 730 ras-related protein Rap-1A [Homo sapiens] >gi|21704066|ref|NP_663516.1| ras-related protein Rap-1A precursor [Mus musculus] >gi|27806115|ref|NP_776873.1| ras-related protein Rap-1A precursor [Bos taurus] >gi|54114993|ref|NP_001005765.1| ras-related protein Rap-1A precursor [Rattus norvegicus] >gi|58331202|ref|NP_001010935.1| ras-related protein Rap-1A [Homo sapiens] >gi|387763528|ref|NP_001248567.1| ras-related protein Rap-1A [Macaca mulatta] >gi|51338596|sp|P62836.1|RAP1A_RAT RecName: Full=Ras-related protein Rap-1A; AltName: Full=Ras-related protein Krev-1; Flags: Precursor >gi|51338606|sp|P62833.1|RAP1A_BOVIN RecName: Full=Ras-related protein Rap-1A; AltName: Full=GTP-binding protein smg p21A; Flags: Precursor >gi|51338607|sp|P62834.1|RAP1A_HUMAN RecName: Full=Ras-related protein Rap-1A; AltName: Full=C21KG; AltName: Full=G-22K; AltName: Full=GTP-binding protein smg p21A; AltName: Full=Ras-related protein Krev-1; Flags: Precursor >gi|51338608|sp|P62835.1|RAP1A_MOUSE RecName: Full=Ras-related protein Rap-1A; AltName: Full=Ras-related protein Krev-1; Flags: Precursor >gi|20147717|gb|AAM12626.1|AF493912_1 Ras family small GTP binding protein RAP1A [Homo sapiens] >gi|162758|gb|AAA30415.1| GTP-binding protein [Bos taurus] >gi|511856|gb|AAA36150.1| ras-related protein [Homo sapiens] >gi|15559438|gb|AAH14086.1| RAP1A, member of RAS oncogene family [Homo sapiens] >gi|37572303|gb|AAH51419.1| RAS-related protein-1a [Mus musculus] >gi|53236951|gb|AAH83128.1| RAS-related protein-1a [Mus musculus] >gi|53733573|gb|AAH83813.1| RAP1A, member of RAS oncogene family [Rattus norvegicus] >gi|56203144|emb|CAI22712.1| RAP1A, member of RAS oncogene family [Homo sapiens] >gi|119576907|gb|EAW56503.1| RAP1A, member of RAS oncogene family [Homo sapiens] >gi|134024651|gb|AAI34567.1| RAP1A, member of RAS oncogene family [Bos taurus] >gi|148675597|gb|EDL07544.1| mCG10748, isoform CRA_a [Mus musculus] >gi|149030393|gb|EDL85430.1| rCG51808, isoform CRA_a [Rattus norvegicus] >gi|197692171|dbj|BAG70049.1| Ras-related protein Rap-1A precursor [Homo sapiens] >gi|197692421|dbj|BAG70174.1| Ras-related protein Rap-1A precursor [Homo sapiens] >gi|296489325|gb|DAA31438.1| ras-related protein Rap-1A precursor [Bos taurus] >gi|344254631|gb|EGW10735.1| Ras-related protein Rap-1A [Cricetulus griseus] >gi|351708065|gb|EHB10984.1| Ras-related protein Rap-1A [Heterocephalus glaber] >gi|380815262|gb|AFE79505.1| ras-related protein Rap-1A precursor [Macaca mulatta] >gi|383410959|gb|AFH28693.1| ras-related protein Rap-1A precursor [Macaca mulatta] >gi|384944334|gb|AFI35772.1| ras-related protein Rap-1A precursor [Macaca mulatta] >gi|387017998|gb|AFJ51117.1| ras-related protein Rap-1A [Crotalus adamanteus]

ATGCGTGAGTACAAGCTAGTGGTCCTTGGTTCAGGAGGCGTGGGGAAGTCTGCTCTGACA

GTTCAGTTTGTTCAGGGAATTTTTGTTGAAAAATATGACCCGACGATAGAGGATTCCTAC

AGAAAGCAAGTTGAAGTAGATTGCCAACAGTGTATGCTGGAAATCCTGGACACAGCAGGA

ACAGAGCAGTTTACAGCGATGAGGGATTTATATATGAAGAATGGCCAAGGATTCGCACTA

GTATATTCTATTACAGCTCAGTCCACGTTTAATGACTTACAGGACTTGAGGGAACAGATT

TTACGGGTTAAGGATACAGAAGATGTTCCAATGATTTTGGTTGGCAATAAATGTGACCTG

GAAGATGAGCGAGTAGTTGGCAAAGAACAGGGCCAGAATTTAGCAAGACAGTGGTGTAAC

TGTGCCTTTTTAGAATCTTCTGCAAAGTCAAAGATCAACGTTAATGAGATATTTTATGAC

CTGGTCAGACAGATAAATAGAAAAACACCAGTGGAAAAGAAGAAGCCTAAAAAGAAATCA

TGCCTGCTGCTC

>CL159.Contig1_All 12 254 Enhancer of mRNA-decapping protein 4 [Heterocephalus glaber]

GTGGCAGCTTTCCCCACAGCCTTTGGAGAGTCTCGGCCTGAACTGGGCTCTGAGGGCCTG

GGATCAACCACTCACGGCTCCCAGCCTGACCTGCGACGCATTGTGGAGCTGCCTGCACCT

GCTGACTTCCTCACCCTGAGCAGTGAAACCAAACCCAAGTTGATGACACCTGATGCCTTT

ATGACACCCACTGCCTCCTTGCAGCAGATCACTGCATCCCCCAGCAGCAGCAGCAGCAGC

AGC

>CL159.Contig2_All 2 1525 minus strand PREDICTED: enhancer of mRNA-decapping protein 4 [Otolemur garnettii]

AAGGTGAAAATCCAGCCTGTTGCCAAGTACGACTGGGAGCAGAAGTACTACTATGGCAAC

CTAATTGCTGTGTCCAACTCTTTCTTAGCCTATGCCATTCGTGCTGCCAACAATGGCTCA

GCAATGGTGCGAGTGATCAGTGTCAGCACTTCAGAGCGGACCCTACTTAAGGGCTTCACA

GGCAGTGTGGCTGATCTGGCCTTTGCACACCTCAACTCTCCACAGCTGGCTTGCCTGGAT

GAGGCAGGCAACCTGTTTGTGTGGCGCTTGGCTCTGGTTAACAGCAAAATTCAAGAAGAG

ATCTTGGTCCATATCCGGCAGCCAGAGGGCACGCCACTGAACCACTTCCGAAGGATCATC

TGGTGCCCCTTTATTCCTGAGGAAAGTGAGGACTGCTGTGAGGAGAGTAGCCCAACAGTG

GCCCTGCTGCATGAAGACCGGGCTGAGGTGTGGGACCTGGACATGCTCCGCTCCAACCAC

AGCTTGTGGCCTGTTGATGTCAGTCAAATCAAGCAGGGGTTCATTGTGGTAAAAGGCCAC

AGCACGTGTCTAAGTGAAGGAGCCCTCTCTCCTGACGGGACTGTCCTAGCTACCGCAAGT

CATGATGGCTATGTCAAGTTCTGGCAGATCTACATTGAGGGGCAGGATGAGCCACGGTGT

CTACATGAGTGGAAGCCTCATGATGGGCGGCCCCTCTCCTGCCTCCTATTCTGTGACAAT

CATAAGAAACAGGACCCTGAGGTCCCATTCTGGAGGTTCCTCATTACTGGTGCTGACCAG

AATCGGGAGTTGAAGATGTGGTGCACAGTGTCCTGGACCTGCCTACAGACCATTCGCTTC

TCCCCAGATATCTTCAGCTCAGTGAGTGTGCCCCCCAGTCTCAAAGTTTGCTTAGACCTC

TCAGCAGAATACTTAATTCTCAGTGATGTACAACGAAAGGTCCTATATGTGATGGAGCTG

CTGCAGAACCAGGAGGAAGGCCGTGCCTGCTTCAGCTCCATTTCAGAGTTCCTGCTTACC

CACCCTGTGCTGAGCTTTGGCATCCAGGTTGTGAGTCGTTGCCGGCTGCGGCACACTGAA

GTGCTGCCTGCTGAGGAGGAAAATGATAGCCTGGGGGCTGAGGGTTCTCATGGAGCTGGT

ACCATGGAGTCTGCAGCTGGCGTACTCATCAAGCTCTTTTGTGTGCATACTAAGGCCTTG

CAAGATGTACAGATCCGTTTCCAGCCACAGCTGAACCCTGATGTGGTGGCCCCACTCCCC

ACTCACACTGCCCATGAGGACTTTGCCTTTGGAGAGTCTCGGCCTGAACTGGGCTCTGAG

GGCCTGGGATCAACCACTCACGGCTCCCAGCCTGACCTGCGACGCATTGTGGAGCTGCCT

GCACCTGCTGACTTCCTCACCCTGAGCAGTGAAACCAAACCCAAGTTGATGACACCTGAT

GCCTTTATGACACCCACTGCCTCCTTGCAGCAGATCACTGCATCCCCCAGCAGCAGCAGC

AGCAGCAGCGCGTTGAAGGCCAGC

>CL160.Contig2_All 2 445 PREDICTED: hypothetical protein LOC715150 [Macaca mulatta]

GCCGAGGCGGGGCCGGAGGACGCGGTGGCGCCCGGGCTCCGGCTGCTGGAGGCGCTGCTG

CGCACAGTGTTCGGCCGCCAGGCGGGTGGCCCGGTGCAGGCAGCCGCCTACTGTCCCAGC

CACCCGGAATCCAGCCTGGCTGTCCAAGCGGCCGCCTGCAGAGCCCTGCAAGCCGCGGGG

CCCGGGCGACCAGAAGAAGGAACTTGGGAGAGATCTGGCCTCCCAGGACTGCTGGCCTGT

TTTACTTGGGGTCCTTGGAGCCAGAGGAAGGACCAAGATGCCACCTCCTCCAGATGCCCA

GCTCAGGAGAACTTCCAAGACTCACAGGAGGAGCTGGCACTCACAACCATATTTCCCAAC

GGAGACTGTGAAGACCGTGGAAGTGGACCCAAAGCCTGTGATGGAGTCGTTCACACGCCC

CCTGAGCCCACTGGAGAAACCAGA

>CL160.Contig3_All 2 445 PREDICTED: hypothetical protein LOC715150 [Macaca mulatta]

GCCGAGGCGGGGCCGGAGGACGCGGTGGCGCCCGGGCTCCGGCTGCTGGAGGCGCTGCTG

CGCACAGTGTTCGGCCGCCAGGCGGGTGGCCCGGTGCAGGCAGCCGCCTACTGTCCCAGC

CACCCGGAATCCAGCCTGGCTGTCCAAGCGGCCGCCTGCAGAGCCCTGCAAGCCGCGGGG

CCCGGGCGACCAGAAGAAGGAACTTGGGAGAGATCTGGCCTCCCAGGACTGCTGGCCTGT

TTTACTTGGGGTCCTTGGAGCCAGAGGAAGGACCAAGATGCCACCTCCTCCAGATGCCCA

GCTCAGGAGAACTTCCAAGACTCACAGGAGGAGCTGGCACTCACAACCATATTTCCCAAC

GGAGACTGTGAAGACCGTGGAAGTGGACCCAAAGCCTGTGATGGAGTCGTTCACACGCCC

CCTGAGCCCACTGGAGAAACCAGA

>CL161.Contig1_All 238 684 minus strand PREDICTED: ribonuclease P protein subunit p20 isoform 2 [Pan troglodytes]

CGGAGGAGGCGGTGGAGGGTGCACAGCATGGCTGAAAACCGAGAGCCCCGCGGGGCAGTC

GACGCTGAGCTGGACCCGGTGGAGTACACCCTTCGGAAAAGGCTTCCCCACCGCCTGCCC

CGGAGGCCCAATGACATTTATGTCAACATGAAGACTGACTTTAAGGCCCAGCTGGCCCGC

TGCCAGAAGCTTCTGGACGGAGGGACACGGGGTCAGAATGCATGCACTGAGATCTACATT

CATGGCTTGGGACTGGCCATCAACCGCGCCATCAACATTGCCCTGCAGTTGCAGGCAGGC

AGCTTCGGGTCCTTGCAGGTGGCTGCCAATACCTCTACTGTGGAGCTTGTGGATGAACTG

GAACCAGAAACTGACACCAGAGAGCCACTGACCCGAATCCGCAACAACTCGGCCATCCAC

ATCCGAGTCTTCAGGGTCACGCCCAAG

>CL161.Contig2_All 47 478 minus strand PREDICTED: ribonuclease P protein subunit p20-like isoform 2 [Nomascus leucogenys]

AGGGTGCACAGCATGGCTGAAAACCGAGAGCCCCGCGGGGCAGTCGACGCTGAGCTGGAC

CCGGTGGAGTACACCCTTCGGAAAAGGCTTCCCCACCGCCTGCCCCGGAGGCCCAATGAC

ATTTATGTCAACATGAAGACTGACTTTAAGGCCCAGCTGGCCCGCTGCCAGAAGCTTCTG

GACGGAGGGACACGGGGTCAGAATGCATGCACTGAGATCTACATTCATGGCTTGGGACTG

GCCATCAACCGCGCCATCAACATTGCCCTGCAGTTGCAGGCAGGCAGCTTCGGGTCCTTG

CAGGTGGCTGCCAATACCTCTACTGTGGAGCTTGTGGATGAACTGGAACCAGAAACTGAC

ACCAGAGAGCCACTGACCCGAATCCGCAACAACTCGGCCATCCACATCCGAGTCTTCAGG

GTCACGCCCAAG

>CL161.Contig3_All 214 648 minus strand PREDICTED: ribonuclease P protein subunit p20-like isoform 2 [Nomascus leucogenys]

AGAGGGGTGCACAGCATGGCTGAAAACCGAGAGCCCCGCGGGGCAGTCGACGCTGAGCTG

GACCCGGTGGAGTACACCCTTCGGAAAAGGCTTCCCCACCGCCTGCCCCGGAGGCCCAAT

GACATTTATGTCAACATGAAGACTGACTTTAAGGCCCAGCTGGCCCGCTGCCAGAAGCTT

CTGGACGGAGGGACACGGGGTCAGAATGCATGCACTGAGATCTACATTCATGGCTTGGGA

CTGGCCATCAACCGCGCCATCAACATTGCCCTGCAGTTGCAGGCAGGCAGCTTCGGGTCC

TTGCAGGTGGCTGCCAATACCTCTACTGTGGAGCTTGTGGATGAACTGGAACCAGAAACT

GACACCAGAGAGCCACTGACCCGAATCCGCAACAACTCGGCCATCCACATCCGAGTCTTC

AGGGTCACGCCCAAG

>CL162.Contig1_All 241 507 Golgin subfamily A member 7 OS=Mus musculus GN=Golga7 PE=2 SV=1

ATGAGGCCGCAGCAGGCGCCGGTGTCCGGGAAGGTATTCATTCAACGAGACTACAGCAGT

GGTACACGCTGCCAGTTCCAGACCAAGTTCCCCGCGGAGCTGGAGAACCGGATTGATAGG

CAGCAGTTTGAAGAGACAGTTCGAACTCTAAATAACCTTTATGCAGAAGCAGAGAAGCTT

GGGGGCCAATCGTATCTTGAAGGCTGTTTGGCTTGTTTAACAGCATATACCATTTTCTTA

TGTATGGAAACTCATTATGAGAAGATC

>CL162.Contig2_All 223 648 golgi autoantigen, golgin subfamily a, 7 [Mustela putorius furo]

GCCGGTCCTGTCCTCGCCATGAGGCCGCAGCAGGCGCCGGTGTCCGGGAAGGTATTCATT

CAACGAGACTACAGCAGTGGTACACGCTGCCAGTTCCAGACCAAGTTCCCCGCGGAGCTG

GAGAACCGGATTGATAGGCAGCAGTTTGAAGAGACAGTTCGAACTCTAAATAACCTTTAT

GCAGAAGCAGAGAAGCTTGGGGGCCAATCGTATCTTGAAGGCTGTTTGGCTTGTTTAACA

GCATATACCATTTTCTTATGTATGGAAACTCATTATGAGAAGGTTCTGAAGAAAGTCTCC

AAGTATATTCAAGAACAGAATGAGAAGATATATGCTCCCCAAGGTCTCCTCCTGACAGAC

CCTATTGAGAGAGGACTTCGAGTTATTGAAATTACCATTTATGAAGACAGAGGCATGAGC

AGTGGA

>CL163.Contig1_All 1 930 minus strand Unc-93 homolog A (C. elegans) [Mus musculus]

CTCCTCTTCACAGCCTACGGAGGTCTGCAGAACCTGCAGAGCAGCCTCTACAGCGAGGAC

GGCATGGGGGTGGCGACGCTCGGCACGCTCTACGGCGCTGTCCTCCTGTCCTCCATGTTC

CTTCCACCCATCCTCATCAAGAGATGTGGCTGCAAGTGGACCATTGTTGGCTCCATGTGC

TGCTACGTGGCCTTCTCCCTGGGCAACTTCTACGCCAGCTGGTTCACCTTGATCCCCACC

TCCATCTTGCTGGGGCTGGGAGCGGCCCCCCTGTGGTCTGCTCAGTGCACCTACCTCACA

GTCCTGGGGAACACGCATGCACAGGAAGTGGGCGAGCTCGGCGAGGACGTGGTGAACCAG

TACTTCGGCATCTTCTTTCTCATCTTCCAGTCCTCTGGTGTGTGGGGCAACTTGATCTCC

TCTCTGGTGTTCGGGCAGACTCCCACTCAAGAGGCCACCCCAGAGGAGCACCTTGAGTCC

TGTGGGGCCAACGACTGCCTGATGGCCACAGCACCCACCAACAGCACCCACCGCCCCTCC

CAGGAGCTGATCTACACACTGCTGGGCATCTACACCGCCATCGGCGTCCTGGCCATCCTG

CTGACAGCCGTGTTTCTGGAAGCCACAGAGGACAGTGAATCCCAGAACGAGGCCGAGACG

CAGCCACCACGCCTTGGGTCCATCTTACTGTCAACCTTCAAGCTCTTCAGGGACAAGCGC

CTGTGCCTCCTGACCCTGCTGCCCATGTACAGTGGACTGCAGCAGGGGTTCCTCTCTGGA

GAGTACACCAGGTCCTTTGTCACCTGCGTCCTGGGCATCCAGTTCGTGGGCTACGTGATG

ATCTGCTTCTCGGCGTCCACTGCGCTCTGCTCTCTGTTGTTTGGGAGGCTCTCCCACTAC

ACGGGCAGGAGAGCCATCTACGGGCTGGGT

>CL163.Contig2_All 1 1062 minus strand PREDICTED: protein unc-93 homolog A isoform 2 [Canis lupus familiaris]

CTCCTCTTCACAGCCTACGGAGGTCTGCAGAACCTGCAGAGCAGCCTCTACAGCGAGGAC

GGCATGGGGGTGGCGACGCTCGGCACGCTCTACGGCGCTGTCCTCCTGTCCTCCATGTTC

CTTCCACCCATCCTCATCAAGAGATGTGGCTGCAAGTGGACCATTGTTGGCTCCATGTGC

TGCTACGTGGCCTTCTCCCTGGGCAACTTCTACGCCAGCTGGTTCACCTTGATCCCCACC

TCCATCTTGCTGGGGCTGGGAGCGGCCCCCCTGTGGTCTGCTCAGTGCACCTACCTCACA

GTCCTGGGGAACACGCATGCACAGGAAGTGGGCGAGCTCGGCGAGGACGTGGTGAACCAG

TACTTCGGCATCTTCTTTCTCATCTTCCAGTCCTCTGGTGTGTGGGGCAACTTGATCTCC

TCTCTGGTGTTCGGGCAGACTCCCACTCAAGAGGCCACCCCAGAGGAGCACCTTGAGTCC

TGTGGGGCCAACGACTGCCTGATGGCCACAGCACCCACCAACAGCACCCACCGCCCCTCC

CAGGAGCTGATCTACACACTGCTGGGCATCTACACCGCCATCGGCGTCCTGGCCATCCTG

CTGACAGCCGTGTTTCTGGAAGCCACAGAGGACAGTGAATCCCAGAACGAGGCCGAGACG

CAGCCACCACGCCTTGGGTCCATCTTACTGTCAACCTTCAAGCTCTTCAGGGACAAGCGC

CTGTGCCTCCTGACCCTGCTGCCCATGTACAGTGGACTGCAGCAGGGGTTCCTCTCTGGA

GAGTACACCAGGTCCTTTGTCACCTGCGTCCTGGGCATCCAGTTCGTGGGCTACGTGATG

ATCTGCTTCTCGGCGTCCACTGCGCTCTGCTCTCTGTTGTTTGGGAGGCTCTCCCACTAC

ACGGGCAGGAGAGCCATCTACGGGCTGGGTGCAGCCACCCACCTCTCCTGCATCGTGGCC

CTGCTGCTGTGGCGTCCACGCTCCTCCCAGATGGCAGTGTTCTTCCTGCTCCCCGGCCTG

TGGGGCATGGGGGATGCTGTCTGGCAGACGCAGAACAATGTT

>CL163.Contig3_All 315 1685 PREDICTED: protein unc-93 homolog A isoform 2 [Canis lupus familiaris]

ATGGAGAGAAGCCTGAGGAACGTCCTCGTGGTCTCCTTTGGATTCCTCCTCCTCTTCACA

GCCTACGGAGGTCTGCAGAACCTGCAGAGCAGCCTCTACAGCGAGGACGGCATGGGGGTG

GCGACGCTCGGCACGCTCTACGGCGCTGTCCTCCTGTCCTCCATGTTCCTTCCACCCATC

CTCATCAAGAGATGTGGCTGCAAGTGGACCATTGTTGGCTCCATGTGCTGCTACGTGGCC

TTCTCCCTGGGCAACTTCTACGCCAGCTGGTTCACCTTGATCCCCACCTCCATCTTGCTG

GGGCTGGGAGCGGCCCCCCTGTGGTCTGCTCAGTGCACCTACCTCACAGTCCTGGGGAAC

ACGCATGCACAGGAAGTGGGCGAGCTCGGCGAGGACGTGGTGAACCAGTACTTCGGCATC

TTCTTTCTCATCTTCCAGTCCTCTGGTGTGTGGGGCAACTTGATCTCCTCTCTGGTGTTC

GGGCAGACTCCCACTCAAGAGGCCACCCCAGAGGAGCACCTTGAGTCCTGTGGGGCCAAC

GACTGCCTGATGGCCACAGCACCCACCAACAGCACCCACCGCCCCTCCCAGGAGCTGATC

TACACACTGCTGGGCATCTACACCGCCATCGGCGTCCTGGCCATCCTGCTGACAGCCGTG

TTTCTGGAAGCCACAGAGGACAGTGAATCCCAGAACGAGGCCGAGATGCAGCCACCACGC

CTTGGGTCCATCTTACTGTCAACCTTCAAGCTCTTCAGGGACAAGCGCCTGTGCCTCCTG

ACCCTGCTGCCCATGTACAGTGGACTGCAGCAGGGGTTCCTCTCTGGAGAGTACACCAGG

TCCTTTGTCACCTGCGTCCTGGGCATCCAGTTCGTGGGCTACGTGATGATCTGCTTCTCG

GCGTCCACTGCGCTCTGCTCTCTGTTGTTTGGGAGGCTCTCCCACTACACGGGCAGGAGA

GCCATCTACGGGCTGGGTGCAGCCACCCACCTCTCCTGCATCGTGGCCCTGCTGCTGTGG

CGTCCACGCTCCTCCCAGATGGCAGTGTTCTTCCTGCTCCCCGGCCTGTGGGGCATGGGG

GATGCTGTCTGGCAGACGCAGAACAATGCTCTGTTTGGCACCCTGTTCGAGAGGAACAAG

GAGGCGGCTTTCGCTGGTTACCGCATGTGGGAGGCCCTCGGCTTCGTCGCCGCCTTCGGC

TACAGCTCCTTTCTCTGTGTCAGTGTCAAGCTCTCCATCCTGCTGGCCGTCCTGGCGGTG

GCCATGGTCTCCTACGGGACTGTTGAGTACCTGGAATCCAGGAGCCCAGTCAAGACCCTC

ACGGCCACACAGACCAGCCAAGCAGTGGAGGAAGAGACGGGGATAGCGATG

>CL164.Contig1_All 42 974 PREDICTED: ADP-ribosyl cyclase 2-like [Sus scrofa]

ATGGCGGCGCGGGGGTGCGTACCGTCGTGGCTGCCTCTGCTCCAGCTGTTGCTGGCTGCG

GGCGCGGGGGGCGCCCAGTGGAGCGGGAAGGGCACCAGCCAACGCCTGCAGAGCATCTTC

CTGGGCCGCTGCGCCGAGTACTGCTCGCTGGTGAGCCCTGAGCTGCGGGACAAGAACTGC

ACAGCCATCTGGGAAGCCTTTAAAGTGGTGCTGGACAAGGACCCTTGTTCTGTGTTCCCC

TCGGACTATGACCTTTTCATTAACCTCTCCAGGCACTCCATTCCCAGAGACAAGTCCCTG

TTCTGGGAAAATAACCACCTCCTTGTTACTAGCTACTCAGAGAACACCCGTCGCTTTACG

TCCCTGAGTGATGTTCTATATGGCCGGGTTGCAGATTTCTTGAGTTGGTGTCGACAGAAA

AATGGGTCTGGGCTGGATTACCAGTCCTGTCCTACATCAGAAGATTGTGAGAACAACCCT

GTGGATTCCTTTTGGAAAAGGGCATCCATTCAGTATTCGAGAGACAGTTCTGGGGTGATC

TATGTCATGCTGAATGGTTCTGAGCCCACAGGAGCCTATCCTGTCAAAGGTTTTTTTGCA

GATTTTGAAATTCCGTACTTCCAGAAGGATAAGATCACACGAATCGAGATCTGGGTCATG

CATGAGATTGGGGGAACCAATGTGGAATCCTGTGGAGAGGGCAGTGTGAAAATCCTGGAG

GAGAGACTGCAGGCCATGGGCTTCCAGTCCAGCTGCGTTGATGACTACCTACCAGTGAAG

CTCTTAAAGTGCGTGGACCACAGTACTCACTCTGATTGTGCCTTAAATTCGGCAGCAGCA

TCTACTCAAAGAGAAGTCTCATCTCTTGATACAGAGCAAAGTGCCAGCTTTATGGTTCCT

CTCTTGGTGGCTTTAGCCTCAGGTTCTCAAATG

>CL164.Contig2_All 926 1027 PREDICTED: ADP-ribosyl cyclase 2-like [Sus scrofa]

GCAGCAGCATCTACTCAAAGAGAAGTCTCATCTCTTGATACAGAGCAAAGTGCCAGCTTT

ATGGTTCCTCTCTTGGTGGCTTTAGCCTCAGGTTCTCAAATG

>CL165.Contig2_All 197 1168 minus strand PREDICTED: CXXC-type zinc finger protein 5 isoform 1 [Saimiri boliviensis boliviensis] >gi|403285282|ref|XP_003933960.1| PREDICTED: CXXC-type zinc finger protein 5 isoform 2 [Saimiri boliviensis boliviensis]

ATGTCGAGCCTCAGCGGTGGCTCCCAAGAGGCCGGCGGCAGTAGTAGCAGCAGCAGCAGC

AGCAGTACCACCACCACCACCAGCAGCAGTGGCAGTGGCCCAAAGGCAGGAGCAACTGAA

AAGGGTGCGGCAGTGGCAACTGCTGCGTCAGCCTCGGTGGCAGATGACACACCACCCCCA

GAGCGTCGGAACAAGAGTGGCATCATCAGCGAACCCCTGAACAAGAGTCTGCGTCGTTCC

CGCCCCCTCTCCCACTACTCTTCCTTTGGTGGCAGTGGTGGCAGTGGCAGCCTGATGGGC

GGGGAGTCTGCTGACAAGGCAGCGGCGGCTGCGGCCGCGGCCTCCCTCTTGGCCAATGGT

CACGACCTGGCGGCAGCCATGGCGGTGGACAAAAGCAACCCTACCTCAAAGCACAAAAGT

GGTGCTGTGGCCAGCCTGCTGAGCAAGGCAGAGCGGGCCTCGGAGCTGGCAGCTGAGGGA

CAGCTGACGCTGCAGCAGTTCGCGCAGTCCACGGAGATGCTGAAGCGCGTGGTGCAGGAG

CACCTACCACTGATGAGCGAGGCGGGCGCTGGCCTGCCCGACATGGAGGCCGTGGCGGGC

GCCGAGGCCCTCAATGGCCAGTCCGACTTCCCCTACCTGGGCGCTTTCCCCATCAACCCG

GGCCTCTTCATCATGACCCCCGCAGGTGTGTTCCTGGCCGAGAGCGCGCTGCACATGGCC

GGCCTGGCCGAGTACCCCATGCAGGGAGAGCTAGCCTCTGCCATCAGCTCGGGCAAGAAG

AAGCGGAAACGCTGCGGCATGTGTGCGCCCTGCCGGCGGCGCATCAACTGTGAGCAGTGC

AGCAGTTGTAGGAACCGAAAGACTGGCCATCAGATTTGCAAATTCAGAAAATGTGAGGAA

CTCAAAAAGAAGCCTTCCGCTGCTCTGGAGAAGGTGATGCTTCCGACGGGAGCCGCCTTC

CGGTGGTTTCAG

>CL166.Contig1_All 2 292 minus strand PREDICTED: T-cell differentiation antigen CD6 [Saimiri boliviensis boliviensis]

GCTAGTGTTCAGCCAGCCACCATGGGGTCGTCTGCGACAGTGAAGGTGGAGGACTGGGGT

TCCCGGGAGCTGACGCTCCTCATCCCCTGCATCCTCCTGGCAGTTCTCCTCCTTGTCTCC

CTTATCTGCATGGCTGTCGTCCTCTTGAGAGTTAAAGGAAAATACGCCCTCCCTGTTCCA

GCGAACCAGCAGCACCTTCCCACCACCATCCCAGCAGGGAGCAATAGCTATCAAGCGGTC

CCCATCACCATCCCCAAAGAAGAAGCTCGTCCCTTTGTGATACTGGCACCT

>CL166.Contig2_All 386 649 minus strand PREDICTED: t-cell differentiation antigen CD6 isoform 1 [Nomascus leucogenys]

CCCATCCACCGAGACCAGGTGAACTGCTCAGGAACTGAGGCCTACCTGTGGGACTGCCCC

GGGCTGCCAGGAGACCAGTACTGTGGCCACAAGGAAGACGCCGGCGCCGTGTGCTCAGAG

CACCAGTCCTGGCGCCTGACGGGGGGCATCGACCCCTGTGAAGGGCAGGTGGAGGTGTAC

TTCCGAGGGGTCTGGAGCACCGTGTGTGACAGTGAGTGGTACTCCTCAGAGGCTGAGGTG

CTGTGCCGGGCCCTGGGCTGTGGA

>CL166.Contig3_All 386 649 minus strand PREDICTED: t-cell differentiation antigen CD6 isoform 2 [Nomascus leucogenys]

CCCATCCACCGAGACCAGGTGAACTGCTCAGGAACTGAGGCCTACCTGTGGGACTGCCCC

GGGCTGCCAGGAGACCAGTACTGTGGCCACAAGGAAGACGCCGGCGCCGTGTGCTCAGAG

CACCAGTCCTGGCGCCTGACGGGGGGCATCGACCCCTGTGAAGGGCAGGTGGAGGTGTAC

TTCCGAGGGGTCTGGAGCACCGTGTGTGACAGTGAGTGGTACTCCTCAGAGGCTGAGGTG

CTGTGCCGGGCCCTGGGCTGTGGA

>CL168.Contig1_All 509 742 PREDICTED: DNA cross-link repair 1A protein-like [Ailuropoda melanoleuca] >gi|281347740|gb|EFB23324.1| hypothetical protein PANDA_001409 [Ailuropoda melanoleuca]

GGATGGACGCACTCTAGCAAGTTCTCCAGCATAGCAGATATTACTCCTCAGACCAAGGGA

AACATTTCAATATATGGAATTCCTTACAGTGAACACAGCAGCTACCTAGAGATGAAGCGT

TTTGTCCAGTGGCTGAAACCGCAGAAAATTATACCTACCGTAAATATTGGCACCTTGAAA

TCTCGGCACACAATGGAGAAATATTTTAAAGAGTGGAAATTGGAAGCTGGATAT

>CL168.Contig2_All 6 809 minus strand DNA cross-link repair 1A protein [Cricetulus griseus]

TTGCCGATGGACACTGAGTGTTTTGTGGATGGTGTCAGGGTTGTTCTACTTGATGCCAAT

CACTGCCCAGGTGCCACCATGATCCTTTTCTACCTTCCTAATGGTGCTGCTGTATTGCAC

ACTGGAGACTTCAGAGCGGATCCCAGCATGGAGCGGTCTCTTCTTGCAGGCCAGAAAATC

CACACGCTGTACCTAGACACCACCTATTGCAGCCCAGAGTACACCTTTCCGTCTCAGCAA

GAAGTTATCCAGTTTGCCATCAACACCGCCTTTGAGGCTGTAACTCTAAACCCCCGTACT

CTGGTTGTCTGTGGCACTTACAGTATCGGAAAAGAGAAAGTCTTCCTAGCTGTTGCTGAT

GTTTTAGGTTCAAAGGTGGGCATGTCCCGAGAAAAGTTTCAAACATTGCAGTGCCTCAAT

ATGCCTGACATTAATCCCCTCATCACCACAGACATGTGGAGTTCATTGGTCCACCTTCTC

CCAATGATGCAAATTAATTTTAAGGGTTTGCAGAATCATCTGAAGAAGTATGATGGGAAA

TATGATCGGATTTTGGCTTTTCGACCCACAGGATGGACGCACTCTAGCAAGTTCTCCAGC

ATAGCAGATATTACTCCTCAGACCAAGGGAAACATTTCAATATATGGAATTCCTTACAGT

GAACACAGCAGCTACCTAGAGATGAAGCGTTTTGTCCAGTGGCTGAAACCGCAGAAAATT

ATACCTACCGTAAATATTGGCACCTTGAAATCTCGGCACACAATGGAGAAATATTTTAAA

GAGTGGAAATTGGAAGCTGGATAT

>CL170.Contig1_All 83 1339 PREDICTED: hyaluronidase-1 isoform 5 [Macaca mulatta]

ATGACACCCTTCAACCCTGAGGTTTCCTCAGACCCACCTACTGCCACTGCAGCCCACCTG

CTTCACATCTGTACCCTCTTGCTGACTTTGGTCTACGTGGCTCAAGGCTCCAGGGGTCCT

GTGGTACCCAACCGGCCCTTCATCACAGTTTGGAATGCAAACACCCAGTGGTGCCTGGAG

ACGCATGGAATAGATGTGGATGTCAGTGTCTTTGATGTGATAGCCAACCGGGGGCAGACC

TTCCGCGGCCCTAACATGACCATTTTCTACAGCACGCAACTGGGGACCTACCCCTACTAC

ACAACTACTGGGGAGCCTATATTTGGTGGCTTGCCCCAGAATGCCAGCCTGCATGCCCAC

CTGGCCCGCACATTTCAGGATATCCAGGATGCCATGCCTGAATCTGACTTCTCAGGGCTG

GCAGTGATTGACTGGGAGGCATGGCGCCCACGCTGGGCCTTCAACTGGGATACCAAGGAC

ATTTACCGGCAGCGTTCTCGGGCACTGGTAAGGGCACAGCATCCTGACTGGCCAGAAACT

TGGGTGGAGGCAGAAGCCCAGGCCCAGTTCCAGGAAGCTGCACAGGCCTGGATGGCAGGC

ACCCTCCAGTTGGGACAGGCACTGCGTCCCCGTGGTCTCTGGGGCTTCTATGGCTTCCCT

GACTGCTACAACTATGACTTTCTAAGGTCCAACTACACAGGCGAATGCCCACCAGGCATC

TGTGCCCAGAACGATCAGCTAGGGTGGCTGTGGAACCAGAGCCGTGCCCTCTATCCCAGT

ATCTATCTGCCTGCAGTGTTGGTGGGCACAGGGAAAGCACAGATGTATGTACGTCATCGT

GTGAGTGAGGCATTTCGTGTGATTATGGCTGCCAGGGACCCCAGTCTGCCGGTGCTGCCC

TACACCCAGATCTTCTATGACATGACAAACCGACTTCTGCCCCTGGAATCATGTCAGGCC

ATCAAGGAATATATGGATACTACCCTGGGGCCCTTCATCCTGAACGTGACCAGTGGAGCT

CTTCTTTGCAGTCAAGCCCTGTGCTCTGGCCATGGCCGTTGTGCCCGTCGCTTCAACCAC

CCTGAAGCTCTCCTTATCCTCAATCCTAACAGTTTCTCCATCCAGCTTATGCCTGGTGGC

AGGTCCTTGACCCTGAAGGGTGCCCTCTCACTTGAGGATCAGGCACAAATGGCTATGGAG

TTCAAATGTCACTGCTATCATGGCTGGCGTGGAGAGTGGTGTGAGCAGCAGGGCATG

>CL170.Contig2_All 83 1429 PREDICTED: hyaluronidase-1 isoform 6 [Macaca mulatta]

ATGACACCCTTCAACCCTGAGGTTTCCTCAGACCCACCTACTGCCACTGCAGCCCACCTG

CTTCACATCTGTACCCTCTTGCTGACTTTGGTCTACGTGGCTCAAGGCTCCAGGGGTCCT

GTGGTACCCAACCGGCCCTTCATCACAGTTTGGAATGCAAACACCCAGTGGTGCCTGGAG

ACGCATGGAATAGATGTGGATGTCAGTGTCTTTGATGTGATAGCCAACCGGGGGCAGACC

TTCCGCGGCCCTAACATGACCATTTTCTACAGCACGCAACTGGGGACCTACCCCTACTAC

ACAACTACTGGGGAGCCTATATTTGGTGGCTTGCCCCAGAATGCCAGCCTGCATGCCCAC

CTGGCCCGCACATTTCAGGATATCCAGGATGCCATGCCTGAATCTGACTTCTCAGGGCTG

GCAGTGATTGACTGGGAGGCATGGCGCCCACGCTGGGCCTTCAACTGGGATACCAAGGAC

ATTTACCGGCAGCGTTCTCGGGCACTGGTAAGGGCACAGCATCCTGACTGGCCAGAAACT

TGGGTGGAGGCAGAAGCCCAGGCCCAGTTCCAGGAAGCTGCACAGGCCTGGATGGCAGGC
[truncated: 25,179,798 more chars]
