# Supplementary material for: Transcriptome Analysis and Comparison of Marmota monax and Marmota himalayana
Source: PLoS One. 2016 Nov 2;11(11):e0165875. doi: 10.1371/journal.pone.0165875 (PMC5091844; doi:10.1371/journal.pone.0165875)
Supplement: S4 File — (DOC) [file pone.0165875.s004.doc]

>CL476.Contig6_C-W 1 202 LEN=202; minus strand

XXTGGAGAGCCTGATGAGGACCTGACTTCCCATCACTTGTCATTGTTCCTGAGCCTCTGC

GTGATCCTCACCACTCTCAAACTCTACCGCTGCCTTGTCTGGACGCTGGTGAGAGCTGCA

ACTCCTCCCTAAGACCCTCTCCTCAACCGGTTGTCCACTCCACGTCAGAAAAAGCCTGCC

TTGGTTCCGGACCTGATCACCGAG

>CL484.Contig1_C-W 1 351 LEN=351; minus strand

AGAGAACCCAGGCTGAGAGCTCAGAGCTCAGAGCACGGGGCCAGGGAGCTGGAGCCCGGA

GTCAGCTTCTGCTTCCGCTTCCTCCAGGCAATAGGAAGCTGTCAGCAGTGGGACTAACTG

GTATATGACATGACAGGAGCTCAAGCAGAAAATGGAATTCTACAGAGAGCAAGGAGGGAG

CAGGCCTCAACAACATTTTCCCGACATCTAGAAAAGTCCCTGGAGCAGAATGGAGGAGGC

TTGAAAACCGTCCGATGGGGAGAAGAAGGTGTGAGCCTGGCAGCGAAAGACGTTGAACAG

GAAXTAGACAGGTTTCTAGCCTGGCTTATCTTGGCTGAAGAAGCCACCTCCCXX

>CL484.Contig3_C-W 1 309 LEN=309; minus strand

AGAGGAGCTGGCCTGAGGGAAGACCGCAGACGCAGACAAGGCAGAAAGCCCTGGAATAAT

GGGGCTGGAGACTAACTGGTATATGACATGACAGGAGCTCAAGCAGAAAATGGAATTCTA

CAGAGAGCAAGGAGGGAGCAGGCCTCAACAACATTTTCCCGACATCTAGAAAAGTCCCTG

GAGCAGAATGGAGGAGGCTTGAAAACCGTCCGATGGGGAGAAGAAGGTGTGAGCCTGGCA

GCGAAAGACGTTGAACAGGAAXTAGACAGGTTTCTAGCCTGGCTTATCTTGGCTGAAGAA

GCCACCTCCCXX

>CL629.Contig3_C-W 1 369 LEN=369; minus strand

XXCATGGAACTTCTGCCAAAAACTGACCTTTGCTTTAATTTTTATCTTCATCCTGTTTTT

GTTCGCTCTGATCTTCTTCAGGTGCTGGTGACXGCCATGTCCAGGCAACGCTTCCAGTAT

TTCCTAGAGTCTCTGTTACAGGACCAGTGGCTGATACCAACAATCTCCTCCATCCAGGAC

TGGTTCGATGCCATCGACAACTTGTGGCTTCAGGGAACTTTCACAGACTTCACCCCTACG

GAAGTAGCTGTCTATAGCCACTGGGTTTTATTTCTGGCTGCCATGATATCCCCAGACCTG

CCCCCCAAGCATTCCTCCACTTCCCTTTCTAGGCCCCACAGCGCCCCCACACAGCAGGAA

GCAGCCGGATCT

>CL629.Contig4_C-W 1 213 LEN=213

XCCATGTCGAGGCAACGCTTCCAATACTTCCTAGAGTCTCTGTTACAAGACCAGTCCTGG

ATGGAGGAGATTGTTGGTATCAGCCACTGGTCCTGTAACAGAGACTCTAGGAAATACTGG

AAGCGTTGCCTGGACATGGCGTCACCAGCACCTGAAGAAGATCAGAGCGAACAAAAACAG

GATGAAGATAAAAATTAAAGCAAAGGTCAGTTTTXX

>CL633.Contig1_C-W 1 561 LEN=561

XAGAAGAAGAAGAAGAAGAAGAAGATGGAGATGGAGATGGAGAGAGGGTTGGAGGTTTCC

CTGGCACAGAATCAAGCTCCAGTCTGTCAAGACTCAGATGCGGGATTGCCAGAGGACCCC

AGGGCCAAGGACGATGCAGTCTATGCACAGCTGGCGGCACAGGATTCTCTGAAGGGCAAG

CGGAAGGTGAAAACTGATATTGATGAGAAACATCTGGAAGGAAAGGGGCCCTGCACCAGT

GTTTACAGCATGGTCCAGAGACCAGGCCAGGCCTTGAAGATGACTTATTAGGAGGAGGGA

GGAGAACCCAGCACTAGGGAGACACCTGCACTCTGCACCCGACACCTGCGGATACCTACT

TCCTCTGACTTCAGTCCAGCTCGAGATGCCACTGGTCTGCATGAGCCTGACCTGGTGGTC

ATCTCTACCTCTGTGGGCTCATGTGGCCCTGTCACTTCTCCTGCCACCTTCCCATCTGCC

CTCACACTCAGAGCTCTCGTGCCATCCAGCCAGGGTTCTTCCTCTGTCACTGCTGTGGCT

TCTGACCCCTCCCTTTCAGAAGXX

>CL633.Contig2_C-W 1 497 LEN=896

XAGAAGAAGAAGAAGAAGAAGAAGATGGAGATGGAGATGGAGAGAGGGTTGGAGGTTTCC

CTGGCACAGAATCAAGCTCCAGTCTGTCAAGACTCAGATGCGGGATTGCCAGAGGACCCC

AGGGCCAAGGACGATGCAGTCTATGCACAGCTGGCGGCACAGGATTCTCTGAAGGGCAAG

CGGAAGGTGAAAACTGTGAGTGGTGACCAGCCAGGTCTCATGGCACCCAGCCCTGGGCTG

CCTGCCCAAAGCCAGGATATTGATGAGAAACATCTGGAAGGAAAGGGGCCCTGCACCAGT

GTTTACAGCATGGTCCAGAGACCAGGCCAGGCCTTGAAGATGACTTATTAGGAGGAGGGA

GGAGAACCCAGCACTAGGGAGACACCTGCACTCTGCACCCGACACCTGCGGATACCTACT

TCCTCTGACTTCAGTCCAGCTCGAGATGCCACTGGTCTGCATGAGCCTGACCTGGTGGTC

ATCTCTACCTCTGTGGGC

>CL874.Contig2_C-W 1 284 LEN=853

XGCGGGCGGGCTCTGCGGGGAAACCTTGGTAGCTGCGGGGTGATCTGGGCTGGTGATCTG

GACCCCGGCAGGGATGAAGGGTCCAGTGCCCGAGATGCATTCGGGAAATGCGAGAAGCAG

CCTGCATCGCTTATCCAGAAAAAGCCGCGGACAGCGGCTCCATGCCGGAATCTAGCAGGC

ATCCCGCGACCCGAGGTTACCGTCGGTGACCAGGAAAGTGCAAGTCCTTCCATGCTGGCT

GGCATGCAGATGAAGTGGCTCGATTTTGAACAACATCTTGTACCA

>CL874.Contig5_C-W 1 280 LEN=554

XXGCGGGCTCTGCGGGGAAACCTTGGTAGCTGGGGGGTGATCTGGGCTGGTGATCTGGAC

CCCGGCAGGGATGAAGGGTCCAGTGCCCGAGATGCATTCGGGAAATGCGAGAAGCAGCCT

GCATCGCTTATCCAGAAAAAGCCGCGGACAGCGGCTCCATGCCGGAATCTAGCAGGCATC

CCGCGACCCGAGGTTACCGTCGGTGACCAGGAAAGTGCAAGTCCTTCCATGCTGGCTGGC

ATGCAGATGAAGTGGCTCGATTTTGAACAACATCTTGTACCA

>CL933.Contig3_C-W 1 279 LEN=279; minus strand

XXTGAGAATGTTGACTGGAAAAATGACTGTGAGGCTTCAATGCGGTCTTGTGACCAGCTT

GGTACCCCCGATTCCTGGTCCGGGTCTCCAGTTTCACCAGACTATCAGCGATTCTGGAGA

TGTTACTGATATAAGACAGCGCTCAAACAGATGTTGGGAAGAGTCAACGAAAGGAGGCAG

GAAAAGTGCTTGGCACAGAGCCTGGCACACAGCAAACGCTTAATAAACGTTCGCGTTACC

GTCTGGGATGACATCTTGAGAAAGAGACTTTTGAGGTGGCCX

>CL977.Contig3_C-W 1 303 LEN=303

CTGCTCATCTCTTCAGTTAAAATGTTCCAGCCTGTATGGTATGACACAACAGAAGTGAAG

GATGTAGACAATGGAGTGTAACAGGACATGGGGGTTTTTGCCATATTTGCGAGTAGTGGA

GCCACCTGCTTCAGAGGGGTGGCTGTGAGGAGATGTCAGCAGTTCTCCTGGGATATCACT

GCCTCCCTTGAGAAAGGTTGCTGTCCTCAGGCAGAGGCAATTGCCAGTCCCAGGTGGCTC

CAGAAGGCAGAAGTCACTTGTGGAAAGCTGAATTATTCTGAGGAAGAACCAATAGAGAAG

AAG

>CL1080.Contig1_C-W 1043 1417 LEN=1417; minus strand

GTGAACAACATGAAAAAACCAGGGAAAAAAGGAGTACAGATAATGCAGGACAACTTAAAT

CTACAGGAGGACCTAGAAGCATCAGAAACATGGACAGGGAAAGAAATCAAGGCATACCTA

ACTCAGATGGAACGGAATTTTAGAGAAGACATGAGACAGCAAATCCAAGAATTGAAAGTA

TATTTTGAAAACCAACTAAACAAACAAATTCAAACTGCAAAGAATGAGCTTTACCAGGAG

ATAGAGATCTTAAAAAAAAAACCAAACAGTAATCCAGAAATGCAAGAAACCATAAACCAG

ATTAAAAACTCTAACGAGAATATTACAAATAGACTAGATCAAGTAGAAGTCAGAACATCA

GATAATGAAGACAAX

>CL1107.Contig1_C-W 1 293 LEN=293; minus strand

XXCTGTTTCATGTGTTCTCTTTCTCTTCCACTATGTTATGATGGATCAGGAAGTCCCTTG

CCAGATGACAGCACCATGCTCTTGGACTTCTGGTCTCAAGAACCATCAGGATATGATATT

GAGATTGGAGGCAGATGTGTGAATTTTATTCATGAAGAAATTCTGCCTGGACCTGCAGAT

GGAAGATTGCCAGATAGTTCTGAGGAGAAAGTGTGCTGTGAAGCAAAAGAGGACTGCAGT

CAAGGAAGACGTCTAATGGCCCCGCTGAGGTGCTGCCAGACAGTCCATCTGGCC

>CL1246.Contig1_C-W 1 95 LEN=341

XCCCGGCCAACTGGAACTGCCCAGCTGATTGTCAACAGTGAGAAGATGGTAATTGGTACC

ATGCTGGTGAAGGATGTTATTCAAGCTCTCACCCAG

>CL1246.Contig2_C-W 1 95 LEN=310

XCCCGGCCAACTGGAACTGCCCAGCTGATTGTCAACAGTGAGAAGATGGTAATTGGTACC

ATGCTGGTGAAGGATGTTATTCAAGCTCTCACCCAG

>CL1250.Contig3_C-W 1 501 LEN=701

XXCCAAATTCAAAATGGCGATAGTGCAGAGTCTGACCATGACTCGCAGTCTGAGGGAGAA

GAATCAGACGTTGAAATTCAGGACTTACAGAGAAATCTTAACAGGCTGCGTTTAACACCG

CCTGCCCAGGCTATTCGAATTCGGCCAGTGCCTGCTAAAAGGACAAAATTTAACAGAAAG

TGCTTTTCXGTCCTATTTGGGTACCTGAACGTGCTATCAGACATGGAAGAGATAGAACCC

AGATTCAAGCGACTGAGGATCGACCCAGATGAGCCCCTATCCAGAAGGAGGAGATATCGG

AAAAGGATGAAGAAAGACCAGATTGACCCAGCCGAAAATCTGATTTCATGGGAAGACGTG

AAGAAGCTAACAACACAGGCTTCCCGAATACTTCGACTCCTAGGAAAGAACAGGACCCCA

GTGATGATGGTAGCAACAGTAATTGCCCTGCTGGGCTGTCAGGATGGCGAGTCTTCAGAA

CAAGAATGCAGAAACTCAAAAGAG

>CL1391.Contig1_C-W 301 684 LEN=941

ATGATTCAGTGTGCTAAAACCTATTTCCCGAACTTGGATGCAAACGAACTGGCTAAACAG

TTGTCATTAGCTGCTGAGCTCGTCTCAACGAACAGAGATACACTTTCTACTGGTCCTGTG

GAAAATATGGTTCAGTGTGCTAAAAGACATTTGCCGAACTTGAACGCGAACGAACTGGCT

GAACGGTTGTCATTTGTCGCTGAGCTCGTCTCAACGAACAAAGATAAATTTTCTCCTGAT

CTTCTGAGAAGTGTTGTTGGTGCCGAAATAAATGTTGCTGGTGCCGTTGATAAACTCAAA

AACAAACTTGCTGGTGCCGGAAACGAACTTGCTAATACCCTTGCTGGTGCCAGAAGCAAA

GTTGGCAATCTCTTAGGCAATGTC

>CL1391.Contig2_C-W 301 684 LEN=941

ATGATTCAGTGTGCTAAAACCTATTTCCCGAACTTGGATGCAAACGAACTGGCTAAACAG

TTGTCATTAGCTGCTGAGCTCGTCTCAACGAACAGAGATACACTTTCTACTGGTCCTGTG

GAAAATATGGTTCAGTGTGCTAAAAGACATTTGCCGAACTTGAACGCGAACGAACTGGCT

GAACGGTTGTCATTTGTCGCTGAGCTCGTCTCAACGAACAAAGATAAATTTTCTCCTGAT

CTTCTGAGAAGTGTTGTTGGTGCCGAAATAAATGTTGCTGGTGCCGTTGATAAACTCAAA

AACAAACTTGCTGGTGCCGGAAACGAACTTGCTAATACCCTTGCTGGTGCCAGAAGCAAA

GTTGGCAATCTCTTAGGCAATGTC

>CL1391.Contig3_C-W 301 717 LEN=730

ATGATTCAGTGTGCTAAAACCTATTTCCCGAACTTGGATGCAAACGAACTGGCTAAACAG

TTGTCATTAGCTGCTGAGCTCGTCTCAACGAACAGAGATACACTTTCTACTGGTCCTGTG

GAAAATATGGTTCAGTGTGCTAAAAGACATTTGCCGAACTTGAACGCGAACGAACTGGCT

GAACGGTTGTCATTTGTCGCTGAGCTCGTCTCAACGAACAAAGATAAATTTTCTCCTGAT

CTTCTGAGAAGTGTTGTTGGTGCCGAAATAAATGTTGCTGGTGCCGTTGATAAACTCAAA

AACAAACTTGCTGGTGCCGGAAACGAACTTGCTAATACCCTTGCTGGTGCCGGAAACGAA

CTTGCTAATACCCTTGCTGGTGCCAGAAGCAAAGTTGGCAATCTCTTAGGCAATGTC

>CL1391.Contig4_C-W 301 620 LEN=620

ATGATTCAGTGTGCTAAAACCTATTTCCCGAACTTGGATGCAAACGAACTGGCTAAACAG

TTGTCATTAGCTGCTGAGCTCGTCTCAACGAACAGAGATACACTTTCTACTGGTCCTGTG

GAAAATATGGTTCAGTGTGCTAAAAGACATTTGCCGAACTTGAACGCGAACGAACTGGCT

GAACGGTTGTCATTAGCTGCTCAGCTCGTCTCAACGAACAGAGATACACTTTCTACTGGT

CCTGTGGAAAATATGGTTCAGTGTGCTAAAAGACATTTGCCGAACTTGAACGCGAACGAA

CTGGCTGAACGGTTGTCATTX

>CL1436.Contig1_C-W 1 238 LEN=238

XXACCTGCTGGGCCTTGGGTGGTTTCACAAAGAGGAGAATATGCACCAGCAAAAATAGCT

GAAGCGTGTGTTGTCGCTAGCATCACTGTCATATTCACTCAGACCAACAAGTTAGAAGAA

CCAAAATTAGTAGCCAAAAAGAAAAATAATGATGATGAAACTGTGAAGCTATTTAACTTC

ATTACAGGTTCAACATCCCTAATCCATCCATCTGCAATCCAAAATGCTCCAAAATCTGAX

>CL1606.Contig2_C-W 1 311 LEN=311; minus strand

XCTAGCAACAAGTTTGTGTGGCGGCAGAAGGGTGTTCACCAAGGCGAGCGCTCAGAXAAG

TCTATCCTGGACCGTACCAGAGGACTTGGGGATCCAACTTTTCACTCACGCTTAACCCTT

CTAAGGGCAATTTATGGCACAGATGACCTGAGGAATGCACTTCATGGGAGTAATGACTTT

ACTGCAGCAGAAAGAGAAATTCGTTTCATGTTTCCTAAAGATTTGGCTGCTGATTGGTTG

CTGCAGAATAATCCCAACAAACCCAAATTTGATTATCATCATATAATTGCAGAAGAGCCT

CAAAATCCTCCA

>CL1709.Contig3_C-W 1 609 LEN=609

GTTGAATTCACAGCACCAAAAAAGAAAAAATTATCTTTACCAAGACTAAAAGGGAAAAAA

AATCTTTTAGGAAAAGAAATCAAGAACTCTGAAGACCCTGAGAGAACACAGCTTCCCCCA

GAACAGGGTCGGTTTATGGAACCTTTAGCCACCAGCGAAGAACAAACTGCGGTCTGTCCC

CAGACTCTTCATGGGCCTCCACCAACATCATCCAGCAGTCCTCCAATCAAGCGACCCAAC

ATTGAGAAGTCTGTATCTTTGGTGAATGAGCAAGAAAAAATTGCTTCAGATCTTCAAGGC

GGGCACGTAAGAGCACAAGAAATGGACAACACAAACACCCAAACACATGATAACATGAAG

GTACAGGAATCTCAGGGACAGCATCAACTTCCAGAACTGTCATCATTTGCCACACAAACA

ACTGTGAGTATGTTCCACACTTTTAXAGATGGCTCCCAGAACACCATCCTGCAGTTTCCA

GACTCTTCAGGTGACTCTAACAGAACCATCCACCAGAATCCAAACTCTGCAGGTGGGTCA

GACCATACTGTCTACCAGTCTCTAGATTCCTCTGATGACTCAAACCCCATTCAGCAGTTG

CTTCCTCAC

>CL1776.Contig1_C-W 1 222 LEN=640

GTGGTCGGTGGCTGTATAGATGCGGTGGACTGCATCTTCCAATCTGACGATTGGTTTGAA

GGGCTGCGGCAGCCCATACGCCTCCCCGAAGACGGGCAACTTCCGGATGGACGACCTAAG

ACAGGGGAGCGTACTTGTCGCTTTGATACATCGGTACCAATACGGCCATCGGCACCTCCG

TATCAAGGGGGTGGAATGACAGGAGTAAATATTGAGGAGGAC

>CL1776.Contig2_C-W 1 821 LEN=1414

AGAAAATTGGTTCATGCAGATATTAGTTGCATTATGAGATGGAATCCTTTTCATTGGCTC

AAGGAAGGATTATTGTATATTTGCTGTTTTGTGTTGTTTGTAATATGCATAAAGATATGT

TTAAAGGTAAATAAGTCTGCAGGAAAGAAAACTTACATTAAAATGGATAAACTTCAACAT

TACAGGAACTATGAGGCAAAAGAAACTAAGACAGCTAAATCCAAGAAGGAGAAGGACGAA

AAGGTCGAAGAATTGCTCTGAGCAGTTGCACTACGCAAAATGTCACCTTTGCATCGAATT

TCATAATTTCTTGAGGAACTXATGTCTGTGGACCAGATAATCGTCAAACGGATATTGGGA

CGCAAGCTGAGGAACGACCTATTATTCAAGAAGGACCGGATGATTCCAATTCCAATGTTT

TTGGAAGATGAAGTTAAATATCCACTTATGATAGTTCATAATTTAGTGATAGATTTATTG

TTAGTTAAGGATTTAGATTTGGATATATATTTGCGTTTGAAAACCCTTGGAGTTACTACT

GTGAGAGCTCCATTCTGCTGTGACATACGTACTTTGCTCCTTCCTCTCCTACGCTCCTGT

GTGGTCGGTGGCTGTATAGATGCGGTGGACTGCATCTTCCAATCTGACGATTGGTTTGAA

GGGCTGCGGCAGCCCATACGCCTCCCCGAAGACGGGCAACTTCCGGATGGACGACCTAAG

ACAGGGGAGCGTACTTGTCGCTTTGATACATCGGTACCAATACGGCCATCGGCACCTCCG

TATCAAGGGGGTGGAATGACAGGAGTAAATATTGAGGAGGAC

>CL1790.Contig1_C-W 1 227 LEN=862

XGGTGCTTTCAAAACGTGAACTTTTCAGGGAACATTAACAATACAAGAGGAAGATGGCGG

CGAATGGAGTGCATCACCCCCGTGTACCACATCACTGCGCGGGTGAATGACGAGTTAGAA

CGGCCAAAAGCTATCTTGTTAGGAATTTCCAGCAAAATTGGGGTGCACCGAAACATAGAG

GAAGGATTTCCATCATCCAAGGATCGGTTACTGGGGATCAACCGTGAG

>CL1891.Contig1_C-W 1 204 LEN=204

XXTGTTAATAAGTGGGTGGATGAAACCTGCAATTTCAGAAGAAAGCAAGCAAACAAGCTG

AACTTTACTCTTATATTCAGAAAAATGATTCAAAAGCCTTATAGGGAAGAGATGGAACAG

GAGTTCCAGAAAGCAATGCTTCTTTGGAGGTTTGGAAGAGCAACTTTTGTCCATGAGCCA

AGCCATGGCATCACCCTGAGAGTTAGX

>CL2052.Contig1_C-W 1 132 LEN=269; minus strand

CAGCCAGCCACCACCAGGAACTACACCCTGCAGGTCTCTTCAAGCTTCAGGGAGCAAGGT

CTGTTGTTTGGCCCCAAGATCCTACTGACAGTCGGTGTCTCTGCCGTCTACATCTACAGG

AAGCAGAAGCCC

>CL2100.Contig1_C-W 1 299 LEN=299; minus strand

XATACACTGGTTGTAGGGAGAAAATCTATTATAACTTTGTATCTGCCACTTTCTCTTGCA

GAAAAACTCCTCGAGACCAGGGGTAACCGGCGCAAAGCCCTAGATTCCAGTCCAGCCCTG

CCACCAACTGGCTGCGCCACTAGGCCTGGGTCACTACTCAGGGGCTCTACTCGCCTGTCT

GCAAAAGAGTCAGTGGGGTTGTCTCAAATAGCCTTTGATTCATATTTACCTAAAACATGA

AAACTCATGAAGCCAGCAAAACAGTCTCAGGCAGCCACCCCAGCAGATGACTTAGTGGAC

>CL2221.Contig2_C-W 1 250 LEN=250

XGCTGGGCCGCAGTCACCGCGCCTCCAGCCTCCCGGATCGCCAACCTGCGGAAAAGGAGC

GTACCTGGTGGAGAGGCGGCTAGAGGGTCAGCGGGCCCAGCCGACCTAGAAGGCGAGAAG

GAGCCTGGCGGCAGTCTGGCCGACCTCAGCCGCTGCCAACTGCATCCACTACTCTCGGTC

AACACCAGCCACATTGACTCTTCAGATTATGCCGGACCCCGAAACCAAAGGCGGAGAGAG

CCGGGGTTTGXX

>CL2326.Contig1_C-W 160 700 LEN=758

GTGGCGGCCCGCGGGAGCGCCCCTTGGTCTTCAGTTCAGGACGTGACGGTGAATCGGGGG

GAGCAGGCTATGTTCTCTTGTGTCGTGGGCTTCCAGCTGCCGCCCGAGGAGGTCACCTAT

ACCTGGAAGTTCCTGCGGGAGATGCGCACTCGGGACGTGTCCTACTTCCGAGAAGTGCCC

CAGGCCCGAGGGTACCTGGCGCGGATCCGGCCAGTGCAACCCAAGCACCGCGGCACCTTC

TCCTGCGTGATCAAGCACGACCAGCGCCCCCTGGCGCGCCTCTACTTCTTCCTGAACGTG

ACGGGCCCGCCCCCGCGCGGCGAGACCGAGCTCCAAGTAGCGTTTCGGGAAGTGGTGAGC

TGGGCGCCGCAGGACGCGGAAATGATCGAGCCCTGGCGGCCCAGCCTGGGCGAACTGCTG

GCCAGGCCCGAGGCTCTGACGCTGGGCAATCTCTGCATGCTCGCAGCTGTAGCCGCCCTG

GCATCTGCGATGGCGACCATGCTGGTGTGGATGTTCTTTCGATGGTACCTCAGTGGCAAC

>CL2674.Contig1_C-W 1 342 LEN=405

GGAAAAAGGAAGAAAGGGAAGAAGAAAGGAGGAATGAAGAAAAAGGTTTTACAGGAGATT

GTTATATTTATGGGAAAAACATCAGATGACTATTAATTGAAGAGGCTGGTTGAGGAGTTG

AAATTCAATAAGGAATTAATTCCTTGTATTGCCATTTCCAGTAATACTGTGAACTGTCTT

CAAGCAACTGCAAATCAAGTATCAACTAAATGGGAGCAATGGGAGATTGAAGGAAGCTGA

AGAGGGAGCAGGCTTAAGGACACTAATGACTTTTCTAAGGCATTTGGGAAGACCCCAAAC

TACACTGGCACCAATTTCCCAACATTCTCCCACTTTCGAGCA

>CL2822.Contig1_C-W 1 235 LEN=235

XXGGCCTTCCGAGCGTCCAGGCGCCCGCGGTGTTGCGGGGAGTCCCGGAACCCGCGAGCC

GGAGCCACCGGGCTCGAGTGGGACTGGCGCGCGGCCGGCGCTTCCCGTGTGGCGACACGA

CTGCTGAGCGCTTGGGGGAGGCTGCGCAGTGGCCGCCGCGGAAGCCGTGCGGGGAGCCGA

GCCTCCATGTTCAACCAGCAGCAGCAGCAGTTCCAGCAGCAGCAGCTGCAGCAGCAG

>CL2846.Contig2_C-W 1 264 LEN=264; minus strand

CCCAAGGTTTTGGTCTTGACCCTCAGTTGTGGGAATTTGCAGCTTCACCTTTTGGTGGCA

GACCTTGGAGGCCCAGAGTTTCTCCAGAATTGGCAAGATAGGTCCAGAAAGCGCCTGCAG

GCAGGAAGAAATATGGCAATCCGGGGCCAGCAGAGAACTAGCGTAATATCAAAGCAGGGC

TTCATGATGTTCCAAAGCAAAGTTCATGATATTCCAAGATCTGGGAACATGCAGACCCTC

AAGGGAGGGCTGTGGACAGCCACA

>CL2931.Contig1_C-W 1 204 LEN=204; minus strand

GCCTTTCCACCTTCAGATGCCAGACCATCGCTCACGTTGTTTCATTTTTCTCCAGGAACA

GATTATGGCCTTTGCCAGCAAATGAGCGCTACTCTTCTGGAGATAATGCTGTTGAAAGAA

GATCCTGCCCTGCAACCACCACATACATTTTCAAGATTTGATTTAATAAACTTACTTTTG

TTCACTGAGGAAGGACTTGCAGAC

>CL2998.Contig2_C-W 1 306 LEN=527; minus strand

AGCAAACCAATATACTGACAGTTACTCTATATTTCCCTAGATTGCTATCAAGTCATAATA

GAGGTGGTATCCATTGATGAGTTAGAAGACCCAAAATATTACATTGTTGAGGTAGCCTCT

GCAACAAATGAGCAAATTCCCCCAAATGACATGGTGGAGTTCATTCCTGGAGAAAGCATG

GGTCAGGAGGCTCCATCAAAAGCCATGGAGGATTCAAGAGGATCCATAATTGGCCACATC

ATCACCATCACGGACATGGTCCACAGAGAAGACATAGAAACTGATGCAGAGGAGGAGGAG

GAGGAA

>CL2998.Contig3_C-W 141 504 LEN=738; minus strand

ATGGCGGAGCAGACCTACTCGTGGCTGTTGCCTCCCATGTTGGAAGAAACCCCCAAATGC

AGAAGACTTACACAGGCCTTGGTTCAGAGGAGGCCTCTGGCCAGAAACAGTGCTGTGCTA

AATGAAAATTCCAACGAAGTTCCCCCAAATGACATGGTGGAGTTCATTCCTGGAGAAAGC

ATGGGTCAGGAGGCTCCATCAAAAGCCATGGAGGATTCAAGAGGATCCATAATTGGTTGG

ACATGGAACAAAGATTATGTTTCAAGCTGTGATGAGGAAGAGCCTGCAGGCCACATCATC

ACCATCACGGACATGGTCCACAGAGAAGACATAGAAACTGATGCAGAGGAGGAGGAGGAG

GAA

>CL3114.Contig4_C-W 1 240 LEN=644

CAGGGAGGCCTGCAGGCAGGGCTGGGCTTCTCCTACCCCGTGGTGTGCACTGAGGGGCAA

CTGCAGGTGCCTGCTGACTGGGACCGTGGAATCCACTGGGCGCCGGTCACAGGTGCACTG

CAGCAGCACCTCGCCTCTCCCCACAGGGAAGCCCTGGGCCGCCTGGCTGCTCTGAGCAGC

GACAGCGACGGTGACAGCAGCGACGGGGACAGCAGTGATGGTGACAGTAGCAAGGGCAGG

>CL3238.Contig1_C-W 1 246 LEN=246

XXCAGGGGGAAATCCATTTCCAGCTTGAACCTGATGTGGAAGTTCCTCTTCATGTCCAAG

GCCAACGTGGGAGTGAAGGTCATTCTTGGCTCGTGAGCGATGGGAGATGGGCATTTTGGT

AGATCACAGCTGTGTCAGTTAGCTTTGCGTTACTGTGTCAAATACCTGACATAACCAGCT

TTACTCCCAAAGAACAAGTTGCAATCTGGAAGGATCTGGCACTGTAGATTAACAGCAGTC

ACCAAGAGX

>CL3314.Contig2_C-W 1 257 LEN=257

XAAGAAAATGGAGAATTACCTCACACTTCTAATGAATTGAAGAATGATGAAGAAACAAAT

GAAACTGAGGAACCTAAACTTAAAACATTACCTGGAAACTGTGATGGTGATATTAAAAAG

AAAAATGTGATTGAAACCACTCCTGTGGAGGAAATCAGTATAAAGAACTATCAGAGTATT

TTTTCTTCCTCAGTTGGCTCAAAGGAAAATGACCAGCTGAACATACCAAAGGTATTCTCT

GATTTTGAAACTGCAAGX

>CL3340.Contig2_C-W 1 302 LEN=302

XCATCCATGTTTGTGAAGGGCAAAAGAGGATGTAGAAATAGTACTAACTATGTGCAGTAT

GAAGAGGTGCTCAGTCACCAGTGCTCTAAGACCGTGGTAGTGAGGTGTCCGTGCTTGTTC

TACTGTGGAATGAAAGAGAAGGCTGAACACAGTCATAACAGATTGGTAGTGGTGGGGGAA

GATACCCAGAGACAGAACAAACAGAGGGACCAGGGAACATTCTGCATTAAATATGTAACC

TCATTAAATAGTCAACATTCAAATTTACATGGGCAAGGCACCAGGAGAGTGACATTGAGA

AAG

>CL3373.Contig1_C-W 1 71 LEN=447

XGCACGAACTTCCCCTACTTCTACGTGTTGGCCTCGGTGATACTCAACGTCCGCCTGCAG

GTGCGGATTGAG

>CL3373.Contig2_C-W 1 71 LEN=736

XGCACGAACTTCCCCTACTTCTACGTGTTGGCCTCGGTGATACTCAACGTCCGCCTGCAG

GTGCGGATTGAG

>CL3425.Contig1_C-W 1 515 LEN=820

XCCCTGTTTGATTTCTCTCTATTTCCTAGTCGTTATTTGGGATTCCATTCATTCAGAGCC

CGAGCTCTGGTTAGCTTTCTCCATGATGTCAGCCCCCAGCACCACTGTGCGGTGACTACA

CTTGTTGATGTTCAAGCTGTCCCTTTCGGCCAAGCCTCTTCGTGTGGTCTCCTGTATCCT

CGTGGATTTGACCCCAGTAGTCGTCGACAGCTGCCTTGCTTCCCGATGACAAGATGTTCC

CAGTTCACCTGCAACATCTCTTGCCGCAGATCTGGGAACTGGCCGCTTCTCCACGGAGCC

CTGAATTCTAATGGACAGTGGTGTTTAGAGACCATAACCCCAGGTGACAGGGTGCTCACT

GCACACACAATGAGTCGCTCTGTCTCACTTCTCCATGGAAGGAGCCAGCAAACACGTGTT

CCTGTCTATAAGGCTCATGAGAGCTTCAGTTCAAGCCACAGGTCTTCACCTCATCTCTGT

GATCTCTCAGGTGCCTCTGCTTTCTTCCATGACACG

>CL3529.Contig3_C-W 1 216 LEN=216

XGTTCTGCAGGAATGCAGGATGCTGAAGTTAATGGGTTGTGGAGGCTTCTGTCAAGATTT

CAAAGGAAGGTCTTGGTGGCCAGGCAAAGTGCAGCAGGGTTGAAGTCCCTATGGGCAGCC

CCTATGAGGGTGATGTATGGAGATGTGAGAAAGCCAAAGCTGCAGCAGAGACCCCCAAGA

TTAAAAAATGGCAGCAATGTAGAACCTCTGCCTAGGAXX

>CL3695.Contig1_C-W 1 784 LEN=784; minus strand

XCCCACACGGGGAGACGGCAGGAAGCTCAGACCGTGGCCCCAACAGCAAGGCGTCATAGT

TTTGGAAACAGCTAGGACCGGAAATTAGTGAGGCTGATTAGGAAAATCAACTTGGCTTGA

CTCGAGAAGTCTCATTGGGAGATGACTGTTGCCACCAAGGACACAGGGAGGACACAGAGT

CGAGACTCACCCTCACCTGCTGAGGCAGGCCCGGATGCCAAGGATGGTGACTGTCATGGA

TCCTCCACCAGTGCCTATTCTGTGGATGAATTAGGCCTGCAAACCCTGAGCTTGGCTCTT

GGGGACAAGGTCAATCCTTCCCCCACCTCCTATGAGGTAGGAAAAAATGGTGTGATTGTT

GGAAATACGTGCATCTATCAAAAGGACATGAAATGCCTGCTCAGAGCCTGCATTCCTTCC

AGAAAATACTTCAGGTCCCAACCCTGCTCCTGTGCTGCCAGGCTAAGCCCCCCACCTGGG

TGGGTCCTAAGTCAACATGACGATGTGCTTACAAGAAGACAGCCATGTGAAGACAGAGAC

GGGGGACCCTGTGAGAAGCTTGAGAAATGCCTCTCCAAGGAAAGGAGTGGCCAGGATTGG

AGGCCATTGCCAGTGCTGACCTCTGCCACAGCCTCAGAAGGAATCAGCACCAGGGAATAC

ATCTCTGTTGTTAAACCCAGCCAGCATGTGGTCACTTGTTACTGCAGACCTAGCCAGCGA

TACCTGGCCTTTCACACCATCAAGAAGGTGTCAAGGGAGAGGACAGAAGAATTTTTTAAA

AACCXX

>CL3721.Contig1_C-W 1 239 LEN=239; minus strand

CATGAATCCTCAAGACCAACGTACACTGCTCACCTGGAGAAGTGGTTCATCCTAAATGTT

TTAGGTCCTTGGAAGGAAATTCAGAGGGTGATCCCTGAAGAGAGCCTTCACTTCACAGCT

GAGGAAATACACTACCTAAGGAGGGAGAAGAAAGCCCAGTGTCTTAGAAGAAAACAAATC

CTGCTCTCCAACCTGCATGGCAACGTAATTAACCAGCATAAAATCAAAACACCAAGGGGX

>CL3836.Contig8_C-W 1 220 LEN=220

GCCAGGCCCCGCTCACCGGTGACGTTCACCACAAAACTGGCTACATGCCGGGTCTGCTGC

TCCCGGGAGCCCTATTTTCTTGAACTACTGGGCACACTCTATCTGTTTGCCATTTCCCCA

GACCCTAAGGTTGTGGAGTGCCAGGCTGGGAACCATCAGCTTATTCTGGCTGCCCTCCGG

TACCCATGCCTCTGGGAGCTGGTGCAAGAGACCTCGGTTGXX

>CL3856.Contig2_C-W 1 323 LEN=627

XTTGACAAGAACAATTTTAGAAAAGTTTTATTTTAGGCCTGCTTCAGAGGGTTTGGTCTG

CAGACAGCTGATTCTGTTGCTCTTGGCCCAAGTGTAGCAGAATATCATGGCAGAAGGGTG

TGGAGGAGGAAATTGGTTCAGGACACCGGCCACCAGAAAGCAGAGGTGGAGAGTTCTCTT

CTTACCCAAGTCATCATGGAGAACTTCAGAGCTCTGGGACAGAGTCTGAAAACTCCACGG

CTCTTTGAGATGTGCTCAGCTTGTTTTTCTAATGAGCTGGATGGCTACTGCATCTATATC

TTTTATGAATCCTCAGACGATGTC

>CL3887.Contig2_C-W 1 268 LEN=268; minus strand

XXCTTCATTGCTGTCTTCAATAGCTCACAAGTGGCTCCTTCTGGAACTGGTGGTGAAGCA

GCCAGTGGAGCAGCCAGTGGGGGTGCTGAGGCAGCAGCGCTGGCAGGAACCCCTGAGGAC

TTTGAAGACCCAGAAAGCTTGAGTCCCTTTCTGGACTGCTTCTTTTTAATTCCCGCCTCG

GCCAAGTCTCCCGGGACTTCAGCATCCGAGATCAAGGTCTCTGCTGGTGGACTCCTATAT

AAAAGGCTCCCGGCTTTCTTCCACTTGTTC

>CL3977.Contig2_C-W 167 467 LEN=467; minus strand

ATGAAGGCCACAGTATCTGGAGACACGGGAGCACTAGCTGCAGCCCAGAAGCCACATCCC

AAGATGACTGGATCTTCCTTCTCTCAGTCCCAGGACAACAGCTCAAGGAGGCTACATATC

CTAGATTTTAGAGATCTGGCTTCTGAGCTGATTCTGCAAAGGCTTCCGAACACAGCTGGA

AGATATGGACAAATCCCTGGGAACAAGAGACCTTTTGCTGTCCTTAGACACACCGAGGCC

CTCATACCCACCACTGCCTTCTTCAGGCTGAGCTCTGGACCCACAAATAAGCCAGACAGA

CXX

>CL4064.Contig3_C-W 1 417 LEN=417; minus strand

XXCTCCATTTTAGCTTGTATTTATAATTTCAAAATTATTCTTGTACAGGAAGTCTTCTCT

CCTGGTGGTCATGTTCAAGAAGAAGGTGGCATCTCCCAGAGTGGTGGCTGTGGTCATCTC

GATCCCGCAATGCAGCCTCCTAGGCCACAGGACAGAGACTCCTTTGCAGGTCCTTTXAGG

GGTGACCTCCTGGGCACCGACTTCCAGGTGCAGACTCTCTCTCGAATGGGCCCAGTTCCT

CCTCTTTCTGGGAGAACAGTGAGCAATCCCCGAGCAGATCTTGCCTCATCTCGGGACCGA

AGGCTCTCAATTACATCTCTGTTCCTGCCACATCAGAGAAGGTGGGAGGGGGGTGGTGGA

ACATCCATGGCCAAGGACGTGGGTGTTTTCATGTGTGGGACCTCAGCGACTGTTGCCAAC

>CL4108.Contig5_C-W 1 259 LEN=259; minus strand

XCAGCCACACCTGATGAGCTGCTGCTCAAACCTGCTTTGCCTGATGAATCATGGTTGGAA

GCTTTATCTGACTTGTGGCACAGTAAGGGAGCTGAGTCCATGGAGCTGGACTGGGGAATG

AGGGATGATCCTGGAGAGACTAAAGGTGATATCTTCTATAGTGTTAGGACACAGCAAGAA

GGCTCTCACCACATGAAGCCCCTCGATCTTGGACTTCCCAGCTTTCAGAATAATGACCAT

AATAAACTTCTAGTATTTAXX

>CL4407.Contig3_C-W 1 225 LEN=225; minus strand

XGAGGGGACCCCACAATCATTGAAGACCCCAGGCCTGAGGACGTTGTTGTGGTTGTGATG

GTCGTGCGGGTCACCGTCACCGCCATGGCTGCGGTCTTAGCTGAGCCGGCCAAAGAGAAA

GATCCGGCAATGAAGGCGATTCAGTCGTCCACCTGGAGAAGACTGGTCAGCGCCCCTCTT

CGGTCAGCGGCGGTTGCGGCTGAAGCAAGCGCGACAGCTGCAGACCXX

>CL4499.Contig3_C-W 1 486 LEN=673; minus strand

XXCCTCAAGCCGCGCTGGCAGCAGAAGAAGGACCCTTTGTGCTCAGCTCTGGAATCCCTG

AATCCCATCGTCTTCCCTAACCCTTACGGCTGCCATGGGTACTTCTCAGGGAGTCTTATT

AAGGAATTTGGGGCCTGCCCCCACGAGATGAAGATGAGGACCAACTTTTCTTCTATTAGA

CGCAGAGTCTCTGGTCTGATTCCTCAGTCTGCCATCTTGAAAGCTGACCGTGCAGAACCC

CTGAAGCTCTCCTGGGAGGAACCATCACCACCTTCAGAAAGGCCACCTCAACTTGGAGAA

GATGACCAAGGCTCATCAGTGGATGTGTCCTGTCTTGTGGAAACCCTCACAGTACTTCTC

TGGGAGCTCGAGGACGGCCTGGTGCCTGCCCTGCAGCAGGGGAACACCCCCTATGTCCAT

GGGGTTTTGTATTTGTACCAAGATTTGGCCAGTACCCAGAAGGCTCTGGAGCTGCTGGGT

AAAAGA

>CL4634.Contig1_C-W 1 312 LEN=312

AAGCCCTACGGACGTCGCAATGAGCAGGAATCCTGTGTTTGGAGTTCCAAAGTCCTTCCG

AATGTTAACACAAAGACCCTGGGGAAGGGGATGTTCACCGTGTGCTTGGCTGTCTCTAAA

CTGGTGTTTACCAGGACCATCATGGAGCACXGTAATATGCCCCTAAGAGTTTATGCCACT

GCCTGGGATGGAGAGGAAGAACTCCATGCACTCAAGACTCCAGAAGCAGCACAGACACCC

AGGAGCAACCTCTCTGCCACTTATCCAATCAGCAGCCTCATCTTGTGCCTCTGCCTGTTG

ATTAAAATTCAGCXX

>CL4638.Contig1_C-W 1 237 LEN=237; minus strand

XTGATGTTAGAAGCAACTGGAGATCTGCTGGGGATATCAGACTCTGCTACTGTGGTAACA

GGTCACTGTTATGTGTACACATTCACTTTTGTATGTCCTTCTTTAAAGGTAATGAACAAT

GCCAGGTACAGTGGTGTAGGCCTGTGATCCCAGCGACTTGAGAGGCTGAAGCAGAAGGAT

CACAAGTTTGAAGCCAGTCTCAGCAACGTGATGAGCCCTAAGCAACTTAGTGAGACCTXX

>CL4648.Contig1_C-W 1 266 LEN=266

CCTGGCATTTTGCCAGAAGGAGCAGTTCCTGCTGCATTGGGTGCCCGCTATTTTTGAGCT

CTCGTGGAGTTCCTAGAGAGTTTGAGTGGGTGGAGTTCGCGGGTTGAATTCCGGTTGAGT

GCTGAAAAGTTCCCGAAGTTCCGGTGGAGCTCTCGGGGAGTTCCTGAAGAGTTCGCGTGG

GCAGCGTGTAGCATGGGTTCTAAAAATAAAGTTCGTTCCTGCTTGAATCTGAAAGTGGCT

CATGATTTGTGCCCAGCCAGACTGCGX

>CL4667.Contig1_C-W 1 231 LEN=231

XXGGGCAGAGTAAATCTGGACATCAAGACAAGGAGAAGCAGGTACCAGGCCTTTTATGGA

CCTTGCTTCCACGATAGCTTCATCTTCTCTAGTGCAGACCACATTTCTCCATGGAACAGA

AAAGAGCCAACATTAAATATTATAACTCCAGAAACTAGAGACAAGAGTCACGCTTTCCAG

AAGCCTTCTAATAGAAAAGATAAAACATACAATGATTACATTGTCACAGTGAGX

>CL4949.Contig2_C-W 1 215 LEN=215; minus strand

TGCCTTAAGCCCAGAGATGACAGGAAAGGGGCGGGGCACCGGGTTACTCTGCCCTGTAGG

CCCCGTCGAGCGTCCACGGAGGATCAAGGCGAAGGCCGAGGTCGGCAACGCCTCCGCTTC

CTCCTCAACCGGAACCCTCCAGAGCTGGCTGTGAGGCGGCCACTCCGGCGGCTGCGCCTA

CCAGAGCGGCCGGGCAGGTTGCAGGCCGGCTCCTCX

>CL5029.Contig2_C-W 1 347 LEN=433

XTTCACACCAAGATGTACACTGACATCAGTGATTCTTACCTTGATATGGAGGAAGAAACA

GGAGTTGGGAGCATCCAGAACTTGCAGGTCCTCAGCAGCAGAGTACGCACACCCAGAACT

CTGTCCCAACCTCTGCAAGTTCCAGCACACTCACTTTTGGAGACATCTGCCCTCCTCCAG

CTTGTCAACCTAAAAAAGTACACCATGTCCCACATTGAACATAACAAGTTTGGGAATGAA

CCCAGGACCTTGTATATGCTAAGCAATTGCTCTACCCCGAGCTTCACCCAGATGGCATGG

TTTATCCACGGTTGGCCTCCAGCCTCTGAGGAGCTCCATGAGAACCTG

>CL5274.Contig1_C-W 1 139 LEN=772

XXTCCAGACGCCAGTAATGGGACCCTGTGGGGTATCAAAAGTAGTTCCAGTGTTGGCAGG

AAAAGCCCAGACAGCAAAGAAGCTAGTAGCAGATGTAGGGATGAGTCACAAGAGGGATCA

AATGTCTCCATCAGCAAGAAG

>CL5276.Contig2_C-W 1 332 LEN=332

TCAAACCACAATACGCAGGCACCACAGTCTGTGGGGAGAGTGTCCATGTACTGGATTCCC

CAGAGGACTAAGGAGTGGCCAGAGTCGTTCTCCACGAAGGGCACCCGGGCCTGTGGAGTT

GCATTTGTGCCTGAGGAGAGGCGAGAAGAGCAGAAGCAAACTCACAAGAGGCAAAAGGAT

GCTCTATGTGGTGCTGTCTATTCCCAGCAGCAGACCCTGTCTTGGGCCATCAGGATTTTA

CTCAGGGATGATAGACCCACACAGAATCTTCACCTTTTAAGGAGAAGAATGAGAGGAGCA

GCCAGAAGCTTGGCATTATGGGAATCAGCCCAX

>CL5339.Contig1_C-W 1 206 LEN=206

XTGAGCCCCTTGGTCACCAATTTCTTGTATACTGTATATAGACTGGACATGTCCTTGCTG

ACTGAGCAAGACCAGCATATCAAATTCCTTGAGTCTGCTCTTTGGCCAGAGGGCACAGGC

TTCACTGTGGGTGTCATCACCACCACCTCTGGTCTTCGTCACCATACAGTGACTCTAAAG

CAGAGCTTGCATGTACCTCACTCCACT

>CL5480.Contig1_C-W 1 277 LEN=277; minus strand

AGCGAAGTTGAGGAAATATCAGAGAATGAGTTCAGAAGGTTCATAATTAAAATGATCTGT

GAATTAAAGAATGACTTAAATGAACAAATACAGGCAAAATTTGATTGCTCCAACAAAGAT

AAGAGAGCAAATACAGGTAGCAAAAAATTACTTCAAAAGAGAGAGATTGAAAAAAAAGTC

AGAAATCCTTGAAATGAAGGATACAATAAATCAAGTAAAAGTTCCACCAGAAAACTTCTT

GAACTAGTAAAACGAATTCAGCAAAGTAGCAGGATATXX

>CL5623.Contig2_C-W 1 218 LEN=280; minus strand

XTCCTGGCTTCCATCGTCGCCTTCAGTAAGAGTCTGAATGAACTGGACCTCAGGGGCAAC

CCCCTGGACAACAGCACCATTCGACTGTTGTGCTTGGCCACGTCACTGCCCGGGCGAGTC

CTGCGGAAGCTCAGAGTGAGCAGGGCTTCAACAGAGGAAGGTTTCTGGTTCGACTTGAGA

CAGGAAAACCCGCTTCCCTGCGAGATCTCCATGGATTAG

>CL5649.Contig1_C-W 1 385 LEN=385

XXGCCTACACATATGCAGTTGTTGTTACACAACAAACATCACAGTGCTGAAGTGACAAAA

TTCAGGGTGCCATCTGCAACTAGAGGACAGGAGCTGGCGCTGAGCACTTGGGGTCCAGGG

ACAGACCACAAGAATGCCTTAGATTTCACTGATGAAAACTCTGCAAATTCTCCATCACAA

AGACGATTGACAACTAAACTAAGAGCTAACAGGAAAATATCAGAAGCCAGTCTGAAAACT

GTACGGAGGACCATCACCGGAGACATTCGAGTCCCGCAGTGTCAGCCCCGTAACCAGGTG

GCACAGGAAATGCTACCCAGTGACAACCTCACCTTGGCCCCGGGCAACAGCAGCGTGTGT

GCCCGGGACTACAAGATCACCCAGGTX

>CL5818.Contig1_C-W 1 214 LEN=214

XXGCAGTGTGGTAGGCCGACACAAAGAAATGCCACAGAACCAGAAAACTTGAGGAAAATG

GTCAATCAGTTGGCAGTTCGTTTCCATCTCCAGGACTTTGATCGGCTGTCCATGAAAATC

CTGATCAAGAAAATTGAAAAGTCTGGATTTTTTGCCTCAGATGGAACTTTGCTTCAGTCA

TTTCATGTTGATCAAAGTAGACTGGCAGTTAAAGAX

>CL5928.Contig2_C-W 1 318 LEN=496; minus strand

CCGGGAGGGAAGGGCAGTGTTAAGGGGCTGGTAGCAACTCAGCGCCTGAGTTTTAGTCAG

GCAGTACGTCGGGCGCCTGGCCAGTTAGACTGTAGGAAAAAGTGGTCTGCTCTTGCCGCT

GGAGATAGAAGCGCTCTCTACTTTCGTCCTCTCCTGTTCAAGATGGTGGCCTCCCGAGCA

ATTGGCAGCCTCAACCGCTTCAACGCCTTCAGGATCCTCCGCTCCCGAGATCTGGAGGAT

CAGGACCCAGTCTGTCTCATCGTTGAGGCCTCACAGCACCTTGGCTTGCAACAAGAGGTG

TCTTACAAGCTTCTCGGA

>CL5989.Contig1_C-W 1 282 LEN=282

TTGCCTCCCAGAGAATCCCCCTCCCTGACCACCCCATCTGAAGAACTGAATTATGACAAG

CATGACAAGTCTGCCAACAGGAAAGAAAGAGGCTTCCACGCAGAGGGACCAGCACAGGCA

GGAAGCACCGGCCGCTACAGACAGTTCAGCTCATTCAAGAACAGCAAGACCGGGTGGCTG

AGAGCGGTTAAGAAAGATCACAGAGAAGGAAGGGGCCAGGTCACATGGCTTCGTGGTGAG

AATTTTAGGATTTTGTTTAAAAAGCAAGGGGAAGCTTTTGAA

>CL6037.Contig1_C-W 1 264 LEN=264; minus strand

TATTCATCTACTGACCAGATGGGGGACTTAAGAAATGGCCAAGGCGTTCAACCATTTTCC

CTGCCTCCACACGACTTCCCTTTCCCTTTATTTCCTCCTCCAGGATTAGAGTCTGTTTCC

TCCACGAAGGAATCCTCCACGAAGGAAGTGCTGTTGATGATGGGGAAAGGCATGAAACAG

GATTTCCACAATACTGATCTTGCTTCCTCAAAGCCTTGCTGCGTGCAGTTCACACAGCGT

GGAGGAGGTCATAAAGGCAGCCTG

>CL6174.Contig2_C-W 1 339 LEN=679; minus strand

GTGGTCCTTGGTAACTGCACAAGAGTAGAACATGTCTGCATCGCCAGTAGTCTGCTAATA

GGGAGGGCTGTGCGATTAAAGGCTTCCATCGATTGGGTAGTATCCTTCAACTCTTTTCGA

CCAGGACCTCTTATTCGGAAGCGTTACAGGAAGACAGCTCTCAACTTAGGGATCAGATCA

CGTTATCAACGCTCTGGGATCGCTGCAACCTGGCACTTCAATAAGTGCACCGATTACGTC

TAGACCGGCAAACACAGATCTAGAGGTGGCCAACTGATCACTGTAGGAGCTGACTGGCAA

AGTCAACCAGGCCCAACCAAGAGTGACCAAGACAACGAT

>CL6302.Contig1_C-W 1 264 LEN=329; minus strand

AACATGAAAAGACAAGGGAAGAAAGTGCCCCAAAGAAATCAAGACACCACATTATTAGAA

TCCAAGGCCAGTACAGCAGAAGAAATTACAGAGAAGGAGTTCAGGATGTACATGGTTAAA

ATGTTCTGTGCACTCAAGGAATATGTAAGAGAGCCAATGCAGGCAGTGAAAGATCTTTTC

AACAAGGAGCTACATAAACAAATACAGGAAGTAAAAGATCACCTCAACAGGGAAATAGAG

CTTCTAAAACAAAACAAAATAAGA

>Unigene95_C-W 1 100 LEN=502; minus strand

XXCGAGAAGGCCGAGCAGGAGAGGCTGGCGCGGCTGCGGCGACAGGAACAAGAGGATCTG

GAGCTGGCCATCGCGCTCAGTAAGGCCGACATGCCGGCCGCC

>Unigene552_C-W 1 963 LEN=963

XXGGACGTACATGCTTCCTACAAGCCTACCATCAAATGCAAAGATACAGCTCCCAAAAAT

GAAAAATGGATGAAGGGAATAAAAGCACTGAAATCAGAGTTACATTGGGACAAGTCATCA

TCTGAAGAGAAGGAATGGCTTGATGTCACAGAAAATCXGTCATCAACAGATATGGAACAA

CTATCTTCACACAAGACAGAAAAAAGCAATTTGATGTTGTTTAAACCAGAGGATACAGGA

AAAGGAAATGCACCATTTTTCCACATGGCAGAGTCACATTTGGACATATCATCAGAGGCA

AAACAGAAGAGGCCTGATGATACTGGACATAGCCAGCAAGAGAGTGATTCTGAGCAAGAT

AATGAGTGTACTTCTTGTCTATTTGGTGTATTCCGTTCACCCACAAAAAAAAGAATAAAA

GGATATGTAAAAGGACAAAAACACAGTGAAGAAACAGCAAATGGAAAAGGCAAAAGAAGT

ACAAATAGTTGTGGAGCATTCCCGAAACCCACAGATGTTGGATCACTTTCTTCACACAAC

AGAGGAAAAATAAAATTGGTATTGGTTGAGCCACCTGATACAGGAAAGGAAGCTATATCA

GTTTCCCACATGGCAGAGTCAAATTTGGACAGATCATCTGAGGCAGAACAGAAGAGGCTT

CATGATATGGATGATACTGCACATCACCAGCAAAAGAGTGATTCTGAGCAAGATAATGAG

TGTACTTCTTGTCTATTTGGTGTATTCCGTTCACCCACAAAAAAAAGAATAAAAGGATAT

GTAAAAGGACAAAAACACAGTGAAGAAACAGCAAATGGAAAAGGCAAAAGAAGTACAAAT

AGTTGTGGAGCATTCCCGAAACCCACAGATGTTGGATCACTTTCTTCACACAACAGAGGA

AAAATAAAATTGGTATTGGTTGAGCCACCTGATACAGGAAAGGAAGCTATATCAGTTTCC

CACATG

>Unigene1985_C-W 1 285 LEN=285

AAGACAGAAGATACCTTTTGTCCCCCAAAGAGAGGCAAAGTCTTTCTTAAGGTTACCTTA

TTTAAAGCCAATGCCAAGGAGTCAACAGGGAGCATCTACCAGGTTTACCAGAAGGAGGGC

AGAGATGACCTGAAATAAGACTCACCAAGCCCAAAAGACCAAGGTGCAGCAATGGAGAAG

GCTGAGGCAAATGTCAGAGCCACACTCAGCTCCAGGACCCTCAGAGACCAGGTACCCTCA

GAGAACCTCGCCAAAGATTTCACTCCAGACACCAGATATGCCAGX

>Unigene2308_C-W 1 229 LEN=229; minus strand

XXAAGAATCGTTGCTATTATTTATGATACTGGTACAAAGCCTCGACAGCAATCTGGGCAG

TCATCACACGCTTGCAACTTATATTTAGAGAATAAACAGCAGAAGAACAGGGAGCTGAAT

AACATTAAGAAACTGAAGAACAAACTTTTCCTCAACTCCAAAGCAAAAATTAGACCCTGG

GTGATCATTTCTTCGCTGGCGGCACAGCCTGTGATTAGCCATGAAAGACCC

>Unigene2351_C-W 1 119 LEN=419; minus strand

XACCAGGCCCAGGCCCAGGCCCAGGCCCAGTCCCAGCAGCCTGACACAGGGACCCTGGAC

AGCAAGGACATGGAGAGCAAAGGCCTGCTTTCTGGCATCAGCGCCTCCACAGAGGACCCC

>Unigene2681_C-W 1 342 LEN=342

GGCATGGCTGGGGAGATGCTGGCATCTGTGTGTGTGAGTCATGGTTGTCCCTCCTCTGTG

ACAATATCCTGTGTGCTTTATGTGCCCATGCACAAAGCTGATCCTGGTTTAGCTGTGCTG

GTCGACTGGTTGGTGCACGATTCCTGTGGGAATGGGGGAGTGAACAGGGTGGAAGGAGAC

GTCTTTCTAATTATTATCAATTCTGTATACGGCTCCTGAAAATCTCTTCTCTCTGAAGAA

CAAAGTATCAACAGCTTAGCTTGCAGACACCTGCCACCTGGAGACCATGGCAAGCTCCCA

GAGACCCTTGCACAAGGTGTTAGGGACAACCTGGTTCTCAAG

>Unigene2702_C-W 1 402 LEN=402; minus strand

CAGGACTCCCGTGACACTATCAGCACAGCAAAGGGAAAAGCAAGACCAAGAAAAAGAGCT

CTGCTACACGTCGCTAGGTGCCGCTGTTCCCAGAGCCTCCCACCACCTCGCCTGTCACCT

GCAAGGCAAGCTCAGAGGTCCCAGGGCCTATACCTGTCACCAGGTGGCCACAGCAGCTCA

AGCAGACCTGGCCCAGCCAAGGAACAGTTGTCAGAAACAGAAAGGGCTCCCCATTTTGAA

GAGGAAAAGGAAGCAGAGAAGGAATTCGCTTGTAAGTGTGAAACTGAACACTTCATCAAA

GACTTCGGAAGTGTACCACCACGGAAATGTAACATATTCTATAAAACTTCTGAATTACTC

GAGCAGGCAGGAGGGCAGCAGCAGGAGGAAGACCACCAAGAX

>Unigene2727_C-W 1 230 LEN=230; minus strand

CCGAAGAGGAACCAGGGCCAGGCGCTGGGGAGCAGCTGCGAGGTGAGGATCGACTTTGGT

AGAAACGAAACAGAACAAAGGCAGAAAGCAGAAACACCTGTCAGCTATTTGGTGTTGACG

GTCCCTGCTGTCCATGTTGACACCCTTGAGATGGGTTATGGCTTCAGTTGTGCTGAACTC

TTGGTGGCTTGTTTGCCCCCACCCTCAGCCACAGAGATCCCTTTTTTGGAX

>Unigene2882_C-W 1 336 LEN=336; minus strand

AAGGCAAACACTCTAACAACTGAGCTATATCCCCAGCCCTTTATTCCAATTGCTATCTGT

CATCATGAAATTTTATCCGTGGTGTCCCTCTTGGTCTTGGACACTCCTGCCTTCTGGTTT

TCGTCTGTCGGTCATTCGTGTCTGAAGCTGCATGCCTTACCATCGCAAGTGTCAACTAGC

TGGAAAACAAAGCCCCCTCCCAGCCCTTCCTCCACCAAGAGAAAACGACCCTTCCGAAAC

TCACTGCTGGCTACCGTCTCACTATGCCAACTCATGGCTAAGAAAGAAGGAGGAGAAAAA

GAGGATGCCAACTCGATTAAATGCAATACTCCATAT

>Unigene3026_C-W 1 206 LEN=206; minus strand

XCACCCACGTTACGACCTCGGCAGTGCTTTGGCTCTCTGAAATTAGAGAATGAGGAGGAG

GGTGAGGTCATGGTGAAAATCCCCCTGCACCGGACACTGCGATGCAGTTTCTTCCCAACA

AGGAAGCAGATAAGCAAACAGATTGAGAACTGCAGCAGGCAAATTTTAGGTTTCCTGCCA

GACTACAGAGGATATGACCAGCAAGAG

>Unigene3381_C-W 1 216 LEN=216

GGCTTTGAACTTGTGATCCTCCTTCCTCAGCCTCCTGAGCTGTTGGGATTACAGGTGTGT

GCCACTGCACCTGGCACATATACTATTTTTCACTCAGCAAATAACCACCCAGTTCTTCAA

TATGCTCAGAACTGCAGGCTCAATCTAAAATCAGAGAAGCCAGACTCCACCATTATGGAA

TTCACGGCAAGTAAAGGAGAAAGATGTTGAATGGAG

>Unigene4292_C-W 1 661 LEN=661

XXCTGGTCTGCATCTGCATGCAGCAATGGACCCCTGATTCCGGTCATCAGTGCCTGGGCC

CAGGTGCCCCTCAACTGTGCAGGTGCACTGTCACAGCTTCTCACCTGGCTGGCTCTGCTG

CAGTCACGTGGCTCCTTGCTACTTGCTCCATCACCAGCCCATGCCTGCCTCAGCCTGAGT

CTGCAAGGTCTTTCTTCTGCTACGCGCATTGAAGATAAGACAAAGAGGAAGAAGATTGGA

CCCTACCTCCAAGGAGACGGCAGTCTTGCTGGGGAAGAAGGATGTACACAAAAACCAAGC

ATTCAGCTGCGATTCAGGGCAGTGGGTAAGAGACCTCXGTGGGATGTGCGTGAGTTCCTG

GTACAGATGGTACGCTATAGATATGTCCAGGAGGAAACAAAGCAAAAGACTAAAACTCCC

CACGCAGATACTGCTTTTCTAATGTTTACAGACACCATTACACTCCCGTCTGCACACAGG

AACACCTTCCAAGAGATTTCTGTGCCTGGCTTTTTATTCCTCATCACACCAGGAATGGAG

ATGGATTTTATTGTCTTTGGGTGGACACCAAAAGGTGATGCTTTGAGCCAACTGCCAACT

ACTAGAGATAACATCTAGGCTGGGACATCCTTCTTCCTCCCTGTCCTGGGTCAATTCAAG

AGGAXX

>Unigene4333_C-W 1 202 LEN=202

CTGGCGTCTGAGGTAAGGGTGGGTGCAGTCTGCCCCTGATATGAATGGCTAACAGGAAGT

GGTGAGCAAGATTCTAGTCCAGAAAACCTGGCTGCTAAACAGTACAGTACAGTAGAACCT

GAGCATTTGAATTTGGCTACAACAGACATGAGAGGAATGGCAGGTGAATTTACTGCAGAG

GACAGTATGGTCTTTACCTGGCXX

>Unigene4633_C-W 1 216 LEN=882; minus strand

XAGGTGGACGCCTCCATCTTCAAGGGCCAGCTAGAAGGGGGAGATGCCATCCGTCCTGAC

TTCGGGCTGGGTGTGAGAGGTGTCCTGAACTTGCAGCAGATGTCTGGACGCAGTCTCTCC

CTGGAGCTGAGCAACCCCATGCCCTCTAGCACTGCCACCGGTAGCACGCCGGCTGGCGTC

CATGGAGAGCCCTTCTGCTCCGGCCTGGCCCACGTG

>Unigene4779_C-W 1 371 LEN=371

XXGATTAAGACTGATTTCATAGGTGTAGCAGTGGGTGTGGGACGGGAAGGTGATACATTG

AAGTGGCCTCCCGGCTGGCTTGGCTCTGCTCAGCTGGTGATTTGGAATTACAAAGTGAAA

GTCCTCCTGGTCACCTGGAACATGGAGAATGAAGTTTGCATCCAGAGTGAATTCACACAG

CCAGTGGTGAGGCAGCCGAGGGGCTGGTGTCTGGGAGTTCTGAAAAGGCAGAGAGAAGAT

GCATGTCCACTTAGAAAGTGGAGGGTGCTGGGGCCCTGGCAGGTCTACAGGACTGTGGAG

GTTTTGTATGCACTGGGACTCAAGTCAGGGGCATGGGAAAGTTCTGCAGCTCTTGCCATG

AGGAAGCTAAGGGXX

>Unigene5575_C-W 1 270 LEN=270

XGCCCCAGATCGCATGTTCTAGAGGCTGGCACTGGTGGCATTGGGTCTCCTACTGATGGG

GGGACTCATTATCACAACACAGCTACTGCTCTTAGGAAGGGAATGTGTTTTAAACTTGGG

AATAGTCATAATCCTGGGAAATTCCACACATTGGCAGCTTTAACCTCTAACAGTCACAGA

ACCTCTTCTCTGGGAGGAGGATCTCTCTGTGGTGTGAGGTACCTCCTCTACCAGCAGAAG

TATGGTGAAATGAGCCAGGCTTATGATTTAGXX

>Unigene5840_C-W 1 299 LEN=299; minus strand

CTCAGTACAGACATCACGGTCTTCCACTCCACTTACCTTTCAGGAACTCTTCCCAGCAGC

ACCTGGGTCTCGGCAAAGACACGGAGTAGGCTTTGGCCTAAGTGTCCCTTCTCAGCTTCA

GGGTCCCCTGCTACCATCATAACTCTGCCTTGCCCTCCCAACTTCTCCATTAAGCTGGAG

AAAAACTGGATTTCGATCTTCCAGAAGCTTTTCAATCCACCTTTACAAAGCCTCATGCTT

CTCACTTCTGCAGCAGCAGAATGCCAATTCCAAATTACTGAAATAAAACAGGAGCACACX

>Unigene6033_C-W 392 587 LEN=587; minus strand

ATGAATCAAGACCCTACTACATTTCCAAAGCCGAAAAGCCATGAGGGTTACAATTTAAAT

GAGCTTGGGAAGGAACGCTCCCGAAGACGTCTGCAGCCTGAAGGGTCAGAGCCACTGGAA

GCCTGGCTGCCTCTGGGCTTTCTCGTCTCAATGCGGCAAGGCACCGCGATCAACCAACCC

GTGATGGGGAAGGCCCXX

>Unigene6050_C-W 1 259 LEN=259

XAGGGTATTACGGTAACGGGGGACGTGTCCCCGGTCTCCTCCTGGCGCCGTAGGCTCGCC

CGGCGTGTCGTCTCTGCACCTCGTCGGGACAACGGGGTGGGGGGCGTGTGCACCAGGTTT

GGCCGCCTCGCAGTACGGTTGGCGGCTCCTCCGGGCGAAGTCCGGAGCGGGGGAGGGGCC

CGAACGGCGGCTGGGGCCACCAAGGGCGACGTCTTAGGACCTGGTTGCCCTATCCCTGCA

GGCGGCCTGGCCGGCGGCATX

>Unigene6184_C-W 1 314 LEN=314; minus strand

XCGGGCAAATTCTGCCTCTCCCTGGAGGAAACAGTCAACCCAGGCCTGTCAGCCCATGGC

CTGCTGAGAATATATCAGATGGACCTCAAACCCCTGGGGATCAGAGTAGTCTATGGCTGG

AAGAGGAGGGTACGACAGTTCCGTGACCTTAACCTGGGTCACACTAAGGGTATGACAGGG

TCTCAACACTCAACAGTGGAGACCCAGCTCTGGGCTTCCCACCCGCTACAGGTGTCTGGG

TTCCCCCTGGAGAAAATAATGCTGGTATTCATTATAATGACAATACCATTAACAAAAGCT

AATTCTACAGAGCAT

>Unigene6197_C-W 1 232 LEN=232; minus strand

CCGACAGCTGCACGCATACAGAAAGAAATGGAGACTGTGAACAGAGGCAAGATCCTTCTC

GGTCTAGAAATCAATCTGGTTCTATTTTATGTCGTGACTGAGCCTGAACAGGAGACAGAG

GGTGAATCAAACAGACCTGGGAGTGAATTGGCCCCTCATCGGGAAACCCAGCTCAACTGA

GCCTCCACAGACACAAAGCCCCAGGAAGCCCCCGGATCCTCCCCAGGTGCTGXX

>Unigene6236_C-W 1 231 LEN=231

XGGAGCGTCGGCCCGGAGCAGAGCGGGAGACCCGCTGTGACACTGCTCTTCCAGCTGTGG

TTTCCCCGTTCCGGTCCCCGGCGATGTGAGCTCGAGGAGCCTACGGTGCCCCCTGGCCCG

GGGGTTGAGGCGGGCGGCAGGTGCCTGTGAGGCGGACGGGTGCTGGGGGACAGCAGGATG

GAGGAGAGCATGGAGGAGGAAGAGGTACTAATCTACGAGGCGATGATGGACGXX

>Unigene6283_C-W 1 258 LEN=258

XGTCTGTATATGTTGGTGCCACGGTGGGGCTCCCCTCCCCGAGTCTGTGAGGTACTGGAA

CACAGAGGCGCTGTGAGCTCATTAGGTAGGAATCCTATGGCTTCCGATATATCCAGAGAT

GGGGAGACCACCAAACAGTATGAACAGGCGAGGGAACAATCCATCCCCAACAGCACAGAA

TACCTCAGCACCCAACTCCATGGATACCACGTGGACAAAGGGTCAGAGATGGAACTCAGA

AAGGTTCATGGTAACGTTGXX

>Unigene6393_C-W 1 214 LEN=214; minus strand

XATCCTGTGCCCTGGGATCGCTTCCCACAGGCCCTGGCGCCCACAGCAGAGCTCCGCACC

AGGAACAAGGCAAGCTCAGCCTGCAGCTTCCCCGTGTCTCGCTATCGCTGGCGCTTCTGT

GATCGTTGCCCCGAGGCTGCAGGGGCACTACCAGATTGTGCAAAGACCCTGTCTCTCTGC

ACAAATACCCCAGGTCTCTCCCACGACAATGTCTTX

>Unigene6442_C-W 1 286 LEN=286

CACATGGCAGAGGAATTTTGTTTACCTCATGGACAGGATATGAAGAAATGGAAGACAAAA

GAGCTGGGGTCACACAACTCACTTCAAGGGCATTCTCCTGTGACCAGAAGACCTCCTACT

AGGCCTCACCTTTTAAAGGTTCTACCACCTCCCAATAGCACTATGGACCGGGCACAAAGC

CATTACCCTCTGGGAACACTTACTCAAACCATAGCATTCTGGCCCTGGAACCCCAAAGCT

CATGACCGCCTCAAGTTGCAAAATACATATAGTCCATCTCTAAAGGXX

>Unigene6539_C-W 1 240 LEN=240; minus strand

CTGCGCTCTTCCCTCCAAACTCCTCCTTTTGTGAAATGCTGCTACCAGTTTACTAACAAA

ATTGAAAAGGAAGGACTGCGCAGAAGGAAGGACGGCAAAGTGAGTTCAGAACTCTATAGC

AAACCAGCTATAAAAAGCGTTAGTCCCCTATCCCGGAAGAGGTGCAGACACTCTCCAGCA

CCTGAACACTGGGACCTCAGTTGCAGGCAGGCACAGGAGAGGTTACGTTTAACACAGCGA

>Unigene6944_C-W 1 271 LEN=271; minus strand

XXCCCCGTCTCCGGCTGGTCGGCGCACTACGCTGCTGCTAGCTGCCGAGCTCGGTCTTGG

GACCCGACGCTTCCTGTGAGCCGGCACCGAGGGGTCGCGGCTCTGAGCTTAGCGGGTCCT

GGCTCCACAGCCCCTCGGACCCGAATCCGCTTCCAGGAGCGCGCCAGGCACCACGCCGTG

CGGTCCCCGGGCGCGATGCCGCTGACTTTCCAGGACGTGGCAGTGTACTTCTCCCCAGCG

GAGGGGCAACAGCTGGGGCCCCAGCAGCGGGCX

>Unigene7564_C-W 680 1151 LEN=1151

GTGGCGAGTGCCAGGCGGTGCAGGAAGGCACACTTTCTACGCTTCCAGGGGAAAACTCGA

ATCAAGATGGCCTGGGCTCCTTCCTGGGACGTGCCATTTGCCGAGAGCTGGGGCTGCAGC

AGGAGACGCTCACTGGGCCTCATCAGAACCCAAGCTGGACTCAGAAATGTGAGCAGAGTT

CGGATGAGGGCTCGTCTCTTTGGGAAGCTGCTACCCAGAAAGGGGCATGGACAGATGGGG

GCACAGCCAGGGGTCAAGATGGCCTCAGCAGACCGGGGAAAACAGATCCATCTCATGTCC

ATCCAGAGCAATCGTCTCCCACACAGGCCTCCAGGAAACCACAGCGACAGCCATCTGCCA

GGCTCCATGATCGTCACTGCTTCCTGCAGCCAGCAGCTCCGAGGAAGCTCGGTTCAATGT

GGGGCTGAACGGCAGAGGGAAATCTCTCCTCTGAAGTCCACCTCTGCGTTCGXX

>Unigene7967_C-W 1 257 LEN=257; minus strand

XXCATGGTGGGCTGCAGTGTCGTCTGCCTCTGCTCCACACCTGAGCGGCCCCCCGAGTCC

CCACCGTGCGAGGCGGCGCCACCCGAGCCCCGTCACGACCCTGCAGTTCTCGGAGAGCCG

AGAAACCAGAAATGCCGCTTCTTTCGAGAGAATAAGATCAGGGGTCGCTTAACTGAGGGA

GACAGACAGGCTGCGCCAGGCCTGCGGGCGTAGCTGGCAGACACCAACCGTCACGAGGTC

CCCACCCGCGAAGAGGCGGXX

>Unigene8076_C-W 1 677 LEN=677

XXATACATCCTTCTTTCTGGAAAAGTTGTTATTCGAATCAGGAAAACTGAAGTGTATCAT

CCAGGAATCCTCTGTGACAATCCCTGTTTCATCATAACGTGTGTCATTGGAATGGACGTC

TTGAATGAAGAAGAAAAAAAAAAAAAAGGCGGGCATTCTCTTCTTAGTGAAGCCGTGCTC

CATCCATTGAATCCGTCAACCAGCGACATTCCACCTGTCGTGGCCGCCCTGGCATCTGAA

ACGGAGTGGAGACAGATTATTAAACCACTCTTAACAAATTCAGAGCAGCGGGTTCATGGC

CCCAGGGAAGGATTGCATTTAGCAAGAGATGTTATTCCTCCAAAGGACCAGGCTCTACTT

CTAGAAGAGCATTGGGAGGGGGCTTTTGTTCTGCGGGGACAACATCTGGATTTGAAGGTG

CCCGTCGGGAGAGGAGATGTCTCATAATGGACCAGACTCGGAACACTCCGAGCACAAGGC

CCGGCGAGCCTTCAAACAAGGCGCCTGGCAGACTGGACTGGTCAGGCCCTGGTGTCGGCT

GTGCGAGCTCCGGAGGTGGGACGGCGCCTGCGTCCTGCGTGTGCCTGTCGCCCTGAAAGA

CTGGCTGCCACCCTATGTCCCCCGCCCCCTCCXGGGAATTTGAGAGCTGAATTCATTTAT

AAAACATTTGTGGTTGCAGGX

>Unigene8120_C-W 1 296 LEN=296; minus strand

XXGCCACCAGGGGTGAAGCAGCATCTGGTTCAACCTGCACCGGAGATGCCCAGCCTGCAG

CCCGTCGTCGCCGTCCCAGGGATGCCCGCGGGCCTGCCACGGGCGGGGGCACGGCCCCGC

GCCCCCGTGGGGAGGGAAGGGGGCGCCGACGCTGTCCAGCTGTCGGTCCACGCCTTTCTC

GCGGGCTGGCTCTTTCCCAACGAGACCCGACCCGAGAGCCGGCAGCCCGAGTCACACCGG

CGCGCGGAACTACCCCCGCGGCGGCCGCCGCACTCACCGCACACCATCAGCAGCAGGAXX

>Unigene8486_C-W 1 397 LEN=397; minus strand

CCCCAGAACAGAAAGTTATAACTCCGTGAAGAGCAAATGGCTTTGAGCAAAAGCCCGAGA

GCCTTGTCAGGAGTGAACTGTGTGTGGCGTGGACGCGGCGGGGAATGGCAATGGCACTGC

TTCCTGAGGCAGTGGACGCTGCGGTTCCCATGTCTGCCCAGGATGCTTTTTGTGAGTTTT

CTCTCCTTCTTGCATCTTGCAAGTGCTGATGACAAATTCCGCGATGCTGATAGGAGACAG

TCACAGTCACAGCAGGCTGTTTTCTCAGCTCCTGGGAGATGCCTTGTGGACTTAATGCCA

AACAAGGGTTCTCCAGATCTCCAGATCAGCCCCCAAGTGCACACATCAGGCAGCTCACCG

ATTAATGATGTAGTTGTGTCAGAGTCTGATAGATACTXX

>Unigene8533_C-W 1 312 LEN=312

XAGGAGGGTCAGAGGCTCTGCTCTGGAAAAGAGATCGAGAGGGGACAGCGAGTGGGGAGG

CAGGGTCAGAGCCAGCTCCCCAAGGCACTGACCATGCAGCGCTTGGCAGGGGTACAGGAG

AACCAGCGTCTGGGCCATGGAGACCAAGGGGGCCAGGAACAGGTAGCTTTGGAGCATGCC

CTGACCCCCACTCTGAAGTTGGTGGAGGGAGGGGACCCTGGTATGCCAGACTTGGCCAGA

GGACCCAGGCCCAAGACCCCCATCTCCCCTCTCTGGGAGCTGGGAGCAGCTGCCCATGAA

GGGAAGGTGGCTGXX

>Unigene8567_C-W 1 280 LEN=280

XXACTGGCGGCTAGATACACCAGCTGCATACACCAGCTGCGGTCAGGTTACAGTCGTACC

GAGCTGTCAGCGTTCGCTGGAGTCGGGTTTCGGTCCTGGGGTACTCGTAAAACACGACTT

TGTTTTAGGTCAATAAAACACCCAACAAAGCCACTAGAACACAACGGTGTGGGGCTTGGA

GGAGAAAGGACAGACCCCTAGCATCCCAGTTTTAAGAAAGCTAAGCAGCGAAATAGGAGG

AAAAAGCAAGTCCAGTCACACACCCGGAACCTCAAGTTACAA

>Unigene8837_C-W 1 323 LEN=323; minus strand

XXATTCTATAAGGACATATCAAAGCTGGCCAAAAGAATGCGTATTTTTAAAAGCAATACC

AGGTTTCCATCTCAGACCGACTTTGATCCCATTCAACACTTGAAATTTCATGTGTTCTTT

TTCGATTGTGGAAGTTCCTCCAATTGGGAATTGTGGAGGGATGGTAAATCTTCTGTAAGA

CCACGGATGAAAAATGAACAAGTCAGAGAAGAAGAGCTTCCCCGAATGTTGGCAGAGCAG

GGCAGCAAGCAGAGGGCATAGAATAGAGAGGGATATCTGCACTTAATCCTAAATGTTTTT

GAAGCAACGTCCATCTTCAGCAAGCXX

>Unigene8839_C-W 1 235 LEN=235; minus strand

XXGCCAGTATTACAGGACACAGCCAAAATATGGAATGGAGGGCTTACTAGCTTCATCAAT

TGATACAACACTTTTGGAGATTCTACAATACACAGGCAGTGTGCGCTCACTGTGAAGAAC

TCTCAGGAAGGTCAGACAGGGGCTTATCACTGTGGACTCCTAGCACAGCAACGGAGAACA

CAGGCCCCAGTACAAGCCTCGGCACAAGAGAACAAGTTCTCGGCGGACACAACATCT

>Unigene8848_C-W 1 293 LEN=293; minus strand

GCCACAGTCCACACCTTCCCTGGTTCTGAGGTTCATAGGGAGCCCCTTGGGAGTATAGGT

CTCTGTTGGACAGTTCTAAGAAGGCTGCAGACCAAATCAGGCAACACACCAGCCCAGCCC

GGGAAGCCATCAAATCCAGCTGTGTGCTACCCAACACAATGGTCCAATCACTGGAAAAAG

CTGCCTTTGTGCCTTAACATCCTAGACTCTGCAATCAGTCAGAATGAGTGCAGCCGGGAT

GCTGCTTTCAAAGCAACCCACGCCCTAAACGTCACCATTTCTATACATCAACCX

>Unigene9084_C-W 1 322 LEN=322

XCCAGAACTTTCAGCAGTCAGGGTGGTGCACCAGTAAGTTCCCAGGACCGGACGGAGCCG

GTGTGGGTTCCAGAACGCCTGACAAGAAGAGTCCCGACATCAATCAACCAACAACAGGAG

GCGCTACAACAATCTCATGATGAGGACCATCCTTCTACTGAGTGTCCTGGCTCTAATGCT

GATATTGATGAAAGCTTCAGGCTCAAGTTCACCCATGGTGATGTTTTGCAAGAAAATCAC

AGTACTGGAGGAGATGGCCAGGCCCAGGATGAAACCCTTTTTGATTTTGGAAAACATTTG

TTTTACATTGCCCATGTGCCCAXX

>Unigene9086_C-W 1 386 LEN=386; minus strand

XCCCGCGAGAGCAGGTTCAGGCGTGGGCTTTCATCTACGCAGCCAGGCAGAAGTGAAATG

CGCCCTCCCCGGGGACCTCGCTTCCCCCGATTCCTCCACGGAGCCGGAGATCAAGGAGCT

GAAGGTCAAGGGAATAACATGTGTCAAGTCAAGTGGCCCTGAAATAGCATGAACAAGAAG

AGGTTTGAGAAAAAATCAGAAGAGGAGTTTTCTGAAAGTGTGGAGACAAGGAAATGGGAC

TTGGCTGGTGGTAGACGGATGACCTCCTCAGCTCATGACTGGCATAGTTCTCTTTGGACT

AGATTCTCTTTCTTCAGCTTTCTCTCTATGGCCATGGGCAACACCTTCTATTACTGGGAC

AAATACACTGTCTGTGAAAGAAAGGCC

>Unigene9143_C-W 1 332 LEN=332

XXTGTCTGGGTTTGGGCTGGTGAACTCCTGCTTCATGTTATTGGACATGGGAATGGTTGG

AATGGCCAGAGATTTTCCATGCCCACTAGGCCTCATCAGGCCCACAATGGACAAGAAATT

GAATTGGACCAGCTACTGGTGAGGGGCCAGGGCTCTGGCCCTGATATCTTTTGGAGTTTT

ACCTTAAGGGATAATTCTGTAGGCTACTTCTTTTTCTTGGTCCCTGCCATGGCTCCATCT

GCCAGTCCACAAACAAGACCCATCATGAGGTATTGTCCTGTTTGTAAAGGAAATTAATTC

TATGCAAGGCTAGGGATACCAATCTCTGACATCGXX

>Unigene9204_C-W 1 259 LEN=259; minus strand

TGGGGTTTTGTGGGTGTTGTTGTTTTACCGTTGCAGGACGTGAACCAGACGGAGCTGTTC

ACGGGTCTGCCAGCTTTGAGACGACAGGATTTCTGTGTTCAGAAGATTAAGTGCTTAATC

AAATCCCGTCACACTAAAGCCAACGTGAGTTCTCTAAGGATCGGAGTCCTGGATTTCAAC

GTTGCGTTGGTTCCAAGAGTAAAAAGTGGTGTGTTTCCCCAGTGTCTAGTGCAACAGGGC

GGTGGTGGCTACGCGATG

>Unigene9231_C-W 1 494 LEN=494

XACATCCACCACCGAGCTTCAATTGTTGCTGAAGCTGGCCTTGACCTTGTGATCTTCCTG

CCTCCAACTACCAAATTGCTGGGATTACAGGTGAGGGCAAAATGAGAAGGTCCCTTTTAT

GAACTGGAAGAGGGCCCTCTTCAGACTGTGACCACTCTGGAAACTXTGACCTTGGACTTC

CCAGCCCCCAAAACTGACCGGATGGAGCCGGTGTGGGTTCCAGAACGCCTGACAAGAAGA

GTCCCGACATCAATCAACCAACAACAGGAGGCGCTACAACAATCTCATGATGAGGACCAT

CCTTCTACTGAGTGTCCTGGCTCTAATGCTGATATTGATGGTACAGGGGACATCAATGCA

GTGAAAGCTTCAGGCTCAAGTTCACCCATGGTGATGTTTTGCATAGAAAATCACAGTACT

GGAGGAGATGGCCAGGCCCAGGATGAAACCCTTTTTGATTTTGGAAAACATTTGTTTTAC

ATTGCCCATGTGCCCAXX

>Unigene9539_C-W 1 269 LEN=269; minus strand

XGCTGTGAGGATCAGGTGGAAAACCTTCAGGACCTGAGCCTCACAAACTGGGTTCTAGGC

TTCATTCTTCACTACCTGATTCTTTCGCAACTGTGGGGGTGCTTGGCACTGTCCCCTGAC

ATCTCAGGAGATGGAAGTTTCAACAAGACAAGGAAGCATAATAAGAACTGTGAAAATGAA

TCGAACCCCGAGGGGGCACCAGAGATGGAGCCATCGGGCTGCACGGAGACAGGCCGTGAG

GCCCAGCTGCACACTCCTCCCACATCCCCC

>Unigene9566_C-W 1 283 LEN=283

ATCAATGCAGGAGAACATTCCAGGTTCCAGTGGCGGGAACTCTGGTCTCCTGCCCAGTTT

GCTATTGGTTTCCCCTTCTCCCAGTCACAGCGCTACAGGAGTGGTCCAAAGCTGGCTGAG

ACTCCTTTCCGGAAAATCCGGACCTTTCAGCCTTTCTCTGCAGATGGCACCTCCAAGCCA

GATCTTCCTCCTGATGGGCCAAGAAATACTGAAGCCTCATCTCCACCCACACTCACAGCC

ACCAGGGTCATTTGGAACTCTTGGGCTGATTATCTGGAAGCA

>Unigene9605_C-W 1 212 LEN=274

XCAGAAGTGGAAAACTGTAGATTTTTTGGTATCCTTTGGGGTTCCTGGAATCAATCCCTT

GTGGATATAATAGATAACGCAGCACCAGCAATCACCATGCGCATCCACAGCAGTCGGTCC

AAGAGCGAGACTATGAGCATCCACATTTCGGGTGGCACTGAGGAATATCTTGAAATGAAG

ATTATAAATGAAGATACTCTTCGTATAATCCAG

>Unigene9636_C-W 1 266 LEN=266; minus strand

CCAGGTGGCATTGTGACTTTTTCAGCTGGTAACAAGGCAGAGGAAGACTGGGTGTGTTTG

ATTCAAGCCTGCACCTCAGAAGGTCATCCAGTCACAGCTAAATGGCAGCCAACTTTCAGC

CTAATAGTGACAGAGATCATTTGAGAGCGACTAGACCCCAGAGGACTCGTCAGCTGGTGG

CAGACATATGAGAGAGCCTGTTCATCTGAGCCCAAGGAGATCAGTAGAGCAATCCAGATA

GGCTTATTGAACTACGATCAATGCTGX

>Unigene9673_C-W 1 315 LEN=315

XXAGGGTACAAACAATAGCCCTCACTAGAAGTTGGACAGATGCAGCCACTTGACCTTGGA

TTTCTAGCCTTCAGAATGTTGGCTGTCCCCAGCTTGAATGAAGAACCTGGAATGAAACTC

ACTTGTCTTGCAATAGATCCTAGTCTCCTGAATCACAGAGTATTGCCTGTACGATTAGGT

TTCTCTGCCAAGGTCTATGTCAATGTGTGGCTTATCAGCCTATGCACAGAGCACAATATT

GGCTATCACAGCATTCTCCAGACCTACACCATAGGCCACCAGCTCAAACATGGCTTTTAG

TTGAAATCTAAGAAGTTX

>Unigene9693_C-W 1 246 LEN=246; minus strand

XTCTCGAATTGGATCCACTCGGAATCCACCGTAGAGTTCCGAAAACTTCCGAGCGACCAC

GGAACCGAGGCTTTCCACGGTCGAGAACACGCCTCCTCCTCTTTACCCGAGGATTTCCXG

AAGGCTCCCGAGCGGTACGCGACTGTTGGCGGAAATAGCCGAAAGCCCCGGAGCCGCCTC

GGAGGCGGGGCCGAAAGGAGGGAGGAAGCGGAGGCCAGCGACTTCAGCCTGCACCATGTC

CGCGCACCX

>Unigene9772_C-W 1 254 LEN=254

XATCAGATCTGGATAAAGCTGAGAGAAGGCACACAGGAACCAAAGGACCAAGCAACGGGG

AACACACGGAGGGAAACTTCAGCACTGCTCAACAGGACACTGAAGTTAACACCCAAATGC

AAACATGTTCTGAACAACAGAAGGCCAGACTGGCCACTAGCTCAGCAAGTTCTTCACTAT

CAAGGAGGAAAAAAGCGAGAACGCCCAATTAATAATTTATCTTTTATTGAAATTGCTGAA

TACTGTATTTTACAG

>Unigene9773_C-W 1 272 LEN=272

GTGGAGAGCCCAGATTTGGCCCATGAGCATGGTTTTCTGACTGCTATTCTAATCTGTGTC

CCCGCTATCAAGAAAAAAAGAAACAAACAATTCCATGCCCAAATAGTCTCAAACCCATCT

CCAGAGTATGAAGACCTGTGTGGGCCTCTTTCCCATGTCCTTTATCCTGACACATGCCAT

ATTTTCATTAGGCTTGAATACAACAAAGAGGTGGCTGCTTTGAGGCCAGATATAGACACA

CAAACTGAGGTGTCTGTGAGTATGCAGATGAGX

>Unigene10844_C-W 1 137 LEN=332; minus strand

XCTCCGTGACCCAAGTCGGAGACGACCATCAAGTGTAGCAAGAGCTGCTCAGACCTCAGC

CCGGGCCGCCTTGGAAGGGACGGCGCTTATAAGGAGGACCGTCATCGCCAGCAGACGGTG

GTGACGTCCAGTCCGGGG

>Unigene11161_C-W 1 651 LEN=651

XXGGGGCTACTCCAAGTCTCTATGCCTGATGATGCAGCCTGTGGACACAGGCACGGCTCT

CAGGGGAGTCTGCCAGGCCAGCGGCCTAAGCTGTGTGCTGAAGCATCTAGTCTTGACCCC

ACAACCTGCAAGTCTCCTGAAGGTGCTGAGGTATCTGTGAAGAAAATCCATGGTGACTCT

GAGGCTTTCATTTGGAAAATGTCATCTGCAGAAGCCAAGTACTGGATCATGCCCTCAGCT

GTGGAATCTACCTGGCAATGGATGAGTGACATTTGGAGGAGCACAAACATTTTTGGACTA

TTTGACTTCTTGCTACTGGAGCTGTCTGTACCCTGCTGCTCCAAAGGTTCTTATGGGCTT

TCTCCACCACAGTGTTGCTCATGGCCGGTGTGGTGGGACTAGGAGCTTAATCCACACAGG

ACTGAAGCAGAATTCAAGGTGGGCCTGGAAGAGGCTGTTGCATATGGGATGACTCATCTG

TGGGGAGGATGGCACCCAATTGTCATGAACCTGACTGTCACAGAAGTGAGGGCACAACTC

CAAGAGGAGGCTGAAXCATCTGTCGTCCATTTAGAGAACAGTAAAGACGTTTTGGCCAGG

AGCAGGATGTCAACCAGGTCACATGTGTGGCTGGACCTGCAGGCAGAGCAGCGG

>Unigene11263_C-W 1 318 LEN=318

XGTAAACATTCAGCCAAATGAGATCTGACCAGTGGTGGTGGTGGCAGCAGAGGAGGAGGA

GTTGGTGAAGGCAGAGGGAACATCTCCATAAAGAATGTCTGGATCACAAGTGGCAGGTGG

GTGGTAGCAGACAGAGGAGGAGGTGTTTGCAATGGGGATCAGTACCAGCTGATGAAAGTC

ATGTATCAGCCTGCTCCAGGTGATAAGCTGCTCCCTTCCCCAATAACATGTCCAGAAGGG

TCCCTGTTTCAGATGTTAAGACACCACATGGGCACCAGCTTTACACAAGTTTCAGGATGG

CTTTCCCCAGTTCTCAAACXX

>Unigene11374_C-W 1 698 LEN=698; minus strand

XCCAGAACCATTTGCGGTTTCCTTTTCCAAAAAATCAACAAGCATTTGGTGAGGCTGGGT

TATGCCCCCGCTTGTGCTGACACTGGGGTCCAAGATGGGTCAGATATGGTACTGGCCACA

CAGAGTACCAGCAGGGAGACAGATGAGAAAGATACAACAATTCAACAGCAAGATACACAC

AATCAGCTTAGTGATAGAGTCAAAGAAAAAAAAAAACATCTCAGGGAAGGCAAGAAACTG

AAATCTGAAGTAGGAGTTACAAGACAGATAAGACAGGGAGATGGAACTCCAGAAAGAGGT

AAAAGCAAAAGTGTGAAGCAAACTGAAGGTACAGGAAATAGCACAGAGTTTCTCAAATTT

ACTGTTCAAGATAAGGGTGGAGAAGAGTCAAAGCTGGATGATCAACAGGGTTTAGAGAAT

CTTGGACATAAGACTAAAGAGCAGCATTTTTCTGTGGGCATGGGGAATAATGAAGGAATT

TTAGCAGAATTATGTCTAGTTATCTTCAAGAGAATGAGTTCCTCTCCTCCCCGCTTAACC

TCTAGATCATAACTGGCTTTTCTATTTTCCTTCCTTATAACGCCGCACAGTGGCTTTACC

ACCACCATCACTATCAGTACCACTACACCACCACCATCATCCCACCAGGTCCTTAGATTC

CCACCTGGACTGACCTTGAAAGAAGAGGACCCCTTCTXX

>Unigene11518_C-W 1 86 LEN=929

XGCCGCACCATGCACGTGCTGGGCCTGGCCAGCCAGGACTCTCTGCACGAAGACTCCGTG

CGGGGCCTGGTGAAGCTCAGCTCCGTG

>Unigene11703_C-W 1 372 LEN=372

XGGTATGGTGCCTTTCCTCCTCTTCCATGTTCTCCAAGGTCAACTGCCACGCTTGGGAGA

GGGGAAGACTTCTTTACCTTACCTCCATCTCTGGGACTGGATCTCCTTCCCTTTCCTGAA

CTCCTCCTTCTCAGCACTCATTCCTGTGGGTGGTGGCTCTCCCTGCCTTCCTGCTCCCTG

CCCTCCCTCTTGGACATGGTTATCCCCATCCCCATCCAGTTTGGAGGAACAGATCTGATT

ATGTTCCCCTGTCATCCCCCATTAGGAAGCCAAGGTCCAAGAAGGAAAACACAGGAAGAA

ATGAAAGGAGTATTATTAGGAGAAAGCAAATTACATGCTCTGTCCGCCACAGCAGCTGGA

GATGGGAAGGACGXX

>Unigene11722_C-W 1 239 LEN=239; minus strand

XCACTGAGCTACATCCCCAGCCCCTACAGTCACATTTCAATGTCCAGGGCCACCCTCAAC

AACTTAGTGAAAACCTACCAAAAACTAAGAAGGGTTGTAGATGCGCTCAGTGGTAAAGCA

CCCTGGGTTCATTACCGCAAGAAAAGAAAAGGAGCAAGTTTACTGAATGCTTGGTATGGA

CCAGCACTGTGCAGCACATCTTATGCATTAATCATTGAGAGGATCGCAATACAAGCTTTA

>Unigene11749_C-W 1 437 LEN=437; minus strand

CTCTACCACTGCGCCACAATCCCAGCCCCTCAATGTACGAATTTTGATGGGGAAACAAAC

ATTCAGCCCGAGCTAGTGGCTTCCTCTCACCACCATGTCCCTGGATTTCCTGCAGCTCCT

TCTACACCCTCCGTCACAGTTTGTAATAAAGGACATGGTCATTCTGTTCCACCTGCCTGT

GGGCTCCTGAGGGCCAGGTGGCTCCTGGATCTTTTTATCAGGGTACAACACAGTGTCTGG

AGTCTCACTGGTGCTTTGGAAGTGACTGAGGAACAAATGAATGGTCCAGAGTCCCTGGAG

TCAGAGCCGCTGGAGTTACAGGATTCCCGACTCCACGTCAGCCACATCACTACTCCTGTC

TGGGCCTCAGTTTCCTCTTCTATGTCTTCATTCATTCATCTAGGGACGCTCATCTCTAGA

TGTGATGATCCCGTGTGX

>Unigene11935_C-W 1 206 LEN=206

GGGGCGCCTCTGCAGGCCAGCTCCAGCCATCCAGTTCACACGCGGAACCTCGAGGCTCTG

CTCACGCACTCCACGGAGGGGAAACTGGACCCAAATGCAAGTCTGAACACACTCCAGGGA

AACATTTCACAGGCAGTGAATCCAAGAGTGACCGAGAGAATCGCGCCGGCTGGCGTCCAT

TGCCTCCTTCAGTACACGCTGCGCAGX

>Unigene11996_C-W 1 229 LEN=229

XAAAAAACAGAGCCCCAGTCCCACAACCCCAAGCAACTAAATGCTGCCAACCCCCAGGAG

CTTGGAAGAGGATCCCAAGCCACAGATGAGAACAAAGCCCCAGCCAATACCTTGATGCAA

GCCCAGTGAGACCTGGAGCAGAAAACCCAGGCCCACAAATCCAGGACTACAAATCCACAG

AACCGTGAGATACAGTCACACATCACTTCACAACGGGGACATCTTCTGAGX

>Unigene12023_C-W 1 270 LEN=270; minus strand

XXGAAAGAAGTCACCATTTGCAGAAATATCACAGTTAGGGAAAAGAATGAGGAATGGCAC

CACCATTTTGCAATTCACTGCTAAATTAATGGAAGTAACTGTCAGGCAATGATCATTAGT

GGTTTCTATCATAATAAAAAGGCTCAACCCTATGGTACTTCCAGTGCCACCACGAAGTGT

CACCAAAAAGTGGAACTTGAACCCGATGGAGCCTCCAGATCTCATTACCATGTACAGGAA

ATAGGAGGGACAGAGTATCATGCTAAAGCAACX

>Unigene12119_C-W 1 257 LEN=257

XXCCGAGGGAGCCTCAGCCTGAGGCTGCAGGACCAACTCTTCGCTTTTGCCATGGAGGCG

TCTCGGTCTCAGGAGCCAATCCTGGATGCTAAATCGCAGGTCACCAACCAGGTGAGTGAG

TGGCTCTCCTGCCTTGTCCTCAGCCGGCCTCTGCTGGTCAAGGGCTGGCGGCCGCTCCCG

GAGAACGGCGTCGCAGTGTGCCGTGCCCTGTCCTTGCATTTCATAACCTGTGTCCTCAAG

CCGGTGTTCTCCAGCCCGCXX

>Unigene12357_C-W 254 327 LEN=327

ATGTCGCTGGTTGCTTACGCCAGCAGCGATGAGAGTGAGCCAGACGAGGCTGAGCCCGAG

CCGGAGGAAGAGGAX

>Unigene12417_C-W 1 571 LEN=571; minus strand

XCAAATCTTCAACGGATGACCTCCATACCTAGGTGCCAAGTGCCCCATGACAGTGTTTAC

ACACCTGGGAATTGTAGTCTGGAAGCTGTAATCTTGGGCCTGGACAAACAAAAGAGACGT

CGTGACCAACCGAGCTGGTGGAGGAAAAGCACAAAACCCAATACCAACCTACACAGTTAG

GATCCCAAAGAGATGCTGACATTTCCGGCCAAAAACAAACAAAAAAACAATAGTGACAAA

TCAAATCTGATTGGAACCGCCAACATCCAGGTTCTCGGAAGAAGGACAGGGAATAAACGA

CATTCTCTAGCTGTGTCCGTTTTTGAATCCCAACCAGGACTGACCGACCTTCATCAGACG

TTTTTAACGGCGCAACAACTCGATTTGGGTCCCAGACTCTCCATTCTGCGCGGCCACAAG

AGAACAACAAGAGAGCATGACAGGATGTTCATGGAGGGGACATTTCCATGGCAAACTCAG

AGACTGAAGTGTTTATATCCTGAATCAGACGAGCTTCAATATGAATTTGCATCTTGCCTC

TGTACTGCTTGCTATTTTTTAAATGATAGGAGX

>Unigene12526_C-W 1 471 LEN=471

XACATCCACCACCGAGCTTCAATTGTTGCTGAAGCTGGCCTTGACCTTGTGATCTTCCTG

CCTCCAACTACCAAATTGCTGGGATTACAGGTGAGGGCAAAATGAGAAGGTCCCTTTTAT

GAACTGGAAGAGGGCCCTCTTCAGACTGTGACCACTCTGGAAACTTGACCTTGGACTTCC

CAGCCCCCAAAACTGACCGGATGGAGCCGGTGTGGGTTCCAGAACGCCTGACAAGAAGAG

TCCCGACATCAATCAACCAACAACAGGAGGCGCTACAACAATCTCATGATGAGGACCATC

CTTCTACTGAGTGTCCTGGCTCTAATGCTGATATTGATGAAAGCTTCAGGCTCAAGTTCA

CCCATGGTGATGTTTTGCATAGAAAATCACAGTACTGGAGGAGATGGCCAGGCCCAGGAT

GAAACCCTTTTTGATTTTGGAAAACATTTGTTTTACATTGCCCATGTGCCCAXX

>Unigene12706_C-W 1 230 LEN=230

GGGGGCTTGATGCCATCAGGCTATAATGAAACTGATAAATGGGAAGGGAGGGTTTCTATA

CTAAGGATGAGGCAGAGGAAAAAGGCTCCAAGATCTCCTGGGAACACCATAAGGAGAGGT

CCACTTCTTGGAAGCACACACTTTTCACCATTGAATGATGGAACAGAATTCAACTCATGC

CAACTAGCCTTTAAGATTCAAAGGTTTGGGCTGGGGAATGTGGCTCAAGCX

>Unigene12779_C-W 1 305 LEN=305; minus strand

XXAGCTAGTCAACTTCCAAGATTAAAAATGTGCTACGGTTATGTCAAACTTTAGAACCTA

TCTGTATCTGAACTTTCTGGAAGAATTGCCTTGGAAAGCAGAAAAAGCCTGGTGGCTGTT

ATAGCCCTGGCTTATTTAAAGGTGGAGTTTTCTCTAAGCTGTCCTGAGTGAGGACGCTGG

CAGACATCAGAAGACAGAGGATGGGCCTGTTACCTGCCCGCAGCATTCTCACCACCACCT

GCCAGCGTTGCCACAGCAGTCACCTATAAGCCCGAGACAGCCACCTTCCAAGGAAATAAA

ACTCGCCXX

>Unigene13556_C-W 1 512 LEN=512; minus strand

CAATGTGTTATTGTTTTGATTTATCTTGTTTTTGTTTCAAGCTGCCTATCCCTAGAAATT

TCTCAGGCAAACTGGAAAAAATGGTTGACTAAAGGTTTGAAGTTTGTCGGCCCTGAGGAA

GAGAAAATTGATACAACGATTTTTTATTCCGTTTTTGTTTCATTCAGTTTCGGTTTTGTT

TTGTGTTATCTTATTGGGTTGCGTTATCTTTATAGTAGATTAGAAATTAGTAAAAAACAA

ACCGAAAAGATGTTAAGTAAATTGTTAGAGGTTCAGACCATGGAGAAAGACATTTTAGAT

CAAGCAAAAGAGAAGGTCTCTCGAGCTAGTCAGACAGAGGAAGAAAATTTAAAGGAAGAA

AGCTTAGAGGAGAAACAGCTATTAGGAAAAAAGCTACAACAGGAGGCTGCTACTAACACC

GTTCTATCACCAGAGGGCGTAATTCAACCAACAGCCCCACCGATGGAGACAGCTGAGTGG

CCCTCAAACCCCGTAGTTGATAGATGGGATCCX

>Unigene14280_C-W 1 92 LEN=610; minus strand

XTCTGGGCTCAACGCAGTCTTCCCTTTCAGATCTCTGCCAATGGCTTTGTTGCCGAGAAA

AGAAGAAGCCGCATTCGAGTGGAAATCGGACCC

>Unigene14499_C-W 1 292 LEN=292; minus strand

XXTGTGTACAAATGTAAGAGCCAGAAACACATCTCCATGCAATTACTAGACTCCGGGGAT

AGAAGTTGGGACTTCCTGGCCATTTTCTCGAACTTTCACACTCCCCAGCGATTCCAGGGC

CCCCGGAAGGAGGTAGAGAAGTACTTATCAGAACTGTGTGAAGACTATTTAAAGCGTTCG

TAGTATTACTTCCATCCAATTCCCCCGCCCCTTTGGCTCCTGATTGTTTTGGCCTTGGGA

CGCGACCTTCCCATTTCTCCAAAGCTCTTGGACCAGACACCACCTATGACCCTC

>Unigene14576_C-W 112 511 LEN=511

AAGACGGTATATGCTCACTTTTTCACCACCAACAGTCTGGCTCCTGCTAAAAACCCATGA

TGCAGTTTCTCCAAGCCTCGAGATCCCATCGCTGAACCCTAAGTATTTACGGTCTGCATC

AGTCAGGCTCCTCCTGATAGTGGCTCCTGGGAGTCCCGAACAAAGAGGGAAGCTGAACCG

GCCCCACCAAGCAAGTTAAGGTGGATTTATGGCGTCCTGCAATGGAGCCTCAATGGTGAA

CCCTTTCTGCGGTTAGCGGACACCTCCACTCCTCCGGGAAGAAGCATACAAAACTCAGAC

GAAATGAAGATCAACATGCCCAACCTCTTCCCAACTTCGGCTCGGCCCAATTTCTCCAGG

ACACCGTGCAACAAAGAAGTCCGAGGCTGCACCTGCCCTGXX

>Unigene14764_C-W 1 268 LEN=268; minus strand

XGTGTAGATAACAGTGAAGGTCAGAAATCATATTATAAAGGACAAGACCAATTAGAAGTG

ACTATTCTTAATAAAAATGTCCTTAATACCATCAACAGTCCTCAAGAAGATTTAATAGCT

CACAAAAGTATTCAATTTATAACTGAAGGACAAGTTTCTGAAAGTGCTGATTTTGAAAAT

GTTTTTACAATCTCTCCACCATTCTGCAATGAACAAAAGATTTCTTCTTCTGCCCAAACA

TACACTTTTAATGATTGTGGAAAGGCCCCX

>Unigene14774_C-W 1 348 LEN=368; minus strand

AATCCAGACCCTCAAAGCCAGGAGATCCTGGAACAACAGATATCAAGCTCTGAAAGAAAA

TGGGTGTCAACCAAGAATCTTAAAAAAACCTTCCATGATCAACACAAGTTAAAAGAATTT

ACAGCTAGGAAGCCTGCACTACAGAACATTCTCAGCAAAATATTCCATGAGGAGAAAATG

GAAATAAAAAATGATGAAAATCACCAAAAGGAGGAAAAGCCAGTCAAAGGAGACAACAAA

ATCAAATTAAAAACCAAAACGACAGGAATACAAATCATATCAATAATAACCCGGAATGTT

AATCGCATATACCTGTCAATTGAACGACATAGACTGGCAGATGGGACA

>Unigene14838_C-W 1 216 LEN=216

XGTCTTGCAAAGTTGTTGAGGTTAGCCTCGAACTCCCAAGTAGCTGGGATTATAGGAATG

AACCTCTGGGTCAAGCTTGAGTCCATAGAGAAAATCCTCCAAGTGGAGCAGGTTTGTGTC

TGGGTAATCTTCTTTGTAACTCAGAAGACCATGAATAAGCAAATGAAGACAGCCCTCTGC

ATCTGGAGATTCAGCTACTCAGGATTCAACCAACCGGXX

>Unigene14875_C-W 1 204 LEN=204

XATATCACAAAGGAACAGGTGAGCGTAGCTCGACCCATGGGGCTGCAGGAAGGCACACCT

AAGCATGAAGATACATTTAGCGACATTTTTACXGACTCACAAGACTGGGCCACCATCTAT

GGCCACTTTGATGTGGTCAGATCACAGGAAGTGACCAGCAGAGCCTCAACCGATCAGGAA

AGGTCACAAGGCTTGGTGGCCTCCCAX

>Unigene15056_C-W 1 319 LEN=319

CCCACTATGCCTTTACAAACAGGTTTCTTTTTGGTTGTCTTAGTGGATGGCATCCAGCTG

AACGCCTTGGCGGTGTTCCTTAAGTAAGCGTCTCCAATTGATGATGATGGGAATGAGTGT

ATGTACATGCCCCGCATTACATGGAATTGTCTGCCAAAGATACTGAATGCTGTGATTCTG

AAGGGAATCATCACAGGGGATAGTGGGCTGATATTACAAAACATCAGCATGATTTTGCAA

TCTCCCAGCAGCAAAAGAAGTGAAAAAGAATTGGGTGAAAAGGAAAGGGAATGGAAACTT

GGGAAAGAACACGAAAATGXX

>Unigene15139_C-W 1 241 LEN=241

CTGCTGTATTATATAAATTAAAAAAGAAAAAATAGCCACAACATGCCACCTGATAGTAGT

GAAAAAGAATCTGGCTTTGCTGTGACTCTTGGAGATGATGCCCAGGCATCCCTGCTGTGA

TTAGCTGCTAATACTAAAAATGGGCTTTCTCAGCTGCAGCGGCCTGCAACTTACACAGCT

CCCACCCACCAGCAGAAGCAGAGAATTGAGTTTCATCTCAAACTCTCGTTTCAAAACCGT

AXX

>Unigene15176_C-W 1 258 LEN=258

GGAGCGCACTCGTTCTGCACGGCCAAGACAGGACAGCCTTCGGAGAATGGCGCCTGCCTG

CCCACGGCACTCTTAACCGTGCTTGACCGGCCTCCAGCCAAGCCCTTAGCAGACACCTGT

CGCCACCGCTCCCCGGCCGCTGAGTGCCCGAGCACCCCGTTCACACGCAGCTCCAGGAGC

ACCTTCCACGGCAGGGTGAATCGCGGGGCCGAACCAGCCACCCTTATGAAACAGGGACCT

AACGGGAAGCTGCGGCGX

>Unigene15206_C-W 1 247 LEN=247; minus strand

XXCTGCCACTCGAGTGCTGTGTGGGGCCAGGGGAGGGCTTATGGCGGTGGTGTACACGCA

CTGCACAGACACACGGACAGTATCACAGAGGACCCACGTAAAGGCCAGCAGGGCTGGGGA

GGGGGGTGGTGGCGAGGCTCTGCTGAGGATGGAGAGCAGGAGATCCAGCTCACCTCAGAA

GCAACAGGTGCTGGAGGCCAGCAGCTAACCGTCATGAAGGGGAAGTCAGACTTCCGAGCT

AGTGTGTCC

>Unigene15337_C-W 1 287 LEN=287; minus strand

XCTTTCATTCATTCCTTTATTGCCCTCTCCATACCTCATCGGGGTATGTGAGGGTTTTGT

GAGCCTGGATTCTCACCCATCCGAGAAAAGGGGCCTTCAGGAGACAAGATGGGGCTCAGG

ACACCCAGCTGTGCTCAAAAGACCACAGGAAAACGAATTCCACGTTTGGCCATGGACCCA

GGAGGAGAGAGGCTATCTGTCCACTTCTTCAGACCTTTTCACACCCCAGCTCCTCTACAG

AGCATCTTTTCCCTGACACTAAACACAGAATTTCTGAAAGCTCCAAAC

>Unigene15377_C-W 1 360 LEN=360; minus strand

XXGAAAATGATTATGTTAAACATGAGGGTTAAGAAAGAAATGAAGAAAACTAAGTTGAGA

AAATGAGATCAGGAAGACAGAATGCAAGGGGGAAAAAGAAATGAAACCAGTAAAAGCATG

GTATTTAAGAAAGATGATGCTCAAAGCAGAATCTACAAGTCATCTGCTCCAGTTTTTATC

CCTGCAGTAACAGAGAGCCACACAGGTGAGCCCTCAGTCTCTCAACATTACCTGGCACTC

CTGCAATTACATCAGCTATTTGGGCAACACGAGCAGCAGAGATCAAGAGCTGCCAATCTA

ACAAGTATTCTCTTGTGTCTACCCTGCCACCTGGAACAGCAGAGGCATTTTCCAGTTGTT

CCX

>Unigene15431_C-W 1 484 LEN=484

GTGCAGATCACCCACATTTATTCCTTTCCATACTTGAATACGGGTCTGGATAAAAAGCCA

TGTTTCTGTCTCTCCTTGAGGCCTCTGTGCTCCCATGCTGGTCTCCGTGGGAAAACCATG

GGAAGCTTAAGAAAAAACACGGTCCTTGCTGTCAAAGACCCCCACAGGTTATTAGGGAAC

TGGCTGCATGGGGAAAATGAGAGTGAGTCATACCAGGAATTGGCAGCAAAAACCAAGTAT

GTAAAGAAGGAGGTGGAGACAAGGGGCTGGTGCCAAGAAGTGGCGAGGTTGGTGGCCTCT

GCAATAATGGCAGAAATACCAGAGCTGGGAGAGCCCAGTGAAGAGCTGGACACCACAGTC

AGCTCCCTCGGACCCAGAGCCAGTGCGGGGGATGGAAAGCGAGGGGAACAAAAGAACCAG

TCTCCTGGCCTGCCCCACTGCAGCAGGGCTCAAGGGCACCAGGATGCCATTGTTTATGCC

CACAXX

>Unigene15489_C-W 1 400 LEN=400

XGGCGGAACACAGATTGAGTTTATTCCATGTACTGCACATTCATAGAAGAAACCCTGGTG

GACACAGGCACTGGGACACAAACGATGCTCAAGTGTCAGCTATGACCCAGGCCCATTGAG

GAGGGCAGGTTCACCACAGAAGATGGATTGTAGTCCAAGACAGCACAAGGGCTGCAAGAG

GATGGTTCTGGAACTGTGTCTTCTGACCCTTCCCTGTTTGTCTGCAAATGGCTCATAGCA

GCAATACATTCACTGGAACACGGAGCTGGTGTTCAAAAAAAGCGGCTCAGACATAGTTTG

TACACAAGCACTCAGCACTCACATGACAAAGAGGGCTCCAGCAGCATGGCCACGAATGTT

CTTATAGCAGTTCACACTCAGCTCTGCTATGCCTGTGGCAGX

>Unigene15513_C-W 1 357 LEN=357

GGGCCAGGGCAGCAGGGGCAGGGGCAGGCACAGCCTGACAGTCGAGGGCTTCTCCAGCAC

ATGCACATTCAAACACTTCTTTCCCTGCCCCGAGACCTGCACCTCAGTCCTCCTGAAGCA

TTGGAGGTCCTGGAGCCCAGCTTGGACCCTGGGGCCCTTCAGTGGCCCTGGACTGAGTGT

GCCCAGCTCACTGAGGGCATCGGGCCCTCGGGCCTGGGCAAGTCCTCCCCGTCTCCAGGC

CCAGAAGCCAGCAAGCCCTGGAGCAGGGACTGGCGTCCTATAAGAGGGGCCCCTGCACGG

CGAAGGACGACAGGGGGCGGACACCGAGTTGCCACAGCCAGGGACAGTGGTTCCGAC

>Unigene15677_C-W 1 241 LEN=359

XXGGGAGATGACCTGGACAATCCAGGTGGACCCAATGTCATTACAAGAGTCGTTAGAAAT

GACAGAGGAAGAACCAGTCAGAGGTGTGTCAAAAGAAAAGGTGTCAGTGTGAACCAGGAG

CGATGTCAGCCACTGCTGGCTCTGCAGGCGGAGGAAGGGGGCATGAGCCAAGGGCTGTTG

GTGGCCTGGACAAGCTGGAAAGCTACAAGACACGTCCCCCAGAGCCTGCAGAAAGCACCG

CCG

>Unigene15681_C-W 1 223 LEN=223; minus strand

XXAGAAAGAAAGAAAGAGAAAGAAAAGGAAAAACTGATACAAGATGGAAATCTTGTCACT

CATGAGCTGGGTAACTATATCAGTTGTCATGGTCATGGTCATCAGCTGACAAGATGGTTT

GTGGGGACTCTGCTCACGAGGAGTAAACAGTCTACTAAGGGAAAAACAAAGAGAGACACT

GGGGTTCAGAACTTAGGAGCTCCCCTGCCAGTGCTCAGGAGAGAC

>Unigene15706_C-W 1 163 LEN=356; minus strand

XXCCCGGCCCATGTGCAGACCGGTGGCAGGAAGGCCCAGATGGAGAAGACTCGGAAGCTC

ATTCCCTCCAAGGAGAAGGAGACTAAACGGCATCTGGGCACCTGCGCCGAGGTCGCCCTG

GTGGACAAGGTTGACCCTCTGCGGCCAGAAGGTGCCTCTACCCAG

>Unigene15812_C-W 1 274 LEN=274

TCGTTCGTCATTCACGCAATAAACGTTTTTGAGCGCCTGCTGTTATGTATGTGCCACAGA

ACTCTGCCCTGGCGCTCCAATGCAATTCTTCATGCCTTCAGAGCCCTTAAGGATTTTACA

AAATACATCCAGGTTTCTTCCTCTTCCCTCAATCCAGTTCAAATCCACTCTCTTGACACT

TACAGTGATCTGTATGACCTTCTTCGCCTCTCTAAGCCTCAGTTTGCTCATGTTGTAAAA

CGGTACACTGCGTCGATAGCACTCTATATCACAGXX

>Unigene15871_C-W 1 108 LEN=266

GTGGAAAAGCACTTTGTTGACAAGGAGAGTGGAATGATGGATGTTAATCGTCAGGAACTG

CTGAATGCCTCGTTAGTTGCCACAAACAAATTCACATATCAGAACGAA

>Unigene16039_C-W 1 159 LEN=401

TGGAACAAGAGAGGAGGATCTTCAGAAAAGGTTATTATGCTAAAAAAGCCAAAGAAACCA

GAGAGTAACACTACAAGTGATAATGATATGACTGAAAAGGAAGTTTCCAATACCCATGTA

GAGGTATCCATTTTTCCAACAGACGACCCCAGTAGGGCC

>Unigene16081_C-W 1 281 LEN=281

XXCTGGAGTGGTCTGGTCGAGATGGGGCCTCACTATGGGCAGCACAGCCTGTTGCCTAGA

ATGGTCAGGAACTCTCTGGCTCAAGCCATCCTCCAGCCCTGGCCTCCCCAGTGGCTGGGA

CTACAGGTACACGACTACAGTGGGACTACAGTATGTGCCCAGCTTATTCAACCATTATTG

ATGGAAGTGTCTCTGAAGTATATCACAGACACTTGTACTTTTCCAAACAAAATAAAACAC

CAACAGCTTCATCGGATGGCCTGCCCCGCAGTGCTTGCCCTCCXX

>Unigene17499_C-W 1 259 LEN=259

GGGATGGGCAAAGGCCCAGGGGTACTTTTGAAGAATCCCAAGCAGCTGAGCCTGGCTGAG

GAGGAGCAATTGAGAGAGTGGCCAGAAATTAGGTGGAAATGTGAGCAGGGGCCTGGTCAT

GCAGGGGGTCTCATGGACATGGTTAGAAAGTTAAGACTTTACACTGTGGTCTGGGATTTG

GAACACAGAAAGCAAATCAGGAGACAAGACAGATCTAAGAGGAGATGGTCAAGACTGGTG

AGTGATCCAAATGATCCCAXX

>Unigene17520_C-W 1 262 LEN=410

XXACCAAGGCGTGCTGAGAACCAATGCAGAGAAGAAAAATGGCACGTGGGCTGGCGGCTG

CTTCCTCAGCCTCCTGACTTACGGCAATGGGCTCCAGCTCCTCACAACCAGAGGGCAGCA

GAATCCAGGAGGCTGGTGGTGCCTTCTCGACACCCAGAGTGGATCAGCTCACTACCCACA

CCTACTCTCAGGGGCCCCAACTTCAAAGACGTGTGGGTTGCTGCCAAAGATGGAGCAGGA

GCTAATGTCACCAGCCAGGAAGAA

>Unigene17797_C-W 1 71 LEN=745

XGCTTCGACCTGCCTGACTGCAGGTCCCGCAAGCAGCCCATCAAGGAGGAGTTCACAGAG

AGTGAGAGCCAC

>Unigene17922_C-W 1 224 LEN=224

CTAAAAGTGACACCTGGTTCCCATGGGAGGCAAGACTGGCTTCTGGCCTGGTTCTCAGGG

ACAAATGCAGGGGCAGTAGATGTACCTTTGCTCCTGGAGAAAAGCGTCCATTTGTATGTG

TTTAAAGTCTCGGCTACCTTCAGACATGGCGTGTTTGCTCTAAACAAGGCAGACAGTTCC

CCAGCCCTCCCTGAGGAGTTAAGTGAATGCACACTGTATCAGAGX

>Unigene18211_C-W 1 226 LEN=226

GGAGTAACCATGTTAACACTTTCCACACCGGGGGAACATAAGGACACTGACACTGAACTC

CTCGACTTTACTGAGCCCAGCCTGCAATTTCAGAAAGCCCTACACTACGTTAAAAATGTA

CATCAATTCACTCACCTTGATTCAAAGAAACTGCAGGCATTCCTGAAAGATCAAGAACAG

AGTTTTCCACTAACCGACTCACAGCGAAAAGAAATAACTGAACGGGXX

>Unigene18277_C-W 1 253 LEN=253

XCGCGGGCCTCTAGGAAGAATATTGCAAGAGAAAAAGAATTATGTGAAGATTTTCAACAA

TCCAGTAAAGATGGAGCAATATCTGACACAGGAACACAAGAAAATAAAACCTTGTTTGAC

AACAGTTGTACTGAGTGTCAAAAGGAAGACGAAGATGAAAACAATCTTGTACAGTTTTTC

AGTATAAAGACCCCTTTCTTTTCAGCAACTGACTCAAAGTCTGAAAACACTGGAGTCTTT

ACTGTGAAGTACCCX

>Unigene18463_C-W 1 206 LEN=485; minus strand

XACTCTACCATTGAGCTACATCCTCAAGCTTTAAATTCAAATCTTAATGCGGTCACTTCC

TCATTGTATGATCTTAATCATAATGCAGAGCTAACAGACCAATATGGAGGATTTTATACT

TTACAAATGAAAGGGGGTACAGTAAAACAGCAGTTGAAAAATATTGCCTTGTCTAATAAA

AAATATAAAGCCAAAAATTATGAGTTC

>Unigene18629_C-W 1 341 LEN=341; minus strand

XCGGTTTTTGTTTCATTCTGTTTCGGTTTTGTTTTATGCTATCTTATTGGGTTGCGTTAC

CTTTATAGTAGATTAGAAACTAGTAAAAAACAAACCGAAAGACTGTTAAGTAAATTGTTA

GAGGTCCAGACTATGGTAGAAAACATGTTAGGTCAAGCGAAAGAGAAGGTCTCTCAAGCT

AGTCAGACAGAGGAAGATTTGAAGAAAGAAAGCTTAGAGGAAAAACAACCATCAGGCGGG

GAATTACAACAGGAGGCTGCTACTAACCCCGTTCTATCACCAGAGGGCGTAATTCAACCA

ACAGCTCCACCTATAGAGACAGCTGAGTGGCCCTCAACCCCC

>Unigene18842_C-W 1 242 LEN=242; minus strand

CGCACTTGCGCAGCGGTTCCTCGAAGCTCAACAACGTTGAAGGTGCTGCTCACATCCGCC

AGTCCTTTCAACTAAACTGGGAGTCTCTTCTTCCAAAATCCACGTCCTGGTGAGCGTTCC

AAAGGAAAACAACAGGTCGCGATGGAGCTTGAAGGGGATGTCAATGAAAAAGAAGGAGAG

AACTCCTTGAAGCAGAGCAACAAAAAAAGTAAAAAGAAGGCGGAAAAGAAACCAACTGTC

AGX

>Unigene20651_C-W 1 98 LEN=415

XTGTTTCTGGGAGACTTCGCCTTTGTGATGAGTGACAACTCCTACATGATGCTGTGAGGT

GGCCCCTCCAAGGCCACCATCGGGATCCACGCTGATGTC

>Unigene20838_C-W 1 732 LEN=732

XXATCAAAGTCAGCAAAGATGTTTAAACTGATAAAACCCCATGCTGGTGAGGATGTGGTG

AAACGGGCATTTTCAAAACTGCTTAGAGGAAGTGTGGGCATGGGCTGGCGAAGCCGTTTG

ACATGCGTGGCACAGGCGAAGGCAGGCCCAGACGGCACTGTCCAGCTCCCAAGAGTCTTC

CCTAAACTTGGAAAAGGCAAGGATGCCCTGAAGATGTTCATTGTCATAGACTCAGAAATT

GAAATGAATCCGTGTTCTCTGTTTGAAAACGGTCCCTCTGCGCTGTTCAGTCACCTGACC

ACGCCTTATGAAGACCTGGGCCGTGGACACAGTGTTCCTGTTGACACTCCATGTCCCTCT

GTGTTGTGTGCGTTAAGAGGGACTTTTTTTAAAAACCTCCAAGAGTCAGTTTTGACCCTG

TTAAGAGAGCTCTGAGCTGTGGTAACGGGGTGGTGCCCGGGGGTTGTCACAGCCTTAAAA

ACCCGCGCAGGTCCAATAAGGAGAAGGCAGAAAACATCAAAATGTGAGAAGGCTCGTCAC

AAAAGGCAGAGCGCGGGCGGTCAGCAGGAACGTGAGAGGCCCAGCCTCACCAGAGGCCGA

GAAGATGTGCGACGAGATCCCGGCGGGGCCTCCGCTTACTCCCCTCTCACTGGCCGCGTG

TTAAAAGTGTCCACCGACGTCGGTGTAGGAGCCCTGGGTGCCCAAAGGACCTCCACACAC

GACCACTTTGAAAXX

>Unigene20850_C-W 1 435 LEN=435

TTTGCAAAAGCAAAGCCCCCAGAGCCACAAGCTGTGCTGCTCCTGCTGCAGGAGGCAGGG

TAGTCTGGGAAAGGCTAACTCCTTCCCGATGATTCTGGAGGCATCGACTGTCATGTGGAG

CAGGAGGCAGGCCAGGCCTTGGCCAGCATGCAGGGCAGACTTCCCAGCGGGGCCGGCTGC

TGTGCGTATGCCTGGGCTGCAGACCACGATGCACTGAGACCACCAAGAAGGGACCTGGTG

AAGGAGTGTGGGCTTTTATCTTCCTCTTTCTCCTCTTCCTTAGACCTCCCTGCCACCGAG

TTGAAGTTTGAGTTGAAGTTTTCAAGCTTACTCATCTGCATACAAATCTTCTTGGAGCCA

TCAGGAGATAAGCCCCAGTTTGAAAAAATAAACTGTTTTTCCAATCTCAACATTTTTCAA

TTCTGCAAACCAAAG

>Unigene20940_C-W 1 222 LEN=222; minus strand

XGTAGATCTAAGTCTCAAAACATCGTTGGCACAGAAGAGGAACGAACTGCTCTTTTGCCT

AATGAAATCTAGCCTTCTGAGTCCTGGATGGATACAGATTGGGAGACAGATGCTGGTGGC

CCTGCGTATCACTGTTGGGAAGGTCTTCTCAAGTTTTGGACACAGAGCTCCATGGGGCCT

CCACCACTCCCTGAAAGAGCCCACATGCTTTTGGATGATCCTGXX

>Unigene21055_C-W 1 383 LEN=383

XXAAGAATTATTCCTCTTACAGCAGCCAAAATATCAGAGTGGAATTCTGCCCTGGAAAAA

ATTTTCAGTGACATGGAAGAATGTGACCCTATGCTTGAACGCAGTCTCACATTTAAGCGC

CTCACCTCCACTGCATTTGTCCCTTATTCAGAAATGCTTAAAGATTTGAGGAGAAAACAG

GCAAACAAAGCTGAAGCAGTTTTTCTAGCCAATTTTAGAGGGAAAGATGCAAAAGATAGA

ACTGCTGGATCACAACATCCCATCTCCTTGACTTTCCTCTCTCCAGGACATGCTGTGAAG

TGGAGTGAAACACAGGTGAAAGGAGCCCACTTGGCCATCCATCCACTGGACAGGTCTTCC

AAATCCATTGGAAAAGAAATGAAG

>Unigene21222_C-W 1 292 LEN=292

XCCTGTAACCGAATGGAAAGAGTAATGGGCTTGGATCCAAAGGACTTGTATTTAGCTAGG

CAGAGGGGCACATGCCCAAGTTCCAGCACTCAGGAAGCTGATTCAAAAGGAGCTCAGGAA

TTTGAGATCTGCCTGGGAAACATAGCAAGACCCCTTCTCAAGCTGGGCACGGTGGCACAT

GACTGTAATTCCAGTGGCGCTCGGGAGGCTGAGGTAGGAGGATCAAGGGTTCAAAGCCAG

CCTCAGCAATGGCAAGGCACTAAGCAACTCAGTGAAACCCTGTCTCTAAATAAX

>Unigene21345_C-W 1 336 LEN=336; minus strand

XGTGGTTCAGCACCCCTGGGTTCAATTCCTGTTACCAAAACAGACAAACAAACAAACAAA

GCTTTGGAGGTATTAGCCCTTTTCTGTGGAGAAGATGCCACATGTGCATTGAATGACCAT

CCTAAGAATGGCTTTTCAGAACCAGTCTTCACTGTATGCAGTGCCCAACTCAAACTACTG

TTCCAGATTCGTACTTCTCAGACTCTTTCTGCAGCCATATGGAAGGCCAAAGAAGGTTCT

GGCTTCTTCTGCCTTCAGTTAGTCTCTGCAATTTTGTTTTGGTTTGTACTTATATCTGGA

TATGTCTTATAAATTGTGTTTGAAAAATATATTGCTGXX

>Unigene21416_C-W 1 217 LEN=217

XTTTTATATCAAAAGAATAAGCCTCCCTTGGGCATGAAAGTTCAGCTGAAAGGACTAAGG

ACTGGGAGCAAGAAGTATGTTCTGGACTTTGGGTTTGGCCAGCAGAAGCCTGTCACAAAA

CCTCTGTATTTTAAAATACAAAATCTAGCAGTGCACAGAGGCCGCCATGACAGTGAGCCT

CGGTTTCTGGTTGGCGGGAAAGGCCTACGAGGATGGGCX

>Unigene21498_C-W 1 228 LEN=228; minus strand

AAATCTCCCAATGAAGACAACTTAGGACTAGATAGTATCACTGGTAAATTCTATCAAATA

TTTGAGAATTCTTGAAAAACTGATACCAGTCTTACTCAAACTTCCAAAAATTTGAAGAGA

AGAGACTATTCCAAAGTTATGAGACTACCAAAGCCAGAAAAAGACACTATAAGAAAATCA

CAGACCACTATCCATGATGAATATACATATAGAATTCCTCAAGAAAAC

>Unigene21776_C-W 1 60 LEN=319; minus strand

GGACAGGATGACATCGCTCATCACGAGAACGAGTCGGTGGAGAAGAAGGGGAGTCAGGCC

>Unigene21857_C-W 1 290 LEN=290

XXGTGGATCAAAACACAGAGTCCAACTGTATGCCGAGACTCATCTCATAGGTGAGGACAC

TCACAGGATGGAGGAAATAGGAAGGAAGAAATCTCCACACAAATGAAGATTGAAAACACC

AGGAGTAGCTTCTTCTCAGACAAGGAGCAGGTGTGCTGGGCAGCTAAGAGAAGGGGACCT

GTTGTCACCACGGTCCTGAACAAAACAGTACAGATCAACCTAAGGAGTGAGTCCACACCT

CTTACGTCTTCACCCACTGTTAGCTCTCATGACACCGCCTTGTCCCCTTCAGXX

>Unigene22070_C-W 1 222 LEN=222; minus strand

XCAATCATGAGTCAGAAATTCAAGCCGCCAGATGCTTTCCAACTAACAGGGACCAAACGG

GACCGTTCTAAGGXTGTCCTAAACGATTTGGAGGTGGGAGTTGGTAAACTAAGAGAATCC

TATCCTAAAGAGGACAAGACCAGACGGCAACAACCGACTCCCGGTTCCTTGGACCTCGGC

ATTCCGTACAAGAAGCCAATATGGCAAGGAAATGATGCCTCCCGX

>Unigene22103_C-W 1 94 LEN=223; minus strand

XXCCCAGGCTCCTCTGAGACTGTGGAGCTAGTGCCCTATGAGCCCGAGCTGCTTCGGCTC

CTAGGGTCTGAGGTGGAATTCCAGTCTTGGAACAGT

>Unigene22155_C-W 1 180 LEN=226; minus strand

CGGTACTCTTAGTTTAAAGGGAAGGGGGGGAATTATTGGGTAGGCAGCTTGCAGTATCCA

GATGAAGAAGATGATGAAGGCGAAGATGAAGACTACAAAGAAGAAGAAGAAGGCGAAGGC

AAAGATGAAGACAAAGAAGAAGAAGGTGCCCATTCTGTGAGTTCAAACTTAAACAACAAT

>Unigene22433_C-W 1 372 LEN=765; minus strand

XXAGCTAGAGGTGGATTTCTTGGAAATGGAAGGCGAAGAAGGACGTGCCAATCTGCGATA

AGCCCGGAGGAGTTGATAAGAGACTTTGATTCCGGGATTTCCGAATGTAGAAATACGATC

AACCGAACGGATGATCATTTTAAGGCCAAXTCAATAACCTTAAAAAGCGAACCTTGTGAA

GTGAAACATCTCAGTAACAAGAGGAAAAGAAAACGAAGCGATTCCCTAAGTAGTGGTGAG

CGAAAGGGGAAAAGGACAAACCGGCTTCGGCCGGGGTTGAAGGACGTCAATGTGGAATCG

AAGTGTATAGAAGAAGTCTTTGGAACAAGACGGCAAAGAGGGCGAACCCCCCGTATTCGA

AATAAACTTAGTACC

>Unigene22654_C-W 1 477 LEN=1634; minus strand

XXTGAAGACACGCTCTCCAAATGGAGTGTTAGAAATACTCCTTCGGTAGAAACTAGAATG

TTGGAGCACTATTCTTTGGGCTCGATTTCAAAAGTCTTACGGGACTTTCTTTCTGGCCAA

GTTTTCCTTCGGTGTCCCTCTGAGCAGTGTCAGCAGGAAGATGACAXCACCCAGTACCTC

TGCTCTCCAACAGAAATTAATCAGCTATGTTTTTTGCCTTTTGGGGAAATGGGTGTAAAC

CAGCCATTCCAACAAGAAACCCTGAAGGAAGCACAGTCGTTCACTAGAGGAAACTTGGGA

GTAATGGTGCAGGTTATGAAGCTGGATGTCACCCAGTTCCTGATTCTCAGCTCAGAGGGG

CAGGAGGTTCAAGGTGAGGTTGAGAGCCTTGCTCCAGAATCCCCACAAACAGATGGCCAG

CTGGCTAGCCATAAGGAGGAATCACCCCCAGCTCAGCTGGACAGTCATGTGATCATAACC

>Unigene24142_C-W 1 504 LEN=504

XXTGGAAAGTTCTGCAGTCTAAAACCTTTGGAAGCAACAGACATTAGTTCTGTTGAAGTC

CTCTGCATAGTTTTTCCTCAGGGAAGTCTACTGTGGATGTCTTTGTTGGTATTGGAGCTG

AATTGGAGAGCTCTCAGGTCTGTGGCTCAGGGCCTTCCTGACATAGGTTCTCCCATGTTG

GGGGTTCTTGTGGTGCACCTATTGAGTGCCACTTTCCATCCTGATACCCCTCCCCCAGAG

CTGAAGATTCCTTATGACATAAATGTTTTCTATGTCATGAACTCTCGCATTCAATATGAC

TTTGTGCTGAGGAAGAGGACCATCCCAAAGGAGAAGGATGAGTGGATCCAGGCCAACTTC

CCCCTTATGATGCAATGGAGGCTCAATCTCTCCACTGGATCCTTGGAAGAACAATTTTCT

GCTGTCTGGCTGGGCAGTGCTGAACATCCCATGATCATTGACATTCACTTCTTTAAGTAT

TTGCTTCAAACTTTTCAAGTTGCCAXX

>Unigene24159_C-W 1 209 LEN=361; minus strand

XCAGGGGACAAGGTTTATGTGGAAGGGAGCATGGAAGGCTGTGAAGTGGGGGGTGTCTGT

GAGAAGTCAAGGTAGAAACACCAGCAGCTGGCTGTGTGGGGAGTGGCCACTGCGCAGCAG

TGTGAGAGCAATAAAAGGCCCAGGACAGAACCCATCGCTGCAGGCATGGTAGAGCCCATC

CAGGAGCATGGCTCCAACTGGGAAGAGGAA

>Unigene24314_C-W 1 278 LEN=278; minus strand

GTAAAGATCACCAGCAGCAGCAAAGACCGTGAAGAGGACATCAGTGCTGGATGGACAGGT

GGCATCTGAGTGGACCTTCAGGGTGGTTGGAGCCACGCAGGCCAAGATGGCATTCCAGAG

GAATGAAGGAGGTTCCGCCAAGGTTTGGAGGTAGAAAATGGTGTCTCAGTGCTTTTAGGT

GGACAGAAAAGAGTGGCCCATTTTAGCTGTAGCAAAGAACTGGAGATTGAAGGGCAGATA

CAAGGAAATCAAGTTGTCAGGCTGTGGCAATGTGGACAX

>Unigene24370_C-W 1 320 LEN=320; minus strand

XXAAGGCTTTCAGGCATGCAAGGGCCAGCTCATCCCCTTGTTAGCAGATGGGGCGACAGT

CCATCTTTCTCCAACTTAGATGAATATCCTGGTCTCCCAAATGCCCCATCCTGTTCCACT

GTAAACCGTCTTCCAAACACTACACATCTGGTCCCGTCATGTGTGTGTTCAGACTTCTCT

GTGTGGCATTCTCCTGTTCCATATCAGCTTTTGGCTGAAGGTTCCATAGCCAAACTGTTG

CTAGAGGTAGAAGGAAAAGAAGATTGGCACCTCCAGAGTTGGAACTGGATGTGGCATGTG

GTAAAGCAGAGTGGAGAGCGTGXX

>Unigene24863_C-W 1 275 LEN=275; minus strand

TTGGGAGATGAAACTGAGGGACCACACAAGGAACAAGGGACATGGTGTGACCCTGAAGGT

GTTTCTGGCTGCTGCCCTAGGACTTCTGCCAGGATAGGACCTGGGAAGGAAGCAGTCATG

CCTGGATGTCAGAACTTCCCACACTTCGGACAATTCTCTCAAAAGCACTCCAGATGGGCC

ATGAAAAAGACCAGGCCCAAAATAGGATCAGACACAGACATGGCCACAGATGTTCTGTGT

TCAGGCCCCTGCCTGCAGCCAGAAGTGGTGGGTAGX

>Unigene24935_C-W 1 288 LEN=288

CATGTCAATATGAGTCTTCGACTCTGTGAGCTGCTTGGATGGGTGGCTGCAGCTGCTACC

ATAGCAACTGTGTCTGGGCCTCTGACCACGCAACGCCACCTACAGACTGTGTTTCGATTC

AACCTCGGCCTCCTCCACCAGATGCCTAGGAGCACGGCTTCCTCACTCCGATTCTCCTTC

TCTGCAAGCAGTCGGATATGGCAGGGTGTTGAGGAAATAGAGTAGGAAAGGAAGCAGAAG

TCTGGAAAACTCAGAGTTGACCCATCTGTTCATTTGCAAATGAACAGA

>Unigene24989_C-W 1 251 LEN=251

XGAAGACCACTTCGAGGGTTACCCCAGTGGACAGCACAGTCGTCCCGGACTTGCCGAGCA

CATCGATTGAGGGACCGAGCCTACCTACGTGAACTATCCAAGTCACAGCTGAGTTGGGAC

CCGAAGAATGCATCTGAGTTACCCAAAACAAGAATGGACGGAAGAGTGTTCCAAGCAGAG

AACTGCGTGGGAAAAGGCCCAGCGATTTGGTTCGATACCCATCCCCGACAAGCAAGCAAA

CAAACAAACAAA

>Unigene25423_C-W 1 355 LEN=355

XGGAGACTGAACCCAAGACCAGGAAAGGTGGAGTCTGCAGATGACGTAGGGGACAAAGAA

GTGGCTCCCCCACCACACGAGTGGAACCTAGGGGAGCTCTCTGGAGTAAAGTCTTCCAGC

GTGGACCAGGAAATACTGGGTGATGACGGAGGTACGCTGATTTATAATCTCCAGACCAAT

TAGCATTCAGCAAAGAGGCTGAGTCCACCTAACCAAACCCATCTATGGGATTACTTCTCT

CACTCCAGCAATCCAGAGGTTGGAGCATCATACTCCAGAAGACCCTACCTAAAACTGCTG

AAAAGAGAAGCTGAGCATATTTTGAAGTCCAACAAAAAGAATTCTTTAACTTTTCAX

>Unigene25436_C-W 1 291 LEN=291

XGCTGGCTCCCTGGGCTTATCTTGAAGGCTCAAATGGAGTCACCCTGCACCAATATCTTC

TCCCTCCTGTGGCACTCGCCCCAGTCTCACTGTCACCTGCAGGATGAGTACCGACAATAC

TGGCTAAAGGAGTATCTTAAAGCATCTTACCAGACGCACCAGGTTTATAAAGGACTTGTG

GGCAAAACCTACTCCATATCAAAAAGAGGTGACACGCGGCTCCCCTCCAGCCCAGTGAGC

CACACCTCAGATCAATCTCACTTCTGCAGCAGTTCAGCTGAGGGGGCACCAGXX

>Unigene25599_C-W 1 323 LEN=323

GAAAGAAAGAAAGAAAGAAAGAAGAGAAGAAAAGAAAAGAAAGACAGACAAGGAAAGAAA

GCAAATTCAGATTCTGCAGCTTTGGAGGATTTTGTCACATGTATTTTAAAGAGCCCATCA

TCCCTGCACTCCAGGGACGTGCCCTTGACCCTGCTGCTGTTCCTTCACAGTCTCTGTCTC

TGCCAGCCACCAGCTCCTTGCTTATCTCAGATGCCTGCCTGGTATACCCGAACTCTCCTT

AACCTCTCCAACTCAGCACCCGAGTCCTGTTTTCTGTCACCAGAGGGACTCGACAGGTAC

CCGGAGGAAAAGCCCACCGCAGGX

>Unigene25675_C-W 1 249 LEN=249

XTGGTTGTTATCAGTAAGATAAAGATTTTGAGATTTGAGTTCAGCACCTTGGTGGTTCCA

TATGATGGCTACCTGTCATACAGGAGTAAATAGAATCATACTGCTTCATCTGACAAAGAC

ATTGCTATAGTGAGCACCATTTATATACTCAAAACAGATTTTAAAAATATTGAAATACAA

ATGAAAGGCTTATACATGAAATTCCATGGGTATCCCAAAAGGAATAAATCAGAGAGAACC

CCAAAACTTAXX

>Unigene26640_C-W 1 397 LEN=397; minus strand

CTAGGACACAAGAGAAGACACCTCCAGCACAACAGATCACACTGGTGCCAGATGAACGAA

GAATATGCTAACATTAAAAGTAGCAATAAAAGTCAATGGCTATTACACCCAGATCTGGGG

GTGCTTTTTATTGCCATGTGCCAGACACTGGGGACACATAAAGGAACTGAGTGTGAGGGG

AAAGAATTCATAATTCTGGTACATCAAAGAAGTACAGTAGCAGAGGGAGCACTGAGCCAG

AGAAGCATGGAGCCTGCCTGGCTAAGCACCAGGGAAACTCGGCTTAACGTGACGCCCGAA

GAGCCTCGCACCGTGATCAGGAATCAGCAGGGCAAGGAAGACCCTTCAAAGAACAGAAGC

GGAAACAACCTGGAGAACATGGGAAATAGCATGTTTCXX

>Unigene26778_C-W 1 664 LEN=664

CAACAAGACAGAAACTTTCAATCAATAACTGCCTCCATCCAGTCAAATATTGCAAGAAAA

ACAAACGCAGCCTTGCCCACACCCCATGAAGCCACAGAAGAACTCAGCAGCGCCAGGCCA

GGGTGGCTCTCTCCCGGTCCTGCAGGTGCAGTCAGAGGCCTGGGGGAAACCGCAGCAGGG

CCTGCACATCCTCTGCAGCTGGTGGTCATGGTCACGTTCCCAAGGTGACCATTCCACTAC

CAGGGTGGCAGAGGAGGCTACTGGGAAAGCCACATCAACCCCAGGGTTTTAGAACACAGT

AATGAAAATGTGGGAGATACCACTACTGATTATGCCAAAACCCAGAAAGTGCACCTCTAC

CCAGGGGACAAGGAAACCAACACTGAGATGACACAGATGTTGGAGCTACATGACAAGGAT

TCAGAACAGCCATCTGGAAATGCTTCAGCGCAAATCACAAACAACACCAAACAGCAAAAA

ACTGGAAAGTCTCGGCAAAGAAATAGAAGATATCAGGAAGAATCAAATGGAAATTTAGAA

CTGGAACATACAACAGCCAAAATAAAAATACTGGGTGGATGGTCTGAGAGCAGAAGAGAG

AGGAAAGAGGAACTAATCCTGGACAGTAATGGGAACCACCCAATCTGAATCAGAGAAAGC

GCX

>Unigene27309_C-W 1 284 LEN=284

XATCCTGCTACTACTCAAAACTCCTGGGCTGGAGGTTTTGGGGGTTTTGATCAGACCTTG

GTAGTCAGTGAGATTTCCACATTGCCTGCGCCAAGATGGATGAGAGCACGGTGGCACTGG

AGCCACCGTGACATTGCCTTGTGGGGAAGTTCAGGCGATCGATCAGTGGTGTGTGCAGTC

GATATTTCTGCTGCCCTACACTGCTTGTCTAAAGGACAAATACAGCTGTGTCACCTGATG

CAGAAAGTCCTTCCTAGGCTCTCTGCCCTCACCCATAACCTTCAG

>Unigene27493_C-W 1 240 LEN=586; minus strand

AATGAAGAACTATTTGAAAGACTCATGAATAAGTTGAGTAAGTTGGAAATGAGAAAGGAA

ATAAACAGAAAGCTTAAAGACATGGCAGTAGAAACTTCCCAAGCTGAAATACAAAGAAAA

TGAATAGAACAAATGGAACAGAATAGCTTAGAACTATGGGACAATTCCAAAAGGAATAAC

ATATACCTTATTGAAATACCAAAAGAAGAAGAGAAAAAGGAGGAGAAAATTTTGGAGTAG

>Unigene27554_C-W 1 345 LEN=345

XTGAAACCTCTTGCAGAATTTTCCTTGGAAGTGAACATAAGGATGCGCCTGCCATCCATC

ACCTTCCGAAGTCTGCAAATCAGCACCTACACCCCTGAATGTACCAAGGATCTCTTGGGA

CAGTGCCAAGCAATTAGTCTCTGCACAGCAGCAGAAGTTGCCTCAGTGTTGGCCAGCATC

CCTGAGGCCCTTAAGGGCTCCTATGGAGCCTACGAGAAACAXCTTGCAGAAAACTTGAGT

CAAAAATTAATTCTGGAACTTGAATTTCAAGTATTAAGGGCCTCCAGAATTGACTTAGCC

CTAGTAATAAAAAGCACTGCCAAAACATCTCAAAGACTCAATCTTGTX

>Unigene27627_C-W 1 221 LEN=437; minus strand

XTGGGAGCTGTAAGGGGCAGTGAAACACTGGCTGTGAGTGAAAGAAAAGAGGAGGCTTCC

AGCACAGATCCTCAGGTGCCAGAGAGGATGGACAAAAAAAGCCATCATCAATGCCAGAAA

CCCAAGGAAGAACAGCAACTAGCTGAAAAGAGGAAACAGAAACCTAGGGACAAGCCAAAA

GTGAAACCAGAGGGCTTAGTGAGGTTTGGCAGCCCACACTTG

>Unigene27689_C-W 1 94 LEN=401

XXGGACCGGCCCCGCTTTGAGCGCGTGCTGGGCCCTTGCTCCGAGATTCTCAAGAGAAAC

ATCCAGCGTTACAACAGCTTCATCTCCCTCACCGTC

>Unigene27854_C-W 1 299 LEN=299

XCTTCCTTGAAGAATCCCACTGGATTGAGATATTCTCGTGAAACTACAGACATAGAGATG

AAGGGTGCCAGGAAGAAGGATGTCAAGACTATTACATCAGAACCACATTTCAATGTGAAG

GCAGAGGAAGCGGGCACAACCCTTGGTGGTTCGAAGAAGCGGAAGCCAAAGAGAGTCTCT

GAGATTTGCTCAGGAGAGAGCTTGGATGGTTCCTCTGAGGATACGACTGAAAAGGAAGCA

CATACTCTGGAAGAACTGGGCCCTCTGTCTTCTCCTGTGATTGCATCAAGCAAGCCCAAG

>Unigene27871_C-W 1 326 LEN=326; minus strand

XGTGTCATCATCTCCGGCCTGCTGCTGGCTGCTGGACACTTAGTAGGGGATCCTTGCAAC

ATGATACCACATGAGTCTGTGAGACCTTTTGCAAAGGATGTCTGGGACAGGGGGCCTGGC

CCTCAGACTGTTCATCACCTACCTGACACAGTATTCCACAGTGTCAATGTCCTAAGGAAA

ATGCCCTGCACAACTAAGGAGCCAGACCAGCCTCTATCTATGAGGAGCCCTTGGTTCCCC

AAGGGTGGTGAAGAGCATTGAAGAGACATCAGGAATCCTAGGCCCATACTGTTCTTCAGC

GTTGAAATACGACTGTCAAAATTGACA

>Unigene27994_C-W 1 264 LEN=264; minus strand

CCAGATTCCTTTTTATGTCCTGCTTCCCTGTTTCTAGGACAAGTAGTGCTGTGTGTGTTA

GCTTTCTATCACTGCAACAAAATACCCAAGAAAATCAATTTAGAGGAGGAAAGATTTATT

TTGGCTTATGGTTTCAGAAGTTTCAATCCATGGTTGACTGGCCCATTGCTATGGGCCTGT

GAATCAGAGTATCATGGCAAAGAATTTGTGGTGCAGCAGAACTTCTCACCTCATGGCAGC

CAGGAAGCAGGCGGGGGAACAAAC

>Unigene28055_C-W 1 263 LEN=263

GTACCACCAAAATTACTCCACGTTGAAAGATCTGTCTCCTGGACCTTTGAGAACCTACTT

CATTGCAGACCCATATAAGAAGTGGGAGAAGGTGAGAGGAAAATCCAAGAGATTCACAGC

TCTGACCCTTCTGAGACCAGCTATACAGAAGAAATAGTGAACTATGTAAATGCCCTACCC

ACAGATGATGAAGTGCTGGGGATAAGCCCAGGAATTGAGAGAGGGGCAGACCAGTGTGGG

CCCGAGGTGCTGAAAGGGAGGCTX

>Unigene28068_C-W 1 456 LEN=456; minus strand

CTGACCCGCGACTCGGTGGACGGCATTTTCTCCAGCCGTAGGATCGAGGAAGGTGAGCGG

CCAGCAGCTCGCCTCGCGGCGGCCTCGTCCTCAACACCTGGGTCGCAGACCGAGGGCAGA

GAAGCAGCGGCCGGCCAACGTGCTGGTGCTAGCCCGCGGCCCTTCCCGCCCTGCGCTGAT

CGTCCCCTGAAGGCAGCCTTTCGCACGAGTGAATCCGTGCGCGGACTCCCGGAACACTGG

TCGCTCTGGCCCCTCAGCTCATCGTGCAGATACAAGTGCGAGTCTCCCGAGTCCGAAAAT

CACGTGCTGCCTGCCGCATCTGCAGCCAGAGATCGCCTGGCCGCAGGCCCTCTGCGCCTC

CAGGGCGGGCACCGGGACGAACCCACGAGCGCGGAACAGCCCGCGTGCAGCCTGACGCCC

CGCCGGCACGCCAGCTTCCGTGCAGCTCCTAGCCCX

>Unigene28154_C-W 1 242 LEN=378; minus strand

XCTACCCTGAGCATCTGGTGAGCAATGGCAGAAGCTGGAACATTCTACAGCATGGAGTTG

CAATCTGAACTTGCAACCCCAACAGACTACAAGGGCTACAAGGTTCTGAGAGGAATGTGT

TCAACATCAGGGACCGAAGAGGCTCATAGGGATTTTTATCTTAGTCTGTTTGCCATTGCT

ATAACAAAATACCTGACACTGGACACAGAAAAGAGGTTTATTCAGCTCAAGAGTTTTGGA

GGT

>Unigene28366_C-W 1 351 LEN=351

XXATTTGCAATTGATCCTCCTGCCTCGGCCTCCCAAGTCACTGGGATTACAAGTGGGTGC

CATCAAGCCTGGCTAGAGAAGAGTCTTAATGGGAATGTGCAGTGAGATGGGAAAGCCTTG

GAGAGGGAAGAAGGGGGCCAGTGCAGGAAGAAGGAGTAGTCAGAAAGGCTGAGGCTGGGA

GCTAGACAGATGCACAAGGAAGAGACAGCAGATGGTATGAAATGCATGGAGCGTAAAGGT

CACTGGAAGGAGAAAAAACTGAAGGCAATTCCAAGAGAAGGAAAGAGTAGTCAATTTCAG

ATTAAGAAGGAAGGAGATGTAAAGGATAAGGCAACAGACCTAGAGCAGGAAGTX

>Unigene28422_C-W 1 345 LEN=345

XCCAGAACTTTCAGCAGTCAGGGTGGTGCACCAGTAAGTTCCCAGGACCGGACGGAGCCG

GTGTGGGTTCCAGAACGCCTGACAAGAAGAGTCCCGACATCAATCAACCAACAACAGGAG

GCGCTACAACAATCTCATGATGAGGACCATCCTTCTACTGAGTGTCCTGGCTCTAATGCT

GATATTGATGGTACAGGGGACATCAATGCAGTGAAAGCTTCAGGCTCAAGTTCACCCATG

GTGATGTTTTGCATAGAAAATCACAGTACTGGAGGAGATGGCCAGGCCCAGGATGAAACC

CTTTTTGATTTTGGAAAACATTTGTTTTACATTGCCCATGTGCCCAXX

>Unigene28605_C-W 1 255 LEN=255; minus strand

XXGTACATGCCACCTCAAGGTCAGTCACAGCTCAGGGAACCCGTCTTCCTCTCCATGTCA

GCTCATTTCCTTGAAACAGCCAAGTGCCAAGAGAGCCTTGGCCAGCAGATCCCAGAAGAG

GCCAGAGGCATGGATCATCACCGTCAGGACACAAGTAAGTGGGACAAGAAGAAAGCTGAT

TTCACCTTGTCCCAAGATGTTTTCACCAGGCAGCCTGTCCCCAAACTGCAAAGGCACAAT

TCCCAATCTCTTCTCTGX

>Unigene28658_C-W 1 340 LEN=340; minus strand

XAGCTGGAGGCCTCTCAACAGAACTCCGAAGAGATGTAGACGCTGAGCTCGATCCTCCGG

TCCGCGTTCCGTGGCAGGCGCGTCAGCAGGCTCACCCTAGAAAGCGCTGTGAGCGCCATT

CCTCTTGGGCGCGAACAGGACTACTAACGTTTCAAGTTTACCAAGTGCAAATCCAAGAAA

AACCAGAACGGCAAAACTTCTCAGACACTGAAGAACTCTGCTGTGAAGCGAAACACCCGA

ACCCCGAAGCGGCTGTCCTGGGAGGCGGGCTGACCGCTCAGGAACATCCACACTGTCTCA

GAAGCCAAGATCACATTTGTGTTGCTGCTGTTTCCCTCCCCX

>Unigene28709_C-W 1 502 LEN=685; minus strand

XXTGGAGAGATACGGAAACCCAAGCCTGAGACCCTGATCTGGGGCTAGCACCCCGAGATC

CAGCCCGCGAAACCCGCAGATCTGGACCCTGAACCCCAATATCTGGAGCTGGAATCCCAA

CATCGGTCCCTGGGACCCCAAGATTTGGAAACTGAATCCCATGATTTGGAGCATGGACCC

CAAGATTTGGTATATAAGCCCCCAAATCCGGAGCTGAACTCACAATTCTGTGCCTGGCAC

CTTGAAATCTGGGACTGGATTCCAAAATCTATATCCTGGAACCCACTATTTGGGAACTGA

ACCCTGGGATTTAGGCCTCAAATTCCAAGATCTGAACCTTGGGATTCCAAGGCACCTGAA

CCTGAATTTGGGCCTGAAGTCCTTCCTGCAGACCTGAATGCTGAAATCTGGGGCTAGAGA

CCTCCAAGTCTTTACTTGGCACCCCAGTATCAGAACACAGAACCCCACTACCAGAATCCA

GGGTTGGATTTGGAACTTAGACCC

>Unigene28859_C-W 1 323 LEN=323

GGAGGGATAGCTGGTCAGATTTTGGCAATAGAAAAGGTGCTTAAAATACCAGGTGATAAG

GTTCGAATGCTTGAAGAAGTAGAGGGAGACAAGAGCAAGAGAGAACTGTGCTCAGTGTAT

ACTGATGGACCAGCAATGTGGGAGTTGGTATCCACTGGGTTCCTTACTGCCCAAATCCAA

ACCCAACAGCAGCTTCCCACAGCTGAGCCAGATACCAAAGTCTGGGTATTAGTCCCCTCT

GTACACTATTTCAACATTCACATGTATATTGAACAATTTCCCATTCGGCTCCTAGATGGA

ACCTGCAGACCCAAGGCAGCAGXX

>Unigene29379_C-W 1 165 LEN=511; minus strand

CCGAGAGCTGGTGCCAGGAAGCCTGTTGAAACTGCGTCAGCGGTGATCAGCTGCACAGTG

AAAGCTCAGCTAGCACGGGCCAGTGGCGCAATGGATAACGCGTCTGACTACGGATCAGAA

GATTCTAGGTTCCCAGATACAAGACCTGAGAAGGCCCAAAACGTT

>Unigene29709_C-W 1 139 LEN=487

XXTCCAGACGCCAGTAATGGGACCCTGTGGGGTATCAAAAGTAGTTCCAGTGTTGGCAGG

AAAAGCCCAGACAGCAAAGAAGCTAGTAGCAGATGTAGGGATGAGTCACAAGAGGGATCA

AATGTCTCCATCAGCAAGAAG

>Unigene29776_C-W 1 374 LEN=374

XXAAAAAAAAAAAAAAAAAAAGATGATGATGATGATGAAAATGATGACGACAACGAGGAC

GACCAGTCAGAATGTCTTAACTGAGCCACAGTGATGCATGCTTTTCGGGAAAACCTTCAT

TTACAAGTATTCCAGACACCACCAGGCTTCAGGCATGGCCATGAGCAAGACCAGCAAATA

ACAGCTTTTTGCCTTGCAGCTCTGACCCCAATGTTGTCTGCTGTCTCCAACATGGCAACT

CTGAACATGGCAACTCTGAACATGGCCTACAGCAGGATGCTGTTGAATAACACCATGGCT

TCATCAGAGGATGTGGXGGTTGTAGTACTTCTGGGTGATGAAGTTGTTTTAGTAAATCCA

TTTTTAAAAATGGATTTX

>Unigene30311_C-W 1 377 LEN=377

TCACCTCTGTTGGCCCCCAAATTTTAGCTGGACGATCATGACAAGCAGAAGGGAGGAAGA

TACACCAAGCCAATTGACGGCTCCTTTTGGAAAAATTGTACCACCGATGACATCATCAGC

AAAAATACCCCAACACTACCACAAGTCGCTGCACCAACAGATAGTTCACAATGCATACAG

GTGAGCTCACAATGTGTCCAGGCAAGTTCTGCAAGCAGTTCAGTGATGGCTATTGCAGAA

GCTGTAGATTGGTTTTATCTTTGACTTCACCAGCACTGGGATGAAGATAGGAATTCTGGC

AATAATGGCTAAAGAAAAAATTATGTAACATTACAGAAGGCACTAAAAGAAAACAGTTTA

CATTATCTTGAACAGAAX

>Unigene30366_C-W 1 662 LEN=662; minus strand

XCTCATTACCTAACCACTTTGGATAGTGTCCTCTATTATTCAAGTCCTCTGATAGGTCAA

TGGCGTTCAACAGGAACTGTCCTCAAGGACACTTATGTCATGGAAAAGACATCAAACAAA

GCAAGCAAGCGCTTCAATGTCTCCACTGTCCCAAGGGTTGGAAAGCACATTTCTACCTGC

CAAGAAGATGCAGTGAGAGAGTCCATGGTCCTGATGAGGTGTCGTCAGGTGACTTGTAGA

CTTGACTTGGCCAXCATCCAAATGACCCTTGGGAACTTCTCAGAATCTCCAGAGAAAGAA

CCAAACCCGCACTTTCCTTCTCAGCTAAAATCACCTCTACTAAAGCTCTCTCCCTTATCT

GTGATCTATGGGCAGGGAATATGTGCAGGTGGGGACATCTGTCACCCTTCTTCTTCTCCA

AGATCGTTGAGGTTATTTGAAACAAACAGATTGGAACAGGAAATAGTTGTACCTGAGGCT

GGTGTTACTCATCGCGCACAGCCTGCTTTCCTGCTGAGCTGGGACCAACCTCCTGTCCCC

AACACAGTTCTCCACGAGGTCACTATCAGTCCATCAGAGATGACATTCTACGCGGTATTT

ATGGACAACCTGTTACTGAAAGCAATGGTGGAAAATGGAGAAGGGTTCATTGGCCATTCC

CTC

>Unigene30691_C-W 1 472 LEN=472; minus strand

CCCCAAAAAAAAAAAGAAAAAAAACAGGTCGGAGTTCTCCTGCTGCCAGCATTCAGCAGG

AAGTGCGCAGTGGGTTGGTGTTTTGCTGTGGAAGTGCGACAGAACGAAGAGGGCCGAGGA

TGGGTGGTGATCCGCTGTAGAACAGACCTGGATAAGACCTTCAGTGATAGCTAAAAGCGG

CTGCCACGAAGAGATGCCATGGCACTGCATCGTGGACCCCAGTGGAATACCGGAACTCCT

CTGGAGCAAAACACATGCTTGAAAAAGGATGAGGAGAAGGTGGTCAGTTTCTTCATATCC

GAGGAAGGTGGAACTGGAGGCACACACCGGTGGCAAACAGGCTCTCTGGAGAGGAGGCCC

AGGAACTCAACCCCACGTGCTGGGGAACTAGCCTTGATAAAGAAGTTTCCTCCCATAATT

GCTCTTGGTGTTTGGGGGAAGAAAGTGTTTGAGAACCAGGTGTTCAAATTC

>Unigene30759_C-W 1 268 LEN=268; minus strand

XCCTCAAGCAAGCTGTTCAAGCTCAGTCGGTTCTTAATGGCACTGCTATTTATTGCTCCT

CCAATTCATGTCATCCCGGTCGACTCCACTGGGAACCAGAGGTCCATTTTTGCTTACCCG

GTACCTGGCCCTTTAAAGTCCGCTTCTCTGGCTAGGAGAGTGGAGATGGGACCTCGCGCC

ACTTTAGGAAATGTCGGTAAAAGGCAGCTTCCGAAAAAGATTGGCAAATTCGGAAAGCCC

ACAGACCGACTCCAGCGGTTGGGTAGAGCX

>Unigene31055_C-W 1 248 LEN=248

TGTGGTGCTGAGGATCGAACAGGGTCTCACACGTGAGAGGCGAGAGCCACAACCCCAGCC

CCCGAAGGATCCGTCTTAAAGGAGCCATGCCAACAGGAACCTGGAATTCCAGAATGTTCT

ATCCACGGCTCACAAGAGCGAGCTCAGGTGTTCCCCAGGGCAATGTTACCTTTAGGAGTC

AAGGCTAGAGGAACAGGCAAGCTTGGACATATTCTCAAATTTGGTAAATGCATGAAGTCC

AGATTTTAX

>Unigene31127_C-W 1 279 LEN=279

XGTCCCACAATAAGGTCATGTGTATTCAAGTGTGATTCTCATCGAAAGAAGGGACCTGGG

AATGGAGCCAAAACAAGTTCAGGTCCCCAAGTAGCTGTTGGTTGGTTCAGCAGCCACCAG

AAGAGGTTCCAGGATGATGGAACAAGAGAGCCTGAGCCACCCAGATGCATTGCTCCCCCA

GAGGCTCAAATGACCGTGGGTGCACAGAGAAATAACCATGTGAGGACACAGCAAGAAGAC

ATCTATACGATCTTGGGCTTCTGGTCTCTTAACCGGAAA

>Unigene31176_C-W 1 331 LEN=331; minus strand

XXAGGACATAGAGAAGCCGCTGCCTGGAGTTCTAGACCCATGTTAAGTGGCCAAGAACAA

CTTACTTGGAAAAGGAGGACACCTTCGAACCCAACTCCCAGGGTGGGTCTAATTGCTACC

TCAGGGAAAGCTGTCTTCAGATCCATGGTTATAACATGGATGGACAGGGATGGGGAAGGA

GAACCAGGCACGCATGATAAGCTAAAGAGACCCAAGGAGGTCAGCACCCCATTGGTGGCA

TCTTTACTTCTAAATGCATATCCTGGGCTACCAGGCCATGCTATCAGAGATGCATATGGT

GTAGGAGTGGACCAAGTCCCCCAAACACTCCTT

>Unigene31234_C-W 1 204 LEN=204

AAACAGGCTGTGGGGGTGAAGCCAAGCAAAGGATACCCAGAAACTCCTGGGGATGAGGAA

CTGGTGATGGCAGGGGCACTTAAACAGGAGTTTCTCATAGGGGATTCTGCAAGCCAAAGT

TCCTGGGAGTACCCTCTTTGTAAGACCACCACTCCCAGCCCCAGGAACACAGAATCCAGG

CACCTTGACCATCACAAGCCTCTT

>Unigene31338_C-W 1 268 LEN=268; minus strand

XCATCACTTCAGGTAGGACCCCAACTTTTAAAGACTGAAGAAGTCAGAGAGCTGGAGGAT

GATGAGGAGGAGTCTTCTAAATGGGGAATCACCAAGGAAACCAGGAAGACTTCAAACTTC

CCTAATGTTCATATTAATTCCCTAGGGAGTTTAGAATCTCAGGGGACCAGTGAAGGACCC

CCCCCTTCTATAAGTTCTATTTCCCAGGGCCAAAAATACTCATGCCTGAAAGCACAGACT

GAGCCAAAGAACCAGCCTCAAGAACTGGCX

>Unigene31418_C-W 1 241 LEN=241

XXTGGTTATCAGTTAACCTTCTCCACTCTTCCCCAGAGGTTGGGAGTTAAGGCTGAAAAA

GTCCCTGCTGTCTCATCATATTTTGGTATTTTAGGTGAACAGCCCATTCTGGGGTCCTCC

AGCCTAACTAGTCATCTCAGTAGCATACTAAAAACATTTTTGTGCCTTCAGAAATTCCAA

GAGATCCCAGATGTGGTGGTGCACACCTATAATCCATTGACTCAGGAGACTGAGGCAGGA

GGA

>Unigene31648_C-W 1 225 LEN=248

CACGCGGAAGGCGAGGACCGGCACCTGCAAGGGACAGTGTTCGGCGTTAACGGGGCGCGG

GTCGTGACCCCGGCTTCCACTCCCAACCCGACATACCGGCTCCAGCACACTCCCGCCTTC

CCCTCCCGCACCCTGGGCGCAAGCCTGGGGTCCCCACAGTCCACCCGGCTGCGGATTGCC

CCGGATTATCACCTCTCCCCGCGGACAGGCGGAGACACTTACAAC

>Unigene31891_C-W 1 297 LEN=297

XXCTGGTCTTCTCACTTAGATCCTGATTCTCTCACGGAAACAGGAATGAACAGTGCAGAG

AGCCTGGAGATCTAGTGGTGCTGCCTAACCTCAAGCAGAGTGCCTGCTTCCAGCTAAGAA

ATTGCCTCCATGATGCCAAAGGAGATGAAGGCTGGAGACAGCAAAACAGGCAGTGACTGG

TTAGTGGAGGCCCAGGCCCGGGACACAGCCTTCTCTGCACTAGACTATTACACGGTCCCT

TCAGCTCAAGCACGGCAACTTGCCCGGGACAGAAAATGCTGTGTCAAAAGCAAGAGGTAX

>Unigene31965_C-W 1 280 LEN=280

XGTTGTTCAAAACTTTACACAGCTAATTTTCTAACTTCTGAATATGAAAAGAAATATCCA

GAGCAGATAGGTTTTGGTGTTGTCATAATTTCACATCTACTGTGCTTGCTTATTGCTTGC

ATTGTCAAGTCCCGACTGGTGACATTTAAAGGATCTGACACTGACCAGGTAGATACTGTT

AACCTGGATGCCCAACCTGTCTCAGAGACATAGTTTCTAACCATGGCAAGCGACATAGCA

GGTACCTATGTTGTTGAGCTGGAAGTGAAATATTCCTTAAGX

>Unigene31970_C-W 1 175 LEN=317; minus strand

XXCTGGCTGACTCCAGAAGCATTTCCAAAATCGAATCGACAGGACTGGGTAGCCCAATGG

AGGGAAGAGGAAAGGGCAGCATCAGAGAAAGCTGTCTCATTGGACTTGTGGGGCCCTCGT

CCAAGATGGGAAATCCAGAAAAAGTCAGAAAAATTAAGTGCCTTGCCTAAGGTCGTA

>Unigene32196_C-W 1 238 LEN=238

XXGTCGGTTTATGCGACAAGAAACTGGATAGTCCACAATGTCCATCTGCGGGCTGGAGAG

CTGGAGGAACCAGTGGCTGCACAGTCCAAGAGGATGAAGCCCCAGAACCGGAGAGTTCAA

CAGTGCTACCCTAGTCTGAGACCAAAGCTTCCGGGGAATCGCTGTGGAGTCTGCTTTGGA

ATAGTGAAGAAGCTGGAGTCTGATATCCTCAGATGATCACAGCAGCCATCAAACTTTCAT

>Unigene32465_C-W 1 213 LEN=213

XCAAATCCAACCTATGGCCATCTTTTCAGCCTCATCTCTAAAAGTGGTCATTTCCATCTT

GGGACACCTCCACTGCCATCTTGGTTTACCCCCTCCTCCACCAACACCACCATCTTCAGT

TATGACAACTTCCACCTCTTGACACTGGCAGAGGACTGGAAGCCCAGCACCAAGGTACTT

ATAGTTTTGACACTACTGCTGCCACCAACAAGGGXX

>Unigene32518_C-W 1 237 LEN=237

GAAGAGAAGCTGAAGTTTAACCACTCTCCTCTCTTATTTCCAAGTCCTGAGGACCCGTAT

CCCCTGGAAGTAGGAAATCCCTACAGCACCCAAGATATTTCTGCTAACAGAGAGGCAGAA

ATCCCCAAGAGGTCAAAAACAATGCAGAAAAATGAACTATTCGTCCTCAACCTCTCTCCT

TACAGTTCAAGGGAATCGAGCCCAAGAACGGAGAAAATCTTTACGGGTGAAGGCTGC

>Unigene32638_C-W 1 284 LEN=284

XGCCCCGCCGAGAACGCGGTGTTGGCTTGTGGCCGCAGGGACCAGGCCGCCTCTCCCACG

GTGCTGCCCGGGACGCGGCCTGCGCCCTTTGCAAGGCCCCAGTTCGGCCATGTTAACCCG

CCCTCCAGCCCCGAGGGGAATACTCGTAGGTTTTTCCAGCTTGCTGGCCTCGGAGTAGCC

AGGCAGCATGTGTGTGCCCAGCCGGAAGTTGCAAACGACCGGGTCCGAGCTAGGAGCCGC

GAGTGGCAGACGCGCATGCTCCGCGGAAGCCCGCACCCGGCCGAG

>Unigene32756_C-W 1 243 LEN=243; minus strand

AAAAGAATGGAAATAGAAAAAGAAAGATTCTTCTCCCCTAAACTATCACCAAGATTCTCA

GTCATTGCACAGGAACAATGCCCAAGTTGTTATGAGGCCCTCTTTATCCACCAACAAGCC

CAGAATCTGTGTCTGAGCTCACCTGTGTCTACTCTGCCTGAGGATAAGACCAATGCCCTC

ATTAAAGCAACTAGTGTAAATGTTGAACCTCTGTGGCCCGGGCAGTGTGCAAATCACCCG

GCC

>Unigene32833_C-W 1 242 LEN=242

GAAAAACCTGGCTGAAGGCAGCAGCTCAACGGGACCTCCGGACAGGACCCAGGACCAGGA

CTTGGGGGTGAACAAAAACCAGCTCCCAGGGTCCAGCCACCAGCAAACCCGAGCCCTCTG

GACCAGCCAGTGGACGCGGAAGGGCCCCCACCCATGGAGTTCTGCCTGGAGACAGAGGCA

GTCAATGGCTCAAAGAATGACTTGAATTTCAACCCCATGAAGGATTACATGGTCCTCAGC

CGX

>Unigene32937_C-W 1 248 LEN=248

XXGGGGTGGTTGGGAATAAAGCAGCCTATAAACAGGGTAAGGCAGCAAGGAGGACTGATT

CTAAAGAAGGAAAAGGAGAACAAATATGGTTTTTGGCAACTTGCAACCAAATACGATTGT

CAGAAAATTCCACAATCAGATACCACGTTTTCTGAGACATGCAGGGAGCACTCAAAAGAC

CAATGGACACTAGGCAGACAGATGTCGGAAACATTATTTCAAGATGATCCTGGCACATGT

GGAACAGATCXX

>Unigene32987_C-W 1 317 LEN=317

XGGGGCGGTGGGCGGCTCCGTCCCAAAGTCAACCAGGGAGCCCACGGGGACCTCGGGGGC

CTCCATGGCCTGGGCGGGTTGTTGAGGACGACGCTGGTGGAACCCAAGGTGGCTTTCCAG

GTGTGTCAGCAGGAGCCTGCCACGTACATGCAGGTGGCCTGGGAGCTGCGGGCCACCACG

GCAGCCCTCTGAGCAGCTCAGCGGCCTTCGGGCATCGTGGCGGGCGGCCGGCAGGCAGAG

GACCGGGCTGCAGGCGGGCGCCGTCCGGTCGGCGGCTGCGACGACTGCGCCGAGCGCTTG

CAGCCGGGTCCTTCCGAG

>Unigene32998_C-W 1 247 LEN=247; minus strand

CTCCTGGGTGGTCTTGTGATCATCGGTCTTCATAATGTGGTGTGTAACAGGCCACTGGCT

GCAACTTGTTGCGTTTTCTTTCAGAAAATGATGGTTTTTCTCCTTGGGACTTGGTCAGAG

CCTTGGAGTGTACGTTGGGATTACCTGGCTGCAGCCAGCAGTATCAAGAGGAACAATACT

GTCAGCCTTCTGACATCCCACCTGGACACTCTTCCACGCTCAGGATCCTCGGGGTCCTTC

ATTCAGCXX

>Unigene33090_C-W 1 263 LEN=263; minus strand

XXGTTGAAAGTTGCTTGGAGACTGCGTCTGCGCAGTCAGGCTTTGCAAGGCCCCGCCTCT

TTGGGGCACCTGAAGTCCTTTGGGGCGGAGGAGGACTGGGACCCCTGGCACCTCTCATCT

GGCGGCTGCTGTGGTGTTCTGTGTCCTCGTGGTGGACTTACTTCGTCGCCCACCTCCTCG

CGAACAACAAAGAATCTGGAAAAGTTGGAGAAGATAAAGTATATCCAGTATCCGAAACAG

GAGATCAATAAAGGAGGAAATGCACXX

>Unigene33428_C-W 1 411 LEN=411

XXGCCTAATAAACCCAAAGGAAAGATGCTCAACAACCCTGATTTTTACCAAAAAGAGTCC

CCTCCTACCCATGGGGGGAGCTACTGTCAAGGACAAGGGTCTCTAAACTCTAACAATGAA

CCGCCCAGAACCATCTCAAGGGAGCCCTCCACCTCAGATCACCTTGACGAGACCCAGCAG

GXGACCGACGTGGTCCAGAAGGTGGGAGGTGTGCACACTGTGGACTGCACAACTTGGTGA

GAGCAACAGAGGGAGGAGCAGCTGGAAGGAAAGACAGCCCTTGTTCATGGACTGTCGAAA

CGCCCAGAGTCTCTGCACACTCGCGGCCGTACCTATCAGAATCCCAAAGGCATTTCTCAC

AGAAATAGGGAGGACCCTCCTAATGGTTCAGATGTAATCCCCAAAGACCTTGAA

>Unigene33440_C-W 1 285 LEN=285

XGGGAGCGGAGCGAGCGGGAGGCGAGACGCCTGGCTGAGGCTGCCCGGCCGGGCGCGGGC

TGCTTCTTCTCGGCTGTGGGGCTGGACGGCGGGGAGCTGAAXGAGAACGCGGGCGACCGC

TTTCGCTCGCTGCGTGTGCGTCGCTTTCTCACTCGGCGGCTGCGGATTGACGCCTCCGCC

TGTTCCCCGGAGGAGAGCGAGCGAGCGAGAAAAAAGACTTTTATTGAAACGATCCAACCA

GCGGCGGCGGCGGAGAAAGGCGACCGGCCGCGGGAGCTTCCCCCACCX

>Unigene33516_C-W 1 237 LEN=237; minus strand

CGCTGGGGCACGGCGTGCGCGGGCCCGGGGCTGCGCGGCGTCTACGAGGGGGGCGCTGCC

TCGGCCCCGCCGCCAGCTGGGTGGGGGCCCGCTCTGTTCGCTTTCATTCCGCACCCATCA

GGGAAGGCACCTGCTGCGTGCAGCGCCCGGAGGGGCCGGGAGCGCTACCGAGTCCTCACC

TGGCGCGTAGGCAGCGACCGGGCCGCGACCTCAGGCGGACGCTTCCTGGCGGCGGCC

>Unigene33622_C-W 1 236 LEN=236

XXGAAAAATAGCTATACGTGCACAGCTGTTTATAGCAACGTTATTTATAAGAGTAAAAGA

GCGGAGCCGGCCAAAGCGCACTGCACGCTGAAGGAAGGAGTGTCGACAGGCAGAATATTT

ATTATGCAGCCCAGAAAAATGGTCAACTTGGAAGATCCTTTGTACATGGACGTGAAGGCA

AGTGAGAACCCAAACCTGAACCTTCTGGCAGGGGCAGAAGGTGACGCAGCGGCCCCAGXX

>Unigene33741_C-W 1 216 LEN=216; minus strand

CTCCAGGAGTTTGGCCCGTCGGCGGTGCGTTTGGGTGTCCCCGACTACAACCCCCGGCAT

GCTCTGTGAGCTGCCTCGGCCTACATTTCCACGCATGCACTGCGGAGCAGGGTGGTGGGC

CATGCGACAGACTATTACCCAGAGAACCCCACGACGGCGCAGGACCCTTGGACTCCATTT

CCCGTCGGCCCGCTCGGGGAGCGCCGGAAGCTCGCC

>Unigene33776_C-W 1 216 LEN=216; minus strand

XXCGCATTCTGTGAGAAGGAAGTTGAGAAGGCACCATTTCTTGTGAAATTACAGAGCCAC

CCAAGGAAAGCTAGCAGAATCCGAGTTATGCCTTCTGTCGTTTGTCACGTCCTCTCTCAT

CGCCTGCCCCGGTCTTCCATCGGAATGCAAACATTTGGGATAAAGCCCTGACAAGTCGAG

GCCCTGCTCTACGCCAACGCGAACCGTTCCCCGTCCGCX

>Unigene33862_C-W 1 214 LEN=214; minus strand

CTGGCCGCCATGCCCGCCCTACGCAGCGTCAACCTCCGCTCCAACCCTCTGAATGCCGAG

ATGAGTGCAGCTGAGCATCTTCTGGGCCTCCGTGGGCTGGAGGCCTTGTTTCTAGTGCTG

AGAGCCAATGGCTTCCCTGAGAGGAGTGGAAGACAACCCTCCACCTGCTGCTTCCTGAGA

ACTGAGGATGAAGCCTTTCGCCATGCCCCTCCTTXX

>Unigene33975_C-W 1 226 LEN=226; minus strand

XXCACTGCCACACAATTTTTTTCTATCTCCAGTATGTTGCAGTATCTTCTGGGGACATGG

AAGCAGGTGCTGCTGAAACATCAAACCATCCTTGTGATGGAACAGGAAAGCATTCTTCCC

TTAGGTATAGTAGGTGAACAGAGGGTGATTGGGAAGCCAGACCTATCGCAAGAGATGCCT

CTTTACATCATGACCAACATCATAGACAGGACAATGTGCACATTGGGA

>Unigene34017_C-W 1 73 LEN=255

XXGTACCAGCAGTACCAGGACGCCACTGCTGACGAACAGGGGGAGTTCGAGGAGGAGGAG

GGCGAGGACGAGGCT

>Unigene34135_C-W 1 268 LEN=268

XXGCTAAGGAGAAAAGCCCGTGCTCTCTGTGTGAGAGGAGAATGGCTGTCCACGTTGCTC

ACTGAGCTCCCTCACGGTGGACGGCCTGTGCGTGTTTTCAGCCCTTGCTTCCTCTGCATA

AATCACATTGGTTCTTTATCATTTCCATCAAATCAGGAGAAAGCAAACGCAGGAGCAATC

CGTAACTGTCAGTCACCTGCCCTGACTTCTCTGATGCTCACAATAAGGGGACATCTTTCT

CACTTGGTCCCTTCTCCCTCTAGGATGAAG

>Unigene34207_C-W 1 267 LEN=267

XXGGTCTATAAGAAGGGGAAGGATAATTTCTACTTCGGTCCTAGTGGTCAAGATGAAACC

ATGTCCCATGGAATGGTTTGTTTTTTGATGGACCCCTCTCCTCAGAGTGTAGGCAGACAG

AGTCAGAGAGGCCTGGAGTGAGGATTTCACCCGTCCACTGCCAGCAATGGACCTGTTGCA

GGGGAAAAGAGAGAGAATCAAGATGACGATGCCCACGAGCATGGCAGGGTTAACACTGTG

AGGAGCAAGTTCAAAGGGTCAGCCTTGCTX

>Unigene34241_C-W 1 227 LEN=227; minus strand

XXAAGGAAAGCCAGGCAGGGTTGTAGTCCCTGCAGGCACCCCCTGAAAAGTCAGTGTGGA

GATGTGAGGAGGAAGCCAAAGGTGCAGTGGAGACCCTCGAGATTAAGAAATGCCAGTAAT

GTGGAACATCATCCTAGGAAAGCTGCAGGAATTGAGCAGAGACAAGCCAAGAAAGAGCCT

GTGTGGGCTGTAACCAACAAGGCCAAAAGGGCAGAGCTGCACAAGGCCCXX

>Unigene34250_C-W 1 208 LEN=208

XAGAAGCAAGTTGAGAGCAGGAGAGCTGCGCTTTTCGGTCTCGCTGCAGTTGTACTTTCA

GCAACTGCGATTGCACCATCAGCGAAAGCCGACATCATTGAGGAGTATCTGGAGAAGAGC

AAGGCCAACAAGGAATTGAACGACAAGAAGAGATTGGCTACGAGCGGTGCGAACTTCGCA

CGTGCTTACACTGTCCAATTCGGCTCCTGX

>Unigene34271_C-W 1 219 LEN=219

XACCTGCAGATGGAGTTCCGGGGGTTGAAGCCATACCATCAAGAGGTCTCATCTACTGAC

GACAGGACCATCCCAGAGGAACTCCAAGAGTCACCTCCTGAACCTCCCCTTTACTCCTGC

TCTCCACACCATCTCATCCCTTTAAGTTGCGGCTCACATGGTGTTCCCCGCAGCCCAGTT

CCTGTTAAAGAAGGCACAATGAGCTCTCCCAACACTCTAAXX

>Unigene34434_C-W 1 247 LEN=247; minus strand

XCAGAGAAATTAAAACATCTGTCCATGAAAGAGGTGCACATGGGTGTGTATACCAGCTTT

ATTGAGAGCAGCCTAAAACTGGAGATAACCCACATAGGAAGTGAAGGCACAAATTGTGGT

ACATCCATCTAACGGAATAGGAATAAAGAGGAAAGAGGTCTTGAAATGTGCAACATGGAC

AAACCTCAAAAATGTCATGTTGAGCCAAAGACTCCATGCACGAAAAGCTTTGATTCCATT

TACATGAAX

>Unigene34538_C-W 1 263 LEN=263; minus strand

GTTGTATTTGTTAGAGACCATAGAAAGGGGTTTGGTTTGGTACCAATTAACCAAGCATAC

ATGGCAATGGCGTCGAGCTCCGCGGTCGTGCTGCAAGGCATCTCCACCCCCTTCGTGTCT

GGCTCGCGCCGCAACCTTCTCAACGTCCCGCTCGTTGGTACCAAGAATGCCGGTGGACGG

AAAGTCGTCGTCATGGCTGCAGTCGCCCCCAAGAAGTCGTGGATTCCCGCGTTCAAGAGC

GACGCCGAGTTCATCAACCCCCCX

>Unigene34629_C-W 1 276 LEN=276; minus strand

AGCACATGCCCAGGACAGGAAGCAGGCGCCCGCTGTCCCCGCACCTCCTCCATTCCCTCT

CACCTCACCCAGCCCAGCCCTGGCACCTTCTGCACCACCAACCAGCACCAGCCACCTGAG

CTCTCTGCAGCAAGGACGGACTCCACAGGTGGCCCTGAGCCCTCGGGGTGTCCTGAGACC

TGGGTCACTGACCACGCCTCACGTAACCAATGCCAGCGTAACAGCTACGCGGCCGTCAAG

GAGGCCAGCGGTGTGCCAGGGAACCCTCTCCTGACT

>Unigene34946_C-W 1 207 LEN=207

GCGCCAATCCTTCCCATGGGACCCTCTCTGGAAGGCTGTGGGCCCAGATCTGGCTCATCC

CTTGAAGAACAGAAGGTTGGGCTCACCAAGCACAAAATGCTGGGCTCGCTGCGCAAGCCT

CCACATCACAAAGTGGTAGGTTACAGCGACTTTATCCTAACTATAAACGGGGTTCAATTG

CACCCTAGCATTTTGTGCTTGGTGAGC

>Unigene35011_C-W 1 377 LEN=377; minus strand

CCTCCTCAGGATGATATGGCTGACAGAGAACAGCTGACCAGTGACAAAACTCCTGAGGCG

TGACAAGGAAAAGCAATGACCAAAAACCCAAACACTGATTCTGTTCAAAGAAAAGAAAGG

ACATAAGGGCTAAATAATGGCTTTCCTGTACCTGCCAGTGACCTAGGAAATGAAGGGCCC

CTGAGTTTATCGCCTCATCTACTCCACACTTTAGGAAAGTTTCCATAGAGCCCAGAAAAC

CTTCTGGAACAAGGAGATGCCAACACTGTAGAAGCAATCAGCATCATGGAAGATTTCTTT

TGCTTTCCTCATTCCATATTTGACAGACATTTTGAAATGGATGAAATGGGAATGAGAATT

CAAGTTGCTGTCCAGGTX

>Unigene35034_C-W 1 289 LEN=289

TTGAGCTCTGAGGCTCCACCTGGGCCTGGTGATGAGGCAGCAGAACTTGGGATACTGGAA

AAGCACATGATAGGGTAGAAGAAAACACAGACCATATCTAAAGGCAAGCAGCATATCTCC

TTGCAGAACCAAAAGTTAGGGCAGCAAGAGGACAGACATGACAGGAAGTCAGAAGTGTCA

AACCTCTCCTGCTGTTCCATCACCCATGAGACCCCTGCAGGCCCAGGTGAAGAGTCAGTG

TTTTCAGCAAAAGCAGACTTCAAGGCCCTGAAAAGTTACCTAGGCAACCXX

>Unigene35058_C-W 1 319 LEN=319

XXGAAGACCCTGCACATGAAGGGCTGGCTCTCCCTGGTGGTCTGCAAGACGCACTCCATC

CTCTTTGAAGTGGATGCTCCCCTCCTGCACATGCTTTAGTGAGGATGGATTCGCCCATGC

AGTCAGCATTTATTAAACACCTTTAGTATCCAGCTCCATGCTGGTGCACCAGGACCAGGT

GCTGGGAAAGAGCTCAGGGACCCCTTGGATAAGCTGCACCCACTGAACCTCCAGAATTTT

AGCAAATACCCAACCTCAAGATGCAGTGCCTTTCAGCATGACCGGAGCCTTCTCCCTTCA

CAAATGTGTCAGGAAATGGAA

>Unigene35144_C-W 1 220 LEN=220; minus strand

CGTCCTTATGCACACTTTGAAAATCTACACTCCACTGTGGTCCTGAAGACTCAGGAAAGT

GAAAGCAAAGTCCCAGGAAAGCAGAAGACCAAACAAATTTCCGGATCACTTAAGGCCTCC

TCTATCCCTCACCCGCAATCTACAGTTAGTGATGTTATCAGCGGGGCCCTCAAATCAGCC

AGCCCCAACCCAGATCTAGGTACTTTGGGGAGAGCATCTTXX

>Unigene35344_C-W 1 305 LEN=305; minus strand

XXTTCCAGGCTTCTAGAACATCTTTCTTCTTCAACTCTTCCTTTGCTCCATACAGTCCCA

CCTCAAGTCCTTACTGCCATCTCTCCAAACACTGTTTCTGATGGGTGGCCCCTCTGTGGT

CGAGACAGCAGTGCTCAGCAGCTGCTCCGCATGGTCTACCTTTCCCAGCTCCCTGCACTT

AGACCAAGGCCTGCAGGTTGGTCAATTGTGACTGGGGGTGACATGCATCATTTTGATCTA

AGGCAATCAGAACACTTTCAAGTTCACACTCTTTTTTACCATCAGTATCACATGAAGGAA

AAAAACCXX

>Unigene35350_C-W 1 237 LEN=237

XXGAATTCAATCCCCAATACCAAAAAGGCCAAACTGAGGTGCTTTCTGCGGGCACAGTCA

AACCTTCTGTACAGAAGGAACCAGAGTGAATATGCCAAATTTACCTATGTCCTGGATTAC

CAGGCAAGTTCCTTACAGAAAAAAGGGTTGTCCATAGCAGCTCAACTGAACTATGTTGAT

CTATAAAAACAAAAACCAGGGTCAGGCCCAGTAGCCACACAGGAAAGGGAATGGTGCCTX

>Unigene35420_C-W 1 308 LEN=308; minus strand

XCCTTCTTCCTTGAAGCCCAGTTCTGGGTTAGTCTGGTTGGAGCCCCGCAGGCTGAAGCC

AGGGCGTGGCGTCGGCTGGCAGCGGCAGCTGGGATCCCCGAGTCTTTGGGGCGGCGGGGG

CTCCGAGAGGAAGTGAAGACGGGAGGGGCGGCTGGCGGTGACAGCGGCGGAGCAGCAGGA

ACCACCCCACCCAGTCGGAGCTCCGAGCTCCAGCTTGAGCGCTCCGGTCACCCTCAGCCC

GCCCGGCTAGCCAAGCTGGGGAGTGCGGATTTTACTCTTCCCTATGTGGACCGGCTGGCC

AGCACAGAC

>Unigene35428_C-W 1 283 LEN=283

XXGGTGCATAAGGAACAGCTGCTTGAATTCTGGCAACACTGTGGGATAGGTGTGGATTAT

GCTGGAATTTGTCACCGGGCTTTTAACGAAGGAGTAGCCAATCCTCAGCCCCGAAAATCC

AATGGATGGAACAACCACACGGTTGAGGCCCTGCCCTTCAGAGATGCTTCTTGGAGGCTA

CAGGGATTGGAAGAGGGTGGGATTTCAGGTGGACACGAGGTGGATGAAGATTCACAACTT

GGACAACTGGATACTGACTCTGTAAGAGTGGACACAATGTCCTAX

>Unigene35451_C-W 1 265 LEN=265; minus strand

TATGGCAAACAATACAGACAAAAGGTGGTGCTGAGGAATGAACCCAAGGCCTCACACGTG

CTAGGATCAAACGAAACTTGGGAATTGGGTTGTGAAGTTCCTGCTGTCCATTCACAATCA

ATAGATCTCATACATGGAGCTGGAGTCCAAGCAGGACCTGTTGGTGTAAGCACAGCAGTC

TATCCTTTTCCCCATGTGATCAGAGGCATAGGTCTTGTCATCCTGGGTCTGGAACCCTAT

ATTTTACATAAATTTTTAGTAATGGXX

>Unigene35484_C-W 1 296 LEN=296; minus strand

XTAGCCGCCACCGTGAGGGCCCAGACGCTTTCATCAATTGCTTGGTCCGAAGGCCTTGCC

ATCGATTCTAAGACACCCCCTCATCCACCCACACAAGCAGGAGACTCCCGCCGGGTCAGC

CGGTGCACCCGACTCTTTTCCTTTGAGTGGCATAGCGGAGAGTGCATCTTCTCCAAAAAG

GATCACAAGAGCAGGCAATTCAGGTCAGAATGGCAATTATCAAGAATACAAGCAACAATA

AGTACTGGCGAGGATGTAGAGAACAAGGTACCTTCATACATTGCTGGTGGGACTGCA

>Unigene35556_C-W 1 220 LEN=220

XXAGCGCCACTAAAGGTCAGAGGAAGAAGCTGTGGAAAGCTCACAGCAGGTATTGGAGTT

TAAGGCAGTGGATTTGGGGCCCTGAGCTCCCTAACCCACAAGGGTGTGAGCCGGACTCTG

CTGAGAATGGAGTGGGCAGGAAAGTACCAGGACTTTCAGGGCTCTGGGTTTGCAGAGAGC

CGAAATGACCATGACTGCCAACAAGAATTCCAGCATCACCCA

>Unigene35710_C-W 1 289 LEN=289; minus strand

ATTATTGTCCCTTGTCTCCAGCTGGGGGAGCTACGGGTGATCAGAGGGTCCTTTGCAAGC

ATCGGGAAACACGTCCATGTTGGTCAGCTGCAGAACTGAGCCTTCTTAGGAAACCTGGTT

TCCCTCTCTCAGATGAAGAACACCTCCCTTCGAGAATGTTCACTCTTCCGCCAAGGGTCC

TTGTCACATTCCTGCAGCTTCTCCCACCTCACTCAGGCACCTTCACGGAACTGGTCCCTT

TCTGGTCCTTCACCTGCAAGCTTTGAGTCTCCCAATCCCAAAAGGCTCCXX

>Unigene35743_C-W 1 298 LEN=298; minus strand

CCTCGGGTACCTGAACGTCCTTCGCAAAGTCTAGAATTAGTGGCCAATGACCAGATTGTC

CTCGTAGGTGTTCATTGCTTCTATCAGGAAGCCAATCCCCACCAGCCCCCCTCAGATAAG

TATCATGTCACCTCTCACAAGACCATCAGGAAGGGACAAATGCAGGTCTGTGTCTTTTCT

ATGAAAAAGGGAGCACAGCACGCCAAGTGTCCTTGAAGAAGAGATCTCAAGGGGCTTGGT

CATCCAAGTCCCCAAGTGCCTGAGATCCGCTGCATTCTGCCCGTGAAGCGGCGAGGTGXX

>Unigene35773_C-W 1 243 LEN=243

XXGAGCAGCTGTGCTCCACCAGCATACAGTCTTCACCAAGAGATCACTAGAGATCTCCTC

CCCTGTCTGAGCACCAAGATGACAGCTGCAGAGGCTTCAGTGGAAATGTTAAAAACAGAC

CTTAGATTCACAGATGGCCTGGAGAAAGCAGGACCTGGTAAAGTAAAAGATGACATTTGA

AAAATACTAAAGGGGATATTTTTTCTCTCCAGCCTCCAAATCACAAAAGGAGCTGAACTT

CAGGCX

>Unigene35803_C-W 1 98 LEN=248; minus strand

XCCATGGTCATCGATCGCATCTTCCTTTGGATGTTCATCATCGTCTGCCTGCTGGGCACC

GTGGGCCTCTTCTTGCCACCCTGGCTGGCCGGCATGATC

>Unigene35914_C-W 1 205 LEN=205; minus strand

XTGAAGTCATCTGATTCAAGGCCAGTTATTGCCCTTTGTAACTCACACTTTGACCTAGAA

AATCTTGGTCACTTGTACAAGTTCTACAGTCAGTCATCTATATACCAGAACCTAGTGAGC

AAAACCTTTAAGTTGGCTGGGTTACTGATCTTGGGATGTAAGAGATCTGGTTTTGAATGT

AAAATTCATATTGCACAAACACCAACX

>Unigene36005_C-W 1 265 LEN=265; minus strand

CCCAAAGTGAAGAAGAAACTTAAGAAGCTTAACAATGGATGTAGAAATATTATCATCAGC

TATCGGAGTAATAGTATCCAGTCATTCCCCACCTACAGGCACAGGATGCAGGCTTAAGCG

GATGAAGAAAACCCTGTAGAAGTGTGGGGACTTAGAGTGTTTAAATATCAGCAAGTGGAG

GGGAAGGACTTTGTTCGCATTAAACCTGAACACATGTCATTCCCTCTTTGTGCTGAATAG

GATGAAACAGTGACAACAGCAAAACXX

>Unigene36586_C-W 1 203 LEN=261

XGTCAGAGGCAAGGGATGAGGATCCCCAGTCGAGGGTATGGGTCGGGGTTCAGAGAGAAC

AAGGCTCCCCGCGCGAAGCCGTACGCCCCCGGCTCCGCGAGGTCAGCGACGTCTTCTGGC

CCGCAGCACCGGAAGCCTCGCGGCTGGACCTTCGGGATCCGGCATTCGGACTACCTGGCC

CCAATGGTGACCAACGTGGACGAC

>Unigene36629_C-W 1 248 LEN=248

XCCAATTTTCAGAAGACCGGTTTGAAGTCGCGCAAGTTCTCGGTTGTGGCGAAGTACGGC

GACAAGAGCGTTTACTTCGATCTTGAGGACATCGGCAACACCACCGGACAGTGGGACTTG

TACGGATCCGACGCTCCGTCGCCTTACAACCCGCTTCAGAGCAAGTTCTTCGAGACATTC

GCGGCGCCATTCACCAAGAGAGGTCTCCTGCTCAAGTTCCTGATGCTCGGCAGCACCTTC

ACCATCGCG

>Unigene36654_C-W 1 178 LEN=287; minus strand

XXCCTCGGTCTTGTTCCTCATCCCTGTGGCCTAGAAGCCCAGAAGATACTTGACAAGGGC

AATGGTATGACGCTTACAGGGGCTGGAAACAGCATCACAGGACGATACCCCAGAGAAGAA

TCCCAGAGTCCATCCATCCATGCCAAAGAACCCGATGCCTCTTTGGACAGGTCACAGGTG

>Unigene36669_C-W 1 205 LEN=205; minus strand

CCCGGGCCGCACGCATGCCAGATGCCCCCCGCAGCCTGGCTTCCAGTGAACCAGTCCAAG

GAGTGGAGGAGTCCATGGGACATGCAGAGGAAGCATCTTCTGAGTGAGATCCTGCACAGG

ACAAGGCGTGATATTGCCCTCATCATTGCTGGGATTGTTGCCCTAGTGGCCGCCATCGCC

ACCATAGCCACTGCCACTGCTGCCCXX

>Unigene36817_C-W 1 226 LEN=226; minus strand

XXATCCCACTTGAGTCTCATTTCAGATGCAAGGACCAGGGCCCCAGAGGATGACCTCGTG

CTTGGGCAGCTGGTTAGTGCAGAGCTGGACTACTATCCACTCTTCGGTGCCTGTGAAGAT

CATTTTTCAGCAGTGGGGGAGAAGAATTTGCTGTTGAGAGGCCTCTCCTCCAAACTTGCC

CATGCTCTACCTTCTCTGCACTCAGGTGACAACATGCCATCTGCCAGC

>Unigene37054_C-W 1 568 LEN=568

XTCCCATAGGAAGCCACAGAGACCAAAATCCAAATCAAAACCCGAGAGCACAACAGACAA

GCCAAAAACCTCTACGACCAACTCTCTGTCATCATGGTGGTGACACAGGGGACTGAAGGA

ACCAGAAGTTAGGAAAAGAAAACCCCTGTTGTCCCTACGTGCTACGAACCAGCTCATCTT

GGGGATGCTGCTTCCCTGACTTCACGGATGGGGCGTGAGCACGTCAATCTCAGTCTCTCA

CAGACCTCCTGGGACCCAGCACAGCAACCTGTGTCCCGGGTGAAGACAATGCTGCCGCCT

CGCACATAACCCCCTTCCCGAGGTCAGCTGGCAGGTCAGCTGGGAAGAATGCAAGCCCAG

GGGACATTTGAGAAGAGCTCTTGGCACCTGCTATTTTCTCTCAGACATTCCCAGGGAGGA

GAAACCACTGAGATGATTTCCATGAAAGCGCTACGATCCCTTCCCCTGGCTCTTGTCTCT

TCAAATCAGGCCAGCAACAAGCTCACCTATAGCAGGAGGTCAGGGTCGAGGACAGGTCCA

GTCAGGACAGCTAATACACGGCACCTGGGX

>Unigene37087_C-W 1 317 LEN=317; minus strand

XCGCTGCGGTTCTGGGTTATAGAGTTCCCACAGACCCGCAGAGATTCAGATCCTCCAGAC

CCCGAGGATGGGGGTCCCGGCTCTGCAAACCCTCCTCCTGCTGCACTCTCAGACCCTGGC

CTTAACCGAGACCTGGATCCCCGGGTCCAGTGCCAGGAGGAGAAXTCTCAGCTGCTGCCT

ATCTTCAGGCTCCACTCCCTTCTGTATTTCACCAAGACTGTGTCCCGGCCCTGCCGCGGG

GAGGCCTGGTACATCTCTATCCGCTACGTGGACGACTTAAATATTTGGCCACTTCGATAG

CGACATGGTGAATCCCAGGXX

>Unigene37123_C-W 1 218 LEN=218; minus strand

CGGAACCCGGAAGCGGAGGAGAGCGGCCCCGGGGCGCCTGGAGTCCCGGACTATCCCCGA

GGGCAGGTGGGCCTCCCCCTGCTTTCCTGCTGCCTCCTCCTGGCGCAGCCGGCTCGCCAG

GGCCCTCTCCCGAAAGGGGATGCGAAGCGGCGTCTGGAGAGGGTCCCGCAGTACCCACGT

GACGACCAGTGCCGGAGCCCGTCGGACTGTCCCCGGCCX

>Unigene37224_C-W 1 60 LEN=287; minus strand

CTGGCCGTGGGAAGCAACGATTACTACATCTCCGTGTACTCGGTGGAGAAGCGCGTCAGA

>Unigene37371_C-W 1 225 LEN=225

XCTCCAAAACCCACTGAACGTTCTAGAGCGAAGAAGAAGCCTGTAGCTCTTTCATCGTTC

CCTATAGCTATTGGAACGTTCTCTATATCTCTTCCAATGGTCCCCGTATTTGTTTATCCA

ACCATGCAGAAAACCTCAACTTTGCTTCCAAAGTATCAAAAAGGAAGAATTCCAACACAA

GAAACTTCAACTCAGCCACAGGTCCCCATAACTCCAGAGCCCAATGXX

>Unigene37429_C-W 1 213 LEN=213; minus strand

ATTGGCAACTTACAAAAGGAGTTAGCTCCCTCTATTTTAGATCTCCCTCTGGATGTTAGT

GAAAAAGAAGAGGTAATAAAAGATACGCAACAATTGACTCATAATTCTTCACAAAATTCT

GCTGATTTAATTGGTACTGCTACGGAAAAGAAAACGAAAGTAAAAACGATGATTGATGAT

CGCTCCGTTTCGGAGCAATTCATACTTGTTATG

>Unigene37455_C-W 1 224 LEN=224; minus strand

ATCCGGGTCATCATGAAGAGCTCCCCCGAGGTGTCCAGCATCAACCAGGAAGCACTGGTG

CTCACGGCCAAAGCCACGCACCACCTATTCTTCAGTATAGATAGAAGAAGTCAGAAACAG

CCCAGAATGAGACCACCAGGAATTTCAGAGCAGCAGGAGGTGTGTGAACAGCCAGGGTGA

GGGGTAAAGGGACTTACCAGTAAGAAACAAGGACAGTTTGGGCXX

>Unigene37472_C-W 1 323 LEN=323; minus strand

TACTGGAAAGAGTCTGCCATCGCACCATATATGCTGGAAGCGGCAGGAGAGAATTCATCT

GAATCAGAATATTTCAGCTGTCTCTCCCCTCCGAGCAAAACCCTTCCTACTGATGAGTAT

GATTTCCAGAAAAGGAAAGGCATTGCTACTGAACCATTTATACTGGAAGCAACCAGAACT

CAGGCAGCAGAAGAGAATTCATGTGATTCAAAATATTTCACCTGTCTCTCCACTTCCAGC

AAACTCATTCCTCCTGAGGAGTGTGAATTCAAGAAGTGTCAAGAAATTGCTCCTGCACCA

TGTAGCATCACCAAGATTCAGACX

>Unigene37473_C-W 1 269 LEN=269

GGGTTCCAGATATTCCAACAGGTTGCTCCTGCCCCATGTAGGATGAACTGGATTCAGGTA

GGAGGAGAAACTTCAACATCTGAAGAAGATTCCAAATATATCTTCTCAGAAAGCAAGCAC

ATTCCTACTGATGAGCATGCTAAAACACCCGATTTCCAGAAGTGGAAAGGCGTTGCTATT

GAAAAATACGAGCTAGAAGCAGCCAGGACTCAGGCAGCAGGACAAAATTCATCTGATTCA

AAATATTTCAGCTGTCTATCCACTACCACX

>Unigene37591_C-W 1 161 LEN=221; minus strand

XTAACAGAGGAAGAAGACGGAACAGAAGAAGACGGCGGTAACCGAAGAAGACCCATTCTC

ACACAGTGTGTTGAACTGCGACTCTTCTACACTGATCTTCAACAAATACTTGCTCCATCT

GGCTTGAGGATTCCATTCTTCGCGTTTGTGGGAGAAGACCTT

>Unigene37754_C-W 1 261 LEN=261

XXGTCACCACAGAACACAAAAATGGTGCACATTCTTAAAAATGCAGGCTCCTTGCAGCTC

CAGTGGCCCACAACAGTAAACAGGAAGCACAGAAAGGCCTAGGTGACTCTGAGCAGCCTG

TGTCTCCGAGATCTCTGGTGTCTGGAGGTTCTCCAGCGAACACTGAGTCCCCAAGGAAGA

CAAAAATGGTTTCTTGGCACCACCATTGCTGCAGGCACGATGAATCTCTTTCACAACGGA

CAACCATTGCACATAAAGCCCAAX

>Unigene37798_C-W 1 217 LEN=217; minus strand

TGGCGGCGAATGGAGTGCATCACCCCCATGTACTGCGTCACTGCGTGGGTGAATGACGAG

TTAGCATGGCCAAAAGATATCTTGTTAGGAATTTCCAGCAAAATTGGGGTGCACCGAAAC

CTACAGGAAGGATTTCCATCACCCAAGGATGGGTTACTGGGGCCCAACCGCGAGTGAACT

GTCCGCCCGGAGATTCAAGCTGTTTATTCAGCTGCCCXX

>Unigene37801_C-W 1 209 LEN=209; minus strand

AGCCAGTACGGCGCCGTCCGGACCCTGGCGGCCATCCCCACGGGCGCGGAGAGCCTGGCG

CCCTTCGGAGTCTCCTCTCTGCTGCCCCCGTCGGCGCGGCGGCCTCGGCGGGTGATGGGG

CTGGAGGTTCGCACGCTGCTAGCAGCGTCCCGGGACGCCCTGCAGACCAGCACCCGGGCG

CTGCCCCGGGCCTGAGCCCGGGACGGACGX

>Unigene37852_C-W 1 241 LEN=241; minus strand

XXGTCTGGCCAGAGTCCTGACCCATTCTCAGCGTGATATGGTGCAGGTCTCTGTGCCTCT

CTGAGCCTGGGTCTCTTCATCCATCCCACCGAGGTGATAATAGTCCCCTGGCTCATCGTC

AGTGCTCAGCCCTTGACCTTTGCAATAAAATTGCTAATTGCCATGGTAGCTAATTACTAT

CAGGAGATCCGGCTTGCAATTTCAGGCACCCAGAAGAAACCACAGAATCAAGCTTGGTCC

AAC

>Unigene37859_C-W 1 209 LEN=209

XXGCGTGGGGAAGGGGTTGAGGACAAGGGTGGGGTTGGGAGAGAAAAGGTTTATGTGATC

AATCCACTTAAAAATGCTAATGCCTTCACTCTCTCTCCCAAGATGCAAAACTCCAAAGTC

AATCAGGAGGCCGAAGAAATTCTTATGAACATTCAGAAAAACTCGATTTTAATCCGGTTA

AAAATCATCAGTGTCATTATCATCATCATCAXX

>Unigene37913_C-W 1 270 LEN=270

XGCCAAGGCAATAGGAAAACCCCGGAGCTTGCGGAGACCCAACGGTTTGAGATTCTAGAA

AGTGTTTGGCGTCATTGGGAAACACCTGCCGAGGGAGAGAGGCGTTGGAGGAATCTTCGA

ATACATGAGATGAAACAAGACAGGCCTTCAAGAATGCAGAATCGGGGAGGTCCTCCTCCA

GACAGACTGCTCAGCACAGACCTGCCGCTGCTAAAGCTTCAGGACCACAGGACCAGGCTG

ATTAAACCAGAACGTTATGACTTCCTGGAG

>Unigene38136_C-W 1 161 LEN=424

XGGGTTATTTTGCTGCTGAAAGTTTTCATTGAAATGACTGATCAGATCTCACATGTCTGC

TTAAAAATGTCTGACTTAGGTCGTTACAAAATGACAGACTATGATAAACTGGATGATAGT

TGGGAGCAAGACAATGAGGCATTGTTTACACAGAAGATTGAG

>Unigene38279_C-W 1 248 LEN=248; minus strand

XXTGGCCTGCAGTCGGGTGGCTGCGGTGAGGTACCTGGGATCCTGAAGGGCGGGCGGTTA

GGTCGTTACCGCCGCCGCCGCTGGCGGACTAAAGCGGGCGGCCAGGAGCGAGCAACCTGG

CGGCTGAGGCGTTCACCGCATAGTGCCTACGGCACCGGGTCATCTCTTTCTGCGACCTCC

GCCTTCGCGGTCCCCACCTTTTTGAGGGCGAAATCACTTTTGGGTTTCGAGACCTTGCGG

CGTGAGGCCCXX

>Unigene38396_C-W 1 343 LEN=343; minus strand

XCCAGCACCCAAAAAGTAGTCTCTGATAGCTTTGTAGAGAGGGAGAGATTCAGACATGGC

TACCATCAACGCCGCCGCGACCACCTCGACTTTTGTCCGCCCCGCCGTGCCAAGACCGGC

TGGGCTCTCTGCATCACCAGTTCTTGGATTGCCCCAGTTCAAAGTGAGTGGGAATGTAAA

ATGCTCAGCCTCAAAAAACGACAAGGCCGCAACCGGCATGGCCAACGGTATGGCTGCCTC

CGTCTTGGCTGCTGCAACCATGTCAGCGGCTCCCGCACTCGCCTTGGTGGACGAAAGAAT

GTCGACCGAGGGAACTGGACTGAGTCTCGGAATAAGCAACAACCX

>Unigene38649_C-W 1 229 LEN=229

GGGGAGACTGCAAAAAGCTGTTGGCATGGGTGTTGTGACAGAGACATCCCTACAGGCCTT

TTAGGCCCAAGAGCCTATGACTTTATGGGATTTCTGACTGAAGGAAGTAGCCCAGGTGGA

ACCAGTGAAGTCGACAGTGTGACTGTTGGGGTGGATTCTCTTAACAATAGAGGAAGGATG

ATTTGGGGGCAGGAGATCCAAGCTATGCCCCAGTACAAGACTGCGGTGGXX

>Unigene38867_C-W 1 232 LEN=232; minus strand

XXCCTCTTCCCGTCCCCTGGGGATAGTGGTGGCCGCAATAATGAAGTCGCACTCCTGGCC

GTCTCTTCCCACACTCTCCGCCTCGAATCCAGGCGTCCGGCCTCCGCTGACGTAGAGGAG

GTGGCGAGAGAGGGCAAGAGAGACTACCACTGTGGGCACAACAGCAGCTCCAGGCAGGGA

GAAGACAGCTTGTTGAAGTGTATGGAGCAAGGAGGCAAAATGAAGGCAGCCCCC

>Unigene38870_C-W 1 223 LEN=223; minus strand

CCCGGGAAGGAGAAGAGGAAGAACCGGGGTGAACATGAAGGCCCCGTCTTCCCACAGCTC

AGGCTCCAGGTGGGACAGATGTGGGGACCGTCTGAGGCCTTGGTCAACAGGGGTGACTGC

CCTCCTTCCATGAGCAGGTTCTGTGCTCAGATCCAGGGAGACAAACCCAACAAGCGTGAC

TTCTGCCCGCCAGGAGCTCTGAGTGGGTGACAGGACAACTGTCXX

>Unigene38919_C-W 1 240 LEN=240

XXTGGCCAAACTGTGCTTTTCATCCAGGTGTGGAATAAGGAAGAGGCCAGCGAGAGCTTA

AAAAGCACCCTCACTTCTAAGTCCAGTCATCATAGAATATCTGTTGTGAAGTTTGTGAGC

TCATGCATACAATGTTCACATTTAAAGTTTGAGAAAATATACTTGTCAGATGCCATCTCG

CTCAATCCTGCCTGGAACATGAATCATCCTTTTGTCCAGTTATCCACAATTTTATCCACC

CGX

>Unigene38959_C-W 1 243 LEN=243; minus strand

XXCTCTGGAAGAGTGAAGAAGCTAGAATCCGATATCCTCAGACAATTGCAGCAGCAATCA

AAAACCCATTCAAGAAGAATTGAGCTTGCATCTACTGCTGCTTCCTTGTTCTTTATTTAT

CCAAACCATCATCCTATTGGACAGTGCTGCCCATTCTTAGAGAGAGTCTCCATGGCTGTC

CACATGCCAATAATTCTTAGATGTACCCTCACTGACACACCCAGAAGCCTCTTAATCATC

CACAAX

>Unigene39198_C-W 1 271 LEN=271; minus strand

XTTGACCTGGAGCACTTAGAATTCAAGGTCATGGTTCCACTTCTTGCTGCCACTGCAGAG

CAATGGCGAGGCTCTGCAGGCGGAGTGTTTGGTCTCACTGGGTACAAAAATGAGGACCAG

CTCCTACAAGGGGCCCCTGACAAGAAAACTGGAACCAGAAAGGCAAAGAACTGGACTCAA

GTCACACAACTGGCAAGATATGGCACCATCTTGGTTCTGGAACCCACATCTTTACAAAAT

ACCACATATCTATCTAAAGGAGTACCTCCCCXX

>Unigene39387_C-W 1 403 LEN=403

XXGGCTTCAGCTGGAGGCCGGGTGCAGGGGCGCATGCCTGTAGTCCCAGCTGCTTCGGAG

GCGGAGGCAGGAGGGTTGCAAGTTCAGGCCCAGTCTCAGCAGTTTAGGGAGACAGCCACA

ACACCCTGCTCTTCCCCTGATGCCTGTCACCACCTCCAGGAGGCCACCCTGTCTCCAGAG

AGACCTGACTGTCCCTCTTTTCCAACTGAAAATCACCAAGGCTAACCTTTGCTGAGCAAT

TACTACATACCAGAGTTTGGAGCTAAACTCCAAACTACACTATCTCCTTTAATCCTTAAA

AGGACATATGAAGTGGTGTCACTGTCACCCCATTTCACAGAAGAGGTCACTGAGGCACAA

AAAGCTAAGAGGCCAACCGAGCTGAACCCTAACTATTCTGCTTCT

>Unigene39453_C-W 1 215 LEN=215

XXGCACAATTCTATCTCTTGGGCAATCTTCACGGGGCTGGCTGCTCTGTGTCTCTTCCAA

GATTTAGAAAATATCAACATTTTCAAGAAAGATAACCGTGTGACTGAAAGACTGAAGGTG

ATTCAGATACAGCCATGCCCAGCACCAATTGTGCATGCTGGAACTTGGAGATCTTCCTTC

TCAGAAATCCTCTTTCTAGCCCCACCTTTCTTCCTCGXX

>Unigene39519_C-W 1 373 LEN=373; minus strand

XCCGCTGTCATTAGACACCTTCAGTCACAGATTCCTGGAGCCCCCCAGTTCAGATGTCCA

GGGCCTCAGGGACACCCTGAGGCTCCCAGTACAACCCACCCCCAGTCAGTTGCACAGGGA

CACAGGGAATGTTCTGAAGAACTGACACTTATCCCCCGATGCTGTCTGATTCTCTCATGT

GCTGCCCTGTTTCTCCACGAAGACGTGGACAAGGTGACCTTGCAGACAGACGGCCCAGGT

TCAAACCTTGATCCAGCACTAAGGAACTGTTGTGTGACCTCAGATAAGTTATCTCACCTC

TCTGTCACTGTCTCCTCAACTGTGAAACAGGATCAGGAAGGGACTTGCCTCACAGAGTTG

TAGGGATTAAAGCAX

>Unigene39548_C-W 1 379 LEN=379

XXGAAAAAGGAAGAAGATATTTGCAGGTCTGTGAGTGAGGCTGCTCCAGAGAACGCTAAA

GTAACATCTGTAGTACGTGACGAAGCCATGGAAAAGCAGCTAAATTTGTGGATTCATGAG

ATGATGATCAACAAAAAAAGTATAGTGGAAAGCATTGTTGTGAGGCTGAAAGCCAAAAAA

ATTTACAGTCACGTAACCCAGGGTCAGGAACATGTTAAATCATTCTCAGCTAGTGCAGGC

TGGCTTGCACGTTTCAAAAGACGATACAGCATGAAAAACTTTAAACTTGCAGTCGAGGAT

GGTTCTGCACACCAGGAAGCCGCAGAAGAATTTAAAAAGTACCXGTTGAGTGTTATACAT

GAAAAGGGTTATGTTGAAGAGCXX

>Unigene39590_C-W 1 246 LEN=246

XXGTCCCACGTGGGCAGTAGGGATGGGGTTCCCCTCATGGAAAGAGTTGGATTAGAAATG

AATACTAGGAAAAATAGGACAGCAAAAGAACCACAGGACACCGAAAATATCTTCAAAAAC

AAGGGCGGGATGGGCAAGCCGATCATATGGATTGGGCTTCTAGACTCTGGCAAAATGGAG

CCTATGCCTGCTTCATTTATATTGGGAAACATCAAAGACCTTCCATAGAATGTGCTTCAC

CAAAAAGGX

>Unigene39705_C-W 1 248 LEN=248

XXAAAGAAACCTGAAACATCTGTCAGAAAATTTGATACAGAATTAAATATTACAGCAGAT

GAATTTAATCAGGTTTCACAGTTGGAAATGACAACCAGTGACCTAGAAAATGAGATTGGT

CAGAAAAGAGATACTCTCAGAGTAAGGACTTTGGTTTCAGAACAAGAAAAAAGACATACA

TATCAAAATGAACAAAGGGAAATGGAAGAACACATCAGAAAGCAGGAATCTATTAGAGAG

AGGAGAATATXX

>Unigene39810_C-W 1 319 LEN=319; minus strand

XXCAAACCTGGAGATTTGATTGAGATTTTTCGGTTTGGCTATCAGCACTGGGTCGTCTAT

GTTGGAAATGGTTATGTGGTCCATCTGACTACCCCAAAAAAGAAATACGAGAGGGAGGCA

GAAGAAATCTAGAATATTCAAAATGTGAACAGGGACAGCAAAAGCGATGCAGCGAGTGAT

GGCGATAAAGAAGGAGGAGGAGACGTGAAAGAAGAAAACAGAGAGGAAGAGAAGGAAAAG

AAAAGAATTTGGCTGTTAGAGGAAGATGAACGCAAAGAATGGAAAACAATTAAAAAAAAA

AACAAAATAAACAAAACCCCX

>Unigene39811_C-W 1 234 LEN=234; minus strand

XXTGGGTGGGTAACAATCATGCACTTTATTCACTTCAGGGTTGGGGTTGTTGGCAGTGCC

AAGCTGGTAGTGTGTATACTTGTTCATTCATTCAATGGATGCAAAGTCAAGCAGATACTC

AAAGCAATAGACTGTGAATACAAGGAGCAAATCATCTTTCAGGTCTGGGAGGACACTGAG

AAAGAACAGGCCAAGGTCAGGACTGTGTTCTCCAAAGACAGGACTGTGGTCACTATX

>Unigene39937_C-W 1 260 LEN=260; minus strand

XXAGGCGCCCTGGGCAGGCGCCTGGGAATGCATGTGACACTCAGGAGCTCTAGCCATCAG

TCACCTGCAGAACACCCAAGAGCATCACCAGAAATCAGTAATTGTGACAAATATGGGGTC

ACTCCATGCAAACTCATGTTCAATGCTGTAAAAACCTCAGTACACGGGAGAAAATACCAC

ATGCTGTCAATACCCCATACTGTCAATACACCATACTGCCAATACCACGTGCTGTCAGTA

CCACACACTGTCTCAATACCACXX

>Unigene40002_C-W 1 236 LEN=236; minus strand

CTTGCTGAAAAACCAAATCAAACCAAAGTGGAAGGAAAGGATAACATACCAAAAACTGAG

GGCAGTGCTCCATGTAGTGAGTGGATGCTCACAGCACTCAGTGTGGAGTGTAAAAAAGAC

AGGCTCCAAACCAGTGGCTTAGCTCCATCCCAAGACAAGAGGACCATGGGAGCAAATGGC

ACACCCACCGAGCAGGAGCCGGGAGCCCATAAAGTTGTGGTCAAGGTCAACAAAGCX

>Unigene40016_C-W 1 313 LEN=313

XXCCCTGGGTTCAGTCCCCAGTACCCCCCTAAAAAAAAAGCAGGTCCTCAAATCTTTGTG

AGGAACGATGGTGGCAGCATTGTTCACAATAGCCAAGAGGTGGCAGCTTCCCAAATATCC

ATGAGAGAGGAGTGGAGAAGCCGACCGTGGTCCATCCACACGGTGGAATATTATTCTGCC

CATAAAAGCAAACCAAGCCCTGATACACGCCACAACACGGACGAACCTCCAATGCAAACA

ACTTCCAAGAGATACACAGGGCGGGGCAATCCACAGAGACGGGGCAGCTTCAAGGTCGTC

AGGGCCACTTGGGGG

>Unigene40084_C-W 1 225 LEN=225; minus strand

XTGCCTCAGCCTTCCAAGCTGCTGGAATTACAGCCTGTTGATGGATGGATTAAATTAATT

GATTTTCAGATGTTAAAGAGAGCTGAGGTACGTTTTATGGCCCAGAATGACATCTATCTT

GGTGAACATTCCATGAGAGTTTGAGAAGAATATGCTGATCTACCTGTTGGTGAGACAGGA

TCTGAATTGCTTAGCACCTCGACATTGCTGAGGCTGGCTTTGAATTXX

>Unigene40151_C-W 1 61 LEN=276

XXTGAGAGGTACAACCGGGAGCTGGAGCAGCAGGAGTACCATGACCTCTTTGAGCAGAAG

TCC

>Unigene40154_C-W 1 219 LEN=219

GACTTTCTCCAGCTCTCCGACTACGAGACAAACGACAAGTATCAGAAGGGTTCTCCTCTT

GGTTTCGGCGAACTAGACTGGTTTTCAGACATCGGTCTCCTGCAAGATCAGCAGACCCCG

ACAAAGGGCTCTCCCGCAACAGCCGAGGTACCTGAGCTGCGGGTTCCGAATTACCCCCAC

CACAACAACAACTCCATTGCACCATCATACAAAGCCCAC

>Unigene40197_C-W 1 272 LEN=272

XXGTTCTCAGGGATCAATGACAAACCAGCACCCACTCAGCCCGTCTTCCAAGTGGACTGC

ACCCTCCAGTACTTTGCCTTTAATCCTCTCTGCCAAGTTCCCTCTGCTTATCCAGATGAG

TGGATTCCATCTGAAAATCGCAGAGTTAATAATCACAGTCCAAGCTGATAAAACCCTGCT

CTGCGTCACGGCCATTTTCTCTCTGGAGTGCGGTTTCCCAGGAAAATAACCAAGCAACTT

CATCAGGCAGGGATTTTAAGTTTAGCCAACGCAGXX

>Unigene40208_C-W 1 231 LEN=231

GCCTGCATCATTGCCAGCTTTGGCCCACTCCTCTCCCGAGACTGTTGTAGTCTCTGTGGC

TGTGGGACTCAGGTGTGGGCCACCTTAGCTTTAATCTTTGTAGCGAAAAGACAGTCTTTA

CCACCTGATCCTCAGTCTCAAGCAGCACAGCTATCCACAACCTCTGAAATAGCCCTCAAA

CTCGGGAGATACAGCCGCAGTCAATTATTACTTCCAGATCAGCCAGCACAT

>Unigene40243_C-W 1 214 LEN=214; minus strand

XXTGCCATCATGGGCACAGGCTCTGATCTGAATGAGGACCTTGGGACCAGACTGCTGGGC

CCCCTGGCCAAGGGGCAGCTTTCTTCCTCCAAAAACCTCCCCAACCCAGAACCAGCAGGG

TGGGCCTGCAGAACAACCCCTGCAGCACCTCCAGAGCTCCTCTCAGATCTGAAGGGCTGC

TCCTCCACCTCACCAGCACCTCACATTGTGGAATCT

>Unigene40280_C-W 1 250 LEN=250

XTGAAAGTTCTGCCAAAGTTAGAGCAAAACATCCCATCCAAACTATATATGCTGATGGCC

ATGGAACGCCGAAGTGTGATCCAACTCTTTCAGATCATTGAGACCATTGAAAATATCCAG

CTXATGGCATACAGCTATGGCATACACCTCATCCTGGATGTTGATGGCATGCAGGAGGAG

GCCAAGAGACCATTCAGGCAGATTGTGAGTGAGATTGAGAAGGGCATTGAGCACCTAGAT

CTGAATGCAGAG

>Unigene40293_C-W 1 212 LEN=212

TTCCTGGAGAGCTTAAAGCACAGGAGGAGAACCAAAATGGAGAGTAAAACAGATGTCATC

AACTATTCTAAAGAATTTCCAGAAGCACTCAATAAGATAGTCAAAATATCAGGCCCCCGT

AAGGACATCATCAGTCTGTCTCAACTCGAAATGTCCTCCAGCATCACTGAGACGAACGAG

AAGAAACAAACCTGCAAAGAAGCCCAGCGCTGX

>Unigene40393_C-W 1 201 LEN=201

XGGAAAAACAGTGATGGCACCCATGAAAGAAAGCTGTTATTTACCATAGTGATCGATGTG

TTCATTTCAGAATCCTACATTTTTCAGCTGGCCACAGTGCAGCCAAATCCTACAGTATGG

AGGGGGATGGGAGGAAGGATAAAAATTGAAAAGAATTACTTAAAAGGATGGGTATTTCTG

ATAGAAGTAAAAGAAGCCCCACXX

>Unigene40522_C-W 1 221 LEN=221; minus strand

XXCCTGAAACCAGTGTGCTTCCGTTACGTGTTGGAAGCCCAAGGGTTTAGGAAAAGACCC

CTGGATGCGACTGTCATCCCTAATACCAAAAGGAGCAGAAAGCAATTACCTCGACATCAG

ATTAGAAGTGTTGATXATAACTTGCAGTCAAAACGCAGAAGTCTCTACAATGGCGAGACT

TTTGCTGTCTCTACAGTGGCGGCCATCCGAGATAATAAAAAGTCX

>Unigene40550_C-W 1 249 LEN=249; minus strand

XXAATTTTACCATTAGTTTTACAATCCAAAGATGACTTAGGATTAGATAATACCAAAGTA

AGATCCATCTATAGATTGTTGGATAGAGACTCAGCTGTGGAAGGAAGTGCTAAATGGTTC

CTGCAGTTTCTACAGGTGAGTAGGGTTTTTATCAAAGACAAGAAGAATTTTTCTGGTAAA

TCAGTATTTTGGTCAAGAGTTAATGACACCATGGATAGCTTGTGGAAGGAATACAATATA

ATAGTGTGGACX

>Unigene40589_C-W 1 334 LEN=334

XGAAGGAAGCAAAAATTTGGTAGAATCCTGAAGGAGAGAATGTGGTGACCCAGCAGAGTA

GAGCGAAAAGTTTTCTTTGAAGAGGGAACTGCACGTGCAAAGGCCCAGAGATTAAAGGAA

TCTGGTGAGTTTGAGGAATTTCCAACTCCTAATGAACTTGTCTCCATACCTAGCAGCCTT

TCCTTCCGGGCATCAGAACTGACTGCTGCTACCAGCCAATCAGCATTTTGTATTTTCCAG

ACTCCTGTTATTGAGTCAGGGATGAGCATATGACCCCAGCCTATCCAGTCAGACTCTCTT

CAGGGACTTCTTGAAAGGGAAGATTGGAAAAGAAGX

>Unigene40604_C-W 1 136 LEN=268; minus strand

XXCGCCAAGCCATCACAGAAGCCGCCGCCTCTGCCTGAGCCACCGCCTGAGCCACCGCCA

GAGCCAGAGCCGGAGCCTCAGCCAATGCCGCCACTGGAGGAGGAGCGGACACTGAGCAGG

AGCAGACGCCAAAGCCGC

>Unigene40695_C-W 1 235 LEN=235

XXGTTGCTGCTGTCAAGCAGAGAAGGTATGAAGAGATTTCTAAATCAAGGAGTGGAGAGA

AGGGCATATGAGTGTGACCCACAGCTCATTCCTCTAGAAAATTCAGGAAGCCCGTTATTA

ACTGCCAGCCTGCTGTATGAGAAAGGTAGAAGGGCTTTTCCAGCACCATCTGTTGTAAGG

CCTCTAAAGAGTACCTTCAGAGCCAGGTACAACGGCAAGTTTGAGGCCAGCCTGGGC

>Unigene40699_C-W 1 233 LEN=233

GATGCTTACTTCAGTGAATGGGACATTAACTCTGCTGCTGGCAGCTACCAACAGGAGCAG

CTTCAGGAGGAAGAATGTTTCTCTAATTTAATTATGAATCTCAGGGAAGCATATCATGAT

CCTCTTCAACCTGCTGTGCTGAGTGGCAGGATGCCACTGGATGTCTACCAGGTAGAGGAA

GGGGCAAGGGAGTGTGTACATCTTTACACTCTGCGACGGAAGCATATCTCCACX

>Unigene40741_C-W 1 256 LEN=256; minus strand

XCTGGCAGGGGTGTAGGAGGACACCTCATCGGAGGGTGGTCATTGGGTTACCTGGACATT

TGGTGTGCTTGTTCCACATCCATATCGACTCTGCAGAGAGACCCTCTCAGCTCCTTGTTC

AAGAAACATGCAGAACCTCATACTGCTATCCTCCTTCATGGACGCCAGCATCCTCCATCT

CATACAGGGAACCTGCAACCTAGATACTATCCTGTTCTCATCGGCCACCAATACCTGCCA

CCCGACATACAGACTCCX

>Unigene40811_C-W 1 330 LEN=330; minus strand

XXCAGGAGACGACTCCTTAACATTCCGACTGGGGTGGGCTTCCGGGAAAAGAATGCTCGG

CCCAGACCCAGCCTGGTTTCCGCGCTTGGCACCCGGCAGGCCGCGCCAGCCAGAATGAGC

CTCTGCCCCAGCAATTCCTGGAGCTTCGGCTCACCCCGGCCTCCCCTGGATGGCAAGGAA

GGCTCCGACCCCGCACCCCCTCAGACACTGGAGCTTAATGGGGTCTCCCTGAGTAAAAGA

ACTTTCCAGCGCACCGGCAGCTCGACCAGCCCCCGCTGCTTCTGCTCCCAGGAAGATGTC

CGAGCGCCCAGACTGCAACCCCAGCGCGAACCX

>Unigene40837_C-W 1 251 LEN=251

XXGCACTCTTCCCCTCCCCTGCGGACCAGCCCCTGGAGCTGCCTCCTCCTGCAGGCCTTG

TCGGGCTTCCCCATGGGTCCAGCAGGGGATCCGACTGTGCTCAGTGGATCTCAGGCAAAC

ACCACCAACACCCCCAGCAGCTCCAGAAGCTCAGTCAGCAGCCGCGGAGGGAAGGCCCAG

GAGCTACCTGCAGCCATGTCAGCCTTCAACCTGGTCATTCTGGGCCTGCTCACCTCAGTG

CCACCTGCCAGCTXX

>Unigene40852_C-W 1 280 LEN=280; minus strand

XCTGCGGCCGCACTCACGCGTAACAAGGATCCAGAGACCGAGCCACGGTCCGGGGCTCTG

CGCTCGGCTCCAGGCTCCGAGCCCCGCCGCCGGGTGGACGTGCACGGAGGCAGCAGGAGT

CGGGGCAGCCGAGCCCAGCCTCGGCTTCCCGCGGGCCCGAGGACGCCGGCTAAGCCACCT

CAGCCCGCGAACCCAGACCCGGAGCGGACAAAGCGCCTCGACCTCCGGACGCGCACTCGC

GAGCCGCTGAGCGCTTCCGGCACAGCCCTCACTCACCGCCTX

>Unigene40919_C-W 1 203 LEN=203; minus strand

XXCCTCGGACCTCGGGGCGCGCCGCTGCTTGTGCCCGTCCGGGGCTGCGGGGTCGCTGGC

CGCCGCCATCGGGCGCGCAGAGCCCAAGCGTTCCTGCCCTCCGAGGGCACGGGCACCGAA

GGCACGGGCACCGAGGGCAACGAGCGAGGCAGGGGCAAGGGACCACTGCTCAGGCGTTGC

CGCCGCTTCCAAGAAAGACAAAAGAXX

>Unigene40930_C-W 1 289 LEN=289; minus strand

XTGAAGCGGGAGTGGGCAGCGCGCTGCACTCAGAGCGCGGAGCGCGGCGCGGCGGCCGGG

CCCACAGGACGGATCAACCCGGTCACTGGATTTCCACTCAGCCACTTGGCCTGCGACTGT

CTCAGGGACCTACTGAAGCCCTCGCCCTGGGGCAGGATGCCCCAGCATGTCACGGTGGAC

AAGGGCTTCCTTTCCATGCCACAGCTCCTCTTTCCCAGCATGCCCTCGGCCACTGAAAAC

GGGATGGACCCCACCCTGCCTGGCATCACTCCCGTCTCCCTGTGCTCCACX

>Unigene40940_C-W 1 244 LEN=244; minus strand

XXCAAGAGGGATGTAGATTGTGTCATTACAATTTTCAAGATGGCTTTGAAGCTAGTGGAG

TTTGTGATTCACTATTCAGTTTATCAGCTCCAGGATTATACAGCTGATATCTAGGAACTC

ACCCAGAAGCAAGTGAGACATAACACTTGAATCAAAGAAGAATTAAGCATGTACAAGACC

CAGGCAGAACTCAAGAAAGCTATTGTTGAAACCCAAAGAGAAAAGGAATATACTAATGTG

TGCCCA

>Unigene40941_C-W 1 125 LEN=242

XGGCTGGGGCTGGAGAGCAGGCGCCTGCGAGAGGCAGAGCGCGAGCGCGAGCGCGAGGGC

GAGGGGGGCGAACGGCGGCAGCGGCGGCGGGAGCGCACAGACACGCACACGCACACGCGC

ACACAC

>Unigene40966_C-W 1 250 LEN=250

GACTGGTTGCATCAGCAGCCTGAAGGTCATTTAGTGGCAGTGGAAAGCATCCGGGTGCTC

TCCACTCCACCCCATTCCTGCTTAGCCTGTCTCCCTGTGGGGTCAGGCCAACAGGCTGAG

CTGCTGGCCCACGGCTGGATCCGAAGAACAAGGAAGAGGGAAAAACTAGCAGTAAACCAC

CTAGGCTTCTCACAGACACCCTTGAATAAGGAAATGCACACGATAAGAAGGAAGAACTTT

TTGTCAGGAAXX

>Unigene40991_C-W 1 209 LEN=209

XGAATGTCCCAAAAGAAGAATAACACGAAGCTGCTAATCGGTCCAGGAAAACATGAAGCA

AAAAATGACCCTGGGAAACCAAAAAATAGAACCTTATCTGACAACAGTGATGCTGAGAGT

CAAAAGGAAGACATGGTTGAAACCACTCTTGTACAACCAATCAACATAAAGAGCAATTAC

AATCCCTTCTTTTCATCAGTTGGTTCAAAG

>Unigene41000_C-W 1 264 LEN=264

XGGAAGGACGGGCTACGGGTGCCGCAGGGTCCAAGCCGCGCCGTCCGGCGGCAGGTGCGG

CGCGGGGCCGAGGACCGCGAGCGGAAGCGACCCCXGCCCTGCCGGAGCCCCTCTGCTGCG

GGCTTCCGGGTGCGGACGAGCGCAGCCCGCGGGTTCCCGGTGCGTGTGCTGCTGGTTCTG

CGGAGGGTAGCGGGTGTCCGGCAGCGCTCGTGCCCGCGGCACGTCGTGGGCTGGAAGAAG

GCGACGGCGGCCGCCCAGGGGCCCGTX

>Unigene41086_C-W 1 237 LEN=237

XXCATTTTTCTAGGAGGACATCCACCTTCATCACATTGTCAAAGAGGGACTGTGACCAAA

CGAAGTGCAGAATCACTGTGCTTCAAGCTAGCTAGACATATTTCCCTCTCTACTTGTGGA

TTAAGGGGAAAGCTTCCCAGAAAGTGGCACTTTAGCTGCCTCTTTGTGGATGAACAGGAG

GAGGCAGGCAGCAGGGCAGAGGGTTTCATGTACAGAGGCATCATGTCTGATAACCGGGGX

>Unigene41139_C-W 1 211 LEN=211

AGTAATGGAAACAGTACAAACCCAGCCCTCTCCAATGCCACGTGTGCACTTCTACCTGAT

TTCTATCTGAAGGTAGAAGAAGGGTATGGGGCTCAAATTGAACCTTCAAAGAATGAGACT

CATCCAAGAATGGATATAGTGGGTCCAACTGGAGGTGGGCAAAGCATTAGGCTGAGTGGG

ACAGTGTTTGAGAAAGGGAACAGGAAGGGCAXX

>Unigene41250_C-W 1 143 LEN=259

XACCTGGGTCCCCAGCACGGACCCCCTTTCCTTTCAAAGGTCTTTCTAGATGGGAAAGAC

GAAAACGAGTACTGGCCCCCCAGCTCCGACTCGCGCACGGAGAACACCGTGCGCCTGAGG

GTCAAGTACCAGCGCAACGAGATC

>Unigene41270_C-W 1 228 LEN=228; minus strand

GAAAATTCCTCAAGTAGGAGAATCCTAACACCTAGTCTGTGCTTCTTCGGTATCTGGATC

CTCTGCATGGGTGTGATTCTTTCTATTGCTGGTCCTATAGAGTACTCACACCATGGAGAT

GGAGCAGGCCAAAATCCTGGACAGATGTGTATGTTTGTCCACATGTTAGTCCCAGCCCAC

CCCAGCCTGGCAGTCCTCCCCCAGATCCCTGCCTTATTTGCGGCATCC

>Unigene41274_C-W 1 409 LEN=409

XXAAGCCAAACCCTCTGGAACAGAGTGGTCACTCACTACTCCAGGCCGCGGGAGCCAGCG

GCGAGACTTCCCGGCATGGGTGCTCGGCCCGGTGCCGCGGAGCGGGTACTCCAGCTCCCC

GGCCGGCGGCAAGAGCACGCTTTCTTCCAACCTGGCCTGCGACGCTGCACAACCCCCGCG

CGCTGCATCCCGATCCGAATCCTACTTCAGCTAAGTGCCCGAAGCTTCAGCGCCGACGAC

CCTTCCCCAGAATGCCCCAGCCTGGGAAGGAACCCGACCCACACCAATCGCCGCCTCCCG

CCAGGCGACAAGCGGAACGAATGGGAGCGTCTCTGCCCGCACGTGCGCGGAAAGCGGGCA

CTACAGGGAGCCATACGGGCTCCAACGCCTCTGTTTTCCATCCGTCCCCGG

>Unigene41292_C-W 1 236 LEN=236; minus strand

XGCCAATGTGCCCCAGAGGTCTTTGGAAAGCTGGAGCCACAGCCTGGCCCGTCCCTGCCC

GGGGAGCACATACCACCCCGTAATACTACTAGTGCTGCAGGCAAAGAGCCTGGCTTCCTG

TATGGCTGCTGGGCAGAACTGGGCAGAACTGGGCAGAACCGGGAAGGGCTGGCGCGGGGT

GCCGCCCTCAGCCCCTCCTACAAGCCAGCTGGTGGCAGCAGTCTGCCTGCCGCAGAC

>Unigene41330_C-W 1 234 LEN=234

XGCCATGAGCAGGTTGAAGAGAGCTCTAACAGGCCTTGGAGGACCGAACCCACGTATGTG

GCAAAATACGGGGATGACTTGTGGCTAGGGGTGAAAGGCCAACCAAGATCGGAGATAGCT

GGTTTTCCXGCGAAATCTATTTCAGTAGAGCGTATGATGTCGATGGCCCGAGGTAGAGCA

CTCAATGGGCTAGGGTGGCCCCCATTTCGCCTTACCAACCCCAGGGAAACTCCGAAX

>Unigene41333_C-W 1 238 LEN=238

XGGAATGCCTGTAGACCAACAAATCTAAACCAGAGGTGTCCTTTAACCATTAGCTTGACA

GGAGCTAGAAACAGTGTGGTTACAAGACTGCCCAGCATAATCTGTTCAGTGTTCCTCATT

GCTCTAAGAAGCAGTGAAGAGATGGATACCTCTCCAAAGATCATTTCTTCATCCATGATG

GTTACATCGATAACACCTCATTATGTAGATATTCTAACAGATGTCCTCTGGATTGCTATX

>Unigene41366_C-W 1 202 LEN=202; minus strand

XXCTGGAATCTAATGTGTGCTGAGATCAAAAAGAAGCAGTGGGTACAACCAGGGCCAAAC

ACTGTCCATTATGTGGAGAGCCGGGAGTGCAGGTTTGCACACAGGCGGCAGGCTGCCTTC

CCACTTACTACATGGGCTGCAGGCATGGCTGTATGTGTCACCAGTGCAGCTTTATCTTTG

TTCCCACCCAGACCTTATACAAAC

>Unigene41489_C-W 1 252 LEN=252

GCTGAGGCTGACTTTGAACTTGCTGTCCCGCTGTCTCAGCCTCTCAAGTTGCTGGAATTA

CAGGTGAATGCCACCCTGCTCAGCCCTCAGCTAGGTCTTATGAGAACCAACAAACAACCA

GATAACTGCAACACTTTAAGACACCTACATATGACTGCCTGTGATGGCACCTCACCCAGC

CAGCCAGAATGGAAAAGCTGCCAGAAGGAAGGTGTGGTTCAAGTGACCAGGTGTCAGAGG

ACTGGACAGATG

>Unigene41518_C-W 1 216 LEN=216

XTGAAGCTTCGGGGAGGCAGAAAAGCAGATACTAATCAGAGGGAACCCCATGTGAGGAGG

GGCAGAAACACACATGATGAAGAGATACTTGGCGTCAGCAAGAAGGCTATCTGGACCAGA

GGAGGGCTACCAATTGGGCAGTGGTCCTTAGTGGAGGGCAAGTTGAACCCTCTGGAAACA

TTGACAACAGACATTTTTAGTTGTCATATTGGGGTTGXX

>Unigene41544_C-W 1 241 LEN=241; minus strand

XCCTCTCCTGCAGGAGATGAAGTCGCAGAAGAAGCAACACCTGCTGCTAGGGACACCTTC

CAAGGCAAGGGAGCTGAGGACCCTCCCGCCTCCCTCCTGCCCCCTGTCCTCTCGGGCCTC

TTGCTGGTTGAACGCAGACCAAAGCCAGCAGACCTGGGGATCCGGACCCCACAGCCAGCC

GTCAGCTCCTCTGCCACCCGCAAGGAGGGCACAGGAGGGCGAGGCATGGAGGGTGACCAG

GCX

>Unigene41646_C-W 1 240 LEN=240

XXGAGGACGAGCCGGGACGCGGCGCCGCGGCACCAGGGCGCGCAGCCGGGCCGGCCCGAC

CCGACCGGCCACACGGTGGAGGCACGGAGGCCCAGACCCACACGCCAACTGAGGCCGCGT

CTCCCCGAGGCTCCCTCTGGGATCTATGCTGAGTCTCAACTCTCGCTGACCAAGGCCACG

ATAATTCCCTCCCCGAAGCCCGGCAGCCCCCCAGCCCCGCGCAGCCCCAGCCTGCCTCCC

ACX

>Unigene41702_C-W 1 205 LEN=205

GGAACTGATACCCAGAGGCTCTGTAACTTCTCCAAGACTGCTCTGAGAACCCAGGATGAA

GATCTGTTACTGGAAAACAGGAATTAGCTTGTCTGCTTGAGGATGTGGTTGGCGCTTTAT

AAAAAGGATATAGAAGAAACTTGCACAGATTGCCAGCTTCGTTATGCTGCCGACAGCTTC

CAGCAAAAGAAAAACCTTCACAGCCXX

>Unigene42076_C-W 1 200 LEN=200

TGGGGAGTCCTGGACAGCCCGTTAGTGCAGCCTGGTTGTGGTTGGAGAGATGGTACTAAA

AGACATCAGAGCCAAGCTCCAGTCTGTGACAAXCCCAAGGACATCCAGGAGCCACTGAAA

GATGATGAGATCAGATTTGCCTTTAGCACCATCACTCTGATCATGGTGTGCAATACAGAT

GGGGGTGGGGACTATGAGAAG

>Unigene42089_C-W 1 217 LEN=217

XTGAGCCGCACCAAGTTGGTTCTGAGGAAAGTGCAAGTCTTCTGTGAGGTGACCTCCCCA

CCCCAGCTGGGCTGGGGCTGGGTGCTCGAGGCGTTGAAGCACATCTTCAACCCTCAAAGA

CGAAAAGGGAAGGCAAGGTCCAAGGCTGCCCCACACGAGGAGGACCGGTCCTCGTCCATC

TTCAAGGACACGGTACCACCAGCTGCAGCAGGAGGAGXX

>Unigene42092_C-W 1 236 LEN=236

XXGTTAAATTGGTAGGGGACATCAAGGCAGAGGGAGGAGCAAGTACAAAGGCCCCGAGGC

AAGGACAAGCTACTGGAGCAGGATCTCAAGAAGTCCTGTATAGATGGAGAAAAGCACAAA

GTGGAAGGAGCAGCAGTAGTGGTGGGCATGAGAATGGAACCAGGGAAACAGGAATTACAC

GGTTTCATAGGCCATTCTGGGACAAATGACACAAACCATATCCAAATCATAGTAAATGXX

>Unigene42110_C-W 1 284 LEN=284

GTCTTCAATCAGATCCTGGGGACCAAGATCGAGAACATCCCCAGCCCCACCGATTTTGTG

CTCAGAGCTCAAGTCTTCCGCTGGCATATGTCCATCCGCGTGCGACAAGTACACGACTTG

GTGACCGTCGTTCTCCGGAATCCTGCCGAACCCCAGGACCACCCGACACCCAGAAACCAC

GATTTCGGCCTCGCGTGTGCCCAGCCGGGTACTCAGGTCCCAGATTTTATCCCGCCCCCT

TCGGCGCTCGGCCTCCGATCCCCGCCCGGTTCCACTCCCTCCGXX

>Unigene42185_C-W 1 278 LEN=278; minus strand

XXTCCTGTGACTGTAGAGCTTCAACTGACTAATCCTCCAAGAAAATTACATCCAAAAATG

GACATTTTATGACAGAATCACTCACTGATCTCAAAAACAGAACAAATAGAAAACAACACC

ATTAAAGGAATCTCAGTATTTTCTGACCAGTATTGTAGAAACACGGAAACGCAGCAGATT

GCAGACATGCAAGAATCAGCACTGTATAGGAAAGAATTAACAAGTCAAACTCAGTACAGC

TTTAACAACTGGATTGAAAGTAGACTCAATAAAACCAAATXX

>Unigene42205_C-W 1 269 LEN=269; minus strand

XCCTCAGAGTCAGTGACATTGGCTGTGGGCAAAAAGAAGACAAATAAGCCCAGGCACTGC

TGGAGAAGAGGGCTTATTTGTGCAGCTGCCAAGGCTACAAAAGCAAAGCCCAAACCAGAC

CTGAGTCAAGGACTGCTGACCCCAGCTCAGGCCTGTAGACCAAGCCTCATTCACCCACCC

TGGAGCCTCCCAGACTCAGCGGTCCCCACTGTCCACGCTGGCAAAGGAAAGCAGTCTTTG

CACCACATTCTTTTTAGAGAATTGCTGAAC

>Unigene42233_C-W 1 227 LEN=227; minus strand

AACAAGTTCAACAAGGCTCCCCTCCTGCCATTGGCCCAGGCAGCCCCGAATGATACACTG

TTGCCCGGGGATGCCCGCCGAGAGCTAATGCACTGTCAGTTCTGCCGGCTGGAGTGGCCC

GAGGGGCACCCGAGCCAGGCCGCTGAGTGCGCGCCCCTAAAGATGCTGGAGGAGCTGATA

CCAGGAGTCAGAGGCACCAACATCTGTAAAGAAGTCATGTCCATGCXX

>Unigene42236_C-W 1 238 LEN=238

GCAAGTGGGGGCTGCTTTGGAGCCGCGGAGACTGCAGCCGGAGCCCAGCGCGCGGACCAG

AAAGTCCCTCGCGTGGATCGGCAAACGTCGCCCCTTCTCCTGGAGCTCACCCTCTTTCTC

TTGCTCCTGCTACTGCTGACCCGGCAAAGGGAGCCGACAGGGCCCCAGGCTGAAGGTAAA

ACCCCACGGAAGGAACATGAAGTTCCCTTGGATATCACTGAAGAAGAAGATGGGAGGGXX

>Unigene42258_C-W 1 236 LEN=236; minus strand

XXGCCGTATCGGAATGATGGTAACCAGAAAAGTAGACAAGCTGAATCCGGAGGAATGGAG

GGAAGTGCCCGGCGTGGGCACCTAGACAAATGAGCTCTTCAAATTGTCTTCGGCTTTATC

CTTAAAGACGGAAAGACGCTCAGATTACCTGGTATAATTTCCTTTGACAGACTGATAAGA

GACCCAAAGAAGAGAAGGGACTTGCCCAAGGTCACAGAAACAAGAAATAAATTCAAGCXX

>Unigene42375_C-W 1 222 LEN=222

GCTAACTGCACTGAAACAAAGAGGCAGAACCAAGGGGACTGTCTGCAGTCACTCCCCTTG

CTCAGCCTGGTTAGTGCTGTTATCACTCAGGCCACGTGTGCTGCAGCATCAAAGGGTGAA

GAATCCAGTGAATTGGAACATTGTCTCTCATCAGCTTCTTTGCAGATGGACAGACCTATA

ACACCACCCTCAGAGACCTTGTCTGGTTTTGACTCACACAGA

>Unigene42391_C-W 1 247 LEN=247; minus strand

TGTTCTCAAGGGACCAAGACCCTGAGGGAAGAACAGGCATCAAAATGCAGTGTGGAACCC

AAGGTCAAGGCCAGTGGTGGGATGCCCAGGGACCATCAGGAAGGAGGACACGTCCTTCCA

GTGGCAAACGATGATTCTGCTGGATATGGCTTCCAAAGAACACCTCAGTGGGAGCACACG

GAAACAGAGAAGACAGAGTCCAAGACATGAAAACCCAAAGGAAGAGATTCTTCCAGAATC

CCAGGGCXX

>Unigene42430_C-W 1 232 LEN=232

XXATTCACCCAACAGATAGTAGTTATAAATAACATACACCTAACATTATATAAAGCAAAC

ATTGTCAGACCTAACAAGGAAGATAGACACAGACACAATAATAGTAGAGAACACTTCACT

ACCCCATTTTCAATAATGAACAGATTACTCAGTCAGAAAATCAATAATGAAATAGCAGGC

TTGAGCACTGTGGACCACATGGCATGTATACAACATCCCATCTTGCAACAGCAG

>Unigene42448_C-W 1 217 LEN=217

XXGGCCTCTGTCACTTGTGCAATTGCACTTCATCCCCTCCTCTCCAGCGCCCCAGAATCT

CTTCGACACAACTACATGTGCAACACTGATGCTAACGAAAGCTCAACAGCTGCCCCAGGG

CTTCCAGAGAACTCTGCTGCCCCAAAGACCGGGCTGACCGATGACCGTCTTTCTGTGTTC

ACCACAATGGGTGGCCTCTACCTCAGAGAGACGGTCTCT

>Unigene42581_C-W 1 244 LEN=244; minus strand

TCCTCAGTAAAATTCAGGAGAAAAGAATTAGGACACATGATACTGAAAATGTCCATTCTC

CCCCAGAGTTTACAAGACATAAAGGGAGCAGATATCAGTCCTCAAAATCTTCCCAAAAAT

AGAAGAGGAAGAAATACTTCCTCATTCCCTGAGATCAGTATTACCCTGATAGCAAAGCCA

GAAAGACAGCTACAAGAGAACTGCAGGCTAACATCCTTTTTGAATACTGGTGCAGGAATC

CGCAXX

>Unigene42629_C-W 1 249 LEN=249; minus strand

XCTGAAGTGGGCGAGGGAAACCCCAAGTCTGGGGGGGGCTCCTTTACCACAATGAGGAGC

TCGACTCGGCCGTTGGAGAGTCAGCCAGCTGGAGCAGGGGCGTGGAGCGCTCTAAGAACA

GACCCCAGTGCACGCCAGTTGGACAAGGCTGACCTAAGCAGGAACCTGCTGATAGCAGGG

TATGACCGCAGGAGACACAGCAAAAGTGACCGTTGCCCGTGGGAGCGACAGGAGGTCCCA

CCCACCCGGCXX

>Unigene42650_C-W 1 220 LEN=220

XXGTGTAAATGTTTGGACACCAAATTTAAACAGAGGGCACAAAAGCAACTCCATGTTAAA

ATTCCCCTGGAAGACAAAGAGTACACAGGGCTCTGTGAAATCCTCCGCAAAGAAGGCACC

ATTTCCCTGGCTGTGCTGCATATATACATAAGAGACCAGGCAACGCCAGCAAGCGCCTCA

GCCTACACCACTGAACGGCCAGTCCAGGACCCTGAGGCTGCT

>Unigene42682_C-W 1 205 LEN=205

XXGTGCTGGACTCGATATGGGAAGTCCTGTTTCATTGTGGTTATTAAGCCTGGGACCATG

CATCTTAAAAATGGTAATAAAATTATCTGCCATTCTTACTTATGGAGCTACTTAGAGAAT

CTAACGAGTCAGTATTCTGTGTGGGAATATGCTCTGAAGACAAAAATTTGTGTTGCATGG

GCTTATTTTCTCCAAGATATCCAGGAA

>Unigene42744_C-W 1 201 LEN=201; minus strand

XGTGGAAGTGACATGATGGTGATCATGGTAGTGATGGAGACGGTAGTGCTAAGGACAGTG

GTGGCAGTAATGATTGTGGTGGTTGAGGGGATGATGATGGTAAAGGAAAATGAGGTGTGT

ATCACTGTCAGATCTAAAATAAGGCTTGGAATAAGATTCAGAGTTGTCCCTGGAGGTACT

TCTGAATTTGAAACCAACTTC

>Unigene42783_C-W 1 227 LEN=227; minus strand

TGTAGAGAAGACACACTTCAATGTGGGTTTTTGTGTGTATGTGTGATGCTTCTGGGGACA

GGAGAGAGAAAGTGCAGCTCCCTAACACAATCTGTTGAGCAGGCGGTGGCATCTGTGATA

ATAGACCACAAGCTCCGAGAAGCCATCACATGTCCAGTGCATGGAAATGTCAATAGCAGT

TCCAGGGCCAATCTTGAACTAAGAGGTCTTTGTGTTTTGAAAGTGGCX

>Unigene42941_C-W 1 193 LEN=255

XXGAGCAAGGCAGAGACGCCAGCGCCCGTCCTCCATGGGCCCCGGGAGGTTGGCCGCGCC

ACCCCTGCTCCTGGTCACCCCGCGTCGATGGATAAGGACGACTTCAGAAGCCCCTGGCGG

GCCCAGGCGTCTGGCGCTGCCATGACTGAGATCAGCGCACGGCATAAATCCGACAGCTCC

GATCCTGGAGTCGCG

>Unigene42970_C-W 1 250 LEN=250

XGTAAAGTTGGCACTGTCCAATGTGAACCAGGTCTGGTCTCAGAGACTACCAAGAACCAA

GACAAGACTCCGATGGGAAAATCCTCCAAGACTGATGGGCAGTACAACTTCCTCCCTCTG

CCTTCATGGAAGAATGACAGGCTGCTCATGGATGCCCTTCATCTGGAGTCATCAACTGGG

ACTTGAATCCCAGTAAGGCAACAGAGAAAAATATCAACAGAGTTTGAAGTCAAACCACTA

AGAAGAGGGGGX

>Unigene43044_C-W 1 278 LEN=278

XXCAGGAATGTGGTGGAGCGCCTGCCCAGCAGGCACGAGGCCCAGGTCCCACACCCAGCG

CTGCAAAAAAGAAATGAGGAGCCCAGACACAGAGGGGCCACGGCCTGTCTGCAATCACAG

GCATCAAGATGCAGGGCTCAGCTGGCCTTAGACGTGGTGATGGAGGCCCGTGGAGCCACC

TTGGGTGACCTGCAGACGGGAGTGGACCATGCCAGGCTCTTACAGAACAGTGAGGCCCAG

AGGGCAGGAGTCTTGCTGGAGGTCAAGTGGCAGAGCCAGGXX

>Unigene43053_C-W 1 205 LEN=205; minus strand

XXAGAGCCTCTTTACCCAAAGCCAGGCCAAGAAGTACACATGTCAGAAAAAGAGCCTCCA

AATCCTGTACCCGATTTGATGGAAACCACCGACCAGAAATCAGACCCCAAAAAGCACGTG

AACACTTTGGGGTTCTCAGACACTCTCCTCCCAAATCCTCAGACTCTGGGGTGCTGTGTA

AGCATCTCAGGGCTCACGGATCACTCC

>Unigene43101_C-W 1 205 LEN=205

XGTCAGCTGGAGCACCCTGATGGTCCTGCAGACCTGCAAGACGGGAACCCCCTAATGAAA

AGAAAACAGGGCTGGCGGGAGCAGCGGCCTCCGAAACCCAAGCCTGCGGCCGGGCACCTG

AATGGGCCCTTTCACGTGGTGGCCGCAGAGCGAGGGCTGGGCGGAGGCGCCCTGGCTGGC

CAGGGAACAGCAAGCCCGGGGCAGGGX

>Unigene43141_C-W 1 248 LEN=248

AATGGCACAGATGTGTTTTCCCGACTCTGGAAGCATAATGGCTCTCTCTTAAACCTGGGA

GCACCAGGTGACCAATTAGACCTATTAACCTCCAGAACAGTAACAACACATTTCCGAGAA

GATGCTGGTGAGCAGAATCTGAAAGCAGACTCCTTTGGCAGTCATATTCTGTGGCTAGAA

ACCCAGCTCTTTGTGGAAACACGGAAGAAATTAGCTCATTTGGAAAAGAAGGATCATGAA

AAAAAAAAX

>Unigene43200_C-W 1 225 LEN=225

XXTATGGTTCTGGAAGCTGAGAGATATGAGGATGAGAGGGACCACCAGTTACTGAAGGCC

CTCTTGCTAGTGGAGATTCATGATTTAGCAACCTCTATAAAGGATCAGAGACTAAATGTT

TTAGGCCCACGGGCCAAATGGTCACTGTGGAAACTGACTCTGCCATTTGCAAAAGCAGCC

AAAGACAATAAATTGAGAGTGGCTATGTTCCAGGATGTGATTGGAGXX

>Unigene43345_C-W 1 229 LEN=229

GCACCACTTCAGGGATTGGAGGTGCTTCATTCAAAGTTCGCGCCATTCACAGGGATTGGA

GGTGACAGTTCACGCACCATTCAGTGCGTCATGGACCTGAGCTCCCAGAAGAGAAGCACT

CTGGGCGCCATCTTTACCAACACTAATAGATTCAACCAAAAAAGATTCCAAGACATGTTA

GAGTCAGACTGTCAAAGTACAAGACAGAGAATTCTAAAGACAACAAGAGXX

>Unigene43438_C-W 1 292 LEN=292; minus strand

XGGCTGACGGCAGGCGGAGGAGGTGGTCTTAGGGAGAAGTCCTGTTCTCACCAGAGTCCA

GAGGCTGCTGTAGAGTTTGAGAGTGGTGAGAACAACACAGAGGATCAGGCAGAGGAGCAG

AGTGGGATGTCGGTCCAGTCCCCATCAGGCTCTCTGGCTTATGTGCACCAATGTCAGCTG

GCTGAATGTCGCTTCATTTGCTCCATCATGTATACAATTTTGGGGGCTTCTGGCCACCCT

TTCAATCCCTATCAGCTGCTACCGGATTTCTCAGTGACATCATTCGTCCTGAGX

>Unigene43603_C-W 1 269 LEN=269

XXGAAATAGGAATGGAAAAGGGAAGAGGTGGCCAGTGAAACCCACAGTTGCTATTTGAAA

ACATCAGTAACACTGACCAGTCTTTAGCTAGCCAACCAAGGGAAAAGGGAGGTCGTCATT

ACCCAAACCAGGAAGGCAAGAGGGGACCTCACAACCGGCTCTCCACAAACTGAAGATTAC

ACGGGAATTCCATGCAAAACTTTATGCCAGCAAATTAAACAAGATGAAAAAGACGAATTT

CTAGAAACACAAATGACTGAAAATGACTCGGXX

>Unigene43685_C-W 1 232 LEN=232

XCAGAGATTCAGTTCATGATTCACTGGCTCCATTACTTTGTGCCTAAGATGATGGCTGAA

CATCATGGCAGAAGAATATGGCAGAGGGAAGGTGCTCCACTGCGGGAGAGCCAGGAGGCA

GAACAGGAAGAGCCAGGGACAGAATATAGAATTCCTAAGGCAAACCCCCAGTTCAGTCAT

GCCCTATCTATAGTTATCACCCAGTAGTTCATTCAAATTATTAATCCATCAAAX

>Unigene43708_C-W 1 255 LEN=255

ACATGTGGCAACAATAATGAGGATTTCAATCACAAAGGCAAAAATATGTTATTAAATCCT

TCCAGGGCACCTAATTCTACACACATGGGCTGGATCAGGATAAGAGTACATCTTTATTGA

GCCCAATCTCTAAAAGAAGAGAAACCTCAGGGAGCTCCTAATGACAAGTACATCCAACAA

TCACTTATTGAACACCCACCACAGAACCCAGAGAGATTCAACAGAGTAATTCATCATTCT

CATGACTCTCAAAAG

>Unigene43711_C-W 1 227 LEN=227; minus strand

GGACAAGAAATGAGGCAGGAAGGAATGAGAGGTGGAACAGGAGTTTATGCTATAGAGTTG

GTCACTAAATGGGTGGTGGTCAGATCTGGGGAAGGAGAGAAGGGAATAAGCTTGGGGAAA

GCTGATGATTTGGTGTGGAACATTTCCCTTTTGAGATGACCCTGGAACATCCTGACGGAG

GTTTCAAACTGGCAAATGGAAAATGTGGGAGAAATTCCCATCTGCCCX

>Unigene43803_C-W 1 238 LEN=238

XGGGTGACAGAGATGAGAGATGAACACTCTAGGAAAGGATCAAAAGGAAATGCTGGAAAT

AACACAGGAACAGAAATGAGGAATGTCTTTGAGCTCATCAGTAGACTGGACACAGCCATA

GACAAAATAAATAAGCTTATGTCAAGAAAAAGCTCCAAAACTGAGCTGCAAAGAGAAAAA

AAAGATGAAGAAATAATCCACCTTCACCCCAAACAAAAAACAATCACAGGACAGCCAAGX

>Unigene43886_C-W 1 236 LEN=236; minus strand

CTCAGAGACGTGGGTGAAAGTAGTTCAAAGTCCAGCCAGATCCGGTTATGTGGTGTCCAG

GCCCTTGGGGATCCCAGGACCCTGGAGGAAGCCCAGGAGAGGGTGTGGCCCAGCCCTTGG

TTCTGTATTGTGCCTTCAGACTCGGAAACATCTGAGACTGAGCCAGAAAAATACATAAAT

AGAGCACAGCTATGCCCCCAAATCCACCATTGCAACTTAAGAGCCAAGACCCCGCGX

>Unigene44283_C-W 1 233 LEN=233

GCTGCAGAAGATTTGTTATTCATTCTATGGGCAGCAGTGGAGGTGGAGGGAGATAAGCCA

GGTATAGAGCCTAGATGTTCTGTTGCTCTGGTGACAACACACGAAGGTGCCCTCAAATTT

AATTCTCGAAGATATAAATCCTCCCTGGCTAAGAGCAGGTCCCAGCAGTACCATCTTAGA

CAAGAAAGTAACCTAGTTCCCTTTGAGTTAGTGGAGCTGCATTCAGTTCAGTGX

>Unigene44523_C-W 1 214 LEN=214; minus strand

XGTGACCCACAGTGGAAAAAGCAGTCAGTAGAAACTGACTTTGGTGGGTTTTGGGAAACT

CCCTGCCAGGAACTGCAAACCATCCTCCATAAGCAGAGAGAGAGCACAGACCGAAGAAAG

AAGCAGACCAGTCTTATTCAGGGAAAAATTAATGAAGCAGACTTGGGTGGCAGCAAGAAC

AGGTACATCTCCCCACTTACAGTGCCCACCTGTCCX

>Unigene44799_C-W 1 220 LEN=220; minus strand

CCTCCATGCAAAGATGTGTCTAGTAGGCATTGCTTGGTATACCTTTGCCTTAAGCTTAAA

GTAGAAACACATCCAGCAAGTACCAAGGCTGACCTCTTAATGGCCAGAGCTGTGTGTTTT

CTGGGTTCTCTGAAGGAGTCTGGAATTCTTGTCCATGTGATAAATGGAGTCTTAGAATTT

CTGACTTATGTGGCAGCTTGGGCCATTGTCTTGAAATTACXX

>Unigene44940_C-W 1 211 LEN=211; minus strand

XXCAGAGAAACAAAGAGGCCTGTGTCACAGAATCGTGAGCTTCAATGCCTCTGGTCACTG

CATACATCTTCTGTGGCTGGAGATAAGCAAGTGCTCTTGAGGCTCAAAGGCAGAAAAGAA

GACGATGATGTCXTCATCACCAAAACCAAACATGGCGAATGGCAGCCTTCATCTATCACG

GACATTCTCTGCAAGGGACTAAAAGGCCACTGCCXX

>Unigene45009_C-W 1 376 LEN=376

AGAAAGAAAGAAAAAGAAATTGTCCACCAAATGTCTGACACAATGAATGAAAAATATGTA

TATCAAAATAATCAATATGAAAGAACACTTATGTCAGAGAGAGGGAGAGAGCTAAAAGCT

TCCAGAGAAAAAGAACAGATCTCAAACAAAGGGCTAGGCAGCAAAAAACAAAAAACTCAG

CATAGTAGCCCACATTTAGAATTCCAGGGACTTGGGAGACTAATGCAAGATCGAAAATTT

AAGACCAGCTTCGTCAATTTAGTGAAACCCTCAGCAACTTATCAAGACCCTGGAATTGGT

GGGGCAAAGTACCCAAGAGAAGAATGCTCACAGAGAAGGGACTGGACTCCAGAATCCCAC

ACAGAGTCACTTGGAGXX

>Unigene45056_C-W 1 146 LEN=282

XGTGCTTCATCTGTCATAGAAGAGGGATTTGGGCGCTTTCACACGAAGCTTCAAGTAACT

AGACAATACCCAGTTTCCTTCGATGCTGCAAATTTATCCAATGGAGATGGCGGTTGCTCT

AGAAAGCACCTTGAGGGCAAATGCGAA

>Unigene45063_C-W 1 237 LEN=237

GATGGCTGTTGCGCTTTTGCCTTTTGTTTGACTGTGTGTTTTCGCCTTTTGTCTCTTAAT

GACGATCACACCATTGCTATATCCAGAGCAGTAAACAGTCCTCTGCGAAGCCGACTTTTT

GCCAACCCAACACTGGAAAATGCAGACGTGGAAGGAAGCAGAAAATGCCATGAGAGTATA

ACTTACAAAAAGGTTCTTGACCGGAGAAGGGGCCATGTCTTGGTACCCCAGCAGAGT

>Unigene45377_C-W 1 224 LEN=224; minus strand

XXCTCTCCGCAGGCCTACAGGAAATGGAAGGTGGACGGGCTGGTGCAGGGAAGATCTTGT

AAGGACCTGGCAGGACATCACCTGGCAGACGGCAGGCTGCACTTACGGATGAATCTTATT

CGTTCTGAGGAGGACCTGTTCCCGTACTGCCCACCACACCCTCGACAGATGCCCGGTAAG

CATGTGGGCCCTGGACTCGTCTGGAAGCAATCTCCTAGGAGGAGTCXX

>Unigene45429_C-W 1 209 LEN=209; minus strand

XCAGGAAAATGTATATACCTACTGCAGGAGTTCAAGAAAAATTCTGTGATGAAAGCCACC

TACATGGAAAGAAATCTGGACCCCAAGGCTGCTTTTCACACACCAGGCCAAGTGTCAGGT

GTCATGGTCCCAAGTGTCATGGTGGAGAGGCAAAGGCACATTCAATCACTCAAACAGATC

CCCCATCTTCTGGAGAAGCACATGTGCCCA

>Unigene45440_C-W 1 299 LEN=299

GTGCCATATTTTAATTGGAGCTCAGAAACTGGAAACAAAAGTACATCTGCTGACAAGGAT

GTTCCAAGAACTGGAAAGCCCGGGGAAAACCAAAAAAGGGAAGCATCAGATAAACAAAGG

AAATGGCGCCTGCCAGCAAGCAAGGGTGAGAAACACACATGTCAAAACAAGCTCCACGTG

AAGCGCAGTGGGGGCTCAGAAGTGTCCAGAGAAGCCGAAGCCGAGGCTGCAGGGGGCAGG

ATGCAGGCGAGCATTTCAGCACACCGACTCCTTTCACCATACCTGGTGCCAACTGCTTGX

>Unigene45591_C-W 1 215 LEN=215; minus strand

XXTAAGGAAGGGCAGGGTACTGGAGGAAAAGGTACTGCTTCAAAAAGACACCTTCCATTT

TTCTTGACAAGAAAGTTCCACGGAGAAACAACACCCAGACACTGTACCGTGTCCAATAAC

TGCACTCCTCAGATGGCTTTGGAGGAACATGAAAGGGTGTCTCATTCAGGAACATCAAGG

AGAAACCATCTCATCCTTCAGCAGGACTGGAATTGGCXX

>Unigene45595_C-W 1 290 LEN=290; minus strand

XXCTCACAGAATCCTCTCCATGAAGAGATGTTTTTGAAAGCCCAGAACTATTTTTCTAAT

GCAAATAAGTTACCTAGCACTTTCGGTAGTTCACCTCCACTGTCTAAGCCCCTGCCAAGA

CAGTCAGTTACACATGTAGGGGTCTCCCATAAAACCACACCCATCTTCACCTCAAGATAC

TCAGGCACCAATGGCTGTCCAGATAGGGGGCAGAGGTCACTAGCCAACAGAGTAGTGCTT

GGCCCCATCAATCAGAGTACAAGACTCAAAACCAGGAACTTAGGATTGTTCTXX

>Unigene45849_C-W 1 224 LEN=224; minus strand

GTGAAAAGTGCAAAACTGAGGTGCACCAGTGAGTCGCCGCCGGGTTCGGAGCTAGGTCCT

CCCGCCAGGTCCGTCCAGGAACTGAACCACGAGCCCCAGAAGTCAGTGCGCCGGCAGCGA

CGGGAGCGCTCGGCTGCTGTGTCTCTGGAGTTAGGCCTGCGCAGCGGCGGCGAGTCGCAC

ACTCGGGCAGACTACAAGTTCCGGGAAGCAGCGCGGCGCAACCXX

>Unigene45862_C-W 1 201 LEN=201

XXGTCTGTCTATGGCTCCCTTTGGGACACGCCCACGTCTGCATTGGTAAATGTAAACAGT

GACGTTTGGGACGCAGTCACCTGTGCTCTGGTGGTTGGCTACGACTCCCTTTGGACACTT

ACACACCTGTACTGGTATTATCTACACTTACGTTTGGGACACAGTCACATCTACATCTTG

ACGTATCTTCAGTGGGCTTGGGAX

>Unigene45963_C-W 1 385 LEN=385

XXGCCCCACAGCAACAAACATCTGTCACTGGCCGTGGACAACATCCTACTTGCCTTCCTG

GACAGGGGCATTGTGACAATGGCTCTGTCCTTCAGCACACGCCACCCTGTCCAGGAAGCA

GCAGTTCCTGGGAAAGACGCCTTCGGCAACACCCTCAGCCAACATGGGCAGCACCCTCCT

CCACTGCACGCAAGACCCAGGGGCTCTCGGGGCCAGCGTGAGAACAGAGTAAGAGCCCAC

AAGTCCACTGGCCCAAGTAAGCCACCAGGGGACTGGTGGGGGCAGGAGAAGAAACCCCTT

CCTCACAACAGAAGTCCCACTAACGAAGGCGGAGGGGGCTGTGAGCCCGGGAGGGAGCGG

CACGGCGACTGCCCCAGACAGACGGCA

>Unigene46009_C-W 1 216 LEN=276; minus strand

XGCCGCGCTGAGAATGAGCCCCGTGCGGTTGGTGCGCGCAAGCACGCACTGCCTGCGTTA

CTAGGAGAGATGACGGACGACGCCCACGCCACACGCCGCTCAGCTCCCCCGTCCAAGCGC

CGGGATCGCTTCTTGGCGGATAAGCCAGTGACTGGCGGGAAGGTCTACGTGCAGGCGCAC

GCGGCACTCTCTGCCGGTGGTCCGGGGACTTTCCCC

>Unigene46047_C-W 1 217 LEN=217; minus strand

XXCGGGGGTGATGAGCAGGAGGAGGCTGTAGAGGACATGGAAGAGCAGGACGGGTGGCAT

GTCAATGCCAAGACAAGAGTTGGCAATGGTAGGGGACCTGCCCACCAGGCCCTCGTTCCT

CAGGGCCAGTGGGGGCTCCTAGCCCTGGAGACTGTCCTCAGCACACCCGAGGTGGTCATC

TTCAGCCACAGAAACGCACAGCTGGTCAAGAGACAGAAG

>Unigene46097_C-W 1 229 LEN=229; minus strand

XXCACCTGGCAGGGAAGTGGCAGAGCTAGAATTAGCAACCAAAATATCCTGACCTCTAGT

TCAGAGCTCAACACCACCAACACCTCATTGGCTCCTCAGAGAAAGAGTGGCACTGGGCAG

CTTGTGCCCCGCATACCAGCAACACTGGGGTTTGATCTCCTCCAGCTGTATTTGTTCCCA

AGAAGTCCACTGAGCAGTCAGAGTTATAGACTGCAGGAAGAAGAGAGTGAC

>Unigene46199_C-W 1 215 LEN=215; minus strand

XXCTGGGAAAGGGAACAGGGGCAGAGATACAGCACTACAGTCACACACTCCAATGGGACC

AATAAGGAAAAAAAGAATGATGACTGTGTGGGTCAAATCCTGGTTCCTTGGAAGAGAAGA

CAGGGGGAGAAGGTCTGGGATGGAGACAGGCTTTTTGGGGTGACAATGGTAGGGAGTCTG

GGCCATGACAGAGCATGGGATGTGGGTTACTCACTCGXX

>Unigene46210_C-W 1 260 LEN=260; minus strand

XXCCCATTTTCTCGACTGCCAGAACTTCATCCACCACCGACCTTCGATTGGAGGGGTCCC

GAAAAGAAAAGCCTTGGCCAAGGTCTCCCACGGCTCCAGCTCCGTCCAGGAAGAGAGCTT

CGACTGTCAGGATTCGGTGATGACCAATCTCTGCAGCTCGAACCTGCCACTAAGGCACTC

CTGATTTTTGGCTCTAGCGGGGACGCCCCTTTTGCCAATAAATCAGTTTCCTCCTTCTCT

CTTATACTACGGTTGCGTCTCCXX

>Unigene46319_C-W 1 209 LEN=209; minus strand

XXTGGAGCAGTAGACTTCTTGGTAAACAGAACTGCATATGCAAAAGGTGAGATGAAGAAG

AACAGAGAAGGTCATGAGATGAGGCTGGAGAGGAGAGCCGTGTTAAGACTTGGAACTGAG

GGATGGCGATATAGCCCAGTTGGTGGAGTGCTTGACTTGCATGCACAAGGGTTCAATCTC

CAGCACCACACATCATCATCATCATCATCATXX

>Unigene46367_C-W 1 206 LEN=206; minus strand

XXAAGAAAGAAAGAAAGAAAGAAAGCAAGCTGGGGATGTAGTTCAGTGGCAAAGCACCCT

GGGTTCAATACCCAGTCCCAAACCAAAAAACAAACAAAAAACGAAGGCACAGAAGAGGAA

GGGAAGAAGAGGAGGGAGGAGGAAGTGGGGAAACCAGAGATGGCCCCTGAGAGACAGATG

CAGAAGGAAAAACGACACGGGAGAGGCCXX

>Unigene46452_C-W 1 100 LEN=306; minus strand

XXCACTGGCAGCATCCTCAGCAATCTTGACTGGAATGCCATTGAAGATATGGTGGCCAGT

GTGGAGGAAAAGAGCCTGTCCGTCCGCTGGGCCATGGACCTG

>Unigene46540_C-W 1 225 LEN=225

XGGCAGCTTGCAGAAAAGGTTTCCCTCTTTCAGGGACATTGGTCTAATGTGATTCTTGTG

ATGGACTTATTCAGCCTCTGTGTTACCTGGCCCAAATACCTACTGCACATTGCTGTTTTG

TGTGATAACAAGGGCTTTATTGATAAAGCCACCTTTAATTTAGTAGACATTTGCTCTGTT

CTCATCCCACTCCTAACAGTGATCAGAACACAGAGATTAAAGGTGGXX

>Unigene46772_C-W 1 414 LEN=414

ATCTCAGCAGAACGTGTGGGGACTCGGTGGGTTTCTTGTACGTTTGATTCTCCACAGGTT

GGAACCCATCGCTGCTCTTTGGCGGTCTCTGCCCTTTGGAGCGTTTGTGAGCTGTACAAT

TCGGGTCCCGCCATGTCTGAGGAAAGCAAGTGCTGTGGTGAGCGCGCAGACAAGCCCGAA

ATCAACCGGGAGGACGGTGCTGGGGACACAAACAAGGATGCAGAAGAGAGTAAAGATCTC

ATTTCCACAATGCAAAGTTCACCAGATAAAGCTAGGACTCCACAAGACTCTGTGGATCCA

CCATCTCCTGATGCGGATAGTTCACAAAAAGAACCTACAAGTGXCAACACTTTAAGACGT

TTTATCACACTGCAAGAATTAGATGAAATTCTTGTCTTCTTTACTTTGTCTTTAAXX

>Unigene46800_C-W 1 293 LEN=293; minus strand

XXCTACTTCCTGCATATGCTACGGAGACCTCATAATGGGCTTGCTGGTTTAGAGCAGCTA

TTAATTAAGACGCAGTGTAGCTTTAGGAATGGGAGTCAAACCTTCAATCTGAAAGCACTG

CTTAGAGTCAACCCTGXAGAGAAGGCAGGCCAGGTGGTCACGGCCAAAGAAGACGGGCAC

TTACCCAACAGCAGCATCCAGCACCACGGTGACTGGGACGTTGAGGTGAGAGAGCGCTTT

GGCCATTTTCTTACTAAAGATGACATTTTGAGAAAATTAGGGAAAGTCCAAGAAAGX

>Unigene46895_C-W 1 268 LEN=268; minus strand

XXGTCTTCCTCATCTCTGGCCCAGAACAATGGAGCCAGGGTTCCATGGACTGAGATGAGG

AAGACAGATCATTATGGCAGAAGGGAGTGTCAGAGGAAAGTAGCTCAAGATAGCACACCA

GAAAGCAGAGAGAAAGCTTCACTCTATCCTTCTCTAGCATATTTTGTACTCACTGTGGAT

AGAATAACACATGATTGTCCAAACTTTAACTCCTTGAGAGTAAAAGGAGGTGGTATTGTT

GATTACTATGGGACTCCAGTTAAAAACCAG

>Unigene47067_C-W 1 295 LEN=295

XGTGAACAACAGAGTGAGCCAGAGGATGAGAGAGGATCTGAGAATGAAGTTTTATTTAAG

ATGGTCAAGGAAGGCATCTTTGATAAGGTCCTATTTGAGGATGCCTTACAGAAAATAAGT

TTTGAGAGAATTCCAGGTCTAGCAACCAGCAAGTGCAAAGGACTTGAGGCAGAAGAGTTG

GACTTGGGAAAAGGAACAGCAAGTAAGTATGTGTAGTTGGAATGGCACCAGTTAGTAAAA

AATGGCAGAGAAGAGCCCAAGGAAGGGGCACAGGGCAGACCATGTGCAGGCAGCAXX

>Unigene47293_C-W 1 242 LEN=242

XCTGGGGATGTGGCTCAGTGGTCGAATGTCTCCAAGTTCAATCCCCAGGACCAAAAAAGA

AAAACAAAAGAAAGAAAGAAAATACCTGGCATGTCCTGTGAGAGAACAACCACGCACAGC

CACTCCTGCCCAGTTGCTGGAGCATCATTGGCTGAGGGAGACCAGGTCATATTCCTCGTA

ATAGGCAATGAGCGGCATGGCCAGGCTTCGGATGCCCCTGGAGGCAGAGGGTCTAGTTTC

CAG

>Unigene47371_C-W 1 309 LEN=309

XXGAAGAAGAAGAAGAAAGAAAGAAAAAGAAGAGGAAAAGATTGTGATCATGTGTGCCGG

TGCCCTAGGAAGCTAACTGGGATGAATGGCAGTTCTAAAGGTGAAATTCTTTCTTCAAGT

CACTTCCAAATGGACTCCCCAGGAGATCAGGTTTTTCAAAACCTTCTGGACAGCTTTCTG

GTCAGCTGCTCTGATGATACTGTGCTGATAAGACCCCCAAACCAGGAAGGAACAGATACA

GGGGAGGTGATCAGGCCCAGGAAACTGCCAGTGTGCACCTCAGGGAAGACATTGGGAACC

CTATAGCCAGAX

>Unigene47809_C-W 1 280 LEN=280

XXGCTGTCCAGTAGCTTGAAGCCTGGCTACAAAGAAAGGAATTAATGAGACCTAAATATA

CAGGGTGGATATTTACCTTTAGGGGCACCAAATGCAGGGATGGTAAAGACAGCACAGGAA

ACAGATGATATCATGCTGGAAACCAAAATATTGTGGAGTGGGATGAAAAACCCAAATGCC

ATGATTTCAACAAAGCAGTGGAAACAGAGGACAGAGTGTATGTGTGTGGCAAATGGGATT

GTGGAACTCAGATCTGGCAAATGGGATTGTGGAACTCAGATC

>Unigene47820_C-W 1 205 LEN=205; minus strand

XTCGAACCCAGTGCTTCATGCATGCAAGGCAAGCGCTCAACCAGTAAGGTACAACCCCAG

CCCCAAAGCATGACAGTATTTTATGCCCTTATTGCTCCTTGGAATGTGAACTATATGAGA

TGCAAGATAAATTCAACTACGGAAAAACCCAGCTCTGAAGATGCCAAAGCACATCAGCAA

TTCAAGCCTCAGGGGCCCCAGGTCCTX

>Unigene47916_C-W 1 255 LEN=255

XGAGAAAAGCAGAGCTGTCATAAGGTGCCAGTATTAGATGCACAAATCCGGGGAGTGGGG

AGCGCCCTGCATTCAAATGCTTACATAAGCCCAGAAACCTTGGCTTACTCAGAAAGTCAA

ACCGGAGAGCCCGCTTGCATTTGCAAACACTGCCCCCCTGGAGCAAAAGGTAAACACACA

AATGCCCCCCTAAGGAACGCTAAGTTAGCTGCAGAGCAAGAAGAGAAAAGGCTCCATGCA

ATTAAGAAACTGAAAGXX

>Unigene48497_C-W 1 269 LEN=269

GGCAAGTGCTCTACCACTGAGCCTGGAAAAGGGTTTGCGCTTTTGCCATTTGGTTATCAG

GTTGGTTTCTTCTTGATATTTGTGAGGAAGGACTGGGAGAAACGAAGGGGAAGGGTGTCA

GAGGGATTAGTACCAGCAACAGTTGCTGCTATACATACCACCCAGATGCAAGTCGATGAG

GTCACTGTAATAAGACTCTCCCCAATAGTCTACAACAAGGAATTGGTGAAGAGAGAGTTA

TTTGACCAGATGACCTTGTTGTCCCCCACX

>Unigene48758_C-W 1 57 LEN=221

GGCGCCCTCTGCGGGGATGAGGTCATCGCCACCTTTGACTCGGTGATCCCAGATAAC

>Unigene48865_C-W 1 211 LEN=211; minus strand

XCTGTGATTCATGTATTCATTCATCGTTTTCTACCATTTGTGTCGGGCACTGCTCCATGC

CAAGAACTTCTCCAGGCATTAGACAAAGGCGTCACCGTCACGGAGTATACAGTCCAGCAT

TCTGGAGGCTGGAAGTCTAAGATCAAGGTGCTGCTAGATCCCACGCTGGGTAAAGGGTTT

GCAGATGGCCACTGTCTCTTTGTAGTTTCATCX

>Unigene48876_C-W 1 229 LEN=229

XGCAGTGTAAAGGTGAAGCAATGGGACAGAGGGGAAGAACATCTGGAGACCGCCACCCAT

GCTCTTTCTTACATGAAAGATCCAAAGATGGCCAGGAGGCAGAATGGCATTGCCCAGGAC

ACAGTTTTGACCACATCAGATCACAGTTTTTTCAGCCAGAAGCTTCCTTCGGGGAACAAA

GTACATAAGAACTTTGCTTTGAAGTCAGACAGAGACGAGTTCAAATTCTGX

>Unigene48953_C-W 1 272 LEN=272

XTGACTATAAGTGACCCAGGGCTTCAGATGACATGGAGAAGAGCAAGCACTCCTGTGGCC

CAGAAAGGTACAAGATGGTGGTATTTGAATTGGGCCTTGACAGGAGACTCAGGCTTGGTC

TCAGAACTGTACCCTGATGGCGTGAACAAAGTGAGCCAGGCTGCGAGGGAATGCAGTAGT

CAGTTTCTGTTAGAGTGGATGAAGTTGTACCCAATATCGGCTCTAACTAAGTCTACTTCT

TCTGATATTTGTACTCATGAGGGTTTCAGCCTG

>Unigene49011_C-W 1 262 LEN=262

XGTTTTAAGAACACAGGGTTTCATCCTTCTTTCAGGAACCTGCTTCTGCAAGATGCAGAG

ACAATCTATTTCTTTGGTTATTACTGCCTTATTTTAATTGAATCCACTTTGTCAGGACAT

GAGGCAAAACTCTTCGCAGCTAACCTTCTTATTACTGACTCCTTCTCCTACGCATGTGTG

TTTGGCTTTGTAGAAAAGAGACAAGATCCCAGGCCTGGTGGCAGGCCTGTAATCCCAGAA

TCTCAGCGACTTGGGAGGCTGAGX

>Unigene49102_C-W 1 90 LEN=241; minus strand

GTGACCACCCGACAGGTGCGCACCATTGTGGAAGAGGTCCAGGATGGAAAGGTCATCTCC

TCCCGCGAGCAGGTCCACCAGACCACCCGC

>Unigene49141_C-W 1 219 LEN=219; minus strand

CCCGGGGAGAGAATGCTTCCAATCCAACAATCCACACATTCCTACACAAAACCAATCACT

GGCATATTTAAAAGCTTGATCTGTGGGGAGAGACAGGCTGGAACTAGCAGGAACAAGCCT

GTGCAAGGTGAATTTATCCTCCACTCCCTCTACTGCAGGTCACCTCAGGATATGGCCCTG

GGTAATCGCCAAGATGGAAAGAATGGATGGATGCCTTGT

>Unigene49269_C-W 1 284 LEN=284; minus strand

XXCTTAATATTTAAAAGTAAAATGGCAAAAAATGTCGTTAXAGCAGAACAAGAAGATCAA

GTAAAAATTAAAAGCAAAAATATCAAAATTAGGAGAGAAGAAACAGAAATAGAGGATCAG

TTCAGCAGCTCTCATATCAAACTAATGAGAGTTCTGGAAAGAGACTACAGAGAAAAGCAA

AGGACAGGGGAGATTATCAAAGAAGTAATACAAGATTTTTCCCCAGAACTGGAGGACTTT

AATTTGAGGATTAGCCAGGAGAATGAGGAAAAAAGACCACAAAAGACX

>Unigene49381_C-W 1 319 LEN=319; minus strand

XXCAAATTCTGCCTTCAGGAACTAGCAAAAGAACAAAATGAAGGCAAGTTAGCAGAAGAA

AGGAACAGAAACAGAGAAAAGAAAAAACAGGGGAAAAACGCCAGTAAACTCGAGGTGGAT

CTTGGAAAACAGAATTTGACAAACCCTGAAGCCACCTAGGGAATGGAGGATGGCTGGAAA

TCAGAGGTGAATGTGAGGACACCCCAGCAGATGCTCCATAATGCTCATGGGGAGCATTAT

GGACAATTAAATCCCAGCAAATTAGACAGCAGAGAGGAAACAGACGAGTCCCCAGAAAAA

TGCAACCTGCTAAATCTCCAX

>Unigene49477_C-W 1 398 LEN=398; minus strand

XXAAACCGCCATGCCGGGTCAGCTGGCAGAGCTCAGGAAGGTAGTGCATCCCGGGCCCTG

GGTGGATCTACTGAGGCCTCTCAGCGGAATCAGGATCCCCGTGGGGTGAGCACCTCAGGG

TCGGGAGACAGGAAAGCAACTGGCCCCCTCCTTCTGAGGCAGATTGTGCCACTAACTTGT

CCAGGGGCCTGTGCCTACCCTCTGGGCTTCCGACACAAACGATCCTTATGTCAATATCCT

TATGTCCATTCCATCTTACTGGAATATTTTGTAGGCACCCTCTTTGGTGTGATGGAAATC

TGGGCCTTCACCATCCTCGAAGCCTTTGGTATCAACAGGGTTCTTTGGTTAGCAGCAAAG

CACAGTCTGACTTCTATTTAAATATCCACTCCTGCTCCCCXX

>Unigene49507_C-W 1 264 LEN=264

GGAAGAAGAGGGGAAAAGACCCTGAGGGGCCGGGGCTGTATTTCTAGCTCACAGAAGGGG

CATTTGCTCCGCATGTGCGAGGCCCTGGGTTCCAGCCTCGGCCCCACCAAACAAGGAAAA

AAAGAAAAAAAGGAACACGGCATAGATATCATTGAGCACAACGTTCATCCTGGCTTCTTT

GGAACATTTGTTACTCACACCCTATCTGTGGAGAACCCTTTCTTCACACCTAAGGGAGAC

GCCATGGTTGGCTATCCAAATGGG

>Unigene49510_C-W 1 202 LEN=202

XXGGGGCATGAAGGACAGGAACTCAGAGACTCCAGGAGGGAGAGGAAAGGTAGATATTCT

TCATCCATAGAGAGGGGACAGTGGATCACGCAGTACTGCCAGTCCCAGGCCAGCTTTCTG

CTCAACCTGCACAAGTCTGCTTTGGGATCATCATGTGAAAAGCCTGAAGCCTGTGTCCCC

AAATCCCAGTTCTCACGGACGCAG

>Unigene49518_C-W 1 262 LEN=262; minus strand

XGAAGAGGAGACACTATGGACCTAGTACATTCAACAGTTCAATTCTCAGTCTTCAAGTAT

AAGAACTACATTACCAGAGAATGTACTAGACATTTGGAGAAACAACAAAGAAAGACCCAG

TTGCTGTCCTCAAGGAGGATACAGACTGGTAAAAGAGTTCTAAAAGCCATAATCCAGGAA

TGCCAAGAATTATTATCTCAGGAGGATAACTTCTTAAATTCTGCATTTGTCTTCACTGTG

CCCTGCTTTGATGGACAACACCCX

>Unigene49601_C-W 1 226 LEN=226; minus strand

CAGGGGATGTGGAAAAGGGAAGTACAGTCAGCAGTGAAGGAAGATGACACAGTGTGGCAC

CAAGTGATGAGCAGGACAGCACAACAGCTAGGGAGCAGAAGGGCAGAGGTAGCCAGAAGC

AGTGACATTAAAGCAGACACACAAGAAGAACCAATGAGTGCTTATGTGTGGACAAAACCA

GTCACAAACCATAATTTAAAACACAGAAAAGGAAGATCTGAGAAGCXX

>Unigene49890_C-W 1 209 LEN=209

GGACGCCATGCCACAAATCACGCCTATGGAGAAGCCCAGGTGGCAAAGAATTGAGGTCTC

TTAACAGTCATGAGAATGAACTTGGAAACAGATCTTCTGAGTGGGCATGGAGTGAGCACA

GAAGTAAATCCTCTCCTGTTGAGCCTCAAGATGACAGCAGCACTGGTTAGCACATTGCTT

ACTGCCTCAAGAGAGATCCTGAACCCCACX

>Unigene49916_C-W 1 208 LEN=208; minus strand

XXAGTCTTTGAAAAATCTACAGTTGATACTTCTGGTACCATCCAGCCCATGACCACACCC

ATGCAGCTACCTTTTGCTGCAGCTGACTGATGTTGCCACCTGGGAATGGGACAACTGGAG

GTCCTGCTGGAACCAACACCCAGGGAGCTGTCCTACTCCTCTACTCCTCGTTCTACAATT

CCATTGGAGATTGGATGGACACAACTCTAC

>Unigene49956_C-W 1 351 LEN=351; minus strand

XXCTCTGGGAACACACGGCGGAAGGAGGACATTCTGGTTGGTGGAACAACTGAGTGGGCC

AGGAATGCTGGACATCCTGCCTGTCACGGGATCAGCCTAGCCCAGGGTCATGGTGACCTT

GAAAGCCAGGATCTGCTGACACTCCGTTTGGGGCCTTTTCTGGACATTCTGCCATTTCGT

GCTTTCAAGGATTGCCTGGGTGCTATGCAACCTGTTTGCTGTTCATTTGCTCGGGCTTAC

GAGTGCCTGAGGTGGAAAAGCAATCCACCAAGATCCTCGCTGAGGACATTCCACTGAGTG

CAGACTGTAGCTCCTAGTTATCACTCTACAGCTACCAAAATTTTCAACTATCCX

>Unigene50054_C-W 1 261 LEN=261

XGCATGTTTGACTTGTTTTAAGAACCTATTAAAAGGATGGTAAAAGAAGAAGAAACTATC

AAAACTGAACAGGTACATGTGAAAATGAATATAATTTCTAGAAATGGAAAAACACGTAAT

GATATTAGAAACCTCAGTGGATTAGACACAGCCGAAGAAAGCATTAATGAGCTGGAAGAA

ATCACCCGGAATGCAGTATATAGCAAGCTACAGAGTAAAAAATGAGAAAGAAAAATTAAA

ACAGACATCATTCTAACAATATXX

>Unigene50132_C-W 1 208 LEN=208

XXAAAAGAAAGAAAGAAAGAAAAGAATAAGAAGATCCCCAAAGTGTTAGTAACCCATCAC

AAATTAATATCTGCTTATTCCAAGAATGATACTGAAACAATCTCTGGAGAGAATAGCCAA

GATAAAGATGTCCCAGTTATTCTAAAACATAGAGATGCAATATTCCCAGCAGTAGCCATC

TTGTTCTCCTACTGTATGGAGTGGATTGTG

>Unigene50229_C-W 1 381 LEN=381; minus strand

CCCAAGCCTGCTCTCCTATGTTCTCCTGCTCTGCAGCTCTATGGACGGAAACCCAGAGGG

CAGATGCAGGAGATAAAGGAAGAAAAAAACTTGTTACTCTCTTCCAAGATCCAGAGGATT

GCTGCTGGCCTCAGGAAATAGGACAGGAAGGTTCCTCCCTTCCACCCAGCCACTAATAAC

CCCAGCTCACCAAGGAATGGAAACAAATTAAAGGACTTCAACCTCCAGGTTGGTGATTCT

TCCAGAAGAAGTGGGCAGAGGAGTACCCTTGAAAAGCACCTGCTGTACCTGGGCCCTATC

ATGTGCCCTGCAAAACAGACAAGACTCACTAACTTGGTACCCAACTTGATCAAGGTCACT

CAACAAGTAAACAAACTAGGC

>Unigene50342_C-W 1 204 LEN=204

XGCGGCAGCATGCTCCAAGGAGCACAAGTGTTTCACAAGGATCTGGGGGGCACAAGGGTC

ATTGCCCAGAAACAAGGTTGGAGCAGGACAACGGACAAGTTCAAGGACTGGTCAGTAAAC

ATGACACAACTGCATGCACTCAGCACAGGAAAACAAGCCTCTCACAAGCTAGCATTAATC

ACTGCCCATCTCATCAGGATGCCTGXX

>Unigene50361_C-W 1 210 LEN=210; minus strand

XAAAAAAGTGATTTTGGAGATGTGATTAAGGTTAAGGACCTTGATGGAGATXTAACCCTG

GATCACCTGAGTGGGCTCAAGGTGATCACCAGTCCTGACCTGCAAAAGATCCTGCCTGAC

CTTACTGGTTGGAGAATGAAGGAAGTGGCCTCTAAGAACTGGGAACAGCCCTCAGTTTAC

AGCCAGCAAGACTATGGGGACCTCAGTCCTACX

>Unigene50404_C-W 1 249 LEN=249

XXAAAAACCCCAAACCAACAACCAATCCTCAAAGAGAAATAACCAGAGAGGGACCTGAGG

TCCTACTCTTGGACATCTTCTCATACATCAAAAACAGAGAAATCCCAGAATAAAAAAGAT

AGCTACACACAAAAGGCCATGCACATAAAAGAGGAAGGGGAATCCATCTCAATTCTGTTC

AAGTCTAAGACTTCTGAAAACATCAGGAAACAGGCAAACAAATCTCCCCCCAAATTCACA

GTCCCCCAGCAX

>Unigene50431_C-W 1 226 LEN=226

XXCGGGCAGAGGGGGTGTTCTGCGGACTCCGGAGCCAGCTGGGGCACGCCACTCTCCAAC

AAACGCGCAAACTCTCCCAACCGGACAAATCACGGTCGCATGGCCCTTGGTACGGGGGCC

GAGATCCGGGACACCCTTGAGGGGAGCCCGACAGCCGCCACGCCCTCGGAAAGAACCCCG

ATTTTGTTCTCGCACGCAGACTTCTACCGCAGGAAGCCTCGGTTCTCG

>Unigene50663_C-W 1 213 LEN=213

XGGGCCGTGTTCTTATTATCCCAGCAGGGAGCATCCCTGCTTCTAGGATCAGTGATGCGA

TTGTTGCAGTGCCCAGTTCCTCCAGGGTTGTTGGCCTGAGGGCTTCAGTTCCTCACCTGC

TGTGGACCAGAAGATGCTCTCAGTTTGTCTCCACCTGGGCCTCTTTATACCATTGCTCAG

AACATGGCAGATAGTCTCCATCAGAGAAATCAAGXX

>Unigene50720_C-W 1 213 LEN=213; minus strand

XTCCCCATCAAACCTTGCCTGACCTGGGTGTTAGTTTCCCTTCTCTCAATAGTTGGTGTC

TGGTGTGGAGAAAACCAAACCAATCTGATACGGGAATTTACAAGCCTTTCTGGATTTATA

GGTGGACAGATTGGCCCAGCCACCTTCTTTAATCCTGCAGACTTCCCAGACAACCAGACA

AAGGATGTCTGGGATCCATCATCGCTGATCCCCCXX

>Unigene50809_C-W 1 92 LEN=322; minus strand

XCAGATGAGCCAGATGGAAGCAGCTACACAGATGTGGTCCACGTGGAGGGGACAGAAGAG

GAAGTTAAGAATGAAACAATGGAGGATACGCAG

>Unigene50906_C-W 1 292 LEN=343; minus strand

CTGGTGTACATATTAGTAAAGTTTAGTCGTCTGTTATTCACTAATCACAACGTGCACCTT

GTTAACATAAAAGGCCAAGCTATTTCCAGCAACATCACTATTTGCACTATTGAGCAGCAA

CTGTTCAGCATGCAAGTGCCTAAAGTCCATGCCAAAGGAGGAGGGAAGGCAAAACAACCA

GTAGAAGCAGCACACGCTGATGTGCACATGCAGGAGGTACAGTACAGTTCAGAAAGGAAA

GGAAAGAAAACGGGGAGAACCAAAGGAAACAATAACTGCTTTGTGATGACG

>Unigene50956_C-W 1 243 LEN=243; minus strand

AATAAGAGCCTTGATGGACAGAAACTAGATGGAGGCTATGGTTATAAAGGCAGGTCCACC

AAACTCCAGGGCCAGGAGAGGGGTTATGAGGATGCAGTTCAACATTTAGATTCTTCCAAT

GTGTTAGATAAAACAGAAGTTTCCAGTCTCCAGTCAGAGGGCCTTTTGGAAGACTGCCTT

ACTCTATTTTCTGTTGCTAAAACTGAATACTACAGATTTGGTGATTTAAAAAGAAAAAAG

TTT

>Unigene51047_C-W 1 303 LEN=303; minus strand

XXAGGAAATCTAAATAAAAGGAAGGAAGCAATAAAGATAAGAGCAGAAATTAATGAAATA

GCAAATTCAGAAACATCAAAAGGAAGTTGGTTCTTTGAAAAGACTGGTAAAATTAGCAAA

GCTCTAATGACATGGATCAAAAATGTGGAAGAAAAATAACAAGAGAGACAAGGAACAGGA

AAGGAATGGAGACACACTACAGATTCTGCAGTCAGTATGCCAACAACACGAGGTTATCTT

GAAAACTTCATTCCAAAACTTTGGAAAACTCGGATGAATAAATTCCTGGGAAAAGACAAC

TTACCX

>Unigene51097_C-W 1 207 LEN=207

GCCCCTTTTTCTTCCATTATAAGTGTAAATTCCAACCCTGTACCAATTCATGTATTGAAA

AGAACTCCAATCAGAAAAGCTGAATCACTGCACACCACTCCAGTGCCCACTATTGTCAAC

AGTAACCCAGATTCTCCAAACTTCCTTAAGATCCTTAAGGAAGATCTCTACCACAAATCT

TCTCAGTGCATTTTGCGTCAGTTTTTA

>Unigene51107_C-W 1 207 LEN=207; minus strand

ACTTTCCCAGAGGCCTGTGAGTGCTCGTCTGCCAGCCGTAGACCGTGGACAACTCACACT

CAGATTCCTGCCCCTGGATCAGCTGAGAACTTAAGCTGCTTACCCATGACCTGCTTACCC

AGGTTTGGTGTAGAGATTACTGATGAAGAGTTTGAGTTGCTACTTGACAGAATCCCCCTT

GATGAAGATGGAAATGTCAGATACCCC

>Unigene51117_C-W 1 257 LEN=257; minus strand

ATCTACACTCTGTTTCTGGAGCATGATCTCAACCTGGGAACCCTGGCCTTGGAGACAGTA

GCCCAGCAGAAAAGAGATTACTACCAGGAAGGGGGTGGGCACTCTGATGTCCTTTGTGTG

AGATTCCCTCCATTCCCAACAATTTGGGAGGAAGTCACTGCTACCATCATCATTGCCATT

ATGCTGATGAGGAAGCTGAGGCCCAGGGATGCTCAGGAACTTGCTCAAGGTTACCCAGCA

GAGATTCAAACCAGGCXX

>Unigene51131_C-W 1 243 LEN=243; minus strand

XAGCTTCCCATTTCTGCTTTACGAATCCAGCGGGCATCAGCAGCTCATTCCCTGGAGGCC

CCTCATCTCTGTCCTGCTATGGTTGATCATTCCATGGCCATCCAATGTGGCTCAACTCAG

CTGCCCAAGAAAGGAGGGACCTTGATACAAATGGATGAGGAAGGGCTTCAGGATGTGGTT

AATGCAGGAGCTGAAGAGGGGCTCCAGAGAGCCTGTCTCAGTGGGGCCAGGACATAGCCT

GATCXX

>Unigene51174_C-W 1 241 LEN=241

GTGGGAATGAGAGGACAGCGGTACACTGGCAAGTACGTTTTATAGAGAGAGTGGAAACAG

GATGCAGTAAGAACCAGCCATGGACGGGGCTGGGAAGCTCTGGTCAGAGAAGGCCTTTCT

GAAGAGGTTAAGGACCTGGCTGACGGAGCTGCAGGTCTCTCTGCCCCTCATCACATCATC

AGGTAAACATCCAACAAACATGAACACCTGTTCAGCCTGAAGGACCTCCCAGGGACCACC

TXX

>Unigene51229_C-W 1 217 LEN=217; minus strand

XCAAGAAAGAATTCAAACAACCTTAAAGAGAAAGGCATCCTGGCCAACAACCATCAAGGA

AGCCAGGCTGTTAGTCCTATGACTACAAGAGACTGAATTCTGCCCATGACCTCAGTGGGC

CTGGAAGTGATGTCCACTCAGAGTCTGCAGATGAAGGCTCAGCTTGGCTCACAGCTTGAG

TCCCTCTTTATGATCCCTGGAACAGAGAATGGAGCGACX

>Unigene51236_C-W 1 206 LEN=206

XXAAAAAAAGCATATGAAAAGAAGTCACTCTATACGTTATGTAACCAAGTAAATGXCAAA

TTTGAAACAACAGTAAGTTATCATTATATACTTATTAAAATGGCCAAAATCGAGAACACT

GACAACATGGATGCAGATGAAGATATGGAGCAATTAGAACACTCAATCATTGGTGTAAAT

GAGAATGGTACCGGGATCTTAGAAGCCAGX

>Unigene51347_C-W 1 207 LEN=207

AAGAGAACTGCCATGACTCCTGGAGGATTTGTTAGCAACATCCTGAACTTCAGTGAAGGT

GGCATTCAGAAAGATGTTTGTCAGTTAATTGCTGCCTCAGAATTCCTACATTTTATTGAC

AAACCTTTACCAGAATTTGTGGCAAAAACTGTAAAGAAGGTTCTTGTTATGGTTTATGAG

CCTGTGTTCAATCTCCTGGCATTTTAX

>Unigene51350_C-W 1 200 LEN=200

XATAAAGATATTGTGTCTATCTACAGTAAAAAAGAAAAAGAAAGAACTTACCAAGTTGCC

ACTGATTGTGAAAAAGCAAGCCATCGTGTGGAGGAAATGTCAATACATGCTTCCAAAGAA

AAGAAGGCCAAXTATCGTGAACAGGCACAGATTTTGAACTGGTGTTTCACAGATATCCAG

TTGGCTTATACGTATTTTGATAXX

>Unigene51378_C-W 1 200 LEN=200

XXCAGGTTTAAGGGCAAGCTCATAAGTACAACCCTGGATATGACTTTGAAATATCTATGT

GGAGATATCCAGGAACTGACTGGAAGAATAGTTGTAGTACTTAGAAGAGAGGCTGGGGAT

AGACTCAGCTCTGATAATGCCTTGGATCCTGTTCAGACCATCCACACACACCCAAGAACA

GGACTCCAGGAATCAAAAGGTGXX

>Unigene51384_C-W 1 212 LEN=212

GGGGAGCCTGAGGCGGGAGCCCCCCAGTCCACAGCACCTACATTCTGCTTCATGGGGCTG

CTTGTTCCATCTTCTCATCTGGCTTTGAGAGGCTGCCCAGTGTTTGCAGCCAGGCAGAAG

GAAGCTCCTTTGAATAAGTCAGTGAATGGGGAAAAGGTGATTGTGTTCATCAATGTCACT

TATCCCAATCTTCCCTATGGATTTTGCACGCAX

>Unigene51406_C-W 1 202 LEN=202; minus strand

XCCAAGTTTGGGGAAGTCACCTACAACCTGACCTACTTACCCACTTCCATCACCATTACC

ACCACCACCATCACCACCACTACCATCATCCCACCACCACCACCACCTCCCACTGGTGAC

AGTTACCACCATCACTGTCACCACCAACAGCATTCCACTAGTACAATCACCACAATCACC

ACTATCCCACCACCACCACCACXX

>Unigene51430_C-W 1 235 LEN=235; minus strand

CCCAGAACTCCCTCTTCTAGTCTTCCGTTGTCGGGTGAAGATTGCACAAACACAGACAAG

GGTGACTCTTGGGGCAATAGTCACCTTTCACTGTCACAACAAGCATTACAATCTCACAAA

TCGTCTTTTGTCCAAACTTATGGCTTTTCTCCTCTCCCCAGGTCCCAGAGTACACTATCC

TACATCAACGCATACCCCTACACAATGGCTGGTCCTCAAGTCCCCAAGGGCACAGXX

>Unigene51455_C-W 1 206 LEN=206

XGGGGAAGTATGTACAATGATACTTTACACTTCATGTATTCAGGAATAAAGAAGCTAGAG

CAGTATTTAGTTGGAGTATCTCAATATGTCACATTATGGCAGGTGTACACATACATCTCC

ACCATGGGTTTCCGGTCAGAGAACGTGGTAATGCTGAATGCTCATGCACAAACCCTCCAC

CTGGAGGAGCTGCTTTGTGCAGAGGCT

>Unigene51523_C-W 1 236 LEN=236

XXGGGGAACATTTTATTTTTAAAACCAATGGGTCCCTGAAAAAAAGTTCAACTCAGGGGA

AAATGGATGGCTTACCACACCTTCATCAATAAAGAAGAAAGACTGAAAATAAAGAAGCTG

AAAACACGCCATGAGACCCGAGGGAAACAGGCCAATGAGGAGAGAACAGATGAAACGGGA

CTTGATCGATTGCCAAAATTAATAACAGAAAATAAGAGGAAGGAAAACACAAATAAACXX

>Unigene51560_C-W 1 236 LEN=236

GGAGACAGAAGCACAGGGTGGGTCTCTCCGAGCACCTTTCACGAGCTGTACACCCTCCAT

CTGAAGGCACAGCTCTTGGTTGTGGGTGCCACGGTCAGCTGGAAAATACCCCGGGAGAGC

AAACACAATGAAACTCCACACAGGGGATGTCGCCGTGGACAGGGCCCCGTGGACGTGGTT

CACTCGAACACAGCCGCTAGAACACGGCAGCCAAAGGATGGGAACAGCCCGGACGCX

>Unigene51572_C-W 1 224 LEN=224

AAAATCATGTTGCCCCTAAGGCTGGGAGCAGCTCAATTGGTAGAGTGTTTAACCTTGTAT

GCACAAGGCCCTGGGGTCAATCTTCAGCACTGGGGGAAAAAAAAAAAAGAAGAACTGGAT

GAATTGGAAGACGGTGGTAACAGAAAGGATGACTGGCCAATTTCTGCCTTGAACACCTGG

GTGGATGACAGGGCCATTCTCAGTGACCTGGCACTTGTGGCGGGX

>Unigene51584_C-W 1 204 LEN=204; minus strand

XGAAAACCTTTCCATCGGACACTTCAAGGCAGCAGTGAAGAGGAGCCCGTGGAGCAGGTC

TGCGTGGAGCCTCCTCCCAGGACTTTCCTCTGACTTCTGTTTGCCTGTGACATCCAGAAA

TGGAACGAGCCTGGTGTGGAACGCAGGTCTCAGGCTGACCTCCCCGCCCAGCAGGACAGT

CCAGGGGCCTCCATGCTGTTCTCCCXX

>Unigene51597_C-W 1 214 LEN=214

XATGAAGAAATAATTGAAGGAATCAATGAAGAAACAGATAGATTGCTTTTCCATGCTGTT

ATTGAATACTTTGAGAATATGGCCAAAGGGACTGGTAGTTCACCAGAAATACCATTAAAT

CTGTGGGTGAAGCATCATTATGCAAAAAGGAGATGTGGTAGAATTGCAGTCATTAGCCTG

TAATCCCAGTGGCTTGGAATGCTGAGGCAGGAGGAX

>Unigene51602_C-W 1 260 LEN=260; minus strand

XCTTACCCATCTGAAAACCACGCCTCTTGCCCAGCCCGGGACCACCTGGACTCCCCTTTT

TTCCCCACCATCACTTTCACCCAGATGGACCGGAATGGTGACTTGAAGACCTCAGAGCAC

ACAAGCTGTAATGCAGCTGGATTTGACAGTGAAATTGGCAACGGATGGGTTTTGGTAAAA

ACCCCACTCGCACCTATACAGGAATTCCATTTTGAGATTTATCCACAAATGGACATGCAC

ACCAAGAATGACGTTATCTCX

>Unigene51603_C-W 1 242 LEN=242

XXGGAGGATTTTGGATTTCAGATCTTTGGATTAGGGACCGCAACCTATAGCAGCGTAAGC

AGAAGTATACTTATAAAAATGGGCAAGGACCAGCTCATGAAAGGCCTTGAAGATAACATT

AAAGATCCTGAGCTTTATTCTGAAGATGATGGGGAGACACTGAAGGGTCTGACTCAGGAG

AGTAATGTGATCAGATTAGAAATTCGAATACTGGTCAGGGGGAAGATAAAGTCTAAAAGA

AGACXX

>Unigene51612_C-W 1 288 LEN=288

XGTCTTGGCAAAGCACAGACCCCTGTGGCTGACCTGCTTTCAACTTCTGAGATTCTCATT

TCTCTCTCATTGGGTGTGTGCGTCCTTGACTTTCAGCTTTCTCGCCCTCCAGTGGCTCCA

GATCATGTTTCTTTGAGAGCATCTTCAGCCTTCTGGTACCTTTCTTCTTCTGCTTGCTTC

CTGGGCTCCCCCATCCGGGCCTCCTGGGGGAAGTGCCTGTTTGCTCTGAGCTTAGGTCTG

AGCATTCATCCCAGCTCTGCTTGGCTCTGGATCTTGTTGGAAAAAGGGGXX

>Unigene51765_C-W 1 214 LEN=214; minus strand

TGTAAATTGGAGTATTGTTCCGTGTTCTATCGTATACCTTTTGACACATTGGGTTCACTT

GTGGAAATTTATCATAAGGAAATCATTACGGGTACATCACCACGATCTATTTACAGAACT

CTTTCATCATCACAAGCAGAAGAATCTCTGTACCCATTAAGCACTAACTCAGCATTCCCC

TGCTTTGCCAAGGTTATCTGCCACCCTTTTATCCXX

>Unigene51774_C-W 1 292 LEN=292

XXTAGATATACTTTTTATTGTTCTCAAAGAACTGGTAAGAGACAGCAATGTCAGGATTCC

ATGATTCCTGGAGGATTTGGTGGTAATATTCTGACTTCAAGTGATGAAGGCATCCAAAAA

GATCTGTGGCATTTAATGGTTGACTTGGATTTTCTCCATTATATAAACAGCCCTATGCCT

GACTTTATAGACAGGAAATGTGAAGAGGGCTCCAACTGTGGCCTGTGGGCCAGTGCTCAA

TCTCCTTATGGTTTAACTTCAAATATGATGACACACATGAATCCATACACCAAG

>Unigene51821_C-W 1 180 LEN=221

GGGGGGGACTTCTACAAACCCTTAGTGGTGGAGGCTGAGGTGAGAGGTTTGAAGCAGTTG

CAGCCACTGGATGCCCCAAGGGAGAGATCTTGGAGTGGCCTGAAGCCTGAAGAAGGACCT

ACTGCAGAGAAATATCCAGTCGTCCATGACATCGTTGAAATTATTGAACCAAGCGAAGCC

>Unigene51882_C-W 1 211 LEN=211; minus strand

XXCTGCTGGGGGCCTCGCCCTCAACGCTGCAGGGGGAGAGAAGTGTCCTGTGAGCGGGCA

TCGGGGACCCGGGCGCCACCTGGCCAGGCACCTGCTGCCCGCTCCCCGTCCCACGATGGC

GTCCCCACGCGCCTGAGGGACCCCGAGGAGACCCTGAGCAACACCATCCTGTGGATCCCG

GCCACCCACGGGCTTCATGTCCTCAGTCAGACC

>Unigene51952_C-W 1 220 LEN=220; minus strand

XCAATTGAGTATAGTTCTTGGAAGAAGACCAACGCACCGTCAACCTTTGAGAAGTCTGAT

CCCTCTCGAAAAACCATACTTAATGCAGAAAACAATGTTGGGAGTTACAGCGAGGAGCAT

TTAAGAAGTTTAGTCTACAGACATGAACATTACTACTCTGAGATGGATGTAACAGGTTAT

CTCAACCTCATGCCACTGAACATCTGATTCCTCCAACACCCX

>Unigene52039_C-W 1 208 LEN=208; minus strand

XCAGCAGAACAACGTGGAACACAAGAGGAGGAACGGCACTGTGTCAGGATGCAGAGTCTG

TCCGGGGATGCTGGAGTCGAGCTTGTCCCTGGTCCTGGGATTCTGGAGGGATGCCCTGAG

GAGTTTTCATCCCTGGTTGTCCAGGAGGAAAATCATGCCAGGCATTTTCACAGACGTCAG

GTTTTGGGGTCTGTGTGTGCATTTATTTAX

>Unigene52056_C-W 1 227 LEN=227; minus strand

CCGGAGCGGCCGGCGTCCCAGTTCGTGTGCGTTGGGCGCTACCGCGAGGCGGGGAAACGA

AAGGACAGCGGCTGTGCGGGTTTTAGGGCGGCGGCTGCCACAGCAGCAGCGCTCCGGGCG

GAGAGGGCCTTGGCGGCCGAGCAAGCTGGGGATGGGGAGAACCGGGTGCCGCACCTCTTT

CGGGTGCGAGTGCGACTCCGGAGTCGCACAGTGACGGCGACGCGCCCX

>Unigene52097_C-W 1 264 LEN=264

XGGATGCTGCATTTTTGTTCCATTCCTCAGAAATTAACTGATGGTAGCCAGGGGAAAGAG

CTCCACAACAAAATCCCAGAACAGGAAAGAGAACGATGCTTGGTGATTGAAACTGCCCCA

TCAGATGATCAGCCACAAACTGATCTGGTGGTTAGAAGGGCAAAGTTGACTGGCAGACCT

TCCATAGCAAATCTCCCAAGCTGTAGCAGCAGTTTCTTTCCATCATGCCTGACCATCAAA

AGGTCAGAATTCAGGGTCTCTCCTCXX

>Unigene52178_C-W 1 206 LEN=206

XXGGACCCAACTGCAGTAAGGAGGGGTCTTATCACCTACTACAGAACAAGAGGAGGTCAG

AGAATTTCTGTACAGCTAACTCTTAGACAAAAAGGCAAGGGAAGATTAGTTTCTGGGACC

CACCTTAGAGAAAGTTCCATTTTTATGGCCCGCCTTGTGGAGGGAGTCATGAGCCAGGAA

CCATGGAAGAAACCCAAAATAAATACAGXX

>Unigene52186_C-W 1 204 LEN=204; minus strand

TGGCTCAGAAATTCCTCCCTGGATATGACCCACATTTTCATGGGAAGACCCAAGACTTCA

GAGTCACATAATCTGTCTTCTGACAACTTCAAAGCGTGGTGGAAACATGAACTCATTCAC

TTGGTCCATATCTCAGAGTCAGGAGAGGACAGAAATAATCTTTCTTCCATTTTCCAGGTG

ATAGAAAAAGCCCAGAGAGGTTTT

>Unigene52217_C-W 1 282 LEN=282

GTGGAGGGATACGTTGATCAAGTATACACACCTAACCCTCAGGGAGACCGCCTCTCGCCT

CCTTTTGTAAGTGGGGACGGCCAGGCAGAGAAGATGGCTTTCTGCCAGCAAGCCCGGATG

CTGGAGGGCATGGTTGGCAAAGGCAGTGCCCAGAGCAGCACCGAGGGCCAGGGTAAAGGC

CGAGGACCCTGTAGACCATTGCTGTGCCCTCACTCCCACAGTGACAGCGAGAGCAAAACC

AGCATCGTGAAGGTGATCCGCTCTATTCTGCGGGAAAACTTX

>Unigene52269_C-W 1 114 LEN=221

ATTAATGATGATGAGGAACTGGGCCAGGTCATGGCAGTGGGAGGCTGTGATGGTCCTGTG

GAGGTCCAGGTCACTGGAGAAGAGGAGGCCATAGAACCCAGAGGTCAGCATGTG

>Unigene52275_C-W 1 149 LEN=276; minus strand

XTGGAGGTTTTCACCGAAAGAAGTGAAAGAGATATTCCTAACCAAATTTGCAGTTCCAGT

TCCTGTTTACATCGTTCCAGAGATTACCTCCATCACGATGCCAGAACTTCACCCAAAGTC

CATGCTTTGAAGGATGCTGAGATCAAAGCA

>Unigene52287_C-W 1 241 LEN=241

XGGCCTGAGGATGGGAAGTCTGGAGCCGCCGTGCATTCCAGCCAAGCGGACAGGGCCGAG

GGGGGAGACCAGGCAGCTGGCACGTGGGTGGTGCTGGGGAGCCACAGGAAGACAGAGCTG

ACAGCCAGGCATGAGGGTCTGACTGGATGCAGCATCCAACCACATAACCTACTGAGTAAG

AAGCAGCTGACCAGCATCTCGGAGGAGGGACGGCAGAAGAGTGGCCAGCCGGGAGGCATG

GCX

>Unigene52328_C-W 1 294 LEN=294

GTGGTTGCTCCAGAAGTGGAATATTATTCAGCTGTGAACAGGAATGAAGCACTGATACCT

CCTCCTGCTCGGATGAGCCTTGGAAGCTTGATGCTGAGTGAAAGAAGCCAGACACAGAGG

ACAAAACAGCATGATTCCACTTACATGTGGCCCCCGAAGAGGCAGAGACAGAAAGTAGAT

AGAGCTTACAGTGATAGATGGAGGGAGATGGAGCTGTTGTTTAACGGGCAAAGCGCTTCT

GTTTGGAATGATGAAAGTGTTCTGGAGATAGCACACTTAAAAGTGATTGAAATX

>Unigene52358_C-W 1 220 LEN=220; minus strand

XTGAAATTCCAATGTAGCGATGTGGCGGTGGAAAAATGGAAAGCTGGGAACTTCTTTCAC

AGCAGTGGCCAGTGTTCCAAAGTGGCCATTGGGGAAGGTAGTCCATTGTACTCACTGCTT

GACTTTTTTACCTGTGGGATGCGATGTTTCCTCAATGCTTTCTCCCTTGTGGTTGGTGGT

GCTTATACAGTGACTGAGAATACAAAGACCACCTGCCCCCCX

>Unigene52387_C-W 1 239 LEN=239

AAGAAAGATAGTGGGGGATTTAGACTCAGAGTAAATAAAAAGAAAATGATAGGAATAAAA

AGGGAAAGAAAAGAAATGAATGAATCAGGGAAAATGATATATGAGGAGTCTAAGTGAAGA

GAAGACTGTGGGGACCTGCACTTGGAGGTACAGAGACTAGAACTAGAGGAAGCCACTCTG

CCATGGTACAGCCAAGAGACCAGAGAATCTCAGAATGGACTCTGTGTCCTTAGAGCCAGX

>Unigene52639_C-W 1 272 LEN=272

XXTCCTGCCTTTGAGTCCTGCCATCTGGGGCTTTGCTGGGTTGGGAAAAATAATCCTGAG

AGACAAGCCAAAGCAAGTCTGGGAGCAGGGAGCTGGCCATATATAACCAAGATAGAGATG

GTAAAGGCTGGAAGTGATGATCCCATGGAGCCTGCAACCTTTCCACTCAGCCTAAGATTT

CTAACCGTGCCATCAGACTATGGAAGGAACCCGGAATCTCAGCCTCAAAGCACACCTCAA

CCAACAATTCTGCTGAGAAAGCTCCCAACTAGAGXX

>Unigene52769_C-W 1 265 LEN=265

XXGGGCAAATGGCAGGGTCCAGGGAGGGAGGCCAGATGGCAGCTGGAGGCGCAGGACAAG

AGGAGTCACGAGGGGGTGCAGGGCAAGTCCTCACAACAGGGCCCAGAAGACACCCAGGCC

AGGGCCACTTGCAGCAGGAGCCACCTGAACAGGGCCCAGGGTATCTCCAAGACGGCAGGC

GCCGAGTTGCAGAGCTGCCCTGAGCAGCAGTGCCTGACAATCCTGCCCCGCAGCTCAGGG

CTGGATGACCACTCGAACTCACTGTTG

>Unigene52795_C-W 1 266 LEN=266; minus strand

CGGGCTCTCCCGCCATGCAGATTAGTGGAGGAACAGCAGAACAACCACTCTGCTGTTTAT

GGAATTAAGAAGGGCCAATTTGGAGGGCCACCTGGCATCAGCCAGACATGTGAGCACTGG

GTGCCATCCATTTGTCGGAGAGAAAAGTGTCCTCACCCGTACTCTGCAGAATTTAGAGGA

AATGCAATGCTTTATAGCAGGATGGCTCACTGCTTTACCAACCCAGTGGCACTGGGCTCC

CAGGTCCCTGCAGTTCAGCCTCCATAX

>Unigene52952_C-W 1 194 LEN=205; minus strand

XCCAAGCCCCTTTCAAGGGCTTGGACCTACAAGGACACGATTATTTTCACAATTCTACTA

CAATATGACCCCTATATTCATGATCAGTCTCTCAGGGGCTATATGAAGCGTGGCACTTGG

CAAATAGAATGCAAGAGCAGATTCGAGAATCCAGCTGTCCTCCATGTTGCTGACATGAAG

AAACCTGCAAACACG

>Unigene52969_C-W 1 216 LEN=216

GTTTCAGAGGTCTTAATCCATAGAAGGCCTTTCCACTCCTCAGGGCTCCAGGTGAAGCTA

AACGTCATGGCAGCAGAGGGTGACAGAAGGATGCAGCTCACTCACATCACGGTGATAAGA

AAATAGAGAGAGGGACTCCACTTGCCAGATACAGATATATACACCAAAGCCATGCCCCAG

TTCCCACCTCTTCCAGTCACAGGCTACCACTCCAGT

>Unigene53140_C-W 1 237 LEN=237; minus strand

GGTCAAGGAAGAGCTAGCTTACAAGGTTTCCAAGAGAAGTCTAAGAAGAATCTTAAGAGA

TTCAAGACATTCACTCAGAAGAAGGATGAACCTATAAAACAAGAGGTCCTTGTAATGAGA

GTTTGTGAGCAGTATTGCAAGTCTTACTATGAAACCGACTGTCATTCAGCCTGGCAGTCT

CAGAGCTGCTTCTGTTGGATGAACCCTGAAAGAATTTCTTGTGCCATGTTCTATTCC

>Unigene53193_C-W 1 204 LEN=204

XXCAAAGTAGGAGAGGAGGCCATCCTTGGTCAAAGGATCATCAGTGAACTTGAGGGACTC

CAGAGCAATGGGAAGGCACGAAAGCAAGAATCCAGTTACGGGCCACAGAGTGGTCAGCGG

GTTCCAGAAGAACTGGACTGATCTACACGTAAAATGTTCTTGACCAGACCTGATGTGAAA

AGGGGAAGGGAAGGAGTACCCTGGGGX

>Unigene53221_C-W 1 263 LEN=263; minus strand

XXCTGGGAGCAGAAGGACAATTACGTGTTTCTCAGTCTCTCCAGTTTAGCTCTTTCTTGG

TCAGAACTGAAACAACAGCAGCCTCTTCTCACCATACACTTTTCTGAGTCTCTGACTCCT

TGTTGCCCGAGGTATCTCAGTTTCCAGAACTTGTGTGAATCTGAATTCAGCTTTGGCCCA

GAATTCTGGCTTCCTTGCCCTGATGTTCTAGCACAGACCTTCACCTCCAACCCTCCCCAG

ACTCCTGAGGAAAGCAAGGCCACACXX

>Unigene53273_C-W 1 214 LEN=214

XGCCCGTGTGTGTTCAGGGCAGATGCGATTTCCCCCCAACTATTTTCAATCGGCTGCTGG

TTGAATCCGCAGAGAGGAGCTGTGGACACGGAGAGCCGGCTGTTTGCTAAGTTAGGAACG

AAGTCCTCCAGCATGGGTCTGAGTGAGAAAGCTAAAGCATGTCACATGATGGAGTCCCAA

ACAAGCATGGAACATCAGGTTTTAGAAATATTTCAX

>Unigene53340_C-W 1 210 LEN=210; minus strand

GTGTCTGTGTGCGAGGTCCAGATCAGGTTAGGGAACTGTCTCCTCATGCGCTTGCCAATT

CTTTATGGTAGAAACATTCGAAGCCTGTTCTTCCAGTTTCTGAGGTTCCAAGCTGACACT

GAGTGTGGCCCAGGGGGGCTCGGAGTCCTTACCTGCTATGAGGCGGATGCCCAGGACTTT

TTCCTCTTGCCAGCTGAAGCTCTGTACCCC

>Unigene53429_C-W 1 218 LEN=218; minus strand

CTTGGCATAGCTCTGTGGGTACAAAGTGAGCAGGGTCAGGTCCATAAGACAGATTACATT

GCAGGTGCCTGGTCCAAGAATATGGTGGTAAGCCAGGCAGAGAGACCTGCTGCCAGGAGG

CACAGAGATCAGGGCTCTGATGGGACAAGCACAAAGTGCTTCGAGATAGAGGAGAAGATA

AAGAAATCAGAGTTCCCTCCTCTGTACCAGAACATCAGX

>Unigene53491_C-W 1 231 LEN=231

XXCGCTGCAGTTATTTTCCCAGGGCGCCTGGTTGCTCTCAGTGGAAGGCAGTGTTTGGAA

ACAGATCTCTGTGTGCCTGTGGCTGTTGGAGGATCATTACTTGCAGGTTTGGCACAAACT

TCACCAGCCCTGGGCACTAAGAATAGCAATTTGTCAAATTTGTCAAATAGCAATGTCACC

TATCACCACCAAAATTGTAACCCGCCTTTTTGTTTGAACACAGTATTTGCCAGX

>Unigene53526_C-W 1 194 LEN=233; minus strand

XAGCTCTTCCTCTACCAGAGGTGGCTGCTGCCCCGCTTCTCCCCCACCTCGCCTCTGCCC

TCTGCGGCCCTGCACCTGCAGCCCCGGGACAGTGGCTTCGGGAAGCAGCCACCCGCTTTC

CAGCTCATCCTGCAGCCACTAGAGCACTTCAGACCCAAGGACTCCTCCCACCCTGACAGC

CCCCCTCCCGTGGCC

>Unigene53599_C-W 1 212 LEN=212

XXGGCTGAGTCACTTACTCTTTCAAATCACCTGGCACACAGCATGGGAGGTGCCATCACA

GACCCCCAAAGTGACAGGAATTCTAGAAGTGACACATTTGCAGTCAACAGCTACTGTGGC

AGCTGCATTGTGCTACCTACGTCACCGTCTGTCATCACCATGCTACGAACTAGTCCTCAA

ACCCAGGAAAGGAAACACATAAGATCAATAAAATXX

>Unigene53631_C-W 1 241 LEN=241; minus strand

XCCTGGATGGCGTTGCACGACTCTGAGCAGCACTGGTCTGCCCAGCACCATTCGGCAACT

CGGGGAGGACTCTGGGGTTGGTTTGTTTTCCTTCAGTGGTTTTCAGGTGTGCGATCAACA

CCGCTAATTCCAGAACTTTCCCTCCAAAAGAAACCTATCCTGAAGACACAGGAATTGATT

TTTAGGATCACACAGAGGTACCTGATTCACAAACTGTCTAAAAAATTAACTAGTGAGCAG

CCX

>Unigene53694_C-W 1 245 LEN=245

XXGGGAGAGGCTAAGCTGGCAGCTTTGCTGTCTCCAGAGGACCAGGGGAGAGAGCGCAAT

AAGGAGGGGCCTGAATGTGTGGAAAGCCAGACGGGGCCCAAGAGTGAGGTCTCTGCATTC

CCCAGATACCAATCAGGACTCTCCCAGGGTGGCCTGGGTGTTAATCAGCAACAGGACAGC

TCCTCCAGCAGGCATTCTAGGCACTGCCAGACCAGCCCCAGCACTTACAACCTGCAGAGA

AGGCTAAXX

>Unigene53759_C-W 1 213 LEN=213

GTTTCTGAACTTCTGGGCTCAAGTAACCCTCCCTCCTCAGCCTTCCATGTAGCTGGGACT

ATAGATGAGTACTACCTTGTCCGACCTAAGGACTTCCTTCTTACTCGTGGTAACAATGAT

GATAGCACCCATCACAGGGGATTGTACATGAAGCCTTCAGCATCAAGTATATACTCAGTT

CTGAGCATTTGCTATCACAGCATTAAACCTGTG

>Unigene53780_C-W 1 217 LEN=217; minus strand

AAGGTCACACATGTACTACTAAGTGGCTATGCAGAGCAAACCCAGATTGAAGGCCATTTC

ATTAGAGAGGAAGACTCAGGAGGATCAGCCTTGAGCTCCAGATCAGAAAGGGAAGGAAGG

GAGGGGGCCGAATTTCAAGCAGTCAGAGCAACCCATGCGGGACCCTACTTGGGACTCTAT

GCTAATAGGGTCCAGCTCACACAATCTGCTGATTCCCXX

>Unigene53832_C-W 1 215 LEN=215; minus strand

XXCTGTTTTCTGAATTATGGCCCAAACATTCATTCAGTAAACCTTTGCTGGGTACTTAGT

GTGCCAGGCATTCTGCTGGATGGTGATGAAACCAAAGTGAAAATGTCAAGTCCCCTTCTC

AAAAGTGGTCAGTGTGCTGGGAGTGTTAGCCAGAAACCAACGCAGTGTGATGAGTGCCAT

AGGAGAATCACAGAGCCAGGTGCAGTGGTGCATGCCTXX

>Unigene53870_C-W 1 243 LEN=243; minus strand

CCAACAAGAACCATTCAGATGAGGAAAAGTTTATCTGGGCTCACAGTTTTGGATATCTTA

GTCCATGGATGGCTGACTACATTGTTCTGGGCCCAAAGTGAGGCAGAAAATAATGGTGGA

CCAAATGAGGTGGGAGAGGAATGCTGCTCCACTCATGACAGCCAGAAAGCAGAGAAAGAG

GGACAAGGGACGGGGAAGGGGCAACAAGGAAGATGAATCCTTCCAGGACATGCTGCCATG

ACC

>Unigene53923_C-W 1 217 LEN=217

XGTTCAGAAGACCAAAATGCTGATAGAAACATGAACAGTAAAGGTTGTGTTGATGAGGTT

TCCAATGGGAATGAGGACTCTACTGGGAACTGGACTATAGGCCATCCATGCTACATTCTG

GAAAAGAACTTGCCTAATTTTTACCCATGCCTTGAGATTATGCAGGATGCTAAATTTAAA

GGCGACAGATTAATTAATCTGGCAAAGGAAATTTCAAGX

>Unigene53977_C-W 1 203 LEN=203

ACCAACACCCCCCCTCACACACACATACAGGATAGAAAAGAGCAGTTTTCTCGAGCACCA

CTGGATCTGGCCCCTTCTCAGACAAATGCCTCTAAATCAATCAATGAACAGGACACTTTT

GCAAAAGGAACCCTGCACAGCACCACAGAACGTGGCACTGACAAGGACCAAGAGCAGGAT

GTGACCTTTCTGACTCGTCATCAX

>Unigene53998_C-W 1 214 LEN=214

XATTCATTGAAAGGACATAAAAATGATGAATGGGTATGCATCTTCAAAATGCAAGAGCTG

AAGCCAGATGTGAAGCGCATGCCTGAAATCCTAGGTATTCAAGAGCTGGAGGTTGGAGGA

TTGTATGTTCAAGGCCAGCCTAGACAACTCAGTGAAAACCTGTCTCAAAAAAGGGTTGGT

TATGTAGCTCAGTGGTTCGATCCCCAGTACCAGAXX

>Unigene54046_C-W 1 210 LEN=210

XXGGTCTATCTGTCCACCCCCACCCCCACTGCACAGTGCTCACACCTGCACCTTCACATC

TCAGCTTGGAGATCACTTCGCCAAAATTCAAAACTTTTGCTCTTCAAAAGACCTCATCAG

GAAATGAAAAGACAAGTAGCCGACTGGAAGGAACAGTTTGCAAAACTTGCATCTGCTAAA

GAGTGGGCTTCTGAGCACCAACAGCAGAAAGAX

>Unigene54094_C-W 1 244 LEN=244; minus strand

XAGTTTATGGGCCAAGGCGATTGCCCTTTCACTGGCTGTAATCAGGCCGTCACCCTTAAT

GTTACTGGATTCAACCGGGCCACCTCATCCCACTTATGTTTAGCAATGCTACACTTCATA

TCAGACCAAACTGAGGAATCTTGTAAGCTCTGGCCTGACACCTATGGTTTGGATCGTACT

CCATTGACTGCGATTCCCATTGATTTTCCACTCAATACCTCATCTAGACCCCAAGGACCT

CCCTCX

>Unigene54096_C-W 1 247 LEN=247; minus strand

XXCCTGGCTGCTGGCTACTTGGGGTTCTGCAAGAAAGAATCCACCACCGATCATTCTAGG

CCTCACAGAGAACGGACTTCATCTAGACACACAGAAGACCACATTGAGAAGATGGCAATC

AAGGAAAAGAGGGTGAAGACCAAGGCCTCCCCCACCATCAAGACTGCATCTGTTAATCAA

GAACTCACACCTGCTGACCCCGGAGCAGAACTGCCAGCAGGCCTGGCTGCAGGTGCAGCT

GATGCAGCX

>Unigene54243_C-W 1 223 LEN=223; minus strand

XCCTTTATGTGCTCCAAGCACCTGCAGTATACACAAGCTCCTACAGGAGGGGCTGCAGGA

GCCCAGCAGGTGGGGAAGGCAAGTGACAGGCAGCTAGAAGCTGTAGTGCCAGAGTGCCAA

CAGGGAAGCAGGTGCCCAGTCACTGCTCACAAGGCTTACTTAGGATATAGTAGCAAGACA

GGGATGGAGAACATAAAGGGCAAAGTTAATGTCCTGCACATGGCX

>Unigene54324_C-W 1 227 LEN=227

XGGAAAGACAGAAGGGATAATGAAGATATTGGGCAAAGCGCCAGACCCGAGTCTGTCTCT

GAAGTCTGCACTATTCATTTCTACACAGAGCAAGAGCAGAAAGCTACAAATAGTCCTCAC

TGACCCACGGGAAATGTTGCCAGGGAAAGATCACAAAGACAATCTACATATATCAGAGAC

CAGTACTTCACAGCTTCACAGCAGGGCAAGGAAACAGCACAACGTCAG

>Unigene54369_C-W 1 257 LEN=257

XXGTGGTTTTGCCCTCTGTATGGACAGGAGCCACATAGTATAAAACCTAATGCTCTGGGA

AAATACCCAGGCTCAGAGATGGCCAATGACCATGCAGACATCTTGGATCAACTGCCTGAA

GCCCTGGGCTGACAGTGTCATGATGGAGTAGCCACATCTGTCATGGGTACAGCTGTAGAA

GGAACCCTCCAGACAGTTACCAAGTACCTACTAACCCGTGACCAAGACGGGCCATTACTG

ATTGTTATTTGTTATGATGXX

>Unigene54490_C-W 1 212 LEN=212; minus strand

XXCTGCTTCTGCCCCAAGATTCTTGCATGTCTCCCCTACAGAAATGAACTACAAGTTCTG

GCTGGAGAGGCATATCAACTTGGAAGGATTCCTTTAGTGATTCTGATACCTTCCCTGTCC

CCACCTGAGTATGCTGATCTTTTTGGAGATGCTCTTCCCAATAATCTCCCTAACTTCCTC

AGGTCTCTGCTCAAATGTCACCTTACCAAAGAGAXX

>Unigene54530_C-W 1 203 LEN=203

XXGAAGGTAGAAGAAGAGAAGGAGGAAGAGGAAAAATAGGATGAGGAGGAGGGAAAAGAA

AAAAGGGGGGAGGAAAAAGAAGAAAAGGGGAAGAGGAGGAAGATCTGGAAAACTATTTCA

AAACTGAAAGAAAAGAAGAAAGAATAATTAGACACTCGGAACTACACAGAGAAGAACCTT

GAGTTGTCGGTGGACACAACATCTTXX

>Unigene54610_C-W 1 201 LEN=201

XATGAAAACTACGATGCCATCTGGTACCTGCAGCAAGCCAGCACCAATCCCCACCACCCA

CTGGGCTACCTGCCACTCCTGGGACAGCTGAACAGGTCCAAGACAAAGCCACAGAGGACT

CTGAGCAGTGAAGGTCCAGGGTTAAACAAAATCAGGTCCCTAAAGGAAGCAAACTATTCT

ACAATTCTAACAACTGCGTCTGXX

>Unigene54695_C-W 1 239 LEN=239; minus strand

XGTGTAACAGGAAGCTAGCTGGTTGTGGTCCACAAAAAGACTGTCACCATCCTCTTCCTA

GAAATATCTACCAAAAAGGTTATAAAGTGTGAATTGGATGTTGTGGTGGTGCACTCCGGT

AATCCAGTGACTGGTGCAACTAAAGCAGAAGAATCACAAGTTTCAGGCCAGCAGGGGCAA

TTTAGCCAAGATCCTCCCTTAACAAAAAGGCTAGAATGTAGCTCACTGGGAAAGCTCCCC

>Unigene54755_C-W 1 207 LEN=207; minus strand

GCCTTTATTGATAGTTCAGCAGGAGTTTCTTCCATGAAAACACAGAATATTAATGGAAAC

CTTGATTTACATTCTAAGTTTAACCCATTTAATAATGATGGGTTTGTTATGAACCAGCCT

TTGTCCCAAAGTATTAATTCTGCTATTTCCCAGCCAGTTGCCTCTAGATCTCCCCAAGGA

ATGAGCAATGATAATATAGCCTGTCCC

>Unigene54756_C-W 1 251 LEN=251; minus strand

XCAGGGAACATCAAAGAAAGACTCCTGAGTGGAGGATATGCCTTCACCCCAGGCTGGCAA

GCTCTGCCCAGGACAACAGAGAGGACTGCAGAACTCAACCCTGGAAACCAGACCCCGAAA

AAGCTCAGGTCGCTAAATCTCAGCCCAGCAAACCTCTCACCTTCTGCCCCAGACTGGAGC

TTGATGGTAATGACTGGTTGTGACTCCACAAGGACGCTCCTTGACTTCCCACAGGGTTGT

ATCCCAACGAAC

>Unigene54800_C-W 1 206 LEN=206; minus strand

XCTTTAGTGGGAGTTATTTCCAAACCTAATTTTCTGTTCATGCTGCATAGTATTAAAGAA

ATTTTAGATAAAAAAGTAAAAGCTGCGGACAAACATGCAAAAATATTAAAATTGGGGGCA

CAATTACAGGTGTCATCACATGTCATTCAGTGGGGACCAACCATGGTACAGGGGAAAGTC

CCAGGCATTAAAGTTGGCTCTGTAGAA

>Unigene54839_C-W 1 202 LEN=202; minus strand

XXTGGGGTTCAGAGGCCCCTTTGTGGGCTAAAGTTCACAAAGGAAGCAGGAGCTACCTGG

GTACTTTCTATGGATTCCACATCATCCAGGGCAGAGGATATCCCAGTGGATGTGGGGAGA

TATTTCCTAAAAGCTCTGGGAAAGTGGACAAGGAAGTCAGGATCTGGAAAGACCCTTCAT

GGACCCAAACAGGGGCCCCAAGGC

>Unigene54926_C-W 1 308 LEN=308; minus strand

XATAAAATAAATCTTGCTGGAAAGTCAAACTGTCTCTATCAGTGTTCCAACAGTTTCTCA

GATGTCTCTGACATCTCTCAGAATCACCAAACCCAGATCAGCCCTCTCGAACCCAATCTC

TATCCCACCAAGCTGGGATTCAGATACATACCTCAGGCTTGGCAACACTGCAGTCCCAAG

ATTTCAGTGCTGCTCCTACTTGCAGAGAGACCACCAGGGATACACTTATGCAAAGGAGCA

ACCCTGAGATTCAGTAGCAAGCCAGGAGCCACCAGCACTCCTGCAGGAAGACCCCAGGCA

CCTCCCAAC

>Unigene54986_C-W 1 201 LEN=201

XXAAAAAAAAAAGATTTATAAACCCTTAGCCAGACTAACCAAAAGATAGAGAGAAGACCC

AAATCAGCAGAATTAGAGATGAAAAAAGAGATATCACCACATGCACATCTGAAATTCAAA

GGATCATTGGAAACTTTTGAAAATTTATACTCCAATAAGCTAGAAAACCTTGAAGATATT

GATAAATTCCTAGAGACCTATGAX

>Unigene55022_C-W 1 214 LEN=214; minus strand

CTGCTGGGTGACAGCTTCCAGCAGGCCTGGGGAAGTGCAGGCAGGCACACAGGCACGGCT

GCCGTCGGGGATGGCGAGGTGCTCACCTCTGCGTCCTGGGTGCAGTTCGGTTTCCCGTTT

TGTACCTGGGTTGCCCACACCCCTTCTGGGATGCTCCTTAACCCTCGCCCACCCCAGATT

GTGTTTGACGAGTGCCACAAGGCCAAGAACGCCAXX

>Unigene55294_C-W 1 203 LEN=203; minus strand

XCAAAAAACTACAAAGTGCCTTATGTTTTTCCCTCCAAATACTACACATGTGCCGCTGTG

AACTGTGAAACATTCAGGACTAAAGGTACAGAGAAATGTGATCACGGTCAAGCAGAGTAT

GTAGCTGAAGTAGAGAAAAACCAGCAGCAGCTCTTGAAAGCTGACATCCACAGGAGCCAC

AGTCCACAACAAAGTCCACAACAC

>Unigene55343_C-W 1 208 LEN=208; minus strand

XXCAAGGAAGCTTTGCACTCCTGTGGTCAAGGGAAGCTTCCTGTGAACCCGTGTTCTCGG

ATTGAGGTCCCATCTTCTGAGTCAGAAAGTGCCAAGACCTTCAGCCCATCCATTGTAAAC

CAAAGCTGCCTAGAGTACTGGAAAATTCCATTAAGAACATTGCTCCTGGCAATCTCATTC

CAAATTCCAGCATACTACATTAAGCCCAAG

>Unigene55374_C-W 1 200 LEN=200; minus strand

XCTTCCAGCTCTCCAGCCTGCAAACAGCCATTGTGGACTATCCAGCTTCTGTTTGTAGGC

TGGAGAGCTGGAAGAACAAGTAGCTACACAGTCCAAGAAGCTGAAGCCACAGAACAAGAG

GGATCAATGGTTCTATCCTGGTCTGAGATGGAAGACTTCTGGAAATTCCCTGGAGAATCA

CTGGTAGAGATCACTTTGGAA

>Unigene55416_C-W 1 258 LEN=258; minus strand

GGAGACAATGAAGAAGAAGGTCAAGATGACTATAAGAAGAAGCATGAGGAGAAAATGAAG

AAGGACAAGAACAAGAAGGAGAAACACAAGCCAAACAACTAATATAACAATAAATCGATT

AAAACTGTGAATGAGAATTATACTGATGAGGAGGAAATGCAGAAGTGGGGAACTCAAGAG

AAACATATTTCTCTGTATGGCACAGCCCTGCTGTGGACATCCTCCACCCACGCCCAACAC

ACCTCCAATCCATACCCC

>Unigene55428_C-W 1 227 LEN=227; minus strand

XXCTGTGGATTAGGACACATCTGTCACCACTGCCATGGAGAATTTACACTCTCCGCTGGC

GGCCATGCTGCCAACCCAACCAAATATCCAAGCCAAAAACCAAACCTGAACCAAAAAGCT

CTGAGAACTTACGCTCTCCACCGAGCCAAAAACCAAAAAGCTCTAAGCATTGCAAAGGAA

AAACAAGAAGGAGAAGAAGGAGGAGAAGAAGAGGAGGAAGAAGAAGAG

>Unigene55485_C-W 1 210 LEN=210

AAAAGAAAGAAAGAAAAGAAGGAAAGGAAGAAAAGGAGGGATCAATTTGGAGGACTTCTA

CTAACTGATTTTGAGAATTGTGATACAACTACAGGAATCATGACGGTGACGTATTGTCGG

CATAAAGATGCATATACAGATCAATGGAACAGAGTAGGGTGTGCTGAAATAGACCTAAAC

AGGTCAAATGAGTTTCAGCAGAAAGGCCAG

>Unigene55562_C-W 1 273 LEN=273

XCACTACTCTACTTTCTAGATCTTCAGATTTGCCAGCTGGGGACATATCTTGTGAATGGA

ATCACACGACATGTGAATTTTGTGTCTGGTTCTTTCAGCATAGTATTTTTGAAGGTCATC

TATATTTTCATACTTCATTCTTGTTCACTGCTGAGTAAGAAGCAAGGTCTCTGGGCCAAG

TTTCCACGAGCAGAATCGGAGGCCCATGCAGTCTCCCCCCCACCCCAGACTCAGCCCTGT

GACTTCTTTCCAGGTGTGGATGACACTGTCTCAGXX

>Unigene55827_C-W 1 396 LEN=396

XCACTGTCCTCCATGGTATTTCAGAATTTCACACATCCTGTTGTGAAGCCTATCTTAGAC

AGGAGGTATTTTCTTCAAAAGAATACAGTGGGGACAACAGAAGAAAGGATATGCAAATCA

GCTGAACCACTTTGGTTTTCATCATCTGAAGAAGAGGAGGAAGGTCTTGATGACACCAAA

AATAGGCAGCCACAGGTATTTTCTGAGGGTGGTCCACAGATGTGCATTTCTCACTCATCT

GGAGAGAAACATTATATAGCAAAAGAGAAAGCAGATGGACATGTCCATGCCTCTAGGGAT

TCATCTGATTTCAGAAAGCGCACCGATCCCATCACAATAAAAGAGGCCCTTCTATGGAAA

GGATTAGGCAAAATGAAAGAAAAGACAGTCAAATTGGXX

>Unigene55953_C-W 1 239 LEN=239

XXGCCGCCAGGGGGCGTGCGCAGGACAGAGAAAACTTTCGCGCTTTGACCCCCGGCGCGG

CCCGTCGCCCGTCGCTGCGGCGGGAAAAATCTGACCGGACCGTCTGGGCCACTGCCTCTG

TCTGCCTTTGAGCGTCTTCCTGAGCTGGTCCGCGTGATGGAGGAGGAGGCGGAGGCCGAG

GAGCAACAGCGATTCTCTTACCAGCAGGTACAGCATAAACCGGGGCCGGGCTGGGCAAGC

AXX

>Unigene55966_C-W 1 212 LEN=212; minus strand

XXAGTCCTGGTCACTCCCATCCAGGACCACATGCTCCATGTCCAGCCTGCACTGGCCACT

CTCATCAAGGCCACGTGCTCCATGTCCAGATCAGTCCTGGTCACTCCCATCCAGGACCAC

ATGCTCCATGTCCAGCCTGCACTGGCCACTCTCATCAAGGCCACGTGCTCCATGTCCAGA

TCTGCTCTGGTCACTCCCATCCAGGACTATGTGGXX

>Unigene55996_C-W 1 311 LEN=311; minus strand

XTGAGTTTGGGACTAAGGCTTCAGGCAAGCAAGAGAAATAGGAAGCTGCCGTCCCTCAGA

GAGCTGTCCCTGAGGAGCAGTGGGTGCCGGGCTAGTGACGTCATGGGTGTGGTGCCACCC

ATTGTGTGGCTCAGGAATGAGACACAGCCACTGGGCAGTGAGGGCTTTTCAGCTCGACAA

AAGCTGCCTTTGTGGATGTCTAATCACGAGTCACACGTGACCATCGTACTCACTTGTACA

AGCCACGACAGCAACGTGAAAAGTAAACTGTAGTCATCCAAAAATGCACACCTAGGAATG

CATCTGACCGGC

>Unigene56004_C-W 1 255 LEN=255; minus strand

XAAAGACCAAAGAGCATAAAAATAGATTTAAAAAAATCTAGGTTTGTTGGCCTGAAAGTG

GCAGTAAAAGCTGTAAAAAATCTTGGCAGAGTTCCAGACCCCTCCATCCTTAAGCAATCT

TCAACAGAAGGCGTGGTACAGTTTGCAGGTACCCTCCCCAACCATATCACCAATGGACTT

CCTTATATTTGTCTTCCATATCTGGCAGCTCCCCTCCCTGAAGTTATCCTAGTCGAAGGA

CAAAGGCCAACTCCCCXX

>Unigene56108_C-W 1 223 LEN=223; minus strand

XCCCAAACTGAGGTGGTGCCTTCTGCCTCTACCCTAGTCATTGAAGACATGGCCAAGCAG

AAGCTTTTCACAGTCATCATGAGGTTTGCTGACTCCTTGTCTTTTTCTCTCCACCAGAAG

CTCACATGTTTTGGAAGGACAGACCGTTCAGCATGCAACCAAGAGGGCACTCTGAGGAGA

CGTGCATCAGCTCCATATCACATGCCTATTTCCAGAGGTCTCTGX

>Unigene56169_C-W 1 315 LEN=315; minus strand

XCTTGTCTTATAGAAGAGGAAATGAAGGCTCTGAGTGACGTGTTGTCAATTTTTGGTCGA

GGTCATATAATTAATAAGCATTCAGACCGGGATTTGGTTCATATCTAGGTTCCTTCCTCT

TTACAAGCTGCCTCCCAGAAAGAGTTCCTCACATCCCAAAGAGAGAAGTCTCGGCATAAA

CAGGATTCACTGCTCCCTATGTCCAGCAAGAAACCAACACAGCCTGGAGCCAATGACAAT

AAAGAGCAGGCCAGAAAATATACCTTGGAGATCATTGAAAGATTTGGAGAGGGGGCTGGG

AATGTGGCTCAAGTG

>Unigene56230_C-W 1 296 LEN=296; minus strand

XGAAAAAGTACAATAAAGAAGAATGATCAAGAGAGAAATGCTTATAATTCCAAGGACACA

CTTCATCAGACACTGCAAATATTACAGCAGAATGAAXTTGACATCATGTCTCCAGTTCAT

CTTAAAAAAAAGAAAAAGAAGGAGAAAAACAAGAAGAAAGAAACTGGAAAATCAAACCTT

ACAGTTCATGATGCAAAGGAATGCTTCTTGAAAGTAAAGTATCCCCTCCAGCAAAATATT

TTACTCGATTTGCCTCAGAGAATTCTCCTGGCAGAGAAAAAAAAGAAAAAGATGCTCCXX

>Unigene56247_C-W 1 259 LEN=259

ACAGTGCTTCCCCGTGACCTGCAGGGCTGGAAGCCAACGGGTGAATTCGCCCCGGCTAAG

ACGGAGGAGGGGAAGAAAGAAGTAACCCCGAGCCCAGAACCGGAGACCACGACTGCCAAG

CACCTGGAGGTTAAGAGTATGCTGAAAGGGCGCATCTATGGCCTGCTTATCTGCTTACTT

ATAATCCTGATAGCAGCCATTATCTTCTGGCTAGTCACTCGGAAAAGGAAGAAAACTAAA

CCAAAGTCCTCACAGGCCAXX

>Unigene56274_C-W 1 209 LEN=209; minus strand

TCAAAAATACAAACAATTATAAAGGTTGAAGAGGATAAGGATAAAAAGGGACACTTTAGT

ATTGTTGGTGGGATTGAAATTAATACAACCGCTATGGAAATCAGTATGGATATTCCTCAA

AGACTAGGTATGGAAACACCATATTACCCAGCTATAAAACTGTTTGGTATTTATCTTAAA

GAATTAGCTGGGGGTATAGCTCAGTTGGTX

>Unigene56366_C-W 1 208 LEN=208

XGCAGAAACAGATTCCTCAGAGATGTAAAATATATGATAATCAAGTACACAGAATATAAG

GAAATGAAATATGATATGTTTAAAGAAATAAAAGACAGTCACAAAAAGAGAAAGCAACAA

GAAAATATAAAAATGATTAGGCCAGATTTGCCCTGGAACCAAATAGAACTTGTAGAAATG

AAAGTGTTTGAACACAAAAACACATCATAX

>Unigene56374_C-W 1 208 LEN=208; minus strand

XCTTTCACAGAGGGGTCTCCAGGGGCCTGGAATTCACGGCTCCTTAATGACAGAGCAAGG

ATTAAGCCCTCACTTGTTCAGTCTCCAAGCCTGGGCTTTTCCTGTAGAGAATCACAGAAT

AAAGGTAGCAAGAAACAGGTCAGCATTGACCCTCTTCAGATGGCAGTTAGGGTAAGACTC

ATAGCAGCAGCATTATTTCTTACAGATCCX

>Unigene56495_C-W 1 216 LEN=216; minus strand

XXGGTGGACAAGCCTGGAGTCATCCTGGACAACTGTGCTCTGGGCAAAGCTAAGGAAGGC

AGCACAGAAGTTCATGCACACAGAGGGATTCCAGCCTATGTGGTACGTGGAAGCTATGAC

ACAGGACCTCCCATCAGTTCCCAAGACACCCTCCCCAACAGAGTGAGGCTTCAAGGGGCC

AATTCGTCTGCAGAGATAATAAAGGTTTTCTAAGTGGCX

>Unigene56633_C-W 1 225 LEN=225; minus strand

XXCTGTATTTGTTACGACCCCACAACCCTCTTTCTTGCTGTATTTTGCCGCTGGAAAACG

ACTGCCGAAGAGCCTCTTCTGATTGCAGACCGCTGTGACATTGAAGCCTGCGCATCACCC

GAGAAACTGGTACGAAAGTCAGTGCCATTACCTGAGTGCTGTGAAAGCTGTGTCAAAGCT

TTGCTCTACCAACTAGAGGACCTCCTAGTAGACAAACTAGTCCACGTX

>Unigene56660_C-W 1 215 LEN=215; minus strand

CTTTCACTCTTTTGGAAGTCATCTGCTCTCCGGCTTCCTTATGATTCTGGCTGGAGTAAG

GGAGGAGAAGGAATTAAAAACAACAGTGATTCAGATTATGAAGATATCAAAGCAAATGAA

ATGTTTGGACCACCTTCTTCCTCTAGCACTTTTCTTCCTCAAACCAGTGAGGGTCTCCCA

CAACTTGTAAGAGTGAAGAGGAAGCTTATGTCCAGX

>Unigene56752_C-W 1 254 LEN=254

XXTGAAACCTCACAGCGGATGAAAAATGGAAGATGTTCCCCTAAGATTGGGAACGAGAAT

GGTGCCCTCTTGGCCAACGCTCCTCAACATGATACCCAGAGTTCAAACCCCAACAGATCA

GCAAGAGGCAGCTATAAAAAGCTTCCAAGTAGAAAGGAAGAAGAAACACGACCTCAATTG

ACAGACGATGTGATCCTAAACAGAGAAGATCCTCAAGAATCGGCAAAGAAATGACTAGAA

CCAAAACACAACTTGGXX

>Unigene56820_C-W 1 263 LEN=263; minus strand

XXTCCGTTCCATGAATATGCAGAGAGGAAAACTTTAAAGGAGACACAGAGAGAGCTCCAC

GTGAGGACACAGGCTGAGAATGGAGTGATGGAGCTTCAGAGTGGGAACACCAAAGATGGA

CAGCCATCAACAGAGCTAGGAGAGAGCAAGAAAGGATCAACCCTTCCTGCACGCTTTTTG

GAGGGAGGATGGTCCTGCAGATGCCTTGATTTCAGACACCCATCCTCTAGAACTGAGACA

ATACATTTCTGTTGTTTTAAGCCACXX

>Unigene56852_C-W 1 256 LEN=256; minus strand

XGCCCCACCTCAAGTGCTCAGTTTTTGTGTGTGACTCAAATAGCAGCAGCAGCAACTCTA

GTAATCTGGGAAAGAGACTCATGAGCCACGACTGTGACCCATCCTGTGCATCCTCTTACT

GTTGGTGGCACTATGGCCTCTTGGGAGCTCTTATCAAATGCCAATGCCCTTTGTCCTGAA

CAGCCCAGTAGAAATGAGAGTCTGAAAACCAAAAAGGAAACCGATAAGATTAGAGACAAC

GTGCTTAGCTTCCTATCX

>Unigene57114_C-W 1 239 LEN=239; minus strand

XCTGATATGACCCAGAGGCAGTACCAGCTGAAGGCCTCTCCAAGGCCAAGAACTCATTTT

AACCCTAGACCAGGAGCAAAGCTTGCTGCTAAATTGTCAGTTCAGCTTTACTTCTTCAGA

GCCATTGTTTTTAGCTATTTAACGAAACACAAAAGCATTGCTGCTCATAAAGCATTTGCT

CCTATGCATCACGAGGGCCACAGCATGAGGACAGGCTCCCATTGGGGCATCTCAGGTCCA

>Unigene57136_C-W 1 209 LEN=209

XGCCGAGGTGTGGTCCACTCTGACCTCCGACTACCTCTTTCCATTTCCTCCACCTCTGTT

CTGTCTCTTGGGTGTGTTTCTTTCTCTCTCAACACTGCCCACCCCGTGAGCCCTGCCCAG

GACAAGCATGCGCTCCTAGACGTGACGCCCTCTGCCATCGAGCGCCTCAACTACGTGCAG

TACTACCCCATTGTGATCTTCTGCGCCCCG

>Unigene57212_C-W 1 215 LEN=215; minus strand

TGGGCCTGGTACCTGAAGCTGACTGTGTCGCTCTGGCGGGAAGCTCTCCACCTACAGCAG

ACCTTCCTGAACGGTCACACGTTGGCCAAGACCCACGTCACCCTGGGACCCGAGCAGCCT

TTCGACCATTCTCAAGTGGGCGAACGAATGCTGCCCCTGGCTTGTGTGCAGAGCTGTGAC

TGTGGTTCCAGAGGGCCGCCTGGGGCAAGCCGGACX

>Unigene57376_C-W 1 263 LEN=263

XGTCTCAGGGTAAAATGGAGTTTGTCTGGTCGAGGCACATATAGTTTGACCCACAACAGT

CAAAAGTGGGATTTGATCCCACATGGGTCCTTCTGGAAAGCAGTGGCAGCTCCTGAGTCC

ATCTGGACAGTGGTCCTGGGGCAGGCTGCGTTGACAGAGGGAGCCCATGAGTTTCAGTCT

TTCCTAGATAGACAGATAGATTTGGGGCCACACCTGGCTAAGGCCAGTGATGTGCTCAGT

CACGCCCAGGTAATACACCTGGGC

>Unigene57625_C-W 1 245 LEN=245

XATGGGGATGAAGTCATCATAACAGATGCAGAAACCAAGGCCCAGACAGCTTTGCGCACT

CAGGATCACACAGATGGTCAACTGCAGGGCCAGGATTTGAACCCAGATGGTCGCTCCAGG

CCCTATGCTCTTAACCACGGCACACCTTGTCTCTCCAAAGGGTGCAGCATGATTCTCACT

TCTGAGGGAGTCCAGAAAGCATAAAATGGGCAGCAGAACAGAAGGACACTGCCAGGGGAA

TGGTCA

>Unigene57645_C-W 1 288 LEN=288

XXGCCACGATTTCAACAGTTCACCAAAGTAAATAAGAACACCAAACAAGCACATGTAAAG

ACACTGAACGTGTTAGTCCTAGGGAAACACAAATTACAACCAAAACGAGATACCACTAAA

GCCTTTGAAACACAACAGATTAAACAAAAACAAAAGGAAACCCTAACACATTTTGGTGAG

AAAGAACTGGGACTCTCATACATTGATGATGGGAATACAAAATGGACAGCCATTTTAGAA

GTTTGTCCATGTTTTGGAAAGTTAAAAATATTTTTACCATACAACCCAGXX

>Unigene57836_C-W 1 288 LEN=288

XGCTGGGATACAAGCAAGAGCCATGGCACCGAGCTCCGTTTTTCAAAAGGCAAGAGGTTA

GACCGTGGGACACAGAATGAAGTCCTCCTTGCTTTAGGGAAGGTAGAGGTCAGGTCCTCA

GGAGGCTGCCTCATACATTCTCCAGGGAAGGCCAGATGCCAACTCACCAGACCCTCTTCC

CACTCAGCTGACAAACTGCTCTTCGGAGAGGTGGAAGGAGAAAAAAAGCAAACATATCTA

CTTGCATTTAAAGCTCCTATTATAGACACAAAACAGATTTCCATGAAAGXX

>Unigene57920_C-W 1 211 LEN=211

XXGAAGAAGAAGAAGAAGAAGAAGATAACAGGTTGGCAAGAATGTGGAGAAGTTGGAACC

TTTGAACACTATGTCAAATGGAGCAACTACTCTGCAAAACAATATGGTGGTTCCTCAAAA

ATAAAAAATAAAATGGCCACATGATCTAGCAATTTTACATCTGGCCATATCCCCACAGAG

CTGAAAGCAGGTCTTAAAAAGATATTCATGCAC

>Unigene57931_C-W 1 254 LEN=254

XTCAGCCTCCCAAGTTGCTGGGATGGGTGGAGGCTGCGCTGTGGTTTTCAGGGAACTGAG

GCAGCCACGGTCATGCTTTTTCTTTGGAGAGGTCAACTGGAATATGGGCTTTGTGATCAT

CTCCAGGAGCACGAATTAACTAAGGATGCCCTTTTTGCAGCACATGTGAGATATCAAGAC

AGATCCAGTCAACAAGGAGCACAGAGATGGTATGAACACATCAATATGGTTTTGGCCCAC

TTAAAAATACAAAGA

>Unigene58023_C-W 1 353 LEN=353

GGAGGCGACGATGGTTCCCTTAAAGAGGCXGGAGGCCAGGAGCTAGAGGAAGGCAGGGTT

CAGATGGACCAGATTAGCCTGGGGATTGCTGGAAGTGCCAAAGCTGAAGGCATAAAAATG

AAAAATGATGATTTGCTGAGAAATGATACACAGCAGAAACCATACGTCCAGGACCCCATG

ATACAGACATGGTGCAAATGGAGAGGAAAACAGACACAGAGCGGGTCCATCCAGAATTAA

TTTCCAATTTGCAGAGCTGCTTGTTTGTGTGTTACATCTGCTACCCAAATTAACATTCCT

GCATCTCCGATTTCCTGTGGCGTCCACTCAATGTACAACGACAACCTGGACCCG

>Unigene58046_C-W 1 252 LEN=252; minus strand

GCTGGTTTAAAAGTTGTACCAACACGACACGTCAACTTGAATTTCTTGTTGTGTGAGCTG

GGAAGCATGGAGCTGATGGTGATGTTGATGATGATGATGGAACTAATTACTGTTATTTTA

AGAAATCCAGAAGAACAGCATCTTCTTATTTTCTTGTCAGGAATAACACATAACCTATCA

TTAAAATTGGATATAACAGCCAAAAAAGAAAGAAAGAAAACCCAAAAATTAATGAAAGGA

ATTCCCCCTCCC

>Unigene58099_C-W 1 209 LEN=209; minus strand

XCAGCAAACAATTCTAGATTAATTCCAAGCACTGCAAACACACTTCATCATGACAGGCTC

ATGACAGGCATTCCCTCTGCATGCAAAGCTCAAGTGTCAGATCAGAAGTCTCAAGTCTTA

GGCTTCAAGTCCAGCAGATGCACACTCACTAAGACCGCGGTGATCACAGATCAGGAACCA

AGGCAAGCTTCTCCTGGGGTGGAGCTGACG

>Unigene58152_C-W 1 264 LEN=264; minus strand

XCCTGAGTTAGGCAACAGGCAACCGGGGTAACTAAGACCCTGAGAAATCAGTCGGCTTAC

TTTGGCCTCTCCATTGGTAAGTGTGGCATGGGGCCAGAGCAGAACTTTACACGCTATTCC

CCTGGGGTGGGAATGCTGGCACTTGGCGAGATGCGGTCAGAAATCCTCAGCATTCGGAAT

TTTAAAATCTGTGGCCCTTGTGCACTGAAATGCTTAGTGCTGAAGTTTTGCAATGCCTGC

AAATTACTTTCCAAGAGGTCAGAAAXX

>Unigene58290_C-W 1 150 LEN=243; minus strand

CTTGTGGACCTGACGTACAAGGTGGAATTCTGGAAGGAGGGCACCACAAACAAGGTGGGC

AGTCCCTCCCCCGCCCCCAGGCCAGGTTCCCGCTACCGCTCCACAGCCCAGTGTGCCTCA

GGCTCCCGCTGCTCCCTGATCCGGGTGTGC

>Unigene58385_C-W 1 201 LEN=201

XXAGTCCTGATGGCCACAGTGAAAAAGGTCATGGGTGGAGAGAAGCAAAGTAGGAAGGAT

CATTTGAAGCACAAACAAATGGGGAAACTGAACAGACAATCTCAGTATCACCACATCTGC

TTCAAAAATAGCACACCAACTCCCTTCCAAAGTGCATCGTTACACTGCACCATCGTGGAA

GAAATGGAAGAGCAGGATGGATTX

>Unigene58397_C-W 1 238 LEN=406; minus strand

XXCACTCCAACACATACTTCCTACCACTACACTCACATCCAGGTTCAAGCTGTGGGAGAC

ACAGGGGGAAAAAAAGATAACAGGGATCTGCCCTACCCTTTTGAAACCACAGCTCCTCCG

ACTGAAGAGGAAAGTTTAAGATCCTCTGTTGTAGCTGTGCCAATGCTGCTGCCAGAGGAT

TTCCTGGGGACTGGAGTACAAGAGAACACAGAAATGGACAAGGTAGAAAAGGAGAGAAAC

>Unigene58405_C-W 1 373 LEN=373

XGCAAGAGAGGGGTAGAGGAGGGAAGAGGAGACCTGGGACTAAGAGGAGGAGAACCAGAA

AAGAAAATGATGAGAGAGTCTCTGAAAAAAATAGAGAAAAGCCAGGATGATGGCTTATGG

AGATATGAAGAATTCTAGGAAGTGGGAGGAGGGCTAGAAATATATGAACTGGGAATGTCC

ACTGGAGCCAACAAGGATAAGCAAGAGCATGAGCCGCACTACAGCTTCATGGAAGTGACT

TTCTCCATTTCAAAAGATTAACTCATGCAGAGGGGAGAGAGCGGGAAGCTGAGACCAAAG

AGGACACATGGGGGTTGTAACTTGAGTGACACCATTGTCCAAGAAGGGGACACAGAAGGA

GAGTTAGATCTGGGX

>Unigene58574_C-W 1 253 LEN=253

XGGGGGAGTCTATTTGAAGGCTACAATTTCCAGGGGAAGGCCTCAATGGTAAACTGTGGT

TGGTTTTGGTCAAATTCAGCTGCAACAGAGTACCAGCTACCCAGTGGACATGCATATATT

TCTGGAGCAGCCTGCATGCAGCTCATGGGAATCAGAGTGGACAGTAAAGACTCCATCCTC

CGACAACTGGGACCTGTGCTCTGGCTGCTGCTGGTCTTGGTGGTATAGACACTCAGGTGG

GCAGCCATCATGACX

>Unigene58610_C-W 1 201 LEN=201; minus strand

XXTTTTTTAGGGACTGGAATCCATGCTGCAGAGAAAATACTTAAACTGGTATCAATTTAT

CAGGATAAAGAAAATAGAGGATCAAGCTGGGTAGGTGAACAAAGCCAGGAAAATGTCAAC

AAAGTATTTGAATATGACTCAAAAAAGAAAAAGAAGAAAAATAGAAAAGATAGCTCTCTG

AAGTTTTTCTGTACCAGGCCTCAX

>Unigene58721_C-W 1 240 LEN=240; minus strand

CTGGGATTAGAGGTGTACACATGATGTCTGGGTAGGCGGCTTGATTTCTTGCAGTTGTTG

TCTTCACCATCAACAGCATTATTAGGCTATGAAGCTAAATATGATCCTTGCTCTTTAGAA

GTTCTGGATAAAGGCATCAACGTTAATAAAACCCAGAGAGAATGCAAACTGAAATGCGAT

CTGGTAGCTGGAGGACTTGGTCTCAGTGGAGGACCCCGAGAGCACCGGGAAACCGAAACX

>Unigene58751_C-W 1 203 LEN=203

XXGCAGGAACAAACTTTATTTCTGATCTCCACCAGCACACTCCCCGGGAACTCTCCCGAA

TACCACCCACATGGCTCCTCCAGGAACACACCACACACCAACCGGAACTCCCTCCTGGAA

CACCACCGGGCTGTGGTGTTCCAGGAGAGAACGCACTCAATCTCGAAACCCCCACGAGAA

TTCAACGGGAACTCCAAAGTAGCGGXX

>Unigene58757_C-W 1 319 LEN=319

TGGGAAGAGAGTCCAAAGATTTCTCAGCATGTTAATAGGATCTATGATGCTTCAATTCAA

GAATTAGGCAGAAAGGAGCAATTAGCCAAGAATAGAACCTTGGGTCTGTTCACTCCTAGC

TGTGTGGTGACAGAGGACTTTGGAGGTGTGGGCACTGCCAGCCTCTTCTGTACCAAGCTC

ATCCAGGCAGAGGCCCCAGAGCTGGAGCTGAGCTGGTCCACTCTTCTTCTCTGCTGCATG

AAGCCTTCACCTCACAAAGGCCACCGAAGTGAAGGATGGTTGGAAAAGTGTGGGGTGTGG

GGACCTGACTGGCTGCTG

>Unigene58769_C-W 154 267 LEN=267; minus strand

CTGGCGCCGTGGCCTGTGGGGCAGCTCCAGGACACCCTCTTCTCCTCAGTGAAGGTCTAC

GTCGGGGTCAAGCAGGAGATCGCCGAGATGCGCATACCCGCCCTCAACGCCTAC

>Unigene58790_C-W 1 225 LEN=225

XXGCCTGATGCCCGAAGGTCACCAAGACTGAAGGGGAGCTGCTGGGGCGCCATGTCACCC

AACTGGGAACGGCGGTGGAACCAGGGACAGCCTGGGGACCAAACCTACATCCGTTTCAGC

TTCCTTGAGGGCCCCCGGCCAACGCTCAAAAAACTGCCCACGACCTTCCTATGCCTGTTG

CGCAACACTCCCAGGGAAGGGGGACAACAAGTGTCCCTGCAGACCAGX

>Unigene58817_C-W 1 261 LEN=261

XGGGAAGTGCAAATCGACTCTCCTGGTGTGAAGATAGGTGATGAGGGGGTTTCACAGCAT

CTTTGGGGTCCTGTTGGGCCCCATGGTTCTGTGAAAGTGCTTTATCTCCTGATTGGTTCT

CTGGTTAGTGCCCTGTCCCCACCCACTGTATTGGAGAAGTCCATTGGCTGGTTTTACTTT

AGCTTCCTGGACACCTGGTGTGGACATCATTCCTACCAGTGGGCACATCTTGTCCTGTGG

CCCCTGCACATCCCTGCCCCCAXX

>Unigene59051_C-W 1 270 LEN=270; minus strand

XXTGTTCTAGAAGAACTGGTCACTGCAGTTAAGTGGGAACAAGAAGTGACGGGCGGAGAC

TGGGAAGGGAGGCAGACGGCTGGCCGCTTCTTGGAGCTGATCTTATTTCGTTGCTGGACT

AACCAAGGAAATCAGCCAACACTTGGAAATGATAATGGATGTAGGAAAGAAATAAAAAAT

ATCAGACGATCCAATAGAAAGTGGCCCACCCTATCATCCAGTGACTCTGGAGGCTGCAGC

AGGAGGACTGCAAGTTCAAGGCTAGCCTCAGCX

>Unigene59080_C-W 1 220 LEN=220; minus strand

GTACTCTTTTCACTGCATGAAGCTGTCTCTCTGGCCAAACTGGATGAATATGTTCAAGCA

GCAGAAAAAGAAGGGTAAAACCATGAAACAGATGGTGTTCACACCTCCAAAGAAGTGACC

ATGGCCATGTGCAGAGATAAGCTTTTGTCAAAGGAACTAGTGAAATCGCCATCCATGGTA

GAAGGAGGGAAGAAGAGGGTAACAAACTCCAAAATTTCTCXX

>Unigene59086_C-W 1 211 LEN=211

GGGCAATGGATAGTGGGCAACCAGGAAGGGGTCAAAAGCAATGAAAGAAACAGGGCATTA

GCAGTATCTCAAAATCCAAGCTTTAGAAGATCATCTCAAGAGAATGGTCAACAAGACCAA

ATGAGACTGAAGGTCGTAGTAAGGTTGGGACGCACGAGGTTTCACGTGTCAAAAGGACCA

AGGTGCGACGGGGGCAACTTCAATCTCAGTGXX

>Unigene59161_C-W 1 254 LEN=254

XGGAAATTCTCTCAACCTGATGAAAAGCTTCTATTTAAAATCTCCACAGTTAACATCAGA

ATTCATGGTAAAAGATAGAATGCTTTTAAGATCAGAGGCCAGGTGAGGATATCCACTTTA

ATCATTTCCAATCACACGTACAATGATGGAGTTCTAACTAGTGCAAAAAGCCAAGGGGAG

AAAAAACCAGAAAAACAGAAAAGAAAAGGCATACAAATTGAAAAGAAAAATAAGCCATCA

GTATGTAAAGACGAC

>Unigene59171_C-W 1 243 LEN=243

CAGGGATGCTATATTATGGAGAAGTTAGGAATGCAGACCACAGCAACTGTGCATTTTCTC

AGTGAAGTCATTTCATTTTCCAATGTTTTGTCAAGTTTAAATAGTTTTGATTTGTGGCTA

CTAAAAGAAAAATGCCCATTTAAGATAAAGTATAGTTACCTGAGCATTGCACAACTTAGA

GGCATGGTAGACCTAGCTAGTGGAAACAGAGATGAGGAAGACTACTTATTAGGCTCCATG

AAG

>Unigene59174_C-W 1 206 LEN=206

GAGCTACATGAAGTATTCAAGGTCATGGTCATGACTGTGACATCCCATCCTGTTACTTCT

CCCCAGGGTGCTCAGTGTCAAAGGGAGCCTGGGATATGTGCCACAGACAGTGGTCTTGCA

ACAACCATGAAGGCCTGTAATGTGGAAATGTTTGCAGTTGCTGGACATTCAGCCAGTTTG

GGACAAAGGAAAGCTAGAGATAGATXX

>Unigene59189_C-W 1 252 LEN=252

XGAAAAGTTTATAAAGGAAGAAGTGAGATGATAATACGGGAGGGCTTGGAAAAATTAAAA

ATCAGTTCCTAGCACACAACCCTGCCTTCTAAGACTTCCATCGAGTTAGAATGTGTGCCC

TGTGATCGTAGGGCCCTGGCACGTTTCTGTAAGATCCGTCTACGAATAAGGCCAGCCGAT

CTTAGAGACAAGGAGACTAAGGATGGCTTTTATTGTTATCCAGGTGACTTGGCCCCCAAA

TGCATAAGGCCCAXX

>Unigene59306_C-W 1 218 LEN=218

AGAGGGAAAAGCTTCGCCTCCACAGAAGGAAGCTGTTAAGAGGAACAGATGGAGATGGTG

TCTAAACAGCTTCAAGAAGGTGTGCTAGTCCCAGAGGTGCTTCCTGTCTCCCAAGAGCTG

TTAGAATTAGGAGCCATTTGGGGACCTGCAGTGCTTCTCCAACCCCGACAAGGACCCTCC

ATCAGCCCAGCACCACTGAAACAACCTGATATCCCCAGX

>Unigene59426_C-W 1 204 LEN=204

XGTTTTTTCTTCTGTTGCACTAAATCAGTCCTGCCGAGCTACCAAAAGAGAGGAGAGAAA

GAGTTTCCAGAATATTCTGAATTTGATTCTCATCAGTTGCTGATTACTGGAGAGCTTAAA

GATGTGGTTAAAGAACAGCTGTCAGTCATCTTTGAAGAATCACTTGTAAAGAAGGGACCC

AAAGGGCTGGGGTTGTTGCTCAGTGXX

>Unigene59427_C-W 1 243 LEN=243

XXGGCTGACAGTCCATGGCCATTTGAGACCCTGTTGCTTTTGGGCAAAGTAGCACATCAT

GGCAGAGAGTATGAGATGGAGCAAGTTGCTCACCTCATAATGACTAGAAAGCATCAAGAG

AAAGCAATTGCATTCCACAACCTTCTTCAAGAACACATTTCCAGTGAGCTAAGACCTCCC

AGTAGGGCCCACCTCTTAAAAGTTCCACCTTCAAATGGTGCCAACCTGAGGACCAAGGCT

ATAACX

>Unigene59429_C-W 1 206 LEN=206

XGAAAAACATACGTTAGAGAAAAGATAGACTTTTAAACAAATGGTGCTGAAAAGCTGGAT

ATCCATATGAAAACAAAGGAAGATTTCTGTCTTTTACCTTGCCCCAAATTCAACCCAAAG

TGGACCAAAGACCTAGGAATTGGACCAGAAACTTTGCAACTGCTAGAAGAAGACAGAGAA

TCAACACTCCAACATATTGGCTCCGAC

>Unigene59497_C-W 1 237 LEN=237; minus strand

ACTGACCACATGTGGTGGTCTGTGATCTCACTTGCTTTGATTGATTTTCATCAGAAAACT

ACAAGATGGCTTCTCTGCCTATACCTTGTTTCTTTGTCAGTAACCTTCATGGAGCCTAGG

CAAGATGTCTCTCTTGATCCTCGGCTCTTTACCATCATTGTTAACCTCCTTTATGCCTCA

CAACTCTTTCATTGGCATCATGCAGATTTGGATGATGCCCTGGTCTTTGTCTCCCGC

>Unigene59555_C-W 1 235 LEN=235

GTGGCAGTAGAGAGAGAGGAGAATAAGTACAGGGATTTGGAAGAAATCTCAGAAATTGCA

CTGCAGGACTTAAGGGGTAGATTGGTTCTGCAGATGGAGCAAGATACAAAAATTAAACAG

CGTAGCAGTGTTGGTTCTTCCTCTCCTGCCCTTACACGCTTTTCAGAGCTGTCACATTCT

TCACAGAATCCTTAGAAAGACCCCATGAGGAAGGCTCCCCATGTGACAGACCTGGXX

>Unigene59592_C-W 1 257 LEN=257

AGGAAGGACCCCAGGGAAATGACTGAGTGGGGTTCAACCATGTTGGATCAGGGCAGGGGC

TGCCGGGGAGGAACCAAAGACATAAATGACATGGCCGGCTTTAGGTGTGAGGCCACACTT

CCTGAAATACTACACAGCACAGCACACAGTCTCAACTGCAGGGACTCCTACATGACAGGA

CTACAAGACTGGACACTGCTCATCTGAAACACGCAAGGTCGCAAGGGTTTCCGATCTCAG

ACATGTGCAGATTCTGGX

>Unigene59602_C-W 1 89 LEN=207; minus strand

XCCGAGGTCTTTGTGGTTCAAAAGCTGAGGGATGCTGCTTCGGAGATGGAGGTCCCTGTG

GATGAAGAGGATAATTCGGATGTAGCTAAC

>Unigene59725_C-W 1 201 LEN=201

XXAAAAAACATGAATGTTATGGATGTCCAGATACAGGAAGCATTTAGAACACCAAATGGA

CATGACCAGAATAGAACTTCTCCACGTTCGTTTATAAAAATTGCCAAAAGTACATTGAAA

GATGTAAAAAAGGCCAAAGCACTTATAGAATAAACTCATAAGAAGAACATAAGAGTTATC

AACAGAAACTTTGAAGGCCAGGGX

>Unigene59756_C-W 1 240 LEN=240

XGTGCTGGTGAAACTAGCAGAATAACAAGCATCATGGGTTGCTGGTTCACATCCTGCAGA

AACAAGAAGAAGCAAACACACTGGAAAGACCAAGAGAAACTCCTTTTTCCTGCAGCGCCC

TTTCAGCTTAACTTGAGTCAGCTGGCTCAAAAGAAAAATTCCCTGGTTGCAACTACAGTA

GTACAAAGCAGGGCAACGAAGGGCAGACTGGGAATAGAGAGGCAGCAGGTTGATAACCAG

CXX

>Unigene59759_C-W 1 160 LEN=230; minus strand

XXGCAGGAGTGTATGGTGGAGCAAAGCTGCTCATCTCGTGGTTATAAGTGGGCTCTTGAG

TATCAGTTCTCTGAAGATGATGTCAGCTTTGGGATGAGGAAGGAGATAATCCATTTGGAA

GCTCTGAAAGATCTTATGCAGGAGAGTGCTTCTGAAACACAG

>Unigene59827_C-W 1 336 LEN=336

XXGTATGAGTCAGATTTGTCATGGATGGAGGAAGAATGGAGGAAAATATCTAGAACTCAG

CGCATTGCCAAAGAGGAGGGACAGGAATGGTGGGCGGAGTCAGACTCAGAGGAACAGGTA

CCTGCTGAGXTGAGAAGAGGCCAGGATAGCAAGCGCAGAGCTCTGTGGGTAGAAGATGCA

GATGGCAGGCAGGCAACGAAAGGCTTTTTGGAGAGGACATTGTTCGCACACTCCTTATTT

GGTTGGGCACTATTTAGGGGCATCCCAACAAAGAGAAATGCACAGGCAGAGAGATTCCAA

GGCTGGAAGGTACAAGCAGAGAGATTCCAAGGCATGATT

>Unigene59841_C-W 1 218 LEN=218; minus strand

XCCAGCTTCCTGGTTGATCCCCCATCTCTACAGGCAACTGCCTCCATTCAGAGGCCACCT

CTCTACTCCATTAACCTCAGACAGAAGATGGAAAGAAACGGTGTACATTTCCTGCTTCTC

TTAGATCCCTCAGATTTGGGCACTGACTCTCCTGCATCTGAGATTAGACATCGTGGTGGC

AGGGCAGGAGGTCTCAGCAGGGAAGTTCTCCACATCACA

>Unigene59860_C-W 1 204 LEN=204

XXTGTGATTCATTCCCAATAATTAACATGTCCTCAGAAAAGACGAATGCTGAAATCTAAA

TCAGACATGGAACTGCAGCAATACCAGATTATCACCACATTCCTTCATGCATTTACTCAG

TCTCAAGTTACATCTCACAACCATCAACCAGTGGAATCAGCTTTTATAACTAGACAAAGA

AAATTCCTCATTGCACACAAAATCAGX

>Unigene59861_C-W 1 225 LEN=225; minus strand

XXCTCCACAGCTCCAAAGCTCCAGAACATCACAGTGATACCTCCTTGGCTCAGATCAGAG

ACAGACTATTTTAGGGAAAAACAAAAATTTGTGTTACTTGCCATTGGCATTCCACACATT

CCTTCCTGCTGGAGAGAACAGCAACACAGTGCCATCTTGGGAGCAGACAGCAGCCCTCAC

CAGACAACTATACCTGCCAGCACCTTGATCTTGAACTTCCTAGCCTCX

>Unigene59933_C-W 1 205 LEN=205; minus strand

XXTGATACTCAAGGTGTGTCCATGAATCACGGCATCGGCATCACCTGGGGCTTTTTAGAG

ACAGACTCTCAGGCCCCACACAGACCTGTGAATCAGAACCCGCATTTAAAAGGATCCTCA

GATGACCTGCATGCACATAAAGTTGGAGAAGCCCTGCTCAAGTCAACCTCCCATTTGACT

CTTGAGAAAACCAGGAAAGTCATAGTX

>Unigene59942_C-W 1 211 LEN=211

XGGGCTAAACTTTTTCATGATACAGCAGATAGGAAGAAAAAGTTACATATTCAGATCTTG

CATCTCCTTGAGAGTGAGTGCCAGCTAAATTTTGCAAAACACTTCACTTTGCCTTGTCCT

AGTCCTGGCCCTAACTGCGAGATTTTACCCAAGTTCTGGAAACTAGCAGTTAATAAGAAC

AGGATACCTAAAGGCAAAGAACAATTTGTGAGX

>Unigene60087_C-W 1 210 LEN=210

XXGGCCGACCCATACTACAATGGCTATACCTTATTCTGGAGCACCAGTGCAAGTGTCCCA

GGAAGTAAGAAGGAAGGCCTGGTGTTATTGAAGTAGGTTTATGGAGACATCAAAGAGGAT

GGCTCCAGGAGGGTACAGTGTGATGATCAGCTTTACAGTGAAAAATGTGATTTTGTCATC

TCATACAAAGATTTTGTCACACTAAATCAGACX

>Unigene60093_C-W 1 252 LEN=252; minus strand

GCCAAGGTTCACGTGCTAAGCCCAGATCACAGTGAGATGACCAGGCTCACAAGCTCGGGG

GCAGATGAGAAATGGCAGCAGGAAGAACTGGGAGTATAGGAAACAGCTTTTATACTCAAA

GGTTCAGAAGAGCAGGAGCACATGAGTCTCAAGCATGTGTCTCGAGAGAACGTCACAGCA

TCTGACGGATATGCCACAAGGTGCAGGTTCCTTCTCACCTCCTCCCCTACAGCACACACA

GCCCAGAGCTCC

>Unigene60122_C-W 1 114 LEN=253

GATGTAATGGTGATGGTGGTGTTGATGATGATCATGGCAGTGATGGTGATAATGATGGTG

GTGGTAGTGGTTGCTGAGGTTGATGCTGATGGTGGTGCTAATGGTGATGGTGCA

>Unigene60166_C-W 1 221 LEN=221

ATCAACCAGCAACTCTCGCACAGTTCAACTTTTGTCGAGGGACGAAGGGAGATCATTTGG

CACTGACGGAGCTTGTACTACGTGTCTGCCACTGAAGATTCCAAATACCTCTTAATTCCA

TCTACCAAGATCATCTGCCCTTCACTGCAGACTAAAAAGCAAGTAGCAATTGGACAGTTC

TTGGCGGACACAACATCTTTATGTGGTGCTGAGGATCGAACX

>Unigene60262_C-W 1 179 LEN=448; minus strand

XCCTCAGTTACCTCTGCTGTTCAGTCTTCTGCCTCTGAAGCACCTGGTAACTTAGACCAA

AAGATTTCGGTTGTGAAAGCTCAGACATCTCAGAAATCTCCAAAGGCTAAACCCCAGACA

TCTCAGAAATCTCTACAGGCTAAATCCCAGGATCCTGAAGAAATAACTGAGCTGGATATC

>Unigene60339_C-W 1 57 LEN=260; minus strand

TGGAACTGCGGCTGCGAGTGGAACCGCACCTGCATGGGCGACCACTGGTTCGACGTG

>Unigene60424_C-W 1 217 LEN=217; minus strand

XXTGATGCTGTTTGTGGGATACACATGCCAGGATTAATTAGTGGAATCAGTAATTGGTCC

TCCTTTTTGTTCTGGAACCCAACTGAGAAGGCTTATCTCCTGGTAGTTGTGGAAATGACA

CCCAACCATCTCCTCCGAGGAATCTTCACGGGCATGGCTTCTCCAAGGTTATCTTGCTCT

CCTATTGGATCAAGGACTTAAGGAGAAATAGATGTGGAT

>Unigene60450_C-W 1 290 LEN=290; minus strand

XXCCGGTGGGAAGATCAGAACCCTCCCTTGCAATCTGTTGAAGGCCATGGTGAGCATCCT

TATCCTTTTGGACAGACCAGGAAGGGCCCGGCCATCCCCACTTCCAGGCTGGACTCCTCA

GAGGTGCAGTTTGACAXCTCTCTGAAGCGGATTCTAAGTTCCGAGTCGGCCGAGCCTGGG

AAGTACTACACCGCGCGGTACAAGTGCAGCCTGGCACACTCGTTCCACACCCGGCTCCTC

GCCAGCAAGCCCATTCCCAGGCTCAAGAAGTATGATGAGCTTTCAAAAAGAAAX

>Unigene60472_C-W 1 234 LEN=234; minus strand

XCCCAGGCCTGGCGAATCCCCGGCGATCCCCGAGGCCGTTCTCACGGCGTGGGGGTCTCC

CAGGCCGTTCCCGCCCCCAGCGGCACCGCCCAGGCTCACAAGCGCCAGCCCTCGCCTCCC

CGGCACGAGCGCCCGGCGCTACGCCCCTTTGTCCAGTCAGTCCTGCCGACGCGCCGCATC

ACTTCGGCCCAAGGCCCGCGGCCCGCGCTCACCGACCCCAGAGCCACCACCACAGXX

>Unigene60530_C-W 1 296 LEN=296

XGCTTGAAGCATTCAGTTTTTCTACCACTGCGTAGCCGAGAGXTGGAAAAAATAACATCT

GAAACAGGATGGGCTCTCGAGATGCCTCGGGTGACAGGCACTTTTCAAACCACTGCAGAA

GTCCTGGACAGGATGCAGAGTGGTGTGGCGGGAAACGCTGTAGGCCTGGGCCTAGAACTT

GAACCTCCAGAGTCTACCGTGGAGCTCACCGTGTGCCAGGAAACTACTAAATTCAGTAAC

AGCCAATATGATGAGCTTGTTCACTCCAGCTTTTGTTCAAGAAGCAACGACCGAGGGAXX

>Unigene60555_C-W 1 201 LEN=201

XGTTCAGTGTGGACATGTTATAATCTGGAAATAATTTTGGCCAGACTAGGAAAGCTACAG

ACTTCATTAGGTAAAAAAATCTTAAAGCAGTTGTATACTGTATTTAGAGAAAAATATGCC

GAGGGCATGGGCCTATTAGACAGCCTGAATACAGCAGGTGCATTTGAATGTGAAAGAGAA

GACGTGAAGACTGAAAAGCCCAXX

>Unigene60578_C-W 1 252 LEN=252

XCACAAGGTGGAGGTAGACGTGGAGGTGAAATGTCGAACATGAATGGCGCTGATGATGAT

GAAGCGATGGACATGTCAAGAGGTAGAAGAAGGGGCGGGCAAAACAACTATGAACGCACA

CAAGGTGGAGGTAGAGGTGGAGGTGAAATGTCGAACATGAATGGCGCTGATGATGATGAA

GCGATGGACATGTCAAGAGGTAGAAGAAGGGGCGGGCAAAACAACTATGAACGCACACAA

GGTGGAGGTAGAGXX

>Unigene60672_C-W 1 212 LEN=212

XCAGGTAACGGGGATAAAAAAGATGGAGGAAAGCAGCAGATCCTTCTCCATCGTAGACCA

TCTTGGGAGCAGGTCCTTCACACCGGGGTGCGGGACTTTCATTTTAGAGTGCAGAGGTGT

GCTTTCACTATCCCCTTCCGTGGGGACTGCATCCAGGGACGTCTTTCCAGGCTGTTTGCC

ACCCAGTACCTAAGTAGCAACAAAGAAGACCAG

>Unigene60861_C-W 1 356 LEN=356

GGCATGATGGATATAATGCCAATATTGCTAGCTGCTAGACGCATCAAATGGGCAGCAGTT

GGCTGGATGAATGAGGCTATATTGAATTCAGACCCTGACCATGGATTTACCTTTXATGCC

AAGTTATACCATACCTACTATGCAAAAGCTTGTGTTATACCACCCTTTGCCATGCTAATA

ATCAATGATACTGATGACATAAATATCACTCATACAGAGATCCCTTGTGCAGTCAATATA

TCCTGCTGGCTCACAAATTGTCTAACCCCTGATATGAAAGCTAAACATATATTTATCCTC

AGCATGCTGCCAGCCACATTTGGCTACAATGAATATGTCCAGAGAATGAAGGAGTCT

>Unigene60895_C-W 1 274 LEN=274; minus strand

CAGAGCCTCTCTGAGCCAACCAGTCTTCTAGAAGAGTTTTTCAGACTTGTATGGGGTTAT

CAGTATGGCAGGGGCACACAGGGAGGTGAAGAGGATCTGGAGGCCACAAAAAATTGGCAT

CAGCTCTACTTGTTAAAGAGACACAGCGCTGCAGAGCCAAAGAGCATTGTGACTACAAGA

GAGGGAGTTTCCTGGGCAGCAATTCCTCGATGCCCTGAATACCCACAGGCAATTGCCCAT

CCAGGGAAAGAAGAAAAACGAGAACACAGAGAAAXX

>Unigene61178_C-W 1 254 LEN=254; minus strand

XTGCCAGAGGATAAAGACCAGGAGGACAGGTCTGAGAATGCCCACGGCATGGCTGGGCCA

CCAGAGAACACCTGAGGTGGAGGTGCACGCGCCACACAAAACGGAACAGCAGAGCTGCCC

GAGGACCAGCTGGTGCCACAGGTGAAGCTGTTGGCTGCCCTGGTTCTGGACTACAGAGCC

TGGTACGGCTCACACACTGCTGGAGCCAGACAGAGCCAGGTGAGCCCAGAGCTGGGACTG

TCACCAAAGGCCTGC

>Unigene61287_C-W 1 266 LEN=266; minus strand

XXTCTTAAACGATTTCAGAATGAGGATGTGACAAGCAGCCATGGCCTGGAGCGGCGGTGC

ACAATCCAGATTTTTTTTTTCCCCGTAAAAAACAAAGAGCAAGAGGAAGAAAGTGAGTCG

AGTGTCATCCGTGTGACAGATGAATGCTGAAGCGATCCTGTGGGTGACAAGGTGATTCCT

GTGAGAGACCAGGACCTGGGGGTGTGGGAGCCGGCCACAGACAAGGACAGACAGGTGACA

AGGGAAGCCTGGAAGAAACCACACCACAXX

>Unigene61500_C-W 1 239 LEN=336; minus strand

XTCATCATGAAGAGCTCCCCCGAGGTGTCCAGCATCAACCAGGAAGCACTGGTGCTCACG

GCCAAAGCCACGCACCACCTATTCTTCAGTATAGATAGAAGAAGTCAGAAACAGCCCAGA

ATGAGACCACCAGGAATTTCAGAGCAGCACGGAGAAATAAGGTCTCCAGCCTCTGATGAC

ATGGAGAAGGGAATCGAGAGCAGCATGAACAGAAAGGATCCGCCACATTGCTGTCTGCCA

>Unigene61544_C-W 485 568 LEN=568

ATGTACCAGAGCCTGGCCATGGCCGCCAACCACGGGCCGCCGCCGGGGGCCTACGAGGCG

GGCGGCCCCGGCGCCTTCATGCAC

>Unigene61592_C-W 1 442 LEN=442; minus strand

GTTCACGGCATAGCCTCCAACTGTGAGTGGATGGTGCACTTTGTGAATAAGGAGATCATG

GCCTCCAACATAACCAGCCAGGAGGAAGGAAGCTTCACAGTCTCGACCAGCCTCACAACG

GAATACGGATCCATGGAGGTGGATATGACCTACAGAAAACAGGAGAACGGGATATACTTG

CAGAAAAGCGAATGGGGGGACAAAATCTTAGAGAAGAGGAAGACGGATTGCAAGACCTAC

ATCATAACCTGCATCAAAGACGTGCATGAAAACAATGGCAAATTTTGCAAGATTGTGTCC

CTCTACGGCAGGACAATGACGGCCTCCCCTTCCATGAAGCAGTCCTTCATAGACTTTGCT

ACGGAATTGCAGATAGACAGAGATCAGATATTCCTTTTGGCCAAGAAAGACGACGACGCA

CAATCTGCCTAGCCACGTCTCAXX

>Unigene61833_C-W 1 280 LEN=280

GTCAACAGCGGTAGAAGAGCAAACATGAATTCCTTCGTGCCCCTCGCTCTTACACTCCTG

GCTGTAGCAGCCAACGCTGCTCCTGCTGCGCAGATTGGTCCACTCTTCGGCGGCACCACC

ACCACCACCACTGAAAGTCCTGTTACCACTAGCGAACGTACACTGAGTGTGGGCAGTCAC

CCAGTGCCACTAAATCATGGCATTAAGGCTCGCAGTGCCCAGAGTCCTTTAGATGACGAT

AACTCATCAACTGAAAAAGATGACAAGACAGAGCCTCCAGXX

>Unigene61834_C-W 1 253 LEN=253

XXTGGGCCGCCCCTGTGGACAAAATACCCAGGGACTCTGAAAGACCAATCATGGCTGTCC

TTTCTGTGTGAAGATGAGTGTGTTCATCATCTCTCTATGCCTGGCATCCAGGGCACAAGT

GACTTCCTGTTGGCTCAGTCAGTGTTTGCTGAATGGAACTTCCTAGACAGGGATGAGCAG

CTTGGGAGGCGCAGGGTCCTTCCCTGGAATGCTGCTGCACAGACCCATGAAGCACCAGTC

AGGACAGGATCACAG

>Unigene61849_C-W 1 89 LEN=273

XGGCAACGCAGGGAAATGGCTGAGTACTTGCGACTGCCCCACTCCCTGGCGCTGATCCGC

CTCCGAAACCCGGCGGTCAACGCGATCAGG

>Unigene61866_C-W 1 326 LEN=326

XXGCTGATGATGATGAAGCGATGGACTAAGAGAACACAAGAAAATAGAGAGACTGAAACA

CAAATAAATGAAATGACGGAAATGACAGAAATTATGGAAATAATTGAATATGCTGCAAAA

GGTCCAAGAATTTTCCCCATATCTGCAAAAAGACTGTTCTCCTCAAGGACATCCAATGAT

CCAGCCTACTCAGCGCTTGATGGTGATGGGGGCAGTAGATACAATCTATGGCTTGATTTT

ACTTTGTTTCCTGTAATAGAATAGTTTGTTGCCCTTGGTGTGGGGAAAAACTTTGTGAAA

ATAAAAAGAAATAAATCCCAATTGCACGXX

>Unigene61871_C-W 1 219 LEN=219; minus strand

CTTGGTATGCCAGCAGGTGGCAGTGCAGGTGGACTTGGTGGTATTACTAATCTGTTCAAA

AACAAGAAAGGCGGAGGTAGAAATAAAATGGGCATGCCACGAATGGACCCGTCAGAAGAT

GGAATGATGGACGGGCAAAACAACTATGAAGGCGTGTCAGGAGGTAGAAGAAGGGGCAGG

CAAAACAACTATGAAAACATGAATGGCGCTGATGATGAT

>Unigene61925_C-W 1 268 LEN=268; minus strand

XXCCGGGAGCCACAGGCCTGCGCTTCCCACAGCAGTGACCACTCCAGCGTCCCCAGCCGG

GCAGCCGGGAGCATGTCAGGGGCCACCGGCTTTGCAGAGCTGGGCCTGGGGCTCCTTGGC

ATCCGCTCACTGGCTGTCACTTGGGGTGGCTCCCACCGAGCCTTGGAGCCACATGCTGTG

ACCCTGAGGCCACCCACATGTCCCAGGGACACTGCTGCCCTGGGCCTCTGGAAAAAGCTG

ACTTTGGAAAGTGGGACTTGGAGACGGAAG

>Unigene62171_C-W 1 317 LEN=317; minus strand

CCCGCTGAGGGGAGGGACCTGGGAGGGCCACTAGTACGCAGGGAGTTGAGTACTGAGACG

GCCGCCGCCCTTCCCGAGCAGCAGCAGCAACAGGTCCTACCCAGGATACTGGGCGCCCGG

AAGCCCACTTTCTGCACCTCCAATCGGGGTCACACCTGTGATACGATCAGCTCTAGGCGC

CTCAGTCTCCCTGCGGACACTGAGACAAGAACGCGAACTGTGCGGAACGGACACCGACCT

ACGGTCACGAATACCACAGGAAGAACTACGCACCTGCGTAGGTGCCGGGTCAACGCAGCC

GCCTGCGCGCACGCGCGX

>Unigene62240_C-W 1 236 LEN=236; minus strand

XXAGCCAAGATGGCGCCGGCGGCGGCCGCGGCGGCGGCTGTTGGCGTCAGCCGGGGCCTC

GAGTCTCTCCTGGGGTGAGCGCTCAAGCGCCTCTCCTCCGGCCGGGTTAGCCCGGGCAGA

GAGGAGACGGCCACGGCGTGAGGGGGCTGTGAGGTTCAGCAAGAGCGCGGCGCCTCCTGC

CTGGGCACCATGGCTGGGATCATCAAGAAACAGATCTTGAAGCACCTCTCCAGCGTTCXX

>Unigene62444_C-W 1 286 LEN=286; minus strand

CCAGCATCCTTTAACAATCTGGGTCAAATGGGCCGTATATGGCTGATCGCTTATATACAA

ACATCTAATAAGTTGGTGGAAAATGGAAGAGGAAGAAAGAGTTGTGAGAGTCTCAGAATT

GCACAGTCTCAGAGATGCCCAATGAGAACAGGCGTGTCGTCTAAGGAGGGAAAGGGGTCG

GGGAGCTTGGTACGGAAACCAGCGGGCAGATGCCAGGCTGCTCGGCTTGGGATTTACCAC

CGCTTTTCTGGTTGGACTTGTGCAGGTAACTTAAGGGCTATCAACCXX

>Unigene62462_C-W 1 201 LEN=201; minus strand

XCTGAAGTTAACTTTAAGGATCAGCAACAGGGAGTTCAACCAACTTTAGCGTTTGATGTG

ACTACAGCTTCCTCTTTAATTACCCTATCCCACTTAAAACCTATGTCCAGGCCAGATGGC

CAGGTCAATTACCGTAACCACCCAAGGGGCAAAGAAGAATTCAGGGGAAAAGCCCATGGG

GCTCAAAGCAAGTGGGACATTTXX

>Unigene62540_C-W 1 262 LEN=262; minus strand

XCTGTGGTCTGCAACCTGGACCAGCCCCCTGGTGGCTGGACAGGGCACTGTGCCGACCAG

GACCGTTCCCCAGTGCCCGGGCCCTGTGTGCACAGCACTGGTGGGCGTGGCTCTCAGAAG

AGTCCTGAACAGTGTGCCTTGGCAGTATCTGAGACGCTGAGCCTGCTGCACCTTTTCCTG

CAGAGGGCCGCCCTGTGTGCCTTGCTGGGTGGCCTTCCGAGCTCCCGCCACGCCCTCCCA

CAGAGCCCAGGACAGAACCCCCGX

>Unigene62547_C-W 1 277 LEN=277

GCTACTCTTGCGCCCTTGCATCTGCATCCTTCTGGGCCTGTGGAGGAAGAAGAGGGAGAG

GAGGTGTGCATCACTGAGTCAGGGGCAGGAGGTGGTCGAGGAGAGCAAGGCAGACGTGAG

CAAGAGGTCAGACAGCCTCTAATCATGGGACCTTCTGCTTTCCACCCAAGAAGGCTCATG

GGCTCCTGTTGCCTTCAGAAGAAAGCCTTAACTGGAAGAGTTAATGATAACGTCAATCAC

CATAAGCTAATTGTCTACAGTGTGACAAGCACTACA

>Unigene62584_C-W 1 260 LEN=260; minus strand

CGTCGCTCAGACTGTGGGGGCACAGCAGTGACAGGTGATCCCTACAGGCCTGCACCCTAT

GCAAAGTGCTCCTCATCAAGACGTCCCCTGTCCTCCCACCAACAGGGGCAGATCGTCTTC

ACCCAGAGGAGCTCAGCTAGCACGTGCCCAGGCCCAGGGACACCTCGCCCAGCAGTGCCA

GGCCCGAGTGGCAACCTCAGCACCATCAAGAAGAGCAAAACTGAAATGTGCTGCAAAGTC

AGCGCCGCCGACTCTTCAACX

>Unigene62593_C-W 1 309 LEN=309; minus strand

XCGCGCGGTGCGTTCCGCGCGGCCCGGCTGGGACCAGAAGCCCCAGCGCGCGGGAAGCGG

CGGGACCCTGCGCGTAGGTCCGGGCGCACGCTGGACTGGCGCGAATGGAAGGCCGGAGGC

CGGCGGAGGAGGCGCGGAGCACAGCCTGAAGCCTGCGCGCCCGCGACATGCGGCGGGAGA

GGTGCGGCGCCCCCCGCCCGCGTGCCGGTCATGGAGGTGCTGCGGCGCTCCTCGGTCTTC

GCCGCCGAGATCATGGACGCCTTTGACCGCTCGCCCACCGACAAGGAGCTGGTGGCCCAG

GCCAAGGCGCXX

>Unigene62713_C-W 1 380 LEN=380

XXAGAACCGGAAAAAGAGGAGGAGAAGCCAACGGTCCCGCCAGGACTGAATTCAACAGGA

TCAGCAATGGGAAAGATGCTAATGCTCCCAGTGGAACCAAATTCGGAAGAACTGGAGGAA

AGAGGGAAGGAATATCGTGAAAGGATGCAGCAGAACAGCGGAGACCACTTCGAATTCATG

ATCTTGAAGCACCAGGCCCTGGAATACTTCAACAAACACAGCGCTGATGCGTTCTACAGA

CCCATAGAAGATACAGACATCAAATTGAAGCGCGCCACTGGCACTATCCTGGCTTTCACG

CAATTTTTGGTGAACACCAACTGTACCAAAGAAGAAGGGCAAAAATTTTCAACCCCACAG

TATGGGATTGATTCCACCGAAGXX

>Unigene62759_C-W 1 236 LEN=236

XXGTCATATAATGGTGGTAAATCATTGTGTGCATTGATTAAAGCATTCGTCCCAGAGGGT

CCTATGGCCACTATGGTAAATAATATGAAGGAGGAAGATATAATAAAAATGCTGAATGCT

TTGGACTCAAGTGACCTTAACATGTTAATGAAAATGGGTTCAGGTCCACCTAATGTTATG

GAATTAATGAAAGTCGGAGCAAAGATATTACCTACTTTGATGAAAGCTAATGGTGGCAXX

>Unigene62777_C-W 1 213 LEN=213; minus strand

XGCCCCGAGGCGGCCCTGCGTCGGGACACCAGGAGCGATCCCTGCCTCCCTGTCCTCCAG

CGCGGGCCCAAGTTCAACCCGACAGATCCCCAGTCCCCGTCGCTGCGGGTGCCACTGCGG

AGCCTGCGACTCCAGGAAAGCAGGGTGGCCACTGCAGTGGGTGCAGACACGCTTGACAAG

ATGAAGAACCGAATCCCTGTGGTGCTCCTGGCC

>Unigene62795_C-W 1 282 LEN=282; minus strand

XGTCAGAAAATGCAAGCAAATTGGGCTTTAGTTTAGTGGTTCCCTGGTTTAGATGAGTTT

CCTTGTGAGCTCAAGGTCGTCTTCTGGTTCACTCCCTTGGGTCTGCTGATTCACCCCAAC

AATGGCAAAGCCCCTGAGGGAGCTGAGGAGAGCCTGAGAAGTGCAGAAATGCTCCACCAA

CTCACCGTGGGAACACAACTGGGGAACGTGCTTAAGGAGATGGATGTTAATGATGCGGTG

CTGAACTGTTCTTCTCTGCCAGGTGCCGCAGAAAGGTGTCCTCXX

>Unigene62817_C-W 1 281 LEN=281

XXGGAAAAAGCAATTGATATATCGGCCAATGCAATACCAAAGCAGAACAGAATCAGAGGA

CTGAGACCACCCTACCTTAACACTTACTGTACACCTGCAGCAATCAGGACAGACTGGGGT

TGGCAAAAGGATAAACAAATAATGGAATGGAACAGAGAGTCTAAAAACAGAATTACACAA

GTAAAATCAACTGATCTTTGGAGAAAGAACAATGGCGATAATGTGAAGAAAAAGTCTTTT

TAACAAATGATGCTGAAATCACTAAATATTCACATGCAACTAAXX

>Unigene62851_C-W 1 206 LEN=206; minus strand

CCTCCACCTGCGAAGGACAGTGGTTTTTTCTCAACTTTTATGTCACTTGCAAAGAAACCA

CTCCAGATGGCGGGTAACATGTTTGACGCTGTCAGGGAACATCTTGGCTGTGATTCTTCC

AGACGTTCTTATCCTGTCAACGGCCAGGTTGGTGCTTCTTCCAATCCTTACAATGAGTAT

TCTGCCAGCAACCAGGTTGGTGTCCCX

>Unigene62930_C-W 1 202 LEN=202; minus strand

XXCCTGGAACTGGAAAAACAAAAGGTGGAAAATACAGACTGCAACAAGTTAAAGGACTTG

GAAAAAGTTGGTGGAAAGTGGCACGGTATACAAGTAAGGTCACTGGGCTTGGGATGGCAA

GGGCGCTGCATTGAAAGAGCATCCACGGGACCAAGTACTCATAATGTCTGGTGGTGTCAT

TCCCTCAACGACTTTAGAATATCT

>Unigene62942_C-W 1 223 LEN=223

XXGCTCCAGATCCCGCGAGAAGACCTCGATCCAAGGGATGTGCCTGTGGTCAGGGATGAC

CACGGGGTGGGTCCAGCGGGTGTGTCCCXGGGCAGTCCCGACCACGGGGTGGGTCCAACG

GAAGTGCCCTGGTCAGCCATGGATGTGCTCGTGGGCATGGCCATCATGATGGGTCCAGCA

GATGTGTCTGTGGTCAACGATGACAATTGGGATGGGTCCAGCGGGGXX

>Unigene63022_C-W 1 210 LEN=210; minus strand

CTGAAAACTGAGCAAAGAGGGACTGCCTCTATTGTGAAAGGGCAGAAAAGTTCTGGAAAG

TTCAAAATAGTACTTAGGCAAGAACTTCCTCCTTTCCCCCAAACACACAAATCTAAAGAG

ATAGCTGCCACACAGTGTGATATGACATCTGTCACCTTACAGAATGTGACTATAATCATG

GCTCCCAGAGCCCCCAAAATTCAAGCATGT

>Unigene63061_C-W 1 239 LEN=239

XCCGGGGCCGGCCGGCACTTTCCTCCCAAGTTGCAGCGCAAGTTCGCTAGTGGCCGTCTG

CGGCGAGGGCACCCGGAGCTTCCCTTGTCTCTGGCCGGCTGGGGCTCCTCAGAGTGCATG

GGCCGGGCCGAGCTGGCCCGGGGGGCTGAGCACCCCGCGCCCAGCGCCATGGAGGGCGCC

GAGCCCCGTGCGCGGCCCGAGCGCCTGGCGGAGGCCGAGGCGCGGGCTGCCGACGGCGGX

>Unigene63083_C-W 1 265 LEN=265

XCTGAGTCCTCCCCGGGGGAAGACCCTCCTGGCAGCGTCTTGCCGGGCTTTCTCCGCCAA

CAGGAGATCCCTGGGTACTGCCGTCCACCCTTGACGGCGCCTGCACTGACCCCGACCCTC

ACACACCACGTGGGACGCCACCAGTCATCCCACACTGAGGXGACCCACGAGAAAGAACTG

AACATGCCAGAGAGATACAGCTCCGATGGAGGCTGCGCAGAGTTGAAGACGGCGGCCATG

GCCACGGCACCCGAGATTCAGCTGAAG

>Unigene63111_C-W 1 266 LEN=266

XXCCTGGTTGCTATTTATGTGGGTGTTGAAAGCAGTTGCTTGAACAGGAACGTTCTGCCT

TCTTGTGTCCAAACGGTGACTGCAAAGCACATCAAAAAGTGTCTCCCACTCAATTTAGTA

AAATACCCCCATCTAGTGGCAGCTGAGCTGTCTGAGCACACATGAGCACAACTTCTGCTG

GTTGATGGAATAGTGGGCTCTGCTCCTGATACCAACCAGCGTCAGGATGCTCCTCCTCCA

CCATCTGCGGGAAAGGGGCAGTCCGAGGXX

>Unigene63148_C-W 1 230 LEN=230; minus strand

TGTGGAGACTTCTGATTCACTGGGTAGACAAAACAAGGGCTGGACAAGGTGATCTGCTCA

CTCATCTCAGATGCTGCTGAGCTCACTGCGTTATCACAGAAGGAAACTCACAAGCTTCGT

TTTTCAAACACCATTCAGGACAAAGGAGGTGAGAGTACACAGCCTCATCTCCATGGCAGC

ATTGTGGAGAATTAGTCAATCAGGCAGGTACGAGGTCTACACAAGTCTACX

>Unigene63155_C-W 1 210 LEN=210

XXGGGCTCCCGGGCGCCGCTGCTGCCGCCGCCTCGGTTCCTTCCCTTTCAGGGACAAGGC

GGGAGGACGCACGATCCCGCGCAGGTCCGGGGCCAGGCGAAGAGAGGACGTGCAACCCTG

GAGAAGGAGAGGCGCTCGGGCCACCGCTGCAGCCCGCCCAAGGAACCGCCTGTCAGCCTG

CGCGCCGCCGCCTTCCACATTCCTCCTCCTCCX

>Unigene63162_C-W 1 222 LEN=222; minus strand

XCTGGATATCTCAGCAGATACCCCACAACACCATCTGCAGGCAAGATAAATGCCTCCACT

CAGCTCTCAGCTGAGCAGGTGTTCCCACCCAACATACCTAAACATCTAACAGTTGATCTC

TGTCTATTGCTTCTAAATTTCTTCACACCTGAAGAAGTGACCACCTCAGCCCCTTCCAAA

TACTTGAACACCACACCTGGTACTGCAAAGTGCATGATGCCAAXX

>Unigene63277_C-W 1 155 LEN=207; minus strand

XCCAAGGAAATGATCTGGGATGGACTAAATATGGAACATAGAGATGCAATGGTACCAGTA

CGAAATGCCACACTGGATGACTGGATTTTGGCCACGCAGGATACAGGGACACAAGCAGCT

CAATCCTCTCTGCTGGCCTCAGCCATGGCAGTTGCA

>Unigene63295_C-W 1 117 LEN=216

CGAACTGCCACTGCCGCTGCCGCCGCCAACATGGCCGAGACAAACGAGGAGGTGGCGGTG

CTGGTGCAGCGAGTGGTGAAGGACATCACCAACGCCTTTAGGAGGAACCCGCACATG
