# Supplementary material for: Transcriptome Analysis and Comparison of Marmota monax and Marmota himalayana
Source: PLoS One. 2016 Nov 2;11(11):e0165875. doi: 10.1371/journal.pone.0165875 (PMC5091844; doi:10.1371/journal.pone.0165875)
Supplement: S1 Table — (DOC) [file pone.0165875.s005.doc]

**S1 Table. The basic information of M. monax and M. himalayana used in the study.**

| **Species** | **Number** | **Age (Year)** | **Sex** | **Duration in laboratory （Month）** | **Date of euthanasia** | **Body weight**  **（Kg）** |
| --- | --- | --- | --- | --- | --- | --- |
| **Mm** | Mm1 | 2-3 | Female | 15 | 2011-09-12 | 5.8 |
|  | Mm2 | 2-3 | Female | 15 | 2011-09-12 | 6.1 |
| **Mh** | Mh1 | 2-3 | Female | 9 | 2011-02-28 | 5.4 |
|  | Mh2 | 2-3 | Male | 8 | 2011-01-17 | 6 |
|  | Mh3 | 2-3 | Male | 8 | 2011-01-17 | 5.3 |
|  | Mh4 | 2-3 | Male | 18 | 2010-11-10 | 5.3 |
