# Supplementary material for: Transcriptome Analysis and Comparison of Marmota monax and Marmota himalayana
Source: PLoS One. 2016 Nov 2;11(11):e0165875. doi: 10.1371/journal.pone.0165875 (PMC5091844; doi:10.1371/journal.pone.0165875)
Supplement: S1 File — (DOC) [file pone.0165875.s001.doc]

>CL1.Contig1_A-W 35 367 minus strand SRA stem-loop-interacting RNA-binding protein, mitochondrial [Bos taurus] >gi|110816417|sp|Q32P59.1|SLIRP_BOVIN RecName: Full=SRA stem-loop-interacting RNA-binding protein, mitochondrial; Flags: Precursor >gi|81294255|gb|AAI08248.1| SRA stem-loop-interacting RNA-binding protein [Bos taurus] >gi|296482906|gb|DAA25021.1| SRA stem-loop-interacting RNA-binding protein, mitochondrial precursor [Bos taurus]

ATGGCAGCCTCGGCGGCGAGAGGTGCAGTGGCCCTGCGTTCGAGTGTTGGTCGTACGGTT

GCTTTTGTCAGAAAAATTCCTTGGACCGCGGCGTCGAGTGAGCTAAGAGAACACTTTTCT

CAGTTTGGTCATATACGGAAGTGCACTGTACCTTTTGACAAAGAGACTGGCTTTCATAAA

GGTATGGGTTGGATTCACTTTTCATCAGAAGAAGAACTTCAGAATGCAATGCAACAGGAA

AATCATATTATTGATGGAGTAAAGGTCTACATTCAAGCTCAAAGACGAAAAATGTTGCAA

GGTGACCAAACATCTGATGAAGAGAAAGATTTC

>CL1.Contig2_A-W 35 136 minus strand SRA stem-loop-interacting RNA-binding protein, mitochondrial [Ovis aries] >gi|222092835|gb|ACM43299.1| SRA stem-loop-interacting RNA-binding protein [Ovis aries]

ATGGCAGCCTCGGCGGCGAGAGGTGCAGTGGCCCTGCGTTCGAGTGTTGGTCGTACGGTT

GCTTTTGTCAGAAAAATTCCTTGGACCGCGGCGTCGAGTGAG

>CL2.Contig1_A-W 3 167 LRRGT00008 [Rattus norvegicus]

CAGCCCAGGAGAGAATTTTTAAAAGCCATGAGAGAAAGGAATCAGATTACATATAGGGGA

AAACCAAGTAGGATAACGGCAGATTTTTCGACACAGACCATAAAAGCTAGAAGATCCTGA

AACAACATATATCAAGCTCTGAAAGGAAATGGATGCCAACCAAGA

>CL2.Contig3_A-W 2 133 minus strand transposase [Sus scrofa]

TCAGACAATGAAGACAAAATATATAATCTTAAAAAGAATGTAGACTACACAGGGATACAG

GGAAAATACCACAAACAGAACATTCAAAAAATATGAGATAACATAAAAAAGACCAAATTA

AAGGGTTTTGGG

>CL3.Contig1_A-W 485 607 PREDICTED: GEM-interacting protein-like [Ailuropoda melanoleuca]

GGGGTGGAGTTGGATTTGAGGCTCATAAGGACCAAAGGGGGCGTGGATGCAGCCCTGGAT

TACGCCAAGACGTGGAGCCGCTATGCCAAGGAGCTGCTGGCCTGGACTGAAAAGAGAGCC

AGC

>CL3.Contig2_A-W 485 1660 PREDICTED: GEM-interacting protein-like [Ailuropoda melanoleuca]

GGGGTGGAGTTGGATTTGAGGCTCATAAGGACCAAAGGGGGCGTGGATGCAGCCCTGGAT

TACGCCAAGACGTGGAGCCGCTATGCCAAGGAGCTGCTGGCCTGGACTGAAAAGAGAGCC

AGCTATGAGCAGGAATTTGCTAAAAGCATCATGAAGATTGCTGAGGCTGGCAAGGTGTCC

ATCCACCAGCAGGTAACCATGAGCAGACTTCCCCAGAGCCCAAACCTTCCGGTTTCCCAT

CTCTGTCCTCTGATCCTCAGCATCCCCTAACCCTACTCCCAGAGCCACATGCCACTGCAG

TATATCTACACTCTGTTTCTGGAGCATGATCTCAACCTGGGAACCCTGGCCTTGGAGACA

GTAGCCCAGCAGAAAAGAGATTACTACCAGCCACTGGCTGCCAAACGGACTGAAATTGAG

AAATGGCGGAAGGAGTTCAAGGAGCAATGGATAAAGGAGCAGAAGCGGATGAACGAGGCA

GTGCAGATATTGCGGCGGGCCCAGCTCCAGTATACGCAGCGCAGTGAGGAACTTTGGGCG

CGCTCCCAGGGGTCCCCTGAGGACCATGCCCCTCAAGCCTCCCCTGGAACCAGCAAGCAG

CAGGAGCGGCGGCGGAGGTCCCGAGAAGAGGCCCAGGCCAAGGCACTGGAGGCTGAGGTG

TTTTATCAGGCCTGCGTCCGCGAAGCCAACGCACGCCAGCAGGACCTGGAAAACACCAAG

CATCGGATTGTGTCCCACGTGCGCAAGTTGGTGTTGCAGGGGGATGAAGTGCTGAGGAGG

GTGACCCTAGGCCTCTTTGGGCTGCGCAGAGCACAGGCTGAACGCGGTCCTCGCGCATTC

GCAGCCTTGGCTGAGTGTTGCGCGCCCTTCGAACCTGGCCAGCGCTACCAGGAGTTTGTG

CGGGCACTGCGGCCTGAGGATCCACCGCCCCCACCTCCGGCCTTCACCTTCCAGGAGTTT

GTACCCATGGTGAACAGCTCCCCTCTGGATTCAAGAAAGAAGTTCTCTGGGCCCCCACCT

CCCAGGCTGGACGAGAGTTCCGCAGAGCAAGGCCCTTGGGAGGACACTGGCATAGGCTGG

CAGGGGACTCCAGGTCCTACTCCTGGCAGTGATGTGGACAGTGTGGGTGGCGGCAGTGAG

TCTCGGTCCTTGGACTCTCCCACTTCCAGCCCAGGT

>CL3.Contig3_A-W 76 438 GEM-interacting protein [Homo sapiens] >gi|212286192|sp|Q9P107.2|GMIP_HUMAN RecName: Full=GEM-interacting protein; Short=GMIP >gi|116496745|gb|AAI26437.1| GEM interacting protein [Homo sapiens] >gi|119605245|gb|EAW84839.1| GEM interacting protein, isoform CRA_b [Homo sapiens] >gi|119605248|gb|EAW84842.1| GEM interacting protein, isoform CRA_b [Homo sapiens]

ATGGATACATCAGAGCCAGGGATCCCCCCAGCTCCTGAGAGCAGGAAGAGGTACAGTGAC

ATCTTCCGCAGCCTGGATAATCTCGAGATCTCACTGGGAAATGTAACCTTCGAGATGTTG

TCTGGAGACCCTGTAATCTCAGAAGACCTGGATCCTGACAAGGTCACCACAGCCACTGTG

ACCAGTGAAGCCAGCCGTTGGAGTGGCCCCTCCCCAGAGGGTCTTGCGCCCCTCACAGGG

GTGGAGTTGGATTTGAGGCTCATAAGGACCAAAGGGGGCGTGGATGCAGCCCTGGATTAC

GCCAAGACGTGGAGCCGCTATGCCAAGGAGCTGCTGGCCTGGACTGAAAAGAGAGCCAGC

TAT

>CL3.Contig4_A-W 485 1570 PREDICTED: GEM-interacting protein-like [Ailuropoda melanoleuca]

GGGGTGGAGTTGGATTTGAGGCTCATAAGGACCAAAGGGGGCGTGGATGCAGCCCTGGAT

TACGCCAAGACGTGGAGCCGCTATGCCAAGGAGCTGCTGGCCTGGACTGAAAAGAGAGCC

AGCTATGAGCAGGAATTTGCTAAAAGCATCATGAAGATTGCTGAGGCTGGCAAGGTGTCC

ATCCACCAGCAGAGCCACATGCCACTGCAGTATATCTACACTCTGTTTCTGGAGCATGAT

CTCAACCTGGGAACCCTGGCCTTGGAGACAGTAGCCCAGCAGAAAAGAGATTACTACCAG

CCACTGGCTGCCAAACGGACTGAAATTGAGAAATGGCGGAAGGAGTTCAAGGAGCAATGG

ATAAAGGAGCAGAAGCGGATGAACGAGGCAGTGCAGATATTGCGGCGGGCCCAGCTCCAG

TATACGCAGCGCAGTGAGGAACTTTGGGCGCGCTCCCAGGGGTCCCCTGAGGACCATGCC

CCTCAAGCCTCCCCTGGAACCAGCAAGCAGCAGGAGCGGCGGCGGAGGTCCCGAGAAGAG

GCCCAGGCCAAGGCACTGGAGGCTGAGGTGTTTTATCAGGCCTGCGTCCGCGAAGCCAAC

GCACGCCAGCAGGACCTGGAAAACACCAAGCATCGGATTGTGTCCCACGTGCGCAAGTTG

GTGTTGCAGGGGGATGAAGTGCTGAGGAGGGTGACCCTAGGCCTCTTTGGGCTGCGCAGA

GCACAGGCTGAACGCGGTCCTCGCGCATTCGCAGCCTTGGCTGAGTGTTGCGCGCCCTTC

GAACCTGGCCAGCGCTACCAGGAGTTTGTGCGGGCACTGCGGCCTGAGGATCCACCGCCC

CCACCTCCGGCCTTCACCTTCCAGGAGTTTGTACCCATGGTGAACAGCTCCCCTCTGGAT

TCAAGAAAGAAGTTCTCTGGGCCCCCACCTCCCAGGCTGGACGAGAGTTCCGCAGAGCAA

GGCCCTTGGGAGGACACTGGCATAGGCTGGCAGGGGACTCCAGGTCCTACTCCTGGCAGT

GATGTGGACAGTGTGGGTGGCGGCAGTGAGTCTCGGTCCTTGGACTCTCCCACTTCCAGC

CCAGGT

>CL3.Contig5_A-W 76 1488 PREDICTED: GEM-interacting protein isoform 1 [Pongo abelii]

ATGGATACATCAGAGCCAGGGATCCCCCCAGCTCCTGAGAGCAGGAAGAGGTACAGTGAC

ATCTTCCGCAGCCTGGATAATCTCGAGATCTCACTGGGAAATGTAACCTTCGAGATGTTG

TCTGGAGACCCTGTAATCTCAGAAGACCTGGATCCTGACAAGGTCACCACAGCCACTGTG

ACCAGTGAAGCCAGCCGTTGGAGTGGCCCCTCCCCAGAGGGTCTTGCGCCCCTCACAGGG

GTGGAGTTGGATTTGAGGCTCATAAGGACCAAAGGGGGCGTGGATGCAGCCCTGGATTAC

GCCAAGACGTGGAGCCGCTATGCCAAGGAGCTGCTGGCCTGGACTGAAAAGAGAGCCAGC

TATGAGCAGGAATTTGCTAAAAGCATCATGAAGATTGCTGAGGCTGGCAAGGTGTCCATC

CACCAGCAGGTAACCATGAGCAGACTTCCCCAGAGCCCAAACCTTCCGGTTTCCCATCTC

TGTCCTCTGATCCTCAGCATCCCCTAACCCTACTCCCAGAGCCACATGCCACTGCAGTAT

ATCTACACTCTGTTTCTGGAGCATGATCTCAACCTGGGAACCCTGGCCTTGGAGACAGTA

GCCCAGCAGAAAAGAGATTACTACCAGCCACTGGCTGCCAAACGGACTGAAATTGAGAAA

TGGCGGAAGGAGTTCAAGGAGCAATGGATAAAGGAGCAGAAGCGGATGAACGAGGCAGTG

CAGATATTGCGGCGGGCCCAGCTCCAGTATACGCAGCGCAGTGAGGAACTTTGGGCGCGC

TCCCAGGGGTCCCCTGAGGACCATGCCCCTCAAGCCTCCCCTGGAACCAGCAAGCAGCAG

GAGCGGCGGCGGAGGTCCCGAGAAGAGGCCCAGGCCAAGGCACTGGAGGCTGAGGTGTTT

TATCAGGCCTGCGTCCGCGAAGCCAACGCACGCCAGCAGGACCTGGAAAACACCAAGCAT

CGGATTGTGTCCCACGTGCGCAAGTTGGTGTTGCAGGGGGATGAAGTGCTGAGGAGGGTG

ACCCTAGGCCTCTTTGGGCTGCGCAGAGCACAGGCTGAACGCGGTCCTCGCGCATTCGCA

GCCTTGGCTGAGTGTTGCGCGCCCTTCGAACCTGGCCAGCGCTACCAGGAGTTTGTGCGG

GCACTGCGGCCTGAGGATCCACCGCCCCCACCTCCGGCCTTCACCTTCCAGGAGTTTGTA

CCCATGGTGAACAGCTCCCCTCTGGATTCAAGAAAGAAGTTCTCTGGGCCCCCACCTCCC

AGGCTGGACGAGAGTTCCGCAGAGCAAGGCCCTTGGGAGGACACTGGCATAGGCTGGCAG

GGGACTCCAGGTCCTACTCCTGGCAGTGATGTGGACAGTGTGGGTGGCGGCAGTGAGTCT

CGGTCCTTGGACTCTCCCACTTCCAGCCCAGGT

>CL3.Contig6_A-W 76 1398 PREDICTED: GEM-interacting protein isoform 1 [Pongo abelii]

ATGGATACATCAGAGCCAGGGATCCCCCCAGCTCCTGAGAGCAGGAAGAGGTACAGTGAC

ATCTTCCGCAGCCTGGATAATCTCGAGATCTCACTGGGAAATGTAACCTTCGAGATGTTG

TCTGGAGACCCTGTAATCTCAGAAGACCTGGATCCTGACAAGGTCACCACAGCCACTGTG

ACCAGTGAAGCCAGCCGTTGGAGTGGCCCCTCCCCAGAGGGTCTTGCGCCCCTCACAGGG

GTGGAGTTGGATTTGAGGCTCATAAGGACCAAAGGGGGCGTGGATGCAGCCCTGGATTAC

GCCAAGACGTGGAGCCGCTATGCCAAGGAGCTGCTGGCCTGGACTGAAAAGAGAGCCAGC

TATGAGCAGGAATTTGCTAAAAGCATCATGAAGATTGCTGAGGCTGGCAAGGTGTCCATC

CACCAGCAGAGCCACATGCCACTGCAGTATATCTACACTCTGTTTCTGGAGCATGATCTC

AACCTGGGAACCCTGGCCTTGGAGACAGTAGCCCAGCAGAAAAGAGATTACTACCAGCCA

CTGGCTGCCAAACGGACTGAAATTGAGAAATGGCGGAAGGAGTTCAAGGAGCAATGGATA

AAGGAGCAGAAGCGGATGAACGAGGCAGTGCAGATATTGCGGCGGGCCCAGCTCCAGTAT

ACGCAGCGCAGTGAGGAACTTTGGGCGCGCTCCCAGGGGTCCCCTGAGGACCATGCCCCT

CAAGCCTCCCCTGGAACCAGCAAGCAGCAGGAGCGGCGGCGGAGGTCCCGAGAAGAGGCC

CAGGCCAAGGCACTGGAGGCTGAGGTGTTTTATCAGGCCTGCGTCCGCGAAGCCAACGCA

CGCCAGCAGGACCTGGAAAACACCAAGCATCGGATTGTGTCCCACGTGCGCAAGTTGGTG

TTGCAGGGGGATGAAGTGCTGAGGAGGGTGACCCTAGGCCTCTTTGGGCTGCGCAGAGCA

CAGGCTGAACGCGGTCCTCGCGCATTCGCAGCCTTGGCTGAGTGTTGCGCGCCCTTCGAA

CCTGGCCAGCGCTACCAGGAGTTTGTGCGGGCACTGCGGCCTGAGGATCCACCGCCCCCA

CCTCCGGCCTTCACCTTCCAGGAGTTTGTACCCATGGTGAACAGCTCCCCTCTGGATTCA

AGAAAGAAGTTCTCTGGGCCCCCACCTCCCAGGCTGGACGAGAGTTCCGCAGAGCAAGGC

CCTTGGGAGGACACTGGCATAGGCTGGCAGGGGACTCCAGGTCCTACTCCTGGCAGTGAT

GTGGACAGTGTGGGTGGCGGCAGTGAGTCTCGGTCCTTGGACTCTCCCACTTCCAGCCCA

GGT

>CL3.Contig7_A-W 76 438 PREDICTED: GEM-interacting protein isoform 1 [Pongo abelii]

ATGGATACATCAGAGCCAGGGATCCCCCCAGCTCCTGAGAGCAGGAAGAGGTACAGTGAC

ATCTTCCGCAGCCTGGATAATCTCGAGATCTCACTGGGAAATGTAACCTTCGAGATGTTG

TCTGGAGACCCTGTAATCTCAGAAGACCTGGATCCTGACAAGGTCACCACAGCCACTGTG

ACCAGTGAAGCCAGCCGTTGGAGTGGCCCCTCCCCAGAGGGTCTTGCGCCCCTCACAGGG

GTGGAGTTGGATTTGAGGCTCATAAGGACCAAAGGGGGCGTGGATGCAGCCCTGGATTAC

GCCAAGACGTGGAGCCGCTATGCCAAGGAGCTGCTGGCCTGGACTGAAAAGAGAGCCAGC

TAT

>CL3.Contig8_A-W 76 2973 GEM-interacting protein [Homo sapiens] >gi|212286192|sp|Q9P107.2|GMIP_HUMAN RecName: Full=GEM-interacting protein; Short=GMIP >gi|116496745|gb|AAI26437.1| GEM interacting protein [Homo sapiens] >gi|119605245|gb|EAW84839.1| GEM interacting protein, isoform CRA_b [Homo sapiens] >gi|119605248|gb|EAW84842.1| GEM interacting protein, isoform CRA_b [Homo sapiens]

ATGGATACATCAGAGCCAGGGATCCCCCCAGCTCCTGAGAGCAGGAAGAGGTACAGTGAC

ATCTTCCGCAGCCTGGATAATCTCGAGATCTCACTGGGAAATGTAACCTTCGAGATGTTG

TCTGGAGACCCTGTAATCTCAGAAGACCTGGATCCTGACAAGGTCACCACAGCCACTGTG

ACCAGTGAAGCCAGCCGTTGGAGTGGCCCCTCCCCAGAGGGTCTTGCGCCCCTCACAGGG

GTGGAGTTGGATTTGAGGCTCATAAGGACCAAAGGGGGCGTGGATGCAGCCCTGGATTAC

GCCAAGACGTGGAGCCGCTATGCCAAGGAGCTGCTGGCCTGGACTGAAAAGAGAGCCAGC

TATGAGCAGGAATTTGCTAAAAGCATCATGAAGATTGCTGAGGCTGGCAAGGTGTCCATC

CACCAGCAGAGCCACATGCCACTGCAGTATATCTACACTCTGTTTCTGGAGCATGATCTC

AACCTGGGAACCCTGGCCTTGGAGACAGTAGCCCAGCAGAAAAGAGATTACTACCAGCCA

CTGGCTGCCAAACGGACTGAAATTGAGAAATGGCGGAAGGAGTTCAAGGAGCAATGGATA

AAGGAGCAGAAGCGGATGAACGAGGCAGTGCAGATATTGCGGCGGGCCCAGCTCCAGTAT

ACGCAGCGCAGTGAGGAACTTTGGGCGCGCTCCCAGGGGTCCCCTGAGGACCATGCCCCT

CAAGCCTCCCCTGGAACCAGCAAGCAGCAGGAGCGGCGGCGGAGGTCCCGAGAAGAGGCC

CAGGCCAAGGCACTGGAGGCTGAGGTGTTTTATCAGGCCTGCGTCCGCGAAGCCAACGCA

CGCCAGCAGGACCTGGAAAACACCAAGCATCGGATTGTGTCCCACGTGCGCAAGTTGGTG

TTGCAGGGGGATGAAGTGCTGAGGAGGGTGACCCTAGGCCTCTTTGGGCTGCGCAGAGCA

CAGGCTGAACGCGGTCCTCGCGCATTCGCAGCCTTGGCTGAGTGTTGCGCGCCCTTCGAA

CCTGGCCAGCGCTACCAGGAGTTTGTGCGGGCACTGCGGCCTGAGGATCCACCGCCCCCA

CCTCCGGCCTTCACCTTCCAGGAGTTTGTACCCATGGTGAACAGCTCCCCTCTGGATTCA

AGAAAGAAGTTCTCTGGGCCCCCACCTCCCAGGCTGGACGAGAGTTCCGCAGAGCAAGGC

CCTTGGGAGGACACTGGCATAGGCTGGCAGGGGACTCCAGGTCCTACTCCTGGCAGTGAT

GTGGACAGTGTGGGTGGCGGCAGTGAGTCTCGGTCCTTGGACTCTCCCACTTCCAGCCCA

GGCCCTGGTACACGGAGGCTGGTGAAGGTCTCATCCACAGGCACTGAATCTTCAGATGAC

TTTGAGGAGCGAGACCCTGACGTGGGAGATGGGCTGGAGAATGGGCTCGGCAGCCCTTTC

AGGAAGTGGACGCTGTCCACTGCGGCTCAGACCCACAGGCTACGGCGGCTGCGGGGTCCA

GCCAAATGTCGAGAGTGTGAAGCCTTCATGGTCAGTGGGACTGAGTGTGAAGAGTGTTTC

CTGACCTGTCATAAGCGATGTCTGGAGACCCTGCTGATCCTCTGCGGACACCAGCGGCTC

CCAGTCCGGACACCTCTCTTTGGGGTCGACTTTCTACAGCTTCCCAGGGACTTCCCAGAG

GAGGTTCCCTTTGTGATCACCAAATGCACAGCTGAGATAGAACACCGTGCCCTGAGTGTT

CAGGGCATTTACCGGGTCAGCGGATCCCGGGTTCGCGTGGAGCGACTGTGCCAAGCCTTT

GAGAATGGCCGAGCATTGGTGGAACTGTCAGGAAACTCACCTCATGACATCTCAAGTGTC

CTTAAGCGGTTTCTCCAGGAGCTCACCGACCCTGTGGTCCCCTTCCACTTCTACGATGCC

TTCATCTCTCTGGCTAAGACCCTGCATGCAGACTCTGGGGACAACCCTGGGACCCCCAGC

CCCAGTCCTGAAGTTATCTGCTCGCTGAAGACTCTCTTGGTACAACTGCCTGACTCTAAC

TACAACACTCTTCGGCACCTGGTGGCCCATCTATTCAGGGTGGCTGCACGGTTTGAAGAA

AACAAGATGTCTGCCAACAACTTGGGAATTGTATTTGGGCCAACGCTTCTGCGGCCACCA

GATGGTCCGTGGGCAGCTGGTACCAGCCCTGTCACCTGCCTGCTAGACTCTGGACACCAG

GCTCAGCTTGTTGAGTTCCTCATTGTGCACTACGAGCAGATCTTCGGGATGGACGAGCTC

CCTCTGGCCACAGAGCCACTGCCCCAAGACCCCAGCCCAGCCCCTGGGCCCCTTACAACC

AGCCCCCAACCTCCAGTCCCATTCCTTGACTCAGAGTCACAGTCCCCAGCCCTAGCCTCG

GACCCTGACCCAGACTCTAAGTCTCATAGTGCCCAGGAGGAGCATTCTGAGTCCACATCC

TCCAAGATTCCAGCTCTGCAGAGTGACCAAAGAGAGGAAGTGGCTGAAGATACCAAAAAT

GGGGAAGGGGAAGTGTCCAGCCAAGGCCCTGAGGACTCACTCCTAGGAACACAGTCCCGC

GGCCATTTCAGCCGCCAGCCAGTGAAGTATCCGCGGGGAGGTGTGCGGCCTGTTACTCAT

CAACTGTCCAGTTTGGCCATGGTGGCTTCCAAGCTGTGTGAGGAGGCCTCTATCTCAATG

CACAGAGGGAGTCTGAGGGGGCGGGGGCCAGGCCCTTCTGCTGCCTCCCCTGACAACAGC

CCCCTGCGACGCACCCCACTGCCTAAGCATTTTGAGATCACCCAGGAGACTGCCCGCTTA

CTCTCTAAGCTGGACAGTGAGGCTGTGCCTAGGACCTGCTGCCCAGACCTCCAGCCTGAG

GAAACCGAGGACCATCTC

>CL3.Contig9_A-W 395 610 PREDICTED: GEM-interacting protein [Equus caballus]

GACATGGGCTACTGGACTAGTCCCAGGGCTCAGGGAAAACTTCTAATGAAGGGCAGTGTT

CAGAGTTAAGGGTTCCACCCATCTGCCCCAGGGGTGGAGTTGGATTTGAGGCTCATAAGG

ACCAAAGGGGGCGTGGATGCAGCCCTGGATTACGCCAAGACGTGGAGCCGCTATGCCAAG

GAGCTGCTGGCCTGGACTGAAAAGAGAGCCAGCTAT

>CL3.Contig10_A-W 121 1983 PREDICTED: GEM-interacting protein [Equus caballus]

CAGGCACTGGAGGCTGAGGTGTTTTATCAGGCCTGCGTCCGCGAAGCCAACGCACGCCAG

CAGGACCTGGAAAACACCAAGCATCGGATTGTGTCCCACGTGCGCAAGTTGGTGTTGCAG

GGGGATGAAGTGCTGAGGAGGGTGACCCTAGGCCTCTTTGGGCTGCGCAGAGCACAGGCT

GAACGCGGTCCTCGCGCATTCGCAGCCTTGGCTGAGTGTTGCGCGCCCTTCGAACCTGGC

CAGCGCTACCAGGAGTTTGTGCGGGCACTGCGGCCTGAGGATCCACCGCCCCCACCTCCG

GCCTTCACCTTCCAGGAGTTTGTACCCATGGTGAACAGCTCCCCTCTGGATTCAAGAAAG

AAGTTCTCTGGGCCCCCACCTCCCAGGCTGGACGAGAGTTCCGCAGAGCAAGGCCCTTGG

GAGGACACTGGCATAGGCTGGCAGGGGACTCCAGGTCCTACTCCTGGCAGTGATGTGGAC

AGTGTGGGTGGCGGCAGTGAGTCTCGGTCCTTGGACTCTCCCACTTCCAGCCCAGGCCCT

GGTACACGGAGGCTGGTGAAGGTCTCATCCACAGGCACTGAATCTTCAGATGACTTTGAG

GAGCGAGACCCTGACGTGGGAGATGGGCTGGAGAATGGGCTCGGCAGCCCTTTCAGGAAG

TGGACGCTGTCCACTGCGGCTCAGACCCACAGGCTACGGCGGCTGCGGGGTCCAGCCAAA

TGTCGAGAGTGTGAAGCCTTCATGGTCAGTGGGACTGAGTGTGAAGAGGTATGTCCTGGA

CTGATGATCCAATGACCTCTGTTGACCCCTAATGACCTAAGATCCCTAAAACCTTCCTGC

CCTCAGGACCCACCCTGATCTGATGATCTCCTACATACCCTCCATTCCCTGAGGATCCCT

ACAACTGTCTTTTTTTCCCACTCACCCCCAATGACCTCACTCACTCTGTGACTTTCTCCC

CAGTGTTTCCTGACCTGTCATAAGCGATGTCTGGAGACCCTGCTGATCCTCTGCGGACAC

CAGCGGCTCCCAGTCCGGACACCTCTCTTTGGGGTCGACTTTCTACAGCTTCCCAGGGAC

TTCCCAGAGGAGGTTCCCTTTGTGATCACCAAATGCACAGCTGAGATAGAACACCGTGCC

CTGAGTGTTCAGGGCATTTACCGGGTCAGCGGATCCCGGGTTCGCGTGGAGCGACTGTGC

CAAGCCTTTGAGAATGGCCGAGCATTGGTGGAACTGTCAGGAAACTCACCTCATGACATC

TCAAGTGTCCTTAAGCGGTTTCTCCAGGAGCTCACCGACCCTGTGGTCCCCTTCCACTTC

TACGATGCCTTCATCTCTCTGGCTAAGACCCTGCATGCAGACTCTGGGGACAACCCTGGG

ACCCCCAGCCCCAGTCCTGAAGTTATCTGCTCGCTGAAGACTCTCTTGGTACAACTGCCT

GACTCTAACTACAACACTCTTCGGCACCTGGTGGCCCATCTATTCAGGGTGGCTGCACGG

TTTGAAGAAAACAAGATGTCTGCCAACAACTTGGGAATTGTATTTGGGCCAACGCTTCTG

CGGCCACCAGATGGTCCGTGGGCAGCTGGTACCAGCCCTGTCACCTGCCTGCTAGACTCT

GGACACCAGGCTCAGCTTGTTGAGTTCCTCATTGTGCACTACGAGCAGATCTTCGGGATG

GACGAGCTCCCTCTGGCCACAGAGCCACTGCCCCAAGACCCCAGCCCAGCCCCTGGGCCC

CTTACAACCAGCCCCCAACCTCCAGTCCCATTCCTTGACTCAGAGTCACAGTCCCCAGCC

CTAGCCTCGGACCCTGACCCAGACTCTAAGTCTCATAGTGCCCAGGAGGAGCATTCTGAG

TCC

>CL3.Contig11_A-W 121 1791 PREDICTED: GEM-interacting protein isoform 1 [Pongo abelii]

CAGGCACTGGAGGCTGAGGTGTTTTATCAGGCCTGCGTCCGCGAAGCCAACGCACGCCAG

CAGGACCTGGAAAACACCAAGCATCGGATTGTGTCCCACGTGCGCAAGTTGGTGTTGCAG

GGGGATGAAGTGCTGAGGAGGGTGACCCTAGGCCTCTTTGGGCTGCGCAGAGCACAGGCT

GAACGCGGTCCTCGCGCATTCGCAGCCTTGGCTGAGTGTTGCGCGCCCTTCGAACCTGGC

CAGCGCTACCAGGAGTTTGTGCGGGCACTGCGGCCTGAGGATCCACCGCCCCCACCTCCG

GCCTTCACCTTCCAGGAGTTTGTACCCATGGTGAACAGCTCCCCTCTGGATTCAAGAAAG

AAGTTCTCTGGGCCCCCACCTCCCAGGCTGGACGAGAGTTCCGCAGAGCAAGGCCCTTGG

GAGGACACTGGCATAGGCTGGCAGGGGACTCCAGGTCCTACTCCTGGCAGTGATGTGGAC

AGTGTGGGTGGCGGCAGTGAGTCTCGGTCCTTGGACTCTCCCACTTCCAGCCCAGGCCCT

GGTACACGGAGGCTGGTGAAGGTCTCATCCACAGGCACTGAATCTTCAGATGACTTTGAG

GAGCGAGACCCTGACGTGGGAGATGGGCTGGAGAATGGGCTCGGCAGCCCTTTCAGGAAG

TGGACGCTGTCCACTGCGGCTCAGACCCACAGGCTACGGCGGCTGCGGGGTCCAGCCAAA

TGTCGAGAGTGTGAAGCCTTCATGGTCAGTGGGACTGAGTGTGAAGAGTGTTTCCTGACC

TGTCATAAGCGATGTCTGGAGACCCTGCTGATCCTCTGCGGACACCAGCGGCTCCCAGTC

CGGACACCTCTCTTTGGGGTCGACTTTCTACAGCTTCCCAGGGACTTCCCAGAGGAGGTT

CCCTTTGTGATCACCAAATGCACAGCTGAGATAGAACACCGTGCCCTGAGTGTTCAGGGC

ATTTACCGGGTCAGCGGATCCCGGGTTCGCGTGGAGCGACTGTGCCAAGCCTTTGAGAAT

GGCCGAGCATTGGTGGAACTGTCAGGAAACTCACCTCATGACATCTCAAGTGTCCTTAAG

CGGTTTCTCCAGGAGCTCACCGACCCTGTGGTCCCCTTCCACTTCTACGATGCCTTCATC

TCTCTGGCTAAGACCCTGCATGCAGACTCTGGGGACAACCCTGGGACCCCCAGCCCCAGT

CCTGAAGTTATCTGCTCGCTGAAGACTCTCTTGGTACAACTGCCTGACTCTAACTACAAC

ACTCTTCGGCACCTGGTGGCCCATCTATTCAGGGTGGCTGCACGGTTTGAAGAAAACAAG

ATGTCTGCCAACAACTTGGGAATTGTATTTGGGCCAACGCTTCTGCGGCCACCAGATGGT

CCGTGGGCAGCTGGTACCAGCCCTGTCACCTGCCTGCTAGACTCTGGACACCAGGCTCAG

CTTGTTGAGTTCCTCATTGTGCACTACGAGCAGATCTTCGGGATGGACGAGCTCCCTCTG

GCCACAGAGCCACTGCCCCAAGACCCCAGCCCAGCCCCTGGGCCCCTTACAACCAGCCCC

CAACCTCCAGTCCCATTCCTTGACTCAGAGTCACAGTCCCCAGCCCTAGCCTCGGACCCT

GACCCAGACTCTAAGTCTCATAGTGCCCAGGAGGAGCATTCTGAGTCCACA

>CL3.Contig12_A-W 351 2384 GEM-interacting protein OS=Homo sapiens GN=GMIP PE=1 SV=2

AACGAGGCAGTGCAGATATTGCGGCGGGCCCAGCTCCAGTATACGCAGCGCAGTGAGGAA

CTTTGGGCGCGCTCCCAGGGGTCCCCTGAGGACCATGCCCCTCAAGCCTCCCCTGGAACC

AGCAAGCAGCAGGAGCGGCGGCGGAGGTCCCGAGAAGAGGCCCAGGCCAAGGCACTGGAG

GCTGAGGTGTTTTATCAGGCCTGCGTCCGCGAAGCCAACGCACGCCAGCAGGACCTGGAA

AACACCAAGCATCGGATTGTGTCCCACGTGCGCAAGTTGGTGTTGCAGGGGGATGAAGTG

CTGAGGAGGGTGACCCTAGGCCTCTTTGGGCTGCGCAGAGCACAGGCTGAACGCGGTCCT

CGCGCATTCGCAGCCTTGGCTGAGTGTTGCGCGCCCTTCGAACCTGGCCAGCGCTACCAG

GAGTTTGTGCGGGCACTGCGGCCTGAGGATCCACCGCCCCCACCTCCGGCCTTCACCTTC

CAGGAGTTTGTACCCATGGTGAACAGCTCCCCTCTGGATTCAAGAAAGAAGTTCTCTGGG

CCCCCACCTCCCAGGCTGGACGAGAGTTCCGCAGAGCAAGGCCCTTGGGAGGACACTGGC

ATAGGCTGGCAGGGGACTCCAGGTCCTACTCCTGGCAGTGATGTGGACAGTGTGGGTGGC

GGCAGTGAGTCTCGGTCCTTGGACTCTCCCACTTCCAGCCCAGGCCCTGGTACACGGAGG

CTGGTGAAGGTCTCATCCACAGGCACTGAATCTTCAGATGACTTTGAGGAGCGAGACCCT

GACGTGGGAGATGGGCTGGAGAATGGGCTCGGCAGCCCTTTCAGGAAGTGGACGCTGTCC

ACTGCGGCTCAGACCCACAGGCTACGGCGGCTGCGGGGTCCAGCCAAATGTCGAGAGTGT

GAAGCCTTCATGGTCAGTGGGACTGAGTGTGAAGAGGTATGTCCTGGACTGATGATCCAA

TGACCTCTGTTGACCCCTAATGACCTAAGATCCCTAAAACCTTCCTGCCCTCAGGACCCA

CCCTGATCTGATGATCTCCTACATACCCTCCATTCCCTGAGGATCCCTACAACTGTCTTT

TTTTCCCACTCACCCCCAATGACCTCACTCACTCTGTGACTTTCTCCCCAGTGTTTCCTG

ACCTGTCATAAGCGATGTCTGGAGACCCTGCTGATCCTCTGCGGACACCAGCGGCTCCCA

GTCCGGACACCTCTCTTTGGGGTCGACTTTCTACAGCTTCCCAGGGACTTCCCAGAGGAG

GTTCCCTTTGTGATCACCAAATGCACAGCTGAGATAGAACACCGTGCCCTGAGTGTTCAG

GGCATTTACCGGGTCAGCGGATCCCGGGTTCGCGTGGAGCGACTGTGCCAAGCCTTTGAG

AATGGCCGAGCATTGGTGGAACTGTCAGGAAACTCACCTCATGACATCTCAAGTGTCCTT

AAGCGGTTTCTCCAGGAGCTCACCGACCCTGTGGTCCCCTTCCACTTCTACGATGCCTTC

ATCTCTCTGGCTAAGACCCTGCATGCAGACTCTGGGGACAACCCTGGGACCCCCAGCCCC

AGTCCTGAAGTTATCTGCTCGCTGAAGACTCTCTTGGTACAACTGCCTGACTCTAACTAC

AACACTCTTCGGCACCTGGTGGCCCATCTATTCAGGGTGGCTGCACGGTTTGAAGAAAAC

AAGATGTCTGCCAACAACTTGGGAATTGTATTTGGGCCAACGCTTCTGCGGCCACCAGAT

GGTCCGTGGGCAGCTGGTACCAGCCCTGTCACCTGCCTGCTAGACTCTGGACACCAGGCT

CAGCTTGTTGAGTTCCTCATTGTGCACTACGAGCAGATCTTCGGGATGGACGAGCTCCCT

CTGGCCACAGAGCCACTGCCCCAAGACCCCAGCCCAGCCCCTGGGCCCCTTACAACCAGC

CCCCAACCTCCAGTCCCATTCCTTGACTCAGAGTCACAGTCCCCAGCCCTAGCCTCGGAC

CCTGACCCAGACTCTAAGTCTCATAGTGCCCAGGAGGAGCATTCTGAGTCCACA

>CL3.Contig13_A-W 407 3430 PREDICTED: GEM-interacting protein isoform 1 [Pongo abelii]

TGGACTAGTCCCAGGGCTCAGGGAAAACTTCTAATGAAGGGCAGTGTTCAGAGTTAAGGG

TTCCACCCATCTGCCCCAGGGGTGGAGTTGGATTTGAGGCTCATAAGGACCAAAGGGGGC

GTGGATGCAGCCCTGGATTACGCCAAGACGTGGAGCCGCTATGCCAAGGAGCTGCTGGCC

TGGACTGAAAAGAGAGCCAGCTATGAGCAGGAATTTGCTAAAAGCATCATGAAGATTGCT

GAGGCTGGCAAGGTGTCCATCCACCAGCAGGTAACCATGAGCAGACTTCCCCAGAGCCCA

AACCTTCCGGTTTCCCATCTCTGTCCTCTGATCCTCAGCATCCCCTAACCCTACTCCCAG

AGCCACATGCCACTGCAGTATATCTACACTCTGTTTCTGGAGCATGATCTCAACCTGGGA

ACCCTGGCCTTGGAGACAGTAGCCCAGCAGAAAAGAGATTACTACCAGCCACTGGCTGCC

AAACGGACTGAAATTGAGAAATGGCGGAAGGAGTTCAAGGAGCAATGGATAAAGGAGCAG

AAGCGGATGAACGAGGCAGTGCAGATATTGCGGCGGGCCCAGCTCCAGTATACGCAGCGC

AGTGAGGAACTTTGGGCGCGCTCCCAGGGGTCCCCTGAGGACCATGCCCCTCAAGCCTCC

CCTGGAACCAGCAAGCAGCAGGAGCGGCGGCGGAGGTCCCGAGAAGAGGCCCAGGCCAAG

GCACTGGAGGCTGAGGTGTTTTATCAGGCCTGCGTCCGCGAAGCCAACGCACGCCAGCAG

GACCTGGAAAACACCAAGCATCGGATTGTGTCCCACGTGCGCAAGTTGGTGTTGCAGGGG

GATGAAGTGCTGAGGAGGGTGACCCTAGGCCTCTTTGGGCTGCGCAGAGCACAGGCTGAA

CGCGGTCCTCGCGCATTCGCAGCCTTGGCTGAGTGTTGCGCGCCCTTCGAACCTGGCCAG

CGCTACCAGGAGTTTGTGCGGGCACTGCGGCCTGAGGATCCACCGCCCCCACCTCCGGCC

TTCACCTTCCAGGAGTTTGTACCCATGGTGAACAGCTCCCCTCTGGATTCAAGAAAGAAG

TTCTCTGGGCCCCCACCTCCCAGGCTGGACGAGAGTTCCGCAGAGCAAGGCCCTTGGGAG

GACACTGGCATAGGCTGGCAGGGGACTCCAGGTCCTACTCCTGGCAGTGATGTGGACAGT

GTGGGTGGCGGCAGTGAGTCTCGGTCCTTGGACTCTCCCACTTCCAGCCCAGGCCCTGGT

ACACGGAGGCTGGTGAAGGTCTCATCCACAGGCACTGAATCTTCAGATGACTTTGAGGAG

CGAGACCCTGACGTGGGAGATGGGCTGGAGAATGGGCTCGGCAGCCCTTTCAGGAAGTGG

ACGCTGTCCACTGCGGCTCAGACCCACAGGCTACGGCGGCTGCGGGGTCCAGCCAAATGT

CGAGAGTGTGAAGCCTTCATGGTCAGTGGGACTGAGTGTGAAGAGGTATGTCCTGGACTG

ATGATCCAATGACCTCTGTTGACCCCTAATGACCTAAGATCCCTAAAACCTTCCTGCCCT

CAGGACCCACCCTGATCTGATGATCTCCTACATACCCTCCATTCCCTGAGGATCCCTACA

ACTGTCTTTTTTTCCCACTCACCCCCAATGACCTCACTCACTCTGTGACTTTCTCCCCAG

TGTTTCCTGACCTGTCATAAGCGATGTCTGGAGACCCTGCTGATCCTCTGCGGACACCAG

CGGCTCCCAGTCCGGACACCTCTCTTTGGGGTCGACTTTCTACAGCTTCCCAGGGACTTC

CCAGAGGAGGTTCCCTTTGTGATCACCAAATGCACAGCTGAGATAGAACACCGTGCCCTG

AGTGTTCAGGGCATTTACCGGGTCAGCGGATCCCGGGTTCGCGTGGAGCGACTGTGCCAA

GCCTTTGAGAATGGCCGAGCATTGGTGGAACTGTCAGGAAACTCACCTCATGACATCTCA

AGTGTCCTTAAGCGGTTTCTCCAGGAGCTCACCGACCCTGTGGTCCCCTTCCACTTCTAC

GATGCCTTCATCTCTCTGGCTAAGACCCTGCATGCAGACTCTGGGGACAACCCTGGGACC

CCCAGCCCCAGTCCTGAAGTTATCTGCTCGCTGAAGACTCTCTTGGTACAACTGCCTGAC

TCTAACTACAACACTCTTCGGCACCTGGTGGCCCATCTATTCAGGGTGGCTGCACGGTTT

GAAGAAAACAAGATGTCTGCCAACAACTTGGGAATTGTATTTGGGCCAACGCTTCTGCGG

CCACCAGATGGTCCGTGGGCAGCTGGTACCAGCCCTGTCACCTGCCTGCTAGACTCTGGA

CACCAGGCTCAGCTTGTTGAGTTCCTCATTGTGCACTACGAGCAGATCTTCGGGATGGAC

GAGCTCCCTCTGGCCACAGAGCCACTGCCCCAAGACCCCAGCCCAGCCCCTGGGCCCCTT

ACAACCAGCCCCCAACCTCCAGTCCCATTCCTTGACTCAGAGTCACAGTCCCCAGCCCTA

GCCTCGGACCCTGACCCAGACTCTAAGTCTCATAGTGCCCAGGAGGAGCATTCTGAGTCC

ACATCCTCCAAGATTCCAGCTCTGCAGAGTGACCAAAGAGAGGAAGTGGCTGAAGATACC

AAAAATGGGGAAGGGGAAGTGTCCAGCCAAGGCCCTGAGGACTCACTCCTAGGAACACAG

TCCCGCGGCCATTTCAGCCGCCAGCCAGTGAAGTATCCGCGGGGAGGTGTGCGGCCTGTT

ACTCATCAACTGTCCAGTTTGGCCATGGTGGCTTCCAAGCTGTGTGAGGAGGCCTCTATC

TCAATGCACAGAGGGAGTCTGAGGGGGCGGGGGCCAGGCCCTTCTGCTGCCTCCCCTGAC

AACAGCCCCCTGCGACGCACCCCACTGCCTAAGCATTTTGAGATCACCCAGGAGACTGCC

CGCTTACTCTCTAAGCTGGACAGTGAGGCTGTGCCTAGGACCTGCTGCCCAGACCTCCAG

CCTGAGGAAACCGAGGACCATCTC

>CL3.Contig14_A-W 175 2013 GEM-interacting protein OS=Homo sapiens GN=GMIP PE=1 SV=2

AACGAGGCAGTGCAGATATTGCGGCGGGCCCAGCTCCAGTATACGCAGCGCAGTGAGGAA

CTTTGGGCGCGCTCCCAGGGGTCCCCTGAGGACCATGCCCCTCAAGCCTCCCCTGGAACC

AGCAAGCAGCAGGAGCGGCGGCGGAGGTCCCGAGAAGAGGCCCAGGCCAAGGCACTGGAG

GCTGAGGTGTTTTATCAGGCCTGCGTCCGCGAAGCCAACGCACGCCAGCAGGACCTGGAA

AACACCAAGCATCGGATTGTGTCCCACGTGCGCAAGTTGGTGTTGCAGGGGGATGAAGTG

CTGAGGAGGGTGACCCTAGGCCTCTTTGGGCTGCGCAGAGCACAGGCTGAACGCGGTCCT

CGCGCATTCGCAGCCTTGGCTGAGTGTTGCGCGCCCTTCGAACCTGGCCAGCGCTACCAG

GAGTTTGTGCGGGCACTGCGGCCTGAGGATCCACCGCCCCCACCTCCGGCCTTCACCTTC

CAGGAGTTTGTACCCATGGTGAACAGCTCCCCTCTGGATTCAAGAAAGAAGTTCTCTGGG

CCCCCACCTCCCAGGCTGGACGAGAGTTCCGCAGAGCAAGGCCCTTGGGAGGACACTGGC

ATAGGCTGGCAGGGGACTCCAGGTCCTACTCCTGGCAGTGATGTGGACAGTGTGGGTGGC

GGCAGTGAGTCTCGGTCCTTGGACTCTCCCACTTCCAGCCCAGGCCCTGGTACACGGAGG

CTGGTGAAGGTCTCATCCACAGGCACTGAATCTTCAGATGACTTTGAGGAGCGAGACCCT

GACGTGGGAGATGGGCTGGAGAATGGGCTCGGCAGCCCTTTCAGGAAGTGGACGCTGTCC

ACTGCGGCTCAGACCCACAGGCTACGGCGGCTGCGGGGTCCAGCCAAATGTCGAGAGTGT

GAAGCCTTCATGGTCAGTGGGACTGAGTGTGAAGAGTGTTTCCTGACCTGTCATAAGCGA

TGTCTGGAGACCCTGCTGATCCTCTGCGGACACCAGCGGCTCCCAGTCCGGACACCTCTC

TTTGGGGTCGACTTTCTACAGCTTCCCAGGGACTTCCCAGAGGAGGTTCCCTTTGTGATC

ACCAAATGCACAGCTGAGATAGAACACCGTGCCCTGAGTGTTCAGGGCATTTACCGGGTC

AGCGGATCCCGGGTTCGCGTGGAGCGACTGTGCCAAGCCTTTGAGAATGGCCGAGCATTG

GTGGAACTGTCAGGAAACTCACCTCATGACATCTCAAGTGTCCTTAAGCGGTTTCTCCAG

GAGCTCACCGACCCTGTGGTCCCCTTCCACTTCTACGATGCCTTCATCTCTCTGGCTAAG

ACCCTGCATGCAGACTCTGGGGACAACCCTGGGACCCCCAGCCCCAGTCCTGAAGTTATC

TGCTCGCTGAAGACTCTCTTGGTACAACTGCCTGACTCTAACTACAACACTCTTCGGCAC

CTGGTGGCCCATCTATTCAGGGTGGCTGCACGGTTTGAAGAAAACAAGATGTCTGCCAAC

AACTTGGGAATTGTATTTGGGCCAACGCTTCTGCGGCCACCAGATGGTCCGTGGGCAGCT

GGTACCAGCCCTGTCACCTGCCTGCTAGACTCTGGACACCAGGCTCAGCTTGTTGAGTTC

CTCATTGTGCACTACGAGCAGATCTTCGGGATGGACGAGCTCCCTCTGGCCACAGAGCCA

CTGCCCCAAGACCCCAGCCCAGCCCCTGGGCCCCTTACAACCAGCCCCCAACCTCCAGTC

CCATTCCTTGACTCAGAGTCACAGTCCCCAGCCCTAGCCTCGGACCCTGACCCAGACTCT

AAGTCTCATAGTGCCCAGGAGGAGCATTCTGAGTCCACA

>CL3.Contig15_A-W 407 3340 PREDICTED: GEM-interacting protein isoform 1 [Pongo abelii]

TGGACTAGTCCCAGGGCTCAGGGAAAACTTCTAATGAAGGGCAGTGTTCAGAGTTAAGGG

TTCCACCCATCTGCCCCAGGGGTGGAGTTGGATTTGAGGCTCATAAGGACCAAAGGGGGC

GTGGATGCAGCCCTGGATTACGCCAAGACGTGGAGCCGCTATGCCAAGGAGCTGCTGGCC

TGGACTGAAAAGAGAGCCAGCTATGAGCAGGAATTTGCTAAAAGCATCATGAAGATTGCT

GAGGCTGGCAAGGTGTCCATCCACCAGCAGAGCCACATGCCACTGCAGTATATCTACACT

CTGTTTCTGGAGCATGATCTCAACCTGGGAACCCTGGCCTTGGAGACAGTAGCCCAGCAG

AAAAGAGATTACTACCAGCCACTGGCTGCCAAACGGACTGAAATTGAGAAATGGCGGAAG

GAGTTCAAGGAGCAATGGATAAAGGAGCAGAAGCGGATGAACGAGGCAGTGCAGATATTG

CGGCGGGCCCAGCTCCAGTATACGCAGCGCAGTGAGGAACTTTGGGCGCGCTCCCAGGGG

TCCCCTGAGGACCATGCCCCTCAAGCCTCCCCTGGAACCAGCAAGCAGCAGGAGCGGCGG

CGGAGGTCCCGAGAAGAGGCCCAGGCCAAGGCACTGGAGGCTGAGGTGTTTTATCAGGCC

TGCGTCCGCGAAGCCAACGCACGCCAGCAGGACCTGGAAAACACCAAGCATCGGATTGTG

TCCCACGTGCGCAAGTTGGTGTTGCAGGGGGATGAAGTGCTGAGGAGGGTGACCCTAGGC

CTCTTTGGGCTGCGCAGAGCACAGGCTGAACGCGGTCCTCGCGCATTCGCAGCCTTGGCT

GAGTGTTGCGCGCCCTTCGAACCTGGCCAGCGCTACCAGGAGTTTGTGCGGGCACTGCGG

CCTGAGGATCCACCGCCCCCACCTCCGGCCTTCACCTTCCAGGAGTTTGTACCCATGGTG

AACAGCTCCCCTCTGGATTCAAGAAAGAAGTTCTCTGGGCCCCCACCTCCCAGGCTGGAC

GAGAGTTCCGCAGAGCAAGGCCCTTGGGAGGACACTGGCATAGGCTGGCAGGGGACTCCA

GGTCCTACTCCTGGCAGTGATGTGGACAGTGTGGGTGGCGGCAGTGAGTCTCGGTCCTTG

GACTCTCCCACTTCCAGCCCAGGCCCTGGTACACGGAGGCTGGTGAAGGTCTCATCCACA

GGCACTGAATCTTCAGATGACTTTGAGGAGCGAGACCCTGACGTGGGAGATGGGCTGGAG

AATGGGCTCGGCAGCCCTTTCAGGAAGTGGACGCTGTCCACTGCGGCTCAGACCCACAGG

CTACGGCGGCTGCGGGGTCCAGCCAAATGTCGAGAGTGTGAAGCCTTCATGGTCAGTGGG

ACTGAGTGTGAAGAGGTATGTCCTGGACTGATGATCCAATGACCTCTGTTGACCCCTAAT

GACCTAAGATCCCTAAAACCTTCCTGCCCTCAGGACCCACCCTGATCTGATGATCTCCTA

CATACCCTCCATTCCCTGAGGATCCCTACAACTGTCTTTTTTTCCCACTCACCCCCAATG

ACCTCACTCACTCTGTGACTTTCTCCCCAGTGTTTCCTGACCTGTCATAAGCGATGTCTG

GAGACCCTGCTGATCCTCTGCGGACACCAGCGGCTCCCAGTCCGGACACCTCTCTTTGGG

GTCGACTTTCTACAGCTTCCCAGGGACTTCCCAGAGGAGGTTCCCTTTGTGATCACCAAA

TGCACAGCTGAGATAGAACACCGTGCCCTGAGTGTTCAGGGCATTTACCGGGTCAGCGGA

TCCCGGGTTCGCGTGGAGCGACTGTGCCAAGCCTTTGAGAATGGCCGAGCATTGGTGGAA

CTGTCAGGAAACTCACCTCATGACATCTCAAGTGTCCTTAAGCGGTTTCTCCAGGAGCTC

ACCGACCCTGTGGTCCCCTTCCACTTCTACGATGCCTTCATCTCTCTGGCTAAGACCCTG

CATGCAGACTCTGGGGACAACCCTGGGACCCCCAGCCCCAGTCCTGAAGTTATCTGCTCG

CTGAAGACTCTCTTGGTACAACTGCCTGACTCTAACTACAACACTCTTCGGCACCTGGTG

GCCCATCTATTCAGGGTGGCTGCACGGTTTGAAGAAAACAAGATGTCTGCCAACAACTTG

GGAATTGTATTTGGGCCAACGCTTCTGCGGCCACCAGATGGTCCGTGGGCAGCTGGTACC

AGCCCTGTCACCTGCCTGCTAGACTCTGGACACCAGGCTCAGCTTGTTGAGTTCCTCATT

GTGCACTACGAGCAGATCTTCGGGATGGACGAGCTCCCTCTGGCCACAGAGCCACTGCCC

CAAGACCCCAGCCCAGCCCCTGGGCCCCTTACAACCAGCCCCCAACCTCCAGTCCCATTC

CTTGACTCAGAGTCACAGTCCCCAGCCCTAGCCTCGGACCCTGACCCAGACTCTAAGTCT

CATAGTGCCCAGGAGGAGCATTCTGAGTCCACATCCTCCAAGATTCCAGCTCTGCAGAGT

GACCAAAGAGAGGAAGTGGCTGAAGATACCAAAAATGGGGAAGGGGAAGTGTCCAGCCAA

GGCCCTGAGGACTCACTCCTAGGAACACAGTCCCGCGGCCATTTCAGCCGCCAGCCAGTG

AAGTATCCGCGGGGAGGTGTGCGGCCTGTTACTCATCAACTGTCCAGTTTGGCCATGGTG

GCTTCCAAGCTGTGTGAGGAGGCCTCTATCTCAATGCACAGAGGGAGTCTGAGGGGGCGG

GGGCCAGGCCCTTCTGCTGCCTCCCCTGACAACAGCCCCCTGCGACGCACCCCACTGCCT

AAGCATTTTGAGATCACCCAGGAGACTGCCCGCTTACTCTCTAAGCTGGACAGTGAGGCT

GTGCCTAGGACCTGCTGCCCAGACCTCCAGCCTGAGGAAACCGAGGACCATCTC

>CL3.Contig16_A-W 407 610 GEM-interacting protein [Homo sapiens] >gi|212286192|sp|Q9P107.2|GMIP_HUMAN RecName: Full=GEM-interacting protein; Short=GMIP >gi|116496745|gb|AAI26437.1| GEM interacting protein [Homo sapiens] >gi|119605245|gb|EAW84839.1| GEM interacting protein, isoform CRA_b [Homo sapiens] >gi|119605248|gb|EAW84842.1| GEM interacting protein, isoform CRA_b [Homo sapiens]

TGGACTAGTCCCAGGGCTCAGGGAAAACTTCTAATGAAGGGCAGTGTTCAGAGTTAAGGG

TTCCACCCATCTGCCCCAGGGGTGGAGTTGGATTTGAGGCTCATAAGGACCAAAGGGGGC

GTGGATGCAGCCCTGGATTACGCCAAGACGTGGAGCCGCTATGCCAAGGAGCTGCTGGCC

TGGACTGAAAAGAGAGCCAGCTAT

>CL3.Contig17_A-W 76 3258 PREDICTED: GEM-interacting protein isoform 1 [Pongo abelii]

ATGGATACATCAGAGCCAGGGATCCCCCCAGCTCCTGAGAGCAGGAAGAGGTACAGTGAC

ATCTTCCGCAGCCTGGATAATCTCGAGATCTCACTGGGAAATGTAACCTTCGAGATGTTG

TCTGGAGACCCTGTAATCTCAGAAGACCTGGATCCTGACAAGGTCACCACAGCCACTGTG

ACCAGTGAAGCCAGCCGTTGGAGTGGCCCCTCCCCAGAGGGTCTTGCGCCCCTCACAGGG

GTGGAGTTGGATTTGAGGCTCATAAGGACCAAAGGGGGCGTGGATGCAGCCCTGGATTAC

GCCAAGACGTGGAGCCGCTATGCCAAGGAGCTGCTGGCCTGGACTGAAAAGAGAGCCAGC

TATGAGCAGGAATTTGCTAAAAGCATCATGAAGATTGCTGAGGCTGGCAAGGTGTCCATC

CACCAGCAGGTAACCATGAGCAGACTTCCCCAGAGCCCAAACCTTCCGGTTTCCCATCTC

TGTCCTCTGATCCTCAGCATCCCCTAACCCTACTCCCAGAGCCACATGCCACTGCAGTAT

ATCTACACTCTGTTTCTGGAGCATGATCTCAACCTGGGAACCCTGGCCTTGGAGACAGTA

GCCCAGCAGAAAAGAGATTACTACCAGCCACTGGCTGCCAAACGGACTGAAATTGAGAAA

TGGCGGAAGGAGTTCAAGGAGCAATGGATAAAGGAGCAGAAGCGGATGAACGAGGCAGTG

CAGATATTGCGGCGGGCCCAGCTCCAGTATACGCAGCGCAGTGAGGAACTTTGGGCGCGC

TCCCAGGGGTCCCCTGAGGACCATGCCCCTCAAGCCTCCCCTGGAACCAGCAAGCAGCAG

GAGCGGCGGCGGAGGTCCCGAGAAGAGGCCCAGGCCAAGGCACTGGAGGCTGAGGTGTTT

TATCAGGCCTGCGTCCGCGAAGCCAACGCACGCCAGCAGGACCTGGAAAACACCAAGCAT

CGGATTGTGTCCCACGTGCGCAAGTTGGTGTTGCAGGGGGATGAAGTGCTGAGGAGGGTG

ACCCTAGGCCTCTTTGGGCTGCGCAGAGCACAGGCTGAACGCGGTCCTCGCGCATTCGCA

GCCTTGGCTGAGTGTTGCGCGCCCTTCGAACCTGGCCAGCGCTACCAGGAGTTTGTGCGG

GCACTGCGGCCTGAGGATCCACCGCCCCCACCTCCGGCCTTCACCTTCCAGGAGTTTGTA

CCCATGGTGAACAGCTCCCCTCTGGATTCAAGAAAGAAGTTCTCTGGGCCCCCACCTCCC

AGGCTGGACGAGAGTTCCGCAGAGCAAGGCCCTTGGGAGGACACTGGCATAGGCTGGCAG

GGGACTCCAGGTCCTACTCCTGGCAGTGATGTGGACAGTGTGGGTGGCGGCAGTGAGTCT

CGGTCCTTGGACTCTCCCACTTCCAGCCCAGGCCCTGGTACACGGAGGCTGGTGAAGGTC

TCATCCACAGGCACTGAATCTTCAGATGACTTTGAGGAGCGAGACCCTGACGTGGGAGAT

GGGCTGGAGAATGGGCTCGGCAGCCCTTTCAGGAAGTGGACGCTGTCCACTGCGGCTCAG

ACCCACAGGCTACGGCGGCTGCGGGGTCCAGCCAAATGTCGAGAGTGTGAAGCCTTCATG

GTCAGTGGGACTGAGTGTGAAGAGGTATGTCCTGGACTGATGATCCAATGACCTCTGTTG

ACCCCTAATGACCTAAGATCCCTAAAACCTTCCTGCCCTCAGGACCCACCCTGATCTGAT

GATCTCCTACATACCCTCCATTCCCTGAGGATCCCTACAACTGTCTTTTTTTCCCACTCA

CCCCCAATGACCTCACTCACTCTGTGACTTTCTCCCCAGTGTTTCCTGACCTGTCATAAG

CGATGTCTGGAGACCCTGCTGATCCTCTGCGGACACCAGCGGCTCCCAGTCCGGACACCT

CTCTTTGGGGTCGACTTTCTACAGCTTCCCAGGGACTTCCCAGAGGAGGTTCCCTTTGTG

ATCACCAAATGCACAGCTGAGATAGAACACCGTGCCCTGAGTGTTCAGGGCATTTACCGG

GTCAGCGGATCCCGGGTTCGCGTGGAGCGACTGTGCCAAGCCTTTGAGAATGGCCGAGCA

TTGGTGGAACTGTCAGGAAACTCACCTCATGACATCTCAAGTGTCCTTAAGCGGTTTCTC

CAGGAGCTCACCGACCCTGTGGTCCCCTTCCACTTCTACGATGCCTTCATCTCTCTGGCT

AAGACCCTGCATGCAGACTCTGGGGACAACCCTGGGACCCCCAGCCCCAGTCCTGAAGTT

ATCTGCTCGCTGAAGACTCTCTTGGTACAACTGCCTGACTCTAACTACAACACTCTTCGG

CACCTGGTGGCCCATCTATTCAGGGTGGCTGCACGGTTTGAAGAAAACAAGATGTCTGCC

AACAACTTGGGAATTGTATTTGGGCCAACGCTTCTGCGGCCACCAGATGGTCCGTGGGCA

GCTGGTACCAGCCCTGTCACCTGCCTGCTAGACTCTGGACACCAGGCTCAGCTTGTTGAG

TTCCTCATTGTGCACTACGAGCAGATCTTCGGGATGGACGAGCTCCCTCTGGCCACAGAG

CCACTGCCCCAAGACCCCAGCCCAGCCCCTGGGCCCCTTACAACCAGCCCCCAACCTCCA

GTCCCATTCCTTGACTCAGAGTCACAGTCCCCAGCCCTAGCCTCGGACCCTGACCCAGAC

TCTAAGTCTCATAGTGCCCAGGAGGAGCATTCTGAGTCCACATCCTCCAAGATTCCAGCT

CTGCAGAGTGACCAAAGAGAGGAAGTGGCTGAAGATACCAAAAATGGGGAAGGGGAAGTG

TCCAGCCAAGGCCCTGAGGACTCACTCCTAGGAACACAGTCCCGCGGCCATTTCAGCCGC

CAGCCAGTGAAGTATCCGCGGGGAGGTGTGCGGCCTGTTACTCATCAACTGTCCAGTTTG

GCCATGGTGGCTTCCAAGCTGTGTGAGGAGGCCTCTATCTCAATGCACAGAGGGAGTCTG

AGGGGGCGGGGGCCAGGCCCTTCTGCTGCCTCCCCTGACAACAGCCCCCTGCGACGCACC

CCACTGCCTAAGCATTTTGAGATCACCCAGGAGACTGCCCGCTTACTCTCTAAGCTGGAC

AGTGAGGCTGTGCCTAGGACCTGCTGCCCAGACCTCCAGCCTGAGGAAACCGAGGACCAT

CTC

>CL3.Contig18_A-W 407 3235 GEM-interacting protein [Homo sapiens] >gi|212286192|sp|Q9P107.2|GMIP_HUMAN RecName: Full=GEM-interacting protein; Short=GMIP >gi|116496745|gb|AAI26437.1| GEM interacting protein [Homo sapiens] >gi|119605245|gb|EAW84839.1| GEM interacting protein, isoform CRA_b [Homo sapiens] >gi|119605248|gb|EAW84842.1| GEM interacting protein, isoform CRA_b [Homo sapiens]

TGGACTAGTCCCAGGGCTCAGGGAAAACTTCTAATGAAGGGCAGTGTTCAGAGTTAAGGG

TTCCACCCATCTGCCCCAGGGGTGGAGTTGGATTTGAGGCTCATAAGGACCAAAGGGGGC

GTGGATGCAGCCCTGGATTACGCCAAGACGTGGAGCCGCTATGCCAAGGAGCTGCTGGCC

TGGACTGAAAAGAGAGCCAGCTATGAGCAGGAATTTGCTAAAAGCATCATGAAGATTGCT

GAGGCTGGCAAGGTGTCCATCCACCAGCAGGTAACCATGAGCAGACTTCCCCAGAGCCCA

AACCTTCCGGTTTCCCATCTCTGTCCTCTGATCCTCAGCATCCCCTAACCCTACTCCCAG

AGCCACATGCCACTGCAGTATATCTACACTCTGTTTCTGGAGCATGATCTCAACCTGGGA

ACCCTGGCCTTGGAGACAGTAGCCCAGCAGAAAAGAGATTACTACCAGCCACTGGCTGCC

AAACGGACTGAAATTGAGAAATGGCGGAAGGAGTTCAAGGAGCAATGGATAAAGGAGCAG

AAGCGGATGAACGAGGCAGTGCAGATATTGCGGCGGGCCCAGCTCCAGTATACGCAGCGC

AGTGAGGAACTTTGGGCGCGCTCCCAGGGGTCCCCTGAGGACCATGCCCCTCAAGCCTCC

CCTGGAACCAGCAAGCAGCAGGAGCGGCGGCGGAGGTCCCGAGAAGAGGCCCAGGCCAAG

GCACTGGAGGCTGAGGTGTTTTATCAGGCCTGCGTCCGCGAAGCCAACGCACGCCAGCAG

GACCTGGAAAACACCAAGCATCGGATTGTGTCCCACGTGCGCAAGTTGGTGTTGCAGGGG

GATGAAGTGCTGAGGAGGGTGACCCTAGGCCTCTTTGGGCTGCGCAGAGCACAGGCTGAA

CGCGGTCCTCGCGCATTCGCAGCCTTGGCTGAGTGTTGCGCGCCCTTCGAACCTGGCCAG

CGCTACCAGGAGTTTGTGCGGGCACTGCGGCCTGAGGATCCACCGCCCCCACCTCCGGCC

TTCACCTTCCAGGAGTTTGTACCCATGGTGAACAGCTCCCCTCTGGATTCAAGAAAGAAG

TTCTCTGGGCCCCCACCTCCCAGGCTGGACGAGAGTTCCGCAGAGCAAGGCCCTTGGGAG

GACACTGGCATAGGCTGGCAGGGGACTCCAGGTCCTACTCCTGGCAGTGATGTGGACAGT

GTGGGTGGCGGCAGTGAGTCTCGGTCCTTGGACTCTCCCACTTCCAGCCCAGGCCCTGGT

ACACGGAGGCTGGTGAAGGTCTCATCCACAGGCACTGAATCTTCAGATGACTTTGAGGAG

CGAGACCCTGACGTGGGAGATGGGCTGGAGAATGGGCTCGGCAGCCCTTTCAGGAAGTGG

ACGCTGTCCACTGCGGCTCAGACCCACAGGCTACGGCGGCTGCGGGGTCCAGCCAAATGT

CGAGAGTGTGAAGCCTTCATGGTCAGTGGGACTGAGTGTGAAGAGTGTTTCCTGACCTGT

CATAAGCGATGTCTGGAGACCCTGCTGATCCTCTGCGGACACCAGCGGCTCCCAGTCCGG

ACACCTCTCTTTGGGGTCGACTTTCTACAGCTTCCCAGGGACTTCCCAGAGGAGGTTCCC

TTTGTGATCACCAAATGCACAGCTGAGATAGAACACCGTGCCCTGAGTGTTCAGGGCATT

TACCGGGTCAGCGGATCCCGGGTTCGCGTGGAGCGACTGTGCCAAGCCTTTGAGAATGGC

CGAGCATTGGTGGAACTGTCAGGAAACTCACCTCATGACATCTCAAGTGTCCTTAAGCGG

TTTCTCCAGGAGCTCACCGACCCTGTGGTCCCCTTCCACTTCTACGATGCCTTCATCTCT

CTGGCTAAGACCCTGCATGCAGACTCTGGGGACAACCCTGGGACCCCCAGCCCCAGTCCT

GAAGTTATCTGCTCGCTGAAGACTCTCTTGGTACAACTGCCTGACTCTAACTACAACACT

CTTCGGCACCTGGTGGCCCATCTATTCAGGGTGGCTGCACGGTTTGAAGAAAACAAGATG

TCTGCCAACAACTTGGGAATTGTATTTGGGCCAACGCTTCTGCGGCCACCAGATGGTCCG

TGGGCAGCTGGTACCAGCCCTGTCACCTGCCTGCTAGACTCTGGACACCAGGCTCAGCTT

GTTGAGTTCCTCATTGTGCACTACGAGCAGATCTTCGGGATGGACGAGCTCCCTCTGGCC

ACAGAGCCACTGCCCCAAGACCCCAGCCCAGCCCCTGGGCCCCTTACAACCAGCCCCCAA

CCTCCAGTCCCATTCCTTGACTCAGAGTCACAGTCCCCAGCCCTAGCCTCGGACCCTGAC

CCAGACTCTAAGTCTCATAGTGCCCAGGAGGAGCATTCTGAGTCCACATCCTCCAAGATT

CCAGCTCTGCAGAGTGACCAAAGAGAGGAAGTGGCTGAAGATACCAAAAATGGGGAAGGG

GAAGTGTCCAGCCAAGGCCCTGAGGACTCACTCCTAGGAACACAGTCCCGCGGCCATTTC

AGCCGCCAGCCAGTGAAGTATCCGCGGGGAGGTGTGCGGCCTGTTACTCATCAACTGTCC

AGTTTGGCCATGGTGGCTTCCAAGCTGTGTGAGGAGGCCTCTATCTCAATGCACAGAGGG

AGTCTGAGGGGGCGGGGGCCAGGCCCTTCTGCTGCCTCCCCTGACAACAGCCCCCTGCGA

CGCACCCCACTGCCTAAGCATTTTGAGATCACCCAGGAGACTGCCCGCTTACTCTCTAAG

CTGGACAGTGAGGCTGTGCCTAGGACCTGCTGCCCAGACCTCCAGCCTGAGGAAACCGAG

GACCATCTC

>CL3.Contig19_A-W 76 3168 PREDICTED: GEM-interacting protein isoform 1 [Pongo abelii]

ATGGATACATCAGAGCCAGGGATCCCCCCAGCTCCTGAGAGCAGGAAGAGGTACAGTGAC

ATCTTCCGCAGCCTGGATAATCTCGAGATCTCACTGGGAAATGTAACCTTCGAGATGTTG

TCTGGAGACCCTGTAATCTCAGAAGACCTGGATCCTGACAAGGTCACCACAGCCACTGTG

ACCAGTGAAGCCAGCCGTTGGAGTGGCCCCTCCCCAGAGGGTCTTGCGCCCCTCACAGGG

GTGGAGTTGGATTTGAGGCTCATAAGGACCAAAGGGGGCGTGGATGCAGCCCTGGATTAC

GCCAAGACGTGGAGCCGCTATGCCAAGGAGCTGCTGGCCTGGACTGAAAAGAGAGCCAGC

TATGAGCAGGAATTTGCTAAAAGCATCATGAAGATTGCTGAGGCTGGCAAGGTGTCCATC

CACCAGCAGAGCCACATGCCACTGCAGTATATCTACACTCTGTTTCTGGAGCATGATCTC

AACCTGGGAACCCTGGCCTTGGAGACAGTAGCCCAGCAGAAAAGAGATTACTACCAGCCA

CTGGCTGCCAAACGGACTGAAATTGAGAAATGGCGGAAGGAGTTCAAGGAGCAATGGATA

AAGGAGCAGAAGCGGATGAACGAGGCAGTGCAGATATTGCGGCGGGCCCAGCTCCAGTAT

ACGCAGCGCAGTGAGGAACTTTGGGCGCGCTCCCAGGGGTCCCCTGAGGACCATGCCCCT

CAAGCCTCCCCTGGAACCAGCAAGCAGCAGGAGCGGCGGCGGAGGTCCCGAGAAGAGGCC

CAGGCCAAGGCACTGGAGGCTGAGGTGTTTTATCAGGCCTGCGTCCGCGAAGCCAACGCA

CGCCAGCAGGACCTGGAAAACACCAAGCATCGGATTGTGTCCCACGTGCGCAAGTTGGTG

TTGCAGGGGGATGAAGTGCTGAGGAGGGTGACCCTAGGCCTCTTTGGGCTGCGCAGAGCA

CAGGCTGAACGCGGTCCTCGCGCATTCGCAGCCTTGGCTGAGTGTTGCGCGCCCTTCGAA

CCTGGCCAGCGCTACCAGGAGTTTGTGCGGGCACTGCGGCCTGAGGATCCACCGCCCCCA

CCTCCGGCCTTCACCTTCCAGGAGTTTGTACCCATGGTGAACAGCTCCCCTCTGGATTCA

AGAAAGAAGTTCTCTGGGCCCCCACCTCCCAGGCTGGACGAGAGTTCCGCAGAGCAAGGC

CCTTGGGAGGACACTGGCATAGGCTGGCAGGGGACTCCAGGTCCTACTCCTGGCAGTGAT

GTGGACAGTGTGGGTGGCGGCAGTGAGTCTCGGTCCTTGGACTCTCCCACTTCCAGCCCA

GGCCCTGGTACACGGAGGCTGGTGAAGGTCTCATCCACAGGCACTGAATCTTCAGATGAC

TTTGAGGAGCGAGACCCTGACGTGGGAGATGGGCTGGAGAATGGGCTCGGCAGCCCTTTC

AGGAAGTGGACGCTGTCCACTGCGGCTCAGACCCACAGGCTACGGCGGCTGCGGGGTCCA

GCCAAATGTCGAGAGTGTGAAGCCTTCATGGTCAGTGGGACTGAGTGTGAAGAGGTATGT

CCTGGACTGATGATCCAATGACCTCTGTTGACCCCTAATGACCTAAGATCCCTAAAACCT

TCCTGCCCTCAGGACCCACCCTGATCTGATGATCTCCTACATACCCTCCATTCCCTGAGG

ATCCCTACAACTGTCTTTTTTTCCCACTCACCCCCAATGACCTCACTCACTCTGTGACTT

TCTCCCCAGTGTTTCCTGACCTGTCATAAGCGATGTCTGGAGACCCTGCTGATCCTCTGC

GGACACCAGCGGCTCCCAGTCCGGACACCTCTCTTTGGGGTCGACTTTCTACAGCTTCCC

AGGGACTTCCCAGAGGAGGTTCCCTTTGTGATCACCAAATGCACAGCTGAGATAGAACAC

CGTGCCCTGAGTGTTCAGGGCATTTACCGGGTCAGCGGATCCCGGGTTCGCGTGGAGCGA

CTGTGCCAAGCCTTTGAGAATGGCCGAGCATTGGTGGAACTGTCAGGAAACTCACCTCAT

GACATCTCAAGTGTCCTTAAGCGGTTTCTCCAGGAGCTCACCGACCCTGTGGTCCCCTTC

CACTTCTACGATGCCTTCATCTCTCTGGCTAAGACCCTGCATGCAGACTCTGGGGACAAC

CCTGGGACCCCCAGCCCCAGTCCTGAAGTTATCTGCTCGCTGAAGACTCTCTTGGTACAA

CTGCCTGACTCTAACTACAACACTCTTCGGCACCTGGTGGCCCATCTATTCAGGGTGGCT

GCACGGTTTGAAGAAAACAAGATGTCTGCCAACAACTTGGGAATTGTATTTGGGCCAACG

CTTCTGCGGCCACCAGATGGTCCGTGGGCAGCTGGTACCAGCCCTGTCACCTGCCTGCTA

GACTCTGGACACCAGGCTCAGCTTGTTGAGTTCCTCATTGTGCACTACGAGCAGATCTTC

GGGATGGACGAGCTCCCTCTGGCCACAGAGCCACTGCCCCAAGACCCCAGCCCAGCCCCT

GGGCCCCTTACAACCAGCCCCCAACCTCCAGTCCCATTCCTTGACTCAGAGTCACAGTCC

CCAGCCCTAGCCTCGGACCCTGACCCAGACTCTAAGTCTCATAGTGCCCAGGAGGAGCAT

TCTGAGTCCACATCCTCCAAGATTCCAGCTCTGCAGAGTGACCAAAGAGAGGAAGTGGCT

GAAGATACCAAAAATGGGGAAGGGGAAGTGTCCAGCCAAGGCCCTGAGGACTCACTCCTA

GGAACACAGTCCCGCGGCCATTTCAGCCGCCAGCCAGTGAAGTATCCGCGGGGAGGTGTG

CGGCCTGTTACTCATCAACTGTCCAGTTTGGCCATGGTGGCTTCCAAGCTGTGTGAGGAG

GCCTCTATCTCAATGCACAGAGGGAGTCTGAGGGGGCGGGGGCCAGGCCCTTCTGCTGCC

TCCCCTGACAACAGCCCCCTGCGACGCACCCCACTGCCTAAGCATTTTGAGATCACCCAG

GAGACTGCCCGCTTACTCTCTAAGCTGGACAGTGAGGCTGTGCCTAGGACCTGCTGCCCA

GACCTCCAGCCTGAGGAAACCGAGGACCATCTC

>CL3.Contig20_A-W 76 438 GEM-interacting protein [Homo sapiens] >gi|212286192|sp|Q9P107.2|GMIP_HUMAN RecName: Full=GEM-interacting protein; Short=GMIP >gi|116496745|gb|AAI26437.1| GEM interacting protein [Homo sapiens] >gi|119605245|gb|EAW84839.1| GEM interacting protein, isoform CRA_b [Homo sapiens] >gi|119605248|gb|EAW84842.1| GEM interacting protein, isoform CRA_b [Homo sapiens]

ATGGATACATCAGAGCCAGGGATCCCCCCAGCTCCTGAGAGCAGGAAGAGGTACAGTGAC

ATCTTCCGCAGCCTGGATAATCTCGAGATCTCACTGGGAAATGTAACCTTCGAGATGTTG

TCTGGAGACCCTGTAATCTCAGAAGACCTGGATCCTGACAAGGTCACCACAGCCACTGTG

ACCAGTGAAGCCAGCCGTTGGAGTGGCCCCTCCCCAGAGGGTCTTGCGCCCCTCACAGGG

GTGGAGTTGGATTTGAGGCTCATAAGGACCAAAGGGGGCGTGGATGCAGCCCTGGATTAC

GCCAAGACGTGGAGCCGCTATGCCAAGGAGCTGCTGGCCTGGACTGAAAAGAGAGCCAGC

TAT

>CL3.Contig21_A-W 407 3145 GEM-interacting protein [Homo sapiens] >gi|212286192|sp|Q9P107.2|GMIP_HUMAN RecName: Full=GEM-interacting protein; Short=GMIP >gi|116496745|gb|AAI26437.1| GEM interacting protein [Homo sapiens] >gi|119605245|gb|EAW84839.1| GEM interacting protein, isoform CRA_b [Homo sapiens] >gi|119605248|gb|EAW84842.1| GEM interacting protein, isoform CRA_b [Homo sapiens]

TGGACTAGTCCCAGGGCTCAGGGAAAACTTCTAATGAAGGGCAGTGTTCAGAGTTAAGGG

TTCCACCCATCTGCCCCAGGGGTGGAGTTGGATTTGAGGCTCATAAGGACCAAAGGGGGC

GTGGATGCAGCCCTGGATTACGCCAAGACGTGGAGCCGCTATGCCAAGGAGCTGCTGGCC

TGGACTGAAAAGAGAGCCAGCTATGAGCAGGAATTTGCTAAAAGCATCATGAAGATTGCT

GAGGCTGGCAAGGTGTCCATCCACCAGCAGAGCCACATGCCACTGCAGTATATCTACACT

CTGTTTCTGGAGCATGATCTCAACCTGGGAACCCTGGCCTTGGAGACAGTAGCCCAGCAG

AAAAGAGATTACTACCAGCCACTGGCTGCCAAACGGACTGAAATTGAGAAATGGCGGAAG

GAGTTCAAGGAGCAATGGATAAAGGAGCAGAAGCGGATGAACGAGGCAGTGCAGATATTG

CGGCGGGCCCAGCTCCAGTATACGCAGCGCAGTGAGGAACTTTGGGCGCGCTCCCAGGGG

TCCCCTGAGGACCATGCCCCTCAAGCCTCCCCTGGAACCAGCAAGCAGCAGGAGCGGCGG

CGGAGGTCCCGAGAAGAGGCCCAGGCCAAGGCACTGGAGGCTGAGGTGTTTTATCAGGCC

TGCGTCCGCGAAGCCAACGCACGCCAGCAGGACCTGGAAAACACCAAGCATCGGATTGTG

TCCCACGTGCGCAAGTTGGTGTTGCAGGGGGATGAAGTGCTGAGGAGGGTGACCCTAGGC

CTCTTTGGGCTGCGCAGAGCACAGGCTGAACGCGGTCCTCGCGCATTCGCAGCCTTGGCT

GAGTGTTGCGCGCCCTTCGAACCTGGCCAGCGCTACCAGGAGTTTGTGCGGGCACTGCGG

CCTGAGGATCCACCGCCCCCACCTCCGGCCTTCACCTTCCAGGAGTTTGTACCCATGGTG

AACAGCTCCCCTCTGGATTCAAGAAAGAAGTTCTCTGGGCCCCCACCTCCCAGGCTGGAC

GAGAGTTCCGCAGAGCAAGGCCCTTGGGAGGACACTGGCATAGGCTGGCAGGGGACTCCA

GGTCCTACTCCTGGCAGTGATGTGGACAGTGTGGGTGGCGGCAGTGAGTCTCGGTCCTTG

GACTCTCCCACTTCCAGCCCAGGCCCTGGTACACGGAGGCTGGTGAAGGTCTCATCCACA

GGCACTGAATCTTCAGATGACTTTGAGGAGCGAGACCCTGACGTGGGAGATGGGCTGGAG

AATGGGCTCGGCAGCCCTTTCAGGAAGTGGACGCTGTCCACTGCGGCTCAGACCCACAGG

CTACGGCGGCTGCGGGGTCCAGCCAAATGTCGAGAGTGTGAAGCCTTCATGGTCAGTGGG

ACTGAGTGTGAAGAGTGTTTCCTGACCTGTCATAAGCGATGTCTGGAGACCCTGCTGATC

CTCTGCGGACACCAGCGGCTCCCAGTCCGGACACCTCTCTTTGGGGTCGACTTTCTACAG

CTTCCCAGGGACTTCCCAGAGGAGGTTCCCTTTGTGATCACCAAATGCACAGCTGAGATA

GAACACCGTGCCCTGAGTGTTCAGGGCATTTACCGGGTCAGCGGATCCCGGGTTCGCGTG

GAGCGACTGTGCCAAGCCTTTGAGAATGGCCGAGCATTGGTGGAACTGTCAGGAAACTCA

CCTCATGACATCTCAAGTGTCCTTAAGCGGTTTCTCCAGGAGCTCACCGACCCTGTGGTC

CCCTTCCACTTCTACGATGCCTTCATCTCTCTGGCTAAGACCCTGCATGCAGACTCTGGG

GACAACCCTGGGACCCCCAGCCCCAGTCCTGAAGTTATCTGCTCGCTGAAGACTCTCTTG

GTACAACTGCCTGACTCTAACTACAACACTCTTCGGCACCTGGTGGCCCATCTATTCAGG

GTGGCTGCACGGTTTGAAGAAAACAAGATGTCTGCCAACAACTTGGGAATTGTATTTGGG

CCAACGCTTCTGCGGCCACCAGATGGTCCGTGGGCAGCTGGTACCAGCCCTGTCACCTGC

CTGCTAGACTCTGGACACCAGGCTCAGCTTGTTGAGTTCCTCATTGTGCACTACGAGCAG

ATCTTCGGGATGGACGAGCTCCCTCTGGCCACAGAGCCACTGCCCCAAGACCCCAGCCCA

GCCCCTGGGCCCCTTACAACCAGCCCCCAACCTCCAGTCCCATTCCTTGACTCAGAGTCA

CAGTCCCCAGCCCTAGCCTCGGACCCTGACCCAGACTCTAAGTCTCATAGTGCCCAGGAG

GAGCATTCTGAGTCCACATCCTCCAAGATTCCAGCTCTGCAGAGTGACCAAAGAGAGGAA

GTGGCTGAAGATACCAAAAATGGGGAAGGGGAAGTGTCCAGCCAAGGCCCTGAGGACTCA

CTCCTAGGAACACAGTCCCGCGGCCATTTCAGCCGCCAGCCAGTGAAGTATCCGCGGGGA

GGTGTGCGGCCTGTTACTCATCAACTGTCCAGTTTGGCCATGGTGGCTTCCAAGCTGTGT

GAGGAGGCCTCTATCTCAATGCACAGAGGGAGTCTGAGGGGGCGGGGGCCAGGCCCTTCT

GCTGCCTCCCCTGACAACAGCCCCCTGCGACGCACCCCACTGCCTAAGCATTTTGAGATC

ACCCAGGAGACTGCCCGCTTACTCTCTAAGCTGGACAGTGAGGCTGTGCCTAGGACCTGC

TGCCCAGACCTCCAGCCTGAGGAAACCGAGGACCATCTC

>CL3.Contig22_A-W 76 3063 GEM-interacting protein [Homo sapiens] >gi|212286192|sp|Q9P107.2|GMIP_HUMAN RecName: Full=GEM-interacting protein; Short=GMIP >gi|116496745|gb|AAI26437.1| GEM interacting protein [Homo sapiens] >gi|119605245|gb|EAW84839.1| GEM interacting protein, isoform CRA_b [Homo sapiens] >gi|119605248|gb|EAW84842.1| GEM interacting protein, isoform CRA_b [Homo sapiens]

ATGGATACATCAGAGCCAGGGATCCCCCCAGCTCCTGAGAGCAGGAAGAGGTACAGTGAC

ATCTTCCGCAGCCTGGATAATCTCGAGATCTCACTGGGAAATGTAACCTTCGAGATGTTG

TCTGGAGACCCTGTAATCTCAGAAGACCTGGATCCTGACAAGGTCACCACAGCCACTGTG

ACCAGTGAAGCCAGCCGTTGGAGTGGCCCCTCCCCAGAGGGTCTTGCGCCCCTCACAGGG

GTGGAGTTGGATTTGAGGCTCATAAGGACCAAAGGGGGCGTGGATGCAGCCCTGGATTAC

GCCAAGACGTGGAGCCGCTATGCCAAGGAGCTGCTGGCCTGGACTGAAAAGAGAGCCAGC

TATGAGCAGGAATTTGCTAAAAGCATCATGAAGATTGCTGAGGCTGGCAAGGTGTCCATC

CACCAGCAGGTAACCATGAGCAGACTTCCCCAGAGCCCAAACCTTCCGGTTTCCCATCTC

TGTCCTCTGATCCTCAGCATCCCCTAACCCTACTCCCAGAGCCACATGCCACTGCAGTAT

ATCTACACTCTGTTTCTGGAGCATGATCTCAACCTGGGAACCCTGGCCTTGGAGACAGTA

GCCCAGCAGAAAAGAGATTACTACCAGCCACTGGCTGCCAAACGGACTGAAATTGAGAAA

TGGCGGAAGGAGTTCAAGGAGCAATGGATAAAGGAGCAGAAGCGGATGAACGAGGCAGTG

CAGATATTGCGGCGGGCCCAGCTCCAGTATACGCAGCGCAGTGAGGAACTTTGGGCGCGC

TCCCAGGGGTCCCCTGAGGACCATGCCCCTCAAGCCTCCCCTGGAACCAGCAAGCAGCAG

GAGCGGCGGCGGAGGTCCCGAGAAGAGGCCCAGGCCAAGGCACTGGAGGCTGAGGTGTTT

TATCAGGCCTGCGTCCGCGAAGCCAACGCACGCCAGCAGGACCTGGAAAACACCAAGCAT

CGGATTGTGTCCCACGTGCGCAAGTTGGTGTTGCAGGGGGATGAAGTGCTGAGGAGGGTG

ACCCTAGGCCTCTTTGGGCTGCGCAGAGCACAGGCTGAACGCGGTCCTCGCGCATTCGCA

GCCTTGGCTGAGTGTTGCGCGCCCTTCGAACCTGGCCAGCGCTACCAGGAGTTTGTGCGG

GCACTGCGGCCTGAGGATCCACCGCCCCCACCTCCGGCCTTCACCTTCCAGGAGTTTGTA

CCCATGGTGAACAGCTCCCCTCTGGATTCAAGAAAGAAGTTCTCTGGGCCCCCACCTCCC

AGGCTGGACGAGAGTTCCGCAGAGCAAGGCCCTTGGGAGGACACTGGCATAGGCTGGCAG

GGGACTCCAGGTCCTACTCCTGGCAGTGATGTGGACAGTGTGGGTGGCGGCAGTGAGTCT

CGGTCCTTGGACTCTCCCACTTCCAGCCCAGGCCCTGGTACACGGAGGCTGGTGAAGGTC

TCATCCACAGGCACTGAATCTTCAGATGACTTTGAGGAGCGAGACCCTGACGTGGGAGAT

GGGCTGGAGAATGGGCTCGGCAGCCCTTTCAGGAAGTGGACGCTGTCCACTGCGGCTCAG

ACCCACAGGCTACGGCGGCTGCGGGGTCCAGCCAAATGTCGAGAGTGTGAAGCCTTCATG

GTCAGTGGGACTGAGTGTGAAGAGTGTTTCCTGACCTGTCATAAGCGATGTCTGGAGACC

CTGCTGATCCTCTGCGGACACCAGCGGCTCCCAGTCCGGACACCTCTCTTTGGGGTCGAC

TTTCTACAGCTTCCCAGGGACTTCCCAGAGGAGGTTCCCTTTGTGATCACCAAATGCACA

GCTGAGATAGAACACCGTGCCCTGAGTGTTCAGGGCATTTACCGGGTCAGCGGATCCCGG

GTTCGCGTGGAGCGACTGTGCCAAGCCTTTGAGAATGGCCGAGCATTGGTGGAACTGTCA

GGAAACTCACCTCATGACATCTCAAGTGTCCTTAAGCGGTTTCTCCAGGAGCTCACCGAC

CCTGTGGTCCCCTTCCACTTCTACGATGCCTTCATCTCTCTGGCTAAGACCCTGCATGCA

GACTCTGGGGACAACCCTGGGACCCCCAGCCCCAGTCCTGAAGTTATCTGCTCGCTGAAG

ACTCTCTTGGTACAACTGCCTGACTCTAACTACAACACTCTTCGGCACCTGGTGGCCCAT

CTATTCAGGGTGGCTGCACGGTTTGAAGAAAACAAGATGTCTGCCAACAACTTGGGAATT

GTATTTGGGCCAACGCTTCTGCGGCCACCAGATGGTCCGTGGGCAGCTGGTACCAGCCCT

GTCACCTGCCTGCTAGACTCTGGACACCAGGCTCAGCTTGTTGAGTTCCTCATTGTGCAC

TACGAGCAGATCTTCGGGATGGACGAGCTCCCTCTGGCCACAGAGCCACTGCCCCAAGAC

CCCAGCCCAGCCCCTGGGCCCCTTACAACCAGCCCCCAACCTCCAGTCCCATTCCTTGAC

TCAGAGTCACAGTCCCCAGCCCTAGCCTCGGACCCTGACCCAGACTCTAAGTCTCATAGT

GCCCAGGAGGAGCATTCTGAGTCCACATCCTCCAAGATTCCAGCTCTGCAGAGTGACCAA

AGAGAGGAAGTGGCTGAAGATACCAAAAATGGGGAAGGGGAAGTGTCCAGCCAAGGCCCT

GAGGACTCACTCCTAGGAACACAGTCCCGCGGCCATTTCAGCCGCCAGCCAGTGAAGTAT

CCGCGGGGAGGTGTGCGGCCTGTTACTCATCAACTGTCCAGTTTGGCCATGGTGGCTTCC

AAGCTGTGTGAGGAGGCCTCTATCTCAATGCACAGAGGGAGTCTGAGGGGGCGGGGGCCA

GGCCCTTCTGCTGCCTCCCCTGACAACAGCCCCCTGCGACGCACCCCACTGCCTAAGCAT

TTTGAGATCACCCAGGAGACTGCCCGCTTACTCTCTAAGCTGGACAGTGAGGCTGTGCCT

AGGACCTGCTGCCCAGACCTCCAGCCTGAGGAAACCGAGGACCATCTC

>CL3.Contig23_A-W 485 607 PREDICTED: GEM-interacting protein-like [Ailuropoda melanoleuca]

GGGGTGGAGTTGGATTTGAGGCTCATAAGGACCAAAGGGGGCGTGGATGCAGCCCTGGAT

TACGCCAAGACGTGGAGCCGCTATGCCAAGGAGCTGCTGGCCTGGACTGAAAAGAGAGCC

AGC

>CL3.Contig24_A-W 485 1570 PREDICTED: GEM-interacting protein-like [Ailuropoda melanoleuca]

GGGGTGGAGTTGGATTTGAGGCTCATAAGGACCAAAGGGGGCGTGGATGCAGCCCTGGAT

TACGCCAAGACGTGGAGCCGCTATGCCAAGGAGCTGCTGGCCTGGACTGAAAAGAGAGCC

AGCTATGAGCAGGAATTTGCTAAAAGCATCATGAAGATTGCTGAGGCTGGCAAGGTGTCC

ATCCACCAGCAGGTAACCATGAGCAGACTTCCCCAGAGCCCAAACCTTCCGGTTTCCCAT

CTCTGTCCTCTGATCCTCAGCATCCCCTAACCCTACTCCCAGAGCCACATGCCACTGCAG

TATATCTACACTCTGTTTCTGGAGCATGATCTCAACCTGGGAACCCTGGCCTTGGAGACA

GTAGCCCAGCAGAAAAGAGATTACTACCAGCCACTGGCTGCCAAACGGACTGAAATTGAG

AAATGGCGGAAGGAGTTCAAGGAGCAATGGATAAAGGAGCAGAAGCGGATGAACGAGGCA

GTGCAGATATTGCGGCGGGCCCAGCTCCAGTATACGCAGCGCAGTGAGGAACTTTGGGCG

CGCTCCCAGGGGTCCCCTGAGGACCATGCCCCTCAAGCCTCCCCTGGAACCAGCAAGCAG

CAGGAGCGGCGGCGGAGGTCCCGAGAAGAGGCCCAGGCCAAGGCACTGGAGGCTGAGGTG

TTTTATCAGGCCTGCGTCCGCGAAGCCAACGCACGCCAGCAGGACCTGGAAAACACCAAG

CATCGGATTGTGTCCCACGTGCGCAAGTTGGTGTTGCAGGGGGATGAAGTGCTGAGGAGG

GTGACCCTAGGCCTCTTTGGGCTGCGCAGAGCACAGGCTGAACGCGGTCCTCGCGCATTC

GCAGCCTTGGCTGAGTGTTGCGCGCCCTTCGAACCTGGCCAGCGCTACCAGGAGTTTGTG

CGGGCACTGCGGCCTGAGGATCCACCGCCCCCACCTCCGGCCTTCACCTTCCAGGAGTTT

GTACCCATGGTGAACAGCTCCCCTCTGGATTCAAGAAAGAAGTTCTCTGGGCCCCCACCT

CCCAGGCTGGACGAGAGTTCCGCAGAGCAAGGCCCTTGGGAGGACACTGGCATAGGCTGG

CAGGGT

>CL3.Contig25_A-W 76 438 GEM-interacting protein [Homo sapiens] >gi|212286192|sp|Q9P107.2|GMIP_HUMAN RecName: Full=GEM-interacting protein; Short=GMIP >gi|116496745|gb|AAI26437.1| GEM interacting protein [Homo sapiens] >gi|119605245|gb|EAW84839.1| GEM interacting protein, isoform CRA_b [Homo sapiens] >gi|119605248|gb|EAW84842.1| GEM interacting protein, isoform CRA_b [Homo sapiens]

ATGGATACATCAGAGCCAGGGATCCCCCCAGCTCCTGAGAGCAGGAAGAGGTACAGTGAC

ATCTTCCGCAGCCTGGATAATCTCGAGATCTCACTGGGAAATGTAACCTTCGAGATGTTG

TCTGGAGACCCTGTAATCTCAGAAGACCTGGATCCTGACAAGGTCACCACAGCCACTGTG

ACCAGTGAAGCCAGCCGTTGGAGTGGCCCCTCCCCAGAGGGTCTTGCGCCCCTCACAGGG

GTGGAGTTGGATTTGAGGCTCATAAGGACCAAAGGGGGCGTGGATGCAGCCCTGGATTAC

GCCAAGACGTGGAGCCGCTATGCCAAGGAGCTGCTGGCCTGGACTGAAAAGAGAGCCAGC

TAT

>CL3.Contig26_A-W 485 1480 PREDICTED: GEM-interacting protein-like [Ailuropoda melanoleuca]

GGGGTGGAGTTGGATTTGAGGCTCATAAGGACCAAAGGGGGCGTGGATGCAGCCCTGGAT

TACGCCAAGACGTGGAGCCGCTATGCCAAGGAGCTGCTGGCCTGGACTGAAAAGAGAGCC

AGCTATGAGCAGGAATTTGCTAAAAGCATCATGAAGATTGCTGAGGCTGGCAAGGTGTCC

ATCCACCAGCAGAGCCACATGCCACTGCAGTATATCTACACTCTGTTTCTGGAGCATGAT

CTCAACCTGGGAACCCTGGCCTTGGAGACAGTAGCCCAGCAGAAAAGAGATTACTACCAG

CCACTGGCTGCCAAACGGACTGAAATTGAGAAATGGCGGAAGGAGTTCAAGGAGCAATGG

ATAAAGGAGCAGAAGCGGATGAACGAGGCAGTGCAGATATTGCGGCGGGCCCAGCTCCAG

TATACGCAGCGCAGTGAGGAACTTTGGGCGCGCTCCCAGGGGTCCCCTGAGGACCATGCC

CCTCAAGCCTCCCCTGGAACCAGCAAGCAGCAGGAGCGGCGGCGGAGGTCCCGAGAAGAG

GCCCAGGCCAAGGCACTGGAGGCTGAGGTGTTTTATCAGGCCTGCGTCCGCGAAGCCAAC

GCACGCCAGCAGGACCTGGAAAACACCAAGCATCGGATTGTGTCCCACGTGCGCAAGTTG

GTGTTGCAGGGGGATGAAGTGCTGAGGAGGGTGACCCTAGGCCTCTTTGGGCTGCGCAGA

GCACAGGCTGAACGCGGTCCTCGCGCATTCGCAGCCTTGGCTGAGTGTTGCGCGCCCTTC

GAACCTGGCCAGCGCTACCAGGAGTTTGTGCGGGCACTGCGGCCTGAGGATCCACCGCCC

CCACCTCCGGCCTTCACCTTCCAGGAGTTTGTACCCATGGTGAACAGCTCCCCTCTGGAT

TCAAGAAAGAAGTTCTCTGGGCCCCCACCTCCCAGGCTGGACGAGAGTTCCGCAGAGCAA

GGCCCTTGGGAGGACACTGGCATAGGCTGGCAGGGT

>CL3.Contig27_A-W 76 1395 PREDICTED: GEM-interacting protein isoform 2 [Pongo abelii]

ATGGATACATCAGAGCCAGGGATCCCCCCAGCTCCTGAGAGCAGGAAGAGGTACAGTGAC

ATCTTCCGCAGCCTGGATAATCTCGAGATCTCACTGGGAAATGTAACCTTCGAGATGTTG

TCTGGAGACCCTGTAATCTCAGAAGACCTGGATCCTGACAAGGTCACCACAGCCACTGTG

ACCAGTGAAGCCAGCCGTTGGAGTGGCCCCTCCCCAGAGGGTCTTGCGCCCCTCACAGGG

GTGGAGTTGGATTTGAGGCTCATAAGGACCAAAGGGGGCGTGGATGCAGCCCTGGATTAC

GCCAAGACGTGGAGCCGCTATGCCAAGGAGCTGCTGGCCTGGACTGAAAAGAGAGCCAGC

TATGAGCAGGAATTTGCTAAAAGCATCATGAAGATTGCTGAGGCTGGCAAGGTGTCCATC

CACCAGCAGGTAACCATGAGCAGACTTCCCCAGAGCCCAAACCTTCCGGTTTCCCATCTC

TGTCCTCTGATCCTCAGCATCCCCTAACCCTACTCCCAGAGCCACATGCCACTGCAGTAT

ATCTACACTCTGTTTCTGGAGCATGATCTCAACCTGGGAACCCTGGCCTTGGAGACAGTA

GCCCAGCAGAAAAGAGATTACTACCAGCCACTGGCTGCCAAACGGACTGAAATTGAGAAA

TGGCGGAAGGAGTTCAAGGAGCAATGGATAAAGGAGCAGAAGCGGATGAACGAGGCAGTG

CAGATATTGCGGCGGGCCCAGCTCCAGTATACGCAGCGCAGTGAGGAACTTTGGGCGCGC

TCCCAGGGGTCCCCTGAGGACCATGCCCCTCAAGCCTCCCCTGGAACCAGCAAGCAGCAG

GAGCGGCGGCGGAGGTCCCGAGAAGAGGCCCAGGCCAAGGCACTGGAGGCTGAGGTGTTT

TATCAGGCCTGCGTCCGCGAAGCCAACGCACGCCAGCAGGACCTGGAAAACACCAAGCAT

CGGATTGTGTCCCACGTGCGCAAGTTGGTGTTGCAGGGGGATGAAGTGCTGAGGAGGGTG

ACCCTAGGCCTCTTTGGGCTGCGCAGAGCACAGGCTGAACGCGGTCCTCGCGCATTCGCA

GCCTTGGCTGAGTGTTGCGCGCCCTTCGAACCTGGCCAGCGCTACCAGGAGTTTGTGCGG

GCACTGCGGCCTGAGGATCCACCGCCCCCACCTCCGGCCTTCACCTTCCAGGAGTTTGTA

CCCATGGTGAACAGCTCCCCTCTGGATTCAAGAAAGAAGTTCTCTGGGCCCCCACCTCCC

AGGCTGGACGAGAGTTCCGCAGAGCAAGGCCCTTGGGAGGACACTGGCATAGGCTGGCAG

>CL3.Contig28_A-W 76 1305 PREDICTED: GEM-interacting protein isoform 2 [Pongo abelii]

ATGGATACATCAGAGCCAGGGATCCCCCCAGCTCCTGAGAGCAGGAAGAGGTACAGTGAC

ATCTTCCGCAGCCTGGATAATCTCGAGATCTCACTGGGAAATGTAACCTTCGAGATGTTG

TCTGGAGACCCTGTAATCTCAGAAGACCTGGATCCTGACAAGGTCACCACAGCCACTGTG

ACCAGTGAAGCCAGCCGTTGGAGTGGCCCCTCCCCAGAGGGTCTTGCGCCCCTCACAGGG

GTGGAGTTGGATTTGAGGCTCATAAGGACCAAAGGGGGCGTGGATGCAGCCCTGGATTAC

GCCAAGACGTGGAGCCGCTATGCCAAGGAGCTGCTGGCCTGGACTGAAAAGAGAGCCAGC

TATGAGCAGGAATTTGCTAAAAGCATCATGAAGATTGCTGAGGCTGGCAAGGTGTCCATC

CACCAGCAGAGCCACATGCCACTGCAGTATATCTACACTCTGTTTCTGGAGCATGATCTC

AACCTGGGAACCCTGGCCTTGGAGACAGTAGCCCAGCAGAAAAGAGATTACTACCAGCCA

CTGGCTGCCAAACGGACTGAAATTGAGAAATGGCGGAAGGAGTTCAAGGAGCAATGGATA

AAGGAGCAGAAGCGGATGAACGAGGCAGTGCAGATATTGCGGCGGGCCCAGCTCCAGTAT

ACGCAGCGCAGTGAGGAACTTTGGGCGCGCTCCCAGGGGTCCCCTGAGGACCATGCCCCT

CAAGCCTCCCCTGGAACCAGCAAGCAGCAGGAGCGGCGGCGGAGGTCCCGAGAAGAGGCC

CAGGCCAAGGCACTGGAGGCTGAGGTGTTTTATCAGGCCTGCGTCCGCGAAGCCAACGCA

CGCCAGCAGGACCTGGAAAACACCAAGCATCGGATTGTGTCCCACGTGCGCAAGTTGGTG

TTGCAGGGGGATGAAGTGCTGAGGAGGGTGACCCTAGGCCTCTTTGGGCTGCGCAGAGCA

CAGGCTGAACGCGGTCCTCGCGCATTCGCAGCCTTGGCTGAGTGTTGCGCGCCCTTCGAA

CCTGGCCAGCGCTACCAGGAGTTTGTGCGGGCACTGCGGCCTGAGGATCCACCGCCCCCA

CCTCCGGCCTTCACCTTCCAGGAGTTTGTACCCATGGTGAACAGCTCCCCTCTGGATTCA

AGAAAGAAGTTCTCTGGGCCCCCACCTCCCAGGCTGGACGAGAGTTCCGCAGAGCAAGGC

CCTTGGGAGGACACTGGCATAGGCTGGCAG

>CL3.Contig29_A-W 130 567 PREDICTED: GEM-interacting protein-like [Ailuropoda melanoleuca]

GAGGCTGAGGTGTTTTATCAGGCCTGCGTCCGCGAAGCCAACGCACGCCAGCAGGACCTG

GAAAACACCAAGCATCGGATTGTGTCCCACGTGCGCAAGTTGGTGTTGCAGGGGGATGAA

GTGCTGAGGAGGGTGACCCTAGGCCTCTTTGGGCTGCGCAGAGCACAGGCTGAACGCGGT

CCTCGCGCATTCGCAGCCTTGGCTGAGTGTTGCGCGCCCTTCGAACCTGGCCAGCGCTAC

CAGGAGTTTGTGCGGGCACTGCGGCCTGAGGATCCACCGCCCCCACCTCCGGCCTTCACC

TTCCAGGAGTTTGTACCCATGGTGAACAGCTCCCCTCTGGATTCAAGAAAGAAGTTCTCT

GGGCCCCCACCTCCCAGGCTGGACGAGAGTTCCGCAGAGCAAGGCCCTTGGGAGGACACT

GGCATAGGCTGGCAGGGT

>CL4.Contig1_A-W 25 753 PREDICTED: aprataxin isoform 6 [Pongo abelii]

AGTAACGTGAACGTTTTGGTCTCGTCAACTCTAGTGATAATGCGGGTGTGCTGGTTGGTG

AGACAAGACAACTTGTACCAGCGAATCAAACTTCCACATTTGGAAGCAGTTATGATTGGG

CGTGGCCCAGAGACCAAGATCACTGATAAGAAATGTTCTCGACAGCAAGTACAGTTGAAA

GCAGAGTGTAACAAGGGATATGTCAAGGTAAAGCAGGTTGGAGTCAATCCCACCAGCATT

GACTCAGTCATAATTGGAAAGGATCAAGAGATGAAGCTGCAGCCTGGTCAGGTTCTCTAC

ATGGTGAATGAACTTTATCCATATATTGTAGAGTTTGAGGAAGAGGCCAAGAACCCTGGC

CCAGAAACACACAGGAAGAGAAAGAGGTCAGGCAATGGTAATTCTGTAGAAAGGGATGCT

GTCCAGGAAGCTGAGCCTGGGACAGGGGTGGAATCTGGGAGCAGCCCCAGACCATGCTCT

ATGGCCCCTCAAAAGGAGAAAGATGCATCTAACAAAAAGGACTCACTGGGCCATTGGAGT

CAAGGCTTGAAAATTTCTATGCAGGACCCCAAAATGCAGGTTTACAAAGATGAGCAGGTA

GTGGTGATTAAGGATAAATATCCTAAGGCTCGTCATCACTGGCTGGTCTTACCATGGGCC

TCCATTTCCAGTCTGAAGGATGTGACTAGGGAACACTTTGAACTCCTCAAGCACATGCAC

ACTGTTGGG

>CL4.Contig2_A-W 25 870 PREDICTED: aprataxin [Pan troglodytes] >gi|397520060|ref|XP_003830165.1| PREDICTED: aprataxin isoform 6 [Pan paniscus]

AGTAACGTGAACGTTTTGGTCTCGTCAACTCTAGTGATAATGCGGGTGTGCTGGTTGGTG

AGACAAGACAACTTGTACCAGCGAATCAAACTTCCACATTTGGAAGCAGTTATGATTGGG

CGTGGCCCAGAGACCAAGATCACTGATAAGAAATGTTCTCGACAGCAAGTACAGTTGAAA

GCAGAGTGTAACAAGGGATATGTCAAGGTAAAGCAGGTTGGAGTCAATCCCACCAGCATT

GACTCAGTCATAATTGGAAAGGATCAAGAGATGAAGCTGCAGCCTGGTCAGGTTCTCTAC

ATGGACTCACTGGGCCATTGGAGTCAAGGCTTGAAAATTTCTATGCAGGACCCCAAAATG

CAGGTTTACAAAGATGAGCAGGTAGTGGTGATTAAGGATAAATATCCTAAGGCTCGTCAT

CACTGGCTGGTCTTACCATGGGCCTCCATTTCCAGTCTGAAGGATGTGACTAGGGAACAC

TTTGAACTCCTCAAGCACATGCACACTGTTGGGGAAAAGGTGATTGCAGATTTTGCTGGA

TCTAGCAAACTCCGCTTCAGAATGGGCTACCATGCCATTCCCAGCATGAGCCATGTACAC

CTACATGTGATCAGCCAAGATTTTGATTCTCCTTGCCTTAAAAACAAAAAACATTGGAAT

TCTTTCAATACAGAATACTTCCTAGAATCAGAAGCTGTGATCAAGATGGTACAAGAGTCT

GGCAGAGTGACTGTTCGAGATGGGATGTCTGAGCTCTTGAAACTGCCCCTTCGTTGTCAT

GAGTGCCAGCAGCTACTGCCTACCATTCCTCAGTTGAAAGAGCATCTCAGGAGGCACTGG

ACAAAG

>CL6.Contig1_A-W 105 1613 PREDICTED: protein notum homolog [Canis lupus familiaris]

ATGGGCCGAAGGGTGTGCGTGCTGCTGCTACTGGGCCTGCTGCACTGGGCCGGGGGCGGC

GAGGGCAGGAAGACCTGGCGGCGCCGCGGCCAGCAGCCTCCTCTGCCGCCGCCCCCGCCG

CCTCCCCCGCAGCGAGCCGAGGCGGCGCCGGCCGCGGGACAACCAGTGGAGAGCTTCCCG

CTGGACTTCACGGCCGTGGAGGGCAACATGGACAGCTTCATGGCTCAGGTCAAGAGCCTG

GCCCAGTCCCTGTACCCCTGCTCGGCGCAACAGCTCAACGAGGACCTACGCCTGCATCTC

CTGCTGAACACATCGGTGACCTGCAACGACGGCAGCCCAGCTGGTTACTACCTAAAGGAG

TCCAAGGGCAGCCGGCGGTGGCTACTCTTTCTGGAAGGCGGCTGGTACTGCTTTAACCGG

GAGAACTGCAACACCAGATATGACACCATGCGTCGCCTTATGAGCTCCAAAGACTGGCCG

CACACGAGGACAGGAACCGGGATCCTGTCTTCTCAGCCGGAGGAGAATCCCCACTGGTGG

AATGCCAACATGGTCTTCATCCCCTACTGCTCCAGTGACGTTTGGAGTGGGGCGTCTTCC

AAGTCTGAGAAGAACGAGTATGCCTTCATGGGCGCCCTCATCATCCAGGAGGTGGTGCGC

GAGCTCCTGGGCCAAGGGCTGAGCGGGGCCAAGGTGCTGCTGCTGGCCGGGAGCAGTGCT

GGGGGCACTGGGGTGCTGTTGAACGTGGACCGTGTGGCCGAGCAGCTGGAGGACCTGGGT

TACCCGGCCATCCAAGTGCGAGGCCTGGCTGACTCAGGCTGGTTCCTGGACAACAAGCAG

TACCGCCGCACGGACTGCATTGACACCATCACCTGCGCCCCCACAGAGGCCATCCGTCGT

GGGATCAGGTACTGGAACGGCATGGTTCCGGAGCGCTGTCGGCGCCAGTTCAAGGAGGGG

GAAGAGTGGAACTGCTTCTTTGGCTACAAAGTTTACCCAACTCTGCGCTGCCCAGTGTTC

GTGGTGCAGTGGCTGTTTGACGAGGCCCAGCTGACCGTGGACAACGTGCACCTCACGGGG

CAGCCAGTGCAGGAGGGGCAGTGGCTGTACATCCAGAACCTGGGCCGCGAGCTGCGCAGC

ACACTCAAGGACGTGCCGGCCAGCTTTGCTCCTGCCTGCCTCTCTCACGAGATCATCATC

CGCAGTCACTGGACAGATGTCCAGGTGAAGGGGACCTCACTGCCTCGGGCACTGCACTGC

TGGGACAGAAGCCTCCATGACAGCCACAAGGCCAGCAAAGCCTCCCTGAAAGGCTGCCCC

GTCCACCTCGTGGACAGCTGCCCTTGGCCTCACTGCAACCCCTCATGTCCCACCATCCGG

GACCAGTTCACGGGGCAGGAGATGAACGTGGCCCAGTTCCTCATGCATATGGGCTTCGAT

GTGCAGACCGTGGCCCAGCAGCAGGGACTGGAGCCCAGCAAGCTGCTGGGGATGCTGAGC

AGTGGAAGC

>CL6.Contig2_A-W 60 1568 PREDICTED: protein notum homolog [Canis lupus familiaris]

ATGGGCCGAAGGGTGTGCGTGCTGCTGCTACTGGGCCTGCTGCACTGGGCCGGGGGCGGC

GAGGGCAGGAAGACCTGGCGGCGCCGCGGCCAGCAGCCTCCTCTGCCGCCGCCCCCGCCG

CCTCCCCCGCAGCGAGCCGAGGCGGCGCCGGCCGCGGGACAACCAGTGGAGAGCTTCCCG

CTGGACTTCACGGCCGTGGAGGGCAACATGGACAGCTTCATGGCTCAGGTCAAGAGCCTG

GCCCAGTCCCTGTACCCCTGCTCGGCGCAACAGCTCAACGAGGACCTACGCCTGCATCTC

CTGCTGAACACATCGGTGACCTGCAACGACGGCAGCCCAGCTGGTTACTACCTAAAGGAG

TCCAAGGGCAGCCGGCGGTGGCTACTCTTTCTGGAAGGCGGCTGGTACTGCTTTAACCGG

GAGAACTGCAACACCAGATATGACACCATGCGTCGCCTTATGAGCTCCAAAGACTGGCCG

CACACGAGGACAGGAACCGGGATCCTGTCTTCTCAGCCGGAGGAGAATCCCCACTGGTGG

AATGCCAACATGGTCTTCATCCCCTACTGCTCCAGTGACGTTTGGAGTGGGGCGTCTTCC

AAGTCTGAGAAGAACGAGTATGCCTTCATGGGCGCCCTCATCATCCAGGAGGTGGTGCGC

GAGCTCCTGGGCCAAGGGCTGAGCGGGGCCAAGGTGCTGCTGCTGGCCGGGAGCAGTGCT

GGGGGCACTGGGGTGCTGTTGAACGTGGACCGTGTGGCCGAGCAGCTGGAGGACCTGGGT

TACCCGGCCATCCAAGTGCGAGGCCTGGCTGACTCAGGCTGGTTCCTGGACAACAAGCAG

TACCGCCGCACGGACTGCATTGACACCATCACCTGCGCCCCCACAGAGGCCATCCGTCGT

GGGATCAGGTACTGGAACGGCATGGTTCCGGAGCGCTGTCGGCGCCAGTTCAAGGAGGGG

GAAGAGTGGAACTGCTTCTTTGGCTACAAAGTTTACCCAACTCTGCGCTGCCCAGTGTTC

GTGGTGCAGTGGCTGTTTGACGAGGCCCAGCTGACCGTGGACAACGTGCACCTCACGGGG

CAGCCAGTGCAGGAGGGGCAGTGGCTGTACATCCAGAACCTGGGCCGCGAGCTGCGCAGC

ACACTCAAGGACGTGCCGGCCAGCTTTGCTCCTGCCTGCCTCTCTCACGAGATCATCATC

CGCAGTCACTGGACAGATGTCCAGGTGAAGGGGACCTCACTGCCTCGGGCACTGCACTGC

TGGGACAGAAGCCTCCATGACAGCCACAAGGCCAGCAAAGCCTCCCTGAAAGGCTGCCCC

GTCCACCTCGTGGACAGCTGCCCTTGGCCTCACTGCAACCCCTCATGTCCCACCATCCGG

GACCAGTTCACGGGGCAGGAGATGAACGTGGCCCAGTTCCTCATGCATATGGGCTTCGAT

GTGCAGACCGTGGCCCAGCAGCAGGGACTGGAGCCCAGCAAGCTGCTGGGGATGCTGAGC

AGTGGAAGC

>CL6.Contig3_A-W 316 1212 PREDICTED: protein notum homolog [Bos taurus]

GACGAGTATGCCTTCATGGGCGCCCTCATCATCCAGGAGGTGGTGCGCGAGCTCCTGGGC

CAAGGGCTGAGCGGGGCCAAGGTGCTGCTGCTGGCCGGGAGCAGTGCTGGGGGCACTGGG

GTGCTGTTGAACGTGGACCGTGTGGCCGAGCAGCTGGAGGACCTGGGTTACCCGGCCATC

CAAGTGCGAGGCCTGGCTGACTCAGGCTGGTTCCTGGACAACAAGCAGTACCGCCGCACG

GACTGCATTGACACCATCACCTGCGCCCCCACAGAGGCCATCCGTCGTGGGATCAGGTAC

TGGAACGGCATGGTTCCGGAGCGCTGTCGGCGCCAGTTCAAGGAGGGGGAAGAGTGGAAC

TGCTTCTTTGGCTACAAAGTTTACCCAACTCTGCGCTGCCCAGTGTTCGTGGTGCAGTGG

CTGTTTGACGAGGCCCAGCTGACCGTGGACAACGTGCACCTCACGGGGCAGCCAGTGCAG

GAGGGGCAGTGGCTGTACATCCAGAACCTGGGCCGCGAGCTGCGCAGCACACTCAAGGAC

GTGCCGGCCAGCTTTGCTCCTGCCTGCCTCTCTCACGAGATCATCATCCGCAGTCACTGG

ACAGATGTCCAGGTGAAGGGGACCTCACTGCCTCGGGCACTGCACTGCTGGGACAGAAGC

CTCCATGACAGCCACAAGGCCAGCAAAGCCTCCCTGAAAGGCTGCCCCGTCCACCTCGTG

GACAGCTGCCCTTGGCCTCACTGCAACCCCTCATGTCCCACCATCCGGGACCAGTTCACG

GGGCAGGAGATGAACGTGGCCCAGTTCCTCATGCATATGGGCTTCGATGTGCAGACCGTG

GCCCAGCAGCAGGGACTGGAGCCCAGCAAGCTGCTGGGGATGCTGAGCAGTGGAAGC

>CL6.Contig4_A-W 217 567 PREDICTED: protein notum homolog [Equus caballus]

AGGCAATGCCCCTCCTCCATGACTCCCACCCGGATTTCTGTCTGCAGTCACTGGACAGAT

GTCCAGGTGAAGGGGACCTCACTGCCTCGGGCACTGCACTGCTGGGACAGAAGCCTCCAT

GACAGCCACAAGGCCAGCAAAGCCTCCCTGAAAGGCTGCCCCGTCCACCTCGTGGACAGC

TGCCCTTGGCCTCACTGCAACCCCTCATGTCCCACCATCCGGGACCAGTTCACGGGGCAG

GAGATGAACGTGGCCCAGTTCCTCATGCATATGGGCTTCGATGTGCAGACCGTGGCCCAG

CAGCAGGGACTGGAGCCCAGCAAGCTGCTGGGGATGCTGAGCAGTGGAAGC

>CL6.Contig5_A-W 33 533 PREDICTED: protein notum homolog [Bos taurus] >gi|296476191|gb|DAA18306.1| hypothetical protein BOS_19422 [Bos taurus]

GGCCCAGTGTTCGTGGTGCAGTGGCTGTTTGACGAGGCCCAGCTGACCGTGGACAACGTG

CACCTCACGGGGCAGCCAGTGCAGGAGGGGCAGTGGCTGTACATCCAGAACCTGGGCCGC

GAGCTGCGCAGCACACTCAAGGACGTGCCGGCCAGCTTTGCTCCTGCCTGCCTCTCTCAC

GAGATCATCATCCGCAGTCACTGGACAGATGTCCAGGTGAAGGGGACCTCACTGCCTCGG

GCACTGCACTGCTGGGACAGAAGCCTCCATGACAGCCACAAGGCCAGCAAAGCCTCCCTG

AAAGGCTGCCCCGTCCACCTCGTGGACAGCTGCCCTTGGCCTCACTGCAACCCCTCATGT

CCCACCATCCGGGACCAGTTCACGGGGCAGGAGATGAACGTGGCCCAGTTCCTCATGCAT

ATGGGCTTCGATGTGCAGACCGTGGCCCAGCAGCAGGGACTGGAGCCCAGCAAGCTGCTG

GGGATGCTGAGCAGTGGAAGC

>CL7.Contig1_A-W 10 276 minus strand PREDICTED: zinc finger protein 12 [Saimiri boliviensis boliviensis]

AAACCTTATGAATGTAGTGAATGTGGGAAAACCTTCTGTCAGAATTCAGCCCTTAATAGA

CATCAGAGAACACATACAGGAGAGAAAGCTTATGAATGTTTTGAATGTGGGAAATGCTTC

TCTCAAATGTCATATCTCACCATACATCATCGAATCCATTCTGGAGAGAAACCATTTGAA

TGTAACGAATGTGGGAAAGCCTTCTCTCGGATGTCATACCTCACTGTGCATTACAGAACC

CATTCAGGAGAGAAGCCCTATGAATGT

>CL7.Contig2_A-W 29 220 RB-associated KRAB zinc finger protein [Heterocephalus glaber]

CATTCAGGAGTGAAACCCTATGAATGTAGTGAATGTGGAAAAACCTTCTACCAGAATTCA

GCCCTTTGTAGACATCGGAGAATACACAGAGGAGAGAAGCCCTATGAATGTTACATATGT

GGAAAGTTCTTCTCTCAGATGTCATACCTCACTATACATCATAGAATTCATTCAGGAGAA

AAACCTTATGAA

>CL8.Contig1_A-W 236 667 minus strand PREDICTED: transcription elongation regulator 1 isoform 1 [Otolemur garnettii]

CCAAGTTCTGCTGTTTCTGTTGCCACGCCTACAGTTAGTGTTTCAACTCCTGCTCCTACA

GCCACACCTGTGCAAACTGTTCCCCAGCCGCATCCCCAGACGTTACCTCCTGCTGTTCCT

CATTCAGTACCTCAGCCAGCAGCAGCAATACCTGCTTTTCCACCAGTAATGGTACCTCCG

TTTCGTGTTCCCCTTCCTGGCATGCCAATTCCACTTCCAGGTGTATTGCCAGGAATGGCC

CCTCCTATTGTACCCATGATACACCCCCAGGTTGCTATTGCAGCTTCACCTGCTACCTTA

GCTGGAGCAACAGCAGTTTCTGAATGGACTGAATATAAAACAGCAGATGGGAAAACATAT

TATTATAACAACAGAACATTAGAATCAACCTGGGAAAAACCCCAAGAACTAAAGGAAAAA

GAAAAATTAGAA

>CL8.Contig2_A-W 2073 2264 PREDICTED: transcription elongation regulator 1 isoform 1 [Otolemur garnettii]

GAGAGGAGAAAGAGAAGCTTTTTAATGAACACATTGAAGCACTCACCAAAAAGAAAAGGG

AGCACTTTAGGCAACTTCTGGATGAAACCACCTGCGATTACCTTAACATCCACGTGGAAA

GAAGTTAAAAAAATCATTAAAGAAGATCCTCGGTGTATTAAGTTTTCTTCTAGTGACAGG

AAAAAAACAAAG

>CL8.Contig3_A-W 236 667 minus strand PREDICTED: transcription elongation regulator 1-like isoform 2 [Ailuropoda melanoleuca]

CCAAGTTCTGCTGTTTCTGTTGCCACGCCTACAGTTAGTGTTTCAACTCCTGCTCCTACA

GCCACACCTGTGCAAACTGTTCCCCAGCCGCATCCCCAGACGTTACCTCCTGCTGTTCCT

CATTCAGTACCTCAGCCAGCAGCAGCAATACCTGCTTTTCCACCAGTAATGGTACCTCCG

TTTCGTGTTCCCCTTCCTGGCATGCCAATTCCACTTCCAGGTGTATTGCCAGGAATGGCC

CCTCCTATTGTACCCATGATACACCCCCAGGTTGCTATTGCAGCTTCACCTGCTACCTTA

GCTGGAGCAACAGCAGTTTCTGAATGGACTGAATATAAAACAGCAGATGGGAAAACATAT

TATTATAACAACAGAACATTAGAATCAACCTGGGAAAAACCCCAAGAACTAAAGGAAAAA

GAAAAATTAGAA

>CL9.Contig1_A-W 3 182 minus strand PREDICTED: solute carrier family 35 member B1 [Otolemur garnettii]

GACCGGCTGCGCCTGCCGCTCTGCTTCCTTGGTGTCTTTGTCTGCTATTTCTACTATGGG

ATCCTGCAGGAAAAGATAACAAGAGGAAAATATGGGGAGGGAGCCAAACAGGAGACGTTC

ACCTTTGCCTTAACCCTAGTCTTCATCCAGTGTGTGATCAATGCTGTGTTTGCCAAGATC

>CL9.Contig2_A-W 3 941 minus strand PREDICTED: solute carrier family 35 member B1 [Otolemur garnettii]

GACCGGCTGCGCCTGCCGCTCTGCTTCCTTGGTGTCTTTGTCTGCTATTTCTACTATGGG

ATCCTGCAGGAAAAGATAACAAGAGGAAAATATGGGGAGGGAGCCAAACAGGAGACGTTC

ACCTTTGCCTTAACCCTAGTCTTCATCCAGTGTGTGATCAATGCTGTGTTTGCCAAGATC

TTGATCCAGTTTTTTGACACTGCCAGGGTGGATCGTACTCGGAGCTGGCTCTATGCTGCC

TGTTCTTTGTCCTATTTGGGTGCTATGGTCTCCAGCAACTCAGCACTACAGTTTGTCAAC

TATCCAACTCAGGTCCTTGGTAAATCCTGCAAGCCAATCCCAGTCATGCTCCTTGGGGTG

ACCCTCTTGAAGAAGAAGTACCCATTGGCCAAGTACCTGTGTGTGTTGCTAATTGTGGCT

GGAGTGGCCCTTTTCATGTACAAACCCAAGAAAGTAGTTGGGATGGAAGAACACACAATC

GGCTATGGAGAATTACTCCTGCTATTGTCTCTGACCTTGGATGGACTGACAGGTGTTTCC

CAGGACCACATGCGGGCTCATTACCAAACAGGCTCCAACCACATGATGTTGAACATCAAC

CTTTGGTCGACATTGCTTCTAGGAGCTGGAATCCTGTTCACTGGGGAGCTCTGGGAGTTC

TTGAGCTTTGCTGAAAGGTACCCAATCATCATCTATAATATCCTGCTCTTTGGCCTGACT

AGTGCCTTGGGTCAGAGCTTCATCTTCATGACAGTTGTGTATTTTGGTCCCCTGACCTGC

TCAATCATCACGACAACTCGGAAGTTCTTCACTATTTTGGCCTCTGTGATCCTCTTTGCC

AACCCCATTAGCCTCATGCAGTGGGTAGGCACTGTGCTTGTGTTCCTGGGTCTTGGTCTT

GATGCCAAGTTTGGGAAGGGAGCAAAGAAGACAACCCAC

>CL10.Contig1_A-W 3 311 PREDICTED: transmembrane protein 184C [Saimiri boliviensis boliviensis]

TTGGTTATAATAAATAACATGTCACAATTGTTTGCCATGTATTGTCTCCTGCTGTTTTAT

AAAGTACTAAAGGAAGAACTGAGTCCTATCCAACCTGTTGGCAAATTTCTTTGTGTAAAG

CTGGTGGTTTTTGTTTCTTTTTGGCAAGCAGTAGTTATTGCTTTGTTGGTAAAAGTTGGC

GTTATTTCTGAAAAGCATACATGGGAATGGCAAACTGTAGAAGCCGTGGCCACAGGACTC

CAGGTAAGTAGTGCTTCACCTCTGAACCCAATAGAACTTCACTATTTATTTGTTAAGCAT

TTTTATATA

>CL10.Contig2_A-W 3 653 PREDICTED: transmembrane protein 184C [Oryctolagus cuniculus]

TTGGTTATAATAAATAACATGTCACAATTGTTTGCCATGTATTGTCTCCTGCTGTTTTAT

AAAGTACTAAAGGAAGAACTGAGTCCTATCCAACCTGTTGGCAAATTTCTTTGTGTAAAG

CTGGTGGTTTTTGTTTCTTTTTGGCAAGCAGTAGTTATTGCTTTGTTGGTAAAAGTTGGC

GTTATTTCTGAAAAGCATACATGGGAATGGCAAACTGTAGAAGCCGTGGCCACAGGACTC

CAGGACTTTATTATCTGTATTGAGATGTTCCTTGCTGCTATTGCTCATCACTACACATTC

TCATATAAACCATATGTCCAAGAAGCAGAAGAGGGCTCATGCTTTGATTCCTTTCTGGCC

ATGTGGGATGTTTCAGATATCAGAGATGACATTTCTGAACAAGTGAGACATGTTGGACGG

ACAGTCAGGGGACATCCTAGGAAAAAATTTTTTCCTGAGGATCAAGATCAAAATGAACAC

ACAAGCTTGTTATCATCATCATCACAAGATGCAATTTCTGTTGCCTCTTCTATGCCACCT

TCACCCATGGGTCACTACCAAGGGTTTGGACACACTGTGACTCCACAGACTACACCTACT

ACAGCTAAGATAAATGAGGAAATACTTAAAGATGCTACAGAAGAGAAAAAA

>CL11.Contig1_A-W 2 562 Oxysterol-binding protein-related protein 1 [Heterocephalus glaber]

TGGAGCATCCTCAGAAAATGTATTGGAATGGAACTATCCAAGATTACGATGCCAGTTATA

TTTAATGAGCCTTTGAGCTTCCTACAACGACTAACTGAGTATATGGAGCATACTTACCTC

ATCCACAAGGCCAGTTCATTTTCTGATCCTGTCGAAAGGATGCAGTGTGTAGCTGCATTT

GCCGTGTCTGCTGTCGCTTCTCAGTGGGAACGCACTGGAAAGCCCTTCAACCCGCTTCTG

GGAGAGACGTATGAATTAGTTCGAGATGACCTTGGATTTAGACTCATTTCTGAACAGGTC

AGCCATCACCCACCAATCAGTGCATTTCATGCAGAAGGGTTAAACAACGATTTCATCTTT

CATGGCTCAATCTATCCCAAACTAAAGTTCTGGGGGAAGAGTGTAGAAGCAGAACCCAAA

GGAACCATCACCTTGGAGCTCCTTGAGCACAATGAAGCATATACATGGACAAATCCTACC

TGCTGTGTGCATAATATCATTGTGGGTAAACTCTGGATCGAACAGTATGGCAATGTGGAA

ATCACAAACCACAAGTAAGGC

>CL11.Contig2_A-W 43 342 Oxysterol-binding protein-related protein 1 [Heterocephalus glaber]

AGAGATGACCTTGGATTTAGACTCATTTCTGAACAGGTCAGCCATCACCCACCAATCAGT

GCATTTCATGCAGAAGGGTTAAACAACGATTTCATCTTTCATGGCTCAATCTATCCCAAA

CTAAAGTTCTGGGGGAAGAGTGTAGAAGCAGAACCCAAAGGAACCATCACCTTGGAGCTC

CTTGAGCACAATGAAGCATATACATGGACAAATCCTACCTGCTGTGTGCATAATATCATT

GTGGGTAAACTCTGGATCGAACAGTATGGCAATGTGGAAATCACAAACCACAAGTAAGGC

>CL11.Contig3_A-W 2 1105 PREDICTED: oxysterol-binding protein-related protein 1-like [Cavia porcellus]

TGGAGCATCCTCAGAAAATGTATTGGAATGGAACTATCCAAGATTACGATGCCAGTTATA

TTTAATGAGCCTTTGAGCTTCCTACAACGACTAACTGAGTATATGGAGCATACTTACCTC

ATCCACAAGGCCAGTTCATTTTCTGATCCTGTCGAAAGGATGCAGTGTGTAGCTGCATTT

GCCGTGTCTGCTGTCGCTTCTCAGTGGGAACGCACTGGAAAGCCCTTCAACCCGCTTCTG

GGAGAGACGTATGAATTAGTTCGAGATGACCTTGGATTTAGACTCATTTCTGAACAGGTC

AGCCATCACCCACCAATCAGTGCATTTCATGCAGAAGGGTTAAACAACGATTTCATCTTT

CATGGCTCAATCTATCCCAAACTAAAGTTCTGGGGGAAGAGTGTAGAAGCAGAACCCAAA

GGAACCATCACCTTGGAGCTCCTTGAGCACAATGAAGCATATACATGGACAAATCCTACC

TGCTGTGTGCATAATATCATTGTGGGTAAACTCTGGATCGAACAGTATGGCAATGTGGAA

ATCACAAACCACAAGACTGGGGACAAATGTGTGTTGAATTTTAAGCCATGTGGCCTTTTT

GGTAAGGAATTACACAAAGTTGAAGGCTACATTCAAGATAAAAGTAAGAAGAAGCTCTGT

GCTCTCTATGGGAAGTGGACTGAGTGTCTATACAGTGTTGACCCTGCCACTTTTGATGCT

TATAAAAAAAATGATAAGAAAAACACTGAAGAGAAGAAGAACAGTAAACAGATGAGCACT

TCTGAAGAGTCTGATGAAATGCCAATGCCAGATTCTGAAAGTGTATTTGTTATCCCTGGA

AGTGTTCTCCTGTGGCGAATAGCCCCACGGCCTCCAAATTCCGCCCAGATGTATAATTTT

ACCAGCTTTGCAATGGTTTTGAATGAAGTTGACAAGGAAATGGAGAGCGTGATTCCTAAG

ACTGACTGCAGGTTACGGCCTGACATCAGAGCCATGGAGAATGGAGAAATAGACCAAGCT

AGTGAAGAAAAAAAACGGCTTGAGGAAAAACAACGAGCAGCCCGTAAAAACAGGTCCAAG

TCAGAAGAAGACTGGAAGACGAGG

>CL12.Contig1_A-W 1 702 PREDICTED: homeodomain-interacting protein kinase 1 isoform 2 [Pan paniscus]

AAGAGAATTACTCCTCTGAAAACTCTTAACCATCAGTTTGTGACAATGACTCACCTTCTG

GATTTTCCACATAGCAATCATGTTAAATCTTGTTTCCAGAACATGGAGATCTGCAAGCGG

AGGGTTCACATGTATGATACAGTGAGTCAGATCAAGAGTCCCTTCACTACACATGTTGCA

CCAAATACAAGCACAAACCTAACCATGAGCTTCAGCAACCAGCTCAATGCAGTGCACAAT

CAGGCCAGTGTTCTAGCTTCCAGTTCTACTGCAGCAGCTGCTACTCTTTCTCTGGCTAAT

TCAGATGTCTCATTACTAAACTACCAGTCAGCTTTGTACCCATCATCTGCTGCACCAGTT

CCTGGAGTTGCACAGCAGGGTGTTTCCTTGCAGCCTGGAACAACTCAGATATGCACACAG

ACGGATCCATTCCAACAAACATTTATAGTATGTCCACCTGCTTTTCAAACTGGCCTGCAA

GCAACAACAAAGCATTCTGGATTCCCTGTGAGGATGGATAATGCTGTGCCAATTGTTCCC

CAGGCGCCAGCTGCTCAGCCGCTACAGATACAGTCAGGAGTTCTTACACAGGGAAGCTGT

ACACCACTAATGGTAGCAACTCTCCACCCTCAAGTAGCCACCATCACACCGCAGTATGCG

GTGCCCTTTACTCTGAGCTGCGCAGCCGGCCGGCCGGCGCTG

>CL13.Contig1_A-W 1 267 PREDICTED: zinc finger protein 653 [Pan paniscus]

GAGGCGCACGGGCCCTGGGTGGACCTGCGGCGCCGCAGCGGCTGGAGCGACGCCAAGCTC

GCCGCCTACCTCATCTCGCTGGAGCGCGGCCAGCGAAGCGGCCGCCACGGGAAGCCTTGG

GAGCAAGTCCCCAAAAAGCCAAAGCGGAAAAAAAGGCGGCGACGCAACGTGAACTGCCTG

AAGAATGTGGTGATCTGGTATGAGGACCACAAGCACCGCTGCCCATATGAGCCACACCTT

GCCGAGCTGGACCCCACCTTTGGCCTG

>CL14.Contig1_A-W 1 660 PREDICTED: 52 kDa repressor of the inhibitor of the protein kinase [Otolemur garnettii]

GCCAGATGCCAGAAGTGGGTAGAGAATTGTAGGAGAGCTGACTTGGAAGATAAAACACCT

GATCAACTAAATAAACATTATCGATTATGTGCCAAACACTTCGAGACCTCTATGATCTGT

AGAACTAGTCCTTATAGGACAGTACTTCGAGATAATGCAATACCAACAATATTTGATCTT

ACCAGTCATTTGAACAATCCACATAGTAGACACAGAAAACGAATAAAAGAATTGAGTGAA

GATGAAATCAGGACACTGAAACAGAAAAAAATTGATGAAACTTCTGAACAGGAACAAAAA

CATAAAGAAACAAACAATAGCAATGCTCAGAACCCCAGTGCAGAAGAAGGGGGTGATGAA

CAGGATGAAGATATTTTGCCTTTAACCCTTGAAGAGAAAGAAAACAAAGAATACTTAAAA

TCTTTATTTGAAATTTTGATTCTTATGGGAAAACAAAATATACCTCTGGACGGGCATGAG

GCTGATGAAATCCCAGAAGGTCTCTTCACTCCTGATAACTTTCAGGCCCTGTTGGAGTGC

CGGATAAATTCTGGTGAAGAGGTCCTGAGAAAACGCTTTGAGACCACAGCAGTTAACACA

TTGTTCTGTTCAAAAACACAACAGAAACAGATGCTGGAGATCTGTGAGAGTTGCATCCGG

>CL14.Contig2_A-W 77 610 PREDICTED: 52 kDa repressor of the inhibitor of the protein kinase [Otolemur garnettii]

AGTCCTTATAGGACAGTACTTCGAGATAATGCAATACCAACAATATTTGATCTTACCAGT

CATTTGAACAATCCACATAGTAGACACAGAAAACGAATAAAAGAATTGAGTGAAGATGAA

ATCAGGACACTGAAACAGAAAAAAATTGATGAAACTTCTGAACAGGAACAAAAACATAAA

GAAACAAACAATAGCAATGCTCAGAACCCCAGTGCAGAAGAAGGGGGTGATGAACAGGAT

GAAGATATTTTGCCTTTAACCCTTGAAGAGAAGGAAAACAAAGAATACTTAAAATCTTTA

TTTGAAATTTTGATTCTTATGGGAAAACAAAATATACCTCTGGACGGGCATGAGGCTGAT

GAAATCCCAGAAGGTCTCTTCACTCCTGATAACTTTCAGGCCCTGTTGGAGTGCCGGATA

AATTCTGGTGAAGAGGTCCTGAGAAAACGCTTTGAGACCACAGCAGTTAACACATTGTTC

TGTTCAAAAACACAACAGAAACAGATGCTGGAGATCTGTGAGAGTTGCATCCGG

>CL14.Contig3_A-W 42 335 minus strand PREDICTED: 52 kDa repressor of the inhibitor of the protein kinase-like isoform 3 [Pan paniscus]

GTAAAATTAACTCTCTGTTTTCCAGTTGATGAAACTTCTGAACAGGAACAAAAACATAAA

GAAACAAACAATAGCAATGCTCAGAACCCCAGTGCAGAAGAAGGGGGTGATGAACAGGAT

GAAGATATTTTGCCTTTAACCCTTGAAGAGAAAGAAAACAAAGAATACTTAAAATCTTTA

TTTGAAATTTTGATTCTTATGGGAAAACAAAATATACCTCTGGACGGGCATGAGGCTGAT

GAAATCCCAGAAGGTCTCTTCACTCCTGATAACTTTCAGGCCCTGTTGGAGTGC

>CL17.Contig1_A-W 360 1022 PREDICTED: prolyl 3-hydroxylase 1 [Pan troglodytes]

TCTGATCACCAATCTCCTTGGCAGCTGGGGCAGGAAGGGAAAGTTCCTCTACAGAGTGCC

CACCTGTACTACAATGTGACGGAGAAGGTGCGGCGTGTCATGGAGTCCTACTTTCGCCTG

GACACCCCTCTCTACTTCTCCTACTCCCACCTGGTGTGCCGCACTGCAATTGAAGAAGCA

CAAGCTGAGAGGAAGGACAGCAGCCATCCAGTCCACGTGGACAACTGCATCCTGAACGCT

GAGACTCTTATGTGCATCAAGGAGCCCCCTGCCTACACCTTCCGGGATTACAGTGCCATC

CTTTACCTAAATGGGGACTTTGATGGAGGAAACTTCTATTTTACAGAACTGGATGCCAAG

ACTGTGACGGCAGAGGTGCAGCCCCAGTGTGGAAGGGCTGTGGGATTTTCTTCAGGCACC

GAAAACCCGCATGGAGTGAAGGCTGTCACCAGGGGGCAACGCTGTGCCATTGCCCTGTGG

TTCACCTTAGACCCTCGACATAGTGAGCGTGACAGGGTGCAGGCAGATGACCTGGTGAAG

ATGCTCTTCAGCCCAGAGGAGATGGACCTGCCCCAGGAGCAGCCCCTGAATGCACAGCAG

GACCCCTCCCAACCTGGAGAAGAGTCTCCCCCAGGCAGTGAGTCGAAGCTCAGGGATGAG

CTA

>CL17.Contig2_A-W 73 219 prolyl 3-hydroxylase 1 isoform 1 precursor [Homo sapiens] >gi|109892809|sp|Q32P28.2|P3H1_HUMAN RecName: Full=Prolyl 3-hydroxylase 1; AltName: Full=Growth suppressor 1; AltName: Full=Leucine- and proline-enriched proteoglycan 1; Short=Leprecan-1; Flags: Precursor

CAGAGTGCCCAGGAGTACCGACAGCGCAGCCTGCTAGAGAAAGAGCTGCTTTTCTTCGCT

TATGATGTATTTGGAATTCCCTTTGTGGATCCGGATTCATGGACTCCAGAAGAGGTTATT

CCCAAGAGACTACAAGAGAAACAGAAG

>CL17.Contig3_A-W 73 1203 unnamed protein product [Homo sapiens]

CAGAGTGCCCAGGAGTACCGACAGCGCAGCCTGCTAGAGAAAGAGCTGCTTTTCTTCGCT

TATGATGTATTTGGAATTCCCTTTGTGGATCCGGATTCATGGACTCCAGAAGAGGTTATT

CCCAAGAGACTACAAGAGAAACAGAAGTCAGAACGGGAAACCGCCGTCCGCATCTCCCAG

GAGATCGGCAACCTTATGAAGGAGATTGAGACTCTTGTGGAAGAGAAGACCAAGGAATCT

TTGGATGTGAGCAGGCTCACCCGGGAAGGAGGTCCCCTGCTATATGAAGGCATCAGTCTC

ACCATGAACTCCAAACTCTTGAATGGTTCCCAGCGAGTGGTGATGGATGGCATGATCTCT

GATGATGAGTGTCGGGAGCTGCAGAGACTGACCAATGCAGCAGCAACTTCAGGAGATGGC

TACCGAGGTCAGACCTCCCCACATACCCCCAGTGAAAAGTTCTATGGAGTCACTGTCTTC

AAAGCCCTCAAGCTGGGGCAGGAAGGGAAAGTTCCTCTACAGAGTGCCCACCTGTACTAC

AATGTGACGGAGAAGGTGCGGCGTGTCATGGAGTCCTACTTTCGCCTGGACACCCCTCTC

TACTTCTCCTACTCCCACCTGGTGTGCCGCACTGCAATTGAAGAAGCACAAGCTGAGAGG

AAGGACAGCAGCCATCCAGTCCACGTGGACAACTGCATCCTGAACGCTGAGACTCTTATG

TGCATCAAGGAGCCCCCTGCCTACACCTTCCGGGATTACAGTGCCATCCTTTACCTAAAT

GGGGACTTTGATGGAGGAAACTTCTATTTTACAGAACTGGATGCCAAGACTGTGACGGCA

GAGGTGCAGCCCCAGTGTGGAAGGGCTGTGGGATTTTCTTCAGGCACCGAAAACCCGCAT

GGAGTGAAGGCTGTCACCAGGGGGCAACGCTGTGCCATTGCCCTGTGGTTCACCTTAGAC

CCTCGACATAGTGAGCGTGACAGGGTGCAGGCAGATGACCTGGTGAAGATGCTCTTCAGC

CCAGAGGAGATGGACCTGCCCCAGGAGCAGCCCCTGAATGCACAGCAGGACCCCTCCCAA

CCTGGAGAAGAGTCTCCCCCAGGCAGTGAGTCGAAGCTCAGGGATGAGCTA

>CL17.Contig4_A-W 73 1371 leprecan 1 [Mus musculus]

CAGAGTGCCCAGGAGTACCGACAGCGCAGCCTGCTAGAGAAAGAGCTGCTTTTCTTCGCT

TATGATGTATTTGGAATTCCCTTTGTGGATCCGGATTCATGGACTCCAGAAGAGGTTATT

CCCAAGAGACTACAAGAGAAACAGAAGTGAGGCCCTTGAAGAAACTGCATGATTGGATCT

GACGAAGCATTTGAGGCTTCCTGCGCCCAGGCAGATGGGAACTCCTGGCCAGGGGTGGGC

AGGTCCAGTCTGGGAAGCTGGGGTGGGAGCCCAGGGCTGGCCCAGCACAGTCCTCAGAGC

CACTGTGCTCACAGGTCAGAACGGGAAACCGCCGTCCGCATCTCCCAGGAGATCGGCAAC

CTTATGAAGGAGATTGAGACTCTTGTGGAAGAGAAGACCAAGGAATCTTTGGATGTGAGC

AGGCTCACCCGGGAAGGAGGTCCCCTGCTATATGAAGGCATCAGTCTCACCATGAACTCC

AAACTCTTGAATGGTTCCCAGCGAGTGGTGATGGATGGCATGATCTCTGATGATGAGTGT

CGGGAGCTGCAGAGACTGACCAATGCAGCAGCAACTTCAGGAGATGGCTACCGAGGTCAG

ACCTCCCCACATACCCCCAGTGAAAAGTTCTATGGAGTCACTGTCTTCAAAGCCCTCAAG

CTGGGGCAGGAAGGGAAAGTTCCTCTACAGAGTGCCCACCTGTACTACAATGTGACGGAG

AAGGTGCGGCGTGTCATGGAGTCCTACTTTCGCCTGGACACCCCTCTCTACTTCTCCTAC

TCCCACCTGGTGTGCCGCACTGCAATTGAAGAAGCACAAGCTGAGAGGAAGGACAGCAGC

CATCCAGTCCACGTGGACAACTGCATCCTGAACGCTGAGACTCTTATGTGCATCAAGGAG

CCCCCTGCCTACACCTTCCGGGATTACAGTGCCATCCTTTACCTAAATGGGGACTTTGAT

GGAGGAAACTTCTATTTTACAGAACTGGATGCCAAGACTGTGACGGCAGAGGTGCAGCCC

CAGTGTGGAAGGGCTGTGGGATTTTCTTCAGGCACCGAAAACCCGCATGGAGTGAAGGCT

GTCACCAGGGGGCAACGCTGTGCCATTGCCCTGTGGTTCACCTTAGACCCTCGACATAGT

GAGCGTGACAGGGTGCAGGCAGATGACCTGGTGAAGATGCTCTTCAGCCCAGAGGAGATG

GACCTGCCCCAGGAGCAGCCCCTGAATGCACAGCAGGACCCCTCCCAACCTGGAGAAGAG

TCTCCCCCAGGCAGTGAGTCGAAGCTCAGGGATGAGCTA

>CL17.Contig5_A-W 47 781 PREDICTED: prolyl 3-hydroxylase 1 [Loxodonta africana]

GCAGCAGCAACTTCAGGAGATGGCTACCGAGGTCAGACCTCCCCACATACCCCCAGTGAA

AAGTTCTATGGAGTCACTGTCTTCAAAGCCCTCAAGCTGGGGCAGGAAGGGAAAGTTCCT

CTACAGAGTGCCCACCTGTACTACAATGTGACGGAGAAGGTGCGGCGTGTCATGGAGTCC

TACTTTCGCCTGGACACCCCTCTCTACTTCTCCTACTCCCACCTGGTGTGCCGCACTGCA

ATTGAAGAAGCACAAGCTGAGAGGAAGGACAGCAGCCATCCAGTCCACGTGGACAACTGC

ATCCTGAACGCTGAGACTCTTATGTGCATCAAGGAGCCCCCTGCCTACACCTTCCGGGAT

TACAGTGCCATCCTTTACCTAAATGGGGACTTTGATGGAGGAAACTTCTATTTTACAGAA

CTGGATGCCAAGACTGTGACGGCAGAGGTGCAGCCCCAGTGTGGAAGGGCTGTGGGATTT

TCTTCAGGCACCGAAAACCCGCATGGAGTGAAGGCTGTCACCAGGGGGCAACGCTGTGCC

ATTGCCCTGTGGTTCACCTTAGACCCTCGACATAGTGAGCGTGACAGGGTGCAGGCAGAT

GACCTGGTGAAGATGCTCTTCAGCCCAGAGGAGATGGACCTGCCCCAGGAGCAGCCCCTG

AATGCACAGCAGGACCCCTCCCAACCTGGAGAAGAGTCTCCCCCAGGCAGTGAGTCGAAG

CTCAGGGATGAGCTA

>CL18.Contig1_A-W 3 299 minus strand PREDICTED: vacuolar protein sorting-associated protein 33B isoform 1 [Nomascus leucogenys]

AAGAGGCTGGCTCGAGACCAACTCATCTATCTGCTTGAGCAGCTTCCTGGAAAAAAGGAT

TTGTTCATTGAGGCAGATCTTATGAGCCCTTTGGATCGAATTGCCAATGTGTCCATACTG

AAGCAACATGAAGTAGACAAGCTGTACAAGGTGGAGAACAAGCCAGCTCTCAGCTCCAAC

GAACAATTGTGCTTCTTGGTCAGACCTCGCATCAAGAATATGCGCTATATTGCCAATCTT

GTCAATGCTGACAAATTGGCTGGCCGAACTCGAAAATACAAAGTGATCTTCAGCCCT

>CL18.Contig2_A-W 40 213 minus strand PREDICTED: vacuolar protein sorting-associated protein 33B [Pan troglodytes]

CAACATGAAGTAGACAAGCTGTACAAGGTGGAGAACAAGCCAGCTCTCAGCTCCAACGAA

CAATTGTGCTTCTTGGTCAGACCTCGCATCAAGAATATGCGCTATATTGCCAATCTTGTC

AATGCTGACAAATTGGCTGGCCGAACTCGAAAATACAAAGTGATCTTCAGCCCT

>CL19.Contig1_A-W 2 721 minus strand PREDICTED: hypothetical protein [Oryctolagus cuniculus]

TGGGCAGATGCAGTCATAGCTGCAGGAGGTGATGGCACAATGCTTCTGGCAGCAAGTAAA

GTCTTGGATAGACTGAAACCAGTTATAGGGGTAAACACTGATCCAGAACGGTCAGAGGGT

CACTTATGTCTGCCTGTTCGATATACACATTCCTTCCCAGAAGCCTTACAGAAGTTCTAT

CGTGGTGAGTTCAGGTGGTTATGGAGGCAGCGTATCAGGTTATACCTTGAAGGGACTGGC

ATAAACCCCATTCCTGTGGATCTTCATGAACAGCAACTAAGTCTGAATCAGCATAGCAGA

GCCTTCAACATTGAAAGAGTTGATGATGAAAGGTCGGAGACTTCAGGACCCCAACTTTTG

CCAGTGAGAGCTCTAAATGAAGTCTTTATTGGGGAGAGTCTATCATCCAGGGCATCCTAC

TACGAAATTTCAGTTGATGATGGCCCGTGGGAAAAACAGAAGAGCTCAGGGCTCAATTTG

TGTACTGGAACGGGATCCAAGGCCTGGTCATTCAATATTAACAGGGTTGCAACTCAGGCT

GTGGAAGATGTTTTAAATATTGCAAAACGACAAGGAAATTTGAATCTTCCATTAAACAGA

GAATTGGTAGAGAAAGTAACAAATGAATATAATGAATCACTGCTGTACAGTCCAGAAGAA

CCAAAAATACTTTTCAGTATTCGAGAACCAATAGCAAACAGAGTTTTCTCAAGCAGTCGT

>CL19.Contig2_A-W 2 343 minus strand PREDICTED: LOW QUALITY PROTEIN: NAD kinase domain-containing protein 1-like [Cavia porcellus]

TTAAGAAGAAGTATACCCACTCTAAAGGGACTGGCATCCTACTACGAAATTTCAGTTGAT

GATGGCCCGTGGGAAAAACAGAAGAGCTCAGGGCTCAATTTGTGTACTGGAACGGGATCC

AAGGCCTGGTCATTCAATATTAACAGGGTTGCAACTCAGGCTGTGGAAGATGTTTTAAAT

ATTGCAAAACGACAAGGAAATTTGAATCTTCCATTAAACAGAGAATTGGTAGAGAAAGTA

ACAAATGAATATAATGAATCACTGCTGTACAGTCCAGAAGAACCAAAAATACTTTTCAGT

ATTCGAGAACCAATAGCAAACAGAGTTTTCTCAAGCAGTCGT

>CL20.Contig1_A-W 2 289 minus strand PREDICTED: WD repeat-containing protein 47 [Bos taurus] >gi|359063940|ref|XP_003585904.1| PREDICTED: WD repeat-containing protein 47 isoform 2 [Bos taurus]

CATTATCCAGGGGTACAAAACCTCAGTAGAAGTCTCATGCTTGAGAATACAGAATGTCAC

AGTATTTATGAGGAATCCCCTGAGCGTGATACACCCGTAGAGGCACAGCAACCTATCAGT

GGTGAAGTCTTGGGCCAGAGTTCAGTCTCAGAAAAAGAGCCTGCAAATGGAGCACAGAAT

CCAGGACCAGCTAAGCAAGAAAAAAATGAGCTTCGAGATTCCACTGAACAATTTCAAGAA

TATTACAGGCAAAGATTACGCTATCAACAGCATTTAGAACAGAAGGAG

>CL20.Contig2_A-W 29 238 minus strand PREDICTED: WD repeat domain 47 isoform 2 [Oryctolagus cuniculus]

GAAAGAAGTGATACACCCGTAGAGGCACAGCAACCTATCAGTGGTGAAGTCTTGGGCCAG

AGTTCAGTCTCAGAAAAAGAGCCTGCAAATGGAGCACAGAATCCAGGACCAGCTAAGCAA

GAAAAAAATGAGCTTCGAGATTCCACTGAACAATTTCAAGAATATTACAGGCAAAGATTA

CGCTATCAACAGCATTTAGAACAGAAGGAG

>CL21.Contig1_A-W 109 279 minus strand Uncharacterized protein KIAA0513 OS=Mus musculus GN=Kiaa0513 PE=2 SV=1

CATAGGGACCGTCCTCAGAGGGAAGAGCAGCCCCTTCCTTGTCCAGCCGCCCTGAAGTCC

GCCAACAGCTGGCTGGCAGAGAAGAAGGACATTGCCGAGCGGCTGCTGAAGAACACGGAG

AACGTGAAGGGCTTCTTTGGGGGGCTGGAGACCAAGCTGAAGGGACCCCTG

>CL21.Contig2_A-W 109 279 minus strand Uncharacterized protein KIAA0513 OS=Mus musculus GN=Kiaa0513 PE=2 SV=1

CATAGGGACCGTCCTCAGAGGGAAGAGCAGCCCCTTCCTTGTCCAGCCGCCCTGAAGTCC

GCCAACAGCTGGCTGGCAGAGAAGAAGGACATTGCCGAGCGGCTGCTGAAGAACACGGAG

AACGTGAAGGGCTTCTTTGGGGGGCTGGAGACCAAGCTGAAGGGACCCCTG

>CL21.Contig3_A-W 418 537 minus strand Uncharacterized protein KIAA0513 OS=Mus musculus GN=Kiaa0513 PE=2 SV=1

CTGAAGTCCGCCAACAGCTGGCTGGCAGAGAAGAAGGACATTGCCGAGCGGCTGCTGAAG

AACACGGAGAACGTGAAGGGCTTCTTTGGGGGGCTGGAGACCAAGCTGAAGGGACCCCTG

>CL21.Contig4_A-W 105 1802 MaSp2b [Deinopis spinosa]

GGGGGTCCAGGGCAGGACCCCGAGGGTCCCGGTTAAGGCCCACCTGCACCCCACAGTGGG

GGTCAGGTCATGTCACTGCTCCTCCCTTCCAGCCTCTGCCCAGGGGACTGGGGCCGCCCC

TCCCTGCCCGGCCCCCTGGTCAGACCCTGCACCCGGGCACCCGGCGGTCGGAGAAGCAGG

TAGAGGAGCCGCTGCAGGTGGGCAAGTTCATTTTAGCCCGTTTCCCCCTAAAAGGGGACT

GGACGCTGAGAGGAAGAGCAAAGGAAGGAGCAGAAACCGCCCGGAGAACAGTCAGGACAA

CAAGGGCCAGGGATGGACGGACAGAAGGACACAGCTCCACTCCTCTCCACCCCTCGGCCC

GAGGCTTCAGGGCTGCAGCACTTCTCCAGCCTCTGAGGGCAGGGCCCAGAGACCCCCAGG

ACGCACTGAGTCCCACCTTATACTCCTCCCCACCGAGTTCCCTCTAATAGTCCCCCTGAG

GGCTGGCAGCCACACAAATCCTGCCCTACCACTGGCCATCAGGAGGCCAGCCCTGCGCAC

TGCACTCCGGCCCGTGGGCTCTGGATGGACTCACACTCGCCCACAGAAGGCCCCTCTGAA

AAGGGGGTCAGAGGTGACAGGAATGACAGTGGAGGTCACTGAAGGAGAGGTCATGTTGCT

GGCCTGGGCATCTGGGGCCTGGGTGGCGCAGGGTGGGTCAGAGGAGTGGAACCCACCAGG

GCTGGGCACGTCGAATCTGTGGGGAGGCCCGAGGCCCCGGAGGCCTAGGAGGCCGGCTGC

GCCTCAGCAGGGCTGGGCAGCGGCACCGGCTCTCAGGGAGGCCAGGCTGGGCCCAGCCTT

CCTGGGCTGTGATGCCAGGAAGCCTGCCAAGCGGGAGGCCATCACCCCGAGGGACCACAG

GACAGGGGCACCACCCCCATTGCCCGCGGGAAGCATCAGGGTCAGGCCCCCCCATGCTCC

CTCTGTCCCCAGGAGGCAGCCACGGAGGCCTCTGTGGTGCCTAAGTGCCCGTCTCAAGGA

CACACTGAGGCTGCGTGGCAGCAGGACTTCTCAGCCACCAGCAAACTCACGAGCTCGCAA

AACCAACACTGAATCAGCAACTAGGTCTCTGTCACCGAGAAGACCTCACAGGGCTCCCAA

CTCGGGCCACTCAGGCTCCAGCCACGGGAGCTCTTGGGGACAGCCCCGCCTGGCTTTTTT

CCAAGGGACCTCCCACCCACACAGCTTCCCCGTCACCCAGGGCTGGAAAAAAGGACACTG

TGCTGCCGAGTGTCCCCAGGCCCATGGCCCAGGCACCTCCCCCCTGAGGGAGGCCCAGCT

GTGGTCTCACCGTCGGCCTGGGCGGGCCAGGTGGGCAGCACAGGTGGACAGAGCCCCCTC

CAAGCCGGGCGGGCTCTCTGCGGTCACTGTTCTTCCTCCCTGAGCCGCGCTGTGCCCTGC

AGGCCCCGCCATCTGCCCAGTCTAGGTCAGGGCCCCCGTGCTCCCTCTGTCCCCAGGAGG

CGGCCACCGACTGCAAGGGCTGGCCGAGGCGGCGCCCTCCTCCCCTCCGGCGTCAGCTGT

CCCTGCACAGAGGGCTCAGTGCCCACCGACTGGTCTCCACACAGACACCTGAGGCCCGCC

GCTCCTCGCCCTGGCTCGCCTCCCCAGGGGCCCTCCCGCCCTGCAGCGGCCTCCGCGGCC

CCCGGTCTGTAAGGCGGA

>CL21.Contig5_A-W 418 537 minus strand Uncharacterized protein KIAA0513 OS=Mus musculus GN=Kiaa0513 PE=2 SV=1

CTGAAGTCCGCCAACAGCTGGCTGGCAGAGAAGAAGGACATTGCCGAGCGGCTGCTGAAG

AACACGGAGAACGTGAAGGGCTTCTTTGGGGGGCTGGAGACCAAGCTGAAGGGACCCCTG

>CL21.Contig6_A-W 1428 2060 minus strand COL17A1; collagen, type XVII, alpha 1; K07603 collagen, type XVII, alpha 1

AGAGGGGATGCTGGACAGCAGGAGAAGAGGTGTGTGTGTCCGCCTTACAGACCGGGGGCC

GCGGAGGCCGCTGCAGGGCGGGAGGGCCCCTGGGGAGGCGAGCCAGGGCGAGGAGCGGCG

GGCCTCAGGTGTCTGTGTGGAGACCAGTCGGTGGGCACTGAGCCCTCTGTGCAGGGACAG

CTGACGCCGGAGGGGAGGAGGGCGCCGCCTCGGCCAGCCCTTGCAGTCGGTGGCCGCCTC

CTGGGGACAGAGGGAGCACGGGGGCCCTGACCTAGACTGGGCAGATGGCGGGGCCTGCAG

GGCACAGCGCGGCTCAGGGAGGAAGAACAGTGACCGCAGAGAGCCCGCCCGGCTTGGAGG

GGGCTCTGTCCACCTGTGCTGCCCACCTGGCCCGCCCAGGCCGACGGTGAGACCACAGCT

GGGCCTCCCTCAGGGGGGAGGTGCCTGGGCCATGGGCCTGGGGACACTCGGCAGCACAGT

GTCCTTTTTTCCAGCCCTGGGTGACGGGGAAGCTGTGTGGGTGGGAGGTCCCTTGGAAAA

AAGCCAGGCGGGGCTGTCCCCAAGAGCTCCCGTGGCTGGAGCCTGAGTGGCCCGAGTTGG

GAGCCCTGTGAGGTCTTCTCGGTGACAGAGACC

>CL21.Contig7_A-W 109 279 minus strand Uncharacterized protein KIAA0513 OS=Mus musculus GN=Kiaa0513 PE=2 SV=1

CATAGGGACCGTCCTCAGAGGGAAGAGCAGCCCCTTCCTTGTCCAGCCGCCCTGAAGTCC

GCCAACAGCTGGCTGGCAGAGAAGAAGGACATTGCCGAGCGGCTGCTGAAGAACACGGAG

AACGTGAAGGGCTTCTTTGGGGGGCTGGAGACCAAGCTGAAGGGACCCCTG

>CL21.Contig8_A-W 960 1592 minus strand COL17A1; collagen, type XVII, alpha 1; K07603 collagen, type XVII, alpha 1

AGAGGGGATGCTGGACAGCAGGAGAAGAGGTGTGTGTGTCCGCCTTACAGACCGGGGGCC

GCGGAGGCCGCTGCAGGGCGGGAGGGCCCCTGGGGAGGCGAGCCAGGGCGAGGAGCGGCG

GGCCTCAGGTGTCTGTGTGGAGACCAGTCGGTGGGCACTGAGCCCTCTGTGCAGGGACAG

CTGACGCCGGAGGGGAGGAGGGCGCCGCCTCGGCCAGCCCTTGCAGTCGGTGGCCGCCTC

CTGGGGACAGAGGGAGCACGGGGGCCCTGACCTAGACTGGGCAGATGGCGGGGCCTGCAG

GGCACAGCGCGGCTCAGGGAGGAAGAACAGTGACCGCAGAGAGCCCGCCCGGCTTGGAGG

GGGCTCTGTCCACCTGTGCTGCCCACCTGGCCCGCCCAGGCCGACGGTGAGACCACAGCT

GGGCCTCCCTCAGGGGGGAGGTGCCTGGGCCATGGGCCTGGGGACACTCGGCAGCACAGT

GTCCTTTTTTCCAGCCCTGGGTGACGGGGAAGCTGTGTGGGTGGGAGGTCCCTTGGAAAA

AAGCCAGGCGGGGCTGTCCCCAAGAGCTCCCGTGGCTGGAGCCTGAGTGGCCCGAGTTGG

GAGCCCTGTGAGGTCTTCTCGGTGACAGAGACC

>CL21.Contig9_A-W 234 866 minus strand COL17A1; collagen, type XVII, alpha 1; K07603 collagen, type XVII, alpha 1

AGAGGGGATGCTGGACAGCAGGAGAAGAGGTGTGTGTGTCCGCCTTACAGACCGGGGGCC

GCGGAGGCCGCTGCAGGGCGGGAGGGCCCCTGGGGAGGCGAGCCAGGGCGAGGAGCGGCG

GGCCTCAGGTGTCTGTGTGGAGACCAGTCGGTGGGCACTGAGCCCTCTGTGCAGGGACAG

CTGACGCCGGAGGGGAGGAGGGCGCCGCCTCGGCCAGCCCTTGCAGTCGGTGGCCGCCTC

CTGGGGACAGAGGGAGCACGGGGGCCCTGACCTAGACTGGGCAGATGGCGGGGCCTGCAG

GGCACAGCGCGGCTCAGGGAGGAAGAACAGTGACCGCAGAGAGCCCGCCCGGCTTGGAGG

GGGCTCTGTCCACCTGTGCTGCCCACCTGGCCCGCCCAGGCCGACGGTGAGACCACAGCT

GGGCCTCCCTCAGGGGGGAGGTGCCTGGGCCATGGGCCTGGGGACACTCGGCAGCACAGT

GTCCTTTTTTCCAGCCCTGGGTGACGGGGAAGCTGTGTGGGTGGGAGGTCCCTTGGAAAA

AAGCCAGGCGGGGCTGTCCCCAAGAGCTCCCGTGGCTGGAGCCTGAGTGGCCCGAGTTGG

GAGCCCTGTGAGGTCTTCTCGGTGACAGAGACC

>CL21.Contig10_A-W 109 279 minus strand Uncharacterized protein KIAA0513 OS=Mus musculus GN=Kiaa0513 PE=2 SV=1

CATAGGGACCGTCCTCAGAGGGAAGAGCAGCCCCTTCCTTGTCCAGCCGCCCTGAAGTCC

GCCAACAGCTGGCTGGCAGAGAAGAAGGACATTGCCGAGCGGCTGCTGAAGAACACGGAG

AACGTGAAGGGCTTCTTTGGGGGGCTGGAGACCAAGCTGAAGGGACCCCTG

>CL21.Contig11_A-W 418 537 minus strand Uncharacterized protein KIAA0513 OS=Mus musculus GN=Kiaa0513 PE=2 SV=1

CTGAAGTCCGCCAACAGCTGGCTGGCAGAGAAGAAGGACATTGCCGAGCGGCTGCTGAAG

AACACGGAGAACGTGAAGGGCTTCTTTGGGGGGCTGGAGACCAAGCTGAAGGGACCCCTG

>CL21.Contig12_A-W 418 537 minus strand Uncharacterized protein KIAA0513 OS=Mus musculus GN=Kiaa0513 PE=2 SV=1

CTGAAGTCCGCCAACAGCTGGCTGGCAGAGAAGAAGGACATTGCCGAGCGGCTGCTGAAG

AACACGGAGAACGTGAAGGGCTTCTTTGGGGGGCTGGAGACCAAGCTGAAGGGACCCCTG

>CL21.Contig13_A-W 105 1802 MaSp2b [Deinopis spinosa]

GGGGGTCCAGGGCAGGACCCCGAGGGTCCCGGTTAAGGCCCACCTGCACCCCACAGTGGG

GGTCAGGTCATGTCACTGCTCCTCCCTTCCAGCCTCTGCCCAGGGGACTGGGGCCGCCCC

TCCCTGCCCGGCCCCCTGGTCAGACCCTGCACCCGGGCACCCGGCGGTCGGAGAAGCAGG

TAGAGGAGCCGCTGCAGGTGGGCAAGTTCATTTTAGCCCGTTTCCCCCTAAAAGGGGACT

GGACGCTGAGAGGAAGAGCAAAGGAAGGAGCAGAAACCGCCCGGAGAACAGTCAGGACAA

CAAGGGCCAGGGATGGACGGACAGAAGGACACAGCTCCACTCCTCTCCACCCCTCGGCCC

GAGGCTTCAGGGCTGCAGCACTTCTCCAGCCTCTGAGGGCAGGGCCCAGAGACCCCCAGG

ACGCACTGAGTCCCACCTTATACTCCTCCCCACCGAGTTCCCTCTAATAGTCCCCCTGAG

GGCTGGCAGCCACACAAATCCTGCCCTACCACTGGCCATCAGGAGGCCAGCCCTGCGCAC

TGCACTCCGGCCCGTGGGCTCTGGATGGACTCACACTCGCCCACAGAAGGCCCCTCTGAA

AAGGGGGTCAGAGGTGACAGGAATGACAGTGGAGGTCACTGAAGGAGAGGTCATGTTGCT

GGCCTGGGCATCTGGGGCCTGGGTGGCGCAGGGTGGGTCAGAGGAGTGGAACCCACCAGG

GCTGGGCACGTCGAATCTGTGGGGAGGCCCGAGGCCCCGGAGGCCTAGGAGGCCGGCTGC

GCCTCAGCAGGGCTGGGCAGCGGCACCGGCTCTCAGGGAGGCCAGGCTGGGCCCAGCCTT

CCTGGGCTGTGATGCCAGGAAGCCTGCCAAGCGGGAGGCCATCACCCCGAGGGACCACAG

GACAGGGGCACCACCCCCATTGCCCGCGGGAAGCATCAGGGTCAGGCCCCCCCATGCTCC

CTCTGTCCCCAGGAGGCAGCCACGGAGGCCTCTGTGGTGCCTAAGTGCCCGTCTCAAGGA

CACACTGAGGCTGCGTGGCAGCAGGACTTCTCAGCCACCAGCAAACTCACGAGCTCGCAA

AACCAACACTGAATCAGCAACTAGGTCTCTGTCACCGAGAAGACCTCACAGGGCTCCCAA

CTCGGGCCACTCAGGCTCCAGCCACGGGAGCTCTTGGGGACAGCCCCGCCTGGCTTTTTT

CCAAGGGACCTCCCACCCACACAGCTTCCCCGTCACCCAGGGCTGGAAAAAAGGACACTG

TGCTGCCGAGTGTCCCCAGGCCCATGGCCCAGGCACCTCCCCCCTGAGGGAGGCCCAGCT

GTGGTCTCACCGTCGGCCTGGGCGGGCCAGGTGGGCAGCACAGGTGGACAGAGCCCCCTC

CAAGCCGGGCGGGCTCTCTGCGGTCACTGTTCTTCCTCCCTGAGCCGCGCTGTGCCCTGC

AGGCCCCGCCATCTGCCCAGTCTAGGTCAGGGCCCCCGTGCTCCCTCTGTCCCCAGGAGG

CGGCCACCGACTGCAAGGGCTGGCCGAGGCGGCGCCCTCCTCCCCTCCGGCGTCAGCTGT

CCCTGCACAGAGGGCTCAGTGCCCACCGACTGGTCTCCACACAGACACCTGAGGCCCGCC

GCTCCTCGCCCTGGCTCGCCTCCCCAGGGGCCCTCCCGCCCTGCAGCGGCCTCCGCGGCC

CCCGGTCTGTAAGGCGGA

>CL21.Contig14_A-W 105 1802 MaSp2b [Deinopis spinosa]

GGGGGTCCAGGGCAGGACCCCGAGGGTCCCGGTTAAGGCCCACCTGCACCCCACAGTGGG

GGTCAGGTCATGTCACTGCTCCTCCCTTCCAGCCTCTGCCCAGGGGACTGGGGCCGCCCC

TCCCTGCCCGGCCCCCTGGTCAGACCCTGCACCCGGGCACCCGGCGGTCGGAGAAGCAGG

TAGAGGAGCCGCTGCAGGTGGGCAAGTTCATTTTAGCCCGTTTCCCCCTAAAAGGGGACT

GGACGCTGAGAGGAAGAGCAAAGGAAGGAGCAGAAACCGCCCGGAGAACAGTCAGGACAA

CAAGGGCCAGGGATGGACGGACAGAAGGACACAGCTCCACTCCTCTCCACCCCTCGGCCC

GAGGCTTCAGGGCTGCAGCACTTCTCCAGCCTCTGAGGGCAGGGCCCAGAGACCCCCAGG

ACGCACTGAGTCCCACCTTATACTCCTCCCCACCGAGTTCCCTCTAATAGTCCCCCTGAG

GGCTGGCAGCCACACAAATCCTGCCCTACCACTGGCCATCAGGAGGCCAGCCCTGCGCAC

TGCACTCCGGCCCGTGGGCTCTGGATGGACTCACACTCGCCCACAGAAGGCCCCTCTGAA

AAGGGGGTCAGAGGTGACAGGAATGACAGTGGAGGTCACTGAAGGAGAGGTCATGTTGCT

GGCCTGGGCATCTGGGGCCTGGGTGGCGCAGGGTGGGTCAGAGGAGTGGAACCCACCAGG

GCTGGGCACGTCGAATCTGTGGGGAGGCCCGAGGCCCCGGAGGCCTAGGAGGCCGGCTGC

GCCTCAGCAGGGCTGGGCAGCGGCACCGGCTCTCAGGGAGGCCAGGCTGGGCCCAGCCTT

CCTGGGCTGTGATGCCAGGAAGCCTGCCAAGCGGGAGGCCATCACCCCGAGGGACCACAG

GACAGGGGCACCACCCCCATTGCCCGCGGGAAGCATCAGGGTCAGGCCCCCCCATGCTCC

CTCTGTCCCCAGGAGGCAGCCACGGAGGCCTCTGTGGTGCCTAAGTGCCCGTCTCAAGGA

CACACTGAGGCTGCGTGGCAGCAGGACTTCTCAGCCACCAGCAAACTCACGAGCTCGCAA

AACCAACACTGAATCAGCAACTAGGTCTCTGTCACCGAGAAGACCTCACAGGGCTCCCAA

CTCGGGCCACTCAGGCTCCAGCCACGGGAGCTCTTGGGGACAGCCCCGCCTGGCTTTTTT

CCAAGGGACCTCCCACCCACACAGCTTCCCCGTCACCCAGGGCTGGAAAAAAGGACACTG

TGCTGCCGAGTGTCCCCAGGCCCATGGCCCAGGCACCTCCCCCCTGAGGGAGGCCCAGCT

GTGGTCTCACCGTCGGCCTGGGCGGGCCAGGTGGGCAGCACAGGTGGACAGAGCCCCCTC

CAAGCCGGGCGGGCTCTCTGCGGTCACTGTTCTTCCTCCCTGAGCCGCGCTGTGCCCTGC

AGGCCCCGCCATCTGCCCAGTCTAGGTCAGGGCCCCCGTGCTCCCTCTGTCCCCAGGAGG

CGGCCACCGACTGCAAGGGCTGGCCGAGGCGGCGCCCTCCTCCCCTCCGGCGTCAGCTGT

CCCTGCACAGAGGGCTCAGTGCCCACCGACTGGTCTCCACACAGACACCTGAGGCCCGCC

GCTCCTCGCCCTGGCTCGCCTCCCCAGGGGCCCTCCCGCCCTGCAGCGGCCTCCGCGGCC

CCCGGTCTGTAAGGCGGA

>CL21.Contig15_A-W 163 732 PREDICTED: bone morphogenetic protein 8B-like [Canis lupus familiaris]

GGGGTCAGGTCATGTCACTGCTCCTCCCTTCCAGCCTCTGCCCAGGGGACTGGGGCCGCC

CCTCCCTGCCCGGCCCCCTGGTCAGACCCTGCACCCGGGCACCCGGCGGTCGGAGAAGCA

GGTAGAGGAGCCGCTGCAGGTGGGCAAGTTCATTTTAGCCCGTTTCCCCCTAAAAGGGGA

CTGGACGCTGAGAGGAAGAGCAAAGGAAGGAGCAGAAACCGCCCGGAGAACAGTCAGGAC

AACAAGGGCCAGGGATGGACGGACAGAAGGACACAGCTCCACTCCTCTCCACCCCTCGGC

CCGAGGCTTCAGGGCTGCAGCACTTCTCCAGCCTCTGAGGGCAGGGCCCAGAGACCCCCA

GGACGCACTGAGTCCCACCTTATACTCCTCCCCACCGAGTTCCCTCTAATAGTCCCCCTG

AGGGCTGGCAGCCACACAAATCCTGCCCTACCACTGGCCATCAGGTACAGTTGTGCAGGG

AGGGCACTGCACACCCCAGGCCACCGGGGAATGGCTCCCCTGAGTGTTCAGCACAGCAGC

CCAGATGCTGCTGTTCTGGACCCACGAGGA

>CL21.Contig16_A-W 2 487 minus strand PREDICTED: uncharacterized protein KIAA0513-like [Cricetulus griseus]

CAGTCCTTCGCGGTGGTGCTGTTCGAGTGTCATCAGATGGATGACTTCGGACCTGCCAAG

AACCTCATGACCATGTGCTTCACCTACTACCACCTGGGCAAGCCGCAGCAGCTCCCCTCC

GAGCCCAGGGAGAAGCCCGCGGGCAGCATCGACTCCTACCTGAAGTCCGCCAACAGCTGG

CTGGCAGAGAAGAAGGACATTGCCGAGCGGCTGCTGAAGAACACGGAGAACGTGAAGGGC

TTCTTTGGGGGGCTGGAGACCAAGCTGAAGGGACCCCTGGTCAGGAAGAATGAGGAAGAT

GAGAACAAGCCCAAGGACAAGCAGCCCAAGACTGTGACCGTGTTCAGCCCCGAGGACGAG

CAGAAGGGGGAGAAGATCTACCTGTACACACACCTGAAGCAGCAGCCCATCTGGCACACG

CTGAGGTTCTGGAACGCAGCCTTTTTTGACGCCGTCCACTGTGAGAGGAGAAAGCGTTCT

CCCACC

>CL22.Contig1_A-W 219 1367 Collagen alpha-1(I) chain OS=Gallus gallus GN=COL1A1 PE=1 SV=3

AAGCCAATAAGAACCCTGGAGAGAAGTCAGCCAGGTTGCTTTAAAAGCTCAAAGAAGACG

GTTCCGGGGTCCCCAAGTAAACCAACGGGAGGTCTAGAGGGTGGCCAGGAGTGAGAAGCA

CCTCAGGAATGACGCGGGGAAGGCGGGTCACCTTCCACCTGGCCCAGAGGACCCAGGGAG

CCCACAGTTGACCAAAAGAAGAGTAGATCCCGCGTCCTCGCCCCACAGGGGTCCAGGAGG

TCGCCCGGCGTTTCTTTTGCAACAAGTGCTTCTCCAGTCTCTGCAAACTTCCGCTGGACT

GTCCAGGTGAGGGGCGGGGCCTGAGGTGCCAGGACCACCACTGGAGAAAAGGGGCTGTTG

TGAGGAAGGGAAGGGGCCCAGGGCACCCGTGGCGGCCCGCGGGAGCGCCCCTTGGTCTTC

AGTTCAGGACGTGACGGTGAATCGGGGGGAGCAGGCTATGTTCTCTTGTGTCGTGGGCTT

CCAGCTGCCGCCCGAGGAGGTCACCTATACCTGGAAGTTCGCTGCGGGAGGTATGCGCAC

TGGGGACGTGTCCTACTTCCGAGAAGTGCCCCAGGCCCGAGGGTACCTGGCGCGGATCCG

GCCAGTGCAGCCCAAGCACCGCGGCACCTTCTCCTGCGTGATCAAGCACGACCAGCGCCC

CCTGGCGCGCCTCTACTTCTTCCTGAACGTGACGGGCCCGCCCCCGCGCGGCGAGACGGA

GCTCCAAGCAGCGTTTCGGGAAGTGGTGAGCTGGGCGCCGCAGGACGCGGAAATGATCGA

GCCCTGGCGGCCCAGCCTGGGCGAACTGCTGGCCAGGCCCGAGGCTCTGACGCTGGGCAA

TCTCTGCATGCTCGCAGCTGTAGCCGCCCTGGCATCTGCGATGGCGACCATGCTGGTGTG

GTGAGTCCCAGCGTCCAGCTGCCCCTCCTGCAGACCCAGGGAGCCAGGCTCAGACCCAGG

ACTCTAGACTCCAGCCCTCCTCCCTCAGACCTAAGAGGTCCAGGTCCCACCAAGCCCCCT

CCCCCGGCCAAGAGACCAGGTCCCCGTCCCTCCCTCAGATGCAGGACTCTAGATCCCAGT

GCCTCCTGCCTCAGACCCAGGGGTCCAGACCCCAGCCCTCCTCCTCAGACCTGGGAGTCA

GGCCCTGCT

>CL22.Contig2_A-W 84 194 PREDICTED: hypothetical protein LOC100735722 [Cavia porcellus]

CTCTCCGCAGTTCAGCCCTGCGTCCCTCCCTGCGGGGTCCAGGAGGTCGCCCGGCGTTTC

TTTTGCAACAAGTGCTTCTCCAGTCTCTGCAAACTTCCGCTGGACTGTCCA

>CL22.Contig3_A-W 219 1367 Collagen alpha-1(I) chain OS=Gallus gallus GN=COL1A1 PE=1 SV=3

AAGCCAATAAGAACCCTGGAGAGAAGTCAGCCAGGTTGCTTTAAAAGCTCAAAGAAGACG

GTTCCGGGGTCCCCAAGTAAACCAACGGGAGGTCTAGAGGGTGGCCAGGAGTGAGAAGCA

CCTCAGGAATGACGCGGGGAAGGCGGGTCACCTTCCACCTGGCCCAGAGGACCCAGGGAG

CCCACAGTTGACCAAAAGAAGAGTAGATCCCGCGTCCTCGCCCCACAGGGGTCCAGGAGG

TCGCCCGGCGTTTCTTTTGCAACAAGTGCTTCTCCAGTCTCTGCAAACTTCCGCTGGACT

GTCCAGGTGAGGGGCGGGGCCTGAGGTGCCAGGACCACCACTGGAGAAAAGGGGCTGTTG

TGAGGAAGGGAAGGGGCCCAGGGCACCCGTGGCGGCCCGCGGGAGCGCCCCTTGGTCTTC

AGTTCAGGACGTGACGGTGAATCGGGGGGAGCAGGCTATGTTCTCTTGTGTCGTGGGCTT

CCAGCTGCCGCCCGAGGAGGTCACCTATACCTGGAAGTTCGCTGCGGGAGGTATGCGCAC

TGGGGACGTGTCCTACTTCCGAGAAGTGCCCCAGGCCCGAGGGTACCTGGCGCGGATCCG

GCCAGTGCAGCCCAAGCACCGCGGCACCTTCTCCTGCGTGATCAAGCACGACCAGCGCCC

CCTGGCGCGCCTCTACTTCTTCCTGAACGTGACGGGCCCGCCCCCGCGCGGCGAGACGGA

GCTCCAAGCAGCGTTTCGGGAAGTGGTGAGCTGGGCGCCGCAGGACGCGGAAATGATCGA

GCCCTGGCGGCCCAGCCTGGGCGAACTGCTGGCCAGGCCCGAGGCTCTGACGCTGGGCAA

TCTCTGCATGCTCGCAGCTGTAGCCGCCCTGGCATCTGCGATGGCGACCATGCTGGTGTG

GTGAGTCCCAGCGTCCAGCTGCCCCTCCTGCAGACCCAGGGAGCCAGGCTCAGACCCAGG

ACTCTAGACTCCAGCCCTCCTCCCTCAGACCTAAGAGGTCCAGGTCCCACCAAGCCCCCT

CCCCCGGCCAAGAGACCAGGTCCCCGTCCCTCCCTCAGATGCAGGACTCTAGATCCCAGT

GCCTCCTGCCTCAGACCCAGGGGTCCAGACCCCAGCCCTCCTCCTCAGACCTGGGAGTCA

GGCCCTGCT

>CL23.Contig1_A-W 3 329 minus strand PREDICTED: partitioning defective 3 homolog [Saimiri boliviensis boliviensis]

GGTACCGAAGGTTTGGGATTCAGCATCACTTCACGAGATGTCACAATAGGTGGCTCAGCT

CCAATTTATGTGAAAAACATCCTCCCCCGAGGTGCTGCCATTCAAGATGGTAGATTGAAG

GCAGGAGATAGACTCATAGAGGTAAATGGAGTAGATTTAGCAGGCAAATCCCAAGAGGAA

GTTGTTTCCCTGTTAAGAAGCACCAAGATGGAAGGGACTGTGAGCCTTCTGGTCTTTCGC

CAGGAAGACGCCTTTCACCCAAGGGAACTGAATGCAGAGCCAAGCCAGATGCAGATTCCA

AAAGAAACGAAAGCAGAAGATGAAGAT

>CL23.Contig2_A-W 3 245 minus strand PREDICTED: partitioning defective 3 homolog isoform 9 [Papio anubis]

GGTACCGAAGGTTTGGGATTCAGCATCACTTCACGAGATGTCACAATAGGTGGCTCAGCT

CCAATTTATGTGAAAAACATCCTCCCCCGAGGTGCTGCCATTCAAGATGGTAGATTGAAG

GCAGGAGATAGACTCATAGAGGTAAATGGAGTAGATTTAGCAGGCAAATCCCAAGAGGAA

GTTGTTTCCCTGTTAAGAAGCACCAAGATGGAAGGGACTGTGAGCCTTCTGGTCTTTCGC

CAG

>CL25.Contig1_A-W 2385 3881 minus strand PREDICTED: myosin-If-like [Cavia porcellus]

AGGGTCAAGCACCAGGTGGAGTACCTGGGCCTGCGGGAGAACATCCGGGTGCGCAGGGCC

GGCTTCGCCTACCGCCGCCAGTTCTCCAAGTTCCTGCAGAGGTACGCCATCCTGACCCCT

GAGACCTGGCCACGGTGGCGTGGGGACGAACGTCAGGGTGTCCAGCACCTGCTTCGGGCG

GTCAACATGGAGCCGGACCAGTACCAGATGGGGAGCACCAAGGTCTTCGTCAAGAACCCA

GAGTCGCTTTTCCTCCTGGAGGAGATGCGAGAGCGGAAGTTCGACGGCTTTGCCCGCACC

ATTCAGAAGGCCTGGAGGCGCCACGTGGCGGTACGGAAGTACCAAGAGATGCGGGAGGAA

GCTTCAAACATCCTGCTCAACAAGAAGGAACGGAGGAGGAACAGCATTAATCGGAACTTC

GTCGGGGACTACCTGGGGCTGGAGGAACGGCCAGAGCTACGTCAGTTCCTGGGCAAGAGG

GAGCGTGTGGACTTTGCAGACTCGGTCACCAAGTATGACCGGCGCTTCAAGCCCATCAAG

CGGGACTTGATCCTGACCCCCAAGTGTGTGTATGTGATTGGGCGAGAGAAGGTGAAGAAA

GGACCAGAGAAGGGCCAAGTGCGTGAGGTCCTGAAGAAGAAGCTGGAGATTCAGGCCCTG

CGGGGAGTCTCCCTCAGCACGCGACAGGACGACTTCTTCATCCTCCAAGAGGACGCGGCT

GACAGCTTCCTGGAGAGCGTCTTCAAGACCGAGTTCGTCAGCCTTCTGTGCAAGCGCTTC

GAGGAGGCGGCACGGAGGCCCCTGCCCCTTACCTTCAGCGACACACTACAGTTCCGGGTG

AAGAAGGAGGGCTGGGGCGGAGGCAGCACTCGCAGCGTCACCTTTTCCCGCGGCTCAGGA

GACATAGCGGTACTCAAGGCTGGCGGCAAAACCCTCACGGTCAGCGTGGGCGACGGACTG

CCCAAGAGCTCCAAGCCTACACGCAAGGGAATGGCCCAGGGAAGACCCCGAAGGTCAGCC

CCGGCCCCTACCCGGGCGGCCCCTGGCCCCCCCAGAGGCCTGGACCGAAATGGTGTGCCT

CCCTCTGCCAGAGGGGGGCCCCTGCCCCTGGAAATCACATCTGGAGGGGGCTCCCAGCGG

CCTCCCCGGGGTCCTCCATCCTCAACCCTGGGGTCCAGTAGACGTCCCCGGGCACGGCCA

CCCTCAGAGCACAACACAGAATTCCTCAATGTGCCTGACCAGGGTGTGGCAGGCATGCAG

AGGAAGCGCAGCGTTGGGCAGAGACCTGTGCCTGGTGTGGGCCGACCCAAGCCCCAACCA

CGTGTACATGGCCCAAGGTGCCGGGCACTATACCAGTACATAGGCCAAGATGTCGATGAG

TTGAGCTTCAATGTGAATGAAGTCATCGAGATCCTCATGGAAGATTCTTCAGGTTGGTGG

AAGGGCCGCCTACATGGCCAGGAGGGTCTTTTCCCCGGAAACTACGTGGAGAAGATC

>CL25.Contig2_A-W 6 1034 minus strand PREDICTED: LOW QUALITY PROTEIN: unconventional myosin-If [Callithrix jacchus]

CGAGGGCCAGGAGCCGGTGAGGAAGGGAGCAGGGCCATGCCCAAGCCGCCCCCAAGAGCC

CTCTGAGCCTGTGCCCCTGCCACAGCCCTTCCGGTGACCCCCCAGGAGCCGGACGCCAAC

CCCACCATGGGCAGCAAGGAGCGCTTCCACTGGCAAAGCCACAACGTGAAGCAAAGCGGC

GTGGACGACATGGTGCTGCTGCCCCAGATCACCGAGGACGCCATCGTGGGCAACCTGCGC

AAGCGCTTCATGGACGACTACATCTTCACCTACATCGGCTCGGTGCTCATCTCCGTAAAT

CCCTTCAAGCAGATGCCCTACTTCACTGACCGTGAGATCGATCTCTACCAGGGCGCGGCC

CAGTACGAGAACCCCCCGCACATCTACGCCCTCACAGACAACATGTACCGCAACATGCTC

ATCGACTGTGAGAACCAGTGCGTCATCATCAGTGGAGAGAGTGGAGCTGGGAAGACAGTG

GCCGCAAAATACATCATGGGCTACATCTCCAAGGTGTCTGGCGGGGGAGAGAAGGTCCAG

CACGTCAAGGACATCATCCTGCAGTCCAACCCGCTGCTGGAGGCCTTCGGCAATGCCAAA

ACCGTGCGCAACAACAATTCCAGCCGATTTGGCAAGTACTTTGAGATCCAGTTTAGCCGT

GGTGGGGAGCCAGATGGGGGCAAGATCTCCAACTTCTTGCTAGAGAAGTCCCGCGTGGTC

ATGCAGAATGAAAATGAGAGGAACTTTCACATCTACTACCAGCTGTTGGAAGGGGCCTCG

CAGGAGCAGCGCCAGAACCTGGGGCTCATGACACCCGACTACTATTACTACCTCAACCAG

TCAGACACCTACAAGGTGGATGGCACCGATGACAGGAGCGACTTCAGTGAGACCCTGAGT

GCCATGCAAGTCATTGGGATCCCACCCAGCGTCCAGCAGCTGGTTCTGCAGCTGGTGGCA

GGGATCCTACACCTGGGGAACATCAGTTTCTGTGAAGAGGGGAATTACGCCCAGGTGGAG

AGTGCAGAC

>CL25.Contig3_A-W 15 3425 minus strand MYO1F variant protein [Homo sapiens]

GGAGCCGGTGAGGAAGGGAGCAGGGCCATGCCCAAGCCGCCCCCAAGAGCCCTCTGAGCC

TGTGCCCCTGCCACAGCCCTTCCGGTGACCCCCCAGGAGCCGGACGCCAACCCCACCATG

GGCAGCAAGGAGCGCTTCCACTGGCAAAGCCACAACGTGAAGCAAAGCGGCGTGGACGAC

ATGGTGCTGCTGCCCCAGATCACCGAGGACGCCATCGTGGGCAACCTGCGCAAGCGCTTC

ATGGACGACTACATCTTCACCTACATCGGCTCGGTGCTCATCTCCGTAAATCCCTTCAAG

CAGATGCCCTACTTCACTGACCGTGAGATCGATCTCTACCAGGGCGCGGCCCAGTACGAG

AACCCCCCGCACATCTACGCCCTCACAGACAACATGTACCGCAACATGCTCATCGACTGT

GAGAACCAGTGCGTCATCATCAGTGGAGAGAGTGGAGCTGGGAAGACAGTGGCCGCAAAA

TACATCATGGGCTACATCTCCAAGGTGTCTGGCGGGGGAGAGAAGGTCCAGCACGTCAAG

GACATCATCCTGCAGTCCAACCCGCTGCTGGAGGCCTTCGGCAATGCCAAAACCGTGCGC

AACAACAATTCCAGCCGATTTGGCAAGTACTTTGAGATCCAGTTTAGCCGTGGTGGGGAG

CCAGATGGGGGCAAGATCTCCAACTTCTTGCTAGAGAAGTCCCGCGTGGTCATGCAGAAT

GAAAATGAGAGGAACTTTCACATCTACTACCAGCTGTTGGAAGGGGCCTCGCAGGAGCAG

CGCCAGAACCTGGGGCTCATGACACCCGACTACTATTACTACCTCAACCAGTCAGACACC

TACAAGGTGGATGGCACCGATGACAGGAGCGACTTCAGTGAGACCCTGAGTGCCATGCAA

GTCATTGGGATCCCACCCAGCGTCCAGCAGCTGGTTCTGCAGCTGGTGGCAGGGATCCTA

CACCTGGGGAACATCAGTTTCTGTGAAGAGGGGAATTACGCCCAGGTGGAGAGTGCAGAC

CTCCTGGCCTTTCCTGCCTACCTGCTGGGCATTGACAGCGGGCGGCTGCAGGAGAAGCTG

ACCAGCCGCAAGATGGACAGCCGCTGGGGCGGACGCAGTGAGTCCATTAATGTGACCCTC

AACGTGGAGCAGGCAGCCTACACCCGTGACGCCCTGGCCAAGGGACTCTATGCCCGCCTC

TTCGACTTCCTGGTGGAGGCCATCAACCGTGCGATGCAGAAGCCCCAGGAGGAGTACAGT

ATCGGTGTCCTTGACATCTATGGCTTCGAGATTTTCCAGAAAAATGGTTTTGAGCAGTTC

TGCATCAACTTCGTCAATGAAAAGCTGCAACAAATCTTCATCGAGCTCACCCTGAAGGCT

GAGCAGGAGGAGTATGTGCAGGAGGGCATCCACTGGACCCCCATCGAGTACTTCAACAAC

AAAGTCGTCTGTGACCTCATTGAAAACAAGCTGAGCCCCCCGGGCATCATGAGCGTGCTG

GACGACGTGTGCGCCACCATGCACGCCACAGGCGGAGGTGCCGACCAGACGCTGCTGCAG

AAGCTGCAGGCGGCCGTGGGCACCCACGAGCACTTCAACAGCTGGAGCGCGGGGTTCGTC

ATCCACCACTACGCCGGCAAAGTCTCCTATGACGTCAGCGGCTTCTGCGAGAGGAACCGG

GATGTCCTCTTCTCTGACCTTATAGAGCTGATGCAGACCAGCGAGCGGGCCTTCCTCCGG

ATGCTCTTTCCTGAGAAGCTGGATGTGGACAAGAAGGGCCGTCCGAGCACTGCTGGCTCC

AAGATCAAGAAACAAGCCAACGACCTGGTGGCCACTCTGAAGAGGTGCACCCCCCACTAC

ATCCGCTGCATCAAACCCAACGAGACCAAGAGGCCCCGAGACTGGGAAGAGAGCAGGGTC

AAGCACCAGGTGGAGTACCTGGGCCTGCGGGAGAACATCCGGGTGCGCAGGGCCGGCTTC

GCCTACCGCCGCCAGTTCTCCAAGTTCCTGCAGAGGTACGCCATCCTGACCCCTGAGACC

TGGCCACGGTGGCGTGGGGACGAACGTCAGGGTGTCCAGCACCTGCTTCGGGCGGTCAAC

ATGGAGCCGGACCAGTACCAGATGGGGAGCACCAAGGTCTTCGTCAAGAACCCAGAGTCG

CTTTTCCTCCTGGAGGAGATGCGAGAGCGGAAGTTCGACGGCTTTGCCCGCACCATTCAG

AAGGCCTGGAGGCGCCACGTGGCGGTACGGAAGTACCAAGAGATGCGGGAGGAAGCTTCA

AACATCCTGCTCAACAAGAAGGAACGGAGGAGGAACAGCATTAATCGGAACTTCGTCGGG

GACTACCTGGGGCTGGAGGAACGGCCAGAGCTACGTCAGTTCCTGGGCAAGAGGGAGCGT

GTGGACTTTGCAGACTCGGTCACCAAGTATGACCGGCGCTTCAAGCCCATCAAGCGGGAC

TTGATCCTGACCCCCAAGTGTGTGTATGTGATTGGGCGAGAGAAGGTGAAGAAAGGACCA

GAGAAGGGCCAAGTGCGTGAGGTCCTGAAGAAGAAGCTGGAGATTCAGGCCCTGCGGGGA

GTCTCCCTCAGCACGCGACAGGACGACTTCTTCATCCTCCAAGAGGACGCGGCTGACAGC

TTCCTGGAGAGCGTCTTCAAGACCGAGTTCGTCAGCCTTCTGTGCAAGCGCTTCGAGGAG

GCGGCACGGAGGCCCCTGCCCCTTACCTTCAGCGACACACTACAGTTCCGGGTGAAGAAG

GAGGGCTGGGGCGGAGGCAGCACTCGCAGCGTCACCTTTTCCCGCGGCTCAGGAGACATA

GCGGTACTCAAGGCTGGCGGCAAAACCCTCACGGTCAGCGTGGGCGACGGACTGCCCAAG

AGCTCCAAGCCTACACGCAAGGGAATGGCCCAGGGAAGACCCCGAAGGTCAGCCCCGGCC

CCTACCCGGGCGGCCCCTGGCCCCCCCAGAGGCCTGGACCGAAATGGTGTGCCTCCCTCT

GCCAGAGGGGGGCCCCTGCCCCTGGAAATCACATCTGGAGGGGGCTCCCAGCGGCCTCCC

CGGGGTCCTCCATCCTCAACCCTGGGGTCCAGTAGACGTCCCCGGGCACGGCCACCCTCA

GAGCACAACACAGAATTCCTCAATGTGCCTGACCAGGGTGTGGCAGGCATGCAGAGGAAG

CGCAGCGTTGGGCAGAGACCTGTGCCTGGTGTGGGCCGACCCAAGCCCCAACCACGTGTA

CATGGCCCAAGGTGCCGGGCACTATACCAGTACATAGGCCAAGATGTCGATGAGTTGAGC

TTCAATGTGAATGAAGTCATCGAGATCCTCATGGAAGATTCTTCAGGTTGGTGGAAGGGC

CGCCTACATGGCCAGGAGGGTCTTTTCCCCGGAAACTACGTGGAGAAGATC

>CL25.Contig4_A-W 52 321 minus strand PREDICTED: myosin-If [Sus scrofa]

CAGCTGTTGGAAGGGGCCTCGCAGGAGCAGCGCCAGAACCTGGGGCTCATGACACCCGAC

TACTATTACTACCTCAACCAGTCAGACACCTACAAGGTGGATGGCACCGATGACAGGAGC

GACTTCAGTGAGACCCTGAGTGCCATGCAAGTCATTGGGATCCCACCCAGCGTCCAGCAG

CTGGTTCTGCAGCTGGTGGCAGGGATCCTACACCTGGGGAACATCAGTTTCTGTGAAGAG

GGGAATTACGCCCAGGTGGAGAGTGCAGAC

>CL25.Contig5_A-W 52 1218 minus strand PREDICTED: myosin-If [Sus scrofa]

CAGCTGTTGGAAGGGGCCTCGCAGGAGCAGCGCCAGAACCTGGGGCTCATGACACCCGAC

TACTATTACTACCTCAACCAGTCAGACACCTACAAGGTGGATGGCACCGATGACAGGAGC

GACTTCAGTGAGACCCTGAGTGCCATGCAAGTCATTGGGATCCCACCCAGCGTCCAGCAG

CTGGTTCTGCAGCTGGTGGCAGGGATCCTACACCTGGGGAACATCAGTTTCTGTGAAGAG

GGGAATTACGCCCAGGTGGAGAGTGCAGACCTCCTGGCCTTTCCTGCCTACCTGCTGGGC

ATTGACAGCGGGCGGCTGCAGGAGAAGCTGACCAGCCGCAAGATGGACAGCCGCTGGGGC

GGACGCAGTGAGTCCATTAATGTGACCCTCAACGTGGAGCAGGCAGCCTACACCCGTGAC

GCCCTGGCCAAGGGACTCTATGCCCGCCTCTTCGACTTCCTGGTGGAGGCCATCAACCGT

GCGATGCAGAAGCCCCAGGAGGAGTACAGTATCGGTGTCCTTGACATCTATGGCTTCGAG

ATTTTCCAGAAAAATGGTTTTGAGCAGTTCTGCATCAACTTCGTCAATGAAAAGCTGCAA

CAAATCTTCATCGAGCTCACCCTGAAGGCTGAGCAGGAGGAGTATGTGCAGGAGGGCATC

CACTGGACCCCCATCGAGTACTTCAACAACAAAGTCGTCTGTGACCTCATTGAAAACAAG

CTGAGCCCCCCGGGCATCATGAGCGTGCTGGACGACGTGTGCGCCACCATGCACGCCACA

GGCGGAGGTGCCGACCAGACGCTGCTGCAGAAGCTGCAGGCGGCCGTGGGCACCCACGAG

CACTTCAACAGCTGGAGCGCGGGGTTCGTCATCCACCACTACGCCGGCAAAGTCTCCTAT

GACGTCAGCGGCTTCTGCGAGAGGAACCGGGATGTCCTCTTCTCTGACCTTATAGAGCTG

ATGCAGACCAGCGAGCGGGCCTTCCTCCGGATGCTCTTTCCTGAGAAGCTGGATGTGGAC

AAGAAGGGCCGTCCGAGCACTGCTGGCTCCAAGATCAAGAAACAAGCCAACGACCTGGTG

GCCACTCTGAAGAGGTGCACCCCCCACTACATCCGCTGCATCAAACCCAACGAGACCAAG

AGGCCCCGAGACTGGGAAGAGAGCAGG

>CL25.Contig6_A-W 52 321 minus strand PREDICTED: LOW QUALITY PROTEIN: unconventional myosin-If [Callithrix jacchus]

CAGCTGTTGGAAGGGGCCTCGCAGGAGCAGCGCCAGAACCTGGGGCTCATGACACCCGAC

TACTATTACTACCTCAACCAGTCAGACACCTACAAGGTGGATGGCACCGATGACAGGAGC

GACTTCAGTGAGACCCTGAGTGCCATGCAAGTCATTGGGATCCCACCCAGCGTCCAGCAG

CTGGTTCTGCAGCTGGTGGCAGGGATCCTACACCTGGGGAACATCAGTTTCTGTGAAGAG

GGGAATTACGCCCAGGTGGAGAGTGCAGAC

>CL25.Contig7_A-W 52 2712 minus strand myosin IF, isoform CRA_c [Homo sapiens]

CAGCTGTTGGAAGGGGCCTCGCAGGAGCAGCGCCAGAACCTGGGGCTCATGACACCCGAC

TACTATTACTACCTCAACCAGTCAGACACCTACAAGGTGGATGGCACCGATGACAGGAGC

GACTTCAGTGAGACCCTGAGTGCCATGCAAGTCATTGGGATCCCACCCAGCGTCCAGCAG

CTGGTTCTGCAGCTGGTGGCAGGGATCCTACACCTGGGGAACATCAGTTTCTGTGAAGAG

GGGAATTACGCCCAGGTGGAGAGTGCAGACCTCCTGGCCTTTCCTGCCTACCTGCTGGGC

ATTGACAGCGGGCGGCTGCAGGAGAAGCTGACCAGCCGCAAGATGGACAGCCGCTGGGGC

GGACGCAGTGAGTCCATTAATGTGACCCTCAACGTGGAGCAGGCAGCCTACACCCGTGAC

GCCCTGGCCAAGGGACTCTATGCCCGCCTCTTCGACTTCCTGGTGGAGGCCATCAACCGT

GCGATGCAGAAGCCCCAGGAGGAGTACAGTATCGGTGTCCTTGACATCTATGGCTTCGAG

ATTTTCCAGAAAAATGGTTTTGAGCAGTTCTGCATCAACTTCGTCAATGAAAAGCTGCAA

CAAATCTTCATCGAGCTCACCCTGAAGGCTGAGCAGGAGGAGTATGTGCAGGAGGGCATC

CACTGGACCCCCATCGAGTACTTCAACAACAAAGTCGTCTGTGACCTCATTGAAAACAAG

CTGAGCCCCCCGGGCATCATGAGCGTGCTGGACGACGTGTGCGCCACCATGCACGCCACA

GGCGGAGGTGCCGACCAGACGCTGCTGCAGAAGCTGCAGGCGGCCGTGGGCACCCACGAG

CACTTCAACAGCTGGAGCGCGGGGTTCGTCATCCACCACTACGCCGGCAAAGTCTCCTAT

GACGTCAGCGGCTTCTGCGAGAGGAACCGGGATGTCCTCTTCTCTGACCTTATAGAGCTG

ATGCAGACCAGCGAGCGGGCCTTCCTCCGGATGCTCTTTCCTGAGAAGCTGGATGTGGAC

AAGAAGGGCCGTCCGAGCACTGCTGGCTCCAAGATCAAGAAACAAGCCAACGACCTGGTG

GCCACTCTGAAGAGGTGCACCCCCCACTACATCCGCTGCATCAAACCCAACGAGACCAAG

AGGCCCCGAGACTGGGAAGAGAGCAGGGTCAAGCACCAGGTGGAGTACCTGGGCCTGCGG

GAGAACATCCGGGTGCGCAGGGCCGGCTTCGCCTACCGCCGCCAGTTCTCCAAGTTCCTG

CAGAGGTACGCCATCCTGACCCCTGAGACCTGGCCACGGTGGCGTGGGGACGAACGTCAG

GGTGTCCAGCACCTGCTTCGGGCGGTCAACATGGAGCCGGACCAGTACCAGATGGGGAGC

ACCAAGGTCTTCGTCAAGAACCCAGAGTCGCTTTTCCTCCTGGAGGAGATGCGAGAGCGG

AAGTTCGACGGCTTTGCCCGCACCATTCAGAAGGCCTGGAGGCGCCACGTGGCGGTACGG

AAGTACCAAGAGATGCGGGAGGAAGCTTCAAACATCCTGCTCAACAAGAAGGAACGGAGG

AGGAACAGCATTAATCGGAACTTCGTCGGGGACTACCTGGGGCTGGAGGAACGGCCAGAG

CTACGTCAGTTCCTGGGCAAGAGGGAGCGTGTGGACTTTGCAGACTCGGTCACCAAGTAT

GACCGGCGCTTCAAGCCCATCAAGCGGGACTTGATCCTGACCCCCAAGTGTGTGTATGTG

ATTGGGCGAGAGAAGGTGAAGAAAGGACCAGAGAAGGGCCAAGTGCGTGAGGTCCTGAAG

AAGAAGCTGGAGATTCAGGCCCTGCGGGGAGTCTCCCTCAGCACGCGACAGGACGACTTC

TTCATCCTCCAAGAGGACGCGGCTGACAGCTTCCTGGAGAGCGTCTTCAAGACCGAGTTC

GTCAGCCTTCTGTGCAAGCGCTTCGAGGAGGCGGCACGGAGGCCCCTGCCCCTTACCTTC

AGCGACACACTACAGTTCCGGGTGAAGAAGGAGGGCTGGGGCGGAGGCAGCACTCGCAGC

GTCACCTTTTCCCGCGGCTCAGGAGACATAGCGGTACTCAAGGCTGGCGGCAAAACCCTC

ACGGTCAGCGTGGGCGACGGACTGCCCAAGAGCTCCAAGCCTACACGCAAGGGAATGGCC

CAGGGAAGACCCCGAAGGTCAGCCCCGGCCCCTACCCGGGCGGCCCCTGGCCCCCCCAGA

GGCCTGGACCGAAATGGTGTGCCTCCCTCTGCCAGAGGGGGGCCCCTGCCCCTGGAAATC

ACATCTGGAGGGGGCTCCCAGCGGCCTCCCCGGGGTCCTCCATCCTCAACCCTGGGGTCC

AGTAGACGTCCCCGGGCACGGCCACCCTCAGAGCACAACACAGAATTCCTCAATGTGCCT

GACCAGGGTGTGGCAGGCATGCAGAGGAAGCGCAGCGTTGGGCAGAGACCTGTGCCTGGT

GTGGGCCGACCCAAGCCCCAACCACGTGTACATGGCCCAAGGTGCCGGGCACTATACCAG

TACATAGGCCAAGATGTCGATGAGTTGAGCTTCAATGTGAATGAAGTCATCGAGATCCTC

ATGGAAGATTCTTCAGGTTGGTGGAAGGGCCGCCTACATGGCCAGGAGGGTCTTTTCCCC

GGAAACTACGTGGAGAAGATC

>CL25.Contig8_A-W 1186 2082 minus strand PREDICTED: myosin-If [Sus scrofa]

GTCCTGGCCTTTCCTGCCTACCTGCTGGGCATTGACAGCGGGCGGCTGCAGGAGAAGCTG

ACCAGCCGCAAGATGGACAGCCGCTGGGGCGGACGCAGTGAGTCCATTAATGTGACCCTC

AACGTGGAGCAGGCAGCCTACACCCGTGACGCCCTGGCCAAGGGACTCTATGCCCGCCTC

TTCGACTTCCTGGTGGAGGCCATCAACCGTGCGATGCAGAAGCCCCAGGAGGAGTACAGT

ATCGGTGTCCTTGACATCTATGGCTTCGAGATTTTCCAGAAAAATGGTTTTGAGCAGTTC

TGCATCAACTTCGTCAATGAAAAGCTGCAACAAATCTTCATCGAGCTCACCCTGAAGGCT

GAGCAGGAGGAGTATGTGCAGGAGGGCATCCACTGGACCCCCATCGAGTACTTCAACAAC

AAAGTCGTCTGTGACCTCATTGAAAACAAGCTGAGCCCCCCGGGCATCATGAGCGTGCTG

GACGACGTGTGCGCCACCATGCACGCCACAGGCGGAGGTGCCGACCAGACGCTGCTGCAG

AAGCTGCAGGCGGCCGTGGGCACCCACGAGCACTTCAACAGCTGGAGCGCGGGGTTCGTC

ATCCACCACTACGCCGGCAAAGTCTCCTATGACGTCAGCGGCTTCTGCGAGAGGAACCGG

GATGTCCTCTTCTCTGACCTTATAGAGCTGATGCAGACCAGCGAGCGGGCCTTCCTCCGG

ATGCTCTTTCCTGAGAAGCTGGATGTGGACAAGAAGGGCCGTCCGAGCACTGCTGGCTCC

AAGATCAAGAAACAAGCCAACGACCTGGTGGCCACTCTGAAGAGGTGCACCCCCCACTAC

ATCCGCTGCATCAAACCCAACGAGACCAAGAGGCCCCGAGACTGGGAAGAGAGCAGG

>CL25.Contig9_A-W 1186 2082 minus strand PREDICTED: myosin-If [Sus scrofa]

GTCCTGGCCTTTCCTGCCTACCTGCTGGGCATTGACAGCGGGCGGCTGCAGGAGAAGCTG

ACCAGCCGCAAGATGGACAGCCGCTGGGGCGGACGCAGTGAGTCCATTAATGTGACCCTC

AACGTGGAGCAGGCAGCCTACACCCGTGACGCCCTGGCCAAGGGACTCTATGCCCGCCTC

TTCGACTTCCTGGTGGAGGCCATCAACCGTGCGATGCAGAAGCCCCAGGAGGAGTACAGT

ATCGGTGTCCTTGACATCTATGGCTTCGAGATTTTCCAGAAAAATGGTTTTGAGCAGTTC

TGCATCAACTTCGTCAATGAAAAGCTGCAACAAATCTTCATCGAGCTCACCCTGAAGGCT

GAGCAGGAGGAGTATGTGCAGGAGGGCATCCACTGGACCCCCATCGAGTACTTCAACAAC

AAAGTCGTCTGTGACCTCATTGAAAACAAGCTGAGCCCCCCGGGCATCATGAGCGTGCTG

GACGACGTGTGCGCCACCATGCACGCCACAGGCGGAGGTGCCGACCAGACGCTGCTGCAG

AAGCTGCAGGCGGCCGTGGGCACCCACGAGCACTTCAACAGCTGGAGCGCGGGGTTCGTC

ATCCACCACTACGCCGGCAAAGTCTCCTATGACGTCAGCGGCTTCTGCGAGAGGAACCGG

GATGTCCTCTTCTCTGACCTTATAGAGCTGATGCAGACCAGCGAGCGGGCCTTCCTCCGG

ATGCTCTTTCCTGAGAAGCTGGATGTGGACAAGAAGGGCCGTCCGAGCACTGCTGGCTCC

AAGATCAAGAAACAAGCCAACGACCTGGTGGCCACTCTGAAGAGGTGCACCCCCCACTAC

ATCCGCTGCATCAAACCCAACGAGACCAAGAGGCCCCGAGACTGGGAAGAGAGCAGG

>CL25.Contig10_A-W 2385 3440 minus strand PREDICTED: myosin-If-like [Cavia porcellus]

AGGGTCAAGCACCAGGTGGAGTACCTGGGCCTGCGGGAGAACATCCGGGTGCGCAGGGCC

GGCTTCGCCTACCGCCGCCAGTTCTCCAAGTTCCTGCAGAGGTACGCCATCCTGACCCCT

GAGACCTGGCCACGGTGGCGTGGGGACGAACGTCAGGGTGTCCAGCACCTGCTTCGGGCG

GTCAACATGGAGCCGGACCAGTACCAGATGGGGAGCACCAAGGTCTTCGTCAAGAACCCA

GAGTCGCTTTTCCTCCTGGAGGAGATGCGAGAGCGGAAGTTCGACGGCTTTGCCCGCACC

ATTCAGAAGGCCTGGAGGCGCCACGTGGCGGTACGGAAGTACCAAGAGATGCGGGAGGAA

GCTTCAAACATCCTGCTCAACAAGAAGGAACGGAGGAGGAACAGCATTAATCGGAACTTC

GTCGGGGACTACCTGGGGCTGGAGGAACGGCCAGAGCTACGTCAGTTCCTGGGCAAGAGG

GAGCGTGTGGACTTTGCAGACTCGGTCACCAAGTATGACCGGCGCTTCAAGCCCATCAAG

CGGGACTTGATCCTGACCCCCAAGTGTGTGTATGTGATTGGGCGAGAGAAGGTGAAGAAA

GGACCAGAGAAGGGCCAAGTGCGTGAGGTCCTGAAGAAGAAGCTGGAGATTCAGGCCCTG

CGGGGAGTCTCCCTCAGCACGCGACAGGACGACTTCTTCATCCTCCAAGAGGACGCGGCT

GACAGCTTCCTGGAGAGCGTCTTCAAGACCGAGTTCGTCAGCCTTCTGTGCAAGCGCTTC

GAGGAGGCGGCACGGAGGCCCCTGCCCCTTACCTTCAGCGACACACTACAGTTCCGGGTG

AAGAAGGAGGGCTGGGGCGGAGGCAGCACTCGCAGCGTCACCTTTTCCCGCGGCTCAGGA

GACATAGCGGTACTCAAGGCTGGCGGCAAAACCCTCACGGTCAGCGTGGGCGACGGACTG

CCCAAGAGCTCCAAGCCTACACGCAAGGGAATGGCCCAGGGAAGACCCCGAAGGTCAGCC

CCGGCCCCTACCCGGGCGGCCCCTGGCCCCCCCAGA

>CL25.Contig11_A-W 132 1034 minus strand PREDICTED: myosin-If [Sus scrofa]

ATGGGCAGCAAGGAGCGCTTCCACTGGCAAAGCCACAACGTGAAGCAAAGCGGCGTGGAC

GACATGGTGCTGCTGCCCCAGATCACCGAGGACGCCATCGTGGGCAACCTGCGCAAGCGC

TTCATGGACGACTACATCTTCACCTACATCGGCTCGGTGCTCATCTCCGTAAATCCCTTC

AAGCAGATGCCCTACTTCACTGACCGTGAGATCGATCTCTACCAGGGCGCGGCCCAGTAC

GAGAACCCCCCGCACATCTACGCCCTCACAGACAACATGTACCGCAACATGCTCATCGAC

TGTGAGAACCAGTGCGTCATCATCAGTGGAGAGAGTGGAGCTGGGAAGACAGTGGCCGCA

AAATACATCATGGGCTACATCTCCAAGGTGTCTGGCGGGGGAGAGAAGGTCCAGCACGTC

AAGGACATCATCCTGCAGTCCAACCCGCTGCTGGAGGCCTTCGGCAATGCCAAAACCGTG

CGCAACAACAATTCCAGCCGATTTGGCAAGTACTTTGAGATCCAGTTTAGCCGTGGTGGG

GAGCCAGATGGGGGCAAGATCTCCAACTTCTTGCTAGAGAAGTCCCGCGTGGTCATGCAG

AATGAAAATGAGAGGAACTTTCACATCTACTACCAGCTGTTGGAAGGGGCCTCGCAGGAG

CAGCGCCAGAACCTGGGGCTCATGACACCCGACTACTATTACTACCTCAACCAGTCAGAC

ACCTACAAGGTGGATGGCACCGATGACAGGAGCGACTTCAGTGAGACCCTGAGTGCCATG

CAAGTCATTGGGATCCCACCCAGCGTCCAGCAGCTGGTTCTGCAGCTGGTGGCAGGGATC

CTACACCTGGGGAACATCAGTTTCTGTGAAGAGGGGAATTACGCCCAGGTGGAGAGTGCA

GAC

>CL25.Contig12_A-W 132 2987 minus strand PREDICTED: myosin-If [Sus scrofa]

ATGGGCAGCAAGGAGCGCTTCCACTGGCAAAGCCACAACGTGAAGCAAAGCGGCGTGGAC

GACATGGTGCTGCTGCCCCAGATCACCGAGGACGCCATCGTGGGCAACCTGCGCAAGCGC

TTCATGGACGACTACATCTTCACCTACATCGGCTCGGTGCTCATCTCCGTAAATCCCTTC

AAGCAGATGCCCTACTTCACTGACCGTGAGATCGATCTCTACCAGGGCGCGGCCCAGTAC

GAGAACCCCCCGCACATCTACGCCCTCACAGACAACATGTACCGCAACATGCTCATCGAC

TGTGAGAACCAGTGCGTCATCATCAGTGGAGAGAGTGGAGCTGGGAAGACAGTGGCCGCA

AAATACATCATGGGCTACATCTCCAAGGTGTCTGGCGGGGGAGAGAAGGTCCAGCACGTC

AAGGACATCATCCTGCAGTCCAACCCGCTGCTGGAGGCCTTCGGCAATGCCAAAACCGTG

CGCAACAACAATTCCAGCCGATTTGGCAAGTACTTTGAGATCCAGTTTAGCCGTGGTGGG

GAGCCAGATGGGGGCAAGATCTCCAACTTCTTGCTAGAGAAGTCCCGCGTGGTCATGCAG

AATGAAAATGAGAGGAACTTTCACATCTACTACCAGCTGTTGGAAGGGGCCTCGCAGGAG

CAGCGCCAGAACCTGGGGCTCATGACACCCGACTACTATTACTACCTCAACCAGTCAGAC

ACCTACAAGGTGGATGGCACCGATGACAGGAGCGACTTCAGTGAGACCCTGAGTGCCATG

CAAGTCATTGGGATCCCACCCAGCGTCCAGCAGCTGGTTCTGCAGCTGGTGGCAGGGATC

CTACACCTGGGGAACATCAGTTTCTGTGAAGAGGGGAATTACGCCCAGGTGGAGAGTGCA

GACCTCCTGGCCTTTCCTGCCTACCTGCTGGGCATTGACAGCGGGCGGCTGCAGGAGAAG

CTGACCAGCCGCAAGATGGACAGCCGCTGGGGCGGACGCAGTGAGTCCATTAATGTGACC

CTCAACGTGGAGCAGGCAGCCTACACCCGTGACGCCCTGGCCAAGGGACTCTATGCCCGC

CTCTTCGACTTCCTGGTGGAGGCCATCAACCGTGCGATGCAGAAGCCCCAGGAGGAGTAC

AGTATCGGTGTCCTTGACATCTATGGCTTCGAGATTTTCCAGAAAAATGGTTTTGAGCAG

TTCTGCATCAACTTCGTCAATGAAAAGCTGCAACAAATCTTCATCGAGCTCACCCTGAAG

GCTGAGCAGGAGGAGTATGTGCAGGAGGGCATCCACTGGACCCCCATCGAGTACTTCAAC

AACAAAGTCGTCTGTGACCTCATTGAAAACAAGCTGAGCCCCCCGGGCATCATGAGCGTG

CTGGACGACGTGTGCGCCACCATGCACGCCACAGGCGGAGGTGCCGACCAGACGCTGCTG

CAGAAGCTGCAGGCGGCCGTGGGCACCCACGAGCACTTCAACAGCTGGAGCGCGGGGTTC

GTCATCCACCACTACGCCGGCAAAGTCTCCTATGACGTCAGCGGCTTCTGCGAGAGGAAC

CGGGATGTCCTCTTCTCTGACCTTATAGAGCTGATGCAGACCAGCGAGCGGGCCTTCCTC

CGGATGCTCTTTCCTGAGAAGCTGGATGTGGACAAGAAGGGCCGTCCGAGCACTGCTGGC

TCCAAGATCAAGAAACAAGCCAACGACCTGGTGGCCACTCTGAAGAGGTGCACCCCCCAC

TACATCCGCTGCATCAAACCCAACGAGACCAAGAGGCCCCGAGACTGGGAAGAGAGCAGG

GTCAAGCACCAGGTGGAGTACCTGGGCCTGCGGGAGAACATCCGGGTGCGCAGGGCCGGC

TTCGCCTACCGCCGCCAGTTCTCCAAGTTCCTGCAGAGGTACGCCATCCTGACCCCTGAG

ACCTGGCCACGGTGGCGTGGGGACGAACGTCAGGGTGTCCAGCACCTGCTTCGGGCGGTC

AACATGGAGCCGGACCAGTACCAGATGGGGAGCACCAAGGTCTTCGTCAAGAACCCAGAG

TCGCTTTTCCTCCTGGAGGAGATGCGAGAGCGGAAGTTCGACGGCTTTGCCCGCACCATT

CAGAAGGCCTGGAGGCGCCACGTGGCGGTACGGAAGTACCAAGAGATGCGGGAGGAAGCT

TCAAACATCCTGCTCAACAAGAAGGAACGGAGGAGGAACAGCATTAATCGGAACTTCGTC

GGGGACTACCTGGGGCTGGAGGAACGGCCAGAGCTACGTCAGTTCCTGGGCAAGAGGGAG

CGTGTGGACTTTGCAGACTCGGTCACCAAGTATGACCGGCGCTTCAAGCCCATCAAGCGG

GACTTGATCCTGACCCCCAAGTGTGTGTATGTGATTGGGCGAGAGAAGGTGAAGAAAGGA

CCAGAGAAGGGCCAAGTGCGTGAGGTCCTGAAGAAGAAGCTGGAGATTCAGGCCCTGCGG

GGAGTCTCCCTCAGCACGCGACAGGACGACTTCTTCATCCTCCAAGAGGACGCGGCTGAC

AGCTTCCTGGAGAGCGTCTTCAAGACCGAGTTCGTCAGCCTTCTGTGCAAGCGCTTCGAG

GAGGCGGCACGGAGGCCCCTGCCCCTTACCTTCAGCGACACACTACAGTTCCGGGTGAAG

AAGGAGGGCTGGGGCGGAGGCAGCACTCGCAGCGTCACCTTTTCCCGCGGCTCAGGAGAC

ATAGCGGTACTCAAGGCTGGCGGCAAAACCCTCACGGTCAGCGTGGGCGACGGACTGCCC

AAGAGCTCCAAGCCTACACGCAAGGGAATGGCCCAGGGAAGACCCCGAAGGTCAGCCCCG

GCCCCTACCCGGGCGGCCCCTGGCCCCCCCAGAGGT

>CL25.Contig13_A-W 52 321 minus strand PREDICTED: myosin-If [Sus scrofa]

CAGCTGTTGGAAGGGGCCTCGCAGGAGCAGCGCCAGAACCTGGGGCTCATGACACCCGAC

TACTATTACTACCTCAACCAGTCAGACACCTACAAGGTGGATGGCACCGATGACAGGAGC

GACTTCAGTGAGACCCTGAGTGCCATGCAAGTCATTGGGATCCCACCCAGCGTCCAGCAG

CTGGTTCTGCAGCTGGTGGCAGGGATCCTACACCTGGGGAACATCAGTTTCTGTGAAGAG

GGGAATTACGCCCAGGTGGAGAGTGCAGAC

>CL25.Contig14_A-W 1672 2727 minus strand PREDICTED: myosin-If-like [Cavia porcellus]

AGGGTCAAGCACCAGGTGGAGTACCTGGGCCTGCGGGAGAACATCCGGGTGCGCAGGGCC

GGCTTCGCCTACCGCCGCCAGTTCTCCAAGTTCCTGCAGAGGTACGCCATCCTGACCCCT

GAGACCTGGCCACGGTGGCGTGGGGACGAACGTCAGGGTGTCCAGCACCTGCTTCGGGCG

GTCAACATGGAGCCGGACCAGTACCAGATGGGGAGCACCAAGGTCTTCGTCAAGAACCCA

GAGTCGCTTTTCCTCCTGGAGGAGATGCGAGAGCGGAAGTTCGACGGCTTTGCCCGCACC

ATTCAGAAGGCCTGGAGGCGCCACGTGGCGGTACGGAAGTACCAAGAGATGCGGGAGGAA

GCTTCAAACATCCTGCTCAACAAGAAGGAACGGAGGAGGAACAGCATTAATCGGAACTTC

GTCGGGGACTACCTGGGGCTGGAGGAACGGCCAGAGCTACGTCAGTTCCTGGGCAAGAGG

GAGCGTGTGGACTTTGCAGACTCGGTCACCAAGTATGACCGGCGCTTCAAGCCCATCAAG

CGGGACTTGATCCTGACCCCCAAGTGTGTGTATGTGATTGGGCGAGAGAAGGTGAAGAAA

GGACCAGAGAAGGGCCAAGTGCGTGAGGTCCTGAAGAAGAAGCTGGAGATTCAGGCCCTG

CGGGGAGTCTCCCTCAGCACGCGACAGGACGACTTCTTCATCCTCCAAGAGGACGCGGCT

GACAGCTTCCTGGAGAGCGTCTTCAAGACCGAGTTCGTCAGCCTTCTGTGCAAGCGCTTC

GAGGAGGCGGCACGGAGGCCCCTGCCCCTTACCTTCAGCGACACACTACAGTTCCGGGTG

AAGAAGGAGGGCTGGGGCGGAGGCAGCACTCGCAGCGTCACCTTTTCCCGCGGCTCAGGA

GACATAGCGGTACTCAAGGCTGGCGGCAAAACCCTCACGGTCAGCGTGGGCGACGGACTG

CCCAAGAGCTCCAAGCCTACACGCAAGGGAATGGCCCAGGGAAGACCCCGAAGGTCAGCC

CCGGCCCCTACCCGGGCGGCCCCTGGCCCCCCCAGA

>CL25.Contig15_A-W 52 321 minus strand PREDICTED: myosin-If [Sus scrofa]

CAGCTGTTGGAAGGGGCCTCGCAGGAGCAGCGCCAGAACCTGGGGCTCATGACACCCGAC

TACTATTACTACCTCAACCAGTCAGACACCTACAAGGTGGATGGCACCGATGACAGGAGC

GACTTCAGTGAGACCCTGAGTGCCATGCAAGTCATTGGGATCCCACCCAGCGTCCAGCAG

CTGGTTCTGCAGCTGGTGGCAGGGATCCTACACCTGGGGAACATCAGTTTCTGTGAAGAG

GGGAATTACGCCCAGGTGGAGAGTGCAGAC

>CL25.Contig16_A-W 52 2274 minus strand PREDICTED: myosin-If [Sus scrofa]

CAGCTGTTGGAAGGGGCCTCGCAGGAGCAGCGCCAGAACCTGGGGCTCATGACACCCGAC

TACTATTACTACCTCAACCAGTCAGACACCTACAAGGTGGATGGCACCGATGACAGGAGC

GACTTCAGTGAGACCCTGAGTGCCATGCAAGTCATTGGGATCCCACCCAGCGTCCAGCAG

CTGGTTCTGCAGCTGGTGGCAGGGATCCTACACCTGGGGAACATCAGTTTCTGTGAAGAG

GGGAATTACGCCCAGGTGGAGAGTGCAGACCTCCTGGCCTTTCCTGCCTACCTGCTGGGC

ATTGACAGCGGGCGGCTGCAGGAGAAGCTGACCAGCCGCAAGATGGACAGCCGCTGGGGC

GGACGCAGTGAGTCCATTAATGTGACCCTCAACGTGGAGCAGGCAGCCTACACCCGTGAC

GCCCTGGCCAAGGGACTCTATGCCCGCCTCTTCGACTTCCTGGTGGAGGCCATCAACCGT

GCGATGCAGAAGCCCCAGGAGGAGTACAGTATCGGTGTCCTTGACATCTATGGCTTCGAG

ATTTTCCAGAAAAATGGTTTTGAGCAGTTCTGCATCAACTTCGTCAATGAAAAGCTGCAA

CAAATCTTCATCGAGCTCACCCTGAAGGCTGAGCAGGAGGAGTATGTGCAGGAGGGCATC

CACTGGACCCCCATCGAGTACTTCAACAACAAAGTCGTCTGTGACCTCATTGAAAACAAG

CTGAGCCCCCCGGGCATCATGAGCGTGCTGGACGACGTGTGCGCCACCATGCACGCCACA

GGCGGAGGTGCCGACCAGACGCTGCTGCAGAAGCTGCAGGCGGCCGTGGGCACCCACGAG

CACTTCAACAGCTGGAGCGCGGGGTTCGTCATCCACCACTACGCCGGCAAAGTCTCCTAT

GACGTCAGCGGCTTCTGCGAGAGGAACCGGGATGTCCTCTTCTCTGACCTTATAGAGCTG

ATGCAGACCAGCGAGCGGGCCTTCCTCCGGATGCTCTTTCCTGAGAAGCTGGATGTGGAC

AAGAAGGGCCGTCCGAGCACTGCTGGCTCCAAGATCAAGAAACAAGCCAACGACCTGGTG

GCCACTCTGAAGAGGTGCACCCCCCACTACATCCGCTGCATCAAACCCAACGAGACCAAG

AGGCCCCGAGACTGGGAAGAGAGCAGGGTCAAGCACCAGGTGGAGTACCTGGGCCTGCGG

GAGAACATCCGGGTGCGCAGGGCCGGCTTCGCCTACCGCCGCCAGTTCTCCAAGTTCCTG

CAGAGGTACGCCATCCTGACCCCTGAGACCTGGCCACGGTGGCGTGGGGACGAACGTCAG

GGTGTCCAGCACCTGCTTCGGGCGGTCAACATGGAGCCGGACCAGTACCAGATGGGGAGC

ACCAAGGTCTTCGTCAAGAACCCAGAGTCGCTTTTCCTCCTGGAGGAGATGCGAGAGCGG

AAGTTCGACGGCTTTGCCCGCACCATTCAGAAGGCCTGGAGGCGCCACGTGGCGGTACGG

AAGTACCAAGAGATGCGGGAGGAAGCTTCAAACATCCTGCTCAACAAGAAGGAACGGAGG

AGGAACAGCATTAATCGGAACTTCGTCGGGGACTACCTGGGGCTGGAGGAACGGCCAGAG

CTACGTCAGTTCCTGGGCAAGAGGGAGCGTGTGGACTTTGCAGACTCGGTCACCAAGTAT

GACCGGCGCTTCAAGCCCATCAAGCGGGACTTGATCCTGACCCCCAAGTGTGTGTATGTG

ATTGGGCGAGAGAAGGTGAAGAAAGGACCAGAGAAGGGCCAAGTGCGTGAGGTCCTGAAG

AAGAAGCTGGAGATTCAGGCCCTGCGGGGAGTCTCCCTCAGCACGCGACAGGACGACTTC

TTCATCCTCCAAGAGGACGCGGCTGACAGCTTCCTGGAGAGCGTCTTCAAGACCGAGTTC

GTCAGCCTTCTGTGCAAGCGCTTCGAGGAGGCGGCACGGAGGCCCCTGCCCCTTACCTTC

AGCGACACACTACAGTTCCGGGTGAAGAAGGAGGGCTGGGGCGGAGGCAGCACTCGCAGC

GTCACCTTTTCCCGCGGCTCAGGAGACATAGCGGTACTCAAGGCTGGCGGCAAAACCCTC

ACGGTCAGCGTGGGCGACGGACTGCCCAAGAGCTCCAAGCCTACACGCAAGGGAATGGCC

CAGGGAAGACCCCGAAGGTCAGCCCCGGCCCCTACCCGGGCGGCCCCTGGCCCCCCCAGA

GGT

>CL26.Contig1_A-W 1 894 minus strand lysine-specific demethylase 5C isoform 1 [Macaca mulatta]

TGGAATCTGAATGTGATGCCAGTGTTGGAGCAGTCTGTATTATGCCACATCAATGCAGAT

ATCTCTGGCATGAAGGTGCCCTGGCTCTATGTGGGCATGGTCTTCTCAGCCTTTTGCTGG

CATATTGAGGATCACTGGAGTTACTCCATTAACTACCTCCACTGGGGTGAGCCGAAGACC

TGGTATGGGGTACCCTCACTTGCAGCAGAACATTTGGAAGAAGTAATGAAGAAGCTGACA

CCTGAGCTCTTTGATAGCCAGCCTGACCTCTTGCACCAACTTGTCACTCTCATGAATCCG

AACACTCTCATGTCCCATGGTGTGCCGGTTGTCCGTACAAACCAGTGTGCAGGAGAATTT

GTCATCACCTTTCCCCGTGCTTACCATAGTGGCTTCAACCAAGGCTACAACTTTGCTGAG

GCTGTCAACTTTTGTACTGCTGACTGGCTACCTGCTGGACGCCAGTGTATTGAACACTAC

CGCCGGCTTCGGCGCTATTGTGTCTTCTCCCATGAGGAGCTTATTTGCAAGATGGCTGCC

TTCCCAGAGAAGTTGGATCTGAATCTAGCAGTGGCTGTGCACAAGGAGATGTTCATTATG

GTTCAGGAGGAGCGACGTCTACGAAAGGCCTTGTTAGAGAAGGGCATCACGGAGGCTGAG

CGAGAGGCTTTTGAGCTGCTTCCAGATGATGAACGTCAGTGCATCAAGTGCAAGACCACG

TGTTTCCTATCAGCTCTAGCATGCTACAACTGCCCAGATGGCCTTGTCTGCCTGTCACAC

ATCAATGATCTTTGCAAGTGTTCAAGTAGCCAGCAGTACCTACGATATCGGTATACCTTG

GATGAGCTACCAACCATGCTGCATAAGCTGAAGGTTAGGGCTGAGTCTTTTGAC

>CL26.Contig2_A-W 1 1107 minus strand PREDICTED: lysine-specific demethylase 5C [Saimiri boliviensis boliviensis]

TGGAATCTGAATGTGATGCCAGTGTTGGAGCAGTCTGTATTATGCCACATCAATGCAGAT

ATCTCTGGCATGAAGGTGCCCTGGCTCTATGTGGGCATGGTCTTCTCAGCCTTTTGCTGG

CATATTGAGGATCACTGGAGTTACTCCATTAACTACCTCCACTGGGGTGAGCCGAAGACC

TGGTATGGGGTACCCTCACTTGCAGCAGAACATTTGGAAGAAGTAATGAAGAAGCTGACA

CCTGAGCTCTTTGATAGCCAGCCTGACCTCTTGCACCAACTTGTCACTCTCATGAATCCG

AACACTCTCATGTCCCATGGTGTGCCGGTTGTCCGTACAAACCAGTGTGCAGGAGAATTT

GTCATCACCTTTCCCCGTGCTTACCATAGTGGCTTCAACCAAGGCTACAACTTTGCTGAG

GCTGTCAACTTTTGCACTGCCGACTGGCTGCCAGCTGGGCGCCAGTGCATTGAGCACTAT

CGCCGGCTCCGAAGGTACTGTGTCTTCTCCCATGAGGAACTCATCTGCAAGATGGCTGCC

TGCCCTGAGAAGCTGGACCTGAACCTGGCAGCAGCTGTGCATAAGGAGATGTTCATCATG

GTGCAAGAGGAGCGGCGTCTACGGAAGGCTCTACTGGAGAAGGGTATCACAGAAGCTGAG

CGAGAGGCTTTTGAGCTGCTCCCAGATGATGAACGCCAATGCATCAAGTGCAAGACTACA

TGCTTCCTTTCTGCCCTGGCCTGCTATGACTGCCCAGATGGCCTTGTCTGCCTTTCCCAT

ATCAATGACCTCTGCAAGTGCTCCAGTAGTCGGCAGTATCTGCGGTATCGGTATACCTTG

GATGAGCTCCCTGCCATGCTGCATAAGCTGAAGGTTCGGGCCGAGTCCTTTGACACCTGG

GCCAACAAAGTGCGAGTGGCCCTGGAGGTGGAGGATGGGCGGAAGCGCAGCCTTGAAGAA

CTGAGAGCACTAGAGTCTGAGGCCCGTGAGCGGAGGTTTCCTAACAGTGAGCTGCTGCAA

CGACTGAAGAATTGCCTGAGTGAGGCAGAAGCTTGCGTGTCCCAGGCTCTGGGGCTGGTC

AGCGGCCAGGAAGCTGGTCCCCACAGG

>CL26.Contig3_A-W 3 482 minus strand lysine-specific demethylase 5D [Canis lupus familiaris] >gi|122138462|sp|Q30DN6.1|KDM5D_CANFA RecName: Full=Lysine-specific demethylase 5D; AltName: Full=Histone demethylase JARID1D; AltName: Full=Jumonji/ARID domain-containing protein 1D; AltName: Full=Protein SmcY >gi|76162896|gb|ABA40834.1| JARID1D [Canis lupus familiaris]

GCTAGCCATATGCAGTTTTAAACACACTTGCAGCTACCTGCTGGACGCCAGTGTATTGAA

CACTACCGCCGGCTTCGGCGCTATTGTGTCTTCTCCCATGAGGAGCTTATTTGCAAGATG

GCTGCCTTCCCAGAGAAGTTGGATCTGAATCTAGCAGTGGCTGTGCACAAGGAGATGTTC

ATTATGGTTCAGGAGGAGCGACGTCTACGAAAGGCCTTGTTAGAGAAGGGCATCACGGAG

GCTGAGCGAGAGGCTTTTGAGCTGCTTCCAGATGATGAACGTCAGTGCATCAAGTGCAAG

ACCACGTGTTTCCTATCAGCTCTAGCATGCTACAACTGCCCAGATGGCCTTGTCTGCCTG

TCACACATCAATGATCTTTGCAAGTGTTCAAGTAGCCAGCAGTACCTACGATATCGGTAT

ACCTTGGATGAGCTACCAACCATGCTGCATAAGCTGAAGGTTAGGGCTGAGTCTTTTGAC

>CL27.Contig1_A-W 43 477 minus strand hypothetical protein [Macaca fascicularis]

CCTGGGCCTGCATCTCCAGTGGCCTCACCCCGAGTCCCCAGGTCCTCCTTCTGTCTCCAT

GCACAGAGCCGAGTGCCTGCCTCTGGCCCGTCCGCCACCTCTGCCCTGAAGGGGACACTC

AGAGTCCCCTCGGGTGTGGGGTGACGGGAGCGCCACCCCTCCAAGCCAGTGACTGCCACC

TAGCCCTGGGCACGGTGTGGGGGGGTGGGGACACTGGTCAGCTGACCTGGCCCACCCTCC

AGGTGCATCCACGGGCTCCTGCAGTGTCCCTCGCACCCCGTGCTGGGGCCGGGCAGGGTC

CCCCGATCCCAGGCTGTCCCCACACCGCCCCCTGCAGCAGAGCCCAGGCCCAACAGGGTC

CCTTCCCCGTGGTGACCTGGATCTGACCCAAGGGCCCCAAAGCCACCTATGCCCAGCGCG

GGCCGGGGACCTCCA

>CL28.Contig1_A-W 712 1173 minus strand Diacylglycerol kinase alpha OS=Sus scrofa GN=DGKA PE=1 SV=1

GGATTTATACACAATCCAGGTCTCCTTCCCTCTGCTTTGCTCCCCTCCCAGGCATCTGAA

CAGGAACGCAAACACAGCAAAACAAGCCAAAAGACCACAGATGACTCAAATTTGTGCATC

TCTGAAGCTCTGCGGATCAACCCTGTTTCTAACACCCACCCACTTCTAGTCTTTGTCAAT

CCCAAGAGTGGTGGAAAGCAGGGACTGAGGGTCCTTTGGAAGTTCCAATATATACTAAAC

CCTCGGCAGGTGATCAACCTCCTAAAGGATGGTCCTGAACAAGGGCTCAGATTCTTCAAA

GATGTTCCTGACAGCCGTATTTTGGTGTGTGGTGGAGATGGCACAGTAGGCTGGATTCTA

GAGGCCATTGACAAAATCAACTTGCCTGTTGTACCTCCTGTTGCTGTGTTGCCCCTGGGC

ACTGGAAATGATCTGGCTCGTTGCCTAAGGTGGGGAGGAGGT

>CL28.Contig2_A-W 1128 1274 minus strand diacylglycerol kinase, alpha 80kDa, isoform CRA_b [Homo sapiens]

GTCCAATCACACGTGTGGGTGCGAGGAGGCTGTGAGTCCGGGCGCTGTGACCGCTGTCAG

AAAAAGATCCGGACCTACCACAGTCTAACCGGGCTGCATTGCGTATGGTGCCACCTAGAG

GTGTGAGAGCTCTCCGTCCAGTCTATA

>CL28.Contig3_A-W 619 789 minus strand diacylglycerol kinase, alpha 80kDa, isoform CRA_b [Homo sapiens]

ATGACACGAGTGGCTGAATATCTGGATTGGGATGTGTCAGAGCTGAGGCCGATTTTTCAG

GAGATGATGAAAGAGATTGACTATGATGGCAGTGGCTCTGTCTCCCTAGCTGAGTGGGTC

CGAGCTGGGGCCACTACGGTGCCGCTGCTAGTACTGCTGGGGCTGGAGATG

>CL28.Contig4_A-W 619 996 minus strand diacylglycerol kinase, alpha 80kDa, isoform CRA_b [Homo sapiens]

ATGACACGAGTGGCTGAATATCTGGATTGGGATGTGTCAGAGCTGAGGCCGATTTTTCAG

GAGATGATGAAAGAGATTGACTATGATGGCAGTGGCTCTGTCTCCCTAGCTGAGTGGGTC

CGAGCTGGGGCCACTACGGTGCCGCTGCTAGTACTGCTGGGGCTGGAGATGACTCTGAAG

GATGATGGGCAGCACATGTGGAGACCCAAGAGATTTCCCAGACCAGTCTACTGCAACCTG

TGCGAGTTGAGCATTGGTCTTGGCAAACAGGGGCTGAGCTGTAACCTCTGTAAGTACACT

GTTCACGACCAGTGTGCCATGAAGGCCCTGCCTTGTGAAGTCAGCACCTATGCCAAGTCT

CGGAAAGACATTGGTGTG

>CL28.Contig5_A-W 517 1215 minus strand Diacylglycerol kinase alpha OS=Homo sapiens GN=DGKA PE=1 SV=3

GGATTTATACACAATCCAGGTCTCCTTCCCTCTGCTTTGCTCCCCTCCCAGGCATCTGAA

CAGGAACGCAAACACAGCAAAACAAGCCAAAAGACCACAGATGACTCAAATTTGTGCATC

TCTGAAGCTCTGCGGATCAACCCTGTTTCTAACACCCACCCACTTCTAGTCTTTGTCAAT

CCCAAGAGTGGTGGAAAGCAGGGACTGAGGGTCCTTTGGAAGTTCCAATATATACTAAAC

CCTCGGCAGGTGATCAACCTCCTAAAGGATGGTCCTGAACAAGGGCTCAGATTCTTCAAA

GATGTTCCTGACAGCCGTATTTTGGTGTGTGGTGGAGATGGCACAGTAGGCTGGATTCTA

GAGGCCATTGACAAAATCAACTTGCCTGTTGTACCTCCTGTTGCTGTGTTGCCCCTGGGC

ACTGGAAATGATCTGGCTCGTTGCCTAAGGTGGGGAGGAGGGTATGAAGGAGAGAATTTG

GCAAAGATTCTCAGGGAATTAGAGATGAGTACGGTGGTATATATAGATCGATGGTCCCTG

GAAGTGATACCCCAACAAACTGAAGAAAAGACTGATCCAGTCCCCTTTCAAATCATCAAT

AACTACTTCTCCATTGGTGTGGATGCTTCTATTGCTCACCGATTTCACATCATGCGAGAG

AAATATCCTGAGAAGTTCAATAGCAGGTGAAGGAAAAAG

>CL28.Contig6_A-W 1 120 minus strand PREDICTED: diacylglycerol kinase alpha isoform 1 [Nomascus leucogenys] >gi|332207605|ref|XP_003252886.1| PREDICTED: diacylglycerol kinase alpha isoform 2 [Nomascus leucogenys] >gi|332207607|ref|XP_003252887.1| PREDICTED: diacylglycerol kinase alpha isoform 3 [Nomascus leucogenys]

ATTTTTCAGGAGATGATGAAAGAGATTGACTATGATGGCAGTGGCTCTGTCTCCCTAGCT

GAGTGGGTCCGAGCTGGGGCCACTACGGTGCCGCTGCTAGTACTGCTGGGGCTGGAGATG

>CL28.Contig7_A-W 712 1353 minus strand Diacylglycerol kinase alpha OS=Homo sapiens GN=DGKA PE=1 SV=3

GGATTTATACACAATCCAGGTCTCCTTCCCTCTGCTTTGCTCCCCTCCCAGGCATCTGAA

CAGGAACGCAAACACAGCAAAACAAGCCAAAAGACCACAGATGACTCAAATTTGTGCATC

TCTGAAGCTCTGCGGATCAACCCTGTTTCTAACACCCACCCACTTCTAGTCTTTGTCAAT

CCCAAGAGTGGTGGAAAGCAGGGACTGAGGGTCCTTTGGAAGTTCCAATATATACTAAAC

CCTCGGCAGGTGATCAACCTCCTAAAGGATGGTCCTGAACAAGGGCTCAGATTCTTCAAA

GATGTTCCTGACAGCCGTATTTTGGTGTGTGGTGGAGATGGCACAGTAGGCTGGATTCTA

GAGGCCATTGACAAAATCAACTTGCCTGTTGTACCTCCTGTTGCTGTGTTGCCCCTGGGC

ACTGGAAATGATCTGGCTCGTTGCCTAAGGTGGGGAGGAGGGTATGAAGGAGAGAATTTG

GCAAAGATTCTCAGGGAATTAGAGATGAGTACGGTGGTATATATAGATCGATGGTCCCTG

GAAGTGATACCCCAACAAACTGAAGAAAAGACTGATCCAGTCCCCTTTCAAATCATCAAT

AACTACTTCTCCATTGGTGTGGTCAGTAGACTGGGGCACAGA

>CL28.Contig8_A-W 416 1048 minus strand Diacylglycerol kinase alpha OS=Homo sapiens GN=DGKA PE=1 SV=3

CAATCCCACCTCCTTTACAGCTGTAGTTGTCCTCGCTCCCAAGGGCTCTCTTTCCAATCC

AGACCCAAGTTGAAAGGTGGTAGAGTCCCTTGCATGATCCTGTATGCCCATCTTGTCTTT

CAGATTCATGATGACTGCCTGCAAGCCGTGGGCTCTGAGTGTGACTGTGGGCTGCTCCGG

GATCACATCCTGCCTCCATCTTCCATCTATCCCAGCGTCCTGGCATCTGAACAGGAACGC

AAACACAGCAAAACAAGCCAAAAGACCACAGATGACTCAAATTTGTGCATCTCTGAAGCT

CTGCGGATCAACCCTGTTTCTAACACCCACCCACTTCTAGTCTTTGTCAATCCCAAGAGT

GGTGGAAAGCAGGGACTGAGGGTCCTTTGGAAGTTCCAATATATACTAAACCCTCGGCAG

GTGATCAACCTCCTAAAGGATGGTCCTGAACAAGGGCTCAGATTCTTCAAAGATGTTCCT

GACAGCCGTATTTTGGTGTGTGGTGGAGATGGCACAGTAGGCTGGATTCTAGAGGCCATT

GACAAAATCAACTTGCCTGTTGTACCTCCTGTTGCTGTGTTGCCCCTGGGCACTGGAAAT

GATCTGGCTCGTTGCCTAAGGTGGGGAGGAGGT

>CL28.Contig9_A-W 1 327 minus strand PREDICTED: diacylglycerol kinase alpha isoform 1 [Nomascus leucogenys] >gi|332207605|ref|XP_003252886.1| PREDICTED: diacylglycerol kinase alpha isoform 2 [Nomascus leucogenys] >gi|332207607|ref|XP_003252887.1| PREDICTED: diacylglycerol kinase alpha isoform 3 [Nomascus leucogenys]

ATTTTTCAGGAGATGATGAAAGAGATTGACTATGATGGCAGTGGCTCTGTCTCCCTAGCT

GAGTGGGTCCGAGCTGGGGCCACTACGGTGCCGCTGCTAGTACTGCTGGGGCTGGAGATG

ACTCTGAAGGATGATGGGCAGCACATGTGGAGACCCAAGAGATTTCCCAGACCAGTCTAC

TGCAACCTGTGCGAGTTGAGCATTGGTCTTGGCAAACAGGGGCTGAGCTGTAACCTCTGT

AAGTACACTGTTCACGACCAGTGTGCCATGAAGGCCCTGCCTTGTGAAGTCAGCACCTAT

GCCAAGTCTCGGAAAGACATTGGTGTG

>CL28.Contig10_A-W 416 1228 minus strand Diacylglycerol kinase alpha OS=Homo sapiens GN=DGKA PE=1 SV=3

CAATCCCACCTCCTTTACAGCTGTAGTTGTCCTCGCTCCCAAGGGCTCTCTTTCCAATCC

AGACCCAAGTTGAAAGGTGGTAGAGTCCCTTGCATGATCCTGTATGCCCATCTTGTCTTT

CAGATTCATGATGACTGCCTGCAAGCCGTGGGCTCTGAGTGTGACTGTGGGCTGCTCCGG

GATCACATCCTGCCTCCATCTTCCATCTATCCCAGCGTCCTGGCATCTGAACAGGAACGC

AAACACAGCAAAACAAGCCAAAAGACCACAGATGACTCAAATTTGTGCATCTCTGAAGCT

CTGCGGATCAACCCTGTTTCTAACACCCACCCACTTCTAGTCTTTGTCAATCCCAAGAGT

GGTGGAAAGCAGGGACTGAGGGTCCTTTGGAAGTTCCAATATATACTAAACCCTCGGCAG

GTGATCAACCTCCTAAAGGATGGTCCTGAACAAGGGCTCAGATTCTTCAAAGATGTTCCT

GACAGCCGTATTTTGGTGTGTGGTGGAGATGGCACAGTAGGCTGGATTCTAGAGGCCATT

GACAAAATCAACTTGCCTGTTGTACCTCCTGTTGCTGTGTTGCCCCTGGGCACTGGAAAT

GATCTGGCTCGTTGCCTAAGGTGGGGAGGAGGGTATGAAGGAGAGAATTTGGCAAAGATT

CTCAGGGAATTAGAGATGAGTACGGTGGTATATATAGATCGATGGTCCCTGGAAGTGATA

CCCCAACAAACTGAAGAAAAGACTGATCCAGTCCCCTTTCAAATCATCAATAACTACTTC

TCCATTGGTGTGGTCAGTAGACTGGGGCACAGA

>CL28.Contig11_A-W 224 445 minus strand PREDICTED: diacylglycerol kinase beta-like, partial [Monodelphis domestica]

GTCCAATCACACGTGTGGGTGCGAGGAGGCTGTGAGTCCGGGCGCTGTGACCGCTGTCAG

AAAAAGATCCGGACCTACCACAGTCTAACCGGGCTGCATTGCGTATGGTGCCACCTAGAG

ATTCATGATGACTGCCTGCAAGCCGTGGGCTCTGAGTGTGACTGTGGGCTGCTCCGGGAT

CACATCCTGCCTCCATCTTCCATCTATCCCAGCGTCCTGGTG

>CL28.Contig12_A-W 517 1158 minus strand Diacylglycerol kinase alpha OS=Homo sapiens GN=DGKA PE=1 SV=3

GGATTTATACACAATCCAGGTCTCCTTCCCTCTGCTTTGCTCCCCTCCCAGGCATCTGAA

CAGGAACGCAAACACAGCAAAACAAGCCAAAAGACCACAGATGACTCAAATTTGTGCATC

TCTGAAGCTCTGCGGATCAACCCTGTTTCTAACACCCACCCACTTCTAGTCTTTGTCAAT

CCCAAGAGTGGTGGAAAGCAGGGACTGAGGGTCCTTTGGAAGTTCCAATATATACTAAAC

CCTCGGCAGGTGATCAACCTCCTAAAGGATGGTCCTGAACAAGGGCTCAGATTCTTCAAA

GATGTTCCTGACAGCCGTATTTTGGTGTGTGGTGGAGATGGCACAGTAGGCTGGATTCTA

GAGGCCATTGACAAAATCAACTTGCCTGTTGTACCTCCTGTTGCTGTGTTGCCCCTGGGC

ACTGGAAATGATCTGGCTCGTTGCCTAAGGTGGGGAGGAGGGTATGAAGGAGAGAATTTG

GCAAAGATTCTCAGGGAATTAGAGATGAGTACGGTGGTATATATAGATCGATGGTCCCTG

GAAGTGATACCCCAACAAACTGAAGAAAAGACTGATCCAGTCCCCTTTCAAATCATCAAT

AACTACTTCTCCATTGGTGTGGTCAGTAGACTGGGGCACAGA

>CL28.Contig13_A-W 224 853 minus strand Diacylglycerol kinase alpha OS=Homo sapiens GN=DGKA PE=1 SV=3

GTCCAATCACACGTGTGGGTGCGAGGAGGCTGTGAGTCCGGGCGCTGTGACCGCTGTCAG

AAAAAGATCCGGACCTACCACAGTCTAACCGGGCTGCATTGCGTATGGTGCCACCTAGAG

ATTCATGATGACTGCCTGCAAGCCGTGGGCTCTGAGTGTGACTGTGGGCTGCTCCGGGAT

CACATCCTGCCTCCATCTTCCATCTATCCCAGCGTCCTGGCATCTGAACAGGAACGCAAA

CACAGCAAAACAAGCCAAAAGACCACAGATGACTCAAATTTGTGCATCTCTGAAGCTCTG

CGGATCAACCCTGTTTCTAACACCCACCCACTTCTAGTCTTTGTCAATCCCAAGAGTGGT

GGAAAGCAGGGACTGAGGGTCCTTTGGAAGTTCCAATATATACTAAACCCTCGGCAGGTG

ATCAACCTCCTAAAGGATGGTCCTGAACAAGGGCTCAGATTCTTCAAAGATGTTCCTGAC

AGCCGTATTTTGGTGTGTGGTGGAGATGGCACAGTAGGCTGGATTCTAGAGGCCATTGAC

AAAATCAACTTGCCTGTTGTACCTCCTGTTGCTGTGTTGCCCCTGGGCACTGGAAATGAT

CTGGCTCGTTGCCTAAGGTGGGGAGGAGGT

>CL28.Contig14_A-W 416 871 minus strand Diacylglycerol kinase alpha OS=Sus scrofa GN=DGKA PE=1 SV=1

CAATCCCACCTCCTTTACAGCTGTAGTTGTCCTCGCTCCCAAGGGCTCTCTTTCCAATCC

AGACCCAAGTTGAAAGGTGGTAGAGTCCCTTGCATGATCCTGTATGCCCATCTTGTCTTT

CAGATTCATGATGACTGCCTGCAAGCCGTGGGCTCTGAGTGTGACTGTGGGCTGCTCCGG

GATCACATCCTGCCTCCATCTTCCATCTATCCCAGCGTCCTGGCATCTGAACAGGAACGC

AAACACAGCAAAACAAGCCAAAAGACCACAGATGACTCAAATTTGTGCATCTCTGAAGCT

CTGCGGATCAACCCTGTTTCTAACACCCACCCACTTCTAGTCTTTGTCAATCCCAAGAGT

GGTGGAAAGCAGGGACTGAGGGTCCTTTGGAAGTTCCAATATATACTAAACCCTCGGCAG

GTGATCAACCTCCTAAAGGATGGTCCTGAACAAGGG

>CL28.Contig15_A-W 224 676 minus strand Diacylglycerol kinase alpha OS=Homo sapiens GN=DGKA PE=1 SV=3

GTCCAATCACACGTGTGGGTGCGAGGAGGCTGTGAGTCCGGGCGCTGTGACCGCTGTCAG

AAAAAGATCCGGACCTACCACAGTCTAACCGGGCTGCATTGCGTATGGTGCCACCTAGAG

ATTCATGATGACTGCCTGCAAGCCGTGGGCTCTGAGTGTGACTGTGGGCTGCTCCGGGAT

CACATCCTGCCTCCATCTTCCATCTATCCCAGCGTCCTGGCATCTGAACAGGAACGCAAA

CACAGCAAAACAAGCCAAAAGACCACAGATGACTCAAATTTGTGCATCTCTGAAGCTCTG

CGGATCAACCCTGTTTCTAACACCCACCCACTTCTAGTCTTTGTCAATCCCAAGAGTGGT

GGAAAGCAGGGACTGAGGGTCCTTTGGAAGTTCCAATATATACTAAACCCTCGGCAGGTG

ATCAACCTCCTAAAGGATGGTCCTGAACAAGGG

>CL28.Contig16_A-W 86 505 minus strand Diacylglycerol kinase alpha [Heterocephalus glaber]

TCCCCAGACAAAATCAACTTGCCTGTTGTACCTCCTGTTGCTGTGTTGCCCCTGGGCACT

GGAAATGATCTGGCTCGTTGCCTAAGGTGGGGAGGAGGGTATGAAGGAGAGAATTTGGCA

AAGATTCTCAGGGAATTAGAGATGAGTACGGTGGTATATATAGATCGATGGTCCCTGGAA

GTGATACCCCAACAAACTGAAGAAAAGACTGATCCAGTCCCCTTTCAAATCATCAATAAC

TACTTCTCCATTGGTGTGGATGCTTCTATTGCTCACCGATTTCACATCATGCGAGAGAAA

TATCCTGAGAAGTTCAATAGCAGAATGAAGAACAAGCTATGGTACTTCGAATTTGCCACA

TCTGAATCCATCTTCTCAACATGCAAAAAGTTGGAGGAGTCTTTGACAGTTGAGGTATGT

>CL28.Contig17_A-W 3 437 minus strand PREDICTED: diacylglycerol kinase alpha-like [Pan paniscus]

ATTCTGCAGAACCAGAGCAGAGGGTTCTTGGAGATTTGCAGATCTGGGGATAAGAGGAGG

GCTCTGGAAGACTCAGGGCTCAGTGCTAATCTGTCTTCTCTTCCACCTTTAGGGTATGAA

GGAGAGAATTTGGCAAAGATTCTCAGGGAATTAGAGATGAGTACGGTGGTATATATAGAT

CGATGGTCCCTGGAAGTGATACCCCAACAAACTGAAGAAAAGACTGATCCAGTCCCCTTT

CAAATCATCAATAACTACTTCTCCATTGGTGTGGATGCTTCTATTGCTCACCGATTTCAC

ATCATGCGAGAGAAATATCCTGAGAAGTTCAATAGCAGAATGAAGAACAAGCTATGGTAC

TTCGAATTTGCCACATCTGAATCCATCTTCTCAACATGCAAAAAGTTGGAGGAGTCTTTG

ACAGTTGAGGTATGT

>CL28.Contig18_A-W 174 458 minus strand Diacylglycerol kinase alpha OS=Sus scrofa GN=DGKA PE=1 SV=1

GGATTTATACACAATCCAGGTCTCCTTCCCTCTGCTTTGCTCCCCTCCCAGGCATCTGAA

CAGGAACGCAAACACAGCAAAACAAGCCAAAAGACCACAGATGACTCAAATTTGTGCATC

TCTGAAGCTCTGCGGATCAACCCTGTTTCTAACACCCACCCACTTCTAGTCTTTGTCAAT

CCCAAGAGTGGTGGAAAGCAGGGACTGAGGGTCCTTTGGAAGTTCCAATATATACTAAAC

CCTCGGCAGGTGATCAACCTCCTAAAGGATGGTCCTGAACAAGGG

>CL28.Contig19_A-W 1193 1339 minus strand PREDICTED: diacylglycerol kinase alpha isoform 6 [Papio anubis]

GTCCAATCACACGTGTGGGTGCGAGGAGGCTGTGAGTCCGGGCGCTGTGACCGCTGTCAG

AAAAAGATCCGGACCTACCACAGTCTAACCGGGCTGCATTGCGTATGGTGCCACCTAGAG

GTGTGAGAGCTCTCCGTCCAGTCTATA

>CL28.Contig20_A-W 1127 1273 minus strand PREDICTED: diacylglycerol kinase alpha isoform 6 [Papio anubis]

GTCCAATCACACGTGTGGGTGCGAGGAGGCTGTGAGTCCGGGCGCTGTGACCGCTGTCAG

AAAAAGATCCGGACCTACCACAGTCTAACCGGGCTGCATTGCGTATGGTGCCACCTAGAG

GTGTGAGAGCTCTCCGTCCAGTCTATA

>CL28.Contig21_A-W 684 854 minus strand diacylglycerol kinase, alpha 80kDa, isoform CRA_b [Homo sapiens]

ATGACACGAGTGGCTGAATATCTGGATTGGGATGTGTCAGAGCTGAGGCCGATTTTTCAG

GAGATGATGAAAGAGATTGACTATGATGGCAGTGGCTCTGTCTCCCTAGCTGAGTGGGTC

CGAGCTGGGGCCACTACGGTGCCGCTGCTAGTACTGCTGGGGCTGGAGATG

>CL28.Contig22_A-W 1080 1226 minus strand diacylglycerol kinase, alpha 80kDa, isoform CRA_b [Homo sapiens]

GTCCAATCACACGTGTGGGTGCGAGGAGGCTGTGAGTCCGGGCGCTGTGACCGCTGTCAG

AAAAAGATCCGGACCTACCACAGTCTAACCGGGCTGCATTGCGTATGGTGCCACCTAGAG

GTGTGAGAGCTCTCCGTCCAGTCTATA

>CL28.Contig23_A-W 1070 1216 minus strand diacylglycerol kinase, alpha 80kDa, isoform CRA_b [Homo sapiens]

GTCCAATCACACGTGTGGGTGCGAGGAGGCTGTGAGTCCGGGCGCTGTGACCGCTGTCAG

AAAAAGATCCGGACCTACCACAGTCTAACCGGGCTGCATTGCGTATGGTGCCACCTAGAG

GTGTGAGAGCTCTCCGTCCAGTCTATA

>CL28.Contig24_A-W 618 788 minus strand diacylglycerol kinase, alpha 80kDa, isoform CRA_b [Homo sapiens]

ATGACACGAGTGGCTGAATATCTGGATTGGGATGTGTCAGAGCTGAGGCCGATTTTTCAG

GAGATGATGAAAGAGATTGACTATGATGGCAGTGGCTCTGTCTCCCTAGCTGAGTGGGTC

CGAGCTGGGGCCACTACGGTGCCGCTGCTAGTACTGCTGGGGCTGGAGATG

>CL28.Contig25_A-W 684 1061 minus strand diacylglycerol kinase, alpha 80kDa, isoform CRA_b [Homo sapiens]

ATGACACGAGTGGCTGAATATCTGGATTGGGATGTGTCAGAGCTGAGGCCGATTTTTCAG

GAGATGATGAAAGAGATTGACTATGATGGCAGTGGCTCTGTCTCCCTAGCTGAGTGGGTC

CGAGCTGGGGCCACTACGGTGCCGCTGCTAGTACTGCTGGGGCTGGAGATGACTCTGAAG

GATGATGGGCAGCACATGTGGAGACCCAAGAGATTTCCCAGACCAGTCTACTGCAACCTG

TGCGAGTTGAGCATTGGTCTTGGCAAACAGGGGCTGAGCTGTAACCTCTGTAAGTACACT

GTTCACGACCAGTGTGCCATGAAGGCCCTGCCTTGTGAAGTCAGCACCTATGCCAAGTCT

CGGAAAGACATTGGTGTG

>CL28.Contig26_A-W 571 741 minus strand diacylglycerol kinase, alpha 80kDa, isoform CRA_b [Homo sapiens]

ATGACACGAGTGGCTGAATATCTGGATTGGGATGTGTCAGAGCTGAGGCCGATTTTTCAG

GAGATGATGAAAGAGATTGACTATGATGGCAGTGGCTCTGTCTCCCTAGCTGAGTGGGTC

CGAGCTGGGGCCACTACGGTGCCGCTGCTAGTACTGCTGGGGCTGGAGATG

>CL28.Contig27_A-W 561 731 minus strand diacylglycerol kinase, alpha 80kDa, isoform CRA_b [Homo sapiens]

ATGACACGAGTGGCTGAATATCTGGATTGGGATGTGTCAGAGCTGAGGCCGATTTTTCAG

GAGATGATGAAAGAGATTGACTATGATGGCAGTGGCTCTGTCTCCCTAGCTGAGTGGGTC

CGAGCTGGGGCCACTACGGTGCCGCTGCTAGTACTGCTGGGGCTGGAGATG

>CL28.Contig28_A-W 618 995 minus strand diacylglycerol kinase, alpha 80kDa, isoform CRA_b [Homo sapiens]

ATGACACGAGTGGCTGAATATCTGGATTGGGATGTGTCAGAGCTGAGGCCGATTTTTCAG

GAGATGATGAAAGAGATTGACTATGATGGCAGTGGCTCTGTCTCCCTAGCTGAGTGGGTC

CGAGCTGGGGCCACTACGGTGCCGCTGCTAGTACTGCTGGGGCTGGAGATGACTCTGAAG

GATGATGGGCAGCACATGTGGAGACCCAAGAGATTTCCCAGACCAGTCTACTGCAACCTG

TGCGAGTTGAGCATTGGTCTTGGCAAACAGGGGCTGAGCTGTAACCTCTGTAAGTACACT

GTTCACGACCAGTGTGCCATGAAGGCCCTGCCTTGTGAAGTCAGCACCTATGCCAAGTCT

CGGAAAGACATTGGTGTG

>CL28.Contig29_A-W 571 948 minus strand diacylglycerol kinase, alpha 80kDa, isoform CRA_b [Homo sapiens]

ATGACACGAGTGGCTGAATATCTGGATTGGGATGTGTCAGAGCTGAGGCCGATTTTTCAG

GAGATGATGAAAGAGATTGACTATGATGGCAGTGGCTCTGTCTCCCTAGCTGAGTGGGTC

CGAGCTGGGGCCACTACGGTGCCGCTGCTAGTACTGCTGGGGCTGGAGATGACTCTGAAG

GATGATGGGCAGCACATGTGGAGACCCAAGAGATTTCCCAGACCAGTCTACTGCAACCTG

TGCGAGTTGAGCATTGGTCTTGGCAAACAGGGGCTGAGCTGTAACCTCTGTAAGTACACT

GTTCACGACCAGTGTGCCATGAAGGCCCTGCCTTGTGAAGTCAGCACCTATGCCAAGTCT

CGGAAAGACATTGGTGTG

>CL28.Contig30_A-W 561 938 minus strand diacylglycerol kinase, alpha 80kDa, isoform CRA_b [Homo sapiens]

ATGACACGAGTGGCTGAATATCTGGATTGGGATGTGTCAGAGCTGAGGCCGATTTTTCAG

GAGATGATGAAAGAGATTGACTATGATGGCAGTGGCTCTGTCTCCCTAGCTGAGTGGGTC

CGAGCTGGGGCCACTACGGTGCCGCTGCTAGTACTGCTGGGGCTGGAGATGACTCTGAAG

GATGATGGGCAGCACATGTGGAGACCCAAGAGATTTCCCAGACCAGTCTACTGCAACCTG

TGCGAGTTGAGCATTGGTCTTGGCAAACAGGGGCTGAGCTGTAACCTCTGTAAGTACACT

GTTCACGACCAGTGTGCCATGAAGGCCCTGCCTTGTGAAGTCAGCACCTATGCCAAGTCT

CGGAAAGACATTGGTGTG

>CL28.Contig31_A-W 719 865 minus strand diacylglycerol kinase, alpha 80kDa, isoform CRA_b [Homo sapiens]

GTCCAATCACACGTGTGGGTGCGAGGAGGCTGTGAGTCCGGGCGCTGTGACCGCTGTCAG

AAAAAGATCCGGACCTACCACAGTCTAACCGGGCTGCATTGCGTATGGTGCCACCTAGAG

GTGTGAGAGCTCTCCGTCCAGTCTATA

>CL28.Contig32_A-W 746 967 minus strand Diacylglycerol kinase alpha [Heterocephalus glaber]

CAATCCCACCTCCTTTACAGCTGTAGTTGTCCTCGCTCCCAAGGGCTCTCTTTCCAATCC

AGACCCAAGTTGAAAGGTGGTAGAGTCCCTTGCATGATCCTGTATGCCCATCTTGTCTTT

CAGATTCATGATGACTGCCTGCAAGCCGTGGGCTCTGAGTGTGACTGTGGGCTGCTCCGG

GATCACATCCTGCCTCCATCTTCCATCTATCCCAGCGTCCTG

>CL28.Contig33_A-W 261 380 minus strand diacylglycerol kinase, alpha 80kDa, isoform CRA_b [Homo sapiens]

ATTTTTCAGGAGATGATGAAAGAGATTGACTATGATGGCAGTGGCTCTGTCTCCCTAGCT

GAGTGGGTCCGAGCTGGGGCCACTACGGTGCCGCTGCTAGTACTGCTGGGGCTGGAGATG

>CL28.Contig34_A-W 651 872 minus strand Diacylglycerol kinase alpha [Heterocephalus glaber]

CAATCCCACCTCCTTTACAGCTGTAGTTGTCCTCGCTCCCAAGGGCTCTCTTTCCAATCC

AGACCCAAGTTGAAAGGTGGTAGAGTCCCTTGCATGATCCTGTATGCCCATCTTGTCTTT

CAGATTCATGATGACTGCCTGCAAGCCGTGGGCTCTGAGTGTGACTGTGGGCTGCTCCGG

GATCACATCCTGCCTCCATCTTCCATCTATCCCAGCGTCCTG

>CL28.Contig35_A-W 554 700 minus strand PREDICTED: diacylglycerol kinase alpha isoform 1 [Nomascus leucogenys] >gi|332207605|ref|XP_003252886.1| PREDICTED: diacylglycerol kinase alpha isoform 2 [Nomascus leucogenys] >gi|332207607|ref|XP_003252887.1| PREDICTED: diacylglycerol kinase alpha isoform 3 [Nomascus leucogenys]

GTCCAATCACACGTGTGGGTGCGAGGAGGCTGTGAGTCCGGGCGCTGTGACCGCTGTCAG

AAAAAGATCCGGACCTACCACAGTCTAACCGGGCTGCATTGCGTATGGTGCCACCTAGAG

GTGTGAGAGCTCTCCGTCCAGTCTATA

>CL28.Contig36_A-W 554 700 minus strand diacylglycerol kinase, alpha 80kDa, isoform CRA_b [Homo sapiens]

GTCCAATCACACGTGTGGGTGCGAGGAGGCTGTGAGTCCGGGCGCTGTGACCGCTGTCAG

AAAAAGATCCGGACCTACCACAGTCTAACCGGGCTGCATTGCGTATGGTGCCACCTAGAG

GTGTGAGAGCTCTCCGTCCAGTCTATA

>CL28.Contig37_A-W 261 587 minus strand diacylglycerol kinase, alpha 80kDa, isoform CRA_b [Homo sapiens]

ATTTTTCAGGAGATGATGAAAGAGATTGACTATGATGGCAGTGGCTCTGTCTCCCTAGCT

GAGTGGGTCCGAGCTGGGGCCACTACGGTGCCGCTGCTAGTACTGCTGGGGCTGGAGATG

ACTCTGAAGGATGATGGGCAGCACATGTGGAGACCCAAGAGATTTCCCAGACCAGTCTAC

TGCAACCTGTGCGAGTTGAGCATTGGTCTTGGCAAACAGGGGCTGAGCTGTAACCTCTGT

AAGTACACTGTTCACGACCAGTGTGCCATGAAGGCCCTGCCTTGTGAAGTCAGCACCTAT

GCCAAGTCTCGGAAAGACATTGGTGTG

>CL28.Contig38_A-W 554 772 minus strand Diacylglycerol kinase alpha [Heterocephalus glaber]

GTCCAATCACACGTGTGGGTGCGAGGAGGCTGTGAGTCCGGGCGCTGTGACCGCTGTCAG

AAAAAGATCCGGACCTACCACAGTCTAACCGGGCTGCATTGCGTATGGTGCCACCTAGAG

ATTCATGATGACTGCCTGCAAGCCGTGGGCTCTGAGTGTGACTGTGGGCTGCTCCGGGAT

CACATCCTGCCTCCATCTTCCATCTATCCCAGCGTCCTG

>CL28.Contig39_A-W 459 605 minus strand PREDICTED: diacylglycerol kinase alpha isoform 1 [Nomascus leucogenys] >gi|332207605|ref|XP_003252886.1| PREDICTED: diacylglycerol kinase alpha isoform 2 [Nomascus leucogenys] >gi|332207607|ref|XP_003252887.1| PREDICTED: diacylglycerol kinase alpha isoform 3 [Nomascus leucogenys]

GTCCAATCACACGTGTGGGTGCGAGGAGGCTGTGAGTCCGGGCGCTGTGACCGCTGTCAG

AAAAAGATCCGGACCTACCACAGTCTAACCGGGCTGCATTGCGTATGGTGCCACCTAGAG

GTGTGAGAGCTCTCCGTCCAGTCTATA

>CL28.Contig40_A-W 459 677 minus strand Diacylglycerol kinase alpha [Heterocephalus glaber]

GTCCAATCACACGTGTGGGTGCGAGGAGGCTGTGAGTCCGGGCGCTGTGACCGCTGTCAG

AAAAAGATCCGGACCTACCACAGTCTAACCGGGCTGCATTGCGTATGGTGCCACCTAGAG

ATTCATGATGACTGCCTGCAAGCCGTGGGCTCTGAGTGTGACTGTGGGCTGCTCCGGGAT

CACATCCTGCCTCCATCTTCCATCTATCCCAGCGTCCTG

>CL28.Contig41_A-W 1 120 minus strand PREDICTED: diacylglycerol kinase alpha isoform 1 [Nomascus leucogenys] >gi|332207605|ref|XP_003252886.1| PREDICTED: diacylglycerol kinase alpha isoform 2 [Nomascus leucogenys] >gi|332207607|ref|XP_003252887.1| PREDICTED: diacylglycerol kinase alpha isoform 3 [Nomascus leucogenys]

ATTTTTCAGGAGATGATGAAAGAGATTGACTATGATGGCAGTGGCTCTGTCTCCCTAGCT

GAGTGGGTCCGAGCTGGGGCCACTACGGTGCCGCTGCTAGTACTGCTGGGGCTGGAGATG

>CL28.Contig42_A-W 712 1410 minus strand Diacylglycerol kinase alpha OS=Homo sapiens GN=DGKA PE=1 SV=3

GGATTTATACACAATCCAGGTCTCCTTCCCTCTGCTTTGCTCCCCTCCCAGGCATCTGAA

CAGGAACGCAAACACAGCAAAACAAGCCAAAAGACCACAGATGACTCAAATTTGTGCATC

TCTGAAGCTCTGCGGATCAACCCTGTTTCTAACACCCACCCACTTCTAGTCTTTGTCAAT

CCCAAGAGTGGTGGAAAGCAGGGACTGAGGGTCCTTTGGAAGTTCCAATATATACTAAAC

CCTCGGCAGGTGATCAACCTCCTAAAGGATGGTCCTGAACAAGGGCTCAGATTCTTCAAA

GATGTTCCTGACAGCCGTATTTTGGTGTGTGGTGGAGATGGCACAGTAGGCTGGATTCTA

GAGGCCATTGACAAAATCAACTTGCCTGTTGTACCTCCTGTTGCTGTGTTGCCCCTGGGC

ACTGGAAATGATCTGGCTCGTTGCCTAAGGTGGGGAGGAGGGTATGAAGGAGAGAATTTG

GCAAAGATTCTCAGGGAATTAGAGATGAGTACGGTGGTATATATAGATCGATGGTCCCTG

GAAGTGATACCCCAACAAACTGAAGAAAAGACTGATCCAGTCCCCTTTCAAATCATCAAT

AACTACTTCTCCATTGGTGTGGATGCTTCTATTGCTCACCGATTTCACATCATGCGAGAG

AAATATCCTGAGAAGTTCAATAGCAGGTGAAGGAAAAAG

>CL28.Contig43_A-W 1 327 minus strand PREDICTED: diacylglycerol kinase alpha isoform 1 [Nomascus leucogenys] >gi|332207605|ref|XP_003252886.1| PREDICTED: diacylglycerol kinase alpha isoform 2 [Nomascus leucogenys] >gi|332207607|ref|XP_003252887.1| PREDICTED: diacylglycerol kinase alpha isoform 3 [Nomascus leucogenys]

ATTTTTCAGGAGATGATGAAAGAGATTGACTATGATGGCAGTGGCTCTGTCTCCCTAGCT

GAGTGGGTCCGAGCTGGGGCCACTACGGTGCCGCTGCTAGTACTGCTGGGGCTGGAGATG

ACTCTGAAGGATGATGGGCAGCACATGTGGAGACCCAAGAGATTTCCCAGACCAGTCTAC

TGCAACCTGTGCGAGTTGAGCATTGGTCTTGGCAAACAGGGGCTGAGCTGTAACCTCTGT

AAGTACACTGTTCACGACCAGTGTGCCATGAAGGCCCTGCCTTGTGAAGTCAGCACCTAT

GCCAAGTCTCGGAAAGACATTGGTGTG

>CL28.Contig44_A-W 547 948 minus strand Diacylglycerol kinase alpha [Heterocephalus glaber]

GAAGTGGAAAGAATCATACTCCAGATGACACGAGTGGCTGAATATCTGGATTGGGATGTG

TCAGAGCTGAGGCCGATTTTTCAGGAGATGATGAAAGAGATTGACTATGATGGCAGTGGC

TCTGTCTCCCTAGCTGAGTGGGTCCGAGCTGGGGCCACTACGGTGCCGCTGCTAGTACTG

CTGGGGCTGGAGATGACTCTGAAGGATGATGGGCAGCACATGTGGAGACCCAAGAGATTT

CCCAGACCAGTCTACTGCAACCTGTGCGAGTTGAGCATTGGTCTTGGCAAACAGGGGCTG

AGCTGTAACCTCTGTAAGTACACTGTTCACGACCAGTGTGCCATGAAGGCCCTGCCTTGT

GAAGTCAGCACCTATGCCAAGTCTCGGAAAGACATTGGTGTG

>CL28.Contig45_A-W 264 938 minus strand Diacylglycerol kinase alpha [Heterocephalus glaber]

CTTCCATCACAGGCCATTGGCTATGAGGGATTTGAGCAATTCCTGAAAATCTATTTGGAA

GTGGATAATGTTCCCCATCACCTAAGCCTGGCACTGTTTCATTCCTTCCACACTGATCAC

TGCTTAAAAGAGAATGTAAGGAAAGCAAATGTGGTATGTCTCAGTGATGTCTCCTGTTAC

TTTTCCCTTCTGGAGGGTGGCCGGCCAGAAGATAAGCTAGAATTCACCTTCAAGCTGTAT

GACACGGACGGAAATGGGATCCTGGACAGCTCAGAAGTGGAAAGAATCATACTCCAGATG

ACACGAGTGGCTGAATATCTGGATTGGGATGTGTCAGAGCTGAGGCCGATTTTTCAGGAG

ATGATGAAAGAGATTGACTATGATGGCAGTGGCTCTGTCTCCCTAGCTGAGTGGGTCCGA

GCTGGGGCCACTACGGTGCCGCTGCTAGTACTGCTGGGGCTGGAGATGACTCTGAAGGAT

GATGGGCAGCACATGTGGAGACCCAAGAGATTTCCCAGACCAGTCTACTGCAACCTGTGC

GAGTTGAGCATTGGTCTTGGCAAACAGGGGCTGAGCTGTAACCTCTGTAAGTACACTGTT

CACGACCAGTGTGCCATGAAGGCCCTGCCTTGTGAAGTCAGCACCTATGCCAAGTCTCGG

AAAGACATTGGTGTG

>CL28.Contig46_A-W 1128 1346 minus strand Diacylglycerol kinase alpha [Heterocephalus glaber]

GTCCAATCACACGTGTGGGTGCGAGGAGGCTGTGAGTCCGGGCGCTGTGACCGCTGTCAG

AAAAAGATCCGGACCTACCACAGTCTAACCGGGCTGCATTGCGTATGGTGCCACCTAGAG

ATTCATGATGACTGCCTGCAAGCCGTGGGCTCTGAGTGTGACTGTGGGCTGCTCCGGGAT

CACATCCTGCCTCCATCTTCCATCTATCCCAGCGTCCTG

>CL28.Contig47_A-W 1223 1441 minus strand Diacylglycerol kinase alpha [Heterocephalus glaber]

GTCCAATCACACGTGTGGGTGCGAGGAGGCTGTGAGTCCGGGCGCTGTGACCGCTGTCAG

AAAAAGATCCGGACCTACCACAGTCTAACCGGGCTGCATTGCGTATGGTGCCACCTAGAG

ATTCATGATGACTGCCTGCAAGCCGTGGGCTCTGAGTGTGACTGTGGGCTGCTCCGGGAT

CACATCCTGCCTCCATCTTCCATCTATCCCAGCGTCCTG

>CL28.Contig48_A-W 156 1061 minus strand Diacylglycerol kinase alpha [Heterocephalus glaber]

GGGCCTTCTGCATCACTTGCTGAGGCCTACCCTCTGAAGAGGACAAACCGCAAGAAGTAC

TACTACGAAGCTATCTTTCTGGCCATCATTCAGAAAAACAGAAAGATGGCCAAGGAAAGG

GGACTATTAAGCCCTGAAGACTTTGCCCAGCTGCAAAAATACATGGAATACTCCAACCAA

AAGGTCAGTGATGTCCTAAAACTCTTCGAGAACGAGAAGATGGCCAAATATCTCCAAGGA

GATGCCATTGGCTATGAGGGATTTGAGCAATTCCTGAAAATCTATTTGGAAGTGGATAAT

GTTCCCCATCACCTAAGCCTGGCACTGTTTCATTCCTTCCACACTGATCACTGCTTAAAA

GAGAATGTAAGGAAAGCAAATGTGGTATGTCTCAGTGATGTCTCCTGTTACTTTTCCCTT

CTGGAGGGTGGCCGGCCAGAAGATAAGCTAGAATTCACCTTCAAGCTGTATGACACGGAC

GGAAATGGGATCCTGGACAGCTCAGAAGTGGAAAGAATCATACTCCAGATGACACGAGTG

GCTGAATATCTGGATTGGGATGTGTCAGAGCTGAGGCCGATTTTTCAGGAGATGATGAAA

GAGATTGACTATGATGGCAGTGGCTCTGTCTCCCTAGCTGAGTGGGTCCGAGCTGGGGCC

ACTACGGTGCCGCTGCTAGTACTGCTGGGGCTGGAGATGACTCTGAAGGATGATGGGCAG

CACATGTGGAGACCCAAGAGATTTCCCAGACCAGTCTACTGCAACCTGTGCGAGTTGAGC

ATTGGTCTTGGCAAACAGGGGCTGAGCTGTAACCTCTGTAAGTACACTGTTCACGACCAG

TGTGCCATGAAGGCCCTGCCTTGTGAAGTCAGCACCTATGCCAAGTCTCGGAAAGACATT

GGTGTG

>CL28.Contig49_A-W 1480 1701 minus strand Diacylglycerol kinase alpha [Heterocephalus glaber]

CAATCCCACCTCCTTTACAGCTGTAGTTGTCCTCGCTCCCAAGGGCTCTCTTTCCAATCC

AGACCCAAGTTGAAAGGTGGTAGAGTCCCTTGCATGATCCTGTATGCCCATCTTGTCTTT

CAGATTCATGATGACTGCCTGCAAGCCGTGGGCTCTGAGTGTGACTGTGGGCTGCTCCGG

GATCACATCCTGCCTCCATCTTCCATCTATCCCAGCGTCCTG

>CL28.Contig50_A-W 1288 1506 minus strand Diacylglycerol kinase alpha [Heterocephalus glaber]

GTCCAATCACACGTGTGGGTGCGAGGAGGCTGTGAGTCCGGGCGCTGTGACCGCTGTCAG

AAAAAGATCCGGACCTACCACAGTCTAACCGGGCTGCATTGCGTATGGTGCCACCTAGAG

ATTCATGATGACTGCCTGCAAGCCGTGGGCTCTGAGTGTGACTGTGGGCTGCTCCGGGAT

CACATCCTGCCTCCATCTTCCATCTATCCCAGCGTCCTG

>CL29.Contig1_A-W 581 703 hypothetical protein [Macaca fascicularis]

CACAGTAACTATGGGCAAGCATCACATGATATGACAGATGAGAAATGGAGAAGAGAGTTG

CTTCCAGAGTACCACAGATATGGTTTACATAAAATCCAGGATCCTGGCTCTGCCAGAGAT

CAG

>CL29.Contig2_A-W 608 856 minus strand PREDICTED: coiled-coil domain-containing protein 15-like [Macaca mulatta]

GATATGACAGATGAGAAATGGAGAAGAGAGTTGCTTCCAGAGTACCACAGATATGGTTTA

CATAAAATCCAGGATCCTGGCTCTGCCAGAGATCAGAGCTTATACTTTAGGCAGCAGCCA

TCTGTTGGAACAGCTGAAAGATGCCGAGAGGACTTGCTTCTGAATCACCATCAGCATCTT

CCACCCAAGCATCAGAGAGACACTTGCAGCAGACGACAGAGGCATTGGCCATGATCCTAT

GACAGGTTT

>CL29.Contig3_A-W 430 540 minus strand Coiled-coil domain-containing protein 15 OS=Mus musculus GN=Ccdc15 PE=2 SV=2

CAGCAGCCATCTGTTGGAACAGCTGAAAGATGCCGAGAGGACTTGCTTCTGAATCACCAT

CAGCATCTTCCACCCAAGCATCAGAGAGACACTTGCAGCAGACGACAGAGG

>CL30.Contig1_A-W 927 1541 minus strand ABI gene family member 3 [Bos taurus] >gi|133777929|gb|AAI14720.1| ABI3 protein [Bos taurus] >gi|296476486|gb|DAA18601.1| NESH protein [Bos taurus]

CAGGACCTGAGCACGCAGCTGTCACGGACTGGGACCCTGTCTCGAAAGAGCATCAAGGCG

CCTGCAACACCTGCCTCCACCACGCTGGGGAGACCACCCCGGATCCCGGAGCCCGTGCAG

CTCCCAGTGGTGCCTGATGGCAAACTCTCGGCCGCCTCCTCCGCCTCTTCCCTGACCTCC

GCCGGCAGCGCCGAAGGTGTCGGTGGGGTCCCTCAGTCCAAGGGACAGGCTCCCCCAACT

CCACCTCCTCCACCCGCCGCCGCCGCCGCCGAGGTGTTTCTGCCGCCCCCTCCACTCGAG

GAGCTGTCCCCGCCCCCACCAGAGCTGCCCCTGCCCCTGGACCTGCCCCCTCCTCCGCCT

CTGGAAGTAGAGGACCTGGGGCTGCCACCTCCACCCCCGCCAGGCTTTGGATCGGATGAA

CCCAGCTGGGTCCCTGAGGCATACTTGGAAAAAGTGGTGACACTGTACCCATACACCCGC

CAGAAGGACAACGAGCTCTCCTTCTCTGAGGGCACGGTCATCTGCGTCACCCGCCGCTAC

TCCGATGGCTGGTGCGAGGGCGTCTGCTCGGAGGGGACTGGATTCTTCCCAGGGAATTAT

GTGGAGCCCAGCAGC

>CL30.Contig2_A-W 927 1394 minus strand ABI gene family member 3 [Bos taurus] >gi|133777929|gb|AAI14720.1| ABI3 protein [Bos taurus] >gi|296476486|gb|DAA18601.1| NESH protein [Bos taurus]

CAGGACCTGAGCACGCAGCTGTCACGGACTGGGACCCTGTCTCGAAAGAGCATCAAGGCG

CCTGCAACACCTGCCTCCACCACGCTGGGGAGACCACCCCGGATCCCGGAGCCCGTGCAG

CTCCCAGTGGTGCCTGATGGCAAACTCTCGGCCGCCTCCTCCGCCTCTTCCCTGACCTCC

GCCGGCAGCGCCGAAGGTGTCGGTGGGGTCCCTCAGTCCAAGGGACAGGCTCCCCCAACT

CCCCCTCCTCCCCCCGCCGCCGCCGCCGCCGAGGTGTTTCTGCCGCCCCCTCCACTCGAG

GAGCTGTCCCCGCCCCCACCAGGTAAGGAGCCCCGCCCTTCCCGGTGTCGCTGTCCCGAG

GTCTCTGTCACACCTCTGCACCCGGTAGCTCTTGGCTGTCACCCTACGAGCTTCTGCGAG

CGCTGCGTGGCTGTTACACCGACCCGAGCGAAAGGCGGCAGAGCGAGT

>CL30.Contig3_A-W 1732 1872 minus strand PREDICTED: ABI gene family member 3 isoform 1 [Canis lupus familiaris]

CCTGTCACAGAGCTGCCCCTGCCCCTGGACCTGCCCCCTCCTCCGCCTCTGGAAGTAGAG

GACCTGGGGCTGCCACCTCCACCCCCGCCAGGCTTTGGATCGGATGAACCCAGCTGGGTC

CCTGAGGCATACTTGGAAAAA

>CL30.Contig4_A-W 1133 1594 minus strand PREDICTED: ABI gene family member 3-like isoform 3 [Sus scrofa]

GGTCCCTCAGTCCAAGGGACAGGCTCCCCCAACTCCACCTCCTCCACCCGCCGCCGCCGC

CGCCGAGGTGTTTCTGCCGCCCCCTCCACTCGAGGAGCTGTCCCCGCCCCCACCAGAGCT

GCCCCTGCCCCTGGACCTGCCCCCTCCTCCGCCTCTGGAAGTAGAGGACCTGGGGCTGCC

ACCTCCACCCCCGCCAGGCTTTGGATCGGATGAACCCAGCTGGGTCCCTGAGGCATACTT

GGAAAAAGGTACCTGAGTCCTAGAACGGAGGGGCACCAGATCCTGCGGCCCTCTTTTGCC

CTGACCCCCATTCTCCCACCCCAAAGGCATTCTCCCCTCCCTCTGCAAAGGTATGAACTT

GTTATCCGCCCCCCCTCCGGTGATGTGATTGGATAGGAAGCAGCTGGTCTAGCATTGAAT

ACTAAGCTGGGGAGGGGGCCTGAAAGCTCGGAAGGCACTCCT

>CL31.Contig1_A-W 171 284 minus strand PREDICTED: methylosome protein 50 [Saimiri boliviensis boliviensis]

ATGCGAAAGGAAACGCCGCCACCGCTAGTAGCCCCGGCTGCCCGGGAGTGGAACCTTCCC

CCTAATGCGCCCGCCTGTATGGAACGGCAGTTGGAGGCTGCGCGGTACCGGTCT

>CL31.Contig2_A-W 171 503 minus strand PREDICTED: methylosome protein 50 [Saimiri boliviensis boliviensis]

ATGCGAAAGGAAACGCCGCCACCGCTAGTAGCCCCGGCTGCCCGGGAGTGGAACCTTCCC

CCTAATGCGCCCGCCTGTATGGAACGGCAGTTGGAGGCTGCGCGGTACCGGTCTGATGGG

GCGCTTCTGCTCGGGGCCTCCAGCTTGAGTGGCCGCTGCTGGGCTGGCTCCCTCTGGCTT

TTCAAGGACCCGTGTGCTGCCCCCAACGAAGGCTTCTGCTCCGCTGGAGTCCAGACGGAG

GCCGGAGTGGCCGACCTCACTTGGGTCGGGGAGAGAGGTATCCTCGTGGCTTCTGATTCA

GGTGCTGTTGAATTATGGGAGCTGGATGAGAAT

>CL32.Contig1_A-W 513 626 minus strand interleukin 8 [Marmota monax]

TCCCATTTCAGTGTCAAGCTTGTTGATGGAAGAGAGCTTTGCCTGAACCCCAAGGAAAAA

TGGGTGCAGAAGGTTGTGGAGCTATTTTTGAAGAGGGCTGAGAATGAAGATCCA

>CL32.Contig2_A-W 119 313 minus strand Interleukin-8 OS=Oryctolagus cuniculus GN=IL8 PE=1 SV=2

CTAGCAGAGCTGTACCTCTTGATGAAATCCCTAACCATTCTTTATTTCTTCTTCACAGCT

GCAGTTCTGGCAAGGATTGGCACAGAACTTCGATGCCAGTGCATAAAAACTCATTCCACA

CCTTTCCACCCCAAATACATCAAAGAACTGAGAGTGATTGAGAGTGGACCACACTGTGCC

AATTCAGAAATCATG

>CL32.Contig3_A-W 78 278 minus strand Interleukin-8 OS=Oryctolagus cuniculus GN=IL8 PE=1 SV=2

ATGACTTCCAAGCTGGCCGTGGCTCTCTTGGTAACCTGCCTACTTTCTGCAACTCTCTGT

GAAGCTGCAGTTCTGGCAAGGATTGGCACAGAACTTCGATGCCAGTGCATAAAAACTCAT

TCCACACCTTTCCACCCCAAATACATCAAAGAACTGAGAGTGATTGAGAGTGGACCACAC

TGTGCCAATTCAGAAATCATG

>CL32.Contig4_A-W 176 415 minus strand interleukin 8 [Marmota monax]

GCTGCAGTTCTGGCAAGGATTGGCACAGAACTTCGATGCCAGTGCATAAAAACTCATTCC

ACACCTTTCCACCCCAAATACATCAAAGAACTGAGAGTGATTGAGAGTGGACCACACTGT

GCCAATTCAGAAATCATTGTCAAGCTTGTTGATGGAAGAGAGCTTTGCCTGAACCCCAAG

GAAAAATGGGTGCAGAAGGTTGTGGAGCTATTTTTGAAGAGGGCTGAGAATGAAGATCCA

>CL32.Contig5_A-W 78 380 minus strand interleukin 8 [Marmota monax]

ATGACTTCCAAGCTGGCCGTGGCTCTCTTGGTAACCTGCCTACTTTCTGCAACTCTCTGT

GAAGCTGCAGTTCTGGCAAGGATTGGCACAGAACTTCGATGCCAGTGCATAAAAACTCAT

TCCACACCTTTCCACCCCAAATACATCAAAGAACTGAGAGTGATTGAGAGTGGACCACAC

TGTGCCAATTCAGAAATCATTGTCAAGCTTGTTGATGGAAGAGAGCTTTGCCTGAACCCC

AAGGAAAAATGGGTGCAGAAGGTTGTGGAGCTATTTTTGAAGAGGGCTGAGAATGAAGAT

CCA

>CL32.Contig6_A-W 176 397 minus strand interleukin 8 [Marmota monax]

GCTGCAGTTCTGGCAAGGATTGGCACAGAACTTCGATGCCAGTGCATAAAAACTCATTCC

ACACCTTTCCACCCCAAATACATCAAAGAACTGAGAGTGATTGAGAGTGGACCACACTGT

GCCAATTCAGAAATCATTGTCAAGCTTGTTGATGGAAGAGAGCTTTGCCTGAACCCCAAG

GAAAAATGGGTGCAGAAGGTTGTGGAGCTATTTTTGAAGAGG

>CL32.Contig7_A-W 78 362 minus strand interleukin 8 [Marmota monax]

ATGACTTCCAAGCTGGCCGTGGCTCTCTTGGTAACCTGCCTACTTTCTGCAACTCTCTGT

GAAGCTGCAGTTCTGGCAAGGATTGGCACAGAACTTCGATGCCAGTGCATAAAAACTCAT

TCCACACCTTTCCACCCCAAATACATCAAAGAACTGAGAGTGATTGAGAGTGGACCACAC

TGTGCCAATTCAGAAATCATTGTCAAGCTTGTTGATGGAAGAGAGCTTTGCCTGAACCCC

AAGGAAAAATGGGTGCAGAAGGTTGTGGAGCTATTTTTGAAGAGG

>CL34.Contig1_A-W 2 727 minus strand PREDICTED: THO complex subunit 3 [Saimiri boliviensis boliviensis]

AGTAATCCTGACCTCTTTGTCACTGCATCTGGCGATAAAACCATTCGCATCTGGGATGTG

AGGACTACAAAATGCATTGCCACTGTAAACACTAAAGGGGAGAACATTAATATCTGCTGG

AGTCCTGATGGGCAGACCATTGCTGTAGGCAACAAGGATGATGTCGTGACCTTTATTGAT

GCCAAGACACACCGTTCCAAAGCAGAAGAGCAGTTCAAGTTCGAGGTCAATGAAATCTCC

TGGAACAATGACAATAATATGTTCTTCCTGACAAATGGCAATGGTTGTATCAACATCCTC

AGCTACCCGGAACTGAAGCCTGTGCAGTCCATCAACGCCCATCCTTCCAATTGCATCTGT

ATCAAATTTGACCCCATGGGGAAATACTTTGCCACAGGAAGTGCAGATGCTTTGGTTAGC

CTCTGGGATGTGGATGAGTTAGTATGTGTACGGTGCTTTTCCAGGCTGGATTGGCCTGTG

AGGACCCTCAGTTTTAGCCATGACGGGAAAATGCTGGCATCAGCATCGGAGGATCATTTT

ATAGACATAGCTGAAGTAGAGACAGGAGACAAACTATGGGAGGTGCAGTGTGAGTCCCCA

ACCTTCACTGTGGCTTGGCACCCCAAAAGGCCTCTGCTTGCATTTGCCTGTGATGACAAA

GATGGAAAATATGACAGCAGCCGAGAAGCTGGGACTGTGAAGTTGTTTGGGCTTCCTAAT

GATTCC

>CL34.Contig2_A-W 44 214 minus strand PREDICTED: THO complex subunit 3 [Saimiri boliviensis boliviensis]

ATTTCTGTAGGAGACAAACTATGGGAGGTGCAGTGTGAGTCCCCAACCTTCACTGTGGCT

TGGCACCCCAAAAGGCCTCTGCTTGCATTTGCCTGTGATGACAAAGATGGAAAATATGAC

AGCAGCCGAGAAGCTGGGACTGTGAAGTTGTTTGGGCTTCCTAATGATTCC

>CL35.Contig1_A-W 3 794 minus strand Mbd3 protein [Rattus norvegicus]

CGGGCCCGGAGGCGGGCCGAGGAGCCGGGCGCGATGGAGCGGAAGAGCCCCAGTGGGAAG

AAGTTCCGCAGCAAGCCCCAGCTGGCGCGCTACCTGGGGGGCTCCATGGACCTGAGCACC

TTTGACTTCCGCACCGGCAAGATGCTGATGAGCAAGATGAACAAGAGCCGCCAACGCGTG

CGCTACGACTCCTCCAACCAGGTCAAGGGCAAGCCCGACCTGAACACAGCGCTGCCTGTG

CGGCAGACAGCGTCCATCTTTAAGCAGCCTGTGACCAAGATCACCAACCACCCCAGCAAC

AAGGTGAAGAGCGACCCACAGAAGGCTGTGGACCAGCCCCGGCAGCTGTTCTGGGAGAAG

AAGCTGAGTGGCCTGAGTGCCTTTGACATCGCCGAGGAGCTTGTCAGGACCATGGACCTG

CCCAAAGGCCTGCAGGGGGTGGGCCCAGGCTGTACGGATGAGACCCTGCTGTCGGCCATC

GCCAGCGCCCTGCACACCAGCACCATGCCCATCACGGGGCAGCTCTCAGCCGCCGTGGAG

AAGAACCCTGGTGTGTGGCTGAACACCACGCAACCCCTGTGCAAAGCATTCATGGTGACT

GACGAGGACATCAGGAAGCAGGAGGAGCTGGTGCAGCAGGTGCGGAAGCGGCTGGAGGAG

GCACTGATGGCTGACATGCTGGCCCACGTGGAGGAGCTGGCCCGTGATGGTGAGGCGCCA

CTGGACAAGGCCTGTGTGGACGACGAGGAGGATGACGAGGAGGACGAGGAGGAGGAGCCA

GAGCCAGACCAG

>CL35.Contig2_A-W 3 794 minus strand Mbd3 protein [Rattus norvegicus]

CGGGCCCGGAGGCGGGCCGAGGAGCCGGGCGCGATGGAGCGGAAGAGCCCCAGTGGGAAG

AAGTTCCGCAGCAAGCCCCAGCTGGCGCGCTACCTGGGGGGCTCCATGGACCTGAGCACC

TTTGACTTCCGCACCGGCAAGATGCTGATGAGCAAGATGAACAAGAGCCGCCAACGCGTG

CGCTACGACTCCTCCAACCAGGTCAAGGGCAAGCCCGACCTGAACACAGCGCTGCCTGTG

CGGCAGACAGCGTCCATCTTTAAGCAGCCTGTGACCAAGATCACCAACCACCCCAGCAAC

AAGGTGAAGAGCGACCCACAGAAGGCTGTGGACCAGCCCCGGCAGCTGTTCTGGGAGAAG

AAGCTGAGTGGCCTGAGTGCCTTTGACATCGCCGAGGAGCTTGTCAGGACCATGGACCTG

CCCAAAGGCCTGCAGGGGGTGGGCCCAGGCTGTACGGATGAGACCCTGCTGTCGGCCATC

GCCAGCGCCCTGCACACCAGCACCATGCCCATCACGGGGCAGCTCTCAGCCGCCGTGGAG

AAGAACCCTGGTGTGTGGCTGAACACCACGCAACCCCTGTGCAAAGCATTCATGGTGACT

GACGAGGACATCAGGAAGCAGGAGGAGCTGGTGCAGCAGGTGCGGAAGCGGCTGGAGGAG

GCACTGATGGCTGACATGCTGGCCCACGTGGAGGAGCTGGCCCGTGATGGTGAGGCGCCA

CTGGACAAGGCCTGTGTGGACGACGAGGAGGATGACGAGGAGGACGAGGAGGAGGAGCCA

GAGCCAGACCAG

>CL35.Contig3_A-W 773 892 minus strand PREDICTED: RING finger protein 126-like isoform 1 [Cavia porcellus]

AAGCACGACAGCTGCCCTGTCTGCCGCAAGAGCCTCACCGGACAGAACACGGCCACCAAC

CCCCCAGGCCTGACTGGGGTGAGCTTCTCCTCCTCCTCCTCGTCCTCCTCCTCGTCGTCC

>CL35.Contig4_A-W 4 804 Mbd3 protein [Rattus norvegicus]

GAGCGGGGGTCGCGGCGGCGGCGGCAGCGGGCCCGGAGGCGGGCCGAGGAGCCGGGCGCG

ATGGAGCGGAAGAGCCCCAGTGGGAAGAAGTTCCGCAGCAAGCCCCAGCTGGCGCGCTAC

CTGGGGGGCTCCATGGACCTGAGCACCTTTGACTTCCGCACCGGCAAGATGCTGATGAGC

AAGATGAACAAGAGCCGCCAACGCGTGCGCTACGACTCCTCCAACCAGGTCAAGGGCAAG

CCCGACCTGAACACAGCGCTGCCTGTGCGGCAGACAGCGTCCATCTTTAAGCAGCCTGTG

ACCAAGATCACCAACCACCCCAGCAACAAGGTGAAGAGCGACCCACAGAAGGCTGTGGAC

CAGCCCCGGCAGCTGTTCTGGGAGAAGAAGCTGAGTGGCCTGAGTGCCTTTGACATCGCC

GAGGAGCTTGTCAGGACCATGGACCTGCCCAAAGGCCTGCAGGGGGTGGGCCCAGGCTGT

ACGGATGAGACCCTGCTGTCGGCCATCGCCAGCGCCCTGCACACCAGCACCATGCCCATC

ACGGGGCAGCTCTCAGCCGCCGTGGAGAAGAACCCTGGTGTGTGGCTGAACACCACGCAA

CCCCTGTGCAAAGCATTCATGGTGACTGACGAGGACATCAGGAAGCAGGAGGAGCTGGTG

CAGCAGGTGCGGAAGCGGCTGGAGGAGGCACTGATGGCTGACATGCTGGCCCACGTGGAG

GAGCTGGCCCGTGATGGTGAGGCGCCACTGGACAAGGCCTGTGTGGACGACGAGGAGGAG

GACGAGGAGGAGGAGGAGAAG

>CL35.Contig5_A-W 3 815 minus strand PREDICTED: RING finger protein 126 [Otolemur garnettii]

CCGCAGCCCGGACGGTACTTCTGCCACTGCTGCTCGGTCGAGATCGTGCCGCGCCTGCCG

GATTACATCTGCCCCAGGTGTGAGTCTGGTTTTATCGAGGAGCTTCCAGAAGAGACCAGG

AATACAGAAAACGGTTCCGCTCCCTCCACGGCCGCCGCTGACCAGAGCCGGCAGCCGTTT

GAGAACGTGGACCAGCACCTGTTCACGCTGCCCCAGGGCTACGGACAATTTGCTTTCGGC

ATCTTTGACGACAGCTTTGAGATCCCCACGTTCCCTCCTGGGGCACAGGCCGATGACAGC

AGGGACCCTGAGAGCCGGAGGGAGAGAGAACACCAGTCCCGGCACCGGATCATCCAGCAG

TTGGTCAACGGCATCATCACTCCGGCCACCATCCCCAACTTGGGCCTGGGCCCCTGGGGC

GTCCTGCACTCCAACCCAATGGACTACGCCTGGGGGGCCAACGGCCTGGATGCCATCATC

ACGCAGCTCCTCAACCAGTTTGAAAACACGGGCCCCCCTCCCGCAGACAAGGAGAAGATC

CAGGCCCTGCCCACCGTCCCCGTCACCGAGGAGCACGTAGGCTCTGGGCTTGAGTGCCCC

GTGTGCAAGGACGACTACGCGCTGGGCGAGAGTGTGCGGCAGCTGCCCTGCAACCACCTG

TTCCACAACGGCTGCATCGTGCCCTGGCTGGAGCAGCACGACAGCTGCCCTGTCTGCCGC

AAGAGCCTCACCGGACAGAACACGGCCACCAACCCCCCAGGCCTGACTGGGGTGAGCTTC

TCCTCCTCCTCCTCGTCCTCCTCCTCGTCGTCC

>CL35.Contig6_A-W 4 804 Mbd3 protein [Rattus norvegicus]

GAGCGGGGGTCGCGGCGGCGGCGGCAGCGGGCCCGGAGGCGGGCCGAGGAGCCGGGCGCG

ATGGAGCGGAAGAGCCCCAGTGGGAAGAAGTTCCGCAGCAAGCCCCAGCTGGCGCGCTAC

CTGGGGGGCTCCATGGACCTGAGCACCTTTGACTTCCGCACCGGCAAGATGCTGATGAGC

AAGATGAACAAGAGCCGCCAACGCGTGCGCTACGACTCCTCCAACCAGGTCAAGGGCAAG

CCCGACCTGAACACAGCGCTGCCTGTGCGGCAGACAGCGTCCATCTTTAAGCAGCCTGTG

ACCAAGATCACCAACCACCCCAGCAACAAGGTGAAGAGCGACCCACAGAAGGCTGTGGAC

CAGCCCCGGCAGCTGTTCTGGGAGAAGAAGCTGAGTGGCCTGAGTGCCTTTGACATCGCC

GAGGAGCTTGTCAGGACCATGGACCTGCCCAAAGGCCTGCAGGGGGTGGGCCCAGGCTGT

ACGGATGAGACCCTGCTGTCGGCCATCGCCAGCGCCCTGCACACCAGCACCATGCCCATC

ACGGGGCAGCTCTCAGCCGCCGTGGAGAAGAACCCTGGTGTGTGGCTGAACACCACGCAA

CCCCTGTGCAAAGCATTCATGGTGACTGACGAGGACATCAGGAAGCAGGAGGAGCTGGTG

CAGCAGGTGCGGAAGCGGCTGGAGGAGGCACTGATGGCTGACATGCTGGCCCACGTGGAG

GAGCTGGCCCGTGATGGTGAGGCGCCACTGGACAAGGCCTGTGTGGACGACGAGGAGGAG

GACGAGGAGGAGGAGGAGAAG

>CL35.Contig7_A-W 856 984 minus strand RING finger protein 126 [Heterocephalus glaber]

GTCCCCACAAAGCACGACAGCTGCCCTGTCTGCCGCAAGAGCCTCACCGGACAGAACACG

GCCACCAACCCCCCAGGCCTGACTGGGGTGAGCTTCTCCTCCTCCTCCTCGTCCTCCTCC

TCGTCGTCC

>CL35.Contig8_A-W 4 804 Mbd3 protein [Rattus norvegicus]

GAGCGGGGGTCGCGGCGGCGGCGGCAGCGGGCCCGGAGGCGGGCCGAGGAGCCGGGCGCG

ATGGAGCGGAAGAGCCCCAGTGGGAAGAAGTTCCGCAGCAAGCCCCAGCTGGCGCGCTAC

CTGGGGGGCTCCATGGACCTGAGCACCTTTGACTTCCGCACCGGCAAGATGCTGATGAGC

AAGATGAACAAGAGCCGCCAACGCGTGCGCTACGACTCCTCCAACCAGGTCAAGGGCAAG

CCCGACCTGAACACAGCGCTGCCTGTGCGGCAGACAGCGTCCATCTTTAAGCAGCCTGTG

ACCAAGATCACCAACCACCCCAGCAACAAGGTGAAGAGCGACCCACAGAAGGCTGTGGAC

CAGCCCCGGCAGCTGTTCTGGGAGAAGAAGCTGAGTGGCCTGAGTGCCTTTGACATCGCC

GAGGAGCTTGTCAGGACCATGGACCTGCCCAAAGGCCTGCAGGGGGTGGGCCCAGGCTGT

ACGGATGAGACCCTGCTGTCGGCCATCGCCAGCGCCCTGCACACCAGCACCATGCCCATC

ACGGGGCAGCTCTCAGCCGCCGTGGAGAAGAACCCTGGTGTGTGGCTGAACACCACGCAA

CCCCTGTGCAAAGCATTCATGGTGACTGACGAGGACATCAGGAAGCAGGAGGAGCTGGTG

CAGCAGGTGCGGAAGCGGCTGGAGGAGGCACTGATGGCTGACATGCTGGCCCACGTGGAG

GAGCTGGCCCGTGATGGTGAGGCGCCACTGGACAAGGCCTGTGTGGACGACGAGGAGGAG

GACGAGGAGGAGGAGGAGAAG

>CL35.Contig9_A-W 4 804 Mbd3 protein [Rattus norvegicus]

GAGCGGGGGTCGCGGCGGCGGCGGCAGCGGGCCCGGAGGCGGGCCGAGGAGCCGGGCGCG

ATGGAGCGGAAGAGCCCCAGTGGGAAGAAGTTCCGCAGCAAGCCCCAGCTGGCGCGCTAC

CTGGGGGGCTCCATGGACCTGAGCACCTTTGACTTCCGCACCGGCAAGATGCTGATGAGC

AAGATGAACAAGAGCCGCCAACGCGTGCGCTACGACTCCTCCAACCAGGTCAAGGGCAAG

CCCGACCTGAACACAGCGCTGCCTGTGCGGCAGACAGCGTCCATCTTTAAGCAGCCTGTG

ACCAAGATCACCAACCACCCCAGCAACAAGGTGAAGAGCGACCCACAGAAGGCTGTGGAC

CAGCCCCGGCAGCTGTTCTGGGAGAAGAAGCTGAGTGGCCTGAGTGCCTTTGACATCGCC

GAGGAGCTTGTCAGGACCATGGACCTGCCCAAAGGCCTGCAGGGGGTGGGCCCAGGCTGT

ACGGATGAGACCCTGCTGTCGGCCATCGCCAGCGCCCTGCACACCAGCACCATGCCCATC

ACGGGGCAGCTCTCAGCCGCCGTGGAGAAGAACCCTGGTGTGTGGCTGAACACCACGCAA

CCCCTGTGCAAAGCATTCATGGTGACTGACGAGGACATCAGGAAGCAGGAGGAGCTGGTG

CAGCAGGTGCGGAAGCGGCTGGAGGAGGCACTGATGGCTGACATGCTGGCCCACGTGGAG

GAGCTGGCCCGTGATGGTGAGGCGCCACTGGACAAGGCCTGTGTGGACGACGAGGAGGAG

GACGAGGAGGAGGAGGAGAAG

>CL35.Contig10_A-W 4 804 Mbd3 protein [Rattus norvegicus]

GAGCGGGGGTCGCGGCGGCGGCGGCAGCGGGCCCGGAGGCGGGCCGAGGAGCCGGGCGCG

ATGGAGCGGAAGAGCCCCAGTGGGAAGAAGTTCCGCAGCAAGCCCCAGCTGGCGCGCTAC

CTGGGGGGCTCCATGGACCTGAGCACCTTTGACTTCCGCACCGGCAAGATGCTGATGAGC

AAGATGAACAAGAGCCGCCAACGCGTGCGCTACGACTCCTCCAACCAGGTCAAGGGCAAG

CCCGACCTGAACACAGCGCTGCCTGTGCGGCAGACAGCGTCCATCTTTAAGCAGCCTGTG

ACCAAGATCACCAACCACCCCAGCAACAAGGTGAAGAGCGACCCACAGAAGGCTGTGGAC

CAGCCCCGGCAGCTGTTCTGGGAGAAGAAGCTGAGTGGCCTGAGTGCCTTTGACATCGCC

GAGGAGCTTGTCAGGACCATGGACCTGCCCAAAGGCCTGCAGGGGGTGGGCCCAGGCTGT

ACGGATGAGACCCTGCTGTCGGCCATCGCCAGCGCCCTGCACACCAGCACCATGCCCATC

ACGGGGCAGCTCTCAGCCGCCGTGGAGAAGAACCCTGGTGTGTGGCTGAACACCACGCAA

CCCCTGTGCAAAGCATTCATGGTGACTGACGAGGACATCAGGAAGCAGGAGGAGCTGGTG

CAGCAGGTGCGGAAGCGGCTGGAGGAGGCACTGATGGCTGACATGCTGGCCCACGTGGAG

GAGCTGGCCCGTGATGGTGAGGCGCCACTGGACAAGGCCTGTGTGGACGACGAGGAGGAG

GACGAGGAGGAGGAGGAGAAG

>CL35.Contig11_A-W 4 645 Mbd3 protein [Rattus norvegicus]

GAGCGGGGGTCGCGGCGGCGGCGGCAGCGGGCCCGGAGGCGGGCCGAGGAGCCGGGCGCG

ATGGAGCGGAAGAGCCCCAGTGGGAAGAAGTTCCGCAGCAAGCCCCAGCTGGCGCGCTAC

CTGGGGGGCTCCATGGACCTGAGCACCTTTGACTTCCGCACCGGCAAGATGCTGATGAGC

AAGATGAACAAGAGCCGCCAACGCGTGCGCTACGACTCCTCCAACCAGGTCAAGGGCAAG

CCCGACCTGAACACAGCGCTGCCTGTGCGGCAGACAGCGTCCATCTTTAAGCAGCCTGTG

ACCAAGATCACCAACCACCCCAGCAACAAGGTGAAGAGCGACCCACAGAAGGCTGTGGAC

CAGCCCCGGCAGCTGTTCTGGGAGAAGAAGCTGAGTGGCCTGAGTGCCTTTGACATCGCC

GAGGAGCTTGTCAGGACCATGGACCTGCCCAAAGGCCTGCAGGGGGTGGGCCCAGGCTGT

ACGGATGAGACCCTGCTGTCGGCCATCGCCAGCGCCCTGCACACCAGCACCATGCCCATC

ACGGGGCAGCTCTCAGCCGCCGTGGAGAAGAACCCTGGTGTGTGGCTGAACACCACGCAA

CCCCTGTGCAAAGCATTCATGGTGACTGACGAGGACATCAGG

>CL35.Contig12_A-W 854 973 minus strand PREDICTED: RING finger protein 126-like isoform 1 [Cavia porcellus]

AAGCACGACAGCTGCCCTGTCTGCCGCAAGAGCCTCACCGGACAGAACACGGCCACCAAC

CCCCCAGGCCTGACTGGGGTGAGCTTCTCCTCCTCCTCCTCGTCCTCCTCCTCGTCGTCC

>CL35.Contig13_A-W 98 907 minus strand PREDICTED: RING finger protein 126 [Otolemur garnettii]

GCTGGCTTCTGCCGACAAGCACCTTCTCTCCCCAGGAATACAGAAAACGGTTCCGCTCCC

TCCACGGCCGCCGCTGACCAGAGCCGGCAGCCGTTTGAGAACGTGGACCAGCACCTGTTC

ACGCTGCCCCAGGGCTACGGACAATTTGCTTTCGGCATCTTTGACGACAGCTTTGAGATC

CCCACGTTCCCTCCTGGGGCACAGGCCGATGACAGCAGGGACCCTGAGAGCCGGAGGGAG

AGAGAACACCAGTCCCGGCACCGGTATGGCGCCCGGCAGCCCCGAGCCCGCCTCACCGCC

AGGCGGGCCACCGGCCGGCACGAAGGCGTCCCCACGCTGGAAGGGATCATCCAGCAGTTG

GTCAACGGCATCATCACTCCGGCCACCATCCCCAACTTGGGCCTGGGCCCCTGGGGCGTC

CTGCACTCCAACCCAATGGACTACGCCTGGGGGGCCAACGGCCTGGATGCCATCATCACG

CAGCTCCTCAACCAGTTTGAAAACACGGGCCCCCCTCCCGCAGACAAGGAGAAGATCCAG

GCCCTGCCCACCGTCCCCGTCACCGAGGAGCACGTAGGCTCTGGGCTTGAGTGCCCCGTG

TGCAAGGACGACTACGCGCTGGGCGAGAGTGTGCGGCAGCTGCCCTGCAACCACCTGTTC

CACAACGGCTGCATCGTGCCCTGGCTGGAGCAGCACGACAGCTGCCCTGTCTGCCGCAAG

AGCCTCACCGGACAGAACACGGCCACCAACCCCCCAGGCCTGACTGGGGTGAGCTTCTCC

TCCTCCTCCTCGTCCTCCTCCTCGTCGTCC

>CL35.Contig14_A-W 4 804 Mbd3 protein [Rattus norvegicus]

GAGCGGGGGTCGCGGCGGCGGCGGCAGCGGGCCCGGAGGCGGGCCGAGGAGCCGGGCGCG

ATGGAGCGGAAGAGCCCCAGTGGGAAGAAGTTCCGCAGCAAGCCCCAGCTGGCGCGCTAC

CTGGGGGGCTCCATGGACCTGAGCACCTTTGACTTCCGCACCGGCAAGATGCTGATGAGC

AAGATGAACAAGAGCCGCCAACGCGTGCGCTACGACTCCTCCAACCAGGTCAAGGGCAAG

CCCGACCTGAACACAGCGCTGCCTGTGCGGCAGACAGCGTCCATCTTTAAGCAGCCTGTG

ACCAAGATCACCAACCACCCCAGCAACAAGGTGAAGAGCGACCCACAGAAGGCTGTGGAC

CAGCCCCGGCAGCTGTTCTGGGAGAAGAAGCTGAGTGGCCTGAGTGCCTTTGACATCGCC

GAGGAGCTTGTCAGGACCATGGACCTGCCCAAAGGCCTGCAGGGGGTGGGCCCAGGCTGT

ACGGATGAGACCCTGCTGTCGGCCATCGCCAGCGCCCTGCACACCAGCACCATGCCCATC

ACGGGGCAGCTCTCAGCCGCCGTGGAGAAGAACCCTGGTGTGTGGCTGAACACCACGCAA

CCCCTGTGCAAAGCATTCATGGTGACTGACGAGGACATCAGGAAGCAGGAGGAGCTGGTG

CAGCAGGTGCGGAAGCGGCTGGAGGAGGCACTGATGGCTGACATGCTGGCCCACGTGGAG

GAGCTGGCCCGTGATGGTGAGGCGCCACTGGACAAGGCCTGTGTGGACGACGAGGAGGAG

GACGAGGAGGAGGAGGAGAAG

>CL35.Contig15_A-W 3 896 minus strand PREDICTED: RING finger protein 126 [Otolemur garnettii]

CCGCAGCCCGGACGGTACTTCTGCCACTGCTGCTCGGTCGAGATCGTGCCGCGCCTGCCG

GATTACATCTGCCCCAGGTGTGAGTCTGGTTTTATCGAGGAGCTTCCAGAAGAGACCAGG

AATACAGAAAACGGTTCCGCTCCCTCCACGGCCGCCGCTGACCAGAGCCGGCAGCCGTTT

GAGAACGTGGACCAGCACCTGTTCACGCTGCCCCAGGGCTACGGACAATTTGCTTTCGGC

ATCTTTGACGACAGCTTTGAGATCCCCACGTTCCCTCCTGGGGCACAGGCCGATGACAGC

AGGGACCCTGAGAGCCGGAGGGAGAGAGAACACCAGTCCCGGCACCGGTATGGCGCCCGG

CAGCCCCGAGCCCGCCTCACCGCCAGGCGGGCCACCGGCCGGCACGAAGGCGTCCCCACG

CTGGAAGGGATCATCCAGCAGTTGGTCAACGGCATCATCACTCCGGCCACCATCCCCAAC

TTGGGCCTGGGCCCCTGGGGCGTCCTGCACTCCAACCCAATGGACTACGCCTGGGGGGCC

AACGGCCTGGATGCCATCATCACGCAGCTCCTCAACCAGTTTGAAAACACGGGCCCCCCT

CCCGCAGACAAGGAGAAGATCCAGGCCCTGCCCACCGTCCCCGTCACCGAGGAGCACGTA

GGCTCTGGGCTTGAGTGCCCCGTGTGCAAGGACGACTACGCGCTGGGCGAGAGTGTGCGG

CAGCTGCCCTGCAACCACCTGTTCCACAACGGCTGCATCGTGCCCTGGCTGGAGCAGCAC

GACAGCTGCCCTGTCTGCCGCAAGAGCCTCACCGGACAGAACACGGCCACCAACCCCCCA

GGCCTGACTGGGGTGAGCTTCTCCTCCTCCTCCTCGTCCTCCTCCTCGTCGTCC

>CL37.Contig1_A-W 29 787 minus strand PREDICTED: nucleolar protein 7-like [Nomascus leucogenys]

ATGGTGCAGCTCCGGCCGCGCGCGTCCCGCACCCCCGCGTCGGCGGAGGCGATGGTGGAT

GAGGGCCAGCCGGCCTCGGAGGAGGCGGAGCACGGCCTGCTGCTCGGGCAGCCCAGCAGC

GGTGCGGCCGCCGAGCCCTTGGAGGATGAGGACGGGGAAGACGAGCTTGACGACGAGGCC

CCAGAGGAGCTGACCTTCGCCAGCGCCCAGGCCGAAGCGAGAGAAGAGGAGCGGCGGGTG

CGCGAGACCGTGCGCAGGGATAAAACGCTGCTGAAGGAGAAGAGGAAGCGGCGCGAGGAG

CTGTTCATCGAGCAGAAGAAAAGAAAACTACTTCCAGATTCTCTTCTAGAACAGTTAACT

GCAGCGCCGCACACCGACATAAAGAAATCACCAGGAAAGTTGAAAGAAGTTAATTTGCAA

AAGAAAAACGAAGAGTGTGAAAAAGGAAGTGACTCCAAGAAAGTTAAAGTACAAAAAGTA

CAGTCTTCCAGCCAGAAAAGCTATTTAGCCCTAAGGCTAAAAGACCAAGATCTGAGAGAT

TCAAGGCAACAAGCCGCCAAAGCCTTTATACATAATTGTCTGTACGGCCCAGGAACCAAC

AGAACTACTGTAAATAAGTTTCTGTCTCTTGACAACAAGAGGTTACCAGTGAAAAAGGCT

GCAGCCCAGTTTTTGAATCATGCTTGGGGAATCCAGAAAAAACAAAACGCCAAGAGGTTT

AAACGACGGTGGATGTTCAGAAAGATGAAAACTAATAAA

>CL37.Contig2_A-W 29 643 minus strand PREDICTED: nucleolar protein 7-like [Nomascus leucogenys]

ATGGTGCAGCTCCGGCCGCGCGCGTCCCGCACCCCCGCGTCGGCGGAGGCGATGGTGGAT

GAGGGCCAGCCGGCCTCGGAGGAGGCGGAGCACGGCCTGCTGCTCGGGCAGCCCAGCAGC

GGTGCGGCCGCCGAGCCCTTGGAGGATGAGGACGGGGAAGACGAGCTTGACGACGAGGCC

CCAGAGGAGCTGACCTTCGCCAGCGCCCAGGCCGAAGCGAGAGAAGAGGAGCGGCGGGTG

CGCGAGACCGTGCGCAGGGATAAAACGCTGCTGAAGGAGAAGAGGAAGCGGCGCGAGGAG

CTGTTCATCGAGCAGAAGAAAAGAAAACTACTTCCAGATTCTCTTCTAGAACAGTTAACT

GCAGCGCCGCACACCGACATAAAGAAATCACCAGGAAAGTTGAAAGAAGTTAATTTGCAA

AAGAAAAACGAAGAGTGTGAAAAAGGAAGTGACTCCAAGAAAGTTAAAGTACAAAAAGTA

CAGTCTTCCAGCCAGAAAAGCTATTTAGCCCTAAGGCTAAAAGACCAAGATCTGAGAGAT

TCAAGGCAACAAGCCGCCAAAGCCTTTATACATAATTGTCTGTACGGCCCAGGAACCAAC

AGAACTACTGGTAAT

>CL37.Contig3_A-W 793 942 minus strand hypothetical protein PANDA_005990 [Ailuropoda melanoleuca]

GTAAATAAGTTTCTGTCTCTTGACAACAAGAGGTTACCAGTGAAAAAGGCTGCAGCCCAG

TTTTTGAATCATGCTTGGGGAATCCAGAAAAAACAAAACGCCAAGAGGTTTAAACGACGG

TGGATGTTCAGAAAGATGAAAACTAATAAA

>CL37.Contig4_A-W 31 276 minus strand hypothetical protein PANDA_005990 [Ailuropoda melanoleuca]

ATGGTGCAGCTCCGGCCGCGCGCGTCCCGCACCCCCGCGTCGGCGGAGGCGATGGTGGAT

GAGGGCCAGCCGGCCTCGGAGGAGGCGGAGCACGGCCTGCTGCTCGGGCAGCCCAGCAGC

GGTGCGGCCGCCGAGCCCTTGGAGGATGAGGACGGGGAAGACGAGCTTGACGACGAGGCC

CCAGAGGAGCTGACCTTCGCCAGCGCCCAGGCCGAAGCGAGAGAAGAGGAGCGGCGGGTG

CGCGAG

>CL38.Contig1_A-W 3 2972 minus strand PREDICTED: extended synaptotagmin-1 isoform 1 [Papio anubis]

AAAGTTCACCCTAGTCAGAGAAAAGACCAGATCTTGCTGGACTTGAACATTAGCTATGTA

GGTGATGTGCAGATTGATGTGGAAGTAAAAAAATATTTCTGCAAAGCAGGAGTAAAAGGC

ATGCAGCTACATGGCATCTTGCGGGTGATTCTTGAGCCACTCGTGGGGGACCTTCCTATT

GTGGGAGCTGTGTCAATGTTCTTTATTCGACGCCCGACCCTGGACATAAACTGGACAGGG

ATGACTAACCTGCTGGATATCCCAGGACTTAGCTCACTCTCTGACACCATGATCATGGAT

TCCATTGCTGCCTTCCTTGTATTGCCCAACCGACTATTGGTGCCTCTTGTACCTGACCTT

CAAGATGTGGCCCAGTTGCGTTCCCCTCTGCCCAGGGGCATTATTCGTATTCATCTGCTG

GCTGCTCAAGGGCTGAGTTCCAAGGACAAATATGTGAAGGGCCTAATTGAAGGCAAGTCA

GATCCCTATGCACTTGTGCGTGTGGGCACCCAGACATTCTGCAGTCGCGTCATCGATGAG

GAACTCAACCCTAAGTGGGGAGAGACATATGAGGTGATAGTACATGAGGTCCCAGGGCAG

GAAATTGAGGTGGAGGTGTTTGACAAGGATCCAGATAAAGATGACTTTCTGGGCAGAATG

AAGCTGGATGTAGGAAAGGTATTACAGGCTGGAGTACTGGATGATTGGTTCCCTCTACAA

GGTGGGCAAGGCCAAGTTCACCTGAGGCTAGAATGGCTGTCACTTTTGCCAGATGCAGAA

AAACTGGAGCAGGTTCTACAGTGGAATCGGGGAGTCTCGTCTCGACCAGATCCCCCATCA

GCTGCCATCCTAGTTGTCTACCTGGATCGGGCCCAAGATCTTCCTCTGAAGAAGATGAAC

AAGGAACCCAACCCCATGGTACAACTGTCAATTCAGGATGTGACTCATGAGAGCAAGACT

GTCTATAGCACCAACAGCCCAATATGGGAGGAAGCTTTCCGGTTCTTCCTGCAAGACCCT

CGAAACCAGGAGCTCGATGTTCAGGTGAAGGATGACTCCAGGGCCCTGACTTTAGGGTCA

CTAACTCTGCCTCTGGCTCGTCTGCTCACTGCCCCTGAACTTACCCTGGACCAGTGGTTC

CAGCTCAGCAGCTCTGGCCTAAACTCCAGGCTCTACATGAAACTAGTCATGCGGATCTTG

TACTTGAATTCATCAGAAATCTGCTTTCCCACTGGGTCTGGTTCTTGGGACCTAGACAAT

GAGAGCCCCCAGACAGGCAGCAGTGTGGACGCTCCGCCTCAGCCCAGTCACACAACTCCT

GACAGCCACTTTGGGACTGAAAATGTGCTTCGGCTCCATGTATTAGAGGCCCAGGACCTG

ATTGCCAAAGACCGTTTCTTGGGGGGATTGGTGAAGGGCAAGTCAGATCCCTATGTCAAG

CTAAAGCTGGCAGGACGAAGTTTCCGGAGCCGTGTTGTTCGGGAAGATCTCAACCCTCGC

TGGAATGAGGTTTTTGAGGTGATTGTCACATCAATTCCAGGCCAAGAGTTAGAGGTTGAA

GTCTTTGACAAGGACTTGGACAAGGATGACTTTCTGGGCAGATGTAAAGTGAGTCTCACC

ACAGTGCTAAACAGTGGCTTCCTTGATGAGTGGCTGACCCTGGAGGATGTCCCATCTGGC

CGTCTGCACTTGCGCCTGGAGCGTCTGACTCCCCGGCCCACTGCTGCTGAGTTAGAGGAG

GTGCTACAGGTGAACAGTCTGATCCAGACACAGAAGAGCGCAGAGCTGGCGGCAGCCCTG

CTGTCAGTCTATGTGGAGCGGGCAGAGGACTTGCCGCTCCGAAAAGGCACCAAACCTCCT

AGTACTTATGCTATTCTTACCGTGGGAGAGACTGCCCATAAAACCAAGACTGTTTCCCAA

ACCTCAGCCCCTGTGTGGGATGAGAATGCCTCCTTTCTCATCAGGAAGCCACACACTGAG

AGCCTGGAGTTGCAGGTTCGAGGCGAGGGGACTGGTGCCTTGGGCTCCTTATTCCTGCCC

CTCTCTGACCTCCTCGAGGCAGATGGGCTCTGCTTGGACCGTTGGTTTGCACTTACCAAT

GGTCAGGGGCAGGTGCTACTGAGAGCACAGCTAGGGGTAAGTGCCAGGAGATGGTGGGTG

GGCCTAGAGGGAGAGGAGAAGGCCTTTCTGGCTGAAGGACACCAAGAGGGTGGGAACAGA

GGGCAGTATCCTTACCGCACGAGAGTTGGGAGAACTGTGCCCAGCTTACAGCTTTCTTAC

TCCCAGATCCTGGCGTCCCAGCACTCAGGAGTGGAAGCTCATAGCCACAGCTCCAGCCAC

AGCTCCTCCTCGCTGAGTGAAGCAGCAGAGCTTGGTGGGGGACCCCCTCACATCACCTCC

TCAGCTCCAGAGCTCCGGCAGCGCCTAACACGTGGTGACAGGTGAAGGGCTGAAACAGAA

GACTGGAGACCAGTAGGAGTATAGAGGGTGAGTTTAAGCCTCCCCAGGCGTTACCTTCTC

CCAGACCTATTTCCATTTCTTCACAGTCCCCTCGAGGCTCCTTCTGGGCCTCTGGGCCAG

GTGAAGTTGACAGTATGGTACTACAGTGAAGAACGGAAGCTGGTCAGCATTATTCACAGC

TGCCGGGCCCTTCGACAAAATGGACGGGATTCCCCTGACCCCTATGTGTCACTGTTGCTA

CTGCCAGACAAGAATCGAGGTACCAAGAGGAAGACCTCGCAGAAGAAGAAGACCCTCAAT

CCTGAATTCAACGAACGGTTTGAGTGGGAACTGCCCCTAGATGAGGCCCTCAGGCGAAAG

CTGGATGTCTTGGTGAAGTCTAACTCCTCCTTCATGTCAAGAGAGCGTGAGCTTCTGGGG

AAGGTGCAGCTGGACCTGGCTGAGATAGACCTTTCCCAGGGTGCAGCCCAGTGGTATGAT

CTGATGGATGACAAAGACAAGGGCAGCTCC

>CL38.Contig2_A-W 3 2867 minus strand PREDICTED: extended synaptotagmin-1 isoform 1 [Papio anubis]

AAAGTTCACCCTAGTCAGAGAAAAGACCAGATCTTGCTGGACTTGAACATTAGCTATGTA

GGTGATGTGCAGATTGATGTGGAAGTAAAAAAATATTTCTGCAAAGCAGGAGTAAAAGGC

ATGCAGCTACATGGCATCTTGCGGGTGATTCTTGAGCCACTCGTGGGGGACCTTCCTATT

GTGGGAGCTGTGTCAATGTTCTTTATTCGACGCCCGACCCTGGACATAAACTGGACAGGG

ATGACTAACCTGCTGGATATCCCAGGACTTAGCTCACTCTCTGACACCATGATCATGGAT

TCCATTGCTGCCTTCCTTGTATTGCCCAACCGACTATTGGTGCCTCTTGTACCTGACCTT

CAAGATGTGGCCCAGTTGCGTTCCCCTCTGCCCAGGGGCATTATTCGTATTCATCTGCTG

GCTGCTCAAGGGCTGAGTTCCAAGGACAAATATGTGAAGGGCCTAATTGAAGGCAAGTCA

GATCCCTATGCACTTGTGCGTGTGGGCACCCAGACATTCTGCAGTCGCGTCATCGATGAG

GAACTCAACCCTAAGTGGGGAGAGACATATGAGGTGATAGTACATGAGGTCCCAGGGCAG

GAAATTGAGGTGGAGGTGTTTGACAAGGATCCAGATAAAGATGACTTTCTGGGCAGAATG

AAGCTGGATGTAGGAAAGGTATTACAGGCTGGAGTACTGGATGATTGGTTCCCTCTACAA

GGTGGGCAAGGCCAAGTTCACCTGAGGCTAGAATGGCTGTCACTTTTGCCAGATGCAGAA

AAACTGGAGCAGGTTCTACAGTGGAATCGGGGAGTCTCGTCTCGACCAGATCCCCCATCA

GCTGCCATCCTAGTTGTCTACCTGGATCGGGCCCAAGATCTTCCTCTGAAGAAGATGAAC

AAGGAACCCAACCCCATGGTACAACTGTCAATTCAGGATGTGACTCATGAGAGCAAGACT

GTCTATAGCACCAACAGCCCAATATGGGAGGAAGCTTTCCGGTTCTTCCTGCAAGACCCT

CGAAACCAGGAGCTCGATGTTCAGGTGAAGGATGACTCCAGGGCCCTGACTTTAGGGTCA

CTAACTCTGCCTCTGGCTCGTCTGCTCACTGCCCCTGAACTTACCCTGGACCAGTGGTTC

CAGCTCAGCAGCTCTGGCCTAAACTCCAGGCTCTACATGAAACTAGTCATGCGGATCTTG

TACTTGAATTCATCAGAAATCTGCTTTCCCACTGGGTCTGGTTCTTGGGACCTAGACAAT

GAGAGCCCCCAGACAGGCAGCAGTGTGGACGCTCCGCCTCAGCCCAGTCACACAACTCCT

GACAGCCACTTTGGGACTGAAAATGTGCTTCGGCTCCATGTATTAGAGGCCCAGGACCTG

ATTGCCAAAGACCGTTTCTTGGGGGGATTGGTGAAGGGCAAGTCAGATCCCTATGTCAAG

CTAAAGCTGGCAGGACGAAGTTTCCGGAGCCGTGTTGTTCGGGAAGATCTCAACCCTCGC

TGGAATGAGGTTTTTGAGGTGATTGTCACATCAATTCCAGGCCAAGAGTTAGAGGTTGAA

GTCTTTGACAAGGACTTGGACAAGGATGACTTTCTGGGCAGATGTAAAGTGAGTCTCACC

ACAGTGCTAAACAGTGGCTTCCTTGATGAGTGGCTGACCCTGGAGGATGTCCCATCTGGC

CGTCTGCACTTGCGCCTGGAGCGTCTGACTCCCCGGCCCACTGCTGCTGAGTTAGAGGAG

GTGCTACAGGTGAACAGTCTGATCCAGACACAGAAGAGCGCAGAGCTGGCGGCAGCCCTG

CTGTCAGTCTATGTGGAGCGGGCAGAGGACTTGCCGCTCCGAAAAGGCACCAAACCTCCT

AGTACTTATGCTATTCTTACCGTGGGAGAGACTGCCCATAAAACCAAGACTGTTTCCCAA

ACCTCAGCCCCTGTGTGGGATGAGAATGCCTCCTTTCTCATCAGGAAGCCACACACTGAG

AGCCTGGAGTTGCAGGTTCGAGGCGAGGGGACTGGTGCCTTGGGCTCCTTATTCCTGCCC

CTCTCTGACCTCCTCGAGGCAGATGGGCTCTGCTTGGACCGTTGGTTTGCACTTACCAAT

GGTCAGGGGCAGGTGCTACTGAGAGCACAGCTAGGGGTAAGTGCCAGGAGATGGTGGGTG

GGCCTAGAGGGAGAGGAGAAGGCCTTTCTGGCTGAAGGACACCAAGAGGGTGGGAACAGA

GGGCAGTATCCTTACCGCACGAGAGTTGGGAGAACTGTGCCCAGCTTACAGCTTTCTTAC

TCCCAGATCCTGGCGTCCCAGCACTCAGGAGTGGAAGCTCATAGCCACAGCTCCAGCCAC

AGCTCCTCCTCGCTGAGTGAAGCAGCAGAGCTTGGTGGGGGACCCCCTCACATCACCTCC

TCAGCTCCAGAGCTCCGGCAGCGCCTAACACGTGGTGACAGTCCCCTCGAGGCTCCTTCT

GGGCCTCTGGGCCAGGTGAAGTTGACAGTATGGTACTACAGTGAAGAACGGAAGCTGGTC

AGCATTATTCACAGCTGCCGGGCCCTTCGACAAAATGGACGGGATTCCCCTGACCCCTAT

GTGTCACTGTTGCTACTGCCAGACAAGAATCGAGGTACCAAGAGGAAGACCTCGCAGAAG

AAGAAGACCCTCAATCCTGAATTCAACGAACGGTTTGAGTGGGAACTGCCCCTAGATGAG

GCCCTCAGGCGAAAGCTGGATGTCTTGGTGAAGTCTAACTCCTCCTTCATGTCAAGAGAG

CGTGAGCTTCTGGGGAAGGTGCAGCTGGACCTGGCTGAGATAGACCTTTCCCAGGGTGCA

GCCCAGTGGTATGATCTGATGGATGACAAAGACAAGGGCAGCTCC

>CL38.Contig3_A-W 3 2822 minus strand PREDICTED: extended synaptotagmin-1 isoform 1 [Papio anubis]

AAAGTTCACCCTAGTCAGAGAAAAGACCAGATCTTGCTGGACTTGAACATTAGCTATGTA

GGTGATGTGCAGATTGATGTGGAAGTAAAAAAATATTTCTGCAAAGCAGGAGTAAAAGGC

ATGCAGCTACATGGCATCTTGCGGGTGATTCTTGAGCCACTCGTGGGGGACCTTCCTATT

GTGGGAGCTGTGTCAATGTTCTTTATTCGACGCCCGACCCTGGACATAAACTGGACAGGG

ATGACTAACCTGCTGGATATCCCAGGACTTAGCTCACTCTCTGACACCATGATCATGGAT

TCCATTGCTGCCTTCCTTGTATTGCCCAACCGACTATTGGTGCCTCTTGTACCTGACCTT

CAAGATGTGGCCCAGTTGCGTTCCCCTCTGCCCAGGGGCATTATTCGTATTCATCTGCTG

GCTGCTCAAGGGCTGAGTTCCAAGGACAAATATGTGAAGGGCCTAATTGAAGGCAAGTCA

GATCCCTATGCACTTGTGCGTGTGGGCACCCAGACATTCTGCAGTCGCGTCATCGATGAG

GAACTCAACCCTAAGTGGGGAGAGACATATGAGGTGATAGTACATGAGGTCCCAGGGCAG

GAAATTGAGGTGGAGGTGTTTGACAAGGATCCAGATAAAGATGACTTTCTGGGCAGAATG

AAGCTGGATGTAGGAAAGGTATTACAGGCTGGAGTACTGGATGATTGGTTCCCTCTACAA

GGTGGGCAAGGCCAAGTTCACCTGAGGCTAGAATGGCTGTCACTTTTGCCAGATGCAGAA

AAACTGGAGCAGGTTCTACAGTGGAATCGGGGAGTCTCGTCTCGACCAGATCCCCCATCA

GCTGCCATCCTAGTTGTCTACCTGGATCGGGCCCAAGATCTTCCTCTGAAGAAGATGAAC

AAGGAACCCAACCCCATGGTACAACTGTCAATTCAGGATGTGACTCATGAGAGCAAGACT

GTCTATAGCACCAACAGCCCAATATGGGAGGAAGCTTTCCGGTTCTTCCTGCAAGACCCT

CGAAACCAGGAGCTCGATGTTCAGGTGAAGGATGACTCCAGGGCCCTGACTTTAGGGTCA

CTAACTCTGCCTCTGGCTCGTCTGCTCACTGCCCCTGAACTTACCCTGGACCAGTGGTTC

CAGCTCAGCAGCTCTGGCCTAAACTCCAGGCTCTACATGAAACTAGTCATGCGGATCTTG

TACTTGAATTCATCAGAAATCTGCTTTCCCACTGGGTCTGGTTCTTGGGACCTAGACAAT

GAGAGCCCCCAGACAGGCAGCAGTGTGGACGCTCCGCCTCAGCCCAGTCACACAACTCCT

GACAGCCACTTTGGGACTGAAAATGTGCTTCGGCTCCATGTATTAGAGGCCCAGGACCTG

ATTGCCAAAGACCGTTTCTTGGGGGGATTGGTGAAGGGCAAGTCAGATCCCTATGTCAAG

CTAAAGCTGGCAGGACGAAGTTTCCGGAGCCGTGTTGTTCGGGAAGATCTCAACCCTCGC

TGGAATGAGGTTTTTGAGGTGATTGTCACATCAATTCCAGGCCAAGAGTTAGAGGTTGAA

GTCTTTGACAAGGACTTGGACAAGGATGACTTTCTGGGCAGATGTAAAGTGAGTCTCACC

ACAGTGCTAAACAGTGGCTTCCTTGATGAGTGGCTGACCCTGGAGGATGTCCCATCTGGC

CGTCTGCACTTGCGCCTGGAGCGTCTGACTCCCCGGCCCACTGCTGCTGAGTTAGAGGAG

GTGCTACAGGTGAACAGTCTGATCCAGACACAGAAGAGCGCAGAGCTGGCGGCAGCCCTG

CTGTCAGTCTATGTGGAGCGGGCAGAGGACTTGCCGCTCCGAAAAGGCACCAAACCTCCT

AGTACTTATGCTATTCTTACCGTGGGAGAGACTGCCCATAAAACCAAGACTGTTTCCCAA

ACCTCAGCCCCTGTGTGGGATGAGAATGCCTCCTTTCTCATCAGGAAGCCACACACTGAG

AGCCTGGAGTTGCAGGTTCGAGGCGAGGGGACTGGTGCCTTGGGCTCCTTATTCCTGCCC

CTCTCTGACCTCCTCGAGGCAGATGGGCTCTGCTTGGACCGTTGGTTTGCACTTACCAAT

GGTCAGGGGCAGGTGCTACTGAGAGCACAGCTAGGGATCCTGGCGTCCCAGCACTCAGGA

GTGGAAGCTCATAGCCACAGCTCCAGCCACAGCTCCTCCTCGCTGAGTGAAGCAGCAGAG

CTTGGTGGGGGACCCCCTCACATCACCTCCTCAGCTCCAGAGCTCCGGCAGCGCCTAACA

CGTGGTGACAGGTGAAGGGCTGAAACAGAAGACTGGAGACCAGTAGGAGTATAGAGGGTG

AGTTTAAGCCTCCCCAGGCGTTACCTTCTCCCAGACCTATTTCCATTTCTTCACAGTCCC

CTCGAGGCTCCTTCTGGGCCTCTGGGCCAGGTGAAGTTGACAGTATGGTACTACAGTGAA

GAACGGAAGCTGGTCAGCATTATTCACAGCTGCCGGGCCCTTCGACAAAATGGACGGGAT

TCCCCTGACCCCTATGTGTCACTGTTGCTACTGCCAGACAAGAATCGAGGTACCAAGAGG

AAGACCTCGCAGAAGAAGAAGACCCTCAATCCTGAATTCAACGAACGGTTTGAGTGGGAA

CTGCCCCTAGATGAGGCCCTCAGGCGAAAGCTGGATGTCTTGGTGAAGTCTAACTCCTCC

TTCATGTCAAGAGAGCGTGAGCTTCTGGGGAAGGTGCAGCTGGACCTGGCTGAGATAGAC

CTTTCCCAGGGTGCAGCCCAGTGGTATGATCTGATGGATGACAAAGACAAGGGCAGCTCC

>CL38.Contig4_A-W 3 2717 minus strand PREDICTED: extended synaptotagmin-1 isoform 1 [Papio anubis]

AAAGTTCACCCTAGTCAGAGAAAAGACCAGATCTTGCTGGACTTGAACATTAGCTATGTA

GGTGATGTGCAGATTGATGTGGAAGTAAAAAAATATTTCTGCAAAGCAGGAGTAAAAGGC

ATGCAGCTACATGGCATCTTGCGGGTGATTCTTGAGCCACTCGTGGGGGACCTTCCTATT

GTGGGAGCTGTGTCAATGTTCTTTATTCGACGCCCGACCCTGGACATAAACTGGACAGGG

ATGACTAACCTGCTGGATATCCCAGGACTTAGCTCACTCTCTGACACCATGATCATGGAT

TCCATTGCTGCCTTCCTTGTATTGCCCAACCGACTATTGGTGCCTCTTGTACCTGACCTT

CAAGATGTGGCCCAGTTGCGTTCCCCTCTGCCCAGGGGCATTATTCGTATTCATCTGCTG

GCTGCTCAAGGGCTGAGTTCCAAGGACAAATATGTGAAGGGCCTAATTGAAGGCAAGTCA

GATCCCTATGCACTTGTGCGTGTGGGCACCCAGACATTCTGCAGTCGCGTCATCGATGAG

GAACTCAACCCTAAGTGGGGAGAGACATATGAGGTGATAGTACATGAGGTCCCAGGGCAG

GAAATTGAGGTGGAGGTGTTTGACAAGGATCCAGATAAAGATGACTTTCTGGGCAGAATG

AAGCTGGATGTAGGAAAGGTATTACAGGCTGGAGTACTGGATGATTGGTTCCCTCTACAA

GGTGGGCAAGGCCAAGTTCACCTGAGGCTAGAATGGCTGTCACTTTTGCCAGATGCAGAA

AAACTGGAGCAGGTTCTACAGTGGAATCGGGGAGTCTCGTCTCGACCAGATCCCCCATCA

GCTGCCATCCTAGTTGTCTACCTGGATCGGGCCCAAGATCTTCCTCTGAAGAAGATGAAC

AAGGAACCCAACCCCATGGTACAACTGTCAATTCAGGATGTGACTCATGAGAGCAAGACT

GTCTATAGCACCAACAGCCCAATATGGGAGGAAGCTTTCCGGTTCTTCCTGCAAGACCCT

CGAAACCAGGAGCTCGATGTTCAGGTGAAGGATGACTCCAGGGCCCTGACTTTAGGGTCA

CTAACTCTGCCTCTGGCTCGTCTGCTCACTGCCCCTGAACTTACCCTGGACCAGTGGTTC

CAGCTCAGCAGCTCTGGCCTAAACTCCAGGCTCTACATGAAACTAGTCATGCGGATCTTG

TACTTGAATTCATCAGAAATCTGCTTTCCCACTGGGTCTGGTTCTTGGGACCTAGACAAT

GAGAGCCCCCAGACAGGCAGCAGTGTGGACGCTCCGCCTCAGCCCAGTCACACAACTCCT

GACAGCCACTTTGGGACTGAAAATGTGCTTCGGCTCCATGTATTAGAGGCCCAGGACCTG

ATTGCCAAAGACCGTTTCTTGGGGGGATTGGTGAAGGGCAAGTCAGATCCCTATGTCAAG

CTAAAGCTGGCAGGACGAAGTTTCCGGAGCCGTGTTGTTCGGGAAGATCTCAACCCTCGC

TGGAATGAGGTTTTTGAGGTGATTGTCACATCAATTCCAGGCCAAGAGTTAGAGGTTGAA

GTCTTTGACAAGGACTTGGACAAGGATGACTTTCTGGGCAGATGTAAAGTGAGTCTCACC

ACAGTGCTAAACAGTGGCTTCCTTGATGAGTGGCTGACCCTGGAGGATGTCCCATCTGGC

CGTCTGCACTTGCGCCTGGAGCGTCTGACTCCCCGGCCCACTGCTGCTGAGTTAGAGGAG

GTGCTACAGGTGAACAGTCTGATCCAGACACAGAAGAGCGCAGAGCTGGCGGCAGCCCTG

CTGTCAGTCTATGTGGAGCGGGCAGAGGACTTGCCGCTCCGAAAAGGCACCAAACCTCCT

AGTACTTATGCTATTCTTACCGTGGGAGAGACTGCCCATAAAACCAAGACTGTTTCCCAA

ACCTCAGCCCCTGTGTGGGATGAGAATGCCTCCTTTCTCATCAGGAAGCCACACACTGAG

AGCCTGGAGTTGCAGGTTCGAGGCGAGGGGACTGGTGCCTTGGGCTCCTTATTCCTGCCC

CTCTCTGACCTCCTCGAGGCAGATGGGCTCTGCTTGGACCGTTGGTTTGCACTTACCAAT

GGTCAGGGGCAGGTGCTACTGAGAGCACAGCTAGGGATCCTGGCGTCCCAGCACTCAGGA

GTGGAAGCTCATAGCCACAGCTCCAGCCACAGCTCCTCCTCGCTGAGTGAAGCAGCAGAG

CTTGGTGGGGGACCCCCTCACATCACCTCCTCAGCTCCAGAGCTCCGGCAGCGCCTAACA

CGTGGTGACAGTCCCCTCGAGGCTCCTTCTGGGCCTCTGGGCCAGGTGAAGTTGACAGTA

TGGTACTACAGTGAAGAACGGAAGCTGGTCAGCATTATTCACAGCTGCCGGGCCCTTCGA

CAAAATGGACGGGATTCCCCTGACCCCTATGTGTCACTGTTGCTACTGCCAGACAAGAAT

CGAGGTACCAAGAGGAAGACCTCGCAGAAGAAGAAGACCCTCAATCCTGAATTCAACGAA

CGGTTTGAGTGGGAACTGCCCCTAGATGAGGCCCTCAGGCGAAAGCTGGATGTCTCGGTG

AAGTCTAACTCCTCCTTCATGTCAAGAGAGCGTGAGCTTCTGGGGAAGGTGCAGCTGGAC

CTGGCTGAGATAGACCTTTCCCAGGGTGCAGCCCAGTGGTATGATCTGATGGATGACAAA

GACAAGGGCAGCTCC

>CL38.Contig5_A-W 81 1058 minus strand PREDICTED: extended synaptotagmin-1 [Equus caballus]

CAGGTTCGAGGCGAGGGGACTGGTGCCTTGGGCTCCTTATTCCTGCCCCTCTCTGACCTC

CTCGAGGCAGATGGGCTCTGCTTGGACCGTTGGTTTGCACTTACCAATGGTCAGGGGCAG

GTGCTACTGAGAGCACAGCTAGGGGTAAGTGCCAGGAGATGGTGGGTGGGCCTAGAGGGA

GAGGAGAAGGCCTTTCTGGCTGAAGGACACCAAGAGGGTGGGAACAGAGGGCAGTATCCT

TACCGCACGAGAGTTGGGAGAACTGTGCCCAGCTTACAGCTTTCTTACTCCCAGATCCTG

GCGTCCCAGCACTCAGGAGTGGAAGCTCATAGCCACAGCTCCAGCCACAGCTCCTCCTCG

CTGAGTGAAGCAGCAGAGCTTGGTGGGGGACCCCCTCACATCACCTCCTCAGCTCCAGAG

CTCCGGCAGCGCCTAACACGTGGTGACAGGTGAAGGGCTGAAACAGAAGACTGGAGACCA

GTAGGAGTATAGAGGGTGAGTTTAAGCCTCCCCAGGCGTTACCTTCTCCCAGACCTATTT

CCATTTCTTCACAGTCCCCTCGAGGCTCCTTCTGGGCCTCTGGGCCAGGTGAAGTTGACA

GTATGGTACTACAGTGAAGAACGGAAGCTGGTCAGCATTATTCACAGCTGCCGGGCCCTT

CGACAAAATGGACGGGATTCCCCTGACCCCTATGTGTCACTGTTGCTACTGCCAGACAAG

AATCGAGGTACCAAGAGGAAGACCTCGCAGAAGAAGAAGACCCTCAATCCTGAATTCAAC

GAACGGTTTGAGTGGGAACTGCCCCTAGATGAGGCCCTCAGGCGAAAGCTGGATGTCTTG

GTGAAGTCTAACTCCTCCTTCATGTCAAGAGAGCGTGAGCTTCTGGGGAAGGTGCAGCTG

GACCTGGCTGAGATAGACCTTTCCCAGGGTGCAGCCCAGTGGTATGATCTGATGGATGAC

AAAGACAAGGGCAGCTCC

>CL38.Contig6_A-W 81 953 minus strand PREDICTED: extended synaptotagmin-1 [Equus caballus]

CAGGTTCGAGGCGAGGGGACTGGTGCCTTGGGCTCCTTATTCCTGCCCCTCTCTGACCTC

CTCGAGGCAGATGGGCTCTGCTTGGACCGTTGGTTTGCACTTACCAATGGTCAGGGGCAG

GTGCTACTGAGAGCACAGCTAGGGGTAAGTGCCAGGAGATGGTGGGTGGGCCTAGAGGGA

GAGGAGAAGGCCTTTCTGGCTGAAGGACACCAAGAGGGTGGGAACAGAGGGCAGTATCCT

TACCGCACGAGAGTTGGGAGAACTGTGCCCAGCTTACAGCTTTCTTACTCCCAGATCCTG

GCGTCCCAGCACTCAGGAGTGGAAGCTCATAGCCACAGCTCCAGCCACAGCTCCTCCTCG

CTGAGTGAAGCAGCAGAGCTTGGTGGGGGACCCCCTCACATCACCTCCTCAGCTCCAGAG

CTCCGGCAGCGCCTAACACGTGGTGACAGTCCCCTCGAGGCTCCTTCTGGGCCTCTGGGC

CAGGTGAAGTTGACAGTATGGTACTACAGTGAAGAACGGAAGCTGGTCAGCATTATTCAC

AGCTGCCGGGCCCTTCGACAAAATGGACGGGATTCCCCTGACCCCTATGTGTCACTGTTG

CTACTGCCAGACAAGAATCGAGGTACCAAGAGGAAGACCTCGCAGAAGAAGAAGACCCTC

AATCCTGAATTCAACGAACGGTTTGAGTGGGAACTGCCCCTAGATGAGGCCCTCAGGCGA

AAGCTGGATGTCTTGGTGAAGTCTAACTCCTCCTTCATGTCAAGAGAGCGTGAGCTTCTG

GGGAAGGTGCAGCTGGACCTGGCTGAGATAGACCTTTCCCAGGGTGCAGCCCAGTGGTAT

GATCTGATGGATGACAAAGACAAGGGCAGCTCC

>CL38.Contig7_A-W 81 908 minus strand PREDICTED: extended synaptotagmin-1 [Equus caballus]

CAGGTTCGAGGCGAGGGGACTGGTGCCTTGGGCTCCTTATTCCTGCCCCTCTCTGACCTC

CTCGAGGCAGATGGGCTCTGCTTGGACCGTTGGTTTGCACTTACCAATGGTCAGGGGCAG

GTGCTACTGAGAGCACAGCTAGGGATCCTGGCGTCCCAGCACTCAGGAGTGGAAGCTCAT

AGCCACAGCTCCAGCCACAGCTCCTCCTCGCTGAGTGAAGCAGCAGAGCTTGGTGGGGGA

CCCCCTCACATCACCTCCTCAGCTCCAGAGCTCCGGCAGCGCCTAACACGTGGTGACAGG

TGAAGGGCTGAAACAGAAGACTGGAGACCAGTAGGAGTATAGAGGGTGAGTTTAAGCCTC

CCCAGGCGTTACCTTCTCCCAGACCTATTTCCATTTCTTCACAGTCCCCTCGAGGCTCCT

TCTGGGCCTCTGGGCCAGGTGAAGTTGACAGTATGGTACTACAGTGAAGAACGGAAGCTG

GTCAGCATTATTCACAGCTGCCGGGCCCTTCGACAAAATGGACGGGATTCCCCTGACCCC

TATGTGTCACTGTTGCTACTGCCAGACAAGAATCGAGGTACCAAGAGGAAGACCTCGCAG

AAGAAGAAGACCCTCAATCCTGAATTCAACGAACGGTTTGAGTGGGAACTGCCCCTAGAT

GAGGCCCTCAGGCGAAAGCTGGATGTCTTGGTGAAGTCTAACTCCTCCTTCATGTCAAGA

GAGCGTGAGCTTCTGGGGAAGGTGCAGCTGGACCTGGCTGAGATAGACCTTTCCCAGGGT

GCAGCCCAGTGGTATGATCTGATGGATGACAAAGACAAGGGCAGCTCC

>CL39.Contig1_A-W 46 1515 C4A variant protein [Homo sapiens]

CTCTTGGATTCTCCAGCCATGAGGCTCCTCTGGGGGCTGATCTGGGCGTCCAGCTTCTTT

GTCTTGTCTCTGCAGAAGCCCAGGTTGCTTCTGTTCTCGCCTTCTGTGGTTCATCTGGGG

GTCCCCCTATCAGTGGGGGTGCAGCTCCAGGATGCCCCCCGGGGACAGGTAGTGAAAGGA

TCACTTTTCCTGAGAAACCCAAGCCGAAATAATCACCGCTGCTCCCCAAAGGAGGACTTT

ACCCTCAGCTCAGAAAAAGACTTTGTGCTCCTCAGTCTCCAGATCCCCCTGAATGATGCA

AAGAGTTGTGGCCTCTTTGAACTACGTAGAAGCCCTGAGGTCCAGCTAGTGGCCCAGTCA

TCATGGCTGAAGGACTCCCTGTCTAGAACAACAGATATTCAGGGTGTTAACCTGCTCTTC

TCCTCTCGTCGGGGACACCTTTTTTTGCAGACTGACCAGCCCATTTACAACCCTGGCCAA

CGGGTTCGGTACCGGGTCTTGTCACTGGATCAAAAGATGCGCCCATCCACTGACACCCTC

ACGATCATGGTTGAGAATTCTTTTGGCCTCCGTGTGCTCAAGAAGGAGGTCCGCGTTCCT

TCACCCATCTTCCAGGATGACTTCACGATTCCAGACATCTCAGAACCAGGGACCTGGAAG

ATCTCAGCCCGATTCTCAGACAGTCTGGAATCCAACAGAAGCACCCTGTTTGAGGTGAAG

AAATATGTCCTTCCCAACTTTGAGGTGAAGATCACCCCTGGAAAGCCCTACATCCTGACT

GCACCTGGCTATCTTGGCGAAATTGAATTAGACATCCAGGCCAGGTACATCTATGGGAAG

CCAGTGCAGGGGGTAGCATATGTGCGCTTTGCCCTCCAGGATGAGGATGGTAAGAAGACT

TTCCTCCGGGGGCTGGAGACTCAGAACAAGTTGGTGGAAGGCCAGAGTCACATTTCCCTC

TCAAAGGATAAGGTCCAGGGTACCCTGGAGAAGCTTAATATTGGTATTGCTGACCTCCAA

GGACTGCGTCTGTATGTTGCAGCAGCCATCATTGAGTCTCCAGGAGGGGAGTTGGAGGAG

GCAGAGCTTACATCCTGGCGTTTTGTGTCATCTGCCTTCTCCTTGGATCTTAGCAAGACC

AAGCGACACCTTGTACCTGGGGCCCCCTTCCTACTGCAGGCCCTGGTCCGTGAAATGTCA

GGCTCCCCAGCTTCTGGCATTCCTGTAAAAGTTTCTGCCACTTTGTCTTCTGAGTCAGTT

TCTAAAATTCAAGACTTTCAACAAAACACAGATGGGACTGGCCACGTCAGCATTCCCATC

GTTGTCCCGAAGACTATCACAGAACTGCAGCTCTTGGTGTCTGCAGGCTCCCTCTATCCA

GCCATAGCCAGGCTCACTGTGCGAGCCCCACCTTCAGGAGGTTCTGGCTTTCTGTCTATT

GAGCGGCTGGATCCTCGACCCCCTCGTGTA

>CL39.Contig2_A-W 43 891 PREDICTED: complement C4-A isoform 2 [Saimiri boliviensis boliviensis]

GTCCCTTCCTTGTCCATCCTCAGACCAGGGACCTGGAAGATCTCAGCCCGATTCTCAGAC

AGTCTGGAATCCAACAGAAGCACCCTGTTTGAGGTGAAGAAATATGTCCTTCCCAACTTT

GAGGTGAAGATCACCCCTGGAAAGCCCTACATCCTGACTGCACCTGGCTATCTTGGCGAA

ATTGAATTAGACATCCAGGCCAGGTACATCTATGGGAAGCCAGTGCAGGGGGTAGCATAT

GTGCGCTTTGCCCTCCAGGATGAGGATGGTAAGAAGACTTTCCTCCGGGGGCTGGAGACT

CAGAACAAGTTGGTGGAAGGCCAGAGTCACATTTCCCTCTCAAAGGATAAGGTCCAGGGT

ACCCTGGAGAAGCTTAATATTGGTATTGCTGACCTCCAAGGACTGCGTCTGTATGTTGCA

GCAGCCATCATTGAGTCTCCAGGAGGGGAGTTGGAGGAGGCAGAGCTTACATCCTGGCGT

TTTGTGTCATCTGCCTTCTCCTTGGATCTTAGCAAGACCAAGCGACACCTTGTACCTGGG

GCCCCCTTCCTACTGCAGGCCCTGGTCCGTGAAATGTCAGGCTCCCCAGCTTCTGGCATT

CCTGTAAAAGTTTCTGCCACTTTGTCTTCTGAGTCAGTTTCTAAAATTCAAGACTTTCAA

CAAAACACAGATGGGACTGGCCACGTCAGCATTCCCATCGTTGTCCCGAAGACTATCACA

GAACTGCAGCTCTTGGTGTCTGCAGGCTCCCTCTATCCAGCCATAGCCAGGCTCACTGTG

CGAGCCCCACCTTCAGGAGGTTCTGGCTTTCTGTCTATTGAGCGGCTGGATCCTCGACCC

CCTCGTGTA

>CL40.Contig1_A-W 881 1090 minus strand PREDICTED: probable O-sialoglycoprotein endopeptidase-like [Ailuropoda melanoleuca] >gi|281345900|gb|EFB21484.1| hypothetical protein PANDA_019763 [Ailuropoda melanoleuca]

AATCTGCGGTTGCAGGAGATGATGGAGACAATGTGCCAGGAACGGGGAGCTAAGCTTTTT

GCCACAGATGAGAGATTCTGCATTGACAATGGAGCCATGATAGCCCACGCTGGCTGGGAG

ATGTTTCAGGCTGGACATAGAACGGCCATCAGTGACTCTGGGATTACACAAAGGTATCGT

ACAGATGAAGTAGAAGTGACCTGGAGGGAC

>CL40.Contig2_A-W 132 1136 PREDICTED: probable O-sialoglycoprotein endopeptidase-like [Ailuropoda melanoleuca] >gi|281345900|gb|EFB21484.1| hypothetical protein PANDA_019763 [Ailuropoda melanoleuca]

ATGCCGACGGTGCTGGGTTTTGAAGGCAGTGCCAACAAGATTGGTGTGGGAGTGGTGCGT

GATGGTAACGTGCTGGCGAACCCGCGACGAACTTATGTTACACCTCCAGGCACGGGGTTC

CTTCCAGGTGATACTGCCAGGCATCACCGGGCTGTTATCCTAGACATTCTGCAGGAGGCC

CTAACAGAAGCTAGAGTAACCTACCAGGATATCGACTGCATTGCTTACACCAAAGGTCCT

GGCATGGGTGCCCCATTGGTTTCTGTAGCCATTGTGGCCCGTACTATGGCCCAGCTATGG

AATAAACCATTGCTAGGTGTGAACCACTGTATAGGCCACATTGAAATGGGCCGTCTCATC

ACTGGAGCCACCAGCCCAACTGTGTTGTATGTCAGTGGAGGAAATACGCAGGTGATTGCC

TACTCAGATCGTCGTTACCGCATATTTGGTGAAACCATCGATATTGCCGTTGGTAATTGT

CTGGATCGTTTTGCTCGAGTGCTGAAGATTTCCAATGATCCTAGTCCAGGCTACAACATT

GAACAGATGGCAAAGCGAGGCAAAAAGCTTGTTGAGCTGCCATATACTGTAAAGGGAATG

GATGTCTCATTCTCAGGGATTCTGTCTTTCATTGAGGATGTAGCCCAACGGATGCTGGCC

ACAGGCGAGTGTACTCCTGAGGATCTATGTTTCTCCTTGCAGGAAACTGTGTTTGCAATG

CTGGTAGAGATCACAGAGCGAGCCATGGTGCATTGTGGTTCCCAGGAAGCCCTCATTGTG

GGAGGTGTGGGGTGTAATCTGCGGTTGCAGGAGATGATGGAGACAATGTGCCAGGAACGG

GGAGCTAAGCTTTTTGCCACAGATGAGAGATTCTGCATTGACAATGGAGCCATGATAGCC

CACGCTGGCTGGGAGATGTTTCAGGCTGGACATAGAACGGCCATCAGTGACTCTGGGATT

ACACAAAGGTATCGTACAGATGAAGTAGAAGTGACCTGGAGGGAC

>CL40.Contig3_A-W 52 261 minus strand PREDICTED: probable tRNA threonylcarbamoyladenosine biosynthesis protein OSGEP-like [Equus caballus]

AATCTGCGGTTGCAGGAGATGATGGAGACAATGTGCCAGGAACGGGGAGCTAAGCTTTTT

GCCACAGATGAGAGATTCTGCATTGACAATGGAGCCATGATAGCCCACGCTGGCTGGGAG

ATGTTTCAGGCTGGACATAGAACGGCCATCAGTGACTCTGGGATTACACAAAGGTATCGT

ACAGATGAAGTAGAAGTGACCTGGAGGGAC

>CL42.Contig1_A-W 67 1506 Dipeptidyl-peptidase 7 [Homo sapiens] >gi|16877430|gb|AAH16961.1| Dipeptidyl-peptidase 7 [Homo sapiens]

GGGGCCCCTTTCTGGGCACAGGTCCTGCTGCTGACACTGGGACTGGGTGACGTCCAGGCT

GGGGCCCACAGAGGCTTGGACGCTGGCTTTTGGGAGAGGTACTTTGAGCAGTTTATGGAC

CATTTCAACTTTGCGAGGTTTGGCAACAAGACCTTTTACCAGCGGGTGCTGGTGTCAGAC

AAGTTCTGGCGAAGGGGCGAGGGGCCCATCTTCTTCTACACGGGGAACGAGGTGGACGTG

TGGACTCTGGCCAACAACACAGGCTTTGTGGTGGAGCTGGCAGCCCAGCAAGGCGCCCTG

CTCATCTTTGCGGAGCATCGCTACTACGGGAAGTCGCTCCCGTTTGGGGAGAAGTCCATG

CAGCCCGGGTACACCGAGCTGCTGACCGTGGAGCAGGCGCTGGCCGACTATGCCGTGCTG

ATCCAGACCCTGCGGCAGAAACTAGGAGCACAGGGTGGCCCTGTCATCACCTTTGGAGGG

AGCTACGGGGGGATGCTCAGCGCCTACATGAGGATGAAGTACCCCCACCTGGTGGCAGGG

GCGCTGGCAGCCAGCGCACCCGTCCTGGCTGTCGCGGGCCTTGGTACCTCCAACCAGTTC

TTCCGGGACGTCACAGCGACCTTCCACAGCCAGAGTCCAAGGTGTGTCCAGGGCGTGCGG

GAGGCCTTCCAGCAGATCAGGGACCTGTTCCTACAGGGAGCCCGTGACAGGGTCAGCCAG

GAGTTTGGCACCTGCCAGCCACTCTTGGACACAAAGGACCTGACTCAGCTCTTTGTGTTT

GCACGCAACGCCTTCACTCTGCTGGCCATGATGGACTACCCGTACCCCACTGACTTCATG

GGGTACCTGCCTGCCAACCCTGTCAAGGTTGGCTGTGCCCGGCTGCTGAGGGAGGCCCAG

AGGATCGTGGGTCTGCGGGCACTGACAGGGCTGGTCTACAACTCCTCGGGCACAGCGCAC

TGCTATGACATCTACCAGCTGTACCACAGCTGTGCGGACCCCACTGGCTGTGGCACTGGC

CCTGATGCCATGGCCTGGGACTATCAGGCCTGCACCGAGATCAACCTGACCTTTGACAGC

AACAACGTGACGGACATGTTCCCTGCCCTTCCGTTCACCGCCACGCTCCGCCAACAGTAC

TGCCTGAATAAGTGGGGCGTGTGGCCTCGGCCAGACTGGCTGCAGACCAGCTTCTGGGGC

GGTGACCTCAGAGCCACCAGCAACATCATCTTCTCCAATGGTGACCTAGACCCCTGGGCA

GGGGGCGGGATTCTGAGGAACGTGAGCGCGTCGGTCATTGCTGTCACCATCCACGGGGGA

GCACACCACCTAGACCTCAGGGCATCCCACCCACAAGACCCCAACTCGGTCCTGGAGGTG

CGCAGGCTGGAGGCTGCTCTCATCCTCGAGTGGGCGAAGGCAGCCCGGAGTAGGCAGCAG

>CL42.Contig2_A-W 40 615 PREDICTED: dipeptidyl peptidase 2 [Pan paniscus]

CAGGTTGGCTGTGCCCGGCTGCTGAGGGAGGCCCAGAGGATCGTGGGTCTGCGGGCACTG

ACAGGGCTGGTCTACAACTCCTCGGGCACAGCGCACTGCTATGACATCTACCAGCTGTAC

CACAGCTGTGCGGACCCCACTGGCTGTGGCACTGGCCCTGATGCCATGGCCTGGGACTAT

CAGGCCTGCACCGAGATCAACCTGACCTTTGACAGCAACAACGTGACGGACATGTTCCCT

GCCCTTCCGTTCACCGCCACGCTCCGCCAACAGTACTGCCTGAATAAGTGGGGCGTGTGG

CCTCGGCCAGACTGGCTGCAGACCAGCTTCTGGGGCGGTGACCTCAGAGCCACCAGCAAC

ATCATCTTCTCCAATGGTGACCTAGACCCCTGGGCAGGGGGCGGGATTCTGAGGAACGTG

AGCGCGTCGGTCATTGCTGTCACCATCCACGGGGGAGCACACCACCTAGACCTCAGGGCA

TCCCACCCACAAGACCCCAACTCGGTCCTGGAGGTGCGCAGGCTGGAGGCTGCTCTCATC

CTCGAGTGGGCGAAGGCAGCCCGGAGTAGGCAGCAG

>CL42.Contig3_A-W 1469 1570 minus strand Dipeptidyl-peptidase 7 [Homo sapiens] >gi|16877430|gb|AAH16961.1| Dipeptidyl-peptidase 7 [Homo sapiens]

AGGGCATCCCACCCACAAGACCCCAACTCGGTCCTGGAGGTGCGCAGGCTGGAGGCTGCT

CTCATCCTCGAGTGGGCGAAGGCAGCCCGGAGTAGGCAGCAG

>CL42.Contig4_A-W 93 218 minus strand Chain A, Human Dipeptidyl Peptidase Dpp7 >gi|261278873|pdb|3JYH|B Chain B, Human Dipeptidyl Peptidase Dpp7 >gi|261278874|pdb|3JYH|C Chain C, Human Dipeptidyl Peptidase Dpp7 >gi|261278875|pdb|3JYH|D Chain D, Human Dipeptidyl Peptidase Dpp7 >gi|301015979|pdb|3N0T|A Chain A, Human Dipeptidil Peptidase Dpp7 Complexed With Inhibitor Gsk237826a >gi|301015980|pdb|3N0T|B Chain B, Human Dipeptidil Peptidase Dpp7 Complexed With Inhibitor Gsk237826a >gi|301015981|pdb|3N0T|C Chain C, Human Dipeptidil Peptidase Dpp7 Complexed With Inhibitor Gsk237826a >gi|301015982|pdb|3N0T|D Chain D, Human Dipeptidil Peptidase Dpp7 Complexed With Inhibitor Gsk237826a

CGCTACTACGGGAAGTCGCTCCCGTTTGGGGAGAAGTCCATGCAGCCCGGGTACACCGAG

CTGCTGACCGTGGAGCAGGCGCTGGCCGACTATGCCGTGCTGATCCAGACCCTGCGGCAG

AAACTT

>CL42.Contig5_A-W 1 126 minus strand PREDICTED: dipeptidyl peptidase 2 isoform 5 [Pan troglodytes]

CGCTACTACGGGAAGTCGCTCCCGTTTGGGGAGAAGTCCATGCAGCCCGGGTACACCGAG

CTGCTGACCGTGGAGCAGGCGCTGGCCGACTATGCCGTGCTGATCCAGACCCTGCGGCAG

AAACTT

>CL42.Contig6_A-W 773 928 minus strand PREDICTED: dipeptidyl peptidase 2 [Callithrix jacchus]

AGCTACGGGGGGATGCTCAGCGCCTACATGAGGATGAAGTACCCCCACCTGGTGGCAGGG

GCGCTGGCAGCCAGCGCACCCGTCCTGGCTGTCGCGGGCCTTGGTACCTCCAACCAGTTC

TTCCGGGACGTCACAGCGGTGAGTGAGGGCCTGGGG

>CL42.Contig7_A-W 1 126 minus strand PREDICTED: dipeptidyl peptidase 2 isoform 5 [Pan troglodytes]

CGCTACTACGGGAAGTCGCTCCCGTTTGGGGAGAAGTCCATGCAGCCCGGGTACACCGAG

CTGCTGACCGTGGAGCAGGCGCTGGCCGACTATGCCGTGCTGATCCAGACCCTGCGGCAG

AAACTT

>CL42.Contig8_A-W 93 218 minus strand PREDICTED: dipeptidyl peptidase 2 isoform 5 [Pan troglodytes]

CGCTACTACGGGAAGTCGCTCCCGTTTGGGGAGAAGTCCATGCAGCCCGGGTACACCGAG

CTGCTGACCGTGGAGCAGGCGCTGGCCGACTATGCCGTGCTGATCCAGACCCTGCGGCAG

AAACTT

>CL42.Contig9_A-W 1069 1323 minus strand PREDICTED: dipeptidyl peptidase 2 isoform 5 [Pan troglodytes]

ACCACTTGGTGTCTTTCAGACCTCAGAGCCACCAGCAACATCATCTTCTCCAATGGTGAC

CTAGACCCCTGGGCAGGGGGCGGGATTCTGAGGAACGTGAGCGCGTCGGTCATTGCTGTC

ACCATCCACGGGGGAGCACACCACCTAGACCTCAGGGCATCCCACCCACAAGACCCCAAC

TCGGTCCTGGAGGTGCGCAGGCTGGAGGCTGCTCTCATCCTCGAGTGGGCGAAGGCAGCC

CGGAGTAGGCAGCAG

>CL42.Contig10_A-W 1 318 minus strand PREDICTED: dipeptidyl peptidase 2 [Callithrix jacchus]

CGCTACTACGGGAAGTCGCTCCCGTTTGGGGAGAAGTCCATGCAGCCCGGGTACACCGAG

CTGCTGACCGTGGAGCAGGCGCTGGCCGACTATGCCGTGCTGATCCAGACCCTGCGGCAG

AAACTAGGAGCACAGGGTGGCCCTGTCATCACCTTTGGAGGGAGCTACGGGGGGATGCTC

AGCGCCTACATGAGGATGAAGTACCCCCACCTGGTGGCAGGGGCGCTGGCAGCCAGCGCA

CCCGTCCTGGCTGTCGCGGGCCTTGGTACCTCCAACCAGTTCTTCCGGGACGTCACAGCG

GTGAGTGAGGGCCTGGGG

>CL42.Contig11_A-W 820 945 minus strand PREDICTED: dipeptidyl peptidase 2 [Callithrix jacchus]

AGCAACAACGTGACGGACATGTTCCCTGCCCTTCCGTTCACCGCCACGCTCCGCCAACAG

TACTGCCTGAATAAGTGGGGCGTGTGGCCTCGGCCGGACTGGCTGCAGACCAGCTTCTGG

GGCGGT

>CL42.Contig12_A-W 773 928 minus strand PREDICTED: dipeptidyl peptidase 2 [Callithrix jacchus]

AGCTACGGGGGGATGCTCAGCGCCTACATGAGGATGAAGTACCCCCACCTGGTGGCAGGG

GCGCTGGCAGCCAGCGCACCCGTCCTGGCTGTCGCGGGCCTTGGTACCTCCAACCAGTTC

TTCCGGGACGTCACAGCGGTGAGTGAGGGCCTGGGG

>CL42.Contig13_A-W 93 410 minus strand PREDICTED: dipeptidyl peptidase 2 [Callithrix jacchus]

CGCTACTACGGGAAGTCGCTCCCGTTTGGGGAGAAGTCCATGCAGCCCGGGTACACCGAG

CTGCTGACCGTGGAGCAGGCGCTGGCCGACTATGCCGTGCTGATCCAGACCCTGCGGCAG

AAACTAGGAGCACAGGGTGGCCCTGTCATCACCTTTGGAGGGAGCTACGGGGGGATGCTC

AGCGCCTACATGAGGATGAAGTACCCCCACCTGGTGGCAGGGGCGCTGGCAGCCAGCGCA

CCCGTCCTGGCTGTCGCGGGCCTTGGTACCTCCAACCAGTTCTTCCGGGACGTCACAGCG

GTGAGTGAGGGCCTGGGG

>CL42.Contig14_A-W 1 318 minus strand PREDICTED: dipeptidyl peptidase 2 [Callithrix jacchus]

CGCTACTACGGGAAGTCGCTCCCGTTTGGGGAGAAGTCCATGCAGCCCGGGTACACCGAG

CTGCTGACCGTGGAGCAGGCGCTGGCCGACTATGCCGTGCTGATCCAGACCCTGCGGCAG

AAACTAGGAGCACAGGGTGGCCCTGTCATCACCTTTGGAGGGAGCTACGGGGGGATGCTC

AGCGCCTACATGAGGATGAAGTACCCCCACCTGGTGGCAGGGGCGCTGGCAGCCAGCGCA

CCCGTCCTGGCTGTCGCGGGCCTTGGTACCTCCAACCAGTTCTTCCGGGACGTCACAGCG

GTGAGTGAGGGCCTGGGG

>CL42.Contig15_A-W 93 410 minus strand PREDICTED: dipeptidyl peptidase 2 [Callithrix jacchus]

CGCTACTACGGGAAGTCGCTCCCGTTTGGGGAGAAGTCCATGCAGCCCGGGTACACCGAG

CTGCTGACCGTGGAGCAGGCGCTGGCCGACTATGCCGTGCTGATCCAGACCCTGCGGCAG

AAACTAGGAGCACAGGGTGGCCCTGTCATCACCTTTGGAGGGAGCTACGGGGGGATGCTC

AGCGCCTACATGAGGATGAAGTACCCCCACCTGGTGGCAGGGGCGCTGGCAGCCAGCGCA

CCCGTCCTGGCTGTCGCGGGCCTTGGTACCTCCAACCAGTTCTTCCGGGACGTCACAGCG

GTGAGTGAGGGCCTGGGG

>CL42.Contig16_A-W 129 494 minus strand Chain A, Human Dipeptidyl Peptidase Dpp7 >gi|261278873|pdb|3JYH|B Chain B, Human Dipeptidyl Peptidase Dpp7 >gi|261278874|pdb|3JYH|C Chain C, Human Dipeptidyl Peptidase Dpp7 >gi|261278875|pdb|3JYH|D Chain D, Human Dipeptidyl Peptidase Dpp7 >gi|301015979|pdb|3N0T|A Chain A, Human Dipeptidil Peptidase Dpp7 Complexed With Inhibitor Gsk237826a >gi|301015980|pdb|3N0T|B Chain B, Human Dipeptidil Peptidase Dpp7 Complexed With Inhibitor Gsk237826a >gi|301015981|pdb|3N0T|C Chain C, Human Dipeptidil Peptidase Dpp7 Complexed With Inhibitor Gsk237826a >gi|301015982|pdb|3N0T|D Chain D, Human Dipeptidil Peptidase Dpp7 Complexed With Inhibitor Gsk237826a

GACGCTGGCTTTTGGGAGAGGTACTTTGAGCAGTTTATGGACCATTTCAACTTTGCGAGG

TTTGGCAACAAGACCTTTTACCAGCGGGTGCTGGTGTCAGACAAGTTCTGGCGAAGGGGC

GAGGGGCCCATCTTCTTCTACACGGGGAACGAGGTGGACGTGTGGACTCTGGCCAACAAC

ACAGGCTTTGTGGTGGAGCTGGCAGCCCAGCAAGGCGCCCTGCTCATCTTTGCGGAGCAT

CGCTACTACGGGAAGTCGCTCCCGTTTGGGGAGAAGTCCATGCAGCCCGGGTACACCGAG

CTGCTGACCGTGGAGCAGGCGCTGGCCGACTATGCCGTGCTGATCCAGACCCTGCGGCAG

AAACTT

>CL42.Contig17_A-W 2 154 minus strand PREDICTED: dipeptidyl peptidase 2 [Callithrix jacchus]

TACGGGGGGATGCTCAGCGCCTACATGAGGATGAAGTACCCCCACCTGGTGGCAGGGGCG

CTGGCAGCCAGCGCACCCGTCCTGGCTGTCGCGGGCCTTGGTACCTCCAACCAGTTCTTC

CGGGACGTCACAGCGGTGAGTGAGGGCCTGGGG

>CL42.Contig18_A-W 93 1214 minus strand Chain A, Human Dipeptidyl Peptidase Dpp7 >gi|261278873|pdb|3JYH|B Chain B, Human Dipeptidyl Peptidase Dpp7 >gi|261278874|pdb|3JYH|C Chain C, Human Dipeptidyl Peptidase Dpp7 >gi|261278875|pdb|3JYH|D Chain D, Human Dipeptidyl Peptidase Dpp7 >gi|301015979|pdb|3N0T|A Chain A, Human Dipeptidil Peptidase Dpp7 Complexed With Inhibitor Gsk237826a >gi|301015980|pdb|3N0T|B Chain B, Human Dipeptidil Peptidase Dpp7 Complexed With Inhibitor Gsk237826a >gi|301015981|pdb|3N0T|C Chain C, Human Dipeptidil Peptidase Dpp7 Complexed With Inhibitor Gsk237826a >gi|301015982|pdb|3N0T|D Chain D, Human Dipeptidil Peptidase Dpp7 Complexed With Inhibitor Gsk237826a

CGCTACTACGGGAAGTCGCTCCCGTTTGGGGAGAAGTCCATGCAGCCCGGGTACACCGAG

CTGCTGACCGTGGAGCAGGCGCTGGCCGACTATGCCGTGCTGATCCAGACCCTGCGGCAG

AAACTAGGAGCACAGGGTGGCCCTGTCATCACCTTTGGAGGGAGCTACGGGGGGATGCTC

AGCGCCTACATGAGGATGAAGTACCCCCACCTGGTGGCAGGGGCGCTGGCAGCCAGCGCA

CCCGTCCTGGCTGTCGCGGGCCTTGGTACCTCCAACCAGTTCTTCCGGGACGTCACAGCG

ACCTTCCACAGCCAGAGTCCAAGGTGTGTCCAGGGCGTGCGGGAGGCCTTCCAGCAGATC

AGGGACCTGTTCCTACAGGGAGCCCGTGACAGGGTCAGCCAGGAGTTTGGCACCTGCCAG

CCACTCTTGGACACAAAGGACCTGACTCAGCTCTTTGTGTTTGCACGCAACGCCTTCACT

CTGCTGGCCATGATGGACTACCCGTACCCCACTGACTTCATGGGGTACCTGCCTGCCAAC

CCTGTCAAGGTTGGCTGTGCCCGGCTGCTGAGGGAGGCCCAGAGGATCGTGGGTCTGCGG

GCACTGACAGGGCTGGTCTACAACTCCTCGGGCACAGCGCACTGCTATGACATCTACCAG

CTGTACCACAGCTGTGCGGACCCCACTGGCTGTGGCACTGGCCCTGATGCCATGGCCTGG

GACTATCAGGCCTGCACCGAGATCAACCTGACCTTTGACAGCAACAACGTGACGGACATG

TTCCCTGCCCTTCCGTTCACCGCCACGCTCCGCCAACAGTACTGCCTGAATAAGTGGGGC

GTGTGGCCTCGGCCGGACTGGCTGCAGACCAGCTTCTGGGGCGGTGACCTCAGAGCCACC

AGCAACATCATCTTCTCCAATGGTGACCTAGACCCCTGGGCAGGGGGCGGGATTCTGAGG

AACGTGAGCGCGTCGGTCATTGCTGTCACCATCCACGGGGGAGCACACCACCTAGACCTC

AGGGCATCCCACCCACAAGACCCCAACTCGGTCCTGGAGGTGCGCAGGCTGGAGGCTGCT

CTCATCCTCGAGTGGGCGAAGGCAGCCCGGAGTAGGCAGCAG

>CL42.Contig19_A-W 51 686 minus strand PREDICTED: dipeptidyl peptidase 2 [Callithrix jacchus]

GGGGCCCCTTTCTGGGCACAGGTCCTGCTGCTGACACTGGGACTGGGTGACGTCCAGGCT

GGGGCCCACAGAGGCTTGGACGCTGGCTTTTGGGAGAGGTACTTTGAGCAGTTTATGGAC

CATTTCAACTTTGCGAGGTTTGGCAACAAGACCTTTTACCAGCGGGTGCTGGTGTCAGAC

AAGTTCTGGCGAAGGGGCGAGGGGCCCATCTTCTTCTACACGGGGAACGAGGTGGACGTG

TGGACTCTGGCCAACAACACAGGCTTTGTGGTGGAGCTGGCAGCCCAGCAAGGCGCCCTG

CTCATCTTTGCGGAGCATCGCTACTACGGGAAGTCGCTCCCGTTTGGGGAGAAGTCCATG

CAGCCCGGGTACACCGAGCTGCTGACCGTGGAGCAGGCGCTGGCCGACTATGCCGTGCTG

ATCCAGACCCTGCGGCAGAAACTAGGAGCACAGGGTGGCCCTGTCATCACCTTTGGAGGG

AGCTACGGGGGGATGCTCAGCGCCTACATGAGGATGAAGTACCCCCACCTGGTGGCAGGG

GCGCTGGCAGCCAGCGCACCCGTCCTGGCTGTCGCGGGCCTTGGTACCTCCAACCAGTTC

TTCCGGGACGTCACAGCGGTGAGTGAGGGCCTGGGG

>CL42.Contig20_A-W 810 1205 minus strand PREDICTED: dipeptidyl peptidase 2 isoform 5 [Pan troglodytes]

CAGGCCTGCACCGAGATCAACCTGACCTTTGACAGCAACAACGTGACGGACATGTTCCCT

GCCCTTCCGTTCACCGCCACGCTCCGCCAACAGTACTGCCTGAATAAGTGGGGCGTGTGG

CCTCGGCCGGACTGGCTGCAGACCAGCTTCTGGGGCGGTGACCTCAGAGCCACCAGCAAC

ATCATCTTCTCCAATGGTGACCTAGACCCCTGGGCAGGGGGCGGGATTCTGAGGAACGTG

AGCGCGTCGGTCATTGCTGTCACCATCCACGGGGGAGCACACCACCTAGACCTCAGGGCA

TCCCACCCACAAGACCCCAACTCGGTCCTGGAGGTGCGCAGGCTGGAGGCTGCTCTCATC

CTCGAGTGGGCGAAGGCAGCCCGGAGTAGGCAGCAG

>CL42.Contig21_A-W 820 1197 minus strand PREDICTED: dipeptidyl peptidase 2 [Callithrix jacchus]

AGCAACAACGTGACGGACATGTTCCCTGCCCTTCCGTTCACCGCCACGCTCCGCCAACAG

TACTGCCTGAATAAGTGGGGCGTGTGGCCTCGGCCGGACTGGCTGCAGACCAGCTTCTGG

GGCGGTGACCTCAGAGCCACCAGCAACATCATCTTCTCCAATGGTGACCTAGACCCCTGG

GCAGGGGGCGGGATTCTGAGGAACGTGAGCGCGTCGGTCATTGCTGTCACCATCCACGGG

GGAGCACACCACCTAGACCTCAGGGCATCCCACCCACAAGACCCCAACTCGGTCCTGGAG

GTGCGCAGGCTGGAGGCTGCTCTCATCCTCGAGTGGGCGAAGGCAGCCCGGAGTAGGCAG

CAGTGTCCTGGACACCCT

>CL42.Contig22_A-W 66 494 minus strand PREDICTED: dipeptidyl peptidase 2 isoform 5 [Pan troglodytes]

GCACAGGTCCTGCTGCTGACACTGGGACTGGGTGACGTCCAGGCTGGGGCCCACAGAGGC

TTGGACGCTGGCTTTTGGGAGAGGTACTTTGAGCAGTTTATGGACCATTTCAACTTTGCG

AGGTTTGGCAACAAGACCTTTTACCAGCGGGTGCTGGTGTCAGACAAGTTCTGGCGAAGG

GGCGAGGGGCCCATCTTCTTCTACACGGGGAACGAGGTGGACGTGTGGACTCTGGCCAAC

AACACAGGCTTTGTGGTGGAGCTGGCAGCCCAGCAAGGCGCCCTGCTCATCTTTGCGGAG

CATCGCTACTACGGGAAGTCGCTCCCGTTTGGGGAGAAGTCCATGCAGCCCGGGTACACC

GAGCTGCTGACCGTGGAGCAGGCGCTGGCCGACTATGCCGTGCTGATCCAGACCCTGCGG

CAGAAACTT

>CL42.Contig23_A-W 646 804 minus strand PREDICTED: dipeptidyl peptidase 2 isoform 5 [Pan troglodytes]

CAGGCCTGCACCGAGATCAACCTGACCTTTGACAGCAACAACGTGACGGACATGTTCCCT

GCCCTTCCGTTCACCGCCACGCTCCGCCAACAGTACTGCCTGAATAAGTGGGGCGTGTGG

CCTCGGCCGGACTGGCTGCAGACCAGCTTCTGGGGCGGT

>CL42.Contig24_A-W 2 154 minus strand PREDICTED: dipeptidyl peptidase 2 [Callithrix jacchus]

TACGGGGGGATGCTCAGCGCCTACATGAGGATGAAGTACCCCCACCTGGTGGCAGGGGCG

CTGGCAGCCAGCGCACCCGTCCTGGCTGTCGCGGGCCTTGGTACCTCCAACCAGTTCTTC

CGGGACGTCACAGCGGTGAGTGAGGGCCTGGGG

>CL42.Contig25_A-W 93 281 minus strand PREDICTED: dipeptidyl peptidase 2 [Callithrix jacchus]

CGCTACTACGGGAAGTCGCTCCCGTTTGGGGAGAAGTCCATGCAGCCCGGGTACACCGAG

CTGCTGACCGTGGAGCAGGCGCTGGCCGACTATGCCGTGCTGATCCAGACCCTGCGGCAG

AAACTTGTGCTGCCTGCTCCAGGAAGCCCTCCCACTGGTCCTGAGCCAGTCCCCAGGCTG

AGCCGAGGA

>CL42.Contig26_A-W 1345 1599 minus strand Dipeptidyl-peptidase 7 [Homo sapiens] >gi|16877430|gb|AAH16961.1| Dipeptidyl-peptidase 7 [Homo sapiens]

ACCACTTGGTGTCTTTCAGACCTCAGAGCCACCAGCAACATCATCTTCTCCAATGGTGAC

CTAGACCCCTGGGCAGGGGGCGGGATTCTGAGGAACGTGAGCGCGTCGGTCATTGCTGTC

ACCATCCACGGGGGAGCACACCACCTAGACCTCAGGGCATCCCACCCACAAGACCCCAAC

TCGGTCCTGGAGGTGCGCAGGCTGGAGGCTGCTCTCATCCTCGAGTGGGCGAAGGCAGCC

CGGAGTAGGCAGCAG

>CL42.Contig27_A-W 2 118 minus strand hypothetical protein EGM_06474, partial [Macaca fascicularis]

CTGGTCTACAACTCCTCGGGCACAGCGCACTGCTATGACATCTACCAGCTGTACCACAGC

TGTGCGGACCCCACTGGCTGTGGCACTGGCCCTGATGCCATGGCCTGGGACTATCAG

>CL42.Contig28_A-W 1 189 minus strand PREDICTED: dipeptidyl peptidase 2 [Callithrix jacchus]

CGCTACTACGGGAAGTCGCTCCCGTTTGGGGAGAAGTCCATGCAGCCCGGGTACACCGAG

CTGCTGACCGTGGAGCAGGCGCTGGCCGACTATGCCGTGCTGATCCAGACCCTGCGGCAG

AAACTTGTGCTGCCTGCTCCAGGAAGCCCTCCCACTGGTCCTGAGCCAGTCCCCAGGCTG

AGCCGAGGA

>CL42.Contig29_A-W 1096 1221 minus strand Dipeptidyl-peptidase 7 [Homo sapiens] >gi|16877430|gb|AAH16961.1| Dipeptidyl-peptidase 7 [Homo sapiens]

AGCAACAACGTGACGGACATGTTCCCTGCCCTTCCGTTCACCGCCACGCTCCGCCAACAG

TACTGCCTGAATAAGTGGGGCGTGTGGCCTCGGCCGGACTGGCTGCAGACCAGCTTCTGG

GGCGGT

>CL42.Contig30_A-W 51 686 minus strand PREDICTED: dipeptidyl peptidase 2 [Callithrix jacchus]

GGGGCCCCTTTCTGGGCACAGGTCCTGCTGCTGACACTGGGACTGGGTGACGTCCAGGCT

GGGGCCCACAGAGGCTTGGACGCTGGCTTTTGGGAGAGGTACTTTGAGCAGTTTATGGAC

CATTTCAACTTTGCGAGGTTTGGCAACAAGACCTTTTACCAGCGGGTGCTGGTGTCAGAC

AAGTTCTGGCGAAGGGGCGAGGGGCCCATCTTCTTCTACACGGGGAACGAGGTGGACGTG

TGGACTCTGGCCAACAACACAGGCTTTGTGGTGGAGCTGGCAGCCCAGCAAGGCGCCCTG

CTCATCTTTGCGGAGCATCGCTACTACGGGAAGTCGCTCCCGTTTGGGGAGAAGTCCATG

CAGCCCGGGTACACCGAGCTGCTGACCGTGGAGCAGGCGCTGGCCGACTATGCCGTGCTG

ATCCAGACCCTGCGGCAGAAACTAGGAGCACAGGGTGGCCCTGTCATCACCTTTGGAGGG

AGCTACGGGGGGATGCTCAGCGCCTACATGAGGATGAAGTACCCCCACCTGGTGGCAGGG

GCGCTGGCAGCCAGCGCACCCGTCCTGGCTGTCGCGGGCCTTGGTACCTCCAACCAGTTC

TTCCGGGACGTCACAGCGGTGAGTGAGGGCCTGGGG

>CL42.Contig31_A-W 1 189 minus strand PREDICTED: dipeptidyl peptidase 2 [Callithrix jacchus]

CGCTACTACGGGAAGTCGCTCCCGTTTGGGGAGAAGTCCATGCAGCCCGGGTACACCGAG

CTGCTGACCGTGGAGCAGGCGCTGGCCGACTATGCCGTGCTGATCCAGACCCTGCGGCAG

AAACTTGTGCTGCCTGCTCCAGGAAGCCCTCCCACTGGTCCTGAGCCAGTCCCCAGGCTG

AGCCGAGGA

>CL42.Contig32_A-W 93 281 minus strand PREDICTED: dipeptidyl peptidase 2 [Callithrix jacchus]

CGCTACTACGGGAAGTCGCTCCCGTTTGGGGAGAAGTCCATGCAGCCCGGGTACACCGAG

CTGCTGACCGTGGAGCAGGCGCTGGCCGACTATGCCGTGCTGATCCAGACCCTGCGGCAG

AAACTTGTGCTGCCTGCTCCAGGAAGCCCTCCCACTGGTCCTGAGCCAGTCCCCAGGCTG

AGCCGAGGA

>CL42.Contig33_A-W 1 126 minus strand PREDICTED: dipeptidyl peptidase 2 isoform 5 [Pan troglodytes]

CGCTACTACGGGAAGTCGCTCCCGTTTGGGGAGAAGTCCATGCAGCCCGGGTACACCGAG

CTGCTGACCGTGGAGCAGGCGCTGGCCGACTATGCCGTGCTGATCCAGACCCTGCGGCAG

AAACTT

>CL42.Contig34_A-W 1096 1458 minus strand Dipeptidyl-peptidase 7 [Homo sapiens] >gi|16877430|gb|AAH16961.1| Dipeptidyl-peptidase 7 [Homo sapiens]

AGCAACAACGTGACGGACATGTTCCCTGCCCTTCCGTTCACCGCCACGCTCCGCCAACAG

TACTGCCTGAATAAGTGGGGCGTGTGGCCTCGGCCGGACTGGCTGCAGACCAGCTTCTGG

GGCGGTGACCTCAGAGCCACCAGCAACATCATCTTCTCCAATGGTGACCTAGACCCCTGG

GCAGGGGGCGGGATTCTGAGGAACGTGAGCGCGTCGGTCATTGCTGTCACCATCCACGGG

GGAGCACACCACCTAGACCTCAGGGCATCCCACCCACAAGACCCCAACTCGGTCCTGGAG

GTGCGCAGGCTGGAGGCTGCTCTCATCCTCGAGTGGGCGAAGGCAGCCCGGAGTAGGCAG

CAG

>CL42.Contig35_A-W 366 467 minus strand hypothetical protein EGM_06474, partial [Macaca fascicularis]

ACCACTTGGTGTCTTTCAGACCTCAGAGCCACCAGCAACATCATCTTCTCCAATGGTGAC

CTAGACCCCTGGGCAGGGGGCGGGGTGAGTCCTGGTCTGGGC

>CL42.Contig36_A-W 334 435 minus strand hypothetical protein EGM_06474, partial [Macaca fascicularis]

ACCACTTGGTGTCTTTCAGACCTCAGAGCCACCAGCAACATCATCTTCTCCAATGGTGAC

CTAGACCCCTGGGCAGGGGGCGGGGTGAGTCCTGGTCTGGGC

>CL42.Contig37_A-W 449 550 minus strand hypothetical protein EGM_06474, partial [Macaca fascicularis]

ACCACTTGGTGTCTTTCAGACCTCAGAGCCACCAGCAACATCATCTTCTCCAATGGTGAC

CTAGACCCCTGGGCAGGGGGCGGGGTGAGTCCTGGTCTGGGC

>CL44.Contig2_A-W 827 958 minus strand hypothetical protein EGM_09865 [Macaca fascicularis]

ATGAAGAGAGCCATGTCCCTAAATGTGCTGAATACAGAGAATGTCAGAGCTCCGGGGGCT

CAGCTCCGCAGTCGGCCCTTGAGCGAGCTCCCTCGGACCCTCAGCGCCAGTATCCAGGAC

ATGAGCCCCCGC

>CL44.Contig3_A-W 3 1283 minus strand TFIIH basal transcription factor complex helicase subunit, partial [Heterocephalus glaber]

CGGCGGCTGCTGGAGTTCGTCAAGTGGCGGCTGCGCGTGCAGCACGTGGTCCAGGAGAGC

CCGCCTGCCTTCCTGAGCAGCCTGGCGCAGCGCGTGTGCATCCAGCGCAAGCCCCTCAGG

TTCTGTGCTGAGCGCCTGCGGTCCCTCCTGCACACCTTGGAGATCGCCGACCTGGCCGAC

TTCTCCCCGCTCACTCTACTTGCTAACTTCGCCACCCTGGTCAGCACCTACGCCAAGGGC

TTTACCATCATCATTGAGCCTTTTGATGACAGGACCCCCACCATCGCCAACCCCATCCTG

CACTTCAGCTGCATGGACGCCTCCCTGGCCATCAAACCTGTGTTTGAGCGCTTCCAATCT

GTCATCATCACCTCCGGGACACTGTCCCCACTGGACATCTACCCCAAGATCCTGGACTTC

CATCCTGTCACCATGGCAACCTTCACAATGACACTGGCCCGGGTCTGTCTGTGCCCCATG

ATTATCGGCAGGGGTAATGACCAGGTGGCCATCAGCTCCAAATTTGAGACCCGGGAAGAT

ATTGCCGTGATCCGGAACTATGGAAACCTCCTGCTGGAGATGTCTGCTGTGGTCCCTGAT

GGCATCGTGGCCTTCTTCACCAGCTACCAGTACATGGAGAGCACTGTGGCCTCCTGGTAC

GAGCAGGGCATCCTTGAGAACATCCAGAGGAACAAGCTGCTCTTCATTGAGACCCAGGAT

GGGGCGGAGACCAGTGTTGCCCTGGAGAAGTATCAGGAGGCCTGCGAGAATGGCCGAGGG

GCCATCCTGCTCTCAGTGGCCCGGGGCAAAGTGTCTGAGGGAATCGACTTCGTGCACCAC

TTCGGGCGGGCAGTCATCATGTTTGGTGTCCCCTATGTCTACACCCAGAGCCGCATTCTG

AAGGCACGGCTGGAATACCTGCGGGACCAGTTCCAGATCCGCGAGAACGACTTCCTCACC

TTTGATGCCATGCGCCACGCGGCCCAGTGTGTAGGCCGGGCCATCCGGGGCAAGACGGAC

TACGGCCTCATGGTCTTCGCCGACAAACGGTTCGCCAGGGCGGACAAGCGGGGGAAGCTG

CCGCGCTGGATCCAGGAGCACCTCACCGAAGCCAACCTCAACCTGACCGTCGACGAGGGC

GTCCAGGTCGCCAAGTACTTCCTGAGGCAGATGGCGCAGCCTTTCCACCGGGAGGATCAG

CTGGGGCTGTCCCTGCTCAGCCTGGAGCAGCTGGAGTCGGAGGAGACGCTGCGGAGGATC

GAGCAGATCGCCCAGCAGATG

>CL44.Contig4_A-W 53 277 PREDICTED: TFIIH basal transcription factor complex helicase XPD subunit isoform 2 [Saimiri boliviensis boliviensis]

GGCTTTACCATCATCATTGAGCCTTTTGATGACAGGACCCCCACCATCGCCAACCCCATC

CTGCACTTCAGCTGCATGGACGCCTCCCTGGCCATCAAACCTGTGTTTGAGCGCTTCCAA

TCTGTCATCATCACCTCCGGGACACTGTCCCCACTGGACATCTACCCCAAGATCCTGGAC

TTCCATCCTGTCACCATGGCAACCTTCACAATGACACTGGCCCGG

>CL46.Contig1_A-W 39 1190 PREDICTED: choline transporter-like protein 2 [Otolemur garnettii]

ATGGAGGACGATCGGAAGGACGGAGCCTACGGAACTCCCCAGAAGTACGATCCTACCTTC

AAAGGACCCATTTACAACAGGGGTTGCACGGATGTCATCTGCTGTGTGCTGCTTCTTCTG

GCCATTGTGGGTTATGTGGCCGTGGGCATCATAGCTTGGACCCATGGGGACCCTCGAAAG

GTGATCTATCCTACTGACAGCCGTGGGGAGTTCTGCGGGCAGAAAGGCACAAAAAATGCG

AACAAACCCTTCCTGTTCTACTTCAACATTGTGAAGTGTGCCAGCCCCCTGGTCCTGCTG

GAATTCCAGTGTCCCACCCCACAGATCTGTGTGGAGAAATGCCCCGACCGCTACCTCACC

TTCCTGAGTGCCCGAAATAGTCGGGACTTCGAGTACTACAAGCAGTTCTGTGTTCCTGGC

TTCCAGAACAATAAGGGTGTGGCAGAGGTGCTTCGAGATGGTGACTGTCCTGCTGTTCTC

ACCCCCAGCAAACCCTTGGCCCGGCGATGCTTCCCAGCCATCCATGCTCACAAAGGGGTC

CTCATGGTGGGCAACGAGACGACCTATGAAGATGGGCACGGCTCTCGGAAGAACATCACA

GAACTGGTGGAGGGTGCCAAAAAGGCCAATGTCGTTCTGGAGGCACGACAGCTGGCCATG

CGGATATTTGAAGATTATACCGTCTCTTGGTACTGGATTGTCATAGGCCTGGTCATCGCC

ATGGTGCTGAGCCTCCTTTTCATCATCCTGCTGCGCTTCTTGGCTGGCATTATGGTCTGG

GTGATGATCGTCATGGTGATTCTGGTGCTGGGCTATGGAATATTTCACTGCTACATGGAG

TATGCCCGGCTGCGTGGGGAGGCCGGCTCTGATGTCTCCCTGGTGGACCTTGGCTTCCAG

ACAGACCTCCGGGTGTACTTGCACTTACGGCAGACCTGGATGGCCTTCATGATCATTCTG

AGCATTGTTGAAGTGGTCATCATCTTGCTGCTCATCTTTCTCCGGAAGAGAATTCTCATC

GCCATTGCACTCATCAAAGAAGCCAGCAGGGCTGTGGGATACGTTATGTGCTCCCTGCTG

TACCCTCTGGTCACCTTCTTCCTGCTGTGTCTTTGCATCGCATACTGGGCCAGCACTGCT

GTGTATCCATCT

>CL46.Contig2_A-W 124 1035 PREDICTED: choline transporter-like protein 2 [Otolemur garnettii]

AACAAACCCTTCCTGTTCTACTTCAACATTGTGAAGTGTGCCAGCCCCCTGGTCCTGCTG

GAATTCCAGTGTCCCACCCCACAGATCTGTGTGGAGAAATGCCCCGACCGCTACCTCACC

TTCCTGAGTGCCCGAAATAGTCGGGACTTCGAGTACTACAAGCAGTTCTGTGTTCCTGGC

TTCCAGAACAATAAGGGTGTGGCAGAGGTGCTTCGAGATGGTGACTGTCCTGCTGTTCTC

ACCCCCAGCAAACCCTTGGCCCGGCGATGCTTCCCAGCCATCCATGCTCACAAAGGGGTC

CTCATGGTGGGCAACGAGACGACCTATGAAGATGGGCACGGCTCTCGGAAGAACATCACA

GAACTGGTGGAGGGTGCCAAAAAGGCCAATGTCGTTCTGGAGGCACGACAGCTGGCCATG

CGGATATTTGAAGATTATACCGTCTCTTGGTACTGGATTGTCATAGGCCTGGTCATCGCC

ATGGTGCTGAGCCTCCTTTTCATCATCCTGCTGCGCTTCTTGGCTGGCATTATGGTCTGG

GTGATGATCGTCATGGTGATTCTGGTGCTGGGCTATGGAATATTTCACTGCTACATGGAG

TATGCCCGGCTGCGTGGGGAGGCCGGCTCTGATGTCTCCCTGGTGGACCTTGGCTTCCAG

ACAGACCTCCGGGTGTACTTGCACTTACGGCAGACCTGGATGGCCTTCATGATCATTCTG

AGCATTGTTGAAGTGGTCATCATCTTGCTGCTCATCTTTCTCCGGAAGAGAATTCTCATC

GCCATTGCACTCATCAAAGAAGCCAGCAGGGCTGTGGGATACGTTATGTGCTCCCTGCTG

TACCCTCTGGTCACCTTCTTCCTGCTGTGTCTTTGCATCGCATACTGGGCCAGCACTGCT

GTGTATCCATCT

>CL46.Contig3_A-W 646 1533 PREDICTED: choline transporter-like protein 2 isoform 2 [Saimiri boliviensis boliviensis]

CAGACCTTCCCTTCCTCCAATGAATCCCGCCTATGCCCCAACGCGCATTGCCAGTTCGCC

TTCTATGGCGGTGAGTCGGGCTACCACCGTGCGCTGCTGGGCCTGCAGATCTTCAATGCC

TTCATGTTCTTTTGGTTGGCCAACTTCGTGCTGGCGCTGGGCCAGGTCACACTGGCTGGG

GCATTTGCCTCCTACTACTGGGCCATGCGCAAGCCAGACGACCTGCCAGCCTTCCCGCTC

TTCTCTGCCTTTGGCCGGGCACTCAGGTACCACACAGGATCCTTGGCCTTTGGTGCCCTC

ATTCTGGCCATTGTGCAGATCATCCGAGTGATGCTTGAGTATTTGGATCAGCGCCTGAAA

GCTGCGGAGAACAAGTTTGCCAAGTTTCTCATGACCTGTCTCAAGTGTTGCTTTTGGTGC

CTGGAGAAGTTCATCAAATTCCTCAACAGGAATGCCTACATCATGATCGCCATCTACGGC

ACCAACTTCTGCACCTCAGCTAGGAATGCCTTCTTCCTGCTCATGAGAAACATCATCAGA

GTGGCCGTCCTAGACAAAGTTACCGACTTCCTCTTCCTGTTGGGCAAACTTCTGATCGTG

GGCAGTGTGGGAATCCTTGCTTTCTTCTTCTTCACCCATCGTATCAGGATCGTGCAAGAT

ACAGCACCACCTCTCAATTATTACTGGGTCCCAATACTGACGGTGATTGTTGGGTCCTAC

TTGATTGCCCATGGTTTCTTCAGCGTCTACGGCATGTGTGTGGATACGTTATTCCTCTGC

TTCTTGGAGGATCTGGAGAGAAACGATGGGTCAGCTGAGAGGCCTTACTTCATGTCTTCC

GCTCTCAAGAAGCTCTTGAACAAGACCAACAAGAAGGTGGCAGAGTCC

>CL46.Contig4_A-W 39 2150 PREDICTED: choline transporter-like protein 2 [Otolemur garnettii]

ATGGAGGACGATCGGAAGGACGGAGCCTACGGAACTCCCCAGAAGTACGATCCTACCTTC

AAAGGACCCATTTACAACAGGGGTTGCACGGATGTCATCTGCTGTGTGCTGCTTCTTCTG

GCCATTGTGGGTTATGTGGCCGTGGGCATCATAGCTTGGACCCATGGGGACCCTCGAAAG

GTGATCTATCCTACTGACAGCCGTGGGGAGTTCTGCGGGCAGAAAGGCACAAAAAATGCG

AACAAACCCTTCCTGTTCTACTTCAACATTGTGAAGTGTGCCAGCCCCCTGGTCCTGCTG

GAATTCCAGTGTCCCACCCCACAGATCTGTGTGGAGAAATGCCCCGACCGCTACCTCACC

TTCCTGAGTGCCCGAAATAGTCGGGACTTCGAGTACTACAAGCAGTTCTGTGTTCCTGGC

TTCCAGAACAATAAGGGTGTGGCAGAGGTGCTTCGAGATGGTGACTGTCCTGCTGTTCTC

ACCCCCAGCAAACCCTTGGCCCGGCGATGCTTCCCAGCCATCCATGCTCACAAAGGGGTC

CTCATGGTGGGCAACGAGACGACCTATGAAGATGGGCACGGCTCTCGGAAGAACATCACA

GAACTGGTGGAGGGTGCCAAAAAGGCCAATGTCGTTCTGGAGGCACGACAGCTGGCCATG

CGGATATTTGAAGATTATACCGTCTCTTGGTACTGGATTGTCATAGGCCTGGTCATCGCC

ATGGTGCTGAGCCTCCTTTTCATCATCCTGCTGCGCTTCTTGGCTGGCATTATGGTCTGG

GTGATGATCGTCATGGTGATTCTGGTGCTGGGCTATGGAATATTTCACTGCTACATGGAG

TATGCCCGGCTGCGTGGGGAGGCCGGCTCTGATGTCTCCCTGGTGGACCTTGGCTTCCAG

ACAGACCTCCGGGTGTACTTGCACTTACGGCAGACCTGGATGGCCTTCATGATCATTCTG

AGCATTGTTGAAGTGGTCATCATCTTGCTGCTCATCTTTCTCCGGAAGAGAATTCTCATC

GCCATTGCACTCATCAAAGAAGCCAGCAGGGCTGTGGGATACGTTATGTGCTCCCTGCTG

TACCCTCTGGTCACCTTCTTCCTGCTGTGTCTTTGCATCGCATACTGGGCCAGCACTGCT

GTCTTCCTGTCCACTTCTAATGAAGCTGTCTATAAGATTTTGGATGATGCCACCTGCTCA

CTAGCTGGGAAAACCTGCAACCCTGAGACCTTCCCTTCCTCCAATGAATCCCGCCTATGC

CCCAACGCGCATTGCCAGTTCGCCTTCTATGGCGGTGAGTCGGGCTACCACCGTGCGCTG

CTGGGCCTGCAGATCTTCAATGCCTTCATGTTCTTTTGGTTGGCCAACTTCGTGCTGGCG

CTGGGCCAGGTCACACTGGCTGGGGCATTTGCCTCCTACTACTGGGCCATGCGCAAGCCA

GACGACCTGCCAGCCTTCCCGCTCTTCTCTGCCTTTGGCCGGGCACTCAGGTACCACACA

GGATCCTTGGCCTTTGGTGCCCTCATTCTGGCCATTGTGCAGATCATCCGAGTGATGCTT

GAGTATTTGGATCAGCGCCTGAAAGCTGCGGAGAACAAGTTTGCCAAGTTTCTCATGACC

TGTCTCAAGTGTTGCTTTTGGTGCCTGGAGAAGTTCATCAAATTCCTCAACAGGAATGCC

TACATCATGATCGCCATCTACGGCACCAACTTCTGCACCTCAGCTAGGAATGCCTTCTTC

CTGCTCATGAGAAACATCATCAGAGTGGCCGTCCTAGACAAAGTTACCGACTTCCTCTTC

CTGTTGGGCAAACTTCTGATCGTGGGCAGTGTGGGAATCCTTGCTTTCTTCTTCTTCACC

CATCGTATCAGGATCGTGCAAGATACAGCACCACCTCTCAATTATTACTGGGTCCCAATA

CTGACGGTGATTGTTGGGTCCTACTTGATTGCCCATGGTTTCTTCAGCGTCTACGGCATG

TGTGTGGATACGTTATTCCTCTGCTTCTTGGAGGATCTGGAGAGAAACGATGGGTCAGCT

GAGAGGCCTTACTTCATGTCTTCCGCTCTCAAGAAGCTCTTGAACAAGACCAACAAGAAG

GTGGCAGAGTCC

>CL47.Contig1_A-W 2 1648 minus strand PREDICTED: zinc finger and BTB domain-containing protein 24 isoform 1 [Pan troglodytes]

TTTGCAGAGGAGGGGGAGATTGGCCAGTCCATTTATATGCTGGAAGGCATGGTTGCAGAC

ACCTTTGGCATCCTGCTAGAATTTATCTACACAGGTTATCTCCATGCTAGTGAGAAAAGT

ACGGAACAAATCCTGGCTACTGCTCAGTTCTTAAAAGTCTACGACTTGATAAAGGCTTAT

ACAGACTTTCAGAATAATCATAACTCCCCAAAGCCAACAACTTTGAACAGAGCTGGTGCT

CCAGTCGTTGTTATTTCTAATAAGAAAAATGATCCTCCAAAACGGAAACGGGGAAGACCA

AGAAAAGTCAATAGTTTGCAGGAGGGGAAATCAGAACAGGCTGCAGAGGAAGAAATCCAG

CTAAGAGTGAACAATTCGGTTCAGAATAGACAAAACTTTGTTGTTAAAGAAGGAGACAAT

GGTGTACTGAATGAACCGATTCCAGTAAAAGAAAAGGAAGGATCTGACCCTTCTTGTGAA

CCAGGTAGAATGGAGGAGATGCCCACTGAAAAAGATGAGAACTGTGATCCCAAGACCCAG

GATGGGCAGGACCACCAGAGTAGATGCAGCAAGCGGAGGATTCGGAGGTCTGTCAAACTT

AAAGATTATAAACTTGTTGGGGATGAGGACGACCAGGCTTCAGCCAAGAGGATATATGGA

AGGAGAAAGCGCCCCAGTGGTCCTGAGGCCCGCTGTAAAGACTGTGGCAAAGTCTTTAAA

TACAATCACTTTTTAGCAATCCATCAGAGGAGCCACACAGGCCACTCATTACCGGAATGC

ACCCACTGTCAACGCAAATTCATGGATGTGTCTCAGCTAAAGAAACATCTACGAACACAC

ACAGGTGAAAAGCCATTTACTTGTGAAATCTGTGGCAAGTCTTTCACAGCAAAGAGTTCT

CTTCAGACCCACATCAGAATTCATCGAGGAGAAAAGCCATACTCCTGTGGCATCTGTGGC

AAATCCTTCTCTGACTCCAGTGCCAAAAGGAGACATTGCATTCTGCACACAGGCAAAAAG

CCTTTCTCCTGCCCAGAGTGTAACTTACAGTTTGCTCGCTTAGACAACTTGAAGGCTCAC

TTGAAAATCCACAGTAAAGAGAAGCATGCATCAGATGCCAGCAGTGTTTCTGGTAGTAGT

AATACAGAAGAGGTCAGAAATATTCTTCAGCTACAGCCATACCAGCTCTCTACCTCTGGA

GAGCAGGAGATTCAGCTTCTGGTAACTGATTCTGTACATAACATCAATTTCATGCCTGGT

CCTAGTCAGGGAATCAGTATCATGACTGCTGAGAGTTCCCAGAACATGACCGCAGACCAG

GCTGCTAATCTCACCCTGCTCACACAGCAGCCAGAGCAACTGCAGAATTTAATTCTTTCA

GCTCAGCAGGAGCAAACAGAACACATTCAGAGTCTCAATATGATTGAAAGCCAGATGGAG

CCCTCACAGACAGAGCCAGTGCATGTAATTACACTGTCCAAGGAAACCCTGGAACATCTT

CATGCCCATCAAGAGCAAACAGAGGAGCTCGATTTAGCGGCAAGTGCTTCAGATCCAGCT

CAGCACCTGCACCTGACTCAGGAGCCTGAATCACCACCACCTACTCACCATGTGCCCCAG

CCCACTCCACTTGTCCAGGAGCGAAGC

>CL47.Contig2_A-W 2 1648 minus strand PREDICTED: zinc finger and BTB domain-containing protein 24 isoform 1 [Pan troglodytes]

TTTGCAGAGGAGGGGGAGATTGGCCAGTCCATTTATATGCTGGAAGGCATGGTTGCAGAC

ACCTTTGGCATCCTGCTAGAATTTATCTACACAGGTTATCTCCATGCTAGTGAGAAAAGT

ACGGAACAAATCCTGGCTACTGCTCAGTTCTTAAAAGTCTACGACTTGATAAAGGCTTAT

ACAGACTTTCAGAATAATCATAACTCCCCAAAGCCAACAACTTTGAACAGAGCTGGTGCT

CCAGTCGTTGTTATTTCTAATAAGAAAAATGATCCTCCAAAACGGAAACGGGGAAGACCA

AGAAAAGTCAATAGTTTGCAGGAGGGGAAATCAGAACAGGCTGCAGAGGAAGAAATCCAG

CTAAGAGTGAACAATTCGGTTCAGAATAGACAAAACTTTGTTGTTAAAGAAGGAGACAAT

GGTGTACTGAATGAACCGATTCCAGTAAAAGAAAAGGAAGGATCTGACCCTTCTTGTGAA

CCAGGTAGAATGGAGGAGATGCCCACTGAAAAAGATGAGAACTGTGATCCCAAGACCCAG

GATGGGCAGGACCACCAGAGTAGATGCAGCAAGCGGAGGATTCGGAGGTCTGTCAAACTT

AAAGATTATAAACTTGTTGGGGATGAGGACGACCAGGCTTCAGCCAAGAGGATATATGGA

AGGAGAAAGCGCCCCAGTGGTCCTGAGGCCCGCTGTAAAGACTGTGGCAAAGTCTTTAAA

TACAATCACTTTTTAGCAATCCATCAGAGGAGCCACACAGGCCACTCATTACCGGAATGC

ACCCACTGTCAACGCAAATTCATGGATGTGTCTCAGCTAAAGAAACATCTACGAACACAC

ACAGGTGAAAAGCCATTTACTTGTGAAATCTGTGGCAAGTCTTTCACAGCAAAGAGTTCT

CTTCAGACCCACATCAGAATTCATCGAGGAGAAAAGCCATACTCCTGTGGCATCTGTGGC

AAATCCTTCTCTGACTCCAGTGCCAAAAGGAGACATTGCATTCTGCACACAGGCAAAAAG

CCTTTCTCCTGCCCAGAGTGTAACTTACAGTTTGCTCGCTTAGACAACTTGAAGGCTCAC

TTGAAAATCCACAGTAAAGAGAAGCATGCATCAGATGCCAGCAGTGTTTCTGGTAGTAGT

AATACAGAAGAGGTCAGAAATATTCTTCAGCTACAGCCATACCAGCTCTCTACCTCTGGA

GAGCAGGAGATTCAGCTTCTGGTAACTGATTCTGTACATAACATCAATTTCATGCCTGGT

CCTAGTCAGGGAATCAGTATCATGACTGCTGAGAGTTCCCAGAACATGACCGCAGACCAG

GCTGCTAATCTCACCCTGCTCACACAGCAGCCAGAGCAACTGCAGAATTTAATTCTTTCA

GCTCAGCAGGAGCAAACAGAACACATTCAGAGTCTCAATATGATTGAAAGCCAGATGGAG

CCCTCACAGACAGAGCCAGTGCATGTAATTACACTGTCCAAGGAAACCCTGGAACATCTT

CATGCCCATCAAGAGCAAACAGAGGAGCTCGATTTAGCGGCAAGTGCTTCAGATCCAGCT

CAGCACCTGCACCTGACTCAGGAGCCTGAATCACCACCACCTACTCACCATGTGCCCCAG

CCCACTCCACTTGTCCAGGAGCGAAGC

>CL47.Contig3_A-W 1 1059 minus strand PREDICTED: zinc finger and BTB domain-containing protein 24 isoform 1 [Pan troglodytes]

ACAGGGGAGCGACCCTTCAAATGTAATGAGTGTGGAAAAGGCTTTGCCCAGAAGCACTCT

TTGCAGGTCCACACCAGGATGCACACAGGCGAGCGGCCATACACCTGCACCGTGTGCAGC

AAGGCTCTGACCACCAAGCACTCACTGCTGGAGCACATGAGCCTGCACTCAGGCCACTCA

TTACCGGAATGCACCCACTGTCAACGCAAATTCATGGATGTGTCTCAGCTAAAGAAACAT

CTACGAACACACACAGGTGAAAAGCCATTTACTTGTGAAATCTGTGGCAAGTCTTTCACA

GCAAAGAGTTCTCTTCAGACCCACATCAGAATTCATCGAGGAGAAAAGCCATACTCCTGT

GGCATCTGTGGCAAATCCTTCTCTGACTCCAGTGCCAAAAGGAGACATTGCATTCTGCAC

ACAGGCAAAAAGCCTTTCTCCTGCCCAGAGTGTAACTTACAGTTTGCTCGCTTAGACAAC

TTGAAGGCTCACTTGAAAATCCACAGTAAAGAGAAGCATGCATCAGATGCCAGCAGTGTT

TCTGGTAGTAGTAATACAGAAGAGGTCAGAAATATTCTTCAGCTACAGCCATACCAGCTC

TCTACCTCTGGAGAGCAGGAGATTCAGCTTCTGGTAACTGATTCTGTACATAACATCAAT

TTCATGCCTGGTCCTAGTCAGGGAATCAGTATCATGACTGCTGAGAGTTCCCAGAACATG

ACCGCAGACCAGGCTGCTAATCTCACCCTGCTCACACAGCAGCCAGAGCAACTGCAGAAT

TTAATTCTTTCAGCTCAGCAGGAGCAAACAGAACACATTCAGAGTCTCAATATGATTGAA

AGCCAGATGGAGCCCTCACAGACAGAGCCAGTGCATGTAATTACACTGTCCAAGGAAACC

CTGGAACATCTTCATGCCCATCAAGAGCAAACAGAGGAGCTCGATTTAGCGGCAAGTGCT

TCAGATCCAGCTCAGCACCTGCACCTGACTCAGGAGCCTGAATCACCACCACCTACTCAC

CATGTGCCCCAGCCCACTCCACTTGTCCAGGAGCGAAGC

>CL47.Contig4_A-W 235 507 minus strand PREDICTED: zinc finger and BTB domain-containing protein 24 isoform 1 [Pan troglodytes]

CGAACACACACAGGTGAAAAGCCATTTACTTGTGAAATCTGTGGCAAGTCTTTCACAGCA

AAGAGTTCTCTTCAGACCCACATCAGAATTCATCGAGGAGAAAAGCCATACTCCTGTGGC

ATCTGTGGCAAATCCTTCTCTGACTCCAGTGCCAAAAGGAGACATTGCATTCTGCACACA

GGCAAAAAGCCTTTCTCCTGCCCAGAGTGTAACTTACAGTTTGCTCGCTTAGACAACTTG

AAGGCTCACTTGAAAATCCACAGTAAAGAGAAG

>CL47.Contig5_A-W 1 1059 minus strand PREDICTED: zinc finger and BTB domain-containing protein 24 isoform 1 [Pan troglodytes]

ACAGGGGAGCGACCCTTCAAATGTAATGAGTGTGGAAAAGGCTTTGCCCAGAAGCACTCT

TTGCAGGTCCACACCAGGATGCACACAGGCGAGCGGCCATACACCTGCACCGTGTGCAGC

AAGGCTCTGACCACCAAGCACTCACTGCTGGAGCACATGAGCCTGCACTCAGGCCACTCA

TTACCGGAATGCACCCACTGTCAACGCAAATTCATGGATGTGTCTCAGCTAAAGAAACAT

CTACGAACACACACAGGTGAAAAGCCATTTACTTGTGAAATCTGTGGCAAGTCTTTCACA

GCAAAGAGTTCTCTTCAGACCCACATCAGAATTCATCGAGGAGAAAAGCCATACTCCTGT

GGCATCTGTGGCAAATCCTTCTCTGACTCCAGTGCCAAAAGGAGACATTGCATTCTGCAC

ACAGGCAAAAAGCCTTTCTCCTGCCCAGAGTGTAACTTACAGTTTGCTCGCTTAGACAAC

TTGAAGGCTCACTTGAAAATCCACAGTAAAGAGAAGCATGCATCAGATGCCAGCAGTGTT

TCTGGTAGTAGTAATACAGAAGAGGTCAGAAATATTCTTCAGCTACAGCCATACCAGCTC

TCTACCTCTGGAGAGCAGGAGATTCAGCTTCTGGTAACTGATTCTGTACATAACATCAAT

TTCATGCCTGGTCCTAGTCAGGGAATCAGTATCATGACTGCTGAGAGTTCCCAGAACATG

ACCGCAGACCAGGCTGCTAATCTCACCCTGCTCACACAGCAGCCAGAGCAACTGCAGAAT

TTAATTCTTTCAGCTCAGCAGGAGCAAACAGAACACATTCAGAGTCTCAATATGATTGAA

AGCCAGATGGAGCCCTCACAGACAGAGCCAGTGCATGTAATTACACTGTCCAAGGAAACC

CTGGAACATCTTCATGCCCATCAAGAGCAAACAGAGGAGCTCGATTTAGCGGCAAGTGCT

TCAGATCCAGCTCAGCACCTGCACCTGACTCAGGAGCCTGAATCACCACCACCTACTCAC

CATGTGCCCCAGCCCACTCCACTTGTCCAGGAGCGAAGC

>CL47.Contig6_A-W 235 507 minus strand PREDICTED: zinc finger and BTB domain-containing protein 24 isoform 1 [Pan troglodytes]

CGAACACACACAGGTGAAAAGCCATTTACTTGTGAAATCTGTGGCAAGTCTTTCACAGCA

AAGAGTTCTCTTCAGACCCACATCAGAATTCATCGAGGAGAAAAGCCATACTCCTGTGGC

ATCTGTGGCAAATCCTTCTCTGACTCCAGTGCCAAAAGGAGACATTGCATTCTGCACACA

GGCAAAAAGCCTTTCTCCTGCCCAGAGTGTAACTTACAGTTTGCTCGCTTAGACAACTTG

AAGGCTCACTTGAAAATCCACAGTAAAGAGAAG

>CL47.Contig7_A-W 328 600 minus strand PREDICTED: zinc finger and BTB domain-containing protein 24 isoform 1 [Pan troglodytes]

CGAACACACACAGGTGAAAAGCCATTTACTTGTGAAATCTGTGGCAAGTCTTTCACAGCA

AAGAGTTCTCTTCAGACCCACATCAGAATTCATCGAGGAGAAAAGCCATACTCCTGTGGC

ATCTGTGGCAAATCCTTCTCTGACTCCAGTGCCAAAAGGAGACATTGCATTCTGCACACA

GGCAAAAAGCCTTTCTCCTGCCCAGAGTGTAACTTACAGTTTGCTCGCTTAGACAACTTG

AAGGCTCACTTGAAAATCCACAGTAAAGAGAAG

>CL48.Contig1_A-W 2 1411 minus strand PREDICTED: cAMP-specific 3&apos;,5&apos;-cyclic phosphodiesterase 4C-like [Equus caballus]

ACCCACCTGTCCGAATCCAGCCGCTCTGGGAACCAGGTGTCCGAGTACATCTCCAGAACC

TTCCTGGACCAGCAGACGGAGGTCGAGCTGCCCAGGGCGGCCCCCGAGGAATCCCAGAGG

CCCATGTCCGAGATCAGTGGCCTCCGTGGGCTCCCCCACAGCGCCAGCCTCTCTGCAGCC

ACTGTCCCACGCTTTGGGGTCCAGACCGACGAGGAGGGGCAGCTGGCCAAGGAGCTGGAA

GACACCAACAGGTGGGGCCTCGATGTGTTCAAGGTGGCGGAGCTGAGCGGGAACAGGCCG

CTGACAGCCGTCATATTCAGCATCTTTCAGGAACGGGACCTGCTCAAGACCTTCCAGATC

CCGGCTGACACCCTGGCCACCTACCTGCTGACGCTGGAGGGTCACTACCACGCCGACGTG

GCCTACCACAACAGCCTGCACGCCGCCGACGTGGCCCAGTCTGCGCATGTGCTGCTGGCC

ACGCCTGCCCTGGAGGCCGTGTTCACAGACCTGGAAGTCCTGGCTGCCATCTTTGCAAGT

GCCATCCACGACGTGGACCACCCCGGGGTCTCCAACCAGTTCCTCATCAACACCAACTCG

GAGCTGGCGCTGATGTACAATGACGCCTCGGTGCTGGAGAACCACCACCTGGCCGTGGGC

TTCAAGCTGCTGCAGGCTCCGAACTGCGACATCTTCCAGAACCTCGGCGCCAAGCAACGG

CTGAGTCTGCGCAGGATGGTCATCGACATGGTGCTGGCCACAGATATGTCCAAACACATG

AATCTCCTGGCGGACCTCAAGACCATGGTAGAAACCAAAAAGGTGACAAGCCTCGGTGTC

CTGCTCCTGGATAACTACTCCGATCGCATCCAGGTCTTACAGAACCTTGTCCACTGTGCT

GACCTCAGCAACCCCACCAAGCCGCTGCCCCTGTACCGCCAGTGGACGGACCGCATCATG

GCAGAGTTCTTCCAGCAGGGTGACCGGGAGCGCGAGTCGGGCCTGGACATCAGCCCCATG

TGCGACAAGCACACGGCCTCGGTGGAGAAGTCCCAGGTGGGCTTCATCGACTACATCGCC

CACCCGCTGTGGGAGACGTGGGCTGACCTGGTCCACCCCGACGCACAGGACCTGCTGGAC

ACGCTGGAGGACAACCGAGAGTGGTACCAAAGCAAGATCCCCCGCAGCCCCGTGGACACC

GCGGTCAGCTCGGAGCGGGGGGCCCCCGACAGGTTCCAGTTCCAGCTCGCTCTGGAGGAA

GCAGAGGAGGAGGAGGAGGAGGAAAAAGGGTCCCTTTGCCCAGAGAAGAGGTTCCTGCGG

CTCAGCGATGGGGCTCTGCTCCTCCGGGTGCTGGGCATCATTTCCCCCAGTTCCCGAGGG

GGACCTCGAATGGTCAGGGGCCACGAAGGA

>CL48.Contig2_A-W 2 877 minus strand PREDICTED: LOW QUALITY PROTEIN: cAMP-specific 3&apos;,5&apos;-cyclic phosphodiesterase 4C [Canis lupus familiaris]

ACCCACCTGTCCGAATCCAGCCGCTCTGGGAACCAGGTGTCCGAGTACATCTCCAGAACC

TTCCTGGACCAGCAGACGGAGGTCGAGCTGCCCAGGGCGGCCCCCGAGGAATCCCAGAGG

CCCATGTCCGAGATCAGTGGCCTCCGTGGGCTCCCCCACAGCGCCAGCCTCTCTGCAGCC

ACTGTCCCACGCTTTGGGGTCCAGACCGACGAGGAGGGGCAGCTGGCCAAGGAGCTGGAA

GACACCAACAGGTGGGGCCTCGATGTGTTCAAGGTGGCGGAGCTGAGCGGGAACAGGCCG

CTGACAGCCGTCATATTCAGCATCTTTCAGGAACGGGACCTGCTCAAGACCTTCCAGATC

CCGGCTGACACCCTGGCCACCTACCTGCTGACGCTGGAGGGTCACTACCACGCCGACGTG

GCCTACCACAACAGCCTGCACGCCGCCGACGTGGCCCAGTCTGCGCATGTGCTGCTGGCC

ACGCCTGCCCTGGAGGCCGTGTTCACAGACCTGGAAGTCCTGGCTGCCATCTTTGCAAGT

GCCATCCACGACGTGGACCACCCCGGGGTCTCCAACCAGTTCCTCATCAACACCAACTCG

GAGCTGGCGCTGATGTACAATGACGCCTCGGTGCTGGAGAACCACCACCTGGCCGTGGGC

TTCAAGCTGCTGCAGGCTCCGAACTGCGACATCTTCCAGAACCTCGGCGCCAAGCAACGG

CTGAGTCTGCGCAGGATGGTCATCGACATGGTGCTGGCCACAGATATGTCCAAACACATG

AATCTCCTGGCGGACCTCAAGACCATGGTAGAAACCAAAAAGGTGACAAGCCTCGGTGTC

CTGCTCCTGGATAACTACTCCGATCGCATCCAGGTA

>CL48.Contig3_A-W 2 856 minus strand phosphodiesterase 4A, cAMP specific, isoform CRA_a [Mus musculus]

ACTGACCAAGAGGACCTCCTGGCCCAAGAACTTGAGAACTTGAGCAAGTGGGGCCTGAAC

ATCTTTTGCGTGTCCGACTATGCCGGAGGCCGCTCGCTCAGCTGTATCATGTACACGATA

TTCCAGGAGCGGGACCTGCTGAAGAAATTCCACATCCCCGTGGACACAATGGTGACCTAC

ATGCTGACTCTGGAGGACCACTACCACGCCGACGTGGCCTACCACAACAGCCTGCATGCA

GCTGACGTGCTGCAGTCCACCCACGTGCTGCTGGCCACGCCTGCCCTGGATGCGGTGTTC

ACTGACCTAGAGATTCTCGCAGCCCTGTTTGCAGCTGCCATCCATGATGTCGACCACCCT

GGGGTCTCCAACCAGTTCCTCATCAACACCAATTCGGAGCTGGCACTCATGTACAATGAC

GAGTCGGTGCTCGAGAACCACCACCTGGCTGTGGGCTTCAAGCTGCTACAGGAGGAGAAC

TGTGACATCTTCCAGAACCTCAGCAAGCGCCAGCGGCAGAGCCTGCGCAAGATGGTTATC

GACATGGTGCTGGCCACAGACATGTCCAAGCACATGACGCTCCTTGCTGATCTAAAAACC

ATGGTGGAAACTAAGAAAGTGACCAGCTCGGGGGTTCTCCTGCTGGACAACTACGCCGAC

CGCATCCAGGTCCTTCGCAATATGGTGCACTGTGCAGACCTCAGCAACCCCACCAAGCCC

CTGGAGCTGTACCGCCAGTGGACGGACCGCATCATGGCAGAGTTCTTCCAGCAGGGTGAC

CGGGAGCGCGAGTCGGGCCTGGACATCAGCCCCATGTGCGACAAGCACACGGCCTCGGTG

GAGAAGTCCCAGGTC

>CL48.Contig4_A-W 54 785 minus strand phosphodiesterase 4A, cAMP specific, isoform CRA_a [Mus musculus]

CAGGAGCGGGACCTGCTGAAGAAATTCCACATCCCCGTGGACACAATGGTGACCTACATG

CTGACTCTGGAGGACCACTACCACGCCGACGTGGCCTACCACAACAGCCTGCATGCAGCT

GACGTGCTGCAGTCCACCCACGTGCTGCTGGCCACGCCTGCCCTGGATGCGGTGTTCACT

GACCTAGAGATTCTCGCAGCCCTGTTTGCAGCTGCCATCCATGATGTCGACCACCCTGGG

GTCTCCAACCAGTTCCTCATCAACACCAATTCGGAGCTGGCACTCATGTACAATGACGAG

TCGGTGCTCGAGAACCACCACCTGGCTGTGGGCTTCAAGCTGCTACAGGAGGAGAACTGT

GACATCTTCCAGAACCTCAGCAAGCGCCAGCGGCAGAGCCTGCGCAAGATGGTTATCGAC

ATGGTGCTGGCCACAGACATGTCCAAGCACATGACGCTCCTTGCTGATCTAAAAACCATG

GTGGAAACTAAGAAAGTGACCAGCTCGGGGGTTCTCCTGCTGGACAACTACGCCGACCGC

ATCCAGGTCCTTCGCAATATGGTGCACTGTGCAGACCTCAGCAACCCCACCAAGCCCCTG

GAGCTGTACCGCCAGTGGACGGACCGCATCATGGCAGAGTTCTTCCAGCAGGGTGACCGG

GAGCGCGAGTCGGGCCTGGACATCAGCCCCATGTGCGACAAGCACACGGCCTCGGTGGAG

AAGTCCCAGGTC

>CL48.Contig5_A-W 2 682 minus strand phosphodiesterase 4A, cAMP specific, isoform CRA_a [Mus musculus]

ACTGACCAAGAGGACCTCCTGGCCCAAGAACTTGAGAACTTGAGCAAGTGGGGCCTGAAC

ATCTTTTGCGTGTCCGACTATGCCGGAGGCCGCTCGCTCAGCTGTATCATGTACACGATA

TTCCAGGAGCGGGACCTGCTGAAGAAATTCCACATCCCCGTGGACACAATGGTGACCTAC

ATGCTGACTCTGGAGGACCACTACCACGCCGACGTGGCCTACCACAACAGCCTGCATGCA

GCTGACGTGCTGCAGTCCACCCACGTGCTGCTGGCCACGCCTGCCCTGGATGCGGTGTTC

ACTGACCTAGAGATTCTCGCAGCCCTGTTTGCAGCTGCCATCCATGATGTCGACCACCCT

GGGGTCTCCAACCAGTTCCTCATCAACACCAATTCGGAGCTGGCACTCATGTACAATGAC

GAGTCGGTGCTCGAGAACCACCACCTGGCTGTGGGCTTCAAGCTGCTACAGGAGGAGAAC

TGTGACATCTTCCAGAACCTCAGCAAGCGCCAGCGGCAGAGCCTGCGCAAGATGGTTATC

GACATGGTGCTGGCCACAGACATGTCCAAGCACATGACGCTCCTTGCTGATCTAAAAACC

ATGGTGGAAACTAAGAAAGTGACCAGCTCGGGGGTTCTCCTGCTGGACAACTACGCCGAC

CGCATCCAGGTGCCCCACAAC

>CL48.Contig6_A-W 54 611 minus strand phosphodiesterase 4A, cAMP specific, isoform CRA_a [Mus musculus]

CAGGAGCGGGACCTGCTGAAGAAATTCCACATCCCCGTGGACACAATGGTGACCTACATG

CTGACTCTGGAGGACCACTACCACGCCGACGTGGCCTACCACAACAGCCTGCATGCAGCT

GACGTGCTGCAGTCCACCCACGTGCTGCTGGCCACGCCTGCCCTGGATGCGGTGTTCACT

GACCTAGAGATTCTCGCAGCCCTGTTTGCAGCTGCCATCCATGATGTCGACCACCCTGGG

GTCTCCAACCAGTTCCTCATCAACACCAATTCGGAGCTGGCACTCATGTACAATGACGAG

TCGGTGCTCGAGAACCACCACCTGGCTGTGGGCTTCAAGCTGCTACAGGAGGAGAACTGT

GACATCTTCCAGAACCTCAGCAAGCGCCAGCGGCAGAGCCTGCGCAAGATGGTTATCGAC

ATGGTGCTGGCCACAGACATGTCCAAGCACATGACGCTCCTTGCTGATCTAAAAACCATG

GTGGAAACTAAGAAAGTGACCAGCTCGGGGGTTCTCCTGCTGGACAACTACGCCGACCGC

ATCCAGGTGCCCCACAAC

>CL48.Contig7_A-W 547 1407 minus strand coiled-coil domain-containing protein 88B precursor [Bos taurus] >gi|296471497|gb|DAA13612.1| coiled-coil domain containing 88B [Bos taurus]

GAAGCAGAGGAGGAGGAGGAGGAGGAAAAAGGGTCCCTTTGCCCAGAGAAGAGGTTCCTG

CGGCTCAGCGATGGGGCTCTGCTCCTCCGGGTGCTGGGCATCATTTCCCCCAGTTCCCGA

GGGGGACCTCGAATGGTCAGGGGCCACGAAGGACCCGCGGCCTGGAGAGTATGGAACCTG

AACCATCTGTGGGTCCGACTGAGGGACTTCTACCAGGAGGAGCTGCAGCTGCTGGTCCTG

TCGCCACCCCCAGACCTCCAGACATTGGGGTTTGACCCCTTCTCAGAGGAGGCGGTGGAG

GAGCTGCAAGGCCTCCTTCGGCTGTTGTTGGGGGCGTCAGTGCAGTGTGACCACCGGGAG

CTCTTCATCCGCCACATCCAGGGCCTCAGCCTCGAGGTCCAGAGCGAGCTGGCCGCCGCC

ATCCAGGAGGTGACCCAGCCTGGGGCCGGTGTAGTGCTTGCGCTGTCTGGGCCTGAGCCA

GGGGAGCTGGAGCCTTCGGAGTTGGAGATGCTGTCCCGGAGCCTGATGGGGGCGCTGTCG

AGGCTGGCCCGGGAGCGTGACCTGGGGGCCCAGAGGCTGGCAGAACTGCTGCTGGAGCGG

GAGCGGGCCCCCTTGCTGCCTGAAGCTCCTACAAAGGCTCCTGCGGAGGGTTCCTCGCAC

CATCTGGCGCTGCAACTGGCCAATGCCAAGGCCCAGCTGCGGCGCCTGCGGCAAGAGCTG

GAGGAGAAGGCCGAAGTGCTGCTAGACTCCCAGGAGGAGGTGCAGGGCTTGGAGGCCGAA

ATTCGAAGGCTCCGCCAGGAGGCCCAGGCGCTGTCGGGACAGGCCAAGCGGGCCGAGCTG

TACCGCGAGGAGGCAGAGGCG

>CL48.Contig8_A-W 329 1189 minus strand coiled-coil domain-containing protein 88B precursor [Bos taurus] >gi|296471497|gb|DAA13612.1| coiled-coil domain containing 88B [Bos taurus]

GAAGCAGAGGAGGAGGAGGAGGAGGAAAAAGGGTCCCTTTGCCCAGAGAAGAGGTTCCTG

CGGCTCAGCGATGGGGCTCTGCTCCTCCGGGTGCTGGGCATCATTTCCCCCAGTTCCCGA

GGGGGACCTCGAATGGTCAGGGGCCACGAAGGACCCGCGGCCTGGAGAGTATGGAACCTG

AACCATCTGTGGGTCCGACTGAGGGACTTCTACCAGGAGGAGCTGCAGCTGCTGGTCCTG

TCGCCACCCCCAGACCTCCAGACATTGGGGTTTGACCCCTTCTCAGAGGAGGCGGTGGAG

GAGCTGCAAGGCCTCCTTCGGCTGTTGTTGGGGGCGTCAGTGCAGTGTGACCACCGGGAG

CTCTTCATCCGCCACATCCAGGGCCTCAGCCTCGAGGTCCAGAGCGAGCTGGCCGCCGCC

ATCCAGGAGGTGACCCAGCCTGGGGCCGGTGTAGTGCTTGCGCTGTCTGGGCCTGAGCCA

GGGGAGCTGGAGCCTTCGGAGTTGGAGATGCTGTCCCGGAGCCTGATGGGGGCGCTGTCG

AGGCTGGCCCGGGAGCGTGACCTGGGGGCCCAGAGGCTGGCAGAACTGCTGCTGGAGCGG

GAGCGGGCCCCCTTGCTGCCTGAAGCTCCTACAAAGGCTCCTGCGGAGGGTTCCTCGCAC

CATCTGGCGCTGCAACTGGCCAATGCCAAGGCCCAGCTGCGGCGCCTGCGGCAAGAGCTG

GAGGAGAAGGCCGAAGTGCTGCTAGACTCCCAGGAGGAGGTGCAGGGCTTGGAGGCCGAA

ATTCGAAGGCTCCGCCAGGAGGCCCAGGCGCTGTCGGGACAGGCCAAGCGGGCCGAGCTG

TACCGCGAGGAGGCAGAGGCG

>CL48.Contig9_A-W 53 577 minus strand Coiled-coil domain containing 88B [Homo sapiens] >gi|187954627|gb|AAI40823.1| Coiled-coil domain containing 88B [Homo sapiens]

TCTGCGCAGTGTGACCACCGGGAGCTCTTCATCCGCCACATCCAGGGCCTCAGCCTCGAG

GTCCAGAGCGAGCTGGCCGCCGCCATCCAGGAGGTGACCCAGCCTGGGGCCGGTGTAGTG

CTTGCGCTGTCTGGGCCTGAGCCAGGGGAGCTGGAGCCTTCGGAGTTGGAGATGCTGTCC

CGGAGCCTGATGGGGGCGCTGTCGAGGCTGGCCCGGGAGCGTGACCTGGGGGCCCAGAGG

CTGGCAGAACTGCTGCTGGAGCGGGAGCGGGCCCCCTTGCTGCCTGAAGCTCCTACAAAG

GCTCCTGCGGAGGGTTCCTCGCACCATCTGGCGCTGCAACTGGCCAATGCCAAGGCCCAG

CTGCGGCGCCTGCGGCAAGAGCTGGAGGAGAAGGCCGAAGTGCTGCTAGACTCCCAGGAG

GAGGTGCAGGGCTTGGAGGCCGAAATTCGAAGGCTCCGCCAGGAGGCCCAGGCGCTGTCG

GGACAGGCCAAGCGGGCCGAGCTGTACCGCGAGGAGGCAGAGGCG

>CL49.Contig2_A-W 1 228 PREDICTED: sugar phosphate exchanger 2 [Saimiri boliviensis boliviensis]

GCTAAGGCCCTCTCCACTGTCTCAGCCATCATCGATGGCACGGGCTCCATAGGTGCTGCT

CTGGGGCCCCTGCTGGCTGGACTCATTTCCCCCACAGGCTGGAACAATGTCTTCTACATG

CTCATCTCTGCTGACATCCTGGCCTGCTTGTTGCTCTGTCGGTTGGTGTACAAAGAGATC

CTGGCTTGGAAGTCATCCCTGAGCAAAGGCAGAGGGTATAAAGAAATA

>CL50.Contig1_A-W 181 591 PREDICTED: geranylgeranyl transferase type-2 subunit beta [Canis lupus familiaris]

TTTTGTGCAGTGGCAACTTTGGCTCTGTTGGGGAAACTTGATGCTATTAATGTTGAAAAG

GCAATCGAATTTGTTTTATCCTGTATGAACTTTGATGGTGGATTTGGTTGTCGACCTGGT

TCTGAGTCCCATGCTGGACAGATCTATTGTTGCACAGGATTTCTGGCTATTACTAGTCAG

TTGCATCAAGTAAATTCTGACTTACTTGGTTGGTGGCTTTGTGAACGTCAGTTACCTTCA

GGAGGACTCAATGGAAGGCCGGAGAAGTTACCAGATGTGTGCTATTCATGGTGGGTATTG

GCTTCCCTAAAGATAATTGGAAGACTTCATTGGATTGACAGAGAGAAACTGCGAGCTTTC

ATTTTGGCATGTCAAGATGAAGAAACTGGAGGATTTGCAGATAGGCCAGGA

>CL50.Contig2_A-W 11 430 PREDICTED: geranylgeranyl transferase type-2 subunit beta [Canis lupus familiaris]

GGCACACCACAGAAGGATGTTATCATCAAGTCTGATGCACCTGTCACCCTTTTACTGGAG

AAACATGCAGATTATATTGCATCCTATGGCTCAAAGAAAGATGACTATGAATACTGTATG

TCTGAGTATTTGAGAATGAGTGGTATCTACTGGGGTCTGACAGTAATGGATCTCATGGGA

CAACTTGATCGGATGAATAGAGAAGAGATTCTGACGTTTATTAAGTCATGCCAACATGAG

TGTGGTGGAATAAGTGCAAGCATTGGACATGATCCTCATCTTTTGTATACTCTTAGTGCT

GTCCAGATTCTTACTCTTTATGATAGTATTAATGTTATTGATGTAAATAAGGTTGTGGAA

TATGTTCAAAGTCTACAGAAAGAAGATGGTTCTTTTGCTGGAGATATTTGGGGTAATGTC

>CL50.Contig3_A-W 2 1012 minus strand PREDICTED: RAB geranylgeranyltransferase, beta subunit [Oryctolagus cuniculus]

TTCTTTTCGCTCTCAGACATGGGCACACCACAGAAGGATGTTATCATCAAGTCTGATGCA

CCTGTCACCCTTTTACTGGAGAAACATGCAGATTATATTGCATCCTATGGCTCAAAGAAA

GATGACTATGAATACTGTATGTCTGAGTATTTGAGAATGAGTGGTATCTACTGGGGTCTG

ACAGTAATGGATCTCATGGGACAACTTGATCGGATGAATAGAGAAGAGATTCTGACGTTT

ATTAAGTCATGCCAACATGAGTGTGGTGGAATAAGTGCAAGCATTGGACATGATCCTCAT

CTTTTGTATACTCTTAGTGCTGTCCAGATTCTTACTCTTTATGATAGTATTAATGTTATT

GATGTAAATAAGGTTGTGGAATATGTTCAAAGTCTACAGAAAGAAGATGGTTCTTTTGCT

GGAGATATTTGGGGAGAAATTGATACAAGATTCTCCTTTTGTGCAGTGGCAACTTTGGCT

CTGTTGGGGAAACTTGATGCTATTAATGTTGAAAAGGCAATCGAATTTGTTTTATCCTGT

ATGAACTTTGATGGTGGATTTGGTTGTCGACCTGGTTCTGAGTCCCATGCTGGACAGATC

TATTGTTGCACAGGATTTCTGGCTATTACTAGTCAGTTGCATCAAGTAAATTCTGACTTA

CTTGGTTGGTGGCTTTGTGAACGTCAGTTACCTTCAGGAGGACTCAATGGAAGGCCGGAG

AAGTTACCAGATGTGTGCTATTCATGGTGGGTATTGGCTTCCCTAAAGATAATTGGAAGA

CTTCATTGGATTGACAGAGAGAAACTGCGAGCTTTCATTTTGGCATGTCAAGATGAAGAA

ACTGGAGGATTTGCAGATAGGCCAGGAGACATGGTGGATCCTTTTCATACATTATTTGGC

ATTGCTGGATTGTCACTTTTGGGAGAAGAACAGATTAAACCTGTTAGTCCTGTTTTTTGC

ATGCCTGAAGAAGTGCTTCGAAGAGTCAATGTTCAGCCTGAACTAGTGAGC

>CL50.Contig4_A-W 8 454 Rab geranylgeranyltransferase, beta subunit [Homo sapiens]

ATGGGCACACCACAGAAGGATGTTATCATCAAGTCTGATGCACCTGTCACCCTTTTACTG

GAGAAACATGCAGATTATATTGCATCCTATGGCTCAAAGAAAGATGACTATGAATACTGT

ATGTCTGAGTATTTGAGAATGAGTGGTATCTACTGGGGTCTGACAGTAATGGATCTCATG

GGACAACTTGATCGGATGAATAGAGAAGAGATTCTGACGTTTATTAAGTCATGCCAACAT

GAGTGTGGTGGAATAAGTGCAAGCATTGGACATGATCCTCATCTTTTGTATACTCTTAGT

GCTGTCCAGATTCTTACTCTTTATGATAGTATTAATGTTATTGATGTAAATAAGGTTGTG

GAATATGTTCAAAGTCTACAGAAAGAAGATGGTTCTTTTGCTGGAGATATTTGGGGTAAT

GTCAGATTTTGTCCATACTACCATAAA

>CL50.Contig5_A-W 8 424 PREDICTED: geranylgeranyl transferase type-2 subunit beta [Otolemur garnettii]

ATGGGCACACCACAGAAGGATGTTATCATCAAGTCTGATGCACCTGTCACCCTTTTACTG

GAGAAACATGCAGATTATATTGCATCCTATGGCTCAAAGAAAGATGACTATGAATACTGT

ATGTCTGAGTATTTGAGAATGAGTGGTATCTACTGGGGTCTGACAGTAATGGATCTCATG

GGACAACTTGATCGGATGAATAGAGAAGAGATTCTGACGTTTATTAAGTCATGCCAACAT

GAGTGTGGTGGAATAAGTGCAAGCATTGGACATGATCCTCATCTTTTGTATACTCTTAGT

GCTGTCCAGATTCTTACTCTTTATGATAGTATTAATGTTATTGATGTAAATAAGGTTGTG

GAATATGTTCAAAGTCTACAGAAAGAAGATGGTTCTTTTGCTGGAGATATTTGGGGT

>CL51.Contig1_A-W 643 747 minus strand PREDICTED: sphingomyelin phosphodiesterase 3 [Bos taurus]

GGTTCCTGCCCCCAGGAAGTGGAACAGGTTATGTTTAGTACAGCCCTGGCGGGGCTCACA

GACCACCTGACCGTGGGCCTGCAGCTCCGAGTTTCGGCGCTCTCC

>CL51.Contig2_A-W 285 452 minus strand PREDICTED: sphingomyelin phosphodiesterase 3 [Bos taurus]

GACCACGCACAGGAACAGGGGCACAAACTCTTTCGCTGTTTCCGGGATCCCTGCCGGCTG

AGCACACGCCAGGATCAGCCTTGGGCCTTGGGAACCATCCTAAGCACCTCCACCCTCCAC

CAATCAGTAGCTAGCTCACCGGAGATGCTGCGGAGGTGAGTGGAGCGC

>CL51.Contig3_A-W 634 738 minus strand PREDICTED: sphingomyelin phosphodiesterase 3 [Bos taurus]

GGTTCCTGCCCCCAGGAAGTGGAACAGGTTATGTTTAGTACAGCCCTGGCGGGGCTCACA

GACCACCTGACCGTGGGCCTGCAGCTCCGAGTTTCGGCGCTCTCC

>CL51.Contig4_A-W 447 674 minus strand PREDICTED: sphingomyelin phosphodiesterase 3 [Bos taurus]

AGGGCCCTAGAGCAGGAGAAAGGGCGCCACCTCTACCTGGCAGGCCCTCCCCGCGGAGGC

CGTCGGGCTAACCCCTGGCTGGGCCGGCGTCTGGACTACATCACCTACCGGGAAATGCCT

GGGAGTGTGCTGATTCCAGAAGTGGAACAGGTTATGTTTAGTACAGCCCTGGCGGGGCTC

ACAGACCACCTGACCGTGGGCCTGCAGCTCCGAGTTTCGGCGCTCTCC

>CL51.Contig5_A-W 2 208 minus strand PREDICTED: sphingomyelin phosphodiesterase 3 [Bos taurus]

CAACTGGGCATCCTGGACGGGCACCGCGTAGTGGGATTCCTGCATTGCACGCATTTACAT

GCACCCACTGAGGATGGGCTCTTGCGCTGCAAACAGCTGACGCTGCTACTGGACTGGGCA

GAGCAGTTTGAGGCCGAGAGCCGCCAGAGTGGTGAGGCTGTGGCCTTTAGTGTGCTTCTG

GGTGACCTAAATTTTGACAACTGCTCA

>CL51.Contig6_A-W 561 665 minus strand PREDICTED: sphingomyelin phosphodiesterase 3 [Bos taurus]

GGTTCCTGCCCCCAGGAAGTGGAACAGGTTATGTTTAGTACAGCCCTGGCGGGGCTCACA

GACCACCTGACCGTGGGCCTGCAGCTCCGAGTTTCGGCGCTCTCC

>CL51.Contig7_A-W 2 592 minus strand PREDICTED: sphingomyelin phosphodiesterase 3 [Bos taurus]

CAACTGGGCATCCTGGACGGGCACCGCGTAGTGGGATTCCTGCATTGCACGCATTTACAT

GCACCCACTGAGGATGGGCTCTTGCGCTGCAAACAGCTGACGCTGCTACTGGACTGGGCA

GAGCAGTTTGAGGCCGAGAGCCGCCAGAGTGGTGAGGCTGTGGCCTTTAGTGTGCTTCTG

GGTGACCTAAATTTTGACAACTGCTCAAAAGACCACGCACAGGAACAGGGGCACAAACTC

TTTCGCTGTTTCCGGGATCCCTGCCGGCTGAGCACACGCCAGGATCAGCCTTGGGCCTTG

GGAACCATCCTAAGCACCTCCACCCTCCACCAATCAGTAGCTAGCTCACCGGAGATGCTG

CGGAGGGCCCTAGAGCAGGAGAAAGGGCGCCACCTCTACCTGGCAGGCCCTCCCCGCGGA

GGCCGTCGGGCTAACCCCTGGCTGGGCCGGCGTCTGGACTACATCACCTACCGGGAAATG

CCTGGGAGTGTGCTGATTCCAGAAGTGGAACAGGTTATGTTTAGTACAGCCCTGGCGGGG

CTCACAGACCACCTGACCGTGGGCCTGCAGCTCCGAGTTTCGGCGCTCTCC

>CL51.Contig8_A-W 1867 1971 minus strand PREDICTED: sphingomyelin phosphodiesterase 3-like [Equus caballus]

GGTTCCTGCCCCCAGGAAGTGGAACAGGTTATGTTTAGTACAGCCCTGGCGGGGCTCACA

GACCACCTGACCGTGGGCCTGCAGCTCCGAGTTTCGGCGCTCTCC

>CL51.Contig9_A-W 1048 1152 minus strand PREDICTED: sphingomyelin phosphodiesterase 3-like [Saimiri boliviensis boliviensis]

GGTTCCTGCCCCCAGGAAGTGGAACAGGTTATGTTTAGTACAGCCCTGGCGGGGCTCACA

GACCACCTGACCGTGGGCCTGCAGCTCCGAGTTTCGGCGCTCTCC

>CL51.Contig10_A-W 3 1079 minus strand PREDICTED: sphingomyelin phosphodiesterase 3-like [Saimiri boliviensis boliviensis]

CCGTGCGCTGAGTTTGCGCGCTGCTTTGTCTTCCTCACTGCCAACCTGTGCCTGCTCCCC

GACGGGCTGGCGCGCTTCAGCAATCTGCCACACAGCCAACAACGCGCAGAGGCCATTGGC

GCTACGCTGTTAAACGGCCTACGGCCCTCGCTTTATGGAGCTACCGGATGCAGCCCACAA

AGGCTTGGGGAGCCAGGCGGGACGCTGACGGCCCAGATGCCCGCGGGGCTGGACTTTGTG

TGCCTGCAGGAGGTGTTCGATATTCGCGCAGCTCGACGCCTGGTGCGCTGCTTGGCTCCG

AATCTGGGCCCGGTGCTGTATGACGTAGGCACACTTGGCCTACAGCCTGGGCCATACTTA

AAACTGCTAGGCAGCGGGCTCCTGCTGGCTTCCCGCTACCCGCTTCTGCGTGCCAACTTC

CGTTGCTTTCCCAATGCGCGTCGCGAGGACGCCTTTGCCTCCAAGGGTCTACTATCCGCT

CAGGTGCAACTGGGCATCCTGGACGGGCACCGCGTAGTGGGATTCCTGCATTGCACGCAT

TTACATGCACCCACTGAGGATGGGCTCTTGCGCTGCAAACAGCTGACGCTGCTACTGGAC

TGGGCAGAGCAGTTTGAGGCCGAGAGCCGCCAGAGTGGTGAGGCTGTGGCCTTTAGTGTG

CTTCTGGGTGACCTAAATTTTGACAACTGCTCAAAAGACCACGCACAGGAACAGGGGCAC

AAACTCTTTCGCTGTTTCCGGGATCCCTGCCGGCTGAGCACACGCCAGGATCAGCCTTGG

GCCTTGGGAACCATCCTAAGCACCTCCACCCTCCACCAATCAGTAGCTAGCTCACCGGAG

ATGCTGCGGAGGGCCCTAGAGCAGGAGAAAGGGCGCCACCTCTACCTGGCAGGCCCTCCC

CGCGGAGGCCGTCGGGCTAACCCCTGGCTGGGCCGGCGTCTGGACTACATCACCTACCGG

GAAATGCCTGGGAGTGTGCTGATTCCAGAAGTGGAACAGGTTATGTTTAGTACAGCCCTG

GCGGGGCTCACAGACCACCTGACCGTGGGCCTGCAGCTCCGAGTTTCGGCGCTCTCC

>CL51.Contig11_A-W 2039 2143 minus strand PREDICTED: sphingomyelin phosphodiesterase 3-like [Equus caballus]

GGTTCCTGCCCCCAGGAAGTGGAACAGGTTATGTTTAGTACAGCCCTGGCGGGGCTCACA

GACCACCTGACCGTGGGCCTGCAGCTCCGAGTTTCGGCGCTCTCC

>CL52.Contig1_A-W 3 446 minus strand PREDICTED: TFIIA alpha, p55 [Oryctolagus cuniculus]

AAATTATATAGATCTGTGATTGAAGATGTTATTAATGATGTGAGAGACATCTTTTTGGAT

GATGGAGTGGATGAACAAGTCCTGATGGAACTAAAAACTTTATGGGAGAATAAACTAATG

CAGTCTAGGGCAGTAGATGGATTTCATTCAGAAGAGCAACAGCTTTTATTGCAAGTTCAA

CAGCAGCATCAACCCCAGCAGCAGCAACACCACCACCACCACCACCATCAGCAAACTCAA

CCTCAGCAGACAGTACCTCAGCAAGCACAGACCCAGCAGGTTCTTATTCCTGCATCACAG

CAAGCCACAGCACCACAAGTTATTGTTCCCGATTCAAAGCTTATACAGCATATGAATGCA

TCAAACATGAGTGCTGCTGCTACAGCTGCTACCTTGGCACTCCCTGCAGGTGTGACTCCT

GTTCAACAGATATTAACAAATTCA

>CL52.Contig2_A-W 3 1097 minus strand PREDICTED: TFIIA alpha, p55 [Oryctolagus cuniculus]

AAATTATATAGATCTGTGATTGAAGATGTTATTAATGATGTGAGAGACATCTTTTTGGAT

GATGGAGTGGATGAACAAGTCCTGATGGAACTAAAAACTTTATGGGAGAATAAACTAATG

CAGTCTAGGGCAGTAGATGGATTTCATTCAGAAGAGCAACAGCTTTTATTGCAAGTTCAA

CAGCAGCATCAACCCCAGCAGCAGCAACACCACCACCACCACCACCATCAGCAAACTCAA

CCTCAGCAGACAGTACCTCAGCAAGCACAGACCCAGCAGGTTCTTATTCCTGCATCACAG

CAAGCCACAGCACCACAAGTTATTGTTCCCGATTCAAAGCTTATACAGCATATGAATGCA

TCAAACATGAGTGCTGCTGCTACAGCTGCTACCTTGGCACTCCCTGCAGGTGTGACTCCT

GTTCAACAGATATTAACAAATTCAGGCCAGCTTCTTCAGGTGGTCAGAGCAGCCAATGGT

GCCCAATATATCTTTCAGCCTCAGCAGTCAGTGGTTCTACAACAGCAGGTTATTCCACAA

ATGCAGCCTGGTGGAGTACAAGCTCCTGTTATACAACAGGTTCTTGCCCCTCTTCCTGGA

GCGATTTCACCACAGACTGGTGTTATCATCCAGCCTCAGCAAATATTATTTACAGGAAAT

AAGACTCAAGTTATACCTACAACAGTGGCAGCGCCCACACCAGCACAAGCACAGATAACT

GCAGCTGGCCAGCAGCAACCACAGGCCCAGCCTGCTCAACAGCAAGCTCCATTGGTATTA

CAAGTTGATGGAACTGGGGATACATCATCTGAAGAAGATGAAGACGAAGAAGAAGACTAT

GATGATGATGAAGAGGAGGATAAAGAGAAAGATGGAGCTGAAGATGGGCAGGTAGAAGAA

GAACCCCTCAATAGTGAAGATGATGTGAGTGATGAGGAAGGACAGGAACTCTTTGACACA

GAAAATGTTGTTGTATGCCAATACGATAAGATACACAGAAGTAAAAACAAATGGAAATTT

CATCTCAAGGATGGTATTATGAATCTTAATGGAAGAGATTATATATTTTCCAAAGCCATT

GGAGATGCTGAATGG

>CL52.Contig3_A-W 3 911 minus strand PREDICTED: TFIIA alpha, p55 [Oryctolagus cuniculus]

AAATTATATAGATCTGTGATTGAAGATGTTATTAATGATGTGAGAGACATCTTTTTGGAT

GATGGAGTGGATGAACAAGTCCTGATGGAACTAAAAACTTTATGGGAGAATAAACTAATG

CAGTCTAGGGCAGTAGATGGATTTCATTCAGAAGAGCAACAGCTTTTATTGCAAGTTCAA

CAGCAGCATCAACCCCAGCAGCAGCAACACCACCACCACCACCACCATCAGCAAACTCAA

CCTCAGCAGACAGTACCTCAGCAAGCACAGACCCAGCAGGTTCTTATTCCTGCATCACAG

CAAGCCACAGCACCACAAGTTATTGTTCCCGATTCAAAGCTTATACAGCATATGAATGCA

TCAAACATGAGTGCTGCTGCTACAGCTGCTACCTTGGCACTCCCTGCAGGTGTGACTCCT

GTTCAACAGATATTAACAAATTCAGGCCAGCTTCTTCAGGTGGTCAGAGCAGCCAATGGT

GCCCAATATATCTTTCAGCCTCAGCAGTCAGTGGTTCTACAACAGCAGGTTATTCCACAA

ATGCAGCCTGGTGGAGTACAAGCTCCTGTTATACAACAGGTTCTTGCCCCTCTTCCTGGA

GCGATTTCACCACAGACTGGTGTTATCATCCAGCCTCAGCAAATATTATTTACAGGAAAT

AAGACTCAAGTTATACCTACAACAGTGGCAGCGCCCACACCAGCACAAGCACAGATAACT

GCAGCTGGCCAGCAGCAACCACAGGCCCAGCCTGCTCAACAGCAAGCTCCATTGGTATTA

CAAGTTGATGGAACTGGGGATACATCATCTGAAGAAGATGAAGACGAAGAAGAAGACTAT

GATGATGATGAAGAGGAGGATAAAGAGAAAGATGGAGCTGAAGATGGGCAGGTAGAAGAA

GTAAGTCTC

>CL52.Contig4_A-W 445 858 minus strand PREDICTED: TFIIA alpha, p55 [Oryctolagus cuniculus]

CAGCCTCAGCAGTCAGTGGTTCTACAACAGCAGGTTATTCCACAAATGCAGCCTGGTGGA

GTACAAGCTCCTGTTATACAACAGGTTCTTGCCCCTCTTCCTGGAGCGATTTCACCACAG

ACTGGTGTTATCATCCAGCCTCAGCAAATATTATTTACAGGAAATAAGACTCAAGTTATA

CCTACAACAGTGGCAGCGCCCACACCAGCACAAGCACAGATAACTGCAGCTGGCCAGCAG

CAACCACAGGCCCAGCCTGCTCAACAGCAAGCTCCATTGGTATTACAAGTTGATGGAACT

GGGGATACATCATCTGAAGAAGATGAAGACGAAGAAGAAGACTATGATGATGATGAAGAG

GAGGATAAAGAGAAAGATGGAGCTGAAGATGGGCAGGTAGAAGAAGTAAGTCTC

>CL53.Contig1_A-W 21 638 minus strand PREDICTED: male-enhanced antigen 1 [Canis lupus familiaris]

GGGGCGGGGCTGGAGGACTGGGAGCCCCGGCACGTCCTGCCCCACCGGAAGTGAAGGTGC

GGGTCCGACGGTGGGCTCTGAGAGGCCCCTGCCCGGATGGCCGCAGTAGTTCTAGGGGGA

GACACCATGGGCCCTGAGCGTATCTTCCCCAATCAGACTGAAGAACTGGGGCCACATCAG

GGCCCTACAGAAGGCACTGGGGATTGGAGCAGTGAGGAGCCTGAAGAAGAGCAGGAGGAA

ACAGGGGCAGGCCCGGCTGGCTACTCCTACCAGCCCCTAAACCAAGATCCTGAACAAGAG

GAGGTGGAGCTCGCACCAGTGGGGGATGGAGAAGATGTAGTTGCTGACATCCAGGATCGG

ATCCAGGCCCTGGGGCTTCATTTGCCAGACCCACCATTAGAGAGTGAGGATGAAGATGAG

GAGGGAGCTACTGCATTGAGCAGCCACAGTTCTATTCCCATGGACCCAGAACATGTAGAG

CTGGTGAAAAGAACAATGGCTGGAGTAAGCCTTCCTGCACCAGGAGTTCCTGCCTGGGCT

CGGGAGATATCAGATGCCCAGTGGGAAGATGTGGTACAGAAAGCCCTTCAAGCCCGGCAG

GCATCTCCTGCTTGGAAG

>CL53.Contig2_A-W 21 638 minus strand PREDICTED: male-enhanced antigen 1 [Canis lupus familiaris]

GGGGCGGGGCTGGAGGACTGGGAGCCCCGGCACGTCCTGCCCCACCGGAAGTGAAGGTGC

GGGTCCGACGGTGGGCTCTGAGAGGCCCCTGCCCGGATGGCCGCAGTAGTTCTAGGGGGA

GACACCATGGGCCCTGAGCGTATCTTCCCCAATCAGACTGAAGAACTGGGGCCACATCAG

GGCCCTACAGAAGGCACTGGGGATTGGAGCAGTGAGGAGCCTGAAGAAGAGCAGGAGGAA

ACAGGGGCAGGCCCGGCTGGCTACTCCTACCAGCCCCTAAACCAAGATCCTGAACAAGAG

GAGGTGGAGCTCGCACCAGTGGGGGATGGAGAAGATGTAGTTGCTGACATCCAGGATCGG

ATCCAGGCCCTGGGGCTTCATTTGCCAGACCCACCATTAGAGAGTGAGGATGAAGATGAG

GAGGGAGCTACTGCATTGAGCAGCCACAGTTCTATTCCCATGGACCCAGAACATGTAGAG

CTGGTGAAAAGAACAATGGCTGGAGTAAGCCTTCCTGCACCAGGAGTTCCTGCCTGGGCT

CGGGAGATATCAGATGCCCAGTGGGAAGATGTGGTACAGAAAGCCCTTCAAGCCCGGCAG

GCATCTCCTGCTTGGAAG

>CL53.Contig3_A-W 2 619 PREDICTED: male-enhanced antigen 1 [Canis lupus familiaris]

GGGGCGGGGCTGGAGGACTGGGAGCCCCGGCACGTCCTGCCCCACCGGAAGTGAAGGTGC

GGGTCCGACGGTGGGCTCTGAGAGGCCCCTGCCCGGATGGCCGCAGTAGTTCTAGGGGGA

GACACCATGGGCCCTGAGCGTATCTTCCCCAATCAGACTGAAGAACTGGGGCCACATCAG

GGCCCTACAGAAGGCACTGGGGATTGGAGCAGTGAGGAGCCTGAAGAAGAGCAGGAGGAA

ACAGGGGCAGGCCCGGCTGGCTACTCCTACCAGCCCCTAAACCAAGATCCTGAACAAGAG

GAGGTGGAGCTCGCACCAGTGGGGGATGGAGAAGATGTAGTTGCTGACATCCAGGATCGG

ATCCAGGCCCTGGGGCTTCATTTGCCAGACCCACCATTAGAGAGTGAGGATGAAGATGAG

GAGGGAGCTACTGCATTGAGCAGCCACAGTTCTATTCCCATGGACCCAGAACATGTAGAG

CTGGTGAAAAGAACAATGGCTGGAGTAAGCCTTCCTGCACCAGGAGTTCCTGCCTGGGCT

CGGGAGATATCAGATGCCCAGTGGGAAGATGTGGTACAGAAAGCCCTTCAAGCCCGGCAG

GCATCTCCTGCTTGGAAG

>CL53.Contig4_A-W 6 590 PREDICTED: male-enhanced antigen-like [Oryctolagus cuniculus]

CGGGGCTGGAGGACTGGGAGCCCCGGCACGTCCTGCCCCACCGGAAGTGAAGCCCCTGCC

CGGATGGCCGCAGTAGTTCTAGGGGGAGACACCATGGGCCCTGAGCGTATCTTCCCCAAT

CAGACTGAAGAACTGGGGCCACATCAGGGCCCTACAGAAGGCACTGGGGATTGGAGCAGT

GAGGAGCCTGAAGAAGAGCAGGAGGAAACAGGGGCAGGCCCGGCTGGCTACTCCTACCAG

CCCCTAAACCAAGATCCTGAACAAGAGGAGGTGGAGCTCGCACCAGTGGGGGATGGAGAA

GATGTAGTTGCTGACATCCAGGATCGGATCCAGGCCCTGGGGCTTCATTTGCCAGACCCA

CCATTAGAGAGTGAGGATGAAGATGAGGAGGGAGCTACTGCATTGAGCAGCCACAGTTCT

ATTCCCATGGACCCAGAACATGTAGAGCTGGTGAAAAGAACAATGGCTGGAGTAAGCCTT

CCTGCACCAGGAGTTCCTGCCTGGGCTCGGGAGATATCAGATGCCCAGTGGGAAGATGTG

GTACAGAAAGCCCTTCAAGCCCGGCAGGCATCTCCTGCTTGGAAG

>CL53.Contig5_A-W 6 590 PREDICTED: male-enhanced antigen-like [Oryctolagus cuniculus]

CGGGGCTGGAGGACTGGGAGCCCCGGCACGTCCTGCCCCACCGGAAGTGAAGCCCCTGCC

CGGATGGCCGCAGTAGTTCTAGGGGGAGACACCATGGGCCCTGAGCGTATCTTCCCCAAT

CAGACTGAAGAACTGGGGCCACATCAGGGCCCTACAGAAGGCACTGGGGATTGGAGCAGT

GAGGAGCCTGAAGAAGAGCAGGAGGAAACAGGGGCAGGCCCGGCTGGCTACTCCTACCAG

CCCCTAAACCAAGATCCTGAACAAGAGGAGGTGGAGCTCGCACCAGTGGGGGATGGAGAA

GATGTAGTTGCTGACATCCAGGATCGGATCCAGGCCCTGGGGCTTCATTTGCCAGACCCA

CCATTAGAGAGTGAGGATGAAGATGAGGAGGGAGCTACTGCATTGAGCAGCCACAGTTCT

ATTCCCATGGACCCAGAACATGTAGAGCTGGTGAAAAGAACAATGGCTGGAGTAAGCCTT

CCTGCACCAGGAGTTCCTGCCTGGGCTCGGGAGATATCAGATGCCCAGTGGGAAGATGTG

GTACAGAAAGCCCTTCAAGCCCGGCAGGCATCTCCTGCTTGGAAG

>CL53.Contig6_A-W 1 1326 minus strand PREDICTED: serine/threonine-protein phosphatase 2A 56 kDa regulatory subunit delta isoform [Saimiri boliviensis boliviensis]

GATGTTGTCACTGAGGCCATCTACCCTGAGGCTGTAACCATGTTTTCAGTGAACCTCTTC

CGGACACTGCCACCTTCATCGAATCCCACAGGGGCTGAATTCGATCCAGAGGAAGATGAG

CCTACCTTGGAAGCTGCCTGGCCTCATCTTCAGCTTGTATATGAGTTTTTCTTACGTTTC

CTTGAGTCTCCTGATTTCCAGCCGAATATAGCCAAGAAGTATATTGACCAGAAGTTTGTC

CTTGCTCTCCTGGACCTATTTGACAGTGAGGATCCTCGAGAGCGGGACTTTCTCAAGACC

ATTTTGCATCGTATCTATGGCAAGTTTTTGGGGCTCCGGGCTTATATCCGTAGGCAGATC

AACCACATCTTCTATAGGTTCATCTATGAGACAGAGCATCACAATGGGATTGCTGAACTC

CTGGAGATCTTGGGCAGTATCATCAATGGCTTTGCCCTACCCCTTAAGGAAGAGCATAAG

ATGTTTCTCATCCGTGTCCTGCTTCCGCTTCACAAAGTCAAGTCCTTGAGTGTATACCAC

CCTCAGTTGGCTTACTGTGTGGTACAATTCCTGGAGAAGGAGAGCAGTCTGACTGAGCCG

GTGATTGTGGGACTTCTTAAGTTTTGGCCCAAGACTCACAGCCCCAAGGAGGTGATGTTC

CTGAATGAACTAGAGGAGATTCTGGATGTCATTGAACCCTCAGAATTTAGCAAAGTGATG

GAACCACTCTTCCGTCAGCTTGCCAAGTGTGTCTCCAGCCCCCATTTCCAGGTGGCAGAG

CGTGCTCTCTATTACTGGAATAATGAATACATCATGAGCCTGATAAGTGACAATGCTGCC

AGAGTCCTCCCCATCATGTTCCCTGCACTCTACAGGAACTCCAAGAGCCACTGGAACAAG

ACAATCCATGGACTGATCTACAATGCCCTGAAGCTGTTTATGGAAATGAATCAGAAGCTC

TTTGATGATTGCACACAACAGTACAAGGCAGAGAAACAGAAGGGCCGGTTCCGAATGAAG

GAAAGAGAAGAGATGTGGCAAAAGATTGAGGAGCTGGCCCGGCTTAATCCTCAGTATCCC

ATGTTCCGTGCTCCTCCACCTCTGCCTCCTGTGTACTCCATGGAGACAGAAACCCCTACA

GCAGAGGACATCCAGCTTCTGAAGAGGACGGTGGAGACAGAGGCTGTGCAGATGCTGAAG

GACATCAAGAAGGAAAAAGTGCTGCTTCGGAGGAAGTCAGAGTTGCCCCAGGACGTCTAC

ACCATCAAGGCACTGGAGGCCCATAAGCGGGCAGAAGAGTTCCTAACTGCCAGCCAAGAG

GCTCTC

>CL54.Contig1_A-W 50 622 NAD-dependent deacetylase sirtuin-3, mitochondrial, partial [Heterocephalus glaber]

ATTACAAGTGGACGAAGGGCCATCTCACTGTCTGTGGGTGCTTCAGGCATCTTTGGAAGT

GGAGGCAACAGTGAGAAAGAGAAACTGTCCCTGCAGGACATCGCTGAGCTGATCCGGGTG

CGAGCCTGTCAGAAGGTGGTGGTCATGGTGGGGGCTGGCATCAGCACACCCAGCGGCATT

CCAGATTTCAGATCTCCAGGGAGCGGCCTCTATAGCAACCTCCAGCAGTACAACATCCCA

TACCCCGAAGCCATTTTTGAACTGGAATTTTTTTTCCACAACCCCAAGCCCTTTTTCACT

TTAGCCAAGGAGCTGTACCCTGGAAACTATAGGCCCAATGCCACTCACTACTTTCTCCGG

TTGCTTCATGACAAGGGACTGCTTCTGCGTCTCTACACACAGAACATCGATGGACTTGAG

AGAGTGTCTGGCATCCCCACCGCGAAGCTAGTTGAAGCTCATGGGACCTTTGCTTCAGCC

ACCTGCACAGTCTGCCGAAGATCCTGCCCCGGGGAGGACTTCTGGGCCGACGTGATGGCC

GACAGGGTGCCCTGCTGCCCAGTCTGCACTGGC

>CL54.Contig2_A-W 50 253 NAD-dependent deacetylase sirtuin-3, mitochondrial, partial [Heterocephalus glaber]

ATTACAAGTGGACGAAGGGCCATCTCACTGTCTGTGGGTGCTTCAGGCATCTTTGGAAGT

GGAGGCAACAGTGAGAAAGAGAAACTGTCCCTGCAGGACATCGCTGAGCTGATCCGGGTG

CGAGCCTGTCAGAAGGTGGTGGTCATGGTGGGGGCTGGCATCAGCACACCCAGCGGCATT

CCAGATTTCAGGTACCACGGTAAC

>CL55.Contig1_A-W 43 1626 minus strand RecName: Full=Nuclear receptor-binding protein 2

ATGGCGGCCCCGGAGCCGGCGCCGAGGCGGAGCCGGGAGCGGGAGCGGGAGGACGAGAGT

GAGGACGAGAGCGACATCCTGGAGGAGAGCCCATGCGGACGCTGGCAGAAGCGGCGCGAG

CAGGTGAACCAGGGGAATATGCCCGGGATCCAGAGCACATTCCTGGCCATGGACACGGAG

GAGGGGGTAGAAGTGGTGTGGAACGAGCTCCACTTTGGCGACAGGAAGGCTTTCGCAGCC

CATGAGGAGAAGATCCAGGCCATGTTTGAGCAGCTGGCGCTGGTGGACCACCCCAACATC

GTCAAGCTGCACAAGTACTGGCTGGATGCCTCTGAGGCCCGTGCAAGGGTCATTTTCATC

ACGGAGTACGTGTCATCAGGCAGCCTCAAGCAATTCCTCAAAAAGACCAAGAAAAACCAC

AAGGCCATGAATGCCCGGGCCTGGAAGAGATGGTGCACGCAGATCCTGTCCGCGCTCAGT

TTTCTGCACGCCTGCAGCCCCCCCATCATCCACGGGAACCTGACCAGCGACACCATCTTC

ATTCAGCATAACGGCCTCATCAAGATCGGCTCTGTGTGGTACCGCATCTTCTCCAATGCG

CTCCCGGATGATCTAAGGAGCCCCATCCTTGCTGAGCGAGAGGAACTTCGGAACTTGCAC

TTCTTCCCACCAGAGTATGGCGAAGTTGCAGATGGGACCGCTGTGGACATCTTCTCTTTT

GGGATGTGTGCACTGGAGATGGCTGTGCTGGAGATCCAGGCTAATGGGGATACCAGAGTC

ACAGAGGAGGCCATTGTTCGAGCCAGGCACTCACTGAATGACCCCAACATGCGAGAGTTC

ATCCTCTCCTGCCTGGCCCGGGACCCTGCCCGACGGCCTTCTGCTCACAACCTCCTCTTC

CACCGAGTGCTCTTCGAGGTACACTCGCTGAAGCTGCTGGCAGCTCACTGCTTTATCCAG

CACCAGTACCTCATGCCTGAGAATGTGGTGGAAGAGAAGACCAAGGCCATGGACCTGCAG

GCTGTCTTGGCAGAGGTCCCCAGGCCCCAGCGGCCCCCAATGCAATGGCGGTACTCAGAG

GTCTCCTTCTTGGAGCTGGACAAATTCCTGGAGGATGTCAGGAATGGGATCTACCCACTG

ATGAATTTTGCTGCTGCTCGGCCCCTGGGGCTGCCCCGTGTATTGGCTCCACCCCTGGAG

GAGGCCCAGAAGGTCAAGACCCCGACTCCAGAGCCCTTTGACTCCGAGACCAGGAAGGTG

ATCCATATGCAGTGCAACCTGGAAAGGAGTGAGGACAAGGCACGCTGGCATCTCACATTG

CTTCTGGTGCTGGAGGACAGACTGCACAGGCAGCTGACCTACGACCTGCTTCCAACGGAC

AGCGCCCAGGACCTCGCGGCTGAGCTGGTGCACTACGGCTTCCTGCGAGAGGTTGGCAGG

GTGGGGCGTGGTGTGGCTTGCTGGGAGGGTGCAGGGCTGCTCTGCCGCGGGTCCCCCATG

CCTCCCTCCCCTCTTTAGGATGACCGGTTGAAGCTGGCCATCTTCCTGGAGACCACCTTC

CTCAAGTACCGTGGGGCCCAGGCC

>CL55.Contig2_A-W 1007 1552 minus strand PREDICTED: nuclear receptor-binding protein 2-like [Nomascus leucogenys]

AGGAATGGGATCTACCCACTGATGAATTTTGCTGCTGCTCGGCCCCTGGGGCTGCCCCGT

GTATTGGCTCCACCCCTGGAGGAGGCCCAGAAGGTCAAGACCCCGACTCCAGAGCCCTTT

GACTCCGAGACCAGGAAGGTGATCCATATGCAGTGCAACCTGGAAAGGAGTGAGGACAAG

GCACGCTGGCATGTGAGCAGGGCCCTGGGCTGGGCTGGGAAAGGAAGGGCCTGCCAACTT

TGACTTGGCACCGACCACACCCTCCTGCCCCAGCTCACATTGCTTCTGGTGCTGGAGGAC

AGACTGCACAGGCAGCTGACCTACGACCTGCTTCCAACGGACAGCGCCCAGGACCTCGCG

GCTGAGCTGGTGCACTACGGCTTCCTGCGAGAGGTTGGCAGGGTGGGGCGTGGTGTGGCT

TGCTGGGAGGGTGCAGGGCTGCTCTGCCGCGGGTCCCCCATGCCTCCCTCCCCTCTTTAG

GATGACCGGTTGAAGCTGGCCATCTTCCTGGAGACCACCTTCCTCAAGTACCGTGGGGCC

CAGGCC

>CL55.Contig3_A-W 479 1870 minus strand PREDICTED: nuclear receptor-binding protein 2-like [Nomascus leucogenys]

GGGTAGGGGGAGTAGGCTGGGGACAGGGAAGGGAGGGTGGCCTCTGGGAAAGGGATGGGA

CTGGACACCGGTCAGCCCCGGAAAGGGGCGGGGCGGCGGGTGGGCTTCGCAGGCCCTGCC

ACTTCTCCTGCGCCCACCCGACGGGAGTCCTGCGTCCGCTGCCAGGCCTGGAAGAGATGG

TGCACGCAGATCCTGTCCGCGCTCAGTTTTCTGCACGCCTGCAGCCCCCCCATCATCCAC

GGGAACCTGACCAGCGACACCATCTTCATTCAGCATAACGGCCTCATCAAGATCGGCTCT

GTGTGGTACCGCATCTTCTCCAATGCGCTCCCGGATGATCTAAGGAGCCCCATCCTTGCT

GAGCGAGAGGAACTTCGGAACTTGCACTTCTTCCCACCAGAGTATGGCGAAGTTGCAGAT

GGGACCGCTGTGGACATCTTCTCTTTTGGGATGTGTGCACTGGAGATGGCTGTGCTGGAG

ATCCAGGCTAATGGGGATACCAGAGTCACAGAGGAGGCCATTGTTCGAGCCAGGCACTCA

CTGAATGACCCCAACATGCGAGAGTTCATCCTCTCCTGCCTGGCCCGGGACCCTGCCCGA

CGGCCTTCTGCTCACAACCTCCTCTTCCACCGAGTGCTCTTCGAGGTACACTCGCTGAAG

CTGCTGGCAGCTCACTGCTTTATCCAGCACCAGTACCTCATGCCTGAGAATGTGGTGGAA

GAGAAGACCAAGGCCATGGACCTGCAGGCTGTCTTGGCAGAGGTCCCCAGGCCCCAGCGG

CCCCCAATGCAATGGCGGTACTCAGAGGTCTCCTTCTTGGAGCTGGACAAATTCCTGGAG

GATGTCAGGAATGGGATCTACCCACTGATGAATTTTGCTGCTGCTCGGCCCCTGGGGCTG

CCCCGTGTATTGGCTCCACCCCTGGAGGAGGCCCAGAAGGTCAAGACCCCGACTCCAGAG

CCCTTTGACTCCGAGACCAGGAAGGTGATCCATATGCAGTGCAACCTGGAAAGGAGTGAG

GACAAGGCACGCTGGCATGTGAGCAGGGCCCTGGGCTGGGCTGGGAAAGGAAGGGCCTGC

CAACTTTGACTTGGCACCGACCACACCCTCCTGCCCCAGCTCACATTGCTTCTGGTGCTG

GAGGACAGACTGCACAGGCAGCTGACCTACGACCTGCTTCCAACGGACAGCGCCCAGGAC

CTCGCGGCTGAGCTGGTGCACTACGGCTTCCTGCGAGAGGTTGGCAGGGTGGGGCGTGGT

GTGGCTTGCTGGGAGGGTGCAGGGCTGCTCTGCCGCGGGTCCCCCATGCCTCCCTCCCCT

CTTTAGGATGACCGGTTGAAGCTGGCCATCTTCCTGGAGACCACCTTCCTCAAGTACCGT

GGGGCCCAGGCC

>CL55.Contig4_A-W 479 1789 minus strand PREDICTED: nuclear receptor-binding protein 2-like [Nomascus leucogenys]

GGGTAGGGGGAGTAGGCTGGGGACAGGGAAGGGAGGGTGGCCTCTGGGAAAGGGATGGGA

CTGGACACCGGTCAGCCCCGGAAAGGGGCGGGGCGGCGGGTGGGCTTCGCAGGCCCTGCC

ACTTCTCCTGCGCCCACCCGACGGGAGTCCTGCGTCCGCTGCCAGGCCTGGAAGAGATGG

TGCACGCAGATCCTGTCCGCGCTCAGTTTTCTGCACGCCTGCAGCCCCCCCATCATCCAC

GGGAACCTGACCAGCGACACCATCTTCATTCAGCATAACGGCCTCATCAAGATCGGCTCT

GTGTGGTACCGCATCTTCTCCAATGCGCTCCCGGATGATCTAAGGAGCCCCATCCTTGCT

GAGCGAGAGGAACTTCGGAACTTGCACTTCTTCCCACCAGAGTATGGCGAAGTTGCAGAT

GGGACCGCTGTGGACATCTTCTCTTTTGGGATGTGTGCACTGGAGATGGCTGTGCTGGAG

ATCCAGGCTAATGGGGATACCAGAGTCACAGAGGAGGCCATTGTTCGAGCCAGGCACTCA

CTGAATGACCCCAACATGCGAGAGTTCATCCTCTCCTGCCTGGCCCGGGACCCTGCCCGA

CGGCCTTCTGCTCACAACCTCCTCTTCCACCGAGTGCTCTTCGAGGTACACTCGCTGAAG

CTGCTGGCAGCTCACTGCTTTATCCAGCACCAGTACCTCATGCCTGAGAATGTGGTGGAA

GAGAAGACCAAGGCCATGGACCTGCAGGCTGTCTTGGCAGAGGTCCCCAGGCCCCAGCGG

CCCCCAATGCAATGGCGGTACTCAGAGGTCTCCTTCTTGGAGCTGGACAAATTCCTGGAG

GATGTCAGGAATGGGATCTACCCACTGATGAATTTTGCTGCTGCTCGGCCCCTGGGGCTG

CCCCGTGTATTGGCTCCACCCCTGGAGGAGGCCCAGAAGGTCAAGACCCCGACTCCAGAG

CCCTTTGACTCCGAGACCAGGAAGGTGATCCATATGCAGTGCAACCTGGAAAGGAGTGAG

GACAAGGCACGCTGGCATCTCACATTGCTTCTGGTGCTGGAGGACAGACTGCACAGGCAG

CTGACCTACGACCTGCTTCCAACGGACAGCGCCCAGGACCTCGCGGCTGAGCTGGTGCAC

TACGGCTTCCTGCGAGAGGTTGGCAGGGTGGGGCGTGGTGTGGCTTGCTGGGAGGGTGCA

GGGCTGCTCTGCCGCGGGTCCCCCATGCCTCCCTCCCCTCTTTAGGATGACCGGTTGAAG

CTGGCCATCTTCCTGGAGACCACCTTCCTCAAGTACCGTGGGGCCCAGGCC

>CL55.Contig5_A-W 1110 1574 minus strand PREDICTED: nuclear receptor-binding protein 2-like [Nomascus leucogenys]

AGGAATGGGATCTACCCACTGATGAATTTTGCTGCTGCTCGGCCCCTGGGGCTGCCCCGT

GTATTGGCTCCACCCCTGGAGGAGGCCCAGAAGGTCAAGACCCCGACTCCAGAGCCCTTT

GACTCCGAGACCAGGAAGGTGATCCATATGCAGTGCAACCTGGAAAGGAGTGAGGACAAG

GCACGCTGGCATCTCACATTGCTTCTGGTGCTGGAGGACAGACTGCACAGGCAGCTGACC

TACGACCTGCTTCCAACGGACAGCGCCCAGGACCTCGCGGCTGAGCTGGTGCACTACGGC

TTCCTGCGAGAGGTTGGCAGGGTGGGGCGTGGTGTGGCTTGCTGGGAGGGTGCAGGGCTG

CTCTGCCGCGGGTCCCCCATGCCTCCCTCCCCTCTTTAGGATGACCGGTTGAAGCTGGCC

ATCTTCCTGGAGACCACCTTCCTCAAGTACCGTGGGGCCCAGGCC

>CL55.Contig6_A-W 651 1874 minus strand PREDICTED: nuclear receptor-binding protein 2-like [Nomascus leucogenys]

GGGTAGGGGGAGTAGGCTGGGGACAGGGAAGGGAGGGTGGCCTCTGGGAAAGGGATGGGA

CTGGACACCGGTCAGCCCCGGAAAGGGGCGGGGCGGCGGGTGGGCTTCGCAGGCCCTGCC

ACTTCTCCTGCGCCCACCCGACGGGAGTCCTGCGTCCGCTGCCAGGCCTGGAAGAGATGG

TGCACGCAGATCCTGTCCGCGCTCAGTTTTCTGCACGCCTGCAGCCCCCCCATCATCCAC

GGGAACCTGACCAGCGACACCATCTTCATTCAGCATAACGGCCTCATCAAGATCGGCTCT

GTGTGGTACCGCATCTTCTCCAATGCGCTCCCGGATGATCTAAGGAGCCCCATCCTTGCT

GAGCGAGAGGAACTTCGGAACTTGCACTTCTTCCCACCAGAGTATGGCGAAGTTGCAGAT

GGGACCGCTGTGGACATCTTCTCTTTTGGGATGTGTGCACTGGAGATGGCTGTGCTGGAG

ATCCAGGCTAATGGGGATACCAGAGTCACAGAGGAGGCCATTGTTCGAGCCAGGCACTCA

CTGAATGACCCCAACATGCGAGAGTTCATCCTCTCCTGCCTGGCCCGGGACCCTGCCCGA

CGGCCTTCTGCTCACAACCTCCTCTTCCACCGAGTGCTCTTCGAGGTACACTCGCTGAAG

CTGCTGGCAGCTCACTGCTTTATCCAGCACCAGTACCTCATGCCTGAGAATGTGGTGGAA

GAGAAGACCAAGGCCATGGACCTGCAGGCTGTCTTGGCAGAGGTCCCCAGGCCCCAGCGG

CCCCCAATGCAATGGCGGTACTCAGAGGTCTCCTTCTTGGAGCTGGACAAATTCCTGGAG

GATGTCAGGAATGGGATCTACCCACTGATGAATTTTGCTGCTGCTCGGCCCCTGGGGCTG

CCCCGTGTATTGGCTCCACCCCTGGAGGAGGCCCAGAAGGTCAAGACCCCGACTCCAGAG

CCCTTTGACTCCGAGACCAGGAAGGTGATCCATATGCAGTGCAACCTGGAAAGGAGTGAG

GACAAGGCACGCTGGCATCTCACATTGCTTCTGGTGCTGGAGGACAGACTGCACAGGCAG

CTGACCTACGACCTGCTTCCAACGGACAGCGCCCAGGACCTCGCGGCTGAGCTGGTGCAC

TACGGCTTCCTGCGAGAGGATGACCGGTTGAAGCTGGCCATCTTCCTGGAGACCACCTTC

CTCAAGTACCGTGGGGCCCAGGCC

>CL55.Contig7_A-W 61 192 RecName: Full=Nuclear receptor-binding protein 2

CTTGTTGCAGCGGACAGCGCCCAGGACCTCGCGGCTGAGCTGGTGCACTACGGCTTCCTG

CGAGAGGATGACCGGTTGAAGCTGGCCATCTTCCTGGAGACCACCTTCCTCAAGTACCGT

GGGGCCCAGGCC

>CL55.Contig8_A-W 295 1686 minus strand PREDICTED: nuclear receptor-binding protein 2-like [Nomascus leucogenys]

GGGTAGGGGGAGTAGGCTGGGGACAGGGAAGGGAGGGTGGCCTCTGGGAAAGGGATGGGA

CTGGACACCGGTCAGCCCCGGAAAGGGGCGGGGCGGCGGGTGGGCTTCGCAGGCCCTGCC

ACTTCTCCTGCGCCCACCCGACGGGAGTCCTGCGTCCGCTGCCAGGCCTGGAAGAGATGG

TGCACGCAGATCCTGTCCGCGCTCAGTTTTCTGCACGCCTGCAGCCCCCCCATCATCCAC

GGGAACCTGACCAGCGACACCATCTTCATTCAGCATAACGGCCTCATCAAGATCGGCTCT

GTGTGGTACCGCATCTTCTCCAATGCGCTCCCGGATGATCTAAGGAGCCCCATCCTTGCT

GAGCGAGAGGAACTTCGGAACTTGCACTTCTTCCCACCAGAGTATGGCGAAGTTGCAGAT

GGGACCGCTGTGGACATCTTCTCTTTTGGGATGTGTGCACTGGAGATGGCTGTGCTGGAG

ATCCAGGCTAATGGGGATACCAGAGTCACAGAGGAGGCCATTGTTCGAGCCAGGCACTCA

CTGAATGACCCCAACATGCGAGAGTTCATCCTCTCCTGCCTGGCCCGGGACCCTGCCCGA

CGGCCTTCTGCTCACAACCTCCTCTTCCACCGAGTGCTCTTCGAGGTACACTCGCTGAAG

CTGCTGGCAGCTCACTGCTTTATCCAGCACCAGTACCTCATGCCTGAGAATGTGGTGGAA

GAGAAGACCAAGGCCATGGACCTGCAGGCTGTCTTGGCAGAGGTCCCCAGGCCCCAGCGG

CCCCCAATGCAATGGCGGTACTCAGAGGTCTCCTTCTTGGAGCTGGACAAATTCCTGGAG

GATGTCAGGAATGGGATCTACCCACTGATGAATTTTGCTGCTGCTCGGCCCCTGGGGCTG

CCCCGTGTATTGGCTCCACCCCTGGAGGAGGCCCAGAAGGTCAAGACCCCGACTCCAGAG

CCCTTTGACTCCGAGACCAGGAAGGTGATCCATATGCAGTGCAACCTGGAAAGGAGTGAG

GACAAGGCACGCTGGCATGTGAGCAGGGCCCTGGGCTGGGCTGGGAAAGGAAGGGCCTGC

CAACTTTGACTTGGCACCGACCACACCCTCCTGCCCCAGCTCACATTGCTTCTGGTGCTG

GAGGACAGACTGCACAGGCAGCTGACCTACGACCTGCTTCCAACGGACAGCGCCCAGGAC

CTCGCGGCTGAGCTGGTGCACTACGGCTTCCTGCGAGAGGTTGGCAGGGTGGGGCGTGGT

GTGGCTTGCTGGGAGGGTGCAGGGCTGCTCTGCCGCGGGTCCCCCATGCCTCCCTCCCCT

CTTTAGGATGACCGGTTGAAGCTGGCCATCTTCCTGGAGACCACCTTCCTCAAGTACCGT

GGGGCCCAGGCC

>CL55.Contig9_A-W 1215 1673 minus strand PREDICTED: nuclear receptor-binding protein 2-like [Nomascus leucogenys]

AGGAATGGGATCTACCCACTGATGAATTTTGCTGCTGCTCGGCCCCTGGGGCTGCCCCGT

GTATTGGCTCCACCCCTGGAGGAGGCCCAGAAGGTCAAGACCCCGACTCCAGAGCCCTTT

GACTCCGAGACCAGGAAGGTGATCCATATGCAGTGCAACCTGGAAAGGAGTGAGGACAAG

GCACGCTGGCATGTGAGCAGGGCCCTGGGCTGGGCTGGGAAAGGAAGGGCCTGCCAACTT

TGACTTGGCACCGACCACACCCTCCTGCCCCAGCTCACATTGCTTCTGGTGCTGGAGGAC

AGACTGCACAGGCAGCTGACCTACGACCTGCTTCCAACGGACAGCGCCCAGGACCTCGCG

GCTGAGCTGGTGCACTACGGCTTCCTGCGAGAGGATGACCGGTTGAAGCTGGCCATCTTC

CTGGAGACCACCTTCCTCAAGTACCGTGGGGCCCAGGCC

>CL55.Contig10_A-W 546 1937 minus strand PREDICTED: nuclear receptor-binding protein 2-like [Nomascus leucogenys]

GGGTAGGGGGAGTAGGCTGGGGACAGGGAAGGGAGGGTGGCCTCTGGGAAAGGGATGGGA

CTGGACACCGGTCAGCCCCGGAAAGGGGCGGGGCGGCGGGTGGGCTTCGCAGGCCCTGCC

ACTTCTCCTGCGCCCACCCGACGGGAGTCCTGCGTCCGCTGCCAGGCCTGGAAGAGATGG

TGCACGCAGATCCTGTCCGCGCTCAGTTTTCTGCACGCCTGCAGCCCCCCCATCATCCAC

GGGAACCTGACCAGCGACACCATCTTCATTCAGCATAACGGCCTCATCAAGATCGGCTCT

GTGTGGTACCGCATCTTCTCCAATGCGCTCCCGGATGATCTAAGGAGCCCCATCCTTGCT

GAGCGAGAGGAACTTCGGAACTTGCACTTCTTCCCACCAGAGTATGGCGAAGTTGCAGAT

GGGACCGCTGTGGACATCTTCTCTTTTGGGATGTGTGCACTGGAGATGGCTGTGCTGGAG

ATCCAGGCTAATGGGGATACCAGAGTCACAGAGGAGGCCATTGTTCGAGCCAGGCACTCA

CTGAATGACCCCAACATGCGAGAGTTCATCCTCTCCTGCCTGGCCCGGGACCCTGCCCGA

CGGCCTTCTGCTCACAACCTCCTCTTCCACCGAGTGCTCTTCGAGGTACACTCGCTGAAG

CTGCTGGCAGCTCACTGCTTTATCCAGCACCAGTACCTCATGCCTGAGAATGTGGTGGAA

GAGAAGACCAAGGCCATGGACCTGCAGGCTGTCTTGGCAGAGGTCCCCAGGCCCCAGCGG

CCCCCAATGCAATGGCGGTACTCAGAGGTCTCCTTCTTGGAGCTGGACAAATTCCTGGAG

GATGTCAGGAATGGGATCTACCCACTGATGAATTTTGCTGCTGCTCGGCCCCTGGGGCTG

CCCCGTGTATTGGCTCCACCCCTGGAGGAGGCCCAGAAGGTCAAGACCCCGACTCCAGAG

CCCTTTGACTCCGAGACCAGGAAGGTGATCCATATGCAGTGCAACCTGGAAAGGAGTGAG

GACAAGGCACGCTGGCATGTGAGCAGGGCCCTGGGCTGGGCTGGGAAAGGAAGGGCCTGC

CAACTTTGACTTGGCACCGACCACACCCTCCTGCCCCAGCTCACATTGCTTCTGGTGCTG

GAGGACAGACTGCACAGGCAGCTGACCTACGACCTGCTTCCAACGGACAGCGCCCAGGAC

CTCGCGGCTGAGCTGGTGCACTACGGCTTCCTGCGAGAGGTTGGCAGGGTGGGGCGTGGT

GTGGCTTGCTGGGAGGGTGCAGGGCTGCTCTGCCGCGGGTCCCCCATGCCTCCCTCCCCT

CTTTAGGATGACCGGTTGAAGCTGGCCATCTTCCTGGAGACCACCTTCCTCAAGTACCGT

GGGGCCCAGGCC

>CL55.Contig11_A-W 43 1644 minus strand RecName: Full=Nuclear receptor-binding protein 2

ATGGCGGCCCCGGAGCCGGCGCCGAGGCGGAGCCGGGAGCGGGAGCGGGAGGACGAGAGT

GAGGACGAGAGCGACATCCTGGAGGAGAGCCCATGCGGACGCTGGCAGAAGCGGCGCGAG

CAGGTGAACCAGGGGAATATGCCCGGGATCCAGAGCACATTCCTGGCCATGGACACGGAG

GAGGGGGTAGAAGTGGTGTGGAACGAGCTCCACTTTGGCGACAGGAAGGCTTTCGCAGCC

CATGAGGAGAAGATCCAGGCCATGTTTGAGCAGCTGGCGCTGGTGGACCACCCCAACATC

GTCAAGCTGCACAAGTACTGGCTGGATGCCTCTGAGGCCCGTGCAAGGGTGAGCTACTGG

GCGGAGTGGGGGCGGGGCGTGGCCTCGGTCTGGGTGGAGCAACTGCGGGGACCCACCCGC

AGTGGCGGAGCCGTGAGCCCGCCCGCCCCACAGGTCATTTTCATCACGGAGTACGTGTCA

TCAGGCAGCCTCAAGCAATTCCTCAAAAAGACCAAGAAAAACCACAAGGCCATGAATGCC

CGGGCCTGGAAGAGATGGTGCACGCAGATCCTGTCCGCGCTCAGTTTTCTGCACGCCTGC

AGCCCCCCCATCATCCACGGGAACCTGACCAGCGACACCATCTTCATTCAGCATAACGGC

CTCATCAAGATCGGCTCTGTGTGGTACCGCATCTTCTCCAATGCGCTCCCGGATGATCTA

AGGAGCCCCATCCTTGCTGAGCGAGAGGAACTTCGGAACTTGCACTTCTTCCCACCAGAG

TATGGCGAAGTTGCAGATGGGACCGCTGTGGACATCTTCTCTTTTGGGATGTGTGCACTG

GAGATGGCTGTGCTGGAGATCCAGGCTAATGGGGATACCAGAGTCACAGAGGAGGCCATT

GTTCGAGCCAGGCACTCACTGAATGACCCCAACATGCGAGAGTTCATCCTCTCCTGCCTG

GCCCGGGACCCTGCCCGACGGCCTTCTGCTCACAACCTCCTCTTCCACCGAGTGCTCTTC

GAGGTACACTCGCTGAAGCTGCTGGCAGCTCACTGCTTTATCCAGCACCAGTACCTCATG

CCTGAGAATGTGGTGGAAGAGAAGACCAAGGCCATGGACCTGCAGGCTGTCTTGGCAGAG

GTCCCCAGGCCCCAGCGGCCCCCAATGCAATGGCGGTACTCAGAGGTCTCCTTCTTGGAG

CTGGACAAATTCCTGGAGGATGTCAGGAATGGGATCTACCCACTGATGAATTTTGCTGCT

GCTCGGCCCCTGGGGCTGCCCCGTGTATTGGCTCCACCCCTGGAGGAGGCCCAGAAGGTC

AAGACCCCGACTCCAGAGCCCTTTGACTCCGAGACCAGGAAGGTGATCCATATGCAGTGC

AACCTGGAAAGGAGTGAGGACAAGGCACGCTGGCATCTCACATTGCTTCTGGTGCTGGAG

GACAGACTGCACAGGCAGCTGACCTACGACCTGCTTCCAACGGACAGCGCCCAGGACCTC

GCGGCTGAGCTGGTGCACTACGGCTTCCTGCGAGAGGATGACCGGTTGAAGCTGGCCATC

TTCCTGGAGACCACCTTCCTCAAGTACCGTGGGGCCCAGGCC

>CL55.Contig12_A-W 43 288 minus strand RecName: Full=Nuclear receptor-binding protein 2

ATGGCGGCCCCGGAGCCGGCGCCGAGGCGGAGCCGGGAGCGGGAGCGGGAGGACGAGAGT

GAGGACGAGAGCGACATCCTGGAGGAGAGCCCATGCGGACGCTGGCAGAAGCGGCGCGAG

CAGGTGAACCAGGGGAATATGCCCGGGATCCAGAGCACATTCCTGGCCATGGACACGGAG

GAGGGGGTAGAAGTGGTGTGGAACGAGCTCCACTTTGGCGACAGGAAGGCTTTCGCAGCC

CATGAG

>CL55.Contig13_A-W 295 1605 minus strand PREDICTED: nuclear receptor-binding protein 2-like [Nomascus leucogenys]

GGGTAGGGGGAGTAGGCTGGGGACAGGGAAGGGAGGGTGGCCTCTGGGAAAGGGATGGGA

CTGGACACCGGTCAGCCCCGGAAAGGGGCGGGGCGGCGGGTGGGCTTCGCAGGCCCTGCC

ACTTCTCCTGCGCCCACCCGACGGGAGTCCTGCGTCCGCTGCCAGGCCTGGAAGAGATGG

TGCACGCAGATCCTGTCCGCGCTCAGTTTTCTGCACGCCTGCAGCCCCCCCATCATCCAC

GGGAACCTGACCAGCGACACCATCTTCATTCAGCATAACGGCCTCATCAAGATCGGCTCT

GTGTGGTACCGCATCTTCTCCAATGCGCTCCCGGATGATCTAAGGAGCCCCATCCTTGCT

GAGCGAGAGGAACTTCGGAACTTGCACTTCTTCCCACCAGAGTATGGCGAAGTTGCAGAT

GGGACCGCTGTGGACATCTTCTCTTTTGGGATGTGTGCACTGGAGATGGCTGTGCTGGAG

ATCCAGGCTAATGGGGATACCAGAGTCACAGAGGAGGCCATTGTTCGAGCCAGGCACTCA

CTGAATGACCCCAACATGCGAGAGTTCATCCTCTCCTGCCTGGCCCGGGACCCTGCCCGA

CGGCCTTCTGCTCACAACCTCCTCTTCCACCGAGTGCTCTTCGAGGTACACTCGCTGAAG

CTGCTGGCAGCTCACTGCTTTATCCAGCACCAGTACCTCATGCCTGAGAATGTGGTGGAA

GAGAAGACCAAGGCCATGGACCTGCAGGCTGTCTTGGCAGAGGTCCCCAGGCCCCAGCGG

CCCCCAATGCAATGGCGGTACTCAGAGGTCTCCTTCTTGGAGCTGGACAAATTCCTGGAG

GATGTCAGGAATGGGATCTACCCACTGATGAATTTTGCTGCTGCTCGGCCCCTGGGGCTG

CCCCGTGTATTGGCTCCACCCCTGGAGGAGGCCCAGAAGGTCAAGACCCCGACTCCAGAG

CCCTTTGACTCCGAGACCAGGAAGGTGATCCATATGCAGTGCAACCTGGAAAGGAGTGAG

GACAAGGCACGCTGGCATCTCACATTGCTTCTGGTGCTGGAGGACAGACTGCACAGGCAG

CTGACCTACGACCTGCTTCCAACGGACAGCGCCCAGGACCTCGCGGCTGAGCTGGTGCAC

TACGGCTTCCTGCGAGAGGTTGGCAGGGTGGGGCGTGGTGTGGCTTGCTGGGAGGGTGCA

GGGCTGCTCTGCCGCGGGTCCCCCATGCCTCCCTCCCCTCTTTAGGATGACCGGTTGAAG

CTGGCCATCTTCCTGGAGACCACCTTCCTCAAGTACCGTGGGGCCCAGGCC

>CL55.Contig14_A-W 1215 1592 minus strand PREDICTED: nuclear receptor-binding protein 2-like [Nomascus leucogenys]

AGGAATGGGATCTACCCACTGATGAATTTTGCTGCTGCTCGGCCCCTGGGGCTGCCCCGT

GTATTGGCTCCACCCCTGGAGGAGGCCCAGAAGGTCAAGACCCCGACTCCAGAGCCCTTT

GACTCCGAGACCAGGAAGGTGATCCATATGCAGTGCAACCTGGAAAGGAGTGAGGACAAG

GCACGCTGGCATCTCACATTGCTTCTGGTGCTGGAGGACAGACTGCACAGGCAGCTGACC

TACGACCTGCTTCCAACGGACAGCGCCCAGGACCTCGCGGCTGAGCTGGTGCACTACGGC

TTCCTGCGAGAGGATGACCGGTTGAAGCTGGCCATCTTCCTGGAGACCACCTTCCTCAAG

TACCGTGGGGCCCAGGCC

>CL55.Contig15_A-W 651 1955 minus strand PREDICTED: nuclear receptor-binding protein 2-like [Nomascus leucogenys]

GGGTAGGGGGAGTAGGCTGGGGACAGGGAAGGGAGGGTGGCCTCTGGGAAAGGGATGGGA

CTGGACACCGGTCAGCCCCGGAAAGGGGCGGGGCGGCGGGTGGGCTTCGCAGGCCCTGCC

ACTTCTCCTGCGCCCACCCGACGGGAGTCCTGCGTCCGCTGCCAGGCCTGGAAGAGATGG

TGCACGCAGATCCTGTCCGCGCTCAGTTTTCTGCACGCCTGCAGCCCCCCCATCATCCAC

GGGAACCTGACCAGCGACACCATCTTCATTCAGCATAACGGCCTCATCAAGATCGGCTCT

GTGTGGTACCGCATCTTCTCCAATGCGCTCCCGGATGATCTAAGGAGCCCCATCCTTGCT

GAGCGAGAGGAACTTCGGAACTTGCACTTCTTCCCACCAGAGTATGGCGAAGTTGCAGAT

GGGACCGCTGTGGACATCTTCTCTTTTGGGATGTGTGCACTGGAGATGGCTGTGCTGGAG

ATCCAGGCTAATGGGGATACCAGAGTCACAGAGGAGGCCATTGTTCGAGCCAGGCACTCA

CTGAATGACCCCAACATGCGAGAGTTCATCCTCTCCTGCCTGGCCCGGGACCCTGCCCGA

CGGCCTTCTGCTCACAACCTCCTCTTCCACCGAGTGCTCTTCGAGGTACACTCGCTGAAG

CTGCTGGCAGCTCACTGCTTTATCCAGCACCAGTACCTCATGCCTGAGAATGTGGTGGAA

GAGAAGACCAAGGCCATGGACCTGCAGGCTGTCTTGGCAGAGGTCCCCAGGCCCCAGCGG

CCCCCAATGCAATGGCGGTACTCAGAGGTCTCCTTCTTGGAGCTGGACAAATTCCTGGAG

GATGTCAGGAATGGGATCTACCCACTGATGAATTTTGCTGCTGCTCGGCCCCTGGGGCTG

CCCCGTGTATTGGCTCCACCCCTGGAGGAGGCCCAGAAGGTCAAGACCCCGACTCCAGAG

CCCTTTGACTCCGAGACCAGGAAGGTGATCCATATGCAGTGCAACCTGGAAAGGAGTGAG

GACAAGGCACGCTGGCATGTGAGCAGGGCCCTGGGCTGGGCTGGGAAAGGAAGGGCCTGC

CAACTTTGACTTGGCACCGACCACACCCTCCTGCCCCAGCTCACATTGCTTCTGGTGCTG

GAGGACAGACTGCACAGGCAGCTGACCTACGACCTGCTTCCAACGGACAGCGCCCAGGAC

CTCGCGGCTGAGCTGGTGCACTACGGCTTCCTGCGAGAGGATGACCGGTTGAAGCTGGCC

ATCTTCCTGGAGACCACCTTCCTCAAGTACCGTGGGGCCCAGGCC

>CL55.Contig16_A-W 3 1523 minus strand RecName: Full=Nuclear receptor-binding protein 2

AAGATCCAGGCCATGTTTGAGCAGCTGGCGCTGGTGGACCACCCCAACATCGTCAAGCTG

CACAAGTACTGGCTGGATGCCTCTGAGGCCCGTGCAAGGGTGAGCTACTGGGCGGAGTGG

GGGCGGGGCGTGGCCTCGGTCTGGGTGGAGCAACTGCGGGGACCCACCCGCAGTGGCGGA

GCCGTGAGCCCGCCCGCCCCACAGGTCATTTTCATCACGGAGTACGTGTCATCAGGCAGC

CTCAAGCAATTCCTCAAAAAGACCAAGAAAAACCACAAGGCCATGAATGCCCGGGCCTGG

AAGAGATGGTGCACGCAGATCCTGTCCGCGCTCAGTTTTCTGCACGCCTGCAGCCCCCCC

ATCATCCACGGGAACCTGACCAGCGACACCATCTTCATTCAGCATAACGGCCTCATCAAG

ATCGGCTCTGTGTGGTACCGCATCTTCTCCAATGCGCTCCCGGATGATCTAAGGAGCCCC

ATCCTTGCTGAGCGAGAGGAACTTCGGAACTTGCACTTCTTCCCACCAGAGTATGGCGAA

GTTGCAGATGGGACCGCTGTGGACATCTTCTCTTTTGGGATGTGTGCACTGGAGATGGCT

GTGCTGGAGATCCAGGCTAATGGGGATACCAGAGTCACAGAGGAGGCCATTGTTCGAGCC

AGGCACTCACTGAATGACCCCAACATGCGAGAGTTCATCCTCTCCTGCCTGGCCCGGGAC

CCTGCCCGACGGCCTTCTGCTCACAACCTCCTCTTCCACCGAGTGCTCTTCGAGGTACAC

TCGCTGAAGCTGCTGGCAGCTCACTGCTTTATCCAGCACCAGTACCTCATGCCTGAGAAT

GTGGTGGAAGAGAAGACCAAGGCCATGGACCTGCAGGCTGTCTTGGCAGAGGTCCCCAGG

CCCCAGCGGCCCCCAATGCAATGGCGGTACTCAGAGGTCTCCTTCTTGGAGCTGGACAAA

TTCCTGGAGGATGTCAGGAATGGGATCTACCCACTGATGAATTTTGCTGCTGCTCGGCCC

CTGGGGCTGCCCCGTGTATTGGCTCCACCCCTGGAGGAGGCCCAGAAGGTCAAGACCCCG

ACTCCAGAGCCCTTTGACTCCGAGACCAGGAAGGTGATCCATATGCAGTGCAACCTGGAA

AGGAGTGAGGACAAGGCACGCTGGCATGTGAGCAGGGCCCTGGGCTGGGCTGGGAAAGGA

AGGGCCTGCCAACTTTGACTTGGCACCGACCACACCCTCCTGCCCCAGCTCACATTGCTT

CTGGTGCTGGAGGACAGACTGCACAGGCAGCTGACCTACGACCTGCTTCCAACGGACAGC

GCCCAGGACCTCGCGGCTGAGCTGGTGCACTACGGCTTCCTGCGAGAGGTTGGCAGGGTG

GGGCGTGGTGTGGCTTGCTGGGAGGGTGCAGGGCTGCTCTGCCGCGGGTCCCCCATGCCT

CCCTCCCCTCTTTAGGATGACCGGTTGAAGCTGGCCATCTTCCTGGAGACCACCTTCCTC

AAGTACCGTGGGGCCCAGGCC

>CL55.Contig17_A-W 584 1888 minus strand PREDICTED: nuclear receptor-binding protein 2-like [Nomascus leucogenys]

GGGTAGGGGGAGTAGGCTGGGGACAGGGAAGGGAGGGTGGCCTCTGGGAAAGGGATGGGA

CTGGACACCGGTCAGCCCCGGAAAGGGGCGGGGCGGCGGGTGGGCTTCGCAGGCCCTGCC

ACTTCTCCTGCGCCCACCCGACGGGAGTCCTGCGTCCGCTGCCAGGCCTGGAAGAGATGG

TGCACGCAGATCCTGTCCGCGCTCAGTTTTCTGCACGCCTGCAGCCCCCCCATCATCCAC

GGGAACCTGACCAGCGACACCATCTTCATTCAGCATAACGGCCTCATCAAGATCGGCTCT

GTGTGGTACCGCATCTTCTCCAATGCGCTCCCGGATGATCTAAGGAGCCCCATCCTTGCT

GAGCGAGAGGAACTTCGGAACTTGCACTTCTTCCCACCAGAGTATGGCGAAGTTGCAGAT

GGGACCGCTGTGGACATCTTCTCTTTTGGGATGTGTGCACTGGAGATGGCTGTGCTGGAG

ATCCAGGCTAATGGGGATACCAGAGTCACAGAGGAGGCCATTGTTCGAGCCAGGCACTCA

CTGAATGACCCCAACATGCGAGAGTTCATCCTCTCCTGCCTGGCCCGGGACCCTGCCCGA

CGGCCTTCTGCTCACAACCTCCTCTTCCACCGAGTGCTCTTCGAGGTACACTCGCTGAAG

CTGCTGGCAGCTCACTGCTTTATCCAGCACCAGTACCTCATGCCTGAGAATGTGGTGGAA

GAGAAGACCAAGGCCATGGACCTGCAGGCTGTCTTGGCAGAGGTCCCCAGGCCCCAGCGG

CCCCCAATGCAATGGCGGTACTCAGAGGTCTCCTTCTTGGAGCTGGACAAATTCCTGGAG

GATGTCAGGAATGGGATCTACCCACTGATGAATTTTGCTGCTGCTCGGCCCCTGGGGCTG

CCCCGTGTATTGGCTCCACCCCTGGAGGAGGCCCAGAAGGTCAAGACCCCGACTCCAGAG

CCCTTTGACTCCGAGACCAGGAAGGTGATCCATATGCAGTGCAACCTGGAAAGGAGTGAG

GACAAGGCACGCTGGCATGTGAGCAGGGCCCTGGGCTGGGCTGGGAAAGGAAGGGCCTGC

CAACTTTGACTTGGCACCGACCACACCCTCCTGCCCCAGCTCACATTGCTTCTGGTGCTG

GAGGACAGACTGCACAGGCAGCTGACCTACGACCTGCTTCCAACGGACAGCGCCCAGGAC

CTCGCGGCTGAGCTGGTGCACTACGGCTTCCTGCGAGAGGATGACCGGTTGAAGCTGGCC

ATCTTCCTGGAGACCACCTTCCTCAAGTACCGTGGGGCCCAGGCC

>CL55.Contig18_A-W 3 1442 minus strand RecName: Full=Nuclear receptor-binding protein 2

AAGATCCAGGCCATGTTTGAGCAGCTGGCGCTGGTGGACCACCCCAACATCGTCAAGCTG

CACAAGTACTGGCTGGATGCCTCTGAGGCCCGTGCAAGGGTGAGCTACTGGGCGGAGTGG

GGGCGGGGCGTGGCCTCGGTCTGGGTGGAGCAACTGCGGGGACCCACCCGCAGTGGCGGA

GCCGTGAGCCCGCCCGCCCCACAGGTCATTTTCATCACGGAGTACGTGTCATCAGGCAGC

CTCAAGCAATTCCTCAAAAAGACCAAGAAAAACCACAAGGCCATGAATGCCCGGGCCTGG

AAGAGATGGTGCACGCAGATCCTGTCCGCGCTCAGTTTTCTGCACGCCTGCAGCCCCCCC

ATCATCCACGGGAACCTGACCAGCGACACCATCTTCATTCAGCATAACGGCCTCATCAAG

ATCGGCTCTGTGTGGTACCGCATCTTCTCCAATGCGCTCCCGGATGATCTAAGGAGCCCC

ATCCTTGCTGAGCGAGAGGAACTTCGGAACTTGCACTTCTTCCCACCAGAGTATGGCGAA

GTTGCAGATGGGACCGCTGTGGACATCTTCTCTTTTGGGATGTGTGCACTGGAGATGGCT

GTGCTGGAGATCCAGGCTAATGGGGATACCAGAGTCACAGAGGAGGCCATTGTTCGAGCC

AGGCACTCACTGAATGACCCCAACATGCGAGAGTTCATCCTCTCCTGCCTGGCCCGGGAC

CCTGCCCGACGGCCTTCTGCTCACAACCTCCTCTTCCACCGAGTGCTCTTCGAGGTACAC

TCGCTGAAGCTGCTGGCAGCTCACTGCTTTATCCAGCACCAGTACCTCATGCCTGAGAAT

GTGGTGGAAGAGAAGACCAAGGCCATGGACCTGCAGGCTGTCTTGGCAGAGGTCCCCAGG

CCCCAGCGGCCCCCAATGCAATGGCGGTACTCAGAGGTCTCCTTCTTGGAGCTGGACAAA

TTCCTGGAGGATGTCAGGAATGGGATCTACCCACTGATGAATTTTGCTGCTGCTCGGCCC

CTGGGGCTGCCCCGTGTATTGGCTCCACCCCTGGAGGAGGCCCAGAAGGTCAAGACCCCG

ACTCCAGAGCCCTTTGACTCCGAGACCAGGAAGGTGATCCATATGCAGTGCAACCTGGAA

AGGAGTGAGGACAAGGCACGCTGGCATCTCACATTGCTTCTGGTGCTGGAGGACAGACTG

CACAGGCAGCTGACCTACGACCTGCTTCCAACGGACAGCGCCCAGGACCTCGCGGCTGAG

CTGGTGCACTACGGCTTCCTGCGAGAGGTTGGCAGGGTGGGGCGTGGTGTGGCTTGCTGG

GAGGGTGCAGGGCTGCTCTGCCGCGGGTCCCCCATGCCTCCCTCCCCTCTTTAGGATGAC

CGGTTGAAGCTGGCCATCTTCCTGGAGACCACCTTCCTCAAGTACCGTGGGGCCCAGGCC

>CL55.Contig19_A-W 3 1436 minus strand RecName: Full=Nuclear receptor-binding protein 2

AAGATCCAGGCCATGTTTGAGCAGCTGGCGCTGGTGGACCACCCCAACATCGTCAAGCTG

CACAAGTACTGGCTGGATGCCTCTGAGGCCCGTGCAAGGGTGAGCTACTGGGCGGAGTGG

GGGCGGGGCGTGGCCTCGGTCTGGGTGGAGCAACTGCGGGGACCCACCCGCAGTGGCGGA

GCCGTGAGCCCGCCCGCCCCACAGGTCATTTTCATCACGGAGTACGTGTCATCAGGCAGC

CTCAAGCAATTCCTCAAAAAGACCAAGAAAAACCACAAGGCCATGAATGCCCGGGCCTGG

AAGAGATGGTGCACGCAGATCCTGTCCGCGCTCAGTTTTCTGCACGCCTGCAGCCCCCCC

ATCATCCACGGGAACCTGACCAGCGACACCATCTTCATTCAGCATAACGGCCTCATCAAG

ATCGGCTCTGTGTGGTACCGCATCTTCTCCAATGCGCTCCCGGATGATCTAAGGAGCCCC

ATCCTTGCTGAGCGAGAGGAACTTCGGAACTTGCACTTCTTCCCACCAGAGTATGGCGAA

GTTGCAGATGGGACCGCTGTGGACATCTTCTCTTTTGGGATGTGTGCACTGGAGATGGCT

GTGCTGGAGATCCAGGCTAATGGGGATACCAGAGTCACAGAGGAGGCCATTGTTCGAGCC

AGGCACTCACTGAATGACCCCAACATGCGAGAGTTCATCCTCTCCTGCCTGGCCCGGGAC

CCTGCCCGACGGCCTTCTGCTCACAACCTCCTCTTCCACCGAGTGCTCTTCGAGGTACAC

TCGCTGAAGCTGCTGGCAGCTCACTGCTTTATCCAGCACCAGTACCTCATGCCTGAGAAT

GTGGTGGAAGAGAAGACCAAGGCCATGGACCTGCAGGCTGTCTTGGCAGAGGTCCCCAGG

CCCCAGCGGCCCCCAATGCAATGGCGGTACTCAGAGGTCTCCTTCTTGGAGCTGGACAAA

TTCCTGGAGGATGTCAGGAATGGGATCTACCCACTGATGAATTTTGCTGCTGCTCGGCCC

CTGGGGCTGCCCCGTGTATTGGCTCCACCCCTGGAGGAGGCCCAGAAGGTCAAGACCCCG

ACTCCAGAGCCCTTTGACTCCGAGACCAGGAAGGTGATCCATATGCAGTGCAACCTGGAA

AGGAGTGAGGACAAGGCACGCTGGCATGTGAGCAGGGCCCTGGGCTGGGCTGGGAAAGGA

AGGGCCTGCCAACTTTGACTTGGCACCGACCACACCCTCCTGCCCCAGCTCACATTGCTT

CTGGTGCTGGAGGACAGACTGCACAGGCAGCTGACCTACGACCTGCTTCCAACGGACAGC

GCCCAGGACCTCGCGGCTGAGCTGGTGCACTACGGCTTCCTGCGAGAGGATGACCGGTTG

AAGCTGGCCATCTTCCTGGAGACCACCTTCCTCAAGTACCGTGGGGCCCAGGCC

>CL55.Contig20_A-W 1052 1429 minus strand RecName: Full=Nuclear receptor-binding protein 2

AGGAATGGGATCTACCCACTGATGAATTTTGCTGCTGCTCGGCCCCTGGGGCTGCCCCGT

GTATTGGCTCCACCCCTGGAGGAGGCCCAGAAGGTCAAGACCCCGACTCCAGAGCCCTTT

GACTCCGAGACCAGGAAGGTGATCCATATGCAGTGCAACCTGGAAAGGAGTGAGGACAAG

GCACGCTGGCATCTCACATTGCTTCTGGTGCTGGAGGACAGACTGCACAGGCAGCTGACC

TACGACCTGCTTCCAACGGACAGCGCCCAGGACCTCGCGGCTGAGCTGGTGCACTACGGC

TTCCTGCGAGAGGATGACCGGTTGAAGCTGGCCATCTTCCTGGAGACCACCTTCCTCAAG

TACCGTGGGGCCCAGGCC

>CL55.Contig21_A-W 546 1856 minus strand PREDICTED: nuclear receptor-binding protein 2-like [Nomascus leucogenys]

GGGTAGGGGGAGTAGGCTGGGGACAGGGAAGGGAGGGTGGCCTCTGGGAAAGGGATGGGA

CTGGACACCGGTCAGCCCCGGAAAGGGGCGGGGCGGCGGGTGGGCTTCGCAGGCCCTGCC

ACTTCTCCTGCGCCCACCCGACGGGAGTCCTGCGTCCGCTGCCAGGCCTGGAAGAGATGG

TGCACGCAGATCCTGTCCGCGCTCAGTTTTCTGCACGCCTGCAGCCCCCCCATCATCCAC

GGGAACCTGACCAGCGACACCATCTTCATTCAGCATAACGGCCTCATCAAGATCGGCTCT

GTGTGGTACCGCATCTTCTCCAATGCGCTCCCGGATGATCTAAGGAGCCCCATCCTTGCT

GAGCGAGAGGAACTTCGGAACTTGCACTTCTTCCCACCAGAGTATGGCGAAGTTGCAGAT

GGGACCGCTGTGGACATCTTCTCTTTTGGGATGTGTGCACTGGAGATGGCTGTGCTGGAG

ATCCAGGCTAATGGGGATACCAGAGTCACAGAGGAGGCCATTGTTCGAGCCAGGCACTCA

CTGAATGACCCCAACATGCGAGAGTTCATCCTCTCCTGCCTGGCCCGGGACCCTGCCCGA

CGGCCTTCTGCTCACAACCTCCTCTTCCACCGAGTGCTCTTCGAGGTACACTCGCTGAAG

CTGCTGGCAGCTCACTGCTTTATCCAGCACCAGTACCTCATGCCTGAGAATGTGGTGGAA

GAGAAGACCAAGGCCATGGACCTGCAGGCTGTCTTGGCAGAGGTCCCCAGGCCCCAGCGG

CCCCCAATGCAATGGCGGTACTCAGAGGTCTCCTTCTTGGAGCTGGACAAATTCCTGGAG

GATGTCAGGAATGGGATCTACCCACTGATGAATTTTGCTGCTGCTCGGCCCCTGGGGCTG

CCCCGTGTATTGGCTCCACCCCTGGAGGAGGCCCAGAAGGTCAAGACCCCGACTCCAGAG

CCCTTTGACTCCGAGACCAGGAAGGTGATCCATATGCAGTGCAACCTGGAAAGGAGTGAG

GACAAGGCACGCTGGCATCTCACATTGCTTCTGGTGCTGGAGGACAGACTGCACAGGCAG

CTGACCTACGACCTGCTTCCAACGGACAGCGCCCAGGACCTCGCGGCTGAGCTGGTGCAC

TACGGCTTCCTGCGAGAGGTTGGCAGGGTGGGGCGTGGTGTGGCTTGCTGGGAGGGTGCA

GGGCTGCTCTGCCGCGGGTCCCCCATGCCTCCCTCCCCTCTTTAGGATGACCGGTTGAAG

CTGGCCATCTTCCTGGAGACCACCTTCCTCAAGTACCGTGGGGCCCAGGCC

>CL55.Contig22_A-W 546 1850 minus strand PREDICTED: nuclear receptor-binding protein 2-like [Nomascus leucogenys]

GGGTAGGGGGAGTAGGCTGGGGACAGGGAAGGGAGGGTGGCCTCTGGGAAAGGGATGGGA

CTGGACACCGGTCAGCCCCGGAAAGGGGCGGGGCGGCGGGTGGGCTTCGCAGGCCCTGCC

ACTTCTCCTGCGCCCACCCGACGGGAGTCCTGCGTCCGCTGCCAGGCCTGGAAGAGATGG

TGCACGCAGATCCTGTCCGCGCTCAGTTTTCTGCACGCCTGCAGCCCCCCCATCATCCAC

GGGAACCTGACCAGCGACACCATCTTCATTCAGCATAACGGCCTCATCAAGATCGGCTCT

GTGTGGTACCGCATCTTCTCCAATGCGCTCCCGGATGATCTAAGGAGCCCCATCCTTGCT

GAGCGAGAGGAACTTCGGAACTTGCACTTCTTCCCACCAGAGTATGGCGAAGTTGCAGAT

GGGACCGCTGTGGACATCTTCTCTTTTGGGATGTGTGCACTGGAGATGGCTGTGCTGGAG

ATCCAGGCTAATGGGGATACCAGAGTCACAGAGGAGGCCATTGTTCGAGCCAGGCACTCA

CTGAATGACCCCAACATGCGAGAGTTCATCCTCTCCTGCCTGGCCCGGGACCCTGCCCGA

CGGCCTTCTGCTCACAACCTCCTCTTCCACCGAGTGCTCTTCGAGGTACACTCGCTGAAG

CTGCTGGCAGCTCACTGCTTTATCCAGCACCAGTACCTCATGCCTGAGAATGTGGTGGAA

GAGAAGACCAAGGCCATGGACCTGCAGGCTGTCTTGGCAGAGGTCCCCAGGCCCCAGCGG

CCCCCAATGCAATGGCGGTACTCAGAGGTCTCCTTCTTGGAGCTGGACAAATTCCTGGAG

GATGTCAGGAATGGGATCTACCCACTGATGAATTTTGCTGCTGCTCGGCCCCTGGGGCTG

CCCCGTGTATTGGCTCCACCCCTGGAGGAGGCCCAGAAGGTCAAGACCCCGACTCCAGAG

CCCTTTGACTCCGAGACCAGGAAGGTGATCCATATGCAGTGCAACCTGGAAAGGAGTGAG

GACAAGGCACGCTGGCATGTGAGCAGGGCCCTGGGCTGGGCTGGGAAAGGAAGGGCCTGC

CAACTTTGACTTGGCACCGACCACACCCTCCTGCCCCAGCTCACATTGCTTCTGGTGCTG

GAGGACAGACTGCACAGGCAGCTGACCTACGACCTGCTTCCAACGGACAGCGCCCAGGAC

CTCGCGGCTGAGCTGGTGCACTACGGCTTCCTGCGAGAGGATGACCGGTTGAAGCTGGCC

ATCTTCCTGGAGACCACCTTCCTCAAGTACCGTGGGGCCCAGGCC

>CL55.Contig23_A-W 43 288 minus strand RecName: Full=Nuclear receptor-binding protein 2

ATGGCGGCCCCGGAGCCGGCGCCGAGGCGGAGCCGGGAGCGGGAGCGGGAGGACGAGAGT

GAGGACGAGAGCGACATCCTGGAGGAGAGCCCATGCGGACGCTGGCAGAAGCGGCGCGAG

CAGGTGAACCAGGGGAATATGCCCGGGATCCAGAGCACATTCCTGGCCATGGACACGGAG

GAGGGGGTAGAAGTGGTGTGGAACGAGCTCCACTTTGGCGACAGGAAGGCTTTCGCAGCC

CATGAG

>CL55.Contig24_A-W 43 288 minus strand RecName: Full=Nuclear receptor-binding protein 2

ATGGCGGCCCCGGAGCCGGCGCCGAGGCGGAGCCGGGAGCGGGAGCGGGAGGACGAGAGT

GAGGACGAGAGCGACATCCTGGAGGAGAGCCCATGCGGACGCTGGCAGAAGCGGCGCGAG

CAGGTGAACCAGGGGAATATGCCCGGGATCCAGAGCACATTCCTGGCCATGGACACGGAG

GAGGGGGTAGAAGTGGTGTGGAACGAGCTCCACTTTGGCGACAGGAAGGCTTTCGCAGCC

CATGAG

>CL55.Contig25_A-W 43 288 minus strand RecName: Full=Nuclear receptor-binding protein 2

ATGGCGGCCCCGGAGCCGGCGCCGAGGCGGAGCCGGGAGCGGGAGCGGGAGGACGAGAGT

GAGGACGAGAGCGACATCCTGGAGGAGAGCCCATGCGGACGCTGGCAGAAGCGGCGCGAG

CAGGTGAACCAGGGGAATATGCCCGGGATCCAGAGCACATTCCTGGCCATGGACACGGAG

GAGGGGGTAGAAGTGGTGTGGAACGAGCTCCACTTTGGCGACAGGAAGGCTTTCGCAGCC

CATGAG

>CL55.Contig26_A-W 43 288 minus strand RecName: Full=Nuclear receptor-binding protein 2

ATGGCGGCCCCGGAGCCGGCGCCGAGGCGGAGCCGGGAGCGGGAGCGGGAGGACGAGAGT

GAGGACGAGAGCGACATCCTGGAGGAGAGCCCATGCGGACGCTGGCAGAAGCGGCGCGAG

CAGGTGAACCAGGGGAATATGCCCGGGATCCAGAGCACATTCCTGGCCATGGACACGGAG

GAGGGGGTAGAAGTGGTGTGGAACGAGCTCCACTTTGGCGACAGGAAGGCTTTCGCAGCC

CATGAG

>CL57.Contig1_A-W 108 2183 minus strand PREDICTED: early endosome antigen 1 [Canis lupus familiaris]

GATCTTCAGAATCATTTGGACACAGCTCAGAATGCATTACAAGATAAACAGCAGGAGTTG

AATAAGATCACCTCTCAGTTGGATCAGGTCACTATAAAGTTACAGGATAAGCAAGAACAC

TGCATTCAGCTGGAAAGTCATCTTAAAGAATATAAAGAGAAACATCTCTCTTTAGAACAA

AAAATTGAAGAGCTAGAAGGCCAAATTAAGAAACTAGAAGCTGATACTCTTGATGTTAAA

GCAAGCAAGGAGCAAGCTTTGCATGATCTTCAACAGCAAAGGCAGCTGAACACAGATTTG

GAGCTTAGAACTATAGAATTGAGTAAACAACTTGAAGCAGAGAAAGAAATTGTATCCACT

ACAAAATTGGATCTACAGAAAAAATCTGAAGCCCTTGAAAATATTAAGCAAACGATTACC

AAGAAAGAAGAAGAAAAAATGATCCTGAAACAAGAAATTGAAAAGTTAAATCAAGATGCA

AAGATGCAGCACAAGGAATTAAATAACAGAATTCAAGCAACAGTAACAGAATTGAAAGAA

GTGAAGATGGAGAAAGAAACTCTAATGACAGAGCTTTCTACAGTGAAAGAGAAATTATCA

AAAGTTTCTGATTCTTTGAAAAACTCCAAAAGTGAATTTGAAAAGGAGAATCAGAAAGGA

AAAGCTGCCATATTAGATTTGGAAAAAACTTGCAACGAATTAAAACATCAACTTCAAGTA

CAAACAGAAAGCACACTAAAGGAACAAAATGAATTGAAAAAGTCACTTGAAAAGGAGAAG

GAGACTTCTCATCAGTTGAAATTGGAACTGAATTCTACTAAGGGAGAAGTCATACAGGCT

CAAAATACTTTAAAACAAAAGGAAAAAGAGGAGCAGCAGCTTCAGGGGAACATAAACGAA

CTAAAGCAATCAACTGAACAAAAGAAAAAGAAAATTGAAGCACTTCAAGGAGAAGTTAAA

ATTGCTCATTCACAGAAGACAGAGCTTGAGAATAAACTACAGCAGCAGTCAGCACAGGCA

GCCCAAGAACTTGCAGCAGAGAAAGAGAAAATATCAGTGTTACAGAACACCTACGAAAAA

TGTCAAGAAAATCTAAAACAACTTCAATCTGATTTCTATGGGAAGGAATCTGAACTTCTG

GCCACAAGACAAGATCTTAAGTCTGTAGAAGAGAAGCTTTCTCTAGCACAGGAGGACTTG

ATATCAAACAGAAATCAAATTGGAAGCCAAAATAAATTGATTCAAGAATTGAAGACTGCC

AAAGCTGCTTTGGAACAGGATTCAGCAAAGAAAGAACAGCAATTGAAGGAGCAGTGTAAA

ACACTCCAAGATACTCAAAAAGAAAAGTCAATAAAAGAAAAAGAACTAGCAAATGAAAAA

TCTAAATTGGCAGAGATAGAGGAAATTAAGTGTAAACAAGAAAAAGAAATCACCAAGCTG

AGTGAAGAACTCAAGTCCCACAAACAAGAAAGCATAAAGGAGGTAACCAATCTCAAGGAT

GCCAAACAACTTTTAATTCAGCAGAAATTAGAACTTCAAGGAAAAGTAGACTCATTAAAG

GCAACTCTTGAACAGGAGAAGAGAAACCTGCAGATACTAACAGAGCAGGTGAAAAAGGAA

GAAGATGAGCTGAAGAAAGAATTTGTTGAGAAAGAAGCTAAACTGCATTCTGAAATAAAA

GAAAAGGAAGTGGGAATGAAGAAACATGAAGAAAATGAGGCTAAACTTACCATGCAGATT

ACAGCATTGAATGAGAATTTGGGCACTGTAAAGAAGGAGTGGCAGTCTAGTCAACGGAGA

GTTAGTGAGCTAGAGAAACAGACTGACGACTTACGTGGTGAGATTGCAGTCTTAGAAGCC

ACGGTTCAGAATAATCAAGACGAAAGGAGAGCACTGCTAGAAAGATGTCTTAAAGGAGAA

GGTGAAATAGAAAAGCTTCAGACCAAAGTCCTAGAATTGCAAAGAAAGCTGGATAATACA

ACTGCAGCAGTGCAGGAGCTGGGCAGAGAAAACCAGTCCCTTCAGATCAAACATACACAA

GCGTTGAATAGAAAGTGGGCTGAGGACAACGAAGTA

>CL57.Contig2_A-W 2759 3892 minus strand early endosome antigen 1 [Macaca mulatta]

AAAGAGGAGCAGCAGCTTCAGGGGAACATAAACGAACTAAAGCAATCAACTGAACAAAAG

AAAAAGAAAATTGAAGCACTTCAAGGAGAAGTTAAAATTGCTCATTCACAGAAGACAGAG

CTTGAGAATAAACTACAGCAGCAGTCAGCACAGGCAGCCCAAGAACTTGCAGCAGAGAAA

GAGAAAATATCAGTGTTACAGAACACCTACGAAAAATGTCAAGAAAATCTAAAACAACTT

CAATCTGATTTCTATGGGAAGGAATCTGAACTTCTGGCCACAAGACAAGATCTTAAGTCT

GTAGAAGAGAAGCTTTCTCTAGCACAGGAGGACTTGATATCAAACAGAAATCAAATTGGA

AGCCAAAATAAATTGATTCAAGAATTGAAGACTGCCAAAGCTGCTTTGGAACAGGATTCA

GCAAAGAAAGAACAGCAATTGAAGGAGCAGTGTAAAACACTCCAAGATACTCAAAAAGAA

AAGTCAATAAAAGAAAAAGAACTAGCAAATGAAAAATCTAAATTGGCAGAGATAGAGGAA

ATTAAGTGTAAACAAGAAAAAGAAATCACCAAGCTGAGTGAAGAACTCAAGTCCCACAAA

CAAGAAAGCATAAAGGAGGTAACCAATCTCAAGGATGCCAAACAACTTTTAATTCAGCAG

AAATTAGAACTTCAAGGAAAAGTAGACTCATTAAAGGCAACTCTTGAACAGGAGAAGAGA

AACCTGCAGATACTAACAGAGCAGGTGAAAAAGGAAGAAGATGAGCTGAAGAAAGAATTT

GTTGAGAAAGAAGCTAAACTGCATTCTGAAATAAAAGAAAAGGAAGTGGGAATGAAGAAA

CATGAAGAAAATGAGGCTAAACTTACCATGCAGATTACAGCATTGAATGAGAATTTGGGC

ACTGTAAAGAAGGAGTGGCAGTCTAGTCAACGGAGAGTTAGTGAGCTAGAGAAACAGACT

GACGACTTACGTGGTGAGATTGCAGTCTTAGAAGCCACGGTTCAGAATAATCAAGACGAA

AGGAGAGCACTGCTAGAAAGATGTCTTAAAGGAGAAGGTGAAATAGAAAAGCTTCAGACC

AAAGTCCTAGAATTGCAAAGAAAGCTGGATAATACAACTGCAGCAGTGCAGGAG

>CL58.Contig1_A-W 1 1839 minus strand PREDICTED: diacylglycerol kinase delta-like [Cricetulus griseus]

CGAGAGCAGCTCATGCTGAGAGCCAACAGCCTGAAGAAAGCGATTCGCCAGATCATAGAA

CACACAGAGAAAGCTGTTGATGAACAGAATGCCCAGACCCAGGAGCAGGAGGGCTTCATG

CTGGGCCTCCCGGAGGCAGAAGAGAAGAAGGACTCAAGGACAGATGGCAGAGTATGCCCA

CCGCTATCCCACAGTGAGAGCTGCGGGGTCCTCAAAGGGAGAAGCCACCGCAAAGCATCC

AGATCCCCATGTGAGAAGCTAATGAGCAAAGGAGGTCTGTCCTTGGGCAACTCTGCCTCT

CTTCCTCCCCAGGCAGGAAGCCGAGATGGCCTGCCTGCACTGAACACCAAGATCCTGTTC

CCAAGTGTTCGGGCTGGGATGTCTGGTTCCTTGCCTGGGGGCTCTGTCATCAGTCGCTTA

TTAATTAACGCTGACCCCTTTAATGCTGAACCCGAAAACCTGGAGTGCTACACAGAGAAG

TGTGTCATGAACAACTATTTTGGCATTGGCCTGGATGCGAAGATCTCCCTGGACTTTAAC

AATAAGCGTGACGAGCACCCGGAGAAGTGCAGGAGTCGGACCAAGAACATGATGTGGTAT

GGAGTCCTGGGCACCAAGGAGCTGCTGCACAGAACCTACAGGAACCTGGAGCAGAAGGTC

TTGCTGGAGTGTGATGGTCGACCAATCCCCCTCCCTAGTCTTCAGGGAATCGCTGTCCTC

AACATTCCCAGCTATGCTGGAGGGACCAACTTCTGGGGGGGCACCAAGGAAGATGATACT

TTTGCAGCTCCGTCGTTTGATGATAAGATTCTGGAGGTGGTCGCTGTGTTTGGCAGCATG

CAGATGGCTGTGTCTCGTGTAATTAAGCTGCAGCATCACCGAATCGCACAGTGTCGCACC

GTGAAGATTTCCATCCTGGGGGATGAGGGTGTACCTGTGCAGGTGGATGGAGAGGCCTGG

ATCCAGCCCCCTGGATACATTCGGATTATCCACAAGAACCGGGCACAGACGCTGACCAGA

GATAGGGCCTTTGAGAACACCCTGAAGTCCTGGGAAGACAAACAGAAGTGTGAGCTGCCC

CGCCCACCATCTTTTTCACTGCACCCAGAGATCCTATCTGAGGAGGAGGCCATCCACATG

GACCAGTTTGGGCAGGCGGCCGGGGTCCTCATCCACAGCATCCGGGAAGTAGCCCAGTGC

CACCGGGATGTGGAGCAGGAGCTCGCTCATGCCGTCAACGCTAGCTCCAAGGCCATGGAC

CGAGTGTATGGCAAGCCTAGGGCTGCGGAGGGGCTGAACTGCAGCTTTGTCCTGGAGATG

GTGAATAACATCAGGGCTCTGCGCAGCGAGACGGAGCTGCTGCTGTCTGGGAAGATGGCC

CTGCAATTGGACCCTCCTCAGAAGGAGCGGCTGGGTGCTGCTCTCGCCGAGATGGACCGG

CAGCTTAGGAAGCTGGCAGATACGCCCTGGCTCTGCCAGCCCACAGAGCCTGGTGATGAA

GAGAATGTGATGTTGGATCTTTCCAAACGCAGCCGCAGTGGTAAATTCCGCCTGGTGACC

AAATTTAAAAAGGAGAAAAACAACAAGAACAAAGAAGCTTACAGTAGCCTGGGAGCCCCG

GTTCACCTCTGGGGGACAGAGGAGGTGGCTGCCTGGCTGGAGCACCTCAGTCTCTGTGAG

TATAAGGACATCTTCACGCGGCACGACATCCGGGGCTCTGAGCTCCTGCACCTGGAGCGG

AGGGACCTCAAGGACCTTGGCGTAACCAAAGTGGGACACATGAAGAGGATCCTGTGTGGA

ATCAAGGAACTGAGCCGGGGCTCCCCTGCCACCGAGGCC

>CL58.Contig2_A-W 24 392 minus strand Diacylglycerol kinase delta [Heterocephalus glaber]

CTGACGCCCAGTGTTTCTCCTTCTCCCCAACAGAATGTGATGTTGGATCTTTCCAAACGC

AGCCGCAGTGGTAAATTCCGCCTGGTGACCAAATTTAAAAAGGAGAAAAACAACAAGAAC

AAAGAAGCTTACAGTAGCCTGGGAGCCCCGGTTCACCTCTGGGGGACAGAGGAGGTGGCT

GCCTGGCTGGAGCACCTCAGTCTCTGTGAGTATAAGGACATCTTCACGCGGCACGACATC

CGGGGCTCTGAGCTCCTGCACCTGGAGCGGAGGGACCTCAAGGACCTTGGCGTAACCAAA

GTGGGACACATGAAGAGGATCCTGTGTGGAATCAAGGAACTGAGCCGGGGCTCCCCTGCC

ACCGAGGCC

>CL59.Contig1_A-W 167 835 PREDICTED: hypothetical protein [Oryctolagus cuniculus]

ATGGCCTGTGAACCGCAGATGGACCCCGGCGGGGCGGCTGGCCCTTTGCCCACCTCCTCC

CCTGGCTGGAGCGCCCTGCAGCCGGGAGGCCCTCCTGGGTGGGGGCAAGAGCTCCACAAC

GGCCAGGTCCTCACCGTCCTCCGGGTCGACAATACCTGTGCACCCATGGCCTTCGACCTG

GGAACCGCAGAGGAGCAACTGCAGACCTGGGGCATTCAGGTCCCTGCTGACCAGTACAGG

AGCTTGGCGGAGAGTGCCCTCTTGGAGCCCCAAGTGAGAAGATACATCATCTACAATTCG

AGGCCCATGCGGCTGGCCTTTGCCGTGGTGTTTTACGTGGTGATCTGGGCCAACATCTAT

TCAACCAGCCAGATGTTTGCCCTGGGCAGCCACTGGGCGGGCGTGCTGCTCGTGACCCTG

GCCGCAGTCAGCCTGACCCTGACTCTCATGCTGGTCTTCGAGAGGCAGCAGAGGAAGGCT

AACACTAATACAGACCTGAGGCTCACGGCTGCCAATAGAGCCCTGCTGAGACACCGGGTG

CTGCTGGGGGTGACGGACACCGTGGAAGGGTGCCAGAGTGTGATCCAGCTCTGGTTTGTC

TACTTCGACCTGGAGGACTGCGTGCAGTTTTTGTCTGACCACGTTCAGGAGATGAAGACT

AACCAAGAG

>CL59.Contig2_A-W 167 1195 transmembrane protein C9orf91 [Macaca mulatta]

ATGGCCTGTGAACCGCAGATGGACCCCGGCGGGGCGGCTGGCCCTTTGCCCACCTCCTCC

CCTGGCTGGAGCGCCCTGCAGCCGGGAGGCCCTCCTGGGTGGGGGCAAGAGCTCCACAAC

GGCCAGGTCCTCACCGTCCTCCGGGTCGACAATACCTGTGCACCCATGGCCTTCGACCTG

GGAACCGCAGAGGAGCAACTGCAGACCTGGGGCATTCAGGTCCCTGCTGACCAGTACAGG

AGCTTGGCGGAGAGTGCCCTCTTGGAGCCCCAAGTGAGAAGATACATCATCTACAATTCG

AGGCCCATGCGGCTGGCCTTTGCCGTGGTGTTTTACGTGGTGATCTGGGCCAACATCTAT

TCAACCAGCCAGATGTTTGCCCTGGGCAGCCACTGGGCGGGCGTGCTGCTCGTGACCCTG

GCCGCAGTCAGCCTGACCCTGACTCTCATGCTGGTCTTCGAGAGGCAGCAGAGGAAGGCT

AACACTAATACAGACCTGAGGCTCACGGCTGCCAATAGAGCCCTGCTGAGACACCGGGTG

CTGCTGGGGGTGACGGACACCGTGGAAGGGTGCCAGAGTGTGATCCAGCTCTGGTTTGTC

TACTTCGACCTGGAGGACTGCGTGCAGTTTTTGTCTGACCACGTTCAGGAGATGAAGACT

AACCAAGAGTCCTTGCTGAGAAGCAGACTGAGCCAGTTATATGTTGTCCTGGAGACGGGG

GTGAGCCCCACGATGGCGGAGGGCCCCGAGAGCTCGGAGGAGGCTCCTCTCCTGCCCAGC

AGTCCTCGTCCTCAGGAGAGACCGCTCGTGCAGACGGAGCTCCACCAGCTTGTTCCCGAG

GCTGAGCCGGAGGAAATGGCCCGACAGCTGCTGGCAGTGTTTGGTGGCTACTACATCCGA

CTCTTGGTGACCTCCCAGCTGCCCCAGGGAGTGGGGACACGGCACACAGACTCTCCCAGG

ATCCCATGCCCCTGCCAGCTCATAGAAGCCCAGGTCCTGGGCACAGGGTGTTGTCCATTC

CTGGCCAGG

>CL60.Contig1_A-W 159 2984 PREDICTED: ATP-dependent RNA helicase DDX42 [Equus caballus]

ATGAACTGGAATAAAGGTGGCCCGGGCACTAAGCGGGGATTTGGTTTCGGAGGTTTTGCC

ATCAGTGCTGGGAAGAAGGAGGAACCTAAACTCCCACAACAGTCCCATAGTGCCTTTGGG

GCAACTAGCTCATCTTCTGGATTTGGGAAGTCAGCTCCACCACAGCTTCCTTCCTTCTAC

AAAATTGGATCTAAGCGAGCCAACTTTGATGAAGAAAATGCCTATTTTGAAGATGAGGAA

GAAGACTCTAGCAATGTTGACTTACCTTACATTCCTGCTGAAAACTCACCTACCCGCCAG

CAGTTTCATTCCAAGCCAGCAGATTCTGACAGTGATGATGATCCCTTGGAAGCATTTATG

GCTGAAGTGGAGGATCAGGCAGCTAGAGACATGAAGAGGCTTGAAGAAAAGGACAAGGAA

AGAAAAAACGTAAAGGGTATTCGAGATGACATTGAAGAGGAAGATGACCAAGAAGCTTAT

TTTCGATACATGGCAGAAAACCCAACTGCCGGTGTGGTTCAAGAGGAAGAAGAAGACAAT

CTGGAATATGATAGTGATGGGAATCCAATCGCACCTTCCAAAAAAATAATCGATCCTCTT

CCGCCCATTGATCATTCAGAGACTAACTTTGAATAATTGGACCTGCAGATTGACTACCCA

CCATTTGAAAAAAATTTTTACAATGAGCATGAGGAGATAACCAACCTCACTCCACAACAG

TTAATAGATCTCCGGCATAAGCTCAATCTTCGGGTTTCTGGTGCTGCACCTCCTAGACCA

GGAAGTAGTTTTGCTCATTTTGGATTTGATGAACAACTTATGCATCAGATTCGGAAATCT

GAGTACACACAGCCCACTCCAATACAATGTCAGGGTGTGCCTGTGGCATTAAGTGGTAGA

GACATGATTGGTATTGCCAAAACAGGCAGTGGAAAAACTGCAGCCTTTATCTGGCCCATG

TTGATTCATATAATGGACCAGAAGGAATTGGAACCAGGTGATGGACCAATTGCAGTGATT

GTGTGTCCTACTAGGGAGCTTTGTCAGCAGATCCATGCAGAATGTAAGCGGTTTGGGAAA

GCATATAATCTTCGATCAGTGGCTGTGTATGGAGGAGGGAGCATGTGGGAGCAAGCCAAG

GCCCTTCAGGAAGGGGCAGAGATTGTTGTGTGTACCCCAGGTCGACTGATTGATCATGTG

AAGAAGAAAGCTACTAATCTTCAAAGAGTCTCTTACCTTGTATTTGATGAAGCAGATCGA

ATGTTTGATATGGGATTTGAGTACCAGGTTCGATCCATAGCAAGTCATGTTCGTCCTGAC

AGACAGACTCTCTTATTCAGTGCAACTTTTCGGAAAAAGATTGAAAAACTGGCAAGAGAT

ATCCTAATTGACCCTATTCGAGTGGTGCAGGGAGATATTGGAGAGGCAAATGAAGATGTG

ACACAGATTGTGGAGATTCTCCATTCTGGGCCTAGTAAATGGAACTGGCTTACCCGGCGT

CTGGTGGAATTTACTTCTTCCGGGAGTGTCCTCCTGTTTGTTACTAAAAAAGCTAATGCC

GAAGAGCTAGCCAATAACCTTAAGCAGGAGGGTCATAATCTTGGGTTGCTTCATGGGGAC

ATGGATCAGAGTGAAAGAAACAAGGTCATTTCCGACTTTAAGAAAAAGGACATCCCAGTC

CTAGTAGCCACAGATGTTGCAGCCCGTGGTCTGGACATTCCTTCAATTAAGACTGTCATT

AACTATGATGTGGCACGAGACATTGATACCCATACTCACAGGATTGGCCGCACAGGAAGA

GCCGGTGAGAAAGGTGTAGCCTATACCCTGTTGACCCCTAAGGACAGCAATTTTGCTGGT

GACCTTGTGCGGAACTTGGAAGGAGCCAATCAGCATGTTTCCAAGGAACTTCTGGACCTG

GCAATGCAGAATGCTTGGTTTCGGAAATCCCGCTTCAAAGGAGGAAAAGGCAAAAAGCTG

AACATTGGTGGAGGAGGCCTAGGCTATAGGGAGCGACCTGGCCTAGGCTCTGAGAACACG

GACCGAGGAAATAACAACAATGTAATGAGCAATTATGAGGCCTATAAGCCTTCCACAGGA

GCCATGGGAGATCGGCTGACAGCAATGAAAGCAGCTTTTCAGTCACAGTACAAGAGTCAC

TTTGTTGCAGCCAGTTTAAGTAATCAGAAGGCTGGAAGCTCTGCTGCTGGGGCAAGTGGA

TGGACTAGTGCAGGGAGCTTGAATTCTGTTCCAACTAATTCAGCACAGCAGGGCCATAAT

AGTCCTGACAACCCCATCACCAGTGCCGCCACCAAGAGCATCCCAGGCTTTGGCAATACA

GGGAACATCACCAGTGCTCCAGTGACCTACCCTTCTGTCGGAGCCCAGGGAGTCAACAAC

ACAGCTTCAGGAAATAACAGCAGAGAAGGACTTGGGGGTGGCAATGGGAAGCGAGAGAGA

TATACTGAGAATCGGGGTGGTGGCCGCCATAGCCATGGAGAGAGTGGCAATCGGCATGGT

GATAGTCCACGGCACGGAGACGGTGGTCGACATGGAGATGGATACCGCTACCCAGAAAGT

AGCAGCAGCCGCCATACTGACAGCCATCGGCATGGGGAGAACAGGCATGGAGGAAGTACA

GGCCGGCATGGGGAAAGCCGGGGTGCAAATGATGGTCGGAATGGAGAAAGCAGGAAAGAA

GGTTGTAATCGTGAAAGCAAGGTGGACAGCAGCAAGATGGACAAGATGGACAGCAAGACA

GATAAGACAGCGGATGGTTTTGCTGTCCCTGAGCCACCCAAACGCAAGAAAAGTAGATGG

GACAGT

>CL60.Contig2_A-W 164 1069 PREDICTED: ATP-dependent RNA helicase DDX42 [Equus caballus]

TCAATGCAGAATGCTTGGTTTCGGAAATCCCGCTTCAAAGGAGGAAAAGGCAAAAAGCTG

AACATTGGTGGAGGAGGCCTAGGCTATAGGGAGCGACCTGGCCTAGGCTCTGAGAACACG

GACCGAGGAAATAACAACAATGTAATGAGCAATTATGAGGCCTATAAGCCTTCCACAGGA

GCCATGGGAGATCGGCTGACAGCAATGAAAGCAGCTTTTCAGTCACAGTACAAGAGTCAC

TTTGTTGCAGCCAGTTTAAGTAATCAGAAGGCTGGAAGCTCTGCTGCTGGGGCAAGTGGA

TGGACTAGTGCAGGGAGCTTGAATTCTGTTCCAACTAATTCAGCACAGCAGGGCCATAAT

AGTCCTGACAACCCCATCACCAGTGCCGCCACCAAGAGCATCCCAGGCTTTGGCAATACA

GGGAACATCACCAGTGCTCCAGTGACCTACCCTTCTGTCGGAGCCCAGGGAGTCAACAAC

ACAGCTTCAGGAAATAACAGCAGAGAAGGACTTGGGGGTGGCAATGGGAAGCGAGAGAGA

TATACTGAGAATCGGGGTGGTGGCCGCCATAGCCATGGAGAGAGTGGCAATCGGCATGGT

GATAGTCCACGGCACGGAGACGGTGGTCGACATGGAGATGGATACCGCTACCCAGAAAGT

AGCAGCAGCCGCCATACTGACAGCCATCGGCATGGGGAGAACAGGCATGGAGGAAGTACA

GGCCGGCATGGGGAAAGCCGGGGTGCAAATGATGGTCGGAATGGAGAAAGCAGGAAAGAA

GGTTGTAATCGTGAAAGCAAGGTGGACAGCAGCAAGATGGACAAGATGGACAGCAAGACA

GATAAGACAGCGGATGGTTTTGCTGTCCCTGAGCCACCCAAACGCAAGAAAAGTAGATGG

GACAGT

>CL60.Contig3_A-W 68 757 PREDICTED: ATP-dependent RNA helicase DDX42 [Equus caballus]

TTTCAGTCACAGTACAAGAGTCACTTTGTTGCAGCCAGTTTAAGTAATCAGAAGGCTGGA

AGCTCTGCTGCTGGGGCAAGTGGATGGACTAGTGCAGGGAGCTTGAATTCTGTTCCAACT

AATTCAGCACAGCAGGGCCATAATAGTCCTGACAACCCCATCACCAGTGCCGCCACCAAG

AGCATCCCAGGCTTTGGCAATACAGGGAACATCACCAGTGCTCCAGTGACCTACCCTTCT

GTCGGAGCCCAGGGAGTCAACAACACAGCTTCAGGAAATAACAGCAGAGAAGGACTTGGG

GGTGGCAATGGGAAGCGAGAGAGATATACTGAGAATCGGGGTGGTGGCCGCCATAGCCAT

GGAGAGAGTGGCAATCGGCATGGTGATAGTCCACGGCACGGAGACGGTGGTCGACATGGA

GATGGATACCGCTACCCAGAAAGTAGCAGCAGCCGCCATACTGACAGCCATCGGCATGGG

GAGAACAGGCATGGAGGAAGTACAGGCCGGCATGGGGAAAGCCGGGGTGCAAATGATGGT

CGGAATGGAGAAAGCAGGAAAGAAGGTTGTAATCGTGAAAGCAAGGTGGACAGCAGCAAG

ATGGACAAGATGGACAGCAAGACAGATAAGACAGCGGATGGTTTTGCTGTCCCTGAGCCA

CCCAAACGCAAGAAAAGTAGATGGGACAGT

>CL61.Contig1_A-W 41 256 PREDICTED: protein kish-A-like [Sus scrofa]

ATGTCTGCCATTTTCAATTTTCAGAGTCTGTTGACTGTAATCTTGCTGCTTATATGTACC

TGTGCTTATATCCGGTCCTTGGCACCCAGCCTCCTGGACAGAAATAAAACTGGACTATTG

GGAATATTTTGGAAGTGTGCCAGAATTGGTGAACGAAAGAGTCCTTATGTTGCAGTATGC

TGTGTAGTGATGGCCTTCAGCATCCTTTTCATACAG

>CL61.Contig2_A-W 43 144 PREDICTED: protein kish-A-like [Sus scrofa]

CTATTGGGAATATTTTGGAAGTGTGCCAGAATTGGTGAACGAAAGAGTCCTTATGTTGCA

GTATGCTGTGTAGTGATGGCCTTCAGCATCCTTTTCATACAG

>CL62.Contig1_A-W 3 1055 minus strand PREDICTED: CWF19-like protein 1 [Equus caballus]

TACGTGCTTGGTGCCAATAACCAGGAAACAGTAAAATATTTCCAAGATGCTGATGGGTGT

GAATTAGCTGAAAACATTACTTATCTGGGTCGGAAAGGTGTCTTCACTGGAAGCTCGGGC

CTGCAGATTGTATATCTCAGTGGGACAGAGTCATTAACTGAGCCAGTTCCAGGTTACAGT

TTTAGTCCTAAGGATGTGTCTTCCCTGAGAACAATGCTGTGTTCAACATCCCAGTTTAAG

GGTGTTGATATCTTGCTCACATCTCCATGGCCCAAGTATGTGGGGAACTTTGGGAATTCT

TCTGGAGAAGTGGATACCAAAAAATGTGGCTCTGCCTTGGTCTCCAGTCTTGCCACAAGC

TTAAAACCAAGATATCATTTCGCTGCCTTGGAAAAGTCATATTATGAGAGGCTTCCATAT

CGAAACCATGTCGTTCTACAAGAAAATGCACAGCATGCCACCCGGTTCATAGCCCTGGCA

AATGTTGGGAATCCAGAAAAGAAAAAGTATCTTTATGCATTCAGTATTGTTCCCATGAAA

CTAATGGATACAGCAGAACTGGTAAAACAGCCTCCAGATGTCACTGAAAATCCTTACAGA

AAATCTGGAAAGGAAGCATCCATAGGAAAGCAAATTCCTATCCCTGAGGAAGATTCAGCC

TGTCAGTTCTTCTTTGATTTAAATGAAAAGCAGGGAAGGAAGCGTTCATCCACAGGCAGA

GATAGCAAATCTCCTCACCCAAAGCAGCCTCGCAAACCTCCTCAGCCTCCAGGACCATGC

TGGTTTTGCCTTGCCAGCCCTGAAGTGGAAAAGCATTTGGTGGTCAACATTGGCACACAT

TGCTACCTTGCCCTGGCTAAAGGAGGCTTATCTGATGACCATGTCCTCATTCTGCCTATT

GGACACTACCAGTCAGTAGTGGAGCTTTCAGCAGAGGTGGTGGAGGAAGTGGAGAAGTAT

AAGGCTACACTGAGACGCTTCTTTAAGAATCGAGGGAAGCGATGTGTTCTTTTTGAGAGA

AATTATAAAAGCCATCATCTCCAGCTACAGGTA

>CL62.Contig2_A-W 3 1412 PREDICTED: CWF19-like protein 1 isoform 1 [Canis lupus familiaris]

TACGTGCTTGGTGCCAATAACCAGGAAACAGTAAAATATTTCCAAGATGCTGATGGGTGT

GAATTAGCTGAAAACATTACTTATCTGGGTCGGAAAGGTGTCTTCACTGGAAGCTCGGGC

CTGCAGATTGTATATCTCAGTGGGACAGAGTCATTAACTGAGCCAGTTCCAGGTTACAGT

TTTAGTCCTAAGGATGTGTCTTCCCTGAGAACAATGCTGTGTTCAACATCCCAGTTTAAG

GGTGTTGATATCTTGCTCACATCTCCATGGCCCAAGTATGTGGGGAACTTTGGGAATTCT

TCTGGAGAAGTGGATACCAAAAAATGTGGCTCTGCCTTGGTCTCCAGTCTTGCCACAAGC

TTAAAACCAAGATATCATTTCGCTGCCTTGGAAAAGTCATATTATGAGAGGCTTCCATAT

CGAAACCATGTCGTTCTACAAGAAAATGCACAGCATGCCACCCGGTTCATAGCCCTGGCA

AATGTTGGGAATCCAGAAAAGAAAAAGTATCTTTATGCATTCAGTATTGTTCCCATGAAA

CTAATGGATACAGCAGAACTGGTAAAACAGCCTCCAGATGTCACTGAAAATCCTTACAGA

AAATCTGGAAAGGAAGCATCCATAGGAAAGCAAATTCCTATCCCTGAGGAAGATTCAGCC

TGTCAGTTCTTCTTTGATTTAAATGAAAAGCAGGGAAGGAAGCGTTCATCCACAGGCAGA

GATAGCAAATCTCCTCACCCAAAGCAGCCTCGCAAACCTCCTCAGCCTCCAGGACCATGC

TGGTTTTGCCTTGCCAGCCCTGAAGTGGAAAAGCATTTGGTGGTCAACATTGGCACACAT

TGCTACCTTGCCCTGGCTAAAGGAGGCTTATCTGATGACCATGTCCTCATTCTGCCTATT

GGACACTACCAGTCAGTAGTGGAGCTTTCAGCAGAGGTGGTGGAGGAAGTGGAGAAGTAT

AAGGCTACACTGAGACGCTTCTTTAAGAATCGAGGGAAGCGATGTGTTCTTTTTGAGAGA

AATTATAAAAGCCATCATCTCCAGCTACAGGTCATTCCTGTGCCACTCAGCTGCTGTGCT

ACTGATGACATTAAAGATGCCTTCATTACCCAGGCTCAAGAGCAACAGATAGAGCTGTTG

GAAATCCCAGAGCACTCTGACATCAAGCAGATTGCACAGCCTGGCGCGGCATATTTTTAT

GTTGAACTTGACACAGGAGAGAAGCTTTTCCACAGAATTAAGAAGAATTTTCCTTTGCAG

TTTGGAAGGGAAGTATTGGCCAGTGAAGCTATTCTTAATATTCCTAACAAGTCTGACTGG

AGACAGTGTCAAATCAGCAAGGAAGAAGAGGAGATTCTGGCTCGTCGCTTCCGGAAAGAC

TTTGAACCCTTTGACTTTACTCTGGATGAC

>CL63.Contig1_A-W 12 2090 PREDICTED: putative ribosomal RNA methyltransferase NOP2 isoform 1 [Nomascus leucogenys] >gi|332249235|ref|XP_003273769.1| PREDICTED: putative ribosomal RNA methyltransferase NOP2 isoform 2 [Nomascus leucogenys]

CCAGACCATAGTAGTGATGAGGAAGAGGATGAGGAAGACCCTGAAGAAGATGGTGTTGTG

AACCAGGGGAACCTCTGGGACTCTGAGGACAGTGATACTGATATGGTAGATGACTATGGA

GCTGACTCCAACTCAGAGGATGAAGGTGAAAAGTTGCTGCCCATTGAAAGAGCTGCTGAG

AAGCAGAAGGCTCAGGAAGCTGCTGTTGGGGGCCAGTGGAGTGAGGAGACTGATGAAGAG

GAAGAGGGGGAAGTGTCCCCTCTATCAGGCCCCACAAAGGATGAGGAGGCAGATGGGGGC

CTGCAGATCAATGTGGATGGGGAAGAGCCATTTGTGCTGCCCCCTGCTGGGGATATGGAG

CAGGATGCCCATGCTCCAGACTTACAACGAGTTCACAAGCGGATTCAGGATATAGTGGGA

GTGTTGCGGGATTTTGGGGCTCAGCGGGAAGAAGGTCGGTCTCGTTCTGAATATCTGAAT

CGGCTTCAGAAGGATCTGGCCACGTATTACTCCTATGGAGACTTCCTTCTTGGCAAGCTC

ATGGAACTCTTCCCTCTGTCTGAGCTGATAGAGTTTTTAGAAGCTAATGAGGTGCCTCGG

CCCATCACCCTCCGGACCAATACCTTGAAAACCCGGCGTAGAGACCTTGCTCAGGCACTG

ATCAATCGTGGGATTAACTTGGATCCTTTGGGCAAGTGGTCAAAGACTGGACTCGTTATA

TATGATTCTTCAGTGCCCATTGGTGCTACTCCTGAATACCTGGCTGGACACTACATGTTG

CAGGGAGCCTCCAGCATGTTGCCTGTCATGGCCTTGGCGCCACAGGAACATGAGCGGATC

TTGGACATGTGTTGTGCCCCTGGAGGAAAGACCAGTTACATAGCCCAACTGATGAAAAAC

ACTGGTGTGATCCTTGCCAATGATGCCAATGCTGTGCGGCTGAAGAGTGTTGTGGGTAAC

CTGCACCGGCTGGGAGTCACTAACACCATCATTAGCCATTATGATGGACGCCAGTTCCCC

AAGGTGGTCGGGGGCTTTGACCGAGTATTGCTGGATGCTCCCTGTAGTGGCACTGGGGTC

ATCTCTAAGGATCCAGCTGTGAAGACTAACAAGGATGAGAAGGACATCCTACGCTGTGCT

CACCTTCAGAAAGAGTTACTCCTGAGTGCTATTGACTCTGTCAATGCCACTTCTAAGACA

GGAGGCTACCTCGTCTACTGCACCTGTTCTATTATGGTGGAAGAGAATGAGTGGGTAGTA

GACTATGCCCTGAAAAAGAGAAATGTGAGGCTTGTGCCCACCGGCCTGGACTTTGGCCAG

GAAGGTTTTACCCGTTTTCGAGAAAGGCGTTTCCATCCCACTCTGCGTTCCACCCGACGA

TTTTACCCTCATACCCACAATATGGATGGTTTCTTTATTGCCAAGTTCAAGAAATTCTCC

AATTGTATCCCTCAGTCCCAAACAGGAAATTCTGCAACAGCCACTCCTACAGACCTAGAC

TTGCCTGACCCAAAGGGTCAGGTCACTCCCAAGTCTGAGAACAACAGCCAACCAGCCAAG

AAAGCCAAAAGGGCTGCAAAGGCAAAGCAGTTCCAGAAACGGCAGCATCCCAAGAATGCC

TCTGTACAGAAGCTGAATGGCATCACCAAAGGCACAGTCTCAGAAGTGTCCACTGTATCT

TCTGTCACAAAGGCCCAAGCTTCCTCCAGGCTCCCAGATAGTACTCAGCCTGCTGGAAAA

GTTGAAGTGATCAGGAAACCAAAGGTGATTGGGAGGCTAAGGCAACAGGCACCTAAATTG

CACTCCTCTAAGAAAGCTGCCTTCCTGAAGCAGAATGCCCCTCCCAAAAGCATGGACACC

GAATCACCTGTTGCGCTGTCCCTTTCTCAGACTCAGGCTACTCCCAGGCTTAAGGACACT

GATCAGCCCCTTGGAAAAGTAAAAGGGGCTGAGACGGTAAAGCAGCCTTTCAAGAAAGCT

ACCTTTCAGAAGGAGAATGGCACCCCCAAGGGATCTCAGATCCCCACTGTGACTCCCCTC

AATTCTGTCCAGCCCCCACCAGCAAAGAAGAGGAAAACT

>CL63.Contig2_A-W 35 883 minus strand PREDICTED: putative ribosomal RNA methyltransferase NOP2 isoform 1 [Nomascus leucogenys] >gi|332249235|ref|XP_003273769.1| PREDICTED: putative ribosomal RNA methyltransferase NOP2 isoform 2 [Nomascus leucogenys]

GTCCAGGTGGAAGAGAATGAGTGGGTAGTAGACTATGCCCTGAAAAAGAGAAATGTGAGG

CTTGTGCCCACCGGCCTGGACTTTGGCCAGGAAGGTTTTACCCGTTTTCGAGAAAGGCGT

TTCCATCCCACTCTGCGTTCCACCCGACGATTTTACCCTCATACCCACAATATGGATGGT

TTCTTTATTGCCAAGTTCAAGAAATTCTCCAATTGTATCCCTCAGTCCCAAACAGGAAAT

TCTGCAACAGCCACTCCTACAGACCTAGACTTGCCTGACCCAAAGGGTCAGGTCACTCCC

AAGTCTGAGAACAACAGCCAACCAGCCAAGAAAGCCAAAAGGGCTGCAAAGGCAAAGCAG

TTCCAGAAACGGCAGCATCCCAAGAATGCCTCTGTACAGAAGCTGAATGGCATCACCAAA

GGCACAGTCTCAGAAGTGTCCACTGTATCTTCTGTCACAAAGGCCCAAGCTTCCTCCAGG

CTCCCAGATAGTACTCAGCCTGCTGGAAAAGTTGAAGTGATCAGGAAACCAAAGGTGATT

GGGAGGCTAAGGCAACAGGCACCTAAATTGCACTCCTCTAAGAAAGCTGCCTTCCTGAAG

CAGAATGCCCCTCCCAAAAGCATGGACACCGAATCACCTGTTGCGCTGTCCCTTTCTCAG

ACTCAGGCTACTCCCAGGCTTAAGGACACTGATCAGCCCCTTGGAAAAGTAAAAGGGGCT

GAGACGGTAAAGCAGCCTTTCAAGAAAGCTACCTTTCAGAAGGAGAATGGCACCCCCAAG

GGATCTCAGATCCCCACTGTGACTCCCCTCAATTCTGTCCAGCCCCCACCAGCAAAGAAG

AGGAAAACT

>CL65.Contig1_A-W 152 1339 minus strand PREDICTED: death domain-associated protein 6-like [Ailuropoda melanoleuca]

GCCCTGGTTAAGCGGCTCAAAGGGGCTCTAATGCTAGAAAATTTACAGAAACATTCAACA

CCCCATGCTGCATTCCAGCCAAATTCCCAGATTGGCGAGGAAATGAGCCAGAACAGTTTC

ATAAAGCAGTATCTGGAAAAGCAGCAGGAGCTACTTAGACAGCGTCTGGAACGTGAAGCT

CGAGAAGCTGCAGAACTTGAAGGAAAAGGCTCCTCTGTTTCTGAAGAGAAAGGTGAATCT

GATGATGAGAAACCAAGAAAAGGAGAAAGACGATCATCTAGGGTCAGACAGGCAAGAGCA

GCTAAATCCTCTGAGTGTAGTCAAGCTGCTGAGGGGGAAGGGAATCAAGAAGTACCTTCT

AGAAATCTGAGGGTCAGAGCAGATCGAAATTTGAAAACTGAAGAGGAAGAAGAAGAGGAG

GAGAGAGCAGGTAAGGATGGAGACAAGAGCTCCATGTCCCCACTACATATTTCCAATGAA

AAGAAGCTGGAATCTGGCAAAGAGATCAGCGGGTCTTCAGGGGAGCAGCAAAACAAAGAA

CTCACACTGTCACCAGCTTCACTGTTGGAAGAACCTCTGGCTCCCTCCAGCATTCATGCA

GAAAGCAGTGGAGAACATCTTGAAGAGTTGCCCCTGGAGGAAGAGAGCCCTATGTCTCAG

ATCTTTGAGCTAGAGATTGAAGCATTGCCGGTGGATACTACCCCTTCCCCTGAAGGCAGC

GATATTTCCTCATCCAGGAAGCAGACAGAGGATCCCCTCACCACTGTCTTGGAGAATGGG

GCAGCCATGGTCACCTCCACGTCCTTCAATGGAGGTGTCTCTCCCCATATTTGGGGAGGT

TCCAGTCCCCCCTGCAAGAAATTTCGGAAGGAGAGGAAGCAAAAAGGATCTGGACCATTA

GAAAACAGCTTTGTAGAAGATCAAAAGACAGTGCATGAGAAGAATGGGAAGAAAATATAT

ACCCTGTCCAGCCCACCTTCCCCCTTGGCTTCCACAGTCCCAGTTGCCGATTCCTCCACG

AGGGTGGACTCTCCCAGTCATGGCCTGGTGACCAGCTCCCTCTGTAGTCCTTCTCCAGCC

CGGTTGTCCCAAATTCCCCAATTACAACCTCCCCGGCCTGGCACTTACAAGATGAGTGTG

GCCACACAGTGTGATCCAGAGGAGATCATCGTACTCTCAGACTCTGAT

>CL65.Contig2_A-W 2 1492 minus strand PREDICTED: apoptotic chromatin condensation inducer in the nucleus [Otolemur garnettii]

GAAGCCGTCGGGACGGACCCGAGTACCTCACGCAAGATGGCGGAGCTGGAGGAGGTGACT

CTGGACGGGAAGCCTCTTCAGGCGCTGCGGGTGACCGACCTGAAGGCCGCACTAGAGCAG

CGAGGCCTAGCCAAGAGCGGGCAGAAGAGTGCCCTGGTTAAGCGGCTCAAAGGGGCTCTA

ATGCTAGAAAATTTACAGAAACATTCAACACCCCATGCTGCATTCCAGCCAAATTCCCAG

ATTGGCGAGGAAATGAGCCAGAACAGTTTCATAAAGCAGTATCTGGAAAAGCAGCAGGAG

CTACTTAGACAGCGTCTGGAACGTGAAGCTCGAGAAGCTGCAGAACTTGAAGAAGCTTCA

GCTGAGTCGGAGGATGAGATGATCCATCCTGGGGGAGTGGCTTCCCTGCTGCCTCCTGAC

TTTCAGAGCAGCCTGGAGAGACCAGAGCTGGAGCTCAGCAGACATTCACCCAGAAAAGGC

TCCTCTGTTTCTGAAGAGAAAGGTGAATCTGATGATGAGAAACCAAGAAAAGGAGAAAGA

CGATCATCTAGGGTCAGACAGGCAAGAGCAGCTAAATCCTCTGAGTGTAGTCAAGCTGCT

GAGGGGGAAGGGAATCAAGAAGTACCTTCTAGAAATCTGAGGGTCAGAGCAGATCGAAAT

TTGAAAACTGAAGAGGAAGAAGAAGAGGAGGAGAGAGCAGGTAAGGATGGAGACAAGAGC

TCCATGTCCCCACTACATATTTCCAATGAAAAGAAGCTGGAATCTGGCAAAGAGATCAGC

GGGTCTTCAGGGGAGCAGCAAAACAAAGAACTCACACTGTCACCAGCTTCACTGTTGGAA

GAACCTCTGGCTCCCTCCAGCATTCATGCAGAAAGCAGTGGAGAACATCTTGAAGAGTTG

CCCCTGGAGGAAGAGAGCCCTATGTCTCAGATCTTTGAGCTAGAGATTGAAGCATTGCCG

GTGGATACTACCCCTTCCCCTGAAGGCAGCGATATTTCCTCATCCAGGAAGCAGACAGAG

GATCCCCTCATCACTGTCTTGGAGAATGGGGCAGCCATGGTCACCTCCACGTCCTTCAAT

GGAGGTGTCTCTCCCCATATTTGGGGAGGTTCCAGTCCCCCCTGCAAGAAATTTCGGAAG

GAGAGGAAGCAAAAAGGATCTGGACCATTAGAAAACAGCTTTGTAGAAGATCAAAAGACA

GTGCATGAGAAGAATGGGAAGAAAATATATACCCTGTCCAGCCCACCTTCCCCCTTGGCT

TCCACAGTCCCAGTTGCCGATTCCTCCACGAGGGTGGACTCTCCCAGTCATGGCCTGGTG

ACCAGCTCCCTCTGTAGCCCTTCTCCAGCCCGGTTGTCCCAAATTCCCCAATTACAACCT

CCCCGGCCTGGCACTTACAAGATGAGTGTGGCCACACAGTGTGATCCAGAGGAGATCATC

GTACTCTCAGACTCTGATTAGCTGCCTCCTCCTCTCCCTGCCTCTAAAATG

>CL65.Contig3_A-W 8 745 PREDICTED: death domain-associated protein 6-like [Ailuropoda melanoleuca]

GAGGAAGAGGAAGAAGAAGAGGAGGAGAGAGCAGGTAAGGATGGAGACAAGAGCTCCATG

TCCCCACTACATATTTCCAATGAAAAGAAGCTGGAATCTGGCAAAGAGATCAGCGGGTCT

TCAGGGGAGCAGCAAAACAAAGAACTCACACTGTCACCAGCTTCACTGTTGGAAGAACCT

CTGGCTCCCTCCAGCATTCATGCAGAAAGCAGTGGAGAACATCTTGAAGAGTTGCCCCTG

GAGGAAGAGAGCCCTATGTCTCAGATCTTTGAGCTAGAGATTGAAGCATTGCCGGTGGAT

ACTACCCCTTCCCCTGAAGGCAGCGATATTTCCTCATCCAGGAAGCAGACAGAGGATCCC

CTCATCACTGTCTTGGAGAATGGGGCAGCCATGGTCACCTCCACGTCCTTCAATGGAGGT

GTCTCTCCCCATATTTGGGGAGGTTCCAGTCCCCCCTGCAAGAAATTTCGGAAGGAGAGG

AAGCAAAAAGGATCTGGACCATTAGAAAACAGCTTTGTAGAAGATCAAAAGACAGTGCAT

GAGAAGAATGGGAAGAAAATATATACCCTGTCCAGCCCACCTTCCCCCTTGGCTTCCACA

GTCCCAGTTGCCGATTCCTCCACGAGGGTGGACTCTCCCAGTCATGGCCTGGTGACCAGC

TCCCTCTGTAGCCCTTCTCCAGCCCGGTTGTCCCAAATTCCCCAATTACAACCTCCCCGG

CCTGGCACTTACAAGGTA

>CL66.Contig1_A-W 87 1022 PREDICTED: coiled-coil domain-containing protein 94-like [Loxodonta africana]

ATGTCGGAGCGCAAAGTTTTAAACAAATACTACCCTCCAGACTTCGACCCTTCAAAGATC

CCCAAACTCAAGCTCCCCAAGGACCGGCAGTACGTAGTGCGGTTGATGGCCCCGTTCAAC

ATGAGGTGTAAGACGTGCGGAGAGTACATCTACAAGGGCAAGAAGTTCAATGCTCGCAAG

GAGACGGTGCAGAACGAGGTCTACCTGGGCCTGCCCATCTTCAGGTTCTATATCAAGTGC

ACGCGCTGCCTGGCCGAGATCACTTTCAAGACAGACCCTGAAAACACCGACTACACCATG

GAGCATGGGGCCACGCGGAACTTCCAGGCCGAGAAACTCCTGGAGGAGGAGGAGAAGCGG

GTGCAGAAGGAGAGGGAGGACGAGGAGCTGAACAACCCCATGAAGGTGCTGGAGAACCGC

ACCAAGGACTCCAAGCTGGAGATGGAGGTGCTGGAGAACCTGCAGGAGCTGAAGGAGCTC

AACCAGCGCCAGGCGCACGTGGACTTCGAGGCCATGCTGCGGCAGCACCGCCTGTCGGAG

GAGGAGCGCCGGCGGCGGCAGCAGGAGGAGGACGAGCAGGAGACCGCGGCCTTGCTGGAG

GAAGCGCGGAGGCGACGGCTGCTGGAGGACTCAGACTCTGAGGAAGAGGCCGCGCCCGCC

CCGCCCCGGCCCGCGCTGCGGCCCCAGCCCACCGCCATCCTGCACGAGGCCCCGAAGGCC

AAGAGGAAGGCAGAGAGCGCGGGCAGGGCCCAGCTGTCGGGCCTGGTCGTGGTGAAGAAG

ACGAAGACGGCCGCAGCGGGGAGCACGGGGCAGGGCCCCAGCCCAGGTGCCCCGGAGAGC

AAGAAGACCACAGACCCTGCACCCCGCACGCCTGGCACCTCCTCCCTGAGCCAGCTGGGC

GCGTACGGGGACAGTGGGGACAGCGACGGCAGCAAC

>CL67.Contig1_A-W 147 2039 PREDICTED: EGF-like module-containing mucin-like hormone receptor-like 3 [Otolemur garnettii]

CTCTGCTTTCTGCTGAGCTTGTCTGGAGCTGAGGCTCAGGTTGCCAAAGGCCTTTGCAAC

TGGTGCCCCTTGCACGGATACTGCGTCAACAGCACTCACTGCACCTGCAACCCTGGATTT

ATTTCTGGATCTGGGAAGCGATTTTTCACATACCCCCTGGAGATATGTAAAGATATCGAT

GAGTGTAAACCGCCCATCAGTATATACTGTGGATTGAATGCTGAGTGTAAGAATGTCGAA

GGAAGTTTTCACTGTCGCTGCGTGCCTGGATATGAGCTCCTCTCCGGAGATGTGCAATTC

AACAATTCCAATGAGAACACTTGTCAGAATATCACCTCCTCAAAGACACCCAACGGCCAC

ACAGAGCTCCAACAGATTGTCAACAACTTTGAGTCACTTCTCACCAATCAGACTTTCTGG

GGAGCAGGAAGCAAGCAAGAAGTCGCCTCAACAGCCTCGTCTATCCTCCAGAATGTGGAA

TCCAAAGTTCTAGAAACCGCCTTGAGAGCCCCAGGACAAAAAATCCAGAAGGTCCAAAAC

AGTACTGTGGCTGTCGAAACTCGAGTGGTTTCAGACAATTGCTCCGAAGAAAGAAAGGCA

TTCCGCTTACACACCCAGATGAACTCCGTGGACATCCACTGCATCGATGTCATCCAGGGA

CGCACACAAGGTCCCAGTGCGGTTGCTTTCATCTCCTATGCTTCTCTGGGAAACATCCTC

AATGCGACTTTTTTTGAAAAGGCCGACGAGAAGGACCCGCTCTACCTGAACTCCCAGGTG

GTGAGTGCCGCGGTGGCACCCAGAAGGAACTCGTCTCTCTCCCAGTCTGTCACTCTGACC

TTCCAGAACCTGAAGATGAAACCCAGCAGCCGAGAGGCCTTCTGCGTCTACTGGAAGATC

ACAGAGGCGGGTGGCCACTGGTCCAGGGATGGCTGTTTCCTCACACAAGTGAACCAAAGT

CACACTGTCTGCAATTCCACGCACCTGTCCAGTTTTGCTGTCCTCGTGGCCTTCACCAGC

CAGGACGACGACCCCGTGCTGAGCCTCATCACCTGCGTGGGGCTGGGCGTGTCGCTGCTG

TGCCTGGTCCTGGCGGCCCTCACCTTCCTCCTGTGCAGAGCCATCCAGAACACCAGCACC

TCCCTGCACCTGCAGCTCTCGCTCTGCCTCCTGCTGGCCCACCTGCTCTTCCTCACGGCC

ATCAACAGAACCCAGCCCAAGGTGCTGTGCGCGGTCACCGCGGGCGCCCTGCACTACCTC

TACCTGGCCTCCTTCACCTGGATGCTGCTGGAGGGGCTGTTCCTCTTCCTCACGGCGCGG

AACCTGACTGTGCTCAGTTCCTCCAGCAGAAACAGGCTCATGAGGAGGCTCATGTTCCCT

GTGGGCTACGGAGTCCCAGCCGCCATCGTGGCCGTGTCTGCAGGAGCCAGACCTCACCTG

TATGGCACCCCTGCTCGGTGCTGGCTCAATCCAGAACAGGGGTTTGTGTGGGGCTTCCTT

GGACCCGCCTATGCCATCTCTTGTGTCAATTTGGTTTTCTTCCTGATGACCCTCTGGATT

GTGAAGACCAAGCTGTCCTCCATCAACAGAGATGTGTCCACCCTCCAGAACACGAGGATG

CTGACATTTAAAGCAATGGCTCATCTCTTCATCTTGGGCTGCACCTGGTGTCTGGGTGTC

CTGCAGGTGGGCCCCATGGCCCCCATCATAGCCTACCTCTTCACCATTGTCAACAGCCTG

CAGGGCGTCTTCATCTTCCTGGTGTACTGCCTCCTCAGCCAACAGGTCCGGGAGCAGTAC

AGGACATGGCTCAAGAAGATCTGGAAATGGACAACTGAACCCGAGGCCTACACTCTGTCT

AGCAAGGCTGTGTCCAACACCTCCTGACCCAGC

>CL67.Contig2_A-W 147 2015 PREDICTED: EGF-like module-containing mucin-like hormone receptor-like 3 [Otolemur garnettii]

CTCTGCTTTCTGCTGAGCTTGTCTGGAGCTGAGGCTCAGGTTGCCAAAGGCCTTTGCAAC

TGGTGCCCCTTGCACGGATACTGCGTCAACAGCACTCACTGCACCTGCAACCCTGGATTT

ATTTCTGGATCTGGGAAGCGATTTTTCACATACCCCCTGGAGATATGTAAAGATATCGAT

GAGTGTAAACCGCCCATCAGTATATACTGTGGATTGAATGCTGAGTGTAAGAATGTCGAA

GGAAGTTTTCACTGTCGCTGCGTGCCTGGATATGAGCTCCTCTCCGGAGATGTGCAATTC

AACAATTCCAATGAGAACACTTGTCAGAATATCACCTCCTCAAAGACACCCAACGGCCAC

ACAGAGCTCCAACAGATTGTCAACAACTTTGAGTCACTTCTCACCAATCAGACTTTCTGG

GGAGCAGGAAGCAAGCAAGAAGTCGCCTCAACAGCCTCGTCTATCCTCCAGAATGTGGAA

TCCAAAGTTCTAGAAACCGCCTTGAGAGCCCCAGGACAAAAAATCCAGAAGGTCCAAAAC

AGTACTGTGGCTGTCGAAACTCGAGTGGTTTCAGACAATTGCTCCGAAGAAAGAAAGGCA

TTCCGCTTACACACCCAGATGAACTCCGTGGACATCCACTGCATCGATGTCATCCAGGGA

CGCACACAAGGTCCCAGTGCGGTTGCTTTCATCTCCTATGCTTCTCTGGGAAACATCCTC

AATGCGACTTTTTTTGAAAAGGCCGACGAGAAGGACCCGCTCTACCTGAACTCCCAGGTG

GTGAGTGCCGCGGTGGCACCCAGAAGGAACTCGTCTCTCTCCCAGTCTGTCACTCTGACC

TTCCAGAACCTGAAGATGAAACCCAGCAGCCGAGAGGCCTTCTGCGTCTACTGGAAGATC

ACAGAGGCGGGTGGCCACTGGTCCAGGGATGGCTGTTTCCTCACACAAGTGAACCAAAGT

CACACTGTCTGCAATTCCACGCACCTGTCCAGTTTTGCTGTCCTCGTGGCCTTCACCAGC

CAGGACGACGACCCCGTGCTGAGCCTCATCACCTGCGTGGGGCTGGGCGTGTCGCTGCTG

TGCCTGGTCCTGGCGGCCCTCACCTTCCTCCTGTGCAGAGCCATCCAGAACACCAGCACC

TCCCTGCACCTGCAGCTCTCGCTCTGCCTCCTGCTGGCCCACCTGCTCTTCCTCACGGCC

ATCAACAGAACCCAGCCCAAGGTGCTGTGCGCGGTCACCGCGGGCGCCCTGCACTACCTC

TACCTGGCCTCCTTCACCTGGATGCTGCTGGAGGGGCTGTTCCTCTTCCTCACGGCGCGG

AACCTGACTGTGCTCAGTTCCTCCAGCAGAAACAGGCTCATGAGGAGGCTCATGTTCCCT

GTGGGCTACGGAGTCCCAGCCGCCATCGTGGCCGTGTCTGCAGGAGCCAGACCTCACCTG

TATGGCACCCCTGCTCGGTGCTGGCTCAATCCAGAACAGGGGTTTGTGTGGGGCTTCCTT

GGACCCGCCTATGCCATCTCTTGTGTCAATTTGGTTTTCTTCCTGATGACCCTCTGGATT

GTGAAGACCAAGCTGTCCTCCATCAACAGAGATGTGTCCACCCTCCAGAACACGAGGATG

CTGACATTTAAAGCAATGGCTCATCTCTTCATCTTGGGCTGCACCTGGTGTCTGGGTGTC

CTGCAGGCCCCCGTCATGGCCTACCTCTTCACCATCGTCAACAGCCTGCAGGGCATCTTC

ATCTTCCTGGTGTACTGCCTCCTCAGCCAACAGGTCCGGGAGCAGTACAGGACATGGCTC

AAGAGTATCAGGAAACAGAAAACTAAACCTGAGTCCTACACTCTGTCTAGCAAGGCTGTT

TCCGATGCC

>CL67.Contig3_A-W 147 1676 PREDICTED: EGF-like module-containing mucin-like hormone receptor-like 3 [Otolemur garnettii]

CTCTGCTTTCTGCTGAGCTTGTCTGGAGCTGAGGCTCAGGTTGCCAAAGGCCTTTGCAAC

TGGTGCCCCTTGCACGGATACTGCGTCAACAGCACTCACTGCACCTGCAACCCTGGATTT

ATTTCTGGATCTGGGAAGCGATTTTTCACATACCCCCTGGAGATATGTAAAGATATCGAT

GAGTGTAAACCGCCCATCAGTATATACTGTGGATTGAATGCTGAGTGTAAGAATGTCGAA

GGAAGTTTTCACTGTCGCTGCGTGCCTGGATATGAGCTCCTCTCCGGAGATGTGCAATTC

AACAATTCCAATGAGAACACTTGTCAGAATATCACCTCCTCAAAGACACCCAACGGCCAC

ACAGAGCTCCAACAGATTGTCAACAACTTTGAGTCACTTCTCACCAATCAGACTTTCTGG

GGAGCAGGAAGCAAGCAAGAAGTCGCCTCAACAGCCTCGTCTATCCTCCAGAATGTGGAA

TCCAAAGTTCTAGAAACCGCCTTGAGAGCCCCAGGACAAAAAATCCAGAAGGTCCAAAAC

AGTACTGTGGCTGTCGAAACTCGAGTGGTTTCAGACAATTGCTCCGAAGAAAGAAAGGCA

TTCCGCTTACACACCCAGATGAACTCCGTGGACATCCACTGCATCGATGTCATCCAGGGA

CGCACACAAGGTCCCAGTGCGGTTGCTTTCATCTCCTATGCTTCTCTGGGAAACATCCTC

AATGCGACTTTTTTTGAAAAGGCCGACGAGAAGGACCCGCTCTACCTGAACTCCCAGGTG

GTGAGTGCCGCGGTGGCACCCAGAAGGAACTCGTCTCTCTCCCAGTCTGTCACTCTGACC

TTCCAGAACCTGAAGATGAAACCCAGCAGCCGAGAGGCCTTCTGCGTCTACTGGAAGATC

ACAGAGGCGGGTGGCCACTGGTCCAGGGATGGCTGTTTCCTCACACAAGTGAACCAAAGT

CACACTGTCTGCAATTCCACGCACCTGTCCAGTTTTGCTGTCCTCGTGGCCTTCACCAGC

CAGGACGACGACCCCGTGCTGAGCCTCATCACCTGCGTGGGGCTGGGCGTGTCGCTGCTG

TGCCTGGTCCTGGCGGCCCTCACCTTCCTCCTGTGCAGAGCCATCCAGAACACCAGCACC

TCCCTGCACCTGCAGCTCTCGCTCTGCCTCCTGCTGGCCCACCTGCTCTTCCTCACGGCC

ATCAACAGAACCCAGCCCAAGGTGCTGTGCGCGGTCACCGCGGGCGCCCTGCACTACCTC

TACCTGGCCTCCTTCACCTGGATGCTGCTGGAGGGGCTGTTCCTCTTCCTCACGGCGCGG

AACCTGACTGTGCTCAGTTCCTCCAGCAGAAACAGGCTCATGAGGAGGCTCATGTTCCCT

GTGGGCTACGGAGTCCCAGCCGCCATCGTGGCCGTGTCTGCAGGAGCCAGACCTCACCTG

TATGGCACCCCTGCTCGGTGCTGGCTCAATCCAGAACAGGGGTTTGTGTGGGGCTTCCTT

GGACCCGCCTATGCCATCTCTTGTGTGAGT

>CL67.Contig4_A-W 147 1370 PREDICTED: EGF-like module-containing mucin-like hormone receptor-like 3 [Otolemur garnettii]

CTCTGCTTTCTGCTGAGCTTGTCTGGAGCTGAGGCTCAGGTTGCCAAAGGCCTTTGCAAC

TGGTGCCCCTTGCACGGATACTGCGTCAACAGCACTCACTGCACCTGCAACCCTGGATTT

ATTTCTGGATCTGGGAAGCGATTTTTCACATACCCCCTGGAGATATGTAAAGATATCGAT

GAGTGTAAACCGCCCATCAGTATATACTGTGGATTGAATGCTGAGTGTAAGAATGTCGAA

GGAAGTTTTCACTGTCGCTGCGTGCCTGGATATGAGCTCCTCTCCGGAGATGTGCAATTC

AACAATTCCAATGAGAACACTTGTCAGAATATCACCTCCTCAAAGACACCCAACGGCCAC

ACAGAGCTCCAACAGATTGTCAACAACTTTGAGTCACTTCTCACCAATCAGACTTTCTGG

GGAGCAGGAAGCAAGCAAGAAGTCGCCTCAACAGCCTCGTCTATCCTCCAGAATGTGGAA

TCCAAAGTTCTAGAAACCGCCTTGAGAGCCCCAGGACAAAAAATCCAGAAGGTCCAAAAC

AGTACTGTGGCTGTCGAAACTCGAGTGGTTTCAGACAATTGCTCCGAAGAAAGAAAGGCA

TTCCGCTTACACACCCAGATGAACTCCGTGGACATCCACTGCATCGATGTCATCCAGGGA

CGCACACAAGGTCCCAGTGCGGTTGCTTTCATCTCCTATGCTTCTCTGGGAAACATCCTC

AATGCGACTTTTTTTGAAAAGGCCGACGAGAAGGACCCGCTCTACCTGAACTCCCAGGTG

GTGAGTGCCGCGGTGGCACCCAGAAGGAACTCGTCTCTCTCCCAGTCTGTCACTCTGACC

TTCCAGAACCTGAAGATGAAACCCAGCAGCCGAGAGGCCTTCTGCGTCTACTGGAAGATC

ACAGAGGCGGGTGGCCACTGGTCCAGGGATGGCTGTTTCCTCACACAAGTGAACCAAAGT

CACACTGTCTGCAATTCCACGCACCTGTCCAGTTTTGCTGTCCTCGTGGCCTTCACCAGC

CAGGACGACGACCCCGTGCTGAGCCTCATCACCTGCGTGGGGCTGGGCGTGTCGCTGCTG

TGCCTGGTCCTGGCGGCCCTCACCTTCCTCCTGTGCAGAGCCATCCAGAACACCAGCACC

TCCCTGCACCTGCAGCTCTCGCTCTGCCTCCTGCTGGCCCACCTGCTCTTCCTCACGGCC

ATCAACAGAACCCAGCCCAAGGTA

>CL67.Contig5_A-W 1997 2122 PREDICTED: EGF-like module-containing mucin-like hormone receptor-like 3 [Otolemur garnettii]

TGTGGGTTGCCTCTGCAGGTCCGGGAGCAGTACAGGACATGGCTCAAGAAGATCTGGAAA

TGGACAACTGAACCCGAGGCCTACACTCTGTCTAGCAAGGCTGTGTCCAACACCTCCTGA

CCCAGC

>CL69.Contig1_A-W 53 319 PREDICTED: serine/threonine-protein phosphatase PP1-gamma catalytic subunit-like [Cricetulus griseus]

AAGGTGGTTGAAGATGGATATGAATTTTTTGCAAAGAGGCAGTTGGTCACTCTGTTTTCT

GCACCCAATTATTGTGGAGAGTTTGACAATGCAGGTGCCATGATGAGTGTGGATGAAACA

CTAATGTGTTCCTTTCAGATTTTAAAGCCTGCAGAGAAAAAGAAGCCGAATGCCACAAGA

CCCGTAACACCTCCAAGGGTTGGATCAGGCCTGAACCCGTCCATTCAGAAAGCTTCAAAT

TATAGAAACAATACTGTTCTATACGAG

>CL69.Contig2_A-W 202 1212 serine/threonine-protein phosphatase PP1-gamma catalytic subunit isoform 2 [Rattus norvegicus] >gi|471976|gb|AAA37526.1| protein phosphatase 1 [Mus musculus] >gi|1280028|gb|AAC53385.1| protein phosphatase 1cgamma [Mus musculus] >gi|50927621|gb|AAH78825.1| Ppp1cc protein [Rattus norvegicus] >gi|148687754|gb|EDL19701.1| mCG129661, isoform CRA_e [Mus musculus] >gi|149063383|gb|EDM13706.1| rCG21849, isoform CRA_c [Rattus norvegicus] >gi|227435|prf||1703469C protein phosphatase 1 gamma2

ATGGCGGATATAGATAAACTCAACATCGACAGCATCATCCAACGGCTGCTGGAAGTGAGA

GGGTCCAAGCCAGGTAAGAATGTCCAGCTACAGGAGAATGAAATCAGAGGACTGTGCTTG

AAGTCTCGTGAGATCTTTCTCAGTCAGCCTATCCTACTAGAACTTGAAGCACCACTCAAA

ATATGTGGTGATATCCATGGGCAGTACTATGATTTGCTTCGACTTTTTGAATACGGTGGT

TTCCCACCAGAAAGCAACTATCTGTTCCTTGGGGACTATGTGGACAGGGGGAAGCAGTCA

CTGGAGACTATCTGCCTCTTACTGGCCTACAAAATCAAATATCCTGAGAATTTTTTTCTT

CTCAGAGGAAACCATGAATGTGCCAGCATCAATAGAATTTATGGATTTTATGATGAGTGT

AAAAGAAGATACAACATTAAACTATGGAAAACTTTCACAGACTGTTTTAACTGTTTACCG

ATAGCAGCCATCGTGGATGAGAAAATATTCTGCTGTCATGGAGGTTTATCACCAGATCTT

CAATCTATGGAGCAGATTCGGCGAATTATGCGACCAACTGATGTACCAGATCAAGGTCTT

CTTTGTGATCTTTTGTGGTCTGACCCCGATAAAGATGTCTTAGGCTGGGGTGAAAATGAC

AGAGGAGTGTCCTTCACATTTGGTGCAGAAGTGGTTGCAAAATTTCTCCATAAGCATGAT

TTGGATCTTATTTGTAGAGCCCATCAGGTGGTTGAAGATGGATATGAATTTTTTGCAAAG

AGGCAGTTGGTCACTCTGTTTTCTGCACCCAATTATTGTGGAGAGTTTGACAATGCAGGT

GCCATGATGAGTGTGGATGAAACACTAATGTGTTCCTTTCAGATTTTAAAGCCTGCAGAG

AAAAAGAAGCCGAATGCCACAAGACCCGTAACACCTCCAAGGGTTGGATCAGGCCTGAAC

CCGTCCATTCAGAAAGCTTCAAATTATAGAAACAATACTGTTCTATACGAG

>CL69.Contig3_A-W 202 1170 serine/threonine-protein phosphatase PP1-gamma catalytic subunit isoform 1 [Rattus norvegicus] >gi|27807051|ref|NP_777006.1| serine/threonine-protein phosphatase PP1-gamma catalytic subunit [Bos taurus] >gi|31980772|ref|NP_038664.2| serine/threonine-protein phosphatase PP1-gamma catalytic subunit [Mus musculus] >gi|113205694|ref|NP_001038025.1| protein phosphatase 1 catalytic subunit gamma isoform [Sus scrofa] >gi|47117094|sp|P61287.1|PP1G_BOVIN RecName: Full=Serine/threonine-protein phosphatase PP1-gamma catalytic subunit; Short=PP-1G; AltName: Full=Protein phosphatase 1C catalytic subunit >gi|52001084|sp|P63088.1|PP1G_RAT RecName: Full=Serine/threonine-protein phosphatase PP1-gamma catalytic subunit; Short=PP-1G; AltName: Full=Protein phosphatase 1C catalytic subunit >gi|52001085|sp|P63087.1|PP1G_MOUSE RecName: Full=Serine/threonine-protein phosphatase PP1-gamma catalytic subunit; Short=PP-1G; AltName: Full=Protein phosphatase 1C catalytic subunit >gi|220871|dbj|BAA14196.1| protein phosphatase 1, catalytic subunit [Rattus norvegicus] >gi|14714914|gb|AAH10613.1| Protein phosphatase 1, catalytic subunit, gamma isoform [Mus musculus] >gi|18203764|gb|AAH21646.1| Protein phosphatase 1, catalytic subunit, gamma isoform [Mus musculus] >gi|18477497|emb|CAD22157.1| protein phosphatase 1C catalytic subunit [Bos taurus] >gi|55250869|gb|AAH85496.1| Protein phosphatase 1, catalytic subunit, gamma isoform [Mus musculus] >gi|87621736|gb|ABD38709.1| protein phosphatase 1 catalytic subunit gamma isoform [Sus scrofa] >gi|115305252|gb|AAI23503.1| Protein phosphatase 1, catalytic subunit, gamma isoform [Bos taurus] >gi|148685233|gb|EDL17180.1| mCG126872 [Mus musculus] >gi|148687750|gb|EDL19697.1| mCG129661, isoform CRA_a [Mus musculus] >gi|149063381|gb|EDM13704.1| rCG21849, isoform CRA_a [Rattus norvegicus] >gi|296478454|gb|DAA20569.1| serine/threonine-protein phosphatase PP1-gamma catalytic subunit [Bos taurus] >gi|227434|prf||1703469B protein phosphatase 1 gamma1

ATGGCGGATATAGATAAACTCAACATCGACAGCATCATCCAACGGCTGCTGGAAGTGAGA

GGGTCCAAGCCAGGTAAGAATGTCCAGCTACAGGAGAATGAAATCAGAGGACTGTGCTTG

AAGTCTCGTGAGATCTTTCTCAGTCAGCCTATCCTACTAGAACTTGAAGCACCACTCAAA

ATATGTGGTGATATCCATGGGCAGTACTATGATTTGCTTCGACTTTTTGAATACGGTGGT

TTCCCACCAGAAAGCAACTATCTGTTCCTTGGGGACTATGTGGACAGGGGGAAGCAGTCA

CTGGAGACTATCTGCCTCTTACTGGCCTACAAAATCAAATATCCTGAGAATTTTTTTCTT

CTCAGAGGAAACCATGAATGTGCCAGCATCAATAGAATTTATGGATTTTATGATGAGTGT

AAAAGAAGATACAACATTAAACTATGGAAAACTTTCACAGACTGTTTTAACTGTTTACCG

ATAGCAGCCATCGTGGATGAGAAAATATTCTGCTGTCATGGAGGTTTATCACCAGATCTT

CAATCTATGGAGCAGATTCGGCGAATTATGCGACCAACTGATGTACCAGATCAAGGTCTT

CTTTGTGATCTTTTGTGGTCTGACCCCGATAAAGATGTCTTAGGCTGGGGTGAAAATGAC

AGAGGAGTGTCCTTCACATTTGGTGCAGAAGTGGTTGCAAAATTTCTCCATAAGCATGAT

TTGGATCTTATTTGTAGAGCCCATCAGGTGGTTGAAGATGGATATGAATTTTTTGCAAAG

AGGCAGTTGGTCACTCTGTTTTCTGCACCCAATTATTGTGGAGAGTTTGACAATGCAGGT

GCCATGATGAGTGTGGATGAAACACTAATGTGTTCCTTTCAGATTTTAAAGCCTGCAGAG

AAAAAGAAGCCGAATGCCACAAGACCCGTAACACCTCCAAGGGGTATGATCACAAAGCAA

GCAAAGAAA

>CL72.Contig1_A-W 19 897 PREDICTED: C-type lectin domain family 4 member G isoform 1 [Pongo abelii]

ATGGACAATGCTGGATACAGCAAGTGGGGCGGCGGGTCCCAGGACCTCCCTGGAGGGCAC

TGGGGACGCTGGGAGCACTGCAAGCAACGATTTTTCTTCCTGGCCCTGGTTCTCCTGGTG

GCCACAATCCTATGGGCTTTCATTCTGAGCATTCTGTTGTCCAAGGCCTCTGTGGAGCGC

CGGGCGCTGGTGGGCCAACAGGACCTGCTGGAGACGAACGCCTCGGAACAGACAGTGGTG

CTGGGTGCCTTGAAGGAGGAGGTCGGAGCCTGCAACAGCTGCTGCTTGGGGACCAAGGCG

AAACTGCAGACCACTCTCACTGAGCTTGGGGAGACACAGGCAAAAGTGCTACAGCAGGAG

AGCGCCCTGAAAGAACTGCAGGAGCGCGTGACCCAGGGCTTGGCTGAAGCCGGCAGGGAC

CGAGAGAACGTCCGCACTGAACTGTTCCGGGTGCTGGAGGGCATCAAGTTCCAGAACGGT

TCCTGTGAGCCATGCCCCAAGTCCTGGCTGACCTTCCAGGGCTCTTGTTACTTCTTCTCG

GAGCCACCCAGCTCGTGGGAGGCGTCGCTGCGCCTCTGTGCAAACCTAAGCGCTCACCTG

GTGATCATCGGGGGCCTGGAGGAGCAGGGCTTCCTGAGTCTGCACACTCGCGGCCTGGGC

TACTGGCTGGGCCTGAGGGCTGTGCGTCACTCTGGCAAGGTTCAGAGCTACAAGTGGATC

GATGGAGTCCCGCTCAGCTTCAGCCACTGGAACACGGGAGAGCCCAATGACTCGAGGAGG

CAAGAAGACTGTGTCATGATGCTGCACTCGGGGCTGTGGAACGACGCACAATGCAGCAGT

AAAATGGACGGCTGCATCTGTGAGAAGAGGCGCAGCTGC

>CL72.Contig2_A-W 124 783 PREDICTED: C-type lectin domain family 4 member G isoform 2 [Pongo abelii]

GCCTCGGAACAGACAGTGGTGCTGGGTGCCTTGAAGGAGGAGGTCGGAGCCTGCAACAGC

TGCTGCTTGGGGACCAAGGCGAAACTGCAGACCACTCTCACTGAGCTTGGGGAGACACAG

GCAAAAGTGCTACAGCAGGAGAGCGCCCTGAAAGAACTGCAGGAGCGCGTGACCCAGGGC

TTGGCTGAAGCCGGCAGGGACCGAGAGAACGTCCGCACTGAACTGTTCCGGGTGCTGGAG

GGCATCAAGTTCCAGAACGGTTCCTGTGAGCCATGCCCCAAGTCCTGGCTGACCTTCCAG

GGCTCTTGTTACTTCTTCTCGGAGCCACCCAGCTCGTGGGAGGCGTCGCTGCGCCTCTGT

GCAAACCTAAGCGCTCACCTGGTGATCATCGGGGGCCTGGAGGAGCAGGGCTTCCTGAGT

CTGCACACTCGCGGCCTGGGCTACTGGCTGGGCCTGAGGGCTGTGCGTCACTCTGGCAAG

GTTCAGAGCTACAAGTGGATCGATGGAGTCCCGCTCAGCTTCAGCCACTGGAACACGGGA

GAGCCCAATGACTCGAGGAGGCAAGAAGACTGTGTCATGATGCTGCACTCGGGGCTGTGG

AACGACGCACAATGCAGCAGTAAAATGGACGGCTGCATCTGTGAGAAGAGGCGCAGCTGC

>CL72.Contig3_A-W 19 897 PREDICTED: C-type lectin domain family 4 member G isoform 1 [Pongo abelii]

ATGGACAATGCTGGATACAGCAAGTGGGGCGGCGGGTCCCAGGACCTCCCTGGAGGGCAC

TGGGGACGCTGGGAGCACTGCAAGCAACGATTTTTCTTCCTGGCCCTGGTTCTCCTGGTG

GCCACAATCCTATGGGCTTTCATTCTGAGCATTCTGTTGTCCAAGGCCTCTGTGGAGCGC

CGGGCGCTGGTGGGCCAACAGGACCTGCTGGAGACGAACGCCTCGGAACAGACAGTGGTG

CTGGGTGCCTTGAAGGAGGAGGTCGGAGCCTGCAACAGCTGCTGCTTGGGGACCAAGGCG

AAACTGCAGACCACTCTCACTGAGCTTGGGGAGACACAGGCAAAAGTGCTACAGCAGGAG

AGCGCCCTGAAAGAACTGCAGGAGCGCGTGACCCAGGGCTTGGCTGAAGCCGGCAGGGAC

CGAGAGAACGTCCGCACTGAACTGTTCCGGGTGCTGGAGGGCATCAAGTTCCAGAACGGT

TCCTGTGAGCCATGCCCCAAGTCCTGGCTGACCTTCCAGGGCTCTTGTTACTTCTTCTCG

GAGCCACCCAGCTCGTGGGAGGCGTCGCTGCGCCTCTGTGCAAACCTAAGCGCTCACCTG

GTGATCATCGGGGGCCTGGAGGAGCAGGGCTTCCTGAGTCTGCACACTCGCGGCCTGGGC

TACTGGCTGGGCCTGAGGGCTGTGCGTCACTCTGGCAAGGTTCAGAGCTACAAGTGGATC

GATGGAGTCCCGCTCAGCTTCAGCCACTGGAACACGGGAGAGCCCAATGACTCGAGGAGG

CAAGAAGACTGTGTCATGATGCTGCACTCGGGGCTGTGGAACGACGCACAATGCAGCAGT

AAAATGGACGGCTGCATCTGTGAGAAGAGGCGCAGCTGC

>CL72.Contig4_A-W 124 783 PREDICTED: C-type lectin domain family 4 member G isoform 2 [Pongo abelii]

GCCTCGGAACAGACAGTGGTGCTGGGTGCCTTGAAGGAGGAGGTCGGAGCCTGCAACAGC

TGCTGCTTGGGGACCAAGGCGAAACTGCAGACCACTCTCACTGAGCTTGGGGAGACACAG

GCAAAAGTGCTACAGCAGGAGAGCGCCCTGAAAGAACTGCAGGAGCGCGTGACCCAGGGC

TTGGCTGAAGCCGGCAGGGACCGAGAGAACGTCCGCACTGAACTGTTCCGGGTGCTGGAG

GGCATCAAGTTCCAGAACGGTTCCTGTGAGCCATGCCCCAAGTCCTGGCTGACCTTCCAG

GGCTCTTGTTACTTCTTCTCGGAGCCACCCAGCTCGTGGGAGGCGTCGCTGCGCCTCTGT

GCAAACCTAAGCGCTCACCTGGTGATCATCGGGGGCCTGGAGGAGCAGGGCTTCCTGAGT

CTGCACACTCGCGGCCTGGGCTACTGGCTGGGCCTGAGGGCTGTGCGTCACTCTGGCAAG

GTTCAGAGCTACAAGTGGATCGATGGAGTCCCGCTCAGCTTCAGCCACTGGAACACGGGA

GAGCCCAATGACTCGAGGAGGCAAGAAGACTGTGTCATGATGCTGCACTCGGGGCTGTGG

AACGACGCACAATGCAGCAGTAAAATGGACGGCTGCATCTGTGAGAAGAGGCGCAGCTGC

>CL73.Contig1_A-W 5 436 minus strand PREDICTED: probable peptide chain release factor C12orf65 homolog, mitochondrial isoform 1 [Papio anubis] >gi|402888032|ref|XP_003907381.1| PREDICTED: probable peptide chain release factor C12orf65 homolog, mitochondrial isoform 2 [Papio anubis] >gi|402888034|ref|XP_003907382.1| PREDICTED: probable peptide chain release factor C12orf65 homolog, mitochondrial isoform 3 [Papio anubis] >gi|402888036|ref|XP_003907383.1| PREDICTED: probable peptide chain release factor C12orf65 homolog, mitochondrial isoform 4 [Papio anubis]

TCAGGTTTATTCCGATTTCCTGCACCACTGACCAGACTATGCAGAGTGCCTCGGGGACTT

GGGCTTTGGGAGAAGCTGACGCTGCTATCCCCAGGAATAGCTGCCACGGTTGTCCAGATG

GCAGGCAAGAAGGACTGTCCTGCTCTGCTTCCCCTGGATGAGAATGAACTTGAAGAGCAG

TTTGTGAAAGGACACGGTCCAGGGGGCCAAGCCACCAACAAAACCAGCAACTGTGTGGTG

CTGAAGCATATCCCCTCTGGCATCGTCGTCAAGTGCCATCAGACAAGATCTGTTGATCAC

AACAGAAAGCTCGCTCGGAAAATCCTACAAGAGAAAGTGGATGTTTTCTACAATGGTGAA

AATAGTCCTGTTTACAAAGAAAAACAGGAGGCAGCAAAGAAAAAGCAAGACAGGAAAAAA

AGAGCAAAGGAA

>CL73.Contig2_A-W 61 204 minus strand PREDICTED: probable peptide chain release factor C12orf65 homolog, mitochondrial isoform 1 [Papio anubis] >gi|402888032|ref|XP_003907381.1| PREDICTED: probable peptide chain release factor C12orf65 homolog, mitochondrial isoform 2 [Papio anubis] >gi|402888034|ref|XP_003907382.1| PREDICTED: probable peptide chain release factor C12orf65 homolog, mitochondrial isoform 3 [Papio anubis] >gi|402888036|ref|XP_003907383.1| PREDICTED: probable peptide chain release factor C12orf65 homolog, mitochondrial isoform 4 [Papio anubis]

CAGTGCCATCAGACAAGATCTGTTGATCACAACAGAAAGCTCGCTCGGAAAATCCTACAA

GAGAAAGTGGATGTTTTCTACAATGGTGAAAATAGTCCTGTTTACAAAGAAAAACAGGAG

GCAGCAAAGAAAAAGCAAGACAGG

>CL74.Contig1_A-W 136 1482 minus strand PREDICTED: peroxisomal acyl-coenzyme A oxidase 2 [Saimiri boliviensis boliviensis]

ATGGGTAGCCCGGTGCACCGCGTGTCCTTGGGAGACAAGTGGAGCAGGCAAGAGCACCCT

GACATAAAAAGTGAGAGGCGTGTGCCGTCCTTCAACGTGGAACGACTCACCAACATCCTT

GACAGAGGTGCCCAGAACACTGCGATCCGGAGGAAAGTCGAAAGCATCATCCACAATGAC

CCAGTGTTTAGCCTTAAGAACAATCATTTCATGACCCAGAATGAGCATTATGAGACCGCC

CTGCGGAAGAGATACCACATACAGATGATAGCACAGCGCCTGGGCTGGCCGGCAGATGGT

TGCGAATTGGCCTATGCTTTCAGGGCCCTTTCTGGAGATGTGGCCTTAACTTTAAATGGT

GTCTTCATGAATGCGCTCAGGAGCCTTGGCTCAGAGGAACAGATTGCTAAATGGATACCA

CTCTGTGAAAACTTCCAGATCATCACAACGTATGCCCAGACAGAGCTGGGACACGGGTCA

TATCTTCAAGGCCTGGAGACTGAAGCCACCTATGATGCAGCCACCCAGGAGTTTGTGATA

CACAGCCCCACCATGACTGCCGCCAAGTGGTGGCCTGGGGACATGGGCCGGTCGGCCACC

CATGCCCTGGTCTTGGCCCAGCTGATCTGTTCGGGAGCCAGGCAGGGAATGCACGCTTTT

ATCGTGCCCATCCGGAGCCTCCAGGACCACACCCCACTGCCAGGAATCACTGTTGGGGAT

ATTGGGCCCAAGATGGACTTGGAAAATGTAGACAATGGCTTCCTGCTTCTAGACCACGTG

CGGGTCCCCAGGGAGAACATGCTGAGTCGCTTTGCACAGGTCTTGCCAGATGGCACCTAC

GTCAAGTTTGGGACGGCACAGAGCAACTATCTTGGCATGGTGGTGACGCGAGTGCGCTTG

CTGTTGACTACCATCCTACCCTTACTGCAGAAGGCTTGTGTCATCGCTGTGCGCTACTCA

GTCGTCCGCCGCCAATCCCGGCTCCAGCCCAGTGGCCCAGAGGCAAAAATTCTGGACTAC

CAGACACAGCAGCAGAAACTCCTTCCTCAGCTGGCCACGAGTTACGCCTTCCACTTCACA

GCGGTCAGCCTCTTGGAATTCTTCCACCGTTCCTACGATGAGATTCTGCGCAAAGATTTC

AGCCTCCTTCCTGAGCTCCACGCACTGAGCACAGGCATGAAGGCCATGATGTCTGAATTG

TGTACCCAGGGGGCCGAGCTGTGTCGCAGGGCCTGCGGGGGACACGGCTACTCAAAACTG

AGTGGTTTGCCATCATTGGTCACCCAAGTGACAGCCTCCTGCACCTATGAGGGTGAGAAT

ACAGTGCTCTACCTGCAAGTGGCCAGG

>CL74.Contig2_A-W 136 1320 minus strand PREDICTED: peroxisomal acyl-coenzyme A oxidase 2 [Saimiri boliviensis boliviensis]

ATGGGTAGCCCGGTGCACCGCGTGTCCTTGGGAGACAAGTGGAGCAGGCAAGAGCACCCT

GACATAAAAAGTGAGAGGCGTGTGCCGTCCTTCAACGTGGAACGACTCACCAACATCCTT

GACAGAGGTGCCCAGAACACTGCGATCCGGAGGAAAGTCGAAAGCATCATCCACAATGAC

CCAGTGTTTAGCCTTAAGAACAATCATTTCATGACCCAGAATGAGCATTATGAGACCGCC

CTGCGGAAGAGATACCACATACAGATGATAGCACAGCGCCTGGGCTGGCCGGCAGATGGT

TGCGAATTGGCCTATGCTTTCAGGGCCCTTTCTGGAGATGTGGCCTTAACTTTAAATGGT

GTCTTCATGAATGCGCTCAGGAGCCTTGGCTCAGAGGAACAGATTGCTAAATGGATACCA

CTCTGTGAAAACTTCCAGATCATCACAACGTATGCCCAGACAGAGCTGGGACACGGGTCA

TATCTTCAAGGCCTGGAGACTGAAGCCACCTATGATGCAGCCACCCAGGAGTTTGTGATA

CACAGCCCCACCATGACTGCCGCCAAGTGGTGGCCTGGGGACATGGGCCGGTCGGCCACC

CATGCCCTGGTCTTGGCCCAGCTGATCTGTTCGGGAGCCAGGCAGGGAATGCACGCTTTT

ATCGTGCCCATCCGGAGCCTCCAGGACCACACCCCACTGCCAGGAATCACTGTTGGGGAT

ATTGGGCCCAAGATGGACTTGGAAAATGTAGACAATGGCTTCCTGCTTCTAGACCACGTG

CGGGTCCCCAGGGAGAACATGCTGAGTCGCTTTGCACAGGTCTTGCCAGATGGCACCTAC

GTCAAGTTTGGGACGGCACAGAGCAACTATCTTGGCATGGTGGTGACGCGAGTGCGCTTG

CTGTTGACTACCATCCTACCCTTACTGCAGAAGGCTTGTGTCATCGCTGTGCGCTACTCA

GTCGTCCGCCGCCAATCCCGGCTCCAGCCCAGTGGCCCAGAGGCAAAAATTCTGGACTAC

CAGACACAGCAGCAGAAACTCCTTCCTCAGCTGGCCACGAGTTACGCCTTCCACTTCACA

GCGGTCAGCCTCTTGGAATTCTTCCACCGTTCCTACGATGAGATTCTGCGCAAAGATTTC

AGCCTCCTTCCTGAGGTATCTACTGTCTGCTGTGGAGCAGCTGCT

>CL74.Contig3_A-W 64 774 minus strand PREDICTED: peroxisomal acyl-coenzyme A oxidase 2 isoform 1 [Otolemur garnettii]

GGGTCATATCTTCAAGGCCTGGAGACTGAAGCCACCTATGATGCAGCCACCCAGGAGTTT

GTGATACACAGCCCCACCATGACTGCCGCCAAGTGGTGGCCTGGGGACATGGGCCGGTCG

GCCACCCATGCCCTGGTCTTGGCCCAGCTGATCTGTTCGGGAGCCAGGCAGGGAATGCAC

GCTTTTATCGTGCCCATCCGGAGCCTCCAGGACCACACCCCACTGCCAGGAATCACTGTT

GGGGATATTGGGCCCAAGATGGACTTGGAAAATGTAGACAATGGCTTCCTGCTTCTAGAC

CACGTGCGGGTCCCCAGGGAGAACATGCTGAGTCGCTTTGCACAGGTCTTGCCAGATGGC

ACCTACGTCAAGTTTGGGACGGCACAGAGCAACTATCTTGGCATGGTGGTGACGCGAGTG

CGCTTGCTGTTGACTACCATCCTACCCTTACTGCAGAAGGCTTGTGTCATCGCTGTGCGC

TACTCAGTCGTCCGCCGCCAATCCCGGCTCCAGCCCAGTGGCCCAGAGGCAAAAATTCTG

GACTACCAGACACAGCAGCAGAAACTCCTTCCTCAGCTGGCCACGAGTTACGCCTTCCAC

TTCACAGCGGTCAGCCTCTTGGAATTCTTCCACCGTTCCTACGATGAGATTCTGCGCAAA

GATTTCAGCCTCCTTCCTGAGGTATCTACTGTCTGCTGTGGAGCAGCTGCT

>CL74.Contig4_A-W 136 2118 minus strand PREDICTED: peroxisomal acyl-coenzyme A oxidase 2 [Saimiri boliviensis boliviensis]

ATGGGTAGCCCGGTGCACCGCGTGTCCTTGGGAGACAAGTGGAGCAGGCAAGAGCACCCT

GACATAAAAAGTGAGAGGCGTGTGCCGTCCTTCAACGTGGAACGACTCACCAACATCCTT

GACAGAGGTGCCCAGAACACTGCGATCCGGAGGAAAGTCGAAAGCATCATCCACAATGAC

CCAGTGTTTAGCCTTAAGAACAATCATTTCATGACCCAGAATGAGCATTATGAGACCGCC

CTGCGGAAGAGATACCACATACAGATGATAGCACAGCGCCTGGGCTGGCCGGCAGATGGT

TGCGAATTGGCCTATGCTTTCAGGGCCCTTTCTGGAGATGTGGCCTTAACTTTAAATGGT

GTCTTCATGAATGCGCTCAGGAGCCTTGGCTCAGAGGAACAGATTGCTAAATGGATACCA

CTCTGTGAAAACTTCCAGATCATCACAACGTATGCCCAGACAGAGCTGGGACACGGGTCA

TATCTTCAAGGCCTGGAGACTGAAGCCACCTATGATGCAGCCACCCAGGAGTTTGTGATA

CACAGCCCCACCATGACTGCCGCCAAGTGGTGGCCTGGGGACATGGGCCGGTCGGCCACC

CATGCCCTGGTCTTGGCCCAGCTGATCTGTTCGGGAGCCAGGCAGGGAATGCACGCTTTT

ATCGTGCCCATCCGGAGCCTCCAGGACCACACCCCACTGCCAGGAATCACTGTTGGGGAT

ATTGGGCCCAAGATGGACTTGGAAAATGTAGACAATGGCTTCCTGCTTCTAGACCACGTG

CGGGTCCCCAGGGAGAACATGCTGAGTCGCTTTGCACAGGTCTTGCCAGATGGCACCTAC

GTCAAGTTTGGGACGGCACAGAGCAACTATCTTGGCATGGTGGTGACGCGAGTGCGCTTG

CTGTTGACTACCATCCTACCCTTACTGCAGAAGGCTTGTGTCATCGCTGTGCGCTACTCA

GTCGTCCGCCGCCAATCCCGGCTCCAGCCCAGTGGCCCAGAGGCAAAAATTCTGGACTAC

CAGACACAGCAGCAGAAACTCCTTCCTCAGCTGGCCACGAGTTACGCCTTCCACTTCACA

GCGGTCAGCCTCTTGGAATTCTTCCACCGTTCCTACGATGAGATTCTGCGCAAAGATTTC

AGCCTCCTTCCTGAGCTCCACGCACTGAGCACAGGCATGAAGGCCATGATGTCTGAATTG

TGTACCCAGGGGGCCGAGCTGTGTCGCAGGGCCTGCGGGGGACACGGCTACTCAAAACTG

AGTGGTTTGCCATCATTGGTCACCCAAGTGACAGCCTCCTGCACCTATGAGGGTGAGAAT

ACAGTGCTCTACCTGCAAGTGGCCAGGTTTCTGGTAAAGAGCTACCTGCAAGCCAAGGTG

TCCCCAGACTCTGCACCACAGAAGCCTCTACCTCAGTCTGTTGAATATTTTGCCAGGCCT

GTCCTGGCCCGTTGCCCAGCCCAGAAGGCAGCCGACTTCCTCTGCCCAGAGTTATACACT

GCAGCCTGGGCACACACGGCCGCCAGGCTCATAGAGGACTCGGTGCATCATCTACAGACC

CTCACACAATCTGGGGCTGACCAGCAGGAGGCTTGGAACCAGACTTCTGTCATGCACCTC

CAGGCTGCTAAGGCACACTGCTACTATATCACTGTGAAGAGTTTCATGGACGCTCTGGAG

AAACTAGACAAGGAACCAGCAATTCAGCAAGTGCTCCAACGCCTCTGTGATCTCTTTGCT

TTACACGGTATCTTGACAAACTCAGGTGACTTTCTCCACGATGGCTTTCTGTCTGGGGCC

CAAGTAGACATGGCAAGAAAAGCTTACCTGGACCTACTCCTCCTGATCAGGAAGGATGCT

ATCTTGTTAACTGATGCATTTGACTTCACCGACCAGTGTTTGAATTCAGCACTTGGCTGT

TATGATGGAAATGTCTATGGACGTCTGTTCCAGTGGGCTCGGGAGTCACCAAGCAATGCT

CAG

>CL75.Contig1_A-W 1 630 minus strand Rab geranylgeranyltransferase, alpha subunit, isoform CRA_b [Homo sapiens]

CCTAAGTCCTATGGCACCTGGCACCACCGCTGCTGGCTGCTAGGCCGCCTGCCTGAGCCC

AACTGGGCCCGGGAACTGGAATTATGTGCCCGCTTTCTTGAAGTCGATGAACGGAACTTT

CACTGCTGGGATTATCGGCGGTTTGTGGCTGCACAGGCAGCTGTGCCCCCTGCAGAGGAG

CTAGCCTTCACTGACAGCCTCATCACCCGAAACTTCTCCAACTACTCCTCCTGGCATTAC

CGTTCTTGCCTCTTGCCCCAGCTGCACCCCCAGCCAGACTCTGGCCCACGGGGGCGCCTC

CCTGAAGATGTGCTGCTCAAAGAGCTGGAGCTGGTGCAGAATGCCTTCTTCACTGACCCC

AATGACCAGAGTGCTTGGTTCTATCACCGCTGGCTTCTGGGCCGAGCTGACCCCCAGGAT

GCGCTGCACTGCCTGCATGTGAGCCGAGATGAGGCCTGCCTGACTGTGTCCTTCTCTCGG

CCCCTCCTAGTAGGCTCCAGTACAGAGACCTTGCTACTCATGGTTGATGAGTCACCCCTG

ATTGTGGAGTGGAGGACCCCAGATGGCAGAAACCGACCCAGCCACGTCTGGGTATCCCAG

GATTGGTGGGGAAGAAATGAGGACTTGGGA

>CL75.Contig2_A-W 3 1562 PREDICTED: geranylgeranyl transferase type-2 subunit alpha [Papio anubis]

GTACTGGAACTGACAAACCAGATTCTGGGAGCCAATCCTGACTTTGCTACCCTCTGGAAT

TGTCGACGAGAGGTGTTCCAGCAGCTGGAGACCCAGAAGTCCCCCGAGGAGTTGGCTGCT

CTGGTGAAGGCAGAACTGGGCTTCCTGGAGAGCTGCCTACGTGTGAACCCTAAGTCCTAT

GGCACCTGGCACCACCGCTGCTGGCTGCTAGGCCGCCTGCCTGAGCCCAACTGGGCCCGG

GAACTGGAATTATGTGCCCGCTTTCTTGAAGTCGATGAACGGAACTTTCACTGCTGGGAT

TATCGGCGGTTTGTGGCTGCACAGGCAGCTGTGCCCCCTGCAGAGGAGCTAGCCTTCACT

GACAGCCTCATCACCCGAAACTTCTCCAACTACTCCTCCTGGCATTACCGATCTTGCCTC

TTGCCCCAGCTGCACCCCCAGCCAGACTCTGGCCCACGGGGGCGCCTCCCTGAAGATGTG

CTGCTCAAAGAGCTGGAGCTGGTGCAGAATGCCTTCTTCACTGACCCCAATGACCAGAGT

GCTTGGTTCTATCACCGCTGGCTTCTGGGCCGAGCTGACCCCCAGGATGCGCTGCACTGC

CTGCATGTGAGCCGAGATGAGGCCTGCCTGACTGTGTCCTTCTCTCGGCCCCTCCTAGTA

GGCTCCAGTACAGAGACCTTGCTACTCATGGTTGATGAGTCACCCCTGATTGTGGAGTGG

AGGACCCCAGATGGCAGAAACCGACCCAGCCACGTCTGGCTCTGTGACCTGCCCACTGCC

TCCCTCAATGACCAGTTGCCCCAACATACATTTCGTGTCATTTGGACAGCAGGCGATGCT

CAGAAGGAGTGTGTGCTTTTAAAAGGCCGCCAAGAGGGCTGGTACCGGGACTCAGCCACT

GATGAGCAACTGTTTAGGTGTGAGCTGTCAGTGGAGAAGTCCACAGTGCTACAATCTGAG

CTCAAATCCTGTAAGGAGCTGCAGGAGCTGGAGCCTGAGAATAAATGGTGCCTGCTTACC

ATCATTCTGCTGATGCGGGCACTGGACCCCCTCTTGTATGAGAAGGAGACACTGCAGTAC

TTCCAAACCCTCAAGGCTGTAGACCCTATGCGAGCAGCCTACCTGGACGACCTGCGCAGC

AAATTCTTGCTAGAGAACAGTGTGCTCAAGATGGAGTATGCCGATGTACGCGTGCTACAC

CTGGCTCACAAGGATCTGACAGTGCTCTGCCATCTGGAACAGCTGCTTTTGGTTACCCAT

CTTGACCTGTCACATAATCGTCTCCGAACCCTGCCCCCTGCCCTGGCTGCTCTGCGTTGC

CTTGAGGTGCTGCAGGCAAACGATAACGCCATAGAGTCCCTGGATGGCATTACCAACTTG

CCCCGGCTGCAGGAACTTTTACTGTGCAACAACCGCCTCCAGCAACCTGCAGCACTCCAG

CCTCTTGCCTCCTGCCCCAGGCTAGTCCTTCTCAACTTGGAGGGCAACCCACTATGCGAA

GAGGGGGGTGTCTTGGAGCACCTGGCTGAATTGCTGCCTTCAGTTAGCAGCATCCTCACC

>CL75.Contig3_A-W 107 1897 PREDICTED: geranylgeranyl transferase type-2 subunit alpha [Papio anubis]

ATGCACGGGCGCCTGAAGGTGAAGACGTCAGAAGAGCAGGCGGAGGCCAAAAGGCTAGAG

AGGGAGCAAAAGCTGAAACTCTACCAGTCAGCCACCCAAGCTGTCTTCCAGAAGCGCCAG

GCTGGAGAGCTAGATGAGTCAGTACTGGAACTGACAAGCCAGATTCTGGGAGCCAATCCT

GACTTTGCTACCCTCTGGAATTGTCGACGAGAGGTGTTCCAGCAGCTGGAGACCCAGAAG

TCCCCCGAGGAGTTGGCTGCTCTGGTGAAGGCAGAACTGGGCTTCCTGGAGAGCTGCCTA

CGTGTGAACCCTAAGTCCTATGGCACCTGGCACCACCGCTGCTGGCTGCTAGGCCGCCTG

CCTGAGCCCAACTGGGCCCGGGAACTGGAATTATGTGCCCGCTTTCTTGAAGTCGATGAA

CGGAACTTTCACTGCTGGGATTATCGGCGGTTTGTGGCTGCACAGGCAGCTGTGCCCCCT

GCAGAGGAGCTAGCCTTCACTGACAGCCTCATCACCCGAAACTTCTCCAACTACTCCTCC

TGGCATTACCGATCTTGCCTCTTGCCCCAGCTGCACCCCCAGCCAGACTCTGGCCCACGG

GGGCGCCTCCCTGAAGATGTGCTGCTCAAAGAGCTGGAGCTGGTGCAGAATGCCTTCTTC

ACTGACCCCAATGACCAGAGTGCTTGGTTCTATCACCGCTGGCTTCTGGGCCGAGCTGAC

CCCCAGGATGCGCTGCACTGCCTGCATGTGAGCCGAGATGAGGCCTGCCTGACTGTGTCC

TTCTCTCGGCCCCTCCTAGTAGGCTCCAGTACAGAGACCTTGCTACTCATGGTTGATGAG

TCACCCCTGATTGTGGAGTGGAGGACCCCAGATGGCAGAAACCGACCCAGCCACGTCTGG

CTCTGTGACCTGCCCACTGCCTCCCTCAATGACCAGTTGCCCCAACATACATTTCGTGTC

ATTTGGACAGCAGGCGATGCTCAGAAGGAGTGTGTGCTTTTAAAAGGTAACGCAACCTGC

ACCCCTGCCCCTTTCAGCAGTCCTCACCCCTCTCCCCCTCTCATGGGTATCTTCTGCTAA

CTCCCCTTTCCCTCAGGCCGCCAAGAGGGCTGGTACCGGGACTCAGCCACTGATGAGCAA

CTGTTTAGGTGTGAGCTGTCAGTGGAGAAGTCCACAGTGCTACAATCTGAGCTCAAATCC

TGTAAGGAGCTGCAGGAGCTGGAGCCTGAGAATAAATGGTGCCTGCTTACCATCATTCTG

CTGATGCGGGCACTGGACCCCCTCTTGTATGAGAAGGAGACACTGCAGTACTTCCAAACC

CTCAAGGCTGTAGACCCTATGCGAGCAGCCTACCTGGACGACCTGCGCAGCAAATTCTTG

CTAGAGAACAGTGTGCTCAAGATGGAGTATGCCGATGTACGCGTGCTACACCTGGCTCAC

AAGGATCTGACAGTGCTCTGCCATCTGGAACAGCTGCTTTTGGTTACCCATCTTGACCTG

TCACATAATCGTCTCCGAACCCTGCCCCCTGCCCTGGCTGTTCTGCGTTGCCTTGAGGTG

CTGCAGGCAAACGATAACGCCATAGAGTCCCTGGATGGCATTACCAACTTGCCCCGGCTG

CAGGAACTTTTACTGTGCAACAACCGCCTCCAGCAACCTGCAGCACTCCAGCCTCTTGCC

TCCTGCCCCAGGCTAGTCCTTCTCAACTTGGAGGGCAACCCACTATGCGAAGAGGGGGGT

GTCTTGGAGCACCTGGCTGAATTGCTGCCTTCAGTTAGCAGCATCCTCACC

>CL75.Contig4_A-W 3 248 Rab geranylgeranyltransferase, alpha subunit, isoform CRA_b [Homo sapiens]

GGAAGAAATGAGGACTTGGGAGACAGCTGGGTGGGATAATCTGAAAAGTGGTAAGGCTAG

GGCATTTCCTTGACCCAATGCTCCCAGCTCTGTGACCTGCCCACTGCCTCCCTCAATGAC

CAGTTGCCCCAACATACATTTCGTGTCATTTGGACAGCAGGCGATGCTCAGAAGGAGTGT

GTGCTTTTAAAAGGCCGCCAAGAGGGCTGGTACCGGGACTCAGCCACTGATGAGCAACTG

TTTAGG

>CL75.Contig5_A-W 80 352 PREDICTED: geranylgeranyl transferase type-2 subunit alpha isoform 2 [Callithrix jacchus]

ACAACTGCCCACTCAATCTTGTGCCTTCCTGCTCCCCAGGTGCTGCAGGCAAACGATAAC

GCCATAGAGTCCCTGGATGGCATTACCAACTTGCCCCGGCTGCAGGAACTTTTACTGTGC

AACAACCGCCTCCAGCAACCTGCAGCACTCCAGCCTCTTGCCTCCTGCCCCAGGCTAGTC

CTTCTCAACTTGGAGGGCAACCCACTATGCGAAGAGGGGGGTGTCTTGGAGCACCTGGCT

GAATTGCTGCCTTCAGTTAGCAGCATCCTCACC

>CL77.Contig1_A-W 505 1179 Tripartite motif-containing protein 11 [Heterocephalus glaber]

TTACAGGACATCAAGGATGCCCTGCGCAGGGTTCAGGATGTGAAGCTGCAGCCCCCGGAG

GTGGTGCCCATGGAGCTGAGGACCGTATGCAGGGTCCCGGGGCTGGTAGAGACTCTGCGG

AGGTTTCGAGGGGATGTGACCCTGGACCCGGACACCGCCAACCCTGAGCTGGTCCTATCT

GAGGACAGGAGGAGCGTGCAGCGAGGTGACCTGCGGCAGGCCCTGCCCGACAGCCCTGAG

CGCTTTGACCCTGGCCCCTGTGTGCTAGGTCAGGAGCGCTTCACCTCGGGCCGCCACTAC

TGGGAGGTGGAGGTGGGGGATCGCACCAGCTGGGCCCTGGGCGTGTGTAGAGAGAACGTC

AACCGCAAGGAGAAGGGGGAGCTGTCCGCTGGCAATGGTTTCTGGATCCTGGTCTTCCTG

GGGAGCTACTTCACCTCCACGGAGCCGGCCTTCTCTCCGCTCCGGGACCCTCCTCGGCGC

GTGGGGATCTTTCTGGACTATGAGGCTGGACACCTCTCATTCTACAGCGTGACTGATGGG

TCCCTGCTGTTCATCTTCCCGGAGACGACCTTCTCTGGGACACTCAGGCCCCTGTTCTCG

CCTCTCTCCAGCAGCCCCACCCCTATGACCATCTGCCAGCTGAAAGGCAGGCCAGGGGAC

ACCCTGGTTCCCCAG

>CL77.Contig2_A-W 111 1514 E3 ubiquitin-protein ligase TRIM11 [Homo sapiens] >gi|26400672|sp|Q96F44.2|TRI11_HUMAN RecName: Full=E3 ubiquitin-protein ligase TRIM11; AltName: Full=Protein BIA1; AltName: Full=RING finger protein 92; AltName: Full=Tripartite motif-containing protein 11 >gi|21591223|gb|AAM63957.1|AF327056_1 BIA1 protein [Homo sapiens] >gi|46621642|gb|AAH69227.1| Tripartite motif-containing 11 [Homo sapiens] >gi|55666433|emb|CAH71671.1| tripartite motif-containing 11 [Homo sapiens]

ATGGCCGCCCCTGACCTGTCCACCAACCTCCAAGAGGAGGCCACCTGCGCCATCTGCCTC

GACTACTTCACTGACCCTGTGATGACCGACTGCGGCCACAACTTCTGCCGCGAGTGCATC

CGGCGCTGCTGGGGTCAGCCCGAAGGCCCGTACGCCTGCCCTGAGTGCCGCGAGTTGTCC

CCGCAGAGGAACCTGCGGCCCAACCGCCCGCTCGCCAAGATGGCCGAGATGGCGCGGCGC

CTGCACCCGCCATCGCCCGTCCCGCAGGGCGTGTGCGCCGCGCACCGCGAGCCGCTGGCC

GCCTTCTGCAGCGACGAGCTGCGCCTGTTGTGCGCGACCTGCGAGCGTTCGGGGGAGCAT

TGGGCACACCGCGTGCGCCCGCTGCAGGACGCGGCCGACGACCTCAAAGGGAAGCTGGAG

AAGTCCCTGGAGCACCTGCGGAAGCAAATGGAAGATGCCCTGCTGTCCCAGGCCCAAGCA

GAGGAGACCTGTGCCTTGTGGCAGAAAATGGTGGAGAGCCAACGGCAGAACGTGCTGGGT

GAGTTTGAGCGCCTGCGCCGCCTGCTGGCCGAGGAGGAGCAGCAGCTGCTGCAGAAGCTG

GAGGAGGAGGAGCTGGAGGTGCTGCCCCGGCTGCATGAGGGCGCTGCAAGGCTGGGCCAG

CAGAGTGCTCAGCTGGCCACGCTCATCACAGAGCTGGAGGGCCGCTGCCAGCTGCCAGCT

CTGGGACTTCTACAGGACATCAAGGATGCCCTGCGCAGGGTTCAGGATGTGAAGCTGCAG

CCCCCGGAGGTGGTGCCCATGGAGCTGAGGACCGTATGCAGGGTCCCGGGGCTGGTAGAG

ACTCTGCGGAGGTTTCGAGGGGATGTGACCCTGGACCCGGACACCGCCAACCCTGAGCTG

GTCCTATCTGAGGACAGGAGGAGCGTGCAGCGAGGTGACCTGCGGCAGGCCCTGCCCGAC

AGCCCTGAGCGCTTTGACCCTGGCCCCTGTGTGCTAGGTCAGGAGCGCTTCACCTCGGGC

CGCCACTACTGGGAGGTGGAGGTGGGGGATCGCACCAGCTGGGCCCTGGGCGTGTGTAGA

GAGAACGTCAACCGCAAGGAGAAGGGGGAGCTGTCCGCTGGCAATGGTTTCTGGATCCTG

GTCTTCCTGGGGAGCTACTTCACCTCCACGGAGCCGGCCTTCTCTCCGCTCCGGGACCCT

CCTCGGCGCGTGGGGATCTTTCTGGACTATGAGGCTGGACACCTCTCATTCTACAGCGTG

ACTGATGGGTCCCTGCTGTTCATCTTCCCGGAGACGACCTTCTCTGGGACACTCAGGCCC

CTGTTCTCGCCTCTCTCCAGCAGCCCCACCCCTATGACCATCTGCCAGCTGAAAGGCAGG

CCAGGGGACACCCTGGTTCCCCAG

>CL78.Contig1_A-W 252 629 minus strand Killer cell immunoglobulin-like receptor 3DL1 [Heterocephalus glaber]

CCACCTAGTCCCCTTGTGGTTGGGGTCTCGGCCGCCGCCGCTGTCCTCCTGGGCCTCTGC

CTCCTCCTCCTGGGCCTCTGCCTATGCCGACACCGAGGGCAGCGTCGGGCCAGACGCAGG

GTGACTGACGGTGGGACCAAGAGCCCAGTGTGGGACGTGAGCTCCAGCCCTGCCTCTGAC

GTCCAGCGGGGAGATGCAGATGACACCGAGCACAGGGAGCATGGACAGCTGGACACACAG

GCCCCTGCGGCAGGAGGCCCACAGGAGGTGACCTATGTCCAGCTGCACCCAAGGACCCTC

ACCAGGAGTGTGGGTCCACTTCTGTCCCGAGCGCCCCAGGACGGGTCAACACAGCCCTGT

GTGTATGCCACGCTCACC

>CL78.Contig2_A-W 172 750 minus strand PREDICTED: hypothetical protein LOC100625763, partial [Sus scrofa]

CCCACCACTCCCTCTCCTCCCAGCCCAAGCCCAGGTGCCCGCTCCACACGTGTGCCTCCC

CGTGGCAGGCCCCTGCGGCAGGAGGCCCACAGGAGGTGACCTATGTCCAGCTGCACCCAA

GGACCCTCACCAGGAGTGTGGGTCCACTTCTGTCCCGAGCGCCCCAGGACGGGTCAACAC

AGCCCTGTGTGTATGCCACGCTCACCTCGCCTTGAGAGGACAGCAGGGCTGGCTGAGTGG

ACGGCAGTGCCCAGGTGCCGCTCCGTGTCCCTCAGCGCCAGCCACCAGGTGGGAGCCCTG

AGACTCGGAGAGAGAAGCTCGTGCACTGCCGGGAACCCCGGGGGCTCTGGCACAGTTCCC

GCTCCTGGCAATTCGCACCAAGTGTTTCAGAGGAGCCTGCTTGGGCTCCCGTGGGGAGGG

GCGGGTGGCCGGGCAGGCCTCCTCTAAGGGACTGGCTGCAGCTGCGGGAGGCCAGCCTGC

CCTCCAGCCCTCAGGAAGCAGACAAGTGGCCTGAGGAGGACCCTGAGTCTTCCGCAGCTG

GACGTTGTGGGTGGCGCTGCAGCACGCTGGTCACCGTGC

>CL78.Contig3_A-W 102 770 minus strand Killer cell immunoglobulin-like receptor 3DL1 [Heterocephalus glaber]

ATGGCCACAGGGGTCTCTGTGCTGCTGGGACTAGTGCTTTTCCTGGAGCAAAGGACCCAG

GCCCAGGAAGGGGTGCCCTCCATCTGGGCAGAGCCAGGATCTCTGGTTCCCCATGGCAGT

GCTGTGACTATCGTCTGCAGGATTCCTCCAGGGGTGACACAGTTGCGTCTGTATCATGTG

GAAACTAAGTTGTGGCGTGACAGAGACCCACAGGGAGCCCCAGAGGTGGCCCAGTTCTCC

CTCCAGCAGGTGACACACAACCAGGCAGGGACTTACCACTGTGAATACTGGACAGAAGGA

CTCCCATCAGGACAAAATGACCCACTAGAGCTGGTGGTGACAGGAATGTACAAGGAGAAG

CCCTCCCTGACTGCTGAGCCTGGTCCCCAGGTGGCCTCAGGGGGACAGGTGACTCTGCTC

TGTCACACAACTTATTCCTATGACGTCTTCACTCTGTGCAGAGACAGGGCAGCCTCCCTG

TCCCAGAAATGCTCCCACCAGACCCACAGCTCGTTCCTCATGTCCCCTGTGAGCCTGGCC

CACGGGGGCACCTACAGGTGCTATTTCTCCTCCAGGAACAGACCCCACCTCTGGTCTCTG

CCCAGTGACCCCCTTGATCTCCTGGTCACAGGTCCACCTAGTCCCCTTGTGGTTGGGGTC

TCGGCCGCC

>CL78.Contig4_A-W 76 306 minus strand PREDICTED: retinol dehydrogenase 13-like [Loxodonta africana]

GACGTGAGCTCCAGCCCTGCCTCTGACGTCCAGCGGGGAGATGCAGATGACACCGAGCAC

AGGGAGCATGGACAGCTGGACACACAGGCCCCTGCGGCAGGAGGCCCACAGGAGGTGACC

TATGTCCAGCTGCACCCAAGGACCCTCACCAGGAGTGTGGGTCCACTTCTGTCCCGAGCG

CCCCAGGACGGGTCAACACAGCCCTGTGTGTATGCCACGCTCACCTCGCCT

>CL78.Contig5_A-W 137 700 minus strand Killer cell immunoglobulin-like receptor 3DL1 [Heterocephalus glaber]

GCTTTTCCTGGAGCAAAGGACCCAGGCCCAGGAAGGATTCCTCCAGGGGTGACACAGTTG

CGTCTGTATCATGTGGAAACTAAGTTGTGGCGTGACAGAGACCCACAGGGAGCCCCAGAG

GTGGCCCAGTTCTCCCTCCAGCAGGTGACACACAACCAGGCAGGGACTTACCACTGTGAA

TACTGGACAGAAGGACTCCCATCAGGACAAAATGACCCACTAGAGCTGGTGGTGACAGGA

ATGTACAAGGAGAAGCCCTCCCTGACTGCTGAGCCTGGTCCCCAGGTGGCCTCAGGGGGA

CAGGTGACTCTGCTCTGTCACACAACTTATTCCTATGACGTCTTCACTCTGTGCAGAGAC

AGGGCAGCCTCCCTGTCCCAGAAATGCTCCCACCAGACCCACAGCTCGTTCCTCATGTCC

CCTGTGAGCCTGGCCCACGGGGGCACCTACAGGTGCTATTTCTCCTCCAGGAACAGACCC

CACCTCTGGTCTCTGCCCAGTGACCCCCTTGATCTCCTGGTCACAGGTCCACCTAGTCCC

CTTGTGGTTGGGGTCTCGGCCGCC

>CL78.Contig6_A-W 102 770 minus strand Killer cell immunoglobulin-like receptor 3DL1 [Heterocephalus glaber]

ATGGCCACAGGGGTCTCTGTGCTGCTGGGACTAGTGCTTTTCCTGGAGCAAAGGACCCAG

GCCCAGGAAGGGGTGCCCTCCATCTGGGCAGAGCCAGGATCTCTGGTTCCCCATGGCAGT

GCTGTGACTATCGTCTGCAGGATTCCTCCAGGGGTGACACAGTTGCGTCTGTATCATGTG

GAAACTAAGTTGTGGCGTGACAGAGACCCACAGGGAGCCCCAGAGGTGGCCCAGTTCTCC

CTCCAGCAGGTGACACACAACCAGGCAGGGACTTACCACTGTGAATACTGGACAGAAGGA

CTCCCATCAGGACAAAATGACCCACTAGAGCTGGTGGTGACAGGAATGTACAAGGAGAAG

CCCTCCCTGACTGCTGAGCCTGGTCCCCAGGTGGCCTCAGGGGGACAGGTGACTCTGCTC

TGTCACACAACTTATTCCTATGACGTCTTCACTCTGTGCAGAGACAGGGCAGCCTCCCTG

TCCCAGAAATGCTCCCACCAGACCCACAGCTCGTTCCTCATGTCCCCTGTGAGCCTGGCC

CACGGGGGCACCTACAGGTGCTATTTCTCCTCCAGGAACAGACCCCACCTCTGGTCTCTG

CCCAGTGACCCCCTTGATCTCCTGGTCACAGGTCCACCTAGTCCCCTTGTGGTTGGGGTC

TCGGCCGCC

>CL78.Contig7_A-W 9 629 minus strand Killer cell immunoglobulin-like receptor 3DL1 [Heterocephalus glaber]

GGGGCTCTTCTGCTTCTCCCAGGGGTGCCCTCCATCTGGGCAGAGCCAGGATCTCTGGTT

CCCCATGGCAGTGCTGTGACTATCGTCTGCAGGATTCCTCCAGGGGTGACACAGTTGCGT

CTGTATCATGTGGAAACTAAGTTGTGGCGTGACAGAGACCCACAGGGAGCCCCAGAGGTG

GCCCAGTTCTCCCTCCAGCAGGTGACACACAACCAGGCAGGGACTTACCACTGTGAATAC

TGGACAGAAGGACTCCCATCAGGACAAAATGACCCACTAGAGCTGGTGGTGACAGGAATG

TACAAGGAGAAGCCCTCCCTGACTGCTGAGCCTGGTCCCCAGGTGGCCTCAGGGGGACAG

GTGACTCTGCTCTGTCACACAACTTATTCCTATGACGTCTTCACTCTGTGCAGAGACAGG

GCAGCCTCCCTGTCCCAGAAATGCTCCCACCAGACCCACAGCTCGTTCCTCATGTCCCCT

GTGAGCCTGGCCCACGGGGGCACCTACAGGTGCTATTTCTCCTCCAGGAACAGACCCCAC

CTCTGGTCTCTGCCCAGTGACCCCCTTGATCTCCTGGTCACAGGTCCACCTAGTCCCCTT

GTGGTTGGGGTCTCGGCCGCC

>CL78.Contig8_A-W 137 700 minus strand Killer cell immunoglobulin-like receptor 3DL1 [Heterocephalus glaber]

GCTTTTCCTGGAGCAAAGGACCCAGGCCCAGGAAGGATTCCTCCAGGGGTGACACAGTTG

CGTCTGTATCATGTGGAAACTAAGTTGTGGCGTGACAGAGACCCACAGGGAGCCCCAGAG

GTGGCCCAGTTCTCCCTCCAGCAGGTGACACACAACCAGGCAGGGACTTACCACTGTGAA

TACTGGACAGAAGGACTCCCATCAGGACAAAATGACCCACTAGAGCTGGTGGTGACAGGA

ATGTACAAGGAGAAGCCCTCCCTGACTGCTGAGCCTGGTCCCCAGGTGGCCTCAGGGGGA

CAGGTGACTCTGCTCTGTCACACAACTTATTCCTATGACGTCTTCACTCTGTGCAGAGAC

AGGGCAGCCTCCCTGTCCCAGAAATGCTCCCACCAGACCCACAGCTCGTTCCTCATGTCC

CCTGTGAGCCTGGCCCACGGGGGCACCTACAGGTGCTATTTCTCCTCCAGGAACAGACCC

CACCTCTGGTCTCTGCCCAGTGACCCCCTTGATCTCCTGGTCACAGGTCCACCTAGTCCC

CTTGTGGTTGGGGTCTCGGCCGCC

>CL78.Contig9_A-W 9 629 minus strand Killer cell immunoglobulin-like receptor 3DL1 [Heterocephalus glaber]

GGGGCTCTTCTGCTTCTCCCAGGGGTGCCCTCCATCTGGGCAGAGCCAGGATCTCTGGTT

CCCCATGGCAGTGCTGTGACTATCGTCTGCAGGATTCCTCCAGGGGTGACACAGTTGCGT

CTGTATCATGTGGAAACTAAGTTGTGGCGTGACAGAGACCCACAGGGAGCCCCAGAGGTG

GCCCAGTTCTCCCTCCAGCAGGTGACACACAACCAGGCAGGGACTTACCACTGTGAATAC

TGGACAGAAGGACTCCCATCAGGACAAAATGACCCACTAGAGCTGGTGGTGACAGGAATG

TACAAGGAGAAGCCCTCCCTGACTGCTGAGCCTGGTCCCCAGGTGGCCTCAGGGGGACAG

GTGACTCTGCTCTGTCACACAACTTATTCCTATGACGTCTTCACTCTGTGCAGAGACAGG

GCAGCCTCCCTGTCCCAGAAATGCTCCCACCAGACCCACAGCTCGTTCCTCATGTCCCCT

GTGAGCCTGGCCCACGGGGGCACCTACAGGTGCTATTTCTCCTCCAGGAACAGACCCCAC

CTCTGGTCTCTGCCCAGTGACCCCCTTGATCTCCTGGTCACAGGTCCACCTAGTCCCCTT

GTGGTTGGGGTCTCGGCCGCC

>CL78.Contig10_A-W 252 512 minus strand Killer cell immunoglobulin-like receptor 3DL1 [Heterocephalus glaber]

CCACCTAGTCCCCTTGTGGTTGGGGTCTCGGCCGCCGCCGCTGTCCTCCTGGGCCTCTGC

CTCCTCCTCCTGGGCCTCTGCCTATGCCGACACCGAGGGCAGCGTCGGGCCAGACGCAGG

GTGACTGACGGTGGGACCAAGAGCCCAGTGTGGGACGTGAGCTCCAGCCCTGCCTCTGAC

GTCCAGCGGGGAGATGCAGATGACACCGAGCACAGGGAGCATGGACAGCTGGACACACAG

GTGAGCCCTCCCACCACTCCC

>CL79.Contig1_A-W 184 333 minus strand hypothetical protein GW7_11805, partial [Heterocephalus glaber]

CTAAAATGCCCAGGCTGGCCTCCAACTTGTGTTCCTCCTGCCTCAGCCTCTCTAGCTGCT

GGGATTACAGATGTGTGCCACCTTGCCCAGCTTGAATTTTCAAAATTCTTCTTCTTCTTC

TTCTTTTTTGCAATGCTGGAGACCTCTGCG

>CL80.Contig1_A-W 92 520 Terminal deoxynucleotidyltransferase-interacting factor 1 [Macaca mulatta] >gi|380787193|gb|AFE65472.1| deoxynucleotidyltransferase terminal-interacting protein 1 [Macaca mulatta] >gi|384944154|gb|AFI35682.1| deoxynucleotidyltransferase terminal-interacting protein 1 [Macaca mulatta]

TGGAAGCCAAAATCCTCTGAACCGATTCGCCGGGAAGGCCCCAAGTGGGACCCAGCTCGG

CTGAATGAAACTACCACCTTCGTGTTGGGCTCTCGAGCCAACAAGGCCCTAGGGATGGGG

GGCACCAGAGGGAGAATCTACATCAAACACCCACATCTCTTTAAGTATGCAGCTGACCCC

CAGGACAAGCACTGGCTGGCTGAGCAGCATCACATGCGGGCAACAGGCGGGAAGATGGCC

TACCTCCTCATCGAGGAGGACATCCGGGATCTTGCTGCCAGTGATGACTACAGAGGATGC

CTGGATCTGAAGTTAGAGGAGCTGAAGTCCTTTGTCCTACCCTCCTGGATGGTTGAGAAG

ATGCGAAAGTACATGGAGACACTACGGACAGAGAATGAGCATCGCACTGCTGAAGCACCT

CCACAGACC

>CL80.Contig2_A-W 52 1041 PREDICTED: deoxynucleotidyltransferase terminal-interacting protein 1 [Sus scrofa]

ATGGGGGCCACTGGCGACGCCGAGCAGCCGCGGGGACCCGGCGGGGCTGAGCGGGGCGGC

ACCGAGCTGGGTGACGCGGGCGCAGCGGGGCAGCTGGTTCTCATGAACCCTTGGAACATA

ATGATAAAGCACCGGCAGGTGCAGCGAAGGGGCCGCCGCTCACAAATGACAACAAGTTTC

ACAGATCCTGCCATCTCCATGGACCTCCTCCGAGCTGTCCTGCAGCCGAGCATCAATGAG

GAGATCCAGACTGTCTTCAACAAGTATATGAAGTTCTTCCAGAAAGCAGCACTGAACGTG

CGAGACAACGTTGGGGAAGAGGTGGATGCAGAGCAGCTGATCCAGGAGGCCTGTCGGAGC

TGCCTTGAGCAGGCTAAGCTACTATTTTCAGATGGAGAAAAAGTAATACCTAGATTGACC

CATGAGCTTCCAGGAATAAAGCGTGGCCGGCAGGCAGAGGAAGAATGTACCCATCGAGGA

AGCCCCGTTCCCAAAAAGAGGAAAGGACGGCCTCCTGGACACATTCTGTCAAATGACCGG

GCAGCAGCCGGCATGGTCAGATGGAAGCCAAAATCCTCTGAACCGATTCGCCGGGAAGGC

CCCAAGTGGGACCCAGCTCGGCTGAATGAAACTACCACCTTCGTGTTGGGCTCTCGAGCC

AACAAGGCCCTAGGGATGGGGGGCACCAGAGGGAGAATCTACATCAAACACCCACATCTC

TTTAAGTATGCAGCTGACCCCCAGGACAAGCACTGGCTGGCTGAGCAGCATCACATGCGG

GCAACAGGCGGGAAGATGGCCTACCTCCTCATCGAGGAGGACATCCGGGATCTTGCTGCC

AGTGATGACTACAGAGGATGCCTGGATCTGAAGTTAGAGGAGCTGAAGTCCTTTGTCCTA

CCCTCCTGGATGGTTGAGAAGATGCGAAAGTACATGGAGACACTACGGACAGAGAATGAG

CATCGCACTGCTGAAGCACCTCCACAGACC

>CL80.Contig3_A-W 52 549 minus strand PREDICTED: deoxynucleotidyltransferase terminal-interacting protein 1 isoform 2 [Canis lupus familiaris]

ATGGGGGCCACTGGCGACGCCGAGCAGCCGCGGGGACCCGGCGGGGCTGAGCGGGGCGGC

ACCGAGCTGGGTGACGCGGGCGCAGCGGGGCAGCTGGTTCTCATGAACCCTTGGAACATA

ATGATAAAGCACCGGCAGGTGCAGCGAAGGGGCCGCCGCTCACAAATGACAACAAGTTTC

ACAGATCCTGCCATCTCCATGGACCTCCTCCGAGCTGTCCTGCAGCCGAGCATCAATGAG

GAGATCCAGACTGTCTTCAACAAGTATATGAAGTTCTTCCAGAAAGCAGCACTGAACGTG

CGAGACAACGTTGGGGAAGAGGTGGATGCAGAGCAGCTGATCCAGGAGGCCTGTCGGAGC

TGCCTTGAGCAGGCTAAGCTACTATTTTCAGATGGAGAAAAAGTAATACCTAGATTGACC

CATGAGCTTCCAGGAATAAAGCGTGGCCGGCAGGCAGAGGAAGAATGTACCCATCGAGGA

AGCCCCGTTCCCAAAAAG

>CL81.Contig1_A-W 221 688 minus strand PREDICTED: ribosomal protein L23a-like [Oryctolagus cuniculus]

ATGGCGCCGAAAGCTAAGAAGGAAGCTCCTGCCCCTCCCAAAGCTGAAGCCAAAGCAAAA

GCTTTGAAAGCCAAGAAAGCAGTGTTGAAGGGTGTCCACAGTCATAAAAAGAAGAAGATC

CGCACGTCACCTACCTTCCGGCGACCCAAGACATTGCGGCTCCGGAGGCAGCCCAAATAT

CCTTGGAAGAGCGCCCCCAGGAGAAACAAGCTTGACCACTATGCCATCATCAAATTCCCT

CTGACCACTGAGTCAGCCATGAAGAAGATTGAAGACAACAACACACTTGTGTTTATTGTG

GATGTCAAGGCAAACAAACACCAGATCAAACAAGCTGTAAAGAAGCTCTATGACATTGAT

GTGGCCAAGGTTAACACCCTGATCAGGCCTGATGGAGAGAAAAAGGCATATGTGCGACTG

GCTCCTGATTATGATGCTTTGGATGTTGCCAATAAAATTGGGATCATC

>CL82.Contig1_A-W 2 163 minus strand PREDICTED: poly [ADP-ribose] polymerase 10 [Papio anubis]

AGCTGCCATGTGCTGAGCCTGAAGCACCCAGGCAGTGCCAGGTTCCTGCTGGGTGTGGAG

GGGCAGCACCTTTTGCAGGGTTTGGAGGCTCAGTTCCAGTGTGTCTTTGGGACAGAGCGT

CTGGCCAGTGCCAACCTGGTCATGGACCCTGAAGAGGTAGAG

>CL82.Contig2_A-W 2 163 minus strand PREDICTED: poly [ADP-ribose] polymerase 10 [Papio anubis]

AGCTGCCATGTGCTGAGCCTGAAGCACCCAGGCAGTGCCAGGTTCCTGCTGGGTGTGGAG

GGGCAGCACCTTTTGCAGGGTTTGGAGGCTCAGTTCCAGTGTGTCTTTGGGACAGAGCGT

CTGGCCAGTGCCAACCTGGTCATGGACCCTGAAGAGGTAGAG

>CL82.Contig3_A-W 246 359 minus strand PREDICTED: poly [ADP-ribose] polymerase 10 [Otolemur garnettii]

GATATCCTTTCCCCACAGGTGGATTCCACTGAGATCCTTCAGGTCCTCCCTGGCCACAGC

CATACCTGGTGGCCCACAGATAGTACAGGCAGTGACCAGGAGAACATGAGCCTG

>CL82.Contig4_A-W 2 253 minus strand PREDICTED: poly [ADP-ribose] polymerase 10 [Otolemur garnettii]

AGCTGCCATGTGCTGAGCCTGAAGCACCCAGGCAGTGCCAGGTTCCTGCTGGGTGTGGAG

GGGCAGCACCTTTTGCAGGGTTTGGAGGCTCAGTTCCAGTGTGTCTTTGGGACAGAGCGT

CTGGCCAGTGCCAACCTGGTCATGGACCCTGAAGAGGTGGATTCCACTGAGATCCTTCAG

GTCCTCCCTGGCCACAGCCATACCTGGTGGCCCACAGATAGTACAGGCAGTGACCAGGAG

AACATGAGCCTG

>CL83.Contig1_A-W 326 952 PREDICTED: regulator of nonsense transcripts 2 [Saimiri boliviensis boliviensis]

GAAAAACAAGAAGGAGAAGAAGAGGAGGAAGAAAATACAGATTACCTTACAGATTCCAAT

AAGGAAAATGAAACTGATGAAGAGAATACTGAGGTAATGATTAAAGGAGGTGGACTTAAG

CATGTACCTTGTGTAGAAGATGAGGACTTCATTCAAGCTCTGGATAAAATGATGCTAGAA

AATCTCCAGCAAAGAAGTGGCGAATCTGTTAAAGTACATCAACTTGATGTTGCTATTCCT

TTGCATCTCAAAAGCCAGCTGAGGAAAGGACCTCCACTTGGTGGCGGGGAAGGAGAGGCA

GAGTCTGCAGATACTATGCCATTTGTCATGTTAACAAGAAAAGGCAATAAACAGCAGTTT

AAAATACTTAATGTGCCCATGTCATCCCAACTTGCTGCAAATCATTGGAACCAGCAACAG

GCAGAACAAGAAGAAAGGATGAGAATGAAAAAGCTCACACTAGATATCAATGAGCGGCAA

GAACAAGAAGATTATCAAGAAATGTTGCAGTCTCTTGCTCAGCGCCCAGCTCCAGCAAAC

ACCAATCGTGAGCGGCGGCCTCGCTACCAGCATCCAAAGGGAGCACCTAATGCAGATCTA

ATCTTTAAGACTGGTGGGAGGAGACGT

>CL84.Contig1_A-W 1321 1521 minus strand THO complex subunit 6 homolog isoform 1 [Macaca mulatta]

AGGACCAGCCTGGAAGTGCCTGAGATCAATGCCTTGCTGCTTGTCCCCAAGGAGAATTCC

CTCATCCTGGCTGGGGGAGACTGTCAGTTGCACACTATGGACCTTGAAACTGGGACTTTC

ACGCGGGCCCTCCGGGGCCACACAGACTACATCCACTGCCTGGCACTGCGGGAACGCAGC

CCTGAGGTGCTGTCAGGTGGC

>CL84.Contig2_A-W 999 2021 minus strand PREDICTED: THO complex subunit 6 homolog isoform 6 [Pan troglodytes] >gi|397465945|ref|XP_003804738.1| PREDICTED: THO complex subunit 6 homolog isoform 1 [Pan paniscus]

ATGGAGCGCGCTGCCCCGCTAGCGGTGCCTCTGGGTCAGACAGAGGTGTTTCAGGCCCTG

CAGCGGCTGCACATGACCATCTTCTCCCAGAGTGTTTCACCCTGTGGGAAGTTCCTGGCG

GCAGGCAACAATTATGGGCAGATCGCTATCTTCAGCCTGTCTGCTGCTTTGAGTTCAGAG

GCCAAAGAGGAAAGCAAGAAACCCGTGGTGACCTTCCAAGCCCATGATGGGCCCGTCTAC

AGCATGGTCTCCACTGATCGACATCTGCTCAGTGCTGGCGATGGGGAGGTGAAGGCCTGG

CTTTGGGCAGAGATCCTCAAGAAGGGCTGTAAGGAACTGTGGCGTCGTCAGCCCCCATAC

AGGACCAGCCTGGAAGTGCCTGAGATCAATGCCTTGCTGCTTGTCCCCAAGGAGAATTCC

CTCATCCTGGCTGGGGGAGACTGTCAGTTGCACACTATGGACCTTGAAACTGGGACTTTC

ACGCGGGCCCTCCGGGGCCACACAGACTACATCCACTGCCTGGCACTGCGGGAACGCAGC

CCTGAGGTGCTGTCAGGTGGCGAGGATGGGGCTGTGCGACTCTGGGATCTCCGAACGGTC

AGAGAGGTCCAGACGATTGAGGTCTATAAGCACGAGGAGTGCTCAAGGCCACACAACGGG

CGCTGGATTGGATGCTTGGCCACTGACTCAGACTGGATGGTCTGTGGAGGGGGCCCAGCC

CTCACCCTCTGGCACCTCCGGTCCTCCACACCCACCACCATCTTTCCAATGCGGGCGCCA

CAGAAGCATGTCACCTTCTACCAGGACCTGATTCTGTCAGCTGGCCAGGGTCGCTGCGTA

AACCACTGGCAGCTGAGCGGAGAGCTTAAGGCCCAGGTGCCTGGATCTTCCCCAGGACTG

CTCAGCCTCAGCCTCAACCAGCAGCCAGCAGCCCCTGAGTGCAAGGTCCTGACAGTCGCT

GGCAGCAGCTGCCGGGTGGATGTTTTCACCAACCTGGGGTACCGAGCCTTCTCCCTGTCC

TTC

>CL84.Contig3_A-W 372 728 host cell factor C1 regulator 1 [Bos taurus] >gi|122136341|sp|Q2M2S6.1|HPIP_BOVIN RecName: Full=Host cell factor C1 regulator 1; AltName: Full=HCF-1 beta-propeller-interacting protein >gi|85057072|gb|AAI11672.1| Host cell factor C1 regulator 1 (XPO1 dependent) [Bos taurus] >gi|296473491|gb|DAA15606.1| host cell factor C1 regulator 1 [Bos taurus]

ATGATCCTTCAGCAGCCCCTGGAGCGGGGCCCCCCACGTCGGGCCCTGCACGATCCGCGG

ACCGCCTCAGGGGCGACTCGAGGCCAGGACGCCAGGGAGCCGTTACGAAAGCAGTTTCTG

TCTGAGGAGAACATGGCCACCCACTTCTCTCGCCTGAGCCTACATAATGACCACCCTTAC

TGCAGCCCCCCCATGGCTTTTCCACCCGCCCTGCCCTCACTGAGGAGTCCTTGCTCAGAA

CTACTTCTCTGGCGCTATCCTGGGAATTTAATCCCTGAGGCTCTCCGGCTGCTGAGGCTG

GGGGATACTCCTAGTCCCCACTATCCTGCAACCCCAGCTGGGGACATAATGGACCTC

>CL84.Contig4_A-W 55 318 host cell factor C1 regulator 1 [Bos taurus] >gi|122136341|sp|Q2M2S6.1|HPIP_BOVIN RecName: Full=Host cell factor C1 regulator 1; AltName: Full=HCF-1 beta-propeller-interacting protein >gi|85057072|gb|AAI11672.1| Host cell factor C1 regulator 1 (XPO1 dependent) [Bos taurus] >gi|296473491|gb|DAA15606.1| host cell factor C1 regulator 1 [Bos taurus]

AGGGAGCCGTTACGAAAGCAGTTTCTGTCTGAGGAGAACATGGCCACCCACTTCTCTCGC

CTGAGCCTACATAATGACCACCCTTACTGCAGCCCCCCCATGGCTTTTCCACCCGCCCTG

CCCTCACTGAGGAGTCCTTGCTCAGAACTACTTCTCTGGCGCTATCCTGGGAATTTAATC

CCTGAGGCTCTCCGGCTGCTGAGGCTGGGGGATACTCCTAGTCCCCACTATCCTGCAACC

CCAGCTGGGGACATAATGGACCTC

>CL84.Contig5_A-W 22 432 PREDICTED: THO complex subunit 6 homolog isoform 6 [Pan troglodytes] >gi|397465945|ref|XP_003804738.1| PREDICTED: THO complex subunit 6 homolog isoform 1 [Pan paniscus]

ATGGAGCGCGCTGCCCCGCTAGCGGTGCCTCTGGGTCAGACAGAGGTGTTTCAGGCCCTG

CAGCGGCTGCACATGACCATCTTCTCCCAGAGTGTTTCACCCTGTGGGAAGTTCCTGGCG

GCAGGCAACAATTATGGGCAGATCGCTATCTTCAGCCTGTCTGCTGCTTTGAGTTCAGAG

GCCAAAGAGGAAAGCAAGAAACCCGTGGTGACCTTCCAAGCCCATGATGGGCCCGTCTAC

AGCATGGTCTCCACTGATCGACATCTGCTCAGTGCTGGCGATGGGGAGGTGAAGGCCTGG

CTTTGGGCAGAGATCCTCAAGAAGGGCTGTAAGGAACTGTGGCGTCGTCAGCCCCCATAC

AGGACCAGCCTGGAAGTGCCTGAGATCAATGCCTTGCTGCTTGTCCCCCAG

>CL85.Contig1_A-W 3 3023 PREDICTED: structural maintenance of chromosomes protein 6 [Canis lupus familiaris]

AGTGCGGTACTCACAGCTCTTATAGTTGGTCTTGGTGGAAAAGCAGTCGCTACTAATAGA

GGATCCTCTTTAAAAGGATTTGTGAAAGATGGACAGAACTCAGCAGATATCACAATAACA

TTAAGGAACAGAGGAGATGATGCCTATAGAGCAAATGTGTATGGTGATTCCATAGTTGTA

CAACAACACATCAGCATGGATGGAAGTCGATCCTATAAACTAAAAAGTGAAACAGGTACT

GTGGTTTCTACTAGGAAAGAAGAACTGATTGCAATTCTGGATCATTTTAATATCCAGGTG

GATAATCCAGTTTCTATTTTAACACAAGAGATGAGTAAGCAGTTCTTGCAGTCTAAAAAT

GAGGGAGACAAATACAAATTCTTCATGAAAGCAACCCAACTTGAACAGATGAAGGAAGAT

TATTCATACATTATGGAAACAAAAGAAAGAACAAAGGAGCAGATAAATCAAGGGGAAGAG

CGACTTACTGAACTAAAGCGCCAGTGTTTGGAGAAAGAAGAACGTTTTCAGAGTATCGCT

GGCTTAAGTACAATGAAGACTAATTTAGAGTATTTGAAACATGAAATGGCTTGGGCAGTG

GTCAACGAAATTGAAAAACAATTGAATGCTATCAGAGATAATATCAAAATTGGAGAAGAT

CGTGCTGCTAGACTTGATAGGAAAATGGAAGAACAACAGGTCAGACTTAATGAAGCAGAG

AAAAAATACAAGGATATTCAAGATAAACTAGAAAAGATTAGTCAGGAGACAAATGCACGA

GCACCAGAATGTATGGCATTGAAAGCAGATGTCACTGCTAAGAAAAGGGCCTATAATGAA

GCTGAGGTTTTATATAACCGTTCTCTAAATGAATATAAAGCATTAAAGAAAGATGATGAG

CAGCTGTGTAAAAGAATTGAAGAACTGAAAAAAAGCACTGATCAGTCTTTGGAACCTGAG

AGGTTGGAAAGGCAAAAAAAAATTTGTTGGTTAAAAGACAGAGTAAAGGCCTTACAGGAC

CAGGAAAATTCAATCAATCAAGAGATTGAACAGTTTCAACAAGCCATAGAAAAGGACAAA

GAAGAATATACTAGAATTAGGGGCGAAGAACTAGATGTGAAGCATGCACTGAACTATAAT

CAGAGGCAATTGAAAGAACTGAAGGATAGTAAAACTGATAGACTGAAAAGATTTGGCTCT

CATGTTCCAGCTCTTTTGGAGGCAATTGATGAAGCTTACAGACGGGGACACTTTACCCAT

AAACCTGTAGGCCCTTTAGGAGCTTGCATTCATCTTCGGGACCCCGAGCATGCTTTGGCT

ATTGAATCCTGTTTAAAAGGACTACTCCAAGCCTATTGTTGCCATAACCATGCTGATGAA

AGAGTACTTCAGGCACTGATGAAAAGGTTTTATCCACCAGGGACTTCACGGCCACAGATA

ATAGTTTCTGAATTTCGGAATGAGATGTATGATGTAAGACACAGAGCTGCTCATCATCCA

GAGTTCCCAACAGTTCTGACAGCTTTAGAAATAGATAATGCAGTTGTTGCTAATAGCCTA

ATCGACATGAGGGGCATAGAGACAGTGCTGCTAATCAAAAATAATTCTGTAGCTCGTGCA

GTTATGCAGTTTCAAAAGCCACCCAAAAATTGTAGAGAAGCTTTTACTGCTGATGGTGAC

CAAGTTTTTGCAGGACGTTATTATTCATCTGAAAATACACGACCTAAGTTCCTAAGCAGA

GATGTGGATTCTGAAATAAGTGACTTGGAAAATGAGGTTGAAAATAAGAAGGCCCAGATA

GTAAATCTTCAGCAGCATCTATCTGCCCTTGAAAAGGATACTCAACACAATGAAGATCTT

CTTAAAAGATGCCAACTACATTATAAAGAGTTAAAGATGAAAATAAGAAAAAGTATTTCC

GAAATTCGGGAACTTGAGAATATAGAAGAACACCAGTCTGTAGATATTGCAACCTTGGAA

GATGAAGCTCAAGAAAATAAAATAAAAATGAAAATGGTTGAGAAAAACATGGAGCAACAA

AAAGAAAATTTGGAACATCATAAAAGTCTTAAAGTAGAAGCAGAAAACAAGTATGATGCA

ATTAAACTAAAAATTAATCAGCTGTCAGAGCTAGCAGATCCACTTAAGGATGAATTAAAC

CTTGCTGATGCTGAAGTGGATAATCAAAAACGAGGGAAGCGGCATTATGAAGAAAAACAA

AAAGAACACTTGGATACCTTAAATAAAAAGAAACGAGAACTGGACATGAAAGTAAAGGAG

CTGGAGGAGAAAATGTCACAAGCAAGACAGATCTGCCCAGAGAGAATAGAAGTAAAAAAG

TCTGCATCAATTCTAGACAAGGAAATTAATCGATTAAGACAAAAGATACAGGCAGAACAT

GCTAGTCATGGAGATCGAGAGGAAATAATGAGGCAGTACCAGGAAGCAAGAGAAACATAC

CTTGATCTGGATAATAAAGTAAGGACTTTAAAAAGATTTATTAAATTACTAGAAGAAATA

ATGACTCATAGATACAAAACATATCAACAGTTTAGAAGGTGTTTGACTTTACGATGCAAA

TTGTACTTTGACAACTTACTATCTCAGCGAGCTTATTGTGGAAAAATGAATTTTGACCAT

AAGAATGAAACTCTTACTATATCAGTTCAGCCTGGAGAGGGAAATAAAGCTGCCTTCAAT

GATATGCGTGCCTTGTCTGGTGGTGAGCGTTCTTTCTCAACAGTCTGCTTCATTCTTTCC

CTGTGGTCCATCGCTGAATCACCTTTCAGATGCCTGGATGAGTTTGATGTTTACATGGAT

ATGGTTAACAGGAGAATTGCAATGGATATGATACTTAAGATGGCAGATTCCCAGCGTTTT

AGACAATTTATCTTGCTTACCCCTCAAAGCATGAGCTCACTTCCTTCAAGTAAACTGATT

AGAATTCTTCGAATGTCTGATCCTGAAAGAGGACAAACTACTTTGCCTTTCCGACCTGTG

ACTCAAGAAGATGATGACAAC

>CL85.Contig2_A-W 37 246 Structural maintenance of chromosomes protein 6 [Cricetulus griseus]

CTCCAGGATATGGTTAACAGGAGAATTGCAATGGATATGATACTTAAGATGGCAGATTCC

CAGCGTTTTAGACAATTTATCTTGCTTACCCCTCAAAGCATGAGCTCACTTCCTTCAAGT

AAACTGATTAGAATTCTTCGAATGTCTGATCCTGAAAGAGGACAAACTACTTTGCCTTTC

CGACCTGTGACTCAAGAAGATGATGACAAC

>CL86.Contig1_A-W 3 833 minus strand Monofunctional C1-tetrahydrofolate synthase, mitochondrial [Heterocephalus glaber]

AGCACAGTCACCATCGGGCTGGTGCAGGCGCTGACCGCCCACCTGCACGTCAACTCCTTT

GCCTGTCTGAGGCAGCCTTCCCAAGGACCGACTTTTGGAGTGAAAGGAGGAGCTGCAGGT

GGCGGATACGCACAGGTCATCCCCATGGAGGAGTTCAACCTGCACTTAACTGGGGACATC

CACGCCATTACTGCTGCCAATAACTTGCTGGCCGCTGCCATCGACACAAGGATTTTGCAT

GAAAACACTCAAACCGATAAGGCTCTTTATAACCGTCTGGTCCCTTCAGTGAATGGTGTC

AGAGAATTTTCAGAAATTCAACTTGCTCGGCTGAAGAAACTGGGGATAAATAAGACTGAC

CCAGGCACACTGACGGAAGAGGAAGTGAGTAAATTCGCCCGTCTCAACATTGACCCCAGT

ACCATCACGTGGCAGAGAGTATTGGATACCAATGACCGATTTCTGCGAAAAATAACCATT

GGGCAGGCCAGCACGGAGAAGGGATATTCCCGGCAGGCGCAATTTGACATTGCGGTGGCC

AGTGAGATCATGGCAGTGTTGGCCCTGACTGACAGCCTTAAGGACATGAAGGAGCGGCTG

GGGAGAATGGTGGTGGCCAGTGACAAAAACGGGCAGCCCGTAACCGCAGACGACTTGGGG

GTGACAGGTGCTTTGACAGTTTTGATGAAAGATGCAATAAAACCAAATCTGATGCAGACC

CTGGAAGGGACACCTGTGTTTGTGCATGCCGGCCCCTTTGCTAACATCGCCCACGGCAAC

TCTTCAGTGTTAGCTGACAAAATTGCCCTGAAACTGGTTGGTGAAGAAGGA

>CL87.Contig1_A-W 57 611 PREDICTED: transcription elongation factor A protein 2 [Otolemur garnettii]

GCCGAGGGTCGCGGCGGCTGCGGCCCTGGTCCGGCCGGGCTGGGTTGCTGAGCTGAGCAG

GGTGCTGGCCCCAAGGCGATGATGGGCAAGGAGGAGGAAATTGCGCGGATCGCCCGGAGG

TTGGACAAGATGGTGACCAGAAAGAGCGCGGAGGGAGCCATGGACCTGCTGCGGGAGCTG

AAGGCCATGCCTATCACTCTACACCTGCTCCAGTCCACCCGTGTTGGGATGTCTGTCAAC

GCCCTACGGAAACAGAGCTCGGACGAGGAGGTCATTGCGCTGGCCAAGTCCCTCATCAAG

TCCTGGAAGAAGCTCCTGGATGCTTCTGATGCCAAAACCAGGAACCGGGGGAAGGGCACA

CCTCTGCCCACATCATCCTCAAAGGATGGCTCAGAGGCCATGGATCCCAGCCGCAAGAGG

CCGGAGCTGCCTAGGATGCTGTCCACCCCAAGGATCACCACGTTTCCCCCAGCGCCTGTT

ACCTGTGATGCTGTGCGCAACAAGTGCCGGGAGCTGCTGACCACTGCCCTGCAAACAGAC

CGTGAGTGCCTGGCA

>CL87.Contig2_A-W 326 562 PREDICTED: transcription elongation factor A protein 2 [Otolemur garnettii]

GATGCTTCTGATGCCAAAACCAGGAACCGGGGGAAGGGCACACCTCTGCCCACATCATCC

TCAAAGGATGGCTCAGAGGCCATGGATCCCAGCCGCAAGAGGCCGGAGCTGCCTAGGATG

CTGTCCACCCCAAGGATCACCACGTTTCCCCCAGCGCCTGTTACCTGTGATGCTGTGCGC

AACAAGTGCCGGGAGCTGCTGACCACTGCCCTGCAAACAGACCGTGAGTGCCTGGCA

>CL87.Contig3_A-W 147 551 PREDICTED: transcription elongation factor A protein 2 [Otolemur garnettii]

GAGGGAGCCATGGACCTGCTGCGGGAGCTGAAGGCCATGCCTATCACTCTACACCTGCTC

CAGTCCACCCGTGTTGGGATGTCTGTCAACGCCCTACGGAAACAGAGCTCGGACGAGGAG

GTCATTGCGCTGGCCAAGTCCCTCATCAAGTCCTGGAAGAAGCTCCTGGATGCTTCTGAT

GCCAAAACCAGGAACCGGGGGAAGGGCACACCTCTGCCCACATCATCCTCAAAGGATGGC

TCAGAGGCCATGGATCCCAGCCGCAAGAGGCCGGAGCTGCCTAGGATGCTGTCCACCCCA

AGGATCACCACGTTTCCCCCAGCGCCTGTTACCTGTGATGCTGTGCGCAACAAGTGCCGG

GAGCTGCTGACCACTGCCCTGCAAACAGACCGTGAGTGCCTGGCA

>CL87.Contig4_A-W 57 206 PREDICTED: transcription elongation factor A protein 2 [Otolemur garnettii]

GCCGAGGGTCGCGGCGGCTGCGGCCCTGGTCCGGCCGGGCTGGGTTGCTGAGCTGAGCAG

GGTGCTGGCCCCAAGGCGATGATGGGCAAGGAGGAGGAAATTGCGCGGATCGCCCGGAGG

TTGGACAAGATGGTGACCAGAAAGAGCGCG

>CL87.Contig5_A-W 57 344 PREDICTED: transcription elongation factor A protein 2 [Otolemur garnettii]

GCCGAGGGTCGCGGCGGCTGCGGCCCTGGTCCGGCCGGGCTGGGTTGCTGAGCTGAGCAG

GGTGCTGGCCCCAAGGCGATGATGGGCAAGGAGGAGGAAATTGCGCGGATCGCCCGGAGG

TTGGACAAGATGGTGACCAGAAAGAGCGCGGAGGGAGCCATGGACCTGCTGCGGGAGCTG

AAGGCCATGCCTATCACTCTACACCTGCTCCAGTCCACCCGTGTTGGGATGTCTGTCAAC

GCCCTACGGAAACAGAGCTCGGACGAGGAGATGCTTCTGATGCCAAAA

>CL87.Contig6_A-W 147 284 PREDICTED: transcription elongation factor A protein 2 isoform 1 [Pongo abelii]

GAGGGAGCCATGGACCTGCTGCGGGAGCTGAAGGCCATGCCTATCACTCTACACCTGCTC

CAGTCCACCCGTGTTGGGATGTCTGTCAACGCCCTACGGAAACAGAGCTCGGACGAGGAG

ATGCTTCTGATGCCAAAA

>CL87.Contig7_A-W 57 1100 PREDICTED: transcription elongation factor A protein 2 [Otolemur garnettii]

GCCGAGGGTCGCGGCGGCTGCGGCCCTGGTCCGGCCGGGCTGGGTTGCTGAGCTGAGCAG

GGTGCTGGCCCCAAGGCGATGATGGGCAAGGAGGAGGAAATTGCGCGGATCGCCCGGAGG

TTGGACAAGATGGTGACCAGAAAGAGCGCGGAGGGAGCCATGGACCTGCTGCGGGAGCTG

AAGGCCATGCCTATCACTCTACACCTGCTCCAGTCCACCCGTGTTGGGATGTCTGTCAAC

GCCCTACGGAAACAGAGCTCGGACGAGGAGGTCATTGCGCTGGCCAAGTCCCTCATCAAG

TCCTGGAAGAAGCTCCTGGATGCTTCTGATGCCAAAACCAGGAACCGGGGGAAGGGCACA

CCTCTGCCCACATCATCCTCAAAGGATGGCTCAGAGGCCATGGATCCCAGCCGCAAGAGG

CCGGAGCTGCCTAGGATGCTGTCCACCCCAAGGATCACCACGTTTCCCCCAGCGCCTGTT

ACCTGTGATGCTGTGCGCAACAAGTGCCGGGAGCTGCTGACCACTGCCCTGCAAACAGAC

CATGACCATGCGGCTGTTGGTGCAGACTGTGAGCGACTGTCGGCCCAGATTGAGGAATGC

ATCTTCCGGGATGTGGGAAACACAGACATGAAGTACAAGAACCGTGTGCGCAGTCGCATT

GCCAACCTGAAGGATGCCAAGAACCCTGACCTCCGGCGGAATGTGCTGTGTGGTGCCATA

ACCCCCCAGCAGATTGCGGTGATGACATCGGAGGTGAGCCCCACTGCAGGGGCTGGGGGC

TGCTTCTGTGGGGCTGCAGGCTGAGTGCTTGGCCCCTCACCCCAGGAGATGGCCAGTGAT

GAGCTGAAGGAGATCCGCAAGGCCATGACCAAGGAGGCCATCCGCGAGCACCAGATGGCA

CGCACAGGTGGCACTCAGACAGACCTGTTCACCTGTGGCAAGTGCAGGAAGAAGAACTGT

ACCTACACGCAGGTACAGACCCGCAGCTCTGATGAGCCCATGACCACCTTTGTTGTTTGC

AATGAATGTGGAAACCGCTGGAAG

>CL87.Contig8_A-W 57 344 PREDICTED: transcription elongation factor A protein 2 [Otolemur garnettii]

GCCGAGGGTCGCGGCGGCTGCGGCCCTGGTCCGGCCGGGCTGGGTTGCTGAGCTGAGCAG

GGTGCTGGCCCCAAGGCGATGATGGGCAAGGAGGAGGAAATTGCGCGGATCGCCCGGAGG

TTGGACAAGATGGTGACCAGAAAGAGCGCGGAGGGAGCCATGGACCTGCTGCGGGAGCTG

AAGGCCATGCCTATCACTCTACACCTGCTCCAGTCCACCCGTGTTGGGATGTCTGTCAAC

GCCCTACGGAAACAGAGCTCGGACGAGGAGATGCTTCTGATGCCAAAA

>CL87.Contig9_A-W 57 1028 PREDICTED: transcription elongation factor A protein 2 [Otolemur garnettii]

GCCGAGGGTCGCGGCGGCTGCGGCCCTGGTCCGGCCGGGCTGGGTTGCTGAGCTGAGCAG

GGTGCTGGCCCCAAGGCGATGATGGGCAAGGAGGAGGAAATTGCGCGGATCGCCCGGAGG

TTGGACAAGATGGTGACCAGAAAGAGCGCGGAGGGAGCCATGGACCTGCTGCGGGAGCTG

AAGGCCATGCCTATCACTCTACACCTGCTCCAGTCCACCCGTGTTGGGATGTCTGTCAAC

GCCCTACGGAAACAGAGCTCGGACGAGGAGGTCATTGCGCTGGCCAAGTCCCTCATCAAG

TCCTGGAAGAAGCTCCTGGATGCTTCTGATGCCAAAACCAGGAACCGGGGGAAGGGCACA

CCTCTGCCCACATCATCCTCAAAGGATGGCTCAGAGGCCATGGATCCCAGCCGCAAGAGG

CCGGAGCTGCCTAGGATGCTGTCCACCCCAAGGATCACCACGTTTCCCCCAGCGCCTGTT

ACCTGTGATGCTGTGCGCAACAAGTGCCGGGAGCTGCTGACCACTGCCCTGCAAACAGAC

CATGACCATGCGGCTGTTGGTGCAGACTGTGAGCGACTGTCGGCCCAGATTGAGGAATGC

ATCTTCCGGGATGTGGGAAACACAGACATGAAGTACAAGAACCGTGTGCGCAGTCGCATT

GCCAACCTGAAGGATGCCAAGAACCCTGACCTCCGGCGGAATGTGCTGTGTGGTGCCATA

ACCCCCCAGCAGATTGCGGTGATGACATCGGAGGAGATGGCCAGTGATGAGCTGAAGGAG

ATCCGCAAGGCCATGACCAAGGAGGCCATCCGCGAGCACCAGATGGCACGCACAGGTGGC

ACTCAGACAGACCTGTTCACCTGTGGCAAGTGCAGGAAGAAGAACTGTACCTACACGCAG

GTACAGACCCGCAGCTCTGATGAGCCCATGACCACCTTTGTTGTTTGCAATGAATGTGGA

AACCGCTGGAAG

>CL87.Contig10_A-W 57 344 PREDICTED: transcription elongation factor A protein 2 [Otolemur garnettii]

GCCGAGGGTCGCGGCGGCTGCGGCCCTGGTCCGGCCGGGCTGGGTTGCTGAGCTGAGCAG

GGTGCTGGCCCCAAGGCGATGATGGGCAAGGAGGAGGAAATTGCGCGGATCGCCCGGAGG

TTGGACAAGATGGTGACCAGAAAGAGCGCGGAGGGAGCCATGGACCTGCTGCGGGAGCTG

AAGGCCATGCCTATCACTCTACACCTGCTCCAGTCCACCCGTGTTGGGATGTCTGTCAAC

GCCCTACGGAAACAGAGCTCGGACGAGGAGATGCTTCTGATGCCAAAA

>CL87.Contig11_A-W 64 1047 PREDICTED: transcription elongation factor A protein 2 [Otolemur garnettii]

GCCGAGGGTCGCGGCGGCTGCGGCCCTGGTCCGGCCGGGCTGGGTTGCTGAGCTGAGCAG

GGTGCTGGCCCCAAGGCGATGATGGGCAAGGAGGAGGAAATTGCGCGGATCGCCCGGAGG

TTGGACAAGATGGTGACCAGAAAGAGCGCGGAGGGAGCCATGGACCTGCTGCGGGAGCTG

AAGGCCATGCCTATCACTCTACACCTGCTCCAGTCCACCCGTGTTGGGATGTCTGTCAAC

GCCCTACGGAAACAGAGCTCGGACGAGGAGGTCATTGCGCTGGCCAAGTCCCTCATCAAG

TCCTGGAAGAAGCTCCTGGGTGTGGATGCTTCTGATGCCAAAACCAGGAACCGGGGGAAG

GGCACACCTCTGCCCACATCATCCTCAAAGGATGGCTCAGAGGCCATGGATCCCAGCCGC

AAGAGGCCGGAGCTGCCTAGGATGCTGTCCACCCCAAGGATCACCACGTTTCCCCCAGCG

CCTGTTACCTGTGATGCTGTGCGCAACAAGTGCCGGGAGCTGCTGACCACTGCCCTGCAA

ACAGACCATGACCATGCGGCTGTTGGTGCAGACTGTGAGCGACTGTCGGCCCAGATTGAG

GAATGCATCTTCCGGGATGTGGGAAACACAGACATGAAGTACAAGAACCGTGTGCGCAGT

CGCATTGCCAACCTGAAGGATGCCAAGAACCCTGACCTCCGGCGGAATGTGCTGTGTGGT

GCCATAACCCCCCAGCAGATTGCGGTGATGACATCGGAGGAGATGGCCAGTGATGAGCTG

AAGGAGATCCGCAAGGCCATGACCAAGGAGGCCATCCGCGAGCACCAGATGGCACGCACA

GGTGGCACTCAGACAGACCTGTTCACCTGTGGCAAGTGCAGGAAGAAGAACTGTACCTAC

ACGCAGGTACAGACCCGCAGCTCTGATGAGCCCATGACCACCTTTGTTGTTTGCAATGAA

TGTGGAAACCGCTGGAAGTTCTGC

>CL87.Contig12_A-W 147 284 transcription elongation factor A (SII), 2, isoform CRA_c [Homo sapiens]

GAGGGAGCCATGGACCTGCTGCGGGAGCTGAAGGCCATGCCTATCACTCTACACCTGCTC

CAGTCCACCCGTGTTGGGATGTCTGTCAACGCCCTACGGAAACAGAGCTCGGACGAGGAG

ATGCTTCTGATGCCAAAA

>CL87.Contig13_A-W 147 284 PREDICTED: transcription elongation factor A protein 2 [Otolemur garnettii]

GAGGGAGCCATGGACCTGCTGCGGGAGCTGAAGGCCATGCCTATCACTCTACACCTGCTC

CAGTCCACCCGTGTTGGGATGTCTGTCAACGCCCTACGGAAACAGAGCTCGGACGAGGAG

ATGCTTCTGATGCCAAAA

>CL89.Contig1_A-W 3 437 unnamed protein product [Mus musculus]

AGCGGCGGCCACGCCATGTGGCTGCTGGGGCCGCTGTGCCTGCTGCTGAGCAGCGCCGCG

GAGAGCCAGCTGCTTCCCGGGAACAACTTCACCAACGAGTGCAACATTCCAGGCAACTTC

ATGTGCGGCAACGGGCGGTGCATCCCGGGCGCCTGGCAGTGCGACGGGCTGCCCGACTGC

TTCGACAAGAGTGACGAGAAGGAGTGCCCAAAAGCTAAGTCCAAGTGCGGCCCCACGTTC

TTTCCCTGCGCCAGCGGCGTCCACTGCATCATCGGCCGCTTCCGGTGCAACGGGTTTGAG

GACTGTCCTGACGGCAGTGACGAGGAGAACTGCACAGCCAACCCCCTGCTCTGCTCCACC

GCCCGCTACCACTGCAGGAACGGCCTCTGCATCGACAAGAGCTTCCTCTGCGACGGGCAG

AACAACTGTCAGGAC

>CL89.Contig2_A-W 3 350 unnamed protein product [Mus musculus]

AGCGGCGGCCACGCCATGTGGCTGCTGGGGCCGCTGTGCCTGCTGCTGAGCAGCGCCGCG

GAGAGCCAGCTGCTTCCCGGGAACAACTTCACCAACGAGTGCAACATTCCAGGCAACTTC

ATGTGCGGCAACGGGCGGTGCATCCCGGGCGCCTGGCAGTGCGACGGGCTGCCCGACTGC

TTCGACAAGAGTGACGAGAAGGAGTGCCCAAAAGCTAAGTCCAAGTGCGGCCCCACGTTC

TTTCCCTGCGCCAGCGGCGTCCACTGCATCATCGGCCGCTTCCGGTGCAACGGGTTTGAG

GACTGTCCTGACGGCAGTGACGAGGAGAACTGCAATTCTCATCTTCTT

>CL90.Contig1_A-W 125 925 PREDICTED: signal transducer and activator of transcription 3 [Saimiri boliviensis boliviensis]

TGTGTCTGTTTACCAGGTAACTGGAAACTTTCCTATTTTACTGGCAGGATCACCTCATTA

GCAGAATCCCAACTTCAGACCCGCCAGCAAATCAAGAAACTGGAGGAGTTGCAGCAAAAA

GTTTCCTACAAAGGGGACCCCATTGTACAGCACCGGCCAATGCTGGAGGAGAGAATCGTG

GAGCTGTTCAGAAACTTAATGAAAAGTGCCTTTGTGGTTGAGCGGCAGCCCTGCATGCCC

ATGCATCCTGACCGGCCGTTAGTCATCAAGACTGGTGTCCAGTTCACCACTAAAGTCAGG

TTGCTGGTCAAATTCCCTGAGTTGAATTATCAGCTTAAGATTAAAGTGTGCATTGACAAA

GACTCTGGGGACGTTGCAGCTCTCAGAGGATCCCGGAAGTTTAACATTCTGGGCACAAAC

ACAAAAGTGATGAACATGGAAGAATCCAACAATGGCAGCCTCTCTGCCGAATTCAAGCAC

TTGACCCTCAGGGAGCAAAGATGTGGAAATGGGGGCCGCGCCAATTGTGATGCCTCCCTG

ATTGTGACCGAGGAGCTGCACCTGATCACCTTTGAGACTGAGGTGTATCACCAAGGCCTC

AAGATCGACCTAGAGACCCACTCCTTGCCAGTTGTGGTGATCTCCAACATCTGTCAGATG

CCAAATGCCTGGGCCTCAATCCTGTGGTATAATATGCTGACCAACAACCCGAAGGTTAGT

GTCCCAACCTCTTGGAAACCACGTTCTCTTGTAACTTCTTGGTACCTGAGGCAGGTCATT

AGCACTACCCTCTCATCTGAA

>CL90.Contig2_A-W 125 799 PREDICTED: signal transducer and activator of transcription 3 [Saimiri boliviensis boliviensis]

TGTGTCTGTTTACCAGGTAACTGGAAACTTTCCTATTTTACTGGCAGGATCACCTCATTA

GCAGAATCCCAACTTCAGACCCGCCAGCAAATCAAGAAACTGGAGGAGTTGCAGCAAAAA

GTTTCCTACAAAGGGGACCCCATTGTACAGCACCGGCCAATGCTGGAGGAGAGAATCGTG

GAGCTGTTCAGAAACTTAATGAAAAGTGCCTTTGTGGTTGAGCGGCAGCCCTGCATGCCC

ATGCATCCTGACCGGCCGTTAGTCATCAAGACTGGTGTCCAGTTCACCACTAAAGTCAGG

TTGCTGGTCAAATTCCCTGAGTTGAATTATCAGCTTAAGATTAAAGTGTGCATTGACAAA

GACTCTGGGGACGTTGCAGCTCTCAGAGGATCCCGGAAGTTTAACATTCTGGGCACAAAC

ACAAAAGTGATGAACATGGAAGAATCCAACAATGGCAGCCTCTCTGCCGAATTCAAGCAC

TTGGTATGTCAGAGGAGCCTGAACCCCTCTTCACATAGAGCCTCTGGCCAAGTGAAGGGC

TGGGACAGCCTCATGTGACTTTTTCTTTTTTTACAGACCCTCAGGGAGCAAAGATGTGGA

AATGGGGGCCGCGCCAATTGTGATGTAAGTTGTTGGGTATGAAAGCCAATGAGGGGGGGT

TCTTTTGAGGGAGAG

>CL90.Contig3_A-W 44 1681 PREDICTED: signal transducer and activator of transcription 3 isoform 3 [Papio anubis]

GGGGAGCTTGGGGGTTCCGACGTCGCAGCGGAGGGAAGGAGCCCCAACCGGATCCTTGAC

GAGCACCCCAGCTCGGCTTTGCCCCTCTGCGTCGCCTAGGAGAGGCCCGGCGGCCCGAGG

GAGCCCGGCCACCCGCACCCGACGCAAGCTCAGCCCCGGCCTGTCGGGCCTCACAGCTGG

AGAAACAGTTGGGACCCCCCTGATTTCAGCAGGATGGCCCAGTGGAATCAGCTTCAGCAG

CTAGACACACGGTACCTGGAGCAGCTTCATCAGCTGTACAGCGACAGCTTCCCAATGGAG

CTCCGGCAGTTTCTTGCACCTTGGATTGAGAGTCAAGACTGGGCATATGCTGCCAGCAAA

GAATCTCATGCCACATTGGTGTTTCATAATCTCTTGGGTGAGATTGACCAGCAATACAGC

CGCTTCCTACAAGAGTCCAATGTTCTCTATCAGCACAATCTGCGAAGAATCAAGCAGTTC

CTTCAGAGTAGGTATCTTGAGAAGCCAATGGAGATCGCTCGAATAGTGGCCAGGTGCCTG

TGGGAAGAGTCCCGCCTCCTCCAGACTGCAGCCACTGCAGCCCAGCAAGGGGGCCAGGCC

AACCACCCCACAGCTGCTGTGGTGACGGAAAAGCAGCAGATGCTGGAGCAGCACCTTCAG

GATGTTCGAAAGCGAGTACAGGATCTAGAACAGAAAATGAAAGTGGTAGAGAATCTCCAG

GATGACTTTGATTTCAACTATAAAACCCTCAAGAGTCAAGGAGACATGCAGGATCTGAAT

GGAAACAACCAGTCTGTGACCAGGCAGAAGATGCAGCAGCTGGAGCAGATGCTCACAGCG

CTGGACCAGATGCGGAGAAGCATCGTGAGTGAACTGGCGGGGCTTTTGTCAGCAATGGAG

TATGTGCAGAAAACACTTACTGACGAAGAGCTGGCTGACTGGAAGAGGCGGCAACAGATT

GCTTGCATTGGAGGCCCTCCTAACATCTGCCTGGATCGTCTGGAAAACTGGATCACCTCA

TTAGCAGAATCCCAACTTCAGACCCGCCAGCAAATCAAGAAACTGGAGGAGTTGCAGCAA

AAAGTTTCCTACAAAGGGGACCCCATTGTACAGCACCGGCCAATGCTGGAGGAGAGAATC

GTGGAGCTGTTCAGAAACTTAATGAAAAGTGCCTTTGTGGTTGAGCGGCAGCCCTGCATG

CCCATGCATCCTGACCGGCCGTTAGTCATCAAGACTGGTGTCCAGTTCACCACTAAAGTC

AGGTTGCTGGTCAAATTCCCTGAGTTGAATTATCAGCTTAAGATTAAAGTGTGCATTGAC

AAAGACTCTGGGGACGTTGCAGCTCTCAGAGGATCCCGGAAGTTTAACATTCTGGGCACA

AACACAAAAGTGATGAACATGGAAGAATCCAACAATGGCAGCCTCTCTGCCGAATTCAAG

CACTTGGTATGTCAGAGGAGCCTGAACCCCTCTTCACATAGAGCCTCTGGCCAAGTGAAG

GGCTGGGACAGCCTCATGTGACTTTTTCTTTTTTTACAGACCCTCAGGGAGCAAAGATGT

GGAAATGGGGGCCGCGCCAATTGTGATGTAAGTTGTTGGGTATGAAAGCCAATGAGGGGG

GGTTCTTTTGAGGGAGAG

>CL90.Contig4_A-W 125 1684 PREDICTED: signal transducer and activator of transcription 3 [Saimiri boliviensis boliviensis]

TGTGTCTGTTTACCAGGTAACTGGAAACTTTCCTATTTTACTGGCAGGATCACCTCATTA

GCAGAATCCCAACTTCAGACCCGCCAGCAAATCAAGAAACTGGAGGAGTTGCAGCAAAAA

GTTTCCTACAAAGGGGACCCCATTGTACAGCACCGGCCAATGCTGGAGGAGAGAATCGTG

GAGCTGTTCAGAAACTTAATGAAAAGTGCCTTTGTGGTTGAGCGGCAGCCCTGCATGCCC

ATGCATCCTGACCGGCCGTTAGTCATCAAGACTGGTGTCCAGTTCACCACTAAAGTCAGG

TTGCTGGTCAAATTCCCTGAGTTGAATTATCAGCTTAAGATTAAAGTGTGCATTGACAAA

GACTCTGGGGACGTTGCAGCTCTCAGAGGATCCCGGAAGTTTAACATTCTGGGCACAAAC

ACAAAAGTGATGAACATGGAAGAATCCAACAATGGCAGCCTCTCTGCCGAATTCAAGCAC

TTGACCCTCAGGGAGCAAAGATGTGGAAATGGGGGCCGCGCCAATTGTGATGCCTCCCTG

ATTGTGACCGAGGAGCTGCACCTGATCACCTTTGAGACTGAGGTGTATCACCAAGGCCTC

AAGATCGACCTAGAGACCCACTCCTTGCCAGTTGTGGTGATCTCCAACATCTGTCAGATG

CCAAATGCCTGGGCCTCAATCCTGTGGTATAATATGCTGACCAACAACCCGAAGAACGTG

AATTTTTTCACCAAGCCCCCAATTGGAACCTGGGATCAAGTGGCTGAGGTGCTGAGCTGG

CAGTTCTCCTCCACCACCAAGCGAGGCCTGAGCATCGAGCAGCTGACCACGCTGGCAGAG

AAACTCTTAGGACCTGGTGTGAACTATTCAGGGTGTCAGATCACATGGGCTAAATTTTGC

AAAGAAAACATGGCTGGCAAGGGCTTCTCCTTCTGGGTCTGGCTAGACAATATCATTGAC

CTTGTGAAGAAGTACATCTTGGCCCTTTGGAATGAAGGGTACATAATGGGCTTCATCAGT

AAGGAGCGGGAGCGGGCCATCCTGAGCACCAAGCCCCCAGGTACCTTCCTGCTGAGATTC

AGTGAAAGCAGCAAAGAAGGAGGAGTCACTTTCACTTGGGTGGAAAAGGACATCAGTGGA

AAGACCCAGATCCAGTCAGTGGAACCATATACCAAGCAGCAACTGAATAATATGTCATTT

GCTGAAATCATCATGGGCTATAAAATCATGGACGCTACCAATATCCTGGTGTCTCCACTG

GTCTATCTCTACCCTGACATTCCTAAGGAGGAGGCATTTGGAAAGTATTGTCGGCCAGAA

AGCCAGGAGCATCCTGAAGCTGACCCAGGTAGTGCTGCCCCATACTTGAAGACCAAGTTT

ATCTGTGTGACACCAACGACCTGCAGCAATACCATTGACCTGCCGATGTCCCCCCGCACT

TTAGATTCATTGATGCAGTTTGGAAATAATGGTGAAGGTGCTGAGCCCTCAGCAGGAGGG

CAGTTTGAGTCCCTCACATTTGACATGGAGTTGACCTCTGAGTGCGCTACCTCCCCCATG

>CL90.Contig5_A-W 1833 2678 PREDICTED: signal transducer and activator of transcription 3 isoform 3 [Papio anubis]

AACGTGAATTTTTTCACCAAGCCCCCAATTGGAACCTGGGATCAAGTGGCTGAGGTGCTG

AGCTGGCAGTTCTCCTCCACCACCAAGCGAGGCCTGAGCATCGAGCAGCTGACCACGCTG

GCAGAGAAACTCTTAGGACCTGGTGTGAACTATTCAGGGTGTCAGATCACATGGGCTAAA

TTTTGCAAAGAAAACATGGCTGGCAAGGGCTTCTCCTTCTGGGTCTGGCTAGACAATATC

ATTGACCTTGTGAAGAAGTACATCTTGGCCCTTTGGAATGAAGGGTACATAATGGGCTTC

ATCAGTAAGGAGCGGGAGCGGGCCATCCTGAGCACCAAGCCCCCAGGTACCTTCCTGCTG

AGATTCAGTGAAAGCAGCAAAGAAGGAGGAGTCACTTTCACTTGGGTGGAAAAGGACATC

AGTGGAAAGACCCAGATCCAGTCAGTGGAACCATATACCAAGCAGCAACTGAATAATATG

TCATTTGCTGAAATCATCATGGGCTATAAAATCATGGACGCTACCAATATCCTGGTGTCT

CCACTGGTCTATCTCTACCCTGACATTCCTAAGGAGGAGGCATTTGGAAAGTATTGTCGG

CCAGAAAGCCAGGAGCATCCTGAAGCTGACCCAGGTAGTGCTGCCCCATACTTGAAGACC

AAGTTTATCTGTGTGACACCAACGACCTGCAGCAATACCATTGACCTGCCGATGTCCCCC

CGCACTTTAGATTCATTGATGCAGTTTGGAAATAATGGTGAAGGTGCTGAGCCCTCAGCA

GGAGGGCAGTTTGAGTCCCTCACATTTGACATGGAGTTGACCTCTGAGTGCGCTACCTCC

CCCATG

>CL90.Contig6_A-W 46 2568 PREDICTED: signal transducer and activator of transcription 3 isoform 2 [Macaca mulatta]

GGGGAGCTTGGGGGTTCCGACGTCGCAGCGGAGGGAAGGAGCCCCAACCGGATCCTTGAC

GAGCACCCCAGCTCGGCTTTGCCCCTCTGCGTCGCCTAGGAGAGGCCCGGCGGCCCGAGG

GAGCCCGGCCACCCGCACCCGACGCAAGCTCAGCCCCGGCCTGTCGGGCCTCACAGCTGG

AGAAACAGTTGGGACCCCCCTGATTTCAGCAGGATGGCCCAGTGGAATCAGCTTCAGCAG

CTAGACACACGGTACCTGGAGCAGCTTCATCAGCTGTACAGCGACAGCTTCCCAATGGAG

CTCCGGCAGTTTCTTGCACCTTGGATTGAGAGTCAAGACTGGGCATATGCTGCCAGCAAA

GAATCTCATGCCACATTGGTGTTTCATAATCTCTTGGGTGAGATTGACCAGCAATACAGC

CGCTTCCTACAAGAGTCCAATGTTCTCTATCAGCACAATCTGCGAAGAATCAAGCAGTTC

CTTCAGAGTAGGTATCTTGAGAAGCCAATGGAGATCGCTCGAATAGTGGCCAGGTGCCTG

TGGGAAGAGTCCCGCCTCCTCCAGACTGCAGCCACTGCAGCCCAGCAAGGGGGCCAGGCC

AACCACCCCACAGCTGCTGTGGTGACGGAAAAGCAGCAGATGCTGGAGCAGCACCTTCAG

GATGTTCGAAAGCGAGTACAGGATCTAGAACAGAAAATGAAAGTGGTAGAGAATCTCCAG

GATGACTTTGATTTCAACTATAAAACCCTCAAGAGTCAAGGAGACATGCAGGATCTGAAT

GGAAACAACCAGTCTGTGACCAGGCAGAAGATGCAGCAGCTGGAGCAGATGCTCACAGCG

CTGGACCAGATGCGGAGAAGCATCGTGAGTGAACTGGCGGGGCTTTTGTCAGCAATGGAG

TATGTGCAGAAAACACTTACTGACGAAGAGCTGGCTGACTGGAAGAGGCGGCAACAGATT

GCTTGCATTGGAGGCCCTCCTAACATCTGCCTGGATCGTCTGGAAAACTGGATCACCTCA

TTAGCAGAATCCCAACTTCAGACCCGCCAGCAAATCAAGAAACTGGAGGAGTTGCAGCAA

AAAGTTTCCTACAAAGGGGACCCCATTGTACAGCACCGGCCAATGCTGGAGGAGAGAATC

GTGGAGCTGTTCAGAAACTTAATGAAAAGTGCCTTTGTGGTTGAGCGGCAGCCCTGCATG

CCCATGCATCCTGACCGGCCGTTAGTCATCAAGACTGGTGTCCAGTTCACCACTAAAGTC

AGGTTGCTGGTCAAATTCCCTGAGTTGAATTATCAGCTTAAGATTAAAGTGTGCATTGAC

AAAGACTCTGGGGACGTTGCAGCTCTCAGAGGATCCCGGAAGTTTAACATTCTGGGCACA

AACACAAAAGTGATGAACATGGAAGAATCCAACAATGGCAGCCTCTCTGCCGAATTCAAG

CACTTGACCCTCAGGGAGCAAAGATGTGGAAATGGGGGCCGCGCCAATTGTGATGCCTCC

CTGATTGTGACCGAGGAGCTGCACCTGATCACCTTTGAGACTGAGGTGTATCACCAAGGC

CTCAAGATCGACCTAGAGACCCACTCCTTGCCAGTTGTGGTGATCTCCAACATCTGTCAG

ATGCCAAATGCCTGGGCCTCAATCCTGTGGTATAATATGCTGACCAACAACCCGAAGAAC

GTGAATTTTTTCACCAAGCCCCCAATTGGAACCTGGGATCAAGTGGCTGAGGTGCTGAGC

TGGCAGTTCTCCTCCACCACCAAGCGAGGCCTGAGCATCGAGCAGCTGACCACGCTGGCA

GAGAAACTCTTAGGACCTGGTGTGAACTATTCAGGGTGTCAGATCACATGGGCTAAATTT

TGCAAAGAAAACATGGCTGGCAAGGGCTTCTCCTTCTGGGTCTGGCTAGACAATATCATT

GACCTTGTGAAGAAGTACATCTTGGCCCTTTGGAATGAAGGGTACATAATGGGCTTCATC

AGTAAGGAGCGGGAGCGGGCCATCCTGAGCACCAAGCCCCCAGGTACCTTCCTGCTGAGA

TTCAGTGAAAGCAGCAAAGAAGGAGGAGTCACTTTCACTTGGGTGGAAAAGGACATCAGT

GGAAAGACCCAGATCCAGTCAGTGGAACCATATACCAAGCAGCAACTGAATAATATGTCA

TTTGCTGAAATCATCATGGGCTATAAAATCATGGACGCTACCAATATCCTGGTGTCTCCA

CTGGTCTATCTCTACCCTGACATTCCTAAGGAGGAGGCATTTGGAAAGTATTGTCGGCCA

GAAAGCCAGGAGCATCCTGAAGCTGACCCAGGTAGTGCTGCCCCATACTTGAAGACCAAG

TTTATCTGTGTGACACCAACGACCTGCAGCAATACCATTGACCTGCCGATGTCCCCCCGC

ACTTTAGATTCATTGATGCAGTTTGGAAATAATGGTGAAGGTGCTGAGCCCTCAGCAGGA

GGGCAGTTTGAGTCCCTCACATTTGACATGGAGTTGACCTCTGAGTGCGCTACCTCCCCC

ATG

>CL90.Contig7_A-W 104 517 PREDICTED: signal transducer and activator of transcription 3 [Saimiri boliviensis boliviensis]

TTTGAAGAAATGCTGGTCTTCTCACCAATCTTAATCTGTGTTCTTAACAGAGACTCTGGG

GACGTTGCAGCTCTCAGAGGGTAAGTTAAGCCTAGAAGCTTCCTTTTGTTTTGTTTAATC

TAACATTATCCTCTGGCCACTCAGTCACCCTTGTGGTTGGTTTTTCTCCTGTGATTCAGA

TCCCGGAAGTTTAACATTCTGGGCACAAACACAAAAGTGATGAACATGGAAGAATCCAAC

AATGGCAGCCTCTCTGCCGAATTCAAGCACTTGGTATGTCAGAGGAGCCTGAACCCCTCT

TCACATAGAGCCTCTGGCCAAGTGAAGGGCTGGGACAGCCTCATGTGACTTTTTCTTTTT

TTACAGACCCTCAGGGAGCAAAGATGTGGAAATGGGGGCCGCGCCAATTGTGAT

>CL90.Contig8_A-W 1 624 PREDICTED: signal transducer and activator of transcription 3 [Saimiri boliviensis boliviensis]

GGTGTCCAGTTCACCACTAAAGTCAGGTTGCTGGTCAAATTCCCTGAGTTGAATTATCAG

CTTAAGATTAAAGTGTGCATTGACAAAGACTCTGGGGACGTTGCAGCTCTCAGAGGATCC

CGGAAGTTTAACATTCTGGGCACAAACACAAAAGTGATGAACATGGAAGAATCCAACAAT

GGCAGCCTCTCTGCCGAATTCAAGCACTTGGTATGTCAGAGGAGCCTGAACCCCTCTTCA

CATAGAGCCTCTGGCCAAGTGAAGGGCTGGGACAGCCTCATGTGACTTTTTCTTTTTTTA

CAGACCCTCAGGGAGCAAAGATGTGGAAATGGGGGCCGCGCCAATTGTGATGCCTCCCTG

ATTGTGACCGAGGAGCTGCACCTGATCACCTTTGAGACTGAGGTGTATCACCAAGGCCTC

AAGATCGACCTAGAGACCCACTCCTTGCCAGTTGTGGTGATCTCCAACATCTGTCAGATG

CCAAATGCCTGGGCCTCAATCCTGTGGTATAATATGCTGACCAACAACCCGAAGAACGTG

AATTTTTTCACCAAGCCCCCAATTGGAACCTGGGATCAAGTGGCTGAGGTGCTGAGCTGG

CAGTTCTCCTCCACCACCAAGCGA

>CL92.Contig1_A-W 346 720 Retrovirus-related Pol polyprotein LINE-1 OS=Mus musculus GN=Pol PE=1 SV=2

TATCTGAATATACCTATAAATAATAGTAAGGCAACTGAATCAGTTATCAAAAGTTCCCTG

ACAAAGAAAACACTTCATGGCTTCATCAGTGAATTCTACCAAACATTTGAAGAATTACCA

TCCTTTGCTCTCTTCCAAAAACTTTTAGAGGAGAGAAAACTACCTAACTCACTCTACAAG

GCCAGCTTTACCCTGATAGCCAAAGCCAATAAAAAAATTCTACAAGAAATCAACAGACCA

ATATCCCTTATAACACTTATAGGAAAATCCCCAACAAAACACAGCAAACCGAATTAAACG

TCACATCAAAACATGATACATCATGAAAAAATGGGATTTATTTCTGGAATTCCAGAACAA

TTCAACAGTAAAAAA

>CL92.Contig2_A-W 138 278 hypothetical protein (L1H 3&apos; region) - human

AGACCAATATCCCTTATAACACTTATAGGAAAATCCCCAACAAAACACAGCAAACCGAAT

TAAACGTCACATCAAAACATGATACATCATGAAAAAATGGGATTTATTTCTGGAATTCCA

GAACAATTCAACAGTAAAAAA

>CL93.Contig1_A-W 226 405 minus strand PREDICTED: protein FAM180A-like [Cavia porcellus]

ATGGATTGGAAGGCACTGGTGCTCCTGCTGCTATATTCCCATGCTCAGGCTACGGTGTCG

CACAGGTGGAGCAGGGCAAAGCTCTTCCCTGCTGCTCATCGGCCAAAGAGGTCGTCATCC

ATGCCATTGAACCCAGTCCTGCAGACCTCCCTGGAGGAAGTGGAGCTGCTCTACCAGGTA

>CL94.Contig1_A-W 340 561 PREDICTED: linker for activation of T-cells family member 2-like [Cricetulus griseus]

TCTGTTTTCTTGGCCAGAGACCCCATTGCCGCAGACTACTACAACTGGGGGTGCTCTACG

AAGTCCCCAGAAGATGACGACGATGCCACCTCCTACGAGAACGTCCTCATCTGCAAGCCC

AGAGCCCTGGAGTCAGAGCAAGGCCTTATAGCAGGACATCCCTGTCCAGCAGGAAGCCCA

GAGGAGGATGCCGGGGAGCCCGACTATGTGAATGGGGACGTG

>CL94.Contig2_A-W 1 444 PREDICTED: linker for activation of T-cells family member 2-like [Equus caballus]

GATCCCGTGTCCCCCAGGTACCAGAACTTCAGCAAAGGTGGGTGGCTGGCTGGGGGCAGG

GGGTGCCCAGCCTGGGGCTCAGGCCTGACTCTCTGCTCCTCTCCAGGGGACGGTGGGGAC

TCGGATGCAGCCTACATAGACCCCATTGCCGCAGACTACTACAACTGGGGGTGCTCTACG

AAGTCCCCAGAAGATGACGACGATGCCACCTCCTACGAGAACGTCCTCATCTGCAAGCCC

AGAGCCCTGGAGTCAGGTTATCCCTGTGTCCCTGTGAGCAGCGAGGAGACTGAGGATTAT

CAGAACTCAGCATCCATTCATCAGTGGCGGGAATCCAGGAGGGCCGTGGAGCAAGGCCTT

ATAGCAGGACATCCCTGTCCAGCAGGAAGCCCAGAGGAGGATGCCGGGGAGCCCGACTAT

GTGAATGGGGACGTGACCATGACT

>CL94.Contig3_A-W 654 1073 Linker for activation of T-cells family member 2 OS=Homo sapiens GN=LAT2 PE=1 SV=1

CCCTGCAGGAATGATAAGCTGCTGCACTTCTCCAGCCCCTTGGACGATCCCGTGTCCCCC

AGGTACCAGAACTTCAGCAAAGGGGACGGTGGGGACTCGGATGCAGCCTACATAGACCCC

ATTGCCGCAGACTACTACAACTGGGGGTGCTCTACGAAGTCCCCAGAAGATGACGACGAT

GCCACCTCCTACGAGAACGTCCTCATCTGCAAGCCCAGAGCCCTGGAGTCAGGTTATCCC

TGTGTCCCTGTGAGCAGCGAGGAGACTGAGGATTATCAGAACTCAGCATCCATTCATCAG

TGGCGGGAATCCAGGAGGGCCGTGGAGCAAGGCCTTATAGCAGGACATCCCTGTCCAGCA

GGAAGCCCAGAGGAGGATGCCGGGGAGCCCGACTATGTGAATGGGGACGTGACCATGACT

>CL94.Contig4_A-W 1 360 Linker for activation of T-cells family member 2 [Heterocephalus glaber]

GATCCCGTGTCCCCCAGGTACCAGAACTTCAGCAAAGGTGGGTGGCTGGCTGGGGGCAGG

GGGTGCCCAGCCTGGGGCTCAGGCCTGACTCTCTGCTCCTCTCCAGGGGACGGTGGGGAC

TCGGATGCAGCCTACATAGACCCCATTGCCGCAGACTACTACAACTGGGGGTGCTCTACG

AAGTCCCCAGAAGATGACGACGATGCCACCTCCTACGAGAACGTCCTCATCTGCAAGCCC

AGAGCCCTGGAGTCAGAGCAAGGCCTTATAGCAGGACATCCCTGTCCAGCAGGAAGCCCA

GAGGAGGATGCCGGGGAGCCCGACTATGTGAATGGGGACGTGACCATGACTGGGGAGGCC

>CL94.Contig5_A-W 305 502 PREDICTED: linker for activation of T-cells family member 2-like [Equus caballus]

TCTCCCGCAGGGCTGAAGAAGGCCGAGAAGATCTACCAGCAGAGGAGCCTGCAGGAGAAC

CGGCAGAGCTTTGCAGTGGCGCGCACGTATTCCTTGGTCAGGCAGCCCTGGCCAGGGCCA

CCGACGGGCATGAACCCTGACATGGCACCTGCAAGGTAGGGCAGGCTTCCTGTCACAAGC

CCTGGGCTCCTGCAGCCC

>CL94.Contig6_A-W 673 1056 Linker for activation of T-cells family member 2 OS=Homo sapiens GN=LAT2 PE=1 SV=1

CCCTGCAGGAATGATAAGCTGCTGCACTTCTCCAGCCCCTTGGACGGGGACGGTGGGGAC

TCGGATGCAGCCTACATAGACCCCATTGCCGCAGACTACTACAACTGGGGGTGCTCTACG

AAGTCCCCAGAAGATGACGACGATGCCACCTCCTACGAGAACGTCCTCATCTGCAAGCCC

AGAGCCCTGGAGTCAGGTTATCCCTGTGTCCCTGTGAGCAGCGAGGAGACTGAGGATTAT

CAGAACTCAGCATCCATTCATCAGTGGCGGGAATCCAGGAGGGCCGTGGAGCAAGGCCTT

ATAGCAGGACATCCCTGTCCAGCAGGAAGCCCAGAGGAGGATGCCGGGGAGCCCGACTAT

GTGAATGGGGACGTGACCATGACT

>CL94.Contig7_A-W 208 483 PREDICTED: linker for activation of T-cells family member 2-like [Equus caballus]

ACCGGGACCGAGCTGCTGTGGCTGGGGGCTGCGCTGCTGCTGCTGCTGGGGACCGCGGCC

GGGCTGTGTGTGCGCTGCTCGCGCCCAGGGCTGAAGAAGGCCGAGAAGATCTACCAGCAG

AGGAGCCTGCAGGAGAACCGGCAGAGCTTTGCAGTGGCGCGCACGTATTCCTTGGTCAGG

CAGCCCTGGCCAGGGCCACCGACGGGCATGAACCCTGACATGGCACCTGCAAGGTAGGGC

AGGCTTCCTGTCACAAGCCCTGGGCTCCTGCAGCCC

>CL94.Contig8_A-W 269 457 Linker for activation of T-cells family member 2 [Heterocephalus glaber]

GGCTGTGTGTGCGCTGCTCGCGCCCAGTACCCCACCTCTCCCGCAGGGCTGAAGAAGGCC

GAGAAGATCTACCAGCAGAGGAGCCTGCAGGAGAACCGGCAGAGCTTTGCAGTGGCGCGC

ACGTATTCCTTGGTCAGGCAGCCCTGGCCAGGGCCACCGACGGGCATGAACCCTGACATG

GCACCTGCA

>CL94.Contig9_A-W 202 492 Linker for activation of T-cells family member 2 OS=Homo sapiens GN=LAT2 PE=1 SV=1

ATGAGCACCGGGACCGAGCTGCTGTGGCTGGGGGCTGCGCTGCTGCTGCTGCTGGGGACC

GCGGCCGGGCTGTGTGTGCGCTGCTCGCGCCCAGGGCTGAAGAAGGCCGAGAAGATCTAC

CAGCAGAGGAGCCTGCAGGAGAACCGGCAGAGCTTTGCAGTGGCGCGCACGTATTCCTTG

GTCAGGCAGCCCTGGCCAGGGCCACCGACGGGCATGAACCCTGACATGGCACCTGCAAGG

TAGGGCAGGCTTCCTGTCACAAGCCCTGGGCTCCTGCAGCCCTGGGGGTCC

>CL94.Contig10_A-W 305 502 PREDICTED: linker for activation of T-cells family member 2-like [Equus caballus]

TCTCCCGCAGGGCTGAAGAAGGCCGAGAAGATCTACCAGCAGAGGAGCCTGCAGGAGAAC

CGGCAGAGCTTTGCAGTGGCGCGCACGTATTCCTTGGTCAGGCAGCCCTGGCCAGGGCCA

CCGACGGGCATGAACCCTGACATGGCACCTGCAAGGTAGGGCAGGCTTCCTGTCACAAGC

CCTGGGCTCCTGCAGCCC

>CL94.Contig11_A-W 654 926 PREDICTED: linker for activation of T-cells family member 2-like isoform 1 [Nomascus leucogenys]

CCCTGCAGGAATGATAAGCTGCTGCACTTCTCCAGCCCCTTGGACGGGGACGGTGGGGAC

TCGGATGCAGCCTACATAGACCCCATTGCCGCAGACTACTACAACTGGGGGTGCTCTACG

AAGTCCCCAGAAGATGACGACGATGCCACCTCCTACGAGAACGTCCTCATCTGCAAGCCC

AGAGCCCTGGAGTCAGAGCAAGGCCTTATAGCAGGACATCCCTGTCCAGCAGGAAGCCCA

GAGGAGGATGCCGGGGAGCCCGACTATGTGAAT

>CL94.Contig12_A-W 305 871 PREDICTED: linker for activation of T-cells family member 2-like [Equus caballus]

TCTCCCGCAGGGCTGAAGAAGGCCGAGAAGATCTACCAGCAGAGGAGCCTGCAGGAGAAC

CGGCAGAGCTTTGCAGTGGCGCGCACGTATTCCTTGGTCAGGCAGCCCTGGCCAGGGCCA

CCGACGGGCATGAACCCTGACATGGCACCTGCAAGGAATGATAAGCTGCTGCACTTCTCC

AGCCCCTTGGACGATCCCGTGTCCCCCAGGTACCAGAACTTCAGCAAAGGGGACGGTGGG

GACTCGGATGCAGCCTACATAGACCCCATTGCCGCAGACTACTACAACTGGGGGTGCTCT

ACGAAGTCCCCAGAAGATGACGACGATGCCACCTCCTACGAGAACGTCCTCATCTGCAAG

CCCAGAGCCCTGGAGTCAGGTTATCCCTGTGTCCCTGTGAGCAGCGAGGAGACTGAGGAT

TATCAGAACTCAGCATCCATTCATCAGTGGCGGGAATCCAGGAGGGCCGTGGAGCAAGGC

CTTATAGCAGGACATCCCTGTCCAGCAGGAAGCCCAGAGGAGGATGCCGGGGAGCCCGAC

TATGTGAATGGGGACGTGACCATGACT

>CL94.Contig13_A-W 208 852 PREDICTED: linker for activation of T-cells family member 2-like [Equus caballus]

ACCGGGACCGAGCTGCTGTGGCTGGGGGCTGCGCTGCTGCTGCTGCTGGGGACCGCGGCC

GGGCTGTGTGTGCGCTGCTCGCGCCCAGGGCTGAAGAAGGCCGAGAAGATCTACCAGCAG

AGGAGCCTGCAGGAGAACCGGCAGAGCTTTGCAGTGGCGCGCACGTATTCCTTGGTCAGG

CAGCCCTGGCCAGGGCCACCGACGGGCATGAACCCTGACATGGCACCTGCAAGGAATGAT

AAGCTGCTGCACTTCTCCAGCCCCTTGGACGATCCCGTGTCCCCCAGGTACCAGAACTTC

AGCAAAGGGGACGGTGGGGACTCGGATGCAGCCTACATAGACCCCATTGCCGCAGACTAC

TACAACTGGGGGTGCTCTACGAAGTCCCCAGAAGATGACGACGATGCCACCTCCTACGAG

AACGTCCTCATCTGCAAGCCCAGAGCCCTGGAGTCAGGTTATCCCTGTGTCCCTGTGAGC

AGCGAGGAGACTGAGGATTATCAGAACTCAGCATCCATTCATCAGTGGCGGGAATCCAGG

AGGGCCGTGGAGCAAGGCCTTATAGCAGGACATCCCTGTCCAGCAGGAAGCCCAGAGGAG

GATGCCGGGGAGCCCGACTATGTGAATGGGGACGTGACCATGACT

>CL94.Contig14_A-W 305 835 PREDICTED: linker for activation of T-cells family member 2-like [Equus caballus]

TCTCCCGCAGGGCTGAAGAAGGCCGAGAAGATCTACCAGCAGAGGAGCCTGCAGGAGAAC

CGGCAGAGCTTTGCAGTGGCGCGCACGTATTCCTTGGTCAGGCAGCCCTGGCCAGGGCCA

CCGACGGGCATGAACCCTGACATGGCACCTGCAAGGAATGATAAGCTGCTGCACTTCTCC

AGCCCCTTGGACGGGGACGGTGGGGACTCGGATGCAGCCTACATAGACCCCATTGCCGCA

GACTACTACAACTGGGGGTGCTCTACGAAGTCCCCAGAAGATGACGACGATGCCACCTCC

TACGAGAACGTCCTCATCTGCAAGCCCAGAGCCCTGGAGTCAGGTTATCCCTGTGTCCCT

GTGAGCAGCGAGGAGACTGAGGATTATCAGAACTCAGCATCCATTCATCAGTGGCGGGAA

TCCAGGAGGGCCGTGGAGCAAGGCCTTATAGCAGGACATCCCTGTCCAGCAGGAAGCCCA

GAGGAGGATGCCGGGGAGCCCGACTATGTGAATGGGGACGTGACCATGACT

>CL94.Contig15_A-W 208 816 PREDICTED: linker for activation of T-cells family member 2-like [Equus caballus]

ACCGGGACCGAGCTGCTGTGGCTGGGGGCTGCGCTGCTGCTGCTGCTGGGGACCGCGGCC

GGGCTGTGTGTGCGCTGCTCGCGCCCAGGGCTGAAGAAGGCCGAGAAGATCTACCAGCAG

AGGAGCCTGCAGGAGAACCGGCAGAGCTTTGCAGTGGCGCGCACGTATTCCTTGGTCAGG

CAGCCCTGGCCAGGGCCACCGACGGGCATGAACCCTGACATGGCACCTGCAAGGAATGAT

AAGCTGCTGCACTTCTCCAGCCCCTTGGACGGGGACGGTGGGGACTCGGATGCAGCCTAC

ATAGACCCCATTGCCGCAGACTACTACAACTGGGGGTGCTCTACGAAGTCCCCAGAAGAT

GACGACGATGCCACCTCCTACGAGAACGTCCTCATCTGCAAGCCCAGAGCCCTGGAGTCA

GGTTATCCCTGTGTCCCTGTGAGCAGCGAGGAGACTGAGGATTATCAGAACTCAGCATCC

ATTCATCAGTGGCGGGAATCCAGGAGGGCCGTGGAGCAAGGCCTTATAGCAGGACATCCC

TGTCCAGCAGGAAGCCCAGAGGAGGATGCCGGGGAGCCCGACTATGTGAATGGGGACGTG

ACCATGACT

>CL94.Contig16_A-W 305 769 Linker for activation of T-cells family member 2 OS=Mus musculus GN=Lat2 PE=1 SV=1

TCTCCCGCAGGGCTGAAGAAGGCCGAGAAGATCTACCAGCAGAGGAGCCTGCAGGAGAAC

CGGCAGAGCTTTGCAGTGGCGCGCACGTATTCCTTGGTCAGGCAGCCCTGGCCAGGGCCA

CCGACGGGCATGAACCCTGACATGGCACCTGCAAGGAATGATAAGCTGCTGCACTTCTCC

AGCCCCTTGGACGATCCCGTGTCCCCCAGGTACCAGAACTTCAGCAAAGGGGACGGTGGG

GACTCGGATGCAGCCTACATAGACCCCATTGCCGCAGACTACTACAACTGGGGGTGCTCT

ACGAAGTCCCCAGAAGATGACGACGATGCCACCTCCTACGAGAACGTCCTCATCTGCAAG

CCCAGAGCCCTGGAGTCAGAGCAAGGCCTTATAGCAGGACATCCCTGTCCAGCAGGAAGC

CCAGAGGAGGATGCCGGGGAGCCCGACTATGTGAATGGGGACGTG

>CL94.Contig17_A-W 208 759 PREDICTED: linker for activation of T-cells family member 2-like [Equus caballus]

ACCGGGACCGAGCTGCTGTGGCTGGGGGCTGCGCTGCTGCTGCTGCTGGGGACCGCGGCC

GGGCTGTGTGTGCGCTGCTCGCGCCCAGGGCTGAAGAAGGCCGAGAAGATCTACCAGCAG

AGGAGCCTGCAGGAGAACCGGCAGAGCTTTGCAGTGGCGCGCACGTATTCCTTGGTCAGG

CAGCCCTGGCCAGGGCCACCGACGGGCATGAACCCTGACATGGCACCTGCAAGGAATGAT

AAGCTGCTGCACTTCTCCAGCCCCTTGGACGATCCCGTGTCCCCCAGGTACCAGAACTTC

AGCAAAGGGGACGGTGGGGACTCGGATGCAGCCTACATAGACCCCATTGCCGCAGACTAC

TACAACTGGGGGTGCTCTACGAAGTCCCCAGAAGATGACGACGATGCCACCTCCTACGAG

AACGTCCTCATCTGCAAGCCCAGAGCCCTGGAGTCAGAGCAAGGCCTTATAGCAGGACAT

CCCTGTCCAGCAGGAAGCCCAGAGGAGGATGCCGGGGAGCCCGACTATGTGAATGGGGAC

GTGACCATGACT

>CL94.Contig18_A-W 305 742 PREDICTED: linker for activation of T-cells family member 2-like [Equus caballus]

TCTCCCGCAGGGCTGAAGAAGGCCGAGAAGATCTACCAGCAGAGGAGCCTGCAGGAGAAC

CGGCAGAGCTTTGCAGTGGCGCGCACGTATTCCTTGGTCAGGCAGCCCTGGCCAGGGCCA

CCGACGGGCATGAACCCTGACATGGCACCTGCAAGGAATGATAAGCTGCTGCACTTCTCC

AGCCCCTTGGACGGGGACGGTGGGGACTCGGATGCAGCCTACATAGACCCCATTGCCGCA

GACTACTACAACTGGGGGTGCTCTACGAAGTCCCCAGAAGATGACGACGATGCCACCTCC

TACGAGAACGTCCTCATCTGCAAGCCCAGAGCCCTGGAGTCAGAGCAAGGCCTTATAGCA

GGACATCCCTGTCCAGCAGGAAGCCCAGAGGAGGATGCCGGGGAGCCCGACTATGTGAAT

GGGGACGTGACCATGACT

>CL94.Contig19_A-W 208 723 PREDICTED: linker for activation of T-cells family member 2-like [Equus caballus]

ACCGGGACCGAGCTGCTGTGGCTGGGGGCTGCGCTGCTGCTGCTGCTGGGGACCGCGGCC

GGGCTGTGTGTGCGCTGCTCGCGCCCAGGGCTGAAGAAGGCCGAGAAGATCTACCAGCAG

AGGAGCCTGCAGGAGAACCGGCAGAGCTTTGCAGTGGCGCGCACGTATTCCTTGGTCAGG

CAGCCCTGGCCAGGGCCACCGACGGGCATGAACCCTGACATGGCACCTGCAAGGAATGAT

AAGCTGCTGCACTTCTCCAGCCCCTTGGACGGGGACGGTGGGGACTCGGATGCAGCCTAC

ATAGACCCCATTGCCGCAGACTACTACAACTGGGGGTGCTCTACGAAGTCCCCAGAAGAT

GACGACGATGCCACCTCCTACGAGAACGTCCTCATCTGCAAGCCCAGAGCCCTGGAGTCA

GAGCAAGGCCTTATAGCAGGACATCCCTGTCCAGCAGGAAGCCCAGAGGAGGATGCCGGG

GAGCCCGACTATGTGAATGGGGACGTGACCATGACT

>CL94.Contig20_A-W 340 654 PREDICTED: linker for activation of T-cells family member 2-like [Cricetulus griseus]

TCTGTTTTCTTGGCCAGAGACCCCATTGCCGCAGACTACTACAACTGGGGGTGCTCTACG

AAGTCCCCAGAAGATGACGACGATGCCACCTCCTACGAGAACGTCCTCATCTGCAAGCCC

AGAGCCCTGGAGTCAGGTTATCCCTGTGTCCCTGTGAGCAGCGAGGAGACTGAGGATTAT

CAGAACTCAGCATCCATTCATCAGTGGCGGGAATCCAGGAGGGCCGTGGAGCAAGGCCTT

ATAGCAGGACATCCCTGTCCAGCAGGAAGCCCAGAGGAGGATGCCGGGGAGCCCGACTAT

GTGAATGGGGACGTG

>CL94.Contig21_A-W 340 477 PREDICTED: linker for activation of T-cells family member 2-like [Cricetulus griseus]

TCTGTTTTCTTGGCCAGAGACCCCATTGCCGCAGACTACTACAACTGGGGGTGCTCTACG

AAGTCCCCAGAAGATGACGACGATGCCACCTCCTACGAGAACGTCCTCATCTGCAAGCCC

AGAGCCCTGGAGTCAGGT

>CL95.Contig3_A-W 1 2550 minus strand PREDICTED: folliculin-interacting protein 1 [Equus caballus]

CGACGCAGCCAAACAACAAGTTTGGAAAATGGGGTATTTCCTAGATGGTCTATAGAAGAA

AGCTTTAATCTGTCAGATGAAAGCTGTGGCCCTAACCCAGGAATTGTGAGGAAAAAGAAG

ATTGCAATTGGGGTAATCTTTTCGTTATCCAAAGATGAAGATGAAAATAACAAATTTAAT

GAATTCTTTTTTTCACATTTTCCTCTCTTTGAAAGCCACATGAACAAATTAAAGAGTGCA

ATAGAACAGGCTATGAAAATGAGCCGGAGATCAGCTGATGCCAGTCAGAGAAGTTTGGCA

TATAATCGGATAGTTGATGCCCTGAATGAATTCAGAACAACAATTTGTAATCTTTACACA

ATGCCACGAATTGGAGAGCCCGTGTGGCTTACAATGATGTCGGGGACTCCAGAAAAGAAC

CAGCTTTGTCATCGATTCATGAAGGAGTTCACTTTTTTAATGGAAAATGCTTCCAAAAAT

CAATTCTTGCCAGCTCTCATAACTGCAGTTTTGACCAATCATCTTGCCTGGGTGCCAACA

GTCATGCCAAATGGACAACCACCTATTAAAATATTTTTAGAAAAACATTCCTCTCAGAGC

GTGGACATGTTAGCAAAGACTCATCCATATAATCCACTCTGGGCACAGCTGGGGGACTTG

TATGGTGCTATTGGCTCTCCTGTACGGCTTGCAAGGACTGTGGTAGTTGGCAAACGACAA

GATATGGTCCAGAGGCTACTTTATTTTCTTACTTACTTTATAAGATGCTCTGAACTTCAA

GAAACCCATCTTTTAGAAAATGGAGAAGATGAAGCCATTGTTATGCCAGGCACAGTGATT

ACTACCACTTTAGAGAAAGGTGAAATAGAAGAATCAGAGTATGTGCTTATCACAATGCAT

AGAAACAAAAGCAGTTTGCTGTTGAAAGAGTCCGAAGAAACAAGAACTTCTAATTGTAAC

TGTAAATACTGTAGTCATCCACTCCTTGGGCAAAATTCAGAAAATGTGTCACAACAAGAG

AAAGAAGATATTCCAAATAAGGAACTGCTAGGCATTTCAGATGAATGCCGAATAATTTCT

CCTTCTGACTGCCAAGAAGAAAATACTGTTGATGTTAAACAGTGTAGAGATAAATTAAGA

ACTTGCCTTGACACCAAGTTAGAGACTGTTGTTTGCACAGGATCTGCTCCGGTAGACAAA

TGTGTATTGTCAGAAACAGACTTAGAGCCAACAGAGGAAACATGGCAGAGTAAGGAATTG

CTGGATCCAGATAATCACACAGGCAAAGTAATGAGATCCACAGGAATAGTTGTGGAAAAG

AAACCTCCAGATAAGACTGTGCCTGCTACATTTTCTTGTGAGGCCATCCAGACAAAGGTT

ACATTTCTGATTGGGGATTCTATGTCACCTGATTCAGATACTGAACTCAGGAGTCAGGCT

GTGGTGGATCAGATTACTAGGCATCACACCAAACCATTGAAGAAAGACAGAGGGGCTAAT

GATTTACATCAAGAAACTAAACAACCAAGCAAGGACCAATCTGTAGAGTCTGATTTACAA

AGCCTGCTTTCTGGAGAGCCCTGTGAACTTCCCTGTTGGAGTCCTTCAGACCCAGAAAGC

CTGAGCTTATTTGATGAATATTTTAATGATGATTCAATTGAAACCAGGACTATTGATGAT

GTTCCAGTTAAAACAAGTACAGACAGTAAAGAACACTGCTGTATGTTAGAGTTTTCAAAA

AGATTGTGTGCAAAAAATAGCAAACAGAACAATGAACTTTGTAAATGTATAGAAACAATT

CACCAAGATTCGTGTAAAACCTGCTTTCCTCAGCAGGACCAAAGAGATACACTCTCCATT

CTTGTCCCCCATGGGGATAAAGAGAGTTCAGAGAAAAAAATTGCTGTAGGAACTGAATGG

GACATTCCAAGAAATGAAAGTTCAGATAGTGCCCTTGGGGATAGTGAAAGTGAAGATACA

GGTCATGATATGCCGAGACAAGTCAGCAGTTACTATGGAGGAGAGCAAGAAGATTGGGCA

GAAGAGGATGAGATACCTTTTCCTGGGTCAAAGTTAATTGAGGTTAGTGCTGTTCAGCCC

AACATTGCCAACTTTGGGAGGTCCTTGCTGGGTGGCTACTGCTCGTCTTATGTGCCTGAC

TTTGTTCTTCAAGGAATTGGGAATGATGAGAAGCTCCGCCAGTGTCTGGTGTCAGATTTG

TCTCATGCTGTGCAGCATCCAGTGTTGGATGAACCAATAGCAGAAGCTGTCTGCATTATA

GCTGATATGGATAAATGGACTGTTCAAGTGGCCAGCAGCCAGAGACGAGTGACAGATAAT

AAATTGGGAAAGGAAGTGTTGGTTTCGAGTCTTGTTTCCAACCTGCTTCATTCCACCCTT

CAGCTTTATAAGCATAACTTGTCTCCAAATTTTTGTGTAATGCATCTTGAAGACCGGCTG

CAGGAACTATACTTCAAGAGCAAAATGCTGTCTGAGTACTTGAGGGGCCAGATGCGTGTT

CATGTCAAGGAGCTGGGTGTGGTTCTTGGG

>CL95.Contig5_A-W 1 333 minus strand PREDICTED: protein FAM170A-like [Otolemur garnettii]

TTCTCCCTCTTTTGTTCAATAATGTTAGATTCAGAAAAATCAACTGCAATGTTGGGACCT

TCAGGGCTGACCCAAGATCACAGGTACACAACACAGGACTCTACCCCTGAAAATATCTGC

AGCACCTTTTCATCAGATAAAGTCACTAACAGAGAGTTTCAGAAGTTTAAAGAGGTTGTT

CCTGTACCATGTGCAGCGGTCCCAGACCGGACTCATCCATCAGGAGGAAATTCATTAGCC

TCAGAATATTTCACCTATGCACCCCCCACCAGGAAGCTTATTCCTACTGGCAAGCATGGG

TTCCAGATATTCCAACAGGTTGCTCCTGCCCCA

>CL95.Contig6_A-W 114 446 minus strand PREDICTED: protein FAM170A-like [Otolemur garnettii]

TTCTCCCTCTTTTGTTCAATAATGTTAGATTCAGAAAAATCAACTGCAATGTTGGGACCT

TCAGGGCTGACCCAAGATCACAGGTACACAACACAGGACTCTACCCCTGAAAATATCTGC

AGCACCTTTTCATCAGATAAAGTCACTAACAGAGAGTTTCAGAAGTTTAAAGAGGTTGTT

CCTGTACCATGTGCAGCGGTCCCAGACCGGACTCATCCATCAGGAGGAAATTCATTAGCC

TCAGAATATTTCACCTATGCACCCCCCACCAGGAAGCTTATTCCTACTGGCAAGCATGGG

TTCCAGATATTCCAACAGGTTGCTCCTGCCCCA

>CL95.Contig7_A-W 1 336 minus strand PREDICTED: folliculin-interacting protein 1 isoform 2 [Pan paniscus]

CGACGCAGCCAAACAACAAGTTTGGAAAATGGGGTATTTCCTAGATGGTCTATAGAAGAA

AGCTTTAATCTGTCAGATGAAAGCTGTGGCCCTAACCCAGGAATTGTGAGGAAAAAGAAG

ATTGCAATTGGGGTAATCTTTTCGTTATCCAAAGATGAAGATGAAAATAACAAATTTAAT

GAATTCTTTTTTTCACATTTTCCTCTCTTTGAAAGCCACATGAACAAATTAAAGAGTGCA

ATAGAACAGGCTATGAAAATGAGCCGGAGATCAGCTGATGCCAGTCAGAGAAGTTTGGCA

TATAATCGGATAGTTGATGCCCTGAATGAATTCAGG

>CL95.Contig10_A-W 45 149 minus strand folliculin-interacting protein 1 [Gallus gallus] >gi|82080030|sp|Q5W4S4.1|FNIP1_CHICK RecName: Full=Folliculin-interacting protein 1

GTAATGATTGTTTTCAAGGCTATGAAAATGAGCCGGAGATCAGCTGATGCCAGTCAGAGA

AGTTTGGCATATAATCGGATAGTTGATGCCCTGAATGAATTCAGG

>CL96.Contig1_A-W 2 2569 PREDICTED: dedicator of cytokinesis protein 10-like [Cavia porcellus]

GATCAAGCAGAAACCCGGAGTCTCCTTATGTGCTTTCTTCACATCATGAAGACAATTTCA

GATGAGACTCTGATTGCCTATTGGCAGAGAGCACCCAGTCCAGAGGTGTCTGACTTCTTC

AGTATCTTGGATGTTTGTCTTCAAAATTTCAGATACCTTGGAAAACGCAATATAATAAGA

AAAATTGCTGCTGCGTTTAAATTTGTGCAATCCACCCAGAACAATGGAACCCTCAAAGGA

TCCAATCCTTCCTGCCAGACATCAGGGCTCTTGTCACAATGGATGCACACCAACTCTGGT

CATGAAGGACATAAGCAGCACAGATCACAAACTTTACCTATAATTCGAGGCAAAAATGCA

CTTTCTAACCCCAAACTCTTACAGATGTTAGACAACACCATGTCCAGCAACTCCAATGAA

ATAGACATCGTGCACCACGTGGACACAGAAGCCAACGTGGCTACAGAGGTCTGCCTCACT

ATCCTCGACCTGCTGTCCCTCTTCACGCACGTACATCAGAGACAACTCCAACAAGCTGAC

TGTCAAAATTCATTGATGAAAAGGGTCTTTGACACCTACATGCTCTTTTTCCAAGTCAAC

CAGTCAGCCACAGCACTGAAGCATGTGTTTGCCTCTTTGAGACTGTTTGTATGCAAGTTT

CCCTCAGCATTCTTTCAAGGGCCTGCTGACCTCTGTGGATCGTTCTGCTATGAAGTCCTA

AAATGCTGTAACCACAGGTCACGGTCAACTCAGATGGAAGCCTCAGCACTTCTGTACTTC

TTCATGAGGAAGAATTTTGAATTTAACAAGCAGAAGTCAATTGTCCGTTCCCATTTACAG

CTCATCAAAGCAGTAAGCCAGTTAATAGCTGATGCAGGGATTGGAGGCTCTCGGTTTCAA

CATTCCCTCGCAATCACCAATAACTTTGCCAATGGAGACAAGCAGATGAAAAACAGCAAT

TTCCCAGCGGAGGTGAAAGACCTAACTAAGCGAATCCGTACAGTTTTGATGGCCACGGCT

CAGATGAAGGAACACGAGAAGGACCCTGAGATGCTGGTGGACCTCCAATACAGCCTGGCG

AATTCCTACGCAAGCACCCCCGAACTACGAAGGACCTGGCTAGAAAGCATGGCCAAGATT

CACGCAAGGAATGGAGACCTGTCCGAGGCTGCTATGTGTTACATCCATATCGCTGCCCTC

ATCGCAGAGTACCTGAAAAGGAAGGGTTACTGGAAAATGGAAAAGATTTGCACATCATCC

CTGCTCCCAGAGGATACCCACCCCTGCGATAGTAATCCATTACTAACAACTCCAGGTGGA

GGAAGCATGTTCTCTATGGGATGGCCAGCTTTCCTGAGCATTACACCAAATATTAAAGAA

GAAGGAGCAATGAAAGAAGATTCAGGAATGCAAGATACTCCATACAATGAGAATATCCTG

GTGGAGCAGCTGTACATGTGTGTGGAGTTTCTTTGGAAGTCGGAGCGATATGAACTCATT

GCTGATGTCAACAAGCCCATCATTGCTGTCTTCGAAAAGCAGCGAGACTTCAAGAAATTG

TCCGATCTCTACTATGACATCCACCGGTCATACTTAAAGGTGGCAGAGGTGGTGAACTCA

GAGAAGCGGCTGTTTGGTCGCTACTATCGTGTGGCATTTTATGGACAGGGTTTTTTTGAA

GAAGAAGAGGGTAAAGAGTATATTTACAAAGAGCCTAAGCTGACGGGTCTCTCTGAGATT

TCCCAAAGACTACTCAAACTCTATGCAGACAAATTTGGAGCAGATAACGTGAAGATAATC

CAAGATTCCAATAAGGTAAACCCCAAGGATTTGGACCCCAAATACGCCTACATCCAGGTG

ACTTATGTCACCCCGTTCTTTGAAGAAAAGGAAATAGAGGACAGGAAGACAGACTTTGAA

ATGCACCACAACATCAACCGCTTTGTCTTCGAGACGCCCTTTACCCTGTCGGGCAAGAAG

CACGGCGGAGTGGCCGAGCAGTGCAAGCGGAGGACGGTGCTGACAACGAGTCATTTGTTC

CCCTATGTGAAGAAGAGGATCCAGGTTGTCAGCCAATCGAGCACAGAACTGAATCCTATT

GAAGTGGCGATTGATGAGATGTCCAGGAAGGTCTCTGAGCTTAATCAGCTTTGCACGATG

GATGAGGTGGACATGATCAGACTACAGCTCAAACTGCAAGGAAGTGTCAGTGTGAAGGTC

AATGCTGGGCCAATGGCCTATGCACGAGCTTTTCTTGAAGAAACTAATGCAAAGAAATAC

CCTGACAACCAAGTAAAACTTTTGAAGGAAATATTCAGACAATTTGCAGATGCGTGTGGG

CAGGCCCTCGATGTGAATGAGCGCCTCATCAAGGAGGACCAGCTGGAGTACCAAGAAGAA

CTGCGGTCCCATTATAAGGACATGTTGAGCGAACTCTCCGCAATCATGAACGAGCAGATT

CCGGGCAGGGACGACCCAGCAAAGCGCGGAGTGGAGCGAACCTACACGCGTGTCATTAGC

AAGGCCCCTCCGGCGCCACCCCTGGTCTTCGTCTCGCAGGGTGCCGAA

>CL96.Contig2_A-W 190 303 Dedicator of cytokinesis protein 10 [Cricetulus griseus]

AAGATTCCGGGCAGGGACGACCCAGCAAAGCGCGGAGTGGAGCGAACCTACACGCGTGTC

ATTAGCAAGGCCCCTCCGGCGCCACCCCTGGTCTTCGTCTCGCAGGGTGCCGAA

>CL97.Contig1_A-W 1120 1476 rCG24330, isoform CRA_c [Rattus norvegicus]

GACTTCCTGCTGTGTGTCAGCAGCTCTGGTGCTGTGCTTCCTGTGAGGACCCAAGACCCC

TTTCTACCTTACCCACCAAGGTCCTGTCCACAGGGACAGGCTGCCTCTAATCCTACGACC

CGTGACCCTGCCCAAAGGGTGGTGCTGATGCTGCGTGAACGCTGGCATTTCTGCCGTGAT

GGCAGGGTGCTGCTGGGCTCCCGTGCCCTGAGGGAGCGGCACCTGGGCCTAATGGGCTAC

CAGCTCCTGCCGCTGCCCTTTGAGGAACTGGAGTCACAGAGAGGCCTGCCCCAGCTCAAG

AGCTACCTTAGGCAGAAGCTCCAGGCCTTAGGCCTCCGCTGGGGGCCTGAAGGAGGG

>CL97.Contig2_A-W 49 165 rCG24330, isoform CRA_c [Rattus norvegicus]

AGTCACAAGGACATAGTAGCTGAGGGGCTGCGCCAGCTGCTGGGGGAGGAGAAATACCGC

CAGGACCTGACTGTGCCTCCCGGCTACTGCACAGGTGAGCTCCAGGTGGGGGGCAGC

>CL97.Contig3_A-W 1550 1654 rCG24330, isoform CRA_c [Rattus norvegicus]

CTGCCCTTTGAGGAACTGGAGTCACAGAGAGGCCTGCCCCAGCTCAAGAGCTACCTTAGG

CAGAAGCTCCAGGCCTTAGGCCTCCGCTGGGGGCCTGAAGGAGGG

>CL97.Contig4_A-W 468 572 PREDICTED: Fas-activated serine/threonine kinase isoform 1 [Oryctolagus cuniculus]

CTGCCCTTTGAGGAACTGGAGTCACAGAGAGGCCTGCCCCAGCTCAAGAGCTACCTTAGG

CAGAAGCTCCAGGCCTTAGGCCTCCGCTGGGGGCCTGAAGGAGGG

>CL97.Contig5_A-W 49 165 rCG24330, isoform CRA_c [Rattus norvegicus]

AGTCACAAGGACATAGTAGCTGAGGGGCTGCGCCAGCTGCTGGGGGAGGAGAAATACCGC

CAGGACCTGACTGTGCCTCCCGGCTACTGCACAGGTGAGCTCCAGGTGGGGGGCAGC

>CL97.Contig6_A-W 49 498 PREDICTED: Fas-activated serine/threonine kinase isoform 2 [Oryctolagus cuniculus]

AGTCACAAGGACATAGTAGCTGAGGGGCTGCGCCAGCTGCTGGGGGAGGAGAAATACCGC

CAGGACCTGACTGTGCCTCCCGGCTACTGCACAGACTTCCTGCTGTGTGTCAGCAGCTCT

GGTGCTGTGCTTCCTGTGAGGACCCAAGACCCCTTTCTACCTTACCCACCAAGGTCCTGT

CCACAGGGACAGGCTGCCTCTAATCCTACGACCCGTGACCCTGCCCAAAGGGTGGTGCTG

ATGCTGCGTGAACGCTGGCATTTCTGCCGTGATGGCAGGGTGCTGCTGGGCTCCCGTGCC

CTGAGGGAGCGGCACCTGGGCCTAATGGGCTACCAGCTCCTGCCGCTGCCCTTTGAGGAA

CTGGAGTCACAGAGAGGCCTGCCCCAGCTCAAGAGCTACCTTAGGCAGAAGCTCCAGGCC

TTAGGCCTCCGCTGGGGGCCTGAAGGAGGG

>CL97.Contig7_A-W 1482 1586 PREDICTED: Fas-activated serine/threonine kinase isoform 1 [Oryctolagus cuniculus]

CTGCCCTTTGAGGAACTGGAGTCACAGAGAGGCCTGCCCCAGCTCAAGAGCTACCTTAGG

CAGAAGCTCCAGGCCTTAGGCCTCCGCTGGGGGCCTGAAGGAGGG

>CL97.Contig8_A-W 904 1179 rCG24330, isoform CRA_c [Rattus norvegicus]

GGCACCCCTCACGCTCTGATTGTTCGACGCTACCTCTCCCTGCTTGACGCGGCTGTGGAG

CTGGAGCTCCCAGGATACCGGGGCCCCCGCCTTCCCCGAAGGCAGCAAGTGCCCATCTTT

CCCCAGCCACTCATCACCGACCGTGCCCGCTGCAAGTACAGTCACAAGGACATAGTAGCT

GAGGGGCTGCGCCAGCTGCTGGGGGAGGAGAAATACCGCCAGGACCTGACTGTGCCTCCC

GGCTACTGCACAGGTGAGCTCCAGGTGGGGGGCAGC

>CL97.Contig9_A-W 1446 1550 rCG24330, isoform CRA_c [Rattus norvegicus]

CTGCCCTTTGAGGAACTGGAGTCACAGAGAGGCCTGCCCCAGCTCAAGAGCTACCTTAGG

CAGAAGCTCCAGGCCTTAGGCCTCCGCTGGGGGCCTGAAGGAGGG

>CL97.Contig10_A-W 901 1512 PREDICTED: Fas-activated serine/threonine kinase isoform 1 [Oryctolagus cuniculus]

ACAGGCACCCCTCACGCTCTGATTGTTCGACGCTACCTCTCCCTGCTTGACGCGGCTGTG

GAGCTGGAGCTCCCAGGATACCGGGGCCCCCGCCTTCCCCGAAGGCAGCAAGTGCCCATC

TTTCCCCAGCCACTCATCACCGACCGTGCCCGCTGCAAGTACAGTCACAAGGACATAGTA

GCTGAGGGGCTGCGCCAGCTGCTGGGGGAGGAGAAATACCGCCAGGACCTGACTGTGCCT

CCCGGCTACTGCACAGACTTCCTGCTGTGTGTCAGCAGCTCTGGTGCTGTGCTTCCTGTG

AGGACCCAAGACCCCTTTCTACCTTACCCACCAAGGTCCTGTCCACAGGGACAGGCTGCC

TCTAATCCTACGACCCGTGACCCTGCCCAAAGGGTGGTGCTGATGCTGCGTGAACGCTGG

CATTTCTGCCGTGATGGCAGGGTGCTGCTGGGCTCCCGTGCCCTGAGGGAGCGGCACCTG

GGCCTAATGGGCTACCAGCTCCTGCCGCTGCCCTTTGAGGAACTGGAGTCACAGAGAGGC

CTGCCCCAGCTCAAGAGCTACCTTAGGCAGAAGCTCCAGGCCTTAGGCCTCCGCTGGGGG

CCTGAAGGAGGG

>CL97.Contig11_A-W 1378 1482 PREDICTED: Fas-activated serine/threonine kinase isoform 1 [Oryctolagus cuniculus]

CTGCCCTTTGAGGAACTGGAGTCACAGAGAGGCCTGCCCCAGCTCAAGAGCTACCTTAGG

CAGAAGCTCCAGGCCTTAGGCCTCCGCTGGGGGCCTGAAGGAGGG

>CL97.Contig12_A-W 2 1408 PREDICTED: Fas-activated serine/threonine kinase isoform 1 [Oryctolagus cuniculus]

CATGTGCAAGGACTGCAGCGGCTTCTGGAACAGGCGAAGAGTCCTGGGGAGCTACTGCGC

TGGCTGGGCCAGAACCCCACCAAGGTTCGCGCCCACCACTATCCTGTGGCACTTCGTCGC

CTGGGCCAGCTCTTGGGGTCTCAGCCTCGGCCCCCTCCTGTGGAGCAGGCCACACTGCAG

GACTTGAGTCAGCTCATCATCCGAAACTGCCCCTCCTTTGACATTCATACCATCCACGTG

TGTCTGCACCTTGCAGTCTTACTTGGCTTTCCATCAGATGGACCTCTGGTGTGTGCCCTG

GAGCAGGAGCGAAGGTTTCGTCTCCCTCCAAAGCCACCTCCCCCTCTGCAGCCTGTCCTC

CGTGGTGGACAAAGGCTAGAAGCTGCTCTGAGCTGCCCCCGTTTCCTGCGGTATCCGAGG

CAGAATCTGATCAGCAGCCTGGCAGAGGCAAGGCCAGAAGAACTGACTCCTCATGTGATG

GTCCTTCTGGCCCAGCATCTCGCTCGGCATCGGTTGCGGGAGCCCCAGCTTCTGGAAGCC

ATTGCCCACTTCCTGGTGGTCCAGGAAGCCCAGCTCAGCAGCAAGGTGGTACAGAAGCTG

GTCTTGCCCTTTGGGCGGCTGAACTACCTACCCCTGGAGCAGCAGTTTATGCCCTGCCTT

GAGAGGATCCTGGCTCGGGAGGCAGGGGTGGCACCCCTGGCCACAGTCAACATTTTGATG

TCACTGTGCCAGTTACGGTGCCTGCCCTTCAGAGCCCTGCACTTTGTCTTCTCCCCGGGT

TTTATCAACCACATCAGTGGCACCCCTCACGCTCTGATTGTTCGACGCTACCTCTCCCTG

CTTGACGCGGCTGTGGAGCTGGAGCTCCCAGGATACCGGGGCCCCCGCCTTCCCCGAAGG

CAGCAAGTGCCCATCTTTCCCCAGCCACTCATCACCGACCGTGCCCGCTGCAAGTACAGT

CACAAGGACATAGTAGCTGAGGGGCTGCGCCAGCTGCTGGGGGAGGAGAAATACCGCCAG

GACCTGACTGTGCCTCCCGGCTACTGCACAGACTTCCTGCTGTGTGTCAGCAGCTCTGGT

GCTGTGCTTCCTGTGAGGACCCAAGACCCCTTTCTACCTTACCCACCAAGGTCCTGTCCA

CAGGGACAGGCTGCCTCTAATCCTACGACCCGTGACCCTGCCCAAAGGGTGGTGCTGATG

CTGCGTGAACGCTGGCATTTCTGCCGTGATGGCAGGGTGCTGCTGGGCTCCCGTGCCCTG

AGGGAGCGGCACCTGGGCCTAATGGGCTACCAGCTCCTGCCGCTGCCCTTTGAGGAACTG

GAGTCACAGAGAGGCCTGCCCCAGCTCAAGAGCTACCTTAGGCAGAAGCTCCAGGCCTTA

GGCCTCCGCTGGGGGCCTGAAGGAGGG

>CL97.Contig13_A-W 441 545 fas-activated serine/threonine kinase [Canis lupus familiaris]

CTGCCCTTTGAGGAACTGGAGTCACAGAGAGGCCTGCCCCAGCTCAAGAGCTACCTTAGG

CAGAAGCTCCAGGCCTTAGGCCTCCGCTGGGGGCCTGAAGGAGGG

>CL97.Contig14_A-W 44 202 fas-activated serine/threonine kinase [Canis lupus familiaris]

GACTTCCTGCTGTGTGTCAGCAGCTCTGGTGCTGTGCTTCCTGTGAGGACCCAAGACCCC

TTTCTACCTTACCCACCAAGGTCCTGTCCACAGGGACAGGCTGCCTCTAATCCTACGACC

CGTGACCCTGCCCAAAGGTAAGAAAGCAGGGTGCGGGAA

>CL99.Contig1_A-W 280 384 minus strand PREDICTED: protein O-mannosyl-transferase 2 [Cavia porcellus]

AGCTTCTACCTCTTCCACCCTCTGGCTTACGGGATGGTTGGTCCCTTAGCCCAGGACCCC

AAGAGTCCAATGGCAGGACTAAGGTGGCTGGAATCATGGGACTTT

>CL99.Contig2_A-W 2 499 minus strand PREDICTED: protein O-mannosyl-transferase 2 [Equus caballus]

ACAGACTTCCGGGTCTATCTGCTTGGCAATCCGGTGATCTGGTGGCTGAATCTGGTGAGC

ATTGCCCTCTACCTCCTCTCAGGGAGCATTGCTGCCATCGCCATACAGAGAGGGGCACAG

CTGCCTGCTGAGGTGGAAGGGCTGACCAAGATCCTGCTGCGAGGAGGAGGCCAGGTGCTG

CTCGGGTGGGTGCTCCATTACTTCCCATTCTTCCTGATGGGCCGGATCCTCTACTTCCAC

CACTACTTCCCAGCCATGCTCTTCTCCAGCATGTTGACAGGCATTCTGTGGGACACCCTC

CTGCGGCTCTGTGCCTGGGCTTTGGCTTCCTCTTGCCTGGCCAGGGGCGTACACGCCTTG

GGAATCCTGAGCCTGCTCCTGGGAGCTGCCTACAGCTTCTACCTCTTCCACCCTCTGGCT

TACGGGATGGTTGGTCCCTTAGCCCAGGACCCCAAGAGTCCAATGGCAGGACTAAGGTGG

CTGGAATCATGGGACTTT

>CL100.Contig1_A-W 2399 2509 KIAA0691 protein [Homo sapiens]

AGGCAGTACCTACCCCGGAACCCTTGCCCGACGCCCCCCTACCACCACCAGATGCCGCCC

CCACACTCGGACACCGTGGAGTTCTATCAGCGCCTGTCGACTGAGACGCTC

>CL100.Contig2_A-W 2299 2409 KIAA0691 protein [Homo sapiens]

AGGCAGTACCTACCCCGGAACCCTTGCCCGACGCCCCCCTACCACCACCAGATGCCGCCC

CCACACTCGGACACCGTGGAGTTCTATCAGCGCCTGTCGACTGAGACGCTC

>CL100.Contig3_A-W 160 282 KIAA0691 protein [Homo sapiens]

AGAGAGTGCGTCTGTAGGGCAGGGAAGATGGCGGACAAGCGCAAACTCCAAGGTGAGATT

GATCGCTGCCTTAAGAAGGTGTCCGAGGGTGTGGAGCAGTTTGAAGATATTTGGCAGAAG

GTT

>CL101.Contig1_A-W 43 2754 serine/arginine repetitive matrix protein 1 [Homo sapiens] >gi|83305833|sp|Q8IYB3.2|SRRM1_HUMAN RecName: Full=Serine/arginine repetitive matrix protein 1; AltName: Full=SR-related nuclear matrix protein of 160 kDa; Short=SRm160; AltName: Full=Ser/Arg-related nuclear matrix protein >gi|55665386|emb|CAH73090.1| serine/arginine repetitive matrix 1 [Homo sapiens] >gi|55959943|emb|CAI14682.1| serine/arginine repetitive matrix 1 [Homo sapiens] >gi|119615544|gb|EAW95138.1| serine/arginine repetitive matrix 1, isoform CRA_a [Homo sapiens]

ATGGACGCGGGATTCTTCCGCGGAACAAGTGCAGAACAGGATAATCGGTTCAGCAACAAA

CAGAAGAAACTACTGAAGCAGCTGAAATTTGCAGAATGCCTAGAAAAAAAGGTGGACATG

AGCAAAGTAAATTTGGAGGTTATAAAGCCTTGGATAACAAAACGAGTAACAGAAATCCTT

GGATTTGAAGATGATGTTGTGATTGAGTTTATATTCAACCAGCTGGAAGTGAAGAATCCA

GACTCCAAAATGATGCAAATCAACCTGACTGGATTTTTGAATGGGAAAAACGCTAGAGAA

TTTATGGGAGAGCTTTGGCCCCTACTGTTAAGTGCACAAGAAAACATCGCTGGAATCCCT

TCTGCTTTCCTAGAACTTAAGAAAGAAGAAATAAAACAGAGACAGATTGAACAAGAAAAA

TTGGCATCTATGAAAAAACAAGATGAAGACAAAGATAAGAGGGATAAGGAAGAAAAAGAA

AGCAGCAGAGAAAAAAGAGAGAGGTCTCGAAGCCCAAGAAGACGCAAATCCAGATCTCCT

TCCCCTAGAAGACGGTCTTCCCCTGTCAGGAGAGAGAGAAAGCGCAGTCATTCTCGATCT

CCCCGTCACAGAACCAAGAGCCGGAGTCCTTCCCCTGCTCCAGAAAAGAAGGAAAAAACT

CCAGAGCTCCCAGAACCTTCAGTGAAAGTAAAAGAACCTTCAGTACAAGAGGCCACATCT

ACAAGTGACATCTTGAAAGTTCCCAAGCCTGAACCTATACCAGAGCCTAAAGAACCTTCT

CCTGAAAAAAATTCCAAAAAAGAAAAGGAAAAGGAAAAGACTCGACCAAGATCTCGGTCA

CGTTCCAAATCAAGATCACGGACACGTTCTCGGTCTCCTTCTCATACTCGCCCAAGACGG

CGCCATAGATCCCGATCAAGATCCTATTCGCCCAGAAGGCGGCCAAGCCCAAGAAGGCGG

CCATCTCCTCGAAGAAGAACTCCACCGAGACGAATGCCTCCTCCACCAAGGCATCGAAGG

AGTAGATCTCCTGTTAGACGAAGAAGGCGTTCATCAGCATCCTTGTCTGGGAGTAGCTCA

TCATCCTCTTCATCTCGTTCCCGGTCACCGCCAAAGAAGCCTCCCAAGAGGACATCTAGC

CCCCCTCGAAAAACTCGTAGGTTATCTCCTTCAGCAAGTCCTCCTCGGAGAAGACACAGG

CCATCACCTCCAGCAACTCCTCCACCAAAAACTCGTCATTCCCCAACACCCCAGCAGTCA

AATCGTACAAGAAAAAGTCGTGTTTCTGTGTCTCCAGGGAGAACTTCAGGTAAAGTGACA

AAACATAAAGGTACTGAGAAAAGAGAGTCACCTTCACCAGCACCAAAGCCTAGGAAAGTA

GAGTTATCTGAATCAGAAGAAGATAAAGGTGGCAAAATGGCTGCAGCAGATTCTGTGCAG

CAGAGACGCCAATACAGACGACAGAACCAGCAGTCTTCTTCTGACTCTGGCTCCTCCTCC

TCTTCAGAAGATGAGCAACCCAAGAGATCACATGTGAAGAATGGCGAGGTAGGCAGGCGG

CGGAGACATTCCCCTTCTCGCAGTGCCTCTCCTTCACCACGGAAGCGCCAAAAAGAGACC

TCTCCTCGTGGTAGACGGAGGAGAAGTCCCTCCCCACCACCCGCCCGAAGGCGACGCTCT

CCTTCTCCCGCTCCTCCTCCTCGGCGGCGCAGATCTCCCACACCACCACCACGGCGAAGG

ACTCCTTCTCCTCCCCCTCGTCGGCGCTCACCTTCCCCAAGAAGATACTCTCCTCCAATA

CAGAGGAGATACTCTCCTTCTCCACCTCCAAAGAGAAGAACAGCCTCTCCTCCACCTCCT

CCTAAACGAAGGGCATCTCCATCTCCACCACCAAAGCGCCGGGTCTCCCATTCTCCTCCT

CCCAAACAGAGGAGCTCTCCAGTCGCCAAGAGACGTTCGCCTTCTTTATCATCAAAGCAT

AGGAAAGGGTCTTCCCCAAGCCGCTCTACCCGGGAGGCTCGGTCGCCACAACCAAACAAA

AGGCATTCTCCCTCGCCACGGCCTCGAGCTCCTCAGACATCTTCAAGTCCTCCACCTGTT

CGACGAGGAACATCAGCATCACCCCAAAGAAGGCAGTCCCCATCTCCAAGTACTAGGCCC

ATTAGGAGAGTCTCCAGGACTCCAGAACCTAAAAAGACAAAAAAGGCTGCCTCACCAAGC

CCACAGTCCGTAAGAAGGGTCTCATCCTCCCGATCTGTCTCTGGATCTCCTGAACCAGCA

GCTAAAAAGCCCCCAGCACCTCCATCCCCTGTCCAGTCTCAGTCACCCTCAACAAACTGG

TCACCAGCGGTACCGGTCAAAAAGGCTAAAAGCCCAACACCGAGCCCATCACCAGCCAGG

AATTCAGATCAAGAAGGAGGTGGAAAGAAAAAGAAGAAAAAGAAGGACAAGAAACACAAA

AAGGATAAGAAACACAAGAAGCACAAAAAACACAAGAAGGAAAAGGCAATGGCCGCAGCT

GCTGCTGCGCCTGTGACCCCTGCAGCCATTGCCGCCGCCACAACCACGTCAGCACAGGAG

GAACCCGAGGCAGCACCGGAGCCCAAGAAGGAGACTGAAAGTGAAGCTGAAGATAACCTT

GATGACTTAGAAAAGCACCTGCGAGAAAAAGCCCTGAGGTCCATGCGGAAGGCTCAAGTG

TCCCCTCAGTCT

>CL101.Contig2_A-W 43 2796 serine/arginine repetitive matrix 1, isoform CRA_c [Homo sapiens]

ATGGACGCGGGATTCTTCCGCGGAACAAGTGCAGAACAGGATAATCGGTTCAGCAACAAA

CAGAAGAAACTACTGAAGCAGCTGAAATTTGCAGAATGCCTAGAAAAAAAGGTGGACATG

AGCAAAGTAAATTTGGAGGTTATAAAGCCTTGGATAACAAAACGAGTAACAGAAATCCTT

GGATTTGAAGATGATGTTGTGATTGAGTTTATATTCAACCAGCTGGAAGTGAAGAATCCA

GACTCCAAAATGATGCAAATCAACCTGACTGGATTTTTGAATGGGAAAAACGCTAGAGAA

TTTATGGGAGAGCTTTGGCCCCTACTGTTAAGTGCACAAGAAAACATCGCTGGAATCCCT

TCTGCTTTCCTAGAACTTAAGAAAGAAGAAATAAAACAGAGACAGATTGAACAAGAAAAA

TTGGCATCTATGAAAAAACAAGATGAAGACAAAGATAAGAGGGATAAGGAAGAAAAAGAA

AGCAGCAGAGAAAAAAGAGAGAGGTCTCGAAGCCCAAGAAGACGCAAATCCAGATCTCCT

TCCCCTAGAAGACGGTCTTCCCCTGTCAGGAGAGAGAGAAAGCGCAGTCATTCTCGATCT

CCCCGTCACAGAACCAAGAGCCGGAGTCCTTCCCCTGCTCCAGAAAAGAAGGAAAAAACT

CCAGAGCTCCCAGAACCTTCAGTGAAAGTAAAAGAACCTTCAGTACAAGAGGCCACATCT

ACAAGTGACATCTTGAAAGTTCCCAAGCCTGAACCTATACCAGAGCCTAAAGAACCTTCT

CCTGAAAAAAATTCCAAAAAAGAAAAGGAAAAGGAAAAGACTCGACCAAGATCTCGGTCA

CGTTCCAAATCAAGATCACGGACACGTTCTCGGTCTCCTTCTCATACTCGCCCAAGACGG

CGCCATAGATCCCGATCAAGATCCTATTCGCCCAGAAGGCGGCCAAGCCCAAGAAGGCGG

CCATCTCCTCGAAGAAGAACTCCACCGAGACGAATGCCTCCTCCACCAAGGCATCGAAGG

AGTAGATCTCCTGTTAGACGAAGAAGGCGTTCATCAGCATCCTTGTCTGGGAGTAGCTCA

TCATCCTCTTCATCTCGTTCCCGGTCACCGCCAAAGAAGCCTCCCAAGAGGACATCTAGC

CCCCCTCGAAAAACTCGTAGGTTATCTCCTTCAGCAAGTCCTCCTCGGAGAAGACACAGG

CCATCACCTCCAGCAACTCCTCCACCAAAAACTCGTCATTCCCCAACACCCCAGCAGTCA

AATCGTACAAGAAAAAGTCGTGTTTCTGTGTCTCCAGGGAGAACTTCAGGTAAAGTGACA

AAACATAAAGGTACTGAGAAAAGAGAGTCACCTTCACCAGCACCAAAGCCTAGGAAAGTA

GAGTTATCTGAATCAGAAGAAGATAAAGGTGGCAAAATGGCTGCAGCAGATTCTGTGCAG

CAGAGACGCCAATACAGACGACAGAACCAGCAGTCTTCTTCTGACTCTGGCTCCTCCTCC

TCTTCAGAAGATGAGCAACCCAAGAGATCACATGTGAAGAATGGCGAGGTAGGCAGGCGG

CGGAGACATTCCCCTTCTCGCAGTGCCTCTCCTTCACCACGGAAGCGCCAAAAAGAGACC

TCTCCTCGGATGCAGATGGGAAAGCGATGGCAATCGCCAGTGACTAAAAGTGGTAGACGG

AGGAGAAGTCCCTCCCCACCACCCGCCCGAAGGCGACGCTCTCCTTCTCCCGCTCCTCCT

CCTCGGCGGCGCAGATCTCCCACACCACCACCACGGCGAAGGACTCCTTCTCCTCCCCCT

CGTCGGCGCTCACCTTCCCCAAGAAGATACTCTCCTCCAATACAGAGGAGATACTCTCCT

TCTCCACCTCCAAAGAGAAGAACAGCCTCTCCTCCACCTCCTCCTAAACGAAGGGCATCT

CCATCTCCACCACCAAAGCGCCGGGTCTCCCATTCTCCTCCTCCCAAACAGAGGAGCTCT

CCAGTCGCCAAGAGACGTTCGCCTTCTTTATCATCAAAGCATAGGAAAGGGTCTTCCCCA

AGCCGCTCTACCCGGGAGGCTCGGTCGCCACAACCAAACAAAAGGCATTCTCCCTCGCCA

CGGCCTCGAGCTCCTCAGACATCTTCAAGTCCTCCACCTGTTCGACGAGGAACATCAGCA

TCACCCCAAAGAAGGCAGTCCCCATCTCCAAGTACTAGGCCCATTAGGAGAGTCTCCAGG

ACTCCAGAACCTAAAAAGACAAAAAAGGCTGCCTCACCAAGCCCACAGTCCGTAAGAAGG

GTCTCATCCTCCCGATCTGTCTCTGGATCTCCTGAACCAGCAGCTAAAAAGCCCCCAGCA

CCTCCATCCCCTGTCCAGTCTCAGTCACCCTCAACAAACTGGTCACCAGCGGTACCGGTC

AAAAAGGCTAAAAGCCCAACACCGAGCCCATCACCAGCCAGGAATTCAGATCAAGAAGGA

GGTGGAAAGAAAAAGAAGAAAAAGAAGGACAAGAAACACAAAAAGGATAAGAAACACAAG

AAGCACAAAAAACACAAGAAGGAAAAGGCAATGGCCGCAGCTGCTGCTGCGCCTGTGACC

CCTGCAGCCATTGCCGCCGCCACAACCACGTCAGCACAGGAGGAACCCGAGGCAGCACCG

GAGCCCAAGAAGGAGACTGAAAGTGAAGCTGAAGATAACCTTGATGACTTAGAAAAGCAC

CTGCGAGAAAAAGCCCTGAGGTCCATGCGGAAGGCTCAAGTGTCCCCTCAGTCT

>CL101.Contig3_A-W 704 2071 serine/arginine repetitive matrix 1, isoform CRA_c [Homo sapiens]

TTATCTGAATCAGAAGAAGATAAAGGTGGCAAAATGGCTGCAGCAGATTCTGTGCAGCAG

AGACGCCAATACAGACGACAGAACCAGCAGTCTTCTTCTGACTCTGGCTCCTCCTCCTCT

TCAGAAGATGAGCAACCCAAGAGATCACATGTGAAGAATGGCGAGGTAGGCAGGCGGCGG

AGACATTCCCCTTCTCGCAGTGCCTCTCCTTCACCACGGAAGCGCCAAAAAGAGACCTCT

CCTCGGATGCAGATGGGAAAGCGATGGCAATCGCCAGTGACTAAAAGTGGTAGACGGAGG

AGAAGTCCCTCCCCACCACCCGCCCGAAGGCGACGCTCTCCTTCTCCCGCTCCTCCTCCT

CGGCGGCGCAGATCTCCCACACCACCACCACGGCGAAGGACTCCTTCTCCTCCCCCTCGT

CGGCGCTCACCTTCCCCAAGAAGATACTCTCCTCCAATACAGAGGAGATACTCTCCTTCT

CCACCTCCAAAGAGAAGAACAGCCTCTCCTCCACCTCCTCCTAAACGAAGGGCATCTCCA

TCTCCACCACCAAAGCGCCGGGTCTCCCATTCTCCTCCTCCCAAACAGAGGAGCTCTCCA

GTCGCCAAGAGACGTTCGCCTTCTTTATCATCAAAGCATAGGAAAGGGTCTTCCCCAAGC

CGCTCTACCCGGGAGGCTCGGTCGCCACAACCAAACAAAAGGCATTCTCCCTCGCCACGG

CCTCGAGCTCCTCAGACATCTTCAAGTCCTCCACCTGTTCGACGAGGAACATCAGCATCA

CCCCAAAGAAGGCAGTCCCCATCTCCAAGTACTAGGCCCATTAGGAGAGTCTCCAGGACT

CCAGAACCTAAAAAGACAAAAAAGGCTGCCTCACCAAGCCCACAGTCCGTAAGAAGGGTC

TCATCCTCCCGATCTGTCTCTGGATCTCCTGAACCAGCAGCTAAAAAGCCCCCAGCACCT

CCATCCCCTGTCCAGTCTCAGTCACCCTCAACAAACTGGTCACCAGCGGTACCGGTCAAA

AAGGCTAAAAGCCCAACACCGAGCCCATCACCAGCCAGGAATTCAGATCAAGAAGGAGGT

GGAAAGAAAAAGAAGAAAAAGAAGGACAAGAAACACAAAAAGGATAAGAAACACAAGAAG

CACAAAAAACACAAGAAGGAAAAGGCAATGGCCGCAGCTGCTGCTGCGCCTGTGACCCCT

GCAGCCATTGCCGCCGCCACAACCACGTCAGCACAGGAGGAACCCGAGGCAGCACCGGAG

CCCAAGAAGGAGACTGAAAGTGAAGCTGAAGATAACCTTGATGACTTAGAAAAGCACCTG

CGAGAAAAAGCCCTGAGGTCCATGCGGAAGGCTCAAGTGTCCCCTCAG

>CL101.Contig4_A-W 704 1930 serine/arginine repetitive matrix protein 1 [Homo sapiens] >gi|83305833|sp|Q8IYB3.2|SRRM1_HUMAN RecName: Full=Serine/arginine repetitive matrix protein 1; AltName: Full=SR-related nuclear matrix protein of 160 kDa; Short=SRm160; AltName: Full=Ser/Arg-related nuclear matrix protein >gi|55665386|emb|CAH73090.1| serine/arginine repetitive matrix 1 [Homo sapiens] >gi|55959943|emb|CAI14682.1| serine/arginine repetitive matrix 1 [Homo sapiens] >gi|119615544|gb|EAW95138.1| serine/arginine repetitive matrix 1, isoform CRA_a [Homo sapiens]

TTATCTGAATCAGAAGAAGATAAAGGTGGCAAAATGGCTGCAGCAGATTCTGTGCAGCAG

AGACGCCAATACAGACGACAGAACCAGCAGTCTTCTTCTGACTCTGGCTCCTCCTCCTCT

TCAGAAGATGAGCAACCCAAGAGATCACATGTGAAGAATGGCGAGGTAGGCAGGCGGCGG

AGACATTCCCCTTCTCGCAGTGCCTCTCCTTCACCACGGAAGCGCCAAAAAGAGACCTCT

CCTCGTGGTAGACGGAGGAGAAGTCCCTCCCCACCACCCGCCCGAAGGCGACGCTCTCCT

TCTCCCGCTCCTCCTCCTCGGCGGCGCAGATCTCCCACACCACCACCACGGCGAAGGACT

CCTTCTCCTCCCCCTCGTCGGCGCTCACCTTCCCCAAGAAGATACTCTCCTCCAATACAG

AGGAGATACTCTCCTTCTCCACCTCCAAAGAGAAGAACAGCCTCTCCTCCACCTCCTCCT

AAACGAAGGGCATCTCCATCTCCACCACCAAAGCGCCGGGTCTCCCATTCTCCTCCTCCC

AAACAGAGGAGCTCTCCAGTCGCCAAGAGACGTTCGCCTTCTTTATCATCAAAGCATAGG

AAAGGGTCTTCCCCAAGCCGCTCTACCCGGGAGGCTCGGTCGCCACAACCAAACAAAAGG

CATTCTCCCTCGCCACGGCCTCGAGCTCCTCAGACATCTTCAAGTCCTCCACCTGTTCGA

CGAGGAACATCAGCATCACCCCAAAGAAGGCAGTCCCCATCTCCAAGTACTAGGCCCATT

AGGAGAGTCTCCAGGACTCCAGAACCTAAAAAGACAAAAAAGGCTGCCTCACCAAGCCCA

CAGTCCGTAAGAAGGGTCTCATCCTCCCGATCTGTCTCTGGATCTCCTGAACCAGCAGCT

AAAAAGCCCCCAGCACCTCCATCCCCTGTCCAGTCTCAGTCACCCTCAACAAACTGGTCA

CCAGCGGTACCGGTCAAAAAGGCTAAAAGCCCAACACCGAGCCCATCACCAGCCAGGAAT

TCAGATCAAGAAGGAGGTGGAAAGAAAAAGAAGAAAAAGAAGGACAAGAAACACAAAAAG

GATAAGAAACACAAGAAGCACAAAAAACACAAGAAGGAAAAGGCAATGGCCGCAGCTGCT

GCTGCGCCTGTGACCCCTGCAGCCATTGCCGCCGCCACAACCACGTCAGCACAGGAGGAA

CCCGAGGCAGCACCGGAGCCCAAGAAG

>CL101.Contig5_A-W 115 426 PREDICTED: serine/arginine repetitive matrix protein 1 isoform 2 [Pan paniscus]

AATTCAGATCAAGAAGGAGGTGGAAAGAAAAAGAAGAAAAAGAAGGACAAGAAACACAAA

AAGGATAAGAAACACAAGAAGCACAAAAAACACAAGAAGGAAAAGGCAATGGCCGCAGCT

GCTGCTGCGCCTGTGACCCCTGCAGCCATTGCCGCCGCCACAACCACGTCAGCACAGGAG

GAACCCGAGGCAGCACCGGAGCCCAAGAAGGAGACTGAAAGTGAAGCTGAAGATAACCTT

GATGACTTAGAAAAGCACCTGCGAGAAAAAGCCCTGAGGTCCATGCGGAAGGCTCAAGTG

TCCCCTCAGTCT

>CL103.Contig1_A-W 1424 2053 minus strand TBC1 domain family member 10A isoform 2 [Macaca mulatta]

GGAGTCAAGATCATCTTCCGGGTGGGGTTGGTGCTACTGAAGCACGCACTGGGCTCCCCA

GAGAAGCTCAAAGCCTGCCAGGGCCAGTACGAGACCATCGAGCAACTGCGGAGCCTCAGT

CCCAAGATCATGCAGGAAGCCTTCCTGGTTCAGGAGGTGGTAGAGTTGCCTGTGACAGAG

CGCCAGATTGAGCGTGAGCATCTCATCCAGCTGCGGCGCTGGCAGGAGACACGGGGTGAG

ATGCAGTGTCGCTCCCCACCCAGGCTGCATGGTGCCAAGGCCATCCTAGATGCAGAGCCC

GGCCCCCGGCCTGCCCTGCAACCTTCACCGTCCATCCGCCTACCCCCAGATGCACCCCTC

CCTGGTCCCAAAGCCAAGCCCAAGCCTCCCAAGCAGGTCCAGAAGGAGCAGCGGAAGCAG

ACAAAAGCAAGTGGGCAACTGGACAAGTCCCTGTCCCCCAACCAAGCCGCGGTAGTGACG

GCTGCAGGAGATGCATGTCTCTCACAGGATGTGCCCTCAAAGGACCTGGCTTCTCAGGAC

CCAGCCCCCCAGGACTCAGCACCCCAGGACTTAGCCCACCACTGCTCCCAGGAGAGCCTG

ACATCCCAGGAGAGTGAAGATACCTACTTG

>CL104.Contig1_A-W 2 253 PREDICTED: ribose-phosphate pyrophosphokinase-like [Sarcophilus harrisii]

GATGACATGGCTGACACATGTGGTACCATCTGCCATGCTGCTGATAAACTGCTCTCAGCT

GGGGCCACTAAAGTTTATGCTATTCTTACTCATGGGATCTTCTCTGGGCCAGCTATATCC

AGAATCAATAATGCTTCCTTTGAGGCTGTTGTTGTCACAAACACAATTCCACAAGAGGAC

AAGATGAAACACTGCTCCAAGATTCAGGTGATTGACATTTCAATGATCCTGGCCGAGGCA

ATCCGAAGGACA

>CL104.Contig2_A-W 2 379 phosphoribosylphosphate synthetase (PRPS2) precursor [Rattus norvegicus]

GACAAGAGTCGTGCTCCAATTTCTGCAAAACTTGTGGCTAACATGCTCTCAGTTGCTGGG

GCTGATCATATCATCACCATGGATCTGCATGCCTCTCAGATACAGGGATTCTTTGATATT

CCTGTGGATAACTTATATGCAGAGCCTGCAGTTCTGCAGTGGATTCGGGAGAATATCACG

GAGTGGAAGAACTGTATCATTGTTTCACCGGATGCAGGGGGAGCCAAGAGGGTCACATCA

ATTGCAGACAGGTTGAATGTGGAATTTGCTTTGATTCACAAAGAGAGGAAGAAAGCAAAT

GAAGTGGACCGGATGGTTCTGGTGGGGGATGTGAAGGACCGCGTGGCCATCCTCGTGGAT

GACATGGCTGACACATGT

>CL104.Contig3_A-W 134 1087 Chain A, Crystal Structure Of Human Phosphoribosyl Pyrophosphate Synthetase 1 >gi|118137848|pdb|2H06|B Chain B, Crystal Structure Of Human Phosphoribosyl Pyrophosphate Synthetase 1 >gi|118137903|pdb|2HCR|A Chain A, Crystal Structure Of Human Phosphoribosyl Pyrophosphate Synthetase 1 In Complex With Amp(Atp), Cadmium And Sulfate Ion >gi|118137904|pdb|2HCR|B Chain B, Crystal Structure Of Human Phosphoribosyl Pyrophosphate Synthetase 1 In Complex With Amp(Atp), Cadmium And Sulfate Ion >gi|208435738|pdb|3EFH|A Chain A, Crystal Structure Of Human Phosphoribosyl Pyrophosphate Synthetase 1 >gi|208435739|pdb|3EFH|B Chain B, Crystal Structure Of Human Phosphoribosyl Pyrophosphate Synthetase 1 >gi|388325646|pdb|3S5J|B Chain B, 2.0a Crystal Structure Of Human Phosphoribosyl Pyrophosphate Synthetase 1 >gi|388325647|pdb|3S5J|A Chain A, 2.0a Crystal Structure Of Human Phosphoribosyl Pyrophosphate Synthetase 1

ATGCCGAATATCAAAATCTTCAGCGGCAGCTCTCATCAGGACTTATCCCAGAAAATTGCT

GACCGCCTGGGCCTGGAGCTAGGCAAGGTGGTGACTAAGAAATTCAGCAACCAGGAGACC

TGTGTGGAAATTGGTGAAAGTGTACGTGGAGAAGATGTCTACATCGTTCAAAGTGGTTGT

GGTGAAATCAACGACAATCTAATGGAGCTTTTGATCATGATTAATGCCTGCAAGATTGCT

TCAGCCAGCCGGGTTACTGCAGTCATCCCATGTTTCCCTTATGCCCGGCAGGATAAGAAG

GATAAGAGCCGGGCACCAATCTCGGCTAAGCTTGTTGCAAATATGCTGTCTGTAGCTGGT

GCAGATCATATTATCACCATGGATCTACATGCTTCTCAAATTCAGGGCTTTTTTGATATC

CCAGTGGACAATTTGTATGCAGAGCCAGCTGTCCTGAAGTGGATTAGGGAGAATATCTCT

GAGTGGAGGAACTGCACTATTGTGTCACCTGATGCTGGTGGAGCCAAGAGAGTGACCTCC

ATTGCAGACCGGTTGAACGTGGACTTTGCCTTGATTCACAAAGAACGGAAGAAGGCCAAT

GAAGTGGACCGCATGGTGCTAGTGGGAGATGTGAAGGATCGAGTCGCTATCCTTGTGGAT

GACATGGCTGACACATGTGGCACAATTTGTCATGCCGCTGACAAACTTCTCTCAGCTGGA

GCGACCAGAGTTTATGCTATCTTGACTCATGGAATCTTTTCTGGCCCAGCCATTTCTCGA

ATCAATAATGCATGTTTTGAAGCAGTAGTGGTCACCAATACCATACCTCAGGAGGACAAG

ATGAAGCATTGCTCTAAAATACAGGTGATTGACATCTCTATGATCCTTGCAGAAGCCATC

AGGAGAACTCATAATGGGGAATCTGTTTCCTACCTATTCAGTCATGTTCCTTTA

>CL106.Contig1_A-W 96 830 minus strand PREDICTED: N-acetylglucosamine-1-phosphotransferase subunit gamma-like [Cavia porcellus]

ATCTGGCAGGAGTGGGAGATCACCAACAACACCTTCACAGGCATGTGGATGCGGGACGGT

GACTCCTGCCGCTCCCGGAGCCGGCAGAGCAAGGTGGGGCTTGAGCGGGTACCCAGGTAG

AGGGCCTAGTCCTCCCTCAGCCACCCACTGAGGTTCCCTCCCCAGGTGGAGCTTACCTGT

GGGAAGAGCAACCGACTGGCCCATGTGTCTGAGCCAAGCACCTGCATCTATGCGTTGACC

TTTGAGACCCCCCTTGTCTGCCACCCCCATTCTTTGTTAGTGTACCCGACCCTGCCAGAG

GCCCTGCAGCAGCGGTGGGGACAGGTGGAGCAGGAACTGGCTGATGACCTGATCACTCCA

CAGGTGAGTGAGGCTCAGTGGGTCCCCCTGCTGGCCCCCAATCGGTGCTCCATCTCTTTC

CTCCACCTTCAGGGCTATGAGAAATCGCTGAGGGCACTTTTTGAGGATGCTGGCTACTTA

AAGGGTCCAGGAGAAAAGGAACCCACCCAGCAAGAAGGAGGGACAACAGGCCTGGGGTTT

GAGACCCTGGACAACTGTAAGAAGGCACACTCAGAACTGTCAAAGGAGATAAAGAGGCTT

GCCAGTCTGCTGAGCCAGCACCACATCCCCCACGCCAAGCCCACAGAAACCTCCCACTCT

GAGCACCTGGGCCAGCACTCTCCCACAGACGGGGTCTCAGAGCAGCTGAGAGGTGATCCA

GGACTGCGTGGGAAC

>CL106.Contig2_A-W 96 758 minus strand PREDICTED: N-acetylglucosamine-1-phosphotransferase subunit gamma-like [Cavia porcellus]

ATCTGGCAGGAGTGGGAGATCACCAACAACACCTTCACAGGCATGTGGATGCGGGACGGT

GACTCCTGCCGCTCCCGGAGCCGGCAGAGCAAGGTGGAGCTTACCTGTGGGAAGAGCAAC

CGACTGGCCCATGTGTCTGAGCCAAGCACCTGCATCTATGCGTTGACCTTTGAGACCCCC

CTTGTCTGCCACCCCCATTCTTTGTTAGTGTACCCGACCCTGCCAGAGGCCCTGCAGCAG

CGGTGGGGACAGGTGGAGCAGGAACTGGCTGATGACCTGATCACTCCACAGGTGAGTGAG

GCTCAGTGGGTCCCCCTGCTGGCCCCCAATCGGTGCTCCATCTCTTTCCTCCACCTTCAG

GGCTATGAGAAATCGCTGAGGGCACTTTTTGAGGATGCTGGCTACTTAAAGGGTCCAGGA

GAAAAGGAACCCACCCAGCAAGAAGGAGGGACAACAGGCCTGGGGTTTGAGACCCTGGAC

AACTGTAAGAAGGCACACTCAGAACTGTCAAAGGAGATAAAGAGGCTTGCCAGTCTGCTG

AGCCAGCACCACATCCCCCACGCCAAGCCCACAGAAACCTCCCACTCTGAGCACCTGGGC

CAGCACTCTCCCACAGACGGGGTCTCAGAGCAGCTGAGAGGTGATCCAGGACTGCGTGGG

AAC

>CL106.Contig3_A-W 66 329 minus strand N-acetylglucosamine-1-phosphotransferase subunit gamma OS=Homo sapiens GN=GNPTG PE=1 SV=1

AGGGACAGAAAGCCAGGGCCTGCGCATCTCTTCAGACTCTCTGGCAAGTGCTTCAGCCTG

GTGGAGTCCACGTACAAGTATGAATTCTGCCCATTCCACAACGTGACCCAGCATGAGCAG

ACCTTCCGCTGGAACGCCTACAGTGGGATCCTCGGCATCTGGCAGGAGTGGGAGATCACC

AACAACACCTTCACAGGCATGTGGATGCGGGACGGTGACTCCTGCCGCTCCCGGAGCCGG

CAGAGCAAGTGTACCCGACCCTGC

>CL106.Contig4_A-W 1094 1267 minus strand T-complex expressed 7 [Mus musculus]

CAGGCACACTCAGAACTGTCAAAGGAGATAAAGAGGCTTGCCAGTCTGCTGAGCCAGCAC

CACATCCCCCACGCCAAGCCCACAGAAACCTCCCACTCTGAGCACCTGGGCCAGCACTCT

CCCACAGACGGGGTCTCAGAGCAGCTGAGAGGTGATCCAGGACTGCGTGGGAAC

>CL106.Contig5_A-W 1036 1227 minus strand PREDICTED: N-acetylglucosamine-1-phosphotransferase subunit gamma-like [Cavia porcellus]

TCACTGGAACTCTTGTTCCAGGCACACTCAGAACTGTCAAAGGAGATAAAGAGGCTTGCC

AGTCTGCTGAGCCAGCACCACATCCCCCACGCCAAGCCCACAGAAACCTCCCACTCTGAG

CACCTGGGCCAGCACTCTCCCACAGACGGGGTCTCAGAGCAGCTGAGAGGTGATCCAGGA

CTGCGTGGGAAC

>CL106.Contig6_A-W 964 1155 minus strand PREDICTED: N-acetylglucosamine-1-phosphotransferase subunit gamma-like [Cavia porcellus]

TCACTGGAACTCTTGTTCCAGGCACACTCAGAACTGTCAAAGGAGATAAAGAGGCTTGCC

AGTCTGCTGAGCCAGCACCACATCCCCCACGCCAAGCCCACAGAAACCTCCCACTCTGAG

CACCTGGGCCAGCACTCTCCCACAGACGGGGTCTCAGAGCAGCTGAGAGGTGATCCAGGA

CTGCGTGGGAAC

>CL106.Contig7_A-W 876 1067 minus strand PREDICTED: N-acetylglucosamine-1-phosphotransferase subunit gamma-like [Cavia porcellus]

TCACTGGAACTCTTGTTCCAGGCACACTCAGAACTGTCAAAGGAGATAAAGAGGCTTGCC

AGTCTGCTGAGCCAGCACCACATCCCCCACGCCAAGCCCACAGAAACCTCCCACTCTGAG

CACCTGGGCCAGCACTCTCCCACAGACGGGGTCTCAGAGCAGCTGAGAGGTGATCCAGGA

CTGCGTGGGAAC

>CL106.Contig8_A-W 149 622 minus strand N-acetylglucosamine-1-phosphotransferase subunit gamma OS=Homo sapiens GN=GNPTG PE=1 SV=1

GGGCCCGCGCCAGCGGCTGCGGCCAAGATGAAGGTGGTGGAGGAGCCCAACGCCTTCGGG

CTCAACAACCCGTTCCTGCCCCAGACCAACCGCCTGCAGCCCAAGCGGGAGCCATCGCCG

CTCTCTGGGCCTGCGCATCTCTTCAGACTCTCTGGCAAGTGCTTCAGCCTGGTGGAGTCC

ACGTACAAGTATGAATTCTGCCCATTCCACAACGTGACCCAGCATGAGCAGACCTTCCGC

TGGAACGCCTACAGTGGGATCCTCGGCATCTGGCAGGAGTGGGAGATCACCAACAACACC

TTCACAGGCATGTGGATGCGGGACGGTGACTCCTGCCGCTCCCGGAGCCGGCAGAGCAAG

GTGGAGCTTACCTGTGGGAAGAGCAACCGACTGGCCCATGTGTCTGAGCCAAGCACCTGC

ATCTATGCGTTGACCTTTGAGACCCCCCTTGTCTGCCACCCCCATTCTTTGTTA

>CL106.Contig9_A-W 975 1166 minus strand PREDICTED: N-acetylglucosamine-1-phosphotransferase subunit gamma-like [Cavia porcellus]

TCACTGGAACTCTTGTTCCAGGCACACTCAGAACTGTCAAAGGAGATAAAGAGGCTTGCC

AGTCTGCTGAGCCAGCACCACATCCCCCACGCCAAGCCCACAGAAACCTCCCACTCTGAG

CACCTGGGCCAGCACTCTCCCACAGACGGGGTCTCAGAGCAGCTGAGAGGTGATCCAGGA

CTGCGTGGGAAC

>CL106.Contig10_A-W 804 995 minus strand PREDICTED: N-acetylglucosamine-1-phosphotransferase subunit gamma-like [Cavia porcellus]

TCACTGGAACTCTTGTTCCAGGCACACTCAGAACTGTCAAAGGAGATAAAGAGGCTTGCC

AGTCTGCTGAGCCAGCACCACATCCCCCACGCCAAGCCCACAGAAACCTCCCACTCTGAG

CACCTGGGCCAGCACTCTCCCACAGACGGGGTCTCAGAGCAGCTGAGAGGTGATCCAGGA

CTGCGTGGGAAC

>CL106.Contig11_A-W 149 1150 minus strand PREDICTED: N-acetylglucosamine-1-phosphotransferase subunit gamma-like [Cavia porcellus]

GGGCCCGCGCCAGCGGCTGCGGCCAAGATGAAGGTGGTGGAGGAGCCCAACGCCTTCGGG

CTCAACAACCCGTTCCTGCCCCAGACCAACCGCCTGCAGCCCAAGCGGGAGCCATCGCCG

CTCTCTGGGCCTGCGCATCTCTTCAGACTCTCTGGCAAGTGCTTCAGCCTGGTGGAGTCC

ACGTACAAGTATGAATTCTGCCCATTCCACAACGTGACCCAGCATGAGCAGACCTTCCGC

TGGAACGCCTACAGTGGGATCCTCGGCATCTGGCAGGAGTGGGAGATCACCAACAACACC

TTCACAGGCATGTGGATGCGGGACGGTGACTCCTGCCGCTCCCGGAGCCGGCAGAGCAAG

GTGGGGCTTGAGCGGGTACCCAGGTAGAGGGCCTAGTCCTCCCTCAGCCACCCACTGAGG

TTCCCTCCCCAGGTGGAGCTTACCTGTGGGAAGAGCAACCGACTGGCCCATGTGTCTGAG

CCAAGCACCTGCATCTATGCGTTGACCTTTGAGACCCCCCTTGTCTGCCACCCCCATTCT

TTGTTAGTGTACCCGACCCTGCCAGAGGCCCTGCAGCAGCGGTGGGGACAGGTGGAGCAG

GAACTGGCTGATGACCTGATCACTCCACAGGTGAGTGAGGCTCAGTGGGTCCCCCTGCTG

GCCCCCAATCGGTGCTCCATCTCTTTCCTCCACCTTCAGGGCTATGAGAAATCGCTGAGG

GCACTTTTTGAGGATGCTGGCTACTTAAAGGGTCCAGGAGAAAAGGAACCCACCCAGCAA

GAAGGAGGGACAACAGGCCTGGGGTTTGAGACCCTGGACAACTGTAAGAAGGCACACTCA

GAACTGTCAAAGGAGATAAAGAGGCTTGCCAGTCTGCTGAGCCAGCACCACATCCCCCAC

GCCAAGCCCACAGAAACCTCCCACTCTGAGCACCTGGGCCAGCACTCTCCCACAGACGGG

GTCTCAGAGCAGCTGAGAGGTGATCCAGGACTGCGTGGGAAC

>CL106.Contig12_A-W 93 911 minus strand PREDICTED: N-acetylglucosamine-1-phosphotransferase subunit gamma-like [Cavia porcellus]

TACAAGTATGAATTCTGCCCATTCCACAACGTGACCCAGCATGAGCAGACCTTCCGCTGG

AACGCCTACAGTGGGATCCTCGGCATCTGGCAGGAGTGGGAGATCACCAACAACACCTTC

ACAGGCATGTGGATGCGGGACGGTGACTCCTGCCGTTCCCGGAGCCGGCAGAGCAAGGTG

GGGCTTGAGCGGGTACCCAGGTAGAGGGCCTAGTCCTCCCTCAGCCACCCACTGAGGTTC

CCTCCCCAGGTGGAGCTTACCTGTGGGAAGAGCAACCGACTGGCCCATGTGTCTGAGCCA

AGCACCTGCATCTATGCGTTGACCTTTGAGACCCCCCTTGTCTGCCACCCCCATTCTTTG

TTAGTGTACCCGACCCTGCCAGAGGCCCTGCAGCAGCGGTGGGGACAGGTGGAGCAGGAA

CTGGCTGATGACCTGATCACTCCACAGGTGAGTGAGGCTCAGTGGGTCCCCCTGCTGGCC

CCCAATCGGTGCTCCATCTCTTTCCTCCACCTTCAGGGCTATGAGAAATCGCTGAGGGCA

CTTTTTGAGGATGCTGGCTACTTAAAGGGTCCAGGAGAAAAGGAACCCACCCAGCAAGAA

GGAGGGACAACAGGCCTGGGGTTTGAGACCCTGGACAACTGTAAGAAGGCACACTCAGAA

CTGTCAAAGGAGATAAAGAGGCTTGCCAGTCTGCTGAGCCAGCACCACATCCCCCACGCC

AAGCCCACAGAAACCTCCCACTCTGAGCACCTGGGCCAGCACTCTCCCACAGACGGGGTC

TCAGAGCAGCTGAGAGGTGATCCAGGACTGCGTGGGAAC

>CL107.Contig1_A-W 205 468 minus strand PREDICTED: t-cell differentiation antigen CD6 isoform 1 [Nomascus leucogenys]

CCCATCCACCGAGACCAGGTGAACTGCTCAGGAACTGAGGCCTACCTGTGGGACTGCCCC

GGGCTGCCAGGAGACCAGTACTGTGGCCACAAGGAAGACGCCGGCGCCGTGTGCTCAGAG

CACCAGTCCTGGCGCCTGACGGGGGGCATCGACCCCTGTGAAGGGCAGGTGGAGGTGTAC

TTCCGAGGGGTCTGGAGCACCGTGTGTGACAGTGAGTGGTACTCCTCAGAGGCTGAGGTG

CTGTGCCGGGCCCTGGGCTGTGGA

>CL107.Contig2_A-W 122 1051 minus strand PREDICTED: T-cell differentiation antigen CD6 [Saimiri boliviensis boliviensis]

GCAGGTTCTCGAAGATTGCAGAATATCTCTACTCCTGAAGTCCCTGCTAGTGTTCAGCCA

GCCACCATGGGGTCGTCTGTGACAGTGAAGGTGGAGGACTGGGGGTCCCGGGAGCTGACG

CTCCTCATCCCCTGCATCCTCCTGGCAGTTCTCCTCCTTGTCTCCCTTATCTGCATGGCT

GTCGTCTTCTTGAGAGTTAAAGGAAAATACGCCCTCCCTGTTCCAGCGAACCAGCAGCAC

CTTCCCACCACCATCCCAGCAGGGAGCAATAGCTATCAAGCGGTCCCCATCACCATCCCC

AAAGAAGAAGTTCCCAAGCTGCCCATCCAAGTCCGGGTCCCGCCCCCCGAGGACTCGAAC

TCCAGCTCGGACTCCGACTATGAGCACTATGACTTCAGCTCCCAGCCTCCCGTGGCCCTG

ACCACCTTCTACAATTCCCAGAGGCACCGGGTCACGGAGGAGGAGGCCCAGCAAAGCAGG

TTCCGGATGCCCCCTTTAGAGGAAGGGGCCGAGGAGCTGCATGTTTCCCACGCCCCAGCA

GCCATCCCCGGACACCACATTGCAGACCGTCCGTCTCTGGTCCCCCAGTTTCACCCAAGG

ATCAACAGTGGGTCCAGCACATCTTCTGGGGAGGATTACTGCAACAGCCCCAGCACCAAG

CCACCTCCGTGGAACCCCCAGGGGTTTTCTTCAGAGAGGAGCCCCTTCCAGGAGCAGCCC

CCCAACTTGGAGCTTGCTGGGTCCCAGGCAACTTTTGCAGCAGGGCCCTCGGCTGACGAC

AGCTCCAGCACCTCATCTGGCGGAGAGTGGTACCAGAACTTCCAGCCGCCACCTCAGCTC

CCCTCGGTGGAACAGCTCGAGTGCCCAGGGACCCACAACCCCCAACGGGACTCCACCGAC

CAGGAAGACTATGATGACATCGGAGCTGCC

>CL107.Contig3_A-W 55 774 minus strand PREDICTED: T-cell differentiation antigen CD6 [Papio anubis]

GCCCTCCCTGTTCCAGCGAACCAGCAGCACCTTCCCACCACCATCCCAGCAGGGAGCAAT

AGCTATCAAGCGGTCCCCATCACCATCCCCAAAGAAGAAGTTCCCAAGCTGCCCATCCAA

GTCCGGGTCCCGCCCCCCGAGGACTCGAACTCCAGCTCGGACTCCGACTATGAGCACTAT

GACTTCAGCTCCCAGCCTCCCGTGGCCCTGACCACCTTCTACAATTCCCAGAGGCACCGG

GTCACGGAGGAGGAGGCCCAGCAAAGCAGGTTCCGGATGCCCCCTTTAGAGGAAGGGGCC

GAGGAGCTGCATGTTTCCCACGCCCCAGCAGCCATCCCCGGACACCACATTGCAGACCGT

CCGTCTCTGGTCCCCCAGTTTCACCCAAGGATCAACAGTGGGTCCAGCACATCTTCTGGG

GAGGATTACTGCAACAGCCCCAGCACCAAGCCACCTCCGTGGAACCCCCAGGGGTTTTCT

TCAGAGAGGAGCCCCTTCCAGGAGCAGCCCCCCAACTTGGAGCTTGCTGGGTCCCAGGCA

ACTTTTGCAGCAGGGCCCTCGGCTGACGACAGCTCCAGCACCTCATCTGGCGGAGAGTGG

TACCAGAACTTCCAGCCGCCACCTCAGCTCCCCTCGGTGGAACAGCTCGAGTGCCCAGGG

ACCCACAACCCCCAACGGGACTCCACCGACCAGGAAGACTATGATGACATCGGAGCTGCC

>CL107.Contig4_A-W 62 583 minus strand T12 [Macaca fascicularis]

CCACTGCCACCTCTGCTTTTTGCAGATTCCCAGAGGCACCGGGTCACGGAGGAGGAGGCC

CAGCAAAGCAGGTTCCGGATGCCCCCTTTAGAGGAAGGGGCCGAGGAGCTGCATGTTTCC

CACGCCCCAGCAGCCATCCCCGGACACCACATTGCAGACCGTCCGTCTCTGGTCCCCCAG

TTTCACCCAAGGATCAACAGTGGGTCCAGCACATCTTCTGGGGAGGATTACTGCAACAGC

CCCAGCACCAAGCCACCTCCGTGGAACCCCCAGGGGTTTTCTTCAGAGAGGAGCCCCTTC

CAGGAGCAGCCCCCCAACTTGGAGCTTGCTGGGTCCCAGGCAACTTTTGCAGCAGGGCCC

TCGGCTGACGACAGCTCCAGCACCTCATCTGGCGGAGAGTGGTACCAGAACTTCCAGCCG

CCACCTCAGCTCCCCTCGGTGGAACAGCTCGAGTGCCCAGGGACCCACAACCCCCAACGG

GACTCCACCGACCAGGAAGACTATGATGACATCGGAGCTGCC

>CL107.Contig5_A-W 205 468 minus strand PREDICTED: t-cell differentiation antigen CD6 isoform 2 [Nomascus leucogenys]

CCCATCCACCGAGACCAGGTGAACTGCTCAGGAACTGAGGCCTACCTGTGGGACTGCCCC

GGGCTGCCAGGAGACCAGTACTGTGGCCACAAGGAAGACGCCGGCGCCGTGTGCTCAGAG

CACCAGTCCTGGCGCCTGACGGGGGGCATCGACCCCTGTGAAGGGCAGGTGGAGGTGTAC

TTCCGAGGGGTCTGGAGCACCGTGTGTGACAGTGAGTGGTACTCCTCAGAGGCTGAGGTG

CTGTGCCGGGCCCTGGGCTGTGGA

>CL108.Contig1_A-W 43 1635 PREDICTED: N-acetylglutamate synthase, mitochondrial [Canis lupus familiaris]

ATGGCGACAGCGCGGGTGGCAGCTACCCTGCGGACCGCTGCTACCGGCGGGAAGTTGCAC

GGCCCACGGGGCACTGGGGGCGCACGGAGACTGAGTGGTAGCGCACGGCGGCGGGCCGCT

GGGGGTGCCAGCCCGGGACGCCGGCTCAGCACCGCGCGGGCACGTGCCCAATCCCAGCCG

GAGGAGGCCGAGGGCGCTGAGGACCGCGTCCCGTCGCCCACTACAACCGAGCCATTGTGG

ACGCCGCCTCCGGCGCCGCCCGAAACCCCCGCGCCCCCGGCTGGCCGTTCTCTGGTGCAG

CGGGACATCCAGGCCTTCCTGAACCAGTGTGGGGCTAGCCCCGGGGAGGCGCGCCACTGG

CTCACCCAGTTCCAGAGCTGTCACTACTCCACAGACAAGCCCTTCGCCGTCATCGAGGTG

GATGAGGAGGTGCTCAAGTGCCCGCGGGGCGTATCCAGCCTGGCCTTCGCCCTGGCCTTT

CTGCAGCGCATGGACATGAAGCCACTGGTGGTTCTGGGGCTGCCGGCCCCCACAGCGCCC

TCGGGCTGTCTTTCATTCTGGGAGGCCAAAGCACAGCTTGCTCAGAGCTGCAAGGTGCTG

GTGGATGAGCTGAGGCACAACGCAGCCACTGCAGTGCCTTTTTTTGGCGGCGGGTCAGTG

CTGAGTGCTGCAGAGCCAGCCCCCCATGCCAGCTATGGTGGCATCGTCTCGGTGGAGACG

GACCTGTTGCAGTGGTGCCTGGAATCCAGCAGCATCCCCATCCTGTGCCCCATTGGGGAG

ACGGCTGCACGCCGCTCAGTGCTTCTCGATTCTCTGGAGGTGACCGCGGCGCTGGCCAAG

GCGCTGCAGCCCACCAAAATCATCTTCCTCAATAACTCAGGCGGCCTGCGCAACAGCAGT

CATAAGGTCCTGAGTAATGTGAACCTGCCTGCGGACCTGGACCTGGTGACCAACGCGGAG

TGGGTGAGCACCAAAGAACGGCAACAGATACGGCTCATTCTGGACGTGCTCAGCCGCCTG

CCCCACCACTCCTCGGCTGTCATCACCGCCGCCAGCACGCTGCTCACGGAGCTCTTCAGC

AACAAGGGCTCAGGGACCCTGTTCAAGAATGCCGAGAGGATGCTGCGAGTTTGCAACCTG

GACAGCCTAGACCAGGGGCGCCTGGTGAACTTGGTCAACGCCAGCTTTGGCAAGAAGCTC

CGGGACGACTACTTGGACTCTCTGCGTCCGCGGCTGCACTCCATCTATGTCTCTGAGGGG

TACAACGCTGCGGCCATTCTGACCCTAGAGCCCGTACTTGGGGGCACCCCGTACCTGGAC

AAATTTGTAGTGAGCTCCAGCCGCCAAGGCCAAGGTTCTGGCCAGATGCTGTGGGAATGC

CTGCGGCGGGACCTGCAGATGCTGTTCTGGCGCTCCAGGGTCACCAACCCCATCAATCCC

TGGTATTTCAAGCACAGTGATGGCAGCTTCTCCAACAAGCAGTGGATCTTCTTCTGGTTT

GGCCTAGCAGATATCCGGGACTCCTATGAACTGGTCAACCATGCCAAGGGGCTGCCAGAC

TCCTTCTGCAAGCCAGATTCTGACCCAGGCAGC

>CL108.Contig2_A-W 43 972 PREDICTED: N-acetylglutamate synthase, mitochondrial [Canis lupus familiaris]

ATGGCGACAGCGCGGGTGGCAGCTACCCTGCGGACCGCTGCTACCGGCGGGAAGTTGCAC

GGCCCACGGGGCACTGGGGGCGCACGGAGACTGAGTGGTAGCGCACGGCGGCGGGCCGCT

GGGGGTGCCAGCCCGGGACGCCGGCTCAGCACCGCGCGGGCACGTGCCCAATCCCAGCCG

GAGGAGGCCGAGGGCGCTGAGGACCGCGTCCCGTCGCCCACTACAACCGAGCCATTGTGG

ACGCCGCCTCCGGCGCCGCCCGAAACCCCCGCGCCCCCGGCTGGCCGTTCTCTGGTGCAG

CGGGACATCCAGGCCTTCCTGAACCAGTGTGGGGCTAGCCCCGGGGAGGCGCGCCACTGG

CTCACCCAGTTCCAGAGCTGTCACTACTCCACAGACAAGCCCTTCGCCGTCATCGAGGTG

GATGAGGAGGTGCTCAAGTGCCCGCGGGGCGTATCCAGCCTGGCCTTCGCCCTGGCCTTT

CTGCAGCGCATGGACATGAAGCCACTGGTGGTTCTGGGGCTGCCGGCCCCCACAGCGCCC

TCGGGCTGTCTTTCATTCTGGGAGGCCAAAGCACAGCTTGCTCAGAGCTGCAAGGTGCTG

GTGGATGAGCTGAGGCACAACGCAGCCACTGCAGTGCCTTTTTTTGGCGGCGGGTCAGTG

CTGAGTGCTGCAGAGCCAGCCCCCCATGCCAGCTATGGTGGCATCGTCTCGGTGGAGACG

GACCTGTTGCAGTGGTGCCTGGAATCCAGCAGCATCCCCATCCTGTGCCCCATTGGGGAG

ACGGCTGCACGCCGCTCAGTGCTTCTCGATTCTCTGGAGGTGACCGCGGCGCTGGCCAAG

GCGCTGCAGCCCACCAAAATCATCTTCCTCAATAACTCAGGCGGCCTGCGCAACAGCAGT

CATAAGGTGCGCTCCTCCTTTTGCACGCCC

>CL108.Contig3_A-W 43 1635 PREDICTED: N-acetylglutamate synthase, mitochondrial [Canis lupus familiaris]

ATGGCGACAGCGCGGGTGGCAGCTACCCTGCGGACCGCTGCTACCGGCGGGAAGTTGCAC

GGCCCACGGGGCACTGGGGGCGCACGGAGACTGAGTGGTAGCGCACGGCGGCGGGCCGCT

GGGGGTGCCAGCCCGGGACGCCGGCTCAGCACCGCGCGGGCACGTGCCCAATCCCAGCCG

GAGGAGGCCGAGGGCGCTGAGGACCGCGTCCCGTCGCCCACTACAACCGAGCCATTGTGG

ACGCCGCCTCCGGCGCCGCCCGAAACCCCCGCGCCCCCGGCTGGCCGTTCTCTGGTGCAG

CGGGACATCCAGGCCTTCCTGAACCAGTGTGGGGCTAGCCCCGGGGAGGCGCGCCACTGG

CTCACCCAGTTCCAGAGCTGTCACTACTCCACAGACAAGCCCTTCGCCGTCATCGAGGTG

GATGAGGAGGTGCTCAAGTGCCCGCGGGGCGTATCCAGCCTGGCCTTCGCCCTGGCCTTT

CTGCAGCGCATGGACATGAAGCCACTGGTGGTTCTGGGGCTGCCGGCCCCCACAGCGCCC

TCGGGCTGTCTTTCATTCTGGGAGGCCAAAGCACAGCTTGCTCAGAGCTGCAAGGTGCTG

GTGGATGAGCTGAGGCACAACGCAGCCACTGCAGTGCCTTTTTTTGGCGGCGGGTCAGTG

CTGAGTGCTGCAGAGCCAGCCCCCCATGCCAGCTATGGTGGCATCGTCTCGGTGGAGACG

GACCTGTTGCAGTGGTGCCTGGAATCCAGCAGCATCCCCATCCTGTGCCCCATTGGGGAG

ACGGCTGCACGCCGCTCAGTGCTTCTCGATTCTCTGGAGGTGACCGCGGCGCTGGCCAAG

GCGCTGCAGCCCACCAAAATCATCTTCCTCAATAACTCAGGCGGCCTGCGCAACAGCAGT

CATAAGGTCCTGAGTAATGTGAACCTGCCTGCGGACCTGGACCTGGTGACCAACGCGGAG

TGGGTGAGCACCAAAGAACGGCAACAGATACGGCTCATTCTGGACGTGCTCAGCCGCCTG

CCCCACCACTCCTCGGCTGTCATCACCGCCGCCAGCACGCTGCTCACGGAGCTCTTCAGC

AACAAGGGCTCAGGGACCCTGTTCAAGAATGCCGAGAGGATGCTGCGAGTTTGCAACCTG

GACAGCCTAGACCAGGGGCGCCTGGTGAACTTGGTCAACGCCAGCTTTGGCAAGAAGCTC

CGGGACGACTACTTGGACTCTCTGCGTCCGCGGCTGCACTCCATCTATGTCTCTGAGGGG

TACAACGCTGCGGCCATTCTGACCCTAGAGCCCGTACTTGGGGGCACCCCGTACCTGGAC

AAATTTGTAGTGAGCTCCAGCCGCCAAGGCCAAGGTTCTGGCCAGATGCTGTGGGAATGC

CTGCGGCGGGACCTGCAGATGCTGTTCTGGCGCTCCAGGGTCACCAACCCCATCAATCCC

TGGTATTTCAAGCACAGTGATGGCAGCTTCTCCAACAAGCAGTGGATCTTCTTCTGGTTT

GGCCTAGCAGATATCCGGGACTCCTATGAACTGGTCAACCATGCCAAGGGGCTGCCAGAC

TCCTTCTGCAAGCCAGATTCTGACCCAGGCAGC

>CL109.Contig1_A-W 3 665 PREDICTED: nuclear receptor corepressor 1-like [Papio anubis]

GGTGGAGGTGACTCTGATATGGCAGCTGCTCAGCCAGGAACTGAGATCTTCAATCTGCCA

GCAGTTACTACATCAGGCTCAGTTAGCTCTAGAAGTCATTCTTTTGCTGATCCTGCGAGT

AATCTTGGTCTAGAAGATATTATAAGGAAGGCTCTCATGGGAAGCTTTGATGACAAAGTT

GAGGATCATGGAGTTGTCATGCCCCAGCCTGTGGGAGTAGTGCCTGGTAGTGCCAGCACC

TCAGTTGTGACCAGCAATGAGACCAGGAGAGATGAAGGAGACCCATCACCTCACTCGGCA

GGAGTTTGCAAACCAAAGCTGATGAGCAAGTCAAACAGCAGAAAGTCTAAATCTCCTATC

CCTGGGCAAGGATATTTAGGAACTGAACGGCCCTCATCAGTATCCTCTGTACATTCAGAA

GGTGATTACCATAGACAGACACCAGGCTGGGCCTGGGAAGACAGGCCCTCCTCGACAGGC

TCAACTCAGTTTCCTTATAACCCTCTGACTATGCGGATGCTCAGCAGTACACCACCAACA

TCCATTGCATGTGCTCCCTCTGCTGTGAACCAAGCAGCTCCTCACCAACAGAACCGGATC

TGGGAGCGAGAGCCAGCCCCTCTGCTCTCTGCACAATATGAGACTCTATCCGATAGTGAT

GAC

>CL109.Contig2_A-W 633 1220 PREDICTED: nuclear receptor corepressor 1-like [Papio anubis]

GGCTCAGTTAGCTCTAGAAGTCATTCTTTTGCTGATCCTGCGAGTAATCTTGGTCTAGAA

GATATTATAAGGAAGGCTCTCATGGGAAGCTTTGATGACAAAGTTGAGGATCATGGAGTT

GTCATGCCCCAGCCTGTGGGAGTAGTGCCTGGTAGTGCCAGCACCTCAGTTGTGACCAGC

AATGAGACCAGGAGAGATGAAGGAGACCCATCACCTCACTCGGCAGGAGTTTGCAAACCA

AAGCTGATGAGCAAGTCAAACAGCAGAAAGTCTAAATCTCCTATCCCTGGGCAAGGATAT

TTAGGAACTGAACGGCCCTCATCAGTATCCTCTGTACATTCAGAAGGTGATTACCATAGA

CAGACACCAGGCTGGGCCTGGGAAGACAGGCCCTCCTCGACAGGCTCAACTCAGTTTCCT

TATAACCCTCTGACTATGCGGATGCTCAGCAGTACACCACCAACATCCATTGCATGTGCT

CCCTCTGCTGTGAACCAAGCAGCTCCTCACCAACAGAACCGGATCTGGGAGCGAGAGCCA

GCCCCTCTGCTCTCTGCACAATATGAGACTCTATCCGATAGTGATGAC

>CL109.Contig3_A-W 633 857 nuclear receptor corepressor 1 [Rattus norvegicus]

GGCTCAGTTAGCTCTAGAAGTCATTCTTTTGCTGATCCTGCGAGTAATCTTGGTCTAGAA

GATATTATAAGGAAGGCTCTCATGGGAAGCTTTGATGACAAAGTTGAGGATCATGGAGTT

GTCATGCCCCAGCCTGTGGGAGTAGTGCCTGGTAGTGCCAGCACCTCAGTTGTGACCAGC

AATGAGACCAGGAGAGATGAAGGAGACCCATCACCTCACTCGGGT

>CL109.Contig4_A-W 3 302 nuclear receptor corepressor 1 [Rattus norvegicus]

GGTGGAGGTGACTCTGATATGGCAGCTGCTCAGCCAGGAACTGAGATCTTCAATCTGCCA

GCAGTTACTACATCAGGCTCAGTTAGCTCTAGAAGTCATTCTTTTGCTGATCCTGCGAGT

AATCTTGGTCTAGAAGATATTATAAGGAAGGCTCTCATGGGAAGCTTTGATGACAAAGTT

GAGGATCATGGAGTTGTCATGCCCCAGCCTGTGGGAGTAGTGCCTGGTAGTGCCAGCACC

TCAGTTGTGACCAGCAATGAGACCAGGAGAGATGAAGGAGACCCATCACCTCACTCGGGT

>CL110.Contig1_A-W 2 1546 PREDICTED: ilvB (bacterial acetolactate synthase)-like isoform 4 [Pan troglodytes] >gi|397484967|ref|XP_003813635.1| PREDICTED: acetolactate synthase-like protein [Pan paniscus]

GCGGCAGTGACGGCAGGTCCTGGCCTCACCAATACAGTGACTGCTGTGAAGAATGCTCAG

GTGGCACAGTCCCCAGTCCTGCTCCTGGGAGGGGCCGCCAGTACCTTGCTGCAGAAACGG

GGTGCTCTCCAGGCCATTGATCAGATGTCCCTATTCCGGCCACTCTGCAAGTTCTGTGCA

TCTGTACGGAGGGTGCGGGACATTATTCCCACCCTGAGGGCTGCAATGGCAGCTGCCCAG

TCGGGCACCCCAGGTCCAGTGTTTGTGGAGCTTCCTGTCGATGTGCTGTACCCCTACTAC

ATGGTCCAGAAGGAGATGGTGCCAGCCAAGTTGCCCAAAGGCCTCATGGGTCGAGTGATC

TCCTGGTACTTAAAGAATTGCCTGGCCAACCTGTTTGTGGGGGCTTGGGAGCCTCAGCCT

GAAGGGCCCCTGCCCCTGGACATCCCCCAGGCATCCCCTCAACAGGTTCAGCGCTGTGTG

GAGATCCTGAGCCGGGCCAAGAAGCCCCTGTTGTTGCTGGGGAGTCAGGCCCTGCTGCCC

CCAGTACCTGCTGACAAGCTTCGGGCTGCTGTGGAGACCCTAGGCGTCCCCTGCTTCCTG

GGGGGGATGGCACGGGGGCTGCTGGGCCGCAACCACCCCCTCCACATCCGGCAGAACCGC

GGTGCAGCCCTGAAGAAAGCAGATGTTGTCCTCCTGGCAGGAGCTGTGTGTGACTTTCGC

CTCTCCTATGGCCGTATCCTCAGCCATAAGAGCAAGATAATTATCATCAATCGGAACCGA

GATGATATGTTGCTCAACTCAGACATCTTCTGGAAGCCCCAGGAGGCTGTGCAGGGAGAT

GTGGCCTCCTTCATGCTGAAGCTGGTAGAAGGCCTTCAGGGCCAGATGTGGGCTCCAGAG

TGGGCAGAAGAGCTGCGGGAAGCGGATCGGCAGAAGGAGCAGACCTTTCGGGAGAAGGCA

GCGATGCCTGTAGCCCAGCACCTGAACCCATTGAGGGTGTTGCAGCTGGTGGAGGAGACT

TTGCCTGACAACTCCTTTCTTGTGGTTGATGGTGGGGACTTCGTAGCAACTGCTGCCTAC

CTGGTACAGCCCCGAGGCCCCCTGCGCTGGCTTGATCCTGGGGCCTTTGGGACTTTGGGA

GTTGGCGCAGGGTTTGCACTTGGGGCCAAGCTGTGCCAGCCAGATGCTGAGGTCTGGTGT

CTGTTTGGGGATGGAGCCTTTGGTTACAGTCTCATTGAGTTTGACACATTTGTCAGACAC

AAGATTCCAGTGATGGCCTTGGTAGGCAATGATGCCGGCTGGACCCAGATTTCTCGGGAG

CAGGTGCCCAGATTGGGCAGCAATGTGGCCTGTGGCCTGGCCTACACTGATTATCACAAA

GCAGCCATGGGTCTGGGGGCCCAGGGCTTGCTACTCTCACGGGAGAATGAAGATCATGTG

GTCAAGGTGCTGCGTGATGGCCAGCAGCAGTGCCGGGATGGCCACCCAGTCGTGATCAAC

GTTCTCATTGGGAGGACGGACTTCCGTGATGGCTCCATTTCTGTA

>CL110.Contig2_A-W 1183 1608 PREDICTED: ilvB (bacterial acetolactate synthase)-like isoform 4 [Pan troglodytes] >gi|397484967|ref|XP_003813635.1| PREDICTED: acetolactate synthase-like protein [Pan paniscus]

GGGGCCTTTGGGACTTTGGGAGTTGGCGCAGGGTTTGCACTTGGGGCCAAGCTGTGCCAG

CCAGATGCTGAGGTCTGGTGTCTGTTTGGGGATGGAGCCTTTGGTTACAGTCTCATTGAG

TTTGACACATTTGTCAGACACAAGATTCCAGTGATGGCCTTGGTAGGCAATGATGCCGGC

TGGACCCAGATTTCTCGGGAGCAGGTGCCCAGATTGGGCAGCAATGTGGCCTGTGGCCTG

GCCTACACTGATTATCACAAAGCAGCCATGGGTCTGGGGGCCCAGGGCTTGCTACTCTCA

CGGGAGAATGAAGATCATGTGGTCAAGGTGCTGCGTGATGGCCAGCAGCAGTGCCGGGAT

GGCCACCCAGTCGTGATCAACATTCTCATTGGGAGGACGGACTTCCGTGATGGCTCCATT

TCTGTA

>CL110.Contig3_A-W 2 937 PREDICTED: acetolactate synthase-like protein [Cricetulus griseus]

GGTCCTGGCCTCACCAATACAGTGACTGCTGTGAAGAATGCTCAGGTGGCACAGTCCCCA

GTCCTGCTCCTGGGAGGGGCCGCCAGTACCTTGCTGCAGAAACGGGGTGCTCTCCAGGCC

ATTGATCAGATGTCCCTATTCCGGCCACTCTGCAAGTTCTGTGCATCTGTACGGAGGGTG

CGGGACATTATTCCCACCCTGAGGGCTGCAATGGCAGCTGCCCAGTCGGGCACCCCAGGT

CCAGTGTTTGTGGAGCTTCCTGTCGATGTGCTGTATCCCTACTACATGGTCCAGAAGGAG

ATGGTGCCAGCCAAGTTGCCCAAAGGCCTCATGGGTCGAGTGATCTCCTGGTACTTAAAG

AATTGCCTGGCCAACCTGTTTGTGGGGGCTTGGGAGCCTCAGCCTGAAGGGCCCCTGCCC

CTGGACATCCCCCAGGCATCCCCTCAACAGGTTCAGCGCTGTGTGGAGATCCTGAGCCGG

GCCAAGAAGCCCCTGTTGTTGCTGGGGAGTCAGGCCCTGCTGCCCCCAGTACCTGCTGAC

AAGCTTCGGGCTGCTGTGGAGACCCTAGGCGTCCCCTGCTTCCTGGGGGGGATGGCACGG

GGGCTGCTGGGCCGCAACCACCCCCTCCACATCCGGCAGAACCGCGGTGCAGCCCTGAAG

AAAGCAGATGTTGTCCTCCTGGCAGGAGCTGTGTGTGACTTTCGCCTCTCCTATGGCCGT

ATCCTCAGCCATAAGAGCAAGATAATTATCATCAATCGGAACCGAGATGATATGTTGCTC

AACTCAGACATCTTCTGGAAGCCCCAGGAGGCTGTGCAGGGAGATGTGGCCTCCTTCATG

CTGAAGCTGGTAGAAGGCCTTCAGGGCCAGATGTGGGCTCCAGAGTGGGCAGAAGAGCTG

CGGGAAGCGGATCGGCAGAAGGAGCAGACCTTTCGG

>CL110.Contig4_A-W 2 826 acetolactate synthase-like protein [Rattus norvegicus]

GGTCCTGGCCTCACCAATACAGTGACTGCTGTGAAGAATGCTCAGGTGGCACAGTCCCCA

GTCCTGCTCCTGGGAGGGGCCGCCAGTACCTTGCTGCAGAAACGGGGTGCTCTCCAGGCC

ATTGATCAGATGTCCCTATTCCGGCCACTCTGCAAGTTCTGTGCATCTGTACGGAGGGTG

CGGGACATTATTCCCACCCTGAGGGCTGCAATGGCAGCTGCCCAGTCGGGCACCCCAGGT

CCAGTGTTTGTGGAGCTTCCTGTCGATGTGCTGTATCCCTACTACATGGTCCAGAAGGAG

ATGGTGCCAGCCAAGTTGCCCAAAGGCCTCATGGGTCGAGTGATCTCCTGGTACTTAAAG

AATTGCCTGGCCAACCTGTTTGTGGGGGCTTGGGAGCCTCAGCCTGAAGGGCCCCTGCCC

CTGGACATCCCCCAGGCATCCCCTCAACAGGTTCAGCGCTGTGTGGAGATCCTGAGCCGG

GCCAAGAAGCCCCTGTTGTTGCTGGGGAGTCAGGCCCTGCTGCCCCCAGTACCTGCTGAC

AAGCTTCGGGCTGCTGTGGAGACCCTAGGCGTCCCCTGCTTCCTGGGGGGGATGGCACGG

GGGCTGCTGGGCCGCAACCACCCCCTCCACATCCGGCAGAACCGCGGTGCAGCCCTGAAG

AAAGCAGATGTTGTCCTCCTGGCAGGAGCTGTGTGTGACTTTCGCCTCTCCTATGGCCGT

ATCCTCAGCCATAAGAGCAAGATAATTATCATCAATCGGAACCGAGATGATATGTTGCTC

AACTCAGACATCTTCTGGAAGCCCCAGGAGGCTGTGCAGGGTGAG

>CL110.Contig5_A-W 2 1663 PREDICTED: ilvB (bacterial acetolactate synthase)-like isoform 4 [Pan troglodytes] >gi|397484967|ref|XP_003813635.1| PREDICTED: acetolactate synthase-like protein [Pan paniscus]

GGTCCTGGCCTCACCAATACAGTGACTGCTGTGAAGAATGCTCAGGTGGCACAGTCCCCA

GTCCTGCTCCTGGGAGGGGCCGCCAGTACCTTGCTGCAGAAACGGGGTGCTCTCCAGGCC

ATTGATCAGATGTCCCTATTCCGGCCACTCTGCAAGTTCTGTGCATCTGTACGGAGGGTG

CGGGACATTATTCCCACCCTGAGGGCTGCAATGGCAGCTGCCCAGTCGGGCACCCCAGGT

CCAGTGTTTGTGGAGCTTCCTGTCGATGTGCTGTATCCCTACTACATGGTCCAGAAGGAG

ATGGTGCCAGCCAAGTTGCCCAAAGGCCTCATGGGTCGAGTGATCTCCTGGTACTTAAAG

AATTGCCTGGCCAACCTGTTTGTGGGGGCTTGGGAGCCTCAGCCTGAAGGGCCCCTGCCC

CTGGACATCCCCCAGGCATCCCCTCAACAGGTTCAGCGCTGTGTGGAGATCCTGAGCCGG

GCCAAGAAGCCCCTGTTGTTGCTGGGGAGTCAGGCCCTGCTGCCCCCAGTACCTGCTGAC

AAGCTTCGGGCTGCTGTGGAGACCCTAGGCGTCCCCTGCTTCCTGGGGGGGATGGCACGG

GGGCTGCTGGGCCGCAACCACCCCCTCCACATCCGGCAGAACCGCGGTGCAGCCCTGAAG

AAAGCAGATGTTGTCCTCCTGGCAGGAGCTGTGTGTGACTTTCGCCTCTCCTATGGCCGT

ATCCTCAGCCATAAGAGCAAGATAATTATCATCAATCGGAACCGAGATGATATGTTGCTC

AACTCAGACATCTTCTGGAAGCCCCAGGAGGCTGTGCAGGGAGATGTGGCCTCCTTCATG

CTGAAGCTGGTAGAAGGCCTTCAGGGCCAGATGTGGGCTCCAGAGTGGGCAGAAGAGCTG

CGGGAAGCGGATCGGCAGAAGGAGCAGACCTTTCGGGAGAAGGCAGCGATGCCTGTAGCC

CAGCACCTGAACCCATTGAGGGTGTTGCAGCTGGTGGAGGAGACTTTGCCTGACAACTCC

TTTCTTGTGGTTGATGGTGGGGACTTCGTAGCAACTGCTGCCTACCTGGTACAGCCCCGA

GGCCCCCTGCGCTGGCTTGATCCTGGGGCCTTTGGGACTTTGGGAGTTGGCGCAGGGTTT

GCACTTGGGGCCAAGCTGTGCCAGCCAGATGCTGAGGTCTGGTGTCTGTTTGGGGATGGA

GCCTTTGGTTACAGTCTCATTGAGTTTGACACATTTGTCAGACACAAGATTCCAGTGATG

GCCTTGGTAGGCAATGATGCCGGCTGGACCCAGATTTCTCGGGAGCAGGTGCCCAGATTG

GGCAGCAATGTGGCCTGTGGCCTGGCCTACACTGGTGAGGGGTCTGGGCAGGGTGTGTGT

GGGTAGGTGGGGTCCTATCTCAGGCCCATCAGGGCTAGTGTTCCTCTCCTGGACTGGCTA

CATTCTGGGCTGGGCCTCTGCCAGTATCTGACTTGCTCTCTTTTAGATTATCACAAAGCA

GCCATGGGTCTGGGGGCCCAGGGCTTGCTACTCTCACGGGAGAATGAAGATCATGTGGTC

AAGGTGCTGCGTGATGGCCAGCAGCAGTGCCGGGATGGCCACCCAGTCGTGATCAACATT

CTCATTGGGAGGACGGACTTCCGTGATGGCTCCATTTCTGTA

>CL110.Contig6_A-W 1183 1740 PREDICTED: ilvB (bacterial acetolactate synthase)-like isoform 4 [Pan troglodytes] >gi|397484967|ref|XP_003813635.1| PREDICTED: acetolactate synthase-like protein [Pan paniscus]

GGGGCCTTTGGGACTTTGGGAGTTGGCGCAGGGTTTGCACTTGGGGCCAAGCTGTGCCAG

CCAGATGCTGAGGTCTGGTGTCTGTTTGGGGATGGAGCCTTTGGTTACAGTCTCATTGAG

TTTGACACATTTGTCAGACACAAGATTCCAGTGATGGCCTTGGTAGGCAATGATGCCGGC

TGGACCCAGATTTCTCGGGAGCAGGTGCCCAGATTGGGCAGCAATGTGGCCTGTGGCCTG

GCCTACACTGGTGAGGGGTCTGGGCAGGGTGTGTGTGGGTAGGTGGGGTCCTATCTCAGG

CCCATCAGGGCTAGTGTTCCTCTCCTGGACTGGCTACATTCTGGGCTGGGCCTCTGCCAG

TATCTGACTTGCTCTCTTTTAGATTATCACAAAGCAGCCATGGGTCTGGGGGCCCAGGGC

TTGCTACTCTCACGGGAGAATGAAGATCATGTGGTCAAGGTGCTGCGTGATGGCCAGCAG

CAGTGCCGGGATGGCCACCCAGTCGTGATCAACATTCTCATTGGGAGGACGGACTTCCGT

GATGGCTCCATTTCTGTA

>CL111.Contig1_A-W 258 704 PREDICTED: spatacsin isoform 3 [Nomascus leucogenys]

CTTTACACAAAGGCCACTAGGGTCCCCCCTATCATCCCTGCCACATGGCTGAAGGATCAG

GTGTGTTTCCTTTTGAAGCTTATGCCACAGCAGTGTCAGACACAGTACGAGTTGGGGAAG

CTCTTACAGCTGTTGGTTGGAGTACAGCATCTCTTCTCTGATGGTCCAGATATGAAGAAG

CTGTGTGTCCTTAGCCAAATTTTGAAGGATACATCCATAGCCATTAATCATGTAATTATT

ACCAGCTACAGCACTGAGAATTTTCAGCATGAATGTAGATTGATTTTGGAAAGACTGCAG

ATGGATGGACAGTTTGCTTTGGCCAGGAAGGTAGCAGAATTAGCTGAGTTACCTGTGGAC

AACTTGGTTATTAAAGAGATCACACAAGAAATGCAGATACTAAAACACATTGAACAGTGG

TCCCTGAAACAAGCAAGAATTGATTTT

>CL111.Contig2_A-W 258 461 PREDICTED: spatacsin [Oryctolagus cuniculus]

CTTTACACAAAGGCCACTAGGGTCCCCCCTATCATCCCTGCCACATGGCTGAAGGATCAG

GTGTGTTTCCTTTTGAAGCTTATGCCACAGCAGTGTCAGACACAGTACGAGTTGGGGAAG

CTCTTACAGCTGTTGGTTGGAGTACAGCATCTCTTCTCTGATGGTAAGAGTTCTTACCCT

GAATTTGCTATTAAGAAATTAGTT

>CL112.Contig1_A-W 1293 1721 PREDICTED: LOW QUALITY PROTEIN: sterol O-acyltransferase 2-like [Sus scrofa]

CAGGACTGGTGGAACTCAACATCCTTCTCCAACTACTACCGCACTTGGAACGTGGTGGTC

CATGACTGGCTGTACAGCTACGTGTATCAGGATGGGCTGTGGCTCCTTGGTGGCCGGGGC

CGAGGGGCAGCCATGCTGGGCGTGTTCCTGGTTTCTGCAGTGGTGCATGAGTACATCTTC

TGCTTTGTCCTGGGATTCTTCTACCCTGTCATGCTGATGCTCTTCCTTGTCATTGGGGGG

CTACTGAACTTCACAATGAATGACAGGCACACGGGCCCCGCGTGGAATGTGTTGATGTGG

ACCTTACTCTTTCTGGGCCAGGGCATCCAAGTCAGCCTGTACTGCCAGGAATGGTATGCA

CGATGGCACTGTCCCCTGCCCCAGACAACCTTCTGGGGGCTGCTGACACCTCGATCTTGG

TCCTGCCAT

>CL112.Contig2_A-W 324 1280 Sterol O-acyltransferase 2 OS=Chlorocebus aethiops GN=SOAT2 PE=2 SV=1

AAGGCAGTAAAGATGCAATTGTTGGAGCAAGCACAGGAAAAGCTGGTGGAGCTGCTGGAT

CGGGCCATATGGGAGGCCATACAATCCCACCCGCCACAAGACAGACCTCTGCCCTCCCCT

CACCCAGCTCCCTTAAGCAAGACCCAGAAGCCATCCTTTGGAAGACGGAAAGTTTTCGTT

GTCCGCAAATCCTTGCTTGATGAGCTGATGGAGGTGCAGCATTTCCGTACCATCTACCAC

ATGTTTATTGCCGGCCTGTGTGTCTTCATCATTAGCACCCTGGCCATCGACTTCATTGAT

GAGGGCAGGCTGGTGCTGGAGTTTGATCTGCTGATCTTCAGCTTCGGACAGCTGCCCCTG

GCGCTGGTGACGTGGGTCCCCATGTTCCTGGCCACTCTGCTGGCGCCCTACCAGGCCCTG

CGGCTGTGGGCCAGGCCCAGGGCAGGGGGCGCCTGGATGCTGAGGGCGGTCCTGGGCTGT

GTGCTGCTGGCTGCCCACGCTGCGGTGCTCTGTGTGCTCCCAATCCACGTGGCAGTGAAG

CATGAGCTCCCGCCAGCCTCGCGTTGCATCCTAGTCTTTGAGCAGGTCAGGCTCCTGATG

AAAAGTTACTCCTTCCTGAGAGAGGCTGTGCCTGGGACCCTTCGTGCTAGAGGACGTGAG

GGCATCTGGGCCCCCAGTTTCTCCAGCTACCTCTACTTCCTTTTCTGCCCCACACTCATC

TACCGGGAGACTTACCCCAGGACACCCAGCGTCAGGTGGAATTACGTGGCCAAGAACTTT

GCCCAGGCCCTGGGCTGTGTGCTCTATGCCTGCTTCATCCTGGGCCGCCTCTGTGTTCCT

ATCTTTGCCAACATGAGCCGGGAGCCCTTCAGCACGCGGGCCCTGCTGCTCTCTATCATG

CATGCCACTCTGCCAGGACTGGTGGAACTCAACATCCTTCTCCAACTACTACCGCAC

>CL112.Contig3_A-W 851 1441 RecName: Full=Sterol O-acyltransferase 2; AltName: Full=Acyl-coenzyme A:cholesterol acyltransferase 2; Short=ACAT-2; AltName: Full=Cholesterol acyltransferase 2 >gi|3582752|gb|AAC62929.1| acyl-CoA:cholesterol acyltransferase 2 [Chlorocebus aethiops]

CTGGGCTGTGTGCTGCTGGCTGCCCACGCTGCGGTGCTCTGTGTGCTCCCAATCCACGTG

GCAGTGAAGCATGAGCTCCCGCCAGCCTCGCGTTGCATCCTAGTCTTTGAGCAGGTCAGG

CTCCTGATGAAAAGTTACTCCTTCCTGAGAGAGGCTGTGCCTGGGACCCTTCGTGCTAGA

GGACGTGAGGGCATCTGGGCCCCCAGTTTCTCCAGCTACCTCTACTTCCTTTTCTGCCCC

ACACTCATCTACCGGGAGACTTACCCCAGGACACCCAGCGTCAGGTGGAATTACGTGGCC

AAGAACTTTGCCCAGGCCCTGGGCTGTGTGCTCTATGCCTGCTTCATCCTGGGCCGCCTC

TGTGTTCCTATCTTTGCCAACATGAGCCGGGAGCCCTTCAGCACGCGGGCCCTGCTGCTC

TCTATCATGCATGCCACTCTGCCAGGGCTACTGAACTTCACAATGAATGACAGGCACACG

GGCCCCGCGTGGAATGTGTTGATGTGGACCTTACTCTTTCTGGGCCAGGGCATCCAAGTC

AGCCTGTACTGCCAGGAATGGTATGCACGATGGCACTGTCCCCTGCCCCAG

>CL112.Contig4_A-W 795 1385 PREDICTED: sterol O-acyltransferase 2 [Macaca mulatta]

CTGGGCTGTGTGCTGCTGGCTGCCCACGCTGCGGTGCTCTGTGTGCTCCCAATCCACGTG

GCAGTGAAGCATGAGCTCCCGCCAGCCTCGCGTTGCATCCTAGTCTTTGAGCAGGTCAGG

CTCCTGATGAAAAGTTACTCCTTCCTGAGAGAGGCTGTGCCTGGGACCCTTCGTGCTAGA

GGACGTGAGGGCATCTGGGCCCCCAGTTTCTCCAGCTACCTCTACTTCCTTTTCTGCCCC

ACACTCATCTACCGGGAGACTTACCCCAGGACACCCAGCGTCAGGTGGAATTACGTGGCC

AAGAACTTTGCCCAGGCCCTGGGCTGTGTGCTCTATGCCTGCTTCATCCTGGGCCGCCTC

TGTGTTCCTATCTTTGCCAACATGAGCCGGGAGCCCTTCAGCACGCGGGCCCTGCTGCTC

TCTATCATGCATGCCACTCTGCCAGGGCTACTGAACTTCACAATGAATGACAGGCACACG

GGCCCCGCGTGGAATGTGTTGATGTGGACCTTACTCTTTCTGGGCCAGGGCATCCAAGTC

AGCCTGTACTGCCAGGAATGGTATGCACGATGGCACTGTCCCCTGCCCCAG

>CL112.Contig5_A-W 851 1486 RecName: Full=Sterol O-acyltransferase 2; AltName: Full=Acyl-coenzyme A:cholesterol acyltransferase 2; Short=ACAT-2; AltName: Full=Cholesterol acyltransferase 2 >gi|3582752|gb|AAC62929.1| acyl-CoA:cholesterol acyltransferase 2 [Chlorocebus aethiops]

CTGGGCTGTGTGCTGCTGGCTGCCCACGCTGCGGTGCTCTGTGTGCTCCCAATCCACGTG

GCAGTGAAGCATGAGCTCCCGCCAGCCTCGCGTTGCATCCTAGTCTTTGAGCAGGTCAGG

CTCCTGATGAAAAGTTACTCCTTCCTGAGAGAGGCTGTGCCTGGGACCCTTCGTGCTAGA

GGACGTGAGGGCATCTGGGCCCCCAGTTTCTCCAGCTACCTCTACTTCCTTTTCTGCCCC

ACACTCATCTACCGGGAGACTTACCCCAGGACACCCAGCGTCAGGTGGAATTACGTGGCC

AAGAACTTTGCCCAGGCCCTGGGCTGTGTGCTCTATGCCTGCTTCATCCTGGGCCGCCTC

TGTGTTCCTATCTTTGCCAACATGAGCCGGGAGCCCTTCAGCACGCGGGCCCTGCTGCTC

TCTATCATGCATGCCACTCTGCCAGGGCTACTGAACTTCACAATGAATGACAGGCACACG

GGCCCCGCGTGGAATGTGTTGATGTGGACCTTACTCTTTCTGGGCCAGGGCATCCAAGTC

AGCCTGTACTGCCAGGAATGGTATGCACGATGGCACTGTCCCCTGCCCCAGACAACCTTC

TGGGGGCTGCTGACACCTCGATCTTGGTCCTGCCAT

>CL112.Contig6_A-W 795 1430 PREDICTED: sterol O-acyltransferase 2 [Macaca mulatta]

CTGGGCTGTGTGCTGCTGGCTGCCCACGCTGCGGTGCTCTGTGTGCTCCCAATCCACGTG

GCAGTGAAGCATGAGCTCCCGCCAGCCTCGCGTTGCATCCTAGTCTTTGAGCAGGTCAGG

CTCCTGATGAAAAGTTACTCCTTCCTGAGAGAGGCTGTGCCTGGGACCCTTCGTGCTAGA

GGACGTGAGGGCATCTGGGCCCCCAGTTTCTCCAGCTACCTCTACTTCCTTTTCTGCCCC

ACACTCATCTACCGGGAGACTTACCCCAGGACACCCAGCGTCAGGTGGAATTACGTGGCC

AAGAACTTTGCCCAGGCCCTGGGCTGTGTGCTCTATGCCTGCTTCATCCTGGGCCGCCTC

TGTGTTCCTATCTTTGCCAACATGAGCCGGGAGCCCTTCAGCACGCGGGCCCTGCTGCTC

TCTATCATGCATGCCACTCTGCCAGGGCTACTGAACTTCACAATGAATGACAGGCACACG

GGCCCCGCGTGGAATGTGTTGATGTGGACCTTACTCTTTCTGGGCCAGGGCATCCAAGTC

AGCCTGTACTGCCAGGAATGGTATGCACGATGGCACTGTCCCCTGCCCCAGACAACCTTC

TGGGGGCTGCTGACACCTCGATCTTGGTCCTGCCAT

>CL112.Contig7_A-W 204 488 PREDICTED: LOW QUALITY PROTEIN: sterol O-acyltransferase 2-like [Sus scrofa]

GGTGAGGGCATCTGGGCCCCCAGTTTCTCCAGCTACCTCTACTTCCTTTTCTGCCCCACA

CTCATCTACCGGGAGACTTACCCCAGGACACCCAGCGTCAGGTGGAATTACGTGGCCAAG

AACTTTGCCCAGGCCCTGGGCTGTGTGCTCTATGCCTGCTTCATCCTGGGCCGCCTCTGT

GTTCCTATCTTTGCCAACATGAGCCGGGAGCCCTTCAGCACGCGGGCCCTGCTGCTCTCT

ATCATGCATGCCACTCTGCCAGGACTGGTGGAACTCAACATCCTT

>CL112.Contig8_A-W 1293 1676 PREDICTED: LOW QUALITY PROTEIN: sterol O-acyltransferase 2-like [Sus scrofa]

CAGGACTGGTGGAACTCAACATCCTTCTCCAACTACTACCGCACTTGGAACGTGGTGGTC

CATGACTGGCTGTACAGCTACGTGTATCAGGATGGGCTGTGGCTCCTTGGTGGCCGGGGC

CGAGGGGCAGCCATGCTGGGCGTGTTCCTGGTTTCTGCAGTGGTGCATGAGTACATCTTC

TGCTTTGTCCTGGGATTCTTCTACCCTGTCATGCTGATGCTCTTCCTTGTCATTGGGGGG

CTACTGAACTTCACAATGAATGACAGGCACACGGGCCCCGCGTGGAATGTGTTGATGTGG

ACCTTACTCTTTCTGGGCCAGGGCATCCAAGTCAGCCTGTACTGCCAGGAATGGTATGCA

CGATGGCACTGTCCCCTGCCCCAG

>CL112.Contig9_A-W 204 488 PREDICTED: LOW QUALITY PROTEIN: sterol O-acyltransferase 2-like [Sus scrofa]

GGTGAGGGCATCTGGGCCCCCAGTTTCTCCAGCTACCTCTACTTCCTTTTCTGCCCCACA

CTCATCTACCGGGAGACTTACCCCAGGACACCCAGCGTCAGGTGGAATTACGTGGCCAAG

AACTTTGCCCAGGCCCTGGGCTGTGTGCTCTATGCCTGCTTCATCCTGGGCCGCCTCTGT

GTTCCTATCTTTGCCAACATGAGCCGGGAGCCCTTCAGCACGCGGGCCCTGCTGCTCTCT

ATCATGCATGCCACTCTGCCAGGACTGGTGGAACTCAACATCCTT

>CL112.Contig10_A-W 435 611 PREDICTED: sterol O-acyltransferase 2 [Callithrix jacchus]

CTGCTCTCTATCATGCATGCCACTCTGCCAGGGCTACTGAACTTCACAATGAATGACAGG

CACACGGGCCCCGCGTGGAATGTGTTGATGTGGACCTTACTCTTTCTGGGCCAGGGCATC

CAAGTCAGCCTGTACTGCCAGGAATGGTATGCACGATGGCACTGTCCCCTGCCCCAG

>CL112.Contig11_A-W 3 449 PREDICTED: sterol O-acyltransferase 2 [Otolemur garnettii]

TTCCTGCACTGCTGGCTCAATGCTTTTGCAGAGATGCTGCGGTTTGGAGACAGGATGTTC

TACAGGGACTGGTGGAACTCAACATCCTTCTCCAACTACTACCGCACTTGGAACGTGGTG

GTCCATGACTGGCTGTACAGCTACGTGTATCAGGATGGGCTGTGGCTCCTTGGTGGCCGG

GGCCGAGGGGCAGCCATGCTGGGCGTGTTCCTGGTTTCTGCAGTGGTGCATGAGTACATC

TTCTGCTTTGTCCTGGGATTCTTCTACCCTGTCATGCTGATGCTCTTCCTTGTCATTGGG

GGGCTACTGAACTTCACAATGAATGACAGGCACACGGGCCCCGCGTGGAATGTGTTGATG

TGGACCTTACTCTTTCTGGGCCAGGGCATCCAAGTCAGCCTGTACTGCCAGGAATGGTAT

GCACGATGGCACTGTCCCCTGCCCCAG

>CL112.Contig12_A-W 435 656 PREDICTED: sterol O-acyltransferase 2 [Callithrix jacchus]

CTGCTCTCTATCATGCATGCCACTCTGCCAGGGCTACTGAACTTCACAATGAATGACAGG

CACACGGGCCCCGCGTGGAATGTGTTGATGTGGACCTTACTCTTTCTGGGCCAGGGCATC

CAAGTCAGCCTGTACTGCCAGGAATGGTATGCACGATGGCACTGTCCCCTGCCCCAGACA

ACCTTCTGGGGGCTGCTGACACCTCGATCTTGGTCCTGCCAT

>CL112.Contig13_A-W 166 312 PREDICTED: sterol O-acyltransferase 2 [Cricetulus griseus]

GGGCTACTGAACTTCACAATGAATGACAGGCACACGGGCCCCGCGTGGAATGTGTTGATG

TGGACCTTACTCTTTCTGGGCCAGGGCATCCAAGTCAGCCTGTACTGCCAGGAATGGTAT

GCACGATGGCACTGTCCCCTGCCCCAG

>CL112.Contig14_A-W 3 494 PREDICTED: sterol O-acyltransferase 2 [Otolemur garnettii]

TTCCTGCACTGCTGGCTCAATGCTTTTGCAGAGATGCTGCGGTTTGGAGACAGGATGTTC

TACAGGGACTGGTGGAACTCAACATCCTTCTCCAACTACTACCGCACTTGGAACGTGGTG

GTCCATGACTGGCTGTACAGCTACGTGTATCAGGATGGGCTGTGGCTCCTTGGTGGCCGG

GGCCGAGGGGCAGCCATGCTGGGCGTGTTCCTGGTTTCTGCAGTGGTGCATGAGTACATC

TTCTGCTTTGTCCTGGGATTCTTCTACCCTGTCATGCTGATGCTCTTCCTTGTCATTGGG

GGGCTACTGAACTTCACAATGAATGACAGGCACACGGGCCCCGCGTGGAATGTGTTGATG

TGGACCTTACTCTTTCTGGGCCAGGGCATCCAAGTCAGCCTGTACTGCCAGGAATGGTAT

GCACGATGGCACTGTCCCCTGCCCCAGACAACCTTCTGGGGGCTGCTGACACCTCGATCT

TGGTCCTGCCAT

>CL112.Contig15_A-W 166 360 PREDICTED: sterol O-acyltransferase 2 [Cricetulus griseus]

GGGCTACTGAACTTCACAATGAATGACAGGCACACGGGCCCCGCGTGGAATGTGTTGATG

TGGACCTTACTCTTTCTGGGCCAGGGCATCCAAGTCAGCCTGTACTGCCAGGAATGGTAT

GCACGATGGCACTGTCCCCTGCCCCAGACAACCTTCTGGGGGCTGCTGACACCTCGATCT

TGGTCCTGCCATGCA

>CL112.Contig16_A-W 324 1280 Sterol O-acyltransferase 2 OS=Chlorocebus aethiops GN=SOAT2 PE=2 SV=1

AAGGCAGTAAAGATGCAATTGTTGGAGCAAGCACAGGAAAAGCTGGTGGAGCTGCTGGAT

CGGGCCATATGGGAGGCCATACAATCCCACCCGCCACAAGACAGACCTCTGCCCTCCCCT

CACCCAGCTCCCTTAAGCAAGACCCAGAAGCCATCCTTTGGAAGACGGAAAGTTTTCGTT

GTCCGCAAATCCTTGCTTGATGAGCTGATGGAGGTGCAGCATTTCCGTACCATCTACCAC

ATGTTTATTGCCGGCCTGTGTGTCTTCATCATTAGCACCCTGGCCATCGACTTCATTGAT

GAGGGCAGGCTGGTGCTGGAGTTTGATCTGCTGATCTTCAGCTTCGGACAGCTGCCCCTG

GCGCTGGTGACGTGGGTCCCCATGTTCCTGGCCACTCTGCTGGCGCCCTACCAGGCCCTG

CGGCTGTGGGCCAGGCCCAGGGCAGGGGGCGCCTGGATGCTGAGGGCGGTCCTGGGCTGT

GTGCTGCTGGCTGCCCACGCTGCGGTGCTCTGTGTGCTCCCAATCCACGTGGCAGTGAAG

CATGAGCTCCCGCCAGCCTCGCGTTGCATCCTAGTCTTTGAGCAGGTCAGGCTCCTGATG

AAAAGTTACTCCTTCCTGAGAGAGGCTGTGCCTGGGACCCTTCGTGCTAGAGGACGTGAG

GGCATCTGGGCCCCCAGTTTCTCCAGCTACCTCTACTTCCTTTTCTGCCCCACACTCATC

TACCGGGAGACTTACCCCAGGACACCCAGCGTCAGGTGGAATTACGTGGCCAAGAACTTT

GCCCAGGCCCTGGGCTGTGTGCTCTATGCCTGCTTCATCCTGGGCCGCCTCTGTGTTCCT

ATCTTTGCCAACATGAGCCGGGAGCCCTTCAGCACGCGGGCCCTGCTGCTCTCTATCATG

CATGCCACTCTGCCAGGACTGGTGGAACTCAACATCCTTCTCCAACTACTACCGCAC

>CL113.Contig1_A-W 9 662 minus strand unnamed protein product [Homo sapiens]

ATGGTGGGGCCCGAGGCCTGGGGATAAGCGGGATGTGGGGCGAGGGGAGGCAGGGGTCGG

GGGCAAAAGGGGCCTGAGGGCTTGAAAGGTTGGCGATGGGGTTGGAAAGTTTAGGGTCCA

GTCCTGCGGGGAAGAGAGTCTCCAGCGTGAACAACAGGAGTTTGTGGGACTGGGATGTTC

GCTTGTGGGACTGGAATTCAGATGCTGACCACTCCCTTCTTCTCCGAGCAGTGTGACTTC

ACGGAGGATCAGACCGCAGAATTCAAGGAGGCCTTCCAGCTGTTTGACCGAACAGGTGAT

GGCAAGATCCTGTACAGCCAGTGTGGGGATGTGATGAGGGCTCTGGGCCAGAACCCCACC

AACGCCGAGGTTCTCAAGGTCCTGGGGAACCCCAAGAGTGATGAGATGAATGTAAAGGTG

CTGGACTTTGAGCACTTCTTGCCCATGCTGCAGACTGTAGCCAAGAACAAGGACCAGGGA

ACCTATGAAGATTATGTTGAAGGTCTTCGGGTGTTTGACAAGGAAGGGAATGGTACTGTC

ATGGGCGCTGAAATCCGTCATGTCCTTGTCACACTGGGTGAGAAGATGACAGAGGAAGAA

GTGGAGATGCTGGTGGCAGGGCATGAGGACAGCAATGGTTGTATCAACTATGAA

>CL113.Contig2_A-W 43 525 minus strand mCG140959, isoform CRA_d [Mus musculus]

GAAAAGGTCCCGCAGAGCTGAGCCGTCAAGATGTGTGACTTCACGGAGGATCAGACCGCA

GAATTCAAGGAGGCCTTCCAGCTGTTTGACCGAACAGGTGATGGCAAGATCCTGTACAGC

CAGTGTGGGGATGTGATGAGGGCTCTGGGCCAGAACCCCACCAACGCCGAGGTTCTCAAG

GTCCTGGGGAACCCCAAGAGTGATGAGATGAATGTAAAGGTGCTGGACTTTGAGCACTTC

TTGCCCATGCTGCAGACTGTAGCCAAGAACAAGGACCAGGGAACCTATGAAGATTATGTT

GAAGGTCTTCGGGTGTTTGACAAGGAAGGGAATGGTACTGTCATGGGCGCTGAAATCCGT

CATGTCCTTGTCACACTGGGTGAGAAGATGACAGAGGAAGAAGTGGAGATGCTGGTGGCA

GGGCATGAGGACAGCAATGGTTGTATCAACTATGAAGAGCTTGTCCGGATGGTGCTGAAT

GGC

>CL113.Contig3_A-W 81 1280 minus strand RecName: Full=SEC14-like protein 3; AltName: Full=45 kDa secretory protein; Short=rsec45 >gi|4164418|emb|CAA10644.1| 45 kDa secretory protein [Rattus norvegicus] >gi|149047538|gb|EDM00208.1| SEC14-like 3 (S. cerevisiae) [Rattus norvegicus]

ATGAGCGGCCGAGTCGGAGACCTGAGCCCCAAGCAGGCAGAGACCCTGGCCAAGTTTCGA

GAAAATGTCCAGGATGTGTTTCCAGTCCTGCCTAACCCTGATGATTATTTCCTTCTTCGT

TGGCTCCGAGCTCGGAATTTCGACCTGCAGAAATCAGAGGCGATGCTCCGCAAGCATATG

GAGTTCCGGAAGACCATGGACATTGAACACATCCTTGATTGGCAGCCCCCAGAGGTGATC

CAGAAGTTCATGCCTGGGGGCCTGTGTGGCTATGATCGTGATGGCTGTCCAGTGTGGTAT

GACATCATTGGGCCACTCGACCCCAAGGGACTACTCTTCTCAGTCACCAAGCAAGACCTG

CTCAAGACCAAGATGAGGGATTGTGAGCGCATCCTACATGAGTGTGATCTGCAGACAGAG

CGGCTGGGGAAGAAGATTGAAACCATTGTGATGATATTTGACTGTGAAGGCCTGGGACTG

AAACACTTCTGGAAACCTCTGGTGGAAGTGTACCAGGAGTTTTTTGGCCTCCTTGAAGAA

AATTACCCAGAGAGATTGAAATTCATGCTCATCGTGAAAGCCACAAAACTCTTCCCTGTG

GGCTACAACCTCATGAAGCCCTTCCTGAGTGAGGACACTCGAAGAAAAATTATAGTGTTG

GGAAACAGCTGGAAGGAAGGTTTGCTGAAACTCATCAGTCCTGAGGAACTGCCTGCCCAT

TTTGGAGGAACCATGACTGACCCAGATGGGAACCCCAAATGCTTAACCAAGATCAACTAT

GGTGGGGAGATCCCCAAGTCCATGTACGTGCGGGACCAGGTGAAGACTCAGTATGAACAC

TCAGTGCAGATCAGCCGTGGCTCCTCACACCAGGTGGAATATGAGATCCTGTTTCCAGGC

TGTGTCCTCAGGTGGCAATTCTCATCTGATGGAGCTGATATTGGCTTTGGGGTTTTCCTG

AAGACCAAGATGGGGGAGCGCCAGAAGGCTGGAGAGATGACAGAGGTGCTGACTAGCCAG

CGCTACAATGCCCACATGGTGCCTGAAGATGGGAGCCTCACCTGCTCGGAGGCTGGTGTC

TATGTTCTGCGTTTTGACAACACCTATAGCTTTGTCCATGCCAAGAAGGTCAGCTTCACA

GTGGAGGTGCTGCTCCCAGATGAGGGCATGCAGAAATACGACGAGGAGCTCACCCCTGTC

>CL113.Contig4_A-W 82 1257 minus strand PREDICTED: SEC14-like protein 4-like [Cavia porcellus]

ATGAGCGGCCGAGTAGGGGACCTGAACCCCCAACAACAGGAGGCGTTGGCCAAGTTCCAG

GAGAACCTCCAGGACCTGCTACCCACCTTGCCCAAGGCTGATGACTACTTCCTCCTGCGC

TGGCTTCGAGCTCGGAACTTCGACCTGCAGAAGTCAGAGGACATGCTCCGGAAGCACATG

GAGTTCCGGAAGCAACAGGACTTGGACAATATCCTTTCATGGCAGCCCTCAGAGGTGATC

CAGCGGTATGACTCGGGCGGTCTGTGTGGCTACGATTATGAAGGCTGTCCTGTGTGGTTT

GACATCATTGGGACCCTCGACCCCAAGGGTCTTCTCCTGTCAGCCTCCAAGCAGGAGCTG

ATCCGGAAACGCATCAAGGCCTTTGAGTTGCTTCTGCAGGAGTGTGAGCTGCAGAGTCAA

AAGCTGGGTAGGAAGATTGAGATGGTGCTGTTGGTGTTTGACATGGAGGGACTGGGCTTG

AAACATCTGTGGAAGCCGGCAGTGGAGGTCTACCAGCAGTTTTTTGCCATGGTGGAAGCA

AATTATCCTGAGACAATGAAGAATTTAATTGTTGTTCGAGCCCCCAGACTTTTCCCAGTG

GCCTTCAACTTGGTTAAATCGTTCATGAGTGAGGAGACACGCAAGAAGATAGTGATTTTG

GGAGACAACTGGAAGCAGGAGCTGTCAAAGTTCATCAGCCCCGACCAGCTGCCTGTGGAG

TTTGGGGGCACTCTGACTGACCCTGATGGCAACCCCAAGTGCATAACTAAGATCAACTAC

GGGGGTGAGGTGCCCAAGAGCTACCACCTGTGCAAGCAGGTGAGGATGCAGTATGAGCAC

ACAGTGACCGTGGGCCGTGGCTCCTCCCAGCATGTGGAAAATGAGATCCTGTTCCCAGGC

TGTGTGCTCAGGTGGCAATTTGCATCGGATGGTGGAGACATTGGCTTTGGGGTTTTCCTG

AAGACCAAGATGGGGGAGCGCCAGAAGGCTGGAGAGATGACAGAGGTGCTGACTAGCCAG

CGCTACAATGCCCACATGGTGCCTGAAGATGGGAGCCTCACCTGCTCGGAGGCTGGTGTC

TATGTTCTGCGTTTTGACAACACCTATAGCTTTGTCCATGCCAAGAAGGTCAGCTTCACA

GTGGAGGTGCTGCTCCCAGATGAGGGCATGCAGAAA

>CL113.Contig5_A-W 82 1257 minus strand PREDICTED: SEC14-like protein 4-like [Cavia porcellus]

ATGAGCGGCCGAGTAGGGGACCTGAACCCCCAACAACAGGAGGCGTTGGCCAAGTTCCAG

GAGAACCTCCAGGACCTGCTACCCACCTTGCCCAAGGCTGATGACTACTTCCTCCTGCGC

TGGCTTCGAGCTCGGAACTTCGACCTGCAGAAGTCAGAGGACATGCTCCGGAAGCACATG

GAGTTCCGGAAGCAACAGGACTTGGACAATATCCTTTCATGGCAGCCCTCAGAGGTGATC

CAGCGGTATGACTCGGGCGGTCTGTGTGGCTACGATTATGAAGGCTGTCCTGTGTGGTTT

GACATCATTGGGACCCTCGACCCCAAGGGTCTTCTCCTGTCAGCCTCCAAGCAGGAGCTG

ATCCGGAAACGCATCAAGGCCTTTGAGTTGCTTCTGCAGGAGTGTGAGCTGCAGAGTCAA

AAGCTGGGTAGGAAGATTGAGATGGTGCTGTTGGTGTTTGACATGGAGGGACTGGGCTTG

AAACATCTGTGGAAGCCGGCAGTGGAGGTCTACCAGCAGTTTTTTGCCATGGTGGAAGCA

AATTATCCTGAGACAATGAAGAATTTAATTGTTGTTCGAGCCCCCAGACTTTTCCCAGTG

GCCTTCAACTTGGTTAAATCGTTCATGAGTGAGGAGACACGCAAGAAGATAGTGATTTTG

GGAGACAACTGGAAGCAGGAGCTGTCAAAGTTCATCAGCCCCGACCAGCTGCCTGTGGAG

TTTGGGGGCACTCTGACTGACCCTGATGGCAACCCCAAGTGCATAACTAAGATCAACTAC

GGGGGTGAGGTGCCCAAGAGCTACCACCTGTGCAAGCAGGTGAGGATGCAGTATGAGCAC

ACAGTGACCGTGGGCCGTGGCTCCTCCCAGCATGTGGAAAATGAGATCCTGTTCCCAGGC

TGTGTGCTCAGGTGGCAATTTGCATCGGATGGTGGAGACATTGGCTTTGGGGTTTTCCTG

AAGACCAAGATGGGGGAGCGCCAGAAGGCTGGAGAGATGACAGAGGTGCTGACTAGCCAG

CGCTACAATGCCCACATGGTGCCTGAAGATGGGAGCCTCACCTGCTCGGAGGCTGGTGTC

TATGTTCTGCGTTTTGACAACACCTATAGCTTTGTCCATGCCAAGAAGGTCAGCTTCACA

GTGGAGGTGCTGCTCCCAGATGAGGGCATGCAGAAA

>CL113.Contig6_A-W 81 1280 minus strand RecName: Full=SEC14-like protein 3; AltName: Full=45 kDa secretory protein; Short=rsec45 >gi|4164418|emb|CAA10644.1| 45 kDa secretory protein [Rattus norvegicus] >gi|149047538|gb|EDM00208.1| SEC14-like 3 (S. cerevisiae) [Rattus norvegicus]

ATGAGCGGCCGAGTCGGAGACCTGAGCCCCAAGCAGGCAGAGACCCTGGCCAAGTTTCGA

GAAAATGTCCAGGATGTGTTTCCAGTCCTGCCTAACCCTGATGATTATTTCCTTCTTCGT

TGGCTCCGAGCTCGGAATTTCGACCTGCAGAAATCAGAGGCGATGCTCCGCAAGCATATG

GAGTTCCGGAAGACCATGGACATTGAACACATCCTTGATTGGCAGCCCCCAGAGGTGATC

CAGAAGTTCATGCCTGGGGGCCTGTGTGGCTATGATCGTGATGGCTGTCCAGTGTGGTAT

GACATCATTGGGCCACTCGACCCCAAGGGACTACTCTTCTCAGTCACCAAGCAAGACCTG

CTCAAGACCAAGATGAGGGATTGTGAGCGCATCCTACATGAGTGTGATCTGCAGACAGAG

CGGCTGGGGAAGAAGATTGAAACCATTGTGATGATATTTGACTGTGAAGGCCTGGGACTG

AAACACTTCTGGAAACCTCTGGTGGAAGTGTACCAGGAGTTTTTTGGCCTCCTTGAAGAA

AATTACCCAGAGAGATTGAAATTCATGCTCATCGTGAAAGCCACAAAACTCTTCCCTGTG

GGCTACAACCTCATGAAGCCCTTCCTGAGTGAGGACACTCGAAGAAAAATTATAGTGTTG

GGAAACAGCTGGAAGGAAGGTTTGCTGAAACTCATCAGTCCTGAGGAACTGCCTGCCCAT

TTTGGAGGAACCATGACTGACCCAGATGGGAACCCCAAATGCTTAACCAAGATCAACTAT

GGTGGGGAGATCCCCAAGTCCATGTACGTGCGGGACCAGGTGAAGACTCAGTATGAACAC

TCAGTGCAGATCAGCCGTGGCTCCTCACACCAGGTGGAATATGAGATCCTGTTTCCAGGC

TGTGTCCTCAGGTGGCAATTCTCATCTGATGGAGCTGATATTGGCTTTGGGGTTTTCCTG

AAGACCAAGATGGGGGAGCGCCAGAAGGCTGGAGAGATGACAGAGGTGCTGACTAGCCAG

CGCTACAATGCCCACATGGTGCCTGAAGATGGGAGCCTCACCTGCTCGGAGGCTGGTGTC

TATGTTCTGCGTTTTGACAACACCTATAGCTTTGTCCATGCCAAGAAGGTCAGCTTCACA

GTGGAGGTGCTGCTCCCAGATGAGGGCATGCAGAAATACGACGAGGAGCTCACCCCTGTC

>CL113.Contig7_A-W 81 1280 minus strand RecName: Full=SEC14-like protein 3; AltName: Full=45 kDa secretory protein; Short=rsec45 >gi|4164418|emb|CAA10644.1| 45 kDa secretory protein [Rattus norvegicus] >gi|149047538|gb|EDM00208.1| SEC14-like 3 (S. cerevisiae) [Rattus norvegicus]

ATGAGCGGCCGAGTCGGAGACCTGAGCCCCAAGCAGGCAGAGACCCTGGCCAAGTTTCGA

GAAAATGTCCAGGATGTGTTTCCAGTCCTGCCTAACCCTGATGATTATTTCCTTCTTCGT

TGGCTCCGAGCTCGGAATTTCGACCTGCAGAAATCAGAGGCGATGCTCCGCAAGCATATG

GAGTTCCGGAAGACCATGGACATTGAACACATCCTTGATTGGCAGCCCCCAGAGGTGATC

CAGAAGTTCATGCCTGGGGGCCTGTGTGGCTATGATCGTGATGGCTGTCCAGTGTGGTAT

GACATCATTGGGCCACTCGACCCCAAGGGACTACTCTTCTCAGTCACCAAGCAAGACCTG

CTCAAGACCAAGATGAGGGATTGTGAGCGCATCCTACATGAGTGTGATCTGCAGACAGAG

CGGCTGGGGAAGAAGATTGAAACCATTGTGATGATATTTGACTGTGAAGGCCTGGGACTG

AAACACTTCTGGAAACCTCTGGTGGAAGTGTACCAGGAGTTTTTTGGCCTCCTTGAAGAA

AATTACCCAGAGAGATTGAAATTCATGCTCATCGTGAAAGCCACAAAACTCTTCCCTGTG

GGCTACAACCTCATGAAGCCCTTCCTGAGTGAGGACACTCGAAGAAAAATTATAGTGTTG

GGAAACAGCTGGAAGGAAGGTTTGCTGAAACTCATCAGTCCTGAGGAACTGCCTGCCCAT

TTTGGAGGAACCATGACTGACCCAGATGGGAACCCCAAATGCTTAACCAAGATCAACTAT

GGTGGGGAGATCCCCAAGTCCATGTACGTGCGGGACCAGGTGAAGACTCAGTATGAACAC

TCAGTGCAGATCAGCCGTGGCTCCTCACACCAGGTGGAATATGAGATCCTGTTTCCAGGC

TGTGTCCTCAGGTGGCAATTCTCATCTGATGGAGCTGATATTGGCTTTGGGGTTTTCCTG

AAGACCAAGATGGGGGAGCGCCAGAAGGCTGGAGAGATGACAGAGGTGCTGACTAGCCAG

CGCTACAATGCCCACATGGTGCCTGAAGATGGGAGCCTCACCTGCTCGGAGGCTGGTGTC

TATGTTCTGCGTTTTGACAACACCTATAGCTTTGTCCATGCCAAGAAGGTCAGCTTCACA

GTGGAGGTGCTGCTCCCAGATGAGGGCATGCAGAAATACGACGAGGAGCTCACCCCTGTC

>CL113.Contig8_A-W 82 1257 minus strand PREDICTED: SEC14-like protein 4-like [Cavia porcellus]

ATGAGCGGCCGAGTAGGGGACCTGAACCCCCAACAACAGGAGGCGTTGGCCAAGTTCCAG

GAGAACCTCCAGGACCTGCTACCCACCTTGCCCAAGGCTGATGACTACTTCCTCCTGCGC

TGGCTTCGAGCTCGGAACTTCGACCTGCAGAAGTCAGAGGACATGCTCCGGAAGCACATG

GAGTTCCGGAAGCAACAGGACTTGGACAATATCCTTTCATGGCAGCCCTCAGAGGTGATC

CAGCGGTATGACTCGGGCGGTCTGTGTGGCTACGATTATGAAGGCTGTCCTGTGTGGTTT

GACATCATTGGGACCCTCGACCCCAAGGGTCTTCTCCTGTCAGCCTCCAAGCAGGAGCTG

ATCCGGAAACGCATCAAGGCCTTTGAGTTGCTTCTGCAGGAGTGTGAGCTGCAGAGTCAA

AAGCTGGGTAGGAAGATTGAGATGGTGCTGTTGGTGTTTGACATGGAGGGACTGGGCTTG

AAACATCTGTGGAAGCCGGCAGTGGAGGTCTACCAGCAGTTTTTTGCCATGGTGGAAGCA

AATTATCCTGAGACAATGAAGAATTTAATTGTTGTTCGAGCCCCCAGACTTTTCCCAGTG

GCCTTCAACTTGGTTAAATCGTTCATGAGTGAGGAGACACGCAAGAAGATAGTGATTTTG

GGAGACAACTGGAAGCAGGAGCTGTCAAAGTTCATCAGCCCCGACCAGCTGCCTGTGGAG

TTTGGGGGCACTCTGACTGACCCTGATGGCAACCCCAAGTGCATAACTAAGATCAACTAC

GGGGGTGAGGTGCCCAAGAGCTACCACCTGTGCAAGCAGGTGAGGATGCAGTATGAGCAC

ACAGTGACCGTGGGCCGTGGCTCCTCCCAGCATGTGGAAAATGAGATCCTGTTCCCAGGC

TGTGTGCTCAGGTGGCAATTTGCATCGGATGGTGGAGACATTGGCTTTGGGGTTTTCCTG

AAGACCAAGATGGGGGAGCGCCAGAAGGCTGGAGAGATGACAGAGGTGCTGACTAGCCAG

CGCTACAATGCCCACATGGTGCCTGAAGATGGGAGCCTCACCTGCTCGGAGGCTGGTGTC

TATGTTCTGCGTTTTGACAACACCTATAGCTTTGTCCATGCCAAGAAGGTCAGCTTCACA

GTGGAGGTGCTGCTCCCAGATGAGGGCATGCAGAAA

>CL113.Contig9_A-W 81 1280 minus strand RecName: Full=SEC14-like protein 3; AltName: Full=45 kDa secretory protein; Short=rsec45 >gi|4164418|emb|CAA10644.1| 45 kDa secretory protein [Rattus norvegicus] >gi|149047538|gb|EDM00208.1| SEC14-like 3 (S. cerevisiae) [Rattus norvegicus]

ATGAGCGGCCGAGTCGGAGACCTGAGCCCCAAGCAGGCAGAGACCCTGGCCAAGTTTCGA

GAAAATGTCCAGGATGTGTTTCCAGTCCTGCCTAACCCTGATGATTATTTCCTTCTTCGT

TGGCTCCGAGCTCGGAATTTCGACCTGCAGAAATCAGAGGCGATGCTCCGCAAGCATATG

GAGTTCCGGAAGACCATGGACATTGAACACATCCTTGATTGGCAGCCCCCAGAGGTGATC

CAGAAGTTCATGCCTGGGGGCCTGTGTGGCTATGATCGTGATGGCTGTCCAGTGTGGTAT

GACATCATTGGGCCACTCGACCCCAAGGGACTACTCTTCTCAGTCACCAAGCAAGACCTG

CTCAAGACCAAGATGAGGGATTGTGAGCGCATCCTACATGAGTGTGATCTGCAGACAGAG

CGGCTGGGGAAGAAGATTGAAACCATTGTGATGATATTTGACTGTGAAGGCCTGGGACTG

AAACACTTCTGGAAACCTCTGGTGGAAGTGTACCAGGAGTTTTTTGGCCTCCTTGAAGAA

AATTACCCAGAGAGATTGAAATTCATGCTCATCGTGAAAGCCACAAAACTCTTCCCTGTG

GGCTACAACCTCATGAAGCCCTTCCTGAGTGAGGACACTCGAAGAAAAATTATAGTGTTG

GGAAACAGCTGGAAGGAAGGTTTGCTGAAACTCATCAGTCCTGAGGAACTGCCTGCCCAT

TTTGGAGGAACCATGACTGACCCAGATGGGAACCCCAAATGCTTAACCAAGATCAACTAT

GGTGGGGAGATCCCCAAGTCCATGTACGTGCGGGACCAGGTGAAGACTCAGTATGAACAC

TCAGTGCAGATCAGCCGTGGCTCCTCACACCAGGTGGAATATGAGATCCTGTTTCCAGGC

TGTGTCCTCAGGTGGCAATTCTCATCTGATGGAGCTGATATTGGCTTTGGGGTTTTCCTG

AAGACCAAGATGGGGGAGCGCCAGAAGGCTGGAGAGATGACAGAGGTGCTGACTAGCCAG

CGCTACAATGCCCACATGGTGCCTGAAGATGGGAGCCTCACCTGCTCGGAGGCTGGTGTC

TATGTTCTGCGTTTTGACAACACCTATAGCTTTGTCCATGCCAAGAAGGTCAGCTTCACA

GTGGAGGTGCTGCTCCCAGATGAGGGCATGCAGAAATACGACGAGGAGCTCACCCCTGTC

>CL113.Contig10_A-W 82 1257 minus strand PREDICTED: SEC14-like protein 4-like [Cavia porcellus]

ATGAGCGGCCGAGTAGGGGACCTGAACCCCCAACAACAGGAGGCGTTGGCCAAGTTCCAG

GAGAACCTCCAGGACCTGCTACCCACCTTGCCCAAGGCTGATGACTACTTCCTCCTGCGC

TGGCTTCGAGCTCGGAACTTCGACCTGCAGAAGTCAGAGGACATGCTCCGGAAGCACATG

GAGTTCCGGAAGCAACAGGACTTGGACAATATCCTTTCATGGCAGCCCTCAGAGGTGATC

CAGCGGTATGACTCGGGCGGTCTGTGTGGCTACGATTATGAAGGCTGTCCTGTGTGGTTT

GACATCATTGGGACCCTCGACCCCAAGGGTCTTCTCCTGTCAGCCTCCAAGCAGGAGCTG

ATCCGGAAACGCATCAAGGCCTTTGAGTTGCTTCTGCAGGAGTGTGAGCTGCAGAGTCAA

AAGCTGGGTAGGAAGATTGAGATGGTGCTGTTGGTGTTTGACATGGAGGGACTGGGCTTG

AAACATCTGTGGAAGCCGGCAGTGGAGGTCTACCAGCAGTTTTTTGCCATGGTGGAAGCA

AATTATCCTGAGACAATGAAGAATTTAATTGTTGTTCGAGCCCCCAGACTTTTCCCAGTG

GCCTTCAACTTGGTTAAATCGTTCATGAGTGAGGAGACACGCAAGAAGATAGTGATTTTG

GGAGACAACTGGAAGCAGGAGCTGTCAAAGTTCATCAGCCCCGACCAGCTGCCTGTGGAG

TTTGGGGGCACTCTGACTGACCCTGATGGCAACCCCAAGTGCATAACTAAGATCAACTAC

GGGGGTGAGGTGCCCAAGAGCTACCACCTGTGCAAGCAGGTGAGGATGCAGTATGAGCAC

ACAGTGACCGTGGGCCGTGGCTCCTCCCAGCATGTGGAAAATGAGATCCTGTTCCCAGGC

TGTGTGCTCAGGTGGCAATTTGCATCGGATGGTGGAGACATTGGCTTTGGGGTTTTCCTG

AAGACCAAGATGGGGGAGCGCCAGAAGGCTGGAGAGATGACAGAGGTGCTGACTAGCCAG

CGCTACAATGCCCACATGGTGCCTGAAGATGGGAGCCTCACCTGCTCGGAGGCTGGTGTC

TATGTTCTGCGTTTTGACAACACCTATAGCTTTGTCCATGCCAAGAAGGTCAGCTTCACA

GTGGAGGTGCTGCTCCCAGATGAGGGCATGCAGAAA

>CL113.Contig11_A-W 81 1280 minus strand RecName: Full=SEC14-like protein 3; AltName: Full=45 kDa secretory protein; Short=rsec45 >gi|4164418|emb|CAA10644.1| 45 kDa secretory protein [Rattus norvegicus] >gi|149047538|gb|EDM00208.1| SEC14-like 3 (S. cerevisiae) [Rattus norvegicus]

ATGAGCGGCCGAGTCGGAGACCTGAGCCCCAAGCAGGCAGAGACCCTGGCCAAGTTTCGA

GAAAATGTCCAGGATGTGTTTCCAGTCCTGCCTAACCCTGATGATTATTTCCTTCTTCGT

TGGCTCCGAGCTCGGAATTTCGACCTGCAGAAATCAGAGGCGATGCTCCGCAAGCATATG

GAGTTCCGGAAGACCATGGACATTGAACACATCCTTGATTGGCAGCCCCCAGAGGTGATC

CAGAAGTTCATGCCTGGGGGCCTGTGTGGCTATGATCGTGATGGCTGTCCAGTGTGGTAT

GACATCATTGGGCCACTCGACCCCAAGGGACTACTCTTCTCAGTCACCAAGCAAGACCTG

CTCAAGACCAAGATGAGGGATTGTGAGCGCATCCTACATGAGTGTGATCTGCAGACAGAG

CGGCTGGGGAAGAAGATTGAAACCATTGTGATGATATTTGACTGTGAAGGCCTGGGACTG

AAACACTTCTGGAAACCTCTGGTGGAAGTGTACCAGGAGTTTTTTGGCCTCCTTGAAGAA

AATTACCCAGAGAGATTGAAATTCATGCTCATCGTGAAAGCCACAAAACTCTTCCCTGTG

GGCTACAACCTCATGAAGCCCTTCCTGAGTGAGGACACTCGAAGAAAAATTATAGTGTTG

GGAAACAGCTGGAAGGAAGGTTTGCTGAAACTCATCAGTCCTGAGGAACTGCCTGCCCAT

TTTGGAGGAACCATGACTGACCCAGATGGGAACCCCAAATGCTTAACCAAGATCAACTAT

GGTGGGGAGATCCCCAAGTCCATGTACGTGCGGGACCAGGTGAAGACTCAGTATGAACAC

TCAGTGCAGATCAGCCGTGGCTCCTCACACCAGGTGGAATATGAGATCCTGTTTCCAGGC

TGTGTCCTCAGGTGGCAATTCTCATCTGATGGAGCTGATATTGGCTTTGGGGTTTTCCTG

AAGACCAAGATGGGGGAGCGCCAGAAGGCTGGAGAGATGACAGAGGTGCTGACTAGCCAG

CGCTACAATGCCCACATGGTGCCTGAAGATGGGAGCCTCACCTGCTCGGAGGCTGGTGTC

TATGTTCTGCGTTTTGACAACACCTATAGCTTTGTCCATGCCAAGAAGGTCAGCTTCACA

GTGGAGGTGCTGCTCCCAGATGAGGGCATGCAGAAATACGACGAGGAGCTCACCCCTGTC

>CL113.Contig12_A-W 82 1257 minus strand PREDICTED: SEC14-like protein 4-like [Cavia porcellus]

ATGAGCGGCCGAGTAGGGGACCTGAACCCCCAACAACAGGAGGCGTTGGCCAAGTTCCAG

GAGAACCTCCAGGACCTGCTACCCACCTTGCCCAAGGCTGATGACTACTTCCTCCTGCGC

TGGCTTCGAGCTCGGAACTTCGACCTGCAGAAGTCAGAGGACATGCTCCGGAAGCACATG

GAGTTCCGGAAGCAACAGGACTTGGACAATATCCTTTCATGGCAGCCCTCAGAGGTGATC

CAGCGGTATGACTCGGGCGGTCTGTGTGGCTACGATTATGAAGGCTGTCCTGTGTGGTTT

GACATCATTGGGACCCTCGACCCCAAGGGTCTTCTCCTGTCAGCCTCCAAGCAGGAGCTG

ATCCGGAAACGCATCAAGGCCTTTGAGTTGCTTCTGCAGGAGTGTGAGCTGCAGAGTCAA

AAGCTGGGTAGGAAGATTGAGATGGTGCTGTTGGTGTTTGACATGGAGGGACTGGGCTTG

AAACATCTGTGGAAGCCGGCAGTGGAGGTCTACCAGCAGTTTTTTGCCATGGTGGAAGCA

AATTATCCTGAGACAATGAAGAATTTAATTGTTGTTCGAGCCCCCAGACTTTTCCCAGTG

GCCTTCAACTTGGTTAAATCGTTCATGAGTGAGGAGACACGCAAGAAGATAGTGATTTTG

GGAGACAACTGGAAGCAGGAGCTGTCAAAGTTCATCAGCCCCGACCAGCTGCCTGTGGAG

TTTGGGGGCACTCTGACTGACCCTGATGGCAACCCCAAGTGCATAACTAAGATCAACTAC

GGGGGTGAGGTGCCCAAGAGCTACCACCTGTGCAAGCAGGTGAGGATGCAGTATGAGCAC

ACAGTGACCGTGGGCCGTGGCTCCTCCCAGCATGTGGAAAATGAGATCCTGTTCCCAGGC

TGTGTGCTCAGGTGGCAATTTGCATCGGATGGTGGAGACATTGGCTTTGGGGTTTTCCTG

AAGACCAAGATGGGGGAGCGCCAGAAGGCTGGAGAGATGACAGAGGTGCTGACTAGCCAG

CGCTACAATGCCCACATGGTGCCTGAAGATGGGAGCCTCACCTGCTCGGAGGCTGGTGTC

TATGTTCTGCGTTTTGACAACACCTATAGCTTTGTCCATGCCAAGAAGGTCAGCTTCACA

GTGGAGGTGCTGCTCCCAGATGAGGGCATGCAGAAA

>CL113.Contig13_A-W 9 668 minus strand unnamed protein product [Homo sapiens]

ATGGTGGGGCCCGAGGCCTGGGGATAAGCGGGATGTGGGGCGAGGGGAGGCAGGGGTCGG

GGGCAAAAGGGGCCTGAGGGCTTGAAAGGTTGGCGATGGGGTTGGAAAGTTTAGGGTCCA

GTCCTGCGGGGAAGAGAGTCTCCAGCGTGAACAACAGGAGTTTGTGGGACTGGGATGTTC

GCTTGTGGGACTGGAATTCAGATGCTGACCACTCCCTTCTTCTCCGAGCAGTGTGACTTC

ACGGAGGATCAGACCGCAGAATTCAAGGAGGCCTTCCAGCTGTTTGACCGAACAGGTGAT

GGCAAGATCCTGTACAGCCAGTGTGGGGATGTGATGAGGGCTCTGGGCCAGAACCCCACC

AACGCCGAGGTTCTCAAGGTCCTGGGGAACCCCAAGAGTGATGAGATGAATGTAAAGGTG

CTGGACTTTGAGCACTTCTTGCCCATGCTGCAGACTGTAGCCAAGAACAAGGACCAGGGA

ACCTATGAAGATTATGTTGAAGGTCTTCGGGTGTTTGACAAGGAAGGGAATGGTACTGTC

ATGGGCGCTGAAATCCGTCATGTCCTTGTCACACTGGGTGAGAAGATGACAGAGGAAGAA

GTGGAGATGCTGGTGGCAGGGCATGAGGACAGCAATGGTTGTATCAACTATGAAGGTGAG

>CL113.Contig14_A-W 43 1251 PREDICTED: SEC14-like protein 2-like [Cavia porcellus]

ATGAGCGGCAGAGTCGGTGATCTGAGCCCCAAACAGAAAGAGGCACTAGCCAAGTTTCGG

GAGAATGTCCAGGACGTGCTGCCTGCCCTGCCGAATCCAGATGACTATTTTCTTCTGCGC

TGGCTCCGAGCAAGAAGCTTCGACCTGCAGAAGTCAGAGGCTATGCTCCGGAAGCATGTG

GAGTTCCGAAAGCAAAAGGACATTGACAACATCATTAGCTGGCAGCCTCCAGAGGTGATC

CAACAGTATCTGTCAGGGGGCAGGTGTGGCTATGACCTGGAGGGCTGCCCTGTCTGGTAC

GATATCATTGGGCCTCTGGATGCCAAGGGTCTGCTGTTGTCAGCCACCAAACAGGACTTG

CTCAGGACCAAGATGCGGGACTGTGAGCTGCTTTTACAGGAGTGTGCCCACCAGAGCACA

AAGTTGGGGAAGAAAATAGAGACCATCACCATGATTTATGACTGTGAGGGGTTGGGCCTC

AAGCATCTCTGGAAACCTGCAGTGGAAGCTTATGGAGAGTTTCTCTGCATGTTTGAGGAA

AATTATCCTGAAACACTGAAGCGTCTATTTGTTGTTAAAGCCCCCAAGCTGTTTCCTGTG

GCCTATAATCTCATCAAACCCTTCCTGAGTGAGGACACTCGGAAGAAAATCATGGTCCTT

GGAGCAAACTGGAAGGAGATTCTACTGAAACATATCAGCCCTGACCAGTTGCCTGTGGAA

TATGGGGGCACCATGACTGACCCTGATGGAAATCCCAAATGCAAATCCAAGATCAACTAT

GGGGGTGACATCCCCAAGAAGTATCATGTGCGAGACCAGGTGAAGCAGCAATATGAACAC

AGTGTGCAGATTTCTCGTGGCTCCTCCCATCAAGTGGAGTATGAGATCCTCTTTCCAGGC

TGCGTCCTCAGGTGGCAGTTTATGTCAGAGGGAGCAGATGTTGGTTTTGGAATTTTCCTA

AAGACCAAGATGGGGGAGCGACAGCGGGCAGGAGAGATGACAGAGGTGCTGCCTAACCAG

AGGTACAACTCCCACCTGGTCCCTGAGGATGGGACCCTCACCTGCAGTGATCCTGGCATT

TATGTCCTGCGGTTTGACAACACCTACAGCTTCATTCATGCCAAGAAGGTCAGTTTCACT

GTGGAGGTCCTGCTTCCAGACAAAGCCTCAGAAGAGAAGATGAATCAGCTGGGGACAGTC

ACCCCCAAA

>CL114.Contig1_A-W 41 799 PREDICTED: nucleolar protein 56-like [Nomascus leucogenys]

ATGGTTTTGCTACACGTGCTGTTCGAGCACGCGGTAGGCTACGCGCTGCTGGCGCTGAAG

GAAGTGGAGGAGATCAGCCTGTTACTGCCGCAGGTGGAGGAATGTGTGCTCAGCCTGGGC

AAGTTCCACAACATTGTCCGTCTTGTGGCCTTCAATCCCTTTTCCTCATCCCAGGTTGCC

TTGGAAAATGCCAATGCGGTGTCTGAAGGCATTGTTCATGAGGACCTCCGCCTGCTCCTG

GAGACCCACCTGCCATCCAAAAAGAAGAAAGTGCTTTTGGGGGTTGGGGATCCCAAGATT

GGTGCTGCTATACAGGAGGAGTTAGGGTACAACTGCCAGACTGGAGGTGTGGTAGCCGAG

ATCCTGCGAGGAGTTCGTCTGCACTTCCATAATCTGGTGAAGGGTCTGACTGACCTGTCA

GCTTGTAAAGCCCAATTAGGGCTGGGACATAGCTATTCTCGTGCCAAAGTTAAGTTTAAT

GTGAACCGGGTGGACAACATGATCATCCAGTCCATTAGCCTCTTGGACCAACTGGATAAA

GACATCAATACTTTCTCTATGCGTGTCAGGGAATGGTACGGGTATCACTTTCCAGAACTG

GTGAAGATCATCAATGACAATGCCACATACTGCCGCCTTGCCCAATTCATTGGAAACCGA

AAAGAACTGAGTGAGGAAAAGTTAGAGAAGCTGGAGGAGCTGACAATGGATGGGGCCAAA

GCTAAAGCTATTCTGGATGCCTCACGGTCCTCCATGGGT

>CL114.Contig2_A-W 41 1825 PREDICTED: nucleolar protein 56-like [Nomascus leucogenys]

ATGGTTTTGCTACACGTGCTGTTCGAGCACGCGGTAGGCTACGCGCTGCTGGCGCTGAAG

GAAGTGGAGGAGATCAGCCTGTTACTGCCGCAGGTGGAGGAATGTGTGCTCAGCCTGGGC

AAGTTCCACAACATTGTCCGTCTTGTGGCCTTCAATCCCTTTTCCTCATCCCAGGTTGCC

TTGGAAAATGCCAATGCGGTGTCTGAAGGCATTGTTCATGAGGACCTCCGCCTGCTCCTG

GAGACCCACCTGCCATCCAAAAAGAAGAAAGTGCTTTTGGGGGTTGGGGATCCCAAGATT

GGTGCTGCTATACAGGAGGAGTTAGGGTACAACTGCCAGACTGGAGGTGTGGTAGCCGAG

ATCCTGCGAGGAGTTCGTCTGCACTTCCATAATCTGGTGAAGGGTCTGACTGACCTGTCA

GCTTGTAAAGCCCAATTAGGGCTGGGACATAGCTATTCTCGTGCCAAAGTTAAGTTTAAT

GTGAACCGGGTGGACAACATGATCATCCAGTCCATTAGCCTCTTGGACCAACTGGATAAA

GACATCAATACTTTCTCTATGCGTGTCAGGGAATGGTACGGGTATCACTTTCCAGAACTG

GTGAAGATCATCAATGACAATGCCACATACTGCCGCCTTGCCCAATTCATTGGAAACCGA

AAAGAACTGAGTGAGGAAAAGTTAGAGAAGCTGGAGGAGCTGACAATGGATGGGGCCAAA

GCTAAAGCTATTCTGGATGCCTCACGGTCCTCCATGGGCATGGACATATCTGCCATCGAC

TTGATAAACATCGAGAGCTTCTCCAGTCGTGTGGTGTCTTTGTCAGAGTACCGCCAGAGC

CTACACACTTACCTGCGCTCCAAGATGAGCCAAGTAGCCCCCAGCCTGTCAGCCCTAATT

GGGGAAGCGGTAGGTGCACGTCTCATTGCTCATGCTGGCAGCCTCACCAACCTGGCCAAG

TATCCAGCATCCACAGTGCAGATCCTTGGGGCTGAAAAGGCCCTATTCAGAGCCCTGAAG

ACAAGGGGTAACACACCAAAATATGGACTCATTTTCCATTCTACCTTCATTGGCCGAGCA

GCTGCCAAGAACAAAGGCCGCATCTCCCGATACCTGGCAAACAAATGCAGTATTGCCTCA

CGAATCGATTGCTTCTCTGAGGTTCCTACAAGTGTATTTGGGGAGAAGCTTCGAGAACAA

GTTGAGGAGCGCCTGTCCTTCTATGAGACTGGAGAGATTCCACGGAAGAATCTGGATGTC

ATGAAGGAAGCAATGGTTCAGGCAGAGGAAGCGGCTGCTGAGATTGCCAGGAAACTGGAG

AAACAGGAGAAGAAGCGCTTGAAGAAGGAAAAAAAGCGATTGGCTGCACTTGCCCTGGCA

TCTTCAGGAAATAGCAGTAGCACTCCAGAGGAGTGTGAGGAGATGAATGAAAAACCCAAA

AAGAAGAAAAAACAAAAGCCCCAGGAGGTTCCTCAGGAGAATGGAATGGAGGAGCCATCT

GTCTCTTACACCAAACCCAAGAAAAAGAGATCTTTTTCCAAGGAGGAATTGGTTAGTAGT

GATCTTGAAGAGACAGCTGGTGGCAGCACAAGTCTTCCGAAGAGGAAGAAGTCTTCACCC

AAGGAAGAAACAGCTAGCGAGCCTGAGGAGGTGAGCAGGAGTGTCTCCAAGAAAAAGAGG

AGATTCTTTTCCAAGGAGGAGCCAGTCAGCAGTGGCCCTGAAGAGGCTGCTAGCAGCAAA

ACCAGCACCAAGAAAAAGAAAAAATTACACAAAGCATCACAAGAT

>CL114.Contig3_A-W 1383 2186 nucleolar protein 56 [Sus scrofa]

ATCTTAAACTCTCCGCTGAATATTCTCAGAGCCCTGAAGACAAGGGGTAACACACCAAAA

TATGGACTCATTTTCCATTCTACCTTCATTGGCCGAGCAGCTGCCAAGAACAAAGGCCGC

ATCTCCCGATACCTGGCAAACAAATGCAGTATTGCCTCACGAATCGATTGCTTCTCTGAG

GTTCCTACAAGTGTATTTGGGGAGAAGCTTCGAGAACAAGTTGAGGAGCGCCTGTCCTTC

TATGAGACTGGAGAGATTCCACGGAAGAATCTGGATGTCATGAAGGAAGCAATGGTTCAG

GCAGAGGAAGCGGCTGCTGAGATTGCCAGGAAACTGGAGAAACAGGAGAAGAAGCGCTTG

AAGAAGGAAAAAAAGCGATTGGCTGCACTTGCCCTGGCATCTTCAGGAAATAGCAGTAGC

ACTCCAGAGGAGTGTGAGGAGATGAATGAAAAACCCAAAAAGAAGAAAAAACAAAAGCCC

CAGGAGGTTCCTCAGGAGAATGGAATGGAGGAGCCATCTGTCTCTTACACCAAACCCAAG

AAAAAGAGATCTTTTTCCAAGGAGGAATTGGTTAGTAGTGATCTTGAAGAGACAGCTGGT

GGCAGCACAAGTCTTCCGAAGAGGAAGAAGTCTTCACCCAAGGAAGAAACAGCTAGCGAG

CCTGAGGAGGTGAGCAGGAGTGTCTCCAAGAAAAAGAGGAGATTCTTTTCCAAGGAGGAG

CCAGTCAGCAGTGGCCCTGAAGAGGCTGCTAGCAGCAAAACCAGCACCAAGAAAAAGAAA

AAATTACACAAAGCATCACAAGAT

>CL114.Contig4_A-W 5 1051 NOP56 ribonucleoprotein-like protein [Mustela putorius furo]

CTAGCCGCATTGCGAGCCGGGCAGGGAGCAAGCGCCATGGTTTTGCTACACGTGCTGTTC

GAGCACGCGGTAGGCTACGCGCTGCTGGCGCTGAAGGAAGTGGAGGAGATCAGCCTGTTA

CTGCCGCAGGTGGAGGAATGTGTGCTCAGCCTGGGCAAGTTCCACAACATTGTCCGTCTT

GTGGCCTTCAATCCCTTTTCCTCATCCCAGGTTGCCTTGGAAAATGCCAATGCGGTGTCT

GAAGGCATTGTTCATGAGGACCTCCGCCTGCTCCTGGAGACCCACCTGCCATCCAAAAAG

AAGAAAGTGCTTTTGGGGGTTGGGGATCCCAAGATTGGTGCTGCTATACAGGAGGAGTTA

GGGTACAACTGCCAGACTGGAGGTGTGGTAGCCGAGATCCTGCGAGGAGTTCGTCTGCAC

TTCCATAATCTGGTGAAGGGTCTGACTGACCTGTCAGCTTGTAAAGCCCAATTAGGGCTG

GGACATAGCTATTCTCGTGCCAAAGTTAAGTTTAATGTGAACCGGGTGGACAACATGATC

ATCCAGTCCATTAGCCTCTTGGACCAACTGGATAAAGACATCAATACTTTCTCTATGCGT

GTCAGGGAATGGTACGGGTATCACTTTCCAGAACTGGTGAAGATCATCAATGACAATGCC

ACATACTGCCGCCTTGCCCAATTCATTGGAAACCGAAAAGAACTGAGTGAGGAAAAGTTA

GAGAAGCTGGAGGAGCTGACAATGGATGGGGCCAAAGCTAAAGCTATTCTGGATGCCTCA

CGGTCCTCCATGGGCATGGACATATCTGCCATCGACTTGATAAACATCGAGAGCTTCTCC

AGTCGTGTGGTGTCTTTGTCAGAGTACCGCCAGAGCCTACACACTTACCTGCGCTCCAAG

ATGAGCCAAGTAGCCCCCAGCCTGTCAGCCCTAATTGGGGAAGCGGTAGGTGCACGTCTC

ATTGCTCATGCTGGCAGCCTCACCAACCTGGCCAAGTATCCAGCATCCACAGTGCAGATC

CTTGGGGCTGAAAAGGCCCTATTCAGG

>CL114.Contig5_A-W 105 296 Nucleolar protein 5A, partial [Macaca mulatta]

AGGGAATGGTACGGGTATCACTTTCCAGAACTGGTGAAGATCATCAATGACAATGCCACA

TACTGCCGCCTTGCCCAATTCATTGGAAACCGAAAAGAACTGAGTGAGGAAAAGTTAGAG

AAGCTGGAGGAGCTGACAATGGATGGGGCCAAAGCTAAAGCTATTCTGGATGCCTCACGG

TCCTCCATGGGT

>CL114.Contig6_A-W 105 956 Nucleolar protein 5A, partial [Macaca mulatta]

AGGGAATGGTACGGGTATCACTTTCCAGAACTGGTGAAGATCATCAATGACAATGCCACA

TACTGCCGCCTTGCCCAATTCATTGGAAACCGAAAAGAACTGAGTGAGGAAAAGTTAGAG

AAGCTGGAGGAGCTGACAATGGATGGGGCCAAAGCTAAAGCTATTCTGGATGCCTCACGG

TCCTCCATGGGCATGGACATATCTGCCATCGACTTGATAAACATCGAGAGCTTCTCCAGT

CGTGTGGTGTCTTTGTCAGAGTACCGCCAGAGCCTACACACTTACCTGCGCTCCAAGATG

AGCCAAGTAGCCCCCAGCCTGTCAGCCCTAATTGGGGAAGCGGTAGGTGCACGTCTCATT

GCTCATGCTGGCAGCCTCACCAACCTGGCCAAGTATCCAGCATCCACAGTGCAGATCCTT

GGGGCTGAAAAGGCCCTATTCAGAGCCCTGAAGACAAGGGGTAACACACCAAAATATGGA

CTCATTTTCCATTCTACCTTCATTGGCCGAGCAGCTGCCAAGAACAAAGGCCGCATCTCC

CGATACCTGGCAAACAAATGCAGTATTGCCTCACGAATCGATTGCTTCTCTGAGGTTCCT

ACAAGTGTATTTGGGGAGAAGCTTCGAGAACAAGTTGAGGAGCGCCTGTCCTTCTATGAG

ACTGGAGAGATTCCACGGAAGAATCTGGATGTCATGAAGGAAGCAATGGTTCAGGCAGAG

GAAGCGGCTGCTGAGATTGCCAGGAAACTGGAGAAACAGGAGAAGAAGCGCTTGAAGAAG

GAAAAAAAGCGATTGGCTGCACTTGCCCTGGCATCTTCAGGAAATAGCAGTAGCACTCCA

GAGGAGTGTGAG

>CL116.Contig1_A-W 3 284 E3 ubiquitin-protein ligase MARCH6 [Macaca mulatta] >gi|383409471|gb|AFH27949.1| E3 ubiquitin-protein ligase MARCH6 [Macaca mulatta] >gi|384939368|gb|AFI33289.1| E3 ubiquitin-protein ligase MARCH6 [Macaca mulatta]

TTGCGAGAGCAGATAGTCCACGGGGGAGCCCCGATTTGGTTGGAACATGCTGCTCCACCC

TTCAATGCTGCTGGACACCACCAAAATGAGGCTCCAGCAGGAGGAAATGGTGCAGAAAAT

GTTGCTCCTGAGCAACCTGCTAACCCACCAGCTGAGAATGCAGTGGTGGGAGAAAACCCC

GATGCGCAGGATGATCAGGCGGAAGAGGAAGAGGAAGACAACGAGGAGGAAGATGATGCA

GGCGTGGAAGAGGAAGAGGAAGACAACGAGGAGGAAGATGAT

>CL116.Contig2_A-W 1 579 minus strand E3 ubiquitin-protein ligase MARCH6 [Macaca mulatta] >gi|383409471|gb|AFH27949.1| E3 ubiquitin-protein ligase MARCH6 [Macaca mulatta] >gi|384939368|gb|AFI33289.1| E3 ubiquitin-protein ligase MARCH6 [Macaca mulatta]

TTGCAGGGTTGTTTTGTGGTGACGTGCACATTGTGTGCGTTCATCAGCCTGGTGTGGTTG

CGAGAGCAGATAGTCCACGGGGGAGCCCCGATTTGGTTGGAACATGCTGCTCCACCCTTC

AATGCTGCTGGACACCACCAAAATGAGGCTCCAGCAGGAGGAAATGGTGCAGAAAATGTT

GCTCCTGAGCAACCTGCTAACCCACCAGCTGAGAATGCAGTGGTGGGAGAAAACCCCGAT

GCGCAGGATGATCAGGCGGAAGAGGAAGAGGAAGACAACGAGGAGGAAGATGATGCAGGC

GTGGAAGATGCAGTGGATGCTAATAATGGAGCCCAGGATGACATGAACTGGAATGCTTTA

GAATGGGACCGAGCTGCAGAAGAACTCACATGGGAAAGAATGCTAGGACTTGATGGATCA

CTCGTTTTTCTGGAACACGTCTTCTGGGTTGTGTCTTTAAATACACTGTTCATTCTTGTT

TTTGCATTTTGCCCTTACCACATTGGTCATTTCTCCCTTGTTGGTTTGGGATTTGAAGAA

CATGTCCAAGCATCTCATTTTGAAGGCCTAATCACAACC

>CL118.Contig1_A-W 60 1403 minus strand annexin A7 isoform 1 [Macaca mulatta] >gi|383417919|gb|AFH32173.1| annexin A7 isoform 1 [Macaca mulatta] >gi|384940472|gb|AFI33841.1| annexin A7 isoform 1 [Macaca mulatta]

CCTGCAGGTCAGGAGTCATCTGTCCCCGCTCCAGGTCAATATCCTTATCCTAGTGGCTTT

CCTCCAATGGGAGGAGGTGCCTACCCACCAGTGCCAAGTGGTGGCTACCCAGGAGCTGGA

GGCTACCCTGCATCTGGAGGTTATCCAGCCCCTGGAGGCTATCCTGGTGCCCCACAGCCA

GGGGGAGCTCCATCCTATCCTGGAGTTCCTCCAGGTCAAGGATATGGAGCCCCACCCAGT

TCAGCGAGCTTTTCTGGTTATCCACAGCCACCTTCACAGACTTATGGTGGTGGTCCAGCC

CAGGTCCCACTACCTGGTGGCTTTCCTGGAGGACAGATACCTTCTCAGTATCCTGGAGGA

CAAGCTCCTTACCCTAGTCAGCCTGCAGCAATGACTCAGGGTACTCAAGGGACCATTCGA

CCAGCTGCCAACTTCGATGCTATGAGAGATGCAGAAATTCTCCGTAAAGCAATGAAAGGA

TTTGGGACAGATGAGCAGGCAATTGTGGATATTGTGGCCAGTCGTTCCAATGATCAGAGG

CAAAAAATTAAAGCAGCTTTTAAGACCATGTATGGCAAGGATTTAATCAAAGATCTCAAG

TCAGAGTTGAGTGGAAATATGGAAGAACTGATTCTTGCCCTGTTCATGCCTCCTACATAT

TATGATGCCTGGAGTTTACGGAATGCAATGCAGGGAGCAGGAACTCAGGAACGAGTATTG

ATAGAGATTTTGTGCACAAGAACAAATCAAGAAATCCGAGACATTGTCAGATGTTATCAA

TCAGAATTTGGACGAGACCTTGAAAAGGACATTAGGTCAGATACATCAGGACATTTTGAA

CGTTTACTTGTATCCATGTGCCAGGGAAATCGTGATGAGAATCAGAGTGTAAACCACCAA

ATGGCTCAAGAAGATGCTCAGCGTCTCTATCAGGCTGGTGAGGGGAGACTAGGGACAGAT

GAATCTTGCTTTAACATGATCCTTGCCACACGAAGCTTTCCTCAGCTGAAAGCTACCATG

GAGGCTTATTCCAGGATGTCTAATCGAGATTTGTTAAGCAGTGTGGGCCGCGAGTTTTCC

GGATATGTTGAAAGTGGTTTGAAGACCATCTTGCAGTGTGCCCTGAACCGTCCTGCCTTC

TTTGCTGAGCGGCTGTACTATTCTATGAAAGGTGCAGGCACAGATGACTCCACTCTGGTC

AGGATTGTGGTCTCTCGAAGTGAGATCGACCTTGTACAAATAAAACAGATGTTCACTCAG

ATGTATCAGAAGACCCTGGGCACAATGATTGCGAGTGACACAAGTGGAGATTACCGAAAG

CTGCTTCTGGCCATTGTGGGTCAG

>CL118.Contig2_A-W 47 280 minus strand PREDICTED: annexin A7-like [Ailuropoda melanoleuca]

GTGCAGTGTGCCCTGAACCGTCCTGCCTTCTTTGCTGAGCGGCTGTACTATTCTATGAAA

GGTGCAGGCACAGATGACTCCACTCTGGTCAGGATTGTGGTCTCTCGAAGTGAGATCGAC

CTTGTACAAATAAAACAGATGTTCACTCAGATGTATCAGAAGACCCTGGGCACAATGATT

GCGAGTGACACAAGTGGAGATTACCGAAAGCTGCTTCTGGCCATTGTGGGTCAG

>CL118.Contig3_A-W 57 1454 minus strand annexin A7 isoform 1 [Macaca mulatta] >gi|383417919|gb|AFH32173.1| annexin A7 isoform 1 [Macaca mulatta] >gi|384940472|gb|AFI33841.1| annexin A7 isoform 1 [Macaca mulatta]

ATGTCATACCCAGGCTATCCCCCCACAGGCTACCCACCTTTCCCTGGATATCCTCCTGCA

GGTCAGGAGTCATCTGTCCCCGCTCCAGGTCAATATCCTTATCCTAGTGGCTTTCCTCCA

ATGGGAGGAGGTGCCTACCCACCAGTGCCAAGTGGTGGCTACCCAGGAGCTGGAGGCTAC

CCTGCATCTGGAGGTTATCCAGCCCCTGGAGGCTATCCTGGTGCCCCACAGCCAGGGGGA

GCTCCATCCTATCCTGGAGTTCCTCCAGGTCAAGGATATGGAGCCCCACCCAGTTCAGCG

AGCTTTTCTGGTTATCCACAGCCACCTTCACAGACTTATGGTGGTGGTCCAGCCCAGGTC

CCACTACCTGGTGGCTTTCCTGGAGGACAGATACCTTCTCAGTATCCTGGAGGACAAGCT

CCTTACCCTAGTCAGCCTGCAGCAATGACTCAGGGTACTCAAGGGACCATTCGACCAGCT

GCCAACTTCGATGCTATGAGAGATGCAGAAATTCTCCGTAAAGCAATGAAAGGATTTGGG

ACAGATGAGCAGGCAATTGTGGATATTGTGGCCAGTCGTTCCAATGATCAGAGGCAAAAA

ATTAAAGCAGCTTTTAAGACCATGTATGGCAAGGATTTAATCAAAGATCTCAAGTCAGAG

TTGAGTGGAAATATGGAAGAACTGATTCTTGCCCTGTTCATGCCTCCTACATATTATGAT

GCCTGGAGTTTACGGAATGCAATGCAGGGAGCAGGAACTCAGGAACGAGTATTGATAGAG

ATTTTGTGCACAAGAACAAATCAAGAAATCCGAGACATTGTCAGATGTTATCAATCAGAA

TTTGGACGAGACCTTGAAAAGGACATTAGGTCAGATACATCAGGACATTTTGAACGTTTA

CTTGTATCCATGTGCCAGGGAAATCGTGATGAGAATCAGAGTGTAAACCACCAAATGGCT

CAAGAAGATGCTCAGCGTCTCTATCAGGCTGGTGAGGGGAGACTAGGGACAGATGAATCT

TGCTTTAACATGATCCTTGCCACACGAAGCTTTCCTCAGCTGAAAGCTACCATGGAGGCT

TATTCCAGGATGTCTAATCGAGATTTGTTAAGCAGTGTGGGCCGCGAGTTTTCCGGATAT

GTTGAAAGTGGTTTGAAGACCATCTTGCAGTGTGCCCTGAACCGTCCTGCCTTCTTTGCT

GAGCGGCTGTACTATTCTATGAAAGGTGCAGGCACAGATGACTCCACTCTGGTCAGGATT

GTGGTCTCTCGAAGTGAGATCGACCTTGTACAAATAAAACAGATGTTCACTCAGATGTAT

CAGAAGACCCTGGGCACAATGATTGCGAGTGACACAAGTGGAGATTACCGAAAGCTGCTT

CTGGCCATTGTGGGTCAG

>CL123.Contig1_A-W 122 505 minus strand PREDICTED: NADH dehydrogenase [ubiquinone] 1 beta subcomplex subunit 6-like [Cavia porcellus]

ATGACGGGGTACACGCCGGATGAAAAACTGCGGCTCCAGCAGTTGCGAGAGCTGAGGCGG

CGATGGCTGAAGGACCAGGAGCTGAGCCCCCGGGAGCCTGTCCTACCTCCCCAGAGGATG

TGGCCTATGGAGGCATTCTGGAATAAGTTTTTGCAGGATAGGGCCCCTTGGAAGAATGTG

ATCTATAAGGTATACAGGACCAGTATCTTTGCTGTTACTTGTGTACTTATACCCACCTGG

ATTGTTCATTATTACGTCAAATATCATGTGTCTAAAAAACCATATGCCATTGTTGAAAGG

AAGCCCAGAATCTTCCCAGGTGATACAATTCTGGAGACTGGAGAAGTAATTCCACCAATG

AAAGAATATCCTGATCAACATCAC

>CL123.Contig2_A-W 118 501 PREDICTED: NADH dehydrogenase [ubiquinone] 1 beta subcomplex subunit 6-like [Cavia porcellus]

ATGACGGGGTACACGCCGGATGAAAAACTGCGGCTCCAGCAGTTGCGAGAGCTGAGGCGG

CGATGGCTGAAGGACCAGGAGCTGAGCCCCCGGGAGCCTGTCCTACCTCCCCAGAGGATG

TGGCCTATGGAGGCATTCTGGAATAAGTTTTTGCAGGATAGGGCCCCTTGGAAGAATGTG

ATCTATAAGGTATACAGGACCAGTATCTTTGCTGTTACTTGTGTACTTATACCCACCTGG

ATTGTTCATTATTACGTCAAATATCATGTGTCTAAAAAACCATATGCCATTGTTGAAAGG

AAGCCCAGAATCTTCCCAGGTGATACAATTCTGGAGACTGGAGAAGTAATTCCACCAATG

AAAGAATATCCTGATCAACATCAC

>CL124.Contig1_A-W 9 1877 DEAD (Asp-Glu-Ala-Asp) box polypeptide 41 [Mus musculus]

AGGATGGAGGAGTCGGAACCCGAGCGGAAGCGCGCCCGCCCGGACGACGCGACCGCAGGA

GGAAGCCGCTCCGAGGATGAGGATGAGGACGATGAGGATTACGTGCCCTATGTGCCGCTG

CGACAGCGCCGACAACTTCTGCTCCAGAAACTGCTGCAGCGAAGACGCAAGGGAGCTGCA

GAGGAAGAGCAGCAGGACAGCAGCAGTGAGCCCCGAGGAGATGAGGACGACATCCCTTTG

GGCCCTCAGTCCAACGTCAGCCTCCTGGATCAGCATCAGCACCTCAAAGAGAAGGCTGAA

GCCCGCAAGGAATCTGCCAAGGAGAAGCAACTAAAGGAAGAAGAGAAGATTCTGGAGAGT

GTGGCTGAAGGCCGAGCCTTGATGTCAGTGAAGGAGATGGCCAAGGGCATTACATATGAT

GACCCAATCAAAACCAGTTGGACACCACCCCGTTACGTCCTGAGCATGTCTGAAGAGCGG

CATGAACGAGTTCGGAAGAAGTACCACATCCTGGTGGAGGGAGATGGTATCCCACCACCC

ATCAAGAGCTTTAAGGAAATGAAGTTTCCTGCAGCCATCCTGCGAGGCCTAAAGAAAAAA

GGCATTCTCCACCCAACACCTATTCAGATCCAAGGTATCCCTACCATTCTTTCGGGCCGG

GACATGATTGGCATTGCCTTCACGGGGTCAGGCAAGACGTTGGTGTTCACACTGCCCGTC

ATCATGTTCTGTCTGGAACAAGAGAAGCGGTTGCCTTTCTCTAAGCGTGAGGGGCCCTAT

GGACTCATCATCTGCCCCTCGCGGGAACTGGCCCGGCAAACCCATGGCATCCTGGAATAT

TACTGCCGCCTGCTGCAGGAGGACAGCTCACCTCTCCTGCGCTGTGCTCTCTGCATTGGG

GGCATGTCTGTCAAAGAGCAGATGGAAACCATCCGACATGGTGTACATATGATGGTGGCC

ACCCCTGGACGCCTCATGGATTTACTACAGAAGAAGATGGTCAGCCTAGACATCTGTCGC

TACCTGGCCCTGGATGAGGCTGACCGCATGATTGACATGGGTTTTGAGGGCGACATCCGC

ACCATCTTTTCCTACTTCAAGGGCCAGCGGCAGACTCTACTCTTCAGTGCTACCATGCCA

AAGAAGATTCAGAACTTTGCCAAGAGTGCCCTGGTAAAGCCTGTTACCATCAATGTGGGG

CGTGCTGGGGCCGCCAGCCTGGATGTCATTCAGGAGGTGGAATACGTGAAGGAAGAGGCC

AAGATGGTATACCTGCTTGAGTGCCTGCAGAAGACCCCCCCTCCTGTGCTCATCTTTGCA

GAGAAGAAGGCCGATGTGGACGCCATCCATGAGTATCTGCTGCTCAAGGGGGTTGAGGCT

GTTGCCATCCATGGGGGCAAAGACCAGGAGGAACGGACCAAAGCCATTGAGGCATTCCGG

GAGGGCAAGAAGGATGTCCTGGTGGCCACAGATGTGGCCTCCAAAGGCCTGGACTTCCCT

GCTATCCAGCACGTCATCAATTACGACATGCCTGAGGAAATCGAGAACTATGTGCACCGA

ATCGGCCGCACAGGGCGCTCAGGAAACACAGGCATTGCTACGACCTTCATTAACAAGGCC

TGTGATGAGTCAGTGCTGATGGATCTCAAGGCCCTGCTGCTAGAAGCCAAGCAGAAGGTG

CCACCTGTACTACAAGTGCTGCACCCTGGGGACGAGTCCATGCTGGACATTGGAGGAGAA

CGTGGTTGTGCCTTCTGTGGGGGCCTGGGCCATCGGATCACTGACTGCCCCAAACTTGAG

GCCATGCAGACCAAACAGGTCAGCAACATTGGCCGCAAGGACTACCTGGCCCACAGCTCC

ATGGACTTC

>CL124.Contig2_A-W 978 1364 DEAD (Asp-Glu-Ala-Asp) box polypeptide 41 [Mus musculus]

CCTGCGTAGGTGCTCATCTTTGCAGAGAAGAAGGCCGATGTGGACGCCATCCATGAGTAT

CTGCTGCTCAAGGGGGTTGAGGCTGTTGCCATCCATGGGGGCAAAGGTCAGGACAGTGCA

TGCATTAGTGTGAACCCTGATTCTATAGCAACCCTTCACCTATCCCTTCCCCTGCCTGAC

CTGCCTGCTTCCAAAACTCCCACCGTAACTCTGATAGCTGCTGCCTGACCCTCCCTAGAC

CAGGAGGAACGGACCAAAGCCATTGAGGCATTCCGGGAGGGCAAGAAGGATGTCCTGGTG

GCCACAGATGTGGCCTCCAAAGGCCTGGACTTCCCTGCTATCCAGCACGTCATCAATTAC

GACATGCCTGAGGAAATCGAGAACTAT

>CL124.Contig3_A-W 9 1229 DEAD (Asp-Glu-Ala-Asp) box polypeptide 41 [Mus musculus]

AGGATGGAGGAGTCGGAACCCGAGCGGAAGCGCGCCCGCCCGGACGACGCGACCGCAGGA

GGAAGCCGCTCCGAGGATGAGGATGAGGACGATGAGGATTACGTGCCCTATGTGCCGCTG

CGACAGCGCCGACAACTTCTGCTCCAGAAACTGCTGCAGCGAAGACGCAAGGGAGCTGCA

GAGGAAGAGCAGCAGGACAGCAGCAGTGAGCCCCGAGGAGATGAGGACGACATCCCTTTG

GGCCCTCAGTCCAACGTCAGCCTCCTGGATCAGCATCAGCACCTCAAAGAGAAGGCTGAA

GCCCGCAAGGAATCTGCCAAGGAGAAGCAACTAAAGGAAGAAGAGAAGATTCTGGAGAGT

GTGGCTGAAGGCCGAGCCTTGATGTCAGTGAAGGAGATGGCCAAGGGCATTACATATGAT

GACCCAATCAAAACCAGGTATGTCTTTCCAGACTGTTCAGTTGCCCTTACTTAGGATGGT

GTGTGGCTGAGACTAGCAGTGACACCCCAAGCCCAGCTGTTCCAAGGCCTCTGTTGAACC

CTACCACCCACCCACAGTTGGACACCACCCCGTTACGTCCTGAGCATGTCTGAAGAGCGG

CATGAACGAGTTCGGAAGAAGTACCACATCCTGGTGGAGGGAGATGGTATCCCACCACCC

ATCAAGAGCTTTAAGGAAATGAAGTTTCCTGCAGCCATCCTGCGAGGCCTAAAGAAAAAA

GGCATTCTCCACCCAACACCTATTCAGATCCAAGGTATCCCTACCATTCTTTCGGGCCGG

GACATGATTGGCATTGCCTTCACGGGGTCAGGCAAGACGTTGGTGTTCACACTGCCCGTC

ATCATGTTCTGTCTGGAACAAGAGAAGCGGTTGCCTTTCTCTAAGCGTGAGGGGCCCTAT

GGACTCATCATCTGCCCCTCGCGGGAACTGGCCCGGCAAACCCATGGCATCCTGGAATAT

TACTGCCGCCTGCTGCAGGAGGACAGCTCACCTCTCCTGCGCTGTGCTCTCTGCATTGGG

GGCATGTCTGTCAAAGAGCAGATGGAAACCATCCGACATGGTGTACATATGATGGTGGCC

ACCCCTGGACGCCTCATGGATTTACTACAGAAGAAGATGGTCAGCCTAGACATCTGTCGC

TACCTGGCCCTGGATGAGGCTGACCGCATGATTGACATGGGTTTTGAGGGCGACATCCGC

ACCATCTTTTCCTACTTCAAG

>CL124.Contig4_A-W 62 1243 PREDICTED: probable ATP-dependent RNA helicase DDX41-like [Cavia porcellus]

AGGTATCCCTACCATGTGAGTATGGACCATGGACTTGGGGTCCCTGAGGAAAGCAGTGAC

AGGGAGAAGCAATAACCAAGTGTCTGACTGGAGAAGTAGTTGCTGTTTCCTGCTGGACCT

GGCCCTCAGGCTATCTTCTCCTACAGTCTTTCGGGCCGGGACATGATTGGCATTGCCTTC

ACGGGGTCAGGCAAGACGTTGGTGTTCACACTGCCCGTCATCATGTTCTGTCTGGAACAA

GAGAAGCGGTTGCCTTTCTCTAAGCGTGAGGGGCCCTATGGACTCATCATCTGCCCCTCG

CGGGAACTGGCCCGGCAAACCCATGGCATCCTGGAATATTACTGCCGCCTGCTGCAGGAG

GACAGCTCACCTCTCCTGCGCTGTGCTCTCTGCATTGGGGGCATGTCTGTCAAAGAGCAG

ATGGAAACCATCCGACATGGTGTACATATGATGGTGGCCACCCCTGGACGCCTCATGGAT

TTACTACAGAAGAAGATGGTCAGCCTAGACATCTGTCGCTACCTGGCCCTGGATGAGGCT

GACCGCATGATTGACATGGGTTTTGAGGGCGACATCCGCACCATCTTTTCCTACTTCAAG

GGCCAGCGGCAGACTCTACTCTTCAGTGCTACCATGCCAAAGAAGATTCAGAACTTTGCC

AAGAGTGCCCTGGTAAAGCCTGTTACCATCAATGTGGGGCGTGCTGGGGCCGCCAGCCTG

GATGTCATTCAGGAGGTGGAATACGTGAAGGAAGAGGCCAAGATGGTATACCTGCTTGAG

TGCCTGCAGAAGACCCCCCCTCCTGTGCTCATCTTTGCAGAGAAGAAGGCCGATGTGGAC

GCCATCCATGAGTATCTGCTGCTCAAGGGGGTTGAGGCTGTTGCCATCCATGGGGGCAAA

GGTCAGGACAGTGCATGCATTAGTGTGAACCCTGATTCTATAGCAACCCTTCACCTATCC

CTTCCCCTGCCTGACCTGCCTGCTTCCAAAACTCCCACCGTAACTCTGATAGCTGCTGCC

TGACCCTCCCTAGACCAGGAGGAACGGACCAAAGCCATTGAGGCATTCCGGGAGGGCAAG

AAGGATGTCCTGGTGGCCACAGATGTGGCCTCCAAAGGCCTGGACTTCCCTGCTATCCAG

CACGTCATCAATTACGACATGCCTGAGGAAATCGAGAACTAT

>CL124.Contig5_A-W 978 1232 DEAD (Asp-Glu-Ala-Asp) box polypeptide 41 [Mus musculus]

CCTGCGTAGGTGCTCATCTTTGCAGAGAAGAAGGCCGATGTGGACGCCATCCATGAGTAT

CTGCTGCTCAAGGGGGTTGAGGCTGTTGCCATCCATGGGGGCAAAGACCAGGAGGAACGG

ACCAAAGCCATTGAGGCATTCCGGGAGGGCAAGAAGGATGTCCTGGTGGCCACAGATGTG

GCCTCCAAAGGCCTGGACTTCCCTGCTATCCAGCACGTCATCAATTACGACATGCCTGAG

GAAATCGAGAACTAT

>CL124.Contig6_A-W 62 1111 PREDICTED: probable ATP-dependent RNA helicase DDX41-like [Cavia porcellus]

AGGTATCCCTACCATGTGAGTATGGACCATGGACTTGGGGTCCCTGAGGAAAGCAGTGAC

AGGGAGAAGCAATAACCAAGTGTCTGACTGGAGAAGTAGTTGCTGTTTCCTGCTGGACCT

GGCCCTCAGGCTATCTTCTCCTACAGTCTTTCGGGCCGGGACATGATTGGCATTGCCTTC

ACGGGGTCAGGCAAGACGTTGGTGTTCACACTGCCCGTCATCATGTTCTGTCTGGAACAA

GAGAAGCGGTTGCCTTTCTCTAAGCGTGAGGGGCCCTATGGACTCATCATCTGCCCCTCG

CGGGAACTGGCCCGGCAAACCCATGGCATCCTGGAATATTACTGCCGCCTGCTGCAGGAG

GACAGCTCACCTCTCCTGCGCTGTGCTCTCTGCATTGGGGGCATGTCTGTCAAAGAGCAG

ATGGAAACCATCCGACATGGTGTACATATGATGGTGGCCACCCCTGGACGCCTCATGGAT

TTACTACAGAAGAAGATGGTCAGCCTAGACATCTGTCGCTACCTGGCCCTGGATGAGGCT

GACCGCATGATTGACATGGGTTTTGAGGGCGACATCCGCACCATCTTTTCCTACTTCAAG

GGCCAGCGGCAGACTCTACTCTTCAGTGCTACCATGCCAAAGAAGATTCAGAACTTTGCC

AAGAGTGCCCTGGTAAAGCCTGTTACCATCAATGTGGGGCGTGCTGGGGCCGCCAGCCTG

GATGTCATTCAGGAGGTGGAATACGTGAAGGAAGAGGCCAAGATGGTATACCTGCTTGAG

TGCCTGCAGAAGACCCCCCCTCCTGTGCTCATCTTTGCAGAGAAGAAGGCCGATGTGGAC

GCCATCCATGAGTATCTGCTGCTCAAGGGGGTTGAGGCTGTTGCCATCCATGGGGGCAAA

GACCAGGAGGAACGGACCAAAGCCATTGAGGCATTCCGGGAGGGCAAGAAGGATGTCCTG

GTGGCCACAGATGTGGCCTCCAAAGGCCTGGACTTCCCTGCTATCCAGCACGTCATCAAT

TACGACATGCCTGAGGAAATCGAGAACTAT

>CL124.Contig7_A-W 381 767 DEAD (Asp-Glu-Ala-Asp) box polypeptide 41, isoform CRA_c [Homo sapiens]

CCTGCGTAGGTGCTCATCTTTGCAGAGAAGAAGGCCGATGTGGACGCCATCCATGAGTAT

CTGCTGCTCAAGGGGGTTGAGGCTGTTGCCATCCATGGGGGCAAAGGTCAGGACAGTGCA

TGCATTAGTGTGAACCCTGATTCTATAGCAACCCTTCACCTATCCCTTCCCCTGCCTGAC

CTGCCTGCTTCCAAAACTCCCACCGTAACTCTGATAGCTGCTGCCTGACCCTCCCTAGAC

CAGGAGGAACGGACCAAAGCCATTGAGGCATTCCGGGAGGGCAAGAAGGATGTCCTGGTG

GCCACAGATGTGGCCTCCAAAGGCCTGGACTTCCCTGCTATCCAGCACGTCATCAATTAC

GACATGCCTGAGGAAATCGAGAACTAT

>CL124.Contig8_A-W 1546 2250 DEAD (Asp-Glu-Ala-Asp) box polypeptide 41 [Mus musculus]

CCTGCGTAGGTGCTCATCTTTGCAGAGAAGAAGGCCGATGTGGACGCCATCCATGAGTAT

CTGCTGCTCAAGGGGGTTGAGGCTGTTGCCATCCATGGGGGCAAAGGTCAGGACAGTGCA

TGCATTAGTGTGAACCCTGATTCTATAGCAACCCTTCACCTATCCCTTCCCCTGCCTGAC

CTGCCTGCTTCCAAAACTCCCACCGTAACTCTGATAGCTGCTGCCTGACCCTCCCTAGAC

CAGGAGGAACGGACCAAAGCCATTGAGGCATTCCGGGAGGGCAAGAAGGATGTCCTGGTG

GCCACAGATGTGGCCTCCAAAGGCCTGGACTTCCCTGCTATCCAGCACGTCATCAATTAC

GACATGCCTGAGGAAATCGAGAACTATGTGCACCGAATCGGCCGCACAGGGCGCTCAGGA

AACACAGGCATTGCTACGACCTTCATTAACAAGGCCTGTGATGAGTCAGTGCTGATGGAT

CTCAAGGCCCTGCTGCTAGAAGCCAAGCAGAAGGTGCCACCTGTACTACAAGTGCTGCAC

CCTGGGGACGAGTCCATGCTGGACATTGGAGGAGAACGTGGTTGTGCCTTCTGTGGGGGC

CTGGGCCATCGGATCACTGACTGCCCCAAACTTGAGGCCATGCAGACCAAACAGGTCAGC

AACATTGGCCGCAAGGACTACCTGGCCCACAGCTCCATGGACTTC

>CL124.Contig9_A-W 62 661 PREDICTED: probable ATP-dependent RNA helicase DDX41-like [Cavia porcellus]

AGGTATCCCTACCATGTGAGTATGGACCATGGACTTGGGGTCCCTGAGGAAAGCAGTGAC

AGGGAGAAGCAATAACCAAGTGTCTGACTGGAGAAGTAGTTGCTGTTTCCTGCTGGACCT

GGCCCTCAGGCTATCTTCTCCTACAGTCTTTCGGGCCGGGACATGATTGGCATTGCCTTC

ACGGGGTCAGGCAAGACGTTGGTGTTCACACTGCCCGTCATCATGTTCTGTCTGGAACAA

GAGAAGCGGTTGCCTTTCTCTAAGCGTGAGGGGCCCTATGGACTCATCATCTGCCCCTCG

CGGGAACTGGCCCGGCAAACCCATGGCATCCTGGAATATTACTGCCGCCTGCTGCAGGAG

GACAGCTCACCTCTCCTGCGCTGTGCTCTCTGCATTGGGGGCATGTCTGTCAAAGAGCAG

ATGGAAACCATCCGACATGGTGTACATATGATGGTGGCCACCCCTGGACGCCTCATGGAT

TTACTACAGAAGAAGATGGTCAGCCTAGACATCTGTCGCTACCTGGCCCTGGATGAGGCT

GACCGCATGATTGACATGGGTTTTGAGGGCGACATCCGCACCATCTTTTCCTACTTCAAG

>CL124.Contig10_A-W 14 646 DEAD (Asp-Glu-Ala-Asp) box polypeptide 41, isoform CRA_c [Homo sapiens]

CCATCCCCACCAGCCCTGCATGCTTCAGCCCCAGCCTCCACCCTTCCTCAGGGCCAGCGG

CAGACTCTACTCTTCAGTGCTACCATGCCAAAGAAGATTCAGAACTTTGCCAAGAGTGCC

CTGGTAAAGCCTGTTACCATCAATGTGGGGCGTGCTGGGGCCGCCAGCCTGGATGTCATT

CAGGAGGTGGAATACGTGAAGGAAGAGGCCAAGATGGTATACCTGCTTGAGTGCCTGCAG

AAGACCCCCCCTCCTGTGCTCATCTTTGCAGAGAAGAAGGCCGATGTGGACGCCATCCAT

GAGTATCTGCTGCTCAAGGGGGTTGAGGCTGTTGCCATCCATGGGGGCAAAGGTCAGGAC

AGTGCATGCATTAGTGTGAACCCTGATTCTATAGCAACCCTTCACCTATCCCTTCCCCTG

CCTGACCTGCCTGCTTCCAAAACTCCCACCGTAACTCTGATAGCTGCTGCCTGACCCTCC

CTAGACCAGGAGGAACGGACCAAAGCCATTGAGGCATTCCGGGAGGGCAAGAAGGATGTC

CTGGTGGCCACAGATGTGGCCTCCAAAGGCCTGGACTTCCCTGCTATCCAGCACGTCATC

AATTACGACATGCCTGAGGAAATCGAGAACTAT

>CL124.Contig11_A-W 14 271 DEAD (Asp-Glu-Ala-Asp) box polypeptide 41, isoform CRA_c [Homo sapiens]

CCATCCCCACCAGCCCTGCATGCTTCAGCCCCAGCCTCCACCCTTCCTCAGGGCCAGCGG

CAGACTCTACTCTTCAGTGCTACCATGCCAAAGAAGATTCAGAACTTTGCCAAGAGTGCC

CTGGTAAAGCCTGTTACCATCAATGTGGGGCGTGCTGGGGCCGCCAGCCTGGATGTCATT

CAGGAGGTGGAATACGTGAAGGAAGAGGCCAAGATGGTATACCTGCTTGAGTGCCTGCAG

AAGACCCCCCCTCCTGTG

>CL124.Contig12_A-W 9 2129 DEAD (Asp-Glu-Ala-Asp) box polypeptide 41 [Mus musculus]

AGGATGGAGGAGTCGGAACCCGAGCGGAAGCGCGCCCGCCCGGACGACGCGACCGCAGGA

GGAAGCCGCTCCGAGGATGAGGATGAGGACGATGAGGATTACGTGCCCTATGTGCCGCTG

CGACAGCGCCGACAACTTCTGCTCCAGAAACTGCTGCAGCGAAGACGCAAGGGAGCTGCA

GAGGAAGAGCAGCAGGACAGCAGCAGTGAGCCCCGAGGAGATGAGGACGACATCCCTTTG

GGCCCTCAGTCCAACGTCAGCCTCCTGGATCAGCATCAGCACCTCAAAGAGAAGGCTGAA

GCCCGCAAGGAATCTGCCAAGGAGAAGCAACTAAAGGAAGAAGAGAAGATTCTGGAGAGT

GTGGCTGAAGGCCGAGCCTTGATGTCAGTGAAGGAGATGGCCAAGGGCATTACATATGAT

GACCCAATCAAAACCAGGTATGTCTTTCCAGACTGTTCAGTTGCCCTTACTTAGGATGGT

GTGTGGCTGAGACTAGCAGTGACACCCCAAGCCCAGCTGTTCCAAGGCCTCTGTTGAACC

CTACCACCCACCCACAGTTGGACACCACCCCGTTACGTCCTGAGCATGTCTGAAGAGCGG

CATGAACGAGTTCGGAAGAAGTACCACATCCTGGTGGAGGGAGATGGTATCCCACCACCC

ATCAAGAGCTTTAAGGAAATGAAGTTTCCTGCAGCCATCCTGCGAGGCCTAAAGAAAAAA

GGCATTCTCCACCCAACACCTATTCAGATCCAAGGTATCCCTACCATTCTTTCGGGCCGG

GACATGATTGGCATTGCCTTCACGGGGTCAGGCAAGACGTTGGTGTTCACACTGCCCGTC

ATCATGTTCTGTCTGGAACAAGAGAAGCGGTTGCCTTTCTCTAAGCGTGAGGGGCCCTAT

GGACTCATCATCTGCCCCTCGCGGGAACTGGCCCGGCAAACCCATGGCATCCTGGAATAT

TACTGCCGCCTGCTGCAGGAGGACAGCTCACCTCTCCTGCGCTGTGCTCTCTGCATTGGG

GGCATGTCTGTCAAAGAGCAGATGGAAACCATCCGACATGGTGTACATATGATGGTGGCC

ACCCCTGGACGCCTCATGGATTTACTACAGAAGAAGATGGTCAGCCTAGACATCTGTCGC

TACCTGGCCCTGGATGAGGCTGACCGCATGATTGACATGGGTTTTGAGGGCGACATCCGC

ACCATCTTTTCCTACTTCAAGGGCCAGCGGCAGACTCTACTCTTCAGTGCTACCATGCCA

AAGAAGATTCAGAACTTTGCCAAGAGTGCCCTGGTAAAGCCTGTTACCATCAATGTGGGG

CGTGCTGGGGCCGCCAGCCTGGATGTCATTCAGGAGGTGGAATACGTGAAGGAAGAGGCC

AAGATGGTATACCTGCTTGAGTGCCTGCAGAAGACCCCCCCTCCTGTGCTCATCTTTGCA

GAGAAGAAGGCCGATGTGGACGCCATCCATGAGTATCTGCTGCTCAAGGGGGTTGAGGCT

GTTGCCATCCATGGGGGCAAAGGTCAGGACAGTGCATGCATTAGTGTGAACCCTGATTCT

ATAGCAACCCTTCACCTATCCCTTCCCCTGCCTGACCTGCCTGCTTCCAAAACTCCCACC

GTAACTCTGATAGCTGCTGCCTGACCCTCCCTAGACCAGGAGGAACGGACCAAAGCCATT

GAGGCATTCCGGGAGGGCAAGAAGGATGTCCTGGTGGCCACAGATGTGGCCTCCAAAGGC

CTGGACTTCCCTGCTATCCAGCACGTCATCAATTACGACATGCCTGAGGAAATCGAGAAC

TATGTGCACCGAATCGGCCGCACAGGGCGCTCAGGAAACACAGGCATTGCTACGACCTTC

ATTAACAAGGCCTGTGATGAGTCAGTGCTGATGGATCTCAAGGCCCTGCTGCTAGAAGCC

AAGCAGAAGGTGCCACCTGTACTACAAGTGCTGCACCCTGGGGACGAGTCCATGCTGGAC

ATTGGAGGAGAACGTGGTTGTGCCTTCTGTGGGGGCCTGGGCCATCGGATCACTGACTGC

CCCAAACTTGAGGCCATGCAGACCAAACAGGTCAGCAACATTGGCCGCAAGGACTACCTG

GCCCACAGCTCCATGGACTTC

>CL124.Contig13_A-W 1546 2118 DEAD (Asp-Glu-Ala-Asp) box polypeptide 41 [Mus musculus]

CCTGCGTAGGTGCTCATCTTTGCAGAGAAGAAGGCCGATGTGGACGCCATCCATGAGTAT

CTGCTGCTCAAGGGGGTTGAGGCTGTTGCCATCCATGGGGGCAAAGACCAGGAGGAACGG

ACCAAAGCCATTGAGGCATTCCGGGAGGGCAAGAAGGATGTCCTGGTGGCCACAGATGTG

GCCTCCAAAGGCCTGGACTTCCCTGCTATCCAGCACGTCATCAATTACGACATGCCTGAG

GAAATCGAGAACTATGTGCACCGAATCGGCCGCACAGGGCGCTCAGGAAACACAGGCATT

GCTACGACCTTCATTAACAAGGCCTGTGATGAGTCAGTGCTGATGGATCTCAAGGCCCTG

CTGCTAGAAGCCAAGCAGAAGGTGCCACCTGTACTACAAGTGCTGCACCCTGGGGACGAG

TCCATGCTGGACATTGGAGGAGAACGTGGTTGTGCCTTCTGTGGGGGCCTGGGCCATCGG

ATCACTGACTGCCCCAAACTTGAGGCCATGCAGACCAAACAGGTCAGCAACATTGGCCGC

AAGGACTACCTGGCCCACAGCTCCATGGACTTC

>CL124.Contig14_A-W 1546 1932 DEAD (Asp-Glu-Ala-Asp) box polypeptide 41 [Mus musculus]

CCTGCGTAGGTGCTCATCTTTGCAGAGAAGAAGGCCGATGTGGACGCCATCCATGAGTAT

CTGCTGCTCAAGGGGGTTGAGGCTGTTGCCATCCATGGGGGCAAAGGTCAGGACAGTGCA

TGCATTAGTGTGAACCCTGATTCTATAGCAACCCTTCACCTATCCCTTCCCCTGCCTGAC

CTGCCTGCTTCCAAAACTCCCACCGTAACTCTGATAGCTGCTGCCTGACCCTCCCTAGAC

CAGGAGGAACGGACCAAAGCCATTGAGGCATTCCGGGAGGGCAAGAAGGATGTCCTGGTG

GCCACAGATGTGGCCTCCAAAGGCCTGGACTTCCCTGCTATCCAGCACGTCATCAATTAC

GACATGCCTGAGGAAATCGAGAACTAT

>CL124.Contig15_A-W 9 1811 DEAD (Asp-Glu-Ala-Asp) box polypeptide 41 [Mus musculus]

AGGATGGAGGAGTCGGAACCCGAGCGGAAGCGCGCCCGCCCGGACGACGCGACCGCAGGA

GGAAGCCGCTCCGAGGATGAGGATGAGGACGATGAGGATTACGTGCCCTATGTGCCGCTG

CGACAGCGCCGACAACTTCTGCTCCAGAAACTGCTGCAGCGAAGACGCAAGGGAGCTGCA

GAGGAAGAGCAGCAGGACAGCAGCAGTGAGCCCCGAGGAGATGAGGACGACATCCCTTTG

GGCCCTCAGTCCAACGTCAGCCTCCTGGATCAGCATCAGCACCTCAAAGAGAAGGCTGAA

GCCCGCAAGGAATCTGCCAAGGAGAAGCAACTAAAGGAAGAAGAGAAGATTCTGGAGAGT

GTGGCTGAAGGCCGAGCCTTGATGTCAGTGAAGGAGATGGCCAAGGGCATTACATATGAT

GACCCAATCAAAACCAGGTATGTCTTTCCAGACTGTTCAGTTGCCCTTACTTAGGATGGT

GTGTGGCTGAGACTAGCAGTGACACCCCAAGCCCAGCTGTTCCAAGGCCTCTGTTGAACC

CTACCACCCACCCACAGTTGGACACCACCCCGTTACGTCCTGAGCATGTCTGAAGAGCGG

CATGAACGAGTTCGGAAGAAGTACCACATCCTGGTGGAGGGAGATGGTATCCCACCACCC

ATCAAGAGCTTTAAGGAAATGAAGTTTCCTGCAGCCATCCTGCGAGGCCTAAAGAAAAAA

GGCATTCTCCACCCAACACCTATTCAGATCCAAGGTATCCCTACCATTCTTTCGGGCCGG

GACATGATTGGCATTGCCTTCACGGGGTCAGGCAAGACGTTGGTGTTCACACTGCCCGTC

ATCATGTTCTGTCTGGAACAAGAGAAGCGGTTGCCTTTCTCTAAGCGTGAGGGGCCCTAT

GGACTCATCATCTGCCCCTCGCGGGAACTGGCCCGGCAAACCCATGGCATCCTGGAATAT

TACTGCCGCCTGCTGCAGGAGGACAGCTCACCTCTCCTGCGCTGTGCTCTCTGCATTGGG

GGCATGTCTGTCAAAGAGCAGATGGAAACCATCCGACATGGTGTACATATGATGGTGGCC

ACCCCTGGACGCCTCATGGATTTACTACAGAAGAAGATGGTCAGCCTAGACATCTGTCGC

TACCTGGCCCTGGATGAGGCTGACCGCATGATTGACATGGGTTTTGAGGGCGACATCCGC

ACCATCTTTTCCTACTTCAAGGGCCAGCGGCAGACTCTACTCTTCAGTGCTACCATGCCA

AAGAAGATTCAGAACTTTGCCAAGAGTGCCCTGGTAAAGCCTGTTACCATCAATGTGGGG

CGTGCTGGGGCCGCCAGCCTGGATGTCATTCAGGAGGTGGAATACGTGAAGGAAGAGGCC

AAGATGGTATACCTGCTTGAGTGCCTGCAGAAGACCCCCCCTCCTGTGCTCATCTTTGCA

GAGAAGAAGGCCGATGTGGACGCCATCCATGAGTATCTGCTGCTCAAGGGGGTTGAGGCT

GTTGCCATCCATGGGGGCAAAGGTCAGGACAGTGCATGCATTAGTGTGAACCCTGATTCT

ATAGCAACCCTTCACCTATCCCTTCCCCTGCCTGACCTGCCTGCTTCCAAAACTCCCACC

GTAACTCTGATAGCTGCTGCCTGACCCTCCCTAGACCAGGAGGAACGGACCAAAGCCATT

GAGGCATTCCGGGAGGGCAAGAAGGATGTCCTGGTGGCCACAGATGTGGCCTCCAAAGGC

CTGGACTTCCCTGCTATCCAGCACGTCATCAATTACGACATGCCTGAGGAAATCGAGAAC

TAT

>CL124.Contig16_A-W 1546 1800 DEAD (Asp-Glu-Ala-Asp) box polypeptide 41 [Mus musculus]

CCTGCGTAGGTGCTCATCTTTGCAGAGAAGAAGGCCGATGTGGACGCCATCCATGAGTAT

CTGCTGCTCAAGGGGGTTGAGGCTGTTGCCATCCATGGGGGCAAAGACCAGGAGGAACGG

ACCAAAGCCATTGAGGCATTCCGGGAGGGCAAGAAGGATGTCCTGGTGGCCACAGATGTG

GCCTCCAAAGGCCTGGACTTCCCTGCTATCCAGCACGTCATCAATTACGACATGCCTGAG

GAAATCGAGAACTAT

>CL124.Contig17_A-W 62 1561 PREDICTED: probable ATP-dependent RNA helicase DDX41-like [Cavia porcellus]

AGGTATCCCTACCATGTGAGTATGGACCATGGACTTGGGGTCCCTGAGGAAAGCAGTGAC

AGGGAGAAGCAATAACCAAGTGTCTGACTGGAGAAGTAGTTGCTGTTTCCTGCTGGACCT

GGCCCTCAGGCTATCTTCTCCTACAGTCTTTCGGGCCGGGACATGATTGGCATTGCCTTC

ACGGGGTCAGGCAAGACGTTGGTGTTCACACTGCCCGTCATCATGTTCTGTCTGGAACAA

GAGAAGCGGTTGCCTTTCTCTAAGCGTGAGGGGCCCTATGGACTCATCATCTGCCCCTCG

CGGGAACTGGCCCGGCAAACCCATGGCATCCTGGAATATTACTGCCGCCTGCTGCAGGAG

GACAGCTCACCTCTCCTGCGCTGTGCTCTCTGCATTGGGGGCATGTCTGTCAAAGAGCAG

ATGGAAACCATCCGACATGGTGTACATATGATGGTGGCCACCCCTGGACGCCTCATGGAT

TTACTACAGAAGAAGATGGTCAGCCTAGACATCTGTCGCTACCTGGCCCTGGATGAGGCT

GACCGCATGATTGACATGGGTTTTGAGGGCGACATCCGCACCATCTTTTCCTACTTCAAG

GGCCAGCGGCAGACTCTACTCTTCAGTGCTACCATGCCAAAGAAGATTCAGAACTTTGCC

AAGAGTGCCCTGGTAAAGCCTGTTACCATCAATGTGGGGCGTGCTGGGGCCGCCAGCCTG

GATGTCATTCAGGAGGTGGAATACGTGAAGGAAGAGGCCAAGATGGTATACCTGCTTGAG

TGCCTGCAGAAGACCCCCCCTCCTGTGCTCATCTTTGCAGAGAAGAAGGCCGATGTGGAC

GCCATCCATGAGTATCTGCTGCTCAAGGGGGTTGAGGCTGTTGCCATCCATGGGGGCAAA

GGTCAGGACAGTGCATGCATTAGTGTGAACCCTGATTCTATAGCAACCCTTCACCTATCC

CTTCCCCTGCCTGACCTGCCTGCTTCCAAAACTCCCACCGTAACTCTGATAGCTGCTGCC

TGACCCTCCCTAGACCAGGAGGAACGGACCAAAGCCATTGAGGCATTCCGGGAGGGCAAG

AAGGATGTCCTGGTGGCCACAGATGTGGCCTCCAAAGGCCTGGACTTCCCTGCTATCCAG

CACGTCATCAATTACGACATGCCTGAGGAAATCGAGAACTATGTGCACCGAATCGGCCGC

ACAGGGCGCTCAGGAAACACAGGCATTGCTACGACCTTCATTAACAAGGCCTGTGATGAG

TCAGTGCTGATGGATCTCAAGGCCCTGCTGCTAGAAGCCAAGCAGAAGGTGCCACCTGTA

CTACAAGTGCTGCACCCTGGGGACGAGTCCATGCTGGACATTGGAGGAGAACGTGGTTGT

GCCTTCTGTGGGGGCCTGGGCCATCGGATCACTGACTGCCCCAAACTTGAGGCCATGCAG

ACCAAACAGGTCAGCAACATTGGCCGCAAGGACTACCTGGCCCACAGCTCCATGGACTTC

>CL124.Contig18_A-W 978 1550 DEAD (Asp-Glu-Ala-Asp) box polypeptide 41 [Mus musculus]

CCTGCGTAGGTGCTCATCTTTGCAGAGAAGAAGGCCGATGTGGACGCCATCCATGAGTAT

CTGCTGCTCAAGGGGGTTGAGGCTGTTGCCATCCATGGGGGCAAAGACCAGGAGGAACGG

ACCAAAGCCATTGAGGCATTCCGGGAGGGCAAGAAGGATGTCCTGGTGGCCACAGATGTG

GCCTCCAAAGGCCTGGACTTCCCTGCTATCCAGCACGTCATCAATTACGACATGCCTGAG

GAAATCGAGAACTATGTGCACCGAATCGGCCGCACAGGGCGCTCAGGAAACACAGGCATT

GCTACGACCTTCATTAACAAGGCCTGTGATGAGTCAGTGCTGATGGATCTCAAGGCCCTG

CTGCTAGAAGCCAAGCAGAAGGTGCCACCTGTACTACAAGTGCTGCACCCTGGGGACGAG

TCCATGCTGGACATTGGAGGAGAACGTGGTTGTGCCTTCTGTGGGGGCCTGGGCCATCGG

ATCACTGACTGCCCCAAACTTGAGGCCATGCAGACCAAACAGGTCAGCAACATTGGCCGC

AAGGACTACCTGGCCCACAGCTCCATGGACTTC

>CL124.Contig19_A-W 9 1679 DEAD (Asp-Glu-Ala-Asp) box polypeptide 41 [Mus musculus]

AGGATGGAGGAGTCGGAACCCGAGCGGAAGCGCGCCCGCCCGGACGACGCGACCGCAGGA

GGAAGCCGCTCCGAGGATGAGGATGAGGACGATGAGGATTACGTGCCCTATGTGCCGCTG

CGACAGCGCCGACAACTTCTGCTCCAGAAACTGCTGCAGCGAAGACGCAAGGGAGCTGCA

GAGGAAGAGCAGCAGGACAGCAGCAGTGAGCCCCGAGGAGATGAGGACGACATCCCTTTG

GGCCCTCAGTCCAACGTCAGCCTCCTGGATCAGCATCAGCACCTCAAAGAGAAGGCTGAA

GCCCGCAAGGAATCTGCCAAGGAGAAGCAACTAAAGGAAGAAGAGAAGATTCTGGAGAGT

GTGGCTGAAGGCCGAGCCTTGATGTCAGTGAAGGAGATGGCCAAGGGCATTACATATGAT

GACCCAATCAAAACCAGGTATGTCTTTCCAGACTGTTCAGTTGCCCTTACTTAGGATGGT

GTGTGGCTGAGACTAGCAGTGACACCCCAAGCCCAGCTGTTCCAAGGCCTCTGTTGAACC

CTACCACCCACCCACAGTTGGACACCACCCCGTTACGTCCTGAGCATGTCTGAAGAGCGG

CATGAACGAGTTCGGAAGAAGTACCACATCCTGGTGGAGGGAGATGGTATCCCACCACCC

ATCAAGAGCTTTAAGGAAATGAAGTTTCCTGCAGCCATCCTGCGAGGCCTAAAGAAAAAA

GGCATTCTCCACCCAACACCTATTCAGATCCAAGGTATCCCTACCATTCTTTCGGGCCGG

GACATGATTGGCATTGCCTTCACGGGGTCAGGCAAGACGTTGGTGTTCACACTGCCCGTC

ATCATGTTCTGTCTGGAACAAGAGAAGCGGTTGCCTTTCTCTAAGCGTGAGGGGCCCTAT

GGACTCATCATCTGCCCCTCGCGGGAACTGGCCCGGCAAACCCATGGCATCCTGGAATAT

TACTGCCGCCTGCTGCAGGAGGACAGCTCACCTCTCCTGCGCTGTGCTCTCTGCATTGGG

GGCATGTCTGTCAAAGAGCAGATGGAAACCATCCGACATGGTGTACATATGATGGTGGCC

ACCCCTGGACGCCTCATGGATTTACTACAGAAGAAGATGGTCAGCCTAGACATCTGTCGC

TACCTGGCCCTGGATGAGGCTGACCGCATGATTGACATGGGTTTTGAGGGCGACATCCGC

ACCATCTTTTCCTACTTCAAGGGCCAGCGGCAGACTCTACTCTTCAGTGCTACCATGCCA

AAGAAGATTCAGAACTTTGCCAAGAGTGCCCTGGTAAAGCCTGTTACCATCAATGTGGGG

CGTGCTGGGGCCGCCAGCCTGGATGTCATTCAGGAGGTGGAATACGTGAAGGAAGAGGCC

AAGATGGTATACCTGCTTGAGTGCCTGCAGAAGACCCCCCCTCCTGTGCTCATCTTTGCA

GAGAAGAAGGCCGATGTGGACGCCATCCATGAGTATCTGCTGCTCAAGGGGGTTGAGGCT

GTTGCCATCCATGGGGGCAAAGACCAGGAGGAACGGACCAAAGCCATTGAGGCATTCCGG

GAGGGCAAGAAGGATGTCCTGGTGGCCACAGATGTGGCCTCCAAAGGCCTGGACTTCCCT

GCTATCCAGCACGTCATCAATTACGACATGCCTGAGGAAATCGAGAACTAT

>CL125.Contig1_A-W 195 2114 PREDICTED: glycosylphosphatidylinositol anchor attachment 1 protein isoform 1 [Papio anubis]

ATGGGCCTCCTGTCGGACCCCGTGCGCCGGCGCGCGCTCGCCCGTCTCGTGCTGCGCCTC

AACGCGCCGCTCTGCGTGCTGAGCTACGTGGCGGGCATCGCCTGGTTCTTGGCTCTGGCC

TTCCCGCCGTTGACCCAGCGTACTTACATGTCGGAGAACGCCATGGGATCCACCATGGTG

GAGGAGCAGTTTGTGGGCGGAGACCGTGCCCGGGGCTTTGCCCGCGATTTCGCTGCCCAC

CGCAGGAAGTCGGGGGCTCTACCAGTGGCCTGGCTGCAGCGGACGATGCGTACAGTAGGG

CTGGAGGTCTACACGCAGAGTTTCTCTCGGAAACTGCCCTTCCCGGATGAGACCCACGAG

CGCTATATGGTATCAGGCACCAACGTGTATGGCATCCTACGGGCCCCACGTGCTGCCAGC

ACCGAGTCTCTGGTGCTCACTGTACCCTGTAACTCTGACTCCACCAATAGCCAGGCTGTT

GGACTGCTGCTGGCACTTGCTGCCCATTTCCGAGGGCAGATTTACTGGGCCAAAGATATC

ATCTTTCTGGTGACAGAACATGATCTTCTGGGCACTGAGGCTTGGCTTGAAGCCTACCAC

GACGTTAATGTAACTGGCATGCAGTCATCCCCATTGCAGGGCCGGGCTGGAGCCATTCAG

GCAGCTGTGGCCCTGGAGTTGAGCAGTGATGTGGTTACCAGCCTTGATGTGACTGTGGAG

GGTCTCAATGGACAGCTGCCCAACCTTGACCTGCTCAACCTTTTCCAGACCTTCTGCCAG

AAAGGGGGGCTGCTATGCACACTTCAGGGCAAGGTTTGACCACTTGCTGAGGTATAGGGC

CTTTGGTGAGGGGTTTATCTAATCCTGTTCCCATCCCTACTCTGCCCTCTCACCATCCCT

CCAAAGCTTCAGCTCCAGGATTGGACAACGCTGGATGGGCCATTGCAGGGCCTGCAGACA

CTGCTGCTGATGGTTCTGCGGCAGGCCTCTGGCCGCCCCCATGGCCCTCATGGCCTCTTT

CTGCGGTACCGGGTAGAGGCCCTGACCCTTCGGGGCATCAATAGCTTCCGGCAGTACAAG

TATGACTTGGTGGCTGTTGGCAAGGCTCTGGAGGGCATGTTCCGAAAGCTCAACCACCTG

CTGGAGCGCCTACACCAGTCATTCTTCTTCTACCTGCTTCCTGCTCTATCTCGCTTTGTC

TCCATTGGTCTCTACATGCCTGCTGCTGGCTTCTTGCTCCTGGTCCTTGGTCTCAAGGCT

CTGGAGCTGTGGATGCAGTTGCATGAAGCTGGAGTGGGCTCTGAAGAGGCTGGGGGTGCT

CCTGGACCGAGTACCCAACTTCCCCCAGCACAGGGTGTGGGGCTGGCCTCGCTTGTAGCA

CCCCTGCTGATCTCTCAGACCATGGGCCTGGCCCTCTACATCCTGCCTGTGTTGGGTCAA

CATGTGGCCGCCCAACACTTCCCAGTGGCTGAGGCTGAGGCTGTGGTGCTGACACTGCTG

GCAATTTATGCAGCTGGCCTGGCTCTTCCTCACAACACCCACCGGGTAATGAGCACACAG

GCCTCAGACAGGGGATGGATGGCACTGAAACTGGTGGCTCTGATCTACCTAGCACTACAG

CTAGGTTGCATCACCCTCACCAACTTCTCCCTGGGTTTCCTGCTAGCTGCCACCATGGTA

CCTGCTGCTGCACTCACCAGACCCTATGGGCCTCGGCCACTCTATGCTGCCCTACTGGTG

CTGACAAGCCCAGCAGCCATGCTCCTGGGCAGCCTGTTCCTGTGGCGGGAGCTACAGGAG

ACACCGCTGTCACTTGCTGAGGGGTGGCAGCTTTTCCTCGTGGCACTGGCCCAGGGCGTG

CTGGAGCACCACACCTATGGTGCCTTGCTCTTCCCACTGCTCTCCTTGGGCCTGTACCCT

>CL125.Contig2_A-W 195 2021 PREDICTED: glycosylphosphatidylinositol anchor attachment 1 protein isoform 1 [Papio anubis]

ATGGGCCTCCTGTCGGACCCCGTGCGCCGGCGCGCGCTCGCCCGTCTCGTGCTGCGCCTC

AACGCGCCGCTCTGCGTGCTGAGCTACGTGGCGGGCATCGCCTGGTTCTTGGCTCTGGCC

TTCCCGCCGTTGACCCAGCGTACTTACATGTCGGAGAACGCCATGGGATCCACCATGGTG

GAGGAGCAGTTTGTGGGCGGAGACCGTGCCCGGGGCTTTGCCCGCGATTTCGCTGCCCAC

CGCAGGAAGTCGGGGGCTCTACCAGTGGCCTGGCTGCAGCGGACGATGCGTACAGTAGGG

CTGGAGGTCTACACGCAGAGTTTCTCTCGGAAACTGCCCTTCCCGGATGAGACCCACGAG

CGCTATATGGTATCAGGCACCAACGTGTATGGCATCCTACGGGCCCCACGTGCTGCCAGC

ACCGAGTCTCTGGTGCTCACTGTACCCTGTAACTCTGACTCCACCAATAGCCAGGCTGTT

GGACTGCTGCTGGCACTTGCTGCCCATTTCCGAGGGCAGATTTACTGGGCCAAAGATATC

ATCTTTCTGGTGACAGAACATGATCTTCTGGGCACTGAGGCTTGGCTTGAAGCCTACCAC

GACGTTAATGTAACTGGCATGCAGTCATCCCCATTGCAGGGCCGGGCTGGAGCCATTCAG

GCAGCTGTGGCCCTGGAGTTGAGCAGTGATGTGGTTACCAGCCTTGATGTGACTGTGGAG

GGTCTCAATGGACAGCTGCCCAACCTTGACCTGCTCAACCTTTTCCAGACCTTCTGCCAG

AAAGGGGGGCTGCTATGCACACTTCAGGGCAAGCTTCAGCTCCAGGATTGGACAACGCTG

GATGGGCCATTGCAGGGCCTGCAGACACTGCTGCTGATGGTTCTGCGGCAGGCCTCTGGC

CGCCCCCATGGCCCTCATGGCCTCTTTCTGCGGTACCGGGTAGAGGCCCTGACCCTTCGG

GGCATCAATAGCTTCCGGCAGTACAAGTATGACTTGGTGGCTGTTGGCAAGGCTCTGGAG

GGCATGTTCCGAAAGCTCAACCACCTGCTGGAGCGCCTACACCAGTCATTCTTCTTCTAC

CTGCTTCCTGCTCTATCTCGCTTTGTCTCCATTGGTCTCTACATGCCTGCTGCTGGCTTC

TTGCTCCTGGTCCTTGGTCTCAAGGCTCTGGAGCTGTGGATGCAGTTGCATGAAGCTGGA

GTGGGCTCTGAAGAGGCTGGGGGTGCTCCTGGACCGAGTACCCAACTTCCCCCAGCACAG

GGTGTGGGGCTGGCCTCGCTTGTAGCACCCCTGCTGATCTCTCAGACCATGGGCCTGGCC

CTCTACATCCTGCCTGTGTTGGGTCAACATGTGGCCGCCCAACACTTCCCAGTGGCTGAG

GCTGAGGCTGTGGTGCTGACACTGCTGGCAATTTATGCAGCTGGCCTGGCTCTTCCTCAC

AACACCCACCGGGTAATGAGCACACAGGCCTCAGACAGGGGATGGATGGCACTGAAACTG

GTGGCTCTGATCTACCTAGCACTACAGCTAGGTTGCATCACCCTCACCAACTTCTCCCTG

GGTTTCCTGCTAGCTGCCACCATGGTACCTGCTGCTGCACTCACCAGACCCTATGGGCCT

CGGCCACTCTATGCTGCCCTACTGGTGCTGACAAGCCCAGCAGCCATGCTCCTGGGCAGC

CTGTTCCTGTGGCGGGAGCTACAGGAGACACCGCTGTCACTTGCTGAGGGGTGGCAGCTT

TTCCTCGTGGCACTGGCCCAGGGCGTGCTGGAGCACCACACCTATGGTGCCTTGCTCTTC

CCACTGCTCTCCTTGGGCCTGTACCCT

>CL125.Contig3_A-W 2026 2235 PREDICTED: glycosylphosphatidylinositol anchor attachment 1 protein isoform 1 [Papio anubis]

CCCAGGCCACTCTATGCTGCCCTACTGGTGCTGACAAGCCCAGCAGCCATGCTCCTGGGC

AGCCTGTTCCTGTGGCGGGAGCTACAGGAGACACCGCTGTCACTTGCTGAGGGGTGGCAG

CTTTTCCTCGTGGCACTGGCCCAGGGCGTGCTGGAGCACCACACCTATGGTGCCTTGCTC

TTCCCACTGCTCTCCTTGGGCCTGTACCCT

>CL125.Contig4_A-W 1933 2142 PREDICTED: glycosylphosphatidylinositol anchor attachment 1 protein isoform 1 [Papio anubis]

CCCAGGCCACTCTATGCTGCCCTACTGGTGCTGACAAGCCCAGCAGCCATGCTCCTGGGC

AGCCTGTTCCTGTGGCGGGAGCTACAGGAGACACCGCTGTCACTTGCTGAGGGGTGGCAG

CTTTTCCTCGTGGCACTGGCCCAGGGCGTGCTGGAGCACCACACCTATGGTGCCTTGCTC

TTCCCACTGCTCTCCTTGGGCCTGTACCCT

>CL125.Contig5_A-W 529 651 Glycosylphosphatidylinositol anchor attachment 1 protein [Cricetulus griseus]

TCCAGGGCTCTACCAGTGGCCTGGCTGCAGCGGACGATGCGTACAGTAGGGCTGGAGGTC

TACACGCAGAGTTTCTCTCGGAAACTGCCCTTCCCGGATGAGACCCACGAGCGCTATGTA

TTG

>CL125.Contig6_A-W 2018 2227 PREDICTED: glycosylphosphatidylinositol anchor attachment 1 protein isoform 1 [Papio anubis]

CCCAGGCCACTCTATGCTGCCCTACTGGTGCTGACAAGCCCAGCAGCCATGCTCCTGGGC

AGCCTGTTCCTGTGGCGGGAGCTACAGGAGACACCGCTGTCACTTGCTGAGGGGTGGCAG

CTTTTCCTCGTGGCACTGGCCCAGGGCGTGCTGGAGCACCACACCTATGGTGCCTTGCTC

TTCCCACTGCTCTCCTTGGGCCTGTACCCT

>CL125.Contig7_A-W 529 651 glycosylphosphatidylinositol anchor attachment protein 1 homolog (yeast), isoform CRA_d [Homo sapiens]

TCCAGGGCTCTACCAGTGGCCTGGCTGCAGCGGACGATGCGTACAGTAGGGCTGGAGGTC

TACACGCAGAGTTTCTCTCGGAAACTGCCCTTCCCGGATGAGACCCACGAGCGCTATGTA

TTG

>CL125.Contig8_A-W 195 449 PREDICTED: glycosylphosphatidylinositol anchor attachment 1 protein isoform 1 [Papio anubis]

ATGGGCCTCCTGTCGGACCCCGTGCGCCGGCGCGCGCTCGCCCGTCTCGTGCTGCGCCTC

AACGCGCCGCTCTGCGTGCTGAGCTACGTGGCGGGCATCGCCTGGTTCTTGGCTCTGGCC

TTCCCGCCGTTGACCCAGCGTACTTACATGTCGGAGAACGCCATGGGATCCACCATGGTG

GAGGAGCAGTTTGTGGGCGGAGACCGTGCCCGGGGCTTTGCCCGCGATTTCGCTGCCCAC

CGCAGGAAGTCGGGG

>CL125.Contig9_A-W 535 651 PREDICTED: glycosylphosphatidylinositol anchor attachment 1 protein [Canis lupus familiaris]

GCTCTACCAGTGGCCTGGCTGCAGCGGACGATGCGTACAGTAGGGCTGGAGGTCTACACG

CAGAGTTTCTCTCGGAAACTGCCCTTCCCGGATGAGACCCACGAGCGCTATGTATTG

>CL125.Contig10_A-W 529 651 PREDICTED: glycosylphosphatidylinositol anchor attachment 1 protein isoform 1 [Papio anubis]

TCCAGGGCTCTACCAGTGGCCTGGCTGCAGCGGACGATGCGTACAGTAGGGCTGGAGGTC

TACACGCAGAGTTTCTCTCGGAAACTGCCCTTCCCGGATGAGACCCACGAGCGCTATGTA

TTG

>CL125.Contig11_A-W 535 651 PREDICTED: glycosylphosphatidylinositol anchor attachment 1 protein [Canis lupus familiaris]

GCTCTACCAGTGGCCTGGCTGCAGCGGACGATGCGTACAGTAGGGCTGGAGGTCTACACG

CAGAGTTTCTCTCGGAAACTGCCCTTCCCGGATGAGACCCACGAGCGCTATGTATTG

>CL125.Contig12_A-W 529 651 glycosylphosphatidylinositol anchor attachment protein 1 homolog (yeast), isoform CRA_d [Homo sapiens]

TCCAGGGCTCTACCAGTGGCCTGGCTGCAGCGGACGATGCGTACAGTAGGGCTGGAGGTC

TACACGCAGAGTTTCTCTCGGAAACTGCCCTTCCCGGATGAGACCCACGAGCGCTATGTA

TTG

>CL125.Contig13_A-W 529 651 PREDICTED: glycosylphosphatidylinositol anchor attachment 1 protein isoform 1 [Papio anubis]

TCCAGGGCTCTACCAGTGGCCTGGCTGCAGCGGACGATGCGTACAGTAGGGCTGGAGGTC

TACACGCAGAGTTTCTCTCGGAAACTGCCCTTCCCGGATGAGACCCACGAGCGCTATGTA

TTG

>CL125.Contig14_A-W 195 449 PREDICTED: glycosylphosphatidylinositol anchor attachment 1 protein isoform 1 [Papio anubis]

ATGGGCCTCCTGTCGGACCCCGTGCGCCGGCGCGCGCTCGCCCGTCTCGTGCTGCGCCTC

AACGCGCCGCTCTGCGTGCTGAGCTACGTGGCGGGCATCGCCTGGTTCTTGGCTCTGGCC

TTCCCGCCGTTGACCCAGCGTACTTACATGTCGGAGAACGCCATGGGATCCACCATGGTG

GAGGAGCAGTTTGTGGGCGGAGACCGTGCCCGGGGCTTTGCCCGCGATTTCGCTGCCCAC

CGCAGGAAGTCGGGG

>CL125.Contig15_A-W 195 566 PREDICTED: glycosylphosphatidylinositol anchor attachment 1 protein [Canis lupus familiaris]

ATGGGCCTCCTGTCGGACCCCGTGCGCCGGCGCGCGCTCGCCCGTCTCGTGCTGCGCCTC

AACGCGCCGCTCTGCGTGCTGAGCTACGTGGCGGGCATCGCCTGGTTCTTGGCTCTGGCC

TTCCCGCCGTTGACCCAGCGTACTTACATGTCGGAGAACGCCATGGGATCCACCATGGTG

GAGGAGCAGTTTGTGGGCGGAGACCGTGCCCGGGGCTTTGCCCGCGATTTCGCTGCCCAC

CGCAGGAAGTCGGGGGCTCTACCAGTGGCCTGGCTGCAGCGGACGATGCGTACAGTAGGG

CTGGAGGTCTACACGCAGAGTTTCTCTCGGAAACTGCCCTTCCCGGATGAGACCCACGAG

CGCTATGTATTG

>CL125.Contig16_A-W 529 651 PREDICTED: glycosylphosphatidylinositol anchor attachment 1 protein isoform 1 [Papio anubis]

TCCAGGGCTCTACCAGTGGCCTGGCTGCAGCGGACGATGCGTACAGTAGGGCTGGAGGTC

TACACGCAGAGTTTCTCTCGGAAACTGCCCTTCCCGGATGAGACCCACGAGCGCTATGTA

TTG

>CL125.Contig17_A-W 529 651 PREDICTED: glycosylphosphatidylinositol anchor attachment 1 protein isoform 1 [Papio anubis]

TCCAGGGCTCTACCAGTGGCCTGGCTGCAGCGGACGATGCGTACAGTAGGGCTGGAGGTC

TACACGCAGAGTTTCTCTCGGAAACTGCCCTTCCCGGATGAGACCCACGAGCGCTATGTA

TTG

>CL125.Contig18_A-W 535 651 PREDICTED: glycosylphosphatidylinositol anchor attachment 1 protein [Canis lupus familiaris]

GCTCTACCAGTGGCCTGGCTGCAGCGGACGATGCGTACAGTAGGGCTGGAGGTCTACACG

CAGAGTTTCTCTCGGAAACTGCCCTTCCCGGATGAGACCCACGAGCGCTATGTATTG

>CL125.Contig19_A-W 529 651 PREDICTED: glycosylphosphatidylinositol anchor attachment 1 protein isoform 1 [Papio anubis]

TCCAGGGCTCTACCAGTGGCCTGGCTGCAGCGGACGATGCGTACAGTAGGGCTGGAGGTC

TACACGCAGAGTTTCTCTCGGAAACTGCCCTTCCCGGATGAGACCCACGAGCGCTATGTA

TTG

>CL125.Contig20_A-W 529 651 Glycosylphosphatidylinositol anchor attachment 1 protein [Cricetulus griseus]

TCCAGGGCTCTACCAGTGGCCTGGCTGCAGCGGACGATGCGTACAGTAGGGCTGGAGGTC

TACACGCAGAGTTTCTCTCGGAAACTGCCCTTCCCGGATGAGACCCACGAGCGCTATGTA

TTG

>CL125.Contig21_A-W 195 566 PREDICTED: glycosylphosphatidylinositol anchor attachment 1 protein isoform 1 [Papio anubis]

ATGGGCCTCCTGTCGGACCCCGTGCGCCGGCGCGCGCTCGCCCGTCTCGTGCTGCGCCTC

AACGCGCCGCTCTGCGTGCTGAGCTACGTGGCGGGCATCGCCTGGTTCTTGGCTCTGGCC

TTCCCGCCGTTGACCCAGCGTACTTACATGTCGGAGAACGCCATGGGATCCACCATGGTG

GAGGAGCAGTTTGTGGGCGGAGACCGTGCCCGGGGCTTTGCCCGCGATTTCGCTGCCCAC

CGCAGGAAGTCGGGGGCTCTACCAGTGGCCTGGCTGCAGCGGACGATGCGTACAGTAGGG

CTGGAGGTCTACACGCAGAGTTTCTCTCGGAAACTGCCCTTCCCGGATGAGACCCACGAG

CGCTATGTATTG

>CL125.Contig22_A-W 195 566 PREDICTED: glycosylphosphatidylinositol anchor attachment 1 protein [Canis lupus familiaris]

ATGGGCCTCCTGTCGGACCCCGTGCGCCGGCGCGCGCTCGCCCGTCTCGTGCTGCGCCTC

AACGCGCCGCTCTGCGTGCTGAGCTACGTGGCGGGCATCGCCTGGTTCTTGGCTCTGGCC

TTCCCGCCGTTGACCCAGCGTACTTACATGTCGGAGAACGCCATGGGATCCACCATGGTG

GAGGAGCAGTTTGTGGGCGGAGACCGTGCCCGGGGCTTTGCCCGCGATTTCGCTGCCCAC

CGCAGGAAGTCGGGGGCTCTACCAGTGGCCTGGCTGCAGCGGACGATGCGTACAGTAGGG

CTGGAGGTCTACACGCAGAGTTTCTCTCGGAAACTGCCCTTCCCGGATGAGACCCACGAG

CGCTATGTATTG

>CL125.Contig23_A-W 529 651 PREDICTED: glycosylphosphatidylinositol anchor attachment 1 protein isoform 1 [Papio anubis]

TCCAGGGCTCTACCAGTGGCCTGGCTGCAGCGGACGATGCGTACAGTAGGGCTGGAGGTC

TACACGCAGAGTTTCTCTCGGAAACTGCCCTTCCCGGATGAGACCCACGAGCGCTATGTA

TTG

>CL125.Contig24_A-W 529 651 PREDICTED: glycosylphosphatidylinositol anchor attachment 1 protein isoform 1 [Papio anubis]

TCCAGGGCTCTACCAGTGGCCTGGCTGCAGCGGACGATGCGTACAGTAGGGCTGGAGGTC

TACACGCAGAGTTTCTCTCGGAAACTGCCCTTCCCGGATGAGACCCACGAGCGCTATGTA

TTG

>CL125.Contig25_A-W 529 651 Glycosylphosphatidylinositol anchor attachment 1 protein [Cricetulus griseus]

TCCAGGGCTCTACCAGTGGCCTGGCTGCAGCGGACGATGCGTACAGTAGGGCTGGAGGTC

TACACGCAGAGTTTCTCTCGGAAACTGCCCTTCCCGGATGAGACCCACGAGCGCTATGTA

TTG

>CL125.Contig26_A-W 529 651 Glycosylphosphatidylinositol anchor attachment 1 protein [Cricetulus griseus]

TCCAGGGCTCTACCAGTGGCCTGGCTGCAGCGGACGATGCGTACAGTAGGGCTGGAGGTC

TACACGCAGAGTTTCTCTCGGAAACTGCCCTTCCCGGATGAGACCCACGAGCGCTATGTA

TTG

>CL125.Contig27_A-W 1769 1957 PREDICTED: glycosylphosphatidylinositol anchor attachment 1 protein [Canis lupus familiaris]

CAGGGTGTGGGGCTGGCCTCGCTTGTAGCACCCCTGCTGATCTCTCAGACCATGGGCCTG

GCCCTCTACATCCTGCCTGTGTTGGGTCAACATGTGGCCGCCCAACACTTCCCAGTGGCT

GAGGCTGAGGCTGTGGTGCTGACACTGCTGGCAATTTATGCAGCTGGCCTGGCTCTTCCT

CACAACACC

>CL125.Contig28_A-W 529 651 PREDICTED: glycosylphosphatidylinositol anchor attachment 1 protein isoform 1 [Papio anubis]

TCCAGGGCTCTACCAGTGGCCTGGCTGCAGCGGACGATGCGTACAGTAGGGCTGGAGGTC

TACACGCAGAGTTTCTCTCGGAAACTGCCCTTCCCGGATGAGACCCACGAGCGCTATGTA

TTG

>CL125.Contig29_A-W 195 566 PREDICTED: glycosylphosphatidylinositol anchor attachment 1 protein isoform 1 [Papio anubis]

ATGGGCCTCCTGTCGGACCCCGTGCGCCGGCGCGCGCTCGCCCGTCTCGTGCTGCGCCTC

AACGCGCCGCTCTGCGTGCTGAGCTACGTGGCGGGCATCGCCTGGTTCTTGGCTCTGGCC

TTCCCGCCGTTGACCCAGCGTACTTACATGTCGGAGAACGCCATGGGATCCACCATGGTG

GAGGAGCAGTTTGTGGGCGGAGACCGTGCCCGGGGCTTTGCCCGCGATTTCGCTGCCCAC

CGCAGGAAGTCGGGGGCTCTACCAGTGGCCTGGCTGCAGCGGACGATGCGTACAGTAGGG

CTGGAGGTCTACACGCAGAGTTTCTCTCGGAAACTGCCCTTCCCGGATGAGACCCACGAG

CGCTATGTATTG

>CL125.Contig30_A-W 195 566 Glycosylphosphatidylinositol anchor attachment 1 protein [Cricetulus griseus]

ATGGGCCTCCTGTCGGACCCCGTGCGCCGGCGCGCGCTCGCCCGTCTCGTGCTGCGCCTC

AACGCGCCGCTCTGCGTGCTGAGCTACGTGGCGGGCATCGCCTGGTTCTTGGCTCTGGCC

TTCCCGCCGTTGACCCAGCGTACTTACATGTCGGAGAACGCCATGGGATCCACCATGGTG

GAGGAGCAGTTTGTGGGCGGAGACCGTGCCCGGGGCTTTGCCCGCGATTTCGCTGCCCAC

CGCAGGAAGTCGGGGGCTCTACCAGTGGCCTGGCTGCAGCGGACGATGCGTACAGTAGGG

CTGGAGGTCTACACGCAGAGTTTCTCTCGGAAACTGCCCTTCCCGGATGAGACCCACGAG

CGCTATGTATTG

>CL125.Contig31_A-W 195 566 Glycosylphosphatidylinositol anchor attachment 1 protein [Cricetulus griseus]

ATGGGCCTCCTGTCGGACCCCGTGCGCCGGCGCGCGCTCGCCCGTCTCGTGCTGCGCCTC

AACGCGCCGCTCTGCGTGCTGAGCTACGTGGCGGGCATCGCCTGGTTCTTGGCTCTGGCC

TTCCCGCCGTTGACCCAGCGTACTTACATGTCGGAGAACGCCATGGGATCCACCATGGTG

GAGGAGCAGTTTGTGGGCGGAGACCGTGCCCGGGGCTTTGCCCGCGATTTCGCTGCCCAC

CGCAGGAAGTCGGGGGCTCTACCAGTGGCCTGGCTGCAGCGGACGATGCGTACAGTAGGG

CTGGAGGTCTACACGCAGAGTTTCTCTCGGAAACTGCCCTTCCCGGATGAGACCCACGAG

CGCTATGTATTG

>CL125.Contig32_A-W 1684 1872 PREDICTED: glycosylphosphatidylinositol anchor attachment 1 protein [Canis lupus familiaris]

CAGGGTGTGGGGCTGGCCTCGCTTGTAGCACCCCTGCTGATCTCTCAGACCATGGGCCTG

GCCCTCTACATCCTGCCTGTGTTGGGTCAACATGTGGCCGCCCAACACTTCCCAGTGGCT

GAGGCTGAGGCTGTGGTGCTGACACTGCTGGCAATTTATGCAGCTGGCCTGGCTCTTCCT

CACAACACC

>CL125.Contig33_A-W 529 1824 Glycosylphosphatidylinositol anchor attachment 1 protein OS=Homo sapiens GN=GPAA1 PE=1 SV=3

TCCAGGGCTCTACCAGTGGCCTGGCTGCAGCGGACGATGCGTACAGTAGGGCTGGAGGTC

TACACGCAGAGTTTCTCTCGGAAACTGCCCTTCCCGGATGAGACCCACGAGCGCTATATG

GTATCAGGCACCAACGTGTATGGCATCCTACGGGCCCCACGTGCTGCCAGCACCGAGTCT

CTGGTGCTCACTGTACCCTGTAACTCTGACTCCACCAATAGCCAGGCTGTTGGACTGCTG

CTGGCACTTGCTGCCCATTTCCGAGGGCAGATTTACTGGGCCAAAGATATCATCTTTCTG

GTGACAGAACATGATCTTCTGGGCACTGAGGCTTGGCTTGAAGCCTACCACGACGTTAAT

GTAACTGGCATGCAGTCATCCCCATTGCAGGGCCGGGCTGGAGCCATTCAGGCAGCTGTG

GCCCTGGAGTTGAGCAGTGATGTGGTTACCAGCCTTGATGTGACTGTGGAGGGTCTCAAT

GGACAGCTGCCCAACCTTGACCTGCTCAACCTTTTCCAGACCTTCTGCCAGAAAGGGGGG

CTGCTATGCACACTTCAGGGCAAGGTTTGACCACTTGCTGAGGTATAGGGCCTTTGGTGA

GGGGTTTATCTAATCCTGTTCCCATCCCTACTCTGCCCTCTCACCATCCCTCCAAAGCTT

CAGCTCCAGGATTGGACAACGCTGGATGGGCCATTGCAGGGCCTGCAGACACTGCTGCTG

ATGGTTCTGCGGCAGGCCTCTGGCCGCCCCCATGGCCCTCATGGCCTCTTTCTGCGGTAC

CGGGTAGAGGCCCTGACCCTTCGGGGCATCAATAGCTTCCGGCAGTACAAGTATGACTTG

GTGGCTGTTGGCAAGGCTCTGGAGGGCATGTTCCGAAAGCTCAACCACCTGCTGGAGCGC

CTACACCAGTCATTCTTCTTCTACCTGCTTCCTGCTCTATCTCGCTTTGTCTCCATTGGT

CTCTACATGCCTGCTGCTGGCTTCTTGCTCCTGGTCCTTGGTCTCAAGGCTCTGGAGCTG

TGGATGCAGTTGCATGAAGCTGGAGTGGGCTCTGAAGAGGCTGGGGGTGCTCCTGGACCG

AGTACCCAACTTCCCCCAGCACAGGGTGTGGGGCTGGCCTCGCTTGTAGCACCCCTGCTG

ATCTCTCAGACCATGGGCCTGGCCCTCTACATCCTGCCTGTGTTGGGTCAACATGTGGCC

GCCCAACACTTCCCAGTGGCTGAGGCTGAGGCTGTGGTGCTGACACTGCTGGCAATTTAT

GCAGCTGGCCTGGCTCTTCCTCACAACACCCACCGC

>CL125.Contig34_A-W 195 566 PREDICTED: glycosylphosphatidylinositol anchor attachment 1 protein isoform 1 [Papio anubis]

ATGGGCCTCCTGTCGGACCCCGTGCGCCGGCGCGCGCTCGCCCGTCTCGTGCTGCGCCTC

AACGCGCCGCTCTGCGTGCTGAGCTACGTGGCGGGCATCGCCTGGTTCTTGGCTCTGGCC

TTCCCGCCGTTGACCCAGCGTACTTACATGTCGGAGAACGCCATGGGATCCACCATGGTG

GAGGAGCAGTTTGTGGGCGGAGACCGTGCCCGGGGCTTTGCCCGCGATTTCGCTGCCCAC

CGCAGGAAGTCGGGGGCTCTACCAGTGGCCTGGCTGCAGCGGACGATGCGTACAGTAGGG

CTGGAGGTCTACACGCAGAGTTTCTCTCGGAAACTGCCCTTCCCGGATGAGACCCACGAG

CGCTATGTATTG

>CL125.Contig35_A-W 1676 1864 PREDICTED: glycosylphosphatidylinositol anchor attachment 1 protein [Canis lupus familiaris]

CAGGGTGTGGGGCTGGCCTCGCTTGTAGCACCCCTGCTGATCTCTCAGACCATGGGCCTG

GCCCTCTACATCCTGCCTGTGTTGGGTCAACATGTGGCCGCCCAACACTTCCCAGTGGCT

GAGGCTGAGGCTGTGGTGCTGACACTGCTGGCAATTTATGCAGCTGGCCTGGCTCTTCCT

CACAACACC

>CL125.Contig36_A-W 195 566 Glycosylphosphatidylinositol anchor attachment 1 protein [Cricetulus griseus]

ATGGGCCTCCTGTCGGACCCCGTGCGCCGGCGCGCGCTCGCCCGTCTCGTGCTGCGCCTC

AACGCGCCGCTCTGCGTGCTGAGCTACGTGGCGGGCATCGCCTGGTTCTTGGCTCTGGCC

TTCCCGCCGTTGACCCAGCGTACTTACATGTCGGAGAACGCCATGGGATCCACCATGGTG

GAGGAGCAGTTTGTGGGCGGAGACCGTGCCCGGGGCTTTGCCCGCGATTTCGCTGCCCAC

CGCAGGAAGTCGGGGGCTCTACCAGTGGCCTGGCTGCAGCGGACGATGCGTACAGTAGGG

CTGGAGGTCTACACGCAGAGTTTCTCTCGGAAACTGCCCTTCCCGGATGAGACCCACGAG

CGCTATGTATTG

>CL125.Contig37_A-W 195 1739 Glycosylphosphatidylinositol anchor attachment 1 protein OS=Homo sapiens GN=GPAA1 PE=1 SV=3

ATGGGCCTCCTGTCGGACCCCGTGCGCCGGCGCGCGCTCGCCCGTCTCGTGCTGCGCCTC

AACGCGCCGCTCTGCGTGCTGAGCTACGTGGCGGGCATCGCCTGGTTCTTGGCTCTGGCC

TTCCCGCCGTTGACCCAGCGTACTTACATGTCGGAGAACGCCATGGGATCCACCATGGTG

GAGGAGCAGTTTGTGGGCGGAGACCGTGCCCGGGGCTTTGCCCGCGATTTCGCTGCCCAC

CGCAGGAAGTCGGGGGCTCTACCAGTGGCCTGGCTGCAGCGGACGATGCGTACAGTAGGG

CTGGAGGTCTACACGCAGAGTTTCTCTCGGAAACTGCCCTTCCCGGATGAGACCCACGAG

CGCTATATGGTATCAGGCACCAACGTGTATGGCATCCTACGGGCCCCACGTGCTGCCAGC

ACCGAGTCTCTGGTGCTCACTGTACCCTGTAACTCTGACTCCACCAATAGCCAGGCTGTT

GGACTGCTGCTGGCACTTGCTGCCCATTTCCGAGGGCAGATTTACTGGGCCAAAGATATC

ATCTTTCTGGTGACAGAACATGATCTTCTGGGCACTGAGGCTTGGCTTGAAGCCTACCAC

GACGTTAATGTAACTGGCATGCAGTCATCCCCATTGCAGGGCCGGGCTGGAGCCATTCAG

GCAGCTGTGGCCCTGGAGTTGAGCAGTGATGTGGTTACCAGCCTTGATGTGACTGTGGAG

GGTCTCAATGGACAGCTGCCCAACCTTGACCTGCTCAACCTTTTCCAGACCTTCTGCCAG

AAAGGGGGGCTGCTATGCACACTTCAGGGCAAGGTTTGACCACTTGCTGAGGTATAGGGC

CTTTGGTGAGGGGTTTATCTAATCCTGTTCCCATCCCTACTCTGCCCTCTCACCATCCCT

CCAAAGCTTCAGCTCCAGGATTGGACAACGCTGGATGGGCCATTGCAGGGCCTGCAGACA

CTGCTGCTGATGGTTCTGCGGCAGGCCTCTGGCCGCCCCCATGGCCCTCATGGCCTCTTT

CTGCGGTACCGGGTAGAGGCCCTGACCCTTCGGGGCATCAATAGCTTCCGGCAGTACAAG

TATGACTTGGTGGCTGTTGGCAAGGCTCTGGAGGGCATGTTCCGAAAGCTCAACCACCTG

CTGGAGCGCCTACACCAGTCATTCTTCTTCTACCTGCTTCCTGCTCTATCTCGCTTTGTC

TCCATTGGTCTCTACATGCCTGCTGCTGGCTTCTTGCTCCTGGTCCTTGGTCTCAAGGCT

CTGGAGCTGTGGATGCAGTTGCATGAAGCTGGAGTGGGCTCTGAAGAGGCTGGGGGTGCT

CCTGGACCGAGTACCCAACTTCCCCCAGCACAGGGTGTGGGGCTGGCCTCGCTTGTAGCA

CCCCTGCTGATCTCTCAGACCATGGGCCTGGCCCTCTACATCCTGCCTGTGTTGGGTCAA

CATGTGGCCGCCCAACACTTCCCAGTGGCTGAGGCTGAGGCTGTGGTGCTGACACTGCTG

GCAATTTATGCAGCTGGCCTGGCTCTTCCTCACAACACCCACCGC

>CL125.Contig38_A-W 1591 1779 PREDICTED: glycosylphosphatidylinositol anchor attachment 1 protein [Canis lupus familiaris]

CAGGGTGTGGGGCTGGCCTCGCTTGTAGCACCCCTGCTGATCTCTCAGACCATGGGCCTG

GCCCTCTACATCCTGCCTGTGTTGGGTCAACATGTGGCCGCCCAACACTTCCCAGTGGCT

GAGGCTGAGGCTGTGGTGCTGACACTGCTGGCAATTTATGCAGCTGGCCTGGCTCTTCCT

CACAACACC

>CL125.Contig39_A-W 529 1731 Glycosylphosphatidylinositol anchor attachment 1 protein OS=Homo sapiens GN=GPAA1 PE=1 SV=3

TCCAGGGCTCTACCAGTGGCCTGGCTGCAGCGGACGATGCGTACAGTAGGGCTGGAGGTC

TACACGCAGAGTTTCTCTCGGAAACTGCCCTTCCCGGATGAGACCCACGAGCGCTATATG

GTATCAGGCACCAACGTGTATGGCATCCTACGGGCCCCACGTGCTGCCAGCACCGAGTCT

CTGGTGCTCACTGTACCCTGTAACTCTGACTCCACCAATAGCCAGGCTGTTGGACTGCTG

CTGGCACTTGCTGCCCATTTCCGAGGGCAGATTTACTGGGCCAAAGATATCATCTTTCTG

GTGACAGAACATGATCTTCTGGGCACTGAGGCTTGGCTTGAAGCCTACCACGACGTTAAT

GTAACTGGCATGCAGTCATCCCCATTGCAGGGCCGGGCTGGAGCCATTCAGGCAGCTGTG

GCCCTGGAGTTGAGCAGTGATGTGGTTACCAGCCTTGATGTGACTGTGGAGGGTCTCAAT

GGACAGCTGCCCAACCTTGACCTGCTCAACCTTTTCCAGACCTTCTGCCAGAAAGGGGGG

CTGCTATGCACACTTCAGGGCAAGCTTCAGCTCCAGGATTGGACAACGCTGGATGGGCCA

TTGCAGGGCCTGCAGACACTGCTGCTGATGGTTCTGCGGCAGGCCTCTGGCCGCCCCCAT

GGCCCTCATGGCCTCTTTCTGCGGTACCGGGTAGAGGCCCTGACCCTTCGGGGCATCAAT

AGCTTCCGGCAGTACAAGTATGACTTGGTGGCTGTTGGCAAGGCTCTGGAGGGCATGTTC

CGAAAGCTCAACCACCTGCTGGAGCGCCTACACCAGTCATTCTTCTTCTACCTGCTTCCT

GCTCTATCTCGCTTTGTCTCCATTGGTCTCTACATGCCTGCTGCTGGCTTCTTGCTCCTG

GTCCTTGGTCTCAAGGCTCTGGAGCTGTGGATGCAGTTGCATGAAGCTGGAGTGGGCTCT

GAAGAGGCTGGGGGTGCTCCTGGACCGAGTACCCAACTTCCCCCAGCACAGGGTGTGGGG

CTGGCCTCGCTTGTAGCACCCCTGCTGATCTCTCAGACCATGGGCCTGGCCCTCTACATC

CTGCCTGTGTTGGGTCAACATGTGGCCGCCCAACACTTCCCAGTGGCTGAGGCTGAGGCT

GTGGTGCTGACACTGCTGGCAATTTATGCAGCTGGCCTGGCTCTTCCTCACAACACCCAC

CGC

>CL125.Contig40_A-W 529 651 PREDICTED: glycosylphosphatidylinositol anchor attachment 1 protein isoform 1 [Papio anubis]

TCCAGGGCTCTACCAGTGGCCTGGCTGCAGCGGACGATGCGTACAGTAGGGCTGGAGGTC

TACACGCAGAGTTTCTCTCGGAAACTGCCCTTCCCGGATGAGACCCACGAGCGCTATGTA

TTG

>CL125.Contig41_A-W 195 449 Glycosylphosphatidylinositol anchor attachment 1 protein [Cricetulus griseus]

ATGGGCCTCCTGTCGGACCCCGTGCGCCGGCGCGCGCTCGCCCGTCTCGTGCTGCGCCTC

AACGCGCCGCTCTGCGTGCTGAGCTACGTGGCGGGCATCGCCTGGTTCTTGGCTCTGGCC

TTCCCGCCGTTGACCCAGCGTACTTACATGTCGGAGAACGCCATGGGATCCACCATGGTG

GAGGAGCAGTTTGTGGGCGGAGACCGTGCCCGGGGCTTTGCCCGCGATTTCGCTGCCCAC

CGCAGGAAGTCGGGG

>CL125.Contig42_A-W 195 566 PREDICTED: glycosylphosphatidylinositol anchor attachment 1 protein isoform 1 [Papio anubis]

ATGGGCCTCCTGTCGGACCCCGTGCGCCGGCGCGCGCTCGCCCGTCTCGTGCTGCGCCTC

AACGCGCCGCTCTGCGTGCTGAGCTACGTGGCGGGCATCGCCTGGTTCTTGGCTCTGGCC

TTCCCGCCGTTGACCCAGCGTACTTACATGTCGGAGAACGCCATGGGATCCACCATGGTG

GAGGAGCAGTTTGTGGGCGGAGACCGTGCCCGGGGCTTTGCCCGCGATTTCGCTGCCCAC

CGCAGGAAGTCGGGGGCTCTACCAGTGGCCTGGCTGCAGCGGACGATGCGTACAGTAGGG

CTGGAGGTCTACACGCAGAGTTTCTCTCGGAAACTGCCCTTCCCGGATGAGACCCACGAG

CGCTATGTATTG

>CL125.Contig43_A-W 195 1646 Glycosylphosphatidylinositol anchor attachment 1 protein OS=Homo sapiens GN=GPAA1 PE=1 SV=3

ATGGGCCTCCTGTCGGACCCCGTGCGCCGGCGCGCGCTCGCCCGTCTCGTGCTGCGCCTC

AACGCGCCGCTCTGCGTGCTGAGCTACGTGGCGGGCATCGCCTGGTTCTTGGCTCTGGCC

TTCCCGCCGTTGACCCAGCGTACTTACATGTCGGAGAACGCCATGGGATCCACCATGGTG

GAGGAGCAGTTTGTGGGCGGAGACCGTGCCCGGGGCTTTGCCCGCGATTTCGCTGCCCAC

CGCAGGAAGTCGGGGGCTCTACCAGTGGCCTGGCTGCAGCGGACGATGCGTACAGTAGGG

CTGGAGGTCTACACGCAGAGTTTCTCTCGGAAACTGCCCTTCCCGGATGAGACCCACGAG

CGCTATATGGTATCAGGCACCAACGTGTATGGCATCCTACGGGCCCCACGTGCTGCCAGC

ACCGAGTCTCTGGTGCTCACTGTACCCTGTAACTCTGACTCCACCAATAGCCAGGCTGTT

GGACTGCTGCTGGCACTTGCTGCCCATTTCCGAGGGCAGATTTACTGGGCCAAAGATATC

ATCTTTCTGGTGACAGAACATGATCTTCTGGGCACTGAGGCTTGGCTTGAAGCCTACCAC

GACGTTAATGTAACTGGCATGCAGTCATCCCCATTGCAGGGCCGGGCTGGAGCCATTCAG

GCAGCTGTGGCCCTGGAGTTGAGCAGTGATGTGGTTACCAGCCTTGATGTGACTGTGGAG

GGTCTCAATGGACAGCTGCCCAACCTTGACCTGCTCAACCTTTTCCAGACCTTCTGCCAG

AAAGGGGGGCTGCTATGCACACTTCAGGGCAAGCTTCAGCTCCAGGATTGGACAACGCTG

GATGGGCCATTGCAGGGCCTGCAGACACTGCTGCTGATGGTTCTGCGGCAGGCCTCTGGC

CGCCCCCATGGCCCTCATGGCCTCTTTCTGCGGTACCGGGTAGAGGCCCTGACCCTTCGG

GGCATCAATAGCTTCCGGCAGTACAAGTATGACTTGGTGGCTGTTGGCAAGGCTCTGGAG

GGCATGTTCCGAAAGCTCAACCACCTGCTGGAGCGCCTACACCAGTCATTCTTCTTCTAC

CTGCTTCCTGCTCTATCTCGCTTTGTCTCCATTGGTCTCTACATGCCTGCTGCTGGCTTC

TTGCTCCTGGTCCTTGGTCTCAAGGCTCTGGAGCTGTGGATGCAGTTGCATGAAGCTGGA

GTGGGCTCTGAAGAGGCTGGGGGTGCTCCTGGACCGAGTACCCAACTTCCCCCAGCACAG

GGTGTGGGGCTGGCCTCGCTTGTAGCACCCCTGCTGATCTCTCAGACCATGGGCCTGGCC

CTCTACATCCTGCCTGTGTTGGGTCAACATGTGGCCGCCCAACACTTCCCAGTGGCTGAG

GCTGAGGCTGTGGTGCTGACACTGCTGGCAATTTATGCAGCTGGCCTGGCTCTTCCTCAC

AACACCCACCGC

>CL125.Contig44_A-W 399 566 PREDICTED: glycosylphosphatidylinositol anchor attachment 1 protein isoform 2 [Papio anubis]

CGTGCCCGGGGCTTTGCCCGCGATTTCGCTGCCCACCGCAGGAAGTCGGGGGCTCTACCA

GTGGCCTGGCTGCAGCGGACGATGCGTACAGTAGGGCTGGAGGTCTACACGCAGAGTTTC

TCTCGGAAACTGCCCTTCCCGGATGAGACCCACGAGCGCTATGTATTG

>CL125.Contig45_A-W 195 449 Glycosylphosphatidylinositol anchor attachment 1 protein [Cricetulus griseus]

ATGGGCCTCCTGTCGGACCCCGTGCGCCGGCGCGCGCTCGCCCGTCTCGTGCTGCGCCTC

AACGCGCCGCTCTGCGTGCTGAGCTACGTGGCGGGCATCGCCTGGTTCTTGGCTCTGGCC

TTCCCGCCGTTGACCCAGCGTACTTACATGTCGGAGAACGCCATGGGATCCACCATGGTG

GAGGAGCAGTTTGTGGGCGGAGACCGTGCCCGGGGCTTTGCCCGCGATTTCGCTGCCCAC

CGCAGGAAGTCGGGG

>CL125.Contig46_A-W 195 566 PREDICTED: glycosylphosphatidylinositol anchor attachment 1 protein isoform 1 [Papio anubis]

ATGGGCCTCCTGTCGGACCCCGTGCGCCGGCGCGCGCTCGCCCGTCTCGTGCTGCGCCTC

AACGCGCCGCTCTGCGTGCTGAGCTACGTGGCGGGCATCGCCTGGTTCTTGGCTCTGGCC

TTCCCGCCGTTGACCCAGCGTACTTACATGTCGGAGAACGCCATGGGATCCACCATGGTG

GAGGAGCAGTTTGTGGGCGGAGACCGTGCCCGGGGCTTTGCCCGCGATTTCGCTGCCCAC

CGCAGGAAGTCGGGGGCTCTACCAGTGGCCTGGCTGCAGCGGACGATGCGTACAGTAGGG

CTGGAGGTCTACACGCAGAGTTTCTCTCGGAAACTGCCCTTCCCGGATGAGACCCACGAG

CGCTATGTATTG

>CL125.Contig47_A-W 195 449 PREDICTED: glycosylphosphatidylinositol anchor attachment 1 protein isoform 1 [Papio anubis]

ATGGGCCTCCTGTCGGACCCCGTGCGCCGGCGCGCGCTCGCCCGTCTCGTGCTGCGCCTC

AACGCGCCGCTCTGCGTGCTGAGCTACGTGGCGGGCATCGCCTGGTTCTTGGCTCTGGCC

TTCCCGCCGTTGACCCAGCGTACTTACATGTCGGAGAACGCCATGGGATCCACCATGGTG

GAGGAGCAGTTTGTGGGCGGAGACCGTGCCCGGGGCTTTGCCCGCGATTTCGCTGCCCAC

CGCAGGAAGTCGGGG

>CL125.Contig48_A-W 895 1383 PREDICTED: glycosylphosphatidylinositol anchor attachment 1 protein isoform 1 [Papio anubis]

ACAGGCATGCAGTCATCCCCATTGCAGGGCCGGGCTGGAGCCATTCAGGCAGCTGTGGCC

CTGGAGTTGAGCAGTGATGTGGTTACCAGCCTTGATGTGACTGTGGAGGGTCTCAATGGA

CAGCTGCCCAACCTTGACCTGCTCAACCTTTTCCAGACCTTCTGCCAGAAAGGGGGGCTG

CTATGCACACTTCAGGGCAAGGTTTGACCACTTGCTGAGGTATAGGGCCTTTGGTGAGGG

GTTTATCTAATCCTGTTCCCATCCCTACTCTGCCCTCTCACCATCCCTCCAAAGCTTCAG

CTCCAGGATTGGACAACGCTGGATGGGCCATTGCAGGGCCTGCAGACACTGCTGCTGATG

GTTCTGCGGCAGGCCTCTGGCCGCCCCCATGGCCCTCATGGCCTCTTTCTGCGGTACCGG

GTAGAGGCCCTGACCCTTCGGGGCATCAATAGCTTCCGGCAGTACAAGTATGACTTGGTG

GCTGTTGGC

>CL125.Contig49_A-W 195 449 PREDICTED: glycosylphosphatidylinositol anchor attachment protein 1 [Oryctolagus cuniculus]

ATGGGCCTCCTGTCGGACCCCGTGCGCCGGCGCGCGCTCGCCCGTCTCGTGCTGCGCCTC

AACGCGCCGCTCTGCGTGCTGAGCTACGTGGCGGGCATCGCCTGGTTCTTGGCTCTGGCC

TTCCCGCCGTTGACCCAGCGTACTTACATGTCGGAGAACGCCATGGGATCCACCATGGTG

GAGGAGCAGTTTGTGGGCGGAGACCGTGCCCGGGGCTTTGCCCGCGATTTCGCTGCCCAC

CGCAGGAAGTCGGGG

>CL125.Contig50_A-W 529 651 glycosylphosphatidylinositol anchor attachment protein 1 homolog (yeast), isoform CRA_d [Homo sapiens]

TCCAGGGCTCTACCAGTGGCCTGGCTGCAGCGGACGATGCGTACAGTAGGGCTGGAGGTC

TACACGCAGAGTTTCTCTCGGAAACTGCCCTTCCCGGATGAGACCCACGAGCGCTATGTA

TTG

>CL125.Contig51_A-W 895 1290 PREDICTED: glycosylphosphatidylinositol anchor attachment 1 protein isoform 1 [Papio anubis]

ACAGGCATGCAGTCATCCCCATTGCAGGGCCGGGCTGGAGCCATTCAGGCAGCTGTGGCC

CTGGAGTTGAGCAGTGATGTGGTTACCAGCCTTGATGTGACTGTGGAGGGTCTCAATGGA

CAGCTGCCCAACCTTGACCTGCTCAACCTTTTCCAGACCTTCTGCCAGAAAGGGGGGCTG

CTATGCACACTTCAGGGCAAGCTTCAGCTCCAGGATTGGACAACGCTGGATGGGCCATTG

CAGGGCCTGCAGACACTGCTGCTGATGGTTCTGCGGCAGGCCTCTGGCCGCCCCCATGGC

CCTCATGGCCTCTTTCTGCGGTACCGGGTAGAGGCCCTGACCCTTCGGGGCATCAATAGC

TTCCGGCAGTACAAGTATGACTTGGTGGCTGTTGGC

>CL125.Contig52_A-W 195 566 PREDICTED: glycosylphosphatidylinositol anchor attachment 1 protein isoform 1 [Papio anubis]

ATGGGCCTCCTGTCGGACCCCGTGCGCCGGCGCGCGCTCGCCCGTCTCGTGCTGCGCCTC

AACGCGCCGCTCTGCGTGCTGAGCTACGTGGCGGGCATCGCCTGGTTCTTGGCTCTGGCC

TTCCCGCCGTTGACCCAGCGTACTTACATGTCGGAGAACGCCATGGGATCCACCATGGTG

GAGGAGCAGTTTGTGGGCGGAGACCGTGCCCGGGGCTTTGCCCGCGATTTCGCTGCCCAC

CGCAGGAAGTCGGGGGCTCTACCAGTGGCCTGGCTGCAGCGGACGATGCGTACAGTAGGG

CTGGAGGTCTACACGCAGAGTTTCTCTCGGAAACTGCCCTTCCCGGATGAGACCCACGAG

CGCTATGTATTG

>CL125.Contig53_A-W 529 651 PREDICTED: glycosylphosphatidylinositol anchor attachment 1 protein isoform 1 [Papio anubis]

TCCAGGGCTCTACCAGTGGCCTGGCTGCAGCGGACGATGCGTACAGTAGGGCTGGAGGTC

TACACGCAGAGTTTCTCTCGGAAACTGCCCTTCCCGGATGAGACCCACGAGCGCTATGTA

TTG

>CL125.Contig54_A-W 2111 2320 PREDICTED: glycosylphosphatidylinositol anchor attachment 1 protein isoform 1 [Papio anubis]

CCCAGGCCACTCTATGCTGCCCTACTGGTGCTGACAAGCCCAGCAGCCATGCTCCTGGGC

AGCCTGTTCCTGTGGCGGGAGCTACAGGAGACACCGCTGTCACTTGCTGAGGGGTGGCAG

CTTTTCCTCGTGGCACTGGCCCAGGGCGTGCTGGAGCACCACACCTATGGTGCCTTGCTC

TTCCCACTGCTCTCCTTGGGCCTGTACCCT

>CL125.Contig55_A-W 535 651 PREDICTED: glycosylphosphatidylinositol anchor attachment 1 protein isoform 2 [Papio anubis]

GCTCTACCAGTGGCCTGGCTGCAGCGGACGATGCGTACAGTAGGGCTGGAGGTCTACACG

CAGAGTTTCTCTCGGAAACTGCCCTTCCCGGATGAGACCCACGAGCGCTATGTATTG

>CL125.Contig56_A-W 535 651 PREDICTED: glycosylphosphatidylinositol anchor attachment 1 protein isoform 2 [Papio anubis]

GCTCTACCAGTGGCCTGGCTGCAGCGGACGATGCGTACAGTAGGGCTGGAGGTCTACACG

CAGAGTTTCTCTCGGAAACTGCCCTTCCCGGATGAGACCCACGAGCGCTATGTATTG

>CL125.Contig57_A-W 535 651 PREDICTED: glycosylphosphatidylinositol anchor attachment 1 protein [Canis lupus familiaris]

GCTCTACCAGTGGCCTGGCTGCAGCGGACGATGCGTACAGTAGGGCTGGAGGTCTACACG

CAGAGTTTCTCTCGGAAACTGCCCTTCCCGGATGAGACCCACGAGCGCTATGTATTG

>CL125.Contig58_A-W 399 566 PREDICTED: glycosylphosphatidylinositol anchor attachment 1 protein isoform 2 [Papio anubis]

CGTGCCCGGGGCTTTGCCCGCGATTTCGCTGCCCACCGCAGGAAGTCGGGGGCTCTACCA

GTGGCCTGGCTGCAGCGGACGATGCGTACAGTAGGGCTGGAGGTCTACACGCAGAGTTTC

TCTCGGAAACTGCCCTTCCCGGATGAGACCCACGAGCGCTATGTATTG

>CL125.Contig59_A-W 195 1820 minus strand glycosylphosphatidylinositol anchor attachment protein 1 homolog (yeast), isoform CRA_d [Homo sapiens]

ATGGGCCTCCTGTCGGACCCCGTGCGCCGGCGCGCGCTCGCCCGTCTCGTGCTGCGCCTC

AACGCGCCGCTCTGCGTGCTGAGCTACGTGGCGGGCATCGCCTGGTTCTTGGCTCTGGCC

TTCCCGCCGTTGACCCAGCGTACTTACATGTCGGAGAACGCCATGGGATCCACCATGGTG

GAGGAGCAGTTTGTGGGCGGAGACCGTGCCCGGGGCTTTGCCCGCGATTTCGCTGCCCAC

CGCAGGAAGTCGGGGGCTCTACCAGTGGCCTGGCTGCAGCGGACGATGCGTACAGTAGGG

CTGGAGGTCTACACGCAGAGTTTCTCTCGGAAACTGCCCTTCCCGGATGAGACCCACGAG

CGCTATATGGTATCAGGCACCAACGTGTATGGCATCCTACGGGCCCCACGTGCTGCCAGC

ACCGAGTCTCTGGTGCTCACTGTACCCTGTAACTCTGACTCCACCAATAGCCAGGCTGTT

GGACTGCTGCTGGCACTTGCTGCCCATTTCCGAGGGCAGATTTACTGGGCCAAAGATATC

ATCTTTCTGGTGACAGAACATGATCTTCTGGGCACTGAGGCTTGGCTTGAAGCCTACCAC

GACGTTAATGTAACTGGCATGCAGTCATCCCCATTGCAGGGCCGGGCTGGAGCCATTCAG

GCAGCTGTGGCCCTGGAGTTGAGCAGTGATGTGGTTACCAGCCTTGATGTGACTGTGGAG

GGTCTCAATGGACAGCTGCCCAACCTTGACCTGCTCAACCTTTTCCAGACCTTCTGCCAG

AAAGGGGGGCTGCTATGCACACTTCAGGGCAAGGTTTGACCACTTGCTGAGGTATAGGGC

CTTTGGTGAGGGGTTTATCTAATCCTGTTCCCATCCCTACTCTGCCCTCTCACCATCCCT

CCAAAGCTTCAGCTCCAGGATTGGACAACGCTGGATGGGCCATTGCAGGGCCTGCAGACA

CTGCTGCTGATGGTTCTGCGGCAGGCCTCTGGCCGCCCCCATGGCCCTCATGGCCTCTTT

CTGCGGTACCGGGTAGAGGCCCTGACCCTTCGGGGCATCAATAGCTTCCGGCAGTACAAG

TATGACTTGGTGGCTGTTGGCAAGGCTCTGGAGGGCATGTTCCGAAAGCTCAACCACCTG

CTGGAGCGCCTACACCAGTCATTCTTCTTCTACCTGCTTCCTGCTCTATCTCGCTTTGTC

TCCATTGGTCTCTACATGCCTGCTGCTGGCTTCTTGCTCCTGGTCCTTGGTCTCAAGATA

TCCTCTGTCCCCCAACTATTCACTCATGGACAGCCTTGGAGCCCCTCAACTCTGCATCAT

CCTACTCTGGGATTGGGGTGGGAAAGTCCTGGGTTCCCCACCTGGCAGATCTCATCACAC

TGTCATTGAGGTCCTTGACTTGGGCCATGCTCTGGAGCTGTGGATGCAGTTGCATGAAGC

TGGAGTGGGCTCTGAAGAGGCTGGGGGTGCTCCTGGACCGAGTACCCAACTTCCCCCAGC

ACAGGGTGTGGGGCTGGCCTCGCTTGTAGCACCCCTGCTGATCTCTCAGACCATGGGCCT

GGCCCTCTACATCCTGCCTGTGTTGGGTCAACATGTGGCCGCCCAACACTTCCCAGTGGC

TGAGGC

>CL125.Contig60_A-W 195 1727 minus strand glycosylphosphatidylinositol anchor attachment protein 1 homolog (yeast), isoform CRA_d [Homo sapiens]

ATGGGCCTCCTGTCGGACCCCGTGCGCCGGCGCGCGCTCGCCCGTCTCGTGCTGCGCCTC

AACGCGCCGCTCTGCGTGCTGAGCTACGTGGCGGGCATCGCCTGGTTCTTGGCTCTGGCC

TTCCCGCCGTTGACCCAGCGTACTTACATGTCGGAGAACGCCATGGGATCCACCATGGTG

GAGGAGCAGTTTGTGGGCGGAGACCGTGCCCGGGGCTTTGCCCGCGATTTCGCTGCCCAC

CGCAGGAAGTCGGGGGCTCTACCAGTGGCCTGGCTGCAGCGGACGATGCGTACAGTAGGG

CTGGAGGTCTACACGCAGAGTTTCTCTCGGAAACTGCCCTTCCCGGATGAGACCCACGAG

CGCTATATGGTATCAGGCACCAACGTGTATGGCATCCTACGGGCCCCACGTGCTGCCAGC

ACCGAGTCTCTGGTGCTCACTGTACCCTGTAACTCTGACTCCACCAATAGCCAGGCTGTT

GGACTGCTGCTGGCACTTGCTGCCCATTTCCGAGGGCAGATTTACTGGGCCAAAGATATC

ATCTTTCTGGTGACAGAACATGATCTTCTGGGCACTGAGGCTTGGCTTGAAGCCTACCAC

GACGTTAATGTAACTGGCATGCAGTCATCCCCATTGCAGGGCCGGGCTGGAGCCATTCAG

GCAGCTGTGGCCCTGGAGTTGAGCAGTGATGTGGTTACCAGCCTTGATGTGACTGTGGAG

GGTCTCAATGGACAGCTGCCCAACCTTGACCTGCTCAACCTTTTCCAGACCTTCTGCCAG

AAAGGGGGGCTGCTATGCACACTTCAGGGCAAGCTTCAGCTCCAGGATTGGACAACGCTG

GATGGGCCATTGCAGGGCCTGCAGACACTGCTGCTGATGGTTCTGCGGCAGGCCTCTGGC

CGCCCCCATGGCCCTCATGGCCTCTTTCTGCGGTACCGGGTAGAGGCCCTGACCCTTCGG

GGCATCAATAGCTTCCGGCAGTACAAGTATGACTTGGTGGCTGTTGGCAAGGCTCTGGAG

GGCATGTTCCGAAAGCTCAACCACCTGCTGGAGCGCCTACACCAGTCATTCTTCTTCTAC

CTGCTTCCTGCTCTATCTCGCTTTGTCTCCATTGGTCTCTACATGCCTGCTGCTGGCTTC

TTGCTCCTGGTCCTTGGTCTCAAGATATCCTCTGTCCCCCAACTATTCACTCATGGACAG

CCTTGGAGCCCCTCAACTCTGCATCATCCTACTCTGGGATTGGGGTGGGAAAGTCCTGGG

TTCCCCACCTGGCAGATCTCATCACACTGTCATTGAGGTCCTTGACTTGGGCCATGCTCT

GGAGCTGTGGATGCAGTTGCATGAAGCTGGAGTGGGCTCTGAAGAGGCTGGGGGTGCTCC

TGGACCGAGTACCCAACTTCCCCCAGCACAGGGTGTGGGGCTGGCCTCGCTTGTAGCACC

CCTGCTGATCTCTCAGACCATGGGCCTGGCCCTCTACATCCTGCCTGTGTTGGGTCAACA

TGTGGCCGCCCAACACTTCCCAGTGGCTGAGGC

>CL126.Contig1_A-W 2 427 unnamed protein product [Mus musculus]

CCCAGCGCCATGACCGATGCTGCCAACTCACAGGCTGCAGCCAAATTGGCCCTTCGCAAA

CAGCTGGAAAAGACACTCCTGGAGATCCCACCTCCAAAACCTCCTGCTCCCTTGCTTCAC

TTCCTGCCTAGTGCAGCCAATAGTGAGTTCATCTACATGGTAGGCTTGGAAGAAGTCGTA

CAGAGTGTCATTGACAGCCAAGGCAAAAGCTGTGCCTCACTTCTGCGGGTCGAACCCTTT

GTATGTGCCCAGTGCCGCACAGATTTCACCCCTCACTGGAAGCAAGAAAAGAATGGTAAG

ATTCTGTGTGAACAGTGTATGACCTCCAACCAGAAGAAGGCTCTAAAAGCTGAACACACC

AACCGGCTGAAAAATGCTTTTGTTAAAGCCCTACAGCAGGAACAGGTAAGAATTCTGATT

TCTACT

>CL126.Contig2_A-W 2 517 PREDICTED: LOW QUALITY PROTEIN: transcriptional repressor p66-beta-like [Cavia porcellus]

CCCAGCGCCATGACCGATGCTGCCAACTCACAGGCTGCAGCCAAATTGGCCCTTCGCAAA

CAGCTGGAAAAGACACTCCTGGAGATCCCACCTCCAAAACCTCCTGCTCCCTTGCTTCAC

TTCCTGCCTAGTGCAGCCAATAGTGAGTTCATCTACATGGTAGGCTTGGAAGAAGTCGTA

CAGAGTGTCATTGACAGCCAAGGCAAAAGCTGTGCCTCACTTCTGCGGGTCGAACCCTTT

GTATGTGCCCAGTGCCGCACAGATTTCACCCCTCACTGGAAGCAAGAAAAGAATGGTAAG

ATTCTGTGTGAACAGTGTATGACCTCCAACCAGAAGAAGGCTCTAAAAGCTGAACACACC

AACCGGCTGAAAAATGCTTTTGTTAAAGCCCTACAGCAGGAACAGGAGATCGAACAGCGA

TTACAGCAGCAGGCAGCCCTTTCCCCTACTACGGCTCCAGCTGTGTCCAGTGTCAGTAAA

CAAGAGACCATCATGAGACATCATACACTTAGGCAG

>CL126.Contig3_A-W 2 766 minus strand PREDICTED: LOW QUALITY PROTEIN: transcriptional repressor p66-beta-like [Cavia porcellus]

CCCAGCGCCATGACCGATGCTGCCAACTCACAGGCTGCAGCCAAATTGGCCCTTCGCAAA

CAGCTGGAAAAGACACTCCTGGAGATCCCACCTCCAAAACCTCCTGCTCCCTTGCTTCAC

TTCCTGCCTAGTGCAGCCAATAGTGAGTTCATCTACATGGTAGGCTTGGAAGAAGTCGTA

CAGAGTGTCATTGACAGCCAAGGCAAAAGCTGTGCCTCACTTCTGCGGGTCGAACCCTTT

GTATGTGCCCAGTGCCGCACAGATTTCACCCCTCACTGGAAGCAAGAAAAGAATGGTAAG

ATTCTGTGTGAACAGTGTATGACCTCCAACCAGAAGAAGGCTCTAAAAGCTGAACACACC

AACCGGCTGAAAAATGCTTTTGTTAAAGCCCTACAGCAGGAACAGGAGATCGAACAGCGA

TTACAGCAGCAGGCAGCCCTTTCCCCTACTACGGCTCCAGCTGTGTCCAGTGTCAGTAAA

CAAGAGACCATCATGAGACATCATACACTTAGGCAGGCTCCACAGCCCCAGAGCAGCCTC

CAGCGTGGCATTCCCACATCTGCCCGCTCCATGCTTTCAAACTTTGCACAGGCTCCCCAG

TTGTCTGTGCCAGGTGGCCTCCTTGGTATGCCAGGTGTCAACATTGCATATTTGAACACT

GGCATTGGAGGACACAAAGCCCCCAGTTTGGCAGACCGACAGCGTGAATACCTTTTAGAC

ATGATCCCTCCCCGGTCTATATCGCAGTCCATCAGTGGACAGAAA

>CL127.Contig1_A-W 73 288 PREDICTED: protein KIAA1731-like [Nomascus leucogenys]

AGACTATATAATCAATTAGCTGAAGTGAAACAACAAAAGGTAGAGAAAGCAAAACAAGAA

GCTTATGCCCAGAACAGAGAAAGGGCAAAAGAATTTCATAAGGTAAACAAGCCCCCTCCC

TTCTATATTTAAGGCATACCACAATAGGAACTGCTGCTTATTTTTTATTTATTCTCTCCA

CAGAAAACACTAGAGAAACTTCGAGCCAAAAATACA

>CL127.Contig2_A-W 73 288 PREDICTED: protein KIAA1731-like [Nomascus leucogenys]

AGACTATATAATCAATTAGCTGAAGTGAAACAACAAAAGGTAGAGAAAGCAAAACAAGAA

GCTTATGCCCAGAACAGAGAAAGGGCAAAAGAATTTCATAAGGTAAACAAGCCCCCTCCC

TTCTATATTTAAGGCATACCACAATAGGAACTGCTGCTTATTTTTTATTTATTCTCTCCA

CAGAAAACACTAGAGAAACTTCGAGCCAAAAATACA

>CL127.Contig3_A-W 3 743 PREDICTED: centrosomal protein KIAA1731-like isoform 2 [Papio anubis]

AATCAGAACTATCCCTCCAAAGAACAGACTGAAATATTACAAAATAAGAAAAAAAGTGTT

CATTTCCAGGTTTCCAGAGGAAATTTAAATTCCACTTACAGTTCATCGGATGAGGCTAAT

GTATTTCATCAGTTACAAGTACAGCATAGTACTCCTTGTAGTTCTTCCTCTAGTGAGTGC

TTAATAAAACATGAACAAGAAAGCAGAAGAGAAAGATGGGACTTTGAAGAACTATCAAAA

AAAGATGTCCTTACAGTGTTACCCAGCCAAGGACTCACTGAAGATGAAAATGAATCTTGT

GGAGTTTTAGCTACAGATCCACATGTTGAAATTGATTCTCAGTTATGTGTAAGAACAGCG

GAGATGGGAACTTCAGTTCAGACACCACATTCATTAAGTATACAAAATGAAAAATATTTT

GAGAATTCAACTAAAACTGAAACTCCACAAGTCATAAACCTGTCTCAAATAGCAAAATCA

GAGTCTCTTAAAAGTTCTGGATCATTTTCATTACAGAGTTCTATTCCAATCTGGGAGACA

GAATCTGGTAATGGTATAATGGAAGAACCAGAACTTACTTTAGTAAGCACCAGTGATATT

AGCATTATTGAAGCTGAATTTGCAAACTTATCTCTAGAAGAGAAGAATGAGGGAAAAGGC

TACCTTCAGGTGAATGAATTTATTCCATCAGAAACAGAAAACTCAGATTATCCAGTCATA

TCAGAGTTTTCTTTGCAGAAG

>CL127.Contig4_A-W 3 1238 PREDICTED: centrosomal protein KIAA1731-like isoform 2 [Papio anubis]

AATCAGAACTATCCCTCCAAAGAACAGACTGAAATATTACAAAATAAGAAAAAAAGTGTT

CATTTCCAGGTTTCCAGAGGAAATTTAAATTCCACTTACAGTTCATCGGATGAGGCTAAT

GTATTTCATCAGTTACAAGTACAGCATAGTACTCCTTGTAGTTCTTCCTCTAGTGAGTGC

TTAATAAAACATGAACAAGAAAGCAGAAGAGAAAGATGGGACTTTGAAGAACTATCAAAA

AAAGATGTCCTTACAGTGTTACCCAGCCAAGGACTCACTGAAGATGAAAATGAATCTTGT

GGAGTTTTAGCTACAGATCCACATGTTGAAATTGATTCTCAGTTATGTGTAAGAACAGCG

GAGATGGGAACTTCAGTTCAGACACCACATTCATTAAGTATACAAAATGAAAAATATTTT

GAGAATTCAACTAAAACTGAAACTCCACAAGTCATAAACCTGTCTCAAATAGCAAAATCA

GAGTCTCTTAAAAGTTCTGGATCATTTTCATTACAGAGTTCTATTCCAATCTGGGAGACA

GAATCTGGTAATGGTATAATGGAAGAACCAGAACTTACTTTAGTAAGCACCAGTGATATT

AGCATTATTGAAGCTGAATTTGCAAACTTATCTCTAGAAGAGAAGAATGAGGGAAAAGGC

TACCTTCAGGTGAATGAATTTATTCCATCAGAAACAGAAAACTCAGATTATCCAGTCATA

TCAGAGTTTTCTTTGCAGAAGTCAACAGAATCTACAGAAACACTACCAAAATGTACAATG

GCAGGAAGCTTACAAGAAGCATTTGTAAAGAGAAAAAAATCATTTTTGGAGAGATCCTAC

CAGAGGCAGAAAGAAATAAGGAATAAGAGTAAACTCACTGAAAATTCCCAAGTCAAAACA

GTTAAGGAGAAACAATGTACAGGTTCATCTGTGAGTCGCCTTAAGGGAGTCAATAAAGTT

AAAATGAATTTTCCAGAAGAAAGAAAGAATGCACACACCCTCATGCACCAAAGGGCTCTA

AGACTATATAATCAATTAGCTGAAGTGAAACAACAAAAGGTAGAGAAAGCAAAACAAGAA

GCTTATGCCCAGAACAGAGAAAGGGCAAAAGAATTTCATAAGGTAAACAAGCCCCCTCCC

TTCTATATTTAAGGCATACCACAATAGGAACTGCTGCTTATTTTTTATTTATTCTCTCCA

CAGAAAACACTAGAGAAACTTCGAGCCAAAAATACA

>CL127.Contig5_A-W 3 1157 PREDICTED: centrosomal protein KIAA1731-like isoform 2 [Papio anubis]

AATCAGAACTATCCCTCCAAAGAACAGACTGAAATATTACAAAATAAGAAAAAAAGTGTT

CATTTCCAGGTTTCCAGAGGAAATTTAAATTCCACTTACAGTTCATCGGATGAGGCTAAT

GTATTTCATCAGTTACAAGTACAGCATAGTACTCCTTGTAGTTCTTCCTCTAGTGAGTGC

TTAATAAAACATGAACAAGAAAGCAGAAGAGAAAGATGGGACTTTGAAGAACTATCAAAA

AAAGATGTCCTTACAGTGTTACCCAGCCAAGGACTCACTGAAGATGAAAATGAATCTTGT

GGAGTTTTAGCTACAGATCCACATGTTGAAATTGATTCTCAGTTATGTGTAAGAACAGCG

GAGATGGGAACTTCAGTTCAGACACCACATTCATTAAGTATACAAAATGAAAAATATTTT

GAGAATTCAACTAAAACTGAAACTCCACAAGTCATAAACCTGTCTCAAATAGCAAAATCA

GAGTCTCTTAAAAGTTCTGGATCATTTTCATTACAGAGTTCTATTCCAATCTGGGAGACA

GAATCTGGTAATGGTATAATGGAAGAACCAGAACTTACTTTAGTAAGCACCAGTGATATT

AGCATTATTGAAGCTGAATTTGCAAACTTATCTCTAGAAGAGAAGAATGAGGGAAAAGGC

TACCTTCAGGTGAATGAATTTATTCCATCAGAAACAGAAAACTCAGATTATCCAGTCATA

TCAGAGTTTTCTTTGCAGAAGTCAACAGAATCTACAGAAACACTACCAAAATGTACAATG

GCAGGAAGCTTACAAGAAGCATTTGTAAAGAGAAAAAAATCATTTTTGGAGAGATCCTAC

CAGAGGCAGAAAGAAATAAGGAATAAGAGTAAACTCACTGAAAATTCCCAAGTCAAAACA

GTTAAGGAGAAACAATGTACAGGTTCATCTGTGAGTCGCCTTAAGGGAGTCAATAAAGTT

AAAATGAATTTTCCAGAAGAAAGAAAGAATGCACACACCCTCATGCACCAAAGGGCTCTA

AGACTATATAATCAATTAGCTGAAGTGAAACAACAAAAGGTAGAGAAAGCAAAACAAGAA

GCTTATGCCCAGAACAGAGAAAGGGCAAAAGAATTTCATAAGAAAACACTAGAGAAACTT

CGAGCCAAAAATACA

>CL127.Contig6_A-W 3 1157 PREDICTED: centrosomal protein KIAA1731-like isoform 2 [Papio anubis]

AATCAGAACTATCCCTCCAAAGAACAGACTGAAATATTACAAAATAAGAAAAAAAGTGTT

CATTTCCAGGTTTCCAGAGGAAATTTAAATTCCACTTACAGTTCATCGGATGAGGCTAAT

GTATTTCATCAGTTACAAGTACAGCATAGTACTCCTTGTAGTTCTTCCTCTAGTGAGTGC

TTAATAAAACATGAACAAGAAAGCAGAAGAGAAAGATGGGACTTTGAAGAACTATCAAAA

AAAGATGTCCTTACAGTGTTACCCAGCCAAGGACTCACTGAAGATGAAAATGAATCTTGT

GGAGTTTTAGCTACAGATCCACATGTTGAAATTGATTCTCAGTTATGTGTAAGAACAGCG

GAGATGGGAACTTCAGTTCAGACACCACATTCATTAAGTATACAAAATGAAAAATATTTT

GAGAATTCAACTAAAACTGAAACTCCACAAGTCATAAACCTGTCTCAAATAGCAAAATCA

GAGTCTCTTAAAAGTTCTGGATCATTTTCATTACAGAGTTCTATTCCAATCTGGGAGACA

GAATCTGGTAATGGTATAATGGAAGAACCAGAACTTACTTTAGTAAGCACCAGTGATATT

AGCATTATTGAAGCTGAATTTGCAAACTTATCTCTAGAAGAGAAGAATGAGGGAAAAGGC

TACCTTCAGGTGAATGAATTTATTCCATCAGAAACAGAAAACTCAGATTATCCAGTCATA

TCAGAGTTTTCTTTGCAGAAGTCAACAGAATCTACAGAAACACTACCAAAATGTACAATG

GCAGGAAGCTTACAAGAAGCATTTGTAAAGAGAAAAAAATCATTTTTGGAGAGATCCTAC

CAGAGGCAGAAAGAAATAAGGAATAAGAGTAAACTCACTGAAAATTCCCAAGTCAAAACA

GTTAAGGAGAAACAATGTACAGGTTCATCTGTGAGTCGCCTTAAGGGAGTCAATAAAGTT

AAAATGAATTTTCCAGAAGAAAGAAAGAATGCACACACCCTCATGCACCAAAGGGCTCTA

AGACTATATAATCAATTAGCTGAAGTGAAACAACAAAAGGTAGAGAAAGCAAAACAAGAA

GCTTATGCCCAGAACAGAGAAAGGGCAAAAGAATTTCATAAGAAAACACTAGAGAAACTT

CGAGCCAAAAATACA

>CL127.Contig7_A-W 3 1238 PREDICTED: centrosomal protein KIAA1731-like isoform 2 [Papio anubis]

AATCAGAACTATCCCTCCAAAGAACAGACTGAAATATTACAAAATAAGAAAAAAAGTGTT

CATTTCCAGGTTTCCAGAGGAAATTTAAATTCCACTTACAGTTCATCGGATGAGGCTAAT

GTATTTCATCAGTTACAAGTACAGCATAGTACTCCTTGTAGTTCTTCCTCTAGTGAGTGC

TTAATAAAACATGAACAAGAAAGCAGAAGAGAAAGATGGGACTTTGAAGAACTATCAAAA

AAAGATGTCCTTACAGTGTTACCCAGCCAAGGACTCACTGAAGATGAAAATGAATCTTGT

GGAGTTTTAGCTACAGATCCACATGTTGAAATTGATTCTCAGTTATGTGTAAGAACAGCG

GAGATGGGAACTTCAGTTCAGACACCACATTCATTAAGTATACAAAATGAAAAATATTTT

GAGAATTCAACTAAAACTGAAACTCCACAAGTCATAAACCTGTCTCAAATAGCAAAATCA

GAGTCTCTTAAAAGTTCTGGATCATTTTCATTACAGAGTTCTATTCCAATCTGGGAGACA

GAATCTGGTAATGGTATAATGGAAGAACCAGAACTTACTTTAGTAAGCACCAGTGATATT

AGCATTATTGAAGCTGAATTTGCAAACTTATCTCTAGAAGAGAAGAATGAGGGAAAAGGC

TACCTTCAGGTGAATGAATTTATTCCATCAGAAACAGAAAACTCAGATTATCCAGTCATA

TCAGAGTTTTCTTTGCAGAAGTCAACAGAATCTACAGAAACACTACCAAAATGTACAATG

GCAGGAAGCTTACAAGAAGCATTTGTAAAGAGAAAAAAATCATTTTTGGAGAGATCCTAC

CAGAGGCAGAAAGAAATAAGGAATAAGAGTAAACTCACTGAAAATTCCCAAGTCAAAACA

GTTAAGGAGAAACAATGTACAGGTTCATCTGTGAGTCGCCTTAAGGGAGTCAATAAAGTT

AAAATGAATTTTCCAGAAGAAAGAAAGAATGCACACACCCTCATGCACCAAAGGGCTCTA

AGACTATATAATCAATTAGCTGAAGTGAAACAACAAAAGGTAGAGAAAGCAAAACAAGAA

GCTTATGCCCAGAACAGAGAAAGGGCAAAAGAATTTCATAAGGTAAACAAGCCCCCTCCC

TTCTATATTTAAGGCATACCACAATAGGAACTGCTGCTTATTTTTTATTTATTCTCTCCA

CAGAAAACACTAGAGAAACTTCGAGCCAAAAATACA

>CL127.Contig8_A-W 45 260 minus strand PREDICTED: protein KIAA1731-like [Nomascus leucogenys]

AGACTATATAATCAATTAGCTGAAGTGAAACAACAAAAGGTAGAGAAAGCAAAACAAGAA

GCTTATGCCCAGAACAGAGAAAGGGCAAAAGAATTTCATAAGGTAAACAAGCCCCCTCCC

TTCTATATTTAAGGCATACCACAATAGGAACTGCTGCTTATTTTTTATTTATTCTCTCCA

CAGAAAACACTAGAGAAACTTCGAGCCAAAAATACA

>CL129.Contig1_A-W 9 1184 GDP-mannose pyrophosphorylase A, isoform CRA_c [Homo sapiens]

AGGTACCTTCAGGAATTTGCCCCCCTGGGCACAGGAGGTGGTCTCTACCATTTTCGGGAC

CAGATCCTAGCTGGAGGCCCTGAAGCATTCTTCGTGCTCAATGCTGACGTCTGCTCTGAC

TTCCCCTTGAGTGCTATGTTGGATGCTCACAGATGCGAGCGCCACCCTTTCTTACTCCTT

GGCACTACGGCTAACAGAACACAGTCCCTCAACTATGGCTGCATCGTTGAGAATCCACAG

ACACATGAGGTCCTGCACTATGTGGAGAAACCCAGCACATTTATTAGTGACATCATCAAC

TGCGGCATCTATCTCTTTTCTCCGGAAGCCCTGAAACCTCTTCGGGACGTCTTCCAGCGT

AATCAGCAGGATGGGCAACTGGAGGATTCTCCAGGTTCATGGCCTGGGGCAGGGACCATC

CGCCTGGAGCAGGATGTGTTCTCAGCCCTGGCTGGGCAAGGCCAGATCTATGTGCACCTC

ACTGATGGCATCTGGAGTCAGATCAAGTCTGCTGGCTCAGCTCTCTATGCCTCTCGCCTC

TATCTGGGCCGATACCAGATCACTCACCCAGAACGTCTGGCCAAGCACACATCAGGGGGT

CCAAGAATCCGAGGTACCCACCCAGCCCCAGTTGTTAACCTTTGCCCATCCCCGTCCCCC

ATTGCTTCCACTCCTGACCTCAGGTCCAGAGGTGAGTCTCAATCCCTGCCCCTTCCCGTC

TAGATAAGGTTTGGGGTATTTGGACCCAGGTCCTCTCTTCTGCTTCTAGGAAACGTTTAC

ATCCACCCCACTGCTAAAGTGGCCCCGTCAGCTGTGCTAGGCCCCAATGTCTCCATCGGG

AAGGGGGTGACTGTGGGTGAAGGTGTGCGGCTCCGAGAGAGCATTGTCCTCCATGGAGCC

ACGCTGCAGGAGCACACATGTATTCTGCACAGTATCGTGGGCTGGGGGAGCACTGTCGGA

CGCTGGGCTCGTGTGGAGGGGACCCCCAATGACCCCAATCCTAATGACCCTCGAGCCCGC

ATGGACAGCGAGAGCCTCTTCAAAGACGGCAAGCTGCTGCCTGCTATCACCATCCTGGGC

TGCCGCGTCCGGATCCCTGCCGAGGTGCTTATCCTGAACTCGATTGTTCTGCCACACAAG

GAATTGAGCCGCAGTTTCACCAACCAGATCATCCTC

>CL129.Contig2_A-W 47 991 GDP-mannose pyrophosphorylase A, isoform CRA_c [Homo sapiens]

AGCCCTCACTGTCCTTAGGTCCTGCACTATGTGGAGAAACCCAGCACATTTATTAGTGAC

ATCATCAACTGCGGCATCTATCTCTTTTCTCCGGAAGCCCTGAAACCTCTTCGGGACGTC

TTCCAGCGTAATCAGCAGGATGGGCAACTGGAGGATTCTCCAGGTTCATGGCCTGGGGCA

GGGACCATCCGCCTGGAGCAGGATGTGTTCTCAGCCCTGGCTGGGCAAGGCCAGATCTAT

GTGCACCTCACTGATGGCATCTGGAGTCAGATCAAGTCTGCTGGCTCAGCTCTCTATGCC

TCTCGCCTCTATCTGGGCCGATACCAGATCACTCACCCAGAACGTCTGGCCAAGCACACA

TCAGGGGGTCCAAGAATCCGAGGTACCCACCCAGCCCCAGTTGTTAACCTTTGCCCATCC

CCGTCCCCCATTGCTTCCACTCCTGACCTCAGGTCCAGAGGTGAGTCTCAATCCCTGCCC

CTTCCCGTCTAGATAAGGTTTGGGGTATTTGGACCCAGGTCCTCTCTTCTGCTTCTAGGA

AACGTTTACATCCACCCCACTGCTAAAGTGGCCCCGTCAGCTGTGCTAGGCCCCAATGTC

TCCATCGGGAAGGGGGTGACTGTGGGTGAAGGTGTGCGGCTCCGAGAGAGCATTGTCCTC

CATGGAGCCACGCTGCAGGAGCACACATGTATTCTGCACAGTATCGTGGGCTGGGGGAGC

ACTGTCGGACGCTGGGCTCGTGTGGAGGGGACCCCCAATGACCCCAATCCTAATGACCCT

CGAGCCCGCATGGACAGCGAGAGCCTCTTCAAAGACGGCAAGCTGCTGCCTGCTATCACC

ATCCTGGGCTGCCGCGTCCGGATCCCTGCCGAGGTGCTTATCCTGAACTCGATTGTTCTG

CCACACAAGGAATTGAGCCGCAGTTTCACCAACCAGATCATCCTC

>CL129.Contig3_A-W 47 835 mannose-1-phosphate guanyltransferase alpha [Rattus norvegicus] >gi|81889856|sp|Q5XIC1.1|GMPPA_RAT RecName: Full=Mannose-1-phosphate guanyltransferase alpha; AltName: Full=GDP-mannose pyrophosphorylase A; AltName: Full=GTP-mannose-1-phosphate guanylyltransferase alpha >gi|53733553|gb|AAH83763.1| GDP-mannose pyrophosphorylase A [Rattus norvegicus] >gi|149016193|gb|EDL75439.1| GDP-mannose pyrophosphorylase A, isoform CRA_a [Rattus norvegicus] >gi|149016194|gb|EDL75440.1| GDP-mannose pyrophosphorylase A, isoform CRA_a [Rattus norvegicus] >gi|149016195|gb|EDL75441.1| GDP-mannose pyrophosphorylase A, isoform CRA_a [Rattus norvegicus]

AGCCCTCACTGTCCTTAGGTCCTGCACTATGTGGAGAAACCCAGCACATTTATTAGTGAC

ATCATCAACTGCGGCATCTATCTCTTTTCTCCGGAAGCCCTGAAACCTCTTCGGGACGTC

TTCCAGCGTAATCAGCAGGATGGGCAACTGGAGGATTCTCCAGGTTCATGGCCTGGGGCA

GGGACCATCCGCCTGGAGCAGGATGTGTTCTCAGCCCTGGCTGGGCAAGGCCAGATCTAT

GTGCACCTCACTGATGGCATCTGGAGTCAGATCAAGTCTGCTGGCTCAGCTCTCTATGCC

TCTCGCCTCTATCTGGGCCGATACCAGATCACTCACCCAGAACGTCTGGCCAAGCACACA

TCAGGGGGTCCAAGAATCCGAGGAAACGTTTACATCCACCCCACTGCTAAAGTGGCCCCG

TCAGCTGTGCTAGGCCCCAATGTCTCCATCGGGAAGGGGGTGACTGTGGGTGAAGGTGTG

CGGCTCCGAGAGAGCATTGTCCTCCATGGAGCCACGCTGCAGGAGCACACATGTATTCTG

CACAGTATCGTGGGCTGGGGGAGCACTGTCGGACGCTGGGCTCGTGTGGAGGGGACCCCC

AATGACCCCAATCCTAATGACCCTCGAGCCCGCATGGACAGCGAGAGCCTCTTCAAAGAC

GGCAAGCTGCTGCCTGCTATCACCATCCTGGGCTGCCGCGTCCGGATCCCTGCCGAGGTG

CTTATCCTGAACTCGATTGTTCTGCCACACAAGGAATTGAGCCGCAGTTTCACCAACCAG

ATCATCCTC

>CL129.Contig4_A-W 362 1000 mannose-1-phosphate guanyltransferase alpha [Rattus norvegicus] >gi|81889856|sp|Q5XIC1.1|GMPPA_RAT RecName: Full=Mannose-1-phosphate guanyltransferase alpha; AltName: Full=GDP-mannose pyrophosphorylase A; AltName: Full=GTP-mannose-1-phosphate guanylyltransferase alpha >gi|53733553|gb|AAH83763.1| GDP-mannose pyrophosphorylase A [Rattus norvegicus] >gi|149016193|gb|EDL75439.1| GDP-mannose pyrophosphorylase A, isoform CRA_a [Rattus norvegicus] >gi|149016194|gb|EDL75440.1| GDP-mannose pyrophosphorylase A, isoform CRA_a [Rattus norvegicus] >gi|149016195|gb|EDL75441.1| GDP-mannose pyrophosphorylase A, isoform CRA_a [Rattus norvegicus]

GAGGATTCTCCAGGTTCATGGCCTGGGGCAGGGACCATCCGCCTGGAGCAGGATGTGTTC

TCAGCCCTGGCTGGGCAAGGCCAGATCTATGTGCACCTCACTGATGGCATCTGGAGTCAG

ATCAAGTCTGCTGGCTCAGCTCTCTATGCCTCTCGCCTCTATCTGGGCCGATACCAGATC

ACTCACCCAGAACGTCTGGCCAAGCACACATCAGGGGGTCCAAGAATCCGAGGAAACGTT

TACATCCACCCCACTGCTAAAGTGGCCCCGTCAGCTGTGCTAGGCCCCAATGTCTCCATC

GGGAAGGGGGTGACTGTGGGTGAAGGTGTGCGGCTCCGAGAGAGCATTGTCCTCCATGGA

GCCACGCTGCAGGAGCACACATGTATTCTGCACAGTATCGTGGGCTGGGGGAGCACTGTC

GGACGCTGGGCTCGTGTGGAGGGGACCCCCAATGACCCCAATCCTAATGACCCTCGAGCC

CGCATGGACAGCGAGAGCCTCTTCAAAGACGGCAAGCTGCTGCCTGCTATCACCATCCTG

GGCTGCCGCGTCCGGATCCCTGCCGAGGTGCTTATCCTGAACTCGATTGTTCTGCCACAC

AAGGAATTGAGCCGCAGTTTCACCAACCAGATCATCCTC

>CL129.Contig5_A-W 413 1207 GDP-mannose pyrophosphorylase A, isoform CRA_c [Homo sapiens]

GAGGATTCTCCAGGTTCATGGCCTGGGGCAGGGACCATCCGCCTGGAGCAGGATGTGTTC

TCAGCCCTGGCTGGGCAAGGCCAGATCTATGTGCACCTCACTGATGGCATCTGGAGTCAG

ATCAAGTCTGCTGGCTCAGCTCTCTATGCCTCTCGCCTCTATCTGGGCCGATACCAGATC

ACTCACCCAGAACGTCTGGCCAAGCACACATCAGGGGGTCCAAGAATCCGAGGTACCCAC

CCAGCCCCAGTTGTTAACCTTTGCCCATCCCCGTCCCCCATTGCTTCCACTCCTGACCTC

AGGTCCAGAGGTGAGTCTCAATCCCTGCCCCTTCCCGTCTAGATAAGGTTTGGGGTATTT

GGACCCAGGTCCTCTCTTCTGCTTCTAGGAAACGTTTACATCCACCCCACTGCTAAAGTG

GCCCCGTCAGCTGTGCTAGGCCCCAATGTCTCCATCGGGAAGGGGGTGACTGTGGGTGAA

GGTGTGCGGCTCCGAGAGAGCATTGTCCTCCATGGAGCCACGCTGCAGGAGCACACATGT

ATTCTGCACAGTATCGTGGGCTGGGGGAGCACTGTCGGACGCTGGGCTCGTGTGGAGGGG

ACCCCCAATGACCCCAATCCTAATGACCCTCGAGCCCGCATGGACAGCGAGAGCCTCTTC

AAAGACGGCAAGCTGCTGCCTGCTATCACCATCCTGGGCTGCCGCGTCCGGATCCCTGCC

GAGGTGCTTATCCTGAACTCGATTGTTCTGCCACACAAGGAATTGAGCCGCAGTTTCACC

AACCAGATCATCCTC

>CL131.Contig1_A-W 1805 2494 minus strand PREDICTED: ATP-binding cassette sub-family A member 7 [Otolemur garnettii]

CTCAGGGACCTGACCAAGGTGTACCATGGGCAGAGGATGCCAGCTGTCCACCGCCTGTGC

CTGGGGATCCCCCCAGGTGAGTGTTTCGGACTGCTCGGTGTGAATGGAGCCGGGAAAACC

ACCACGTTCCGCATGCTGACCGGGGACACACTGCCCAGCATGGGCGAGGCTGTGCTGGCA

GGTCACAGCGTGGCCCAGGAACCAGCCGCCGCACACTGCCACATGGGCTACTGCCCGCAG

TCAGACGCCATCTTCGAGCTGCTGACTGGCCGCGAACACCTAGAGCTGTTTGCACGCCTG

CGAGGCGTCCCTGAGGCCCTGGTGGCAGGGACTGCCAGTTGGGGCCTGGCGCGCCTGGGG

CTCCCGCGATACGCCGACAGACTCGCGGGCACCTACAGTGGGGGCAACAAACGCAAGCTG

GCCACAGCCGTGGCGCTCGTTGGGGATCCCGCTGTGGTCTTCCTGGATGAGCCCACCACA

GGCATGGACCCCAGCGCGCGGCGCTTCCTCTGGAACAGCCTTCTGGCCGTGGTGCGCCAG

GGCCGCTCCGTGGTGCTCACCTCGCACAGCATGGAGGAGTGCGAGGCGCTCTGCACGCGC

CTGGCCATCATGGTGAACGGGCGGTTCCGCTGCCTGGGCAGCACGCAGCACCTCAAGCGC

AGATTCGGTGCTGGCCACACACTGACCCTG

>CL131.Contig2_A-W 770 1960 minus strand PREDICTED: ATP-binding cassette sub-family A member 7 [Papio anubis]

CTGATGGCCTCGTCCGTGGACGTGCTCGTCTCCATCTGTGTGGTCTTCGCCATGTCCTTC

GTCCCGGCCAGCTTCACCCTCGTCCTCATTGAGGAGCGCGTCACGCGAGCCAAACACCTG

CAGCTCGTCGGGGGTCTGCCCCCCGCCCTCTACTGGCTGGGCAACTTTGTCTGGGACATG

TGTAACTACTTGGTGGCTGTGTGCATCGTGGTGCTCATCTTCCTGGTCTTCCAGCACAAG

GCGTATGTGGCCCCTGCCAACCTGCCTGCCCTCCTGCTGCTGCTGCTACTGTATGGCTGG

TCAATCACGCCACTCATGTACCCGGCCTCCTTCTTCTTCTCCGTGCCCAGCACGGCTTAC

GTCGTGCTCACCTGCATCAACCTCTTTGTGGGCATCAACGGCAGCATGGCTACCTTCGTG

CTGGAGCTCTTCTCGGATCAGAAGCTGAAGGAGGTGAGCCGCATCCTGAAACGCATCTTC

CTGGTCTTCCCCCACTTCTGCCTGGGCCGGGGGCTCATCGATCTGGTGCGGAACCAGGCC

ACGGCTGACGCTTTTGAGCGCCTGGGAGACGGGCAATTCCAGTCGCCCCTGCGCTGGGAG

GTGGTGGGCAAGAACCTGCTGGCCATGTCTGTGCAGGGGCCCCTCTTCCTCCTGCTCACG

CTCCTGCTGCAGCACCTCGGCCGCCTCCTGCCACAACCCCAGCTGAGGACACGGCCGCCC

CTGGGAGAGGAGGATGAGGATGTGGCCCACGAGCGGCAGCGGGTGGCCAGAGGGGCCACC

GAGGGGGATGTGTTGGTTCTCAGGGACCTGACCAAGGTGTACCATGGGCAGAGGATGCCA

GCTGTCCACCGCCTGTGCCTGGGGATCCCCCCAGGTGAGTGTTTCGGACTGCTCGGTGTG

AATGGAGCCGGGAAAACCACCACGTTCCGCATGCTGACCGGGGACACACTGCCCAGCATG

GGCGAGGCTGTGCTGGCAGGTCACAGCGTGGCCCAGGAACCAGCCGCCGCACACTGCCAC

ATGGGCTACTGCCCGCAGTCAGACGCCATCTTCGAGCTGCTGACTGGCCGCGAACACCTA

GAGCTGTTTGCACGCCTGCGAGGCGTCCCTGAGGCCCTGGTGGCAGGGACTGCCAGTTGG

GGCCTGGCGCGCCTGGGGCTCCCGCGATACGCCGACAGACTCGCGGGCACC

>CL131.Contig3_A-W 1141 2046 minus strand PREDICTED: ATP-binding cassette sub-family A member 7 isoform 1 [Pan paniscus] >gi|397485327|ref|XP_003813802.1| PREDICTED: ATP-binding cassette sub-family A member 7 isoform 2 [Pan paniscus]

CTCAGGGACCTGACCAAGGTGTACCATGGGCAGAGGATGCCAGCTGTCCACCGCCTGTGC

CTGGGGATCCCCCCAGGTGAGTGTTTCGGACTGCTCGGTGTGAATGGAGCCGGGAAAACC

ACCACGTTCCGCATGCTGACCGGGGACACACTGCCCAGCATGGGCGAGGCTGTGCTGGCA

GGTCACAGCGTGGCCCAGGAACCAGCCGCCGCACACTGCCACATGGGCTACTGCCCGCAG

TCAGACGCCATCTTCGAGCTGCTGACTGGCCGCGAACACCTAGAGCTGTTTGCACGCCTG

CGAGGCGTCCCTGAGGCCCTGGTGGCAGGGACTGCCAGTTGGGGCCTGGCGCGCCTGGGG

CTCCCGCGATACGCCGACAGACTCGCGGGCACCTACAGTGGGGGCAACAAACGCAAGCTG

GCCACAGCCGTGGCGCTCGTTGGGGATCCCGCTGTGGTCTTCCTGGATGAGCCCACCACA

GGCATGGACCCCAGCGCGCGGCGCTTCCTCTGGAACAGCCTTCTGGCCGTGGTGCGCCAG

GGCCGCTCCGTGGTGCTCACCTCGCACAGCATGGAGGAGTGCGAGGCGCTCTGCACGCGC

CTGGCCATCATGGTGAACGGGCGGTTCCGCTGCCTGGGCAGCACGCAGCACCTCAAGCGC

AGATTCGGTGCTGGCCACACACTGACCCTGAGGGTGCCCGTGGACCGGCACGAGGGAGCG

ATAGCCTTTGTGGTGGCGGCATTCCCGGGGGCCGAGCTGCGAGAAGCGCATGGGGGTCGC

CTACGCTTCCAGCTGCCACAGGGAGAGCGCGGCGCCCTGGCACACATTTTTGGAGAGCTG

GCAGCCCATGGTTCAGACCATGGCATGGAGGACTTCTCTGTGAGCCAGACCACACTGGAG

GAGGTG

>CL131.Contig4_A-W 77 442 minus strand PREDICTED: LOW QUALITY PROTEIN: ATP-binding cassette sub-family A member 7 [Callithrix jacchus]

GGGATGCCCCTCCAGATTGTGCTGCCCGCCCTCTTTGTGGGCCTGGCCCTGCTGTTCAGC

CTCATCGTGCCTCCTTTTGGGCACTACCCGGCTCTGCGGCTCAGTCCTGCCATGTACGGT

GCCCAGGTGTCCTTTTTCAGGTGAGTGCAGAGGAGAGGGGCTGGTGGAGGGAGGGCTTGC

CACCTGGGGACACAGCTCTGATCTGCACACACAGTGAGGACACCCAGGGGGACCCCAAGT

ATGGCCACCTGCTCAAGGCGCTGCTGGGGCAGGCAGGACTGGAGGAGCCCCACCTGCAGA

ACACCTCCAACAGGCCGCCCGAGTGCCCAGGGGCCCTCTCCTGCCTGTTCTCAGTCCCTG

AGGCCA

>CL131.Contig5_A-W 77 214 minus strand PREDICTED: ATP-binding cassette sub-family A member 7 [Otolemur garnettii]

GGGATGCCCCTCCAGATTGTGCTGCCCGCCCTCTTTGTGGGCCTGGCCCTGCTGTTCAGC

CTCATCGTGCCTCCTTTTGGGCACTACCCGGCTCTGCGGCTCAGTCCTGCCATGTACGGT

GCCCAGGTGTCCTTTTTC

>CL131.Contig6_A-W 77 214 minus strand PREDICTED: ATP-binding cassette sub-family A member 7 [Otolemur garnettii]

GGGATGCCCCTCCAGATTGTGCTGCCCGCCCTCTTTGTGGGCCTGGCCCTGCTGTTCAGC

CTCATCGTGCCTCCTTTTGGGCACTACCCGGCTCTGCGGCTCAGTCCTGCCATGTACGGT

GCCCAGGTGTCCTTTTTC

>CL131.Contig7_A-W 842 2032 minus strand PREDICTED: ATP-binding cassette sub-family A member 7 [Papio anubis]

CTGATGGCCTCGTCCGTGGACGTGCTCGTCTCCATCTGTGTGGTCTTCGCCATGTCCTTC

GTCCCGGCCAGCTTCACCCTCGTCCTCATTGAGGAGCGCGTCACGCGAGCCAAACACCTG

CAGCTCGTCGGGGGTCTGCCCCCCGCCCTCTACTGGCTGGGCAACTTTGTCTGGGACATG

TGTAACTACTTGGTGGCTGTGTGCATCGTGGTGCTCATCTTCCTGGTCTTCCAGCACAAG

GCGTATGTGGCCCCTGCCAACCTGCCTGCCCTCCTGCTGCTGCTGCTACTGTATGGCTGG

TCAATCACGCCACTCATGTACCCGGCCTCCTTCTTCTTCTCCGTGCCCAGCACGGCTTAC

GTCGTGCTCACCTGCATCAACCTCTTTGTGGGCATCAACGGCAGCATGGCTACCTTCGTG

CTGGAGCTCTTCTCGGATCAGAAGCTGAAGGAGGTGAGCCGCATCCTGAAACGCATCTTC

CTGGTCTTCCCCCACTTCTGCCTGGGCCGGGGGCTCATCGATCTGGTGCGGAACCAGGCC

ACGGCTGACGCTTTTGAGCGCCTGGGAGACGGGCAATTCCAGTCGCCCCTGCGCTGGGAG

GTGGTGGGCAAGAACCTGCTGGCCATGTCTGTGCAGGGGCCCCTCTTCCTCCTGCTCACG

CTCCTGCTGCAGCACCTCGGCCGCCTCCTGCCACAACCCCAGCTGAGGACACGGCCGCCC

CTGGGAGAGGAGGATGAGGATGTGGCCCACGAGCGGCAGCGGGTGGCCAGAGGGGCCACC

GAGGGGGATGTGTTGGTTCTCAGGGACCTGACCAAGGTGTACCATGGGCAGAGGATGCCA

GCTGTCCACCGCCTGTGCCTGGGGATCCCCCCAGGTGAGTGTTTCGGACTGCTCGGTGTG

AATGGAGCCGGGAAAACCACCACGTTCCGCATGCTGACCGGGGACACACTGCCCAGCATG

GGCGAGGCTGTGCTGGCAGGTCACAGCGTGGCCCAGGAACCAGCCGCCGCACACTGCCAC

ATGGGCTACTGCCCGCAGTCAGACGCCATCTTCGAGCTGCTGACTGGCCGCGAACACCTA

GAGCTGTTTGCACGCCTGCGAGGCGTCCCTGAGGCCCTGGTGGCAGGGACTGCCAGTTGG

GGCCTGGCGCGCCTGGGGCTCCCGCGATACGCCGACAGACTCGCGGGCACC

>CL131.Contig8_A-W 767 1957 minus strand PREDICTED: ATP-binding cassette sub-family A member 7 [Papio anubis]

CTGATGGCCTCGTCCGTGGACGTGCTCGTCTCCATCTGTGTGGTCTTCGCCATGTCCTTC

GTCCCGGCCAGCTTCACCCTCGTCCTCATTGAGGAGCGCGTCACGCGAGCCAAACACCTG

CAGCTCGTCGGGGGTCTGCCCCCCGCCCTCTACTGGCTGGGCAACTTTGTCTGGGACATG

TGTAACTACTTGGTGGCTGTGTGCATCGTGGTGCTCATCTTCCTGGTCTTCCAGCACAAG

GCGTATGTGGCCCCTGCCAACCTGCCTGCCCTCCTGCTGCTGCTGCTACTGTATGGCTGG

TCAATCACGCCACTCATGTACCCGGCCTCCTTCTTCTTCTCCGTGCCCAGCACGGCTTAC

GTCGTGCTCACCTGCATCAACCTCTTTGTGGGCATCAACGGCAGCATGGCTACCTTCGTG

CTGGAGCTCTTCTCGGATCAGAAGCTGAAGGAGGTGAGCCGCATCCTGAAACGCATCTTC

CTGGTCTTCCCCCACTTCTGCCTGGGCCGGGGGCTCATCGATCTGGTGCGGAACCAGGCC

ACGGCTGACGCTTTTGAGCGCCTGGGAGACGGGCAATTCCAGTCGCCCCTGCGCTGGGAG

GTGGTGGGCAAGAACCTGCTGGCCATGTCTGTGCAGGGGCCCCTCTTCCTCCTGCTCACG

CTCCTGCTGCAGCACCTCGGCCGCCTCCTGCCACAACCCCAGCTGAGGACACGGCCGCCC

CTGGGAGAGGAGGATGAGGATGTGGCCCACGAGCGGCAGCGGGTGGCCAGAGGGGCCACC

GAGGGGGATGTGTTGGTTCTCAGGGACCTGACCAAGGTGTACCATGGGCAGAGGATGCCA

GCTGTCCACCGCCTGTGCCTGGGGATCCCCCCAGGTGAGTGTTTCGGACTGCTCGGTGTG

AATGGAGCCGGGAAAACCACCACGTTCCGCATGCTGACCGGGGACACACTGCCCAGCATG

GGCGAGGCTGTGCTGGCAGGTCACAGCGTGGCCCAGGAACCAGCCGCCGCACACTGCCAC

ATGGGCTACTGCCCGCAGTCAGACGCCATCTTCGAGCTGCTGACTGGCCGCGAACACCTA

GAGCTGTTTGCACGCCTGCGAGGCGTCCCTGAGGCCCTGGTGGCAGGGACTGCCAGTTGG

GGCCTGGCGCGCCTGGGGCTCCCGCGATACGCCGACAGACTCGCGGGCACC

>CL131.Contig9_A-W 1450 2139 minus strand PREDICTED: ATP-binding cassette sub-family A member 7 [Otolemur garnettii]

CTCAGGGACCTGACCAAGGTGTACCATGGGCAGAGGATGCCAGCTGTCCACCGCCTGTGC

CTGGGGATCCCCCCAGGTGAGTGTTTCGGACTGCTCGGTGTGAATGGAGCCGGGAAAACC

ACCACGTTCCGCATGCTGACCGGGGACACACTGCCCAGCATGGGCGAGGCTGTGCTGGCA

GGTCACAGCGTGGCCCAGGAACCAGCCGCCGCACACTGCCACATGGGCTACTGCCCGCAG

TCAGACGCCATCTTCGAGCTGCTGACTGGCCGCGAACACCTAGAGCTGTTTGCACGCCTG

CGAGGCGTCCCTGAGGCCCTGGTGGCAGGGACTGCCAGTTGGGGCCTGGCGCGCCTGGGG

CTCCCGCGATACGCCGACAGACTCGCGGGCACCTACAGTGGGGGCAACAAACGCAAGCTG

GCCACAGCCGTGGCGCTCGTTGGGGATCCCGCTGTGGTCTTCCTGGATGAGCCCACCACA

GGCATGGACCCCAGCGCGCGGCGCTTCCTCTGGAACAGCCTTCTGGCCGTGGTGCGCCAG

GGCCGCTCCGTGGTGCTCACCTCGCACAGCATGGAGGAGTGCGAGGCGCTCTGCACGCGC

CTGGCCATCATGGTGAACGGGCGGTTCCGCTGCCTGGGCAGCACGCAGCACCTCAAGCGC

AGATTCGGTGCTGGCCACACACTGACCCTG

>CL131.Contig10_A-W 1379 2068 minus strand PREDICTED: ATP-binding cassette sub-family A member 7 [Otolemur garnettii]

CTCAGGGACCTGACCAAGGTGTACCATGGGCAGAGGATGCCAGCTGTCCACCGCCTGTGC

CTGGGGATCCCCCCAGGTGAGTGTTTCGGACTGCTCGGTGTGAATGGAGCCGGGAAAACC

ACCACGTTCCGCATGCTGACCGGGGACACACTGCCCAGCATGGGCGAGGCTGTGCTGGCA

GGTCACAGCGTGGCCCAGGAACCAGCCGCCGCACACTGCCACATGGGCTACTGCCCGCAG

TCAGACGCCATCTTCGAGCTGCTGACTGGCCGCGAACACCTAGAGCTGTTTGCACGCCTG

CGAGGCGTCCCTGAGGCCCTGGTGGCAGGGACTGCCAGTTGGGGCCTGGCGCGCCTGGGG

CTCCCGCGATACGCCGACAGACTCGCGGGCACCTACAGTGGGGGCAACAAACGCAAGCTG

GCCACAGCCGTGGCGCTCGTTGGGGATCCCGCTGTGGTCTTCCTGGATGAGCCCACCACA

GGCATGGACCCCAGCGCGCGGCGCTTCCTCTGGAACAGCCTTCTGGCCGTGGTGCGCCAG

GGCCGCTCCGTGGTGCTCACCTCGCACAGCATGGAGGAGTGCGAGGCGCTCTGCACGCGC

CTGGCCATCATGGTGAACGGGCGGTTCCGCTGCCTGGGCAGCACGCAGCACCTCAAGCGC

AGATTCGGTGCTGGCCACACACTGACCCTG

>CL131.Contig11_A-W 1213 1605 minus strand PREDICTED: ATP-binding cassette sub-family A member 7 [Papio anubis]

CTCAGGGACCTGACCAAGGTGTACCATGGGCAGAGGATGCCAGCTGTCCACCGCCTGTGC

CTGGGGATCCCCCCAGGTGAGTGTTTCGGACTGCTCGGTGTGAATGGAGCCGGGAAAACC

ACCACGTTCCGCATGCTGACCGGGGACACACTGCCCAGCATGGGCGAGGCTGTGCTGGCA

GGTCACAGCGTGGCCCAGGAACCAGCCGCCGCACACTGCCACATGGGCTACTGCCCGCAG

TCAGACGCCATCTTCGAGCTGCTGACTGGCCGCGAACACCTAGAGCTGTTTGCACGCCTG

CGAGGCGTCCCTGAGGCCCTGGTGGCAGGGACTGCCAGTTGGGGCCTGGCGCGCCTGGGG

CTCCCGCGATACGCCGACAGACTCGCGGGCACC

>CL131.Contig12_A-W 109 225 minus strand PREDICTED: ATP-binding cassette sub-family A member 7-like [Cavia porcellus]

TGTAACTACTTGGTGGCTGTGTGCATCGTGGTGCTCATCTTCCTGGTCTTCCAGCACAAG

GCGTATGTGGCCCCTGCCAACCTGCCTGCCCTCCTGCTGCTGCTGCTACTGTATGGG

>CL131.Contig13_A-W 109 225 minus strand PREDICTED: ATP-binding cassette sub-family A member 7-like [Cavia porcellus]

TGTAACTACTTGGTGGCTGTGTGCATCGTGGTGCTCATCTTCCTGGTCTTCCAGCACAAG

GCGTATGTGGCCCCTGCCAACCTGCCTGCCCTCCTGCTGCTGCTGCTACTGTATGGG

>CL131.Contig14_A-W 558 1703 minus strand PREDICTED: ATP-binding cassette sub-family A member 7-like [Cavia porcellus]

TGTGCAGGGGCCCCTCTTCCTCCTGCTCACGCTCCTGCTGCAGCACCTCGGCCGCCTCCT

GCCACAGTCAGTGGGGAGCAGGGTGGGGTGGCAGGGCCAGGGACCTGCCTGGGACGCACG

CGTGTCTCTGTCTTCAGACCCCAGCTGAGGACACGGCCGCCCCTGGGAGAGGAGGATGAG

GATGTGGCCCACGAGCGGCAGCGGGTGGCCAGAGGGGCCACCGAGGGGGATGTGTTGGTT

CTCAGGGACCTGACCAAGGTGTACCATGGGCAGAGGATGCCAGCTGTCCACCGCCTGTGC

CTGGGGATCCCCCCAGGTGAGTGTTTCGGACTGCTCGGTGTGAATGGAGCCGGGAAAACC

ACCACGTTCCGCATGCTGACCGGGGACACACTGCCCAGCATGGGCGAGGCTGTGCTGGCA

GGTCACAGCGTGGCCCAGGAACCAGCCGCCGCACACTGCCACATGGGCTACTGCCCGCAG

TCAGACGCCATCTTCGAGCTGCTGACTGGCCGCGAACACCTAGAGCTGTTTGCACGCCTG

CGAGGCGTCCCTGAGGCCCTGGTGGCAGGGACTGCCAGTTGGGGCCTGGCGCGCCTGGGG

CTCCCGCGATACGCCGACAGACTCGCGGGCACCTACAGTGGGGGCAACAAACGCAAGCTG

GCCACAGCCGTGGCGCTCGTTGGGGATCCCGCTGTGGTCTTCCTGGATGAGCCCACCACA

GGCATGGACCCCAGCGCGCGGCGCTTCCTCTGGAACAGCCTTCTGGCCGTGGTGCGCCAG

GGCCGCTCCGTGGTGCTCACCTCGCACAGCATGGAGGAGTGCGAGGCGCTCTGCACGCGC

CTGGCCATCATGGTGAACGGGCGGTTCCGCTGCCTGGGCAGCACGCAGCACCTCAAGCGC

AGATTCGGTGCTGGCCACACACTGACCCTGAGGGTGCCCGTGGACCGGCACGAGGGAGCG

ATAGCCTTTGTGGTGGCGGCATTCCCGGGGGCCGAGCTGCGAGAAGCGCATGGGGGTCGC

CTACGCTTCCAGCTGCCACAGGGAGAGCGCGGCGCCCTGGCACACATTTTTGGAGAGCTG

GCAGCCCATGGTTCAGACCATGGCATGGAGGACTTCTCTGTGAGCCAGACCACACTGGAG

GAGGTG

>CL131.Contig15_A-W 643 1632 minus strand PREDICTED: ATP-binding cassette sub-family A member 7-like [Cavia porcellus]

CCGCCCCTGGGAGAGGAGGATGAGGATGTGGCCCACGAGCGGCAGCGGGTGGCCAGAGGG

GCCACCGAGGGGGATGTGTTGGTTCTCAGGGACCTGACCAAGGTGTACCATGGGCAGAGG

ATGCCAGCTGTCCACCGCCTGTGCCTGGGGATCCCCCCAGGTGAGTGTTTCGGACTGCTC

GGTGTGAATGGAGCCGGGAAAACCACCACGTTCCGCATGCTGACCGGGGACACACTGCCC

AGCATGGGCGAGGCTGTGCTGGCAGGTCACAGCGTGGCCCAGGAACCAGCCGCCGCACAC

TGCCACATGGGCTACTGCCCGCAGTCAGACGCCATCTTCGAGCTGCTGACTGGCCGCGAA

CACCTAGAGCTGTTTGCACGCCTGCGAGGCGTCCCTGAGGCCCTGGTGGCAGGGACTGCC

AGTTGGGGCCTGGCGCGCCTGGGGCTCCCGCGATACGCCGACAGACTCGCGGGCACCTAC

AGTGGGGGCAACAAACGCAAGCTGGCCACAGCCGTGGCGCTCGTTGGGGATCCCGCTGTG

GTCTTCCTGGATGAGCCCACCACAGGCATGGACCCCAGCGCGCGGCGCTTCCTCTGGAAC

AGCCTTCTGGCCGTGGTGCGCCAGGGCCGCTCCGTGGTGCTCACCTCGCACAGCATGGAG

GAGTGCGAGGCGCTCTGCACGCGCCTGGCCATCATGGTGAACGGGCGGTTCCGCTGCCTG

GGCAGCACGCAGCACCTCAAGCGCAGATTCGGTGCTGGCCACACACTGACCCTGAGGGTG

CCCGTGGACCGGCACGAGGGAGCGATAGCCTTTGTGGTGGCGGCATTCCCGGGGGCCGAG

CTGCGAGAAGCGCATGGGGGTCGCCTACGCTTCCAGCTGCCACAGGGAGAGCGCGGCGCC

CTGGCACACATTTTTGGAGAGCTGGCAGCCCATGGTTCAGACCATGGCATGGAGGACTTC

TCTGTGAGCCAGACCACACTGGAGGAGGTG

>CL131.Contig16_A-W 1213 1779 minus strand PREDICTED: LOW QUALITY PROTEIN: ATP-binding cassette sub-family A member 7 [Callithrix jacchus]

CTCAGGGACCTGACCAAGGTGTACCATGGGCAGAGGATGCCAGCTGTCCACCGCCTGTGC

CTGGGGATCCCCCCAGGTGAGTGTTTCGGACTGCTCGGTGTGAATGGAGCCGGGAAAACC

ACCACGTTCCGCATGCTGACCGGGGACACACTGCCCAGCATGGGCGAGGCTGTGCTGGCA

GGTCACAGCGTGGCCCAGGAACCAGCCGCCGCACACTGCCACATGGGCTACTGCCCGCAG

TCAGACGCCATCTTCGAGCTGCTGACTGGCCGCGAACACCTAGAGCTGTTTGCACGCCTG

CGAGGCGTCCCTGAGGCCCTGGTGGCAGGGACTGCCAGTTGGGGCCTGGCGCGCCTGGGG

CTCCCGCGATACGCCGACAGACTCGCGGGCACCTACAGTGGGGGCAACAAACGCAAGCTG

GCCACAGCCGTGGCGCTCGTTGGGGATCCCGCTGTGGTCTTCCTGGATGAGCCCACCACA

GGCATGGACCCCAGCGCGCGGCGCTTCCTCTGGAACAGCCTTCTGGCCGTGGTGCGCCAG

GGCCGCTCCGTGGTGCTCACCTCGCAC

>CL131.Contig17_A-W 269 1414 minus strand PREDICTED: ATP-binding cassette sub-family A member 7-like [Cavia porcellus]

TGTGCAGGGGCCCCTCTTCCTCCTGCTCACGCTCCTGCTGCAGCACCTCGGCCGCCTCCT

GCCACAGTCAGTGGGGAGCAGGGTGGGGTGGCAGGGCCAGGGACCTGCCTGGGACGCACG

CGTGTCTCTGTCTTCAGACCCCAGCTGAGGACACGGCCGCCCCTGGGAGAGGAGGATGAG

GATGTGGCCCACGAGCGGCAGCGGGTGGCCAGAGGGGCCACCGAGGGGGATGTGTTGGTT

CTCAGGGACCTGACCAAGGTGTACCATGGGCAGAGGATGCCAGCTGTCCACCGCCTGTGC

CTGGGGATCCCCCCAGGTGAGTGTTTCGGACTGCTCGGTGTGAATGGAGCCGGGAAAACC

ACCACGTTCCGCATGCTGACCGGGGACACACTGCCCAGCATGGGCGAGGCTGTGCTGGCA

GGTCACAGCGTGGCCCAGGAACCAGCCGCCGCACACTGCCACATGGGCTACTGCCCGCAG

TCAGACGCCATCTTCGAGCTGCTGACTGGCCGCGAACACCTAGAGCTGTTTGCACGCCTG

CGAGGCGTCCCTGAGGCCCTGGTGGCAGGGACTGCCAGTTGGGGCCTGGCGCGCCTGGGG

CTCCCGCGATACGCCGACAGACTCGCGGGCACCTACAGTGGGGGCAACAAACGCAAGCTG

GCCACAGCCGTGGCGCTCGTTGGGGATCCCGCTGTGGTCTTCCTGGATGAGCCCACCACA

GGCATGGACCCCAGCGCGCGGCGCTTCCTCTGGAACAGCCTTCTGGCCGTGGTGCGCCAG

GGCCGCTCCGTGGTGCTCACCTCGCACAGCATGGAGGAGTGCGAGGCGCTCTGCACGCGC

CTGGCCATCATGGTGAACGGGCGGTTCCGCTGCCTGGGCAGCACGCAGCACCTCAAGCGC

AGATTCGGTGCTGGCCACACACTGACCCTGAGGGTGCCCGTGGACCGGCACGAGGGAGCG

ATAGCCTTTGTGGTGGCGGCATTCCCGGGGGCCGAGCTGCGAGAAGCGCATGGGGGTCGC

CTACGCTTCCAGCTGCCACAGGGAGAGCGCGGCGCCCTGGCACACATTTTTGGAGAGCTG

GCAGCCCATGGTTCAGACCATGGCATGGAGGACTTCTCTGTGAGCCAGACCACACTGGAG

GAGGTG

>CL131.Contig18_A-W 354 1343 minus strand PREDICTED: ATP-binding cassette sub-family A member 7-like [Cavia porcellus]

CCGCCCCTGGGAGAGGAGGATGAGGATGTGGCCCACGAGCGGCAGCGGGTGGCCAGAGGG

GCCACCGAGGGGGATGTGTTGGTTCTCAGGGACCTGACCAAGGTGTACCATGGGCAGAGG

ATGCCAGCTGTCCACCGCCTGTGCCTGGGGATCCCCCCAGGTGAGTGTTTCGGACTGCTC

GGTGTGAATGGAGCCGGGAAAACCACCACGTTCCGCATGCTGACCGGGGACACACTGCCC

AGCATGGGCGAGGCTGTGCTGGCAGGTCACAGCGTGGCCCAGGAACCAGCCGCCGCACAC

TGCCACATGGGCTACTGCCCGCAGTCAGACGCCATCTTCGAGCTGCTGACTGGCCGCGAA

CACCTAGAGCTGTTTGCACGCCTGCGAGGCGTCCCTGAGGCCCTGGTGGCAGGGACTGCC

AGTTGGGGCCTGGCGCGCCTGGGGCTCCCGCGATACGCCGACAGACTCGCGGGCACCTAC

AGTGGGGGCAACAAACGCAAGCTGGCCACAGCCGTGGCGCTCGTTGGGGATCCCGCTGTG

GTCTTCCTGGATGAGCCCACCACAGGCATGGACCCCAGCGCGCGGCGCTTCCTCTGGAAC

AGCCTTCTGGCCGTGGTGCGCCAGGGCCGCTCCGTGGTGCTCACCTCGCACAGCATGGAG

GAGTGCGAGGCGCTCTGCACGCGCCTGGCCATCATGGTGAACGGGCGGTTCCGCTGCCTG

GGCAGCACGCAGCACCTCAAGCGCAGATTCGGTGCTGGCCACACACTGACCCTGAGGGTG

CCCGTGGACCGGCACGAGGGAGCGATAGCCTTTGTGGTGGCGGCATTCCCGGGGGCCGAG

CTGCGAGAAGCGCATGGGGGTCGCCTACGCTTCCAGCTGCCACAGGGAGAGCGCGGCGCC

CTGGCACACATTTTTGGAGAGCTGGCAGCCCATGGTTCAGACCATGGCATGGAGGACTTC

TCTGTGAGCCAGACCACACTGGAGGAGGTG

>CL131.Contig19_A-W 1 117 minus strand PREDICTED: LOW QUALITY PROTEIN: ATP-binding cassette sub-family A member 7 [Callithrix jacchus]

TGTAACTACTTGGTGGCTGTGTGCATCGTGGTGCTCATCTTCCTGGTCTTCCAGCACAAG

GCGTATGTGGCCCCTGCCAACCTGCCTGCCCTCCTGCTGCTGCTGCTACTGTATGGG

>CL131.Contig20_A-W 798 1364 minus strand PREDICTED: LOW QUALITY PROTEIN: ATP-binding cassette sub-family A member 7 [Callithrix jacchus]

CTCAGGGACCTGACCAAGGTGTACCATGGGCAGAGGATGCCAGCTGTCCACCGCCTGTGC

CTGGGGATCCCCCCAGGTGAGTGTTTCGGACTGCTCGGTGTGAATGGAGCCGGGAAAACC

ACCACGTTCCGCATGCTGACCGGGGACACACTGCCCAGCATGGGCGAGGCTGTGCTGGCA

GGTCACAGCGTGGCCCAGGAACCAGCCGCCGCACACTGCCACATGGGCTACTGCCCGCAG

TCAGACGCCATCTTCGAGCTGCTGACTGGCCGCGAACACCTAGAGCTGTTTGCACGCCTG

CGAGGCGTCCCTGAGGCCCTGGTGGCAGGGACTGCCAGTTGGGGCCTGGCGCGCCTGGGG

CTCCCGCGATACGCCGACAGACTCGCGGGCACCTACAGTGGGGGCAACAAACGCAAGCTG

GCCACAGCCGTGGCGCTCGTTGGGGATCCCGCTGTGGTCTTCCTGGATGAGCCCACCACA

GGCATGGACCCCAGCGCGCGGCGCTTCCTCTGGAACAGCCTTCTGGCCGTGGTGCGCCAG

GGCCGCTCCGTGGTGCTCACCTCGCAC

>CL131.Contig21_A-W 1 117 minus strand PREDICTED: LOW QUALITY PROTEIN: ATP-binding cassette sub-family A member 7-like [Ailuropoda melanoleuca]

TGTAACTACTTGGTGGCTGTGTGCATCGTGGTGCTCATCTTCCTGGTCTTCCAGCACAAG

GCGTATGTGGCCCCTGCCAACCTGCCTGCCCTCCTGCTGCTGCTGCTACTGTATGGG

>CL131.Contig22_A-W 690 1007 minus strand PREDICTED: LOW QUALITY PROTEIN: ATP-binding cassette sub-family A member 7-like [Ailuropoda melanoleuca]

CTCAGGGACCTGACCAAGGTGTACCATGGGCAGAGGATGCCAGCTGTCCACCGCCTGTGC

CTGGGGATCCCCCCAGGTGAGTGTTTCGGACTGCTCGGTGTGAATGGAGCCGGGAAAACC

ACCACGTTCCGCATGCTGACCGGGGACACACTGCCCAGCATGGGCGAGGCTGTGCTGGCA

GGTCACAGCGTGGCCCAGGAACCAGCCGCCGCACACTGCCACATGGGCTACTGCCCGCAG

TCAGACGCCATCTTCGAGCTGCTGACTGGCCGCGAACACCTAGAGCTGTTTGCACGCCTG

CGAGGCGTCCCTGAGGCC

>CL131.Contig23_A-W 92 793 minus strand PREDICTED: ATP-binding cassette sub-family A member 7-like [Cavia porcellus]

GCCGCCGCACACTGCCACATGGGCTACTGCCCGCAGTCAGACGCCATCTTCGAGCTGCTG

ACTGGCCGCGAACACCTAGAGCTGTTTGCACGCCTGCGAGGCGTCCCTGAGGCCCTGGTG

GCAGGGACTGCCAGTTGGGGCCTGGCGCGCCTGGGGCTCCCGCGATACGCCGACAGACTC

GCGGGCACCTACAGTGGGGGCAACAAACGCAAGCTGGCCACAGCCGTGGCGCTCGTTGGG

GATCCCGCTGTGGTCTTCCTGGATGAGCCCACCACAGGCATGGACCCCAGCGCGCGGCGC

TTCCTCTGGAACAGCCTTCTGGCCGTGGTGCGCCAGGGCCGCTCCGTGGTGCTCACCTCG

CACAGCATGGAGGAGTGCGAGGCGCTCTGCACGCGCCTGGCCATCATGGTGAACGGGCGG

TTCCGCTGCCTGGGCAGCACGCAGCACCTCAAGCGCAGATTCGGTGCTGGCCACACACTG

ACCCTGAGGGTGCCCGTGGACCGGCACGAGGGAGCGATAGCCTTTGTGGTGGCGGCATTC

CCGGGGGCCGAGCTGCGAGAAGCGCATGGGGGTCGCCTACGCTTCCAGCTGCCACAGGGA

GAGCGCGGCGCCCTGGCACACATTTTTGGAGAGCTGGCAGCCCATGGTTCAGACCATGGC

ATGGAGGACTTCTCTGTGAGCCAGACCACACTGGAGGAGGTG

>CL131.Contig24_A-W 509 1075 minus strand PREDICTED: LOW QUALITY PROTEIN: ATP-binding cassette sub-family A member 7 [Callithrix jacchus]

CTCAGGGACCTGACCAAGGTGTACCATGGGCAGAGGATGCCAGCTGTCCACCGCCTGTGC

CTGGGGATCCCCCCAGGTGAGTGTTTCGGACTGCTCGGTGTGAATGGAGCCGGGAAAACC

ACCACGTTCCGCATGCTGACCGGGGACACACTGCCCAGCATGGGCGAGGCTGTGCTGGCA

GGTCACAGCGTGGCCCAGGAACCAGCCGCCGCACACTGCCACATGGGCTACTGCCCGCAG

TCAGACGCCATCTTCGAGCTGCTGACTGGCCGCGAACACCTAGAGCTGTTTGCACGCCTG

CGAGGCGTCCCTGAGGCCCTGGTGGCAGGGACTGCCAGTTGGGGCCTGGCGCGCCTGGGG

CTCCCGCGATACGCCGACAGACTCGCGGGCACCTACAGTGGGGGCAACAAACGCAAGCTG

GCCACAGCCGTGGCGCTCGTTGGGGATCCCGCTGTGGTCTTCCTGGATGAGCCCACCACA

GGCATGGACCCCAGCGCGCGGCGCTTCCTCTGGAACAGCCTTCTGGCCGTGGTGCGCCAG

GGCCGCTCCGTGGTGCTCACCTCGCAC

>CL131.Contig25_A-W 438 1004 minus strand PREDICTED: LOW QUALITY PROTEIN: ATP-binding cassette sub-family A member 7 [Callithrix jacchus]

CTCAGGGACCTGACCAAGGTGTACCATGGGCAGAGGATGCCAGCTGTCCACCGCCTGTGC

CTGGGGATCCCCCCAGGTGAGTGTTTCGGACTGCTCGGTGTGAATGGAGCCGGGAAAACC

ACCACGTTCCGCATGCTGACCGGGGACACACTGCCCAGCATGGGCGAGGCTGTGCTGGCA

GGTCACAGCGTGGCCCAGGAACCAGCCGCCGCACACTGCCACATGGGCTACTGCCCGCAG

TCAGACGCCATCTTCGAGCTGCTGACTGGCCGCGAACACCTAGAGCTGTTTGCACGCCTG

CGAGGCGTCCCTGAGGCCCTGGTGGCAGGGACTGCCAGTTGGGGCCTGGCGCGCCTGGGG

CTCCCGCGATACGCCGACAGACTCGCGGGCACCTACAGTGGGGGCAACAAACGCAAGCTG

GCCACAGCCGTGGCGCTCGTTGGGGATCCCGCTGTGGTCTTCCTGGATGAGCCCACCACA

GGCATGGACCCCAGCGCGCGGCGCTTCCTCTGGAACAGCCTTCTGGCCGTGGTGCGCCAG

GGCCGCTCCGTGGTGCTCACCTCGCAC

>CL131.Contig26_A-W 509 826 minus strand PREDICTED: LOW QUALITY PROTEIN: ATP-binding cassette sub-family A member 7-like [Ailuropoda melanoleuca]

CTCAGGGACCTGACCAAGGTGTACCATGGGCAGAGGATGCCAGCTGTCCACCGCCTGTGC

CTGGGGATCCCCCCAGGTGAGTGTTTCGGACTGCTCGGTGTGAATGGAGCCGGGAAAACC

ACCACGTTCCGCATGCTGACCGGGGACACACTGCCCAGCATGGGCGAGGCTGTGCTGGCA

GGTCACAGCGTGGCCCAGGAACCAGCCGCCGCACACTGCCACATGGGCTACTGCCCGCAG

TCAGACGCCATCTTCGAGCTGCTGACTGGCCGCGAACACCTAGAGCTGTTTGCACGCCTG

CGAGGCGTCCCTGAGGCC

>CL131.Contig27_A-W 438 755 minus strand PREDICTED: LOW QUALITY PROTEIN: ATP-binding cassette sub-family A member 7-like [Ailuropoda melanoleuca]

CTCAGGGACCTGACCAAGGTGTACCATGGGCAGAGGATGCCAGCTGTCCACCGCCTGTGC

CTGGGGATCCCCCCAGGTGAGTGTTTCGGACTGCTCGGTGTGAATGGAGCCGGGAAAACC

ACCACGTTCCGCATGCTGACCGGGGACACACTGCCCAGCATGGGCGAGGCTGTGCTGGCA

GGTCACAGCGTGGCCCAGGAACCAGCCGCCGCACACTGCCACATGGGCTACTGCCCGCAG

TCAGACGCCATCTTCGAGCTGCTGACTGGCCGCGAACACCTAGAGCTGTTTGCACGCCTG

CGAGGCGTCCCTGAGGCC

>CL131.Contig28_A-W 182 637 minus strand PREDICTED: ATP-binding cassette sub-family A member 7-like [Cavia porcellus]

GCCAACAGCATGGAGGAGTGCGAGGCGCTCTGCACGCGCCTGGCCATCATGGTGAACGGG

CGGTTCCGCTGCCTGGGCAGCACGCAGCACCTCAAGCGCAGATTCGGTGCTGGCCACACA

CTGACCCTGAGGGTGCCCGTGGACCGGCACGAGGGAGCGATAGCCTTTGTGGTGGCGGCA

TTCCCGGGGGCCGAGCTGCGAGAAGCGCATGGGGGTCGCCTACGCTTCCAGCTGCCACAG

GGAGAGCGCGGCGCCCTGGCACACATTTTTGGAGAGCTGGCAGCCCATGGTTCAGACCAT

GGCATGGAGGACTTCTCTGTGAGCCAGACCACACTGGAGGAGGTGACCACAGTGTCGGGC

TGGGACTGGGGAGGGGCTGGGGCCAGGGTCCTGGGCTGACAGCCCCTCCTGTCCATCCTG

TCCTCTCTGGACCACCCACCTGCCAGGTCTTCCTGC

>CL131.Contig29_A-W 92 454 minus strand PREDICTED: ATP-binding cassette sub-family A member 7-like [Cavia porcellus]

GCCGCCGCACACTGCCACATGGGCTACTGCCCGCAGTCAGACGCCATCTTCGAGCTGCTG

ACTGGCCGCGAACACCTAGAGCTGTTTGCACGCCTGCGAGGCGTCCCTGAGGCCCTGGTG

GCAGGGACTGCCAGTTGGGGCCTGGCGCGCCTGGGGCTCCCGCGATACGCCGACAGACTC

GCGGGCACCTACAGTGGGGGCAACAAACGCAAGCTGGCCACAGCCGTGGCGCTCGTTGGG

GATCCCGCTGTGGTCTTCCTGGATGAGCCCACCACAGGCATGGACCCCAGCGCGCGGCGC

TTCCTCTGGAACAGCCTTCTGGCCGTGGTGCGCCAGGGCCGCTCCGTGGTGCTCACCTCG

CAC

>CL132.Contig1_A-W 1 285 PREDICTED: regulation of nuclear pre-mRNA domain-containing protein 2 [Equus caballus]

GATAATCGTGATGTGGAAGATATGGAACTCTCAGATGTAGAAGATGATGGGTCAAAAATC

ATTGTGGAGGACAGGAAGGAAAAACCTGTGGAGAAATCAGCTGTCTCTACTTCTGTACCT

ACAAAGCCAACAGAAACTCTCCCAAAGGCCTCTTCCTGTACCCCAGTGCCTGTGACCATG

ACAGCAACCCCACCTCTTCCAAAGCCTGTGAATACTTCTCTTCTGTCCCCGTCCCCAGCT

TTGGCTTTGCCCAACCTGGCTAACGTGGATCTGGCAAAGATTAGT

>CL132.Contig2_A-W 48 269 PREDICTED: regulation of nuclear pre-mRNA domain-containing protein 2 [Equus caballus]

GTGGAGGACAGGAAGGAAAAACCTGTGGAGAAATCAGCTGTCTCTACTTCTGTACCTACA

AAGCCAACAGAAACTCTCCCAAAGGCCTCTTCCTGTACCCCAGTGCCTGTGACCATGACA

GCAACCCCACCTCTTCCAAAGCCTGTGAATACTTCTCTTCTGTCCCCGTCCCCAGCTTTG

GCTTTGCCCAACCTGGCTAACGTGGATCTGGCAAAGATTAGT

>CL135.Contig1_A-W 2 1057 minus strand sirtuin 2 (silent mating type information regulation 2, homolog) 2 (S. cerevisiae), isoform CRA_c [Mus musculus]

AGAGCAGTCGGTGACAGCCCCGAGGGCCCCCGCCCCGCGCCCATGGCCGAGCCGGACCCC

TCTGACCCTCTGGAGAACCAGGCAGGGAAGGTGCAGGAGGCTCAGGACTCCGACTCAGAC

CCTGAAGGAGGAGCTGCTGGCGGAGAAGCAGAGATGGATTTCCTGCGGAACTTCTTCTCC

CAGACCCTGGGCCTGGGCACCCAAAAGGAGCGTCTGCTGGATGAGCTGACCCTGGAAGGG

GTGGCCCGCTACATGCAGAGTGAGCGCTGCCGCAGGGTGATCTGTTTGGTGGGAGCCGGA

ATCTCCACAGCTGCGGGCATCCCCGACTTCCGCTCTCCATCCACTGGCCTCTACGCCAAC

CTGGAGAAATACCATCTCCCCTACCCGGAGGCCATCTTTGAGATTGGCTACTTTAAGAAA

CATCCAGAACCCTTCTTTGCCCTTGCCAAGGAACTCTATCCTGGGCAATTCAAGCCGACC

ATCTGTCACTACTTCATTCGCCTGATGAAGGAGAAGGGGCTGCTCCTGCGCTGCTACACC

CAGAACATAGACACCCTGGAGCGCGTGGCAGGGCTGGAGCCCGAGGACCTGGTGGAGGCC

CATGGCACCTTCTACACCTCCCACTGCATCAGCCCCCTGTGCAGGCGCGAGTACCCGCTG

AGCTGGATGAAAGAGAAGCTCTTCACGGAGGTGACTCCACGGTGCGAGAAGTGCCAGAGT

GTGGTGAAACCTGACATCATTTTTTTTGGCGAGAACCTCCCGGCGCGGTTCTTCTCCTGC

ATGCAGTCAGACTTCCTGAAGGTGGACCTGCTCATCATCATGGGCACCTCCCTGCAGGTG

CAGCCCTTCGCCTCCCTCGTCAGCAAGGCACCCCTCTCCACCCCGCGCCTGCTCATCAAC

AAGGAGAAGACGGGCCAGACGGACCCCTTCCTGGGCATGATGATGGGCCTGGGAAGAGGC

ATGGACTTCGACTCCAAGAAGGCCTACAGGGACGTGGCCTGGCTGGGTGACTGTGACCAG

GGCTGCCTGGCCCTCGCTGACCTCCTCGGATGGAAG

>CL135.Contig2_A-W 52 1104 minus strand PREDICTED: LOW QUALITY PROTEIN: NAD-dependent deacetylase sirtuin-2-like [Equus caballus]

TCTGACCCTCTGGAGAACCAGGCAGGGAAGGTGCAGGAGGCTCAGGACTCCGACTCAGAC

CCTGAAGGAGGAGCTGCTGGCGGAGAAGCAGAGATGGATTTCCTGCGGAACTTCTTCTCC

CAGACCCTGGGCCTGGGCACCCAAAAGGAGCGTCTGCTGGATGAGCTGACCCTGGAAGGG

GTGGCCCGCTACATGCAGAGTGAGCGCTGCCGCAGGGTGATCTGTTTGGTGGGAGCCGGA

ATCTCCACAGCTGCGGGCATCCCCGACTTCCGCTCTCCATCCACTGGCCTCTACGCCAAC

CTGGAGAAATACCATCTCCCCTACCCGGAGGCCATCTTTGAGATTGGCTACTTTAAGAAA

CATCCAGAACCCTTCTTTGCCCTTGCCAAGGAACTCTATCCTGGGCAATTCAAGCCGACC

ATCTGTCACTACTTCATTCGCCTGATGAAGGAGAAGGGGCTGCTCCTGCGCTGCTACACC

CAGAACATAGACACCCTGGAGCGCGTGGCAGGGCTGGAGCCCGAGGACCTGGTGGAGGCC

CATGGCACCTTCTACACCTCCCACTGCATCAGCCCCCTGTGCAGGCGCGAGTACCCGCTG

AGCTGGATGAAAGAGAAGCTCTTCACGGAGGTGACTCCACGGTGCGAGAAGTGCCAGAGT

GTGGTGAAACCTGACATCATTTTTTTTGGCGAGAACCTCCCGGCGCGGTTCTTCTCCTGC

ATGCAGTCAGACTTCCTGAAGGTGGACCTGCTCATCATCATGGGCACCTCCCTGCAGGTG

CAGCCCTTCGCCTCCCTCGTCAGCAAGGCACCCCTCTCCACCCCGCGCCTGCTCATCAAC

AAGGAGAAGACGGGCCAGACGGACCCCTTCCTGGGCATGATGATGGGCCTGGGAAGAGGC

ATGGACTTCGACTCCAAGAAGGCCTACAGGGACGTGGCCTGGCTGGGTGACTGTGACCAG

GGCTGCCTGGCCCTCGCTGACCTCCTCGGATGGAAGGTGAGGGGCTGGGGGACCCCAGCC

TACCCTGTTCCCAGTCCTTGGACCCCACCCAAC

>CL135.Contig3_A-W 48 1055 minus strand PREDICTED: LOW QUALITY PROTEIN: NAD-dependent deacetylase sirtuin-2-like [Equus caballus]

GACTCCGACTCAGACCCTGAAGGAGGAGCTGCTGGCGGAGAAGCAGAGATGGATTTCCTG

CGGAACTTCTTCTCCCAGACCCTGGGCCTGGGCACCCAAAAGGAGCGTCTGCTGGATGAG

CTGACCCTGGAAGGGGTGGCCCGCTACATGCAGAGTGAGCGCTGCCGCAGGGTGATCTGT

TTGGTGGGAGCCGGAATCTCCACAGCTGCGGGCATCCCCGACTTCCGCTCTCCATCCACT

GGCCTCTACGCCAACCTGGAGAAATACCATCTCCCCTACCCGGAGGCCATCTTTGAGATT

GGCTACTTTAAGAAACATCCAGAACCCTTCTTTGCCCTTGCCAAGGAACTCTATCCTGGG

CAATTCAAGCCGACCATCTGTCACTACTTCATTCGCCTGATGAAGGAGAAGGGGCTGCTC

CTGCGCTGCTACACCCAGAACATAGACACCCTGGAGCGCGTGGCAGGGCTGGAGCCCGAG

GACCTGGTGGAGGCCCATGGCACCTTCTACACCTCCCACTGCATCAGCCCCCTGTGCAGG

CGCGAGTACCCGCTGAGCTGGATGAAAGAGAAGCTCTTCACGGAGGTGACTCCACGGTGC

GAGAAGTGCCAGAGTGTGGTGAAACCTGACATCATTTTTTTTGGCGAGAACCTCCCGGCG

CGGTTCTTCTCCTGCATGCAGTCAGACTTCCTGAAGGTGGACCTGCTCATCATCATGGGC

ACCTCCCTGCAGGTGCAGCCCTTCGCCTCCCTCGTCAGCAAGGCACCCCTCTCCACCCCG

CGCCTGCTCATCAACAAGGAGAAGACGGGCCAGACGGACCCCTTCCTGGGCATGATGATG

GGCCTGGGAAGAGGCATGGACTTCGACTCCAAGAAGGCCTACAGGGACGTGGCCTGGCTG

GGTGACTGTGACCAGGGCTGCCTGGCCCTCGCTGACCTCCTCGGATGGAAGGTGAGGGGC

TGGGGGACCCCAGCCTACCCTGTTCCCAGTCCTTGGACCCCACCCAAC

>CL135.Contig4_A-W 45 1148 minus strand NAD-dependent deacetylase sirtuin-2 [Sus scrofa] >gi|164653929|gb|ABY65333.1| sirtuin 2 [Sus scrofa]

ACCGACTCCGACTCAGACCCTGAAGGAGGAGCTGCTGGCGGAGAAGCAGAGATGGATTTC

CTGCGGAACTTCTTCTCCCAGACCCTGGGCCTGGGCACCCAAAAGGAGCGTCTGCTGGAT

GAGCTGACCCTGGAAGGGGTGGCCCGCTACATGCAGAGTGAGCGCTGCCGCAGGGTGATC

TGTTTGGTGGGAGCCGGAATCTCCACAGCTGCGGGCATCCCCGACTTCCGCTCTCCATCC

ACTGGCCTCTACGCCAACCTGGAGAAATACCATCTCCCCTACCCGGAGGCCATCTTTGAG

ATTGGCTACTTTAAGAAACATCCAGAACCCTTCTTTGCCCTTGCCAAGGAACTCTATCCT

GGGCAATTCAAGCCGACCATCTGTCACTACTTCATTCGCCTGATGAAGGAGAAGGGGCTG

CTCCTGCGCTGCTACACCCAGAACATAGACACCCTGGAGCGCGTGGCAGGGCTGGAGCCC

GAGGACCTGGTGGAGGCCCATGGCACCTTCTACACCTCCCACTGCATCAGCCCCCTGTGC

AGGCGCGAGTACCCGCTGAGCTGGATGAAAGAGAAGCTCTTCACGGAGGTGACTCCACGG

TGCGAGAAGTGCCAGAGTGTGGTGAAACCTGACATCATTTTTTTTGGCGAGAACCTCCCG

GCGCGGTTCTTCTCCTGCATGCAGTCAGACTTCCTGAAGGTGGACCTGCTCATCATCATG

GGCACCTCCCTGCAGGTGCAGCCCTTCGCCTCCCTCGTCAGCAAGGCACCCCTCTCCACC

CCGCGCCTGCTCATCAACAAGGAGAAGACGGGCCAGACGGACCCCTTCCTGGGCATGATG

ATGGGCCTGGGAAGAGGCATGGACTTCGACTCCAAGAAGGCCTACAGGGACGTGGCCTGG

CTGGGTGACTGTGACCAGGGCTGCCTGGCCCTCGCTGACCTCCTCGGATGGAAGAAAGAG

CTGGAGGACCTCGTTCGGAGGGAGCACGCCAGCATAGATGCCCAGTCTGGGTCAGGGGCC

CCCAACCCCAACACCGTAGCTTCCCCCAGGAGGTCCCCACCTCCTACCAAGGACGGGGCC

AGGACCGCAGAGCCGGAAAAGCCC

>CL135.Contig5_A-W 2 1204 minus strand sirtuin 2 (silent mating type information regulation 2, homolog) 2 (S. cerevisiae), isoform CRA_c [Mus musculus]

AGAGCAGTCGGTGACAGCCCCGAGGGCCCCCGCCCCGCGCCCATGGCCGAGCCGGACCCC

TCTGACCCTCTGGAGAACCAGGCAGGGAAGGTGCAGGAGGCTCAGGACTCCGACTCAGAC

CCTGAAGGAGGAGCTGCTGGCGGAGAAGCAGAGATGGATTTCCTGCGGAACTTCTTCTCC

CAGACCCTGGGCCTGGGCACCCAAAAGGAGCGTCTGCTGGATGAGCTGACCCTGGAAGGG

GTGGCCCGCTACATGCAGAGTGAGCGCTGCCGCAGGGTGATCTGTTTGGTGGGAGCCGGA

ATCTCCACAGCTGCGGGCATCCCCGACTTCCGCTCTCCATCCACTGGCCTCTACGCCAAC

CTGGAGAAATACCATCTCCCCTACCCGGAGGCCATCTTTGAGATTGGCTACTTTAAGAAA

CATCCAGAACCCTTCTTTGCCCTTGCCAAGGAACTCTATCCTGGGCAATTCAAGCCGACC

ATCTGTCACTACTTCATTCGCCTGATGAAGGAGAAGGGGCTGCTCCTGCGCTGCTACACC

CAGAACATAGACACCCTGGAGCGCGTGGCAGGGCTGGAGCCCGAGGACCTGGTGGAGGCC

CATGGCACCTTCTACACCTCCCACTGCATCAGCCCCCTGTGCAGGCGCGAGTACCCGCTG

AGCTGGATGAAAGAGAAGCTCTTCACGGAGGTGACTCCACAGTGCGAGAAGTGCCAGAGT

GTGGTGAAACCTGACATCATTTTTTTTGGCGAGAACCTCCCGGCGCGGTTCTTCTCCTGC

ATGCAGTCAGACTTCCTGAAGGTGGACCTGCTCATCATCATGGGCACCTCCCTGCAGGTG

CAGCCCTTCGCCTCCCTCGTCAGCAAGGCACCCCTCTCCACCCCGCGCCTGCTCATCAAC

AAGGAGAAGACGGGCCAGACGGACCCCTTCCTGGGCATGATGATGGGCCTGGGAAGAGGC

ATGGACTTCGACTCCAAGAAGGCCTACAGGGACGTGGCCTGGCTGGGTGACTGTGACCAG

GGCTGCCTGGCCCTCGCTGACCTCCTCGGATGGAAGAAAGAGCTGGAGGACCTCGTTCGG

AGGGAGCACGCCAGCATAGATGCCCAGTCTGGGTCAGGGGCCCCCAACCCCAACACCATA

GCTTCCCCCAGGAGGTCCCCACCTCCTACCAAGGACGGGGCCAGGACCGCAGAGCCGGAA

AAG

>CL135.Contig6_A-W 3 674 minus strand sirtuin 2 (silent mating type information regulation 2, homolog) 2 (S. cerevisiae), isoform CRA_b [Mus musculus]

ACAGCCCAGAGGGCCCCCGCCCCGCGCCCATGGCCGAGCCGGACCGACTCCGACTCAGAC

CCTGAAGGAGGAGCTGCTGGCGGAGAAGCAGAGATGGATTTCCTGCGGAACTTCTTCTCC

CAGACCCTGGGCCTGGGCACCCAAAAGGAGCGTCTGCTGGATGAGCTGACCCTGGAAGGG

GTGGCCCGCTACATGCAGAGTGAGCGCTGCCGCAGGGTGATCTGTTTGGTGGGAGCCGGA

ATCTCCACAGCTGCGGGCATCCCCGACTTCCGCTCTCCATCCACTGGCCTCTACGCCAAC

CTGGAGAAATACCATCTCCCCTACCCGGAGGCCATCTTTGAGATTGGCTACTTTAAGAAA

CATCCAGAACCCTTCTTTGCCCTTGCCAAGGAACTCTATCCTGGGCAATTCAAGCCGACC

ATCTGTCACTACTTCATTCGCCTGATGAAGGAGAAGGGGCTGCTCCTGCGCTGCTACACC

CAGAACATAGACACCCTGGAGCGCGTGGCAGGGCTGGAGCCCGAGGACCTGGTGGAGGCC

CATGGCACCTTCTACACCTCCCACTGCATCAGCCCCCTGTGCAGGCGCGAGTACCCGCTG

AGCTGGATGAAAGAGAAGCTCTTCACGGAGGTGACTCCACGGTGCGAGAAGTGCCAGAGT

GTGGTGAAACCT

>CL135.Contig7_A-W 2 733 minus strand sirtuin 2 (silent mating type information regulation 2, homolog) 2 (S. cerevisiae), isoform CRA_c [Mus musculus]

AGAGCAGTCGGTGACAGCCCCGAGGGCCCCCGCCCCGCGCCCATGGCCGAGCCGGACCCC

TCTGACCCTCTGGAGAACCAGGCAGGGAAGGTGCAGGAGGCTCAGGACTCCGACTCAGAC

CCTGAAGGAGGAGCTGCTGGCGGAGAAGCAGAGATGGATTTCCTGCGGAACTTCTTCTCC

CAGACCCTGGGCCTGGGCACCCAAAAGGAGCGTCTGCTGGATGAGCTGACCCTGGAAGGG

GTGGCCCGCTACATGCAGAGTGAGCGCTGCCGCAGGGTGATCTGTTTGGTGGGAGCCGGA

ATCTCCACAGCTGCGGGCATCCCCGACTTCCGCTCTCCATCCACTGGCCTCTACGCCAAC

CTGGAGAAATACCATCTCCCCTACCCGGAGGCCATCTTTGAGATTGGCTACTTTAAGAAA

CATCCAGAACCCTTCTTTGCCCTTGCCAAGGAACTCTATCCTGGGCAATTCAAGCCGACC

ATCTGTCACTACTTCATTCGCCTGATGAAGGAGAAGGGGCTGCTCCTGCGCTGCTACACC

CAGAACATAGACACCCTGGAGCGCGTGGCAGGGCTGGAGCCCGAGGACCTGGTGGAGGCC

CATGGCACCTTCTACACCTCCCACTGCATCAGCCCCCTGTGCAGGCGCGAGTACCCGCTG

AGCTGGATGAAAGAGAAGCTCTTCACGGAGGTGACTCCACAGTGCGAGAAGTGCCAGAGT

GTGGTGAAACCT

>CL135.Contig8_A-W 55 393 minus strand PREDICTED: LOW QUALITY PROTEIN: NAD-dependent deacetylase sirtuin-2-like [Ailuropoda melanoleuca]

TCTGTCTCCACCTCAGACATCATTTTTTTTGGCGAGAACCTCCCGGCGCGGTTCTTCTCC

TGCATGCAGTCAGACTTCCTGAAGGTGGACCTGCTCATCATCATGGGCACCTCCCTGCAG

GTGCAGCCCTTCGCCTCCCTCGTCAGCAAGGCACCCCTCTCCACCCCGCGCCTGCTCATC

AACAAGGAGAAGACGGGCCAGACGGACCCCTTCCTGGGCATGATGATGGGCCTGGGAAGA

GGCATGGACTTCGACTCCAAGAAGGCCTACAGGGACGTGGCCTGGCTGGGTGACTGTGAC

CAGGGCTGCCTGGCCCTCGCTGACCTCCTCGGATGGAAG

>CL136.Contig1_A-W 2 1000 PREDICTED: transient receptor potential cation channel, subfamily V, member 2 [Oryctolagus cuniculus]

GGAAACTCTATGCTGCTGCTGGGCCACATCTTGATCCTGCTTGGGGGAATCTACCTCCTC

TTGGGCCAGCTGTGGTACTTCTGGCGCCGACGTCTGTTCATCTGGATCTCCTTCGTGGAC

AGTTACTTTGAAATTCTCTTTCTGCTCCAGGCTCTGCTCACGGTGCTGTCCCAGGTGCTG

TGTTTCATGGCCATCGAGTGGTACCTGCCCCTACTTGTGTCCTCGCTGGTTCTGGGCTGG

CTGAACCTGCTTTACTATACACGTGGCTTCCAGCACACAGGCATCTACAGCGTCATGATC

CAGAAGGTCATCCTGAGGGACCTACTCCGTTTCCTTCTGGTCTACTTAGTCTTCCTTTTC

GGCTTTGCTGTAGCCCTGGTGAGCCTAAGCCGAGAGGCCCGGGACTCTGGAACCCCTGCA

GGAAACAATGCCACCGAGGTAGCTCAGCCTGGGACAGAGCAGGAGGATGAGGCAACACCA

TACAGGGGCATCATGGATGCCTCCCTGGAGCTCTTCAAGTTCACCATTGGCATGGGCGAG

CTGGCTTTCCAGGAACAGCTGCGCTTCCGTGGGGTGGTCCTGCTGCTGCTGCTGGCCTAC

GTGCTGCTCACCTACGTCCTTCTTCTCAACATGCTCATCGCTCTCATGAGCGAGACCGTC

AACAACGTTGCCACGGATAGCTGGAGCATTTGGAAGCTGCAGAAAGCCATCTCTGTCTTG

GAGATGGAGAATGGCTACTGGTGGTGCAGAAGGAAGAAGCAACGGGCAGGTGTGCTACTG

AAAGTTGGCACCAGGCCAGATGGCACCCCTGATGAACGCTGGTGCTTCAGGGTAGAGGAA

GTGAACTGGGCTTCGTGGGAGCAGACACTATCCACAGTGCGTGAGGAACCATCAGAAGGC

AATGCCCCTGGTTCCATGAAGAACCCCATTCTGGCTTCCCAGCCTGGGGAGGATCATGCC

TCAGAGGAAGACCATCTTCCTCTTCAGGTGCTTCAGTCT

>CL136.Contig2_A-W 135 992 PREDICTED: transient receptor potential cation channel, subfamily V, member 2 [Oryctolagus cuniculus]

CTGCTCCAGGCTCTGCTCACGGTGCTGTCCCAGGTGCTGTGTTTCATGGCCATCGAGTGG

TACCTGCCCCTACTTGTGTCCTCGCTGGTTCTGGGCTGGCTGAACCTGCTTTACTATACA

CGTGGCTTCCAGCACACAGGCATCTACAGCGTCATGATCCAGAAGGTCATCCTGAGGGAC

CTACTCCGTTTCCTTCTGGTCTACTTAGTCTTCCTTTTCGGCTTTGCTGTAGCCCTGGTG

AGCCTAAGCCGAGAGGCCCGGGACTCTGGAACCCCTGCAGGAAACAATGCCACCGAGGTA

GCTCAGCCTGGGACAGAGCAGGAGGATGAGGCAACACCATACAGGGGCATCATGGATGCC

TCCCTGGAGCTCTTCAAGTTCACCATTGGCATGGGCGAGCTGGCTTTCCAGGAACAGCTG

CGCTTCCGTGGGGTGGTCCTGCTGCTGCTGCTGGCCTACGTGCTGCTCACCTACGTCCTT

CTTCTCAACATGCTCATCGCTCTCATGAGCGAGACCGTCAACAACGTTGCCACGGATAGC

TGGAGCATTTGGAAGCTGCAGAAAGCCATCTCTGTCTTGGAGATGGAGAATGGCTACTGG

TGGTGCAGAAGGAAGAAGCAACGGGCAGGTGTGCTACTGAAAGTTGGCACCAGGCCAGAT

GGCACCCCTGATGAACGCTGGTGCTTCAGGGTAGAGGAAGTGAACTGGGCTTCGTGGGAG

CAGACACTATCCACAGTGCGTGAGGAACCATCAGAAGGCAATGCCCCTGGTTCCATGAAG

AACCCCATTCTGGCTTCCCAGCCTGGGGAGGATCATGCCTCAGAGGAAGACCATCTTCCT

CTTCAGGTGCTTCAGTCT

>CL137.Contig1_A-W 573 1892 PREDICTED: lipolysis-stimulated lipoprotein receptor [Canis lupus familiaris]

GGTTTTTCCCGGCTCCCGGCTCAGGCCTCCTCTGGTCTCCCTGCAGACTGGCTCTTCGTG

GTCGTGGTCTGCCTGGCTGCCTTCCTTGTCTTTCTCCTCCTGGGCATTTGCTGGTGTCAG

TGCTGCCCACATACCTGCTGCTGTTACGTCAGGTGCCCCTGCTGCCCGGAGAAGTGCTGC

TGTCCCGAGGCCCTGTATGCAGCTGGCAAAGCAGCCACATCAGGCGTCCCAAGCATCTAC

GCCCCAAGCACCTACGCCCACCTCTCCCCGACCAAGACCCCACCTCCACCGCCAGCCATG

ATTCCCATGGGCCCTGTCTACAATGGATACCCTGGAGGGTACGCTGGAGAATTTGACAGG

AATAGTTCAGTTGGCCACAGCTCCCAGGTACCCCTGCTTCGGGACACAGACAGCAACATG

ACCTCTGAAGTCCGCAGTGGGTACAGGATCCAAGCCAGCCAGCAGGATGACTCCATGAGA

GTCCTGTACTACATGGAGAAAGAGCTGGCCAACTTCGACCCCTCTCGACCGGGACCTGGC

CCCCCCAATGGTCGTTTAGAACGGGCTATGAGTGAAGTCACCTCCCTCCATGAAGACGAC

TGGAGATCCCGGCCTTCCCGGGGTCCTGCCCTCACCCCAATCCGGGATGAGGAGTGGGGT

CGCCACTCCCCCCGGAGTCCCAGGAGATGGGAGCAGGAGCCCCCCACAGAGCGGCCAGGG

GGTGGCTGGGGGGGCAGGCGGCCCCGGGCCCACTCTGTGGATGCTCTGGATGATCTAACC

CGGCCGGGTTCTGCTGAGTCTGGGAGGAGGTCTCCCCCAAGTAGTGGGAGGAGAGGACGT

GCCTACGCACCCCCACGAAGCCGTAGCCGTGATGACCTCTACGACCCAGATGACTCGAGG

GACTTTCCACATTCGCGGGATCCTCACTACGATGACTTCAGGTCTAGGGACCGCCCTCAG

GTGGACCCCAGATCCCGCCAGCACCGATCCCGAGACTCCCGGGATGAAGGCTCTAGGTCT

GGGGACCCGCATTATGATGAGCGTCTACTAGAAGAAGCCTTAAGGAAAAAGGGGCCAGGG

GAGAGGAGGAAACATTACAGGGAGGAAGAGGAAGAAGAGGAGGCCTACTACCCTCCACCG

CCTCCCCCGTACTCGGAGACGGACTCCCAGGCCTCACGGGAGCGGAGGCTAAAGAAGGTG

AGCGAATGGTCCTCCCCGAGGTTAGACCCTGCCCAGGCCCCTCTTCTCAGTCCCTGCCCA

GTTTACACTCTTCTTTCTCCTTTGCAGAACTTGGCCCTGAGTCGGGAAAGTTTAGTCGTC

>CL137.Contig2_A-W 573 1802 PREDICTED: lipolysis-stimulated lipoprotein receptor [Canis lupus familiaris]

GGTTTTTCCCGGCTCCCGGCTCAGGCCTCCTCTGGTCTCCCTGCAGACTGGCTCTTCGTG

GTCGTGGTCTGCCTGGCTGCCTTCCTTGTCTTTCTCCTCCTGGGCATTTGCTGGTGTCAG

TGCTGCCCACATACCTGCTGCTGTTACGTCAGGTGCCCCTGCTGCCCGGAGAAGTGCTGC

TGTCCCGAGGCCCTGTATGCAGCTGGCAAAGCAGCCACATCAGGCGTCCCAAGCATCTAC

GCCCCAAGCACCTACGCCCACCTCTCCCCGACCAAGACCCCACCTCCACCGCCAGCCATG

ATTCCCATGGGCCCTGTCTACAATGGATACCCTGGAGGGTACGCTGGAGAATTTGACAGG

AATAGTTCAGTTGGCCACAGCTCCCAGGTACCCCTGCTTCGGGACACAGACAGCAACATG

ACCTCTGAAGTCCGCAGTGGGTACAGGATCCAAGCCAGCCAGCAGGATGACTCCATGAGA

GTCCTGTACTACATGGAGAAAGAGCTGGCCAACTTCGACCCCTCTCGACCGGGACCTGGC

CCCCCCAATGGTCGTTTAGAACGGGCTATGAGTGAAGTCACCTCCCTCCATGAAGACGAC

TGGAGATCCCGGCCTTCCCGGGGTCCTGCCCTCACCCCAATCCGGGATGAGGAGTGGGGT

CGCCACTCCCCCCGGAGTCCCAGGAGATGGGAGCAGGAGCCCCCCACAGAGCGGCCAGGG

GGTGGCTGGGGGGGCAGGCGGCCCCGGGCCCACTCTGTGGATGCTCTGGATGATCTAACC

CGGCCGGGTTCTGCTGAGTCTGGGAGGAGGTCTCCCCCAAGTAGTGGGAGGAGAGGACGT

GCCTACGCACCCCCACGAAGCCGTAGCCGTGATGACCTCTACGACCCAGATGACTCGAGG

GACTTTCCACATTCGCGGGATCCTCACTACGATGACTTCAGGTCTAGGGACCGCCCTCAG

GTGGACCCCAGATCCCGCCAGCACCGATCCCGAGACTCCCGGGATGAAGGCTCTAGGTCT

GGGGACCCGCATTATGATGAGCGTCTACTAGAAGAAGCCTTAAGGAAAAAGGGGCCAGGG

GAGAGGAGGAAACATTACAGGGAGGAAGAGGAAGAAGAGGAGGCCTACTACCCTCCACCG

CCTCCCCCGTACTCGGAGACGGACTCCCAGGCCTCACGGGAGCGGAGGCTAAAGAAGAAC

TTGGCCCTGAGTCGGGAAAGTTTAGTCGTC

>CL137.Contig3_A-W 1169 1639 PREDICTED: LOW QUALITY PROTEIN: lipolysis-stimulated lipoprotein receptor-like [Cavia porcellus]

CCCCCACGAAGCCGTAGCCGTGATGACCTCTACGACCCAGATGACTCGAGGGACTTTCCA

CATTCGCGGGATCCTCACTACGATGACTTCAGGTCTAGGGACCGCCCTCAGGTGGACCCC

AGATCCCGCCAGCACCGATCCCGAGACTCCCGGGATGAAGGCTCTAGGTCTGGGGACCCG

CATTATGATGAGCGTCTACTAGAAGAAGCCTTAAGGAAAAAGGGGCCAGGGGAGAGGAGG

AAACATTACAGGGAGGAAGAGGAAGAAGAGGAGGCCTACTACCCTCCACCGCCTCCCCCG

TACTCGGAGACGGACTCCCAGGCCTCACGGGAGCGGAGGCTAAAGAAGGTGAGCGAATGG

TCCTCCCCGAGGTTAGACCCTGCCCAGGCCCCTCTTCTCAGTCCCTGCCCAGTTTACACT

CTTCTTTCTCCTTTGCAGAACTTGGCCCTGAGTCGGGAAAGTTTAGTCGTC

>CL137.Contig4_A-W 922 1113 PREDICTED: lipolysis-stimulated lipoprotein receptor isoform 2 [Papio anubis]

GGTTTTTCCCAGCTCCCAGCTCAGGCCTCCTCTGGTCTCCCTGCAGACTGGCTCTTCGTG

GTCGTGGTCTGCCTGGCTGCCTTCCTTGTCTTTCTCCTCCTGGGCATTTGCTGGTGTCAG

TGCTGCCCACATACCTGCTGCTGTTACGTCAGGTGCCCCTGCTGCCCGGAGAAGTGCTGC

TGTCCCGAGGCC

>CL137.Contig5_A-W 573 1802 PREDICTED: lipolysis-stimulated lipoprotein receptor [Canis lupus familiaris]

GGTTTTTCCCGGCTCCCGGCTCAGGCCTCCTCTGGTCTCCCTGCAGACTGGCTCTTCGTG

GTCGTGGTCTGCCTGGCTGCCTTCCTTGTCTTTCTCCTCCTGGGCATTTGCTGGTGTCAG

TGCTGCCCACATACCTGCTGCTGTTACGTCAGGTGCCCCTGCTGCCCGGAGAAGTGCTGC

TGTCCCGAGGCCCTGTATGCAGCTGGCAAAGCAGCCACATCAGGCGTCCCAAGCATCTAC

GCCCCAAGCACCTACGCCCACCTCTCCCCGACCAAGACCCCACCTCCACCGCCAGCCATG

ATTCCCATGGGCCCTGTCTACAATGGATACCCTGGAGGGTACGCTGGAGAATTTGACAGG

AATAGTTCAGTTGGCCACAGCTCCCAGGTACCCCTGCTTCGGGACACAGACAGCAACATG

ACCTCTGAAGTCCGCAGTGGGTACAGGATCCAAGCCAGCCAGCAGGATGACTCCATGAGA

GTCCTGTACTACATGGAGAAAGAGCTGGCCAACTTCGACCCCTCTCGACCGGGACCTGGC

CCCCCCAATGGTCGTTTAGAACGGGCTATGAGTGAAGTCACCTCCCTCCATGAAGACGAC

TGGAGATCCCGGCCTTCCCGGGGTCCTGCCCTCACCCCAATCCGGGATGAGGAGTGGGGT

CGCCACTCCCCCCGGAGTCCCAGGAGATGGGAGCAGGAGCCCCCCACAGAGCGGCCAGGG

GGTGGCTGGGGGGGCAGGCGGCCCCGGGCCCACTCTGTGGATGCTCTGGATGATCTAACC

CGGCCGGGTTCTGCTGAGTCTGGGAGGAGGTCTCCCCCAAGTAGTGGGAGGAGAGGACGT

GCCTACGCACCCCCACGAAGCCGTAGCCGTGATGACCTCTACGACCCAGATGACTCGAGG

GACTTTCCACATTCGCGGGATCCTCACTACGATGACTTCAGGTCTAGGGACCGCCCTCAG

GTGGACCCCAGATCCCGCCAGCACCGATCCCGAGACTCCCGGGATGAAGGCTCTAGGTCT

GGGGACCCGCATTATGATGAGCGTCTACTAGAAGAAGCCTTAAGGAAAAAGGGGCCAGGG

GAGAGGAGGAAACATTACAGGGAGGAAGAGGAAGAAGAGGAGGCCTACTACCCTCCACCG

CCTCCCCCGTACTCGGAGACGGACTCCCAGGCCTCACGGGAGCGGAGGCTAAAGAAGAAC

TTGGCCCTGAGTCGGGAAAGTTTAGTCGTC

>CL137.Contig6_A-W 573 1022 PREDICTED: lipolysis-stimulated lipoprotein receptor-like [Ailuropoda melanoleuca]

GGTTTTTCCCGGCTCCCGGCTCAGGCCTCCTCTGGTCTCCCTGCAGACTGGCTCTTCGTG

GTCGTGGTCTGCCTGGCTGCCTTCCTTGTCTTTCTCCTCCTGGGCATTTGCTGGTGTCAG

TGCTGCCCACATACCTGCTGCTGTTACGTCAGGTGCCCCTGCTGCCCGGAGAAGTGCTGC

TGTCCCGAGGCCCGTAAGTGCTCCTCATGCCACCTTCATGCAGCCTCCCTGCTGGGCAGT

GGCTGCCTACCCAGGACAGTGCCCTGATCTGGAGGTTAGGAGGTTAGCACTGAGCTGATG

TAGGGTTTTTTCCTAGGTGGGGCTCAGCACTTATTCTTCTGCTTTTGAACCAAATCAGAG

TAGGGCGTCTCCCTGGGCCACCAATCTCCTTCAGTTGGCCACAGCTCCCAGGTACCCCTG

CTTCGGGACACAGACAGCAACATGACCTCT

>CL137.Contig7_A-W 931 2250 PREDICTED: lipolysis-stimulated lipoprotein receptor [Canis lupus familiaris]

GGTTTTTCCCAGCTCCCAGCTCAGGCCTCCTCTGGTCTCCCTGCAGACTGGCTCTTCGTG

GTCGTGGTCTGCCTGGCTGCCTTCCTTGTCTTTCTCCTCCTGGGCATTTGCTGGTGTCAG

TGCTGCCCACATACCTGCTGCTGTTACGTCAGGTGCCCCTGCTGCCCGGAGAAGTGCTGC

TGTCCCGAGGCCCTGTATGCAGCTGGCAAAGCAGCCACATCAGGCGTCCCAAGCATCTAC

GCCCCAAGCACCTACGCCCACCTCTCCCCGACCAAGACCCCACCTCCACCGCCAGCCATG

ATTCCCATGGGCCCTGTCTACAATGGATACCCTGGAGGGTACGCTGGAGAATTTGACAGG

AATAGTTCAGTTGGCCACAGCTCCCAGGTACCCCTGCTTCGGGACACAGACAGCAACATG

ACCTCTGAAGTCCGCAGTGGGTACAGGATCCAAGCCAGCCAGCAGGATGACTCCATGAGA

GTCCTGTACTACATGGAGAAAGAGCTGGCCAACTTCGACCCCTCTCGACCGGGACCTGGC

CCCCCCAATGGTCGTTTAGAACGGGCTATGAGTGAAGTCACCTCCCTCCATGAAGACGAC

TGGAGATCCCGGCCTTCCCGGGGTCCTGCCCTCACCCCAATCCGGGATGAGGAGTGGGGT

CGCCACTCCCCCCGGAGTCCCAGGAGATGGGAGCAGGAGCCCCCCACAGAGCGGCCAGGG

GGTGGCTGGGGGGGCAGGCGGCCCCGGGCCCACTCTGTGGATGCTCTGGATGATCTAACC

CGGCCGGGTTCTGCTGAGTCTGGGAGGAGGTCTCCCCCAAGTAGTGGGAGGAGAGGACGT

GCCTACGCACCCCCACGAAGCCGTAGCCGTGATGACCTCTACGACCCAGATGACTCGAGG

GACTTTCCACATTCGCGGGATCCTCACTACGATGACTTCAGGTCTAGGGACCGCCCTCAG

GTGGACCCCAGATCCCGCCAGCACCGATCCCGAGACTCCCGGGATGAAGGCTCTAGGTCT

GGGGACCCGCATTATGATGAGCGTCTACTAGAAGAAGCCTTAAGGAAAAAGGGGCCAGGG

GAGAGGAGGAAACATTACAGGGAGGAAGAGGAAGAAGAGGAGGCCTACTACCCTCCACCG

CCTCCCCCGTACTCGGAGACGGACTCCCAGGCCTCACGGGAGCGGAGGCTAAAGAAGGTG

AGCGAATGGTCCTCCCCGAGGTTAGACCCTGCCCAGGCCCCTCTTCTCAGTCCCTGCCCA

GTTTACACTCTTCTTTCTCCTTTGCAGAACTTGGCCCTGAGTCGGGAAAGTTTAGTCGTC

>CL137.Contig8_A-W 573 1136 PREDICTED: LOW QUALITY PROTEIN: lipolysis-stimulated lipoprotein receptor-like [Cavia porcellus]

GGTTTTTCCCGGCTCCCGGCTCAGGCCTCCTCTGGTCTCCCTGCAGACTGGCTCTTCGTG

GTCGTGGTCTGCCTGGCTGCCTTCCTTGTCTTTCTCCTCCTGGGCATTTGCTGGTGTCAG

TGCTGCCCACATACCTGCTGCTGTTACGTCAGGTGCCCCTGCTGCCCGGAGAAGTGCTGC

TGTCCCGAGGCCCTGTATGCAGCTGGCAAAGCAGCCACATCAGGCGTCCCAAGCATCTAC

GCCCCAAGCACCTACGCCCACCTCTCCCCGACCAAGACCCCACCTCCACCGCCAGCCATG

ATTCCCATGGGCCCTGTCTACAATGGATACCCTGGAGGGTACGCTGGAGAATTTGACAGG

AATAGTTCAGGTGAGGGCTGACCTAAGAGGAGCTGGACTGGGCACTGGACACAAAGGGGG

CTGGGGCTGGGAGGAAGAGTGGGTGGGAGGTACTGGGAATGCAAGGCTGGGGGGGTGGGG

GGAGCCACTGACCACCAATCTCCTTCAGTTGGCCACAGCTCCCAGGTACCCCTGCTTCGG

GACACAGACAGCAACATGACCTCT

>CL137.Contig9_A-W 573 764 PREDICTED: lipolysis-stimulated lipoprotein receptor isoform 2 [Papio anubis]

GGTTTTTCCCGGCTCCCGGCTCAGGCCTCCTCTGGTCTCCCTGCAGACTGGCTCTTCGTG

GTCGTGGTCTGCCTGGCTGCCTTCCTTGTCTTTCTCCTCCTGGGCATTTGCTGGTGTCAG

TGCTGCCCACATACCTGCTGCTGTTACGTCAGGTGCCCCTGCTGCCCGGAGAAGTGCTGC

TGTCCCGAGGCC

>CL137.Contig10_A-W 573 1022 PREDICTED: LOW QUALITY PROTEIN: lipolysis-stimulated lipoprotein receptor-like [Cavia porcellus]

GGTTTTTCCCGGCTCCCGGCTCAGGCCTCCTCTGGTCTCCCTGCAGACTGGCTCTTCGTG

GTCGTGGTCTGCCTGGCTGCCTTCCTTGTCTTTCTCCTCCTGGGCATTTGCTGGTGTCAG

TGCTGCCCACATACCTGCTGCTGTTACGTCAGGTGCCCCTGCTGCCCGGAGAAGTGCTGC

TGTCCCGAGGCCCGTAAGTGCTCCTCATGCCACCTTCATGCAGCCTCCCTGCTGGGCAGT

GGCTGCCTACCCAGGACAGTGCCCTGATCTGGAGGTTAGGAGGTTAGCACTGAGCTGATG

TAGGGTTTTTTCCTAGGTGGGGCTCAGCACTTATTCTTCTGCTTTTGAACCAAATCAGAG

TAGGGCGTCTCCCTGGGCCACCAATCTCCTTCAGTTGGCCACAGCTCCCAGGTACCCCTG

CTTCGGGACACAGACAGCAACATGACCTCT

>CL137.Contig11_A-W 573 764 PREDICTED: lipolysis-stimulated lipoprotein receptor isoform 2 [Papio anubis]

GGTTTTTCCCGGCTCCCGGCTCAGGCCTCCTCTGGTCTCCCTGCAGACTGGCTCTTCGTG

GTCGTGGTCTGCCTGGCTGCCTTCCTTGTCTTTCTCCTCCTGGGCATTTGCTGGTGTCAG

TGCTGCCCACATACCTGCTGCTGTTACGTCAGGTGCCCCTGCTGCCCGGAGAAGTGCTGC

TGTCCCGAGGCC

>CL140.Contig1_A-W 345 602 DNA-directed RNA polymerase I subunit RPA43 [Heterocephalus glaber]

AAGAAAAAGAAAAAGAAAAAGAAAGATCCAGAAATTTATGAAGTAAATGATGGTGTCACA

GAGCCAGCAGATTTTACAGGTATCACTCCAAAGGAAGAGGAAGAGCTACAGGTTAGTAAT

GACATGAATGGCCTCTGTGAGGAAGAGCCAAAGAAAAAGAAGAAGAAGAAGAAGCACCAG

GAAGATCAGGATCCTATTTTTCAGTGCAGTGACTCCAGTGGTTACCAAAGTGACCATAAA

AAGAAAAAAAAGAAAAGA

>CL140.Contig2_A-W 72 476 DNA-directed RNA polymerase I subunit RPA43 [Heterocephalus glaber]

ATTTATAGTTTGCAGTCCAGGCACCCTAATGTTTCTGAAGAAGTAACAGAAACTCTAACT

GAAGAATCTGTTGAAAAAATTCCAAAGAAAAAGAAAAAGAAAAAGAAAGATCCAGAAATT

TATGAAGTAAATGATGGTGTCACAGAGCCAGCAGATTTTACAGGTATCACTCCAAAGGAA

GAGGAAGAGCTACAGGTTAGTAATGACATGAATGGCCTCTGTGAGGAAGAGCCAAAGAAA

AAGAAGAAGAAGAAGAAGCACCAGGAAGATCAGGATCCTATTTTTCAGTGCAGTGACTCC

AGTGGTTACCAAAGTGACCATAAAAAGAAAAAAAAGAAAAGAAAACATAGTGAAGAGGCT

GAACTTACACCAGTTCTGGAATGCTCACCTAAGAAGAAACAAAAA

>CL140.Contig4_A-W 345 665 DNA-directed RNA polymerase I subunit RPA43 [Heterocephalus glaber]

AAGAAAAAGAAAAAGAAAAAGAAAGATCCAGAAATTTATGAAGTAAATGATGGTGTCACA

GAGCCAGCAGATTTTACAGGTATCACTCCAAAGGAAGAGGAAGAGCTACAGGTTAGTAAT

GACATGAATGGCCTCTGTGAGGAAGAGCCAAAGAAAAAGAAGAAGAAGAAGAAGCACCAG

GAAGATCAGGATCCTATTTTTCAGTGCAGTGACTCCAGTGGTTACCAAAGTGACCATAAA

AAGAAAAAAAAGAAAAGAAAACATAGTGAAGAGGCTGAACTTACACCAGTTCTGGAATGC

TCACCTAAGAAGAAACAAAAA

>CL141.Contig1_A-W 87 1919 KIAA0010 [Homo sapiens]

GCGGCGAAGATGTTCAGCTTCGAAGGGGACTTCAAAACGAGGCCCAAGGTGTCGCTCGGC

GGCGCGAGCAGGAAGGAAGAAAAGGCTTCTCTTTTACATCGCACTCAGGAAGAAAGAAGG

AAAAGAGAGGAAGAAAGGCGAAGATTAAAAAATGCAATAATTATTCAGTCATTTATTCGA

GGCTACAGAGACAGAAAACAGCAATATTCCCTCCAGAGAAGTGCATTCGATCGCTGCGCA

CACTCGGCACAGTCTGGTGGCACTTTCTCCATCACTAGTGCCCCCAATCTTACCCTCCTG

GTAAGGCAGCTTCTGTTTTTTTACAAACAAAGCGAAGATGCAAAACGATTGATATGGCTG

TATCAGAACTTAATTAAACACAGCTCCCTGTTTGTCAAGCAACTGGACGGATCTGAGAGA

CCGACATGCTTATTTCAGATAAAGAGGCTCATGAGCCTCTGTTGCAGGTTGCTGCAGAGC

TGTAATGATGACAGTTTGAATGTGGCACTTCCAATGCGAATGCTTGAGGTCTTTTCCTCT

GAGAATACTTACTTGCCTGTTTTACAAGATGCTAGCTATGTGGTGTCCGTAATTGAACAG

ATCTTGCACTACATGATCCACAGTGGGTATTATAGGTCTCTCTATTTGTTGATCAACAGC

AAGCTTCCATCAAGTATTGAATATTCTGATTTATCTCGAGTTCCTATAGCAAAAATTTTG

CTGGAGAATGTCCTAAAACCGTTGCACTTTACCTACAGCTCCTGCCCAGAAGGTGCAAGG

CAGCAAGTTTTCTCAGCCTTCACAGAGGAGTTTCTGGCAGCACCTTTTACAGATCAGATT

TTCCGCTTCATCATCCCGGCCTTAGCAGATGTACAGACCGCTTTCCCTTACGAGCCCTTT

CTGAATGCACTGTTGTTACTAGAGAGTAGATGTTCAAAAAGGAGCCGTGGAGCACCGTGG

CTTTTCTATTTTGTTTTAACTGTTGGCGAAAATTACTTGGGGGTGCTCTCTGAGGAGGGC

CTGCTGGTGTATCTGCACGTGCTCCAGACTTTCCTCTCCCAGTTACCTGCCTCCCCTGCC

GGCGCCAGCTCCCAGGACTCCACCAGCGACTCTGAGGACGAGAGCGAGGAGGCCGACAAG

CCCGCCAGCCCCGAGGACGGCAGAGTCTCTGCCCCATACATCACCGAGGAATGTCTAAAG

AAGCTGGACACGAAGCAGCAGACCAGTGCCCTGCTGAACCTGGTGTGGAGGGACTCGGCC

AGCGAGGAGGTCTTCACCCGGATGGCCTCCATCTGCCACACGCTGATGGTGCAGCACCGC

ATGATGGTGCCCAAAGTCAGGCTTCTCTACAGTCTGGCCTTCAATGCCAGGTTTCTGCGG

CACCTTTGGGTCCTCATCTCTTCCATGACAACACGGATGATCACAGGGTCGATGGTGCCG

CTGCTCCAGGTGATATCCAGGGGCTCTCCCATGTCCTTCGAGGACTCGGGCCGCATCATC

CCGCTCTTTTACCTCTTCAGCTCCCTCTTCAGCCACTCACTGATTTCCACCCACGATAAT

GAGTTCTTTGGCGACCCCATAGAAGTTGTAGGGCAGAGGCGGTCGTCCATGATGCCCTTC

ACGCTGGAGGAGCTGGTCGCCCTGTCCCGCTGCCTGCGCGACGCTTGCCTGGGCATCATC

AAGCTGGCCTACCCGGAAACAAAGCCCGAGGTGCGGGAGGAGTACGTCACCGCCTTTCAG

AGCATAGGCGTCACCACCAGCGCTGAGATGCAGCAATGTATACAGATGGAGCAGAAGCGT

TGGATTCAGTTGTTTAAGGTTTTAATTAAACTC

>CL141.Contig2_A-W 87 3290 KIAA0010 [Homo sapiens]

GCGGCGAAGATGTTCAGCTTCGAAGGGGACTTCAAAACGAGGCCCAAGGTGTCGCTCGGC

GGCGCGAGCAGGAAGGAAGAAAGGCGAAGATTAAAAAATGCAATAATTATTCAGTCATTT

ATTCGAGGCTACAGAGACAGAAAACAGCAATATTCCCTCCAGAGAAGTGCATTCGATCGC

TGCGCACACTCGGCACAGTCTGGTGGCACTTTCTCCATCACTAGTGCCCCCAATCTTACC

CTCCTGGTAAGGCAGCTTCTGTTTTTTTACAAACAAAGCGAAGATGCAAAACGATTGATA

TGGCTGTATCAGAACTTAATTAAACACAGCTCCCTGTTTGTCAAGCAACTGGACGGATCT

GAGAGACCGACATGCTTATTTCAGATAAAGAGGCTCATGAGCCTCTGTTGCAGGTTGCTG

CAGAGCTGTAATGATGACAGTTTGAATGTGGCACTTCCAATGCGAATGCTTGAGGTCTTT

TCCTCTGAGAATACTTACTTGCCTGTTTTACAAGATGCTAGCTATGTGGTGTCCGTAATT

GAACAGATCTTGCACTACATGATCCACAGTGGGTATTATAGGTCTCTCTATTTGTTGATC

AACAGCAAGCTTCCATCAAGTATTGAATATTCTGATTTATCTCGAGTTCCTATAGCAAAA

ATTTTGCTGGAGAATGTCCTAAAACCGTTGCACTTTACCTACAGCTCCTGCCCAGAAGGT

GCAAGGCAGCAAGTTTTCTCAGCCTTCACAGAGGAGTTTCTGGCAGCACCTTTTACAGAT

CAGATTTTCCGCTTCATCATCCCGGCCTTAGCAGATGTACAGACCGCTTTCCCTTACGAG

CCCTTTCTGAATGCACTGTTGTTACTAGAGAGTAGATGTTCAAAAAGGAGCCGTGGAGCA

CCGTGGCTTTTCTATTTTGTTTTAACTGTTGGCGAAAATTACTTGGGGGTGCTCTCTGAG

GAGGGCCTGCTGGTGTATCTGCACGTGCTCCAGACTTTCCTCTCCCAGTTACCTGCCTCC

CCTGCCGGCGCCAGCTCCCAGGACTCCACCAGCGACTCTGAGGACGAGAGCGAGGAGGCC

GACAAGCCCGCCAGCCCCGAGGACGGCAGAGTCTCTGCCCCATACATCACCGAGGAATGT

CTAAAGAAGCTGGACACGAAGCAGCAGACCAGTGCCCTGCTGAACCTGGTGTGGAGGGAC

TCGGCCAGCGAGGAGGTCTTCACCCGGATGGCCTCCATCTGCCACACGCTGATGGTGCAG

CACCGCATGATGGTGCCCAAAGTCAGGCTTCTCTACAGTCTGGCCTTCAATGCCAGGTTT

CTGCGGCACCTTTGGGTCCTCATCTCTTCCATGACAACACGGATGATCACAGGGTCGATG

GTGCCGCTGCTCCAGGTGATATCCAGGGGCTCTCCCATGTCCTTCGAGGACTCGGGCCGC

ATCATCCCGCTCTTTTACCTCTTCAGCTCCCTCTTCAGCCACTCACTGATTTCCACCCAC

GATAATGAGTTCTTTGGCGACCCCATAGAAGTTGTAGGGCAGAGGCGGTCGTCCATGATG

CCCTTCACGCTGGAGGAGCTGGTCGCCCTGTCCCGCTGCCTGCGCGACGCTTGCCTGGGC

ATCATCAAGCTGGCCTACCCGGAAACAAAGCCCGAGGTGCGGGAGGAGTACGTCACCGCC

TTTCAGAGCATAGGCGTCACCACCAGCGCTGAGATGCAGCAATGTATACAGATGGAGCAG

AAGCGTTGGATTCAGTTGTTTAAGGTCATCACCAACCTAGTGAAAATGCTGAAGTCCAGA

GACACCAGGAGAAACTTTTGTCCTCCAAATCACTGGCTGTCAGAACAAGAAGATATTAAA

GCAGATAAGGTCACCCAGCTCTACGTGCCCGCATCCAGACATGTGTGGAGGTTCCGTCGG

ATGGGGAGGATCGGCCCGCTGCAGTCCACCCTGGAGGTGGGCCTGGAGTCCCCACCGCTG

ACTGTGTCTGAGGAGCGGCAGCTGGCTGTCCTGACGGAGCTGCCCTTTGTGGTCCCCTTT

GAGGAGCGAGTGAAGATCTTTCAAAGGTTGATTTATGCAGATAAGCAAGAAGTTCAAGGA

GATGGCCCATTTCTGGATGGAATTAATGTCACAATAAGAAGAAACTATATTTACGAGGAT

GCTTATGACAAACTTTCCCCAGAAAATGAGCCTGATTTGAAAAAGCGGATCCGTGTGCAC

TTGCTCAACGCCCATGGCCTGGATGAAGCTGGCATCGATGGTGGTGGTATTTTCAGGGAG

TTTTTAAATGAACTACTAAAATCAGGATTTAACCCCAACCAGGGGTTCTTTAAGACTACT

AATGAAGGGCTTCTGTACCCCAACCCGGCTGCTCAGATGCTCGTGGGAGATTCTTTTGCC

AGACATTACTACTTCCTAGGCAGGATGCTTGGAAAGGCTCTCTATGAAAACATGCTGGTG

GAGCTGCCCTTTGCCGGCTTCTTCCTCTCCAAGCTGCTGGGCACCAGTGCCGATGTGGAC

ATCCACCATCTGGCCTCCTTGGACCCCGAGGTCTACAGGAACCTGCTGTTCCTCAAGAGC

TACGAGGAGGATGTGGAGGAGCTGGGGCTGAACTTCACCGTGGTGAACAACGACCTGGGA

GAGGCCCAGGTGGTTGAACTCAAGCTTGGTGGGAAGGATATCCCCGTCACCAGTGCCAAC

CGCATCGCGTACATCCACCTGGTGGCAGACTACAGGCTGAACAAGCAGATCCGCCCACAC

TGCCTGGCTTTCCGGCAGGGCCTGGCTAACGTCGTCAGCCTGGAGTGGCTCCGAATGTTC

GACCAGCAGGAAATTCAGGTATTAATTTCTGGTGCACAAGTTCCCATAAGTCTGGAGGAC

CTAAAATCCTTTACGAACTATTCAGGAGGCTATTCTGCCGATCACCCTGTCATTAAAATC

TTCTGGAGAGTCGTAGAAGGTTTCACTGATGAAGAGAAGCGCAAGCTGCTCAAGTTTGTG

ACGAGCTGTTCCCGACCCCCTCTCCTGGGGTTCAAGGAGCTGTACCCGGCCTTTTGCATT

CACAACGGAGGCTCTGACCTCGAGCGGCTCCCCACAGCCAGCACCTGCATGAACCTACTG

AAGCTCCCAGAGTTTTACGATGAGACCCTTCTGCGAAGCAAGCTGCTCTATGCCATCGAG

TGTGCTGCTGGCTTTGAGCTGAGC

>CL141.Contig3_A-W 1904 2920 KIAA0010 [Homo sapiens]

GAGCCTGATTTGAAAAAGCGGATCCGTGTGCACTTGCTCAACGCCCATGGCCTGGATGAA

GCTGGCATCGATGGTGGTGGTATTTTCAGGGAGTTTTTAAATGAACTACTAAAATCAGGA

TTTAACCCCAACCAGGGGTTCTTTAAGACTACTAATGAAGGGCTTCTGTACCCCAACCCG

GCTGCTCAGATGCTCGTGGGAGATTCTTTTGCCAGACATTACTACTTCCTAGGCAGGATG

CTTGGAAAGGCTCTCTATGAAAACATGCTGGTGGAGCTGCCCTTTGCCGGCTTCTTCCTC

TCCAAGCTGCTGGGCACCAGTGCCGATGTGGACATCCACCATCTGGCCTCCTTGGACCCC

GAGGTCTACAGGAACCTGCTGTTCCTCAAGAGCTACGAGGAGGATGTGGAGGAGCTGGGG

CTGAACTTCACCGTGGTGAACAACGACCTGGGAGAGGCCCAGGTGGTTGAACTCAAGCTT

GGTGGGAAGGATATCCCCGTCACCAGTGCCAACCGCATCGCGTACATCCACCTGGTGGCA

GACTACAGGCTGAACAAGCAGATCCGCCCACACTGCCTGGCTTTCCGGCAGGGCCTGGCT

AACGTCGTCAGCCTGGAGTGGCTCCGAATGTTCGACCAGCAGGAAATTCAGGTATTAATT

TCTGGTGCACAAGTTCCCATAAGTCTGGAGGACCTAAAATCCTTTACGAACTATTCAGGA

GGCTATTCTGCCGATCACCCTGTCATTAAAATCTTCTGGAGAGTCGTAGAAGGTTTCACT

GATGAAGAGAAGCGCAAGCTGCTCAAGTTTGTGACGAGCTGTTCCCGACCCCCTCTCCTG

GGGTTCAAGGAGCTGTACCCGGCCTTTTGCATTCACAACGGAGGCTCTGACCTCGAGCGG

CTCCCCACAGCCAGCACCTGCATGAACCTACTGAAGCTCCCAGAGTTTTACGATGAGACC

CTTCTGCGAAGCAAGCTGCTCTATGCCATCGAGTGTGCTGCTGGCTTTGAGCTGAGC

>CL141.Contig4_A-W 87 1865 KIAA0010 [Homo sapiens]

GCGGCGAAGATGTTCAGCTTCGAAGGGGACTTCAAAACGAGGCCCAAGGTGTCGCTCGGC

GGCGCGAGCAGGAAGGAAGAAAGGCGAAGATTAAAAAATGCAATAATTATTCAGTCATTT

ATTCGAGGCTACAGAGACAGAAAACAGCAATATTCCCTCCAGAGAAGTGCATTCGATCGC

TGCGCACACTCGGCACAGTCTGGTGGCACTTTCTCCATCACTAGTGCCCCCAATCTTACC

CTCCTGGTAAGGCAGCTTCTGTTTTTTTACAAACAAAGCGAAGATGCAAAACGATTGATA

TGGCTGTATCAGAACTTAATTAAACACAGCTCCCTGTTTGTCAAGCAACTGGACGGATCT

GAGAGACCGACATGCTTATTTCAGATAAAGAGGCTCATGAGCCTCTGTTGCAGGTTGCTG

CAGAGCTGTAATGATGACAGTTTGAATGTGGCACTTCCAATGCGAATGCTTGAGGTCTTT

TCCTCTGAGAATACTTACTTGCCTGTTTTACAAGATGCTAGCTATGTGGTGTCCGTAATT

GAACAGATCTTGCACTACATGATCCACAGTGGGTATTATAGGTCTCTCTATTTGTTGATC

AACAGCAAGCTTCCATCAAGTATTGAATATTCTGATTTATCTCGAGTTCCTATAGCAAAA

ATTTTGCTGGAGAATGTCCTAAAACCGTTGCACTTTACCTACAGCTCCTGCCCAGAAGGT

GCAAGGCAGCAAGTTTTCTCAGCCTTCACAGAGGAGTTTCTGGCAGCACCTTTTACAGAT

CAGATTTTCCGCTTCATCATCCCGGCCTTAGCAGATGTACAGACCGCTTTCCCTTACGAG

CCCTTTCTGAATGCACTGTTGTTACTAGAGAGTAGATGTTCAAAAAGGAGCCGTGGAGCA

CCGTGGCTTTTCTATTTTGTTTTAACTGTTGGCGAAAATTACTTGGGGGTGCTCTCTGAG

GAGGGCCTGCTGGTGTATCTGCACGTGCTCCAGACTTTCCTCTCCCAGTTACCTGCCTCC

CCTGCCGGCGCCAGCTCCCAGGACTCCACCAGCGACTCTGAGGACGAGAGCGAGGAGGCC

GACAAGCCCGCCAGCCCCGAGGACGGCAGAGTCTCTGCCCCATACATCACCGAGGAATGT

CTAAAGAAGCTGGACACGAAGCAGCAGACCAGTGCCCTGCTGAACCTGGTGTGGAGGGAC

TCGGCCAGCGAGGAGGTCTTCACCCGGATGGCCTCCATCTGCCACACGCTGATGGTGCAG

CACCGCATGATGGTGCCCAAAGTCAGGCTTCTCTACAGTCTGGCCTTCAATGCCAGGTTT

CTGCGGCACCTTTGGGTCCTCATCTCTTCCATGACAACACGGATGATCACAGGGTCGATG

GTGCCGCTGCTCCAGGTGATATCCAGGGGCTCTCCCATGTCCTTCGAGGACTCGGGCCGC

ATCATCCCGCTCTTTTACCTCTTCAGCTCCCTCTTCAGCCACTCACTGATTTCCACCCAC

GATAATGAGTTCTTTGGCGACCCCATAGAAGTTGTAGGGCAGAGGCGGTCGTCCATGATG

CCCTTCACGCTGGAGGAGCTGGTCGCCCTGTCCCGCTGCCTGCGCGACGCTTGCCTGGGC

ATCATCAAGCTGGCCTACCCGGAAACAAAGCCCGAGGTGCGGGAGGAGTACGTCACCGCC

TTTCAGAGCATAGGCGTCACCACCAGCGCTGAGATGCAGCAATGTATACAGATGGAGCAG

AAGCGTTGGATTCAGTTGTTTAAGGTTTTAATTAAACTC

>CL141.Contig5_A-W 87 3344 KIAA0010 [Homo sapiens]

GCGGCGAAGATGTTCAGCTTCGAAGGGGACTTCAAAACGAGGCCCAAGGTGTCGCTCGGC

GGCGCGAGCAGGAAGGAAGAAAAGGCTTCTCTTTTACATCGCACTCAGGAAGAAAGAAGG

AAAAGAGAGGAAGAAAGGCGAAGATTAAAAAATGCAATAATTATTCAGTCATTTATTCGA

GGCTACAGAGACAGAAAACAGCAATATTCCCTCCAGAGAAGTGCATTCGATCGCTGCGCA

CACTCGGCACAGTCTGGTGGCACTTTCTCCATCACTAGTGCCCCCAATCTTACCCTCCTG

GTAAGGCAGCTTCTGTTTTTTTACAAACAAAGCGAAGATGCAAAACGATTGATATGGCTG

TATCAGAACTTAATTAAACACAGCTCCCTGTTTGTCAAGCAACTGGACGGATCTGAGAGA

CCGACATGCTTATTTCAGATAAAGAGGCTCATGAGCCTCTGTTGCAGGTTGCTGCAGAGC

TGTAATGATGACAGTTTGAATGTGGCACTTCCAATGCGAATGCTTGAGGTCTTTTCCTCT

GAGAATACTTACTTGCCTGTTTTACAAGATGCTAGCTATGTGGTGTCCGTAATTGAACAG

ATCTTGCACTACATGATCCACAGTGGGTATTATAGGTCTCTCTATTTGTTGATCAACAGC

AAGCTTCCATCAAGTATTGAATATTCTGATTTATCTCGAGTTCCTATAGCAAAAATTTTG

CTGGAGAATGTCCTAAAACCGTTGCACTTTACCTACAGCTCCTGCCCAGAAGGTGCAAGG

CAGCAAGTTTTCTCAGCCTTCACAGAGGAGTTTCTGGCAGCACCTTTTACAGATCAGATT

TTCCGCTTCATCATCCCGGCCTTAGCAGATGTACAGACCGCTTTCCCTTACGAGCCCTTT

CTGAATGCACTGTTGTTACTAGAGAGTAGATGTTCAAAAAGGAGCCGTGGAGCACCGTGG

CTTTTCTATTTTGTTTTAACTGTTGGCGAAAATTACTTGGGGGTGCTCTCTGAGGAGGGC

CTGCTGGTGTATCTGCACGTGCTCCAGACTTTCCTCTCCCAGTTACCTGCCTCCCCTGCC

GGCGCCAGCTCCCAGGACTCCACCAGCGACTCTGAGGACGAGAGCGAGGAGGCCGACAAG

CCCGCCAGCCCCGAGGACGGCAGAGTCTCTGCCCCATACATCACCGAGGAATGTCTAAAG

AAGCTGGACACGAAGCAGCAGACCAGTGCCCTGCTGAACCTGGTGTGGAGGGACTCGGCC

AGCGAGGAGGTCTTCACCCGGATGGCCTCCATCTGCCACACGCTGATGGTGCAGCACCGC

ATGATGGTGCCCAAAGTCAGGCTTCTCTACAGTCTGGCCTTCAATGCCAGGTTTCTGCGG

CACCTTTGGGTCCTCATCTCTTCCATGACAACACGGATGATCACAGGGTCGATGGTGCCG

CTGCTCCAGGTGATATCCAGGGGCTCTCCCATGTCCTTCGAGGACTCGGGCCGCATCATC

CCGCTCTTTTACCTCTTCAGCTCCCTCTTCAGCCACTCACTGATTTCCACCCACGATAAT

GAGTTCTTTGGCGACCCCATAGAAGTTGTAGGGCAGAGGCGGTCGTCCATGATGCCCTTC

ACGCTGGAGGAGCTGGTCGCCCTGTCCCGCTGCCTGCGCGACGCTTGCCTGGGCATCATC

AAGCTGGCCTACCCGGAAACAAAGCCCGAGGTGCGGGAGGAGTACGTCACCGCCTTTCAG

AGCATAGGCGTCACCACCAGCGCTGAGATGCAGCAATGTATACAGATGGAGCAGAAGCGT

TGGATTCAGTTGTTTAAGGTCATCACCAACCTAGTGAAAATGCTGAAGTCCAGAGACACC

AGGAGAAACTTTTGTCCTCCAAATCACTGGCTGTCAGAACAAGAAGATATTAAAGCAGAT

AAGGTCACCCAGCTCTACGTGCCCGCATCCAGACATGTGTGGAGGTTCCGTCGGATGGGG

AGGATCGGCCCGCTGCAGTCCACCCTGGAGGTGGGCCTGGAGTCCCCACCGCTGACTGTG

TCTGAGGAGCGGCAGCTGGCTGTCCTGACGGAGCTGCCCTTTGTGGTCCCCTTTGAGGAG

CGAGTGAAGATCTTTCAAAGGTTGATTTATGCAGATAAGCAAGAAGTTCAAGGAGATGGC

CCATTTCTGGATGGAATTAATGTCACAATAAGAAGAAACTATATTTACGAGGATGCTTAT

GACAAACTTTCCCCAGAAAATGAGCCTGATTTGAAAAAGCGGATCCGTGTGCACTTGCTC

AACGCCCATGGCCTGGATGAAGCTGGCATCGATGGTGGTGGTATTTTCAGGGAGTTTTTA

AATGAACTACTAAAATCAGGATTTAACCCCAACCAGGGGTTCTTTAAGACTACTAATGAA

GGGCTTCTGTACCCCAACCCGGCTGCTCAGATGCTCGTGGGAGATTCTTTTGCCAGACAT

TACTACTTCCTAGGCAGGATGCTTGGAAAGGCTCTCTATGAAAACATGCTGGTGGAGCTG

CCCTTTGCCGGCTTCTTCCTCTCCAAGCTGCTGGGCACCAGTGCCGATGTGGACATCCAC

CATCTGGCCTCCTTGGACCCCGAGGTCTACAGGAACCTGCTGTTCCTCAAGAGCTACGAG

GAGGATGTGGAGGAGCTGGGGCTGAACTTCACCGTGGTGAACAACGACCTGGGAGAGGCC

CAGGTGGTTGAACTCAAGCTTGGTGGGAAGGATATCCCCGTCACCAGTGCCAACCGCATC

GCGTACATCCACCTGGTGGCAGACTACAGGCTGAACAAGCAGATCCGCCCACACTGCCTG

GCTTTCCGGCAGGGCCTGGCTAACGTCGTCAGCCTGGAGTGGCTCCGAATGTTCGACCAG

CAGGAAATTCAGGTATTAATTTCTGGTGCACAAGTTCCCATAAGTCTGGAGGACCTAAAA

TCCTTTACGAACTATTCAGGAGGCTATTCTGCCGATCACCCTGTCATTAAAATCTTCTGG

AGAGTCGTAGAAGGTTTCACTGATGAAGAGAAGCGCAAGCTGCTCAAGTTTGTGACGAGC

TGTTCCCGACCCCCTCTCCTGGGGTTCAAGGAGCTGTACCCGGCCTTTTGCATTCACAAC

GGAGGCTCTGACCTCGAGCGGCTCCCCACAGCCAGCACCTGCATGAACCTACTGAAGCTC

CCAGAGTTTTACGATGAGACCCTTCTGCGAAGCAAGCTGCTCTATGCCATCGAGTGTGCT

GCTGGCTTTGAGCTGAGC

>CL141.Contig6_A-W 162 1694 PREDICTED: ubiquitin-protein ligase E3C, partial [Cricetulus griseus]

GAAGAAAAGGCTTCTCTTTTACATCGCACTCAGGAAGAAAGAAGGAAAAGAGAGGAAGAA

AGGCGAAGATTAAAAAATGCAATAATTATTCAGTCATTTATTCGAGGCTACAGAGACAGA

AAACAGCAATATTCCCTCCAGAGAAGTGCATTCGATCGCTGCGCACACTCGGCACAGTCT

GGTGGCACTTTCTCCATCACTAGTGCCCCCAATCTTACCCTCCTGGTAAGGCAGCTTCTG

TTTTTTTACAAACAAAGCGAAGATGCAAAACGATTGATATGGCTGTATCAGAACTTAATT

AAACACAGCTCCCTGTTTGTCAAGCAACTGGACGGATCTGAGAGACCGACATGCTTATTT

CAGATAAAGAGGCTCATGAGCCTCTGTTGCAGGTTGCTGCAGAGCTGTAATGATGACAGT

TTGAATGTGGCACTTCCAATGCGAATGCTTGAGGTCTTTTCCTCTGAGAATACTTACTTG

CCTGTTTTACAAGATGCTAGCTATGTGGTGTCCGTAATTGAACAGATCTTGCACTACATG

ATCCACAGTGGGTATTATAGGTCTCTCTATTTGTTGATCAACAGCAAGCTTCCATCAAGT

ATTGAATATTCTGATTTATCTCGAGTTCCTATAGCAAAAATTTTGCTGGAGAATGTCCTA

AAACCGTTGCACTTTACCTACAGCTCCTGCCCAGAAGGTGCAAGGCAGCAAGTTTTCTCA

GCCTTCACAGAGGAGTTTCTGGCAGCACCTTTTACAGATCAGATTTTCCGCTTCATCATC

CCGGCCTTAGCAGATGTACAGACCGCTTTCCCTTACGAGCCCTTTCTGAATGCACTGTTG

TTACTAGAGAGTAGATGTTCAAAAAGGAGCCGTGGAGCACCGTGGCTTTTCTATTTTGTT

TTAACTGTTGGCGAAAATTACTTGGGGGTGCTCTCTGAGGAGGGCCTGCTGGTGTATCTG

CACGTGCTCCAGACTTTCCTCTCCCAGTTACCTGCCTCCCCTGCCGGCGCCAGCTCCCAG

GACTCCACCAGCGACTCTGAGGACGAGAGCGAGGAGGCCGACAAGCCCGCCAGCCCCGAG

GACGGCAGAGTCTCTGCCCCATACATCACCGAGGAATGTCTAAAGAAGCTGGACACGAAG

CAGCAGACCAGTGCCCTGCTGAACCTGGTGTGGAGGGACTCGGCCAGCGAGGAGGTCTTC

ACCCGGATGGCCTCCATCTGCCACACGCTGATGGTGCAGCACCGCATGATGGTGCCCAAA

GTCAGGCTTCTCTACAGTCTGGCCTTCAATGCCAGGTTTCTGCGGCACCTTTGGGTCCTC

ATCTCTTCCATGACAACACGGATGATCACAGGGTCGATGGTGCCGCTGCTCCAGGTGATA

TCCAGGGGCTCTCCCATGTCCTTCGAGGACTCGGGCCGCATCATCCCGCTCTTTTACCTC

TTCAGCTCCCTCTTCAGCCACTCACTGATTTCCACCCACGATAATGAGTTCTTTGGCGAC

CCCATAGAAGGTGAGCGTTCTGAGGAGCTCCTT

>CL141.Contig7_A-W 87 500 KIAA0010 [Homo sapiens]

GCGGCGAAGATGTTCAGCTTCGAAGGGGACTTCAAAACGAGGCCCAAGGTGTCGCTCGGC

GGCGCGAGCAGGAAGGAAGAAAGGCGAAGATTAAAAAATGCAATAATTATTCAGTCATTT

ATTCGAGGCTACAGAGACAGAAAACAGCAATATTCCCTCCAGAGAAGTGCATTCGATCGC

TGCGCACACTCGGCACAGTCTGGTGGCACTTTCTCCATCACTAGTGCCCCCAATCTTACC

CTCCTGGTAAGGCAGCTTCTGTTTTTTTACAAACAAAGCGAAGATGCAAAACGATTGATA

TGGCTGTATCAGAACTTAATTAAACACAGCTCCCTGTTTGTCAAGCAACTGGACGGATCT

GAGAGACCGACATGCTTATTTCAGATAAAGAGGCTCATGAGCCTCTGTTGCAGG

>CL142.Contig1_A-W 2 253 minus strand Ahnak protein [Mus musculus]

CTTAACCTGAAAGGCCCCAAAGTGAAGGGCGATGTGGACGTGTCTCTGCCCAAAGTGGAA

GGTGAGATTAAAGTTCCTGAAGTGGACATCAAAGGCCCCAAAGTGGACGTTGATGTTCCA

GATGTGGATGTTCATGGCCCAGACTGGCACTTGAAGATGCCCAAAATAAAAATGCCCAAG

TTCAGCATGCCAGGCTTCAAGGGAGAAGGACCAGAGGTTGATGTGAACTTGCCCAAAGCT

GACATTGATGTC

>CL142.Contig2_A-W 629 3457 minus strand PREDICTED: neuroblast differentiation-associated protein AHNAK [Pan paniscus]

GATATGGACTTCAAGGGGCCCAAAGTTGATTTGGAAGCTCCAAGCTTAGATGTACACATG

GAAAGCCCAGATATTAACATTGAGGGGCCAGATGTTAAGATTCCTAAGTTTAAGAAACCC

AAGTTTGGATTTGGGGCAAAAAGTCCAAAAGCTGACATCAAGTCACCTTCACTCGATGTG

ACTCTTCCTGAGGCAGAACTGAATGTTGAAGCTCCTGAAATTAGTGTTGGTGGCAAGGGC

AAGAAAAGTAAATTTAAAATGCCTAAAATTCACATGAGTGGTCCTAAAGTTAAGGCCAAA

AAGCAGGGATTTGATTTGAATGTTCCTGGGGCTGAGATCGATGCCAGTCTCAAAGCTCCT

GATGTAGATGTCAATGTCACGGGGCCAGATGCTGCACTCAAAGTCGATGTAAAATCTCCC

AAAACCAAGAAGACTATGTTTGGAAAAATGTACTTCCCAGATGTAGAGTTTGACATCAAA

TCACCTAAATTTAAAACTGAGGCCCCGCTCCCTAGCCCCAAGCTGGAGGGTGAAATCCAG

GCACCTGATCTAAATATTTCTTCACCAGGAATTAATGTGGAAGGTCCCAAGTTCAAGATG

CCAGGTGTGGATGTGTCAGGGCCAAAAATAGAGGGCGACTTGAAAGGCCCCAGGGTACAG

GCCAACTTAGGCACACCTGATATCAACATCGAAGGCCCAGATGCTAAAATCAAAGCACCA

TCTTTTGGCATTTCTGCCCCTCAAGTCTCCATGCCTGATGTGAATGTTAACTTGAAAGGA

CCAAAGATAAAGGGTGATGTCCCCAGTGTGGGACTGGAAGGACCAGATGTAGATCTGCAT

GGTCCAGAAGCAAAAATCAAGTTCCCCCACTTTTCGATGCCCAAGATCAGCGTCCCTGGT

GTGAAAATGGAGGGTGGAGCAGCCGAGCTCAATGCCCAGCTGCCCTCTCTTGAAGGAGGC

TTGAGCACACCAGATGTTAAGCTTGAAGGGCCTGATGTCTCTCTGAAGGGGCCAGGAATA

GACTTGCCTTCAGTCAACCTCTCTATGCCAAAAGTCTCTGGGCCTGATCTTGGCCTGAAC

TTGAAAGGACCAAGTTTGAAGGGAGACCTGGATGCATCGGCTCCAAGCATAAAGGTGCAG

GCCCCAGGGCTCGACCTCAGAGGTGTCGGTGGAAAGGTGCAGGTGGGAGGAGATGGTATG

AAAGTGCCGGGGATCAACGCCACAACGCTTAATGTCGGGGCACCAGACGTGACACTGAAG

GGACCATGCCTCCAGGGAGATCTGGCTGTCTCTGGTGACATCAAGGGCCCTAAAATATCC

GTAGGTGCTCCTGATCTAAGCTTGGAGGCACCCGAAGGTGGTATTAAACTTCCCCAAATG

AAGCTGCCCCAGTTTGACATCTCCACTCCAGGGTCTGACTTGGACATTAACATCAAGGGG

CCACAAGTTGGTGGAGAGCTAAAGGGGCCAGGCCTGGATGTGAACCTGAAAGGGCCTCAG

ATCTCAGCACCAGATGTGGACTTGAACTTGGAAGGACCAAAAGTGAAAGGGAGCCTCGGG

GGCATTGGTGAGATGAAAGGCCCCACAGTCGCAGGAAGTCTGCCGGGTGTCAGTGTTCAG

GGCCTGGACGGCAATCTCCACATGCCTGGAATCAAGTCCCCTGGATGTGCTATGGACCTG

CCAGGTGTGAATGTCAAACTCCCAACTGGGCAGATTTCGGGTCCTGAAATCAAAGGTGAT

CTGAAAGGTTCAGGAATGGGTTTCCATGGAGCTGTTCCTGACATCAATGTAAAAGGGCCT

TCCTTTAATGTGGCATCTCCTGAGTCAGATTTTGGTGTCAGCTTGAAGGGCCCAAAAATC

AAAGGCGGTGTGGATGTTTCAGGTGGTATCAGTGCCCCAGATATCAGCCTGGGCGAAGGG

CATATGAGTGTTAAAGGATCTGGGGTGGAGTGGAAGGGACCCCAGGCCTCCTCTGCTCTC

AACCTGGATGCATCTAAACTTGCTGGGGGCCTTCATTTCTCAGGACCAAAGATAGAAGGA

GGAGTGAAAGGAGGTCAGATTGGACTCCAGGGTCCTGGGCTGAGTGTGTCTGGGCCTCAA

GGTCACTTGGAAAGTGGATCTGGAAAAGTAACATTCCCCAAGATGAAGATCCCCAAATTT

ACCTTCTCTGGCCGTGAGCTGGTTGGCAGAGAAGTGGGGGTGGATATCAACTTCCCGAAA

GTGGAGGGCAATGTGCAGGCCTGTGCTGGAGAAGGCGAGTGGGAAGAATCCGAAGTAAAA

CTTAAAAAGTCCAAAATCAAAATGCCCAAGTTTAATTTTTCCAAACCTAAAGGGAAAGGT

GGCGTCACGGGTTCACCAGAAGCATCCATCTCTGGGTCCAAAGGTGACCTGAAAAGTTCC

AAGGCCAGCCTGGGCTCCCTGGAAGGAGAAGCAGAGGCCGAGGCATCTTCACCCAAAGGC

AAATTCTCCCTATTTAAAAGTAAGAAGCCACGGCACCGCTCCAATTCTTTCAGTGATGAA

AGGGAGTTCTCCGCCCCTTCCACCCCCACGGGGACTTTGGAGTTTGAAGGTGGAGAAATG

TCCCTGGAAGGTGGGAAAGTTAAAGGGAAGCATGGGAAGCTGAAGTTTGGTACCTTTGGT

GGAATAGGGTCAAAGAGCAAAGGTCATTACGAGGTGACCGGGAGCGATGATGAGGCAGGC

AAGTTACAGGGGAGTGGAGTGTCCCTGGCCTCTAAGAAGTCCCGACTGTCCTCTTCTTCT

AGCAACGACAGTGGTACTAAGGTCGGCATCCAGCTTCCTGAGGTTGAGCTGGCGGTTTCC

ACAAAGAAA

>CL142.Contig3_A-W 26 364 minus strand PREDICTED: neuroblast differentiation-associated protein AHNAK [Papio anubis]

AAAGGTGATATGGATATTTCTCCACCCAAACTTGAAGGGGATCTGAAAGGGCCAGAAGTT

GATATCAAAGGCCCTAAAGTGGACATCAGTGCCCCAGAAGTGGATGTTCATGGTCCAGAC

TGGAACCTGAAGATGCCCAAGATGAAAATGCCCAAATTTGGTGTTCCTGGCTTAAAAGCA

GAAGGTCCAGAAATGGCTGTGGATCTACCAAAAGGAGACATCAACATAGAAGGTCCCACT

CTGAATGTTGAGGGCCCAGAACTCAATGTGGAAGGTCCAGAAGGAAGCATAAAAGGTCCC

AAATTCAAGATGCCTGACATGAACATAAAAGCTCCTAAG

>CL143.Contig1_A-W 62 625 minus strand PREDICTED: 1-phosphatidylinositol-4,5-bisphosphate phosphodiesterase beta-3-like [Ailuropoda melanoleuca]

GAGGCCCAGGCGGACATAGAGGCTGAGCGGAGGCTAGAGCACCTGAGACAGGCTCAGCAG

CGGCTCAGGGAGATCGTCCTGGATGTGCACACAACTCAGTTCAAAAGGCTGAAGGAGATG

AACGAGAGGGAGAAGAAGGAGCTGCAGAAGATCCTGGACAGGAAGCGCCACAACAGCATC

TCTGAGGCCAAGACAAGAGAGAAACATAAGAAAGAAGTGGAACTGACGGAGATTAACCGT

CGGCACATCACCGAGTCCGTCAACTCCATCCGTCGGCTGGAGGAGGCCCAGAAGCAGCGA

CATGACCGCCTAGTGGCTGGGCAGCAGCAGGTGCTACAGCAACTGGCAGAAGAAGAGCCC

AAACTGCTGGCCCAGCTGACCCAGGAGTGTCAGGAGCAGCGGGCGAGGCTGCCTCAGGAG

ATCCGACGGAGCCTGCTGGGAGAGACACCAGAGGGGCTAAGGGACGGGCCCCTGGTGGCC

TGTGCCAGCAATGGCCATGCACCCGGGAGCAGTGGGCACCTGTCTGGTGTTGACTCGGAG

AGCCAGGAGGAGAACACGCAGCTC

>CL143.Contig2_A-W 2 280 PREDICTED: 1-phosphatidylinositol-4,5-bisphosphate phosphodiesterase beta-3-like [Ailuropoda melanoleuca]

GAGGAGGAGGCCAAGAGGTATCGAGAGTTCCAGAACAGACAGGTGCAAAGCTTGCTGGAG

CTGAGGGAGGCCCAGGCGGACATAGAGGCTGAGCGGAGGCTAGAGCACCTGAGACAGGCT

CAGCAGCGGCTCAGGGAGATCGTCCTGGATGTGCACACAACTCAGTTCAAAAGGCTGAAG

GAGATGAACGAGAGGGAGAAGAAGGAGCTGCAGAAGATCCTGGACAGGAAGCGCCACAAC

AGCATCTCTGAGGCCAAGACAAGAGAGAAACATAAGAAA

>CL144.Contig1_A-W 3 1388 minus strand PREDICTED: vacuolar protein sorting-associated protein 33B [Cricetulus griseus]

GAACTGCCAGAATTTTTCAGGGACTATTTCCTGGAAGGAGATCAGCGTTGGACCAACACT

GTGGCTCAGGCGTTGCACCTTCTCAGCACTCTCTATGGATCTTTTCCAAACTGCTATGGA

GTTGGCAGATGTGCCAGGATGTCATATGAACTATGGAGGAAACTGGAGGAAGAGGAGGAT

AGTGAAACCAAAGGCCGAAGACCAGAAATTGGTCATATCTTTCTCCTGGACAGAGACATG

GACTTTGTGACGGCACTTTGCTCCCAAGTGGTTTATGAGGGCCTGGTAGACGACACCTTC

CGCATCAAATGTGGAAGTGTGGACTTTGGCCCAGAAGTTACATCCTCTGACAAAAGCCTG

AAGGTGCTACTCAATGCTGAGGACAAGGTATTTAATGAGATTCGTAATGAGCACTTCTCC

AATGTCTTTGGCTTCTTGAGCCAGAAGGCCCGGAACTTGCAGGCCCAGTATGATCGCCGG

AGAGGCATGGACATTAAGCAGATGAAGAACTTTGTGTCTCAGGAGCTCAAAGGACTGAAA

CAGGAGCATCGCCTGCTGAGCCTCCATATTGGGGCCTGTGAATCTATCATGAAGAAGAAA

ACTAAGCAGGATTTCCAAGAGCTAATCAAGACTGAGCATGCACTGCTAGAGGGGTTCAAC

ATTCGGGAGAGCACCAACTACATTGAAGAACACATTGACCGGCAGGTGTCACCTATAGAA

AGCCTTCGCCTCATGTGCCTTTTGTCCATCACTGAGAATGGTTTGATCCCCAAGGATTAC

CGATCTCTGAAAACACAGTATCTGCAAAGCTATGGCCCTGAGCATCTGCTCACCTTCTCT

AATCTGCGGCGAGCTGGGCTCCTAACAGAGCAGGCCCCAGGGGACACTCTCACAGCAGTG

GAGAGTAAAGTAAGCAAGCTGGTGACTGACAAGGCTGCAGGAAAGATTACTGATGCCTTC

AGTTCTCTGGCCAAGAGGAGCAATTTTCGGGCCATCAGCAAAAAGCTGAATTTGATTCCA

CGCGTGGACGGAGAGTATGATCTGAAAGTGCCCCGAGACATGGCTTATGTCTTCAGTGGT

GCTTATGTGCCCCTGAGCTGCCGAATCATTGAGCAGGTGTTAGAGCGGCGAAGCTGGCAG

GGTCTTGATGAGGTGGTACGGCTACTCAATTGCAGTGAGTTTGCATTCACAGACATGACC

AAGGAAGACAAGACTTCCAGTGAATCCCTGCGCCTTATCTTGGTGGTATTCCTGGGTGGC

TGCACATTCTCTGAGATCTCAGCCCTTCGGTTCTTGGGCAAAGAGAAAGGGTACAGGTTC

ATTTTTCTGACAACAGCTATCACAAATAGTGCTCGCCTTATGGAGGCCATGAGTGAGGTG

AAAGCC

>CL144.Contig2_A-W 2 1438 minus strand PREDICTED: vacuolar protein sorting-associated protein 33B [Cricetulus griseus]

AGCCCTCAGAAGTTTTATGCATGTGAGATGGTGCTTGAGGAAGAGGGAATCTATGGAGAT

GTAAGCTGCGATGAATGGGCCTTCTCTTTGCTGCCTCTCGATGTGGATCTGCTGAGCATG

GAACTGCCAGAATTTTTCAGGGACTATTTCCTGGAAGGAGATCAGCGTTGGACCAACACT

GTGGCTCAGGCGTTGCACCTTCTCAGCACTCTCTATGGATCTTTTCCAAACTGCTATGGA

GTTGGCAGATGTGCCAGGATGTCATATGAACTATGGAGGAAACTGGAGGAAGAGGAGGAT

AGTGAAACCAAAGGCCGAAGACCAGAAATTGGTCATATCTTTCTCCTGGACAGAGACATG

GACTTTGTGACGGCACTTTGCTCCCAAGTGGTTTATGAGGGCCTGGTAGACGACACCTTC

CGCATCAAATGTGGAAGTGTGGACTTTGGCCCAGAAGTTACATCCTCTGACAAAAGCCTG

AAGGTGCTACTCAATGCTGAGGACAAGGTATTTAATGAGATTCGTAATGAGCACTTCTCC

AATGTCTTTGGCTTCTTGAGCCAGAAGGCCCGGAACTTGCAGGCCCAGTATGATCGCCGG

AGAGGCATGGACATTAAGCAGATGAAGAACTTTGTGTCTCAGGAGCTCAAAGGACTGAAA

CAGGAGCATCGCCTGCTGAGCCTCCATATTGGGGCCTGTGAATCTATCATGAAGAAGAAA

ACTAAGCAGGATTTCCAAGAGCTAATCAAGACTGAGCATGCACTGCTAGAGGGGTTCAAC

ATTCGGGAGAGCACCAACTACATTGAAGAACACATTGACCGGCAGGTGTCACCTATAGAA

AGCCTTCGCCTCATGTGCCTTTTGTCCATCACTGAGAATGGTTTGATCCCCAAGGATTAC

CGATCTCTGAAAACACAGTATCTGCAAAGCTATGGCCCTGAGCATCTGCTCACCTTCTCT

AATCTGCGGCGAGCTGGGCTCCTAACAGAGCAGGCCCCAGGGGACACTCTCACAGCAGTG

GAGAGTAAAGTAAGCAAGCTGGTGACTGACAAGGCTGCAGGAAAGATTACTGATGCCTTC

AGTTCTCTGGCCAAGAGGAGCAATTTTCGGGCCATCAGCAAAAAGCTGAATTTGATTCCA

CGCGTGGACGGAGAGTATGATCTGAAAGTGCCCCGAGACATGGCTTATGTCTTCAGTGGT

GCTTATGTGCCCCTGAGCTGCCGAATCATTGAGCAGGTGTTAGAGCGGCGAAGCTGGCAG

GGTCTTGATGAGGTGGTACGGCTACTCAATTGCAGTGAGTTTGCATTCACAGACATGACC

AAGGAAGACAAGACTTCCAGTGAATCCCTGCGCCTTATCTTGGTGGTATTCCTGGGTGGC

TGCACATTCTCTGAGATCTCAGCCCTTCGGTTCTTGGGCAAAGAGAAAGGTAAAAGA

>CL144.Contig3_A-W 19 537 minus strand PREDICTED: vacuolar protein sorting-associated protein 33B [Pan troglodytes]

TTTCTTCAGAGCTATGGCCCTGAGCATCTGCTCACCTTCTCTAATCTGCGGCGAGCTGGG

CTCCTAACAGAGCAGGCCCCAGGGGACACTCTCACAGCAGTGGAGAGTAAAGTAAGCAAG

CTGGTGACTGACAAGGCTGCAGGAAAGATTACTGATGCCTTCAGTTCTCTGGCCAAGAGG

AGCAATTTTCGGGCCATCAGCAAAAAGCTGAATTTGATTCCACGCGTGGACGGAGAGTAT

GATCTGAAAGTGCCCCGAGACATGGCTTATGTCTTCAGTGGTGCTTATGTGCCCCTGAGC

TGCCGAATCATTGAGCAGGTGTTAGAGCGGCGAAGCTGGCAGGGTCTTGATGAGGTGGTA

CGGCTACTCAATTGCAGTGAGTTTGCATTCACAGACATGACCAAGGAAGACAAGACTTCC

AGTGAATCCCTGCGCCTTATCTTGGTGGTATTCCTGGGTGGCTGCACATTCTCTGAGATC

TCAGCCCTTCGGTTCTTGGGCAAAGAGAAAGGTAAAAGA

>CL144.Contig4_A-W 47 349 minus strand PREDICTED: vacuolar protein sorting-associated protein 33B [Pan troglodytes]

ATTCCACGCGTGGACGGAGAGTATGATCTGAAAGTGCCCCGAGACATGGCTTATGTCTTC

AGTGGTGCTTATGTGCCCCTGAGCTGCCGAATCATTGAGCAGGTGTTAGAGCGGCGAAGC

TGGCAGGGTCTTGATGAGGTGGTACGGCTACTCAATTGCAGTGAGTTTGCATTCACAGAC

ATGACCAAGGAAGACAAGACTTCCAGTGAATCCCTGCGCCTTATCTTGGTGGTATTCCTG

GGTGGCTGCACATTCTCTGAGATCTCAGCCCTTCGGTTCTTGGGCAAAGAGAAAGGTAAA

AGA

>CL144.Contig5_A-W 47 418 minus strand PREDICTED: vacuolar protein sorting-associated protein 33B isoform 3 [Papio anubis]

ATTCCACGCGTGGACGGAGAGTATGATCTGAAAGTGCCCCGAGACATGGCTTATGTCTTC

AGTGGTGCTTATGTGCCCCTGAGCTGCCGAATCATTGAGCAGGTGTTAGAGCGGCGAAGC

TGGCAGGGTCTTGATGAGGTGGTACGGCTACTCAATTGCAGTGAGTTTGCATTCACAGAC

ATGACCAAGGAAGACAAGACTTCCAGTGAATCCCTGCGCCTTATCTTGGTGGTATTCCTG

GGTGGCTGCACATTCTCTGAGATCTCAGCCCTTCGGTTCTTGGGCAAAGAGAAAGGGTAC

AGGTTCATTTTTCTGACAACAGCTATCACAAATAGTGCTCGCCTTATGGAGGCCATGAGT

GAGGTGAAAGCC

>CL144.Contig6_A-W 19 441 minus strand Vacuolar protein sorting-associated protein 33B [Heterocephalus glaber]

TTTCTTCAGAGCTATGGCCCTGAGCATCTGCTCACCTTCTCTAATCTGCGGCGAGCTGGG

CTCCTAACAGAGCAGGCCCCAGGGGACACTCTCACAGCAGTGGAGAGTAAAGTAAGCAAG

CTGGTGACTGACAAGGCTGCAGGAAAGATTACTGATGCCTTCAGTTCTCTGGCCAAGAGG

AGCAATTTTCGGGCCATCAGCAAAAAGCTGAATTTGATTCCACGCGTGGACGGAGAGTAT

GATCTGAAAGTGCCCCGAGACATGGCTTATGTCTTCAGTGGTGCTTATGTGCCCCTGAGC

TGCCGAATCATTGAGCAGGTGTTAGAGCGGCGAAGCTGGCAGGGTCTTGATGAGGTGGTA

CGGCTACTCAATTGCAGTGAGTTTGCATTCACAGGTGTGTGGCATTGCCAGGTGGGGCAG

GGT

>CL145.Contig1_A-W 3 365 protein YIPF1 [Pongo abelii] >gi|75042045|sp|Q5RBL0.1|YIPF1_PONAB RecName: Full=Protein YIPF1; AltName: Full=YIP1 family member 1

AGAAACAGCAAAGTTATGAACATCGTTTCCTATTCGTTTCTGGAGATTGTGTGTGTCTAT

GGCTATTCGCTCTTCATTTATATTCCCACAGCAATACTGTGGATTATCCCTCAGAAAGCT

GTTCGTTGGATTCTAGTCATGATTGCCCTGGCCATCTCAGGATCTGTCTTGGCAATGACG

TTTTGGCCAGCTGTTCGTGAGGATAACCGGCGCATTGCATTGGCCACAATTGTGACGATT

GTGTTGCTTCATATGCTTCTTTCTGTGGGCTGCTTGGCATACTTTTTTGATGCACCAGAG

ATGGACCATCTTCCACCAGCTACAGCTACTCCAAACCAAACAGTCGCAGCAGCCAAGTCC

AGC

>CL146.Contig1_A-W 1378 1542 minus strand interleukin 1 beta precursor [Marmota monax]

GAGCCCATCTTCTGTGACTCCTGGGATGACGACGGATACGAGTATGACGCGGCCGTCCCG

GTGCTGAACTGCCGGCTGCGTGACGTCCAGCAGAAGAGCCTGGTGCTGTCCGACCCGTGC

GAGCTCAAAGCTCTGCACCTCAATGGACCGAATCTGAGGCAACAA

>CL146.Contig2_A-W 1580 1876 minus strand interleukin 1 beta precursor [Marmota monax]

GAGCCCATCTTCTGTGACTCCTGGGATGACGACGGATACGAGTATGACGCGGCCGTCCCG

GTGCTGAACTGCCGGCTGCGTGACGTCCAGCAGAAGAGCCTGGTGCTGTCCGACCCGTGC

GAGCTCAAAGCTCTGCACCTCAATGGACCGAATCTGAGGCAACAAGTGGTGTTCTCCATG

AGCTTTGTGCTGGGAGAAGGGAATGACAGCAAGACACCCGTGGCCTTGGGCATCAAGGGG

AAGAACCTGTACCTGACCTGTGTGATGAAGGGGGACAAGCCCACCCTGCAGCTGGAG

>CL146.Contig3_A-W 367 498 minus strand interleukin 1 beta precursor [Sigmodon hispidus]

GTGGTGTTCTCCATGAGCTTTGTGCTGGGAGAAGGGAATGACAGCAAGACACCCGTGGCC

TTGGGCATCAAGGGGAAGAACCTGTACCTGACCTGTGTGATGAAGGGGGACAAGCCCACC

CTGCAGCTGGAG

>CL146.Contig4_A-W 642 1409 minus strand PREDICTED: interleukin-1 beta-like [Cricetulus griseus]

AGCCACGTCTATCTTTATTGCAGTGACGAGAACGACCTGTTCTTTGAGGTGGACGGCCCC

AAACACATGAAGTGCAACTGGCAGGACCTTTACCTCGGCTCCGTGGATGAGGGCATCCAG

CTGCAAATCTCCCAGCTGCACCTCAACAAGAGCTTGCAGCAGGTGGTGTCACTCGTCGTG

GCCGTGGAGAAGCTGCGGATCCTTCCGGGCGCACAGGCCCCCCGGGGGGAGGACCTGCAC

ACCTGCCTTTCCCTCATCTTTGAAGAAGAGCCCATCTTCTGTGACTCCTGGGATGACGAC

GGATACGAGTATGACGCGGCCGTCCCGGTGCTGAACTGCCGGCTGCGTGACGTCCAGCAG

AAGAGCCTGGTGCTGTCCGACCCGTGCGAGCTCAAAGCTCTGCACCTCAATGGACCGAAT

CTGAGGCAACAAGTGGTGTTCTCCATGAGCTTTGTGCTGGGAGAAGGGAATGACAGCAAG

ACACCCGTGGCCTTGGGCATCAAGGGGAAGAACCTGTACCTGACCTGTGTGATGAAGGGG

GACAAGCCCACCCTGCAGCTGGAGAGTGTAGACCCCAAAAGTTACCCCAAGAAGAGGATG

GAGCCGAGGTTCGTGTTCAACAAGATAGAGGTCAAAGACAAGGTGGAGTTTGAGTCGGCT

CAGTTCCCCAACTGGTACATCAGCACCTCCCAGGCGGAGCACCTGCCCGTCTTCCTGGGG

AACAACAGCGGCCAGGATATAACAGACTTCACCATGGAATTCGTGTCC

>CL146.Contig5_A-W 116 406 minus strand PREDICTED: interleukin-1 beta-like [Cricetulus griseus]

ATGGCAGCAGTACCCGAACTGGCCAGTGAGATGATGGCTTACCACAGTGACGAGAACGAC

CTGTTCTTTGAGGTGGACGGCCCCAAACACATGAAGTGCAACTGGCAGGACCTTTACCTC

GGCTCCGTGGATGAGGGCATCCAGCTGCAAATCTCCCAGCTGCACCTCAACAAGAGCTTG

CAGCAGGTGGTGTCACTCGTCGTGGCCGTGGAGAAGCTGCGGATCCTTCCGGGCGCACAG

GCCCCCCGGGGGGAGGACCTGCACACCTGCCTTTCCCTCATCTTTGAAGAA

>CL146.Contig6_A-W 1078 1242 minus strand interleukin 1, beta, isoform CRA_c [Homo sapiens]

GAGCCCATCTTCTGTGACTCCTGGGATGACGACGGATACGAGTATGACGCGGCCGTCCCG

GTGCTGAACTGCCGGCTGCGTGACGTCCAGCAGAAGAGCCTGGTGCTGTCCGACCCGTGC

GAGCTCAAAGCTCTGCACCTCAATGGACCGAATCTGAGGCAACAA

>CL146.Contig7_A-W 318 1109 minus strand PREDICTED: interleukin-1 beta-like [Cricetulus griseus]

ATGGCAGCAGTACCCGAACTGGCCAGTGAGATGATGGCTTACCACAGTGACGAGAACGAC

CTGTTCTTTGAGGTGGACGGCCCCAAACACATGAAGTGCAACTGGCAGGACCTTTACCTC

GGCTCCGTGGATGAGGGCATCCAGCTGCAAATCTCCCAGCTGCACCTCAACAAGAGCTTG

CAGCAGGTGGTGTCACTCGTCGTGGCCGTGGAGAAGCTGCGGATCCTTCCGGGCGCACAG

GCCCCCCGGGGGGAGGACCTGCACACCTGCCTTTCCCTCATCTTTGAAGAAGAGCCCATC

TTCTGTGACTCCTGGGATGACGACGGATACGAGTATGACGCGGCCGTCCCGGTGCTGAAC

TGCCGGCTGCGTGACGTCCAGCAGAAGAGCCTGGTGCTGTCCGACCCGTGCGAGCTCAAA

GCTCTGCACCTCAATGGACCGAATCTGAGGCAACAAGTGGTGTTCTCCATGAGCTTTGTG

CTGGGAGAAGGGAATGACAGCAAGACACCCGTGGCCTTGGGCATCAAGGGGAAGAACCTG

TACCTGACCTGTGTGATGAAGGGGGACAAGCCCACCCTGCAGCTGGAGAGTGTAGACCCC

AAAAGTTACCCCAAGAAGAGGATGGAGCCGAGGTTCGTGTTCAACAAGATAGAGGTCAAA

GACAAGGTGGAGTTTGAGTCGGCTCAGTTCCCCAACTGGTACATCAGCACCTCCCAGGCG

GAGCACCTGCCCGTCTTCCTGGGGAACAACAGCGGCCAGGATATAACAGACTTCACCATG

GAATTCGTGTCC

>CL146.Contig8_A-W 116 907 minus strand PREDICTED: interleukin-1 beta-like [Cricetulus griseus]

ATGGCAGCAGTACCCGAACTGGCCAGTGAGATGATGGCTTACCACAGTGACGAGAACGAC

CTGTTCTTTGAGGTGGACGGCCCCAAACACATGAAGTGCAACTGGCAGGACCTTTACCTC

GGCTCCGTGGATGAGGGCATCCAGCTGCAAATCTCCCAGCTGCACCTCAACAAGAGCTTG

CAGCAGGTGGTGTCACTCGTCGTGGCCGTGGAGAAGCTGCGGATCCTTCCGGGCGCACAG

GCCCCCCGGGGGGAGGACCTGCACACCTGCCTTTCCCTCATCTTTGAAGAAGAGCCCATC

TTCTGTGACTCCTGGGATGACGACGGATACGAGTATGACGCGGCCGTCCCGGTGCTGAAC

TGCCGGCTGCGTGACGTCCAGCAGAAGAGCCTGGTGCTGTCCGACCCGTGCGAGCTCAAA

GCTCTGCACCTCAATGGACCGAATCTGAGGCAACAAGTGGTGTTCTCCATGAGCTTTGTG

CTGGGAGAAGGGAATGACAGCAAGACACCCGTGGCCTTGGGCATCAAGGGGAAGAACCTG

TACCTGACCTGTGTGATGAAGGGGGACAAGCCCACCCTGCAGCTGGAGAGTGTAGACCCC

AAAAGTTACCCCAAGAAGAGGATGGAGCCGAGGTTCGTGTTCAACAAGATAGAGGTCAAA

GACAAGGTGGAGTTTGAGTCGGCTCAGTTCCCCAACTGGTACATCAGCACCTCCCAGGCG

GAGCACCTGCCCGTCTTCCTGGGGAACAACAGCGGCCAGGATATAACAGACTTCACCATG

GAATTCGTGTCC

>CL146.Contig9_A-W 1378 1674 minus strand interleukin 1 beta precursor [Marmota monax]

GAGCCCATCTTCTGTGACTCCTGGGATGACGACGGATACGAGTATGACGCGGCCGTCCCG

GTGCTGAACTGCCGGCTGCGTGACGTCCAGCAGAAGAGCCTGGTGCTGTCCGACCCGTGC

GAGCTCAAAGCTCTGCACCTCAATGGACCGAATCTGAGGCAACAAGTGGTGTTCTCCATG

AGCTTTGTGCTGGGAGAAGGGAATGACAGCAAGACACCCGTGGCCTTGGGCATCAAGGGG

AAGAACCTGTACCTGACCTGTGTGATGAAGGGGGACAAGCCCACCCTGCAGCTGGAG

>CL146.Contig10_A-W 367 702 minus strand PREDICTED: interleukin-1 beta-like [Cricetulus griseus]

GTGGTGTTCTCCATGAGCTTTGTGCTGGGAGAAGGGAATGACAGCAAGACACCCGTGGCC

TTGGGCATCAAGGGGAAGAACCTGTACCTGACCTGTGTGATGAAGGGGGACAAGCCCACC

CTGCAGCTGGAGAGTGTAGACCCCAAAAGTTACCCCAAGAAGAGGATGGAGCCGAGGTTC

GTGTTCAACAAGATAGAGGTCAAAGACAAGGTGGAGTTTGAGTCGGCTCAGTTCCCCAAC

TGGTACATCAGCACCTCCCAGGCGGAGCACCTGCCCGTCTTCCTGGGGAACAACAGCGGC

CAGGATATAACAGACTTCACCATGGAATTCGTGTCC

>CL146.Contig11_A-W 1754 2050 minus strand interleukin 1 beta precursor [Marmota monax]

GAGCCCATCTTCTGTGACTCCTGGGATGACGACGGATACGAGTATGACGCGGCCGTCCCG

GTGCTGAACTGCCGGCTGCGTGACGTCCAGCAGAAGAGCCTGGTGCTGTCCGACCCGTGC

GAGCTCAAAGCTCTGCACCTCAATGGACCGAATCTGAGGCAACAAGTGGTGTTCTCCATG

AGCTTTGTGCTGGGAGAAGGGAATGACAGCAAGACACCCGTGGCCTTGGGCATCAAGGGG

AAGAACCTGTACCTGACCTGTGTGATGAAGGGGGACAAGCCCACCCTGCAGCTGGAG

>CL146.Contig12_A-W 1273 1581 minus strand interleukin 1 beta precursor [Marmota monax]

ATCTTTGAAGAAGAGCCCATCTTCTGTGACTCCTGGGATGACGACGGATACGAGTATGAC

GCGGCCGTCCCGGTGCTGAACTGCCGGCTGCGTGACGTCCAGCAGAAGAGCCTGGTGCTG

TCCGACCCGTGCGAGCTCAAAGCTCTGCACCTCAATGGACCGAATCTGAGGCAACAAGTG

GTGTTCTCCATGAGCTTTGTGCTGGGAGAAGGGAATGACAGCAAGACACCCGTGGCCTTG

GGCATCAAGGGGAAGAACCTGTACCTGACCTGTGTGATGAAGGGGGACAAGCCCACCCTG

CAGCTGGAG

>CL146.Contig13_A-W 2142 2351 minus strand Interleukin-1 beta [Heterocephalus glaber]

CTGCAGAGTGTAGACCCCAAAAGTTACCCCAAGAAGAGGATGGAGCCGAGGTTCGTGTTC

AACAAGATAGAGGTCAAAGACAAGGTGGAGTTTGAGTCGGCTCAGTTCCCCAACTGGTAC

ATCAGCACCTCCCAGGCGGAGCACCTGCCCGTCTTCCTGGGGAACAACAGCGGCCAGGAT

ATAACAGACTTCACCATGGAATTCGTGTCC

>CL146.Contig14_A-W 1078 1374 minus strand interleukin 1 beta precursor [Marmota monax]

GAGCCCATCTTCTGTGACTCCTGGGATGACGACGGATACGAGTATGACGCGGCCGTCCCG

GTGCTGAACTGCCGGCTGCGTGACGTCCAGCAGAAGAGCCTGGTGCTGTCCGACCCGTGC

GAGCTCAAAGCTCTGCACCTCAATGGACCGAATCTGAGGCAACAAGTGGTGTTCTCCATG

AGCTTTGTGCTGGGAGAAGGGAATGACAGCAAGACACCCGTGGCCTTGGGCATCAAGGGG

AAGAACCTGTACCTGACCTGTGTGATGAAGGGGGACAAGCCCACCCTGCAGCTGGAG

>CL146.Contig15_A-W 1090 1284 minus strand PREDICTED: interleukin-1 beta-like [Cricetulus griseus]

TGCAACTGGCAGGACCTTTACCTCGGCTCCGTGGATGAGGGCATCCAGCTGCAAATCTCC

CAGCTGCACCTCAACAAGAGCTTGCAGCAGGTGGTGTCACTCGTCGTGGCCGTGGAGAAG

CTGCGGATCCTTCCGGGCGCACAGGCCCCCCGGGGGGAGGACCTGCACACCTGCCTTTCC

CTCATCTTTGAAGAA

>CL146.Contig16_A-W 1940 2149 minus strand Interleukin-1 beta [Heterocephalus glaber]

CTGCAGAGTGTAGACCCCAAAAGTTACCCCAAGAAGAGGATGGAGCCGAGGTTCGTGTTC

AACAAGATAGAGGTCAAAGACAAGGTGGAGTTTGAGTCGGCTCAGTTCCCCAACTGGTAC

ATCAGCACCTCCCAGGCGGAGCACCTGCCCGTCTTCCTGGGGAACAACAGCGGCCAGGAT

ATAACAGACTTCACCATGGAATTCGTGTCC

>CL146.Contig17_A-W 876 1172 minus strand interleukin 1 beta precursor [Marmota monax]

GAGCCCATCTTCTGTGACTCCTGGGATGACGACGGATACGAGTATGACGCGGCCGTCCCG

GTGCTGAACTGCCGGCTGCGTGACGTCCAGCAGAAGAGCCTGGTGCTGTCCGACCCGTGC

GAGCTCAAAGCTCTGCACCTCAATGGACCGAATCTGAGGCAACAAGTGGTGTTCTCCATG

AGCTTTGTGCTGGGAGAAGGGAATGACAGCAAGACACCCGTGGCCTTGGGCATCAAGGGG

AAGAACCTGTACCTGACCTGTGTGATGAAGGGGGACAAGCCCACCCTGCAGCTGGAG

>CL146.Contig18_A-W 844 1110 minus strand PREDICTED: interleukin-1 beta-like [Cricetulus griseus]

AGCCACGTCTATCTTTATTGCAGTGACGAGAACGACCTGTTCTTTGAGGTGGACGGCCCC

AAACACATGAAGTGCAACTGGCAGGACCTTTACCTCGGCTCCGTGGATGAGGGCATCCAG

CTGCAAATCTCCCAGCTGCACCTCAACAAGAGCTTGCAGCAGGTGGTGTCACTCGTCGTG

GCCGTGGAGAAGCTGCGGATCCTTCCGGGCGCACAGGCCCCCCGGGGGGAGGACCTGCAC

ACCTGCCTTTCCCTCATCTTTGAAGAA

>CL146.Contig19_A-W 642 908 minus strand PREDICTED: interleukin-1 beta-like [Cricetulus griseus]

AGCCACGTCTATCTTTATTGCAGTGACGAGAACGACCTGTTCTTTGAGGTGGACGGCCCC

AAACACATGAAGTGCAACTGGCAGGACCTTTACCTCGGCTCCGTGGATGAGGGCATCCAG

CTGCAAATCTCCCAGCTGCACCTCAACAAGAGCTTGCAGCAGGTGGTGTCACTCGTCGTG

GCCGTGGAGAAGCTGCGGATCCTTCCGGGCGCACAGGCCCCCCGGGGGGAGGACCTGCAC

ACCTGCCTTTCCCTCATCTTTGAAGAA

>CL146.Contig20_A-W 1640 1849 minus strand Interleukin-1 beta [Heterocephalus glaber]

CTGCAGAGTGTAGACCCCAAAAGTTACCCCAAGAAGAGGATGGAGCCGAGGTTCGTGTTC

AACAAGATAGAGGTCAAAGACAAGGTGGAGTTTGAGTCGGCTCAGTTCCCCAACTGGTAC

ATCAGCACCTCCCAGGCGGAGCACCTGCCCGTCTTCCTGGGGAACAACAGCGGCCAGGAT

ATAACAGACTTCACCATGGAATTCGTGTCC

>CL146.Contig21_A-W 1580 1744 minus strand interleukin 1 beta precursor [Marmota monax]

GAGCCCATCTTCTGTGACTCCTGGGATGACGACGGATACGAGTATGACGCGGCCGTCCCG

GTGCTGAACTGCCGGCTGCGTGACGTCCAGCAGAAGAGCCTGGTGCTGTCCGACCCGTGC

GAGCTCAAAGCTCTGCACCTCAATGGACCGAATCTGAGGCAACAA

>CL146.Contig22_A-W 1438 1647 minus strand Interleukin-1 beta [Heterocephalus glaber]

CTGCAGAGTGTAGACCCCAAAAGTTACCCCAAGAAGAGGATGGAGCCGAGGTTCGTGTTC

AACAAGATAGAGGTCAAAGACAAGGTGGAGTTTGAGTCGGCTCAGTTCCCCAACTGGTAC

ATCAGCACCTCCCAGGCGGAGCACCTGCCCGTCTTCCTGGGGAACAACAGCGGCCAGGAT

ATAACAGACTTCACCATGGAATTCGTGTCC

>CL146.Contig23_A-W 844 1611 minus strand PREDICTED: interleukin-1 beta-like [Cricetulus griseus]

AGCCACGTCTATCTTTATTGCAGTGACGAGAACGACCTGTTCTTTGAGGTGGACGGCCCC

AAACACATGAAGTGCAACTGGCAGGACCTTTACCTCGGCTCCGTGGATGAGGGCATCCAG

CTGCAAATCTCCCAGCTGCACCTCAACAAGAGCTTGCAGCAGGTGGTGTCACTCGTCGTG

GCCGTGGAGAAGCTGCGGATCCTTCCGGGCGCACAGGCCCCCCGGGGGGAGGACCTGCAC

ACCTGCCTTTCCCTCATCTTTGAAGAAGAGCCCATCTTCTGTGACTCCTGGGATGACGAC

GGATACGAGTATGACGCGGCCGTCCCGGTGCTGAACTGCCGGCTGCGTGACGTCCAGCAG

AAGAGCCTGGTGCTGTCCGACCCGTGCGAGCTCAAAGCTCTGCACCTCAATGGACCGAAT

CTGAGGCAACAAGTGGTGTTCTCCATGAGCTTTGTGCTGGGAGAAGGGAATGACAGCAAG

ACACCCGTGGCCTTGGGCATCAAGGGGAAGAACCTGTACCTGACCTGTGTGATGAAGGGG

GACAAGCCCACCCTGCAGCTGGAGAGTGTAGACCCCAAAAGTTACCCCAAGAAGAGGATG

GAGCCGAGGTTCGTGTTCAACAAGATAGAGGTCAAAGACAAGGTGGAGTTTGAGTCGGCT

CAGTTCCCCAACTGGTACATCAGCACCTCCCAGGCGGAGCACCTGCCCGTCTTCCTGGGG

AACAACAGCGGCCAGGATATAACAGACTTCACCATGGAATTCGTGTCC

>CL146.Contig24_A-W 318 608 minus strand PREDICTED: interleukin-1 beta-like [Cricetulus griseus]

ATGGCAGCAGTACCCGAACTGGCCAGTGAGATGATGGCTTACCACAGTGACGAGAACGAC

CTGTTCTTTGAGGTGGACGGCCCCAAACACATGAAGTGCAACTGGCAGGACCTTTACCTC

GGCTCCGTGGATGAGGGCATCCAGCTGCAAATCTCCCAGCTGCACCTCAACAAGAGCTTG

CAGCAGGTGGTGTCACTCGTCGTGGCCGTGGAGAAGCTGCGGATCCTTCCGGGCGCACAG

GCCCCCCGGGGGGAGGACCTGCACACCTGCCTTTCCCTCATCTTTGAAGAA

>CL147.Contig1_A-W 4 1359 MAU2 chromatid cohesion factor homolog OS=Mus musculus GN=Mau2 PE=2 SV=3

GAGGCCAAAATGGCGGCTCAGGCGGCAGCGGCAGCACAAGCGGCGGCGGCCCAGGCAGCA

CAGGCTGAAGCGGCCGAGTCTTGGTATCTGGCGCTTCTGGGCTTCGCTGAGCACTTCCGC

ACTTCCAGTCCACCCAAGATCCGCCTATGCGTGCACTGCCTACAGGCCGTGTTTCCCTTC

AAGCCGCCGCAGCGTATCGAGGCCCGCACGCATCTGCAGCTAGGCTCTGTGCTCTATCAC

CATACCAAGAACAGCGAGCAGGCGCGCAGTCACCTGGAGAAGGCGTGGTTGATATCACAG

CAAATCCCACAGTTTGAAGATGTTAAATTTGAAGCAGCAAGTCTGTTGTCTGAATTGTAC

TGTCAAGAGAATTCTGTTGATGCAGCAAAGCCACTGCTGAGGAAAGCGATTCAGATCTCA

CAGCAGACCCCATACTGGCACTGCCGCCTGCTCTTCCAGCTTGCTCAACTGCACACACTT

GAGAAGGACTTGGTGTCAGCATGTGACCTCCTGGGTGTAGGGGCCGAATACGCCAGGGTG

GTGGGATCCGAGTACACACGGGCGCTGTTCCTGCTCAGCAAGGGGATGCTGCTGCTGATG

GAGCGCAAGTTGCAGGAAGTACACCCACTGCTGACCCTGTGTGGACAGATCGTGGAGAAC

TGGCAGGGGAACCCCATCCAGAAGGAATCACTGCGAGTATTCTTCCTGGTGCTCCAGGTG

ACCCACTACCTGGATGCTGGGCAGGTAAAGAGTGTGAAGCCATGCCTGAAGCAGCTGCAG

CAGTGCATCCAGACCATCTCCACACTGCATGATGATGAGATCCTGCCCAGCAACCCCGCT

GACCTCTTCCACTGGCTACCCAAGGAGCACATGTGTGTGCTTGTTTACCTGGTGACAGTG

ATGCACTCCATGCAGGCGGGCTACCTGGAGAAGGCACAGAAGTACACAGACAAGGCCCTC

ATGCAGCTGGAGAAGCTCAAGATGCTCGACTGCAGCCCCATCCTGTCCTCTTTCCAAGTG

ATCCTGCTGGAGCATATCATCATGTGCCGACTCGTCACAGGCCACAAGGCTACTGCACTA

CAGGAGATCTCCCAGGTCTGCCAACTGTGCCAGCAATCTCCCCGACTCTTCTCCAACCAT

GCAGCACAGCTACACACACTGCTGGGTTTGTACTGCGTCTCTGTCAACTGCATGGACAAT

GCGGAAGCACAGTTCACCACTGCCCTGCGGCTCACCAACCACCAGGAGCTGTGGGCCTTC

ATAGTGACCAACCTGGCGAGTGTGTATATACGGGAAGGAAATAGACACCAAGAGGTAGTA

GCTGTACAGTTTATTAGAGAGGATAAATCCAGACCA

>CL147.Contig2_A-W 1 900 PREDICTED: Scc4-like protein [Oryctolagus cuniculus]

GTAGAGGCCAAAATGGCGGCTCAGGCGGCAGCGGCAGCACAAGCGGCGGCGGCCCAGGCA

GCACAGGCTGAAGCGGCCGAGTCTTGGTATCTGGCGCTTCTGGGCTTCGCTGAGCACTTC

CGCACTTCCAGTCCACCCAAGATCCGCCTATGCGTGCACTGCCTACAGGCCGTGTTTCCC

TTCAAGCCGCCGCAGCGTATCGAGGCCCGCACGCATCTGCAGCTAGGCTCTGTGCTCTAT

CACCATACCAAGAACAGCGAGCAGGCGCGCAGTCACCTGGAGAAGGCGTGGTTGATATCA

CAGCAAATCCCACAGTTTGAAGATGTTAAATTTGAAGCAGCAAGTCTGTTGTCTGAATTG

TACTGTCAAGAGAATTCTGTTGATGCAGCAAAGCCACTGCTGAGGAAAGCGATTCAGATC

TCACAGCAGACCCCATACTGGCACTGCCGCCTGCTCTTCCAGCTTGCTCAACTGCACACA

CTTGAGAAGGACTTGGTGTCAGCATGTGACCTCCTGGGTGTAGGGGCCGAATACGCCAGG

GTGGTGGGATCCGAGTACACACGGGCGCTGTTCCTGCTCAGCAAGGGGATGCTGCTGCTG

ATGGAGCGCAAGTTGCAGGAAGTACACCCACTGCTGACCCTGTGTGGACAGATCGTGGAG

AACTGGCAGGGGAACCCCATCCAGAAGGAATCACTGCGAGTATTCTTCCTGGTGCTCCAG

GTGACCCACTACCTGGATGCTGGGCAGGTAAAGAGTGTGAAGCCATGCCTGAAGCAGCTG

CAGCAGTGCATCCAGACCATCTCCACACTGCATGATGATGAGATCCTGCCCAGCAACCCC

GCTGACCTCTTCCACTGGCTACCCAAGGAGCACATGTGTGTGCTTGTTTACCTGGTGAGT

>CL148.Contig1_A-W 199 1515 minus strand PREDICTED: myc box-dependent-interacting protein 1 isoform 1 [Saimiri boliviensis boliviensis]

ATGGCAGAGATGGGCAGTAAGGGGGTGACGGCGGGGAAGATCGCCAGCAATGTACAGAAG

AAGCTCACCCGCGCGCAGGAGAAGGTCCTTCAGAAACTGGGGAAGGCAGATGAGACCAAG

GATGAGCAGTTTGAACAGTGCGTCCAGAATTTCAACAAGCAGCTGACTGAGGGCACCCGG

CTGCAGAAGGATCTCCGGACTTACCTGGCTTCAGTCAAAGCCATGCACGAAGCCTCCAAG

AAGCTGAACGAGTGTCTACAGGAGGTGTATGAGCCCGACTGGCCCGGCAGGGACGAGGCC

AATAAGATCGCCGAGAACAATGACCTGCTGTGGATGGACTACCACCAGAAACTGGTGGAC

CAGGCGCTGCTGACCATGGACACGTACTTGGGCCAGTTCCCTGACATCAAGTCACGCATT

GCCAAGCGAGGGCGGAAATTGGTGGACTACGACAGCGCCCGGCACCACTATGAGTCCCTT

CAAACTGCCAAAAAGAAAGATGAAGTCAAAATCGCCAAGGCCGAGGAGGAGCTCATCAAG

GCTCAGAAGGTGTTTGAGGAGATGAATGTGGACCTCCAGGAGGAGCTCCCATCCCTGTGG

AACAGCCGAGTAGGTTTCTATGTCAATACATTTCAGAGCATCGCGGGCCTGGAGGAGAAC

TTCCACAAGGAGATGAGCAAGCTCAACCAGAACCTCAACGATGTGCTGGTCAGCCTGGAG

AAGCACCACGGCAGCAACACCTTCACAGTCAAGGCCCAGCCCAGTGACAATGCCCCTGCC

AAAGGGAACAAGAGCCCATCGCCTCCACCAGATGGCTCCCCTGCAGCCACCCCCGAGATC

AGAGTGAACCATGAGCCAGAGGCAGCCAGCGGGGCCACACCCGGGGCCTCCATCCCCAAG

TCCCCATCTCAGCCCGCAGAGGCCTTGGAGGTGGCAGGTGGGACCCAACCTGCGCCTGGA

GCCCAGGAGCCTGGGGAGACGGCGGCAAGTGAAGCAGCCTCCAGTTCTCTTCCGGCTGTG

GTGGTGGAAACGTTCTCAGCAACTGTGAATGGCACCGTGGAAGGCAGCAGTGGGGCCGGC

CGTTTGGACCTCCCTCCGGGCTTCATGTTCAAGGTGCAGGCTCAACACGATTACACGGCC

ACTGACACCGACGAGCTGCAGCTCAAGGCTGGTGACGTGGTGCTGGTGATCCCCTTTCAG

AACCCGGAGGAACAGGATGAAGGCTGGCTCATGGGCGTAAAGGAGAGTGACTGGAACCAG

CACAAGGAGTTGGAAAAATGCCGGGGCGTCTTCCCTGAGAACTTCACGGAACGGGTG

>CL148.Contig2_A-W 199 1623 minus strand PREDICTED: myc box-dependent-interacting protein 1 isoform 4 [Papio anubis]

ATGGCAGAGATGGGCAGTAAGGGGGTGACGGCGGGGAAGATCGCCAGCAATGTACAGAAG

AAGCTCACCCGCGCGCAGGAGAAGGTCCTTCAGAAACTGGGGAAGGCAGATGAGACCAAG

GATGAGCAGTTTGAACAGTGCGTCCAGAATTTCAACAAGCAGCTGACTGAGGGCACCCGG

CTGCAGAAGGATCTCCGGACTTACCTGGCTTCAGTCAAAGCCATGCACGAAGCCTCCAAG

AAGCTGAACGAGTGTCTACAGGAGGTGTATGAGCCCGACTGGCCCGGCAGGGACGAGGCC

AATAAGATCGCCGAGAACAATGACCTGCTGTGGATGGACTACCACCAGAAACTGGTGGAC

CAGGCGCTGCTGACCATGGACACGTACTTGGGCCAGTTCCCTGACATCAAGTCACGCATT

GCCAAGCGAGGGCGGAAATTGGTGGACTACGACAGCGCCCGGCACCACTATGAGTCCCTT

CAAACTGCCAAAAAGAAAGATGAAGTCAAAATCGCCAAGGCCGAGGAGGAGCTCATCAAG

GCTCAGAAGGTGTTTGAGGAGATGAATGTGGACCTCCAGGAGGAGCTCCCATCCCTGTGG

AACAGCCGAGTAGGTTTCTATGTCAATACATTTCAGAGCATCGCGGGCCTGGAGGAGAAC

TTCCACAAGGAGATGAGCAAGCTCAACCAGAACCTCAACGATGTGCTGGTCAGCCTGGAG

AAGCACCACGGCAGCAACACCTTCACAGTCAAGGCCCAGCCCAGTGACAATGCCCCTGCC

AAAGGGAACAAGAGCCCATCGCCTCCACCAGATGGCTCCCCTGCAGCCACCCCCGAGATC

AGAGTGAACCATGAGCCAGAGGCAGCCAGCGGGGCCACACCCGGGGCCTCCATCCCCAAG

TCCCCATCTCAGCCCACAGAGAGTCCAGCTGGCAGCCTGCCATCCGGGGAGCCTGGTGCT

GCTGAGGGCACATTTGCTGTGTCCTGGCCCAACCAGACGGCCGAGCAGGGGTCTGCCCAA

CCCGCAGAGGCCTTGGAGGTGGCAGGTGGGACCCAACCTGCGCCTGGAGCCCAGGAGCCT

GGGGAGACGGCGGCAAGTGAAGCAGCCTCCAGTTCTCTTCCGGCTGTGGTGGTGGAAACG

TTCTCAGCAACTGTGAATGGCACCGTGGAAGGCAGCAGTGGGGCCGGCCGTTTGGACCTC

CCTCCGGGCTTCATGTTCAAGGTGCAGGCTCAACACGATTACACGGCCACTGACACCGAC

GAGCTGCAGCTCAAGGCTGGTGACGTGGTGCTGGTGATCCCCTTTCAGAACCCGGAGGAA

CAGGATGAAGGCTGGCTCATGGGCGTAAAGGAGAGTGACTGGAACCAGCACAAGGAGTTG

GAAAAATGCCGGGGCGTCTTCCCTGAGAACTTCACGGAACGGGTG

>CL148.Contig3_A-W 110 748 minus strand PREDICTED: myc box-dependent-interacting protein 1 isoform 7 [Saimiri boliviensis boliviensis]

CAGCTCAACCAGAACCTCAACGATGTGCTGGTCAGCCTGGAGAAGCACCATGGCAGCAAC

ACCTTCACAGTCAAGGCCCAGCCCAGTGACAATGCCCCTGCCAAAGGGAACAAGAGCCCA

TCGCCTCCACCAGATGGCTCCCCTGCAGCCACCCCCGAGATCAGAGTGAACCATGAGCCA

GAGGCAGCCAGCGGGGCCACACCCGGGGCCTCCATCCCCAAGTCCCCATCTCAGCCCGCA

GAGGCCTTGGAGGTGGCAGGTGGGACCCAACCTGCGCCTGGAGCCCAGGATCCTGGGGAG

ACGGCGGCAAGTGAAGCAGCCTCCAGTTCTCTTCCGGCTGTGGTGGTGGAAACGTTCTCA

GCAACTGTGAATGGCACCGTGGAAGGCAGCAGTGGGGCCGGCCGTTTGGACCTCCCTCCG

GGCTTCATGTTCAAGGTGCAGGCTCAACACGATTACACGGCCACTGACACCGACGAGCTG

CAGCTCAAGGCTGGTGACGTGGTGCTGGTGATCCCCTTTCAGAACCCGGAGGAACAGGAT

GAAGGCTGGCTCATGGGCGTAAAGGAGAGTGACTGGAACCAGCACAAGGAGTTGGAAAAA

TGCCGGGGCGTCTTCCCTGAGAACTTCACGGAACGGGTG

>CL148.Contig4_A-W 183 731 minus strand hypothetical protein PANDA_016718 [Ailuropoda melanoleuca]

CCTGGCAATGCTAGAACTGGAGACCAGCCAGGCCAATATCCCTGGCCTTCCCACTCAGGT

GCTGCCCTCCCCTGGCCTGTTCCCTGTGTCCTGGTTGCAGCATCCTGTCCTGTGTCTGAC

CCCTTGACGGCATTTATGTTGCAGCCCGCAGAGGCCTTGGAGGTGGCAGGTGGGACCCAA

CCTGCGCCTGGAGCCCAGGATCCTGGGGAGACGGCGGCAAGTGAAGCAGCCTCCAGTTCT

CTTCCGGCTGTGGTGGTGGAAACGTTCTCAGCAACTGTGAATGGCACCGTGGAAGGCAGC

AGTGGGGCCGGCCGTTTGGACCTCCCTCCGGGCTTCATGTTCAAGGTGCAGGCTCAACAC

GATTACACGGCCACTGACACCGACGAGCTGCAGCTCAAGGCTGGTGACGTGGTGCTGGTG

ATCCCCTTTCAGAACCCGGAGGAACAGGATGAAGGCTGGCTCATGGGCGTAAAGGAGAGT

GACTGGAACCAGCACAAGGAGTTGGAAAAATGCCGGGGCGTCTTCCCTGAGAACTTCACG

GAACGGGTG

>CL148.Contig5_A-W 110 661 minus strand Bin1 protein [Rattus norvegicus]

CAGCTCAACCAGAACCTCAACGATGTGCTGGTCAGCCTGGAGAAGCACCATGGCAGCAAC

ACCTTCACAGTCAAGGCCCAGCCCAGTGACAATGCCCCTGCCAAAGGGAACAAGAGCCCA

TCGCCTCCACCAGATGGCTCCCCTGCAGCCACCCCCGAGATCAGAGTGAACCATGAGCCA

GAGGCAGCCAGCGGGGCCACACCCGGGGCCTCCATCCCCAAGTCCCCATCTCAGAGTTCT

CTTCCGGCTGTGGTGGTGGAAACGTTCTCAGCAACTGTGAATGGCACCGTGGAAGGCAGC

AGTGGGGCCGGCCGTTTGGACCTCCCTCCGGGCTTCATGTTCAAGGTGCAGGCTCAACAC

GATTACACGGCCACTGACACCGACGAGCTGCAGCTCAAGGCTGGTGACGTGGTGCTGGTG

ATCCCCTTTCAGAACCCGGAGGAACAGGATGAAGGCTGGCTCATGGGCGTAAAGGAGAGT

GACTGGAACCAGCACAAGGAGTTGGAAAAATGCCGGGGCGTCTTCCCTGAGAACTTCACG

GAACGGGTGCAG

>CL148.Contig6_A-W 43 150 minus strand PREDICTED: myc box-dependent-interacting protein 1 isoform 5 [Otolemur garnettii]

CAGGATGAAGGCTGGCTCATGGGCGTAAAGGAGAGTGACTGGAACCAGCACAAGGAGTTG

GAAAAATGCCGGGGCGTCTTCCCTGAGAACTTCACGGAACGGGTGCAG

>CL148.Contig7_A-W 138 1478 minus strand PREDICTED: myc box-dependent-interacting protein 1 isoform 4 [Papio anubis]

GTCCTTCAGAAACTGGGGAAGGCAGATGAGACCAAGGATGAGCAGTTTGAACAGTGCGTC

CAGAATTTCAACAAGCAGCTGACTGAGGGCACCCGGCTGCAGAAGGATCTCCGGACTTAC

CTGGCTTCAGTCAAAGCCATGCACGAAGCCTCCAAGAAGCTGAACGAGTGTCTACAGGAG

GTGTATGAGCCCGACTGGCCCGGCAGGGACGAGGCCAATAAGATCGCCGAGAACAATGAC

CTGCTGTGGATGGACTACCACCAGAAACTGGTGGACCAGGCGCTGCTGACCATGGACACG

TACTTGGGCCAGTTCCCTGACATCAAGTCACGCATTGCCAAGCGAGGGCGGAAATTGGTG

GACTACGACAGCGCCCGGCACCACTATGAGTCCCTTCAAACTGCCAAAAAGAAAGATGAA

GTCAAAATCGCCAAGGCCGAGGAGGAGCTCATCAAGGCTCAGAAGGTGTTTGAGGAGATG

AATGTGGACCTCCAGGAGGAGCTCCCATCCCTGTGGAACAGCCGAGTAGGTTTCTATGTC

AATACATTTCAGAGCATCGCGGGCCTGGAGGAGAACTTCCACAAGGAGATGAGCAAGCTC

AACCAGAACCTCAACGATGTGCTGGTCAGCCTGGAGAAGCACCATGGCAGCAACACCTTC

ACAGTCAAGGCCCAGCCCAGTGACAATGCCCCTGCCAAAGGGAACAAGAGCCCATCGCCT

CCACCAGATGGCTCCCCTGCAGCCACCCCCGAGATCAGAGTGAACCATGAGCCAGAGGCA

GCCAGCGGGGCCACACCCGGGGCCTCCATCCCCAAGTCCCCATCTCAGCCCACAGAGAGT

CCAGCTGGCAGCCTGCCATCCGGGGAGCCTGGTGCTGCTGAGGGCACATTTGCTGTGTCC

TGGCCCAACCAGACGGCCGAGCAGGGGTCTGCCCAACCCGCAGAGGCCTTGGAGGTGGCA

GGTGGGACCCAACCTGCGCCTGGAGCCCAGGATCCTGGGGAGACGGCGGCAAGTGAAGCA

GCCTCCAGTTCTCTTCCGGCTGTGGTGGTGGAAACGTTCTCAGCAACTGTGAATGGCACC

GTGGAAGGCAGCAGTGGGGCCGGCCGTTTGGACCTCCCTCCGGGCTTCATGTTCAAGGTG

CAGGCTCAACACGATTACACGGCCACTGACACCGACGAGCTGCAGCTCAAGGCTGGTGAC

GTGGTGCTGGTGATCCCCTTTCAGAACCCGGAGGAACAGGATGAAGGCTGGCTCATGGGC

GTAAAGGAGAGTGACTGGAACCAGCACAAGGAGTTGGAAAAATGCCGGGGCGTCTTCCCT

GAGAACTTCACGGAACGGGTG

>CL148.Contig8_A-W 199 1428 minus strand Bin1 protein [Rattus norvegicus]

ATGGCAGAGATGGGCAGTAAGGGGGTGACGGCGGGGAAGATCGCCAGCAATGTACAGAAG

AAGCTCACCCGCGCGCAGGAGAAGGTCCTTCAGAAACTGGGGAAGGCAGATGAGACCAAG

GATGAGCAGTTTGAACAGTGCGTCCAGAATTTCAACAAGCAGCTGACTGAGGGCACCCGG

CTGCAGAAGGATCTCCGGACTTACCTGGCTTCAGTCAAAGCCATGCACGAAGCCTCCAAG

AAGCTGAACGAGTGTCTACAGGAGGTGTATGAGCCCGACTGGCCCGGCAGGGACGAGGCC

AATAAGATCGCCGAGAACAATGACCTGCTGTGGATGGACTACCACCAGAAACTGGTGGAC

CAGGCGCTGCTGACCATGGACACGTACTTGGGCCAGTTCCCTGACATCAAGTCACGCATT

GCCAAGCGAGGGCGGAAATTGGTGGACTACGACAGCGCCCGGCACCACTATGAGTCCCTT

CAAACTGCCAAAAAGAAAGATGAAGTCAAAATCGCCAAGGCCGAGGAGGAGCTCATCAAG

GCTCAGAAGGTGTTTGAGGAGATGAATGTGGACCTCCAGGAGGAGCTCCCATCCCTGTGG

AACAGCCGAGTAGGTTTCTATGTCAATACATTTCAGAGCATCGCGGGCCTGGAGGAGAAC

TTCCACAAGGAGATGAGCAAGCTCAACCAGAACCTCAACGATGTGCTGGTCAGCCTGGAG

AAGCACCATGGCAGCAACACCTTCACAGTCAAGGCCCAGCCCAGTGACAATGCCCCTGCC

AAAGGGAACAAGAGCCCATCGCCTCCACCAGATGGCTCCCCTGCAGCCACCCCCGAGATC

AGAGTGAACCATGAGCCAGAGGCAGCCAGCGGGGCCACACCCGGGGCCTCCATCCCCAAG

TCCCCATCTCAGAGTTCTCTTCCGGCTGTGGTGGTGGAAACGTTCTCAGCAACTGTGAAT

GGCACCGTGGAAGGCAGCAGTGGGGCCGGCCGTTTGGACCTCCCTCCGGGCTTCATGTTC

AAGGTGCAGGCTCAACACGATTACACGGCCACTGACACCGACGAGCTGCAGCTCAAGGCT

GGTGACGTGGTGCTGGTGATCCCCTTTCAGAACCCGGAGGAACAGGATGAAGGCTGGCTC

ATGGGCGTAAAGGAGAGTGACTGGAACCAGCACAAGGAGTTGGAAAAATGCCGGGGCGTC

TTCCCTGAGAACTTCACGGAACGGGTGCAG

>CL148.Contig9_A-W 138 1370 minus strand PREDICTED: myc box-dependent-interacting protein 1 isoform 1 [Saimiri boliviensis boliviensis]

GTCCTTCAGAAACTGGGGAAGGCAGATGAGACCAAGGATGAGCAGTTTGAACAGTGCGTC

CAGAATTTCAACAAGCAGCTGACTGAGGGCACCCGGCTGCAGAAGGATCTCCGGACTTAC

CTGGCTTCAGTCAAAGCCATGCACGAAGCCTCCAAGAAGCTGAACGAGTGTCTACAGGAG

GTGTATGAGCCCGACTGGCCCGGCAGGGACGAGGCCAATAAGATCGCCGAGAACAATGAC

CTGCTGTGGATGGACTACCACCAGAAACTGGTGGACCAGGCGCTGCTGACCATGGACACG

TACTTGGGCCAGTTCCCTGACATCAAGTCACGCATTGCCAAGCGAGGGCGGAAATTGGTG

GACTACGACAGCGCCCGGCACCACTATGAGTCCCTTCAAACTGCCAAAAAGAAAGATGAA

GTCAAAATCGCCAAGGCCGAGGAGGAGCTCATCAAGGCTCAGAAGGTGTTTGAGGAGATG

AATGTGGACCTCCAGGAGGAGCTCCCATCCCTGTGGAACAGCCGAGTAGGTTTCTATGTC

AATACATTTCAGAGCATCGCGGGCCTGGAGGAGAACTTCCACAAGGAGATGAGCAAGCTC

AACCAGAACCTCAACGATGTGCTGGTCAGCCTGGAGAAGCACCATGGCAGCAACACCTTC

ACAGTCAAGGCCCAGCCCAGTGACAATGCCCCTGCCAAAGGGAACAAGAGCCCATCGCCT

CCACCAGATGGCTCCCCTGCAGCCACCCCCGAGATCAGAGTGAACCATGAGCCAGAGGCA

GCCAGCGGGGCCACACCCGGGGCCTCCATCCCCAAGTCCCCATCTCAGCCCGCAGAGGCC

TTGGAGGTGGCAGGTGGGACCCAACCTGCGCCTGGAGCCCAGGATCCTGGGGAGACGGCG

GCAAGTGAAGCAGCCTCCAGTTCTCTTCCGGCTGTGGTGGTGGAAACGTTCTCAGCAACT

GTGAATGGCACCGTGGAAGGCAGCAGTGGGGCCGGCCGTTTGGACCTCCCTCCGGGCTTC

ATGTTCAAGGTGCAGGCTCAACACGATTACACGGCCACTGACACCGACGAGCTGCAGCTC

AAGGCTGGTGACGTGGTGCTGGTGATCCCCTTTCAGAACCCGGAGGAACAGGATGAAGGC

TGGCTCATGGGCGTAAAGGAGAGTGACTGGAACCAGCACAAGGAGTTGGAAAAATGCCGG

GGCGTCTTCCCTGAGAACTTCACGGAACGGGTG

>CL148.Contig10_A-W 138 1280 minus strand unnamed protein product [Homo sapiens]

GTCCTTCAGAAACTGGGGAAGGCAGATGAGACCAAGGATGAGCAGTTTGAACAGTGCGTC

CAGAATTTCAACAAGCAGCTGACTGAGGGCACCCGGCTGCAGAAGGATCTCCGGACTTAC

CTGGCTTCAGTCAAAGCCATGCACGAAGCCTCCAAGAAGCTGAACGAGTGTCTACAGGAG

GTGTATGAGCCCGACTGGCCCGGCAGGGACGAGGCCAATAAGATCGCCGAGAACAATGAC

CTGCTGTGGATGGACTACCACCAGAAACTGGTGGACCAGGCGCTGCTGACCATGGACACG

TACTTGGGCCAGTTCCCTGACATCAAGTCACGCATTGCCAAGCGAGGGCGGAAATTGGTG

GACTACGACAGCGCCCGGCACCACTATGAGTCCCTTCAAACTGCCAAAAAGAAAGATGAA

GTCAAAATCGCCAAGGCCGAGGAGGAGCTCATCAAGGCTCAGAAGGTGTTTGAGGAGATG

AATGTGGACCTCCAGGAGGAGCTCCCATCCCTGTGGAACAGCCGAGTAGGTTTCTATGTC

AATACATTTCAGAGCATCGCGGGCCTGGAGGAGAACTTCCACAAGGAGATGAGCAAGCTC

AACCAGAACCTCAACGATGTGCTGGTCAGCCTGGAGAAGCACCATGGCAGCAACACCTTC

ACAGTCAAGGCCCAGCCCAGTGACAATGCCCCTGCCAAAGGGAACAAGAGCCCATCGCCT

CCACCAGATGGCTCCCCTGCAGCCACCCCCGAGATCAGAGTGAACCATGAGCCAGAGGCA

GCCAGCGGGGCCACACCCGGGGCCTCCATCCCCAAGTCCCCATCTCAGAGTTCTCTTCCG

GCTGTGGTGGTGGAAACGTTCTCAGCAACTGTGAATGGCACCGTGGAAGGCAGCAGTGGG

GCCGGCCGTTTGGACCTCCCTCCGGGCTTCATGTTCAAGGTGCAGGCTCAACACGATTAC

ACGGCCACTGACACCGACGAGCTGCAGCTCAAGGCTGGTGACGTGGTGCTGGTGATCCCC

TTTCAGAACCCGGAGGAACAGGATGAAGGCTGGCTCATGGGCGTAAAGGAGAGTGACTGG

AACCAGCACAAGGAGTTGGAAAAATGCCGGGGCGTCTTCCCTGAGAACTTCACGGAACGG

GTG

>CL148.Contig11_A-W 110 856 minus strand PREDICTED: myc box-dependent-interacting protein 1 isoform 4 [Papio anubis]

CAGCTCAACCAGAACCTCAACGATGTGCTGGTCAGCCTGGAGAAGCACCATGGCAGCAAC

ACCTTCACAGTCAAGGCCCAGCCCAGTGACAATGCCCCTGCCAAAGGGAACAAGAGCCCA

TCGCCTCCACCAGATGGCTCCCCTGCAGCCACCCCCGAGATCAGAGTGAACCATGAGCCA

GAGGCAGCCAGCGGGGCCACACCCGGGGCCTCCATCCCCAAGTCCCCATCTCAGCCCACA

GAGAGTCCAGCTGGCAGCCTGCCATCCGGGGAGCCTGGTGCTGCTGAGGGCACATTTGCT

GTGTCCTGGCCCAACCAGACGGCCGAGCAGGGGTCTGCCCAACCCGCAGAGGCCTTGGAG

GTGGCAGGTGGGACCCAACCTGCGCCTGGAGCCCAGGATCCTGGGGAGACGGCGGCAAGT

GAAGCAGCCTCCAGTTCTCTTCCGGCTGTGGTGGTGGAAACGTTCTCAGCAACTGTGAAT

GGCACCGTGGAAGGCAGCAGTGGGGCCGGCCGTTTGGACCTCCCTCCGGGCTTCATGTTC

AAGGTGCAGGCTCAACACGATTACACGGCCACTGACACCGACGAGCTGCAGCTCAAGGCT

GGTGACGTGGTGCTGGTGATCCCCTTTCAGAACCCGGAGGAACAGGATGAAGGCTGGCTC

ATGGGCGTAAAGGAGAGTGACTGGAACCAGCACAAGGAGTTGGAAAAATGCCGGGGCGTC

TTCCCTGAGAACTTCACGGAACGGGTG

>CL149.Contig1_A-W 986 1135 PREDICTED: surfeit locus protein 1-like [Equus caballus]

GAGAGCACAGTCCCTGGAGGACCCATCGGAGGTCAAACCAGAGTCACTCTGCGGAATGAA

CACATGCAGTACATCATTACCTGGTATGGACTATGTGCAGCTACATCATACCTGTGGTTT

AAGAAATTCCTGCGCCGGACACCTGGCATG

>CL149.Contig2_A-W 4 708 Surfeit locus protein 1 OS=Homo sapiens GN=SURF1 PE=1 SV=1

GCTGCCTGGACGCGCGTCGCAGTCGCCTCGCGCGCGGGGGTGGCCTGGGGGCCGAGCAGG

TGTGGCAGTTCTATGGCAGAAGCACCTGCCACAAAAACGGAAGATGACTCCTTTCTCCAG

TGGTTTCTGCTTCTCATCCCTGTGACTGCCTTTGGCCTGGGGACTTGGCAGGTCCAGCGT

CGGAAATGGAAGCTGAAATTGATTGCAGAATTAGAGTCTAGAGTTATGGCTGATCCCGTC

CCTCTGCCAGCAGACCCAATAGAACTGCAAAATCTGGAGTATAGACCAGTTAAGGTCAGG

GGGCGCTTTGATCACTCCAAAGAGCTGTACATGATGCCCCGGACCATGGTGGATCCTGCC

CGGGAGGCCCGGGAAGCTGGTCGGATCTCCTCCTCAACTGAGAGTGGGGCCTATGTAGTC

ACCCCCTTCCTCTGCACCGACCTGGGAGTCACTATTCTGGTAAACCGAGGGTTTGTCCCA

AGGAAGAAAGTGAATCCTGAAACTCGGCAGAAAGGCCAGGTTGAGGGAGAAGTGGACCTA

GTCGGGATGGTGAGGCTTACAGAAACCAGGAAGCCCTTTGTTCCCGAGAATAATCCAGAA

AGGAACCATTGGCATTACCGGGACCTGGAGGCCATGGCTAGGGTCACAGGCACAGACCCC

ATTTTTATTGATGCCGACTTCCGTATGTTGAAACCAGCCCTCCCC

>CL149.Contig3_A-W 4 525 Surfeit locus protein 1 OS=Homo sapiens GN=SURF1 PE=1 SV=1

GCTGCCTGGACGCGCGTCGCAGTCGCCTCGCGCGCGGGGGTGGCCTGGGGGCCGAGCAGG

TGTGGCAGTTCTATGGCAGAAGCACCTGCCACAAAAACGGAAGATGACTCCTTTCTCCAG

TGGTTTCTGCTTCTCATCCCTGTGACTGCCTTTGGCCTGGGGACTTGGCAGGTCCAGCGT

CGGAAATGGAAGCTGAAATTGATTGCAGAATTAGAGTCTAGAGTTATGGCTGATCCCGTC

CCTCTGCCAGCAGACCCAATAGAACTGCAAAATCTGGAGTATAGACCAGTTAAGGTCAGG

GGGCGCTTTGATCACTCCAAAGAGCTGTACATGATGCCCCGGACCATGGTGGATCCTGCC

CGGGAGGCCCGGGAAGCTGGTCGGATCTCCTCCTCAACTGAGAGTGGGGCCTATGTAGTC

ACCCCCTTCCTCTGCACCGACCTGGGAGTCACTATTCTGGTAAACCGAGGGTTTGTCCCA

AGGAAGAAAGTGAATCCTGAAACTCGGCAGAAAGGCCAGGTA

>CL149.Contig4_A-W 474 623 PREDICTED: surfeit locus protein 1-like [Equus caballus]

GAGAGCACAGTCCCTGGAGGACCCATCGGAGGTCAAACCAGAGTCACTCTGCGGAATGAA

CACATGCAGTACATCATTACCTGGTATGGACTATGTGCAGCTACATCATACCTGTGGTTT

AAGAAATTCCTGCGCCGGACACCTGGCATG

>CL149.Contig5_A-W 600 911 PREDICTED: surfeit locus protein 1-like [Equus caballus]

GTTGAGGGAGAAGTGGACCTAGTCGGGATGGTGAGGCTTACAGAAACCAGGAAGCCCTTT

GTTCCCGAGAATAATCCAGAAAGGAACCATTGGCATTACCGGGACCTGGAGGCCATGGCT

AGGGTCACAGGCACAGACCCCATTTTTATTGATGCCGACTTCCAGAGCACAGTCCCTGGA

GGACCCATCGGAGGTCAAACCAGAGTCACTCTGCGGAATGAACACATGCAGTACATCATT

ACCTGGTATGGACTATGTGCAGCTACATCATACCTGTGGTTTAAGAAATTCCTGCGCCGG

ACACCTGGCATG

>CL149.Contig6_A-W 4 768 Surfeit locus protein 1 OS=Homo sapiens GN=SURF1 PE=1 SV=1

GCTGCCTGGACGCGCGTCGCAGTCGCCTCGCGCGCGGGGGTGGCCTGGGGGCCGAGCAGG

TGTGGCAGTTCTATGGCAGAAGCACCTGCCACAAAAACGGAAGATGACTCCTTTCTCCAG

TGGTTTCTGCTTCTCATCCCTGTGACTGCCTTTGGCCTGGGGACTTGGCAGGTCCAGCGT

CGGAAATGGAAGCTGAAATTGATTGCAGAATTAGAGTCTAGAGTTATGGCTGATCCCGTC

CCTCTGCCAGCAGACCCAATAGAACTGCAAAATCTGGAGTATAGACCAGTTAAGGTCAGG

GGGCGCTTTGATCACTCCAAAGAGCTGTACATGATGCCCCGGACCATGGTGGATCCTGCC

CGGGAGGCCCGGGAAGCTGGTCGGATCTCCTCCTCAACTGAGAGTGGGGCCTATGTAGTC

ACCCCCTTCCTCTGCACCGACCTGGGAGTCACTATTCTGGTAAACCGAGGGTTTGTCCCA

AGGAAGAAAGTGAATCCTGAAACTCGGCAGAAAGGCCAGGTTGAGGGAGAAGTGGACCTA

GTCGGGATGGTGAGGCTTACAGAAACCAGGAAGCCCTTTGTTCCCGAGAATAATCCAGAA

AGGAACCATTGGCATTACCGGGACCTGGAGGCCATGGCTAGGGTCACAGGCACAGACCCC

ATTTTTATTGATGCCGACTTCCAGAGCACAGTCCCTGGAGGACCCATCGGAGGTCAAACC

AGAGTCACTCTGCGGAATGAACACATGCAGTACATCATTACCTGG

>CL149.Contig7_A-W 1 834 PREDICTED: surfeit locus protein 1-like [Equus caballus]

CGCGCTGCCTGGACGCGCGTCGCAGTCGCCTCGCGCGCGGGGGTGGCCTGGGGGCCGAGC

AGGTGTGGCAGTTCTATGGCAGAAGCACCTGCCACAAAAACGGAAGATGACTCCTTTCTC

CAGTGGTTTCTGCTTCTCATCCCTGTGACTGCCTTTGGCCTGGGGACTTGGCAGGTCCAG

CGTCGGAAATGGAAGCTGAAATTGATTGCAGAATTAGAGTCTAGAGTTATGGCTGATCCC

GTCCCTCTGCCAGCAGACCCAATAGAACTGCAAAATCTGGAGTATAGACCAGTTAAGGTC

AGGGGGCGCTTTGATCACTCCAAAGAGCTGTACATGATGCCCCGGACCATGGTGGATCCT

GCCCGGGAGGCCCGGGAAGCTGGTCGGATCTCCTCCTCAACTGAGAGTGGGGCCTATGTA

GTCACCCCCTTCCTCTGCACCGACCTGGGAGTCACTATTCTGGTAAACCGAGGGTTTGTC

CCAAGGAAGAAAGTGAATCCTGAAACTCGGCAGAAAGGCCAGGTTGAGGGAGAAGTGGAC

CTAGTCGGGATGGTGAGGCTTACAGAAACCAGGAAGCCCTTTGTTCCCGAGAATAATCCA

GAAAGGAACCATTGGCATTACCGGGACCTGGAGGCCATGGCTAGGGTCACAGGCACAGAC

CCCATTTTTATTGATGCCGACTTCCAGAGCACAGTCCCTGGAGGACCCATCGGAGGTCAA

ACCAGAGTCACTCTGCGGAATGAACACATGCAGTACATCATTACCTGGTATGGACTATGT

GCAGCTACATCATACCTGTGGTTTAAGAAATTCCTGCGCCGGACACCTGGCATG

>CL149.Contig8_A-W 395 640 Surfeit locus protein 1 OS=Homo sapiens GN=SURF1 PE=1 SV=1

GTTGAGGGAGAAGTGGACCTAGTCGGGATGGTGAGGCTTACAGAAACCAGGAAGCCCTTT

GTTCCCGAGAATAATCCAGAAAGGAACCATTGGCATTACCGGGACCTGGAGGCCATGGCT

AGGGTCACAGGCGCAGACCCCATTTTTATTGATGCCGACTTCCAGAGCACAGTCCCTGGA

GGACCCATCGGAGGTCAAACCAGAGTCACTCTGCGGAATGAACACATGCAGTACATCATT

ACCTGG

>CL149.Contig9_A-W 395 706 Surfeit locus protein 1 OS=Homo sapiens GN=SURF1 PE=1 SV=1

GTTGAGGGAGAAGTGGACCTAGTCGGGATGGTGAGGCTTACAGAAACCAGGAAGCCCTTT

GTTCCCGAGAATAATCCAGAAAGGAACCATTGGCATTACCGGGACCTGGAGGCCATGGCT

AGGGTCACAGGCGCAGACCCCATTTTTATTGATGCCGACTTCCAGAGCACAGTCCCTGGA

GGACCCATCGGAGGTCAAACCAGAGTCACTCTGCGGAATGAACACATGCAGTACATCATT

ACCTGGTATGGACTATGTGCAGCTACATCATACCTGTGGTTTAAGAAATTCCTGCGCCGG

ACACCTGGCATG

>CL149.Contig10_A-W 1 699 PREDICTED: surfeit locus protein 1-like [Equus caballus]

TGGGGGCCGAGCAGGTGTGGCAGTTCTATGGCAGAAGCACCTGCCACAAAAACGGAAGAT

GACTCCTTTCTCCAGTGGTTTCTGCTTCTCATCCCTGTGACTGCCTTTGGCCTGGGGACT

TGGCAGGTCCAGCGTCGGAAATGGAAGCTGAAATTGATTGCAGAATTAGAGTCTAGAGTT

ATGGCTGATCCCGTCCCTCTGCCAGCAGACCCAATAGAACTGCAAAATCTGGAGTATAGA

CCAGTTAAGGTCAGGGGGCGCTTTGATCACTCCAAAGAGCTGTACATGATGCCCCGGACC

ATGGTGGATCCTGCCCGGGAGGCCCGGGAAGCTGGTCGGATCTCCTCCTCAACTGAGAGT

GGGGCCTATGTAGTCACCCCCTTCCTCTGCACCGACCTGGGAGTCACTATTCTGGTAAAC

CGAGGGTTTGTCCCAAGGAAGAAAGTGAATCCTGAAACTCGGCAGAAAGGCCAGAGAGCA

CAGTCCCTGGAGGACCCATCGGAGGTCAAACCAGAGTCACTCTGCGGAATGAACACATGC

AGTACATCATTACCTGGTCAGTTGTACTGCCCCTGGTCCCCTCTTTTTAGTGCACTTCAC

AGCCTAACAGCTTAATTTTCTTTCAACCTAAGGTATGGACTATGTGCAGCTACATCATAC

CTGTGGTTTAAGAAATTCCTGCGCCGGACACCTGGCATG

>CL149.Contig11_A-W 246 629 PREDICTED: surfeit locus protein 1-like [Equus caballus]

GTCACTATTCTGGTAAACCGAGGGTTTGTCCCAAGGAAGAAAGTGAATCCTGAAACTCGG

CAGAAAGGCCAGGTTGAGGGAGAAGTGGACCTAGTCGGGATGGTGAGGCTTACAGAAACC

AGGAAGCCCTTTGTTCCCGAGAATAATCCAGAAAGGAACCATTGGCATTACCGGGACCTG

GAGGCCATGGCTAGGGTCACAGGCGCAGACCCCATTTTTATTGATGCCGACTTCCAGAGC

ACAGTCCCTGGAGGACCCATCGGAGGTCAAACCAGAGTCACTCTGCGGAATGAACACATG

CAGTACATCATTACCTGGTATGGACTATGTGCAGCTACATCATACCTGTGGTTTAAGAAA

TTCCTGCGCCGGACACCTGGCATG

>CL149.Contig12_A-W 781 930 PREDICTED: surfeit locus protein 1 [Canis lupus familiaris]

GAGAGCACAGTCCCTGGAGGACCCATCGGAGGTCAAACCAGAGTCACTCTGCGGAATGAA

CACATGCAGTACATCATTACCTGGTATGGACTATGTGCAGCTACATCATACCTGTGGTTT

AAGAAATTCCTGCGCCGGACACCTGGCATG

>CL149.Contig13_A-W 395 580 Surfeit locus protein 1 OS=Homo sapiens GN=SURF1 PE=1 SV=1

GTTGAGGGAGAAGTGGACCTAGTCGGGATGGTGAGGCTTACAGAAACCAGGAAGCCCTTT

GTTCCCGAGAATAATCCAGAAAGGAACCATTGGCATTACCGGGACCTGGAGGCCATGGCT

AGGGTCACAGGCGCAGACCCCATTTTTATTGATGCCGACTTCCGTATGTTGAAACCAGCC

CTCCCC

>CL149.Contig14_A-W 704 853 PREDICTED: surfeit locus protein 1 [Canis lupus familiaris]

GAGAGCACAGTCCCTGGAGGACCCATCGGAGGTCAAACCAGAGTCACTCTGCGGAATGAA

CACATGCAGTACATCATTACCTGGTATGGACTATGTGCAGCTACATCATACCTGTGGTTT

AAGAAATTCCTGCGCCGGACACCTGGCATG

>CL149.Contig15_A-W 246 503 Surfeit locus protein 1 OS=Homo sapiens GN=SURF1 PE=1 SV=1

GTCACTATTCTGGTAAACCGAGGGTTTGTCCCAAGGAAGAAAGTGAATCCTGAAACTCGG

CAGAAAGGCCAGGTTGAGGGAGAAGTGGACCTAGTCGGGATGGTGAGGCTTACAGAAACC

AGGAAGCCCTTTGTTCCCGAGAATAATCCAGAAAGGAACCATTGGCATTACCGGGACCTG

GAGGCCATGGCTAGGGTCACAGGCGCAGACCCCATTTTTATTGATGCCGACTTCCGTATG

TTGAAACCAGCCCTCCCC

>CL149.Contig17_A-W 909 1058 PREDICTED: surfeit locus protein 1-like [Equus caballus]

GAGAGCACAGTCCCTGGAGGACCCATCGGAGGTCAAACCAGAGTCACTCTGCGGAATGAA

CACATGCAGTACATCATTACCTGGTATGGACTATGTGCAGCTACATCATACCTGTGGTTT

AAGAAATTCCTGCGCCGGACACCTGGCATG

>CL149.Contig18_A-W 4 525 Surfeit locus protein 1 OS=Homo sapiens GN=SURF1 PE=1 SV=1

GCTGCCTGGACGCGCGTCGCAGTCGCCTCGCGCGCGGGGGTGGCCTGGGGGCCGAGCAGG

TGTGGCAGTTCTATGGCAGAAGCACCTGCCACAAAAACGGAAGATGACTCCTTTCTCCAG

TGGTTTCTGCTTCTCATCCCTGTGACTGCCTTTGGCCTGGGGACTTGGCAGGTCCAGCGT

CGGAAATGGAAGCTGAAATTGATTGCAGAATTAGAGTCTAGAGTTATGGCTGATCCCGTC

CCTCTGCCAGCAGACCCAATAGAACTGCAAAATCTGGAGTATAGACCAGTTAAGGTCAGG

GGGCGCTTTGATCACTCCAAAGAGCTGTACATGATGCCCCGGACCATGGTGGATCCTGCC

CGGGAGGCCCGGGAAGCTGGTCGGATCTCCTCCTCAACTGAGAGTGGGGCCTATGTAGTC

ACCCCCTTCCTCTGCACCGACCTGGGAGTCACTATTCTGGTAAACCGAGGGTTTGTCCCA

AGGAAGAAAGTGAATCCTGAAACTCGGCAGAAAGGCCAGGTA

>CL149.Contig19_A-W 246 563 Surfeit locus protein 1 OS=Homo sapiens GN=SURF1 PE=1 SV=1

GTCACTATTCTGGTAAACCGAGGGTTTGTCCCAAGGAAGAAAGTGAATCCTGAAACTCGG

CAGAAAGGCCAGGTTGAGGGAGAAGTGGACCTAGTCGGGATGGTGAGGCTTACAGAAACC

AGGAAGCCCTTTGTTCCCGAGAATAATCCAGAAAGGAACCATTGGCATTACCGGGACCTG

GAGGCCATGGCTAGGGTCACAGGCGCAGACCCCATTTTTATTGATGCCGACTTCCAGAGC

ACAGTCCCTGGAGGACCCATCGGAGGTCAAACCAGAGTCACTCTGCGGAATGAACACATG

CAGTACATCATTACCTGG

>CL150.Contig1_A-W 52 1266 PREDICTED: ubiquitin-associated domain-containing protein 1 [Papio anubis]

ATGTTCGTGCAGGAGGAGAAGATCTTCGCGGGCAAGGTGCTGCGGCTGCACATCTGTGCC

GCGGACGGCGCCGAGTGGCTGGAGGAGGCGACCGAGGACACCTCGGTGGAGAAGCTCAAG

GAGCGCTGCCTCAAGCACTGTGCTCATGGGAGCTTAGAGGATCCCAAAAGTGTGACCCAT

CATAAGTTAATCCACGCTGCTTCAGAGAGGGTGCTGAGTGACACCAGGACAATCTTAGAG

GAGAATGTCCAAGACAAAGATGTCCTATTATTGATAAAAAAGCGTGCTCCAGAGCCACTT

CCCAAGATGGCTGATGTCTCCGCAGAAGAAAAGAAGAAACAAGAACAGAAGGCTCCAGAC

AGAGACGCCATACATCGGGCCACAGCCAACCTGCCCGCCTGTAGCACGGACCGGGCCGCA

GTCCAGACCACCATGAGGGATTTCCAGACAGAGCTCCGGAAAATCTTGGTGTCTCTCATC

GAGGTGGCACAGAAGTTGTTAGCACTGAACCCTGACGCAGTTGAATTGTTTAAGAAGGCA

AATGCCATGTTGGACGAGGATGAGGACGAGCGAGTGGATGAGGCCGCCCTGCGGCAGCTC

ACAGAGATGGGCTTTCCTGAGAGCAGGGCTGCAAAGGCCCTTCGGCTGAACCACATGTCG

GTGCCTCAAGCCATGGAGTGGCTGATAGAGCACGCCGAGGACCCGGCCATCGATGCGCCT

CTTCCAGGCCAGGCCTCACCAGCAGCAGCAGGTGCTGCGGCCGCCACTCCAGAAGCTGCT

GCTGGGACTAGCGAGGGAGACCAGGAGGTCCGGGACGAGCTCACGGATATCTTCAAGAAG

ATTCGCAGGAAAAGGGAATTCCGGGCCGATGCTCGGGCCGTCGTTTCCCTGATGGAGATG

GGCTTTGATGAGAAGGAGGTGGTGGACGCCCTCAGAGTGAGCAACAACCAGCAGAATGCC

GCCTGCGAGTGGCTGCTGGGAGACCGCAAGCCATCCCCTGAGGAGCTGGACCAGGGCATC

GACCCCAACAGCCCTCTCTTTCAGGCTATTCTGGACAACCCGGTGGTGCAGCTGGGGCTT

ACCAACCCCAAAACGTTGCTCGCATTTGAAGACATGCTGGAGAACCCACTGAACAGCACC

CAGTGGATGAATGACCCAGAAACCGGCCCAGTCATGCTGCAGATTTCTAGAATCTTCCAG

ACGCTGAACCGCACG

>CL150.Contig2_A-W 52 600 Ubiquitin associated domain containing 1 [Mus musculus]

ATGTTCGTGCAGGAGGAGAAGATCTTCGCGGGCAAGGTGCTGCGGCTGCACATCTGTGCC

GCGGACGGCGCCGAGTGGCTGGAGGAGGCGACCGAGGACACCTCGGTGGAGAAGCTCAAG

GAGCGCTGCCTCAAGCACTGTGCTCATGGGAGCTTAGAGGATCCCAAAAGTGTGACCCAT

CATAAGTTAATCCACGCTGCTTCAGAGAGGGTGCTGAGTGACACCAGGACAATCTTAGAG

GAGAATGTCCAAGACAAAGATGTCCTATTATTGATAAAAAAGCGTGCTCCAGAGCCACTT

CCCAAGATGGCTGATGTCTCCGCAGAAGAAAAGAAGAAACAAGAACAGAAGGCTCCAGAC

AGAGACGCCATACATCGGGCCACAGCCAACCTGCCCGCCTGTAGCACGGACCGGGCCGCA

GTCCAGACCACCATGAGGGATTTCCAGACAGAGCTCCGGAAAATCTTGGTGTCTCTCATC

GAGGTGGCACAGAAGTTGTTAGCACTGAACCCTGACGCAGTTGAATTGTTTAAGAAGGCA

AATGGTATG

>CL151.Contig1_A-W 1 1488 minus strand PREDICTED: prostate tumor-overexpressed gene 1 protein-like [Macaca mulatta]

CCCAGCCCTTCTTGGTACGGCCCGACCACGCCCCCTCGCGGACGCCGTTGGTACGCGCCG

AGCTCCCCCGCTTGCCGCAGTGGTTTGGCCGCGGCGACCCCTCTGGTAGCGCGTCCCCCG

CGCTTGGTACGGCTCAGCCCGTCTCCCCTGAAGCCGCGCGCCCGCGCCCCTCAGTCGGTG

GATCCCGCAGCCCCCCGTGTGGCCCGTGGCAGCCGTCAGCCCGCTCGGCCCTCGTTCGCC

ATGGTCCGTCCGCGCCGCGCCCCGCATCGCTCTGGCGTTGGGGGCCCCCTCGGGGGTCGC

GGTCGCCCCCCGCGGCCTCTCGTGGTGCGTGCGGTCCGCTCGCGCTCCTGGCCAGCCAGC

CCTCGAGGCCCTCAGCCACCGAGGATCAGGGCCCGCTCGGCCCCTCCCATGGAAGGTGCT

CGGGTCTTCGGGGCTCTGGGTCCCATCGGGCCTTCCTCACCTGGCCTCTCCCTTGGGGGT

CTGGCTGTAAGTGAACACCGTCTCAGCAATAAGCTGCTGGCCTGGAGCGGCGTCCTGGAA

TGGCAGGAGAAACGCAGACCCTACTCTGACTCTACTGCAAAGTTGAAACGGGCCCTGCCC

TGCCAGGCCTACGTGAACCAGGGCGAGAACCTGGAGACTGACCAGTGGCCACAGAAGTTG

ATCATGCAGCTGATCCCGCAGCAGCTGCTGACCACATTGGGCCCCCTGTTTCGCAACTCC

CAGCTGGCACAGTTCCACTTCACCAACAGAGACTGCGACTCCCTCAAGGGGCTTTGCCGC

ATCATGGGCAACGGCTTTGCGGGCTGCATGCTTTTTCCCCACATCTCCCCCTGCGAGGTG

CGCGTGCTCATGCTCCTGTACTCGTCCAAGAAGAAGATCTTCATGGGCCTCATCCCCTAT

GACCAGAGCGGCTTCGTCAATGCCATACGGCAGGTCATCACTACTCGCAAACAGGTGGTG

GGACCTGGTGGAGTCCACTCAGGCCCTGTCCAAATTGTCAACAACAAATTCCTGGCATGG

AGTGGTGTTATGGAGTGGCAGGAGCCTAGGCCTGAGCCCAACAGTCGATCCAAGAGGTGG

TTGCCATCCCATGTCTACGTGAACCAAGGAGAGATCTTGAGGACTGAGCAGTGGCCGAGG

AAGCTGTACATGCAGCTCATCCCACAGCAGCTCCTGACCACTTTGGTGCCACTGTTTCGG

AACTCCCGCCTGGTGCAGTTTCACTTCACCAAGGACATGGAGACGCTGAAGAGCCTGTGC

CGGATCATGGACAACGGCTTTGCTGGCTGCGTGCACTTTTCCTATAAAGCCTCATGTGAG

ATCCGCGTGCTCATGCTCCTGTACTCCTCGGAGAAGAAGATCTTCATTGGCCTCATCCCC

CACGACCAGAGCAACTTCGTCAACGGTATCCGGCGTGTCATAGCCAACCAGCAGCAGGTC

CTTCAGCGGAATCTGGAGCAGGAGCAGCAGCAGCGAGGGATGGGGGGG

>CL151.Contig2_A-W 1 1488 minus strand PREDICTED: prostate tumor-overexpressed gene 1 protein-like [Macaca mulatta]

CCCAGCCCTTCTTGGTACGGCCCGACCACGCCCCCTCGCGGACGCCGTTGGTACGCGCCG

AGCTCCCCCGCTTGCCGCAGTGGTTTGGCCGCGGCGACCCCTCTGGTAGCGCGTCCCCCG

CGCTTGGTACGGCTCAGCCCGTCTCCCCTGAAGCCGCGCGCCCGCGCCCCTCAGTCGGTG

GATCCCGCAGCCCCCCGTGTGGCCCGTGGCAGCCGTCAGCCCGCTCGGCCCTCGTTCGCC

ATGGTCCGTCCGCGCCGCGCCCCGCATCGCTCTGGCGTTGGGGGCCCCCTCGGGGGTCGC

GGTCGCCCCCCGCGGCCTCTCGTGGTGCGTGCGGTCCGCTCGCGCTCCTGGCCAGCCAGC

CCTCGAGGCCCTCAGCCACCGAGGATCAGGGCCCGCTCGGCCCCTCCCATGGAAGGTGCT

CGGGTCTTCGGGGCTCTGGGTCCCATCGGGCCTTCCTCACCTGGCCTCTCCCTTGGGGGT

CTGGCTGTAAGTGAACACCGTCTCAGCAATAAGCTGCTGGCCTGGAGCGGCGTCCTGGAA

TGGCAGGAGAAACGCAGACCCTACTCTGACTCTACTGCAAAGTTGAAACGGGCCCTGCCC

TGCCAGGCCTACGTGAACCAGGGCGAGAACCTGGAGACTGACCAGTGGCCACAGAAGTTG

ATCATGCAGCTGATCCCGCAGCAGCTGCTGACCACATTGGGCCCCCTGTTTCGCAACTCC

CAGCTGGCACAGTTCCACTTCACCAACAGAGACTGCGACTCCCTCAAGGGGCTTTGCCGC

ATCATGGGCAACGGCTTTGCGGGCTGCATGCTTTTTCCCCACATCTCCCCCTGCGAGGTG

CGCGTGCTCATGCTCCTGTACTCGTCCAAGAAGAAGATCTTCATGGGCCTCATCCCCTAT

GACCAGAGCGGCTTCGTCAATGCCATACGGCAGGTCATCACTACTCGCAAACAGGTGGTG

GGACCTGGTGGAGTCCACTCAGGCCCTGTCCAAATTGTCAACAACAAATTCCTGGCATGG

AGTGGTGTTATGGAGTGGCAGGAGCCTAGGCCTGAGCCCAACAGTCGATCCAAGAGGTGG

TTGCCATCCCATGTCTACGTGAACCAAGGAGAGATCTTGAGGACTGAGCAGTGGCCGAGG

AAGCTGTACATGCAGCTCATCCCACAGCAGCTCCTGACCACTTTGGTGCCACTGTTTCGG

AACTCCCGCCTGGTGCAGTTTCACTTCACCAAGGACATGGAGACGCTGAAGAGCCTGTGC

CGGATCATGGACAACGGCTTTGCTGGCTGCGTGCACTTTTCCTATAAAGCCTCATGTGAG

ATCCGCGTGCTCATGCTCCTGTACTCCTCGGAGAAGAAGATCTTCATTGGCCTCATCCCC

CACGACCAGAGCAACTTCGTCAACGGTATCCGGCGTGTCATAGCCAACCAGCAGCAGGTC

CTTCAGCGGAATCTGGAGCAGGAGCAGCAGCAGCGAGGGATGGGGGGG

>CL151.Contig3_A-W 1 402 PREDICTED: prostate tumor-overexpressed gene 1 protein-like [Macaca mulatta]

CGGACGCCGTTGGTACGCGCCGAGCTCCCCCGCTTGCCGCAGTGGTTTGGCCGCGGCGAC

CCCTCTGGTAGCGCGTCCCCCGCGCTTGGTACGGCTCAGCCCGTCTCCCCTGAAGCCGCG

CGCCCGCGCCCCTCAGTCGGTGGATCCCGCAGCCCCCCGTGTGGCCCGTGGCAGCCGTCA

GCCCGCTCGGCCCTCGTTCGCCATGGTCCGTCCGCGCCGCGCCCCGCATCGCTCTGGCGT

TGGGGGCCCCCTCGGGGGTCGCGGTCGCCCCCCGCGGCCTCTCGTGGTGCGTGCGGTCCG

CTCGCGCTCCTGGCCAGCCAGCCCTCGAGGCCCTCAGCCACCGAGGATCAGGGCCCGCTC

GGCCCCTCCCATGGAAGGTGCTCGGGTCTTCGGGGCTCTGGG

>CL151.Contig4_A-W 216 395 Prostate tumor overexpressed gene 1 protein, partial [Heterocephalus glaber]

CTGAAGAGCCTCTGTCTGCAGGCGGGCTGCGTGCACTTCCCTCACACGGCCCCCTGCGAG

GTGCGCGTGCTCATGCTCCTGTACTCGTCCAAGAAGAAGATCTTCATGGGCCTCATCCCC

TATGACCAGAGCGGCTTCGTCAATGCCATACGGCAGGTCATCACTACTCGCAAACAGGTC

>CL151.Contig5_A-W 65 391 Mediator of RNA polymerase II transcription subunit 25 OS=Mus musculus GN=Med25 PE=1 SV=1

AAGACTGAGCAGTGGCCCCAGAAGCTGATCATGCAGCTTATCCCCCAGCAACTGCTGACC

ACCCTGGGCCCTCTGTTCCGGAACTCGAGGATGGTCCAGTTCCACTTCACCAACAAGGAC

CTGGAGTCCCTCAAAGGCCTCTACCGCATTATGGGCAATGGCTTTGCGGGCTGCGTGCAC

TTCCCTCACACGGCCCCCTGCGAGGTGCGCGTGCTCATGCTCCTGTACTCGTCCAAGAAG

AAGATCTTCATGGGCCTCATCCCCTATGACCAGAGCGGCTTCGTCAATGCCATACGGCAG

GTCATCACTACTCGCAAACAGGTCAGG

>CL151.Contig6_A-W 1 402 PREDICTED: prostate tumor-overexpressed gene 1 protein-like [Macaca mulatta]

CGGACGCCGTTGGTACGCGCCGAGCTCCCCCGCTTGCCGCAGTGGTTTGGCCGCGGCGAC

CCCTCTGGTAGCGCGTCCCCCGCGCTTGGTACGGCTCAGCCCGTCTCCCCTGAAGCCGCG

CGCCCGCGCCCCTCAGTCGGTGGATCCCGCAGCCCCCCGTGTGGCCCGTGGCAGCCGTCA

GCCCGCTCGGCCCTCGTTCGCCATGGTCCGTCCGCGCCGCGCCCCGCATCGCTCTGGCGT

TGGGGGCCCCCTCGGGGGTCGCGGTCGCCCCCCGCGGCCTCTCGTGGTGCGTGCGGTCCG

CTCGCGCTCCTGGCCAGCCAGCCCTCGAGGCCCTCAGCCACCGAGGATCAGGGCCCGCTC

GGCCCCTCCCATGGAAGGTGCTCGGGTCTTCGGGGCTCTGGG

>CL151.Contig7_A-W 902 1060 Prostate tumor overexpressed gene 1 protein, partial [Heterocephalus glaber]

GCGGGCTGCATGCTTTTTCCCCACATCTCCCCCTGCGAGGTGCGCGTGCTCATGCTCCTG

TACTCGTCCAAGAAGAAGATCTTCATGGGCCTCATCCCCTATGACCAGAGCGGCTTCGTC

AATGCCATACGGCAGGTCATCACTACTCGCAAACAGGTC

>CL151.Contig8_A-W 5 496 PREDICTED: mediator of RNA polymerase II transcription subunit 25 [Saimiri boliviensis boliviensis]

GGGGCTCCAGCCCTCGGTGGGCAGCAGTCGGTCTCTAATAAACTCCTGGCCTGGAGTGGG

GTTCTCGAGTGGCAGGAGAAACCCAAGCCTGCCTCGGTGGATGCCAACACCAAGCTGACA

CGGTCACTGCCCTGCCAGGTCTACGTGAATCATGGAGAGAACCTGAAGACTGAGCAGTGG

CCCCAGAAGCTGATCATGCAGCTTATCCCCCAGCAACTGCTGACCACCCTGGGCCCTCTG

TTCCGGAACTCGAGGATGGTCCAGTTCCACTTCACCAACAAGGACCTGGAGTCCCTCAAA

GGCCTCTACCGCATTATGGGCAATGGCTTTGCGGGCTGCGTGCACTTCCCTCACACGGCC

CCCTGCGAGGTGCGCGTGCTCATGCTCCTGTACTCGTCCAAGAAGAAGATCTTCATGGGC

CTCATCCCCTATGACCAGAGCGGCTTCGTCAATGCCATACGGCAGGTCATCACTACTCGC

AAACAGGTGGTG

>CL151.Contig9_A-W 846 1169 PREDICTED: mediator of RNA polymerase II transcription subunit 25 [Saimiri boliviensis boliviensis]

AGGACTGAGCAGTGGCCGAGGAAGCTGTACATGCAGCTCATCCCACAGCAGCTCCTGACC

ACTTTGGTGCCACTGTTTCGGAACTCCCGCCTGGTGCAGTTTCACTTCACCAAGGACATG

GAGACGCTGAAGAGCCTGTGCCGGATCATGGACAACGGCTTTGCTGGCTGCGTGCACTTT

TCCTATAAAGCCTCATGTGAGATCCGCGTGCTCATGCTCCTGTACTCCTCGGAGAAGAAG

ATCTTCATTGGCCTCATCCCCCACGACCAGAGCAACTTCGTCAACGGTATCCGGCGTGTC

ATAGCCAACCAGCAGCAGGTCCTT

>CL151.Contig10_A-W 216 398 Prostate tumor overexpressed gene 1 protein, partial [Heterocephalus glaber]

CTGAAGAGCCTCTGTCTGCAGGCGGGCTGCGTGCACTTCCCTCACACGGCCCCCTGCGAG

GTGCGCGTGCTCATGCTCCTGTACTCGTCCAAGAAGAAGATCTTCATGGGCCTCATCCCC

TATGACCAGAGCGGCTTCGTCAATGCCATACGGCAGGTCATCACTACTCGCAAACAGGTG

GTG

>CL151.Contig11_A-W 902 1060 PREDICTED: mediator of RNA polymerase II transcription subunit 25 [Pan paniscus]

GCGGGCTGCATGCTTTTTCCCCACATCTCCCCCTGCGAGGTGCGCGTGCTCATGCTCCTG

TACTCGTCCAAGAAGAAGATCTTCATGGGCCTCATCCCCTATGACCAGAGCGGCTTCGTC

AATGCCATACGGCAGGTCATCACTACTCGCAAACAGGTC

>CL151.Contig12_A-W 216 398 PREDICTED: mediator of RNA polymerase II transcription subunit 25 [Pan paniscus]

CTGAAGAGCCTCTGTCTGCAGGCGGGCTGCGTGCACTTCCCTCACACGGCCCCCTGCGAG

GTGCGCGTGCTCATGCTCCTGTACTCGTCCAAGAAGAAGATCTTCATGGGCCTCATCCCC

TATGACCAGAGCGGCTTCGTCAATGCCATACGGCAGGTCATCACTACTCGCAAACAGGTG

GTG

>CL151.Contig13_A-W 902 1063 PREDICTED: mediator of RNA polymerase II transcription subunit 25 [Pan paniscus]

GCGGGCTGCATGCTTTTTCCCCACATCTCCCCCTGCGAGGTGCGCGTGCTCATGCTCCTG

TACTCGTCCAAGAAGAAGATCTTCATGGGCCTCATCCCCTATGACCAGAGCGGCTTCGTC

AATGCCATACGGCAGGTCATCACTACTCGCAAACAGGTGGTG

>CL151.Contig14_A-W 902 1063 Prostate tumor overexpressed gene 1 protein, partial [Heterocephalus glaber]

GCGGGCTGCATGCTTTTTCCCCACATCTCCCCCTGCGAGGTGCGCGTGCTCATGCTCCTG

TACTCGTCCAAGAAGAAGATCTTCATGGGCCTCATCCCCTATGACCAGAGCGGCTTCGTC

AATGCCATACGGCAGGTCATCACTACTCGCAAACAGGTGGTG

>CL152.Contig1_A-W 44 742 minus strand unknown [Homo sapiens]

GGAGCGGCACCCGCTTTCGCCCGCTCGCGGACTGGCTCTCCTCCCGCCGCAGGAGCGGCG

CACCGCGCTCCCCGGGCCATGCAGCGCTTAGCTATGGACCTGCGGATGCTCTCCCGAGAG

CTCTCCCTCTACCTGGAGCACCAAATCCGGGTTGGATTTTTCGGTTCGGGGGTGGGTTTA

TCCCTGATCCTGGGCTTCAGCGTCGCGTACGCCTGCTACTACCTGAGCAGCATTGCCAAG

AAACCCCAGTTAGTGACGGGAGGGGAGAGTTTCAACCGCTTCCTGCAGAACCATTGTCCG

GTGGTGACAGAAACGTATTACCCTACGGTCTGGTGCTGGGAGAGTCGAGGACAGACACTG

CTGAGACCTTTCATCACTTCCAAGCCGCCGGTGCAGTACAGGAATGAACTTATTAAAACT

GCTGATGGAGGACAGATTTCACTGGACTGGTTTGATAATGATAACAGTGTGTGTTATACG

GATGGCAGTACCAGACCTACTATCTTATTGTTACCTGGCCTCACTGGAACAAGCAAGGAA

TCCTATATCCTTCATATGATCCATCTCAGTGAAGAATTGGGATACAGATGTGTGGTTTTT

AACAACAGAGGAGTAGCAGGAGAGAATCTCTTGACGCCAAGGACTTACTGTTGTGCTAAC

ACTGAAGACTTGGAGACAGTTATTCACCATGTCCACAGC

>CL152.Contig2_A-W 5 1018 PREDICTED: LOW QUALITY PROTEIN: abhydrolase domain-containing protein 3 [Callithrix jacchus]

GCTCCCCGCGCAGGAGACGCTCTAGTTCAGGGCGCCGGCGGAGCGGCACCCGCTTTCGCC

CGCTCGCGGACTGGCTCTCCTCCCGCCGCAGGAGCGGCGCACCGCGCTCCCCGGGCCATG

CAGCGCTTAGCTATGGACCTGCGGATGCTCTCCCGAGAGCTCTCCCTCTACCTGGAGCAC

CAAATCCGGGTTGGATTTTTCGGTTCGGGGGTGGGTTTATCCCTGATCCTGGGCTTCAGC

GTCGCGTACGCCTGCTACTACCTGAGCAGCATTGCCAAGAAACCCCAGTTAGTGACGGGA

GGGGAGAGTTTCAACCGCTTCCTGCAGAACCATTGTCCGGTGGTGACAGAAACGTATTAC

CCTACGGTCTGGTGCTGGGAGAGTCGAGGACAGACACTGCTGAGACCTTTCATCACTTCC

AAGCCGCCGGTGCAGTACAGGAATGAACTTATTAAAACTGCTGATGGAGGACAGATTTCA

CTGGACTGGTTTGATAATGATAACAGTGTGTGTTATACGGATGGCAGTACCAGACCTACT

ATCTTATTGTTACCTGGCCTCACTGGAACAAGCAAGGAATCCTATATCCTTCATATGATC

CATCTCAGTGAAGAATTGGGATACAGAATGCTGCTTCTAAATTACCTGGGGAAAATTGGG

CCCAAAACTCCTTTGATGGCAGCTGCCACTTTTTCAGTTGGTTGGAATACTTTTGCTTGC

TCAGAGTCATTGGAGAAACCACTGAACTGGCTGCTTTTTAATTACTATCTGACAACCTGC

CTCCAGTCTTCAGTTAATAAGCACCGCCATATGTTTGTAAAACAAATTGATATGGATCAT

GTCATGAAGGCTAAATCTATCAGAGAATTTGATAAGCGATTCACTTCTGTCATGTTTGGA

TACCGAACAATCGATGATTATTATACTGATGCCAGCCCAAATCGTAGATTGAATTCAGTA

GGAATTCCAGTATTGTGCCTGAATTCTGTGGATGATGTTTTCTCACCAAGTCAT

>CL152.Contig3_A-W 298 570 minus strand PREDICTED: abhydrolase domain-containing protein 3 [Loxodonta africana]

AATTGGGATACAGATGTGTGGTTTTTAACAACAGAGGAGTAGCAGGAGAGAATCTCTTGG

CACCGCCATATGTTTGTAAAACAAATTGATATGGATCATGTCATGAAGGCTAAATCTATC

AGAGAATTTGATAAGCGATTCACTTCTGTCATGTTTGGATACCGAACAATCGATGATTAT

TATACTGATGCCAGCCCAAATCGTAGATTGAATTCAGTAGGAATTCCAGTATTGTGCCTG

AATTCTGTGGATGATGTTTTCTCACCAAGTCAT

>CL152.Contig4_A-W 3 356 minus strand PREDICTED: abhydrolase domain-containing protein 3 [Loxodonta africana]

TTCCTGCAGAACCATTGTCCGGTGGTGACAGAAACGTATTACCCTACGGTCTGGTGCTGG

GAGAGTCGAGGACAGACACTGCTGAGACCTTTCATCACTTCCAAGCCGCCGGTGCAGTAC

AGGAATGAACTTATTAAAACTGCTGATGGAGGACAGATTTCACTGGACTGGTTTGATAAT

GATAACAGTGTGTGTTATACGGATGGCAGTACCAGACCTACTATCTTATTGTTACCTGGC

CTCACTGGAACAAGCAAGGAATCCTATATCCTTCATATGATCCATCTCAGTGAAGAATTG

GGATACAGATGTGTGGTTTTTAACAACAGAGGAGTAGCAGGAGAGAATCTCTTG

>CL152.Contig5_A-W 58 486 PREDICTED: LOW QUALITY PROTEIN: abhydrolase domain-containing protein 3 [Callithrix jacchus]

ATGCTGCTTCTAAATTACCTGGGGAAAATTGGGCCCAAAACTCCTTTGATGGCAGCTGCC

ACTTTTTCAGTTGGTTGGAATACTTTTGCTTGCTCAGAGTCATTGGAGAAACCACTGAAC

TGGCTGCTTTTTAATTACTATCTGACAACCTGCCTCCAGTCTTCAGTTAATAAGCACCGC

CATATGTTTGTAAAACAAATTGATATGGATCATGTCATGAAGGCTAAATCTATCAGAGAA

TTTGATAAGCGATTCACTTCTGTCATGTTTGGATACCGAACAATCGATGATTATTATACT

GATGCCAGCCCAAATCGTAGATTGAATTCAGTAGGAATTCCAGTATTGTGCCTGAATTCT

GTGGATGATGTTTTCTCACCAAGTCATGCTATTCCAATAGAAACTGCTAAGCAAAATCCT

AATGTTGCT

>CL152.Contig6_A-W 231 395 Abhydrolase domain-containing protein 3 [Heterocephalus glaber]

GCTAAATCTATCAGAGAATTTGATAAGCGATTCACTTCTGTCATGTTTGGATACCGAACA

ATCGATGATTATTATACTGATGCCAGCCCAAATCGTAGATTGAATTCAGTAGGAATTCCA

GTATTGTGCCTGAATTCTGTGGATGATGTTTTCTCACCAAGTCAT

>CL152.Contig7_A-W 3 482 PREDICTED: LOW QUALITY PROTEIN: abhydrolase domain-containing protein 3 [Callithrix jacchus]

AGCCTGTACCCTTCTGCTCCTTTTCTGGCAGCTGGGGTTTCAATGGGAGGAATGCTGCTT

CTAAATTACCTGGGGAAAATTGGGCCCAAAACTCCTTTGATGGCAGCTGCCACTTTTTCA

GTTGGTTGGAATACTTTTGCTTGCTCAGAGTCATTGGAGAAACCACTGAACTGGCTGCTT

TTTAATTACTATCTGACAACCTGCCTCCAGTCTTCAGTTAATAAGCACCGCCATATGTTT

GTAAAACAAATTGATATGGATCATGTCATGAAGGCTAAATCTATCAGAGAATTTGATAAG

CGATTCACTTCTGTCATGTTTGGATACCGAACAATCGATGATTATTATACTGATGCCAGC

CCAAATCGTAGATTGAATTCAGTAGGAATTCCAGTATTGTGCCTGAATTCTGTGGATGAT

GTTTTCTCACCAAGTCATGCTATTCCAATAGAAACTGCTAAGCAAAATCCTAATGTTGCT

>CL152.Contig8_A-W 227 391 abhydrolase domain-containing protein 3 [Bos taurus] >gi|122143460|sp|Q0VC00.1|ABHD3_BOVIN RecName: Full=Abhydrolase domain-containing protein 3 >gi|111305457|gb|AAI20422.1| Abhydrolase domain containing 3 [Bos taurus]

GCTAAATCTATCAGAGAATTTGATAAGCGATTCACTTCTGTCATGTTTGGATACCGAACA

ATCGATGATTATTATACTGATGCCAGCCCAAATCGTAGATTGAATTCAGTAGGAATTCCA

GTATTGTGCCTGAATTCTGTGGATGATGTTTTCTCACCAAGTCAT

>CL152.Contig9_A-W 58 231 PREDICTED: LOW QUALITY PROTEIN: abhydrolase domain-containing protein 3 [Callithrix jacchus]

ATGCTGCTTCTAAATTACCTGGGGAAAATTGGGCCCAAAACTCCTTTGATGGCAGCTGCC

ACTTTTTCAGTTGGTTGGAATACTTTTGCTTGCTCAGAGTCATTGGAGAAACCACTGAAC

TGGCTGCTTTTTAATTACTATCTGACAACCTGCCTCCAGTCTTCAGTTAATAAG

>CL152.Contig10_A-W 227 433 abhydrolase domain-containing protein 3 [Bos taurus] >gi|122143460|sp|Q0VC00.1|ABHD3_BOVIN RecName: Full=Abhydrolase domain-containing protein 3 >gi|111305457|gb|AAI20422.1| Abhydrolase domain containing 3 [Bos taurus]

GCTAAATCTATCAGAGAATTTGATAAGCGATTCACTTCTGTCATGTTTGGATACCGAACA

ATCGATGATTATTATACTGATGCCAGCCCAAATCGTAGATTGAATTCAGTAGGAATTCCA

GTATTGTGCCTGAATTCTGTGGATGATGTTTTCTCACCAAGTCATGCTATTCCAATAGAA

ACTGCTAAGCAAAATCCTAATGTTGCT

>CL152.Contig11_A-W 5 1060 PREDICTED: LOW QUALITY PROTEIN: abhydrolase domain-containing protein 3 [Callithrix jacchus]

GCTCCCCGCGCAGGAGACGCTCTAGTTCAGGGCGCCGGCGGAGCGGCACCCGCTTTCGCC

CGCTCGCGGACTGGCTCTCCTCCCGCCGCAGGAGCGGCGCACCGCGCTCCCCGGGCCATG

CAGCGCTTAGCTATGGACCTGCGGATGCTCTCCCGAGAGCTCTCCCTCTACCTGGAGCAC

CAAATCCGGGTTGGATTTTTCGGTTCGGGGGTGGGTTTATCCCTGATCCTGGGCTTCAGC

GTCGCGTACGCCTGCTACTACCTGAGCAGCATTGCCAAGAAACCCCAGTTAGTGACGGGA

GGGGAGAGTTTCAACCGCTTCCTGCAGAACCATTGTCCGGTGGTGACAGAAACGTATTAC

CCTACGGTCTGGTGCTGGGAGAGTCGAGGACAGACACTGCTGAGACCTTTCATCACTTCC

AAGCCGCCGGTGCAGTACAGGAATGAACTTATTAAAACTGCTGATGGAGGACAGATTTCA

CTGGACTGGTTTGATAATGATAACAGTGTGTGTTATACGGATGGCAGTACCAGACCTACT

ATCTTATTGTTACCTGGCCTCACTGGAACAAGCAAGGAATCCTATATCCTTCATATGATC

CATCTCAGTGAAGAATTGGGATACAGAATGCTGCTTCTAAATTACCTGGGGAAAATTGGG

CCCAAAACTCCTTTGATGGCAGCTGCCACTTTTTCAGTTGGTTGGAATACTTTTGCTTGC

TCAGAGTCATTGGAGAAACCACTGAACTGGCTGCTTTTTAATTACTATCTGACAACCTGC

CTCCAGTCTTCAGTTAATAAGCACCGCCATATGTTTGTAAAACAAATTGATATGGATCAT

GTCATGAAGGCTAAATCTATCAGAGAATTTGATAAGCGATTCACTTCTGTCATGTTTGGA

TACCGAACAATCGATGATTATTATACTGATGCCAGCCCAAATCGTAGATTGAATTCAGTA

GGAATTCCAGTATTGTGCCTGAATTCTGTGGATGATGTTTTCTCACCAAGTCATGCTATT

CCAATAGAAACTGCTAAGCAAAATCCTAATGTTGCT

>CL152.Contig12_A-W 805 969 PREDICTED: LOW QUALITY PROTEIN: abhydrolase domain-containing protein 3 [Callithrix jacchus]

GCTAAATCTATCAGAGAATTTGATAAGCGATTCACTTCTGTCATGTTTGGATACCGAACA

ATCGATGATTATTATACTGATGCCAGCCCAAATCGTAGATTGAATTCAGTAGGAATTCCA

GTATTGTGCCTGAATTCTGTGGATGATGTTTTCTCACCAAGTCAT

>CL152.Contig13_A-W 805 1011 PREDICTED: LOW QUALITY PROTEIN: abhydrolase domain-containing protein 3 [Callithrix jacchus]

GCTAAATCTATCAGAGAATTTGATAAGCGATTCACTTCTGTCATGTTTGGATACCGAACA

ATCGATGATTATTATACTGATGCCAGCCCAAATCGTAGATTGAATTCAGTAGGAATTCCA

GTATTGTGCCTGAATTCTGTGGATGATGTTTTCTCACCAAGTCATGCTATTCCAATAGAA

ACTGCTAAGCAAAATCCTAATGTTGCT

>CL153.Contig1_A-W 2 268 minus strand PREDICTED: dnaJ homolog subfamily A member 4 [Papio anubis]

GGAGAACAGGCAATTAAAGAAGGAGGCTCGGGTAGCCCCAGTTTCTCTTCACCCATGGAC

ATCTTTGACATGTTCTTTGGTGGTGGAGGACGGATGGCTAGAGAGAGAAGAGGCAAGAAT

GTGGTACATCAATTGTCTGTAACTCTTGAAGATTTATATAATGGTGTCACAAAGAAATTG

GCCCTCCAGAAAAATGTAATTTGTGAGAAATGTGAAGGTATTGGTGGGAAGAAGGGATCA

GTGGAGAAATGCCCACTGTGCAAGGGG

>CL154.Contig1_A-W 30 1091 minus strand PREDICTED: LOW QUALITY PROTEIN: mitochondrial inner membrane protein OXA1L-like [Equus caballus]

TGGCTTCCTTTCACATAGGTCCAAGTACCTCCTGTTGTTCCTGCAACTCCTTCACCCACA

GCAGTACCTGAGGTGGCTTCTGGAGGAACTACAGATACAATCCAAGCTGTTGCAGAACAG

AGCTTCGCTGAACTGGGACTAGGGTCATACACCCCAGTGGGATTGATCCAGAACCTCCTG

GAATTTATGCATGTTAACCTGGGCCTTCCTTGGTGGGGGGCCATTGCAGCATGTACAGTC

CTTGCCCGCTGCCTCGTTTTTCCTCTCATCGTGAAGGGCCAGCGAGAGGCAGCCAAAATT

CACAACCACTTGCCAGAGATCCAGAAGTTTTCCACTAGAATCAGAGAGGCCAAGTTGGCA

GGAGACAATGCTGAGTTTTACAGGGCCTCCTCGGAGATGACACTTTACCAGAAAAAGCAC

GATATTAAATTCTTTAAACCCCTCATTCTGCCTCTGACTCAGGCACCAGTCTTCATCTCC

TTCTTCATTGCCTTGAGAGAGATGGCCAACCTTCCTGTGCCCAGTATGCAGACAGGTGGC

CTCTGGTGGTTCCAGGATCTCACTGTATCTGATCCCATCTACATGTTACCGATAGTAGTC

ACTGCTACAATGTGGGGTGTCCTGGAGCTAGGTGCTGACACAGGTGTGCAAAGTTCTGAC

CTTCAGTGGATGAGAAATGTTATCAGAGTGATGCCCCTGGCAGTCTTGCCCATAACCATC

CACTTCCCCTCGGCAGTATTCATGTACTGGCTCTCCTCCAATATGTTTTCCCTGGGCCAA

GTAGCCTGCCTCCGGATTCCAGCTGTACGCACTGTACTTAAAATACCCCAGCGTATTGTG

CATGACCCTGACAAATTACCTCCCCGGGAAGGCTTCATAAAGAGCTTCAAACAAGGCTGG

AAGAATGCTGAAATTGCACATCAGCTCCGTGAGCGTGAACGACGCATGCAGAACCACTTG

GAGCTAGCAGCCAGGGGTCCCTTACGGCAGACCTTTACCCACAACCCTCTACTACAGCAT

GAAAAGAATCACCCTCCCAAAACCCCCAGCAGCAGCAGCAGC

>CL154.Contig2_A-W 30 1007 minus strand PREDICTED: LOW QUALITY PROTEIN: mitochondrial inner membrane protein OXA1L-like [Equus caballus]

TGGCTTCCTTTCACATAGGTCCAAGTACCTCCTGTTGTTCCTGCAACTCCTTCACCCACA

GCAGTACCTGAGGTGGCTTCTGGAGGAACTACAGATACAATCCAAGCTGTTGCAGAACAG

AGCTTCGCTGAACTGGGACTAGGGTCATACACCCCAGTGGGATTGATCCAGAACCTCCTG

GAATTTATGCATGTTAACCTGGGCCTTCCTTGGTGGGGGGCCATTGCAGCATGTACAGTC

CTTGCCCGCTGCCTCGTTTTTCCTCTCATCGTGAAGGGCCAGCGAGAGGCAGCCAAAATT

CACAACCACTTGCCAGAGATCCAGAAGTTTTCCACTAGAATCAGAGAGGCCAAGTTGGCA

GGAGACAATGCTGAGTTTTACAGGGCCTCCTCGGAGATGACACTTTACCAGAAAAAGCAC

GATATTAAATTCTTTAAACCCCTCATTCTGCCTCTGACTCAGGCACCAGTCTTCATCTCC

TTCTTCATTGCCTTGAGAGAGATGGCCAACCTTCCTGTGCCCAGTATGCAGACAGGTGGC

CTCTGGTGGTTCCAGGATCTCACTGTATCTGATCCCATCTACATGTTACCGATAGTAGTC

ACTGCTACAATGTGGGGTGTCCTGGAGCTAGGTGCTGACACAGGTGTGCAAAGTTCTGAC

CTTCAGTGGATGAGAAATGTTATCAGAGTGATGCCCCTGGCAGTCTTGCCCATAACCATC

CACTTCCCCTCGGCAGTATTCATGTACTGGCTCTCCTCCAATATGTTTTCCCTGGGCCAA

GTAGCCTGCCTCCGGATTCCAGCTGTACGCACTGTACTTAAAATACCCCAGCGTATTGTG

CATGACCCTGACAAATTACCTCCCCGGGAAGGCTTCATAAAGAGCTTCAAACAAGGCTGG

AAGAATGCTGAAATTGCACATCAGCTCCGTGAGCGTGAACGACGCATGCAGAACCACTTG

GAGCTAGCAGCCAGGGGT

>CL154.Contig3_A-W 3 773 minus strand PREDICTED: LOW QUALITY PROTEIN: mitochondrial inner membrane protein OXA1L-like [Equus caballus]

CCTCTTCCGGGCAAAATGGCGATGGGCTTGATGTGCGGACGCCGGGAGCTTGTGCGCCTG

CTACGGCCCCAGCGTCAGTTCCACAGTATCGTAGGGTCCTCGCAGTGGCCGCGGAAACCG

CTGAGAGAGGGGCTCCAGGTCCCAAGCCACCCGGGCTGCAGGCAGCCGCATTATGTCCTC

CTCACAACCTCCAGCCACCGCTGCCTCAGTACCTCTGGCATTTCATTGGCAGAAGCCCAG

GTCCAAGTACCTCCTGTTGTTCCTGCAACTCCTTCACCCACAGCAGTACCTGAGGTGGCT

TCTGGAGGAACTACAGATACAATCCAAGCTGTTGCAGAACAGAGCTTCGCTGAACTGGGA

CTAGGGTCATACACCCCAGTGGGATTGATCCAGAACCTCCTGGAATTTATGCATGTTAAC

CTGGGCCTTCCTTGGTGGGGGGCCATTGCAGCATGTACAGTCCTTGCCCGCTGCCTCGTT

TTTCCTCTCATCGTGAAGGGCCAGCGAGAGGCAGCCAAAATTCACAACCACTTGCCAGAG

ATCCAGAAGTTTTCCACTAGAATCAGAGAGGCCAAGTTGGCAGGAGACAATGCTGAGTTT

TACAGGGCCTCCTCGGAGATGACACTTTACCAGAAAAAGCACGATATTAAATTCTTTAAA

CCCCTCATTCTGCCTCTGACTCAGGTGAGCAAAAACATTTCCCTCCTTATTTCATCAAAT

GGCCTTCTCTGTATTTTCCATGCCCCAGACCTCCACAAAAGCAGTTGGTGG

>CL154.Contig4_A-W 3 1286 PREDICTED: LOW QUALITY PROTEIN: mitochondrial inner membrane protein OXA1L-like [Equus caballus]

CCTCTTCCGGGCAAAATGGCGATGGGCTTGATGTGCGGACGCCGGGAGCTTGTGCGCCTG

CTACGGCCCCAGCGTCAGTTCCACAGTATCGTAGGGTCCTCGCAGTGGCCGCGGAAACCG

CTGAGAGAGGGGCTCCAGGTCCCAAGCCACCCGGGCTGCAGGCAGCCGCATTATGTCCTC

CTCACAACCTCCAGCCACCGCTGCCTCAGTACCTCTGGCATTTCATTGGCAGAAGCCCAG

GTCCAAGTACCTCCTGTTGTTCCTGCAACTCCTTCACCCACAGCAGTACCTGAGGTGGCT

TCTGGAGGAACTACAGATACAATCCAAGCTGTTGCAGAACAGAGCTTCGCTGAACTGGGA

CTAGGGTCATACACCCCAGTGGGATTGATCCAGAACCTCCTGGAATTTATGCATGTTAAC

CTGGGCCTTCCTTGGTGGGGGGCCATTGCAGCATGTACAGTCCTTGCCCGCTGCCTCGTT

TTTCCTCTCATCGTGAAGGGCCAGCGAGAGGCAGCCAAAATTCACAACCACTTGCCAGAG

ATCCAGAAGTTTTCCACTAGAATCAGAGAGGCCAAGTTGGCAGGAGACAATGCTGAGTTT

TACAGGGCCTCCTCGGAGATGACACTTTACCAGAAAAAGCACGATATTAAATTCTTTAAA

CCCCTCATTCTGCCTCTGACTCAGGCACCAGTCTTCATCTCCTTCTTCATTGCCTTGAGA

GAGATGGCCAACCTTCCTGTGCCCAGTATGCAGACAGGTGGCCTCTGGTGGTTCCAGGAT

CTCACTGTATCTGATCCCATCTACATGTTACCGATAGTAGTCACTGCTACAATGTGGGGT

GTCCTGGAGCTAGGTGCTGACACAGGTGTGCAAAGTTCTGACCTTCAGTGGATGAGAAAT

GTTATCAGAGTGATGCCCCTGGCAGTCTTGCCCATAACCATCCACTTCCCCTCGGCAGTA

TTCATGTACTGGCTCTCCTCCAATATGTTTTCCCTGGGCCAAGTAGCCTGCCTCCGGATT

CCAGCTGTACGCACTGTACTTAAAATACCCCAGCGTATTGTGCATGACCCTGACAAATTA

CCTCCCCGGGAAGGCTTCATAAAGAGCTTCAAACAAGGCTGGAAGAATGCTGAAATTGCA

CATCAGCTCCGTGAGCGTGAACGACGCATGCAGAACCACTTGGAGCTAGCAGCCAGGGGT

CCCTTACGGCAGACCTTTACCCACAACCCTCTACTACAGCATGAAAAGAATCACCCTCCC

AAAACCCCCAGCAGCAGCAGCAGC

>CL154.Contig5_A-W 3 1202 minus strand PREDICTED: LOW QUALITY PROTEIN: mitochondrial inner membrane protein OXA1L-like [Equus caballus]

CCTCTTCCGGGCAAAATGGCGATGGGCTTGATGTGCGGACGCCGGGAGCTTGTGCGCCTG

CTACGGCCCCAGCGTCAGTTCCACAGTATCGTAGGGTCCTCGCAGTGGCCGCGGAAACCG

CTGAGAGAGGGGCTCCAGGTCCCAAGCCACCCGGGCTGCAGGCAGCCGCATTATGTCCTC

CTCACAACCTCCAGCCACCGCTGCCTCAGTACCTCTGGCATTTCATTGGCAGAAGCCCAG

GTCCAAGTACCTCCTGTTGTTCCTGCAACTCCTTCACCCACAGCAGTACCTGAGGTGGCT

TCTGGAGGAACTACAGATACAATCCAAGCTGTTGCAGAACAGAGCTTCGCTGAACTGGGA

CTAGGGTCATACACCCCAGTGGGATTGATCCAGAACCTCCTGGAATTTATGCATGTTAAC

CTGGGCCTTCCTTGGTGGGGGGCCATTGCAGCATGTACAGTCCTTGCCCGCTGCCTCGTT

TTTCCTCTCATCGTGAAGGGCCAGCGAGAGGCAGCCAAAATTCACAACCACTTGCCAGAG

ATCCAGAAGTTTTCCACTAGAATCAGAGAGGCCAAGTTGGCAGGAGACAATGCTGAGTTT

TACAGGGCCTCCTCGGAGATGACACTTTACCAGAAAAAGCACGATATTAAATTCTTTAAA

CCCCTCATTCTGCCTCTGACTCAGGCACCAGTCTTCATCTCCTTCTTCATTGCCTTGAGA

GAGATGGCCAACCTTCCTGTGCCCAGTATGCAGACAGGTGGCCTCTGGTGGTTCCAGGAT

CTCACTGTATCTGATCCCATCTACATGTTACCGATAGTAGTCACTGCTACAATGTGGGGT

GTCCTGGAGCTAGGTGCTGACACAGGTGTGCAAAGTTCTGACCTTCAGTGGATGAGAAAT

GTTATCAGAGTGATGCCCCTGGCAGTCTTGCCCATAACCATCCACTTCCCCTCGGCAGTA

TTCATGTACTGGCTCTCCTCCAATATGTTTTCCCTGGGCCAAGTAGCCTGCCTCCGGATT

CCAGCTGTACGCACTGTACTTAAAATACCCCAGCGTATTGTGCATGACCCTGACAAATTA

CCTCCCCGGGAAGGCTTCATAAAGAGCTTCAAACAAGGCTGGAAGAATGCTGAAATTGCA

CATCAGCTCCGTGAGCGTGAACGACGCATGCAGAACCACTTGGAGCTAGCAGCCAGGGGT

>CL154.Contig6_A-W 236 847 minus strand mitochondrial inner membrane protein OXA1L [Bos taurus] >gi|122064647|sp|Q3SYV3.1|OXA1L_BOVIN RecName: Full=Mitochondrial inner membrane protein OXA1L; AltName: Full=Oxidase assembly 1-like protein; Short=OXA1-like protein; Flags: Precursor >gi|74268195|gb|AAI03368.1| Oxidase (cytochrome c) assembly 1-like [Bos taurus] >gi|296483636|gb|DAA25751.1| mitochondrial inner membrane protein OXA1L precursor [Bos taurus]

CACCTCCCAGTTTACAGGGCCTCCTCGGAGATGACACTTTACCAGAAAAAGCACGATATT

AAATTCTTTAAACCCCTCATTCTGCCTCTGACTCAGGCACCAGTCTTCATCTCCTTCTTC

ATTGCCTTGAGAGAGATGGCCAACCTTCCTGTGCCCAGTATGCAGACAGGTGGCCTCTGG

TGGTTCCAGGATCTCACTGTATCTGATCCCATCTACATGTTACCGATAGTAGTCACTGCT

ACAATGTGGGGTGTCCTGGAGCTAGGTGCTGACACAGGTGTGCAAAGTTCTGACCTTCAG

TGGATGAGAAATGTTATCAGAGTGATGCCCCTGGCAGTCTTGCCCATAACCATCCACTTC

CCCTCGGCAGTATTCATGTACTGGCTCTCCTCCAATATGTTTTCCCTGGGCCAAGTAGCC

TGCCTCCGGATTCCAGCTGTACGCACTGTACTTAAAATACCCCAGCGTATTGTGCATGAC

CCTGACAAATTACCTCCCCGGGAAGGCTTCATAAAGAGCTTCAAACAAGGCTGGAAGAAT

GCTGAAATTGCACATCAGCTCCGTGAGCGTGAACGACGCATGCAGAACCACTTGGAGCTA

GCAGCCAGGGGT

>CL155.Contig1_A-W 3006 3680 minus strand PREDICTED: myosin-Ig [Cricetulus griseus]

TCCAGGTGGCGGGCCCGGCAGCTGGTAAAGAACATTCCACCTTCAGATATGGCTCAGATC

AAGGCCAAAGTGGCTGCCATGGGGGCCCTGCAGGGTCTACGTCAGGACTGGGGCTGCCAG

AGAGCCTGGGCTCAAGACTACCTGTCCTCTGGCACTGACAACCCCACAGCCTCAGGCTTG

TTTGCTCAGCAACTGAAGACCCTTCGAGAGAAGGATGGCTTTGGGGCTGTGCTCTTTTCT

AGCCATGTCCGTAAGGTGAACCGCTTCCGCAAGTGCCGGGACCGGGCCCTTCTGCTTACA

GACCGGCACCTCTACAAACTGGAACCTGGCCGTCAGTACCGGGTAATGCGGGCTGTGCCC

CTGGATGCGGTAACAGGGGTGAGTGTGACCAGTGGACGGGACCAGCTGGTGGTACTGCAT

GCCCGGGGCCATGATGACCTCGTGGTATGTCTGCACCGCTCCCAGCCACCTCTGGACAAT

CGTATCGGGGAGCTGGTGGGTGTACTGGCTGCACACTGCCAGAGGGAGGGAAGAAACCTA

GAGGTCCGTGTCTCTGACTGCATTTCACTGAGCCAGCGTGGGGCCCAACGCCTTGTTTCC

GTGGAGTCCAGGCCAGAGCAACCAGAACCAGATTTCTGCTGTAGCCGTGGCAACTTTACC

CTCCTCTGGCCAAAC

>CL155.Contig2_A-W 2994 3638 minus strand PREDICTED: unconventional myosin-Ig [Pongo abelii]

AGGTGGCGGGCCCGGCAGCTGGTAAAGAACATTCCACCTTCAGATATGGCTCAGATCAAG

GCCAAAGTGGCTGCCATGGGGGCCCTGCAGGGTCTACGTCAGGACTGGGGCTGCCAGAGA

GCCTGGGCTCAAGACTACCTGTCCTCTGGCACTGACAACCCCACAGCCTCAGGCTTGTTT

GCTCAGCAACTGAAGACCCTTCGAGAGAAGGATGGCTTTGGGGCTGTGCTCTTTTCTAGC

CATGTCCGTAAGGTGAACCGCTTCCGCAAGTGCCGGGACCGGGCCCTTCTGCTTACAGAC

CGGCACCTCTACAAACTGGAACCTGGCCGTCAGTACCGGGTAATGCGGGCTGTGCCCCTG

GATGCGGTAACAGGGGTGAGTGTGACCAGTGGACGGGACCAGCTGGTGGTACTGCATGCC

CGGGGCCATGATGACCTCGTGGTATGTCTGCACCGCTCCCAGCCACCTCTGGACAATCGT

ATCGGGGAGCTGGTGGGTGTACTGGCTGCACACTGCCAGAGGGAGGGAAGAAACCTAGAG

GTCCGTGTCTCTGACTGCATTTCACTGAGCCAGCGTGGGGCCCAACGCCTTGTTTCCGTG

GAGTCCAGGCCAGAGCAACCAGAACCAGATTTCTGCTGTAGCCGT

>CL155.Contig3_A-W 3360 3665 minus strand PREDICTED: myosin-Ig [Cricetulus griseus]

GTAACAGGGGTGAGTGTGACCAGTGGACGGGACCAGCTGGTGGTACTGCATGCCCGGGGC

CATGATGACCTCGTGGTATGTCTGCACCGCTCCCAGCCACCTCTGGACAATCGTATCGGG

GAGCTGGTGGGTGTACTGGCTGCACACTGCCAGAGGGAGGGAAGAAACCTAGAGGTCCGT

GTCTCTGACTGCATTTCACTGAGCCAGCGTGGGGCCCAACGCCTTGTTTCCGTGGAGTCC

AGGCCAGAGCAACCAGAACCAGATTTCTGCTGTAGCCGTGGCAACTTTACCCTCCTCTGG

CCAAAC

>CL155.Contig4_A-W 3353 3658 minus strand PREDICTED: myosin-Ig [Cricetulus griseus]

GTAACAGGGGTGAGTGTGACCAGTGGACGGGACCAGCTGGTGGTACTGCATGCCCGGGGC

CATGATGACCTCGTGGTATGTCTGCACCGCTCCCAGCCACCTCTGGACAATCGTATCGGG

GAGCTGGTGGGTGTACTGGCTGCACACTGCCAGAGGGAGGGAAGAAACCTAGAGGTCCGT

GTCTCTGACTGCATTTCACTGAGCCAGCGTGGGGCCCAACGCCTTGTTTCCGTGGAGTCC

AGGCCAGAGCAACCAGAACCAGATTTCTGCTGTAGCCGTGGCAACTTTACCCTCCTCTGG

CCAAAC

>CL155.Contig5_A-W 3345 3650 minus strand PREDICTED: myosin-Ig [Cricetulus griseus]

GTAACAGGGGTGAGTGTGACCAGTGGACGGGACCAGCTGGTGGTACTGCATGCCCGGGGC

CATGATGACCTCGTGGTATGTCTGCACCGCTCCCAGCCACCTCTGGACAATCGTATCGGG

GAGCTGGTGGGTGTACTGGCTGCACACTGCCAGAGGGAGGGAAGAAACCTAGAGGTCCGT

GTCTCTGACTGCATTTCACTGAGCCAGCGTGGGGCCCAACGCCTTGTTTCCGTGGAGTCC

AGGCCAGAGCAACCAGAACCAGATTTCTGCTGTAGCCGTGGCAACTTTACCCTCCTCTGG

CCAAAC

>CL155.Contig6_A-W 3259 3564 minus strand PREDICTED: myosin-Ig [Cricetulus griseus]

GTAACAGGGGTGAGTGTGACCAGTGGACGGGACCAGCTGGTGGTACTGCATGCCCGGGGC

CATGATGACCTCGTGGTATGTCTGCACCGCTCCCAGCCACCTCTGGACAATCGTATCGGG

GAGCTGGTGGGTGTACTGGCTGCACACTGCCAGAGGGAGGGAAGAAACCTAGAGGTCCGT

GTCTCTGACTGCATTTCACTGAGCCAGCGTGGGGCCCAACGCCTTGTTTCCGTGGAGTCC

AGGCCAGAGCAACCAGAACCAGATTTCTGCTGTAGCCGTGGCAACTTTACCCTCCTCTGG

CCAAAC

>CL155.Contig7_A-W 4255 4560 minus strand PREDICTED: myosin-Ig [Cricetulus griseus]

GTAACAGGGGTGAGTGTGACCAGTGGACGGGACCAGCTGGTGGTACTGCATGCCCGGGGC

CATGATGACCTCGTGGTATGTCTGCACCGCTCCCAGCCACCTCTGGACAATCGTATCGGG

GAGCTGGTGGGTGTACTGGCTGCACACTGCCAGAGGGAGGGAAGAAACCTAGAGGTCCGT

GTCTCTGACTGCATTTCACTGAGCCAGCGTGGGGCCCAACGCCTTGTTTCCGTGGAGTCC

AGGCCAGAGCAACCAGAACCAGATTTCTGCTGTAGCCGTGGCAACTTTACCCTCCTCTGG

CCAAAC

>CL155.Contig8_A-W 3244 3549 minus strand PREDICTED: myosin-Ig [Cricetulus griseus]

GTAACAGGGGTGAGTGTGACCAGTGGACGGGACCAGCTGGTGGTACTGCATGCCCGGGGC

CATGATGACCTCGTGGTATGTCTGCACCGCTCCCAGCCACCTCTGGACAATCGTATCGGG

GAGCTGGTGGGTGTACTGGCTGCACACTGCCAGAGGGAGGGAAGAAACCTAGAGGTCCGT

GTCTCTGACTGCATTTCACTGAGCCAGCGTGGGGCCCAACGCCTTGTTTCCGTGGAGTCC

AGGCCAGAGCAACCAGAACCAGATTTCTGCTGTAGCCGTGGCAACTTTACCCTCCTCTGG

CCAAAC

>CL155.Contig9_A-W 3242 3547 minus strand myosin IG [Homo sapiens] >gi|109658840|gb|AAI17242.1| Myosin IG [Homo sapiens] >gi|109731069|gb|AAI13545.1| Myosin IG [Homo sapiens] >gi|119581472|gb|EAW61068.1| myosin IG, isoform CRA_a [Homo sapiens] >gi|166788570|dbj|BAG06733.1| MYO1G variant protein [Homo sapiens]

GTAACAGGGGTGAGTGTGACCAGTGGACGGGACCAGCTGGTGGTACTGCATGCCCGGGGC

CATGATGACCTCGTGGTATGTCTGCACCGCTCCCAGCCACCTCTGGACAATCGTATCGGG

GAGCTGGTGGGTGTACTGGCTGCACACTGCCAGAGGGAGGGAAGAAACCTAGAGGTCCGT

GTCTCTGACTGCATTTCACTGAGCCAGCGTGGGGCCCAACGCCTTGTTTCCGTGGAGTCC

AGGCCAGAGCAACCAGAACCAGATTTCTGCTGTAGCCGTGGCAACTTTACCCTCCTCTGG

CCAAAC

>CL155.Contig10_A-W 24 365 minus strand PREDICTED: unconventional myosin-Ig [Pongo abelii]

ATCCACAGGGTTAAAGATGTACTGCTCAAATCCACCTGTGTGCTAGAGGCCTTTGGCAAT

GCCCGCACCAACCGCAATCACAACTCCAGCCGCTTTGGCAAGTACATGGATATTAACTTT

GACTTCAAGGGAGATCCTGTTGGAGGACACATCCACAGTTACCTGCTGGAAAAGTCCAGG

GTCCTCAAGCAACATGTGGGCGAAAGGAACTTCCATGCATTCTACCAGTTGCTTAGGGGC

TGTAAGGACCAAGAGCTACAAGGACTGTACCTGGAGAGAAACCCTGCTGTGTATAATTTC

ACACGCCAGGGAGCCGGGCTCAACGTGGGTGTGCACAATGCG

>CL155.Contig11_A-W 103 834 minus strand PREDICTED: myosin-Ig [Cricetulus griseus]

ATGGAAGATGAGGAGGGTCCTGAGTATGGCAAGCCTGACTTTGTGCTCTTGGACCAAGTG

TCCATGGAGGACTTCATGAAGAACCTGCAGCTCAGGTTTGAGAAAGGCCGCATCTACACC

TACATCGGTGAGGTACTGGTGTCCGTGAATCCCTACCAGGAGCTGCCTCTATATGGGCCT

GAGGCCATTGCCAGGTACCAGGGCCGAGAGCTCTACGAGCGACCACCTCACCTCTATGCT

GTAGCCAATGCTGCCTACAAGGCGATGAAGCGCAGGTCCAGGGACACCTGCATCGTCATC

TCAGGGGAGAGTGGGGCAGGGAAGACAGAAGCCAGCAAGCACATCATGCAGTACATCGCT

GCTGTCACCAATCCAAGCCAGAGAGCTGAGGTGGAGAGGGTTAAAGATGTACTGCTCAAA

TCCACCTGTGTGCTAGAGGCCTTTGGCAATGCCCGCACCAACCGCAATCACAACTCCAGC

CGCTTTGGCAAGTACATGGATATTAACTTTGACTTCAAGGGAGATCCTGTTGGAGGACAC

ATCCACAGTTACCTGCTGGAAAAGTCCAGGGTCCTCAAGCAACATGTGGGCGAAAGGAAC

TTCCATGCATTCTACCAGTTGCTTAGGGGCTGTAAGGACCAAGAGCTACAAGGACTGTAC

CTGGAGAGAAACCCTGCTGTGTATAATTTCACACGCCAGGGAGCCGGGCTCAACGTGGGT

GTGCACAATGCG

>CL155.Contig12_A-W 3112 3417 minus strand myosin IG, isoform CRA_d [Homo sapiens]

GTAACAGGGGTGAGTGTGACCAGTGGACGGGACCAGCTGGTGGTACTGCATGCCCGGGGC

CATGATGACCTCGTGGTATGTCTGCACCGCTCCCAGCCACCTCTGGACAATCGTATCGGG

GAGCTGGTGGGTGTACTGGCTGCACACTGCCAGAGGGAGGGAAGAAACCTAGAGGTCCGT

GTCTCTGACTGCATTTCACTGAGCCAGCGTGGGGCCCAACGCCTTGTTTCCGTGGAGTCC

AGGCCAGAGCAACCAGAACCAGATTTCTGCTGTAGCCGTGGCAACTTTACCCTCCTCTGG

CCAAAC

>CL155.Contig13_A-W 2537 3211 minus strand PREDICTED: myosin-Ig [Cricetulus griseus]

TCCAGGTGGCGGGCCCGGCAGCTGGTAAAGAACATTCCACCTTCAGATATGGCTCAGATC

AAGGCCAAAGTGGCTGCCATGGGGGCCCTGCAGGGTCTACGTCAGGACTGGGGCTGCCAG

AGAGCCTGGGCTCAAGACTACCTGTCCTCTGGCACTGACAACCCCACAGCCTCAGGCTTG

TTTGCTCAGCAACTGAAGACCCTTCGAGAGAAGGATGGCTTTGGGGCTGTGCTCTTTTCT

AGCCATGTCCGTAAGGTGAACCGCTTCCGCAAGTGCCGGGACCGGGCCCTTCTGCTTACA

GACCGGCACCTCTACAAACTGGAACCTGGCCGTCAGTACCGGGTAATGCGGGCTGTGCCC

CTGGATGCGGTAACAGGGGTGAGTGTGACCAGTGGACGGGACCAGCTGGTGGTACTGCAT

GCCCGGGGCCATGATGACCTCGTGGTATGTCTGCACCGCTCCCAGCCACCTCTGGACAAT

CGTATCGGGGAGCTGGTGGGTGTACTGGCTGCACACTGCCAGAGGGAGGGAAGAAACCTA

GAGGTCCGTGTCTCTGACTGCATTTCACTGAGCCAGCGTGGGGCCCAACGCCTTGTTTCC

GTGGAGTCCAGGCCAGAGCAACCAGAACCAGATTTCTGCTGTAGCCGTGGCAACTTTACC

CTCCTCTGGCCAAAC

>CL155.Contig14_A-W 2522 3196 minus strand PREDICTED: myosin-Ig [Cricetulus griseus]

TCCAGGTGGCGGGCCCGGCAGCTGGTAAAGAACATTCCACCTTCAGATATGGCTCAGATC

AAGGCCAAAGTGGCTGCCATGGGGGCCCTGCAGGGTCTACGTCAGGACTGGGGCTGCCAG

AGAGCCTGGGCTCAAGACTACCTGTCCTCTGGCACTGACAACCCCACAGCCTCAGGCTTG

TTTGCTCAGCAACTGAAGACCCTTCGAGAGAAGGATGGCTTTGGGGCTGTGCTCTTTTCT

AGCCATGTCCGTAAGGTGAACCGCTTCCGCAAGTGCCGGGACCGGGCCCTTCTGCTTACA

GACCGGCACCTCTACAAACTGGAACCTGGCCGTCAGTACCGGGTAATGCGGGCTGTGCCC

CTGGATGCGGTAACAGGGGTGAGTGTGACCAGTGGACGGGACCAGCTGGTGGTACTGCAT

GCCCGGGGCCATGATGACCTCGTGGTATGTCTGCACCGCTCCCAGCCACCTCTGGACAAT

CGTATCGGGGAGCTGGTGGGTGTACTGGCTGCACACTGCCAGAGGGAGGGAAGAAACCTA

GAGGTCCGTGTCTCTGACTGCATTTCACTGAGCCAGCGTGGGGCCCAACGCCTTGTTTCC

GTGGAGTCCAGGCCAGAGCAACCAGAACCAGATTTCTGCTGTAGCCGTGGCAACTTTACC

CTCCTCTGGCCAAAC

>CL155.Contig15_A-W 103 3153 minus strand PREDICTED: myosin-Ig [Cricetulus griseus]

ATGGAAGATGAGGAGGGTCCTGAGTATGGCAAGCCTGACTTTGTGCTCTTGGACCAAGTG

TCCATGGAGGACTTCATGAAGAACCTGCAGCTCAGGTTTGAGAAAGGCCGCATCTACACC

TACATCGGTGAGGTACTGGTGTCCGTGAATCCCTACCAGGAGCTGCCTCTATATGGGCCT

GAGGCCATTGCCAGGTACCAGGGCCGAGAGCTCTACGAGCGACCACCTCACCTCTATGCT

GTAGCCAATGCTGCCTACAAGGCGATGAAGCGCAGGTCCAGGGACACCTGCATCGTCATC

TCAGGGGAGAGTGGGGCAGGGAAGACAGAAGCCAGCAAGCACATCATGCAGTACATCGCT

GCTGTCACCAATCCAAGCCAGAGAGCTGAGGTGGAGAGGGTTAAAGATGTACTGCTCAAA

TCCACCTGTGTGCTAGAGGCCTTTGGCAATGCCCGCACCAACCGCAATCACAACTCCAGC

CGCTTTGGCAAGTACATGGATATTAACTTTGACTTCAAGGGAGATCCTGTTGGAGGACAC

ATCCACAGTTACCTGCTGGAAAAGTCCAGGGTCCTCAAGCAACATGTGGGCGAAAGGAAC

TTCCATGCATTCTACCAGTTGCTTAGGGGCTGTAAGGACCAAGAGCTACAAGGACTGTAC

CTGGAGAGAAACCCTGCTGTGTATAATTTCACACGCCAGGGAGCCGGGCTCAACGTGGGT

GTGCACAATGGCTTGGACAGTGATGAGAGGAGCCACCAGGCAGTGACTGAGGCCATGAGG

GTCATTGGCTTCAGTCCTGAGGAGGTGGCATCAGTACACCGCATCCTGGCTGCCATATTG

CACCTGGGAAACATCGAGTTCATGGAGAGAGAAGAAGATGGGCCACAGAAAGGGAGCCTA

GCAGTGGCTGAGGAGGCATTGGTGGACCATGTGGCTGAGCTGACTGCCACACCCAGGGAC

CTGGTGCTCCGCTCCCTGCTGGCTCGCACTGTGGCTTCTGGAGGCCGGGAACTCATAGAG

AAAGGCCATACTGTGGCGGAGGCCAGCTACGCCCGGGATGCCTGTGCCAAGGCAGTCTAC

CAGCGGCTGTTTGAGTGGGTGGTGGACAGGATCAATGGTATCATGGAAGCCCGGGGCCGT

GACCCTCGTCGTGATGGCAAGGACACAGTCATTGGTGTGCTGGACATCTACGGCTTTGAG

GTGTTCCCTGTCAACAGCTTTGAGCAGTTCTGCATCAACTACTGCAATGAGAAGCTGCAG

CAGCTCTTCATCCGGCTCATCCTGAAGCAGGAGCAGGAAGAGTATGAGCGTGAGGGCATC

ACCTGGCAGACTGTTGAATACTTCAACAATGCCACCATCGTGGAGCTGGTGGAGCGGCCC

CATCGAGGCATCCTGGCTGTGCTGGATGAGGCCTGTAGCACTGCGGGACCCATTACTGAC

CGAATCTTCCTGCAGACCCTGGACACACACCATCGCCACCATCCACACTACTCCAGCCGC

CAGCTTTGCCCCACTGACAAGACAATGGAGTTTGGCCGAGACTTCCGGATCAAGCATTAT

GCAGGAGATGTCACGTACTCTGTGGAGGGTTTCATTGACAAGAACAGAGACTCCCTCTTC

CAGGACTTCAAGCGGCTGTTCTACAATAGCTCAGATCCCACATTGCGGGCTATGTGGCCA

GATGGACAGCAGGACATCACAGAGGTAACCAAGCGCCCCCTGACAGCTGGCACACTATTC

AAGAACTCCATGGTGGCCCTAGTGGAGAACCTGGCCTCCAAGGAACCCTTCTATGTCCGC

TGCATCAAACCCAATGAGGACAAGGTGGCTGGAAGGCTGGATGAGGACCACTGCCGCCAC

CAGGTCGCATACCTGGGGCTGCTGGAGAATGTGAGAGTCCGCAGGGCTGGCTTTGCTTCC

CGCCAGCCATACTCTCGATTTCTCCTCAGGTACAAGATGACCTGTGAGTACACATGGCCC

AACCACCTGCTGGGCTCTGACAAGGCAGCTGTGAGCGCCCTCCTGGAGCAGCACAGGCTG

CAAGGGGATGTGGCCTTTGGCCACAGCAAGCTGTTCATCCGCTCACCCCGGACGCTGGTC

ACACTGGAGCAGAGCCGAGCCCGCCTCATCCCCATCATTGTGCTGCTGCTACAGAAGGCA

TGGAGAGGTACCCTGGCTAGATGGCGCTGCAGGCGGCTGCGAGCCATCTACACTATCATG

CGCTGGTTCCGGAGGCATAAGGTGCGTGCTTACTTGGCAGAGCTGCAGCGGAGGTTTCAG

GCTGCACGGCAGCCCCCACTCTATGGCCGGGACATTGAGTGGCCGCTGCCCCCTGCTGTG

CTGCAGCCCTTCCAGGACACCTGCCACATGCTCTTCTGCAGGTGGCGGGCCCGGCAGCTG

GTAAAGAACATTCCACCTTCAGATATGGCTCAGATCAAGGCCAAAGTGGCTGCCATGGGG

GCCCTGCAGGGTCTACGTCAGGACTGGGGCTGCCAGAGAGCCTGGGCTCAAGACTACCTG

TCCTCTGGCACTGACAACCCCACAGCCTCAGGCTTGTTTGCTCAGCAACTGAAGACCCTT

CGAGAGAAGGATGGCTTTGGGGCTGTGCTCTTTTCTAGCCATGTCCGTAAGGTGAACCGC

TTCCGCAAGTGCCGGGACCGGGCCCTTCTGCTTACAGACCGGCACCTCTACAAACTGGAA

CCTGGCCGTCAGTACCGGGTAATGCGGGCTGTGCCCCTGGATGCGGTAACAGGGGTGAGT

GTGACCAGTGGACGGGACCAGCTGGTGGTACTGCATGCCCGGGGCCATGATGACCTCGTG

GTATGTCTGCACCGCTCCCAGCCACCTCTGGACAATCGTATCGGGGAGCTGGTGGGTGTA

CTGGCTGCACACTGCCAGAGGGAGGGAAGAAACCTAGAGGTCCGTGTCTCTGACTGCATT

TCACTGAGCCAGCGTGGGGCCCAACGCCTTGTTTCCGTGGAGTCCAGGCCAGAGCAACCA

GAACCAGATTTCTGCTGTAGCCGTGGCAACTTTACCCTCCTCTGGCCAAAC

>CL155.Contig16_A-W 4098 4376 minus strand PREDICTED: unconventional myosin-Ig [Pongo abelii]

GTAACAGGGGTGAGTGTGACCAGTGGACGGGACCAGCTGGTGGTACTGCATGCCCGGGGC

CATGATGACCTCGTGGTATGTCTGCACCGCTCCCAGCCACCTCTGGACAATCGTATCGGG

GAGCTGGTGGGTGTACTGGCTGCACACTGCCAGAGGGAGGGAAGAAACCTAGAGGTCCGT

GTCTCTGACTGCATTTCACTGAGCCAGCGTGGGGCCCAACGCCTTGTTTCCGTGGAGTCC

AGGCCAGAGCAACCAGAACCAGATTTCTGCTGTAGCCGT

>CL155.Contig17_A-W 103 3138 minus strand PREDICTED: myosin-Ig [Cricetulus griseus]

ATGGAAGATGAGGAGGGTCCTGAGTATGGCAAGCCTGACTTTGTGCTCTTGGACCAAGTG

TCCATGGAGGACTTCATGAAGAACCTGCAGCTCAGGTTTGAGAAAGGCCGCATCTACACC

TACATCGGTGAGGTACTGGTGTCCGTGAATCCCTACCAGGAGCTGCCTCTATATGGGCCT

GAGGCCATTGCCAGGTACCAGGGCCGAGAGCTCTACGAGCGACCACCTCACCTCTATGCT

GTAGCCAATGCTGCCTACAAGGCGATGAAGCGCAGGTCCAGGGACACCTGCATCGTCATC

TCAGGGGAGAGTGGGGCAGGGAAGACAGAAGCCAGCAAGCACATCATGCAGTACATCGCT

GCTGTCACCAATCCAAGCCAGAGAGCTGAGGTGGAGAGGGTTAAAGATGTACTGCTCAAA

TCCACCTGTGTGCTAGAGGCCTTTGGCAATGCCCGCACCAACCGCAATCACAACTCCAGC

CGCTTTGGCAAGTACATGGATATTAACTTTGACTTCAAGGGAGATCCTGTTGGAGGACAC

ATCCACAGTTACCTGCTGGAAAAGTCCAGGGTCCTCAAGCAACATGTGGGCGAAAGGAAC

TTCCATGCATTCTACCAGTTGCTTAGGGGCTGTAAGGACCAAGAGCTACAAGGACTGTAC

CTGGAGAGAAACCCTGCTGTGTATAATTTCACACGCCAGGGAGCCGGGCTCAACGGCTTA

GACAGTGATGAGAGGAGCCACCAGGCAGTGACTGAGGCCATGAGGGTCATTGGCTTCAGT

CCTGAGGAGGTGGCATCAGTACACCGCATCCTGGCTGCCATATTGCACCTGGGAAACATC

GAGTTCATGGAGAGAGAAGAAGATGGGCCACAGAAAGGGAGCCTAGCAGTGGCTGAGGAG

GCATTGGTGGACCATGTGGCTGAGCTGACTGCCACACCCAGGGACCTGGTGCTCCGCTCC

CTGCTGGCTCGCACTGTGGCTTCTGGAGGCCGGGAACTCATAGAGAAAGGCCATACTGTG

GCGGAGGCCAGCTACGCCCGGGATGCCTGTGCCAAGGCAGTCTACCAGCGGCTGTTTGAG

TGGGTGGTGGACAGGATCAATGGTATCATGGAAGCCCGGGGCCGTGACCCTCGTCGTGAT

GGCAAGGACACAGTCATTGGTGTGCTGGACATCTACGGCTTTGAGGTGTTCCCTGTCAAC

AGCTTTGAGCAGTTCTGCATCAACTACTGCAATGAGAAGCTGCAGCAGCTCTTCATCCGG

CTCATCCTGAAGCAGGAGCAGGAAGAGTATGAGCGTGAGGGCATCACCTGGCAGACTGTT

GAATACTTCAACAATGCCACCATCGTGGAGCTGGTGGAGCGGCCCCATCGAGGCATCCTG

GCTGTGCTGGATGAGGCCTGTAGCACTGCGGGACCCATTACTGACCGAATCTTCCTGCAG

ACCCTGGACACACACCATCGCCACCATCCACACTACTCCAGCCGCCAGCTTTGCCCCACT

GACAAGACAATGGAGTTTGGCCGAGACTTCCGGATCAAGCATTATGCAGGAGATGTCACG

TACTCTGTGGAGGGTTTCATTGACAAGAACAGAGACTCCCTCTTCCAGGACTTCAAGCGG

CTGTTCTACAATAGCTCAGATCCCACATTGCGGGCTATGTGGCCAGATGGACAGCAGGAC

ATCACAGAGGTAACCAAGCGCCCCCTGACAGCTGGCACACTATTCAAGAACTCCATGGTG

GCCCTAGTGGAGAACCTGGCCTCCAAGGAACCCTTCTATGTCCGCTGCATCAAACCCAAT

GAGGACAAGGTGGCTGGAAGGCTGGATGAGGACCACTGCCGCCACCAGGTCGCATACCTG

GGGCTGCTGGAGAATGTGAGAGTCCGCAGGGCTGGCTTTGCTTCCCGCCAGCCATACTCT

CGATTTCTCCTCAGGTACAAGATGACCTGTGAGTACACATGGCCCAACCACCTGCTGGGC

TCTGACAAGGCAGCTGTGAGCGCCCTCCTGGAGCAGCACAGGCTGCAAGGGGATGTGGCC

TTTGGCCACAGCAAGCTGTTCATCCGCTCACCCCGGACGCTGGTCACACTGGAGCAGAGC

CGAGCCCGCCTCATCCCCATCATTGTGCTGCTGCTACAGAAGGCATGGAGAGGTACCCTG

GCTAGATGGCGCTGCAGGCGGCTGCGAGCCATCTACACTATCATGCGCTGGTTCCGGAGG

CATAAGGTGCGTGCTTACTTGGCAGAGCTGCAGCGGAGGTTTCAGGCTGCACGGCAGCCC

CCACTCTATGGCCGGGACATTGAGTGGCCGCTGCCCCCTGCTGTGCTGCAGCCCTTCCAG

GACACCTGCCACATGCTCTTCTGCAGGTGGCGGGCCCGGCAGCTGGTAAAGAACATTCCA

CCTTCAGATATGGCTCAGATCAAGGCCAAAGTGGCTGCCATGGGGGCCCTGCAGGGTCTA

CGTCAGGACTGGGGCTGCCAGAGAGCCTGGGCTCAAGACTACCTGTCCTCTGGCACTGAC

AACCCCACAGCCTCAGGCTTGTTTGCTCAGCAACTGAAGACCCTTCGAGAGAAGGATGGC

TTTGGGGCTGTGCTCTTTTCTAGCCATGTCCGTAAGGTGAACCGCTTCCGCAAGTGCCGG

GACCGGGCCCTTCTGCTTACAGACCGGCACCTCTACAAACTGGAACCTGGCCGTCAGTAC

CGGGTAATGCGGGCTGTGCCCCTGGATGCGGTAACAGGGGTGAGTGTGACCAGTGGACGG

GACCAGCTGGTGGTACTGCATGCCCGGGGCCATGATGACCTCGTGGTATGTCTGCACCGC

TCCCAGCCACCTCTGGACAATCGTATCGGGGAGCTGGTGGGTGTACTGGCTGCACACTGC

CAGAGGGAGGGAAGAAACCTAGAGGTCCGTGTCTCTGACTGCATTTCACTGAGCCAGCGT

GGGGCCCAACGCCTTGTTTCCGTGGAGTCCAGGCCAGAGCAACCAGAACCAGATTTCTGC

TGTAGCCGTGGCAACTTTACCCTCCTCTGGCCAAAC

>CL155.Contig18_A-W 1 336 minus strand PREDICTED: myosin-Ig [Cricetulus griseus]

AGGGTTAAAGATGTACTGCTCAAATCCACCTGTGTGCTAGAGGCCTTTGGCAATGCCCGC

ACCAACCGCAATCACAACTCCAGCCGCTTTGGCAAGTACATGGATATTAACTTTGACTTC

AAGGGAGATCCTGTTGGAGGACACATCCACAGTTACCTGCTGGAAAAGTCCAGGGTCCTC

AAGCAACATGTGGGCGAAAGGAACTTCCATGCATTCTACCAGTTGCTTAGGGGCTGTAAG

GACCAAGAGCTACAAGGACTGTACCTGGAGAGAAACCCTGCTGTGTATAATTTCACACGC

CAGGGAGCCGGGCTCAACGTGGGTGTGCACAATGCG

>CL155.Contig19_A-W 2715 3020 minus strand PREDICTED: myosin-Ig [Cricetulus griseus]

GTAACAGGGGTGAGTGTGACCAGTGGACGGGACCAGCTGGTGGTACTGCATGCCCGGGGC

CATGATGACCTCGTGGTATGTCTGCACCGCTCCCAGCCACCTCTGGACAATCGTATCGGG

GAGCTGGTGGGTGTACTGGCTGCACACTGCCAGAGGGAGGGAAGAAACCTAGAGGTCCGT

GTCTCTGACTGCATTTCACTGAGCCAGCGTGGGGCCCAACGCCTTGTTTCCGTGGAGTCC

AGGCCAGAGCAACCAGAACCAGATTTCTGCTGTAGCCGTGGCAACTTTACCCTCCTCTGG

CCAAAC

>CL155.Contig20_A-W 24 365 minus strand PREDICTED: myosin-Ig [Cricetulus griseus]

ATCCACAGGGTTAAAGATGTACTGCTCAAATCCACCTGTGTGCTAGAGGCCTTTGGCAAT

GCCCGCACCAACCGCAATCACAACTCCAGCCGCTTTGGCAAGTACATGGATATTAACTTT

GACTTCAAGGGAGATCCTGTTGGAGGACACATCCACAGTTACCTGCTGGAAAAGTCCAGG

GTCCTCAAGCAACATGTGGGCGAAAGGAACTTCCATGCATTCTACCAGTTGCTTAGGGGC

TGTAAGGACCAAGAGCTACAAGGACTGTACCTGGAGAGAAACCCTGCTGTGTATAATTTC

ACACGCCAGGGAGCCGGGCTCAACGTGGGTGTGCACAATGCG

>CL155.Contig21_A-W 2634 3278 minus strand PREDICTED: unconventional myosin-Ig [Pongo abelii]

AGGTGGCGGGCCCGGCAGCTGGTAAAGAACATTCCACCTTCAGATATGGCTCAGATCAAG

GCCAAAGTGGCTGCCATGGGGGCCCTGCAGGGTCTACGTCAGGACTGGGGCTGCCAGAGA

GCCTGGGCTCAAGACTACCTGTCCTCTGGCACTGACAACCCCACAGCCTCAGGCTTGTTT

GCTCAGCAACTGAAGACCCTTCGAGAGAAGGATGGCTTTGGGGCTGTGCTCTTTTCTAGC

CATGTCCGTAAGGTGAACCGCTTCCGCAAGTGCCGGGACCGGGCCCTTCTGCTTACAGAC

CGGCACCTCTACAAACTGGAACCTGGCCGTCAGTACCGGGTAATGCGGGCTGTGCCCCTG

GATGCGGTAACAGGGGTGAGTGTGACCAGTGGACGGGACCAGCTGGTGGTACTGCATGCC

CGGGGCCATGATGACCTCGTGGTATGTCTGCACCGCTCCCAGCCACCTCTGGACAATCGT

ATCGGGGAGCTGGTGGGTGTACTGGCTGCACACTGCCAGAGGGAGGGAAGAAACCTAGAG

GTCCGTGTCTCTGACTGCATTTCACTGAGCCAGCGTGGGGCCCAACGCCTTGTTTCCGTG

GAGTCCAGGCCAGAGCAACCAGAACCAGATTTCTGCTGTAGCCGT

>CL155.Contig22_A-W 2590 2895 minus strand PREDICTED: unconventional myosin-Ig [Pan paniscus]

GTAACAGGGGTGAGTGTGACCAGTGGACGGGACCAGCTGGTGGTACTGCATGCCCGGGGC

CATGATGACCTCGTGGTATGTCTGCACCGCTCCCAGCCACCTCTGGACAATCGTATCGGG

GAGCTGGTGGGTGTACTGGCTGCACACTGCCAGAGGGAGGGAAGAAACCTAGAGGTCCGT

GTCTCTGACTGCATTTCACTGAGCCAGCGTGGGGCCCAACGCCTTGTTTCCGTGGAGTCC

AGGCCAGAGCAACCAGAACCAGATTTCTGCTGTAGCCGTGGCAACTTTACCCTCCTCTGG

CCAAAC

>CL155.Contig23_A-W 2585 2890 minus strand PREDICTED: myosin-Ig [Cricetulus griseus]

GTAACAGGGGTGAGTGTGACCAGTGGACGGGACCAGCTGGTGGTACTGCATGCCCGGGGC

CATGATGACCTCGTGGTATGTCTGCACCGCTCCCAGCCACCTCTGGACAATCGTATCGGG

GAGCTGGTGGGTGTACTGGCTGCACACTGCCAGAGGGAGGGAAGAAACCTAGAGGTCCGT

GTCTCTGACTGCATTTCACTGAGCCAGCGTGGGGCCCAACGCCTTGTTTCCGTGGAGTCC

AGGCCAGAGCAACCAGAACCAGATTTCTGCTGTAGCCGTGGCAACTTTACCCTCCTCTGG

CCAAAC

>CL155.Contig24_A-W 1 939 minus strand PREDICTED: myosin-Ig [Cricetulus griseus]

AGGGTTAAAGATGTACTGCTCAAATCCACCTGTGTGCTAGAGGCCTTTGGCAATGCCCGC

ACCAACCGCAATCACAACTCCAGCCGCTTTGGCAAGTACATGGATATTAACTTTGACTTC

AAGGGAGATCCTGTTGGAGGACACATCCACAGTTACCTGCTGGAAAAGTCCAGGGTCCTC

AAGCAACATGTGGGCGAAAGGAACTTCCATGCATTCTACCAGTTGCTTAGGGGCTGTAAG

GACCAAGAGCTACAAGGACTGTACCTGGAGAGAAACCCTGCTGTGTATAATTTCACACGC

CAGGGAGCCGGGCTCAACGTGGGTGTGCACAATGGCTTGGACAGTGATGAGAGGAGCCAC

CAGGCAGTGACTGAGGCCATGAGGGTCATTGGCTTCAGTCCTGAGGAGGTGGCATCAGTA

CACCGCATCCTGGCTGCCATATTGCACCTGGGAAACATCGAGTTCATGGAGAGAGAAGAA

GATGGGCCACAGAAAGGGAGCCTAGCAGTGGCTGAGGAGGCATTGGTGGACCATGTGGCT

GAGCTGACTGCCACACCCAGGGACCTGGTGCTCCGCTCCCTGCTGGCTCGCACTGTGGCT

TCTGGAGGCCGGGAACTCATAGAGAAAGGCCATACTGTGGCGGAGGCCAGCTACGCCCGG

GATGCCTGTGCCAAGGCAGTCTACCAGCGGCTGTTTGAGTGGGTGGTGGACAGGATCAAT

GGTATCATGGAAGCCCGGGGCCGTGACCCTCGTCGTGATGGCAAGGACACAGTCATTGGT

GTGCTGGACATCTACGGCTTTGAGGTGTTCCCTGTCAACAGCTTTGAGCAGTTCTGCATC

AACTACTGCAATGAGAAGCTGCAGCAGCTCTTCATCCGGCTCATCCTGAAGCAGGAGCAG

GAAGAGTATGAGCGTGAGGGCATCACCTGGCAGACTGTG

>CL155.Contig25_A-W 1 924 minus strand PREDICTED: myosin-Ig [Cricetulus griseus]

AGGGTTAAAGATGTACTGCTCAAATCCACCTGTGTGCTAGAGGCCTTTGGCAATGCCCGC

ACCAACCGCAATCACAACTCCAGCCGCTTTGGCAAGTACATGGATATTAACTTTGACTTC

AAGGGAGATCCTGTTGGAGGACACATCCACAGTTACCTGCTGGAAAAGTCCAGGGTCCTC

AAGCAACATGTGGGCGAAAGGAACTTCCATGCATTCTACCAGTTGCTTAGGGGCTGTAAG

GACCAAGAGCTACAAGGACTGTACCTGGAGAGAAACCCTGCTGTGTATAATTTCACACGC

CAGGGAGCCGGGCTCAACGGCTTAGACAGTGATGAGAGGAGCCACCAGGCAGTGACTGAG

GCCATGAGGGTCATTGGCTTCAGTCCTGAGGAGGTGGCATCAGTACACCGCATCCTGGCT

GCCATATTGCACCTGGGAAACATCGAGTTCATGGAGAGAGAAGAAGATGGGCCACAGAAA

GGGAGCCTAGCAGTGGCTGAGGAGGCATTGGTGGACCATGTGGCTGAGCTGACTGCCACA

CCCAGGGACCTGGTGCTCCGCTCCCTGCTGGCTCGCACTGTGGCTTCTGGAGGCCGGGAA

CTCATAGAGAAAGGCCATACTGTGGCGGAGGCCAGCTACGCCCGGGATGCCTGTGCCAAG

GCAGTCTACCAGCGGCTGTTTGAGTGGGTGGTGGACAGGATCAATGGTATCATGGAAGCC

CGGGGCCGTGACCCTCGTCGTGATGGCAAGGACACAGTCATTGGTGTGCTGGACATCTAC

GGCTTTGAGGTGTTCCCTGTCAACAGCTTTGAGCAGTTCTGCATCAACTACTGCAATGAG

AAGCTGCAGCAGCTCTTCATCCGGCTCATCCTGAAGCAGGAGCAGGAAGAGTATGAGCGT

GAGGGCATCACCTGGCAGACTGTG

>CL155.Contig26_A-W 24 2684 minus strand PREDICTED: myosin-Ig [Cricetulus griseus]

ATCCACAGGGTTAAAGATGTACTGCTCAAATCCACCTGTGTGCTAGAGGCCTTTGGCAAT

GCCCGCACCAACCGCAATCACAACTCCAGCCGCTTTGGCAAGTACATGGATATTAACTTT

GACTTCAAGGGAGATCCTGTTGGAGGACACATCCACAGTTACCTGCTGGAAAAGTCCAGG

GTCCTCAAGCAACATGTGGGCGAAAGGAACTTCCATGCATTCTACCAGTTGCTTAGGGGC

TGTAAGGACCAAGAGCTACAAGGACTGTACCTGGAGAGAAACCCTGCTGTGTATAATTTC

ACACGCCAGGGAGCCGGGCTCAACGTGGGTGTGCACAATGGCTTGGACAGTGATGAGAGG

AGCCACCAGGCAGTGACTGAGGCCATGAGGGTCATTGGCTTCAGTCCTGAGGAGGTGGCA

TCAGTACACCGCATCCTGGCTGCCATATTGCACCTGGGAAACATCGAGTTCATGGAGAGA

GAAGAAGATGGGCCACAGAAAGGGAGCCTAGCAGTGGCTGAGGAGGCATTGGTGGACCAT

GTGGCTGAGCTGACTGCCACACCCAGGGACCTGGTGCTCCGCTCCCTGCTGGCTCGCACT

GTGGCTTCTGGAGGCCGGGAACTCATAGAGAAAGGCCATACTGTGGCGGAGGCCAGCTAC

GCCCGGGATGCCTGTGCCAAGGCAGTCTACCAGCGGCTGTTTGAGTGGGTGGTGGACAGG

ATCAATGGTATCATGGAAGCCCGGGGCCGTGACCCTCGTCGTGATGGCAAGGACACAGTC

ATTGGTGTGCTGGACATCTACGGCTTTGAGGTGTTCCCTGTCAACAGCTTTGAGCAGTTC

TGCATCAACTACTGCAATGAGAAGCTGCAGCAGCTCTTCATCCGGCTCATCCTGAAGCAG

GAGCAGGAAGAGTATGAGCGTGAGGGCATCACCTGGCAGACTGTTGAATACTTCAACAAT

GCCACCATCGTGGAGCTGGTGGAGCGGCCCCATCGAGGCATCCTGGCTGTGCTGGATGAG

GCCTGTAGCACTGCGGGACCCATTACTGACCGAATCTTCCTGCAGACCCTGGACACACAC

CATCGCCACCATCCACACTACTCCAGCCGCCAGCTTTGCCCCACTGACAAGACAATGGAG

TTTGGCCGAGACTTCCGGATCAAGCATTATGCAGGAGATGTCACGTACTCTGTGGAGGGT

TTCATTGACAAGAACAGAGACTCCCTCTTCCAGGACTTCAAGCGGCTGTTCTACAATAGC

TCAGATCCCACATTGCGGGCTATGTGGCCAGATGGACAGCAGGACATCACAGAGGTAACC

AAGCGCCCCCTGACAGCTGGCACACTATTCAAGAACTCCATGGTGGCCCTAGTGGAGAAC

CTGGCCTCCAAGGAACCCTTCTATGTCCGCTGCATCAAACCCAATGAGGACAAGGTGGCT

GGAAGGCTGGATGAGGACCACTGCCGCCACCAGGTCGCATACCTGGGGCTGCTGGAGAAT

GTGAGAGTCCGCAGGGCTGGCTTTGCTTCCCGCCAGCCATACTCTCGATTTCTCCTCAGG

TACAAGATGACCTGTGAGTACACATGGCCCAACCACCTGCTGGGCTCTGACAAGGCAGCT

GTGAGCGCCCTCCTGGAGCAGCACAGGCTGCAAGGGGATGTGGCCTTTGGCCACAGCAAG

CTGTTCATCCGCTCACCCCGGACGCTGGTCACACTGGAGCAGAGCCGAGCCCGCCTCATC

CCCATCATTGTGCTGCTGCTACAGAAGGCATGGAGAGGTACCCTGGCTAGATGGCGCTGC

AGGCGGCTGCGAGCCATCTACACTATCATGCGCTGGTTCCGGAGGCATAAGGTGCGTGCT

TACTTGGCAGAGCTGCAGCGGAGGTTTCAGGCTGCACGGCAGCCCCCACTCTATGGCCGG

GACATTGAGTGGCCGCTGCCCCCTGCTGTGCTGCAGCCCTTCCAGGACACCTGCCACATG

CTCTTCTGCAGGTGGCGGGCCCGGCAGCTGGTAAAGAACATTCCACCTTCAGATATGGCT

CAGATCAAGGCCAAAGTGGCTGCCATGGGGGCCCTGCAGGGTCTACGTCAGGACTGGGGC

TGCCAGAGAGCCTGGGCTCAAGACTACCTGTCCTCTGGCACTGACAACCCCACAGCCTCA

GGCTTGTTTGCTCAGCAACTGAAGACCCTTCGAGAGAAGGATGGCTTTGGGGCTGTGCTC

TTTTCTAGCCATGTCCGTAAGGTGAACCGCTTCCGCAAGTGCCGGGACCGGGCCCTTCTG

CTTACAGACCGGCACCTCTACAAACTGGAACCTGGCCGTCAGTACCGGGTAATGCGGGCT

GTGCCCCTGGATGCGGTAACAGGGGTGAGTGTGACCAGTGGACGGGACCAGCTGGTGGTA

CTGCATGCCCGGGGCCATGATGACCTCGTGGTATGTCTGCACCGCTCCCAGCCACCTCTG

GACAATCGTATCGGGGAGCTGGTGGGTGTACTGGCTGCACACTGCCAGAGGGAGGGAAGA

AACCTAGAGGTCCGTGTCTCTGACTGCATTTCACTGAGCCAGCGTGGGGCCCAACGCCTT

GTTTCCGTGGAGTCCAGGCCAGAGCAACCAGAACCAGATTTCTGCTGTAGCCGTGGCAAC

TTTACCCTCCTCTGGCCAAAC

>CL155.Contig27_A-W 4040 4345 minus strand PREDICTED: myosin-Ig [Cricetulus griseus]

GTAACAGGGGTGAGTGTGACCAGTGGACGGGACCAGCTGGTGGTACTGCATGCCCGGGGC

CATGATGACCTCGTGGTATGTCTGCACCGCTCCCAGCCACCTCTGGACAATCGTATCGGG

GAGCTGGTGGGTGTACTGGCTGCACACTGCCAGAGGGAGGGAAGAAACCTAGAGGTCCGT

GTCTCTGACTGCATTTCACTGAGCCAGCGTGGGGCCCAACGCCTTGTTTCCGTGGAGTCC

AGGCCAGAGCAACCAGAACCAGATTTCTGCTGTAGCCGTGGCAACTTTACCCTCCTCTGG

CCAAAC

>CL155.Contig28_A-W 24 2669 minus strand PREDICTED: myosin-Ig [Cricetulus griseus]

ATCCACAGGGTTAAAGATGTACTGCTCAAATCCACCTGTGTGCTAGAGGCCTTTGGCAAT

GCCCGCACCAACCGCAATCACAACTCCAGCCGCTTTGGCAAGTACATGGATATTAACTTT

GACTTCAAGGGAGATCCTGTTGGAGGACACATCCACAGTTACCTGCTGGAAAAGTCCAGG

GTCCTCAAGCAACATGTGGGCGAAAGGAACTTCCATGCATTCTACCAGTTGCTTAGGGGC

TGTAAGGACCAAGAGCTACAAGGACTGTACCTGGAGAGAAACCCTGCTGTGTATAATTTC

ACACGCCAGGGAGCCGGGCTCAACGGCTTAGACAGTGATGAGAGGAGCCACCAGGCAGTG

ACTGAGGCCATGAGGGTCATTGGCTTCAGTCCTGAGGAGGTGGCATCAGTACACCGCATC

CTGGCTGCCATATTGCACCTGGGAAACATCGAGTTCATGGAGAGAGAAGAAGATGGGCCA

CAGAAAGGGAGCCTAGCAGTGGCTGAGGAGGCATTGGTGGACCATGTGGCTGAGCTGACT

GCCACACCCAGGGACCTGGTGCTCCGCTCCCTGCTGGCTCGCACTGTGGCTTCTGGAGGC

CGGGAACTCATAGAGAAAGGCCATACTGTGGCGGAGGCCAGCTACGCCCGGGATGCCTGT

GCCAAGGCAGTCTACCAGCGGCTGTTTGAGTGGGTGGTGGACAGGATCAATGGTATCATG

GAAGCCCGGGGCCGTGACCCTCGTCGTGATGGCAAGGACACAGTCATTGGTGTGCTGGAC

ATCTACGGCTTTGAGGTGTTCCCTGTCAACAGCTTTGAGCAGTTCTGCATCAACTACTGC

AATGAGAAGCTGCAGCAGCTCTTCATCCGGCTCATCCTGAAGCAGGAGCAGGAAGAGTAT

GAGCGTGAGGGCATCACCTGGCAGACTGTTGAATACTTCAACAATGCCACCATCGTGGAG

CTGGTGGAGCGGCCCCATCGAGGCATCCTGGCTGTGCTGGATGAGGCCTGTAGCACTGCG

GGACCCATTACTGACCGAATCTTCCTGCAGACCCTGGACACACACCATCGCCACCATCCA

CACTACTCCAGCCGCCAGCTTTGCCCCACTGACAAGACAATGGAGTTTGGCCGAGACTTC

CGGATCAAGCATTATGCAGGAGATGTCACGTACTCTGTGGAGGGTTTCATTGACAAGAAC

AGAGACTCCCTCTTCCAGGACTTCAAGCGGCTGTTCTACAATAGCTCAGATCCCACATTG

CGGGCTATGTGGCCAGATGGACAGCAGGACATCACAGAGGTAACCAAGCGCCCCCTGACA

GCTGGCACACTATTCAAGAACTCCATGGTGGCCCTAGTGGAGAACCTGGCCTCCAAGGAA

CCCTTCTATGTCCGCTGCATCAAACCCAATGAGGACAAGGTGGCTGGAAGGCTGGATGAG

GACCACTGCCGCCACCAGGTCGCATACCTGGGGCTGCTGGAGAATGTGAGAGTCCGCAGG

GCTGGCTTTGCTTCCCGCCAGCCATACTCTCGATTTCTCCTCAGGTACAAGATGACCTGT

GAGTACACATGGCCCAACCACCTGCTGGGCTCTGACAAGGCAGCTGTGAGCGCCCTCCTG

GAGCAGCACAGGCTGCAAGGGGATGTGGCCTTTGGCCACAGCAAGCTGTTCATCCGCTCA

CCCCGGACGCTGGTCACACTGGAGCAGAGCCGAGCCCGCCTCATCCCCATCATTGTGCTG

CTGCTACAGAAGGCATGGAGAGGTACCCTGGCTAGATGGCGCTGCAGGCGGCTGCGAGCC

ATCTACACTATCATGCGCTGGTTCCGGAGGCATAAGGTGCGTGCTTACTTGGCAGAGCTG

CAGCGGAGGTTTCAGGCTGCACGGCAGCCCCCACTCTATGGCCGGGACATTGAGTGGCCG

CTGCCCCCTGCTGTGCTGCAGCCCTTCCAGGACACCTGCCACATGCTCTTCTGCAGGTGG

CGGGCCCGGCAGCTGGTAAAGAACATTCCACCTTCAGATATGGCTCAGATCAAGGCCAAA

GTGGCTGCCATGGGGGCCCTGCAGGGTCTACGTCAGGACTGGGGCTGCCAGAGAGCCTGG

GCTCAAGACTACCTGTCCTCTGGCACTGACAACCCCACAGCCTCAGGCTTGTTTGCTCAG

CAACTGAAGACCCTTCGAGAGAAGGATGGCTTTGGGGCTGTGCTCTTTTCTAGCCATGTC

CGTAAGGTGAACCGCTTCCGCAAGTGCCGGGACCGGGCCCTTCTGCTTACAGACCGGCAC

CTCTACAAACTGGAACCTGGCCGTCAGTACCGGGTAATGCGGGCTGTGCCCCTGGATGCG

GTAACAGGGGTGAGTGTGACCAGTGGACGGGACCAGCTGGTGGTACTGCATGCCCGGGGC

CATGATGACCTCGTGGTATGTCTGCACCGCTCCCAGCCACCTCTGGACAATCGTATCGGG

GAGCTGGTGGGTGTACTGGCTGCACACTGCCAGAGGGAGGGAAGAAACCTAGAGGTCCGT

GTCTCTGACTGCATTTCACTGAGCCAGCGTGGGGCCCAACGCCTTGTTTCCGTGGAGTCC

AGGCCAGAGCAACCAGAACCAGATTTCTGCTGTAGCCGTGGCAACTTTACCCTCCTCTGG

CCAAAC

>CL155.Contig29_A-W 1996 2667 minus strand myosin IG [Homo sapiens] >gi|109658840|gb|AAI17242.1| Myosin IG [Homo sapiens] >gi|109731069|gb|AAI13545.1| Myosin IG [Homo sapiens] >gi|119581472|gb|EAW61068.1| myosin IG, isoform CRA_a [Homo sapiens] >gi|166788570|dbj|BAG06733.1| MYO1G variant protein [Homo sapiens]

AGGTGGCGGGCCCGGCAGCTGGTAAAGAACATTCCACCTTCAGATATGGCTCAGATCAAG

GCCAAAGTGGCTGCCATGGGGGCCCTGCAGGGTCTACGTCAGGACTGGGGCTGCCAGAGA

GCCTGGGCTCAAGACTACCTGTCCTCTGGCACTGACAACCCCACAGCCTCAGGCTTGTTT

GCTCAGCAACTGAAGACCCTTCGAGAGAAGGATGGCTTTGGGGCTGTGCTCTTTTCTAGC

CATGTCCGTAAGGTGAACCGCTTCCGCAAGTGCCGGGACCGGGCCCTTCTGCTTACAGAC

CGGCACCTCTACAAACTGGAACCTGGCCGTCAGTACCGGGTAATGCGGGCTGTGCCCCTG

GATGCGGTAACAGGGGTGAGTGTGACCAGTGGACGGGACCAGCTGGTGGTACTGCATGCC

CGGGGCCATGATGACCTCGTGGTATGTCTGCACCGCTCCCAGCCACCTCTGGACAATCGT

ATCGGGGAGCTGGTGGGTGTACTGGCTGCACACTGCCAGAGGGAGGGAAGAAACCTAGAG

GTCCGTGTCTCTGACTGCATTTCACTGAGCCAGCGTGGGGCCCAACGCCTTGTTTCCGTG

GAGTCCAGGCCAGAGCAACCAGAACCAGATTTCTGCTGTAGCCGTGGCAACTTTACCCTC

CTCTGGCCAAAC

>CL155.Contig30_A-W 1866 2537 minus strand myosin IG, isoform CRA_d [Homo sapiens]

AGGTGGCGGGCCCGGCAGCTGGTAAAGAACATTCCACCTTCAGATATGGCTCAGATCAAG

GCCAAAGTGGCTGCCATGGGGGCCCTGCAGGGTCTACGTCAGGACTGGGGCTGCCAGAGA

GCCTGGGCTCAAGACTACCTGTCCTCTGGCACTGACAACCCCACAGCCTCAGGCTTGTTT

GCTCAGCAACTGAAGACCCTTCGAGAGAAGGATGGCTTTGGGGCTGTGCTCTTTTCTAGC

CATGTCCGTAAGGTGAACCGCTTCCGCAAGTGCCGGGACCGGGCCCTTCTGCTTACAGAC

CGGCACCTCTACAAACTGGAACCTGGCCGTCAGTACCGGGTAATGCGGGCTGTGCCCCTG

GATGCGGTAACAGGGGTGAGTGTGACCAGTGGACGGGACCAGCTGGTGGTACTGCATGCC

CGGGGCCATGATGACCTCGTGGTATGTCTGCACCGCTCCCAGCCACCTCTGGACAATCGT

ATCGGGGAGCTGGTGGGTGTACTGGCTGCACACTGCCAGAGGGAGGGAAGAAACCTAGAG

GTCCGTGTCTCTGACTGCATTTCACTGAGCCAGCGTGGGGCCCAACGCCTTGTTTCCGTG

GAGTCCAGGCCAGAGCAACCAGAACCAGATTTCTGCTGTAGCCGTGGCAACTTTACCCTC

CTCTGGCCAAAC

>CL155.Contig31_A-W 2063 2368 minus strand PREDICTED: unconventional myosin-Ig [Saimiri boliviensis boliviensis]

GTAACAGGGGTGAGTGTGACCAGTGGACGGGACCAGCTGGTGGTACTGCATGCCCGGGGC

CATGATGACCTCGTGGTATGTCTGCACCGCTCCCAGCCACCTCTGGACAATCGTATCGGG

GAGCTGGTGGGTGTACTGGCTGCACACTGCCAGAGGGAGGGAAGAAACCTAGAGGTCCGT

GTCTCTGACTGCATTTCACTGAGCCAGCGTGGGGCCCAACGCCTTGTTTCCGTGGAGTCC

AGGCCAGAGCAACCAGAACCAGATTTCTGCTGTAGCCGTGGCAACTTTACCCTCCTCTGG

CCAAAC

>CL155.Contig32_A-W 3880 4158 minus strand PREDICTED: unconventional myosin-Ig [Pongo abelii]

GTAACAGGGGTGAGTGTGACCAGTGGACGGGACCAGCTGGTGGTACTGCATGCCCGGGGC

CATGATGACCTCGTGGTATGTCTGCACCGCTCCCAGCCACCTCTGGACAATCGTATCGGG

GAGCTGGTGGGTGTACTGGCTGCACACTGCCAGAGGGAGGGAAGAAACCTAGAGGTCCGT

GTCTCTGACTGCATTTCACTGAGCCAGCGTGGGGCCCAACGCCTTGTTTCCGTGGAGTCC

AGGCCAGAGCAACCAGAACCAGATTTCTGCTGTAGCCGT

>CL155.Contig33_A-W 22 294 minus strand PREDICTED: myosin-Ig [Cricetulus griseus]

TGCCCCCAGGCAGTCTACCAGCGGCTGTTTGAGTGGGTGGTGGACAGGATCAATGGTATC

ATGGAAGCCCGGGGCCGTGACCCTCGTCGTGATGGCAAGGACACAGTCATTGGTGTGCTG

GACATCTACGGCTTTGAGGTGTTCCCTGTCAACAGCTTTGAGCAGTTCTGCATCAACTAC

TGCAATGAGAAGCTGCAGCAGCTCTTCATCCGGCTCATCCTGAAGCAGGAGCAGGAAGAG

TATGAGCGTGAGGGCATCACCTGGCAGACTGTG

>CL155.Contig34_A-W 193 822 minus strand PREDICTED: myosin-Ig [Bos taurus] >gi|359064818|ref|XP_002686927.2| PREDICTED: myosin-Ig [Bos taurus]

TCTCTAACCCCCACTCTGTCCCTATAGGAACCCTTCTATGTCCGCTGCATCAAACCCAAT

GAGGACAAGGTGGCTGGAAGGCTGGATGAGGACCACTGCCGCCACCAGGTCGCATACCTG

GGGCTGCTGGAGAATGTGAGAGTCCGCAGGGCTGGCTTTGCTTCCCGCCAGCCATACTCT

CGATTTCTCCTCAGGTACAAGATGACCTGTGAGTACACATGGCCCAACCACCTGCTGGGC

TCTGACAAGGCAGCTGTGAGCGCCCTCCTGGAGCAGCACAGGCTGCAAGGGGATGTGGCC

TTTGGCCACAGCAAGCTGTTCATCCGCTCACCCCGGACGCTGGTCACACTGGAGCAGAGC

CGAGCCCGCCTCATCCCCATCATTGTGCTGCTGCTACAGAAGGCATGGAGAGGTACCCTG

GCTAGATGGCGCTGCAGGCGGCTGCGAGCCATCTACACTATCATGCGCTGGTTCCGGAGG

CATAAGGTGCGTGCTTACTTGGCAGAGCTGCAGCGGAGGTTTCAGGCTGCACGGCAGCCC

CCACTCTATGGCCGGGACATTGAGTGGCCGCTGCCCCCTGCTGTGCTGCAGCCCTTCCAG

GACACCTGCCACATGCTCTTCTGCAGGTAC

>CL155.Contig35_A-W 22 2010 minus strand PREDICTED: myosin-Ig [Cricetulus griseus]

TGCCCCCAGGCAGTCTACCAGCGGCTGTTTGAGTGGGTGGTGGACAGGATCAATGGTATC

ATGGAAGCCCGGGGCCGTGACCCTCGTCGTGATGGCAAGGACACAGTCATTGGTGTGCTG

GACATCTACGGCTTTGAGGTGTTCCCTGTCAACAGCTTTGAGCAGTTCTGCATCAACTAC

TGCAATGAGAAGCTGCAGCAGCTCTTCATCCGGCTCATCCTGAAGCAGGAGCAGGAAGAG

TATGAGCGTGAGGGCATCACCTGGCAGACTGTTGAATACTTCAACAATGCCACCATCGTG

GAGCTGGTGGAGCGGCCCCATCGAGGCATCCTGGCTGTGCTGGATGAGGCCTGTAGCACT

GCGGGACCCATTACTGACCGAATCTTCCTGCAGACCCTGGACACACACCATCGCCACCAT

CCACACTACTCCAGCCGCCAGCTTTGCCCCACTGACAAGACAATGGAGTTTGGCCGAGAC

TTCCGGATCAAGCATTATGCAGGAGATGTCACGTACTCTGTGGAGGGTTTCATTGACAAG

AACAGAGACTCCCTCTTCCAGGACTTCAAGCGGCTGTTCTACAATAGCTCAGATCCCACA

TTGCGGGCTATGTGGCCAGATGGACAGCAGGACATCACAGAGGTAACCAAGCGCCCCCTG

ACAGCTGGCACACTATTCAAGAACTCCATGGTGGCCCTAGTGGAGAACCTGGCCTCCAAG

GAACCCTTCTATGTCCGCTGCATCAAACCCAATGAGGACAAGGTGGCTGGAAGGCTGGAT

GAGGACCACTGCCGCCACCAGGTCGCATACCTGGGGCTGCTGGAGAATGTGAGAGTCCGC

AGGGCTGGCTTTGCTTCCCGCCAGCCATACTCTCGATTTCTCCTCAGGTACAAGATGACC

TGTGAGTACACATGGCCCAACCACCTGCTGGGCTCTGACAAGGCAGCTGTGAGCGCCCTC

CTGGAGCAGCACAGGCTGCAAGGGGATGTGGCCTTTGGCCACAGCAAGCTGTTCATCCGC

TCACCCCGGACGCTGGTCACACTGGAGCAGAGCCGAGCCCGCCTCATCCCCATCATTGTG

CTGCTGCTACAGAAGGCATGGAGAGGTACCCTGGCTAGATGGCGCTGCAGGCGGCTGCGA

GCCATCTACACTATCATGCGCTGGTTCCGGAGGCATAAGGTGCGTGCTTACTTGGCAGAG

CTGCAGCGGAGGTTTCAGGCTGCACGGCAGCCCCCACTCTATGGCCGGGACATTGAGTGG

CCGCTGCCCCCTGCTGTGCTGCAGCCCTTCCAGGACACCTGCCACATGCTCTTCTGCAGG

TGGCGGGCCCGGCAGCTGGTAAAGAACATTCCACCTTCAGATATGGCTCAGATCAAGGCC

AAAGTGGCTGCCATGGGGGCCCTGCAGGGTCTACGTCAGGACTGGGGCTGCCAGAGAGCC

TGGGCTCAAGACTACCTGTCCTCTGGCACTGACAACCCCACAGCCTCAGGCTTGTTTGCT

CAGCAACTGAAGACCCTTCGAGAGAAGGATGGCTTTGGGGCTGTGCTCTTTTCTAGCCAT

GTCCGTAAGGTGAACCGCTTCCGCAAGTGCCGGGACCGGGCCCTTCTGCTTACAGACCGG

CACCTCTACAAACTGGAACCTGGCCGTCAGTACCGGGTAATGCGGGCTGTGCCCCTGGAT

GCGGTAACAGGGGTGAGTGTGACCAGTGGACGGGACCAGCTGGTGGTACTGCATGCCCGG

GGCCATGATGACCTCGTGGTATGTCTGCACCGCTCCCAGCCACCTCTGGACAATCGTATC

GGGGAGCTGGTGGGTGTACTGGCTGCACACTGCCAGAGGGAGGGAAGAAACCTAGAGGTC

CGTGTCTCTGACTGCATTTCACTGAGCCAGCGTGGGGCCCAACGCCTTGTTTCCGTGGAG

TCCAGGCCAGAGCAACCAGAACCAGATTTCTGCTGTAGCCGTGGCAACTTTACCCTCCTC

TGGCCAAAC

>CL155.Contig36_A-W 193 1488 minus strand PREDICTED: unconventional myosin-Ig [Papio anubis]

TCTCTAACCCCCACTCTGTCCCTATAGGAACCCTTCTATGTCCGCTGCATCAAACCCAAT

GAGGACAAGGTGGCTGGAAGGCTGGATGAGGACCACTGCCGCCACCAGGTCGCATACCTG

GGGCTGCTGGAGAATGTGAGAGTCCGCAGGGCTGGCTTTGCTTCCCGCCAGCCATACTCT

CGATTTCTCCTCAGGTACAAGATGACCTGTGAGTACACATGGCCCAACCACCTGCTGGGC

TCTGACAAGGCAGCTGTGAGCGCCCTCCTGGAGCAGCACAGGCTGCAAGGGGATGTGGCC

TTTGGCCACAGCAAGCTGTTCATCCGCTCACCCCGGACGCTGGTCACACTGGAGCAGAGC

CGAGCCCGCCTCATCCCCATCATTGTGCTGCTGCTACAGAAGGCATGGAGAGGTACCCTG

GCTAGATGGCGCTGCAGGCGGCTGCGAGCCATCTACACTATCATGCGCTGGTTCCGGAGG

CATAAGGTGCGTGCTTACTTGGCAGAGCTGCAGCGGAGGTTTCAGGCTGCACGGCAGCCC

CCACTCTATGGCCGGGACATTGAGTGGCCGCTGCCCCCTGCTGTGCTGCAGCCCTTCCAG

GACACCTGCCACATGCTCTTCTGCAGGTGGCGGGCCCGGCAGCTGGTAAAGAACATTCCA

CCTTCAGATATGGCTCAGATCAAGGCCAAAGTGGCTGCCATGGGGGCCCTGCAGGGTCTA

CGTCAGGACTGGGGCTGCCAGAGAGCCTGGGCTCAAGACTACCTGTCCTCTGGCACTGAC

AACCCCACAGCCTCAGGCTTGTTTGCTCAGCAACTGAAGACCCTTCGAGAGAAGGATGGC

TTTGGGGCTGTGCTCTTTTCTAGCCATGTCCGTAAGGTGAACCGCTTCCGCAAGTGCCGG

GACCGGGCCCTTCTGCTTACAGACCGGCACCTCTACAAACTGGAACCTGGCCGTCAGTAC

CGGGTAATGCGGGCTGTGCCCCTGGATGCGGTAACAGGGGTGAGTGTGACCAGTGGACGG

GACCAGCTGGTGGTACTGCATGCCCGGGGCCATGATGACCTCGTGGTATGTCTGCACCGC

TCCCAGCCACCTCTGGACAATCGTATCGGGGAGCTGGTGGGTGTACTGGCTGCACACTGC

CAGAGGGAGGGAAGAAACCTAGAGGTCCGTGTCTCTGACTGCATTTCACTGAGCCAGCGT

GGGGCCCAACGCCTTGTTTCCGTGGAGTCCAGGCCAGAGCAACCAGAACCAGATTTCTGC

TGTAGCCGTGGCAACTTTACCCTCCTCTGGCCAAAC

>CL155.Contig37_A-W 3786 4091 minus strand PREDICTED: myosin-Ig [Cricetulus griseus]

GTAACAGGGGTGAGTGTGACCAGTGGACGGGACCAGCTGGTGGTACTGCATGCCCGGGGC

CATGATGACCTCGTGGTATGTCTGCACCGCTCCCAGCCACCTCTGGACAATCGTATCGGG

GAGCTGGTGGGTGTACTGGCTGCACACTGCCAGAGGGAGGGAAGAAACCTAGAGGTCCGT

GTCTCTGACTGCATTTCACTGAGCCAGCGTGGGGCCCAACGCCTTGTTTCCGTGGAGTCC

AGGCCAGAGCAACCAGAACCAGATTTCTGCTGTAGCCGTGGCAACTTTACCCTCCTCTGG

CCAAAC

>CL155.Contig38_A-W 3742 4047 minus strand PREDICTED: myosin-Ig [Cricetulus griseus]

GTAACAGGGGTGAGTGTGACCAGTGGACGGGACCAGCTGGTGGTACTGCATGCCCGGGGC

CATGATGACCTCGTGGTATGTCTGCACCGCTCCCAGCCACCTCTGGACAATCGTATCGGG

GAGCTGGTGGGTGTACTGGCTGCACACTGCCAGAGGGAGGGAAGAAACCTAGAGGTCCGT

GTCTCTGACTGCATTTCACTGAGCCAGCGTGGGGCCCAACGCCTTGTTTCCGTGGAGTCC

AGGCCAGAGCAACCAGAACCAGATTTCTGCTGTAGCCGTGGCAACTTTACCCTCCTCTGG

CCAAAC

>CL155.Contig39_A-W 3728 4033 minus strand PREDICTED: myosin-Ig [Cricetulus griseus]

GTAACAGGGGTGAGTGTGACCAGTGGACGGGACCAGCTGGTGGTACTGCATGCCCGGGGC

CATGATGACCTCGTGGTATGTCTGCACCGCTCCCAGCCACCTCTGGACAATCGTATCGGG

GAGCTGGTGGGTGTACTGGCTGCACACTGCCAGAGGGAGGGAAGAAACCTAGAGGTCCGT

GTCTCTGACTGCATTTCACTGAGCCAGCGTGGGGCCCAACGCCTTGTTTCCGTGGAGTCC

AGGCCAGAGCAACCAGAACCAGATTTCTGCTGTAGCCGTGGCAACTTTACCCTCCTCTGG

CCAAAC

>CL155.Contig40_A-W 4567 4845 minus strand PREDICTED: unconventional myosin-Ig [Pongo abelii]

GTAACAGGGGTGAGTGTGACCAGTGGACGGGACCAGCTGGTGGTACTGCATGCCCGGGGC

CATGATGACCTCGTGGTATGTCTGCACCGCTCCCAGCCACCTCTGGACAATCGTATCGGG

GAGCTGGTGGGTGTACTGGCTGCACACTGCCAGAGGGAGGGAAGAAACCTAGAGGTCCGT

GTCTCTGACTGCATTTCACTGAGCCAGCGTGGGGCCCAACGCCTTGTTTCCGTGGAGTCC

AGGCCAGAGCAACCAGAACCAGATTTCTGCTGTAGCCGT

>CL155.Contig41_A-W 3713 4018 minus strand PREDICTED: myosin-Ig [Cricetulus griseus]

GTAACAGGGGTGAGTGTGACCAGTGGACGGGACCAGCTGGTGGTACTGCATGCCCGGGGC

CATGATGACCTCGTGGTATGTCTGCACCGCTCCCAGCCACCTCTGGACAATCGTATCGGG

GAGCTGGTGGGTGTACTGGCTGCACACTGCCAGAGGGAGGGAAGAAACCTAGAGGTCCGT

GTCTCTGACTGCATTTCACTGAGCCAGCGTGGGGCCCAACGCCTTGTTTCCGTGGAGTCC

AGGCCAGAGCAACCAGAACCAGATTTCTGCTGTAGCCGTGGCAACTTTACCCTCCTCTGG

CCAAAC

>CL155.Contig42_A-W 3321 3965 minus strand PREDICTED: unconventional myosin-Ig [Pongo abelii]

AGGTGGCGGGCCCGGCAGCTGGTAAAGAACATTCCACCTTCAGATATGGCTCAGATCAAG

GCCAAAGTGGCTGCCATGGGGGCCCTGCAGGGTCTACGTCAGGACTGGGGCTGCCAGAGA

GCCTGGGCTCAAGACTACCTGTCCTCTGGCACTGACAACCCCACAGCCTCAGGCTTGTTT

GCTCAGCAACTGAAGACCCTTCGAGAGAAGGATGGCTTTGGGGCTGTGCTCTTTTCTAGC

CATGTCCGTAAGGTGAACCGCTTCCGCAAGTGCCGGGACCGGGCCCTTCTGCTTACAGAC

CGGCACCTCTACAAACTGGAACCTGGCCGTCAGTACCGGGTAATGCGGGCTGTGCCCCTG

GATGCGGTAACAGGGGTGAGTGTGACCAGTGGACGGGACCAGCTGGTGGTACTGCATGCC

CGGGGCCATGATGACCTCGTGGTATGTCTGCACCGCTCCCAGCCACCTCTGGACAATCGT

ATCGGGGAGCTGGTGGGTGTACTGGCTGCACACTGCCAGAGGGAGGGAAGAAACCTAGAG

GTCCGTGTCTCTGACTGCATTTCACTGAGCCAGCGTGGGGCCCAACGCCTTGTTTCCGTG

GAGTCCAGGCCAGAGCAACCAGAACCAGATTTCTGCTGTAGCCGT

>CL155.Contig43_A-W 3571 3876 minus strand PREDICTED: myosin-Ig [Cricetulus griseus]

GTAACAGGGGTGAGTGTGACCAGTGGACGGGACCAGCTGGTGGTACTGCATGCCCGGGGC

CATGATGACCTCGTGGTATGTCTGCACCGCTCCCAGCCACCTCTGGACAATCGTATCGGG

GAGCTGGTGGGTGTACTGGCTGCACACTGCCAGAGGGAGGGAAGAAACCTAGAGGTCCGT

GTCTCTGACTGCATTTCACTGAGCCAGCGTGGGGCCCAACGCCTTGTTTCCGTGGAGTCC

AGGCCAGAGCAACCAGAACCAGATTTCTGCTGTAGCCGTGGCAACTTTACCCTCCTCTGG

CCAAAC

>CL155.Contig44_A-W 330 1982 minus strand Unconventional myosin-Ig OS=Homo sapiens GN=MYO1G PE=1 SV=2

TTGGACAGTGATGAGAGGAGCCACCAGGCAGTGACTGAGGCCATGAGGGTCATTGGCTTC

AGTCCTGAGGAGGTGGCATCAGTACACCGCATCCTGGCTGCCATATTGCACCTGGGAAAC

ATCGAGTTCATGGAGAGAGAAGAAGATGGGCCACAGAAAGGGAGCCTAGCAGTGGCTGAG

GAGGCATTGGTGGACCATGTGGCTGAGCTGACTGCCACACCCAGGGACCTGGTGCTCCGC

TCCCTGCTGGCTCGCACTGTGGCTTCTGGAGGCCGGGAACTCATAGAGAAAGGCCATACT

GTGGCGGAGGCCAGCTACGCCCGGGATGCCTGTGCCAAGGCAGTCTACCAGCGGCTGTTT

GAGTGGGTGGTGGACAGGATCAATGGTATCATGGAAGCCCGGGGCCGTGACCCTCGTCGT

GATGGCAAGGACACAGTCATTGGTGTGCTGGACATCTACGGCTTTGAGGTGTTCCCTGTC

AACAGCTTTGAGCAGTTCTGCATCAACTACTGCAATGAGAAGCTGCAGCAGCTCTTCATC

CGGCTCATCCTGAAGCAGGAGCAGGAAGAGTATGAGCGTGAGGGCATCACCTGGCAGACT

GTTGAATACTTCAACAATGCCACCATCGTGGAGCTGGTGGAGCGGCCCCATCGAGGCATC

CTGGCTGTGCTGGATGAGGCCTGTAGCACTGCGGGACCCATTACTGACCGAATCTTCCTG

CAGACCCTGGACACACACCATCGCCACCATCCACACTACTCCAGCCGCCAGCTTTGCCCC

ACTGACAAGACAATGGAGTTTGGCCGAGACTTCCGGATCAAGCATTATGCAGGAGATGTC

ACGTACTCTGTGGAGGGTTTCATTGACAAGAACAGAGACTCCCTCTTCCAGGACTTCAAG

CGGCTGTTCTACAATAGCTCAGATCCCACATTGCGGGCTATGTGGCCAGATGGACAGCAG

GACATCACAGAGGTAACCAAGCGCCCCCTGACAGCTGGCACACTATTCAAGAACTCCATG

GTGGCCCTAGTGGAGAACCTGGCCTCCAAGGAACCCTTCTATGTCCGCTGCATCAAACCC

AATGAGGACAAGGTGGCTGGAAGGCTGGATGAGGACCACTGCCGCCACCAGGTCGCATAC

CTGGGGCTGCTGGAGAATGTGAGAGTCCGCAGGGCTGGCTTTGCTTCCCGCCAGCCATAC

TCTCGATTTCTCCTCAGGTACAAGATGACCTGTGAGTACACATGGCCCAACCACCTGCTG

GGCTCTGACAAGGCAGCTGTGAGCGCCCTCCTGGAGCAGCACAGGCTGCAAGGGGATGTG

GCCTTTGGCCACAGCAAGCTGTTCATCCGCTCACCCCGGACGCTGGTCACACTGGAGCAG

AGCCGAGCCCGCCTCATCCCCATCATTGTGCTGCTGCTACAGAAGGCATGGAGAGGTACC

CTGGCTAGATGGCGCTGCAGGCGGCTGCGAGCCATCTACACTATCATGCGCTGGTTCCGG

AGGCATAAGGTGCGTGCTTACTTGGCAGAGCTGCAGCGGAGGTTTCAGGCTGCACGGCAG

CCCCCACTCTATGGCCGGGACATTGAGTGGCCGCTGCCCCCTGCTGTGCTGCAGCCCTTC

CAGGACACCTGCCACATGCTCTTCTGCAGGTAC

>CL155.Contig45_A-W 640 870 minus strand PREDICTED: unconventional myosin-Ig [Otolemur garnettii]

TCTCAGGGCACTGACAACCCCACAGCCTCAGGCTTGTTTGCTCAGCAACTGAAGACCCTT

CGAGAGAAGGATGGCTTTGGGGCTGTGCTCTTTTCTAGCCATGTCCGTAAGGTGAACCGC

TTCCGCAAGTGCCGGGACCGGGCCCTTCTGCTTACAGACCGGCACCTCTACAAACTGGAA

CCTGGCCGTCAGTACCGGGTAATGCGGGCTGTGCCCCTGGATGCGGTGAGT

>CL155.Contig46_A-W 249 368 minus strand PREDICTED: unconventional myosin-Ig [Otolemur garnettii]

GTGAACCGCTTCCGCAAGTGCCGGGACCGGGCCCTTCTGCTTACAGACCGGCACCTCTAC

AAACTGGAACCTGGCCGTCAGTACCGGGTAATGCGGGCTGTGCCCCTGGATGCGGTGAGT

>CL155.Contig47_A-W 640 1173 minus strand PREDICTED: myosin-Ig [Bos taurus] >gi|359064818|ref|XP_002686927.2| PREDICTED: myosin-Ig [Bos taurus]

TCTCAGGGCACTGACAACCCCACAGCCTCAGGCTTGTTTGCTCAGCAACTGAAGACCCTT

CGAGAGAAGGATGGCTTTGGGGCTGTGCTCTTTTCTAGCCATGTCCGTAAGGTGAACCGC

TTCCGCAAGTGCCGGGACCGGGCCCTTCTGCTTACAGACCGGCACCTCTACAAACTGGAA

CCTGGCCGTCAGTACCGGGTAATGCGGGCTGTGCCCCTGGATGCGGTAACAGGGGTGAGT

GTGACCAGTGGACGGGACCAGCTGGTGGTACTGCATGCCCGGGGCCATGATGACCTCGTG

GTATGTCTGCACCGCTCCCAGCCACCTCTGGACAATCGTATCGGGGAGCTGGTGGGTGTA

CTGGCTGCACACTGCCAGAGGGAGGGAAGAAACCTAGAGGTCCGTGTCTCTGACTGCATT

TCACTGAGCCAGCGTGGGGCCCAACGCCTTGTTTCCGTGGAGTCCAGGCCAGAGCAACCA

GAACCAGATTTCTGCTGTAGCCGTGGCAACTTTACCCTCCTCTGGCCAAACTGC

>CL155.Contig48_A-W 249 671 minus strand PREDICTED: myosin-Ig [Bos taurus] >gi|359064818|ref|XP_002686927.2| PREDICTED: myosin-Ig [Bos taurus]

GTGAACCGCTTCCGCAAGTGCCGGGACCGGGCCCTTCTGCTTACAGACCGGCACCTCTAC

AAACTGGAACCTGGCCGTCAGTACCGGGTAATGCGGGCTGTGCCCCTGGATGCGGTAACA

GGGGTGAGTGTGACCAGTGGACGGGACCAGCTGGTGGTACTGCATGCCCGGGGCCATGAT

GACCTCGTGGTATGTCTGCACCGCTCCCAGCCACCTCTGGACAATCGTATCGGGGAGCTG

GTGGGTGTACTGGCTGCACACTGCCAGAGGGAGGGAAGAAACCTAGAGGTCCGTGTCTCT

GACTGCATTTCACTGAGCCAGCGTGGGGCCCAACGCCTTGTTTCCGTGGAGTCCAGGCCA

GAGCAACCAGAACCAGATTTCTGCTGTAGCCGTGGCAACTTTACCCTCCTCTGGCCAAAC

TGC

>CL155.Contig49_A-W 640 750 minus strand Unconventional myosin-Ig OS=Mus musculus GN=Myo1g PE=2 SV=1

TCTCAGGGCACTGACAACCCCACAGCCTCAGGCTTGTTTGCTCAGCAACTGAAGACCCTT

CGAGAGAAGGATGGCTTTGGGGCTGTGCTCTTTTCTAGCCATGTCCGTAAG

>CL156.Contig1_A-W 1 645 PREDICTED: pre-mRNA 3&apos;-end-processing factor FIP1-like [Cavia porcellus]

CCACCACCAGGTATTCCAATAACTGTACCACCTCCAGGTTTTCCTCCTCCACCAGGTGCT

CCTCCTCCATCTCTTATACCAACAATAGAAAGCGGACATTCCTCTGGTTATGATAGCCGT

TCTGCACGTGCATTTCCATATGGCAATGTTGCCTTTCCCCATCTTACTGGTTCTGCTCCT

TCATGGCCTAGTCTTGTGGACACCACCAAGCAATGGGACTATTATTCAAGAAGAGAAAAA

GACCGAGATAGAGAAAGAGACAGAGACAGAGAGCGAGATCGTGATAGGGACAGAGAACGA

GAACGCACCAGAGAGAGAGAGAGGGAGCGTGATCACAGTCCTACCCCAAGTGTTTTCAAC

AGTGATGAAGAGCGATACAGATACAGGGAATATGCAGAAAGAGGCTACGAGCGTCACAGG

GCAAGTCGAGAGAAAGAAGAACGACATAGAGAAAGACGACACAGAGAGAAAGAGGAAACC

AGACACAAGTCATCTCGCAGTAATAGTAGACGTCGCCATGAAAGTGAAGAAGGAGACAGT

CACAGGAGACATAAGCACAAAAAGTCTAAAAGAAGCAAAGAAGGAAAAGAAGCTGGCAGT

GAGCCTGCCCCCGAACAGGAGAGCACTGAAGCTGCACCTGCGGAG

>CL156.Contig2_A-W 76 393 PREDICTED: pre-mRNA 3&apos;-end-processing factor FIP1 [Saimiri boliviensis boliviensis]

AGTGTCAAGAAAACTTGTTTCCATTTCTGTTGCAGTGATGAAGAGCGATACAGATACAGG

GAATATGCAGAAAGAGGCTACGAGCGTCACAGGGCAAGTCGAGAGAAAGAAGAACGACAT

AGAGAAAGACGACACAGAGAGAAAGAGGAAACCAGACACAAGTCATCTCGCAGTAATAGT

AGACGTCGCCATGAAAGTGAAGAAGGAGACAGTCACAGGAGACATAAGCACAAAAAGTCT

AAAAGAAGCAAAGAAGGAAAAGAAGCTGGCAGTGAGCCTGCCCCCGAACAGGAGAGCACT

GAAGCTGCACCTGCGGAG

>CL156.Contig3_A-W 2 1300 pre-mRNA 3&apos;-end-processing factor FIP1 isoform 1 [Macaca mulatta] >gi|384949614|gb|AFI38412.1| pre-mRNA 3&apos;-end-processing factor FIP1 isoform 1 [Macaca mulatta]

GTAGATCTTGATGCACCTGGAAGCATTAATGGAGTTCCACTCTTGGAGGTAGATTTGGAT

TCCTTTGAAGATAAACCGTGGCGTAAACCTGGTGCTGATCTTTCTGATTACTTTAATTAT

GGGTTCAATGAAGATACTTGGAAAGCTTACTGTGAAAAACAAAAGAGGATACGAATGGGA

CTTGAAGTTATACCAGTTACTTCTACTACAAATAAAATTACGGTACAGCAGGGCAGAACT

GGAAATTCAGAGAAAGAAACAGCACTTCCATCTACAAAAGCCGAGTTTACTTCTCCTCCA

TCTTTGTTCAAGACTGGACTCCCACCAAGCAGAAACAGCACCTCTTCTCAGTCTCAGACA

AGTACTGCCTCCAGAAAAGCCAATTCAAGCGTTGGGAAGTGGCAGGATCGATATGGGAGG

GCCGAATCACCTGATCTAAGGAGATTACCTGGAGCAATTGATGTTATTGGTCAGACTATA

ACTATTAGCCGAGTAGAGGGAAGGCGACGGGCAAATGAAAACAGCAACATACAGGTCCTT

TCTGAAAGATCTGCTACTGAAGTAGACAACAATTTTAGCAAACCACCTCCGTTTTTCCCT

CCAGGAGCTCCTCCCACCCACCTTCCACCACCTCCATTTCTTCCACCACCTCCAACTGTC

AGCACTGCTCCACCTTTGATTCCACCACCAGGTTTTCCTCCTCCACCAGGTGCTCCTCCT

CCATCTCTTATACCAACAATAGAAAGCGGACATTCCTCTGGTTATGATAGCCGTTCTGCA

CGTGCATTTCCATATGGCAATGTTGCCTTTCCCCATCTTACTGGTTCTGCTCCTTCATGG

CCTAGTCTTGTGGACACCACCAAGCAATGGGACTATTATTCAAGAAGAGAAAAAGACCGA

GATAGAGAAAGAGACAGAGACAGAGAGCGAGATCGTGATAGGGACAGAGAACGAGAACGC

ACCAGAGAGAGAGAGAGGGAGCGTGATCACAGTCCTACCCCAAGTGTTTTCAACAGTGAT

GAAGAGCGATACAGATACAGGGAATATGCAGAAAGAGGCTACGAGCGTCACAGGGCAAGT

CGAGAGAAAGAAGAACGACATAGAGAAAGACGACACAGAGAGAAAGAGGAAACCAGACAC

AAGTCATCTCGCAGTAATAGTAGACGTCGCCATGAAAGTGAAGAAGGAGACAGTCACAGG

AGACATAAGCACAAAAAGTCTAAAAGAAGCAAAGAAGGAAAAGAAGCTGGCAGTGAGCCT

GCCCCCGAACAGGAGAGCACTGAAGCTGCACCTGCGGAG

>CL157.Contig2_A-W 3 527 minus strand PREDICTED: condensin complex subunit 1 isoform 2 [Pan troglodytes]

GCCTACTGCATGTCACAGCTTCCCATCACAGAGCGAGGCCTACGCAAGATGATGGACAAT

TTTGACTGCTTTGGAGATAAATTGTCAGATGAGTCCATCTTCGGAGCTCTTTTGTCCATT

GTGGGCAAATTGCGGCGAGGGGCCAAGCCAGAGGGCAAGGCTATAATAGATGAATTTGAG

CAGAAGCTTCGGGCCTATCACACCAAAGGATCAGATGGAATAGAGGAGCTTGAGATTGGC

CAAGGAGGTAGCCAGAGAGCCCCATCAGCCAGGAACCAATCTTCTGTCCCTAGGAAGCAG

CGCTTGGCCTCTGCCTCCTCGGACAATGACTTTGTAACACCCAAGCCTCGGCGAACTACT

CGCCAACTTCCAAAAACTCAGCAGCGCACTTCGAAAAAGAAACCCAAGGTTGTCTTTTCA

AGCGATGAGTCCAGTGAGGAAGAGCTTTCAGCAGAGATGACAGAAGATGAGACACCCAAG

AAAACAACCCCCCTCCACAGAGCATCCACTCTCAGGCACAGATCC

>CL158.Contig1_A-W 3 1535 minus strand PREDICTED: switch-associated protein 70 isoform 2 [Canis lupus familiaris]

AAGTTCATTTTGGAAAAGGTCCAAGATAACTTTGACAAGATTGAATTCAATAGAATGTGT

TGGACCCTCTGTGTGAAAAAAAACCTCACAAAGAGCCCTCTGCTTATTACAGAAGAAGAT

GCATTTAAAGTATGGGTTATTTTCAACTTTTTATCTGAGGACAAGTATCCATTAATTATT

GTTCCTGAAGAGATTGAATACCTGCTTAAGAAACTTACAGAAGCTATGGGAGGAGGCTGG

CAACAAGAACAATTTGAACATTATAAAATCAATTTTGATGACAGTAAAGATGGCCTTTCT

GCGTGGGAACTTATTGAGCTTATTGGAAATGGACAGTTTAGTAAAGGCATGGATCGGCAG

ACGATATCTATGGCAATTAATGAAGTGTTTAATGAGCTTATATTAGATGTGTTAAAACAG

GGTTACATGATGAAAAAAGGCCATAGACGGAAAAACTGGACTGAACGCTGGTTTGTACTA

AAACCCAACATAATTTCTTATTATGTAAGTGAGGATCTGAAAGATAAGAAAGGAGACATT

CTCTTGGATGAAAACTGCTGTGTAGAGTCTCTGCCTGACAAAGATGGAAAGAAATGCCTC

TTTATAATAAAATGTTTTGATAAGACCTTTGAAATCAGTGCTTCAGATAAGAAAAAGAAA

CAAGAGTGGATTCAGGCCATTCATTCTACTATCCACTTGTTGAAGCTGGGCAGCCCTCCA

CCACACAAAGAAGCCCGTCAGCGTCGGAAAGAAATGCGAAAGAAGCTATTGGCTGAGCAA

GAGGAGCTGGAGCGGCAGATGAAGGAGCTCCAGGCCGCCAATGAAAGCAAGCAGGAGGAG

CTGGAGACTGTGAGGAAGAAACTGGAGGAAGCAGCATCTCGTGCAGCAGAAGAGGAAAAG

AAACGCCTGCAAACTCAAGTGGAGCTGCAAGCCAGGTTCAGCACAGAGCTGGAGCGTGAG

AAGCTGATCAGACAGCAGATGGAAGAACAGGTTGCCCAAAAGTCCTCTGAACTGGAACAG

TATCTACAGCGAGTACGGGAGCTGGAAGACATGTACCTAAAGCTGCAGGAGGCTCTTGAG

GATGAGAGGCAGGCCCGGCAAGATGAAGAGATGGTGCGGAAACTTCAGGCCAGGTTGTTG

GAGGAAGAGTCTTCCAAGAGGGCAGAACTGGAAAAGTGGCACTTGGTACAGCAGCAGGCC

ATTCAGACAACAGAGGCAGAGAAGCAGGAGTTGGAGCACCAGCGTGTCATGAAGGAGCAG

GCCCTTCAGGAGGCCATGGCACAGCTGGAGCAGCTTGAGCTTGAGCGGAAGCAGGCTTTG

GAGCAGTATGAGGGGGTTAAAAAGAAGCTAGAAATGGCAACTAATAAGACCAAGAGCTGG

AAGGACAAAGTGGCCCATCATGAAGGATTAATTCGATTGATAGAACCAGGTTCAAAGAAC

CCTCACCTGATCACTAATTGGGGACCTGCCGCTTTTACCCAGGCAGAACTGGAGGAAAGA

GAAAAGAACTGGAAAGAAAAAAAGACCACGGAG

>CL158.Contig2_A-W 3 635 minus strand SWA-70 protein, isoform CRA_b [Mus musculus]

AAGTTCATTTTGGAAAAGGTCCAAGATAACTTTGACAAGATTGAATTCAATAGAATGTGT

TGGACCCTCTGTGTGAAAAAAAACCTCACAAAGAGCCCTCTGCTTATTACAGAAGAAGAT

GCATTTAAAGTATGGGTTATTTTCAACTTTTTATCTGAGGACAAGTATCCATTAATTATT

GTTCCTGAAGAGATTGAATACCTGCTTAAGAAACTTACAGAAGCTATGGGAGGAGGCTGG

CAACAAGAACAATTTGAACATTATAAAATCAATTTTGATGACAGTAAAGATGGCCTTTCT

GCGTGGGAACTTATTGAGCTTATTGGAAATGGACAGTTTAGTAAAGGCATGGATCGGCAG

ACGATATCTATGGCAATTAATGAAGTGTTTAATGAGCTTATATTAGATGTGTTAAAACAG

GGTTACATGATGAAAAAAGGCCATAGACGGAAAAACTGGACTGAACGCTGGTTTGTACTA

AAACCCAACATAATTTCTTATTATGTAAGTGAGGATCTGAAAGATAAGAAAGGAGACATT

CTCTTGGATGAAAACTGCTGTGTAGAGGTACGTCTACTGTTACTTCTTTGCAGGATTACC

TGCTGCTGCCCCCTGCTGTCAAAGGAGTTTCCT

>CL160.Contig1_A-W 1 972 PREDICTED: serine/threonine-protein phosphatase 6 regulatory subunit 3 isoform 5 [Papio anubis]

CAGATGCAACAAATGACGTCCAATTTTATTGACCAGTTTGGCTTCAACGATGAGAAGTTT

GCAGATCAAGATGACATTGGCAATGTTTCTTTTGATCGGGTGTCAGACATCAACTTTACT

CTCAATACAAATGAAAGTGGTAATATTGCTCTGTTTGAAGCGTGTTGTAAGGAGAGAATA

CAACAGTTTGATGATGGTGGCTCTGACGAGGAGGACATCTGGGAGGAAAAGCACATTGCA

TGTACGCCAGAGTCCCAGAGGCGATCCAGCTCGGGGAGTACAGACAGCGAAGAAAGTACA

GATTCTGAAGAAGAAGATGGAGCAAAGCAGGACTTGTTTGAGTCCAGTAGCGCCAATACA

GAGGATAAAATGGAGGTAGACCTGAATGAACCACCCAGCTGGTCGGCCAACTTTGATGTC

CCAATGGAGACCACCCATGGCACTCCGTTAGACTCAGTGGGCTCTGATGTCTGGAGCACA

GAGGAGCCAATGCCAACTAAGGAGACGGGCTGGGCTTCTTTTTCAGAGTTCACGTCATCT

CTGAGCACTAAAGATTCCTTAAGGAGTAATTCTCCAGTGGAAATGGAAACCAGCACTGAA

GCCATGGACCCCCTGACTCCCAGTGTCACTGCCTTGGCAGCACAGCCAGAGGCGCCAAGC

AGTGTGGCCATGGAAGCCAGCTCCGATGGAGAGGAGGATGCCGAGAGTGCAGACAAGGTA

ACTGAGACGGTGATGAATGGCGGCATGAAGGAAACACTCAGCCTCACCGTGGATGCCAAG

ACGGAGACCGCAGTCTTCAAAAGTGAGGAAGGAAAACTGTCTACCTCTCAAGATGCTGCT

TGTAAAGAAGTGGAGGAGAGCCCTGAGCCAGCGGAGGCCAAGTGCGCGGCCCCCCGGCCC

CCCAGCAGTAGCCCAGAGCAGAGGAGTGACCAGCCAAGTGTGCCAGGTGACACGTCAGTC

AATGGCCCTGTA

>CL160.Contig2_A-W 1 921 PREDICTED: serine/threonine-protein phosphatase 6 regulatory subunit 3 isoform 5 [Papio anubis]

CAGATGCAACAAATGACGTCCAATTTTATTGACCAGTTTGGCTTCAACGATGAGAAGTTT

GCAGATCAAGATGACATTGGCAATGTTTCTTTTGATCGGGTGTCAGACATCAACTTTACT

CTCAATACAAATGAAAGTGGTAATATTGCTCTGTTTGAAGCGTGTTGTAAGGAGAGAATA

CAACAGTTTGATGATGGTGGCTCTGACGAGGAGGACATCTGGGAGGAAAAGCACATTGCA

TGTACGCCAGAGTCCCAGAGGCGATCCAGCTCGGGGAGTACAGACAGCGAAGAAAGTACA

GATTCTGAAGAAGAAGATGGAGCAAAGCAGGACTTGTTTGAGTCCAGTAGCGCCAATACA

GAGGATAAAATGGAGGTAGACCTGAATGAACCACCCAGCTGGTCGGCCAACTTTGATGTC

CCAATGGAGACCACCCATGGCACTCCGTTAGACTCAGTGGGCTCTGATGTCTGGAGCACA

GAGGAGCCAATGCCAACTAAGGAGACGGGCTGGGCTTCTTTTTCAGAGTTCACGTCATCT

CTGAGCACTAAAGATTCCTTAAGGAGTAATTCTCCAGTGGAAATGGAAACCAGCACTGAA

GCCATGGACCCCCTGACTCCCAGTGTCACTGCCTTGGCAGCACAGCCAGAGGCGCCAAGC

AGTGTGGCCATGGAAGCCAGCTCCGATGGAGAGGAGGATGCCGAGAGTGCAGACAAGGTA

ACTGAGACGGTGATGAATGGCGGCATGAAGGAAACACTCAGCCTCACCGTGGATGCCAAG

ACGGAGACCGCAGTCTTCAAAAGTGAGGAAGGAAAACTGTCTACCTCTCAAGATGCTGCT

TGTAAAGAAGTGGAGGAGAGCCCTGAGCCAGCGGAGGCCAAGTGCGCGGCCCCCCGGCCC

CCCAGCAGTAGCCCAGAGCAG

>CL160.Contig3_A-W 1 957 PREDICTED: serine/threonine-protein phosphatase 6 regulatory subunit 3 isoform 3 [Saimiri boliviensis boliviensis]

CTAGTTACAACCTGCCATATTCATTCATCCAGTGATGATGAAATTGACTTTAAAGAAACG

GGTTTCTCACAGGATTCCTCTTTGCAGCAAGCCTTTTCTGATTATCAGATGCAACAAATG

ACGTCCAATTTTATTGACCAGTTTGGCTTCAACGATGAGAAGTTTGCAGATCAAGATGAC

ATTGGCAATGTTTCTTTTGATCGGGTGTCAGACATCAACTTTACTCTCAATACAAATGAA

AGTGGTAATATTGCTCTGTTTGAAGCGTGTTGTAAGGAGAGAATACAACAGTTTGATGAT

GGTGGCTCTGACGAGGAGGACATCTGGGAGGAAAAGCACATTGCATGTACGCCAGAGTCC

CAGAGGCGATCCAGCTCGGGGAGTACAGACAGCGAAGAAAGTACAGATTCTGAAGAAGAA

GATGGAGCAAAGCAGGACTTGTTTGAGTCCAGTAGCGCCAATACAGAGGATAAAATGGAG

GTAGACCTGAATGAACCACCCAGCTGGTCGGCCAACTTTGATGTCCCAATGGAGACCACC

CATGGCACTCCGTTAGACTCAGTGGGCTCTGATGTCTGGAGCACAGAGGAGCCAATGCCA

ACTAAGGAGACGGGCTGGGCTTCTTTTTCAGAGTTCACGTCATCTCTGAGCACTAAAGAT

TCCTTAAGGAGTAATTCTCCAGTGGAAATGGAAACCAGCACTGAAGCCATGGACCCCCTG

ACTCCCAGTGTCACTGCCTTGGCAGCACAGCCAGAGGCGCCAAGCAGTGTGGCCATGGAA

GCCAGCTCCGATGGAGAGGAGGATGCCGAGAGTGCAGACAAGGTAACTGAGACGGTGATG

AATGGCGGCATGAAGGAAACACTCAGCCTCACCGTGGATGCCAAGACGGAGACCGCAGTC

TTCAAAAGGAGTGACCAGCCAAGTGTGCCAGGTGACACGTCAGTCAATGGCCCTGTA

>CL160.Contig4_A-W 1 1077 minus strand PREDICTED: serine/threonine-protein phosphatase 6 regulatory subunit 3 isoform 1 [Papio anubis] >gi|402892577|ref|XP_003909487.1| PREDICTED: serine/threonine-protein phosphatase 6 regulatory subunit 3 isoform 6 [Papio anubis]

CTAGTTACAACCTGCCATATTCATTCATCCAGTGATGATGAAATTGACTTTAAAGAAACG

GGTTTCTCACAGGATTCCTCTTTGCAGCAAGCCTTTTCTGATTATCAGATGCAACAAATG

ACGTCCAATTTTATTGACCAGTTTGGCTTCAACGATGAGAAGTTTGCAGATCAAGATGAC

ATTGGCAATGTTTCTTTTGATCGGGTGTCAGACATCAACTTTACTCTCAATACAAATGAA

AGTGGTAATATTGCTCTGTTTGAAGCGTGTTGTAAGGAGAGAATACAACAGTTTGATGAT

GGTGGCTCTGACGAGGAGGACATCTGGGAGGAAAAGCACATTGCATGTACGCCAGAGTCC

CAGAGGCGATCCAGCTCGGGGAGTACAGACAGCGAAGAAAGTACAGATTCTGAAGAAGAA

GATGGAGCAAAGCAGGACTTGTTTGAGTCCAGTAGCGCCAATACAGAGGATAAAATGGAG

GTAGACCTGAATGAACCACCCAGCTGGTCGGCCAACTTTGATGTCCCAATGGAGACCACC

CATGGCACTCCGTTAGACTCAGTGGGCTCTGATGTCTGGAGCACAGAGGAGCCAATGCCA

ACTAAGGAGACGGGCTGGGCTTCTTTTTCAGAGTTCACGTCATCTCTGAGCACTAAAGAT

TCCTTAAGGAGTAATTCTCCAGTGGAAATGGAAACCAGCACTGAAGCCATGGACCCCCTG

ACTCCCAGTGTCACTGCCTTGGCAGCACAGCCAGAGGCGCCAAGCAGTGTGGCCATGGAA

GCCAGCTCCGATGGAGAGGAGGATGCCGAGAGTGCAGACAAGGTAACTGAGACGGTGATG

AATGGCGGCATGAAGGAAACACTCAGCCTCACCGTGGATGCCAAGACGGAGACCGCAGTC

TTCAAAAGTGAGGAAGGAAAACTGTCTACCTCTCAAGATGCTGCTTGTAAAGAAGTGGAG

GAGAGCCCTGAGCCAGCGGAGGCCAAGTGCGCGGCCCCCCGGCCCCCCAGCAGTAGCCCA

GAGCAGAGGAGTGACCAGCCAAGTGTGCCAGGTGACACGTCAGTCAATGGCCCTGTA

>CL160.Contig5_A-W 1 1077 PREDICTED: serine/threonine-protein phosphatase 6 regulatory subunit 3 isoform 1 [Papio anubis] >gi|402892577|ref|XP_003909487.1| PREDICTED: serine/threonine-protein phosphatase 6 regulatory subunit 3 isoform 6 [Papio anubis]

CTAGTTACAACCTGCCATATTCATTCATCCAGTGATGATGAAATTGACTTTAAAGAAACG

GGTTTCTCACAGGATTCCTCTTTGCAGCAAGCCTTTTCTGATTATCAGATGCAACAAATG

ACGTCCAATTTTATTGACCAGTTTGGCTTCAACGATGAGAAGTTTGCAGATCAAGATGAC

ATTGGCAATGTTTCTTTTGATCGGGTGTCAGACATCAACTTTACTCTCAATACAAATGAA

AGTGGTAATATTGCTCTGTTTGAAGCGTGTTGTAAGGAGAGAATACAACAGTTTGATGAT

GGTGGCTCTGACGAGGAGGACATCTGGGAGGAAAAGCACATTGCATGTACGCCAGAGTCC

CAGAGGCGATCCAGCTCGGGGAGTACAGACAGCGAAGAAAGTACAGATTCTGAAGAAGAA

GATGGAGCAAAGCAGGACTTGTTTGAGTCCAGTAGCGCCAATACAGAGGATAAAATGGAG

GTAGACCTGAATGAACCACCCAGCTGGTCGGCCAACTTTGATGTCCCAATGGAGACCACC

CATGGCACTCCGTTAGACTCAGTGGGCTCTGATGTCTGGAGCACAGAGGAGCCAATGCCA

ACTAAGGAGACGGGCTGGGCTTCTTTTTCAGAGTTCACGTCATCTCTGAGCACTAAAGAT

TCCTTAAGGAGTAATTCTCCAGTGGAAATGGAAACCAGCACTGAAGCCATGGACCCCCTG

ACTCCCAGTGTCACTGCCTTGGCAGCACAGCCAGAGGCGCCAAGCAGTGTGGCCATGGAA

GCCAGCTCCGATGGAGAGGAGGATGCCGAGAGTGCAGACAAGGTAACTGAGACGGTGATG

AATGGCGGCATGAAGGAAACACTCAGCCTCACCGTGGATGCCAAGACGGAGACCGCAGTC

TTCAAAAGTGAGGAAGGAAAACTGTCTACCTCTCAAGATGCTGCTTGTAAAGAAGTGGAG

GAGAGCCCTGAGCCAGCGGAGGCCAAGTGCGCGGCCCCCCGGCCCCCCAGCAGTAGCCCA

GAGCAGAGGAGTGACCAGCCAAGTGTGCCAGGTGACACGTCAGTCAATGGCCCTGTA

>CL160.Contig6_A-W 1 957 PREDICTED: serine/threonine-protein phosphatase 6 regulatory subunit 3 isoform 3 [Saimiri boliviensis boliviensis]

CTAGTTACAACCTGCCATATTCATTCATCCAGTGATGATGAAATTGACTTTAAAGAAACG

GGTTTCTCACAGGATTCCTCTTTGCAGCAAGCCTTTTCTGATTATCAGATGCAACAAATG

ACGTCCAATTTTATTGACCAGTTTGGCTTCAACGATGAGAAGTTTGCAGATCAAGATGAC

ATTGGCAATGTTTCTTTTGATCGGGTGTCAGACATCAACTTTACTCTCAATACAAATGAA

AGTGGTAATATTGCTCTGTTTGAAGCGTGTTGTAAGGAGAGAATACAACAGTTTGATGAT

GGTGGCTCTGACGAGGAGGACATCTGGGAGGAAAAGCACATTGCATGTACGCCAGAGTCC

CAGAGGCGATCCAGCTCGGGGAGTACAGACAGCGAAGAAAGTACAGATTCTGAAGAAGAA

GATGGAGCAAAGCAGGACTTGTTTGAGTCCAGTAGCGCCAATACAGAGGATAAAATGGAG

GTAGACCTGAATGAACCACCCAGCTGGTCGGCCAACTTTGATGTCCCAATGGAGACCACC

CATGGCACTCCGTTAGACTCAGTGGGCTCTGATGTCTGGAGCACAGAGGAGCCAATGCCA

ACTAAGGAGACGGGCTGGGCTTCTTTTTCAGAGTTCACGTCATCTCTGAGCACTAAAGAT

TCCTTAAGGAGTAATTCTCCAGTGGAAATGGAAACCAGCACTGAAGCCATGGACCCCCTG

ACTCCCAGTGTCACTGCCTTGGCAGCACAGCCAGAGGCGCCAAGCAGTGTGGCCATGGAA

GCCAGCTCCGATGGAGAGGAGGATGCCGAGAGTGCAGACAAGGTAACTGAGACGGTGATG

AATGGCGGCATGAAGGAAACACTCAGCCTCACCGTGGATGCCAAGACGGAGACCGCAGTC

TTCAAAAGGAGTGACCAGCCAAGTGTGCCAGGTGACACGTCAGTCAATGGCCCTGTA

>CL161.Contig1_A-W 3 2246 C-Maf-inducing protein isoform C-Mip, partial [Macaca mulatta]

GACGTGTCGGCCCCCGAGGGCACGAAGATGGGCGCCGTGCCCTGCCGCCGGGCCCTGCTG

CTCTGCAACGGGATGAGGTACAAACTGCTGCAGGAGGGCGACATCCAGGTCTGTGTCATC

CGGCACCCGCGGACCTTTCTCAGCAAGATCCTCACCTCGAAATTCCTGAGGCGCTGGGAG

CCGCACCACCTAACGCTGGCCGACAACAGCCTGGCGTCCGCCACGCCAACTGGGTACATG

GAAAACTCAGTCTCCTACAGCGCCATCGAAGACGTTCAGCTGCTGTCCTGGGAGAATGCC

CCGAAGTACTGTTTACAGCTCACAATCCCCGGGGGGACCGTCCTGCTGCAGGCTGCCAAC

AGCTACCTGCGAGACCAGTGGTTCCATTCTCTGCAGTGGAAGAAAAAGATTTACAAGTAC

AAGAAAGTCCTGAGTAACCCAAGCCGTTGGGAAGTTGTCTTGAAGGAGATTCGGACGCTG

GTGGACATGGCCCTCACATCCCCCCTGCAGGATGACTCCATCAACCAAGCCCCACTGGAA

ATTGTCTCAAAACTGCTCTCAGAAAACACAAACTTGACCACCCAGGAGCATGAGAACATC

ATTGTGGCCATCGCTCCTCTGCTGGAAAACAACCATCCCCCTCCAGACCTCTGTGAATTC

TTTTGCAAGCACTGCAGAGAGCGGCCCCGGTCCATGGTGGTCATCGAGGTGTTCACCCCC

GTGGTCCAGAGAATCCTCAAGCATAACATGGACTTTGGGAAGTGCCCGCGACTGAGGCTC

TTTACTCAGGAGTACATCCTCGCCTTGAACGAACTCAACGCGGGGATGGAAGTGGTGAAG

AAGTTTATTCAGAGCATGCATGGCCCCACAGGGCACTGTCCCCACCCCCGGGTCCTGCCC

AACCTGGTGGCCGTGTGCCTGGCTGCCATCTACTCCTGCTACGAAGAATTCATCAACAGC

CGCGACAACTCCCCCAGCCTGAAGGAGATCCGGAATGGCTGTCAGCAGCCCTGCGACCGG

AAGCCCACCTTACCTCTGCGCCTTCTGCACCCCAGCCCGGACCTGGTGTCTCAGGAAGCC

ACGCTGTCCGAGGCGCGGCTCAAGTCGGTGGTCGTGGCCTCCAGTGAGATCCACGTGGAG

GTGGAACGCACCAGCACTGCCAAGCCAGCCCTGACGGCCAGCGCGGGCAACGACAGCGAG

CCCAACCTCATCGACTGCCTCATGGTCAGCCCCGCCTGCAGCACCATGAGCATCGAGCTG

GGCCCCCAGGCTGACCGCACGCTTGGCTGCTACGTGGAGATCCTCAAGCTGCTGTCGGAC

TATGATGACTGGAGACCGTCTCTGGCCAGCTTGCTTCAACCCATTCCATTCCCCAAAGAA

GCGCTTGCGCACGAGAAGTTCACCAAGGAGCTGAAGTATGTGATTCAGAGGTTTGCGGAA

GACCCGCGGCAAGAGGTGCATTCCTGCCTGCTCAGCGTGCGGGCTGGCAAGGACGGCTGG

TTTCAGCTCTACAGCCCCGGAGGGGTGGCCTGTGACGATGACGGGGAGCTGTTTGCCAGC

ATGGTGCACATCCTCATGGGCTCCTGTTACAAGACCAAAAAGTTTCTGCTCTCCTTGGCG

GAAAACAAGCTGGGCCCCTGCATGCTCCTGGCGCTGAGGGGGAACCAGACCATGGTGGAG

ATCCTGTGCTTGATGCTGGAGTACAACATCATCGACAACAACGACACGCAGCTGCAGATC

ATCTCCACGCTGGAGAGCACGGACGTGGGCAAGCGCATGTACGAGCAGCTGTGCGACCGG

CAGCGGGAGCTGAAGGAGCTGCAAAGGAAAGGCGGGCCCACCAGACTGACGCTGCCCTCC

AAGTCCACGGACGCTGACCTGGCTCGCTTGCTGAGCTCGGGCTCCTTTGGAAACCTGGAG

AACCTCAGTTTGGCCTTCACCAACGTAACCAGTGCCTGCGCTGAGCACCTCATCAAACTG

CCTTCTCTCAAGCAGCTGAACCTGTGGTCCACTCAGTTTGGAGACGCCGGCCTGCGGCTC

CTGTCGGAACACCTCACCATGCTCCAGGTGCTGAACCTGTGCGAGACCCCGGTCACCGAC

GCCGGCCTGCTGGCCCTCAGCTCCATGAAGAGTCTCTGCAGTTTGAACATGAACAGCACC

AAGCTCTCAGCCGACACCTACGAGGACCTGAAGGCCAAGCTTCCTAATCTGAAGGAGGTG

GACGTCCGCTACACAGAAGCCTGG

>CL162.Contig1_A-W 46 1113 PREDICTED: surfeit locus protein 6-like isoform 1 [Cavia porcellus]

ATGGCCTCCCTGCAAGCCAAGGACGCCTACTTGCAGGACCTGGCCAGGAAGATCTGCGCC

CCGCACGGCCCCGAGCCACCGCGGCGCTCGCGGGTTAGTAAAATTCAAGGCTCAGAAGCT

TCTGGACTCCGAAAAAAGAAAAGAAAGAAAACACCAAAAAAATTCCGGGAACAGGAACAG

AAGGCTTCAGAGCATAAAGCCAAGACCCTTGGGGAGAAGAAGTCTCCAGTGGCTCCTGGG

ACCAAGAAGCCAACAGCACACAAAGAGGAGCCTTCCATCTCCACAGGGGCCCCTGCAGGT

GGCCAGGCTACAGCACCTGATTCTGTCTTTGCCCTGGATGTCCTGCGCCAGCGGCTGCAT

GAGAAGATCCAGCAGGCTCGAGGCCAGGATGGCACGCTCTCTGCTGCCAGTCTGGAGAAA

AGGCAGCGGAGAAAGCAGGAGCGAGAGCGGAAGAAGAGGAAGCGGAGGGAGCTGCTAGCG

AAGGAGAAGGAGAAGGCGGCGGCGGCACAGGCCGGAGGCACTGCAGCGGTGACGGCTCCC

TCTGCGGCGGTCTGCGCAGGGCCGCAGGAGCCAGCGCTGATCTTCAATAAGGTGGAGGTC

AGTGAGGAGGAGTCGGCCACCAAGGCCCAGCGGCGGAAGGAGAAGCGGCAGAAGGTGAAG

GGGAACCTGACCCCTCTGACGGGCAAGAACTACCGGCAGCTGCTGGAGCGCCTGCAGGCG

CGGCAAGGCCGGCTGGAGGAGCTGCGGGAGCAGGACTCGGGGAAAGCCCGGGAGCTGGAG

GCCAAGATGAAGTGGACCAACCTGCTGTACAAGGCCGAGGGGGTCAAGATCCGCGACAAC

GAGCGGCTGCTGCAGGAGGCCCTGAAGCGCAAGGAGAAGCGCCGGGCTCAGCGGCAGCGC

CAGTGGGAGAAGCGCTCGGCCCACGTCGTGGAGAAAATGCAGCAGCGGCAGGACCGGCGG

CGGCAGAACCTGCGCAGGAAGAAGGCGGCCAAGGCTGAGCGGCGCCTGCAGAAGGCCCGC

AGGAAGGGCCGCGTGCTGCCCCAGGACCTGGAGCGGGCGGGCCTCGCC

>CL162.Contig2_A-W 38 526 PREDICTED: surfeit locus protein 6-like isoform 1 [Cavia porcellus]

ATGGCCTCCCTGCAAGCCAAGGACGCCTACTTGCAGGACCTGGCCAGGAAGATCTGCGCC

CCGCACGGCCCCGAGCCACCGCGGCGCTCGCGGGTTAGTAAAATTCAAGGCTCAGAAGCT

TCTGGACTCCGAAAAAAGAAAAGAAAGAAAACACCAAAAAAATTCCGGGAACAGGAACAG

AAGGCTTCAGAGCATAAAGCCAAGACCCTTGGGGAGAAGAAGTCTCCAGTGGCTCCTGGG

ACCAAGAAGCCAACAGCACACAAAGAGGAGCCTTCCATCTCCACAGGGGCCCCTGCAGGT

GGCCAGGCTACAGCACCTGATTCTGTCTTTGCCCTGGATGTCCTGCGCCAGCGGCTGCAT

GAGAAGATCCAGCAGGCTCGAGGCCAGGATGGCACGCTCTCTGCTGCCAGTCTGGAGAAA

AGGCAGCGGAGAAAGCAGGAGCGAGAGCGGAAGAAGAGGAAGCGGAGGGAGCTGCTAGCG

AAGGAGAAG

>CL163.Contig1_A-W 79 1947 PREDICTED: TAO kinase 2-like [Oryctolagus cuniculus]

TCTGATTATTTTCGCAACTTTGTAGATTCTTGCCTCCAGAAAATCCCTCAAGATCGTCCA

ACATCAGAGGAACTTTTAAAGCACATGTTTGTTCTTCGAGAGCGCCCTGAAACAGTGTTA

ATAGATCTTATTCAGAGGACAAAGGATGCAGTAAGAGAGCTGGACAATCTGCAGTATCGA

AAAATGAAGAAACTTCTTTTCCAGGAGGCACATAATGGACCAACAGTGGAAGCACAGGAA

GAAGAAGAGGAACAAGATCATGGTGTTGGCCGGACTGGAACAGTCAATAGTGTTGGAAGT

AATCAGTCTATTCCCAGTATGTCTATCAGTGCCAGCAGCCAAAGCAGTAGTGTTAATAGT

CTTCCAGATGCCTCAGATGATAAAAGTGAACTAGACATGATGGAAGGAGACCATACGGTG

ATGTCCAACAGTTCTGTCATCCATTTAAAACCTGAAGAAGAAAATTACAGAGAAGAAGGA

GATCCTAGAACAAGAGTATCAGATCCACAATCTCCTCCCCAAGTATCTCGCCACAAATCA

CATTATCGTAATCGAGAACACTTTGCAACCATAAGGACGGCATCACTGGTTACAAGGCAA

ATGCAAGAACATGAGCAGGACTCTGAGCTTAGAGAACAGATGTCTGGCTATAAGCGAATG

AGACGGCAGCACCAAAAGCAACTGATGACTCTGGAAAATAAGCTAAAGGCTGAGATGGAT

GAACACCGCCTAAGATTAGACAAAGATCTTGAAACTCAGCGTAATAATTTTGCTGCAGAA

ATGGAGAAACTTATCAAGAAACATCAAGCTGCTATGGAAAAAGAGGCTAAAGTGATGGCC

AATGAGGAGAAAAAATTTCAGCAACATATTCAGGCCCAACAGAAGAAAGAACTGAATAGC

TTTTTGGAGTCCCAGAAAAGAGAATATAAACTTCGAAAAGAACAGCTTAAAGAGGAGCTG

AATGAAAACCAGAGCACCCCTAAAAAAGAAAAACAAGAGTGGCTTTCAAAGCAGAAGGAG

AATATACAACATTTCCAAGCAGAAGAAGAAGCTAACCTTCTTCGACGTCAGAGACAATAC

CTAGAGCTGGAATGCCGTCGCTTCAAGAGAAGAATGTTACTCGGACGGCATAACTTGGAG

CAGGACCTTGTCAGGGAGGAGTTAAACAAAAGACAGACTCAAAAGGACTTAGAGCATGCC

ATGTTACTACGACAGCATGAATCCATGCAAGAACTGGAGTTTCGCCACCTCAACACAATT

CAGAAGATGCGCTGTGAGTTGATCAGATTGCAGCATCAAACTGAGCTCACTAATCAGCTG

GAATATAATAAGCGAAGGGAACGAGAACTAAGGCGAAAGCATGTCATGGAAGTTCGACAA

CAGCCTAAGAGTTTGAAGTCTAAAGAACTCCAAATAAAAAAGCAGTTTCAGGATACCTGC

AAAATCCAAACCAGACAGTACAAAGCATTAAGAAATCACCTACTGGAGACTACACCAAAG

AGTGAGCATAAAGCTGTTCTGAAAAGGCTCAAGGAGGAGCAGACCCGCAAGTTAGCCATC

TTGGCTGAACAGTATGATCACAGCATTAATGAAATGCTCTCCACACAGGCTCTGCGTTTG

GATGAAGCACAGGAAGCAGAGTGCCAGGTTTTGAAGATGCAGCTGCAGCAGGAACTGGAG

CTGTTGAATGCATATCAGAGCAAAATCAAGATGCAGGCTGAGGCACAACATGATCGAGAG

CTTCGGGAGCTTGAACAGAGGGTCTCCCTCCGCAGGGCACTCTTAGAACAAAAGATTGAA

GAAGAAATGTTGGCTTTGCAGAATGAACGAACAGAACGAATACGGAGCCTGCTAGAGCGT

CAAGCGAGA

>CL163.Contig2_A-W 104 1384 PREDICTED: serine/threonine-protein kinase TAO1 [Otolemur garnettii]

GTTACAAGGCAAATGCAAGAACATGAGCAGGACTCTGAGCTTAGAGAACAGATGTCTGGC

TATAAGCGAATGAGACGGCAGCACCAAAAGCAACTGATGACTCTGGAAAATAAGCTAAAG

GCTGAGATGGATGAACACCGCCTAAGATTAGACAAAGATCTTGAAACTCAGCGTAATAAT

TTTGCTGCAGAAATGGAGAAACTTATCAAGAAACATCAAGCTGCTATGGAAAAAGAGGCT

AAAGTGATGGCCAATGAGGAGAAAAAATTTCAGCAACATATTCAGGCCCAACAGAAGAAA

GAACTGAATAGCTTTTTGGAGTCCCAGAAAAGAGAATATAAACTTCGAAAAGAACAGCTT

AAAGAGGAGCTGAATGAAAACCAGAGCACCCCTAAAAAAGAAAAACAAGAGTGGCTTTCA

AAGCAGAAGGAGAATATACAACATTTCCAAGCAGAAGAAGAAGCTAACCTTCTTCGACGT

CAGAGACAATACCTAGAGCTGGAATGCCGTCGCTTCAAGAGAAGAATGTTACTCGGACGG

CATAACTTGGAGCAGGACCTTGTCAGGGAGGAGTTAAACAAAAGACAGACTCAAAAGGAC

TTAGAGCATGCCATGTTACTACGACAGCATGAATCCATGCAAGAACTGGAGTTTCGCCAC

CTCAACACAATTCAGAAGATGCGCTGTGAGTTGATCAGATTGCAGCATCAAACTGAGCTC

ACTAATCAGCTGGAATATAATAAGCGAAGGGAACGAGAACTAAGGCGAAAGCATGTCATG

GAAGTTCGACAACAGCCTAAGAGTTTGAAGTCTAAAGAACTCCAAATAAAAAAGCAGTTT

CAGGATACCTGCAAAATCCAAACCAGACAGTACAAAGCATTAAGAAATCACCTACTGGAG

ACTACACCAAAGAGTGAGCATAAAGCTGTTCTGAAAAGGCTCAAGGAGGAGCAGACCCGC

AAGTTAGCCATCTTGGCTGAACAGTATGATCACAGCATTAATGAAATGCTCTCCACACAG

GCTCTGCGTTTGGATGAAGCACAGGAAGCAGAGTGCCAGGTTTTGAAGATGCAGCTGCAG

CAGGAACTGGAGCTGTTGAATGCATATCAGAGCAAAATCAAGATGCAGGCTGAGGCACAA

CATGATCGAGAGCTTCGGGAGCTTGAACAGAGGGTCTCCCTCCGCAGGGCACTCTTAGAA

CAAAAGATTGAAGAAGAAATGTTGGCTTTGCAGAATGAACGAACAGAACGAATACGGAGC

CTGCTAGAGCGTCAAGCGAGA

>CL163.Contig3_A-W 104 913 PREDICTED: serine/threonine-protein kinase TAO1 [Otolemur garnettii]

GTTACAAGGCAAATGCAAGAACATGAGCAGGACTCTGAGCTTAGAGAACAGATGTCTGGC

TATAAGCGAATGAGACGGCAGCACCAAAAGCAACTGATGACTCTGGAAAATAAGCTAAAG

GCTGAGATGGATGAACACCGCCTAAGATTAGACAAAGATCTTGAAACTCAGCGTAATAAT

TTTGCTGCAGAAATGGAGAAACTTATCAAGAAACATCAAGCTGCTATGGAAAAAGAGGCT

AAAGTGATGGCCAATGAGGAGAAAAAATTTCAGCAACATATTCAGGCCCAACAGAAGAAA

GAACTGAATAGCTTTTTGGAGTCCCAGAAAAGAGAATATAAACTTCGAAAAGAACAGCTT

AAAGAGGAGCTGAATGAAAACCAGAGCACCCCTAAAAAAGAAAAACAAGAGTGGCTTTCA

AAGCAGAAGGAGAATATACAACATTTCCAAGCAGAAGAAGAAGCTAACCTTCTTCGACGT

CAGAGACAATACCTAGAGCTGGAATGCCGTCGCTTCAAGAGAAGAATGTTACTCGGACGG

CATAACTTGGAGCAGGACCTTGTCAGGGAGGAGTTAAACAAAAGACAGACTCAAAAGGAC

TTAGAGCATGCCATGTTACTACGACAGCATGAATCCATGCAAGAACTGGAGTTTCGCCAC

CTCAACACAATTCAGAAGATGCGCTGTGAGTTGATCAGATTGCAGCATCAAACTGAGCTC

ACTAATCAGCTGGAATATAATAAGCGAAGGGAACGAGAACTAAGGCGAAAGCATGTCATG

GAAGTTCGACAACAGCCTAAGAGTTTGAAG

>CL163.Contig4_A-W 42 614 PREDICTED: serine/threonine-protein kinase TAO1 [Otolemur garnettii]

GCTAAAGTGATGGCCAATGAGGAGAAAAAATTTCAGCAACATATTCAGGCCCAACAGAAG

AAAGAACTGAATAGCTTTTTGGAGTCCCAGAAAAGAGAATATAAACTTCGAAAAGAACAG

CTTAAAGAGGAGCTGAATGAAAACCAGAGCACCCCTAAAAAAGAAAAACAAGAGTGGCTT

TCAAAGCAGAAGGAGAATATACAACATTTCCAAGCAGAAGAAGAAGCTAACCTTCTTCGA

CGTCAGAGACAATACCTAGAGCTGGAATGCCGTCGCTTCAAGAGAAGAATGTTACTCGGA

CGGCATAACTTGGAGCAGGACCTTGTCAGGGAGGAGTTAAACAAAAGACAGACTCAAAAG

GACTTAGAGCATGCCATGTTACTACGACAGCATGAATCCATGCAAGAACTGGAGTTTCGC

CACCTCAACACAATTCAGAAGATGCGCTGTGAGTTGATCAGATTGCAGCATCAAACTGAG

CTCACTAATCAGCTGGAATATAATAAGCGAAGGGAACGAGAACTAAGGCGAAAGCATGTC

ATGGAAGTTCGACAACAGCCTAAGAGTTTGAAG

>CL164.Contig1_A-W 597 938 minus strand PREDICTED: ras-related protein Rab-4B-like [Pan troglodytes]

AGGTCGGTGACACGGAGTTATTACCGAGGGGCGGCTGGAGCCCTGCTGGTGTATGACATC

ACAAGGTGGGTGTGGGCTACTCAGCATGAGCAGCGTGGTTGGGGTGGGACATGGGTGGCC

TCTCTCCTGTGCCACCTTCCTTCTCTGTCCCTTCTCCATAGCCGGGAGACATACAATTCA

CTGGCTGCCTGGCTGACGGATGCCCGCACACTGGCCAGCCCCAACATTGTGGTCATCCTC

TGTGGCAACAAGAAGGACCTGGACCCTGAGCGTGAGGTCACTTTCCTGGAGGCTTCCCGC

TTTGCCCAGGAGAATGGTGAGGGCTCTGCTGTGGACTGGGTG

>CL164.Contig2_A-W 597 842 minus strand PREDICTED: ras-related protein Rab-4B-like [Pan troglodytes]

AGGTCGGTGACACGGAGTTATTACCGAGGGGCGGCTGGAGCCCTGCTGGTGTATGACATC

ACAAGCCGGGAGACATACAATTCACTGGCTGCCTGGCTGACGGATGCCCGCACACTGGCC

AGCCCCAACATTGTGGTCATCCTCTGTGGCAACAAGAAGGACCTGGACCCTGAGCGTGAG

GTCACTTTCCTGGAGGCTTCCCGCTTTGCCCAGGAGAATGGTGAGGGCTCTGCTGTGGAC

TGGGTG

>CL164.Contig3_A-W 597 1121 minus strand PREDICTED: ras-related protein Rab-4B-like [Macaca mulatta]

AGGTCGGTGACACGGAGTTATTACCGAGGGGCGGCTGGAGCCCTGCTGGTGTATGACATC

ACAAGGTGGGTGTGGGCTACTCAGCATGAGCAGCGTGGTTGGGGTGGGACATGGGTGGCC

TCTCTCCTGTGCCACCTTCCTTCTCTGTCCCTTCTCCATAGCCGGGAGACATACAATTCA

CTGGCTGCCTGGCTGACGGATGCCCGCACACTGGCCAGCCCCAACATTGTGGTCATCCTC

TGTGGCAACAAGAAGGACCTGGACCCTGAGCGTGAGGTCACTTTCCTGGAGGCTTCCCGC

TTTGCCCAGGAGAATGAGCTAATGTTCCTGGAGACCAGCGCTCTCACAGGCGAGAACGTG

GAGGAGGCCTTCCTGAAGTGTGCCCGCACCATCCTCAACAAGATCGACTCAGGTGAGCTA

GACCCTGAGAGGATGGGCTCAGGCATTCAGTATGGTGATGCTTCTCTCCGCCAGCTGCGG

CAGCCTCGGAGTGCCCAGGCCGTGGCCCCTCAGCCCTGTGGCTGC

>CL164.Contig4_A-W 597 1025 minus strand PREDICTED: ras-related protein Rab-4B-like [Macaca mulatta]

AGGTCGGTGACACGGAGTTATTACCGAGGGGCGGCTGGAGCCCTGCTGGTGTATGACATC

ACAAGCCGGGAGACATACAATTCACTGGCTGCCTGGCTGACGGATGCCCGCACACTGGCC

AGCCCCAACATTGTGGTCATCCTCTGTGGCAACAAGAAGGACCTGGACCCTGAGCGTGAG

GTCACTTTCCTGGAGGCTTCCCGCTTTGCCCAGGAGAATGAGCTAATGTTCCTGGAGACC

AGCGCTCTCACAGGCGAGAACGTGGAGGAGGCCTTCCTGAAGTGTGCCCGCACCATCCTC

AACAAGATCGACTCAGGTGAGCTAGACCCTGAGAGGATGGGCTCAGGCATTCAGTATGGT

GATGCTTCTCTCCGCCAGCTGCGGCAGCCTCGGAGTGCCCAGGCCGTGGCCCCTCAGCCC

TGTGGCTGC

>CL164.Contig5_A-W 186 824 ras-related protein Rab-4B [Canis lupus familiaris] >gi|82659107|ref|NP_057238.3| ras-related protein Rab-4B [Homo sapiens] >gi|190360735|ref|NP_001121979.1| ras-related protein Rab-4B [Bos taurus] >gi|46577634|sp|P61017.1|RAB4B_CANFA RecName: Full=Ras-related protein Rab-4B >gi|46577635|sp|P61018.1|RAB4B_HUMAN RecName: Full=Ras-related protein Rab-4B >gi|5640004|gb|AAD45923.1|AF165522_1 ras-related GTP-binding protein 4b [Homo sapiens] >gi|20379046|gb|AAM21083.1|AF498935_1 small GTP binding protein RAB4B [Homo sapiens] >gi|919|emb|CAA39800.1| rab4b [Canis lupus familiaris] >gi|28422140|gb|AAH46927.1| RAB4B, member RAS oncogene family [Homo sapiens] >gi|119577405|gb|EAW57001.1| hCG2044074, isoform CRA_b [Homo sapiens] >gi|119577410|gb|EAW57006.1| hCG1995540, isoform CRA_c [Homo sapiens] >gi|158455121|gb|AAI22835.2| RAB4B protein [Bos taurus] >gi|380785613|gb|AFE64682.1| ras-related protein Rab-4B [Macaca mulatta] >gi|383419263|gb|AFH32845.1| ras-related protein Rab-4B [Macaca mulatta] >gi|384947736|gb|AFI37473.1| ras-related protein Rab-4B [Macaca mulatta]

ATGGCCGAGACCTACGACTTCCTCTTCAAATTCCTGGTGATCGGCAGTGCAGGAACTGGC

AAATCATGCCTCCTTCATCAGTTCATTGAGAATAAGTTCAAACAGGACTCCAACCACACA

ATCGGTGTGGAGTTTGGATCCCGGGTGGTCAACGTGGGTGGGAAGACTGTGAAGCTACAG

ATTTGGGACACTGCTGGCCAGGAGCGGTTTCGGTCGGTGACACGGAGTTATTACCGAGGG

GCGGCTGGAGCCCTGCTGGTGTATGACATCACAAGCCGGGAGACATACAATTCACTGGCT

GCCTGGCTGACGGATGCCCGCACACTGGCCAGCCCCAACATTGTGGTCATCCTCTGTGGC

AACAAGAAGGACCTGGACCCTGAGCGTGAGGTCACTTTCCTGGAGGCTTCCCGCTTTGCC

CAGGAGAATGAGCTAATGTTCCTGGAGACCAGCGCTCTCACAGGCGAGAACGTGGAGGAG

GCCTTCCTGAAGTGTGCCCGCACCATCCTCAACAAGATCGACTCAGGTGAGCTAGACCCT

GAGAGGATGGGCTCAGGCATTCAGTATGGTGATGCTTCTCTCCGCCAGCTGCGGCAGCCT

CGGAGTGCCCAGGCCGTGGCCCCTCAGCCCTGTGGCTGC

>CL164.Contig6_A-W 494 1057 minus strand unnamed protein product [Mus musculus]

CCTCTCTCCTTCCCATTCCCAGTCAAACAGGACTCCAACCACACAATCGGTGTGGAGTTT

GGATCCCGGGTGGTCAACGTGGGTGGGAAGACTGTGAAGCTACAGATTTGGGACACTGCT

GGCCAGGAGCGGTTTCGGTCGGTGACACGGAGTTATTACCGAGGGGCGGCTGGAGCCCTG

CTGGTGTATGACATCACAAGCCGGGAGACATACAATTCACTGGCTGCCTGGCTGACGGAT

GCCCGCACACTGGCCAGCCCCAACATTGTGGTCATCCTCTGTGGCAACAAGAAGGACCTG

GACCCTGAGCGTGAGGTCACTTTCCTGGAGGCTTCCCGCTTTGCCCAGGAGAATGAGCTA

ATGTTCCTGGAGACCAGCGCTCTCACAGGCGAGAACGTGGAGGAGGCCTTCCTGAAGTGT

GCCCGCACCATCCTCAACAAGATCGACTCAGGTGAGCTAGACCCTGAGAGGATGGGCTCA

GGCATTCAGTATGGTGATGCTTCTCTCCGCCAGCTGCGGCAGCCTCGGAGTGCCCAGGCC

GTGGCCCCTCAGCCCTGTGGCTGC

>CL164.Contig7_A-W 518 847 minus strand Ras-related protein Rab-4B OS=Rattus norvegicus GN=Rab4b PE=2 SV=1

AAACAGGACTCCAACCACACAATCGGTGTGGAGTTTGGATCCCGGGTGGTCAACGTGGGT

GGGAAGACTGTGAAGCTACAGATTTGGGACACTGCTGGCCAGGAGCGGTTTCGGTCGGTG

ACACGGAGTTATTACCGAGGGGCGGCTGGAGCCCTGCTGGTGTATGACATCACAAGCCGG

GAGACATACAATTCACTGGCTGCCTGGCTGACGGATGCCCGCACACTGGCCAGCCCCAAC

ATTGTGGTCATCCTCTGTGGCAACAAGAAGGACCTGGACCCTGAGCGTGAGGTCACTTTC

CTGGAGGCTTCCCGCTTTGCCCAGGAGAAT

>CL164.Contig8_A-W 186 641 minus strand PREDICTED: ras-related protein Rab-4B-like [Pan troglodytes]

ATGGCCGAGACCTACGACTTCCTCTTCAAATTCCTGGTGATCGGCAGTGCAGGAACTGGC

AAATCATGCCTCCTTCATCAGTTCATTGAGAATAAGTTCAAACAGGACTCCAACCACACA

ATCGGTGTGGAGTTTGGATCCCGGGTGGTCAACGTGGGTGGGAAGACTGTGAAGCTACAG

ATTTGGGACACTGCTGGCCAGGAGCGGTTTCGGTCGGTGACACGGAGTTATTACCGAGGG

GCGGCTGGAGCCCTGCTGGTGTATGACATCACAAGCCGGGAGACATACAATTCACTGGCT

GCCTGGCTGACGGATGCCCGCACACTGGCCAGCCCCAACATTGTGGTCATCCTCTGTGGC

AACAAGAAGGACCTGGACCCTGAGCGTGAGGTCACTTTCCTGGAGGCTTCCCGCTTTGCC

CAGGAGAATGGTGAGGGCTCTGCTGTGGACTGGGTG

>CL165.Contig1_A-W 130 1440 PREDICTED: serine/threonine-protein kinase STK11 [Otolemur garnettii]

ATGGACGTGGCGGACCCGCAGCAGCTGGGCATGTTCACGGAGGGCGAGCTGATGTCCGTG

GGCATGGACACCTTCATCCACCGCATCGACTCCACGGAGGTCATCTACCAGCCCCGCCGC

AAGCGGGCCAAACTCATCGGCAAGTACCTGATGGGGGACTTGCTGGGGGAGGGCTCCTAC

GGCAAGGTGAAGGAGGTGCTGGACTCGGAGACGCTGTGCCGGAGGGCCGTCAAGATCCTC

AAGAAGAAGAAGTTGCGGCGGATCCCCAACGGGGAGGCCAACGTGAAGAAGGAGATCCAG

CTCCTGAGGCGTCTGCGGCACAGAAACGTCATCCAGCTGGTAGACGTGCTATACAACGAG

GAGAAGCAAAAGATGTATATGGTGATGGAGTACTGCGTTTGTGGCATGCAGGAGATGCTG

GACAGTGTGCCCGAGAAGCGCTTCCCTGTGTGCCAAGCCCATGGGTACTTCTGCCAGCTC

ATCGACGGACTGGAGTACCTGCACAGCCAGGGTATCGTGCACAAGGACATCAAACCCGGC

AATCTGCTGCTCACCACCAGTGGCACACTCAAGATCTCCGACCTGGGTGTCGCGGAGGCC

CTGCACCCTTTTGCTGCGGATGACACTTGCCGGACAAGCCAGGGCTCCCCAGCATTCCAG

CCACCTGAAATTGCCAATGGCCTGGACACCTTCTCTGGCTTCAAGGTGGACATCTGGTCA

GCTGGAGTCACACTCTACAACATCACAACGGGCCTGTACCCATTTGAAGGGGACAACATC

TACAAGCTGTTCGAGAACATTGGGAAGGGGGACTACACGATCCCGGGTGACTGTGGCCCC

CCGCTCTCTGACCTGCTCAAAGGGATGCTGGAGTACGAGCCCACCAAGAGGTTCTCCATA

CGGCAGATCCGGCAGCACAGCTGGTTCCGGAAGAAGCATCCATTGGCAGAGGCCTTGGTG

CCCATCCCGCCCAGCCCAGACACCAAGGACCGCTGGCGCAGCATGACAGTCGTGCCCTAC

CTGGAGGACCTACATGGCCGCACTGATGAGGATGAGGATGAGGACCTCTTTGACATTGAG

GATGGCATCATCTACACCCAGGACTTCACCGTGCCTGGACAGGTCCCAGAAGAGGAGGCC

AGTCAGAATGGACAGAGCCACAGCCTGCCCAAGGCTGTGTGCATGAATGGCACAGAGTCG

GCGCAGCTGAGCTCCAAGACCAAGGTGGAGTGCCGGGCCAGTGCCACCAACCCTGCCCGC

AAGGCCTGCTCCAGCAGCAAGATCCGCCGACTCTCAGCCTGCAAGCAGCAG

>CL165.Contig2_A-W 104 298 PREDICTED: serine/threonine-protein kinase STK11 [Otolemur garnettii]

GGACAGGTCCCAGAAGAGGAGGCCAGTCAGAATGGACAGAGCCACAGCCTGCCCAAGGCT

GTGTGCATGAATGGCACAGAGTCGGCGCAGCTGAGCTCCAAGACCAAGGTGGAGTGCCGG

GCCAGTGCCACCAACCCTGCCCGCAAGGCCTGCTCCAGCAGCAAGATCCGCCGACTCTCA

GCCTGCAAGCAGCAG

>CL165.Contig4_A-W 43 777 serine/threonine protein kinase 11 variant [Homo sapiens]

GGGAAGTGGGGGTCGGAGAAGAGCCGCCCACCGAGGGGACAAGGGGGACCAGGGCTCCCC

GGCGCCGGGTCCAGGATGGACGTGGCGGACCCGCAGCAGCTGGGCATGTTCACGGAGGGC

GAGCTGATGTCCGTGGGCATGGACACCTTCATCCACCGCATCGACTCCACGGAGGTCATC

TACCAGCCCCGCCGCAAGCGGGCCAAACTCATCGGCAAGTACCTGATGGGGGACTTGCTG

GGGGAGGGCTCCTACGGCAAGGTGAAGGAGGTGCTGGACTCGGAGACGCTGTGCCGGAGG

GCCGTCAAGATCCTCAAGAAGAAGAAGTTGCGGCGGATCCCCAACGGGGAGGCCAACGTG

AAGAAGGAGATCCAGCTCCTGAGGCGTCTGCGGCACAGAAACGTCATCCAGCTGGTAGAC

GTGCTATACAACGAGGAGAAGCAAAAGATGTATATGGTGATGGAGTACTGCGTGTGTGGC

ATGCAGGAGATGCTGGACAGTGTGCCCGAGAAGCGCTTCCCTGTGTGCCAAGCCCATGGG

TACTTCTGCCAGCTCATCGACGGACTGGAGTACCTGCACAGCCAGGGTATCGTGCACAAG

GACATCAAACCCGGCAATCTGCTGCTCACCACCAGTGGCACACTCAAGATCTCCGACCTG

GGTGTCGCGGAGGCCCTGCACCCTTTTGCTGCGGATGACACTTGCCGGACAAGCCAGGCC

ATTGGCAATTTCAGG

>CL166.Contig1_A-W 2 1288 minus strand LIM domain kinase 2 [Heterocephalus glaber]

TGCCATGGGTGCTCCCTGCTAATGACAGGACCTGTCATGGTGGCTGGGGAATTCAAGTAC

CATCCAGAGTGTTTTGCTTGTATGAGCTGCAAGGTGATCATCGAGGATGGGGATGCATAT

GCCCTGGTACAGCATGCCACACTCTACTGTGGAAAATGCCACAATGAGGTGGTGTTGGCA

CCCATGTTTGAGAGGCTCTCCACAGAATCTGTTCAGGACCAGCTGCCCTACTCTGTCACG

CTCATTTCCATGCCAGCTACCACTGAGGGCAGGCGGGGCTTCTCCGTGTCTGTAGAAAGT

GCCTGCTCCAACTATGCCACCACTGTGCAAGTGAAAGAGGTCAACCGGATGCACATCAGT

CCCAACAACCGCAATGCCATCCACCCTGGGGACCGCATCCTGGAGATCAATGGCACCCCT

GTCCGCACACTCCAAGTGGAGGAGGTGGAAGATGCAATTAGCCAAACAAGCCAGACACTT

CAGCTGTTGATTGAACATGACCCCGTCTCCCAGCGCCTGGACCAGCTGCGGTTAGATGCC

CGGCTTTCTCCCCACATACAGAATGATGGACATTCTCACACCCTCAGCACTCTGGACACC

AAGGACAATCTGGAGGGGACACTGAGGAGACGCTCCCTGAGGCGCAGTAATAGCATCTCT

AAGTCCCCTGGCCCAAGCTCCCCCAAGGAGCCCCTGCTGCTCAGCCGAGACATCAGCCGC

TCAGAATCCCTCCGCTGTTCTAGCAGCTACTCACAGCAGATCTTCCGACCATGTGACCTG

ATCCATGGGGAGGTCCTGGGAAAGGGCTTCTTTGGGCAGGCCATCAAGGTGACACACAAA

GCCACAGGCAAAGTGATGGTCATGAAGGAGTTAATTCGATGTGATGAGGAGACACAGAAG

ACTTTCCTGACTGAGGTGAAAGTGATGCGCAGCCTAGACCACCCCAACGTGCTCAAGTTC

ATTGGTGTGCTGTACAAGGACAAGAAGCTGAACCTGCTGACAGAATACATTGAGGGGGGT

ACACTGAAGGATTTTCTGCGCAGTGTGGACCCATTCCCCTGGCAGCAGAAGGTCAGATTT

GCAAAAGGCATCGCCTCCGGAATGGCCTATTTGCACTCCATGTGCATCATCCACCGGGAT

CTGAATTCACACAACTGCCTCATCAAGTTGGACAAGACTGTGGTGGTAGCAGACTTTGGG

CTGTCGCGGCTTATAGTGGAAGAGAGGAAAAGGCCCCCAGTAGAGAAGGCCACCACCAAG

AAACGCACCTTGCGCAAGAATGACCGC

>CL166.Contig2_A-W 2 922 minus strand LIM domain kinase 2 [Heterocephalus glaber]

TGCCATGGGTGCTCCCTGCTAATGACAGGACCTGTCATGGTGGCTGGGGAATTCAAGTAC

CATCCAGAGTGTTTTGCTTGTATGAGCTGCAAGGTGATCATCGAGGATGGGGATGCATAT

GCCCTGGTACAGCATGCCACACTCTACTGTGGAAAATGCCACAATGAGGTGGTGTTGGCA

CCCATGTTTGAGAGGCTCTCCACAGAATCTGTTCAGGACCAGCTGCCCTACTCTGTCACG

CTCATTTCCATGCCAGCTACCACTGAGGGCAGGCGGGGCTTCTCCGTGTCTGTAGAAAGT

GCCTGCTCCAACTATGCCACCACTGTGCAAGTGAAAGAGGTCAACCGGATGCACATCAGT

CCCAACAACCGCAATGCCATCCACCCTGGGGACCGCATCCTGGAGATCAATGGCACCCCT

GTCCGCACACTCCAAGTGGAGGAGGTGGAAGATGCAATTAGCCAAACAAGCCAGACACTT

CAGCTGTTGATTGAACATGACCCCGTCTCCCAGCGCCTGGACCAGCTGCGGTTAGATGCC

CGGCTTTCTCCCCACATACAGAATGATGGACATTCTCACACCCTCAGCACTCTGGACACC

AAGGACAATCTGGAGGGGACACTGAGGAGACGCTCCCTGAGGCGCAGTAATAGCATCTCT

AAGTCCCCTGGCCCAAGCTCCCCCAAGGAGCCCCTGCTGCTCAGCCGAGACATCAGCCGC

TCAGAATCCCTCCGCTGTTCTAGCAGCTACTCACAGCAGATCTTCCGACCATGTGACCTG

ATCCATGGGGAGGTCCTGGGAAAGGGCTTCTTTGGGCAGGCCATCAAGGTGACACACAAA

GCCACAGGCAAAGTGATGGTCATGAAGGAGTTAATTCGATGTGATGAGGAGACACAGAAG

ACTTTCCTGACTGAGGTAAGA

>CL166.Contig3_A-W 2 1480 minus strand PREDICTED: LIM domain kinase 2 isoform 2 [Equus caballus]

TGCCATGGGTGCTCCCTGCTAATGACAGGACCTGTCATGGTGGCTGGGGAATTCAAGTAC

CATCCAGAGTGTTTTGCTTGTATGAGCTGCAAGGTGATCATCGAGGATGGGGATGCATAT

GCCCTGGTACAGCATGCCACACTCTACTGTGGAAAATGCCACAATGAGGTGGTGTTGGCA

CCCATGTTTGAGAGGCTCTCCACAGAATCTGTTCAGGACCAGCTGCCCTACTCTGTCACG

CTCATTTCCATGCCAGCTACCACTGAGGGCAGGCGGGGCTTCTCCGTGTCTGTAGAAAGT

GCCTGCTCCAACTATGCCACCACTGTGCAAGTGAAAGAGGTCAACCGGATGCACATCAGT

CCCAACAACCGCAATGCCATCCACCCTGGGGACCGCATCCTGGAGATCAATGGCACCCCT

GTCCGCACACTCCAAGTGGAGGAGGTGGAAGATGCAATTAGCCAAACAAGCCAGACACTT

CAGCTGTTGATTGAACATGACCCCGTCTCCCAGCGCCTGGACCAGCTGCGGTTAGATGCC

CGGCTTTCTCCCCACATACAGAATGATGGACATTCTCACACCCTCAGCACTCTGGACACC

AAGGACAATCTGGAGGGGACACTGAGGAGACGCTCCCTGAGGCGCAGTAATAGCATCTCT

AAGTCCCCTGGCCCAAGCTCCCCCAAGGAGCCCCTGCTGCTCAGCCGAGACATCAGCCGC

TCAGAATCCCTCCGCTGTTCTAGCAGCTACTCACAGCAGATCTTCCGACCATGTGACCTG

ATCCATGGGGAGGTCCTGGGAAAGGGCTTCTTTGGGCAGGCCATCAAGGTGACACACAAA

GCCACAGGCAAAGTGATGGTCATGAAGGAGTTAATTCGATGTGATGAGGAGACACAGAAG

ACTTTCCTGACTGAGGTGAAAGTGATGCGCAGCCTAGACCACCCCAACGTGCTCAAGTTC

ATTGGTGTGCTGTACAAGGACAAGAAGCTGAACCTGCTGACAGAATACATTGAGGGGGGT

ACACTGAAGGATTTTCTGCGCAGTGTGGTGAGCATACTGCCCCATAGTCACCAAGATCCT

TGGCAGGCTGTTCAGAGTGACACCTGTGTCCCCAAAGATCATGTTGGGGGGAACAAGCAC

AGACAGGGAGGCTGCACTGGCATATGCATACTATGCTGACCCATGGAGCCTGCACCATTT

GTGGCCACAGCCTCTGACTGGCATTCCCCCACCCTGCAGGACCCATTCCCCTGGCAGCAG

AAGGTCAGATTTGCAAAAGGCATCGCCTCCGGAATGGCCTATTTGCACTCCATGTGCATC

ATCCACCGGGATCTGAATTCACACAACTGCCTCATCAAGTTGGACAAGACTGTGGTGGTA

GCAGACTTTGGGCTGTCGCGGCTTATAGTGGAAGAGAGGAAAAGGCCCCCAGTAGAGAAG

GCCACCACCAAGAAACGCACCTTGCGCAAGAATGACCGC

>CL167.Contig1_A-W 122 493 minus strand PREDICTED: dnaJ homolog subfamily C member 7 isoform 1 [Callithrix jacchus] >gi|403304474|ref|XP_003942821.1| PREDICTED: dnaJ homolog subfamily C member 7 [Saimiri boliviensis boliviensis]

AAGGAACAAGGAAATGCATACTATGCCAAGAAAGATTACAATGAAGCTTATAACTATTAT

ACAAAAGCCATAGATATGTGTCCTAAAAATGCTAGCTATTATGGTAATCGAGCAGCCACG

TTGATGATGCTTGGAAGGTTCCGGGAAGCTCTTGGAGATGCACAACAGTCAGTGAGGTTG

GATGACAGTTTTGTCCGGGGTCATCTACGAGAAGGCAAGTGCCACCTCTCCCTAGGGAAT

GCCATGGCAGCATGTCGTAGTTTCCAGAGAGCCCTAGAACTGGATCACAAAAATGCTCAG

GCACAACAAGAGTTCAAGAATGCTAATGCAGTCATGGAATATGAAAAAATAGCAGAAATG

GATTTTGAGAAG

>CL167.Contig2_A-W 120 590 minus strand PREDICTED: dnaJ homolog subfamily C member 7 [Sarcophilus harrisii]

TACATGGACACAGAGCAGTATGAAGAAGCAGTGCGGGACTATGAAAAAGTGTATCAGACG

GAGAAAACAAAAGAACACAAACAGCTCCTAAAAAATGCACAACTGGAACTGAAGAAGAGT

AAGAGGAAAGATTACTACAAGATTCTGGGAGTGGACAAGAATGCCTCTGAGGATGAGATC

AAGAAAGCATATCGGAAACGGGCCTTGATGCACCATCCAGATCGGCACAGTGGAGCCAGT

GCCGAGGTTCAGAAAGAAGAGGAGAAGAAGTTCAAGGAAGTTGGAGAGGCCTTTACCATC

CTTTCTGATCCCAAGAAAAAAACTCGTTATGACAGTGGACAGGACCTGGATGAAGAGGGC

ATGAATATGGGTGATTTTGATGCAAACAATATCTTCAAGGCATTCTTTGGAGGTCCTGGG

GGCTTCAGCTTTGAAGCCTCTGGTCCAGGGAATTTCTTCTTTCAGTTTGGC

>CL167.Contig3_A-W 29 1510 minus strand PREDICTED: dnaJ homolog subfamily C member 7 isoform 1 [Callithrix jacchus] >gi|403304474|ref|XP_003942821.1| PREDICTED: dnaJ homolog subfamily C member 7 [Saimiri boliviensis boliviensis]

ATGGCGGCTGCCGCGGAGTGCGATGTGGTAATGGCGGCGACCGAATCAGAGCTGCTCGAC

GACGAAGAAGCAAAAAGGGAAGCAGAATGCTTCAAGGAACAAGGAAATGCATACTATGCC

AAGAAAGATTACAATGAAGCTTATAACTATTATACAAAAGCCATAGATATGTGTCCTAAA

AATGCTAGCTATTATGGTAATCGAGCAGCCACGTTGATGATGCTTGGAAGGTTCCGGGAA

GCTCTTGGAGATGCACAACAGTCAGTGAGGTTGGATGACAGTTTTGTCCGGGGTCATCTA

CGAGAAGGCAAGTGCCACCTCTCCCTAGGGAATGCCATGGCAGCATGTCGTAGTTTCCAG

AGAGCCCTAGAACTGGATCACAAAAATGCTCAGGCACAACAAGAGTTCAAGAATGCTAAT

GCAGTCATGGAATATGAAAAAATAGCAGAAATGGATTTTGAGAAGCGAGATTTTCGGAAG

GTTGTTTTCTGCATGGACCGTGCCCTAGAATTTGCCCCTGCTTGCCATCGCTTCAAAATC

CTCAAAGCAGAATGTTTAGCAATGCTGGGTCGATATCCAGAAGCACAGTCTGTAGCCAGT

GACATTTTACGAATGGATTCCACCAATGCAGATGCTCTGTATGTACGGGGTCTTTGCCTT

TATTATGAAGATTGTATTGAGAAGGCGGTTCAGTTTTTTGTACAGGCTCTCAGGATGGCT

CCTGACCATGAGAAGGCTTGCATTGCTTGCAGAAATGCGAAAGCACTCAAAGCAAAGAAA

GAAGATGGAAATAAAGCATTTAAGGAAGGAAATTACAAGCTAGCATATGAACTGTACACA

GAAGCCCTCGGGATAGACCCCAACAATATAAAAACAAATGCTAAACTCTACTGTAATCGG

GGTACGGTTAATTCCAAGCTTAGGAAACTAGATGATGCAATAGAAGACTGCACACATGCA

GTGAAGCTCGATGACACATATATAAAAGCCTACTTGAGAAGAGCTCAGTGTTACATGGAC

ACAGAGCAGTATGAAGAAGCAGTGCGGGACTATGAAAAAGTGTATCAGACGGAGAAAACA

AAAGAACACAAACAGCTCCTAAAAAATGCACAACTGGAACTGAAGAAGAGTAAGAGGAAA

GATTACTACAAGATTCTGGGAGTGGACAAGAATGCCTCTGAGGATGAGATCAAGAAAGCA

TATCGGAAACGGGCCTTGATGCACCATCCAGATCGGCACAGTGGAGCCAGTGCCGAGGTT

CAGAAAGAAGAGGAGAAGAAGTTCAAGGAAGTTGGAGAGGCCTTTACCATCCTTTCTGAT

CCCAAGAAAAAAACTCGTTATGACAGTGGACAGGACCTGGATGAAGAGGGCATGAATATG

GGTGATTTTGATGCAAACAATATCTTCAAGGCATTCTTTGGAGGTCCTGGGGGCTTCAGC

TTTGAAGCCTCTGGTCCAGGGAATTTCTTCTTTCAGTTTGGC

>CL167.Contig4_A-W 1469 1720 minus strand PREDICTED: dnaJ homolog subfamily C member 7 isoform 1 [Callithrix jacchus] >gi|403304474|ref|XP_003942821.1| PREDICTED: dnaJ homolog subfamily C member 7 [Saimiri boliviensis boliviensis]

GATCGGCACAGTGGAGCCAGTGCCGAGGTTCAGAAAGAAGAGGAGAAGAAGTTCAAGGAA

GTTGGAGAGGCCTTTACCATCCTTTCTGATCCCAAGAAAAAAACTCGTTATGACAGTGGA

CAGGACCTGGATGAAGAGGGCATGAATATGGGTGATTTTGATGCAAACAATATCTTCAAG

GCATTCTTTGGAGGTCCTGGGGGCTTCAGCTTTGAAGCCTCTGGTCCAGGGAATTTCTTC

TTTCAGTTTGGC

>CL167.Contig5_A-W 931 1182 minus strand PREDICTED: dnaJ homolog subfamily C member 7 isoform 2 [Sus scrofa] >gi|335297638|ref|XP_003358088.1| PREDICTED: dnaJ homolog subfamily C member 7 isoform 3 [Sus scrofa]

GATCGGCACAGTGGAGCCAGTGCCGAGGTTCAGAAAGAAGAGGAGAAGAAGTTCAAGGAA

GTTGGAGAGGCCTTTACCATCCTTTCTGATCCCAAGAAAAAAACTCGTTATGACAGTGGA

CAGGACCTGGATGAAGAGGGCATGAATATGGGTGATTTTGATGCAAACAATATCTTCAAG

GCATTCTTTGGAGGTCCTGGGGGCTTCAGCTTTGAAGCCTCTGGTCCAGGGAATTTCTTC

TTTCAGTTTGGC

>CL167.Contig6_A-W 886 1137 minus strand PREDICTED: dnaJ homolog subfamily C member 7 isoform 2 [Sus scrofa] >gi|335297638|ref|XP_003358088.1| PREDICTED: dnaJ homolog subfamily C member 7 isoform 3 [Sus scrofa]

GATCGGCACAGTGGAGCCAGTGCCGAGGTTCAGAAAGAAGAGGAGAAGAAGTTCAAGGAA

GTTGGAGAGGCCTTTACCATCCTTTCTGATCCCAAGAAAAAAACTCGTTATGACAGTGGA

CAGGACCTGGATGAAGAGGGCATGAATATGGGTGATTTTGATGCAAACAATATCTTCAAG

GCATTCTTTGGAGGTCCTGGGGGCTTCAGCTTTGAAGCCTCTGGTCCAGGGAATTTCTTC

TTTCAGTTTGGC

>CL167.Contig7_A-W 88 972 minus strand PREDICTED: dnaJ homolog subfamily C member 7 isoform 2 [Sus scrofa] >gi|335297638|ref|XP_003358088.1| PREDICTED: dnaJ homolog subfamily C member 7 isoform 3 [Sus scrofa]

AGTGACATTTTACGAATGGATTCCACCAATGCAGATGCTCTGTATGTACGGGGTCTTTGC

CTTTATTATGAAGATTGTATTGAGAAGGCGGTTCAGTTTTTTGTACAGGCTCTCAGGATG

GCTCCTGACCATGAGAAGGCTTGCATTGCTTGCAGAAATGCGAAAGCACTCAAAGCAAAG

AAAGAAGATGGAAATAAAGCATTTAAGGAAGGAAATTACAAGCTAGCATATGAACTGTAC

ACAGAAGCCCTCGGGATAGACCCCAACAATATAAAAACAAATGCTAAACTCTACTGTAAT

CGGGGTACGGTTAATTCCAAGCTTAGGAAACTAGATGATGCAATAGAAGACTGCACACAT

GCAGTGAAGCTCGATGACACATATATAAAAGCCTACTTGAGAAGAGCTCAGTGTTACATG

GACACAGAGCAGTATGAAGAAGCAGTGCGGGACTATGAAAAAGTGTATCAGACGGAGAAA

ACAAAAGAACACAAACAGCTCCTAAAAAATGCACAACTGGAACTGAAGAAGAGTAAGAGG

AAAGATTACTACAAGATTCTGGGAGTGGACAAGAATGCCTCTGAGGATGAGATCAAGAAA

GCATATCGGAAACGGGCCTTGATGCACCATCCAGATCGGCACAGTGGAGCCAGTGCCGAG

GTTCAGAAAGAAGAGGAGAAGAAGTTCAAGGAAGTTGGAGAGGCCTTTACCATCCTTTCT

GATCCCAAGAAAAAAACTCGTTATGACAGTGGACAGGACCTGGATGAAGAGGGCATGAAT

ATGGGTGATTTTGATGCAAACAATATCTTCAAGGCATTCTTTGGAGGTCCTGGGGGCTTC

AGCTTTGAAGCCTCTGGTCCAGGGAATTTCTTCTTTCAGTTTGGC

>CL167.Contig8_A-W 199 927 minus strand PREDICTED: dnaJ homolog subfamily C member 7 isoform 2 [Sus scrofa] >gi|335297638|ref|XP_003358088.1| PREDICTED: dnaJ homolog subfamily C member 7 isoform 3 [Sus scrofa]

AATGCGAAAGCACTCAAAGCAAAGAAAGAAGATGGAAATAAAGCATTTAAGGAAGGAAAT

TACAAGCTAGCATATGAACTGTACACAGAAGCCCTCGGGATAGACCCCAACAATATAAAA

ACAAATGCTAAACTCTACTGTAATCGGGGTACGGTTAATTCCAAGCTTAGGAAACTAGAT

GATGCAATAGAAGACTGCACACATGCAGTGAAGCTCGATGACACATATATAAAAGCCTAC

TTGAGAAGAGCTCAGTGTTACATGGACACAGAGCAGTATGAAGAAGCAGTGCGGGACTAT

GAAAAAGTGTATCAGACGGAGAAAACAAAAGAACACAAACAGCTCCTAAAAAATGCACAA

CTGGAACTGAAGAAGAGTAAGAGGAAAGATTACTACAAGATTCTGGGAGTGGACAAGAAT

GCCTCTGAGGATGAGATCAAGAAAGCATATCGGAAACGGGCCTTGATGCACCATCCAGAT

CGGCACAGTGGAGCCAGTGCCGAGGTTCAGAAAGAAGAGGAGAAGAAGTTCAAGGAAGTT

GGAGAGGCCTTTACCATCCTTTCTGATCCCAAGAAAAAAACTCGTTATGACAGTGGACAG

GACCTGGATGAAGAGGGCATGAATATGGGTGATTTTGATGCAAACAATATCTTCAAGGCA

TTCTTTGGAGGTCCTGGGGGCTTCAGCTTTGAAGCCTCTGGTCCAGGGAATTTCTTCTTT

CAGTTTGGC

>CL167.Contig9_A-W 122 514 minus strand PREDICTED: DnaJ (Hsp40) homolog, subfamily C, member 7 [Oryctolagus cuniculus]

AAGGAACAAGGAAATGCATACTATGCCAAGAAAGATTACAATGAAGCTTATAACTATTAT

ACAAAAGCCATAGATATGTGTCCTAAAAATGCTAGCTATTATGGTAATCGAGCAGCCACG

TTGATGATGCTTGGAAGGTTCCGGGAAGCTCTTGGAGATGCACAACAGTCAGTGAGGTTG

GATGACAGTTTTGTCCGGGGTCATCTACGAGAAGGCAAGTGCCACCTCTCCCTAGGGAAT

GCCATGGCAGCATGTCGTAGTTTCCAGAGAGCCCTAGAACTGGATCACAAAAATGCTCAG

GCACAACAAGAGTTCAAGAATGCTAATGCAGTCATGGAATATGAAAAAATAGCAGAAATG

GATTTTGAGAAGCGAGATTTTCGGAAGGTTGTT

>CL168.Contig1_A-W 139 312 CAAX prenyl protease 1-like protein [Heterocephalus glaber]

TATTCATGGGTTATTCTGACCTATTTTCAGGTTCTAGTCACAATCTATGCTGATTATATT

GCCCCCTTATTTGACAAATTCACACCTCTGCCTGAGGGAAAGCTTAAACAAGAAATTGAA

GTAATGGCAAAGAATATTGACTTTCCTTTGACTAAGGTGTATGTTGTTGAAGGT

>CL168.Contig2_A-W 2 265 PREDICTED: CAAX prenyl protease 1 homolog [Cavia porcellus]

AAATTTATTGTGACTCAGTGTATTTTATTGCCTGTGTCTTCACTTCTGCTTTACATTATT

AAAATTGGAGGTGACTATTTTTTTATATATGCCTGGCTCTTCACATTAGTTGTTTCTCTG

GTTCTAGTCACAATCTATGCTGATTATATTGCCCCCTTATTTGACAAATTCACACCTCTG

CCTGAGGGAAAGCTTAAACAAGAAATTGAAGTAATGGCAAAGAATATTGACTTTCCTTTG

ACTAAGGTGTATGTTGTTGAAGGT

>CL169.Contig1_A-W 2 883 PREDICTED: fatty acid desaturase 3-like [Oryctolagus cuniculus]

ATCGTCTACCTCCTGGGCCCTGGCTGGGTGCCCAGCACCCTTGCTGCCCTCATCCTGGCC

GTCTCCCAGGCCCAGTGCTGGTGCCTGCAGCATGACCTGGGCCACGCCTCCATCTTCAGG

AAGTCCCGGTGGAACCACGTGGCCCAGCAGTTTGTGATGGGACAGCTGAAGGGCTTCTCG

GCCCACTGGTGGAACTTCCGCCACTTCCAGCACCACGCCAAGCCCAACATCTTCCACAAG

GACCCAGACGTGACCGTGGCACCTGTCTTCCTCCTGGGGGAGTCGTCTGTCGAGTACGGC

AAGAAGAAACGCAGATACCTTCCTTACAACCACCAGCACCTGTACTTCTTCCTGATTGGC

CCGCCGCTGCTCACCTTGGTGAACTTTGAAGTGGAGAATCTGGCGTACATGCTGGTGTGC

CTGCAGTGGACAGACTTGCTCTGGGCTGCCAGCTTCTACTCCCGCTTCTTCTTGTCCTAC

GTCCCCTTCTACGGTGTCCCCGGGGCCCTGCTTCTCTTTGTTGCCGTCAGGGTCCTGGAG

AGCCACTGGTTCGTGTGGATCACACAGATGAACCACATCCCCAAGGAGATCGGCCACGAG

AAGCACCGGGACTGGGCCAGCTCTCAGCTGGCAGCCACCTGCAACGTGGAGCCCTCGCTC

TTCATCGACTGGTTCAGCGGGCACCTCAACTTCCAGATCGAGCACCACCTCTTCCCCACC

ATGCCAAGGCACAACTACCGCAGGGTGGCCCCTCTGGTCAAGGCGCTGTGTGCCAAGCAT

GGCCTGCACTATGAGGTGAAGCCCTTCCTCACTGCCCTGCTGGACATCGTCGGGTCCCTG

AAGAAGTCTGGCGACATCTGGCTGGATGCCTACCTCCACCAG

>CL169.Contig2_A-W 44 754 PREDICTED: LOW QUALITY PROTEIN: fatty acid desaturase 3 [Canis lupus familiaris]

GGCTTCTCGGCCCACTGGTGGAACTTCCGCCACTTCCAGCACCACGCCAAGCCCAACATC

TTCCACAAGGACCCAGACGTGACCGTGGCACCTGTCTTCCTCCTGGGGGAGTCGTCTGTC

GAGTACGGCAAGAAGAAACGCAGATACCTTCCTTACAACCACCAGCACCTGTACTTCTTC

CTGATTGGCCCGCCGCTGCTCACCTTGGTGAACTTTGAAGTGGAGAATCTGGCGTACATG

CTGGTGTGCCTGCAGTGGACAGACTTGCTCTGGGCTGCCAGCTTCTACTCCCGCTTCTTC

TTGTCCTACGTCCCCTTCTACGGTGTCCCCGGGGCCCTGCTTCTCTTTGTTGCCGTCAGG

GTCCTGGAGAGCCACTGGTTCGTGTGGATCACACAGATGAACCACATCCCCAAGGAGATC

GGCCACGAGAAGCACCGGGACTGGGCCAGCTCTCAGCTGGCAGCCACCTGCAACGTGGAG

CCCTCGCTCTTCATCGACTGGTTCAGCGGGCACCTCAACTTCCAGATCGAGCACCACCTC

TTCCCCACCATGCCAAGGCACAACTACCGCAGGGTGGCCCCTCTGGTCAAGGCGCTGTGT

GCCAAGCATGGCCTGCACTATGAGGTGAAGCCCTTCCTCACTGCCCTGCTGGACATCGTC

GGGTCCCTGAAGAAGTCTGGCGACATCTGGCTGGATGCCTACCTCCACCAG

>CL169.Contig3_A-W 2 433 PREDICTED: fatty acid desaturase 3-like [Oryctolagus cuniculus]

ATCGTCTACCTCCTGGGCCCTGGCTGGGTGCCCAGCACCCTTGCTGCCCTCATCCTGGCC

GTCTCCCAGGCCCAGTGCTGGTGCCTGCAGCATGACCTGGGCCACGCCTCCATCTTCAGG

AAGTCCCGGTGGAACCACGTGGCCCAGCAGTTTGTGATGGGACAGCTGAAGGGCTTCTCG

GCCCACTGGTGGAACTTCCGCCACTTCCAGCACCACGCCAAGCCCAACATCTTCCACAAG

GACCCAGACGTGACCGTGGCACCTGTCTTCCTCCTGGGGGAGTCGTCTGTCGAGTACGGC

AAGAAGAAACGCAGATACCTTCCTTACAACCACCAGCACCTGTACTTCTTCCTGATTGGC

CCGCCGCTGCTCACCTTGGTGAACTTTGAAGTGGAGAATCTGGCGTACATGCTGGTGTGC

CTGCAGTGGACA

>CL169.Contig4_A-W 44 328 PREDICTED: fatty acid desaturase 3 [Pongo abelii]

GGCTTCTCGGCCCACTGGTGGAACTTCCGCCACTTCCAGCACCACGCCAAGCCCAACATC

TTCCACAAGGACCCAGACGTGACCGTGGCACCTGTCTTCCTCCTGGGGGAGTCGTCTGTC

GAGTACGGCAAGAAGAAACGCAGATACCTTCCTTACAACCACCAGCACCTGTACTTCTTC

CTGATTGGCCCGCCGCTGCTCACCTTGGTGAACTTTGAAGTGGAGAATCTGGCGTACATG

CTGGTGTGCCTGCAGTGGACAGTGAGTGGGGGATGCTCAGGACCC

>CL169.Contig5_A-W 2 295 PREDICTED: fatty acid desaturase 3-like [Oryctolagus cuniculus]

ATCGTCTACCTCCTGGGCCCTGGCTGGGTGCCCAGCACCCTTGCTGCCCTCATCCTGGCC

GTCTCCCAGGCCCAGTGCTGGTGCCTGCAGCATGACCTGGGCCACGCCTCCATCTTCAGG

AAGTCCCGGTGGAACCACGTGGCCCAGCAGTTTGTGATGGGACAGCTGAAGGGCTTCTCG

GCCCACTGGTGGAACTTCCGCCACTTCCAGCACCACGCCAAGCCCAACATCTTCCACAAG

GACCCAGACGTGACCGTGGCACCTGTCTTCCTCCTGGGGGAGTCGTCTGTCGAG

>CL169.Contig6_A-W 44 166 PREDICTED: LOW QUALITY PROTEIN: fatty acid desaturase 3 [Canis lupus familiaris]

GGCTTCTCGGCCCACTGGTGGAACTTCCGCCACTTCCAGCACCACGCCAAGCCCAACATC

TTCCACAAGGACCCAGACGTGACCGTGGCACCTGTCTTCCTCCTGGGGGAGTCGTCTGTC

GAG

>CL169.Contig7_A-W 41 490 PREDICTED: LOW QUALITY PROTEIN: fatty acid desaturase 3 [Canis lupus familiaris]

GACTTGCTCTGGGCTGCCAGCTTCTACTCCCGCTTCTTCTTGTCCTACGTCCCCTTCTAC

GGTGTCCCCGGGGCCCTGCTTCTCTTTGTTGCCGTCAGGGTCCTGGAGAGCCACTGGTTC

GTGTGGATCACACAGATGAACCACATCCCCAAGGAGATCGGCCACGAGAAGCACCGGGAC

TGGGCCAGCTCTCAGCTGGCAGCCACCTGCAACGTGGAGCCCTCGCTCTTCATCGACTGG

TTCAGCGGGCACCTCAACTTCCAGATCGAGCACCACCTCTTCCCCACCATGCCAAGGCAC

AACTACCGCAGGGTGGCCCCTCTGGTCAAGGCGCTGTGTGCCAAGCATGGCCTGCACTAT

GAGGTGAAGCCCTTCCTCACTGCCCTGCTGGACATCGTCGGGTCCCTGAAGAAGTCTGGC

GACATCTGGCTGGATGCCTACCTCCACCAG

>CL170.Contig1_A-W 2 841 minus strand Nuclear inhibitor of protein phosphatase 1 [Heterocephalus glaber]

TTGTGTGACTTTACCATTGACCATCAATCTTGCTCTCGGGTCCATGCTGCATTGGTCTAC

CACAAGCATCTGAAGAGAGTTTTCCTGATAGATCTCAACAGTACACATGGCACTTTCTTA

GGTCACATTCGGTTGGAACCTCACAAGCCACAACAAATTCCCATCGATTCCACAGTCTCA

TTTGGCGCTTCCACAAGGGCATACACTCTGCGCGAGAAGCCTCAGACGTTGCCATCAGCT

GTCAAAGGAGATGAAAAGATGAGTGGAGAGGATGATGAACTCAAGGGCCTGTTGGGGCTT

CCAGAGGAAGAAACTGAGCTTGATAATCTGACAGAGTTCAACACTGCCCACAACAAACGG

ATTTCTACCCTCACCATTGAGGAAGGAAATCTGGACATTCAGAGACCAAAGAGGAAGAGG

AAGAACTCACGGGTGACTTTCAGCGAAGATGATGAGATCATCAACCCAGAGGATGTGGAT

CCCTCAGTTGGTCGCTTCCGGAACATGGTGCAAACTGCAGTAGTCCCAGTGAAGAAAAAG

TGAGTGGAGGGCCCTGGATCCCTAGCCTGGAGGAATGAGGGAGCAGGCACATGCAGAATT

TTGCCTCTAGTGGAGGATTCTATGGGGGATTTCCCCCAACACAGTGAAGCAGGCTCCCAG

CCGCATGGCATTCACAGGACAGCACTTGTGGGTGGCTTGCCCATGCCATACCCGAACCTT

GCTCCTGATGTGGACTTGACTCCCATCGTGCCATCAGCAGTGAAAATGAACCACACACCA

AATCCTGCAGTCTATAACCCTGAAGCTGTTCATGAGCCCAAGAAGAAAAAATATGCAGAG

>CL170.Contig2_A-W 22 246 Nuclear inhibitor of protein phosphatase 1 OS=Mus musculus GN=Ppp1r8 PE=1 SV=1

AAGCCACAACAAATTCCACAGTCTCATTTGGCACTTCCACAAAGGCACACACTGCACAAG

AAACCTCAGACATTGCCATCAGCTGTGAAAGGAGAGGAAAAGATGAATGAAGATTATGAA

TTCAAGGACCCATTGGGACTTCCAGAGAAAGAAACTGAGCTTGATAACCTGACAGAGCTC

AACACTGTCCACAAGAAGTGGATTTCTATCCTCACATTAAGGAAG

>CL170.Contig3_A-W 1 321 minus strand PREDICTED: nuclear inhibitor of protein phosphatase 1 [Pan paniscus]

CAAATTCCCATCGATTCCACAGTCTCATTTGGCGCTTCCACAAGGGCATACACTCTGCGC

GAGAAGCCTCAGACGTTGCCATCAGCTGTCAAAGGAGATGAAAAGATGAGTGGAGAGGAT

GATGAACTCAAGGGCCTGTTGGGGCTTCCAGAGGAAGAAACTGAGCTTGATAATCTGACA

GAGTTCAACACTGCCCACAACAAACGGATTTCTACCCTCACCATTGAGGAAGGAAATCTG

GACATTCAGAGACCAAAGAGGAAGAGGAAGAACTCACGGGTGACTTTCAGCGAAGATGAT

GAGATCATCAACCCAGGTGAG

>CL170.Contig4_A-W 3 329 minus strand Nuclear inhibitor of protein phosphatase 1 [Heterocephalus glaber]

GTAAAGAAGAAGCGGGTGGAGGGCCCTGGCTCCCTGGGCCTGGAGGAATCAGGGAGCAGG

CGCATGCAGAACTTTGCCTTCAGTGGAGGACTCTACGGGGGCCTGCCCCCCACACACAGT

GAAGCAGGCTCCCAGCCGCATGGCATTCATGGGACAGCACTCATCGGTGGCTTGCCCATG

CCATACCCGAACCTCGCCCCTGATGTGGACTTGACTCCCGTCGTACCATCAGCAGTGAAC

ATGAACCCCACACCAAATCCTGCAGTCTATAACCCTGAAGCTGTTAATGAACCCAAGAAG

AAGAAATATGCAAAAGAGGCTTGGCCG

>CL171.Contig1_A-W 3 2000 minus strand PREDICTED: far upstream element-binding protein 2-like [Sus scrofa]

CGCCAGATTGCAGCCAAAATTGGAGGTGATGCTGCTACAACCGTGAATAACAGCACTCCT

GATTTTGGTTTTGGGGGCCAAAAGAGACAGTTGGAAGATGGAGATCAGCCGGAGAGCAAG

AAGCTGGCTTCCCAGGGAGACTCAATCAGTTCTCAACTTGGACCTATCCATCCTCCCCCA

AGGACTTCAATGACAGAAGAGTACAGGGTCCCAGATGGCATGGTGGGCCTAATCATTGGC

AGAGGAGGCGAACAAATTAACAAAATCCAGCAAGATTCAGGCTGCAAAGTACAGATCTCT

CCAGACAGTGGTGGCCTACCTGAGCGCAGCGTGTCCCTGACAGGAGCCCCTGAGTCTGTC

CAGAAAGCGAAGATGATGCTGGATGACATTGTGTCTCGGGGTCGTGGGGGTCCCCCAGGA

CAGTTCCACGACAATGCCAATGGGGGCCAGAATGGCACTGTGCAGGAGATCATGATCCCT

GCAGGCAAGGCTGGCCTGGTCATCGGCAAAGGCGGGGAGACAATTAAGCAGCTACAGGAA

CGTGCTGGAGTGAAGATGATCTTGATTCAGGACGGTTCCCAGAATACGAATGTGGACAAG

CCCCTCCGTATCATTGGGGATCCTTATAAAGTGCAGCAAGCTTGTGAGATGGTGATGGAC

ATCCTCCGAGAACGTGACCAAGGAGGCTTTGGGGATCGAAATGAGTATGGATCCCGGATT

GGTGGGGGAATTGATGTGCCTGTGCCCAGGCATTCTGTTGGGGTGGTCATTGGCCGGAGT

GGAGAAATGATCAAGAAGATTCAGAATGATGCTGGTGTGCGGATACAGTTCAAGCAAGAT

GACGGGACGGGACCTGAAAAGATCGCTCACATCATGGGTCCCCCAGACAGATGTGAGCAT

GCTGCCCGGATTATCAATGATCTCCTCCAGAGCCTCAGGAGTGGTCCCCCGGGCCCTCCT

GGGGGCCCTGGCATGCCCCCCGGGGGCCGGGGCCGAGGACGAGGCCAAGGCAGCTGGGGC

CCTCCTGGTGGGGAGATGACCTTCTCCATTCCCACCCACAAATGTGGGCTGGTCATTGGC

AGAGGCGGCGAGAATGTGAAAGCCATAAACCAACAAACGGGCGCCTTTGTGGAGATCTCT

CGGCAGCTGCCACCCAATGGAGACCCCAACTTCAAACTGTTCATCATCCGGGGCTCACCC

CAGCAGATTGACCATGCCAAGCAGCTCATTGAGGAAAAGATTGAGGGTCCGCTCTGCCCA

GTTGGACCAGGCCCTGGGGGACCAGGCCCTGCTGGCCCCATGGGGCCCTTCAACCCTGGG

CCCTTCAATCAGGGGCCACCAGGGGCTCCCCCACATGCTGGTGGCCCCCCTCCTCACCAG

TACCCGCCCCAAGGCTGGGGCAATACCTATCCCCAATGGCAACCGCCTGCTCCTCATGAC

CCAAATAAGGCTGCTGCAGCAGCTGCAGACCCCAACGCCGCCTGGGCCGCCTACTACTCA

CACTACTACCAGCAGCCCCCAGGCCCTGTTCCTGGCCCCGCCCCGGCTCCTGCAGCACCG

CCCACTCAGGGGGAGCCTCCGCAGCCCCCACCCACTGGCCAATCAGACTACACTAAGGCC

TGGGAAGAGTATTACAAAAAGATAGGCCAGCAGCCCCAGCAGCCCGGAGCACCCCCGCAG

CAAGACTACACGAAGGCCTGGGAGGAGTACTACAAGAAGCAAGCGCAAGTGGCCACCGGA

GGGGGTCCCGGAGCACCCCCAGGATCCCAGCCAGACTATAGTGCCGCCTGGGCGGAGTAT

TACAGACAGCAGGCCGCTTACTACGGACAGACTCCAGGTCCTGGTGGCCCCCAGCCGCCT

CCCACCCAGCAGGGACAGCAGCAGGCAAGTGGGAATTGCCACCCTCCTCCTCCTCCTTTC

TCCTTCCAACCCCCGGCCACCGTCCATCCTGCCTTAGTGGGTAGCGCCGGAAACCCCTTC

CCCTGCGGGGTGTGCCCT

>CL171.Contig2_A-W 3 2321 minus strand PREDICTED: far upstream element-binding protein 2-like [Bos taurus]

CGCCAGATTGCAGCCAAAATTGGAGGTGATGCTGCTACAACCGTGAATAACAGCACTCCT

GATTTTGGTTTTGGGGGCCAAAAGAGACAGTTGGAAGATGGAGATCAGCCGGAGAGCAAG

AAGCTGGCTTCCCAGGGAGACTCAATCAGTTCTCAACTTGGACCTATCCATCCTCCCCCA

AGGACTTCAATGACAGAAGAGTACAGGGTCCCAGATGGCATGGTGGGCCTAATCATTGGC

AGAGGAGGCGAACAAATTAACAAAATCCAGCAAGATTCAGGCTGCAAAGTACAGATCTCT

CCAGACAGTGGTGGCCTACCTGAGCGCAGCGTGTCCCTGACAGGAGCCCCTGAGTCTGTC

CAGAAAGCGAAGATGATGCTGGATGACATTGTGTCTCGGGGTCGTGGGGGTCCCCCAGGA

CAGTTCCACGACAATGCCAATGGGGGCCAGAATGGCACTGTGCAGGAGATCATGATCCCT

GCAGGCAAGGCTGGCCTGGTCATCGGCAAAGGCGGGGAGACAATTAAGCAGCTACAGGAA

CGTGCTGGAGTGAAGATGATCTTGATTCAGGACGGTTCCCAGAATACGAATGTGGACAAG

CCCCTCCGTATCATTGGGGATCCTTATAAAGTGCAGCAAGCTTGTGAGATGGTGATGGAC

ATCCTCCGAGAACGTGACCAAGGAGGCTTTGGGGATCGAAATGAGTATGGATCCCGGATT

GGTGGGGGAATTGATGTGCCTGTGCCCAGGCATTCTGTTGGGGTGGTCATTGGCCGGAGT

GGAGAAATGATCAAGAAGATTCAGAATGATGCTGGTGTGCGGATACAGTTCAAGCAAGAT

GACGGGACGGGACCTGAAAAGATCGCTCACATCATGGGTCCCCCAGACAGATGTGAGCAT

GCTGCCCGGATTATCAATGATCTCCTCCAGAGCCTCAGGAGTGGTCCCCCGGGCCCTCCT

GGGGGCCCTGGCATGCCCCCCGGGGGCCGGGGCCGAGGACGAGGCCAAGGCAGCTGGGGC

CCTCCTGGTGGGGAGATGACCTTCTCCATTCCCACCCACAAATGTGGGCTGGTCATTGGC

AGAGGCGGCGAGAATGTGAAAGCCATAAACCAACAAACGGGCGCCTTTGTGGAGATCTCT

CGGCAGCTGCCACCCAATGGAGACCCCAACTTCAAACTGTTCATCATCCGGGGCTCACCC

CAGCAGATTGACCATGCCAAGCAGCTCATTGAGGAAAAGATTGAGGGTCCGCTCTGCCCA

GTTGGACCAGGCCCTGGGGGACCAGGCCCTGCTGGCCCCATGGGGCCCTTCAACCCTGGG

CCCTTCAATCAGGGGCCACCAGGGGCTCCCCCACATGCTGGTGGCCCCCCTCCTCACCAG

TACCCGCCCCAAGGCTGGGGCAATACCTATCCCCAATGGCAACCGCCTGCTCCTCATGAC

CCAAATAAGGCTGCTGCAGCAGCTGCAGACCCCAACGCCGCCTGGGCCGCCTACTACTCA

CACTACTACCAGCAGCCCCCAGGCCCTGTTCCTGGCCCCGCCCCGGCTCCTGCAGCACCG

CCCACTCAGGGGGAGCCTCCGCAGCCCCCACCCACTGGCCAATCAGACTACACTAAGGCC

TGGGAAGAGTATTACAAAAAGATAGGTGAGTCTGTAGTGCAGCTGGGGTGGTTCCAGGCC

CACCCAGGAAGGGCATCCTCTGACCTCACTTGGCCCCCACAGGCCAGCAGCCCCAGCAGC

CCGGAGCACCCCCGCAGCAAGACTACACGAAGGCCTGGGAGGAGTACTACAAGAAGCAAG

CGCAAGTGGCCACCGGAGGGGGTCCCGGAGCACCCCCAGGATCCCAGCCAGACTATAGTG

CCGCCTGGGCGGAGTATTACAGACAGCAGGCCGCTTACTACGGACAGACTCCAGGTCCTG

GTGGCCCCCAGCCGCCTCCCACCCAGCAGGGACAGCAGCAGGCAAGTGGGAATTGCCACC

CTCCTCCTCCTCCTTTCTCCTTCCAACCCCCGGCCACCGTCCATCCTGCCTTAGTGGGTA

GCGCCGGAAACCCCTTCCCCTGCGGGGTGTGCCCTTGATGCCTGCAGCGGGGCCGTGTGG

CAGAGGTCTCCGGGAGTGCCCACGCGCCCGCCGGGAAGCGCACTCTGGGTCCAGAGGTTA

ACGAAGAGGCCTCCCTCCGCCAGCTGCTTGTTCCTGTGTAATGCTGTCGCGATGCTGGGG

GAGCGCGAATCACACAAAGGTGGAACTTGTGAACTGGTGGGTAGTCCTGGGGGTGGGGGA

CAAGCAAGCAGCACCCGTGTTGGTCGCCATCCCGGCTGC

>CL171.Contig3_A-W 90 977 minus strand PREDICTED: far upstream element-binding protein 2-like [Anolis carolinensis]

CGAGGACGAGGCCAAGGCAGCTGGGGCCCTCCTGGTGGGGAGATGACCTTCTCCATTCCC

ACCCACAAATGTGGGCTGGTCATTGGCAGAGGCGGCGAGAATGTGAAAGCCATAAACCAA

CAAACGGGCGCCTTTGTGGAGATCTCTCGGCAGCTGCCACCCAATGGAGACCCCAACTTC

AAACTGTTCATCATCCGGGGCTCACCCCAGCAGATTGACCATGCCAAGCAGCTCATTGAG

GAAAAGATTGAGGGTCCGCTCTGCCCAGTTGGACCAGGCCCTGGGGGACCAGGCCCTGCT

GGCCCCATGGGGCCCTTCAACCCTGGGCCCTTCAATCAGGGGCCACCAGGGGCTCCCCCA

CATGCTGGTGGCCCCCCTCCTCACCAGTACCCGCCCCAAGGCTGGGGCAATACCTATCCC

CAATGGCAACCGCCTGCTCCTCATGACCCAAATAAGGCTGCTGCAGCAGCTGCAGACCCC

AACGCCGCCTGGGCCGCCTACTACTCACACTACTACCAGCAGCCCCCAGGCCCTGTTCCT

GGCCCCGCCCCGGCTCCTGCAGCACCGCCCACTCAGGGGGAGCCTCCGCAGCCCCCACCC

ACTGGCCAATCAGACTACACTAAGGCCTGGGAAGAGTATTACAAAAAGATAGGTGAGTCT

GTAGTGCAGCTGGGGTGGTTCCAGGCCCACCCAGGAAGGGCATCCTCTGACCTCACTTGG

CCCCCACAGGCCAGCAGCCCCAGCAGCCCGGAGCACCCCCGCAGCAAGACTACACGAAGG

CCTGGGAGGAGTACTACAAGAAGCAAGCGCAAGTGGCCACCGGAGGGGGTCCCGGAGCAC

CCCCAGGATCCCAGCCAGACTATAGTGCCGCCTGGGCGGAGTATTACA

>CL171.Contig4_A-W 18 1094 minus strand PREDICTED: far upstream element-binding protein 2 [Equus caballus]

CTCCTCCCTGTCTGGCAGAGTGGTCCCCCGGGCCCTCCTGGGGGCCCTGGCATGCCCCCC

GGGGGCCGGGGCCGAGGACGAGGCCAAGGCAGCTGGGGCCCTCCTGGTGGGGAGATGACC

TTCTCCATTCCCACCCACAAATGTGGGCTGGTCATTGGCAGAGGCGGCGAGAATGTGAAA

GCCATAAACCAACAAACGGGCGCCTTTGTGGAGATCTCTCGGCAGCTGCCACCCAATGGA

GACCCCAACTTCAAACTGTTCATCATCCGGGGCTCACCCCAGCAGATTGACCATGCCAAG

CAGCTCATTGAGGAAAAGATTGAGGGTCCGCTCTGCCCAGTTGGACCAGGCCCTGGGGGA

CCAGGCCCTGCTGGCCCCATGGGGCCCTTCAACCCTGGGCCCTTCAATCAGGGGCCACCA

GGGGCTCCCCCACATGCTGGTGGCCCCCCTCCTCACCAGTACCCGCCCCAAGGCTGGGGC

AATACCTATCCCCAATGGCAACCGCCTGCTCCTCATGACCCAAATAAGGCTGCTGCAGCA

GCTGCAGACCCCAACGCCGCCTGGGCCGCCTACTACTCACACTACTACCAGCAGCCCCCA

GGCCCTGTTCCTGGCCCCGCCCCGGCTCCTGCAGCACCGCCCACTCAGGGGGAGCCTCCG

CAGCCCCCACCCACTGGCCAATCAGACTACACTAAGGCCTGGGAAGAGTATTACAAAAAG

ATAGGCCAGCAGCCCCAGCAGCCCGGAGCACCCCCGCAGCAAGACTACACGAAGGCCTGG

GAGGAGTACTACAAGAAGCAAGCGCAAGTGGCCACCGGAGGGGGTCCCGGAGCACCCCCA

GGATCCCAGCCAGACTATAGTGCCGCCTGGGCGGAGTATTACAGACAGCAGGCCGCTTAC

TACGGACAGACTCCAGGTCCTGGTGGCCCCCAGCCGCCTCCCACCCAGCAGGGACAGCAG

CAGGCAAGTGGGAATTGCCACCCTCCTCCTCCTCCTTTCTCCTTCCAACCCCCGGCCACC

GTCCATCCTGCCTTAGTGGGTAGCGCCGGAAACCCCTTCCCCTGCGGGGTGTGCCCT

>CL171.Contig5_A-W 90 977 minus strand PREDICTED: far upstream element-binding protein 2-like [Anolis carolinensis]

CGAGGACGAGGCCAAGGCAGCTGGGGCCCTCCTGGTGGGGAGATGACCTTCTCCATTCCC

ACCCACAAATGTGGGCTGGTCATTGGCAGAGGCGGCGAGAATGTGAAAGCCATAAACCAA

CAAACGGGCGCCTTTGTGGAGATCTCTCGGCAGCTGCCACCCAATGGAGACCCCAACTTC

AAACTGTTCATCATCCGGGGCTCACCCCAGCAGATTGACCATGCCAAGCAGCTCATTGAG

GAAAAGATTGAGGGTCCGCTCTGCCCAGTTGGACCAGGCCCTGGGGGACCAGGCCCTGCT

GGCCCCATGGGGCCCTTCAACCCTGGGCCCTTCAATCAGGGGCCACCAGGGGCTCCCCCA

CATGCTGGTGGCCCCCCTCCTCACCAGTACCCGCCCCAAGGCTGGGGCAATACCTATCCC

CAATGGCAACCGCCTGCTCCTCATGACCCAAATAAGGCTGCTGCAGCAGCTGCAGACCCC

AACGCCGCCTGGGCCGCCTACTACTCACACTACTACCAGCAGCCCCCAGGCCCTGTTCCT

GGCCCCGCCCCGGCTCCTGCAGCACCGCCCACTCAGGGGGAGCCTCCGCAGCCCCCACCC

ACTGGCCAATCAGACTACACTAAGGCCTGGGAAGAGTATTACAAAAAGATAGGTGAGTCT

GTAGTGCAGCTGGGGTGGTTCCAGGCCCACCCAGGAAGGGCATCCTCTGACCTCACTTGG

CCCCCACAGGCCAGCAGCCCCAGCAGCCCGGAGCACCCCCGCAGCAAGACTACACGAAGG

CCTGGGAGGAGTACTACAAGAAGCAAGCGCAAGTGGCCACCGGAGGGGGTCCCGGAGCAC

CCCCAGGATCCCAGCCAGACTATAGTGCCGCCTGGGCGGAGTATTACA

>CL171.Contig6_A-W 18 983 minus strand PREDICTED: far upstream element-binding protein 2-like [Loxodonta africana]

CTCCTCCCTGTCTGGCAGAGTGGTCCCCCGGGCCCTCCTGGGGGCCCTGGCATGCCCCCC

GGGGGCCGGGGCCGAGGACGAGGCCAAGGCAGCTGGGGCCCTCCTGGTGGGGAGATGACC

TTCTCCATTCCCACCCACAAATGTGGGCTGGTCATTGGCAGAGGCGGCGAGAATGTGAAA

GCCATAAACCAACAAACGGGCGCCTTTGTGGAGATCTCTCGGCAGCTGCCACCCAATGGA

GACCCCAACTTCAAACTGTTCATCATCCGGGGCTCACCCCAGCAGATTGACCATGCCAAG

CAGCTCATTGAGGAAAAGATTGAGGGTCCGCTCTGCCCAGTTGGACCAGGCCCTGGGGGA

CCAGGCCCTGCTGGCCCCATGGGGCCCTTCAACCCTGGGCCCTTCAATCAGGGGCCACCA

GGGGCTCCCCCACATGCTGGTGGCCCCCCTCCTCACCAGTACCCGCCCCAAGGCTGGGGC

AATACCTATCCCCAATGGCAACCGCCTGCTCCTCATGACCCAAATAAGGCTGCTGCAGCA

GCTGCAGACCCCAACGCCGCCTGGGCCGCCTACTACTCACACTACTACCAGCAGCCCCCA

GGCCCTGTTCCTGGCCCCGCCCCGGCTCCTGCAGCACCGCCCACTCAGGGGGAGCCTCCG

CAGCCCCCACCCACTGGCCAATCAGACTACACTAAGGCCTGGGAAGAGTATTACAAAAAG

ATAGGCCAGCAGCCCCAGCAGCCCGGAGCACCCCCGCAGCAAGACTACACGAAGGCCTGG

GAGGAGTACTACAAGAAGCAAGCGCAAGTGGCCACCGGAGGGGGTCCCGGAGCACCCCCA

GGATCCCAGCCAGACTATAGTGCCGCCTGGGCGGAGTATTACAGACAGCAGGCCGCTTAC

TACGGACAGACTCCAGGTCCTGGTGGCCCCCAGCCGCCTCCCACCCAGCAGGGACAGCAG

CAGGCT

>CL172.Contig1_A-W 53 1021 PREDICTED: phosphatidylinositol 4,5-bisphosphate 3-kinase catalytic subunit delta isoform [Papio anubis]

CAGGGGGAAGCACTCAGTAAGCTGAAGGCACTGAATGACTTTGTGAAGGTGAGCTCTCAG

AAGACCACCAAACCCCAAACCAAGGAGCTCATGCACTTGTGCATGCGCCAGGAGACCTAC

CTGGAGGCCCTCTCACACCTGCAATCCCCACTGGACCCCAGCACACTTCTGGCAGAAGTC

TGTGTGGAGCAGTGCACCTTTATGGACTCCAAGATGAAACCTTTGTGGATCATGTACAGC

AGCGAGGAGGCGGGCAGCGCTGGCAGCGTGGGCATCATCTTTAAGAATGGGGATGACCTC

CGCCAGGACATGCTGACTCTGCAGATGATCCAGCTCATGGACGTCCTGTGGAAGCAGGAG

GGCCTGGACCTAAGGATGACCCCCTACGGCTGCCTGCCCACTGGTGACCGCACGGGGCTC

ATTGAGGTGGTGCTGCACTCGGACACCATTGCCAACATCCAGCTGAACAAGAGCAACATG

GCCGCCACGGCCGCCTTCAACAAGGACGCCCTGCTCAACTGGCTCAAGTCCAAGAACCCT

GGAGAGGCCCTAGATCGAGCCATTGAGGAGTTCACTCTCTCCTGTGCTGGCTACTGTGTG

GCCACCTACGTGTTGGGCATCGGCGATCGGCACAGTGACAACATCATGATCCGTGAGAGT

GGGCAGCTCTTCCACATTGACTTTGGCCACTTTCTGGGGAATTTCAAGACCAAGTTTGGA

ATCAACCGTGAGCGAGTCCCATTCATCCTCACCTACGACTTTGTCCACGTGATTCAGCAG

GGCAAGACTAGTAACAGTGAGAAGTTTGAAAGGTTCCGGGGCTACTGTGAGCGGGCCTAC

ACCATCCTGCGGCGCCACGGGCTCCTCTTCCTCCACCTCTTTGCTCTGATGCGGGCAGCA

GGCCTGCCTGAGCTCAGCTGCTCCAAAGACATCCAGTATTTGAAGGTATGCCAGCCCAGG

GCCACACCA

>CL172.Contig2_A-W 1 1860 Chain A, The Crystal Structure Of The Murine Class Ia Pi 3-Kinase P110delta In Complex With Pik-39. >gi|283806782|pdb|2WXG|A Chain A, The Crystal Structure Of The Murine Class Ia Pi 3-Kinase P110delta In Complex With Sw13. >gi|283806783|pdb|2WXH|A Chain A, The Crystal Structure Of The Murine Class Ia Pi 3-Kinase P110delta In Complex With Sw14. >gi|283806784|pdb|2WXI|A Chain A, The Crystal Structure Of The Murine Class Ia Pi 3-Kinase P110delta In Complex With Sw30. >gi|283806785|pdb|2WXJ|A Chain A, The Crystal Structure Of The Murine Class Ia Pi 3-Kinase P110delta In Complex With Ink654. >gi|283806786|pdb|2WXK|A Chain A, The Crystal Structure Of The Murine Class Ia Pi 3-Kinase P110delta In Complex With Ink666. >gi|283806787|pdb|2WXL|A Chain A, The Crystal Structure Of The Murine Class Ia Pi 3-Kinase P110delta In Complex With Zstk474. >gi|283806788|pdb|2WXM|A Chain A, The Crystal Structure Of The Murine Class Ia Pi 3-Kinase P110delta In Complex With Dl06. >gi|283806789|pdb|2WXN|A Chain A, The Crystal Structure Of The Murine Class Ia Pi 3-Kinase P110delta In Complex With Dl07. >gi|283806790|pdb|2WXO|A Chain A, The Crystal Structure Of The Murine Class Ia Pi 3-Kinase P110delta In Complex With As5. >gi|283806791|pdb|2WXP|A Chain A, The Crystal Structure Of The Murine Class Ia Pi 3-Kinase P110delta In Complex With Gdc-0941. >gi|283806792|pdb|2WXQ|A Chain A, The Crystal Structure Of The Murine Class Ia Pi 3-Kinase P110delta In Complex With As15. >gi|283806793|pdb|2WXR|A Chain A, The Crystal Structure Of The Murine Class Ia Pi 3-Kinase P110delta. >gi|285803182|pdb|2X38|A Chain A, The Crystal Structure Of The Murine Class Ia Pi 3-Kinase P110delta In Complex With Ic87114

GTGTACTACCCTGCCCTGGAGAAGATCCTGGAGCTGGGGCGTCACGGGGAGCGTGGGCGT

GCCACTGAGGAGGAGCAGCTGCAGCTGCGGGAAATCCTGGAGCGGCGGGGGTCGGGGGAG

CTATACGAGCACGAGAAGGACCTGGTGTGGAAGATGCGACATGAAGTGCAGGAGCGCTTC

CCAGAGGCGCTGGCCCGCCTGCTGCTGGTCACCAAGTGGAACAAGCACGAGGATGTGGCC

CAGATGCTCTACCTGCTGTGCTCCTGGCCCGAGCTGCCCGTCCTGAGTGCCCTGGAGCTG

CTGGACTTCAGCTTCCCCGACTGCCACGTGGGCTCCTTCGCCATCAAGTCCCTGCGGAAA

CTGACGGACGACGAGCTTTTCCAGTACTTGCTGCAGCTGGTGCAGGTCCTCAAGTATGAG

TCCTACCTGGACTGCGAGCTGACCAAATTCTTGCTGGACCGGGCCCTGGCCAACCGCAAG

ATCGGCCACTTCCTCTTCTGGCATCTCCGCTCTGAGATGCACGTGCCGTCAGTGGCCTTG

CGCTTTGGCCTCATCATGGAGGCCTACTGCAGGGGCAGCACCCACCACATGAAGGTGCTG

ATGAAGCAGGGGGAAGCACTCAGTAAGCTGAAGGCACTGAATGACTTTGTGAAGGTGAGC

TCTCAGAAGACCACCAAACCCCAAACCAAGGAGCTCATGCACTTGTGCATGCGCCAGGAG

ACCTACCTGGAGGCCCTCTCACACCTGCAATCCCCACTGGACCCCAGCACACTTCTGGCA

GAAGTCTGTGTGGAGCAGTGCACCTTTATGGACTCCAAGATGAAACCTTTGTGGATCATG

TACAGCAGCGAGGAGGCGGGCAGCGCTGGCAGCGTGGGCATCATCTTTAAGAATGGGGAT

GGTGAGCCAGGCTGGGCAGGGGACATGCTGAGGCCTTGGCCTGCCCGCCCAGGCCTGTAC

TACCCTGCGGGAGGGTCCCAGGGGGTACCCTGAGAAGACAGAGCTCAGGGACTCTGAGCA

TTGCAGGGGTGGATAGCCCACCAGGAACTCCCGCCCTTCCTCCTGGCCCCTGCAGACCTC

CGCCAGGACATGCTGACTCTGCAGATGATCCAGCTCATGGACGTCCTGTGGAAGCAGGAG

GGCCTGGACCTAAGGATGACCCCCTACGGCTGCCTGCCCACTGGTGACCGCACGGGGCTC

ATTGAGGTGGTGCTGCACTCGGACACCATTGCCAACATCCAGCTGAACAAGAGCAACATG

GCCGCCACGGCCGCCTTCAACAAGGACGCCCTGCTCAACTGGCTCAAGTCCAAGAACCCT

GGAGAGGCCCTAGATCGAGCCATTGAGGAGTTCACTCTCTCCTGTGCTGGCTACTGTGTG

GCCACCTACGTGTTGGGCATCGGCGATCGGCACAGTGACAACATCATGATCCGTGAGAGT

GGGCAGCTCTTCCACATTGACTTTGGCCACTTTCTGGGGAATTTCAAGACCAAGTTTGGA

ATCAACCGTGAGCGAGTCCCATTCATCCTCACCTACGACTTTGTCCACGTGATTCAGCAG

GGCAAGACTAGTAACAGTGAGAAGTTTGAAAGGTTCCGGGGCTACTGTGAGCGGGCCTAC

ACCATCCTGCGGCGCCACGGGCTCCTCTTCCTCCACCTCTTTGCTCTGATGCGGGCAGCA

GGCCTGCCTGAGCTCAGCTGCTCCAAAGACATCCAGTATTTGAAGGACTCTCTGGCGCTG

GGCAAGACGGAGGAGGAAGCGCTCAAGCACTTCCGGGTGAAGTTCAACGAAGCTCTGAGG

GAGAGCTGGAAGACCAAAGTGAACTGGCTGGCACACAACGTGTCCAAGGACAACCGGCAA

>CL172.Contig3_A-W 1 990 Chain A, The Crystal Structure Of The Murine Class Ia Pi 3-Kinase P110delta In Complex With Pik-39. >gi|283806782|pdb|2WXG|A Chain A, The Crystal Structure Of The Murine Class Ia Pi 3-Kinase P110delta In Complex With Sw13. >gi|283806783|pdb|2WXH|A Chain A, The Crystal Structure Of The Murine Class Ia Pi 3-Kinase P110delta In Complex With Sw14. >gi|283806784|pdb|2WXI|A Chain A, The Crystal Structure Of The Murine Class Ia Pi 3-Kinase P110delta In Complex With Sw30. >gi|283806785|pdb|2WXJ|A Chain A, The Crystal Structure Of The Murine Class Ia Pi 3-Kinase P110delta In Complex With Ink654. >gi|283806786|pdb|2WXK|A Chain A, The Crystal Structure Of The Murine Class Ia Pi 3-Kinase P110delta In Complex With Ink666. >gi|283806787|pdb|2WXL|A Chain A, The Crystal Structure Of The Murine Class Ia Pi 3-Kinase P110delta In Complex With Zstk474. >gi|283806788|pdb|2WXM|A Chain A, The Crystal Structure Of The Murine Class Ia Pi 3-Kinase P110delta In Complex With Dl06. >gi|283806789|pdb|2WXN|A Chain A, The Crystal Structure Of The Murine Class Ia Pi 3-Kinase P110delta In Complex With Dl07. >gi|283806790|pdb|2WXO|A Chain A, The Crystal Structure Of The Murine Class Ia Pi 3-Kinase P110delta In Complex With As5. >gi|283806791|pdb|2WXP|A Chain A, The Crystal Structure Of The Murine Class Ia Pi 3-Kinase P110delta In Complex With Gdc-0941. >gi|283806792|pdb|2WXQ|A Chain A, The Crystal Structure Of The Murine Class Ia Pi 3-Kinase P110delta In Complex With As15. >gi|283806793|pdb|2WXR|A Chain A, The Crystal Structure Of The Murine Class Ia Pi 3-Kinase P110delta. >gi|285803182|pdb|2X38|A Chain A, The Crystal Structure Of The Murine Class Ia Pi 3-Kinase P110delta In Complex With Ic87114

GTGTACTACCCTGCCCTGGAGAAGATCCTGGAGCTGGGGCGTCACGGGGAGCGTGGGCGT

GCCACTGAGGAGGAGCAGCTGCAGCTGCGGGAAATCCTGGAGCGGCGGGGGTCGGGGGAG

CTATACGAGCACGAGAAGGACCTGGTGTGGAAGATGCGACATGAAGTGCAGGAGCGCTTC

CCAGAGGCGCTGGCCCGCCTGCTGCTGGTCACCAAGTGGAACAAGCACGAGGATGTGGCC

CAGATGCTCTACCTGCTGTGCTCCTGGCCCGAGCTGCCCGTCCTGAGTGCCCTGGAGCTG

CTGGACTTCAGCTTCCCCGACTGCCACGTGGGCTCCTTCGCCATCAAGTCCCTGCGGAAA

CTGACGGACGACGAGCTTTTCCAGTACTTGCTGCAGCTGGTGCAGGTCCTCAAGTATGAG

TCCTACCTGGACTGCGAGCTGACCAAATTCTTGCTGGACCGGGCCCTGGCCAACCGCAAG

ATCGGCCACTTCCTCTTCTGGCATCTCCGCTCTGAGATGCACGTGCCGTCAGTGGCCTTG

CGCTTTGGCCTCATCATGGAGGCCTACTGCAGGGGCAGCACCCACCACATGAAGGTGCTG

ATGAAGCAGGGGGAAGCACTCAGTAAGCTGAAGGCACTGAATGACTTTGTGAAGGTGAGC

TCTCAGAAGACCACCAAACCCCAAACCAAGGAGCTCATGCACTTGTGCATGCGCCAGGAG

ACCTACCTGGAGGCCCTCTCACACCTGCAATCCCCACTGGACCCCAGCACACTTCTGGCA

GAAGTCTGTGTGGAGCAGTGCACCTTTATGGACTCCAAGATGAAACCTTTGTGGATCATG

TACAGCAGCGAGGAGGCGGGCAGCGCTGGCAGCGTGGGCATCATCTTTAAGAATGGGGAT

GACCTCCGCCAGGACATGCTGACTCTGCAGATGATCCAGCTCATGGACGTCCTGTGGAAG

CAGGAGGGCCTGGACCTAAGGTGGGGGCCT

>CL172.Contig4_A-W 206 502 PREDICTED: phosphatidylinositol 4,5-bisphosphate 3-kinase catalytic subunit delta isoform [Papio anubis]

AGGATGACCCCCTACGGCTGCCTGCCCACTGGTGACCGCACGGGGCTCATTGAGGTGGTG

CTGCACTCGGACACCATTGCCAACATCCAGCTGAACAAGAGCAACATGGCCGCCACGGCC

GCCTTCAACAAGGACGCCCTGCTCAACTGGCTCAAGTCCAAGAACCCTGGAGAGGCCCTA

GATCGAGCCATTGAGGAGTTCACTCTCTCCTGTGCTGGCTACTGTGTGGCCACCTACGTG

TTGGGCATCGGCGATCGGCACAGTGACAACATCATGATCCGTGAGAGTGGGCAGGTA

>CL172.Contig5_A-W 53 610 PREDICTED: phosphatidylinositol 4,5-bisphosphate 3-kinase catalytic subunit delta isoform [Pan paniscus]

CAGGGGGAAGCACTCAGTAAGCTGAAGGCACTGAATGACTTTGTGAAGGTGAGCTCTCAG

AAGACCACCAAACCCCAAACCAAGGAGCTCATGCACTTGTGCATGCGCCAGGAGACCTAC

CTGGAGGCCCTCTCACACCTGCAATCCCCACTGGACCCCAGCACACTTCTGGCAGAAGTC

TGTGTGGAGCAGTGCACCTTTATGGACTCCAAGATGAAACCTTTGTGGATCATGTACAGC

AGCGAGGAGGCGGGCAGCGCTGGCAGCGTGGGCATCATCTTTAAGAATGGGGATGGTGAG

CCAGGCTGGGCAGGGGACATGCTGAGGCCTTGGCCTGCCCGCCCAGGCCTGTACTACCCT

GCGGGAGGGTCCCAGGGGGTACCCTGAGAAGACAGAGCTCAGGGACTCTGAGCATTGCAG

GGGTGGATAGCCCACCAGGAACTCCCGCCCTTCCTCCTGGCCCCTGCAGACCTCCGCCAG

GACATGCTGACTCTGCAGATGATCCAGCTCATGGACGTCCTGTGGAAGCAGGAGGGCCTG

GACCTAAGGTGGGGGCCT

>CL172.Contig6_A-W 53 436 PREDICTED: phosphatidylinositol-4,5-bisphosphate 3-kinase catalytic subunit delta isoform [Cavia porcellus]

CAGGGGGAAGCACTCAGTAAGCTGAAGGCACTGAATGACTTTGTGAAGGTGAGCTCTCAG

AAGACCACCAAACCCCAAACCAAGGAGCTCATGCACTTGTGCATGCGCCAGGAGACCTAC

CTGGAGGCCCTCTCACACCTGCAATCCCCACTGGACCCCAGCACACTTCTGGCAGAAGTC

TGTGTGGAGCAGTGCACCTTTATGGACTCCAAGATGAAACCTTTGTGGATCATGTACAGC

AGCGAGGAGGCGGGCAGCGCTGGCAGCGTGGGCATCATCTTTAAGAATGGGGATGACCTC

CGCCAGGACATGCTGACTCTGCAGATGATCCAGCTCATGGACGTCCTGTGGAAGCAGGAG

GGCCTGGACCTAAGGTGGGGGCCT

>CL172.Contig7_A-W 1434 1856 Chain A, The Crystal Structure Of The Murine Class Ia Pi 3-Kinase P110delta In Complex With Pik-39. >gi|283806782|pdb|2WXG|A Chain A, The Crystal Structure Of The Murine Class Ia Pi 3-Kinase P110delta In Complex With Sw13. >gi|283806783|pdb|2WXH|A Chain A, The Crystal Structure Of The Murine Class Ia Pi 3-Kinase P110delta In Complex With Sw14. >gi|283806784|pdb|2WXI|A Chain A, The Crystal Structure Of The Murine Class Ia Pi 3-Kinase P110delta In Complex With Sw30. >gi|283806785|pdb|2WXJ|A Chain A, The Crystal Structure Of The Murine Class Ia Pi 3-Kinase P110delta In Complex With Ink654. >gi|283806786|pdb|2WXK|A Chain A, The Crystal Structure Of The Murine Class Ia Pi 3-Kinase P110delta In Complex With Ink666. >gi|283806787|pdb|2WXL|A Chain A, The Crystal Structure Of The Murine Class Ia Pi 3-Kinase P110delta In Complex With Zstk474. >gi|283806788|pdb|2WXM|A Chain A, The Crystal Structure Of The Murine Class Ia Pi 3-Kinase P110delta In Complex With Dl06. >gi|283806789|pdb|2WXN|A Chain A, The Crystal Structure Of The Murine Class Ia Pi 3-Kinase P110delta In Complex With Dl07. >gi|283806790|pdb|2WXO|A Chain A, The Crystal Structure Of The Murine Class Ia Pi 3-Kinase P110delta In Complex With As5. >gi|283806791|pdb|2WXP|A Chain A, The Crystal Structure Of The Murine Class Ia Pi 3-Kinase P110delta In Complex With Gdc-0941. >gi|283806792|pdb|2WXQ|A Chain A, The Crystal Structure Of The Murine Class Ia Pi 3-Kinase P110delta In Complex With As15. >gi|283806793|pdb|2WXR|A Chain A, The Crystal Structure Of The Murine Class Ia Pi 3-Kinase P110delta. >gi|285803182|pdb|2X38|A Chain A, The Crystal Structure Of The Murine Class Ia Pi 3-Kinase P110delta In Complex With Ic87114

TCCCCTCAGCTCTTCCACATTGACTTTGGCCACTTTCTGGGGAATTTCAAGACCAAGTTT

GGAATCAACCGTGAGCGAGTCCCATTCATCCTCACCTACGACTTTGTCCACGTGATTCAG

CAGGGCAAGACTAGTAACAGTGAGAAGTTTGAAAGGTTCCGGGGCTACTGTGAGCGGGCC

TACACCATCCTGCGGCGCCACGGGCTCCTCTTCCTCCACCTCTTTGCTCTGATGCGGGCA

GCAGGCCTGCCTGAGCTCAGCTGCTCCAAAGACATCCAGTATTTGAAGGACTCTCTGGCG

CTGGGCAAGACGGAGGAGGAAGCGCTCAAGCACTTCCGGGTGAAGTTCAACGAAGCTCTG

AGGGAGAGCTGGAAGACCAAAGTGAACTGGCTGGCACACAACGTGTCCAAGGACAACCGG

CAA

>CL172.Contig8_A-W 1 1686 Chain A, The Crystal Structure Of The Murine Class Ia Pi 3-Kinase P110delta In Complex With Pik-39. >gi|283806782|pdb|2WXG|A Chain A, The Crystal Structure Of The Murine Class Ia Pi 3-Kinase P110delta In Complex With Sw13. >gi|283806783|pdb|2WXH|A Chain A, The Crystal Structure Of The Murine Class Ia Pi 3-Kinase P110delta In Complex With Sw14. >gi|283806784|pdb|2WXI|A Chain A, The Crystal Structure Of The Murine Class Ia Pi 3-Kinase P110delta In Complex With Sw30. >gi|283806785|pdb|2WXJ|A Chain A, The Crystal Structure Of The Murine Class Ia Pi 3-Kinase P110delta In Complex With Ink654. >gi|283806786|pdb|2WXK|A Chain A, The Crystal Structure Of The Murine Class Ia Pi 3-Kinase P110delta In Complex With Ink666. >gi|283806787|pdb|2WXL|A Chain A, The Crystal Structure Of The Murine Class Ia Pi 3-Kinase P110delta In Complex With Zstk474. >gi|283806788|pdb|2WXM|A Chain A, The Crystal Structure Of The Murine Class Ia Pi 3-Kinase P110delta In Complex With Dl06. >gi|283806789|pdb|2WXN|A Chain A, The Crystal Structure Of The Murine Class Ia Pi 3-Kinase P110delta In Complex With Dl07. >gi|283806790|pdb|2WXO|A Chain A, The Crystal Structure Of The Murine Class Ia Pi 3-Kinase P110delta In Complex With As5. >gi|283806791|pdb|2WXP|A Chain A, The Crystal Structure Of The Murine Class Ia Pi 3-Kinase P110delta In Complex With Gdc-0941. >gi|283806792|pdb|2WXQ|A Chain A, The Crystal Structure Of The Murine Class Ia Pi 3-Kinase P110delta In Complex With As15. >gi|283806793|pdb|2WXR|A Chain A, The Crystal Structure Of The Murine Class Ia Pi 3-Kinase P110delta. >gi|285803182|pdb|2X38|A Chain A, The Crystal Structure Of The Murine Class Ia Pi 3-Kinase P110delta In Complex With Ic87114

GTGTACTACCCTGCCCTGGAGAAGATCCTGGAGCTGGGGCGTCACGGGGAGCGTGGGCGT

GCCACTGAGGAGGAGCAGCTGCAGCTGCGGGAAATCCTGGAGCGGCGGGGGTCGGGGGAG

CTATACGAGCACGAGAAGGACCTGGTGTGGAAGATGCGACATGAAGTGCAGGAGCGCTTC

CCAGAGGCGCTGGCCCGCCTGCTGCTGGTCACCAAGTGGAACAAGCACGAGGATGTGGCC

CAGATGCTCTACCTGCTGTGCTCCTGGCCCGAGCTGCCCGTCCTGAGTGCCCTGGAGCTG

CTGGACTTCAGCTTCCCCGACTGCCACGTGGGCTCCTTCGCCATCAAGTCCCTGCGGAAA

CTGACGGACGACGAGCTTTTCCAGTACTTGCTGCAGCTGGTGCAGGTCCTCAAGTATGAG

TCCTACCTGGACTGCGAGCTGACCAAATTCTTGCTGGACCGGGCCCTGGCCAACCGCAAG

ATCGGCCACTTCCTCTTCTGGCATCTCCGCTCTGAGATGCACGTGCCGTCAGTGGCCTTG

CGCTTTGGCCTCATCATGGAGGCCTACTGCAGGGGCAGCACCCACCACATGAAGGTGCTG

ATGAAGCAGGGGGAAGCACTCAGTAAGCTGAAGGCACTGAATGACTTTGTGAAGGTGAGC

TCTCAGAAGACCACCAAACCCCAAACCAAGGAGCTCATGCACTTGTGCATGCGCCAGGAG

ACCTACCTGGAGGCCCTCTCACACCTGCAATCCCCACTGGACCCCAGCACACTTCTGGCA

GAAGTCTGTGTGGAGCAGTGCACCTTTATGGACTCCAAGATGAAACCTTTGTGGATCATG

TACAGCAGCGAGGAGGCGGGCAGCGCTGGCAGCGTGGGCATCATCTTTAAGAATGGGGAT

GACCTCCGCCAGGACATGCTGACTCTGCAGATGATCCAGCTCATGGACGTCCTGTGGAAG

CAGGAGGGCCTGGACCTAAGGATGACCCCCTACGGCTGCCTGCCCACTGGTGACCGCACG

GGGCTCATTGAGGTGGTGCTGCACTCGGACACCATTGCCAACATCCAGCTGAACAAGAGC

AACATGGCCGCCACGGCCGCCTTCAACAAGGACGCCCTGCTCAACTGGCTCAAGTCCAAG

AACCCTGGAGAGGCCCTAGATCGAGCCATTGAGGAGTTCACTCTCTCCTGTGCTGGCTAC

TGTGTGGCCACCTACGTGTTGGGCATCGGCGATCGGCACAGTGACAACATCATGATCCGT

GAGAGTGGGCAGCTCTTCCACATTGACTTTGGCCACTTTCTGGGGAATTTCAAGACCAAG

TTTGGAATCAACCGTGAGCGAGTCCCATTCATCCTCACCTACGACTTTGTCCACGTGATT

CAGCAGGGCAAGACTAGTAACAGTGAGAAGTTTGAAAGGTTCCGGGGCTACTGTGAGCGG

GCCTACACCATCCTGCGGCGCCACGGGCTCCTCTTCCTCCACCTCTTTGCTCTGATGCGG

GCAGCAGGCCTGCCTGAGCTCAGCTGCTCCAAAGACATCCAGTATTTGAAGGACTCTCTG

GCGCTGGGCAAGACGGAGGAGGAAGCGCTCAAGCACTTCCGGGTGAAGTTCAACGAAGCT

CTGAGGGAGAGCTGGAAGACCAAAGTGAACTGGCTGGCACACAACGTGTCCAAGGACAAC

CGGCAA

>CL172.Contig9_A-W 1054 1476 Chain A, Discovery And Optimization Of New Benzimidazole- And Benzoxazole- Pyrimidone Selective Pi3kbeta Inhibitors For The Treatment Of Phosphatase And Tensin Homologue (Pten)-Deficient Cancers >gi|387766252|pdb|4AJW|B Chain B, Discovery And Optimization Of New Benzimidazole- And Benzoxazole- Pyrimidone Selective Pi3kbeta Inhibitors For The Treatment Of Phosphatase And Tensin Homologue (Pten)-Deficient Cancers

TCCCCTCAGCTCTTCCACATTGACTTTGGCCACTTTCTGGGGAATTTCAAGACCAAGTTT

GGAATCAACCGTGAGCGAGTCCCATTCATCCTCACCTACGACTTTGTCCACGTGATTCAG

CAGGGCAAGACTAGTAACAGTGAGAAGTTTGAAAGGTTCCGGGGCTACTGTGAGCGGGCC

TACACCATCCTGCGGCGCCACGGGCTCCTCTTCCTCCACCTCTTTGCTCTGATGCGGGCA

GCAGGCCTGCCTGAGCTCAGCTGCTCCAAAGACATCCAGTATTTGAAGGACTCTCTGGCG

CTGGGCAAGACGGAGGAGGAAGCGCTCAAGCACTTCCGGGTGAAGTTCAACGAAGCTCTG

AGGGAGAGCTGGAAGACCAAAGTGAACTGGCTGGCACACAACGTGTCCAAGGACAACCGG

CAA

>CL172.Contig10_A-W 1608 2030 Chain A, The Crystal Structure Of The Murine Class Ia Pi 3-Kinase P110delta In Complex With Pik-39. >gi|283806782|pdb|2WXG|A Chain A, The Crystal Structure Of The Murine Class Ia Pi 3-Kinase P110delta In Complex With Sw13. >gi|283806783|pdb|2WXH|A Chain A, The Crystal Structure Of The Murine Class Ia Pi 3-Kinase P110delta In Complex With Sw14. >gi|283806784|pdb|2WXI|A Chain A, The Crystal Structure Of The Murine Class Ia Pi 3-Kinase P110delta In Complex With Sw30. >gi|283806785|pdb|2WXJ|A Chain A, The Crystal Structure Of The Murine Class Ia Pi 3-Kinase P110delta In Complex With Ink654. >gi|283806786|pdb|2WXK|A Chain A, The Crystal Structure Of The Murine Class Ia Pi 3-Kinase P110delta In Complex With Ink666. >gi|283806787|pdb|2WXL|A Chain A, The Crystal Structure Of The Murine Class Ia Pi 3-Kinase P110delta In Complex With Zstk474. >gi|283806788|pdb|2WXM|A Chain A, The Crystal Structure Of The Murine Class Ia Pi 3-Kinase P110delta In Complex With Dl06. >gi|283806789|pdb|2WXN|A Chain A, The Crystal Structure Of The Murine Class Ia Pi 3-Kinase P110delta In Complex With Dl07. >gi|283806790|pdb|2WXO|A Chain A, The Crystal Structure Of The Murine Class Ia Pi 3-Kinase P110delta In Complex With As5. >gi|283806791|pdb|2WXP|A Chain A, The Crystal Structure Of The Murine Class Ia Pi 3-Kinase P110delta In Complex With Gdc-0941. >gi|283806792|pdb|2WXQ|A Chain A, The Crystal Structure Of The Murine Class Ia Pi 3-Kinase P110delta In Complex With As15. >gi|283806793|pdb|2WXR|A Chain A, The Crystal Structure Of The Murine Class Ia Pi 3-Kinase P110delta. >gi|285803182|pdb|2X38|A Chain A, The Crystal Structure Of The Murine Class Ia Pi 3-Kinase P110delta In Complex With Ic87114

TCCCCTCAGCTCTTCCACATTGACTTTGGCCACTTTCTGGGGAATTTCAAGACCAAGTTT

GGAATCAACCGTGAGCGAGTCCCATTCATCCTCACCTACGACTTTGTCCACGTGATTCAG

CAGGGCAAGACTAGTAACAGTGAGAAGTTTGAAAGGTTCCGGGGCTACTGTGAGCGGGCC

TACACCATCCTGCGGCGCCACGGGCTCCTCTTCCTCCACCTCTTTGCTCTGATGCGGGCA

GCAGGCCTGCCTGAGCTCAGCTGCTCCAAAGACATCCAGTATTTGAAGGACTCTCTGGCG

CTGGGCAAGACGGAGGAGGAAGCGCTCAAGCACTTCCGGGTGAAGTTCAACGAAGCTCTG

AGGGAGAGCTGGAAGACCAAAGTGAACTGGCTGGCACACAACGTGTCCAAGGACAACCGG

CAA

>CL172.Contig11_A-W 53 1306 Chain A, Discovery And Optimization Of New Benzimidazole- And Benzoxazole- Pyrimidone Selective Pi3kbeta Inhibitors For The Treatment Of Phosphatase And Tensin Homologue (Pten)-Deficient Cancers >gi|387766252|pdb|4AJW|B Chain B, Discovery And Optimization Of New Benzimidazole- And Benzoxazole- Pyrimidone Selective Pi3kbeta Inhibitors For The Treatment Of Phosphatase And Tensin Homologue (Pten)-Deficient Cancers

CAGGGGGAAGCACTCAGTAAGCTGAAGGCACTGAATGACTTTGTGAAGGTGAGCTCTCAG

AAGACCACCAAACCCCAAACCAAGGAGCTCATGCACTTGTGCATGCGCCAGGAGACCTAC

CTGGAGGCCCTCTCACACCTGCAATCCCCACTGGACCCCAGCACACTTCTGGCAGAAGTC

TGTGTGGAGCAGTGCACCTTTATGGACTCCAAGATGAAACCTTTGTGGATCATGTACAGC

AGCGAGGAGGCGGGCAGCGCTGGCAGCGTGGGCATCATCTTTAAGAATGGGGATGGTGAG

CCAGGCTGGGCAGGGGACATGCTGAGGCCTTGGCCTGCCCGCCCAGGCCTGTACTACCCT

GCGGGAGGGTCCCAGGGGGTACCCTGAGAAGACAGAGCTCAGGGACTCTGAGCATTGCAG

GGGTGGATAGCCCACCAGGAACTCCCGCCCTTCCTCCTGGCCCCTGCAGACCTCCGCCAG

GACATGCTGACTCTGCAGATGATCCAGCTCATGGACGTCCTGTGGAAGCAGGAGGGCCTG

GACCTAAGGATGACCCCCTACGGCTGCCTGCCCACTGGTGACCGCACGGGGCTCATTGAG

GTGGTGCTGCACTCGGACACCATTGCCAACATCCAGCTGAACAAGAGCAACATGGCCGCC

ACGGCCGCCTTCAACAAGGACGCCCTGCTCAACTGGCTCAAGTCCAAGAACCCTGGAGAG

GCCCTAGATCGAGCCATTGAGGAGTTCACTCTCTCCTGTGCTGGCTACTGTGTGGCCACC

TACGTGTTGGGCATCGGCGATCGGCACAGTGACAACATCATGATCCGTGAGAGTGGGCAG

CTCTTCCACATTGACTTTGGCCACTTTCTGGGGAATTTCAAGACCAAGTTTGGAATCAAC

CGTGAGCGAGTCCCATTCATCCTCACCTACGACTTTGTCCACGTGATTCAGCAGGGCAAG

ACTAGTAACAGTGAGAAGTTTGAAAGGTTCCGGGGCTACTGTGAGCGGGCCTACACCATC

CTGCGGCGCCACGGGCTCCTCTTCCTCCACCTCTTTGCTCTGATGCGGGCAGCAGGCCTG

CCTGAGCTCAGCTGCTCCAAAGACATCCAGTATTTGAAGGACTCTCTGGCGCTGGGCAAG

ACGGAGGAGGAAGCGCTCAAGCACTTCCGGGTGAAGTTCAACGAAGCTCTGAGGGAGAGC

TGGAAGACCAAAGTGAACTGGCTGGCACACAACGTGTCCAAGGACAACCGGCAA

>CL172.Contig12_A-W 53 718 PREDICTED: phosphatidylinositol-4,5-bisphosphate 3-kinase catalytic subunit delta isoform, partial [Cricetulus griseus]

CAGGGGGAAGCACTCAGTAAGCTGAAGGCACTGAATGACTTTGTGAAGGTGAGCTCTCAG

AAGACCACCAAACCCCAAACCAAGGAGCTCATGCACTTGTGCATGCGCCAGGAGACCTAC

CTGGAGGCCCTCTCACACCTGCAATCCCCACTGGACCCCAGCACACTTCTGGCAGAAGTC

TGTGTGGAGCAGTGCACCTTTATGGACTCCAAGATGAAACCTTTGTGGATCATGTACAGC

AGCGAGGAGGCGGGCAGCGCTGGCAGCGTGGGCATCATCTTTAAGAATGGGGATGACCTC

CGCCAGGACATGCTGACTCTGCAGATGATCCAGCTCATGGACGTCCTGTGGAAGCAGGAG

GGCCTGGACCTAAGGATGACCCCCTACGGCTGCCTGCCCACTGGTGACCGCACGGGGCTC

ATTGAGGTGGTGCTGCACTCGGACACCATTGCCAACATCCAGCTGAACAAGAGCAACATG

GCCGCCACGGCCGCCTTCAACAAGGACGCCCTGCTCAACTGGCTCAAGTCCAAGAACCCT

GGAGAGGCCCTAGATCGAGCCATTGAGGAGTTCACTCTCTCCTGTGCTGGCTACTGTGTG

GCCACCTACGTGTTGGGCATCGGCGATCGGCACAGTGACAACATCATGATCCGTGAGAGT

GGGCAG

>CL172.Contig13_A-W 53 1132 Chain A, Discovery And Optimization Of New Benzimidazole- And Benzoxazole- Pyrimidone Selective Pi3kbeta Inhibitors For The Treatment Of Phosphatase And Tensin Homologue (Pten)-Deficient Cancers >gi|387766252|pdb|4AJW|B Chain B, Discovery And Optimization Of New Benzimidazole- And Benzoxazole- Pyrimidone Selective Pi3kbeta Inhibitors For The Treatment Of Phosphatase And Tensin Homologue (Pten)-Deficient Cancers

CAGGGGGAAGCACTCAGTAAGCTGAAGGCACTGAATGACTTTGTGAAGGTGAGCTCTCAG

AAGACCACCAAACCCCAAACCAAGGAGCTCATGCACTTGTGCATGCGCCAGGAGACCTAC

CTGGAGGCCCTCTCACACCTGCAATCCCCACTGGACCCCAGCACACTTCTGGCAGAAGTC

TGTGTGGAGCAGTGCACCTTTATGGACTCCAAGATGAAACCTTTGTGGATCATGTACAGC

AGCGAGGAGGCGGGCAGCGCTGGCAGCGTGGGCATCATCTTTAAGAATGGGGATGACCTC

CGCCAGGACATGCTGACTCTGCAGATGATCCAGCTCATGGACGTCCTGTGGAAGCAGGAG

GGCCTGGACCTAAGGATGACCCCCTACGGCTGCCTGCCCACTGGTGACCGCACGGGGCTC

ATTGAGGTGGTGCTGCACTCGGACACCATTGCCAACATCCAGCTGAACAAGAGCAACATG

GCCGCCACGGCCGCCTTCAACAAGGACGCCCTGCTCAACTGGCTCAAGTCCAAGAACCCT

GGAGAGGCCCTAGATCGAGCCATTGAGGAGTTCACTCTCTCCTGTGCTGGCTACTGTGTG

GCCACCTACGTGTTGGGCATCGGCGATCGGCACAGTGACAACATCATGATCCGTGAGAGT

GGGCAGCTCTTCCACATTGACTTTGGCCACTTTCTGGGGAATTTCAAGACCAAGTTTGGA

ATCAACCGTGAGCGAGTCCCATTCATCCTCACCTACGACTTTGTCCACGTGATTCAGCAG

GGCAAGACTAGTAACAGTGAGAAGTTTGAAAGGTTCCGGGGCTACTGTGAGCGGGCCTAC

ACCATCCTGCGGCGCCACGGGCTCCTCTTCCTCCACCTCTTTGCTCTGATGCGGGCAGCA

GGCCTGCCTGAGCTCAGCTGCTCCAAAGACATCCAGTATTTGAAGGACTCTCTGGCGCTG

GGCAAGACGGAGGAGGAAGCGCTCAAGCACTTCCGGGTGAAGTTCAACGAAGCTCTGAGG

GAGAGCTGGAAGACCAAAGTGAACTGGCTGGCACACAACGTGTCCAAGGACAACCGGCAA

>CL172.Contig14_A-W 1608 1895 Chain A, The Crystal Structure Of The Murine Class Ia Pi 3-Kinase P110delta In Complex With Pik-39. >gi|283806782|pdb|2WXG|A Chain A, The Crystal Structure Of The Murine Class Ia Pi 3-Kinase P110delta In Complex With Sw13. >gi|283806783|pdb|2WXH|A Chain A, The Crystal Structure Of The Murine Class Ia Pi 3-Kinase P110delta In Complex With Sw14. >gi|283806784|pdb|2WXI|A Chain A, The Crystal Structure Of The Murine Class Ia Pi 3-Kinase P110delta In Complex With Sw30. >gi|283806785|pdb|2WXJ|A Chain A, The Crystal Structure Of The Murine Class Ia Pi 3-Kinase P110delta In Complex With Ink654. >gi|283806786|pdb|2WXK|A Chain A, The Crystal Structure Of The Murine Class Ia Pi 3-Kinase P110delta In Complex With Ink666. >gi|283806787|pdb|2WXL|A Chain A, The Crystal Structure Of The Murine Class Ia Pi 3-Kinase P110delta In Complex With Zstk474. >gi|283806788|pdb|2WXM|A Chain A, The Crystal Structure Of The Murine Class Ia Pi 3-Kinase P110delta In Complex With Dl06. >gi|283806789|pdb|2WXN|A Chain A, The Crystal Structure Of The Murine Class Ia Pi 3-Kinase P110delta In Complex With Dl07. >gi|283806790|pdb|2WXO|A Chain A, The Crystal Structure Of The Murine Class Ia Pi 3-Kinase P110delta In Complex With As5. >gi|283806791|pdb|2WXP|A Chain A, The Crystal Structure Of The Murine Class Ia Pi 3-Kinase P110delta In Complex With Gdc-0941. >gi|283806792|pdb|2WXQ|A Chain A, The Crystal Structure Of The Murine Class Ia Pi 3-Kinase P110delta In Complex With As15. >gi|283806793|pdb|2WXR|A Chain A, The Crystal Structure Of The Murine Class Ia Pi 3-Kinase P110delta. >gi|285803182|pdb|2X38|A Chain A, The Crystal Structure Of The Murine Class Ia Pi 3-Kinase P110delta In Complex With Ic87114

TCCCCTCAGCTCTTCCACATTGACTTTGGCCACTTTCTGGGGAATTTCAAGACCAAGTTT

GGAATCAACCGTGAGCGAGTCCCATTCATCCTCACCTACGACTTTGTCCACGTGATTCAG

CAGGGCAAGACTAGTAACAGTGAGAAGTTTGAAAGGTTCCGGGGCTACTGTGAGCGGGCC

TACACCATCCTGCGGCGCCACGGGCTCCTCTTCCTCCACCTCTTTGCTCTGATGCGGGCA

GCAGGCCTGCCTGAGCTCAGCTGCTCCAAAGACATCCAGTATTTGAAG

>CL172.Contig15_A-W 206 502 PREDICTED: phosphatidylinositol 4,5-bisphosphate 3-kinase catalytic subunit delta isoform [Saimiri boliviensis boliviensis]

AGGATGACCCCCTACGGCTGCCTGCCCACTGGTGACCGCACGGGGCTCATTGAGGTGGTG

CTGCACTCGGACACCATTGCCAACATCCAGCTGAACAAGAGCAACATGGCCGCCACGGCC

GCCTTCAACAAGGACGCCCTGCTCAACTGGCTCAAGTCCAAGAACCCTGGAGAGGCCCTA

GATCGAGCCATTGAGGAGTTCACTCTCTCCTGTGCTGGCTACTGTGTGGCCACCTACGTG

TTGGGCATCGGCGATCGGCACAGTGACAACATCATGATCCGTGAGAGTGGGCAGGTA

>CL172.Contig16_A-W 1434 1721 Chain A, The Crystal Structure Of The Murine Class Ia Pi 3-Kinase P110delta In Complex With Pik-39. >gi|283806782|pdb|2WXG|A Chain A, The Crystal Structure Of The Murine Class Ia Pi 3-Kinase P110delta In Complex With Sw13. >gi|283806783|pdb|2WXH|A Chain A, The Crystal Structure Of The Murine Class Ia Pi 3-Kinase P110delta In Complex With Sw14. >gi|283806784|pdb|2WXI|A Chain A, The Crystal Structure Of The Murine Class Ia Pi 3-Kinase P110delta In Complex With Sw30. >gi|283806785|pdb|2WXJ|A Chain A, The Crystal Structure Of The Murine Class Ia Pi 3-Kinase P110delta In Complex With Ink654. >gi|283806786|pdb|2WXK|A Chain A, The Crystal Structure Of The Murine Class Ia Pi 3-Kinase P110delta In Complex With Ink666. >gi|283806787|pdb|2WXL|A Chain A, The Crystal Structure Of The Murine Class Ia Pi 3-Kinase P110delta In Complex With Zstk474. >gi|283806788|pdb|2WXM|A Chain A, The Crystal Structure Of The Murine Class Ia Pi 3-Kinase P110delta In Complex With Dl06. >gi|283806789|pdb|2WXN|A Chain A, The Crystal Structure Of The Murine Class Ia Pi 3-Kinase P110delta In Complex With Dl07. >gi|283806790|pdb|2WXO|A Chain A, The Crystal Structure Of The Murine Class Ia Pi 3-Kinase P110delta In Complex With As5. >gi|283806791|pdb|2WXP|A Chain A, The Crystal Structure Of The Murine Class Ia Pi 3-Kinase P110delta In Complex With Gdc-0941. >gi|283806792|pdb|2WXQ|A Chain A, The Crystal Structure Of The Murine Class Ia Pi 3-Kinase P110delta In Complex With As15. >gi|283806793|pdb|2WXR|A Chain A, The Crystal Structure Of The Murine Class Ia Pi 3-Kinase P110delta. >gi|285803182|pdb|2X38|A Chain A, The Crystal Structure Of The Murine Class Ia Pi 3-Kinase P110delta In Complex With Ic87114

TCCCCTCAGCTCTTCCACATTGACTTTGGCCACTTTCTGGGGAATTTCAAGACCAAGTTT

GGAATCAACCGTGAGCGAGTCCCATTCATCCTCACCTACGACTTTGTCCACGTGATTCAG

CAGGGCAAGACTAGTAACAGTGAGAAGTTTGAAAGGTTCCGGGGCTACTGTGAGCGGGCC

TACACCATCCTGCGGCGCCACGGGCTCCTCTTCCTCCACCTCTTTGCTCTGATGCGGGCA

GCAGGCCTGCCTGAGCTCAGCTGCTCCAAAGACATCCAGTATTTGAAG

>CL172.Contig17_A-W 206 913 Chain A, Discovery And Optimization Of New Benzimidazole- And Benzoxazole- Pyrimidone Selective Pi3kbeta Inhibitors For The Treatment Of Phosphatase And Tensin Homologue (Pten)-Deficient Cancers >gi|387766252|pdb|4AJW|B Chain B, Discovery And Optimization Of New Benzimidazole- And Benzoxazole- Pyrimidone Selective Pi3kbeta Inhibitors For The Treatment Of Phosphatase And Tensin Homologue (Pten)-Deficient Cancers

AGGATGACCCCCTACGGCTGCCTGCCCACTGGTGACCGCACGGGGCTCATTGAGGTGGTG

CTGCACTCGGACACCATTGCCAACATCCAGCTGAACAAGAGCAACATGGCCGCCACGGCC

GCCTTCAACAAGGACGCCCTGCTCAACTGGCTCAAGTCCAAGAACCCTGGAGAGGCCCTA

GATCGAGCCATTGAGGAGTTCACTCTCTCCTGTGCTGGCTACTGTGTGGCCACCTACGTG

TTGGGCATCGGCGATCGGCACAGTGACAACATCATGATCCGTGAGAGTGGGCAGCTCTTC

CACATTGACTTTGGCCACTTTCTGGGGAATTTCAAGACCAAGTTTGGAATCAACCGTGAG

CGAGTCCCATTCATCCTCACCTACGACTTTGTCCACGTGATTCAGCAGGGCAAGACTAGT

AACAGTGAGAAGTTTGAAAGGTTCCGGGGCTACTGTGAGCGGGCCTACACCATCCTGCGG

CGCCACGGGCTCCTCTTCCTCCACCTCTTTGCTCTGATGCGGGCAGCAGGCCTGCCTGAG

CTCAGCTGCTCCAAAGACATCCAGTATTTGAAGGACTCTCTGGCGCTGGGCAAGACGGAG

GAGGAAGCGCTCAAGCACTTCCGGGTGAAGTTCAACGAAGCTCTGAGGGAGAGCTGGAAG

ACCAAAGTGAACTGGCTGGCACACAACGTGTCCAAGGACAACCGGCAA

>CL172.Contig18_A-W 1 1551 Chain A, The Crystal Structure Of The Murine Class Ia Pi 3-Kinase P110delta In Complex With Pik-39. >gi|283806782|pdb|2WXG|A Chain A, The Crystal Structure Of The Murine Class Ia Pi 3-Kinase P110delta In Complex With Sw13. >gi|283806783|pdb|2WXH|A Chain A, The Crystal Structure Of The Murine Class Ia Pi 3-Kinase P110delta In Complex With Sw14. >gi|283806784|pdb|2WXI|A Chain A, The Crystal Structure Of The Murine Class Ia Pi 3-Kinase P110delta In Complex With Sw30. >gi|283806785|pdb|2WXJ|A Chain A, The Crystal Structure Of The Murine Class Ia Pi 3-Kinase P110delta In Complex With Ink654. >gi|283806786|pdb|2WXK|A Chain A, The Crystal Structure Of The Murine Class Ia Pi 3-Kinase P110delta In Complex With Ink666. >gi|283806787|pdb|2WXL|A Chain A, The Crystal Structure Of The Murine Class Ia Pi 3-Kinase P110delta In Complex With Zstk474. >gi|283806788|pdb|2WXM|A Chain A, The Crystal Structure Of The Murine Class Ia Pi 3-Kinase P110delta In Complex With Dl06. >gi|283806789|pdb|2WXN|A Chain A, The Crystal Structure Of The Murine Class Ia Pi 3-Kinase P110delta In Complex With Dl07. >gi|283806790|pdb|2WXO|A Chain A, The Crystal Structure Of The Murine Class Ia Pi 3-Kinase P110delta In Complex With As5. >gi|283806791|pdb|2WXP|A Chain A, The Crystal Structure Of The Murine Class Ia Pi 3-Kinase P110delta In Complex With Gdc-0941. >gi|283806792|pdb|2WXQ|A Chain A, The Crystal Structure Of The Murine Class Ia Pi 3-Kinase P110delta In Complex With As15. >gi|283806793|pdb|2WXR|A Chain A, The Crystal Structure Of The Murine Class Ia Pi 3-Kinase P110delta. >gi|285803182|pdb|2X38|A Chain A, The Crystal Structure Of The Murine Class Ia Pi 3-Kinase P110delta In Complex With Ic87114

GTGTACTACCCTGCCCTGGAGAAGATCCTGGAGCTGGGGCGTCACGGGGAGCGTGGGCGT

GCCACTGAGGAGGAGCAGCTGCAGCTGCGGGAAATCCTGGAGCGGCGGGGGTCGGGGGAG

CTATACGAGCACGAGAAGGACCTGGTGTGGAAGATGCGACATGAAGTGCAGGAGCGCTTC

CCAGAGGCGCTGGCCCGCCTGCTGCTGGTCACCAAGTGGAACAAGCACGAGGATGTGGCC

CAGATGCTCTACCTGCTGTGCTCCTGGCCCGAGCTGCCCGTCCTGAGTGCCCTGGAGCTG

CTGGACTTCAGCTTCCCCGACTGCCACGTGGGCTCCTTCGCCATCAAGTCCCTGCGGAAA

CTGACGGACGACGAGCTTTTCCAGTACTTGCTGCAGCTGGTGCAGGTCCTCAAGTATGAG

TCCTACCTGGACTGCGAGCTGACCAAATTCTTGCTGGACCGGGCCCTGGCCAACCGCAAG

ATCGGCCACTTCCTCTTCTGGCATCTCCGCTCTGAGATGCACGTGCCGTCAGTGGCCTTG

CGCTTTGGCCTCATCATGGAGGCCTACTGCAGGGGCAGCACCCACCACATGAAGGTGCTG

ATGAAGCAGGGGGAAGCACTCAGTAAGCTGAAGGCACTGAATGACTTTGTGAAGGTGAGC

TCTCAGAAGACCACCAAACCCCAAACCAAGGAGCTCATGCACTTGTGCATGCGCCAGGAG

ACCTACCTGGAGGCCCTCTCACACCTGCAATCCCCACTGGACCCCAGCACACTTCTGGCA

GAAGTCTGTGTGGAGCAGTGCACCTTTATGGACTCCAAGATGAAACCTTTGTGGATCATG

TACAGCAGCGAGGAGGCGGGCAGCGCTGGCAGCGTGGGCATCATCTTTAAGAATGGGGAT

GACCTCCGCCAGGACATGCTGACTCTGCAGATGATCCAGCTCATGGACGTCCTGTGGAAG

CAGGAGGGCCTGGACCTAAGGATGACCCCCTACGGCTGCCTGCCCACTGGTGACCGCACG

GGGCTCATTGAGGTGGTGCTGCACTCGGACACCATTGCCAACATCCAGCTGAACAAGAGC

AACATGGCCGCCACGGCCGCCTTCAACAAGGACGCCCTGCTCAACTGGCTCAAGTCCAAG

AACCCTGGAGAGGCCCTAGATCGAGCCATTGAGGAGTTCACTCTCTCCTGTGCTGGCTAC

TGTGTGGCCACCTACGTGTTGGGCATCGGCGATCGGCACAGTGACAACATCATGATCCGT

GAGAGTGGGCAGCTCTTCCACATTGACTTTGGCCACTTTCTGGGGAATTTCAAGACCAAG

TTTGGAATCAACCGTGAGCGAGTCCCATTCATCCTCACCTACGACTTTGTCCACGTGATT

CAGCAGGGCAAGACTAGTAACAGTGAGAAGTTTGAAAGGTTCCGGGGCTACTGTGAGCGG

GCCTACACCATCCTGCGGCGCCACGGGCTCCTCTTCCTCCACCTCTTTGCTCTGATGCGG

GCAGCAGGCCTGCCTGAGCTCAGCTGCTCCAAAGACATCCAGTATTTGAAG

>CL172.Contig19_A-W 1054 1341 Chain A, Discovery And Optimization Of New Benzimidazole- And Benzoxazole- Pyrimidone Selective Pi3kbeta Inhibitors For The Treatment Of Phosphatase And Tensin Homologue (Pten)-Deficient Cancers >gi|387766252|pdb|4AJW|B Chain B, Discovery And Optimization Of New Benzimidazole- And Benzoxazole- Pyrimidone Selective Pi3kbeta Inhibitors For The Treatment Of Phosphatase And Tensin Homologue (Pten)-Deficient Cancers

TCCCCTCAGCTCTTCCACATTGACTTTGGCCACTTTCTGGGGAATTTCAAGACCAAGTTT

GGAATCAACCGTGAGCGAGTCCCATTCATCCTCACCTACGACTTTGTCCACGTGATTCAG

CAGGGCAAGACTAGTAACAGTGAGAAGTTTGAAAGGTTCCGGGGCTACTGTGAGCGGGCC

TACACCATCCTGCGGCGCCACGGGCTCCTCTTCCTCCACCTCTTTGCTCTGATGCGGGCA

GCAGGCCTGCCTGAGCTCAGCTGCTCCAAAGACATCCAGTATTTGAAG

>CL172.Contig20_A-W 53 1171 Chain A, Discovery And Optimization Of New Benzimidazole- And Benzoxazole- Pyrimidone Selective Pi3kbeta Inhibitors For The Treatment Of Phosphatase And Tensin Homologue (Pten)-Deficient Cancers >gi|387766252|pdb|4AJW|B Chain B, Discovery And Optimization Of New Benzimidazole- And Benzoxazole- Pyrimidone Selective Pi3kbeta Inhibitors For The Treatment Of Phosphatase And Tensin Homologue (Pten)-Deficient Cancers

CAGGGGGAAGCACTCAGTAAGCTGAAGGCACTGAATGACTTTGTGAAGGTGAGCTCTCAG

AAGACCACCAAACCCCAAACCAAGGAGCTCATGCACTTGTGCATGCGCCAGGAGACCTAC

CTGGAGGCCCTCTCACACCTGCAATCCCCACTGGACCCCAGCACACTTCTGGCAGAAGTC

TGTGTGGAGCAGTGCACCTTTATGGACTCCAAGATGAAACCTTTGTGGATCATGTACAGC

AGCGAGGAGGCGGGCAGCGCTGGCAGCGTGGGCATCATCTTTAAGAATGGGGATGGTGAG

CCAGGCTGGGCAGGGGACATGCTGAGGCCTTGGCCTGCCCGCCCAGGCCTGTACTACCCT

GCGGGAGGGTCCCAGGGGGTACCCTGAGAAGACAGAGCTCAGGGACTCTGAGCATTGCAG

GGGTGGATAGCCCACCAGGAACTCCCGCCCTTCCTCCTGGCCCCTGCAGACCTCCGCCAG

GACATGCTGACTCTGCAGATGATCCAGCTCATGGACGTCCTGTGGAAGCAGGAGGGCCTG

GACCTAAGGATGACCCCCTACGGCTGCCTGCCCACTGGTGACCGCACGGGGCTCATTGAG

GTGGTGCTGCACTCGGACACCATTGCCAACATCCAGCTGAACAAGAGCAACATGGCCGCC

ACGGCCGCCTTCAACAAGGACGCCCTGCTCAACTGGCTCAAGTCCAAGAACCCTGGAGAG

GCCCTAGATCGAGCCATTGAGGAGTTCACTCTCTCCTGTGCTGGCTACTGTGTGGCCACC

TACGTGTTGGGCATCGGCGATCGGCACAGTGACAACATCATGATCCGTGAGAGTGGGCAG

CTCTTCCACATTGACTTTGGCCACTTTCTGGGGAATTTCAAGACCAAGTTTGGAATCAAC

CGTGAGCGAGTCCCATTCATCCTCACCTACGACTTTGTCCACGTGATTCAGCAGGGCAAG

ACTAGTAACAGTGAGAAGTTTGAAAGGTTCCGGGGCTACTGTGAGCGGGCCTACACCATC

CTGCGGCGCCACGGGCTCCTCTTCCTCCACCTCTTTGCTCTGATGCGGGCAGCAGGCCTG

CCTGAGCTCAGCTGCTCCAAAGACATCCAGTATTTGAAG

>CL172.Contig21_A-W 53 718 PREDICTED: phosphatidylinositol-4,5-bisphosphate 3-kinase catalytic subunit delta isoform, partial [Cricetulus griseus]

CAGGGGGAAGCACTCAGTAAGCTGAAGGCACTGAATGACTTTGTGAAGGTGAGCTCTCAG

AAGACCACCAAACCCCAAACCAAGGAGCTCATGCACTTGTGCATGCGCCAGGAGACCTAC

CTGGAGGCCCTCTCACACCTGCAATCCCCACTGGACCCCAGCACACTTCTGGCAGAAGTC

TGTGTGGAGCAGTGCACCTTTATGGACTCCAAGATGAAACCTTTGTGGATCATGTACAGC

AGCGAGGAGGCGGGCAGCGCTGGCAGCGTGGGCATCATCTTTAAGAATGGGGATGACCTC

CGCCAGGACATGCTGACTCTGCAGATGATCCAGCTCATGGACGTCCTGTGGAAGCAGGAG

GGCCTGGACCTAAGGATGACCCCCTACGGCTGCCTGCCCACTGGTGACCGCACGGGGCTC

ATTGAGGTGGTGCTGCACTCGGACACCATTGCCAACATCCAGCTGAACAAGAGCAACATG

GCCGCCACGGCCGCCTTCAACAAGGACGCCCTGCTCAACTGGCTCAAGTCCAAGAACCCT

GGAGAGGCCCTAGATCGAGCCATTGAGGAGTTCACTCTCTCCTGTGCTGGCTACTGTGTG

GCCACCTACGTGTTGGGCATCGGCGATCGGCACAGTGACAACATCATGATCCGTGAGAGT

GGGCAG

>CL175.Contig1_A-W 547 1824 integrin alpha-M isoform 2 precursor [Mus musculus] >gi|148685679|gb|EDL17626.1| integrin alpha M, isoform CRA_a [Mus musculus]

AGAGAACTGTTTCAGAGTGCCAATGGAGCCCGGGAAAATGCTGTTAAGATCCTAGTTGTC

ATCACAGATGGAGAAAAGTATCAAGATCCCCTGAATTATGAGGATGTCATCCCTGAGGCC

GACAGAAAGGGGGTCATTCGCTATGTCATTGGGGTGGGAGGTGCCTTCAACAATGACAGA

TCCCGTGAAGAGCTTAACACCATTGCCTCTAAGCCAGCTCGTGATCATGTGTTCCGGGTG

AATAACTTTGAAGCTCTAAAGACCATTCAAAACCAGCTTCAGGAAAAGATCTTTGCAATT

GAAGGAACTCAGACAGGAAGTACCAGCTCCTTTGAGCATGAGATGTCTCAGGAAGGCTTC

AGTGCTGCCATCACCTCTAATGGTCCTTTGCTGGGCTCCGTGGGGAGCTTTGACTGGGCT

GGTGGGGCCTTTTTGCATAGGTCCAAGAATGAAATCACCTTCATCAACACAACCAGGGTA

GACTCAGACATGAATGATGCTTACTTGGGTTATGCATCTGCAGTCATCTTGAGGAACCGG

GTGCAAAGCCTGGTTCTGGGTGCACCTCGATATCAACACATTGGCCTGGTGGCGATGTTC

AGACAGAATGCTGGTGCCTGGGAGACCAACACTATTATCAAGGGCAACCAGATTGGCTCC

TACTTTGGGGCTTCCCTCTGCTCGGTGGACATGGACGGTGATGGAAACACCAACCTGGTC

CTCATTGGGGCACCCCATTTCTATGAGCAGACCCGAGGGGGCCAGGTGTCCGTGTGTCCC

TTGCCTAAGGGGAGGGCTCGGTGGCGGTGTGAGGCTGTTCTCCACGGTGAGCAGGGCCAT

CCCTGGGGCCGCTTTGGGGCAGCTCTGACAGTGCTGGGAGATGTGAATGGGGACAAGCTG

ACAGACGTGGCTATTGGAGCCCCAGGAGAACAGGAGAATCGGGGTGCTATTTACATTTTT

CATGGAATATCAGTATTTGGCATCAGCTCCTCCCACAGCCAGCGGATTACAGGCTCCCAG

CTTTCCCCCCAGCTCCAGTATTTTGGTCAGTCACTGAGTGGCGGTCAGGACCTCACAATG

GATGGATTGGCGGACCTGGCTGTAGGGGCCCAGGGACACATACTGCTGCTCAGATCCCAG

CCAGTGCTAAGAGTTGTGGTGACCATGGAGTTCACACCCAAGGAAGTGGCCAGGGATGTA

TTTGAGTGTCATGAACAGGTGGTGACAAGCAAGGCTGCTGGGGAGGTCAGAGTCTGCCTC

CTTGTCCGCAAGAGCACA

>CL175.Contig2_A-W 93 1976 PREDICTED: integrin alpha-M [Otolemur garnettii]

TTCAGCCATGACTTGTAGAGTTCTTCTGTTAACAGGATTGTGGTTGGAGCCCCCCAGGAG

ATAAAAGCTGACAACCAAACAGGAGGCCTCTATCAATGTGAAATCAACACCCGCAAGTGT

GAGCCCATCCACTTTCAGGTCCCCCTGGAGGCTGTGAACATGTCCTTGGGCCTCTCCCTG

GCGACTGCCATCAACCCCTCACAGCTGCTGGCCTGTGGCCCCACTGTGCACCAAACTTGC

AAGGAGAACACTTATGTGAATGGCTTCTGCTTCCTATTTGGATCCAACCTACTGCAGCAG

CCCCAGAGGTTTCCAGAGGCCCTCAGAGAATGTCCTCAGCAGGAGAGCGACATTGCCTTT

TTGATTGATGGCTCTGGTAGCATCAATGAAAACGACTTTAGGAAGATGAAGGACTTTGTG

TCAACAGTGATGGACCAATTCAAAAAGTCTAAAACCTTGTTCTCTTTGATGCAGTACTCA

AATGACTTCCAGACTCATTTTACCTTCAATTATTTCAAGACAAATCCTAACCCAAGATTT

CTGGTAAACCAAATAAGACAACTGCATGGGACAACATACACTGCCACAGGAATCCGCAAA

GTAGTAAGAGAACTGTTTCAGAGTGCCAATGGAGCCCGGGAAAATGCTGTTAAGATCCTA

GTTGTCATCACAGATGGAGAAAAGTATCAAGATCCCCTGAATTATGAGGATGTCATCCCT

GAGGCCGACAGAAAGGGGGTCATTCGCTATGTCATTGGGGTGGGAGGTGCCTTCAACAAT

GACAGATCCCGTGAAGAGCTTAACACCATTGCCTCTAAGCCAGCTCGTGATCATGTGTTC

CGGGTGAATAACTTTGAAGCTCTAAAGACCATTCAAAACCAGCTTCAGGAAAAGATCTTT

GCAATTGAAGGAACTCAGACAGGAAGTACCAGCTCCTTTGAGCATGAGATGTCTCAGGAA

GGCTTCAGTGCTGCCATCACCTCTAATGGTCCTTTGCTGGGCTCCGTGGGGAGCTTTGAC

TGGGCTGGTGGGGCCTTTTTGCATAGGTCCAAGAATGAAATCACCTTCATCAACACAACC

AGGGTAGACTCAGACATGAATGATGCTTACTTGGGTTATGCATCTGCAGTCATCTTGAGG

AACCGGGTGCAAAGCCTGGTTCTGGGTGCACCTCGATATCAACACATTGGCCTGGTGGCG

ATGTTCAGACAGAATGCTGGTGCCTGGGAGACCAACACTATTATCAAGGGCAACCAGATT

GGCTCCTACTTTGGGGCTTCCCTCTGCTCGGTGGACATGGACGGTGATGGAAACACCAAC

CTGGTCCTCATTGGGGCACCCCATTTCTATGAGCAGACCCGAGGGGGCCAGGTGTCCGTG

TGTCCCTTGCCTAAGGGGAGGGCTCGGTGGCGGTGTGAGGCTGTTCTCCACGGTGAGCAG

GGCCATCCCTGGGGCCGCTTTGGGGCAGCTCTGACAGTGCTGGGAGATGTGAATGGGGAC

AAGCTGACAGACGTGGCTATTGGAGCCCCAGGAGAACAGGAGAATCGGGGTGCTATTTAC

ATTTTTCATGGAATATCAGTATTTGGCATCAGCTCCTCCCACAGCCAGCGGATTACAGGC

TCCCAGCTTTCCCCCCAGCTCCAGTATTTTGGTCAGTCACTGAGTGGCGGTCAGGACCTC

ACAATGGATGGATTGGCGGACCTGGCTGTAGGGGCCCAGGGACACATACTGCTGCTCAGA

TCCCAGCCAGTGCTAAGAGTTGTGGTGACCATGGAGTTCACACCCAAGGAAGTGGCCAGG

GATGTATTTGAGTGTCATGAACAGGTGGTGACAAGCAAGGCTGCTGGGGAGGTCAGAGTC

TGCCTCCTTGTCCGCAAGAGCACA

>CL175.Contig3_A-W 1495 2265 PREDICTED: integrin alpha-M-like [Ailuropoda melanoleuca]

GGTTATGCATCTGCAGTCATCTTGAGGAACCGGGTGCAAAGCCTGGTTCTGGGTGCACCT

CGATATCAACACATTGGCCTGGTGGCGATGTTCAGACAGAATGCTGGTGCCTGGGAGACC

AACACTATTATCAAGGGCAACCAGATTGGCTCCTACTTTGGGGCTTCCCTCTGCTCGGTG

GACATGGACGGTGATGGAAACACCAACCTGGTCCTCATTGGGGCACCCCATTTCTATGAG

CAGACCCGAGGGGGCCAGGTGTCCGTGTGTCCCTTGCCTAAGGGGAGGGCTCGGTGGCGG

TGTGAGGCTGTTCTCCACGGTGAGCAGGGCCATCCCTGGGGCCGCTTTGGGGCAGCTCTG

ACAGTGCTGGGAGATGTGAATGGGGACAAGCTGACAGACGTGGCTATTGGAGCCCCAGGA

GAACAGGAGAATCGGGGTGCTATTTACATTTTTCATGGAATATCAGTATTTGGCATCAGC

TCCTCCCACAGCCAGCGGATTACAGGCTCCCAGCTTTCCCCCCAGCTCCAGTATTTTGGT

CAGTCACTGAGTGGCGGTCAGGACCTCACAATGGATGGATTGGCGGACCTGGCTGTAGGG

GCCCAGGGACACATACTGCTGCTCAGATCCCAGCCAGTGCTAAGAGTTGTGGTGACCATG

GAGTTCACACCCAAGGAAGTGGCCAGGGATGTATTTGAGTGTCATGAACAGGTGGTGACA

AGCAAGGCTGCTGGGGAGGTCAGAGTCTGCCTCCTTGTCCGCAAGAGCACA

>CL175.Contig4_A-W 127 2082 Integrin alpha-M, partial [Heterocephalus glaber]

GCCTTGGCTTTGTGTCGTGCATTCAACCTGGACACTGAAAATCCAATGACCTTCCAAGAG

AACGCAAGGGGGTTTGGGCAGAGTGTGGTCCAGTTTGAGGGATCCCGGATTGTGGTTGGA

GCCCCCCAGGAGATAAAAGCTGACAACCAAACAGGAGGCCTCTATCAATGTGAAATCAAC

ACCCGCAAGTGTGAGCCCATCCACTTTCAGGTCCCCCTGGAGGCTGTGAACATGTCCTTG

GGCCTCTCCCTGGCGACTGCCATCAACCCCTCACAGCTGCTGGCCTGTGGCCCCACTGTG

CACCAAACTTGCAAGGAGAACACTTATGTGAATGGCTTCTGCTTCCTATTTGGATCCAAC

CTACTGCAGCAGCCCCAGAGGTTTCCAGAGGCCCTCAGAGAATGTCCTCAGCAGGAGAGC

GACATTGCCTTTTTGATTGATGGCTCTGGTAGCATCAATGAAAACGACTTTAGGAAGATG

AAGGACTTTGTGTCAACAGTGATGGACCAATTCAAAAAGTCTAAAACCTTGTTCTCTTTG

ATGCAGTACTCAAATGACTTCCAGACTCATTTTACCTTCAATTATTTCAAGACAAATCCT

AACCCAAGATTTCTGGTAAACCAAATAAGACAACTGCATGGGACAACATACACTGCCACA

GGAATCCGCAAAGTAGTAAGAGAACTGTTTCAGAGTGCCAATGGAGCCCGGGAAAATGCT

GTTAAGATCCTAGTTGTCATCACAGATGGAGAAAAGTATCAAGATCCCCTGAATTATGAG

GATGTCATCCCTGAGGCCGACAGAAAGGGGGTCATTCGCTATGTCATTGGGGTGGGAGGT

GCCTTCAACAATGACAGATCCCGTGAAGAGCTTAACACCATTGCCTCTAAGCCAGCTCGT

GATCATGTGTTCCGGGTGAATAACTTTGAAGCTCTAAAGACCATTCAAAACCAGCTTCAG

GAAAAGATCTTTGCAATTGAAGGAACTCAGACAGGAAGTACCAGCTCCTTTGAGCATGAG

ATGTCTCAGGAAGGCTTCAGTGCTGCCATCACCTCTAATGGTCCTTTGCTGGGCTCCGTG

GGGAGCTTTGACTGGGCTGGTGGGGCCTTTTTGCATAGGTCCAAGAATGAAATCACCTTC

ATCAACACAACCAGGGTAGACTCAGACATGAATGATGCTTACTTGGGTTATGCATCTGCA

GTCATCTTGAGGAACCGGGTGCAAAGCCTGGTTCTGGGTGCACCTCGATATCAACACATT

GGCCTGGTGGCGATGTTCAGACAGAATGCTGGTGCCTGGGAGACCAACACTATTATCAAG

GGCAACCAGATTGGCTCCTACTTTGGGGCTTCCCTCTGCTCGGTGGACATGGACGGTGAT

GGAAACACCAACCTGGTCCTCATTGGGGCACCCCATTTCTATGAGCAGACCCGAGGGGGC

CAGGTGTCCGTGTGTCCCTTGCCTAAGGGGAGGGCTCGGTGGCGGTGTGAGGCTGTTCTC

CACGGTGAGCAGGGCCATCCCTGGGGCCGCTTTGGGGCAGCTCTGACAGTGCTGGGAGAT

GTGAATGGGGACAAGCTGACAGACGTGGCTATTGGAGCCCCAGGAGAACAGGAGAATCGG

GGTGCTATTTACATTTTTCATGGAATATCAGTATTTGGCATCAGCTCCTCCCACAGCCAG

CGGATTACAGGCTCCCAGCTTTCCCCCCAGCTCCAGTATTTTGGTCAGTCACTGAGTGGC

GGTCAGGACCTCACAATGGATGGATTGGCGGACCTGGCTGTAGGGGCCCAGGGACACATA

CTGCTGCTCAGATCCCAGCCAGTGCTAAGAGTTGTGGTGACCATGGAGTTCACACCCAAG

GAAGTGGCCAGGGATGTATTTGAGTGTCATGAACAGGTGGTGACAAGCAAGGCTGCTGGG

GAGGTCAGAGTCTGCCTCCTTGTCCGCAAGAGCACA

>CL175.Contig5_A-W 1601 2371 PREDICTED: LOW QUALITY PROTEIN: integrin alpha-M [Cavia porcellus]

GGTTATGCATCTGCAGTCATCTTGAGGAACCGGGTGCAAAGCCTGGTTCTGGGTGCACCT

CGATATCAACACATTGGCCTGGTGGCGATGTTCAGACAGAATGCTGGTGCCTGGGAGACC

AACACTATTATCAAGGGCAACCAGATTGGCTCCTACTTTGGGGCTTCCCTCTGCTCGGTG

GACATGGACGGTGATGGAAACACCAACCTGGTCCTCATTGGGGCACCCCATTTCTATGAG

CAGACCCGAGGGGGCCAGGTGTCCGTGTGTCCCTTGCCTAAGGGGAGGGCTCGGTGGCGG

TGTGAGGCTGTTCTCCACGGTGAGCAGGGCCATCCCTGGGGCCGCTTTGGGGCAGCTCTG

ACAGTGCTGGGAGATGTGAATGGGGACAAGCTGACAGACGTGGCTATTGGAGCCCCAGGA

GAACAGGAGAATCGGGGTGCTATTTACATTTTTCATGGAATATCAGTATTTGGCATCAGC

TCCTCCCACAGCCAGCGGATTACAGGCTCCCAGCTTTCCCCCCAGCTCCAGTATTTTGGT

CAGTCACTGAGTGGCGGTCAGGACCTCACAATGGATGGATTGGCGGACCTGGCTGTAGGG

GCCCAGGGACACATACTGCTGCTCAGATCCCAGCCAGTGCTAAGAGTTGTGGTGACCATG

GAGTTCACACCCAAGGAAGTGGCCAGGGATGTATTTGAGTGTCATGAACAGGTGGTGACA

AGCAAGGCTGCTGGGGAGGTCAGAGTCTGCCTCCTTGTCCGCAAGAGCACA

>CL175.Contig6_A-W 164 1588 PREDICTED: integrin alpha-M [Cricetulus griseus]

TTCTCTTTGATGCAGTACTCAAATGACTTCCAGACTCATTTTACCTTCAATTATTTCAAG

ACAAATCCTAACCCAAGATTTCTGGTAAACCAAATAAGACAACTGCATGGGACAACATAC

ACTGCCACAGGAATCCGCAAAGTAGTAAGAGAACTGTTTCAGAGTGCCAATGGAGCCCGG

GAAAATGCTGTTAAGATCCTAGTTGTCATCACAGATGGAGAAAAGTATCAAGATCCCCTG

AATTATGAGGATGTCATCCCTGAGGCCGACAGAAAGGGGGTCATTCGCTATGTCATTGGG

GTGGGAGGTGCCTTCAACAATGACAGATCCCGTGAAGAGCTTAACACCATTGCCTCTAAG

CCAGCTCGTGATCATGTGTTCCGGGTGAATAACTTTGAAGCTCTAAAGACCATTCAAAAC

CAGCTTCAGGAAAAGATCTTTGCAATTGAAGGAACTCAGACAGGAAGTACCAGCTCCTTT

GAGCATGAGATGTCTCAGGAAGGCTTCAGTGCTGCCATCACCTCTAATGGTCCTTTGCTG

GGCTCCGTGGGGAGCTTTGACTGGGCTGGTGGGGCCTTTTTGCATAGGTCCAAGAATGAA

ATCACCTTCATCAACACAACCAGGGTAGACTCAGACATGAATGATGCTTACTTGGGTTAT

GCATCTGCAGTCATCTTGAGGAACCGGGTGCAAAGCCTGGTTCTGGGTGCACCTCGATAT

CAACACATTGGCCTGGTGGCGATGTTCAGACAGAATGCTGGTGCCTGGGAGACCAACACT

ATTATCAAGGGCAACCAGATTGGCTCCTACTTTGGGGCTTCCCTCTGCTCGGTGGACATG

GACGGTGATGGAAACACCAACCTGGTCCTCATTGGGGCACCCCATTTCTATGAGCAGACC

CGAGGGGGCCAGGTGTCCGTGTGTCCCTTGCCTAAGGGGAGGGCTCGGTGGCGGTGTGAG

GCTGTTCTCCACGGTGAGCAGGGCCATCCCTGGGGCCGCTTTGGGGCAGCTCTGACAGTG

CTGGGAGATGTGAATGGGGACAAGCTGACAGACGTGGCTATTGGAGCCCCAGGAGAACAG

GAGAATCGGGGTGCTATTTACATTTTTCATGGAATATCAGTATTTGGCATCAGCTCCTCC

CACAGCCAGCGGATTACAGGCTCCCAGCTTTCCCCCCAGCTCCAGTATTTTGGTCAGTCA

CTGAGTGGCGGTCAGGACCTCACAATGGATGGATTGGCGGACCTGGCTGTAGGGGCCCAG

GGACACATACTGCTGCTCAGATCCCAGCCAGTGCTAAGAGTTGTGGTGACCATGGAGTTC

ACACCCAAGGAAGTGGCCAGGGATGTATTTGAGTGTCATGAACAGGTGGTGACAAGCAAG

GCTGCTGGGGAGGTCAGAGTCTGCCTCCTTGTCCGCAAGAGCACA

>CL175.Contig7_A-W 103 234 PREDICTED: integrin alpha-M [Otolemur garnettii]

AATGGTCCTTTGCTGGGCTCCGTGGGGAGCTTTGACTGGGCTGGTGGGGCCTTTTTGCAT

AGGTCCAAGAATGAAATCACCTTCATCAACACAACCAGGGTAGACTCAGACATGAATGAT

GCTTACTTGGGT

>CL175.Contig8_A-W 103 1002 integrin alpha-M isoform 2 precursor [Mus musculus] >gi|148685679|gb|EDL17626.1| integrin alpha M, isoform CRA_a [Mus musculus]

AATGGTCCTTTGCTGGGCTCCGTGGGGAGCTTTGACTGGGCTGGTGGGGCCTTTTTGCAT

AGGTCCAAGAATGAAATCACCTTCATCAACACAACCAGGGTAGACTCAGACATGAATGAT

GCTTACTTGGGTTATGCATCTGCAGTCATCTTGAGGAACCGGGTGCAAAGCCTGGTTCTG

GGTGCACCTCGATATCAACACATTGGCCTGGTGGCGATGTTCAGACAGAATGCTGGTGCC

TGGGAGACCAACACTATTATCAAGGGCAACCAGATTGGCTCCTACTTTGGGGCTTCCCTC

TGCTCGGTGGACATGGACGGTGATGGAAACACCAACCTGGTCCTCATTGGGGCACCCCAT

TTCTATGAGCAGACCCGAGGGGGCCAGGTGTCCGTGTGTCCCTTGCCTAAGGGGAGGGCT

CGGTGGCGGTGTGAGGCTGTTCTCCACGGTGAGCAGGGCCATCCCTGGGGCCGCTTTGGG

GCAGCTCTGACAGTGCTGGGAGATGTGAATGGGGACAAGCTGACAGACGTGGCTATTGGA

GCCCCAGGAGAACAGGAGAATCGGGGTGCTATTTACATTTTTCATGGAATATCAGTATTT

GGCATCAGCTCCTCCCACAGCCAGCGGATTACAGGCTCCCAGCTTTCCCCCCAGCTCCAG

TATTTTGGTCAGTCACTGAGTGGCGGTCAGGACCTCACAATGGATGGATTGGCGGACCTG

GCTGTAGGGGCCCAGGGACACATACTGCTGCTCAGATCCCAGCCAGTGCTAAGAGTTGTG

GTGACCATGGAGTTCACACCCAAGGAAGTGGCCAGGGATGTATTTGAGTGTCATGAACAG

GTGGTGACAAGCAAGGCTGCTGGGGAGGTCAGAGTCTGCCTCCTTGTCCGCAAGAGCACA

>CL175.Contig9_A-W 547 1056 PREDICTED: integrin alpha-M [Otolemur garnettii]

AGAGAACTGTTTCAGAGTGCCAATGGAGCCCGGGAAAATGCTGTTAAGATCCTAGTTGTC

ATCACAGATGGAGAAAAGTATCAAGATCCCCTGAATTATGAGGATGTCATCCCTGAGGCC

GACAGAAAGGGGGTCATTCGCTATGTCATTGGGGTGGGAGGTGCCTTCAACAATGACAGA

TCCCGTGAAGAGCTTAACACCATTGCCTCTAAGCCAGCTCGTGATCATGTGTTCCGGGTG

AATAACTTTGAAGCTCTAAAGACCATTCAAAACCAGCTTCAGGAAAAGATCTTTGCAATT

GAAGGAACTCAGACAGGAAGTACCAGCTCCTTTGAGCATGAGATGTCTCAGGAAGGCTTC

AGTGCTGCCATCACCTCTAATGGTCCTTTGCTGGGCTCCGTGGGGAGCTTTGACTGGGCT

GGTGGGGCCTTTTTGCATAGGTCCAAGAATGAAATCACCTTCATCAACACAACCAGGGTA

GACTCAGACATGAATGATGCTTACTTGGGT

>CL175.Contig10_A-W 164 820 PREDICTED: integrin alpha-M [Otolemur garnettii]

TTCTCTTTGATGCAGTACTCAAATGACTTCCAGACTCATTTTACCTTCAATTATTTCAAG

ACAAATCCTAACCCAAGATTTCTGGTAAACCAAATAAGACAACTGCATGGGACAACATAC

ACTGCCACAGGAATCCGCAAAGTAGTAAGAGAACTGTTTCAGAGTGCCAATGGAGCCCGG

GAAAATGCTGTTAAGATCCTAGTTGTCATCACAGATGGAGAAAAGTATCAAGATCCCCTG

AATTATGAGGATGTCATCCCTGAGGCCGACAGAAAGGGGGTCATTCGCTATGTCATTGGG

GTGGGAGGTGCCTTCAACAATGACAGATCCCGTGAAGAGCTTAACACCATTGCCTCTAAG

CCAGCTCGTGATCATGTGTTCCGGGTGAATAACTTTGAAGCTCTAAAGACCATTCAAAAC

CAGCTTCAGGAAAAGATCTTTGCAATTGAAGGAACTCAGACAGGAAGTACCAGCTCCTTT

GAGCATGAGATGTCTCAGGAAGGCTTCAGTGCTGCCATCACCTCTAATGGTCCTTTGCTG

GGCTCCGTGGGGAGCTTTGACTGGGCTGGTGGGGCCTTTTTGCATAGGTCCAAGAATGAA

ATCACCTTCATCAACACAACCAGGGTAGACTCAGACATGAATGATGCTTACTTGGGT

>CL176.Contig2_A-W 3 194 ORF2 [Mus musculus domesticus] >gi|3599323|gb|AAC72795.1| ORF2 [Mus musculus domesticus]

CCTAGATGCCCTTCAATAGATGGTTAAAAAAAAAAAATGTGGCATATATACACAATGGAA

TTTTACTCAGCAATAAAGGAGAATAAAATCATGGCATTTGCAAGTAAATGGATGGAGTTG

GAGAATACCAAGCTCAGTGAAGTAAACCAATCCCAAAACACCAAAGGCCAAATGTTCTTT

CTGATAAGTGGA

>CL177.Contig1_A-W 98 652 minus strand PREDICTED: LOW QUALITY PROTEIN: transmembrane and coiled-coil domain-containing protein 6-like [Cavia porcellus]

CTCGCATGCCCTGTGCTCCGGTGTCTAAGCAACCTGCTGACAGAGGTGGCAGTAGAGGCT

GTGGGAGGGCAAATGCAACTCAAAGATGAACGTATCGTGGCAGCCTTATTTATCCTCTTC

CAGTTCTTCCTTCAGAAACAGCCCAGCCTGCTTCCAGAGGGCCTCTGGCTACTCAATAAT

CTCACTGCAAACAGTCCTACTTTGTGTACCTCCTTGCTTTCCTTGGATCTGATTGAGCCC

CTCTTGCAGCTGTTGCCACTATCTAATGTGGTTAGTGTACTGGTACTCACAGTTCTTTGC

AATGTTGCAGAGAAGGGTCCAGCTTACTGCCGGCAGCTGTGGCCAGGACCCCTGCTTCCC

TGCTTGCTGAACATGTTGGCCCTCTCTGATACTGAAGTAGTAGGCCAGAGTTTGGAGCTG

CTACAGCTGCTATTCCTGTATCGTCCAGAGGCTGTCCAGGCCTTCCTGCAGCAATCAGGA

CTGCAGGCTCTGGAAAGACATCAGGAGGAGGTCCAGCTTCAGGATCGGGTGCATGCCCTC

CAGCAGATAGCTCTT

>CL177.Contig2_A-W 349 630 minus strand PREDICTED: transmembrane and coiled-coil domain-containing protein 6 isoform 2 [Nomascus leucogenys]

GTTGCCACTATCTAATGTGGTTAGTGTACTGAGAAGGGTCCAGCTTACTGCCGGCAGCTG

TGGCCAGGACCCCTGCTTCCCTGCTTGCTGAACATGTTGGCCCTCTCTGATACTGAAGTA

GTAGGCCAGAGTTTGGAGCTGCTACAGCTGCTATTCCTGTATCGTCCAGAGGCTGTCCAG

GCCTTCCTGCAGCAATCAGGACTGCAGGCTCTGGAAAGACATCAGGAGGAGGTCCAGCTT

CAGGATCGGGTGCATGCCCTCCAGCAGATAGCTCTTCGTGGC

>CL177.Contig3_A-W 484 1119 PREDICTED: transmembrane and coiled-coil domain-containing protein 6 isoform 1 [Nomascus leucogenys]

TGTGTGTTCTGTCTTTGTTCTGGGCTTCAGGTCCAGGAGTTCTTGCGGCTAGCCCAGCGG

GGTACAGAGGAAAAGGAGAGAGAGAAGGCTCTGGTCAGTCTTCGTCGAGGCTTGCAGCAC

CCTGAGACACAGCAAATTTTCATCAGGCTGGAGGGCAGCATGCGGACCTTGGTTGGGCTC

CTGACCAGCAACAGAGCCCTGTTGCAGCTTGAGGCAGCTCGGTGCCTGCATGAGCTTTCT

CATTCTGAGCAGTCTGTGGTGGCTGAGGCCTGCCTGCCAGCTACTTCCTACCTCCTCACC

TACCTTTCTGGTCACAGCTCAGATTTCATAGAGCTCTGTTTGTATACACTGGGGAATCTG

ATCGTGGAGAGTGAGGTTGTGAGACAGCAGCTCCTGCCACAGGGCATTGTTTCAGCCTTG

GCTGCCTGCATCCAGTCCCCTCATGTGGCTGTGCTGGAAGCCCTTGGATATGCCTTGTCC

CAGCTTCTGCAGGCTAAGGAAGCTCCAGAGAAGATCATTCCCTCTATCCTGGACTCCCTC

CTCCCCCAGCACATGCTGCGATTGATGCAACCTGGCCCCAAACTGAACCCTGGGGTTGCT

GTGGAGTTTGCTTGGTGCCTTCACTACATCATCTGT

>CL177.Contig4_A-W 484 1794 minus strand PREDICTED: transmembrane and coiled-coil domain-containing protein 6 isoform 1 [Nomascus leucogenys]

TGTGTGTTCTGTCTTTGTTCTGGGCTTCAGGTCCAGGAGTTCTTGCGGCTAGCCCAGCGG

GGTACAGAGGAAAAGGAGAGAGAGAAGGCTCTGGTCAGTCTTCGTCGAGGCTTGCAGCAC

CCTGAGACACAGCAAATTTTCATCAGGCTGGAGGGCAGCATGCGGACCTTGGTTGGGCTC

CTGACCAGCAACAGAGCCCTGTTGCAGCTTGAGGCAGCTCGGTGCCTGCATGAGCTTTCT

CATTCTGAGCAGTCTGTGGTGGCTGAGGCCTGCCTGCCAGCTACTTCCTACCTCCTCACC

TACCTTTCTGGTCACAGCTCAGATTTCATAGAGCTCTGTTTGTATACACTGGGGAATCTG

ATCGTGGAGAGTGAGGTTGTGAGACAGCAGCTCCTGCCACAGGGCATTGTTTCAGCCTTG

GCTGCCTGCATCCAGTCCCCTCATGTGGCTGTGCTGGAAGCCCTTGGATATGCCTTGTCC

CAGCTTCTGCAGGCTAAGGAAGCTCCAGAGAAGATCATTCCCTCTATCCTGGACTCCCTC

CTCCCCCAGCACATGCTGCGATTGATGCAACCTGGCCCCAAACTGAACCCTGGGGTTGCT

GTGGAGTTTGCTTGGTGCCTTCACTACATCATCTGTAGTCAGGTCAATAATGCTCTGCTC

ATCACCCATGGGGCTCTGTCTACTCTGGGGCTACTGCTGTTGGACTTGGCGGGGGCTGTC

CAGAAAACGGAGGAGGCAGGACTGGAGCTGCTCGCATGCCCTGTGCTCCGGTGTCTAAGC

AACCTGCTGACAGAGGTGGCAGTAGAGGCTGTGGGAGGGCAAATGCAACTCAAAGATGAA

CGTATCGTGGCAGCCTTATTTATCCTCTTCCAGTTCTTCCTTCAGAAACAGCCCAGCCTG

CTTCCAGAGGGCCTCTGGCTACTCAATAATCTCACTGCAAACAGTCCTACTTTGTGTACC

TCCTTGCTTTCCTTGGATCTGATTGAGCCCCTCTTGCAGCTGTTGCCACTATCTAATGTG

GTTAGTGTACTGGTACTCACAGTTCTTTGCAATGTTGCAGAGAAGGGTCCAGCTTACTGC

CGGCAGCTGTGGCCAGGACCCCTGCTTCCCTGCTTGCTGAACATGTTGGCCCTCTCTGAT

ACTGAAGTAGTAGGCCAGAGTTTGGAGCTGCTACAGCTGCTATTCCTGTATCGTCCAGAG

GCTGTCCAGGCCTTCCTGCAGCAATCAGGACTGCAGGCTCTGGAAAGACATCAGGAGGAG

GTCCAGCTTCAGGATCGGGTGCATGCCCTCCAGCAGATAGCTCTTCGTGGC

>CL177.Contig5_A-W 1485 1766 minus strand PREDICTED: transmembrane and coiled-coil domain-containing protein 6 isoform 1 [Nomascus leucogenys]

GTTGCCACTATCTAATGTGGTTAGTGTACTGAGAAGGGTCCAGCTTACTGCCGGCAGCTG

TGGCCAGGACCCCTGCTTCCCTGCTTGCTGAACATGTTGGCCCTCTCTGATACTGAAGTA

GTAGGCCAGAGTTTGGAGCTGCTACAGCTGCTATTCCTGTATCGTCCAGAGGCTGTCCAG

GCCTTCCTGCAGCAATCAGGACTGCAGGCTCTGGAAAGACATCAGGAGGAGGTCCAGCTT

CAGGATCGGGTGCATGCCCTCCAGCAGATAGCTCTTCGTGGC

>CL179.Contig1_A-W 451 636 mature T-cell proliferation 1 neighbor [Bos taurus] >gi|122142382|sp|Q0VBY0.1|MTCNB_BOVIN RecName: Full=Mature T-cell proliferation 1 neighbor protein >gi|111307594|gb|AAI20454.1| Mature T-cell proliferation 1 [Bos taurus] >gi|296471118|gb|DAA13233.1| mature T-cell proliferation 1 [Bos taurus]

ATGCCGCAAAAGGATCCGTGCCAGAAACAAGCCTGTGCAATACAGAAATGTTTACAAGCC

AACAACTACATCGAATCAAAGTGTCAGGCTGTTATCGAAGAACTGCGTAAGTGTTGTGCT

CGATATCCCAAGGGAAGATCTCTCGTCTGTTCAGGATTTGAAAAAGAAGAAAACCAGACA

CTGAAG

>CL179.Contig2_A-W 91 222 minus strand hypothetical protein PANDA_020691 [Ailuropoda melanoleuca]

TTTGCAGCCAACAACTACATCGAATCAAAGTGTCAGGCTGTTATCGAAGAACTGCGTAAG

TGTTGTGCTCGATATCCCAAGGGAAGATCTCTCGTCTGTTCAGGATTTGAAAAAGAAGAA

AACCAGACACTG

>CL180.Contig1_A-W 2021 2311 PREDICTED: LOW QUALITY PROTEIN: very long-chain specific acyl-CoA dehydrogenase, mitochondrial, partial [Pongo abelii]

GTGAATGAGCAGTTTGTGCTGCAGCGTCTGGCAGATGGGGCCATTGACCTCTATGCCATG

GTGGTGGTTCTCTCCAGGGCCTCAAGATCCCTGAGTGAGGGCTACCCTACAGCCCAGCAT

GAGAAAATGCTCTGTGACAGCTGGTGTATTGAGGCTGCAGCCCGGATCCAAGAGAACATG

ACTGCTCTGCTGTCTGACCCTCGGCAGCAAGAGCTCTTCCGTAACTTCAAAAGCATCTCC

AAGGCCTTGGTGGAGCGGGGTGGTGTAGTCACCAGCAATCCCCTTGGATTC

>CL180.Contig2_A-W 1766 2056 PREDICTED: LOW QUALITY PROTEIN: very long-chain specific acyl-CoA dehydrogenase, mitochondrial [Callithrix jacchus]

GTGAATGAGCAGTTTGTGCTGCAGCGTCTGGCAGATGGGGCCATTGACCTCTATGCCATG

GTGGTGGTTCTCTCCAGGGCCTCAAGATCCCTGAGTGAGGGCTACCCTACAGCCCAGCAT

GAGAAAATGCTCTGTGACAGCTGGTGTATTGAGGCTGCAGCCCGGATCCAAGAGAACATG

ACTGCTCTGCTGTCTGACCCTCGGCAGCAAGAGCTCTTCCGTAACTTCAAAAGCATCTCC

AAGGCCTTGGTGGAGCGGGGTGGTGTAGTCACCAGCAATCCCCTTGGATTC

>CL180.Contig3_A-W 1865 2155 PREDICTED: LOW QUALITY PROTEIN: very long-chain specific acyl-CoA dehydrogenase, mitochondrial [Callithrix jacchus]

GTGAATGAGCAGTTTGTGCTGCAGCGTCTGGCAGATGGGGCCATTGACCTCTATGCCATG

GTGGTGGTTCTCTCCAGGGCCTCAAGATCCCTGAGTGAGGGCTACCCTACAGCCCAGCAT

GAGAAAATGCTCTGTGACAGCTGGTGTATTGAGGCTGCAGCCCGGATCCAAGAGAACATG

ACTGCTCTGCTGTCTGACCCTCGGCAGCAAGAGCTCTTCCGTAACTTCAAAAGCATCTCC

AAGGCCTTGGTGGAGCGGGGTGGTGTAGTCACCAGCAATCCCCTTGGATTC

>CL180.Contig4_A-W 2120 2410 PREDICTED: LOW QUALITY PROTEIN: very long-chain specific acyl-CoA dehydrogenase, mitochondrial, partial [Pongo abelii]

GTGAATGAGCAGTTTGTGCTGCAGCGTCTGGCAGATGGGGCCATTGACCTCTATGCCATG

GTGGTGGTTCTCTCCAGGGCCTCAAGATCCCTGAGTGAGGGCTACCCTACAGCCCAGCAT

GAGAAAATGCTCTGTGACAGCTGGTGTATTGAGGCTGCAGCCCGGATCCAAGAGAACATG

ACTGCTCTGCTGTCTGACCCTCGGCAGCAAGAGCTCTTCCGTAACTTCAAAAGCATCTCC

AAGGCCTTGGTGGAGCGGGGTGGTGTAGTCACCAGCAATCCCCTTGGATTC

>CL180.Contig5_A-W 251 955 RecName: Full=Very long-chain specific acyl-CoA dehydrogenase, mitochondrial; Short=VLCAD; Flags: Precursor >gi|74267810|gb|AAI03105.1| ACADVL protein [Bos taurus] >gi|146231822|gb|ABQ12986.1| acyl-Coenzyme A dehydrogenase, very long chain isoform 1 precursor [Bos taurus] >gi|296476717|gb|DAA18832.1| very long-chain specific acyl-CoA dehydrogenase, mitochondrial precursor [Bos taurus]

CAGTATGCTCGTTTGGCAGAGATCGTGGGCATGCATGACCTTGGTGTGGGTGTTACCCTG

GGGGCTCATCAGAGCATCGGTTTCAAAGGCATCCTACTCTTTGGCACAAAGGCTCAGAAA

GAAAAATACCTCCCTAAAGTGGCATCTGGGGAGACTTTGGCTGCTTTCTGTCTAACGGAG

CCCTCAAGTGGATCAGATGCAGCTTCCATCCGAAGCTCTGCTGTCCCCAGCCCCTGTGGA

AAATATTATACCCTAAATGGAAGCAAGATTTGGATCAGGCAATCTCCTATTCCTACCTAC

TTCTTCTCAGCCCAATTCTACTCCCCACTGCTCTGAGCTCTCCCCACTCTGACCACCACC

CCCTCATTCTCCCACAGTAATGGGGGCCTGGCAGATATTTTCACAGTTTTTGCCAAGACA

CCAGTTACAGATGCAGCCACAGGGGCTGTAAAGGAGAAGATCACAGCTTTTGTGGTGGAG

AGGAGCTTTGGAGGTGTAACCCATGGGCCCCCTGAAAAGAAGATGGGCATCAAAGCCTCA

AACACAGCAGAGGTGTACTTTGATGGAGTACAGGTGCCATCGGAAAATGTTCTGGGGGAG

GTAGGGGGAGGCTTCAAGGTTGCTGTGCATATCCTCAACAATGGAAGGTTTGGCATGGCT

GCAACCCTTGCAGGCACCATGAAAGCCATGATTTCGAAGGCGGTG

>CL180.Contig6_A-W 10 564 PREDICTED: LOW QUALITY PROTEIN: very long-chain specific acyl-CoA dehydrogenase, mitochondrial, partial [Pongo abelii]

CGGGCACCCAGACTTGGGCAGGCGGGACGAGAGACCGAGGTCACCGCTCCAAACAGTGGC

GGCCGGAGCGGATTGGACATGCAGACGTCTCGGATGACTCCGAGCATAGGGCGGCAGCTG

CTGAGGTTGGGGAGTCGAAGCTTGCGGCCCAGTGTACTCCAGGGAACGCCCCGGCCTAGC

ACTGCTCAGCGACCCTATGCCAGTGGAGCCGCCCAGGCTGTTCTGGACAAGTCAGATTCC

CTATCCTCTGATGCTTCCACTAGGGAAAAACAGACCAAGGCGGAATCTAAGTCCTTTGCT

GTGGGGATGTTCAAAGGCCAGCTCACCACTGATCAGGTGTTCCCATACCCATCCGTACTC

AATGAAGAGCAGACACAGTTTCTCAAAGAGCTGGTAGGGCCTGTGGCCCGTTTCTTCAAG

GAGGTGAATGATCCTGCGAAGAATGATACTCTAGAGAGGGTGGAGGAAACTACTTTGCAG

GGTCTCAAGGAGTTGGGGGCCTTTGGTCTGCAAGTGCCCAGTGAGCTGGGTGGTGTGGGC

CTCTGCAACACCCAG

>CL180.Contig7_A-W 1520 1660 minus strand RecName: Full=Very long-chain specific acyl-CoA dehydrogenase, mitochondrial; Short=VLCAD; Flags: Precursor >gi|74267810|gb|AAI03105.1| ACADVL protein [Bos taurus] >gi|146231822|gb|ABQ12986.1| acyl-Coenzyme A dehydrogenase, very long chain isoform 1 precursor [Bos taurus] >gi|296476717|gb|DAA18832.1| very long-chain specific acyl-CoA dehydrogenase, mitochondrial precursor [Bos taurus]

CAGGCTGCAGCCCGGATCCAAGAGAACATGACTGCTCTGCTGTCTGACCCTCGGCAGCAA

GAGCTCTTCCGTAACTTCAAAAGCATCTCCAAGGCCTTGGTGGAGCGGGGTGGTGTAGTC

ACCAGCAATCCCCTTGGATTC

>CL180.Contig8_A-W 1495 1635 minus strand RecName: Full=Very long-chain specific acyl-CoA dehydrogenase, mitochondrial; Short=VLCAD; Flags: Precursor >gi|74267810|gb|AAI03105.1| ACADVL protein [Bos taurus] >gi|146231822|gb|ABQ12986.1| acyl-Coenzyme A dehydrogenase, very long chain isoform 1 precursor [Bos taurus] >gi|296476717|gb|DAA18832.1| very long-chain specific acyl-CoA dehydrogenase, mitochondrial precursor [Bos taurus]

CAGGCTGCAGCCCGGATCCAAGAGAACATGACTGCTCTGCTGTCTGACCCTCGGCAGCAA

GAGCTCTTCCGTAACTTCAAAAGCATCTCCAAGGCCTTGGTGGAGCGGGGTGGTGTAGTC

ACCAGCAATCCCCTTGGATTC

>CL180.Contig9_A-W 1421 1561 minus strand RecName: Full=Very long-chain specific acyl-CoA dehydrogenase, mitochondrial; Short=VLCAD; Flags: Precursor >gi|74267810|gb|AAI03105.1| ACADVL protein [Bos taurus] >gi|146231822|gb|ABQ12986.1| acyl-Coenzyme A dehydrogenase, very long chain isoform 1 precursor [Bos taurus] >gi|296476717|gb|DAA18832.1| very long-chain specific acyl-CoA dehydrogenase, mitochondrial precursor [Bos taurus]

CAGGCTGCAGCCCGGATCCAAGAGAACATGACTGCTCTGCTGTCTGACCCTCGGCAGCAA

GAGCTCTTCCGTAACTTCAAAAGCATCTCCAAGGCCTTGGTGGAGCGGGGTGGTGTAGTC

ACCAGCAATCCCCTTGGATTC

>CL180.Contig10_A-W 1073 1225 minus strand Very long-chain specific acyl-CoA dehydrogenase, mitochondrial [Heterocephalus glaber]

AGGCGGGCAGGGCTGGGAAGTGGCCTGAGTCTCAGTGGAGTGGTCCACCCAGAGCTGAGT

CGGAGCGGTGAGCTGGCAGTGCAGGCTCTGGAGCAGTTTGCCACTGTGGTGGAAGCCAAG

CTAATAAAGCACAAGAAGGGGATTGTCAGTGAA

>CL180.Contig11_A-W 784 1461 minus strand unnamed protein product [Macaca fascicularis]

TCCCTCCAGTTTAAGCCTAACCCCTCTCCTGGATACAGCAGACAACAGTGGAACAGGTGT

GCCCTCTTCCAGGTGGATCATGCTGCTAATCGTACCCAGTTTGGGGACAAAATTCACAAC

TTTGGGCTGATCCAGGAGAAGCTGGCCCGAATGGCTATGCTGCAGTATGTGACTGAGTCC

ATGGCTTACATGCTGAGTGCCAACATGGACCAGGGATCCACAGACTTCCAGATAGAGGCT

GCCATCAGCAAAATCTTTGGCTCGGAAGCAGCCTGGAAAGTGACAGATGAATGCATCCAA

ATCATGGGTGGCATGGGCTTCATGAAGGAGCCCGGGGTAGAGCGAGTGCTCCGAGATATT

CGCATCTTCCGGATCTTTGAGGGGACAAACGACATTCTTCGGCTCTTTGTGGCTCTGCAG

GGCTGTATGGACAAAGGAAAGGAACTTTCTGGGCTTAGCAGTGCCCTAAAGAATCCTTTT

GGGAATGCTGGCCTTCTGCTAGGAGAGGCAGGAAAACAGCTGAGACGGCGGGCAGGGCTG

GGAAGTGGCCTGAGTCTCAGTGGAGTGGTCCACCCAGAGCTGAGTCGGAGCGGTGAGCTG

GCAGTGCAGGCTCTGGAGCAGTTTGCCACTGTGGTGGAAGCCAAGCTAATAAAGCACAAG

AAGGGGATTGTCAGTGAG

>CL180.Contig12_A-W 1066 1206 minus strand RecName: Full=Very long-chain specific acyl-CoA dehydrogenase, mitochondrial; Short=VLCAD; Flags: Precursor >gi|74267810|gb|AAI03105.1| ACADVL protein [Bos taurus] >gi|146231822|gb|ABQ12986.1| acyl-Coenzyme A dehydrogenase, very long chain isoform 1 precursor [Bos taurus] >gi|296476717|gb|DAA18832.1| very long-chain specific acyl-CoA dehydrogenase, mitochondrial precursor [Bos taurus]

CAGGCTGCAGCCCGGATCCAAGAGAACATGACTGCTCTGCTGTCTGACCCTCGGCAGCAA

GAGCTCTTCCGTAACTTCAAAAGCATCTCCAAGGCCTTGGTGGAGCGGGGTGGTGTAGTC

ACCAGCAATCCCCTTGGATTC

>CL180.Contig13_A-W 1012 1260 minus strand unnamed protein product [Macaca fascicularis]

GACAAAGGAAAGGAACTTTCTGGGCTTAGCAGTGCCCTAAAGAATCCTTTTGGGAATGCT

GGCCTTCTGCTAGGAGAGGCAGGAAAACAGCTGAGACGGCGGGCAGGGCTGGGAAGTGGC

CTGAGTCTCAGTGGAGTGGTCCACCCAGAGCTGAGTCGGAGCGGTGAGCTGGCAGTGCAG

GCTCTGGAGCAGTTTGCCACTGTGGTGGAAGCCAAGCTAATAAAGCACAAGAAGGGGATT

GTCAGTGAG

>CL180.Contig14_A-W 954 1106 minus strand unnamed protein product [Macaca fascicularis]

AGGCGGGCAGGGCTGGGAAGTGGCCTGAGTCTCAGTGGAGTGGTCCACCCAGAGCTGAGT

CGGAGCGGTGAGCTGGCAGTGCAGGCTCTGGAGCAGTTTGCCACTGTGGTGGAAGCCAAG

CTAATAAAGCACAAGAAGGGGATTGTCAGTGAG

>CL180.Contig15_A-W 757 1005 minus strand RecName: Full=Very long-chain specific acyl-CoA dehydrogenase, mitochondrial; Short=VLCAD; Flags: Precursor >gi|74267810|gb|AAI03105.1| ACADVL protein [Bos taurus] >gi|146231822|gb|ABQ12986.1| acyl-Coenzyme A dehydrogenase, very long chain isoform 1 precursor [Bos taurus] >gi|296476717|gb|DAA18832.1| very long-chain specific acyl-CoA dehydrogenase, mitochondrial precursor [Bos taurus]

GACAAAGGAAAGGAACTTTCTGGGCTTAGCAGTGCCCTAAAGAATCCTTTTGGGAATGCT

GGCCTTCTGCTAGGAGAGGCAGGAAAACAGCTGAGACGGCGGGCAGGGCTGGGAAGTGGC

CTGAGTCTCAGTGGAGTGGTCCACCCAGAGCTGAGTCGGAGCGGTGAGCTGGCAGTGCAG

GCTCTGGAGCAGTTTGCCACTGTGGTGGAAGCCAAGCTAATAAAGCACAAGAAGGGGATT

GTCAGTGAG

>CL180.Contig16_A-W 1947 2195 minus strand PREDICTED: LOW QUALITY PROTEIN: very long-chain specific acyl-CoA dehydrogenase, mitochondrial, partial [Pongo abelii]

GACAAAGGAAAGGAACTTTCTGGGCTTAGCAGTGCCCTAAAGAATCCTTTTGGGAATGCT

GGCCTTCTGCTAGGAGAGGCAGGAAAACAGCTGAGACGGCGGGCAGGGCTGGGAAGTGGC

CTGAGTCTCAGTGGAGTGGTCCACCCAGAGCTGAGTCGGAGCGGTGAGCTGGCAGTGCAG

GCTCTGGAGCAGTTTGCCACTGTGGTGGAAGCCAAGCTAATAAAGCACAAGAAGGGGATT

GTCAGTGAA

>CL180.Contig17_A-W 2 931 minus strand RecName: Full=Very long-chain specific acyl-CoA dehydrogenase, mitochondrial; Short=VLCAD; Flags: Precursor >gi|74267810|gb|AAI03105.1| ACADVL protein [Bos taurus] >gi|146231822|gb|ABQ12986.1| acyl-Coenzyme A dehydrogenase, very long chain isoform 1 precursor [Bos taurus] >gi|296476717|gb|DAA18832.1| very long-chain specific acyl-CoA dehydrogenase, mitochondrial precursor [Bos taurus]

AATGGGGGCCTGGCAGATATTTTCACAGTTTTTGCCAAGACACCAGTTACAGATGCAGCC

ACAGGGGCTGTAAAGGAGAAGATCACAGCTTTTGTGGTGGAGAGGAGCTTTGGAGGTGTA

ACCCATGGGCCCCCTGAAAAGAAGATGGGCATCAAAGCCTCAAACACAGCAGAGGTGTAC

TTTGATGGAGTACAGGTGCCATCGGAAAATGTTCTGGGGGAGGTAGGGGGAGGCTTCAAG

GTTGCTGTGCATATCCTCAACAATGGAAGGTTTGGCATGGCTGCAACCCTTGCAGGCACC

ATGAAAGCCATGATTTCGAAGGCGGTGGATCATGCTGCTAATCGTACCCAGTTTGGGGAC

AAAATTCACAACTTTGGGCTGATCCAGGAGAAGCTGGCCCGAATGGCTATGCTGCAGTAT

GTGACTGAGTCCATGGCTTACATGCTGAGTGCCAACATGGACCAGGGATCCACAGACTTC

CAGATAGAGGCTGCCATCAGCAAAATCTTTGGCTCGGAAGCAGCCTGGAAAGTGACAGAT

GAATGCATCCAAATCATGGGTGGCATGGGCTTCATGAAGGAGCCCGGGGTAGAGCGAGTG

CTCCGAGATATTCGCATCTTCCGGATCTTTGAGGGGACAAACGACATTCTTCGGCTCTTT

GTGGCTCTGCAGGGCTGTATGGACAAAGGAAAGGAACTTTCTGGGCTTAGCAGTGCCCTA

AAGAATCCTTTTGGGAATGCTGGCCTTCTGCTAGGAGAGGCAGGAAAACAGCTGAGACGG

CGGGCAGGGCTGGGAAGTGGCCTGAGTCTCAGTGGAGTGGTCCACCCAGAGCTGAGTCGG

AGCGGTGAGCTGGCAGTGCAGGCTCTGGAGCAGTTTGCCACTGTGGTGGAAGCCAAGCTA

ATAAAGCACAAGAAGGGGATTGTCAGTGAG

>CL180.Contig18_A-W 699 851 minus strand Very long-chain specific acyl-CoA dehydrogenase, mitochondrial [Heterocephalus glaber]

AGGCGGGCAGGGCTGGGAAGTGGCCTGAGTCTCAGTGGAGTGGTCCACCCAGAGCTGAGT

CGGAGCGGTGAGCTGGCAGTGCAGGCTCTGGAGCAGTTTGCCACTGTGGTGGAAGCCAAG

CTAATAAAGCACAAGAAGGGGATTGTCAGTGAG

>CL180.Contig19_A-W 1848 2096 minus strand PREDICTED: LOW QUALITY PROTEIN: very long-chain specific acyl-CoA dehydrogenase, mitochondrial, partial [Pongo abelii]

GACAAAGGAAAGGAACTTTCTGGGCTTAGCAGTGCCCTAAAGAATCCTTTTGGGAATGCT

GGCCTTCTGCTAGGAGAGGCAGGAAAACAGCTGAGACGGCGGGCAGGGCTGGGAAGTGGC

CTGAGTCTCAGTGGAGTGGTCCACCCAGAGCTGAGTCGGAGCGGTGAGCTGGCAGTGCAG

GCTCTGGAGCAGTTTGCCACTGTGGTGGAAGCCAAGCTAATAAAGCACAAGAAGGGGATT

GTCAGTGAA

>CL180.Contig20_A-W 1889 2041 minus strand PREDICTED: LOW QUALITY PROTEIN: very long-chain specific acyl-CoA dehydrogenase, mitochondrial, partial [Pongo abelii]

AGGCGGGCAGGGCTGGGAAGTGGCCTGAGTCTCAGTGGAGTGGTCCACCCAGAGCTGAGT

CGGAGCGGTGAGCTGGCAGTGCAGGCTCTGGAGCAGTTTGCCACTGTGGTGGAAGCCAAG

CTAATAAAGCACAAGAAGGGGATTGTCAGTGAA

>CL180.Contig21_A-W 1790 1942 minus strand PREDICTED: LOW QUALITY PROTEIN: very long-chain specific acyl-CoA dehydrogenase, mitochondrial, partial [Pongo abelii]

AGGCGGGCAGGGCTGGGAAGTGGCCTGAGTCTCAGTGGAGTGGTCCACCCAGAGCTGAGT

CGGAGCGGTGAGCTGGCAGTGCAGGCTCTGGAGCAGTTTGCCACTGTGGTGGAAGCCAAG

CTAATAAAGCACAAGAAGGGGATTGTCAGTGAA

>CL180.Contig22_A-W 1692 1940 minus strand PREDICTED: LOW QUALITY PROTEIN: very long-chain specific acyl-CoA dehydrogenase, mitochondrial, partial [Pongo abelii]

GACAAAGGAAAGGAACTTTCTGGGCTTAGCAGTGCCCTAAAGAATCCTTTTGGGAATGCT

GGCCTTCTGCTAGGAGAGGCAGGAAAACAGCTGAGACGGCGGGCAGGGCTGGGAAGTGGC

CTGAGTCTCAGTGGAGTGGTCCACCCAGAGCTGAGTCGGAGCGGTGAGCTGGCAGTGCAG

GCTCTGGAGCAGTTTGCCACTGTGGTGGAAGCCAAGCTAATAAAGCACAAGAAGGGGATT

GTCAGTGAA

>CL180.Contig23_A-W 1593 1841 minus strand PREDICTED: LOW QUALITY PROTEIN: very long-chain specific acyl-CoA dehydrogenase, mitochondrial, partial [Pongo abelii]

GACAAAGGAAAGGAACTTTCTGGGCTTAGCAGTGCCCTAAAGAATCCTTTTGGGAATGCT

GGCCTTCTGCTAGGAGAGGCAGGAAAACAGCTGAGACGGCGGGCAGGGCTGGGAAGTGGC

CTGAGTCTCAGTGGAGTGGTCCACCCAGAGCTGAGTCGGAGCGGTGAGCTGGCAGTGCAG

GCTCTGGAGCAGTTTGCCACTGTGGTGGAAGCCAAGCTAATAAAGCACAAGAAGGGGATT

GTCAGTGAA

>CL180.Contig24_A-W 1 702 minus strand RecName: Full=Very long-chain specific acyl-CoA dehydrogenase, mitochondrial; Short=VLCAD; Flags: Precursor >gi|74267810|gb|AAI03105.1| ACADVL protein [Bos taurus] >gi|146231822|gb|ABQ12986.1| acyl-Coenzyme A dehydrogenase, very long chain isoform 1 precursor [Bos taurus] >gi|296476717|gb|DAA18832.1| very long-chain specific acyl-CoA dehydrogenase, mitochondrial precursor [Bos taurus]

TACGCTCGTTTGGCAGAGATCGTGGGCATGCATGACCTTGGTGTGGGTGTTACCCTGGGG

GCTCATCAGAGCATCGGTTTCAAAGGCATCCTACTCTTTGGCACAAAGGCTCAGAAAGAA

AAATACCTCCCTAAAGTGGCATCTGGGGAGACTTTGGCTGCTTTCTGTCTAACGGAGCCC

TCAAGTGGATCAGATGCAGCTTCCATCCGAAGCTCTGCTGTCCCCAGCCCCTGTGGAAAA

TATTATACCCTAAATGGAAGCAAGATTTGGATCAGGCAATCTCCTATTCCTACCTACTTC

TTCTCAGCCCAATTCTACTCCCCACTGCTCTGAGCTCTCCCCACTCTGACCACCACCCCC

TCATTCTCCCACAGTAATGGGGGCCTGGCAGATATTTTCACAGTTTTTGCCAAGACACCA

GTTACAGATGCAGCCACAGGGGCTGTAAAGGAGAAGATCACAGCTTTTGTGGTGGAGAGG

AGCTTTGGAGGTGTAACCCATGGGCCCCCTGAAAAGAAGATGGGCATCAAAGCCTCAAAC

ACAGCAGAGGTGTACTTTGATGGAGTACAGGTGCCATCGGAAAATGTTCTGGGGGAGGTA

GGGGGAGGCTTCAAGGTTGCTGTGCATATCCTCAACAATGGAAGGTTTGGCATGGCTGCA

ACCCTTGCAGGCACCATGAAAGCCATGATTTCGAAGGCGGTG

>CL180.Contig25_A-W 1535 1687 minus strand PREDICTED: LOW QUALITY PROTEIN: very long-chain specific acyl-CoA dehydrogenase, mitochondrial [Callithrix jacchus]

AGGCGGGCAGGGCTGGGAAGTGGCCTGAGTCTCAGTGGAGTGGTCCACCCAGAGCTGAGT

CGGAGCGGTGAGCTGGCAGTGCAGGCTCTGGAGCAGTTTGCCACTGTGGTGGAAGCCAAG

CTAATAAAGCACAAGAAGGGGATTGTCAGTGAA

>CL180.Contig26_A-W 1 702 minus strand RecName: Full=Very long-chain specific acyl-CoA dehydrogenase, mitochondrial; Short=VLCAD; Flags: Precursor >gi|74267810|gb|AAI03105.1| ACADVL protein [Bos taurus] >gi|146231822|gb|ABQ12986.1| acyl-Coenzyme A dehydrogenase, very long chain isoform 1 precursor [Bos taurus] >gi|296476717|gb|DAA18832.1| very long-chain specific acyl-CoA dehydrogenase, mitochondrial precursor [Bos taurus]

TACGCTCGTTTGGCAGAGATCGTGGGCATGCATGACCTTGGTGTGGGTGTTACCCTGGGG

GCTCATCAGAGCATCGGTTTCAAAGGCATCCTACTCTTTGGCACAAAGGCTCAGAAAGAA

AAATACCTCCCTAAAGTGGCATCTGGGGAGACTTTGGCTGCTTTCTGTCTAACGGAGCCC

TCAAGTGGATCAGATGCAGCTTCCATCCGAAGCTCTGCTGTCCCCAGCCCCTGTGGAAAA

TATTATACCCTAAATGGAAGCAAGATTTGGATCAGGCAATCTCCTATTCCTACCTACTTC

TTCTCAGCCCAATTCTACTCCCCACTGCTCTGAGCTCTCCCCACTCTGACCACCACCCCC

TCATTCTCCCACAGTAATGGGGGCCTGGCAGATATTTTCACAGTTTTTGCCAAGACACCA

GTTACAGATGCAGCCACAGGGGCTGTAAAGGAGAAGATCACAGCTTTTGTGGTGGAGAGG

AGCTTTGGAGGTGTAACCCATGGGCCCCCTGAAAAGAAGATGGGCATCAAAGCCTCAAAC

ACAGCAGAGGTGTACTTTGATGGAGTACAGGTGCCATCGGAAAATGTTCTGGGGGAGGTA

GGGGGAGGCTTCAAGGTTGCTGTGCATATCCTCAACAATGGAAGGTTTGGCATGGCTGCA

ACCCTTGCAGGCACCATGAAAGCCATGATTTCGAAGGCGGTG

>CL180.Contig27_A-W 1 603 minus strand RecName: Full=Very long-chain specific acyl-CoA dehydrogenase, mitochondrial; Short=VLCAD; Flags: Precursor >gi|74267810|gb|AAI03105.1| ACADVL protein [Bos taurus] >gi|146231822|gb|ABQ12986.1| acyl-Coenzyme A dehydrogenase, very long chain isoform 1 precursor [Bos taurus] >gi|296476717|gb|DAA18832.1| very long-chain specific acyl-CoA dehydrogenase, mitochondrial precursor [Bos taurus]

TACGCTCGTTTGGCAGAGATCGTGGGCATGCATGACCTTGGTGTGGGTGTTACCCTGGGG

GCTCATCAGAGCATCGGTTTCAAAGGCATCCTACTCTTTGGCACAAAGGCTCAGAAAGAA

AAATACCTCCCTAAAGTGGCATCTGGGGAGACTTTGGCTGCTTTCTGTCTAACGGAGCCC

TCAAGTGGATCAGATGCAGCTTCCATCCGAAGCTCTGCTGTCCCCAGCCCCTGTGGAAAA

TATTATACCCTAAATGGAAGCAAGATTTGGATCAGTAATGGGGGCCTGGCAGATATTTTC

ACAGTTTTTGCCAAGACACCAGTTACAGATGCAGCCACAGGGGCTGTAAAGGAGAAGATC

ACAGCTTTTGTGGTGGAGAGGAGCTTTGGAGGTGTAACCCATGGGCCCCCTGAAAAGAAG

ATGGGCATCAAAGCCTCAAACACAGCAGAGGTGTACTTTGATGGAGTACAGGTGCCATCG

GAAAATGTTCTGGGGGAGGTAGGGGGAGGCTTCAAGGTTGCTGTGCATATCCTCAACAAT

GGAAGGTTTGGCATGGCTGCAACCCTTGCAGGCACCATGAAAGCCATGATTTCGAAGGCG

GTG

>CL180.Contig28_A-W 1 702 minus strand RecName: Full=Very long-chain specific acyl-CoA dehydrogenase, mitochondrial; Short=VLCAD; Flags: Precursor >gi|74267810|gb|AAI03105.1| ACADVL protein [Bos taurus] >gi|146231822|gb|ABQ12986.1| acyl-Coenzyme A dehydrogenase, very long chain isoform 1 precursor [Bos taurus] >gi|296476717|gb|DAA18832.1| very long-chain specific acyl-CoA dehydrogenase, mitochondrial precursor [Bos taurus]

TACGCTCGTTTGGCAGAGATCGTGGGCATGCATGACCTTGGTGTGGGTGTTACCCTGGGG

GCTCATCAGAGCATCGGTTTCAAAGGCATCCTACTCTTTGGCACAAAGGCTCAGAAAGAA

AAATACCTCCCTAAAGTGGCATCTGGGGAGACTTTGGCTGCTTTCTGTCTAACGGAGCCC

TCAAGTGGATCAGATGCAGCTTCCATCCGAAGCTCTGCTGTCCCCAGCCCCTGTGGAAAA

TATTATACCCTAAATGGAAGCAAGATTTGGATCAGGCAATCTCCTATTCCTACCTACTTC

TTCTCAGCCCAATTCTACTCCCCACTGCTCTGAGCTCTCCCCACTCTGACCACCACCCCC

TCATTCTCCCACAGTAATGGGGGCCTGGCAGATATTTTCACAGTTTTTGCCAAGACACCA

GTTACAGATGCAGCCACAGGGGCTGTAAAGGAGAAGATCACAGCTTTTGTGGTGGAGAGG

AGCTTTGGAGGTGTAACCCATGGGCCCCCTGAAAAGAAGATGGGCATCAAAGCCTCAAAC

ACAGCAGAGGTGTACTTTGATGGAGTACAGGTGCCATCGGAAAATGTTCTGGGGGAGGTA

GGGGGAGGCTTCAAGGTTGCTGTGCATATCCTCAACAATGGAAGGTTTGGCATGGCTGCA

ACCCTTGCAGGCACCATGAAAGCCATGATTTCGAAGGCGGTG

>CL180.Contig29_A-W 1 603 minus strand RecName: Full=Very long-chain specific acyl-CoA dehydrogenase, mitochondrial; Short=VLCAD; Flags: Precursor >gi|74267810|gb|AAI03105.1| ACADVL protein [Bos taurus] >gi|146231822|gb|ABQ12986.1| acyl-Coenzyme A dehydrogenase, very long chain isoform 1 precursor [Bos taurus] >gi|296476717|gb|DAA18832.1| very long-chain specific acyl-CoA dehydrogenase, mitochondrial precursor [Bos taurus]

TACGCTCGTTTGGCAGAGATCGTGGGCATGCATGACCTTGGTGTGGGTGTTACCCTGGGG

GCTCATCAGAGCATCGGTTTCAAAGGCATCCTACTCTTTGGCACAAAGGCTCAGAAAGAA

AAATACCTCCCTAAAGTGGCATCTGGGGAGACTTTGGCTGCTTTCTGTCTAACGGAGCCC

TCAAGTGGATCAGATGCAGCTTCCATCCGAAGCTCTGCTGTCCCCAGCCCCTGTGGAAAA

TATTATACCCTAAATGGAAGCAAGATTTGGATCAGTAATGGGGGCCTGGCAGATATTTTC

ACAGTTTTTGCCAAGACACCAGTTACAGATGCAGCCACAGGGGCTGTAAAGGAGAAGATC

ACAGCTTTTGTGGTGGAGAGGAGCTTTGGAGGTGTAACCCATGGGCCCCCTGAAAAGAAG

ATGGGCATCAAAGCCTCAAACACAGCAGAGGTGTACTTTGATGGAGTACAGGTGCCATCG

GAAAATGTTCTGGGGGAGGTAGGGGGAGGCTTCAAGGTTGCTGTGCATATCCTCAACAAT

GGAAGGTTTGGCATGGCTGCAACCCTTGCAGGCACCATGAAAGCCATGATTTCGAAGGCG

GTG

>CL180.Contig30_A-W 1594 1734 minus strand RecName: Full=Very long-chain specific acyl-CoA dehydrogenase, mitochondrial; Short=VLCAD; Flags: Precursor >gi|74267810|gb|AAI03105.1| ACADVL protein [Bos taurus] >gi|146231822|gb|ABQ12986.1| acyl-Coenzyme A dehydrogenase, very long chain isoform 1 precursor [Bos taurus] >gi|296476717|gb|DAA18832.1| very long-chain specific acyl-CoA dehydrogenase, mitochondrial precursor [Bos taurus]

CAGGCTGCAGCCCGGATCCAAGAGAACATGACTGCTCTGCTGTCTGACCCTCGGCAGCAA

GAGCTCTTCCGTAACTTCAAAAGCATCTCCAAGGCCTTGGTGGAGCGGGGTGGTGTAGTC

ACCAGCAATCCCCTTGGATTC

>CL180.Contig31_A-W 33 287 minus strand RecName: Full=Very long-chain specific acyl-CoA dehydrogenase, mitochondrial; Short=VLCAD; Flags: Precursor >gi|74267810|gb|AAI03105.1| ACADVL protein [Bos taurus] >gi|146231822|gb|ABQ12986.1| acyl-Coenzyme A dehydrogenase, very long chain isoform 1 precursor [Bos taurus] >gi|296476717|gb|DAA18832.1| very long-chain specific acyl-CoA dehydrogenase, mitochondrial precursor [Bos taurus]

CTGACAGATGAGCAGTTTGTGCTGCAGCGTCTGGCAGATGGGGCCATTGACCTCTATGCC

ATGGTGGTGGTTCTCTCCAGGTGAGGAGGCAGAGGTGGGGTGGGAGGCATGAGCCACAGT

GGGGCTGGGCCAGGATCGTAGCCCAGGGTTATATTCTCTTGCTTCCTCACCAGGGCCTCA

AGATCCCTGAGTGAGGGCTACCCTACAGCCCAGCATGAGAAAATGCTCTGTGACAGCTGG

TGTATTGAGGTAAGC

>CL181.Contig1_A-W 101 370 minus strand PREDICTED: methyl-CpG-binding domain protein 4 [Equus caballus]

CGGCCTGTCGCTGCAGCTGGACCCCGCTTGACTGGCTCAGCCTTGATGGAGACGCCGAGC

CTCGGGGACGGCGGACCCGCCCCCTCGATGGCCTCCAGCGACCTCCCAGTCCCAGAGCAG

CAGCGTGACCTCCGCGAGGAAGATGTGGCTGTGGGGTCGGAAAGAATGGGAGAAGATGAA

AGACAAATAGCAATAAAAAGAATCAGTGAGTGTCAGCCCCTGCTACAAGAAGCCATCGCC

TCTTCTCAGTTGGGTGACACCACAGTGACC

>CL181.Contig2_A-W 101 238 minus strand MBD4; methyl-CpG binding domain protein 4; K10801 methyl-CpG-binding domain protein 4 [EC:3.2.2.-]

CGGCCTGTCGCTGCAGCTGGACCCCGCTTGACTGGCTCAGCCTTGATGGAGACGCCGAGC

CTCGGGGACGGCGGACCCGCCCCCTCGATGGCCTCCAGCGACCTCCCAGTCCCAGAGCAG

CAGCGTGACCTCCGGCAA

>CL182.Contig1_A-W 76 1842 minus strand Putative G-protein coupled receptor 97 [Heterocephalus glaber]

ATGGCGACACCTTGGGGCATGGGGACCCTGCTCCTCCTGCTCCTGGCCTCAGGCGAGGGG

GCAGGCCCTGAAGAGCCCCGCAACTTCTGCCTGGGGCTGGACATCCAGGACCAGTACGAC

CCCTTCTACTTGAACCGCACCGGGGAGTGCTTTGCCAGGTGCCTGCAGCTGGGGAATGTC

TCCTGCAACCCTGAAAAGCTGGAGAGATACTGGCTACACTACGAGTTCTACCTGGTGGAG

AAGAATTTGGAGGACAGAGTGGATATGCCTTTTGTGAAGGCTTTGGTCCAGAACTTCAGC

ACCGACACCTCGGAAGATCTGCTTTTCTCTCTGAAACCCTCTCAGATTCCGAGGCAGGTG

ATGATGGGCGAGGACAAGCCCCCTGACAGAGTCCGACTTCCCAGGAGCTTTTTCGCCTCC

CTGCCGGGCAGCAGGTCTGCAGTCCGGTTGGCCATATCTGTTCTGGACATCGGTGCTGGA

GACCTCTTCAAGGGCCCCCAGCTTCGCCTGGAAGATGGCAGCAGTGTGTTGAACAACCAC

ATGGTGGGCTTGAGTGTGGGTCGCACCCCTGTCACCAGGCTGCCAGAGCCTCTGGAGATC

ACCTTCTCCCACCAACGTCAGCCCCCGAACGCGACCCTCACCTGTGTGTTCTGGGATGTG

AATAAAGGGCCTGCTGGGGACTGGGACTCCAAGGGCTGCTCCACCGAGCTCGGAGCTGAG

GGGACTGTCTGCCACTGTGACCACCTGACCTTCTTCGCCTTGCTGTTGAGGCCCGTCTTG

GACCGTGCCACCGCGCAGGTCCTTACTCACATCTCCCAGGCAGGCTGTGGAGTCTCCATG

ATCTTCCTGGCCTTCACCATTGTCCTCTATCTTACCCTGAGAGCCTGTGAGCTGGGCTTG

GAATACCAGGAAGAATGTCACCAAGATGGGGCCTGGCAACTTAGGAGAGGTGGACATTTA

TCCCTCATGCCCCCTCCAGGAAGCCCCACACTCCCATTCCTCAGGTTCTCCCTGCAGAGA

TTCAAGTCCGAAGATGCCCCCAAGATCCACGTGGCCCTGAGTAGTAGCTTGTTCCTCCTG

AATCTGGCCTTCCTGGTCAGCGTGGGCAGCGGCTCGGCGGGGTCCCGTGCTGCATGCTGG

ATCCGGGGAGCCATCTTCCACTACTTCCTGCTCTGCACCTTCACCTGGATGGGCCTGGAA

GCCTTCCACCTCTACCTCCTCGTCATCAAGGTCTTCAACACCTACTTCGGCCATTACTAC

CTGAAGCTGAGCCTGGTGGGCTGGGGCCTGCCTGCCCTCATGGTCATTGGCACCGGCAGT

GCCAACAGCTATGGCCTTTACACCATCCGCGACCAGGAGAACCGCACCTCTCTGGAGCTG

TGCTGGTTCCAGAAAGAGCCTGCCCTCTACGCCACCGTCCACGGTTACTTCCTCATCACC

TTCCTCTTTGGTGCCGTGGTGCTGGCCCTGGTGGCCTGGAAGATCTTCACCCTGAGCAGT

GTCACAGCCGGCAAGGAGCAGGGGCAGAGCTGGAAGGCCGTCCTCACCATCCTGGGCCTC

TCAAGCCTGGTGGGTGTGACCTGGGGGCTGGCTGTCCTCACGCCCCTGGGTCTGTCCACC

ATCTATATCTTCGCCCTCTTCAACTCCCTGCAAGGTGTCTTCATCTTCTGCTGGTTTGCC

ATCCTCTACCTCCCGGGTCAGATCTCCACGCCTTCTTCCTCGGGGGGCACTGGCCGGCTG

GACCAGGCCCCCTCTGTGTCCCATGAG

>CL182.Contig2_A-W 1644 1757 minus strand Putative G-protein coupled receptor 97 [Heterocephalus glaber]

GGTGTCTTCATCTTCTGCTGGTTTGCCATCCTCTACCTCCCGGGTCAGATCTCCACGCCT

TCTTCCTCGGGGGGCACTGGCCGGCTGGACCAGGCCCCCTCTGTGTCCCATGAG

>CL182.Contig3_A-W 76 1719 minus strand G-protein coupled receptor PGR26 [Macaca fascicularis]

ATGGCGACACCTTGGGGCATGGGGACCCTGCTCCTCCTGCTCCTGGCCTCAGGCGAGGGG

GCAGGCCCTGAAGAGCCCCGCAACTTCTGCCTGGGGCTGGACATCCAGGACCAGTACGAC

CCCTTCTACTTGAACCGCACCGGGGAGTGCTTTGCCAGGTGCCTGCAGCTGGGGAATGTC

TCCTGCAACCCTGAAAAGCTGGAGAGATACTGGCTACACTACGAGTTCTACCTGGTGGAG

AAGAATTTGGAGGACAGAGTGGATATGCCTTTTGTGAAGGCTTTGGTCCAGAACTTCAGC

ACCGACACCTCGGAAGATCTGCTTTTCTCTCTGAAACCCTCTCAGATTCCGAGGCAGGTG

ATGATGGGCGAGGACAAGCCCCCTGACAGAGTCCGACTTCCCAGGAGCTTTTTCGCCTCC

CTGCCGGGCAGCAGGTCTGCAGTCCGGTTGGCCATATCTGTTCTGGACATCGGTGCTGGA

GACCTCTTCAAGGGCCCCCAGCTTCGCCTGGAAGATGGCAGCAGTGTGTTGAACAACCAC

ATGGTGGGCTTGAGTGTGGGTCGCACCCCTGTCACCAGGCTGCCAGAGCCTCTGGAGATC

ACCTTCTCCCACCAACGTCAGCCCCCGAACGCGACCCTCACCTGTGTGTTCTGGGATGTG

AATAAAGGGCCTGCTGGGGACTGGGACTCCAAGGGCTGCTCCACCGAGCTCGGAGCTGAG

GGGACTGTCTGCCACTGTGACCACCTGACCTTCTTCGCCTTGCTGTTGAGGCCCGTCTTG

GACCGTGCCACCGCGCAGGTCCTTACTCACATCTCCCAGGCAGGCTGTGGAGTCTCCATG

ATCTTCCTGGCCTTCACCATTGTCCTCTATCTTACCCTGAGGTTCTCCCTGCAGAGATTC

AAGTCCGAAGATGCCCCCAAGATCCACGTGGCCCTGAGTAGTAGCTTGTTCCTCCTGAAT

CTGGCCTTCCTGGTCAGCGTGGGCAGCGGCTCGGCGGGGTCCCGTGCTGCATGCTGGATC

CGGGGAGCCATCTTCCACTACTTCCTGCTCTGCACCTTCACCTGGATGGGCCTGGAAGCC

TTCCACCTCTACCTCCTCGTCATCAAGGTCTTCAACACCTACTTCGGCCATTACTACCTG

AAGCTGAGCCTGGTGGGCTGGGGCCTGCCTGCCCTCATGGTCATTGGCACCGGCAGTGCC

AACAGCTATGGCCTTTACACCATCCGCGACCAGGAGAACCGCACCTCTCTGGAGCTGTGC

TGGTTCCAGAAAGAGCCTGCCCTCTACGCCACCGTCCACGGTTACTTCCTCATCACCTTC

CTCTTTGGTGCCGTGGTGCTGGCCCTGGTGGCCTGGAAGATCTTCACCCTGAGCAGTGTC

ACAGCCGGCAAGGAGCAGGGGCAGAGCTGGAAGGCCGTCCTCACCATCCTGGGCCTCTCA

AGCCTGGTGGGTGTGACCTGGGGGCTGGCTGTCCTCACGCCCCTGGGTCTGTCCACCATC

TATATCTTCGCCCTCTTCAACTCCCTGCAAGGTGTCTTCATCTTCTGCTGGTTTGCCATC

CTCTACCTCCCGGGTCAGATCTCCACGCCTTCTTCCTCGGGGGGCACTGGCCGGCTGGAC

CAGGCCCCCTCTGTGTCCCATGAG

>CL182.Contig4_A-W 1521 1634 minus strand G-protein coupled receptor PGR26 [Macaca fascicularis]

GGTGTCTTCATCTTCTGCTGGTTTGCCATCCTCTACCTCCCGGGTCAGATCTCCACGCCT

TCTTCCTCGGGGGGCACTGGCCGGCTGGACCAGGCCCCCTCTGTGTCCCATGAG

>CL182.Contig5_A-W 198 584 minus strand Putative G-protein coupled receptor 97 [Heterocephalus glaber]

TGCTGGTTCCAGAAAGAGCCTGCCCTCTACGCCACCGTCCACGGTTACTTCCTCATCACC

TTCCTCTTTGGTGCCGTGGTGCTGGCCCTGGTGGCCTGGAAGATCTTCACCCTGAGCAGT

GTCACAGCCGGCAAGGAGCAGGGGCAGAGCTGGAAGGCCGTCCTCACCATCCTGGGCCTC

TCAAGCCTGGTGGGTGTGACCTGGGGGCTGGCTGTCCTCACGCCCCTGGGTCTGTCCACC

ATCTATATCTTCGCCCTCTTCAACTCCCTGCAAGGTGTCTTCATCTTCTGCTGGTTTGCC

ATCCTCTACCTCCCGGGTCAGATCTCCACGCCTTCTTCCTCGGGGGGCACTGGCCGGCTG

GACCAGGCCCCCTCTGTGTCCCATGAG

>CL182.Contig6_A-W 386 499 minus strand Putative G-protein coupled receptor 97 [Heterocephalus glaber]

GGTGTCTTCATCTTCTGCTGGTTTGCCATCCTCTACCTCCCGGGTCAGATCTCCACGCCT

TCTTCCTCGGGGGGCACTGGCCGGCTGGACCAGGCCCCCTCTGTGTCCCATGAG

>CL183.Contig1_A-W 27 263 minus strand PREDICTED: muscleblind-like protein 1 isoform 2 [Sarcophilus harrisii]

GTCACCGTCTGTATGGATTACATCAAAGGGAGATGCTCTCGGGAAAAGTGCAAATACTTT

CACCCTCCTGCACATCTGCAAGCCAAGATCAAGGCTGCCCAATACCAGGTCAACCAGGCT

GCAGCAGCACAGGCTGCAGCAACCGCAGCTGCCATGGGAATTCCTCAAGCTGTACTTCCC

CCATTACCAAAGAGGCCTGCTCTTGAAAAAACCAACGGTGCCACCGCAGTCTTTAAC

>CL183.Contig2_A-W 506 955 minus strand Muscleblind-like protein 1 [Cricetulus griseus]

CTGGAGGTATGTCGAGAGTACCAGCGTGGCAACTGCAACCGAGGAGAGAATGATTGCCGG

TTTGCCCACCCTGCTGACAGTACGATGATTGACACCAATGACAACACAGTCACCGTCTGT

ATGGATTACATCAAAGGGAGATGCTCTCGGGAAAAGTGCAAATACTTTCACCCTCCTGCA

CATCTGCAAGCCAAGATCAAGGCTGCCCAATACCAGGTCAACCAGGCTGCAGCAGCACAG

GCTGCAGCAACCGCAGCTGCCATGGGAATTCCTCAAGCTGTACTTCCCCCATTACCAAAG

AGGCCTGCTCTTGAAAAAACCAACGGTGCCACCGCAGTCTTTAACACTGGCATTTTCCAG

TACCAACAGGCTCTGGCCAACATGCAGTTACAGCAGCACACGGCGTTCCTCCCACCAGGC

TCAATATTGTGCATGACACCCGCTACAAGT

>CL183.Contig3_A-W 7 1008 minus strand mKIAA0428 protein [Mus musculus]

AATCTAAACATGGCTGTTAGTGTCACACCAATTCGGGACACAAAATGGCTAACACTGGAA

GTATGTAGAGAGTTTCAAAGGGGGACTTGCTCACGGCCAGACACGGAATGTAAATTTGCA

CATCCTTCGAAAAGCTGCCAAGTTGAAAATGGACGAGTAATCGCCTGCTTTGATTCATTG

AAAGGTCGTTGCTCCAGGGAGAACTGCAAGTATCTTCACCCACCCCCACACTTAAAAACA

CAGTTGGAGATCAATGGGCGCAATAACTTGATTCAGCAGAAGAACATGGCCATGCTGGCC

CAGCAGATGCAACTAGCCAACGCCATGATGCCCGGCACCCCGCTGCAGCCCGTGCCAATG

TTTTCAGTTGCACCAAGCTTAGCCACCAATGCATCAGCAGCCTTTAATCCCTACCTGGGA

CCTGTCTCCCCCAGCCTGGTTCCAGCAGAGATCTTGCCCACGGCACCCATGTTGGTTACA

GGGAATCCCGGAGTGCCCGTGCCTGCGGCTGCCGCTGCTGCTGCCCAAAAACTGATGCGC

ACAGACAGACTGGAGGTATGTCGAGAGTACCAGCGTGGCAACTGCAACCGAGGAGAGAAT

GATTGCCGGTTTGCCCACCCTGCTGACAGTACGATGATTGACACCAATGACAACACAGTC

ACCGTCTGTATGGATTACATCAAAGGGAGATGCTCTCGGGAAAAGTGCAAATACTTTCAC

CCTCCTGCACATCTGCAAGCCAAGATCAAGGCTGCCCAATACCAGGTCAACCAGGCTGCA

GCAGCACAGGCTGCAGCAACCGCAGCTGCCATGGGAATTCCTCAAGCTGTACTTCCCCCA

TTACCAAAGAGGCCTGCTCTTGAAAAAACCAACGGTGCCACCGCAGTCTTTAACACTGGC

ATTTTCCAGTACCAACAGGCTCTGGCCAACATGCAGTTACAGCAGCACACGGCGTTCCTC

CCACCAGGCTCAATATTGTGCATGACACCCGCTACAAGTGTT

>CL183.Contig4_A-W 438 902 minus strand rCG50114, isoform CRA_b [Rattus norvegicus] >gi|383418423|gb|AFH32425.1| muscleblind-like protein 1 isoform g [Macaca mulatta]

CTGGAGGTATGTCGAGAGTACCAGCGTGGCAACTGCAACCGAGGAGAGAATGATTGCCGG

TTTGCCCACCCTGCTGACAGTACGATGATTGACACCAATGACAACACAGTCACCGTCTGT

ATGGATTACATCAAAGGGAGATGCTCTCGGGAAAAGTGCAAATACTTTCACCCTCCTGCA

CATCTGCAAGCCAAGATCAAGGCTGCCCAATACCAGGTCAACCAGGCTGCAGCAGCACAG

GCTGCAGCAACCGCAGCTGCCATGGGAATTCCTCAAGCTGTACTTCCCCCATTACCAAAG

AGGCCTGCTCTTGAAAAAACCAACGGTGCCACCGCAGTCTTTAACACTGGCATTTTCCAG

TACCAACAGGCTCTGGCCAACATGCAGTTACAGCAGCACACGGCGTTCCTCCCACCAGGC

TCAATATTGTGCATGACACCCGCTACAAGTGTTGGTAGGTGCCAG

>CL183.Contig5_A-W 417 881 minus strand rCG50114, isoform CRA_b [Rattus norvegicus] >gi|383418423|gb|AFH32425.1| muscleblind-like protein 1 isoform g [Macaca mulatta]

CTGGAGGTATGTCGAGAGTACCAGCGTGGCAACTGCAACCGAGGAGAGAATGATTGCCGG

TTTGCCCACCCTGCTGACAGTACGATGATTGACACCAATGACAACACAGTCACCGTCTGT

ATGGATTACATCAAAGGGAGATGCTCTCGGGAAAAGTGCAAATACTTTCACCCTCCTGCA

CATCTGCAAGCCAAGATCAAGGCTGCCCAATACCAGGTCAACCAGGCTGCAGCAGCACAG

GCTGCAGCAACCGCAGCTGCCATGGGAATTCCTCAAGCTGTACTTCCCCCATTACCAAAG

AGGCCTGCTCTTGAAAAAACCAACGGTGCCACCGCAGTCTTTAACACTGGCATTTTCCAG

TACCAACAGGCTCTGGCCAACATGCAGTTACAGCAGCACACGGCGTTCCTCCCACCAGGC

TCAATATTGTGCATGACACCCGCTACAAGTGTTGGTAGGTGCCAG

>CL183.Contig6_A-W 7 1158 minus strand mKIAA0428 protein [Mus musculus]

AATCTAAACATGGCTGTTAGTGTCACACCAATTCGGGACACAAAATGGCTAACACTGGAA

GTATGTAGAGAGTTTCAAAGGGGGACTTGCTCACGGCCAGACACGGAATGTAAATTTGCA

CATCCTTCGAAAAGCTGCCAAGTTGAAAATGGACGAGTAATCGCCTGCTTTGATTCATTG

AAAGGTCGTTGCTCCAGGGAGAACTGCAAGTATCTTCACCCACCCCCACACTTAAAAACA

CAGTTGGAGATCAATGGGCGCAATAACTTGATTCAGCAGAAGAACATGGCCATGCTGGCC

CAGCAGATGCAACTAGCCAACGCCATGATGCCCGGCACCCCGCTGCAGCCCGTGCCAATG

TTTTCAGTTGCACCAAGCTTAGCCACCAATGCATCAGCAGCCTTTAATCCCTACCTGGGA

CCTGTCTCCCCCAGCCTGGTTCCAGCAGAGATCTTGCCCACGGCACCCATGTTGGTTACA

GGGAATCCCGGAGTGCCCGTGCCTGCGGCTGCCGCTGCTGCTGCCCAAAAACTGATGCGC

ACAGACAGACTGGAGGTATGTCGAGAGTACCAGCGTGGCAACTGCAACCGAGGAGAGAAT

GATTGCCGGTTTGCCCACCCTGCTGACAGTACGATGATTGACACCAATGACAACACAGTC

ACCGTCTGTATGGATTACATCAAAGGGAGATGCTCTCGGGAAAAGTGCAAATACTTTCAC

CCTCCTGCACATCTGCAAGCCAAGATCAAGGCTGCCCAATACCAGGTCAACCAGGCTGCA

GCAGCACAGGCTGCAGCAACCGCAGCTGCCATGGGAATTCCTCAAGCTGTACTTCCCCCA

TTACCAAAGAGGCCTGCTCTTGAAAAAACCAACGGTGCCACCGCAGTCTTTAACACTGGC

ATTTTCCAGTACCAACAGGCTCTGGCCAACATGCAGTTACAGCAGCACACGGCGTTCCTC

CCACCAGGCTCAATATTGTGCATGACACCCGCTACAAGTGTTGTTCCCATGGTGCACGGT

GCTACGCCAGCCACTGTGTCCGCAGCAACAACATCTGCCACAAGTGTTCCCTTCGCTGCA

ACAGCCACAGCCAACCAGATACCCATAATATCTGCCGAACATCTGACTAGCCACAAGTAC

GTTACCCAGATG

>CL183.Contig7_A-W 438 887 minus strand Muscleblind-like protein 1 [Cricetulus griseus]

CTGGAGGTATGTCGAGAGTACCAGCGTGGCAACTGCAACCGAGGAGAGAATGATTGCCGG

TTTGCCCACCCTGCTGACAGTACGATGATTGACACCAATGACAACACAGTCACCGTCTGT

ATGGATTACATCAAAGGGAGATGCTCTCGGGAAAAGTGCAAATACTTTCACCCTCCTGCA

CATCTGCAAGCCAAGATCAAGGCTGCCCAATACCAGGTCAACCAGGCTGCAGCAGCACAG

GCTGCAGCAACCGCAGCTGCCATGGGAATTCCTCAAGCTGTACTTCCCCCATTACCAAAG

AGGCCTGCTCTTGAAAAAACCAACGGTGCCACCGCAGTCTTTAACACTGGCATTTTCCAG

TACCAACAGGCTCTGGCCAACATGCAGTTACAGCAGCACACGGCGTTCCTCCCACCAGGC

TCAATATTGTGCATGACACCCGCTACAAGT

>CL183.Contig8_A-W 417 866 minus strand Muscleblind-like protein 1 [Cricetulus griseus]

CTGGAGGTATGTCGAGAGTACCAGCGTGGCAACTGCAACCGAGGAGAGAATGATTGCCGG

TTTGCCCACCCTGCTGACAGTACGATGATTGACACCAATGACAACACAGTCACCGTCTGT

ATGGATTACATCAAAGGGAGATGCTCTCGGGAAAAGTGCAAATACTTTCACCCTCCTGCA

CATCTGCAAGCCAAGATCAAGGCTGCCCAATACCAGGTCAACCAGGCTGCAGCAGCACAG

GCTGCAGCAACCGCAGCTGCCATGGGAATTCCTCAAGCTGTACTTCCCCCATTACCAAAG

AGGCCTGCTCTTGAAAAAACCAACGGTGCCACCGCAGTCTTTAACACTGGCATTTTCCAG

TACCAACAGGCTCTGGCCAACATGCAGTTACAGCAGCACACGGCGTTCCTCCCACCAGGC

TCAATATTGTGCATGACACCCGCTACAAGT

>CL183.Contig9_A-W 243 707 minus strand rCG50114, isoform CRA_b [Rattus norvegicus] >gi|383418423|gb|AFH32425.1| muscleblind-like protein 1 isoform g [Macaca mulatta]

CTGGAGGTATGTCGAGAGTACCAGCGTGGCAACTGCAACCGAGGAGAGAATGATTGCCGG

TTTGCCCACCCTGCTGACAGTACGATGATTGACACCAATGACAACACAGTCACCGTCTGT

ATGGATTACATCAAAGGGAGATGCTCTCGGGAAAAGTGCAAATACTTTCACCCTCCTGCA

CATCTGCAAGCCAAGATCAAGGCTGCCCAATACCAGGTCAACCAGGCTGCAGCAGCACAG

GCTGCAGCAACCGCAGCTGCCATGGGAATTCCTCAAGCTGTACTTCCCCCATTACCAAAG

AGGCCTGCTCTTGAAAAAACCAACGGTGCCACCGCAGTCTTTAACACTGGCATTTTCCAG

TACCAACAGGCTCTGGCCAACATGCAGTTACAGCAGCACACGGCGTTCCTCCCACCAGGC

TCAATATTGTGCATGACACCCGCTACAAGTGTTGGTAGGTGCCAG

>CL183.Contig10_A-W 64 321 minus strand PREDICTED: muscleblind-like protein 1 [Papio anubis]

CGACTGATTTCTCTTTTTTCTTTTTCTCTTTTTACTGGTATTTGTTTTTTATACTCATTT

ACTAAACAGGGAATTCCTCAAGCTGTACTTCCCCCATTACCAAAGAGGCCTGCTCTTGAA

AAAACCAACGGTGCCACCGCAGTCTTTAACACTGGCATTTTCCAGTACCAACAGGCTCTG

GCCAACATGCAGTTACAGCAGCACACGGCGTTCCTCCCACCAGGCTCAATATTGTGCATG

ACACCCGCTACAAGTGTT

>CL184.Contig1_A-W 364 576 minus strand Pax transcription activation domain interacting protein PTIP [Mus musculus]

GTTGTAAAGCCTTCTTGGGTGACTTTGTCCGTACAGTGTGGAGCTCTTCTGCCAGTCAAT

GGATTTTCTTGAGAGTCATGTCAGATTTTTTTTGGAATCACTGCCTGCCTTTCTCAGGTG

TCATCTGAAGACAGAAGCACCCTGTGGGCTTTGCTCACGTTCCATGGCGGGAGCTGCCAG

CTGAACCTCAACAGGAAATGCACACACTTGGTC

>CL184.Contig2_A-W 762 1187 PREDICTED: ankyrin repeat domain-containing protein 26-like [Bos taurus]

CCAAATGCTCACAACAACCAAAATGAAACTCCTCTTCACTTTGCTCTGTCTCTGAAGAAA

ACATCACTAGCAGAAAAACTGCTTTCACTCAATGCAGATATAGAAGCCAGAACTACGAGG

GGCTATACACCACTTTTACTTGCCATAGAGCAAAACAGACAGCAGATGGTTGAAACATTA

ATTCAGAACAAAGCAAATATACATGCAGTTGACCAGAAGGGAAGAACAGCCCTCATGTTT

GCTTTGGAACAAAAATCAAAACACGTAGTTGAGATGCTTCTTCAGAGTGGTATTGATGTC

TCTGCTGCAGATAACTTTGGGCAGATGGCATTAGCTTATGCTATTGCTAGTGGTGATACT

AACCTGAAGGAATTAATTCTTCAATATATGAAAGTAGAATCTAAACATCCTGAAAATATC

AACCCA

>CL184.Contig3_A-W 870 1295 PREDICTED: ankyrin repeat domain-containing protein 26-like [Bos taurus]

CCAAATGCTCACAACAACCAAAATGAAACTCCTCTTCACTTTGCTCTGTCTCTGAAGAAA

ACATCACTAGCAGAAAAACTGCTTTCACTCAATGCAGATATAGAAGCCAGAACTACGAGG

GGCTATACACCACTTTTACTTGCCATAGAGCAAAACAGACAGCAGATGGTTGAAACATTA

ATTCAGAACAAAGCAAATATACATGCAGTTGACCAGAAGGGAAGAACAGCCCTCATGTTT

GCTTTGGAACAAAAATCAAAACACGTAGTTGAGATGCTTCTTCAGAGTGGTATTGATGTC

TCTGCTGCAGATAACTTTGGGCAGATGGCATTAGCTTATGCTATTGCTAGTGGTGATACT

AACCTGAAGGAATTAATTCTTCAATATATGAAAGTAGAATCTAAACATCCTGAAAATATC

AACCCA

>CL184.Contig4_A-W 308 421 Dual specificity protein kinase CLK4 OS=Homo sapiens GN=CLK4 PE=1 SV=1

ATGCGACATTCCCAACGAACACACTGCCCTGACTGGGATAGCAGAGAAAGCTGGGGTCAT

GAAAGCTACAGTGGAAGTCACAAACGGAAGAGGAGATCTCACACAAGAGAACAG

>CL184.Contig5_A-W 217 474 PREDICTED: dual specificity protein kinase CLK4-like [Cavia porcellus]

ATGCGACATTCCCAACGAACACACTGCCCTGACTGGGATAGCAGAGAAAGCTGGGGTCAT

GAAAGCTACAGTGGAAGTCACAAACGGAAGAGGAGATCTCACACAAGAGAACAGGCATTG

TAAACCGCATCACCAGTTTAAAGAATCTGATTGGATGAAAGCGCTTATCGAATCCACTGC

AGTAAATCCTCAGTCCGAAGCAGGAGAAGCAGTCCTAAAAGGAAGCGTAATAGACACTGT

TCAAGTCATCAGTTACGT

>CL184.Contig6_A-W 523 702 PREDICTED: dual specificity protein kinase CLK4-like [Cavia porcellus]

GAAATCGTGGACACTTTGGGTGAAGGGGCCTTTGGCAAAGTTGTAGAATGCATTGATCAT

GGCATGGATGGCTTACATGTAGCAGTGAAAATAGTAAAAAATGTAGGCCGTTACCGTGAA

GCAGCTCGTTCAGAAATCCAAGTATTGGAGCATTTAAATAGTACAGATCCTAATAGTGTC

>CL184.Contig7_A-W 110 223 Dual specificity protein kinase CLK4 OS=Homo sapiens GN=CLK4 PE=1 SV=1

ATGCGACATTCCCAACGAACACACTGCCCTGACTGGGATAGCAGAGAAAGCTGGGGTCAT

GAAAGCTACAGTGGAAGTCACAAACGGAAGAGGAGATCTCACACAAGAGAACAG

>CL184.Contig8_A-W 818 1243 PREDICTED: ankyrin repeat domain-containing protein 26-like [Bos taurus]

CCAAATGCTCACAACAACCAAAATGAAACTCCTCTTCACTTTGCTCTGTCTCTGAAGAAA

ACATCACTAGCAGAAAAACTGCTTTCACTCAATGCAGATATAGAAGCCAGAACTACGAGG

GGCTATACACCACTTTTACTTGCCATAGAGCAAAACAGACAGCAGATGGTTGAAACATTA

ATTCAGAACAAAGCAAATATACATGCAGTTGACCAGAAGGGAAGAACAGCCCTCATGTTT

GCTTTGGAACAAAAATCAAAACACGTAGTTGAGATGCTTCTTCAGAGTGGTATTGATGTC

TCTGCTGCAGATAACTTTGGGCAGATGGCATTAGCTTATGCTATTGCTAGTGGTGATACT

AACCTGAAGGAATTAATTCTTCAATATATGAAAGTAGAATCTAAACATCCTGAAAATATC

AACCCA

>CL184.Contig9_A-W 710 1135 PREDICTED: ankyrin repeat domain-containing protein 26-like [Bos taurus]

CCAAATGCTCACAACAACCAAAATGAAACTCCTCTTCACTTTGCTCTGTCTCTGAAGAAA

ACATCACTAGCAGAAAAACTGCTTTCACTCAATGCAGATATAGAAGCCAGAACTACGAGG

GGCTATACACCACTTTTACTTGCCATAGAGCAAAACAGACAGCAGATGGTTGAAACATTA

ATTCAGAACAAAGCAAATATACATGCAGTTGACCAGAAGGGAAGAACAGCCCTCATGTTT

GCTTTGGAACAAAAATCAAAACACGTAGTTGAGATGCTTCTTCAGAGTGGTATTGATGTC

TCTGCTGCAGATAACTTTGGGCAGATGGCATTAGCTTATGCTATTGCTAGTGGTGATACT

AACCTGAAGGAATTAATTCTTCAATATATGAAAGTAGAATCTAAACATCCTGAAAATATC

AACCCA

>CL184.Contig10_A-W 217 474 Dual specificity protein kinase CLK4 OS=Homo sapiens GN=CLK4 PE=1 SV=1

ATGCGACATTCCCAACGAACACACTGCCCTGACTGGGATAGCAGAGAAAGCTGGGGTCAT

GAAAGCTACAGTGGAAGTCACAAACGGAAGAGGAGATCTCACACAAGAGAACAGGCATTG

TAAACCGCATCACCAGTTTAAAGAATCTGATTGGATGAAAGCGCTTATCGAATCCACTGC

AGTAAATCCTCAGTCCGAAGCAGGAGAAGCAGTCCTAAAAGGAAGCGTAATAGACACTGT

TCAAGTCATCAGTTACGT

>CL184.Contig11_A-W 110 223 Dual specificity protein kinase CLK4 OS=Homo sapiens GN=CLK4 PE=1 SV=1

ATGCGACATTCCCAACGAACACACTGCCCTGACTGGGATAGCAGAGAAAGCTGGGGTCAT

GAAAGCTACAGTGGAAGTCACAAACGGAAGAGGAGATCTCACACAAGAGAACAG

>CL184.Contig12_A-W 94 351 Dual specificity protein kinase CLK4 OS=Homo sapiens GN=CLK4 PE=1 SV=1

ATGCGACATTCCCAACGAACACACTGCCCTGACTGGGATAGCAGAGAAAGCTGGGGTCAT

GAAAGCTACAGTGGAAGTCACAAACGGAAGAGGAGATCTCACACAAGAGAACAGGCATTG

TAAACCGCATCACCAGTTTAAAGAATCTGATTGGATGAAAGCGCTTATCGAATCCACTGC

AGTAAATCCTCAGTCCGAAGCAGGAGAAGCAGTCCTAAAAGGAAGCGTAATAGACACTGT

TCAAGTCATCAGTTACGT

>CL184.Contig13_A-W 94 207 Dual specificity protein kinase CLK4 OS=Homo sapiens GN=CLK4 PE=1 SV=1

ATGCGACATTCCCAACGAACACACTGCCCTGACTGGGATAGCAGAGAAAGCTGGGGTCAT

GAAAGCTACAGTGGAAGTCACAAACGGAAGAGGAGATCTCACACAAGAGAACAG

>CL184.Contig14_A-W 94 207 Dual specificity protein kinase CLK4 OS=Mus musculus GN=Clk4 PE=1 SV=1

ATGCGACATTCCCAACGAACACACTGCCCTGACTGGGATAGCAGAGAAAGCTGGGGTCAT

GAAAGCTACAGTGGAAGTCACAAACGGAAGAGGAGATCTCACACAAGAGAACAG

>CL184.Contig15_A-W 309 443 Dual specificity protein kinase CLK4 OS=Homo sapiens GN=CLK4 PE=1 SV=1

GAAAGCGCTTATCGAATCCACTGCAGTAAATCCTCAGTCCGAAGCAGGAGAAGCAGTCCT

AAAAGGAAGCGTAATAGACACTGTTCAAGTCATCAGTCACGTTCGAAGAGCCACCGAAGG

AAAAGATCCAGGAGT

>CL184.Contig16_A-W 94 207 Dual specificity protein kinase CLK4 OS=Mus musculus GN=Clk4 PE=1 SV=1

ATGCGACATTCCCAACGAACACACTGCCCTGACTGGGATAGCAGAGAAAGCTGGGGTCAT

GAAAGCTACAGTGGAAGTCACAAACGGAAGAGGAGATCTCACACAAGAGAACAG

>CL184.Contig17_A-W 94 207 Dual specificity protein kinase CLK4 OS=Mus musculus GN=Clk4 PE=1 SV=1

ATGCGACATTCCCAACGAACACACTGCCCTGACTGGGATAGCAGAGAAAGCTGGGGTCAT

GAAAGCTACAGTGGAAGTCACAAACGGAAGAGGAGATCTCACACAAGAGAACAG

>CL184.Contig18_A-W 94 207 Dual specificity protein kinase CLK4 OS=Homo sapiens GN=CLK4 PE=1 SV=1

ATGCGACATTCCCAACGAACACACTGCCCTGACTGGGATAGCAGAGAAAGCTGGGGTCAT

GAAAGCTACAGTGGAAGTCACAAACGGAAGAGGAGATCTCACACAAGAGAACAG

>CL184.Contig19_A-W 94 207 Dual specificity protein kinase CLK4 OS=Homo sapiens GN=CLK4 PE=1 SV=1

ATGCGACATTCCCAACGAACACACTGCCCTGACTGGGATAGCAGAGAAAGCTGGGGTCAT

GAAAGCTACAGTGGAAGTCACAAACGGAAGAGGAGATCTCACACAAGAGAACAG

>CL184.Contig20_A-W 94 351 Dual specificity protein kinase CLK4 OS=Homo sapiens GN=CLK4 PE=1 SV=1

ATGCGACATTCCCAACGAACACACTGCCCTGACTGGGATAGCAGAGAAAGCTGGGGTCAT

GAAAGCTACAGTGGAAGTCACAAACGGAAGAGGAGATCTCACACAAGAGAACAGGCATTG

TAAACCGCATCACCAGTTTAAAGAATCTGATTGGATGAAAGCGCTTATCGAATCCACTGC

AGTAAATCCTCAGTCCGAAGCAGGAGAAGCAGTCCTAAAAGGAAGCGTAATAGACACTGT

TCAAGTCATCAGTTACGT

>CL184.Contig21_A-W 94 351 Dual specificity protein kinase CLK4 OS=Homo sapiens GN=CLK4 PE=1 SV=1

ATGCGACATTCCCAACGAACACACTGCCCTGACTGGGATAGCAGAGAAAGCTGGGGTCAT

GAAAGCTACAGTGGAAGTCACAAACGGAAGAGGAGATCTCACACAAGAGAACAGGCATTG

TAAACCGCATCACCAGTTTAAAGAATCTGATTGGATGAAAGCGCTTATCGAATCCACTGC

AGTAAATCCTCAGTCCGAAGCAGGAGAAGCAGTCCTAAAAGGAAGCGTAATAGACACTGT

TCAAGTCATCAGTTACGT

>CL184.Contig22_A-W 94 207 Dual specificity protein kinase CLK4 OS=Homo sapiens GN=CLK4 PE=1 SV=1

ATGCGACATTCCCAACGAACACACTGCCCTGACTGGGATAGCAGAGAAAGCTGGGGTCAT

GAAAGCTACAGTGGAAGTCACAAACGGAAGAGGAGATCTCACACAAGAGAACAG

>CL184.Contig23_A-W 94 207 Dual specificity protein kinase CLK4 OS=Mus musculus GN=Clk4 PE=1 SV=1

ATGCGACATTCCCAACGAACACACTGCCCTGACTGGGATAGCAGAGAAAGCTGGGGTCAT

GAAAGCTACAGTGGAAGTCACAAACGGAAGAGGAGATCTCACACAAGAGAACAG

>CL184.Contig24_A-W 937 1683 ankyrin repeat domain 26-like [Bos taurus]

CAGGCCCTACAATATCTGGAAGAGAAATGTGCAACTATCCTTCTAGAAAATGGTGCTGAT

CCAAATGCTCACAACAACCAAAATGAAACTCCTCTTCACTTTGCTCTGTCTCTGAAGAAA

ACATCACTAGCAGAAAAACTGCTTTCACTCAATGCAGATATAGAAGCCAGAACTACGAGG

GGCTATACACCACTTTTACTTGCCATAGAGCAAAACAGACAGCAGATGGTTGAAACATTA

ATTCAGAACAAAGCAAATATACATGCAGTTGACCAGAAGGGAAGAACAGCCCTCATGTTT

GCTTTGGAACAAAAATCAAAACACGTAGTTGAGATGCTTCTTCAGAGTGGTATTGATGTC

TCTGCTGCAGATAACTTTGGGCAGATGGCATTAGCTTATGCTATTGCTAGTGGTGATACT

AACCTGAAGGAATTAATTCTTCAATATATGAAAGTAGAATCTAAACATCCTGAAAATATC

AACCCAGTGGATGAAGATTCTACAGATGACCATTTAAAAAGGATTTCTAATAAAACTGAT

CCTGATGATCATTGTCTCACCTTGGATGAGGCCAAATATAAATATTATGACAAGGATATC

CCCAAAGAAAATGGAGAATTACCTCACACTTCTAATGAATCGAAGAATGATGAAGAAACA

AATGAAACTGAGGAACCTAAACTTAAAACATTACCTGGAAACTGTGATGGTGATATTAAA

AAGAAAAATGTGATTGAAACCACTCCT

>CL184.Contig25_A-W 94 351 PREDICTED: dual specificity protein kinase CLK4-like [Cavia porcellus]

ATGCGACATTCCCAACGAACACACTGCCCTGACTGGGATAGCAGAGAAAGCTGGGGTCAT

GAAAGCTACAGTGGAAGTCACAAACGGAAGAGGAGATCTCACACAAGAGAACAGGCATTG

TAAACCGCATCACCAGTTTAAAGAATCTGATTGGATGAAAGCGCTTATCGAATCCACTGC

AGTAAATCCTCAGTCCGAAGCAGGAGAAGCAGTCCTAAAAGGAAGCGTAATAGACACTGT

TCAAGTCATCAGTTACGT

>CL184.Contig26_A-W 94 351 Dual specificity protein kinase CLK4 OS=Homo sapiens GN=CLK4 PE=1 SV=1

ATGCGACATTCCCAACGAACACACTGCCCTGACTGGGATAGCAGAGAAAGCTGGGGTCAT

GAAAGCTACAGTGGAAGTCACAAACGGAAGAGGAGATCTCACACAAGAGAACAGGCATTG

TAAACCGCATCACCAGTTTAAAGAATCTGATTGGATGAAAGCGCTTATCGAATCCACTGC

AGTAAATCCTCAGTCCGAAGCAGGAGAAGCAGTCCTAAAAGGAAGCGTAATAGACACTGT

TCAAGTCATCAGTTACGT

>CL184.Contig27_A-W 94 384 PREDICTED: dual specificity protein kinase CLK4 isoform 1 [Canis lupus familiaris]

ATGCGACATTCCCAACGAACACACTGCCCTGACTGGGATAGCAGAGAAAGCTGGGGTCAT

GAAAGCTACAGTGGAAGTCACAAACGGAAGAGGAGATCTCACACAAGAGAACAGGCATTG

TAAACCGCATCACCAGTTTAAAGAATCTGATTGGATGAAAGCGCTTATCGAATCCACTGC

AGTAAATCCTCAGTCCGAAGCAGGAGAAGCAGTCCTAAAAGGAAGCGTAATAGACACTGT

TCAAGTCATCAGTCACGTTCGAAGAGCCACCGAAGGAAAAGATCCAGGAGT

>CL184.Contig28_A-W 19 144 minus strand dual specificity protein kinase CLK1 [Bos taurus] >gi|151556155|gb|AAI49370.1| CLK1 protein [Bos taurus] >gi|157279322|gb|AAI49004.1| CDC-like kinase 1 [Bos taurus] >gi|296490446|gb|DAA32559.1| CDC-like kinase 1 [Bos taurus]

ATTTATACTTAGTTACTTATACCTCTTTTTCTCTCCCTCACTTTAGATGAAATCGTGGAC

ACTTTGGGTGAAGGGGCCTTTGGCAAAGTTGTAGAATGCATTGATCATGGCATACCTGGC

CCTCAC

>CL186.Contig1_A-W 5 148 PREDICTED: serine/threonine kinase 40 [Oryctolagus cuniculus]

GCGGCTGTTCGCGCGGCTGTGCTGGGAGGCGGCGGTGAGCGGCTCCCACGCCCCCGGCCC

GGCCCTGGCCCTCCGGGACGGAGCGCCGAGCAGCCCCGGCTCCGGGCTGCGGACTATGGG

CGAGCAGCGCTGATTACCCGGGGA

>CL186.Contig2_A-W 5 148 PREDICTED: serine/threonine kinase 40 [Oryctolagus cuniculus]

GCGGCTGTTCGCGCGGCTGTGCTGGGAGGCGGCGGTGAGCGGCTCCCACGCCCCCGGCCC

GGCCCTGGCCCTCCGGGACGGAGCGCCGAGCAGCCCCGGCTCCGGGCTGCGGACTATGGG

CGAGCAGCGCTGATTACCCGGGGA

>CL188.Contig1_A-W 76 798 minus strand PREDICTED: glutaminyl-tRNA synthetase-like [Equus caballus]

ATGGCGGCTCTAGACTCTCTGGCGCTTTTCACCGGCTTGGGCCTGAGCGAGCATAAGGCC

CGTGAGACGCTCAAGAACGCTGCTCTGAGCACGCAGCTGCGCGACGCGGCGACTCAGGCA

CAGCAGACTCTGGGCTCCACCATCGACAAGGCTACCGGGACCCTTCTATATGGCTTAGCT

TCCCGACTCAGGGATACCAGGCGTCTCTCTTTCCTCGTGAGCTATATAGCCAGTAAGAAG

ATCCACACTGAGCCTCAGCTGAGCGCTGCCCTTGAATATGTGCGGAGTCACCCCCTGGAC

CCTATTGACACCATGGACTTTGAGCAGGAATGTGGTGTGGGTGTCATGGTGACCCCAGAG

CAGATTGAGGAGGCTGTGGAGGCCACCATAAATAAGCACCGGGCTCAGATTCTGGTGGAG

CGTTACCGGTTCAACATGGGGCTGCTGATGGGAGAGGCTCGGGCTGCGCTTAAGTGGGCA

GATGGCAAAATGATCAAGCACGAAGTGGACATGCAGGTCCTCCACCTTCTGGGTCCCAAG

ATGGAAGCTGACCTAGAAAAGAAACCCAAGGTGGCCAAGGCTCGTGTGGAAGAAACAGAC

CAGAGGACAGCAAAAGATGTGGTGGAGAATGCAGTTGAGGCTGCTGGACAAACCTTGTCT

CTGATGGAGCAGCTCCGGGGTGAAGCCCTAAAGTTCCATAAACCTGGTACGTACCCGGTT

CCC

>CL188.Contig2_A-W 76 1242 minus strand PREDICTED: glutamine--tRNA ligase isoform 1 [Pan paniscus]

ATGGCGGCTCTAGACTCTCTGGCGCTTTTCACCGGCTTGGGCCTGAGCGAGCATAAGGCC

CGTGAGACGCTCAAGAACGCTGCTCTGAGCACGCAGCTGCGCGACGCGGCGACTCAGGCA

CAGCAGACTCTGGGCTCCACCATCGACAAGGCTACCGGGACCCTTCTATATGGCTTAGCT

TCCCGACTCAGGGATACCAGGCGTCTCTCTTTCCTCGTGAGCTATATAGCCAGTAAGAAG

ATCCACACTGAGCCTCAGCTGAGCGCTGCCCTTGAATATGTGCGGAGTCACCCCCTGGAC

CCTATTGACACCATGGACTTTGAGCAGGAATGTGGTGTGGGTGTCATGGTGACCCCAGAG

CAGATTGAGGAGGCTGTGGAGGCCACCATAAATAAGCACCGGGCTCAGATTCTGGTGGAG

CGTTACCGGTTCAACATGGGGCTGCTGATGGGAGAGGCTCGGGCTGCGCTTAAGTGGGCA

GATGGCAAAATGATCAAGCACGAAGTGGACATGCAGGTCCTCCACCTTCTGGGTCCCAAG

ATGGAAGCTGACCTAGAAAAGAAACCCAAGGTGGCCAAGGCTCGTGTGGAAGAAACAGAC

CAGAGGACAGCAAAAGATGTGGTGGAGAATGCAGTTGAGGCTGCTGGACAAACCTTGTCT

CTGATGGAGCAGCTCCGGGGTGAAGCCCTAAAGTTCCATAAACCTGGTGAAAACTACAAA

ACCCCAGGCTATGTGATCACTCCACACACCATGGATCTACTGAGGCACCACCTGGAGATC

ACTGGGGGGCAGGTACGTACCCGGTTCCCGCCAGAACCCAACGGAATCCTGCATATTGGA

CATGCCAAAGCCATCAATTTCAACTTTGGCTATGCCAAGGCCAACAATGGTATCTGTTTT

CTGCGCTTTGATGACACCAATCCTGAGAAAGAGGAAGAAAAATTCTTCACTGCTATCTGT

GACATGGTGGCTTGGTTAGGTTATACACCTTACAAGGTGACATATGCGTCTGACTATTTT

GACCAGCTCTATGCTTGGGCTGTGGAGCTCATCCACAGGGGTCAGGCTTACGTGTGCCAT

CAGCGAGGGGAGGAGCTCAAAGGCCACAACCCTCTGCCCTCACCCTGGAGAGACCGTCCC

TTAGAGGAGTCATTGCTGCTCTTTGAG

>CL188.Contig3_A-W 521 1339 minus strand Glutamine--tRNA ligase OS=Homo sapiens GN=QARS PE=1 SV=1

CAGGCAATGCGCAAGGGTAAATTTTCAGAGGGAGAGGCTACACTGCGTATGAAGTTGGTG

ATGGAAGACGGCAAGATGGACCCTGTGGCCTATCGAGTCAAGTATACACCACACCACCGT

ACAGGGGACAAATGGTGCATCTATCCCACGTATGACTACACACACTGCCTCTGTGACTCC

ATTGAGCATATCACCCACTCACTCTGCACTAAGGAATTCCAGGCCCGACGTTCTTCTTAT

TTCTGGCTGTGCAATGCACTGGATGTATATTGCCCTGTGCAGTGGGAGTATGGCCGTCTC

AATCTGCACTATGCTGTTGTCTCTAAGAGGAAGATCCTTCAGCTTGTAGCTGCTGGTGCT

GTGCGGGACTGGGATGACCCACGACTCTTCACACTGACTGCTCTGCAACGGCGGGGCTTT

CCGCCTGAGGCCATCAACAACTTCTGTGCTCGGGTGGGAGTGACAGTAGCACAGACCACA

ATGGAGCCACATCTTCTTGAAGCCTGTGTACGTGATGTGCTGAACGATACAGCTCCACGG

GCTATGGCTGTGCTGGAGTCACTACGGGTTGTCATTACCAACTTTCCTGCTGCCAAGCCC

TTGGAAATCCAGGTGCCCAACTTTCCAGCTGATGAAACCAAGGGCTTCCACCAGGTTCCT

TTTGCACCCATAGTCTTCATTGAGAGGACTGACTTCAAGGAGGAGCCAGAGCCAGGCTAT

AAGCGTCTAGCTTGGGGCCAGCCTGTGGGCCTGAGACATACAGGCTATGTCATTGAGCTG

CGGCATGTTATCAAGGTGAGAGGCTGCGTTCAGGATTTG

>CL188.Contig4_A-W 66 566 minus strand PREDICTED: glutaminyl-tRNA synthetase [Oryctolagus cuniculus]

GGGACTAGATGGTATAACCCACTGCCTATGCCCCATAGGTGCATCTATCCCACGTATGAC

TACACACACTGCCTCTGTGACTCCATTGAGCATATCACCCACTCACTCTGCACTAAGGAA

TTCCAGGCCCGACGTTCTTCTTATTTCTGGCTGTGCAATGCACTGGATGTATATTGCCCT

GTGCAGTGGGAGTATGGCCGTCTCAATCTGCACTATGCTGTTGTCTCTAAGAGGAAGATC

CTTCAGCTTGTAGCTGCTGGTGCTGTGCGGGACTGGGATGACCCACGACTCTTCACACTG

ACTGCTCTGCAACGGCGGGGCTTTCCGCCTGAGGCCATCAACAACTTCTGTGCTCGGGTG

GGAGTGACAGTAGCACAGACCACAATGGAGCCACATCTTCTTGAAGCCTGTGTACGTGAT

GTGCTGAACGATACAGCTCCACGGGCTATGGCTGTGCTGGAGTCACTACGGGTTGTCATT

ACCAACTTTCCTGCTGCCAAG

>CL188.Contig5_A-W 51 788 minus strand Glutamine--tRNA ligase OS=Homo sapiens GN=QARS PE=1 SV=1

GTACGTACCCGGTTCCCGCCAGAACCCAACGGAATCCTGCATATTGGACATGCCAAAGCC

ATCAATTTCAACTTTGGCTATGCCAAGGCCAACAATGGTATCTGTTTTCTGCGCTTTGAT

GACACCAATCCTGAGAAAGAGGAAGAAAAATTCTTCACTGCTATCTGTGACATGGTGGCT

TGGTTAGGTTATACACCTTACAAGGTGACATATGCGTCTGACTATTTTGACCAGCTCTAT

GCTTGGGCTGTGGAGCTCATCCACAGGGGTCAGGCTTACGTGTGCCATCAGCGAGGGGAG

GAGCTCAAAGGCCACAACCCTCTGCCCTCACCCTGGAGAGACCGTCCCTTAGAGGAGTCA

TTGCTGCTCTTTGAGGCAATGCGCAAGGGTAAATTTTCAGAGGGAGAGGCTACACTGCGT

ATGAAGTTGGTGATGGAAGACGGCAAGATGGACCCTGTGGCCTATCGAGTCAAGTATACA

CCACACCACCGTACAGGGGACAAATGGTGCATCTATCCCACGTATGACTACACACACTGC

CTCTGTGACTCCATTGAGCATATCACCCACTCACTCTGCACTAAGGAATTCCAGGCCCGA

CGTTCTTCTTATTTCTGGCTGTGCAATGCACTGGATGTATATTGCCCTGTGCAGTGGGAG

TATGGCCGTCTCAATCTGCACTATGCTGTTGTCTCTAAGAGGAAGATCCTTCAGCTTGTA

GCTGCTGGTGCTGTGCGG

>CL188.Contig6_A-W 105 335 minus strand PREDICTED: glutaminyl-tRNA synthetase-like [Loxodonta africana]

TGCATCTATCCCACGTATGACTACACACACTGCCTCTGTGACTCCATTGAGCATATCACC

CACTCACTCTGCACTAAGGAATTCCAGGCCCGACGTTCTTCTTATTTCTGGCTGTGCAAT

GCACTGGATGTATATTGCCCTGTGCAGTGGGAGTATGGCCGTCTCAATCTGCACTATGCT

GTTGTCTCTAAGAGGAAGATCCTTCAGCTTGTAGCTGCTGGTGCTGTGCGG

>CL188.Contig7_A-W 51 1241 minus strand Glutamine--tRNA ligase OS=Homo sapiens GN=QARS PE=1 SV=1

GTACGTACCCGGTTCCCGCCAGAACCCAACGGAATCCTGCATATTGGACATGCCAAAGCC

ATCAATTTCAACTTTGGCTATGCCAAGGCCAACAATGGTATCTGTTTTCTGCGCTTTGAT

GACACCAATCCTGAGAAAGAGGAAGAAAAATTCTTCACTGCTATCTGTGACATGGTGGCT

TGGTTAGGTTATACACCTTACAAGGTGACATATGCGTCTGACTATTTTGACCAGCTCTAT

GCTTGGGCTGTGGAGCTCATCCACAGGGGTCAGGCTTACGTGTGCCATCAGCGAGGGGAG

GAGCTCAAAGGCCACAACCCTCTGCCCTCACCCTGGAGAGACCGTCCCTTAGAGGAGTCA

TTGCTGCTCTTTGAGGCAATGCGCAAGGGTAAATTTTCAGAGGGAGAGGCTACACTGCGT

ATGAAGTTGGTGATGGAAGACGGCAAGATGGACCCTGTGGCCTATCGAGTCAAGTATACA

CCACACCACCGTACAGGGGACAAATGGTGCATCTATCCCACGTATGACTACACACACTGC

CTCTGTGACTCCATTGAGCATATCACCCACTCACTCTGCACTAAGGAATTCCAGGCCCGA

CGTTCTTCTTATTTCTGGCTGTGCAATGCACTGGATGTATATTGCCCTGTGCAGTGGGAG

TATGGCCGTCTCAATCTGCACTATGCTGTTGTCTCTAAGAGGAAGATCCTTCAGCTTGTA

GCTGCTGGTGCTGTGCGGGACTGGGATGACCCACGACTCTTCACACTGACTGCTCTGCAA

CGGCGGGGCTTTCCGCCTGAGGCCATCAACAACTTCTGTGCTCGGGTGGGAGTGACAGTA

GCACAGACCACAATGGAGCCACATCTTCTTGAAGCCTGTGTACGTGATGTGCTGAACGAT

ACAGCTCCACGGGCTATGGCTGTGCTGGAGTCACTACGGGTTGTCATTACCAACTTTCCT

GCTGCCAAGCCCTTGGAAATCCAGGTGCCCAACTTTCCAGCTGATGAAACCAAGGGCTTC

CACCAGGTTCCTTTTGCACCCATAGTCTTCATTGAGAGGACTGACTTCAAGGAGGAGCCA

GAGCCAGGCTATAAGCGTCTAGCTTGGGGCCAGCCTGTGGGCCTGAGACATACAGGCTAT

GTCATTGAGCTGCGGCATGTTATCAAGGTGAGAGGCTGCGTTCAGGATTTG

>CL188.Contig8_A-W 1 108 minus strand PREDICTED: glutaminyl-tRNA synthetase [Oryctolagus cuniculus]

GGTCAGGCTTACGTGTGCCATCAGCGAGGGGAGGAGCTCAAAGGCCACAACCCTCTGCCC

TCACCCTGGAGAGACCGTCCCTTAGAGGAGTCATTGCTGCTCTTTGAG

>CL188.Contig9_A-W 105 1133 minus strand PREDICTED: glutaminyl-tRNA synthetase-like [Loxodonta africana]

TGCATCTATCCCACGTATGACTACACACACTGCCTCTGTGACTCCATTGAGCATATCACC

CACTCACTCTGCACTAAGGAATTCCAGGCCCGACGTTCTTCTTATTTCTGGCTGTGCAAT

GCACTGGATGTATATTGCCCTGTGCAGTGGGAGTATGGCCGTCTCAATCTGCACTATGCT

GTTGTCTCTAAGAGGAAGATCCTTCAGCTTGTAGCTGCTGGTGCTGTGCGGGACTGGGAT

GACCCACGACTCTTCACACTGACTGCTCTGCAACGGCGGGGCTTTCCGCCTGAGGCCATC

AACAACTTCTGTGCTCGGGTGGGAGTGACAGTAGCACAGACCACAATGGAGCCACATCTT

CTTGAAGCCTGTGTACGTGATGTGCTGAACGATACAGCTCCACGGGCTATGGCTGTGCTG

GAGTCACTACGGGTTGTCATTACCAACTTTCCTGCTGCCAAGCCCTTGGAAATCCAGGTG

CCCAACTTTCCAGCTGATGAAACCAAGGGCTTCCACCAGGTTCCTTTTGCACCCATAGTC

TTCATTGAGAGGACTGACTTCAAGGAGGAGCCAGAGCCAGGCTATAAGCGTCTAGCTTGG

GGCCAGCCTGTGGGCCTGAGACATACAGGCTATGTCATTGAGCTGCGGCATGTTATCAAG

GATCCTAGTGGCTGTGTGGAGAGCCTGGAGGTGACCTGTAGACGGGCTGATGCTGGAGAG

AAGCCCAAGGCTTTTATTCACTGGGTTTCACAGCCTTTGATATGCGAAATTCGCCTTTAT

GATCGACTATTCCAGCACAAGAACCCTGAAGATCCTGCTGAGGTGCCAGGTGGATTCTTA

AGTGATCTGAACCCGGCATCACTACAAGTGGTGGAGGCAGCATTAGTGGATTGTTCTGTG

GCCCTGGCAAAACCCTTTGACAAGTTCCAGTTTGAGCGCCTCGGGTACTTCTCCGTGGAT

CCGGATAGCCGCCAAGGACAGCTTGTCTTCAACCGAACAGTCACACTAAAGGAGGACCCA

GGAAAGGTG

>CL188.Contig10_A-W 1 108 minus strand PREDICTED: glutaminyl-tRNA synthetase [Oryctolagus cuniculus]

GGTCAGGCTTACGTGTGCCATCAGCGAGGGGAGGAGCTCAAAGGCCACAACCCTCTGCCC

TCACCCTGGAGAGACCGTCCCTTAGAGGAGTCATTGCTGCTCTTTGAG

>CL188.Contig11_A-W 896 1117 minus strand PREDICTED: glutaminyl-tRNA synthetase [Oryctolagus cuniculus]

CCCTTGGAAATCCAGGTGCCCAACTTTCCAGCTGATGAAACCAAGGGCTTCCACCAGGTT

CCTTTTGCACCCATAGTCTTCATTGAGAGGACTGACTTCAAGGAGGAGCCAGAGCCAGGC

TATAAGCGTCTAGCTTGGGGCCAGCCTGTGGGCCTGAGACATACAGGCTATGTCATTGAG

CTGCGGCATGTTATCAAGGTGAGAGGCTGCGTTCAGGATTTG

>CL188.Contig12_A-W 1 108 minus strand PREDICTED: glutaminyl-tRNA synthetase isoform 1 [Pan troglodytes]

GGTCAGGCTTACGTGTGCCATCAGCGAGGGGAGGAGCTCAAAGGCCACAACCCTCTGCCC

TCACCCTGGAGAGACCGTCCCTTAGAGGAGTCATTGCTGCTCTTTGAG

>CL188.Contig13_A-W 825 1043 minus strand PREDICTED: glutaminyl-tRNA synthetase isoform 1 [Pan troglodytes]

TTGGAAATCCAGGTGCCCAACTTTCCAGCTGATGAAACCAAGGGCTTCCACCAGGTTCCT

TTTGCACCCATAGTCTTCATTGAGAGGACTGACTTCAAGGAGGAGCCAGAGCCAGGCTAT

AAGCGTCTAGCTTGGGGCCAGCCTGTGGGCCTGAGACATACAGGCTATGTCATTGAGCTG

CGGCATGTTATCAAGGTGAGAGGCTGCGTTCAGGATTTG

>CL188.Contig14_A-W 760 981 minus strand PREDICTED: glutaminyl-tRNA synthetase [Oryctolagus cuniculus]

CCCTTGGAAATCCAGGTGCCCAACTTTCCAGCTGATGAAACCAAGGGCTTCCACCAGGTT

CCTTTTGCACCCATAGTCTTCATTGAGAGGACTGACTTCAAGGAGGAGCCAGAGCCAGGC

TATAAGCGTCTAGCTTGGGGCCAGCCTGTGGGCCTGAGACATACAGGCTATGTCATTGAG

CTGCGGCATGTTATCAAGGTGAGAGGCTGCGTTCAGGATTTG

>CL188.Contig15_A-W 686 907 minus strand PREDICTED: glutaminyl-tRNA synthetase [Oryctolagus cuniculus]

CCCTTGGAAATCCAGGTGCCCAACTTTCCAGCTGATGAAACCAAGGGCTTCCACCAGGTT

CCTTTTGCACCCATAGTCTTCATTGAGAGGACTGACTTCAAGGAGGAGCCAGAGCCAGGC

TATAAGCGTCTAGCTTGGGGCCAGCCTGTGGGCCTGAGACATACAGGCTATGTCATTGAG

CTGCGGCATGTTATCAAGGTGAGAGGCTGCGTTCAGGATTTG

>CL188.Contig16_A-W 105 335 minus strand PREDICTED: glutaminyl-tRNA synthetase isoform 1 [Pan troglodytes]

TGCATCTATCCCACGTATGACTACACACACTGCCTCTGTGACTCCATTGAGCATATCACC

CACTCACTCTGCACTAAGGAATTCCAGGCCCGACGTTCTTCTTATTTCTGGCTGTGCAAT

GCACTGGATGTATATTGCCCTGTGCAGTGGGAGTATGGCCGTCTCAATCTGCACTATGCT

GTTGTCTCTAAGAGGAAGATCCTTCAGCTTGTAGCTGCTGGTGCTGTGCGG

>CL188.Contig17_A-W 105 788 minus strand PREDICTED: glutamine--tRNA ligase isoform 1 [Pan paniscus]

TGCATCTATCCCACGTATGACTACACACACTGCCTCTGTGACTCCATTGAGCATATCACC

CACTCACTCTGCACTAAGGAATTCCAGGCCCGACGTTCTTCTTATTTCTGGCTGTGCAAT

GCACTGGATGTATATTGCCCTGTGCAGTGGGAGTATGGCCGTCTCAATCTGCACTATGCT

GTTGTCTCTAAGAGGAAGATCCTTCAGCTTGTAGCTGCTGGTGCTGTGCGGGACTGGGAT

GACCCACGACTCTTCACACTGACTGCTCTGCAACGGCGGGGCTTTCCGCCTGAGGCCATC

AACAACTTCTGTGCTCGGGTGGGAGTGACAGTAGCACAGACCACAATGGAGCCACATCTT

CTTGAAGCCTGTGTACGTGATGTGCTGAACGATACAGCTCCACGGGCTATGGCTGTGCTG

GAGTCACTACGGGTTGTCATTACCAACTTTCCTGCTGCCAAGCCCTTGGAAATCCAGGTG

CCCAACTTTCCAGCTGATGAAACCAAGGGCTTCCACCAGGTTCCTTTTGCACCCATAGTC

TTCATTGAGAGGACTGACTTCAAGGAGGAGCCAGAGCCAGGCTATAAGCGTCTAGCTTGG

GGCCAGCCTGTGGGCCTGAGACATACAGGCTATGTCATTGAGCTGCGGCATGTTATCAAG

GTGAGAGGCTGCGTTCAGGATTTG

>CL188.Contig18_A-W 50 415 minus strand glutamine--tRNA ligase [Bos taurus] >gi|110287978|sp|Q3MHH4.1|SYQ_BOVIN RecName: Full=Glutamine--tRNA ligase; AltName: Full=Glutaminyl-tRNA synthetase; Short=GlnRS >gi|75948195|gb|AAI05238.1| Glutamyl-prolyl-tRNA synthetase [Bos taurus] >gi|95769540|gb|ABF57443.1| glutaminyl-tRNA synthetase [Bos taurus]

CCTAGTGGCTGTGTGGAGAGCCTGGAGGTGACCTGTAGACGGGCTGATGCTGGAGAGAAG

CCCAAGGCTTTTATTCACTGGGTTTCACAGCCTTTGATATGCGAAATTCGCCTTTATGAT

CGACTATTCCAGCACAAGAACCCTGAAGATCCTGCTGAGGTGCCAGGTGGATTCTTAAGT

GATCTGAACCCGGCATCACTACAAGTGGTGGAGGCAGCATTAGTGGATTGTTCTGTGGCC

CTGGCAAAACCCTTTGACAAGTTCCAGTTTGAGCGCCTCGGGTACTTCTCCGTGGATCCG

GATAGCCGCCAAGGACAGCTTGTCTTCAACCGAACAGTCACACTAAAGGAGGACCCAGGA

AAGGTG

>CL188.Contig19_A-W 76 783 minus strand PREDICTED: glutaminyl-tRNA synthetase-like [Loxodonta africana]

ATGGCGGCTCTAGACTCTCTGGCGCTTTTCACCGGCTTGGGCCTGAGCGAGCATAAGGCC

CGTGAGACGCTCAAGAACGCTGCTCTGAGCACGCAGCTGCGCGACGCGGCGACTCAGGCA

CAGCAGACTCTGGGCTCCACCATCGACAAGGCTACCGGGACCCTTCTATATGGCTTAGCT

TCCCGACTCAGGGATACCAGGCGTCTCTCTTTCCTCGTGAGCTATATAGCCAGTAAGAAG

ATCCACACTGAGCCTCAGCTGAGCGCTGCCCTTGAATATGTGCGGAGTCACCCCCTGGAC

CCTATTGACACCATGGACTTTGAGCAGGAATGTGGTGTGGGTGTCATGGTGACCCCAGAG

CAGATTGAGGAGGCTGTGGAGGCCACCATAAATAAGCACCGGGCTCAGATTCTGGTGGAG

CGTTACCGGTTCAACATGGGGCTGCTGATGGGAGAGGCTCGGGCTGCGCTTAAGTGGGCA

GATGGCAAAATGATCAAGCACGAAGTGGACATGCAGGTCCTCCACCTTCTGGGTCCCAAG

ATGGAAGCTGACCTAGAAAAGAAACCCAAGGTGGCCAAGGCTCGTGTGGAAGAAACAGAC

CAGAGGACAGCAAAAGATGTGGTGGAGAATGCAGTTGAGGCTGCTGGACAAACCTTGTCT

CTGATGGAGCAGCTCCGGGGTGAAGCCCTAAAGTTCCATAAACCTGGT

>CL188.Contig20_A-W 77 247 minus strand PREDICTED: glutaminyl-tRNA synthetase-like [Equus caballus]

TCACTACAAGTGGTGGAGGCAGCATTAGTGGATTGTTCTGTGGCCCTGGCAAAACCCTTT

GACAAGTTCCAGTTTGAGCGCCTCGGGTACTTCTCCGTGGATCCGGATAGCCGCCAAGGA

CAGCTTGTCTTCAACCGAACAGTCACACTAAAGGAGGACCCAGGAAAGGTG

>CL188.Contig21_A-W 1870 2436 minus strand PREDICTED: glutaminyl-tRNA synthetase [Oryctolagus cuniculus]

CCCTTGGAAATCCAGGTGCCCAACTTTCCAGCTGATGAAACCAAGGGCTTCCACCAGGTT

CCTTTTGCACCCATAGTCTTCATTGAGAGGACTGACTTCAAGGAGGAGCCAGAGCCAGGC

TATAAGCGTCTAGCTTGGGGCCAGCCTGTGGGCCTGAGACATACAGGCTATGTCATTGAG

CTGCGGCATGTTATCAAGGATCCTAGTGGCTGTGTGGAGAGCCTGGAGGTGACCTGTAGA

CGGGCTGATGCTGGAGAGAAGCCCAAGGCTTTTATTCACTGGGTTTCACAGCCTTTGATA

TGCGAAATTCGCCTTTATGATCGACTATTCCAGCACAAGAACCCTGAAGATCCTGCTGAG

GTGCCAGGTGGATTCTTAAGTGATCTGAACCCGGCATCACTACAAGTGGTGGAGGCAGCA

TTAGTGGATTGTTCTGTGGCCCTGGCAAAACCCTTTGACAAGTTCCAGTTTGAGCGCCTC

GGGTACTTCTCCGTGGATCCGGATAGCCGCCAAGGACAGCTTGTCTTCAACCGAACAGTC

ACACTAAAGGAGGACCCAGGAAAGGTG

>CL188.Contig22_A-W 782 1156 minus strand PREDICTED: glutamine--tRNA ligase isoform 1 [Pan paniscus]

GTACGTACCCGGTTCCCGCCAGAACCCAACGGAATCCTGCATATTGGACATGCCAAAGCC

ATCAATTTCAACTTTGGCTATGCCAAGGCCAACAATGGTATCTGTTTTCTGCGCTTTGAT

GACACCAATCCTGAGAAAGAGGAAGAAAAATTCTTCACTGCTATCTGTGACATGGTGGCT

TGGTTAGGTTATACACCTTACAAGGTGACATATGCGTCTGACTATTTTGACCAGCTCTAT

GCTTGGGCTGTGGAGCTCATCCACAGGGGTCAGGCTTACGTGTGCCATCAGCGAGGGGAG

GAGCTCAAAGGCCACAACCCTCTGCCCTCACCCTGGAGAGACCGTCCCTTAGAGGAGTCA

TTGCTGCTCTTTGAG

>CL188.Contig23_A-W 1314 1877 minus strand Glutamine--tRNA ligase OS=Homo sapiens GN=QARS PE=1 SV=1

TTGGAAATCCAGGTGCCCAACTTTCCAGCTGATGAAACCAAGGGCTTCCACCAGGTTCCT

TTTGCACCCATAGTCTTCATTGAGAGGACTGACTTCAAGGAGGAGCCAGAGCCAGGCTAT

AAGCGTCTAGCTTGGGGCCAGCCTGTGGGCCTGAGACATACAGGCTATGTCATTGAGCTG

CGGCATGTTATCAAGGATCCTAGTGGCTGTGTGGAGAGCCTGGAGGTGACCTGTAGACGG

GCTGATGCTGGAGAGAAGCCCAAGGCTTTTATTCACTGGGTTTCACAGCCTTTGATATGC

GAAATTCGCCTTTATGATCGACTATTCCAGCACAAGAACCCTGAAGATCCTGCTGAGGTG

CCAGGTGGATTCTTAAGTGATCTGAACCCGGCATCACTACAAGTGGTGGAGGCAGCATTA

GTGGATTGTTCTGTGGCCCTGGCAAAACCCTTTGACAAGTTCCAGTTTGAGCGCCTCGGG

TACTTCTCCGTGGATCCGGATAGCCGCCAAGGACAGCTTGTCTTCAACCGAACAGTCACA

CTAAAGGAGGACCCAGGAAAGGTG

>CL188.Contig24_A-W 521 1117 minus strand Glutamine--tRNA ligase OS=Homo sapiens GN=QARS PE=1 SV=1

CAGGCAATGCGCAAGGGTAAATTTTCAGAGGGAGAGGCTACACTGCGTATGAAGTTGGTG

ATGGAAGACGGCAAGATGGACCCTGTGGCCTATCGAGTCAAGTATACACCACACCACCGT

ACAGGGGACAAATGGTGCATCTATCCCACGTATGACTACACACACTGCCTCTGTGACTCC

ATTGAGCATATCACCCACTCACTCTGCACTAAGGAATTCCAGGCCCGACGTTCTTCTTAT

TTCTGGCTGTGCAATGCACTGGATGTATATTGCCCTGTGCAGTGGGAGTATGGCCGTCTC

AATCTGCACTATGCTGTTGTCTCTAAGAGGAAGATCCTTCAGCTTGTAGCTGCTGGTGCT

GTGCGGGACTGGGATGACCCACGACTCTTCACACTGACTGCTCTGCAACGGCGGGGCTTT

CCGCCTGAGGCCATCAACAACTTCTGTGCTCGGGTGGGAGTGACAGTAGCACAGACCACA

ATGGAGCCACATCTTCTTGAAGCCTGTGTACGTGATGTGCTGAACGATACAGCTCCACGG

GCTATGGCTGTGCTGGAGTCACTACGGGTTGTCATTACCAACTTTCCTGCTGCCAAG

>CL188.Contig25_A-W 51 788 minus strand Glutamine--tRNA ligase OS=Homo sapiens GN=QARS PE=1 SV=1

GTACGTACCCGGTTCCCGCCAGAACCCAACGGAATCCTGCATATTGGACATGCCAAAGCC

ATCAATTTCAACTTTGGCTATGCCAAGGCCAACAATGGTATCTGTTTTCTGCGCTTTGAT

GACACCAATCCTGAGAAAGAGGAAGAAAAATTCTTCACTGCTATCTGTGACATGGTGGCT

TGGTTAGGTTATACACCTTACAAGGTGACATATGCGTCTGACTATTTTGACCAGCTCTAT

GCTTGGGCTGTGGAGCTCATCCACAGGGGTCAGGCTTACGTGTGCCATCAGCGAGGGGAG

GAGCTCAAAGGCCACAACCCTCTGCCCTCACCCTGGAGAGACCGTCCCTTAGAGGAGTCA

TTGCTGCTCTTTGAGGCAATGCGCAAGGGTAAATTTTCAGAGGGAGAGGCTACACTGCGT

ATGAAGTTGGTGATGGAAGACGGCAAGATGGACCCTGTGGCCTATCGAGTCAAGTATACA

CCACACCACCGTACAGGGGACAAATGGTGCATCTATCCCACGTATGACTACACACACTGC

CTCTGTGACTCCATTGAGCATATCACCCACTCACTCTGCACTAAGGAATTCCAGGCCCGA

CGTTCTTCTTATTTCTGGCTGTGCAATGCACTGGATGTATATTGCCCTGTGCAGTGGGAG

TATGGCCGTCTCAATCTGCACTATGCTGTTGTCTCTAAGAGGAAGATCCTTCAGCTTGTA

GCTGCTGGTGCTGTGCGG

>CL188.Contig26_A-W 958 1758 minus strand Glutamine--tRNA ligase OS=Homo sapiens GN=QARS PE=1 SV=1

AGGGACTGGGATGACCCACGACTCTTCACACTGACTGCTCTGCAACGGCGGGGCTTTCCG

CCTGAGGCCATCAACAACTTCTGTGCTCGGGTGGGAGTGACAGTAGCACAGACCACAATG

GAGCCACATCTTCTTGAAGCCTGTGTACGTGATGTGCTGAACGATACAGCTCCACGGGCT

ATGGCTGTGCTGGAGTCACTACGGGTTGTCATTACCAACTTTCCTGCTGCCAAGCCCTTG

GAAATCCAGGTGCCCAACTTTCCAGCTGATGAAACCAAGGGCTTCCACCAGGTTCCTTTT

GCACCCATAGTCTTCATTGAGAGGACTGACTTCAAGGAGGAGCCAGAGCCAGGCTATAAG

CGTCTAGCTTGGGGCCAGCCTGTGGGCCTGAGACATACAGGCTATGTCATTGAGCTGCGG

CATGTTATCAAGGATCCTAGTGGCTGTGTGGAGAGCCTGGAGGTGACCTGTAGACGGGCT

GATGCTGGAGAGAAGCCCAAGGCTTTTATTCACTGGGTTTCACAGCCTTTGATATGCGAA

ATTCGCCTTTATGATCGACTATTCCAGCACAAGAACCCTGAAGATCCTGCTGAGGTGCCA

GGTGGATTCTTAAGTGATCTGAACCCGGCATCACTACAAGTGGTGGAGGCAGCATTAGTG

GATTGTTCTGTGGCCCTGGCAAAACCCTTTGACAAGTTCCAGTTTGAGCGCCTCGGGTAC

TTCTCCGTGGATCCGGATAGCCGCCAAGGACAGCTTGTCTTCAACCGAACAGTCACACTA

AAGGAGGACCCAGGAAAGGTG

>CL189.Contig1_A-W 4 207 minus strand Collagen triple helix repeat protein [Polysphondylium pallidum PN500]

TGGACCACGAAGGGCACGTGGACCATGAAGGGCACGTGGACCATGAGCACCACGTGGACG

AGGACCACGTGGACCACGAGCACCACGTGGACCAGCAGGAGCAGCAGCCGCAGCAGCAGC

AGCAGCCGCAGCGCGTGGACCAGCAGGAGCCTCGCGAGCAGCACGTGGTCCACGAGACAC

ACGTGGATGTGGTCCACGAGAGGC

>CL190.Contig1_A-W 54 1349 splicing factor, arginine/serine-rich 11 [Homo sapiens]

ATGATAAATAATCATATAGAGATCAAGCTTTATATTTTAGCGAACAATAAGTACTTTCAA

CAAACTCAGGTGGTGTATCAGGGAGACATTTTCTGGGTGTTTTTGTGTGTTTTCTGTTTT

ACAAAAAAATGTGTTCTCATGCAAGGATATTTCTCTGCAGGAGTTATTCCTGATGAGACT

AAGGCTTTGTCTCTGTTGGCACCAGCAAATGCAGTGGCAGGTCTTCTGCCTGGTGGTGGA

CTCCTGCCTACACCTAACCCACTTACCCAGATTGGCGCTGTTCCATTGGCTGCTTTGGGA

GCTCCTACTCTTGATCCTGCCCTTGCTGCACTTGGGCTTCCTGGAGCAAACTTGAACTCT

CAGTCTCTTGCCGCAGATCAGTTGCTGAAGCTTATGAGCACTGTTGATCCCAAGTTGAAT

CATGTAGCTGCTGGTCTGGTTTCACCAAATCTGAAATCAGATACCTCTAGTAAAGAAATA

GAGGAAGCTATGAAAAGAGTACGTGAAGCACAGTCCTTGATTTCTGCTGCTATTGAACCA

GATAAGAAAGAAGAAAAAAGAAGGCATTCAAGATCAAGATCACGTTCTAGGAGGAGGCGG

ACTCCCTCATCTTCTAGACACAGGCGATCAAGAAGCAGGTCAAGGAGGCGATCACATTCT

AAGTCAAGGAGTAGACGACGATCCAAAAGTCCAAGACGGAGAAGATCTCATTCCAGAGAA

AGAGGTAGAAGGTCAAGAAGCACATCAAAAACCAGAGATAAAAAGAAGGAAGACAAAGAA

AAGAAACGTTCCAAAACACCACCAAAAAGTTACAGCACAGCCAGACGTTCGAGAAGTGCA

AGCAGTTTGCACGTTTGTGTTTCTAGAGAGAGACGACGAAGAAGAAGCAGGAGTGGAACA

AGATCTCCTAAAAAGCCTAGGTCTCCTAAAAGAAAATTATCTCGCTCACCATCCCCTAGG

AGACATAAAAAGGAGAAGAAGAAAGATAAAGACAAAGAAAGAAGCAGAGATGAACGGGAA

CGATCAACAAGCAAAAAGAAAAAGAGTAAAGATAAGGAGAAGGACCGGGAAAGAAAATCA

GAGAGTGATAAAGATGTAAAACAGGTTACACGGGATTACGATGAAGAGGAACAGGGGTAT

GACAGCGAGAAAGAGAAAAAAGAGGAGAAGAAACCAACAGAAACAGGTTCCCCTAAAACA

AAGGAATGTTCTGTAGAAAAGGGAACCGGTGATTCACTAAGAGAATCCAAAGTGAATGGG

GACGATCATCATGAAGAAGACATGGATATGAGTGAC

>CL190.Contig2_A-W 279 1745 serine/arginine-rich splicing factor 11 isoform 1 [Macaca mulatta] >gi|383422895|gb|AFH34661.1| serine/arginine-rich splicing factor 11 isoform 1 [Macaca mulatta] >gi|384950356|gb|AFI38783.1| serine/arginine-rich splicing factor 11 isoform 1 [Macaca mulatta]

ATGAGCAACACTACCGTCGTCCCCAGCACTGCGGGCCCGGGCCCCAGCGGCGGGCCGGGT

GGCGGAGGTGGCGGCGGAGGCGGTGGCACCGAGGTAATCCAGGTGACTAATGTCTCCCCG

AGCGCTAGCTCTGAGCAGATGCGGACTCTCTTCGGTTTCCTAGGCAAGATCGACGAACTG

CGCCTCTTCCCGCCGGATGATTCACCTTTGCCAGTCTCATCTCGTGTCTGCTTTGTTAAG

TTCCATGATCCGGACTCAGCAGTTGTGGCACAGCATCTGACAAATACTGTATTCGTTGAC

AGAGCTTTGATAGTCGTACCATATGCAGAAGGAGTTATTCCTGATGAGACTAAGGCTTTG

TCTCTGTTGGCACCAGCAAATGCAGTGGCAGGTCTTCTGCCTGGTGGTGGACTCCTGCCT

ACACCTAACCCACTTACCCAGATTGGCGCTGTTCCATTGGCTGCTTTGGGAGCTCCTACT

CTTGATCCTGCCCTTGCTGCACTTGGGCTTCCTGGAGCAAACTTGAACTCTCAGTCTCTT

GCCGCAGATCAGTTGCTGAAGCTTATGAGCACTGTTGATCCCAAGTTGAATCATGTAGCT

GCTGGTCTGGTTTCACCAAATCTGAAATCAGATACCTCTAGTAAAGAAATAGAGGAAGCT

ATGAAAAGAGTACGTGAAGCACAGTCCTTGATTTCTGCTGCTATTGAACCAGATAAGAAA

GAAGAAAAAAGAAGGCATTCAAGATCAAGATCACGTTCTAGGAGGAGGCGGACTCCCTCA

TCTTCTAGACACAGGCGATCAAGAAGCAGGTCAAGGAGGCGATCACATTCTAAGTCAAGG

AGTAGACGACGATCCAAAAGTCCAAGACGGAGAAGATCTCATTCCAGAGAAAGAGGTAGA

AGGTCAAGAAGCACATCAAAAACCAGAGATAAAAAGAAGGAAGACAAAGAAAAGAAACGT

TCCAAAACACCACCAAAAAGTTACAGCACAGCCAGACGTTCGAGAAGTGCAAGCAGTTTG

CACGTTTGTGTTTCTAGAGAGAGACGACGAAGAAGAAGCAGGAGTGGAACAAGATCTCCT

AAAAAGCCTAGGTCTCCTAAAAGAAAATTATCTCGCTCACCATCCCCTAGGAGACATAAA

AAGGAGAAGAAGAAAGATAAAGACAAAGAAAGAAGCAGAGATGAACGGGAACGATCAACA

AGCAAAAAGAAAAAGAGTAAAGATAAGGAGAAGGACCGGGAAAGAAAATCAGAGAGTGAT

AAAGATGTAAAACAGGTTACACGGGATTACGATGAAGAGGAACAGGGGTATGACAGCGAG

AAAGAGAAAAAAGAGGAGAAGAAACCAACAGAAACAGGTTCCCCTAAAACAAAGGAATGT

TCTGTAGAAAAGGGAACCGGTGATTCACTAAGAGAATCCAAAGTGAATGGGGACGATCAT

CATGAAGAAGACATGGATATGAGTGAC

>CL190.Contig3_A-W 274 1410 PREDICTED: serine/arginine-rich splicing factor 11 [Equus caballus]

GGAGTTATTCCTGATGAGACTAAGGCTTTGTCTCTGTTGGCACCAGCAAATGCAGTGGCA

GGTCTTCTGCCTGGTGGTGGACTCCTGCCTACACCTAACCCACTTACCCAGATTGGCGCT

GTTCCATTGGCTGCTTTGGGAGCTCCTACTCTTGATCCTGCCCTTGCTGCACTTGGGCTT

CCTGGAGCAAACTTGAACTCTCAGTCTCTTGCCGCAGATCAGTTGCTGAAGCTTATGAGC

ACTGTTGATCCCAAGTTGAATCATGTAGCTGCTGGTCTGGTTTCACCAAATCTGAAATCA

GATACCTCTAGTAAAGAAATAGAGGAAGCTATGAAAAGAGTACGTGAAGCACAGTCCTTG

ATTTCTGCTGCTATTGAACCAGATAAGAAAGAAGAAAAAAGAAGGCATTCAAGATCAAGA

TCACGTTCTAGGAGGAGGCGGACTCCCTCATCTTCTAGACACAGGCGATCAAGAAGCAGG

TCAAGGAGGCGATCACATTCTAAGTCAAGGAGTAGACGACGATCCAAAAGTCCAAGACGG

AGAAGATCTCATTCCAGAGAAAGAGGTAGAAGGTCAAGAAGCACATCAAAAACCAGAGAT

AAAAAGAAGGAAGACAAAGAAAAGAAACGTTCCAAAACACCACCAAAAAGTTACAGCACA

GCCAGACGTTCGAGAAGTGCAAGCAGTTTGCACGTTTGTGTTTCTAGAGAGAGACGACGA

AGAAGAAGCAGGAGTGGAACAAGATCTCCTAAAAAGCCTAGGTCTCCTAAAAGAAAATTA

TCTCGCTCACCATCCCCTAGGAGACATAAAAAGGAGAAGAAGAAAGATAAAGACAAAGAA

AGAAGCAGAGATGAACGGGAACGATCAACAAGCAAAAAGAAAAAGAGTAAAGATAAGGAG

AAGGACCGGGAAAGAAAATCAGAGAGTGATAAAGATGTAAAACAGGTTACACGGGATTAC

GATGAAGAGGAACAGGGGTATGACAGCGAGAAAGAGAAAAAAGAGGAGAAGAAACCAACA

GAAACAGGTTCCCCTAAAACAAAGGAATGTTCTGTAGAAAAGGGAACCGGTGATTCACTA

AGAGAATCCAAAGTGAATGGGGACGATCATCATGAAGAAGACATGGATATGAGTGAC

>CL190.Contig4_A-W 189 338 Serine/arginine-rich splicing factor 11 OS=Homo sapiens GN=SRSF11 PE=1 SV=1

ATGAGCAACACTACCGTCGTCCCCAGCACTGCGGGCCCGGGCCCCAGCGGCGGGCCGGGT

GGCGGAGGTGGCGGCGGAGGCGGTGGCACCGAGGTAATCCAGGTGACTAATGTCTCCCCG

AGCGCTAGCTCTGAGCAGATGCGGACTCTC

>CL190.Contig5_A-W 2 1435 minus strand serine/arginine-rich splicing factor 11 isoform 1 [Macaca mulatta] >gi|383422895|gb|AFH34661.1| serine/arginine-rich splicing factor 11 isoform 1 [Macaca mulatta] >gi|384950356|gb|AFI38783.1| serine/arginine-rich splicing factor 11 isoform 1 [Macaca mulatta]

TTCGGTTTCCTAGGCAAGATCGACGAACTGCGCCTCTTCCCGCCGGATGATTCACCTTTG

CCAGTCTCATCTCGTGTCTGCTTTGTTAAGTTCCATGATCCGGACTCAGCAGTTGTGGCA

CAGCATCTGACAAATACTGTATTCGTTGACAGAGCTTTGATAGTCGTACCATATGCAGAA

GTGAAGCAGCCGACACGAGTCGTTGTTCATAAAACAGCTTTTGAAAGTTGAGAGCACACC

CCTGGAGAACCGACTGTGCTTGCTTACGTTTGGTTCATGACTTAAAAATCGAGTACAGGA

GTTATTCCTGATGAGACTAAGGCTTTGTCTCTGTTGGCACCAGCAAATGCAGTGGCAGGT

CTTCTGCCTGGTGGTGGACTCCTGCCTACACCTAACCCACTTACCCAGATTGGCGCTGTT

CCATTGGCTGCTTTGGGAGCTCCTACTCTTGATCCTGCCCTTGCTGCACTTGGGCTTCCT

GGAGCAAACTTGAACTCTCAGTCTCTTGCCGCAGATCAGTTGCTGAAGCTTATGAGCACT

GTTGATCCCAAGTTGAATCATGTAGCTGCTGGTCTGGTTTCACCAAATCTGAAATCAGAT

ACCTCTAGTAAAGAAATAGAGGAAGCTATGAAAAGAGTACGTGAAGCACAGTCCTTGATT

TCTGCTGCTATTGAACCAGATAAGAAAGAAGAAAAAAGAAGGCATTCAAGATCAAGATCA

CGTTCTAGGAGGAGGCGGACTCCCTCATCTTCTAGACACAGGCGATCAAGAAGCAGGTCA

AGGAGGCGATCACATTCTAAGTCAAGGAGTAGACGACGATCCAAAAGTCCAAGACGGAGA

AGATCTCATTCCAGAGAAAGAGGTAGAAGGTCAAGAAGCACATCAAAAACCAGAGATAAA

AAGAAGGAAGACAAAGAAAAGAAACGTTCCAAAACACCACCAAAAAGTTACAGCACAGCC

AGACGTTCGAGAAGTGCAAGCAGTTTGCACGTTTGTGTTTCTAGAGAGAGACGACGAAGA

AGAAGCAGGAGTGGAACAAGATCTCCTAAAAAGCCTAGGTCTCCTAAAAGAAAATTATCT

CGCTCACCATCCCCTAGGAGACATAAAAAGGAGAAGAAGAAAGATAAAGACAAAGAAAGA

AGCAGAGATGAACGGGAACGATCAACAAGCAAAAAGAAAAAGAGTAAAGATAAGGAGAAG

GACCGGGAAAGAAAATCAGAGAGTGATAAAGATGTAAAACAGGTTACACGGGATTACGAT

GAAGAGGAACAGGGGTATGACAGCGAGAAAGAGAAAAAAGAGGAGAAGAAACCAACAGAA

ACAGGTTCCCCTAAAACAAAGGAATGTTCTGTAGAAAAGGGAACCGGTGATTCACTAAGA

GAATCCAAAGTGAATGGGGACGATCATCATGAAGAAGACATGGATATGAGTGAC

>CL190.Contig6_A-W 2 1414 minus strand serine/arginine-rich splicing factor 11 isoform 1 [Macaca mulatta] >gi|383422895|gb|AFH34661.1| serine/arginine-rich splicing factor 11 isoform 1 [Macaca mulatta] >gi|384950356|gb|AFI38783.1| serine/arginine-rich splicing factor 11 isoform 1 [Macaca mulatta]

TTCGGTTTCCTAGGCAAGATCGACGAACTGCGCCTCTTCCCGCCGGATGATTCACCTTTG

CCAGTCTCATCTCGTGTCTGCTTTGTTAAGTTCCATGATCCGGACTCAGCAGTTGTGGCA

CAGCATCTGACAAATACTGTATTCGTTGACAGAGCTTTGATAGTCGTACCATATGCAGAA

GTGAAGCAGCCGACACGAGTCGTTGTTCATAAAACAGCTTTTGAAAGTTGAGAGCACACC

CCTGGAGAACCGACTGTGCTTGCTTACGTTTGGTTCATGACTTAAAAATCGAGTACAGGA

GTTATTCCTGATGAGACTAAGGCTTTGTCTCTGTTGGCACCAGCAAATGCAGTGGCAGGT

CTTCTGCCTGGTGGTGGACTCCTGCCTACACCTAACCCACTTACCCAGATTGGCGCTGTT

CCATTGGCTGCTTTGGGAGCTCCTACTCTTGATCCTGCCCTTGCTGCACTTGGGCTTCCT

GGAGCAAACTTGAACTCTCAGTCTCTTGCCGCAGATCAGTTGCTGAAGCTTATGAGCACT

GTTGATCCCAAGTTGAATCATGTAGCTGCTGGTCTGGTTTCACCAAATCTGAAATCAGAT

ACCTCTAGTAAAGAAATAGAGGAAGCTATGAAAAGAGTACGTGAAGCACAGTCCTTGATT

TCTGCTGCTATTGAACCAGATAAGAAAGAAGAAAAAAGAAGGCATTCAAGATCAAGATCA

CGTTCTAGGAGGAGGCGGACTCCCTCATCTTCTAGACACAGGCGATCAAGAAGCAGGTCA

AGGAGGCGATCACATTCTAAGTCAAGGAGTAGACGACGATCCAAAAGTCCAAGACGGAGA

AGATCTCATTCCAGAGAAAGAGGTAGAAGGTCAAGAAGCACATCAAAAACCAGAGATAAA

AAGAAGGAAGACAAAGAAAAGAAACGTTCCAAAACACCACCAAAAAGTTACAGCACAGCC

AGACGTTCGAGAAGTGCAAGCAGAGAGAGACGACGAAGAAGAAGCAGGAGTGGAACAAGA

TCTCCTAAAAAGCCTAGGTCTCCTAAAAGAAAATTATCTCGCTCACCATCCCCTAGGAGA

CATAAAAAGGAGAAGAAGAAAGATAAAGACAAAGAAAGAAGCAGAGATGAACGGGAACGA

TCAACAAGCAAAAAGAAAAAGAGTAAAGATAAGGAGAAGGACCGGGAAAGAAAATCAGAG

AGTGATAAAGATGTAAAACAGGTTACACGGGATTACGATGAAGAGGAACAGGGGTATGAC

AGCGAGAAAGAGAAAAAAGAGGAGAAGAAACCAACAGAAACAGGTTCCCCTAAAACAAAG

GAATGTTCTGTAGAAAAGGGAACCGGTGATTCACTAAGAGAATCCAAAGTGAATGGGGAC

GATCATCATGAAGAAGACATGGATATGAGTGAC

>CL191.Contig2_A-W 76 225 Retrovirus-related Pol polyprotein LINE-1 OS=Mus musculus GN=Pol PE=1 SV=2

TTCTACAAATCATGCTGGGAAACCTGCCTGTCTACATGCAAAATAATGATGTTGGACTCC

TACCTAACATCATATATAAAAATTAATTCAAAATGGATCAGAGACCTAAATTTAAAACTT

CAAATTGTAGAACTCTTACTAGAAAACATG

>CL192.Contig1_A-W 138 1487 PREDICTED: CAP-Gly domain-containing linker protein 1 [Otolemur garnettii]

GCAGCCTCTGTCCAGAGAAGTATGCAAGAAACTGTAAATAAATTGCACCAAAAGGAAGAA

GAGTTTAACATGCTGTCTTCTGAATTGGAGAAGTTAAGAGAAAATTTATCAGATATGGAG

GCGAAATTTAGAGAACGAGATGAAAAAGAAGAGCAGTTGATAAAGGCAAAGGAAAAATTG

GAAAATGACATTGCCGAAATAATGAAGATGTCAGGAGATAATTCTTCTCAGCTGACGAAA

ATGAATGATGAATTACGTCTGAAAGAAAGATGTGTGGAAGAATTACAGCTAAAACTTTCA

AAGGCCAATGAAAATGCGAGTTTTCTTCAGAAAGGTATTGGGGAAATAACTCTTAAAGCT

GAACAGAGCCAGCAGGAAGCAGCCAGAAAGCACAAGGAAGAAAGGAAAGAATTGGAGGAA

AAATTGTTAGACCTGGAAAAGAAGGTGGAAATGAGCCACAACCAGTGTCAGGATCTGAAA

TCCAGGTATGAAAAGGCCAGTTCTGAGACGAAAACAAAGCATGAAGAAATTCTGCAGAAC

CTACAGAAGGTGCTGCTGGCCACGGAGGAGAAGCTGAAGGCTGCCCAGGAGGAGAACAGA

GGCCTGATGCAGGAGATGGAGGACCTGAAAGTACAGGCTGACAAAGCCAAAGCTGCTTGG

ACTGCGGAAGATGCCATGCAGATAATGGAGCAGATGACCAAGGAGAAGTCCGAGACTTTG

GCCTCCCTGGAGGACACCAGGCAAACAAACGCAAAACTGCAAAGCGAATTGGACACGCTT

AAAGAAAACAACTTGAAAAATGTGGAAGAGCTGAACAAATCAAAAGAACTTCTGACTGTA

GAGAATCAAAAAATGGAAGAATTCAGGAAAGAAATAGAAACCCTAAAGCAGGCGGCAGCT

CAGAAGTCCCAGCAGCTTTCAGCACTGCAAGAAGAGAACGTCAAACTTGCCGAGGAGCTG

GGGAGAAGCAGGGACGAAGTCACAAGTCATCAAAAGCTGGAAGAAGAACGATCTGTGCTC

AATAATCAGTTATTAGAAATGAAAAAGAGAGAATCCAAGTACATAAAAGAGACAGATGAA

GAGAAAGCTTCCTTACAGAAATCCATCAGTATTACTAGTGCTTTACTCACAGAGAAGGAT

GCAGAACTGGAAAAGCTGAGGAATGAGGTCACAGTGCTCAGGGGAGAAAACGCTTCTGCC

AAGTCTCTGCATTCGGTTGTTCAGACTCTAGAGTCTGATAAGGTGAAGCTCGAGCTCAAG

GTAAAGAACTTGGAGCTTCAACTCAAAGAAAACAAGAGGCAGCTCGGCAGCTCCTCAGGT

AATACTGATACTCAGGCAGAAGAGGATGAG

>CL192.Contig2_A-W 6 1136 minus strand PREDICTED: CAP-Gly domain-containing linker protein 1 [Otolemur garnettii]

AGAGAGCTCCAGGGAAAAGAGCTAAAGCTTACTAACCTTGAGGAAAACTTGAGTGAAGTC

AGTCGAGTGAAAGAGGCTTTGGAAAAAGAACTTCATATTTTGAAAGAAAAATTTGCTGAT

GCTTCGGAGGAGGCAGCCTCTGTCCAGAGAAGTATGCAAGAAACTGTAAATAAATTGCAC

CAAAAGGAAGAAGAGTTTAACATGCTGTCTTCTGAATTGGAGAAGTTAAGAGAAAATTTA

TCAGATATGGAGGCGAAATTTAGAGAACGAGATGAAAAAGAAGAGCAGTTGATAAAGGCA

AAGGAAAAATTGGAAAATGACATTGCCGAAATAATGAAGATGTCAGGAGATAATTCTTCT

CAGCTGACGAAAATGAATGATGAATTACGTCTGAAAGAAAGATGTGTGGAAGAATTACAG

CTAAAACTTTCAAAGGCCAATGAAAATGCGAGTTTTCTTCAGAAAGGTATTGGGGAAATA

ACTCTTAAAGCTGAACAGAGCCAGCAGGAAGCAGCCAGAAAGCACAAGGAAGAAAGGAAA

GAATTGGAGGAAAAATTGTTAGACCTGGAAAAGAAGGTGGAAATGAGCCACAACCAGTGT

CAGGATCTGAAATCCAGGTATGAAAAGGCCAGTTCTGAGACGAAAACAAAGCATGAAGAA

ATTCTGCAGAACCTACAGAAGGTGCTGCTGGCCACGGAGGAGAAGCTGAAGGCTGCCCAG

GAGGAGAACAGAGGCCTGATGCAGGAGATGGAGGACCTGAAAGTACAGGCTGACAAAGCC

AAAGCTGCTTGGACTGCGGAAGATGCCATGCAGATAATGGAGCAGATGACCAAGGAGAAG

TCCGAGACTTTGGCCTCCCTGGAGGACACCAGGCAAACAAACGCAAAACTGCAAAGCGAA

TTGGACACGCTTAAAGAAAACAACTTGAAAAATGTGGAAGAGCTGAACAAATCAAAAGAA

CTTCTGACTGTAGAGAATCAAAAAATGGAAGAATTCAGGAAAGAAATAGAAACCCTAAAG

CAGGCGGCAGCTCAGAAGTCCCAGCAGCTTTCAGCACTGCAAGAAGAGAACGTCAAACTT

GCCGAGGAGCTGGGGAGAAGCAGGGACGAAGTCACAAGTCATCAAAAGGTC

>CL192.Contig3_A-W 46 918 minus strand PREDICTED: CAP-Gly domain-containing linker protein 1 [Otolemur garnettii]

ATAGAAACTGTAAATAAATTGCACCAAAAGGAAGAAGAGTTTAACATGCTGTCTTCTGAA

TTGGAGAAGTTAAGAGAAAATTTATCAGATATGGAGGCGAAATTTAGAGAACGAGATGAA

AAAGAAGAGCAGTTGATAAAGGCAAAGGAAAAATTGGAAAATGACATTGCCGAAATAATG

AAGATGTCAGGAGATAATTCTTCTCAGCTGACGAAAATGAATGATGAATTACGTCTGAAA

GAAAGATGTGTGGAAGAATTACAGCTAAAACTTTCAAAGGCCAATGAAAATGCGAGTTTT

CTTCAGAAAGGTATTGGGGAAATAACTCTTAAAGCTGAACAGAGCCAGCAGGAAGCAGCC

AGAAAGCACAAGGAAGAAAGGAAAGAATTGGAGGAAAAATTGTTAGACCTGGAAAAGAAG

GTGGAAATGAGCCACAACCAGTGTCAGGATCTGAAATCCAGGTATGAAAAGGCCAGTTCT

GAGACGAAAACAAAGCATGAAGAAATTCTGCAGAACCTACAGAAGGTGCTGCTGGCCACG

GAGGAGAAGCTGAAGGCTGCCCAGGAGGAGAACAGAGGCCTGATGCAGGAGATGGAGGAC

CTGAAAGTACAGGCTGACAAAGCCAAAGCTGCTTGGACTGCGGAAGATGCCATGCAGATA

ATGGAGCAGATGACCAAGGAGAAGTCCGAGACTTTGGCCTCCCTGGAGGACACCAGGCAA

ACAAACGCAAAACTGCAAAGCGAATTGGACACGCTTAAAGAAAACAACTTGAAAAATGTG

GAAGAGCTGAACAAATCAAAAGAACTTCTGACTGTAGAGAATCAAAAAATGGAAGAATTC

AGGAAAGAAATAGAAACCCTAAAGCAGGCGGCA

>CL192.Contig4_A-W 46 918 minus strand PREDICTED: CAP-Gly domain-containing linker protein 1 [Otolemur garnettii]

ATAGAAACTGTAAATAAATTGCACCAAAAGGAAGAAGAGTTTAACATGCTGTCTTCTGAA

TTGGAGAAGTTAAGAGAAAATTTATCAGATATGGAGGCGAAATTTAGAGAACGAGATGAA

AAAGAAGAGCAGTTGATAAAGGCAAAGGAAAAATTGGAAAATGACATTGCCGAAATAATG

AAGATGTCAGGAGATAATTCTTCTCAGCTGACGAAAATGAATGATGAATTACGTCTGAAA

GAAAGATGTGTGGAAGAATTACAGCTAAAACTTTCAAAGGCCAATGAAAATGCGAGTTTT

CTTCAGAAAGGTATTGGGGAAATAACTCTTAAAGCTGAACAGAGCCAGCAGGAAGCAGCC

AGAAAGCACAAGGAAGAAAGGAAAGAATTGGAGGAAAAATTGTTAGACCTGGAAAAGAAG

GTGGAAATGAGCCACAACCAGTGTCAGGATCTGAAATCCAGGTATGAAAAGGCCAGTTCT

GAGACGAAAACAAAGCATGAAGAAATTCTGCAGAACCTACAGAAGGTGCTGCTGGCCACG

GAGGAGAAGCTGAAGGCTGCCCAGGAGGAGAACAGAGGCCTGATGCAGGAGATGGAGGAC

CTGAAAGTACAGGCTGACAAAGCCAAAGCTGCTTGGACTGCGGAAGATGCCATGCAGATA

ATGGAGCAGATGACCAAGGAGAAGTCCGAGACTTTGGCCTCCCTGGAGGACACCAGGCAA

ACAAACGCAAAACTGCAAAGCGAATTGGACACGCTTAAAGAAAACAACTTGAAAAATGTG

GAAGAGCTGAACAAATCAAAAGAACTTCTGACTGTAGAGAATCAAAAAATGGAAGAATTC

AGGAAAGAAATAGAAACCCTAAAGCAGGCGGCA

>CL193.Contig1_A-W 142 660 PREDICTED: nesprin-2 [Papio anubis]

TCAGGTAAATCCATTTCTGATGGCCACTCGTGGCATGTTCCTGACAGCCCTTCCCATCCC

AAGCATCACTACAAACAAATGGAAGGTGACAGGAATGTACCACCCATCCCCTCGGGTTCC

AGCACCCCTTACAAATCAGCCTATGGAAAGCTGCTATTACATCCAGGCACTGATGGTGGC

AAAGAAAGTCCAAGAGTCCTGAATGGCAGCCCTCAGCAGGAAGACGAGCATCTGGCCACC

CTAACAGGACAGCCATCAGGTGCCTTCGACAGATGGGAACTGATTCAGGCACAAGAGCTT

CACAGTAAACTCAGAATGAAACAAAACATGCAGCAGCTGAACTCTAATATCAGCGCCATC

ACTGCTTGGCTGGGAAAAACTGAAGCAGAGCTTGAAACTTTAAAGATGGCAAAGCCTCCC

TCCAACATCCAGGAAATGGAACCCAGAGTGAAGAGATTGCAGGAGATATTAAAAGCTTTT

GACAACTACAAGGCATTAATGGTCTCTGTCAATGTGAGC

>CL193.Contig2_A-W 5 652 PREDICTED: nesprin-2 isoform 5 [Pan troglodytes]

CATGAGCGGTCTGGCTGTGAGACCCCTGTTAGTGTGGACTCCATCCCTCTGGAGTGGGAT

CACACAGGTGACGTGGGGGGCTCCTCCTCTCACGAAGAAGATGAGGAAGGTCCATACTAC

AGTGCACTCTCAGGTAAATCCATTTCTGATGGCCACTCGTGGCATGTTCCTGACAGCCCT

TCCCATCCCAAGCATCACTACAAACAAATGGAAGGTGACAGGAATGTACCACCCATCCCC

TCGGGTTCCAGCACCCCTTACAAATCAGCCTATGGAAAGCTGCTATTACATCCAGGCACT

GATGGTGGCAAAGAAAGTCCAAGAGTCCTGAATGGCAGCCCTCAGCAGGAAGACGAGCAT

CTGGCCACCCTAACAGGACAGCCATCAGGTGCCTTCGACAGATGGGAACTGATTCAGGCA

CAAGAGCTTCACAGTAAACTCAGAATGAAACAAAACATGCAGCAGCTGAACTCTAATATC

AGCGCCATCACTGCTTGGCTGGGAAAAACTGAAGCAGAGCTTGAAACTTTAAAGATGGCA

AAGCCTCCCTCCAACATCCAGGAAATGGAACCCAGAGTGAAGAGATTGCAGGAGATATTA

AAAGCTTTTGACAACTACAAGGCATTAATGGTCTCTGTCAATGTGAGC

>CL195.Contig1_A-W 595 1725 PREDICTED: lymphocyte transmembrane adapter 1 isoform 1 [Pongo abelii]

AAAGCCAGCCGGAGCATGGCACTTGGTTTTCCTCCTAGAGATAACGACAAGAGCAGCATC

ATCTTTTCTGGGTTAGTGGGACTCCTGGCTGTCCTCCTGCTCATCACAGTGGCCTGCATC

CTGTGGAACTGGAACAAACGGAAGAAGCGGCAGGTTCCTTACCTCCCAGTGACTGCCATG

CCCTCATTGACTCTGCCTCGACCCAGACCACGAGCCAAAAATATTTATGATCTCTTGCCT

CAGCGGCAAGAAGAGCTGGGGAGACATCAGCCAAGGAATATCCGCATGTTCAGTGCCGAG

AGCCTCCTGTCCAGAAATTCCGACAGCCCGGAGCATATGCCCTCCCAAGCAGAGAGCACC

CTCCAGATGCACACAGCCCATATCCACACCATGGGATATGCAGTTGGTGTCTACGACAAT

GCAACGGTGCCCCAGGTGTGCAGGAACGTCACTCCCTCGACACACTATGTCAATGTCAGA

GCTTCCAGGGATGTCTCGAGCACTTCTTCAGAGGATTCAAATGATTATGTCAATGTCCCC

ACAGCAGAAGAGATGGCTGAGACTCTAACTCCTAACAACAGCCTGCCTGAAAGTCTCCTT

GTTCTTTCAAGCGCCCAGCAGTCGGACTTAACTGAGAAAAGACACAAGGGCTGTGGGGAT

GCCAGTGACTGTACCAGCTTTTGGGCTCCAGGAACTAAGGACAGCGATCCACTCAGCGAT

GGGGAGGATTCTTCTCAGACCTCAAATGACTATGTCAACATGACAGGTTTGGATCTCGAG

GACATCCAAGAGGAGTTGCCCTGGGTGGCTTTTCAATGCTGCAGAGATTATGAGAATGTC

CCACCAGCAGATCCCAAGGGAAGCCAGCAGCAAACTGAAGAAGAAGTGACATCTTCAAAC

ACAGACCATGAAGAGGGAAAGACAGATGGCCCGGGGACTGACATCCATCCTCTCACAAGG

AATTCCCTATCTTCAGGGCACTGTGTATCCCCACTGAGTGAGGACTGTCAGATGAAACAT

GAAGAAGAAATGTCACACGAAGACACTCATGACTATGAGAATGTGCTACCTGCCAAGTTA

GGAGGCAGAGACAGGGAGCAGGGGCCTGGTGCTCAGCTCCCTCCTGATGAA

>CL195.Contig2_A-W 522 1691 PREDICTED: lymphocyte transmembrane adapter 1 isoform 3 [Pan troglodytes]

ACCAACTCAACCCTACGGGATAGCAAGAGTACCTTGCAGCCCAGCACTCCACGGGTGATT

CTCGGCAGCCAGGAAAGAGATAACGACAAGAGCAGCATCATCTTTTCTGGGTTAGTGGGA

CTCCTGGCTGTCCTCCTGCTCATCACAGTGGCCTGCATCCTGTGGAACTGGAACAAACGG

AAGAAGCGGCAGGTTCCTTACCTCCCAGTGACTGCCATGCCCTCATTGACTCTGCCTCGA

CCCAGACCACGAGCCAAAAATATTTATGATCTCTTGCCTCAGCGGCAAGAAGAGCTGGGG

AGACATCAGCCAAGGAATATCCGCATGTTCAGTGCCGAGAGCCTCCTGTCCAGAAATTCC

GACAGCCCGGAGCATATGCCCTCCCAAGCAGAGAGCACCCTCCAGATGCACACAGCCCAT

ATCCACACCATGGGATATGCAGTTGGTGTCTACGACAATGCAACGGTGCCCCAGGTGTGC

AGGAACGTCACTCCCTCGACACACTATGTCAATGTCAGAGCTTCCAGGGATGTCTCGAGC

ACTTCTTCAGAGGATTCAAATGATTATGTCAATGTCCCCACAGCAGAAGAGATGGCTGAG

ACTCTAACTCCTAACAACAGCCTGCCTGAAAGTCTCCTTGTTCTTTCAAGCGCCCAGCAG

TCGGACTTAACTGAGAAAAGACACAAGGGCTGTGGGGATGCCAGTGACTGTACCAGCTTT

TGGGCTCCAGGAACTAAGGACAGCGATCCACTCAGCGATGGGGAGGATTCTTCTCAGACC

TCAAATGACTATGTCAACATGACAGGTTTGGATCTCGAGGACATCCAAGAGGAGTTGCCC

TGGGTGGCTTTTCAATGCTGCAGAGATTATGAGAATGTCCCACCAGCAGATCCCAAGGGA

AGCCAGCAGCAAACTGAAGAAGAAGTGACATCTTCAAACACAGACCATGAAGAGGGAAAG

ACAGATGGCCCGGGGACTGACATCCATCCTCTCACAAGGAATTCCCTATCTTCAGGGCAC

TGTGTATCCCCACTGAGTGAGGACTGTCAGATGAAACATGAAGAAGAAATGTCACACGAA

GACACTCATGACTATGAGAATGTGCTACCTGCCAAGTTAGGAGGCAGAGACAGGGAGCAG

GGGCCTGGTGCTCAGCTCCCTCCTGATGAA

>CL195.Contig3_A-W 278 1258 PREDICTED: lymphocyte transmembrane adapter 1 isoform 2 [Pongo abelii]

CAGGTTCCTTACCTCCCAGTGACTGCCATGCCCTCATTGACTCTGCCTCGACCCAGACCA

CGAGCCAAAAATATTTATGATCTCTTGCCTCAGCGGCAAGAAGAGCTGGGGAGACATCAG

CCAAGGAATATCCGCATGTTCAGTGCCGAGAGCCTCCTGTCCAGAAATTCCGACAGCCCG

GAGCATATGCCCTCCCAAGCAGAGAGCACCCTCCAGATGCACACAGCCCATATCCACACC

ATGGGATATGCAGTTGGTGTCTACGACAATGCAACGGTGCCCCAGGTGTGCAGGAACGTC

ACTCCCTCGACACACTATGTCAATGTCAGAGCTTCCAGGGATGTCTCGAGCACTTCTTCA

GAGGATTCAAATGATTATGTCAATGTCCCCACAGCAGAAGAGATGGCTGAGACTCTAACT

CCTAACAACAGCCTGCCTGAAAGTCTCCTTGTTCTTTCAAGCGCCCAGCAGTCGGACTTA

ACTGAGAAAAGACACAAGGGCTGTGGGGATGCCAGTGACTGTACCAGCTTTTGGGCTCCA

GGAACTAAGGACAGCGATCCACTCAGCGATGGGGAGGATTCTTCTCAGACCTCAAATGAC

TATGTCAACATGACAGGTTTGGATCTCGAGGACATCCAAGAGGAGTTGCCCTGGGTGGCT

TTTCAATGCTGCAGAGATTATGAGAATGTCCCACCAGCAGATCCCAAGGGAAGCCAGCAG

CAAACTGAAGAAGAAGTGACATCTTCAAACACAGACCATGAAGAGGGAAAGACAGATGGC

CCGGGGACTGACATCCATCCTCTCACAAGGAATTCCCTATCTTCAGGGCACTGTGTATCC

CCACTGAGTGAGGACTGTCAGATGAAACATGAAGAAGAAATGTCACACGAAGACACTCAT

GACTATGAGAATGTGCTACCTGCCAAGTTAGGAGGCAGAGACAGGGAGCAGGGGCCTGGT

GCTCAGCTCCCTCCTGATGAA

>CL195.Contig4_A-W 286 1158 PREDICTED: lymphocyte transmembrane adapter 1 isoform 2 [Pongo abelii]

GGGAGACATCAGCCAAGGAATATCCGCATGTTCAGTGCCGAGAGCCTCCTGTCCAGAAAT

TCCGACAGCCCGGAGCATATGCCCTCCCAAGCAGAGAGCACCCTCCAGATGCACACAGCC

CATATCCACACCATGGGATATGCAGTTGGTGTCTACGACAATGCAACGGTGCCCCAGGTG

TGCAGGAACGTCACTCCCTCGACACACTATGTCAATGTCAGAGCTTCCAGGGATGTCTCG

AGCACTTCTTCAGAGGATTCAAATGATTATGTCAATGTCCCCACAGCAGAAGAGATGGCT

GAGACTCTAACTCCTAACAACAGCCTGCCTGAAAGTCTCCTTGTTCTTTCAAGCGCCCAG

CAGTCGGACTTAACTGAGAAAAGACACAAGGGCTGTGGGGATGCCAGTGACTGTACCAGC

TTTTGGGCTCCAGGAACTAAGGACAGCGATCCACTCAGCGATGGGGAGGATTCTTCTCAG

ACCTCAAATGACTATGTCAACATGACAGGTTTGGATCTCGAGGACATCCAAGAGGAGTTG

CCCTGGGTGGCTTTTCAATGCTGCAGAGATTATGAGAATGTCCCACCAGCAGATCCCAAG

GGAAGCCAGCAGCAAACTGAAGAAGAAGTGACATCTTCAAACACAGACCATGAAGAGGGA

AAGACAGATGGCCCGGGGACTGACATCCATCCTCTCACAAGGAATTCCCTATCTTCAGGG

CACTGTGTATCCCCACTGAGTGAGGACTGTCAGATGAAACATGAAGAAGAAATGTCACAC

GAAGACACTCATGACTATGAGAATGTGCTACCTGCCAAGTTAGGAGGCAGAGACAGGGAG

CAGGGGCCTGGTGCTCAGCTCCCTCCTGATGAA

>CL195.Contig5_A-W 278 466 Lymphocyte transmembrane adapter 1 [Heterocephalus glaber]

CAGGTTCCTTACCTCCCAGTGACTGCCATGCCCTCATTGACTCTGCCTCGACCCAGACCA

CGAGCCAAAAATATTTATGATCTCTTGCCTCAGCGGCAAGAAGAGCTGGGGAGACATCAG

CCAAGGAATATCCGCATGTTCAGTGCCGAGAGCCTCCTGTCCAGAAATTCCGACAGCCCG

GAGCATATG

>CL197.Contig1_A-W 106 1104 minus strand PREDICTED: serine/Arginine-related protein 53 isoform 1 [Nomascus leucogenys]

ATGGGACGCCGGTCTTCAGATACCGAAGAGGAGAGCAGAAGCAAGAGAAAAAAGAAACAC

CGTAGACGTTCATCTTCAAGCAGTTCGTCAGACAGTAGAACTTATAGCAGAAAGAAAGGA

GGAAGGAAATCAAGATCAAAGTCAAGATCATGGTCGAGAGACCTTCAGCCACGCTCACAT

TCTTATGATAGAAGACGCAGACATCGGTCAAGCAGTAGCTCTTCTTACGGCTCTAGAAGA

AAAAGAAGTAGAAGTCGTTCAAGGGGTCGAGGGAAATCTTACAGAATCCAGAGATCTAGA

TCAAAAAGCAGAACAAGAAGGTCCAGGTCAAGACCTCGACCACGTTCCCACAGTCGTAGC

AGTGAGAGGTCCAGTCACAGAAGAACCCGCAGCCGTTCCAGGGATCGAGAGAGACGGAAA

GGCAGAGACAAAGAGAAGCGGGAGAAGGAGAAAGATAAGGGCAAGGACAAAGAGCCACAC

AGCAGCAAACGAGGGGAATCTGGAAACATCAAAGCTGGATTAGAACATCTGCCACCAGCT

GAGCAGGCCAAAGCCAGACTACAACTTGTTCTTGAAGCTGCTGCAAAAGCTGATGAAGCA

TTGAAAGCCAAAGAAAGAAACGAGGAAGAAGCAAAGAGAAGAAAGGAGGGAGACCAAGCC

ACCTTGGGAGAACAAGTAAAACGAGTGAAAGAAATTGAAGCCATCGAAAGTGATTCTTTC

ATCCAGCAGACATTCAGATCAAGTAAGGAAATCAGAAAGGTGGTGGAGCCTAGTGAAGTG

ACACATGAGACTTCAACAGCAGGCCCAGCATCAGTAGTTGCTGACCCACCCAGTGCTGAA

CGGGAAGTAGACCCTGCCATCCCCACTGCTATCAAGTACCAAGACGACAACTCTCTGGCT

CATCCAAATTTATTTATAGAGAAAGCAGAAGCTGAGGAAAAGTGGTTCAAGAGATTAATT

GCTCTCCGACAGGAACGACTAATGGGCAGTCCTGTGGCC

>CL197.Contig2_A-W 34 540 minus strand PREDICTED: serine/Arginine-related protein 53 isoform 2 [Nomascus leucogenys]

AAGGAATCTGGAAACATCAAAGCTGGATTAGAACATCTGCCACCAGCTGAGCAGGCCAAA

GCCAGACTACAACTTGTTCTTGAAGCTGCTGCAAAAGCTGATGAAGCATTGAAAGCCAAA

GAAAGAAACGAGGAAGAAGCAAAGAGAAGAAAGGAGGGAGACCAAGCCACCTTGGGAGAA

CAAGTAAAACGAGTGAAAGAAATTGAAGCCATCGAAAGTGATTCTTTCATCCAGCAGACA

TTCAGATCAAGTAAGGAAATCAGAAAGGTGGTGGAGCCTAGTGAAGTGACACATGAGACT

TCAACAGCAGGCCCAGCATCAGTAGTTGCTGACCCACCCAGTGCTGAACGGGAAGTAGAC

CCTGCCATCCCCACTGCTATCAAGTACCAAGACGACAACTCTCTGGCTCATCCAAATTTA

TTTATAGAGAAAGCAGAAGCTGAGGAAAAGTGGTTCAAGAGATTAATTGCTCTCCGACAG

GAACGACTAATGGGCAGTCCTGTGGCC

>CL197.Contig3_A-W 99 341 minus strand PREDICTED: serine/Arginine-related protein 53-like [Cavia porcellus]

CAGGTGGTGGAGCCTAGTGAAGTGACACATGAGACTTCAACAGCAGGCCCAGCATCAGTA

GTTGCTGACCCACCCAGTGCTGAACGGGAAGTAGACCCTGCCATCCCCACTGCTATCAAG

TACCAAGACGACAACTCTCTGGCTCATCCAAATTTATTTATAGAGAAAGCAGAAGCTGAG

GAAAAGTGGTTCAAGAGATTAATTGCTCTCCGACAGGAACGACTAATGGGCAGTCCTGTG

GCC

>CL200.Contig1_A-W 54 197 minus strand PREDICTED: transcription elongation factor SPT5 isoform 2 [Saimiri boliviensis boliviensis]

CAGGTAAAGGTGATCCTGGGTGAGGATCGGGAGGCCACCGGAGTCCTGCTGAGCATCGAT

GGTGAGGATGGCATCGTCCGCATGGACCTTGACGAGCAGCTCAAGATCCTCAACCTCCGC

TTCCTGGGGAAGCTCCTGGAGGCC

>CL200.Contig2_A-W 72 3332 PREDICTED: transcription elongation factor SPT5-like isoform 1 [Cavia porcellus]

ATGTCGGACAGCGAAGACAGCAACTTCTCCGAGGAGGAGGACAGTGAACGCAGCAGTGAC

GGCGAGGAGGCCGAGGTAGAGGAAGAGCGGCGGAGTGCAGCAGGCAGTGAGAAGGAAGAG

GAGCAGGAGGAGGAAGAGGAGGAAGAAGAGGAGGAGGAATATGATGAAGAGGAGGAAGAA

GAAGATGATGACCGCCCCCCCAAGAAACCCCGCCATGGTGGCTTCATTCTGGATGAAGCT

GATGTTGACGACGAGTATGAGGATGAGGACCAGTGGGAGGACGGAGCTGAAGACATCCTG

GAGAAAGAAGAGATTGAAGCCTCCAATATTGATAATGTTGTCCTGGATGAAGACCGTTCT

GGAGCCCGCCGCCTACAAAACCTGTGGAGGGACCAGCGAGAAGAAGAACTGGGCGAGTAT

TACATGAAGAAATACGCCAAGTCATCTGTGGGAGAGACGGTGTATGGAGGGTCTGATGAG

CTCTCAGATGACATCACCCAGCAGCAGCTGCTCCCAGGAGTCAAGGATCCCAATCTGTGG

ACAGTCAAATGTAAGATTGGGGAGGAGCGAGCCACAGCCATCTCCCTGATGCGCAAGTTC

ATTGCCTACCAATTCACAGACACGCCCCTGCAGATCAAGTCAGTGGTGGCACCAGAGCAC

GTGAAGGGCTATATCTACGTGGAAGCCTACAAGCAGACCCATGTGAAGCAGGCCATCGAG

GGTGTGGGCAACCTGAGGCTCGGCTACTGGAACCAACAGATGGTGCCCATTAAGGAGATG

ACAGATGTGCTCAAAGTGGTGAAGGAGGTGGCCAACCTGAAGCCGAAGTCCTGGGTCCGC

CTCAAGCGAGGCATCTATAAGGATGACATTGCTCAGGTGGACTACGTGGAGCCCAGCCAG

AACACCATCTCTTTGAAGATGATTCCACGCATTGACTATGACCGCATCAAGGCCCGCATG

AGCTTGAAAGACTGGTTTGCCAAAAGGAAGAAGTTCAAGCGGCCTCCACAGAGGCTCTTT

GATGCTGAGAAGATTAGGTCCCTGGGGGGCGACGTTGCCTCCGATGGTGACTTCCTCATC

TTCGAGGGGAACCGGTACAGCCGAAAGGGGTTTCTGTTCAAAAGCTTCGCCATGTCAGCT

GTGATCACAGAGGGTGTGAAGCCCACGCTGTCTGAACTAGAGAAGTTCGAGGACCAGCCA

GAGGGCATCGACCTGGAGGTGGTGACAGAAAGCACAGGGAAGGAGCGGGAGCACAACTTC

CAGCCCGGGGACAATGTAGAGGTGTGTGAAGGCGAGCTCATCAACCTGCAGGGCAAGGTC

CTCAGCGTGGACGGCAACAAAATCACCATCATGCCCAAGCACGAGGACCTCAAGGACATG

CTCGAGTTCCCCGCCCAGGAACTTCGGAAGTACTTCAAGATGGGGGACCACGTGAAGGTG

ATCGCTGGCCGATTTGAGGGCGACACAGGCCTCATTGTGCGGGTGGAGGAGAACTTCATC

ATTCTCTTCTCTGACCTCACCATGCATGAGCTGAAGGTGCTACCCCGGGACCTGCAGCTG

TGCTCCGAGACGGCCTCAGGTGTGGACGTGGGGGGCCAGCATGAATGGGGTGAGCTGGTG

CAACTGGACCCCCGGACCGTAGGTGTCATCGTGCGACTAGAGCGGGAGACCTTTCAGGTG

CTGAACATGTATGGGAAGGTGGTGACTGTCAGGCACCAGGCTGTGACTCAGAAGAAGGAC

AAACGCTTCGCTGTGGCCCTGGATTCTGAACAGAACAACATCCACGTGAAAGACATCGTC

AAGGTCATCGACGGACCCCACTCAGACCGGGAGGGTGAGATCCGTCATCTGTACCACAGC

TTTGCCTTCCTCCATTGCAAGAAGCTGGTGGAGAATGGAGGCATGTTTGTCTGCAAGACC

CGCCACCTGGTGCTGGCTGGGGGTTTGAAGCCCCGAGATGTGACCAACCTCACTGTGGGC

GGCTTTGTCCCTATGAGCCCCCGGATCAGCAGTCCCATGCACCCCAGTGCTGGAGGTCAA

CATGGAGGCTTCGGTAGCCCAGGTGGAGGTGGTGGCGGCATGAGCAGGGGCCGAGGGAGG

AGAGACAATGAGCTCATTGGTCAGACAGTGCGCATCTCCCAGGGACCCTACAAAGGTTAC

ATTGGTGTGGTGAAGGATGCCACCGAGTCCACAGCCCGTGTGGAGCTGCACTCCACCTGC

CAGACCATCTCGGTGGATCGCCAGCGTCTCACCACGGTCGGCTCTCGGCGTCCTGGTGGC

ATGACCACAACTTATGGGCGGACACCCATGTATGGCTCCCAGACACCCATGTATGGCTCT

GGCTCCCGCACACCCATGTACGGCTCGCAGACACCTCTCCAGGACGGCAGCCGCACTCCA

CATTATGGCTCACAGACACCCCTACATGATGGCAGCCGCACTCCTGCCCAGAGTGGGGCA

TGGGACCCCAACAACCCCAACACACCATCACGGGCTGAAGAAGAGTATGAGTATGCCTTC

GACGATGAGCCTACTCCGTCTCCACAGGCCTATGGGGGGACCCCCAACCCCCAAACACCT

GGCTATCCAGATCCTTCCTCCCCCCAGGTCAACCCACAGTATAACCCACAGACTCCAGGG

ACGCCAGCCATGTACAACACAGACCAGTTCTCCCCCTATGCCGCCCCCTCCCCTCAAGGC

TCCTACCAGCCCAGCCCCAGTCCCCAGAGCTACCACCAGGTGGCACCAAGCCCAGCAGGC

TACCAAAACACCCATTCGCCAGCCAGCTACCACCCCACACCCTCGCCCATGGCCTATCAG

GCAAGCCCTAGCCCTAGCCCAGTTGGCTATAGTCCGATGACACCTGGAGCTCCCTCCCCT

GGTGGCTACAACCCGCACACACCTGGCTCAGGCATTGAGCAGAACTCTAGCGACTGGGTA

ACCACTGACATCCAGGTGAAGGTGCGGGACACCTACCTGGACACACAGGTGGTGGGTCAG

ACAGGAGTCATCCGTAGTGTCACGGGAGGCATGTGCTCTGTGTACCTGAAGGACAGTGAG

AAGGTCGTCAGCATTTCCAGTGAGCACCTGGAACCCATCACCCCCACCAAGAACAACAAG

GTAAAGGTGATCCTGGGTGAGGATCGGGAGGCCACCGGAGTCCTGCTGAGCATCGATGGT

GAGGATGGCATCGTCCGCATGGACCTTGACGAGCAGCTCAAGATCCTCAACCTCCGCTTC

CTGGGGAAGCTCCTGGAGGCC

>CL201.Contig1_A-W 97 222 minus strand Probable crossover junction endonuclease EME2 OS=Mus musculus GN=Eme2 PE=2 SV=2

GCCGTGCTGGAGGACACTCGTGCTGACGTCCTAATGGAGGCTCTGGGCGCCCTGGACTGT

GAATGCCGCATCGAGCCGCAGCGCCGGGCCCGCAGCCTGCGGTGGAGCGGAGTGAGGCAC

GACCCC

>CL201.Contig2_A-W 2 289 minus strand PREDICTED: probable crossover junction endonuclease EME2-like [Cavia porcellus]

GACGGCTCTGCAGAGGCCGGAGCGCGGGCTCGGGGCGTGGCGGGGGAGCGCAGGGCGGCG

GCGTGGCGGCCCGAGCAGGCTCTGCAGCGCCTGGTGGTGCGCGTGGACCCAGCCGTGCTG

GAGGACACTCGTGCTGACGTCCTAATGGAGGCTCTGGGCGCCCTGGACTGTGAATGCCGC

ATCGAGCCGCAGCGCCGGGCCCGCAGCCTGCGGTGGAGCGGAGTGAGGCACGACCCCGGC

AGAGTGAGTGGCTGGTTCTACCCTCCCAGACCATGTCCCGAGTCCTGG

>CL201.Contig3_A-W 2 1183 PREDICTED: probable crossover junction endonuclease EME2-like [Cavia porcellus]

TCTGAGGCGGAGGGCGACGGCTCTGCAGAGGCCGGAGCGCGGGCTCGGGGCGTGGCGGGG

GAGCGCAGGGCGGCGGCGTGGCGGCCCGAGCAGGCTCTGCAGCGCCTGGTGGTGCGCGTG

GACCCAGCCGTGCTGGAGGACACTCGTGCTGACGTCCTAATGGAGGCTCTGGGCGCCCTG

GACTGTGAATGCCGCATCGAGCCGCAGCGCCGGGCCCGCAGCCTGCGGTGGAGCGGAGTG

AGGCACGACCCCGGCAGAGTGGCTCCTGAGGTGTGGGCTACAGAGGAGCAGGAACTACTA

CTGCTGCTGCTGCTGGAGCCTGAGGAGTTTCTGCAGGGCACTGCCCAGCTGACCCAGACA

GAAGGCCCACCCTGTCCGGTGCCTTGGATCTCTCCTGAGAGCCCTGCCCACCCCCACCTG

GCTGTCATAGGGCTGGATGCCCACCTATGGTACCATTCACCTCAAGGCCCTTAGTGGGGC

TGGCTGGAGTGGGCAGGGAGAGGTAGGGGTGCCTCTGGAGGCTGCTGTCACAGCTGATTC

ACTGCCCTGGGGGCCCTGGCAGAGGCAGAACTCAGGAAGAAGGGGTGGGGAACTGGGGGG

TTGCCTCTGCTCAGGTCTTGCCAGCCCAGTGCCCAGGGGATGCAGCAGCTGCAGAATTCA

GAGGTGGTCCAGGCCAAGGAGGCTGTCAGCTGGCCGGAAGTGGAGGAGGCTCTGGTGCTC

CTTCAGCTCCGTGCAGACCTGGATGTGCTACTGGTGGCCTCCTGGCAAGAGCTGACTCAG

CATGTGTGTGCCTACACCAAGGCCCTTGCCCAGCTCCCCTCTAAGCAGTGCTGGGACTCC

CAGGCCTTTTCCTTCTGCACAGCAGGTCGCTGGGCTGCAGGCCAGCAGGTGGCCAGAGAT

GGCTCTGGGTTGCGTGGGGTTTGGTGGAGACAGATCAGGCAGTTTAACCGGGTCAGCCCT

GCTGTGGCAGATGCCATTGTCACCGCCTTCCCCTCCCCACGCCTTCTGCAGCAGGCGCTC

ATGGCCTGCAGCACAGAACAGGAACGTCTGGGCCTCCTGGCTGACCTCCCTGTGGAGGCC

AGCAAAGGCAGACAGTCCCGAAGGGTGGGGCCTGACCTTTCCCGTCGCATCTGCCTGTTC

CTGACCACCACCAGCCCTGACCTGCTACTGGACCTGGGCTCC

>CL201.Contig4_A-W 2 1165 PREDICTED: probable crossover junction endonuclease EME2-like [Cavia porcellus]

TCTGAGGCGGAGGGCGACGGCTCTGCAGAGGCCGGAGCGCGGGCTCGGGGCGTGGCGGGG

GAGCGCAGGGCGGCGGCGTGGCGGCCCGAGCAGGCTCTGCAGCGCCTGGTGGTGCGCGTG

GACCCAGCCGTGCTGGAGGACACTCGTGCTGACGTCCTAATGGAGGCTCTGGGCGCCCTG

GACTGTGAATGCCGCATCGAGCCGCAGCGCCGGGCCCGCAGCCTGCGGTGGAGCGGAGTG

AGGCACGACCCCGGCAGAGTGGCTCCTGAGGTGTGGGCTACAGAGGAGCAGGAACTACTA

CTGCTGCTGCTGCTGGAGCCTGAGGAGTTTCTGCAGGGCACTGCCCAGCTGACCCAGACA

GAAGGCCCACCCTGTCCGGTGCCTTGGATCTCTCCTGAGAGCCCTGCCCACCCCCACCTG

GCTGTCATAGGGCTGGATGCCCACCTATGGTACCATTCACCTCAAGGCCCTTAGTGGGGC

TGGCTGGAGTGGGCAGGGAGAGGTAGGGGTGCCTCTGGAGGCTGCTGTCACAGCTGATTC

ACTGCCCTGGGGGCCCTGGCAGAGGCAGAACTCAGGAAGAAGGGGTGGGGAACTGGGGGG

TTGCCTCTGCTCAGGTCTTGCCAGCCCAGTGCCCAGGGGATGCAGCAGCTGCAGAATTCA

GAGGTGGTCCAGGCCAAGGAGGCTGTCAGCTGGCCGGAAGTGGAGGAGCTCCGTGCAGAC

CTGGATGTGCTACTGGTGGCCTCCTGGCAAGAGCTGACTCAGCATGTGTGTGCCTACACC

AAGGCCCTTGCCCAGCTCCCCTCTAAGCAGTGCTGGGACTCCCAGGCCTTTTCCTTCTGC

ACAGCAGGTCGCTGGGCTGCAGGCCAGCAGGTGGCCAGAGATGGCTCTGGGTTGCGTGGG

GTTTGGTGGAGACAGATCAGGCAGTTTAACCGGGTCAGCCCTGCTGTGGCAGATGCCATT

GTCACCGCCTTCCCCTCCCCACGCCTTCTGCAGCAGGCGCTCATGGCCTGCAGCACAGAA

CAGGAACGTCTGGGCCTCCTGGCTGACCTCCCTGTGGAGGCCAGCAAAGGCAGACAGTCC

CGAAGGGTGGGGCCTGACCTTTCCCGTCGCATCTGCCTGTTCCTGACCACCACCAGCCCT

GACCTGCTACTGGACCTGGGCTCC

>CL201.Contig5_A-W 2 1018 PREDICTED: probable crossover junction endonuclease EME2-like [Cavia porcellus]

TCTGAGGCGGAGGGCGACGGCTCTGCAGAGGCCGGAGCGCGGGCTCGGGGCGTGGCGGGG

GAGCGCAGGGCGGCGGCGTGGCGGCCCGAGCAGGCTCTGCAGCGCCTGGTGGTGCGCGTG

GACCCAGCCGTGCTGGAGGACACTCGTGCTGACGTCCTAATGGAGGCTCTGGGCGCCCTG

GACTGTGAATGCCGCATCGAGCCGCAGCGCCGGGCCCGCAGCCTGCGGTGGAGCGGAGTG

AGGCACGACCCCGGCAGAGTGGCTCCTGAGGTGTGGGCTACAGAGGAGCAGGAACTACTA

CTGCTGCTGCTGCTGGAGCCTGAGGAGTTTCTGCAGGGCACTGCCCAGCTGACCCAGACA

GAAGGCCCACCCTGTCCGGTGCCTTGGATCTCTCCTGAGAGCCCTGCCCACCCCCACCTG

GCTGTCATAGGGCTGGATGCCCACCTATGGTCTTGCCAGCCCAGTGCCCAGGGGATGCAG

CAGCTGCAGAATTCAGAGGTGGTCCAGGCCAAGGAGGCTGTCAGCTGGCCGGAAGTGGAG

GAGGCTCTGGTGCTCCTTCAGCTCCGTGCAGACCTGGATGTGCTACTGGTGGCCTCCTGG

CAAGAGCTGACTCAGCATGTGTGTGCCTACACCAAGGCCCTTGCCCAGCTCCCCTCTAAG

CAGTGCTGGGACTCCCAGGCCTTTTCCTTCTGCACAGCAGGTCGCTGGGCTGCAGGCCAG

CAGGTGGCCAGAGATGGCTCTGGGTTGCGTGGGGTTTGGTGGAGACAGATCAGGCAGTTT

AACCGGGTCAGCCCTGCTGTGGCAGATGCCATTGTCACCGCCTTCCCCTCCCCACGCCTT

CTGCAGCAGGCGCTCATGGCCTGCAGCACAGAACAGGAACGTCTGGGCCTCCTGGCTGAC

CTCCCTGTGGAGGCCAGCAAAGGCAGACAGTCCCGAAGGGTGGGGCCTGACCTTTCCCGT

CGCATCTGCCTGTTCCTGACCACCACCAGCCCTGACCTGCTACTGGACCTGGGCTCC

>CL201.Contig6_A-W 2 1000 PREDICTED: probable crossover junction endonuclease EME2-like [Cavia porcellus]

TCTGAGGCGGAGGGCGACGGCTCTGCAGAGGCCGGAGCGCGGGCTCGGGGCGTGGCGGGG

GAGCGCAGGGCGGCGGCGTGGCGGCCCGAGCAGGCTCTGCAGCGCCTGGTGGTGCGCGTG

GACCCAGCCGTGCTGGAGGACACTCGTGCTGACGTCCTAATGGAGGCTCTGGGCGCCCTG

GACTGTGAATGCCGCATCGAGCCGCAGCGCCGGGCCCGCAGCCTGCGGTGGAGCGGAGTG

AGGCACGACCCCGGCAGAGTGGCTCCTGAGGTGTGGGCTACAGAGGAGCAGGAACTACTA

CTGCTGCTGCTGCTGGAGCCTGAGGAGTTTCTGCAGGGCACTGCCCAGCTGACCCAGACA

GAAGGCCCACCCTGTCCGGTGCCTTGGATCTCTCCTGAGAGCCCTGCCCACCCCCACCTG

GCTGTCATAGGGCTGGATGCCCACCTATGGTCTTGCCAGCCCAGTGCCCAGGGGATGCAG

CAGCTGCAGAATTCAGAGGTGGTCCAGGCCAAGGAGGCTGTCAGCTGGCCGGAAGTGGAG

GAGCTCCGTGCAGACCTGGATGTGCTACTGGTGGCCTCCTGGCAAGAGCTGACTCAGCAT

GTGTGTGCCTACACCAAGGCCCTTGCCCAGCTCCCCTCTAAGCAGTGCTGGGACTCCCAG

GCCTTTTCCTTCTGCACAGCAGGTCGCTGGGCTGCAGGCCAGCAGGTGGCCAGAGATGGC

TCTGGGTTGCGTGGGGTTTGGTGGAGACAGATCAGGCAGTTTAACCGGGTCAGCCCTGCT

GTGGCAGATGCCATTGTCACCGCCTTCCCCTCCCCACGCCTTCTGCAGCAGGCGCTCATG

GCCTGCAGCACAGAACAGGAACGTCTGGGCCTCCTGGCTGACCTCCCTGTGGAGGCCAGC

AAAGGCAGACAGTCCCGAAGGGTGGGGCCTGACCTTTCCCGTCGCATCTGCCTGTTCCTG

ACCACCACCAGCCCTGACCTGCTACTGGACCTGGGCTCC

>CL201.Contig7_A-W 2 1138 SPRY domain-containing SOCS box protein 3 isoform 2 [Mus musculus]

GGGGGCGGGGATTGTTTACGTCCCGGTCGGAGACGAAAGTGGGTCACAGCCTGCTAAGCG

CGGCTGTGGCGGCTGCAGGTCCTCTCTAGCATGGCCAGACGTCCGAGGAGTAGCAGGGCA

TGGCATTTTGTCTTGAGCGCAGCCCGCCGGGATGCTGATGCCCGGGCTGTGGCCCTGGCA

GGCACCACTAACCGGGGCTATGACTCTGATGGGCAGCACAGTGACTCCGATTCTGACCCT

GAGTCCTCGTCCCTGCCACCATCCATCCCCAGTGCTGTGCCTGTGACAGGGGAGTCCTTC

TGTGACTGTGAGAGCCAGAGCGAGGCCCCCTTCTGCAGCAGCCCACATACCACGCACCGT

GGCAAGGACTGCCGCTGTGGAGAAGAGGATGAGTACTTTGACTGGGTCTGGGATGACCTG

AATAAGTCCTCAGCTGCCCTGCTAAGCTGTGACAACCGCAAGGTCAGCTTCCATATGGAG

TATAGCTGTGGTACCGCAGCCATCCGGGGCACCAAGGAGCTGGGGGATGGCCAACACTTC

TGGGAAATTAAGATGACCTCACCTGTCTATGGCACTGATATGATGGTGGGCATTGGGACG

TCAGACGTGGACCTGGACAAGTACCATCACACCTTCTGCAGCCTGCTTGGCAGGGACGAG

GACAGCTGGGGCCTCTCCTACACGGGGCTCCTCCACCATAAGGGTGACAAGACCAGCTTC

TCATCACGGTTTGGCCAAGGCTCCATCATTGGTGTGCACCTGGATGCCTGGCACGGGACG

CTGACTTTCTTTAAGAACAGGAAATGCATAGGAGTGGCAGCCACCAGACTGAGGAACAGG

AGGTTCTACCCAATGGTGTGCTCCACAGCGGCCAGAAGCAGCATGAAGGTGATCCGTGCC

TGTGCCACCGCCACCTCCCTGCAGTACTTGTGCTGCTCCCGCCTGCGCCAGTTGCGCCCT

GACTCAGGGGACACCCTTGAGGGCCTGCCCCTGCCACCTGGTCTCAAGCAGGTGCTGCGT

GACAAGTTGGGCTGGGTCTTGAGCATGAACTGCAGTCGGCGCAAGCCCCCAGCGTCCCCA

CCTGGTACCCCCAGCCTAGAGGCCAGGCCCTGCCGAAGGAAGAGGTGCCGACGGACC

>CL201.Contig8_A-W 2 1138 SPRY domain-containing SOCS box protein 3 isoform 2 [Mus musculus]

GGGGGCGGGGATTGTTTACGTCCCGGTCGGAGACGAAAGTGGGTCACAGCCTGCTAAGCG

CGGCTGTGGCGGCTGCAGGTCCTCTCTAGCATGGCCAGACGTCCGAGGAGTAGCAGGGCA

TGGCATTTTGTCTTGAGCGCAGCCCGCCGGGATGCTGATGCCCGGGCTGTGGCCCTGGCA

GGCACCACTAACCGGGGCTATGACTCTGATGGGCAGCACAGTGACTCCGATTCTGACCCT

GAGTCCTCGTCCCTGCCACCATCCATCCCCAGTGCTGTGCCTGTGACAGGGGAGTCCTTC

TGTGACTGTGAGAGCCAGAGCGAGGCCCCCTTCTGCAGCAGCCCACATACCACGCACCGT

GGCAAGGACTGCCGCTGTGGAGAAGAGGATGAGTACTTTGACTGGGTCTGGGATGACCTG

AATAAGTCCTCAGCTGCCCTGCTAAGCTGTGACAACCGCAAGGTCAGCTTCCATATGGAG

TATAGCTGTGGTACCGCAGCCATCCGGGGCACCAAGGAGCTGGGGGATGGCCAACACTTC

TGGGAAATTAAGATGACCTCACCTGTCTATGGCACTGATATGATGGTGGGCATTGGGACG

TCAGACGTGGACCTGGACAAGTACCATCACACCTTCTGCAGCCTGCTTGGCAGGGACGAG

GACAGCTGGGGCCTCTCCTACACGGGGCTCCTCCACCATAAGGGTGACAAGACCAGCTTC

TCATCACGGTTTGGCCAAGGCTCCATCATTGGTGTGCACCTGGATGCCTGGCACGGGACG

CTGACTTTCTTTAAGAACAGGAAATGCATAGGAGTGGCAGCCACCAGACTGAGGAACAGG

AGGTTCTACCCAATGGTGTGCTCCACAGCGGCCAGAAGCAGCATGAAGGTGATCCGTGCC

TGTGCCACCGCCACCTCCCTGCAGTACTTGTGCTGCTCCCGCCTGCGCCAGTTGCGCCCT

GACTCAGGGGACACCCTTGAGGGCCTGCCCCTGCCACCTGGTCTCAAGCAGGTGCTGCGT

GACAAGTTGGGCTGGGTCTTGAGCATGAACTGCAGTCGGCGCAAGCCCCCAGCGTCCCCA

CCTGGTACCCCCAGCCTAGAGGCCAGGCCCTGCCGAAGGAAGAGGTGCCGACGGACC

>CL202.Contig1_A-W 227 421 IMP1 inner mitochondrial membrane peptidase-like [Sus scrofa]

TTTTTATGGTATCCAAAGGTGCCAACAGGTCATGTTTGGTTAGAAGGTGATAATTTACAG

AATTCTACAGATTCCAGGTATTATGGACCTATTCCATATGGACTAATAAGAGGACGAATC

TTCTTTAAGATCTGGCCTCTGAGTGATTTTGGATTTCTACGAGACAGCCCAAATGGCCAC

AGATTTTCTGATAAT

>CL202.Contig2_A-W 21 548 PREDICTED: mitochondrial inner membrane protease subunit 1-like [Ailuropoda melanoleuca]

GGCCAATGGTGGCAGAGTCTACATAGAACTATGCTTCGTGGTGTTCTGGGAAAAACCTTC

CGACTTGTTGGCTATACTATTCAGTATGGCTGTATAGCCCATTGTGCTTTTGAATACGTT

GGTGGTGTTGTAATGTGTTCTGGACCATCAATGGAACCAACAATTCAAAATTCAGATATT

GTCTTTGCAGAAAATGTTAGCCGACATTTTTATGGTATCCAAAGAGGTGACATTGTGATT

GCAAAAAGCCCGAGTGATCCAAAATCAAATATTTGTAAAAGAGTAATTGGTTTGGAAGGA

GACAAAATCCTCACCACTAGTCCATCAGATTTCTTTAAAAGCCATATTTATGTGCCAACA

GGTCATGTTTGGTTAGAAGGTGATAATTTACAGAATTCTACAGATTCCAGGTATTATGGA

CCTATTCCATATGGACTAATAAGAGGACGAATCTTCTTTAAGATCTGGCCTCTGAGTGAT

TTTGGATTTCTACGAGACAGCCCAAATGGCCACAGATTTTCTGATAAT

>CL203.Contig1_A-W 133 351 PREDICTED: zinc finger protein 76 isoform 2 [Otolemur garnettii]

CAGGTCACAATCATTACCTCTGGGGCTGTGGTGGCCGAGAACTCAAGTGTAGCATCCCTT

CATCATCAACAGGTGGCGCTGTTGGCCACAGCCAACGGAACACACATAGCAGTGCAGCTG

GAGGAGCAGCAGACCTTGGAAGAGGCTATCAGCGTTGCCACTGCTGCCATGCAGCAAGGG

GCCATGACTCTGGAGACAGCAGTGTCGGAGAGTGGTTGC

>CL203.Contig2_A-W 142 537 PREDICTED: zinc finger protein 76 [Equus caballus]

CACCTGTCAGAAGTGAAGGAAGAGGGTGATGACATCCCAGCTCAAGTGGCCATGGTGACT

GAGGAGGATGGGGCTCCCCAGGTGGCTCTCATCACTCAGGATGGTGCCCAGCAGGTCAGC

CTGTCCCCAGAAGACCTGCAGGCCCTGGGGAGTGCCATCAGTGTGGTAACCCAGCACGGC

AGCACCACCCTCACCATCCCCAGCCACAACGATGACCTGGCCACATCTGGCACACATACA

GTCACCATGGTCAGCGCTGATGGCACCCAGACGCAGCCCGTCACAATCATTACCTCTGGG

GCTGTGGTGGCCGAGAACTCAAGTGTAGCATCCCTTCATCATCAACAGGTGGCGCTGTTG

GCCACAGCCAACGGAACACACATAGCAGTGCAGGTG

>CL203.Contig3_A-W 142 258 PREDICTED: zinc finger protein 76 [Loxodonta africana]

CACCTGTCAGAAGTGAAGGAAGAGGGTGATGACATCCCAGCTCAAGTGGCCATGGTGACT

GAGGAGGATGGGGCTCCCCAGGTGGCTCTCATCACTCAGGATGGTGCCCAGCAGGTA

>CL203.Contig4_A-W 133 252 PREDICTED: zinc finger protein 76 isoform 2 [Otolemur garnettii]

CAGGTCACAATCATTACCTCTGGGGCTGTGGTGGCCGAGAACTCAAGTGTAGCATCCCTT

CATCATCAACAGGTGGCGCTGTTGGCCACAGCCAACGGAACACACATAGCAGTGCAGGTG

>CL203.Contig5_A-W 142 636 PREDICTED: LOW QUALITY PROTEIN: zinc finger protein 76-like [Nomascus leucogenys]

CACCTGTCAGAAGTGAAGGAAGAGGGTGATGACATCCCAGCTCAAGTGGCCATGGTGACT

GAGGAGGATGGGGCTCCCCAGGTGGCTCTCATCACTCAGGATGGTGCCCAGCAGGTCAGC

CTGTCCCCAGAAGACCTGCAGGCCCTGGGGAGTGCCATCAGTGTGGTAACCCAGCACGGC

AGCACCACCCTCACCATCCCCAGCCACAACGATGACCTGGCCACATCTGGCACACATACA

GTCACCATGGTCAGCGCTGATGGCACCCAGACGCAGCCCGTCACAATCATTACCTCTGGG

GCTGTGGTGGCCGAGAACTCAAGTGTAGCATCCCTTCATCATCAACAGGTGGCGCTGTTG

GCCACAGCCAACGGAACACACATAGCAGTGCAGCTGGAGGAGCAGCAGACCTTGGAAGAG

GCTATCAGCGTTGCCACTGCTGCCATGCAGCAAGGGGCCATGACTCTGGAGACAGCAGTG

TCGGAGAGTGGTTGC

>CL204.Contig1_A-W 152 403 minus strand PREDICTED: trinucleotide repeat-containing gene 6A protein [Callithrix jacchus]

AGGGATTTAGTGCAAGAAGAAGAACAGTTGATGGAAGAAAAGAAAAAGAAAAAAGACGAC

AAGAAAAAGAAGGAAGCTGCTCAAAAGAAGGTAGCCACTGAACAAAAAATCAAAGTGCCA

GAACAGATAAAGCCCAGTGTAAGCCAGCCTCAGCCTGCCAACTCTAATAACGGCACTTCC

ACAGCAACCAGCACTAATAATAATGCCAAGCGAGCTACAGCCAACAATCAGCAGCAGCAG

CAGCAACAGCAG

>CL204.Contig2_A-W 131 262 minus strand mCG20982, isoform CRA_d [Mus musculus]

TTAATTGCAACTGTGTGGTTCAGGGATTTAGTGCAAGAAGAAGAACAGTTGATGGAAGAA

AAGAAAAAGAAAAAAGACGACAAGAAAAAGAAGGAAGCTGCTCAAAAGAAGGCCACTGAA

CAAAAAATCAAA

>CL204.Contig3_A-W 120 257 mCG20982, isoform CRA_d [Mus musculus]

TTAATTGCAACTGTGTGGTTCAGGGATTTAGTGCAAGAAGAAGAACAGTTGATGGAAGAA

AAGAAAAAGAAAAAAGACGACAAGAAAAAGAAGGAAGCTGCTCAAAAGAAGGCCACTGAA

CAAAAAATCAAAGGTCCA

>CL205.Contig1_A-W 217 807 minus strand PREDICTED: hypothetical protein LOC707643 isoform 1 [Macaca mulatta]

CAGGTTAAGGCTCGCGATCAGGCCGCCTCTTCTGTCAGTGAAAAAACTAAGTCTTTACTG

CATAAATTAGAGGAAGCAATTTCAGAACAACGGAACCTCCAAACTATAAATAGTGAGTTA

TCGAACACTTGCCAGAAGCTTCAGGAGAAGACAAGAAAACTGAAAACAACTATTGAGGTA

CTTCGAAAGCAGTTAACTGAAGGACAACACCATGGCTTTCTGTTTCAGAGGTTTTTAGAT

GAGGAATTTTCTCACGATGATCCCCCGTCCTGCCCAGAGAAAATTCAATCATCTCTTCAA

GAAAAATTGAAGCAGTGTTACCACAAACCACACAGTAGTCAAGAGGCAAGAATTCATCAC

ATACCCCTGACCTTGAAAAACACATGCTGGTATACGCCTCTTCTGGATGCCTTGTCTCTG

GATAGTCTTACAGTGATTCCAAGGCCACAATTGACATCTTCCTTATGTGTAGCCAACCCA

ACTGAGGCTTCAAGTGAAAGATCCAAATATGGAGAAATAAAGGATGGTGCTTTGGATGTG

ACGAGACAACACAAGTGTCCTGTAGGTGGTCCAGACCCAGGAGCAGATCTT

>CL205.Contig2_A-W 208 387 minus strand PREDICTED: lymphoid-restricted membrane protein [Pan paniscus]

GTGCATATCCAGGTTAAGGCTCGCGATCAGGCCGCCTCTTCTGTCAGTGAAAAAACTAAG

TCTTTACTGCATAAATTAGAGGAAGCAATTTCAGAACAACGGAACCTCCAAACTATAAAT

AGTGAGTTATCGAACACTTGCCAGAAGCTTCAGGAGAAGACAAGAAAACTGAAAAAGGTT

>CL205.Contig3_A-W 522 710 minus strand PREDICTED: hypothetical protein LOC707643 isoform 1 [Macaca mulatta]

ATTGATGCCTTGTCTCTGGATAGTCTTACAGTGATTCCAAGGCCACAATTGACATCTTCC

TTATGTGTAGCCAACCCAACTGAGGCTTCAAGTGAAAGATCCAAATATGGAGAAATAAAG

GATGGTGCTTTGGATGTGACGAGACAACACAAGTGTCCTGTAGGTGGTCCAGACCCAGGA

GCAGATCTT

>CL205.Contig4_A-W 526 648 minus strand Lymphoid-restricted membrane protein OS=Homo sapiens GN=LRMP PE=1 SV=3

GTAGCCAACCCAACTGAGGCTTCAAGTGAAAGATCCAAATATGGAGAAATAAAGGATGGT

GCTTTGGATGTGACGAGACAACACAAGTGTCCTGTAGGTGGTCCAGACCCAGGAGCAGAT

CTT

>CL206.Contig1_A-W 1427 3220 minus strand nuclear receptor subfamily 4, group A, member 1, isoform CRA_b [Homo sapiens]

ATGCCCTGTATCCAAGCCCAATATGGGACACCAGCACCAAGTCCAGGACCCCGTGACCAC

CTGGCAAGCGACGCCCTGACCCCTGAACTCAGCAAGCCCACCATGGACCTGGCCAGCCCT

GAGGCGGCCCCTGCTGCCCCCACTGCCCTGCCCAGCTTCAGCACCTTCATGGATGGCTAC

ACGGGAGAGTTTGATACCTTCCTCTACCAGCTGCCCGGGACAGCCCAGCCGTGCTCCTCG

GCTTCCTCCTCCGCCTCCTCCACATCCTCCTCCTCGGCCACCTCCCCTGCCTCTGCTTCT

TTCAAATTTGAAGACTTCCAGGTGTACGGCTGCTACCCTGGCACCCTGAGTGGTCCACTG

GATGAGACCTTATCCTCCAGTGGCTCTGACTACTATGGCAGCCCCTGCTCTGCCCCATCG

CCGTCCACACCCAGCTTCCAGCCACCCCAGCTCTCTCCCTGGGATGGCTCATTTGGCCAC

TTCTCCCCCAGCCAGACTTACGAAGGCCTGAGGGCATGGACAGAGCAGCTGCCCAAGGCT

TCTGGGCCCCCGCAACCACCAACCTTCTTCTCTTTCAGTCCTCCCCCTGGTCCTAGCCCC

AGTCTGGCCCAAAGCTCCCTGAAGTTACTCCCCTCACAGGCTGCCCACCAGCTGGGGGAG

GGAGAGAGCTATTCCATGCCAGTGACTTTCCCAAGCCTGGCGCCCACTTCCCCACACCTG

GACGGCCCAGGAATGCTGGATGTGCCTGTGACCTCAGCCAAGGCCCGGAGCGGGGCTCCA

GGTGGAAATGAGGGCCGCTGTGCTGTGTGTGGGGACAATGCTTCATGTCAGCATTATGGT

GTACGCACCTGTGAAGGCTGCAAGGGCTTCTTTAAGCGCACAGTGCAGAAAAGCGCCAAG

TACATCTGCCTGGCAAACAAGGACTGCCCTGTGGACAAGAGGCGGCGGAACCGCTGCCAG

TTCTGCCGCTTCCAGAAGTGCCTGGCTGTGGGCATGGTGAAGGAAGTTGTCCGAACAGAC

AGCCTGAAGGGGCGGCGGGGCCGGCTACCTTCAAAACCCAAGCAGCCTCCAGATGCCTCC

CCTGCCAATCTCCTCACCTCCCTTGTCCGAGCACACCTGGACTCGGGCCCCAGCATGGCG

AAACTGGACTACTCCAAGTTCCAGGAGCTGGTGCTGCCCCACTTTGGGAAGGAAGATGCT

GGTGATGTGCAGCAGTTCTACGATCTTCTGTCGGGTTCCCTGGAGGTTATCCGCAAGTGG

GCCGAGAAGATCCCTGGCTTTACTGAGCTGTCTCCAGGTGACCAGGACCTGCTGCTGGAG

TCAGCCTTCTTGGAGCTCTTCATCCTCCGCCTGGCCTACCGGTCTAAGCCAGGTGAGGGG

AAGCTCATCTTCTGCTCAGGCCTGGTGCTGCACCGACTCCAGTGTGCCCGCGGCTTTGGT

GACTGGATTGACAGTATCCTGGCGTTCTCACGGTCCCTGCATAGCTTGGTAGTCGATGTC

CCTGCCTTTGCTTGCCTTTCTGCTCTTGTCCTCATCACTGACCGGCATGGGCTACAGGAG

CCACGGCGGGTAGAGGAGCTACAGAACCGCATTGCCAGCTGCCTAAAGGAGCACATGGCA

GCCGTAGCCGGTGAGCCCCAGCCAGCCAGCTGCCTGTCACGTCTGCTGGGCAAATTGCCC

GAGTTGCGAACCCTGTGCACCCAGGGCCTACAGCGCATCTTCTACCTCAAGCTGGAGGAC

TTGGTGCCTCCTCCACCCATTGTTGACAAGATTTTTATGGACACACTGCCCTTC

>CL206.Contig2_A-W 181 1725 minus strand nuclear receptor subfamily 4, group A, member 1, isoform CRA_b [Homo sapiens]

ATGCCCTGTATCCAAGCCCAATATGGGACACCAGCACCAAGTCCAGGACCCCGTGACCAC

CTGGCAAGCGACGCCCTGACCCCTGAACTCAGCAAGCCCACCATGGACCTGGCCAGCCCT

GAGGCGGCCCCTGCTGCCCCCACTGCCCTGCCCAGCTTCAGCACCTTCATGGATGGCTAC

ACGGGAGAGTTTGATACCTTCCTCTACCAGCTGCCCGGGACAGCCCAGCCGTGCTCCTCG

GCTTCCTCCTCCGCCTCCTCCACATCCTCCTCCTCGGCCACCTCCCCTGCCTCTGCTTCT

TTCAAATTTGAAGACTTCCAGGTGTACGGCTGCTACCCTGGCACCCTGAGTGGTCCACTG

GATGAGACCTTATCCTCCAGTGGCTCTGACTACTATGGCAGCCCCTGCTCTGCCCCATCG

CCGTCCACACCCAGCTTCCAGCCACCCCAGCTCTCTCCCTGGGATGGCTCATTTGGCCAC

TTCTCCCCCAGCCAGACTTACGAAGGCCTGAGGGCATGGACAGAGCAGCTGCCCAAGGCT

TCTGGGCCCCCGCAACCACCAACCTTCTTCTCTTTCAGTCCTCCCCCTGGTCCTAGCCCC

AGTCTGGCCCAAAGCTCCCTGAAGTTACTCCCCTCACAGGCTGCCCACCAGCTGGGGGAG

GGAGAGAGCTATTCCATGCCAGTGACTTTCCCAAGCCTGGCGCCCACTTCCCCACACCTG

GACGGCCCAGGAATGCTGGATGTGCCTGTGACCTCAGCCAAGGCCCGGAGCGGGGCTCCA

GGTGGAAATGAGGGCCGCTGTGCTGTGTGTGGGGACAATGCTTCATGTCAGCATTATGGT

GTACGCACCTGTGAAGGCTGCAAGGGCTTCTTTAAGCGCACAGTGCAGAAAAGCGCCAAG

TACATCTGCCTGGCAAACAAGGACTGCCCTGTGGACAAGAGGCGGCGGAACCGCTGCCAG

TTCTGCCGCTTCCAGAAGTGCCTGGCTGTGGGCATGGTGAAGGAAGTTGTCCGAACAGAC

AGCCTGAAGGGGCGGCGGGGCCGGCTACCTTCAAAACCCAAGCAGCCTCCAGATGCCTCC

CCTGCCAATCTCCTCACCTCCCTTGTCCGAGCACACCTGGACTCGGGCCCCAGCATGGCG

AAACTGGACTACTCCAAGTTCCAGGAGCTGGTGCTGCCCCACTTTGGGAAGGAAGATGCT

GGTGATGTGCAGCAGTTCTACGATCTTCTGTCGGGTTCCCTGGAGGTTATCCGCAAGTGG

GCCGAGAAGATCCCTGGCTTTACTGAGCTGTCTCCAGGTGACCAGGACCTGCTGCTGGAG

TCAGCCTTCTTGGAGCTCTTCATCCTCCGCCTGGCCTACCGGTCTAAGCCAGGTGAGGGG

AAGCTCATCTTCTGCTCAGGCCTGGTGCTGCACCGACTCCAGTGTGCCCGCGGCTTTGGT

GACTGGATTGACAGTATCCTGGCGTTCTCACGGTCCCTGCATAGCTTGGTAGTCGATGTC

CCTGCCTTTGCTTGCCTTTCTGCTCTTGTCCTCATCACTGGTAAG

>CL206.Contig3_A-W 1427 2971 minus strand nuclear receptor subfamily 4, group A, member 1, isoform CRA_b [Homo sapiens]

ATGCCCTGTATCCAAGCCCAATATGGGACACCAGCACCAAGTCCAGGACCCCGTGACCAC

CTGGCAAGCGACGCCCTGACCCCTGAACTCAGCAAGCCCACCATGGACCTGGCCAGCCCT

GAGGCGGCCCCTGCTGCCCCCACTGCCCTGCCCAGCTTCAGCACCTTCATGGATGGCTAC

ACGGGAGAGTTTGATACCTTCCTCTACCAGCTGCCCGGGACAGCCCAGCCGTGCTCCTCG

GCTTCCTCCTCCGCCTCCTCCACATCCTCCTCCTCGGCCACCTCCCCTGCCTCTGCTTCT

TTCAAATTTGAAGACTTCCAGGTGTACGGCTGCTACCCTGGCACCCTGAGTGGTCCACTG

GATGAGACCTTATCCTCCAGTGGCTCTGACTACTATGGCAGCCCCTGCTCTGCCCCATCG

CCGTCCACACCCAGCTTCCAGCCACCCCAGCTCTCTCCCTGGGATGGCTCATTTGGCCAC

TTCTCCCCCAGCCAGACTTACGAAGGCCTGAGGGCATGGACAGAGCAGCTGCCCAAGGCT

TCTGGGCCCCCGCAACCACCAACCTTCTTCTCTTTCAGTCCTCCCCCTGGTCCTAGCCCC

AGTCTGGCCCAAAGCTCCCTGAAGTTACTCCCCTCACAGGCTGCCCACCAGCTGGGGGAG

GGAGAGAGCTATTCCATGCCAGTGACTTTCCCAAGCCTGGCGCCCACTTCCCCACACCTG

GACGGCCCAGGAATGCTGGATGTGCCTGTGACCTCAGCCAAGGCCCGGAGCGGGGCTCCA

GGTGGAAATGAGGGCCGCTGTGCTGTGTGTGGGGACAATGCTTCATGTCAGCATTATGGT

GTACGCACCTGTGAAGGCTGCAAGGGCTTCTTTAAGCGCACAGTGCAGAAAAGCGCCAAG

TACATCTGCCTGGCAAACAAGGACTGCCCTGTGGACAAGAGGCGGCGGAACCGCTGCCAG

TTCTGCCGCTTCCAGAAGTGCCTGGCTGTGGGCATGGTGAAGGAAGTTGTCCGAACAGAC

AGCCTGAAGGGGCGGCGGGGCCGGCTACCTTCAAAACCCAAGCAGCCTCCAGATGCCTCC

CCTGCCAATCTCCTCACCTCCCTTGTCCGAGCACACCTGGACTCGGGCCCCAGCATGGCG

AAACTGGACTACTCCAAGTTCCAGGAGCTGGTGCTGCCCCACTTTGGGAAGGAAGATGCT

GGTGATGTGCAGCAGTTCTACGATCTTCTGTCGGGTTCCCTGGAGGTTATCCGCAAGTGG

GCCGAGAAGATCCCTGGCTTTACTGAGCTGTCTCCAGGTGACCAGGACCTGCTGCTGGAG

TCAGCCTTCTTGGAGCTCTTCATCCTCCGCCTGGCCTACCGGTCTAAGCCAGGTGAGGGG

AAGCTCATCTTCTGCTCAGGCCTGGTGCTGCACCGACTCCAGTGTGCCCGCGGCTTTGGT

GACTGGATTGACAGTATCCTGGCGTTCTCACGGTCCCTGCATAGCTTGGTAGTCGATGTC

CCTGCCTTTGCTTGCCTTTCTGCTCTTGTCCTCATCACTGGTAAG

>CL206.Contig4_A-W 1100 2644 minus strand nuclear receptor subfamily 4, group A, member 1, isoform CRA_b [Homo sapiens]

ATGCCCTGTATCCAAGCCCAATATGGGACACCAGCACCAAGTCCAGGACCCCGTGACCAC

CTGGCAAGCGACGCCCTGACCCCTGAACTCAGCAAGCCCACCATGGACCTGGCCAGCCCT

GAGGCGGCCCCTGCTGCCCCCACTGCCCTGCCCAGCTTCAGCACCTTCATGGATGGCTAC

ACGGGAGAGTTTGATACCTTCCTCTACCAGCTGCCCGGGACAGCCCAGCCGTGCTCCTCG

GCTTCCTCCTCCGCCTCCTCCACATCCTCCTCCTCGGCCACCTCCCCTGCCTCTGCTTCT

TTCAAATTTGAAGACTTCCAGGTGTACGGCTGCTACCCTGGCACCCTGAGTGGTCCACTG

GATGAGACCTTATCCTCCAGTGGCTCTGACTACTATGGCAGCCCCTGCTCTGCCCCATCG

CCGTCCACACCCAGCTTCCAGCCACCCCAGCTCTCTCCCTGGGATGGCTCATTTGGCCAC

TTCTCCCCCAGCCAGACTTACGAAGGCCTGAGGGCATGGACAGAGCAGCTGCCCAAGGCT

TCTGGGCCCCCGCAACCACCAACCTTCTTCTCTTTCAGTCCTCCCCCTGGTCCTAGCCCC

AGTCTGGCCCAAAGCTCCCTGAAGTTACTCCCCTCACAGGCTGCCCACCAGCTGGGGGAG

GGAGAGAGCTATTCCATGCCAGTGACTTTCCCAAGCCTGGCGCCCACTTCCCCACACCTG

GACGGCCCAGGAATGCTGGATGTGCCTGTGACCTCAGCCAAGGCCCGGAGCGGGGCTCCA

GGTGGAAATGAGGGCCGCTGTGCTGTGTGTGGGGACAATGCTTCATGTCAGCATTATGGT

GTACGCACCTGTGAAGGCTGCAAGGGCTTCTTTAAGCGCACAGTGCAGAAAAGCGCCAAG

TACATCTGCCTGGCAAACAAGGACTGCCCTGTGGACAAGAGGCGGCGGAACCGCTGCCAG

TTCTGCCGCTTCCAGAAGTGCCTGGCTGTGGGCATGGTGAAGGAAGTTGTCCGAACAGAC

AGCCTGAAGGGGCGGCGGGGCCGGCTACCTTCAAAACCCAAGCAGCCTCCAGATGCCTCC

CCTGCCAATCTCCTCACCTCCCTTGTCCGAGCACACCTGGACTCGGGCCCCAGCATGGCG

AAACTGGACTACTCCAAGTTCCAGGAGCTGGTGCTGCCCCACTTTGGGAAGGAAGATGCT

GGTGATGTGCAGCAGTTCTACGATCTTCTGTCGGGTTCCCTGGAGGTTATCCGCAAGTGG

GCCGAGAAGATCCCTGGCTTTACTGAGCTGTCTCCAGGTGACCAGGACCTGCTGCTGGAG

TCAGCCTTCTTGGAGCTCTTCATCCTCCGCCTGGCCTACCGGTCTAAGCCAGGTGAGGGG

AAGCTCATCTTCTGCTCAGGCCTGGTGCTGCACCGACTCCAGTGTGCCCGCGGCTTTGGT

GACTGGATTGACAGTATCCTGGCGTTCTCACGGTCCCTGCATAGCTTGGTAGTCGATGTC

CCTGCCTTTGCTTGCCTTTCTGCTCTTGTCCTCATCACTGGTAAG

>CL206.Contig5_A-W 1100 2893 minus strand nuclear receptor subfamily 4, group A, member 1, isoform CRA_b [Homo sapiens]

ATGCCCTGTATCCAAGCCCAATATGGGACACCAGCACCAAGTCCAGGACCCCGTGACCAC

CTGGCAAGCGACGCCCTGACCCCTGAACTCAGCAAGCCCACCATGGACCTGGCCAGCCCT

GAGGCGGCCCCTGCTGCCCCCACTGCCCTGCCCAGCTTCAGCACCTTCATGGATGGCTAC

ACGGGAGAGTTTGATACCTTCCTCTACCAGCTGCCCGGGACAGCCCAGCCGTGCTCCTCG

GCTTCCTCCTCCGCCTCCTCCACATCCTCCTCCTCGGCCACCTCCCCTGCCTCTGCTTCT

TTCAAATTTGAAGACTTCCAGGTGTACGGCTGCTACCCTGGCACCCTGAGTGGTCCACTG

GATGAGACCTTATCCTCCAGTGGCTCTGACTACTATGGCAGCCCCTGCTCTGCCCCATCG

CCGTCCACACCCAGCTTCCAGCCACCCCAGCTCTCTCCCTGGGATGGCTCATTTGGCCAC

TTCTCCCCCAGCCAGACTTACGAAGGCCTGAGGGCATGGACAGAGCAGCTGCCCAAGGCT

TCTGGGCCCCCGCAACCACCAACCTTCTTCTCTTTCAGTCCTCCCCCTGGTCCTAGCCCC

AGTCTGGCCCAAAGCTCCCTGAAGTTACTCCCCTCACAGGCTGCCCACCAGCTGGGGGAG

GGAGAGAGCTATTCCATGCCAGTGACTTTCCCAAGCCTGGCGCCCACTTCCCCACACCTG

GACGGCCCAGGAATGCTGGATGTGCCTGTGACCTCAGCCAAGGCCCGGAGCGGGGCTCCA

GGTGGAAATGAGGGCCGCTGTGCTGTGTGTGGGGACAATGCTTCATGTCAGCATTATGGT

GTACGCACCTGTGAAGGCTGCAAGGGCTTCTTTAAGCGCACAGTGCAGAAAAGCGCCAAG

TACATCTGCCTGGCAAACAAGGACTGCCCTGTGGACAAGAGGCGGCGGAACCGCTGCCAG

TTCTGCCGCTTCCAGAAGTGCCTGGCTGTGGGCATGGTGAAGGAAGTTGTCCGAACAGAC

AGCCTGAAGGGGCGGCGGGGCCGGCTACCTTCAAAACCCAAGCAGCCTCCAGATGCCTCC

CCTGCCAATCTCCTCACCTCCCTTGTCCGAGCACACCTGGACTCGGGCCCCAGCATGGCG

AAACTGGACTACTCCAAGTTCCAGGAGCTGGTGCTGCCCCACTTTGGGAAGGAAGATGCT

GGTGATGTGCAGCAGTTCTACGATCTTCTGTCGGGTTCCCTGGAGGTTATCCGCAAGTGG

GCCGAGAAGATCCCTGGCTTTACTGAGCTGTCTCCAGGTGACCAGGACCTGCTGCTGGAG

TCAGCCTTCTTGGAGCTCTTCATCCTCCGCCTGGCCTACCGGTCTAAGCCAGGTGAGGGG

AAGCTCATCTTCTGCTCAGGCCTGGTGCTGCACCGACTCCAGTGTGCCCGCGGCTTTGGT

GACTGGATTGACAGTATCCTGGCGTTCTCACGGTCCCTGCATAGCTTGGTAGTCGATGTC

CCTGCCTTTGCTTGCCTTTCTGCTCTTGTCCTCATCACTGACCGGCATGGGCTACAGGAG

CCACGGCGGGTAGAGGAGCTACAGAACCGCATTGCCAGCTGCCTAAAGGAGCACATGGCA

GCCGTAGCCGGTGAGCCCCAGCCAGCCAGCTGCCTGTCACGTCTGCTGGGCAAATTGCCC

GAGTTGCGAACCCTGTGCACCCAGGGCCTACAGCGCATCTTCTACCTCAAGCTGGAGGAC

TTGGTGCCTCCTCCACCCATTGTTGACAAGATTTTTATGGACACACTGCCCTTC

>CL206.Contig6_A-W 1311 3104 minus strand nuclear receptor subfamily 4, group A, member 1, isoform CRA_b [Homo sapiens]

ATGCCCTGTATCCAAGCCCAATATGGGACACCAGCACCAAGTCCAGGACCCCGTGACCAC

CTGGCAAGCGACGCCCTGACCCCTGAACTCAGCAAGCCCACCATGGACCTGGCCAGCCCT

GAGGCGGCCCCTGCTGCCCCCACTGCCCTGCCCAGCTTCAGCACCTTCATGGATGGCTAC

ACGGGAGAGTTTGATACCTTCCTCTACCAGCTGCCCGGGACAGCCCAGCCGTGCTCCTCG

GCTTCCTCCTCCGCCTCCTCCACATCCTCCTCCTCGGCCACCTCCCCTGCCTCTGCTTCT

TTCAAATTTGAAGACTTCCAGGTGTACGGCTGCTACCCTGGCACCCTGAGTGGTCCACTG

GATGAGACCTTATCCTCCAGTGGCTCTGACTACTATGGCAGCCCCTGCTCTGCCCCATCG

CCGTCCACACCCAGCTTCCAGCCACCCCAGCTCTCTCCCTGGGATGGCTCATTTGGCCAC

TTCTCCCCCAGCCAGACTTACGAAGGCCTGAGGGCATGGACAGAGCAGCTGCCCAAGGCT

TCTGGGCCCCCGCAACCACCAACCTTCTTCTCTTTCAGTCCTCCCCCTGGTCCTAGCCCC

AGTCTGGCCCAAAGCTCCCTGAAGTTACTCCCCTCACAGGCTGCCCACCAGCTGGGGGAG

GGAGAGAGCTATTCCATGCCAGTGACTTTCCCAAGCCTGGCGCCCACTTCCCCACACCTG

GACGGCCCAGGAATGCTGGATGTGCCTGTGACCTCAGCCAAGGCCCGGAGCGGGGCTCCA

GGTGGAAATGAGGGCCGCTGTGCTGTGTGTGGGGACAATGCTTCATGTCAGCATTATGGT

GTACGCACCTGTGAAGGCTGCAAGGGCTTCTTTAAGCGCACAGTGCAGAAAAGCGCCAAG

TACATCTGCCTGGCAAACAAGGACTGCCCTGTGGACAAGAGGCGGCGGAACCGCTGCCAG

TTCTGCCGCTTCCAGAAGTGCCTGGCTGTGGGCATGGTGAAGGAAGTTGTCCGAACAGAC

AGCCTGAAGGGGCGGCGGGGCCGGCTACCTTCAAAACCCAAGCAGCCTCCAGATGCCTCC

CCTGCCAATCTCCTCACCTCCCTTGTCCGAGCACACCTGGACTCGGGCCCCAGCATGGCG

AAACTGGACTACTCCAAGTTCCAGGAGCTGGTGCTGCCCCACTTTGGGAAGGAAGATGCT

GGTGATGTGCAGCAGTTCTACGATCTTCTGTCGGGTTCCCTGGAGGTTATCCGCAAGTGG

GCCGAGAAGATCCCTGGCTTTACTGAGCTGTCTCCAGGTGACCAGGACCTGCTGCTGGAG

TCAGCCTTCTTGGAGCTCTTCATCCTCCGCCTGGCCTACCGGTCTAAGCCAGGTGAGGGG

AAGCTCATCTTCTGCTCAGGCCTGGTGCTGCACCGACTCCAGTGTGCCCGCGGCTTTGGT

GACTGGATTGACAGTATCCTGGCGTTCTCACGGTCCCTGCATAGCTTGGTAGTCGATGTC

CCTGCCTTTGCTTGCCTTTCTGCTCTTGTCCTCATCACTGACCGGCATGGGCTACAGGAG

CCACGGCGGGTAGAGGAGCTACAGAACCGCATTGCCAGCTGCCTAAAGGAGCACATGGCA

GCCGTAGCCGGTGAGCCCCAGCCAGCCAGCTGCCTGTCACGTCTGCTGGGCAAATTGCCC

GAGTTGCGAACCCTGTGCACCCAGGGCCTACAGCGCATCTTCTACCTCAAGCTGGAGGAC

TTGGTGCCTCCTCCACCCATTGTTGACAAGATTTTTATGGACACACTGCCCTTC

>CL206.Contig7_A-W 1311 2855 minus strand nuclear receptor subfamily 4, group A, member 1, isoform CRA_b [Homo sapiens]

ATGCCCTGTATCCAAGCCCAATATGGGACACCAGCACCAAGTCCAGGACCCCGTGACCAC

CTGGCAAGCGACGCCCTGACCCCTGAACTCAGCAAGCCCACCATGGACCTGGCCAGCCCT

GAGGCGGCCCCTGCTGCCCCCACTGCCCTGCCCAGCTTCAGCACCTTCATGGATGGCTAC

ACGGGAGAGTTTGATACCTTCCTCTACCAGCTGCCCGGGACAGCCCAGCCGTGCTCCTCG

GCTTCCTCCTCCGCCTCCTCCACATCCTCCTCCTCGGCCACCTCCCCTGCCTCTGCTTCT

TTCAAATTTGAAGACTTCCAGGTGTACGGCTGCTACCCTGGCACCCTGAGTGGTCCACTG

GATGAGACCTTATCCTCCAGTGGCTCTGACTACTATGGCAGCCCCTGCTCTGCCCCATCG

CCGTCCACACCCAGCTTCCAGCCACCCCAGCTCTCTCCCTGGGATGGCTCATTTGGCCAC

TTCTCCCCCAGCCAGACTTACGAAGGCCTGAGGGCATGGACAGAGCAGCTGCCCAAGGCT

TCTGGGCCCCCGCAACCACCAACCTTCTTCTCTTTCAGTCCTCCCCCTGGTCCTAGCCCC

AGTCTGGCCCAAAGCTCCCTGAAGTTACTCCCCTCACAGGCTGCCCACCAGCTGGGGGAG

GGAGAGAGCTATTCCATGCCAGTGACTTTCCCAAGCCTGGCGCCCACTTCCCCACACCTG

GACGGCCCAGGAATGCTGGATGTGCCTGTGACCTCAGCCAAGGCCCGGAGCGGGGCTCCA

GGTGGAAATGAGGGCCGCTGTGCTGTGTGTGGGGACAATGCTTCATGTCAGCATTATGGT

GTACGCACCTGTGAAGGCTGCAAGGGCTTCTTTAAGCGCACAGTGCAGAAAAGCGCCAAG

TACATCTGCCTGGCAAACAAGGACTGCCCTGTGGACAAGAGGCGGCGGAACCGCTGCCAG

TTCTGCCGCTTCCAGAAGTGCCTGGCTGTGGGCATGGTGAAGGAAGTTGTCCGAACAGAC

AGCCTGAAGGGGCGGCGGGGCCGGCTACCTTCAAAACCCAAGCAGCCTCCAGATGCCTCC

CCTGCCAATCTCCTCACCTCCCTTGTCCGAGCACACCTGGACTCGGGCCCCAGCATGGCG

AAACTGGACTACTCCAAGTTCCAGGAGCTGGTGCTGCCCCACTTTGGGAAGGAAGATGCT

GGTGATGTGCAGCAGTTCTACGATCTTCTGTCGGGTTCCCTGGAGGTTATCCGCAAGTGG

GCCGAGAAGATCCCTGGCTTTACTGAGCTGTCTCCAGGTGACCAGGACCTGCTGCTGGAG

TCAGCCTTCTTGGAGCTCTTCATCCTCCGCCTGGCCTACCGGTCTAAGCCAGGTGAGGGG

AAGCTCATCTTCTGCTCAGGCCTGGTGCTGCACCGACTCCAGTGTGCCCGCGGCTTTGGT

GACTGGATTGACAGTATCCTGGCGTTCTCACGGTCCCTGCATAGCTTGGTAGTCGATGTC

CCTGCCTTTGCTTGCCTTTCTGCTCTTGTCCTCATCACTGGTAAG

>CL207.Contig1_A-W 126 710 PREDICTED: cytochrome P450 19A1-like [Loxodonta africana]

ATGGTTTTGGAAATGCTGAATCCAATGCACTATAATGTCACTGGCATGGTGCCAGAAGTC

ATGCTTGTCGGCACCATGCTGATTCTGCTGCTCATGGGCCTTCTTCTCTTGATTTGGAAT

TATGAGAACACATCTTCAATACCAGGTCCTGGCTACTGTATGGGAATTGGGCCCCTCATT

TCCCATGGTAGATTCCTGTGGATGGGGATTGGCAGTGCCTGCAACTACTACAACAAGATG

TATGGAGAATTCATGAGAGTCTGGATAGGTGGAGAAGAAACACTCATTATCAGCAAGTCC

TCAAGTATGTTCCATGTAATGAAGCACAGTCACTACATCTCCCGATTTGGCAGCAAACTT

GGGTTGCAGTGCATTGGTATGCATGAGAATGGCATCATATTTAACAATAATCCAGACCTC

TGGAGAGCAGTACGACCTTTTTTTATGAAAGCTTTAACAGGCCCTGGCCTTGTTCGCATG

GTGGCAGTTTGTGCTGAATCCATCATAAGGCACCTGGACAGACTGGAGGAGGTCACCAAT

ATGTCGGGCTACATTGACGTGTTGACCCTCATGCGGCGCATCATG

>CL207.Contig2_A-W 97 681 PREDICTED: cytochrome P450 19A1-like [Loxodonta africana]

ATGGTTTTGGAAATGCTGAATCCAATGCACTATAATGTCACTGGCATGGTGCCAGAAGTC

ATGCTTGTCGGCACCATGCTGATTCTGCTGCTCATGGGCCTTCTTCTCTTGATTTGGAAT

TATGAGAACACATCTTCAATACCAGGTCCTGGCTACTGTATGGGAATTGGGCCCCTCATT

TCCCATGGTAGATTCCTGTGGATGGGGATTGGCAGTGCCTGCAACTACTACAACAAGATG

TATGGAGAATTCATGAGAGTCTGGATAGGTGGAGAAGAAACACTCATTATCAGCAAGTCC

TCAAGTATGTTCCATGTAATGAAGCACAGTCACTACATCTCCCGATTTGGCAGCAAACTT

GGGTTGCAGTGCATTGGTATGCATGAGAATGGCATCATATTTAACAATAATCCAGACCTC

TGGAGAGCAGTACGACCTTTTTTTATGAAAGCTTTAACAGGCCCTGGCCTTGTTCGCATG

GTGGCAGTTTGTGCTGAATCCATCATAAGGCACCTGGACAGACTGGAGGAGGTCACCAAT

ATGTCGGGCTACATTGACGTGTTGACCCTCATGCGGCGCATCATG

>CL208.Contig1_A-W 3 2006 PREDICTED: PAB-dependent poly(A)-specific ribonuclease subunit 3 isoform 2 [Otolemur garnettii]

TTTATTGGAGTCAATGGCTTTGGAAGCCCAGTAGAAACAAAGTACCCCTTGATGCAGAGG

ATGACTAACAGTAGCAGCTCCCCAAGCCTTCTAAACGACAGTGCCAAGCCATATGCAGGC

CATGATGCTCTAAGTTCACCTGCTTCATCCTTGTTTAATGACTTTGGTGCCCTCAACATC

TCTCAGAGGAGAAAGACACCAAATCCTACTGCAAGCGAGTTTATTCCTAAAGGAGGATCA

ACCTCCAGGCTGAGTAACGTGTCCCAGTCAAATATGTCTGCCTTCTCTCAAGTTTTCTCT

CACCCTTCCATGGGAAGCCCTGCTTCTGCTGGATTAGCACCAGGAATGTCATTGTCTGCT

GGATCTTCCCCTCTTCATTCCCCCAAGATTACTCCACATACTTCTCCTGCTCCCAGAAGA

AGAAGTCACACTCCAAATCCAGCAAGTTATATGGTGCCTTCTAGTGCCTCTACACCTGTT

AATAATGCTGTTTCTCAGACTCCATCTTCTGGTCAGGTTATCCAGAAGGAAACTGTTGGT

GGGACCACTTACTTCTATACGGACACAACTCCAGCACCTTTGACTGGAATGGTATTTCCA

AACTACCATATTTATCCTCCAACTGCACCTCATGTTGCGTATATGCAACCAAAAGCAAAT

GCACCTTCCTTCTTCATGGCTGATGAACTCCGACAGGAGCTGATCAACAGACATTTAATA

ACAATGGCTCAAATCGATCAAGCAGATATGCCAGCAGTCCCCACAGAAGTTGACAGCTAC

CATAGCCTATTCCCTCTAGAACCACTGCCACCCCCCAACCGGATACAGAAATCAAGTAAT

TTTGGATATATTACATCTTGCTACAAAGCTGTAAATAGCAAAGATGATCTGCCATATTGC

CTTCGGAGGATACATGGTTTTCGTCTTGTTAACACAAAGTGCATGGTGTTGGTTGATATG

TGGAAAAAAATTCAGCACTCAAATATTGTAACCCTGCGTGAAGTATTTACCACTAAAGCA

TTCTCCGAGCCTTCTCTTGTGTTTGCATATGATTTCCATGCTGGAGGAGAAACTATGATG

AGCAGACACTTTAATGACCCTAATGCTGATTCCTATTTCACAAAGAGAAAATGGGGCCAA

CACGATGGACCGTTGCCTAGACAGCATGCTGGATTATTACCAGAATCTCTTATTTGGGCA

TACATTGTCCAGCTGAGTTCTGCATTGCGAACCATTCACACAGCAGGTTTGGCATGTCGA

GTTATGGACCCAACAAAGATTCTGATAACGGGCAAAACAAGGTTACGAGTAAATTGTGTT

GGAGTTTTTGATGTTTTGACATTTGATAACAGTCAAAATAGTAATCCATTGGCATTGATG

GCTCAGTATCAGCAAGCAGATCTGATATCATTAGGAAAAGTTGTGTTGGCTTTGGCTTGC

AACTCTTTGGCAGGAATACAGCGAGAGAATTTACAGAAAGCCATGGAATTAGTGACAATC

AACTACTCCTCTGACCTGAAGAATCTGATCTTGTATTTGTTGACTGACCAAAATAGGATG

CGAAGTGTAAATGACATCATGCCCATGATTGGTGCTCGATTTTATACTCAATTGGATGCT

GCTCAAATGAGAAATGATGTCATTGAAGAAGACCTTGCAAAGGAAGTTCAAAATGGAAGA

CTGTTTAGGCTTCTAGCAAAATTGGGAACAATCAATGAGAGGCCGGAGTTTCAGAAGGAT

CCTACTTGGTCAGAGACTGGAGACCGTTATCTGTTGAAACTCTTTAGGGATCATCTTTTT

CATCAGGTGACAGAAGCAGGTGCTCCCTGGATTGACCTCAGTCATATCATTTCTTGTCTT

AACAAGCTAGATGCTGGTGTGCCAGAAAAAATAAGCCTGATTTCCAGAGATGAGAAGAGT

GTACTTGTGGTGACTTACAGTGACTTAAAGCGCTGCTTTGAAAATACTTTTCAAGAACTG

ATTGCAGCTGCAAATGGTCAGTTG

>CL208.Contig2_A-W 51 1859 PREDICTED: PAB-dependent poly(A)-specific ribonuclease subunit 3 [Canis lupus familiaris]

ACACCAAATCCTACTGCAAGCGAGTTTATTCCTAAAGGAGGATCAACCTCCAGGCTGAGT

AACGTGTCCCAGTCAAATATGTCTGCCTTCTCTCAAGTTTTCTCTCACCCTTCCATGGGA

AGCCCTGCTTCTGCTGGATTAGCACCAGGAATGTCATTGTCTGCTGGATCTTCCCCTCTT

CATTCCCCCAAGATTACTCCACATACTTCTCCTGCTCCCAGAAGAAGAAGTCACACTCCA

AATCCAGCAAGTTATATGGTGCCTTCTAGTGCCTCTACACCTGTTAATAATGCTGTTTCT

CAGACTCCATCTTCTGGTCAGGTTATCCAGAAGGAAACTGTTGGTGGGACCACTTACTTC

TATACGGACACAACTCCAGCACCTTTGACTGGAATGGTATTTCCAAACTACCATATTTAT

CCTCCAACTGCACCTCATGTTGCGTATATGCAACCAAAAGCAAATGCACCTTCCTTCTTC

ATGGCTGATGAACTCCGACAGGAGCTGATCAACAGACATTTAATAACAATGGCTCAAATC

GATCAAGCAGATATGCCAGCAGTCCCCACAGAAGTTGACAGCTACCATAGCCTATTCCCT

CTAGAACCACTGCCACCCCCCAACCGGATACAGAAATCAAGTAATTTTGGATATATTACA

TCTTGCTACAAAGCTGTAAATAGCAAAGATGATCTGCCATATTGCCTTCGGAGGATACAT

GGTTTTCGTCTTGTTAACACAAAGTGCATGGTGTTGGTTGATATGTGGAAAAAAATTCAG

CACTCAAATATTGTAACCCTGCGTGAAGTATTTACCACTAAAGCATTCTCCGAGCCTTCT

CTTGTGTTTGCATATGATTTCCATGCTGGAGGAGAAACTATGATGAGCAGACACTTTAAT

GACCCTAATGCTGATTCCTATTTCACAAAGAGAAAATGGGGCCAACACGATGGACCGTTG

CCTAGACAGCATGCTGGATTATTACCAGAATCTCTTATTTGGGCATACATTGTCCAGCTG

AGTTCTGCATTGCGAACCATTCACACAGCAGGTTTGGCATGTCGAGTTATGGACCCAACA

AAGATTCTGATAACGGGCAAAACAAGGTTACGAGTAAATTGTGTTGGAGTTTTTGATGTT

TTGACATTTGATAACAGTCAAAATAGTAATCCATTGGCATTGATGGCTCAGTATCAGCAA

GCAGATCTGATATCATTAGGAAAAGTTGTGTTGGCTTTGGCTTGCAACTCTTTGGCAGGA

ATACAGCGAGAGAATTTACAGAAAGCCATGGAATTAGTGACAATCAACTACTCCTCTGAC

CTGAAGAATCTGATCTTGTATTTGTTGACTGACCAAAATAGGATGCGAAGTGTAAATGAC

ATCATGCCCATGATTGGTGCTCGATTTTATACTCAATTGGATGCTGCTCAAATGAGAAAT

GATGTCATTGAAGAAGACCTTGCAAAGGAAGTTCAAAATGGAAGACTGTTTAGGCTTCTA

GCAAAATTGGGAACAATCAATGAGAGGCCGGAGTTTCAGAAGGATCCTACTTGGTCAGAG

ACTGGAGACCGTTATCTGTTGAAACTCTTTAGGGATCATCTTTTTCATCAGGTGACAGAA

GCAGGTGCTCCCTGGATTGACCTCAGTCATATCATTTCTTGTCTTAACAAGCTAGATGCT

GGTGTGCCAGAAAAAATAAGCCTGATTTCCAGAGATGAGAAGAGTGTACTTGTGGTGACT

TACAGTGACTTAAAGCGCTGCTTTGAAAATACTTTTCAAGAACTGATTGCAGCTGCAAAT

GGTCAGTTG

>CL208.Contig3_A-W 52 1713 PREDICTED: PAB-dependent poly(A)-specific ribonuclease subunit 3 [Canis lupus familiaris]

GGAATGTCATTGTCTGCTGGATCTTCCCCTCTTCATTCCCCCAAGATTACTCCACATACT

TCTCCTGCTCCCAGAAGAAGAAGTCACACTCCAAATCCAGCAAGTTATATGGTGCCTTCT

AGTGCCTCTACACCTGTTAATAATGCTGTTTCTCAGACTCCATCTTCTGGTCAGGTTATC

CAGAAGGAAACTGTTGGTGGGACCACTTACTTCTATACGGACACAACTCCAGCACCTTTG

ACTGGAATGGTATTTCCAAACTACCATATTTATCCTCCAACTGCACCTCATGTTGCGTAT

ATGCAACCAAAAGCAAATGCACCTTCCTTCTTCATGGCTGATGAACTCCGACAGGAGCTG

ATCAACAGACATTTAATAACAATGGCTCAAATCGATCAAGCAGATATGCCAGCAGTCCCC

ACAGAAGTTGACAGCTACCATAGCCTATTCCCTCTAGAACCACTGCCACCCCCCAACCGG

ATACAGAAATCAAGTAATTTTGGATATATTACATCTTGCTACAAAGCTGTAAATAGCAAA

GATGATCTGCCATATTGCCTTCGGAGGATACATGGTTTTCGTCTTGTTAACACAAAGTGC

ATGGTGTTGGTTGATATGTGGAAAAAAATTCAGCACTCAAATATTGTAACCCTGCGTGAA

GTATTTACCACTAAAGCATTCTCCGAGCCTTCTCTTGTGTTTGCATATGATTTCCATGCT

GGAGGAGAAACTATGATGAGCAGACACTTTAATGACCCTAATGCTGATTCCTATTTCACA

AAGAGAAAATGGGGCCAACACGATGGACCGTTGCCTAGACAGCATGCTGGATTATTACCA

GAATCTCTTATTTGGGCATACATTGTCCAGCTGAGTTCTGCATTGCGAACCATTCACACA

GCAGGTTTGGCATGTCGAGTTATGGACCCAACAAAGATTCTGATAACGGGCAAAACAAGG

TTACGAGTAAATTGTGTTGGAGTTTTTGATGTTTTGACATTTGATAACAGTCAAAATAGT

AATCCATTGGCATTGATGGCTCAGTATCAGCAAGCAGATCTGATATCATTAGGAAAAGTT

GTGTTGGCTTTGGCTTGCAACTCTTTGGCAGGAATACAGCGAGAGAATTTACAGAAAGCC

ATGGAATTAGTGACAATCAACTACTCCTCTGACCTGAAGAATCTGATCTTGTATTTGTTG

ACTGACCAAAATAGGATGCGAAGTGTAAATGACATCATGCCCATGATTGGTGCTCGATTT

TATACTCAATTGGATGCTGCTCAAATGAGAAATGATGTCATTGAAGAAGACCTTGCAAAG

GAAGTTCAAAATGGAAGACTGTTTAGGCTTCTAGCAAAATTGGGAACAATCAATGAGAGG

CCGGAGTTTCAGAAGGATCCTACTTGGTCAGAGACTGGAGACCGTTATCTGTTGAAACTC

TTTAGGGATCATCTTTTTCATCAGGTGACAGAAGCAGGTGCTCCCTGGATTGACCTCAGT

CATATCATTTCTTGTCTTAACAAGCTAGATGCTGGTGTGCCAGAAAAAATAAGCCTGATT

TCCAGAGATGAGAAGAGTGTACTTGTGGTGACTTACAGTGACTTAAAGCGCTGCTTTGAA

AATACTTTTCAAGAACTGATTGCAGCTGCAAATGGTCAGTTG

>CL208.Contig4_A-W 3 1544 PREDICTED: PAB-dependent poly(A)-specific ribonuclease subunit 3 isoform 2 [Otolemur garnettii]

TTTATTGGAGTCAATGGCTTTGGAAGCCCAGTAGAAACAAAGTACCCCTTGATGCAGAGG

ATGACTAACAGTAGCAGCTCCCCAAGCCTTCTAAACGACAGTGCCAAGCCATATGCAGGC

CATGATGCTCTAAGTTCACCTGCTTCATCCTTGTTTAATGACTTTGGTGCCCTCAACATC

TCTCAGAGGAGAAAGACACCAAATCCTACTGCAAGCGAGTTTATTCCTAAAGGAGGATCA

ACCTCCAGGCTGAGTAACGTGTCCCAGTCAAATATGTCTGCCTTCTCTCAAGTTTTCTCT

CACCCTTCCATGGGAAGCCCTGCTTCTGCTGGATTAGCACCAGGAATGTCATTGTCTGCT

GGATCTTCCCCTCTTCATTCCCCCAAGATTACTCCACATACTTCTCCTGCTCCCAGAAGA

AGAAGTCACACTCCAAATCCAGCAAGTTATATGGTGCCTTCTAGTGCCTCTACACCTGTT

AATAATGCTGTTTCTCAGACTCCATCTTCTGGTCAGGTTATCCAGAAGGAAACTGTTGGT

GGGACCACTTACTTCTATACGGACACAACTCCAGCACCTTTGACTGGAATGGTATTTCCA

AACTACCATATTTATCCTCCAACTGCACCTCATGTTGCGTATATGCAACCAAAAGCAAAT

GCACCTTCCTTCTTCATGGCTGATGAACTCCGACAGGAGCTGATCAACAGACATTTAATA

ACAATGGCTCAAATCGATCAAGCAGATATGCCAGCAGTCCCCACAGAAGTTGACAGCTAC

CATAGCCTATTCCCTCTAGAACCACTGCCACCCCCCAACCGGATACAGAAATCAAGTAAT

TTTGGATATATTACATCTTGCTACAAAGCTGTAAATAGCAAAGATGATCTGCCATATTGC

CTTCGGAGGATACATGGTTTTCGTCTTGTTAACACAAAGTGCATGGTGTTGGTTGATATG

TGGAAAAAAATTCAGCACTCAAATATTGTAACCCTGCGTGAAGTATTTACCACTAAAGCA

TTCTCCGAGCCTTCTCTTGTGTTTGCATATGATTTCCATGCTGGAGGAGAAACTATGATG

AGCAGACACTTTAATGACCCTAATGCTGATTCCTATTTCACAAAGAGAAAATGGGGCCAA

CACGATGGACCGTTGCCTAGACAGCATGCTGGATTATTACCAGAATCTCTTATTTGGGCA

TACATTGTCCAGCTGAGTTCTGCATTGCGAACCATTCACACAGCAGGTTTGGCATGTCGA

GTTATGGACCCAACAAAGATTCTGATAACGGGCAAAACAAGGTTACGAGTAAATTGTGTT

GGAGTTTTTGATGTTTTGACATTTGATAACAGTCAAAATAGTAATCCATTGGCATTGATG

GCTCAGTATCAGCAAGCAGATCTGATATCATTAGGAAAAGTTGTGTTGGCTTTGGCTTGC

AACTCTTTGGCAGGAATACAGCGAGAGAATTTACAGAAAGCCATGGAATTAGTGACAATC

AACTACTCCTCTGACCTGAAGAATCTGATCTTGTAAGTTTTA

>CL208.Contig5_A-W 51 1397 PREDICTED: PAB-dependent poly(A)-specific ribonuclease subunit 3 [Canis lupus familiaris]

ACACCAAATCCTACTGCAAGCGAGTTTATTCCTAAAGGAGGATCAACCTCCAGGCTGAGT

AACGTGTCCCAGTCAAATATGTCTGCCTTCTCTCAAGTTTTCTCTCACCCTTCCATGGGA

AGCCCTGCTTCTGCTGGATTAGCACCAGGAATGTCATTGTCTGCTGGATCTTCCCCTCTT

CATTCCCCCAAGATTACTCCACATACTTCTCCTGCTCCCAGAAGAAGAAGTCACACTCCA

AATCCAGCAAGTTATATGGTGCCTTCTAGTGCCTCTACACCTGTTAATAATGCTGTTTCT

CAGACTCCATCTTCTGGTCAGGTTATCCAGAAGGAAACTGTTGGTGGGACCACTTACTTC

TATACGGACACAACTCCAGCACCTTTGACTGGAATGGTATTTCCAAACTACCATATTTAT

CCTCCAACTGCACCTCATGTTGCGTATATGCAACCAAAAGCAAATGCACCTTCCTTCTTC

ATGGCTGATGAACTCCGACAGGAGCTGATCAACAGACATTTAATAACAATGGCTCAAATC

GATCAAGCAGATATGCCAGCAGTCCCCACAGAAGTTGACAGCTACCATAGCCTATTCCCT

CTAGAACCACTGCCACCCCCCAACCGGATACAGAAATCAAGTAATTTTGGATATATTACA

TCTTGCTACAAAGCTGTAAATAGCAAAGATGATCTGCCATATTGCCTTCGGAGGATACAT

GGTTTTCGTCTTGTTAACACAAAGTGCATGGTGTTGGTTGATATGTGGAAAAAAATTCAG

CACTCAAATATTGTAACCCTGCGTGAAGTATTTACCACTAAAGCATTCTCCGAGCCTTCT

CTTGTGTTTGCATATGATTTCCATGCTGGAGGAGAAACTATGATGAGCAGACACTTTAAT

GACCCTAATGCTGATTCCTATTTCACAAAGAGAAAATGGGGCCAACACGATGGACCGTTG

CCTAGACAGCATGCTGGATTATTACCAGAATCTCTTATTTGGGCATACATTGTCCAGCTG

AGTTCTGCATTGCGAACCATTCACACAGCAGGTTTGGCATGTCGAGTTATGGACCCAACA

AAGATTCTGATAACGGGCAAAACAAGGTTACGAGTAAATTGTGTTGGAGTTTTTGATGTT

TTGACATTTGATAACAGTCAAAATAGTAATCCATTGGCATTGATGGCTCAGTATCAGCAA

GCAGATCTGATATCATTAGGAAAAGTTGTGTTGGCTTTGGCTTGCAACTCTTTGGCAGGA

ATACAGCGAGAGAATTTACAGAAAGCCATGGAATTAGTGACAATCAACTACTCCTCTGAC

CTGAAGAATCTGATCTTGTAAGTTTTA

>CL208.Contig6_A-W 52 1251 PREDICTED: PAB-dependent poly(A)-specific ribonuclease subunit 3 [Canis lupus familiaris]

GGAATGTCATTGTCTGCTGGATCTTCCCCTCTTCATTCCCCCAAGATTACTCCACATACT

TCTCCTGCTCCCAGAAGAAGAAGTCACACTCCAAATCCAGCAAGTTATATGGTGCCTTCT

AGTGCCTCTACACCTGTTAATAATGCTGTTTCTCAGACTCCATCTTCTGGTCAGGTTATC

CAGAAGGAAACTGTTGGTGGGACCACTTACTTCTATACGGACACAACTCCAGCACCTTTG

ACTGGAATGGTATTTCCAAACTACCATATTTATCCTCCAACTGCACCTCATGTTGCGTAT

ATGCAACCAAAAGCAAATGCACCTTCCTTCTTCATGGCTGATGAACTCCGACAGGAGCTG

ATCAACAGACATTTAATAACAATGGCTCAAATCGATCAAGCAGATATGCCAGCAGTCCCC

ACAGAAGTTGACAGCTACCATAGCCTATTCCCTCTAGAACCACTGCCACCCCCCAACCGG

ATACAGAAATCAAGTAATTTTGGATATATTACATCTTGCTACAAAGCTGTAAATAGCAAA

GATGATCTGCCATATTGCCTTCGGAGGATACATGGTTTTCGTCTTGTTAACACAAAGTGC

ATGGTGTTGGTTGATATGTGGAAAAAAATTCAGCACTCAAATATTGTAACCCTGCGTGAA

GTATTTACCACTAAAGCATTCTCCGAGCCTTCTCTTGTGTTTGCATATGATTTCCATGCT

GGAGGAGAAACTATGATGAGCAGACACTTTAATGACCCTAATGCTGATTCCTATTTCACA

AAGAGAAAATGGGGCCAACACGATGGACCGTTGCCTAGACAGCATGCTGGATTATTACCA

GAATCTCTTATTTGGGCATACATTGTCCAGCTGAGTTCTGCATTGCGAACCATTCACACA

GCAGGTTTGGCATGTCGAGTTATGGACCCAACAAAGATTCTGATAACGGGCAAAACAAGG

TTACGAGTAAATTGTGTTGGAGTTTTTGATGTTTTGACATTTGATAACAGTCAAAATAGT

AATCCATTGGCATTGATGGCTCAGTATCAGCAAGCAGATCTGATATCATTAGGAAAAGTT

GTGTTGGCTTTGGCTTGCAACTCTTTGGCAGGAATACAGCGAGAGAATTTACAGAAAGCC

ATGGAATTAGTGACAATCAACTACTCCTCTGACCTGAAGAATCTGATCTTGTAAGTTTTA

>CL208.Contig7_A-W 33 878 PREDICTED: PAB-dependent poly(A)-specific ribonuclease subunit 3 isoform 2 [Saimiri boliviensis boliviensis]

GAGCTGATCAACAGACATTTAATAACAATGGCTCAAATCGATCAAGCAGATATGCCAGCA

GTCCCCACAGAAGTTGACAGCTACCATAGCCTATTCCCTCTAGAACCACTGCCACCCCCC

AACCGGATACAGAAATCAAGTAATTTTGGATATATTACATCTTGCTACAAAGCTGTAAAT

AGCAAAGATGATCTGCCATATTGCCTTCGGAGGATACATGGTTTTCGTCTTGTTAACACA

AAGTGCATGGTGTTGGTTGATATGTGGAAAAAAATTCAGCACTCAAATATTGTAACCCTG

CGTGAAGTATTTACCACTAAAGCATTCTCCGAGCCTTCTCTTGTGTTTGCATATGATTTC

CATGCTGGAGGAGAAACTATGATGAGCAGACACTTTAATGACCCTAATGCTGATTCCTAT

TTCACAAAGAGAAAATGGGGCCAACACGATGGACCGTTGCCTAGACAGCATGCTGGATTA

TTACCAGAATCTCTTATTTGGGCATACATTGTCCAGCTGAGTTCTGCATTGCGAACCATT

CACACAGCAGGTTTGGCATGTCGAGTTATGGACCCAACAAAGATTCTGATAACGGGCAAA

ACAAGGTTACGAGTAAATTGTGTTGGAGTTTTTGATGTTTTGACATTTGATAACAGTCAA

AATAGTAATCCATTGGCATTGATGGCTCAGTATCAGCAAGCAGATCTGATATCATTAGGA

AAAGTTGTGTTGGCTTTGGCTTGCAACTCTTTGGCAGGAATACAGCGAGAGAATTTACAG

AAAGCCATGGAATTAGTGACAATCAACTACTCCTCTGACCTGAAGAATCTGATCTTGTAA

GTTTTA

>CL208.Contig8_A-W 3 347 PREDICTED: PAB-dependent poly(A)-specific ribonuclease subunit 3 isoform 2 [Otolemur garnettii]

TTTATTGGAGTCAATGGCTTTGGAAGCCCAGTAGAAACAAAGTACCCCTTGATGCAGAGG

ATGACTAACAGTAGCAGCTCCCCAAGCCTTCTAAACGACAGTGCCAAGCCATATGCAGGC

CATGATGCTCTAAGTTCACCTGCTTCATCCTTGTTTAATGACTTTGGTGCCCTCAACATC

TCTCAGAGGAGAAAGACACCAAATCCTACTGCAAGCGAGTTTATTCCTAAAGGAGGATCA

ACCTCCAGGCTGAGTAACGTGTCCCAGTCAAATATGTCTGCCTTCTCTCAAGTTTTCTCT

CACCCTTCCATGGGAAGCCCTGCTTCTGCTGGATTAGCACCAGGT

>CL209.Contig1_A-W 2 259 PREDICTED: IQ motif and SEC7 domain-containing protein 2 [Saimiri boliviensis boliviensis]

TTCAACAGAGACGTGTTGGACTGTGTTGTGGATGAGATGGATTTTTCTTCCATGGATCTG

GATGACGCGCTCCGGAAGTTCCAATCCCATATCCGGGTTCAGGGTGAGGCCCAGAAAGTG

GAGCGACTCATCGAAGCTTTTAGCCAGAGGTACTGTGTCTGTAACCCAGCCCTCGTGCGC

CAGTTCCGGAACCCAGACACCATCTTCATCCTTGCTTTTGCCATCATCCTCCTCAATACC

GACATGTACAGTCCTAGC

>CL209.Contig2_A-W 6 1589 PREDICTED: IQ motif and SEC7 domain-containing protein 1 [Canis lupus familiaris]

GGCGCCTACGAGCGCGGCCGGGTCGGGCCACAGGGCAGCCCCAAGCGCATCGCGCACGGC

GTCAAGGGGACCCGCGACGAGCCCGAGCCCCGCACCCGGCCGCCGCGGCCCTTGGAGGGC

CACCTGGCCATCAATGGCTCGGCCAACCGGCAGAGCAAGTCGGAGTCGGACTACTCGGAC

GGCGACAACGACAGCATCAACAGCACGTCCAACTCGAACGACACCATCCACTGCAGCTCC

GAGTCGTCGTCGCGCGACAGCCTGCGGGAGCAGGCGCTGAGCAAGCCCTCCTACCACAAG

GAGGCGCGCCACAGCTGGGACTCGCCCGCCTTCAGCCACGACGTGGTGCGCAAGAGGCAC

TACCGCATCGGCCTCAACCTCTTCAACAAGAAGCCTGAGAAGGGCGTGCAGTACCTCACT

GAACGCGGCTTCGTCCCCGACACCCCCGTGGGCGTGGCCCACTTCCTGCTGCAGCGCAAG

GGTCTCAGCCGCCAGATGATCGGCGAGTTCCTGGGCAACCGGCAGAAGCAGTTCAACCGC

GACGTGCTCGACTGCGTGGTGGACGAGATGGACTTCTCTGCCATGGAGCTGGACGAGGCC

CTCAGGAAGTTCCAGGCCCACATCCGGGTCCAGGGCGAGGCCCAGAAAGTGGAGCGGCTC

ATCGAGGCCTTCAGCCAGCGGTACTGCATCTGCAACCCCGGGGTGGTGCGCCAGTTCCGG

AACCCGGACACCATCTTCATCCTGGCCTTCGCCATCATCCTGCTCAACACGGACATGTAC

AGCCCCAACGTCAAGCCTGAGCGCAAGATGAAGCTCGAGGACTTCGTCAAGAACCTGCGA

GGAGTGGACGACGGCGAGGACATCCCCCGGGAGATGCTGATCGGCATCTACGAGCGCATC

CGGAAGCGGGAGCTCAAGACCAACGAGGACCACGTGTCCCAGGTGCAGAAGGTGGAGAAG

CTCATCGTGGGGAAGAAGCCGATCGGATCCCTGCACCACGGGCTGGGCTGTGTGCTGTCC

CTGCCCCACCGGCGGCTGGTGTGCTACTGCAGGCTCTTCGAGGTCCCCGACCCCAGCAAG

CCCCAGAAGCTGGGCCTGCACCAGAGGGAGATCTTCCTCTTCAACGACCTGCTGGTGGTC

ACCAAGATCTTCCAGAAGAAGAAGAACTCGGTGACGTACAGCTTCCGACAGTCCTTCTCC

CTGTGCGGCCTGCAGGTCCTGCTCTTCGAGAACCAGTACTACCCCAACGGCATCCGGCTC

ACGTCGGCTGTCCCCGGGGCGGACATCAAAGTGCTCATCAACTTCAACGCGCCCAACCCC

CAGGACCGGAAGAAGTTCACCGATGACCTGCGGGAGTCGGTGGCCGAGGTGCAGGAGATG

GAGAAACACCGCATCGAATCGGAGCTGGAGAAGCAGAAGGGCGTCGTGCGGCCCAGCATG

TCCCAGTGCTCCAGCCTCAAGAAGGAGCCGGGCAACGGGGCGCTGGGCAGGGCCTGCTTG

GACGACAGCTACGCCGGCGGGGAGGGCCTCAAGCGCAGCGCCCTCAGCAGCTCCCTGCGC

GACCTCTCGGAAGCGGGGGTCCGT

>CL209.Contig3_A-W 6 395 PREDICTED: IQ motif and SEC7 domain-containing protein 1 [Canis lupus familiaris]

GGCGCCTACGAGCGCGGCCGGGTCGGGCCACAGGGCAGCCCCAAGCGCATCGCGCACGGC

GTCAAGGGGACCCGCGACGAGCCCGAGCCCCGCACCCGGCCGCCGCGGCCCTTGGAGGGC

CACCTGGCCATCAATGGCTCGGCCAACCGGCAGAGCAAGTCGGAGTCGGACTACTCGGAC

GGCGACAACGACAGCATCAACAGCACGTCCAACTCGAACGACACCATCCACTGCAGCTCC

GAGTCGTCGTCGCGCGACAGCCTGCGGGAGCAGGCGCTGAGCAAGCCCTCCTACCACAAG

GAGGCGCGCCACAGCTGGGACTCGCCCGCCTTCAGCCACGACGTGGTGCGCAAGAGGCAC

TACCGCATCGGCCTCAACCTCTTCAACAAG

>CL211.Contig1_A-W 3 749 bisphosphate 3&apos;-nucleotidase 1, isoform CRA_b [Mus musculus]

GAGGATCTCCCTCCTGAAGAAGTGGATCAAGAGCTGATTGAAGATGGTCAGTGGGAGGAA

ATATTGAAGCAACCATGCCCATCACAGTACAGTGCTATTAAAGAGGAAGACCTTGTGGTT

TGGGTTGATCCTCTGGATGGTACCAAAGAATATACTGAAGGTCTTCTTGACAATGTAACG

GTTCTTATTGGAATTGCCTATGAAGGAAAAGCCATAGCAGGCATTATTAACCAACCATAT

TACAACTACCAGAACAATGAAAAGCAGAAGTTAAGGGAACACAGGAATGAAGCAAAGGCA

GGACCAGATGCTGTGTTGGGGAGGACAATCTGGGGAGTTTTAGGTTTAGGTGCCTTTGGG

TTTCAGCTGAAAGAAGTCCCTGCTGGGAAACACATTATCACAACTACCCGATCCCATAGC

AACAAGTTGGTTACAGACTGTGTTTCTGCTATGAACCCTGATGATGTGCTGCGAGTAGGA

GGAGCAGGAAATAAGATCATTCAGCTGATCGAAGGCAAAGCTTCTGCTTATGTATTTGCA

AGCCCTGGATGTAAGAAATGGGACACTTGTGCTCCCGAAGTTATCTTACATGCTGTGGGA

GGCAAGTTAACCGATATCCATGGAAATGCCCTTCAGTACAATAAGGAAGTGAAGCATATG

AACTCTGCAGGGGTCCTGGCCACACTGAGGAATTATGACTACTATGCGAGCCGAGTTCCA

GAATCTGTTAAAAATGCACTTGTTCCT

>CL211.Contig2_A-W 3 704 PREDICTED: 3&apos;(2&apos;),5&apos;-bisphosphate nucleotidase 1-like isoform 1 [Sus scrofa]

GAGGATCTCCCTCCTGAAGAAGTGGATCAAGAGCTGATTGAAGATGGTCAGTGGGAGGAA

ATATTGAAGCAACCATGCCCATCACAGTACAGTGCTATTAAAGAGGAAGACCTTGTGGTT

TGGGTTGATCCTCTGGATGGTACCAAAGAATATACTGAAGGTCTTCTTGACAATGTAACG

GTTCTTATTGGAATTGCCTATGAAGGAAAAGCCATAGCAGGCATTATTAACCAACCATAT

TACAACTACCAGGCAGGACCAGATGCTGTGTTGGGGAGGACAATCTGGGGAGTTTTAGGT

TTAGGTGCCTTTGGGTTTCAGCTGAAAGAAGTCCCTGCTGGGAAACACATTATCACAACT

ACCCGATCCCATAGCAACAAGTTGGTTACAGACTGTGTTTCTGCTATGAACCCTGATGAT

GTGCTGCGAGTAGGAGGAGCAGGAAATAAGATCATTCAGCTGATCGAAGGCAAAGCTTCT

GCTTATGTATTTGCAAGCCCTGGATGTAAGAAATGGGACACTTGTGCTCCCGAAGTTATC

TTACATGCTGTGGGAGGCAAGTTAACCGATATCCATGGAAATGCCCTTCAGTACAATAAG

GAAGTGAAGCATATGAACTCTGCAGGGGTCCTGGCCACACTGAGGAATTATGACTACTAT

GCGAGCCGAGTTCCAGAATCTGTTAAAAATGCACTTGTTCCT

>CL212.Contig1_A-W 185 1063 SAGA-associated factor 29 homolog [Bos taurus]

ATGGCCCTCGTGTCTGCCGACTCCCGCATTGCAGAACTTCTCACAGAGCTCCACCAGCTC

ATCAAGCAAACCCAGGAAGAGCGATCGAGGAGTGAACACAACCTAGTGAACATCCAGAAG

ACCCATGAGCGGATGCAGACAGAGAATAAGATTTCTCCTTATTACCGGACAAAACTGCGA

GGCCTCTACACCACCGCCAAGGCCGATGCGGAGGCCGAGTGCAACATCCTCCGCAAAGCC

CTGGATAAGATTGCTGAGATCAAGTCGCTGTTGGAAGAGAGGCGGATTGCGGCCAAGATC

GCAGGTTTGTACAATGACTCGGAGCCGCCTCGCAAGACCATGCGCAGAGGGGTGCTGATG

ACCTTGCTGCAGCAGTCGGCCATGACCCTGCCCCTGTGGATCGGGAAGCCTGGTGACAAG

CCCCCACCCCTCTGTGGAGCCATTCCAGCCTCAGGGGACTATGTGGCCAAGCCTGGAGAC

AAGGTGGCTGCGCGGGTCAAGGCTGTGGATGGGGATGAGCAGTGGATCCTGGCAGAGGTG

GTCAGTTACAGCCATGCCACCAACAAGTATGAGGTAGATGACATTGATGAAGAAGGCAAA

GAGAGACACACCCTGAGCCGCCGGCGCATCATCCCACTGCCCCAGTGGAAAGCCAACCCT

GAGACGGACCCTGAGGCCTTATTCCAGAAGGAGCAGCTTGTATTGGCCTTGTATCCCCAG

ACCACCTGCTTCTACCGTGCCCTGATCCATACGCCCCCACAGCGGCCCCAAGATGACTAC

TCGGTTCTCTTTGAAGACACCTCCTATGCAGATGGCTACTCCCCTCCCCTCAATGTGGCC

CAGAGGTACGTGGTGGCTTGTAAGGAACCCAAGAAAAAG

>CL212.Contig2_A-W 49 639 SAGA-associated factor 29-like protein [Heterocephalus glaber]

GCGGCCAAGATCGCAGGTTTGTACAATGACTCGGAGCCGCCTCGCAAGACCATGCGCAGA

GGGGTGCTGATGACCTTGCTGCAGCAGTCGGCCATGACCCTGCCCCTGTGGATCGGGAAG

CCTGGTGACAAGCCCCCACCCCTCTGTGGAGCCATTCCAGCCTCAGGGGACTATGTGGCC

AAGCCTGGAGACAAGGTGGCTGCGCGGGTCAAGGCTGTGGATGGGGATGAGCAGTGGATC

CTGGCAGAGGTGGTCAGTTACAGCCATGCCACCAACAAGTATGAGGTAGATGACATTGAT

GAAGAAGGCAAAGAGAGACACACCCTGAGCCGCCGGCGCATCATCCCACTGCCCCAGTGG

AAAGCCAACCCTGAGACGGACCCTGAGGCCTTATTCCAGAAGGAGCAGCTTGTATTGGCC

TTGTATCCCCAGACCACCTGCTTCTACCGTGCCCTGATCCATACGCCCCCACAGCGGCCC

CAAGATGACTACTCGGTTCTCTTTGAAGACACCTCCTATGCAGATGGCTACTCCCCTCCC

CTCAATGTGGCCCAGAGGTACGTGGTGGCTTGTAAGGAACCCAAGAAAAAG

>CL212.Contig3_A-W 309 1118 SAGA-associated factor 29 homolog OS=Rattus norvegicus GN=Ccdc101 PE=1 SV=1

TCACAGGAAGAGCGATCGAGGAGTGAACACAACCTAGTGAACATCCAGAAGACCCATGAG

CGGATGCAGACAGAGAATAAGATTTCTCCTTATTACCGGACAAAACTGCGAGGCCTCTAC

ACCACCGCCAAGGCCGATGCGGAGGCCGAGTGCAACATCCTCCGCAAAGCCCTGGATAAG

ATTGCTGAGATCAAGTCGCTGTTGGAAGAGAGGCGGATTGCGGCCAAGATCGCAGGTTTG

TACAATGACTCGGAGCCGCCTCGCAAGACCATGCGCAGAGGGGTGCTGATGACCTTGCTG

CAGCAGTCGGCCATGACCCTGCCCCTGTGGATCGGGAAGCCTGGTGACAAGCCCCCACCC

CTCTGTGGAGCCATTCCAGCCTCAGGGGACTATGTGGCCAAGCCTGGAGACAAGGTGGCT

GCGCGGGTCAAGGCTGTGGATGGGGATGAGCAGTGGATCCTGGCAGAGGTGGTCAGTTAC

AGCCATGCCACCAACAAGTATGAGGTAGATGACATTGATGAAGAAGGCAAAGAGAGACAC

ACCCTGAGCCGCCGGCGCATCATCCCACTGCCCCAGTGGAAAGCCAACCCTGAGACGGAC

CCTGAGGCCTTATTCCAGAAGGAGCAGCTTGTATTGGCCTTGTATCCCCAGACCACCTGC

TTCTACCGTGCCCTGATCCATACGCCCCCACAGCGGCCCCAAGATGACTACTCGGTTCTC

TTTGAAGACACCTCCTATGCAGATGGCTACTCCCCTCCCCTCAATGTGGCCCAGAGGTAC

GTGGTGGCTTGTAAGGAACCCAAGAAAAAG

>CL212.Contig4_A-W 182 643 Coiled-coil domain-containing protein 101 [Macaca mulatta]

ATGGCCCTCGTGTCTGCCGACTCCCGCATTGCAGAACTTCTCACAGAGCTCCACCAGCTC

ATCAAGCAAACCCAGGAAGAGCGATCGAGGAGTGAACACAACCTAGTGAACATCCAGAAG

ACCCATGAGCGGATGCAGACAGAGAATAAGATTTCTCCTTATTACCGGACAAAACTGCGA

GGCCTCTACACCACCGCCAAGGCCGATGCGGAGGCCGAGTGCAACATCCTCCGCAAAGCC

CTGGATAAGATTGCTGAGATCAAGTCGCTGTTGGAAGAGAGGCGGATTGCGGCCAAGATC

GCAGGTTTGTACAATGACTCGGAGCCGCCTCGCAAGACCATGCGCAGAGGGGTGCTGATG

ACCTTGCTGCAGCAGTCGGCCATGACCCTGCCCCTGTGGATCGGGAAGCCTGGTGACAAG

TGAGGGTGGCAGCCAGCAGGGGGGGCCTGGTGTCCAGGCCCA

>CL214.Contig1_A-W 644 913 minus strand PREDICTED: protein FAM173A-like isoform 2 [Pan troglodytes]

ATGGAGCACGACGACCCTGCCGAGGCGCTGACGGAGCTGCGTGAGCGGCGGCTGGGCGCG

CTGGAGCTGCTGCAGGCGGCGGCGGGCTCGGGGCTGGCCGCCTACGCCGTGTGGGCGCTG

CTGTTGCAGCCAGGCTTCCGCCGAGTGCCGCTGCGGCTGCAGGTACCCTATGTAGGCGCC

AGTGCACGGCAGGTTGAGCATGTGTTGTCGTTGCTGCGTGGTCGCCCGGGAAAAATGGTG

GATCTGGGCTCTGGAGACGGCAGAATTGTA

>CL214.Contig2_A-W 1002 1634 minus strand PREDICTED: protein FAM173A-like isoform 2 [Pan troglodytes]

CGGCAGGTGTTGGCGGCCCACAGGTGCGGTCTCCGCCCAGCTGTGGGCTACGAACTGAAC

CCATGGCTTGTGGGGCTTGCGAGGTTGCATGCCTGGAGGGCCGGTTGTGCTGGCAGCGTC

TGCTACCTTCGTGAGGATCTCTGGAAGGTAACCTAGGGGACCTAGACCCCTGAGATCCAC

ACCTGATTATGCTTCAGATTCTGCCTTGGTCAGCCTTCTGGCAGCCCCAGGTTTCTCTGA

CTCCTGGGTGGGCCAGGGGTCAGGTCTGAAACTGCAGGGACCCGGGCCGCCAGCCCCCTC

CCCCTGCCGGTGACTCTCCTTCTCACAGGTGAGCCTGAGGGACTGCCGAAACGTGTCCGT

GTTCCTGGCCCCCAGCGTGGTAGGTCTGAGCTCCCCTTGCTGGAAGATAAACTGCTGGCG

GAGCTGCCTGCTGGGGCTCGTGTGGTATCTGGACGCTTCCCCCTACCCACCTGGCAGCCT

GTGGCTGTGATCGGTGAAGGCCTAGATCGGGTCTGGGCCTACGATGTCGACATGGACGTC

CACCATGCCAGACCTGCTGCCACATCCTGTCCCATCCTGCCCACCCCAGGACCTGGTTCT

GCCTTGGCCCCCAGCCTCCCAAGTTCTCAGGCT

>CL214.Contig3_A-W 1230 1487 minus strand PREDICTED: protein FAM173A-like [Cavia porcellus]

CAGGTGAGCCTGAGGGACTGCCGAAACGTGTCCGTGTTCCTGGCCCCCAGCGTGCTCCCC

TTGCTGGAAGATAAACTGCTGGCGGAGCTGCCTGCTGGGGCTCGTGTGGTATCTGGACGC

TTCCCCCTACCCACCTGGCAGCCTGTGGCTGTGATCGGTGAAGGCCTAGATCGGGTCTGG

GCCTACGATGTCGACATGGACGTCCACCATGCCAGACCTGCTGCCACATCCTGTCCCATC

CTGCCCACCCCAGGACCT

>CL214.Contig4_A-W 644 1345 minus strand PREDICTED: protein FAM173A-like isoform 2 [Pan troglodytes]

ATGGAGCACGACGACCCTGCCGAGGCGCTGACGGAGCTGCGTGAGCGGCGGCTGGGCGCG

CTGGAGCTGCTGCAGGCGGCGGCGGGCTCGGGGCTGGCCGCCTACGCCGTGTGGGCGCTG

CTGTTGCAGCCAGGCTTCCGCCGAGTGCCGCTGCGGCTGCAGGTACCCTATGTAGGCGCC

AGTGCACGGCAGGTTGAGCATGTGTTGTCGTTGCTGCGTGGTCGCCCGGGAAAAATGGTG

GATCTGGGCTCTGGAGACGGCAGAATTGTGTTGGCGGCCCACAGGTGCGGTCTCCGCCCA

GCTGTGGGCTACGAACTGAACCCATGGCTTGTGGGGCTTGCGAGGTTGCATGCCTGGAGG

GCCGGTTGTGCTGGCAGCGTCTGCTACCTTCGTGAGGATCTCTGGAAGGTGAGCCTGAGG

GACTGCCGAAACGTGTCCGTGTTCCTGGCCCCCAGCGTGCTCCCCTTGCTGGAAGATAAA

CTGCTGGCGGAGCTGCCTGCTGGGGCTCGTGTGGTATCTGGACGCTTCCCCCTACCCACC

TGGCAGCCTGTGGCTGTGATCGGTGAAGGCCTAGATCGGGTCTGGGCCTACGATGTCGAC

ATGGACGTCCACCATGCCAGACCTGCTGCCACATCCTGTCCCATCCTGCCCACCCCAGGA

CCTGGTTCTGCCTTGGCCCCCAGCCTCCCAAGTTCTCAGGCT

>CL215.Contig1_A-W 58 342 PREDICTED: serine/threonine-protein phosphatase 2A activator isoform 2 [Saimiri boliviensis boliviensis]

GACCACCCGTACCTGGAGCCCAGGCACTTTGTGGACGAGAAGGCCGTGAACGAGAACCAC

AAGGACTATATGTTCCTGGAGTGTATCCTGTTTATCACTGAGATGAAGACCGGCCCATTT

GCAGAGCACTCCAACCAGCTGTGGAACATCAGCGCCGTCCCCTCCTGGTCCAAGGTGAAC

CAGGGCCTCATCCGCATGTATAAGGCTGAGTGCCTAGAGAAGTTCCCTGTGATCCAGCAC

TTCAAGTTCGGAAGCCTGCTGCCCATTCATCCTGTCACATCTGGC

>CL215.Contig2_A-W 119 1207 protein phosphatase 2A, regulatory subunit B&apos; (PR 53), isoform CRA_f [Homo sapiens]

GGCTGTAGCCGCCCGGCGCTCGCTCCTCTTCCGGACCTCGGAGCCCGCGCAGCGGAGGGC

AGGTGCGGCGTGCCAAGCTAGGGGGCACCGCGGGCGCCGGCCGGCCGCTGGCGGGAGAAG

ATGGCTGAGGGCGAGCGGCAGCCGCCGCCAGATTCTTCAGAGGAGACCCCTTCAGGCACT

CAGAACTTCATCATTCCGAAAAAGGAGATCCACACAGTTCCAGACATGGGCAAATGGAAG

CGTTCTCAGGCCTACGCTGACTACATTGGTTTCATCCTCACCCTCAACGAAGGCGTGAAG

GGGAAGAAGCTGACCTTTGAGTACAGAGTCTCTGAGGCCATTGAGAAGCTGGTGGCCCTT

CTCAACACACTCGACAGGTGGATTGATGAGACCCCTCCGGTGGACCAGCCCTCTCGATTT

GGGAACAAGGCCTACAGGACCTGGTATGCCAAGCTTGATGAGGAAGCAGAAAACTTGGTA

TCCACAGTCGTGCCCCCTCAGCTGGCCGCAGCCGTGCCTGAAGTGGCTGTCTACCTGAAG

GAGTCTGTGGGGAACTCCACGCGCATCGACTACGGCACAGGGCACGAGGCGGCCTTTGCT

GCCTTCCTCTGCTGTCTCTGCAAGATCGGGGTGCTCCGGGTGGATGACCAGATAGCTATT

GTCTTCAAGGTGTTCAATCGGTATCTGGAGGTTATGAGGAAGCTCCAGAAAACATACCGG

ATGGAACCGGCTGGCAGCCAGGGTGTGTGGGGCCTGGATGATTTCCAGTTTCTGCCCTTC

ATCTGGGGCAGTTCACAGCTGATAGACCACCCGTACCTGGAGCCCAGGCACTTTGTGGAC

GAGAAGGCCGTGAACGAGAACCACAAGGACTATATGTTCCTGGAGTGTATCCTGTTTATC

ACTGAGATGAAGACCGGCCCATTTGCAGAGCACTCCAACCAGCTGTGGAACATCAGCGCC

GTCCCCTCCTGGTCCAAGGTGAACCAGGGCCTCATCCGCATGTATAAGGCTGAGTGCCTA

GAGAAGTTCCCTGTGATCCAGCACTTCAAGTTCGGAAGCCTGCTGCCCATTCATCCTGTC

ACATCTGGC

>CL216.Contig1_A-W 590 745 CLEC10A protein [Homo sapiens]

AGAACCTCTTGTCTTTAGTTCCCAAGACATGTCTCATTTAGGGAAATGGTTAACAACCTT

GACTATACATTGGAACCACCCCTGAGCCTTAGTAACTATTTCCATCTGAACCATGCCCTC

AGAAATTTAATTGGCCTGAGCATTGGAATAAATATG

>CL216.Contig2_A-W 543 698 unnamed protein product [Homo sapiens]

AGAACCTCTTGTCTTTAGTTCCCAAGACATGTCTCATTTAGGGAAATGGTTAACAACCTT

GACTATACATTGGAACCACCCCTGAGCCTTAGTAACTATTTCCATCTGAACCATGCCCTC

AGAAATTTAATTGGCCTGAGCATTGGAATAAATATG

>CL216.Contig3_A-W 14 118 CLEC10A protein [Homo sapiens]

GATCTCAATTCCCTGTTTCCTCCAGGTCAGAGCTTGCAAGAAACCATAACCTCTCTGAAA

GCTGAGGTGGAGGATCACAGGCAGGAACTGCTGGCAGCCCACAGC

>CL216.Contig4_A-W 467 622 unnamed protein product [Homo sapiens]

AGAACCTCTTGTCTTTAGTTCCCAAGACATGTCTCATTTAGGGAAATGGTTAACAACCTT

GACTATACATTGGAACCACCCCTGAGCCTTAGTAACTATTTCCATCTGAACCATGCCCTC

AGAAATTTAATTGGCCTGAGCATTGGAATAAATATG

>CL216.Contig5_A-W 420 575 unnamed protein product [Homo sapiens]

AGAACCTCTTGTCTTTAGTTCCCAAGACATGTCTCATTTAGGGAAATGGTTAACAACCTT

GACTATACATTGGAACCACCCCTGAGCCTTAGTAACTATTTCCATCTGAACCATGCCCTC

AGAAATTTAATTGGCCTGAGCATTGGAATAAATATG

>CL216.Contig6_A-W 14 118 CLEC10A protein [Homo sapiens]

GATCTCAATTCCCTGTTTCCTCCAGGTCAGAGCTTGCAAGAAACCATAACCTCTCTGAAA

GCTGAGGTGGAGGATCACAGGCAGGAACTGCTGGCAGCCCACAGC

>CL216.Contig7_A-W 467 622 unnamed protein product [Homo sapiens]

AGAACCTCTTGTCTTTAGTTCCCAAGACATGTCTCATTTAGGGAAATGGTTAACAACCTT

GACTATACATTGGAACCACCCCTGAGCCTTAGTAACTATTTCCATCTGAACCATGCCCTC

AGAAATTTAATTGGCCTGAGCATTGGAATAAATATG

>CL216.Contig8_A-W 420 575 unnamed protein product [Homo sapiens]

AGAACCTCTTGTCTTTAGTTCCCAAGACATGTCTCATTTAGGGAAATGGTTAACAACCTT

GACTATACATTGGAACCACCCCTGAGCCTTAGTAACTATTTCCATCTGAACCATGCCCTC

AGAAATTTAATTGGCCTGAGCATTGGAATAAATATG

>CL216.Contig9_A-W 14 118 unnamed protein product [Homo sapiens]

GATCTCAATTCCCTGTTTCCTCCAGGTCAGAGCTTGCAAGAAACCATAACCTCTCTGAAA

GCTGAGGTGGAGGATCACAGGCAGGAACTGCTGGCAGCCCACAGC

>CL216.Contig10_A-W 14 118 unnamed protein product [Homo sapiens]

GATCTCAATTCCCTGTTTCCTCCAGGTCAGAGCTTGCAAGAAACCATAACCTCTCTGAAA

GCTGAGGTGGAGGATCACAGGCAGGAACTGCTGGCAGCCCACAGC

>CL217.Contig1_A-W 46 348 IQ motif containing GTPase activating protein 2, isoform CRA_a [Homo sapiens]

CATGTGAACCCATCCTCAGGATTGGCTGCCGTGGACCACATCAATGCTGTCATTCGGGAA

GGTGACCCTGCAAATACACTGCTTGCACTGAAGAAACCGGAGGCCCAGCTGCCTGAGGTC

TATCCCTTTGCTGCTACTATGTATCAGAATGAACTCCTCAACCTCCAGAAACAGAACACC

ATGAACTATTTGGCCCATGAGGACCTCTTGTTTGCGGTGGAAATGTTGTCTGCTGTTGCT

TTACTAAACCAGGCCTTGGAGAGCAATGATCTTGTGTCTGTGCAGAATCAACTCAGAAGC

CCC

>CL217.Contig2_A-W 1 258 PREDICTED: ras GTPase-activating-like protein IQGAP2 [Papio anubis]

GGAGAAAGGAAACCTGCTGGGGGAGGCGGCGGCCGGTGAGGGAACGAGGGAGGAGAGTTC

ACTTTTGCTCCAGTGTCAGCCCGCGGCGGCTGCAGCGGACTCTGGGGTGGAAGCACCGAG

GGAGCAGGTCGCCCATCTCAGAGCCGGCGGGCGAGGGCAGAGAGGCGGGATCCCAGCGCG

CCGGAGGGGCGCGAAGCCCGCGAGCCTGGCCAGCGAGGGGAGCCGTGGGGGGCGCGCCCC

GGGAGGGCCCCCGGAGGC

>CL217.Contig3_A-W 1 258 PREDICTED: ras GTPase-activating-like protein IQGAP2 [Papio anubis]

GGAGAAAGGAAACCTGCTGGGGGAGGCGGCGGCCGGTGAGGGAACGAGGGAGGAGAGTTC

ACTTTTGCTCCAGTGTCAGCCCGCGGCGGCTGCAGCGGACTCTGGGGTGGAAGCACCGAG

GGAGCAGGTCGCCCATCTCAGAGCCGGCGGGCGAGGGCAGAGAGGCGGGATCCCAGCGCG

CCGGAGGGGCGCGAAGCCCGCGAGCCTGGCCAGCGAGGGGAGCCGTGGGGGGCGCGCCCC

GGGAGGGCCCCCGGAGGC

>CL218.Contig1_A-W 74 454 minus strand PREDICTED: uridine-cytidine kinase-like 1 isoform 2 [Cavia porcellus]

TCGTCCCCGCCGCCTCCTGCGGCCCCGGACGCGCCCGGAGCCTGCGCGGGAGGACAGAGC

GAGACCGCGTGCGAGGACCGCAGCCATGCAGGGTCCCTGGACAGGCTTCTCCCCACCGTG

GGCACCAGGCGCTCGCCCCGGAAGCGCACCACCAGCCAGTGCAAGTCGGAGCCACCCCTG

CTGCGCACCAGCAAGCGCACCATCTACACGGCAGGGCGGCCACCCTGGTACAATGAGCAT

GGCACACAGTCCAAGGAGGCCTTTGCCATCGGCCTGGGAGGTGGCAGTGCCTCGGGGAAG

ACCACCGTGGCCAGGATGATCATTGAGGCTCTAGATGTGCCCTGGGTGGTCTTGCTGTCC

ATGGACTCCTTCTACAAGGTG

>CL218.Contig2_A-W 74 454 minus strand PREDICTED: uridine-cytidine kinase-like 1 isoform 2 [Cavia porcellus]

TCGTCCCCGCCGCCTCCTGCGGCCCCGGACGCGCCCGGAGCCTGCGCGGGAGGACAGAGC

GAGACCGCGTGCGAGGACCGCAGCCATGCAGGGTCCCTGGACAGGCTTCTCCCCACCGTG

GGCACCAGGCGCTCGCCCCGGAAGCGCACCACCAGCCAGTGCAAGTCGGAGCCACCCCTG

CTGCGCACCAGCAAGCGCACCATCTACACGGCAGGGCGGCCACCCTGGTACAATGAGCAT

GGCACACAGTCCAAGGAGGCCTTTGCCATCGGCCTGGGAGGTGGCAGTGCCTCGGGGAAG

ACCACCGTGGCCAGGATGATCATTGAGGCTCTAGATGTGCCCTGGGTGGTCTTGCTGTCC

ATGGACTCCTTCTACAAGGTG

>CL218.Contig3_A-W 38 1687 minus strand PREDICTED: uridine-cytidine kinase-like 1 isoform 1 [Cavia porcellus]

ATGGCTGCGCCACCGGCCTCCGCGGACGCCGCCCCCTCGTCCCCGCCGCCTCCTGCGGCC

CCGGACGCGCCCGGAGCCTGCGCGGGAGGACAGAGCGAGACCGCGTGCGAGGACCGCAGC

CATGCAGGGTCCCTGGACAGGCTTCTCCCCACCGTGGGCACCAGGCGCTCGCCCCGGAAG

CGCACCACCAGCCAGTGCAAGTCGGAGCCACCCCTGCTGCGCACCAGCAAGCGCACCATC

TACACGGCAGGGCGGCCACCCTGGTACAATGAGCATGGCACACAGTCCAAGGAGGCCTTT

GCCATCGGCCTGGGAGGTGGCAGTGCCTCGGGGAAGACCACCGTGGCCAGGATGATCATT

GAGGCTCTAGATGTGCCCTGGGTGGTCTTGCTGTCCATGGACTCCTTCTACAAGGTGCTG

ACCCAGCAGCAGCAGGAGCAGGCCGCCCACAACGACTTCAACTTTGACCACCCCGACGCC

TTCGACTTCGACCTCATCATCTCCACCCTCAAGAAGCTGAAGCAGGGCAGGAGCGTCCAA

GTGCCCATCTATGACTTCACCACCCACAGCCGGAAGAAGGACTGGAAAACGCTCTATGGT

GCAAACGTCATCATCTTCGAGGGTATCATGGCCTTCGCTGACAAGACGCTGCTGGAGCTC

CTGGACATGAGAATCTTTGTGGACACGGACTCCGACATCCGCTTAGTGCGGCGGCTGCGC

CGGGACATCAGTGAACGAGGCCGGGACATCGAGGGTGTCATCAAGCAGTACCACAAGTTT

GTCAAGCCTGCCTTTGACCAATACATCCAGCCCACCATGCGCCTGGCAGACATTGTGGTG

CCCAGAGGGAGTGGGAACGCGGTGGCCATTGACCTGATCGTGCAGCATGTGCACAGCCAG

CTGGAGGAGAGGGAGCTGCGCTGGGATATGGCGGCCCTGGCCTCAGCGCACCAGTGCCAC

CCACTTCCCCAAACGCTGAGCGTCCTCAAGAGCACCCCGCAAGTGCGCGGTATGCACACC

ATCATCAGGGACAGGGAGACTAGTCGGGACGAGTTCATCTTCTACTCCAAGAGACTGATG

CGGCTGCTCATCGAGCATGCGCTTTCCTTCCTGCCCTTCCAGGACTGTGTGGTGCAGACC

CCACAGGGGCAGGACTACGCAGGCAAGTGCTATGCGGGAAAGCAGATCACTGGAGTATCC

ATCCTGCGTGCTGGGGAGACCATGGAGCCTGCTCTGCGTGCTGTGTGCAAAGACGTGCGC

ATTGGCACCATCCTCATCCAGACCAACCAGCTCACGGGGGAGCCCGAGCTCCACTATCTT

CGGCTGCCCAAGGACATCAGTGACGACCACGTGATCCTGATGGACTGTACGGTGTCTACT

GGCGCTGCGGCCATGATGGCCGTCCGTGTCCTCCTGGACCACGATGTGCCCGAGGACAAG

ATCTTCTTGCTGTCACTGCTGATGGCGGAGATGGGTGTCCACTCCGTGGCCTATGCTTTC

CCACGAGTGAGAATCATCACCACAGCTGTGGACAAGCGGGTCAATGACCTTTTCCGCATC

ATCCCAGGCATAGGGAACTTTGGGGATCGCTACTTTGGGACAGATGCGGTCCCTGATGGC

AGCGACGAGGAAGAGGTGGCTTCCACTGGT

>CL218.Contig4_A-W 38 1759 minus strand PREDICTED: uridine-cytidine kinase-like 1 isoform 1 [Cavia porcellus]

ATGGCTGCGCCACCGGCCTCCGCGGACGCCGCCCCCTCGTCCCCGCCGCCTCCTGCGGCC

CCGGACGCGCCCGGAGCCTGCGCGGGAGGACAGAGCGAGACCGCGTGCGAGGACCGCAGC

CATGCAGGGTCCCTGGACAGGCTTCTCCCCACCGTGGGCACCAGGCGCTCGCCCCGGAAG

CGCACCACCAGCCAGTGCAAGTCGGAGCCACCCCTGCTGCGCACCAGCAAGCGCACCATC

TACACGGCAGGGCGGCCACCCTGGTACAATGAGCATGGCACACAGTCCAAGGAGGCCTTT

GCCATCGGCCTGGGAGGTGGCAGTGCCTCGGGGAAGACCACCGTGGCCAGGATGATCATT

GAGGCTCTAGATGTGCCCTGGGTGGTCTTGCTGTCCATGGACTCCTTCTACAAGGTGCTG

ACCCAGCAGCAGCAGGAGCAGGCCGCCCACAACGACTTCAACTTTGACCACCCCGACGCC

TTCGACTTCGACCTCATCATCTCCACCCTCAAGAAGCTGAAGCAGGGCAGGAGCGTCCAA

GTGCCCATCTATGACTTCACCACCCACAGCCGGAAGAAGGACTGGAAAACGCTCTATGGT

GCAAACGTCATCATCTTCGAGGGTATCATGGCCTTCGCTGACAAGACGCTGCTGGAGCTC

CTGGACATGAGAATCTTTGTGGACACGGACTCCGACATCCGCTTAGTGCGGCGGCTGCGC

CGGGACATCAGTGAACGAGGCCGGGACATCGAGGGTGTCATCAAGCAGTACCACAAGTTT

GTCAAGCCTGCCTTTGACCAATACATCCAGCCCACCATGCGCCTGGCAGACATTGTGGTG

CCCAGAGGGAGTGGGAACGCGGTGGCCATTGACCTGATCGTGCAGCATGTGCACAGCCAG

CTGGAGGAGAGGGAGCTGCGCTGGGATATGGCGGCCCTGGCCTCAGCGCACCAGTGCCAC

CCACTTCCCCAAACGCTGAGCGTCCTCAAGAGCACCCCGCAAGTGCGCGGTATGCACACC

ATCATCAGGGACAGGGAGACTAGTCGGGACGAGTTCATCTTCTACTCCAAGAGACTGATG

CGGCTGCTCATCGAGCATGCGCTTTCCTTCCTGCCCTTCCAGGACTGTGTGGTGCAGACC

CCACAGGGGCAGGACTACGCAGGCAAGTGCTATGCGGGAAAGCAGGTATGTGCACCCTGG

GAGGGCGATACCTTAGCTGGGCTGGGTGTCCCATTGGCTCAGCCCTCCCTCCCCTAGATC

ACTGGAGTATCCATCCTGCGTGCTGGGGAGACCATGGAGCCTGCTCTGCGTGCTGTGTGC

AAAGACGTGCGCATTGGCACCATCCTCATCCAGACCAACCAGCTCACGGGGGAGCCCGAG

CTCCACTATCTTCGGCTGCCCAAGGACATCAGTGACGACCACGTGATCCTGATGGACTGT

ACGGTGTCTACTGGCGCTGCGGCCATGATGGCCGTCCGTGTCCTCCTGGACCACGATGTG

CCCGAGGACAAGATCTTCTTGCTGTCACTGCTGATGGCGGAGATGGGTGTCCACTCCGTG

GCCTATGCTTTCCCACGAGTGAGAATCATCACCACAGCTGTGGACAAGCGGGTCAATGAC

CTTTTCCGCATCATCCCAGGCATAGGGAACTTTGGGGATCGCTACTTTGGGACAGATGCG

GTCCCTGATGGCAGCGACGAGGAAGAGGTGGCTTCCACTGGT

>CL218.Contig5_A-W 3 302 minus strand PREDICTED: uridine-cytidine kinase 1-like 1 isoform 1 [Pongo abelii]

AGCCATGCAGGGTCCCTGGACAGGCTTCTCCCCACCGTGGGCACCAGGCGCTCGCCCCGG

AAGCGCACCACCAGCCAGTGCAAGTCGGAGCCACCCCTGCTGCGCACCAGCAAGCGCACC

ATCTACACGGCAGGGCGGCCACCCTGGTACAATGAGCATGGCACACAGTCCAAGGAGGCC

TTTGCCATCGGCCTGGGAGGTGGCAGTGCCTCGGGGAAGACCACCGTGGCCAGGATGATC

ATTGAGGCTCTAGATGTGCCCTGGGTGGTCTTGCTGTCCATGGACTCCTTCTACAAGGTG

>CL218.Contig6_A-W 3 302 minus strand PREDICTED: uridine-cytidine kinase-like 1 isoform 2 [Cavia porcellus]

AGCCATGCAGGGTCCCTGGACAGGCTTCTCCCCACCGTGGGCACCAGGCGCTCGCCCCGG

AAGCGCACCACCAGCCAGTGCAAGTCGGAGCCACCCCTGCTGCGCACCAGCAAGCGCACC

ATCTACACGGCAGGGCGGCCACCCTGGTACAATGAGCATGGCACACAGTCCAAGGAGGCC

TTTGCCATCGGCCTGGGAGGTGGCAGTGCCTCGGGGAAGACCACCGTGGCCAGGATGATC

ATTGAGGCTCTAGATGTGCCCTGGGTGGTCTTGCTGTCCATGGACTCCTTCTACAAGGTG

>CL218.Contig7_A-W 35 451 minus strand PREDICTED: uridine-cytidine kinase-like 1 isoform 1 [Cavia porcellus]

ATGGCTGCGCCACCGGCCTCCGCGGACGCCGCCCCCTCGTCCCCGCCGCCTCCTGCGGCC

CCGGACGCGCCCGGAGCCTGCGCGGGAGGACAGAGCGAGACCGCGTGCGAGGACCGCAGC

CATGCAGGGTCCCTGGACAGGCTTCTCCCCACCGTGGGCACCAGGCGCTCGCCCCGGAAG

CGCACCACCAGCCAGTGCAAGTCGGAGCCACCCCTGCTGCGCACCAGCAAGCGCACCATC

TACACGGCAGGGCGGCCACCCTGGTACAATGAGCATGGCACACAGTCCAAGGAGGCCTTT

GCCATCGGCCTGGGAGGTGGCAGTGCCTCGGGGAAGACCACCGTGGCCAGGATGATCATT

GAGGCTCTAGATGTGCCCTGGGTGGTCTTGCTGTCCATGGACTCCTTCTACAAGGTG

>CL218.Contig8_A-W 942 1679 minus strand PREDICTED: uridine-cytidine kinase-like 1 isoform 1 [Cavia porcellus]

AGGGCGGCCCTGGCCTCAGCGCACCAGTGCCACCCACTTCCCCAAACGCTGAGCGTCCTC

AAGAGCACCCCGCAAGTGCGCGGTATGCACACCATCATCAGGGACAGGGAGACTAGTCGG

GACGAGTTCATCTTCTACTCCAAGAGACTGATGCGGCTGCTCATCGAGCATGCGCTTTCC

TTCCTGCCCTTCCAGGACTGTGTGGTGCAGACCCCACAGGGGCAGGACTACGCAGGCAAG

TGCTATGCGGGAAAGCAGCCCTCCCTCCCCTAGATCACTGGAGTATCCATCCTGCGTGCT

GGGGAGACCATGGAGCCTGCTCTGCGTGCTGTGTGCAAAGACGTGCGCATTGGCACCATC

CTCATCCAGACCAACCAGCTCACGGGGGAGCCCGAGCTCCACTATCTTCGGCTGCCCAAG

GACATCAGTGACGACCACGTGATCCTGATGGACTGTACGGTGTCTACTGGCGCTGCGGCC

ATGATGGCCGTCCGTGTCCTCCTGGACCACGATGTGCCCGAGGACAAGATCTTCTTGCTG

TCACTGCTGATGGCGGAGATGGGTGTCCACTCCGTGGCCTATGCTTTCCCACGAGTGAGA

ATCATCACCACAGCTGTGGACAAGCGGGTCAATGACCTTTTCCGCATCATCCCAGGCATA

GGGAACTTTGGGGATCGCTACTTTGGGACAGATGCGGTCCCTGATGGCAGCGACGAGGAA

GAGGTGGCTTCCACTGGT

>CL218.Contig9_A-W 3 302 minus strand PREDICTED: uridine-cytidine kinase-like 1 isoform 1 [Cavia porcellus]

AGCCATGCAGGGTCCCTGGACAGGCTTCTCCCCACCGTGGGCACCAGGCGCTCGCCCCGG

AAGCGCACCACCAGCCAGTGCAAGTCGGAGCCACCCCTGCTGCGCACCAGCAAGCGCACC

ATCTACACGGCAGGGCGGCCACCCTGGTACAATGAGCATGGCACACAGTCCAAGGAGGCC

TTTGCCATCGGCCTGGGAGGTGGCAGTGCCTCGGGGAAGACCACCGTGGCCAGGATGATC

ATTGAGGCTCTAGATGTGCCCTGGGTGGTCTTGCTGTCCATGGACTCCTTCTACAAGGTG

>CL218.Contig10_A-W 942 1664 minus strand PREDICTED: uridine-cytidine kinase-like 1 isoform 1 [Cavia porcellus]

AGGGCGGCCCTGGCCTCAGCGCACCAGTGCCACCCACTTCCCCAAACGCTGAGCGTCCTC

AAGAGCACCCCGCAAGTGCGCGGTATGCACACCATCATCAGGGACAGGGAGACTAGTCGG

GACGAGTTCATCTTCTACTCCAAGAGACTGATGCGGCTGCTCATCGAGCATGCGCTTTCC

TTCCTGCCCTTCCAGGACTGTGTGGTGCAGACCCCACAGGGGCAGGACTACGCAGGCAAG

TGCTATGCGGGAAAGCAGATCACTGGAGTATCCATCCTGCGTGCTGGGGAGACCATGGAG

CCTGCTCTGCGTGCTGTGTGCAAAGACGTGCGCATTGGCACCATCCTCATCCAGACCAAC

CAGCTCACGGGGGAGCCCGAGCTCCACTATCTTCGGCTGCCCAAGGACATCAGTGACGAC

CACGTGATCCTGATGGACTGTACGGTGTCTACTGGCGCTGCGGCCATGATGGCCGTCCGT

GTCCTCCTGGACCACGATGTGCCCGAGGACAAGATCTTCTTGCTGTCACTGCTGATGGCG

GAGATGGGTGTCCACTCCGTGGCCTATGCTTTCCCACGAGTGAGAATCATCACCACAGCT

GTGGACAAGCGGGTCAATGACCTTTTCCGCATCATCCCAGGCATAGGGAACTTTGGGGAT

CGCTACTTTGGGACAGATGCGGTCCCTGATGGCAGCGACGAGGAAGAGGTGGCTTCCACT

GGT

>CL218.Contig11_A-W 53 523 minus strand PREDICTED: uridine-cytidine kinase-like 1 isoform 2 [Cavia porcellus]

AGCAGCCATGCAGGGTCCCTGGACAGGCTTCTCCCCACCGTGGGCACCAGGCGCTCGCCC

CGGAAGCGCACCACCAGCCAGTGCAAGTCGGAGCCACCCCTGCTGCGCACCAGCAAGCGC

ACCATCTACACGGCAGGGCGGCCACCCTGGTACAATGAGCATGGCACACAGTCCAAGGAG

GCCTTTGCCATCGGCCTGGGAGGTGGCAGTGCCTCGGGGAAGACCACCGTGGCCAGGATG

ATCATTGAGGCTCTAGATGTGCCCTGGGTGGTCTTGCTGTCCATGGACTCCTTCTACAAG

GTGCTGACCCAGCAGCAGCAGGAGCAGGCCGCCCACAACGACTTCAACTTTGACCACCCC

GACGCCTTCGACTTCGACCTCATCATCTCCACCCTCAAGAAGCTGAAGCAGGGCAGGAGC

GTCCAAGTGCCCATCTATGACTTCACCACCCACAGCCGGAAGAAGGACTGG

>CL218.Contig12_A-W 545 868 minus strand PREDICTED: uridine-cytidine kinase-like 1 isoform 1 [Cavia porcellus]

AAAACGCTCTATGGTGCAAACGTCATCATCTTCGAGGGTATCATGGCCTTCGCTGACAAG

ACGCTGCTGGAGCTCCTGGACATGAGAATCTTTGTGGACACGGACTCCGACATCCGCTTA

GTGCGGCGGCTGCGCCGGGACATCAGTGAACGAGGCCGGGACATCGAGGGTGTCATCAAG

CAGTACCACAAGTTTGTCAAGCCTGCCTTTGACCAATACATCCAGCCCACCATGCGCCTG

GCAGACATTGTGGTGCCCAGAGGGAGTGGGAACGCGGTGGCCATTGACCTGATCGTGCAG

CATGTGCACAGCCAGCTGGAGGAG

>CL218.Contig13_A-W 598 921 minus strand PREDICTED: uridine-cytidine kinase-like 1 isoform 2 [Cavia porcellus]

AAAACGCTCTATGGTGCAAACGTCATCATCTTCGAGGGTATCATGGCCTTCGCTGACAAG

ACGCTGCTGGAGCTCCTGGACATGAGAATCTTTGTGGACACGGACTCCGACATCCGCTTA

GTGCGGCGGCTGCGCCGGGACATCAGTGAACGAGGCCGGGACATCGAGGGTGTCATCAAG

CAGTACCACAAGTTTGTCAAGCCTGCCTTTGACCAATACATCCAGCCCACCATGCGCCTG

GCAGACATTGTGGTGCCCAGAGGGAGTGGGAACGCGGTGGCCATTGACCTGATCGTGCAG

CATGTGCACAGCCAGCTGGAGGAG

>CL218.Contig14_A-W 1 903 minus strand PREDICTED: uridine-cytidine kinase-like 1 isoform 2 [Cavia porcellus]

AAAACGCTCTATGGTGCAAACGTCATCATCTTCGAGGGTATCATGGCCTTCGCTGACAAG

ACGCTGCTGGAGCTCCTGGACATGAGAATCTTTGTGGACACGGACTCCGACATCCGCTTA

GTGCGGCGGCTGCGCCGGGACATCAGTGAACGAGGCCGGGACATCGAGGGTGTCATCAAG

CAGTACCACAAGTTTGTCAAGCCTGCCTTTGACCAATACATCCAGCCCACCATGCGCCTG

GCAGACATTGTGGTGCCCAGAGGGAGTGGGAACGCGGTGGCCATTGACCTGATCGTGCAG

CATGTGCACAGCCAGCTGGAGGAGAGGGAGCTGCGCTGGGATATGGCGGCCCTGGCCTCA

GCGCACCAGTGCCACCCACTTCCCCAAACGCTGAGCGTCCTCAAGAGCACCCCGCAAGTG

CGCGGTATGCACACCATCATCAGGGACAGGGAGACTAGTCGGGACGAGTTCATCTTCTAC

TCCAAGAGACTGATGCGGCTGCTCATCGAGCATGCGCTTTCCTTCCTGCCCTTCCAGGAC

TGTGTGGTGCAGACCCCACAGGGGCAGGACTACGCAGGCAAGTGCTATGCGGGAAAGCAG

GTATGTGCACCCTGGGAGGGCGATACCTTAGCTGGGCTGGGTGTCCCATTGGCTCAGCCC

TCCCTCCCCTAGATCACTGGAGTATCCATCCTGCGTGCTGGGGAGACCATGGAGCCTGCT

CTGCGTGCTGTGTGCAAAGACGTGCGCATTGGCACCATCCTCATCCAGACCAACCAGCTC

ACGGGGGAGCCCGAGCTCCACTATCTTCGGCTGCCCAAGGACATCAGTGACGACCACGTG

ATCCTGATGGACTGTACGGTGTCTACTGGCGCTGCGGCCATGATGGCCGTCCGTGTCCTC

CTG

>CL218.Contig15_A-W 598 921 minus strand PREDICTED: uridine-cytidine kinase-like 1 isoform 2 [Cavia porcellus]

AAAACGCTCTATGGTGCAAACGTCATCATCTTCGAGGGTATCATGGCCTTCGCTGACAAG

ACGCTGCTGGAGCTCCTGGACATGAGAATCTTTGTGGACACGGACTCCGACATCCGCTTA

GTGCGGCGGCTGCGCCGGGACATCAGTGAACGAGGCCGGGACATCGAGGGTGTCATCAAG

CAGTACCACAAGTTTGTCAAGCCTGCCTTTGACCAATACATCCAGCCCACCATGCGCCTG

GCAGACATTGTGGTGCCCAGAGGGAGTGGGAACGCGGTGGCCATTGACCTGATCGTGCAG

CATGTGCACAGCCAGCTGGAGGAG

>CL218.Contig16_A-W 846 1640 minus strand PREDICTED: uridine-cytidine kinase-like 1 isoform 2 [Cavia porcellus]

AGGGCGGCCCTGGCCTCAGCGCACCAGTGCCACCCACTTCCCCAAACGCTGAGCGTCCTC

AAGAGCACCCCGCAAGTGCGCGGTATGCACACCATCATCAGGGACAGGGAGACTAGTCGG

GACGAGTTCATCTTCTACTCCAAGAGACTGATGCGGCTGCTCATCGAGCATGCGCTTTCC

TTCCTGCCCTTCCAGGACTGTGTGGTGCAGACCCCACAGGGGCAGGACTACGCAGGCAAG

TGCTATGCGGGAAAGCAGGTATGTGCACCCTGGGAGGGCGATACCTTAGCTGGGCTGGGT

GTCCCATTGGCTCAGCCCTCCCTCCCCTAGATCACTGGAGTATCCATCCTGCGTGCTGGG

GAGACCATGGAGCCTGCTCTGCGTGCTGTGTGCAAAGACGTGCGCATTGGCACCATCCTC

ATCCAGACCAACCAGCTCACGGGGGAGCCCGAGCTCCACTATCTTCGGCTGCCCAAGGAC

ATCAGTGACGACCACGTGATCCTGATGGACTGTACGGTGTCTACTGGCGCTGCGGCCATG

ATGGCCGTCCGTGTCCTCCTGGACCACGATGTGCCCGAGGACAAGATCTTCTTGCTGTCA

CTGCTGATGGCGGAGATGGGTGTCCACTCCGTGGCCTATGCTTTCCCACGAGTGAGAATC

ATCACCACAGCTGTGGACAAGCGGGTCAATGACCTTTTCCGCATCATCCCAGGCATAGGG

AACTTTGGGGATCGCTACTTTGGGACAGATGCGGTCCCTGATGGCAGCGACGAGGAAGAG

GTGGCTTCCACTGGT

>CL218.Contig17_A-W 53 1588 minus strand PREDICTED: uridine-cytidine kinase-like 1 isoform 2 [Cavia porcellus]

AGCAGCCATGCAGGGTCCCTGGACAGGCTTCTCCCCACCGTGGGCACCAGGCGCTCGCCC

CGGAAGCGCACCACCAGCCAGTGCAAGTCGGAGCCACCCCTGCTGCGCACCAGCAAGCGC

ACCATCTACACGGCAGGGCGGCCACCCTGGTACAATGAGCATGGCACACAGTCCAAGGAG

GCCTTTGCCATCGGCCTGGGAGGTGGCAGTGCCTCGGGGAAGACCACCGTGGCCAGGATG

ATCATTGAGGCTCTAGATGTGCCCTGGGTGGTCTTGCTGTCCATGGACTCCTTCTACAAG

GTGCTGACCCAGCAGCAGCAGGAGCAGGCCGCCCACAACGACTTCAACTTTGACCACCCC

GACGCCTTCGACTTCGACCTCATCATCTCCACCCTCAAGAAGCTGAAGCAGGGCAGGAGC

GTCCAAGTGCCCATCTATGACTTCACCACCCACAGCCGGAAGAAGGACTGGAAAACGCTC

TATGGTGCAAACGTCATCATCTTCGAGGGTATCATGGCCTTCGCTGACAAGACGCTGCTG

GAGCTCCTGGACATGAGAATCTTTGTGGACACGGACTCCGACATCCGCTTAGTGCGGCGG

CTGCGCCGGGACATCAGTGAACGAGGCCGGGACATCGAGGGTGTCATCAAGCAGTACCAC

AAGTTTGTCAAGCCTGCCTTTGACCAATACATCCAGCCCACCATGCGCCTGGCAGACATT

GTGGTGCCCAGAGGGAGTGGGAACGCGGTGGCCATTGACCTGATCGTGCAGCATGTGCAC

AGCCAGCTGGAGGAGAGGGAGCTGCGCTGGGATATGGCGGCCCTGGCCTCAGCGCACCAG

TGCCACCCACTTCCCCAAACGCTGAGCGTCCTCAAGAGCACCCCGCAAGTGCGCGGTATG

CACACCATCATCAGGGACAGGGAGACTAGTCGGGACGAGTTCATCTTCTACTCCAAGAGA

CTGATGCGGCTGCTCATCGAGCATGCGCTTTCCTTCCTGCCCTTCCAGGACTGTGTGGTG

CAGACCCCACAGGGGCAGGACTACGCAGGCAAGTGCTATGCGGGAAAGCAGATCACTGGA

GTATCCATCCTGCGTGCTGGGGAGACCATGGAGCCTGCTCTGCGTGCTGTGTGCAAAGAC

GTGCGCATTGGCACCATCCTCATCCAGACCAACCAGCTCACGGGGGAGCCCGAGCTCCAC

TATCTTCGGCTGCCCAAGGACATCAGTGACGACCACGTGATCCTGATGGACTGTACGGTG

TCTACTGGCGCTGCGGCCATGATGGCCGTCCGTGTCCTCCTGGACCACGATGTGCCCGAG

GACAAGATCTTCTTGCTGTCACTGCTGATGGCGGAGATGGGTGTCCACTCCGTGGCCTAT

GCTTTCCCACGAGTGAGAATCATCACCACAGCTGTGGACAAGCGGGTCAATGACCTTTTC

CGCATCATCCCAGGCATAGGGAACTTTGGGGATCGCTACTTTGGGACAGATGCGGTCCCT

GATGGCAGCGACGAGGAAGAGGTGGCTTCCACTGGT

>CL218.Contig18_A-W 846 1583 minus strand PREDICTED: uridine-cytidine kinase-like 1 isoform 2 [Cavia porcellus]

AGGGCGGCCCTGGCCTCAGCGCACCAGTGCCACCCACTTCCCCAAACGCTGAGCGTCCTC

AAGAGCACCCCGCAAGTGCGCGGTATGCACACCATCATCAGGGACAGGGAGACTAGTCGG

GACGAGTTCATCTTCTACTCCAAGAGACTGATGCGGCTGCTCATCGAGCATGCGCTTTCC

TTCCTGCCCTTCCAGGACTGTGTGGTGCAGACCCCACAGGGGCAGGACTACGCAGGCAAG

TGCTATGCGGGAAAGCAGCCCTCCCTCCCCTAGATCACTGGAGTATCCATCCTGCGTGCT

GGGGAGACCATGGAGCCTGCTCTGCGTGCTGTGTGCAAAGACGTGCGCATTGGCACCATC

CTCATCCAGACCAACCAGCTCACGGGGGAGCCCGAGCTCCACTATCTTCGGCTGCCCAAG

GACATCAGTGACGACCACGTGATCCTGATGGACTGTACGGTGTCTACTGGCGCTGCGGCC

ATGATGGCCGTCCGTGTCCTCCTGGACCACGATGTGCCCGAGGACAAGATCTTCTTGCTG

TCACTGCTGATGGCGGAGATGGGTGTCCACTCCGTGGCCTATGCTTTCCCACGAGTGAGA

ATCATCACCACAGCTGTGGACAAGCGGGTCAATGACCTTTTCCGCATCATCCCAGGCATA

GGGAACTTTGGGGATCGCTACTTTGGGACAGATGCGGTCCCTGATGGCAGCGACGAGGAA

GAGGTGGCTTCCACTGGT

>CL218.Contig19_A-W 846 1568 minus strand PREDICTED: uridine-cytidine kinase-like 1 isoform 2 [Cavia porcellus]

AGGGCGGCCCTGGCCTCAGCGCACCAGTGCCACCCACTTCCCCAAACGCTGAGCGTCCTC

AAGAGCACCCCGCAAGTGCGCGGTATGCACACCATCATCAGGGACAGGGAGACTAGTCGG

GACGAGTTCATCTTCTACTCCAAGAGACTGATGCGGCTGCTCATCGAGCATGCGCTTTCC

TTCCTGCCCTTCCAGGACTGTGTGGTGCAGACCCCACAGGGGCAGGACTACGCAGGCAAG

TGCTATGCGGGAAAGCAGATCACTGGAGTATCCATCCTGCGTGCTGGGGAGACCATGGAG

CCTGCTCTGCGTGCTGTGTGCAAAGACGTGCGCATTGGCACCATCCTCATCCAGACCAAC

CAGCTCACGGGGGAGCCCGAGCTCCACTATCTTCGGCTGCCCAAGGACATCAGTGACGAC

CACGTGATCCTGATGGACTGTACGGTGTCTACTGGCGCTGCGGCCATGATGGCCGTCCGT

GTCCTCCTGGACCACGATGTGCCCGAGGACAAGATCTTCTTGCTGTCACTGCTGATGGCG

GAGATGGGTGTCCACTCCGTGGCCTATGCTTTCCCACGAGTGAGAATCATCACCACAGCT

GTGGACAAGCGGGTCAATGACCTTTTCCGCATCATCCCAGGCATAGGGAACTTTGGGGAT

CGCTACTTTGGGACAGATGCGGTCCCTGATGGCAGCGACGAGGAAGAGGTGGCTTCCACT

GGT

>CL218.Contig20_A-W 833 1552 minus strand PREDICTED: uridine-cytidine kinase 1-like 1 isoform 1 [Pongo abelii]

GCGGCCCTGGCCTCAGCGCACCAGTGCCACCCACTTCCCCAAACGCTGAGCGTCCTCAAG

AGCACCCCGCAAGTGCGCGGTATGCACACCATCATCAGGGACAGGGAGACTAGTCGGGAC

GAGTTCATCTTCTACTCCAAGAGACTGATGCGGCTGCTCATCGAGCATGCGCTTTCCTTC

CTGCCCTTCCAGGACTGTGTGGTGCAGACCCCACAGGGGCAGGACTACGCAGGCAAGTGC

TATGCGGGAAAGCAGATCACTGGAGTATCCATCCTGCGTGCTGGGGAGACCATGGAGCCT

GCTCTGCGTGCTGTGTGCAAAGACGTGCGCATTGGCACCATCCTCATCCAGACCAACCAG

CTCACGGGGGAGCCCGAGCTCCACTATCTTCGGCTGCCCAAGGACATCAGTGACGACCAC

GTGATCCTGATGGACTGTACGGTGTCTACTGGCGCTGCGGCCATGATGGCCGTCCGTGTC

CTCCTGGACCACGATGTGCCCGAGGACAAGATCTTCTTGCTGTCACTGCTGATGGCGGAG

ATGGGTGTCCACTCCGTGGCCTATGCTTTCCCACGAGTGAGAATCATCACCACAGCTGTG

GACAAGCGGGTCAATGACCTTTTCCGCATCATCCCAGGCATAGGGAACTTTGGGGATCGC

TACTTTGGGACAGATGCGGTCCCTGATGGCAGCGACGAGGAAGAGGTGGCTTCCACTGGT

>CL218.Contig21_A-W 3 1550 minus strand PREDICTED: uridine-cytidine kinase-like 1 isoform 2 [Cavia porcellus]

AGCCATGCAGGGTCCCTGGACAGGCTTCTCCCCACCGTGGGCACCAGGCGCTCGCCCCGG

AAGCGCACCACCAGCCAGTGCAAGTCGGAGCCACCCCTGCTGCGCACCAGCAAGCGCACC

ATCTACACGGCAGGGCGGCCACCCTGGTACAATGAGCATGGCACACAGTCCAAGGAGGCC

TTTGCCATCGGCCTGGGAGGTGGCAGTGCCTCGGGGAAGACCACCGTGGCCAGGATGATC

ATTGAGGCTCTAGATGTGCCCTGGGTGGTCTTGCTGTCCATGGACTCCTTCTACAAGGTG

CTGACCCAGCAGCAGCAGGAGCAGGCCGCCCACAACGACTTCAACTTTGACCACCCCGAC

GCCTTCGACTTCGACCTCATCATCTCCACCCTCAAGAAGCTGAAGCAGGGCAGGAGCGTC

CAAGTGCCCATCTATGACTTCACCACCCACAGCCGGAAGAAGGACTGGAAAACGCTCTAT

GGTGCAAACGTCATCATCTTCGAGGGTATCATGGCCTTCGCTGACAAGACGCTGCTGGAG

CTCCTGGACATGAGAATCTTTGTGGACACGGACTCCGACATCCGCTTAGTGCGGCGGCTG

CGCCGGGACATCAGTGAACGAGGCCGGGACATCGAGGGTGTCATCAAGCAGTACCACAAG

TTTGTCAAGCCTGCCTTTGACCAATACATCCAGCCCACCATGCGCCTGGCAGACATTGTG

GTGCCCAGAGGGAGTGGGAACGCGGTGGCCATTGACCTGATCGTGCAGCATGTGCACAGC

CAGCTGGAGGAGAGGGAGCTGCGCTGGGATATGGCGGCCCTGGCCTCAGCGCACCAGTGC

CACCCACTTCCCCAAACGCTGAGCGTCCTCAAGAGCACCCCGCAAGTGCGCGGTATGCAC

ACCATCATCAGGGACAGGGAGACTAGTCGGGACGAGTTCATCTTCTACTCCAAGAGACTG

ATGCGGCTGCTCATCGAGCATGCGCTTTCCTTCCTGCCCTTCCAGGACTGTGTGGTGCAG

ACCCCACAGGGGCAGGACTACGCAGGCAAGTGCTATGCGGGAAAGCAGCCCTCCCTCCCC

TAGATCACTGGAGTATCCATCCTGCGTGCTGGGGAGACCATGGAGCCTGCTCTGCGTGCT

GTGTGCAAAGACGTGCGCATTGGCACCATCCTCATCCAGACCAACCAGCTCACGGGGGAG

CCCGAGCTCCACTATCTTCGGCTGCCCAAGGACATCAGTGACGACCACGTGATCCTGATG

GACTGTACGGTGTCTACTGGCGCTGCGGCCATGATGGCCGTCCGTGTCCTCCTGGACCAC

GATGTGCCCGAGGACAAGATCTTCTTGCTGTCACTGCTGATGGCGGAGATGGGTGTCCAC

TCCGTGGCCTATGCTTTCCCACGAGTGAGAATCATCACCACAGCTGTGGACAAGCGGGTC

AATGACCTTTTCCGCATCATCCCAGGCATAGGGAACTTTGGGGATCGCTACTTTGGGACA

GATGCGGTCCCTGATGGCAGCGACGAGGAAGAGGTGGCTTCCACTGGT

>CL218.Contig22_A-W 1 168 minus strand PREDICTED: uridine-cytidine kinase 1-like 1 isoform 1 [Pongo abelii]

CTGACCCAGCAGCAGCAGGAGCAGGCCGCCCACAACGACTTCAACTTTGACCACCCCGAC

GCCTTCGACTTCGACCTCATCATCTCCACCCTCAAGAAGCTGAAGCAGGGCAGGAGCGTC

CAAGTGCCCATCTATGACTTCACCACCCACAGCCGGAAGAAGGACTGG

>CL218.Contig23_A-W 35 451 minus strand PREDICTED: uridine-cytidine kinase-like 1 isoform 1 [Cavia porcellus]

ATGGCTGCGCCACCGGCCTCCGCGGACGCCGCCCCCTCGTCCCCGCCGCCTCCTGCGGCC

CCGGACGCGCCCGGAGCCTGCGCGGGAGGACAGAGCGAGACCGCGTGCGAGGACCGCAGC

CATGCAGGGTCCCTGGACAGGCTTCTCCCCACCGTGGGCACCAGGCGCTCGCCCCGGAAG

CGCACCACCAGCCAGTGCAAGTCGGAGCCACCCCTGCTGCGCACCAGCAAGCGCACCATC

TACACGGCAGGGCGGCCACCCTGGTACAATGAGCATGGCACACAGTCCAAGGAGGCCTTT

GCCATCGGCCTGGGAGGTGGCAGTGCCTCGGGGAAGACCACCGTGGCCAGGATGATCATT

GAGGCTCTAGATGTGCCCTGGGTGGTCTTGCTGTCCATGGACTCCTTCTACAAGGTG

>CL218.Contig24_A-W 1 168 minus strand PREDICTED: uridine-cytidine kinase-like 1 isoform 1 [Cavia porcellus]

CTGACCCAGCAGCAGCAGGAGCAGGCCGCCCACAACGACTTCAACTTTGACCACCCCGAC

GCCTTCGACTTCGACCTCATCATCTCCACCCTCAAGAAGCTGAAGCAGGGCAGGAGCGTC

CAAGTGCCCATCTATGACTTCACCACCCACAGCCGGAAGAAGGACTGG

>CL218.Contig25_A-W 1 168 minus strand PREDICTED: uridine-cytidine kinase 1-like 1 isoform 1 [Pongo abelii]

CTGACCCAGCAGCAGCAGGAGCAGGCCGCCCACAACGACTTCAACTTTGACCACCCCGAC

GCCTTCGACTTCGACCTCATCATCTCCACCCTCAAGAAGCTGAAGCAGGGCAGGAGCGTC

CAAGTGCCCATCTATGACTTCACCACCCACAGCCGGAAGAAGGACTGG

>CL218.Contig26_A-W 1 168 minus strand PREDICTED: uridine-cytidine kinase 1-like 1 isoform 1 [Pongo abelii]

CTGACCCAGCAGCAGCAGGAGCAGGCCGCCCACAACGACTTCAACTTTGACCACCCCGAC

GCCTTCGACTTCGACCTCATCATCTCCACCCTCAAGAAGCTGAAGCAGGGCAGGAGCGTC

CAAGTGCCCATCTATGACTTCACCACCCACAGCCGGAAGAAGGACTGG

>CL218.Contig27_A-W 1 522 minus strand PREDICTED: uridine-cytidine kinase 1-like 1 isoform 1 [Pongo abelii]

CTGACCCAGCAGCAGCAGGAGCAGGCCGCCCACAACGACTTCAACTTTGACCACCCCGAC

GCCTTCGACTTCGACCTCATCATCTCCACCCTCAAGAAGCTGAAGCAGGGCAGGAGCGTC

CAAGTGCCCATCTATGACTTCACCACCCACAGCCGGAAGAAGGACTGGAAAACGCTCTAT

GGTGCAAACGTCATCATCTTCGAGGGTATCATGGCCTTCGCTGACAAGACGCTGCTGGAG

CTCCTGGACATGAGAATCTTTGTGGACACGGACTCCGACATCCGCTTAGTGCGGCGGCTG

CGCCGGGACATCAGTGAACGAGGCCGGGACATCGAGGGTGTCATCAAGCAGTACCACAAG

TTTGTCAAGCCTGCCTTTGACCAATACATCCAGCCCACCATGCGCCTGGCAGACATTGTG

GTGCCCAGAGGGAGTGGGAACGCGGTGGCCATTGACCTGATCGTGCAGCATGTGCACAGC

CAGCTGGAGGAGCGTGAACTCAGCGTCAGAGGGAGCTGCGCT

>CL218.Contig28_A-W 1 168 minus strand PREDICTED: uridine-cytidine kinase-like 1 isoform 1 [Cavia porcellus]

CTGACCCAGCAGCAGCAGGAGCAGGCCGCCCACAACGACTTCAACTTTGACCACCCCGAC

GCCTTCGACTTCGACCTCATCATCTCCACCCTCAAGAAGCTGAAGCAGGGCAGGAGCGTC

CAAGTGCCCATCTATGACTTCACCACCCACAGCCGGAAGAAGGACTGG

>CL218.Contig29_A-W 35 451 minus strand PREDICTED: uridine-cytidine kinase-like 1 isoform 1 [Cavia porcellus]

ATGGCTGCGCCACCGGCCTCCGCGGACGCCGCCCCCTCGTCCCCGCCGCCTCCTGCGGCC

CCGGACGCGCCCGGAGCCTGCGCGGGAGGACAGAGCGAGACCGCGTGCGAGGACCGCAGC

CATGCAGGGTCCCTGGACAGGCTTCTCCCCACCGTGGGCACCAGGCGCTCGCCCCGGAAG

CGCACCACCAGCCAGTGCAAGTCGGAGCCACCCCTGCTGCGCACCAGCAAGCGCACCATC

TACACGGCAGGGCGGCCACCCTGGTACAATGAGCATGGCACACAGTCCAAGGAGGCCTTT

GCCATCGGCCTGGGAGGTGGCAGTGCCTCGGGGAAGACCACCGTGGCCAGGATGATCATT

GAGGCTCTAGATGTGCCCTGGGTGGTCTTGCTGTCCATGGACTCCTTCTACAAGGTG

>CL218.Contig30_A-W 1 522 minus strand PREDICTED: uridine-cytidine kinase 1-like 1 isoform 1 [Pongo abelii]

CTGACCCAGCAGCAGCAGGAGCAGGCCGCCCACAACGACTTCAACTTTGACCACCCCGAC

GCCTTCGACTTCGACCTCATCATCTCCACCCTCAAGAAGCTGAAGCAGGGCAGGAGCGTC

CAAGTGCCCATCTATGACTTCACCACCCACAGCCGGAAGAAGGACTGGAAAACGCTCTAT

GGTGCAAACGTCATCATCTTCGAGGGTATCATGGCCTTCGCTGACAAGACGCTGCTGGAG

CTCCTGGACATGAGAATCTTTGTGGACACGGACTCCGACATCCGCTTAGTGCGGCGGCTG

CGCCGGGACATCAGTGAACGAGGCCGGGACATCGAGGGTGTCATCAAGCAGTACCACAAG

TTTGTCAAGCCTGCCTTTGACCAATACATCCAGCCCACCATGCGCCTGGCAGACATTGTG

GTGCCCAGAGGGAGTGGGAACGCGGTGGCCATTGACCTGATCGTGCAGCATGTGCACAGC

CAGCTGGAGGAGCGTGAACTCAGCGTCAGAGGGAGCTGCGCT

>CL218.Contig31_A-W 1 168 minus strand PREDICTED: uridine-cytidine kinase-like 1 [Saimiri boliviensis boliviensis]

CTGACCCAGCAGCAGCAGGAGCAGGCCGCCCACAACGACTTCAACTTTGACCACCCCGAC

GCCTTCGACTTCGACCTCATCATCTCCACCCTCAAGAAGCTGAAGCAGGGCAGGAGCGTC

CAAGTGCCCATCTATGACTTCACCACCCACAGCCGGAAGAAGGACTGG

>CL218.Contig32_A-W 1 168 minus strand PREDICTED: uridine-cytidine kinase-like 1 [Saimiri boliviensis boliviensis]

CTGACCCAGCAGCAGCAGGAGCAGGCCGCCCACAACGACTTCAACTTTGACCACCCCGAC

GCCTTCGACTTCGACCTCATCATCTCCACCCTCAAGAAGCTGAAGCAGGGCAGGAGCGTC

CAAGTGCCCATCTATGACTTCACCACCCACAGCCGGAAGAAGGACTGG

>CL218.Contig33_A-W 1 999 minus strand PREDICTED: uridine-cytidine kinase-like 1 [Saimiri boliviensis boliviensis]

CTGACCCAGCAGCAGCAGGAGCAGGCCGCCCACAACGACTTCAACTTTGACCACCCCGAC

GCCTTCGACTTCGACCTCATCATCTCCACCCTCAAGAAGCTGAAGCAGGGCAGGAGCGTC

CAAGTGCCCATCTATGACTTCACCACCCACAGCCGGAAGAAGGACTGGAAAACGCTCTAT

GGTGCAAACGTCATCATCTTCGAGGGTATCATGGCCTTCGCTGACAAGACGCTGCTGGAG

CTCCTGGACATGAGAATCTTTGTGGACACGGACTCCGACATCCGCTTAGTGCGGCGGCTG

CGCCGGGACATCAGTGAACGAGGCCGGGACATCGAGGGTGTCATCAAGCAGTACCACAAG

TTTGTCAAGCCTGCCTTTGACCAATACATCCAGCCCACCATGCGCCTGGCAGACATTGTG

GTGCCCAGAGGGAGTGGGAACGCGGTGGCCATTGACCTGATCGTGCAGCATGTGCACAGC

CAGCTGGAGGAGAGGGAGCTGCGCTGGGATATGGCGGCCCTGGCCTCAGCGCACCAGTGC

CACCCACTTCCCCAAACGCTGAGCGTCCTCAAGAGCACCCCGCAAGTGCGCGGTATGCAC

ACCATCATCAGGGACAGGGAGACTAGTCGGGACGAGTTCATCTTCTACTCCAAGAGACTG

ATGCGGCTGCTCATCGAGCATGCGCTTTCCTTCCTGCCCTTCCAGGACTGTGTGGTGCAG

ACCCCACAGGGGCAGGACTACGCAGGCAAGTGCTATGCGGGAAAGCAGATCACTGGAGTA

TCCATCCTGCGTGCTGGGGAGACCATGGAGCCTGCTCTGCGTGCTGTGTGCAAAGACGTG

CGCATTGGCACCATCCTCATCCAGACCAACCAGCTCACGGGGGAGCCCGAGCTCCACTAT

CTTCGGCTGCCCAAGGACATCAGTGACGACCACGTGATCCTGATGGACTGTACGGTGTCT

ACTGGCGCTGCGGCCATGATGGCCGTCCGTGTCCTCCTG

>CL218.Contig34_A-W 1 492 minus strand PREDICTED: uridine-cytidine kinase-like 1 [Saimiri boliviensis boliviensis]

CTGACCCAGCAGCAGCAGGAGCAGGCCGCCCACAACGACTTCAACTTTGACCACCCCGAC

GCCTTCGACTTCGACCTCATCATCTCCACCCTCAAGAAGCTGAAGCAGGGCAGGAGCGTC

CAAGTGCCCATCTATGACTTCACCACCCACAGCCGGAAGAAGGACTGGAAAACGCTCTAT

GGTGCAAACGTCATCATCTTCGAGGGTATCATGGCCTTCGCTGACAAGACGCTGCTGGAG

CTCCTGGACATGAGAATCTTTGTGGACACGGACTCCGACATCCGCTTAGTGCGGCGGCTG

CGCCGGGACATCAGTGAACGAGGCCGGGACATCGAGGGTGTCATCAAGCAGTACCACAAG

TTTGTCAAGCCTGCCTTTGACCAATACATCCAGCCCACCATGCGCCTGGCAGACATTGTG

GTGCCCAGAGGGAGTGGGAACGCGGTGGCCATTGACCTGATCGTGCAGCATGTGCACAGC

CAGCTGGAGGAG

>CL218.Contig35_A-W 87 905 minus strand PREDICTED: uridine-cytidine kinase-like 1 isoform 2 [Cavia porcellus]

GAGCAGAGGGAGCTGCGCTGGGATATGGCGGCCCTGGCCTCAGCGCACCAGTGCCACCCA

CTTCCCCAAACGCTGAGCGTCCTCAAGAGCACCCCGCAAGTGCGCGGTATGCACACCATC

ATCAGGGACAGGGAGACTAGTCGGGACGAGTTCATCTTCTACTCCAAGAGACTGATGCGG

CTGCTCATCGAGCATGCGCTTTCCTTCCTGCCCTTCCAGGACTGTGTGGTGCAGACCCCA

CAGGGGCAGGACTACGCAGGCAAGTGCTATGCGGGAAAGCAGGTATGTGCACCCTGGGAG

GGCGATACCTTAGCTGGGCTGGGTGTCCCATTGGCTCAGCCCTCCCTCCCCTAGATCACT

GGAGTATCCATCCTGCGTGCTGGGGAGACCATGGAGCCTGCTCTGCGTGCTGTGTGCAAA

GACGTGCGCATTGGCACCATCCTCATCCAGACCAACCAGCTCACGGGGGAGCCCGAGCTC

CACTATCTTCGGCTGCCCAAGGACATCAGTGACGACCACGTGATCCTGATGGACTGTACG

GTGTCTACTGGCGCTGCGGCCATGATGGCCGTCCGTGTCCTCCTGGACCACGATGTGCCC

GAGGACAAGATCTTCTTGCTGTCACTGCTGATGGCGGAGATGGGTGTCCACTCCGTGGCC

TATGCTTTCCCACGAGTGAGAATCATCACCACAGCTGTGGACAAGCGGGTCAATGACCTT

TTCCGCATCATCCCAGGCATAGGGAACTTTGGGGATCGCTACTTTGGGACAGATGCGGTC

CCTGATGGCAGCGACGAGGAAGAGGTGGCTTCCACTGGT

>CL218.Contig36_A-W 70 885 minus strand PREDICTED: uridine-cytidine kinase-like 1 isoform 2 [Cavia porcellus]

GAACACTCTTCACTGGCGAGCAGGGCGGCCCTGGCCTCAGCGCACCAGTGCCACCCACTT

CCCCAAACGCTGAGCGTCCTCAAGAGCACCCCGCAAGTGCGCGGTATGCACACCATCATC

AGGGACAGGGAGACTAGTCGGGACGAGTTCATCTTCTACTCCAAGAGACTGATGCGGCTG

CTCATCGAGCATGCGCTTTCCTTCCTGCCCTTCCAGGACTGTGTGGTGCAGACCCCACAG

GGGCAGGACTACGCAGGCAAGTGCTATGCGGGAAAGCAGGTATGTGCACCCTGGGAGGGC

GATACCTTAGCTGGGCTGGGTGTCCCATTGGCTCAGCCCTCCCTCCCCTAGATCACTGGA

GTATCCATCCTGCGTGCTGGGGAGACCATGGAGCCTGCTCTGCGTGCTGTGTGCAAAGAC

GTGCGCATTGGCACCATCCTCATCCAGACCAACCAGCTCACGGGGGAGCCCGAGCTCCAC

TATCTTCGGCTGCCCAAGGACATCAGTGACGACCACGTGATCCTGATGGACTGTACGGTG

TCTACTGGCGCTGCGGCCATGATGGCCGTCCGTGTCCTCCTGGACCACGATGTGCCCGAG

GACAAGATCTTCTTGCTGTCACTGCTGATGGCGGAGATGGGTGTCCACTCCGTGGCCTAT

GCTTTCCCACGAGTGAGAATCATCACCACAGCTGTGGACAAGCGGGTCAATGACCTTTTC

CGCATCATCCCAGGCATAGGGAACTTTGGGGATCGCTACTTTGGGACAGATGCGGTCCCT

GATGGCAGCGACGAGGAAGAGGTGGCTTCCACTGGT

>CL218.Contig37_A-W 87 848 minus strand PREDICTED: uridine-cytidine kinase-like 1 isoform 2 [Cavia porcellus]

GAGCAGAGGGAGCTGCGCTGGGATATGGCGGCCCTGGCCTCAGCGCACCAGTGCCACCCA

CTTCCCCAAACGCTGAGCGTCCTCAAGAGCACCCCGCAAGTGCGCGGTATGCACACCATC

ATCAGGGACAGGGAGACTAGTCGGGACGAGTTCATCTTCTACTCCAAGAGACTGATGCGG

CTGCTCATCGAGCATGCGCTTTCCTTCCTGCCCTTCCAGGACTGTGTGGTGCAGACCCCA

CAGGGGCAGGACTACGCAGGCAAGTGCTATGCGGGAAAGCAGCCCTCCCTCCCCTAGATC

ACTGGAGTATCCATCCTGCGTGCTGGGGAGACCATGGAGCCTGCTCTGCGTGCTGTGTGC

AAAGACGTGCGCATTGGCACCATCCTCATCCAGACCAACCAGCTCACGGGGGAGCCCGAG

CTCCACTATCTTCGGCTGCCCAAGGACATCAGTGACGACCACGTGATCCTGATGGACTGT

ACGGTGTCTACTGGCGCTGCGGCCATGATGGCCGTCCGTGTCCTCCTGGACCACGATGTG

CCCGAGGACAAGATCTTCTTGCTGTCACTGCTGATGGCGGAGATGGGTGTCCACTCCGTG

GCCTATGCTTTCCCACGAGTGAGAATCATCACCACAGCTGTGGACAAGCGGGTCAATGAC

CTTTTCCGCATCATCCCAGGCATAGGGAACTTTGGGGATCGCTACTTTGGGACAGATGCG

GTCCCTGATGGCAGCGACGAGGAAGAGGTGGCTTCCACTGGT

>CL218.Contig38_A-W 56 355 minus strand PREDICTED: uridine-cytidine kinase-like 1 isoform 1 [Cavia porcellus]

AGCCATGCAGGGTCCCTGGACAGGCTTCTCCCCACCGTGGGCACCAGGCGCTCGCCCCGG

AAGCGCACCACCAGCCAGTGCAAGTCGGAGCCACCCCTGCTGCGCACCAGCAAGCGCACC

ATCTACACGGCAGGGCGGCCACCCTGGTACAATGAGCATGGCACACAGTCCAAGGAGGCC

TTTGCCATCGGCCTGGGAGGTGGCAGTGCCTCGGGGAAGACCACCGTGGCCAGGATGATC

ATTGAGGCTCTAGATGTGCCCTGGGTGGTCTTGCTGTCCATGGACTCCTTCTACAAGGTG

>CL218.Contig39_A-W 1 354 minus strand PREDICTED: uridine-cytidine kinase-like 1 [Saimiri boliviensis boliviensis]

AAAACGCTCTATGGTGCAAACGTCATCATCTTCGAGGGTATCATGGCCTTCGCTGACAAG

ACGCTGCTGGAGCTCCTGGACATGAGAATCTTTGTGGACACGGACTCCGACATCCGCTTA

GTGCGGCGGCTGCGCCGGGACATCAGTGAACGAGGCCGGGACATCGAGGGTGTCATCAAG

CAGTACCACAAGTTTGTCAAGCCTGCCTTTGACCAATACATCCAGCCCACCATGCGCCTG

GCAGACATTGTGGTGCCCAGAGGGAGTGGGAACGCGGTGGCCATTGACCTGATCGTGCAG

CATGTGCACAGCCAGCTGGAGGAGCGTGAACTCAGCGTCAGAGGGAGCTGCGCT

>CL218.Contig40_A-W 87 833 minus strand PREDICTED: uridine-cytidine kinase-like 1 isoform 2 [Cavia porcellus]

GAGCAGAGGGAGCTGCGCTGGGATATGGCGGCCCTGGCCTCAGCGCACCAGTGCCACCCA

CTTCCCCAAACGCTGAGCGTCCTCAAGAGCACCCCGCAAGTGCGCGGTATGCACACCATC

ATCAGGGACAGGGAGACTAGTCGGGACGAGTTCATCTTCTACTCCAAGAGACTGATGCGG

CTGCTCATCGAGCATGCGCTTTCCTTCCTGCCCTTCCAGGACTGTGTGGTGCAGACCCCA

CAGGGGCAGGACTACGCAGGCAAGTGCTATGCGGGAAAGCAGATCACTGGAGTATCCATC

CTGCGTGCTGGGGAGACCATGGAGCCTGCTCTGCGTGCTGTGTGCAAAGACGTGCGCATT

GGCACCATCCTCATCCAGACCAACCAGCTCACGGGGGAGCCCGAGCTCCACTATCTTCGG

CTGCCCAAGGACATCAGTGACGACCACGTGATCCTGATGGACTGTACGGTGTCTACTGGC

GCTGCGGCCATGATGGCCGTCCGTGTCCTCCTGGACCACGATGTGCCCGAGGACAAGATC

TTCTTGCTGTCACTGCTGATGGCGGAGATGGGTGTCCACTCCGTGGCCTATGCTTTCCCA

CGAGTGAGAATCATCACCACAGCTGTGGACAAGCGGGTCAATGACCTTTTCCGCATCATC

CCAGGCATAGGGAACTTTGGGGATCGCTACTTTGGGACAGATGCGGTCCCTGATGGCAGC

GACGAGGAAGAGGTGGCTTCCACTGGT

>CL218.Contig41_A-W 70 828 minus strand PREDICTED: uridine-cytidine kinase-like 1 isoform 2 [Cavia porcellus]

GAACACTCTTCACTGGCGAGCAGGGCGGCCCTGGCCTCAGCGCACCAGTGCCACCCACTT

CCCCAAACGCTGAGCGTCCTCAAGAGCACCCCGCAAGTGCGCGGTATGCACACCATCATC

AGGGACAGGGAGACTAGTCGGGACGAGTTCATCTTCTACTCCAAGAGACTGATGCGGCTG

CTCATCGAGCATGCGCTTTCCTTCCTGCCCTTCCAGGACTGTGTGGTGCAGACCCCACAG

GGGCAGGACTACGCAGGCAAGTGCTATGCGGGAAAGCAGCCCTCCCTCCCCTAGATCACT

GGAGTATCCATCCTGCGTGCTGGGGAGACCATGGAGCCTGCTCTGCGTGCTGTGTGCAAA

GACGTGCGCATTGGCACCATCCTCATCCAGACCAACCAGCTCACGGGGGAGCCCGAGCTC

CACTATCTTCGGCTGCCCAAGGACATCAGTGACGACCACGTGATCCTGATGGACTGTACG

GTGTCTACTGGCGCTGCGGCCATGATGGCCGTCCGTGTCCTCCTGGACCACGATGTGCCC

GAGGACAAGATCTTCTTGCTGTCACTGCTGATGGCGGAGATGGGTGTCCACTCCGTGGCC

TATGCTTTCCCACGAGTGAGAATCATCACCACAGCTGTGGACAAGCGGGTCAATGACCTT

TTCCGCATCATCCCAGGCATAGGGAACTTTGGGGATCGCTACTTTGGGACAGATGCGGTC

CCTGATGGCAGCGACGAGGAAGAGGTGGCTTCCACTGGT

>CL218.Contig42_A-W 1 354 minus strand PREDICTED: uridine-cytidine kinase-like 1 [Saimiri boliviensis boliviensis]

AAAACGCTCTATGGTGCAAACGTCATCATCTTCGAGGGTATCATGGCCTTCGCTGACAAG

ACGCTGCTGGAGCTCCTGGACATGAGAATCTTTGTGGACACGGACTCCGACATCCGCTTA

GTGCGGCGGCTGCGCCGGGACATCAGTGAACGAGGCCGGGACATCGAGGGTGTCATCAAG

CAGTACCACAAGTTTGTCAAGCCTGCCTTTGACCAATACATCCAGCCCACCATGCGCCTG

GCAGACATTGTGGTGCCCAGAGGGAGTGGGAACGCGGTGGCCATTGACCTGATCGTGCAG

CATGTGCACAGCCAGCTGGAGGAGCGTGAACTCAGCGTCAGAGGGAGCTGCGCT

>CL218.Contig43_A-W 1 846 minus strand PREDICTED: uridine-cytidine kinase-like 1 isoform 2 [Cavia porcellus]

AAAACGCTCTATGGTGCAAACGTCATCATCTTCGAGGGTATCATGGCCTTCGCTGACAAG

ACGCTGCTGGAGCTCCTGGACATGAGAATCTTTGTGGACACGGACTCCGACATCCGCTTA

GTGCGGCGGCTGCGCCGGGACATCAGTGAACGAGGCCGGGACATCGAGGGTGTCATCAAG

CAGTACCACAAGTTTGTCAAGCCTGCCTTTGACCAATACATCCAGCCCACCATGCGCCTG

GCAGACATTGTGGTGCCCAGAGGGAGTGGGAACGCGGTGGCCATTGACCTGATCGTGCAG

CATGTGCACAGCCAGCTGGAGGAGAGGGAGCTGCGCTGGGATATGGCGGCCCTGGCCTCA

GCGCACCAGTGCCACCCACTTCCCCAAACGCTGAGCGTCCTCAAGAGCACCCCGCAAGTG

CGCGGTATGCACACCATCATCAGGGACAGGGAGACTAGTCGGGACGAGTTCATCTTCTAC

TCCAAGAGACTGATGCGGCTGCTCATCGAGCATGCGCTTTCCTTCCTGCCCTTCCAGGAC

TGTGTGGTGCAGACCCCACAGGGGCAGGACTACGCAGGCAAGTGCTATGCGGGAAAGCAG

CCCTCCCTCCCCTAGATCACTGGAGTATCCATCCTGCGTGCTGGGGAGACCATGGAGCCT

GCTCTGCGTGCTGTGTGCAAAGACGTGCGCATTGGCACCATCCTCATCCAGACCAACCAG

CTCACGGGGGAGCCCGAGCTCCACTATCTTCGGCTGCCCAAGGACATCAGTGACGACCAC

GTGATCCTGATGGACTGTACGGTGTCTACTGGCGCTGCGGCCATGATGGCCGTCCGTGTC

CTCCTG

>CL218.Contig44_A-W 3 302 minus strand PREDICTED: uridine-cytidine kinase-like 1 isoform 1 [Cavia porcellus]

AGCCATGCAGGGTCCCTGGACAGGCTTCTCCCCACCGTGGGCACCAGGCGCTCGCCCCGG

AAGCGCACCACCAGCCAGTGCAAGTCGGAGCCACCCCTGCTGCGCACCAGCAAGCGCACC

ATCTACACGGCAGGGCGGCCACCCTGGTACAATGAGCATGGCACACAGTCCAAGGAGGCC

TTTGCCATCGGCCTGGGAGGTGGCAGTGCCTCGGGGAAGACCACCGTGGCCAGGATGATC

ATTGAGGCTCTAGATGTGCCCTGGGTGGTCTTGCTGTCCATGGACTCCTTCTACAAGGTG

>CL218.Contig45_A-W 70 813 minus strand PREDICTED: uridine-cytidine kinase-like 1 isoform 2 [Cavia porcellus]

GAACACTCTTCACTGGCGAGCAGGGCGGCCCTGGCCTCAGCGCACCAGTGCCACCCACTT

CCCCAAACGCTGAGCGTCCTCAAGAGCACCCCGCAAGTGCGCGGTATGCACACCATCATC

AGGGACAGGGAGACTAGTCGGGACGAGTTCATCTTCTACTCCAAGAGACTGATGCGGCTG

CTCATCGAGCATGCGCTTTCCTTCCTGCCCTTCCAGGACTGTGTGGTGCAGACCCCACAG

GGGCAGGACTACGCAGGCAAGTGCTATGCGGGAAAGCAGATCACTGGAGTATCCATCCTG

CGTGCTGGGGAGACCATGGAGCCTGCTCTGCGTGCTGTGTGCAAAGACGTGCGCATTGGC

ACCATCCTCATCCAGACCAACCAGCTCACGGGGGAGCCCGAGCTCCACTATCTTCGGCTG

CCCAAGGACATCAGTGACGACCACGTGATCCTGATGGACTGTACGGTGTCTACTGGCGCT

GCGGCCATGATGGCCGTCCGTGTCCTCCTGGACCACGATGTGCCCGAGGACAAGATCTTC

TTGCTGTCACTGCTGATGGCGGAGATGGGTGTCCACTCCGTGGCCTATGCTTTCCCACGA

GTGAGAATCATCACCACAGCTGTGGACAAGCGGGTCAATGACCTTTTCCGCATCATCCCA

GGCATAGGGAACTTTGGGGATCGCTACTTTGGGACAGATGCGGTCCCTGATGGCAGCGAC

GAGGAAGAGGTGGCTTCCACTGGT

>CL218.Contig46_A-W 1 324 minus strand PREDICTED: uridine-cytidine kinase-like 1 isoform 1 [Cavia porcellus]

AAAACGCTCTATGGTGCAAACGTCATCATCTTCGAGGGTATCATGGCCTTCGCTGACAAG

ACGCTGCTGGAGCTCCTGGACATGAGAATCTTTGTGGACACGGACTCCGACATCCGCTTA

GTGCGGCGGCTGCGCCGGGACATCAGTGAACGAGGCCGGGACATCGAGGGTGTCATCAAG

CAGTACCACAAGTTTGTCAAGCCTGCCTTTGACCAATACATCCAGCCCACCATGCGCCTG

GCAGACATTGTGGTGCCCAGAGGGAGTGGGAACGCGGTGGCCATTGACCTGATCGTGCAG

CATGTGCACAGCCAGCTGGAGGAG

>CL218.Contig47_A-W 84 614 minus strand hypothetical protein EGK_02170 [Macaca mulatta]

GGCGAGCAGAGGGAGCTGCGCTGGGATATGGCGGCCCTGGCCTCAGCGCACCAGTGCCAC

CCACTTCCCCAAACGCTGAGCGTCCTCAAGAGCACCCCGCAAGTGCGCGGTATGCACACC

ATCATCAGGGACAGGGAGACTAGTCGGGACGAGTTCATCTTCTACTCCAAGAGACTGATG

CGGCTGCTCATCGAGCATGCGCTTTCCTTCCTGCCCTTCCAGGACTGTGTGGTGCAGACC

CCACAGGGGCAGGACTACGCAGGCAAGTGCTATGCGGGAAAGCAGCCCTCCCTCCCCTAG

ATCACTGGAGTATCCATCCTGCGTGCTGGGGAGACCATGGAGCCTGCTCTGCGTGCTGTG

TGCAAAGACGTGCGCATTGGCACCATCCTCATCCAGACCAACCAGCTCACGGGGGAGCCC

GAGCTCCACTATCTTCGGCTGCCCAAGGACATCAGTGACGACCACGTGATCCTGATGGAC

TGTACGGTGTCTACTGGCGCTGCGGCCATGATGGCCGTCCGTGTCCTCCTG

>CL218.Contig48_A-W 84 599 minus strand hypothetical protein EGK_02170 [Macaca mulatta]

GGCGAGCAGAGGGAGCTGCGCTGGGATATGGCGGCCCTGGCCTCAGCGCACCAGTGCCAC

CCACTTCCCCAAACGCTGAGCGTCCTCAAGAGCACCCCGCAAGTGCGCGGTATGCACACC

ATCATCAGGGACAGGGAGACTAGTCGGGACGAGTTCATCTTCTACTCCAAGAGACTGATG

CGGCTGCTCATCGAGCATGCGCTTTCCTTCCTGCCCTTCCAGGACTGTGTGGTGCAGACC

CCACAGGGGCAGGACTACGCAGGCAAGTGCTATGCGGGAAAGCAGATCACTGGAGTATCC

ATCCTGCGTGCTGGGGAGACCATGGAGCCTGCTCTGCGTGCTGTGTGCAAAGACGTGCGC

ATTGGCACCATCCTCATCCAGACCAACCAGCTCACGGGGGAGCCCGAGCTCCACTATCTT

CGGCTGCCCAAGGACATCAGTGACGACCACGTGATCCTGATGGACTGTACGGTGTCTACT

GGCGCTGCGGCCATGATGGCCGTCCGTGTCCTCCTG

>CL218.Contig49_A-W 3 302 minus strand PREDICTED: uridine-cytidine kinase-like 1 isoform 1 [Cavia porcellus]

AGCCATGCAGGGTCCCTGGACAGGCTTCTCCCCACCGTGGGCACCAGGCGCTCGCCCCGG

AAGCGCACCACCAGCCAGTGCAAGTCGGAGCCACCCCTGCTGCGCACCAGCAAGCGCACC

ATCTACACGGCAGGGCGGCCACCCTGGTACAATGAGCATGGCACACAGTCCAAGGAGGCC

TTTGCCATCGGCCTGGGAGGTGGCAGTGCCTCGGGGAAGACCACCGTGGCCAGGATGATC

ATTGAGGCTCTAGATGTGCCCTGGGTGGTCTTGCTGTCCATGGACTCCTTCTACAAGGTG

>CL218.Contig50_A-W 70 594 minus strand Uridine-cytidine kinase-like 1 [Heterocephalus glaber]

GAACACTCTTCACTGGCGAGCAGGGCGGCCCTGGCCTCAGCGCACCAGTGCCACCCACTT

CCCCAAACGCTGAGCGTCCTCAAGAGCACCCCGCAAGTGCGCGGTATGCACACCATCATC

AGGGACAGGGAGACTAGTCGGGACGAGTTCATCTTCTACTCCAAGAGACTGATGCGGCTG

CTCATCGAGCATGCGCTTTCCTTCCTGCCCTTCCAGGACTGTGTGGTGCAGACCCCACAG

GGGCAGGACTACGCAGGCAAGTGCTATGCGGGAAAGCAGCCCTCCCTCCCCTAGATCACT

GGAGTATCCATCCTGCGTGCTGGGGAGACCATGGAGCCTGCTCTGCGTGCTGTGTGCAAA

GACGTGCGCATTGGCACCATCCTCATCCAGACCAACCAGCTCACGGGGGAGCCCGAGCTC

CACTATCTTCGGCTGCCCAAGGACATCAGTGACGACCACGTGATCCTGATGGACTGTACG

GTGTCTACTGGCGCTGCGGCCATGATGGCCGTCCGTGTCCTCCTG

>CL218.Contig51_A-W 70 579 minus strand Uridine-cytidine kinase-like 1 [Heterocephalus glaber]

GAACACTCTTCACTGGCGAGCAGGGCGGCCCTGGCCTCAGCGCACCAGTGCCACCCACTT

CCCCAAACGCTGAGCGTCCTCAAGAGCACCCCGCAAGTGCGCGGTATGCACACCATCATC

AGGGACAGGGAGACTAGTCGGGACGAGTTCATCTTCTACTCCAAGAGACTGATGCGGCTG

CTCATCGAGCATGCGCTTTCCTTCCTGCCCTTCCAGGACTGTGTGGTGCAGACCCCACAG

GGGCAGGACTACGCAGGCAAGTGCTATGCGGGAAAGCAGATCACTGGAGTATCCATCCTG

CGTGCTGGGGAGACCATGGAGCCTGCTCTGCGTGCTGTGTGCAAAGACGTGCGCATTGGC

ACCATCCTCATCCAGACCAACCAGCTCACGGGGGAGCCCGAGCTCCACTATCTTCGGCTG

CCCAAGGACATCAGTGACGACCACGTGATCCTGATGGACTGTACGGTGTCTACTGGCGCT

GCGGCCATGATGGCCGTCCGTGTCCTCCTG

>CL218.Contig52_A-W 56 355 minus strand PREDICTED: uridine-cytidine kinase-like 1 isoform 1 [Cavia porcellus]

AGCCATGCAGGGTCCCTGGACAGGCTTCTCCCCACCGTGGGCACCAGGCGCTCGCCCCGG

AAGCGCACCACCAGCCAGTGCAAGTCGGAGCCACCCCTGCTGCGCACCAGCAAGCGCACC

ATCTACACGGCAGGGCGGCCACCCTGGTACAATGAGCATGGCACACAGTCCAAGGAGGCC

TTTGCCATCGGCCTGGGAGGTGGCAGTGCCTCGGGGAAGACCACCGTGGCCAGGATGATC

ATTGAGGCTCTAGATGTGCCCTGGGTGGTCTTGCTGTCCATGGACTCCTTCTACAAGGTG

>CL218.Contig53_A-W 35 619 minus strand PREDICTED: uridine-cytidine kinase-like 1 isoform 1 [Cavia porcellus]

ATGGCTGCGCCACCGGCCTCCGCGGACGCCGCCCCCTCGTCCCCGCCGCCTCCTGCGGCC

CCGGACGCGCCCGGAGCCTGCGCGGGAGGACAGAGCGAGACCGCGTGCGAGGACCGCAGC

CATGCAGGGTCCCTGGACAGGCTTCTCCCCACCGTGGGCACCAGGCGCTCGCCCCGGAAG

CGCACCACCAGCCAGTGCAAGTCGGAGCCACCCCTGCTGCGCACCAGCAAGCGCACCATC

TACACGGCAGGGCGGCCACCCTGGTACAATGAGCATGGCACACAGTCCAAGGAGGCCTTT

GCCATCGGCCTGGGAGGTGGCAGTGCCTCGGGGAAGACCACCGTGGCCAGGATGATCATT

GAGGCTCTAGATGTGCCCTGGGTGGTCTTGCTGTCCATGGACTCCTTCTACAAGGTGCTG

ACCCAGCAGCAGCAGGAGCAGGCCGCCCACAACGACTTCAACTTTGACCACCCCGACGCC

TTCGACTTCGACCTCATCATCTCCACCCTCAAGAAGCTGAAGCAGGGCAGGAGCGTCCAA

GTGCCCATCTATGACTTCACCACCCACAGCCGGAAGAAGGACTGG

>CL218.Contig54_A-W 694 1017 minus strand PREDICTED: uridine-cytidine kinase-like 1 isoform 1 [Cavia porcellus]

AAAACGCTCTATGGTGCAAACGTCATCATCTTCGAGGGTATCATGGCCTTCGCTGACAAG

ACGCTGCTGGAGCTCCTGGACATGAGAATCTTTGTGGACACGGACTCCGACATCCGCTTA

GTGCGGCGGCTGCGCCGGGACATCAGTGAACGAGGCCGGGACATCGAGGGTGTCATCAAG

CAGTACCACAAGTTTGTCAAGCCTGCCTTTGACCAATACATCCAGCCCACCATGCGCCTG

GCAGACATTGTGGTGCCCAGAGGGAGTGGGAACGCGGTGGCCATTGACCTGATCGTGCAG

CATGTGCACAGCCAGCTGGAGGAG

>CL218.Contig55_A-W 694 1017 minus strand PREDICTED: uridine-cytidine kinase-like 1 isoform 1 [Cavia porcellus]

AAAACGCTCTATGGTGCAAACGTCATCATCTTCGAGGGTATCATGGCCTTCGCTGACAAG

ACGCTGCTGGAGCTCCTGGACATGAGAATCTTTGTGGACACGGACTCCGACATCCGCTTA

GTGCGGCGGCTGCGCCGGGACATCAGTGAACGAGGCCGGGACATCGAGGGTGTCATCAAG

CAGTACCACAAGTTTGTCAAGCCTGCCTTTGACCAATACATCCAGCCCACCATGCGCCTG

GCAGACATTGTGGTGCCCAGAGGGAGTGGGAACGCGGTGGCCATTGACCTGATCGTGCAG

CATGTGCACAGCCAGCTGGAGGAG

>CL218.Contig56_A-W 942 1736 minus strand PREDICTED: uridine-cytidine kinase-like 1 isoform 1 [Cavia porcellus]

AGGGCGGCCCTGGCCTCAGCGCACCAGTGCCACCCACTTCCCCAAACGCTGAGCGTCCTC

AAGAGCACCCCGCAAGTGCGCGGTATGCACACCATCATCAGGGACAGGGAGACTAGTCGG

GACGAGTTCATCTTCTACTCCAAGAGACTGATGCGGCTGCTCATCGAGCATGCGCTTTCC

TTCCTGCCCTTCCAGGACTGTGTGGTGCAGACCCCACAGGGGCAGGACTACGCAGGCAAG

TGCTATGCGGGAAAGCAGGTATGTGCACCCTGGGAGGGCGATACCTTAGCTGGGCTGGGT

GTCCCATTGGCTCAGCCCTCCCTCCCCTAGATCACTGGAGTATCCATCCTGCGTGCTGGG

GAGACCATGGAGCCTGCTCTGCGTGCTGTGTGCAAAGACGTGCGCATTGGCACCATCCTC

ATCCAGACCAACCAGCTCACGGGGGAGCCCGAGCTCCACTATCTTCGGCTGCCCAAGGAC

ATCAGTGACGACCACGTGATCCTGATGGACTGTACGGTGTCTACTGGCGCTGCGGCCATG

ATGGCCGTCCGTGTCCTCCTGGACCACGATGTGCCCGAGGACAAGATCTTCTTGCTGTCA

CTGCTGATGGCGGAGATGGGTGTCCACTCCGTGGCCTATGCTTTCCCACGAGTGAGAATC

ATCACCACAGCTGTGGACAAGCGGGTCAATGACCTTTTCCGCATCATCCCAGGCATAGGG

AACTTTGGGGATCGCTACTTTGGGACAGATGCGGTCCCTGATGGCAGCGACGAGGAAGAG

GTGGCTTCCACTGGT

>CL218.Contig57_A-W 53 355 minus strand PREDICTED: uridine-cytidine kinase-like 1 isoform 2 [Cavia porcellus]

AGCAGCCATGCAGGGTCCCTGGACAGGCTTCTCCCCACCGTGGGCACCAGGCGCTCGCCC

CGGAAGCGCACCACCAGCCAGTGCAAGTCGGAGCCACCCCTGCTGCGCACCAGCAAGCGC

ACCATCTACACGGCAGGGCGGCCACCCTGGTACAATGAGCATGGCACACAGTCCAAGGAG

GCCTTTGCCATCGGCCTGGGAGGTGGCAGTGCCTCGGGGAAGACCACCGTGGCCAGGATG

ATCATTGAGGCTCTAGATGTGCCCTGGGTGGTCTTGCTGTCCATGGACTCCTTCTACAAG

GTG

>CL218.Contig58_A-W 3 302 minus strand PREDICTED: uridine-cytidine kinase-like 1 isoform 1 [Cavia porcellus]

AGCCATGCAGGGTCCCTGGACAGGCTTCTCCCCACCGTGGGCACCAGGCGCTCGCCCCGG

AAGCGCACCACCAGCCAGTGCAAGTCGGAGCCACCCCTGCTGCGCACCAGCAAGCGCACC

ATCTACACGGCAGGGCGGCCACCCTGGTACAATGAGCATGGCACACAGTCCAAGGAGGCC

TTTGCCATCGGCCTGGGAGGTGGCAGTGCCTCGGGGAAGACCACCGTGGCCAGGATGATC

ATTGAGGCTCTAGATGTGCCCTGGGTGGTCTTGCTGTCCATGGACTCCTTCTACAAGGTG

>CL218.Contig59_A-W 56 355 minus strand PREDICTED: uridine-cytidine kinase-like 1 isoform 1 [Cavia porcellus]

AGCCATGCAGGGTCCCTGGACAGGCTTCTCCCCACCGTGGGCACCAGGCGCTCGCCCCGG

AAGCGCACCACCAGCCAGTGCAAGTCGGAGCCACCCCTGCTGCGCACCAGCAAGCGCACC

ATCTACACGGCAGGGCGGCCACCCTGGTACAATGAGCATGGCACACAGTCCAAGGAGGCC

TTTGCCATCGGCCTGGGAGGTGGCAGTGCCTCGGGGAAGACCACCGTGGCCAGGATGATC

ATTGAGGCTCTAGATGTGCCCTGGGTGGTCTTGCTGTCCATGGACTCCTTCTACAAGGTG

>CL218.Contig60_A-W 242 964 minus strand PREDICTED: uridine-cytidine kinase-like 1 isoform 2 [Cavia porcellus]

AGGGCGGCCCTGGCCTCAGCGCACCAGTGCCACCCACTTCCCCAAACGCTGAGCGTCCTC

AAGAGCACCCCGCAAGTGCGCGGTATGCACACCATCATCAGGGACAGGGAGACTAGTCGG

GACGAGTTCATCTTCTACTCCAAGAGACTGATGCGGCTGCTCATCGAGCATGCGCTTTCC

TTCCTGCCCTTCCAGGACTGTGTGGTGCAGACCCCACAGGGGCAGGACTACGCAGGCAAG

TGCTATGCGGGAAAGCAGATCACTGGAGTATCCATCCTGCGTGCTGGGGAGACCATGGAG

CCTGCTCTGCGTGCTGTGTGCAAAGACGTGCGCATTGGCACCATCCTCATCCAGACCAAC

CAGCTCACGGGGGAGCCCGAGCTCCACTATCTTCGGCTGCCCAAGGACATCAGTGACGAC

CACGTGATCCTGATGGACTGTACGGTGTCTACTGGCGCTGCGGCCATGATGGCCGTCCGT

GTCCTCCTGGACCACGATGTGCCCGAGGACAAGATCTTCTTGCTGTCACTGCTGATGGCG

GAGATGGGTGTCCACTCCGTGGCCTATGCTTTCCCACGAGTGAGAATCATCACCACAGCT

GTGGACAAGCGGGTCAATGACCTTTTCCGCATCATCCCAGGCATAGGGAACTTTGGGGAT

CGCTACTTTGGGACAGATGCGGTCCCTGATGGCAGCGACGAGGAAGAGGTGGCTTCCACT

GGT

>CL218.Contig61_A-W 462 1256 minus strand PREDICTED: uridine-cytidine kinase-like 1 isoform 2 [Cavia porcellus]

AGGGCGGCCCTGGCCTCAGCGCACCAGTGCCACCCACTTCCCCAAACGCTGAGCGTCCTC

AAGAGCACCCCGCAAGTGCGCGGTATGCACACCATCATCAGGGACAGGGAGACTAGTCGG

GACGAGTTCATCTTCTACTCCAAGAGACTGATGCGGCTGCTCATCGAGCATGCGCTTTCC

TTCCTGCCCTTCCAGGACTGTGTGGTGCAGACCCCACAGGGGCAGGACTACGCAGGCAAG

TGCTATGCGGGAAAGCAGGTATGTGCACCCTGGGAGGGCGATACCTTAGCTGGGCTGGGT

GTCCCATTGGCTCAGCCCTCCCTCCCCTAGATCACTGGAGTATCCATCCTGCGTGCTGGG

GAGACCATGGAGCCTGCTCTGCGTGCTGTGTGCAAAGACGTGCGCATTGGCACCATCCTC

ATCCAGACCAACCAGCTCACGGGGGAGCCCGAGCTCCACTATCTTCGGCTGCCCAAGGAC

ATCAGTGACGACCACGTGATCCTGATGGACTGTACGGTGTCTACTGGCGCTGCGGCCATG

ATGGCCGTCCGTGTCCTCCTGGACCACGATGTGCCCGAGGACAAGATCTTCTTGCTGTCA

CTGCTGATGGCGGAGATGGGTGTCCACTCCGTGGCCTATGCTTTCCCACGAGTGAGAATC

ATCACCACAGCTGTGGACAAGCGGGTCAATGACCTTTTCCGCATCATCCCAGGCATAGGG

AACTTTGGGGATCGCTACTTTGGGACAGATGCGGTCCCTGATGGCAGCGACGAGGAAGAG

GTGGCTTCCACTGGT

>CL218.Contig62_A-W 74 454 minus strand PREDICTED: uridine-cytidine kinase-like 1 isoform 2 [Cavia porcellus]

TCGTCCCCGCCGCCTCCTGCGGCCCCGGACGCGCCCGGAGCCTGCGCGGGAGGACAGAGC

GAGACCGCGTGCGAGGACCGCAGCCATGCAGGGTCCCTGGACAGGCTTCTCCCCACCGTG

GGCACCAGGCGCTCGCCCCGGAAGCGCACCACCAGCCAGTGCAAGTCGGAGCCACCCCTG

CTGCGCACCAGCAAGCGCACCATCTACACGGCAGGGCGGCCACCCTGGTACAATGAGCAT

GGCACACAGTCCAAGGAGGCCTTTGCCATCGGCCTGGGAGGTGGCAGTGCCTCGGGGAAG

ACCACCGTGGCCAGGATGATCATTGAGGCTCTAGATGTGCCCTGGGTGGTCTTGCTGTCC

ATGGACTCCTTCTACAAGGTG

>CL219.Contig1_A-W 3 626 minus strand PREDICTED: COBW domain-containing protein 3-like isoform 3 [Macaca mulatta]

GTTGTAGATTCAAAATATGGATTAAAACATTTAACAGAAGAAAAACCTGATGGCCTAATC

AGTGAAGCTACTAGGCAAGTTGCTTTGGCAGATATCATTCTCATCAATAAAACAGACTTG

ATTTCAGAAGAGGAGCTAAACAAATTAAGAGCAATCATTAGATCAATAAATGGACTGGGA

AAAATTTTAGAAACACAAAGATCAAGTTTGCAGAAAAAACTTCAACATGTGTCAGCCACA

CAATCTCACCTTGATCAGAGTATTGTTACAATCACATTTGAAGTACCAGGAAACGCGAAG

GAAGACAGTCTAAATATATTTATTCAGAATCTCCTGTGGGAAAAGAATGTAAGAAACAAG

GACAATCGCTGCATGGAAGTCATACGACTTAAGGGGTTGGTGTCGATCAAAGACAAACCA

CAACAAGTGATTGTCCAAGGTGTCCATGAACTCTATGACCTGGAAGAGACTCCAGTGAGC

TGGGAAGATGACACTGAGAGAACCAATCGATTAGTCTTTATTGGCAGGAATTTAGATAAG

AATATTCTTAAACAACTATTTATTGCTACAGTGACAGAAACAGAAAAGCAGTGGACAACA

CAGTTCAAAGAAGATCAAGTATGT

>CL219.Contig2_A-W 3 806 minus strand Cobalamin synthase W domain-containing protein 3 [Macaca fascicularis]

AAGGGGAAATTTGATTACATACTGTTAGAGACTACTGGGTTAGCAGATCCTGGTGCTGTG

GCTTCTATGTTTTGGGTTGATGCTGAATTAGGGAGTGACATTTACCTTGATGGTATCATA

ACTGTTGTAGATTCAAAATATGGATTAAAACATTTAACAGAAGAAAAACCTGATGGCCTA

ATCAGTGAAGCTACTAGGCAAGTTGCTTTGGCAGATATCATTCTCATCAATAAAACAGAC

TTGATTTCAGAAGAGGAGCTAAACAAATTAAGAGCAATCATTAGATCAATAAATGGACTG

GGAAAAATTTTAGAAACACAAAGATCAAGAGTTGATCTCTCTAATGTATTAGATCTTCAT

GCCTTTGATAGTCTCTCTGGAATAAGTTTGCAGAAAAAACTTCAACATGTGTCAGCCACA

CAATCTCACCTTGATCAGAGTATTGTTACAATCACATTTGAAGTACCAGGAAACGCGAAG

GAAGACAGTCTAAATATATTTATTCAGAATCTCCTGTGGGAAAAGAATGTAAGAAACAAG

GACAATCGCTGCATGGAAGTCATACGACTTAAGGGGTTGGTGTCGATCAAAGACAAACCA

CAACAAGTGATTGTCCAAGGTGTCCATGAACTCTATGACCTGGAAGAGACTCCAGTGAGC

TGGGAAGATGACACTGAGAGAACCAATCGATTAGTCTTTATTGGCAGGAATTTAGATAAG

AATATTCTTAAACAACTATTTATTGCTACAGTGACAGAAACAGAAAAGCAGTGGACAACA

CAGTTCAAAGAAGATCAAGTATGT

>CL223.Contig1_A-W 3 458 minus strand seipin [Macaca mulatta] >gi|384946418|gb|AFI36814.1| seipin isoform 1 [Macaca mulatta]

ATGCAGTGGGTGTGGGGGGGCATCTGGCCCCGACATCGCTTCTCTTTGCAGGTTAATATC

CGAAAAAGAGAGACTTCCCAGAAGGAAGGCCAACGAAGGATCTCTACTCATCAGCCTGGT

AAAGGGCCTGAAGGACAGGAGGAGTCAACTCAACTGTCAGATGTGACAGAAGATGGTGAG

AGCCCTGAAGATCCCTCAGGGACAGAGGGCCAGCTGTCTGAGGAGGAGAAGCCAGACCAG

CAGCCCCTGAGTGGAGAGGAGGAGCTGGAGCGGGAGGCCAGTGATGGTTCAGGCTCCTGG

GAAGATGCTGCTCTGCTGACTGACGCCAACCTGCCTGCTTCTGCCTCTGCCTCTGCTTCT

GCCCCTGCGTCTGCCCCTGCCCCTGCCCCTGAGACTCTGGGCAGCTCTGAGCCCTCTATG

GGTGCTCTGAGACAGCGCCCCACCTGCTCCAGCTCC

>CL223.Contig2_A-W 69 239 minus strand seipin [Macaca mulatta] >gi|384946418|gb|AFI36814.1| seipin isoform 1 [Macaca mulatta]

GGTTCAGGCTCCTGGGAAGATGCTGCTCTGCTGACTGACGCCAACCTGCCTGCTTCTGCC

TCTGCCTCTGCTTCTGCCCCTGCGTCTGCCCCTGCCCCTGCCCCTGAGACTCTGGGCAGC

TCTGAGCCCTCTATGGGTGCTCTGAGACAGCGCCCCACCTGCTCCAGCTCC

>CL224.Contig1_A-W 40 351 minus strand Phosphoacetylglucosamine mutase [Heterocephalus glaber]

CAGGCAGCTGGTGATGCTATTGCTGACATGCTGGTGATTGAGGCAATCCTGGCTTTAAAG

AACCTGACTATAGAACAGTGGGATGCTCTCTATACTGACCTTCCGAATAGACAACTCAAA

GTTAAGGTTGCTGACAGGAAAGTTATTAGCACCACAGATGCTGAAAGACAAGCAGTTACA

CCGCCTGGACTACAGGAGGCAATTAATGACCTGGTAAAGAAGTACAGGCTCTCTAGAGCG

TTTGTCCGGCCCTCTGGTACAGAAGATGTCATCCGAGTGTATGCAGAAGCAGATTCACAA

GTAAGCTGGGAT

>CL224.Contig2_A-W 2 778 minus strand PREDICTED: phosphoacetylglucosamine mutase [Sus scrofa]

TATTACTACTGTGATGCTGATGGTCATTTTCATCTCATAGATGGAGACAAGATAGCAACA

TTAATTAGCAGCTTCCTTAAAGAGCTCCTGTTGGAGATTGGAGAAAATTTGAATGTTGGG

GTTGTACAAACTGCATATGCAAATGGAAGTTCAACACGCTATCTTGAAGAAGTTATGAAG

GTACCTGTTTACTGCACTAAAACCGGTGTAAAACATTTGCACCACAAGGCTCAAGAGTTT

GACATTGGAGTTTATTTTGAAGCAAATGGGCATGGAACAGCGCTGTTTAGTAAAGCTGTT

GAAGACAAGATAAACCAACTTGCAAGAGAATTAGAAGATAAGAAAGGAAAAGCTGCTAAG

ATTCTTAGAAATATCATTGACTTGTTTAACCAGGCAGCTGGTGATGCTATTGCTGACATG

CTGGTGATTGAGGCAATCCTGGCTTTAAAGAACCTGACTATAGAACAGTGGGATGCTCTC

TATACTGACCTTCCGAATAGACAACTCAAAGTTAAGGTTGCTGACAGGAAAGTTATTAGC

ACCACAGATGCTGAAAGACAAGCAGTTACACCGCCTGGACTACAGGAGGCAATTAATGAC

CTGGTAAAGAAGTACAGGCTCTCTAGAGCGTTTGTCCGGCCCTCTGGTACAGAAGATGTC

ATCCGAGTGTATGCAGAAGCAGATTCACAAGAAAGTGCTGATAGCCTCGCGCATGAAGTG

AGCTTGGCAGTGTTTGATCTGGCTGGAGGAATTGGAGAAAGACCTCAACCAGGTTTC

>CL225.Contig1_A-W 44 322 minus strand NADH dehydrogenase, quinone 2 [Mustela putorius furo]

CTGCAGGATAAACTAGCCCTCCTTTCCTTAACCACGGGAGGCACAGCCGAGATGTACTCA

AAAGCAGGGGTCAGTGGAGATTTTCGGTACTTTCTGTGGCCACTCCAGCATGGCGCTTTG

CACTTCTGTGGATTTAAAATTCTTGCCCCTCAGATCAGTTTTGCTCCTGAGTTTTCATCA

GAAGAAGAAAGAAAAGCAATGGTGGCATCTTGGGCCCAACGATTAAAGAGCATCTGGGAG

GAAGAGCCCATCCGCTGCACACCCCCTTGGTACTTTGGG

>CL225.Contig2_A-W 209 985 minus strand PREDICTED: ribosyldihydronicotinamide dehydrogenase [quinone] [Otolemur garnettii]

TGGGATCACCAAAGAGAGTGACTGCACCTGAAAGCCTGGGGCCGCCACACCAAATCAGAG

GAAAAAAATCAGTCTTCTTCGGGCATGGCAGGTAAGAAAGTGCTCATTGTCTATGCACAC

CAAGAACCCAAGTCTTTCAATGGCTCCTTGAAGAAAGTGGCAGTGGATGAATTGAGCCAG

CAGGGATGCACCGTCACCGTGTCTGATTTGTATGCCATGAACTTTGAGCCAAGGGCCACA

AGGAAAGATATCACTGGTGACCTTTCTAATCCTGAGGTCTTCAGTTATGGGGTGGAAGCC

TATGAAGCCTACAAGAAAAACTGTCTAACCAGTGACATCACTGAGGAGCAGAAAAAGGTG

CAGGAAGCTGATCTCGTGATATTCCAGTTTCCGCTGTACTGGTTCAGCGTGCCGGCTATC

CTGAAGGGCTGGATGGACAGGGTGCTATGCCAAGGGTTCGCCTTCGACATCCCAGGGTTC

TATGATTCTGGTTTTCTCAAGGATAAACTAGCCCTCCTTTCCTTAACCACGGGAGGCACA

GCCGAGATGTACTCAAAAGCAGGGGTCAGTGGAGATTTTCGGTACTTTCTGTGGCCACTC

CAGCATGGCGCTTTGCACTTCTGTGGATTTAAAATTCTTGCCCCTCAGATCAGTTTTGCT

CCTGAGTTTTCATCAGAAGAAGAAAGAAAAGCAATGGTGGCATCTTGGGCCCAACGATTA

AAGAGCATCTGGGAGGAAGAGCCCATCCGCTGCACACCCCCTTGGTACTTTGGGGAA

>CL225.Contig3_A-W 187 963 minus strand PREDICTED: ribosyldihydronicotinamide dehydrogenase [quinone] [Otolemur garnettii]

TGGGATCACCAAAGAGAGTGACTGCACCTGAAAGCCTGGGGCCGCCACACCAAATCAGAG

GAAAAAAATCAGTCTTCTTCGGGCATGGCAGGTAAGAAAGTGCTCATTGTCTATGCACAC

CAAGAACCCAAGTCTTTCAATGGCTCCTTGAAGAAAGTGGCAGTGGATGAATTGAGCCAG

CAGGGATGCACCGTCACCGTGTCTGATTTGTATGCCATGAACTTTGAGCCAAGGGCCACA

AGGAAAGATATCACTGGTGACCTTTCTAATCCTGAGGTCTTCAGTTATGGGGTGGAAGCC

TATGAAGCCTACAAGAAAAACTGTCTAACCAGTGACATCACTGAGGAGCAGAAAAAGGTG

CAGGAAGCTGATCTCGTGATATTCCAGTTTCCGCTGTACTGGTTCAGCGTGCCGGCTATC

CTGAAGGGCTGGATGGACAGGGTGCTATGCCAAGGGTTCGCCTTCGACATCCCAGGGTTC

TATGATTCTGGTTTTCTCAAGGATAAACTAGCCCTCCTTTCCTTAACCACGGGAGGCACA

GCCGAGATGTACTCAAAAGCAGGGGTCAGTGGAGATTTTCGGTACTTTCTGTGGCCACTC

CAGCATGGCGCTTTGCACTTCTGTGGATTTAAAATTCTTGCCCCTCAGATCAGTTTTGCT

CCTGAGTTTTCATCAGAAGAAGAAAGAAAAGCAATGGTGGCATCTTGGGCCCAACGATTA

AAGAGCATCTGGGAGGAAGAGCCCATCCGCTGCACACCCCCTTGGTACTTTGGGGAA

>CL226.Contig1_A-W 282 617 minus strand solute carrier family 35, member C2, isoform CRA_c [Homo sapiens]

ATGGGGAGGTGGGCCCTTGACGTGGCCTTTGTGTGGAAGGCAGTGTTGACCCTGGGGCTG

GTCCTTCTCTATTACTGCTTCTCCATCGGCATCACCTTCTACAACAAATGGCTGACAAAG

AGCTTTCACTTCCCCCTCTTCATGACGATGCTGCACCTGGCCGTGATCTTCCTCTTCTCC

GCCCTGTCCAGGACGCTGGTTCAGTGCTCCAGCCACAGGGCCCGTGTGGTGCTGAGCTGG

ACCGACTACCTCAAAAGAGTGGCCCCCACAGCACTGGCAACAGCACTTGACGTGGGCTTG

TCCAACTGGAGCTTCCTCTACATCACCGTCTCGCTG

>CL226.Contig2_A-W 243 1253 minus strand PREDICTED: solute carrier family 35 member C2 [Pan paniscus]

GTCCCGTGGCCCAGGAGCAGGGGCACACAGCCTCCGCGGATGGGGAGGTGGGCCCTTGAC

GTGGCCTTTGTGTGGAAGGCAGTGTTGACCCTGGGGCTGGTCCTTCTCTATTACTGCTTC

TCCATCGGCATCACCTTCTACAACAAATGGCTGACAAAGAGCTTTCACTTCCCCCTCTTC

ATGACGATGCTGCACCTGGCCGTGATCTTCCTCTTCTCCGCCCTGTCCAGGACGCTGGTT

CAGTGCTCCAGCCACAGGGCCCGTGTGGTGCTGAGCTGGACCGACTACCTCAAAAGAGTG

GCCCCCACAGCACTGGCAACAGCACTTGACGTGGGCTTGTCCAACTGGAGCTTCCTCTAC

ATCACCGTCTCGCTGTACACAATGACCAAGTCCTCGGCTGTGCTGTTCATCTTGATCTTC

TCTCTGATCTTCAAGCTGGAGGAGCTGCGTGCGGCCCTGGTCCTGGTGGTCCTCCTCATC

GCTGGGGGCCTCTTCATGTTCACCTACAAGTCCACCCAGTTCAACATGAAGGGCTTCTCC

CTGGTGCTGGGGGCCTCGTTCATCGGTGGAATTCGCTGGACCCTCACCCAGATGCTCCTG

CAGAAGGCTGAGCTGGGCCTCCAGAACCCCATCGACACCATGTTCCACCTGCAGCCACTC

ATGTTTCTGGGGCTCTTCCCTCTGTTTGCCATATTTGAAGGTCTGCACTTGTCCACATCT

GAGAAAATCTTCCGCTTCCAGGACACGGGGCTGCTCCTGTGGGTACTTGGGAGTCTCTTC

CTTGGCGGGATTCTCGCCTTCGGTTTGGGTTTCTCGGAGTTCCTCCTGGTTTCCCGAACC

TCCAGCCTCACTCTCTCCATTGCTGGCATTTTTAAGGAAGTCTGCACTTTGCTGCTGGCA

GCTCACCTGCTGGGCGATCAGATCAGCCTCCTGAACTGGCTGGGCTTTGCCCTTTGCCTC

TCGGGAATATCTCTGCACGTGGCCCTCAAAACTCTACACTCTAGAGGTAAC

>CL226.Contig3_A-W 282 605 minus strand hypothetical protein PANDA_021105 [Ailuropoda melanoleuca]

ATGGGGAGGTGGGCCCTTGACGTGGCCTTTGTGTGGAAGGCAGTGTTGACCCTGGGGCTG

GTCCTTCTCTATTACTGCTTCTCCATCGGCATCACCTTCTACAACAAATGGCTGACAAAG

AGCTTTCACTTCCCCCTCTTCATGACGATGCTGCACCTGGCCGTGATCTTCCTCTTCTCC

GCCCTGTCCAGGACGCTGGTTCAGTGCTCCAGCCACAGGGCCCGTGTGGTGCTGAGCTGG

ACCGACTACCTCAAAAGAGTGGCCCCCACAGCACTGGCAACAGCACTTGACGTGGGCTTG

TCCAACTGGAGCTTCCTCTACATC

>CL226.Contig4_A-W 243 1118 minus strand PREDICTED: solute carrier family 35 member C2 [Pan paniscus]

GTCCCGTGGCCCAGGAGCAGGGGCACACAGCCTCCGCGGATGGGGAGGTGGGCCCTTGAC

GTGGCCTTTGTGTGGAAGGCAGTGTTGACCCTGGGGCTGGTCCTTCTCTATTACTGCTTC

TCCATCGGCATCACCTTCTACAACAAATGGCTGACAAAGAGCTTTCACTTCCCCCTCTTC

ATGACGATGCTGCACCTGGCCGTGATCTTCCTCTTCTCCGCCCTGTCCAGGACGCTGGTT

CAGTGCTCCAGCCACAGGGCCCGTGTGGTGCTGAGCTGGACCGACTACCTCAAAAGAGTG

GCCCCCACAGCACTGGCAACAGCACTTGACGTGGGCTTGTCCAACTGGAGCTTCCTCTAC

ATCACCGTCTCGCTGTACACAATGACCAAGTCCTCGGCTGTGCTGTTCATCTTGATCTTC

TCTCTGATCTTCAAGCTGGAGGAGCTGCGTGCGGCCCTGGTCCTGGTGGTCCTCCTCATC

GCTGGGGGCCTCTTCATGTTCACCTACAAGTCCACCCAGTTCAACATGAAGGGCTTCTCC

CTGGTGCTGGGGGCCTCGTTCATCGGTGGAATTCGCTGGACCCTCACCCAGATGCTCCTG

CAGAAGGCTGAGCTGGGCCTCCAGAACCCCATCGACACCATGTTCCACCTGCAGCCACTC

ATGTTTCTGGGGCTCTTCCCTCTGTTTGCCATATTTGAAGGTCTGCACTTGTCCACATCT

GAGAAAATCTTCCGCTTCCAGGACACGGGGCTGCTCCTGTGGGTACTTGGGAGTCTCTTC

CTTGGCGGGATTCTCGCCTTCGGTTTGGGTTTCTCGGAGTTCCTCCTGGTTTCCCGAACC

TCCAGCCTCACTCTCTCCATTGCTGGCATTTTTAAG

>CL226.Contig5_A-W 243 551 minus strand PREDICTED: solute carrier family 35 member C2 [Callithrix jacchus]

GTCCCGTGGCCCAGGAGCAGGGGCACACAGCCTCCGCGGATGGGGAGGTGGGCCCTTGAC

GTGGCCTTTGTGTGGAAGGCAGTGTTGACCCTGGGGCTGGTCCTTCTCTATTACTGCTTC

TCCATCGGCATCACCTTCTACAACAAATGGCTGACAAAGAGCTTTCACTTCCCCCTCTTC

ATGACGATGCTGCACCTGGCCGTGATCTTCCTCTTCTCCGCCCTGTCCAGGACGCTGGTT

CAGTGCTCCAGCCACAGGGCCCGTGTGGTGCTGAGCTGGACCGACTACCTCAAAAGAGTG

GCCCCCACA

>CL226.Contig6_A-W 452 1126 minus strand PREDICTED: solute carrier family 35 member C2 [Otolemur garnettii]

GAGAGCTTTCACTTCCCCCTCTTCATGACGATGCTGCACCTGGCCGTGATCTTCCTCTTC

TCCGCCCTGTCCAGGACGCTGGTTCAGTGCTCCAGCCACAGGGCCCGTGTGGTGCTGAGC

TGGACCGACTACCTCAAAAGAGTGGCCCCCACAGCACTGGCAACAGCACTTGACGTGGGC

TTGTCCAACTGGAGCTTCCTCTACATCACCGTCTCGCTGTACACAATGACCAAGTCCTCG

GCTGTGCTGTTCATCTTGATCTTCTCTCTGATCTTCAAGCTGGAGGAGCTGCGTGCGGCC

CTGGTCCTGGTGGTCCTCCTCATCGCTGGGGGCCTCTTCATGTTCACCTACAAGTCCACC

CAGTTCAACATGAAGGGCTTCTCCCTGGTGCTGGGGGCCTCGTTCATCGGTGGAATTCGC

TGGACCCTCACCCAGATGCTCCTGCAGAAGGCTGAGCTGGGCCTCCAGAACCCCATCGAC

ACCATGTTCCACCTGCAGCCACTCATGTTTCTGGGGCTCTTCCCTCTGTTTGCCGTATTT

GAAGGTCTGCACTTGTCCACATCTGAGAAAATCTTCCGCTTCCAGGACACGGGGCTGCTC

CTGTGGGTACTTGGGAGTCTCTTCCTTGGCGGGATTCTCGCCTTCGGTTTGGGTTTCTCG

GAGTTCCTCCTGGTT

>CL226.Contig7_A-W 336 1322 minus strand PREDICTED: solute carrier family 35 member C2 isoform 1 [Loxodonta africana]

TGGGCTCTGCAGAGCTTTCACTTCCCCCTCTTCATGACGATGCTGCACCTGGCCGTGATC

TTCCTCTTCTCCGCCCTGTCCAGGACGCTGGTTCAGTGCTCCAGCCACAGGGCCCGTGTG

GTGCTGAGCTGGACCGACTACCTCAAAAGAGTGGCCCCCACAGCACTGGCAACAGCACTT

GACGTGGGCTTGTCCAACTGGAGCTTCCTCTACATCACCGTCTCGCTGTACACAATGACC

AAGTCCTCGGCTGTGCTGTTCATCTTGATCTTCTCTCTGATCTTCAAGCTGGAGGAGCTG

CGTGCGGCCCTGGTCCTGGTGGTCCTCCTCATCGCTGGGGGCCTCTTCATGTTCACCTAC

AAGTCCACCCAGTTCAACATGAAGGGCTTCTCCCTGGTGCTGGGGGCCTCGTTCATCGGT

GGAATTCGCTGGACCCTCACCCAGATGCTCCTGCAGAAGGCTGAGCTGGGCCTCCAGAAC

CCCATCGACACCATGTTCCACCTGCAGCCACTCATGTTTCTGGGGCTCTTCCCTCTGTTT

GCCATATTTGAAGGTCTGCACTTGTCCACATCTGAGAAAATCTTCCGCTTCCAGGACACG

GGGCTGCTCCTGTGGGTACTTGGGAGTCTCTTCCTTGGCGGGATTCTCGCCTTCGGTTTG

GGTTTCTCGGAGTTCCTCCTGGTTTCCCGAACCTCCAGCCTCACTCTCTCCATTGCTGGC

ATTTTTAAGGAAGTCTGCACTTTGCTGCTGGCAGCTCACCTGCTGGGCGATCAGATCAGC

CTCCTGAACTGGCTGGGCTTTGCCCTTTGCCTCTCGGGAATATCTCTGCACGTGGCCCTC

AAAACTCTACACTCTAGAGGTGAAGGTGGATCCAGACCCCTGAAGGGGCTGAGCTCCAGC

CCTGACCTGGAGCTGCTGCTCCGGAGCAGCCAGCCAGAGGATGAGGACAATGAGGAGGAA

GACTACTTCGTGGCCCAGGGGCAGCAG

>CL226.Contig8_A-W 481 1491 minus strand PREDICTED: solute carrier family 35 member C2 [Pan paniscus]

GTCCCGTGGCCCAGGAGCAGGGGCACACAGCCTCCGCGGATGGGGAGGTGGGCCCTTGAC

GTGGCCTTTGTGTGGAAGGCAGTGTTGACCCTGGGGCTGGTCCTTCTCTATTACTGCTTC

TCCATCGGCATCACCTTCTACAACAAATGGCTGACAAAGAGCTTTCACTTCCCCCTCTTC

ATGACGATGCTGCACCTGGCCGTGATCTTCCTCTTCTCCGCCCTGTCCAGGACGCTGGTT

CAGTGCTCCAGCCACAGGGCCCGTGTGGTGCTGAGCTGGACCGACTACCTCAAAAGAGTG

GCCCCCACAGCACTGGCAACAGCACTTGACGTGGGCTTGTCCAACTGGAGCTTCCTCTAC

ATCACCGTCTCGCTGTACACAATGACCAAGTCCTCGGCTGTGCTGTTCATCTTGATCTTC

TCTCTGATCTTCAAGCTGGAGGAGCTGCGTGCGGCCCTGGTCCTGGTGGTCCTCCTCATC

GCTGGGGGCCTCTTCATGTTCACCTACAAGTCCACCCAGTTCAACATGAAGGGCTTCTCC

CTGGTGCTGGGGGCCTCGTTCATCGGTGGAATTCGCTGGACCCTCACCCAGATGCTCCTG

CAGAAGGCTGAGCTGGGCCTCCAGAACCCCATCGACACCATGTTCCACCTGCAGCCACTC

ATGTTTCTGGGGCTCTTCCCTCTGTTTGCCATATTTGAAGGTCTGCACTTGTCCACATCT

GAGAAAATCTTCCGCTTCCAGGACACGGGGCTGCTCCTGTGGGTACTTGGGAGTCTCTTC

CTTGGCGGGATTCTCGCCTTCGGTTTGGGTTTCTCGGAGTTCCTCCTGGTTTCCCGAACC

TCCAGCCTCACTCTCTCCATTGCTGGCATTTTTAAGGAAGTCTGCACTTTGCTGCTGGCA

GCTCACCTGCTGGGCGATCAGATCAGCCTCCTGAACTGGCTGGGCTTTGCCCTTTGCCTC

TCGGGAATATCTCTGCACGTGGCCCTCAAAACTCTACACTCTAGAGGTAAC

>CL226.Contig9_A-W 426 1232 minus strand solute carrier family 35, member C2, isoform CRA_c [Homo sapiens]

AATGGCCACCTCCAGGACGTAGCGGCCACCTGGCTGGATGTTCCTGTGCCACCTCAGCCC

CGGGCGCCCCTTATCTCCACAGCACTGGCAACAGCACTTGACGTGGGCTTGTCCAACTGG

AGCTTCCTCTACATCACCGTCTCGCTGTACACAATGACCAAGTCCTCGGCTGTGCTGTTC

ATCTTGATCTTCTCTCTGATCTTCAAGCTGGAGGAGCTGCGTGCGGCCCTGGTCCTGGTG

GTCCTCCTCATCGCTGGGGGCCTCTTCATGTTCACCTACAAGTCCACCCAGTTCAACATG

AAGGGCTTCTCCCTGGTGCTGGGGGCCTCGTTCATCGGTGGAATTCGCTGGACCCTCACC

CAGATGCTCCTGCAGAAGGCTGAGCTGGGCCTCCAGAACCCCATCGACACCATGTTCCAC

CTGCAGCCACTCATGTTTCTGGGGCTCTTCCCTCTGTTTGCCATATTTGAAGGTCTGCAC

TTGTCCACATCTGAGAAAATCTTCCGCTTCCAGGACACGGGGCTGCTCCTGTGGGTACTT

GGGAGTCTCTTCCTTGGCGGGATTCTCGCCTTCGGTTTGGGTTTCTCGGAGTTCCTCCTG

GTTTCCCGAACCTCCAGCCTCACTCTCTCCATTGCTGGCATTTTTAAGGAAGTCTGCACT

TTGCTGCTGGCAGCTCACCTGCTGGGCGATCAGATCAGCCTCCTGAACTGGCTGGGCTTT

GCCCTTTGCCTCTCGGGAATATCTCTGCACGTGGCCCTCAAAACTCTACACTCTAGAGGT

AACTGGAGCCTTCCAGAGGCCTCCATT

>CL226.Contig10_A-W 399 1331 minus strand PREDICTED: solute carrier family 35 member C2 [Pongo abelii]

CCCAGAGAGGAAGCCAGAGTGGGGTGAAATGGCCACCTCCAGGACGTAGCGGCCACCTGG

CTGGATGTTCCTGTGCCACCTCAGCCCCGGGCGCCCCTTATCTCCACAGCACTGGCAACA

GCACTTGACGTGGGCTTGTCCAACTGGAGCTTCCTCTACATCACCGTCTCGCTGTACACA

ATGACCAAGTCCTCGGCTGTGCTGTTCATCTTGATCTTCTCTCTGATCTTCAAGCTGGAG

GAGCTGCGTGCGGCCCTGGTCCTGGTGGTCCTCCTCATCGCTGGGGGCCTCTTCATGTTC

ACCTACAAGTCCACCCAGTTCAACATGAAGGGCTTCTCCCTGGTGCTGGGGGCCTCGTTC

ATCGGTGGAATTCGCTGGACCCTCACCCAGATGCTCCTGCAGAAGGCTGAGCTGGGCCTC

CAGAACCCCATCGACACCATGTTCCACCTGCAGCCACTCATGTTTCTGGGGCTCTTCCCT

CTGTTTGCCATATTTGAAGGTCTGCACTTGTCCACATCTGAGAAAATCTTCCGCTTCCAG

GACACGGGGCTGCTCCTGTGGGTACTTGGGAGTCTCTTCCTTGGCGGGATTCTCGCCTTC

GGTTTGGGTTTCTCGGAGTTCCTCCTGGTTTCCCGAACCTCCAGCCTCACTCTCTCCATT

GCTGGCATTTTTAAGGAAGTCTGCACTTTGCTGCTGGCAGCTCACCTGCTGGGCGATCAG

ATCAGCCTCCTGAACTGGCTGGGCTTTGCCCTTTGCCTCTCGGGAATATCTCTGCACGTG

GCCCTCAAAACTCTACACTCTAGAGGTGAAGGTGGATCCAGACCCCTGAAGGGGCTGAGC

TCCAGCCCTGACCTGGAGCTGCTGCTCCGGAGCAGCCAGCCAGAGGATGAGGACAATGAG

GAGGAAGACTACTTCGTGGCCCAGGGGCAGCAG

>CL226.Contig11_A-W 481 1614 minus strand PREDICTED: solute carrier family 35 member C2 [Pan paniscus]

GTCCCGTGGCCCAGGAGCAGGGGCACACAGCCTCCGCGGATGGGGAGGTGGGCCCTTGAC

GTGGCCTTTGTGTGGAAGGCAGTGTTGACCCTGGGGCTGGTCCTTCTCTATTACTGCTTC

TCCATCGGCATCACCTTCTACAACAAATGGCTGACAAAGAGCTTTCACTTCCCCCTCTTC

ATGACGATGCTGCACCTGGCCGTGATCTTCCTCTTCTCCGCCCTGTCCAGGACGCTGGTT

CAGTGCTCCAGCCACAGGGCCCGTGTGGTGCTGAGCTGGACCGACTACCTCAAAAGAGTG

GCCCCCACAGCACTGGCAACAGCACTTGACGTGGGCTTGTCCAACTGGAGCTTCCTCTAC

ATCACCGTCTCGCTGTACACAATGACCAAGTCCTCGGCTGTGCTGTTCATCTTGATCTTC

TCTCTGATCTTCAAGCTGGAGGAGCTGCGTGCGGCCCTGGTCCTGGTGGTCCTCCTCATC

GCTGGGGGCCTCTTCATGTTCACCTACAAGTCCACCCAGTTCAACATGAAGGGCTTCTCC

CTGGTGCTGGGGGCCTCGTTCATCGGTGGAATTCGCTGGACCCTCACCCAGATGCTCCTG

CAGAAGGCTGAGCTGGGCCTCCAGAACCCCATCGACACCATGTTCCACCTGCAGCCACTC

ATGTTTCTGGGGCTCTTCCCTCTGTTTGCCATATTTGAAGGTCTGCACTTGTCCACATCT

GAGAAAATCTTCCGCTTCCAGGACACGGGGCTGCTCCTGTGGGTACTTGGGAGTCTCTTC

CTTGGCGGGATTCTCGCCTTCGGTTTGGGTTTCTCGGAGTTCCTCCTGGTTTCCCGAACC

TCCAGCCTCACTCTCTCCATTGCTGGCATTTTTAAGGAAGTCTGCACTTTGCTGCTGGCA

GCTCACCTGCTGGGCGATCAGATCAGCCTCCTGAACTGGCTGGGCTTTGCCCTTTGCCTC

TCGGGAATATCTCTGCACGTGGCCCTCAAAACTCTACACTCTAGAGGTGAAGGTGGATCC

AGACCCCTGAAGGGGCTGAGCTCCAGCCCTGACCTGGAGCTGCTGCTCCGGAGCAGCCAG

CCAGAGGATGAGGACAATGAGGAGGAAGACTACTTCGTGGCCCAGGGGCAGCAG

>CL226.Contig12_A-W 520 855 minus strand PREDICTED: solute carrier family 35 member C2 isoform 1 [Loxodonta africana]

ATGGGGAGGTGGGCCCTTGACGTGGCCTTTGTGTGGAAGGCAGTGTTGACCCTGGGGCTG

GTCCTTCTCTATTACTGCTTCTCCATCGGCATCACCTTCTACAACAAATGGCTGACAAAG

AGCTTTCACTTCCCCCTCTTCATGACGATGCTGCACCTGGCCGTGATCTTCCTCTTCTCC

GCCCTGTCCAGGACGCTGGTTCAGTGCTCCAGCCACAGGGCCCGTGTGGTGCTGAGCTGG

ACCGACTACCTCAAAAGAGTGGCCCCCACAGCACTGGCAACAGCACTTGACGTGGGCTTG

TCCAACTGGAGCTTCCTCTACATCACCGTCTCGCTG

>CL226.Contig13_A-W 520 855 minus strand solute carrier family 35, member C2, isoform CRA_c [Homo sapiens]

ATGGGGAGGTGGGCCCTTGACGTGGCCTTTGTGTGGAAGGCAGTGTTGACCCTGGGGCTG

GTCCTTCTCTATTACTGCTTCTCCATCGGCATCACCTTCTACAACAAATGGCTGACAAAG

AGCTTTCACTTCCCCCTCTTCATGACGATGCTGCACCTGGCCGTGATCTTCCTCTTCTCC

GCCCTGTCCAGGACGCTGGTTCAGTGCTCCAGCCACAGGGCCCGTGTGGTGCTGAGCTGG

ACCGACTACCTCAAAAGAGTGGCCCCCACAGCACTGGCAACAGCACTTGACGTGGGCTTG

TCCAACTGGAGCTTCCTCTACATCACCGTCTCGCTG

>CL226.Contig14_A-W 520 843 minus strand hypothetical protein PANDA_021105 [Ailuropoda melanoleuca]

ATGGGGAGGTGGGCCCTTGACGTGGCCTTTGTGTGGAAGGCAGTGTTGACCCTGGGGCTG

GTCCTTCTCTATTACTGCTTCTCCATCGGCATCACCTTCTACAACAAATGGCTGACAAAG

AGCTTTCACTTCCCCCTCTTCATGACGATGCTGCACCTGGCCGTGATCTTCCTCTTCTCC

GCCCTGTCCAGGACGCTGGTTCAGTGCTCCAGCCACAGGGCCCGTGTGGTGCTGAGCTGG

ACCGACTACCTCAAAAGAGTGGCCCCCACAGCACTGGCAACAGCACTTGACGTGGGCTTG

TCCAACTGGAGCTTCCTCTACATC

>CL226.Contig15_A-W 336 1223 minus strand solute carrier family 35, member C2, isoform CRA_c [Homo sapiens]

TGGGCTCTGCAGAGCTTTCACTTCCCCCTCTTCATGACGATGCTGCACCTGGCCGTGATC

TTCCTCTTCTCCGCCCTGTCCAGGACGCTGGTTCAGTGCTCCAGCCACAGGGCCCGTGTG

GTGCTGAGCTGGACCGACTACCTCAAAAGAGTGGCCCCCACAGCACTGGCAACAGCACTT

GACGTGGGCTTGTCCAACTGGAGCTTCCTCTACATCACCGTCTCGCTGTACACAATGACC

AAGTCCTCGGCTGTGCTGTTCATCTTGATCTTCTCTCTGATCTTCAAGCTGGAGGAGCTG

CGTGCGGCCCTGGTCCTGGTGGTCCTCCTCATCGCTGGGGGCCTCTTCATGTTCACCTAC

AAGTCCACCCAGTTCAACATGAAGGGCTTCTCCCTGGTGCTGGGGGCCTCGTTCATCGGT

GGAATTCGCTGGACCCTCACCCAGATGCTCCTGCAGAAGGCTGAGCTGGGCCTCCAGAAC

CCCATCGACACCATGTTCCACCTGCAGCCACTCATGTTTCTGGGGCTCTTCCCTCTGTTT

GCCATATTTGAAGGTCTGCACTTGTCCACATCTGAGAAAATCTTCCGCTTCCAGGACACG

GGGCTGCTCCTGTGGGTACTTGGGAGTCTCTTCCTTGGCGGGATTCTCGCCTTCGGTTTG

GGTTTCTCGGAGTTCCTCCTGGTTTCCCGAACCTCCAGCCTCACTCTCTCCATTGCTGGC

ATTTTTAAGGAAGTCTGCACTTTGCTGCTGGCAGCTCACCTGCTGGGCGATCAGATCAGC

CTCCTGAACTGGCTGGGCTTTGCCCTTTGCCTCTCGGGAATATCTCTGCACGTGGCCCTC

AAAACTCTACACTCTAGAGGTAACTGGAGCCTTCCAGAGGCCTCCATT

>CL226.Contig16_A-W 336 551 minus strand hypothetical protein PANDA_021105 [Ailuropoda melanoleuca]

TGGGCTCTGCAGAGCTTTCACTTCCCCCTCTTCATGACGATGCTGCACCTGGCCGTGATC

TTCCTCTTCTCCGCCCTGTCCAGGACGCTGGTTCAGTGCTCCAGCCACAGGGCCCGTGTG

GTGCTGAGCTGGACCGACTACCTCAAAAGAGTGGCCCCCACAGCACTGGCAACAGCACTT

GACGTGGGCTTGTCCAACTGGAGCTTCCTCTACATC

>CL226.Contig17_A-W 336 1064 minus strand PREDICTED: solute carrier family 35 member C2 [Otolemur garnettii]

TGGGCTCTGCAGAGCTTTCACTTCCCCCTCTTCATGACGATGCTGCACCTGGCCGTGATC

TTCCTCTTCTCCGCCCTGTCCAGGACGCTGGTTCAGTGCTCCAGCCACAGGGCCCGTGTG

GTGCTGAGCTGGACCGACTACCTCAAAAGAGTGGCCCCCACAGCACTGGCAACAGCACTT

GACGTGGGCTTGTCCAACTGGAGCTTCCTCTACATCACCGTCTCGCTGTACACAATGACC

AAGTCCTCGGCTGTGCTGTTCATCTTGATCTTCTCTCTGATCTTCAAGCTGGAGGAGCTG

CGTGCGGCCCTGGTCCTGGTGGTCCTCCTCATCGCTGGGGGCCTCTTCATGTTCACCTAC

AAGTCCACCCAGTTCAACATGAAGGGCTTCTCCCTGGTGCTGGGGGCCTCGTTCATCGGT

GGAATTCGCTGGACCCTCACCCAGATGCTCCTGCAGAAGGCTGAGCTGGGCCTCCAGAAC

CCCATCGACACCATGTTCCACCTGCAGCCACTCATGTTTCTGGGGCTCTTCCCTCTGTTT

GCCATATTTGAAGGTCTGCACTTGTCCACATCTGAGAAAATCTTCCGCTTCCAGGACACG

GGGCTGCTCCTGTGGGTACTTGGGAGTCTCTTCCTTGGCGGGATTCTCGCCTTCGGTTTG

GGTTTCTCGGAGTTCCTCCTGGTTTCCCGAACCTCCAGCCTCACTCTCTCCATTGCTGGC

ATTTTTAAG

>CL227.Contig1_A-W 1868 2791 PREDICTED: complement C4-A [Papio anubis]

GGCTACATGCGGATCCAGCAGTTTCGGAAGACAGATGGTTCCTTTGGGGCTTGGTTACAC

AGGGACAGCAGCACCTGGCTCACAGCCTTTGTGCTGAAGGTTCTAAGTTTGGCTCAGGAG

CAGGTGGGCGGCTCACCTGAAAAACTGCAGGAGACAGCCAGTTGGTTGCTGTCCCAGCAA

CAGGCAGACGGCTCATTCCAGGACCCATGTCCAGTTATCCACAGGGCCATGCAGGGGGGT

TTGGTGGGAAATGATGAGACTGTGGCTCTTACTGCTTTCGTGGTCATTGCCCTTCAGCAT

GGGCTGGCTGTCTTCCAGGACCAGAGTGCACAGCAATTGAAGCAGAGAGTGGAAACCTCC

ATCTCAAAGGCAAACTCATTCTTGGGGGAGAAAGCAAGTGCTGGGCTCCTGGGTGCCCAT

GCAGCTGCCATCACGGCCTATGCCCTAACTCTGTCCAAGGCTTCCCAGAACCTGCGGGAT

GTTGCCCACAACAACCTTATGGCCATGGCCCAGGAGACTGGTGATTACTTGTACTGGGGC

TCAGTCACTGGTTCTCAGAACAATGTCGTGTCACCCACCCTGGCTCCTCGCAGTCCAGCA

GACCCCATGCCCCAGGCCCCAGCCCTGTGGATTGAAACCACAGCCTATGGCCTGCTGCAC

CTGCTGCTTCGAGAGGGCAAAGCAGAGCTGGCCAATCAGGCGGCATCCTGGCTCACCCAT

CAGGGCAGCTTCCAAGGGGGATTCCGCAGCACTCAGGACACAGTGATTGCGCTGGATGCC

CTGTCTGCATACTGGATCGCTGCCCACACCACTGAGGAACGGGGACTCAATGTGACTCTC

AGTTCCATAGGCCGCAGTGGGTTCAAATCCTATGTGCTTCAGCTGAACAACCACCAAATT

AAAGGGATAGAGGAGGAGCTGCAG

>CL227.Contig2_A-W 3136 3357 PREDICTED: complement C4-A [Papio anubis]

AAGTTTTCTCTGAGCAACAAGATTAATGTGGAAGTGGGAGGAAACAGCAAGGGAACCTTG

AAGGTCCTGCGTACCTACAATGTCCTGGACATGAAGAACACAACCTGCACAGACCTTCAG

ATTGAGGTGTCAGTCATAGGCCATGTAGACTACACAAGGGAAGCAAATGAGGATTATGAG

GATTATGAGTATGAGGAATTTCCAGCCAGTGATGACCCAGGC

>CL227.Contig3_A-W 2976 4133 PREDICTED: complement C4-A isoform 2 [Otolemur garnettii]

AAGTTTTCTCTGAGCAACAAGATTAATGTGGAAGTGGGAGGAAACAGCAAGGGAACCTTG

AAGGTCCTGCGTACCTACAATGTCCTGGACATGAAGAACACAACCTGCACAGACCTTCAG

ATTGAGGTGTCAGTCATAGGCCATGTAGACTACACAAGGGAAGCAAATGAGGATTATGAG

GATTATGAGTATGAGGAATTTCCAGCCAGTGATGACCCAGGTGTGTATACCCATCCTGTG

ACACCCCTGCAGCTGTTTGAGGGACGGAGGAACCGCCGTAGGAGGGAGGTGCCCAAGGTG

GCAGAGGAACAGGAATCCAGAGTGCAGTATACTGTGTGCATCTGGCGAAATGGCAAGGTG

GGGCTGTCTGGTATGGCCATTGCGGATATTACTCTTCTGAGTGGATTCCATGCCCTGCGG

GCTGACCTAGAGAAGCTGACCTCCCTCTCTGACCGTTATGTGAGTCACTTTGAGACGGAT

GGACCCCATGTCTTACTGTACTTTGACTCGGTCCCCACCTCCCGGGAGTGTGTGGGCTTT

GGAGCTGTGCAGGAGGTGGCCGTGGGGCTGGTGCAGCCAGCTAGCGCCGTCCTATATGAC

TACTACAGCCCTGAGCACAAATGTTCTGTGTTTTATGGGGCACCAACTAAGAGCAAACTT

CTGTCCACATTGTGCTCTGCTGATGTCTGCCAGTGTGCTGAGGGGAAATGCCCTCGACAG

CGTCGTGCCCTGGAGCGGGGGCTGCAGGATGAGGATGGTTATAGGATGAAGTTTGCCTGC

TACTATCCCCGTGTGGAATATGGCTTCCAGGTTCATGTTCTCCGAGAAGATAGCAGAACT

GCTTTCCGCCTCTTTGAAACCAGGATCACCCAAGTCCTGCACTTCACCAAGGATTCCAAG

GCTGCTGCTGGTCAGACCCGCAACTTCCTGGTTCGAGCTTCTTGCCGCCTTCACTTGGAG

CCTGAGAAAGAATATTTGATCATGGGTCTGGATGGGGCCACATATGACCTCAAAGGAGAC

CCCCAATACCTGCTGGATTCAAATAGCTGGATTGAAGAAATGCCCTCTGAACGCCTGTGC

CGGAGCACCCGCCAGCGGGCAGCCTGTGCCCAGCTCAGTGACTTCCTCCAGGAGTATGGC

ACCCAGGGGTGCCAAGTG

>CL227.Contig4_A-W 2976 3197 PREDICTED: complement C4-A isoform 2 [Otolemur garnettii]

AAGTTTTCTCTGAGCAACAAGATTAATGTGGAAGTGGGAGGAAACAGCAAGGGAACCTTG

AAGGTCCTGCGTACCTACAATGTCCTGGACATGAAGAACACAACCTGCACAGACCTTCAG

ATTGAGGTGTCAGTCATAGGCCATGTAGACTACACAAGGGAAGCAAATGAGGATTATGAG

GATTATGAGTATGAGGAATTTCCAGCCAGTGATGACCCAGGC

>CL227.Contig5_A-W 4 1710 PREDICTED: complement C4-A isoform 1 [Otolemur garnettii]

GTAGGGGACACTCTCAACTTGAACCTGCAAGCCATGGGCATCAGTGGGGACACCTTCTCT

CATTATTACTACATGATCCTATCCCGGGGAGAGATCGTGTCTGTGAATCGAGAGGACAGG

AGGGCCCTGACCTCGGTCTCTGTGTTTGTGGACCATCGCCTAGCACCCTCCTTCTACTTC

GTGGCCTTCTATTATCATCGAGGCCTCCCAGTGGCCAACTCCCTGCGAGTGGATGTCCAG

GCTGAGGCCTGCGAGGGCAAGCTGGAACTGAACCTGGATGGTGCCAAGGACTATCGTAAT

GGGGAGAATGTAAAGCTGCATCTGCAAACAGATTCTCAAGCCCTGGTGGCCCTGGGAGCT

GTGGACACAGCTCTGTATGCTGTGGGTGGCAGGTCCTATAAGCCCCTGGACATGAGCAAG

GTCTTTGAAGTTATGAATAGCTATGACCTTGGGTGTGGTGCTGGAGGTGGGGACAGTGCC

CTTCAGGTGTTCCAGGCAGCGGGTCTGGCCTTTTCTGATGGAGAGAAGTTGACGTCCGTT

AGAAAGAGTCTGAGCTGTCCCAAGGAGAATAAAATCCGGAAAAAGAGAAATGTGAACTTC

CAAAAGGCATTTAGTGAGAAGTTGGGCCAGTATTCTTCACCTGAAGCCAAGCGCTGCTGT

CAGGATGGGCTGACACGGCTACCCATGGCACGCTCCTGTGAGCAGCGAGTAGCTCGTGTG

TTGCAGCTGGCCTGCCGGGAGCCCTTCCTGTCCTGCTGCCAGTTTGCTGAGAGCCTGCGC

AAGAAGAACAGGACAAGGGGCCAGGTGGGCCTCGCCCGAGCCCTGGAGGTCCTACAGGAA

GAGGAACTGATGCATGAAGATGATATTGTGGTGCGCAGCTTCTTCCCAGAGAACTGGCTC

TGGAGAGTGGAGACAGTGGACCGTTCCAAAATATTGTCATTGTGGCTCCCTGACTCTCTG

ACCACGTGGGAGATCCATGGCGTGAGCTTGTCCAAAAGCACAGGTTTGTGTGTGGCTACA

CCGGTCCATCTCCGAGTGTTCCGCGAGTTCCACCTGCACCTCCGCCTGCCCGTCTCTGTC

CGCCGCTTTGAGCAACTTGAGCTGCGGCCTGTCCTCTATAACTACCTGGATAAAAACCTG

ACTGTGAGCGTCCATGTGTCCCCAGTGGAGGGGCTGTGTCTGGCTGGGGGCGGAGGGCTG

GCCCAGCAGGTGCTGGTGCCTGCAGGCTCTGCCCGGCCCGTTGCCTTCTCTATGGTGCCC

ATGGCAGCTGCTGCTATGTCCCTGAAGGTGGTGGCTCGAGGATCCTTTGAATTCCCTGTA

GGGGATGCAGTGTCCAAGGTTCTGCAAATTGAGAAGGAAGGGGCCATCCACAGGGAGGAG

ATTGTCTATGAACTCAACCCCCTGGACCACCGAGGCCGGAGCTTGGAAATTCCTGGCAAC

TCTGATCCAAATATTATCCCTGATGGAGATTTCAGCAGCATCGTGAGAGTTACAGCCTCG

GATCCATTGGAGACCCTTGGCTCTGAGGGGGCCTTATCCCCAGGGGGCCTAGCCGCCCTC

CTGAGGCTTCCCTATGGCTGTGCTGAACAAACCATGATCTACTTGGCTCCCACACTGGCA

GCCTCCCACTATCTGGACAAGACAGAACAATGGAGCAAACTGCCCCCTGAGACCAAGGAT

CGTGCCGTGGATCTGATCCAGAAAGGT

>CL227.Contig6_A-W 4 1710 PREDICTED: complement C4-A isoform 2 [Otolemur garnettii]

GTAGGGGACACTCTCAACTTGAACCTGCAAGCCATGGGCATCAGTGGGGACACCTTCTCT

CATTATTACTACATGATCCTATCCCGGGGAGAGATCGTGTCTGTGAATCGAGAGGACAGG

AGGGCCCTGACCTCGGTCTCTGTGTTTGTGGACCATCGCCTAGCACCCTCCTTCTACTTC

GTGGCCTTCTATTATCATCGAGGCCTCCCAGTGGCCAACTCCCTGCGAGTGGATGTCCAG

GCTGAGGCCTGCGAGGGCAAGCTGGAACTGAACCTGGATGGTGCCAAGGACTATCGTAAT

GGGGAGAATGTAAAGCTGCATCTGCAAACAGATTCTCAAGCCCTGGTGGCCCTGGGAGCT

GTGGACACAGCTCTGTATGCTGTGGGTGGCAGGTCCTATAAGCCCCTGGACATGAGCAAG

GTCTTTGAAGTTATGAATAGCTATGACCTTGGGTGTGGTGCTGGAGGTGGGGACAGTGCC

CTTCAGGTGTTCCAGGCAGCGGGTCTGGCCTTTTCTGATGGAGAGAAGTTGACGTCCGTT

AGAAAGAGTCTGAGCTGTCCCAAGGAGAATAAAATCCGGAAAAAGAGAAATGTGAACTTC

CAAAAGGCATTTAGTGAGAAGTTGGGCCAGTATTCTTCACCTGAAGCCAAGCGCTGCTGT

CAGGATGGGCTGACACGGCTACCCATGGCACGCTCCTGTGAGCAGCGAGTAGCTCGTGTG

TTGCAGCTGGCCTGCCGGGAGCCCTTCCTGTCCTGCTGCCAGTTTGCTGAGAGCCTGCGC

AAGAAGAACAGGACAAGGGGCCAGGTGGGCCTCGCCCGAGCCCTGGAGGTCCTACAGGAA

GAGGAACTGATGCATGAAGATGATATTGTGGTGCGCAGCTTCTTCCCAGAGAACTGGCTC

TGGAGAGTGGAGACAGTGGACCGTTCCAAAATATTGTCATTGTGGCTCCCTGACTCTCTG

ACCACGTGGGAGATCCATGGCGTGAGCTTGTCCAAAAGCACAGGTTTGTGTGTGGCTACA

CCGGTCCATCTCCGAGTGTTCCGCGAGTTCCACCTGCACCTCCGCCTGCCCGTCTCTGTC

CGCCGCTTTGAGCAACTTGAGCTGCGGCCTGTCCTCTATAACTACCTGGATAAAAACCTG

ACTGTGAGCGTCCATGTGTCCCCAGTGGAGGGGCTGTGTCTGGCTGGGGGCGGAGGGCTG

GCCCAGCAGGTGCTGGTGCCTGCAGGCTCTGCCCGGCCCGTTGCCTTCTCTATGGTGCCC

ATGGCAGCTGCTGCTATGTCCCTGAAGGTGGTGGCTCGAGGATCCTTTGAATTCCCTGTA

GGGGATGCAGTGTCCAAGGTTCTGCAAATTGAGAAGGAAGGGGCCATCCACAGGGAGGAG

ATTGTCTATGAACTCAACCCCCTGGACCACCGAGGCCGGAGCTTGGAAATTCCTGGCAAC

TCTGATCCAAATATTATCCCTGATGGAGATTTCAGCAGCATCGTGAGAGTTACAGCCTCG

GATCCATTGGAGACCCTTGGCTCTGAGGGGGCCTTATCCCCAGGGGGCCTAGCCGCCCTC

CTGAGGCTTCCCTATGGCTGTGCTGAACAAACCATGATCTACTTGGCTCCCACACTGGCA

GCCTCCCACTATCTGGACAAGACAGAACAATGGAGCAAACTGCCCCCTGAGACCAAGGAT

CGTGCCGTGGATCTGATCCAGAAAGGT

>CL227.Contig7_A-W 4 3786 PREDICTED: complement C4-A isoform 1 [Otolemur garnettii]

GTAGGGGACACTCTCAACTTGAACCTGCAAGCCATGGGCATCAGTGGGGACACCTTCTCT

CATTATTACTACATGATCCTATCCCGGGGAGAGATCGTGTCTGTGAATCGAGAGGACAGG

AGGGCCCTGACCTCGGTCTCTGTGTTTGTGGACCATCGCCTAGCACCCTCCTTCTACTTC

GTGGCCTTCTATTATCATCGAGGCCTCCCAGTGGCCAACTCCCTGCGAGTGGATGTCCAG

GCTGAGGCCTGCGAGGGCAAGCTGGAACTGAACCTGGATGGTGCCAAGGACTATCGTAAT

GGGGAGAATGTAAAGCTGCATCTGCAAACAGATTCTCAAGCCCTGGTGGCCCTGGGAGCT

GTGGACACAGCTCTGTATGCTGTGGGTGGCAGGTCCTATAAGCCCCTGGACATGAGCAAG

GTCTTTGAAGTTATGAATAGCTATGACCTTGGGTGTGGTGCTGGAGGTGGGGACAGTGCC

CTTCAGGTGTTCCAGGCAGCGGGTCTGGCCTTTTCTGATGGAGAGAAGTTGACGTCCGTT

AGAAAGAGTCTGAGCTGTCCCAAGGAGAATAAAATCCGGAAAAAGAGAAATGTGAACTTC

CAAAAGGCATTTAGTGAGAAGTTGGGCCAGTATTCTTCACCTGAAGCCAAGCGCTGCTGT

CAGGATGGGCTGACACGGCTACCCATGGCACGCTCCTGTGAGCAGCGAGTAGCTCGTGTG

TTGCAGCTGGCCTGCCGGGAGCCCTTCCTGTCCTGCTGCCAGTTTGCTGAGAGCCTGCGC

AAGAAGAACAGGACAAGGGGCCAGGTGGGCCTCGCCCGAGCCCTGGAGGTCCTACAGGAA

GAGGAACTGATGCATGAAGATGATATTGTGGTGCGCAGCTTCTTCCCAGAGAACTGGCTC

TGGAGAGTGGAGACAGTGGACCGTTCCAAAATATTGTCATTGTGGCTCCCTGACTCTCTG

ACCACGTGGGAGATCCATGGCGTGAGCTTGTCCAAAAGCACAGGTTTGTGTGTGGCTACA

CCGGTCCATCTCCGAGTGTTCCGCGAGTTCCACCTGCACCTCCGCCTGCCCGTCTCTGTC

CGCCGCTTTGAGCAACTTGAGCTGCGGCCTGTCCTCTATAACTACCTGGATAAAAACCTG

ACTGTGAGCGTCCATGTGTCCCCAGTGGAGGGGCTGTGTCTGGCTGGGGGCGGAGGGCTG

GCCCAGCAGGTGCTGGTGCCTGCAGGCTCTGCCCGGCCCGTTGCCTTCTCTATGGTGCCC

ATGGCAGCTGCTGCTATGTCCCTGAAGGTGGTGGCTCGAGGATCCTTTGAATTCCCTGTA

GGGGATGCAGTGTCCAAGGTTCTGCAAATTGAGAAGGAAGGGGCCATCCACAGGGAGGAG

ATTGTCTATGAACTCAACCCCCTGGACCACCGAGGCCGGAGCTTGGAAATTCCTGGCAAC

TCTGATCCAAATATTATCCCTGATGGAGATTTCAGCAGCATCGTGAGAGTTACAGCCTCG

GATCCATTGGAGACCCTTGGCTCTGAGGGGGCCTTATCCCCAGGGGGCCTAGCCGCCCTC

CTGAGGCTTCCCTATGGCTGTGCTGAACAAACCATGATCTACTTGGCTCCCACACTGGCA

GCCTCCCACTATCTGGACAAGACAGAACAATGGAGCAAACTGCCCCCTGAGACCAAGGAT

CGTGCCGTGGATCTGATCCAGAAAGGCTACATGCGGATCCAGCAGTTTCGGAAGACAGAT

GGTTCCTTTGGGGCTTGGTTACACAGGGACAGCAGCACCTGGCTCACAGCCTTTGTGCTG

AAGGTTCTAAGTTTGGCTCAGGAGCAGGTGGGCGGCTCACCTGAAAAACTGCAGGAGACA

GCCAGTTGGTTGCTGTCCCAGCAACAGGCAGACGGCTCATTCCAGGACCCATGTCCAGTT

ATCCACAGGGCCATGCAGGGGGGTTTGGTGGGAAATGATGAGACTGTGGCTCTTACTGCT

TTCGTGGTCATTGCCCTTCAGCATGGGCTGGCTGTCTTCCAGGACCAGAGTGCACAGCAA

TTGAAGCAGAGAGTGGAAACCTCCATCTCAAAGGCAAACTCATTCTTGGGGGAGAAAGCA

AGTGCTGGGCTCCTGGGTGCCCATGCAGCTGCCATCACGGCCTATGCCCTAACTCTGTCC

AAGGCTTCCCAGAACCTGCGGGATGTTGCCCACAACAACCTTATGGCCATGGCCCAGGAG

ACTGGTGATTACTTGTACTGGGGCTCAGTCACTGGTTCTCAGAACAATGTCGTGTCACCC

ACCCTGGCTCCTCGCAGTCCAGCAGACCCCATGCCCCAGGCCCCAGCCCTGTGGATTGAA

ACCACAGCCTATGGCCTGCTGCACCTGCTGCTTCGAGAGGGCAAAGCAGAGCTGGCCAAT

CAGGCGGCATCCTGGCTCACCCATCAGGGCAGCTTCCAAGGGGGATTCCGCAGCACTCAG

GACACAGTGATTGCGCTGGATGCCCTGTCTGCATACTGGATCGCTGCCCACACCACTGAG

GAACGGGGACTCAATGTGACTCTCAGTTCCATAGGCCGCAGTGGGTTCAAATCCTATGTG

CTTCAGCTGAACAACCACCAAATTAAAGGGATAGAGGAGGAGCTGCAGTTTTCTCTGAGC

AACAAGATTAATGTGGAAGTGGGAGGAAACAGCAAGGGAACCTTGAAGGTCCTGCGTACC

TACAATGTCCTGGACATGAAGAACACAACCTGCACAGACCTTCAGATTGAGGTGTCAGTC

ATAGGCCATGTAGACTACACAAGGGAAGCAAATGAGGATTATGAGGATTATGAGTATGAG

GAATTTCCAGCCAGTGATGACCCAGGTGTGTATACCCATCCTGTGACACCCCTGCAGCTG

TTTGAGGGACGGAGGAACCGCCGTAGGAGGGAGGTGCCCAAGGTGGCAGAGGAACAGGAA

TCCAGAGTGCAGTATACTGTGTGCATCTGGCGAAATGGCAAGGTGGGGCTGTCTGGTATG

GCCATTGCGGATATTACTCTTCTGAGTGGATTCCATGCCCTGCGGGCTGACCTAGAGAAG

CTGACCTCCCTCTCTGACCGTTATGTGAGTCACTTTGAGACGGATGGACCCCATGTCTTA

CTGTACTTTGACTCGGTCCCCACCTCCCGGGAGTGTGTGGGCTTTGGAGCTGTGCAGGAG

GTGGCCGTGGGGCTGGTGCAGCCAGCTAGCGCCGTCCTATATGACTACTACAGCCCTGAG

CACAAATGTTCTGTGTTTTATGGGGCACCAACTAAGAGCAAACTTCTGTCCACATTGTGC

TCTGCTGATGTCTGCCAGTGTGCTGAGGGGAAATGCCCTCGACAGCGTCGTGCCCTGGAG

CGGGGGCTGCAGGATGAGGATGGTTATAGGATGAAGTTTGCCTGCTACTATCCCCGTGTG

GAATATGGCTTCCAGGTTCATGTTCTCCGAGAAGATAGCAGAACTGCTTTCCGCCTCTTT

GAAACCAGGATCACCCAAGTCCTGCACTTCACCAAGGATTCCAAGGCTGCTGCTGGTCAG

ACCCGCAACTTCCTGGTTCGAGCTTCTTGCCGCCTTCACTTGGAGCCTGAGAAAGAATAT

TTGATCATGGGTCTGGATGGGGCCACATATGACCTCAAAGGAGACCCCCAATACCTGCTG

GATTCAAATAGCTGGATTGAAGAAATGCCCTCTGAACGCCTGTGCCGGAGCACCCGCCAG

CGGGCAGCCTGTGCCCAGCTCAGTGACTTCCTCCAGGAGTATGGCACCCAGGGGTGCCAA

GTG

>CL227.Contig8_A-W 2922 3662 PREDICTED: complement C4-A isoform 2 [Otolemur garnettii]

CGGGCTGACCTAGAGAAGCTGACCTCCCTCTCTGACCGTTATGTGAGTCACTTTGAGACG

GATGGACCCCATGTCTTACTGTACTTTGACTCGGTCCCCACCTCCCGGGAGTGTGTGGGC

TTTGGAGCTGTGCAGGAGGTGGCCGTGGGGCTGGTGCAGCCAGCTAGCGCCGTCCTATAT

GACTACTACAGCCCTGAGCACAAATGTTCTGTGTTTTATGGGGCACCAACTAAGAGCAAA

CTTCTGTCCACATTGTGCTCTGCTGATGTCTGCCAGTGTGCTGAGGGGAAATGCCCTCGA

CAGCGTCGTGCCCTGGAGCGGGGGCTGCAGGATGAGGATGGTTATAGGATGAAGTTTGCC

TGCTACTATCCCCGTGTGGAATATGGCTTCCAGGTTCATGTTCTCCGAGAAGATAGCAGA

ACTGCTTTCCGCCTCTTTGAAACCAGGATCACCCAAGTCCTGCACTTCACCAAGGATTCC

AAGGCTGCTGCTGGTCAGACCCGCAACTTCCTGGTTCGAGCTTCTTGCCGCCTTCACTTG

GAGCCTGAGAAAGAATATTTGATCATGGGTCTGGATGGGGCCACATATGACCTCAAAGGA

GACCCCCAATACCTGCTGGATTCAAATAGCTGGATTGAAGAAATGCCCTCTGAACGCCTG

TGCCGGAGCACCCGCCAGCGGGCAGCCTGTGCCCAGCTCAGTGACTTCCTCCAGGAGTAT

GGCACCCAGGGGTGCCAAGTG

>CL227.Contig9_A-W 115 429 PREDICTED: complement C4-A [Loxodonta africana]

TTCCCTACCTCTCTAATGGCTCTTGCACATATTATCCCTTCAGCCAAGGATTCCAAGGCT

GCTGCTGGTCAGACCCGCAACTTCCTGGTTCGAGCTTCTTGCCGCCTTCACTTGGAGCCT

GAGAAAGAATATTTGATCATGGGTCTGGATGGGGCCACATATGACCTCAAAGGAGACCCC

CAATACCTGCTGGATTCAAATAGCTGGATTGAAGAAATGCCCTCTGAACGCCTGTGCCGG

AGCACCCGCCAGCGGGCAGCCTGTGCCCAGCTCAGTGACTTCCTCCAGGAGTATGGCACC

CAGGGGTGCCAAGTG

>CL228.Contig1_A-W 14 850 PREDICTED: peflin [Papio anubis]

ATGGCCAGCTATCCGTATGGGCAGGGCTGCCCAGGAGCTACAGGACAGGCACCTGGAGCC

CCTCCGGGTAGCTACTACCCTGGACCTCCACATGGTGGAGGGCAGTATGGCAGCGGAGGT

TATGGGGGTCCTGCCCCTGGAGGGCCTTATGGACCACCAGCTGGTGGAGGGCCCTATGGA

TACCCCAATGCTGGAGGGCTCCCCCCTGGAACCCCAGGAGGACCATATGGTGGTGCCGCC

CCAGGTGGCCCCTATAGTCAGCCACCTCCAAGTTCCTATGGTGCCCAGCAGCCTGGGCCT

TATGGACAGGGTGGTGTCCCACCCAATGTGGATCCTGAGGCCTACTCCTGGTTCCAGTCA

GTGGACTCAGATCACAGTGGCTACATCTCCAAGAAGGAGCTAAAGCAGGCCCTGGTCAAC

TCCAACTGGTCTTCATTCAATGACGAGACATGCCTCATGATGATAAACATGTTTGACAAG

ACCAAGTCAGGCCGCATTGATATCTATGGTTTCTCAGCACTGTGGAAATTCATCCAGCAG

TGGAAGAACCTCTTCCAGCAGTATGACCGGGACCACTCAGGTTCCATCAGCTACACAGAG

CTGCAGCAAGCTCTGTCCCAAATGGGCTACAACCTGAGCCCCCAGTTCACCCAGCTCCTG

GTCTCCCGCTACTGCCCACGCTCTGCCAGTCCCGCCATGCAGCTCGACCGCTTCATTCAG

GTGTGCACCCAGCTGCAGGTGCTGACCGAGGCCTTCCGAGAGAAGGATACCGCGGTGCAG

GGCAACATTCGGCTCAGCTTCGAGGACTTCGTCACCATGACAGCTTCTCGGATGCTA

>CL228.Contig2_A-W 159 386 minus strand PREDICTED: peflin [Saimiri boliviensis boliviensis]

GCTCTGTCCCAAATGGGCTACAACCTGAGCCCCCAGTTCACCCAGCTCCTGGTCTCCCGC

TACTGCCCACGCTCTGCCAGTCCCGCCATGCAGCTCGACCGCTTCATTCAGGTGTGCACC

CAGCTGCAGGTGCTGACCGAGGCCTTCCGAGAGAAGGATACCGCGGTGCAGGGCAACATT

CGGCTCAGCTTCGAGGACTTCGTCACCATGACAGCTTCTCGGATGCTA

>CL228.Contig3_A-W 37 564 PREDICTED: peflin-like isoform 2 [Sus scrofa]

GGTGGTGTCCCACCCAATGTGGATCCTGAGGCCTACTCCTGGTTCCAGTCAGTGGACTCA

GATCACAGTGGCTACATCTCCAAGAAGGAGCTAAAGCAGGCCCTGGTCAACTCCAACTGG

TCTTCATTCAATGACGAGACATGCCTCATGATGATAAACATGTTTGACAAGACCAAGTCA

GGCCGCATTGATATCTATGGTTTCTCAGCACTGTGGAAATTCATCCAGCAGTGGAAGAAC

CTCTTCCAGCAGTATGACCGGGACCACTCAGGTTCCATCAGCTACACAGAGCTGCAGCAA

GCTCTGTCCCAAATGGGCTACAACCTGAGCCCCCAGTTCACCCAGCTCCTGGTCTCCCGC

TACTGCCCACGCTCTGCCAGTCCCGCCATGCAGCTCGACCGCTTCATTCAGGTGTGCACC

CAGCTGCAGGTGCTGACCGAGGCCTTCCGAGAGAAGGATACCGCGGTGCAGGGCAACATT

CGGCTCAGCTTCGAGGACTTCGTCACCATGACAGCTTCTCGGATGCTA

>CL229.Contig2_A-W 918 1079 hCG1988652 [Homo sapiens]

TTGTCAAATGTTGCCAATACATGCCTTTTGCCTTTTCACATGGTTATGATCACTCTTGTA

TGGAGTATGAGGTCCCACTGCTCCCACTTCTATAGCCTTTTCACAGCTCTCTGCAGCCTA

GTTTCTGGCTTTGACTTTAGGATGCTACATTGGGTGGGAAAA

>CL230.Contig1_A-W 2 442 PREDICTED: DENN domain-containing protein 4C-like [Canis lupus familiaris]

GAACTTGAATCTTTGCTAGAAAATGAAGGTGATCAGGTGATTCATACATCCTCTTTCATC

AATCAACATCCAATCATTTTCTGGAACCTTGTTTGGTATTTCAGACGTTTAGACCTTCCT

AGTAACTTGCCAGGACTTATTCTCACATCTGAACACTGTAACGAAGGTATACAGCTTCCT

TTATCATCTCTGTCCCAGGATAGCAAACTTGTCTATATTCAACTCTTATGGGATAATATC

AACCTTCATCAAGAACCAGGAGAACCTCTGTATGTCTCTTGGAGGAATTTTAATTCTGAA

AAGAAATTATCCCTCCTGTCAGAGGAACAACAAGCAACAAGCACTTTAGTTGAAACTATT

AGGCAGAGTATTCAGCATAATGATGTTCTTAAGCCTATCAATCTGCTGTCTGAGCAAATG

AAGCCAGAAATGAAAAGACAA

>CL231.Contig1_A-W 2 1177 minus strand PREDICTED: EF-hand domain-containing protein KIAA0494-like [Sus scrofa]

TCAGACTCTGATTCCAGCTCAGAGGAGGAAGAGGAATTCGGTGTTGTTGGAAATCGCTCT

CGCTTTGTCAAGGGAGACTACTTAAGATGCTGCAAGATCTGTTATCCTCTCTGTGCTTTT

GTCATCCTCGCGGCCTGTGTCGTGGCCTGTGTTGGCTTGGTGTGGATGCAAGTTGCTCTG

AAGGAGGATCTGGATATTCTCAAGGAAAGATTTAGAACAATGGAATCTAATCAGAAAAGC

TCATTCCAGGAAATACCCAAACTTAATGAAGAACTACTCAGTGAACAGAAACAACTTGAG

AAGATTGAGTCTGGAGAGCTGGGCTTGAATGATGTCTGGCTCAACATCACAGAAATGAAT

AAGCAGATTTCTCTGTTGACGTCTGCAGTAAACCACCTTAAAGCCAATGTTAAGTCAGCT

GCAGACTTAATTAGCCTGCCTACCACTGTAGAAGGACTTCAGAAAAGTGTAGCTTCCATT

GGCAATACTTTAAACAGTGTCCATCTAGCTATGGAGGCAATACAGAAAACTGTGGATGAA

CACAAGAAAACCGTGGAATTGCTGCAAATTGATATGAATCGGCACTTCTCAAATTGGACC

ATTGGAAGCAACAAAAGCATTCCAACACCCTCAGCTACATCAGAACTTGATAATAAAACC

CACAGTGAGCATTTGAAACAGATGAGTGATAGAACTGCTATTCTGAAAAGAGAGTCTTTG

GATCAAGGGGCCAACAGAACTGATATCATAAAAACCCAAAACATAAAGAAGGAAGATAGT

CCAAATTCTCAGGTATCCAAGCTGCGAGAGAAACTGCAGCTGATCAGTGCTCTCACAAAC

AAACCTGAGAGCAACAGGCCTCCAGAGATTGCAGACGAAGAACAAGTACAGAGTTTCACA

TCAAAGCCATCAACATTGCCAAAATTCTCACAATTTCTTGGAGACCAAATTGAGAAAGCT

GCCCAACTAAGGCCCGTCTCCCTACCAGGAATTTCTAGCATTGAAGATCTTCAGGATTTA

TTCCACAAGACTGGCCAGGATGTGGATGGGAAGCTGACCTACCAGGAAATCTGGAACTCT

CTAGGTTCTACCTTGCCAGATCCAGAGAGCTTGAAAGCATTTGATTCCAATGGAGATGGA

AGATACTCATTCCTGGAGCTAAGAGTAGCTTTAGGT

>CL231.Contig2_A-W 2 1375 minus strand PREDICTED: EF-hand domain-containing protein KIAA0494 homolog [Pan paniscus]

TCAGACTCTGATTCCAGCTCAGAGGAGGAAGAGGAATTCGGTGTTGTTGGAAATCGCTCT

CGCTTTGTCAAGGGAGACTACTTAAGATGCTGCAAGATCTGTTATCCTCTCTGTGCTTTT

GTCATCCTCGCGGCCTGTGTCGTGGCCTGTGTTGGCTTGGTGTGGATGCAAGTTGCTCTG

AAGGAGGATCTGGATATTCTCAAGGAAAGATTTAGAACAATGGAATCTAATCAGAAAAGC

TCATTCCAGGAAATACCCAAACTTAATGAAGAACTACTCAGTGAACAGAAACAACTTGAG

AAGATTGAGTCTGGAGAGCTGGGCTTGAATGATGTCTGGCTCAACATCACAGAAATGAAT

AAGCAGATTTCTCTGTTGACGTCTGCAGTAAACCACCTTAAAGCCAATGTTAAGTCAGCT

GCAGACTTAATTAGCCTGCCTACCACTGTAGAAGGACTTCAGAAAAGTGTAGCTTCCATT

GGCAATACTTTAAACAGTGTCCATCTAGCTATGGAGGCAATACAGAAAACTGTGGATGAA

CACAAGAAAACCGTGGAATTGCTGCAAATTGATATGAATCGGCACTTCTCAAATTGGACC

ATTGGAAGCAACAAAAGCATTCCAACACCCTCAGCTACATCAGAACTTGATAATAAAACC

CACAGTGAGCATTTGAAACAGGATATCCTGTACCTTCATAACTCTTTAGAGGCGGTAAAC

AGTACCCTAGTAGCATACCAGAGACAGAATGATCTTAAACTCGAGGGGATGAATGAGACA

CTCAGTAATCTCATTCAGAGAACCAACCTGATAGAAAGCAATGTGGTTTTTGCTCTGGGT

GAAGTAGAAAACACAACAAACCTGACCTCCAGCATGATGAGTGATAGAACTGCTATTCTG

AAAAGAGAGTCTTTGGATCAAGGGGCCAACAGAACTGATATCATAAAAACCCAAAACATA

AAGAAGGAAGATAGTCCAAATTCTCAGGTATCCAAGCTGCGAGAGAAACTGCAGCTGATC

AGTGCTCTCACAAACAAACCTGAGAGCAACAGGCCTCCAGAGATTGCAGACGAAGAACAA

GTACAGAGTTTCACATCAAAGCCATCAACATTGCCAAAATTCTCACAATTTCTTGGAGAC

CAAATTGAGAAAGCTGCCCAACTAAGGCCCGTCTCCCTACCAGGAATTTCTAGCATTGAA

GATCTTCAGGATTTATTCCACAAGACTGGCCAGGATGTGGATGGGAAGCTGACCTACCAG

GAAATCTGGAACTCTCTAGGTTCTACCTTGCCAGATCCAGAGAGCTTGAAAGCATTTGAT

TCCAATGGAGATGGAAGATACTCATTCCTGGAGCTAAGAGTAGCTTTAGGTGTC

>CL233.Contig1_A-W 10 156 PREDICTED: uncharacterized protein C15orf43-like [Pan troglodytes]

ATGTTTCAAGGGCAGCGCGGTTGGTTTAGCGGTAGCGTCAGCCGAGAGCTGAGGCAGTTC

TGGGTGGCCGAAGGGGGGACAGTCAGCGACTCGCGGGCGGCAGACTTCTTGTTCAGCTGT

GACGCCTCGCACCCGGACACCCTGAGA

>CL233.Contig2_A-W 137 499 Uncharacterized protein C15orf43 OS=Homo sapiens GN=C15orf43 PE=2 SV=1

GAAATAAGAAGAAAAATTGGTAGTTTTATTTGGGAACAAGACCAGGATTTTCTGATAGAA

AAGCCTGAGGAAATAGCATCAAATAAAATAAAAATCAGTGAACTAGCAACAGAGAACAAA

AAAGAATTATCCAAAAGCATAGAAAAGAATTTTATAAGGACTCCAGTTGTAGAAAAGCAA

ATGTACTTCCCTCTTCAGAATTATCCAGTGAACAACATGGTAACAGGTTATATATCAATT

GATGCTATGAAGAAATTCCTTGGGGAGTTACATGACTTCATTCCTGGAAGCTCAGGATAT

TTGGCATATCATGTTCAAAATGAAATTAACATGTCTGCTATAAAAAACAAATTGAAGAGG

AAG

>CL233.Contig3_A-W 71 466 PREDICTED: uncharacterized protein C15orf43-like [Pan troglodytes]

AAAAATAGTATTGGACTTGTTTTTCTCACCACAGAAATAAGAAGAAAAATTGGTAGTTTT

ATTTGGGAACAAGACCAGGATTTTCTGATAGAAAAGCCTGAGGAAATAGCATCAAATAAA

ATAAAAATCAGTGAACTAGCAACAGAGAACAAAAAAGAATTATCCAAAAGCATAGAAAAG

AATTTTATAAGGACTCCAGTTGTAGAAAAGCAAATGTACTTCCCTCTTCAGAATTATCCA

GTGAACAACATGGTAACAGGTTATATATCAATTGATGCTATGAAGAAATTCCTTGGGGAG

TTACATGACTTCATTCCTGGAAGCTCAGGATATTTGGCATATCATGTTCAAAATGAAATT

AACATGTCTGCTATAAAAAACAAATTGAAGAGGAAG

>CL233.Contig4_A-W 264 416 PREDICTED: uncharacterized protein C15orf43-like [Pan troglodytes]

AAGAATTATCCAAAAGGTTATATATCAATTGATGCTATGAAGAAATTCCTTGGGGAGTTA

CATGACTTCATTCCTGGAAGCTCAGGATATTTGGCATATCATGTTCAAAATGAAATTAAC

ATGTCTGCTATAAAAAACAAATTGAAGAGGAAG

>CL234.Contig1_A-W 3 1616 PREDICTED: lanosterol synthase [Papio anubis]

TTCTGGCTGGCGGTCCTGAACGTCTACAGCTGGGAAGGCCTCAACACCCTGTTCCCTGAG

ATGTGGCTGTTTCCTGACTGGATGCCCGCGCACCCGTCCACACTCTGGTGCCACTGTCGG

CAGGTCTACCTCCCCATGAGCTACTGCTACGCCATCCGGCTAAGTGCCTCAGAGGACCCA

CTGGTCCAGAGCCTCCGCCAGGAGCTCTATGTGGAGGACTATGCCAGCATCAGCTGGCCA

GCACAGAGGAACAAAGTGGCCCATGAAGAGCAGTACACACCACACAGCTGGCTGCTGCGC

GTCACATATGCCCTCCTCAACCTGTATGAGCGCTACCACAGCCCCAGCCTGCGGCGGCGG

GCAGTCCAACTGCTATACGAGCACATCGCTGCAGACGACCGCTTCACCAAGTGCATCAGC

ATTGGCCCGATCTCAAAGACCATCAACATGCTTGTGCGCTGGTCTGTGGAAGGGCCGGCC

TCCTCTGCTTTCCAGGAGCATGTCTCTAGGATCCCAGATTATCTCTGGCTGGGTCTGGAT

GGCATGAAAATGCAGGGTACCAATGGCTCACAGATCTGGGACACCGCATTTGCCATCCAG

GCTCTGCTTGAGGCAGGTGCTCACCACAGGCCAGAGTTTATGTCCTGCCTGCAGAAGGCA

CATGAGTTCCTGAGGCTGTCACAGGTCCCCGACAACCCTCCTGACTACAAGAAGTACTAC

CGCCAAATGAACAAGGGCGGCTTCTGCTTCAGCACTCTGGACTGCGGCTGGATTGTCGCT

GACTGCACAGCCGAGGCCTTGAAGTCTGTGCTGCTCCTGCAGGAGAAGTGTCCCTCCATC

ACCCAGCACATCCCCCGACAGAGGCTCTGCGATGCTGTGGCTGTGCTGTTGAACATGAGG

AATCCTGACGGAGGATTCGCCACCTATGAGACCAAGCGTGGGGGGCACTTGCTGGAGCTG

CTGAACCCATCTGAGGTCTTTGGGGATGTCATGATCGACTACACGTACGTGGAGTGCACC

TCGGCAGTGATGCAGGCACTGAGGCTTTTCCACAAGCACTGTCCAGACTACAGAGCAGTG

GAAATCAGGGAGACACTGGAGCGGGGCCTGGCGTTCTGTCAGCAGAAGCAGAGGGCTGAC

GGCTCCTGGGAGGGTTCCTGGGGGGTTTGTTTCACCTATGGCACCTGGTTTGGCCTGGAA

GCTTTCGCCTGCATGGGGCATACCTACCGTGATGGGGCTGCCTGTGAGGAGGTCTCCCGG

GCCTGTGACTTCCTGCTGTCCCGGCAGATGGTGGATGGAGGCTGGGGGGAGGACTTTGAG

TCCTGTGAGCAGCAGCGTTACGTGCAGAGTGCCCAGTCCCAGATCCACAACACATGCTGG

GCCCTGATGGGGCTGATGGCTGTCAGGCACCCTGACATCACAGCCCAGGAGAGAGGGGTC

CAGTGTCTGCTGGACAAGCAGCTTCCCAATGGTGACTGGCCCCAGGAGAACATCTCTGGG

GTCTTCAACAAGTCCTGTGCCATCAGCTACACCAGCTACAGGAATATCTTCCCCATCTGG

GTCCTGGGCCGCTTCGCCAACCTGTACCCCGAGAGCACCCTCACTGGCCGCCCT

>CL234.Contig2_A-W 3 1406 minus strand PREDICTED: lanosterol synthase [Papio anubis]

AAGTCCACAGTGTTTGGGACTGCACTCAACTATGTGGCTCTCAGAATCCTGGGCATTGGG

CCTGATGATCCTGACCTGGTGCGAGCCCGGAACCTTCTTCACCAGAAAGGTGGTGCTGTG

GCTATCCCTTCCTGGGGGAAGTTCTGGCTGGCGGTCCTGAACGTCTACAGCTGGGAAGGC

CTCAACACCCTGTTCCCTGAGATGTGGCTGTTTCCTGACTGGATGCCCGCGCACCCGTCC

ACACTCTGGTGCCACTGTCGGCAGGTCTACCTCCCCATGAGCTACTGCTACGCCATCCGG

CTAAGTGCCTCAGAGGACCCACTGGTCCAGAGCCTCCGCCAGGAGCTCTATGTGGAGGAC

TATGCCAGCATCAGCTGGCCAGCACAGAGGAACAAAGTGGCCCATGAAGAGCAGTACACA

CCACACAGCTGGCTGCTGCGCGTCACATATGCCCTCCTCAACCTGTATGAGCGCTACCAC

AGCCCCAGCCTGCGGCGGCGGGCAGTCCAACTGCTATACGAGCACATCGCTGCAGACGAC

CGCTTCACCAAGTGCATCAGCATTGGCCCGATCTCAAAGACCATCAACATGCTTGTGCGC

TGGTCTGTGGAAGGGCCGGCCTCCTCTGCTTTCCAGGAGCATGTCTCTAGGATCCCAGAT

TATCTCTGGCTGGGTCTGGATGGCATGAAAATGCAGGGTACCAATGGCTCACAGATCTGG

GACACCGCATTTGCCATCCAGGCTCTGCTTGAGGCAGGTGCTCACCACAGGCCAGAGTTT

ATGTCCTGCCTGCAGAAGGCACATGAGTTCCTGAGGCTGTCACAGGTCCCCGACAACCCT

CCTGACTACAAGAAGTACTACCGCCAAATGAACAAGGGCGGCTTCTGCTTCAGCACTCTG

GACTGCGGCTGGATTGTCGCTGACTGCACAGCCGAGGCCTTGAAGTCTGTGCTGCTCCTG

CAGGAGAAGTGTCCCTCCATCACCCAGCACATCCCCCGACAGAGGCTCTGCGATGCTGTG

GCTGTGCTGTTGAACATGAGGAATCCTGACGGAGGATTCGCCACCTATGAGACCAAGCGT

GGGGGGCACTTGCTGGAGCTGCTGAACCCATCTGAGGTCTTTGGGGATGTCATGATCGAC

TACACGTACGTGGAGTGCACCTCGGCAGTGATGCAGGCACTGAGGCTTTTCCACAAGCAC

TGTCCAGACTACAGAGCAGTGGAAATCAGGGAGACACTGGAGCGGGGCCTGGCGTTCTGT

CAGCAGAAGCAGAGGGCTGACGGCTCCTGGGAGGGTTCCTGGGGGGTTTGTTTCACCTAT

GGCACCTGGTTTGGCCTGGAAGCTTTCGCCTGCATGGGGCATACCTACCGTGATGGGGCT

GCCTGTGAGGAGGTCTCCCCAGCC

>CL234.Contig3_A-W 3 1055 minus strand PREDICTED: lanosterol synthase [Papio anubis]

AAGTCCACAGTGTTTGGGACTGCACTCAACTATGTGGCTCTCAGAATCCTGGGCATTGGG

CCTGATGATCCTGACCTGGTGCGAGCCCGGAACCTTCTTCACCAGAAAGGTGGTGCTGTG

GCTATCCCTTCCTGGGGGAAGTTCTGGCTGGCGGTCCTGAACGTCTACAGCTGGGAAGGC

CTCAACACCCTGTTCCCTGAGATGTGGCTGTTTCCTGACTGGATGCCCGCGCACCCGTCC

ACACTCTGGTGCCACTGTCGGCAGGTCTACCTCCCCATGAGCTACTGCTACGCCATCCGG

CTAAGTGCCTCAGAGGACCCACTGGTCCAGAGCCTCCGCCAGGAGCTCTATGTGGAGGAC

TATGCCAGCATCAGCTGGCCAGCACAGAGGAACAAAGTGGCCCATGAAGAGCAGTACACA

CCACACAGCTGGCTGCTGCGCGTCACATATGCCCTCCTCAACCTGTATGAGCGCTACCAC

AGCCCCAGCCTGCGGCGGCGGGCAGTCCAACTGCTATACGAGCACATCGCTGCAGACGAC

CGCTTCACCAAGTGCATCAGCATTGGCCCGATCTCAAAGACCATCAACATGCTTGTGCGC

TGGTCTGTGGAAGGGCCGGCCTCCTCTGCTTTCCAGGAGCATGTCTCTAGGATCCCAGAT

TATCTCTGGCTGGGTCTGGATGGCATGAAAATGCAGGGTACCAATGGCTCACAGATCTGG

GACACCGCATTTGCCATCCAGGCTCTGCTTGAGGCAGGTGCTCACCACAGGCCAGAGTTT

ATGTCCTGCCTGCAGAAGGCACATGAGTTCCTGAGGCTGTCACAGGTCCCCGACAACCCT

CCTGACTACAAGAAGTACTACCGCCAAATGAACAAGGGCGGCTTCTGCTTCAGCACTCTG

GACTGCGGCTGGATTGTCGCTGACTGCACAGCCGAGGCCTTGAAGTCTGTGCTGCTCCTG

CAGGAGAAGTGTCCCTCCATCACCCAGCACATCCCCCGACAGAGGCTCTGCGATGCTGTG

GCTGTGGTGAGGCTCTGCCGCATCCCGCCAGGA

>CL235.Contig1_A-W 364 501 PREDICTED: rho GTPase-activating protein 17 [Macaca mulatta]

ATATGTAATGACACCAATTCTAGGGCTTCAGAACCGCTTCGCAGCATCTTTTCTGAAATG

CACTCAGACTCCGCAAACAAAGACCTGCCCGGCCACATTCTGCTGGATATAGACAATGAC

ACGGAAAGTACCGCCTTG

>CL235.Contig2_A-W 670 927 MSTP038 [Homo sapiens]

AACGAGAAGCAACGCGCCGTCTATTTAATAAAACCCTCAGCCATGCAGGTCAGTGCTGCT

TGCTACTGGGGCACTCACCCTCGTGTACCAGGAGGGAGCCCCTCCTGCAGACTTGCTGCA

GAACCGTTTTGTATTAAGTCTGGTCTGTGTCTTCCCAGTCCCCTCAGAACCGTGAGCATC

GATGGTGACTGCTGGATCTGTCACCTCATCAAACTGGACGTGACTTGTGCCTTGTCAGAT

TGCCAGAATGTAGAGAAA

>CL235.Contig3_A-W 1 1692 rho GTPase-activating protein 17 isoform 2 [Macaca mulatta] >gi|383419573|gb|AFH33000.1| rho GTPase-activating protein 17 isoform 2 [Macaca mulatta] >gi|384947938|gb|AFI37574.1| rho GTPase-activating protein 17 isoform 2 [Macaca mulatta]

GAGCACCTGAAGCGGAGTGGGCGTGAAATCGCGCTTCCCATCGAAGCCTGTGTCATGCTG

CTCCTGGAGACGGGCATGAAGGAGGAGGGCCTTTTCCGAATTGGGGCTGGAGCCTCCAAG

TTAAAGAAGCTGAAAGCCGCTTTGGATTGTTCTACTTCTCACCTGGACGAGTTCTACTCA

GACCCCCATGCTGTAGCAGGTGCTTTGAAGTCCTACCTGCGGGAATTGCCTGAACCTCTG

ATGACTTTTAATCTGTATGAGGAGTGGACACAAGTGGCAAGTGTGCAGGATCAGGACAAA

AAACTTCAAGATTTATGGAGAACATGTCAGAAGTTGCCACCACAGAATTTTGTTAATTTT

AGGTATTTAATCAAGTTCCTTGCGAAGCTCGCTCAGACTAGTGACATTAATAAAATGACT

CCTAGCAACATTGCAATCGTGTTAGGCCCTAATTTGTTATGGGCCAAAAATGAAGGAACA

CTGGCTGAAATGGCAGCGGCCACATCCGTCCATGTGGTTGCAGTGATTGAGCCCATCATC

CAGCACGCAGACTGGTTCTTCCCTGAAGAGGTGGAGTTTAATGTATCAGAAGCCTTCGTG

CCTCTCGCCACCCCAAATTCCAATCATTCATCCCACACTGGAAACGACTCTGACTCGGGG

ACTCTGGAGAGGAAGCGGCCGGCCAGCATGGCGGTGATGGAAGGGGACTTGGTGAAGAAG

GAGAGTCCTCCAAAACCCAAGGATTCTGTATCTGCAGCTGTCCCTGCACCAGGGAGAAAT

AGCAGTCAAATGACCAGTGGCCAAAGCCAGGCCCAGGCAGGTGCCAGCTCCCATCAGCTC

TCTGTTGGCCCAGCTCACAACACTGCTGGTCCCAGCCCTCACACGCTGCGCCGGGCAGTT

AAAAAACCTGCTCCAGCACCCCCGAAACCAGGAAACCCGCCTCCTGGCCATCACGGGGGC

CCGAGTGCTCCAGGAGCGTCTCAGCTTCCACCCAGCCTGTCACCAAAGCCATCCGCTCGA

AGTCCGTCTCCTCCCCCACAGCACACAGGCCCAGCCCCAGGCCAGCCCTCGGCCACCTCC

CAGCTCTCTGCACCCCGGAGGTACTCCAGCAGCCTGTCTCCAATACAAGCACCCAGCCAC

CCGCCGCCACAGCCCCCCACCCAGGCCACGCCACAGGGGCACAGCAAGCTGAGCGGCCAG

GGCCCACCCACCTCCATGACACTACCCAGTGAGCACGGACTCGAGCAGCCACCGCACACC

CCTCCCCAGACGCCAACACCCCCCAGTACTCCACCCCTAGGAAAGCAGAACTCCAACCTG

CCAGCTGCTCAGACCCAGGCGGTGAGTAACCCTGAGGCTGCGCAGACACATGCTGGAACC

CTGCCGAGACCCAGGCCAGTACCAAAGCCGAGGAACCGACCCAGCATGCCCCCGCCCCCC

CATCCTCCTGGAGCCCACTCTGCCGGGGACAGCAGCCTCAGCAGCGCAGCACCAACAGCT

TCCAAAATAGTGACAGATGTCCTTCCTAGTGCCCTCACAGGTGGGGAAAGTGTCCAGGAC

TAAGACACCAATTCTAGGGCTTCAGAACCGCTTCGCAGCATCTTTTCTGAAATGCACTCA

GACTCCGCAAACAAAGACCTGCCCGGCCACATTCTGCTGGATATAGACAATGACACGGAA

AGTACCGCCTTG

>CL235.Contig4_A-W 1 1644 rho GTPase-activating protein 17 isoform 2 [Macaca mulatta] >gi|383419573|gb|AFH33000.1| rho GTPase-activating protein 17 isoform 2 [Macaca mulatta] >gi|384947938|gb|AFI37574.1| rho GTPase-activating protein 17 isoform 2 [Macaca mulatta]

GAGCACCTGAAGCGGAGTGGGCGTGAAATCGCGCTTCCCATCGAAGCCTGTGTCATGCTG

CTCCTGGAGACGGGCATGAAGGAGGAGGGCCTTTTCCGAATTGGGGCTGGAGCCTCCAAG

TTAAAGAAGCTGAAAGCCGCTTTGGATTGTTCTACTTCTCACCTGGACGAGTTCTACTCA

GACCCCCATGCTGTAGCAGGTGCTTTGAAGTCCTACCTGCGGGAATTGCCTGAACCTCTG

ATGACTTTTAATCTGTATGAGGAGTGGACACAAGTGGCAAGTGTGCAGGATCAGGACAAA

AAACTTCAAGATTTATGGAGAACATGTCAGAAGTTGCCACCACAGAATTTTGTTAATTTT

AGGTATTTAATCAAGTTCCTTGCGAAGCTCGCTCAGACTAGTGACATTAATAAAATGACT

CCTAGCAACATTGCAATCGTGTTAGGCCCTAATTTGTTATGGGCCAAAAATGAAGGAACA

CTGGCTGAAATGGCAGCGGCCACATCCGTCCATGTGGTTGCAGTGATTGAGCCCATCATC

CAGCACGCAGACTGGTTCTTCCCTGAAGAGGTGGAGTTTAATGTATCAGAAGCCTTCGTG

CCTCTCGCCACCCCAAATTCCAATCATTCATCCCACACTGGAAACGACTCTGACTCGGGG

ACTCTGGAGAGGAAGCGGCCGGCCAGCATGGCGGTGATGGAAGGGGACTTGGTGAAGAAG

GAGAGTCCTCCAAAACCCAAGGATTCTGTATCTGCAGCTGTCCCTGCACCAGGGAGAAAT

AGCAGTCAAATGACCAGTGGCCAAAGCCAGGCCCAGGCAGGTGCCAGCTCCCATCAGCTC

TCTGTTGGCCCAGCTCACAACACTGCTGGTCCCAGCCCTCACACGCTGCGCCGGGCAGTT

AAAAAACCTGCTCCAGCACCCCCGAAACCAGGAAACCCGCCTCCTGGCCATCACGGGGGC

CCGAGTGCTCCAGGAGCGTCTCAGCTTCCACCCAGCCTGTCACCAAAGCCATCCGCTCGA

AGTCCGTCTCCTCCCCCACAGCACACAGGCCCAGCCCCAGGCCAGCCCTCGGCCACCTCC

CAGCTCTCTGCACCCCGGAGGTACTCCAGCAGCCTGTCTCCAATACAAGCACCCAGCCAC

CCGCCGCCACAGCCCCCCACCCAGGCCACGCCGCAGGGGCACAGCAAGCTGAGCGGCCAG

GGCCCACCCACCTCCATGACACTACCCAGTGAGCACGGACTCGAGCAGCCACCGCACACC

CCTCCCCAGACGCCAACACCCCCCAGTACTCCACCCCTAGGAAAGCAGAACTCCAACCTG

CCAGCTGCTCAGACCCAGGCGGTGAGTAACCCTGAGGCTGCGCAGACACATGCTGGAACC

CTGCCGAGACCCAGGCCAGTACCAAAGCCGAGGAACCGACCCAGCATGCCCCCGCCCCCC

CATCCTCCTGGAGCCCACTCTGCCGGGGACAGCAGCCTCAGCAGCGCAGCACCAACAGCT

TCCAAAATAGTGACAGACACCAATTCTAGGGCTTCAGAACCGCTTCGCAGCATCTTTTCT

GAAATGCACTCAGACTCCGCAAACAAAGACCTGCCCGGCCACATTCTGCTGGATATAGAC

AATGACACGGAAAGTACCGCCTTG

>CL236.Contig1_A-W 1 228 minus strand eukaryotic translation initiation factor 2C, 1 [Mustela putorius furo]

CTGGCCATTCGCGATGCCTGCATCAAACTGGAAAAGGACTACCAGCCTGGGATCACTTAT

ATTGTGGTGCAGAAACGCCATCACACCCGCCTTTTTTGTGCTGACAAGAATGAGCGAATT

GGGAAGAGTGGTAACATCCCAGCTGGGACCACTGTGGACACCAACATCACCCACCCATTT

GAGTTTGACTTCTATCTGTGCAGCCATGCAGGCATCCAGGGTACCAGC

>CL236.Contig2_A-W 2 445 minus strand PREDICTED: protein argonaute-2 [Papio anubis]

CAGTTCCAGCAGGTCCTTCACCACGAGCTGCTGGCCATCCGTGAGGCCTGTATTAAGCTG

GAAAAGGACTACCAGCCGGGGATTACGTTCATCGTGGTGCAGAAGAGGCACCACACACGG

CTCTTCTGCACTGACAAGAACGAGCGGGTTGGGAAGAGTGGGAACATTCCAGCAGGCACA

ACTGTGGACACGAAGATCACCCACCCAACGGAGTTTGACTTCTACCTATGTAGTCATGCC

GGCATCCAGGGGACAAGCAGGCCGTCCCACTACCATGTCCTCTGGGATGACAATCGTTTC

TCTTCTGACGAGCTGCAGATTCTCACCTACCAGCTGTGTCACACCTACGTGCGCTGCACA

CGCTCTGTGTCCATCCCAGCGCCAGCCTACTATGCCCACCTGGTGGCCTTCCGGGCCAGG

TACCACCTGGTAGACAAAGAACAC

>CL238.Contig1_A-W 223 963 Myeloid leukemia factor 2 [Heterocephalus glaber]

ATGTTCCGCTTCATGAGGGACGTGGAGCCTGAGGATCCCATGTTCCTGATGGACCCCTTT

GCTATTCACCGTCAGCATATGAGCCGCATGTTGTCAGGTGGCTTTGGCTATAGCCCCTTC

CTTAGCATCACAGATGGCAACATGCCAGGGACCAGGCCTGCCAGCCGCAGGATGCAGGCT

GGGGCTGTCTCCCCCTTTGGGATGCTGGGAATGTCGGGTGGCTTCATGGACATGTTTGGG

ATGATGAATGACATGATTGGAAATATGGAGCATATGACAGCTGGGGGCAATTGCCAGACC

TTTTCATCCTCTACCGTCATCTCCTACTCCAATACTGGTGATGGTGCCCCCAAGGTCTAT

CAGGAGACATCAGAGATGCGCTCAGCACCAGGCGGGATCCGGGAGACACGGAGGACTGTC

CGCGACTCAGACAGTGGACTAGAGCAGATGTCCATTGGGCATCACATCCGAGACAGGGCT

CACATCCTTCAGCGTTCCCGAAATCACCGCACAGGAGACCAGGAGGAACGGCAGGACTAT

ATCAACCTGGATGAGAGTGAGGCAGCAGCATTTGATGATGAGTGGCGAAGAGAGACATCC

AGATTCCGGCAGCAGCGCCCTCTGGAATTTCGAAGGCATGAGGCATCTGTGAGTGGGGGT

CGAAGGGCCGAGGGGCCTCCCCGCCTGGCCATCCAGGGACCTGAGGACTCCCCCTCCCGA

CAGTCCCGCCGCTATGACTGG

>CL238.Contig2_A-W 199 357 unnamed protein product [Macaca fascicularis]

CCCTGAAAAAACTTTTTCCTGTCCAACTCCCACCCCCAATTTAATATTAAATTAACAGGC

AAGCTGGCCCCCACCTCTCCCTGGGGGTCTCAGGGAGAACCTTACACTGCCCTCGATACC

TACATTTTCTGTCTTTAATCCCCCTACCACACAATGACT

>CL239.Contig1_A-W 831 1229 PREDICTED: protein FAM190B-like [Ailuropoda melanoleuca]

CCTCAAGTACTACAGCCTTCCAGCAACCTGCCCAGACCCACAGACCACGCCCAGGGAAAA

CTAATAAAGCCACAACATATCGAGGCCCACAGTGAATGTGCAGTCCAGGGCATGCACCAG

AGCAGTGCCCTTCCAGATGGAGGCTTTACACATGGCTTGCAACAAGAAAACAATTATGAT

TTGGAAAACCGGCCTTCTTCATCAAGCCCACTGTTCAAAATGGATGTAACTAAGAGTGCA

CCTTCTGAAGCGAACTTGAACATGACCATGAATGTTCAAGAGCCTTATCGTTTGACAAAG

AATCAGATTAGTGACATGCAGTTTATACCTACTTCTCTACAGACACCTCCCCAGTTAAGT

ATAGTAGACCAAGCTAAGAGAGTTGGAAGAGATCAGTCT

>CL239.Contig2_A-W 2 1147 PREDICTED: protein FAM190B-like [Ailuropoda melanoleuca]

GTTGGCAGCTACGATTCTTCTGAAATGAATAGCATTGATATTCTTAATAATCTTGAATCA

TGTGACCTTGAGGATGATGATCTTATGCTTGATGTGGATCTGCCTGAGGATGCACCTCTT

GACAATGTGGAGTGTGACAATATGAACCGCTTTGACCGACCAGACAGAAATGTTCGGCAG

TCTCAGGAAGGATTTTGGAAAAGACCACCCCAGAGGTGGAGTGGACAGGAACATTACCAC

CTTAGCCATCCTGACCACTATCATCACCATGGAAAAAGTGACTTGAGCAGAGGCTCTCCT

TATAGAGAATCTCCTTTGGGTCATTTTGAAAGCTATGGAGGGACCCCCTTTTTCCAGGCC

CAGAAGATGTTTGCAGATGTACCGGAAAATACAGTGATACTGGATGAGATGACTCTCCGA

CACATGGTCCAGGATTGCACTGCTGTAAAAACTCAGTTACTCAAACTCAAACGTCTGCTG

CATCAGCATGATGGAAGTGGGTCATTGCATGATATTCAGCTATCATTGCCATCTAGTCCA

GAACCAGAAGAAGGTGATCAGATATATAAGAATGAAGATTTATTAAATGAAATGAAACAA

CTTAAAGAAGAAATAAGGAAAAAAGATGAAAAAATACAACTATTAGAACATCAACTTGCA

ACCCGGTGTAACTGCCACCAAAAATCTAAAGAGGAAAAATGCTCATATGCTGATAAATAT

ACCCAAACACCCTGGAGAAGAATTCCTCCTCAAGTACTACAGCCTTCCAGCAACCTGCCC

AGACCCACAGACCACGCCCAGGGAAAACTAATAAAGCCACAACATATCGAGGCCCACAGT

GAATGTGCAGTCCAGGGCATGCACCAGAGCAGTGCCCTTCCAGATGGAGGCTTTACACAT

GGCTTGCAACAAGAAAACAATTATGATTTGGAAAACCGGCCTTCTTCATCAAGCCCACTG

TTCAAAATGGATGTAACTAAGAGTGCACCTTCTGAAGCGAACTTGAACATGACCATGAAT

GTTCAAGAGCCTTATCGTTTGACAAAGAATCAGATTAGTGACATGCAGTTTATACCTACT

TCTCTACAGACACCTCCCCAGTTAAGTATAGTAGACCAAGCTAAGAGAGTTGGAAGAGAT

CAGTCT

>CL240.Contig1_A-W 266 418 minus strand PREDICTED: LOW QUALITY PROTEIN: serine/threonine-protein kinase 32B-like [Nomascus leucogenys]

CCTGTTTTCTTCCCACTTCAGAAAGGGAGGTTGAACTGTGACCCCACGTTTGAACTTGAA

GAGATGATTCTAGAATCCAAGCCCCTTCACAAAAAGAAGAAGAGGTTGGCAAAGCACAGG

TCCAAAGACGGCACCAAGGACAGCTGCCCCCTG

>CL240.Contig2_A-W 64 216 minus strand PREDICTED: LOW QUALITY PROTEIN: serine/threonine-protein kinase 32B-like [Nomascus leucogenys]

CCTGTTTTCTTCCCACTTCAGAAAGGGAGGTTGAACTGTGACCCCACGTTTGAACTTGAA

GAGATGATTCTAGAATCCAAGCCCCTTCACAAAAAGAAGAAGAGGTTGGCAAAGCACAGG

TCCAAAGACGGCACCAAGGACAGCTGCCCCCTG

>CL240.Contig3_A-W 266 418 minus strand PREDICTED: LOW QUALITY PROTEIN: serine/threonine-protein kinase 32B-like [Nomascus leucogenys]

CCTGTTTTCTTCCCACTTCAGAAAGGGAGGTTGAACTGTGACCCCACGTTTGAACTTGAA

GAGATGATTCTAGAATCCAAGCCCCTTCACAAAAAGAAGAAGAGGTTGGCAAAGCACAGG

TCCAAAGACGGCACCAAGGACAGCTGCCCCCTG

>CL240.Contig4_A-W 64 216 minus strand PREDICTED: LOW QUALITY PROTEIN: serine/threonine-protein kinase 32B-like [Nomascus leucogenys]

CCTGTTTTCTTCCCACTTCAGAAAGGGAGGTTGAACTGTGACCCCACGTTTGAACTTGAA

GAGATGATTCTAGAATCCAAGCCCCTTCACAAAAAGAAGAAGAGGTTGGCAAAGCACAGG

TCCAAAGACGGCACCAAGGACAGCTGCCCCCTG

>CL240.Contig5_A-W 64 216 minus strand PREDICTED: LOW QUALITY PROTEIN: serine/threonine-protein kinase 32B-like [Nomascus leucogenys]

CCTGTTTTCTTCCCACTTCAGAAAGGGAGGTTGAACTGTGACCCCACGTTTGAACTTGAA

GAGATGATTCTAGAATCCAAGCCCCTTCACAAAAAGAAGAAGAGGTTGGCAAAGCACAGG

TCCAAAGACGGCACCAAGGACAGCTGCCCCCTG

>CL240.Contig6_A-W 64 216 minus strand PREDICTED: LOW QUALITY PROTEIN: serine/threonine-protein kinase 32B-like [Nomascus leucogenys]

CCTGTTTTCTTCCCACTTCAGAAAGGGAGGTTGAACTGTGACCCCACGTTTGAACTTGAA

GAGATGATTCTAGAATCCAAGCCCCTTCACAAAAAGAAGAAGAGGTTGGCAAAGCACAGG

TCCAAAGACGGCACCAAGGACAGCTGCCCCCTG

>CL240.Contig8_A-W 1465 1593 uncharacterized protein C6orf132 [Homo sapiens] >gi|327478596|sp|Q5T0Z8.4|CF132_HUMAN RecName: Full=Uncharacterized protein C6orf132

CCCTGCCCTGGCAGCCGCCCCGGGAGCAGCCAGGTACGCAGCGAGGCGGACACAGGAGCA

GGACGGCAGTCCCGACTCACTGGTGCCCTCTGGCCACCTCATCCATGCTGCCTGTGTGTG

CCAACGCCG

>CL240.Contig11_A-W 266 418 minus strand PREDICTED: LOW QUALITY PROTEIN: serine/threonine-protein kinase 32B-like [Nomascus leucogenys]

CCTGTTTTCTTCCCACTTCAGAAAGGGAGGTTGAACTGTGACCCCACGTTTGAACTTGAA

GAGATGATTCTAGAATCCAAGCCCCTTCACAAAAAGAAGAAGAGGTTGGCAAAGCACAGG

TCCAAAGACGGCACCAAGGACAGCTGCCCCCTG

>CL240.Contig12_A-W 1572 2441 Uncharacterized protein C6orf132 OS=Homo sapiens GN=C6orf132 PE=1 SV=4

CTGCCCTGGCAGCCGCCCCGGGAGCAGCCAGCGCCTGCAGTCGGTGCCAGCCTCACTGCA

GAGCCATGACCGCAAGTCTCCAAGGGCCGCTCGGCACACCGGGCAAGTCCACGTTTTCCT

AATCGATTTCCCTCTCCACGTCGGTGGGTGGGGGGAGCATTGAATTGGGTGAAGCTAAAA

GCAGCCAGACTCAACCCCTAAATGACCCCAGGGGAGGAGCCCATTCCCATTTGCTTTCAG

AGGGACACACTGTGGAATCCAAATTGAAGCATCTTCTGGGACACAGCAAAACGCCAAATG

CTCTTGCTGCCGGCTCGGGCTCTGCTCTGCTCAGCCTTCGCGGGCTCCCATTGCCAGCCG

GGCACTCTAGGTGCAGCTGGGCCATTCAGAGCAGATGCCTGACCCAGGAGGCTGCCTGAG

CCAGCGGTCACAGACGGTTTCTGAGCTGAAGCTCCTGAGCCCGGTTGTCTAGGGCTGGGG

AAGGGCTGGGAGCTTGCAGCAGCCTGGACTGCCCAGGATCTGCAGTGGCCCGTCACTATG

CATGGTGTCACTCCCCTTCTGCAGACCTCACTGGGAGCAGACCCTGCCCTGCCCTGTCCT

TCACCTCCCTGCCCCCTTTGGGAACTCAAGCGCCTGCCCATCGGCAGGTCCCCACCTGCC

CTGCAGCCTCCCTTCTCTGTGGCTTGTCCCCGTGTCTCACCTGGATGTCTTTCCCCTCTG

CCTTGCTCAGACTCAGGTGGGCACAGTCCCCCCAGAAGCTTCCCTGATCCCCAGGCTGGG

CCGGCACCTGTGTGGAAGCCACAGCCCAGGGCTGCCTTTCCTGTCAATGACCCCTGTGCT

GCTGTCATCTGTTGAAAATGGATGGACCCT

>CL240.Contig13_A-W 1556 2425 Uncharacterized protein C6orf132 OS=Homo sapiens GN=C6orf132 PE=1 SV=4

CTGCCCTGGCAGCCGCCCCGGGAGCAGCCAGCGCCTGCAGTCGGTGCCAGCCTCACTGCA

GAGCCATGACCGCAAGTCTCCAAGGGCCGCTCGGCACACCGGGCAAGTCCACGTTTTCCT

AATCGATTTCCCTCTCCACGTCGGTGGGTGGGGGGAGCATTGAATTGGGTGAAGCTAAAA

GCAGCCAGACTCAACCCCTAAATGACCCCAGGGGAGGAGCCCATTCCCATTTGCTTTCAG

AGGGACACACTGTGGAATCCAAATTGAAGCATCTTCTGGGACACAGCAAAACGCCAAATG

CTCTTGCTGCCGGCTCGGGCTCTGCTCTGCTCAGCCTTCGCGGGCTCCCATTGCCAGCCG

GGCACTCTAGGTGCAGCTGGGCCATTCAGAGCAGATGCCTGACCCAGGAGGCTGCCTGAG

CCAGCGGTCACAGACGGTTTCTGAGCTGAAGCTCCTGAGCCCGGTTGTCTAGGGCTGGGG

AAGGGCTGGGAGCTTGCAGCAGCCTGGACTGCCCAGGATCTGCAGTGGCCCGTCACTATG

CATGGTGTCACTCCCCTTCTGCAGACCTCACTGGGAGCAGACCCTGCCCTGCCCTGTCCT

TCACCTCCCTGCCCCCTTTGGGAACTCAAGCGCCTGCCCATCGGCAGGTCCCCACCTGCC

CTGCAGCCTCCCTTCTCTGTGGCTTGTCCCCGTGTCTCACCTGGATGTCTTTCCCCTCTG

CCTTGCTCAGACTCAGGTGGGCACAGTCCCCCCAGAAGCTTCCCTGATCCCCAGGCTGGG

CCGGCACCTGTGTGGAAGCCACAGCCCAGGGCTGCCTTTCCTGTCAATGACCCCTGTGCT

GCTGTCATCTGTTGAAAATGGATGGACCCT

>CL240.Contig15_A-W 266 418 minus strand PREDICTED: LOW QUALITY PROTEIN: serine/threonine-protein kinase 32B-like [Nomascus leucogenys]

CCTGTTTTCTTCCCACTTCAGAAAGGGAGGTTGAACTGTGACCCCACGTTTGAACTTGAA

GAGATGATTCTAGAATCCAAGCCCCTTCACAAAAAGAAGAAGAGGTTGGCAAAGCACAGG

TCCAAAGACGGCACCAAGGACAGCTGCCCCCTG

>CL240.Contig19_A-W 1467 2336 Uncharacterized protein C6orf132 OS=Homo sapiens GN=C6orf132 PE=1 SV=4

CTGCCCTGGCAGCCGCCCCGGGAGCAGCCAGCGCCTGCAGTCGGTGCCAGCCTCACTGCA

GAGCCATGACCGCAAGTCTCCAAGGGCCGCTCGGCACACCGGGCAAGTCCACGTTTTCCT

AATCGATTTCCCTCTCCACGTCGGTGGGTGGGGGGAGCATTGAATTGGGTGAAGCTAAAA

GCAGCCAGACTCAACCCCTAAATGACCCCAGGGGAGGAGCCCATTCCCATTTGCTTTCAG

AGGGACACACTGTGGAATCCAAATTGAAGCATCTTCTGGGACACAGCAAAACGCCAAATG

CTCTTGCTGCCGGCTCGGGCTCTGCTCTGCTCAGCCTTCGCGGGCTCCCATTGCCAGCCG

GGCACTCTAGGTGCAGCTGGGCCATTCAGAGCAGATGCCTGACCCAGGAGGCTGCCTGAG

CCAGCGGTCACAGACGGTTTCTGAGCTGAAGCTCCTGAGCCCGGTTGTCTAGGGCTGGGG

AAGGGCTGGGAGCTTGCAGCAGCCTGGACTGCCCAGGATCTGCAGTGGCCCGTCACTATG

CATGGTGTCACTCCCCTTCTGCAGACCTCACTGGGAGCAGACCCTGCCCTGCCCTGTCCT

TCACCTCCCTGCCCCCTTTGGGAACTCAAGCGCCTGCCCATCGGCAGGTCCCCACCTGCC

CTGCAGCCTCCCTTCTCTGTGGCTTGTCCCCGTGTCTCACCTGGATGTCTTTCCCCTCTG

CCTTGCTCAGACTCAGGTGGGCACAGTCCCCCCAGAAGCTTCCCTGATCCCCAGGCTGGG

CCGGCACCTGTGTGGAAGCCACAGCCCAGGGCTGCCTTTCCTGTCAATGACCCCTGTGCT

GCTGTCATCTGTTGAAAATGGATGGACCCT

>CL240.Contig22_A-W 64 216 minus strand PREDICTED: LOW QUALITY PROTEIN: serine/threonine-protein kinase 32B-like [Nomascus leucogenys]

CCTGTTTTCTTCCCACTTCAGAAAGGGAGGTTGAACTGTGACCCCACGTTTGAACTTGAA

GAGATGATTCTAGAATCCAAGCCCCTTCACAAAAAGAAGAAGAGGTTGGCAAAGCACAGG

TCCAAAGACGGCACCAAGGACAGCTGCCCCCTG

>CL240.Contig25_A-W 1343 2212 Uncharacterized protein C6orf132 OS=Homo sapiens GN=C6orf132 PE=1 SV=4

CTGCCCTGGCAGCCGCCCCGGGAGCAGCCAGCGCCTGCAGTCGGTGCCAGCCTCACTGCA

GAGCCATGACCGCAAGTCTCCAAGGGCCGCTCGGCACACCGGGCAAGTCCACGTTTTCCT

AATCGATTTCCCTCTCCACGTCGGTGGGTGGGGGGAGCATTGAATTGGGTGAAGCTAAAA

GCAGCCAGACTCAACCCCTAAATGACCCCAGGGGAGGAGCCCATTCCCATTTGCTTTCAG

AGGGACACACTGTGGAATCCAAATTGAAGCATCTTCTGGGACACAGCAAAACGCCAAATG

CTCTTGCTGCCGGCTCGGGCTCTGCTCTGCTCAGCCTTCGCGGGCTCCCATTGCCAGCCG

GGCACTCTAGGTGCAGCTGGGCCATTCAGAGCAGATGCCTGACCCAGGAGGCTGCCTGAG

CCAGCGGTCACAGACGGTTTCTGAGCTGAAGCTCCTGAGCCCGGTTGTCTAGGGCTGGGG

AAGGGCTGGGAGCTTGCAGCAGCCTGGACTGCCCAGGATCTGCAGTGGCCCGTCACTATG

CATGGTGTCACTCCCCTTCTGCAGACCTCACTGGGAGCAGACCCTGCCCTGCCCTGTCCT

TCACCTCCCTGCCCCCTTTGGGAACTCAAGCGCCTGCCCATCGGCAGGTCCCCACCTGCC

CTGCAGCCTCCCTTCTCTGTGGCTTGTCCCCGTGTCTCACCTGGATGTCTTTCCCCTCTG

CCTTGCTCAGACTCAGGTGGGCACAGTCCCCCCAGAAGCTTCCCTGATCCCCAGGCTGGG

CCGGCACCTGTGTGGAAGCCACAGCCCAGGGCTGCCTTTCCTGTCAATGACCCCTGTGCT

GCTGTCATCTGTTGAAAATGGATGGACCCT

>CL240.Contig26_A-W 266 418 minus strand PREDICTED: LOW QUALITY PROTEIN: serine/threonine-protein kinase 32B-like [Nomascus leucogenys]

CCTGTTTTCTTCCCACTTCAGAAAGGGAGGTTGAACTGTGACCCCACGTTTGAACTTGAA

GAGATGATTCTAGAATCCAAGCCCCTTCACAAAAAGAAGAAGAGGTTGGCAAAGCACAGG

TCCAAAGACGGCACCAAGGACAGCTGCCCCCTG

>CL240.Contig28_A-W 1570 1698 uncharacterized protein C6orf132 [Homo sapiens] >gi|327478596|sp|Q5T0Z8.4|CF132_HUMAN RecName: Full=Uncharacterized protein C6orf132

CCCTGCCCTGGCAGCCGCCCCGGGAGCAGCCAGGTACGCAGCGAGGCGGACACAGGAGCA

GGACGGCAGTCCCGACTCACTGGTGCCCTCTGGCCACCTCATCCATGCTGCCTGTGTGTG

CCAACGCCG

>CL240.Contig32_A-W 1465 1593 uncharacterized protein C6orf132 [Homo sapiens] >gi|327478596|sp|Q5T0Z8.4|CF132_HUMAN RecName: Full=Uncharacterized protein C6orf132

CCCTGCCCTGGCAGCCGCCCCGGGAGCAGCCAGGTACGCAGCGAGGCGGACACAGGAGCA

GGACGGCAGTCCCGACTCACTGGTGCCCTCTGGCCACCTCATCCATGCTGCCTGTGTGTG

CCAACGCCG

>CL240.Contig33_A-W 266 418 minus strand PREDICTED: LOW QUALITY PROTEIN: serine/threonine-protein kinase 32B-like [Nomascus leucogenys]

CCTGTTTTCTTCCCACTTCAGAAAGGGAGGTTGAACTGTGACCCCACGTTTGAACTTGAA

GAGATGATTCTAGAATCCAAGCCCCTTCACAAAAAGAAGAAGAGGTTGGCAAAGCACAGG

TCCAAAGACGGCACCAAGGACAGCTGCCCCCTG

>CL240.Contig34_A-W 1554 1682 uncharacterized protein C6orf132 [Homo sapiens] >gi|327478596|sp|Q5T0Z8.4|CF132_HUMAN RecName: Full=Uncharacterized protein C6orf132

CCCTGCCCTGGCAGCCGCCCCGGGAGCAGCCAGGTACGCAGCGAGGCGGACACAGGAGCA

GGACGGCAGTCCCGACTCACTGGTGCCCTCTGGCCACCTCATCCATGCTGCCTGTGTGTG

CCAACGCCG

>CL240.Contig37_A-W 1572 2441 Uncharacterized protein C6orf132 OS=Homo sapiens GN=C6orf132 PE=1 SV=4

CTGCCCTGGCAGCCGCCCCGGGAGCAGCCAGCGCCTGCAGTCGGTGCCAGCCTCACTGCA

GAGCCATGACCGCAAGTCTCCAAGGGCCGCTCGGCACACCGGGCAAGTCCACGTTTTCCT

AATCGATTTCCCTCTCCACGTCGGTGGGTGGGGGGAGCATTGAATTGGGTGAAGCTAAAA

GCAGCCAGACTCAACCCCTAAATGACCCCAGGGGAGGAGCCCATTCCCATTTGCTTTCAG

AGGGACACACTGTGGAATCCAAATTGAAGCATCTTCTGGGACACAGCAAAACGCCAAATG

CTCTTGCTGCCGGCTCGGGCTCTGCTCTGCTCAGCCTTCGCGGGCTCCCATTGCCAGCCG

GGCACTCTAGGTGCAGCTGGGCCATTCAGAGCAGATGCCTGACCCAGGAGGCTGCCTGAG

CCAGCGGTCACAGACGGTTTCTGAGCTGAAGCTCCTGAGCCCGGTTGTCTAGGGCTGGGG

AAGGGCTGGGAGCTTGCAGCAGCCTGGACTGCCCAGGATCTGCAGTGGCCCGTCACTATG

CATGGTGTCACTCCCCTTCTGCAGACCTCACTGGGAGCAGACCCTGCCCTGCCCTGTCCT

TCACCTCCCTGCCCCCTTTGGGAACTCAAGCGCCTGCCCATCGGCAGGTCCCCACCTGCC

CTGCAGCCTCCCTTCTCTGTGGCTTGTCCCCGTGTCTCACCTGGATGTCTTTCCCCTCTG

CCTTGCTCAGACTCAGGTGGGCACAGTCCCCCCAGAAGCTTCCCTGATCCCCAGGCTGGG

CCGGCACCTGTGTGGAAGCCACAGCCCAGGGCTGCCTTTCCTGTCAATGACCCCTGTGCT

GCTGTCATCTGTTGAAAATGGATGGACCCT

>CL240.Contig38_A-W 266 418 minus strand PREDICTED: LOW QUALITY PROTEIN: serine/threonine-protein kinase 32B-like [Nomascus leucogenys]

CCTGTTTTCTTCCCACTTCAGAAAGGGAGGTTGAACTGTGACCCCACGTTTGAACTTGAA

GAGATGATTCTAGAATCCAAGCCCCTTCACAAAAAGAAGAAGAGGTTGGCAAAGCACAGG

TCCAAAGACGGCACCAAGGACAGCTGCCCCCTG

>CL240.Contig40_A-W 1556 2425 Uncharacterized protein C6orf132 OS=Homo sapiens GN=C6orf132 PE=1 SV=4

CTGCCCTGGCAGCCGCCCCGGGAGCAGCCAGCGCCTGCAGTCGGTGCCAGCCTCACTGCA

GAGCCATGACCGCAAGTCTCCAAGGGCCGCTCGGCACACCGGGCAAGTCCACGTTTTCCT

AATCGATTTCCCTCTCCACGTCGGTGGGTGGGGGGAGCATTGAATTGGGTGAAGCTAAAA

GCAGCCAGACTCAACCCCTAAATGACCCCAGGGGAGGAGCCCATTCCCATTTGCTTTCAG

AGGGACACACTGTGGAATCCAAATTGAAGCATCTTCTGGGACACAGCAAAACGCCAAATG

CTCTTGCTGCCGGCTCGGGCTCTGCTCTGCTCAGCCTTCGCGGGCTCCCATTGCCAGCCG

GGCACTCTAGGTGCAGCTGGGCCATTCAGAGCAGATGCCTGACCCAGGAGGCTGCCTGAG

CCAGCGGTCACAGACGGTTTCTGAGCTGAAGCTCCTGAGCCCGGTTGTCTAGGGCTGGGG

AAGGGCTGGGAGCTTGCAGCAGCCTGGACTGCCCAGGATCTGCAGTGGCCCGTCACTATG

CATGGTGTCACTCCCCTTCTGCAGACCTCACTGGGAGCAGACCCTGCCCTGCCCTGTCCT

TCACCTCCCTGCCCCCTTTGGGAACTCAAGCGCCTGCCCATCGGCAGGTCCCCACCTGCC

CTGCAGCCTCCCTTCTCTGTGGCTTGTCCCCGTGTCTCACCTGGATGTCTTTCCCCTCTG

CCTTGCTCAGACTCAGGTGGGCACAGTCCCCCCAGAAGCTTCCCTGATCCCCAGGCTGGG

CCGGCACCTGTGTGGAAGCCACAGCCCAGGGCTGCCTTTCCTGTCAATGACCCCTGTGCT

GCTGTCATCTGTTGAAAATGGATGGACCCT

>CL240.Contig41_A-W 1661 2530 Uncharacterized protein C6orf132 OS=Homo sapiens GN=C6orf132 PE=1 SV=4

CTGCCCTGGCAGCCGCCCCGGGAGCAGCCAGCGCCTGCAGTCGGTGCCAGCCTCACTGCA

GAGCCATGACCGCAAGTCTCCAAGGGCCGCTCGGCACACCGGGCAAGTCCACGTTTTCCT

AATCGATTTCCCTCTCCACGTCGGTGGGTGGGGGGAGCATTGAATTGGGTGAAGCTAAAA

GCAGCCAGACTCAACCCCTAAATGACCCCAGGGGAGGAGCCCATTCCCATTTGCTTTCAG

AGGGACACACTGTGGAATCCAAATTGAAGCATCTTCTGGGACACAGCAAAACGCCAAATG

CTCTTGCTGCCGGCTCGGGCTCTGCTCTGCTCAGCCTTCGCGGGCTCCCATTGCCAGCCG

GGCACTCTAGGTGCAGCTGGGCCATTCAGAGCAGATGCCTGACCCAGGAGGCTGCCTGAG

CCAGCGGTCACAGACGGTTTCTGAGCTGAAGCTCCTGAGCCCGGTTGTCTAGGGCTGGGG

AAGGGCTGGGAGCTTGCAGCAGCCTGGACTGCCCAGGATCTGCAGTGGCCCGTCACTATG

CATGGTGTCACTCCCCTTCTGCAGACCTCACTGGGAGCAGACCCTGCCCTGCCCTGTCCT

TCACCTCCCTGCCCCCTTTGGGAACTCAAGCGCCTGCCCATCGGCAGGTCCCCACCTGCC

CTGCAGCCTCCCTTCTCTGTGGCTTGTCCCCGTGTCTCACCTGGATGTCTTTCCCCTCTG

CCTTGCTCAGACTCAGGTGGGCACAGTCCCCCCAGAAGCTTCCCTGATCCCCAGGCTGGG

CCGGCACCTGTGTGGAAGCCACAGCCCAGGGCTGCCTTTCCTGTCAATGACCCCTGTGCT

GCTGTCATCTGTTGAAAATGGATGGACCCT

>CL240.Contig44_A-W 64 216 minus strand PREDICTED: LOW QUALITY PROTEIN: serine/threonine-protein kinase 32B-like [Nomascus leucogenys]

CCTGTTTTCTTCCCACTTCAGAAAGGGAGGTTGAACTGTGACCCCACGTTTGAACTTGAA

GAGATGATTCTAGAATCCAAGCCCCTTCACAAAAAGAAGAAGAGGTTGGCAAAGCACAGG

TCCAAAGACGGCACCAAGGACAGCTGCCCCCTG

>CL240.Contig46_A-W 1467 2336 Uncharacterized protein C6orf132 OS=Homo sapiens GN=C6orf132 PE=1 SV=4

CTGCCCTGGCAGCCGCCCCGGGAGCAGCCAGCGCCTGCAGTCGGTGCCAGCCTCACTGCA

GAGCCATGACCGCAAGTCTCCAAGGGCCGCTCGGCACACCGGGCAAGTCCACGTTTTCCT

AATCGATTTCCCTCTCCACGTCGGTGGGTGGGGGGAGCATTGAATTGGGTGAAGCTAAAA

GCAGCCAGACTCAACCCCTAAATGACCCCAGGGGAGGAGCCCATTCCCATTTGCTTTCAG

AGGGACACACTGTGGAATCCAAATTGAAGCATCTTCTGGGACACAGCAAAACGCCAAATG

CTCTTGCTGCCGGCTCGGGCTCTGCTCTGCTCAGCCTTCGCGGGCTCCCATTGCCAGCCG

GGCACTCTAGGTGCAGCTGGGCCATTCAGAGCAGATGCCTGACCCAGGAGGCTGCCTGAG

CCAGCGGTCACAGACGGTTTCTGAGCTGAAGCTCCTGAGCCCGGTTGTCTAGGGCTGGGG

AAGGGCTGGGAGCTTGCAGCAGCCTGGACTGCCCAGGATCTGCAGTGGCCCGTCACTATG

CATGGTGTCACTCCCCTTCTGCAGACCTCACTGGGAGCAGACCCTGCCCTGCCCTGTCCT

TCACCTCCCTGCCCCCTTTGGGAACTCAAGCGCCTGCCCATCGGCAGGTCCCCACCTGCC

CTGCAGCCTCCCTTCTCTGTGGCTTGTCCCCGTGTCTCACCTGGATGTCTTTCCCCTCTG

CCTTGCTCAGACTCAGGTGGGCACAGTCCCCCCAGAAGCTTCCCTGATCCCCAGGCTGGG

CCGGCACCTGTGTGGAAGCCACAGCCCAGGGCTGCCTTTCCTGTCAATGACCCCTGTGCT

GCTGTCATCTGTTGAAAATGGATGGACCCT

>CL240.Contig48_A-W 1537 2406 Uncharacterized protein C6orf132 OS=Homo sapiens GN=C6orf132 PE=1 SV=4

CTGCCCTGGCAGCCGCCCCGGGAGCAGCCAGCGCCTGCAGTCGGTGCCAGCCTCACTGCA

GAGCCATGACCGCAAGTCTCCAAGGGCCGCTCGGCACACCGGGCAAGTCCACGTTTTCCT

AATCGATTTCCCTCTCCACGTCGGTGGGTGGGGGGAGCATTGAATTGGGTGAAGCTAAAA

GCAGCCAGACTCAACCCCTAAATGACCCCAGGGGAGGAGCCCATTCCCATTTGCTTTCAG

AGGGACACACTGTGGAATCCAAATTGAAGCATCTTCTGGGACACAGCAAAACGCCAAATG

CTCTTGCTGCCGGCTCGGGCTCTGCTCTGCTCAGCCTTCGCGGGCTCCCATTGCCAGCCG

GGCACTCTAGGTGCAGCTGGGCCATTCAGAGCAGATGCCTGACCCAGGAGGCTGCCTGAG

CCAGCGGTCACAGACGGTTTCTGAGCTGAAGCTCCTGAGCCCGGTTGTCTAGGGCTGGGG

AAGGGCTGGGAGCTTGCAGCAGCCTGGACTGCCCAGGATCTGCAGTGGCCCGTCACTATG

CATGGTGTCACTCCCCTTCTGCAGACCTCACTGGGAGCAGACCCTGCCCTGCCCTGTCCT

TCACCTCCCTGCCCCCTTTGGGAACTCAAGCGCCTGCCCATCGGCAGGTCCCCACCTGCC

CTGCAGCCTCCCTTCTCTGTGGCTTGTCCCCGTGTCTCACCTGGATGTCTTTCCCCTCTG

CCTTGCTCAGACTCAGGTGGGCACAGTCCCCCCAGAAGCTTCCCTGATCCCCAGGCTGGG

CCGGCACCTGTGTGGAAGCCACAGCCCAGGGCTGCCTTTCCTGTCAATGACCCCTGTGCT

GCTGTCATCTGTTGAAAATGGATGGACCCT

>CL240.Contig50_A-W 266 418 minus strand PREDICTED: LOW QUALITY PROTEIN: serine/threonine-protein kinase 32B-like [Nomascus leucogenys]

CCTGTTTTCTTCCCACTTCAGAAAGGGAGGTTGAACTGTGACCCCACGTTTGAACTTGAA

GAGATGATTCTAGAATCCAAGCCCCTTCACAAAAAGAAGAAGAGGTTGGCAAAGCACAGG

TCCAAAGACGGCACCAAGGACAGCTGCCCCCTG

>CL240.Contig51_A-W 1343 2212 Uncharacterized protein C6orf132 OS=Homo sapiens GN=C6orf132 PE=1 SV=4

CTGCCCTGGCAGCCGCCCCGGGAGCAGCCAGCGCCTGCAGTCGGTGCCAGCCTCACTGCA

GAGCCATGACCGCAAGTCTCCAAGGGCCGCTCGGCACACCGGGCAAGTCCACGTTTTCCT

AATCGATTTCCCTCTCCACGTCGGTGGGTGGGGGGAGCATTGAATTGGGTGAAGCTAAAA

GCAGCCAGACTCAACCCCTAAATGACCCCAGGGGAGGAGCCCATTCCCATTTGCTTTCAG

AGGGACACACTGTGGAATCCAAATTGAAGCATCTTCTGGGACACAGCAAAACGCCAAATG

CTCTTGCTGCCGGCTCGGGCTCTGCTCTGCTCAGCCTTCGCGGGCTCCCATTGCCAGCCG

GGCACTCTAGGTGCAGCTGGGCCATTCAGAGCAGATGCCTGACCCAGGAGGCTGCCTGAG

CCAGCGGTCACAGACGGTTTCTGAGCTGAAGCTCCTGAGCCCGGTTGTCTAGGGCTGGGG

AAGGGCTGGGAGCTTGCAGCAGCCTGGACTGCCCAGGATCTGCAGTGGCCCGTCACTATG

CATGGTGTCACTCCCCTTCTGCAGACCTCACTGGGAGCAGACCCTGCCCTGCCCTGTCCT

TCACCTCCCTGCCCCCTTTGGGAACTCAAGCGCCTGCCCATCGGCAGGTCCCCACCTGCC

CTGCAGCCTCCCTTCTCTGTGGCTTGTCCCCGTGTCTCACCTGGATGTCTTTCCCCTCTG

CCTTGCTCAGACTCAGGTGGGCACAGTCCCCCCAGAAGCTTCCCTGATCCCCAGGCTGGG

CCGGCACCTGTGTGGAAGCCACAGCCCAGGGCTGCCTTTCCTGTCAATGACCCCTGTGCT

GCTGTCATCTGTTGAAAATGGATGGACCCT

>CL240.Contig52_A-W 1448 2317 Uncharacterized protein C6orf132 OS=Homo sapiens GN=C6orf132 PE=1 SV=4

CTGCCCTGGCAGCCGCCCCGGGAGCAGCCAGCGCCTGCAGTCGGTGCCAGCCTCACTGCA

GAGCCATGACCGCAAGTCTCCAAGGGCCGCTCGGCACACCGGGCAAGTCCACGTTTTCCT

AATCGATTTCCCTCTCCACGTCGGTGGGTGGGGGGAGCATTGAATTGGGTGAAGCTAAAA

GCAGCCAGACTCAACCCCTAAATGACCCCAGGGGAGGAGCCCATTCCCATTTGCTTTCAG

AGGGACACACTGTGGAATCCAAATTGAAGCATCTTCTGGGACACAGCAAAACGCCAAATG

CTCTTGCTGCCGGCTCGGGCTCTGCTCTGCTCAGCCTTCGCGGGCTCCCATTGCCAGCCG

GGCACTCTAGGTGCAGCTGGGCCATTCAGAGCAGATGCCTGACCCAGGAGGCTGCCTGAG

CCAGCGGTCACAGACGGTTTCTGAGCTGAAGCTCCTGAGCCCGGTTGTCTAGGGCTGGGG

AAGGGCTGGGAGCTTGCAGCAGCCTGGACTGCCCAGGATCTGCAGTGGCCCGTCACTATG

CATGGTGTCACTCCCCTTCTGCAGACCTCACTGGGAGCAGACCCTGCCCTGCCCTGTCCT

TCACCTCCCTGCCCCCTTTGGGAACTCAAGCGCCTGCCCATCGGCAGGTCCCCACCTGCC

CTGCAGCCTCCCTTCTCTGTGGCTTGTCCCCGTGTCTCACCTGGATGTCTTTCCCCTCTG

CCTTGCTCAGACTCAGGTGGGCACAGTCCCCCCAGAAGCTTCCCTGATCCCCAGGCTGGG

CCGGCACCTGTGTGGAAGCCACAGCCCAGGGCTGCCTTTCCTGTCAATGACCCCTGTGCT

GCTGTCATCTGTTGAAAATGGATGGACCCT

>CL240.Contig53_A-W 1432 2301 Uncharacterized protein C6orf132 OS=Homo sapiens GN=C6orf132 PE=1 SV=4

CTGCCCTGGCAGCCGCCCCGGGAGCAGCCAGCGCCTGCAGTCGGTGCCAGCCTCACTGCA

GAGCCATGACCGCAAGTCTCCAAGGGCCGCTCGGCACACCGGGCAAGTCCACGTTTTCCT

AATCGATTTCCCTCTCCACGTCGGTGGGTGGGGGGAGCATTGAATTGGGTGAAGCTAAAA

GCAGCCAGACTCAACCCCTAAATGACCCCAGGGGAGGAGCCCATTCCCATTTGCTTTCAG

AGGGACACACTGTGGAATCCAAATTGAAGCATCTTCTGGGACACAGCAAAACGCCAAATG

CTCTTGCTGCCGGCTCGGGCTCTGCTCTGCTCAGCCTTCGCGGGCTCCCATTGCCAGCCG

GGCACTCTAGGTGCAGCTGGGCCATTCAGAGCAGATGCCTGACCCAGGAGGCTGCCTGAG

CCAGCGGTCACAGACGGTTTCTGAGCTGAAGCTCCTGAGCCCGGTTGTCTAGGGCTGGGG

AAGGGCTGGGAGCTTGCAGCAGCCTGGACTGCCCAGGATCTGCAGTGGCCCGTCACTATG

CATGGTGTCACTCCCCTTCTGCAGACCTCACTGGGAGCAGACCCTGCCCTGCCCTGTCCT

TCACCTCCCTGCCCCCTTTGGGAACTCAAGCGCCTGCCCATCGGCAGGTCCCCACCTGCC

CTGCAGCCTCCCTTCTCTGTGGCTTGTCCCCGTGTCTCACCTGGATGTCTTTCCCCTCTG

CCTTGCTCAGACTCAGGTGGGCACAGTCCCCCCAGAAGCTTCCCTGATCCCCAGGCTGGG

CCGGCACCTGTGTGGAAGCCACAGCCCAGGGCTGCCTTTCCTGTCAATGACCCCTGTGCT

GCTGTCATCTGTTGAAAATGGATGGACCCT

>CL240.Contig55_A-W 64 216 minus strand PREDICTED: LOW QUALITY PROTEIN: serine/threonine-protein kinase 32B-like [Nomascus leucogenys]

CCTGTTTTCTTCCCACTTCAGAAAGGGAGGTTGAACTGTGACCCCACGTTTGAACTTGAA

GAGATGATTCTAGAATCCAAGCCCCTTCACAAAAAGAAGAAGAGGTTGGCAAAGCACAGG

TCCAAAGACGGCACCAAGGACAGCTGCCCCCTG

>CL240.Contig62_A-W 64 216 minus strand PREDICTED: LOW QUALITY PROTEIN: serine/threonine-protein kinase 32B-like [Nomascus leucogenys]

CCTGTTTTCTTCCCACTTCAGAAAGGGAGGTTGAACTGTGACCCCACGTTTGAACTTGAA

GAGATGATTCTAGAATCCAAGCCCCTTCACAAAAAGAAGAAGAGGTTGGCAAAGCACAGG

TCCAAAGACGGCACCAAGGACAGCTGCCCCCTG

>CL240.Contig66_A-W 64 216 minus strand PREDICTED: LOW QUALITY PROTEIN: serine/threonine-protein kinase 32B-like [Nomascus leucogenys]

CCTGTTTTCTTCCCACTTCAGAAAGGGAGGTTGAACTGTGACCCCACGTTTGAACTTGAA

GAGATGATTCTAGAATCCAAGCCCCTTCACAAAAAGAAGAAGAGGTTGGCAAAGCACAGG

TCCAAAGACGGCACCAAGGACAGCTGCCCCCTG

>CL240.Contig74_A-W 266 418 minus strand PREDICTED: LOW QUALITY PROTEIN: serine/threonine-protein kinase 32B-like [Nomascus leucogenys]

CCTGTTTTCTTCCCACTTCAGAAAGGGAGGTTGAACTGTGACCCCACGTTTGAACTTGAA

GAGATGATTCTAGAATCCAAGCCCCTTCACAAAAAGAAGAAGAGGTTGGCAAAGCACAGG

TCCAAAGACGGCACCAAGGACAGCTGCCCCCTG

>CL240.Contig77_A-W 64 216 minus strand PREDICTED: LOW QUALITY PROTEIN: serine/threonine-protein kinase 32B-like [Nomascus leucogenys]

CCTGTTTTCTTCCCACTTCAGAAAGGGAGGTTGAACTGTGACCCCACGTTTGAACTTGAA

GAGATGATTCTAGAATCCAAGCCCCTTCACAAAAAGAAGAAGAGGTTGGCAAAGCACAGG

TCCAAAGACGGCACCAAGGACAGCTGCCCCCTG

>CL240.Contig85_A-W 266 418 minus strand PREDICTED: LOW QUALITY PROTEIN: serine/threonine-protein kinase 32B-like [Nomascus leucogenys]

CCTGTTTTCTTCCCACTTCAGAAAGGGAGGTTGAACTGTGACCCCACGTTTGAACTTGAA

GAGATGATTCTAGAATCCAAGCCCCTTCACAAAAAGAAGAAGAGGTTGGCAAAGCACAGG

TCCAAAGACGGCACCAAGGACAGCTGCCCCCTG

>CL240.Contig88_A-W 64 216 minus strand PREDICTED: LOW QUALITY PROTEIN: serine/threonine-protein kinase 32B-like [Nomascus leucogenys]

CCTGTTTTCTTCCCACTTCAGAAAGGGAGGTTGAACTGTGACCCCACGTTTGAACTTGAA

GAGATGATTCTAGAATCCAAGCCCCTTCACAAAAAGAAGAAGAGGTTGGCAAAGCACAGG

TCCAAAGACGGCACCAAGGACAGCTGCCCCCTG

>CL240.Contig91_A-W 266 418 minus strand PREDICTED: LOW QUALITY PROTEIN: serine/threonine-protein kinase 32B-like [Nomascus leucogenys]

CCTGTTTTCTTCCCACTTCAGAAAGGGAGGTTGAACTGTGACCCCACGTTTGAACTTGAA

GAGATGATTCTAGAATCCAAGCCCCTTCACAAAAAGAAGAAGAGGTTGGCAAAGCACAGG

TCCAAAGACGGCACCAAGGACAGCTGCCCCCTG

>CL241.Contig1_A-W 71 304 gamma-glutamyltransferase-like activity 1, isoform CRA_b [Mus musculus]

CCAGTTCCAGGTGAGCGGCCTCCATCATCCATGGTACCCTCCATCTTGGTCAACAAAAAC

CAAGGGTCAAAGCTAGTGATTGGTGGAGCTGGTGGGGAACTCATCATCTCTGCTGTGGTC

CAGACAATCATGAACAAGCTGTGGCTTGGCTTTGACCTGACAAAAGCCATTGCGGCCCCC

ATCCTGCACGTCAACAACAACGGCCATGTGGAGTATGAGCCCAAATTCAGCCAG

>CL241.Contig2_A-W 2 670 PREDICTED: gamma-glutamyltransferase 5 isoform 1 [Sus scrofa]

AGAGGGTTCAACTTCTCTGCAGAGTCAGTGGCTAGACCTGAAGGGAGAGTGAGCATGTAC

CATCACCTTGTGGAAACACTCAAGTTTGCCAGAGGACAAAGGTGGCGGCTGTGGGACCCT

CTCAGCCACCCAGAGGTCAAGAATGCCTCTCAGGACCTGCTAGGGGAGGCTCTGGCCCAG

CACATCCGCCAGCAGATTGATGAACGTGGTGACCACCAGCTCAGCCACTACAACCTGGTC

AGGACCTGGGACCACAGGATGGGCACCTCTCATGTGTCTGTGCTGGGAGAAGATGGGAGT

GCTGTGTCTGCCACCAGCACCATCAACACACCCTTTGGAGCGATGGTGTACTCGCCGAGC

ACAGGCATCCTCCTCAACAATGAGCTACTGGACTTATGCTGGAGATATTCACAAGGTTCC

AATGTCACCCCGCCACCAGTTCCAGGTGAGCGGCCTCCATCATCCATGGTACCCTCCATC

TTGGTCAACAAAAACCAAGGGTCAAAGCTAGTGATTGGTGGAGCTGGTGGGGAACTCATC

ATCTCTGCTGTGGTCCAGACAATCATGAACAAGCTGTGGCTTGGCTTTGACCTGACAAAA

GCCATTGCGGCCCCCATCCTGCACGTCAACAACAACGGCCATGTGGAGTATGAGCCCAAA

TTCAGCCAG

>CL241.Contig3_A-W 46 435 gamma-glutamyltransferase-like activity 1, isoform CRA_b [Mus musculus]

GTGCTCTATGTTGATGGGAGCCCAAGCCTAGGCTCCGTCTCCCCTGCCTGCAGCTTTGGA

GCGATGGTGTACTCGCCGAGCACAGGCATCCTCCTCAACAATGAGCTACTGGACTTATGC

TGGAGATATTCACAAGGTTCCAATGTCACCCCGCCACCAGTTCCAGGTGAGCGGCCTCCA

TCATCCATGGTACCCTCCATCTTGGTCAACAAAAACCAAGGGTCAAAGCTAGTGATTGGT

GGAGCTGGTGGGGAACTCATCATCTCTGCTGTGGTCCAGACAATCATGAACAAGCTGTGG

CTTGGCTTTGACCTGACAAAAGCCATTGCGGCCCCCATCCTGCACGTCAACAACAACGGC

CATGTGGAGTATGAGCCCAAATTCAGCCAG

>CL243.Contig1_A-W 95 1216 arginine/serine-rich coiled-coil protein 2 [Bos taurus] >gi|151556312|gb|AAI48067.1| RSRC2 protein [Bos taurus] >gi|296478523|gb|DAA20638.1| arginine/serine-rich coiled-coil protein 2 [Bos taurus]

AGAACCAACTTCTTCTTAAAACAGGCAAGAAGACATGAATCCAAAGATAAATCTTCTAAG

AAACACAAGTCTGAGGAACATAATGACAAAGAACATTCTTCTGATAAAGGAAGAGAGCGA

CTAAATTCCTCTGAAAATGGTGAGGACAGACACAAACGCAAAGAAAGAAAATCATCACGA

GGCAGAAGTCACTCAAGATCTAGGTCCCGTGAAAGGCGCCATCGTAGTAGAAGCAGGGAG

CGGAAGAAGTCTCGATCCAGGAGTAGGGAGCGGAAGAAGTCACGATCCAGAAGCAGAGAG

AGGAAGAAGTCTCGATCCAGAAGCAGGGAAAGAAAACGGCGGATCAGATCTCGTTCCCGC

TCAAGATCAAGACACAGGCATAGGACTAGAAGCAGGAGTAGGACACGGAGTAGAAGTCGA

GATAGAAAGAAGAGAATTGAAAAACCAAGAAGATTTAGCAGAAGTTTAAGCCGGACTCCC

AGTCCTCCTCCCTTCAGAGGCAGAAATACAGCAATGGATGCACAAGAAGCTTTAGCTAGG

AGATTGGAAAGGGCAAAGAAATTACAAGAACAACGAGAAAAGGAAATGGTTGAAAAACAA

AAACAGCAAGAAATAGCTGCAGCAGCTGCAGCCACTGGTGGTTCTGTTCTTAATGTTGCT

GCCCTATTGGCATCAGGAACACAAGTAACTCCTCAGATAGCTATGGCAGCTCAAATGGCA

GCCCTGCAGGCTAAAGCCTTGGCTGAGACTGGAATAGCTGTACCTAGCTACTATAACCCA

GCAGCTGTGAATCCAATGAAATTTGCTGAACAAGAGAAAAAAAGGAAGATGCTTTGGCAA

GGCAAAAAGGAAGGGGACAAATCCCAGTCTGCTGAAATATGGGAGAAATTGAACTTTGGA

AACAAGGACCAAAACGTCAAATTTAGGAAATTAATGGGTATTAAGAGTGAAGATGAAGCT

GGATGTAGCTCTGTTGATGAAGAAAGTTATAAGACTTTGAAGCAACAGGAAGAAGTATTT

AGAAATCTAGATGCTCAGTATGAAATGGCAAGATCACAGACCCACACACAAAGAGGAATG

GGTTTGGGTTTTACATCTTCAATGCGAGGAATGGATGCAGTT

>CL243.Contig2_A-W 34 738 similar to splicing factor, arginine/serine-rich 4, isoform CRA_b [Homo sapiens]

AGAGATAGAAAGAAGAGAATTGAAAAACCAAGAAGATTTAGCAGAAGTTTAAGCCGGACT

CCCAGTCCTCCTCCCTTCAGAGGCAGAAATACAGCAATGGATGCACAAGAAGCTTTAGCT

AGGAGATTGGAAAGGGCAAAGAAATTACAAGAACAACGAGAAAAGGAAATGGTTGAAAAA

CAAAAACAGCAAGAAATAGCTGCAGCAGCTGCAGCCACTGGTGGTTCTGTTCTTAATGTT

GCTGCCCTATTGGCATCAGGAACACAAGTAACTCCTCAGATAGCTATGGCAGCTCAAATG

GCAGCCCTGCAGGCTAAAGCCTTGGCTGAGACTGGAATAGCTGTACCTAGCTACTATAAC

CCAGCAGCTGTGAATCCAATGAAATTTGCTGAACAAGAGAAAAAAAGGAAGATGCTTTGG

CAAGGCAAAAAGGAAGGGGACAAATCCCAGTCTGCTGAAATATGGGAGAAATTGAACTTT

GGAAACAAGGACCAAAACGTCAAATTTAGGAAATTAATGGGTATTAAGAGTGAAGATGAA

GCTGGATGTAGCTCTGTTGATGAAGAAAGTTATAAGACTTTGAAGCAACAGGAAGAAGTA

TTTAGAAATCTAGATGCTCAGTATGAAATGGCAAGATCACAGACCCACACACAAAGAGGA

ATGGGTTTGGGTTTTACATCTTCAATGCGAGGAATGGATGCAGTT

>CL243.Contig3_A-W 11 1444 PREDICTED: hypothetical protein LOC100713251 [Cavia porcellus]

GGGCCTCATCCCCTTTTTGCCATCCGGGTCTCTCGCGCGAGCGCTTTAGTCTGTGGCGAA

GCTTCGGGGCGGCCGGTACTGTTGAAAGCGGCAAGTGAAGGTGCCGCCTTAGCGGCCGGG

TTCTGGACTATGGCGGCTAGTGATACAGAACGAGATGGACTAGCCCCAGAAAAGACAACC

CCAGATAGAGATAAGAAAAAAGAGCAGTCAGATGTATCTGTTTCTCCTAGAGCCTCAAAA

CATCATTATTCAAGATCACGATCAAGGTCAAGAGAAAGAAAACGAAAATCGGATAATGAA

GGAAGAAAACACAGGAGCCGGAGCAGAAGCAAAGAGGCAAGAAGACATGAATCCAAAGAT

AAATCTTCTAAGAAACACAAGTCTGAGGAACATAATGACAAAGAACATTCTTCTGATAAA

GGAAGAGAGCGACTAAATTCCTCTGAAAATGGTGAGGACAGACACAAACGCAAAGAAAGA

AAATCATCACGAGGCAGAAGTCACTCAAGATCTAGGTCCCGTGAAAGGCGCCATCGTAGT

AGAAGCAGGGAGCGGAAGAAGTCTCGATCCAGGAGTAGGGAGCGGAAGAAGTCACGATCC

AGAAGCAGAGAGAGGAAGAAGTCTCGATCCAGAAGCAGGGAAAGAAAACGGCGGATCAGA

TCTCGTTCCCGCTCAAGATCAAGACACAGGCATAGGACTAGAAGCAGGAGTAGGACACGG

AGTAGAAGTCGAGATAGAAAGAAGAGAATTGAAAAACCAAGAAGATTTAGCAGAAGTTTA

AGCCGGACTCCCAGTCCTCCTCCCTTCAGAGGCAGAAATACAGCAATGGATGCACAAGAA

GCTTTAGCTAGGAGATTGGAAAGGGCAAAGAAATTACAAGAACAACGAGAAAAGGAAATG

GTTGAAAAACAAAAACAGCAAGAAATAGCTGCAGCAGCTGCAGCCACTGGTGGTTCTGTT

CTTAATGTTGCTGCCCTATTGGCATCAGGAACACAAGTAACTCCTCAGATAGCTATGGCA

GCTCAAATGGCAGCCCTGCAGGCTAAAGCCTTGGCTGAGACTGGAATAGCTGTACCTAGC

TACTATAACCCAGCAGCTGTGAATCCAATGAAATTTGCTGAACAAGAGAAAAAAAGGAAG

ATGCTTTGGCAAGGCAAAAAGGAAGGGGACAAATCCCAGTCTGCTGAAATATGGGAGAAA

TTGAACTTTGGAAACAAGGACCAAAACGTCAAATTTAGGAAATTAATGGGTATTAAGAGT

GAAGATGAAGCTGGATGTAGCTCTGTTGATGAAGAAAGTTATAAGACTTTGAAGCAACAG

GAAGAAGTATTTAGAAATCTAGATGCTCAGTATGAAATGGCAAGATCACAGACCCACACA

CAAAGAGGAATGGGTTTGGGTTTTACATCTTCAATGCGAGGAATGGATGCAGTT

>CL243.Contig4_A-W 47 1423 similar to splicing factor, arginine/serine-rich 4, isoform CRA_b [Homo sapiens]

GCTAGTGATACAGAACGAGATGGACTAGCCCCAGAAAAGACAACCCCAGATAGAGATAAG

AAAAAAGAGCAGTCAGATGTATCTGTTTCTCCTAGAGCCTCAAAACATCATTATTCAAGA

TCACGATCAAGGTCAAGAGAAAGAAAACGAAAATCGGATAATGAAGGAAGAAAACACAGG

AGCCGGAGCAGAAGCAAAGAGCGTGCTAATGCGCGAAGAGACTGAACTGAAGACGCTGCA

GACTCAGATAGCAAAATAATAAGCCTACTTCATGATAAGGCAAGAAGACATGAATCCAAA

GATAAATCTTCTAAGAAACACAAGTCTGAGGAACATAATGACAAAGAACATTCTTCTGAT

AAAGGAAGAGAGCGACTAAATTCCTCTGAAAATGGTGAGGACAGACACAAACGCAAAGAA

AGAAAATCATCACGAGGCAGAAGTCACTCAAGATCTAGGTCCCGTGAAAGGCGCCATCGT

AGTAGAAGCAGGGAGCGGAAGAAGTCTCGATCCAGGAGTAGGGAGCGGAAGAAGTCACGA

TCCAGAAGCAGAGAGAGGAAGAAGTCTCGATCCAGAAGCAGGGAAAGAAAACGGCGGATC

AGATCTCGTTCCCGCTCAAGATCAAGACACAGGCATAGGACTAGAAGCAGGAGTAGGACA

CGGAGTAGAAGTCGAGATAGAAAGAAGAGAATTGAAAAACCAAGAAGATTTAGCAGAAGT

TTAAGCCGGACTCCCAGTCCTCCTCCCTTCAGAGGCAGAAATACAGCAATGGATGCACAA

GAAGCTTTAGCTAGGAGATTGGAAAGGGCAAAGAAATTACAAGAACAACGAGAAAAGGAA

ATGGTTGAAAAACAAAAACAGCAAGAAATAGCTGCAGCAGCTGCAGCCACTGGTGGTTCT

GTTCTTAATGTTGCTGCCCTATTGGCATCAGGAACACAAGTAACTCCTCAGATAGCTATG

GCAGCTCAAATGGCAGCCCTGCAGGCTAAAGCCTTGGCTGAGACTGGAATAGCTGTACCT

AGCTACTATAACCCAGCAGCTGTGAATCCAATGAAATTTGCTGAACAAGAGAAAAAAAGG

AAGATGCTTTGGCAAGGCAAAAAGGAAGGGGACAAATCCCAGTCTGCTGAAATATGGGAG

AAATTGAACTTTGGAAACAAGGACCAAAACGTCAAATTTAGGAAATTAATGGGTATTAAG

AGTGAAGATGAAGCTGGATGTAGCTCTGTTGATGAAGAAAGTTATAAGACTTTGAAGCAA

CAGGAAGAAGTATTTAGAAATCTAGATGCTCAGTATGAAATGGCAAGATCACAGACCCAC

ACACAAAGAGGAATGGGTTTGGGTTTTACATCTTCAATGCGAGGAATGGATGCAGTT

>CL243.Contig5_A-W 11 1522 PREDICTED: hypothetical protein LOC100713251 [Cavia porcellus]

GGGCCTCATCCCCTTTTTGCCATCCGGGTCTCTCGCGCGAGCGCTTTAGTCTGTGGCGAA

GCTTCGGGGCGGCCGGTACTGTTGAAAGCGGCAAGTGAAGGTGCCGCCTTAGCGGCCGGG

TTCTGGACTATGGCGGCTAGTGATACAGAACGAGATGGACTAGCCCCAGAAAAGACAACC

CCAGATAGAGATAAGAAAAAAGAGCAGTCAGATGTATCTGTTTCTCCTAGAGCCTCAAAA

CATCATTATTCAAGATCACGATCAAGGTCAAGAGAAAGAAAACGAAAATCGGATAATGAA

GGAAGAAAACACAGGAGCCGGAGCAGAAGCAAAGAGCGTGCTAATGCGCGAAGAGACTGA

ACTGAAGACGCTGCAGACTCAGATAGCAAAATAATAAGCCTACTTCATGATAAGGCAAGA

AGACATGAATCCAAAGATAAATCTTCTAAGAAACACAAGTCTGAGGAACATAATGACAAA

GAACATTCTTCTGATAAAGGAAGAGAGCGACTAAATTCCTCTGAAAATGGTGAGGACAGA

CACAAACGCAAAGAAAGAAAATCATCACGAGGCAGAAGTCACTCAAGATCTAGGTCCCGT

GAAAGGCGCCATCGTAGTAGAAGCAGGGAGCGGAAGAAGTCTCGATCCAGGAGTAGGGAG

CGGAAGAAGTCACGATCCAGAAGCAGAGAGAGGAAGAAGTCTCGATCCAGAAGCAGGGAA

AGAAAACGGCGGATCAGATCTCGTTCCCGCTCAAGATCAAGACACAGGCATAGGACTAGA

AGCAGGAGTAGGACACGGAGTAGAAGTCGAGATAGAAAGAAGAGAATTGAAAAACCAAGA

AGATTTAGCAGAAGTTTAAGCCGGACTCCCAGTCCTCCTCCCTTCAGAGGCAGAAATACA

GCAATGGATGCACAAGAAGCTTTAGCTAGGAGATTGGAAAGGGCAAAGAAATTACAAGAA

CAACGAGAAAAGGAAATGGTTGAAAAACAAAAACAGCAAGAAATAGCTGCAGCAGCTGCA

GCCACTGGTGGTTCTGTTCTTAATGTTGCTGCCCTATTGGCATCAGGAACACAAGTAACT

CCTCAGATAGCTATGGCAGCTCAAATGGCAGCCCTGCAGGCTAAAGCCTTGGCTGAGACT

GGAATAGCTGTACCTAGCTACTATAACCCAGCAGCTGTGAATCCAATGAAATTTGCTGAA

CAAGAGAAAAAAAGGAAGATGCTTTGGCAAGGCAAAAAGGAAGGGGACAAATCCCAGTCT

GCTGAAATATGGGAGAAATTGAACTTTGGAAACAAGGACCAAAACGTCAAATTTAGGAAA

TTAATGGGTATTAAGAGTGAAGATGAAGCTGGATGTAGCTCTGTTGATGAAGAAAGTTAT

AAGACTTTGAAGCAACAGGAAGAAGTATTTAGAAATCTAGATGCTCAGTATGAAATGGCA

AGATCACAGACCCACACACAAAGAGGAATGGGTTTGGGTTTTACATCTTCAATGCGAGGA

ATGGATGCAGTT

>CL243.Contig6_A-W 47 1345 similar to splicing factor, arginine/serine-rich 4, isoform CRA_b [Homo sapiens]

GCTAGTGATACAGAACGAGATGGACTAGCCCCAGAAAAGACAACCCCAGATAGAGATAAG

AAAAAAGAGCAGTCAGATGTATCTGTTTCTCCTAGAGCCTCAAAACATCATTATTCAAGA

TCACGATCAAGGTCAAGAGAAAGAAAACGAAAATCGGATAATGAAGGAAGAAAACACAGG

AGCCGGAGCAGAAGCAAAGAGGCAAGAAGACATGAATCCAAAGATAAATCTTCTAAGAAA

CACAAGTCTGAGGAACATAATGACAAAGAACATTCTTCTGATAAAGGAAGAGAGCGACTA

AATTCCTCTGAAAATGGTGAGGACAGACACAAACGCAAAGAAAGAAAATCATCACGAGGC

AGAAGTCACTCAAGATCTAGGTCCCGTGAAAGGCGCCATCGTAGTAGAAGCAGGGAGCGG

AAGAAGTCTCGATCCAGGAGTAGGGAGCGGAAGAAGTCACGATCCAGAAGCAGAGAGAGG

AAGAAGTCTCGATCCAGAAGCAGGGAAAGAAAACGGCGGATCAGATCTCGTTCCCGCTCA

AGATCAAGACACAGGCATAGGACTAGAAGCAGGAGTAGGACACGGAGTAGAAGTCGAGAT

AGAAAGAAGAGAATTGAAAAACCAAGAAGATTTAGCAGAAGTTTAAGCCGGACTCCCAGT

CCTCCTCCCTTCAGAGGCAGAAATACAGCAATGGATGCACAAGAAGCTTTAGCTAGGAGA

TTGGAAAGGGCAAAGAAATTACAAGAACAACGAGAAAAGGAAATGGTTGAAAAACAAAAA

CAGCAAGAAATAGCTGCAGCAGCTGCAGCCACTGGTGGTTCTGTTCTTAATGTTGCTGCC

CTATTGGCATCAGGAACACAAGTAACTCCTCAGATAGCTATGGCAGCTCAAATGGCAGCC

CTGCAGGCTAAAGCCTTGGCTGAGACTGGAATAGCTGTACCTAGCTACTATAACCCAGCA

GCTGTGAATCCAATGAAATTTGCTGAACAAGAGAAAAAAAGGAAGATGCTTTGGCAAGGC

AAAAAGGAAGGGGACAAATCCCAGTCTGCTGAAATATGGGAGAAATTGAACTTTGGAAAC

AAGGACCAAAACGTCAAATTTAGGAAATTAATGGGTATTAAGAGTGAAGATGAAGCTGGA

TGTAGCTCTGTTGATGAAGAAAGTTATAAGACTTTGAAGCAACAGGAAGAAGTATTTAGA

AATCTAGATGCTCAGTATGAAATGGCAAGATCACAGACCCACACACAAAGAGGAATGGGT

TTGGGTTTTACATCTTCAATGCGAGGAATGGATGCAGTT

>CL244.Contig1_A-W 1298 1498 PREDICTED: protein kinase C delta type [Pan troglodytes]

TCCCCCCAGATCTTGCAGGGCCTGAAGTACACATTCTCTGTGGACTGGTGGTCCTTTGGG

GTCCTCCTCTACGAAATGCTGATTGGTCAGTCCCCCTTCCATGGTGATGATGAGGACGAA

CTCTTCGAGTCCATACGTTTGGACACACCGCATTATCCCCGTTGGATCACCAAAGAGTCC

AAGGACATCCTGGAGAAGGTG

>CL244.Contig2_A-W 1881 2204 PREDICTED: protein kinase C delta type [Pan troglodytes]

TTCCCGAGAATACTAACCAGGGCCCCTGCTGGGTTGCAGTTATTTGAAAGGGACCCACCC

AAGAGGCTGGGAATGACAGGAAACATCAGACTCCACCCCTTCTTCAAGACCATCAACTGG

ACTCTGCTGGAGAAGAGGAAGGTGGAGCCGCCCTTCAGGCCCAAAGTGAAGCACCCAGGA

GACTACAGCAACTTCGACTTGGAGTTCCTGAAGGAGAAGCCACGCCTCTCCTTTAGTGAC

AAGAACCTCATCGACTCCATGGACCAGACAGCATTCGCCGGCTTCTCCTTTGTAAACCCC

AGATTTGAGCACCTCTTGGAAAAC

>CL244.Contig3_A-W 696 896 PREDICTED: LOW QUALITY PROTEIN: protein kinase C delta type-like [Equus caballus]

TCCCCCCAGATCTTGCAGGGCCTGAAGTACACATTCTCTGTGGACTGGTGGTCCTTTGGG

GTCCTCCTCTACGAAATGCTGATTGGTCAGTCCCCCTTCCATGGTGATGATGAGGACGAA

CTCTTCGAGTCCATACGTTTGGACACACCGCATTATCCCCGTTGGATCACCAAAGAGTCC

AAGGACATCCTGGAGAAGGTG

>CL244.Contig4_A-W 2047 2247 PREDICTED: protein kinase C delta type [Cavia porcellus]

TCCCCCCAGATCTTGCAGGGCCTGAAGTACACATTCTCTGTGGACTGGTGGTCCTTTGGG

GTCCTCCTCTACGAAATGCTGATTGGTCAGTCCCCCTTCCATGGTGATGATGAGGACGAA

CTCTTCGAGTCCATACGTTTGGACACACCGCATTATCCCCGTTGGATCACCAAAGAGTCC

AAGGACATCCTGGAGAAGGTG

>CL244.Contig5_A-W 1279 1599 PREDICTED: LOW QUALITY PROTEIN: protein kinase C delta type-like [Equus caballus]

TTCCCGAGAATACTAACCAGGGCCCCTGCTGGGTTGCAGTTATTTGAAAGGGACCCACCC

AAGAGGCTGGGAATGACAGGAAACATCAGACTCCACCCCTTCTTCAAGACCATCAACTGG

ACTCTGCTGGAGAAGAGGAAGGTGGAGCCGCCCTTCAGGCCCAAAGTGAAGCACCCAGGA

GACTACAGCAACTTCGACTTGGAGTTCCTGAAGGAGAAGCCACGCCTCTCCTTTAGTGAC

AAGAACCTCATCGACTCCATGGACCAGACAGCATTCGCCGGCTTCTCCTTTGTAAACCCC

AGATTTGAGCACCTCTTGGAA

>CL244.Contig6_A-W 1241 1405 PREDICTED: protein kinase C delta type [Cricetulus griseus]

TTCCCGAGAATACTAACCAGGGCCCCTGCTGGGTTGCAGTTATTTGAAAGGGACCCACCC

AAGAGGCTGGGAATGACAGGAAACATCAGACTCCACCCCTTCTTCAAGACCATCAACTGG

ACTCTGCTGGAGAAGAGGAAGGTGGAGCCGCCCTTCAGGCCCAAA

>CL244.Contig7_A-W 1140 1304 Protein kinase C delta type [Heterocephalus glaber]

TTCCCGAGAATACTAACCAGGGCCCCTGCTGGGTTGCAGTTATTTGAAAGGGACCCACCC

AAGAGGCTGGGAATGACAGGAAACATCAGACTCCACCCCTTCTTCAAGACCATCAACTGG

ACTCTGCTGGAGAAGAGGAAGGTGGAGCCGCCCTTCAGGCCCAAA

>CL244.Contig8_A-W 1083 1247 PREDICTED: protein kinase C delta type [Cavia porcellus]

TTCCCGAGAATACTAACCAGGGCCCCTGCTGGGTTGCAGTTATTTGAAAGGGACCCACCC

AAGAGGCTGGGAATGACAGGAAACATCAGACTCCACCCCTTCTTCAAGACCATCAACTGG

ACTCTGCTGGAGAAGAGGAAGGTGGAGCCGCCCTTCAGGCCCAAA

>CL244.Contig9_A-W 982 1146 Protein kinase C delta type [Heterocephalus glaber]

TTCCCGAGAATACTAACCAGGGCCCCTGCTGGGTTGCAGTTATTTGAAAGGGACCCACCC

AAGAGGCTGGGAATGACAGGAAACATCAGACTCCACCCCTTCTTCAAGACCATCAACTGG

ACTCTGCTGGAGAAGAGGAAGGTGGAGCCGCCCTTCAGGCCCAAA

>CL244.Contig10_A-W 2630 2950 PREDICTED: protein kinase C delta type [Cavia porcellus]

TTCCCGAGAATACTAACCAGGGCCCCTGCTGGGTTGCAGTTATTTGAAAGGGACCCACCC

AAGAGGCTGGGAATGACAGGAAACATCAGACTCCACCCCTTCTTCAAGACCATCAACTGG

ACTCTGCTGGAGAAGAGGAAGGTGGAGCCGCCCTTCAGGCCCAAAGTGAAGCACCCAGGA

GACTACAGCAACTTCGACTTGGAGTTCCTGAAGGAGAAGCCACGCCTCTCCTTTAGTGAC

AAGAACCTCATCGACTCCATGGACCAGACAGCATTCGCCGGCTTCTCCTTTGTAAACCCC

AGATTTGAGCACCTCTTGGAA

>CL244.Contig11_A-W 2047 2526 PREDICTED: protein kinase C delta type [Cavia porcellus]

TCCCCCCAGATCTTGCAGGGCCTGAAGTACACATTCTCTGTGGACTGGTGGTCCTTTGGG

GTCCTCCTCTACGAAATGCTGATTGGTCAGTCCCCCTTCCATGGTGATGATGAGGACGAA

CTCTTCGAGTCCATACGTTTGGACACACCGCATTATCCCCGTTGGATCACCAAAGAGTCC

AAGGACATCCTGGAGAAGTTATTTGAAAGGGACCCACCCAAGAGGCTGGGAATGACAGGA

AACATCAGACTCCACCCCTTCTTCAAGACCATCAACTGGACTCTGCTGGAGAAGAGGAAG

GTGGAGCCGCCCTTCAGGCCCAAAGTGAAGCACCCAGGAGACTACAGCAACTTCGACTTG

GAGTTCCTGAAGGAGAAGCCACGCCTCTCCTTTAGTGACAAGAACCTCATCGACTCCATG

GACCAGACAGCATTCGCCGGCTTCTCCTTTGTAAACCCCAGATTTGAGCACCTCTTGGAA

>CL245.Contig1_A-W 128 499 PREDICTED: coronin-7 [Otolemur garnettii]

GCCAAGTACCTGGCCCAAATCATTGTGATGGGCGTACAGGTGGTAGGCAGAGCCTTTGCT

CGGGCCCTGCGGCAGGAGTTTGCAGCCAGCCGAGCCGCAGCTGATGCCCGGGGACGAGCT

GGGCACCAGTCTGCAGCTGCATCCAACCTCTCTGGCCTCAGTCTTCAGGAGGCCCAGCAG

ATTCTCAACGTCTCCAAACTGAGTCCCGAGGAGGTCCAGAAGAACTATGAACATCTATTT

AAGGTGAATGATAAATCCGTGGGCGGCTCCTTCTACCTACAATCCAAGGTTGTCCGGGCA

AAGGAGCGCCTGGATGAGGAACTCAGAATCCAGGCCCAAGAGGAGAAAGAGAAAGAGCAG

GTGCCCAGAACG

>CL245.Contig2_A-W 378 533 RecName: Full=Mitochondrial import inner membrane translocase subunit TIM16; AltName: Full=Mitochondria-associated granulocyte macrophage CSF-signaling molecule; AltName: Full=Presequence translocated-associated motor subunit PAM16 >gi|149054466|gb|EDM06283.1| rCG63469 [Rattus norvegicus]

CTGCAGAACTATGAACATCTATTTAAGGTGAATGATAAATCCGTGGGCGGCTCCTTCTAC

CTACAATCCAAGGTTGTCCGGGCAAAGGAGCGCCTGGATGAGGAACTCAGAATCCAGGCC

CAAGAGGAGAAAGAGAAAGAGCAGGTGCCCAGAACG

>CL245.Contig3_A-W 125 286 hCG15164, isoform CRA_a [Homo sapiens]

ATGGCCAAGTACCTGGCCCAAATCATTGTGATGGGCGTACAGGTGGTAGGCAGAGCCTTT

GCTCGGGCCCTGCGGCAGGAGTTTGCAGGGGTGAGCCACCCATCCGGAAGGCTCAGGGGG

AGCTTTGGGCCATGGAGGGCTGAGCGATCACCCATGTGTCCA

>CL246.Contig1_A-W 228 1652 PREDICTED: HIV Tat-specific factor 1 homolog [Ailuropoda melanoleuca]

ATGAGCGGCAACAACTTGGATGCGAACGATGAGTTTGATGAGCAGTTGCGAATGCAAGAA

TTGTACGGAGACACCAAGGACGGTGACACCCAGAATGATCCCAGCGGAGAAGCCGATTCT

TTTGAGCAGCAGCCGGCCGACACCCCCTATGAGTGGGACCTGGACAAGAAGGCATGGTTC

CCCAAGATCACTGAAGATTTCATTGCTACCTATCAAGCCAATTATGGCTTCTCTAATGAT

GGCGCATCTAGTTCTACTGCAAATGTCCAAGATGCCAACACTAGGACTGCAGAGGAACCG

CCACAAAGAAAAACCCCTGAACCTTCTGATCCTAGAAAGAAGGGAGAAAAAAGAAAGGCT

GAATCAGGGTGGTTTCATGTTGAAGAAGACAGAAATACAAATGTATATGTGTCTGGTTTA

CCTCCTGACATTACAGTGGATGAATTTATACAGCTTATGTCTAAATTTGGTATTATTATG

AGGGATCCTCAGACAGAAGAATTTAAGGTCAAACTTTACAAAGATAATCAAGGAAATCTT

AAAGGAGATGGGCTTTGCTGTTATTTGAAGAGGGAATCTGTGGAACTTGCATTAAAACTT

TTGGATGAAGATGAAATTAGAGGCTACAAATTACATGTCGAGGTAGCAAAGTTTCAGCTG

AAGGGGGAATATGACGCCTCAAAGAAGAAAAAGAAGTGCAAAGACTATAAGAAGAAGCTG

TCTCTGCAACAGAAGCAGTTGGATTGGAGACCTGAGAGGAGAGCTGGGCCATCCCGGATG

CGCCATGAGCGAGTTGTCATCATCAAAAATATGTTTCACCCTATGGATTTTGAGGACGAT

CCATTGGTGCTGAATGAGATCAGAGAAGATCTTCGAGTAGAGTGCTCCAAGTTTGGACAA

ATTAGGAAGCTCCTTCTCTTTGATAGGCACCCAGATGGTGTGGCCTCTGTGTCCTTTAGG

GATCCCGAGGAAGCTGATCATTGTATTCAGACCCTTGATGGAAGATGGTTTGGTGGTCGA

CAGATCACCGCCCAAGCATGGGATGGGACTACAGATTATCAGGTGGAGGAGACCTCAAGA

GAAAGAGAGGAAAGGCTGAGAGGATGGGAGGCATTTCTCAATGCTCCGGAGGCCAACAGA

GGCCTTCAGCGTTCAAATTCTGTCTGTGCTTCAGAAAGGGCAGGGCCTTCTAGAATAAGG

CATTTTTCAGAGCACCCAAGCACATCTAAAATGAATGCTCAAGAAGCTGCAACTGGAGTG

GCATTTGAAGAACCTATTGATGAGAAGAAGTTTGAAAAAGCAGAAGATGTGGAAGAAGTT

GAAGGAGAGGCTTCTGGAAAAGATGCTAAAGAAGGTGGGCCTGAAGAAGAGGTCAAAGAA

GGCTGCCCTGAAAAAGAGTCTGAAGAGAGCTGCCTTGAGAAAGAG

>CL246.Contig2_A-W 205 1629 PREDICTED: HIV Tat-specific factor 1 homolog [Ailuropoda melanoleuca]

ATGAGCGGCAACAACTTGGATGCGAACGATGAGTTTGATGAGCAGTTGCGAATGCAAGAA

TTGTACGGAGACACCAAGGACGGTGACACCCAGAATGATCCCAGCGGAGAAGCCGATTCT

TTTGAGCAGCAGCCGGCCGACACCCCCTATGAGTGGGACCTGGACAAGAAGGCATGGTTC

CCCAAGATCACTGAAGATTTCATTGCTACCTATCAAGCCAATTATGGCTTCTCTAATGAT

GGCGCATCTAGTTCTACTGCAAATGTCCAAGATGCCAACACTAGGACTGCAGAGGAACCG

CCACAAAGAAAAACCCCTGAACCTTCTGATCCTAGAAAGAAGGGAGAAAAAAGAAAGGCT

GAATCAGGGTGGTTTCATGTTGAAGAAGACAGAAATACAAATGTATATGTGTCTGGTTTA

CCTCCTGACATTACAGTGGATGAATTTATACAGCTTATGTCTAAATTTGGTATTATTATG

AGGGATCCTCAGACAGAAGAATTTAAGGTCAAACTTTACAAAGATAATCAAGGAAATCTT

AAAGGAGATGGGCTTTGCTGTTATTTGAAGAGGGAATCTGTGGAACTTGCATTAAAACTT

TTGGATGAAGATGAAATTAGAGGCTACAAATTACATGTCGAGGTAGCAAAGTTTCAGCTG

AAGGGGGAATATGACGCCTCAAAGAAGAAAAAGAAGTGCAAAGACTATAAGAAGAAGCTG

TCTCTGCAACAGAAGCAGTTGGATTGGAGACCTGAGAGGAGAGCTGGGCCATCCCGGATG

CGCCATGAGCGAGTTGTCATCATCAAAAATATGTTTCACCCTATGGATTTTGAGGACGAT

CCATTGGTGCTGAATGAGATCAGAGAAGATCTTCGAGTAGAGTGCTCCAAGTTTGGACAA

ATTAGGAAGCTCCTTCTCTTTGATAGGCACCCAGATGGTGTGGCCTCTGTGTCCTTTAGG

GATCCCGAGGAAGCTGATCATTGTATTCAGACCCTTGATGGAAGATGGTTTGGTGGTCGA

CAGATCACCGCCCAAGCATGGGATGGGACTACAGATTATCAGGTGGAGGAGACCTCAAGA

GAAAGAGAGGAAAGGCTGAGAGGATGGGAGGCATTTCTCAATGCTCCGGAGGCCAACAGA

GGCCTTCAGCGTTCAAATTCTGTCTGTGCTTCAGAAAGGGCAGGGCCTTCTAGAATAAGG

CATTTTTCAGAGCACCCAAGCACATCTAAAATGAATGCTCAAGAAGCTGCAACTGGAGTG

GCATTTGAAGAACCTATTGATGAGAAGAAGTTTGAAAAAGCAGAAGATGTGGAAGAAGTT

GAAGGAGAGGCTTCTGGAAAAGATGCTAAAGAAGGTGGGCCTGAAGAAGAGGTCAAAGAA

GGCTGCCCTGAAAAAGAGTCTGAAGAGAGCTGCCTTGAGAAAGAG

>CL246.Contig3_A-W 56 1384 PREDICTED: HIV Tat-specific factor 1 homolog [Ailuropoda melanoleuca]

ATGAGCGGCAACAACTTGGATGCGAACGATGAGTTTGATGAGCAGTTGCGAATGCAAGAA

TTGTACGGAGACACCAAGGACGGTGACACCCAGAATGATCCCAGCGGAGAAGCCGATTCT

TTTGAGCAGCAGCCGGCCGACACCCCCTATGAGTGGGACCTGGACAAGAAGGCATGGTTC

CCCAAGATCACTGAAGATTTCATTGCTACCTATCAAGCCAATTATGGCTTCTCTAATGAT

GGCGCATCTAGTTCTACTGCAAATGTCCAAGATGCCAACACTAGGACTGCAGAGGAACCG

CCACAAAGAAAAACCCCTGAACCTTCTGATCCTAGAAAGAAGGGAGAAAAAAGAAAGGCT

GAATCAGGGTGGTTTCATGTTGAAGAAGACAGAAATACAAATGTATATGTGTCTGGTTTA

CCTCCTGACATTACAGTGGATGAATTTATACAGCTTATGTCTAAATTTGGTATTATTATG

AGGGATCCTCAGACAGAAGAATTTAAGGTCAAACTTTACAAAGATAATCAAGGAAATCTT

AAAGGAGATGGGCTTTGCTGTTATTTGAAGAGGGAATCTGTGGAACTTGCATTAAAACTT

TTGGATGAAGATGAAATTAGAGGCTACAAATTACATGTCGAGGTAGCAAAGTTTCAGCTG

AAGGGGGAATATGACGCCTCAAAGAAGAAAAAGAAGTGCAAAGACTATAAGAAGAAGCTG

TCTCTGCAACAGAAGCAGTTGGATTGGAGACCTGAGAGGAGAGCTGGGCCATCCCGGATG

CGCCATGAGCGAGTTGTCATCATCAAAAATATGTTTCACCCTATGGATTTTGAGGACGAT

CCATTGGTGCTGAATGAGATCAGAGAAGATCTTCGAGTAGAGTGCTCCAAGTTTGGACAA

ATTAGGAAGCTCCTTCTCTTTGATAGGCACCCAGATGGTGTGGCCTCTGTGTCCTTTAGG

GATCCCGAGGAAGCTGATCATTGTATTCAGACCCTTGATGGAAGATGGTTTGGTGGTCGA

CAGATCACCGCCCAAGCATGGGATGGGACTACAGATTATCAGGTGGAGGAGACCTCAAGA

GAAAGAGAGGAAAGGCTGAGAGGATGGGAGGCATTTCTCAATGCTCCGGAGGCCAACAGA

GGCCTTCAGCGTTCAAATTCTGTCTGTGCTTCAGAAAGGGCAGGGCCTTCTAGAATAAGG

CATTTTTCAGAGCACCCAAGCACATCTAAAATGAATGCTCAAGAAGCTGCAACTGGAGTG

GCATTTGAAGAACCTATTGATGAGAAGAAGTTTGAAAAAGCAGAAGATGTGGAAGAAGTT

GAAGGAGAG

>CL247.Contig1_A-W 2 652 PREDICTED: LOW QUALITY PROTEIN: histone acetyltransferase KAT2A-like [Loxodonta africana]

GGGTCCTTGGGCAGCCCTCCTTTTGAGAAGCCTAACATTGAACAGGGTGTGCTGAACTTT

GTGCAGTACAAGTTTAGTCACCTGGCTCCCCGAGAGCGGCAGACAATGTTCGAGCTCTCA

AAGATGTTCCTGCTCTGCCTTAACTACTGGAAGCTTGAGACACCTGCCCAATTTCGGCAG

AGGTCCCAGTCAGAGGATGTGGCTACCTACAAGGTCAATTACACCAGATGGCTGTGTTAC

TGCCATGTACCCCAGAGCTGCGATAGCCTCCCCCGCTATGAGACCACCCACGTCTTTGGA

CGAAGCCTTCTACGGTCCATTTTCACTGTTACTCGCCGGCAGCTGCTGGAAAAGTTCCGA

GTAGAAAAGGACAAGCTGGTGCCTGAGAAGAGGACCCTCATCCTTACTCACTTTCCCAAA

TTCCTGTCCATGCTTGAGGAGGAGATCTACGGGGCAAACTCTCCTATCTGGGAGTCAGGC

TTCACCATGCCACCTTCAGAAGGGACACAGTTGGTTCCCCGGCCAGCTACAGTCAGTGCA

GCAGTTGTTCCCAGCACCCCCATCTTCAGCCCCACCATGGGCGGGGGCAGCAACAGCTCC

CTGAGTCTGGATTCTGCAGGGGCCGAGCCCATGCCAGGCGAGAAGAGGAAG

>CL247.Contig2_A-W 65 298 minus strand PREDICTED: LOW QUALITY PROTEIN: histone acetyltransferase KAT2A-like [Loxodonta africana]

AGATTCCTGTCCATGCTTGAGGAGGAGATCTACGGGGCAAACTCTCCTATCTGGGAGTCA

GGCTTCACCATGCCACCTTCAGAAGGGACACAGTTGGTTCCCCGGCCAGCTACAGTCAGT

GCAGCAGTTGTTCCCAGCACCCCCATCTTCAGCCCCACCATGGGCGGGGGCAGCAACAGC

TCCCTGAGTCTGGATTCTGCAGGGGCCGAGCCCATGCCAGGCGAGAAGAGGAAG

>CL247.Contig3_A-W 33 266 minus strand PREDICTED: LOW QUALITY PROTEIN: histone acetyltransferase KAT2A-like [Loxodonta africana]

AGATTCCTGTCCATGCTTGAGGAGGAGATCTACGGGGCAAACTCTCCTATCTGGGAGTCA

GGCTTCACCATGCCACCTTCAGAAGGGACACAGTTGGTTCCCCGGCCAGCTACAGTCAGT

GCAGCAGTTGTTCCCAGCACCCCCATCTTCAGCCCCACCATGGGCGGGGGCAGCAACAGC

TCCCTGAGTCTGGATTCTGCAGGGGCCGAGCCCATGCCAGGCGAGAAGAGGAAG

>CL248.Contig1_A-W 4 159 minus strand hypothetical protein EGM_01560, partial [Macaca fascicularis]

AAAGCAGTATGGAGATTCCTCAAAAAACTAGAAATGGAACCACCGTTTGACCCTGTTATC

CCACTCCTCGGGAAATAACTGAAAAATTTTAAATTGTCATACTACAGTAAATCAGCCACA

TTAATGTTTATAGTGGTGCAGTTCACAATAGCTAAG

>CL249.Contig1_A-W 1 735 speckle targeted PIP5K1A-regulated poly(A) polymerase [Rattus norvegicus] >gi|118595569|sp|Q3MHT4.1|STPAP_RAT RecName: Full=Speckle targeted PIP5K1A-regulated poly(A) polymerase; Short=Star-PAP; AltName: Full=RNA-binding motif protein 21; Short=RNA-binding protein 21; AltName: Full=U6 snRNA-specific terminal uridylyltransferase 1; Short=U6-TUTase >gi|75773232|gb|AAI04696.1| Terminal uridylyl transferase 1, U6 snRNA-specific [Rattus norvegicus] >gi|149062339|gb|EDM12762.1| similar to RNA binding motif protein 21 [Rattus norvegicus]

GTGGTGCTGAGAATCAAACCTTTGTTCTCTGCTTGGCACTTAGGCTGTGTGGTCCATCCT

TTTGGCTCGTCCATAAACAGCTTTGATGTCCATGGCTGTGATCTTGACCTCTTCTTGGAT

CTGGGTGACTTGGAAGAGCCCCAGTCAGTCTTAAAGGCTCCCGAGTCTCCATCATTGGAC

TCAGCCCTTGCTTCTCCACTGGATCCTCAAGCCCTGGCCTGTGCCCTAGCCTCCCCTCTA

GGCTCACAGTCTCCAGCTTCTCCCCAAGATTCTGAAGCCCTGGACTGTGGAACTCCTTCC

TCCTCCCTGGCACCCCAGACTCCGGACTCGGCTTTGGCCTCTGAGACTGTTGCCTCTCCC

CAGTCCCTGCCTCCAGCCTCACCACTGCAGGAAGACAGGGGAGAGCAGGACCTGGGAAAG

GCCCTGGAACTAGCAGAGGCCCCAAAGGGGGAGAAGCCAGAGGGAGCAGCAATGCTGGAG

CTCGTGGGATCCATTCTCCGGGGCTGTGTCCCTGGGGTGTACCGAGTCCAAACTGTGCCT

TCTGCCCGGCGCCCTGTTGTCAAGTTCTGCCATCGACCTTCTGGTCTTCATGGTGACGTC

TCCCTCAGTAACCGGCTAGCCCTACATAATTCCCGTTTTTTGAGTCTCTGCTCTGAGCTG

GATGGGCGAGTCCGGCCCCTTGTGTATACTCTCCGCTGCTGGGCTCAGGGTCGAGGGCTG

TCAGACCAGGGACCC

>CL249.Contig2_A-W 1 849 PREDICTED: speckle targeted PIP5K1A-regulated poly(A) polymerase, partial [Papio anubis]

GTGGTGCTGAGAATCAAACCTTTGTTCTCTGCTTGGCACTTAGGCTGTGTGGTCCATCCT

TTTGGCTCGTCCATAAACAGCTTTGATGTCCATGGCTGTGATCTTGACCTCTTCTTGGAT

CTGGGTGACTTGGAAGAGCCCCAGTCAGTCTTAAAGGCTCCCGAGTCTCCATCATTGGAC

TCAGCCCTTGCTTCTCCACTGGATCCTCAAGCCCTGGCCTGTGCCCTAGCCTCCCCTCTA

GGCTCACAGTCTCCAGCTTCTCCCCAAGATTCTGAAGCCCTGGACTGTGGAACTCCTTCC

TCCTCCCTGGCACCCCAGACTCCGGACTCGGCTTTGGCCTCTGAGACTGTTGCCTCTCCC

CAGTCCCTGCCTCCAGCCTCACCACTGCAGGAAGACAGGGGAGAGCAGGACCTGGGAAAG

GCCCTGGAACTAGCAGAGGCCCCAAAGGGGGAGAAGCCAGAGGGAGCAGCAATGCTGGAG

CTCGTGGGATCCATTCTCCGGGGCTGTGTCCCTGGGGTGTACCGAGTCCAAACTGTGCCT

TCTGCCCGGCGCCCTGTTGTCAAGTTCTGCCATCGACCTTCTGGTCTTCATGGTGACGTC

TCCCTCAGTAACCGGCTAGCCCTACATAATTCCCGTTTTTTGAGTCTCTGCTCTGAGCTG

GATGGGCGAGTCCGGCCCCTTGTGTATACTCTCCGCTGCTGGGCTCAGGGTCGAGGGCTG

TCAGGGAGTGGCCCGCTTCTGAATAACTACGCCTTGACCTTGCTTGTGATCTATTTCCTT

CAGACCAGGGACCCTCCTGTGTTGCCCACTGTGTCCCAACTCACCCAAAAAGCAGGTATT

TCAGCCCAG

>CL249.Contig3_A-W 65 775 PREDICTED: LOW QUALITY PROTEIN: speckle targeted PIP5K1A-regulated poly(A) polymerase-like [Loxodonta africana]

CCACAGTCAGTCTTAAAGGCTCCCGAGTCTCCATCATTGGACTCAGCCCTTGCTTCTCCA

CTGGATCCTCAAGCCCTGGCCTGTGCCCTAGCCTCCCCTCTAGGCTCACAGTCTCCAGCT

TCTCCCCAAGATTCTGAAGCCCTGGACTGTGGAACTCCTTCCTCCTCCCTGGCACCCCAG

ACTCCGGACTCGGCTTTGGCCTCTGAGACTGTTGCCTCTCCCCAGTCCCTGCCTCCAGCC

TCACCACTGCAGGAAGACAGGGGAGAGCAGGACCTGGGAAAGGCCCTGGAACTAGCAGAG

GCCCCAAAGGGGGAGAAGCCAGAGGGAGCAGCAATGCTGGAGCTCGTGGGATCCATTCTC

CGGGGCTGTGTCCCTGGGGTGTACCGAGTCCAAACTGTGCCTTCTGCCCGGCGCCCTGTT

GTCAAGTTCTGCCATCGACCTTCTGGTCTTCATGGTGACGTCTCCCTCAGTAACCGGCTA

GCCCTACATAATTCCCGTTTTTTGAGTCTCTGCTCTGAGCTGGATGGGCGAGTCCGGCCC

CTTGTGTATACTCTCCGCTGCTGGGCTCAGGGTCGAGGGCTGTCAGGGAGTGGCCCGCTT

CTGAATAACTACGCCTTGACCTTGCTTGTGATCTATTTCCTTCAGACCAGGGACCCTCCT

GTGTTGCCCACTGTGTCCCAACTCACCCAAAAAGCAGGTATTTCAGCCCAG

>CL249.Contig4_A-W 65 541 PREDICTED: speckle targeted PIP5K1A-regulated poly(A) polymerase, partial [Papio anubis]

CCACAGTCAGTCTTAAAGGCTCCCGAGTCTCCATCATTGGACTCAGCCCTTGCTTCTCCA

CTGGATCCTCAAGCCCTGGCCTGTGCCCTAGCCTCCCCTCTAGGCTCACAGTCTCCAGCT

TCTCCCCAAGATTCTGAAGCCCTGGACTGTGGAACTCCTTCCTCCTCCCTGGCACCCCAG

ACTCCGGACTCGGCTTTGGCCTCTGAGACTGTTGCCTCTCCCCAGTCCCTGCCTCCAGCC

TCACCACTGCAGGAAGACAGGGGAGAGCAGGACCTGGGAAAGGCCCTGGAACTAGCAGAG

GCCCCAAAGGGGGAGAAGCCAGAGGGAGCAGCAATGCTGGAGCTCGTGGGATCCATTCTC

CGGGGCTGTGTCCCTGGGGTGTACCGAGTCCAAACTGTGCCTTCTGCCCGGCGCCCTGTT

GTCAAGTTCTGCCATCGACCTTCTGGTCTTCATGGTGACGTCTCCCTCAGTAACCGG

>CL251.Contig1_A-W 19 831 PREDICTED: glucose-6-phosphatase 3-like, partial [Pan paniscus]

AGGTTTCTGTTTGGAGACAGGCCCTTTTGGTGGGTCCATGAGTCTGGGTACTACAGCCAG

ACTCCTGCCCGGGTTCACCAGTTCCCCTCTTCTTGTGAAACTGGTCCAGGCAGCCCTTCT

GGACACTGCATGGTCACAGGAGCAGCTTTCTGGCCAATAATGACAGCCGTCTCTTCTCAA

GTAGCTGCTCGGACCCGCAGCCGCTGGGTAAGGGTGATTCCTAGCCTGGCTTATTGCACC

TTCCTATTGGCAGTTGGCCTGTCCCGGGTCTTCCTCTTAGCACATTTCCCTCACCAAGTG

TTGGCTGGCCTAATAACTGGTGCTGTCCTGGGCTGGCTGATGACTCCGCGGGTCCCTATG

GAGCGGGAATTAAGTTTCTATGGGTTGACTGCACTGGCCCTCATGCTGGGTGCTAGTCTC

ATCTATTGGACTCTCTTTTCACTGGGCCTAGATCTTTCTTGGTCCATCAACCTAGCCTCC

AAGTGGTGTGAGCGACCTGAGTGGGTACACTTGGACAGCCGGCCCTTTGCTTCCCTGAGT

CGTGACTCAGGGGCTGCCCTGGGGCTGGGCATTGCCCTGCACTCTCCCTGCTATGCTCAG

ATACGACGGGCACAACTGGGAAATGGCCAGAAGGTAGCCTGCCTTGTGCTGGCCATGGTG

CTTCTGGGTCTCCTAGACTGGCTGGGCCACCCACCTCAGATCAGCCTCTTCTACATCTTC

AACTTCCTTAAGTACACCCTCTGGCCATGTCTGGTCCTTGCCCTCGTGCCCTGGCTTGTA

CATATGTTCAGTACCCAGGAAATACCACCCATC

>CL251.Contig2_A-W 19 228 PREDICTED: glucose-6-phosphatase 3-like, partial [Pan paniscus]

AGGTTTCTGTTTGGAGACAGGCCCTTTTGGTGGGTCCATGAGTCTGGGTACTACAGCCAG

ACTCCTGCCCGGGTTCACCAGTTCCCCTCTTCTTGTGAAACTGGTCCAGGCAGCCCTTCT

GGACACTGCATGGTCACAGGAGCAGCTTTCTGGCCAATAATGACAGCCGTCTCTTCTCAA

GTAGCTGCTCGGACCCGCAGCCGCTGGGTG

>CL251.Contig3_A-W 22 1050 minus strand PREDICTED: glucose-6-phosphatase 3 [Papio anubis]

TTTCTGTTTGGAGACAGGCCCTTTTGGTGGGTCCATGAGTCTGGGTACTACAGCCAGACT

CCTGCCCGGGTTCACCAGTTCCCCTCTTCTTGTGAAACTGGTCCAGGCAGCCCTTCTGGA

CACTGCATGGTCACAGGAGCAGCTTTCTGGCCAATAATGACAGCCGTCTCTTCTCAAGTA

GCTGCTCGGACCCGCAGCCGCTGGGTAAGGGTGATTCCTAGCCTGGCTTATTGCACCTTC

CTATTGGCAGTTGGCCTGTCCCGGGTCTTCCTCTTAGCACATTTCCCTCACCAAGTGTTG

GCTGGCCTAATAACTGGTGAGCAATAGGGGCCATAGGGTGGGCCAAGAGGGTGCCTTTGG

CAGTGGGATGACCAGTCTAAGATCTTCCCATCATTCCCAGTTTCTATAGAGTCAACCCCA

AGTGGTCTTAGTCTGTCCTTCCACCAAGCTGCACTGCAGTTCAGGGGGTGGGGGTGGGGA

TAATTTCCCCAAACTTCTTGAAGCTGCTGTCATCCCACTCTCCTAGGTGCTGTCCTGGGC

TGGCTGATGACTCCGCGGGTCCCTATGGAGCGGGAATTAAGTTTCTATGGGTTGACTGCA

CTGGCCCTCATGCTGGGTGCTAGTCTCATCTATTGGACTCTCTTTTCACTGGGCCTAGAT

CTTTCTTGGTCCATCAACCTAGCCTCCAAGTGGTGTGAGCGACCTGAGTGGGTACACTTG

GACAGCCGGCCCTTTGCTTCCCTGAGTCGTGACTCAGGGGCTGCCCTGGGGCTGGGCATT

GCCCTGCACTCTCCCTGCTATGCTCAGATACGACGGGCACAACTGGGAAATGGCCAGAAG

GTAGCCTGCCTTGTGCTGGCCATGGTGCTTCTGGGTCTCCTAGACTGGCTGGGCCACCCA

CCTCAGATCAGCCTCTTCTACATCTTCAACTTCCTTAAGTACACCCTCTGGCCATGTCTG

GTCCTTGCCCTCGTGCCCTGGCTTGTACATATGTTCAGTGCCCAGGAAATACCACCCATC

CACTCTTCT

>CL252.Contig1_A-W 1628 1759 hypothetical protein [Monosiga brevicollis MX1] >gi|163775182|gb|EDQ88807.1| predicted protein [Monosiga brevicollis MX1]

TCACCGGGTCGGGAGAGAGTTGGTCTGGCTGTCAGTTGCCTGGCTGGCTGCTGTGTGTGT

GTGTGTGAGTGTGAGTGTTTGTGTCTCTCTGTGTGTGTGTGTGTGTGTGTGAGTGTGTGC

GCACGCATGTCT

>CL253.Contig1_A-W 12 659 hypothetical protein FLJ13305, isoform CRA_a [Homo sapiens]

CACAAGGATTCAAAACTCTTAACTGGCAGTAAACCATCTGATCTTCATACATCCTCAAGT

GCATCTGCTAAAAGAGAACCCCTTTTGACAGACATTAGAGCAGATGAAGAAAATTTGAAA

GAAACACATTGGCCTTATCAGTCTCTAAGACACAAGTCACCAGTAAGAAGTACAAGTGCA

AAGATCATGCCTTGTAACTGCAACCCTCCAGCGCCCACAGTATCTTCCAGAGGAAGGGAA

CAAGCCATAAGAAAGAGTGAAAAGGAAAGAATGAGAGAATACCGACAAGAAATAGAAGAA

AGAGAAGAAAAATTAAAAAAGAGGCCACTACTATTTGAAAGAGTTGCTCAGAAAAATGCA

AGAATGGCAGCAGAAAAGCATTATTCTAACACCCTAAAAGCACTAGGAATATCTGATGAG

TTTGTTTCAAAGAAAGGCCAAAGTGGAAAAGTATTTGAAAAGTTCGACAATCAAGAGATG

AAAAGTTCCACTGAAGATAAAGAAAGCTTTAATGAAGAAGAAAAAATAGAAGAGAGACAG

AATGAAAAAGAAAATTATTTTATTGATACCAGCAGCCAGGATTCTTGCAAGGAAGAAAAC

GAAAATGATGAAGAAAGTGCAGAAGAAAACTCTGTCGAAGAACAAAAC

>CL253.Contig2_A-W 1 570 PREDICTED: protein FAM161A isoform 2 [Callithrix jacchus]

ATAAGGAGATCACTTGAGGAAAAGAAAATGTTGGAAGAAGAGAGAAATCGGATCCTAACT

AAGCAGAAGCAAAGAATCAAAGAATTGCAGAAACTCCTGACAACCCGGGCTAAGGCTTAT

GACTCACATCAAAGTTTAGCTCAAGTTTTTAAGTCCAAAGTAAAATATCTCAGAAAGAGT

GAAAAGGAAAGAATGAGAGAATACCGACAAGAAATAGAAGAAAGAGAAGAAAAATTAAAA

AAGAGGCCACTACTATTTGAAAGAGTTGCTCAGAAAAATGCAAGAATGGCAGCAGAAAAG

CATTATTCTAACACCCTAAAAGCACTAGGAATATCTGATGAGTTTGTTTCAAAGAAAGGC

CAAAGTGGAAAAGTATTTGAAAAGTTCGACAATCAAGAGATGAAAAGTTCCACTGAAGAT

AAAGAAAGCTTTAATGAAGAAGAAAAAATAGAAGAGAGACAGAATGAAAAAGAAAATTAT

TTTATTGATACCAGCAGCCAGGATTCTTGCAAGGAAGAAAACGAAAATGATGAAGAAAGT

GCAGAAGAAAACTCTGTCGAAGAACAAAAC

>CL254.Contig1_A-W 596 829 Eukaryotic translation initiation factor 2 subunit 2 [Heterocephalus glaber]

GCAAAAAAAGATGATGGGATCTCATTCAGTAACCAGACAGGTCCTGCTTGGGCAGGCTCA

GAAAGAGACTACACCTATGAGGAGCTACTGAATCGAGTGTTCAACATCATGAGGGAAAAG

AATCCAGATATGGTTGCTGGAGAAAAAAGAAAATTTGTTATGAAACCTCCACAGGTTGTC

CGAGTAGGAACCAAGAAAACTTCTTTTGTCAACTTTACAGATATCTGTAAACTG

>CL254.Contig2_A-W 187 603 Eukaryotic translation initiation factor 2 subunit 2 [Heterocephalus glaber]

AAGAAAAAGAAGAAGAAGAAGCCCTTTATGTTAGATGAGGAAGGGGATGCCCAGACAGAA

GAAACCCAGCCCTCTGAAACAAAAGAAGTGGAACCAGAACCAACTGAGGATAAAGATGTG

GAAGCTGATGAAGAAGACAGTAGGAAAAAAGATGCTTCCGATGATCTAGATGATTTGAAC

TTCTTTAATCAAAAGAAAAAGAAGAAAAAAACAAAAAAGATATTTGATATTGATGAAGCT

GAAGAAGGTGTAAAGGATCTTAAGATTGAAAGTGATGTTCAAGAGCCAGCTGAACCAGAG

GATGATCTTGATATTATGCTTGGCAATAAAAAGAAGAAAAAGAAGAATGTCAAGTTCCCA

GATGAGGATGAAATATTAGAGAAAGATGAAGCTGTAGAAGATGAAGATAGCAAAAAA

>CL256.Contig1_A-W 3 509 similar to intermediate filament-like protein MGC:2625 isoform 2; HOM-TES-103 tumor antigen-like, isoform CRA_d [Rattus norvegicus]

CCCACGCTGGTGCAAGGGCCTGGCCTGTCGTGGGTGCACCCTGATGGGGTGGGCGTCCAG

ATCGACACCATCACCCCGGAGATCCGCGCACTCTACAACGTGTTGGCCAAAGTGAAGCGG

GAGCGGGATGAGTACAAGAGGAGGTGGGAAGAGGAATACACAGTGCGGATACAGCTGCAG

GAGCGAGTTTCTGAGCTCCAGGAGGAAGCCCAAGAGGCTGATGCCTGCCAAGAGGAGCTA

GCCATGAAGGTGGAACAGTTGAAAGCTGAGTTGGTCGTTTTCAAGGGGCTCATGAGTAAT

AACCTGTCAGAGCTGGACACCAAAATCCAGGAAAAGGCCATGAAGGTAGATATGGACATC

TGCCGCCGCATTGACATCACCGCCAAACTGTGTGATGTGGCTCAGCAGCGCAACTGTGAG

GACATGATTCAGATGTTCCAGAAGCTGGTTAGTTTGCTTTCATGCAAGTTTATTAATGTC

CCAGTCCCAGTCTCTGAACCACACAGG

>CL256.Contig2_A-W 3 458 intermediate filament family orphan 1 isoform 5 [Homo sapiens]

CCCACGCTGGTGCAAGGGCCTGGCCTGTCGTGGGTGCACCCTGATGGGGTGGGCGTCCAG

ATCGACACCATCACCCCGGAGATCCGCGCACTCTACAACGTGTTGGCCAAAGTGAAGCGG

GAGCGGGATGAGTACAAGAGGAGGTGGGAAGAGGAATACACAGTGCGGATACAGCTGCAG

GAGCGAGTTTCTGAGCTCCAGGAGGAAGCCCAAGAGGCTGATGCCTGCCAAGAGGAGCTA

GCCATGAAGGTGGAACAGTTGAAAGCTGAGTTGGTCGTTTTCAAGGGGCTCATGAGTAAT

AACCTGTCAGAGCTGGACACCAAAATCCAGGAAAAGGCCATGAAGGTAGATATGGACATC

TGCCGCCGCATTGACATCACCGCCAAACTGTGTGATGTGGCTCAGCAGCGCAACTGTGAG

GACATGATTCAGATGTTCCAGAAGCTGGTTAGTTTG

>CL256.Contig3_A-W 255 491 RIKEN cDNA A930037G23, isoform CRA_c [Mus musculus]

CAGCTGGAGTTGGCCACAGCGAAGAACGACATGAACCGGCACCTGCATGAGTACATGGAG

ATGTGCAGCATGAAGCGGGGCCTGGATGTGCAGATGGAGACCTGCCGCCGTCTCATCACC

CAGTCTGGAGACCGAAAGTCTCCTGCTTTCACTGCGGTCCCGCTTAGCGACCCGCCGCCA

CCGCCAAGCGAGACTGAGGACTCCGATCGCGATGTCTCATCTGATAGCTCCATGAGA

>CL256.Contig4_A-W 315 419 RIKEN cDNA A930037G23, isoform CRA_c [Mus musculus]

AGAAAGTCTCCTGCTTTCACTGCGGTCCCGCTTAGCGACCCGCCGCCACCGCCAAGCGAG

ACTGAGGACTCCGATCGCGATGTCTCATCTGATAGCTCCATGAGA

>CL256.Contig5_A-W 93 194 RIKEN cDNA A930037G23, isoform CRA_c [Mus musculus]

AAGTCTCCTGCTTTCACTGCGGTCCCGCTTAGCGACCCGCCGCCACCGCCAAGCGAGACT

GAGGACTCCGATCGCGATGTCTCATCTGATAGCTCCATGAGA

>CL256.Contig6_A-W 1 498 PREDICTED: intermediate filament family orphan 2 [Bos taurus]

GAGGCCGAGGCCATCCAGGAGGAGATGAACGAGAAGATCGAGAGGCTCAAGGCCGAGCTG

GTGGTGTTCAAGGGGCTCATGAGTGACCCCATGACAGACCTGGACACAAAGATCCAAGAA

AAGGCCATGAAGGTGGACATGGACATCTGCCGCCGAATCGATATCACGGCCAAGCTGTGC

GATGTCGCGCAGCAGCGGAACTCGGAAGACGTGTCCAAGATCTTCCAGGTGGTCCCCAAG

AAGAAAGAGCGTAAGGTGACTTCGGACGATGACATCTCTGAACAGGATGGAGAGGTGAAC

CGGTTCTCGGACGACGAGGTTGGCTCCATGAACATCACGGACGAGATGAAGCGCATGTTT

AACCAACTGCGGGAGACCTTTGACTTCGATGACGACTGTGACAGCCTGACGTGGGAGGAG

AACGAGGACACGCTGCTGCTCTGGGAAGATTTCACCAACTGCAACCCCAGCATCGACCTC

CAGGGCGAGCAAGAGGAG

>CL257.Contig1_A-W 34 966 PREDICTED: cyclic AMP-dependent transcription factor ATF-7 isoform 1 [Saimiri boliviensis boliviensis]

ATGGGAGACGACAGACCGTTTGTGTGCAATGCCCCGGGCTGTGGACAGAGATTTACAAAT

GAGGACCACCTGGCAGTTCATAAACACAAGCATGAGATGACATTGAAATTTGGCCCAGCC

CGAACTGACTCAGTCATCATCGCAGATCAAACTCCTACTCCAACTAGATTCCTGAAGAAC

TGTGAGGAGGTGGGACTCTTCAATGAACTAGCTAGCTCCTTTGAACATGAATTCAAGAAA

GCTGCAGATGAGGATGAGAAAAAGGGTGCTGCTGGGCCCCTTGACATGTCTCTGCCTTCC

ACACCAGACGTCAAAATCAAAGAAGAAGAGCCAGTAGAGGTAGACTCATCCCCACCAGAA

AGTCCTGCCTCTAGCTCCTGTTCCCCACCCCTGAAGGAGAAGGAAGTTGCCCCAAAGCCT

GTTGTGATCTCTACCCCTACGCCCACCATCGTCCGTCCTGGTTCCTTGCCTCTCCACTTA

GGCTATGATCCACTTCATCCAACCCTTCCTTCCCCAACCTCAGTCATCACACAGGCTCCA

CCATCCAACAGGCAAATGGGGTCTCCCACTGGCTCCCTTCCTCTTGTCATGCATCTTGCT

AATGGACAGACCATGCCTGTGCTGCCGGGACCTCCAGTTCAGATGCCTTCTGTTATATCG

CTGGCCAGACCTGTGTCCATGGTGCCCAACATTCCTGGTATCCCTGGCCCGCCAGTTAAT

AGCAGTGGCTCCATTTCTCCCTCTGGCCATCCTGTACCATCAGAAGCCAAAATGAGACTA

AAAGCCACCCTAACCCACCAAGTCTCCTCAATCAATGGTGGTTGTGGAATGGTGGTAGGT

ACTGCCAGTACCATGGTAACCTCCCGTCCAGAGCAGAGCCAGATTCTCATCCAGCATCCT

GATGCCCCATCCCCAGCCCAGCCACAGGTCAGT

>CL257.Contig2_A-W 60 431 PREDICTED: cyclic AMP-dependent transcription factor ATF-7 isoform 2 [Otolemur garnettii]

TCTCCCACTGGCTCCCTTCCTCTTGTCATGCATCTTGCTAATGGACAGACCATGCCTGTG

CTGCCGGGACCTCCAGTTCAGATGCCTTCTGTTATATCGCTGGCCAGACCTGTGTCCATG

GTGCCCAACATTCCTGGTATCCCTGGCCCGCCAGTTAATAGCAGTGGCTCCATTTCTCCC

TCTGGCCATCCTGTACCATCAGAAGCCAAAATGAGACTAAAAGCCACCCTAACCCACCAA

GTCTCCTCAATCAATGGTGGTTGTGGAATGGTGGTAGGTACTGCCAGTACCATGGTAACC

TCCCGTCCAGAGCAGAGCCAGATTCTCATCCAGCATCCTGATGCCCCATCCCCAGCCCAG

CCACAGGTCAGT

>CL257.Contig3_A-W 75 341 PREDICTED: cyclic AMP-dependent transcription factor ATF-7 isoform 2 [Saimiri boliviensis boliviensis]

CAGGAAGTTGCCCCAAAGCCTGTTGTGATCTCTACCCCTACGCCCACCATCGTCCGTCCT

GGTTCCTTGCCTCTCCACTTAGGCTATGATCCACTTCATCCAACCCTTCCTTCCCCAACC

TCAGTCATCACACAGGCTCCACCATCCAACAGGCAAATGGGGTCTCCCACTGGCTCCCTT

CCTCTTGTCATGCATCTTGCTAATGGACAGACCATGCCTGTGCTGCCGGGACCTCCAGTT

CAGATGCCTTCTGTTATATCGGTGAGC

>CL257.Contig4_A-W 54 788 PREDICTED: cyclic AMP-dependent transcription factor ATF-7 isoform 2 [Otolemur garnettii]

GATATGTTGGTCTCTTCTCCCCAGGAAGTTGCCCCAAAGCCTGTTGTGATCTCTACCCCT

ACGCCCACCATCGTCCGTCCTGGTTCCTTGCCTCTCCACTTAGGCTATGATCCACTTCAT

CCAACCCTTCCTTCCCCAACCTCAGTCATCACACAGGCTCCACCATCCAACAGGCAAATG

GGGTCTCCCACTGGCTCCCTTCCTCTTGTCATGCATCTTGCTAATGGACAGACCATGCCT

GTGCTGCCGGGACCTCCAGTTCAGATGCCTTCTGTTATATCGCTGGCCAGACCTGTGTCC

ATGGTGCCCAACATTCCTGGTATCCCTGGCCCGCCAGTTAATAGCAGTGGCTCCATTTCT

CCCTCTGGCCATCCTGTACCATCAGAAGCCAAAATGAGACTAAAAGCCACCCTAACCCAC

CAAGTCTCCTCAATCAATGGTGGTTGTGGAATGGTGGTAGGTACTGCCAGTACCATGGTA

ACCTCCCGTCCAGAGCAGAGCCAGATTCTCATCCAGCATCCTGATGCCCCATCCCCAGCC

CAGCCACAGGTCTCTCCAGCCCAGCCTACTCCTAGTACTGGGGGGCGGCGGCGGCGCACA

GTAGATGAAGATCCAGACGAACGGCGGCAACGTTTTCTGGAGCGCAACCGGGCTGCAGCC

TCCCGCTGCCGCCAGAAGCGGAAGCTATGGGTGTCTTCCCTAGAGAAAAAGGCAGAAGAA

CTCACTTCTCAGAAC

>CL257.Contig5_A-W 62 859 PREDICTED: cyclic AMP-dependent transcription factor ATF-7 isoform 1 [Saimiri boliviensis boliviensis]

ATGTTTACAGATCAAACTCCTACTCCAACTAGATTCCTGAAGAACTGTGAGGAGGTGGGA

CTCTTCAATGAACTAGCTAGCTCCTTTGAACATGAATTCAAGAAAGCTGCAGATGAGGAT

GAGAAAAAGGGTGCTGCTGGGCCCCTTGACATGTCTCTGCCTTCCACACCAGACGTCAAA

ATCAAAGAAGAAGAGCCAGTAGAGGTAGACTCATCCCCACCAGAAAGTCCTGCCTCTAGC

TCCTGTTCCCCACCCCTGAAGGAGAAGGAAGTTGCCCCAAAGCCTGTTGTGATCTCTACC

CCTACGCCCACCATCGTCCGTCCTGGTTCCTTGCCTCTCCACTTAGGCTATGATCCACTT

CATCCAACCCTTCCTTCCCCAACCTCAGTCATCACACAGGCTCCACCATCCAACAGGCAA

ATGGGGTCTCCCACTGGCTCCCTTCCTCTTGTCATGCATCTTGCTAATGGACAGACCATG

CCTGTGCTGCCGGGACCTCCAGTTCAGATGCCTTCTGTTATATCGCTGGCCAGACCTGTG

TCCATGGTGCCCAACATTCCTGGTATCCCTGGCCCGCCAGTTAATAGCAGTGGCTCCATT

TCTCCCTCTGGCCATCCTGTACCATCAGAAGCCAAAATGAGACTAAAAGCCACCCTAACC

CACCAAGTCTCCTCAATCAATGGTGGTTGTGGAATGGTGGTAGGTACTGCCAGTACCATG

GTAACCTCCCGTCCAGAGCAGAGCCAGATTCTCATCCAGCATCCTGATGCCCCATCCCCA

GCCCAGCCACAGGTCAGT

>CL257.Contig6_A-W 54 608 PREDICTED: cyclic AMP-dependent transcription factor ATF-7 isoform 2 [Otolemur garnettii]

GATATGTTGGTCTCTTCTCCCCAGGAAGTTGCCCCAAAGCCTGTTGTGATCTCTACCCCT

ACGCCCACCATCGTCCGTCCTGGTTCCTTGCCTCTCCACTTAGGCTATGATCCACTTCAT

CCAACCCTTCCTTCCCCAACCTCAGTCATCACACAGGCTCCACCATCCAACAGGCAAATG

GGGTCTCCCACTGGCTCCCTTCCTCTTGTCATGCATCTTGCTAATGGACAGACCATGCCT

GTGCTGCCGGGACCTCCAGTTCAGATGCCTTCTGTTATATCGCTGGCCAGACCTGTGTCC

ATGGTGCCCAACATTCCTGGTATCCCTGGCCCGCCAGTTAATAGCAGTGGCTCCATTTCT

CCCTCTGGCCATCCTGTACCATCAGAAGCCAAAATGAGACTAAAAGCCACCCTAACCCAC

CAAGTCTCCTCAATCAATGGTGGTTGTGGAATGGTGGTAGGTACTGCCAGTACCATGGTA

ACCTCCCGTCCAGAGCAGAGCCAGATTCTCATCCAGCATCCTGATGCCCCATCCCCAGCC

CAGCCACAGGTCAGT

>CL257.Contig7_A-W 34 315 PREDICTED: cyclic AMP-dependent transcription factor ATF-7 isoform 2 [Otolemur garnettii]

TTGATACAGCTGGCCAGACCTGTGTCCATGGTGCCCAACATTCCTGGTATCCCTGGCCCG

CCAGTTAATAGCAGTGGCTCCATTTCTCCCTCTGGCCATCCTGTACCATCAGAAGCCAAA

ATGAGACTAAAAGCCACCCTAACCCACCAAGTCTCCTCAATCAATGGTGGTTGTGGAATG

GTGGTAGGTACTGCCAGTACCATGGTAACCTCCCGTCCAGAGCAGAGCCAGATTCTCATC

CAGCATCCTGATGCCCCATCCCCAGCCCAGCCACAGGTCAGT

>CL257.Contig8_A-W 116 274 PREDICTED: cyclic AMP-dependent transcription factor ATF-7 isoform 2 [Otolemur garnettii]

AGACTAAAAGCCACCCTAACCCACCAAGTCTCCTCAATCAATGGTGGTTGTGGAATGGTG

GTAGGTACTGCCAGTACCATGGTAACCTCCCGTCCAGAGCAGAGCCAGATTCTCATCCAG

CATCCTGATGCCCCATCCCCAGCCCAGCCACAGGTCAGT

>CL257.Contig9_A-W 75 236 PREDICTED: cyclic AMP-dependent transcription factor ATF-7 isoform 2 [Saimiri boliviensis boliviensis]

CAGGAAGTTGCCCCAAAGCCTGTTGTGATCTCTACCCCTACGCCCACCATCGTCCGTCCT

GGTTCCTTGCCTCTCCACTTAGGCTATGATCCACTTCATCCAACCCTTCCTTCCCCAACC

TCAGTCATCACACAGGCTCCACCATCCAACAGGCAAATGGGG

>CL258.Contig1_A-W 2 451 minus strand PREDICTED: nuclear valosin-containing protein-like isoform 2 [Saimiri boliviensis boliviensis]

TTTGAGCAAGCTGTGTCAAATGCACCCTGTATTGTTTTCATCGATGAAATTGATGCTATT

ACTCCCAAAAGAGAAGTTGCTTCAAAAGATATGGAACGAAGAATTGTAGCCCAGCTCCTA

ACCTGCATGGATGATTTGAATAACGTGGCTGCTACAGCTCAAGTCCTGGTTATTGGAGCT

ACTAATCGACCAGATTCGTTAGACCCTGCTTTGAGACGTGCAGGAAGGTTCGACCGAGAA

ATATGCTTAGGTATCCCTGATGAAGCATCCAGAGAAAGGATACTTCAAACTTTGTGCCGA

AAACTAAGACTTCCTGAGACTTTTAACTTTTGTCACTTAGCACACTTAACGCCAGGCTTT

GTTGGTGCTGATCTGATGGCACTCTGCCGAGAGGCAGCTATGTGTGCAGTCAACAGGGTC

TTGATGAAGCTACATGAACAGCAGAAGAAA

>CL259.Contig1_A-W 605 736 PHYHD1 protein (predicted) [Callithrix jacchus]

GCTCTGCACGCCCATGACCCTGTCTTCAGGAGTGTCACACATTCCCCCAAGGTGCAGGCC

TTGGCCAGAAGTCTGGGCCTCCAGATGCCTGTGGTGGTGCAGAGCATGTACATCTTTAAG

GTGAGCGATGTG

>CL259.Contig2_A-W 605 1162 PREDICTED: phytanoyl-CoA dioxygenase domain-containing protein 1 [Saimiri boliviensis boliviensis]

GCTCTGCACGCCCATGACCCTGTCTTCAGGAGTGTCACACATTCCCCCAAGGTGCAGGCC

TTGGCCAGAAGTCTGGGCCTCCAGATGCCTGTGGTGGTGCAGAGCATGTACATCTTTAAG

CAACCTCACTTTGGTGGTGAAGCCTCCCCTCACCAGGATGCCTCGTTCCTGTACACGGAG

CCTCTGGGCCGGGTGCTGGGCATGTGGATGGCACTGGAGGATGCCACGCTGGAGAATGGC

TGCCTTTGGTTTATCCCTGGCTCCCACACCAGTGGAGTGTCAAGAAGGATGATCCGGGCC

CCTGCTGGCTCATGGCCTGCTACCAGCTTCCTCGGGGCAGAGCCAGACTGGGATAACAGC

CTCTTTGTGCCCACACCAGTGCAGAAAGGGGCCCTGGTCTTGATCCATGGAGAGGTGGTA

CACAAGAGTGAGCAGAACCTTTCAGACCACTCACGCCAGGCCTACACTTTCCACCTCATG

GAAGCCACTGGCACCGTCTGGAGCCCAGAGAACTGGCTTCAGCCGACAGCTGAGCTGCCG

TTTCCTGCCTTGTACACC

>CL259.Contig3_A-W 63 545 phytanoyl-CoA dioxygenase domain containing 1, isoform CRA_c [Homo sapiens]

ATGGCCTGCCTGAGCCCCTTGCAGCTCCAGAAGTTCCAGGAGGATGGATTCCTGGTGCTG

GAAGGATTCTTGTCTGTGGATGAGTGTGAGGCTATGAAACAAAGGATTGGTGAGATCGTG

GCCGAGATGGATGTCCCTCCCCACTGCCGCACAGAATTTTCCACCCAACATGAGGAACAG

CTTCGAGCCCAGGGCAGCACAGACTATTTCTTGAGCAGTGGTGACAAGATTCGATTCTTC

TTTGAGAAAGGTGTTTTGGACAATAAAGGAAATTTCTTGGTGCCTCCAGAGCAATCAGTC

AACAAAATCGGTCATGCTCTGCACGCCCATGACCCTGTCTTCAGGAGTGTCACACATTCC

CCCAAGGTGCAGGCCTTGGCCAGAAGTCTGGGCCTCCAGATGCCTGTGGTGGTGCAGAGC

ATGTACATCTTTAAGCAACCTCACTTTGGTGGTGAAGGTAAGCTGTGAGCAGCTGTGGGG

CCC

>CL259.Contig4_A-W 605 754 phytanoyl-CoA dioxygenase domain containing 1, isoform CRA_d [Rattus norvegicus]

GCTCTGCACGCCCATGACCCTGTCTTCAGGAGTGTCACACATTCCCCCAAGGTGCAGGCC

TTGGCCAGAAGTCTGGGCCTCCAGATGCCTGTGGTGGTGCAGAGCATGTACATCTTTAAG

CAACCTCACTTTGGTGGTGAAGGTAAGCTG

>CL259.Contig5_A-W 58 888 minus strand phytanoyl-CoA dioxygenase domain-containing protein 1 isoform a [Macaca mulatta]

ATGGCCTGCCTGAGCCCCTTGCAGCTCCAGAAGTTCCAGGAGGATGGATTCCTGGTGCTG

GAAGGATTCTTGTCTGTGGATGAGTGTGAGGCTATGAAACAAAGGATTGGTGAGATCGTG

GCCGAGATGGATGTCCCTCCCCACTGCCGCACAGAATTTTCCACCCAACACGAGGAACAG

CTTCGAGCCCAGGGCAGCACAGACTATTTCTTGAGCAGTGGTGACAAGATTCGATTCTTC

TTTGAGAAAGGTGTTTTGGACAATAAAGGAAATTTCTTGGTGCCTCCAGAGCAATCAGTC

AACAAAATCGGTCATGCTCTGCACGCCCATGACCCTGTCTTCAGGAGTGTCACACATTCC

CCCAAGGTGCAGGCCTTGGCCAGAAGTCTGGGCCTCCAGATGCCTGTGGTGGTGCAGAGC

ATGTACATCTTTAAGCAACCTCACTTTGGTGGTGAAGCCTCCCCTCACCAGGATGCCTCG

TTCCTGTACACGGAGCCTCTGGGCCGGGTGCTGGGCATGTGGATGGCACTGGAGGATGCC

ACGCTGGAGAATGGCTGCCTTTGGTTTATCCCTGGCTCCCACACCAGTGGAGTGTCAAGA

AGGATGATCCGGGCCCCTGCTGGCTCATGGCCTGCTACCAGCTTCCTCGGGGCAGAGCCA

GACTGGGATAACAGCCTCTTTGTGCCCACACCAGTGCAGAAAGGGGCCCTGGTCTTGATC

CATGGAGAGGTGGTACACAAGAGTGAGCAGAACCTTTCAGACCACTCACGCCAGGCCTAC

ACTTTCCACCTCATGGAAGCCACTGGCACCGTCTGGAGCCCAGAGAACTGG

>CL259.Contig6_A-W 58 540 minus strand phytanoyl-CoA dioxygenase domain containing 1, isoform CRA_c [Homo sapiens]

ATGGCCTGCCTGAGCCCCTTGCAGCTCCAGAAGTTCCAGGAGGATGGATTCCTGGTGCTG

GAAGGATTCTTGTCTGTGGATGAGTGTGAGGCTATGAAACAAAGGATTGGTGAGATCGTG

GCCGAGATGGATGTCCCTCCCCACTGCCGCACAGAATTTTCCACCCAACACGAGGAACAG

CTTCGAGCCCAGGGCAGCACAGACTATTTCTTGAGCAGTGGTGACAAGATTCGATTCTTC

TTTGAGAAAGGTGTTTTGGACAATAAAGGAAATTTCTTGGTGCCTCCAGAGCAATCAGTC

AACAAAATCGGTCATGCTCTGCACGCCCATGACCCTGTCTTCAGGAGTGTCACACATTCC

CCCAAGGTGCAGGCCTTGGCCAGAAGTCTGGGCCTCCAGATGCCTGTGGTGGTGCAGAGC

ATGTACATCTTTAAGCAACCTCACTTTGGTGGTGAAGGTAAGCTGTGAGCAGCTGTGGGG

CCC

>CL259.Contig7_A-W 63 935 phytanoyl-CoA dioxygenase domain-containing protein 1 isoform a [Macaca mulatta]

ATGGCCTGCCTGAGCCCCTTGCAGCTCCAGAAGTTCCAGGAGGATGGATTCCTGGTGCTG

GAAGGATTCTTGTCTGTGGATGAGTGTGAGGCTATGAAACAAAGGATTGGTGAGATCGTG

GCCGAGATGGATGTCCCTCCCCACTGCCGCACAGAATTTTCCACCCAACATGAGGAACAG

CTTCGAGCCCAGGGCAGCACAGACTATTTCTTGAGCAGTGGTGACAAGATTCGATTCTTC

TTTGAGAAAGGTGTTTTGGACAATAAAGGAAATTTCTTGGTGCCTCCAGAGCAATCAGTC

AACAAAATCGGTCATGCTCTGCACGCCCATGACCCTGTCTTCAGGAGTGTCACACATTCC

CCCAAGGTGCAGGCCTTGGCCAGAAGTCTGGGCCTCCAGATGCCTGTGGTGGTGCAGAGC

ATGTACATCTTTAAGCAACCTCACTTTGGTGGTGAAGCCTCCCCTCACCAGGATGCCTCG

TTCCTGTACACGGAGCCTCTGGGCCGGGTGCTGGGCATGTGGATGGCACTGGAGGATGCC

ACGCTGGAGAATGGCTGCCTTTGGTTTATCCCTGGCTCCCACACCAGTGGAGTGTCAAGA

AGGATGATCCGGGCCCCTGCTGGCTCATGGCCTGCTACCAGCTTCCTCGGGGCAGAGCCA

GACTGGGATAACAGCCTCTTTGTGCCCACACCAGTGCAGAAAGGGGCCCTGGTCTTGATC

CATGGAGAGGTGGTACACAAGAGTGAGCAGAACCTTTCAGACCACTCACGCCAGGCCTAC

ACTTTCCACCTCATGGAAGCCACTGGCACCGTCTGGAGCCCAGAGAACTGGCTTCAGCCG

ACAGCTGAGCTGCCGTTTCCTGCCTTGTACACC

>CL259.Contig8_A-W 58 540 minus strand phytanoyl-CoA dioxygenase domain containing 1, isoform CRA_c [Homo sapiens]

ATGGCCTGCCTGAGCCCCTTGCAGCTCCAGAAGTTCCAGGAGGATGGATTCCTGGTGCTG

GAAGGATTCTTGTCTGTGGATGAGTGTGAGGCTATGAAACAAAGGATTGGTGAGATCGTG

GCCGAGATGGATGTCCCTCCCCACTGCCGCACAGAATTTTCCACCCAACACGAGGAACAG

CTTCGAGCCCAGGGCAGCACAGACTATTTCTTGAGCAGTGGTGACAAGATTCGATTCTTC

TTTGAGAAAGGTGTTTTGGACAATAAAGGAAATTTCTTGGTGCCTCCAGAGCAATCAGTC

AACAAAATCGGTCATGCTCTGCACGCCCATGACCCTGTCTTCAGGAGTGTCACACATTCC

CCCAAGGTGCAGGCCTTGGCCAGAAGTCTGGGCCTCCAGATGCCTGTGGTGGTGCAGAGC

ATGTACATCTTTAAGCAACCTCACTTTGGTGGTGAAGGTAAGCTGTGAGCAGCTGTGGGG

CCC

>CL259.Contig9_A-W 58 540 minus strand phytanoyl-CoA dioxygenase domain containing 1, isoform CRA_c [Homo sapiens]

ATGGCCTGCCTGAGCCCCTTGCAGCTCCAGAAGTTCCAGGAGGATGGATTCCTGGTGCTG

GAAGGATTCTTGTCTGTGGATGAGTGTGAGGCTATGAAACAAAGGATTGGTGAGATCGTG

GCCGAGATGGATGTCCCTCCCCACTGCCGCACAGAATTTTCCACCCAACACGAGGAACAG

CTTCGAGCCCAGGGCAGCACAGACTATTTCTTGAGCAGTGGTGACAAGATTCGATTCTTC

TTTGAGAAAGGTGTTTTGGACAATAAAGGAAATTTCTTGGTGCCTCCAGAGCAATCAGTC

AACAAAATCGGTCATGCTCTGCACGCCCATGACCCTGTCTTCAGGAGTGTCACACATTCC

CCCAAGGTGCAGGCCTTGGCCAGAAGTCTGGGCCTCCAGATGCCTGTGGTGGTGCAGAGC

ATGTACATCTTTAAGCAACCTCACTTTGGTGGTGAAGGTAAGCTGTGAGCAGCTGTGGGG

CCC

>CL259.Contig10_A-W 197 346 minus strand phytanoyl-CoA dioxygenase domain containing 1, isoform CRA_d [Rattus norvegicus]

GCTCTGCACGCCCATGACCCTGTCTTCAGGAGTGTCACACATTCCCCCAAGGTGCAGGCC

TTGGCCAGAAGTCTGGGCCTCCAGATGCCTGTGGTGGTGCAGAGCATGTACATCTTTAAG

CAACCTCACTTTGGTGGTGAAGGTAAGCTG

>CL259.Contig11_A-W 197 337 minus strand PREDICTED: phytanoyl-CoA dioxygenase domain-containing protein 1 [Saimiri boliviensis boliviensis]

GCTCTGCACGCCCATGACCCTGTCTTCAGGAGTGTCACACATTCCCCCAAGGTGCAGGCC

TTGGCCAGAAGTCTGGGCCTCCAGATGCCTGTGGTGGTGCAGAGCATGTACATCTTTAAG

CAACCTCACTTTGGTGGTGAA

>CL259.Contig12_A-W 56 349 minus strand PREDICTED: phytanoyl-CoA dioxygenase domain-containing protein 1 isoform 1 [Sus scrofa]

GAAGCCGTGGTCAAATCCGTACTGACTCTGCACGCCCATGACCCTGTCTTCAGGAGTGTC

ACACATTCCCCCAAGGTGCAGGCCTTGGCCAGAAGTCTGGGCCTCCAGATGCCTGTGGTG

GTGCAGAGCATGTACATCTTTAAGCAACCTCACTTTGGTGGTGAAGCCTCCCCTCACCAG

GATGCCTCGTTCCTGTACACGGAGCCTCTGGGCCGGGTGCTGGGCATGTGGATGGCACTG

GAGGATGCCACGCTGGAGAATGGCTGCCTTTGGTTTATCCCTGGCTCCCACACC

>CL259.Contig13_A-W 83 220 minus strand PREDICTED: phytanoyl-CoA dioxygenase domain-containing protein 1 [Saimiri boliviensis boliviensis]

CTGCACGCCCATGACCCTGTCTTCAGGAGTGTCACACATTCCCCCAAGGTGCAGGCCTTG

GCCAGAAGTCTGGGCCTCCAGATGCCTGTGGTGGTGCAGAGCATGTACATCTTTAAGCAA

CCTCACTTTGGTGGTGAA

>CL259.Contig15_A-W 83 229 minus strand phytanoyl-CoA dioxygenase domain containing 1, isoform CRA_d [Rattus norvegicus]

CTGCACGCCCATGACCCTGTCTTCAGGAGTGTCACACATTCCCCCAAGGTGCAGGCCTTG

GCCAGAAGTCTGGGCCTCCAGATGCCTGTGGTGGTGCAGAGCATGTACATCTTTAAGCAA

CCTCACTTTGGTGGTGAAGGTAAGCTG

>CL259.Contig16_A-W 83 637 minus strand PREDICTED: phytanoyl-CoA dioxygenase domain-containing protein 1 [Saimiri boliviensis boliviensis]

CTGCACGCCCATGACCCTGTCTTCAGGAGTGTCACACATTCCCCCAAGGTGCAGGCCTTG

GCCAGAAGTCTGGGCCTCCAGATGCCTGTGGTGGTGCAGAGCATGTACATCTTTAAGCAA

CCTCACTTTGGTGGTGAAGCCTCCCCTCACCAGGATGCCTCGTTCCTGTACACGGAGCCT

CTGGGCCGGGTGCTGGGCATGTGGATGGCACTGGAGGATGCCACGCTGGAGAATGGCTGC

CTTTGGTTTATCCCTGGCTCCCACACCAGTGGAGTGTCAAGAAGGATGATCCGGGCCCCT

GCTGGCTCATGGCCTGCTACCAGCTTCCTCGGGGCAGAGCCAGACTGGGATAACAGCCTC

TTTGTGCCCACACCAGTGCAGAAAGGGGCCCTGGTCTTGATCCATGGAGAGGTGGTACAC

AAGAGTGAGCAGAACCTTTCAGACCACTCACGCCAGGCCTACACTTTCCACCTCATGGAA

GCCACTGGCACCGTCTGGAGCCCAGAGAACTGGCTTCAGCCGACAGCTGAGCTGCCGTTT

CCTGCCTTGTACACC

>CL261.Contig1_A-W 44 379 minus strand phosphoribosylglycinamide formyltransferase (predicted) [Rhinolophus ferrumequinum]

GCTCTGCTTTGTAGTGCGGACTTCCCTGCTTTGGTTGTGAAGGCCAGTGGTCTTGCAGCT

GGGAAAGGAGTGATTGTTGCAAAGAGCAAAGAGGAAGCCTGCAAAGCTGTACAGGAGATC

ATGCAGGATAAAGCTTTTGGAGCAGCTGGAGAAACAATTGTTGTTGAAGAACTTCTTGAA

GGAGAAGAGGTGTCTTGTCTGTGTTTCACTGATGGGAGGACAGTGGCCCCCATGCCTCCA

GCACAGGACCATAAACGATTACTGGAGGGAGATCAAGGCCCCAACACAGGGGGAATGGGA

GCCTATTGTCCCACACCTCAGGTACTTCCCAGGGAC

>CL261.Contig2_A-W 38 256 minus strand phosphoribosylglycinamide formyltransferase (predicted) [Rhinolophus ferrumequinum]

ATAATTTAGGATAAAGCTTTTGGAGCAGCTGGAGAAACAATTGTTGTTGAAGAACTTCTT

GAAGGAGAAGAGGTGTCTTGTCTGTGTTTCACTGATGGGAGGACAGTGGCCCCCATGCCT

CCAGCACAGGACCATAAACGATTACTGGAGGGAGATCAAGGCCCCAACACAGGGGGAATG

GGAGCCTATTGTCCCACACCTCAGGTACTTCCCAGGGAC

>CL261.Contig3_A-W 50 178 minus strand Trifunctional purine biosynthetic protein adenosine-3 [Cricetulus griseus]

TGTCTGTGTTTCACTGATGGGAGGACAGTGGCCCCCATGCCTCCAGCACAGGACCATAAA

CGATTACTGGAGGGAGATCAAGGCCCCAACACAGGGGGAATGGGAGCCTATTGTCCCACA

CCTCAGGTA

>CL262.Contig1_A-W 949 1101 minus strand PREDICTED: tyrosine-protein kinase Fes/Fps isoform 4 [Cricetulus griseus]

CAGGGGAACTTCGGTGAAGTATTCAGTGGACGCCTGAGAGCTGATAATACTCTGGTGGCA

GTGAAATCTTGTCGAGAGACACTCCCCCCTGACCTCAAGGCCAAGTTTCTGCAGGAAGCA

AGGTGGGTAACAAAATAAGGACAACCAGAGGTT

>CL262.Contig2_A-W 2298 2450 minus strand PREDICTED: tyrosine-protein kinase Fes/Fps isoform 1 [Cavia porcellus]

CAGGGGAACTTCGGTGAAGTATTCAGTGGACGCCTGAGAGCTGATAATACTCTGGTGGCA

GTGAAATCTTGTCGAGAGACACTCCCCCCTGACCTCAAGGCCAAGTTTCTGCAGGAAGCA

AGGTGGGTAACAAAATAAGGACAACCAGAGGTT

>CL262.Contig3_A-W 949 1101 minus strand tyrosine-protein kinase Fes/Fps [Bos taurus] >gi|59858259|gb|AAX08964.1| V-FES feline sarcoma viral/V-FPS fujinami avian sarcoma viral oncogene homolog [Bos taurus] >gi|296475535|gb|DAA17650.1| feline sarcoma oncogene [Bos taurus]

CAGGGGAACTTCGGTGAAGTATTCAGTGGACGCCTGAGAGCTGATAATACTCTGGTGGCA

GTGAAATCTTGTCGAGAGACACTCCCCCCTGACCTCAAGGCCAAGTTTCTGCAGGAAGCA

AGGTGGGTAACAAAATAAGGACAACCAGAGGTT

>CL262.Contig4_A-W 1750 2049 minus strand PREDICTED: tyrosine-protein kinase Fes/Fps isoform 2 [Cavia porcellus]

TGGACAGCTCTCTCATGCTGCCTCACCTCCTCTGCAGGCCGCTACTCCTCTGAGAGTGAT

GTATGGAGCTTTGGCATCTTGCTCTGGGAGACCTTTAGCCTGGGGGCCTCGCCCTATCCC

AACCTCAGCAATCAGCAAACACGAGAGTTTGTGGAAACGGGGGGTCGCCTGCCCTGCCCA

GAGCTGTGCCCTGATGCTGTGTTCAGGCTCATGGAGCAGTGCTGGGCCTATGAGCCTGGG

CAGCGACCCAGCTTCAGCACCATCTACCAGGAGCTGCAGAGCATTCGAAAGCGGCATCGA

>CL262.Contig5_A-W 2298 2450 minus strand PREDICTED: tyrosine-protein kinase Fes/Fps isoform 1 [Cavia porcellus]

CAGGGGAACTTCGGTGAAGTATTCAGTGGACGCCTGAGAGCTGATAATACTCTGGTGGCA

GTGAAATCTTGTCGAGAGACACTCCCCCCTGACCTCAAGGCCAAGTTTCTGCAGGAAGCA

AGGTGGGTAACAAAATAAGGACAACCAGAGGTT

>CL262.Contig6_A-W 933 1109 minus strand PREDICTED: tyrosine-protein kinase Fes/Fps isoform 4 [Cricetulus griseus]

TGGACAGCTCTCTCATGCTGCCTCACCTCCTCTGCAGGCCGCTACTCCTCTGAGAGTGAT

GTATGGAGCTTTGGCATCTTGCTCTGGGAGACCTTTAGCCTGGGGGCCTCGCCCTATCCC

AACCTCAGCAATCAGCAAACACGAGAGTTTGTGGAAACGGGTAAGGCAAGCCTGTGT

>CL262.Contig7_A-W 3 206 minus strand tyrosine-protein kinase Fes/Fps [Bos taurus] >gi|59858259|gb|AAX08964.1| V-FES feline sarcoma viral/V-FPS fujinami avian sarcoma viral oncogene homolog [Bos taurus] >gi|296475535|gb|DAA17650.1| feline sarcoma oncogene [Bos taurus]

GACAAGTGGGTACTGAACCATGAGGACCTGGTGTTAGGCGAGCAGATTGGGCGGGGGAAC

TTCGGTGAAGTATTCAGTGGACGCCTGAGAGCTGATAATACTCTGGTGGCAGTGAAATCT

TGTCGAGAGACACTCCCCCCTGACCTCAAGGCCAAGTTTCTGCAGGAAGCAAGGTGGGTA

ACAAAATAAGGACAACCAGAGGTT

>CL262.Contig8_A-W 455 631 minus strand proto-oncogene c-fes variant 2 [Homo sapiens]

TGGACAGCTCTCTCATGCTGCCTCACCTCCTCTGCAGGCCGCTACTCCTCTGAGAGTGAT

GTATGGAGCTTTGGCATCTTGCTCTGGGAGACCTTTAGCCTGGGGGCCTCGCCCTATCCC

AACCTCAGCAATCAGCAAACACGAGAGTTTGTGGAAACGGGTAAGGCAAGCCTGTGT

>CL262.Contig9_A-W 455 754 minus strand proto-oncogene c-fes variant 2 [Homo sapiens]

TGGACAGCTCTCTCATGCTGCCTCACCTCCTCTGCAGGCCGCTACTCCTCTGAGAGTGAT

GTATGGAGCTTTGGCATCTTGCTCTGGGAGACCTTTAGCCTGGGGGCCTCGCCCTATCCC

AACCTCAGCAATCAGCAAACACGAGAGTTTGTGGAAACGGGGGGTCGCCTGCCCTGCCCA

GAGCTGTGCCCTGATGCTGTGTTCAGGCTCATGGAGCAGTGCTGGGCCTATGAGCCTGGG

CAGCGACCCAGCTTCAGCACCATCTACCAGGAGCTGCAGAGCATTCGAAAGCGGCATCGA

>CL262.Contig10_A-W 2649 2948 minus strand PREDICTED: tyrosine-protein kinase Fes/Fps isoform 1 [Cavia porcellus]

TGGACAGCTCTCTCATGCTGCCTCACCTCCTCTGCAGGCCGCTACTCCTCTGAGAGTGAT

GTATGGAGCTTTGGCATCTTGCTCTGGGAGACCTTTAGCCTGGGGGCCTCGCCCTATCCC

AACCTCAGCAATCAGCAAACACGAGAGTTTGTGGAAACGGGGGGTCGCCTGCCCTGCCCA

GAGCTGTGCCCTGATGCTGTGTTCAGGCTCATGGAGCAGTGCTGGGCCTATGAGCCTGGG

CAGCGACCCAGCTTCAGCACCATCTACCAGGAGCTGCAGAGCATTCGAAAGCGGCATCGA

>CL262.Contig11_A-W 1399 1551 minus strand PREDICTED: tyrosine-protein kinase Fes/Fps isoform 2 [Cavia porcellus]

CAGGGGAACTTCGGTGAAGTATTCAGTGGACGCCTGAGAGCTGATAATACTCTGGTGGCA

GTGAAATCTTGTCGAGAGACACTCCCCCCTGACCTCAAGGCCAAGTTTCTGCAGGAAGCA

AGGTGGGTAACAAAATAAGGACAACCAGAGGTT

>CL262.Contig12_A-W 1399 1551 minus strand PREDICTED: tyrosine-protein kinase Fes/Fps isoform 2 [Cavia porcellus]

CAGGGGAACTTCGGTGAAGTATTCAGTGGACGCCTGAGAGCTGATAATACTCTGGTGGCA

GTGAAATCTTGTCGAGAGACACTCCCCCCTGACCTCAAGGCCAAGTTTCTGCAGGAAGCA

AGGTGGGTAACAAAATAAGGACAACCAGAGGTT

>CL262.Contig13_A-W 949 1101 minus strand PREDICTED: tyrosine-protein kinase Fes/Fps isoform 4 [Cricetulus griseus]

CAGGGGAACTTCGGTGAAGTATTCAGTGGACGCCTGAGAGCTGATAATACTCTGGTGGCA

GTGAAATCTTGTCGAGAGACACTCCCCCCTGACCTCAAGGCCAAGTTTCTGCAGGAAGCA

AGGTGGGTAACAAAATAAGGACAACCAGAGGTT

>CL262.Contig14_A-W 2869 3045 minus strand PREDICTED: tyrosine-protein kinase Fes/Fps isoform 1 [Cavia porcellus]

TGGACAGCTCTCTCATGCTGCCTCACCTCCTCTGCAGGCCGCTACTCCTCTGAGAGTGAT

GTATGGAGCTTTGGCATCTTGCTCTGGGAGACCTTTAGCCTGGGGGCCTCGCCCTATCCC

AACCTCAGCAATCAGCAAACACGAGAGTTTGTGGAAACGGGTAAGGCAAGCCTGTGT

>CL262.Contig15_A-W 91 522 minus strand proto-oncogene c-fes variant 2 [Homo sapiens]

CCACCTCCAGGAGGAGATTTCCTGACCTTCCTACGGACAGAGGGAGCCCGCCTGAGGGTG

AAGACTCTGCTGCAGATGGTGGGGGATGCAGCTGCAGGCATGGAATACTTAGAAAGCAAG

TGTTGCATCCACCGGGACTTGGCTGCTCGAAACTGCCTGGTGACAGAGAAGAACGTTCTG

AAGATCAGTGATTTTGGGATGTCCCGAGAGGAAGCTGATGGAATCTATGCAGCCTCAGGG

GGTCTCAGACAAGTCCCAGTGAAGTGGACCGCACCGGAGGCCCTTAACTATGGCCGCTAC

TCCTCTGAGAGTGATGTATGGAGCTTTGGCATCTTGCTCTGGGAGACCTTTAGCCTGGGG

GCCTCGCCCTATCCCAACCTCAGCAATCAGCAAACACGAGAGTTTGTGGAAACGGGTAAG

GCAAGCCTGTGT

>CL263.Contig1_A-W 79 207 scribbled homolog (Drosophila), isoform CRA_d [Mus musculus]

ATCGTGCGCCAGACTGGGGGCCTTGGCATCAGCATCGCAGGGGGCAAGGGTTCCACCCCC

TACAAGGGAGATGACGAGGGTGTCTTCATTTCTCGGGTGTCTGAGGAGGGCCCTGCGGCC

CGGGCTGGA

>CL263.Contig2_A-W 66 278 PREDICTED: protein scribble homolog isoform 3 [Otolemur garnettii]

CTACAGGTGAACGGCGTGGCTTTGCAGGACGCTGAGCACCATGAGGCTGTGGAGGCCCTC

CGAGGGGCAGGCACAGCCGTGCAGATGCGTGTGTGGCGGGAACGCATGGTAGAACCTGAG

AACGCAGTCACCATCACACCCCTGCGGCCCGAGGACGACTACAGCCCCCGAGAGCGGCGG

GGAGGCGGCCTGCGCCTGCCCCTGCTGCAGCCG

>CL264.Contig1_A-W 334 450 minus strand PREDICTED: ubiquitin-conjugating enzyme E2 E3 isoform 2 [Saimiri boliviensis boliviensis]

CAAATGGCCAGTGATAAGCAAATGTCCGATGATGACAGCCCCAGCACCAGCAGTGGCAGT

TCAAAGTCGGACCAGGAAGACCCTGCTGCTCCAGAGCCTGAAGATCATGAAGAAAGA

>CL264.Contig2_A-W 167 373 minus strand PREDICTED: nuclear pore membrane glycoprotein 210 [Loxodonta africana]

TTGAGCCTGGTCCTGCACACCCAGGACAAGGAGGCCCTGGCGGCCCTGCCTCTGGGGATG

ACCGTGACCTTCACCGTCCACTTCCATGACCACTTTGGAGATGTCTTCCACGCGCACAGT

TCGGTCCTCAGCTTTGCCACCAACAGAGAGGACTTCGTGCAGGTCACCAAGGGCACCACC

AACAACATCTGCGTACTGCGCACGGTC

>CL264.Contig3_A-W 1 174 minus strand PREDICTED: heterogeneous nuclear ribonucleoprotein M-like [Macaca mulatta]

CGCCGCGCAGCCCGCTCCCTCACACAAAGCCCAGACGCGGAGAAAATGGCGGCAGGGGTC

GAAGCGGCGGCAGAGGTGGCAGCGACGGAGCCCAAAATGGAGGAGGAGAGCGGCGCGCCC

GGCGTGCCGAGTGGCAACGGGGCTCCGGGCCCGAAGGGGTCTTTGGTTGAGCCT

>CL264.Contig4_A-W 172 399 minus strand PREDICTED: nuclear pore membrane glycoprotein 210 [Loxodonta africana]

ATATTCTATATATGTCTTTGGTTGAGCCTGGTCCTGCACACCCAGGACAAGGAGGCCCTG

GCGGCCCTGCCTCTGGGGATGACCGTGACCTTCACCGTCCACTTCCATGACCACTTTGGA

GATGTCTTCCACGCGCACAGTTCGGTCCTCAGCTTTGCCACCAACAGAGAGGACTTCGTG

CAGGTCACCAAGGGCACCACCAACAACATCTGCGTACTGCGCACGGTC

>CL264.Contig5_A-W 360 476 minus strand PREDICTED: ubiquitin-conjugating enzyme E2 E3 isoform 2 [Saimiri boliviensis boliviensis]

CAAATGGCCAGTGATAAGCAAATGTCCGATGATGACAGCCCCAGCACCAGCAGTGGCAGT

TCAAAGTCGGACCAGGAAGACCCTGCTGCTCCAGAGCCTGAAGATCATGAAGAAAGA

>CL264.Contig6_A-W 88 2649 minus strand PREDICTED: nuclear pore membrane glycoprotein 210 [Equus caballus]

GGGCGGCTGCCCCAGGGGCCTGAGCACTGTGGTGGCGTCCGGGTGAAGGCCGAGGCTCGG

GGCTCCACCACACTCCTGGTGAGCTACACGCATGGCCACGTCCACCTGAGCGCCAGCCTC

ACCATCACCGCCTACCTGCCCCTCAGGGCCGTGGACCCGTCCTCCGTAGCTGTGGTCACC

CTGGGCTCCTCAAAGGACATGCTGTTTGAAGGAGGCCCCCGGCCCTGGGTCCTGGAGCCT

GCCAGGTTCTTCCGGAACCTCACCTCTGAGGACGGGGACAGCATCAGCCTGGCCCTCCTG

GGCCCGCCCGCCTCCCGGAGTCACCAGCAGCACAGCATCCGCGCCACCTGCCGGGCCCTG

GGCGACCAGGTCATCGCCCTCTCCGTGGGGAACCAGCCCAGCCTCACCAACCCTTTCCCG

GCCGTGGAGCCCGCCGTGGTGAAGGTCGTCTGCGCCCCGCCATCCAGGATCAGCCTCACG

CCTGTCCACGCCGGTCCCCCGCTGGACCCGTCCTGTCCACGGCTGCAGCACGACAAGCAG

GTGGTTCCCGTCTCCAGCCACCGCAACCCCCTGCTGGACCTCGCCGCCTATGACCAGCAG

GGCCGCCAATTCGACAACTTCAGCTCCCTGAGCGTCCAGTGGGAATCCACCCGGCCCTCG

CTGGCCAGCATCGAGCTGGACCTGCCCATGCAGCTGGTGTCCCGGGACGACGGGAGTGGC

CAGAGGAAGTTTCACGGATTGCAAGCCATTGCTGTCCATGAGGCATCAGGGACCATCTCC

ATCTCTGCCACGGTGACCGGCTACCAGCTGTCCCACCTCAGTGCAGCCACAGTGAAGCCA

CTGCCTGGCCCACCTGCGCCCGTGTCCGCCTCGGTGGAGCTGGTCCTGGTGGAGGACGTG

CGTGTGGATCCGGACATGGTGACCCTCTACAACCACCCTGATGTCCAGGCGGAGTTGCAC

GTCAAGGAGGGCTCCGGCTACTTCTTCCTCAACACGAGCGCTGCCAACGTGGTCAGGGTG

GCCTACCAGGAGGCCAGGGGCATCGCCACGGTGCACCCTTTGCTGCCAGGCTCGGCCACC

GTCCTGATCCATGACCTGTGCCTGCCCTTCCTGGCCCCAGCCAAGGCCACCGTCCACGTC

TCGGACATCCACGAGCTGTACGTCCGTGTGGTCGACAAGGTGGAGATTGGGAAGACGGTC

AAGGCCCACGTCCGAGTGCTGGACTTTTACAAGAAGTCTTTCCTGGCCAAGTACTTTGCC

TTCATGGACCTCAAACTGCGGGCGGCCTCCCAGATCATCACCCTGGTGGCCCTGGACGAA

GCCCTGGACAGCTACACCGCTGCTTTCCTGGTGCACGGCACGGCCATCGGCCAGACCAGC

CTGACCGCAAGCGTGACCGATACCGCCGGACAGAGGATCAGCTCGACCCCGCAGCAGATT

GAAGTCTTCCCCCCGTTCAGGCTGATTCCCAGGAGGGTGACGCTGATCATCGGGGCCACG

ATGCAGGTCACCCCTGAGGGCGGTCCCCAGCCGCAGTCCCACATCCTCTTCTCCATCAGC

AATGCCAGCGTGGCCACCGTGGGCAGCACAGGGCTGGTCCGTGGCCTGGCCGTGGGCAGC

GGCACCGTGTCGGGCGTCGTGCAAGCGGCGGACGCGGACACCGGCAGGGTCATCGTGGTC

TCCCAGGACCTGGTGGAGCTAGAGGTGGTGCAGCTGCGGGCGGTGAGGATCCGCGCTCCC

ATCACGCGCATGAGGACGGGCACCCAGATGCCCGTCTATGTCACCGGGATCACCAACAAC

CAGAGCCCTTTCTCCTTTGCAAACGCGGTGCCAGGCCTGACCTTCCACTGGTCGGTCACC

AAGCGCGACATCTTGGAGCTCCGTGGGCGGCACCATGAGGCGTCCATTCAGCTGCCCTCG

CAGTACAACTTCGCCATGAGCCTGCTGGGCCGGGCGAAGGGCCGCACGGGGCTGAGGGTG

GTGGTCAAGGCCCTGGACCCCTCGGCGGGACAGCTGCACGGCCTTGCCAAGGAGCTCTCG

GACGAGATCCAGGTCCAGGTTTTCGAGAAGCTGCGGCTGCTGAACCCTGAGATAGAAGCA

GAACAAATATTAATGTCCCCCAACTCGTTTATCAAGCTCCAGACAAACAGGGACGGTGCC

GCGGCTCTTTGCTACCGTGTCCTGGAGGGGCCTGAGAAGGTTCCCGTCGTGCATGCCGAT

GAGAGGGGCTTCCTGGTGTCCGGCTCTGTGATCGGGACGTCCACTGTCAAAGTGACCGCT

CAGGAGCCTTTCGGGGCCAACCAGACCATCATCATTGCCGTGAAGGTGTCCCCCATCTCC

TACCTGAGGATGTCCATGAGCCTGGTCCTGCACACCCAGGACAAGGAGGCCCTGGCGGCC

CTGCCTCTGGGGATGACCGTGACCTTCACCGTCCACTTCCATGACCACTTTGGAGATGTC

TTCCACGCGCACAGTTCGGTCCTCAGCTTTGCCACCAACAGAGAGGACTTCGTGCAGGTC

ACCAAGGGCACCACCAACAACATCTGCGTACTGCGCACGGTC

>CL264.Contig7_A-W 167 373 minus strand PREDICTED: nuclear pore membrane glycoprotein 210 [Loxodonta africana]

TTGAGCCTGGTCCTGCACACCCAGGACAAGGAGGCCCTGGCGGCCCTGCCTCTGGGGATG

ACCGTGACCTTCACCGTCCACTTCCATGACCACTTTGGAGATGTCTTCCACGCGCACAGT

TCGGTCCTCAGCTTTGCCACCAACAGAGAGGACTTCGTGCAGGTCACCAAGGGCACCACC

AACAACATCTGCGTACTGCGCACGGTC

>CL264.Contig8_A-W 646 762 minus strand PREDICTED: ubiquitin-conjugating enzyme E2 E3 isoform 2 [Saimiri boliviensis boliviensis]

CAAATGGCCAGTGATAAGCAAATGTCCGATGATGACAGCCCCAGCACCAGCAGTGGCAGT

TCAAAGTCGGACCAGGAAGACCCTGCTGCTCCAGAGCCTGAAGATCATGAAGAAAGA

>CL264.Contig9_A-W 109 336 minus strand PREDICTED: nuclear pore membrane glycoprotein 210 [Loxodonta africana]

ATATTCTATATATGTCTTTGGTTGAGCCTGGTCCTGCACACCCAGGACAAGGAGGCCCTG

GCGGCCCTGCCTCTGGGGATGACCGTGACCTTCACCGTCCACTTCCATGACCACTTTGGA

GATGTCTTCCACGCGCACAGTTCGGTCCTCAGCTTTGCCACCAACAGAGAGGACTTCGTG

CAGGTCACCAAGGGCACCACCAACAACATCTGCGTACTGCGCACGGTC

>CL264.Contig10_A-W 249 350 minus strand PREDICTED: ubiquitin-conjugating enzyme E2 E3 isoform 2 [Saimiri boliviensis boliviensis]

AAGAAAGAACCTTCTGCCACCCAGCAGAAGAAGAGCACCAAACTCTCTAGCAAAACCACT

GCTAAGTTATCCACTAGTGCTAAAAGACTTGAGATTCATCTG

>CL264.Contig11_A-W 78 1127 minus strand PREDICTED: nuclear pore membrane glycoprotein 210 [Equus caballus]

GGGCGGCTGCCCCAGGGGCCTGAGCACTGTGGTGGCGTCCGGGTGAAGGCCGAGGCTCGG

GGCTCCACCACACTCCTGGTGAGCTACACGCATGGCCACGTCCACCTGAGCGCCAGCCTC

ACCATCACCGCCTACCTGCCCCTCAGGGCCGTGGACCCGTCCTCCGTAGCTGTGGTCACC

CTGGGCTCCTCAAAGGACATGCTGTTTGAAGGAGGCCCCCGGCCCTGGGTCCTGGAGCCT

GCCAAGTTCTTCCGGAACCTCACCTCTGAGGACGGGGACAGCATCAGCTTGGCCCTCCTT

GGCCCGCCCGCCTCCCGGAGTCACCAGCAGCACAGCATCTGCTCCACCTGCCAGGCCCTG

GGCGAGCAGGTCATCGCCCTCTCCGTGGGGAACCAGGCTGGCCTCACCAACCCTTTCCCC

GCCGTGGAGCCTGCCGTGGTGAAGGTCGTCTGCGCCCCGCCATCCAGGATCAGCCTCACG

CCTGTCCACACCGCTCCCCCGCTGGACCCGTCATATCCACGGCTGCAGCACGACAAGCAG

GTGGTTCCCGTCTCCAGCCACCGCAACCCCCTGCTGGACCTGGCCGCCTATGACCAGCAG

GGCCGCCAATTCGACAACTTCAGCTCCCTGGGCGTCCAGTGGGAATCCACCTGGCCCTTG

CTGGCCAGCATCGAGCTGGACCTGCCCATGCAGCTGGTGTCCCGGGACGACGGGAGTGGC

CAGAGGAAGTTTCACGGATTGCAAGCCATTGCTGTCCATGAGGCATCAGGGACCATCTCC

ATCTCTGCCACGGTGACCGGCTACCAGCTGTCCCACCTCAGTGCAGCCACAGTGAAGCCA

CTGCCTGGCCCACCTGCGCCCGTGTCCGCCTCGGTGGAGCTGGTCCTGGTGGAGGACGTG

CGTGTGGATCCGGACATGGTGACCCTCTACAACCACCCTGATGTCCAGGCAGAGTTGCAC

GTCAAGGAGGGCTCCGGCTACTTCTTCCTCAACACGAGCGCTGCCAACGTGGTCAGGGTG

GCCTACCAGGAGGCCAGGGGCATCGCCACG

>CL264.Contig12_A-W 78 1127 minus strand PREDICTED: nuclear pore membrane glycoprotein 210 [Equus caballus]

GGGCGGCTGCCCCAGGGGCCTGAGCACTGTGGTGGCGTCCGGGTGAAGGCCGAGGCTCGG

GGCTCCACCACACTCCTGGTGAGCTACACGCATGGCCACGTCCACCTGAGCGCCAGCCTC

ACCATCACCGCCTACCTGCCCCTCAGGGCCGTGGACCCGTCCTCCGTAGCTGTGGTCACC

CTGGGCTCCTCAAAGGACATGCTGTTTGAAGGAGGCCCCCGGCCCTGGGTCCTGGAGCCT

GCCAAGTTCTTCCGGAACCTCACCTCTGAGGACGGGGACAGCATCAGCTTGGCCCTCCTT

GGCCCGCCCGCCTCCCGGAGTCACCAGCAGCACAGCATCTGCTCCACCTGCCAGGCCCTG

GGCGAGCAGGTCATCGCCCTCTCCGTGGGGAACCAGGCTGGCCTCACCAACCCTTTCCCC

GCCGTGGAGCCTGCCGTGGTGAAGGTCGTCTGCGCCCCGCCATCCAGGATCAGCCTCACG

CCTGTCCACACCGCTCCCCCGCTGGACCCGTCATATCCACGGCTGCAGCACGACAAGCAG

GTGGTTCCCGTCTCCAGCCACCGCAACCCCCTGCTGGACCTGGCCGCCTATGACCAGCAG

GGCCGCCAATTCGACAACTTCAGCTCCCTGGGCGTCCAGTGGGAATCCACCTGGCCCTTG

CTGGCCAGCATCGAGCTGGACCTGCCCATGCAGCTGGTGTCCCGGGACGACGGGAGTGGC

CAGAGGAAGTTTCACGGATTGCAAGCCATTGCTGTCCATGAGGCATCAGGGACCATCTCC

ATCTCTGCCACGGTGACCGGCTACCAGCTGTCCCACCTCAGTGCAGCCACAGTGAAGCCA

CTGCCTGGCCCACCTGCGCCCGTGTCCGCCTCGGTGGAGCTGGTCCTGGTGGAGGACGTG

CGTGTGGATCCGGACATGGTGACCCTCTACAACCACCCTGATGTCCAGGCAGAGTTGCAC

GTCAAGGAGGGCTCCGGCTACTTCTTCCTCAACACGAGCGCTGCCAACGTGGTCAGGGTG

GCCTACCAGGAGGCCAGGGGCATCGCCACG

>CL264.Contig13_A-W 140 256 minus strand PREDICTED: ubiquitin-conjugating enzyme E2 E3 isoform 2 [Saimiri boliviensis boliviensis]

CAAATGGCCAGTGATAAGCAAATGTCCGATGATGACAGCCCCAGCACCAGCAGTGGCAGT

TCAAAGTCGGACCAGGAAGACCCTGCTGCTCCAGAGCCTGAAGATCATGAAGAAAGA

>CL265.Contig1_A-W 1665 2180 minus strand PREDICTED: regulator of G-protein signaling 19, partial [Pan troglodytes]

TTCCTCTCTGCTGCCAGGAACCAAGAGCGGCGGCGAGCATGGCAGGCCTCACAGGAGAGC

AAGCTGCAGCCCCTCCCCAGCTGTGATGCCTGCACCCCCCCAAGCCCTGAGGAGGTACAG

AGCTGGGCGCAGTCCTTCGACAAGCTGATGCACAGCCCCGCGGGCCGCAGTGTGTTCCGC

GCCTTCCTGCGGACAGAGTACAGCGAGGAGAACATGCTCTTCTGGCTGGCCTGCGAGGAG

CTGAAGGCAGAGGCTAACCAGCACGTGGTGGATGAGAAGGCGCGGCTCATCTATGAGGAC

TACGTGTCCATCCTGTCCCCCAAGGAGGTGAGCCTGGACTCCCGAGTGCGGGAGGGCATC

AACAGGAAGATGCAGGAGCCGTCAGCACACACATTTGACGACGCGCAGCTGCAGATCTAC

ACGCTCATGCACCGGGACTCGTACCCACGCTTCCTCAGCTCCCCAGCCTACCGCACCCTG

CTGCTCCGGGGGTCCCCACAGTCCTCTTCGGAGGCC

>CL265.Contig2_A-W 1593 2108 minus strand PREDICTED: regulator of G-protein signaling 19, partial [Pan troglodytes]

TTCCTCTCTGCTGCCAGGAACCAAGAGCGGCGGCGAGCATGGCAGGCCTCACAGGAGAGC

AAGCTGCAGCCCCTCCCCAGCTGTGATGCCTGCACCCCCCCAAGCCCTGAGGAGGTACAG

AGCTGGGCGCAGTCCTTCGACAAGCTGATGCACAGCCCCGCGGGCCGCAGTGTGTTCCGC

GCCTTCCTGCGGACAGAGTACAGCGAGGAGAACATGCTCTTCTGGCTGGCCTGCGAGGAG

CTGAAGGCAGAGGCTAACCAGCACGTGGTGGATGAGAAGGCGCGGCTCATCTATGAGGAC

TACGTGTCCATCCTGTCCCCCAAGGAGGTGAGCCTGGACTCCCGAGTGCGGGAGGGCATC

AACAGGAAGATGCAGGAGCCGTCAGCACACACATTTGACGACGCGCAGCTGCAGATCTAC

ACGCTCATGCACCGGGACTCGTACCCACGCTTCCTCAGCTCCCCAGCCTACCGCACCCTG

CTGCTCCGGGGGTCCCCACAGTCCTCTTCGGAGGCC

>CL265.Contig3_A-W 1471 2058 minus strand PREDICTED: regulator of G-protein signaling 19, partial [Pan troglodytes]

TTCCTCTCTGCTGCCAGGAACCAAGAGCGGCGGCGAGCATGGCAGGCCTCACAGGAGAGC

AAGCTGCAGCCCCTCCCCAGCTGTGATGCCTGCACCCCCCCAAGCCCTGAGGAGGTACAG

AGCTGGGCGCAGTCCTTCGACAAGCTGATGCACAGCCCCGCGGGCCGCAGTGTGTTCCGC

GCCTTCCTGCGGACAGAGTACAGCGAGGAGAACATGCTCTTCTGGCTGGCCTGCGAGGAG

CTGAAGGCAGAGGCTAACCAGCACGTGGTGGATGAGAAGGCGCGGCTCATCTATGAGGAC

TACGTGTCCATCCTGTCCCCCAAGGAGGTGCGCCAGCTGCTGGCACGGGTTGGGGTGGCC

TGGGGAGGGTGCCTCTGACCTCGGTGCCCCGTCCCACAGGTGAGCCTGGACTCCCGAGTG

CGGGAGGGCATCAACAGGAAGATGCAGGAGCCGTCAGCACACACATTTGACGACGCGCAG

CTGCAGATCTACACGCTCATGCACCGGGACTCGTACCCACGCTTCCTCAGCTCCCCAGCC

TACCGCACCCTGCTGCTCCGGGGGTCCCCACAGTCCTCTTCGGAGGCC

>CL265.Contig4_A-W 1471 1986 minus strand PREDICTED: regulator of G-protein signaling 19, partial [Pan troglodytes]

TTCCTCTCTGCTGCCAGGAACCAAGAGCGGCGGCGAGCATGGCAGGCCTCACAGGAGAGC

AAGCTGCAGCCCCTCCCCAGCTGTGATGCCTGCACCCCCCCAAGCCCTGAGGAGGTACAG

AGCTGGGCGCAGTCCTTCGACAAGCTGATGCACAGCCCCGCGGGCCGCAGTGTGTTCCGC

GCCTTCCTGCGGACAGAGTACAGCGAGGAGAACATGCTCTTCTGGCTGGCCTGCGAGGAG

CTGAAGGCAGAGGCTAACCAGCACGTGGTGGATGAGAAGGCGCGGCTCATCTATGAGGAC

TACGTGTCCATCCTGTCCCCCAAGGAGGTGAGCCTGGACTCCCGAGTGCGGGAGGGCATC

AACAGGAAGATGCAGGAGCCGTCAGCACACACATTTGACGACGCGCAGCTGCAGATCTAC

ACGCTCATGCACCGGGACTCGTACCCACGCTTCCTCAGCTCCCCAGCCTACCGCACCCTG

CTGCTCCGGGGGTCCCCACAGTCCTCTTCGGAGGCC

>CL265.Contig5_A-W 1787 2374 minus strand PREDICTED: regulator of G-protein signaling 19, partial [Pan troglodytes]

TTCCTCTCTGCTGCCAGGAACCAAGAGCGGCGGCGAGCATGGCAGGCCTCACAGGAGAGC

AAGCTGCAGCCCCTCCCCAGCTGTGATGCCTGCACCCCCCCAAGCCCTGAGGAGGTACAG

AGCTGGGCGCAGTCCTTCGACAAGCTGATGCACAGCCCCGCGGGCCGCAGTGTGTTCCGC

GCCTTCCTGCGGACAGAGTACAGCGAGGAGAACATGCTCTTCTGGCTGGCCTGCGAGGAG

CTGAAGGCAGAGGCTAACCAGCACGTGGTGGATGAGAAGGCGCGGCTCATCTATGAGGAC

TACGTGTCCATCCTGTCCCCCAAGGAGGTGCGCCAGCTGCTGGCACGGGTTGGGGTGGCC

TGGGGAGGGTGCCTCTGACCTCGGTGCCCCGTCCCACAGGTGAGCCTGGACTCCCGAGTG

CGGGAGGGCATCAACAGGAAGATGCAGGAGCCGTCAGCACACACATTTGACGACGCGCAG

CTGCAGATCTACACGCTCATGCACCGGGACTCGTACCCACGCTTCCTCAGCTCCCCAGCC

TACCGCACCCTGCTGCTCCGGGGGTCCCCACAGTCCTCTTCGGAGGCC

>CL265.Contig6_A-W 985 1482 minus strand Regulator of G-protein signaling 19 OS=Homo sapiens GN=RGS19 PE=1 SV=1

AACCAAGAGCGGCGGCGAGCATGGCAGGCCTCACAGGAGAGCAAGCTGCAGCCCCTCCCC

AGCTGTGATGCCTGCACCCCCCCAAGCCCTGAGGAGGTACAGAGCTGGGCGCAGTCCTTC

GACAAGCTGATGCACAGCCCCGCGGGCCGCAGTGTGTTCCGCGCCTTCCTGCGGACAGAG

TACAGCGAGGAGAACATGCTCTTCTGGCTGGCCTGCGAGGAGCTGAAGGCAGAGGCTAAC

CAGCACGTGGTGGATGAGAAGGCGCGGCTCATCTATGAGGACTACGTGTCCATCCTGTCC

CCCAAGGAGGTGAGCCTGGACTCCCGAGTGCGGGAGGGCATCAACAGGAAGATGCAGGAG

CCGTCAGCACACACATTTGACGACGCGCAGCTGCAGATCTACACGCTCATGCACCGGGAC

TCGTACCCACGCTTCCTCAGCTCCCCAGCCTACCGCACCCTGCTGCTCCGGGGGTCCCCA

CAGTCCTCTTCGGAGGCC

>CL265.Contig7_A-W 894 1391 minus strand Regulator of G-protein signaling 19 OS=Homo sapiens GN=RGS19 PE=1 SV=1

AACCAAGAGCGGCGGCGAGCATGGCAGGCCTCACAGGAGAGCAAGCTGCAGCCCCTCCCC

AGCTGTGATGCCTGCACCCCCCCAAGCCCTGAGGAGGTACAGAGCTGGGCGCAGTCCTTC

GACAAGCTGATGCACAGCCCCGCGGGCCGCAGTGTGTTCCGCGCCTTCCTGCGGACAGAG

TACAGCGAGGAGAACATGCTCTTCTGGCTGGCCTGCGAGGAGCTGAAGGCAGAGGCTAAC

CAGCACGTGGTGGATGAGAAGGCGCGGCTCATCTATGAGGACTACGTGTCCATCCTGTCC

CCCAAGGAGGTGAGCCTGGACTCCCGAGTGCGGGAGGGCATCAACAGGAAGATGCAGGAG

CCGTCAGCACACACATTTGACGACGCGCAGCTGCAGATCTACACGCTCATGCACCGGGAC

TCGTACCCACGCTTCCTCAGCTCCCCAGCCTACCGCACCCTGCTGCTCCGGGGGTCCCCA

CAGTCCTCTTCGGAGGCC

>CL265.Contig8_A-W 890 1387 minus strand Regulator of G-protein signaling 19 OS=Homo sapiens GN=RGS19 PE=1 SV=1

AACCAAGAGCGGCGGCGAGCATGGCAGGCCTCACAGGAGAGCAAGCTGCAGCCCCTCCCC

AGCTGTGATGCCTGCACCCCCCCAAGCCCTGAGGAGGTACAGAGCTGGGCGCAGTCCTTC

GACAAGCTGATGCACAGCCCCGCGGGCCGCAGTGTGTTCCGCGCCTTCCTGCGGACAGAG

TACAGCGAGGAGAACATGCTCTTCTGGCTGGCCTGCGAGGAGCTGAAGGCAGAGGCTAAC

CAGCACGTGGTGGATGAGAAGGCGCGGCTCATCTATGAGGACTACGTGTCCATCCTGTCC

CCCAAGGAGGTGAGCCTGGACTCCCGAGTGCGGGAGGGCATCAACAGGAAGATGCAGGAG

CCGTCAGCACACACATTTGACGACGCGCAGCTGCAGATCTACACGCTCATGCACCGGGAC

TCGTACCCACGCTTCCTCAGCTCCCCAGCCTACCGCACCCTGCTGCTCCGGGGGTCCCCA

CAGTCCTCTTCGGAGGCC

>CL265.Contig9_A-W 867 1364 minus strand Regulator of G-protein signaling 19 OS=Homo sapiens GN=RGS19 PE=1 SV=1

AACCAAGAGCGGCGGCGAGCATGGCAGGCCTCACAGGAGAGCAAGCTGCAGCCCCTCCCC

AGCTGTGATGCCTGCACCCCCCCAAGCCCTGAGGAGGTACAGAGCTGGGCGCAGTCCTTC

GACAAGCTGATGCACAGCCCCGCGGGCCGCAGTGTGTTCCGCGCCTTCCTGCGGACAGAG

TACAGCGAGGAGAACATGCTCTTCTGGCTGGCCTGCGAGGAGCTGAAGGCAGAGGCTAAC

CAGCACGTGGTGGATGAGAAGGCGCGGCTCATCTATGAGGACTACGTGTCCATCCTGTCC

CCCAAGGAGGTGAGCCTGGACTCCCGAGTGCGGGAGGGCATCAACAGGAAGATGCAGGAG

CCGTCAGCACACACATTTGACGACGCGCAGCTGCAGATCTACACGCTCATGCACCGGGAC

TCGTACCCACGCTTCCTCAGCTCCCCAGCCTACCGCACCCTGCTGCTCCGGGGGTCCCCA

CAGTCCTCTTCGGAGGCC

>CL265.Contig10_A-W 863 1360 minus strand Regulator of G-protein signaling 19 OS=Homo sapiens GN=RGS19 PE=1 SV=1

AACCAAGAGCGGCGGCGAGCATGGCAGGCCTCACAGGAGAGCAAGCTGCAGCCCCTCCCC

AGCTGTGATGCCTGCACCCCCCCAAGCCCTGAGGAGGTACAGAGCTGGGCGCAGTCCTTC

GACAAGCTGATGCACAGCCCCGCGGGCCGCAGTGTGTTCCGCGCCTTCCTGCGGACAGAG

TACAGCGAGGAGAACATGCTCTTCTGGCTGGCCTGCGAGGAGCTGAAGGCAGAGGCTAAC

CAGCACGTGGTGGATGAGAAGGCGCGGCTCATCTATGAGGACTACGTGTCCATCCTGTCC

CCCAAGGAGGTGAGCCTGGACTCCCGAGTGCGGGAGGGCATCAACAGGAAGATGCAGGAG

CCGTCAGCACACACATTTGACGACGCGCAGCTGCAGATCTACACGCTCATGCACCGGGAC

TCGTACCCACGCTTCCTCAGCTCCCCAGCCTACCGCACCCTGCTGCTCCGGGGGTCCCCA

CAGTCCTCTTCGGAGGCC

>CL265.Contig11_A-W 1791 2378 minus strand PREDICTED: regulator of G-protein signaling 19, partial [Pan troglodytes]

TTCCTCTCTGCTGCCAGGAACCAAGAGCGGCGGCGAGCATGGCAGGCCTCACAGGAGAGC

AAGCTGCAGCCCCTCCCCAGCTGTGATGCCTGCACCCCCCCAAGCCCTGAGGAGGTACAG

AGCTGGGCGCAGTCCTTCGACAAGCTGATGCACAGCCCCGCGGGCCGCAGTGTGTTCCGC

GCCTTCCTGCGGACAGAGTACAGCGAGGAGAACATGCTCTTCTGGCTGGCCTGCGAGGAG

CTGAAGGCAGAGGCTAACCAGCACGTGGTGGATGAGAAGGCGCGGCTCATCTATGAGGAC

TACGTGTCCATCCTGTCCCCCAAGGAGGTGCGCCAGCTGCTGGCACGGGTTGGGGTGGCC

TGGGGAGGGTGCCTCTGACCTCGGTGCCCCGTCCCACAGGTGAGCCTGGACTCCCGAGTG

CGGGAGGGCATCAACAGGAAGATGCAGGAGCCGTCAGCACACACATTTGACGACGCGCAG

CTGCAGATCTACACGCTCATGCACCGGGACTCGTACCCACGCTTCCTCAGCTCCCCAGCC

TACCGCACCCTGCTGCTCCGGGGGTCCCCACAGTCCTCTTCGGAGGCC

>CL265.Contig12_A-W 791 1360 minus strand Regulator of G-protein signaling 19 OS=Homo sapiens GN=RGS19 PE=1 SV=1

AACCAAGAGCGGCGGCGAGCATGGCAGGCCTCACAGGAGAGCAAGCTGCAGCCCCTCCCC

AGCTGTGATGCCTGCACCCCCCCAAGCCCTGAGGAGGTACAGAGCTGGGCGCAGTCCTTC

GACAAGCTGATGCACAGCCCCGCGGGCCGCAGTGTGTTCCGCGCCTTCCTGCGGACAGAG

TACAGCGAGGAGAACATGCTCTTCTGGCTGGCCTGCGAGGAGCTGAAGGCAGAGGCTAAC

CAGCACGTGGTGGATGAGAAGGCGCGGCTCATCTATGAGGACTACGTGTCCATCCTGTCC

CCCAAGGAGGTGCGCCAGCTGCTGGCACGGGTTGGGGTGGCCTGGGGAGGGTGCCTCTGA

CCTCGGTGCCCCGTCCCACAGGTGAGCCTGGACTCCCGAGTGCGGGAGGGCATCAACAGG

AAGATGCAGGAGCCGTCAGCACACACATTTGACGACGCGCAGCTGCAGATCTACACGCTC

ATGCACCGGGACTCGTACCCACGCTTCCTCAGCTCCCCAGCCTACCGCACCCTGCTGCTC

CGGGGGTCCCCACAGTCCTCTTCGGAGGCC

>CL265.Contig13_A-W 791 1288 minus strand Regulator of G-protein signaling 19 OS=Homo sapiens GN=RGS19 PE=1 SV=1

AACCAAGAGCGGCGGCGAGCATGGCAGGCCTCACAGGAGAGCAAGCTGCAGCCCCTCCCC

AGCTGTGATGCCTGCACCCCCCCAAGCCCTGAGGAGGTACAGAGCTGGGCGCAGTCCTTC

GACAAGCTGATGCACAGCCCCGCGGGCCGCAGTGTGTTCCGCGCCTTCCTGCGGACAGAG

TACAGCGAGGAGAACATGCTCTTCTGGCTGGCCTGCGAGGAGCTGAAGGCAGAGGCTAAC

CAGCACGTGGTGGATGAGAAGGCGCGGCTCATCTATGAGGACTACGTGTCCATCCTGTCC

CCCAAGGAGGTGAGCCTGGACTCCCGAGTGCGGGAGGGCATCAACAGGAAGATGCAGGAG

CCGTCAGCACACACATTTGACGACGCGCAGCTGCAGATCTACACGCTCATGCACCGGGAC

TCGTACCCACGCTTCCTCAGCTCCCCAGCCTACCGCACCCTGCTGCTCCGGGGGTCCCCA

CAGTCCTCTTCGGAGGCC

>CL265.Contig14_A-W 669 1238 minus strand Regulator of G-protein signaling 19 OS=Homo sapiens GN=RGS19 PE=1 SV=1

AACCAAGAGCGGCGGCGAGCATGGCAGGCCTCACAGGAGAGCAAGCTGCAGCCCCTCCCC

AGCTGTGATGCCTGCACCCCCCCAAGCCCTGAGGAGGTACAGAGCTGGGCGCAGTCCTTC

GACAAGCTGATGCACAGCCCCGCGGGCCGCAGTGTGTTCCGCGCCTTCCTGCGGACAGAG

TACAGCGAGGAGAACATGCTCTTCTGGCTGGCCTGCGAGGAGCTGAAGGCAGAGGCTAAC

CAGCACGTGGTGGATGAGAAGGCGCGGCTCATCTATGAGGACTACGTGTCCATCCTGTCC

CCCAAGGAGGTGCGCCAGCTGCTGGCACGGGTTGGGGTGGCCTGGGGAGGGTGCCTCTGA

CCTCGGTGCCCCGTCCCACAGGTGAGCCTGGACTCCCGAGTGCGGGAGGGCATCAACAGG

AAGATGCAGGAGCCGTCAGCACACACATTTGACGACGCGCAGCTGCAGATCTACACGCTC

ATGCACCGGGACTCGTACCCACGCTTCCTCAGCTCCCCAGCCTACCGCACCCTGCTGCTC

CGGGGGTCCCCACAGTCCTCTTCGGAGGCC

>CL265.Contig15_A-W 1818 2333 minus strand PREDICTED: regulator of G-protein signaling 19, partial [Pan troglodytes]

TTCCTCTCTGCTGCCAGGAACCAAGAGCGGCGGCGAGCATGGCAGGCCTCACAGGAGAGC

AAGCTGCAGCCCCTCCCCAGCTGTGATGCCTGCACCCCCCCAAGCCCTGAGGAGGTACAG

AGCTGGGCGCAGTCCTTCGACAAGCTGATGCACAGCCCCGCGGGCCGCAGTGTGTTCCGC

GCCTTCCTGCGGACAGAGTACAGCGAGGAGAACATGCTCTTCTGGCTGGCCTGCGAGGAG

CTGAAGGCAGAGGCTAACCAGCACGTGGTGGATGAGAAGGCGCGGCTCATCTATGAGGAC

TACGTGTCCATCCTGTCCCCCAAGGAGGTGAGCCTGGACTCCCGAGTGCGGGAGGGCATC

AACAGGAAGATGCAGGAGCCGTCAGCACACACATTTGACGACGCGCAGCTGCAGATCTAC

ACGCTCATGCACCGGGACTCGTACCCACGCTTCCTCAGCTCCCCAGCCTACCGCACCCTG

CTGCTCCGGGGGTCCCCACAGTCCTCTTCGGAGGCC

>CL265.Contig16_A-W 669 1166 minus strand Regulator of G-protein signaling 19 OS=Homo sapiens GN=RGS19 PE=1 SV=1

AACCAAGAGCGGCGGCGAGCATGGCAGGCCTCACAGGAGAGCAAGCTGCAGCCCCTCCCC

AGCTGTGATGCCTGCACCCCCCCAAGCCCTGAGGAGGTACAGAGCTGGGCGCAGTCCTTC

GACAAGCTGATGCACAGCCCCGCGGGCCGCAGTGTGTTCCGCGCCTTCCTGCGGACAGAG

TACAGCGAGGAGAACATGCTCTTCTGGCTGGCCTGCGAGGAGCTGAAGGCAGAGGCTAAC

CAGCACGTGGTGGATGAGAAGGCGCGGCTCATCTATGAGGACTACGTGTCCATCCTGTCC

CCCAAGGAGGTGAGCCTGGACTCCCGAGTGCGGGAGGGCATCAACAGGAAGATGCAGGAG

CCGTCAGCACACACATTTGACGACGCGCAGCTGCAGATCTACACGCTCATGCACCGGGAC

TCGTACCCACGCTTCCTCAGCTCCCCAGCCTACCGCACCCTGCTGCTCCGGGGGTCCCCA

CAGTCCTCTTCGGAGGCC

>CL265.Contig17_A-W 209 964 minus strand regulator of G-protein signaling 19 [Mus musculus]

GAGTTGGCACCACAGAGCCTGGGCAGTCCCCTCCACCTACCCATGCCCACCCCGCATGAG

GCTGAGAAGCAGCACACAGGGCCAGAGGAGGCGGACAGGCCCCCCCCAATGTCCAGCCAT

GATGTGGTCCCCCCGGCGGCCCCCAGCCGCAACCCCTGCTGTTTGTGCTGGTGTTGCTGC

TGCAGCTGCTCCTGGAACCAAGAGCGGCGGCGAGCATGGCAGGCCTCACAGGAGAGCAAG

CTGCAGCCCCTCCCCAGCTGTGATGCCTGCACCCCCCCAAGCCCTGAGGAGGTACAGAGC

TGGGCGCAGTCCTTCGACAAGCTGATGCACAGCCCCGCGGGCCGCAGTGTGTTCCGCGCC

TTCCTGCGGACAGAGTACAGCGAGGAGAACATGCTCTTCTGGCTGGCCTGCGAGGAGCTG

AAGGCAGAGGCTAACCAGCACGTGGTGGATGAGAAGGCGCGGCTCATCTATGAGGACTAC

GTGTCCATCCTGTCCCCCAAGGAGGTGCGCCAGCTGCTGGCACGGGTTGGGGTGGCCTGG

GGAGGGTGCCTCTGACCTCGGTGCCCCGTCCCACAGGTGAGCCTGGACTCCCGAGTGCGG

GAGGGCATCAACAGGAAGATGCAGGAGCCGTCAGCACACACATTTGACGACGCGCAGCTG

CAGATCTACACGCTCATGCACCGGGACTCGTACCCACGCTTCCTCAGCTCCCCAGCCTAC

CGCACCCTGCTGCTCCGGGGGTCCCCACAGTCCTCT

>CL265.Contig18_A-W 700 1269 minus strand Regulator of G-protein signaling 19 OS=Homo sapiens GN=RGS19 PE=1 SV=1

AACCAAGAGCGGCGGCGAGCATGGCAGGCCTCACAGGAGAGCAAGCTGCAGCCCCTCCCC

AGCTGTGATGCCTGCACCCCCCCAAGCCCTGAGGAGGTACAGAGCTGGGCGCAGTCCTTC

GACAAGCTGATGCACAGCCCCGCGGGCCGCAGTGTGTTCCGCGCCTTCCTGCGGACAGAG

TACAGCGAGGAGAACATGCTCTTCTGGCTGGCCTGCGAGGAGCTGAAGGCAGAGGCTAAC

CAGCACGTGGTGGATGAGAAGGCGCGGCTCATCTATGAGGACTACGTGTCCATCCTGTCC

CCCAAGGAGGTGCGCCAGCTGCTGGCACGGGTTGGGGTGGCCTGGGGAGGGTGCCTCTGA

CCTCGGTGCCCCGTCCCACAGGTGAGCCTGGACTCCCGAGTGCGGGAGGGCATCAACAGG

AAGATGCAGGAGCCGTCAGCACACACATTTGACGACGCGCAGCTGCAGATCTACACGCTC

ATGCACCGGGACTCGTACCCACGCTTCCTCAGCTCCCCAGCCTACCGCACCCTGCTGCTC

CGGGGGTCCCCACAGTCCTCTTCGGAGGCC

>CL265.Contig19_A-W 209 892 minus strand regulator of G-protein signaling 19 [Mus musculus]

GAGTTGGCACCACAGAGCCTGGGCAGTCCCCTCCACCTACCCATGCCCACCCCGCATGAG

GCTGAGAAGCAGCACACAGGGCCAGAGGAGGCGGACAGGCCCCCCCCAATGTCCAGCCAT

GATGTGGTCCCCCCGGCGGCCCCCAGCCGCAACCCCTGCTGTTTGTGCTGGTGTTGCTGC

TGCAGCTGCTCCTGGAACCAAGAGCGGCGGCGAGCATGGCAGGCCTCACAGGAGAGCAAG

CTGCAGCCCCTCCCCAGCTGTGATGCCTGCACCCCCCCAAGCCCTGAGGAGGTACAGAGC

TGGGCGCAGTCCTTCGACAAGCTGATGCACAGCCCCGCGGGCCGCAGTGTGTTCCGCGCC

TTCCTGCGGACAGAGTACAGCGAGGAGAACATGCTCTTCTGGCTGGCCTGCGAGGAGCTG

AAGGCAGAGGCTAACCAGCACGTGGTGGATGAGAAGGCGCGGCTCATCTATGAGGACTAC

GTGTCCATCCTGTCCCCCAAGGAGGTGAGCCTGGACTCCCGAGTGCGGGAGGGCATCAAC

AGGAAGATGCAGGAGCCGTCAGCACACACATTTGACGACGCGCAGCTGCAGATCTACACG

CTCATGCACCGGGACTCGTACCCACGCTTCCTCAGCTCCCCAGCCTACCGCACCCTGCTG

CTCCGGGGGTCCCCACAGTCCTCT

>CL265.Contig20_A-W 1814 2329 minus strand PREDICTED: regulator of G-protein signaling 19, partial [Pan troglodytes]

TTCCTCTCTGCTGCCAGGAACCAAGAGCGGCGGCGAGCATGGCAGGCCTCACAGGAGAGC

AAGCTGCAGCCCCTCCCCAGCTGTGATGCCTGCACCCCCCCAAGCCCTGAGGAGGTACAG

AGCTGGGCGCAGTCCTTCGACAAGCTGATGCACAGCCCCGCGGGCCGCAGTGTGTTCCGC

GCCTTCCTGCGGACAGAGTACAGCGAGGAGAACATGCTCTTCTGGCTGGCCTGCGAGGAG

CTGAAGGCAGAGGCTAACCAGCACGTGGTGGATGAGAAGGCGCGGCTCATCTATGAGGAC

TACGTGTCCATCCTGTCCCCCAAGGAGGTGAGCCTGGACTCCCGAGTGCGGGAGGGCATC

AACAGGAAGATGCAGGAGCCGTCAGCACACACATTTGACGACGCGCAGCTGCAGATCTAC

ACGCTCATGCACCGGGACTCGTACCCACGCTTCCTCAGCTCCCCAGCCTACCGCACCCTG

CTGCTCCGGGGGTCCCCACAGTCCTCTTCGGAGGCC

>CL265.Contig21_A-W 158 646 minus strand Regulator of G-protein signaling 19 OS=Homo sapiens GN=RGS19 PE=1 SV=1

CCAAGCCCTGAGGAGGTACAGAGCTGGGCGCAGTCCTTCGACAAGCTGATGCACAGCCCC

GCGGGCCGCAGTGTGTTCCGCGCCTTCCTGCGGACAGAGTACAGCGAGGAGAACATGCTC

TTCTGGCTGGCCTGCGAGGAGCTGAAGGCAGAGGCTAACCAGCACGTGGTGGATGAGAAG

GCGCGGCTCATCTATGAGGACTACGTGTCCATCCTGTCCCCCAAGGAGGTGCGCCAGCTG

CTGGCACGGGTTGGGGTGGCCTGGGGAGGGTGCCTCTGACCTCGGTGCCCCGTCCCACAG

GTGAGCCTGGACTCCCGAGTGCGGGAGGGCATCAACAGGAAGATGCAGGAGCCGTCAGCA

CACACATTTGACGACGCGCAGCTGCAGATCTACACGCTCATGCACCGGGACTCGTACCCA

CGCTTCCTCAGCTCCCCAGCCTACCGCACCCTGCTGCTCCGGGGGTCCCCACAGTCCTCT

TCGGAGGCC

>CL265.Contig22_A-W 158 574 minus strand Regulator of G-protein signaling 19 OS=Homo sapiens GN=RGS19 PE=1 SV=1

CCAAGCCCTGAGGAGGTACAGAGCTGGGCGCAGTCCTTCGACAAGCTGATGCACAGCCCC

GCGGGCCGCAGTGTGTTCCGCGCCTTCCTGCGGACAGAGTACAGCGAGGAGAACATGCTC

TTCTGGCTGGCCTGCGAGGAGCTGAAGGCAGAGGCTAACCAGCACGTGGTGGATGAGAAG

GCGCGGCTCATCTATGAGGACTACGTGTCCATCCTGTCCCCCAAGGAGGTGAGCCTGGAC

TCCCGAGTGCGGGAGGGCATCAACAGGAAGATGCAGGAGCCGTCAGCACACACATTTGAC

GACGCGCAGCTGCAGATCTACACGCTCATGCACCGGGACTCGTACCCACGCTTCCTCAGC

TCCCCAGCCTACCGCACCCTGCTGCTCCGGGGGTCCCCACAGTCCTCTTCGGAGGCC

>CL265.Contig23_A-W 1791 2306 minus strand PREDICTED: regulator of G-protein signaling 19, partial [Pan troglodytes]

TTCCTCTCTGCTGCCAGGAACCAAGAGCGGCGGCGAGCATGGCAGGCCTCACAGGAGAGC

AAGCTGCAGCCCCTCCCCAGCTGTGATGCCTGCACCCCCCCAAGCCCTGAGGAGGTACAG

AGCTGGGCGCAGTCCTTCGACAAGCTGATGCACAGCCCCGCGGGCCGCAGTGTGTTCCGC

GCCTTCCTGCGGACAGAGTACAGCGAGGAGAACATGCTCTTCTGGCTGGCCTGCGAGGAG

CTGAAGGCAGAGGCTAACCAGCACGTGGTGGATGAGAAGGCGCGGCTCATCTATGAGGAC

TACGTGTCCATCCTGTCCCCCAAGGAGGTGAGCCTGGACTCCCGAGTGCGGGAGGGCATC

AACAGGAAGATGCAGGAGCCGTCAGCACACACATTTGACGACGCGCAGCTGCAGATCTAC

ACGCTCATGCACCGGGACTCGTACCCACGCTTCCTCAGCTCCCCAGCCTACCGCACCCTG

CTGCTCCGGGGGTCCCCACAGTCCTCTTCGGAGGCC

>CL265.Contig24_A-W 1787 2302 minus strand PREDICTED: regulator of G-protein signaling 19, partial [Pan troglodytes]

TTCCTCTCTGCTGCCAGGAACCAAGAGCGGCGGCGAGCATGGCAGGCCTCACAGGAGAGC

AAGCTGCAGCCCCTCCCCAGCTGTGATGCCTGCACCCCCCCAAGCCCTGAGGAGGTACAG

AGCTGGGCGCAGTCCTTCGACAAGCTGATGCACAGCCCCGCGGGCCGCAGTGTGTTCCGC

GCCTTCCTGCGGACAGAGTACAGCGAGGAGAACATGCTCTTCTGGCTGGCCTGCGAGGAG

CTGAAGGCAGAGGCTAACCAGCACGTGGTGGATGAGAAGGCGCGGCTCATCTATGAGGAC

TACGTGTCCATCCTGTCCCCCAAGGAGGTGAGCCTGGACTCCCGAGTGCGGGAGGGCATC

AACAGGAAGATGCAGGAGCCGTCAGCACACACATTTGACGACGCGCAGCTGCAGATCTAC

ACGCTCATGCACCGGGACTCGTACCCACGCTTCCTCAGCTCCCCAGCCTACCGCACCCTG

CTGCTCCGGGGGTCCCCACAGTCCTCTTCGGAGGCC

>CL265.Contig25_A-W 1665 2252 minus strand PREDICTED: regulator of G-protein signaling 19, partial [Pan troglodytes]

TTCCTCTCTGCTGCCAGGAACCAAGAGCGGCGGCGAGCATGGCAGGCCTCACAGGAGAGC

AAGCTGCAGCCCCTCCCCAGCTGTGATGCCTGCACCCCCCCAAGCCCTGAGGAGGTACAG

AGCTGGGCGCAGTCCTTCGACAAGCTGATGCACAGCCCCGCGGGCCGCAGTGTGTTCCGC

GCCTTCCTGCGGACAGAGTACAGCGAGGAGAACATGCTCTTCTGGCTGGCCTGCGAGGAG

CTGAAGGCAGAGGCTAACCAGCACGTGGTGGATGAGAAGGCGCGGCTCATCTATGAGGAC

TACGTGTCCATCCTGTCCCCCAAGGAGGTGCGCCAGCTGCTGGCACGGGTTGGGGTGGCC

TGGGGAGGGTGCCTCTGACCTCGGTGCCCCGTCCCACAGGTGAGCCTGGACTCCCGAGTG

CGGGAGGGCATCAACAGGAAGATGCAGGAGCCGTCAGCACACACATTTGACGACGCGCAG

CTGCAGATCTACACGCTCATGCACCGGGACTCGTACCCACGCTTCCTCAGCTCCCCAGCC

TACCGCACCCTGCTGCTCCGGGGGTCCCCACAGTCCTCTTCGGAGGCC

>CL265.Contig26_A-W 1696 2211 minus strand PREDICTED: regulator of G-protein signaling 19, partial [Pan troglodytes]

TTCCTCTCTGCTGCCAGGAACCAAGAGCGGCGGCGAGCATGGCAGGCCTCACAGGAGAGC

AAGCTGCAGCCCCTCCCCAGCTGTGATGCCTGCACCCCCCCAAGCCCTGAGGAGGTACAG

AGCTGGGCGCAGTCCTTCGACAAGCTGATGCACAGCCCCGCGGGCCGCAGTGTGTTCCGC

GCCTTCCTGCGGACAGAGTACAGCGAGGAGAACATGCTCTTCTGGCTGGCCTGCGAGGAG

CTGAAGGCAGAGGCTAACCAGCACGTGGTGGATGAGAAGGCGCGGCTCATCTATGAGGAC

TACGTGTCCATCCTGTCCCCCAAGGAGGTGAGCCTGGACTCCCGAGTGCGGGAGGGCATC

AACAGGAAGATGCAGGAGCCGTCAGCACACACATTTGACGACGCGCAGCTGCAGATCTAC

ACGCTCATGCACCGGGACTCGTACCCACGCTTCCTCAGCTCCCCAGCCTACCGCACCCTG

CTGCTCCGGGGGTCCCCACAGTCCTCTTCGGAGGCC

>CL265.Contig27_A-W 1669 2256 minus strand PREDICTED: regulator of G-protein signaling 19, partial [Pan troglodytes]

TTCCTCTCTGCTGCCAGGAACCAAGAGCGGCGGCGAGCATGGCAGGCCTCACAGGAGAGC

AAGCTGCAGCCCCTCCCCAGCTGTGATGCCTGCACCCCCCCAAGCCCTGAGGAGGTACAG

AGCTGGGCGCAGTCCTTCGACAAGCTGATGCACAGCCCCGCGGGCCGCAGTGTGTTCCGC

GCCTTCCTGCGGACAGAGTACAGCGAGGAGAACATGCTCTTCTGGCTGGCCTGCGAGGAG

CTGAAGGCAGAGGCTAACCAGCACGTGGTGGATGAGAAGGCGCGGCTCATCTATGAGGAC

TACGTGTCCATCCTGTCCCCCAAGGAGGTGCGCCAGCTGCTGGCACGGGTTGGGGTGGCC

TGGGGAGGGTGCCTCTGACCTCGGTGCCCCGTCCCACAGGTGAGCCTGGACTCCCGAGTG

CGGGAGGGCATCAACAGGAAGATGCAGGAGCCGTCAGCACACACATTTGACGACGCGCAG

CTGCAGATCTACACGCTCATGCACCGGGACTCGTACCCACGCTTCCTCAGCTCCCCAGCC

TACCGCACCCTGCTGCTCCGGGGGTCCCCACAGTCCTCTTCGGAGGCC

>CL265.Contig28_A-W 989 1486 minus strand Regulator of G-protein signaling 19 OS=Homo sapiens GN=RGS19 PE=1 SV=1

AACCAAGAGCGGCGGCGAGCATGGCAGGCCTCACAGGAGAGCAAGCTGCAGCCCCTCCCC

AGCTGTGATGCCTGCACCCCCCCAAGCCCTGAGGAGGTACAGAGCTGGGCGCAGTCCTTC

GACAAGCTGATGCACAGCCCCGCGGGCCGCAGTGTGTTCCGCGCCTTCCTGCGGACAGAG

TACAGCGAGGAGAACATGCTCTTCTGGCTGGCCTGCGAGGAGCTGAAGGCAGAGGCTAAC

CAGCACGTGGTGGATGAGAAGGCGCGGCTCATCTATGAGGACTACGTGTCCATCCTGTCC

CCCAAGGAGGTGAGCCTGGACTCCCGAGTGCGGGAGGGCATCAACAGGAAGATGCAGGAG

CCGTCAGCACACACATTTGACGACGCGCAGCTGCAGATCTACACGCTCATGCACCGGGAC

TCGTACCCACGCTTCCTCAGCTCCCCAGCCTACCGCACCCTGCTGCTCCGGGGGTCCCCA

CAGTCCTCTTCGGAGGCC

>CL265.Contig29_A-W 578 1147 minus strand Regulator of G-protein signaling 19 OS=Homo sapiens GN=RGS19 PE=1 SV=1

AACCAAGAGCGGCGGCGAGCATGGCAGGCCTCACAGGAGAGCAAGCTGCAGCCCCTCCCC

AGCTGTGATGCCTGCACCCCCCCAAGCCCTGAGGAGGTACAGAGCTGGGCGCAGTCCTTC

GACAAGCTGATGCACAGCCCCGCGGGCCGCAGTGTGTTCCGCGCCTTCCTGCGGACAGAG

TACAGCGAGGAGAACATGCTCTTCTGGCTGGCCTGCGAGGAGCTGAAGGCAGAGGCTAAC

CAGCACGTGGTGGATGAGAAGGCGCGGCTCATCTATGAGGACTACGTGTCCATCCTGTCC

CCCAAGGAGGTGCGCCAGCTGCTGGCACGGGTTGGGGTGGCCTGGGGAGGGTGCCTCTGA

CCTCGGTGCCCCGTCCCACAGGTGAGCCTGGACTCCCGAGTGCGGGAGGGCATCAACAGG

AAGATGCAGGAGCCGTCAGCACACACATTTGACGACGCGCAGCTGCAGATCTACACGCTC

ATGCACCGGGACTCGTACCCACGCTTCCTCAGCTCCCCAGCCTACCGCACCCTGCTGCTC

CGGGGGTCCCCACAGTCCTCTTCGGAGGCC

>CL265.Contig30_A-W 118 873 minus strand regulator of G-protein signaling 19 [Mus musculus]

GAGTTGGCACCACAGAGCCTGGGCAGTCCCCTCCACCTACCCATGCCCACCCCGCATGAG

GCTGAGAAGCAGCACACAGGGCCAGAGGAGGCGGACAGGCCCCCCCCAATGTCCAGCCAT

GATGTGGTCCCCCCGGCGGCCCCCAGCCGCAACCCCTGCTGTTTGTGCTGGTGTTGCTGC

TGCAGCTGCTCCTGGAACCAAGAGCGGCGGCGAGCATGGCAGGCCTCACAGGAGAGCAAG

CTGCAGCCCCTCCCCAGCTGTGATGCCTGCACCCCCCCAAGCCCTGAGGAGGTACAGAGC

TGGGCGCAGTCCTTCGACAAGCTGATGCACAGCCCCGCGGGCCGCAGTGTGTTCCGCGCC

TTCCTGCGGACAGAGTACAGCGAGGAGAACATGCTCTTCTGGCTGGCCTGCGAGGAGCTG

AAGGCAGAGGCTAACCAGCACGTGGTGGATGAGAAGGCGCGGCTCATCTATGAGGACTAC

GTGTCCATCCTGTCCCCCAAGGAGGTGCGCCAGCTGCTGGCACGGGTTGGGGTGGCCTGG

GGAGGGTGCCTCTGACCTCGGTGCCCCGTCCCACAGGTGAGCCTGGACTCCCGAGTGCGG

GAGGGCATCAACAGGAAGATGCAGGAGCCGTCAGCACACACATTTGACGACGCGCAGCTG

CAGATCTACACGCTCATGCACCGGGACTCGTACCCACGCTTCCTCAGCTCCCCAGCCTAC

CGCACCCTGCTGCTCCGGGGGTCCCCACAGTCCTCT

>CL265.Contig31_A-W 1692 2207 minus strand PREDICTED: regulator of G-protein signaling 19, partial [Pan troglodytes]

TTCCTCTCTGCTGCCAGGAACCAAGAGCGGCGGCGAGCATGGCAGGCCTCACAGGAGAGC

AAGCTGCAGCCCCTCCCCAGCTGTGATGCCTGCACCCCCCCAAGCCCTGAGGAGGTACAG

AGCTGGGCGCAGTCCTTCGACAAGCTGATGCACAGCCCCGCGGGCCGCAGTGTGTTCCGC

GCCTTCCTGCGGACAGAGTACAGCGAGGAGAACATGCTCTTCTGGCTGGCCTGCGAGGAG

CTGAAGGCAGAGGCTAACCAGCACGTGGTGGATGAGAAGGCGCGGCTCATCTATGAGGAC

TACGTGTCCATCCTGTCCCCCAAGGAGGTGAGCCTGGACTCCCGAGTGCGGGAGGGCATC

AACAGGAAGATGCAGGAGCCGTCAGCACACACATTTGACGACGCGCAGCTGCAGATCTAC

ACGCTCATGCACCGGGACTCGTACCCACGCTTCCTCAGCTCCCCAGCCTACCGCACCCTG

CTGCTCCGGGGGTCCCCACAGTCCTCTTCGGAGGCC

>CL265.Contig32_A-W 1669 2184 minus strand PREDICTED: regulator of G-protein signaling 19, partial [Pan troglodytes]

TTCCTCTCTGCTGCCAGGAACCAAGAGCGGCGGCGAGCATGGCAGGCCTCACAGGAGAGC

AAGCTGCAGCCCCTCCCCAGCTGTGATGCCTGCACCCCCCCAAGCCCTGAGGAGGTACAG

AGCTGGGCGCAGTCCTTCGACAAGCTGATGCACAGCCCCGCGGGCCGCAGTGTGTTCCGC

GCCTTCCTGCGGACAGAGTACAGCGAGGAGAACATGCTCTTCTGGCTGGCCTGCGAGGAG

CTGAAGGCAGAGGCTAACCAGCACGTGGTGGATGAGAAGGCGCGGCTCATCTATGAGGAC

TACGTGTCCATCCTGTCCCCCAAGGAGGTGAGCCTGGACTCCCGAGTGCGGGAGGGCATC

AACAGGAAGATGCAGGAGCCGTCAGCACACACATTTGACGACGCGCAGCTGCAGATCTAC

ACGCTCATGCACCGGGACTCGTACCCACGCTTCCTCAGCTCCCCAGCCTACCGCACCCTG

CTGCTCCGGGGGTCCCCACAGTCCTCTTCGGAGGCC

>CL265.Contig33_A-W 1593 2180 minus strand PREDICTED: regulator of G-protein signaling 19, partial [Pan troglodytes]

TTCCTCTCTGCTGCCAGGAACCAAGAGCGGCGGCGAGCATGGCAGGCCTCACAGGAGAGC

AAGCTGCAGCCCCTCCCCAGCTGTGATGCCTGCACCCCCCCAAGCCCTGAGGAGGTACAG

AGCTGGGCGCAGTCCTTCGACAAGCTGATGCACAGCCCCGCGGGCCGCAGTGTGTTCCGC

GCCTTCCTGCGGACAGAGTACAGCGAGGAGAACATGCTCTTCTGGCTGGCCTGCGAGGAG

CTGAAGGCAGAGGCTAACCAGCACGTGGTGGATGAGAAGGCGCGGCTCATCTATGAGGAC

TACGTGTCCATCCTGTCCCCCAAGGAGGTGCGCCAGCTGCTGGCACGGGTTGGGGTGGCC

TGGGGAGGGTGCCTCTGACCTCGGTGCCCCGTCCCACAGGTGAGCCTGGACTCCCGAGTG

CGGGAGGGCATCAACAGGAAGATGCAGGAGCCGTCAGCACACACATTTGACGACGCGCAG

CTGCAGATCTACACGCTCATGCACCGGGACTCGTACCCACGCTTCCTCAGCTCCCCAGCC

TACCGCACCCTGCTGCTCCGGGGGTCCCCACAGTCCTCTTCGGAGGCC

>CL265.Contig34_A-W 476 718 minus strand PREDICTED: regulator of G-protein signaling 19-like isoform 1 [Cricetulus griseus]

ATGCCCACCCCGCATGAGGCTGAGAAGCAGCACACAGGGCCAGAGGAGGCGGACAGGCCC

CCCCCAATGTCCAGCCATGATGTGGTCCCCCCGGCGGCCCCCAGCCGCAACCCCTGCTGT

TTGTGCTGGTGTTGCTGCTGCAGCTGCTGCCAGGAACCAAGAGCGGCGGCGAGCATGGCA

GGCCTCACAGGAGAGCAAGCTGCAGCCCCTCCCCAGCTGTGATGCCTGCACCCCCCCAAG

CCC

>CL266.Contig1_A-W 2 1486 minus strand PREDICTED: EH domain-containing protein 1 [Canis lupus familiaris]

TACCGCTTCCACGAGTTCCACTCGCCCGCCCTGGAGGATGCTGACTTCGACAACAAGCCC

ATGGTGCTTCTCGTGGGCCAGTACAGCACGGGCAAGACCACCTTCATCCGGCACCTGATC

GAGCAGGACTTCCCGGGGATGCGCATCGGGCCCGAGCCCACCACCGACTCTTTTATCGCG

GTCATGCACGGCCCCACCGAGGGCGTGGTGCCCGGCAACGCGCTCGTCGTGGACCCACGG

CGCCCCTTCCGCAAGCTCAACGCCTTCGGCAACGCCTTCCTCAACAGGTTCATGTGTGCC

CAGCTGCCCAACCCTGTCCTGGACAGCATCAGCATCATTGACACTCCCGGGATCCTCTCT

GGAGAGAAGCAACGCATCAGCAGAGGTTACGACTTTGCTGCGGTCCTGGAGTGGTTCGCT

GAGCGGGTGGACCGCATCATCCTGCTCTTCGATGCCCACAAGCTGGACATCTCCGACGAG

TTCTCTGAGGTCATTAAGGCACTCAAGAACCACGAGGACAAGATCCGCGTGGTACTGAAC

AAGGCCGACCAGATCGAGACGCAGCAGCTGATGCGGGTGTACGGGGCCCTCATGTGGTCC

CTGGGCAAGATCATCAACACCCCCGAGGTGGTCCGCGTCTACATTGGTTCTTTCTGGTCC

CACCCGCTGCTCATCCCCGACAACCGAAAGCTCTTCGAGGCCGAGGAGCAGGACCTCTTC

AAAGACATCCAGTCTCTGCCCCGAAACGCTGCCCTCAGGAAGCTCAATGACCTGATCAAG

CGGGCCAGGCTGGCCAAGGTCCATGCCTACATCATCAGCTCCCTCAAGAAGGAGATGCCC

AACGTCTTTGGTAAAGAGAGCAAGAAGAAGGAACTGGTGAACAACCTGGGTGAGATCTAC

CAGAAGATTGAGCGGGAGCATCAGATCTCCCCCGGAGACTTCCCGAGCCTCCGCAAGATG

CAGGAACTACTGCAGACCCAGGACTTCAGCAAGTTCCAGGCCTTGAAGCCCAAGCTGCTG

GACACAGTGGACGACATGCTTGCCAACGACATTGCTCGACTGATGGTGATGGTGCGCCAG

GAAGAGTCCCTGATGCCCTCCCAGGCCGTGAAGGGCGGTGCCTTCGATGGCACCATGAAC

GGGCCGTTTGGGCACGGCTATGGCGAGGGGGCCAGTGAGGGCATCGACGACGTTGAGTGG

GTAGTGGGCAAGGACAAGCCCACCTACGATGAGATCTTCTACACACTGTCACCTGTCAAT

GGCAAGATCACAGGAGCCAACGCCAAGAAGGAGATGGTGAAGTCCAAGCTACCCAACACG

GTGCTGGGGAAGATCTGGAAGCTGGCCGACGTGGACAAGGACGGGCTGCTGGACGACGAG

GAGTTCGCCCTGGCCAACCACCTCATCAAGGTCAAGCTGGAGGGCCACGAGCTGCCCGCT

GACCTGCCCCCACACCTCGTCCCGCCCTCCAAGCGCAGGCATGAG

>CL266.Contig2_A-W 2 1492 PREDICTED: EH domain-containing protein 1 [Canis lupus familiaris]

GAGCACTACCGCTTCCACGAGTTCCACTCGCCCGCCCTGGAGGATGCTGACTTCGACAAC

AAGCCCATGGTGCTTCTCGTGGGCCAGTACAGCACGGGCAAGACCACCTTCATCCGGCAC

CTGATCGAGCAGGACTTCCCGGGGATGCGCATCGGGCCCGAGCCCACCACCGACTCTTTT

ATCGCGGTCATGCACGGCCCCACCGAGGGCGTGGTGCCCGGCAACGCGCTCGTCGTGGAC

CCACGGCGCCCCTTCCGCAAGCTCAACGCCTTCGGCAACGCCTTCCTCAACAGGTTCATG

TGTGCCCAGCTGCCCAACCCTGTCCTGGACAGCATCAGCATCATTGACACTCCCGGGATC

CTCTCTGGAGAGAAGCAACGCATCAGCAGAGGTTACGACTTTGCTGCGGTCCTGGAGTGG

TTCGCTGAGCGGGTGGACCGCATCATCCTGCTCTTCGATGCCCACAAGCTGGACATCTCC

GACGAGTTCTCTGAGGTCATTAAGGCACTCAAGAACCACGAGGACAAGATCCGCGTGGTA

CTGAACAAGGCCGACCAGATCGAGACGCAGCAGCTGATGCGGGTGTACGGGGCCCTCATG

TGGTCCCTGGGCAAGATCATCAACACCCCCGAGGTGGTCCGCGTCTACATTGGTTCTTTC

TGGTCCCACCCGCTGCTCATCCCCGACAACCGAAAGCTCTTCGAGGCCGAGGAGCAGGAC

CTCTTCAAAGACATCCAGTCTCTGCCCCGAAACGCTGCCCTCAGGAAGCTCAATGACCTG

ATCAAGCGGGCCAGGCTGGCCAAGGTCCATGCCTACATCATCAGCTCCCTCAAGAAGGAG

ATGCCCAACGTCTTTGGTAAAGAGAGCAAGAAGAAGGAACTGGTGAACAACCTGGGTGAG

ATCTACCAGAAGATTGAGCGGGAGCATCAGATCTCCCCCGGAGACTTCCCGAGCCTCCGC

AAGATGCAGGAACTACTGCAGACCCAGGACTTCAGCAAGTTCCAGGCCTTGAAGCCCAAG

CTGCTGGACACAGTGGACGACATGCTTGCCAACGACATTGCTCGACTGATGGTGATGGTG

CGCCAGGAAGAGTCCCTGATGCCCTCCCAGGCCGTGAAGGGCGGTGCCTTCGATGGCACC

ATGAACGGGCCGTTTGGGCACGGCTATGGCGAGGGGGCCGGTGAGGGCATCGACGACGTT

GAGTGGGTAGTGGGCAAGGACAAGCCCACCTACGATGAGATCTTCTACACACTGTCACCT

GTCAATGGCAAGATCACAGGAGCCAACGCCAAGAAGGAGATGGTGAAGTCCAAGCTACCC

AACACGGTGCTGGGGAAGATCTGGAAGCTGGCCGACGTGGACAAGGACGGGCTGCTGGAC

GACGAGGAGTTCGCCCTGGCCAACCACCTCATCAAGGTCAAGCTGGAGGGCCACGAGCTG

CCCGCTGACCTGCCCCCACACCTCGTCCCGCCCTCCAAGCGCAGGCATGAG

>CL266.Contig3_A-W 135 830 minus strand PREDICTED: EH domain-containing protein 1 [Canis lupus familiaris]

CTGTTACAGGTCCATGCCTACATCATCAGCTCCCTCAAGAAGGAGATGCCCAACGTCTTT

GGTAAAGAGAGCAAGAAGAAGGAACTGGTGAACAACCTGGGTGAGATCTACCAGAAGATT

GAGCGGGAGCATCAGATCTCCCCCGGAGACTTCCCGAGCCTCCGCAAGATGCAGGAACTA

CTGCAGACCCAGGACTTCAGCAAGTTCCAGGCCTTGAAGCCCAAGCTGCTGGACACAGTG

GACGACATGCTTGCCAACGACATTGCTCGACTGATGGTGATGGTGCGCCAGGAAGAGTCC

CTGATGCCCTCCCAGGCCGTGAAGGGCGGTGCCTTCGATGGCACCATGAACGGGCCGTTT

GGGCACGGCTATGGCGAGGGGGCCAGTGAGGGCATCGACGACGTTGAGTGGGTAGTGGGC

AAGGACAAGCCCACCTACGATGAGATCTTCTACACACTGTCACCTGTCAATGGCAAGATC

ACAGGAGCCAACGCCAAGAAGGAGATGGTGAAGTCCAAGCTACCCAACACGGTGCTGGGG

AAGATCTGGAAGCTGGCCGACGTGGACAAGGACGGGCTGCTGGACGACGAGGAGTTCGCC

CTGGCCAACCACCTCATCAAGGTCAAGCTGGAGGGCCACGAGCTGCCCGCTGACCTGCCC

CCACACCTCGTCCCGCCCTCCAAGCGCAGGCATGAG

>CL267.Contig1_A-W 152 448 protein dpy-30 homolog [Homo sapiens] >gi|118150944|ref|NP_001071384.1| protein dpy-30 homolog [Bos taurus] >gi|349732201|ref|NP_001231854.1| dpy-30 homolog [Sus scrofa] >gi|386780676|ref|NP_001248022.1| protein dpy-30 homolog [Macaca mulatta] >gi|14916555|sp|Q9C005.1|DPY30_HUMAN RecName: Full=Protein dpy-30 homolog; AltName: Full=Dpy-30-like protein; Short=Dpy-30L >gi|110808210|sp|Q2NKU6.1|DPY30_BOVIN RecName: Full=Protein dpy-30 homolog; AltName: Full=Dpy-30-like protein; Short=Dpy-30L >gi|12655886|gb|AAK00640.1|AF226998_1 dpy-30-like protein [Homo sapiens] >gi|16359014|gb|AAH15970.1| DPY30 protein [Homo sapiens] >gi|84201627|gb|AAI11635.1| Dpy-30 homolog (C. elegans) [Bos taurus] >gi|119620868|gb|EAX00463.1| dpy-30-like protein, isoform CRA_a [Homo sapiens] >gi|119620871|gb|EAX00466.1| dpy-30-like protein, isoform CRA_a [Homo sapiens] >gi|296482690|gb|DAA24805.1| protein dpy-30 homolog [Bos taurus] >gi|380784207|gb|AFE63979.1| protein dpy-30 homolog [Macaca mulatta] >gi|383414173|gb|AFH30300.1| protein dpy-30 homolog [Macaca mulatta] >gi|384949868|gb|AFI38539.1| protein dpy-30 homolog [Macaca mulatta]

ATGGAGCCAGAGCAGATGCTGGAAGGACAGACGCAGGTTGCAGAAAACCCTCACTCTGAG

TACGGTCTGACCGACAACGTTGAGAGGATAGTAGAAAATGAGAAGATGAATGCAGAAAAG

TCATCAAAACAGAAGGTGGATTTACAGTCTTTGCCAACTCGTGCCTACCTGGATCAGACG

GTTGTGCCTATCTTATTACAGGGACTTGCTGTGCTTGCAAAGGAAAGACCACCAAATCCC

ATTGAATTTCTAGCATCCTATCTTTTAAAAAACAAGGCACAATTTGAAGATCGAAAC

>CL267.Contig2_A-W 123 419 protein dpy-30 homolog [Homo sapiens] >gi|118150944|ref|NP_001071384.1| protein dpy-30 homolog [Bos taurus] >gi|349732201|ref|NP_001231854.1| dpy-30 homolog [Sus scrofa] >gi|386780676|ref|NP_001248022.1| protein dpy-30 homolog [Macaca mulatta] >gi|14916555|sp|Q9C005.1|DPY30_HUMAN RecName: Full=Protein dpy-30 homolog; AltName: Full=Dpy-30-like protein; Short=Dpy-30L >gi|110808210|sp|Q2NKU6.1|DPY30_BOVIN RecName: Full=Protein dpy-30 homolog; AltName: Full=Dpy-30-like protein; Short=Dpy-30L >gi|12655886|gb|AAK00640.1|AF226998_1 dpy-30-like protein [Homo sapiens] >gi|16359014|gb|AAH15970.1| DPY30 protein [Homo sapiens] >gi|84201627|gb|AAI11635.1| Dpy-30 homolog (C. elegans) [Bos taurus] >gi|119620868|gb|EAX00463.1| dpy-30-like protein, isoform CRA_a [Homo sapiens] >gi|119620871|gb|EAX00466.1| dpy-30-like protein, isoform CRA_a [Homo sapiens] >gi|296482690|gb|DAA24805.1| protein dpy-30 homolog [Bos taurus] >gi|380784207|gb|AFE63979.1| protein dpy-30 homolog [Macaca mulatta] >gi|383414173|gb|AFH30300.1| protein dpy-30 homolog [Macaca mulatta] >gi|384949868|gb|AFI38539.1| protein dpy-30 homolog [Macaca mulatta]

ATGGAGCCAGAGCAGATGCTGGAAGGACAGACGCAGGTTGCAGAAAACCCTCACTCTGAG

TACGGTCTGACCGACAACGTTGAGAGGATAGTAGAAAATGAGAAGATGAATGCAGAAAAG

TCATCAAAACAGAAGGTGGATTTACAGTCTTTGCCAACTCGTGCCTACCTGGATCAGACG

GTTGTGCCTATCTTATTACAGGGACTTGCTGTGCTTGCAAAGGAAAGACCACCAAATCCC

ATTGAATTTCTAGCATCCTATCTTTTAAAAAACAAGGCACAATTTGAAGATCGAAAC

>CL269.Contig1_A-W 1 3015 PREDICTED: protein phosphatase 1 regulatory subunit 12A isoform 1 [Pan paniscus] >gi|397525975|ref|XP_003832919.1| PREDICTED: protein phosphatase 1 regulatory subunit 12A isoform 2 [Pan paniscus]

CCTCCCGTGGTGAAGCGCCAGAAGACCAAGGTGAAGTTCGACGATGGCGCCGTCTTCCTG

GCTGCTTGCTCCAGCGGCGACACGGACGAGGTTCTCAAGCTGCTGCACCGCGGCGCCGAC

ATCAATTACGCCAATGTGGACGGACTTACTGCCCTCCATCAGGCCTGCATTGATGACAAT

GTTGATATGGTGAAGTTTCTGGTAGAAAATGGAGCAAATATCAATCAACCTGATAATGAA

GGCTGGATACCACTACATGCAGCTGCTTCCTGTGGATATCTTGATATTGCAGAGTTTTTG

ATTGGTCAGGGAGCACATGTTGGTGCTGTCAACAGTGAAGGAGATACACCTTTAGATATT

GCAGAGGAGGAAGCAATGGAAGAACTACTTCAAAATGAAGTTAATCGGCAAGGGGTTGAT

ATAGAGGCAGCTCGAAAGGAAGAAGAACGAATAATGCTTAGAGATGCAAGGCAGTGGTTA

AATAGTGGTCATATAAATGATGTACGGCATGCAAAATCTGGAGGTACAGCGCTTCATGTT

GCAGCCGCTAAGGGCTATACAGAAGTTTTAAAACTTTTAATACAGGCAGGCTATGATGTT

AATATTAAAGATTATGATGGCTGGACACCTCTTCATGCTGCAGCTCACTGGGGTAAAGAA

GAAGCATGTCGAATTTTAGTGGACAATCTGTGTGATATGGAGATGGTCAACAAAGTGGGC

CAGACAGCCTTTGATGTAGCAGATGAAGACATTTTAGGATATTTAGAAGAGTTACAAAAG

AAACAAAATCTGCTCCATAGTGAAAAACGGGATAAGAAATCTCCACTAATAGAATCAACA

GCAAATATGGAAAACAATCAGTCACAGAAGACTTTTAAAAACAAAGAGACTTTGATTATT

GAGCCAGAGAAAAATGCATCCCGTATTGAATCTCTGGAACAGGAAAAGGTTGATGAAGAA

GAAGAAGGAAAGAAAGATGAATCTAGTTGTTCCAGTGAAGAAGATGAAGAAGATGACTCA

GAATCAGAAGCTGAAACAGATAAGACAAAACCTGTGACTTCTGTAACTACTGCCAACACT

TCTAGTACACAAGCAGCTCCTATAGCTGTTACAACACCTACTCTGTCATCAAGTCAGGCA

ACACCCACATCACCTATTAAAAAGTTTCCAACTTCAACTACAAAAATTTCTCCCAAAGAA

GAAGAGAGAAAAGATGAATCTCCTGCATCTTGGAGGTTAGGACTTAGAAAGACTGGCAGT

TATGGTGCACTTGCTGAAATCACAGCATCTAAAGAGACTCAGAAAGAAAAAGACACTGCA

GGTGTTATACGTTCAGCTTCAAGTCCCAGACTTTCCTCCTCTTTGGATAATAAAGAAAAG

GAGAGAGAGAGTAAAGGAGCAAGGCTTGCATATGTTGCACCTACTATACCAAGACGTCTA

GCTAGCACATCTGACATTGAAGAGAAAGAAAACAGAGACTCTTTGAGTTTGCGAACAAGT

AGCTCTTATACAAGAAGAAAATGGGAAGATGATCTTAAAAAAAACAGCTCAATTAATGAA

GGATCAACTTATCATAAAAGTTGCTCCTTTGGTAGAAGACAAGATGATTTGATTAGTTCT

AGTGTTCCAAGCACCACATCAACACCAACAGTTACCTCTGCAGCTGGGCTTCAGAAAAGC

CTGCTTTCCAGCACAAGCACTACTACAAAGATTACAACGGGTTCTTCCTCAGCAGGCACA

CAAAGCAGTACCTCAAATCGTTTGTGGGCTGATGATAGTACTGAAAAAGAAAAGGACAGT

GTTCCTACGGCAGTGACCATTCCTGTTGCTCCAACTGTTGTAAATGCTGCAGCTTCTACC

ACAACCCTGACTACAACTACTGCTGGCACTGTCTCCTCCACATCAGAGGTCAGGGAGAGA

CGCAGATCATACCTCACTCCTGTTAGAGATGAAGAATCTGAATCCCAAAGAAAAGCAAGA

TCTAGACAAGCAAGACAGTCTAGAAGATCAACACAGGGGGTGACATTGACTGATCTTCAG

GAAGCTGAAAAAACTATAGGAAGAAGTCGTTCTACCAGAACCAGAGAGCAAGAAAATGAA

GAAAAAGAAAAGGAGGAAAAAGAAAAACAGGATAAGGAGAAACAAGAAGAAAAGAAGGAG

TCAGAAACATCTAGAGAAGATGAATATAAGCAAAAATACTCTAGAACATATGATGAGACT

TTTCCACGTTATAGACCAGTGTCAACTTCAAGTTCAACCACTGCATCCTCTGCCCTTCCT

ACAATGACCAGTTCACTCTATGCTTCAAGTCAACTAAACAGGCCAAATAGTCTTGTAGGT

ATAACTTCTGCTTACTCCAGAGGATTAACTAAAGAAAATGAAAGAGAGGGAGAAAAAAAA

GAAGAGGAGAAGGAAGGTGAAGATAAATCACAGCCTAAATCCATCAGAGAACGACGGAGA

CCAAGAGAGAAAAGAAGATCTACGGGAGTGTCTTTTTGGACACAAGATAGTGATGAAAAT

GAACAAGAACAACAATCAGATACAGAAGAAGGATCCAATAAAAAAGAAACTCAGACGGAT

TCCATTTCTAGATATGAAACCAGTTCCACTTCAGCTAGTGATCGGTATGATTCCTTGCTG

GGTCGCTCCGGATCGTATAGTTACTTAGAAGAAAGAAAACCATATAGTAGCAGGCTAGAA

AAGGACGACTCAACTGACTTTAAAAAGCTTTATGAACAAATTTTAGCTGAAAACGAAAAG

TTGAAGGCACAGCTACATGATACAAATATGGAATTAACAGATCTTAAATTGCAGTTGGAA

AAGGCCACCCAGAGACAAGAAAGATTTGCTGATAGGTCACTATTGGAAATGGAAAAAAGG

GAACGAAGAGCTCTAGAAAGAAGAATATCTGAAATGGAAGAAGAACTCAAAATGTTACCA

GACCTAAAAGCAGACAACCAGAGACTAAAGGATGAAAATGGGGCCTTGATCAGAGTTATA

AGCAAACTTTCCAAA

>CL269.Contig2_A-W 311 598 protein phosphatase 1 regulatory subunit 12A isoform d [Homo sapiens] >gi|119617762|gb|EAW97356.1| protein phosphatase 1, regulatory (inhibitor) subunit 12A, isoform CRA_e [Homo sapiens]

GATGTACGGCATGCAAAATCTGGAGGTACAGCGCTTCATGTTGCAGCCGCTAAGGGCTAT

ACAGAAGTTTTAAAACTTTTAATACAGGCAGGCTATGATGTTAATATTAAAGATTATGAT

GGCTGGACACCTCTTCATGCTGCAGCTCACTGGGGTAAAGAAGAAGCATGTCGAATTTTA

GTGGACAATCTGTGTGATATGGAGATGGTCAACAAAGTGGGCCAGACAGCCTTTGATGTA

GCAGATGAAGACATTTTAGGATATTTAGAAGAGTTACAAAAGAAACAA

>CL269.Contig3_A-W 334 963 PREDICTED: protein phosphatase 1 regulatory subunit 12A isoform 4 [Pan paniscus]

GAGGGAGAAAAAAAAGAAGAGGAGAAGGAAGGTGAAGATAAATCACAGCCTAAATCCATC

AGAGAACGACGGAGACCAAGAGAGAAAAGAAGATCTACGGGAGTGTCTTTTTGGACACAA

GATAGTGATGAAAATGAACAAGAACAACAATCAGATACAGAAGAAGGATCCAATAAAAAA

GAAACTCAGACGGATTCCATTTCTAGATATGAAACCAGTTCCACTTCAGCTAGTGATCGG

TATGATTCCTTGCTGGGTCGCTCCGGATCGTATAGTTACTTAGAAGAAAGAAAACCATAT

AGTAGCAGGCTAGAAAAGGACGACTCAACTGACTTTAAAAAGCTTTATGAACAAATTTTA

GCTGAAAACGAAAAGTTGAAGGCACAGCTACATGATACAAATATGGAATTAACAGATCTT

AAATTGCAGTTGGAAAAGGCCACCCAGAGACAAGAAAGATTTGCTGATAGGTCACTATTG

GAAATGGAAAAAAGGGAACGAAGAGCTCTAGAAAGAAGAATATCTGAAATGGAAGAAGAA

CTCAAAATGTTACCAGACCTAAAAGCAGACAACCAGAGACTAAAGGATGAAAATGGGGCC

TTGATCAGAGTTATAAGCAAACTTTCCAAA

>CL269.Contig4_A-W 1 2838 protein phosphatase 1, regulatory (inhibitor) subunit 12A, isoform CRA_e [Rattus norvegicus]

CCTCCCGTGGTGAAGCGCCAGAAGACCAAGGTGAAGTTCGACGATGGCGCCGTCTTCCTG

GCTGCTTGCTCCAGCGGCGACACGGACGAGGTTCTCAAGCTGCTGCACCGCGGCGCCGAC

ATCAATTACGCCAATGTGGACGGACTTACTGCCCTCCATCAGGCCTGCATTGATGACAAT

GTTGATATGGTGAAGTTTCTGGTAGAAAATGGAGCAAATATCAATCAACCTGATAATGAA

GGCTGGATACCACTACATGCAGCTGCTTCCTGTGGATATCTTGATATTGCAGAGTTTTTG

ATTGGTCAGGGAGCACATGTTGGTGCTGTCAACAGTGAAGGAGATACACCTTTAGATATT

GCAGAGGAGGAAGCAATGGAAGAACTACTTCAAAATGAAGTTAATCGGCAAGGGGTTGAT

ATAGAGGCAGCTCGAAAGGAAGAAGAACGAATAATGCTTAGAGATGCAAGGCAGTGGTTA

AATAGTGGTCATATAAATGATGTACGGCATGCAAAATCTGGAGGTACAGCGCTTCATGTT

GCAGCCGCTAAGGGCTATACAGAAGTTTTAAAACTTTTAATACAGGCAGGCTATGATGTT

AATATTAAAGATTATGATGGCTGGACACCTCTTCATGCTGCAGCTCACTGGGGTAAAGAA

GAAGCATGTCGAATTTTAGTGGACAATCTGTGTGATATGGAGATGGTCAACAAAGTGGGC

CAGACAGCCTTTGATGTAGCAGATGAAGACATTTTAGGATATTTAGAAGAGTTACAAAAG

AAACAAAATCTGCTCCATAGTGAAAAACGGGATAAGAAATCTCCACTAATAGAATCAACA

GCAAATATGGAAAACAATCAGTCACAGAAGACTTTTAAAAACAAAGAGACTTTGATTATT

GAGCCAGAGAAAAATGCATCCCGTATTGAATCTCTGGAACAGGAAAAGGTTGATGAAGAA

GAAGAAGGAAAGAAAGATGAATCTAGTTGTTCCAGTGAAGAAGATGAAGAAGATGACTCA

GAATCAGAAGCTGAAACAGATAAGACAAAACCTGTGACTTCTGTAACTACTGCCAACACT

TCTAGTACACAAGCAGCTCCTATAGCTGTTACAACACCTACTCTGTCATCAAGTCAGGCA

ACACCCACATCACCTATTAAAAAGTTTCCAACTTCAACTACAAAAATTTCTCCCAAAGAA

GAAGAGAGAAAAGATGAATCTCCTGCATCTTGGAGGTTAGGACTTAGAAAGACTGGCAGT

TATGGTGCACTTGCTGAAATCACAGCATCTAAAGAGACTCAGAAAGAAAAAGACACTGCA

GGTGTTATACGTTCAGCTTCAAGTCCCAGACTTTCCTCCTCTTTGGATAATAAAGAAAAG

GAGAGAGAGAGTAAAGGAGCAAGGCTTGCATATGTTGCACCTACTATACCAAGACGTCTA

GCTAGCACATCTGACATTGAAGAGAAAGAAAACAGAGACTCTTTGAGTTTGCGAACAAGT

AGCTCTTATACAAGAAGAAAATGGGAAGATGATCTTAAAAAAAACAGCTCAATTAATGAA

GGATCAACTTATCATAAAAGTTGCTCCTTTGGTAGAAGACAAGATGATTTGATTAGTTCT

AGTGTTCCAAGCACCACATCAACACCAACAGTTACCTCTGCAGCTGGGCTTCAGAAAAGC

CTGCTTTCCAGCACAAGCACTACTACAAAGATTACAACGGGTTCTTCCTCAGCAGGCACA

CAAAGCAGATCATACCTCACTCCTGTTAGAGATGAAGAATCTGAATCCCAAAGAAAAGCA

AGATCTAGACAAGCAAGACAGTCTAGAAGATCAACACAGGGGGTGACATTGACTGATCTT

CAGGAAGCTGAAAAAACTATAGGAAGAAGTCGTTCTACCAGAACCAGAGAGCAAGAAAAT

GAAGAAAAAGAAAAGGAGGAAAAAGAAAAACAGGATAAGGAGAAACAAGAAGAAAAGAAG

GAGTCAGAAACATCTAGAGAAGATGAATATAAGCAAAAATACTCTAGAACATATGATGAG

ACTTTTCCACGTTATAGACCAGTGTCAACTTCAAGTTCAACCACTGCATCCTCTGCCCTT

CCTACAATGACCAGTTCACTCTATGCTTCAAGTCAACTAAACAGGCCAAATAGTCTTGTA

GGTATAACTTCTGCTTACTCCAGAGGATTAACTAAAGAAAATGAAAGAGAGGGAGAAAAA

AAAGAAGAGGAGAAGGAAGGTGAAGATAAATCACAGCCTAAATCCATCAGAGAACGACGG

AGACCAAGAGAGAAAAGAAGATCTACGGGAGTGTCTTTTTGGACACAAGATAGTGATGAA

AATGAACAAGAACAACAATCAGATACAGAAGAAGGATCCAATAAAAAAGAAACTCAGACG

GATTCCATTTCTAGATATGAAACCAGTTCCACTTCAGCTAGTGATCGGTATGATTCCTTG

CTGGGTCGCTCCGGATCGTATAGTTACTTAGAAGAAAGAAAACCATATAGTAGCAGGCTA

GAAAAGGACGACTCAACTGACTTTAAAAAGCTTTATGAACAAATTTTAGCTGAAAACGAA

AAGTTGAAGGCACAGCTACATGATACAAATATGGAATTAACAGATCTTAAATTGCAGTTG

GAAAAGGCCACCCAGAGACAAGAAAGATTTGCTGATAGGTCACTATTGGAAATGGAAAAA

AGGGAACGAAGAGCTCTAGAAAGAAGAATATCTGAAATGGAAGAAGAACTCAAAATGTTA

CCAGACCTAAAAGCAGACAACCAGAGACTAAAGGATGAAAATGGGGCCTTGATCAGAGTT

ATAAGCAAACTTTCCAAA

>CL269.Contig5_A-W 1 2670 protein phosphatase 1 regulatory subunit 12A isoform d [Homo sapiens] >gi|119617762|gb|EAW97356.1| protein phosphatase 1, regulatory (inhibitor) subunit 12A, isoform CRA_e [Homo sapiens]

CCTCCCGTGGTGAAGCGCCAGAAGACCAAGGTGAAGTTCGACGATGGCGCCGTCTTCCTG

GCTGCTTGCTCCAGCGGCGACACGGACGAGGTTCTCAAGCTGCTGCACCGCGGCGCCGAC

ATCAATTACGCCAATGTGGACGGACTTACTGCCCTCCATCAGGCCTGCATTGATGACAAT

GTTGATATGGTGAAGTTTCTGGTAGAAAATGGAGCAAATATCAATCAACCTGATAATGAA

GGCTGGATACCACTACATGCAGCTGCTTCCTGTGGATATCTTGATATTGCAGAGTTTTTG

ATTGGTCAGGGAGCACATGTTGGTGCTGTCAACAGTGAAGGAGATACACCTTTAGATATT

GCAGAGGAGGAAGCAATGGAAGAACTACTTCAAAATGAAGTTAATCGGCAAGGGGTTGAT

ATAGAGGCAGCTCGAAAGGAAGAAGAACGAATAATGCTTAGAGATGCAAGGCAGTGGTTA

AATAGTGGTCATATAAATGATGTACGGCATGCAAAATCTGGAGGTACAGCGCTTCATGTT

GCAGCCGCTAAGGGCTATACAGAAGTTTTAAAACTTTTAATACAGGCAGGCTATGATGTT

AATATTAAAGATTATGATGGCTGGACACCTCTTCATGCTGCAGCTCACTGGGGTAAAGAA

GAAGCATGTCGAATTTTAGTGGACAATCTGTGTGATATGGAGATGGTCAACAAAGTGGGC

CAGACAGCCTTTGATGTAGCAGATGAAGACATTTTAGGATATTTAGAAGAGTTACAAAAG

AAACAAAATCTGCTCCATAGTGAAAAACGGGATAAGAAATCTCCACTAATAGAATCAACA

GCAAATATGGAAAACAATCAGTCACAGAAGACTTTTAAAAACAAAGAGACTTTGATTATT

GAGCCAGAGAAAAATGCATCCCGTATTGAATCTCTGGAACAGGAAAAGGTTGATGAAGAA

GAAGAAGGAAAGAAAGATGAATCTAGTTGTTCCAGTGAAGAAGATGAAGAAGATGACTCA

GAATCAGAAGCTGAAACAGATAAGACAAAACCTGTGACTTCTGTAACTACTGCCAACACT

TCTAGTACACAAGCAGCTCCTATAGCTGTTACAACACCTACTCTGTCATCAAGTCAGGCA

ACACCCACATCACCTATTAAAAAGTTTCCAACTTCAACTACAAAAATTTCTCCCAAAGAA

GAAGAGAGAAAAGATGAATCTCCTGCATCTTGGAGGTTAGGACTTAGAAAGACTGGCAGT

TATGGTGCACTTGCTGAAATCACAGCATCTAAAGAGACTCAGAAAGAAAAAGACACTGCA

GGTGTTATACGTTCAGCTTCAAGTCCCAGACTTTCCTCCTCTTTGGATAATAAAGAAAAG

GAGAGAGAGAGTAAAGGAGCAAGGCTTGCATATGTTGCACCTACTATACCAAGACGTCTA

GCTAGCACATCTGACATTGAAGAGAAAGAAAACAGAGACTCTTTGAGTTTGCGAACAAGT

AGCTCTTATACAAGAAGAAAATGGGAAGATGATCTTAAAAAAAACAGCTCAATTAATGAA

GGATCAACTTATCATAAAAGATCATACCTCACTCCTGTTAGAGATGAAGAATCTGAATCC

CAAAGAAAAGCAAGATCTAGACAAGCAAGACAGTCTAGAAGATCAACACAGGGGGTGACA

TTGACTGATCTTCAGGAAGCTGAAAAAACTATAGGAAGAAGTCGTTCTACCAGAACCAGA

GAGCAAGAAAATGAAGAAAAAGAAAAGGAGGAAAAAGAAAAACAGGATAAGGAGAAACAA

GAAGAAAAGAAGGAGTCAGAAACATCTAGAGAAGATGAATATAAGCAAAAATACTCTAGA

ACATATGATGAGACTTTTCCACGTTATAGACCAGTGTCAACTTCAAGTTCAACCACTGCA

TCCTCTGCCCTTCCTACAATGACCAGTTCACTCTATGCTTCAAGTCAACTAAACAGGCCA

AATAGTCTTGTAGGTATAACTTCTGCTTACTCCAGAGGATTAACTAAAGAAAATGAAAGA

GAGGGAGAAAAAAAAGAAGAGGAGAAGGAAGGTGAAGATAAATCACAGCCTAAATCCATC

AGAGAACGACGGAGACCAAGAGAGAAAAGAAGATCTACGGGAGTGTCTTTTTGGACACAA

GATAGTGATGAAAATGAACAAGAACAACAATCAGATACAGAAGAAGGATCCAATAAAAAA

GAAACTCAGACGGATTCCATTTCTAGATATGAAACCAGTTCCACTTCAGCTAGTGATCGG

TATGATTCCTTGCTGGGTCGCTCCGGATCGTATAGTTACTTAGAAGAAAGAAAACCATAT

AGTAGCAGGCTAGAAAAGGACGACTCAACTGACTTTAAAAAGCTTTATGAACAAATTTTA

GCTGAAAACGAAAAGTTGAAGGCACAGCTACATGATACAAATATGGAATTAACAGATCTT

AAATTGCAGTTGGAAAAGGCCACCCAGAGACAAGAAAGATTTGCTGATAGGTCACTATTG

GAAATGGAAAAAAGGGAACGAAGAGCTCTAGAAAGAAGAATATCTGAAATGGAAGAAGAA

CTCAAAATGTTACCAGACCTAAAAGCAGACAACCAGAGACTAAAGGATGAAAATGGGGCC

TTGATCAGAGTTATAAGCAAACTTTCCAAA

>CL269.Contig6_A-W 1 2217 PREDICTED: protein phosphatase 1 regulatory subunit 12A-like isoform 3 [Cavia porcellus]

CCTCCCGTGGTGAAGCGCCAGAAGACCAAGGTGAAGTTCGACGATGGCGCCGTCTTCCTG

GCTGCTTGCTCCAGCGGCGACACGGACGAGGTTCTCAAGCTGCTGCACCGCGGCGCCGAC

ATCAATTACGCCAATGTGGACGGACTTACTGCCCTCCATCAGGCCTGCATTGATGACAAT

GTTGATATGGTGAAGTTTCTGGTAGAAAATGGAGCAAATATCAATCAACCTGATAATGAA

GGCTGGATACCACTACATGCAGCTGCTTCCTGTGGATATCTTGATATTGCAGAGTTTTTG

ATTGGTCAGGGAGCACATGTTGGTGCTGTCAACAGTGAAGGAGATACACCTTTAGATATT

GCAGAGGAGGAAGCAATGGAAGAACTACTTCAAAATGAAGTTAATCGGCAAGGGGTTGAT

ATAGAGGCAGCTCGAAAGGAAGAAGAACGAATAATGCTTAGAGATGCAAGGCAGTGGTTA

AATAGTGGTCATATAAATGATGTACGGCATGCAAAATCTGGAGGTACAGCGCTTCATGTT

GCAGCCGCTAAGGGCTATACAGAAGTTTTAAAACTTTTAATACAGGCAGGCTATGATGTT

AATATTAAAGATTATGATGGCTGGACACCTCTTCATGCTGCAGCTCACTGGGGTAAAGAA

GAAGCATGTCGAATTTTAGTGGACAATCTGTGTGATATGGAGATGGTCAACAAAGTGGGC

CAGACAGCCTTTGATGTAGCAGATGAAGACATTTTAGGATATTTAGAAGAGTTACAAAAG

AAACAAAATCTGCTCCATAGTGAAAAACGGGATAAGAAATCTCCACTAATAGAATCAACA

GCAAATATGGAAAACAATCAGTCACAGAAGACTTTTAAAAACAAAGAGACTTTGATTATT

GAGCCAGAGAAAAATGCATCCCGTATTGAATCTCTGGAACAGGAAAAGGTTGATGAAGAA

GAAGAAGGAAAGAAAGATGAATCTAGTTGTTCCAGTGAAGAAGATGAAGAAGATGACTCA

GAATCAGAAGCTGAAACAGATAAGACAAAACCTGTGACTTCTGTAACTACTGCCAACACT

TCTAGTACACAAGCAGCTCCTATAGCTGTTACAACACCTACTCTGTCATCAAGTCAGGCA

ACACCCACATCACCTATTAAAAAGTTTCCAACTTCAACTACAAAAATTTCTCCCAAAGAA

GAAGAGAGAAAAGATGAATCTCCTGCATCTTGGAGGTTAGGACTTAGAAAGACTGGCAGT

TATGGTGCACTTGCTGAAATCACAGCATCTAAAGAGACTCAGAAAGAAAAAGACACTGCA

GGTGTTATACGTTCAGCTTCAAGTCCCAGACTTTCCTCCTCTTTGGATAATAAAGAAAAG

GAGAGAGAGAGTAAAGGAGCAAGGCTTGCATATGTTGCACCTACTATACCAAGACGTCTA

GCTAGCACATCTGACATTGAAGAGAAAGAAAACAGAGACTCTTTGAGTTTGCGAACAAGT

AGCTCTTATACAAGAAGAAAATGGGAAGATGATCTTAAAAAAAACAGCTCAATTAATGAA

GGATCAACTTATCATAAAAGTTGCTCCTTTGGTAGAAGACAAGATGATTTGATTAGTTCT

AGTGTTCCAAGCACCACATCAACACCAACAGTTACCTCTGCAGCTGGGCTTCAGAAAAGC

CTGCTTTCCAGCACAAGCACTACTACAAAGATTACAACGGGTTCTTCCTCAGCAGGCACA

CAAAGCAGTACCTCAAATCGTTTGTGGGCTGATGATAGTACTGAAAAAGAAAAGGACAGT

GTTCCTACGGCAGTGACCATTCCTGTTGCTCCAACTGTTGTAAATGCTGCAGCTTCTACC

ACAACCCTGACTACAACTACTGCTGGCACTGTCTCCTCCACATCAGAGGTCAGGGAGAGA

CGCAGATCATACCTCACTCCTGTTAGAGATGAAGAATCTGAATCCCAAAGAAAAGCAAGA

TCTAGACAAGCAAGACAGTCTAGAAGATCAACACAGGGGGTGACATTGACTGATCTTCAG

GAAGCTGAAAAAACTATAGGAAGAAGTCGTTCTACCAGAACCAGAGAGCAAGAAAATGAA

GAAAAAGAAAAGGAGGAAAAAGAAAAACAGGATAAGGAGAAACAAGAAGAAAAGAAGGAG

TCAGAAACATCTAGAGAAGATGAATATAAGCAAAAATACTCTAGAACATATGATGAG

>CL269.Contig7_A-W 1 2040 protein phosphatase 1, regulatory (inhibitor) subunit 12A, isoform CRA_e [Rattus norvegicus]

CCTCCCGTGGTGAAGCGCCAGAAGACCAAGGTGAAGTTCGACGATGGCGCCGTCTTCCTG

GCTGCTTGCTCCAGCGGCGACACGGACGAGGTTCTCAAGCTGCTGCACCGCGGCGCCGAC

ATCAATTACGCCAATGTGGACGGACTTACTGCCCTCCATCAGGCCTGCATTGATGACAAT

GTTGATATGGTGAAGTTTCTGGTAGAAAATGGAGCAAATATCAATCAACCTGATAATGAA

GGCTGGATACCACTACATGCAGCTGCTTCCTGTGGATATCTTGATATTGCAGAGTTTTTG

ATTGGTCAGGGAGCACATGTTGGTGCTGTCAACAGTGAAGGAGATACACCTTTAGATATT

GCAGAGGAGGAAGCAATGGAAGAACTACTTCAAAATGAAGTTAATCGGCAAGGGGTTGAT

ATAGAGGCAGCTCGAAAGGAAGAAGAACGAATAATGCTTAGAGATGCAAGGCAGTGGTTA

AATAGTGGTCATATAAATGATGTACGGCATGCAAAATCTGGAGGTACAGCGCTTCATGTT

GCAGCCGCTAAGGGCTATACAGAAGTTTTAAAACTTTTAATACAGGCAGGCTATGATGTT

AATATTAAAGATTATGATGGCTGGACACCTCTTCATGCTGCAGCTCACTGGGGTAAAGAA

GAAGCATGTCGAATTTTAGTGGACAATCTGTGTGATATGGAGATGGTCAACAAAGTGGGC

CAGACAGCCTTTGATGTAGCAGATGAAGACATTTTAGGATATTTAGAAGAGTTACAAAAG

AAACAAAATCTGCTCCATAGTGAAAAACGGGATAAGAAATCTCCACTAATAGAATCAACA

GCAAATATGGAAAACAATCAGTCACAGAAGACTTTTAAAAACAAAGAGACTTTGATTATT

GAGCCAGAGAAAAATGCATCCCGTATTGAATCTCTGGAACAGGAAAAGGTTGATGAAGAA

GAAGAAGGAAAGAAAGATGAATCTAGTTGTTCCAGTGAAGAAGATGAAGAAGATGACTCA

GAATCAGAAGCTGAAACAGATAAGACAAAACCTGTGACTTCTGTAACTACTGCCAACACT

TCTAGTACACAAGCAGCTCCTATAGCTGTTACAACACCTACTCTGTCATCAAGTCAGGCA

ACACCCACATCACCTATTAAAAAGTTTCCAACTTCAACTACAAAAATTTCTCCCAAAGAA

GAAGAGAGAAAAGATGAATCTCCTGCATCTTGGAGGTTAGGACTTAGAAAGACTGGCAGT

TATGGTGCACTTGCTGAAATCACAGCATCTAAAGAGACTCAGAAAGAAAAAGACACTGCA

GGTGTTATACGTTCAGCTTCAAGTCCCAGACTTTCCTCCTCTTTGGATAATAAAGAAAAG

GAGAGAGAGAGTAAAGGAGCAAGGCTTGCATATGTTGCACCTACTATACCAAGACGTCTA

GCTAGCACATCTGACATTGAAGAGAAAGAAAACAGAGACTCTTTGAGTTTGCGAACAAGT

AGCTCTTATACAAGAAGAAAATGGGAAGATGATCTTAAAAAAAACAGCTCAATTAATGAA

GGATCAACTTATCATAAAAGTTGCTCCTTTGGTAGAAGACAAGATGATTTGATTAGTTCT

AGTGTTCCAAGCACCACATCAACACCAACAGTTACCTCTGCAGCTGGGCTTCAGAAAAGC

CTGCTTTCCAGCACAAGCACTACTACAAAGATTACAACGGGTTCTTCCTCAGCAGGCACA

CAAAGCAGATCATACCTCACTCCTGTTAGAGATGAAGAATCTGAATCCCAAAGAAAAGCA

AGATCTAGACAAGCAAGACAGTCTAGAAGATCAACACAGGGGGTGACATTGACTGATCTT

CAGGAAGCTGAAAAAACTATAGGAAGAAGTCGTTCTACCAGAACCAGAGAGCAAGAAAAT

GAAGAAAAAGAAAAGGAGGAAAAAGAAAAACAGGATAAGGAGAAACAAGAAGAAAAGAAG

GAGTCAGAAACATCTAGAGAAGATGAATATAAGCAAAAATACTCTAGAACATATGATGAG

>CL269.Contig8_A-W 1 1872 protein phosphatase 1, regulatory (inhibitor) subunit 12A, isoform CRA_e [Rattus norvegicus]

CCTCCCGTGGTGAAGCGCCAGAAGACCAAGGTGAAGTTCGACGATGGCGCCGTCTTCCTG

GCTGCTTGCTCCAGCGGCGACACGGACGAGGTTCTCAAGCTGCTGCACCGCGGCGCCGAC

ATCAATTACGCCAATGTGGACGGACTTACTGCCCTCCATCAGGCCTGCATTGATGACAAT

GTTGATATGGTGAAGTTTCTGGTAGAAAATGGAGCAAATATCAATCAACCTGATAATGAA

GGCTGGATACCACTACATGCAGCTGCTTCCTGTGGATATCTTGATATTGCAGAGTTTTTG

ATTGGTCAGGGAGCACATGTTGGTGCTGTCAACAGTGAAGGAGATACACCTTTAGATATT

GCAGAGGAGGAAGCAATGGAAGAACTACTTCAAAATGAAGTTAATCGGCAAGGGGTTGAT

ATAGAGGCAGCTCGAAAGGAAGAAGAACGAATAATGCTTAGAGATGCAAGGCAGTGGTTA

AATAGTGGTCATATAAATGATGTACGGCATGCAAAATCTGGAGGTACAGCGCTTCATGTT

GCAGCCGCTAAGGGCTATACAGAAGTTTTAAAACTTTTAATACAGGCAGGCTATGATGTT

AATATTAAAGATTATGATGGCTGGACACCTCTTCATGCTGCAGCTCACTGGGGTAAAGAA

GAAGCATGTCGAATTTTAGTGGACAATCTGTGTGATATGGAGATGGTCAACAAAGTGGGC

CAGACAGCCTTTGATGTAGCAGATGAAGACATTTTAGGATATTTAGAAGAGTTACAAAAG

AAACAAAATCTGCTCCATAGTGAAAAACGGGATAAGAAATCTCCACTAATAGAATCAACA

GCAAATATGGAAAACAATCAGTCACAGAAGACTTTTAAAAACAAAGAGACTTTGATTATT

GAGCCAGAGAAAAATGCATCCCGTATTGAATCTCTGGAACAGGAAAAGGTTGATGAAGAA

GAAGAAGGAAAGAAAGATGAATCTAGTTGTTCCAGTGAAGAAGATGAAGAAGATGACTCA

GAATCAGAAGCTGAAACAGATAAGACAAAACCTGTGACTTCTGTAACTACTGCCAACACT

TCTAGTACACAAGCAGCTCCTATAGCTGTTACAACACCTACTCTGTCATCAAGTCAGGCA

ACACCCACATCACCTATTAAAAAGTTTCCAACTTCAACTACAAAAATTTCTCCCAAAGAA

GAAGAGAGAAAAGATGAATCTCCTGCATCTTGGAGGTTAGGACTTAGAAAGACTGGCAGT

TATGGTGCACTTGCTGAAATCACAGCATCTAAAGAGACTCAGAAAGAAAAAGACACTGCA

GGTGTTATACGTTCAGCTTCAAGTCCCAGACTTTCCTCCTCTTTGGATAATAAAGAAAAG

GAGAGAGAGAGTAAAGGAGCAAGGCTTGCATATGTTGCACCTACTATACCAAGACGTCTA

GCTAGCACATCTGACATTGAAGAGAAAGAAAACAGAGACTCTTTGAGTTTGCGAACAAGT

AGCTCTTATACAAGAAGAAAATGGGAAGATGATCTTAAAAAAAACAGCTCAATTAATGAA

GGATCAACTTATCATAAAAGATCATACCTCACTCCTGTTAGAGATGAAGAATCTGAATCC

CAAAGAAAAGCAAGATCTAGACAAGCAAGACAGTCTAGAAGATCAACACAGGGGGTGACA

TTGACTGATCTTCAGGAAGCTGAAAAAACTATAGGAAGAAGTCGTTCTACCAGAACCAGA

GAGCAAGAAAATGAAGAAAAAGAAAAGGAGGAAAAAGAAAAACAGGATAAGGAGAAACAA

GAAGAAAAGAAGGAGTCAGAAACATCTAGAGAAGATGAATATAAGCAAAAATACTCTAGA

ACATATGATGAG

>CL269.Contig9_A-W 109 1200 PREDICTED: protein phosphatase 1 regulatory subunit 12A isoform 4 [Pan paniscus]

AGATCATACCTCACTCCTGTTAGAGATGAAGAATCTGAATCCCAAAGAAAAGCAAGATCT

AGACAAGCAAGACAGTCTAGAAGATCAACACAGGGGGTGACATTGACTGATCTTCAGGAA

GCTGAAAAAACTATAGGAAGAAGTCGTTCTACCAGAACCAGAGAGCAAGAAAATGAAGAA

AAAGAAAAGGAGGAAAAAGAAAAACAGGATAAGGAGAAACAAGAAGAAAAGAAGGAGTCA

GAAACATCTAGAGAAGATGAATATAAGCAAAAATACTCTAGAACATATGATGAGACTTTT

CCACGTTATAGACCAGTGTCAACTTCAAGTTCAACCACTGCATCCTCTGCCCTTCCTACA

ATGACCAGTTCACTCTATGCTTCAAGTCAACTAAACAGGCCAAATAGTCTTGTAGGTATA

ACTTCTGCTTACTCCAGAGGATTAACTAAAGAAAATGAAAGAGAGGGAGAAAAAAAAGAA

GAGGAGAAGGAAGGTGAAGATAAATCACAGCCTAAATCCATCAGAGAACGACGGAGACCA

AGAGAGAAAAGAAGATCTACGGGAGTGTCTTTTTGGACACAAGATAGTGATGAAAATGAA

CAAGAACAACAATCAGATACAGAAGAAGGATCCAATAAAAAAGAAACTCAGACGGATTCC

ATTTCTAGATATGAAACCAGTTCCACTTCAGCTAGTGATCGGTATGATTCCTTGCTGGGT

CGCTCCGGATCGTATAGTTACTTAGAAGAAAGAAAACCATATAGTAGCAGGCTAGAAAAG

GACGACTCAACTGACTTTAAAAAGCTTTATGAACAAATTTTAGCTGAAAACGAAAAGTTG

AAGGCACAGCTACATGATACAAATATGGAATTAACAGATCTTAAATTGCAGTTGGAAAAG

GCCACCCAGAGACAAGAAAGATTTGCTGATAGGTCACTATTGGAAATGGAAAAAAGGGAA

CGAAGAGCTCTAGAAAGAAGAATATCTGAAATGGAAGAAGAACTCAAAATGTTACCAGAC

CTAAAAGCAGACAACCAGAGACTAAAGGATGAAAATGGGGCCTTGATCAGAGTTATAAGC

AAACTTTCCAAA

>CL269.Contig10_A-W 103 933 PREDICTED: protein phosphatase 1 regulatory subunit 12A isoform 4 [Pan paniscus]

TTTCAAGTGGATTTTAATTTTAACGTTTCACAGACTTTTCCACGTTATAGACCAGTGTCA

ACTTCAAGTTCAACCACTGCATCCTCTGCCCTTCCTACAATGACCAGTTCACTCTATGCT

TCAAGTCAACTAAACAGGCCAAATAGTCTTGTAGGTATAACTTCTGCTTACTCCAGAGGA

TTAACTAAAGAAAATGAAAGAGAGGGAGAAAAAAAAGAAGAGGAGAAGGAAGGTGAAGAT

AAATCACAGCCTAAATCCATCAGAGAACGACGGAGACCAAGAGAGAAAAGAAGATCTACG

GGAGTGTCTTTTTGGACACAAGATAGTGATGAAAATGAACAAGAACAACAATCAGATACA

GAAGAAGGATCCAATAAAAAAGAAACTCAGACGGATTCCATTTCTAGATATGAAACCAGT

TCCACTTCAGCTAGTGATCGGTATGATTCCTTGCTGGGTCGCTCCGGATCGTATAGTTAC

TTAGAAGAAAGAAAACCATATAGTAGCAGGCTAGAAAAGGACGACTCAACTGACTTTAAA

AAGCTTTATGAACAAATTTTAGCTGAAAACGAAAAGTTGAAGGCACAGCTACATGATACA

AATATGGAATTAACAGATCTTAAATTGCAGTTGGAAAAGGCCACCCAGAGACAAGAAAGA

TTTGCTGATAGGTCACTATTGGAAATGGAAAAAAGGGAACGAAGAGCTCTAGAAAGAAGA

ATATCTGAAATGGAAGAAGAACTCAAAATGTTACCAGACCTAAAAGCAGACAACCAGAGA

CTAAAGGATGAAAATGGGGCCTTGATCAGAGTTATAAGCAAACTTTCCAAA

>CL271.Contig1_A-W 180 557 minus strand similar to FLJ00364 protein (predicted) [Rattus norvegicus]

GGTCTCCAGAGTTCTCGGAGTAACCCTTCCATCCAAGCCACGCTCAATAAGATCGCGCTT

TCCTCTTCCCTAAATAGCCACCCACAGACATCAGTTGCCAACGCGTCTGCTCTTCACCCT

TCACTCCGATTATTTTCCCTTAGCAACCCATCTCTCCCCACCACAAATCTAAGTGGCCCT

TCTCGGCGCAGGCAGCCTCCCGTCAGCCCTCTCACACTTTCTCCTGGCCCTGAAGCACAT

CAAGGTTTCAGCAGACAGCTCTCTTCAACCAGCCCACTGAACCCATATCCTGCCTCCCAG

ATGGTGTCCTCAGAGCGAAGCCCACTTTCCTTCTTGCCCACAGAAGCTCAAGCTCAAGTA

TCCCCACCACCCCCTTAC

>CL271.Contig2_A-W 2 499 minus strand similar to FLJ00364 protein (predicted) [Rattus norvegicus]

TCGGTGCCCCTGCCTGCCTCCCTGGATACCAGTGACAACCTCTTTGGTAGTATGAGTGTG

GGGAATAGTGTGGGAAACCTCCCAGCTGCTATGACTCACCTGGGGATAAGAAGCTCCTCT

GGTCTCCAGAGTTCTCGGAGTAACCCTTCCATCCAAGCCACGCTCAATAAGATCGCGCTT

TCCTCTTCCCTAAATAGCCACCCACAGACATCAGTTGCCAACGCGTCTGCTCTTCACCCT

TCACTCCGATTATTTTCCCTTAGCAACCCATCTCTCCCCACCACAAATCTAAGTGGCCCT

TCTCGGCGCAGGCAGCCTCCCGTCAGCCCTCTCACACTTTCTCCTGGCCCTGAAGCACAT

CAAGGTTTCAGCAGACAGCTCTCTTCAACCAGCCCACTGAACCCATATCCTGCCTCCCAG

ATGGTGTCCTCAGAGCGAAGCCCACTTTCCTTCTTGCCCACAGAAGCTCAAGCTCAAGTA

TCCCCACCACCCCCTTAC

>CL272.Contig1_A-W 3 1187 minus strand PHD finger protein 10 isoform a [Homo sapiens] >gi|296439276|sp|Q8WUB8.3|PHF10_HUMAN RecName: Full=PHD finger protein 10; AltName: Full=BRG1-associated factor 45a; Short=BAF45a; AltName: Full=XAP135 >gi|119567827|gb|EAW47442.1| PHD finger protein 10, isoform CRA_a [Homo sapiens]

CGGCCGTGCGACAGCGACCCGGCCACTCCCGGAGCGCAGTCCCCGAAGGAAGATAATGAA

GATAATTCCAATGATGGGACCCAACCATCCAAAAGGAGGCGAATGGGCTCGGGAGACAGC

TCCAGAAGCTGCGAGACTTCGAGCCAGGACCTTGGTTTCAGTTACTATCCAGCTGAAAAT

CTGATAGAGTACAAATGGCCGCCTGACGAAACCGGAGAATATTATATGCTTCAGGAGCAA

GTCAGCGAGTACCTGGGTGTGACCTCCTTCAAGCGGAAATATCCAGATTTAGAGAGACGA

GATTTGTCTCACAAGGAGAAGCTGTACCTGAGGGAGCTGAATGTCATCACAGAGACTCAG

TGCACTTTAGGTTTAACAGCATTGCGCAGTGATGAAGTGATTGACTTAATGATAAAAGAG

TATCCAGCCAAACATGCTGAGTACTCTGTCATTCTACAGGAGAAGGAACGTCAGCGAATT

ACAGATCATTACAAAGAGTATTCTCAAATGCAACAACAGAACACTCAGAAAGTTGAAGCC

AGCAAAGTGCCTGAGTACATTAAGAAGGCTGCTAAAAAAGCAGCTGAGTTTAACAGCAAC

CTGAACCGGGAACGCATGGAAGAAAGAAGAGCTTACTTTGACCTGCAGACCCACGTCATC

CAGGTTCCACAAGGGAAGTACAGAGTGTTGCCAACAGAGCGGACAAAGGTCAGTTCTTAC

CCCGTGGCGCTCATCCCCGGACAGTTCCAGGAGTATTACAAGAGGTACTCACCAGACGAG

CTTCGGTATTTGCCACTGAACACGGCGCTGTACGAGCCCCCCCTGGACCCCGAGCTCCCT

GCCCTGGATAGTGATGGCGACTCGGATGATGGTGAAGATGGTCGAGGTGATGAGAAACGG

AAAAATAAAGGCACCTCGGACAGCTCCTCGGGCAACGTGTCTGAAGGGGAGAGTCCCCCT

GACAGCCAGGAGGACGCCTTCCAGGGAAGACAGAAGTCAAGGGACAAGGCTGCCACTCCG

AGAAAAGACGGCTCCAAGCGTTCTGTTCTGTCCAAGTCAGTTCCTGGGTACAAGCCAAAG

GTCATTCCAAATGCTCTATGTGGGATTTGTCTGAAGGGTAAGGAGTCCAACAAGAAAGGA

AAGGCTGAGTCACTTATACACTGCTCCCAGTGTGATAACAGTGGT

>CL272.Contig2_A-W 1 705 minus strand PHD finger protein 10 isoform a [Homo sapiens] >gi|296439276|sp|Q8WUB8.3|PHF10_HUMAN RecName: Full=PHD finger protein 10; AltName: Full=BRG1-associated factor 45a; Short=BAF45a; AltName: Full=XAP135 >gi|119567827|gb|EAW47442.1| PHD finger protein 10, isoform CRA_a [Homo sapiens]

TGCGACAGCGACCCGGCCACTCCCGGAGCGCAGTCCCCGAAGGAAGATAATGAAGATAAT

TCCAATGATGGGACCCAACCATCCAAAAGGAGGCGAATGGGCTCGGGAGACAGCTCCAGA

AGCTGCGAGACTTCGAGCCAGGACCTTGGTTTCAGTTACTATCCAGCTGAAAATCTGATA

GAGTACAAATGGCCGCCTGACGAAACCGGAGAATATTATATGCTTCAGGAGCAAGTCAGC

GAGTACCTGGGTGTGACCTCCTTCAAGCGGAAATATCCAGATTTAGAGAGACGAGATTTG

TCTCACAAGGAGAAGCTGTACCTGAGGGAGCTGAATGTCATCACAGAGACTCAGTGCACT

TTAGGTTTAACAGCATTGCGCAGTGATGAAGTGATTGACTTAATGATAAAAGAGTATCCA

GCCAAACATGCTGAGTACTCTGTCATTCTACAGGAGAAGGAACGTCAGCGAATTACAGAT

CATTACAAAGAGTATTCTCAAATGCAACAACAGAACACTCAGAAAGTTGAAGCCAGCAAA

GTGCCTGAGTACATTAAGAAGGCTGCTAAAAAAGCAGCTGAGTTTAACAGCAACCTGAAC

CGGGAACGCATGGAAGAAAGAAGAGCTTACTTTGACCTGCAGACCCACGTCATCCAGGTT

CCACAAGGGAAGTACAGAGTGTTGCCAACAGAGCGGACAAAGGTA

>CL272.Contig3_A-W 1 1068 minus strand PREDICTED: PHD finger protein 10 [Papio anubis]

TGCGACAGCGACCCGGCCACTCCCGGAGCGCAGTCCCCGAAGGAAGATAATGAAGATAAT

TCCAATGATGGGACCCAACCATCCAAAAGGAGGCGAATGGGCTCGGGAGACAGCTCCAGA

AGCTGCGAGACTTCGAGCCAGGACCTTGGTTTCAGTTACTATCCAGCTGAAAATCTGATA

GAGTACAAATGGCCGCCTGACGAAACCGGAGAATATTATATGCTTCAGGAGCAAGTCAGC

GAGTACCTGGGTGTGACCTCCTTCAAGCGGAAATATCCAGATTTAGAGAGACGAGATTTG

TCTCACAAGGAGAAGCTGTACCTGAGGGAGCTGAATGTCATCACAGAGACTCAGTGCACT

TTAGGTTTAACAGCATTGCGCAGTGATGAAGTGATTGACTTAATGATAAAAGAGTATCCA

GCCAAACATGCTGAGTACTCTGTCATTCTACAGGAGAAGGAACGTCAGCGAATTACAGAT

CATTACAAAGAGTATTCTCAAATGCAACAACAGAACACTCAGAAAGTTGAAGCCAGCAAA

GTGCCTGAGTACATTAAGAAGGCTGCTAAAAAAGCAGCTGAGTTTAACAGCAACCTGAAC

CGGGAACGCATGGAAGAAAGAAGAGCTTACTTTGACCTGCAGACCCACGTCATCCAGGTT

CCACAAGGGAAGTACAGAGTGTTGCCAACAGAGCGGACAAAGGTCAGTTCTTACCCCGTG

GCGCTCATCCCCGGACAGTTCCAGGAGTATTACAAGAGGTACTCACCAGACGAGCTTCGG

TATTTGCCACTGAACACGGCGCTGTACGAGCCCCCCCTGGACCCCGAGCTCCCTGCCCTG

GATAGTGATGGCGACTCGGATGATGGTGAAGATGGTCGAGGTGATGAGAAACGGAAAAAT

AAAGGCACCTCGGACAGCTCCTCGGGCAACGTGTCTGAAGGGGAGAGTCCCCCTGACAGC

CAGGAGGACGCCTTCCAGGGAAGACAGAAGTCAAGGGACAAGGCTGCCACTCCGAGAAAA

GACGGCTCCAAGCGTTCTGTTCTGTCCAAGTCAGTTCCTGGGTACAAG

>CL272.Contig4_A-W 686 1012 minus strand PREDICTED: PHD finger protein 10 [Papio anubis]

CAACAGAGCGGACAAAGGTACTCACCAGACGAGCTTCGGTATTTGCCACTGAACACGGCG

CTGTACGAGCCCCCCCTGGACCCCGAGCTCCCTGCCCTGGATAGTGATGGCGACTCGGAT

GATGGTGAAGATGGTCGAGGTGATGAGAAACGGAAAAATAAAGGCACCTCGGACAGCTCC

TCGGGCAACGTGTCTGAAGGGGAGAGTCCCCCTGACAGCCAGGAGGACGCCTTCCAGGGA

AGACAGAAGTCAAGGGACAAGGCTGCCACTCCGAGAAAAGACGGCTCCAAGCGTTCTGTT

CTGTCCAAGTCAGTTCCTGGGTACAAG

>CL272.Contig5_A-W 686 1123 minus strand PHD finger protein 10 isoform a [Homo sapiens] >gi|296439276|sp|Q8WUB8.3|PHF10_HUMAN RecName: Full=PHD finger protein 10; AltName: Full=BRG1-associated factor 45a; Short=BAF45a; AltName: Full=XAP135 >gi|119567827|gb|EAW47442.1| PHD finger protein 10, isoform CRA_a [Homo sapiens]

CAACAGAGCGGACAAAGGTACTCACCAGACGAGCTTCGGTATTTGCCACTGAACACGGCG

CTGTACGAGCCCCCCCTGGACCCCGAGCTCCCTGCCCTGGATAGTGATGGCGACTCGGAT

GATGGTGAAGATGGTCGAGGTGATGAGAAACGGAAAAATAAAGGCACCTCGGACAGCTCC

TCGGGCAACGTGTCTGAAGGGGAGAGTCCCCCTGACAGCCAGGAGGACGCCTTCCAGGGA

AGACAGAAGTCAAGGGACAAGGCTGCCACTCCGAGAAAAGACGGCTCCAAGCGTTCTGTT

CTGTCCAAGTCAGTTCCTGGGTACAAGCCAAAGGTCATTCCAAATGCTCTATGTGGGATT

TGTCTGAAGGGTAAGGAGTCCAACAAGAAAGGAAAGGCTGAGTCACTTATACACTGCTCC

CAGTGTGATAACAGTGGT

>CL272.Contig6_A-W 1 1449 minus strand PHD finger protein 10 isoform a [Homo sapiens] >gi|296439276|sp|Q8WUB8.3|PHF10_HUMAN RecName: Full=PHD finger protein 10; AltName: Full=BRG1-associated factor 45a; Short=BAF45a; AltName: Full=XAP135 >gi|119567827|gb|EAW47442.1| PHD finger protein 10, isoform CRA_a [Homo sapiens]

TGCGACAGCGACCCGGCCACTCCCGGAGCGCAGTCCCCGAAGGAAGATAATGAAGATAAT

TCCAATGATGGGACCCAACCATCCAAAAGGAGGCGAATGGGCTCGGGAGACAGCTCCAGA

AGCTGCGAGACTTCGAGCCAGGACCTTGGTTTCAGTTACTATCCAGCTGAAAATCTGATA

GAGTACAAATGGCCGCCTGACGAAACCGGAGAATATTATATGCTTCAGGAGCAAGTCAGC

GAGTACCTGGGTGTGACCTCCTTCAAGCGGAAATATCCAGATTTAGAGAGACGAGATTTG

TCTCACAAGGAGAAGCTGTACCTGAGGGAGCTGAATGTCATCACAGAGACTCAGTGCACT

TTAGGTTTAACAGCATTGCGCAGTGATGAAGTGATTGACTTAATGATAAAAGAGTATCCA

GCCAAACATGCTGAGTACTCTGTCATTCTACAGGAGAAGGAACGTCAGCGAATTACAGAT

CATTACAAAGAGTATTCTCAAATGCAACAACAGAACACTCAGAAAGTTGAAGCCAGCAAA

GTGCCTGAGTACATTAAGAAGGCTGCTAAAAAAGCAGCTGAGTTTAACAGCAACCTGAAC

CGGGAACGCATGGAAGAAAGAAGAGCTTACTTTGACCTGCAGACCCACGTCATCCAGGTT

CCACAAGGGAAGTACAGAGTGTTGCCAACAGAGCGGACAAAGGTCAGTTCTTACCCCGTG

GCGCTCATCCCCGGACAGTTCCAGGAGTATTACAAGAGGTACTCACCAGACGAGCTTCGG

TATTTGCCACTGAACACGGCGCTGTACGAGCCCCCCCTGGACCCCGAGCTCCCTGCCCTG

GATAGTGATGGCGACTCGGATGATGGTGAAGATGGTCGAGGTGATGAGAAACGGAAAAAT

AAAGGCACCTCGGACAGCTCCTCGGGCAACGTGTCTGAAGGGGAGAGTCCCCCTGACAGC

CAGGAGGACGCCTTCCAGGGAAGACAGAAGTCAAGGGACAAGGCTGCCACTCCGAGAAAA

GACGGCTCCAAGCGTTCTGTTCTGTCCAAGTCAGTTCCTGGGTACAAGCCAAAGGTCATT

CCAAATGCTCTATGTGGGATTTGTCTGAAGGGTAAGGAGTCCAACAAGAAAGGAAAGGCT

GAGTCACTTATACACTGCTCCCAGTGTGATAACAGTGGCCATCCTTCTTGCCTGGACATG

ACCATGGAGCTTGTTTCTATCATTAAGACCTACCCATGGCAGTGTATGGAGTGTAAGACA

TGCATTGTATGTGGACAGCCCCACCATGAAGAAGAAATGATGTTCTGTGATGTGTGTGAC

AGAGGTTATCATACTTTTTGTGTGGGCCTTGGTGCTATTCCATCAGGTCGCTGGATTTGT

GACTGTTGTCAGCGAGCCCCCCCAACACCCAGGAAAGTGGGCAGAAGGGGGAAAAACAGC

AAAGAGGGA

>CL272.Contig7_A-W 286 672 PHD finger protein 10 [Cricetulus griseus]

TTCCAGCCAAAGGTCATTCCAAATGCTCTATGTGGGATTTGTCTGAAGGGTAAGGAGTCC

AACAAGAAAGGAAAGGCTGAGTCACTTATACACTGCTCCCAGTGTGATAACAGTGGCCAT

CCTTCTTGCCTGGACATGACCATGGAGCTTGTTTCTATCATTAAGACCTACCCATGGCAG

TGTATGGAGTGTAAGACATGCATTGTATGTGGACAGCCCCACCACGAAGAAGAAATGATG

TTCTGTGATGTGTGTGACAGAGGTTATCATACTTTTTGTGTGGGCCTTGGTGCTATTCCA

TCAGGTCGCTGGATTTGTGACTGTTGTCAGCGAGCCCCCCCAACACCCAGGAAAGTGGGC

AGAAGGGGGAAAAACAGCAAAGAGGGA

>CL272.Contig8_A-W 146 838 PREDICTED: PHD finger protein 10 [Pan troglodytes]

AGGTACTCACCAGACGAGCTTCGGTATTTGCCACTGAACACGGCGCTGTACGAGCCCCCC

CTGGACCCCGAGCTCCCTGCCCTGGATAGTGATGGCGACTCGGATGATGGTGAAGATGGT

CGAGGTGATGAGAAACGGAAAAATAAAGGCACCTCGGACAGCTCCTCGGGCAACGTGTCT

GAAGGGGAGAGTCCCCCTGACAGCCAGGAGGACGCCTTCCAGGGAAGACAGAAGTCAAGG

GACAAGGCTGCCACTCCGAGAAAAGACGGCTCCAAGCGTTCTGTTCTGTCCAAGTCAGTT

CCTGGGTACAAGCCAAAGGTCATTCCAAATGCTCTATGTGGGATTTGTCTGAAGGGTAAG

GAGTCCAACAAGAAAGGAAAGGCTGAGTCACTTATACACTGCTCCCAGTGTGATAACAGT

GGCCATCCTTCTTGCCTGGACATGACCATGGAGCTTGTTTCTATCATTAAGACCTACCCA

TGGCAGTGTATGGAGTGTAAGACATGCATTGTATGTGGACAGCCCCACCATGAAGAAGAA

ATGATGTTCTGTGATGTGTGTGACAGAGGTTATCATACTTTTTGTGTGGGCCTTGGTGCT

ATTCCATCAGGTCGCTGGATTTGTGACTGTTGTCAGCGAGCCCCCCCAACACCCAGGAAA

GTGGGCAGAAGGGGGAAAAACAGCAAAGAGGGA

>CL272.Contig9_A-W 29 451 PREDICTED: PHD finger protein 10 [Pan troglodytes]

AGGTACTCACCAGACGAGCTTCGGTATTTGCCACTGAACACGGCGCTGTACGAGCCCCCC

CTGGACCCCGAGCTCCCTGCCCTGGATAGTGATGGCGACTCGGATGATGGTGAAGATGGT

CGAGGTGATGAGAAACGGAAAAATAAAGGCACCTCGGACAGCTCCTCGGGCAACGTGTCT

GAAGGGGAGAGTCCCCCTGACAGCCAGGAGGACGCCTTCCAGGGAAGACAGAAGTCAAGG

GACAAGGCTGCCACTCCGAGAAAAGACGGCTCCAAGCGTTCTGTTCTGTCCAAGTCAGTT

CCTGGGTACAAGCCAAAGGTCATTCCAAATGCTCTATGTGGGATTTGTCTGAAGGGTAAG

GAGTCCAACAAGAAAGGAAAGGCTGAGTCACTTATACACTGCTCCCAGTGTGATAACAGT

GGT

>CL272.Contig10_A-W 142 414 minus strand PHD finger protein 10 [Cricetulus griseus]

GGCCATCCTTCTTGCCTGGACATGACCATGGAGCTTGTTTCTATCATTAAGACCTACCCA

TGGCAGTGTATGGAGTGTAAGACATGCATTGTATGTGGACAGCCCCACCATGAAGAAGAA

ATGATGTTCTGTGATGTGTGTGACAGAGGTTATCATACTTTTTGTGTGGGCCTTGGTGCT

ATTCCATCAGGTCGCTGGATTTGTGACTGTTGTCAGCGAGCCCCCCCAACACCCAGGAAA

GTGGGCAGAAGGGGGAAAAACAGCAAAGAGGGA

>CL273.Contig1_A-W 1 120 minus strand PREDICTED: acid sphingomyelinase-like phosphodiesterase 3a [Papio anubis]

AGACAATACTACAATGAGAAATTGATAGATATTTTTAGAAGATATAGCAATATCATTGCA

GGACAATTCTATGGACATAATCACAAAGACAGCTTTATGGTTCTTTCAGATAAAAAAGGA

>CL273.Contig2_A-W 1 1047 minus strand PREDICTED: acid sphingomyelinase-like phosphodiesterase 3a-like isoform 1 [Macaca mulatta]

GATGTCCTGTGTGATTCTCCATATCAACTTATTTTGTCAGCATTTGATTTTATTAAGAAT

TCAGGACAAGAAGCATCTTTCATGATATGGACGGGGGATAGTCCACCTCATGTTCCAGTA

CCTGAGCTCTCAACAGACACTGTTATCAAGGTGATCTCTAACATGACAATGACCATCCAG

AGTCTCTTTCCAAATCTCCAAGTTTTCCCTGCCCTGGGCAATCATGACTACTGGCCACAG

GATCAGCTGCCTATAGTCACCAGTGAAGTGTACAATGCAGTGGCCAACCTCTGGAAACCA

TGGCTAGATGAAGAAGCTATTAGTACTTTAAGGAAAGGTGGTTTTTATTCACAGAAAGTC

ACAAGTAATCCAAACCTTAGGATCATCAGTCTAAACACAAACTTGTACTATGGTCCAAAT

GTCATGACCCTGAACAAGACTGACCCAGCAAGTCAATTTGAATGGCTAGAAGATACTCTG

AACAGCTCGCAGCAAAATAAGGAAAAGGTGTACATCATTGCACATATTCCAGTAGGATAT

CTGCCTTTTTCGAGTGGTATCACAGCAATGAGACAATACTACAATGAGAAATTGATAGAT

ATTTTTAGAAGATATAGCAATATCATTGCAGGACAATTCTATGGACATAATCACAAAGAC

AGCTTTATGGTTCTTTCAGATAAAAAAGGAAGTCCAGTAAATTCTTTGTTTGTGGCTCCT

GCTGTTACACCAGTAAAGGGTGTTTTAGAAAAAGAAACTAATAATCCTGGTGTCCGATTA

TTTCAGTATAAGCCTTGTGATTATACATTATTGGATATGTTGCAGTATTATCTGAATCTG

ACAGATGCAAATCTAAAGGGAGATGCCAATTGGAAGCTGGAATATATCCTAACTCAGACC

TATGACATTGAGGATTTGCAGCCAAAAAGTTTATATGGATTAGCTAAACAATTTGCAGTC

CCTGACAGTAAGCAGTTCATAAAATACTACAATTACTTCTTTGTGAATTATGACAGCAGT

ATAATTTGTGATAAGAAATGTAAGGCC

>CL274.Contig1_A-W 1412 1765 PREDICTED: mitogen-activated protein kinase kinase kinase 6 [Papio anubis]

AGACTTCGAGAGGTCCTGGCAGAGAAGGAACGGGAGTGCCAGGCCCTGATATACCAGACT

CTACACCAGATGAATGCAGAGGCCAGGACCTGTGCCCTCGTCTCAGAGCCCCAAGATGCC

CTCCCAAAGGACCAGGGCTTGGTGCAGTGGTTACAGGGGCTAAAGGTGGATTCAGGCACC

ATCCAAACGCTGCTGAACCACAGTTTCACCCTCCGTGCCCTGCTCACCTGTGCCACTCGA

GATGACCTCCTTTATACCGGCATCAGGGGAGGGATGGTATGCCGCATCTGGAGAGGCATC

TTGGCACAGCGAGCAGGATCTGCATCAGTTACCCCTGGACCCCGGGAGGCTGAA

>CL274.Contig2_A-W 3 1682 PREDICTED: mitogen-activated protein kinase kinase kinase 6 [Papio anubis]

AGGTCAGTATGGGGACCCCTGAAGGACAATGAGAGTACTATCAGTTTCTACACCCGCCAG

ATCTTGCAGGGACTCAGCTATCTGCATGACAATCGGATTGTGCACCGAGACATCAAGGGG

GACAATGTGCTCATCAACACCTTCAGTGGTCTGCTCAAAATTTCTGACTTTGGTACCTCC

AAACGACTGGCGGGCATCACACCCTGCACTGAGACCTTCACAGGAACCCTGCAGTATATG

GCCCCAGAAATCATTGACCAGGGCCCACGAGGATATGGGAAGGCAGCTGACATCTGGTCT

TTGGGCTGCACTGTAATTGAGATGGCCACAGGTCGCCCACCCTTCCATGAACTAGGGAGC

CCACAGGCTGCCATGTTTCAGGTGGGCATGTACAAGGTACACCCGCCAATGCCCAGTTCT

CTGTCAGCTGAGGCCCAAGCCTTCCTCCTCAGAACATTTGAGCCAGACCCCCATCTCCGA

GCCAGTGCTCAAGCACTGCTTGGGGATCCCTTCCTGCAGCCTGGGAAGAGGAGCCGCAGC

CCTGCCTCCCCCCATCATGCTCCTCGGCCCTCAGATGCCCCTGCAGCCAGTCCCATTCCT

TTGGCTGACCCCACCACTCAGTCCCAGACATTCCCGCAGCCTCAGGCACCCTCTCAGCAC

CCACCCAGCCCCCCAAAACGCTGTCTCAGTTATGGGGACACCAGCCAGCTCCGGGTACCG

GAGGAGCCTGGGGTCGAGGAGCCTACGTCCCCGGAAGAGAGTTCAGGGCTGAGCCTGCTA

CATCAGGAGAGCAAGCGCCGAGCCATGCTGGCCGAGGTGGTGGAACAGGAGCTGCCAACG

CTCGCCGAGAACCTGTGCCTGGAGCAGGAACAGGGTTCCCGACTGAGCAGGAATCATGTG

GAACATCTGCTCCGCTGCCTGGGGGCACACATCCATACTCCCAATCGTCGGCAGCTGGCC

CAGGAGCTGCGGACACTGCAAGCGCTTCTGCGGGCGCAGGGCCTTGGGTCTACACTTCTG

AATGCACCGCTCTTCGCCTTTCCAGATGCGGTGAAGCAGATCCTCCGCAGGCGCCAGATC

CGCCCACACTGGATGTTCGTGCTGGACTCACTGCTCAGCCGCGCTGTGCGGGCAGCCTTG

GCGGTGCTGGACCCTGAGATAGAGAAGAAGGAGGGCTCACTGAGGTCAGAGGAATTGAGT

AAAGAAGGGGAGTCTCAGCAGAAGCAACAAGAATCTCCTGTCTTGCGGAGTAGCGGACTC

ACAGAAGGAGAGCAAGACTCGCCTCCTCTGATAGTTCAGCTGGGCCTCTTGCGGGCAGAG

ACTGACAGACTTCGAGAGGTCCTGGCAGAGAAGGAACGGGAGTGCCAGGCCCTGATATAC

CAGACTCTACACCAGATGAATGCAGAGGCCAGGACCTGTGCCCTCGTCTCAGAGCCCCAA

GATGCCCTCCCAAAGGACCAGGGCTTGGTGCAGTGGTTACAGGGGCTAAAGGTGGATTCA

GGCACCATCCAAACGCTGCTGAACCACAGTTTCACCCTCCGTGCCCTGCTCACCTGTGCC

ACTCGAGATGACCTCCTTTATACCGGCATCAGGGGAGGGATGGTATGCCGCATCTGGAGA

GGCATCTTGGCACAGCGAGCAGGATCTGCATCAGTTACCCCTGGACCCCGGGAGGCTGAA

>CL274.Contig3_A-W 1056 1220 minus strand PREDICTED: synaptotagmin-like 1 isoform 2 [Equus caballus]

CAGTACTCCATCCCGCAGGCCGAGCTCTCGGGCCGCGTCCTGAGCCTGTCGGTGTGGCAC

CGGGAAAGCCTGGGTCGCAACATCTTCCTGGGCGAAGCCGAAGTGTCCTTGGACACGTGG

AACTGGGACTCGGAGCCTACCTGGCTCCCCCTGCAACCCCAAGTA

>CL274.Contig4_A-W 486 899 minus strand PREDICTED: synaptotagmin-like 1 isoform 2 [Equus caballus]

ATGAGCCTGTCGGGGGAGGTGGAGGCGGGCGCGGTGCAGGTGCAGGTGCGCGGCTCTGTG

CACTTCGCGCTGCACTTCGAACCCAGTGCCTCCGAGCTGCGCGTGCACGTGATCCAATGT

CAGGGCCTGGCTGCCGCCCGGCGCGGCCGCTCCGACCCCTACGTCAAAAGCTACCTCCTC

CCTGATAAACAGAGCAAGCGCAAGACCGCGGTGAAGAAGAGGAATCTGAACCCGGTCTTC

AACGAGACTCTCCGGTACTCCATCCCGCAGGCCGAGCTCTCGGGCCGCGTCCTGAGCCTG

TCGGTGTGGCACCGGGAAAGCCTGGGTCGCAACATCTTCCTGGGCGAAGCCGAAGTGTCC

TTGGACACGTGGAACTGGGACTCGGAGCCTACCTGGCTCCCCCTGCAACCCCAA

>CL275.Contig2_A-W 152 400 Tigger transposable element-derived protein 1 OS=Homo sapiens GN=TIGD1 PE=2 SV=1

GAGGGCTTCCGGATTCCAGTGGAGGAGGAGCTGCAGGTGCGGTGGGAACAGCAGGGGGAC

TGGAAGTGGAGCCGGAAGTGGGGCCTGACTGCTGCCATCTCAAGGGACAAGGAGCTGCCT

CTTACAGATGAGCTACGAGAGCGCTGTCTGGAGATGGAATCCACTGAGGGTGCAGGGAAC

GCTGCTGAGATGACGACAGAGGCTTGAGAATGTCACACAAAAGCTGAAAGGGCTGACCTC

AATTTTGAA

>CL277.Contig1_A-W 80 559 Creatine kinase B-type OS=Homo sapiens GN=CKB PE=1 SV=1

ATGCCCTTCTCCAATGGCCACAACGCGTTGAAGCTGCGCTTCCCTGCCGAGGATGAGTTC

CCCGACCTGAGCGTCCACAACAACCACATGGCCAAAGTGCTGACCCCCGAGCTGTACGCA

GAGCTGCGCGCCAAGAGCACGCCAAGCGGCTTCACGCTGGACGACGTCATTCAGACTGGC

GTGGACAACCCGGGCCACCCGTACATCATGACCGTGGGCTGCGTGGCGGGCGACGAGGAG

TCGTACGAGGTGTTCAAGGCACTTTTCGACCCCATAATCGAGGACCGGCACGGCGGCTAC

CAGCCCAGCGATGAACACAAGACCGACCTAAACCCGGACAACCTGCAGGGCGGCGATGAC

CTGGACCCCAACTACGTGCTGAGCTCGCGGGTGCGCACGGGCCGCAGCATCCGCGGCTTC

TGCCTTCCCCCGCACTGCAGCCGCGGGGAGCGCCGGGCCATCGAGAAGCTCGCGGTGGAA

>CL277.Contig2_A-W 41 1222 Ckb protein, partial [Rattus norvegicus]

CCCGCCCCTCCAGGCCGCCCGCCCGCTGCCGCCGCCGTCATGCCCTTCTCCAATGGCCAC

AACGCGTTGAAGCTGCGCTTCCCTGCCGAGGATGAGTTCCCCGACCTGAGCGTCCACAAC

AACCACATGGCCAAAGTGCTGACCCCCGAGCTGTACGCAGAGCTGCGCGCCAAGAGCACG

CCAAGCGGCTTCACGCTGGACGACGTCATTCAGACTGGCGTGGACAACCCGGGCCACCCG

TACATCATGACCGTGGGCTGCGTGGCGGGCGACGAGGAGTCGTACGAGGTGTTCAAGGCA

CTTTTCGACCCCATAATCGAGGACCGGCACGGCGGCTACCAGCCCAGCGATGAACACAAG

ACCGACCTAAACCCGGACAACCTGCAGGGCGGCGATGACCTGGACCCCAACTACGTGCTG

AGCTCGCGGGTGCGCACGGGCCGCAGCATCCGCGGCTTCTGCCTTCCCCCGCACTGCAGC

CGCGGGGAGCGCCGGGCCATCGAGAAGCTCGCGGTGGAAGCCCTGTCGAGCCTAGACGGC

GACCTGGCGGGCAGGTACTACGCGCTCAAGAGCATGACCGAGGCGGAGCAGCAGCAACTC

ATCGACGACCACTTCCTCTTCGACAAGCCGGTGTCGCCCCTGCTGCTGGCCTCGGGCATG

GCCCGCGACTGGCCGGACGCCCGCGGCATCTGGCACAATGACAATAAGACTTTCCTGGTG

TGGATCAATGAGGAGGACCACCTGAGGGTCATCTCCATGCAGAAGGGGGGCAACATGAAG

GAGGTGTTCACTCGCTTCTGCACTGGCCTCACCCAGATTGAAACTCTCTTCAAGTCCAAG

AACTACGAGTTCATGTGGAACCCTCACCTGGGCTACATCCTTACCTGCCCTTCCAACCTG

GGCACAGGGCTGAGGGCAGGTGTGCACATCAAGCTGCCCCACCTGGGCAAGCACGAGAAG

TTCTCTGAGGTGCTCAAGCGGCTTCGGCTTCAGAAGCGGGGCACAGGTGGTGTGGACACG

GCTGCTGTGGGCGGGGTTTTTGACGTCTCCAATGCAGACCGCCTGGGCTTCTCCGAGGTA

GAGCTGGTGCAGATGGTGGTGGATGGAGTGAAGCTGCTCATTGAGATGGAGCAGCGGCTG

GAGCAGGGCCAGGCCATTGACGACCTCATGCCTGCCCAGAAG

>CL278.Contig1_A-W 3 380 minus strand PREDICTED: Kv channel-interacting protein 2-like isoform 1 [Loxodonta africana]

ACAGACAGCGTAGAGGATGAATTTGAACTGTCCACTGTGTGTCACCGACCAGAAGGTCTG

GAGCAGCTGCAAGAGCAAACCAAGTTTACGCGCAAGGAGTTGCAGGTCCTGTACCGGGGC

TTCAAGAACGAATGTCCCAGCGGAATTGTCAATGAAGAGAACTTCAAGCAAATTTACTCC

CAGTTCTTTCCTCAAGGAGACTCCAGCACTTACGCTACTTTTCTCTTCAACGCCTTCGAC

ACCAACCATGATGGCTCTGTCAGTTTTGAGGACTTTGTTGCTGGTTTGTCGGTGATTCTT

CGGGGAACCATAGATGACAGGTTGAATTGGGCCTTCAACTTGTATGACCTCAACAAAGAT

GGCTGTATCACCAAGGAG

>CL278.Contig2_A-W 3 380 minus strand PREDICTED: Kv channel-interacting protein 2-like isoform 1 [Loxodonta africana]

ACAGACAGCGTAGAGGATGAATTTGAACTGTCCACTGTGTGTCACCGACCAGAAGGTCTG

GAGCAGCTGCAAGAGCAAACCAAGTTTACGCGCAAGGAGTTGCAGGTCCTGTACCGGGGC

TTCAAGAACGAATGTCCCAGCGGAATTGTCAATGAAGAGAACTTCAAGCAAATTTACTCC

CAGTTCTTTCCTCAAGGAGACTCCAGCACTTACGCTACTTTTCTCTTCAACGCCTTCGAC

ACCAACCATGATGGCTCTGTCAGTTTTGAGGACTTTGTTGCTGGTTTGTCGGTGATTCTT

CGGGGAACCATAGATGACAGGTTGAATTGGGCCTTCAACTTGTATGACCTCAACAAAGAT

GGCTGTATCACCAAGGAG

>CL278.Contig3_A-W 3 485 minus strand PREDICTED: Kv channel-interacting protein 2 isoform 2 [Equus caballus]

ACAGACAGCGTAGAGGATGAATTTGAACTGTCCACTGTGTGTCACCGACCAGAAGGTCTG

GAGCAGCTGCAAGAGCAAACCAAGTTTACGCGCAAGGAGTTGCAGGTCCTGTACCGGGGC

TTCAAGAACGAATGTCCCAGCGGAATTGTCAATGAAGAGAACTTCAAGCAAATTTACTCC

CAGTTCTTTCCTCAAGGAGACTCCAGCACTTACGCTACTTTTCTCTTCAACGCCTTCGAC

ACCAACCATGATGGCTCTGTCAGTTTTGAGGACTTTGTTGCTGGTTTGTCGGTGATTCTT

CGGGGAACCATAGATGACAGGTTGAATTGGGCCTTCAACTTGTATGACCTCAACAAAGAT

GGCTGTATCACCAAGGAGGAAATGCTTGACATCATGAAGTCCATTTATGACATGATGGGC

AAGTACACATACCCTGCACTCCGAGAGGAGGCCCCAAGGGAACACGTGGAGAGCTTCTTC

CAG

>CL278.Contig4_A-W 6 581 minus strand unnamed protein product [Homo sapiens]

GACAGCGTAGAGGATGAATTTGAACTGTCCACTGTGTGTCACCGACCAGAAGGTCTGGAG

CAGCTGCAAGAGCAAACCAAGTTTACGCGCAAGGAGTTGCAGGTCCTGTACCGGGGCTTC

AAGAACGAATGTCCCAGCGGAATTGTCAATGAAGAGAACTTCAAGCAAATTTACTCCCAG

TTCTTTCCTCAAGGAGACTCCAGCACTTACGCTACTTTTCTCTTCAACGCCTTCGACACC

AACCATGATGGCTCTGTCAGTTTTGAGGACTTTGTTGCTGGTTTGTCGGTGATTCTTCGG

GGAACCATAGATGACAGGTTGAATTGGGCCTTCAACTTGTATGACCTCAACAAAGATGGC

TGTATCACCAAGGAGGAAATGCTTGACATCATGAAGTCCATTTATGACATGATGGGCAAG

TACACATACCCTGCACTCCGAGAGGAGGCCCCAAGGGAACACGTGGAGAGCTTCTTCCAG

AAGATGGACAGAAACAAGGATGGTGTGGTGACCATTGAGGAATTCATTGAATCTTGTCAA

AAGGTACAGGCCCTGCCCTCTACATTTCCCTGGTCT

>CL279.Contig1_A-W 392 1408 mCG67940, isoform CRA_b [Mus musculus]

TCTTGGCATCAGGGAAGGGAGCATTCAGGCACATTTCACCATCAGCATCCCAGGATGGCA

GCCATTGATTTGTCCCATGGCCTTCTTTCCACGGAACCAGTCTGCTTTTATGAAGAAAAG

ACAAACGTAGAAAGAATGGTGACTGACTACCAGGCAAATTATTACCAGGATTCAGTGACC

TTTGATGATGTGGCTGTGGAGTTCACCCTAGAGGAGTGGACTTTACTGGACACAACTCAG

AGAAACCTATACAAAGATGTGATGCTGGAGAACTTTGAGAACCTGACCTCAGTAGGATAT

CAGTTCTTCATACCTAGTCTGATCTCCTCGTTAAAAGAAGAACAGTTGAGGACAATGGAA

AGTGAAGTTCTTCAATCTTCAGAATTGGAACTCCAACCTAGCACCAAAGGGTCAGAGCTT

CAGAATGACTGTATGACACAAACTTCCATTGGGATACAAACGGCAAGCCTCAGTGATGGA

GGGACACTGTGTGACTATAAGCCATGTGGAGAAGTACCCATTGAACAGTCATGCCTTATG

ACAGATGTGAGAATGCAAAATCAAGGATACATTTCTGAGGGTAATGGGTATGGAGAAAAC

ATCCTTACTCTACATAAACAAACCTCTACTGAACAGAACCTTTCTGAGTTGAATCAGTGT

GAACAAGTCTTCAGCCTGACTCCAGATATGATGTACCAGGGAACTAGCATGACAGAGAAA

CCCTTTGAATACAGAGACAGTGGGGAAGCCTTCTTTAATCAATCATACCTTCAGGCAAAT

ATGGGAACTCACAATGGAGGAAATCCGTATGAATGGGAGAAATATGGGAACGAACCTATT

CATGCCACAGACCTTGATGTACATCTTCAAATTCTCAATGCAACAAAACCCTACAAATGG

GAGGAATGTGGAAATGGCTTTCCATGTTTTGTATGCCTTAATAATCCCATGGGAACCCAC

ACTGGAAATAAACTCTGTGAATGCAAGGAATGTTGGAGAGCCTTTGCAGTTTCCTCA

>CL279.Contig2_A-W 392 1330 mCG67940, isoform CRA_b [Mus musculus]

TCTTGGCATCAGGGAAGGGAGCATTCAGGCACATTTCACCATCAGCATCCCAGGATGGCA

GCCATTGATTTGTCCCATGGCCTTCTTTCCACGGAACCAGTCTGCTTTTATGAAGAAAAG

ACAAACGTAGAAAGAATGGTGACTGACTACCAGGCAAATTATTACCAGGATTCAGTGACC

TTTGATGATGTGGCTGTGGAGTTCACCCTAGAGGAGTGGACTTTACTGGACACAACTCAG

AGAAACCTATACAAAGATGTGATGCTGGAGAACTTTGAGAACCTGACCTCAGTAGCTTCA

GAATTGGAACTCCAACCTAGCACCAAAGGGTCAGAGCTTCAGAATGACTGTATGACACAA

ACTTCCATTGGGATACAAACGCAGGCAAGCCTCAGTGATGGAGGGACACTGTGTGACTAT

AAGCCATGTGGAGAAGTACCCATTGAACAGTCATGCCTTATGACAGATGTGAGAATGCAA

AATCAAGGATACATTTCTGAGGGTAATGGGTATGGAGAAAACATCCTTACTCTACATAAA

CAAACCTCTACTGAACAGAACCTTTCTGAGTTGAATCAGTGTGAACAAGTCTTCAGCCTG

ACTCCAGATATGATGTACCAGGGAACTAGCATGACAGAGAAACCCTTTGAATACAGAGAC

AGTGGGGAAGCCTTCTTTAATCAATCATACCTTCAGGCAAATATGGGAACTCACAATGGA

GGAAATCCGTATGAATGGGAGAAATATGGGAACGAACCTATTCATGCCACAGACCTTGAT

GTACATCTTCAAATTCTCAATGCAACAAAACCCTACAAATGGGAGGAATGTGGAAATGGC

TTTCCATGTTTTGTATGCCTTAATAATCCCATGGGAACCCACACTGGAAATAAACTCTGT

GAATGCAAGGAATGTTGGAGAGCCTTTGCAGTTTCCTCA

>CL280.Contig1_A-W 153 4082 minus strand PREDICTED: ATP-dependent RNA helicase A-like [Ailuropoda melanoleuca]

ATGGGTGACGTTAAAAATTTTCTGTATGCCTGGTGTGGCAAAAGGAAGATGACCCCAGCG

TATGAAATTAGAGCAGTAGGGAACAAAAACAGGCAGAAATTCATGTGTGAGGTTCGAGTA

GAAGGGTATAATTACACTGGCATGGGCAACTCTACCAATAAAAAAGATGCACAGAGCAAT

GCTGCCAGAGACTTTGTTAACTATTTGGTTCGAATAAATGAAGTAAAGAGTGAAGAAGTC

CCGGCTATGGGGATTGCCCCTCCTCCACCCACTGTTCCTGCTGAGACATCTGACACCACA

GCAAGTACTGGAGGAGAATTACCAACAACCATGGGAGGACCTCTTCCTCCACATTTGGCT

CTCAAGGCAGAAAATAATTCTGGGGGAGCATCTGGCTTTGGTGCTCCTGGACCAACCTGG

GACCGGGGAGCCAACTTGAAAGATTACTACTCAAGAAAGGAGGAACAAGAAGTACAAGCG

ACTCTAGAGTCAGAAGAAGTAGATTTAAATGCTGGACTTCATGGAAATTGGACCTTGGAA

AATGCTAAGGCTCGCCTGAACCAGTATTTCCAAAAAGAAAAGATCCAAGGAGAATATAAA

TACACCCAAGTGGGTCCGGATCACAACAGGAGCTTTATTGCAGAAATGACCATTTATATC

AAGCAGCTGGGCAGAAGGATTTTTGCACGTGAACATGGATCAAATAAGAAATTGGCAGCA

CAGTCCTGTGCCCTATCACTTGTCAGACAGCTCTATCATCTTGGAGTGATTGAAGCTTAC

TCTGGACTTACAAAGAAGAAAGAAGGAGAGACAGTGGAGCCTTACAGAGTTAACATCTCT

CAAGATTTAGAGCATCAACTTCAAAATGTGATTCAAGAGCTAAATCTTGAAATTGTGCCC

CCACCTATTGATCCTTCTATGCCAGTTATGCTCAACATTGGAAAATTGGCTCATTTTGAA

CCATCTCAGCGTCAAAACCAAGTGGGTGTGGTTCCTTGGTCACCTCCACAATCCAACTGG

AATCCTTGGACTAGTAGCAACATTGATGAGGGTCCCCTGGCTTATGCTACTCCAGAACAA

ATAAGTTTGGACCTCAAGAATGAATTGATGTACCAGCTGGAACAGGATCATGACTTGCAA

GCAGTTTTGCAGGAGAGAGAGTTACTACCTGTGAAGAAATTTGAAAGTGAAATCCTGGAA

GCAATTAGTCAAAATTCAGTTGTCATTATCCGAGGGGCTACTGGATGTGGTAAAACCACA

CAGGTTCCACAGTTCATTCTGGATGACTTTATCCAGAACGACCGAGCAGCAGAGTGTAAT

ATCGTAGTAACTCAACCCAGGAGAATTAGTGCAGTTTCTGTGGCAGAGCGAGTTGCATAT

GAGAGAGGAGAAGAACCCGGAAAAAGCTGTGGCTATAGTGTTCGATTTGAGTCTATACTT

CCCCGTCCTCATGCCAGTATAATGTTTTGTACTGTGGGAGTGCTTCTCAGAAAATTAGAA

GCAGGCATTAGAGGAATCAGTCATGTAATTGTAGATGAAATACATGAACGAGACATCAAT

ACTGACTTCCTCTTGGTAGTACTGCGTGATGTGGTTTTGGCTTATCCTGAAGTTCGCATA

GTTCTTATGTCTGCTACTATTGATACCAGCATGTTCTGTGAATATTTCTTCAACTGCCCC

ATAATTGAAGTTTATGGGAGGACTTATCCAGTTCAAGAATATTTTCTGGAAGATTGTATT

CAGATGACCCAGTTTGTTCCTCCACCAAAGGACAAGAAAAAAAAGGACAAGGAGGATGAT

GGTGGTGAGGATGATGATGCAAATTGCAACCTGATCTGTGGAGATGAATATGGTCCAGAA

ACAAAAATGAGCATGTCTCAGTTGAATGAAAAGGAAACTCCTTTTGAACTCATTGAAGCT

CTGCTTAAGTACATTGAAACGCTCAATGTTCCTGGAGCTGTTTTGGTTTTTTTACCTGGC

TGGAATTTGATTTATACTATGCAAAAGCATTTGGAAATGAATCCACATTTTGGAACACAT

AGGTATCAAATTCTACCTCTGCATTCTCAGATACCTCGAGAGGAACAGCGCAAAGTATTT

GATCCTGTACCAGTTGGTGTAACCAAGGTCATTTTGTCCACAAATATTGCTGAGACAAGC

ATTACTATAAATGATGTTGTTTATGTCATTGATTCCTGCAAGCAAAAAGTGAAACTGTTC

ACTGCTCATAATAACATGACCAACTATGCTACAGTATGGGCATCCAAAACAAATCTTGAG

CAGCGGAAAGGCCGAGCTGGACGCGTACGGCCTGGATTCTGTTTTCACCTCTGTAGCCGA

GCTAGATTTGAGAGACTTGAAACACACATGACACCAGAGATGTTTCGAACACCATTGCAT

GAAATTGCTTTGAGCATAAAACTTCTGCGTCTAGGAGGAATTGGCCAATTCCTGGCCAAG

GCAATTGAACCTCCACCTTTGGATGCTGTGATTGAAGCCGAGCACACTCTTAGAGAACTT

GATGCATTGGATGTCAATGATGAGTTGACTCCTTTGGGACGAATCCTGGCTAAGCTCCCC

ATCGAGCCTCGCTTTGGCAAAATGATGATAATGGGGTGTATTTTCTATGTGGGTGATGCT

GTCTGTACCATCTCTGCTGCTACCTGCTTTCCTGAGCCTTTCATTAGTGAGGGAAAGCGC

CTGGGGTATATCCATCGGAACTTTGCTGGAAATAGATTTTCTGATCATGTGGCCCTTTTG

TCAGTGTTTCAAGCCTGGGATGATGCGAGAATGAGTGGAGAAGAAGCAGAGATTCGTTTT

TGTGAACATAAACGTCTCAATATGGCTACACTGAGAATGACTTGGGAAGCCAAAGTTCAG

CTTAAAGAGATTCTGATTAATTCTGGATTTCCAGAAGATTGTTTGTTGACACAAGTGTTT

ACTAACACTGGACCAGATAATAATTTGGATGTTGTTATCTCTCTCCTGGCTTTTGGCGTG

TACCCTAATGTATGCTATCATAAGGAAAAGAGAAAGATTCTTACTACCGAAGGGCGTAAT

GCACTTATCCACAAATCATCTGTTAATTGTCCATTTAGCAGCCAAGACATGAAGTACCCT

TCTCCCTTCTTTGTATTTGGTGAAAAGATTCGAACCCGAGCCATCTCTGCTAAAGGCATG

ACCTTAGTCACCCCCTTGCAGTTGCTTCTCTTTGCGTCCAAGAGAGTCCAATCTGACGGG

CAGATTGTGTTTGTAGATGACTGGATCAGACTACAAATATCCCATGAAGCCGCTGCCTGT

ATTACTGCCCTCAGAGCAGCAATGGAAGCTCTGGTTGTTGAAGTAAGCAAACAACCTAAT

ATCATCAGCCAGTTGGACCCTGTAAATGAACATATGCTTAACATGATTCGCCAAATCTCT

AGGCCATCAGCTGCTGGTATAAACCTTATGATTGGCAGTGCACGGTATGGAGATGGTCCA

CGACCTCCCAAGATGGCCCGATATGATAATGGCAGTGGATATAGAAGGGGAGTTTCTGGA

TATGGTGGTGGAGGCTATGGCACTGGAGGCTATGGTGGTGGCTATAACAGTGGAGGTTTT

GGTGGGGGAGGCTATGGAGGTGGTGGTGGTTATGGTGGTGGCTCCAACTCTTATCGAGGA

GGATATGGTGGAGGTGGTGGTGGAGGTTACAGAGGAGGTTTCCGAGGAGGGTATAGGGGA

AACTTTGGTGGGGACTACAGAGGATCTGGTGGAGACTACAGAGGATCCGGTGGAGACTAT

AGAGGATCTGGGGGAGACTACAGAGGATGTGGTGGAGATTATAGAGGATCTGGAGGGGGC

TACCGAGGATCTGGGGGATTCCATCGAGGTGGTGGTAGGGGGGGCTATGGTGGTGGCTAC

TTTGGACAGGGAAGAGGAGGTGGTGGTTAT

>CL281.Contig7_A-W 3 461 minus strand ENV bovine endogenous retrovirus K1 envelope precursor protein [Bos taurus]

GTTATAGACATAGGTAATGAACTTTCAGCTTTAAAATTTAAAGAAAGGCTTAAATGCCAT

GCAAATTATAAATATGTGTGTGTTACAGCTGCCAAGTACAATGCTTCTCTCTGGGATTGG

GAGAAGGTTAAAAACCATTTGTTAGGTATTTGGCAAGATAGTAATAATAGCTTGTATATT

CTGGAACTTCATCACCATATCCAAGACATTCAAAATAATAGAGCTGATTTTTCAGATCCT

TCTTCTCTGGCAAACCAAATATTAAAGGAGTTGAATGGATTCAACCCTGGAAATATTCTT

AAACACTCGGCCTGGTACATTATTGGATTATTCTCTTTGCTGTTAATTCTCTTTTGTGTT

GTAATTATAGGATGGCGAGTCTTCGGAACAAGAATACAGAAACTCCAAAGAGTGAACCGC

CGCCTCATTTTTAAGAATAGAAAGGGGGAATATGTGGGA

>CL281.Contig9_A-W 1 969 PREDICTED: cholesteryl ester transfer protein [Pan paniscus]

CTCCAAGGAGAGCATAAGCCCAGCTGGATCCAGCAGCTGTTCACAAACGTCATCTCCTTT

ACTCTGAGGCTGGTCCTGAAGGGGCAGATCTGCAAAGAGATCAACGCCATCTCCAACATC

ATGGCCGACTTTGTCCAGACAAGGGCCGCCAGCATCCTCTCCTCTGGAGACATCCGGGTG

GACATTTCCCTGACAGGACCCCCTGACATCACAGCAGCCCACCTGGAGTCACACCACAAG

GGTCATTTCATCTACAAGAACGTCTCCGAGGACCTCCCGCTGCCCACCTTCTCGCCCAGC

CTCCTGGGGGACGCCCGCATGCTCTACTTCTGGTTCTCGGAGCAAGTCCTCGACTCCCTG

GCCAGGGCCGCCTTCCAGGATGGACGCCTCAGGCTCAGCCTGACCGGGGAGGAGTTCAAG

GCAGTGCTGGAGACCCACGGCTTCAACACCAACCAGGACATGTTTCTGGAGCTATTCGGC

AATATTCCCACCAGCCAGACCCAAGTGATCGTCTACTGCCCGAAGAGGCCCAAGATCTCT

TGTCAGAACAAGGGCGTCGTGGTCACCTCCTCCGTGGTGGTGAAATTCCTCGTCCCCCAC

CCGGATGGCCAAACTGCTCTGGCTCATACCTTTGAAGAGGACATCGTCACCACCATCCAG

GCCTCCTATGCCAAGAAGAAGCTGTCCTTGCACCTCCTGGATCTCCAGACTCAACCCAAG

TCTACTTCCAGCATCGATGAGAGCAACTTCAAGTCCGCCCAGAACTTCCTGTGGTCGATG

GTCTCCACGGTGGGCATCCCTGAGGTCATGACTCGGCTCGAGGTGACATTTACGGCGCTC

ATGAACAGCAAGGGCCTGGACCTCTTCGACATCATCAACCCTGAGATCATCTCTCGTGAT

GGCTTTCTGCTGCTGCAAATGGACTTTGGCTTCCCGGAGCACCTGCTGGTGGACTTCCTG

CAGAGCCTG

>CL281.Contig10_A-W 879 1046 minus strand hypothetical protein DDB_G0279459 [Dictyostelium discoideum AX4] >gi|60469680|gb|EAL67668.1| hypothetical protein DDB_G0279459 [Dictyostelium discoideum AX4]

GAGGAAGAGAAGGAGGAAGAAAAGTAAAAAGAAAAGAAGAAGAAGAAAGAAAAACAAGAA

AAAATGGGGGGAAGAGATTGAGAAAAGGGAATGAAAAAAGAGAGAGAGAAAAGGAGAGAA

GAAAAAGATAAGAAGAAAAAAGGAAGGAGGGAAAAAGAAAGAAAAAAA

>CL283.Contig1_A-W 1215 1802 minus strand PREDICTED: protein Shroom1-like [Macaca mulatta]

GTGGAGCTAGCCGGCCTTCTCCAAGAGATGCTGCAGGACCTTCACATCCAGGAGGAGCGG

CTGCGAGGTGCTGCCCAAGAATGGACCAGACGCAGGGTGGCTCTGGAATCCGCAGTGGGC

CAGGCCTGTGCACCCAGGGAACTGGAGCAGTTCAGCCGGTTCTTGGCTGACCTGGAGCGT

GTCCTTGGCCTCCTGCTGCTACTCGGCAGTCGCCTGGCCCGTGTGCACCATGCCTTAGCC

CGGATGGGCCCAGATGGTGACCCTGATGAGCGGGACTCTCTGTTGCAGCGACTTGGGCTT

CTGCAACGGCAGCAGGAAGATGCCAAGGAGCTGAAGGAGCATGTGGCACGACGTGAGCGG

GCCCTGCGCGAGGTGCTGGCGAGGGCACTGTCTGCAGAGGAGTTGTGCTCCTACAGTGAG

CTGCTGGTGGGCAAGGCCACCGTTCTGGCCCAGCAGCGCTACCTGGAGGAACATGTCCAG

CTCTTTCAGAACCAACTAGACGCCCTTGGGAGCAACCTTGGCCTTCGTCCCGTCTCTCCC

AGGCCGTCCTGGCCCCCAGAGACCTGCCCTCGGGATAAACCACCCTTC

>CL283.Contig2_A-W 214 1695 minus strand protein Shroom1 isoform 1 [Macaca mulatta]

TCCAGATTCTTGACTCAGAAGGAAGCTGCACTGGTGCATCCTGCAGAGGACCCCCAGAGC

AGCCCTGCCAAGTGTGAGCAGGAGGTCTCAGAGATCTGCATGGAGTCTCCATATGCCCTC

CTTCCCTCCCTTCCTGATGATGAAGTGTTCCTGGAAGAAACCCTGCATGTTGGAATGAGA

TCACTTCCAGACTCCCATGTTCCCCAGGAGTTTCCAATCAGTGTCCATGCCTCTGACCAG

CAGTGTGGAACTGGCTTGGGCCAAAGGGCTGGCCAGGCTTCAGTCCCCCCAGAGTGCACC

CTGCATGAATGTCCGGGGACTGCAGGGGCAGGTGACTGCTGGCAGGAGATGAATGGTTCT

GTGGGTATCTGCAGGCCCACAAGCTATATTCCCCTTGGGACTGCAAATGGTGACATCTCA

ATCATTGATACCAGTGGACTGCTTACCACTGACCACCCTGCAGTTGATGCCCTGGGACCT

TCAGGCAATGATAAACTAGGGTCCCCTCACCATACTTCCCTGGCCTGGGGTACTGGACAG

CCTGGTTCCAAGCCAACATGGCCATGTCAACGTCTTGAGGAGCTGGTTCAGGAGCTGGCC

AGACTGGATCCCTCTCTAAGTGACACTCTTACCTCCCAGCCATGCCCAGAGCCACCCCTG

GGCCTGCTGGATGGGCTGATTCCTTTAGCAGAAGTCTGGGCTGCAATGAAGCCAGGCTGT

GGTGAAGCTGGAGAAGAGGGTCCTGGTACTTCTGAGCCAGGGTCCTATCAATTCAACTTT

ACCCAGCTCCTGCCAACTTCTCAGGAGATGACAAGGCCTGAAAACTCTCCCTCCTGTCCT

GTGCCTGACTGTCCTAGTGCCCAAACATCAAACAACAGCATCCAGGCTAAGAAAGTGGAG

CTAGCCGGCCTTCTCCAAGAGATGCTGCAGGACCTTCACATCCAGGAGGAGCGGCTGCGA

GGTGCTGCCCAAGAATGGACCAGACGCAGGGTGGCTCTGGAATCCGCAGTGGGCCAGGCC

TGTGCACCCAGGGAACTGGAGCAGTTCAGCCGGTTCTTGGCTGACCTGGAGCGTGTCCTT

GGCCTCCTGCTGCTACTCGGCAGTCGCCTGGCCCGTGTGCACCATGCCTTAGCCCGGATG

GGCCCAGATGGTGACCCTGATGAGCGGGACTCTCTGTTGCAGCGACTTGGGCTTCTGCAA

CGGCAGCAGGAAGATGCCAAGGAGCTGAAGGAGCATGTGGCACGACGTGAGCGGGCCCTG

CGCGAGGTGCTGGCGAGGGCACTGTCTGCAGAGGAGTTGTGCTCCTACAGTGAGCTGCTG

GTGGGCAAGGCCACCGTTCTGGCCCAGCAGCGCTACCTGGAGGAACATGTCCAGCTCTTT

CAGAACCAACTAGACGCCCTTGGGAGCAACCTTGGCCTTCGTCCCGTCTCTCCCAGGCCG

TCCTGGCCCCCAGAGACCTGCCCTCGGGATAAACCACCCTTC

>CL283.Contig3_A-W 2258 2845 minus strand KIAA1960 protein [Homo sapiens]

GTGGAGCTAGCCGGCCTTCTCCAAGAGATGCTGCAGGACCTTCACATCCAGGAGGAGCGG

CTGCGAGGTGCTGCCCAAGAATGGACCAGACGCAGGGTGGCTCTGGAATCCGCAGTGGGC

CAGGCCTGTGCACCCAGGGAACTGGAGCAGTTCAGCCGGTTCTTGGCTGACCTGGAGCGT

GTCCTTGGCCTCCTGCTGCTACTCGGCAGTCGCCTGGCCCGTGTGCACCATGCCTTAGCC

CGGATGGGCCCAGATGGTGACCCTGATGAGCGGGACTCTCTGTTGCAGCGACTTGGGCTT

CTGCAACGGCAGCAGGAAGATGCCAAGGAGCTGAAGGAGCATGTGGCACGACGTGAGCGG

GCCCTGCGCGAGGTGCTGGCGAGGGCACTGTCTGCAGAGGAGTTGTGCTCCTACAGTGAG

CTGCTGGTGGGCAAGGCCACCGTTCTGGCCCAGCAGCGCTACCTGGAGGAACATGTCCAG

CTCTTTCAGAACCAACTAGACGCCCTTGGGAGCAACCTTGGCCTTCGTCCCGTCTCTCCC

AGGCCGTCCTGGCCCCCAGAGACCTGCCCTCGGGATAAACCACCCTTC

>CL283.Contig4_A-W 2174 2761 minus strand KIAA1960 protein [Homo sapiens]

GTGGAGCTAGCCGGCCTTCTCCAAGAGATGCTGCAGGACCTTCACATCCAGGAGGAGCGG

CTGCGAGGTGCTGCCCAAGAATGGACCAGACGCAGGGTGGCTCTGGAATCCGCAGTGGGC

CAGGCCTGTGCACCCAGGGAACTGGAGCAGTTCAGCCGGTTCTTGGCTGACCTGGAGCGT

GTCCTTGGCCTCCTGCTGCTACTCGGCAGTCGCCTGGCCCGTGTGCACCATGCCTTAGCC

CGGATGGGCCCAGATGGTGACCCTGATGAGCGGGACTCTCTGTTGCAGCGACTTGGGCTT

CTGCAACGGCAGCAGGAAGATGCCAAGGAGCTGAAGGAGCATGTGGCACGACGTGAGCGG

GCCCTGCGCGAGGTGCTGGCGAGGGCACTGTCTGCAGAGGAGTTGTGCTCCTACAGTGAG

CTGCTGGTGGGCAAGGCCACCGTTCTGGCCCAGCAGCGCTACCTGGAGGAACATGTCCAG

CTCTTTCAGAACCAACTAGACGCCCTTGGGAGCAACCTTGGCCTTCGTCCCGTCTCTCCC

AGGCCGTCCTGGCCCCCAGAGACCTGCCCTCGGGATAAACCACCCTTC

>CL283.Contig5_A-W 90 2738 minus strand KIAA1960 protein [Homo sapiens]

AGATCCACGGAACCAGCCTGCTCCTTCTGCACAGCCATGGAAGCCCTGGGTCCCAGGGAC

GACAGCGCCTCCCCAGCCTCATCCACACCAAGCCTAGACTTACGGCGGCTGTCCACTCGC

GCCGACTCGGCCTACGGTTCTTTCTCGGCCCCTGAGGCACGCACGCCGTCGCCTCGCGCG

GATCTCCACCCTTACCTCGACTGGGAATACGTGCGCGTGGTTTGGGGCGGCCCGGCCCCA

GCCCCCCCTGATGCCGATCCTCCCACCACGCAGCGGCCTCGGCCAGCTGTTGCTGCGTGC

TGTGAGCCATGGCCCCCAGAGGCCCAGGGAACCCCGGGGCCACTCAGCAGACAGGCAACC

CCGCTGCTATACGCGCAGGCTGCAGAGGCTGAGGCCGGGGCGCGGGCCGCCGAGCCACCC

AGCCCACCAGCTTCCTGGGCCGCCTACCGCCAGCGATTGCAGGGCGCGCAGCGGCGAGTG

CTCCGGGAGACATCCTTCCAGCGCAAGGAGCTCCGCATGAGCCTGCCCACCCGCCTGCGA

CCAGCTGTCCCCGCGCGACCCCCCACTGCGCACCCGCGCTCCTCCTCGCTTAGCCACCCG

GGCGGGGAGGTAGGACCCACGTGCTCCGGAGCTCCAGCGCCAGGATCCACTGGTCCGGGG

CGCCTCGCCAATCAGCAGCGGAAATGGTGCTTCTCAGAGCCAGGAAAGTTGGATTGCATG

GGTCGGGGTGGTGGGCTGGCGGGGGAGTGCTTGAGCTTGGCCTGCTCCAGCTCTGGCCTC

AACAGTCCTGAGCCCCGGGAGTTGCAGGCTGAGGTCAAAGGGTTGCAGTGCTGGGCCCAA

CCCCAAAGCACAGCGGAGCTGAACTCCGGGTCCACGAAGCTCGGCAATGGCTGTAGGCCT

GCCAGTCGGAGTCAGAGCCCTTCAGGAGAGGTCTTGGGCCCCTGGAAAGGTCCAGGAGGG

GCCGTGGCCCCTGTCCAGGTATGGAGATAGAGGATGCAGGCTTGTGGTGGAGGGGAGCAT

GTGGGAGATGGCCACATTTCAGTCACCTACTCACCTTTCCAGGCTGTTTCCCAAGCAGCA

GAAAACCCCAGACTGCTGTTTCAGACCAAACTTTCCAGCACGAAGACTTTCCTTCCTGGT

CACTGTGGTCAGTGTGGGATCCCTGCCTCCAGATTCTTGACTCAGAAGGAAGCTGCACTG

GTGCATCCTGCAGAGGACCCCCAGAGCAGCCCTGCCAAGTGTGAGCAGGAGGTCTCAGAG

ATCTGCATGGAGTCTCCATATGCCCTCCTTCCCTCCCTTCCTGATGATGAAGTGTTCCTG

GAAGAAACCCTGCATGTTGGAATGAGATCACTTCCAGACTCCCATGTTCCCCAGGAGTTT

CCAATCAGTGTCCATGCCTCTGACCAGCAGTGTGGAACTGGCTTGGGCCAAAGGGCTGGC

CAGGCTTCAGTCCCCCCAGAGTGCACCCTGCATGAATGTCCGGGGACTGCAGGGGCAGGT

GACTGCTGGCAGGAGATGAATGGTTCTGTGGGTATCTGCAGGCCCACAAGCTATATTCCC

CTTGGGACTGCAAATGGTGACATCTCAATCATTGATACCAGTGGACTGCTTACCACTGAC

CACCCTGCAGTTGATGCCCTGGGACCTTCAGGCAATGATAAACTAGGGTCCCCTCACCAT

ACTTCCCTGGCCTGGGGTACTGGACAGCCTGGTTCCAAGCCAACATGGCCATGTCAACGT

CTTGAGGAGCTGGTTCAGGAGCTGGCCAGACTGGATCCCTCTCTAAGTGACACTCTTACC

TCCCAGCCATGCCCAGAGCCACCCCTGGGCCTGCTGGATGGGCTGATTCCTTTAGCAGAA

GTCTGGGCTGCAATGAAGCCAGGCTGTGGTGAAGCTGGAGAAGAGGGTCCTGGTACTTCT

GAGCCAGGGTCCTATCAATTCAACTTTACCCAGCTCCTGCCAACTTCTCAGGAGATGACA

AGGCCTGAAAACTCTCCCTCCTGTCCTGTGCCTGACTGTCCTAGTGCCCAAACATCAAAC

AACAGCATCCAGGCTAAGAAAGTGGAGCTAGCCGGCCTTCTCCAAGAGATGCTGCAGGAC

CTTCACATCCAGGAGGAGCGGCTGCGAGGTGCTGCCCAAGAATGGACCAGACGCAGGGTG

GCTCTGGAATCCGCAGTGGGCCAGGCCTGTGCACCCAGGGAACTGGAGCAGTTCAGCCGG

TTCTTGGCTGACCTGGAGCGTGTCCTTGGCCTCCTGCTGCTACTCGGCAGTCGCCTGGCC

CGTGTGCACCATGCCTTAGCCCGGATGGGCCCAGATGGTGACCCTGATGAGCGGGACTCT

CTGTTGCAGCGACTTGGGCTTCTGCAACGGCAGCAGGAAGATGCCAAGGAGCTGAAGGAG

CATGTGGCACGACGTGAGCGGGCCCTGCGCGAGGTGCTGGCGAGGGCACTGTCTGCAGAG

GAGTTGTGCTCCTACAGTGAGCTGCTGGTGGGCAAGGCCACCGTTCTGGCCCAGCAGCGC

TACCTGGAGGAACATGTCCAGCTCTTTCAGAACCAACTAGACGCCCTTGGGAGCAACCTT

GGCCTTCGTCCCGTCTCTCCCAGGCCGTCCTGGCCCCCAGAGACCTGCCCTCGGGATAAA

CCACCCTTC

>CL283.Contig6_A-W 90 2654 minus strand KIAA1960 protein [Homo sapiens]

AGATCCACGGAACCAGCCTGCTCCTTCTGCACAGCCATGGAAGCCCTGGGTCCCAGGGAC

GACAGCGCCTCCCCAGCCTCATCCACACCAAGCCTAGACTTACGGCGGCTGTCCACTCGC

GCCGACTCGGCCTACGGTTCTTTCTCGGCCCCTGAGGCACGCACGCCGTCGCCTCGCGCG

GATCTCCACCCTTACCTCGACTGGGAATACGTGCGCGTGGTTTGGGGCGGCCCGGCCCCA

GCCCCCCCTGATGCCGATCCTCCCACCACGCAGCGGCCTCGGCCAGCTGTTGCTGCGTGC

TGTGAGCCATGGCCCCCAGAGGCCCAGGGAACCCCGGGGCCACTCAGCAGACAGGCAACC

CCGCTGCTATACGCGCAGGCTGCAGAGGCTGAGGCCGGGGCGCGGGCCGCCGAGCCACCC

AGCCCACCAGCTTCCTGGGCCGCCTACCGCCAGCGATTGCAGGGCGCGCAGCGGCGAGTG

CTCCGGGAGACATCCTTCCAGCGCAAGGAGCTCCGCATGAGCCTGCCCACCCGCCTGCGA

CCAGCTGTCCCCGCGCGACCCCCCACTGCGCACCCGCGCTCCTCCTCGCTTAGCCACCCG

GGCGGGGAGGTAGGACCCACGTGCTCCGGAGCTCCAGCGCCAGGATCCACTGGTCCGGGG

CGCCTCGCCAATCAGCAGCGGAAATGGTGCTTCTCAGAGCCAGGAAAGTTGGATTGCATG

GGTCGGGGTGGTGGGCTGGCGGGGGAGTGCTTGAGCTTGGCCTGCTCCAGCTCTGGCCTC

AACAGTCCTGAGCCCCGGGAGTTGCAGGCTGAGGTCAAAGGGTTGCAGTGCTGGGCCCAA

CCCCAAAGCACAGCGGAGCTGAACTCCGGGTCCACGAAGCTCGGCAATGGCTGTAGGCCT

GCCAGTCGGAGTCAGAGCCCTTCAGGAGAGGTCTTGGGCCCCTGGAAAGGTCCAGGAGGG

GCCGTGGCCCCTGTCCAGGCTGTTTCCCAAGCAGCAGAAAACCCCAGACTGCTGTTTCAG

ACCAAACTTTCCAGCACGAAGACTTTCCTTCCTGGTCACTGTGGTCAGTGTGGGATCCCT

GCCTCCAGATTCTTGACTCAGAAGGAAGCTGCACTGGTGCATCCTGCAGAGGACCCCCAG

AGCAGCCCTGCCAAGTGTGAGCAGGAGGTCTCAGAGATCTGCATGGAGTCTCCATATGCC

CTCCTTCCCTCCCTTCCTGATGATGAAGTGTTCCTGGAAGAAACCCTGCATGTTGGAATG

AGATCACTTCCAGACTCCCATGTTCCCCAGGAGTTTCCAATCAGTGTCCATGCCTCTGAC

CAGCAGTGTGGAACTGGCTTGGGCCAAAGGGCTGGCCAGGCTTCAGTCCCCCCAGAGTGC

ACCCTGCATGAATGTCCGGGGACTGCAGGGGCAGGTGACTGCTGGCAGGAGATGAATGGT

TCTGTGGGTATCTGCAGGCCCACAAGCTATATTCCCCTTGGGACTGCAAATGGTGACATC

TCAATCATTGATACCAGTGGACTGCTTACCACTGACCACCCTGCAGTTGATGCCCTGGGA

CCTTCAGGCAATGATAAACTAGGGTCCCCTCACCATACTTCCCTGGCCTGGGGTACTGGA

CAGCCTGGTTCCAAGCCAACATGGCCATGTCAACGTCTTGAGGAGCTGGTTCAGGAGCTG

GCCAGACTGGATCCCTCTCTAAGTGACACTCTTACCTCCCAGCCATGCCCAGAGCCACCC

CTGGGCCTGCTGGATGGGCTGATTCCTTTAGCAGAAGTCTGGGCTGCAATGAAGCCAGGC

TGTGGTGAAGCTGGAGAAGAGGGTCCTGGTACTTCTGAGCCAGGGTCCTATCAATTCAAC

TTTACCCAGCTCCTGCCAACTTCTCAGGAGATGACAAGGCCTGAAAACTCTCCCTCCTGT

CCTGTGCCTGACTGTCCTAGTGCCCAAACATCAAACAACAGCATCCAGGCTAAGAAAGTG

GAGCTAGCCGGCCTTCTCCAAGAGATGCTGCAGGACCTTCACATCCAGGAGGAGCGGCTG

CGAGGTGCTGCCCAAGAATGGACCAGACGCAGGGTGGCTCTGGAATCCGCAGTGGGCCAG

GCCTGTGCACCCAGGGAACTGGAGCAGTTCAGCCGGTTCTTGGCTGACCTGGAGCGTGTC

CTTGGCCTCCTGCTGCTACTCGGCAGTCGCCTGGCCCGTGTGCACCATGCCTTAGCCCGG

ATGGGCCCAGATGGTGACCCTGATGAGCGGGACTCTCTGTTGCAGCGACTTGGGCTTCTG

CAACGGCAGCAGGAAGATGCCAAGGAGCTGAAGGAGCATGTGGCACGACGTGAGCGGGCC

CTGCGCGAGGTGCTGGCGAGGGCACTGTCTGCAGAGGAGTTGTGCTCCTACAGTGAGCTG

CTGGTGGGCAAGGCCACCGTTCTGGCCCAGCAGCGCTACCTGGAGGAACATGTCCAGCTC

TTTCAGAACCAACTAGACGCCCTTGGGAGCAACCTTGGCCTTCGTCCCGTCTCTCCCAGG

CCGTCCTGGCCCCCAGAGACCTGCCCTCGGGATAAACCACCCTTC

>CL283.Contig7_A-W 90 1472 minus strand KIAA1960 protein [Homo sapiens]

AGATCCACGGAACCAGCCTGCTCCTTCTGCACAGCCATGGAAGCCCTGGGTCCCAGGGAC

GACAGCGCCTCCCCAGCCTCATCCACACCAAGCCTAGACTTACGGCGGCTGTCCACTCGC

GCCGACTCGGCCTACGGTTCTTTCTCGGCCCCTGAGGCACGCACGCCGTCGCCTCGCGCG

GATCTCCACCCTTACCTCGACTGGGAATACGTGCGCGTGGTTTGGGGCGGCCCGGCCCCA

GCCCCCCCTGATGCCGATCCTCCCACCACGCAGCGGCCTCGGCCAGCTGTTGCTGCGTGC

TGTGAGCCATGGCCCCCAGAGGCCCAGGGAACCCCGGGGCCACTCAGCAGACAGGCAACC

CCGCTGCTATACGCGCAGGCTGCAGAGGCTGAGGCCGGGGCGCGGGCCGCCGAGCCACCC

AGCCCACCAGCTTCCTGGGCCGCCTACCGCCAGCGATTGCAGGGCGCGCAGCGGCGAGTG

CTCCGGGAGACATCCTTCCAGCGCAAGGAGCTCCGCATGAGCCTGCCCACCCGCCTGCGA

CCAGCTGTCCCCGCGCGACCCCCCACTGCGCACCCGCGCTCCTCCTCGCTTAGCCACCCG

GGCGGGGAGGTAGGACCCACGTGCTCCGGAGCTCCAGCGCCAGGATCCACTGGTCCGGGG

CGCCTCGCCAATCAGCAGCGGAAATGGTGCTTCTCAGAGCCAGGAAAGTTGGATTGCATG

GGTCGGGGTGGTGGGCTGGCGGGGGAGTGCTTGAGCTTGGCCTGCTCCAGCTCTGGCCTC

AACAGTCCTGAGCCCCGGGAGTTGCAGGCTGAGGTCAAAGGGTTGCAGTGCTGGGCCCAA

CCCCAAAGCACAGCGGAGCTGAACTCCGGGTCCACGAAGCTCGGCAATGGCTGTAGGCCT

GCCAGTCGGAGTCAGAGCCCTTCAGGAGAGGTCTTGGGCCCCTGGAAAGGTCCAGGAGGG

GCCGTGGCCCCTGTCCAGGTATGGAGATAGAGGATGCAGGCTTGTGGTGGAGGGGAGCAT

GTGGGAGATGGCCACATTTCAGTCACCTACTCACCTTTCCAGGCTGTTTCCCAAGCAGCA

GAAAACCCCAGACTGCTGTTTCAGACCAAACTTTCCAGCACGAAGACTTTCCTTCCTGGT

CACTGTGGTCAGTGTGGGATCCCTGCCTCCAGATTCTTGACTCAGAAGGAAGCTGCACTG

GTGCATCCTGCAGAGGACCCCCAGAGCAGCCCTGCCAAGTGTGAGCAGGAGGTCTCAGAG

ATCTGCATGGAGTCTCCATATGCCCTCCTTCCCTCCCTTCCTGATGATGAAGTGTTCCTG

GAAGAAACCCTGCATGTTGGAATGAGATCACTTCCAGACTCCCATGTTCCCCAGGAGTTT

CCA

>CL283.Contig8_A-W 90 1388 minus strand KIAA1960 protein [Homo sapiens]

AGATCCACGGAACCAGCCTGCTCCTTCTGCACAGCCATGGAAGCCCTGGGTCCCAGGGAC

GACAGCGCCTCCCCAGCCTCATCCACACCAAGCCTAGACTTACGGCGGCTGTCCACTCGC

GCCGACTCGGCCTACGGTTCTTTCTCGGCCCCTGAGGCACGCACGCCGTCGCCTCGCGCG

GATCTCCACCCTTACCTCGACTGGGAATACGTGCGCGTGGTTTGGGGCGGCCCGGCCCCA

GCCCCCCCTGATGCCGATCCTCCCACCACGCAGCGGCCTCGGCCAGCTGTTGCTGCGTGC

TGTGAGCCATGGCCCCCAGAGGCCCAGGGAACCCCGGGGCCACTCAGCAGACAGGCAACC

CCGCTGCTATACGCGCAGGCTGCAGAGGCTGAGGCCGGGGCGCGGGCCGCCGAGCCACCC

AGCCCACCAGCTTCCTGGGCCGCCTACCGCCAGCGATTGCAGGGCGCGCAGCGGCGAGTG

CTCCGGGAGACATCCTTCCAGCGCAAGGAGCTCCGCATGAGCCTGCCCACCCGCCTGCGA

CCAGCTGTCCCCGCGCGACCCCCCACTGCGCACCCGCGCTCCTCCTCGCTTAGCCACCCG

GGCGGGGAGGTAGGACCCACGTGCTCCGGAGCTCCAGCGCCAGGATCCACTGGTCCGGGG

CGCCTCGCCAATCAGCAGCGGAAATGGTGCTTCTCAGAGCCAGGAAAGTTGGATTGCATG

GGTCGGGGTGGTGGGCTGGCGGGGGAGTGCTTGAGCTTGGCCTGCTCCAGCTCTGGCCTC

AACAGTCCTGAGCCCCGGGAGTTGCAGGCTGAGGTCAAAGGGTTGCAGTGCTGGGCCCAA

CCCCAAAGCACAGCGGAGCTGAACTCCGGGTCCACGAAGCTCGGCAATGGCTGTAGGCCT

GCCAGTCGGAGTCAGAGCCCTTCAGGAGAGGTCTTGGGCCCCTGGAAAGGTCCAGGAGGG

GCCGTGGCCCCTGTCCAGGCTGTTTCCCAAGCAGCAGAAAACCCCAGACTGCTGTTTCAG

ACCAAACTTTCCAGCACGAAGACTTTCCTTCCTGGTCACTGTGGTCAGTGTGGGATCCCT

GCCTCCAGATTCTTGACTCAGAAGGAAGCTGCACTGGTGCATCCTGCAGAGGACCCCCAG

AGCAGCCCTGCCAAGTGTGAGCAGGAGGTCTCAGAGATCTGCATGGAGTCTCCATATGCC

CTCCTTCCCTCCCTTCCTGATGATGAAGTGTTCCTGGAAGAAACCCTGCATGTTGGAATG

AGATCACTTCCAGACTCCCATGTTCCCCAGGAGTTTCCA

>CL283.Contig9_A-W 80 238 minus strand PREDICTED: protein Shroom1 [Sus scrofa]

CCCAGGTCCTATCAATTCAACTTTACCCAGCTCCTGCCAACTTCTCAGGAGATGACAAGG

CCTGAAAACTCTCCCTCCTGTCCTGTGCCTGACTGTCCTAGTGCCCAAACATCAAACAAC

AGCATCCAGGCTAAGAAAGTGAGTGTGGAGAGGTTGGTG

>CL283.Contig10_A-W 47 820 minus strand protein Shroom1 [Bos taurus] >gi|154757402|gb|AAI51640.1| SHROOM1 protein [Bos taurus] >gi|296485385|gb|DAA27500.1| shroom family member 1 [Bos taurus]

GGACAGGACAACCCAGGGAATTCTAACTTTGTCCCCAGGTCCTATCAATTCAACTTTACC

CAGCTCCTGCCAACTTCTCAGGAGATGACAAGGCCTGAAAACTCTCCCTCCTGTCCTGTG

CCTGACTGTCCTAGTGCCCAAACATCAAACAACAGCATCCAGGCTAAGAAAGTGGAGCTA

GCCGGCCTTCTCCAAGAGATGCTGCAGGACCTTCACATCCAGGAGGAGCGGCTGCGAGGT

GCTGCCCAAGAATGGACCAGACGCAGGGTGGCTCTGGAATCCGCAGTGGGCCAGGCCTGT

GCACCCAGGGAACTGGAGCAGTTCAGCCGGTTCTTGGCTGACCTGGAGCGTGTCCTTGGC

CTCCTGCTGCTACTCGGCAGTCGCCTGGCCCGTGTGCACCATGCCTTAGCCCGGATGGGC

CCAGATGGTGACCCTGATGAGCGGGACTCTCTGTTGCAGCGACTTGGGCTTCTGCAACGG

CAGCAGGAAGATGCCAAGGAGCTGAAGGAGCATGTGGCACGACGTGAGCGGGCCCTGCGC

GAGGTGCTGGCGAGGGCACTGTCTGCAGAGGAGTTGTGCTCCTACAGTGAGCTGCTGGTG

GGCAAGGCCACCGTTCTGGCCCAGCAGCGCTACCTGGAGGAACATGTCCAGCTCTTTCAG

AACCAACTAGACGCCCTTGGGAGCAACCTTGGCCTTCGTCCCGTCTCTCCCAGGCCGTCC

TGGCCCCCAGAGACCTGCCCTCGGGATAAACCACCCTTCCTTCCTCCCTCTATC

>CL285.Contig1_A-W 330 1175 minus strand MAF1 homolog (S. cerevisiae), isoform CRA_d [Mus musculus]

TCCGGCCCGATCTGCCAGGCCCATACAGGCAGCACTAGCCCTTCTGGAGCAAGGAGTCTT

TTCCCCAAGGACATGAAGCTGTTGGAGAACTCGAGCTTTGAGGCCATCAACTCACAACTG

ACAGTAGAGACTGGAGATGCCCACATCATTGGCAGGATTGAGAGCTACTCGTGTAAGATG

GCAGGAGATGACAAACACATGTTCAAGCAGTTTTGCCAGGAGGGTCAGCCCCATGTGCTG

GAGGCACTATCCCCACCCCAGACTTCGGGCCTCAGCCCCAGCAGACTGAGCAAAAGCCAG

GGTGGTGAAGATGAGGGTCCTCTCAGTGACAAGTGCAGTCGCAAGACCCTCTTCTATCTG

ATTGCCACACTCAATGAGTCCTTCAGGCCAGACTATGACTTCAGCACAGCCCGAAGCCAC

GAATTCAGCCGGGAGCCCAGCCTGAGCTGGGTGGTGAATGCAGTCAACTGCAGCCTGTTC

TCAGCTGTGCGGGAAGACTTCAAGGCCCTCAAGCCGCAGCTGTGGGACGCAGTGGATGAG

GAGATCTGTCTAGCTGAATGTGACATCTACAGCTATAACCCAGACCTAGACTCGGACCCC

TTTGGGGAAGATGGCAGCCTCTGGTCTTTCAATTATTTCTTCTACAACAAGCGGCTCAAG

CGAATTGTCTTCTTCAGCTGCCGCTCCATCAGTGGTTCCACTTACACACCCTCAGAAGCA

GGCAATGAGCTGGACATGGAACTGGGGGAGGAAGAAGTGGAGGAAGAGAGTGGAGGCGGA

GGCAGTGAGGGCAGGGCTGAAGAGACCAGCACCATGGAGGAGGACAGGGTCCCTGTGATC

TGTATG

>CL285.Contig2_A-W 330 854 minus strand MAF1 homolog (S. cerevisiae), isoform CRA_d [Mus musculus]

TCCGGCCCGATCTGCCAGGCCCATACAGGCAGCACTAGCCCTTCTGGAGCAAGGAGTCTT

TTCCCCAAGGACATGAAGCTGTTGGAGAACTCGAGCTTTGAGGCCATCAACTCACAACTG

ACAGTAGAGACTGGAGATGCCCACATCATTGGCAGGATTGAGAGCTACTCGTGTAAGATG

GCAGGAGATGACAAACACATGTTCAAGCAGTTTTGCCAGGAGGGTCAGCCCCATGTGCTG

GAGGCACTATCCCCACCCCAGACTTCGGGCCTCAGCCCCAGCAGGTGAGCTGAGATAGGA

CCTGCCTCTTTGGGTCTGTGGGATGGCAGTCTAGGCTGATGTTTGTCTCTGTGCAGACTG

AGCAAAAGCCAGGGTGGTGAAGATGAGGGTCCTCTCAGTGACAAGTGCAGTCGCAAGACC

CTCTTCTATCTGATTGCCACACTCAATGAGTCCTTCAGGCCAGACTATGACTTCAGCACA

GCCCGAAGCCACGAATTCAGCCGGGAGCCCAGCCTGAGCTGGGTG

>CL285.Contig3_A-W 1334 1489 minus strand MAF1 homolog (S. cerevisiae), isoform CRA_d [Mus musculus]

AGTGGTTCCACTTACACACCCTCAGAAGCAGGCAATGAGCTGGACATGGAACTGGGGGAG

GAAGAAGTGGAGGAAGAGAGTGGAGGCGGAGGCAGTGAGGGCAGGGCTGAAGAGACCAGC

ACCATGGAGGAGGACAGGGTCCCTGTGATCTGTATG

>CL285.Contig4_A-W 330 782 minus strand MAF1 homolog (S. cerevisiae), isoform CRA_d [Mus musculus]

TCCGGCCCGATCTGCCAGGCCCATACAGGCAGCACTAGCCCTTCTGGAGCAAGGAGTCTT

TTCCCCAAGGACATGAAGCTGTTGGAGAACTCGAGCTTTGAGGCCATCAACTCACAACTG

ACAGTAGAGACTGGAGATGCCCACATCATTGGCAGGATTGAGAGCTACTCGTGTAAGATG

GCAGGAGATGACAAACACATGTTCAAGCAGTTTTGCCAGGAGGGTCAGCCCCATGTGCTG

GAGGCACTATCCCCACCCCAGACTTCGGGCCTCAGCCCCAGCAGACTGAGCAAAAGCCAG

GGTGGTGAAGATGAGGGTCCTCTCAGTGACAAGTGCAGTCGCAAGACCCTCTTCTATCTG

ATTGCCACACTCAATGAGTCCTTCAGGCCAGACTATGACTTCAGCACAGCCCGAAGCCAC

GAATTCAGCCGGGAGCCCAGCCTGAGCTGGGTG

>CL285.Contig5_A-W 1262 1417 minus strand MAF1 homolog (S. cerevisiae), isoform CRA_d [Mus musculus]

AGTGGTTCCACTTACACACCCTCAGAAGCAGGCAATGAGCTGGACATGGAACTGGGGGAG

GAAGAAGTGGAGGAAGAGAGTGGAGGCGGAGGCAGTGAGGGCAGGGCTGAAGAGACCAGC

ACCATGGAGGAGGACAGGGTCCCTGTGATCTGTATG

>CL285.Contig6_A-W 1253 1408 minus strand MAF1 homolog (S. cerevisiae), isoform CRA_d [Mus musculus]

AGTGGTTCCACTTACACACCCTCAGAAGCAGGCAATGAGCTGGACATGGAACTGGGGGAG

GAAGAAGTGGAGGAAGAGAGTGGAGGCGGAGGCAGTGAGGGCAGGGCTGAAGAGACCAGC

ACCATGGAGGAGGACAGGGTCCCTGTGATCTGTATG

>CL285.Contig7_A-W 1181 1336 minus strand MAF1 homolog (S. cerevisiae), isoform CRA_d [Mus musculus]

AGTGGTTCCACTTACACACCCTCAGAAGCAGGCAATGAGCTGGACATGGAACTGGGGGAG

GAAGAAGTGGAGGAAGAGAGTGGAGGCGGAGGCAGTGAGGGCAGGGCTGAAGAGACCAGC

ACCATGGAGGAGGACAGGGTCCCTGTGATCTGTATG

>CL285.Contig8_A-W 330 1328 minus strand MAF1 homolog (S. cerevisiae), isoform CRA_d [Mus musculus]

TCCGGCCCGATCTGCCAGGCCCATACAGGCAGCACTAGCCCTTCTGGAGCAAGGAGTCTT

TTCCCCAAGGACATGAAGCTGTTGGAGAACTCGAGCTTTGAGGCCATCAACTCACAACTG

ACAGTAGAGACTGGAGATGCCCACATCATTGGCAGGATTGAGAGCTACTCGTGTAAGATG

GCAGGAGATGACAAACACATGTTCAAGCAGTTTTGCCAGGAGGGTCAGCCCCATGTGCTG

GAGGCACTATCCCCACCCCAGACTTCGGGCCTCAGCCCCAGCAGGTGAGCTGAGATAGGA

CCTGCCTCTTTGGGTCTGTGGGATGGCAGTCTAGGCTGATGTTTGTCTCTGTGCAGACTG

AGCAAAAGCCAGGGTGGTGAAGATGAGGGTCCTCTCAGTGACAAGTGCAGTCGCAAGACC

CTCTTCTATCTGATTGCCACACTCAATGAGTCCTTCAGGCCAGACTATGACTTCAGCACA

GCCCGAAGCCACGAATTCAGCCGGGAGCCCAGCCTGAGCTGGGTGGTGAATGCAGTCAAC

TGCAGCCTGTTCTCAGCTGTGCGGGAAGACTTCAAGGCCCTCAAGCCGCAGCTGTGGGAC

GCAGTGGATGAGGAGATCTGTCTAGCTGAATGTGACATCTACAGGTGGGCCTGTATCTTC

ACAGGTAGACCCCTAGGTATATGTGACACGGAGAGTCTCATACTATGTCTTGGCCTACTC

TGCAGCTATAACCCAGACCTAGACTCGGACCCCTTTGGGGAAGATGGCAGCCTCTGGTCT

TTCAATTATTTCTTCTACAACAAGCGGCTCAAGCGAATTGTCTTCTTCAGCTGCCGCTCC

ATCAGTGGTTCCACTTACACACCCTCAGAAGCAGGCAATGAGCTGGACATGGAACTGGGG

GAGGAAGAAGTGGAGGAAGAGAGTGGAGGCGGAGGCAGTGAGGGCAGGGCTGAAGAGACC

AGCACCATGGAGGAGGACAGGGTCCCTGTGATCTGTATG

>CL285.Contig9_A-W 330 1256 minus strand MAF1 homolog (S. cerevisiae), isoform CRA_d [Mus musculus]

TCCGGCCCGATCTGCCAGGCCCATACAGGCAGCACTAGCCCTTCTGGAGCAAGGAGTCTT

TTCCCCAAGGACATGAAGCTGTTGGAGAACTCGAGCTTTGAGGCCATCAACTCACAACTG

ACAGTAGAGACTGGAGATGCCCACATCATTGGCAGGATTGAGAGCTACTCGTGTAAGATG

GCAGGAGATGACAAACACATGTTCAAGCAGTTTTGCCAGGAGGGTCAGCCCCATGTGCTG

GAGGCACTATCCCCACCCCAGACTTCGGGCCTCAGCCCCAGCAGACTGAGCAAAAGCCAG

GGTGGTGAAGATGAGGGTCCTCTCAGTGACAAGTGCAGTCGCAAGACCCTCTTCTATCTG

ATTGCCACACTCAATGAGTCCTTCAGGCCAGACTATGACTTCAGCACAGCCCGAAGCCAC

GAATTCAGCCGGGAGCCCAGCCTGAGCTGGGTGGTGAATGCAGTCAACTGCAGCCTGTTC

TCAGCTGTGCGGGAAGACTTCAAGGCCCTCAAGCCGCAGCTGTGGGACGCAGTGGATGAG

GAGATCTGTCTAGCTGAATGTGACATCTACAGGTGGGCCTGTATCTTCACAGGTAGACCC

CTAGGTATATGTGACACGGAGAGTCTCATACTATGTCTTGGCCTACTCTGCAGCTATAAC

CCAGACCTAGACTCGGACCCCTTTGGGGAAGATGGCAGCCTCTGGTCTTTCAATTATTTC

TTCTACAACAAGCGGCTCAAGCGAATTGTCTTCTTCAGCTGCCGCTCCATCAGTGGTTCC

ACTTACACACCCTCAGAAGCAGGCAATGAGCTGGACATGGAACTGGGGGAGGAAGAAGTG

GAGGAAGAGAGTGGAGGCGGAGGCAGTGAGGGCAGGGCTGAAGAGACCAGCACCATGGAG

GAGGACAGGGTCCCTGTGATCTGTATG

>CL285.Contig10_A-W 330 1247 minus strand MAF1 homolog (S. cerevisiae), isoform CRA_d [Mus musculus]

TCCGGCCCGATCTGCCAGGCCCATACAGGCAGCACTAGCCCTTCTGGAGCAAGGAGTCTT

TTCCCCAAGGACATGAAGCTGTTGGAGAACTCGAGCTTTGAGGCCATCAACTCACAACTG

ACAGTAGAGACTGGAGATGCCCACATCATTGGCAGGATTGAGAGCTACTCGTGTAAGATG

GCAGGAGATGACAAACACATGTTCAAGCAGTTTTGCCAGGAGGGTCAGCCCCATGTGCTG

GAGGCACTATCCCCACCCCAGACTTCGGGCCTCAGCCCCAGCAGGTGAGCTGAGATAGGA

CCTGCCTCTTTGGGTCTGTGGGATGGCAGTCTAGGCTGATGTTTGTCTCTGTGCAGACTG

AGCAAAAGCCAGGGTGGTGAAGATGAGGGTCCTCTCAGTGACAAGTGCAGTCGCAAGACC

CTCTTCTATCTGATTGCCACACTCAATGAGTCCTTCAGGCCAGACTATGACTTCAGCACA

GCCCGAAGCCACGAATTCAGCCGGGAGCCCAGCCTGAGCTGGGTGGTGAATGCAGTCAAC

TGCAGCCTGTTCTCAGCTGTGCGGGAAGACTTCAAGGCCCTCAAGCCGCAGCTGTGGGAC

GCAGTGGATGAGGAGATCTGTCTAGCTGAATGTGACATCTACAGCTATAACCCAGACCTA

GACTCGGACCCCTTTGGGGAAGATGGCAGCCTCTGGTCTTTCAATTATTTCTTCTACAAC

AAGCGGCTCAAGCGAATTGTCTTCTTCAGCTGCCGCTCCATCAGTGGTTCCACTTACACA

CCCTCAGAAGCAGGCAATGAGCTGGACATGGAACTGGGGGAGGAAGAAGTGGAGGAAGAG

AGTGGAGGCGGAGGCAGTGAGGGCAGGGCTGAAGAGACCAGCACCATGGAGGAGGACAGG

GTCCCTGTGATCTGTATG

>CL286.Contig1_A-W 2 904 PREDICTED: solute carrier family 2, facilitated glucose transporter member 3-like [Loxodonta africana]

TTAATCCTGGGCCGCTTGATTATTGGACTTTTCTGTGGACTCTGTACAGGTTTTGTGCCC

ATGTACATTGGCGAAGTATCTCCTACTGCTCTGCGGGGTGCCTTTGGCACTCTCAACCAG

CTGGGGATCGTCATTGGGATTCTGGTGGCTCAGATATTTGGTCTGAAATTTATCCTGGGA

TCTGAAGATCTGTGGCCTGTGCTGTTGGGCTTAACAATTATTCCAGCTGTCCTACAAAGT

ATAGCCCTTCCATTTTGCCCTGAAAGTCCTAGATTCTTGCTCATTAACAGAAAAGAAGAG

GAGAATGCTAAGAGGATCCTCCAACGGTTATGGGGCACACCAGATGTGAACCAGGATATC

CTGGAAATGAAAGAAGAGAGTGTTAGAATGTCACAAGAAAAGCAAGTCTCTGTGCTGCAG

CTCTTTAAATCACCCAACTACCAACAACCCCTCCTCATTTCCATTGTGCTCCAGCTCTCC

CAGCAGCTCTCTGGGATCAATGCTGTGTTCTATTACTCAACAGGAATCTTTAAGGAAGCA

GGTGTTGATGAGCCAATATATGCTACCATTGGAGCCGGTGTTGTTAATACTATTTTCACT

GTAGTTTCTCTGTTTCTGGTGGAAAGGGCAGGAAGAAGGACTTTACATATGATAGGCCTT

GGAGGAATGGCTATATGTTCCATCATCATGACTATTTCTTTGTTATTGAAGGATAACTAT

AATGCAATGAGCTTTGTCTGTATCACAGCTATCTTGATCTATGTGGCCTTCTTTGAGATT

GGACCAGGCCCTATTCCATGGTTTATTGTGGCTGAACTCTTTGGACAGGGACCACGCCCA

GCGGCCATGGCAGTGGCTGGTTGTTCTAACTGGACCTCCAACTTTTTGGTTGGACTGCTC

TTT

>CL286.Contig2_A-W 2 613 PREDICTED: solute carrier family 2, facilitated glucose transporter member 3-like [Loxodonta africana]

TTAATCCTGGGCCGCTTGATTATTGGACTTTTCTGTGGACTCTGTACAGGTTTTGTGCCC

ATGTACATTGGCGAAGTATCTCCTACTGCTCTGCGGGGTGCCTTTGGCACTCTCAACCAG

CTGGGGATCGTCATTGGGATTCTGGTGGCTCAGATATTTGGTCTGAAATTTATCCTGGGA

TCTGAAGATCTGTGGCCTGTGCTGTTGGGCTTAACAATTATTCCAGCTGTCCTACAAAGT

ATAGCCCTTCCATTTTGCCCTGAAAGTCCTAGATTCTTGCTCATTAACAGAAAAGAAGAG

GAGAATGCTAAGAGGATCCTCCAACGGTTATGGGGCACACCAGATGTGAACCAGGATATC

CTGGAAATGAAAGAAGAGAGTGTTAGAATGTCACAAGAAAAGCAAGTCTCTGTGCTGCAG

CTCTTTAAATCACCCAACTACCAACAACCCCTCCTCATTTCCATTGTGCTCCAGCTCTCC

CAGCAGCTCTCTGGGATCAATGCTGTGTTCTATTACTCAACAGGAATCTTTAAGGAAGCA

GGTGTTGATGAGCCAATATATGCTACCATTGGAGCCGGTGTTGTTAATACTATTTTCACT

GTAGTTTCTGTA

>CL286.Contig3_A-W 2 517 PREDICTED: solute carrier family 2, facilitated glucose transporter member 3-like [Loxodonta africana]

TTAATCCTGGGCCGCTTGATTATTGGACTTTTCTGTGGACTCTGTACAGGTTTTGTGCCC

ATGTACATTGGCGAAGTATCTCCTACTGCTCTGCGGGGTGCCTTTGGCACTCTCAACCAG

CTGGGGATCGTCATTGGGATTCTGGTGGCTCAGATATTTGGTCTGAAATTTATCCTGGGA

TCTGAAGATCTGTGGCCTGTGCTGTTGGGCTTAACAATTATTCCAGCTGTCCTACAAAGT

ATAGCCCTTCCATTTTGCCCTGAAAGTCCTAGATTCTTGCTCATTAACAGAAAAGAAGAG

GAGAATGCTAAGAGGATCCTCCAACGGTTATGGGGCACACCAGATGTGAACCAGGATATC

CTGGAAATGAAAGAAGAGAGTGTTAGAATGTCACAAGAAAAGCAAGTCTCTGTGCTGCAG

CTCTTTAAATCACCCAACTACCAACAACCCCTCCTCATTTCCATTGTGCTCCAGCTCTCC

CAGCAGCTCTCTGGGATCAATGCTGTGAGTATCTAC

>CL286.Contig4_A-W 35 670 solute carrier family 2 (facilitated glucose transporter), member 3 variant [Homo sapiens]

TGGGGGCAGCACAATGTCTTTATCCTTTCCATTTCTTTCTTTTTACCAGTCCTCCAACGG

TTATGGGGCACACCAGATGTGAACCAGGATATCCTGGAAATGAAAGAAGAGAGTGTTAGA

ATGTCACAAGAAAAGCAAGTCTCTGTGCTGCAGCTCTTTAAATCACCCAACTACCAACAA

CCCCTCCTCATTTCCATTGTGCTCCAGCTCTCCCAGCAGCTCTCTGGGATCAATGCTGTG

TTCTATTACTCAACAGGAATCTTTAAGGAAGCAGGTGTTGATGAGCCAATATATGCTACC

ATTGGAGCCGGTGTTGTTAATACTATTTTCACTGTAGTTTCTCTGTTTCTGGTGGAAAGG

GCAGGAAGAAGGACTTTACATATGATAGGCCTTGGAGGAATGGCTATATGTTCCATCATC

ATGACTATTTCTTTGTTATTGAAGGATAACTATAATGCAATGAGCTTTGTCTGTATCACA

GCTATCTTGATCTATGTGGCCTTCTTTGAGATTGGACCAGGCCCTATTCCATGGTTTATT

GTGGCTGAACTCTTTGGACAGGGACCACGCCCAGCGGCCATGGCAGTGGCTGGTTGTTCT

AACTGGACCTCCAACTTTTTGGTTGGACTGCTCTTT

>CL287.Contig1_A-W 1680 1979 minus strand membrane-associated phosphatidylinositol transfer protein 1 isoform a [Macaca mulatta]

AGCCTGAGCCCCTACAGCCATGATGGTGACAGCCTGTCCCGCTCCCAGGACCACATTCCA

CTGGCTGCCCTGCCACTGCTGGCCACCTCATCTTCCCGCTACCAGGGCGCTGTGGCCACT

GTCATTGCCCGCACCAACCAGGCCTACGCAGCTTTTCTGCGCTCATCTGAGGGTGCTGGC

TTCTGTGGGCAGGTGGTGCTGATTGGAGACGGCGTTGGTGGCATCCTGGGCTTTGATGCG

CTCTGCCACAGTGCCAACGTGGGCACAGGGAGTCGGGGCAGCAGCCGCCGTGGGAGCATG

>CL287.Contig2_A-W 1680 1874 minus strand membrane-associated phosphatidylinositol transfer protein 1 isoform a [Macaca mulatta]

AGCCTGAGCCCCTACAGCCATGATGGTGACAGCCTGTCCCGCTCCCAGGACCACATTCCA

CTGGCTGCCCTGCCACTGCTGGCCACCTCATCTTCCCGCTACCAGGGCGCTGTGGCCACT

GTCATTGCCCGCACCAACCAGGCCTACGCAGCTTTTCTGCGCTCATCTGAGGGTGCTGGC

TTCTGTGGGCAGGTC

>CL287.Contig3_A-W 152 1933 minus strand membrane-associated phosphatidylinositol transfer protein 1 isoform a [Macaca mulatta]

ATGCTCATCAAGGAGTACCACATCCTGCTGCCCATGAGCCTGGACGAGTACCAGGTGGCT

CAGCTCTACATGATCCAGAAAAAGAGTCGGGAGGAGTCCAGTGGTGAGGGCAGCGGTGTG

GAGATCCTGGCCAACCGGCCCTACACTGATGGGCCCGGGGGGAGCGGGCAGTACACACAC

AAAGTGTACCATGTGGGCTCCCACATCCCAGGCTGGTTCCGGGCACTGCTGCCCAAAGCC

GCCCTGCAGGTAGAGGAGGAGTCTTGGAATGCATACCCCTACACCCGAACCCGGTACACC

TGCCCCTTCGTGGAGAAATTCTCCATTGAAATAGAAACCTATTACCTCCCTGATGGGGGG

CAGCAGCCAAACGTCTTCAACCTAAGTGGAGCTGAGAGGAGACAGCGCATCCTCGACACC

ATCGACATTGTGCGAGATGCAGTGGCTCCAGGTGAATACAAAGCTGAAGAGGACCCCCGG

CTGTACCGTTCAGTCAAAACAGGCCGAGGGCCATTGACTGATGACTGGGCGCGGACAGCC

GCCAAGACGGGACCCCTCATGTGTGCCTACAAGCTGTGCAAGGTGGAGTTCCGATACTGG

GGCATGCAGGCCAAGATCGAGCAGTTCATCCATGATGTTGGTCTGCGAAGGGTGATGCTG

CGGGCCCACCGCCAGGCCTGGTGCTGGCAGGACGAGTGGACAGAGCTGAGCATGGCTGAC

ATCCGGGCACTGGAGGAGGAGACCGCCCGCATGCTGGCCCAGCGCATGGCCAAGTGCAAC

ATCGGCAGTGAGGGGCCAGAGGCCCAGCCCCCTGGGAAACCCAACACCGAGAACCGGTCG

GGGGCCAGCAGTGCAGGCACCCCAGATGGGCCAGAGGCCCCTGCTGGCCCTGATGCCTCA

CCTGATGCCAGTTTTGGGAAGCAGTGGTCCTCATCCTCCCGTTCCTCCTACTCATCCCAA

CATGGAGGGGGCGTGTCTCCCCAGAGCTTATCTGAGTGGCGCATGCAGAACATCGCCCGA

GACTCTGAGAACAGCTCCGAGGACGAGTTCTTTGATGCCCATGAAGGCTTCTCGGACAGT

GATGAGGTCTTCCCCAAGGAAATGACCAAGTGGAACTCAAATGACTTCATTGATGCCTTT

GCCTCCCCAACTGAAGCAGAGGGGACACCAGACCCTGGAGTCGAGGCAACTAAAGACATT

GAGGACAGGGCGCGAGTACCCAGGGACTCTGAGGGCCCAGATGGAGCTGGGGACCTTGGG

GCTGAGGCATGCGCGGTCCATGCCCTCTTCCTCATCCTGCACAGCGGCAACATCCTGGAC

TCTGGCCCAGGGGATGCCAACTCCAAGCAGGCCGACGTGCAGACGCTGAGCTCCAGCTTC

GAGGCCGTCACCCGCATCCACTTCCCTGAGGCCCTGGGCCACGTGGCCCTGCGACTGGTG

CCCTGCCCACCCATCTGTGCTGCCGCCTATGCCCTTGTCTCCAACCTGAGCCCCTACAGC

CATGATGGTGACAGCCTGTCCCGCTCCCAGGACCACATTCCACTGGCTGCCCTGCCACTG

CTGGCCACCTCATCTTCCCGCTACCAGGGCGCTGTGGCCACTGTCATTGCCCGCACCAAC

CAGGCCTACGCAGCTTTTCTGCGCTCATCTGAGGGTGCTGGCTTCTGTGGGCAGGTGGTG

CTGATTGGAGACGGCGTTGGTGGCATCCTGGGCTTTGATGCGCTCTGCCACAGTGCCAAC

GTGGGCACAGGGAGTCGGGGCAGCAGCCGCCGTGGGAGCATG

>CL287.Contig4_A-W 152 1828 minus strand membrane-associated phosphatidylinositol transfer protein 1 isoform a [Macaca mulatta]

ATGCTCATCAAGGAGTACCACATCCTGCTGCCCATGAGCCTGGACGAGTACCAGGTGGCT

CAGCTCTACATGATCCAGAAAAAGAGTCGGGAGGAGTCCAGTGGTGAGGGCAGCGGTGTG

GAGATCCTGGCCAACCGGCCCTACACTGATGGGCCCGGGGGGAGCGGGCAGTACACACAC

AAAGTGTACCATGTGGGCTCCCACATCCCAGGCTGGTTCCGGGCACTGCTGCCCAAAGCC

GCCCTGCAGGTAGAGGAGGAGTCTTGGAATGCATACCCCTACACCCGAACCCGGTACACC

TGCCCCTTCGTGGAGAAATTCTCCATTGAAATAGAAACCTATTACCTCCCTGATGGGGGG

CAGCAGCCAAACGTCTTCAACCTAAGTGGAGCTGAGAGGAGACAGCGCATCCTCGACACC

ATCGACATTGTGCGAGATGCAGTGGCTCCAGGTGAATACAAAGCTGAAGAGGACCCCCGG

CTGTACCGTTCAGTCAAAACAGGCCGAGGGCCATTGACTGATGACTGGGCGCGGACAGCC

GCCAAGACGGGACCCCTCATGTGTGCCTACAAGCTGTGCAAGGTGGAGTTCCGATACTGG

GGCATGCAGGCCAAGATCGAGCAGTTCATCCATGATGTTGGTCTGCGAAGGGTGATGCTG

CGGGCCCACCGCCAGGCCTGGTGCTGGCAGGACGAGTGGACAGAGCTGAGCATGGCTGAC

ATCCGGGCACTGGAGGAGGAGACCGCCCGCATGCTGGCCCAGCGCATGGCCAAGTGCAAC

ATCGGCAGTGAGGGGCCAGAGGCCCAGCCCCCTGGGAAACCCAACACCGAGAACCGGTCG

GGGGCCAGCAGTGCAGGCACCCCAGATGGGCCAGAGGCCCCTGCTGGCCCTGATGCCTCA

CCTGATGCCAGTTTTGGGAAGCAGTGGTCCTCATCCTCCCGTTCCTCCTACTCATCCCAA

CATGGAGGGGGCGTGTCTCCCCAGAGCTTATCTGAGTGGCGCATGCAGAACATCGCCCGA

GACTCTGAGAACAGCTCCGAGGACGAGTTCTTTGATGCCCATGAAGGCTTCTCGGACAGT

GATGAGGTCTTCCCCAAGGAAATGACCAAGTGGAACTCAAATGACTTCATTGATGCCTTT

GCCTCCCCAACTGAAGCAGAGGGGACACCAGACCCTGGAGTCGAGGCAACTAAAGACATT

GAGGACAGGGCGCGAGTACCCAGGGACTCTGAGGGCCCAGATGGAGCTGGGGACCTTGGG

GCTGAGGCATGCGCGGTCCATGCCCTCTTCCTCATCCTGCACAGCGGCAACATCCTGGAC

TCTGGCCCAGGGGATGCCAACTCCAAGCAGGCCGACGTGCAGACGCTGAGCTCCAGCTTC

GAGGCCGTCACCCGCATCCACTTCCCTGAGGCCCTGGGCCACGTGGCCCTGCGACTGGTG

CCCTGCCCACCCATCTGTGCTGCCGCCTATGCCCTTGTCTCCAACCTGAGCCCCTACAGC

CATGATGGTGACAGCCTGTCCCGCTCCCAGGACCACATTCCACTGGCTGCCCTGCCACTG

CTGGCCACCTCATCTTCCCGCTACCAGGGCGCTGTGGCCACTGTCATTGCCCGCACCAAC

CAGGCCTACGCAGCTTTTCTGCGCTCATCTGAGGGTGCTGGCTTCTGTGGGCAGGTC

>CL287.Contig5_A-W 1919 2452 minus strand membrane-associated phosphatidylinositol transfer protein 1 isoform a [Macaca mulatta]

AGCCTGAGCCCCTACAGCCATGATGGTGACAGCCTGTCCCGCTCCCAGGACCACATTCCA

CTGGCTGCCCTGCCACTGCTGGCCACCTCATCTTCCCGCTACCAGGGCGCTGTGGCCACT

GTCATTGCCCGCACCAACCAGGCCTACGCAGCTTTTCTGCGCTCATCTGAGGGTGCTGGC

TTCTGTGGGCAGGTGGTGCTGATTGGAGACGGCGTTGGTGGCATCCTGGGCTTTGATGCG

CTCTGCCACAGTGCCAACGTGGGCACAGGGAGTCGGGGCAGCAGCCGCCGTGGGAGCATG

AACAACGAGCTGCTCTCTCCAGAGGTCGGCCCAGCACGAGACCCACTGGCAGATGGGGCG

GAGACACTAGGCCGAGCCAGTCCGGAACCCTCAGCCCTGCTTGCCCAGCGCAACCCCAGC

GACATGGCCAGTTCTGAGCCTGAGGGCTCTCAGAACAGCCTGCAGGCGGTCCCTGCACTC

ACCTCCTCTGGGGAGCCCCGGCGGCCTAGCACAGCCTCCTGCCCACCCGCTGCC

>CL287.Contig6_A-W 152 793 minus strand PREDICTED: membrane-associated phosphatidylinositol transfer protein 1 [Pan paniscus]

ATGCTCATCAAGGAGTACCACATCCTGCTGCCCATGAGCCTGGACGAGTACCAGGTGGCT

CAGCTCTACATGATCCAGAAAAAGAGTCGGGAGGAGTCCAGTGGTGAGGGCAGCGGTGTG

GAGATCCTGGCCAACCGGCCCTACACTGATGGGCCCGGGGGGAGCGGGCAGTACACACAC

AAAGTGTACCATGTGGGCTCCCACATCCCAGGCTGGTTCCGGGCACTGCTGCCCAAAGCC

GCCCTGCAGGTAGAGGAGGAGTCTTGGAATGCATACCCCTACACCCGAACCCGGTACACC

TGCCCCTTCGTGGAGAAATTCTCCATTGAAATAGAAACCTATTACCTCCCTGATGGGGGG

CAGCAGCCAAACGTCTTCAACCTAAGTGGAGCTGAGAGGAGACAGCGCATCCTCGACACC

ATCGACATTGTGCGAGATGCAGTGGCTCCAGGTGAATACAAAGCTGAAGAGGACCCCCGG

CTGTACCGTTCAGTCAAAACAGGCCGAGGGCCATTGACTGATGACTGGGCGCGGACAGCC

GCCAAGACGGGACCCCTCATGTGTGCCTACAAGCTGTGCAAGGTGGAGTTCCGATACTGG

GGCATGCAGGCCAAGATCGAGCAGTTCATCCATGATGTTGGT

>CL288.Contig1_A-W 235 357 minus strand endonuclease/reverse transcriptase [Rattus norvegicus]

AAAAAAGAAAATACAATACCTATGGCAAAAATAGGAAAGAAAGAAAACAAATTATCACTG

TTTGTGGATGATATAATCCTATGCCTAGAAGATCCAAAAAGTTCCACCAAAAGACTGCTT

AGA

>CL288.Contig2_A-W 235 354 minus strand endonuclease reverse transcriptase [Bos taurus]

AAAAAAGAAAATACAATACCTATGGCAAAAATAGGAAAGAAAGAAAACAAATTATCACTG

TTTGTGGATGATATAATCCTATGCCTAGAAGATCCAAAAAGTTCCACCAAAAGACTGCTT

>CL290.Contig1_A-W 1626 1745 minus strand PREDICTED: acyl-CoA synthetase family member 2 [Oryctolagus cuniculus]

CAGATCTCCCACTTCAAGATCCCCCGCTACATCGTCTTTGTCAGGGACTACCCCCTCACG

GTCTCAGGAAAGATCCAGAAATACAAACTCCGAGAGCAGATGGAAAGACATCTGAAACTG

>CL290.Contig2_A-W 927 1046 minus strand Acyl-CoA synthetase family member 2, mitochondrial [Cricetulus griseus]

CAGATCTCCCACTTCAAGATCCCCCGCTACATCGTCTTTGTCAGGGACTACCCCCTCACG

GTCTCAGGAAAGATCCAGAAATACAAACTCCGAGAGCAGATGGAAAGACATCTGAAACTG

>CL290.Contig3_A-W 329 856 minus strand PREDICTED: acyl-CoA synthetase family member 2, mitochondrial-like [Cavia porcellus]

TCCCAGGCCCGGATCGTGAACGTGAATACAGGGGAGCTGGCCGAGCTGAACACGCCCGGG

GAGCTGTGCATCCGAGGGTACTGCGTCATGCAGGGATACTGGGGAGAGCCTCAGAAGACC

TTTGAAGCAGTTGGGCAGGACAAGTGGTATCGAACAGGAGACATCGCCTCCATAGACGAG

CAGGGCTTCTGCAAGATCGTGGGTCGCTCTAAGGACATGATCATCCGGGGCGGTGAGAAC

ATCTACCCAGCAGAACTTGAGGATTTCTTCCACACACACCCACAGGTGCAGGAAGTGCAG

GTGGTGGGAGTGAAGGACCACAGGATGGGGGAGGAGATTTGTGCCTGCATCCGGCTAAAG

AGTGGGGAGACCACCACTGAGGAAGAGATCAAAGCTTTCTGCAAAGGGAAGATCTCCCAC

TTCAAGATCCCCCGCTACATCGTCTTTGTCAGGGACTACCCCCTCACGGTCTCAGGAAAG

ATCCAGAAATACAAACTCCGAGAGCAGATGGAAAGACATCTGAAACTG

>CL290.Contig4_A-W 521 640 minus strand PREDICTED: acyl-CoA synthetase family member 2 [Oryctolagus cuniculus]

CAGATCTCCCACTTCAAGATCCCCCGCTACATCGTCTTTGTCAGGGACTACCCCCTCACG

GTCTCAGGAAAGATCCAGAAATACAAACTCCGAGAGCAGATGGAAAGACATCTGAAACTG

>CL290.Contig5_A-W 346 465 minus strand Acyl-CoA synthetase family member 2, mitochondrial [Heterocephalus glaber]

CAGATCTCCCACTTCAAGATCCCCCGCTACATCGTCTTTGTCAGGGACTACCCCCTCACG

GTCTCAGGAAAGATCCAGAAATACAAACTCCGAGAGCAGATGGAAAGACATCTGAAACTG

>CL290.Contig6_A-W 82 450 minus strand PREDICTED: acyl-CoA synthetase family member 2 [Oryctolagus cuniculus]

GACATCGCCTCCATAGACGAGCAGGGCTTCTGCAAGATCGTGGGTCGCTCTAAGGACATG

ATCATCCGGGGCGGTGAGAACATCTACCCAGCAGAACTTGAGGATTTCTTCCACACACAC

CCACAGGTGCAGGAAGTGCAGGTGGTGGGAGTGAAGGACCACAGGATGGGGGAGGAGATT

TGTGCCTGCATCCGGCTAAAGAGTGGGGAGACCACCACTGAGGAAGAGATCAAAGCTTTC

TGCAAAGGGAAGATCTCCCACTTCAAGATCCCCCGCTACATCGTCTTTGTCAGGGACTAC

CCCCTCACGGTCTCAGGAAAGATCCAGAAATACAAACTCCGAGAGCAGATGGAAAGACAT

CTGAAACTG

>CL290.Contig7_A-W 45 275 minus strand Acyl-CoA synthetase family member 2, mitochondrial [Heterocephalus glaber]

AAGGTGGTGGGAGTGAAGGACCACAGGATGGGGGAGGAGATTTGTGCCTGCATCCGGCTA

AAGAGTGGGGAGACCACCACTGAGGAAGAGATCAAAGCTTTCTGCAAAGGGAAGATCTCC

CACTTCAAGATCCCCCGCTACATCGTCTTTGTCAGGGACTACCCCCTCACGGTCTCAGGA

AAGATCCAGAAATACAAACTCCGAGAGCAGATGGAAAGACATCTGAAACTG

>CL290.Contig8_A-W 63 1904 minus strand PREDICTED: acyl-CoA synthetase family member 2 [Oryctolagus cuniculus]

GCTGTCTCTGCCGGCATGCTGCGCCTCCGGAGGCTGTGTGCCATGAGCCTGGCGGTGCAG

GGGCCCCGGCCTGTCCTCTCTCCGGGATGGCAGGAAGCGAATTCGCAGCACGTCCGTCCG

CTCAGCTCCCAAGAGCTGGAACGCACCGCCCCTCTGCCCGTTGGGGGCTTCAGCTACCTT

CAGGGACACACCAGTTTCCCTCTTATCAACAAGACTGTGGGGCAGTGCCTTGATGCCACA

GCACAGAGGTTCCCCGACCGAGAGGCCTTGGTTGTCCTCCATGAAAACGTCAGGCTGAAC

TTCGCCCAACTCAAGGAAGAGGTGGATAAAGCTGCTTCTGGTCTTCTGAGCATTGGCCTC

TGCAAGGGTGACCGGTTGGGCATGTGGGGACCCAACTCCTATGCATGGGTGCTCATGCAG

TTGGCCACTGCCCAGGCAGGCATCATTCTGGTGTCTGTGAACCCAGCCTACCAGGCTATG

GAACTGGAGTATGTCCTCAAAAAGGTGGGCTGCAAAGCCCTCGTATTCCCCAAGCAATTC

AAGACCCAGCAATACTACAACATCTTGAAGCAGATCTGTCCGGAGCTTGAAAAGGCCCAG

CCAGGGGCCTTAAAGAGTGAGAGTCTCCCAGATCTGACCACAGTCATCTCGGTGGACGAC

CCTTTGCCGGGGACCCTGCTGCTGGATGATGTGGTGGCAGCTGGTAGCACAGAGCAGCAT

CTGGCCCAGCTTCGACACACCCAGCAGTTCCTGTCCTGCCATGACCCCATCAACATCCAG

TTCACCTCGGGAACCACAGGCAGCCCCAAGGGGGCCACCCTCTCCCACTACAACATTGTG

AACAACTCCAACATGATAGGCCAGCGCCTTAAAATGCATGTGAAGCTGCCAGAGGAGTTG

CGGGTGGTCATGCCCTGCCCCCTGTACCACTGCCTGGGCTCCGTGGGGGGCACGATGATG

TGTTTGATGCACGGTGCCACTCTCCTTCTGTCCTCTCCGAGCTTCAATGGCAAGAAGGCG

CTGGAGGCCATCACCAGAGAGAAAGGCTCCTTCATATATGGCACCCCAACGATGTTCGTG

GACATTATGAACCAGGCCGACTTCTCCAGTTACGACTTATCATCCTTATGTGGAGGTGTG

ATCGCCGGGTCCCCTGCACCTCCAGAGCTGGTCCGAGCCATCATCCACAAAATGAACATG

AAGGAGCTGGTGGTTGTTTATGGAACTACAGAGAATAGTCCTGTGACCTTCATGAACTTC

CCTGAGGACACTCTGGAGCAGAAGGCAGAGAGTGTGGGCAGAGTTATGCCTCACACGGAG

GCCCGGATCGTGAACGTGAATACAGGGGAGCTGGCCGAGCTGAACACGCCCGGGGAGCTG

TGCATCCGAGGGTACTGCGTCATGCAGGGATACTGGGGAGAGCCTCAGAAGACCTTTGAA

GCAGTTGGGCAGGACAAGTGGTATCGAACAGGAGACATCGCCTCCATAGACGAGCAGGGC

TTCTGCAAGATCGTGGGTCGCTCTAAGGACATGATCATCCGGGGCGGTGAGAACATCTAC

CCAGCAGAACTTGAGGATTTCTTCCACACACACCCACAGGTGCAGGAAGTGCAGGTGGTG

GGAGTGAAGGACCACAGGATGGGGGAGGAGATTTGTGCCTGCATCCGGCTAAAGAGTGGG

GAGACCACCACTGAGGAAGAGATCAAAGCTTTCTGCAAAGGGAAGATCTCCCACTTCAAG

ATCCCCCGCTACATCGTCTTTGTCAGGGACTACCCCCTCACGGTCTCAGGAAAGATCCAG

AAATACAAACTCCGAGAGCAGATGGAAAGACATCTGAAACTG

>CL291.Contig1_A-W 27 1130 Glucagon receptor [Heterocephalus glaber]

GGACCAGTCAGAGGCAGGGAGTGGCCCCCAGCCCTGAGCAGCCACTGTGGTTCACAGAAG

GAGGTAGCCAAGATGTACAGCAGCTTCCAGGTGATGTACACCGTGGGCTACTCCCTGTCC

CTGGGGGCCCTGCTGCTCGCCCTGGCCATCATGCTGGGCCTCAGCAAACTCCACTGCACC

CGCAACTACATCCACGTGAACCTGTTTGCATCCTTTGTGCTGAAGGCCAGCTCTGTGCTG

GTCATCGACTGGCTGCTGAAGACCCGTTACAGCCAGAAGATCGGGGATGACCTCAGTGTC

AGCATCTGGCTCAGCGATGGGGCGGTGGCCGGCTGCCGTGTGGCCGCAGTGATCATGCAG

TATGGCGTGGTGGCCAACTACTGCTGGCTGCTGGTGGAGGGCGTGTACCTGCATGGCCTG

CTGGGCCTGGCCTCCTTCCCTGAGCGAAGCTTCTTCACCCTCTACCTGGGCATTGGCTGG

GGTGCGCCCCTGCTGTTCATCATCCCCTGGGCAGTGGTCAAGTGTCTGTTTGAGAACATT

CAGTGCTGGACCAGCAATACCAACATGGGCGTCTGGTGGATCTTGCGAGCCCCGGTCTTC

CTGGCCATCCTGATCAACTCCTTCATCTTTGTCCGCGTGGTCCACCTTCTCATCACCAAG

CTTCAGGCCCGCCAGATGCACTACGCTGACTACAAGTTCCGGCTGGCCAGATCCACCCTG

ACCCTCATCCCCCTGCTGGGGGTCCACGAAGTGGTCTTCGCCTTTGTGACCGATGAGCAG

GCTCAGGGGGCCCTGCGCTCCGCCAAGCTCTTCTTTGACCTCTTCCTCAGCTCCTTCCAG

GGCCTGCTGGTGGCTGTCCTCTACTGCTTCCTCAGCAAAGAGGTACAGGCAGAGCTGCTG

CAGCGCTGGCGGCGCTGGCACGAGGGCAGAGCGCTGCAGGACCCGCGACGCAGCAGCAGC

CCCTCGGCCCCAGCTAGGCCTTGCCGTGGCCCCTCCTGCGAGAAGCTTCGACTTGTGAAG

GGTGGCAGCAGCAATGGGGCTGGCCAGGACCCCTCTGCAGAGACCTCCTTGGCCTGTGGC

CTCCCTGAGTTGGCTGAGAGCCCC

>CL291.Contig2_A-W 589 1104 Glucagon receptor [Heterocephalus glaber]

AGGTGCTGGACCAGCAATACCAACATGGGCGTCTGGTGGATCTTGCGAGCCCCGGTCTTC

CTGGCCATCCTGCTTCAGGCCCGCCAGATGCACTACGCTGACTACAAGTTCCGGCTGGCC

AGATCCACCCTGACCCTCATCCCCCTGCTGGGGGTCCACGAAGTGGTCTTCGCCTTTGTG

ACCGATGAGCAGGCTCAGGGGGCCCTGCGCTCCGCCAAGCTCTTCTTTGACCTCTTCCTC

AGCTCCTTCCAGGGCCTGCTGGTGGCTGTCCTCTACTGCTTCCTCAGCAAAGAGGTACAG

GCAGAGCTGCTGCAGCGCTGGCGGCGCTGGCACGAGGGCAGAGCGCTGCAGGACCCGCGA

CGCAGCAGCAGCCCCTCGGCCCCAGCTAGGCCTTGCCGTGGCCCCTCCTGCGAGAAGCTT

CGACTTGTGAAGGGTGGCAGCAGCAATGGGGCTGGCCAGGACCCCTCTGCAGAGACCTCC

TTGGCCTGTGGCCTCCCTGAGTTGGCTGAGAGCCCC

>CL291.Contig3_A-W 544 1008 Glucagon receptor [Heterocephalus glaber]

AGCAAACTCCACTGCACCCGCAACTACATCCACGTGAACCTGTTTGCATCCTTTGTGCTG

AAGGCCAGCTCTGTGCTGGTCATCGACTGGCTGCTGAAGACCCGTTACAGCCAGAAGATC

GGGGATGACCTCAGTGTCAGCATCTGGCTCAGCGATGGGGTGAGTGCCAGAGCAGACCAG

CCTAGACTGCTGCCGCCTTGGGCAGGCAGGGTGCTGACAGCTGCCCACACTGGCCCTGCC

CAGGCGGTGGCCGGCTGCCGTGTGGCCGCAGTGATCATGCAGTATGGCGTGGTGGCCAAC

TACTGCTGGCTGCTGGTGGAGGGCGTGTACCTGCATGGCCTGCTGGGCCTGGCCTCCTTC

CCTGAGCGAAGCTTCTTCACCCTCTACCTGGGCATTGGCTGGGGTGCGCCCCTGCTGTTC

ATCATCCCCTGGGCAGTGGTCAAGTGTCTGTTTGAGAACATTCAG

>CL291.Contig4_A-W 544 1008 PREDICTED: glucagon receptor [Equus caballus]

AGCAAACTCCACTGCACCCGCAACTACATCCACGTGAACCTGTTTGCATCCTTTGTGCTG

AAGGCCAGCTCTGTGCTGGTCATCGACTGGCTGCTGAAGACCCGTTACAGCCAGAAGATC

GGGGATGACCTCAGTGTCAGCATCTGGCTCAGCGATGGGGTGAGTGCCAGAGCAGACCAG

CCTAGACTGCTGCCGCCTTGGGCAGGCAGGGTGCTGACAGCTGCCCACACTGGCCCTGCC

CAGGCGGTGGCCGGCTGCCGTGTGGCCGCAGTGATCATGCAGTATGGCGTGGTGGCCAAC

TACTGCTGGCTGCTGGTGGAGGGCGTGTACCTGCATGGCCTGCTGGGCCTGGCCTCCTTC

CCTGAGCGAAGCTTCTTCACCCTCTACCTGGGCATTGGCTGGGGTGCGCCCCTGCTGTTC

ATCATCCCCTGGGCAGTGGTCAAGTGTCTGTTTGAGAACATTCAG

>CL291.Contig5_A-W 544 948 Glucagon receptor [Heterocephalus glaber]

AGCAAACTCCACTGCACCCGCAACTACATCCACGTGAACCTGTTTGCATCCTTTGTGCTG

AAGGCCAGCTCTGTGCTGGTCATCGACTGGCTGCTGAAGACCCGTTACAGCCAGAAGATC

GGGGATGACCTCAGTGTCAGCATCTGGCTCAGCGATGGGGTGAGTGCCAGAGCAGACCAG

CCTAGACTGCTGCCGCCTTGGGCAGGCAGGGTGCTGACAGCTGCCCACACTGGCCCTGCC

CAGGCGGTGGCCGGCTGCCGTGTGGCCGCAGTGATCATGCAGTATGGCGTGGTGGCCAAC

TACTGCTGGCTGCTGGTGGAGGGCGTGTACCTGCATGGCCTGCTGGGCCTGGCCTCCTTC

CCTGAGCGAAGCTTCTTCACCCTCTACCTGGGCATTGGCTGGGGT

>CL291.Contig6_A-W 3 935 Glucagon receptor [Heterocephalus glaber]

CTGCTGCTGCTGCTGCTGCTGGCCTGCCAGCCGCAGGCCCTCTCTGCCCAGGTAATGGAC

TTCTTGTTCGAGAAGTGGAAGCTCTACAGTGACCAGTGCCACCGCAACATGAGCCTGCTG

CCTCCCCCAAATGACCTGGTCTGCAACAGGACCTTCGACAAGTACTCCTGCTGGCCAGAC

ACCCCTCCCAATACCACGGCCAACATCTCCTGCCCCTGGTACCTGCCTTGGTATCACAAA

GTGCAGCACCGCCTGGTCTTCAAGAAGTGTGGGCCGGATGGGCAGTGGGTGCGTGGGCCT

CGGGGACAGCCATGGCGCAACGCTTCCCAGTGCCAGATGGAGGACGAGGAGCTCGAGGGC

CAGAAGGAGGTAGCCAAGATGTACAGCAGCTTCCAGGTGATGTACACCGTGGGCTACTCC

CTGTCCCTGGGGGCCCTGCTGCTCGCCCTGGCCATCATGCTGGGCCTCAGCAAACTCCAC

TGCACCCGCAACTACATCCACGTGAACCTGTTTGCATCCTTTGTGCTGAAGGCCAGCTCT

GTGCTGGTCATCGACTGGCTGCTGAAGACCCGTTACAGCCAGAAGATCGGGGATGACCTC

AGTGTCAGCATCTGGCTCAGCGATGGGGTGAGTGCCAGAGCAGACCAGCCTAGACTGCTG

CCGCCTTGGGCAGGCAGGGTGCTGACAGCTGCCCACACTGGCCCTGCCCAGGCGGTGGCC

GGCTGCCGTGTGGCCGCAGTGATCATGCAGTATGGCGTGGTGGCCAACTACTGCTGGCTG

CTGGTGGAGGGCGTGTACCTGCATGGCCTGCTGGGCCTGGCCTCCTTCCCTGAGCGAAGC

TTCTTCACCCTCTACCTGGGCATTGGCTGGGGTGCGCCCCTGCTGTTCATCATCCCCTGG

GCAGTGGTCAAGTGTCTGTTTGAGAACATTCAG

>CL291.Contig7_A-W 3 875 Glucagon receptor [Heterocephalus glaber]

CTGCTGCTGCTGCTGCTGCTGGCCTGCCAGCCGCAGGCCCTCTCTGCCCAGGTAATGGAC

TTCTTGTTCGAGAAGTGGAAGCTCTACAGTGACCAGTGCCACCGCAACATGAGCCTGCTG

CCTCCCCCAAATGACCTGGTCTGCAACAGGACCTTCGACAAGTACTCCTGCTGGCCAGAC

ACCCCTCCCAATACCACGGCCAACATCTCCTGCCCCTGGTACCTGCCTTGGTATCACAAA

GTGCAGCACCGCCTGGTCTTCAAGAAGTGTGGGCCGGATGGGCAGTGGGTGCGTGGGCCT

CGGGGACAGCCATGGCGCAACGCTTCCCAGTGCCAGATGGAGGACGAGGAGCTCGAGGGC

CAGAAGGAGGTAGCCAAGATGTACAGCAGCTTCCAGGTGATGTACACCGTGGGCTACTCC

CTGTCCCTGGGGGCCCTGCTGCTCGCCCTGGCCATCATGCTGGGCCTCAGCAAACTCCAC

TGCACCCGCAACTACATCCACGTGAACCTGTTTGCATCCTTTGTGCTGAAGGCCAGCTCT

GTGCTGGTCATCGACTGGCTGCTGAAGACCCGTTACAGCCAGAAGATCGGGGATGACCTC

AGTGTCAGCATCTGGCTCAGCGATGGGGTGAGTGCCAGAGCAGACCAGCCTAGACTGCTG

CCGCCTTGGGCAGGCAGGGTGCTGACAGCTGCCCACACTGGCCCTGCCCAGGCGGTGGCC

GGCTGCCGTGTGGCCGCAGTGATCATGCAGTATGGCGTGGTGGCCAACTACTGCTGGCTG

CTGGTGGAGGGCGTGTACCTGCATGGCCTGCTGGGCCTGGCCTCCTTCCCTGAGCGAAGC

TTCTTCACCCTCTACCTGGGCATTGGCTGGGGT

>CL291.Contig8_A-W 3 851 Glucagon receptor [Heterocephalus glaber]

CTGCTGCTGCTGCTGCTGCTGGCCTGCCAGCCGCAGGCCCTCTCTGCCCAGGTAATGGAC

TTCTTGTTCGAGAAGTGGAAGCTCTACAGTGACCAGTGCCACCGCAACATGAGCCTGCTG

CCTCCCCCAAATGACCTGGTCTGCAACAGGACCTTCGACAAGTACTCCTGCTGGCCAGAC

ACCCCTCCCAATACCACGGCCAACATCTCCTGCCCCTGGTACCTGCCTTGGTATCACAAA

GTGCAGCACCGCCTGGTCTTCAAGAAGTGTGGGCCGGATGGGCAGTGGGTGCGTGGGCCT

CGGGGACAGCCATGGCGCAACGCTTCCCAGTGCCAGATGGAGGACGAGGAGCTCGAGGGC

CAGAAGGAGGTAGCCAAGATGTACAGCAGCTTCCAGGTGATGTACACCGTGGGCTACTCC

CTGTCCCTGGGGGCCCTGCTGCTCGCCCTGGCCATCATGCTGGGCCTCAGCAAACTCCAC

TGCACCCGCAACTACATCCACGTGAACCTGTTTGCATCCTTTGTGCTGAAGGCCAGCTCT

GTGCTGGTCATCGACTGGCTGCTGAAGACCCGTTACAGCCAGAAGATCGGGGATGACCTC

AGTGTCAGCATCTGGCTCAGCGATGGGGCGGTGGCCGGCTGCCGTGTGGCCGCAGTGATC

ATGCAGTATGGCGTGGTGGCCAACTACTGCTGGCTGCTGGTGGAGGGCGTGTACCTGCAT

GGCCTGCTGGGCCTGGCCTCCTTCCCTGAGCGAAGCTTCTTCACCCTCTACCTGGGCATT

GGCTGGGGTGCGCCCCTGCTGTTCATCATCCCCTGGGCAGTGGTCAAGTGTCTGTTTGAG

AACATTCAG

>CL291.Contig9_A-W 3 791 Glucagon receptor [Heterocephalus glaber]

CTGCTGCTGCTGCTGCTGCTGGCCTGCCAGCCGCAGGCCCTCTCTGCCCAGGTAATGGAC

TTCTTGTTCGAGAAGTGGAAGCTCTACAGTGACCAGTGCCACCGCAACATGAGCCTGCTG

CCTCCCCCAAATGACCTGGTCTGCAACAGGACCTTCGACAAGTACTCCTGCTGGCCAGAC

ACCCCTCCCAATACCACGGCCAACATCTCCTGCCCCTGGTACCTGCCTTGGTATCACAAA

GTGCAGCACCGCCTGGTCTTCAAGAAGTGTGGGCCGGATGGGCAGTGGGTGCGTGGGCCT

CGGGGACAGCCATGGCGCAACGCTTCCCAGTGCCAGATGGAGGACGAGGAGCTCGAGGGC

CAGAAGGAGGTAGCCAAGATGTACAGCAGCTTCCAGGTGATGTACACCGTGGGCTACTCC

CTGTCCCTGGGGGCCCTGCTGCTCGCCCTGGCCATCATGCTGGGCCTCAGCAAACTCCAC

TGCACCCGCAACTACATCCACGTGAACCTGTTTGCATCCTTTGTGCTGAAGGCCAGCTCT

GTGCTGGTCATCGACTGGCTGCTGAAGACCCGTTACAGCCAGAAGATCGGGGATGACCTC

AGTGTCAGCATCTGGCTCAGCGATGGGGCGGTGGCCGGCTGCCGTGTGGCCGCAGTGATC

ATGCAGTATGGCGTGGTGGCCAACTACTGCTGGCTGCTGGTGGAGGGCGTGTACCTGCAT

GGCCTGCTGGGCCTGGCCTCCTTCCCTGAGCGAAGCTTCTTCACCCTCTACCTGGGCATT

GGCTGGGGT

>CL291.Contig10_A-W 3 470 Glucagon receptor [Heterocephalus glaber]

CTGCTGCTGCTGCTGCTGCTGGCCTGCCAGCCGCAGGCCCTCTCTGCCCAGGTAATGGAC

TTCTTGTTCGAGAAGTGGAAGCTCTACAGTGACCAGTGCCACCGCAACATGAGCCTGCTG

CCTCCCCCAAATGACCTGGTCTGCAACAGGACCTTCGACAAGTACTCCTGCTGGCCAGAC

ACCCCTCCCAATACCACGGCCAACATCTCCTGCCCCTGGTACCTGCCTTGGTATCACAAA

GTGCAGCACCGCCTGGTCTTCAAGAAGTGTGGGCCGGATGGGCAGTGGGTGCGTGGGCCT

CGGGGACAGCCATGGCGCAACGCTTCCCAGTGCCAGATGGAGGACGAGGAGCTCGAGGGC

CAGAAGGAGGTAGCCAAGATGTACAGCAGCTTCCAGGTGATGTACACCGTGGGCTACTCC

CTGTCCCTGGGGGCCCTGCTGCTCGCCCTGGCCATCATGCTGGGCCTC

>CL291.Contig11_A-W 27 188 Glucagon receptor [Heterocephalus glaber]

GGACCAGTCAGAGGCAGGGAGTGGCCCCCAGCCCTGAGCAGCCACTGTGGTTCACAGAAG

GAGGTAGCCAAGATGTACAGCAGCTTCCAGGTGATGTACACCGTGGGCTACTCCCTGTCC

CTGGGGGCCCTGCTGCTCGCCCTGGCCATCATGCTGGGCCTC

>CL291.Contig12_A-W 955 1518 Glucagon receptor [Heterocephalus glaber]

AGGTGCTGGACCAGCAATACCAACATGGGCGTCTGGTGGATCTTGCGAGCCCCGGTCTTC

CTGGCCATCCTGATCAACTCCTTCATCTTTGTCCGCGTGGTCCACCTTCTCATCACCAAG

CTTCAGGCCCGCCAGATGCACTACGCTGACTACAAGTTCCGGCTGGCCAGATCCACCCTG

ACCCTCATCCCCCTGCTGGGGGTCCACGAAGTGGTCTTCGCCTTTGTGACCGATGAGCAG

GCTCAGGGGGCCCTGCGCTCCGCCAAGCTCTTCTTTGACCTCTTCCTCAGCTCCTTCCAG

GGCCTGCTGGTGGCTGTCCTCTACTGCTTCCTCAGCAAAGAGGTACAGGCAGAGCTGCTG

CAGCGCTGGCGGCGCTGGCACGAGGGCAGAGCGCTGCAGGACCCGCGACGCAGCAGCAGC

CCCTCGGCCCCAGCTAGGCCTTGCCGTGGCCCCTCCTGCGAGAAGCTTCGACTTGTGAAG

GGTGGCAGCAGCAATGGGGCTGGCCAGGACCCCTCTGCAGAGACCTCCTTGGCCTGTGGC

CTCCCTGAGTTGGCTGAGAGCCCC

>CL291.Contig13_A-W 27 188 Glucagon receptor [Heterocephalus glaber]

GGACCAGTCAGAGGCAGGGAGTGGCCCCCAGCCCTGAGCAGCCACTGTGGTTCACAGAAG

GAGGTAGCCAAGATGTACAGCAGCTTCCAGGTGATGTACACCGTGGGCTACTCCCTGTCC

CTGGGGGCCCTGCTGCTCGCCCTGGCCATCATGCTGGGCCTC

>CL291.Contig14_A-W 27 653 Glucagon receptor [Heterocephalus glaber]

GGACCAGTCAGAGGCAGGGAGTGGCCCCCAGCCCTGAGCAGCCACTGTGGTTCACAGAAG

GAGGTAGCCAAGATGTACAGCAGCTTCCAGGTGATGTACACCGTGGGCTACTCCCTGTCC

CTGGGGGCCCTGCTGCTCGCCCTGGCCATCATGCTGGGCCTCAGCAAACTCCACTGCACC

CGCAACTACATCCACGTGAACCTGTTTGCATCCTTTGTGCTGAAGGCCAGCTCTGTGCTG

GTCATCGACTGGCTGCTGAAGACCCGTTACAGCCAGAAGATCGGGGATGACCTCAGTGTC

AGCATCTGGCTCAGCGATGGGGTGAGTGCCAGAGCAGACCAGCCTAGACTGCTGCCGCCT

TGGGCAGGCAGGGTGCTGACAGCTGCCCACACTGGCCCTGCCCAGGCGGTGGCCGGCTGC

CGTGTGGCCGCAGTGATCATGCAGTATGGCGTGGTGGCCAACTACTGCTGGCTGCTGGTG

GAGGGCGTGTACCTGCATGGCCTGCTGGGCCTGGCCTCCTTCCCTGAGCGAAGCTTCTTC

ACCCTCTACCTGGGCATTGGCTGGGGTGCGCCCCTGCTGTTCATCATCCCCTGGGCAGTG

GTCAAGTGTCTGTTTGAGAACATTCAG

>CL291.Contig15_A-W 27 188 Glucagon receptor [Heterocephalus glaber]

GGACCAGTCAGAGGCAGGGAGTGGCCCCCAGCCCTGAGCAGCCACTGTGGTTCACAGAAG

GAGGTAGCCAAGATGTACAGCAGCTTCCAGGTGATGTACACCGTGGGCTACTCCCTGTCC

CTGGGGGCCCTGCTGCTCGCCCTGGCCATCATGCTGGGCCTC

>CL291.Contig16_A-W 27 593 Glucagon receptor [Heterocephalus glaber]

GGACCAGTCAGAGGCAGGGAGTGGCCCCCAGCCCTGAGCAGCCACTGTGGTTCACAGAAG

GAGGTAGCCAAGATGTACAGCAGCTTCCAGGTGATGTACACCGTGGGCTACTCCCTGTCC

CTGGGGGCCCTGCTGCTCGCCCTGGCCATCATGCTGGGCCTCAGCAAACTCCACTGCACC

CGCAACTACATCCACGTGAACCTGTTTGCATCCTTTGTGCTGAAGGCCAGCTCTGTGCTG

GTCATCGACTGGCTGCTGAAGACCCGTTACAGCCAGAAGATCGGGGATGACCTCAGTGTC

AGCATCTGGCTCAGCGATGGGGTGAGTGCCAGAGCAGACCAGCCTAGACTGCTGCCGCCT

TGGGCAGGCAGGGTGCTGACAGCTGCCCACACTGGCCCTGCCCAGGCGGTGGCCGGCTGC

CGTGTGGCCGCAGTGATCATGCAGTATGGCGTGGTGGCCAACTACTGCTGGCTGCTGGTG

GAGGGCGTGTACCTGCATGGCCTGCTGGGCCTGGCCTCCTTCCCTGAGCGAAGCTTCTTC

ACCCTCTACCTGGGCATTGGCTGGGGT

>CL291.Contig17_A-W 27 188 Glucagon receptor [Heterocephalus glaber]

GGACCAGTCAGAGGCAGGGAGTGGCCCCCAGCCCTGAGCAGCCACTGTGGTTCACAGAAG

GAGGTAGCCAAGATGTACAGCAGCTTCCAGGTGATGTACACCGTGGGCTACTCCCTGTCC

CTGGGGGCCCTGCTGCTCGCCCTGGCCATCATGCTGGGCCTC

>CL291.Contig18_A-W 27 569 Glucagon receptor [Heterocephalus glaber]

GGACCAGTCAGAGGCAGGGAGTGGCCCCCAGCCCTGAGCAGCCACTGTGGTTCACAGAAG

GAGGTAGCCAAGATGTACAGCAGCTTCCAGGTGATGTACACCGTGGGCTACTCCCTGTCC

CTGGGGGCCCTGCTGCTCGCCCTGGCCATCATGCTGGGCCTCAGCAAACTCCACTGCACC

CGCAACTACATCCACGTGAACCTGTTTGCATCCTTTGTGCTGAAGGCCAGCTCTGTGCTG

GTCATCGACTGGCTGCTGAAGACCCGTTACAGCCAGAAGATCGGGGATGACCTCAGTGTC

AGCATCTGGCTCAGCGATGGGGCGGTGGCCGGCTGCCGTGTGGCCGCAGTGATCATGCAG

TATGGCGTGGTGGCCAACTACTGCTGGCTGCTGGTGGAGGGCGTGTACCTGCATGGCCTG

CTGGGCCTGGCCTCCTTCCCTGAGCGAAGCTTCTTCACCCTCTACCTGGGCATTGGCTGG

GGTGCGCCCCTGCTGTTCATCATCCCCTGGGCAGTGGTCAAGTGTCTGTTTGAGAACATT

CAG

>CL291.Contig19_A-W 27 509 Glucagon receptor [Heterocephalus glaber]

GGACCAGTCAGAGGCAGGGAGTGGCCCCCAGCCCTGAGCAGCCACTGTGGTTCACAGAAG

GAGGTAGCCAAGATGTACAGCAGCTTCCAGGTGATGTACACCGTGGGCTACTCCCTGTCC

CTGGGGGCCCTGCTGCTCGCCCTGGCCATCATGCTGGGCCTCAGCAAACTCCACTGCACC

CGCAACTACATCCACGTGAACCTGTTTGCATCCTTTGTGCTGAAGGCCAGCTCTGTGCTG

GTCATCGACTGGCTGCTGAAGACCCGTTACAGCCAGAAGATCGGGGATGACCTCAGTGTC

AGCATCTGGCTCAGCGATGGGGCGGTGGCCGGCTGCCGTGTGGCCGCAGTGATCATGCAG

TATGGCGTGGTGGCCAACTACTGCTGGCTGCTGGTGGAGGGCGTGTACCTGCATGGCCTG

CTGGGCCTGGCCTCCTTCCCTGAGCGAAGCTTCTTCACCCTCTACCTGGGCATTGGCTGG

GGT

>CL291.Contig20_A-W 544 924 Glucagon receptor [Heterocephalus glaber]

AGCAAACTCCACTGCACCCGCAACTACATCCACGTGAACCTGTTTGCATCCTTTGTGCTG

AAGGCCAGCTCTGTGCTGGTCATCGACTGGCTGCTGAAGACCCGTTACAGCCAGAAGATC

GGGGATGACCTCAGTGTCAGCATCTGGCTCAGCGATGGGGCGGTGGCCGGCTGCCGTGTG

GCCGCAGTGATCATGCAGTATGGCGTGGTGGCCAACTACTGCTGGCTGCTGGTGGAGGGC

GTGTACCTGCATGGCCTGCTGGGCCTGGCCTCCTTCCCTGAGCGAAGCTTCTTCACCCTC

TACCTGGGCATTGGCTGGGGTGCGCCCCTGCTGTTCATCATCCCCTGGGCAGTGGTCAAG

TGTCTGTTTGAGAACATTCAG

>CL291.Contig21_A-W 3 1412 Glucagon receptor [Heterocephalus glaber]

CTGCTGCTGCTGCTGCTGCTGGCCTGCCAGCCGCAGGCCCTCTCTGCCCAGGTAATGGAC

TTCTTGTTCGAGAAGTGGAAGCTCTACAGTGACCAGTGCCACCGCAACATGAGCCTGCTG

CCTCCCCCAAATGACCTGGTCTGCAACAGGACCTTCGACAAGTACTCCTGCTGGCCAGAC

ACCCCTCCCAATACCACGGCCAACATCTCCTGCCCCTGGTACCTGCCTTGGTATCACAAA

GTGCAGCACCGCCTGGTCTTCAAGAAGTGTGGGCCGGATGGGCAGTGGGTGCGTGGGCCT

CGGGGACAGCCATGGCGCAACGCTTCCCAGTGCCAGATGGAGGACGAGGAGCTCGAGGGC

CAGAAGGAGGTAGCCAAGATGTACAGCAGCTTCCAGGTGATGTACACCGTGGGCTACTCC

CTGTCCCTGGGGGCCCTGCTGCTCGCCCTGGCCATCATGCTGGGCCTCAGCAAACTCCAC

TGCACCCGCAACTACATCCACGTGAACCTGTTTGCATCCTTTGTGCTGAAGGCCAGCTCT

GTGCTGGTCATCGACTGGCTGCTGAAGACCCGTTACAGCCAGAAGATCGGGGATGACCTC

AGTGTCAGCATCTGGCTCAGCGATGGGGCGGTGGCCGGCTGCCGTGTGGCCGCAGTGATC

ATGCAGTATGGCGTGGTGGCCAACTACTGCTGGCTGCTGGTGGAGGGCGTGTACCTGCAT

GGCCTGCTGGGCCTGGCCTCCTTCCCTGAGCGAAGCTTCTTCACCCTCTACCTGGGCATT

GGCTGGGGTGCGCCCCTGCTGTTCATCATCCCCTGGGCAGTGGTCAAGTGTCTGTTTGAG

AACATTCAGTGCTGGACCAGCAATACCAACATGGGCGTCTGGTGGATCTTGCGAGCCCCG

GTCTTCCTGGCCATCCTGATCAACTCCTTCATCTTTGTCCGCGTGGTCCACCTTCTCATC

ACCAAGCTTCAGGCCCGCCAGATGCACTACGCTGACTACAAGTTCCGGCTGGCCAGATCC

ACCCTGACCCTCATCCCCCTGCTGGGGGTCCACGAAGTGGTCTTCGCCTTTGTGACCGAT

GAGCAGGCTCAGGGGGCCCTGCGCTCCGCCAAGCTCTTCTTTGACCTCTTCCTCAGCTCC

TTCCAGGGCCTGCTGGTGGCTGTCCTCTACTGCTTCCTCAGCAAAGAGGTACAGGCAGAG

CTGCTGCAGCGCTGGCGGCGCTGGCACGAGGGCAGAGCGCTGCAGGACCCGCGACGCAGC

AGCAGCCCCTCGGCCCCAGCTAGGCCTTGCCGTGGCCCCTCCTGCGAGAAGCTTCGACTT

GTGAAGGGTGGCAGCAGCAATGGGGCTGGCCAGGACCCCTCTGCAGAGACCTCCTTGGCC

TGTGGCCTCCCTGAGTTGGCTGAGAGCCCC

>CL291.Contig22_A-W 3 1496 Glucagon receptor [Heterocephalus glaber]

CTGCTGCTGCTGCTGCTGCTGGCCTGCCAGCCGCAGGCCCTCTCTGCCCAGGTAATGGAC

TTCTTGTTCGAGAAGTGGAAGCTCTACAGTGACCAGTGCCACCGCAACATGAGCCTGCTG

CCTCCCCCAAATGACCTGGTCTGCAACAGGACCTTCGACAAGTACTCCTGCTGGCCAGAC

ACCCCTCCCAATACCACGGCCAACATCTCCTGCCCCTGGTACCTGCCTTGGTATCACAAA

GTGCAGCACCGCCTGGTCTTCAAGAAGTGTGGGCCGGATGGGCAGTGGGTGCGTGGGCCT

CGGGGACAGCCATGGCGCAACGCTTCCCAGTGCCAGATGGAGGACGAGGAGCTCGAGGGC

CAGAAGGAGGTAGCCAAGATGTACAGCAGCTTCCAGGTGATGTACACCGTGGGCTACTCC

CTGTCCCTGGGGGCCCTGCTGCTCGCCCTGGCCATCATGCTGGGCCTCAGCAAACTCCAC

TGCACCCGCAACTACATCCACGTGAACCTGTTTGCATCCTTTGTGCTGAAGGCCAGCTCT

GTGCTGGTCATCGACTGGCTGCTGAAGACCCGTTACAGCCAGAAGATCGGGGATGACCTC

AGTGTCAGCATCTGGCTCAGCGATGGGGTGAGTGCCAGAGCAGACCAGCCTAGACTGCTG

CCGCCTTGGGCAGGCAGGGTGCTGACAGCTGCCCACACTGGCCCTGCCCAGGCGGTGGCC

GGCTGCCGTGTGGCCGCAGTGATCATGCAGTATGGCGTGGTGGCCAACTACTGCTGGCTG

CTGGTGGAGGGCGTGTACCTGCATGGCCTGCTGGGCCTGGCCTCCTTCCCTGAGCGAAGC

TTCTTCACCCTCTACCTGGGCATTGGCTGGGGTGCGCCCCTGCTGTTCATCATCCCCTGG

GCAGTGGTCAAGTGTCTGTTTGAGAACATTCAGTGCTGGACCAGCAATACCAACATGGGC

GTCTGGTGGATCTTGCGAGCCCCGGTCTTCCTGGCCATCCTGATCAACTCCTTCATCTTT

GTCCGCGTGGTCCACCTTCTCATCACCAAGCTTCAGGCCCGCCAGATGCACTACGCTGAC

TACAAGTTCCGGCTGGCCAGATCCACCCTGACCCTCATCCCCCTGCTGGGGGTCCACGAA

GTGGTCTTCGCCTTTGTGACCGATGAGCAGGCTCAGGGGGCCCTGCGCTCCGCCAAGCTC

TTCTTTGACCTCTTCCTCAGCTCCTTCCAGGGCCTGCTGGTGGCTGTCCTCTACTGCTTC

CTCAGCAAAGAGGTACAGGCAGAGCTGCTGCAGCGCTGGCGGCGCTGGCACGAGGGCAGA

GCGCTGCAGGACCCGCGACGCAGCAGCAGCCCCTCGGCCCCAGCTAGGCCTTGCCGTGGC

CCCTCCTGCGAGAAGCTTCGACTTGTGAAGGGTGGCAGCAGCAATGGGGCTGGCCAGGAC

CCCTCTGCAGAGACCTCCTTGGCCTGTGGCCTCCCTGAGTTGGCTGAGAGCCCC

>CL291.Contig23_A-W 3 470 Glucagon receptor [Heterocephalus glaber]

CTGCTGCTGCTGCTGCTGCTGGCCTGCCAGCCGCAGGCCCTCTCTGCCCAGGTAATGGAC

TTCTTGTTCGAGAAGTGGAAGCTCTACAGTGACCAGTGCCACCGCAACATGAGCCTGCTG

CCTCCCCCAAATGACCTGGTCTGCAACAGGACCTTCGACAAGTACTCCTGCTGGCCAGAC

ACCCCTCCCAATACCACGGCCAACATCTCCTGCCCCTGGTACCTGCCTTGGTATCACAAA

GTGCAGCACCGCCTGGTCTTCAAGAAGTGTGGGCCGGATGGGCAGTGGGTGCGTGGGCCT

CGGGGACAGCCATGGCGCAACGCTTCCCAGTGCCAGATGGAGGACGAGGAGCTCGAGGGC

CAGAAGGAGGTAGCCAAGATGTACAGCAGCTTCCAGGTGATGTACACCGTGGGCTACTCC

CTGTCCCTGGGGGCCCTGCTGCTCGCCCTGGCCATCATGCTGGGCCTC

>CL291.Contig24_A-W 955 1470 Glucagon receptor [Heterocephalus glaber]

AGGTGCTGGACCAGCAATACCAACATGGGCGTCTGGTGGATCTTGCGAGCCCCGGTCTTC

CTGGCCATCCTGCTTCAGGCCCGCCAGATGCACTACGCTGACTACAAGTTCCGGCTGGCC

AGATCCACCCTGACCCTCATCCCCCTGCTGGGGGTCCACGAAGTGGTCTTCGCCTTTGTG

ACCGATGAGCAGGCTCAGGGGGCCCTGCGCTCCGCCAAGCTCTTCTTTGACCTCTTCCTC

AGCTCCTTCCAGGGCCTGCTGGTGGCTGTCCTCTACTGCTTCCTCAGCAAAGAGGTACAG

GCAGAGCTGCTGCAGCGCTGGCGGCGCTGGCACGAGGGCAGAGCGCTGCAGGACCCGCGA

CGCAGCAGCAGCCCCTCGGCCCCAGCTAGGCCTTGCCGTGGCCCCTCCTGCGAGAAGCTT

CGACTTGTGAAGGGTGGCAGCAGCAATGGGGCTGGCCAGGACCCCTCTGCAGAGACCTCC

TTGGCCTGTGGCCTCCCTGAGTTGGCTGAGAGCCCC

>CL291.Contig25_A-W 544 924 Glucagon receptor [Heterocephalus glaber]

AGCAAACTCCACTGCACCCGCAACTACATCCACGTGAACCTGTTTGCATCCTTTGTGCTG

AAGGCCAGCTCTGTGCTGGTCATCGACTGGCTGCTGAAGACCCGTTACAGCCAGAAGATC

GGGGATGACCTCAGTGTCAGCATCTGGCTCAGCGATGGGGCGGTGGCCGGCTGCCGTGTG

GCCGCAGTGATCATGCAGTATGGCGTGGTGGCCAACTACTGCTGGCTGCTGGTGGAGGGC

GTGTACCTGCATGGCCTGCTGGGCCTGGCCTCCTTCCCTGAGCGAAGCTTCTTCACCCTC

TACCTGGGCATTGGCTGGGGTGCGCCCCTGCTGTTCATCATCCCCTGGGCAGTGGTCAAG

TGTCTGTTTGAGAACATTCAG

>CL291.Contig26_A-W 871 1434 Glucagon receptor [Heterocephalus glaber]

AGGTGCTGGACCAGCAATACCAACATGGGCGTCTGGTGGATCTTGCGAGCCCCGGTCTTC

CTGGCCATCCTGATCAACTCCTTCATCTTTGTCCGCGTGGTCCACCTTCTCATCACCAAG

CTTCAGGCCCGCCAGATGCACTACGCTGACTACAAGTTCCGGCTGGCCAGATCCACCCTG

ACCCTCATCCCCCTGCTGGGGGTCCACGAAGTGGTCTTCGCCTTTGTGACCGATGAGCAG

GCTCAGGGGGCCCTGCGCTCCGCCAAGCTCTTCTTTGACCTCTTCCTCAGCTCCTTCCAG

GGCCTGCTGGTGGCTGTCCTCTACTGCTTCCTCAGCAAAGAGGTACAGGCAGAGCTGCTG

CAGCGCTGGCGGCGCTGGCACGAGGGCAGAGCGCTGCAGGACCCGCGACGCAGCAGCAGC

CCCTCGGCCCCAGCTAGGCCTTGCCGTGGCCCCTCCTGCGAGAAGCTTCGACTTGTGAAG

GGTGGCAGCAGCAATGGGGCTGGCCAGGACCCCTCTGCAGAGACCTCCTTGGCCTGTGGC

CTCCCTGAGTTGGCTGAGAGCCCC

>CL291.Contig27_A-W 871 1386 Glucagon receptor [Heterocephalus glaber]

AGGTGCTGGACCAGCAATACCAACATGGGCGTCTGGTGGATCTTGCGAGCCCCGGTCTTC

CTGGCCATCCTGCTTCAGGCCCGCCAGATGCACTACGCTGACTACAAGTTCCGGCTGGCC

AGATCCACCCTGACCCTCATCCCCCTGCTGGGGGTCCACGAAGTGGTCTTCGCCTTTGTG

ACCGATGAGCAGGCTCAGGGGGCCCTGCGCTCCGCCAAGCTCTTCTTTGACCTCTTCCTC

AGCTCCTTCCAGGGCCTGCTGGTGGCTGTCCTCTACTGCTTCCTCAGCAAAGAGGTACAG

GCAGAGCTGCTGCAGCGCTGGCGGCGCTGGCACGAGGGCAGAGCGCTGCAGGACCCGCGA

CGCAGCAGCAGCCCCTCGGCCCCAGCTAGGCCTTGCCGTGGCCCCTCCTGCGAGAAGCTT

CGACTTGTGAAGGGTGGCAGCAGCAATGGGGCTGGCCAGGACCCCTCTGCAGAGACCTCC

TTGGCCTGTGGCCTCCCTGAGTTGGCTGAGAGCCCC

>CL291.Contig28_A-W 27 188 Glucagon receptor [Heterocephalus glaber]

GGACCAGTCAGAGGCAGGGAGTGGCCCCCAGCCCTGAGCAGCCACTGTGGTTCACAGAAG

GAGGTAGCCAAGATGTACAGCAGCTTCCAGGTGATGTACACCGTGGGCTACTCCCTGTCC

CTGGGGGCCCTGCTGCTCGCCCTGGCCATCATGCTGGGCCTC

>CL291.Contig29_A-W 27 188 Glucagon receptor [Heterocephalus glaber]

GGACCAGTCAGAGGCAGGGAGTGGCCCCCAGCCCTGAGCAGCCACTGTGGTTCACAGAAG

GAGGTAGCCAAGATGTACAGCAGCTTCCAGGTGATGTACACCGTGGGCTACTCCCTGTCC

CTGGGGGCCCTGCTGCTCGCCCTGGCCATCATGCTGGGCCTC

>CL291.Contig30_A-W 27 1214 Glucagon receptor [Heterocephalus glaber]

GGACCAGTCAGAGGCAGGGAGTGGCCCCCAGCCCTGAGCAGCCACTGTGGTTCACAGAAG

GAGGTAGCCAAGATGTACAGCAGCTTCCAGGTGATGTACACCGTGGGCTACTCCCTGTCC

CTGGGGGCCCTGCTGCTCGCCCTGGCCATCATGCTGGGCCTCAGCAAACTCCACTGCACC

CGCAACTACATCCACGTGAACCTGTTTGCATCCTTTGTGCTGAAGGCCAGCTCTGTGCTG

GTCATCGACTGGCTGCTGAAGACCCGTTACAGCCAGAAGATCGGGGATGACCTCAGTGTC

AGCATCTGGCTCAGCGATGGGGTGAGTGCCAGAGCAGACCAGCCTAGACTGCTGCCGCCT

TGGGCAGGCAGGGTGCTGACAGCTGCCCACACTGGCCCTGCCCAGGCGGTGGCCGGCTGC

CGTGTGGCCGCAGTGATCATGCAGTATGGCGTGGTGGCCAACTACTGCTGGCTGCTGGTG

GAGGGCGTGTACCTGCATGGCCTGCTGGGCCTGGCCTCCTTCCCTGAGCGAAGCTTCTTC

ACCCTCTACCTGGGCATTGGCTGGGGTGCGCCCCTGCTGTTCATCATCCCCTGGGCAGTG

GTCAAGTGTCTGTTTGAGAACATTCAGTGCTGGACCAGCAATACCAACATGGGCGTCTGG

TGGATCTTGCGAGCCCCGGTCTTCCTGGCCATCCTGATCAACTCCTTCATCTTTGTCCGC

GTGGTCCACCTTCTCATCACCAAGCTTCAGGCCCGCCAGATGCACTACGCTGACTACAAG

TTCCGGCTGGCCAGATCCACCCTGACCCTCATCCCCCTGCTGGGGGTCCACGAAGTGGTC

TTCGCCTTTGTGACCGATGAGCAGGCTCAGGGGGCCCTGCGCTCCGCCAAGCTCTTCTTT

GACCTCTTCCTCAGCTCCTTCCAGGGCCTGCTGGTGGCTGTCCTCTACTGCTTCCTCAGC

AAAGAGGTACAGGCAGAGCTGCTGCAGCGCTGGCGGCGCTGGCACGAGGGCAGAGCGCTG

CAGGACCCGCGACGCAGCAGCAGCCCCTCGGCCCCAGCTAGGCCTTGCCGTGGCCCCTCC

TGCGAGAAGCTTCGACTTGTGAAGGGTGGCAGCAGCAATGGGGCTGGCCAGGACCCCTCT

GCAGAGACCTCCTTGGCCTGTGGCCTCCCTGAGTTGGCTGAGAGCCCC

>CL291.Contig31_A-W 27 188 Glucagon receptor [Heterocephalus glaber]

GGACCAGTCAGAGGCAGGGAGTGGCCCCCAGCCCTGAGCAGCCACTGTGGTTCACAGAAG

GAGGTAGCCAAGATGTACAGCAGCTTCCAGGTGATGTACACCGTGGGCTACTCCCTGTCC

CTGGGGGCCCTGCTGCTCGCCCTGGCCATCATGCTGGGCCTC

>CL291.Contig32_A-W 673 1188 Glucagon receptor [Heterocephalus glaber]

AGGTGCTGGACCAGCAATACCAACATGGGCGTCTGGTGGATCTTGCGAGCCCCGGTCTTC

CTGGCCATCCTGCTTCAGGCCCGCCAGATGCACTACGCTGACTACAAGTTCCGGCTGGCC

AGATCCACCCTGACCCTCATCCCCCTGCTGGGGGTCCACGAAGTGGTCTTCGCCTTTGTG

ACCGATGAGCAGGCTCAGGGGGCCCTGCGCTCCGCCAAGCTCTTCTTTGACCTCTTCCTC

AGCTCCTTCCAGGGCCTGCTGGTGGCTGTCCTCTACTGCTTCCTCAGCAAAGAGGTACAG

GCAGAGCTGCTGCAGCGCTGGCGGCGCTGGCACGAGGGCAGAGCGCTGCAGGACCCGCGA

CGCAGCAGCAGCCCCTCGGCCCCAGCTAGGCCTTGCCGTGGCCCCTCCTGCGAGAAGCTT

CGACTTGTGAAGGGTGGCAGCAGCAATGGGGCTGGCCAGGACCCCTCTGCAGAGACCTCC

TTGGCCTGTGGCCTCCCTGAGTTGGCTGAGAGCCCC

>CL291.Contig33_A-W 27 188 Glucagon receptor [Heterocephalus glaber]

GGACCAGTCAGAGGCAGGGAGTGGCCCCCAGCCCTGAGCAGCCACTGTGGTTCACAGAAG

GAGGTAGCCAAGATGTACAGCAGCTTCCAGGTGATGTACACCGTGGGCTACTCCCTGTCC

CTGGGGGCCCTGCTGCTCGCCCTGGCCATCATGCTGGGCCTC

>CL291.Contig34_A-W 544 924 Glucagon receptor [Heterocephalus glaber]

AGCAAACTCCACTGCACCCGCAACTACATCCACGTGAACCTGTTTGCATCCTTTGTGCTG

AAGGCCAGCTCTGTGCTGGTCATCGACTGGCTGCTGAAGACCCGTTACAGCCAGAAGATC

GGGGATGACCTCAGTGTCAGCATCTGGCTCAGCGATGGGGCGGTGGCCGGCTGCCGTGTG

GCCGCAGTGATCATGCAGTATGGCGTGGTGGCCAACTACTGCTGGCTGCTGGTGGAGGGC

GTGTACCTGCATGGCCTGCTGGGCCTGGCCTCCTTCCCTGAGCGAAGCTTCTTCACCCTC

TACCTGGGCATTGGCTGGGGTGCGCCCCTGCTGTTCATCATCCCCTGGGCAGTGGTCAAG

TGTCTGTTTGAGAACATTCAG

>CL291.Contig35_A-W 544 864 Glucagon receptor [Heterocephalus glaber]

AGCAAACTCCACTGCACCCGCAACTACATCCACGTGAACCTGTTTGCATCCTTTGTGCTG

AAGGCCAGCTCTGTGCTGGTCATCGACTGGCTGCTGAAGACCCGTTACAGCCAGAAGATC

GGGGATGACCTCAGTGTCAGCATCTGGCTCAGCGATGGGGCGGTGGCCGGCTGCCGTGTG

GCCGCAGTGATCATGCAGTATGGCGTGGTGGCCAACTACTGCTGGCTGCTGGTGGAGGGC

GTGTACCTGCATGGCCTGCTGGGCCTGGCCTCCTTCCCTGAGCGAAGCTTCTTCACCCTC

TACCTGGGCATTGGCTGGGGT

>CL292.Contig1_A-W 90 1061 PIN2/TERF1-interacting telomerase inhibitor 1 [Macaca mulatta]

ATGTCGATGCTGGCGGAGCGTCGGCGGAAGCAGAAGTGGGCTGTGGATCCAAGAAACACT

GCGTGGAGTAATGATGATTCCAAATTTGGCCAGAGGATGCTAGAGAAGATGGGGTGGTCT

AAAGGAAAGGGTTTGGGGGCTCAGGAGCAAGGAGCCACAGATCATATTAAAGTTAAAGTT

AAAAATAACCACCTGGGACTTGGAGCTACCATCAATAATGAAGACAACTGGATTGCTCAT

CAGGATGATTTTAACCAGCTTCTGGCTGAACTGAACACTTGCCACGGGCAGGAAATAGCA

GACTCCTCAGACAATAAGGAAAAGAAATCTTTTAATCTTGAGGAAAAGTCCAAGATCTCT

AAAAACCGGGTGCATTACATGAAATTCACGAAAGGGAAGGATCTGTCGTCGAGGAGCAGC

ACAGATCTTGACTGCATTTTTGGGAAAAGGCAAAGCAAGAAGACTGCTGAGCAGGACGGT

CCCAGCCCCTCCACTGCAGACGGGAGCGAGACCTCCACGACGACCACCAGCGCCTTCACC

ATCCAGGAGTACTTTGCCCTCCGGATGGCCCAGCTCCAGAGCAGGCCCCGGCTCCCAGCT

CCCCAGCCTGGCCTCTTGGAGACCCAGGTGGAACGGAAAAGCAGAAAGAAGAAAAACAAA

GAGGCGGCAGACAACCATGACGAAAATTACTCCCAGCCCAAGGCCAAGCGGTCTAAAAAG

GACGAGAGCCAGGGCTGTGTGGCCAAGAAGAGAGGGCCAGCCGAGTGGCAGCCCAGAGGG

CCCCGTGGGGATGAGCACTCAGAGGTCCCTGCTGAGGGCTGTGTGCAACCTCCTGGTGGC

CAGGACTGTACCCTAAAGCCCAAAAAGAGGAGAGCAAAGGACAAGCCACAGAGGCCAGCA

GAGGTGGCTGCAGATGCCACACTGGACAATGAAGGTCCCACGGTGAAAAAAAGGAAAAAG

AAGAAGAAGAAA

>CL292.Contig2_A-W 90 614 PINY1 [Homo sapiens] >gi|119585999|gb|EAW65595.1| PIN2-interacting protein 1, isoform CRA_a [Homo sapiens]

ATGTCGATGCTGGCGGAGCGTCGGCGGAAGCAGAAGTGGGCTGTGGATCCAAGAAACACT

GCGTGGAGTAATGATGATTCCAAATTTGGCCAGAGGATGCTAGAGAAGATGGGGTGGTCT

AAAGGAAAGGGTTTGGGGGCTCAGGAGCAAGGAGCCACAGATCATATTAAAGTTAAAGTT

AAAAATAACCACCTGGGACTTGGAGCTACCATCAATAATGAAGACAACTGGATTGCTCAT

CAGGATGATTTTAACCAGCTTCTGGCTGAACTGAACACTTGCCACGGGCAGGAAATAGCA

GACTCCTCAGACAATAAGGAAAAGAAATCTTTTAATCTTGAGGAAAAGTCCAAGATCTCT

AAAAACCGGGTGCATTACATGAAATTCACGAAAGCAGGACGGTCCCAGCCCCTCCACTGC

AGACGGGAGCGAGACCTCCACGACGACCACCAGCGCCTTCACCATCCAGGAGTACTTTGC

CCTCCGGATGGCCCAGCTCCAGAGCAGGCCCCGGCTCCCAGCTCC

>CL294.Contig1_A-W 317 856 PREDICTED: EGF-like module-containing mucin-like hormone receptor-like 2-like [Nomascus leucogenys]

CAATCCCACCAGGATTACATGAAGGACGACGACCCCGTGCTGAGCGTCATCACCTGCGTG

GGGCTGGGCGTGTCGCTGCTGTGCCTGGTCCTGGCGGCCCTCACCTTCCTCCTGTGCAGA

GCCATCCAGAACACCAGCACCTCCCTGCACCTGCAGCTCTCGCTCTGCCTCCTGCTGGCC

CACCTGCTCTTCCTCACGGCCATCAACAGAACCCAGCCCAAGGTGCTGTGCGCGGTCACC

GCGGGCGCCCTGCACTACCTCTACCTGGCCTCCTTCACCTGGATGCTGCTGGAGGGGCTG

TTCCTCTTCCTCACGGCGCGGAACCTGACTGTGCTCAGTTCCTCCAGCAGAAACAGGCTC

ATGAGGAGGCTCATGTTCCCTGTGGGCTACGGAGTCCCAGCCGCCATCGTGGCCGTGTCT

GCAGGAGCCAGACCTCACCTGTATGGCACCCCTGCTCGGTGCTGGCTCAATCCAGAACAG

GGGTTTGTGTGGGGCTTCCTTGGACCCGCCTATGCCATCTCTTGTGTGAGTGATGTCATC

>CL294.Contig2_A-W 320 541 PREDICTED: egf-like module containing, mucin-like, hormone receptor-like 2 [Canis lupus familiaris]

TCCCACCAGGATTACATGAAGGACGACGACCCCGTGCTGAGCGTCATCACCTGCGTGGGG

CTGGGCGTGTCGCTGCTGTGCCTGGTCCTGGCGGCCCTCACCTTCCTCCTGTGCAGAGCC

ATCCAGAACACCAGCACCTCCCTGCACCTGCAGCTCTCGCTCTGCCTCCTGCTGGCCCAC

CTGCTCTTCCTCACGGCCATCAACAGAACCCAGCCCAAGGTA

>CL296.Contig4_A-W 320 448 PREDICTED: solute carrier family 25 member 51 [Otolemur garnettii]

AAACAAATCAGAGTAGAATAGGACAGGAGACAGACAAATCTTTTCAGAGGTGTCCACCTG

AATGACCATGGGTCCCTTATCTCTTGGCGCATAATCAATGCCACTTACGAGTTCTTGTTG

AAGGTTATA

>CL297.Contig1_A-W 68 1057 minus strand mannose-P-dolichol utilization defect 1, isoform CRA_f [Homo sapiens]

ATGGCGGCAGAGGCAGACGGGCCACTCAAGCGAGTGCTGGTGCCGATTTTTTTACCCGAG

GAATGCTACGATCAGCTTTTCGTCCAATGGGACTTGCTTCATGTTCCCTGTCTCAAAATT

CTTCTCAGCAAAGGCCTTGGGCTGGCCATTGTGGCTGGGTCACTTCTTGTAAAGCTGCCC

CAGGTATTTAAAATCCTGAGAGCCAAGAGTGCAGAAGGGCTGAGTCTCCAGTCAGTAATG

CTGGAGCTAGTGGCACTGACTGGGACCATGGTCTACAGCATCACCAACAACTTCCCCTTC

AGCTCTTGGGGTGAAGCCCTGTTCCTGATGCTCCAGACGATCACCATCTGCTTCCTGATC

ATGCACTACAGAGGACAGACTGTGAAAGGAGTAGCTTTCCTAGCCTGTTATGCACTGGTC

CTGCTGGTCCTGCTGTCACCACTCACACCCTTGGTAGTGATTACCCTGCTCCAGGCTTCC

AATGTACCTGCTGTGGTGGTGGGAAAGCTGCTCCAGGCAGCCACCAACTACCACAATGGG

CACACAGGCCAGCTCTCGGCCATCACAGTCTTTCTACTGTTTGGGGGTTCCCTGGCCCGA

ATATTCACTTCCATTCAGGTAAGTGCACTTTCTGCCCTCCAGAGGGCACTCTAACCTCAT

CTACCCTTCTCCACTGGAGACTTATGTTGCCCCCTGAGGCTTTTGTCCTTAGGGGAACCT

TTCTTGATTTATGCTGAAGGGTGGCCTTCTCTGTCCTTTACAACCAGGAAACCGGAGACC

TTCTCATGGCTGGAATCTTTGTGATCTCCTCCCTCTGCAATGGCCTCATTGCTGCCCAGG

TCCTCTTCTACTGGAACGCAAAACCTCCCCACAAGAAGAAAAAGGAGGAATAGGGTTGAG

CCAGCTGTTTGAGTTGGGAGTCATTTTGTGTTTCCATCCATCCACCCAACCTCAGGGTTC

TCCCCATGTGAGCTAGTCTGTTGATGTGAC

>CL297.Contig2_A-W 699 821 minus strand PREDICTED: mannose-P-dolichol utilization defect 1 protein-like [Sus scrofa]

CAGGAAACCGGAGACCTTCTCATGGCTGGAATCTTTGTGATCTCCTCCCTCTGCAATGGC

CTCATTGCTGCCCAGGTCCTCTTCTACTGGAACGCAAAACCTCCCCACAAGAAGAAAAAG

GAG

>CL297.Contig3_A-W 68 808 minus strand mannose-P-dolichol utilization defect 1 protein [Homo sapiens] >gi|215274025|sp|O75352.2|MPU1_HUMAN RecName: Full=Mannose-P-dolichol utilization defect 1 protein; AltName: Full=Suppressor of Lec15 and Lec35 glycosylation mutation homolog; Short=SL15 >gi|119610567|gb|EAW90161.1| mannose-P-dolichol utilization defect 1, isoform CRA_d [Homo sapiens]

ATGGCGGCAGAGGCAGACGGGCCACTCAAGCGAGTGCTGGTGCCGATTTTTTTACCCGAG

GAATGCTACGATCAGCTTTTCGTCCAATGGGACTTGCTTCATGTTCCCTGTCTCAAAATT

CTTCTCAGCAAAGGCCTTGGGCTGGCCATTGTGGCTGGGTCACTTCTTGTAAAGCTGCCC

CAGGTATTTAAAATCCTGAGAGCCAAGAGTGCAGAAGGGCTGAGTCTCCAGTCAGTAATG

CTGGAGCTAGTGGCACTGACTGGGACCATGGTCTACAGCATCACCAACAACTTCCCCTTC

AGCTCTTGGGGTGAAGCCCTGTTCCTGATGCTCCAGACGATCACCATCTGCTTCCTGATC

ATGCACTACAGAGGACAGACTGTGAAAGGAGTAGCTTTCCTAGCCTGTTATGCACTGGTC

CTGCTGGTCCTGCTGTCACCACTCACACCCTTGGTAGTGATTACCCTGCTCCAGGCTTCC

AATGTACCTGCTGTGGTGGTGGGAAAGCTGCTCCAGGCAGCCACCAACTACCACAATGGG

CACACAGGCCAGCTCTCGGCCATCACAGTCTTTCTACTGTTTGGGGGTTCCCTGGCCCGA

ATATTCACTTCCATTCAGGAAACCGGAGACCTTCTCATGGCTGGAATCTTTGTGATCTCC

TCCCTCTGCAATGGCCTCATTGCTGCCCAGGTCCTCTTCTACTGGAACGCAAAACCTCCC

CACAAGAAGAAAAAGGAGGAA

>CL297.Contig4_A-W 68 235 minus strand PREDICTED: mannose-P-dolichol utilization defect 1 protein-like [Sus scrofa]

ATGGCGGCAGAGGCAGACGGGCCACTCAAGCGAGTGCTGGTGCCGATTTTTTTACCCGAG

GAATGCTACGATCAGCTTTTCGTCCAATGGGACTTGCTTCATGTTCCCTGTCTCAAAATT

CTTCTCAGCAAAGGCCTTGGGCTGGCCATTGTGGCTGGGTCACTTCTT

>CL297.Contig5_A-W 62 670 PREDICTED: mannose-P-dolichol utilization defect 1 protein [Pongo abelii]

GTAAAGCTGCCCCAGGTATTTAAAATCCTGAGAGCCAAGAGTGCAGAAGGGCTGAGTCTC

CAGTCAGTAATGCTGGAGCTAGTGGCACTGACTGGGACCATGGTCTACAGCATCACCAAC

AACTTCCCCTTCAGGCCCTCCTTTCTTCCTCCCAACTCTTGACTCTGCAGCTCTTGGGGT

GAAGCCCTGTTCCTGATGCTCCAGACGATCACCATCTGCTTCCTGATCATGCACTACAGA

GGACAGACTGTGAAAGGAGTAGCTTTCCTAGCCTGTTATGCACTGGTCCTGCTGGTCCTG

CTGTCACCACTCACACCCTTGGTAGTGATTACCCTGCTCCAGGCTTCCAATGTACCTGCT

GTGGTGGTGGGAAAGCTGCTCCAGGCAGCCACCAACTACCACAATGGGCACACAGGCCAG

CTCTCGGCCATCACAGTCTTTCTACTGTTTGGGGGTTCCCTGGCCCGAATATTCACTTCC

ATTCAGGAAACCGGAGACCTTCTCATGGCTGGAATCTTTGTGATCTCCTCCCTCTGCAAT

GGCCTCATTGCTGCCCAGGTCCTCTTCTACTGGAACGCAAAACCTCCCCACAAGAAGAAA

AAGGAGGAA

>CL297.Contig6_A-W 573 1175 mannose-P-dolichol utilization defect 1, isoform CRA_f [Homo sapiens]

GGAGTAGCTTTCCTAGCCTGTTATGCACTGGTCCTGCTGGTCCTGCTGTCACCACTCACA

CCCTTGGTAGTGATTACCCTGCTCCAGGCTTCCAATGTACCTGCTGTGGTGGTGGGAAAG

CTGCTCCAGGCAGCCACCAACTACCACAATGGGCACACAGGCCAGCTCTCGGCCATCACA

GTCTTTCTACTGTTTGGGGGTTCCCTGGCCCGAATATTCACTTCCATTCAGGTAAGTGCA

CTTTCTGCCCTCCAGAGGGCACTCTAACCTCATCTACCCTTCTCCACTGGAGACTTATGT

TGCCCCCTGAGGCTTTTGTCCTTAGGGGAACCTTTCTTGATTTATGCTGAAGGGTGGCCT

TCTCTGTCCTTTACAACCAGGAAACCGGAGACCTTCTCATGGCTGGAATCTTTGTGATCT

CCTCCCTCTGCAATGGCCTCATTGCTGCCCAGGTCCTCTTCTACTGGAACGCAAAACCTC

CCCACAAGAAGAAAAAGGAGGAATAGGGTTGAGCCAGCTGTTTGAGTTGGGAGTCATTTT

GTGTTTCCATCCATCCACCCAACCTCAGGGTTCTCCCCATGTGAGCTAGTCTGTTGATGT

GAC

>CL297.Contig7_A-W 62 634 PREDICTED: mannose-P-dolichol utilization defect 1 protein [Pongo abelii]

GTAAAGCTGCCCCAGGTATTTAAAATCCTGAGAGCCAAGAGTGCAGAAGGGCTGAGTCTC

CAGTCAGTAATGCTGGAGCTAGTGGCACTGACTGGGACCATGGTCTACAGCATCACCAAC

AACTTCCCCTTCAGCTCTTGGGGTGAAGCCCTGTTCCTGATGCTCCAGACGATCACCATC

TGCTTCCTGATCATGCACTACAGAGGACAGACTGTGAAAGGAGTAGCTTTCCTAGCCTGT

TATGCACTGGTCCTGCTGGTCCTGCTGTCACCACTCACACCCTTGGTAGTGATTACCCTG

CTCCAGGCTTCCAATGTACCTGCTGTGGTGGTGGGAAAGCTGCTCCAGGCAGCCACCAAC

TACCACAATGGGCACACAGGCCAGCTCTCGGCCATCACAGTCTTTCTACTGTTTGGGGGT

TCCCTGGCCCGAATATTCACTTCCATTCAGGAAACCGGAGACCTTCTCATGGCTGGAATC

TTTGTGATCTCCTCCCTCTGCAATGGCCTCATTGCTGCCCAGGTCCTCTTCTACTGGAAC

GCAAAACCTCCCCACAAGAAGAAAAAGGAGGAA

>CL297.Contig8_A-W 180 662 mannose-P-dolichol utilization defect 1, isoform CRA_f [Homo sapiens]

CTGCTCCAGGCAGCCACCAACTACCACAATGGGCACACAGGCCAGCTCTCGGCCATCACA

GTCTTTCTACTGTTTGGGGGTTCCCTGGCCCGAATATTCACTTCCATTCAGGTAAGTGCA

CTTTCTGCCCTCCAGAGGGCACTCTAACCTCATCTACCCTTCTCCACTGGAGACTTATGT

TGCCCCCTGAGGCTTTTGTCCTTAGGGGAACCTTTCTTGATTTATGCTGAAGGGTGGCCT

TCTCTGTCCTTTACAACCAGGAAACCGGAGACCTTCTCATGGCTGGAATCTTTGTGATCT

CCTCCCTCTGCAATGGCCTCATTGCTGCCCAGGTCCTCTTCTACTGGAACGCAAAACCTC

CCCACAAGAAGAAAAAGGAGGAATAGGGTTGAGCCAGCTGTTTGAGTTGGGAGTCATTTT

GTGTTTCCATCCATCCACCCAACCTCAGGGTTCTCCCCATGTGAGCTAGTCTGTTGATGT

GAC

>CL297.Contig9_A-W 180 413 Lec35 protein [Cricetulus griseus] >gi|344237785|gb|EGV93888.1| Mannose-P-dolichol utilization defect 1 protein [Cricetulus griseus]

CTGCTCCAGGCAGCCACCAACTACCACAATGGGCACACAGGCCAGCTCTCGGCCATCACA

GTCTTTCTACTGTTTGGGGGTTCCCTGGCCCGAATATTCACTTCCATTCAGGAAACCGGA

GACCTTCTCATGGCTGGAATCTTTGTGATCTCCTCCCTCTGCAATGGCCTCATTGCTGCC

CAGGTCCTCTTCTACTGGAACGCAAAACCTCCCCACAAGAAGAAAAAGGAGGAA

>CL297.Contig10_A-W 68 1093 mannose-P-dolichol utilization defect 1, isoform CRA_f [Homo sapiens]

ATGGCGGCAGAGGCAGACGGGCCACTCAAGCGAGTGCTGGTGCCGATTTTTTTACCCGAG

GAATGCTACGATCAGCTTTTCGTCCAATGGGACTTGCTTCATGTTCCCTGTCTCAAAATT

CTTCTCAGCAAAGGCCTTGGGCTGGCCATTGTGGCTGGGTCACTTCTTGTAAAGCTGCCC

CAGGTATTTAAAATCCTGAGAGCCAAGAGTGCAGAAGGGCTGAGTCTCCAGTCAGTAATG

CTGGAGCTAGTGGCACTGACTGGGACCATGGTCTACAGCATCACCAACAACTTCCCCTTC

AGGCCCTCCTTTCTTCCTCCCAACTCTTGACTCTGCAGCTCTTGGGGTGAAGCCCTGTTC

CTGATGCTCCAGACGATCACCATCTGCTTCCTGATCATGCACTACAGAGGACAGACTGTG

AAAGGAGTAGCTTTCCTAGCCTGTTATGCACTGGTCCTGCTGGTCCTGCTGTCACCACTC

ACACCCTTGGTAGTGATTACCCTGCTCCAGGCTTCCAATGTACCTGCTGTGGTGGTGGGA

AAGCTGCTCCAGGCAGCCACCAACTACCACAATGGGCACACAGGCCAGCTCTCGGCCATC

ACAGTCTTTCTACTGTTTGGGGGTTCCCTGGCCCGAATATTCACTTCCATTCAGGTAAGT

GCACTTTCTGCCCTCCAGAGGGCACTCTAACCTCATCTACCCTTCTCCACTGGAGACTTA

TGTTGCCCCCTGAGGCTTTTGTCCTTAGGGGAACCTTTCTTGATTTATGCTGAAGGGTGG

CCTTCTCTGTCCTTTACAACCAGGAAACCGGAGACCTTCTCATGGCTGGAATCTTTGTGA

TCTCCTCCCTCTGCAATGGCCTCATTGCTGCCCAGGTCCTCTTCTACTGGAACGCAAAAC

CTCCCCACAAGAAGAAAAAGGAGGAATAGGGTTGAGCCAGCTGTTTGAGTTGGGAGTCAT

TTTGTGTTTCCATCCATCCACCCAACCTCAGGGTTCTCCCCATGTGAGCTAGTCTGTTGA

TGTGAC

>CL297.Contig11_A-W 609 962 mannose-P-dolichol utilization defect 1 protein [Homo sapiens] >gi|215274025|sp|O75352.2|MPU1_HUMAN RecName: Full=Mannose-P-dolichol utilization defect 1 protein; AltName: Full=Suppressor of Lec15 and Lec35 glycosylation mutation homolog; Short=SL15 >gi|119610567|gb|EAW90161.1| mannose-P-dolichol utilization defect 1, isoform CRA_d [Homo sapiens]

GGAGTAGCTTTCCTAGCCTGTTATGCACTGGTCCTGCTGGTCCTGCTGTCACCACTCACA

CCCTTGGTAGTGATTACCCTGCTCCAGGCTTCCAATGTACCTGCTGTGGTGGTGGGAAAG

CTGCTCCAGGCAGCCACCAACTACCACAATGGGCACACAGGCCAGCTCTCGGCCATCACA

GTCTTTCTACTGTTTGGGGGTTCCCTGGCCCGAATATTCACTTCCATTCAGGAAACCGGA

GACCTTCTCATGGCTGGAATCTTTGTGATCTCCTCCCTCTGCAATGGCCTCATTGCTGCC

CAGGTCCTCTTCTACTGGAACGCAAAACCTCCCCACAAGAAGAAAAAGGAGGAA

>CL297.Contig12_A-W 817 939 PREDICTED: mannose-P-dolichol utilization defect 1 protein-like [Sus scrofa]

CAGGAAACCGGAGACCTTCTCATGGCTGGAATCTTTGTGATCTCCTCCCTCTGCAATGGC

CTCATTGCTGCCCAGGTCCTCTTCTACTGGAACGCAAAACCTCCCCACAAGAAGAAAAAG

GAG

>CL297.Contig13_A-W 609 1211 mannose-P-dolichol utilization defect 1, isoform CRA_f [Homo sapiens]

GGAGTAGCTTTCCTAGCCTGTTATGCACTGGTCCTGCTGGTCCTGCTGTCACCACTCACA

CCCTTGGTAGTGATTACCCTGCTCCAGGCTTCCAATGTACCTGCTGTGGTGGTGGGAAAG

CTGCTCCAGGCAGCCACCAACTACCACAATGGGCACACAGGCCAGCTCTCGGCCATCACA

GTCTTTCTACTGTTTGGGGGTTCCCTGGCCCGAATATTCACTTCCATTCAGGTAAGTGCA

CTTTCTGCCCTCCAGAGGGCACTCTAACCTCATCTACCCTTCTCCACTGGAGACTTATGT

TGCCCCCTGAGGCTTTTGTCCTTAGGGGAACCTTTCTTGATTTATGCTGAAGGGTGGCCT

TCTCTGTCCTTTACAACCAGGAAACCGGAGACCTTCTCATGGCTGGAATCTTTGTGATCT

CCTCCCTCTGCAATGGCCTCATTGCTGCCCAGGTCCTCTTCTACTGGAACGCAAAACCTC

CCCACAAGAAGAAAAAGGAGGAATAGGGTTGAGCCAGCTGTTTGAGTTGGGAGTCATTTT

GTGTTTCCATCCATCCACCCAACCTCAGGGTTCTCCCCATGTGAGCTAGTCTGTTGATGT

GAC

>CL297.Contig14_A-W 62 343 mannose-P-dolichol utilization defect 1, isoform CRA_f [Homo sapiens]

GTAAAGCTGCCCCAGGTATTTAAAATCCTGAGAGCCAAGAGTGCAGAAGGGCTGAGTCTC

CAGTCAGTAATGCTGGAGCTAGTGGCACTGACTGGGACCATGGTCTACAGCATCACCAAC

AACTTCCCCTTCAGGCCCTCCTTTCTTCCTCCCAACTCTTGACTCTGCAGCTCTTGGGGT

GAAGCCCTGTTCCTGATGCTCCAGACGATCACCATCTGCTTCCTGATCATGCACTACAGA

GGACAGACTGTGAAAGGTGCTGGTAATTTGCCAGGAATAGGC

>CL297.Contig15_A-W 573 926 mannose-P-dolichol utilization defect 1 protein [Homo sapiens] >gi|215274025|sp|O75352.2|MPU1_HUMAN RecName: Full=Mannose-P-dolichol utilization defect 1 protein; AltName: Full=Suppressor of Lec15 and Lec35 glycosylation mutation homolog; Short=SL15 >gi|119610567|gb|EAW90161.1| mannose-P-dolichol utilization defect 1, isoform CRA_d [Homo sapiens]

GGAGTAGCTTTCCTAGCCTGTTATGCACTGGTCCTGCTGGTCCTGCTGTCACCACTCACA

CCCTTGGTAGTGATTACCCTGCTCCAGGCTTCCAATGTACCTGCTGTGGTGGTGGGAAAG

CTGCTCCAGGCAGCCACCAACTACCACAATGGGCACACAGGCCAGCTCTCGGCCATCACA

GTCTTTCTACTGTTTGGGGGTTCCCTGGCCCGAATATTCACTTCCATTCAGGAAACCGGA

GACCTTCTCATGGCTGGAATCTTTGTGATCTCCTCCCTCTGCAATGGCCTCATTGCTGCC

CAGGTCCTCTTCTACTGGAACGCAAAACCTCCCCACAAGAAGAAAAAGGAGGAA

>CL297.Contig16_A-W 62 307 mannose-P-dolichol utilization defect 1, isoform CRA_f [Homo sapiens]

GTAAAGCTGCCCCAGGTATTTAAAATCCTGAGAGCCAAGAGTGCAGAAGGGCTGAGTCTC

CAGTCAGTAATGCTGGAGCTAGTGGCACTGACTGGGACCATGGTCTACAGCATCACCAAC

AACTTCCCCTTCAGCTCTTGGGGTGAAGCCCTGTTCCTGATGCTCCAGACGATCACCATC

TGCTTCCTGATCATGCACTACAGAGGACAGACTGTGAAAGGTGCTGGTAATTTGCCAGGA

ATAGGC

>CL297.Contig17_A-W 68 844 mannose-P-dolichol utilization defect 1 protein [Homo sapiens] >gi|215274025|sp|O75352.2|MPU1_HUMAN RecName: Full=Mannose-P-dolichol utilization defect 1 protein; AltName: Full=Suppressor of Lec15 and Lec35 glycosylation mutation homolog; Short=SL15 >gi|119610567|gb|EAW90161.1| mannose-P-dolichol utilization defect 1, isoform CRA_d [Homo sapiens]

ATGGCGGCAGAGGCAGACGGGCCACTCAAGCGAGTGCTGGTGCCGATTTTTTTACCCGAG

GAATGCTACGATCAGCTTTTCGTCCAATGGGACTTGCTTCATGTTCCCTGTCTCAAAATT

CTTCTCAGCAAAGGCCTTGGGCTGGCCATTGTGGCTGGGTCACTTCTTGTAAAGCTGCCC

CAGGTATTTAAAATCCTGAGAGCCAAGAGTGCAGAAGGGCTGAGTCTCCAGTCAGTAATG

CTGGAGCTAGTGGCACTGACTGGGACCATGGTCTACAGCATCACCAACAACTTCCCCTTC

AGGCCCTCCTTTCTTCCTCCCAACTCTTGACTCTGCAGCTCTTGGGGTGAAGCCCTGTTC

CTGATGCTCCAGACGATCACCATCTGCTTCCTGATCATGCACTACAGAGGACAGACTGTG

AAAGGAGTAGCTTTCCTAGCCTGTTATGCACTGGTCCTGCTGGTCCTGCTGTCACCACTC

ACACCCTTGGTAGTGATTACCCTGCTCCAGGCTTCCAATGTACCTGCTGTGGTGGTGGGA

AAGCTGCTCCAGGCAGCCACCAACTACCACAATGGGCACACAGGCCAGCTCTCGGCCATC

ACAGTCTTTCTACTGTTTGGGGGTTCCCTGGCCCGAATATTCACTTCCATTCAGGAAACC

GGAGACCTTCTCATGGCTGGAATCTTTGTGATCTCCTCCCTCTGCAATGGCCTCATTGCT

GCCCAGGTCCTCTTCTACTGGAACGCAAAACCTCCCCACAAGAAGAAAAAGGAGGAA

>CL297.Contig18_A-W 62 919 mannose-P-dolichol utilization defect 1, isoform CRA_f [Homo sapiens]

GTAAAGCTGCCCCAGGTATTTAAAATCCTGAGAGCCAAGAGTGCAGAAGGGCTGAGTCTC

CAGTCAGTAATGCTGGAGCTAGTGGCACTGACTGGGACCATGGTCTACAGCATCACCAAC

AACTTCCCCTTCAGGCCCTCCTTTCTTCCTCCCAACTCTTGACTCTGCAGCTCTTGGGGT

GAAGCCCTGTTCCTGATGCTCCAGACGATCACCATCTGCTTCCTGATCATGCACTACAGA

GGACAGACTGTGAAAGGAGTAGCTTTCCTAGCCTGTTATGCACTGGTCCTGCTGGTCCTG

CTGTCACCACTCACACCCTTGGTAGTGATTACCCTGCTCCAGGCTTCCAATGTACCTGCT

GTGGTGGTGGGAAAGCTGCTCCAGGCAGCCACCAACTACCACAATGGGCACACAGGCCAG

CTCTCGGCCATCACAGTCTTTCTACTGTTTGGGGGTTCCCTGGCCCGAATATTCACTTCC

ATTCAGGTAAGTGCACTTTCTGCCCTCCAGAGGGCACTCTAACCTCATCTACCCTTCTCC

ACTGGAGACTTATGTTGCCCCCTGAGGCTTTTGTCCTTAGGGGAACCTTTCTTGATTTAT

GCTGAAGGGTGGCCTTCTCTGTCCTTTACAACCAGGAAACCGGAGACCTTCTCATGGCTG

GAATCTTTGTGATCTCCTCCCTCTGCAATGGCCTCATTGCTGCCCAGGTCCTCTTCTACT

GGAACGCAAAACCTCCCCACAAGAAGAAAAAGGAGGAATAGGGTTGAGCCAGCTGTTTGA

GTTGGGAGTCATTTTGTGTTTCCATCCATCCACCCAACCTCAGGGTTCTCCCCATGTGAG

CTAGTCTGTTGATGTGAC

>CL297.Contig19_A-W 226 336 PREDICTED: mannose-P-dolichol utilization defect 1 protein-like [Sus scrofa]

GTCACTTCTTGCTCTTGGGGTGAAGCCCTGTTCCTGATGCTCCAGACGATCACCATCTGC

TTCCTGATCATGCACTACAGAGGACAGACTGTGAAAGGTGCTGGTAATTTG

>CL297.Contig20_A-W 62 343 PREDICTED: mannose-P-dolichol utilization defect 1 protein [Pongo abelii]

GTAAAGCTGCCCCAGGTATTTAAAATCCTGAGAGCCAAGAGTGCAGAAGGGCTGAGTCTC

CAGTCAGTAATGCTGGAGCTAGTGGCACTGACTGGGACCATGGTCTACAGCATCACCAAC

AACTTCCCCTTCAGGCCCTCCTTTCTTCCTCCCAACTCTTGACTCTGCAGCTCTTGGGGT

GAAGCCCTGTTCCTGATGCTCCAGACGATCACCATCTGCTTCCTGATCATGCACTACAGA

GGACAGACTGTGAAAGGTGCTGGTAATTTGCCAGGAATAGGC

>CL297.Contig21_A-W 62 883 mannose-P-dolichol utilization defect 1, isoform CRA_f [Homo sapiens]

GTAAAGCTGCCCCAGGTATTTAAAATCCTGAGAGCCAAGAGTGCAGAAGGGCTGAGTCTC

CAGTCAGTAATGCTGGAGCTAGTGGCACTGACTGGGACCATGGTCTACAGCATCACCAAC

AACTTCCCCTTCAGCTCTTGGGGTGAAGCCCTGTTCCTGATGCTCCAGACGATCACCATC

TGCTTCCTGATCATGCACTACAGAGGACAGACTGTGAAAGGAGTAGCTTTCCTAGCCTGT

TATGCACTGGTCCTGCTGGTCCTGCTGTCACCACTCACACCCTTGGTAGTGATTACCCTG

CTCCAGGCTTCCAATGTACCTGCTGTGGTGGTGGGAAAGCTGCTCCAGGCAGCCACCAAC

TACCACAATGGGCACACAGGCCAGCTCTCGGCCATCACAGTCTTTCTACTGTTTGGGGGT

TCCCTGGCCCGAATATTCACTTCCATTCAGGTAAGTGCACTTTCTGCCCTCCAGAGGGCA

CTCTAACCTCATCTACCCTTCTCCACTGGAGACTTATGTTGCCCCCTGAGGCTTTTGTCC

TTAGGGGAACCTTTCTTGATTTATGCTGAAGGGTGGCCTTCTCTGTCCTTTACAACCAGG

AAACCGGAGACCTTCTCATGGCTGGAATCTTTGTGATCTCCTCCCTCTGCAATGGCCTCA

TTGCTGCCCAGGTCCTCTTCTACTGGAACGCAAAACCTCCCCACAAGAAGAAAAAGGAGG

AATAGGGTTGAGCCAGCTGTTTGAGTTGGGAGTCATTTTGTGTTTCCATCCATCCACCCA

ACCTCAGGGTTCTCCCCATGTGAGCTAGTCTGTTGATGTGAC

>CL297.Contig22_A-W 62 307 PREDICTED: mannose-P-dolichol utilization defect 1 protein [Pongo abelii]

GTAAAGCTGCCCCAGGTATTTAAAATCCTGAGAGCCAAGAGTGCAGAAGGGCTGAGTCTC

CAGTCAGTAATGCTGGAGCTAGTGGCACTGACTGGGACCATGGTCTACAGCATCACCAAC

AACTTCCCCTTCAGCTCTTGGGGTGAAGCCCTGTTCCTGATGCTCCAGACGATCACCATC

TGCTTCCTGATCATGCACTACAGAGGACAGACTGTGAAAGGTGCTGGTAATTTGCCAGGA

ATAGGC

>CL298.Contig1_A-W 5 529 minus strand PREDICTED: activating NK receptor-like [Oryctolagus cuniculus]

GGATTCTACGAAGCCCAGATAAACACAGAACCTTCTCTAACGGTTTCCTGTTACACTCTG

AGGATCTTCAGACCACTGAGTAACTTACAAGTTACCCATCACACTCAACTGTCTGGGAAT

AGCACCTGTGAGATCCACCTGACCTGCTCTCTGGAGAATCCAAATGATACTGTCTCATTC

GGGTGGAAGCTCTCAGAAAACAGCTCTCTGCGAGAACCAAACCTCACTATCTCCTGGGAC

CCCATGAGTTCCAGTGAACAGAACTACACCTGCATAGCTGAGAACCCGGTCAGCAATTTA

TCATCCTTAGTCACTGCCCAGAGTCTCTGCACAGGCTCCCTCCCTTTGCATACTCAGCAA

ACTCAGCATCCTGCAGAGCCTCTACAGAACACAGAGTGCACATCAGTCTCTCCAGGGAGC

ACCGTGTACGCACAGGTCACGTATCCAAACCTGGAAATGAAAATCCCAGCACCCACAAAA

CAGGACTCCATTACAATTTACTCCACAATTAATCATTCCAGGGAG

>CL298.Contig2_A-W 3 362 minus strand PREDICTED: SLAM family member 6 [Callithrix jacchus]

CAGAGTCTCTGCACAGGTGTTAAGAAAAATGTAAACTGGGTCAGAACATTGATTATGATT

CTGGTTGCTTTGTTTCTCCTCGCCATTGCGTGTGCACTGGCTTCACGGGCTTGGAAGAAC

AGAACAGGCTCCCTCCCTTTGCATACTCAGCAAACTCAGCATCCTGCAGAGCCTCTACAG

AACACAGAGTGCACATCAGTCTCTCCAGGGAGCACCGTGTACGCACAGGTCACGTATCCA

AACCTGGAAATGAAAATCCCAGCACCCACAAAACAGGACTCCATTACAATTTACTCCACA

ATTAATCATTCCAGGGAGACTAAGACCACTTCTTCCAGGGCAACTGCCCTTAACAACATC

>CL299.Contig1_A-W 151 1506 minus strand Integrin-linked protein kinase [Heterocephalus glaber]

ATGGACGACATTTTCACTCAGTGCCGGGAGGGCAACGCGGTTGCCGTGCGCCTGTGGCTG

GACAACACGGAGAACGATCTCAACCAGGGGGATGATCATGGCTTTTCTCCCTTGCACTGG

GCCTGCCGAGAGGGCCGCTCTGCAGTGGTTGAGATGCTGATCATGAGGGGGGCACGGATC

AATGTGATGAACCGTGGGGACGACACACCCCTGCATCTGGCAGCTAGTCACGGACATCGT

GATATTGTACAAAAGCTGTTGCAGTATAAGGCCGACATCAATGCAGTGAATGAGCATGGG

AATGTGCCTCTGCACTATGCCTGTTTTTGGGGCCAGGATCAAGTGGCAGAGGATCTGGTG

GCTAATGGAGCCCTTGTCAGCATCTGTAACAAGTATGGCGAGATGCCTGTAGACAAAGCC

AAGGCACCCTTGAGAGAGCTTCTCCGAGAACGGGCAGAGAAGATGGGTCAGAATCTCAAC

CGTATTCCATACAAGGACACATTCTGGAAGGGGACCACCCGTACTCGGCCCCGAAACGGG

ACCCTGAACAAACACTCGGGCATTGACTTTAAACAGCTCAACTTTCTGGCGAAGCTCAAT

GAGAATCACTCTGGAGAGCTATGGAAGGGCCGCTGGCAGGGAAATGACATTGTCGTGAAG

GTGCTGAAGGTTCGAGACTGGAGTACAAGGAAAAGCAGGGACTTCAATGAGGAGTGTCCC

CGGCTCAGGATTTTCTCACACCCGAACGTGCTCCCAGTGTTAGGTGCCTGCCAGTCTCCA

CCTGCTCCTCACCCTACACTCATCACACACTGGATGCCGTATGGATCCCTCTACAATGTA

CTACATGAAGGCACCAATTTTGTCGTGGATCAGAGCCAAGCTGTGAAGTTTGCTTTAGAT

ATGGCAAGAGGCATGGCTTTCCTGCACACACTAGAGCCCCTCATCCCACGACATGCACTC

AACAGTCGAAGTGTAATGATTGACGAGGACATGACTGCCCGAATCAGCATGGCCGATGTC

AAGTTTTCTTTCCAATGCCCTGGGCGCATGTATGCACCTGCCTGGGTGGCCCCTGAAGCT

CTGCAGAAGAAGCCTGAAGACACAAACAGACGTTCAGCAGACATGTGGAGTTTTGCAGTA

CTTCTATGGGAACTGGTGACACGAGAGGTACCTTTTGCTGACCTCTCCAATATGGAGATT

GGAATGAAGGTGGCACTAGAAGGCCTTAGGCCTACCATTCCACCAGGTATTTCCCCCCAT

GTGTGTAAGCTCATGAAGATTTGCATGAATGAAGACCCTGCCAAGCGGCCCAAGTTTGAC

ATGATTGTGCCTATTCTGGAAAAGATGCAGGACAAA

>CL299.Contig2_A-W 327 1682 minus strand Integrin-linked protein kinase [Heterocephalus glaber]

ATGGACGACATTTTCACTCAGTGCCGGGAGGGCAACGCGGTTGCCGTGCGCCTGTGGCTG

GACAACACGGAGAACGATCTCAACCAGGGGGATGATCATGGCTTTTCTCCCTTGCACTGG

GCCTGCCGAGAGGGCCGCTCTGCAGTGGTTGAGATGCTGATCATGAGGGGGGCACGGATC

AATGTGATGAACCGTGGGGACGACACACCCCTGCATCTGGCAGCTAGTCACGGACATCGT

GATATTGTACAAAAGCTGTTGCAGTATAAGGCCGACATCAATGCAGTGAATGAGCATGGG

AATGTGCCTCTGCACTATGCCTGTTTTTGGGGCCAGGATCAAGTGGCAGAGGATCTGGTG

GCTAATGGAGCCCTTGTCAGCATCTGTAACAAGTATGGCGAGATGCCTGTAGACAAAGCC

AAGGCACCCTTGAGAGAGCTTCTCCGAGAACGGGCAGAGAAGATGGGTCAGAATCTCAAC

CGTATTCCATACAAGGACACATTCTGGAAGGGGACCACCCGTACTCGGCCCCGAAACGGG

ACCCTGAACAAACACTCGGGCATTGACTTTAAACAGCTCAACTTTCTGGCGAAGCTCAAT

GAGAATCACTCTGGAGAGCTATGGAAGGGCCGCTGGCAGGGAAATGACATTGTCGTGAAG

GTGCTGAAGGTTCGAGACTGGAGTACAAGGAAAAGCAGGGACTTCAATGAGGAGTGTCCC

CGGCTCAGGATTTTCTCACACCCGAACGTGCTCCCAGTGTTAGGTGCCTGCCAGTCTCCA

CCTGCTCCTCACCCTACACTCATCACACACTGGATGCCGTATGGATCCCTCTACAATGTA

CTACATGAAGGCACCAATTTTGTCGTGGATCAGAGCCAAGCTGTGAAGTTTGCTTTAGAT

ATGGCAAGAGGCATGGCTTTCCTGCACACACTAGAGCCCCTCATCCCACGACATGCACTC

AACAGTCGAAGTGTAATGATTGACGAGGACATGACTGCCCGAATCAGCATGGCCGATGTC

AAGTTTTCTTTCCAATGCCCTGGGCGCATGTATGCACCTGCCTGGGTGGCCCCTGAAGCT

CTGCAGAAGAAGCCTGAAGACACAAACAGACGTTCAGCAGACATGTGGAGTTTTGCAGTA

CTTCTATGGGAACTGGTGACACGAGAGGTACCTTTTGCTGACCTCTCCAATATGGAGATT

GGAATGAAGGTGGCACTAGAAGGCCTTAGGCCTACCATTCCACCAGGTATTTCCCCCCAT

GTGTGTAAGCTCATGAAGATTTGCATGAATGAAGACCCTGCCAAGCGGCCCAAGTTTGAC

ATGATTGTGCCTATTCTGGAAAAGATGCAGGACAAA

>CL299.Contig3_A-W 196 1551 minus strand Integrin-linked protein kinase [Heterocephalus glaber]

ATGGACGACATTTTCACTCAGTGCCGGGAGGGCAACGCGGTTGCCGTGCGCCTGTGGCTG

GACAACACGGAGAACGATCTCAACCAGGGGGATGATCATGGCTTTTCTCCCTTGCACTGG

GCCTGCCGAGAGGGCCGCTCTGCAGTGGTTGAGATGCTGATCATGAGGGGGGCACGGATC

AATGTGATGAACCGTGGGGACGACACACCCCTGCATCTGGCAGCTAGTCACGGACATCGT

GATATTGTACAAAAGCTGTTGCAGTATAAGGCCGACATCAATGCAGTGAATGAGCATGGG

AATGTGCCTCTGCACTATGCCTGTTTTTGGGGCCAGGATCAAGTGGCAGAGGATCTGGTG

GCTAATGGAGCCCTTGTCAGCATCTGTAACAAGTATGGCGAGATGCCTGTAGACAAAGCC

AAGGCACCCTTGAGAGAGCTTCTCCGAGAACGGGCAGAGAAGATGGGTCAGAATCTCAAC

CGTATTCCATACAAGGACACATTCTGGAAGGGGACCACCCGTACTCGGCCCCGAAACGGG

ACCCTGAACAAACACTCGGGCATTGACTTTAAACAGCTCAACTTTCTGGCGAAGCTCAAT

GAGAATCACTCTGGAGAGCTATGGAAGGGCCGCTGGCAGGGAAATGACATTGTCGTGAAG

GTGCTGAAGGTTCGAGACTGGAGTACAAGGAAAAGCAGGGACTTCAATGAGGAGTGTCCC

CGGCTCAGGATTTTCTCACACCCGAACGTGCTCCCAGTGTTAGGTGCCTGCCAGTCTCCA

CCTGCTCCTCACCCTACACTCATCACACACTGGATGCCGTATGGATCCCTCTACAATGTA

CTACATGAAGGCACCAATTTTGTCGTGGATCAGAGCCAAGCTGTGAAGTTTGCTTTAGAT

ATGGCAAGAGGCATGGCTTTCCTGCACACACTAGAGCCCCTCATCCCACGACATGCACTC

AACAGTCGAAGTGTAATGATTGACGAGGACATGACTGCCCGAATCAGCATGGCCGATGTC

AAGTTTTCTTTCCAATGCCCTGGGCGCATGTATGCACCTGCCTGGGTGGCCCCTGAAGCT

CTGCAGAAGAAGCCTGAAGACACAAACAGACGTTCAGCAGACATGTGGAGTTTTGCAGTA

CTTCTATGGGAACTGGTGACACGAGAGGTACCTTTTGCTGACCTCTCCAATATGGAGATT

GGAATGAAGGTGGCACTAGAAGGCCTTAGGCCTACCATTCCACCAGGTATTTCCCCCCAT

GTGTGTAAGCTCATGAAGATTTGCATGAATGAAGACCCTGCCAAGCGGCCCAAGTTTGAC

ATGATTGTGCCTATTCTGGAAAAGATGCAGGACAAA

>CL299.Contig4_A-W 277 1632 minus strand Integrin-linked protein kinase [Heterocephalus glaber]

ATGGACGACATTTTCACTCAGTGCCGGGAGGGCAACGCGGTTGCCGTGCGCCTGTGGCTG

GACAACACGGAGAACGATCTCAACCAGGGGGATGATCATGGCTTTTCTCCCTTGCACTGG

GCCTGCCGAGAGGGCCGCTCTGCAGTGGTTGAGATGCTGATCATGAGGGGGGCACGGATC

AATGTGATGAACCGTGGGGACGACACACCCCTGCATCTGGCAGCTAGTCACGGACATCGT

GATATTGTACAAAAGCTGTTGCAGTATAAGGCCGACATCAATGCAGTGAATGAGCATGGG

AATGTGCCTCTGCACTATGCCTGTTTTTGGGGCCAGGATCAAGTGGCAGAGGATCTGGTG

GCTAATGGAGCCCTTGTCAGCATCTGTAACAAGTATGGCGAGATGCCTGTAGACAAAGCC

AAGGCACCCTTGAGAGAGCTTCTCCGAGAACGGGCAGAGAAGATGGGTCAGAATCTCAAC

CGTATTCCATACAAGGACACATTCTGGAAGGGGACCACCCGTACTCGGCCCCGAAACGGG

ACCCTGAACAAACACTCGGGCATTGACTTTAAACAGCTCAACTTTCTGGCGAAGCTCAAT

GAGAATCACTCTGGAGAGCTATGGAAGGGCCGCTGGCAGGGAAATGACATTGTCGTGAAG

GTGCTGAAGGTTCGAGACTGGAGTACAAGGAAAAGCAGGGACTTCAATGAGGAGTGTCCC

CGGCTCAGGATTTTCTCACACCCGAACGTGCTCCCAGTGTTAGGTGCCTGCCAGTCTCCA

CCTGCTCCTCACCCTACACTCATCACACACTGGATGCCGTATGGATCCCTCTACAATGTA

CTACATGAAGGCACCAATTTTGTCGTGGATCAGAGCCAAGCTGTGAAGTTTGCTTTAGAT

ATGGCAAGAGGCATGGCTTTCCTGCACACACTAGAGCCCCTCATCCCACGACATGCACTC

AACAGTCGAAGTGTAATGATTGACGAGGACATGACTGCCCGAATCAGCATGGCCGATGTC

AAGTTTTCTTTCCAATGCCCTGGGCGCATGTATGCACCTGCCTGGGTGGCCCCTGAAGCT

CTGCAGAAGAAGCCTGAAGACACAAACAGACGTTCAGCAGACATGTGGAGTTTTGCAGTA

CTTCTATGGGAACTGGTGACACGAGAGGTACCTTTTGCTGACCTCTCCAATATGGAGATT

GGAATGAAGGTGGCACTAGAAGGCCTTAGGCCTACCATTCCACCAGGTATTTCCCCCCAT

GTGTGTAAGCTCATGAAGATTTGCATGAATGAAGACCCTGCCAAGCGGCCCAAGTTTGAC

ATGATTGTGCCTATTCTGGAAAAGATGCAGGACAAA

>CL299.Contig5_A-W 1203 1943 minus strand Integrin-linked protein kinase OS=Homo sapiens GN=ILK PE=1 SV=2

CAGCTATGGAAGGGCCGCTGGCAGGGAAATGACATTGTCGTGAAGGTGCTGAAGGTTCGA

GACTGGAGTACAAGGAAAAGCAGGGACTTCAATGAGGAGTGTCCCCGGCTCAGGATTTTC

TCACACCCGAACGTGCTCCCAGTGTTAGGTGCCTGCCAGTCTCCACCTGCTCCTCACCCT

ACACTCATCACACACTGGATGCCGTATGGATCCCTCTACAATGTACTACATGAAGGCACC

AATTTTGTCGTGGATCAGAGCCAAGCTGTGAAGTTTGCTTTAGATATGGCAAGAGGCATG

GCTTTCCTGCACACACTAGAGCCCCTCATCCCACGACATGCACTCAACAGTCGAAGTGTA

ATGATTGACGAGGACATGACTGCCCGAATCAGCATGGCCGATGTCAAGTTTTCTTTCCAA

TGCCCTGGGCGCATGTATGCACCTGCCTGGGTGGCCCCTGAAGCTCTGCAGAAGAAGCCT

GAAGACACAAACAGACGTTCAGCAGACATGTGGAGTTTTGCAGTACTTCTATGGGAACTG

GTGACACGAGAGGTACCTTTTGCTGACCTCTCCAATATGGAGATTGGAATGAAGGTGGCA

CTAGAAGGCCTTAGGCCTACCATTCCACCAGGTATTTCCCCCCATGTGTGTAAGCTCATG

AAGATTTGCATGAATGAAGACCCTGCCAAGCGGCCCAAGTTTGACATGATTGTGCCTATT

CTGGAAAAGATGCAGGACAAA

>CL299.Contig6_A-W 581 1936 minus strand Integrin-linked protein kinase [Heterocephalus glaber]

ATGGACGACATTTTCACTCAGTGCCGGGAGGGCAACGCGGTTGCCGTGCGCCTGTGGCTG

GACAACACGGAGAACGATCTCAACCAGGGGGATGATCATGGCTTTTCTCCCTTGCACTGG

GCCTGCCGAGAGGGCCGCTCTGCAGTGGTTGAGATGCTGATCATGAGGGGGGCACGGATC

AATGTGATGAACCGTGGGGACGACACACCCCTGCATCTGGCAGCTAGTCACGGACATCGT

GATATTGTACAAAAGCTGTTGCAGTATAAGGCCGACATCAATGCAGTGAATGAGCATGGG

AATGTGCCTCTGCACTATGCCTGTTTTTGGGGCCAGGATCAAGTGGCAGAGGATCTGGTG

GCTAATGGAGCCCTTGTCAGCATCTGTAACAAGTATGGCGAGATGCCTGTAGACAAAGCC

AAGGCACCCTTGAGAGAGCTTCTCCGAGAACGGGCAGAGAAGATGGGTCAGAATCTCAAC

CGTATTCCATACAAGGACACATTCTGGAAGGGGACCACCCGTACTCGGCCCCGAAACGGG

ACCCTGAACAAACACTCGGGCATTGACTTTAAACAGCTCAACTTTCTGGCGAAGCTCAAT

GAGAATCACTCTGGAGAGCTATGGAAGGGCCGCTGGCAGGGAAATGACATTGTCGTGAAG

GTGCTGAAGGTTCGAGACTGGAGTACAAGGAAAAGCAGGGACTTCAATGAGGAGTGTCCC

CGGCTCAGGATTTTCTCACACCCGAACGTGCTCCCAGTGTTAGGTGCCTGCCAGTCTCCA

CCTGCTCCTCACCCTACACTCATCACACACTGGATGCCGTATGGATCCCTCTACAATGTA

CTACATGAAGGCACCAATTTTGTCGTGGATCAGAGCCAAGCTGTGAAGTTTGCTTTAGAT

ATGGCAAGAGGCATGGCTTTCCTGCACACACTAGAGCCCCTCATCCCACGACATGCACTC

AACAGTCGAAGTGTAATGATTGACGAGGACATGACTGCCCGAATCAGCATGGCCGATGTC

AAGTTTTCTTTCCAATGCCCTGGGCGCATGTATGCACCTGCCTGGGTGGCCCCTGAAGCT

CTGCAGAAGAAGCCTGAAGACACAAACAGACGTTCAGCAGACATGTGGAGTTTTGCAGTA

CTTCTATGGGAACTGGTGACACGAGAGGTACCTTTTGCTGACCTCTCCAATATGGAGATT

GGAATGAAGGTGGCACTAGAAGGCCTTAGGCCTACCATTCCACCAGGTATTTCCCCCCAT

GTGTGTAAGCTCATGAAGATTTGCATGAATGAAGACCCTGCCAAGCGGCCCAAGTTTGAC

ATGATTGTGCCTATTCTGGAAAAGATGCAGGACAAA

>CL299.Contig7_A-W 1284 1502 minus strand PREDICTED: integrin-linked protein kinase-like isoform 2 [Equus caballus] >gi|345788486|ref|XP_003433078.1| PREDICTED: integrin-linked protein kinase [Canis lupus familiaris]

ATGCCTGTAGACAAAGCCAAGGCACCCTTGAGAGAGCTTCTCCGAGAACGGGCAGAGAAG

ATGGGTCAGAATCTCAACCGTATTCCATACAAGGACACATTCTGGAAGGGGACCACCCGT

ACTCGGCCCCGAAACGGGACCCTGAACAAACACTCGGGCATTGACTTTAAACAGCTCAAC

TTTCTGGCGAAGCTCAATGAGAATCACTCTGGAGAGGTG

>CL299.Contig8_A-W 1182 1922 minus strand Integrin-linked protein kinase OS=Homo sapiens GN=ILK PE=1 SV=2

CAGCTATGGAAGGGCCGCTGGCAGGGAAATGACATTGTCGTGAAGGTGCTGAAGGTTCGA

GACTGGAGTACAAGGAAAAGCAGGGACTTCAATGAGGAGTGTCCCCGGCTCAGGATTTTC

TCACACCCGAACGTGCTCCCAGTGTTAGGTGCCTGCCAGTCTCCACCTGCTCCTCACCCT

ACACTCATCACACACTGGATGCCGTATGGATCCCTCTACAATGTACTACATGAAGGCACC

AATTTTGTCGTGGATCAGAGCCAAGCTGTGAAGTTTGCTTTAGATATGGCAAGAGGCATG

GCTTTCCTGCACACACTAGAGCCCCTCATCCCACGACATGCACTCAACAGTCGAAGTGTA

ATGATTGACGAGGACATGACTGCCCGAATCAGCATGGCCGATGTCAAGTTTTCTTTCCAA

TGCCCTGGGCGCATGTATGCACCTGCCTGGGTGGCCCCTGAAGCTCTGCAGAAGAAGCCT

GAAGACACAAACAGACGTTCAGCAGACATGTGGAGTTTTGCAGTACTTCTATGGGAACTG

GTGACACGAGAGGTACCTTTTGCTGACCTCTCCAATATGGAGATTGGAATGAAGGTGGCA

CTAGAAGGCCTTAGGCCTACCATTCCACCAGGTATTTCCCCCCATGTGTGTAAGCTCATG

AAGATTTGCATGAATGAAGACCCTGCCAAGCGGCCCAAGTTTGACATGATTGTGCCTATT

CTGGAAAAGATGCAGGACAAA

>CL299.Contig9_A-W 1158 1898 minus strand Integrin-linked protein kinase OS=Homo sapiens GN=ILK PE=1 SV=2

CAGCTATGGAAGGGCCGCTGGCAGGGAAATGACATTGTCGTGAAGGTGCTGAAGGTTCGA

GACTGGAGTACAAGGAAAAGCAGGGACTTCAATGAGGAGTGTCCCCGGCTCAGGATTTTC

TCACACCCGAACGTGCTCCCAGTGTTAGGTGCCTGCCAGTCTCCACCTGCTCCTCACCCT

ACACTCATCACACACTGGATGCCGTATGGATCCCTCTACAATGTACTACATGAAGGCACC

AATTTTGTCGTGGATCAGAGCCAAGCTGTGAAGTTTGCTTTAGATATGGCAAGAGGCATG

GCTTTCCTGCACACACTAGAGCCCCTCATCCCACGACATGCACTCAACAGTCGAAGTGTA

ATGATTGACGAGGACATGACTGCCCGAATCAGCATGGCCGATGTCAAGTTTTCTTTCCAA

TGCCCTGGGCGCATGTATGCACCTGCCTGGGTGGCCCCTGAAGCTCTGCAGAAGAAGCCT

GAAGACACAAACAGACGTTCAGCAGACATGTGGAGTTTTGCAGTACTTCTATGGGAACTG

GTGACACGAGAGGTACCTTTTGCTGACCTCTCCAATATGGAGATTGGAATGAAGGTGGCA

CTAGAAGGCCTTAGGCCTACCATTCCACCAGGTATTTCCCCCCATGTGTGTAAGCTCATG

AAGATTTGCATGAATGAAGACCCTGCCAAGCGGCCCAAGTTTGACATGATTGTGCCTATT

CTGGAAAAGATGCAGGACAAA

>CL299.Contig10_A-W 750 968 minus strand PREDICTED: integrin-linked protein kinase-like isoform 2 [Equus caballus] >gi|345788486|ref|XP_003433078.1| PREDICTED: integrin-linked protein kinase [Canis lupus familiaris]

ATGCCTGTAGACAAAGCCAAGGCACCCTTGAGAGAGCTTCTCCGAGAACGGGCAGAGAAG

ATGGGTCAGAATCTCAACCGTATTCCATACAAGGACACATTCTGGAAGGGGACCACCCGT

ACTCGGCCCCGAAACGGGACCCTGAACAAACACTCGGGCATTGACTTTAAACAGCTCAAC

TTTCTGGCGAAGCTCAATGAGAATCACTCTGGAGAGGTG

>CL299.Contig11_A-W 327 584 minus strand Integrin-linked protein kinase [Heterocephalus glaber]

ATGGACGACATTTTCACTCAGTGCCGGGAGGGCAACGCGGTTGCCGTGCGCCTGTGGCTG

GACAACACGGAGAACGATCTCAACCAGGGGGATGATCATGGCTTTTCTCCCTTGCACTGG

GCCTGCCGAGAGGGCCGCTCTGCAGTGGTTGAGATGCTGATCATGAGGGGGGCACGGATC

AATGTGATGAACCGTGGGGACGACACACCCCTGCATCTGGCAGCTAGTCACGGACATCGT

GATATTGTACAAAAGGTA

>CL299.Contig12_A-W 729 947 minus strand PREDICTED: integrin-linked protein kinase-like isoform 2 [Equus caballus] >gi|345788486|ref|XP_003433078.1| PREDICTED: integrin-linked protein kinase [Canis lupus familiaris]

ATGCCTGTAGACAAAGCCAAGGCACCCTTGAGAGAGCTTCTCCGAGAACGGGCAGAGAAG

ATGGGTCAGAATCTCAACCGTATTCCATACAAGGACACATTCTGGAAGGGGACCACCCGT

ACTCGGCCCCGAAACGGGACCCTGAACAAACACTCGGGCATTGACTTTAAACAGCTCAAC

TTTCTGGCGAAGCTCAATGAGAATCACTCTGGAGAGGTG

>CL299.Contig13_A-W 209 466 minus strand Integrin-linked protein kinase [Heterocephalus glaber]

ATGGACGACATTTTCACTCAGTGCCGGGAGGGCAACGCGGTTGCCGTGCGCCTGTGGCTG

GACAACACGGAGAACGATCTCAACCAGGGGGATGATCATGGCTTTTCTCCCTTGCACTGG

GCCTGCCGAGAGGGCCGCTCTGCAGTGGTTGAGATGCTGATCATGAGGGGGGCACGGATC

AATGTGATGAACCGTGGGGACGACACACCCCTGCATCTGGCAGCTAGTCACGGACATCGT

GATATTGTACAAAAGGTA

>CL299.Contig14_A-W 196 453 minus strand Integrin-linked protein kinase [Heterocephalus glaber]

ATGGACGACATTTTCACTCAGTGCCGGGAGGGCAACGCGGTTGCCGTGCGCCTGTGGCTG

GACAACACGGAGAACGATCTCAACCAGGGGGATGATCATGGCTTTTCTCCCTTGCACTGG

GCCTGCCGAGAGGGCCGCTCTGCAGTGGTTGAGATGCTGATCATGAGGGGGGCACGGATC

AATGTGATGAACCGTGGGGACGACACACCCCTGCATCTGGCAGCTAGTCACGGACATCGT

GATATTGTACAAAAGGTA

>CL299.Contig15_A-W 1737 2477 minus strand Integrin-linked protein kinase OS=Homo sapiens GN=ILK PE=1 SV=2

CAGCTATGGAAGGGCCGCTGGCAGGGAAATGACATTGTCGTGAAGGTGCTGAAGGTTCGA

GACTGGAGTACAAGGAAAAGCAGGGACTTCAATGAGGAGTGTCCCCGGCTCAGGATTTTC

TCACACCCGAACGTGCTCCCAGTGTTAGGTGCCTGCCAGTCTCCACCTGCTCCTCACCCT

ACACTCATCACACACTGGATGCCGTATGGATCCCTCTACAATGTACTACATGAAGGCACC

AATTTTGTCGTGGATCAGAGCCAAGCTGTGAAGTTTGCTTTAGATATGGCAAGAGGCATG

GCTTTCCTGCACACACTAGAGCCCCTCATCCCACGACATGCACTCAACAGTCGAAGTGTA

ATGATTGACGAGGACATGACTGCCCGAATCAGCATGGCCGATGTCAAGTTTTCTTTCCAA

TGCCCTGGGCGCATGTATGCACCTGCCTGGGTGGCCCCTGAAGCTCTGCAGAAGAAGCCT

GAAGACACAAACAGACGTTCAGCAGACATGTGGAGTTTTGCAGTACTTCTATGGGAACTG

GTGACACGAGAGGTACCTTTTGCTGACCTCTCCAATATGGAGATTGGAATGAAGGTGGCA

CTAGAAGGCCTTAGGCCTACCATTCCACCAGGTATTTCCCCCCATGTGTGTAAGCTCATG

AAGATTTGCATGAATGAAGACCCTGCCAAGCGGCCCAAGTTTGACATGATTGTGCCTATT

CTGGAAAAGATGCAGGACAAA

>CL299.Contig16_A-W 705 923 minus strand PREDICTED: integrin-linked protein kinase-like isoform 2 [Equus caballus] >gi|345788486|ref|XP_003433078.1| PREDICTED: integrin-linked protein kinase [Canis lupus familiaris]

ATGCCTGTAGACAAAGCCAAGGCACCCTTGAGAGAGCTTCTCCGAGAACGGGCAGAGAAG

ATGGGTCAGAATCTCAACCGTATTCCATACAAGGACACATTCTGGAAGGGGACCACCCGT

ACTCGGCCCCGAAACGGGACCCTGAACAAACACTCGGGCATTGACTTTAAACAGCTCAAC

TTTCTGGCGAAGCTCAATGAGAATCACTCTGGAGAGGTG

>CL299.Contig17_A-W 1051 1791 minus strand Integrin-linked protein kinase OS=Homo sapiens GN=ILK PE=1 SV=2

CAGCTATGGAAGGGCCGCTGGCAGGGAAATGACATTGTCGTGAAGGTGCTGAAGGTTCGA

GACTGGAGTACAAGGAAAAGCAGGGACTTCAATGAGGAGTGTCCCCGGCTCAGGATTTTC

TCACACCCGAACGTGCTCCCAGTGTTAGGTGCCTGCCAGTCTCCACCTGCTCCTCACCCT

ACACTCATCACACACTGGATGCCGTATGGATCCCTCTACAATGTACTACATGAAGGCACC

AATTTTGTCGTGGATCAGAGCCAAGCTGTGAAGTTTGCTTTAGATATGGCAAGAGGCATG

GCTTTCCTGCACACACTAGAGCCCCTCATCCCACGACATGCACTCAACAGTCGAAGTGTA

ATGATTGACGAGGACATGACTGCCCGAATCAGCATGGCCGATGTCAAGTTTTCTTTCCAA

TGCCCTGGGCGCATGTATGCACCTGCCTGGGTGGCCCCTGAAGCTCTGCAGAAGAAGCCT

GAAGACACAAACAGACGTTCAGCAGACATGTGGAGTTTTGCAGTACTTCTATGGGAACTG

GTGACACGAGAGGTACCTTTTGCTGACCTCTCCAATATGGAGATTGGAATGAAGGTGGCA

CTAGAAGGCCTTAGGCCTACCATTCCACCAGGTATTTCCCCCCATGTGTGTAAGCTCATG

AAGATTTGCATGAATGAAGACCCTGCCAAGCGGCCCAAGTTTGACATGATTGTGCCTATT

CTGGAAAAGATGCAGGACAAA

>CL299.Contig18_A-W 327 872 minus strand Integrin-linked protein kinase [Heterocephalus glaber]

ATGGACGACATTTTCACTCAGTGCCGGGAGGGCAACGCGGTTGCCGTGCGCCTGTGGCTG

GACAACACGGAGAACGATCTCAACCAGGGGGATGATCATGGCTTTTCTCCCTTGCACTGG

GCCTGCCGAGAGGGCCGCTCTGCAGTGGTTGAGATGCTGATCATGAGGGGGGCACGGATC

AATGTGATGAACCGTGGGGACGACACACCCCTGCATCTGGCAGCTAGTCACGGACATCGT

GATATTGTACAAAAGCTGTTGCAGTATAAGGCCGACATCAATGCAGTGAATGAGCATGGG

AATGTGCCTCTGCACTATGCCTGTTTTTGGGGCCAGGATCAAGTGGCAGAGGATCTGGTG

GCTAATGGAGCCCTTGTCAGCATCTGTAACAAGTATGGCGAGATGCCTGTAGACAAAGCC

AAGGCACCCTTGAGAGAGCTTCTCCGAGAACGGGCAGAGAAGATGGGTCAGAATCTCAAC

CGTATTCCATACAAGGACACATTCTGGAAGGGGACCACCCGTACTCGGCCCCGTGAGTCA

CTGTTG

>CL299.Contig19_A-W 820 987 minus strand Integrin-linked protein kinase [Heterocephalus glaber]

GATGATCATGGCTTTTCTCCCTTGCACTGGGCCTGCCGAGAGGGCCGCTCTGCAGTGGTT

GAGATGCTGATCATGAGGGGGGCACGGATCAATGTGATGAACCGTGGGGACGACACACCC

CTGCATCTGGCAGCTAGTCACGGACATCGTGATATTGTACAAAAGGTA

>CL299.Contig20_A-W 151 408 minus strand Integrin-linked protein kinase [Heterocephalus glaber]

ATGGACGACATTTTCACTCAGTGCCGGGAGGGCAACGCGGTTGCCGTGCGCCTGTGGCTG

GACAACACGGAGAACGATCTCAACCAGGGGGATGATCATGGCTTTTCTCCCTTGCACTGG

GCCTGCCGAGAGGGCCGCTCTGCAGTGGTTGAGATGCTGATCATGAGGGGGGCACGGATC

AATGTGATGAACCGTGGGGACGACACACCCCTGCATCTGGCAGCTAGTCACGGACATCGT

GATATTGTACAAAAGGTA

>CL299.Contig21_A-W 1006 1746 minus strand Integrin-linked protein kinase OS=Homo sapiens GN=ILK PE=1 SV=2

CAGCTATGGAAGGGCCGCTGGCAGGGAAATGACATTGTCGTGAAGGTGCTGAAGGTTCGA

GACTGGAGTACAAGGAAAAGCAGGGACTTCAATGAGGAGTGTCCCCGGCTCAGGATTTTC

TCACACCCGAACGTGCTCCCAGTGTTAGGTGCCTGCCAGTCTCCACCTGCTCCTCACCCT

ACACTCATCACACACTGGATGCCGTATGGATCCCTCTACAATGTACTACATGAAGGCACC

AATTTTGTCGTGGATCAGAGCCAAGCTGTGAAGTTTGCTTTAGATATGGCAAGAGGCATG

GCTTTCCTGCACACACTAGAGCCCCTCATCCCACGACATGCACTCAACAGTCGAAGTGTA

ATGATTGACGAGGACATGACTGCCCGAATCAGCATGGCCGATGTCAAGTTTTCTTTCCAA

TGCCCTGGGCGCATGTATGCACCTGCCTGGGTGGCCCCTGAAGCTCTGCAGAAGAAGCCT

GAAGACACAAACAGACGTTCAGCAGACATGTGGAGTTTTGCAGTACTTCTATGGGAACTG

GTGACACGAGAGGTACCTTTTGCTGACCTCTCCAATATGGAGATTGGAATGAAGGTGGCA

CTAGAAGGCCTTAGGCCTACCATTCCACCAGGTATTTCCCCCCATGTGTGTAAGCTCATG

AAGATTTGCATGAATGAAGACCCTGCCAAGCGGCCCAAGTTTGACATGATTGTGCCTATT

CTGGAAAAGATGCAGGACAAA

>CL299.Contig22_A-W 196 453 minus strand Integrin-linked protein kinase [Heterocephalus glaber]

ATGGACGACATTTTCACTCAGTGCCGGGAGGGCAACGCGGTTGCCGTGCGCCTGTGGCTG

GACAACACGGAGAACGATCTCAACCAGGGGGATGATCATGGCTTTTCTCCCTTGCACTGG

GCCTGCCGAGAGGGCCGCTCTGCAGTGGTTGAGATGCTGATCATGAGGGGGGCACGGATC

AATGTGATGAACCGTGGGGACGACACACCCCTGCATCTGGCAGCTAGTCACGGACATCGT

GATATTGTACAAAAGGTA

>CL299.Contig23_A-W 598 816 minus strand PREDICTED: integrin-linked protein kinase-like isoform 2 [Equus caballus] >gi|345788486|ref|XP_003433078.1| PREDICTED: integrin-linked protein kinase [Canis lupus familiaris]

ATGCCTGTAGACAAAGCCAAGGCACCCTTGAGAGAGCTTCTCCGAGAACGGGCAGAGAAG

ATGGGTCAGAATCTCAACCGTATTCCATACAAGGACACATTCTGGAAGGGGACCACCCGT

ACTCGGCCCCGAAACGGGACCCTGAACAAACACTCGGGCATTGACTTTAAACAGCTCAAC

TTTCTGGCGAAGCTCAATGAGAATCACTCTGGAGAGGTG

>CL299.Contig24_A-W 151 408 minus strand Integrin-linked protein kinase [Heterocephalus glaber]

ATGGACGACATTTTCACTCAGTGCCGGGAGGGCAACGCGGTTGCCGTGCGCCTGTGGCTG

GACAACACGGAGAACGATCTCAACCAGGGGGATGATCATGGCTTTTCTCCCTTGCACTGG

GCCTGCCGAGAGGGCCGCTCTGCAGTGGTTGAGATGCTGATCATGAGGGGGGCACGGATC

AATGTGATGAACCGTGGGGACGACACACCCCTGCATCTGGCAGCTAGTCACGGACATCGT

GATATTGTACAAAAGGTA

>CL299.Contig25_A-W 581 838 minus strand Integrin-linked protein kinase [Heterocephalus glaber]

ATGGACGACATTTTCACTCAGTGCCGGGAGGGCAACGCGGTTGCCGTGCGCCTGTGGCTG

GACAACACGGAGAACGATCTCAACCAGGGGGATGATCATGGCTTTTCTCCCTTGCACTGG

GCCTGCCGAGAGGGCCGCTCTGCAGTGGTTGAGATGCTGATCATGAGGGGGGCACGGATC

AATGTGATGAACCGTGGGGACGACACACCCCTGCATCTGGCAGCTAGTCACGGACATCGT

GATATTGTACAAAAGGTA

>CL299.Contig26_A-W 196 741 minus strand Integrin-linked protein kinase [Heterocephalus glaber]

ATGGACGACATTTTCACTCAGTGCCGGGAGGGCAACGCGGTTGCCGTGCGCCTGTGGCTG

GACAACACGGAGAACGATCTCAACCAGGGGGATGATCATGGCTTTTCTCCCTTGCACTGG

GCCTGCCGAGAGGGCCGCTCTGCAGTGGTTGAGATGCTGATCATGAGGGGGGCACGGATC

AATGTGATGAACCGTGGGGACGACACACCCCTGCATCTGGCAGCTAGTCACGGACATCGT

GATATTGTACAAAAGCTGTTGCAGTATAAGGCCGACATCAATGCAGTGAATGAGCATGGG

AATGTGCCTCTGCACTATGCCTGTTTTTGGGGCCAGGATCAAGTGGCAGAGGATCTGGTG

GCTAATGGAGCCCTTGTCAGCATCTGTAACAAGTATGGCGAGATGCCTGTAGACAAAGCC

AAGGCACCCTTGAGAGAGCTTCTCCGAGAACGGGCAGAGAAGATGGGTCAGAATCTCAAC

CGTATTCCATACAAGGACACATTCTGGAAGGGGACCACCCGTACTCGGCCCCGTGAGTCA

CTGTTG

>CL299.Contig27_A-W 553 771 minus strand PREDICTED: integrin-linked protein kinase-like isoform 2 [Equus caballus] >gi|345788486|ref|XP_003433078.1| PREDICTED: integrin-linked protein kinase [Canis lupus familiaris]

ATGCCTGTAGACAAAGCCAAGGCACCCTTGAGAGAGCTTCTCCGAGAACGGGCAGAGAAG

ATGGGTCAGAATCTCAACCGTATTCCATACAAGGACACATTCTGGAAGGGGACCACCCGT

ACTCGGCCCCGAAACGGGACCCTGAACAAACACTCGGGCATTGACTTTAAACAGCTCAAC

TTTCTGGCGAAGCTCAATGAGAATCACTCTGGAGAGGTG

>CL299.Contig28_A-W 151 696 minus strand Integrin-linked protein kinase [Heterocephalus glaber]

ATGGACGACATTTTCACTCAGTGCCGGGAGGGCAACGCGGTTGCCGTGCGCCTGTGGCTG

GACAACACGGAGAACGATCTCAACCAGGGGGATGATCATGGCTTTTCTCCCTTGCACTGG

GCCTGCCGAGAGGGCCGCTCTGCAGTGGTTGAGATGCTGATCATGAGGGGGGCACGGATC

AATGTGATGAACCGTGGGGACGACACACCCCTGCATCTGGCAGCTAGTCACGGACATCGT

GATATTGTACAAAAGCTGTTGCAGTATAAGGCCGACATCAATGCAGTGAATGAGCATGGG

AATGTGCCTCTGCACTATGCCTGTTTTTGGGGCCAGGATCAAGTGGCAGAGGATCTGGTG

GCTAATGGAGCCCTTGTCAGCATCTGTAACAAGTATGGCGAGATGCCTGTAGACAAAGCC

AAGGCACCCTTGAGAGAGCTTCTCCGAGAACGGGCAGAGAAGATGGGTCAGAATCTCAAC

CGTATTCCATACAAGGACACATTCTGGAAGGGGACCACCCGTACTCGGCCCCGTGAGTCA

CTGTTG

>CL299.Contig29_A-W 1585 2325 minus strand Integrin-linked protein kinase OS=Homo sapiens GN=ILK PE=1 SV=2

CAGCTATGGAAGGGCCGCTGGCAGGGAAATGACATTGTCGTGAAGGTGCTGAAGGTTCGA

GACTGGAGTACAAGGAAAAGCAGGGACTTCAATGAGGAGTGTCCCCGGCTCAGGATTTTC

TCACACCCGAACGTGCTCCCAGTGTTAGGTGCCTGCCAGTCTCCACCTGCTCCTCACCCT

ACACTCATCACACACTGGATGCCGTATGGATCCCTCTACAATGTACTACATGAAGGCACC

AATTTTGTCGTGGATCAGAGCCAAGCTGTGAAGTTTGCTTTAGATATGGCAAGAGGCATG

GCTTTCCTGCACACACTAGAGCCCCTCATCCCACGACATGCACTCAACAGTCGAAGTGTA

ATGATTGACGAGGACATGACTGCCCGAATCAGCATGGCCGATGTCAAGTTTTCTTTCCAA

TGCCCTGGGCGCATGTATGCACCTGCCTGGGTGGCCCCTGAAGCTCTGCAGAAGAAGCCT

GAAGACACAAACAGACGTTCAGCAGACATGTGGAGTTTTGCAGTACTTCTATGGGAACTG

GTGACACGAGAGGTACCTTTTGCTGACCTCTCCAATATGGAGATTGGAATGAAGGTGGCA

CTAGAAGGCCTTAGGCCTACCATTCCACCAGGTATTTCCCCCCATGTGTGTAAGCTCATG

AAGATTTGCATGAATGAAGACCCTGCCAAGCGGCCCAAGTTTGACATGATTGTGCCTATT

CTGGAAAAGATGCAGGACAAA

>CL299.Contig30_A-W 544 1284 minus strand Integrin-linked protein kinase OS=Homo sapiens GN=ILK PE=1 SV=2

CAGCTATGGAAGGGCCGCTGGCAGGGAAATGACATTGTCGTGAAGGTGCTGAAGGTTCGA

GACTGGAGTACAAGGAAAAGCAGGGACTTCAATGAGGAGTGTCCCCGGCTCAGGATTTTC

TCACACCCGAACGTGCTCCCAGTGTTAGGTGCCTGCCAGTCTCCACCTGCTCCTCACCCT

ACACTCATCACACACTGGATGCCGTATGGATCCCTCTACAATGTACTACATGAAGGCACC

AATTTTGTCGTGGATCAGAGCCAAGCTGTGAAGTTTGCTTTAGATATGGCAAGAGGCATG

GCTTTCCTGCACACACTAGAGCCCCTCATCCCACGACATGCACTCAACAGTCGAAGTGTA

ATGATTGACGAGGACATGACTGCCCGAATCAGCATGGCCGATGTCAAGTTTTCTTTCCAA

TGCCCTGGGCGCATGTATGCACCTGCCTGGGTGGCCCCTGAAGCTCTGCAGAAGAAGCCT

GAAGACACAAACAGACGTTCAGCAGACATGTGGAGTTTTGCAGTACTTCTATGGGAACTG

GTGACACGAGAGGTACCTTTTGCTGACCTCTCCAATATGGAGATTGGAATGAAGGTGGCA

CTAGAAGGCCTTAGGCCTACCATTCCACCAGGTATTTCCCCCCATGTGTGTAAGCTCATG

AAGATTTGCATGAATGAAGACCCTGCCAAGCGGCCCAAGTTTGACATGATTGTGCCTATT

CTGGAAAAGATGCAGGACAAA

>CL299.Contig31_A-W 503 1243 minus strand Integrin-linked protein kinase OS=Homo sapiens GN=ILK PE=1 SV=2

CAGCTATGGAAGGGCCGCTGGCAGGGAAATGACATTGTCGTGAAGGTGCTGAAGGTTCGA

GACTGGAGTACAAGGAAAAGCAGGGACTTCAATGAGGAGTGTCCCCGGCTCAGGATTTTC

TCACACCCGAACGTGCTCCCAGTGTTAGGTGCCTGCCAGTCTCCACCTGCTCCTCACCCT

ACACTCATCACACACTGGATGCCGTATGGATCCCTCTACAATGTACTACATGAAGGCACC

AATTTTGTCGTGGATCAGAGCCAAGCTGTGAAGTTTGCTTTAGATATGGCAAGAGGCATG

GCTTTCCTGCACACACTAGAGCCCCTCATCCCACGACATGCACTCAACAGTCGAAGTGTA

ATGATTGACGAGGACATGACTGCCCGAATCAGCATGGCCGATGTCAAGTTTTCTTTCCAA

TGCCCTGGGCGCATGTATGCACCTGCCTGGGTGGCCCCTGAAGCTCTGCAGAAGAAGCCT

GAAGACACAAACAGACGTTCAGCAGACATGTGGAGTTTTGCAGTACTTCTATGGGAACTG

GTGACACGAGAGGTACCTTTTGCTGACCTCTCCAATATGGAGATTGGAATGAAGGTGGCA

CTAGAAGGCCTTAGGCCTACCATTCCACCAGGTATTTCCCCCCATGTGTGTAAGCTCATG

AAGATTTGCATGAATGAAGACCCTGCCAAGCGGCCCAAGTTTGACATGATTGTGCCTATT

CTGGAAAAGATGCAGGACAAA

>CL299.Contig32_A-W 91 309 minus strand PREDICTED: integrin-linked protein kinase-like isoform 2 [Equus caballus] >gi|345788486|ref|XP_003433078.1| PREDICTED: integrin-linked protein kinase [Canis lupus familiaris]

ATGCCTGTAGACAAAGCCAAGGCACCCTTGAGAGAGCTTCTCCGAGAACGGGCAGAGAAG

ATGGGTCAGAATCTCAACCGTATTCCATACAAGGACACATTCTGGAAGGGGACCACCCGT

ACTCGGCCCCGAAACGGGACCCTGAACAAACACTCGGGCATTGACTTTAAACAGCTCAAC

TTTCTGGCGAAGCTCAATGAGAATCACTCTGGAGAGGTG

>CL299.Contig33_A-W 37 234 minus strand Integrin-linked protein kinase [Heterocephalus glaber]

CAGGATCTGGTGGCTAATGGAGCCCTTGTCAGCATCTGTAACAAGTATGGCGAGATGCCT

GTAGACAAAGCCAAGGCACCCTTGAGAGAGCTTCTCCGAGAACGGGCAGAGAAGATGGGT

CAGAATCTCAACCGTATTCCATACAAGGACACATTCTGGAAGGGGACCACCCGTACTCGG

CCCCGTGAGTCACTGTTG

>CL299.Contig34_A-W 820 2085 minus strand Integrin-linked protein kinase [Heterocephalus glaber]

GATGATCATGGCTTTTCTCCCTTGCACTGGGCCTGCCGAGAGGGCCGCTCTGCAGTGGTT

GAGATGCTGATCATGAGGGGGGCACGGATCAATGTGATGAACCGTGGGGACGACACACCC

CTGCATCTGGCAGCTAGTCACGGACATCGTGATATTGTACAAAAGCTGTTGCAGTATAAG

GCCGACATCAATGCAGTGAATGAGCATGGGAATGTGCCTCTGCACTATGCCTGTTTTTGG

GGCCAGGATCAAGTGGCAGAGGATCTGGTGGCTAATGGAGCCCTTGTCAGCATCTGTAAC

AAGTATGGCGAGATGCCTGTAGACAAAGCCAAGGCACCCTTGAGAGAGCTTCTCCGAGAA

CGGGCAGAGAAGATGGGTCAGAATCTCAACCGTATTCCATACAAGGACACATTCTGGAAG

GGGACCACCCGTACTCGGCCCCGAAACGGGACCCTGAACAAACACTCGGGCATTGACTTT

AAACAGCTCAACTTTCTGGCGAAGCTCAATGAGAATCACTCTGGAGAGCTATGGAAGGGC

CGCTGGCAGGGAAATGACATTGTCGTGAAGGTGCTGAAGGTTCGAGACTGGAGTACAAGG

AAAAGCAGGGACTTCAATGAGGAGTGTCCCCGGCTCAGGATTTTCTCACACCCGAACGTG

CTCCCAGTGTTAGGTGCCTGCCAGTCTCCACCTGCTCCTCACCCTACACTCATCACACAC

TGGATGCCGTATGGATCCCTCTACAATGTACTACATGAAGGCACCAATTTTGTCGTGGAT

CAGAGCCAAGCTGTGAAGTTTGCTTTAGATATGGCAAGAGGCATGGCTTTCCTGCACACA

CTAGAGCCCCTCATCCCACGACATGCACTCAACAGTCGAAGTGTAATGATTGACGAGGAC

ATGACTGCCCGAATCAGCATGGCCGATGTCAAGTTTTCTTTCCAATGCCCTGGGCGCATG

TATGCACCTGCCTGGGTGGCCCCTGAAGCTCTGCAGAAGAAGCCTGAAGACACAAACAGA

CGTTCAGCAGACATGTGGAGTTTTGCAGTACTTCTATGGGAACTGGTGACACGAGAGGTA

CCTTTTGCTGACCTCTCCAATATGGAGATTGGAATGAAGGTGGCACTAGAAGGCCTTAGG

CCTACCATTCCACCAGGTATTTCCCCCCATGTGTGTAAGCTCATGAAGATTTGCATGAAT

GAAGACCCTGCCAAGCGGCCCAAGTTTGACATGATTGTGCCTATTCTGGAAAAGATGCAG

GACAAA

>CL299.Contig35_A-W 95 268 minus strand PREDICTED: integrin-linked protein kinase-like isoform 2 [Equus caballus] >gi|345788486|ref|XP_003433078.1| PREDICTED: integrin-linked protein kinase [Canis lupus familiaris]

GAACGGGCAGAGAAGATGGGTCAGAATCTCAACCGTATTCCATACAAGGACACATTCTGG

AAGGGGACCACCCGTACTCGGCCCCGAAACGGGACCCTGAACAAACACTCGGGCATTGAC

TTTAAACAGCTCAACTTTCTGGCGAAGCTCAATGAGAATCACTCTGGAGAGGTG

>CL299.Contig36_A-W 283 1104 minus strand Integrin-linked protein kinase OS=Rattus norvegicus GN=Ilk PE=2 SV=1

AACGGGACCCTGAACAAACACTCGGGCATTGACTTTAAACAGCTCAACTTTCTGGCGAAG

CTCAATGAGAATCACTCTGGAGAGCTATGGAAGGGCCGCTGGCAGGGAAATGACATTGTC

GTGAAGGTGCTGAAGGTTCGAGACTGGAGTACAAGGAAAAGCAGGGACTTCAATGAGGAG

TGTCCCCGGCTCAGGATTTTCTCACACCCGAACGTGCTCCCAGTGTTAGGTGCCTGCCAG

TCTCCACCTGCTCCTCACCCTACACTCATCACACACTGGATGCCGTATGGATCCCTCTAC

AATGTACTACATGAAGGCACCAATTTTGTCGTGGATCAGAGCCAAGCTGTGAAGTTTGCT

TTAGATATGGCAAGAGGCATGGCTTTCCTGCACACACTAGAGCCCCTCATCCCACGACAT

GCACTCAACAGTCGAAGTGTAATGATTGACGAGGACATGACTGCCCGAATCAGCATGGCC

GATGTCAAGTTTTCTTTCCAATGCCCTGGGCGCATGTATGCACCTGCCTGGGTGGCCCCT

GAAGCTCTGCAGAAGAAGCCTGAAGACACAAACAGACGTTCAGCAGACATGTGGAGTTTT

GCAGTACTTCTATGGGAACTGGTGACACGAGAGGTACCTTTTGCTGACCTCTCCAATATG

GAGATTGGAATGAAGGTGGCACTAGAAGGCCTTAGGCCTACCATTCCACCAGGTATTTCC

CCCCATGTGTGTAAGCTCATGAAGATTTGCATGAATGAAGACCCTGCCAAGCGGCCCAAG

TTTGACATGATTGTGCCTATTCTGGAAAAGATGCAGGACAAA

>CL299.Contig37_A-W 37 1044 minus strand Integrin-linked protein kinase [Heterocephalus glaber]

CAGGATCTGGTGGCTAATGGAGCCCTTGTCAGCATCTGTAACAAGTATGGCGAGATGCCT

GTAGACAAAGCCAAGGCACCCTTGAGAGAGCTTCTCCGAGAACGGGCAGAGAAGATGGGT

CAGAATCTCAACCGTATTCCATACAAGGACACATTCTGGAAGGGGACCACCCGTACTCGG

CCCCGAAACGGGACCCTGAACAAACACTCGGGCATTGACTTTAAACAGCTCAACTTTCTG

GCGAAGCTCAATGAGAATCACTCTGGAGAGCTATGGAAGGGCCGCTGGCAGGGAAATGAC

ATTGTCGTGAAGGTGCTGAAGGTTCGAGACTGGAGTACAAGGAAAAGCAGGGACTTCAAT

GAGGAGTGTCCCCGGCTCAGGATTTTCTCACACCCGAACGTGCTCCCAGTGTTAGGTGCC

TGCCAGTCTCCACCTGCTCCTCACCCTACACTCATCACACACTGGATGCCGTATGGATCC

CTCTACAATGTACTACATGAAGGCACCAATTTTGTCGTGGATCAGAGCCAAGCTGTGAAG

TTTGCTTTAGATATGGCAAGAGGCATGGCTTTCCTGCACACACTAGAGCCCCTCATCCCA

CGACATGCACTCAACAGTCGAAGTGTAATGATTGACGAGGACATGACTGCCCGAATCAGC

ATGGCCGATGTCAAGTTTTCTTTCCAATGCCCTGGGCGCATGTATGCACCTGCCTGGGTG

GCCCCTGAAGCTCTGCAGAAGAAGCCTGAAGACACAAACAGACGTTCAGCAGACATGTGG

AGTTTTGCAGTACTTCTATGGGAACTGGTGACACGAGAGGTACCTTTTGCTGACCTCTCC

AATATGGAGATTGGAATGAAGGTGGCACTAGAAGGCCTTAGGCCTACCATTCCACCAGGT

ATTTCCCCCCATGTGTGTAAGCTCATGAAGATTTGCATGAATGAAGACCCTGCCAAGCGG

CCCAAGTTTGACATGATTGTGCCTATTCTGGAAAAGATGCAGGACAAA

>CL299.Contig38_A-W 95 1003 minus strand PREDICTED: integrin-linked protein kinase-like isoform 2 [Equus caballus] >gi|345788486|ref|XP_003433078.1| PREDICTED: integrin-linked protein kinase [Canis lupus familiaris]

GAACGGGCAGAGAAGATGGGTCAGAATCTCAACCGTATTCCATACAAGGACACATTCTGG

AAGGGGACCACCCGTACTCGGCCCCGAAACGGGACCCTGAACAAACACTCGGGCATTGAC

TTTAAACAGCTCAACTTTCTGGCGAAGCTCAATGAGAATCACTCTGGAGAGCTATGGAAG

GGCCGCTGGCAGGGAAATGACATTGTCGTGAAGGTGCTGAAGGTTCGAGACTGGAGTACA

AGGAAAAGCAGGGACTTCAATGAGGAGTGTCCCCGGCTCAGGATTTTCTCACACCCGAAC

GTGCTCCCAGTGTTAGGTGCCTGCCAGTCTCCACCTGCTCCTCACCCTACACTCATCACA

CACTGGATGCCGTATGGATCCCTCTACAATGTACTACATGAAGGCACCAATTTTGTCGTG

GATCAGAGCCAAGCTGTGAAGTTTGCTTTAGATATGGCAAGAGGCATGGCTTTCCTGCAC

ACACTAGAGCCCCTCATCCCACGACATGCACTCAACAGTCGAAGTGTAATGATTGACGAG

GACATGACTGCCCGAATCAGCATGGCCGATGTCAAGTTTTCTTTCCAATGCCCTGGGCGC

ATGTATGCACCTGCCTGGGTGGCCCCTGAAGCTCTGCAGAAGAAGCCTGAAGACACAAAC

AGACGTTCAGCAGACATGTGGAGTTTTGCAGTACTTCTATGGGAACTGGTGACACGAGAG

GTACCTTTTGCTGACCTCTCCAATATGGAGATTGGAATGAAGGTGGCACTAGAAGGCCTT

AGGCCTACCATTCCACCAGGTATTTCCCCCCATGTGTGTAAGCTCATGAAGATTTGCATG

AATGAAGACCCTGCCAAGCGGCCCAAGTTTGACATGATTGTGCCTATTCTGGAAAAGATG

CAGGACAAA

>CL299.Contig39_A-W 820 987 minus strand Integrin-linked protein kinase [Heterocephalus glaber]

GATGATCATGGCTTTTCTCCCTTGCACTGGGCCTGCCGAGAGGGCCGCTCTGCAGTGGTT

GAGATGCTGATCATGAGGGGGGCACGGATCAATGTGATGAACCGTGGGGACGACACACCC

CTGCATCTGGCAGCTAGTCACGGACATCGTGATATTGTACAAAAGGTA

>CL299.Contig40_A-W 1135 1353 minus strand PREDICTED: integrin-linked protein kinase-like isoform 2 [Equus caballus] >gi|345788486|ref|XP_003433078.1| PREDICTED: integrin-linked protein kinase [Canis lupus familiaris]

ATGCCTGTAGACAAAGCCAAGGCACCCTTGAGAGAGCTTCTCCGAGAACGGGCAGAGAAG

ATGGGTCAGAATCTCAACCGTATTCCATACAAGGACACATTCTGGAAGGGGACCACCCGT

ACTCGGCCCCGAAACGGGACCCTGAACAAACACTCGGGCATTGACTTTAAACAGCTCAAC

TTTCTGGCGAAGCTCAATGAGAATCACTCTGGAGAGGTG

>CL299.Contig41_A-W 1132 1350 minus strand PREDICTED: integrin-linked protein kinase-like isoform 2 [Equus caballus] >gi|345788486|ref|XP_003433078.1| PREDICTED: integrin-linked protein kinase [Canis lupus familiaris]

ATGCCTGTAGACAAAGCCAAGGCACCCTTGAGAGAGCTTCTCCGAGAACGGGCAGAGAAG

ATGGGTCAGAATCTCAACCGTATTCCATACAAGGACACATTCTGGAAGGGGACCACCCGT

ACTCGGCCCCGAAACGGGACCCTGAACAAACACTCGGGCATTGACTTTAAACAGCTCAAC

TTTCTGGCGAAGCTCAATGAGAATCACTCTGGAGAGGTG

>CL299.Contig42_A-W 1470 2210 minus strand Integrin-linked protein kinase OS=Homo sapiens GN=ILK PE=1 SV=2

CAGCTATGGAAGGGCCGCTGGCAGGGAAATGACATTGTCGTGAAGGTGCTGAAGGTTCGA

GACTGGAGTACAAGGAAAAGCAGGGACTTCAATGAGGAGTGTCCCCGGCTCAGGATTTTC

TCACACCCGAACGTGCTCCCAGTGTTAGGTGCCTGCCAGTCTCCACCTGCTCCTCACCCT

ACACTCATCACACACTGGATGCCGTATGGATCCCTCTACAATGTACTACATGAAGGCACC

AATTTTGTCGTGGATCAGAGCCAAGCTGTGAAGTTTGCTTTAGATATGGCAAGAGGCATG

GCTTTCCTGCACACACTAGAGCCCCTCATCCCACGACATGCACTCAACAGTCGAAGTGTA

ATGATTGACGAGGACATGACTGCCCGAATCAGCATGGCCGATGTCAAGTTTTCTTTCCAA

TGCCCTGGGCGCATGTATGCACCTGCCTGGGTGGCCCCTGAAGCTCTGCAGAAGAAGCCT

GAAGACACAAACAGACGTTCAGCAGACATGTGGAGTTTTGCAGTACTTCTATGGGAACTG

GTGACACGAGAGGTACCTTTTGCTGACCTCTCCAATATGGAGATTGGAATGAAGGTGGCA

CTAGAAGGCCTTAGGCCTACCATTCCACCAGGTATTTCCCCCCATGTGTGTAAGCTCATG

AAGATTTGCATGAATGAAGACCCTGCCAAGCGGCCCAAGTTTGACATGATTGTGCCTATT

CTGGAAAAGATGCAGGACAAA

>CL299.Contig43_A-W 820 1275 minus strand Integrin-linked protein kinase [Heterocephalus glaber]

GATGATCATGGCTTTTCTCCCTTGCACTGGGCCTGCCGAGAGGGCCGCTCTGCAGTGGTT

GAGATGCTGATCATGAGGGGGGCACGGATCAATGTGATGAACCGTGGGGACGACACACCC

CTGCATCTGGCAGCTAGTCACGGACATCGTGATATTGTACAAAAGCTGTTGCAGTATAAG

GCCGACATCAATGCAGTGAATGAGCATGGGAATGTGCCTCTGCACTATGCCTGTTTTTGG

GGCCAGGATCAAGTGGCAGAGGATCTGGTGGCTAATGGAGCCCTTGTCAGCATCTGTAAC

AAGTATGGCGAGATGCCTGTAGACAAAGCCAAGGCACCCTTGAGAGAGCTTCTCCGAGAA

CGGGCAGAGAAGATGGGTCAGAATCTCAACCGTATTCCATACAAGGACACATTCTGGAAG

GGGACCACCCGTACTCGGCCCCGTGAGTCACTGTTG

>CL299.Contig44_A-W 983 1201 minus strand PREDICTED: integrin-linked protein kinase-like isoform 2 [Equus caballus] >gi|345788486|ref|XP_003433078.1| PREDICTED: integrin-linked protein kinase [Canis lupus familiaris]

ATGCCTGTAGACAAAGCCAAGGCACCCTTGAGAGAGCTTCTCCGAGAACGGGCAGAGAAG

ATGGGTCAGAATCTCAACCGTATTCCATACAAGGACACATTCTGGAAGGGGACCACCCGT

ACTCGGCCCCGAAACGGGACCCTGAACAAACACTCGGGCATTGACTTTAAACAGCTCAAC

TTTCTGGCGAAGCTCAATGAGAATCACTCTGGAGAGGTG

>CL299.Contig45_A-W 463 720 minus strand Integrin-linked protein kinase [Heterocephalus glaber]

ATGGACGACATTTTCACTCAGTGCCGGGAGGGCAACGCGGTTGCCGTGCGCCTGTGGCTG

GACAACACGGAGAACGATCTCAACCAGGGGGATGATCATGGCTTTTCTCCCTTGCACTGG

GCCTGCCGAGAGGGCCGCTCTGCAGTGGTTGAGATGCTGATCATGAGGGGGGCACGGATC

AATGTGATGAACCGTGGGGACGACACACCCCTGCATCTGGCAGCTAGTCACGGACATCGT

GATATTGTACAAAAGGTA

>CL299.Contig46_A-W 1318 2058 minus strand Integrin-linked protein kinase OS=Homo sapiens GN=ILK PE=1 SV=2

CAGCTATGGAAGGGCCGCTGGCAGGGAAATGACATTGTCGTGAAGGTGCTGAAGGTTCGA

GACTGGAGTACAAGGAAAAGCAGGGACTTCAATGAGGAGTGTCCCCGGCTCAGGATTTTC

TCACACCCGAACGTGCTCCCAGTGTTAGGTGCCTGCCAGTCTCCACCTGCTCCTCACCCT

ACACTCATCACACACTGGATGCCGTATGGATCCCTCTACAATGTACTACATGAAGGCACC

AATTTTGTCGTGGATCAGAGCCAAGCTGTGAAGTTTGCTTTAGATATGGCAAGAGGCATG

GCTTTCCTGCACACACTAGAGCCCCTCATCCCACGACATGCACTCAACAGTCGAAGTGTA

ATGATTGACGAGGACATGACTGCCCGAATCAGCATGGCCGATGTCAAGTTTTCTTTCCAA

TGCCCTGGGCGCATGTATGCACCTGCCTGGGTGGCCCCTGAAGCTCTGCAGAAGAAGCCT

GAAGACACAAACAGACGTTCAGCAGACATGTGGAGTTTTGCAGTACTTCTATGGGAACTG

GTGACACGAGAGGTACCTTTTGCTGACCTCTCCAATATGGAGATTGGAATGAAGGTGGCA

CTAGAAGGCCTTAGGCCTACCATTCCACCAGGTATTTCCCCCCATGTGTGTAAGCTCATG

AAGATTTGCATGAATGAAGACCCTGCCAAGCGGCCCAAGTTTGACATGATTGTGCCTATT

CTGGAAAAGATGCAGGACAAA

>CL299.Contig47_A-W 581 1126 minus strand Integrin-linked protein kinase [Heterocephalus glaber]

ATGGACGACATTTTCACTCAGTGCCGGGAGGGCAACGCGGTTGCCGTGCGCCTGTGGCTG

GACAACACGGAGAACGATCTCAACCAGGGGGATGATCATGGCTTTTCTCCCTTGCACTGG

GCCTGCCGAGAGGGCCGCTCTGCAGTGGTTGAGATGCTGATCATGAGGGGGGCACGGATC

AATGTGATGAACCGTGGGGACGACACACCCCTGCATCTGGCAGCTAGTCACGGACATCGT

GATATTGTACAAAAGCTGTTGCAGTATAAGGCCGACATCAATGCAGTGAATGAGCATGGG

AATGTGCCTCTGCACTATGCCTGTTTTTGGGGCCAGGATCAAGTGGCAGAGGATCTGGTG

GCTAATGGAGCCCTTGTCAGCATCTGTAACAAGTATGGCGAGATGCCTGTAGACAAAGCC

AAGGCACCCTTGAGAGAGCTTCTCCGAGAACGGGCAGAGAAGATGGGTCAGAATCTCAAC

CGTATTCCATACAAGGACACATTCTGGAAGGGGACCACCCGTACTCGGCCCCGTGAGTCA

CTGTTG

>CL299.Contig48_A-W 881 1099 minus strand PREDICTED: integrin-linked protein kinase-like isoform 2 [Equus caballus] >gi|345788486|ref|XP_003433078.1| PREDICTED: integrin-linked protein kinase [Canis lupus familiaris]

ATGCCTGTAGACAAAGCCAAGGCACCCTTGAGAGAGCTTCTCCGAGAACGGGCAGAGAAG

ATGGGTCAGAATCTCAACCGTATTCCATACAAGGACACATTCTGGAAGGGGACCACCCGT

ACTCGGCCCCGAAACGGGACCCTGAACAAACACTCGGGCATTGACTTTAAACAGCTCAAC

TTTCTGGCGAAGCTCAATGAGAATCACTCTGGAGAGGTG

>CL299.Contig49_A-W 1216 1956 minus strand Integrin-linked protein kinase OS=Homo sapiens GN=ILK PE=1 SV=2

CAGCTATGGAAGGGCCGCTGGCAGGGAAATGACATTGTCGTGAAGGTGCTGAAGGTTCGA

GACTGGAGTACAAGGAAAAGCAGGGACTTCAATGAGGAGTGTCCCCGGCTCAGGATTTTC

TCACACCCGAACGTGCTCCCAGTGTTAGGTGCCTGCCAGTCTCCACCTGCTCCTCACCCT

ACACTCATCACACACTGGATGCCGTATGGATCCCTCTACAATGTACTACATGAAGGCACC

AATTTTGTCGTGGATCAGAGCCAAGCTGTGAAGTTTGCTTTAGATATGGCAAGAGGCATG

GCTTTCCTGCACACACTAGAGCCCCTCATCCCACGACATGCACTCAACAGTCGAAGTGTA

ATGATTGACGAGGACATGACTGCCCGAATCAGCATGGCCGATGTCAAGTTTTCTTTCCAA

TGCCCTGGGCGCATGTATGCACCTGCCTGGGTGGCCCCTGAAGCTCTGCAGAAGAAGCCT

GAAGACACAAACAGACGTTCAGCAGACATGTGGAGTTTTGCAGTACTTCTATGGGAACTG

GTGACACGAGAGGTACCTTTTGCTGACCTCTCCAATATGGAGATTGGAATGAAGGTGGCA

CTAGAAGGCCTTAGGCCTACCATTCCACCAGGTATTTCCCCCCATGTGTGTAAGCTCATG

AAGATTTGCATGAATGAAGACCCTGCCAAGCGGCCCAAGTTTGACATGATTGTGCCTATT

CTGGAAAAGATGCAGGACAAA

>CL300.Contig1_A-W 2 1387 ATP-dependent RNA helicase DDX39 [Macaca mulatta]

AGGAGGCAGGAAGCGCCGCAGCTCGTGTCGCGGCGCGTGCTGGGTAACCGGAGTCAGGCG

GCTCTCCTCGTAGCACGCGGAGGCGAACGGAGACCCAGTACCATGGCAGAGCAGGATGTG

GAGAACGAGCTCTTGGATTATGAAGAAGATGAAGAGCCCCAGGCTCCTCCAGAGAGTACT

CCAGCTCCACCTAAGAAAGACGTCAAGGGGTCCTACGTCTCCATCCATAGCTCTGGCTTC

CGAGACTTTTTGCTGAAGCCAGAGCTCCTGAGGGCCATAGTGGACTGTGGTTTTGAACAC

CCATCCGAGGTCCAGCATGAGTGTATCCCCCAGGCCATCCTGGGCATGGACGTCCTGTGC

CAGGCCAAGTCTGGGATGGGCAAGACGGCTGTCTTCGTGCTGGCTACTCTGCAGCAGATC

GAGCCAGTTAATGGACAGGTGACAGTCCTGGTCATGTGCCATACGAGGGAGTTGGCCTTC

CAAATCAGCAAGGAGTATGAGCGCTTCTCCAAATACATGCCCAGTGTCAAGGTGTCTGTG

TTCTTTGGGGGCCTCTCCATCAAGAAAGATGAAGAAGTGTTGAAGAAGAATTGTCCCCAC

GTAGTGGTGGGGACACCAGGCAGGATCCTGGCCCTCGTGCGGAACAGGAGCCTCAACTTA

AAAAACGTGAAGCACTTTGTGTTGGACGAGTGTGACAAGATGCTGGAGCAGCTGGACATG

CGGCGGGATGTGCAGGAGATCTTCCGCCTGACGCCTCATGAGAAGCAGTGCATGATGTTC

AGTGCCACCCTGAGCAAGGAGATCCGGCCAGTCTGCAGGAAGTTCATGCAGGATCCCATG

GAGGTGTTTGTGGACGACGAGACCAAGCTCACGCTGCATGGGCTGCAGCAGTACTATGTC

AAGCTCAAGGACAGCGAGAAGAACCGAAAGCTCTTTGACCTCCTGGATGTGCTGGAGTTT

AACCAGGTGATGCCCCCACAGCCTGTCCTGGGCTTTGGGCCTCAGGAGCGGGTACCCGGC

CAGGCCTAACCGCTGCCTCTCCTACAGGTGGTGATCTTCGTCAAATCCGTGCAGCGCTGC

ATGGCCCTGGCCCAGCTCCTTGTGGAGCAGAACTTCCCAGCCATCGCCATTCACCGGGGC

ATGGCCCAGGAGGAGCGGTGAGTGCACGCCACCTGCCGTGGCCAGAGGGAGCAGCGCCCA

GGAGCCCGTGTCTGACAGCCTCCACTTATTTCTCCTGCACCCAGTCTGTCACGCTACCAG

CAGTTCAAGGACTTCCAGCGGCGGATCTTGGTGGCCACCAATCTCTTTGGCCGAGGGATG

GATATTGAGCGCGTCAACATCGTCTTCAACTATGACATGCCCGAGGACTCGGACACCTAC

CTCCAC

>CL300.Contig2_A-W 1203 1838 ATP-dependent RNA helicase DDX39 [Macaca mulatta]

CCCATGGAGGTGTTTGTGGACGACGAGACCAAGCTCACGCTGCATGGGCTGCAGCAGTAC

TATGTCAAGCTCAAGGACAGCGAGAAGAACCGAAAGCTCTTTGACCTCCTGGATGTGCTG

GAGTTTAACCAGGTGGTGATCTTCGTCAAATCCGTGCAGCGCTGCATGGCCCTGGCCCAG

CTCCTTGTGGAGCAGAACTTCCCAGCCATCGCCATTCACCGGGGCATGGCCCAGGAGGAG

CGGTGAGTGCACGCCACCTGCCGTGGCCAGAGGGAGCAGCGCCCAGGAGCCCGTGTCTGA

CAGCCTCCACTTATTTCTCCTGCACCCAGTCTGTCACGCTACCAGCAGTTCAAGGACTTC

CAGCGGCGGATCTTGGTGGCCACCAATCTCTTTGGCCGAGGGATGGATATTGAGCGCGTC

AACATCGTCTTCAACTATGACATGCCCGAGGACTCGGACACCTACCTCCACCGGGTGGCC

CGTGCAGGTCGCTTTGGTACCAAAGGCCTGGCCATCACTTTTGTGTCTGATGAAAATGAT

GCCAAAATTCTCAATGATGTCCAGGACCGGTTTGAAGTGAATGTGGCTGAGCTTCCGGAA

GAAATCGATATCTCCACATACATCGAGCAGAGCCGG

>CL300.Contig3_A-W 180 1382 PREDICTED: ATP-dependent RNA helicase DDX39A [Canis lupus familiaris] >gi|395850755|ref|XP_003797941.1| PREDICTED: ATP-dependent RNA helicase DDX39A [Otolemur garnettii]

ATGGCAGAGCAGGATGTGGAGAACGAGCTCTTGGATTATGAAGAAGATGAAGAGCCCCAG

GCTCCTCCAGAGAGTACTCCAGCTCCACCTAAGAAAGACGTCAAGGGGTCCTACGTCTCC

ATCCATAGCTCTGGCTTCCGAGACTTTTTGCTGAAGCCAGAGCTCCTGAGGGCCATAGTG

GACTGTGGTTTTGAACACCCATCCGAGGTCCAGCATGAGTGTATCCCCCAGGCCATCCTG

GGCATGGACGTCCTGTGCCAGGCCAAGTCTGGGATGGGCAAGACGGCTGTCTTCGTGCTG

GCTACTCTGCAGCAGATCGAGCCAGTTAATGGACAGGTGACAGTCCTGGTCATGTGCCAT

ACGAGGGAGTTGGCCTTCCAAATCAGCAAGGAGTATGAGCGCTTCTCCAAATACATGCCC

AGTGTCAAGGTGTCTGTGTTCTTTGGGGGCCTCTCCATCAAGAAAGATGAAGAAGTGTTG

AAGAAGAATTGTCCCCACGTAGTGGTGGGGACACCAGGCAGGATCCTGGCCCTCGTGCGG

AACAGGAGCCTCAACTTAAAAAACGTGAAGCACTTTGTGTTGGACGAGTGTGACAAGATG

CTGGAGCAGCTGGACATGCGGCGGGATGTGCAGGAGATCTTCCGCCTGACGCCTCATGAG

AAGCAGTGCATGATGTTCAGTGCCACCCTGAGCAAGGAGATCCGGCCAGTCTGCAGGAAG

TTCATGCAGGATCCCATGGAGGTGTTTGTGGACGACGAGACCAAGCTCACGCTGCATGGG

CTGCAGCAGTACTATGTCAAGCTCAAGGACAGCGAGAAGAACCGAAAGCTCTTTGACCTC

CTGGATGTGCTGGAGTTTAACCAGGTGGTGATCTTCGTCAAATCCGTGCAGCGCTGCATG

GCCCTGGCCCAGCTCCTTGTGGAGCAGAACTTCCCAGCCATCGCCATTCACCGGGGCATG

GCCCAGGAGGAGCGGTGAGTGCACGCCACCTGCCGTGGCCAGAGGGAGCAGCGCCCAGGA

GCCCGTGTCTGACAGCCTCCACTTATTTCTCCTGCACCCAGTCTGTCACGCTACCAGCAG

TTCAAGGACTTCCAGCGGCGGATCTTGGTGGCCACCAATCTCTTTGGCCGAGGGATGGAT

ATTGAGCGCGTCAACATCGTCTTCAACTATGACATGCCCGAGGACTCGGACACCTACCTC

CAC

>CL300.Contig4_A-W 180 1376 PREDICTED: ATP-dependent RNA helicase DDX39A [Canis lupus familiaris] >gi|395850755|ref|XP_003797941.1| PREDICTED: ATP-dependent RNA helicase DDX39A [Otolemur garnettii]

ATGGCAGAGCAGGATGTGGAGAACGAGCTCTTGGATTATGAAGAAGATGAAGAGCCCCAG

GCTCCTCCAGAGAGTACTCCAGCTCCACCTAAGAAAGACGTCAAGGGGTCCTACGTCTCC

ATCCATAGCTCTGGCTTCCGAGACTTTTTGCTGAAGCCAGAGCTCCTGAGGGCCATAGTG

GACTGTGGTTTTGAACACCCATCCGAGGTCCAGCATGAGTGTATCCCCCAGGCCATCCTG

GGCATGGACGTCCTGTGCCAGGCCAAGTCTGGGATGGGCAAGACGGCTGTCTTCGTGCTG

GCTACTCTGCAGCAGATCGAGCCAGTTAATGGACAGGTGACAGTCCTGGTCATGTGCCAT

ACGAGGGAGTTGGCCTTCCAAATCAGCAAGGAGTATGAGCGCTTCTCCAAATACATGCCC

AGTGTCAAGGTGTCTGTGTTCTTTGGGGGCCTCTCCATCAAGAAAGATGAAGAAGTGTTG

AAGAAGAATTGTCCCCACGTAGTGGTGGGGACACCAGGCAGGATCCTGGCCCTCGTGCGG

AACAGGAGCCTCAACTTAAAAAACGTGAAGCACTTTGTGTTGGACGAGTGTGACAAGATG

CTGGAGCAGCTGGACATGCGGCGGGATGTGCAGGAGATCTTCCGCCTGACGCCTCATGAG

AAGCAGTGCATGATGTTCAGTGCCACCCTGAGCAAGGAGATCCGGCCAGTCTGCAGGAAG

TTCATGCAGGATCCCATGGAGGTGTTTGTGGACGACGAGACCAAGCTCACGCTGCATGGG

CTGCAGCAGTACTATGTCAAGCTCAAGGACAGCGAGAAGAACCGAAAGCTCTTTGACCTC

CTGGATGTGCTGGAGTTTAACCAGGTGATGCCCCCACAGCCTGTCCTGGGCTTTGGGCCT

CAGGAGCGGGTACCCGGCCAGGCCTAACCGCTGCCTCTCCTACAGGTGGTGATCTTCGTC

AAATCCGTGCAGCGCTGCATGGCCCTGGCCCAGCTCCTTGTGGAGCAGAACTTCCCAGCC

ATCGCCATTCACCGGGGCATGGCCCAGGAGGAGCGTCTGTCACGCTACCAGCAGTTCAAG

GACTTCCAGCGGCGGATCTTGGTGGCCACCAATCTCTTTGGCCGAGGGATGGATATTGAG

CGCGTCAACATCGTCTTCAACTATGACATGCCCGAGGACTCGGACACCTACCTCCAC

>CL300.Contig5_A-W 1279 1914 PREDICTED: ATP-dependent RNA helicase DDX39A [Canis lupus familiaris] >gi|395850755|ref|XP_003797941.1| PREDICTED: ATP-dependent RNA helicase DDX39A [Otolemur garnettii]

CCCATGGAGGTGTTTGTGGACGACGAGACCAAGCTCACGCTGCATGGGCTGCAGCAGTAC

TATGTCAAGCTCAAGGACAGCGAGAAGAACCGAAAGCTCTTTGACCTCCTGGATGTGCTG

GAGTTTAACCAGGTGGTGATCTTCGTCAAATCCGTGCAGCGCTGCATGGCCCTGGCCCAG

CTCCTTGTGGAGCAGAACTTCCCAGCCATCGCCATTCACCGGGGCATGGCCCAGGAGGAG

CGGTGAGTGCACGCCACCTGCCGTGGCCAGAGGGAGCAGCGCCCAGGAGCCCGTGTCTGA

CAGCCTCCACTTATTTCTCCTGCACCCAGTCTGTCACGCTACCAGCAGTTCAAGGACTTC

CAGCGGCGGATCTTGGTGGCCACCAATCTCTTTGGCCGAGGGATGGATATTGAGCGCGTC

AACATCGTCTTCAACTATGACATGCCCGAGGACTCGGACACCTACCTCCACCGGGTGGCC

CGTGCAGGTCGCTTTGGTACCAAAGGCCTGGCCATCACTTTTGTGTCTGATGAAAATGAT

GCCAAAATTCTCAATGATGTCCAGGACCGGTTTGAAGTGAATGTGGCTGAGCTTCCGGAA

GAAATCGATATCTCCACATACATCGAGCAGAGCCGG

>CL300.Contig6_A-W 1203 1832 ATP-dependent RNA helicase DDX39 [Macaca mulatta]

CCCATGGAGGTGTTTGTGGACGACGAGACCAAGCTCACGCTGCATGGGCTGCAGCAGTAC

TATGTCAAGCTCAAGGACAGCGAGAAGAACCGAAAGCTCTTTGACCTCCTGGATGTGCTG

GAGTTTAACCAGGTGATGCCCCCACAGCCTGTCCTGGGCTTTGGGCCTCAGGAGCGGGTA

CCCGGCCAGGCCTAACCGCTGCCTCTCCTACAGGTGGTGATCTTCGTCAAATCCGTGCAG

CGCTGCATGGCCCTGGCCCAGCTCCTTGTGGAGCAGAACTTCCCAGCCATCGCCATTCAC

CGGGGCATGGCCCAGGAGGAGCGTCTGTCACGCTACCAGCAGTTCAAGGACTTCCAGCGG

CGGATCTTGGTGGCCACCAATCTCTTTGGCCGAGGGATGGATATTGAGCGCGTCAACATC

GTCTTCAACTATGACATGCCCGAGGACTCGGACACCTACCTCCACCGGGTGGCCCGTGCA

GGTCGCTTTGGTACCAAAGGCCTGGCCATCACTTTTGTGTCTGATGAAAATGATGCCAAA

ATTCTCAATGATGTCCAGGACCGGTTTGAAGTGAATGTGGCTGAGCTTCCGGAAGAAATC

GATATCTCCACATACATCGAGCAGAGCCGG

>CL300.Contig7_A-W 1279 1827 PREDICTED: ATP-dependent RNA helicase DDX39A [Canis lupus familiaris] >gi|395850755|ref|XP_003797941.1| PREDICTED: ATP-dependent RNA helicase DDX39A [Otolemur garnettii]

CCCATGGAGGTGTTTGTGGACGACGAGACCAAGCTCACGCTGCATGGGCTGCAGCAGTAC

TATGTCAAGCTCAAGGACAGCGAGAAGAACCGAAAGCTCTTTGACCTCCTGGATGTGCTG

GAGTTTAACCAGGTGGTGATCTTCGTCAAATCCGTGCAGCGCTGCATGGCCCTGGCCCAG

CTCCTTGTGGAGCAGAACTTCCCAGCCATCGCCATTCACCGGGGCATGGCCCAGGAGGAG

CGTCTGTCACGCTACCAGCAGTTCAAGGACTTCCAGCGGCGGATCTTGGTGGCCACCAAT

CTCTTTGGCCGAGGGATGGATATTGAGCGCGTCAACATCGTCTTCAACTATGACATGCCC

GAGGACTCGGACACCTACCTCCACCGGGTGGCCCGTGCAGGTCGCTTTGGTACCAAAGGC

CTGGCCATCACTTTTGTGTCTGATGAAAATGATGCCAAAATTCTCAATGATGTCCAGGAC

CGGTTTGAAGTGAATGTGGCTGAGCTTCCGGAAGAAATCGATATCTCCACATACATCGAG

CAGAGCCGG

>CL300.Contig8_A-W 1279 1830 PREDICTED: ATP-dependent RNA helicase DDX39A [Canis lupus familiaris] >gi|395850755|ref|XP_003797941.1| PREDICTED: ATP-dependent RNA helicase DDX39A [Otolemur garnettii]

CCCATGGAGGTGTTTGTGGACGACGAGACCAAGCTCACGCTGCATGGGCTGCAGCAGTAC

TATGTCAAGCTCAAGGACAGCGAGAAGAACCGAAAGCTCTTTGACCTCCTGGATGTGCTG

GAGTTTAACCAGGTGATGCCCCCACAGCCTGTCCTGGGCTTTGGGCCTCAGGAGCGGGTA

CCCGGCCAGGCCTAACCGCTGCCTCTCCTACAGGTGGTGATCTTCGTCAAATCCGTGCAG

CGCTGCATGGCCCTGGCCCAGCTCCTTGTGGAGCAGAACTTCCCAGCCATCGCCATTCAC

CGGGGCATGGCCCAGGAGGAGCGGTGAGTGCACGCCACCTGCCGTGGCCAGAGGGAGCAG

CGCCCAGGAGCCCGTGTCTGACAGCCTCCACTTATTTCTCCTGCACCCAGTCTGTCACGC

TACCAGCAGTTCAAGGACTTCCAGCGGCGGATCTTGGTGGCCACCAATCTCTTTGGCCGA

GGGATGGATATTGAGCGCGTCAACATCGTCTTCAACTATGACATGCCCGAGGACTCGGAC

ACCTACCTCCAC

>CL300.Contig9_A-W 1203 1754 ATP-dependent RNA helicase DDX39 [Macaca mulatta]

CCCATGGAGGTGTTTGTGGACGACGAGACCAAGCTCACGCTGCATGGGCTGCAGCAGTAC

TATGTCAAGCTCAAGGACAGCGAGAAGAACCGAAAGCTCTTTGACCTCCTGGATGTGCTG

GAGTTTAACCAGGTGATGCCCCCACAGCCTGTCCTGGGCTTTGGGCCTCAGGAGCGGGTA

CCCGGCCAGGCCTAACCGCTGCCTCTCCTACAGGTGGTGATCTTCGTCAAATCCGTGCAG

CGCTGCATGGCCCTGGCCCAGCTCCTTGTGGAGCAGAACTTCCCAGCCATCGCCATTCAC

CGGGGCATGGCCCAGGAGGAGCGGTGAGTGCACGCCACCTGCCGTGGCCAGAGGGAGCAG

CGCCCAGGAGCCCGTGTCTGACAGCCTCCACTTATTTCTCCTGCACCCAGTCTGTCACGC

TACCAGCAGTTCAAGGACTTCCAGCGGCGGATCTTGGTGGCCACCAATCTCTTTGGCCGA

GGGATGGATATTGAGCGCGTCAACATCGTCTTCAACTATGACATGCCCGAGGACTCGGAC

ACCTACCTCCAC

>CL300.Contig10_A-W 1279 1743 PREDICTED: ATP-dependent RNA helicase DDX39A [Canis lupus familiaris] >gi|395850755|ref|XP_003797941.1| PREDICTED: ATP-dependent RNA helicase DDX39A [Otolemur garnettii]

CCCATGGAGGTGTTTGTGGACGACGAGACCAAGCTCACGCTGCATGGGCTGCAGCAGTAC

TATGTCAAGCTCAAGGACAGCGAGAAGAACCGAAAGCTCTTTGACCTCCTGGATGTGCTG

GAGTTTAACCAGGTGATGCCCCCACAGCCTGTCCTGGGCTTTGGGCCTCAGGAGCGGGTA

CCCGGCCAGGCCTAACCGCTGCCTCTCCTACAGGTGGTGATCTTCGTCAAATCCGTGCAG

CGCTGCATGGCCCTGGCCCAGCTCCTTGTGGAGCAGAACTTCCCAGCCATCGCCATTCAC

CGGGGCATGGCCCAGGAGGAGCGTCTGTCACGCTACCAGCAGTTCAAGGACTTCCAGCGG

CGGATCTTGGTGGCCACCAATCTCTTTGGCCGAGGGATGGATATTGAGCGCGTCAACATC

GTCTTCAACTATGACATGCCCGAGGACTCGGACACCTACCTCCAC

>CL300.Contig11_A-W 2 1471 ATP-dependent RNA helicase DDX39 [Macaca mulatta]

AGGAGGCAGGAAGCGCCGCAGCTCGTGTCGCGGCGCGTGCTGGGTAACCGGAGTCAGGCG

GCTCTCCTCGTAGCACGCGGAGGCGAACGGAGACCCAGTACCATGGCAGAGCAGGATGTG

GAGAACGAGCTCTTGGATTATGAAGAAGATGAAGAGCCCCAGGCTCCTCCAGAGAGTACT

CCAGCTCCACCTAAGAAAGACGTCAAGGGGTCCTACGTCTCCATCCATAGCTCTGGCTTC

CGAGACTTTTTGCTGAAGCCAGAGCTCCTGAGGGCCATAGTGGACTGTGGTTTTGAACAC

CCATCCGAGGTCCAGCATGAGTGTATCCCCCAGGCCATCCTGGGCATGGACGTCCTGTGC

CAGGCCAAGTCTGGGATGGGCAAGACGGCTGTCTTCGTGCTGGCTACTCTGCAGCAGATC

GAGCCAGTTAATGGACAGGTGACAGTCCTGGTCATGTGCCATACGAGGGAGTTGGCCTTC

CAAATCAGCAAGGAGTATGAGCGCTTCTCCAAATACATGCCCAGTGTCAAGGTGTCTGTG

TTCTTTGGGGGCCTCTCCATCAAGAAAGATGAAGAAGTGTTGAAGAAGAATTGTCCCCAC

GTAGTGGTGGGGACACCAGGCAGGATCCTGGCCCTCGTGCGGAACAGGAGCCTCAACTTA

AAAAACGTGAAGCACTTTGTGTTGGACGAGTGTGACAAGATGCTGGAGCAGCTGGACATG

CGGCGGGATGTGCAGGAGATCTTCCGCCTGACGCCTCATGAGAAGCAGTGCATGATGTTC

AGTGCCACCCTGAGCAAGGAGATCCGGCCAGTCTGCAGGAAGTTCATGCAGGATCCCATG

GAGGTGTTTGTGGACGACGAGACCAAGCTCACGCTGCATGGGCTGCAGCAGTACTATGTC

AAGCTCAAGGACAGCGAGAAGAACCGAAAGCTCTTTGACCTCCTGGATGTGCTGGAGTTT

AACCAGGTGGTGATCTTCGTCAAATCCGTGCAGCGCTGCATGGCCCTGGCCCAGCTCCTT

GTGGAGCAGAACTTCCCAGCCATCGCCATTCACCGGGGCATGGCCCAGGAGGAGCGGTGA

GTGCACGCCACCTGCCGTGGCCAGAGGGAGCAGCGCCCAGGAGCCCGTGTCTGACAGCCT

CCACTTATTTCTCCTGCACCCAGTCTGTCACGCTACCAGCAGTTCAAGGACTTCCAGCGG

CGGATCTTGGTGGCCACCAATCTCTTTGGCCGAGGGATGGATATTGAGCGCGTCAACATC

GTCTTCAACTATGACATGCCCGAGGACTCGGACACCTACCTCCACCGGGTGGCCCGTGCA

GGTCGCTTTGGTACCAAAGGCCTGGCCATCACTTTTGTGTCTGATGAAAATGATGCCAAA

ATTCTCAATGATGTCCAGGACCGGTTTGAAGTGAATGTGGCTGAGCTTCCGGAAGAAATC

GATATCTCCACATACATCGAGCAGAGCCGG

>CL300.Contig12_A-W 2 1465 ATP-dependent RNA helicase DDX39 [Macaca mulatta]

AGGAGGCAGGAAGCGCCGCAGCTCGTGTCGCGGCGCGTGCTGGGTAACCGGAGTCAGGCG

GCTCTCCTCGTAGCACGCGGAGGCGAACGGAGACCCAGTACCATGGCAGAGCAGGATGTG

GAGAACGAGCTCTTGGATTATGAAGAAGATGAAGAGCCCCAGGCTCCTCCAGAGAGTACT

CCAGCTCCACCTAAGAAAGACGTCAAGGGGTCCTACGTCTCCATCCATAGCTCTGGCTTC

CGAGACTTTTTGCTGAAGCCAGAGCTCCTGAGGGCCATAGTGGACTGTGGTTTTGAACAC

CCATCCGAGGTCCAGCATGAGTGTATCCCCCAGGCCATCCTGGGCATGGACGTCCTGTGC

CAGGCCAAGTCTGGGATGGGCAAGACGGCTGTCTTCGTGCTGGCTACTCTGCAGCAGATC

GAGCCAGTTAATGGACAGGTGACAGTCCTGGTCATGTGCCATACGAGGGAGTTGGCCTTC

CAAATCAGCAAGGAGTATGAGCGCTTCTCCAAATACATGCCCAGTGTCAAGGTGTCTGTG

TTCTTTGGGGGCCTCTCCATCAAGAAAGATGAAGAAGTGTTGAAGAAGAATTGTCCCCAC

GTAGTGGTGGGGACACCAGGCAGGATCCTGGCCCTCGTGCGGAACAGGAGCCTCAACTTA

AAAAACGTGAAGCACTTTGTGTTGGACGAGTGTGACAAGATGCTGGAGCAGCTGGACATG

CGGCGGGATGTGCAGGAGATCTTCCGCCTGACGCCTCATGAGAAGCAGTGCATGATGTTC

AGTGCCACCCTGAGCAAGGAGATCCGGCCAGTCTGCAGGAAGTTCATGCAGGATCCCATG

GAGGTGTTTGTGGACGACGAGACCAAGCTCACGCTGCATGGGCTGCAGCAGTACTATGTC

AAGCTCAAGGACAGCGAGAAGAACCGAAAGCTCTTTGACCTCCTGGATGTGCTGGAGTTT

AACCAGGTGATGCCCCCACAGCCTGTCCTGGGCTTTGGGCCTCAGGAGCGGGTACCCGGC

CAGGCCTAACCGCTGCCTCTCCTACAGGTGGTGATCTTCGTCAAATCCGTGCAGCGCTGC

ATGGCCCTGGCCCAGCTCCTTGTGGAGCAGAACTTCCCAGCCATCGCCATTCACCGGGGC

ATGGCCCAGGAGGAGCGTCTGTCACGCTACCAGCAGTTCAAGGACTTCCAGCGGCGGATC

TTGGTGGCCACCAATCTCTTTGGCCGAGGGATGGATATTGAGCGCGTCAACATCGTCTTC

AACTATGACATGCCCGAGGACTCGGACACCTACCTCCACCGGGTGGCCCGTGCAGGTCGC

TTTGGTACCAAAGGCCTGGCCATCACTTTTGTGTCTGATGAAAATGATGCCAAAATTCTC

AATGATGTCCAGGACCGGTTTGAAGTGAATGTGGCTGAGCTTCCGGAAGAAATCGATATC

TCCACATACATCGAGCAGAGCCGG

>CL300.Contig13_A-W 180 1460 PREDICTED: ATP-dependent RNA helicase DDX39A [Canis lupus familiaris] >gi|395850755|ref|XP_003797941.1| PREDICTED: ATP-dependent RNA helicase DDX39A [Otolemur garnettii]

ATGGCAGAGCAGGATGTGGAGAACGAGCTCTTGGATTATGAAGAAGATGAAGAGCCCCAG

GCTCCTCCAGAGAGTACTCCAGCTCCACCTAAGAAAGACGTCAAGGGGTCCTACGTCTCC

ATCCATAGCTCTGGCTTCCGAGACTTTTTGCTGAAGCCAGAGCTCCTGAGGGCCATAGTG

GACTGTGGTTTTGAACACCCATCCGAGGTCCAGCATGAGTGTATCCCCCAGGCCATCCTG

GGCATGGACGTCCTGTGCCAGGCCAAGTCTGGGATGGGCAAGACGGCTGTCTTCGTGCTG

GCTACTCTGCAGCAGATCGAGCCAGTTAATGGACAGGTGACAGTCCTGGTCATGTGCCAT

ACGAGGGAGTTGGCCTTCCAAATCAGCAAGGAGTATGAGCGCTTCTCCAAATACATGCCC

AGTGTCAAGGTGTCTGTGTTCTTTGGGGGCCTCTCCATCAAGAAAGATGAAGAAGTGTTG

AAGAAGAATTGTCCCCACGTAGTGGTGGGGACACCAGGCAGGATCCTGGCCCTCGTGCGG

AACAGGAGCCTCAACTTAAAAAACGTGAAGCACTTTGTGTTGGACGAGTGTGACAAGATG

CTGGAGCAGCTGGACATGCGGCGGGATGTGCAGGAGATCTTCCGCCTGACGCCTCATGAG

AAGCAGTGCATGATGTTCAGTGCCACCCTGAGCAAGGAGATCCGGCCAGTCTGCAGGAAG

TTCATGCAGGATCCCATGGAGGTGTTTGTGGACGACGAGACCAAGCTCACGCTGCATGGG

CTGCAGCAGTACTATGTCAAGCTCAAGGACAGCGAGAAGAACCGAAAGCTCTTTGACCTC

CTGGATGTGCTGGAGTTTAACCAGGTGGTGATCTTCGTCAAATCCGTGCAGCGCTGCATG

GCCCTGGCCCAGCTCCTTGTGGAGCAGAACTTCCCAGCCATCGCCATTCACCGGGGCATG

GCCCAGGAGGAGCGTCTGTCACGCTACCAGCAGTTCAAGGACTTCCAGCGGCGGATCTTG

GTGGCCACCAATCTCTTTGGCCGAGGGATGGATATTGAGCGCGTCAACATCGTCTTCAAC

TATGACATGCCCGAGGACTCGGACACCTACCTCCACCGGGTGGCCCGTGCAGGTCGCTTT

GGTACCAAAGGCCTGGCCATCACTTTTGTGTCTGATGAAAATGATGCCAAAATTCTCAAT

GATGTCCAGGACCGGTTTGAAGTGAATGTGGCTGAGCTTCCGGAAGAAATCGATATCTCC

ACATACATCGAGCAGAGCCGG

>CL300.Contig14_A-W 180 1463 PREDICTED: ATP-dependent RNA helicase DDX39A [Canis lupus familiaris] >gi|395850755|ref|XP_003797941.1| PREDICTED: ATP-dependent RNA helicase DDX39A [Otolemur garnettii]

ATGGCAGAGCAGGATGTGGAGAACGAGCTCTTGGATTATGAAGAAGATGAAGAGCCCCAG

GCTCCTCCAGAGAGTACTCCAGCTCCACCTAAGAAAGACGTCAAGGGGTCCTACGTCTCC

ATCCATAGCTCTGGCTTCCGAGACTTTTTGCTGAAGCCAGAGCTCCTGAGGGCCATAGTG

GACTGTGGTTTTGAACACCCATCCGAGGTCCAGCATGAGTGTATCCCCCAGGCCATCCTG

GGCATGGACGTCCTGTGCCAGGCCAAGTCTGGGATGGGCAAGACGGCTGTCTTCGTGCTG

GCTACTCTGCAGCAGATCGAGCCAGTTAATGGACAGGTGACAGTCCTGGTCATGTGCCAT

ACGAGGGAGTTGGCCTTCCAAATCAGCAAGGAGTATGAGCGCTTCTCCAAATACATGCCC

AGTGTCAAGGTGTCTGTGTTCTTTGGGGGCCTCTCCATCAAGAAAGATGAAGAAGTGTTG

AAGAAGAATTGTCCCCACGTAGTGGTGGGGACACCAGGCAGGATCCTGGCCCTCGTGCGG

AACAGGAGCCTCAACTTAAAAAACGTGAAGCACTTTGTGTTGGACGAGTGTGACAAGATG

CTGGAGCAGCTGGACATGCGGCGGGATGTGCAGGAGATCTTCCGCCTGACGCCTCATGAG

AAGCAGTGCATGATGTTCAGTGCCACCCTGAGCAAGGAGATCCGGCCAGTCTGCAGGAAG

TTCATGCAGGATCCCATGGAGGTGTTTGTGGACGACGAGACCAAGCTCACGCTGCATGGG

CTGCAGCAGTACTATGTCAAGCTCAAGGACAGCGAGAAGAACCGAAAGCTCTTTGACCTC

CTGGATGTGCTGGAGTTTAACCAGGTGATGCCCCCACAGCCTGTCCTGGGCTTTGGGCCT

CAGGAGCGGGTACCCGGCCAGGCCTAACCGCTGCCTCTCCTACAGGTGGTGATCTTCGTC

AAATCCGTGCAGCGCTGCATGGCCCTGGCCCAGCTCCTTGTGGAGCAGAACTTCCCAGCC

ATCGCCATTCACCGGGGCATGGCCCAGGAGGAGCGGTGAGTGCACGCCACCTGCCGTGGC

CAGAGGGAGCAGCGCCCAGGAGCCCGTGTCTGACAGCCTCCACTTATTTCTCCTGCACCC

AGTCTGTCACGCTACCAGCAGTTCAAGGACTTCCAGCGGCGGATCTTGGTGGCCACCAAT

CTCTTTGGCCGAGGGATGGATATTGAGCGCGTCAACATCGTCTTCAACTATGACATGCCC

GAGGACTCGGACACCTACCTCCAC

>CL300.Contig15_A-W 2 1384 ATP-dependent RNA helicase DDX39 [Macaca mulatta]

AGGAGGCAGGAAGCGCCGCAGCTCGTGTCGCGGCGCGTGCTGGGTAACCGGAGTCAGGCG

GCTCTCCTCGTAGCACGCGGAGGCGAACGGAGACCCAGTACCATGGCAGAGCAGGATGTG

GAGAACGAGCTCTTGGATTATGAAGAAGATGAAGAGCCCCAGGCTCCTCCAGAGAGTACT

CCAGCTCCACCTAAGAAAGACGTCAAGGGGTCCTACGTCTCCATCCATAGCTCTGGCTTC

CGAGACTTTTTGCTGAAGCCAGAGCTCCTGAGGGCCATAGTGGACTGTGGTTTTGAACAC

CCATCCGAGGTCCAGCATGAGTGTATCCCCCAGGCCATCCTGGGCATGGACGTCCTGTGC

CAGGCCAAGTCTGGGATGGGCAAGACGGCTGTCTTCGTGCTGGCTACTCTGCAGCAGATC

GAGCCAGTTAATGGACAGGTGACAGTCCTGGTCATGTGCCATACGAGGGAGTTGGCCTTC

CAAATCAGCAAGGAGTATGAGCGCTTCTCCAAATACATGCCCAGTGTCAAGGTGTCTGTG

TTCTTTGGGGGCCTCTCCATCAAGAAAGATGAAGAAGTGTTGAAGAAGAATTGTCCCCAC

GTAGTGGTGGGGACACCAGGCAGGATCCTGGCCCTCGTGCGGAACAGGAGCCTCAACTTA

AAAAACGTGAAGCACTTTGTGTTGGACGAGTGTGACAAGATGCTGGAGCAGCTGGACATG

CGGCGGGATGTGCAGGAGATCTTCCGCCTGACGCCTCATGAGAAGCAGTGCATGATGTTC

AGTGCCACCCTGAGCAAGGAGATCCGGCCAGTCTGCAGGAAGTTCATGCAGGATCCCATG

GAGGTGTTTGTGGACGACGAGACCAAGCTCACGCTGCATGGGCTGCAGCAGTACTATGTC

AAGCTCAAGGACAGCGAGAAGAACCGAAAGCTCTTTGACCTCCTGGATGTGCTGGAGTTT

AACCAGGTGGTGATCTTCGTCAAATCCGTGCAGCGCTGCATGGCCCTGGCCCAGCTCCTT

GTGGAGCAGAACTTCCCAGCCATCGCCATTCACCGGGGCATGGCCCAGGAGGAGCGTCTG

TCACGCTACCAGCAGTTCAAGGACTTCCAGCGGCGGATCTTGGTGGCCACCAATCTCTTT

GGCCGAGGGATGGATATTGAGCGCGTCAACATCGTCTTCAACTATGACATGCCCGAGGAC

TCGGACACCTACCTCCACCGGGTGGCCCGTGCAGGTCGCTTTGGTACCAAAGGCCTGGCC

ATCACTTTTGTGTCTGATGAAAATGATGCCAAAATTCTCAATGATGTCCAGGACCGGTTT

GAAGTGAATGTGGCTGAGCTTCCGGAAGAAATCGATATCTCCACATACATCGAGCAGAGC

CGG

>CL300.Contig16_A-W 1203 1751 ATP-dependent RNA helicase DDX39 [Macaca mulatta]

CCCATGGAGGTGTTTGTGGACGACGAGACCAAGCTCACGCTGCATGGGCTGCAGCAGTAC

TATGTCAAGCTCAAGGACAGCGAGAAGAACCGAAAGCTCTTTGACCTCCTGGATGTGCTG

GAGTTTAACCAGGTGGTGATCTTCGTCAAATCCGTGCAGCGCTGCATGGCCCTGGCCCAG

CTCCTTGTGGAGCAGAACTTCCCAGCCATCGCCATTCACCGGGGCATGGCCCAGGAGGAG

CGTCTGTCACGCTACCAGCAGTTCAAGGACTTCCAGCGGCGGATCTTGGTGGCCACCAAT

CTCTTTGGCCGAGGGATGGATATTGAGCGCGTCAACATCGTCTTCAACTATGACATGCCC

GAGGACTCGGACACCTACCTCCACCGGGTGGCCCGTGCAGGTCGCTTTGGTACCAAAGGC

CTGGCCATCACTTTTGTGTCTGATGAAAATGATGCCAAAATTCTCAATGATGTCCAGGAC

CGGTTTGAAGTGAATGTGGCTGAGCTTCCGGAAGAAATCGATATCTCCACATACATCGAG

CAGAGCCGG

>CL300.Contig17_A-W 2 715 minus strand ATP-dependent RNA helicase DDX39 [Macaca mulatta]

AGGAGGCAGGAAGCGCCGCAGCTCGTGTCGCGGCGCGTGCTGGGTAACCGGAGTCAGGCG

GCTCTCCTCGTAGCACGCGGAGGCGAACGGAGACCCAGTACCATGGCAGAGCAGGATGTG

GAGAACGAGCTCTTGGATTATGAAGAAGATGAAGAGCCCCAGGCTCCTCCAGAGAGTACT

CCAGCTCCACCTAAGAAAGACGTCAAGGGGTCCTACGTCTCCATCCATAGCTCTGGCTTC

CGAGACTTTTTGCTGAAGCCAGAGCTCCTGAGGGCCATAGTGGACTGTGGTTTTGAACAC

CCATCCGAGGTCCAGCATGAGTGTATCCCCCAGGCCATCCTGGGCATGGACGTCCTGTGC

CAGGCCAAGTCTGGGATGGGCAAGACGGCTGTCTTCGTGCTGGCTACTCTGCAGCAGATC

GAGCCAGTTAATGGACAGGTGACAGTCCTGGTCATGTGCCATACGAGGGAGTTGGCCTTC

CAAATCAGCAAGGAGTATGAGCGCTTCTCCAAATACATGCCCAGTGTCAAGGTGTCTGTG

TTCTTTGGGGGCCTCTCCATCAAGAAAGATGAAGAAGTGTTGAAGAAGAATTGTCCCCAC

GTAGTGGTGGGGACACCAGGCAGGATCCTGGCCCTCGTGCGGAACAGGAGCCTCAACTTA

AAAAACGTGAAGCACTTTGTGTTGGACGAGTGTGACAAGATGCTGGAGCAGCTG

>CL300.Contig18_A-W 180 791 PREDICTED: ATP-dependent RNA helicase DDX39A [Canis lupus familiaris] >gi|395850755|ref|XP_003797941.1| PREDICTED: ATP-dependent RNA helicase DDX39A [Otolemur garnettii]

ATGGCAGAGCAGGATGTGGAGAACGAGCTCTTGGATTATGAAGAAGATGAAGAGCCCCAG

GCTCCTCCAGAGAGTACTCCAGCTCCACCTAAGAAAGACGTCAAGGGGTCCTACGTCTCC

ATCCATAGCTCTGGCTTCCGAGACTTTTTGCTGAAGCCAGAGCTCCTGAGGGCCATAGTG

GACTGTGGTTTTGAACACCCATCCGAGGTCCAGCATGAGTGTATCCCCCAGGCCATCCTG

GGCATGGACGTCCTGTGCCAGGCCAAGTCTGGGATGGGCAAGACGGCTGTCTTCGTGCTG

GCTACTCTGCAGCAGATCGAGCCAGTTAATGGACAGGTGACAGTCCTGGTCATGTGCCAT

ACGAGGGAGTTGGCCTTCCAAATCAGCAAGGAGTATGAGCGCTTCTCCAAATACATGCCC

AGTGTCAAGGTGTCTGTGTTCTTTGGGGGCCTCTCCATCAAGAAAGATGAAGAAGTGTTG

AAGAAGAATTGTCCCCACGTAGTGGTGGGGACACCAGGCAGGATCCTGGCCCTCGTGCGG

AACAGGAGCCTCAACTTAAAAAACGTGAAGCACTTTGTGTTGGACGAGTGTGACAAGATG

CTGGAGCAGCTG

>CL300.Contig19_A-W 180 617 PREDICTED: ATP-dependent RNA helicase DDX39A [Canis lupus familiaris] >gi|395850755|ref|XP_003797941.1| PREDICTED: ATP-dependent RNA helicase DDX39A [Otolemur garnettii]

ATGGCAGAGCAGGATGTGGAGAACGAGCTCTTGGATTATGAAGAAGATGAAGAGCCCCAG

GCTCCTCCAGAGAGTACTCCAGCTCCACCTAAGAAAGACGTCAAGGGGTCCTACGTCTCC

ATCCATAGCTCTGGCTTCCGAGACTTTTTGCTGAAGCCAGAGCTCCTGAGGGCCATAGTG

GACTGTGGTTTTGAACACCCATCCGAGGTCCAGCATGAGTGTATCCCCCAGGCCATCCTG

GGCATGGACGTCCTGTGCCAGGCCAAGTCTGGGATGGGCAAGACGGCTGTCTTCGTGCTG

GCTACTCTGCAGCAGATCGAGCCAGTTAATGGACAGGTGACAGTCCTGGTCATGTGCCAT

ACGAGGGAGTTGGCCTTCCAAATCAGCAAGGAGTATGAGCGCTTCTCCAAATACATGCCC

AGTGTCAAGGTGAGCCTC

>CL301.Contig1_A-W 567 1226 minus strand KIAA0324 protein [Homo sapiens]

GGCATCAGTGATTCCTACGTGGATGGCAGCTCTTTTGATCCTCAGCGTCGTGCTCGAGAA

GCTAAACAACCAGCTCCCGAGCCTCCCAAACCTTACAGCCTTGTTAGGGAGTCCAGCAGT

TCTCGTTCACCGACCCCAAAGCAAAAGAAGAAGAAAAAGAAGAAAGACAGAGGACGCAGG

TCAGAGAGTAGCTCTCCTCGACGGGAGAGAAAAAAGAGCTCAAAAAAGAAGAAGCACAGG

TCGGAATCTGAGTCCAAGAAACGAAAGCACAGGTCTCCCACTCCAAAGAGTAAACGTAAA

TCTAAAGACAAGAAACGGAAGCGGTCTCGTAGTACAACACCAGCTCCCAAGAGCCGCCGG

GCCCATCGGTCAACCTCTGCTGATTCTGCTTCCTCCTCTGACACTTCTCGCAGTCGGTCT

CGAAGTGCTGCAGCTAAAATACATACAACTGCTTTGACTGGGCGAAGTCCTTCCCCTGCT

CCAGGGCGTCGAGGGGAGGGAGATGCGCCTTCTAGTGAACCAGGTACCACCAATATACAA

CGGCCTAGCAGCCCAGAGACTTCTACAAAGCAGCCTAGCAGTCCTTACGAGGACAAAGAC

AAGAAGGAGAAACCTGCAGTTCGACCTAGCCCCTCTCCGGAAAGGAGCAGCACAGGCCCT

>CL301.Contig2_A-W 462 3356 minus strand KIAA0324 protein [Homo sapiens]

CCTGGGGGCAAGGAGGAAACCCCAGGGCAGAGGCCAGCGGTAACTGAGACGCATCAGTTG

GCAGAATTGAATGAGAAGAAGAATGAGCGGCTCCGAGCTGCCTTTGGCATCAGTGATTCC

TACGTGGATGGCAGCTCTTTTGATCCTCAGCGTCGTGCTCGAGAAGCTAAACAACCAGCT

CCCGAGCCTCCCAAACCTTACAGCCTTGTTAGGGAGTCCAGCAGTTCTCGTTCACCGACC

CCAAAGCAAAAGAAGAAGAAAAAGAAGAAAGACAGAGGACGCAGGTCAGAGAGTAGCTCT

CCTCGACGGGAGAGAAAAAAGAGCTCAAAAAAGAAGAAGCACAGGTCGGAATCTGAGTCC

AAGAAACGAAAGCACAGGTCTCCCACTCCAAAGAGTAAACGTAAATCTAAAGACAAGAAA

CGGAAGCGGTCTCGTAGTACAACACCAGCTCCCAAGAGCCGCCGGGCCCATCGGTCAACC

TCTGCTGATTCTGCTTCCTCCTCTGACACTTCTCGCAGTCGGTCTCGAAGTGCTGCAGCT

AAAATACATACAACTGCTTTGACTGGGCGAAGTCCTTCCCCTGCTCCAGGGCGTCGAGGG

GAGGGAGATGCGCCTTCTAGTGAACCAGGTACCACCAATATACAACGGCCTAGCAGCCCA

GAGACTTCTACAAAGCAGCCTAGCAGTCCTTACGAGGACAAAGACAAGAAGGAGAAACCT

GCAGTTCGACCTAGCCCCTCTCCGGAAAGGAGCAGCACAGGCCCTGAACCACCTGTTCCC

ACTCCGCTCCTTGCTGAGCAGCATGGCGGCTCCCCACAGCCCCTTGCAACAACCTCCTTA

AGTCAGGAGCCAGTGAACCCCCCATCTGAGGCCTCCCCAACCCGAGGCCCTTCACCACCT

AAGTCTCCTGAGAAACCACCTCAGTCTTCTTCCTCAGAGAGCTGCCCACCATCCCCACAG

CCTACCAAAGTTTCTCGGCATGCCAGCTCTTCCCCTGAAAGTCCTAAACCCACACCAGCT

CCTGGTGCCCGCCGAGAGATTTCTTCTTCCCCCACATCCAAGAATCGGTCACATGGCCGA

GCAAAGCGGGATAAATCACACTCTCATACTCCTTCCCATAGGATGGGGAGATCACGCAGC

CCTGCCACTGCTAAGAGGGGGAGATCACGGTCTCGAACCCCTACCAAAAGGGGTCATTCT

CGTTCCCGGTCCCCTCAGTGGCGTAGGTCACGGTCTGCACAGAGGTGGGGAAGATCTAGA

AGCCCCCAACGGCGTGGCCGTTCTAGGTCCCCTCAGCGACCAGGTTGGTCCAGGAGCAGA

AATACTCAGCGAAGAGGCAGGTCTAGGTCAACAAGGCGAGGCAGATCACACTCTAGATCC

CCAGCCACCAGGGGCAGATCACGTTCTAGAACACCAGCTCGGCGGGGTAGGTCCCGCTCT

AGAACACCTGCCAGGCGGAGATCACGATCTAGAACACCCGCCAGGCGCAGGTCCAGGTCA

AGAACACCAGCCCGCAGGGGCAGGTCTCGGTCAAGAACACCTGCTAGACGCAGGTCTAGG

ACCCGATCACCAGTACGAAGGAGATCTCGCAGTAGATCTCCAGCCAGGAGAAGTGGTCGG

TCACGTTCTAGAACCCCAGCTAGACGGGGCCGATCGCGTTCTAGAACCCCAGCTAGACGT

AGTGGCCGATCACGCTCTAGAACCCCAGCTAGGAGAGGGAGGTCTCGGTCTAGAACCCCA

GCAAGACGAAGATCCCGCAGTAGAAGTATAGTTAGGCGAGGAAGATCTCACTCTAGGACA

CCACAAAGAAGAGGCAGGTCTGGTTCGTCATCAGAGCGGAAGAACAAATCCAGAACATCT

CAGAGGAGGAGCAGGTCTAACTCAAGTCCAGAAATGAAAAAATCTCACATTTCCTCAAGG

CGGAGCAGGTCTCTCTCTTCACCAAGATCTAAGGCAAAATCTCGCTTGTCTTTGAGACGC

AGTCTTTCTGGGTCCTCTCCGTGCCCTAAACAAAAGTCACAGACACCACCTAGGCGCAGT

CGCTCTGGATCATCACAACCTAAAGCAAAATCTAGAACACCAAGGCGGAGTCGTTCCGGT

TCTTCACCACCTAAACAGAAATCTAAAACACCATCAAGACAAAGCCATTCCAGTTCATCT

CCTCACCCAAAAGTGAAATCTGGAACACCACCAAGACAAGGTTCCATCACAAGTCCCCAG

ACCATCGAACAATCTGCAACACCACAAAGACGGAGCCATTCTGAATCATCACCTGATCCC

GAGGTAAAATCTAGGACTCCTTCGAGGCACAGCTGTTCTAGGTCCTCTCCTCCTCAAGTG

AAGTCTAGTACACCTCCAAGACAGACCCCATCTAGGTCATTGTCTCCACAGCCCAAAGTG

AAGGCAATGTCACCAGGACAAAGAAGCCATTCTGGCTCCTCTTCTCCAAGTCCTAGTAGG

ATGACTTCTAGAACACCTCCAAGGCAAAGCAGATCAGTGTCTCCTTGCACCAAGGTGGAA

TCTAGATCGTTGCCAAGAGAGAGCCATTCTAGATCCTCCTCACCAGATACCAAAGTGAAA

CCTGAAACACCACCAAGACAAAGTCACTCAGGGTCTGTGTCACCATACCCTAAAGTAAAG

CCTCAAACACCACCAGGGCAAAGTCAGTCTGGGTCAAAATCACCATGTCCCCAAGAGAAG

TCTATAGAAATTCAAGCACAGAGTTGCTCTGGATCATTTTCACTCTGTCCAGGAGTACCG

TCTAACACACCACCCGGAGAAAGCTACTTTGGCTCCTCATCTCTGCAACATAAAGGACAA

TCTCAAACTTCACCAGACCCCAGATCTGATACTTCAAGTCCAGAAATGAGACAGAGTCAC

TTGGAGTCTTCACCT

>CL301.Contig3_A-W 591 1016 minus strand KIAA0324 protein [Homo sapiens]

GGCAGCTCTTTTGATCCTCAGCGTCGTGCTCGAGAAGCTAAACAACCAGCTCCCGAGCCT

CCCAAACCTTACAGCCTTGTTAGGGAGTCCAGCAGTTCTCGTTCACCGACCCCAAAGCAA

AAGAAGAAGAAAAAGAAGAAAGACAGAGGACGCAGGTCAGAGAGTAGCTCTCCTCGACGG

GAGAGAAAAAAGAGCTCAAAAAAGAAGAAGCACAGGTCGGAATCTGAGTCCAAGAAACGA

AAGCACAGGTCTCCCACTCCAAAGAGTAAACGTAAATCTAAAGACAAGAAACGGAAGCGG

TCTCGTAGTACAACACCAGCTCCCAAGAGCCGCCGGGCCCATCGGTCAACCTCTGCTGAT

TCTGCTTCCTCCTCTGACACTTCTCGCAGTCGGTAAGGGGTAGTCCAGAAGGAAGGGAGG

GAGAGG

>CL301.Contig4_A-W 42 998 minus strand KIAA0324 protein [Homo sapiens]

GTGACTCGGGAGCCCGGGCAGCGGCAGCGGCAGCGGCAGGACCTCTCCCCCTCACAGGCG

GCGGGCGGAGGCGGCGGCGGGAGCGGTGGTGCCCCCGGGCACAGGGCCATGTACAACGGG

ATCGGGCTGCCGACGCCCCGGGGCAGCGGCACCAACGGCTACGTCCAGCGCAACCTGTCC

CTGGTGCGGGGCCGCCGGGGTGAGCGGCCTGACTACAAGGGAGAGGAGGAACTGCGGCGC

CTGGAGGCTGCCCTGGTGAAGCGGCCTAATCCTGACATCCTGGACCACGAGCGCAAGCGG

CGCGTGGAGCTGCGATGCCTCGAGCTGGAGGAGATGATGGAAGAGCAGGGGTACGAGGAA

CAGCAAATCCAAGAGAAAGTGGCGACCTTTCGACTCATGTTGCTGGAGAAGGATGTGAAC

CCTGGGGGCAAGGAGGAAACCCCAGGGCAGAGGCCAGCGGTAACTGAGACGCATCAGTTG

GCAGAATTGAATGAGAAGAAGAATGAGCGGCTCCGAGCTGCCTTTGGCATCAGTGATTCC

TACGTGGATGGCAGCTCTTTTGATCCTCAGCGTCGTGCTCGAGAAGCTAAACAACCAGCT

CCCGAGCCTCCCAAACCTTACAGCCTTGTTAGGGAGTCCAGCAGTTCTCGTTCACCGACC

CCAAAGCAAAAGAAGAAGAAAAAGAAGAAAGACAGAGGACGCAGGTCAGAGAGTAGCTCT

CCTCGACGGGAGAGAAAAAAGAGCTCAAAAAAGAAGAAGCACAGGTCGGAATCTGAGTCC

AAGAAACGAAAGCACAGGTCTCCCACTCCAAAGAGTAAACGTAAATCTAAAGACAAGAAA

CGGAAGCGGTCTCGTAGTACAACACCAGCTCCCAAGAGCCGCCGGGCCCATCGGTCAACC

TCTGCTGATTCTGCTTCCTCCTCTGACACTTCTCGCAGTCGGCGCTGCACAGACCAT

>CL301.Contig5_A-W 120 713 minus strand Serine/arginine repetitive matrix protein 2 OS=Homo sapiens GN=SRRM2 PE=1 SV=2

GGGTACGAGGAACAGCAAATCCAAGAGAAAGTGGCGACCTTTCGACTCATGTTGCTGGAG

AAGGATGTGAACCCTGGGGGCAAGGAGGAAACCCCAGGGCAGAGGCCAGCGGTAACTGAG

ACGCATCAGTTGGCAGAATTGAATGAGAAGAAGAATGAGCGGCTCCGAGCTGCCTTTGGC

ATCAGTGATTCCTACGTGGATGGCAGCTCTTTTGATCCTCAGCGTCGTGCTCGAGAAGCT

AAACAACCAGCTCCCGAGCCTCCCAAACCTTACAGCCTTGTTAGGGAGTCCAGCAGTTCT

CGTTCACCGACCCCAAAGCAAAAGAAGAAGAAAAAGAAGAAAGACAGAGGACGCAGGTCA

GAGAGTAGCTCTCCTCGACGGGAGAGAAAAAAGAGCTCAAAAAAGAAGAAGCACAGGTCG

GAATCTGAGTCCAAGAAACGAAAGCACAGGTCTCCCACTCCAAAGAGTAAACGTAAATCT

AAAGACAAGAAACGGAAGCGGTCTCGTAGTACAACACCAGCTCCCAAGAGCCGCCGGGCC

CATCGGTCAACCTCTGCTGATTCTGCTTCCTCCTCTGACACTTCTCGCAGTCGG

>CL301.Contig6_A-W 207 851 minus strand RNA binding protein [Homo sapiens]

GAAACCCCAGGGCAGAGGCCAGCGGTAACTGAGACGCATCAGTTGGCAGAATTGAATGAG

AAGAAGAATGAGCGGCTCCGAGCTGCCTTTGGCATCAGTGATTCCTACGTGGATGGCAGC

TCTTTTGATCCTCAGCGTCGTGCTCGAGAAGCTAAACAACCAGCTCCCGAGCCTCCCAAA

CCTTACAGCCTTGTTAGGGAGTCCAGCAGTTCTCGTTCACCGACCCCAAAGCAAAAGAAG

AAGAAAAAGAAGAAAGACAGAGGACGCAGGTCAGAGAGTAGCTCTCCTCGACGGGAGAGA

AAAAAGAGCTCAAAAAAGAAGAAGCACAGGTCGGAATCTGAGTCCAAGAAACGAAAGCAC

AGGTCTCCCACTCCAAAGAGTAAACGTAAATCTAAAGACAAGAAACGGAAGCGGTCTCGT

AGTACAACACCAGCTCCCAAGAGCCGCCGGGCCCATCGGTCAACCTCTGCTGATTCTGCT

TCCTCCTCTGACACTTCTCGCAGTCGGTCTCGAAGTGCTGCAGCTAAAATACATACAACT

GCTTTGACTGGGCGAAGTCCTTCCCCTGCTCCAGGGCGTCGAGGGGAGGGAGATGCGCCT

TCTAGTGAACCAGGTACCACCAATATACAACGGCCTAGCAGCCCA

>CL301.Contig7_A-W 120 752 minus strand serine/arginine repetitive matrix 2 [Bos taurus]

GGGTACGAGGAACAGCAAATCCAAGAGAAAGTGGCGACCTTTCGACTCATGTTGCTGGAG

AAGGATGTGAACCCTGGGGGCAAGGAGGAAACCCCAGGGCAGAGGCCAGCGGTAACTGAG

ACGCATCAGTTGGCAGAATTGAATGAGAAGAAGAATGAGCGGCTCCGAGCTGCCTTTGGC

ATCAGTGATTCCTACGTGGATGGCAGCTCTTTTGATCCTCAGCGTCGTGCTCGAGAAGCT

AAACAACCAGCTCCCGAGCCTCCCAAACCTTACAGCCTTGTTAGGGAGTCCAGCAGTTCT

CGTTCACCGACCCCAAAGCAAAAGAAGAAGAAAAAGAAGAAAGACAGAGGACGCAGGTCA

GAGAGTAGCTCTCCTCGACGGGAGAGAAAAAAGAGCTCAAAAAAGAAGAAGCACAGGTCG

GAATCTGAGTCCAAGAAACGAAAGCACAGGTCTCCCACTCCAAAGAGTAAACGTAAATCT

AAAGACAAGAAACGGAAGCGGTCTCGTAGTACAACACCAGCTCCCAAGAGCCGCCGGGCC

CATCGGTCAACCTCTGCTGATTCTGCTTCCTCCTCTGACACTTCTCGCAGTCGGCGCTGC

ACAGACCATTCGGAAGACACGGTCCCTGCCCTC

>CL302.Contig1_A-W 1 918 minus strand PREDICTED: nuclear receptor corepressor 1-like [Papio anubis]

CAATCTGCCATCAAACACAATGTCAAATCCTTAATAACGGGGCCTAGCAAACTACCCCGT

GGATTGCCTGCACTGGAAATTGTACCAGAGAATATAAAAGTGGTGGAAAGGGGAAAATAT

GAAGATGTGAAAGCAGGTGAGCCAGTGCGTTCCCGGCACACGTCGGTGGTGAGCTCCGGC

CCCTCCGTTCTCAGGTCCACACTTCATGAAGCTCCAAAAGCACAGCTGAGCCCGGGAATT

TACGATGACTCCAGTGCTCGGAGGACCCCTGTGAGTTATCAGAACACCATGTCCAGAGGC

TCGCCTATGATGAACAGAACTTCTGAAGTTACGATCTCTTCTAGCAAGTCTGCCAATCAT

GAAAGGAAGTCAACACTGACCCCTACCCAAAGGGAAAGTATACCAGCTAAGTCTCCAGTG

CCTGGGGTAGATCCTGTTGTGAGCCACAGCCCATTTGACCCTCATCACCGGAGCAGCGCC

GCAGGGGAAGTTTATCGGAGCCACCTTTCCACACACCTGGATCCAGCCATGCCTTTTCAC

AGGGCTCTGGATCCTGCAGCTGCTGCTTACCTGTTTCAGAGACAGCTTTCACCAACTCCA

GGCTACCCAAGTCAGTATCAGCTTTATGCAATGGAAAACACAAGGCAGACAATCCTAAAT

GATTACATTACCTCACAGCAGATGCAAGTGAACTTGCGCCCAGATGTAGCCAGAGGACTC

TCCCCAAGAGAGCAACCACTGGGTCTCCCATACCCAGCCACAAGAGGAATCATTGATCTG

ACCAATATGCCTCCAACGATATTAGTGCCTCATCCAGGGGGAACAAGCACTCCTCCCATG

GACAGAATCACTTATATTCCTGGTACACAGATTACTTTCCCTCCCAGGCCATACAATTCT

GCTTCTATGTCTCCAGGT

>CL302.Contig2_A-W 2 658 minus strand NCOR isoform c [Homo sapiens]

AGATGGATAAATAACCATACTTTGCAAGTTTTCTTAAGGCTTTGTGGCATTGGCCCCTTT

TTTCCAGTTACGATCTCTTCTAGCAAGTCTGCCAATCATGAAAGGAAGTCAACACTGACC

CCTACCCAAAGGGAAAGTATACCAGCTAAGTCTCCAGTGCCTGGGGTAGATCCTGTTGTG

AGCCACAGCCCATTTGACCCTCATCACCGGAGCAGCGCCGCAGGGGAAGTTTATCGGAGC

CACCTTTCCACACACCTGGATCCAGCCATGCCTTTTCACAGGGCTCTGGATCCTGCAGCT

GCTGCTTACCTGTTTCAGAGACAGCTTTCACCAACTCCAGGCTACCCAAGTCAGTATCAG

CTTTATGCAATGGAAAACACAAGGCAGACAATCCTAAATGATTACATTACCTCACAGCAG

ATGCAAGTGAACTTGCGCCCAGATGTAGCCAGAGGACTCTCCCCAAGAGAGCAACCACTG

GGTCTCCCATACCCAGCCACAAGAGGAATCATTGATCTGACCAATATGCCTCCAACGATA

TTAGTGCCTCATCCAGGGGGAACAAGCACTCCTCCCATGGACAGAATCACTTATATTCCT

GGTACACAGATTACTTTCCCTCCCAGGCCATACAATTCTGCTTCTATGTCTCCAGGT

>CL302.Contig3_A-W 76 255 minus strand PREDICTED: nuclear receptor corepressor 1-like [Ailuropoda melanoleuca]

GGAATCATTGATCTGACCAATATGCCTCCAACGATATTAGTGCCTCATCCAGGGGGAACA

AGCACTCCTCCCATGGACAGAATCACTTATATTCCTGGTACACAGATTACTTTCCCTCCC

AGGCCATACAATTCTGCTTCTATGTCTCCAGGTAAGATGCGGCAGACATATACTTCTCCA

>CL303.Contig1_A-W 2 1588 minus strand PREDICTED: LOW QUALITY PROTEIN: ribonucleoprotein PTB-binding 1 isoform 1 [Equus caballus]

GCTGGGCAGCTAACACCTGCCCTCCTCCACTCCCGCTGCCTCTGTGTTGACCGCCTGCCA

CCAGGCTTCAGTGATGTGGATGCCCTGCGTCGGGCGTTGTCTACAGTCCACACACCCACC

TTCTGCCAGCTGGCGTGTGGCCAGGATGGGCAGCTGAAGGGCTTCGCAGTGCTGGAGTAC

GAGACAGCGGAGATGGCGGAGGCAGCACAACAGAGGGCAGATGGCCTGGCCCTGGGGGGC

AGCCACTTGCGAGTCTCCTTCTGTGCCCCAGGGCCTCCTGGCCGCAGTATGCTGGCTGCG

CTCATCGCTGCCCAGGCCACAGCCCTCAATCGTGGCAAAGGGCTCTTGCCTGAGCCAAAC

ATCCTACAGCTGCTGAACAACCTGGGCCCGTCTGCCTCACTTCAGCTGCTGCTCAACCCC

CTGCTCCATGGCAGCACAGGGGGCAAGCAGGGCCTCCTGGGCGCCCCCCCAGCCATGCCA

CTGCTCAGCGGGCCTGCCCTGTCTACAGCACTGCTGCAGCTTGCCCTGCAGACCCAGAGC

CAGAAGAAGCCTGGGATCCTGGGAGACTCACCCCTGGGCACCCTCCAGCCTGGGGCCCAG

CCCACCAACCCCCTCCTTGGAGAGCTATCTGCAGGAGGGGGCCTACCCTCGGAGCTGCCA

CCCCGGCGAGGGAAGCCACCACCCCTGCTACCACCACTGCTGGGCCCCTCTGGGGGTGAC

CGGGAGACCATGGGCCTGGGTCCTCCAGCAGCCCAGCTTACTCCACCCCCTGCCCCTGCG

GGACTCCGTGGCACAGGCCTCAGAGGCCTTCAGAAGGACAGTGGGCCCCTGCCAACTCCC

CCTGGGGTATCGCTACTAGGGGAGCCCCCCAAGGACTACCGGATCCCCTTGAATCCCTAC

CTGAACCTACACAGCCTGCTCCCAGCCAGTAACCTAGCGGGTAAGGAGGCCCGTAGCTGG

GGAGGCGCTGGGAGAGGCCGCCGCCCAGCTGAGGGCCCCCTGCCCAATCCCCCCACCCCT

GGTGGAGGTGGTGGTGGCAGCAAAGCCTTCCAGCTCAAGTCCCGCCTGCTCAGCCCCCTC

ACCAGCGCCCGCCTGCCCCCTGAACCAGGGTTGCCTGACAGCTATGGCTTCGACTACCCC

TCGGATATGGGACCTCGGAGGCTCTTTTCTCACCCACGGGAGCCAGCCCTAGGGCCTCAT

GGACCTAGCCGACACAAGACGTCTCCCCCACCCAGTGGCTTTGGAGAGCGGAGTGGTGGA

GGTGGCGGGGGGCCCCTTTCTCACTTCTATTCGGGCTCGCCCACTTCCTACTTCACCAGC

GGCCTGCAGGCTGGCCTCAAGCAGAGCCATCTCAGCAAGGCAGTCGGCTCCTCCCCTTTG

GGCTCCAGTGAAGGACTCCTGGGTCTGGGCCCTGGGCCCAATGGCCACAGCCACCTCTTG

AAGACCCCACTGGGCGGCCAGAAACGCAGCTTTGCTCACCTGCTCCCCTCACCTGAGCCC

AGTCCAGAGGGTAGCTACGTGGGCCAACACTCCCAGGGCCTTGGTGGCCACTACGCAGAC

TCCTACCTGAAGCGGAAGAGGATTTTC

>CL303.Contig2_A-W 2 1423 minus strand PREDICTED: LOW QUALITY PROTEIN: ribonucleoprotein PTB-binding 1 isoform 1 [Equus caballus]

GCTGGGCAGCTAACACCTGCCCTCCTCCACTCCCGCTGCCTCTGTGTTGACCGCCTGCCA

CCAGGCTTCAGTGATGTGGATGCCCTGCGTCGGGCGTTGTCTACAGTCCACACACCCACC

TTCTGCCAGCTGGCGTGTGGCCAGGATGGGCAGCTGAAGGGCTTCGCAGTGCTGGAGTAC

GAGACAGCGGAGATGGCGGAGGCAGCACAACAGAGGGCAGATGGCCTGGCCCTGGGGGGC

AGCCACTTGCGAGTCTCCTTCTGTGCCCCAGGGCCTCCTGGCCGCAGTATGCTGGCTGCG

CTCATCGCTGCCCAGGCCACAGCCCTCAATCGTGGCAAAGGGCTCTTGCCTGAGCCAAAC

ATCCTACAGCTGCTGAACAACCTGGGCCCGTCTGCCTCACTTCAGCTGCTGCTCAACCCC

CTGCTCCATGGCAGCACAGGGGGCAAGCAGGGCCTCCTGGGCGCCCCCCCAGCCATGCCA

CTGCTCAGCGGGCCTGCCCTGTCTACAGCACTGCTGCAGCTTGCCCTGCAGACCCAGAGC

CAGAAGAAGCCTGGGATCCTGGGAGACTCACCCCTGGGCACCCTCCAGCCTGGGGCCCAG

CCCACCAACCCCCTCCTTGGAGAGCTATCTGCAGGAGGGGGCCTACCCTCGGAGCTGCCA

CCCCGGCGAGGGAAGCCACCACCCCTGCTACCACCACTGCTGGGCCCCTCTGGGGGTGAC

CGGGAGACCATGGGCCTGGGTCCTCCAGCAGCCCAGCTTACTCCACCCCCTGCCCCTGCG

GGACTCCGTGGCACAGGCCTCAGAGGCCTTCAGAAGGACAGTGGGCCCCTGCCAACTCCC

CCTGGGGTATCGCTACTAGGGGAGCCCCCCAAGGACTACCGGATCCCCTTGAATCCCTAC

CTGAACCTACACAGCCTGCTCCCAGCCAGTAACCTAGCGGGTAAGGAGGCCCGTAGCTGG

GGAGGCGCTGGGAGAGGCCGCCGCCCAGCTGAGGGCCCCCTGCCCAATCCCCCCACCCCT

GGTGGAGGTGGTGGTGGCAGCAAAGCCTTCCAGCTCAAGTCCCGCCTGCTCAGCCCCCTC

ACCAGCGCCCGCCTGCCCCCTGAACCAGGGTTGCCTGACAGCTATGGCTTCGACTACCCC

TCGGATATGGGACCTCGGAGGCTCTTTTCTCACCCACGGGAGCCAGCCCTAGGGCCTCAT

GGACCTAGCCGACACAAGACGTCTCCCCCACCCAGTGGCTTTGGAGAGCGGAGTGGTGGA

GGTGGCGGGGGGCCCCTTTCTCACTTCTATTCGGGCTCGCCCACTTCCTACTTCACCAGC

GGCCTGCAGGCTGGCCTCAAGCAGAGCCATCTCAGCAAGGTGAGGAGCCTCCCCAGCCAA

GGACAACCAGGATGTGGGGGTTGGGGGTGTCTGCCTCCAGGG

>CL304.Contig1_A-W 1 600 PREDICTED: COMM domain-containing protein 9 isoform 1 [Pan paniscus]

GGAAAAATGGCCGCCGTGACAGCGGAGCACTTCGCGGCTCTCCAGAGTTTGCTGAAGGCC

TCCTCAAAAGATGTTGTCAGGCAGCTGTGTCAAGAAAGCTTTTCCAGTTCAGCCCTTGGC

TCAAAAAAGCTCTTGGATATTACGTGTTCCAGCTTGTCTGTGACCCAGGAGGAGGCAGAG

CAACTCCTCCAGGCTCTGCACCGCCTCACTCGGCTGGTGGCATTCCGTGACCTGTCGTCC

GCTGAGGCAATTCTGGCTCTCTTTCCAGAAAATTTCCACCAAAACCTCAAAAACCTGCTG

ACAAAAATCATCCTAGAACATGTATCTACTTGGAGAACTGAAGCCCAAGCAAATCAGATC

TCTCTGCCACGCCTGGTGGACCTGGACTGGAGAGTGGATATCAAAACCTCCTCAGACAGC

ATCAGCCGCATGGCCATCCCTACCTGCCTGCTCCAGATGAAGATCCAAGAAGATCCCAGT

CTGTGCGGGGACAAACCCTCCATCTCAGCTGTGACTGTGGAGTTGAGCAAAGAAACACTA

GACACCATGTTAGATGGGCTGGGCCGCATCCGAGACCAGCTTTCTGCTGTGGCCAGTAAA

>CL304.Contig2_A-W 89 532 COMM domain-containing protein 9 isoform 2 [Homo sapiens]

TACAGGGCTGTCTCCTCTATCCTGCAGCTCCTCCAGGCTCTGCACCGCCTCACTCGGCTG

GTGGCATTCCGTGACCTGTCGTCCGCTGAGGCAATTCTGGCTCTCTTTCCAGAAAATTTC

CACCAAAACCTCAAAAACCTGCTGACAAAAATCATCCTAGAACATGTATCTACTTGGAGA

ACTGAAGCCCAAGCAAATCAGATCTCTCTGCCACGCCTGGTGGACCTGGACTGGAGAGTG

GATATCAAAACCTCCTCAGACAGCATCAGCCGCATGGCCATCCCTACCTGCCTGCTCCAG

ATGAAGATCCAAGAAGATCCCAGTCTGTGCGGGGACAAACCCTCCATCTCAGCTGTGACT

GTGGAGTTGAGCAAAGAAACACTAGACACCATGTTAGATGGGCTGGGCCGCATCCGAGAC

CAGCTTTCTGCTGTGGCCAGTAAA

>CL304.Contig3_A-W 73 516 PREDICTED: COMM domain-containing protein 9 isoform 2 [Pan paniscus]

TTCAGGAATCTTCCACAGAAGATGGAGCTCCTCCAGGCTCTGCACCGCCTCACTCGGCTG

GTGGCATTCCGTGACCTGTCGTCCGCTGAGGCAATTCTGGCTCTCTTTCCAGAAAATTTC

CACCAAAACCTCAAAAACCTGCTGACAAAAATCATCCTAGAACATGTATCTACTTGGAGA

ACTGAAGCCCAAGCAAATCAGATCTCTCTGCCACGCCTGGTGGACCTGGACTGGAGAGTG

GATATCAAAACCTCCTCAGACAGCATCAGCCGCATGGCCATCCCTACCTGCCTGCTCCAG

ATGAAGATCCAAGAAGATCCCAGTCTGTGCGGGGACAAACCCTCCATCTCAGCTGTGACT

GTGGAGTTGAGCAAAGAAACACTAGACACCATGTTAGATGGGCTGGGCCGCATCCGAGAC

CAGCTTTCTGCTGTGGCCAGTAAA

>CL304.Contig4_A-W 67 219 PREDICTED: COMM domain-containing protein 9 isoform 1 [Saimiri boliviensis boliviensis] >gi|403254590|ref|XP_003920045.1| PREDICTED: COMM domain-containing protein 9 isoform 2 [Saimiri boliviensis boliviensis]

ATGTTCTCTTTACAGATCCAAGAAGATCCCAGTCTGTGCGGGGACAAACCCTCCATCTCA

GCTGTGACTGTGGAGTTGAGCAAAGAAACACTAGACACCATGTTAGATGGGCTGGGCCGC

ATCCGAGACCAGCTTTCTGCTGTGGCCAGTAAA

>CL304.Contig5_A-W 2 466 COMM domain-containing protein 9 isoform 2 [Homo sapiens]

GCCGCCGTGACAGCGGAGCACTTCGCGGCTCTCCAGAGTTTGCTGAAGCTCCTCCAGGCT

CTGCACCGCCTCACTCGGCTGGTGGCATTCCGTGACCTGTCGTCCGCTGAGGCAATTCTG

GCTCTCTTTCCAGAAAATTTCCACCAAAACCTCAAAAACCTGCTGACAAAAATCATCCTA

GAACATGTATCTACTTGGAGAACTGAAGCCCAAGCAAATCAGATCTCTCTGCCACGCCTG

GTGGACCTGGACTGGAGAGTGGATATCAAAACCTCCTCAGACAGCATCAGCCGCATGGCC

ATCCCTACCTGCCTGCTCCAGATGAAGATCCAAGAAGATCCCAGTCTGTGCGGGGACAAA

CCCTCCATCTCGGCTGTGACTGTGGAGTTGAGCAAAGAAACACTAGACACCATGTTAGAT

GGGCTGGGCCGCATCCGAGACCAGCTTTCTGCTGTGGCCAGTAAA

>CL305.Contig1_A-W 27 191 ORF2 [Rattus norvegicus]

CAGAGAGAGAGATATATATACACAAAAAAAATGTAGTTCTACTCAGCCATTAAAAAGAAT

GAAAATATGGCATTTGTCAGTAAATGGGTGGAAATGGAGAATATCATTTTAAGTGAAAAT

AGCCAAACTCAGAAGCTCAAAGGTCAAATATTTTCTCTCATATGC

>CL305.Contig3_A-W 82 189 minus strand Retrovirus-related Pol polyprotein LINE-1 OS=Mus musculus GN=Pol PE=1 SV=2

CAAAATGTTGTATACATATATACAATAGAGTTCTATTCAGCCATAAAGAAGAATGAAGTT

ATGGCATTTGCCTGTAAATGGATGGAAATGGAGAATATCATGCTAAGT

>CL305.Contig4_A-W 167 379 minus strand uncharacterized protein LOC100361645 [Rattus norvegicus] >gi|37361836|gb|AAQ91031.1| LRRGT00075 [Rattus norvegicus] >gi|149047772|gb|EDM00388.1| rCG65901 [Rattus norvegicus]

GAGACAGCCATCCTGCTCCTTGGTATTTTCCTAAAAGATCTAAAAACTTTTTAGATTATC

TTTTTATACAGCAGTGATACAGTCACCTCGTTGTTCACACCAGCACAATTCACAATAGTT

AAATTATAGAATAAACCCAGGTGCCCATCAATAGAAGAATGGATTAAGAAATTGTAGTAT

ATGTGTACAATGGAGTTCTACTCAGTCATAAAA

>CL305.Contig5_A-W 176 382 minus strand LINE-1 reverse transcriptase homolog OS=Nycticebus coucang PE=1 SV=1

ATCCTGCTCCTTGGTATTTTCCTAAAAGATCTAAAAACTTTTTAGATTATCTTTTTATAC

AGCAGTGATACAGTCACCTCGTTGTTCACACCAGCACAATTCACAATAGTTAAATTATAG

AATAAACCCAGGTGCCCATCAATAGAAGAATGGATTAAGAAATTGTAGTATATGTGTACA

ATGGAGTTCTACTCAGTCATAAAAAAA

>CL306.Contig1_A-W 2 1663 minus strand PREDICTED: glycine dehydrogenase (decarboxylating) [Oryctolagus cuniculus]

ACACTTGGTATTTCTCTTGATGAAACAGTCAACGAAAAAGATCTCGATGATTTGTTATGG

ATCTTTGGCTGTGAGTCATCTGCAGAACTGGTGGCTGAAAGCATGGGAGAGGAATGGAGA

GGTATTCCAGGATCTGCATTCAAGAGAACTAGCCCATTCCTCACACATCAAGTGTTCAAC

AGCTATCACTCAGAAACAAATATTGTCCGGTACATGAAGAAACTAGAGAACAAAGACATT

TCCCTTGTTCACAGCATGATTCCACTGGGATCCTGTACCATGAAGCTCAATAGCTCATCT

GAACTCACACCCATCACATGGAAAGAATTTGCAAACATCCACCCCTTTGTGCCTCTGGAC

CAAGCTCAAGGATACCAGCAACTATTCCGAGAGCTTGAGAAAGATTTGTGTGAACTCACA

GGTTATGATCAAGTCTCTTTCCAGCCTAACAGCGGAGCCCAGGGGGAATATGCTGGACTG

GCCACAATCCGAGCATACTTAGACCGGAAAGGAGAGAGGCACAGAACGGTTTGCCTCATT

CCGAAATCAGCACATGGTACCAATCCAGCAAGTGCCCACATGGCAGGCATGAAGATTCAG

CCTGTAGAAGTGGATAAATATGGCAATATCGACGCTGCTCACCTCAAGGCCATGGTGGAT

AAACACAAGGAGAACCTGGCTGCCATCATGATCACGTACCCATCCACTAACGGGGTGTTT

GAAGAGAACATCAGTGATGTGTGTGCTCTGATCCACGAACATGGAGGGCAGGTCTATCTC

GACGGGGCCAATATGAATGCTCAGGTGGGAATCTGTCGTCCTGGAGACTTTGGGTCTGAT

GTCTCACACCTAAATCTTCACAAGACCTTCTGTATTCCCCATGGAGGAGGTGGCCCTGGC

ATGGGGCCCATTGGAGTGAAGAAGCATCTTGTCCCCTTTCTGCCCAATCATCCCATCATT

GCAGTAAAGCCGAATGAGGACACCTGGCCTGTGGGGACCGTCAGTGCAGCCCCGTGGGGA

TCTAGTTCCATCTTGCCCATTTCCTGGGCTTATATTAAGATGATGGGAGGCAAAGGCCTT

AAACAGGCCACAGAAATTGCTATATTAAATGCCAACTACATGGCGAAGCGATTAGAAAAA

CACTATAGAGTCCTTTTTAGGGGTGCAAGAGGTTTTGTGGCTCATGAGTTTATTTTGGAC

ACAAGACCCTTCAAAAAGTCAGCAAATATTGATGCTGTGGATGTGGCCAAGAGGCTTCAA

GATTATGGATTTCATGCCCCCACCATGTCCTGGCCTGTGGCAGGAACCCTCATGGTTGAG

CCCACTGAGTCAGAAGACAAGGCAGAGCTGGACAGATTCTGTGATGCCATGATCAACATT

CGGCAGGAAATTGCTGACATTGAAGAGGGCCGCATCGACCCTAGGGTCAATCCATTAAAG

ATGTCTCCACACTCCTTGACCTGTGTCACATCCTCCCACTGGGATCGTCCATATTCCAGA

GAGGTGGCGGCATTCCCACTTCCCTTTGTGAAACCAGAGAACAAATTCTGGCCAACCATT

GCCCGGATTGATGACATATATGGAGATCAGCACCTGGTCTGTACCTGCCCACCCATGGAA

GTTTATGAGTCCCCATTTTCTGAACAGAAGAGAGCTTCTTCT

>CL306.Contig2_A-W 2 1528 minus strand PREDICTED: glycine dehydrogenase (decarboxylating) [Oryctolagus cuniculus]

ACACTTGGTATTTCTCTTGATGAAACAGTCAACGAAAAAGATCTCGATGATTTGTTATGG

ATCTTTGGCTGTGAGTCATCTGCAGAACTGGTGGCTGAAAGCATGGGAGAGGAATGGAGA

GGTATTCCAGGATCTGCATTCAAGAGAACTAGCCCATTCCTCACACATCAAGTGTTCAAC

AGCTATCACTCAGAAACAAATATTGTCCGGTACATGAAGAAACTAGAGAACAAAGACATT

TCCCTTGTTCACAGCATGATTCCACTGGGATCCTGTACCATGAAGCTCAATAGCTCATCT

GAACTCACACCCATCACATGGAAAGAATTTGCAAACATCCACCCCTTTGTGCCTCTGGAC

CAAGCTCAAGGATACCAGCAACTATTCCGAGAGCTTGAGAAAGATTTGTGTGAACTCACA

GGTTATGATCAAGTCTCTTTCCAGCCTAACAGCGGAGCCCAGGGGGAATATGCTGGACTG

GCCACAATCCGAGCATACTTAGACCGGAAAGGAGAGAGGCACAGAACGGTTTGCCTCATT

CCGAAATCAGCACATGGTACCAATCCAGCAAGTGCCCACATGGCAGGCATGAAGATTCAG

CCTGTAGAAGTGGATAAATATGGCAATATCGACGCTGCTCACCTCAAGGCCATGGTGGAT

AAACACAAGGAGAACCTGGCTGCCATCATGATCACGTACCCATCCACTAACGGGGTGTTT

GAAGAGAACATCAGTGATGTGTGTGCTCTGATCCACGAACATGGAGGGCAGGTCTATCTC

GACGGGGCCAATATGAATGCTCAGGTGGGAATCTGTCGTCCTGGAGACTTTGGGTCTGAT

GTCTCACACCTAAATCTTCACAAGACCTTCTGTATTCCCCATGGAGGAGGTGGCCCTGGC

ATGGGGCCCATTGGAGTGAAGAAGCATCTTGTCCCCTTTCTGCCCAATCATCCCATCATT

GCAGTAAAGCCGAATGAGGACACCTGGCCTGTGGGGACCGTCAGTGCAGCCCCGTGGGGA

TCTAGTTCCATCTTGCCCATTTCCTGGGCTTATATTAAGATGATGGGAGGCAAAGGCCTT

AAACAGGCCACAGAAATTGCTATATTAAATGCCAACTACATGGCGAAGCGATTAGAAAAA

CACTATAGAGTCCTTTTTAGGGGTGCAAGAGGTTTTGTGGCTCATGAGTTTATTTTGGAC

ACAAGACCCTTCAAAAAGTCAGCAAATATTGATGCTGTGGATGTGGCCAAGAGGCTTCAA

GATTATGGATTTCATGCCCCCACCATGTCCTGGCCTGTGGCAGGAACCCTCATGGTTGAG

CCCACTGAGTCAGAAGACAAGGCAGAGCTGGACAGATTCTGTGATGCCATGATCAACATT

CGGCAGGAAATTGCTGACATTGAAGAGGGCCGCATCGACCCTAGGGTCAATCCATTAAAG

ATGTCTCCACACTCCTTGACCTGTGTCACATCCTCCCACTGGGATCGTCCATATTCCAGA

GAGGTGGCGGCATTCCCACTTTATTTT

>CL307.Contig1_A-W 16 435 minus strand PREDICTED: 40S ribosomal protein S15a [Bos taurus] >gi|359075221|ref|XP_002694888.2| PREDICTED: 40S ribosomal protein S15a [Bos taurus]

CTCTTTCCGCCATTTTTTCGTGCCGCCATAATGGTGCGCATGAACGTCCTGGCTGACGCT

CTCAAGAGCATCAACAATGCCGAAAAGAGAGGCAAACGCCAGGTCCTTATTAGGCCGTGC

TCCAAAGTCATTGTCCGGTTTCTAACTGTGATGATGAAGCATGGTTACATTGGCGAATTT

GAAATCATTGATGATCACAGAGCTGGGAAAATTGTTGTCAACCTCACAGGCAGGTTAAAC

AAGTGTGGAGTGATAAGCCCCAGATTTGATGTGCAGCTCAAAGACCTGGAAAAATGGCAG

AATAATCTGCTCCCATCCCGTCAGTTTGGTTTCATTGTATTGACAACCTCTGCTGGCATC

ATGGACCACGAAGAAGCAAGAAGAAAACACACAGGAGGAAAAATCCTGGGATTCTTTTTC

>CL308.Contig1_A-W 146 781 mCG1492, isoform CRA_d [Mus musculus]

AATGTAAAACACAGGAGCTGTCAGCAGCCAAGTGTCCTACCATATGGAACCAGCTGGTCT

GCAGTGAAAAAGAAGCCAACATGCAAAAAGCAGCAGAGAACCATGTGTGCTCACTACTGC

ATCTCCTTTGCTGATGTTGAAAAAGCTCACATCAACATTCGAGATTACATCCATCTCACA

CCAGTGCTAACAAGCTCTATTTTGAATCAAGTAGCAGGACGCAATCTTTTCTTCAAATGT

GAAGTCTTCCAGAAAACTGGATCTTTTAAGATTCGTGGGGCACTTAATGCCATCAGAGGC

TTAATTCCTGCCACCTTAGAAGGGAAGCCCAAAGCTGTTGTTACTCACAGCAGTGGAAAT

CATGGCCAGGCTCTCACTTACGCTGCTAAACTGGAAGGGATTCCTGCTTATATTGTGGTA

CCCCACACAGCTCCCAACTGTAAAAAACTGGCAATACAAGCCTATGGAGCCTCTATAGTT

TACAGTGAACCGAGCGACGAGTCCAGAGAAAAGGTTACAAAAAAAATTTTGGAAGAAACA

GAAGGCATCATGGTACATCCCAACCAGGAGCCTGCGGTGATAGCTGGGCAAGGCACAATT

GCCTTAGAAGTGCTGAGTCAGGTAAAACAGCCTAGT

>CL308.Contig2_A-W 44 886 PREDICTED: serine racemase isoform 1 [Macaca mulatta]

GTCCTACCATATGGAACCAGCTGGTCTGCAGTGAAAAAGAAGCCAACATGCAAAAAGCAG

CAGAGAACCATGTGTGCTCACTACTGCATCTCCTTTGCTGATGTTGAAAAAGCTCACATC

AACATTCGAGATTACATCCATCTCACACCAGTGCTAACAAGCTCTATTTTGAATCAAGTA

GCAGGACGCAATCTTTTCTTCAAATGTGAAGTCTTCCAGAAAACTGGATCTTTTAAGATT

CGTGGGGCACTTAATGCCATCAGAGGCTTAATTCCTGCCACCTTAGAAGGGAAGCCCAAA

GCTGTTGTTACTCACAGCAGTGGAAATCATGGCCAGGCTCTCACTTACGCTGCTAAACTG

GAAGGTACTTGATAACTTCTCAAGGTAGTCATCAGATTGAGTGGAAAAGTGTCCTTAATT

TTCATTCAGAATGGTGATAGCTATGGGGTCAATTTCCAAAGGAGTCTCTGATTACAAGCA

AAATGAACAAAAGTTTCTATCCAAAATTCTCCTGACTTGTGCTCACATGTTTTTCCTCTC

CCAGGGATTCCTGCTTATATTGTGGTACCCCACACAGCTCCCAACTGTAAAAAACTGGCA

ATACAAGCCTATGGAGCCTCTATAGTTTACAGTGAACCGAGCGACGAGTCCAGAGAAAAG

GTTACAAAAAAAATTTTGGAAGAAACAGAAGGCATCATGGTACATCCCAACCAGGAGCCT

GCGGTGATAGCTGGGCAAGGCACAATTGCCTTAGAAGTGCTGAGTCAGGTTCCCTTGGTG

GATGCACTAGTGGTTCCTGTAGGGGGAGGAGGAATGGTTGCTGGAATAGCAATTACTGTT

AAG

>CL308.Contig3_A-W 44 814 PREDICTED: serine racemase isoform 1 [Macaca mulatta]

GTCCTACCATATGGAACCAGCTGGTCTGCAGTGAAAAAGAAGCCAACATGCAAAAAGCAG

CAGAGAACCATGTGTGCTCACTACTGCATCTCCTTTGCTGATGTTGAAAAAGCTCACATC

AACATTCGAGATTACATCCATCTCACACCAGTGCTAACAAGCTCTATTTTGAATCAAGTA

GCAGGACGCAATCTTTTCTTCAAATGTGAAGTCTTCCAGAAAACTGGATCTTTTAAGATT

CGTGGGGCACTTAATGCCATCAGAGGCTTAATTCCTGCCACCTTAGAAGGGAAGCCCAAA

GCTGTTGTTACTCACAGCAGTGGAAATCATGGCCAGGCTCTCACTTACGCTGCTAAACTG

GAAGGTACTTGATAACTTCTCAAGGTAGTCATCAGATTGAGTGGAAAAGTGTCCTTAATT

TTCATTCAGAATGGTGATAGCTATGGGGTCAATTTCCAAAGGAGTCTCTGATTACAAGCA

AAATGAACAAAAGTTTCTATCCAAAATTCTCCTGACTTGTGCTCACATGTTTTTCCTCTC

CCAGGGATTCCTGCTTATATTGTGGTACCCCACACAGCTCCCAACTGTAAAAAACTGGCA

ATACAAGCCTATGGAGCCTCTATAGTTTACAGTGAACCGAGCGACGAGTCCAGAGAAAAG

GTTACAAAAAAAATTTTGGAAGAAACAGAAGGCATCATGGTACATCCCAACCAGGAGCCT

GCGGTGATAGCTGGGCAAGGCACAATTGCCTTAGAAGTGCTGAGTCAGGTA

>CL308.Contig4_A-W 548 706 mCG1492, isoform CRA_d [Mus musculus]

AATTCTCCTGACTTGTGCTCACATGTTTTTCCTCTCCCAGGGATTCCTGCTTATATTGTG

GTACCCCACACAGCTCCCAACTGTAAAAAACTGGCAATACAAGCCTATGGAGCCTCTATA

GTTTACAGTGAACCGAGCGACGAGGTAAAGAAAGCAGTG

>CL308.Contig5_A-W 44 706 PREDICTED: serine racemase isoform 1 [Macaca mulatta]

GTCCTACCATATGGAACCAGCTGGTCTGCAGTGAAAAAGAAGCCAACATGCAAAAAGCAG

CAGAGAACCATGTGTGCTCACTACTGCATCTCCTTTGCTGATGTTGAAAAAGCTCACATC

AACATTCGAGATTACATCCATCTCACACCAGTGCTAACAAGCTCTATTTTGAATCAAGTA

GCAGGACGCAATCTTTTCTTCAAATGTGAAGTCTTCCAGAAAACTGGATCTTTTAAGATT

CGTGGGGCACTTAATGCCATCAGAGGCTTAATTCCTGCCACCTTAGAAGGGAAGCCCAAA

GCTGTTGTTACTCACAGCAGTGGAAATCATGGCCAGGCTCTCACTTACGCTGCTAAACTG

GAAGGGATTCCTGCTTATATTGTGGTACCCCACACAGCTCCCAACTGTAAAAAACTGGCA

ATACAAGCCTATGGAGCCTCTATAGTTTACAGTGAACCGAGCGACGAGTCCAGAGAAAAG

GTTACAAAAAAAATTTTGGAAGAAACAGAAGGCATCATGGTACATCCCAACCAGGAGCCT

GCGGTGATAGCTGGGCAAGGCACAATTGCCTTAGAAGTGCTGAGTCAGGTTCCCTTGGTG

GATGCACTAGTGGTTCCTGTAGGGGGAGGAGGAATGGTTGCTGGAATAGCAATTACTGTT

AAG

>CL308.Contig6_A-W 11 526 mCG1492, isoform CRA_d [Mus musculus]

AATGTAAAACACAGGAGCTGTCAGCAGCCAAGTGTCCTACCATATGGAACCAGCTGGTCT

GCAGTGAAAAAGAAGCCAACATGCAAAAAGCAGCAGAGAACCATGTGTGCTCACTACTGC

ATCTCCTTTGCTGATGTTGAAAAAGCTCACATCAACATTCGAGATTACATCCATCTCACA

CCAGTGCTAACAAGCTCTATTTTGAATCAAGTAGCAGGACGCAATCTTTTCTTCAAATGT

GAAGTCTTCCAGAAAACTGGATCTTTTAAGATTCGTGGGGCACTTAATGCCATCAGAGGC

TTAATTCCTGCCACCTTAGAAGGGAAGCCCAAAGCTGTTGTTACTCACAGCAGTGGAAAT

CATGGCCAGGCTCTCACTTACGCTGCTAAACTGGAAGGGATTCCTGCTTATATTGTGGTA

CCCCACACAGCTCCCAACTGTAAAAAACTGGCAATACAAGCCTATGGAGCCTCTATAGTT

TACAGTGAACCGAGCGACGAGGTAAAGAAAGCAGTG

>CL308.Contig7_A-W 209 1264 PREDICTED: serine racemase isoform 2 [Otolemur garnettii]

GTGAAAAAGAAGCCAACATGCAAAAAGCAGCAGAGAACCATGTGTGCTCACTACTGCATC

TCCTTTGCTGATGTTGAAAAAGCTCACATCAACATTCGAGATTACATCCATCTCACACCA

GTGCTAACAAGCTCTATTTTGAATCAAGTAGCAGGACGCAATCTTTTCTTCAAATGTGAA

GTCTTCCAGAAAACTGGATCTTTTAAGATTCGTGGGGCACTTAATGCCATCAGAGGCTTA

ATTCCTGCCACCTTAGAAGGGAAGCCCAAAGCTGTTGTTACTCACAGCAGTGGAAATCAT

GGCCAGGCTCTCACTTACGCTGCTAAACTGGAAGGGATTCCTGCTTATATTGTGGTACCC

CACACAGCTCCCAACTGTAAAAAACTGGCAATACAAGCCTATGGAGCCTCTATAGTTTAC

AGTGAACCGAGCGACGAGTCCAGAGAAAAGGTTACAAAAAAAATTTTGGAAGAAACAGAA

GGCATCATGGTACATCCCAACCAGGAGCCTGCGGTGATAGCTGGGCAAGGCACAATTGCC

TTAGAAGTGCTGAGTCAGGTTCCCTTGGTGGATGCACTAGTGGTTCCTGTAGGGGGAGGA

GGAATGGTTGCTGGAATAGCAATTACTGTTAAGGCTCTGAAACCTAGCGTGAAGGTATAT

GCTGCTGAACCCTTGAATGCAGATGATTGCTACAAGTCCAAACTGAAAGGGGAGCTGACC

CCCAATCCTTATCCTCCAGAAACTATAGCAGATGGTGTCAAATCCAGCATTGGCTTAAAC

ACCTGGCCTATTATAAGGGACCTTGTGGATGATGTCTTCACTGTCACAGAGGATGAAATC

AAGTATGCAACCCAACTGGTTTGGGAAAGGATGAAACTACTCATTGAACCTACAGCTGGT

GTTGGAGTGGCTGCTGTGCTGTCTCGGAATTTTCAAGCAGTTTCTCCAGAAGTAAAGAAC

ATTTGTATTGTTCTCAGTGGTGGAAATGTAGACTTAACCTCCTTAAGTTGGGTGAAGCAC

GCTGAAAGGCCAGCTCCTTATCAGTCTGTTTCTGTT

>CL308.Contig8_A-W 209 1063 PREDICTED: serine racemase isoform 2 [Otolemur garnettii]

GTGAAAAAGAAGCCAACATGCAAAAAGCAGCAGAGAACCATGTGTGCTCACTACTGCATC

TCCTTTGCTGATGTTGAAAAAGCTCACATCAACATTCGAGATTACATCCATCTCACACCA

GTGCTAACAAGCTCTATTTTGAATCAAGTAGCAGGACGCAATCTTTTCTTCAAATGTGAA

GTCTTCCAGAAAACTGGATCTTTTAAGATTCGTGGGGCACTTAATGCCATCAGAGGCTTA

ATTCCTGCCACCTTAGAAGGGAAGCCCAAAGCTGTTGTTACTCACAGCAGTGGAAATCAT

GGCCAGGCTCTCACTTACGCTGCTAAACTGGAAGGGATTCCTGCTTATATTGTGGTACCC

CACACAGCTCCCAACTGTAAAAAACTGGCAATACAAGCCTATGGAGCCTCTATAGTTTAC

AGTGAACCGAGCGACGAGTCCAGAGAAAAGGTTACAAAAAAAATTTTGGAAGAAACAGAA

GGCATCATGGTACATCCCAACCAGGAGCCTGCGGTGATAGCTGGGCAAGGCACAATTGCC

TTAGAAGTGCTGAGTCAGGTTCCCTTGGTGGATGCACTAGTGGTTCCTGTAGGGGGAGGA

GGAATGGTTGCTGGAATAGCAATTACTGTTAAGGCTCTGAAACCTAGCGTGAAGGTATAT

GCTGCTGAACCCTTGAATGCAGATGATTGCTACAAGTCCAAACTGAAAGGGGAGCTGACC

CCCAATCCTTATCCTCCAGAAACTATAGCAGATGGTGTCAAATCCAGCATTGGCTTAAAC

ACCTGGCCTATTATAAGGGACCTTGTGGATGATGTCTTCACTGTCACAGAGGATGAAATC

AAGGTAAGGCTCCAA

>CL309.Contig1_A-W 70 2529 PREDICTED: probable ATP-dependent RNA helicase DDX23 isoform 2 [Canis lupus familiaris]

ATGGCAGGAGAACTGGCTGATAAAAAGGACCGAGATGCATCACCTTCCAAGGAGGAAAGG

AAGCGATCACGGACTCCTGACAGAGAGCGGGATAGAGACCGGGACCGGAAGTCCTCCCCA

TCTAAAGATAGGAAGCGACATCGTTCAAGGGATAGACGTCGAGGAGGCAGCCGCTCCCGC

TCTCGTTCCCGTTCTAAGTCTACAGAGAGAGAACGACGGCACAAAGAACGAGAACGAGAT

AAGGACCGAGATCGGAATAAGAAGGACCGCGATCGAGACAAGGATGGGCACAGACGAGAC

AAGGACCGTAAACGATCCAGTTTATCGCCTGGCCGAGGAAAAGATTTTAAATCTCGGAAG

GACAGAGACTCTAAAAAGGATGAAGATGATGAGCATGGTGATAAGAAGCCTAAGGCCCAG

CCACTATCTCTGGAGGAACTTTTAGCCAAGAAAAAGGCTGAGGAAGAAGCTGAGGCTAAG

CCTAAGTTCCTCTCCAAAGCAGAGCGGGAAGCTGAAGCTCTAAAGCGACGGCAGCAGGAA

GTGGAAGAGCGGCAGAGAATGCTTGAAGAAGAAAGGAAGAAAAGGAAACAGTTCCAAGAC

TTGGGCAGGAAGATGTTGGAAGACCCTCAGGAAAGGGAACGCCGGGAACGAAGGGAGAGA

ATGGAGCGGGAGACCAATGGAAATGAGGATGAGGAAGGTCGTCAGAAGATCCGGGAAGAG

AAGGATAAGAGCAAGGAACTGCACGCTATTAAGGAACGTTACCTGGGTGGCATCAAAAAG

CGGCGTCGAACAAGGCATCTCAATGATCGCAAGTTTGTCTTTGAGTGGGATGCATCTGAG

GACACATCCATCGATTACAACCCCCTATACAAAGAACGGCACCAGGTGCAGTTGTTGGGG

CGAGGCTTCATTGCAGGCATTGACCTAAAGCAGCAGAAACGAGAGCAGTCACGCTTCTAT

GGAGACCTAATGGAGAAGAGGCGAACACTGGAAGAAAAGGAGCAGGAGGAAGCAAGACTC

CGAAAACTTCGAAAGAAAGAAGCCAAACAGCGCTGGGATGATCGTCATTGGTCGCAGAAG

AAGTTAGATGAGATGACAGACAGGGACTGGCGGATCTTCCGTGAGGACTATAGCATTACC

ACCAAAGGTGGCAAAATCCCTAATCCCATTCGATCCTGGAAAGACTCTTCTCTGCCCCCA

CACATCTTGGAGGTCATTGATAAATGTGGCTATAAGGAACCAACCCCTATCCAGCGCCAG

GCAATTCCAATTGGGTTACAAAATCGTGACATCATTGGTGTGGCTGAGACTGGCAGTGGT

AAAACTGCAGCCTTCCTCATCCCGTTGCTGGTCTGGATCACCACACTTCCCAAAATTGAC

AGGATCGAAGAGTCAGACCAGGGCCCTTATGCCATCATCCTGGCTCCTACTCGTGAGTTG

GCTCAGCAGATTGAGGAAGAGACCATCAAATTTGGGAAGCCGCTAGGCATCCGCACTGTG

GCTGTCATTGGTGGTATCTCCAGAGAAGACCAGGGCTTCAGGCTGCGCATGGGTTGTGAG

ATCGTGATCGCTACCCCGGGACGTCTGATTGATGTACTGGAAAACCGTTACCTAGTGTTG

AGCCGCTGTACCTATGTGGTTCTGGATGAGGCAGATAGGATGATTGACATGGGCTTTGAG

CCAGATGTCCAGAAGATCCTGGAGCACATGCCTGTCAGCAACCAGAAACCAGACACAGAT

GAGGCTGAGGATCCTGAGAAGATGTTGGCCAACTTTGAGTCAGGAAAACATAAGTACCGC

CAAACAGTCATGTTCACTGCCACCATGCCCCCAGCGGTGGAGCGTCTGGCCAGGAGCTAT

CTTCGGCGACCTGCTGTGGTATACATTGGCTCGGCAGGCAAGCCCCATGAGCGTGTGGAA

CAGAAGGTCTTCCTCATGTCAGAGTCAGAAAAGAGGAAAAAGCTGCTGGCAATATTGGAA

CAAGGCTTTGATCCACCCATCATCATTTTTGTCAATCAAAAGAAGGGTTGTGATGTGTTG

GCCAAGTCCCTGGAGAAAATGGGGTACAATGCTTGTACACTGCATGGTGGAAAAGGTCAG

GAGCAGCGAGAATTTGCGCTGTCCAACCTCAAGGCTGGGGCCAAGGATATTTTGGTGGCT

ACAGATGTGGCTGGTCGTGGTATCGACATCCAGGATGTGTCTATGGTTGTCAACTATGAT

ATGGCCAAAAACATTGAAGATTATATCCACCGCATTGGCCGTACGGGACGAGCAGGCAAG

AGTGGTGTGGCCATCACCTTCCTCACCAAAGAGGACTCAGCTGTGTTCTATGAGCTGAAG

CAAGCCATCCTGGAGAGCCCAGTGTCTTCCTGTCCCCCAGAGCTAGCCAACCACCCAGAT

GCTCAGCACAAGCCAGGCACCATCCTCACCAAGAAGCGCCGAGAAGAGACCATCTTTGCT

>CL309.Contig2_A-W 58 1572 PREDICTED: probable ATP-dependent RNA helicase DDX23 isoform 2 [Canis lupus familiaris]

ATGGCAGGAGAACTGGCTGATAAAAAGGACCGAGATGCATCACCTTCCAAGGAGGAAAGG

AAGCGATCACGGACTCCTGACAGAGAGCGGGATAGAGACCGGGACCGGAAGTCCTCCCCA

TCTAAAGATAGGAAGCGACATCGTTCAAGGGATAGACGTCGAGGAGGCAGCCGCTCCCGC

TCTCGTTCCCGTTCTAAGTCTACAGAGAGAGAACGACGGCACAAAGAACGAGAACGACGG

CACAAAGAACGAGAACGAGATAAGGACCGAGATCGGAATAAGAAGGACCGCGATCGAGAC

AAGGATGGGCACAGACGAGACAAGGACCGTAAACGATCCAGTTTATCGCCTGGCCGAGGA

AAAGATTTTAAATCTCGGAAGGACAGAGACTCTAAAAAGGATGAAGATGATGAGCATGGT

GATAAGAAGCCTAAGGCCCAGCCACTATCTCTGGAGGAACTTTTAGCCAAGAAAAAGGCT

GAGGAAGAAGCTGAGGCTAAGCCTAAGTTCCTCTCCAAAGCAGAGCGGGAAGCTGAAGCT

CTAAAGCGACGGCAGCAGGAAGTGGAAGAGCGGCAGAGAATGCTTGAAGAAGAAAGGAAG

AAAAGGAAACAGTTCCAAGACTTGGGCAGGAAGATGTTGGAAGACCCTCAGGAAAGGGAA

CGCCGGGAACGAAGGGAGAGAATGGAGCGGGAGACCAATGGAAATGAGGATGAGGAAGGT

CGTCAGAAGATCCGGGAAGAGAAGGATAAGAGCAAGGAACTGCACGCTATTAAGGAACGT

TACCTGGGTGGCATCAAAAAGCGGCGTCGAACAAGGCATCTCAATGATCGCAAGTTTGTC

TTTGAGTGGGATGCATCTGAGGACACATCCATCGATTACAACCCCCTATACAAAGAACGG

CACCAGGTGCAGTTGTTGGGGCGAGGCTTCATTGCAGGCATTGACCTAAAGCAGCAGAAA

CGAGAGCAGTCACGCTTCTATGGAGACCTAATGGAGAAGAGGCGAACACTGGAAGAAAAG

GAGCAGGAGGAAGCAAGACTCCGAAAACTTCGAAAGAAAGAAGCCAAACAGCGCTGGGAT

GATCGTCATTGGTCGCAGAAGAAGTTAGATGAGATGACAGACAGGGACTGGCGGATCTTC

CGTGAGGACTATAGCATTACCACCAAAGGTGGCAAAATCCCTAATCCCATTCGATCCTGG

AAAGACTCTTCTCTGCCCCCACACATCTTGGAGGTCATTGATAAATGTGGCTATAAGGAA

CCAACCCCTATCCAGCGCCAGGCAATTCCAATTGGGTTACAAAATCGTGACATCATTGGT

GTGGCTGAGACTGGCAGTGGTAAAACTGCAGCCTTCCTCATCCCGTTGCTGGTCTGGATC

ACCACACTTCCCAAAATTGACAGGATCGAAGAGTCAGACCAGGGCCCTTATGCCATCATC

CTGGCTCCTACTCGTGAGTTGGCTCAGCAGATTGAGGAAGAGACCATCAAATTTGGGAAG

CCGCTAGGCATCCGC

>CL309.Contig3_A-W 55 717 DEAD (Asp-Glu-Ala-Asp) box polypeptide 23, isoform CRA_c [Homo sapiens]

GTGATGGCAGGAGAACTGGCTGATAAAAAGGACCGAGATGCATCACCTTCCAAGGAGGAA

AGGAAGCGATCACGGACTCCTGACAGAGAGCGGGATAGAGACCGGGACCGGAAGTCCTCC

CCATCTAAAGATAGGAAGCGACATCGTTCAAGGGATAGACGTCGAGGAGGCAGCCGCTCC

CGCTCTCGTTCCCGTTCTAAGTCTACAGAGAGAGAACGACGGCACAAAGAACGAGAACGA

CGGCACAAAGAACGAGAACGAGATAAGGACCGAGATCGGAATAAGAAGGACCGCGATCGA

GACAAGGATGGGCACAGACGAGACAAGGACCGTAAACGATCCAGTTTATCGCCTGGCCGA

GGAAAAGATTTTAAATCTCGGAAGGACAGAGACTCTAAAAAGGATGAAGATGATGAGCAT

GGTGATAAGAAGCCTAAGGCCCAGCCACTATCTCTGGAGGAACTTTTAGCCAAGAAAAAG

GCTGAGGAAGAAGCTGAGGCTAAGCCTAAGTTCCTCTCCAAAGCAGAGCGGGAAGCTGAA

GCTCTAAAGCGACGGCAGCAGGAAGTGGAAGAGCGGCAGAGAATGCTTGAAGAAGAAAGG

AAGAAAAGGAAACAGTTCCAAGACTTGGGCAGGAAGATGTTGGGTTTGTTTTTCTATCCT

GTG

>CL309.Contig4_A-W 55 696 DEAD (Asp-Glu-Ala-Asp) box polypeptide 23, isoform CRA_c [Homo sapiens]

GTGATGGCAGGAGAACTGGCTGATAAAAAGGACCGAGATGCATCACCTTCCAAGGAGGAA

AGGAAGCGATCACGGACTCCTGACAGAGAGCGGGATAGAGACCGGGACCGGAAGTCCTCC

CCATCTAAAGATAGGAAGCGACATCGTTCAAGGGATAGACGTCGAGGAGGCAGCCGCTCC

CGCTCTCGTTCCCGTTCTAAGTCTACAGAGAGAGAACGACGGCACAAAGAACGAGAACGA

GATAAGGACCGAGATCGGAATAAGAAGGACCGCGATCGAGACAAGGATGGGCACAGACGA

GACAAGGACCGTAAACGATCCAGTTTATCGCCTGGCCGAGGAAAAGATTTTAAATCTCGG

AAGGACAGAGACTCTAAAAAGGATGAAGATGATGAGCATGGTGATAAGAAGCCTAAGGCC

CAGCCACTATCTCTGGAGGAACTTTTAGCCAAGAAAAAGGCTGAGGAAGAAGCTGAGGCT

AAGCCTAAGTTCCTCTCCAAAGCAGAGCGGGAAGCTGAAGCTCTAAAGCGACGGCAGCAG

GAAGTGGAAGAGCGGCAGAGAATGCTTGAAGAAGAAAGGAAGAAAAGGAAACAGTTCCAA

GACTTGGGCAGGAAGATGTTGGGTTTGTTTTTCTATCCTGTG

>CL310.Contig1_A-W 3 1034 Non-structural maintenance of chromosomes element 4 homolog A OS=Bos taurus GN=NSMCE4A PE=2 SV=1

CGGGGCCGCGACCCGCACCGGGACCGCACCCGTTCTCGCTCCCGCTCGCGGTCCCCGTTG

TCCCGCCGCGGCGCCGCGCCGGAGCGCAGGGAGGCCCCGGAGCGCCCGAGCTTGGAGGAG

GCAGAGCCGTCGGATTCCGGGGACGAGATGGTGGACCCGGCTAGCTTGGAGGCGGAGACC

GACCACGGCCTGTGCCGCCAGATCCGCCATCAGTACCGGGCGCTCATCAACTCCGTCCAA

CAAAACCGGGAGGATATTCTGAACGCCAGCGACAAATTAACAGAGGTCCTTGAAGAGGCC

AACACTCTGTTTAATGGAGTGTCCCGAGCAAGAGAAGCAGTCCTAGATGCCCAGTTTCTT

GTTCTGGCTTCAGATTTGGGCAAAGAGAAAGCAAAGCAGCTGCGCTCAGATTTGAACTCG

TTTGATATGTTAAGATATGTTGAAACTCTACTGACACATATGGGTGTAAATCCGCTAGAA

GCTGAAGAACTCATCCGTGATGAAGATAGTTCTGATTTTGAATTCATAGTCTATGACTCC

TGGAAAATATCAGGCAAAACAGCAGAAAACACCTTTAATAAAACCCATACATTCCACTTT

CTGTTGGGTTCAATACAAGGAGAGCTCCCTGTGCCAAAGCCACGAATCGATCGTCCAAGA

AAACATCATATGATAGAAGAGCAGAGAGCAATGCCTGCTCAGTTGAAAAGAATGGAAGAA

TCTCATCAAGAAGCAACAGAAAAAGAAGTGGAAAGAATCTTGGGATTGTTGCAAACATAT

TTTCGAGAAGACCCTGATACTCCAATGTCCTTTTTTGACTTTGTGGTTGATCCACATTCT

TTCCCCCGTACAGTGGAAAACATCTTTCATGTTTCCTTCATCATACGGGATGGTTTTGCA

AGAATAAGACTTGACCAGGACCGACTGCCAATAATAGAACCTGTTAATATTAATGAAGAA

AGTGAGGGAATTGACCAAAACACCCAAATTAGGAATCAAGGAATTATAGCTTTGAGTTAT

CGAGACTGGGAG

>CL310.Contig2_A-W 3 794 PREDICTED: non-structural maintenance of chromosomes element 4 homolog A [Otolemur garnettii]

CGGGGCCGCGACCCGCACCGGGACCGCACCCGTTCTCGCTCCCGCTCGCGGTCCCCGTTG

TCCCGCCGCGGCGCCGCGCCGGAGCGCAGGGAGGCCCCGGAGCGCCCGAGCTTGGAGGAG

GCAGAGCCGTCGGATTCCGGGGACGAGATGGTGGACCCGGCTAGCTTGGAGGCGGAGACC

GACCACGGCCTGTGCCGCCAGATCCGCCATCAGTACCGGGCGCTCATCAACTCCGTCCAA

CAAAACCGGGAGGATATTCTGAACGCCAGCGACAAATTAACAGAGGTCCTTGAAGAGGCC

AACACTCTGTTTAATGGAGTGTCCCGAGCAAGAGAAGCAGTCCTAGATGCCCAGTTTCTT

GTTCTGGCTTCAGATTTGGGCAAAGAGAAAGCAAAGCAGCTGCGCTCAGATTTGAACTCG

TTTGATATGTTAAGATATGTTGAAACTCTACTGACACATATGGGTGTAAATCCGCTAGAA

GCTGAAGAACTCATCCGTGATGAAGATAGTTCTGATTTTGAATTCATAGTCTATGACTCC

TGGAAAATATCAGGCAAAACAGCAGAAAACACCTTTAATAAAACCCATACATTCCACTTT

CTGTTGGGTTCAATACAAGGAGAGCTCCCTGTGCCAAAGCCACGAATCGATCGTCCAAGA

AAACATCATATGATAGAAGAGCAGAGAGCAATGCCTGCTCAGTTGAAAAGAATGGAAGAA

TCTCATCAAGAAGCAACAGAAAAAGAAGTGGAAAGAATCTTGGGATTGTTGCAAACATAT

TTTCGAGAAGAC

>CL310.Contig3_A-W 3 605 PREDICTED: non-structural maintenance of chromosomes element 4 homolog A [Sus scrofa]

CGGGGCCGCGACCCGCACCGGGACCGCACCCGTTCTCGCTCCCGCTCGCGGTCCCCGTTG

TCCCGCCGCGGCGCCGCGCCGGAGCGCAGGGAGGCCCCGGAGCGCCCGAGCTTGGAGGAG

GCAGAGCCGTCGGATTCCGGGGACGAGATGGTGGACCCGGCTAGCTTGGAGGCGGAGACC

GACCACGGCCTGTGCCGCCAGATCCGCCATCAGTACCGGGCGCTCATCAACTCCGTCCAA

CAAAACCGGGAGGATATTCTGAACGCCAGCGACAAATTAACAGAGGTCCTTGAAGAGGCC

AACACTCTGTTTAATGGAGTGTCCCGAGCAAGAGAAGCAGTCCTAGATGCCCAGTTTCTT

GTTCTGGCTTCAGATTTGGGCAAAGAGAAAGCAAAGCAGCTGCGCTCAGATTTGAACTCG

TTTGATATGTTAAGATATGTTGAAACTCTACTGACACATATGGGTGTAAATCCGCTAGAA

GCTGAAGAACTCATCCGTGATGAAGATAGTTCTGATTTTGAATTCATAGTCTATGACTCC

TGGAAAATATCAGGCAAAACAGCAGAAAACACCTTTAATAAAACCCATACATTCCACTTT

CTG

>CL310.Contig4_A-W 3 1100 PREDICTED: non-structural maintenance of chromosomes element 4 homolog A [Otolemur garnettii]

CGGGGCCGCGACCCGCACCGGGACCGCACCCGTTCTCGCTCCCGCTCGCGGTCCCCGTTG

TCCCGCCGCGGCGCCGCGCCGGAGCGCAGGGAGGCCCCGGAGCGCCCGAGCTTGGAGGAG

GCAGAGCCGTCGGATTCCGGGGACGAGATGGTGGACCCGGCTAGCTTGGAGGCGGAGACC

GACCACGGCCTGTGCCGCCAGATCCGCCATCAGTACCGGGCGCTCATCAACTCCGTCCAA

CAAAACCGGGAGGATATTCTGAACGCCAGCGACAAATTAACAGAGGTCCTTGAAGAGGCC

AACACTCTGTTTAATGGAGTGTCCCGAGCAAGAGAAGCAGTCCTAGATGCCCAGTTTCTT

GTTCTGGCTTCAGATTTGGGCAAAGAGAAAGCAAAGCAGCTGCGCTCAGATTTGAACTCG

TTTGATATGTTAAGATATGTTGAAACTCTACTGACACATATGGGTGTAAATCCGCTAGAA

GCTGAAGAACTCATCCGTGATGAAGATAGTTCTGATTTTGAATTCATAGTCTATGACTCC

TGGAAAATATCAGGCAAAACAGCAGAAAACACCTTTAATAAAACCCATACATTCCACTTT

CTGTTGGGTTCAATACAAGGAGAGCTCCCTGTGCCAAAGCCACGAATCGATCGTCCAAGA

AAACATCATATGATAGAAGAGCAGAGAGCAATGCCTGCTCAGTTGAAAAGAATGGAAGAA

TCTCATCAAGAAGCAACAGAAAAAGAAGTGGAAAGAATCTTGGGATTGTTGCAAACATAT

TTTCGAGAAGACCCTGATACTCCAATGTCCTTTTTTGACTTTGTGGTTGATCCACATTCT

TTCCCCCGTACAGTGGAAAACATCTTTCATGTTTCCTTCATCATACGGGATGGTTTTGCA

AGAATAAGACTTGACCAGGACCGACTGCCAATAATAGAACCTGTTAATATTAATGAAGAA

AGTGAGGGAATTGACCAAAACACCCAAATTAGGAATCAAGGAATTATAGCTTTGAGTTAT

CGAGACTGGGAGGAGATTGTGAAGACCTTCGAGATTGCCGAGCCTGTGATTGCTTCGGGT

CAGAGCCGGCAGAGGCTG

>CL310.Contig5_A-W 6 482 Non-structural maintenance of chromosomes element 4 homolog A OS=Bos taurus GN=NSMCE4A PE=2 SV=1

AAAAAAAAAAAAAACAGAAAAAGACATGAAAGCATATTTTTCATTAGGTTGGGTTCAATA

CAAGGAGAGCTCCCTGTGCCAAAGCCACGAATCGATCGTCCAAGAAAACATCATATGATA

GAAGAGCAGAGAGCAATGCCTGCTCAGTTGAAAAGAATGGAAGAATCTCATCAAGAAGCA

ACAGAAAAAGAAGTGGAAAGAATCTTGGGATTGTTGCAAACATATTTTCGAGAAGACCCT

GATACTCCAATGTCCTTTTTTGACTTTGTGGTTGATCCACATTCTTTCCCCCGTACAGTG

GAAAACATCTTTCATGTTTCCTTCATCATACGGGATGGTTTTGCAAGAATAAGACTTGAC

CAGGACCGACTGCCAATAATAGAACCTGTTAATATTAATGAAGAAAGTGAGGGAATTGAC

CAAAACACCCAAATTAGGAATCAAGGAATTATAGCTTTGAGTTATCGAGACTGGGAG

>CL310.Contig6_A-W 6 554 PREDICTED: non-structural maintenance of chromosomes element 4 homolog A-like [Equus caballus]

AAAAAAAAAAAAAACAGAAAAAGACATGAAAGCATATTTTTCATTAGGTTGGGTTCAATA

CAAGGAGAGCTCCCTGTGCCAAAGCCACGAATCGATCGTCCAAGAAAACATCATATGATA

GAAGAGCAGAGAGCAATGCCTGCTCAGTTGAAAAGAATGGAAGAATCTCATCAAGAAGCA

ACAGAAAAAGAAGTGGAAAGAATCTTGGGATTGTTGCAAACATATTTTCGAGAAGACCCT

GATACTCCAATGTCCTTTTTTGACTTTGTGGTTGATCCACATTCTTTCCCCCGTACAGTG

GAAAACATCTTTCATGTTTCCTTCATCATACGGGATGGTTTTGCAAGAATAAGACTTGAC

CAGGACCGACTGCCAATAATAGAACCTGTTAATATTAATGAAGAAAGTGAGGGAATTGAC

CAAAACACCCAAATTAGGAATCAAGGAATTATAGCTTTGAGTTATCGAGACTGGGAGGAG

ATTGTGAAGACCTTCGAGATTGCCGAGCCTGTGATTGCTTCGGGTCAGAGCCGGCAGAGG

CTGAGTGCT

>CL311.Contig1_A-W 2316 3092 minus strand PREDICTED: protein SCO2 homolog, mitochondrial-like [Ailuropoda melanoleuca]

ATGCTGTTGCTGGCTCGGGGGCCCAAGGCCAAGGCTTGGCTTTGGCTTTGTCAGCTTAGG

CCACCAGTACCCTCTATGACCCCTGGAAATGAAGCCCTGCACATGAGGCCCCGGCACTTC

TCGTGGCAGGGCCCACCTCAAGGCCCTAGGCTTCGAACCAGGCTGCTCATCACTGCCCTA

TTTGGGGCTGGGCTGGGTGGGGCCTGGCTGGCTGCCAGGGCTGAGAAGGAACAGCGGCGG

CAGCAACAGCGGACGGAAGCCCTGCGCCAGGCTGCTGTGGGTCAGGGCGACTTCAGCCTA

TTGGACCACCAAGGCCAGGCTCGCTGCAAGGCCGACTTCCGGGGCCAGTGGGTACTGATG

TACTTTGGCTTCACACACTGCCCAGACATCTGCCCAGACGAGCTGGAGAAGCTGGTACAG

GTGGTACAACAGCTGGAAGCCAAGCCTGGACTGCCCCCTGTGCAGCCCATCTTCATTACT

GTGGACCCTGAGCGGGATGATGTGGCAGCTATGGCCCGATATGTGCGAGACTTCCACCCA

AGACTGCTGGGTCTAACGGGCTCTGTTGAGCAGGTGGCCCAGGCTAGCCGCAGCTACCGT

GTGTACTACAGTGCTGGCCCCAAGGATGAGGACCAGGACTACATTGTCGACCACTCCATT

GCTATCTACTTGCTAAACCCTGATGGCCTCTTCACGGACTACTATGGTAGGGGCAGGTCC

GCGGAGCAGATTGCAGACAGTGTGCAGCGGCACATGGCTGGCTTTCGCAGTGTCCTC

>CL311.Contig2_A-W 282 497 minus strand PREDICTED: thymidine phosphorylase [Saimiri boliviensis boliviensis]

ATGGCAGCCCCAGGAACTCCAGCCCCACCAACCCCAATCGCACCAGGTGATGGTTTGCTG

GGAGGACGGGGGGGACCTCCTGACTCAATGCCAGGGGCCAAGCAGCTCCCAGTGCTGATC

CGCAGGAAGCGAGATGGAGGCCGCTTAAGTGAGGAGGACATCAAGGGCTTCATCAGTGCT

GTGGTGGAGGGGAGTGCACAGGGGGCACAGATTGGT

>CL311.Contig3_A-W 2849 3625 minus strand PREDICTED: protein SCO2 homolog, mitochondrial-like [Ailuropoda melanoleuca]

ATGCTGTTGCTGGCTCGGGGGCCCAAGGCCAAGGCTTGGCTTTGGCTTTGTCAGCTTAGG

CCACCAGTACCCTCTATGACCCCTGGAAATGAAGCCCTGCACATGAGGCCCCGGCACTTC

TCGTGGCAGGGCCCACCTCAAGGCCCTAGGCTTCGAACCAGGCTGCTCATCACTGCCCTA

TTTGGGGCTGGGCTGGGTGGGGCCTGGCTGGCTGCCAGGGCTGAGAAGGAACAGCGGCGG

CAGCAACAGCGGACGGAAGCCCTGCGCCAGGCTGCTGTGGGTCAGGGCGACTTCAGCCTA

TTGGACCACCAAGGCCAGGCTCGCTGCAAGGCCGACTTCCGGGGCCAGTGGGTACTGATG

TACTTTGGCTTCACACACTGCCCAGACATCTGCCCAGACGAGCTGGAGAAGCTGGTACAG

GTGGTACAACAGCTGGAAGCCAAGCCTGGACTGCCCCCTGTGCAGCCCATCTTCATTACT

GTGGACCCTGAGCGGGATGATGTGGCAGCTATGGCCCGATATGTGCGAGACTTCCACCCA

AGACTGCTGGGTCTAACGGGCTCTGTTGAGCAGGTGGCCCAGGCTAGCCGCAGCTACCGT

GTGTACTACAGTGCTGGCCCCAAGGATGAGGACCAGGACTACATTGTCGACCACTCCATT

GCTATCTACTTGCTAAACCCTGATGGCCTCTTCACGGACTACTATGGTAGGGGCAGGTCC

GCGGAGCAGATTGCAGACAGTGTGCAGCGGCACATGGCTGGCTTTCGCAGTGTCCTC

>CL311.Contig4_A-W 2018 2794 minus strand PREDICTED: protein SCO2 homolog, mitochondrial-like [Ailuropoda melanoleuca]

ATGCTGTTGCTGGCTCGGGGGCCCAAGGCCAAGGCTTGGCTTTGGCTTTGTCAGCTTAGG

CCACCAGTACCCTCTATGACCCCTGGAAATGAAGCCCTGCACATGAGGCCCCGGCACTTC

TCGTGGCAGGGCCCACCTCAAGGCCCTAGGCTTCGAACCAGGCTGCTCATCACTGCCCTA

TTTGGGGCTGGGCTGGGTGGGGCCTGGCTGGCTGCCAGGGCTGAGAAGGAACAGCGGCGG

CAGCAACAGCGGACGGAAGCCCTGCGCCAGGCTGCTGTGGGTCAGGGCGACTTCAGCCTA

TTGGACCACCAAGGCCAGGCTCGCTGCAAGGCCGACTTCCGGGGCCAGTGGGTACTGATG

TACTTTGGCTTCACACACTGCCCAGACATCTGCCCAGACGAGCTGGAGAAGCTGGTACAG

GTGGTACAACAGCTGGAAGCCAAGCCTGGACTGCCCCCTGTGCAGCCCATCTTCATTACT

GTGGACCCTGAGCGGGATGATGTGGCAGCTATGGCCCGATATGTGCGAGACTTCCACCCA

AGACTGCTGGGTCTAACGGGCTCTGTTGAGCAGGTGGCCCAGGCTAGCCGCAGCTACCGT

GTGTACTACAGTGCTGGCCCCAAGGATGAGGACCAGGACTACATTGTCGACCACTCCATT

GCTATCTACTTGCTAAACCCTGATGGCCTCTTCACGGACTACTATGGTAGGGGCAGGTCC

GCGGAGCAGATTGCAGACAGTGTGCAGCGGCACATGGCTGGCTTTCGCAGTGTCCTC

>CL311.Contig5_A-W 282 497 minus strand PREDICTED: thymidine phosphorylase [Saimiri boliviensis boliviensis]

ATGGCAGCCCCAGGAACTCCAGCCCCACCAACCCCAATCGCACCAGGTGATGGTTTGCTG

GGAGGACGGGGGGGACCTCCTGACTCAATGCCAGGGGCCAAGCAGCTCCCAGTGCTGATC

CGCAGGAAGCGAGATGGAGGCCGCTTAAGTGAGGAGGACATCAAGGGCTTCATCAGTGCT

GTGGTGGAGGGGAGTGCACAGGGGGCACAGATTGGT

>CL311.Contig6_A-W 115 330 minus strand PREDICTED: thymidine phosphorylase [Saimiri boliviensis boliviensis]

ATGGCAGCCCCAGGAACTCCAGCCCCACCAACCCCAATCGCACCAGGTGATGGTTTGCTG

GGAGGACGGGGGGGACCTCCTGACTCAATGCCAGGGGCCAAGCAGCTCCCAGTGCTGATC

CGCAGGAAGCGAGATGGAGGCCGCTTAAGTGAGGAGGACATCAAGGGCTTCATCAGTGCT

GTGGTGGAGGGGAGTGCACAGGGGGCACAGATTGGT

>CL311.Contig7_A-W 115 330 minus strand PREDICTED: thymidine phosphorylase [Saimiri boliviensis boliviensis]

ATGGCAGCCCCAGGAACTCCAGCCCCACCAACCCCAATCGCACCAGGTGATGGTTTGCTG

GGAGGACGGGGGGGACCTCCTGACTCAATGCCAGGGGCCAAGCAGCTCCCAGTGCTGATC

CGCAGGAAGCGAGATGGAGGCCGCTTAAGTGAGGAGGACATCAAGGGCTTCATCAGTGCT

GTGGTGGAGGGGAGTGCACAGGGGGCACAGATTGGT

>CL311.Contig8_A-W 313 1089 minus strand PREDICTED: protein SCO2 homolog, mitochondrial-like [Ailuropoda melanoleuca]

ATGCTGTTGCTGGCTCGGGGGCCCAAGGCCAAGGCTTGGCTTTGGCTTTGTCAGCTTAGG

CCACCAGTACCCTCTATGACCCCTGGAAATGAAGCCCTGCACATGAGGCCCCGGCACTTC

TCGTGGCAGGGCCCACCTCAAGGCCCTAGGCTTCGAACCAGGCTGCTCATCACTGCCCTA

TTTGGGGCTGGGCTGGGTGGGGCCTGGCTGGCTGCCAGGGCTGAGAAGGAACAGCGGCGG

CAGCAACAGCGGACGGAAGCCCTGCGCCAGGCTGCTGTGGGTCAGGGCGACTTCAGCCTA

TTGGACCACCAAGGCCAGGCTCGCTGCAAGGCCGACTTCCGGGGCCAGTGGGTACTGATG

TACTTTGGCTTCACACACTGCCCAGACATCTGCCCAGACGAGCTGGAGAAGCTGGTACAG

GTGGTACAACAGCTGGAAGCCAAGCCTGGACTGCCCCCTGTGCAGCCCATCTTCATTACT

GTGGACCCTGAGCGGGATGATGTGGCAGCTATGGCCCGATATGTGCGAGACTTCCACCCA

AGACTGCTGGGTCTAACGGGCTCTGTTGAGCAGGTGGCCCAGGCTAGCCGCAGCTACCGT

GTGTACTACAGTGCTGGCCCCAAGGATGAGGACCAGGACTACATTGTCGACCACTCCATT

GCTATCTACTTGCTAAACCCTGATGGCCTCTTCACGGACTACTATGGTAGGGGCAGGTCC

GCGGAGCAGATTGCAGACAGTGTGCAGCGGCACATGGCTGGCTTTCGCAGTGTCCTC

>CL311.Contig9_A-W 2553 3329 minus strand PREDICTED: protein SCO2 homolog, mitochondrial-like [Ailuropoda melanoleuca]

ATGCTGTTGCTGGCTCGGGGGCCCAAGGCCAAGGCTTGGCTTTGGCTTTGTCAGCTTAGG

CCACCAGTACCCTCTATGACCCCTGGAAATGAAGCCCTGCACATGAGGCCCCGGCACTTC

TCGTGGCAGGGCCCACCTCAAGGCCCTAGGCTTCGAACCAGGCTGCTCATCACTGCCCTA

TTTGGGGCTGGGCTGGGTGGGGCCTGGCTGGCTGCCAGGGCTGAGAAGGAACAGCGGCGG

CAGCAACAGCGGACGGAAGCCCTGCGCCAGGCTGCTGTGGGTCAGGGCGACTTCAGCCTA

TTGGACCACCAAGGCCAGGCTCGCTGCAAGGCCGACTTCCGGGGCCAGTGGGTACTGATG

TACTTTGGCTTCACACACTGCCCAGACATCTGCCCAGACGAGCTGGAGAAGCTGGTACAG

GTGGTACAACAGCTGGAAGCCAAGCCTGGACTGCCCCCTGTGCAGCCCATCTTCATTACT

GTGGACCCTGAGCGGGATGATGTGGCAGCTATGGCCCGATATGTGCGAGACTTCCACCCA

AGACTGCTGGGTCTAACGGGCTCTGTTGAGCAGGTGGCCCAGGCTAGCCGCAGCTACCGT

GTGTACTACAGTGCTGGCCCCAAGGATGAGGACCAGGACTACATTGTCGACCACTCCATT

GCTATCTACTTGCTAAACCCTGATGGCCTCTTCACGGACTACTATGGTAGGGGCAGGTCC

GCGGAGCAGATTGCAGACAGTGTGCAGCGGCACATGGCTGGCTTTCGCAGTGTCCTC

>CL311.Contig10_A-W 2478 3254 minus strand PREDICTED: protein SCO2 homolog, mitochondrial-like [Ailuropoda melanoleuca]

ATGCTGTTGCTGGCTCGGGGGCCCAAGGCCAAGGCTTGGCTTTGGCTTTGTCAGCTTAGG

CCACCAGTACCCTCTATGACCCCTGGAAATGAAGCCCTGCACATGAGGCCCCGGCACTTC

TCGTGGCAGGGCCCACCTCAAGGCCCTAGGCTTCGAACCAGGCTGCTCATCACTGCCCTA

TTTGGGGCTGGGCTGGGTGGGGCCTGGCTGGCTGCCAGGGCTGAGAAGGAACAGCGGCGG

CAGCAACAGCGGACGGAAGCCCTGCGCCAGGCTGCTGTGGGTCAGGGCGACTTCAGCCTA

TTGGACCACCAAGGCCAGGCTCGCTGCAAGGCCGACTTCCGGGGCCAGTGGGTACTGATG

TACTTTGGCTTCACACACTGCCCAGACATCTGCCCAGACGAGCTGGAGAAGCTGGTACAG

GTGGTACAACAGCTGGAAGCCAAGCCTGGACTGCCCCCTGTGCAGCCCATCTTCATTACT

GTGGACCCTGAGCGGGATGATGTGGCAGCTATGGCCCGATATGTGCGAGACTTCCACCCA

AGACTGCTGGGTCTAACGGGCTCTGTTGAGCAGGTGGCCCAGGCTAGCCGCAGCTACCGT

GTGTACTACAGTGCTGGCCCCAAGGATGAGGACCAGGACTACATTGTCGACCACTCCATT

GCTATCTACTTGCTAAACCCTGATGGCCTCTTCACGGACTACTATGGTAGGGGCAGGTCC

GCGGAGCAGATTGCAGACAGTGTGCAGCGGCACATGGCTGGCTTTCGCAGTGTCCTC

>CL311.Contig11_A-W 2408 3184 minus strand PREDICTED: protein SCO2 homolog, mitochondrial-like [Ailuropoda melanoleuca]

ATGCTGTTGCTGGCTCGGGGGCCCAAGGCCAAGGCTTGGCTTTGGCTTTGTCAGCTTAGG

CCACCAGTACCCTCTATGACCCCTGGAAATGAAGCCCTGCACATGAGGCCCCGGCACTTC

TCGTGGCAGGGCCCACCTCAAGGCCCTAGGCTTCGAACCAGGCTGCTCATCACTGCCCTA

TTTGGGGCTGGGCTGGGTGGGGCCTGGCTGGCTGCCAGGGCTGAGAAGGAACAGCGGCGG

CAGCAACAGCGGACGGAAGCCCTGCGCCAGGCTGCTGTGGGTCAGGGCGACTTCAGCCTA

TTGGACCACCAAGGCCAGGCTCGCTGCAAGGCCGACTTCCGGGGCCAGTGGGTACTGATG

TACTTTGGCTTCACACACTGCCCAGACATCTGCCCAGACGAGCTGGAGAAGCTGGTACAG

GTGGTACAACAGCTGGAAGCCAAGCCTGGACTGCCCCCTGTGCAGCCCATCTTCATTACT

GTGGACCCTGAGCGGGATGATGTGGCAGCTATGGCCCGATATGTGCGAGACTTCCACCCA

AGACTGCTGGGTCTAACGGGCTCTGTTGAGCAGGTGGCCCAGGCTAGCCGCAGCTACCGT

GTGTACTACAGTGCTGGCCCCAAGGATGAGGACCAGGACTACATTGTCGACCACTCCATT

GCTATCTACTTGCTAAACCCTGATGGCCTCTTCACGGACTACTATGGTAGGGGCAGGTCC

GCGGAGCAGATTGCAGACAGTGTGCAGCGGCACATGGCTGGCTTTCGCAGTGTCCTC

>CL311.Contig12_A-W 282 497 minus strand PREDICTED: thymidine phosphorylase [Saimiri boliviensis boliviensis]

ATGGCAGCCCCAGGAACTCCAGCCCCACCAACCCCAATCGCACCAGGTGATGGTTTGCTG

GGAGGACGGGGGGGACCTCCTGACTCAATGCCAGGGGCCAAGCAGCTCCCAGTGCTGATC

CGCAGGAAGCGAGATGGAGGCCGCTTAAGTGAGGAGGACATCAAGGGCTTCATCAGTGCT

GTGGTGGAGGGGAGTGCACAGGGGGCACAGATTGGT

>CL311.Contig13_A-W 2083 2859 minus strand PREDICTED: protein SCO2 homolog, mitochondrial-like [Ailuropoda melanoleuca]

ATGCTGTTGCTGGCTCGGGGGCCCAAGGCCAAGGCTTGGCTTTGGCTTTGTCAGCTTAGG

CCACCAGTACCCTCTATGACCCCTGGAAATGAAGCCCTGCACATGAGGCCCCGGCACTTC

TCGTGGCAGGGCCCACCTCAAGGCCCTAGGCTTCGAACCAGGCTGCTCATCACTGCCCTA

TTTGGGGCTGGGCTGGGTGGGGCCTGGCTGGCTGCCAGGGCTGAGAAGGAACAGCGGCGG

CAGCAACAGCGGACGGAAGCCCTGCGCCAGGCTGCTGTGGGTCAGGGCGACTTCAGCCTA

TTGGACCACCAAGGCCAGGCTCGCTGCAAGGCCGACTTCCGGGGCCAGTGGGTACTGATG

TACTTTGGCTTCACACACTGCCCAGACATCTGCCCAGACGAGCTGGAGAAGCTGGTACAG

GTGGTACAACAGCTGGAAGCCAAGCCTGGACTGCCCCCTGTGCAGCCCATCTTCATTACT

GTGGACCCTGAGCGGGATGATGTGGCAGCTATGGCCCGATATGTGCGAGACTTCCACCCA

AGACTGCTGGGTCTAACGGGCTCTGTTGAGCAGGTGGCCCAGGCTAGCCGCAGCTACCGT

GTGTACTACAGTGCTGGCCCCAAGGATGAGGACCAGGACTACATTGTCGACCACTCCATT

GCTATCTACTTGCTAAACCCTGATGGCCTCTTCACGGACTACTATGGTAGGGGCAGGTCC

GCGGAGCAGATTGCAGACAGTGTGCAGCGGCACATGGCTGGCTTTCGCAGTGTCCTC

>CL311.Contig14_A-W 1555 2331 PREDICTED: protein SCO2 homolog, mitochondrial-like [Ailuropoda melanoleuca]

ATGCTGTTGCTGGCTCGGGGGCCCAAGGCCAAGGCTTGGCTTTGGCTTTGTCAGCTTAGG

CCACCAGTACCCTCTATGACCCCTGGAAATGAAGCCCTGCACATGAGGCCCCGGCACTTC

TCGTGGCAGGGCCCACCTCAAGGCCCTAGGCTTCGAACCAGGCTGCTCATCACTGCCCTA

TTTGGAGCTGGGCTGGGTGGGGCCTGGCTGGCTGCCAGGGCTGAGAAGGAACAGCGGCGG

CAGCAACAGCGGACGGAAGCCCTGCGCCAGGCTGCTGTGGGTCAGGGCGACTTCAGCCTA

TTGGACCACCAAGGCCAGGCTCGCTGCAAGGCCGACTTCCGGGGCCAGTGGGTACTGATG

TACTTTGGCTTCACACACTGCCCAGACATCTGCCCAGACGAGCTGGAGAAGCTGGTACAG

GTGGTACAACAGCTGGAAGCCAAGCCTGGACTGCCCCCTGTGCAGCCCATCTTCATTACT

GTGGACCCTGAGCGGGATGATGTGGCAGCTATGGCCCGATATGTGCGAGACTTCCACCCA

AGACTGCTGGGTCTAACGGGCTCCGTTGAGCAGGTGGCCCAGGCTAGCCGCAGCTACCGT

GTGTACTACAGTGCTGGCCCCAAGGATGAGGACCAGGACTACATTGTCGACCACTCCATT

GCTATCTACTTGCTAAACCCTGATGGCCTCTTCACGGACTACTATGGTAGGGGCAGGTCC

GCGGAGCAGATTGCAGACAGTGTGCAGCGGCACATGGCTGGCTTTCGCAGTGTCCTC

>CL311.Contig15_A-W 252 455 PREDICTED: LOW QUALITY PROTEIN: thymidine phosphorylase [Callithrix jacchus]

GGAACTCCAGCCCCACCAACCCCAATCGCACCAGGTGATGGTTTGCTGGGAGGACGGGGG

GTACCTCCTGACTCAATGCCAGGGGCCAAGCAGCTCCCAGTGCTGATCCGCAGGAAGCGA

GATGGAGGCCGCTTAAGTGAGGAGGACATCAAGGGCTTCATCAGTGCTGTGGTGGAGGGG

AGTGCACAGGGGGCACAGATTGGT

>CL311.Contig16_A-W 183 1106 PREDICTED: thymidine phosphorylase [Saimiri boliviensis boliviensis]

ATGCAAGTGCTGTTGGAGCAAGTGGGCTGCTGTATTGTGGGTCAAAGCAAGGAGCTGGTT

CCTGCAGATGGAATCCTGTATGCTGTACGAGATGTGACAGCCACTGTGGATAGCCTGCCA

CTCATCACAGCCTCCATCCTCAGTAAAAAGGCGGTGGAAGGGCTCTCTGCCCTGGTGATA

GACGTTAAGTTTGGTGGCGCTGCAGTCTTCCCAGACCAGGAGAAAGCTCGGGAATTGGCA

AGAGCACTGGTTGGCGTGGGGGCGGATCTGGGGCTTCGGGTGGCAGCAGCCCTGACAGCC

ATGGATAACCCTCTGGGTCGCAACGTGGGCCATACCCTGGAAGTGGAGGAAGCGCTGCTC

TGCCTGGATGGCACGGGGCCACCGGACCTGCGGGACCTGGTCACTAGACTCGGGGGTGTC

CTATTATGGCTCAGTGGACATGCGGGTAGCTCGGCCCAGGGCACCGCCCGGATCGCTACG

GCACTGGACGACGGCTCCGCGCGGCGCCGCTTCGAGCAGATGCTTGAAGCACAGGGCGTG

GATTCGAACCTGGCCCGATCACTGTGCTCCGGGACCCCGGCGCAGCGTCGTCAGTTGCTG

CCCCACGCCCGGGAGCAAGAGGAGTTACACGCGCCTGCGGACGGCACCGTGGAGAGCGTC

CAAGCGCTGCCACTAGCGCTCGTGCTGCACGAGCTCGGGGCCGGACGCAGCCGTGCAGGG

GAGCCAATCCGCCTGGGCGTGGGCGCCGAGCTGTTGGTGGACGTGGGTCAAAGCCTAAGC

CGAGGGACGCCCTGGCTCCGCGTGCATCTGGACGGCCCTGCGCTAAGCGATTCGCAGCGC

CGAGCACTGCAGGCAGCGCTTGTGCTGTCTGATCGCGCGCCCTTCACGGCCCCCTCGCCC

TTTGCGGAGCTGATCCTGCCGCCC

>CL311.Contig17_A-W 264 455 PREDICTED: endothelial cell growth factor 1 (platelet-derived)-like [Oryctolagus cuniculus]

CCACCAACCCCAATCGCACCAGGTGATGGTTTGCTGGGAGGACGGGGGGTACCTCCTGAC

TCAATGCCAGGGGCCAAGCAGCTCCCAGTGCTGATCCGCAGGAAGCGAGATGGAGGCCGC

TTAAGTGAGGAGGACATCAAGGGCTTCATCAGTGCTGTGGTGGAGGGGAGTGCACAGGGG

GCACAGATTGGT

>CL311.Contig18_A-W 183 434 PREDICTED: endothelial cell growth factor 1 (platelet-derived)-like [Oryctolagus cuniculus]

ATGCAAGTGCTGTTGGAGCAAGTGGGCTGCTGTATTGTGGGTCAAAGCAAGGAGCTGGTT

CCTGCAGATGGAATCCTGTATGCTGTACGAGATGTGACAGCCACTGTGGATAGCCTGCCA

CTCATCACAGCCTCCATCCTCAGTAAAAAGGCGGTGGAAGGGCTCTCTGCCCTGGTGATA

GACGTTAAGTTTGGTGGCGCTGCAGTCTTCCCAGACCAGGAGAAAGCTCGGGAATTGGCA

AGAGCACTGGTG

>CL311.Contig19_A-W 183 827 PREDICTED: thymidine phosphorylase [Saimiri boliviensis boliviensis]

ATGCAAGTGCTGTTGGAGCAAGTGGGCTGCTGTATTGTGGGTCAAAGCAAGGAGCTGGTT

CCTGCAGATGGAATCCTGTATGCTGTACGAGATGTGACAGCCACTGTGGATAGCCTGCCA

CTCATCACAGCCTCCATCCTCAGTAAAAAGGCGGTGGAAGGGCTCTCTGCCCTGGTGATA

GACGTTAAGTTTGGTGGCGCTGCAGTCTTCCCAGACCAGGAGAAAGCTCGGGAATTGGCA

AGAGCACTGGTTGGCGTGGGGGCGGATCTGGGGCTTCGGGTGGCAGCAGCCCTGACAGCC

ATGGATAACCCTCTGGGTCGCAACGTGGGCCATACCCTGGAAGTGGAGGAAGCGCTGCTC

TGCCTGGATGGCACGGGGCCACCGGACCTGCGGGACCTGGTCACTAGACTCGGGGGTGTC

CTATTATGGCTCAGTGGACATGCGGGTAGCTCGGCCCAGGGCACCGCCCGGATCGCTACG

GCACTGGACGACGGCTCCGCGCGGCGCCGCTTCGAGCAGATGCTTGAAGCACAGGGCGTG

GATTCGAACCTGGCCCGATCACTGTGCTCCGGGACCCCGGCGCAGCGTCGTCAGTTGCTG

CCCCACGCCCGGGAGCAAGAGGAGTTACACGCGCCTGCGGACGGT

>CL312.Contig1_A-W 359 739 minus strand PREDICTED: baculoviral IAP repeat-containing protein 2 isoform 3 [Papio anubis]

AGATTTCTTACTTATAATATGTGGCCCTTAACCTTTTTGTCACCATTGGAATTGGCGAGA

GCTGGCTTTTACTATGTAGGACCTGGAGATAGGGTAGCCTGCTTTGCCTGTGGCGGGAAG

CTAAGTAACTGGGAACCAAAGGATGATGCTATGTCAGAACACAGGAGACATTTTCCCAAC

TGTCCGTTTTTGGAAAATTCTCTAGAAACGCTGAGGTTTAGTATTTCAAATCTGAGCATG

CAGACACACACAGCTCGAATGAGAACATTTATGTACTGGCCAGCTAGTGTTCCAGTTCAG

CCTGAGCAGCTTGCAAGTGCTGGATTTTATTACGTGGATCGCAATGATGATGTCAAATGC

TTTTGTTGTGATGGTGGCTTG

>CL312.Contig2_A-W 738 2549 Baculoviral IAP repeat-containing protein 3 [Heterocephalus glaber]

ATGAATATAGTAGAAAATAGCATCTTCTTATCAAATTTGATGAAGAATGCCAACTCATTT

AAAATGAAGTACGACTTTTCATGTGAACTGTACCGGATGTCCACATATTCCACTTTCCCC

TCAGGAGTTCCCGTCTCGGAAAGGAGTCTTGCTCGTGCTGGTTTCTATTACACTGGTGTG

AAAGACAAGGTCAAATGCTTCTGTTGTGGCCTCATGCTGGATAACTGGAAACACGGAGAC

AATCCTATCGAAAAGCATAAAAAATTGTATCCTAGTTGCAGCTTTGTTCAGAATCTAAAT

TCAGTTAACAATTCGAAAGCCACTTCTCAGCCTTCTTTTTCTTCTTCAGTAACAAATTCC

ACACATTCATTACTTCCAAGTTTGGAAAACAGTGGATATTTTAGTGGCTCTTATTCAAGC

TTTCCATCAAATCCTGTCAACTCCAGAGCAAATCAAGATTTTTCTGCCTTGAGGATAAAT

CCCTATCATTGTTCAATGAATACTGAAAAGGCCAGATTACTCACTTATCAAATGTGGCCA

TTGACCTTTCTGTCACCTACAGAACTAGCCAAAGCAGGCTTTTACTACATAGGGCCTGGA

GACAGAGTGGCTTGCTTTGCCTGCGGTGGAAAATTGAGCAATTGGGAACCAAAGGACGAT

GCTATGTCAGAACACCTAAGACATTTTCCCAACTGCCCGTTTGTAGAAAATCAGTTTCAA

GATACTTCAAGATACACCGTTTCTAATCTGAGCATGCAGACACATGCAGCCCGCTTTAAA

ACGTTCTTTAACTGGCCTTCCAGTGTTCTGGTTCATCCTGAGCAACTTGCAAGTGCAGGT

TTTTATTATGTGGGTCACAGTGATGATGTCAAATGCTTTTGCTGTGATGGTGGACTGAGG

TGTTGGGAGTTGGGAGATGATCCATGGGTGGAACATGCCAAGTGGTTTCCAAGGTGTGAG

TACTTGATAAGAATTAAAGGACAAGAATTCATCAGTCAAGTTCAAGCCAGTTATCCCCAT

CTGCTTGAACAGCTGTTATCCACATCAGATACCCCAGAAGATGAAAATGCAGAGTCACCA

ATTGTTCATTTTGGACCTGGAGAAAACCATTCAGAAGATGCTGTCATGATGAATACACCT

GTGGTTAAAGCTGCTTTGGAAATGGGCTTCAGTAGAAACCTGGTAAAACAGACAGTTCAG

AGTAAAATCCTAACCGCTGGAGAGAATTATAAAACCATCAGTGATCTTGTATTAGATTTG

CTTAATGCAGAAGATGAAATAAGGGAAGAGGCAAAAGAAAGAGCAACTGAGGAAAAAGAA

TCAGAAGACCTATTATTAATTCGGAAGAATAGAATGGCACTTTTCCAACATCTGACTTGT

GTAATTCCAATTCTGGATAGTCTACTAATTGCCAGAGTGATTAATGAACAAGAGCATGAT

GTAATTAAGCAGAAAACACAGACTTCTTTACAAGCAAGAGAACTGATTGACACCATTTTA

GTAAAAGGAAATACCGCAGCCACTGTATTCAAAAACTCTCTACAAGAAACTGACCCTATG

TTATACAAGCATTTATTTGTGCAACAGGACATAAGGTGTATTCGCACAGAAGATACTTCA

GATCTACCAATGGAAGAACAATTGCGGAGACTACAAGAAGAAAGAACATGTAAAGTGTGC

ATGGACAAAGAAGTGTCCATAGTGTTCATTCCTTGTGGTCATCTAGTAGTATGCAAAGAT

TGTGCCCCTTCTCTAAGAAAATGTCCGATTTGTAGGGGTACCATCAAGGGTACAGTTCGT

ACATTTCTTTCA

>CL312.Contig3_A-W 2 925 minus strand PREDICTED: baculoviral IAP repeat-containing protein 2 isoform 3 [Papio anubis]

GATGGTGGCTTGAGATGTTGGGAATCTGGAGATGATCCATGGGTAGAACATGCCAAATGG

TTTCCAAGGTGTGAGTTCTTGATACGAATGAAAGGGCAAGAGTTTGTTGATGAGATTCAA

GCTAGATATCCTCATCTTCTTGAACAGCTGTTGTCAACTTCAGATACTACTGGAGAAGAA

AATGCTGAACCAATTGTTCATTTGGGACCTGGAGAAAGTTCTTCAGAAGATGCAGTCATG

ATGAATACACCTGTGGTTAAATCTGCTTTGGAAATGGGCTTTAGTAGAAGTCTGGTAAAA

CAGACAGTTCAAAGTAAAATCCTAACCACTGGAGAGAATTATAAAACAGTTAATGATATT

GTATCAGCACTTCTTGATGCTGAAGATGAAAGAAGAGAAGAGGAGAAGGAAAGACAAGCT

GAAGAAATGGCATCAGATGATTTGTCATTAATACGGAAGAATAGAATGGCTCTTTTTCAA

CAGTTGACATGTGTCCTTCCTATCTTGGATAATCTTTTAAAGGCCAATGTAATTAATAAA

CAGGAACATGATATTATTAAACAAAAAACACAGATACCTTTACAAGCAAGAGAACTGATT

GATACCATTTTAGTTAAAGGAAATGCTGCAGCCAACATCTTCAAAAACTGTCTAAAGGAA

ATTGACTCTACATTATATGAGAACTTATTTGTGGGAAAGAATATGAAGTATATTCCAACA

GAAGATGTTTCAGGTCTGTCACTAGAAGAACAGTTGAGGAGATTACAAGAAGAAAGAACC

TGCAAAGTGTGTATGGACAAAGAAGTTTCTATTGTGTTCATTCCTTGTGGTCATCTGGTA

GTATGCCAGGAGTGTGCCCCATCTCTAAGAAAATGTCCTATTTGCAGGGGTATAATCAAG

GGTACTGTTCGAACATTTCTCTCA

>CL313.Contig1_A-W 19 2292 minus strand mitochondrial inner membrane protein isoform 1 [Homo sapiens] >gi|29427676|sp|Q16891.1|IMMT_HUMAN RecName: Full=Mitochondrial inner membrane protein; AltName: Full=Cell proliferation-inducing gene 4/52 protein; AltName: Full=Mitofilin; AltName: Full=p87/89 >gi|516768|dbj|BAA04656.1| motor protein [Homo sapiens] >gi|12803209|gb|AAH02412.1| Inner membrane protein, mitochondrial (mitofilin) [Homo sapiens] >gi|59877349|gb|AAX10024.1| cell proliferation-inducing protein 52 [Homo sapiens] >gi|745495|prf||2016304A motor protein

ATGCTGCGGGCCTGTCAGCTGTCTGGCGTGAGCGCCGCCGCCCAGACTTGTCTCTGTGGA

AAGCTTGTCCTCCGTCCATTGCGACCATGCCGAAGATACTCAACTTCTAGCAGTTCTGGG

TTGACTGCAGGCAGGATCGCTGGAGCGGGTCTGCTGTTTGTTGGTGGTGGTATTGGAGGT

ACCATCTTATATGCCAAGTGGGACTCCCACTTCCGAGAAAGTGTAGAGAAAACCATTCCT

TACTCAGACAGACTCTTTGAGATGGTTCTTGGTTCTGCACCTTATAACGTTCCATTGCCA

AAGAAGCCGATTCAATCCGGTCCACTAAAAATCTCTAGTGTGTCAGAGGTAATGAAAGAA

TCCAAACAGCCTGCGTCACAGCTCCAAAAGCAGAAGGGAGACACTCCAGCCTCGACAACA

GCACCTACAGAGGCAGCTCAAATTATTTCTGCAGCAGGTGATACACTTTCCGTCCCAGCC

CCTGCGGTTCAACATGAGGAATCTGTAAAAACTGATCGCCCTGAAATTGGTGGAGGAAAA

CCCACACCTGTAACCTCAGAGGAAGCACCCTCATCCCCTGTAAGGGAGCGGCCACCCGAA

GAAGTCGCAGCTCGCCTGGCGCAGCAGGAAAAGCACGAGCAAGTTGAGATTGCGTCTCTA

GCCAAGAGCTTAGAAGATGCTCTGAGCCAAACTGCTTCCGTCACTCTGCAGGCTATTGCA

GCCCAGAACGCTGCCGTCCAGGCCGTGAATGCCCACTCCAGCGTCCTGAAGGCAGCCATG

GACGATGCCGAGGTTGCAGGTGAAAAGAAGTCTGCCCAGTGGCGCACGGTGGAAGGCGCC

CTGAAGGAGCGCAGGAAGGCCGTGGATGAAGCTGCTGATGCCCTTCTCAAAGCCAAAGAG

GAGTTAGAGAAGATGAAAAGTGTCATTGAAAATGCAAAGAGAAAAGAGGTTGCTGGAGCC

AAGCCTCACATTACAGCCGCAGAGGGGAAGCTCCGCGACATGATCGTCGGTCTGGACAAT

GTGGTCCAGAAGGTCCAGGCTGCTCAGTCCGAGGCTAAGGTGGTGTCGCAGTACCATGAG

TTGGTGGTCCAAGCCCGGGATGACTTCAGACGAGAACTAGAGAGTATCACTCCAGAGGTC

CTTCCTGGGTGGAAAGGGATGAGTATTTCTGACCTAGCTGACAAGCTGTCTACCGATGAC

CTGAACTCTCTGATTGCGCATGCACACCGTCGCATTGACCAGCTGAACAGAGAGCTAGCA

GAGCAGAAGGCCACAGAGAAGCAGCATATCGCGTTGGCCTTGGAGAAACAGAAGCTGGAA

GAAAAGCGGGCGTTTGATTCTGCAGTAGCAAAGGCATTAGAGCATCACCGAAGCGAAGTG

CAGGCTGAGCAGGACCGAAAGGTGGAGGAAGTCAGAGATGCTATGGAGAATGAGATGAGG

ACCCAACTCCGCCGCCAGGCGGCCGCGCACACTGACCACTTACGAGATGTCCTCAGGGTG

CAAGAACAAGAGCTCAAGTTTGAGTTTGAGCAGGACCTGTCTGAGAAACTCTCGGAACAA

GAGTTAGAGTTCCGTCGCCTCAGTCAAGAGCAAGTTGACAACTTCACCCTGGACATCAAT

ACGGCCTATGCTAGACTGAGAGGAATCGAGCAGGCTGTGCAGAGCCACGCAGTTGCTGAA

GAAGAAGCCAGAAAGGCCCACCAGCTCTGGCTTTCCGTGGAGGCACTGAAGTACAGCATG

AAGACCTCGTCCGCAGACACGCCCACCGTCCCGCTGGGCGCTGCGGTGGAGGCCATAAGA

GCCAACTGCTCTGACAGTGAATTTGCCCAAGCCTTAACTGCGGCCATCCCTCCAGAGTCC

CTGACCAGAGGGGTCTACAGTGAAGAGACCCTGAGGGCCCGTTTCTACGCTGTTCAGAAA

CAAGCCCGCAGGGTAGCCATGATTGACGAAACCAGAAACAGCTTATACCAGTACTTCCTG

TCCTACCTGCAGTCGCTGCTCCTGTTCCCACCTCAGCAGCTGAAGCCGCCAGCAGAGCTC

TACCCCGAGGACATGAGCACCTTTAAACTACTCTCATACGCCTCCTACTGCATCGAGCAC

GGTGACCTGGAGCTGGCGGCCAAGTTTGTCAACCAGCTGAAGGGGGAGTCCCGACGAGTG

GCCCAGGACTGGCTGAAGGAGGCCCGGATGACGCTAGAGACGAAGCAGATAGTAGAGATC

CTGACAGCGTATGCCAGTGCCGTGGGCATAGGGACCACTCAGGTACAGCCAGAG

>CL313.Contig2_A-W 19 2277 minus strand inner membrane protein, mitochondrial (mitofilin), isoform CRA_b [Homo sapiens]

ATGCTGCGGGCCTGTCAGCTGTCTGGCGTGAGCGCCGCCGCCCAGACTTGTCTCTGTGGA

AAGCTTGTCCTCCGTCCATTGCGACCATGCCGAAGATACTCAACTTCTAGCAGTTCTGGG

TTGACTGCAGGCAGGATCGCTGGAGCGGGTCTGCTGTTTGTTGGTGGTGGTATTGGAGGT

ACCATCTTATATGCCAAGTGGGACTCCCACTTCCGAGAAAGTGTAGAGAAAACCATTCCT

TACTCAGACAGACTCTTTGAGATGGTTCTTGGTTCTGCACCTTATAACGTTCCATTGCCA

AAGAAGCCGATTCAATCCGGTCCACTAAAAATCTCTAGTGTGTCAGAGGTAATGAAAGAA

TCCAAACAGCCTGCGTCACAGCTCCAAAAGCAGAAGGGAGACACTCCAGCCTCGACAACA

GCACCTACAGAGGCAGCTCAAATTATTTCTGCAGCAGGTGATACACTTTCCGTCCCAGCC

CCTGCGGTTCAACATGAGGAATCTGTAAAAACTGATCGCCCTGAAATTGGTGGAGGAAAA

CCCACACCTGTAACCTCAGAGGAAGCACCCTCATCCCCTGTAAGGGAGCGGCCACCCGAA

GAAGTCGCAGCTCGCCTGGCGCAGCAGGAAAAGCACGAGCAAGTTGAGATTGCGTCTCTA

GCCAAGAGCTTAGAAGATGCTCTGAGCCAAACTGCTTCCGTCACTCTGCAGGCTATTGCA

GCCCAGAACGCTGCCGTCCAGGCCGTGAATGCCCACTCCAGCGTCCTGAAGGCAGCCATG

GACGATGCCGAGGTTGCAGGTGAAAAGAAGTCTGCCCAGTGGCGCACGGTGGAAGGCGCC

CTGAAGGAGCGCAGGAAGGCCGTGGATGAAGCTGCTGATGCCCTTCTCAAAGCCAAAGAG

GAGTTAGAGAAGATGAAAAGTGTCATTGAAAATGCAAAGAGAAAAGAGGTTGCTGGAGCC

AAGCCTCACATTACAGCCGCAGAGGGGAAGCTCCGCGACATGATCGTCGGTCTGGACAAT

GTGGTCCAGAAGGTCCAGGCTGCTCAGTCCGAGGCTAAGGTGGTGTCGCAGTACCATGAG

TTGGTGGTCCAAGCCCGGGATGACTTCAGACGAGAACTAGAGAGTATCACTCCAGAGGTC

CTTCCTGGGTGGAAAGGGATGACTGACAAGCTGTCTACCGATGACCTGAACTCTCTGATT

GCGCATGCACACCGTCGCATTGACCAGCTGAACAGAGAGCTAGCAGAGCAGAAGGCCACA

GAGAAGCAGCATATCGCGTTGGCCTTGGAGAAACAGAAGCTGGAAGAAAAGCGGGCGTTT

GATTCTGCAGTAGCAAAGGCATTAGAGCATCACCGAAGCGAAGTGCAGGCTGAGCAGGAC

CGAAAGGTGGAGGAAGTCAGAGATGCTATGGAGAATGAGATGAGGACCCAACTCCGCCGC

CAGGCGGCCGCGCACACTGACCACTTACGAGATGTCCTCAGGGTGCAAGAACAAGAGCTC

AAGTTTGAGTTTGAGCAGGACCTGTCTGAGAAACTCTCGGAACAAGAGTTAGAGTTCCGT

CGCCTCAGTCAAGAGCAAGTTGACAACTTCACCCTGGACATCAATACGGCCTATGCTAGA

CTGAGAGGAATCGAGCAGGCTGTGCAGAGCCACGCAGTTGCTGAAGAAGAAGCCAGAAAG

GCCCACCAGCTCTGGCTTTCCGTGGAGGCACTGAAGTACAGCATGAAGACCTCGTCCGCA

GACACGCCCACCGTCCCGCTGGGCGCTGCGGTGGAGGCCATAAGAGCCAACTGCTCTGAC

AGTGAATTTGCCCAAGCCTTAACTGCGGCCATCCCTCCAGAGTCCCTGACCAGAGGGGTC

TACAGTGAAGAGACCCTGAGGGCCCGTTTCTACGCTGTTCAGAAACAAGCCCGCAGGGTA

GCCATGATTGACGAAACCAGAAACAGCTTATACCAGTACTTCCTGTCCTACCTGCAGTCG

CTGCTCCTGTTCCCACCTCAGCAGCTGAAGCCGCCAGCAGAGCTCTACCCCGAGGACATG

AGCACCTTTAAACTACTCTCATACGCCTCCTACTGCATCGAGCACGGTGACCTGGAGCTG

GCGGCCAAGTTTGTCAACCAGCTGAAGGGGGAGTCCCGACGAGTGGCCCAGGACTGGCTG

AAGGAGGCCCGGATGACGCTAGAGACGAAGCAGATAGTAGAGATCCTGACAGCGTATGCC

AGTGCCGTGGGCATAGGGACCACTCAGGTACAGCCAGAG

>CL313.Contig3_A-W 19 2244 minus strand mitochondrial inner membrane protein isoform 3 [Macaca mulatta]

ATGCTGCGGGCCTGTCAGCTGTCTGGCGTGAGCGCCGCCGCCCAGACTTGTCTCTGTGGA

AAGCTTGTCCTCCGTCCATTGCGACCATGCCGAAGATACTCAACTTCTAGCAGTTCTGGG

TTGACTGCAGGCAGGATCGCTGGAGCGGGTCTGCTGTTTGTTGGTGGTGGTATTGGAGGT

ACCATCTTATATGCCAAGTGGGACTCCCACTTCCGAGAAAGTGTAGAGAAAACCATTCCT

TACTCAGACAGACTCTTTGAGATGGTTCTTGGTTCTGCACCTTATAACGTTCCATTGCCA

AAGAAGCCGATTCAATCCGGTCCACTAAAAATCTCTAGTGTGTCAGAGGTAATGAAAGAA

TCCAAACAGCCTGCGTCACAGCTCCAAAAGCAGAAGGGAGACACTCCAGCCTCGACAACA

GCAGGTGATACACTTTCCGTCCCAGCCCCTGCGGTTCAACATGAGGAATCTGTAAAAACT

GATCGCCCTGAAATTGGTGGAGGAAAACCCACACCTGTAACCTCAGAGGAAGCACCCTCA

TCCCCTGTAAGGGAGCGGCCACCCGAAGAAGTCGCAGCTCGCCTGGCGCAGCAGGAAAAG

CACGAGCAAGTTGAGATTGCGTCTCTAGCCAAGAGCTTAGAAGATGCTCTGAGCCAAACT

GCTTCCGTCACTCTGCAGGCTATTGCAGCCCAGAACGCTGCCGTCCAGGCCGTGAATGCC

CACTCCAGCGTCCTGAAGGCAGCCATGGACGATGCCGAGGTTGCAGGTGAAAAGAAGTCT

GCCCAGTGGCGCACGGTGGAAGGCGCCCTGAAGGAGCGCAGGAAGGCCGTGGATGAAGCT

GCTGATGCCCTTCTCAAAGCCAAAGAGGAGTTAGAGAAGATGAAAAGTGTCATTGAAAAT

GCAAAGAGAAAAGAGGTTGCTGGAGCCAAGCCTCACATTACAGCCGCAGAGGGGAAGCTC

CGCGACATGATCGTCGGTCTGGACAATGTGGTCCAGAAGGTCCAGGCTGCTCAGTCCGAG

GCTAAGGTGGTGTCGCAGTACCATGAGTTGGTGGTCCAAGCCCGGGATGACTTCAGACGA

GAACTAGAGAGTATCACTCCAGAGGTCCTTCCTGGGTGGAAAGGGATGACTGACAAGCTG

TCTACCGATGACCTGAACTCTCTGATTGCGCATGCACACCGTCGCATTGACCAGCTGAAC

AGAGAGCTAGCAGAGCAGAAGGCCACAGAGAAGCAGCATATCGCGTTGGCCTTGGAGAAA

CAGAAGCTGGAAGAAAAGCGGGCGTTTGATTCTGCAGTAGCAAAGGCATTAGAGCATCAC

CGAAGCGAAGTGCAGGCTGAGCAGGACCGAAAGGTGGAGGAAGTCAGAGATGCTATGGAG

AATGAGATGAGGACCCAACTCCGCCGCCAGGCGGCCGCGCACACTGACCACTTACGAGAT

GTCCTCAGGGTGCAAGAACAAGAGCTCAAGTTTGAGTTTGAGCAGGACCTGTCTGAGAAA

CTCTCGGAACAAGAGTTAGAGTTCCGTCGCCTCAGTCAAGAGCAAGTTGACAACTTCACC

CTGGACATCAATACGGCCTATGCTAGACTGAGAGGAATCGAGCAGGCTGTGCAGAGCCAC

GCAGTTGCTGAAGAAGAAGCCAGAAAGGCCCACCAGCTCTGGCTTTCCGTGGAGGCACTG

AAGTACAGCATGAAGACCTCGTCCGCAGACACGCCCACCGTCCCGCTGGGCGCTGCGGTG

GAGGCCATAAGAGCCAACTGCTCTGACAGTGAATTTGCCCAAGCCTTAACTGCGGCCATC

CCTCCAGAGTCCCTGACCAGAGGGGTCTACAGTGAAGAGACCCTGAGGGCCCGTTTCTAC

GCTGTTCAGAAACAAGCCCGCAGGGTAGCCATGATTGACGAAACCAGAAACAGCTTATAC

CAGTACTTCCTGTCCTACCTGCAGTCGCTGCTCCTGTTCCCACCTCAGCAGCTGAAGCCG

CCAGCAGAGCTCTACCCCGAGGACATGAGCACCTTTAAACTACTCTCATACGCCTCCTAC

TGCATCGAGCACGGTGACCTGGAGCTGGCGGCCAAGTTTGTCAACCAGCTGAAGGGGGAG

TCCCGACGAGTGGCCCAGGACTGGCTGAAGGAGGCCCGGATGACGCTAGAGACGAAGCAG

ATAGTAGAGATCCTGACAGCGTATGCCAGTGCCGTGGGCATAGGGACCACTCAGGTACAG

CCAGAG

>CL313.Contig4_A-W 312 1412 minus strand PREDICTED: LOW QUALITY PROTEIN: mitochondrial inner membrane protein-like [Cavia porcellus]

TTAGCTGACAAGCTGTCTACCGATGACCTGAACTCTCTGATTGCGCATGCACACCGTCGC

ATTGACCAGCTGAACAGAGAGCTAGCAGAGCAGAAGGCCACAGAGAAGCAGCATATCGCG

TTGGCCTTGGAGAAACAGAAGCTGGAAGAAAAGCGGGCGTTTGATTCTGCAGTAGCAAAG

GCATTAGAGCATCACCGAAGCGAAGTGCAGGCTGAGCAGGACCGAAAGGTGGAGGAAGTC

AGAGATGCTATGGAGAATGAGATGAGGACCCAACTCCGCCGCCAGGCGGCCGCGCACACT

GACCACTTACGAGATGTCCTCAGGGTGCAAGAACAAGAGCTCAAGTTTGAGTTTGAGCAG

GACCTGTCTGAGAAACTCTCGGAACAAGAGTTAGAGTTCCGTCGCCTCAGTCAAGAGCAA

GTTGACAACTTCACCCTGGACATCAATACGGCCTATGCTAGACTGAGAGGAATCGAGCAG

GCTGTGCAGAGCCACGCAGTTGCTGAAGAAGAAGCCAGAAAGGCCCACCAGCTCTGGCTT

TCCGTGGAGGCACTGAAGTACAGCATGAAGACCTCGTCCGCAGACACGCCCACCGTCCCG

CTGGGCGCTGCGGTGGAGGCCATAAGAGCCAACTGCTCTGACAGTGAATTTGCCCAAGCC

TTAACTGCGGCCATCCCTCCAGAGTCCCTGACCAGAGGGGTCTACAGTGAAGAGACCCTG

AGGGCCCGTTTCTACGCTGTTCAGAAACAAGCCCGCAGGGTAGCCATGATTGACGAAACC

AGAAACAGCTTATACCAGTACTTCCTGTCCTACCTGCAGTCGCTGCTCCTGTTCCCACCT

CAGCAGCTGAAGCCGCCAGCAGAGCTCTACCCCGAGGACATGAGCACCTTTAAACTACTC

TCATACGCCTCCTACTGCATCGAGCACGGTGACCTGGAGCTGGCGGCCAAGTTTGTCAAC

CAGCTGAAGGGGGAGTCCCGACGAGTGGCCCAGGACTGGCTGAAGGAGGCCCGGATGACG

CTAGAGACGAAGCAGATAGTAGAGATCCTGACAGCGTATGCCAGTGCCGTGGGCATAGGG

ACCACTCAGGTACAGCCAGAG

>CL313.Contig5_A-W 19 2259 minus strand mitochondrial inner membrane protein isoform 3 [Homo sapiens]

ATGCTGCGGGCCTGTCAGCTGTCTGGCGTGAGCGCCGCCGCCCAGACTTGTCTCTGTGGA

AAGCTTGTCCTCCGTCCATTGCGACCATGCCGAAGATACTCAACTTCTAGCAGTTCTGGG

TTGACTGCAGGCAGGATCGCTGGAGCGGGTCTGCTGTTTGTTGGTGGTGGTATTGGAGGT

ACCATCTTATATGCCAAGTGGGACTCCCACTTCCGAGAAAGTGTAGAGAAAACCATTCCT

TACTCAGACAGACTCTTTGAGATGGTTCTTGGTTCTGCACCTTATAACGTTCCATTGCCA

AAGAAGCCGATTCAATCCGGTCCACTAAAAATCTCTAGTGTGTCAGAGGTAATGAAAGAA

TCCAAACAGCCTGCGTCACAGCTCCAAAAGCAGAAGGGAGACACTCCAGCCTCGACAACA

GCAGGTGATACACTTTCCGTCCCAGCCCCTGCGGTTCAACATGAGGAATCTGTAAAAACT

GATCGCCCTGAAATTGGTGGAGGAAAACCCACACCTGTAACCTCAGAGGAAGCACCCTCA

TCCCCTGTAAGGGAGCGGCCACCCGAAGAAGTCGCAGCTCGCCTGGCGCAGCAGGAAAAG

CACGAGCAAGTTGAGATTGCGTCTCTAGCCAAGAGCTTAGAAGATGCTCTGAGCCAAACT

GCTTCCGTCACTCTGCAGGCTATTGCAGCCCAGAACGCTGCCGTCCAGGCCGTGAATGCC

CACTCCAGCGTCCTGAAGGCAGCCATGGACGATGCCGAGGTTGCAGGTGAAAAGAAGTCT

GCCCAGTGGCGCACGGTGGAAGGCGCCCTGAAGGAGCGCAGGAAGGCCGTGGATGAAGCT

GCTGATGCCCTTCTCAAAGCCAAAGAGGAGTTAGAGAAGATGAAAAGTGTCATTGAAAAT

GCAAAGAGAAAAGAGGTTGCTGGAGCCAAGCCTCACATTACAGCCGCAGAGGGGAAGCTC

CGCGACATGATCGTCGGTCTGGACAATGTGGTCCAGAAGGTCCAGGCTGCTCAGTCCGAG

GCTAAGGTGGTGTCGCAGTACCATGAGTTGGTGGTCCAAGCCCGGGATGACTTCAGACGA

GAACTAGAGAGTATCACTCCAGAGGTCCTTCCTGGGTGGAAAGGGATGAGTATTTCTGAC

CTAGCTGACAAGCTGTCTACCGATGACCTGAACTCTCTGATTGCGCATGCACACCGTCGC

ATTGACCAGCTGAACAGAGAGCTAGCAGAGCAGAAGGCCACAGAGAAGCAGCATATCGCG

TTGGCCTTGGAGAAACAGAAGCTGGAAGAAAAGCGGGCGTTTGATTCTGCAGTAGCAAAG

GCATTAGAGCATCACCGAAGCGAAGTGCAGGCTGAGCAGGACCGAAAGGTGGAGGAAGTC

AGAGATGCTATGGAGAATGAGATGAGGACCCAACTCCGCCGCCAGGCGGCCGCGCACACT

GACCACTTACGAGATGTCCTCAGGGTGCAAGAACAAGAGCTCAAGTTTGAGTTTGAGCAG

GACCTGTCTGAGAAACTCTCGGAACAAGAGTTAGAGTTCCGTCGCCTCAGTCAAGAGCAA

GTTGACAACTTCACCCTGGACATCAATACGGCCTATGCTAGACTGAGAGGAATCGAGCAG

GCTGTGCAGAGCCACGCAGTTGCTGAAGAAGAAGCCAGAAAGGCCCACCAGCTCTGGCTT

TCCGTGGAGGCACTGAAGTACAGCATGAAGACCTCGTCCGCAGACACGCCCACCGTCCCG

CTGGGCGCTGCGGTGGAGGCCATAAGAGCCAACTGCTCTGACAGTGAATTTGCCCAAGCC

TTAACTGCGGCCATCCCTCCAGAGTCCCTGACCAGAGGGGTCTACAGTGAAGAGACCCTG

AGGGCCCGTTTCTACGCTGTTCAGAAACAAGCCCGCAGGGTAGCCATGATTGACGAAACC

AGAAACAGCTTATACCAGTACTTCCTGTCCTACCTGCAGTCGCTGCTCCTGTTCCCACCT

CAGCAGCTGAAGCCGCCAGCAGAGCTCTACCCCGAGGACATGAGCACCTTTAAACTACTC

TCATACGCCTCCTACTGCATCGAGCACGGTGACCTGGAGCTGGCGGCCAAGTTTGTCAAC

CAGCTGAAGGGGGAGTCCCGACGAGTGGCCCAGGACTGGCTGAAGGAGGCCCGGATGACG

CTAGAGACGAAGCAGATAGTAGAGATCCTGACAGCGTATGCCAGTGCCGTGGGCATAGGG

ACCACTCAGGTACAGCCAGAG

>CL313.Contig6_A-W 5 1504 minus strand mitochondrial inner membrane protein isoform 3 [Homo sapiens]

ATGCTGCGGGCCTGTCAGCTGTCTGGCGTGAGCGCCGCCGCCCAGACTTGTCTCTGTGGA

AAGCTTGTCCTCCGTCCATTGCGACCATGCCGAAGATACTCAACTTCTAGCAGTTCTGGG

TTGACTGCAGGCAGGATCGCTGGAGCGGGTCTGCTGTTTGTTGGTGGTGGTATTGGAGGT

ACCATCTTATATGCCAAGTGGGACTCCCACTTCCGAGAAAGTGTAGAGAAAACCATTCCT

TACTCAGACAGACTCTTTGAGATGGTTCTTGGTTCTGCACCTTATAACGTTCCATTGCCA

AAGAAGCCGATTCAATCCGGTCCACTAAAAATCTCTAGTGTGTCAGAGGTAATGAAAGAA

TCCAAACAGCCTGCGTCACAGCTCCAAAAGCAGAAGGGAGACACTCCAGCCTCGACAACA

GCAGGTGATACACTTTCCGTCCCAGCCCCTGCGGTTCAACATGAGGAATCTGTAAAAACT

GATCGCCCTGAAATTGGTGGAGGAAAACCCACACCTGTAACCTCAGAGGAAGCACCCTCA

TCCCCTGTAAGGGAGCGGCCACCCGAAGAAGTCGCAGCTCGCCTGGCGCAGCAGGAAAAG

CACGAGCAAGTTGAGATTGCGTCTCTAGCCAAGAGCTTAGAAGATGCTCTGAGCCAAACT

GCTTCCGTCACTCTGCAGGCTATTGCAGCCCAGAACGCTGCCGTCCAGGCCGTGAATGCC

CACTCCAGCGTCCTGAAGGCAGCCATGGACGATGCCGAGGTTGCAGGTGAAAAGAAGTCT

GCCCAGTGGCGCACGGTGGAAGGCGCCCTGAAGGAGCGCAGGAAGGCCGTGGATGAAGCT

GCTGATGCCCTTCTCAAAGCCAAAGAGGAGTTAGAGAAGATGAAAAGTGTCATTGAAAAT

GCAAAGAGAAAAGAGGTTGCTGGAGCCAAGCCTCACATTACAGCCGCAGAGGGGAAGCTC

CGCGACATGATCGTCGGTCTGGACAATGTGGTCCAGAAGGTCCAGGCTGCTCAGTCCGAG

GCTAAGGTGGTGTCGCAGTACCATGAGTTGGTGGTCCAAGCCCGGGATGACTTCAGACGA

GAACTAGAGAGTATCACTCCAGAGGTCCTTCCTGGGTGGAAAGGGATGAGTATTTCTGAC

CTAGCTGACAAGCTGTCTACCGATGACCTGAACTCTCTGATTGCGCATGCACACCGTCGC

ATTGACCAGCTGAACAGAGAGCTAGCAGAGCAGAAGGCCACAGAGAAGCAGCATATCGCG

TTGGCCTTGGAGAAACAGAAGCTGGAAGAAAAGCGGGCGTTTGATTCTGCAGTAGCAAAG

GCATTAGAGCATCACCGAAGCGAAGTGCAGGCTGAGCAGGACCGAAAGGTGGAGGAAGTC

AGAGATGCTATGGAGAATGAGATGAGGACCCAACTCCGCCGCCAGGCGGCCGCGCACACT

GACCACTTACGAGATGTCCTCAGGGTGCAAGAACAAGAGCTCAAGTTTGAGTTTGAGCAG

>CL316.Contig1_A-W 9 236 pol protein [Sus scrofa]

AAAAAAAAAACAACAATGGTGCAAATGGTGGTCAAGAATATCCTGAAAGATATTTTTCCA

AGATATGGCCTGCCTAAGGTAATAGGGTCTGATAATGGACCAGTGTTCATGGCCCAGGTA

AGTCAGGGGTTAGTCAGGACCCTGGGGATTAATTGGAAGTTACATTGTGCTTATAGACCC

CAGAGCTCAGGACAGGTAAAAAAATAAATAGAACCATTAAAGAGAACT

>CL316.Contig2_A-W 2288 2653 MLV-related proviral Env polyprotein OS=Mus musculus PE=2 SV=3

GTAATATTGTAGATGTTTCCCAGAGTGCTCTATCATGACACAGGATCATTTAAATTTCAA

ATAGGGGGACATACAACCCAGTTCTGCCAGAAACCTGTATCCCTCACTTTAGCCATCCTA

TTAGGAATAGGAGTTGCTGCAGGGTTGGGCACAGGAACCACTGCCCTAGTCCATGGGACA

CAACAAATGACCCAATTAGAAACAGCAATGGACCAAAACTTAAAGATATTAAAAACCTCC

ATCACAGCTTTACAGGAATCTTTAACCTCTCTCTCTGAAGTTGTTTTACAGAACAGGCGA

GGGTTAGAACTCCTTTTCATGAGAAAAGGGGGCCTTTATGCTGCATTGAGGGAAAAAATG

TTGTTT

>CL316.Contig4_A-W 5 658 minus strand env protein [Sus scrofa]

TCTGTAAATTATTGTGTAATGGTACAAATATTTCCCAGAGTGCTCTATCATGACGCAAAT

ACATTTGAAGATCAAATAAGGGGATATACCACCCGATTCCGCCGGGAACCTGTGTCCCTC

ACTTTAGCCATATTATTAGAAATAGGAGTTGCTACAGGAGTGGGTACAGGAACCGCTGCC

CTGGTCCATGGGTCTCAACAAATGGCCCAATTACAAGCAGCAATAGATCAGGACTTAAAA

ACATTAGAAACCTCCATCACAGCCTTACAAGAGTCCCTAACCTCTCTCTCTGAAGTTGTT

TTACAAAATAGACGAGGGTTAGACCTCCTCTTCATGAGGGAAGGGGGACTCTGTGCTGCC

CTAAGGGAGGAATGTTGCTTCTATGCTGATCATAGTGGGGTAGTCAAAGAATCAATGACT

AAATTAAGAAAACGCCTTGAGGAGCGACAACGAGAAAGAGAGGCTCAAAAAGGATGGTTT

GAGTCTTGGTTCACACAATCCCCCTGGCTAACTACACTCTTATCAGCCCTAGCTGGTCCT

TTAATAATCCTCATGTTATTGTTAACTGTGGGACCCTGTTTACTCAACCGTGTGGTCTCC

TTTCTCCAAGCTCAAATTGGACAAGTTAAACTTATGGTAATAAGACAACAGTAT

>CL316.Contig5_A-W 3 266 minus strand BC035947 protein [Mus musculus] >gi|187951869|gb|AAI38136.1| BC035947 protein [Mus musculus]

CAGGTGTTTGACAGTTCTGTAAACTATTGTGTCATGGTGCAAATACTCCCCAGGGTGCTT

TATCATGATGCAGAATAATTTGAAGGTCTAGTAGGGGGACAAACAACCCGTTTCCACCGG

GAATCAGTGTCCCTCACTCTAGCCATCCTATTAGGAATAGGAGCTGCTGCAGGAGTAGGT

ACGGGAGCAGCCGCTATGATCCGGGGGTCACAACAAATGGCCTAGTTAGAAACAGCAATA

GATCAGGACTTGAAGACATTAGAG

>CL317.Contig1_A-W 2 676 minus strand malonyl CoA:ACP acyltransferase [Mustela putorius furo]

GCTGTCTTCGTGGCTTCGCTGGCCGCTGTCGAGAAGCTCCATCACCTGCAGCCCGCGGTT

ATTGAAAATTGTATTGCTGCTGCTGGATTCAGTGTAGGAGAATTTGCAGCCCTAGTGTTT

GCTGGAGCCATGGAATTTTCTGAAGGTCTGTATGCAGTGAAGGTTCGAGCTGAGGCCATG

CAGGAAGCTTCAGAAGCTGTTCCCAGTGGGATGTTATCTGTCCTCGGCCAGCCTCGGTCC

AAGTTCGACTTTGCCTGTCTGGAAGCCCAGAAGCACTGCAAGTCATTGGGCATAGAGAGT

CCTGTGTGTGAGGTGGCCAACTATCTCTTTCCTGATTGCAGGGTGATTTCAGGACACCTA

GAGGCCCTGCGGTTTCTCCAGAAGAATTCCTCCAGGTACCACTTCAGACGCACCAAGATG

CTGCCTGTGAGCGGCGGCTTCCACACCCGCCTCATGGGGCCCGCTGTGCAGCCCCTGGCA

CAGGTTTTAAAGACAGTGGCCATCAGGAAGCCGCTGGTGGCCGTCTACTCTAACGTCAGC

GGGAGCAGGTACCTCCACCCCACGCACATCCGGCAGCTGCTGGGGCAGCAGGTGGTGTCC

CCAGTGAAGTGGGAGCAGACCATGCACGCCATATACGAGAGGAGGAGGGGCACCGAGTTC

CCCAGCACGTGGGAA

>CL317.Contig2_A-W 2 148 minus strand malonyl-CoA-acyl carrier protein transacylase, mitochondrial [Bos taurus]

GCTGTCTTCGTGGCTTCGCTGGCCGCTGTCGAGAAGCTCCATCACCTGCAGCCCGCGGTT

ATTGAAAATTGTATTGCTGCTGCTGGATTCAGTGTAGGAGAATTTGCAGCCCTAGTGTTT

GCTGGAGCCATGGAATTTTCTGAAGGC

>CL318.Contig1_A-W 33 359 minus strand PREDICTED: rab GTPase-binding effector protein 2-like [Equus caballus]

GCCTCCCTGTGTAGCCTGAGGACCGAGATGGACCGGATCCAGCAGGAACAGAGCCAGGCC

CAGCGCCAGGAAGGCCTCCTGCAACAGCCAGGCTCTGGCCGGACAGAAGAGGCCCAGCTC

AAAGACCTCCTCTCAGAGCAGAGGGCCAAGGTGCTGCGGCTGCAGGCTGAGCTGGAGACC

AGCGAACAGGTGCAGAGGGACTTCGTGCGACTGTCCCAGGCCCTGCAGGTGCGCCTAGAG

CGGATCCGCCAGGCTGGGACTCTGGAGCAGGTGCGCTGCATCCTGGATGAGGGATCCCTC

AAGGATGTGAGGGACATCAAGGACACC

>CL318.Contig2_A-W 80 352 minus strand PREDICTED: rab GTPase-binding effector protein 2-like [Equus caballus]

CAGGCCCAGCGCCAGGAAGGCCTCCTGCAACAGCCAGGCTCTGGCCGGACAGAAGAGGCC

CAGCTCAAAGACCTCCTCTCAGAGCAGAGGGCCAAGGTGCTGCGGCTGCAGGCTGAGCTG

GAGACCAGCGAACAGGTGCAGAGGGACTTCGTGCGACTGTCCCAGGCCCTGCAGGTGCGC

CTAGAGCGGATCCGCCAGGCTGGGACTCTGGAGCAGGTGCGCTGCATCCTGGATGAGGGA

TCCCTCAAGGATGTGAGGGACATCAAGGACACC

>CL319.Contig1_A-W 577 756 minus strand PREDICTED: leucine-rich repeat-containing protein 57 isoform 1 [Pan paniscus]

GTTCTTCGCCTGGAAGAGAACTGTCTTGAGCTCAGCATGCTTCCACAGAGCATCCTCAGT

GACTCACAGATCTGTCTGCTTGCTGTGGAAGGCAATCTTTTTGAAATAAAGAAACTTCGA

GAACTAGAAGGCTATGATAAGTACATGGAGAGGTTCACAGCCACCAAGAAGAAGTTTGCA

>CL319.Contig2_A-W 722 901 minus strand leucine-rich repeat-containing protein 57 isoform a [Mus musculus]

GTTCTTCGCCTGGAAGAGAACTGTCTTGAGCTCAGCATGCTTCCACAGAGCATCCTCAGT

GACTCACAGATCTGTCTGCTTGCTGTGGAAGGCAATCTTTTTGAAATAAAGAAACTTCGA

GAACTAGAAGGCTATGATAAGTACATGGAGAGGTTCACAGCCACCAAGAAGAAGTTTGCA

>CL319.Contig3_A-W 11 235 minus strand PREDICTED: leucine-rich repeat-containing protein 57 [Otolemur garnettii]

ATATCTCAGATCTCAGTAAAGATATCTTGCTGTCCTCGCCTTAAAGTTCTTCGCCTGGAA

GAGAACTGTCTTGAGCTCAGCATGCTTCCACAGAGCATCCTCAGTGACTCACAGATCTGT

CTGCTTGCTGTGGAAGGCAATCTTTTTGAAATAAAGAAACTTCGAGAACTAGAAGGCTAT

GATAAGTACATGGAGAGGTTCACAGCCACCAAGAAGAAGTTTGCA

>CL320.Contig1_A-W 2186 2926 minus strand SAFB-like, transcription modulator, isoform CRA_b [Homo sapiens] >gi|119597959|gb|EAW77553.1| SAFB-like, transcription modulator, isoform CRA_b [Homo sapiens]

AAGAAAAAAAGGAATTCTTTGAAACGCCCACATGATGTAGATCATAGGCGAGATGACCCT

TACTGGAGCGAGAATAAAAAGTTGTCTCTAGATACAGATGCACGATTCAGTCATGGATCT

GACTACTCTCGGCAACAGAACAGATTTAATGACTTTGATCACCGGGAAAGGGGCAGGTTT

CCCGAGAGTTCAGCTGTACAGTCTTCATCTTTTGAAAGGAGAGAACGCTTTGTAGGTCAA

AGTGAAGGGAAAAAAGCACGACCAACTGCACGAAGAGAAGATCCAAGCTTTGAAAGGTAT

CCCAAAAATTTCAGTGATTCCAGAAGAAATGAGCCTCCACCACCAAGAAACGAACTTAGA

GAAACAGACAGACGAGAGGTACGAGGGGAACGAGATGAGAGGAGAACAGTGATCATTCAT

GACAGGCCTGATATTGCTCACACTAGACACCCTCGAGAAGCAGGGCCCAATCCTTCTAGA

CCAACCTCCTGGAAAAGTGAAGGAAGCATGTCTGCAGATAAACGTGAGGCAAGAGTTGAA

AGGCCGGAACGGTCCGGTAGAGAAGTATCAGGACACAGTGTGAGAGGCGCTCCCCCTGGA

AATCGCAGTAGTGCCACAGGTTATGGAAGCAGAGAAGGAGACAGAGGAATCATCTCAGAT

CGAGGGAGTGGAGCCCAGCACTATCCTGAAGAACGACATGTGGTTGAACGCCATGGACGG

GACACAAGTGGACCAAGGAAA

>CL320.Contig2_A-W 1 2745 minus strand SAFB-like, transcription modulator, isoform CRA_b [Homo sapiens] >gi|119597959|gb|EAW77553.1| SAFB-like, transcription modulator, isoform CRA_b [Homo sapiens]

CCGAGGCCTGGGTGGAAGTTGGCGGTGCTGCCGCCGCCCTGCAGCCCACTCGCTGCCTCG

GCAGCGCGCTGCTCTTCTAAGATGGCTGCCGCTACCGGTGCTGTGGCAGCCTCGGCTGCC

CCGGGTCAGGCGGAAGGTAAAAAGATCACCGATCTGCGGGTCATCGATCTCAAGTCCGAG

CTGAAGCGGCGGAACTTAGACATCACCGGAGTCAAGACTGTGCTGGTCTCCCGACTCAAG

CAGGCTATTGAAGAGGAAGGAGGCGATCCAGATAATATTGAATTAACTGTTTCAACTGAT

ACTCCAAACAAGAAACCAACCAAAGGCAAAGGTAAAAAACAAGAAGCAGATGAGTTGAGT

GGAGATGCTTCTGTGGAAGATGATGCTTTTGTCAAGGACTGTGAATTGGAGAATCAAGAG

GCACATGAACAAGACGGAAATGATGAACTAAAGGACTCTGAAGAATTTGGTGAAAATGAA

GAAGACTGTGTGCATTCCAAGGAGTTACTTTCTGCAGAAGAAAACAAGAGAGCTCATGAA

TTAATAGAGGCAGAAGCAATAGAAGATATAGAAAAAGAGGACATCGAAAGTCAGGAAATT

GAAGCTCAAGAAGGTGAAGATGATACCTTTCTAACAGCCCAAGATGGTGAGGAAGAAGAA

AATGAGAAAGAAGGGAACCTAGCTGAGGCTGATCACACAGCTCATGAAGAGATGGAAGCT

AATGCGACTGTGAAAGAAGCTGAGGATGACAACATCTCGGTCACAATCCAGGCTGAAGAT

GCCATCACTCTGGATTTTGATGGTGATGACCTCCTAGAAACAGGTAAAAATGTGAAAATT

ACAGATTCTGAAGCAAGTAAGCCAAAAGATGGGCAGGACGCCATTGCACAGAGCCCGGAG

AAGGAAACCAAGGATTATGAGATGAATGCGAACCATAAAGATGGTAAGAAGGAAGACTGC

GTGAAGGGTGATCCTGTCGAGAAGGAAGCCAGAGAAAGTTCTAAGAAAGCAGAATCTGGA

GAAAAAGAAAAGGATACTTTGAAGAAAGGGCCCTCGTCTACTGGGGCCTCTGGTCAAGCA

AAGAGCTCTTCAAAGGAATCTAAAGACAGCAAGACATCATCTAAAGATGACAAAGGAAGT

ACAAGTAGTACTAGTGGTAGCAGTGGAAGCTCAACTAAAAATATCTGGGTTAGTGGACTT

TCTTCTAATACCAAAGCTGCTGATTTGAAGAACCTCTTTGGCAAATATGGAAAGGTTCTT

AGTGCAAAGGTAGTTACAAATGCTCGAAGTCCTGGGGCAAAATGCTATGGCATTGTAACT

ATGTCATCAAGCACAGAGGTGGCCAGATGTATTGCACATCTTCATCGCACTGAGCTGCAT

GGTCAGCTGATTTCTGTTGAAAAAGTAAAAGGTGATCCTTCTAAGAAAGAAATGAAGAAA

GAAAATGATGAAAAGAGTAGTTCAAGAAGTTCTGGAGATAAAAAAAATATGAGTGATAGA

AGTAGCAAGATACAAGCCTCTGTCAAAAAAGAAGAGAAAAGGTCATCTGAAAAATCTGAA

AAAAAAGAAAGCAAGGATGCTAAGAAAATAGAAGGTAAAGATGAGAAGAATGATAATGGA

GGAAGTGGCCAAACTTCAGAATCGATTAAAAAAAGTGAAGAAAAGAAGCGAATAAGTTCA

AAGAGTCCAGGACACATGGTAATATTAGACAAAACTAAGGGAGATCATTGTAGGCCATCA

AGAAGAGGAAGATATGAAAAAATTCATGGAAGAAGCAAGGAAAAGGAGAGAGCTAGCCTA

GATAAAAAAAGGGACAAAGACTACAGAAGGAAAGAGATTTTGCCTTTTGAAAAGATGAAG

GAACAACGATTGAGAGAACATTTAGTTCGTTTTGAAAGACTGCGACGAGCAATTTCCCTC

CGAAGGAACGTCGTAAGGAAGCTGAACGGATTGCTCGAGAACGAGAGGAACTCAGAAGGC

AGCAGCAGCAGCTACGTTATGAACAAGAAAAAAAGGAATTCTTTGAAACGCCCACATGAT

GTAGATCATAGGCGAGATGACCCTTACTGGAGCGAGAATAAAAAGTTGTCTCTAGATACA

GATGCACGATTCAGTCATGGATCTGACTACTCTCGGCAACAGAACAGATTTAATGACTTT

GATCACCGGGAAAGGGGCAGGTTTCCCGAGAGTTCAGCTGTACAGTCTTCATCTTTTGAA

AGGAGAGAACGCTTTGTAGGTCAAAGTGAAGGGAAAAAAGCACGACCAACTGCACGAAGA

GAAGATCCAAGCTTTGAAAGGTATCCCAAAAATTTCAGTGATTCCAGAAGAAATGAGCCT

CCACCACCAAGAAACGAACTTAGAGAAACAGACAGACGAGAGGTACGAGGGGAACGAGAT

GAGAGGAGAACAGTGATCATTCATGACAGGCCTGATATTGCTCACACTAGACACCCTCGA

GAAGCAGGGCCCAATCCTTCTAGACCAACCTCCTGGAAAAGTGAAGGAAGCATGTCTGCA

GATAAACGTGAGGCAAGAGTTGAAAGGCCGGAACGGTCCGGTAGAGAAGTATCAGGACAC

AGTGTGAGAGGCGCTCCCCCTGGAAATCGCAGTAGTGCCACAGGTTATGGAAGCAGAGAA

GGAGACAGAGGAATCATCTCAGATCGAGGGAGTGGAGCCCAGCACTATCCTGAAGAACGA

CATGTGGTTGAACGCCATGGACGGGACACAAGTGGACCAAGGAAA

>CL320.Contig3_A-W 1853 2593 minus strand SAFB-like transcription modulator isoform b [Macaca mulatta] >gi|383421657|gb|AFH34042.1| SAFB-like transcription modulator isoform b [Macaca mulatta] >gi|384949486|gb|AFI38348.1| SAFB-like transcription modulator isoform b [Macaca mulatta]

AAGAAAAAAAGGAATTCTTTGAAACGCCCACATGATGTAGATCATAGGCGAGATGACCCT

TACTGGAGCGAGAATAAAAAGTTGTCTCTAGATACAGATGCACGATTCAGTCATGGATCT

GACTACTCTCGGCAACAGAACAGATTTAATGACTTTGATCACCGGGAAAGGGGCAGGTTT

CCCGAGAGTTCAGCTGTACAGTCTTCATCTTTTGAAAGGAGAGAACGCTTTGTAGGTCAA

AGTGAAGGGAAAAAAGCACGACCAACTGCACGAAGAGAAGATCCAAGCTTTGAAAGGTAT

CCCAAAAATTTCAGTGATTCCAGAAGAAATGAGCCTCCACCACCAAGAAACGAACTTAGA

GAAACAGACAGACGAGAGGTACGAGGGGAACGAGATGAGAGGAGAACAGTGATCATTCAT

GACAGGCCTGATATTGCTCACACTAGACACCCTCGAGAAGCAGGGCCCAATCCTTCTAGA

CCAACCTCCTGGAAAAGTGAAGGAAGCATGTCTGCAGATAAACGTGAGGCAAGAGTTGAA

AGGCCGGAACGGTCCGGTAGAGAAGTATCAGGACACAGTGTGAGAGGCGCTCCCCCTGGA

AATCGCAGTAGTGCCACAGGTTATGGAAGCAGAGAAGGAGACAGAGGAATCATCTCAGAT

CGAGGGAGTGGAGCCCAGCACTATCCTGAAGAACGACATGTGGTTGAACGCCATGGACGG

GACACAAGTGGACCAAGGAAA

>CL320.Contig4_A-W 287 2617 minus strand SAFB-like transcription modulator isoform b [Homo sapiens]

GAAATTGAAGCTCAAGAAGGTGAAGATGATACCTTTCTAACAGCCCAAGATGGTGAGGAA

GAAGAAAATGAGAAAGAAGGGAACCTAGCTGAGGCTGATCACACAGCTCATGAAGAGATG

GAAGCTAATGCGACTGTGAAAGAAGCTGAGGATGACAACATCTCGGTCACAATCCAGGCT

GAAGATGCCATCACTCTGGATTTTGATGGTGATGACCTCCTAGAAACAGGTAAAAATGTG

AAAATTACAGATTCTGAAGCAAGTAAGCCAAAAGATGGGCAGGACGCCATTGCACAGAGC

CCGGAGAAGGAAACCAAGGATTATGAGATGAATGCGAACCATAAAGATGGTAAGAAGGAA

GACTGCGTGAAGGGTGATCCTGTCGAGAAGGAAGCCAGAGAAAGTTCTAAGAAAGCAGAA

TCTGGAGAAAAAGAAAAGGATACTTTGAAGAAAGGGCCCTCGTCTACTGGGGCCTCTGGT

CAAGCAAAGAGCTCTTCAAAGGAATCTAAAGACAGCAAGACATCATCTAAAGATGACAAA

GGAAGTACAAGTAGTACTAGTGGTAGCAGTGGAAGCTCAACTAAAAATATCTGGGTTAGT

GGACTTTCTTCTAATACCAAAGCTGCTGATTTGAAGAACCTCTTTGGCAAATATGGAAAG

GTTCTTAGTGCAAAGGTAGTTACAAATGCTCGAAGTCCTGGGGCAAAATGCTATGGCATT

GTAACTATGTCATCAAGCACAGAGGTGGCCAGATGTATTGCACATCTTCATCGCACTGAG

CTGCATGGTCAGCTGATTTCTGTTGAAAAAGTAAAAGGTGATCCTTCTAAGAAAGAAATG

AAGAAAGAAAATGATGAAAAGAGTAGTTCAAGAAGTTCTGGAGATAAAAAAAATATGAGT

GATAGAAGTAGCAAGATACAAGCCTCTGTCAAAAAAGAAGAGAAAAGGTCATCTGAAAAA

TCTGAAAAAAAAGAAAGCAAGGATGCTAAGAAAATAGAAGGTAAAGATGAGAAGAATGAT

AATGGAGGAAGTGGCCAAACTTCAGAATCGATTAAAAAAAGTGAAGAAAAGAAGCGAATA

AGTTCAAAGAGTCCAGGACACATGGTAATATTAGACAAAACTAAGGGAGATCATTGTAGG

CCATCAAGAAGAGGAAGATATGAAAAAATTCATGGAAGAAGCAAGGAAAAGGAGAGAGCT

AGCCTAGATAAAAAAAGGGACAAAGACTACAGAAGGAAAGAGATTTTGCCTTTTGAAAAG

ATGAAGGAACAACGATTGAGAGAACATTTAGTTCGTTTTGAAAGACTGCGACGAGCAATT

TCCCTCCGAAGACGAAGAGAGATTGCAGAAAGAGAGCGTCGAGAGCGAGAACGCATTAGA

ATAATTCGTGAACGGGAAGAACGGGAACGCTTACAGAGAGAGAGAGAGCGCCTAGAAATT

GAAAGGCAAAAACTAGAGAGAGAGAGAATGGAACGCGAACGCTTGGAAAGGGAACGCATT

CGTATTGAACAGGAACGTCGTAAGGAAGCTGAACGGATTGCTCGAGAACGAGAGGAACTC

AGAAGGCAGCAGCAGCAGCTACGTTATGAACAAGAAAAAAGGAATTCTTTGAAACGCCCA

CATGATGTAGATCATAGGCGAGATGACCCTTACTGGAGCGAGAATAAAAAGTTGTCTCTA

GATACAGATGCACGATTCAGTCATGGATCTGACTACTCTCGGCAACAGAACAGATTTAAT

GACTTTGATCACCGGGAAAGGGGCAGGTTTCCCGAGAGTTCAGCTGTACAGTCTTCATCT

TTTGAAAGGAGAGAACGCTTTGTAGGTCAAAGTGAAGGGAAAAAAGCACGACCAACTGCA

CGAAGAGAAGATCCAAGCTTTGAAAGGTATCCCAAAAATTTCAGTGATTCCAGAAGAAAT

GAGCCTCCACCACCAAGAAACGAACTTAGAGAAACAGACAGACGAGAGGTACGAGGGGAA

CGAGATGAGAGGAGAACAGTGATCATTCATGACAGGCCTGATATTGCTCACACTAGACAC

CCTCGAGAAGCAGGGCCCAATCCTTCTAGACCAACCTCCTGGAAAAGTGAAGGAAGCATG

TCTGCAGATAAACGTGAGGCAAGAGTTGAAAGGCCGGAACGGTCCGGTAGAGAAGTATCA

GGACACAGTGTGAGAGGCGCTCCCCCTGGAAATCGCAGTAGTGCCACAGGTTATGGAAGC

AGAGAAGGAGACAGAGGAATCATCTCAGATCGAGGGAGTGGAGCCCAGCACTATCCTGAA

GAACGACATGTGGTTGAACGCCATGGACGGGACACAAGTGGACCAAGGAAA

>CL320.Contig5_A-W 1615 2436 minus strand SAFB-like transcription modulator isoform b [Macaca mulatta] >gi|383421657|gb|AFH34042.1| SAFB-like transcription modulator isoform b [Macaca mulatta] >gi|384949486|gb|AFI38348.1| SAFB-like transcription modulator isoform b [Macaca mulatta]

AAGGAACGTCGTAAGGAAGCTGAACGGATTGCTCGAGAACGAGAGGAACTCAGAAGGCAG

CAGCAGCAGCTACGTTATGAACAAGAAAAAAGGAATTCTTTGAAACGCCCACATGATGTA

GATCATAGGCGAGATGACCCTTACTGGAGCGAGAATAAAAAGTTGTCTCTAGATACAGAT

GCACGATTCAGTCATGGATCTGACTACTCTCGGCAACAGAACAGATTTAATGACTTTGAT

CACCGGGAAAGGGGCAGGTTTCCCGAGAGTTCAGCTGTACAGTCTTCATCTTTTGAAAGG

AGAGAACGCTTTGTAGGTCAAAGTGAAGGGAAAAAAGCACGACCAACTGCACGAAGAGAA

GATCCAAGCTTTGAAAGGTATCCCAAAAATTTCAGTGATTCCAGAAGAAATGAGCCTCCA

CCACCAAGAAACGAACTTAGAGAAACAGACAGACGAGAGGTACGAGGGGAACGAGATGAG

AGGAGAACAGTGATCATTCATGACAGGCCTGATATTGCTCACACTAGACACCCTCGAGAA

GCAGGGCCCAATCCTTCTAGACCAACCTCCTGGAAAAGTGAAGGAAGCATGTCTGCAGAT

AAACGTGAGGCAAGAGTTGAAAGGCCGGAACGGTCCGGTAGAGAAGTATCAGGACACAGT

GTGAGAGGCGCTCCCCCTGGAAATCGCAGTAGTGCCACAGGTTATGGAAGCAGAGAAGGA

GACAGAGGAATCATCTCAGATCGAGGGAGTGGAGCCCAGCACTATCCTGAAGAACGACAT

GTGGTTGAACGCCATGGACGGGACACAAGTGGACCAAGGAAA

>CL320.Contig6_A-W 1 2226 minus strand SAFB-like, transcription modulator, isoform CRA_b [Homo sapiens] >gi|119597959|gb|EAW77553.1| SAFB-like, transcription modulator, isoform CRA_b [Homo sapiens]

CCTGGGTGGAAGTTGGCGGTGCTGCCGCCGCCCTGCAGCCCACTCGCTGCCTCGGCAGCG

CGCTGCTCTTCTAAGATGGCTGCCGCTACCGGTGCTGTGGCAGCCTCGGCTGCCCCGGGT

CAGGCGGAAGGTAAAAAGATCACCGATCTGCGGGTCATCGATCTCAAGTCCGAGCTGAAG

CGGCGGAACTTAGACATCACCGGAGTCAAGACTGTGCTGGTCTCCCGACTCAAGCAGGCT

ATTGAAGAGGAAGGAGGCGATCCAGATAATATTGAATTAACTGTTTCAACTGATACTCCA

AACAAGAAACCAACCAAAGGCAAAGGTAAAAAACAAGAAGCAGATGAGTTGAGTGGAGAT

GCTTCTGTGGAAGATGATGCTTTTGTCAAGGACTGTGAATTGGAGAATCAAGAGGCACAT

GAACAAGACGGAAATGATGAACTAAAGGACTCTGAAGAATTTGGTGAAAATGAAGAAGAC

TGTGTGCATTCCAAGGAGTTACTTTCTGCAGAAGAAAACAAGAGAGCTCATGAATTAATA

GAGGCAGAAGCAATAGAAGATATAGAAAAAGAGGACATCGAAAGTCAGGAAATTGAAGCT

CAAGAAGGTGAAGATGATACCTTTCTAACAGCCCAAGATGGTGAGGAAGAAGAAAATGAG

AAAGAAGGGAACCTAGCTGAGGCTGATCACACAGCTCATGAAGAGATGGAAGCTAATGCG

ACTGTGAAAGAAGCTGAGGATGACAACATCTCGGTCACAATCCAGGCTGAAGATGCCATC

ACTCTGGATTTTGATGGTGATGACCTCCTAGAAACAGGTAAAAATGTGAAAATTACAGAT

TCTGAAGCAAGTAAGCCAAAAGATGGGCAGGACGCCATTGCACAGAGCCCGGAGAAGGAA

ACCAAGGATTATGAGATGAATGCGAACCATAAAGATGGTAAGAAGGAAGACTGCGTGAAG

GGTGATCCTGTCGAGAAGGAAGCCAGAGAAAGTTCTAAGAAAGCAGAATCTGGAGAAAAA

GAAAAGGATACTTTGAAGAAAGGGCCCTCGTCTACTGGGGCCTCTGGTCAAGCAAAGAGC

TCTTCAAAGGAATCTAAAGACAGCAAGACATCATCTAAAGATGACAAAGGAAGTACAAGT

AGTACTAGTGGTAGCAGTGGAAGCTCAACTAAAAATATCTGGGTTAGTGGACTTTCTTCT

AATACCAAAGCTGCTGATTTGAAGAACCTCTTTGGCAAATATGGAAAGGTTCTTAGTGCA

AAGGTAGTTACAAATGCTCGAAGTCCTGGGGCAAAATGCTATGGCATTGTAACTATGTCA

TCAAGCACAGAGGTGGCCAGATGTATTGCACATCTTCATCGCACTGAGCTGCATGGTCAG

CTGATTTCTGTTGAAAAAGTAAAAGGTGATCCTTCTAAGAAAGAAATGAAGAAAGAAAAT

GATGAAAAGAGTAGTTCAAGAAGTTCTGGAGATAAAAAAAATATGAGTGATAGAAGTAGC

AAGATACAAGCCTCTGTCAAAAAAGAAGAGAAAAGGTCATCTGAAAAATCTGAAAAAAAA

GAAAGCAAGGATGCTAAGAAAATAGAAGGTAAAGATGAGAAGAATGATAATGGAGGAAGT

GGCCAAACTTCAGAATCGATTAAAAAAAGTGAAGAAAAGAAGCGAATAAGTTCAAAGAGT

CCAGGACACATGGTAATATTAGACAAAACTAAGGGAGATCATTGTAGGCCATCAAGAAGA

GGAAGATATGAAAAAATTCATGGAAGAAGCAAGGAAAAGGAGAGAGCTAGCCTAGATAAA

AAAAGGGACAAAGACTACAGAAGGAAAGAGATTTTGCCTTTTGAAAAGATGAAGGAACAA

CGATTGAGAGAACATTTAGTTCGTTTTGAAAGACTGCGACGAGCAATTTCCCTCCGAAGA

CGAAGAGAGATTGCAGAAAGAGAGCGTCGAGAGCGAGAACGCATTAGAATAATTCGTGAA

CGGGAAGAACGGGAACGCTTACAGAGAGAGAGAGAGCGCCTAGAAATTGAAAGGCAAAAA

CTAGAGAGAGAGAGAATGGAACGCGAACGCTTGGAAAGGGAACGCATTCGTATTGAACAG

GAACGTCGTAAGGAAGCTGAACGGATTGCTCGAGAACGAGAGGAACTCAGAAGGCAGCAG

CAGCAGCTACGTTATGAACAAGAAAAAAGGAATTCTTTGAAACGCCCACATGATGTAGAT

CATAGG

>CL320.Contig7_A-W 1917 2045 minus strand SAFB-like, transcription modulator, isoform CRA_b [Homo sapiens] >gi|119597959|gb|EAW77553.1| SAFB-like, transcription modulator, isoform CRA_b [Homo sapiens]

AAGGAACGTCGTAAGGAAGCTGAACGGATTGCTCGAGAACGAGAGGAACTCAGAAGGCAG

CAGCAGCAGCTACGTTATGAACAAGAAAAAAGGAATTCTTTGAAACGCCCACATGATGTA

GATCATAGG

>CL320.Contig8_A-W 287 1924 minus strand SAFB-like transcription modulator isoform b [Macaca mulatta] >gi|383421657|gb|AFH34042.1| SAFB-like transcription modulator isoform b [Macaca mulatta] >gi|384949486|gb|AFI38348.1| SAFB-like transcription modulator isoform b [Macaca mulatta]

GAAATTGAAGCTCAAGAAGGTGAAGATGATACCTTTCTAACAGCCCAAGATGGTGAGGAA

GAAGAAAATGAGAAAGAAGGGAACCTAGCTGAGGCTGATCACACAGCTCATGAAGAGATG

GAAGCTAATGCGACTGTGAAAGAAGCTGAGGATGACAACATCTCGGTCACAATCCAGGCT

GAAGATGCCATCACTCTGGATTTTGATGGTGATGACCTCCTAGAAACAGGTAAAAATGTG

AAAATTACAGATTCTGAAGCAAGTAAGCCAAAAGATGGGCAGGACGCCATTGCACAGAGC

CCGGAGAAGGAAACCAAGGATTATGAGATGAATGCGAACCATAAAGATGGTAAGAAGGAA

GACTGCGTGAAGGGTGATCCTGTCGAGAAGGAAGCCAGAGAAAGTTCTAAGAAAGCAGAA

TCTGGAGAAAAAGAAAAGGATACTTTGAAGAAAGGGCCCTCGTCTACTGGGGCCTCTGGT

CAAGCAAAGAGCTCTTCAAAGGAATCTAAAGACAGCAAGACATCATCTAAAGATGACAAA

GGAAGTACAAGTAGTACTAGTGGTAGCAGTGGAAGCTCAACTAAAAATATCTGGGTTAGT

GGACTTTCTTCTAATACCAAAGCTGCTGATTTGAAGAACCTCTTTGGCAAATATGGAAAG

GTTCTTAGTGCAAAGGTAGTTACAAATGCTCGAAGTCCTGGGGCAAAATGCTATGGCATT

GTAACTATGTCATCAAGCACAGAGGTGGCCAGATGTATTGCACATCTTCATCGCACTGAG

CTGCATGGTCAGCTGATTTCTGTTGAAAAAGTAAAAGGTGATCCTTCTAAGAAAGAAATG

AAGAAAGAAAATGATGAAAAGAGTAGTTCAAGAAGTTCTGGAGATAAAAAAAATATGAGT

GATAGAAGTAGCAAGATACAAGCCTCTGTCAAAAAAGAAGAGAAAAGGTCATCTGAAAAA

TCTGAAAAAAAAGAAAGCAAGGATGCTAAGAAAATAGAAGGTAAAGATGAGAAGAATGAT

AATGGAGGAAGTGGCCAAACTTCAGAATCGATTAAAAAAAGTGAAGAAAAGAAGCGAATA

AGTTCAAAGAGTCCAGGACACATGGTAATATTAGACAAAACTAAGGGAGATCATTGTAGG

CCATCAAGAAGAGGAAGATATGAAAAAATTCATGGAAGAAGCAAGGAAAAGGAGAGAGCT

AGCCTAGATAAAAAAAGGGACAAAGACTACAGAAGGAAAGAGATTTTGCCTTTTGAAAAG

ATGAAGGAACAACGATTGAGAGAACATTTAGTTCGTTTTGAAAGACTGCGACGAGCAATT

TCCCTCCGAAGACGAAGAGAGATTGCAGAAAGAGAGCGTCGAGAGCGAGAACGCATTAGA

ATAATTCGTGAACGGGAAGAACGGGAACGCTTACAGAGAGAGAGAGAGCGCCTAGAAATT

GAAAGGCAAAAACTAGAGAGAGAGAGAATGGAACGCGAACGCTTGGAAAGGGAACGCATT

CGTATTGAACAGGAACGTCGTAAGGAAGCTGAACGGATTGCTCGAGAACGAGAGGAACTC

AGAAGGCAGCAGCAGCAGCTACGTTATGAACAAGAAAAAAGGAATTCTTTGAAACGCCCA

CATGATGTAGATCATAGG

>CL320.Contig9_A-W 61 1899 minus strand SAFB-like transcription modulator isoform b [Macaca mulatta] >gi|383421657|gb|AFH34042.1| SAFB-like transcription modulator isoform b [Macaca mulatta] >gi|384949486|gb|AFI38348.1| SAFB-like transcription modulator isoform b [Macaca mulatta]

AAGGACTGTGAATTGGAGAATCAAGAGGCACATGAACAAGACGGAAATGATGAACTAAAG

GACTCTGAAGAATTTGGTGAAAATGAAGAAGACTGTGTGCATTCCAAGGAGTTACTTTCT

GCAGAAGAAAACAAGAGAGCTCATGAATTAATAGAGGCAGAAGCAATAGAAGATATAGAA

AAAGAGGACATCGAAAGTCAGGAAATTGAAGCTCAAGAAGGTGAAGATGATACCTTTCTA

ACAGCCCAAGATGGTGAGGAAGAAGAAAATGAGAAAGAAGGGAACCTAGCTGAGGCTGAT

CACACAGCTCATGAAGAGATGGAAGCTAATGCGACTGTGAAAGAAGCTGAGGATGACAAC

ATCTCGGTCACAATCCAGGCTGAAGATGCCATCACTCTGGATTTTGATGGTGATGACCTC

CTAGAAACAGGTAAAAATGTGAAAATTACAGATTCTGAAGCAAGTAAGCCAAAAGATGGG

CAGGACGCCATTGCACAGAGCCCGGAGAAGGAAACCAAGGATTATGAGATGAATGCGAAC

CATAAAGATGGTAAGAAGGAAGACTGCGTGAAGGGTGATCCTGTCGAGAAGGAAGCCAGA

GAAAGTTCTAAGAAAGCAGAATCTGGAGAAAAAGAAAAGGATACTTTGAAGAAAGGGCCC

TCGTCTACTGGGGCCTCTGGTCAAGCAAAGAGCTCTTCAAAGGAATCTAAAGACAGCAAG

ACATCATCTAAAGATGACAAAGGAAGTACAAGTAGTACTAGTGGTAGCAGTGGAAGCTCA

ACTAAAAATATCTGGGTTAGTGGACTTTCTTCTAATACCAAAGCTGCTGATTTGAAGAAC

CTCTTTGGCAAATATGGAAAGGTTCTTAGTGCAAAGGTAGTTACAAATGCTCGAAGTCCT

GGGGCAAAATGCTATGGCATTGTAACTATGTCATCAAGCACAGAGGTGGCCAGATGTATT

GCACATCTTCATCGCACTGAGCTGCATGGTCAGCTGATTTCTGTTGAAAAAGTAAAAGGT

GATCCTTCTAAGAAAGAAATGAAGAAAGAAAATGATGAAAAGAGTAGTTCAAGAAGTTCT

GGAGATAAAAAAAATATGAGTGATAGAAGTAGCAAGATACAAGCCTCTGTCAAAAAAGAA

GAGAAAAGGTCATCTGAAAAATCTGAAAAAAAAGAAAGCAAGGATGCTAAGAAAATAGAA

GGTAAAGATGAGAAGAATGATAATGGAGGAAGTGGCCAAACTTCAGAATCGATTAAAAAA

AGTGAAGAAAAGAAGCGAATAAGTTCAAAGAGTCCAGGACACATGGTAATATTAGACAAA

ACTAAGGGAGATCATTGTAGGCCATCAAGAAGAGGAAGATATGAAAAAATTCATGGAAGA

AGCAAGGAAAAGGAGAGAGCTAGCCTAGATAAAAAAAGGGACAAAGACTACAGAAGGAAA

GAGATTTTGCCTTTTGAAAAGATGAAGGAACAACGATTGAGAGAACATTTAGTTCGTTTT

GAAAGACTGCGACGAGCAATTTCCCTCCGAAGACGAAGAGAGATTGCAGAAAGAGAGCGT

CGAGAGCGAGAACGCATTAGAATAATTCGTGAACGGGAAGAACGGGAACGCTTACAGAGA

GAGAGAGAGCGCCTAGAAATTGAAAGGCAAAAACTAGAGAGAGAGAGAATGGAACGCGAA

CGCTTGGAAAGGGAACGCATTCGTATTGAACAGGAACGTCGTAAGGAAGCTGAACGGATT

GCTCGAGAACGAGAGGAACTCAGAAGGCAGCAGCAGCAGCTACGTTATGAACAAGAAAAA

AGGAATTCTTTGAAACGCCCACATGATGTAGATCATAGG

>CL320.Contig10_A-W 1615 1743 minus strand SAFB-like transcription modulator isoform b [Macaca mulatta] >gi|383421657|gb|AFH34042.1| SAFB-like transcription modulator isoform b [Macaca mulatta] >gi|384949486|gb|AFI38348.1| SAFB-like transcription modulator isoform b [Macaca mulatta]

AAGGAACGTCGTAAGGAAGCTGAACGGATTGCTCGAGAACGAGAGGAACTCAGAAGGCAG

CAGCAGCAGCTACGTTATGAACAAGAAAAAAGGAATTCTTTGAAACGCCCACATGATGTA

GATCATAGG

>CL320.Contig11_A-W 1 2598 SAFB-like, transcription modulator, isoform CRA_b [Homo sapiens] >gi|119597959|gb|EAW77553.1| SAFB-like, transcription modulator, isoform CRA_b [Homo sapiens]

CCGAGGCCTGGGTGGAAGTTGGCGGTGCTGCCGCCGCCCTGCAGCCCACTCGCTGCCTCG

GCAGCGCGCTGCTCTTCTAAGATGGCTGCCGCTACCGGTGCTGTGGCAGCCTCGGCTGCC

CCGGGTCAGGCGGAAGGTAAAAAGATCACCGATCTGCGGGTCATCGATCTCAAGTCCGAG

CTGAAGCGGCGGAACTTAGACATCACCGGAGTCAAGACTGTGCTGGTCTCCCGACTCAAG

CAGGCTATTGAAGAGGAAGGAGGCGATCCAGATAATATTGAATTAACTGTTTCAACTGAT

ACTCCAAACAAGAAACCAACCAAAGGCAAAGGTAAAAAACAAGAAGCAGATGAGTTGAGT

GGAGATGCTTCTGTGGAAGATGATGCTTTTGTCAAGGAAATTGAAGCTCAAGAAGGTGAA

GATGATACCTTTCTAACAGCCCAAGATGGTGAGGAAGAAGAAAATGAGAAAGATATAGCA

GGTTCTGGTGATGGTACACAAGAAGTATCTAAACCTCTTCCTTCAGAAGGGAACCTAGCT

GAGGCTGATCACACAGCTCATGAAGAGATGGAAGCTAATGCGACTGTGAAAGAAGCTGAG

GATGACAACATCTCGGTCACAATCCAGGCTGAAGATGCCATCACTCTGGATTTTGATGGT

GATGACCTCCTAGAAACAGGTAAAAATGTGAAAATTACAGATTCTGAAGCAAGTAAGCCA

AAAGATGGGCAGGACGCCATTGCACAGAGCCCGGAGAAGGAAACCAAGGATTATGAGATG

AATGCGAACCATAAAGATGGTAAGAAGGAAGACTGCGTGAAGGGTGATCCTGTCGAGAAG

GAAGCCAGAGAAAGTTCTAAGAAAGCAGAATCTGGAGAAAAAGAAAAGGATACTTTGAAG

AAAGGGCCCTCGTCTACTGGGGCCTCTGGTCAAGCAAAGAGCTCTTCAAAGGAATCTAAA

GACAGCAAGACATCATCTAAAGATGACAAAGGAAGTACAAGTAGTACTAGTGGTAGCAGT

GGAAGCTCAACTAAAAATATCTGGGTTAGTGGACTTTCTTCTAATACCAAAGCTGCTGAT

TTGAAGAACCTCTTTGGCAAATATGGAAAGGTTCTTAGTGCAAAGGTAGTTACAAATGCT

CGAAGTCCTGGGGCAAAATGCTATGGCATTGTAACTATGTCATCAAGCACAGAGGTGGCC

AGATGTATTGCACATCTTCATCGCACTGAGCTGCATGGTCAGCTGATTTCTGTTGAAAAA

GTAAAAGGTGATCCTTCTAAGAAAGAAATGAAGAAAGAAAATGATGAAAAGAGTAGTTCA

AGAAGTTCTGGAGATAAAAAAAATATGAGTGATAGAAGTAGCAAGATACAAGCCTCTGTC

AAAAAAGAAGAGAAAAGGTCATCTGAAAAATCTGAAAAAAAAGAAAGCAAGGATGCTAAG

AAAATAGAAGGTAAAGATGAGAAGAATGATAATGGAGGAAGTGGCCAAACTTCAGAATCG

ATTAAAAAAAGTGAAGAAAAGAAGCGAATAAGTTCAAAGAGTCCAGGACACATGGTAATA

TTAGACAAAACTAAGGGAGATCATTGTAGGCCATCAAGAAGAGGAAGATATGAAAAAATT

CATGGAAGAAGCAAGGAAAAGGAGAGAGCTAGCCTAGATAAAAAAAGGGACAAAGACTAC

AGAAGGAAAGAGATTTTGCCTTTTGAAAAGATGAAGGAACAACGATTGAGAGAACATTTA

GTTCGTTTTGAAAGACTGCGACGAGCAATTTCCCTCCGAAGGAACGTCGTAAGGAAGCTG

AACGGATTGCTCGAGAACGAGAGGAACTCAGAAGGCAGCAGCAGCAGCTACGTTATGAAC

AAGAAAAAAAGGAATTCTTTGAAACGCCCACATGATGTAGATCATAGGCGAGATGACCCT

TACTGGAGCGAGAATAAAAAGTTGTCTCTAGATACAGATGCACGATTCAGTCATGGATCT

GACTACTCTCGGCAACAGAACAGATTTAATGACTTTGATCACCGGGAAAGGGGCAGGTTT

CCCGAGAGTTCAGCTGTACAGTCTTCATCTTTTGAAAGGAGAGAACGCTTTGTAGGTCAA

AGTGAAGGGAAAAAAGCACGACCAACTGCACGAAGAGAAGATCCAAGCTTTGAAAGGTAT

CCCAAAAATTTCAGTGATTCCAGAAGAAATGAGCCTCCACCACCAAGAAACGAACTTAGA

GAAACAGACAGACGAGAGGTACGAGGGGAACGAGATGAGAGGAGAACAGTGATCATTCAT

GACAGGCCTGATATTGCTCACACTAGACACCCTCGAGAAGCAGGGCCCAATCCTTCTAGA

CCAACCTCCTGGAAAAGTGAAGGAAGCATGTCTGCAGATAAACGTGAGGCAAGAGTTGAA

AGGCCGGAACGGTCCGGTAGAGAAGTATCAGGACACAGTGTGAGAGGCGCTCCCCCTGGA

AATCGCAGTAGTGCCACAGGTTATGGAAGCAGAGAAGGAGACAGAGGAATCATCTCAGAT

CGAGGGAGTGGAGCCCAGCACTATCCTGAAGAACGACATGTGGTTGAACGCCATGGACGG

GACACAAGTGGACCAAGG

>CL320.Contig12_A-W 1 2796 SAFB-like, transcription modulator, isoform CRA_b [Homo sapiens] >gi|119597959|gb|EAW77553.1| SAFB-like, transcription modulator, isoform CRA_b [Homo sapiens]

CCGAGGCCTGGGTGGAAGTTGGCGGTGCTGCCGCCGCCCTGCAGCCCACTCGCTGCCTCG

GCAGCGCGCTGCTCTTCTAAGATGGCTGCCGCTACCGGTGCTGTGGCAGCCTCGGCTGCC

CCGGGTCAGGCGGAAGGTAAAAAGATCACCGATCTGCGGGTCATCGATCTCAAGTCCGAG

CTGAAGCGGCGGAACTTAGACATCACCGGAGTCAAGACTGTGCTGGTCTCCCGACTCAAG

CAGGCTATTGAAGAGGAAGGAGGCGATCCAGATAATATTGAATTAACTGTTTCAACTGAT

ACTCCAAACAAGAAACCAACCAAAGGCAAAGGTAAAAAACAAGAAGCAGATGAGTTGAGT

GGAGATGCTTCTGTGGAAGATGATGCTTTTGTCAAGGACTGTGAATTGGAGAATCAAGAG

GCACATGAACAAGACGGAAATGATGAACTAAAGGACTCTGAAGAATTTGGTGAAAATGAA

GAAGACTGTGTGCATTCCAAGGAGTTACTTTCTGCAGAAGAAAACAAGAGAGCTCATGAA

TTAATAGAGGCAGAAGCAATAGAAGATATAGAAAAAGAGGACATCGAAAGTCAGGAAATT

GAAGCTCAAGAAGGTGAAGATGATACCTTTCTAACAGCCCAAGATGGTGAGGAAGAAGAA

AATGAGAAAGATATAGCAGGTTCTGGTGATGGTACACAAGAAGTATCTAAACCTCTTCCT

TCAGAAGGGAACCTAGCTGAGGCTGATCACACAGCTCATGAAGAGATGGAAGCTAATGCG

ACTGTGAAAGAAGCTGAGGATGACAACATCTCGGTCACAATCCAGGCTGAAGATGCCATC

ACTCTGGATTTTGATGGTGATGACCTCCTAGAAACAGGTAAAAATGTGAAAATTACAGAT

TCTGAAGCAAGTAAGCCAAAAGATGGGCAGGACGCCATTGCACAGAGCCCGGAGAAGGAA

ACCAAGGATTATGAGATGAATGCGAACCATAAAGATGGTAAGAAGGAAGACTGCGTGAAG

GGTGATCCTGTCGAGAAGGAAGCCAGAGAAAGTTCTAAGAAAGCAGAATCTGGAGAAAAA

GAAAAGGATACTTTGAAGAAAGGGCCCTCGTCTACTGGGGCCTCTGGTCAAGCAAAGAGC

TCTTCAAAGGAATCTAAAGACAGCAAGACATCATCTAAAGATGACAAAGGAAGTACAAGT

AGTACTAGTGGTAGCAGTGGAAGCTCAACTAAAAATATCTGGGTTAGTGGACTTTCTTCT

AATACCAAAGCTGCTGATTTGAAGAACCTCTTTGGCAAATATGGAAAGGTTCTTAGTGCA

AAGGTAGTTACAAATGCTCGAAGTCCTGGGGCAAAATGCTATGGCATTGTAACTATGTCA

TCAAGCACAGAGGTGGCCAGATGTATTGCACATCTTCATCGCACTGAGCTGCATGGTCAG

CTGATTTCTGTTGAAAAAGTAAAAGGTGATCCTTCTAAGAAAGAAATGAAGAAAGAAAAT

GATGAAAAGAGTAGTTCAAGAAGTTCTGGAGATAAAAAAAATATGAGTGATAGAAGTAGC

AAGATACAAGCCTCTGTCAAAAAAGAAGAGAAAAGGTCATCTGAAAAATCTGAAAAAAAA

GAAAGCAAGGATGCTAAGAAAATAGAAGGTAAAGATGAGAAGAATGATAATGGAGGAAGT

GGCCAAACTTCAGAATCGATTAAAAAAAGTGAAGAAAAGAAGCGAATAAGTTCAAAGAGT

CCAGGACACATGGTAATATTAGACAAAACTAAGGGAGATCATTGTAGGCCATCAAGAAGA

GGAAGATATGAAAAAATTCATGGAAGAAGCAAGGAAAAGGAGAGAGCTAGCCTAGATAAA

AAAAGGGACAAAGACTACAGAAGGAAAGAGATTTTGCCTTTTGAAAAGATGAAGGAACAA

CGATTGAGAGAACATTTAGTTCGTTTTGAAAGACTGCGACGAGCAATTTCCCTCCGAAGG

AACGTCGTAAGGAAGCTGAACGGATTGCTCGAGAACGAGAGGAACTCAGAAGGCAGCAGC

AGCAGCTACGTTATGAACAAGAAAAAAAGGAATTCTTTGAAACGCCCACATGATGTAGAT

CATAGGCGAGATGACCCTTACTGGAGCGAGAATAAAAAGTTGTCTCTAGATACAGATGCA

CGATTCAGTCATGGATCTGACTACTCTCGGCAACAGAACAGATTTAATGACTTTGATCAC

CGGGAAAGGGGCAGGTTTCCCGAGAGTTCAGCTGTACAGTCTTCATCTTTTGAAAGGAGA

GAACGCTTTGTAGGTCAAAGTGAAGGGAAAAAAGCACGACCAACTGCACGAAGAGAAGAT

CCAAGCTTTGAAAGGTATCCCAAAAATTTCAGTGATTCCAGAAGAAATGAGCCTCCACCA

CCAAGAAACGAACTTAGAGAAACAGACAGACGAGAGGTACGAGGGGAACGAGATGAGAGG

AGAACAGTGATCATTCATGACAGGCCTGATATTGCTCACACTAGACACCCTCGAGAAGCA

GGGCCCAATCCTTCTAGACCAACCTCCTGGAAAAGTGAAGGAAGCATGTCTGCAGATAAA

CGTGAGGCAAGAGTTGAAAGGCCGGAACGGTCCGGTAGAGAAGTATCAGGACACAGTGTG

AGAGGCGCTCCCCCTGGAAATCGCAGTAGTGCCACAGGTTATGGAAGCAGAGAAGGAGAC

AGAGGAATCATCTCAGATCGAGGGAGTGGAGCCCAGCACTATCCTGAAGAACGACATGTG

GTTGAACGCCATGGACGGGACACAAGTGGACCAAGG

>CL320.Contig13_A-W 2042 2779 PREDICTED: SAFB-like transcription modulator-like [Macaca mulatta]

AAGAAAAAAAGGAATTCTTTGAAACGCCCACATGATGTAGATCATAGGCGAGATGACCCT

TACTGGAGCGAGAATAAAAAGTTGTCTCTAGATACAGATGCACGATTCAGTCATGGATCT

GACTACTCTCGGCAACAGAACAGATTTAATGACTTTGATCACCGGGAAAGGGGCAGGTTT

CCCGAGAGTTCAGCTGTACAGTCTTCATCTTTTGAAAGGAGAGAACGCTTTGTAGGTCAA

AGTGAAGGGAAAAAAGCACGACCAACTGCACGAAGAGAAGATCCAAGCTTTGAAAGGTAT

CCCAAAAATTTCAGTGATTCCAGAAGAAATGAGCCTCCACCACCAAGAAACGAACTTAGA

GAAACAGACAGACGAGAGGTACGAGGGGAACGAGATGAGAGGAGAACAGTGATCATTCAT

GACAGGCCTGATATTGCTCACACTAGACACCCTCGAGAAGCAGGGCCCAATCCTTCTAGA

CCAACCTCCTGGAAAAGTGAAGGAAGCATGTCTGCAGATAAACGTGAGGCAAGAGTTGAA

AGGCCGGAACGGTCCGGTAGAGAAGTATCAGGACACAGTGTGAGAGGCGCTCCCCCTGGA

AATCGCAGTAGTGCCACAGGTTATGGAAGCAGAGAAGGAGACAGAGGAATCATCTCAGAT

CGAGGGAGTGGAGCCCAGCACTATCCTGAAGAACGACATGTGGTTGAACGCCATGGACGG

GACACAAGTGGACCAAGG

>CL320.Contig14_A-W 2240 2980 SAFB-like, transcription modulator, isoform CRA_b [Homo sapiens] >gi|119597959|gb|EAW77553.1| SAFB-like, transcription modulator, isoform CRA_b [Homo sapiens]

AAGAAAAAAAGGAATTCTTTGAAACGCCCACATGATGTAGATCATAGGCGAGATGACCCT

TACTGGAGCGAGAATAAAAAGTTGTCTCTAGATACAGATGCACGATTCAGTCATGGATCT

GACTACTCTCGGCAACAGAACAGATTTAATGACTTTGATCACCGGGAAAGGGGCAGGTTT

CCCGAGAGTTCAGCTGTACAGTCTTCATCTTTTGAAAGGAGAGAACGCTTTGTAGGTCAA

AGTGAAGGGAAAAAAGCACGACCAACTGCACGAAGAGAAGATCCAAGCTTTGAAAGGTAT

CCCAAAAATTTCAGTGATTCCAGAAGAAATGAGCCTCCACCACCAAGAAACGAACTTAGA

GAAACAGACAGACGAGAGGTACGAGGGGAACGAGATGAGAGGAGAACAGTGATCATTCAT

GACAGGCCTGATATTGCTCACACTAGACACCCTCGAGAAGCAGGGCCCAATCCTTCTAGA

CCAACCTCCTGGAAAAGTGAAGGAAGCATGTCTGCAGATAAACGTGAGGCAAGAGTTGAA

AGGCCGGAACGGTCCGGTAGAGAAGTATCAGGACACAGTGTGAGAGGCGCTCCCCCTGGA

AATCGCAGTAGTGCCACAGGTTATGGAAGCAGAGAAGGAGACAGAGGAATCATCTCAGAT

CGAGGGAGTGGAGCCCAGCACTATCCTGAAGAACGACATGTGGTTGAACGCCATGGACGG

GACACAAGTGGACCAAGGAAA

>CL320.Contig15_A-W 82 1701 SAFB-like transcription modulator OS=Homo sapiens GN=SLTM PE=1 SV=2

ATGGCTGCCGCTACCGGTGCTGTGGCAGCCTCGGCTGCCCCGGGTCAGGCGGAAGGTAAA

AAGATCACCGATCTGCGGGTCATCGATCTCAAGTCCGAGCTGAAGCGGCGGAACTTAGAC

ATCACCGGAGTCAAGACTGTGCTGGTCTCCCGACTCAAGCAGGCTATTGAAGAGGAAGGA

GGCGATCCAGATAATATTGAATTAACTGTTTCAACTGATACTCCAAACAAGAAACCAACC

AAAGGCAAAGGTAAAAAACAAGAAGCAGATGAGTTGAGTGGAGATGCTTCTGTGGAAGAT

GATGCTTTTGTCAAGGAAATTGAAGCTCAAGAAGGTGAAGATGATACCTTTCTAACAGCC

CAAGATGGTGAGGAAGAAGAAAATGAGAAAGATATAGCAGGTTCTGGTGATGGTACACAA

GAAGTATCTAAACCTCTTCCTTCAGAAGGGAACCTAGCTGAGGCTGATCACACAGCTCAT

GAAGAGATGGAAGCTAATGCGACTGTGAAAGAAGCTGAGGATGACAACATCTCGGTCACA

ATCCAGGCTGAAGATGCCATCACTCTGGATTTTGATGGTGATGACCTCCTAGAAACAGGT

AAAAATGTGAAAATTACAGATTCTGAAGCAAGTAAGCCAAAAGATGGGCAGGACGCCATT

GCACAGAGCCCGGAGAAGGAAACCAAGGATTATGAGATGAATGCGAACCATAAAGATGGT

AAGAAGGAAGACTGCGTGAAGGGTGATCCTGTCGAGAAGGAAGCCAGAGAAAGTTCTAAG

AAAGCAGAATCTGGAGAAAAAGAAAAGGATACTTTGAAGAAAGGGCCCTCGTCTACTGGG

GCCTCTGGTCAAGCAAAGAGCTCTTCAAAGGAATCTAAAGACAGCAAGACATCATCTAAA

GATGACAAAGGAAGTACAAGTAGTACTAGTGGTAGCAGTGGAAGCTCAACTAAAAATATC

TGGGTTAGTGGACTTTCTTCTAATACCAAAGCTGCTGATTTGAAGAACCTCTTTGGCAAA

TATGGAAAGGTTCTTAGTGCAAAGGTAGTTACAAATGCTCGAAGTCCTGGGGCAAAATGC

TATGGCATTGTAACTATGTCATCAAGCACAGAGGTGGCCAGATGTATTGCACATCTTCAT

CGCACTGAGCTGCATGGTCAGCTGATTTCTGTTGAAAAAGTAAAAGGTGATCCTTCTAAG

AAAGAAATGAAGAAAGAAAATGATGAAAAGAGTAGTTCAAGAAGTTCTGGAGATAAAAAA

AATATGAGTGATAGAAGTAGCAAGATACAAGCCTCTGTCAAAAAAGAAGAGAAAAGGTCA

TCTGAAAAATCTGAAAAAAAAGAAAGCAAGGATGCTAAGAAAATAGAAGGTTCAAAGAGT

CCAGGACACATGGTAATATTAGACAAAACTAAGGGAGATCATTGTAGGCCATCAAGAAGA

GGAAGATATGAAAAAATTCATGGAAGAAGCAAGGAAAAGGAGAGAGCTAGCCTAGATAAA

AAAAGGGACAAAGACTACAGAAGGAAAGAGATTTTGCCTTTTGAAAAGATGAAGGAACAA

CGATTGAGAGAACATTTAGTTCGTTTTGAAAGACTGCGACGAGCAATTTCCCTCCGAAGG

>CL320.Contig16_A-W 82 1647 SAFB-like transcription modulator OS=Homo sapiens GN=SLTM PE=1 SV=2

ATGGCTGCCGCTACCGGTGCTGTGGCAGCCTCGGCTGCCCCGGGTCAGGCGGAAGGTAAA

AAGATCACCGATCTGCGGGTCATCGATCTCAAGTCCGAGCTGAAGCGGCGGAACTTAGAC

ATCACCGGAGTCAAGACTGTGCTGGTCTCCCGACTCAAGCAGGCTATTGAAGAGGAAGGA

GGCGATCCAGATAATATTGAATTAACTGTTTCAACTGATACTCCAAACAAGAAACCAACC

AAAGGCAAAGGTAAAAAACAAGAAGCAGATGAGTTGAGTGGAGATGCTTCTGTGGAAGAT

GATGCTTTTGTCAAGGAAATTGAAGCTCAAGAAGGTGAAGATGATACCTTTCTAACAGCC

CAAGATGGTGAGGAAGAAGAAAATGAGAAAGAAGGGAACCTAGCTGAGGCTGATCACACA

GCTCATGAAGAGATGGAAGCTAATGCGACTGTGAAAGAAGCTGAGGATGACAACATCTCG

GTCACAATCCAGGCTGAAGATGCCATCACTCTGGATTTTGATGGTGATGACCTCCTAGAA

ACAGGTAAAAATGTGAAAATTACAGATTCTGAAGCAAGTAAGCCAAAAGATGGGCAGGAC

GCCATTGCACAGAGCCCGGAGAAGGAAACCAAGGATTATGAGATGAATGCGAACCATAAA

GATGGTAAGAAGGAAGACTGCGTGAAGGGTGATCCTGTCGAGAAGGAAGCCAGAGAAAGT

TCTAAGAAAGCAGAATCTGGAGAAAAAGAAAAGGATACTTTGAAGAAAGGGCCCTCGTCT

ACTGGGGCCTCTGGTCAAGCAAAGAGCTCTTCAAAGGAATCTAAAGACAGCAAGACATCA

TCTAAAGATGACAAAGGAAGTACAAGTAGTACTAGTGGTAGCAGTGGAAGCTCAACTAAA

AATATCTGGGTTAGTGGACTTTCTTCTAATACCAAAGCTGCTGATTTGAAGAACCTCTTT

GGCAAATATGGAAAGGTTCTTAGTGCAAAGGTAGTTACAAATGCTCGAAGTCCTGGGGCA

AAATGCTATGGCATTGTAACTATGTCATCAAGCACAGAGGTGGCCAGATGTATTGCACAT

CTTCATCGCACTGAGCTGCATGGTCAGCTGATTTCTGTTGAAAAAGTAAAAGGTGATCCT

TCTAAGAAAGAAATGAAGAAAGAAAATGATGAAAAGAGTAGTTCAAGAAGTTCTGGAGAT

AAAAAAAATATGAGTGATAGAAGTAGCAAGATACAAGCCTCTGTCAAAAAAGAAGAGAAA

AGGTCATCTGAAAAATCTGAAAAAAAAGAAAGCAAGGATGCTAAGAAAATAGAAGGTTCA

AAGAGTCCAGGACACATGGTAATATTAGACAAAACTAAGGGAGATCATTGTAGGCCATCA

AGAAGAGGAAGATATGAAAAAATTCATGGAAGAAGCAAGGAAAAGGAGAGAGCTAGCCTA

GATAAAAAAAGGGACAAAGACTACAGAAGGAAAGAGATTTTGCCTTTTGAAAAGATGAAG

GAACAACGATTGAGAGAACATTTAGTTCGTTTTGAAAGACTGCGACGAGCAATTTCCCTC

CGAAGG

>CL320.Contig17_A-W 48 1550 PREDICTED: SAFB-like transcription modulator [Papio anubis]

AAGGACTGTGAATTGGAGAATCAAGAGGCACATGAACAAGACGGAAATGATGAACTAAAG

GACTCTGAAGAATTTGGTGAAAATGAAGAAGACTGTGTGCATTCCAAGGAGTTACTTTCT

GCAGAAGAAAACAAGAGAGCTCATGAATTAATAGAGGCAGAAGCAATAGAAGATATAGAA

AAAGAGGACATCGAAAGTCAGGAAATTGAAGCTCAAGAAGGTGAAGATGATACCTTTCTA

ACAGCCCAAGATGGTGAGGAAGAAGAAAATGAGAAAGATATAGCAGGTTCTGGTGATGGT

ACACAAGAAGTATCTAAACCTCTTCCTTCAGAAGGGAACCTAGCTGAGGCTGATCACACA

GCTCATGAAGAGATGGAAGCTAATGCGACTGTGAAAGAAGCTGAGGATGACAACATCTCG

GTCACAATCCAGGCTGAAGATGCCATCACTCTGGATTTTGATGGTGATGACCTCCTAGAA

ACAGGTAAAAATGTGAAAATTACAGATTCTGAAGCAAGTAAGCCAAAAGATGGGCAGGAC

GCCATTGCACAGAGCCCGGAGAAGGAAACCAAGGATTATGAGATGAATGCGAACCATAAA

GATGGTAAGAAGGAAGACTGCGTGAAGGGTGATCCTGTCGAGAAGGAAGCCAGAGAAAGT

TCTAAGAAAGCAGAATCTGGAGAAAAAGAAAAGGATACTTTGAAGAAAGGGCCCTCGTCT

ACTGGGGCCTCTGGTCAAGCAAAGAGCTCTTCAAAGGAATCTAAAGACAGCAAGACATCA

TCTAAAGATGACAAAGGAAGTACAAGTAGTACTAGTGGTAGCAGTGGAAGCTCAACTAAA

AATATCTGGGTTAGTGGACTTTCTTCTAATACCAAAGCTGCTGATTTGAAGAACCTCTTT

GGCAAATATGGAAAGGTTCTTAGTGCAAAGGTAGTTACAAATGCTCGAAGTCCTGGGGCA

AAATGCTATGGCATTGTAACTATGTCATCAAGCACAGAGGTGGCCAGATGTATTGCACAT

CTTCATCGCACTGAGCTGCATGGTCAGCTGATTTCTGTTGAAAAAGTAAAAGGTGATCCT

TCTAAGAAAGAAATGAAGAAAGAAAATGATGAAAAGAGTAGTTCAAGAAGTTCTGGAGAT

AAAAAAAATATGAGTGATAGAAGTAGCAAGATACAAGCCTCTGTCAAAAAAGAAGAGAAA

AGGTCATCTGAAAAATCTGAAAAAAAAGAAAGCAAGGATGCTAAGAAAATAGAAGGTTCA

AAGAGTCCAGGACACATGGTAATATTAGACAAAACTAAGGGAGATCATTGTAGGCCATCA

AGAAGAGGAAGATATGAAAAAATTCATGGAAGAAGCAAGGAAAAGGAGAGAGCTAGCCTA

GATAAAAAAAGGGACAAAGACTACAGAAGGAAAGAGATTTTGCCTTTTGAAAAGATGAAG

GAACAACGATTGAGAGAACATTTAGTTCGTTTTGAAAGACTGCGACGAGCAATTTCCCTC

CGA

>CL320.Contig18_A-W 191 1501 PREDICTED: SAFB-like transcription modulator-like [Macaca mulatta]

TTTACTTAGGAAATTGAAGCTCAAGAAGGTGAAGATGATACCTTTCTAACAGCCCAAGAT

GGTGAGGAAGAAGAAAATGAGAAAGATATAGCAGGTTCTGGTGATGGTACACAAGAAGTA

TCTAAACCTCTTCCTTCAGAAGGGAACCTAGCTGAGGCTGATCACACAGCTCATGAAGAG

ATGGAAGCTAATGCGACTGTGAAAGAAGCTGAGGATGACAACATCTCGGTCACAATCCAG

GCTGAAGATGCCATCACTCTGGATTTTGATGGTGATGACCTCCTAGAAACAGGTAAAAAT

GTGAAAATTACAGATTCTGAAGCAAGTAAGCCAAAAGATGGGCAGGACGCCATTGCACAG

AGCCCGGAGAAGGAAACCAAGGATTATGAGATGAATGCGAACCATAAAGATGGTAAGAAG

GAAGACTGCGTGAAGGGTGATCCTGTCGAGAAGGAAGCCAGAGAAAGTTCTAAGAAAGCA

GAATCTGGAGAAAAAGAAAAGGATACTTTGAAGAAAGGGCCCTCGTCTACTGGGGCCTCT

GGTCAAGCAAAGAGCTCTTCAAAGGAATCTAAAGACAGCAAGACATCATCTAAAGATGAC

AAAGGAAGTACAAGTAGTACTAGTGGTAGCAGTGGAAGCTCAACTAAAAATATCTGGGTT

AGTGGACTTTCTTCTAATACCAAAGCTGCTGATTTGAAGAACCTCTTTGGCAAATATGGA

AAGGTTCTTAGTGCAAAGGTAGTTACAAATGCTCGAAGTCCTGGGGCAAAATGCTATGGC

ATTGTAACTATGTCATCAAGCACAGAGGTGGCCAGATGTATTGCACATCTTCATCGCACT

GAGCTGCATGGTCAGCTGATTTCTGTTGAAAAAGTAAAAGGTGATCCTTCTAAGAAAGAA

ATGAAGAAAGAAAATGATGAAAAGAGTAGTTCAAGAAGTTCTGGAGATAAAAAAAATATG

AGTGATAGAAGTAGCAAGATACAAGCCTCTGTCAAAAAAGAAGAGAAAAGGTCATCTGAA

AAATCTGAAAAAAAAGAAAGCAAGGATGCTAAGAAAATAGAAGGTTCAAAGAGTCCAGGA

CACATGGTAATATTAGACAAAACTAAGGGAGATCATTGTAGGCCATCAAGAAGAGGAAGA

TATGAAAAAATTCATGGAAGAAGCAAGGAAAAGGAGAGAGCTAGCCTAGATAAAAAAAGG

GACAAAGACTACAGAAGGAAAGAGATTTTGCCTTTTGAAAAGATGAAGGAACAACGATTG

AGAGAACATTTAGTTCGTTTTGAAAGACTGCGACGAGCAATTTCCCTCCGA

>CL320.Contig19_A-W 48 1496 SAFB-like transcription modulator isoform b [Macaca mulatta] >gi|383421657|gb|AFH34042.1| SAFB-like transcription modulator isoform b [Macaca mulatta] >gi|384949486|gb|AFI38348.1| SAFB-like transcription modulator isoform b [Macaca mulatta]

AAGGACTGTGAATTGGAGAATCAAGAGGCACATGAACAAGACGGAAATGATGAACTAAAG

GACTCTGAAGAATTTGGTGAAAATGAAGAAGACTGTGTGCATTCCAAGGAGTTACTTTCT

GCAGAAGAAAACAAGAGAGCTCATGAATTAATAGAGGCAGAAGCAATAGAAGATATAGAA

AAAGAGGACATCGAAAGTCAGGAAATTGAAGCTCAAGAAGGTGAAGATGATACCTTTCTA

ACAGCCCAAGATGGTGAGGAAGAAGAAAATGAGAAAGAAGGGAACCTAGCTGAGGCTGAT

CACACAGCTCATGAAGAGATGGAAGCTAATGCGACTGTGAAAGAAGCTGAGGATGACAAC

ATCTCGGTCACAATCCAGGCTGAAGATGCCATCACTCTGGATTTTGATGGTGATGACCTC

CTAGAAACAGGTAAAAATGTGAAAATTACAGATTCTGAAGCAAGTAAGCCAAAAGATGGG

CAGGACGCCATTGCACAGAGCCCGGAGAAGGAAACCAAGGATTATGAGATGAATGCGAAC

CATAAAGATGGTAAGAAGGAAGACTGCGTGAAGGGTGATCCTGTCGAGAAGGAAGCCAGA

GAAAGTTCTAAGAAAGCAGAATCTGGAGAAAAAGAAAAGGATACTTTGAAGAAAGGGCCC

TCGTCTACTGGGGCCTCTGGTCAAGCAAAGAGCTCTTCAAAGGAATCTAAAGACAGCAAG

ACATCATCTAAAGATGACAAAGGAAGTACAAGTAGTACTAGTGGTAGCAGTGGAAGCTCA

ACTAAAAATATCTGGGTTAGTGGACTTTCTTCTAATACCAAAGCTGCTGATTTGAAGAAC

CTCTTTGGCAAATATGGAAAGGTTCTTAGTGCAAAGGTAGTTACAAATGCTCGAAGTCCT

GGGGCAAAATGCTATGGCATTGTAACTATGTCATCAAGCACAGAGGTGGCCAGATGTATT

GCACATCTTCATCGCACTGAGCTGCATGGTCAGCTGATTTCTGTTGAAAAAGTAAAAGGT

GATCCTTCTAAGAAAGAAATGAAGAAAGAAAATGATGAAAAGAGTAGTTCAAGAAGTTCT

GGAGATAAAAAAAATATGAGTGATAGAAGTAGCAAGATACAAGCCTCTGTCAAAAAAGAA

GAGAAAAGGTCATCTGAAAAATCTGAAAAAAAAGAAAGCAAGGATGCTAAGAAAATAGAA

GGTTCAAAGAGTCCAGGACACATGGTAATATTAGACAAAACTAAGGGAGATCATTGTAGG

CCATCAAGAAGAGGAAGATATGAAAAAATTCATGGAAGAAGCAAGGAAAAGGAGAGAGCT

AGCCTAGATAAAAAAAGGGACAAAGACTACAGAAGGAAAGAGATTTTGCCTTTTGAAAAG

ATGAAGGAACAACGATTGAGAGAACATTTAGTTCGTTTTGAAAGACTGCGACGAGCAATT

TCCCTCCGA

>CL320.Contig20_A-W 71 913 PREDICTED: SAFB-like transcription modulator [Papio anubis]

GAAAGCTCTTCAAAGGAATCTAAAGACAGCAAGACATCATCTAAAGATGACAAAGGAAGT

ACAAGTAGTACTAGTGGTAGCAGTGGAAGCTCAACTAAAAATATCTGGGTTAGTGGACTT

TCTTCTAATACCAAAGCTGCTGATTTGAAGAACCTCTTTGGCAAATATGGAAAGGTTCTT

AGTGCAAAGGTAGTTACAAATGCTCGAAGTCCTGGGGCAAAATGCTATGGCATTGTAACT

ATGTCATCAAGCACAGAGGTGGCCAGATGTATTGCACATCTTCATCGCACTGAGCTGCAT

GGTCAGCTGATTTCTGTTGAAAAAGTAAAAGGTGATCCTTCTAAGAAAGAAATGAAGAAA

GAAAATGATGAAAAGAGTAGTTCAAGAAGTTCTGGAGATAAAAAAAATATGAGTGATAGA

AGTAGCAAGATACAAGCCTCTGTCAAAAAAGAAGAGAAAAGGTCATCTGAAAAATCTGAA

AAAAAAGAAAGCAAGGATGCTAAGAAAATAGAAGGTAAAGATGAGAAGAATGATAATGGA

GGAAGTGGCCAAACTTCAGAATCGATTAAAAAAAGTGAAGAAAAGAAGCGAATAAGTTCA

AAGAGTCCAGGACACATGGTAATATTAGACAAAACTAAGGGAGATCATTGTAGGCCATCA

AGAAGAGGAAGATATGAAAAAATTCATGGAAGAAGCAAGGAAAAGGAGAGAGCTAGCCTA

GATAAAAAAAGGGACAAAGACTACAGAAGGAAAGAGATTTTGCCTTTTGAAAAGATGAAG

GAACAACGATTGAGAGAACATTTAGTTCGTTTTGAAAGACTGCGACGAGCAATTTCCCTC

CGA

>CL320.Contig21_A-W 71 832 PREDICTED: SAFB-like transcription modulator [Papio anubis]

GAAAGCTCTTCAAAGGAATCTAAAGACAGCAAGACATCATCTAAAGATGACAAAGGAAGT

ACAAGTAGTACTAGTGGTAGCAGTGGAAGCTCAACTAAAAATATCTGGGTTAGTGGACTT

TCTTCTAATACCAAAGCTGCTGATTTGAAGAACCTCTTTGGCAAATATGGAAAGGTTCTT

AGTGCAAAGGTAGTTACAAATGCTCGAAGTCCTGGGGCAAAATGCTATGGCATTGTAACT

ATGTCATCAAGCACAGAGGTGGCCAGATGTATTGCACATCTTCATCGCACTGAGCTGCAT

GGTCAGCTGATTTCTGTTGAAAAAGTAAAAGGTGATCCTTCTAAGAAAGAAATGAAGAAA

GAAAATGATGAAAAGAGTAGTTCAAGAAGTTCTGGAGATAAAAAAAATATGAGTGATAGA

AGTAGCAAGATACAAGCCTCTGTCAAAAAAGAAGAGAAAAGGTCATCTGAAAAATCTGAA

AAAAAAGAAAGCAAGGATGCTAAGAAAATAGAAGGTTCAAAGAGTCCAGGACACATGGTA

ATATTAGACAAAACTAAGGGAGATCATTGTAGGCCATCAAGAAGAGGAAGATATGAAAAA

ATTCATGGAAGAAGCAAGGAAAAGGAGAGAGCTAGCCTAGATAAAAAAAGGGACAAAGAC

TACAGAAGGAAAGAGATTTTGCCTTTTGAAAAGATGAAGGAACAACGATTGAGAGAACAT

TTAGTTCGTTTTGAAAGACTGCGACGAGCAATTTCCCTCCGA

>CL320.Contig22_A-W 2105 2842 SAFB-like, transcription modulator, isoform CRA_b [Homo sapiens] >gi|119597959|gb|EAW77553.1| SAFB-like, transcription modulator, isoform CRA_b [Homo sapiens]

AAGAAAAAAAGGAATTCTTTGAAACGCCCACATGATGTAGATCATAGGCGAGATGACCCT

TACTGGAGCGAGAATAAAAAGTTGTCTCTAGATACAGATGCACGATTCAGTCATGGATCT

GACTACTCTCGGCAACAGAACAGATTTAATGACTTTGATCACCGGGAAAGGGGCAGGTTT

CCCGAGAGTTCAGCTGTACAGTCTTCATCTTTTGAAAGGAGAGAACGCTTTGTAGGTCAA

AGTGAAGGGAAAAAAGCACGACCAACTGCACGAAGAGAAGATCCAAGCTTTGAAAGGTAT

CCCAAAAATTTCAGTGATTCCAGAAGAAATGAGCCTCCACCACCAAGAAACGAACTTAGA

GAAACAGACAGACGAGAGGTACGAGGGGAACGAGATGAGAGGAGAACAGTGATCATTCAT

GACAGGCCTGATATTGCTCACACTAGACACCCTCGAGAAGCAGGGCCCAATCCTTCTAGA

CCAACCTCCTGGAAAAGTGAAGGAAGCATGTCTGCAGATAAACGTGAGGCAAGAGTTGAA

AGGCCGGAACGGTCCGGTAGAGAAGTATCAGGACACAGTGTGAGAGGCGCTCCCCCTGGA

AATCGCAGTAGTGCCACAGGTTATGGAAGCAGAGAAGGAGACAGAGGAATCATCTCAGAT

CGAGGGAGTGGAGCCCAGCACTATCCTGAAGAACGACATGTGGTTGAACGCCATGGACGG

GACACAAGTGGACCAAGG

>CL320.Contig23_A-W 1988 2725 PREDICTED: SAFB-like transcription modulator-like [Macaca mulatta]

AAGAAAAAAAGGAATTCTTTGAAACGCCCACATGATGTAGATCATAGGCGAGATGACCCT

TACTGGAGCGAGAATAAAAAGTTGTCTCTAGATACAGATGCACGATTCAGTCATGGATCT

GACTACTCTCGGCAACAGAACAGATTTAATGACTTTGATCACCGGGAAAGGGGCAGGTTT

CCCGAGAGTTCAGCTGTACAGTCTTCATCTTTTGAAAGGAGAGAACGCTTTGTAGGTCAA

AGTGAAGGGAAAAAAGCACGACCAACTGCACGAAGAGAAGATCCAAGCTTTGAAAGGTAT

CCCAAAAATTTCAGTGATTCCAGAAGAAATGAGCCTCCACCACCAAGAAACGAACTTAGA

GAAACAGACAGACGAGAGGTACGAGGGGAACGAGATGAGAGGAGAACAGTGATCATTCAT

GACAGGCCTGATATTGCTCACACTAGACACCCTCGAGAAGCAGGGCCCAATCCTTCTAGA

CCAACCTCCTGGAAAAGTGAAGGAAGCATGTCTGCAGATAAACGTGAGGCAAGAGTTGAA

AGGCCGGAACGGTCCGGTAGAGAAGTATCAGGACACAGTGTGAGAGGCGCTCCCCCTGGA

AATCGCAGTAGTGCCACAGGTTATGGAAGCAGAGAAGGAGACAGAGGAATCATCTCAGAT

CGAGGGAGTGGAGCCCAGCACTATCCTGAAGAACGACATGTGGTTGAACGCCATGGACGG

GACACAAGTGGACCAAGG

>CL320.Contig24_A-W 1 2544 SAFB-like, transcription modulator, isoform CRA_b [Homo sapiens] >gi|119597959|gb|EAW77553.1| SAFB-like, transcription modulator, isoform CRA_b [Homo sapiens]

CCGAGGCCTGGGTGGAAGTTGGCGGTGCTGCCGCCGCCCTGCAGCCCACTCGCTGCCTCG

GCAGCGCGCTGCTCTTCTAAGATGGCTGCCGCTACCGGTGCTGTGGCAGCCTCGGCTGCC

CCGGGTCAGGCGGAAGGTAAAAAGATCACCGATCTGCGGGTCATCGATCTCAAGTCCGAG

CTGAAGCGGCGGAACTTAGACATCACCGGAGTCAAGACTGTGCTGGTCTCCCGACTCAAG

CAGGCTATTGAAGAGGAAGGAGGCGATCCAGATAATATTGAATTAACTGTTTCAACTGAT

ACTCCAAACAAGAAACCAACCAAAGGCAAAGGTAAAAAACAAGAAGCAGATGAGTTGAGT

GGAGATGCTTCTGTGGAAGATGATGCTTTTGTCAAGGAAATTGAAGCTCAAGAAGGTGAA

GATGATACCTTTCTAACAGCCCAAGATGGTGAGGAAGAAGAAAATGAGAAAGAAGGGAAC

CTAGCTGAGGCTGATCACACAGCTCATGAAGAGATGGAAGCTAATGCGACTGTGAAAGAA

GCTGAGGATGACAACATCTCGGTCACAATCCAGGCTGAAGATGCCATCACTCTGGATTTT

GATGGTGATGACCTCCTAGAAACAGGTAAAAATGTGAAAATTACAGATTCTGAAGCAAGT

AAGCCAAAAGATGGGCAGGACGCCATTGCACAGAGCCCGGAGAAGGAAACCAAGGATTAT

GAGATGAATGCGAACCATAAAGATGGTAAGAAGGAAGACTGCGTGAAGGGTGATCCTGTC

GAGAAGGAAGCCAGAGAAAGTTCTAAGAAAGCAGAATCTGGAGAAAAAGAAAAGGATACT

TTGAAGAAAGGGCCCTCGTCTACTGGGGCCTCTGGTCAAGCAAAGAGCTCTTCAAAGGAA

TCTAAAGACAGCAAGACATCATCTAAAGATGACAAAGGAAGTACAAGTAGTACTAGTGGT

AGCAGTGGAAGCTCAACTAAAAATATCTGGGTTAGTGGACTTTCTTCTAATACCAAAGCT

GCTGATTTGAAGAACCTCTTTGGCAAATATGGAAAGGTTCTTAGTGCAAAGGTAGTTACA

AATGCTCGAAGTCCTGGGGCAAAATGCTATGGCATTGTAACTATGTCATCAAGCACAGAG

GTGGCCAGATGTATTGCACATCTTCATCGCACTGAGCTGCATGGTCAGCTGATTTCTGTT

GAAAAAGTAAAAGGTGATCCTTCTAAGAAAGAAATGAAGAAAGAAAATGATGAAAAGAGT

AGTTCAAGAAGTTCTGGAGATAAAAAAAATATGAGTGATAGAAGTAGCAAGATACAAGCC

TCTGTCAAAAAAGAAGAGAAAAGGTCATCTGAAAAATCTGAAAAAAAAGAAAGCAAGGAT

GCTAAGAAAATAGAAGGTAAAGATGAGAAGAATGATAATGGAGGAAGTGGCCAAACTTCA

GAATCGATTAAAAAAAGTGAAGAAAAGAAGCGAATAAGTTCAAAGAGTCCAGGACACATG

GTAATATTAGACAAAACTAAGGGAGATCATTGTAGGCCATCAAGAAGAGGAAGATATGAA

AAAATTCATGGAAGAAGCAAGGAAAAGGAGAGAGCTAGCCTAGATAAAAAAAGGGACAAA

GACTACAGAAGGAAAGAGATTTTGCCTTTTGAAAAGATGAAGGAACAACGATTGAGAGAA

CATTTAGTTCGTTTTGAAAGACTGCGACGAGCAATTTCCCTCCGAAGGAACGTCGTAAGG

AAGCTGAACGGATTGCTCGAGAACGAGAGGAACTCAGAAGGCAGCAGCAGCAGCTACGTT

ATGAACAAGAAAAAAAGGAATTCTTTGAAACGCCCACATGATGTAGATCATAGGCGAGAT

GACCCTTACTGGAGCGAGAATAAAAAGTTGTCTCTAGATACAGATGCACGATTCAGTCAT

GGATCTGACTACTCTCGGCAACAGAACAGATTTAATGACTTTGATCACCGGGAAAGGGGC

AGGTTTCCCGAGAGTTCAGCTGTACAGTCTTCATCTTTTGAAAGGAGAGAACGCTTTGTA

GGTCAAAGTGAAGGGAAAAAAGCACGACCAACTGCACGAAGAGAAGATCCAAGCTTTGAA

AGGTATCCCAAAAATTTCAGTGATTCCAGAAGAAATGAGCCTCCACCACCAAGAAACGAA

CTTAGAGAAACAGACAGACGAGAGGTACGAGGGGAACGAGATGAGAGGAGAACAGTGATC

ATTCATGACAGGCCTGATATTGCTCACACTAGACACCCTCGAGAAGCAGGGCCCAATCCT

TCTAGACCAACCTCCTGGAAAAGTGAAGGAAGCATGTCTGCAGATAAACGTGAGGCAAGA

GTTGAAAGGCCGGAACGGTCCGGTAGAGAAGTATCAGGACACAGTGTGAGAGGCGCTCCC

CCTGGAAATCGCAGTAGTGCCACAGGTTATGGAAGCAGAGAAGGAGACAGAGGAATCATC

TCAGATCGAGGGAGTGGAGCCCAGCACTATCCTGAAGAACGACATGTGGTTGAACGCCAT

GGACGGGACACAAGTGGACCAAGG

>CL320.Contig25_A-W 1 2160 SAFB-like, transcription modulator, isoform CRA_b [Homo sapiens] >gi|119597959|gb|EAW77553.1| SAFB-like, transcription modulator, isoform CRA_b [Homo sapiens]

CCGAGGCCTGGGTGGAAGTTGGCGGTGCTGCCGCCGCCCTGCAGCCCACTCGCTGCCTCG

GCAGCGCGCTGCTCTTCTAAGATGGCTGCCGCTACCGGTGCTGTGGCAGCCTCGGCTGCC

CCGGGTCAGGCGGAAGGTAAAAAGATCACCGATCTGCGGGTCATCGATCTCAAGTCCGAG

CTGAAGCGGCGGAACTTAGACATCACCGGAGTCAAGACTGTGCTGGTCTCCCGACTCAAG

CAGGCTATTGAAGAGGAAGGAGGCGATCCAGATAATATTGAATTAACTGTTTCAACTGAT

ACTCCAAACAAGAAACCAACCAAAGGCAAAGGTAAAAAACAAGAAGCAGATGAGTTGAGT

GGAGATGCTTCTGTGGAAGATGATGCTTTTGTCAAGGACTGTGAATTGGAGAATCAAGAG

GCACATGAACAAGACGGAAATGATGAACTAAAGGACTCTGAAGAATTTGGTGAAAATGAA

GAAGACTGTGTGCATTCCAAGGAGTTACTTTCTGCAGAAGAAAACAAGAGAGCTCATGAA

TTAATAGAGGCAGAAGCAATAGAAGATATAGAAAAAGAGGACATCGAAAGTCAGGAAATT

GAAGCTCAAGAAGGTGAAGATGATACCTTTCTAACAGCCCAAGATGGTGAGGAAGAAGAA

AATGAGAAAGATATAGCAGGTTCTGGTGATGGTACACAAGAAGTATCTAAACCTCTTCCT

TCAGAAGGGAACCTAGCTGAGGCTGATCACACAGCTCATGAAGAGATGGAAGCTAATGCG

ACTGTGAAAGAAGCTGAGGATGACAACATCTCGGTCACAATCCAGGCTGAAGATGCCATC

ACTCTGGATTTTGATGGTGATGACCTCCTAGAAACAGGTAAAAATGTGAAAATTACAGAT

TCTGAAGCAAGTAAGCCAAAAGATGGGCAGGACGCCATTGCACAGAGCCCGGAGAAGGAA

ACCAAGGATTATGAGATGAATGCGAACCATAAAGATGGTAAGAAGGAAGACTGCGTGAAG

GGTGATCCTGTCGAGAAGGAAGCCAGAGAAAGTTCTAAGAAAGCAGAATCTGGAGAAAAA

GAAAAGGATACTTTGAAGAAAGGGCCCTCGTCTACTGGGGCCTCTGGTCAAGCAAAGAGC

TCTTCAAAGGAATCTAAAGACAGCAAGACATCATCTAAAGATGACAAAGGAAGTACAAGT

AGTACTAGTGGTAGCAGTGGAAGCTCAACTAAAAATATCTGGGTTAGTGGACTTTCTTCT

AATACCAAAGCTGCTGATTTGAAGAACCTCTTTGGCAAATATGGAAAGGTTCTTAGTGCA

AAGGTAGTTACAAATGCTCGAAGTCCTGGGGCAAAATGCTATGGCATTGTAACTATGTCA

TCAAGCACAGAGGTGGCCAGATGTATTGCACATCTTCATCGCACTGAGCTGCATGGTCAG

CTGATTTCTGTTGAAAAAGTAAAAGGTGATCCTTCTAAGAAAGAAATGAAGAAAGAAAAT

GATGAAAAGAGTAGTTCAAGAAGTTCTGGAGATAAAAAAAATATGAGTGATAGAAGTAGC

AAGATACAAGCCTCTGTCAAAAAAGAAGAGAAAAGGTCATCTGAAAAATCTGAAAAAAAA

GAAAGCAAGGATGCTAAGAAAATAGAAGGTTCAAAGAGTCCAGGACACATGGTAATATTA

GACAAAACTAAGGGAGATCATTGTAGGCCATCAAGAAGAGGAAGATATGAAAAAATTCAT

GGAAGAAGCAAGGAAAAGGAGAGAGCTAGCCTAGATAAAAAAAGGGACAAAGACTACAGA

AGGAAAGAGATTTTGCCTTTTGAAAAGATGAAGGAACAACGATTGAGAGAACATTTAGTT

CGTTTTGAAAGACTGCGACGAGCAATTTCCCTCCGAAGACGAAGAGAGATTGCAGAAAGA

GAGCGTCGAGAGCGAGAACGCATTAGAATAATTCGTGAACGGGAAGAACGGGAACGCTTA

CAGAGAGAGAGAGAGCGCCTAGAAATTGAAAGGCAAAAACTAGAGAGAGAGAGAATGGAA

CGCGAACGCTTGGAAAGGGAACGCATTCGTATTGAACAGGAACGTCGTAAGGAAGCTGAA

CGGATTGCTCGAGAACGAGAGGAACTCAGAAGGCAGCAGCAGCAGCTACGTTATGAACAA

>CL320.Contig26_A-W 82 1899 SAFB-like transcription modulator OS=Homo sapiens GN=SLTM PE=1 SV=2

ATGGCTGCCGCTACCGGTGCTGTGGCAGCCTCGGCTGCCCCGGGTCAGGCGGAAGGTAAA

AAGATCACCGATCTGCGGGTCATCGATCTCAAGTCCGAGCTGAAGCGGCGGAACTTAGAC

ATCACCGGAGTCAAGACTGTGCTGGTCTCCCGACTCAAGCAGGCTATTGAAGAGGAAGGA

GGCGATCCAGATAATATTGAATTAACTGTTTCAACTGATACTCCAAACAAGAAACCAACC

AAAGGCAAAGGTAAAAAACAAGAAGCAGATGAGTTGAGTGGAGATGCTTCTGTGGAAGAT

GATGCTTTTGTCAAGGACTGTGAATTGGAGAATCAAGAGGCACATGAACAAGACGGAAAT

GATGAACTAAAGGACTCTGAAGAATTTGGTGAAAATGAAGAAGACTGTGTGCATTCCAAG

GAGTTACTTTCTGCAGAAGAAAACAAGAGAGCTCATGAATTAATAGAGGCAGAAGCAATA

GAAGATATAGAAAAAGAGGACATCGAAAGTCAGGAAATTGAAGCTCAAGAAGGTGAAGAT

GATACCTTTCTAACAGCCCAAGATGGTGAGGAAGAAGAAAATGAGAAAGATATAGCAGGT

TCTGGTGATGGTACACAAGAAGTATCTAAACCTCTTCCTTCAGAAGGGAACCTAGCTGAG

GCTGATCACACAGCTCATGAAGAGATGGAAGCTAATGCGACTGTGAAAGAAGCTGAGGAT

GACAACATCTCGGTCACAATCCAGGCTGAAGATGCCATCACTCTGGATTTTGATGGTGAT

GACCTCCTAGAAACAGGTAAAAATGTGAAAATTACAGATTCTGAAGCAAGTAAGCCAAAA

GATGGGCAGGACGCCATTGCACAGAGCCCGGAGAAGGAAACCAAGGATTATGAGATGAAT

GCGAACCATAAAGATGGTAAGAAGGAAGACTGCGTGAAGGGTGATCCTGTCGAGAAGGAA

GCCAGAGAAAGTTCTAAGAAAGCAGAATCTGGAGAAAAAGAAAAGGATACTTTGAAGAAA

GGGCCCTCGTCTACTGGGGCCTCTGGTCAAGCAAAGAGCTCTTCAAAGGAATCTAAAGAC

AGCAAGACATCATCTAAAGATGACAAAGGAAGTACAAGTAGTACTAGTGGTAGCAGTGGA

AGCTCAACTAAAAATATCTGGGTTAGTGGACTTTCTTCTAATACCAAAGCTGCTGATTTG

AAGAACCTCTTTGGCAAATATGGAAAGGTTCTTAGTGCAAAGGTAGTTACAAATGCTCGA

AGTCCTGGGGCAAAATGCTATGGCATTGTAACTATGTCATCAAGCACAGAGGTGGCCAGA

TGTATTGCACATCTTCATCGCACTGAGCTGCATGGTCAGCTGATTTCTGTTGAAAAAGTA

AAAGGTGATCCTTCTAAGAAAGAAATGAAGAAAGAAAATGATGAAAAGAGTAGTTCAAGA

AGTTCTGGAGATAAAAAAAATATGAGTGATAGAAGTAGCAAGATACAAGCCTCTGTCAAA

AAAGAAGAGAAAAGGTCATCTGAAAAATCTGAAAAAAAAGAAAGCAAGGATGCTAAGAAA

ATAGAAGGTTCAAAGAGTCCAGGACACATGGTAATATTAGACAAAACTAAGGGAGATCAT

TGTAGGCCATCAAGAAGAGGAAGATATGAAAAAATTCATGGAAGAAGCAAGGAAAAGGAG

AGAGCTAGCCTAGATAAAAAAAGGGACAAAGACTACAGAAGGAAAGAGATTTTGCCTTTT

GAAAAGATGAAGGAACAACGATTGAGAGAACATTTAGTTCGTTTTGAAAGACTGCGACGA

GCAATTTCCCTCCGAAGG

>CL320.Contig27_A-W 82 1962 PREDICTED: SAFB-like transcription modulator-like [Macaca mulatta]

ATGGCTGCCGCTACCGGTGCTGTGGCAGCCTCGGCTGCCCCGGGTCAGGCGGAAGGTAAA

AAGATCACCGATCTGCGGGTCATCGATCTCAAGTCCGAGCTGAAGCGGCGGAACTTAGAC

ATCACCGGAGTCAAGACTGTGCTGGTCTCCCGACTCAAGCAGGCTATTGAAGAGGAAGGA

GGCGATCCAGATAATATTGAATTAACTGTTTCAACTGATACTCCAAACAAGAAACCAACC

AAAGGCAAAGGTAAAAAACAAGAAGCAGATGAGTTGAGTGGAGATGCTTCTGTGGAAGAT

GATGCTTTTGTCAAGGAAATTGAAGCTCAAGAAGGTGAAGATGATACCTTTCTAACAGCC

CAAGATGGTGAGGAAGAAGAAAATGAGAAAGATATAGCAGGTTCTGGTGATGGTACACAA

GAAGTATCTAAACCTCTTCCTTCAGAAGGGAACCTAGCTGAGGCTGATCACACAGCTCAT

GAAGAGATGGAAGCTAATGCGACTGTGAAAGAAGCTGAGGATGACAACATCTCGGTCACA

ATCCAGGCTGAAGATGCCATCACTCTGGATTTTGATGGTGATGACCTCCTAGAAACAGGT

AAAAATGTGAAAATTACAGATTCTGAAGCAAGTAAGCCAAAAGATGGGCAGGACGCCATT

GCACAGAGCCCGGAGAAGGAAACCAAGGATTATGAGATGAATGCGAACCATAAAGATGGT

AAGAAGGAAGACTGCGTGAAGGGTGATCCTGTCGAGAAGGAAGCCAGAGAAAGTTCTAAG

AAAGCAGAATCTGGAGAAAAAGAAAAGGATACTTTGAAGAAAGGGCCCTCGTCTACTGGG

GCCTCTGGTCAAGCAAAGAGCTCTTCAAAGGAATCTAAAGACAGCAAGACATCATCTAAA

GATGACAAAGGAAGTACAAGTAGTACTAGTGGTAGCAGTGGAAGCTCAACTAAAAATATC

TGGGTTAGTGGACTTTCTTCTAATACCAAAGCTGCTGATTTGAAGAACCTCTTTGGCAAA

TATGGAAAGGTTCTTAGTGCAAAGGTAGTTACAAATGCTCGAAGTCCTGGGGCAAAATGC

TATGGCATTGTAACTATGTCATCAAGCACAGAGGTGGCCAGATGTATTGCACATCTTCAT

CGCACTGAGCTGCATGGTCAGCTGATTTCTGTTGAAAAAGTAAAAGGTGATCCTTCTAAG

AAAGAAATGAAGAAAGAAAATGATGAAAAGAGTAGTTCAAGAAGTTCTGGAGATAAAAAA

AATATGAGTGATAGAAGTAGCAAGATACAAGCCTCTGTCAAAAAAGAAGAGAAAAGGTCA

TCTGAAAAATCTGAAAAAAAAGAAAGCAAGGATGCTAAGAAAATAGAAGGTTCAAAGAGT

CCAGGACACATGGTAATATTAGACAAAACTAAGGGAGATCATTGTAGGCCATCAAGAAGA

GGAAGATATGAAAAAATTCATGGAAGAAGCAAGGAAAAGGAGAGAGCTAGCCTAGATAAA

AAAAGGGACAAAGACTACAGAAGGAAAGAGATTTTGCCTTTTGAAAAGATGAAGGAACAA

CGATTGAGAGAACATTTAGTTCGTTTTGAAAGACTGCGACGAGCAATTTCCCTCCGAAGA

CGAAGAGAGATTGCAGAAAGAGAGCGTCGAGAGCGAGAACGCATTAGAATAATTCGTGAA

CGGGAAGAACGGGAACGCTTACAGAGAGAGAGAGAGCGCCTAGAAATTGAAAGGCAAAAA

CTAGAGAGAGAGAGAATGGAACGCGAACGCTTGGAAAGGGAACGCATTCGTATTGAACAG

GAACGTCGTAAGGAAGCTGAACGGATTGCTCGAGAACGAGAGGAACTCAGAAGGCAGCAG

CAGCAGCTACGTTATGAACAA

>CL320.Contig28_A-W 82 1845 SAFB-like transcription modulator OS=Homo sapiens GN=SLTM PE=1 SV=2

ATGGCTGCCGCTACCGGTGCTGTGGCAGCCTCGGCTGCCCCGGGTCAGGCGGAAGGTAAA

AAGATCACCGATCTGCGGGTCATCGATCTCAAGTCCGAGCTGAAGCGGCGGAACTTAGAC

ATCACCGGAGTCAAGACTGTGCTGGTCTCCCGACTCAAGCAGGCTATTGAAGAGGAAGGA

GGCGATCCAGATAATATTGAATTAACTGTTTCAACTGATACTCCAAACAAGAAACCAACC

AAAGGCAAAGGTAAAAAACAAGAAGCAGATGAGTTGAGTGGAGATGCTTCTGTGGAAGAT

GATGCTTTTGTCAAGGACTGTGAATTGGAGAATCAAGAGGCACATGAACAAGACGGAAAT

GATGAACTAAAGGACTCTGAAGAATTTGGTGAAAATGAAGAAGACTGTGTGCATTCCAAG

GAGTTACTTTCTGCAGAAGAAAACAAGAGAGCTCATGAATTAATAGAGGCAGAAGCAATA

GAAGATATAGAAAAAGAGGACATCGAAAGTCAGGAAATTGAAGCTCAAGAAGGTGAAGAT

GATACCTTTCTAACAGCCCAAGATGGTGAGGAAGAAGAAAATGAGAAAGAAGGGAACCTA

GCTGAGGCTGATCACACAGCTCATGAAGAGATGGAAGCTAATGCGACTGTGAAAGAAGCT

GAGGATGACAACATCTCGGTCACAATCCAGGCTGAAGATGCCATCACTCTGGATTTTGAT

GGTGATGACCTCCTAGAAACAGGTAAAAATGTGAAAATTACAGATTCTGAAGCAAGTAAG

CCAAAAGATGGGCAGGACGCCATTGCACAGAGCCCGGAGAAGGAAACCAAGGATTATGAG

ATGAATGCGAACCATAAAGATGGTAAGAAGGAAGACTGCGTGAAGGGTGATCCTGTCGAG

AAGGAAGCCAGAGAAAGTTCTAAGAAAGCAGAATCTGGAGAAAAAGAAAAGGATACTTTG

AAGAAAGGGCCCTCGTCTACTGGGGCCTCTGGTCAAGCAAAGAGCTCTTCAAAGGAATCT

AAAGACAGCAAGACATCATCTAAAGATGACAAAGGAAGTACAAGTAGTACTAGTGGTAGC

AGTGGAAGCTCAACTAAAAATATCTGGGTTAGTGGACTTTCTTCTAATACCAAAGCTGCT

GATTTGAAGAACCTCTTTGGCAAATATGGAAAGGTTCTTAGTGCAAAGGTAGTTACAAAT

GCTCGAAGTCCTGGGGCAAAATGCTATGGCATTGTAACTATGTCATCAAGCACAGAGGTG

GCCAGATGTATTGCACATCTTCATCGCACTGAGCTGCATGGTCAGCTGATTTCTGTTGAA

AAAGTAAAAGGTGATCCTTCTAAGAAAGAAATGAAGAAAGAAAATGATGAAAAGAGTAGT

TCAAGAAGTTCTGGAGATAAAAAAAATATGAGTGATAGAAGTAGCAAGATACAAGCCTCT

GTCAAAAAAGAAGAGAAAAGGTCATCTGAAAAATCTGAAAAAAAAGAAAGCAAGGATGCT

AAGAAAATAGAAGGTTCAAAGAGTCCAGGACACATGGTAATATTAGACAAAACTAAGGGA

GATCATTGTAGGCCATCAAGAAGAGGAAGATATGAAAAAATTCATGGAAGAAGCAAGGAA

AAGGAGAGAGCTAGCCTAGATAAAAAAAGGGACAAAGACTACAGAAGGAAAGAGATTTTG

CCTTTTGAAAAGATGAAGGAACAACGATTGAGAGAACATTTAGTTCGTTTTGAAAGACTG

CGACGAGCAATTTCCCTCCGAAGG

>CL320.Contig29_A-W 82 1908 PREDICTED: SAFB-like transcription modulator-like [Macaca mulatta]

ATGGCTGCCGCTACCGGTGCTGTGGCAGCCTCGGCTGCCCCGGGTCAGGCGGAAGGTAAA

AAGATCACCGATCTGCGGGTCATCGATCTCAAGTCCGAGCTGAAGCGGCGGAACTTAGAC

ATCACCGGAGTCAAGACTGTGCTGGTCTCCCGACTCAAGCAGGCTATTGAAGAGGAAGGA

GGCGATCCAGATAATATTGAATTAACTGTTTCAACTGATACTCCAAACAAGAAACCAACC

AAAGGCAAAGGTAAAAAACAAGAAGCAGATGAGTTGAGTGGAGATGCTTCTGTGGAAGAT

GATGCTTTTGTCAAGGAAATTGAAGCTCAAGAAGGTGAAGATGATACCTTTCTAACAGCC

CAAGATGGTGAGGAAGAAGAAAATGAGAAAGAAGGGAACCTAGCTGAGGCTGATCACACA

GCTCATGAAGAGATGGAAGCTAATGCGACTGTGAAAGAAGCTGAGGATGACAACATCTCG

GTCACAATCCAGGCTGAAGATGCCATCACTCTGGATTTTGATGGTGATGACCTCCTAGAA

ACAGGTAAAAATGTGAAAATTACAGATTCTGAAGCAAGTAAGCCAAAAGATGGGCAGGAC

GCCATTGCACAGAGCCCGGAGAAGGAAACCAAGGATTATGAGATGAATGCGAACCATAAA

GATGGTAAGAAGGAAGACTGCGTGAAGGGTGATCCTGTCGAGAAGGAAGCCAGAGAAAGT

TCTAAGAAAGCAGAATCTGGAGAAAAAGAAAAGGATACTTTGAAGAAAGGGCCCTCGTCT

ACTGGGGCCTCTGGTCAAGCAAAGAGCTCTTCAAAGGAATCTAAAGACAGCAAGACATCA

TCTAAAGATGACAAAGGAAGTACAAGTAGTACTAGTGGTAGCAGTGGAAGCTCAACTAAA

AATATCTGGGTTAGTGGACTTTCTTCTAATACCAAAGCTGCTGATTTGAAGAACCTCTTT

GGCAAATATGGAAAGGTTCTTAGTGCAAAGGTAGTTACAAATGCTCGAAGTCCTGGGGCA

AAATGCTATGGCATTGTAACTATGTCATCAAGCACAGAGGTGGCCAGATGTATTGCACAT

CTTCATCGCACTGAGCTGCATGGTCAGCTGATTTCTGTTGAAAAAGTAAAAGGTGATCCT

TCTAAGAAAGAAATGAAGAAAGAAAATGATGAAAAGAGTAGTTCAAGAAGTTCTGGAGAT

AAAAAAAATATGAGTGATAGAAGTAGCAAGATACAAGCCTCTGTCAAAAAAGAAGAGAAA

AGGTCATCTGAAAAATCTGAAAAAAAAGAAAGCAAGGATGCTAAGAAAATAGAAGGTTCA

AAGAGTCCAGGACACATGGTAATATTAGACAAAACTAAGGGAGATCATTGTAGGCCATCA

AGAAGAGGAAGATATGAAAAAATTCATGGAAGAAGCAAGGAAAAGGAGAGAGCTAGCCTA

GATAAAAAAAGGGACAAAGACTACAGAAGGAAAGAGATTTTGCCTTTTGAAAAGATGAAG

GAACAACGATTGAGAGAACATTTAGTTCGTTTTGAAAGACTGCGACGAGCAATTTCCCTC

CGAAGACGAAGAGAGATTGCAGAAAGAGAGCGTCGAGAGCGAGAACGCATTAGAATAATT

CGTGAACGGGAAGAACGGGAACGCTTACAGAGAGAGAGAGAGCGCCTAGAAATTGAAAGG

CAAAAACTAGAGAGAGAGAGAATGGAACGCGAACGCTTGGAAAGGGAACGCATTCGTATT

GAACAGGAACGTCGTAAGGAAGCTGAACGGATTGCTCGAGAACGAGAGGAACTCAGAAGG

CAGCAGCAGCAGCTACGTTATGAACAA

>CL320.Contig30_A-W 1590 2411 minus strand SAFB-like transcription modulator isoform b [Macaca mulatta] >gi|383421657|gb|AFH34042.1| SAFB-like transcription modulator isoform b [Macaca mulatta] >gi|384949486|gb|AFI38348.1| SAFB-like transcription modulator isoform b [Macaca mulatta]

AAGGAACGTCGTAAGGAAGCTGAACGGATTGCTCGAGAACGAGAGGAACTCAGAAGGCAG

CAGCAGCAGCTACGTTATGAACAAGAAAAAAGGAATTCTTTGAAACGCCCACATGATGTA

GATCATAGGCGAGATGACCCTTACTGGAGCGAGAATAAAAAGTTGTCTCTAGATACAGAT

GCACGATTCAGTCATGGATCTGACTACTCTCGGCAACAGAACAGATTTAATGACTTTGAT

CACCGGGAAAGGGGCAGGTTTCCCGAGAGTTCAGCTGTACAGTCTTCATCTTTTGAAAGG

AGAGAACGCTTTGTAGGTCAAAGTGAAGGGAAAAAAGCACGACCAACTGCACGAAGAGAA

GATCCAAGCTTTGAAAGGTATCCCAAAAATTTCAGTGATTCCAGAAGAAATGAGCCTCCA

CCACCAAGAAACGAACTTAGAGAAACAGACAGACGAGAGGTACGAGGGGAACGAGATGAG

AGGAGAACAGTGATCATTCATGACAGGCCTGATATTGCTCACACTAGACACCCTCGAGAA

GCAGGGCCCAATCCTTCTAGACCAACCTCCTGGAAAAGTGAAGGAAGCATGTCTGCAGAT

AAACGTGAGGCAAGAGTTGAAAGGCCGGAACGGTCCGGTAGAGAAGTATCAGGACACAGT

GTGAGAGGCGCTCCCCCTGGAAATCGCAGTAGTGCCACAGGTTATGGAAGCAGAGAAGGA

GACAGAGGAATCATCTCAGATCGAGGGAGTGGAGCCCAGCACTATCCTGAAGAACGACAT

GTGGTTGAACGCCATGGACGGGACACAAGTGGACCAAGGAAA

>CL321.Contig1_A-W 107 559 PREDICTED: indoleamine 2,3-dioxygenase 2 [Loxodonta africana]

ATGGAGCCTCAAACAGAAAATTTGAAGACAGCATGGCAGTTGTCTTTGGGAAGCTATTAC

ATTTCTGAGGAGTATGGCTTTCTACTTCCAAATCCTCTGATGCCCCTCCTGAGCTGCCAG

TTCCTAAAGAGTTACCGGCAACAACGCTTGGCCCATTTAGTCCTGAGCTCCATTACCATG

GGATATGTCTGGCAGGAAGGAGAGACACAGCCTAAAGAGGTGCTGCCAAGAAATCTTGCC

ATTCCCTTTGTTGAAGTCTCCAGGAACTTGGGGCTCCCTCCTATCCTGGTACACTCAGAC

TTGGTGCTGGCAAACTGGACGAAAAGGAACTCAGAAGGACCCCTGGAAATCAGCAACCTG

GACACCATCATCCTGTTTCCTGGGGGAGAGAGTCTGCGTGGCTTCATACTGGTGACTGTT

TTAGTGGAGAAAGCAGCTGTGCCTGGAATTAAG

>CL321.Contig2_A-W 483 836 PREDICTED: indoleamine 2,3-dioxygenase 2 [Papio anubis]

ATGCCCCTCCTGAGCTGCCAGTTCCTAAAGAGTTACCGGCAACAACGCTTGGCCCATTTA

GTCCTGAGCTCCATTACCATGGGATATGTCTGGCAGGAAGGAGAGACACAGCCTAAAGAG

GTGCTGCCAAGAAATCTTGCCATTCCCTTTGTTGAAGTCTCCAGGAACTTGGGGCTCCCT

CCTATCCTGGTACACTCAGACTTGGTGCTGGCAAACTGGACGAAAAGGAACTCAGAAGGA

CCCCTGGAAATCAGCAACCTGGACACCATCATCCTGTTTCCTGGGGGAGAGAGTCTGCGT

GGCTTCATACTGGTGACTGTTTTAGTGGAGAAAGCAGCTGTGCCTGGAATTAAG

>CL321.Contig3_A-W 483 968 PREDICTED: indoleamine 2,3-dioxygenase 2 [Pongo abelii]

ATGCCCCTCCTGAGCTGCCAGTTCCTAAAGAGTTACCGGCAACAACGCTTGGCCCATTTA

GTCCTGAGCTCCATTACCATGGGATATGTCTGGCAGGAAGGAGAGACACAGCCTAAAGAG

GTGCTGCCAAGAAATCTTGCCATTCCCTTTGTTGAAGTCTCCAGGAACTTGGGGCTCCCT

CCTATCCTGGTACACTCAGACTTGGTGCTGGCAAACTGGACGAAAAGGAACTCAGAAGGA

CCCCTGGAAATCAGCAACCTGGACACCATCATCCTGTTTCCTGGGGGAGAGAGTCTGCGT

GGCTTCATACTGGTGACTGTTTTAGTGGAGAAAGCAGCTGTGCCTGGAATTAAGGCACTT

GCTCAAGCAATGCATGCTGTCCTGCAGCCCAGCCAGGATGCCCTGCTTAAGGCCCTGCAG

CAACTGAAGCTCTGCATCCAGGACATGACCAGAACCTTAGGACGAATGCATGGTATAATG

CCCCCT

>CL321.Contig4_A-W 237 569 PREDICTED: similar to indoleamine-pyrrole 2,3 dioxygenase [Taeniopygia guttata]

GAACTTCCAGATCATTACAGGCCTTGGATGGAAATTGCCAAAAAGCTTCCTCACTTGATT

GAAAGTCACCAGCTTCGAGCTCATGTCAACAAGATGCCCCTCCTGAGCTGCCAGTTCCTA

AAGAGTTACCGGCAACAACGCTTGGCCCATTTAGTCCTGAGCTCCATTACCATGGGATAT

GTCTGGCAGGAAGGAGAGACACAGCCTAAAGAGGTGCTGCCAAGAAATCTTGCCATTCCC

TTTGTTGAAGTCTCCAGGAACTTGGGGCTCCCTCCTATCCTGGTACACTCAGACTTGGTG

CTGGCAAACTGGACGAAAAGGAACTCAGAAGGA

>CL321.Contig5_A-W 234 815 PREDICTED: indoleamine 2,3-dioxygenase 2 [Pongo abelii]

AGGGAACTTCCAGATCATTACAGGCCTTGGATGGAAATTGCCAAAAAGCTTCCTCACTTG

ATTGAAAGTCACCAGCTTCGAGCTCATGTCAACAAGATGCCCCTCCTGAGCTGCCAGTTC

CTAAAGAGTTACCGGCAACAACGCTTGGCCCATTTAGTCCTGAGCTCCATTACCATGGGA

TATGTCTGGCAGGAAGGAGAGACACAGCCTAAAGAGGTGCTGCCAAGAAATCTTGCCATT

CCCTTTGTTGAAGTCTCCAGGAACTTGGGGCTCCCTCCTATCCTGGTACACTCAGACTTG

GTGCTGGCAAACTGGACGAAAAGGAACTCAGAAGGACCCCTGGAAATCAGCAACCTGGAC

ACCATCATCCTGTTTCCTGGGGGAGAGAGTCTGCGTGGCTTCATACTGGTGACTGTTTTA

GTGGAGAAAGCAGCTGTGCCTGGAATTAAGGCACTTGCTCAAGCAATGCATGCTGTCCTG

CAGCCCAGCCAGGATGCCCTGCTTAAGGCCCTGCAGCAACTGAAGCTCTGCATCCAGGAC

ATGACCAGAACCTTAGGACGAATGCATGGTATAATGCCCCCT

>CL321.Contig6_A-W 289 804 PREDICTED: indoleamine 2,3-dioxygenase 2 [Papio anubis]

TTACTTCTGCTTAGATGGAAAGATAACCCAGCAATGCCCATGGGGCTAATATATGAAGGA

ATCTCCAAAGAACCCTTGAAATACTCTGGAGGGAGTGCAGCTCAGAGCTCAGTACTTCAT

GCCTTTGATGAGTTCCTGGGCATTCATCACAGCGAGGAAAGTGCTGACTTTCTGTACAGA

ATGAGAGACTACATGCCTCCTTCCCATAAGGCCTTCATAGAAGAAATCCACTCAGCTCCT

TCACTGAGGGACTTCATCCTATCCTCTGGCCATGGTCACTGTCTGACAGCCTATAACCAG

TGTCTGGAGGCCCTGGCAGAACTGCGCAGTTATCACATCACCGTGGTGACCAGATATCTC

GTCACAGCTGCAGCAATAGCAAGGAGCAGGAGGCTAGACCATCTCCCAGGGCCACCTCAG

GCTTTAGAAGACAGGGGCACTGGAGGAACTGCAGTTCTGAGCTTCCTGAAGAATGTCAGG

GATAAGACCTTAGAGACAACCCTCCACCCAAGTGGT

>CL321.Contig7_A-W 129 359 PREDICTED: indoleamine 2,3-dioxygenase 2 [Saimiri boliviensis boliviensis]

AACCTGGACACCATCATCCTGTTTCCTGGGGGAGAGAGTCTGCGTGGCTTCATACTGGTG

ACTGTTTTAGTGGAGAAAGCAGCTGTGCCTGGAATTAAGGCACTTGCTCAAGCAATGCAT

GCTGTCCTGCAGCCCAGCCAGGATGCCCTGCTTAAGGCCCTGCAGCAACTGAAGCTCTGC

ATCCAGGACATGACCAGAACCTTAGGACGAATGCATGGTATAATGCCCCCT

>CL321.Contig8_A-W 229 729 Indoleamine 2,3-dioxygenase 2 OS=Homo sapiens GN=IDO2 PE=1 SV=3

TGGAAAGATAACCCAGCAATGCCCATGGGGCTAATATATGAAGGAATCTCCAAAGAACCC

TTGAAATACTCTGGAGGGAGTGCAGCTCAGAGCTCAGTACTTCATGCCTTTGATGAGTTC

CTGGGCATTCATCACAGCGAGGAAAGTGCTGACTTTCTGTACAGAATGAGAGACTACATG

CCTCCTTCCCATAAGGCCTTCATAGAAGAAATCCACTCAGCTCCTTCACTGAGGGACTTC

ATCCTATCCTCTGGCCATGGTCACTGTCTGACAGCCTATAACCAGTGTCTGGAGGCCCTG

GCAGAACTGCGCAGTTATCACATCACCGTGGTGACCAGATATCTCGTCACAGCTGCAGCA

ATAGCAAGGAGCAGGAGGCTAGACCATCTCCCAGGGCCACCTCAGGCTTTAGAAGACAGG

GGCACTGGAGGAACTGCAGTTCTGAGCTTCCTGAAGAATGTCAGGGATAAGACCTTAGAG

ACAACCCTCCACCCAAGTGGT

>CL321.Contig9_A-W 107 691 PREDICTED: indoleamine 2,3-dioxygenase 2 [Pongo abelii]

ATGGAGCCTCAAACAGAAAATTTGAAGACAGCATGGCAGTTGTCTTTGGGAAGCTATTAC

ATTTCTGAGGAGTATGGCTTTCTACTTCCAAATCCTCTGATGCCCCTCCTGAGCTGCCAG

TTCCTAAAGAGTTACCGGCAACAACGCTTGGCCCATTTAGTCCTGAGCTCCATTACCATG

GGATATGTCTGGCAGGAAGGAGAGACACAGCCTAAAGAGGTGCTGCCAAGAAATCTTGCC

ATTCCCTTTGTTGAAGTCTCCAGGAACTTGGGGCTCCCTCCTATCCTGGTACACTCAGAC

TTGGTGCTGGCAAACTGGACGAAAAGGAACTCAGAAGGACCCCTGGAAATCAGCAACCTG

GACACCATCATCCTGTTTCCTGGGGGAGAGAGTCTGCGTGGCTTCATACTGGTGACTGTT

TTAGTGGAGAAAGCAGCTGTGCCTGGAATTAAGGCACTTGCTCAAGCAATGCATGCTGTC

CTGCAGCCCAGCCAGGATGCCCTGCTTAAGGCCCTGCAGCAACTGAAGCTCTGCATCCAG

GACATGACCAGAACCTTAGGACGAATGCATGGTATAATGCCCCCT

>CL321.Contig10_A-W 107 559 PREDICTED: indoleamine 2,3-dioxygenase 2 [Papio anubis]

ATGGAGCCTCAAACAGAAAATTTGAAGACAGCATGGCAGTTGTCTTTGGGAAGCTATTAC

ATTTCTGAGGAGTATGGCTTTCTACTTCCAAATCCTCTGATGCCCCTCCTGAGCTGCCAG

TTCCTAAAGAGTTACCGGCAACAACGCTTGGCCCATTTAGTCCTGAGCTCCATTACCATG

GGATATGTCTGGCAGGAAGGAGAGACACAGCCTAAAGAGGTGCTGCCAAGAAATCTTGCC

ATTCCCTTTGTTGAAGTCTCCAGGAACTTGGGGCTCCCTCCTATCCTGGTACACTCAGAC

TTGGTGCTGGCAAACTGGACGAAAAGGAACTCAGAAGGACCCCTGGAAATCAGCAACCTG

GACACCATCATCCTGTTTCCTGGGGGAGAGAGTCTGCGTGGCTTCATACTGGTGACTGTT

TTAGTGGAGAAAGCAGCTGTGCCTGGAATTAAG

>CL321.Contig11_A-W 864 1415 PREDICTED: indoleamine 2,3-dioxygenase 2 [Loxodonta africana]

GATTATGTAGATCCAGACATATTTTATGCAGTCATCCGGATCTTTCTCTCTGGATGGAAA

GATAACCCAGCAATGCCCATGGGGCTAATATATGAAGGAATCTCCAAAGAACCCTTGAAA

TACTCTGGAGGGAGTGCAGCTCAGAGCTCAGTACTTCATGCCTTTGATGAGTTCCTGGGC

ATTCATCACAGCGAGGAAAGTGCTGACTTTCTGTACAGAATGAGAGACTACATGCCTCCT

TCCCATAAGGCCTTCATAGAAGAAATCCACTCAGCTCCTTCACTGAGGGACTTCATCCTA

TCCTCTGGCCATGGTCACTGTCTGACAGCCTATAACCAGTGTCTGGAGGCCCTGGCAGAA

CTGCGCAGTTATCACATCACCGTGGTGACCAGATATCTCGTCACAGCTGCAGCAATAGCA

AGGAGCAGGAGGCTAGACCATCTCCCAGGGCCACCTCAGGCTTTAGAAGACAGGGGCACT

GGAGGAACTGCAGTTCTGAGCTTCCTGAAGAATGTCAGGGATAAGACCTTAGAGACAACC

CTCCACCCAAGT

>CL321.Contig12_A-W 107 541 PREDICTED: similar to indoleamine-pyrrole 2,3 dioxygenase [Taeniopygia guttata]

ATGGAGCCTCAAACAGAAAATTTGAAGACAGCATGGCAGTTGTCTTTGGGAAGCTATTAC

ATTTCTGAGGAGTATGGCTTTCTACTTCCAAATCCTCTGAGGGAACTTCCAGATCATTAC

AGGCCTTGGATGGAAATTGCCAAAAAGCTTCCTCACTTGATTGAAAGTCACCAGCTTCGA

GCTCATGTCAACAAGATGCCCCTCCTGAGCTGCCAGTTCCTAAAGAGTTACCGGCAACAA

CGCTTGGCCCATTTAGTCCTGAGCTCCATTACCATGGGATATGTCTGGCAGGAAGGAGAG

ACACAGCCTAAAGAGGTGCTGCCAAGAAATCTTGCCATTCCCTTTGTTGAAGTCTCCAGG

AACTTGGGGCTCCCTCCTATCCTGGTACACTCAGACTTGGTGCTGGCAAACTGGACGAAA

AGGAACTCAGAAGGA

>CL322.Contig1_A-W 3 263 kinectin isoform a [Macaca mulatta] >gi|383418273|gb|AFH32350.1| kinectin isoform a [Macaca mulatta] >gi|384946990|gb|AFI37100.1| kinectin isoform a [Macaca mulatta]

GAAAGAAAGAAGAAGGAAAAGAAACAAAAACCTTTACTTGAAGAGCAAGTCATCAAAGAA

AGTGATGTATCAAAGGTGCCTGGCAAAAAAAGTAGAACCTATCCCAGTTACAAAACAACC

CACCCTGCCCTCCGAAGCAACTTCCTCAAAGAAGAAACCAGGGCAGAAGAAGTCAAAAAA

TGGAAGCGATGATCAGGACAAAAAGGTGGAAACTCTCATGGCACCATCAAAAAAGCAAGA

AGTATTACCCTTCCATCAAGA

>CL322.Contig2_A-W 3 263 kinectin isoform c [Macaca mulatta] >gi|383418275|gb|AFH32351.1| kinectin isoform c [Macaca mulatta] >gi|384946992|gb|AFI37101.1| kinectin isoform c [Macaca mulatta]

GAAAGAAAGAAGAAGGAAAAGAAACAAAAACCTTTACTTGAAGAGCAAGTCATCAAAGAA

AGTGATGTATCAAAGGTGCCTGGCAAAAAAAGTAGAACCTATCCCAGTTACAAAACAACC

CACCCTGCCCTCCGAAGCAACTTCCTCAAAGAAGAAACCAGGGCAGAAGAAGTCAAAAAA

TGGAAGCGATGATCAGGACAAAAAGGTGGAAACTCTCATGGCACCATCAAAAAAGCAAGA

AGTATTACCCTTCCATCAAGA

>CL322.Contig3_A-W 4 3417 kinectin 1 (kinesin receptor) [Sus scrofa]

AAAGAAAGAAGAAGGAAAAGAAACAAAAACCTTTACTTGAAGAGCAAGTCATCAAAGAAA

GTGATGTATCAAAGGTGCCTGGCAAAAAAAGTAGAACCTATCCCAGTTACAAAACAACCC

ACCCTGCCCTCCGAAGCAACTTCCTCAAAGAAGAAACCAGGGCAGAAGAAGTCAAAAAAT

GGAAGCGATGATCAGGACAAAAAGGTGGAAACTCTCATGGCACCATCAAAAAAGCAAGAA

GTATTACCCTTCCATCAAGAGACTAAACAAGAAAGTGGATCAGGAAAAAAGAAGGCTTCA

TCAAAGAAGCAGAAGACAGAAAATGTCTTGGTAGATGAACCCCCTATTCATGCAACTACT

TATATTCCTTTGATGGATAATGCTAACTCAAATCTTGTGGTAGATAAGAGAGAAGTTATT

GATCTGATTAAATCTGACCAAGTAGAAGGAATCCAGAAATCTGGGACAAAAAAGTTGAAG

ATTGAAACTGACAAAGAAAATGCTGAAGTGAAATTTAAAGATTTTCTTCTGTCATTGAAG

ACTATGATGTTTTCTGAAGATGAGGCCCTTTGTGTTGTAGACTTGCTGAAAGAAAAGTCT

GGTGTGTTACAGGATGCTTTAAAGAAGTCAAGTAAAGGAGAATTGGCTGCACTTCTGCAT

CAGCTCCAGGAAAAGGACAAGTTGCTTGCTGCTATAAAAGAAGATGCTGCTGCTACAAAG

GATCGGTGTAAGCAGTTAACCCAGGAAATGATGACAGAGAAAGAAAGAAGCAGCGTAGTT

ATAGCAAGGATGAAAGATCGGATTGGAACATTAGAAAAGGAACATAATGTATTTCAGAAC

AAAATGCATGTCAGTTATCAAGAGACTCAACAGATGCAGATGAAGTTTCAGCAAGTTCGT

GAGCAGATGGAAGCAGAGATAGCACACTTGAAGCAGGAAAATGGTATACTGAGAGATGCT

GTCAGCAACACTACAAACCAACTAGAAAGCAAGCAGTCTGCAGAACTCAATAAACTGCGC

CAGGATTATGCTAGGTTGGTGAATGAGCTGGCTGAGAAGACAGGAAAGCTACAGCAAGAG

GAAGTCCAAAAAAAGAATGCAGAGCAAGCAGTTACTCAGTTGAAGGTTCAACTACAAGAA

GCTGAGAGAAGGTGGGAAGAAGTTCAAAGCTACATCAGGAAGAGAACAGCAGAACATGAG

GCAGCCCAGCAAGATTTACAGAGTAAATTTGTGGCCAAAGAAAATGAAGTACAGAGTCTG

CATAGTAAGCTTACAGATACGTTGGTATCAAAACAACAGTTGGAGCAAAGACTAATGCAG

TTAATGGAATCAGAACAAAAAAGGGTAAACAAAGAAGAGTCTCTACAAATGCAAGTTCAG

GATATTTTGGAGCAGAATGAGGCTTTGAAAGCTCAAATTCAACAGTTCCATTCCCAGATG

GCAGCCCAGACCTCCGCTTCAGTTCTAGCAGAAGAATTACATAAAGTGATTGCAGAAAAG

GATAAGCAGATAAAACAGACTGAAGACTCTTTAGCAAATGAACATGATCATTTAGCAAGT

AAAGAGGAGGAACTTAAGGATATACAGAATATGAATTTCTTATTAAAAGCTGAAGTACAG

AAATTACAGGCCCTGGCAAATGAGCAGGCTGCTGCAGCACATGAATTAGAGAAGATTCAA

AAAAGTGTTCATGTTAAGGATGATAAAATACGATTGCTGGAAGAGCAGCTACAGTGTGAA

ATTTCAAACAAAATTGAAGAATTTAAGATTCTAAATGACCAAAACAAAGCATTACAATTA

GAAGTTCAGAAACTACAGACTCTTCTTTCTGAACAGCCTAATAAAGATGTTGTGGAACAA

ATGGAAAAATGCATTCAAGAAAAAGATGAAAAGTTAAAGACCGTAGAAGAATTACTTGAA

ACTGGACTTATTCAGGCAATAAGAACAGAAAATTCATCTCTGACGAAAGAAGTTCAAGAC

TTAAAGGCTAAGCAAAATGATCAGGTTTCTTTTGCATCTTTAGTTGAAGAACTTAAGAAA

GTGATCCATGAGAAAGATGGAAAGATCAGATCTGTGGAAGAGCTTCTGGAAGCTGAAGTT

CTCAAAGTTGCTAATAAGGAGAAAACTGTTCAGGATTTGAAACAGGAAATAGAGACTCTA

AAAGAAGAAATAGGAAATATCCAGCTTGAAAAGGCTCAACAGTTATCTATCACTTCTCAA

GTTCAGGAGCTTCAGAAGTTATTAAAAGGAAAGGAGGAACAGATGAATACCATGAAGGTT

CTTATAGAAGAGAAGGAGAAAGACCTAGCCAATAGAGAGAAATGGTTACAGGATCTGCAA

GAAGAAAACGAATCTTTGAAGACTTATATGCAGGAAGCAGCACAACATAACTTGCAGGAG

GCGTATTCTACATCACAATTTGAAGAGCTTGAAATTATGTTGAAAGAGAAGGAAAATGAA

ATGAAGAGAGTAGAAGCTGTGCTAAAAGAGAGGGAGAGTGATCTTTCTAGCAAAACAAAG

CTGTTACAGGAGGTACAAGATGAAAACAAATTGTTTCAATCCCAGATTGAGCAGCTTAAG

CAACAAAATTACCAACAGGTATCTTCTTTCCCCCCTCATGAGGAATTATTGAAAGTAATT

TCAGACAGAGAGAAGGAAATAACTGGTCTCCAGAATGAGTTAGATTCTTTGAAAGATGCT

GTTGAACACCAAAGGAAGAAAAATAATGACCTTCGGGAGAAAAACTGGGAAGCAATGGAA

GCATTGGCATCAACTGAAAAAATGCTGCAGGACAAAGTGAACAAGACTTCCAAGGAAAGA

CAGCAACATGTAGAAGCTGTTGAGTTGAAGGCTAAAGAAGTTCTCAAAAAATTATTTCCA

AAGGTGTCTGTCCCTTCTAATTTGAATTACAGTGAATGGTTGTGTGGATTTGAACAGAAG

GCCAAAGAATGTATAGCTGGAACTTCAGGTTCAGAGGAGGTTAAGGTTCTAGAACACAAG

TTGAAAGAAGCTGATGAAATGCACACACTTTTACAGCTGGAGTGTGAAAAATATAAATCT

GTCCTTGCAGAAACAGAAGGAATTTTACAGAAGCTTCAGAGAAGTGTGGAGCAAGAAGAA

AATAAATGGAAAGTTAAGGTTGATGAATCACAAAAGACTATTAAGCAGATGCAGTCATCA

TTTACATCTTCAGAGCAAGAGCTAGAGCGATTAAGAAGAGAAAACAAAGATATTGAAAAT

CTGAGGAGAGAACGAGAACATTTGGAAATGGAACTAGAGAAGGCAGAAATTGAACGATCT

ACCTATGTTACAGAAGTCAGAGAGCTGAAAGATCTGTTGACTGAATTGCAGAAAAAACTT

GATGATTCATATTCTGAAGCAGTAAGACAGAATGAAGAGCTAAATTTGTTGAAG

>CL322.Contig4_A-W 4 3330 kinectin 1 (kinesin receptor) [Sus scrofa]

AAAGAAAGAAGAAGGAAAAGAAACAAAAACCTTTACTTGAAGAGCAAGTCATCAAAGAAA

GTGATGTATCAAAGGTGCCTGGCAAAAAAAGTAGAACCTATCCCAGTTACAAAACAACCC

ACCCTGCCCTCCGAAGCAACTTCCTCAAAGAAGAAACCAGGGCAGAAGAAGTCAAAAAAT

GGAAGCGATGATCAGGACAAAAAGGTGGAAACTCTCATGGCACCATCAAAAAAGCAAGAA

GTATTACCCTTCCATCAAGAGACTAAACAAGAAAGTGGATCAGGAAAAAAGAAGGCTTCA

TCAAAGAAGCAGAAGACAGAAAATGTCTTGGTAGATGAACCCCCTATTCATGCAACTACT

TATATTCCTTTGATGGATAATGCTAACTCAAATCTTGTGGTAGATAAGAGAGAAGTTATT

GATCTGATTAAATCTGACCAAGTAGAAGGAATCCAGAAATCTGGGACAAAAAAGTTGAAG

ATTGAAACTGACAAAGAAAATGCTGAAGTGAAATTTAAAGATTTTCTTCTGTCATTGAAG

ACTATGATGTTTTCTGAAGATGAGGCCCTTTGTGTTGTAGACTTGCTGAAAGAAAAGTCT

GGTGTGTTACAGGATGCTTTAAAGAAGTCAAGTAAAGGAGAATTGGCTGCACTTCTGCAT

CAGCTCCAGGAAAAGGACAAGTTGCTTGCTGCTATAAAAGAAGATGCTGCTGCTACAAAG

GATCGGTGTAAGCAGTTAACCCAGGAAATGATGACAGAGAAAGAAAGAAGCAGCGTAGTT

ATAGCAAGGATGAAAGATCGGATTGGAACATTAGAAAAGGAACATAATGTATTTCAGAAC

AAAATGCATGTCAGTTATCAAGAGACTCAACAGATGCAGATGAAGTTTCAGCAAGTTCGT

GAGCAGATGGAAGCAGAGATAGCACACTTGAAGCAGGAAAATGGTATACTGAGAGATGCT

GTCAGCAACACTACAAACCAACTAGAAAGCAAGCAGTCTGCAGAACTCAATAAACTGCGC

CAGGATTATGCTAGGTTGGTGAATGAGCTGGCTGAGAAGACAGGAAAGCTACAGCAAGAG

GAAGTCCAAAAAAAGAATGCAGAGCAAGCAGTTACTCAGTTGAAGGTTCAACTACAAGAA

GCTGAGAGAAGGTGGGAAGAAGTTCAAAGCTACATCAGGAAGAGAACAGCAGAACATGAG

GCAGCCCAGCAAGATTTACAGAGTAAATTTGTGGCCAAAGAAAATGAAGTACAGAGTCTG

CATAGTAAGCTTACAGATACGTTGGTATCAAAACAACAGTTGGAGCAAAGACTAATGCAG

TTAATGGAATCAGAACAAAAAAGGGTAAACAAAGAAGAGTCTCTACAAATGCAAGTTCAG

GATATTTTGGAGCAGAATGAGGCTTTGAAAGCTCAAATTCAACAGTTCCATTCCCAGATG

GCAGCCCAGACCTCCGCTTCAGTTCTAGCAGAAGAATTACATAAAGTGATTGCAGAAAAG

GATAAGCAGATAAAACAGACTGAAGACTCTTTAGCAAATGAACATGATCATTTAGCAAGT

AAAGAGGAGGAACTTAAGGATATACAGAATATGAATTTCTTATTAAAAGCTGAAGTACAG

AAATTACAGGCCCTGGCAAATGAGCAGGCTGCTGCAGCACATGAATTAGAGAAGATTCAA

AAAAGTGTTCATGTTAAGGATGATAAAATACGATTGCTGGAAGAGCAGCTACAGTGTGAA

ATTTCAAACAAAATTGAAGAATTTAAGATTCTAAATGACCAAAACAAAGCATTACAATTA

GAAGTTCAGAAACTACAGACTCTTCTTTCTGAACAGCCTAATAAAGATGTTGTGGAACAA

ATGGAAAAATGCATTCAAGAAAAAGATGAAAAGTTAAAGACCGTAGAAGAATTACTTGAA

ACTGGACTTATTCAGGCAATAAGAACAGAAAATTCATCTCTGACGAAAGAAGTTCAAGAC

TTAAAGGCTAAGCAAAATGATCAGGTTTCTTTTGCATCTTTAGTTGAAGAACTTAAGAAA

GTGATCCATGAGAAAGATGGAAAGATCAGATCTGTGGAAGAGCTTCTGGAAGCTGAAGTT

CTCAAAGTTGCTAATAAGGAGAAAACTGTTCAGGATTTGAAACAGGAAATAGAGACTCTA

AAAGAAGAAATAGGAAATATCCAGCTTGAAAAGGCTCAACAGTTATCTATCACTTCTCAA

GTTCAGGAGCTTCAGAAGTTATTAAAAGGAAAGGAGGAACAGATGAATACCATGAAGGTT

CTTATAGAAGAGAAGGAGAAAGACCTAGCCAATAGAGAGAAATGGTTACAGGATCTGCAA

GAAGAAAACGAATCTTTGAAGACTTATATGCAGGAAGCAGCACAACATAACTTGCAGGAG

GCGTATTCTACATCACAATTTGAAGAGCTTGAAATTATGTTGAAAGAGAAGGAAAATGAA

ATGAAGAGAGTAGAAGCTGTGCTAAAAGAGAGGGAGAGTGATCTTTCTAGCAAAACAAAG

CTGTTACAGGAGGTACAAGATGAAAACAAATTGTTTCAATCCCAGATTGAGCAGCTTAAG

CAACAAAATTACCAACAGGTATCTTCTTTCCCCCCTCATGAGGAATTATTGAAAGTAATT

TCAGACAGAGAGAAGGAAATAACTGGTCTCCAGAATGAGTTAGATTCTTTGAAAGATGCT

GTTGAACACCAAAGGAAGAAAAATAATGAAAGACAGCAACATGTAGAAGCTGTTGAGTTG

AAGGCTAAAGAAGTTCTCAAAAAATTATTTCCAAAGGTGTCTGTCCCTTCTAATTTGAAT

TACAGTGAATGGTTGTGTGGATTTGAACAGAAGGCCAAAGAATGTATAGCTGGAACTTCA

GGTTCAGAGGAGGTTAAGGTTCTAGAACACAAGTTGAAAGAAGCTGATGAAATGCACACA

CTTTTACAGCTGGAGTGTGAAAAATATAAATCTGTCCTTGCAGAAACAGAAGGAATTTTA

CAGAAGCTTCAGAGAAGTGTGGAGCAAGAAGAAAATAAATGGAAAGTTAAGGTTGATGAA

TCACAAAAGACTATTAAGCAGATGCAGTCATCATTTACATCTTCAGAGCAAGAGCTAGAG

CGATTAAGAAGAGAAAACAAAGATATTGAAAATCTGAGGAGAGAACGAGAACATTTGGAA

ATGGAACTAGAGAAGGCAGAAATTGAACGATCTACCTATGTTACAGAAGTCAGAGAGCTG

AAAGATCTGTTGACTGAATTGCAGAAAAAACTTGATGATTCATATTCTGAAGCAGTAAGA

CAGAATGAAGAGCTAAATTTGTTGAAG

>CL322.Contig5_A-W 76 1506 PREDICTED: kinectin isoform 4 [Papio anubis]

TCTCAAGTTCAGGAGCTTCAGAAGTTATTAAAAGGAAAGGAGGAACAGATGAATACCATG

AAGGTTCTTATAGAAGAGAAGGAGAAAGACCTAGCCAATAGAGAGAAATGGTTACAGGAT

CTGCAAGAAGAAAACGAATCTTTGAAGACTTATATGCAGGAAGCAGCACAACATAACTTG

CAGGAGGCGTATTCTACATCACAATTTGAAGAGCTTGAAATTATGTTGAAAGAGAAGGAA

AATGAAATGAAGAGAGTAGAAGCTGTGCTAAAAGAGAGGGAGAGTGATCTTTCTAGCAAA

ACAAAGCTGTTACAGGAGGTACAAGATGAAAACAAATTGTTTCAATCCCAGATTGAGCAG

CTTAAGCAACAAAATTACCAACAGGTATCTTCTTTCCCCCCTCATGAGGAATTATTGAAA

GTAATTTCAGACAGAGAGAAGGAAATAACTGGTCTCCAGAATGAGTTAGATTCTTTGAAA

GATGCTGTTGAACACCAAAGGAAGAAAAATAATGACCTTCGGGAGAAAAACTGGGAAGCA

ATGGAAGCATTGGCATCAACTGAAAAAATGCTGCAGGACAAAGTGAACAAGACTTCCAAG

GAAAGACAGCAACATGTAGAAGCTGTTGAGTTGAAGGCTAAAGAAGTTCTCAAAAAATTA

TTTCCAAAGGTGTCTGTCCCTTCTAATTTGAATTACAGTGAATGGTTGTGTGGATTTGAA

CAGAAGGCCAAAGAATGTATAGCTGGAACTTCAGGTTCAGAGGAGGTTAAGGTTCTAGAA

CACAAGTTGAAAGAAGCTGATGAAATGCACACACTTTTACAGCTGGAGTGTGAAAAATAT

AAATCTGTCCTTGCAGAAACAGAAGGAATTTTACAGAAGCTTCAGAGAAGTGTGGAGCAA

GAAGAAAATAAATGGAAAGTTAAGGTTGATGAATCACAAAAGACTATTAAGCAGATGCAG

TCATCATTTACATCTTCAGAGCAAGAGCTAGAGCGATTAAGAAGAGAAAACAAAGATATT

GAAAATCTGAGGAGAGAACGAGAACATTTGGAAATGGAACTAGAGAAGGCAGAAATTGAA

CGATCTACCTATGTTACAGAAGTCAGAGAGTTGAAGACCCAGTTAAATGAAACAGTCATA

AAACTTAAAACTGAACAAAGTGAAAGACAGAAGGTAGCTGGTGATTTATATAAGGCCCAA

CAGTCACTGGATCTTATCCAGTCCAAAATAGTAAAAGCTGCTGGAGACACTATTGTTATA

GAGAATAGTGAAGTTTCCTCAGAAACGGAGTCTTCTGAAAAGGAGAAAATGTTTATAAGT

CTAAATCAGACTCTCACACAGTTACAGCAGTTGCTACAGTCAGCAAACCAACAGCTCACA

AAGGAGAAATAACACTACCAGGCCTTGGAGTGAAGTGACTGGGAAACCATT

>CL322.Contig6_A-W 76 1239 PREDICTED: kinectin isoform 3 [Papio anubis] >gi|402876263|ref|XP_003901895.1| PREDICTED: kinectin isoform 5 [Papio anubis]

TCTCAAGTTCAGGAGCTTCAGAAGTTATTAAAAGGAAAGGAGGAACAGATGAATACCATG

AAGGTTCTTATAGAAGAGAAGGAGAAAGACCTAGCCAATAGAGAGAAATGGTTACAGGAT

CTGCAAGAAGAAAACGAATCTTTGAAGACTTATATGCAGGAAGCAGCACAACATAACTTG

CAGGAGGCGTATTCTACATCACAATTTGAAGAGCTTGAAATTATGTTGAAAGAGAAGGAA

AATGAAATGAAGAGAGTAGAAGCTGTGCTAAAAGAGAGGGAGAGTGATCTTTCTAGCAAA

ACAAAGCTGTTACAGGAGGTACAAGATGAAAACAAATTGTTTCAATCCCAGATTGAGCAG

CTTAAGCAACAAAATTACCAACAGGTATCTTCTTTCCCCCCTCATGAGGAATTATTGAAA

GTAATTTCAGACAGAGAGAAGGAAATAACTGGTCTCCAGAATGAGTTAGATTCTTTGAAA

GATGCTGTTGAACACCAAAGGAAGAAAAATAATGACCTTCGGGAGAAAAACTGGGAAGCA

ATGGAAGCATTGGCATCAACTGAAAAAATGCTGCAGGACAAAGTGAACAAGACTTCCAAG

GAAAGACAGCAACATGTAGAAGCTGTTGAGTTGAAGGCTAAAGAAGTTCTCAAAAAATTA

TTTCCAAAGGTGTCTGTCCCTTCTAATTTGAATTACAGTGAATGGTTGTGTGGATTTGAA

CAGAAGGCCAAAGAATGTATAGCTGGAACTTCAGGTTCAGAGGAGGTTAAGGTTCTAGAA

CACAAGTTGAAAGAAGCTGATGAAATGCACACACTTTTACAGCTGGAGTGTGAAAAATAT

AAATCTGTCCTTGCAGAAACAGAAGGAATTTTACAGAAGCTTCAGAGAAGTGTGGAGCAA

GAAGAAAATAAATGGAAAGTTAAGGTTGATGAATCACAAAAGACTATTAAGCAGATGCAG

TCATCATTTACATCTTCAGAGCAAGAGCTAGAGCGATTAAGAAGAGAAAACAAAGATATT

GAAAATCTGAGGAGAGAACGAGAACATTTGGAAATGGAACTAGAGAAGGCAGAAATTGAA

CGATCTACCTATGTTACAGAAGTCAGAGAGCTGAAAGATCTGTTGACTGAATTGCAGAAA

AAACTTGATGATTCATATTCTGAA

>CL322.Contig7_A-W 76 1182 PREDICTED: kinectin isoform 3 [Papio anubis] >gi|402876263|ref|XP_003901895.1| PREDICTED: kinectin isoform 5 [Papio anubis]

TCTCAAGTTCAGGAGCTTCAGAAGTTATTAAAAGGAAAGGAGGAACAGATGAATACCATG

AAGGTTCTTATAGAAGAGAAGGAGAAAGACCTAGCCAATAGAGAGAAATGGTTACAGGAT

CTGCAAGAAGAAAACGAATCTTTGAAGACTTATATGCAGGAAGCAGCACAACATAACTTG

CAGGAGGCGTATTCTACATCACAATTTGAAGAGCTTGAAATTATGTTGAAAGAGAAGGAA

AATGAAATGAAGAGAGTAGAAGCTGTGCTAAAAGAGAGGGAGAGTGATCTTTCTAGCAAA

ACAAAGCTGTTACAGGAGGTACAAGATGAAAACAAATTGTTTCAATCCCAGATTGAGCAG

CTTAAGCAACAAAATTACCAACAGGTATCTTCTTTCCCCCCTCATGAGGAATTATTGAAA

GTAATTTCAGACAGAGAGAAGGAAATAACTGGTCTCCAGAATGAGTTAGATTCTTTGAAA

GATGCTGTTGAACACCAAAGGAAGAAAAATAATGAAAGACAGCAACATGTAGAAGCTGTT

GAGTTGAAGGCTAAAGAAGTTCTCAAAAAATTATTTCCAAAGGTGTCTGTCCCTTCTAAT

TTGAATTACAGTGAATGGTTGTGTGGATTTGAACAGAAGGCCAAAGAATGTATAGCTGGA

ACTTCAGGTTCAGAGGAGGTTAAGGTTCTAGAACACAAGTTGAAAGAAGCTGATGAAATG

CACACACTTTTACAGCTGGAGTGTGAAAAATATAAATCTGTCCTTGCAGAAACAGAAGGA

ATTTTACAGAAGCTTCAGAGAAGTGTGGAGCAAGAAGAAAATAAATGGAAAGTTAAGGTT

GATGAATCACAAAAGACTATTAAGCAGATGCAGTCATCATTTACATCTTCAGAGCAAGAG

CTAGAGCGATTAAGAAGAGAAAACAAAGATATTGAAAATCTGAGGAGAGAACGAGAACAT

TTGGAAATGGAACTAGAGAAGGCAGAAATTGAACGATCTACCTATGTTACAGAAGTCAGA

GAGCTGAAAGATCTGTTGACTGAATTGCAGAAAAAACTTGATGATTCATATTCTGAAGCA

GTAAGACAGAATGAAGAGCTAAATTTG

>CL322.Contig8_A-W 2 3736 kinectin isoform a [Macaca mulatta] >gi|383418271|gb|AFH32349.1| kinectin isoform a [Macaca mulatta] >gi|384946988|gb|AFI37099.1| kinectin isoform a [Macaca mulatta]

GAAAGAAAGAAGAAGGAAAAGAAACAAAAACCTTTACTTGAAGAGCAAGTCATCAAAGAA

AGTGATGTATCAAAGGTGCCTGGCAAAAAAGTAGAACCTATCCCAGTTACAAAACAACCC

ACCCTGCCCTCCGAAGCAACTTCCTCAAAGAAGAAACCAGGGCAGAAGAAGTCAAAAAAT

GGAAGCGATGATCAGGACAAAAAGGTGGAAACTCTCATGGCACCATCAAAAAAGCAAGAA

GTATTACCCTTCCATCAAGAGACTAAACAAGAAAGTGGATCAGGAAAAAAGAAGGCTTCA

TCAAAGAAGCAGAAGACAGAAAATGTCTTGGTAGATGAACCCCCTATTCATGCAACTACT

TATATTCCTTTGATGGATAATGCTAACTCAAATCTTGTGGTAGATAAGAGAGAAGTTATT

GATCTGATTAAATCTGACCAAGTAGAAGGAATCCAGAAATCTGGGACAAAAAAGTTGAAG

ATTGAAACTGACAAAGAAAATGCTGAAGTGAAATTTAAAGATTTTCTTCTGTCATTGAAG

ACTATGATGTTTTCTGAAGATGAGGCCCTTTGTGTTGTAGACTTGCTGAAAGAAAAGTCT

GGTGTGTTACAGGATGCTTTAAAGAAGTCAAGTAAAGGAGAATTGGCTGCACTTCTGCAT

CAGCTCCAGGAAAAGGACAAGTTGCTTGCTGCTATAAAAGAAGATGCTGCTGCTACAAAG

GATCGGTGTAAGCAGTTAACCCAGGAAATGATGACAGAGAAAGAAAGAAGCAGCGTAGTT

ATAGCAAGGATGAAAGATCGGATTGGAACATTAGAAAAGGAACATAATGTATTTCAGAAC

AAAATGCATGTCAGTTATCAAGAGACTCAACAGATGCAGATGAAGTTTCAGCAAGTTCGT

GAGCAGATGGAAGCAGAGATAGCACACTTGAAGCAGGAAAATGGTATACTGAGAGATGCT

GTCAGCAACACTACAAACCAACTAGAAAGCAAGCAGTCTGCAGAACTCAATAAACTGCGC

CAGGATTATGCTAGGTTGGTGAATGAGCTGGCTGAGAAGACAGGAAAGCTACAGCAAGAG

GAAGTCCAAAAAAAGAATGCAGAGCAAGCAGTTACTCAGTTGAAGGTTCAACTACAAGAA

GCTGAGAGAAGGTGGGAAGAAGTTCAAAGCTACATCAGGAAGAGAACAGCAGAACATGAG

GCAGCCCAGCAAGATTTACAGAGTAAATTTGTGGCCAAAGAAAATGAAGTACAGAGTCTG

CATAGTAAGCTTACAGATACGTTGGTATCAAAACAACAGTTGGAGCAAAGACTAATGCAG

TTAATGGAATCAGAACAAAAAAGGGTAAACAAAGAAGAGTCTCTACAAATGCAAGTTCAG

GATATTTTGGAGCAGAATGAGGCTTTGAAAGCTCAAATTCAACAGTTCCATTCCCAGATG

GCAGCCCAGACCTCCGCTTCAGTTCTAGCAGAAGAATTACATAAAGTGATTGCAGAAAAG

GATAAGCAGATAAAACAGACTGAAGACTCTTTAGCAAATGAACATGATCATTTAGCAAGT

AAAGAGGAGGAACTTAAGGATATACAGAATATGAATTTCTTATTAAAAGCTGAAGTACAG

AAATTACAGGCCCTGGCAAATGAGCAGGCTGCTGCAGCACATGAATTAGAGAAGATTCAA

AAAAGTGTTCATATTAAGGATGATAAAATACGATTGCTGGAAGAGCAGCTACAGTGTGAA

ATTTCAAACAAAATTGAAGAATTTAAGATTCTAAATGACCAAAACAAAGCATTACAATTA

GAAGTTCAGAAACTACAGACTCTTCTTTCTGAACAGCCTAATAAAGATGTTGTGGAACAA

ATGGAAAAATGCATTCAAGAAAAAGATGAAAAGTTAAAGACCGTAGAAGAATTACTTGAA

ACTGGACTTATTCAGGTGGCCACTAAAGAGGAGGAACTGAATGCAATAAGAACAGAAAAT

TCATCTCTGACGAAAGAAGTTCAAGACTTAAAGGCTAAGCAAAATGATCAGGTTTCTTTT

GCATCTTTAGTTGAAGAACTTAAGAAAGTGATCCATGAGAAAGATGGAAAGATCAGATCT

GTGGAAGAGCTTCTGGAAGCTGAAGTTCTCAAAGTTGCTAATAAGGAGAAAACTGTTCAG

GATTTGAAACAGGAAATAGAGACTCTAAAAGAAGAAATAGGAAATATCCAGCTTGAAAAG

GCTCAACAGTTATCTATCACTTCTCAAGTTCAGGAGCTTCAGAAGTTATTAAAAGGAAAG

GAGGAACAGATGAATACCATGAAGGTTCTTATAGAAGAGAAGGAGAAAGACCTAGCCAAT

AGAGAGAAATGGTTACAGGATCTGCAAGAAGAAAACGAATCTTTGAAGACTTATATGCAG

GAAGCAGCACAACATAACTTGCAGGAGGCGTATTCTGCATCACAATTTGAAGAGCTTGAA

ATTATGTTGAAAGAGAAGGAAAATGAAATGAAGAGAGTAGAAGCTGTGCTAAAAGAGAGG

GAGAGCGATCTTTCTAGCAAAACAAAGCTGTTACAGGAGGTACAAGATGAAAACAAATTG

TTTCAATCCCAGATTGAGCAGCTTAAGCAACAAAATTACCAACAGGTATCTTCTTTCCCC

CCTCATGAGGAATTATTGAAAGTAATTTCAGACAGAGAGAAGGAAATAACTGGTCTCCAG

AATGAGTTAGATTCTTTGAAAGATGCTGTTGAACACCAAAGGAAGAAAAATAATGACCTT

CGGGAGAAAAACTGGGAAGCAATGGAAGCATTGGCATCAACTGAAAAAATGCTGCAGGAC

AAAGTGAACAAGACTTCCAAGGAAAGACAGCAACATGTAGAAGCTGTTGAGTTGAAGGCT

AAAGAAGTTCTCAAAAAATTATTTCCAAAGGTGTCTGTCCCTTCTAATTTGAATTACAGT

GAATGGTTGTGTGGATTTGAACAGAAGGCCAAAGAATGTATAGCTGGAACTTCAGGTTCA

GAGGAGGTTAAGGTTCTAGAACACAAGTTGAAAGAAGCTGATGAAATGCACACACTTTTA

CAGCTGGAGTGTGAAAAATATAAATCTGTCCTTGCAGAAACAGAAGGAATTTTACAGAAG

CTTCAGAGAAGTGTGGAGCAAGAAGAAAATAAATGGAAAGTTAAGGTTGATGAATCACAA

AAGACTATTAAGCAGATGCAGTCATCATTTACATCTTCAGAGCAAGAGCTAGAGCGATTA

AGAAGAGAAAACAAAGATATTGAAAATCTGAGGAGAGAACGAGAACATTTGGAAATGGAA

CTAGAGAAGGCAGAAATTGAACGATCTACCTATGTTACAGAAGTCAGAGAGCTGAAAGAT

CTGTTGACTGAATTGCAGAAAAAACTTGATGATTCATATTCTGAAGCAGTAAGACAGAAT

GAAGAGCTAAATTTGTTGAAGACCCAGTTAAATGAAACAGTCATAAAACTTAAAACTGAA

CAAAGTGAAAGACAGAAGGTAGCTGGTGATTTATATAAGGCCCAACAGTCACTGGATCTT

ATCCAGTCCAAAATAGTAAAAGCTGCTGGAGACACTATTGTTATAGAGAATAGTGAAGTT

TCCTCAGAAACGGAGTCTTCTGAAAAGGAGAAAATGTTTATAAGTCTAAATCAGACTCTC

ACACAGTTACAGCAGTTGCTACAGTCAGCAAACCAACAGCTCACAAAGGAGAAATAACAC

TACCAGGCCTTGGAG

>CL322.Contig9_A-W 3 263 PREDICTED: kinectin-like [Ailuropoda melanoleuca]

GAAAGAAAGAAGAAGGAAAAGAAACAAAAACCTTTACTTGAAGAGCAAGTCATCAAAGAA

AGTGATGTATCAAAGGTGCCTGGCAAAAAAAGTAGAACCTATCCCAGTTACAAAACAACC

CACCCTGCCCTCCGAAGCAACTTCCTCAAAGAAGAAACCAGGGCAGAAGAAGTCAAAAAA

TGGAAGCGATGATCAGGACAAAAAGGTGGAAACTCTCATGGCACCATCAAAAAAGCAAGA

AGTATTACCCTTCCATCAAGA

>CL325.Contig1_A-W 16 603 PREDICTED: mitochondrial ribosomal protein S18A [Oryctolagus cuniculus]

ATGGCGTCCTTCATGGCGCTGGTATCCGGCTGTGGGCGGCTGCTCCGAGGGCTCCTGGGG

GTCCCAGTCACGACCAGTTGCCCTCGGGTTCCAGCTCGAGGATTCAGGGAAGTGGTAGAG

ATCCAAGAAGGGAAGACAACTATAATCGAAGGCCGTATCATAGAGACTCCCAAGGAGAGT

CCACATCCCCCCAACCCCTCAGGCCAGTGCCCCATCTGCCGTTGGAACCTGAAGCACAAG

TATACCTATGAGGATGTTCTGCTACTAAGCCAGTTCATCCGGCCTCATGGAGGCATGCTG

CCCCGAAAAATCACAGGCTTATGCCAGGAAGAACACCGCAAGATTGAGGAGTGTGTGAAG

ATGGCCCACCGAGCAGGTCTGCTCCCAAATCACAGGCCTCGGCTTCCTGAAGGATTTGTT

CCAAAGAGCAAACCCCAACTCAACCGTTACCTGACCCGCTGGTCTCCCCACTCCGTCAAG

CCAATCTACAAAAGAGGTGCCCGCTGGAACAAGGTGGGCATGGCTGTGGGGTCACCACTC

CTAAAGGACAACATCTGCTACTCAAGAACGCCTTTGAAATTCTATCAT

>CL325.Contig2_A-W 45 488 PREDICTED: 28S ribosomal protein S18a, mitochondrial [Otolemur garnettii]

ATCGAAGGCCGTATCATAGAGACTCCCAAGGAGAGTCCACATCCCCCCAACCCCTCAGGC

CAGTGCCCCATCTGCCGTTGGAACCTGAAGCACAAGTATACCTATGAGGATGTTCTGCTA

CTAAGCCAGTTCATCCGGCCTCATGGAGGCATGCTGCCCCGAAAAATCACAGGCTTATGC

CAGGAAGAACACCGCAAGATTGAGGAGTGTGTGAAGATGGCCCACCGAGCAGGTCTGCTC

CCAAATCACAGGCCTCGGCTTCCTGAAGGATTTGTTCCAAAGAGCAAACCCCAACTCAAC

CGTTACCTGACCCGCTGGTCTCCCCACTCCGTCAAGCCAATCTACAAAAGAGGTGCCCGC

TGGAACAAGGTGGGCATGGCTGTGGGGTCACCACTCCTAAAGGACAACATCTGCTACTCA

AGAACGCCTTTGAAATTCTATCAT

>CL326.Contig1_A-W 169 441 minus strand PREDICTED: serine/arginine-rich splicing factor 6 isoform 2 [Nomascus leucogenys]

ATGCCTCGCGTCTACATAGGACGCCTGAGCTACAACGTCCGGGAGAAGGACATCCAGCGC

TTTTTCAGCGGCTATGGCCGCCTCCTCGAAGTAGACCTCAAAAATGGGTACGGCTTCGTG

GAGTTCGAGGACTCCCGCGACGCCGACGACGCCGTTTACGAGCTGAACGGCAAGGAGCTC

TGCGGTGAGCGCGTGATCGTGGAGCACGCCCGGGGCCCGCGTCGCGATCGCGACGGCTAC

AGCTACGGAAGCCGCATGACCAATGGGGCTGAG

>CL326.Contig2_A-W 767 1474 minus strand PREDICTED: serine/arginine-rich splicing factor 6 isoform 2 [Nomascus leucogenys]

AGGCTTATTGTAGAAAATCTTTCTAGTCGTTGCAGTTGGCAAGATTTAAAGGATTTCATG

CGGCAAGCAGGTGAAGTAACATATGCAGATGCTCACAAAGAACGTACAAATGAAGGTGTA

ATTGAATTTCGCTCCTACTCTGACATGAAGCGTGCTTTGGATAAACTAGATGGTACAGAG

ATAAATGGCAGGAATATTAGACTTATTGAAGATAAGCCACGAGCAAGCCATAGGAGGTCT

TATTCTGGAAGCAGATCTAGGTCACGTTCAAGAAGGAGGTCACGAAGTAGGAGTCGCAGG

AGCAGCCGCAGTAGATCTCGAAGTGTCTCAAAAAGTCGCTCCCGATCCAGGTCACGGAGC

AAAGGTCGATCACGTTCTCGATCAAAAGGCAGGAAATCTAGATCAAAAAGCAAATCTAAG

CCCAAGTCTGATCGGGGTTCCCATTCACATTCTCGAAGCAGATCTAAGGATGAGTATGAA

AAGTCCCGAAGCAGGTCTCGATCTCGGTCCCCCAAAGAAAATGGAAAAGGTGATATAAAG

TCAAAATCCAGATCAAGGAGCCAGTCTCGTTCCAATTCACCCCTTCCTGTTCCACCCTCA

AAGGCTCGTTCTGTGTCCCCTCCACCAAAAAGAGCTACTTCAAGATCCCGTTCTAGATCT

CGCTCAAGGTCAAGGTCCAGATCAAGTTCCAGAGATTAACCCAGAACT

>CL326.Contig3_A-W 169 1320 minus strand serine/arginine-rich splicing factor 6 [Homo sapiens] >gi|307078161|ref|NP_001182497.1| splicing factor, arginine/serine-rich 6 [Pongo abelii] >gi|20981728|sp|Q13247.2|SRSF6_HUMAN RecName: Full=Serine/arginine-rich splicing factor 6; AltName: Full=Pre-mRNA-splicing factor SRP55; AltName: Full=Splicing factor, arginine/serine-rich 6 >gi|4972266|emb|CAB43960.1| splicing factor, arginine/serine-rich 6 [Homo sapiens] >gi|13905094|gb|AAH06832.1| Splicing factor, arginine/serine-rich 6 [Homo sapiens] >gi|119596370|gb|EAW75964.1| splicing factor, arginine/serine-rich 6, isoform CRA_a [Homo sapiens] >gi|119596373|gb|EAW75967.1| splicing factor, arginine/serine-rich 6, isoform CRA_a [Homo sapiens]

ATGCCTCGCGTCTACATAGGACGCCTGAGCTACAACGTCCGGGAGAAGGACATCCAGCGC

TTTTTCAGCGGCTATGGCCGCCTCCTCGAAGTAGACCTCAAAAATGGGTACGGCTTCGTG

GAGTTCGAGGACTCCCGCGACGCCGACGACGCCGTTTACGAGCTGAACGGCAAGGAGCTC

TGCGGTGAGCGCGTGATCGTGGAGCACGCCCGGGGCCCGCGTCGCGATCGCGACGGCTAC

AGCTACGGAAGCCGCAGTGGTGGAGGTGGATACAGCAGTCGGAGAACCTCTGGCAGAGAC

AAATATGGACCTCCTGTTCGTACAGAGTACAGGCTTATTGTAGAAAATCTTTCTAGTCGT

TGCAGTTGGCAAGATTTAAAGGTATGTCTTATAATTTGAGTTGAGATTTGATTAATTCTT

AAAAGGTAGGCCCTTTTGGTACCTTTTTTTCTGAGAACTAAAACCAGCATGCCCAGCTTG

TGAGTCTGATAAGTCTTATTGTTTCAGGATTTCATGCGGCAAGCAGGTGAAGTAACATAT

GCAGATGCTCACAAAGAACGTACAAATGAAGGTGTAATTGAATTTCGCTCCTACTCTGAC

ATGAAGCGTGCTTTGGATAAACTAGATGGTACAGAGATAAATGGCAGGAATATTAGACTT

ATTGAAGATAAGCCACGAGCAAGCCATAGGAGGTCTTATTCTGGAAGCAGATCTAGGTCA

CGTTCAAGAAGGAGGTCACGAAGTAGGAGTCGCAGGAGCAGCCGCAGTAGATCTCGAAGT

GTCTCAAAAAGTCGCTCCCGATCCAGGTCACGGAGCAAAGGTCGATCACGTTCTCGATCA

AAAGGCAGGAAATCTAGATCAAAAAGCAAATCTAAGCCCAAGTCTGATCGGGGTTCCCAT

TCACATTCTCGAAGCAGATCTAAGGATGAGTATGAAAAGTCCCGAAGCAGGTCTCGATCT

CGGTCCCCCAAAGAAAATGGAAAAGGTGATATAAAGTCAAAATCCAGATCAAGGAGCCAG

TCTCGTTCCAATTCACCCCTTCCTGTTCCACCCTCAAAGGCTCGTTCTGTGTCCCCTCCA

CCAAAAAGAGCTACTTCAAGATCCCGTTCTAGATCTCGCTCAAGGTCAAGGTCCAGATCA

AGTTCCAGAGAT

>CL326.Contig4_A-W 345 1244 minus strand splicing factor, arginine/serine-rich 6, isoform CRA_c [Homo sapiens]

AAAGGTGGTGGAGGTGGATACAGCAGTCGGAGAACCTCTGGCAGAGACAAATATGGACCT

CCTGTTCGTACAGAGTACAGGCTTATTGTAGAAAATCTTTCTAGTCGTTGCAGTTGGCAA

GATTTAAAGGTATGTCTTATAATTTGAGTTGAGATTTGATTAATTCTTAAAAGGTAGGCC

CTTTTGGTACCTTTTTTTCTGAGAACTAAAACCAGCATGCCCAGCTTGTGAGTCTGATAA

GTCTTATTGTTTCAGGATTTCATGCGGCAAGCAGGTGAAGTAACATATGCAGATGCTCAC

AAAGAACGTACAAATGAAGGTGTAATTGAATTTCGCTCCTACTCTGACATGAAGCGTGCT

TTGGATAAACTAGATGGTACAGAGATAAATGGCAGGAATATTAGACTTATTGAAGATAAG

CCACGAGCAAGCCATAGGAGGTCTTATTCTGGAAGCAGATCTAGGTCACGTTCAAGAAGG

AGGTCACGAAGTAGGAGTCGCAGGAGCAGCCGCAGTAGATCTCGAAGTGTCTCAAAAAGT

CGCTCCCGATCCAGGTCACGGAGCAAAGGTCGATCACGTTCTCGATCAAAAGGCAGGAAA

TCTAGATCAAAAAGCAAATCTAAGCCCAAGTCTGATCGGGGTTCCCATTCACATTCTCGA

AGCAGATCTAAGGATGAGTATGAAAAGTCCCGAAGCAGGTCTCGATCTCGGTCCCCCAAA

GAAAATGGAAAAGGTGATATAAAGTCAAAATCCAGATCAAGGAGCCAGTCTCGTTCCAAT

TCACCCCTTCCTGTTCCACCCTCAAAGGCTCGTTCTGTGTCCCCTCCACCAAAAAGAGCT

ACTTCAAGATCCCGTTCTAGATCTCGCTCAAGGTCAAGGTCCAGATCAAGTTCCAGAGAT

>CL326.Contig5_A-W 67 426 minus strand arginine/serine-rich splicing factor 6 variant [Homo sapiens]

GGCGCGTGTTCGGGCCCTCGCCGTCCCCGCACCCGCACCGCGGCTACTGCCTTGCTGTCC

GCCGTCCGACAGCCAGCCTTCGGGACCTCGCCCACCACGGACATGCCTCGCGTCTACATA

GGACGCCTGAGCTACAACGTCCGGGAGAAGGACATCCAGCGCTTTTTCAGCGGCTATGGC

CGCCTCCTCGAAGTAGACCTCAAAAATGGGTACGGCTTCGTGGAGTTCGAGGACTCCCGC

GACGCCGACGACGCCGTTTACGAGCTGAACGGCAAGGAGCTCTGCGGTGAGCGCGTGATC

GTGGAGCACGCCCGGGGCCCGCGTCGCGATCGCGACGGCTACAGCTACGGAAGCCGCAGT

>CL326.Contig6_A-W 345 1118 minus strand splicing factor, arginine/serine-rich 6, isoform CRA_c [Homo sapiens]

AAAGGTGGTGGAGGTGGATACAGCAGTCGGAGAACCTCTGGCAGAGACAAATATGGACCT

CCTGTTCGTACAGAGTACAGGCTTATTGTAGAAAATCTTTCTAGTCGTTGCAGTTGGCAA

GATTTAAAGGATTTCATGCGGCAAGCAGGTGAAGTAACATATGCAGATGCTCACAAAGAA

CGTACAAATGAAGGTGTAATTGAATTTCGCTCCTACTCTGACATGAAGCGTGCTTTGGAT

AAACTAGATGGTACAGAGATAAATGGCAGGAATATTAGACTTATTGAAGATAAGCCACGA

GCAAGCCATAGGAGGTCTTATTCTGGAAGCAGATCTAGGTCACGTTCAAGAAGGAGGTCA

CGAAGTAGGAGTCGCAGGAGCAGCCGCAGTAGATCTCGAAGTGTCTCAAAAAGTCGCTCC

CGATCCAGGTCACGGAGCAAAGGTCGATCACGTTCTCGATCAAAAGGCAGGAAATCTAGA

TCAAAAAGCAAATCTAAGCCCAAGTCTGATCGGGGTTCCCATTCACATTCTCGAAGCAGA

TCTAAGGATGAGTATGAAAAGTCCCGAAGCAGGTCTCGATCTCGGTCCCCCAAAGAAAAT

GGAAAAGGTGATATAAAGTCAAAATCCAGATCAAGGAGCCAGTCTCGTTCCAATTCACCC

CTTCCTGTTCCACCCTCAAAGGCTCGTTCTGTGTCCCCTCCACCAAAAAGAGCTACTTCA

AGATCCCGTTCTAGATCTCGCTCAAGGTCAAGGTCCAGATCAAGTTCCAGAGAT

>CL326.Contig7_A-W 169 1194 minus strand serine/arginine-rich splicing factor 6 [Homo sapiens] >gi|307078161|ref|NP_001182497.1| splicing factor, arginine/serine-rich 6 [Pongo abelii] >gi|20981728|sp|Q13247.2|SRSF6_HUMAN RecName: Full=Serine/arginine-rich splicing factor 6; AltName: Full=Pre-mRNA-splicing factor SRP55; AltName: Full=Splicing factor, arginine/serine-rich 6 >gi|4972266|emb|CAB43960.1| splicing factor, arginine/serine-rich 6 [Homo sapiens] >gi|13905094|gb|AAH06832.1| Splicing factor, arginine/serine-rich 6 [Homo sapiens] >gi|119596370|gb|EAW75964.1| splicing factor, arginine/serine-rich 6, isoform CRA_a [Homo sapiens] >gi|119596373|gb|EAW75967.1| splicing factor, arginine/serine-rich 6, isoform CRA_a [Homo sapiens]

ATGCCTCGCGTCTACATAGGACGCCTGAGCTACAACGTCCGGGAGAAGGACATCCAGCGC

TTTTTCAGCGGCTATGGCCGCCTCCTCGAAGTAGACCTCAAAAATGGGTACGGCTTCGTG

GAGTTCGAGGACTCCCGCGACGCCGACGACGCCGTTTACGAGCTGAACGGCAAGGAGCTC

TGCGGTGAGCGCGTGATCGTGGAGCACGCCCGGGGCCCGCGTCGCGATCGCGACGGCTAC

AGCTACGGAAGCCGCAGTGGTGGAGGTGGATACAGCAGTCGGAGAACCTCTGGCAGAGAC

AAATATGGACCTCCTGTTCGTACAGAGTACAGGCTTATTGTAGAAAATCTTTCTAGTCGT

TGCAGTTGGCAAGATTTAAAGGATTTCATGCGGCAAGCAGGTGAAGTAACATATGCAGAT

GCTCACAAAGAACGTACAAATGAAGGTGTAATTGAATTTCGCTCCTACTCTGACATGAAG

CGTGCTTTGGATAAACTAGATGGTACAGAGATAAATGGCAGGAATATTAGACTTATTGAA

GATAAGCCACGAGCAAGCCATAGGAGGTCTTATTCTGGAAGCAGATCTAGGTCACGTTCA

AGAAGGAGGTCACGAAGTAGGAGTCGCAGGAGCAGCCGCAGTAGATCTCGAAGTGTCTCA

AAAAGTCGCTCCCGATCCAGGTCACGGAGCAAAGGTCGATCACGTTCTCGATCAAAAGGC

AGGAAATCTAGATCAAAAAGCAAATCTAAGCCCAAGTCTGATCGGGGTTCCCATTCACAT

TCTCGAAGCAGATCTAAGGATGAGTATGAAAAGTCCCGAAGCAGGTCTCGATCTCGGTCC

CCCAAAGAAAATGGAAAAGGTGATATAAAGTCAAAATCCAGATCAAGGAGCCAGTCTCGT

TCCAATTCACCCCTTCCTGTTCCACCCTCAAAGGCTCGTTCTGTGTCCCCTCCACCAAAA

AGAGCTACTTCAAGATCCCGTTCTAGATCTCGCTCAAGGTCAAGGTCCAGATCAAGTTCC

AGAGAT

>CL326.Contig8_A-W 514 1221 minus strand PREDICTED: serine/arginine-rich splicing factor 6 isoform 2 [Nomascus leucogenys]

AGGCTTATTGTAGAAAATCTTTCTAGTCGTTGCAGTTGGCAAGATTTAAAGGATTTCATG

CGGCAAGCAGGTGAAGTAACATATGCAGATGCTCACAAAGAACGTACAAATGAAGGTGTA

ATTGAATTTCGCTCCTACTCTGACATGAAGCGTGCTTTGGATAAACTAGATGGTACAGAG

ATAAATGGCAGGAATATTAGACTTATTGAAGATAAGCCACGAGCAAGCCATAGGAGGTCT

TATTCTGGAAGCAGATCTAGGTCACGTTCAAGAAGGAGGTCACGAAGTAGGAGTCGCAGG

AGCAGCCGCAGTAGATCTCGAAGTGTCTCAAAAAGTCGCTCCCGATCCAGGTCACGGAGC

AAAGGTCGATCACGTTCTCGATCAAAAGGCAGGAAATCTAGATCAAAAAGCAAATCTAAG

CCCAAGTCTGATCGGGGTTCCCATTCACATTCTCGAAGCAGATCTAAGGATGAGTATGAA

AAGTCCCGAAGCAGGTCTCGATCTCGGTCCCCCAAAGAAAATGGAAAAGGTGATATAAAG

TCAAAATCCAGATCAAGGAGCCAGTCTCGTTCCAATTCACCCCTTCCTGTTCCACCCTCA

AAGGCTCGTTCTGTGTCCCCTCCACCAAAAAGAGCTACTTCAAGATCCCGTTCTAGATCT

CGCTCAAGGTCAAGGTCCAGATCAAGTTCCAGAGATTAACCCAGAACT

>CL326.Contig9_A-W 442 1335 minus strand PREDICTED: serine/arginine-rich splicing factor 6 isoform 2 [Nomascus leucogenys]

GGTGGAGGTGGATACAGCAGTCGGAGAACCTCTGGCAGAGACAAATATGGACCTCCTGTT

CGTACAGAGTACAGGCTTATTGTAGAAAATCTTTCTAGTCGTTGCAGTTGGCAAGATTTA

AAGGTATGTCTTATAATTTGAGTTGAGATTTGATTAATTCTTAAAAGGTAGGCCCTTTTG

GTACCTTTTTTTCTGAGAACTAAAACCAGCATGCCCAGCTTGTGAGTCTGATAAGTCTTA

TTGTTTCAGGATTTCATGCGGCAAGCAGGTGAAGTAACATATGCAGATGCTCACAAAGAA

CGTACAAATGAAGGTGTAATTGAATTTCGCTCCTACTCTGACATGAAGCGTGCTTTGGAT

AAACTAGATGGTACAGAGATAAATGGCAGGAATATTAGACTTATTGAAGATAAGCCACGA

GCAAGCCATAGGAGGTCTTATTCTGGAAGCAGATCTAGGTCACGTTCAAGAAGGAGGTCA

CGAAGTAGGAGTCGCAGGAGCAGCCGCAGTAGATCTCGAAGTGTCTCAAAAAGTCGCTCC

CGATCCAGGTCACGGAGCAAAGGTCGATCACGTTCTCGATCAAAAGGCAGGAAATCTAGA

TCAAAAAGCAAATCTAAGCCCAAGTCTGATCGGGGTTCCCATTCACATTCTCGAAGCAGA

TCTAAGGATGAGTATGAAAAGTCCCGAAGCAGGTCTCGATCTCGGTCCCCCAAAGAAAAT

GGAAAAGGTGATATAAAGTCAAAATCCAGATCAAGGAGCCAGTCTCGTTCCAATTCACCC

CTTCCTGTTCCACCCTCAAAGGCTCGTTCTGTGTCCCCTCCACCAAAAAGAGCTACTTCA

AGATCCCGTTCTAGATCTCGCTCAAGGTCAAGGTCCAGATCAAGTTCCAGAGAT

>CL328.Contig1_A-W 2 1060 unnamed protein product [Homo sapiens]

GAGGACACAGCCACCTATTACTGCGCGAGTAGCGGGTCCTACTGGGGCCAGGGCACCCTG

GTCACCGTCTCCTCAGCTTCGACCACGGCCCCATCAGTCTTCCCCCTGGCCCCCAGCTCT

AGGGACACATCTGGCTCCTCAGTGAACCTGGGCTGCCTGGTCAAGGGCTACTTCCCTGAG

CCGGTGACCGTGACATGGAACTCAGGATCCCTGTCCAGTGGAGTCCGCACCTTCCCAGCT

GTCCTGCAGTCAGGGCTCTACTCTGTCACCAGCATGGTGACTGTGCCCACCAGCACCTGG

CCCAGCCAGACCGTCACCTGCAATGTAGCCCACCCGGCCAGCAGCACCAAGGTGGACAAG

ACCATTGCTCCCTGTGGTCGTGAACCTACCCCATGTCCACCATGTCCACCTCCTGAAATC

CTGGGGGGACCATCCGTCTTCATCTTCCCCCCAAAGCCCAAGGACACGCTCATGATCTCC

CTGACCCCCAAGGTCACATGCGTGGTGGTAGATGTGAGCCAGGATGACCCCGAGGTCCAG

TTCAGCTGGTATGTAGACAACGTGGAGGTGAGCACAGCTGAGACGAAGCCACAGGAGAGG

CAGTACAACAGCACATTCCGCGTGGTCAGTGTCCTCCCCATCAAGCACCAGGACTGGCTG

AATGGCAAGAAGTTCAAGTGCAAGGTCAACAACAAAGCCCTGCCAGCCCCCATCGAGAGG

ACCATCTCCAAAGTCGCAGGGCAACCCCGGGCGCCAGAGGTGTACGTCTTGCCCCCGGCC

CAGGAGGAGCTGACCAAGAACACCGTCAGTCTGACCTGCTACATCCAAGGCTTCTACCCT

GCCGACATCTCTGTGGAGTGGGAGAAACAGGGGCAGCCGGAGCAGGACTCCAAGACCACC

GAGCCCGTCCTGGACTCCGACGGGACCTACTTCCTCTACAGCAAGCTCAGGGTGGACAGG

AACAGATGGCAGCAAGGAGACCTCTACACATGCTCAGTGATGCACGAGGCTCTGCACAAC

CACTTCACCCAGAAGACCCTGTCCCGGTCCCCGGGTAAA

>CL328.Contig2_A-W 31 1062 anti-HBs antibody heavy chain [Homo sapiens]

AATGCATTTGATGTCTGGGGCCAGGGAACCCTGGTGACCGTCAGCTCAGCTTCGACCACG

GCCCCATCAGTCTTCCCCCTGGCCCCCAGCTCTAGGGACACATCTGGCTCCTCAGTGAAC

CTGGGCTGCCTGGTCAAGGGCTACTTCCCTGAGCCGGTGACCGTGACATGGAACTCAGGA

TCCCTGTCCAGTGGAGTCCGCACCTTCCCAGCTGTCCTGCAGTCAGGGCTCTACTCTGTC

ACCAGCATGGTGACTGTGCCCACCAGCACCTGGCCCAGCCAGACCGTCACCTGCAATGTA

GCCCACCCGGCCAGCAGCACCAAGGTGGACAAGACCATTGCTCCCTGTGGTCGTGAACCT

ACCCCATGTCCACCATGTCCACCTCCTGAAATCCTGGGGGGACCATCCGTCTTCATCTTC

CCCCCAAAGCCCAAGGACACGCTCATGATCTCCCTGACCCCCAAGGTCACATGCGTGGTG

GTAGATGTGAGCCAGGATGACCCCGAGGTCCAGTTCAGCTGGTATGTAGACAACGTGGAG

GTGAGCACAGCTGAGACGAAGCCACAGGAGAGGCAGTACAACAGCACATTCCGCGTGGTC

AGTGTCCTCCCCATCAAGCACCAGGACTGGCTGAATGGCAAGAAGTTCAAGTGCAAGGTC

AACAACAAAGCCCTGCCAGCCCCCATCGAGAGGACCATCTCCAAAGTCGCAGGGCAACCC

CGGGCGCCAGAGGTGTACGTCTTGCCCCCGGCCCAGGAGGAGCTGACCAAGAACACCGTC

AGTCTGACCTGCTACATCCAAGGCTTCTACCCTGCCGACATCTCTGTGGAGTGGGAGAAA

CAGGGGCAGCCGGAGCAGGACTCCAAGACCACCGAGCCCGTCCTGGACTCCGACGGGACC

TACTTCCTCTACAGCAAGCTCAGGGTGGACAGGAACAGATGGCAGCAAGGAGACCTCTAC

ACATGCTCAGTGATGCACGAGGCTCTGCACAACCACTTCACCCAGAAGACCCTGTCCCGG

TCCCCGGGTAAA

>CL329.Contig1_A-W 1030 1149 minus strand uncharacterized protein C17orf62 homolog isoform 3 [Mus musculus]

AGTGACGTGGAAGCTGTAGCCAGGCTCGTCACCAACTTCCTGGAGCTGCACCGCCTGGAG

AGCCCTTCGGAACGGTCTCAGAGCAGCGACAGTGAGGCTGACAGCCCTGGCGGCCAGAGC

>CL329.Contig2_A-W 286 855 minus strand uncharacterized protein C17orf62 homolog isoform 3 [Mus musculus]

GTTCCAGGGATGTACATGCAGGTAGAGACTCGCACCAACTCCTGCCTCCATCTGAAGAGG

GCTCCGGGCATCCGGTCCTGGTCCCTCTTGGTCGGAATCTTGTCAACTGGCCTGGCTGCT

GCTTACTACAGTGGAGATAGCCTGGGCTGGAAGCTCTTCTATGTCACAGGCTGCCTGTTT

GTGGCTGTGCAGAACTTGGAAGACTGGGAGGAAGCCATCTTCAACAAGAGCACCGGGAAG

GTGGTTCTGAAAACATTCAGCCTCTACAGGAAGCTGCTGACTCTTCTCAGAGCAGGCCAT

GACCAAGTGGTGGTCCTGCTAAATGACATCCGGGATGTGAATGTGGAGGAGGAGAAGGTC

CGGTATTTTGGGAAGGGCTACATGGTGGTGCTCCGGTTTGCCACAGGCTTCTCCCACCCC

CTCACCCAGAGTGCAGTCATGGGCCGTCGCAGTGACGTGGAAGCTGTAGCCAGGCTCGTC

ACCAACTTCCTGGAGCTGCACCGCCTGGAGAGCCCTTCGGAACGGTCTCAGAGCAGCGAC

AGTGAGGCTGACAGCCCTGGCGGCCAGAGC

>CL329.Contig3_A-W 192 1385 hexosaminidase D [Rattus norvegicus]

GAGTTTGTGCTGAAGCACGCGGCCTTTGCCCACCTCCGGGAAGTGGCGCCCTTCCCCAAC

ACCCTGAACCCCCACGAGGCAGAGTCCCTGGCGCTCGTGGGAGCCATGATCGACCAGGTC

CTGGAGCTGCACAGAGGTGCCCGATGGCTCCATGTCGGGTGTGATGAGGTCTACTACCTG

GGTGAGGGGGAGGCCTCAAGACGGTGGCTGCAGCAGGAGCAGAACAGCCAGGCAAAACTC

TGTCTGTCCCACATGCGGGCGGTGGCCAGCCACGTGCTGGCCCGACACCCCGGCACCACT

CCCCTGGTGTGGGACGACATGCTGCGGGACATACCCCAGGACCAGCTCACAGCATCCGGG

GTGCCGCAGCTGGTGGAGCCTGTACTCTGGGACTACGGGGCTGACCTGGACATCCACAGT

AAGGTCCTCCTCATGGAGAAGTACCGGGAGTGCGGCTTCCCACGGCTGTGGGCTGCCAGT

GCCTTCAAGGGGGCCACAGGGGCCAGCCAGGCCCTGCCCCACATCGAGCACCACCTCAGG

AACCATGCGCAGTGGCTGCAGGTGGCAGGCAGTGGGCCAGCGGACATGCTGCAGGGCATC

ATCCTGACTGGATGGCAGAGGTACGACCACTTCTCCGTGCTGTGCGAGCTGCTGCCTGTG

GGGATCCCCTCGCTGGCTGCCTGTCTGCAGTTGCTGCTCCATGGAGGATTCACTGAGCAC

GTGAGACTGAGGGTGGAGAACCTCCTCGGGCTTCCCAGCCTGGAAGCAGCAGCTATCCCG

AGTGAGGGGACCGGCTCCTTCCCTGGCAGTGACATTCATGCCCTCGTCACTCAAGTCAGC

TTCTACCTGCGCAGTTCTGTGGACGCACTGCTAGAGAGGAACAGGTATGTCACTGGCTGG

TTCAGCCCCTACCATCGCAGGCGGAAGCTCATCCACCCGGTCATGGTCCAGCACATCCAG

CCTGAGGTGCTCAGCCTCCTGGGCAGGTGGAGTGCCCTGGTGCAGCAGCTGGAGACAGCC

CTGCAGCTCGCCTTCTACCCAGATGCTGTGGAGGAGTGGTTGGAGGAGAACGTGCGCCCA

AGCATGCAGCAACTGCAGGACCTACTACAGGACCTCAGTGAGGCGGCCACCACTGGCCCC

TGCCCACCAGCCCTGGCTGGGACGCTGGTGGGGACTGCCGAGGGGCAGGGTCCA

>CL329.Contig4_A-W 739 1308 minus strand uncharacterized protein C17orf62 homolog isoform 3 [Mus musculus]

GTTCCAGGGATGTACATGCAGGTAGAGACTCGCACCAACTCCTGCCTCCATCTGAAGAGG

GCTCCGGGCATCCGGTCCTGGTCCCTCTTGGTCGGAATCTTGTCAACTGGCCTGGCTGCT

GCTTACTACAGTGGAGATAGCCTGGGCTGGAAGCTCTTCTATGTCACAGGCTGCCTGTTT

GTGGCTGTGCAGAACTTGGAAGACTGGGAGGAAGCCATCTTCAACAAGAGCACCGGGAAG

GTGGTTCTGAAAACATTCAGCCTCTACAGGAAGCTGCTGACTCTTCTCAGAGCAGGCCAT

GACCAAGTGGTGGTCCTGCTAAATGACATCCGGGATGTGAATGTGGAGGAGGAGAAGGTC

CGGTATTTTGGGAAGGGCTACATGGTGGTGCTCCGGTTTGCCACAGGCTTCTCCCACCCC

CTCACCCAGAGTGCAGTCATGGGCCGTCGCAGTGACGTGGAAGCTGTAGCCAGGCTCGTC

ACCAACTTCCTGGAGCTGCACCGCCTGGAGAGCCCTTCGGAACGGTCTCAGAGCAGCGAC

AGTGAGGCTGACAGCCCTGGCGGCCAGAGC

>CL329.Contig5_A-W 739 1308 minus strand uncharacterized protein C17orf62 homolog isoform 3 [Mus musculus]

GTTCCAGGGATGTACATGCAGGTAGAGACTCGCACCAACTCCTGCCTCCATCTGAAGAGG

GCTCCGGGCATCCGGTCCTGGTCCCTCTTGGTCGGAATCTTGTCAACTGGCCTGGCTGCT

GCTTACTACAGTGGAGATAGCCTGGGCTGGAAGCTCTTCTATGTCACAGGCTGCCTGTTT

GTGGCTGTGCAGAACTTGGAAGACTGGGAGGAAGCCATCTTCAACAAGAGCACCGGGAAG

GTGGTTCTGAAAACATTCAGCCTCTACAGGAAGCTGCTGACTCTTCTCAGAGCAGGCCAT

GACCAAGTGGTGGTCCTGCTAAATGACATCCGGGATGTGAATGTGGAGGAGGAGAAGGTC

CGGTATTTTGGGAAGGGCTACATGGTGGTGCTCCGGTTTGCCACAGGCTTCTCCCACCCC

CTCACCCAGAGTGCAGTCATGGGCCGTCGCAGTGACGTGGAAGCTGTAGCCAGGCTCGTC

ACCAACTTCCTGGAGCTGCACCGCCTGGAGAGCCCTTCGGAACGGTCTCAGAGCAGCGAC

AGTGAGGCTGACAGCCCTGGCGGCCAGAGC

>CL329.Contig6_A-W 1044 1163 minus strand uncharacterized protein C17orf62 homolog isoform 3 [Mus musculus]

AGTGACGTGGAAGCTGTAGCCAGGCTCGTCACCAACTTCCTGGAGCTGCACCGCCTGGAG

AGCCCTTCGGAACGGTCTCAGAGCAGCGACAGTGAGGCTGACAGCCCTGGCGGCCAGAGC

>CL329.Contig7_A-W 1044 1163 minus strand uncharacterized protein C17orf62 homolog isoform 3 [Mus musculus]

AGTGACGTGGAAGCTGTAGCCAGGCTCGTCACCAACTTCCTGGAGCTGCACCGCCTGGAG

AGCCCTTCGGAACGGTCTCAGAGCAGCGACAGTGAGGCTGACAGCCCTGGCGGCCAGAGC

>CL329.Contig8_A-W 286 855 minus strand uncharacterized protein C17orf62 homolog isoform 3 [Mus musculus]

GTTCCAGGGATGTACATGCAGGTAGAGACTCGCACCAACTCCTGCCTCCATCTGAAGAGG

GCTCCGGGCATCCGGTCCTGGTCCCTCTTGGTCGGAATCTTGTCAACTGGCCTGGCTGCT

GCTTACTACAGTGGAGATAGCCTGGGCTGGAAGCTCTTCTATGTCACAGGCTGCCTGTTT

GTGGCTGTGCAGAACTTGGAAGACTGGGAGGAAGCCATCTTCAACAAGAGCACCGGGAAG

GTGGTTCTGAAAACATTCAGCCTCTACAGGAAGCTGCTGACTCTTCTCAGAGCAGGCCAT

GACCAAGTGGTGGTCCTGCTAAATGACATCCGGGATGTGAATGTGGAGGAGGAGAAGGTC

CGGTATTTTGGGAAGGGCTACATGGTGGTGCTCCGGTTTGCCACAGGCTTCTCCCACCCC

CTCACCCAGAGTGCAGTCATGGGCCGTCGCAGTGACGTGGAAGCTGTAGCCAGGCTCGTC

ACCAACTTCCTGGAGCTGCACCGCCTGGAGAGCCCTTCGGAACGGTCTCAGAGCAGCGAC

AGTGAGGCTGACAGCCCTGGCGGCCAGAGC

>CL329.Contig9_A-W 591 710 minus strand hypothetical protein GW7_04121 [Heterocephalus glaber]

AGTGACGTGGAAGCTGTAGCCAGGCTCGTCACCAACTTCCTGGAGCTGCACCGCCTGGAG

AGCCCTTCGGAACGGTCTCAGAGCAGCGACAGTGAGGCTGACAGCCCTGGCGGCCAGAGC

>CL329.Contig10_A-W 591 710 minus strand hypothetical protein GW7_04121 [Heterocephalus glaber]

AGTGACGTGGAAGCTGTAGCCAGGCTCGTCACCAACTTCCTGGAGCTGCACCGCCTGGAG

AGCCCTTCGGAACGGTCTCAGAGCAGCGACAGTGAGGCTGACAGCCCTGGCGGCCAGAGC

>CL329.Contig11_A-W 739 1188 minus strand uncharacterized protein C17orf62 homolog isoform 3 [Mus musculus]

GTTCCAGGGATGTACATGCAGGTAGAGACTCGCACCAACTCCTGCCTCCATCTGAAGAGG

GCTCCGGGCATCCGGTCCTGGTCCCTCTTGGTCGGAATCTTGTCAACTGGCCTGGCTGCT

GCTTACTACAGTGGAGATAGCCTGGGCTGGAAGCTCTTCTATGTCACAGGCTGCCTGTTT

GTGGCTGTGCAGAACTTGGAAGACTGGGAGGAAGCCATCTTCAACAAGAGCACCGGGAAG

GTGGTTCTGAAAACATTCAGCCTCTACAGGAAGCTGCTGACTCTTCTCAGAGCAGGCCAT

GACCAAGTGGTGGTCCTGCTAAATGACATCCGGGATGTGAATGTGGAGGAGGAGAAGGTC

CGGTATTTTGGGAAGGGCTACATGGTGGTGCTCCGGTTTGCCACAGGCTTCTCCCACCCC

CTCACCCAGAGTGCAGTCATGGGCCGTCGC

>CL330.Contig1_A-W 367 2790 mKIAA0885 protein [Mus musculus]

CTTGGTAGATCATTTTCTCTCGCTCTTTTTTTTTAATACTAGCAACTTTCATCCTTTGAA

ACGTGTGCTGAAAAAGAAGAATCAGCAAATACTACTGAAAGTGCAATATTTGATTATCAC

TGCGAGATGAGCTTTGATCCAAACCTTCTCCACAACAATGGACACAATGGGTACCCCAAT

GGTACTTCAGCAGCACTGCGTGAAACTGGGGTTATTGAAAAACTGTTAACCTCTTATGGA

TTTATTCAGTGTTCAGAACGTCAAGCTAGACTTTTCTTCCACTGTTCACAATATAATGGC

AACCTGCAGGATTTAAAAGTAGGAGATGATGTTGAATTTGAAGTATCATCTGACCGGCGG

ACTGGGAAACCCATTGCTATTAAATTGGTGAAGATTAAACCAGAAATCCATCCTGAAGAA

CGAATGAATGGACAAGAAGTGTTTTATCTGACATACACCCCTGAAGATGTTGAAGGGAAT

GTTCAGCTGGAAACCGGAGATAAAATAAATTTTGTAATTGATAACAATAAACATACTGGT

GCTGTAAGTGCTCGTAACATTATGCTGTTGAAAAAGAAACAAGCCCGCTGTCAGGGAGTA

GTTTGTGCCATGAAGGAGGCGTTTGGCTTTATCGAAAGAGGTGATGTTGTAAAAGAGATA

TTCTTTCACTATAGTGAATTTAAAGGTGACCTAGAAACCTTACAGCCTGGAGATGATGTG

GAATTCACAATCAAGGACAGAAATGGTAAAGAAGTTGCAACAGATGTCAGACTTTTGCCT

CAAGGAACAGTCATTTTTGAAGACATCAGCATTGAACATTTTGAAGGAACTGTAACCAAA

GTTATCCCAAAAGTACCCAGTAAAAACCAGAATGACCCATTGCCAGGACGAATCAAAGTT

GACTTTGTGATTCCTAAAGAACTTCCCTTTGGAGACAAAGATACAAAATCCAAGGTGACC

CTATTGGAAGGTGACCATGTTAGGTTTAATATTTCAACAGACCGACGTGACAAATTAGAA

CGAGCAACTAACATAGAAGTTCTATCAAATACATTTCAGTTCACTAATGAAGCCAGAGAA

ATGGGTGTGATTGCTGCCATGAGAGATGGTTTTGGTTTCATCAAGTGTGTGGATCGTGAT

GCTCGTATGTTCTTCCACTTCAGTGAAATTCTGGATGGGAACCAGCTCCATATTGCAGAT

GAAGTAGAGTTTACTGTGGTTCCTGATATGCTCTCTGCCCAAAGAAATCATGCTATTAGG

ATTAAAAAACTTCCCAAGGGCACGGTTTCATTCCATTCCCATTCAGATCATCGTTTTCTG

GGCACTGTAGAGAAAGAAGCCACTTTTTCCAATCCTAAAACCACTAGCCCAAATAAAGGC

AAAGAGAAGGAGGCTGAGGATGGCATTATTGCTTACGATGATTGTGGGGTGAAACTGACC

ATTGCTTTTCAAGCCAAGGATGTGGAAGGATCTACTTCTCCTCAAATAGGAGATAAGGTT

GAATTTAGTATTAGTGACAAACAGAGGCCTGGACAGCAGATTGCAACTTGTGTGCGACTT

TTAGGTCGTAATTCCAACAAGAGGCTCTTGGGTTATGTGGCAACTCTGAAAGATAATTTT

GGGTTTATTGAAACAGCCAATCATGATAAGGAAATCTTTTTCCATTACAGTGAGTTCTCT

GGTGATGTCGATAGCCTGGAGCTGGGGGACATGGTCGAATACAGCTTGTCCAAAGGCAAA

GGCAACAAAGTCAGTGCTGAAAAAGTAAACAAAACTCACTCAGTGAATGGTATTACTGAA

GAAGCTGATCCCACTATCTATTCTGGTAAAGTCATTCGCCCCCTCAGAGGTGTTGATCCA

ACACAGATTGAGTATCAAGGAATGATTGAGATCGTGGATGAGGGGGATATGAAAGGTGAG

GTGTATCCATTTGGCATAGTCGGAATGGCCAATAAAGGGGATTGCCTGCAGAAAGGGGAG

AGTGTCAAGTTCCAATTGTGTGTCCTGGGCCAAAATGCACAGACTATGGCCTATAACATC

ACGCCCCTGCGCAGGGCCACAGTAGAGTGTGTGAAAGATCAGTTTGGCTTCATTAACTAC

GAAGTAGGAGATAGCAAGAAGCTCTTTTTCCATGTGAAAGAAGTTCAAGATGGCATTGAG

CTACAGGCAGGAGATGAAGTGGAGTTCTCAGTGATTCTTAATCAGCGTACTGGCAAGTGC

AGTGCCTGTAATGTTTGGCGAGTCTGTGAGGGCCCCAAGGCCGTTGCAGCTCCTCGACCT

GATCGGTTGGTCAATCGCTTGAAGAACATCACCCTGGATGATGCTAGTGCTCCTCGCCTA

ATGGTTCTTCGTCAGCCAAGGGGACCAGATAACTCAATGGGGTTTGGTGCAGAAAGAAAG

ATTCGTCAAGCTGGTGTCATTGAC

>CL330.Contig2_A-W 409 2883 KIAA0885 protein [Homo sapiens]

CAACTTTCATCCTTTGAAACGTGTGCTGAAAAAGAAGAATCAGCAAATACTACTGAAAGT

GCAATATTTGATTATCACTGCGAGATGAGCTTTGATCCAAACCTTCTCCACAACAATGGA

CACAATGGGTACCCCAATGGTACTTCAGCAGCACTGCGTGAAACTGGGGTTATTGAAAAA

CTGTTAACCTCTTATGGATTTATTCAGTGTTCAGAACGTCAAGCTAGACTTTTCTTCCAC

TGTTCACAATATAATGGCAACCTGCAGGATTTAAAAGTAGGAGATGATGTTGAATTTGAA

GTATCATCTGACCGGCGGACTGGGAAACCCATTGCTATTAAATTGGTGAAGATTAAACCA

GAAATCCATCCTGAAGAACGAATGAATGGACAAGTTGTGTGCGCTGTTCCTCACAACTTA

GAGAGTAAATCTCCAGCTGCCCCGGGTCAGAGTCCAACAGGGAGTGTATGCTACGAACGT

AATGGGGAAGTGTTTTATCTGACATACACCCCTGAAGATGTTGAAGGGAATGTTCAGCTG

GAAACCGGAGATAAAATAAATTTTGTAATTGATAACAATAAACATACTGGTGCTGTAAGT

GCTCGTAACATTATGCTGTTGAAAAAGAAACAAGCCCGCTGTCAGGGAGTAGTTTGTGCC

ATGAAGGAGGCGTTTGGCTTTATCGAAAGAGGTGATGTTGTAAAAGAGATATTCTTTCAC

TATAGTGAATTTAAAGGTGACCTAGAAACCTTACAGCCTGGAGATGATGTGGAATTCACA

ATCAAGGACAGAAATGGTAAAGAAGTTGCAACAGATGTCAGACTTTTGCCTCAAGGAACA

GTCATTTTTGAAGACATCAGCATTGAACATTTTGAAGGAACTGTAACCAAAGTTATCCCA

AAAGTACCCAGTAAAAACCAGAATGACCCATTGCCAGGACGAATCAAAGTTGACTTTGTG

ATTCCTAAAGAACTTCCCTTTGGAGACAAAGATACAAAATCCAAGGTGACCCTATTGGAA

GGTGACCATGTTAGGTTTAATATTTCAACAGACCGACGTGACAAATTAGAACGAGCAACT

AACATAGAAGTTCTATCAAATACATTTCAGTTCACTAATGAAGCCAGAGAAATGGGTGTG

ATTGCTGCCATGAGAGATGGTTTTGGTTTCATCAAGTGTGTGGATCGTGATGCTCGTATG

TTCTTCCACTTCAGTGAAATTCTGGATGGGAACCAGCTCCATATTGCAGATGAAGTAGAG

TTTACTGTGGTTCCTGATATGCTCTCTGCCCAAAGAAATCATGCTATTAGGATTAAAAAA

CTTCCCAAGGGCACGGTTTCATTCCATTCCCATTCAGATCATCGTTTTCTGGGCACTGTA

GAGAAAGAAGCCACTTTTTCCAATCCTAAAACCACTAGCCCAAATAAAGGCAAAGAGAAG

GAGGCTGAGGATGGCATTATTGCTTACGATGATTGTGGGGTGAAACTGACCATTGCTTTT

CAAGCCAAGGATGTGGAAGGATCTACTTCTCCTCAAATAGGAGATAAGGTTGAATTTAGT

ATTAGTGACAAACAGAGGCCTGGACAGCAGATTGCAACTTGTGTGCGACTTTTAGGTCGT

AATTCCAACAAGAGGCTCTTGGGTTATGTGGCAACTCTGAAAGATAATTTTGGGTTTATT

GAAACAGCCAATCATGATAAGGAAATCTTTTTCCATTACAGTGAGTTCTCTGGTGATGTC

GATAGCCTGGAGCTGGGGGACATGGTCGAATACAGCTTGTCCAAAGGCAAAGGCAACAAA

GTCAGTGCTGAAAAAGTAAACAAAACTCACTCAGTGAATGGTATTACTGAAGAAGCTGAT

CCCACTATCTATTCTGGTAAAGTCATTCGCCCCCTCAGAGGTGTTGATCCAACACAGATT

GAGTATCAAGGAATGATTGAGATCGTGGATGAGGGGGATATGAAAGGTGAGGTGTATCCA

TTTGGCATAGTCGGAATGGCCAATAAAGGGGATTGCCTGCAGAAAGGGGAGAGTGTCAAG

TTCCAATTGTGTGTCCTGGGCCAAAATGCACAGACTATGGCCTATAACATCACGCCCCTG

CGCAGGGCCACAGTAGAGTGTGTGAAAGATCAGTTTGGCTTCATTAACTACGAAGTAGGA

GATAGCAAGAAGCTCTTTTTCCATGTGAAAGAAGTTCAAGATGGCATTGAGCTACAGGCA

GGAGATGAAGTGGAGTTCTCAGTGATTCTTAATCAGCGTACTGGCAAGTGCAGTGCCTGT

AATGTTTGGCGAGTCTGTGAGGGCCCCAAGGCCGTTGCAGCTCCTCGACCTGATCGGTTG

GTCAATCGCTTGAAGAACATCACCCTGGATGATGCTAGTGCTCCTCGCCTAATGGTTCTT

CGTCAGCCAAGGGGACCAGATAACTCAATGGGGTTTGGTGCAGAAAGAAAGATTCGTCAA

GCTGGTGTCATTGAC

>CL330.Contig3_A-W 160 336 PREDICTED: cold shock domain-containing protein E1 isoform 5 [Saimiri boliviensis boliviensis]

GAGGGCCCCAAGGCCGTTGCAGCTCCTCGACCTGATCGGTTGGTCAATCGCTTGAAGAAC

ATCACCCTGGATGATGCTAGTGCTCCTCGCCTAATGGTTCTTCGTCAGCCAAGGGGACCA

GATAACTCAATGGGGTTTGGTGCAGAAAGAAAGATTCGTCAAGCTGGTGTCATTGAC

>CL331.Contig1_A-W 24 1523 suppressor of SWI4 1 homolog [Sus scrofa]

ATGGGGCAATCGGGGAGGTCCCGGCACCAGAAGCGCGCGCGCGCCCAGGCGCGGCTCCGC

AACCTCGAGGCTTACGCCGCGCAACCGCACTCGTTCGTGTTCACGCGGGGCAGCGCGGGT

CGCAGCGTCCGGCAGCTCAGCCTGGACGTGCGTCGGGTCATGGAGCCGCTCACCGCCACC

CGTCTGCAGATCCGCAAGAAGAACTCGCTGAAGGACTGTGTGGCAGTGGCTGGGCCCCTT

GGGGTCACACACTTCCTGATCCTAAGCAAAACAGAGACCAGTGTCTATTTTAAACTGATG

CGGCTCCCAGGAGGCCCCACTTTGACCTTCCAGATCAACAAGTACACACTGGTCCGCGAT

GTGGTCTCCTCGCTGCGCCGGCACCGCATGCATGAACAGCAGTTTGCCCACCCTCCCCTC

CTGGTACTCAACAACTTTGGACCTCACGGCATGCATGTGAAGCTTATGGCCACCATGTTC

CAGAACCTGTTCCCCTCCATCAACGTGCACAAGGTGAACCTGAACACCATCAAGCGCTGC

CTTCTCATCAATTACAACCCTGACTCCCAGGAGCTGGACTTCCGCCATTATAGCATCAAA

GTGGTTCCCGTGGGTGCAAGTCGGGGGATGAAGAAGCTCCTGCAGGAGAAGTTCCCCAAC

ATGAGTCGCCTGCAGGACATCAGTGAGCTGCTGGCCACGTGAGGAGGGCTCAGGGTGGGA

GGTCACTGCAATCCAAGGGCCTCAGAACTGTCCCTGGAATCTGATCACCTGCTGAGTCCC

ATGTGTCCCCACAGGGGTGCAGGGCTTTCAGAAAGTGAGGCGGAGCCTGATGGCGAACAC

AACATTACTGAGCTACCCCAGGCCATTGCAGGACGCGGCAACATGCGGGCCCAGCAGAGC

GCAGTGCGGCTCACAGAGATTGGTCCCCGGATGACGCTGCAGCTCATTAAGATCCAAGAG

GGCGTCGGGGAGGGCAACGTGCTGTTCCACAGTTTTGTGCACAAGACGGAGGAGGAGCTG

CAGGCCATCCTGGCAGCCAAGGAGGAGAAGCTGCGGCTCAAGGCCCAGAGGCAGGATCAG

CAGAACCAGAATGTCCAGCGCAAGCGGGAGCAGCGGGAGACACACAGGAAGAAGAGCCTG

GCAGGCATGAAGAGAGCTCGTGCTGATGGGGACAGTGATGCTGAGGACCCAGGGGTGCCC

CCAGAGGCCGAGAGGGCTGGCCAGCAGGAGGAAGAAGATGAAGCGGAGTATTTCCGCCAG

GCAGTGGGCGAGGAGCCTGATGAGGACATGTTCCCCAGGGTGACCAAGCGGAGACTGCAC

ACGGGGCCTCCAGGCAAGAAGCAGCGGGTGAAGGAGCGGAGGCCTAACCGAGACAGGGGT

CAGGATGGTGGCCGGAGGCCTCTGAAGCCCAAGAGTAGACCCACGGGAGTCCAGGGCAAG

CAGGGGCACAGAGGGACTGCCCGAGAGCGCAGCCGGGGAAGGCCACTGAAGAGAAGGGCT

>CL331.Contig2_A-W 1286 1501 suppressor of SWI4 1 homolog [Bos taurus]

GACATGTTCCCCAGGGTGACCAAGCGGAGACTGCACACGGGGCCTCCAGGCAAGAAGCAG

CGGGTGAAAGAGCGGAGGCCTAACCGAGACAGGGGTCAGGATGGTGGCCGGAGGCCTCTG

AAGCCCAAGAGTAGACCCACGGGAGTCCAGGGCAAGCAGGGGCACAGAGGGACTGCCCGA

GAGCGCAGCCGGGGAAGGCCACTGAAGAGAAGGGCT

>CL331.Contig3_A-W 1221 1421 PREDICTED: suppressor of SWI4 1 homolog [Cricetulus griseus]

GACATGTTCCCCAGGGTGACCAAGCGGAGACTGCACACGGGGCCTCCAGGCAAGAAGCAG

CGGGTGAAAGAGCGGAGGCCTAACCGAGACAGGGGTCAGGATGGTGGCCGGAGGCCTCTG

AAGCCCAAGAGTAGACCCACGGGAGTCCAGGGCAAGCAGGGGCACAGAGGGACTGCCCGA

GAGCGCAGCCGGGGAAGGCCA

>CL331.Contig4_A-W 919 1422 suppressor of SWI4 1 homolog [Sus scrofa]

GTGCACAAGACGGAGGAGGAGCTGCAGGCCATCCTGGCAGCCAAGGAGGAGAAGCTGCGG

CTCAAGGCCCAGAGGCAGGATCAGCAGAACCAGAATGTCCAGCGCAAGCGGGAGCAGCGG

GAGACACACAGGAAGAAGAGCCTGGCAGGCATGAAGAGAGCTCGTGCTGATGGGGACAGT

GATGCTGAGGACCCAGGGGTGCCCCCAGAGGCCGAGAGGGCTGGCCAGCAGGAGGAAGAA

GATGAAGCGGAGTATTTCCGCCAGGCAGTGGGCGAGGAGCCTGATGAGGACATGTTCCCC

AGGGTGACCAAGCGGAGACTGCACACGGGGCCTCCAGGCAAGAAGCAGCGGGTGAAAGAG

CGGAGGCCTAACCGAGACAGGGGTCAGGATGGTGGCCGGAGGCCTCTGAAGCCCAAGAGT

AGACCCACGGGAGTCCAGGGCAAGCAGGGGCACAGAGGGACTGCCCGAGAGCGCAGCCGG

GGAAGGCCACTGAAGAGAAGGGCT

>CL331.Contig5_A-W 2 1135 peter pan-like protein [Mustela putorius furo]

TCGTTCGTGTTCACGCGGGGCAGCGCGGGTCGCAGCGTCCGGCAGCTCAGCCTGGACGTG

CGTCGGGTCATGGAGCCGCTCACCGCCACCCGTCTGCAGATCCGCAAGAAGAACTCGCTG

AAGGACTGTGTGGCAGTGGCTGGGCCCCTTGGGGTCACACACTTCCTGATCCTAAGCAAA

ACAGAGACCAGTGTCTATTTTAAACTGATGCGGCTCCCAGGAGGCCCCACTTTGACCTTC

CAGATCAACAAGTACACACTGGTCCGCGATGTGGTCTCCTCGCTGCGCCGGCACCGCATG

CATGAACAGCAGTTTGCCCACCCTCCCCTCCTGGTACTCAACAACTTTGGACCTCACGGC

ATGCATGTGAAGCTTATGGCCACCATGTTCCAGAACCTGTTCCCCTCCATCAACGTGCAC

AAGGTGAACCTGAACACCATCAAGCGCTGCCTTCTCATCAATTACAACCCTGACTCCCAG

GAGCTGGACTTCCGCCATTATAGCATCAAAGTGGTTCCCGTGGGTGCAAGTCGGGGGATG

AAGAAGCTCCTGCAGGAGAAGTTCCCCAACATGAGTCGCCTGCAGGACATCAGTGAGCTG

CTGGCCACGGGTGCAGGGCTTTCAGAAAGTGAGGCGGAGCCTGATGGCGAACACAACATT

ACTGAGCTACCCCAGGCCATTGCAGGACGCGGCAACATGCGGGCCCAGCAGAGCGCAGTG

CGGCTCACAGAGATTGGTCCCCGGATGACGCTGCAGCTCATTAAGATCCAAGAGGGCGTC

GGGGAGGGCAACGTGCTGTTCCACAGTTTTGTGCACAAGACGGAGGAGGAGCTGCAGGCC

ATCCTGGCAGCCAAGGAGGAGAAGCTGCGGCTCAAGGCCCAGAGGCAGGATCAGCAGAAC

CAGAATGTCCAGCGCAAGCGGGAGCAGCGGGAGACACACAGGAAGAAGAGCCTGGCAGGC

ATGAAGAGAGCTCGTGCTGATGGGGACAGTGATGCTGAGGACCCAGGGGTGCCCCCAGAG

GCCGAGAGGGCTGGCCAGCAGGAGGAAGAAGATGAAGCGGAGTATTTCCGCCAGGCAGTG

GGCGAGGAGCCTGATGAGGGTGTGTGGCAGGGTCCTGCAGCTGGGCACCCAGGA

>CL331.Contig6_A-W 854 1357 suppressor of SWI4 1 homolog [Sus scrofa]

GTGCACAAGACGGAGGAGGAGCTGCAGGCCATCCTGGCAGCCAAGGAGGAGAAGCTGCGG

CTCAAGGCCCAGAGGCAGGATCAGCAGAACCAGAATGTCCAGCGCAAGCGGGAGCAGCGG

GAGACACACAGGAAGAAGAGCCTGGCAGGCATGAAGAGAGCTCGTGCTGATGGGGACAGT

GATGCTGAGGACCCAGGGGTGCCCCCAGAGGCCGAGAGGGCTGGCCAGCAGGAGGAAGAA

GATGAAGCGGAGTATTTCCGCCAGGCAGTGGGCGAGGAGCCTGATGAGGACATGTTCCCC

AGGGTGACCAAGCGGAGACTGCACACGGGGCCTCCAGGCAAGAAGCAGCGGGTGAAAGAG

CGGAGGCCTAACCGAGACAGGGGTCAGGATGGTGGCCGGAGGCCTCTGAAGCCCAAGAGT

AGACCCACGGGAGTCCAGGGCAAGCAGGGGCACAGAGGGACTGCCCGAGAGCGCAGCCGG

GGAAGGCCACTGAAGAGAAGGGCT

>CL331.Contig7_A-W 1114 1314 PREDICTED: suppressor of SWI4 1 homolog [Cricetulus griseus]

GACATGTTCCCCAGGGTGACCAAGCGGAGACTGCACACGGGGCCTCCAGGCAAGAAGCAG

CGGGTGAAAGAGCGGAGGCCTAACCGAGACAGGGGTCAGGATGGTGGCCGGAGGCCTCTG

AAGCCCAAGAGTAGACCCACGGGAGTCCAGGGCAAGCAGGGGCACAGAGGGACTGCCCGA

GAGCGCAGCCGGGGAAGGCCA

>CL331.Contig8_A-W 36 1250 suppressor of SWI4 1 homolog [Sus scrofa]

ATCCGCAAGAAGAACTCGCTGAAGGACTGTGTGGCAGTGGCTGGGCCCCTTGGGGTCACA

CACTTCCTGATCCTAAGCAAAACAGAGACCAGTGTCTATTTTAAACTGATGCGGCTCCCA

GGAGGCCCCACTTTGACCTTCCAGATCAACAAGTACACACTGGTCCGCGATGTGGTCTCC

TCGCTGCGCCGGCACCGCATGCATGAACAGCAGTTTGCCCACCCTCCCCTCCTGGTACTC

AACAACTTTGGACCTCACGGCATGCATGTGAAGCTTATGGCCACCATGTTCCAGAACCTG

TTCCCCTCCATCAACGTGCACAAGGTGAACCTGAACACCATCAAGCGCTGCCTTCTCATC

AATTACAACCCTGACTCCCAGGAGCTGGACTTCCGCCATTATAGCATCAAAGTGGTTCCC

GTGGGTGCAAGTCGGGGGATGAAGAAGCTCCTGCAGGAGAAGTTCCCCAACATGAGTCGC

CTGCAGGACATCAGTGAGCTGCTGGCCACGGGTGCAGGGCTTTCAGAAAGTGAGGCGGAG

CCTGATGGCGAACACAACATTACTGAGCTACCCCAGGCCATTGCAGGACGCGGCAACATG

CGGGCCCAGCAGAGCGCAGTGCGGCTCACAGAGATTGGTCCCCGGATGACGCTGCAGCTC

ATTAAGATCCAAGAGGGCGTCGGGGAGGGCAACGTGCTGTTCCACAGTTTTGTGCACAAG

ACGGAGGAGGAGCTGCAGGCCATCCTGGCAGCCAAGGAGGAGAAGCTGCGGCTCAAGGCC

CAGAGGCAGGATCAGCAGAACCAGAATGTCCAGCGCAAGCGGGAGCAGCGGGAGACACAC

AGGAAGAAGAGCCTGGCAGGCATGAAGAGAGCTCGTGCTGATGGGGACAGTGATGCTGAG

GACCCAGGGGTGCCCCCAGAGGCCGAGAGGGCTGGCCAGCAGGAGGAAGAAGATGAAGCG

GAGTATTTCCGCCAGGCAGTGGGCGAGGAGCCTGATGAGGACATGTTCCCCAGGGTGACC

AAGCGGAGACTGCACACGGGGCCTCCAGGCAAGAAGCAGCGGGTGAAAGAGCGGAGGCCT

AACCGAGACAGGGGTCAGGATGGTGGCCGGAGGCCTCTGAAGCCCAAGAGTAGACCCACG

GGAGTCCAGGGCAAGCAGGGGCACAGAGGGACTGCCCGAGAGCGCAGCCGGGGAAGGCCA

CTGAAGAGAAGGGCT

>CL331.Contig9_A-W 24 1427 suppressor of SWI4 1 homolog [Sus scrofa]

ATGGGGCAATCGGGGAGGTCCCGGCACCAGAAGCGCGCGCGCGCCCAGGCGCGGCTCCGC

AACCTCGAGGCTTACGCCGCGCAACCGCACTCGTTCGTGTTCACGCGGGGCAGCGCGGGT

CGCAGCGTCCGGCAGCTCAGCCTGGACGTGCGTCGGGTCATGGAGCCGCTCACCGCCACC

CGTCTGCAGATCCGCAAGAAGAACTCGCTGAAGGACTGTGTGGCAGTGGCTGGGCCCCTT

GGGGTCACACACTTCCTGATCCTAAGCAAAACAGAGACCAGTGTCTATTTTAAACTGATG

CGGCTCCCAGGAGGCCCCACTTTGACCTTCCAGATCAACAAGTACACACTGGTCCGCGAT

GTGGTCTCCTCGCTGCGCCGGCACCGCATGCATGAACAGCAGTTTGCCCACCCTCCCCTC

CTGGTACTCAACAACTTTGGACCTCACGGCATGCATGTGAAGCTTATGGCCACCATGTTC

CAGAACCTGTTCCCCTCCATCAACGTGCACAAGGTGAACCTGAACACCATCAAGCGCTGC

CTTCTCATCAATTACAACCCTGACTCCCAGGAGCTGGACTTCCGCCATTATAGCATCAAA

GTGGTTCCCGTGGGTGCAAGTCGGGGGATGAAGAAGCTCCTGCAGGAGAAGTTCCCCAAC

ATGAGTCGCCTGCAGGACATCAGTGAGCTGCTGGCCACGGGTGCAGGGCTTTCAGAAAGT

GAGGCGGAGCCTGATGGCGAACACAACATTACTGAGCTACCCCAGGCCATTGCAGGACGC

GGCAACATGCGGGCCCAGCAGAGCGCAGTGCGGCTCACAGAGATTGGTCCCCGGATGACG

CTGCAGCTCATTAAGATCCAAGAGGGCGTCGGGGAGGGCAACGTGCTGTTCCACAGTTTT

GTGCACAAGACGGAGGAGGAGCTGCAGGCCATCCTGGCAGCCAAGGAGGAGAAGCTGCGG

CTCAAGGCCCAGAGGCAGGATCAGCAGAACCAGAATGTCCAGCGCAAGCGGGAGCAGCGG

GAGACACACAGGAAGAAGAGCCTGGCAGGCATGAAGAGAGCTCGTGCTGATGGGGACAGT

GATGCTGAGGACCCAGGGGTGCCCCCAGAGGCCGAGAGGGCTGGCCAGCAGGAGGAAGAA

GATGAAGCGGAGTATTTCCGCCAGGCAGTGGGCGAGGAGCCTGATGAGGACATGTTCCCC

AGGGTGACCAAGCGGAGACTGCACACGGGGCCTCCAGGCAAGAAGCAGCGGGTGAAGGAG

CGGAGGCCTAACCGAGACAGGGGTCAGGATGGTGGCCGGAGGCCTCTGAAGCCCAAGAGT

AGACCCACGGGAGTCCAGGGCAAGCAGGGGCACAGAGGGACTGCCCGAGAGCGCAGCCGG

GGAAGGCCACTGAAGAGAAGGGCT

>CL332.Contig1_A-W 53 772 PREDICTED: LOW QUALITY PROTEIN: transcription factor GATA-4 [Callithrix jacchus]

CACCCAGTAGACATGTTTGATGACTTCTCAGAAGGCAGAGAGTGTGTCAACTGTGGGGCC

ATGTCCACCCCACTCTGGAGGCGGGACGGGACCGGGCACTACCTGTGTAACGCCTGTGGC

CTCTACCACAAGATGAACGGCATCAACCGGCCCCTCATTAAGCCTCAGCGCCGGCTGTCC

GCCTCCCGCCGAGTGGGCCTCTCCTGTGCCAACTGCCAGACCACGACCACCACGTTGTGG

CGCCGCAATGCTGAGGGTGAGCCGGTGTGCAACGCCTGTGGTCTCTACATGAAGCTCCAC

GGGGTCCCCAGGCCTCTCGCGATGCGGAAAGAGGGGATTCAAACCAGAAAACGGAAGCCC

AAGAACCTGAATAAATCCAAGACAGCAGCAGGTCCTTCCGGCAGTGAGAGCCTTCCTCCC

ACCAGCAGTGCTTCCAGCAGCACCAGCAGCACCACCACCACCAGCAGCGAGGAGATGCGC

CCCATCAAGACAGAGCCCGGCTTGTCATCTCACTATGGGCACAGCAGCTCCATGTCCCAG

ACGTTCTCCGTCAGTGCCGTGTCTGGCCACGGGCCCTCCATCCACCCGGTCCTCTCTGCC

CTGAAGCTCTCCCCACAAGGCTACGCATCTCCTGTCAGCCAGTCACCACAGGCCAGCTCC

AAGCAGGACTCTTGGAACAGTCTGGTCTTGGCTGACAGTCACGGGGACATAATCACTGCC

>CL332.Contig2_A-W 86 244 PREDICTED: transcription factor GATA-4-like [Equus caballus]

GGCAGAGAGTGTGTCAACTGTGGGGCCATGTCCACCCCACTCTGGAGGCGGGACGGGACC

GGGCACTACCTGTGTAACGCCTGTGGCCTCTACCACAAGATGAACGGCATCAACCGGCCC

CTCATTAAGCCTCAGCGCCGGCTGTCCGCCTCCCGCCGA

>CL333.Contig1_A-W 12 467 minus strand Ubiquitin carboxyl-terminal hydrolase 32 [Heterocephalus glaber]

GATGACAGTATGGGCTATCAATATCCATTCACTCTACGAGTTGTGCAGAAAGATGGGAAC

TCCTGTGCTTGGTGCCCGTGGTATAGATTTTGCAGAGGCTGTAAAATTGATTGTGGGGAA

GATAGAGCTTTCATTGGAAATGCATATATTGCTGTGGATTGGGATCCCACAGCCCTTCAT

CTCCGCTATCAAACATCACAGGAAAGAGTTGTAGATGAGCACGAAAGTGTGGAGCAGAGT

CGGCGAGCACAAGCTGAGCCCATCAACCTGGACAGTTGTCTCCGTGCTTTCACCAGTGAG

GAGGAACTAGGGGAAAATGAGATGTACTACTGTTCCAAGTGTAAGACCCACTGCTTAGCA

ACCAAAAAGCTGGATCTCTGGAGGCTTCCCCCTATCTTGATTATTCACCTTAAACGATTT

CAGTTTGTAAACGGTCGATGGATAAAATCACAAAAA

>CL333.Contig2_A-W 12 467 minus strand Ubiquitin carboxyl-terminal hydrolase 32 [Heterocephalus glaber]

GATGACAGTATGGGCTATCAATATCCATTCACTCTACGAGTTGTGCAGAAAGATGGGAAC

TCCTGTGCTTGGTGCCCGTGGTATAGATTTTGCAGAGGCTGTAAAATTGATTGTGGGGAA

GATAGAGCTTTCATTGGAAATGCATATATTGCTGTGGATTGGGATCCCACAGCCCTTCAT

CTCCGCTATCAAACATCACAGGAAAGAGTTGTAGATGAGCACGAAAGTGTGGAGCAGAGT

CGGCGAGCACAAGCTGAGCCCATCAACCTGGACAGTTGTCTCCGTGCTTTCACCAGTGAG

GAGGAACTAGGGGAAAATGAGATGTACTACTGTTCCAAGTGTAAGACCCACTGCTTAGCA

ACCAAAAAGCTGGATCTCTGGAGGCTTCCCCCTATCTTGATTATTCACCTTAAACGATTT

CAGTTTGTAAACGGTCGATGGATAAAATCACAAAAA

>CL334.Contig1_A-W 3 1235 minus strand PREDICTED: histone acetyltransferase p300 [Saimiri boliviensis boliviensis]

GTTGACTTGTGCTTCTTTGGCATGCATGTTCAAGAGTATGGCTCTGACTGCCCTCCACCC

AACCAGAGGAGAGTATATATATCTTACCTCGATAGTGTTCATTTCTTCCGTCCTAAATGC

TTGAGGACTGCAGTCTATCATGAAATTCTAATTGGATATTTAGAATATGTCAAGAAATTA

GGATATACAACAGGGCATATTTGGGCATGTCCACCAAGTGAGGGGGATGACTACATCTTC

CATTGCCATCCTCCTGACCAGAAGATACCCAAACCTAAGCGACTGCAGGAGTGGTACAAA

AAGATGCTTGACAAGGCTGTGTCAGAGCGTATTGTCCATGACTACAAGGATATTTTTAAA

CAAGCTACTGAAGATCGATTGACAAGTGCAAAGGAATTGCCATACTTTGAGGGTGATTTT

TGGCCCAATGTTTTGGAAGAGAGTATTAAGGAACTGGAACAAGAGGAAGAAGAGAGAAAG

CGGGAAGAAAACACCAGCAATGAAAGCACTGATGTGACAAAAGGAGACAGCAAAAATGCT

AAAAAGAAAAATAATAAGAAAACCAGCAAAAACAAGAGCAGCTTGAGTAGGGGTAACAAA

AAGAAGCCTGGTATGCCCAATGTATCTAATGACCTCTCTCAGAAACTATATGCAACCATG

GAGAAACATAAAGAGGTCTTCTTTGTGATCCGCCTCATCGCCGGCCCTGCTGCCAACTCC

CTGCCTCCCATCGTTGATCCTGACCCTCTCATCCCTTGTGATCTGATGGATGGTCGGGAT

GCATTCCTTACCCTGGCCAGGGACAAGCATCTGGAGTTCTCTTCACTCCGACGAGCCCAG

TGGTCCACCATGTGCATGCTGGTGGAACTGCACACACAGAGCCAGGACCGCTTTGTCTAC

ACCTGCAATGAGTGCAAGCACCACGTGGAGACACGCTGGCACTGCACCGTATGTGAGGAT

TATGACTTGTGTATCACCTGCTATAACACTAAAAACCATGATCACAAAATGGAGAAATTA

GGTCTCGGCTTAGATGATGAGAGCAACAACCAGCAGGCTGCAGCCACCCAGAGCCCAGGA

GATTCTCGTCGCCTGAGCATCCAGCGCTGTATCCAGTCTCTGGTCCATGCCTGCCAGTGC

CGCAATGCCAACTGCTCTCTGCCCTCTTGCCAGAAGATGAAGCGGGTTGTGCAGCATACC

AAGGGCTGCAAACGGAAAACCAATGGTGGGTGC

>CL334.Contig2_A-W 141 590 CREB-binding protein [Heterocephalus glaber]

AGGCGTGTTTACATATCTTATTTGGACAGTATTCATTTCTTCCGGCCGCGATGCCTCCGG

ACAGCTGTTTACCACGAGATCCTTATTGGATACCTAGAATATGTGAAGAAATTGGGGTAT

GTAACAGGACACATCTGGGCCTGTCCCCCAAGTGAAGGAGATGACTACATCTTCCATTGC

CACCCCCCTGATCAAAAAATCCCCAAACCAAAACGACTGCAGGAGTGGTATAAAAAGATG

CTGGACAAGGCATTTGCAGAGCGGATCATTCATGACTATAAGGATATCTTCAAACAAGCA

ACCGAAGACAGGCTCACAAGTGCCAAGGAGTTGCCCTATTTTGAAGGAGATTTTTGGCCC

AATGTGTTGGAAGAGAGCATTAAGGAACTAGAACAAGAAGAAGAAGAGAGGAAGAAAGAG

GAGAGCACGGCAGCTAGCGAGACCCCCGAG

>CL334.Contig3_A-W 2 1147 PREDICTED: CREB-binding protein isoform 2 [Saimiri boliviensis boliviensis]

AAGCGGAAGCTGGACACAGGGCAGTATCAGGAGCCCTGGCAGTATGTGGATGATGTCTGG

CTCATGTTCAATAATGCCTGGCTCTACAACCGCAAGACATCCCGTGTCTATAAGTTTTGC

AGTAAACTTGCAGAGGTCTTTGAGCAAGAAATTGACCCTGTCATGCAGTCCCTTGGATAT

TGCTGTGGACGCAAGTATGAGTTTTCCCCACAGACTTTGTGCTGCTATGGAAAGCAGCTG

TGTACCATTCCTCGAGATGCAGCCTACTACAGCTATCAGAATAGGTATCATTTCTGTGAG

AAGTGTTTCACAGAGATCCAGGGCGAGAATGTGACCCTGGGTGACGACCCTTCACAGCCC

CAGACGACAATTTCCAAGGATCAGTTTGAAAAGAAGAAAAATGATACCTTAGACCCTGAA

CCTTTCGTTGATTGCAAGGAGTGTGGCCGCAAGATGCATCAGATTTGTGTCCTGCACTAT

GACATCATTTGGCCTTCAGGCTTTGTGTGTGACAACTGCTTGAAGAAAACTGGCAGACCT

CGGAAAGAAAACAAATTCAGCGCTAAGCGACTGCAGACCACAAGATTGGGAAACCACTTA

GAAGACCGAGTGAACAAGTTTTTGCGGCGCCAGAATCACCCTGAAGCTGGGGAGGTTTTT

GTCAGAGTCGTGGCCAGCTCGGACAAGACTGTGGAGGTCAAACCTGGGATGAAGTCAAGG

TTTGTGGATTCTGGAGAAATGTCTGAGTCTTTTCCCTATCGAACCAAAGCACTCTTTGCT

TTCGAGGAGATCGATGGAGTGGATGTGTGCTTTTTTGGAATGCACGTCCAGGAGTACGGC

TCTGATTGTCCCCCGCCAAACACAAGGCGTGTTTACATATCTTATTTGGACAGTATTCAT

TTCTTCCGGCCGCGATGCCTCCGGACAGCTGTTTACCACGAGATCCTTATTGGATACCTA

GAATATGTGAAGAAATTGGGGTATGTAACAGGACACATCTGGGCCTGTCCCCCAAGTGAA

GGAGATGACTACATCTTCCATTGCCACCCCCCTGATCAAAAAATCCCCAAACCAAAACGA

CTGCAGGAGTGGTATAAAAAGATGCTGGACAAGGCATTTGCAGAGCGGATCATTCATGAC

TATAAG

>CL334.Contig4_A-W 177 1019 PREDICTED: histone acetyltransferase p300 [Saimiri boliviensis boliviensis]

GTGACAAAAGGAGACAGCAAAAATGCTAAAAAGAAAAATAATAAGAAAACCAGCAAAAAC

AAGAGCAGCTTGAGTAGGGGTAACAAAAAGAAGCCTGGTATGCCCAATGTATCTAATGAC

CTCTCTCAGAAACTATATGCAACCATGGAGAAACATAAAGAGGTCTTCTTTGTGATCCGC

CTCATCGCCGGCCCTGCTGCCAACTCCCTGCCTCCCATCGTTGATCCTGACCCTCTCATC

CCTTGTGATCTGATGGATGGTCGGGATGCATTCCTTACCCTGGCCAGGGACAAGCATCTG

GAGTTCTCTTCACTCCGACGAGCCCAGTGGTCCACCATGTGCATGCTGGTGGAACTGCAC

ACACAGAGCCAGGACCGCTTTGTCTACACCTGCAATGAGTGCAAGCACCACGTGGAGACA

CGCTGGCACTGCACCGTATGTGAGGATTATGACTTGTGTATCACCTGCTATAACACTAAA

AACCATGATCACAAAATGGAGAAATTAGGTCTCGGCTTAGATGATGAGAGCAACAACCAG

CAGGCTGCAGCCACCCAGAGCCCAGGAGATTCTCGTCGCCTGAGCATCCAGCGCTGTATC

CAGTCTCTGGTCCATGCCTGCCAGTGCCGCAATGCCAACTGCTCTCTGCCCTCTTGCCAG

AAGATGAAGCGGGTTGTGCAGCATACCAAGGGCTGCAAACGGAAAACCAATGGTGGGTGC

CCTATCTGCAAGCAGCTCATTGCCCTCTGCTGCTACCATGCCAAGCACTGCCAGGAGAAC

AAGTGCCCTGTGCCGTTCTGCCTCAACATCAAGCAAAAGCTCCGGCAACAGCAACTGCAG

CAC

>CL334.Contig5_A-W 2 2710 EP300, partial [Equus caballus]

AAGCGGAAGCTGGACACAGGGCAGTATCAGGAGCCCTGGCAGTATGTGGATGATGTCTGG

CTCATGTTCAATAATGCCTGGCTCTACAACCGCAAGACATCCCGTGTCTATAAGTTTTGC

AGTAAACTTGCAGAGGTCTTTGAGCAAGAAATTGACCCTGTCATGCAGTCCCTTGGATAT

TGCTGTGGACGCAAGTATGAGTTTTCCCCACAGACTTTGTGCTGCTATGGAAAGCAGCTG

TGTACCATTCCTCGAGATGCAGCCTACTACAGCTATCAGAATAGGTATCATTTCTGTGAG

AAGTGTTTCACAGAGATCCAGGGCGAGAATGTGACCCTGGGTGACGACCCTTCACAGCCC

CAGACGACAATTTCCAAGGATCAGTTTGAAAAGAAGAAAAATGATACCTTAGACCCTGAA

CCTTTCGTTGATTGCAAGGAGTGTGGCCGCAAGATGCATCAGATTTGTGTCCTGCACTAT

GACATCATTTGGCCTTCAGGCTTTGTGTGTGACAACTGCTTGAAGAAAACTGGCAGACCT

CGGAAAGAAAACAAATTCAGCGCTAAGCGACTGCAGACCACAAGATTGGGAAACCACTTA

GAAGACCGAGTGAACAAGTTTTTGCGGCGCCAGAATCACCCTGAAGCTGGGGAGGTTTTT

GTCAGAGTCGTGGCCAGCTCGGACAAGACTGTGGAGGTCAAACCTGGGATGAAGTCAAGG

TTTGTGGATTCTGGAGAAATGTCTGAGTCTTTTCCCTATCGAACCAAAGCACTCTTTGCT

TTCGAGGAGATCGATGGAGTGGATGTGTGCTTTTTTGGAATGCACGTCCAGGAGTACGGC

TCTGATTGTCCCCCGCCAAACACAAGGCGTGTTTACATATCTTATTTGGACAGTATTCAT

TTCTTCCGGCCGCGATGCCTCCGGACAGCTGTTTACCACGAGATCCTTATTGGATACCTA

GAATATGTGAAGAAATTGGGGTATGTAACAGGACACATCTGGGCCTGTCCCCCAAGTGAA

GGAGATGACTACATCTTCCATTGCCACCCCCCTGATCAAAAAATCCCCAAACCAAAACGA

CTGCAGGAGTGGTATAAAAAGATGCTGGACAAGGCATTTGCAGAGCGGATCATTCATGAC

TATAAGGATATCTTCAAACAAGCAACCGAAGACAGGCTCACAAGTGCCAAGGAGTTGCCC

TATTTTGAAGGAGATTTTTGGCCCAATGTGTTGGAAGAGAGCATTAAGGAACTAGAACAA

GAAGAAGAAGAGAGGAAGAAAGAGGAGAGCACGGCAGCTAGCGAGACCCCCGAGGGCAGT

CAGGGTGACAGCAAGAATGCCAAGAAAAAGAACAACAAGAAGACGAACAAGAACAAAAGT

AGCATTAGCCGCGCCAACAAGAAGAAGCCCAGCATGCCCAATGTGTCCAACGACCTGTCC

CAGAAGCTCTATGCTACCATGGAGAAGCACAAAGAGGTCTTCTTTGTGATTCACCTGCAT

GCTGGGCCTGTCATCAACACCCTGCCCCCCATCGTGGACCCTGACCCCCTGCTCAGCTGC

GACCTCATGGATGGACGTGATGCCTTCCTCACCTTGGCCAGAGACAAGCACTGGGAGTTC

TCCTCCTTGCGCCGCTCCAAGTGGTCCACACTGTGCATGCTGGTGGAGCTGCACACCCAG

GGCCAGGACCGCTTTGTCTATACCTGCAACGAGTGCAAACACCATGTGGAGACCCGCTGG

CACTGTACTGTGTGTGAGGACTATGATCTTTGCATCAACTGCTACAACACAAAGAGCCAC

ACCCACAAGATGGTGAAGTGGGGCCTGGGCCTGGATGATGAGGGCAGCAGCCAGGGTGAG

CCACAGTCCAAGAGCCCCCAGGAGTCCCGGCGTCTGAGCATCCAACGCTGCATCCAGTCC

CTGGTGCATGCTTGCCAGTGCCGCAATGCCAACTGCTCTCTGCCCTCTTGCCAGAAGATG

AAGCGGGTTGTGCAGCATACCAAGGGCTGCAAACGGAAAACCAATGGTGGGTGCCCTATC

TGCAAGCAGCTCATTGCCCTCTGCTGCTACCATGCCAAGCACTGCCAGGAGAACAAGTGC

CCTGTGCCGTTCTGCCTCAACATCAAGCAAAAGCTCCGGCAACAGCAACTGCAGCACCGG

CTCCAGCAGGCTCAGATGCTCCGCAGGAGGATGGCCAGCATGCAGCGGACTGGTGTGGTG

GGGCAACAGGGCCTGCCCTCCCCAACTCCTGCCACTCCAACCACACCAACTGGCCAACAG

CCAACCACACCACAGACACCCCAGCCCCAGCCCACCTCTCAGCCCCAGCCTACCCCTCCC

AACAGCATGCCCCCCTACTTACCCAGGACTCAGGCTGCTGGCCCTGTGTCCCAGGGTAAG

GCAGCAGGCCAGGTGACCCCTCCAACCCCCCCTCAGACTGCTCAACCACCCCTTCCAGGC

CCCCCACCTGCAGCAGTAGAAATGGCAATGCAAATTCAGAGGGCAGCTGAGACGCAGCGC

CAGATGGCCCATGTTCAAATTTTCCAAAGGCCAATCCAACACCAGATGCCCCAGATGACT

CCCATGGGAATGAACCCACCTCCCATGGCCAGAGGTCCCACTGGGCATCTGGAGCCAGGA

ATGGGACCTACAGGAATGCAGCAACAGCCACCCTGGGCCCAAGGAGGACTGCCTCAGCCC

CAGCAACTC

>CL334.Contig6_A-W 2 1315 PREDICTED: CREB-binding protein-like isoform 2 [Cavia porcellus]

AAGCGGAAGCTGGACACAGGGCAGTATCAGGAGCCCTGGCAGTATGTGGATGATGTCTGG

CTCATGTTCAATAATGCCTGGCTCTACAACCGCAAGACATCCCGTGTCTATAAGTTTTGC

AGTAAACTTGCAGAGGTCTTTGAGCAAGAAATTGACCCTGTCATGCAGTCCCTTGGATAT

TGCTGTGGACGCAAGTATGAGTTTTCCCCACAGACTTTGTGCTGCTATGGAAAGCAGCTG

TGTACCATTCCTCGAGATGCAGCCTACTACAGCTATCAGAATAGGTATCATTTCTGTGAG

AAGTGTTTCACAGAGATCCAGGGCGAGAATGTGACCCTGGGTGACGACCCTTCACAGCCC

CAGACGACAATTTCCAAGGATCAGTTTGAAAAGAAGAAAAATGATACCTTAGACCCTGAA

CCTTTCGTTGATTGCAAGGAGTGTGGCCGCAAGATGCATCAGATTTGTGTCCTGCACTAT

GACATCATTTGGCCTTCAGGCTTTGTGTGTGACAACTGCTTGAAGAAAACTGGCAGACCT

CGGAAAGAAAACAAATTCAGCGCTAAGCGACTGCAGACCACAAGATTGGGAAACCACTTA

GAAGACCGAGTGAACAAGTTTTTGCGGCGCCAGAATCACCCTGAAGCTGGGGAGGTTTTT

GTCAGAGTCGTGGCCAGCTCGGACAAGACTGTGGAGGTCAAACCTGGGATGAAGTCAAGG

TTTGTGGATTCTGGAGAAATGTCTGAGTCTTTTCCCTATCGAACCAAAGCACTCTTTGCT

TTCGAGGAGATCGATGGAGTGGATGTGTGCTTTTTTGGAATGCACGTCCAGGAGTACGGC

TCTGATTGTCCCCCGCCAAACACAAGGCGTGTTTACATATCTTATTTGGACAGTATTCAT

TTCTTCCGGCCGCGATGCCTCCGGACAGCTGTTTACCACGAGATCCTTATTGGATACCTA

GAATATGTGAAGAAATTGGGGTATGTAACAGGACACATCTGGGCCTGTCCCCCAAGTGAA

GGAGATGACTACATCTTCCATTGCCACCCCCCTGATCAAAAAATCCCCAAACCAAAACGA

CTGCAGGAGTGGTATAAAAAGATGCTGGACAAGGCATTTGCAGAGCGGATCATTCATGAC

TATAAGGATATCTTCAAACAAGCAACCGAAGACAGGCTCACAAGTGCCAAGGAGTTGCCC

TATTTTGAAGGAGATTTTTGGCCCAATGTGTTGGAAGAGAGCATTAAGGAACTAGAACAA

GAAGAAGAAGAGAGGAAGAAAGAGGAGAGCACGGCAGCTAGCGAGACCCCCGAG

>CL334.Contig7_A-W 2 409 PREDICTED: histone acetyltransferase p300 [Saimiri boliviensis boliviensis]

AAACCTAAGCGACTGCAGGAGTGGTACAAAAAGATGCTTGACAAGGCTGTGTCAGAGCGT

ATTGTCCATGACTACAAGGATATTTTTAAACAAGCTACTGAAGATCGATTGACAAGTGCA

AAGGAATTGCCATACTTTGAGGGTGATTTTTGGCCCAATGTTTTGGAAGAGAGTATTAAG

GAACTGGAACAAGAGGAAGAAGAGAGAAAGCGGGAAGAAAACACCAGCAATGAAAGCACT

GATGTGACAAAAGGAGACAGCAAAAATGCTAAAAAGAAAAATAATAAGAAAACCAGCAAA

AACAAGAGCAGCTTGAGTAGGGGTAACAAAAAGAAGCCTGGTATGCCCAATGTATCTAAT

GACCTCTCTCAGAAACTATATGCAACCATGGAGAAACATAAAGAGGTA

>CL334.Contig8_A-W 117 449 CREB-binding protein [Heterocephalus glaber]

TATGTAACAGGACACATCTGGGCCTGTCCCCCAAGTGAAGGAGATGACTACATCTTCCAT

TGCCACCCCCCTGATCAAAAAATCCCCAAACCAAAACGACTGCAGGAGTGGTATAAAAAG

ATGCTGGACAAGGCATTTGCAGAGCGGATCATTCATGACTATAAGGATATCTTCAAACAA

GCAACCGAAGACAGGCTCACAAGTGCCAAGGAGTTGCCCTATTTTGAAGGAGATTTTTGG

CCCAATGTGTTGGAAGAGAGCATTAAGGAACTAGAACAAGAAGAAGAAGAGAGGAAGAAA

GAGGAGAGCACGGCAGCTAGCGAGACCCCCGAG

>CL335.Contig1_A-W 217 738 minus strand PREDICTED: pre-rRNA-processing protein TSR1 homolog [Cricetulus griseus]

TTGTTTTCCTTTTCTCCTTTAGGAATGCACAGCCTTGTTGCCACAGGCCATCTGTTGTCA

GTGGATCCAGATAGAATGGTCATCAAGAGAGTTGTTCTGAGTGGTCATCCTTTCAAAATT

TTCACTAAGATGGCAGTAGTACGTTATATGTTCTTTAATAGAGGTAGGTACAAAGGAAAA

GGATGGGATCTGATTGCTTTTATGGTGTGAGGGTTGTGTTTACATTATGAGTAAATGTTT

CATTTTGGTCTTTTGTATGCTCCATTCTAAGAGGATGTGCTATGGTTTAAACCAGTGGAA

CTGAGAACAAAGTGGGGCCGTAGAGGACACATCAGGGAGCCTTTAGGTACCCATGGCCAT

ATGAAATGCAGTTTTGATGGGAAGCTAAAATCTCAGGACACAGTACTGATGAATCTTTAT

AAACGAGTTTTCCCTAAATGGACTTACGATCCATATGTACCAGAACCAGTACCTTGGGTG

AAAAGTGAGATTTCTTCAACAGTGCCTGAATTGGACATGGAG

>CL335.Contig2_A-W 689 868 minus strand PREDICTED: pre-rRNA-processing protein TSR1 homolog [Saimiri boliviensis boliviensis]

CTAGGTACCCATGGCCATATGAAATGCAGTTTTGATGGGAAGCTAAAATCTCAGGACACA

GTACTGATGAATCTTTATAAACGAGTTTTCCCTAAATGGACTTACGATCCATATGTACCA

GAACCAGTACCTTGGGTGAAAAGTGAGATTTCTTCAACAGTGCCTGAATTGGACATGGAG

>CL335.Contig3_A-W 1 528 minus strand PREDICTED: pre-rRNA-processing protein TSR1 homolog [Saimiri boliviensis boliviensis]

GCCTTGGTGGTGACGGTATATGCACCAATTACTTTTCCTCCTGCATCTGTGCTGCTTTTC

AAGCAGAAAAGCAACGGAATGCACAGCCTTGTTGCCACAGGCCATCTGTTGTCAGTGGAT

CCAGATAGAATGGTCATCAAGAGAGTTGTTCTGAGTGGTCATCCTTTCAAAATTTTCACT

AAGATGGCAGTAGTACGTTATATGTTCTTTAATAGAGGTAGGTACAAAGGAAAAGGATGG

GATCTGATTGCTTTTATGGTGTGAGGGTTGTGTTTACATTATGAGTAAATGTTTCATTTT

GGTCTTTTGTATGCTCCATTCTAAGAGGATGTGCTATGGTTTAAACCAGTGGAACTGAGA

ACAAAGTGGGGCCGTAGAGGACACATCAGGGAGCCTTTAGGTACCCATGGCCATATGAAA

TGCAGTTTTGATGGGAAGCTAAAATCTCAGGACACAGTACTGATGAATCTTTATAAACGA

GTTTTCCCTAAATGGACTTACGATCCATATGTACCAGAACCAGTACCT

>CL335.Contig4_A-W 1 402 minus strand PREDICTED: pre-rRNA-processing protein TSR1 homolog [Saimiri boliviensis boliviensis]

GCCTTGGTGGTGACGGTATATGCACCAATTACTTTTCCTCCTGCATCTGTGCTGCTTTTC

AAGCAGAAAAGCAACGGAATGCACAGCCTTGTTGCCACAGGCCATCTGTTGTCAGTGGAT

CCAGATAGAATGGTCATCAAGAGAGTTGTTCTGAGTGGTCATCCTTTCAAAATTTTCACT

AAGATGGCAGTAGTACGTTATATGTTCTTTAATAGAGGTAGGTACAAAGGAAAAGGATGG

GATCTGATTGCTTTTATGGTGTGAGGGTTGTGTTTACATTATGAGTAAATGTTTCATTTT

GGTCTTTTGTATGCTCCATTCTAAGAGGATGTGCTATGGTTTAAACCAGTGGAACTGAGA

ACAAAGTGGGGCCGTAGAGGACACATCAGGGAGCCTTTAGGT

>CL335.Contig5_A-W 1 420 minus strand PREDICTED: pre-rRNA-processing protein TSR1 homolog [Saimiri boliviensis boliviensis]

GCCTTGGTGGTGACGGTATATGCACCAATTACTTTTCCTCCTGCATCTGTGCTGCTTTTC

AAGCAGAAAAGCAACGGAATGCACAGCCTTGTTGCCACAGGCCATCTGTTGTCAGTGGAT

CCAGATAGAATGGTCATCAAGAGAGTTGTTCTGAGTGGTCATCCTTTCAAAATTTTCACT

AAGATGGCAGTAGTACGTTATATGTTCTTTAATAGAGAGGATGTGCTATGGTTTAAACCA

GTGGAACTGAGAACAAAGTGGGGCCGTAGAGGACACATCAGGGAGCCTTTAGGTACCCAT

GGCCATATGAAATGCAGTTTTGATGGGAAGCTAAAATCTCAGGACACAGTACTGATGAAT

CTTTATAAACGAGTTTTCCCTAAATGGACTTACGATCCATATGTACCAGAACCAGTACCT

>CL335.Contig6_A-W 1 294 minus strand PREDICTED: pre-rRNA-processing protein TSR1 homolog [Saimiri boliviensis boliviensis]

GCCTTGGTGGTGACGGTATATGCACCAATTACTTTTCCTCCTGCATCTGTGCTGCTTTTC

AAGCAGAAAAGCAACGGAATGCACAGCCTTGTTGCCACAGGCCATCTGTTGTCAGTGGAT

CCAGATAGAATGGTCATCAAGAGAGTTGTTCTGAGTGGTCATCCTTTCAAAATTTTCACT

AAGATGGCAGTAGTACGTTATATGTTCTTTAATAGAGAGGATGTGCTATGGTTTAAACCA

GTGGAACTGAGAACAAAGTGGGGCCGTAGAGGACACATCAGGGAGCCTTTAGGT

>CL336.Contig1_A-W 41 835 PREDICTED: U3 small nucleolar ribonucleoprotein protein MPP10-like [Cavia porcellus]

TTTAATGTTGTGTTTCATTTAGCACCTGTGATCACAGAGGAAACCACCTTCCAACTAGAA

GATATCATTAAGCAGAGAATACGAGATCAGGCTTGGGATGATGTTGTACGTAAAGAAAAA

CCTAAAGAAGATGCATATGAGTATAAAAAGCGTTTAACATTAGACCATGAGAAGAGTAAA

TTGAGCCTTGCTGAAATATATGAACAAGAATACATCAAACTTAACCAGCAAAAAACAGAA

GAAGAAGAAAATCCAGAACATGTAGAAATTCAGAAGATGATGGATTCTCTCTTTTTAAAA

TTGGATGCCCTGTCAAACTTCCACTTCATTCCTAAACCACCTGTACCAGAGATTAAGGTT

GTGTCAAATCTGCCAGCTATAACCATGGAAGAAGTAGCTCCAGTGAGTGTCAGTGATGCA

GCCCTCCTGGCCCCAGAGGAAATCAAGGAGAAAAATAAAGCTGGAGATCTAAAAACAGCT

GCTGAGAAAACAGCTACAGACAAGAAACGAGAACGGAGGAAAAAGAAATATCAAAAACGT

TTGAAAATAAAGGAGAAGGAGAAACGGAGAAAATTGCTTGAAAAGAGCAACCCAGATCAA

ACAGGGAAATACACGAAAGCAGTAGCATCAGAGAAGTTGAAACAACTGACCAAAACTGGC

AGAGTTTCCTTATTAAAGGATGAAGGAAAAGACAAGGCCCTAAAGTCATCTCAGGCATTC

TTTTCTAAATTACAAGATCAAGTAAAAATGCAAATCAGTGATGCCAAAAAAACAGAAAAG

AAAAAGAAGAAAAAA

>CL336.Contig2_A-W 21 2027 PREDICTED: U3 small nucleolar ribonucleoprotein protein MPP10 [Pongo abelii]

ATGGCTCCTCGGGTCTGGCGTCGGCGGACCCTGGAGCGGTGTCTGAGAGAGGTCGGTAAA

GCCACCAGCCAACCCGAACGCTTTCTCACGATTCAAGATGGATTAGCATCAAACTTCACT

TCTTTAACAAAAGTTCTTTATGACTTCAATAAAATACTAGAGAATGGTAGGATTCTTGGA

AGTCCTTTACAAAAACTTGTGATAAACAATTTTGATGATGAGCAAATTTGGCAACAACTG

GAATTGCAAAATGAACCAGTTTTACAATACTTCCAGAAGGCAGTTAGTGAAACAATTAAA

AATGAAGACATCACTCTTCTTCCAAAGAATATAGAGCAGGATTGTGAAGAGAATGGCTCA

GAGATGGAGGCTGATAACCAGGAGAACCTAGAAGATTTGGAAGAGGAGGAGAAAGTGTCA

GATGTGGGTGATAATGATCCTAAAGCTGGTGAAAAGCCTAAAAACTCAAGCAAATTTGAT

CAGAGGAAAAGCTCAGTTTTCAGTGATGAGGATTCTGATCTTGACTTTGATATTAGCAAA

TTGGAACAGCAGAGCAAGGTGCAAAATAAACAGCCAGAGAAGTCAAGGGAAAAGTCTATA

GTAGATGACAAATTCTTTAAACTATCTGAAATGGAGACCTTTTTGGAGAACATAGAAAAA

GAAGAACAAAAAAAAGATGAAGAGGAGAAAGAAGATATTGACTTTTTTGAAGATATTGAT

TCTGATGAAGAAGATGAGGAAGAATTGTTTAGAAATCAAAAACTTAAGTCAGATAAAAGT

TCAAGAAATCTGAAATACAAAGATTTTTTTGATCCAGTTGAAAGTGATGAAGACATGGTA

AATGGTGATGATGGGCTGGATTCAAACAAAGAAGAGGAAGAAATTGCTGAAGAAGAAGCA

GAAGAAAGTATTTCTGAAACGGATGAGGATAATGACCTTGAAGAAAGTGAAGATAGTGAA

CAGCATAAAGGCTCAAAAAGCGTGACCTTTGCTTTGCCAGATGATAAAGAAACTGAAGAT

ACAGATGTGTTAAATGTAAAGAAAGATCCTGATGAAGTTAAATCCTCTTTTGAAAAAAGA

CAGGAGAAGATGAATGAAAAAATTGCATCTTTAGAAAAAGAGTTGTTGGAAAAAAAGCCT

TGGCAGCTTCAAGGGGAAGTGACAGCACAAAAGCGACCAGAGAACAGTCTCCTAGAGGAG

AACCTGCACTTTGATCATGCTGTCCGGATGGCACCTGTGATCACAGAGGAAACCACCTTC

CAACTAGAAGATATCATTAAGCAGAGAATACGAGATCAGGCTTGGGATGATGTTGTACGT

AAAGAAAAACCTAAAGAAGATGCATATGAGTATAAAAAGCGTTTAACATTAGACCATGAG

AAGAGTAAATTGAGCCTTGCTGAAATATATGAACAAGAATACATCAAACTTAACCAGCAA

AAAACAGAAGAAGAAGAAAATCCAGAACATGTAGAAATTCAGAAGATGATGGATTCTCTC

TTTTTAAAATTGGATGCCCTGTCAAACTTCCACTTCATTCCTAAACCACCTGTACCAGAG

ATTAAGGTTGTGTCAAATCTGCCAGCTATAACCATGGAAGAAGTAGCTCCAGTGAGTGTC

AGTGATGCAGCCCTCCTGGCCCCAGAGGAAATCAAGGAGAAAAATAAAGCTGGAGATCTA

AAAACAGCTGCTGAGAAAACAGCTACAGACAAGAAACGAGAACGGAGGAAAAAGAAATAT

CAAAAACGTTTGAAAATAAAGGAGAAGGAGAAACGGAGAAAATTGCTTGAAAAGAGCAAC

CCAGATCAAACAGGGAAATACACGAAAGCAGTAGCATCAGAGAAGTTGAAACAACTGACC

AAAACTGGCAGAGTTTCCTTATTAAAGGATGAAGGAAAAGACAAGGCCCTAAAGTCATCT

CAGGCATTCTTTTCTAAATTACAAGATCAAGTAAAAATGCAAATCAGTGATGCCAAAAAA

ACAGAAAAGAAAAAGAAGAAAAAACAG

>CL337.Contig2_A-W 604 726 minus strand nuclear factor I/X [Mustela putorius furo]

CACACACACGAGAGAAAATAAGGAGCAATAAAGTCACGAGAACTTTTGTCCCCCAATCGA

GAGCCCGAGGGGCACCCCAGCCCCACCTCTGCTGCCCCCATCCCACCCCCCCTTGGGGCG

CCC

>CL338.Contig1_A-W 402 545 minus strand PREDICTED: autophagy-related protein 16-2 isoform 2 [Nomascus leucogenys]

CAGGAAACACTGTCTGGACACAAGGACAAGGTGACAGCTGCCAAATTCAAGCTAACAAGA

CACCAGGCGGTGACTGGGAGCAGAGACCGGACAGTGAAGGAGTGGGACCTTGGCCGTGCC

TACTGTGAGGCTCAGCTCCAACTC

>CL338.Contig2_A-W 87 617 minus strand PREDICTED: autophagy-related protein 16-2 isoform 2 [Nomascus leucogenys]

CCACACACTCTACATGATGGGATCAGAGAGCAGACAGAGACTCTGGCTCTGGCTATTAAG

CCTGAGTCCCTGGAGAGTGAGGCTTGTGGGAAGTGGAAAAGGCCTTTCAGGTCTGCCTCA

GCCACCTCTCTGACTCTGTCCCACTGTGTGGATGTGGTGAAGGGGCTGCTAGATTTCAAG

AAGAGGAGAGGTCACTCAATTGGGGGTGCCCCTGAGCAGCGATACCAGAGCATCCCTGTG

TGTGTGGCTGCCCGACTTCCTACTTGGGCTCAAGATGTCCTGGATGCCCACCTTTCTGAG

GTCAATGCTGTATGTTTTGGCCCTAACAGCAGTCTCCTGGCCACTGGTGGGGCTGACTGT

CTTATCCACCTCTGGAATGTGGTGGGAGGTCGTCTGGAGGCCAACCAGACCCTTGAGGGA

GCTGGTGGCAGCATCACCAGTGTGGACTTCGACCCCTCGGGCTCCCAGGTTTTGGCAGCT

ACTTACAATCAGGCTGCCCAGCTCTGGAAGGTGGGAGAGGCACGGTCCAAG

>CL338.Contig3_A-W 1748 2245 minus strand PREDICTED: autophagy-related protein 16-2 isoform 1 [Saimiri boliviensis boliviensis]

TGGTCCCTAGGTTCCAGAACTATCAATGTCCTTTCCTATTGTAACGACGTGGTGTGCGGG

GACCACATCATCATTAGTGGCCACAATGACCAGAAGATCCGGTTCTGGGATAGCAGGGGT

CCCCGCTGCACCCAGGTCATTCCTGTGCAGGGCCGGGTCACCTCCCTGAGCCTCAGCCAT

GACCAGCTGCACCTGCTCATCTGCTCCAGAGATAATACACTCAAGGTCATTGACCTCCGG

GTCAGCAATATCCGCCAGGTGTTCAGGGCTGATGGCTTCAAATGTGGTTCCGACTGGACC

AAAGCTGTGTTCAGCCCTGACAGAAGCTATGCACTGGCAGGCTCCTCGAATGGAGCCCTT

TTCATCTGGGATGTTGACACTGGGAAACTGGAGAGCAGCCTTCAGGGCCCCCACTGTGCT

GCTATCAACGCTGTGGCCTGGTGCTGCTCCGGGAGCCACGTGGTGAGCGTGGACCAGGGC

AGGAAGGTTGTGCTCTGG

>CL338.Contig4_A-W 22 528 minus strand PREDICTED: autophagy-related protein 16-2 isoform 2 [Nomascus leucogenys]

CTCTGCAGTTTCAAGAAGAGGAGAGGTCACTCAATTGGGGGTGCCCCTGAGCAGCGATAC

CAGAGCATCCCTGTGTGTGTGGCTGCCCGACTTCCTACTTGGGCTCAAGATGTCCTGGAT

GCCCACCTTTCTGAGGTCAATGCTGTATGTTTTGGCCCTAACAGCAGTCTCCTGGCCACT

GGTGGGGCTGACTGTCTTATCCACCTCTGGAATGTGGTGGGAGGTCGTCTGGAGGCCAAC

CAGACCCTTGAGGGAGCTGGTGGCAGCATCACCAGTGTGGACTTCGACCCCTCGGGCTCC

CAGGTTTTGGCAGCTACTTACAATCAGGCTGCCCAGCTCTGGAAGGTGGGAGAGGCACGG

TCCAAGGAAACACTGTCTGGACACAAGGACAAGGTGACAGCTGCCAAATTCAAGCTAACA

AGACACCAGGCGGTGACTGGGAGCAGAGACCGGACAGTGAAGGAGTGGGACCTTGGCCGT

GCCTACTGTGAGGCTCAGCTCCAACTC

>CL338.Contig5_A-W 402 563 minus strand mCG6774, isoform CRA_b [Mus musculus]

CAGGAAACACTGTCTGGACACAAGGACAAGGTGACAGCTGCCAAATTCAAGCTAACAAGA

CACCAGGCGGTGACTGGGAGCAGAGACCGGACAGTGAAGGAGTGGGACCTTGGCCGTGCC

TACTGTGAGGCTCAGCTCCAACTCCACCTCCCATCCCCCCTT

>CL338.Contig6_A-W 87 617 minus strand PREDICTED: autophagy-related protein 16-2 isoform 2 [Pan paniscus]

CCACACACTCTACATGATGGGATCAGAGAGCAGACAGAGACTCTGGCTCTGGCTATTAAG

CCTGAGTCCCTGGAGAGTGAGGCTTGTGGGAAGTGGAAAAGGCCTTTCAGGTCTGCCTCA

GCCACCTCTCTGACTCTGTCCCACTGTGTGGATGTGGTGAAGGGGCTGCTAGATTTCAAG

AAGAGGAGAGGTCACTCAATTGGGGGTGCCCCTGAGCAGCGATACCAGAGCATCCCTGTG

TGTGTGGCTGCCCGACTTCCTACTTGGGCTCAAGATGTCCTGGATGCCCACCTTTCTGAG

GTCAATGCTGTATGTTTTGGCCCTAACAGCAGTCTCCTGGCCACTGGTGGGGCTGACTGT

CTTATCCACCTCTGGAATGTGGTGGGAGGTCGTCTGGAGGCCAACCAGACCCTTGAGGGA

GCTGGTGGCAGCATCACCAGTGTGGACTTCGACCCCTCGGGCTCCCAGGTTTTGGCAGCT

ACTTACAATCAGGCTGCCCAGCTCTGGAAGGTGGGAGAGGCACGGTCCAAG

>CL338.Contig7_A-W 28 546 minus strand mCG6774, isoform CRA_b [Mus musculus]

AGTTTCAAGAAGAGGAGAGGTCACTCAATTGGGGGTGCCCCTGAGCAGCGATACCAGAGC

ATCCCTGTGTGTGTGGCTGCCCGACTTCCTACTTGGGCTCAAGATGTCCTGGATGCCCAC

CTTTCTGAGGTCAATGCTGTATGTTTTGGCCCTAACAGCAGTCTCCTGGCCACTGGTGGG

GCTGACTGTCTTATCCACCTCTGGAATGTGGTGGGAGGTCGTCTGGAGGCCAACCAGACC

CTTGAGGGAGCTGGTGGCAGCATCACCAGTGTGGACTTCGACCCCTCGGGCTCCCAGGTT

TTGGCAGCTACTTACAATCAGGCTGCCCAGCTCTGGAAGGTGGGAGAGGCACGGTCCAAG

GAAACACTGTCTGGACACAAGGACAAGGTGACAGCTGCCAAATTCAAGCTAACAAGACAC

CAGGCGGTGACTGGGAGCAGAGACCGGACAGTGAAGGAGTGGGACCTTGGCCGTGCCTAC

TGTGAGGCTCAGCTCCAACTCCACCTCCCATCCCCCCTT

>CL338.Contig8_A-W 87 638 minus strand PREDICTED: autophagy-related protein 16-2 isoform 1 [Nomascus leucogenys]

CCACACACTCTACATGATGGGATCAGAGAGCAGACAGAGACTCTGGCTCTGGCTATTAAG

CCTGAGTCCCTGGAGAGTGAGGCTTGTGGGAAGTGGAAAAGGCCTTTCAGGTCTGCCTCA

GCCACCTCTCTGACTCTGTCCCACTGTGTGGATGTGGTGAAGGGGCTGCTAGATTTCAAG

AAGAGGAGAGGTCACTCAATTGGGGGTGCCCCTGAGCAGCGATACCAGAGCATCCCTGTG

TGTGTGGCTGCCCGACTTCCTACTTGGGCTCAAGATGTCCTGGATGCCCACCTTTCTGAG

GTCAATGCTGTATGTTTTGGCCCTAACAGCAGTCTCCTGGCCACTGGTGGGGCTGACTGT

CTTATCCACCTCTGGAATGTGGTGGGAGGTCGTCTGGAGGCCAACCAGACCCTTGAGGGA

GCTGGTGGCAGCATCACCAGTGTGGACTTCGACCCCTCGGGCTCCCAGGTTTTGGCAGCT

ACTTACAATCAGGCTGCCCAGCTCTGGAAGGTGGGAGAGGCACGGTCCAAGACTTGGGAT

GGGGGCAGGAAA

>CL338.Contig9_A-W 87 1229 minus strand PREDICTED: autophagy-related protein 16-2 isoform 2 [Nomascus leucogenys]

CCACACACTCTACATGATGGGATCAGAGAGCAGACAGAGACTCTGGCTCTGGCTATTAAG

CCTGAGTCCCTGGAGAGTGAGGCTTGTGGGAAGTGGAAAAGGCCTTTCAGGTCTGCCTCA

GCCACCTCTCTGACTCTGTCCCACTGTGTGGATGTGGTGAAGGGGCTGCTAGATTTCAAG

AAGAGGAGAGGTCACTCAATTGGGGGTGCCCCTGAGCAGCGATACCAGAGCATCCCTGTG

TGTGTGGCTGCCCGACTTCCTACTTGGGCTCAAGATGTCCTGGATGCCCACCTTTCTGAG

GTCAATGCTGTATGTTTTGGCCCTAACAGCAGTCTCCTGGCCACTGGTGGGGCTGACTGT

CTTATCCACCTCTGGAATGTGGTGGGAGGTCGTCTGGAGGCCAACCAGACCCTTGAGGGA

GCTGGTGGCAGCATCACCAGTGTGGACTTCGACCCCTCGGGCTCCCAGGTTTTGGCAGCT

ACTTACAATCAGGCTGCCCAGCTCTGGAAGGTGGGAGAGGCACGGTCCAAGGAAACACTG

TCTGGACACAAGGACAAGGTGACAGCTGCCAAATTCAAGCTAACAAGACACCAGGCGGTG

ACTGGGAGCAGAGACCGGACAGTGAAGGAGTGGGACCTTGGCCGTGCCTACTGTTCCAGA

ACTATCAATGTCCTTTCCTATTGTAACGACGTGGTGTGCGGGGACCACATCATCATTAGT

GGCCACAATGACCAGAAGATCCGGTTCTGGGATAGCAGGGGTCCCCGCTGCACCCAGGTC

ATTCCTGTGCAGGGCCGGGTCACCTCCCTGAGCCTCAGCCATGACCAGCTGCACCTGCTC

ATCTGCTCCAGAGATAATACACTCAAGGTCATTGACCTCCGGGTCAGCAATATCCGCCAG

GTGTTCAGGGCTGATGGCTTCAAATGTGGTTCCGACTGGACCAAAGCTGTGTTCAGCCCT

GACAGAAGCTATGCACTGGCAGGCTCCTCGAATGGAGCCCTTTTCATCTGGGATGTTGAC

ACTGGGAAACTGGAGAGCAGCCTTCAGGGCCCCCACTGTGCTGCTATCAACGCTGTGGCC

TGGTGCTGCTCCGGGAGCCACGTGGTGAGCGTGGACCAGGGCAGGAAGGTTGTGCTCTGG

CGC

>CL338.Contig10_A-W 87 638 minus strand autophagy-related protein 16-2 [Homo sapiens] >gi|189027648|sp|Q8NAA4.2|A16L2_HUMAN RecName: Full=Autophagy-related protein 16-2; AltName: Full=APG16-like 2; AltName: Full=WD repeat-containing protein 80 >gi|119595280|gb|EAW74874.1| ATG16 autophagy related 16-like 2 (S. cerevisiae), isoform CRA_a [Homo sapiens] >gi|187950669|gb|AAI37490.1| ATG16 autophagy related 16-like 2 (S. cerevisiae) [Homo sapiens] >gi|187952629|gb|AAI37491.1| ATG16 autophagy related 16-like 2 (S. cerevisiae) [Homo sapiens]

CCACACACTCTACATGATGGGATCAGAGAGCAGACAGAGACTCTGGCTCTGGCTATTAAG

CCTGAGTCCCTGGAGAGTGAGGCTTGTGGGAAGTGGAAAAGGCCTTTCAGGTCTGCCTCA

GCCACCTCTCTGACTCTGTCCCACTGTGTGGATGTGGTGAAGGGGCTGCTAGATTTCAAG

AAGAGGAGAGGTCACTCAATTGGGGGTGCCCCTGAGCAGCGATACCAGAGCATCCCTGTG

TGTGTGGCTGCCCGACTTCCTACTTGGGCTCAAGATGTCCTGGATGCCCACCTTTCTGAG

GTCAATGCTGTATGTTTTGGCCCTAACAGCAGTCTCCTGGCCACTGGTGGGGCTGACTGT

CTTATCCACCTCTGGAATGTGGTGGGAGGTCGTCTGGAGGCCAACCAGACCCTTGAGGGA

GCTGGTGGCAGCATCACCAGTGTGGACTTCGACCCCTCGGGCTCCCAGGTTTTGGCAGCT

ACTTACAATCAGGCTGCCCAGCTCTGGAAGGTGGGAGAGGCACGGTCCAAGACTTGGGAT

GGGGGCAGGAAA

>CL338.Contig11_A-W 1345 1533 minus strand PREDICTED: autophagy-related protein 16-2 isoform 1 [Saimiri boliviensis boliviensis]

TTATGGGGTCTTGGCCATGCCTTTGATCCAGACTTGGGATGGGGGCAGGAAACACTGTCT

GGACACAAGGACAAGGTGACAGCTGCCAAATTCAAGCTAACAAGACACCAGGCGGTGACT

GGGAGCAGAGACCGGACAGTGAAGGAGTGGGACCTTGGCCGTGCCTACTGTGAGGCTCAG

CTCCAACTC

>CL338.Contig12_A-W 87 1142 minus strand PREDICTED: autophagy-related protein 16-2 [Pan troglodytes]

CCACACACTCTACATGATGGGATCAGAGAGCAGACAGAGACTCTGGCTCTGGCTATTAAG

CCTGAGTCCCTGGAGAGTGAGGCTTGTGGGAAGTGGAAAAGGCCTTTCAGGTCTGCCTCA

GCCACCTCTCTGACTCTGTCCCACTGTGTGGATGTGGTGAAGGGGCTGCTAGATTTCAAG

AAGAGGAGAGGTCACTCAATTGGGGGTGCCCCTGAGCAGCGATACCAGAGCATCCCTGTG

TGTGTGGCTGCCCGACTTCCTACTTGGGCTCAAGATGTCCTGGATGCCCACCTTTCTGAG

GTCAATGCTGTATGTTTTGGCCCTAACAGCAGTCTCCTGGCCACTGGTGGGGCTGACTGT

CTTATCCACCTCTGGAATGTGGTGGGAGGTCGTCTGGAGGCCAACCAGACCCTTGAGGGA

GCTGGTGGCAGCATCACCAGTGTGGACTTCGACCCCTCGGGCTCCCAGGTTTTGGCAGCT

ACTTACAATCAGGCTGCCCAGCTCTGGAAGGTGGGAGAGGCACGGTCCAAGGAAACACTG

TCTGGACACAAGGACAAGGTGACAGCTGCCAAATTCAAGCTAACAAGACACCAGGCGGTG

ACTGGGAGCAGAGACCGGACAGTGAAGGAGTGGGACCTTGGCCGTGCCTACTGTTCCAGA

ACTATCAATGTCCTTTCCTATTGTAACGACGTGGTGTGCGGGGACCACATCATCATTAGT

GGCCACAATGACCAGAAGATCCGGTTCTGGGATAGCAGGGGTCCCCGCTGCACCCAGGTC

ATTCCTGTGCAGGGCCGGGTCACCTCCCTGAGCCTCAGCCATGACCAGCTGCACCTGCTC

ATCTGCTCCAGAGATAATACACTCAAGGTCATTGACCTCCGGGTCAGCAATATCCGCCAG

GTGTTCAGGGCTGATGGCTTCAAATGTGGTTCCGACTGGACCAAAGCTGTGTTCAGCCCT

GACAGAAGCTATGCACTGGCAGGCTCCTCGAATGGAGCCCTTTTCATCTGGGATGTTGAC

ACTGGGAAACTGGAGAGCAGCCTTCAGGGCCCCCAC

>CL338.Contig13_A-W 22 387 minus strand PREDICTED: autophagy-related protein 16-2 isoform 2 [Nomascus leucogenys]

CTCTGCAGTTTCAAGAAGAGGAGAGGTCACTCAATTGGGGGTGCCCCTGAGCAGCGATAC

CAGAGCATCCCTGTGTGTGTGGCTGCCCGACTTCCTACTTGGGCTCAAGATGTCCTGGAT

GCCCACCTTTCTGAGGTCAATGCTGTATGTTTTGGCCCTAACAGCAGTCTCCTGGCCACT

GGTGGGGCTGACTGTCTTATCCACCTCTGGAATGTGGTGGGAGGTCGTCTGGAGGCCAAC

CAGACCCTTGAGGGAGCTGGTGGCAGCATCACCAGTGTGGACTTCGACCCCTCGGGCTCC

CAGGTTTTGGCAGCTACTTACAATCAGGCTGCCCAGCTCTGGAAGGTGGGAGAGGCACGG

TCCAAG

>CL338.Contig14_A-W 136 324 minus strand PREDICTED: autophagy-related protein 16-2 isoform 2 [Pan paniscus]

TTATGGGGTCTTGGCCATGCCTTTGATCCAGACTTGGGATGGGGGCAGGAAACACTGTCT

GGACACAAGGACAAGGTGACAGCTGCCAAATTCAAGCTAACAAGACACCAGGCGGTGACT

GGGAGCAGAGACCGGACAGTGAAGGAGTGGGACCTTGGCCGTGCCTACTGTGAGGCTCAG

CTCCAACTC

>CL338.Contig15_A-W 33 1421 minus strand PREDICTED: autophagy-related protein 16-2 isoform 1 [Saimiri boliviensis boliviensis]

ATGGCAGGGCCCGAAGCATCCTTCGACCCCACCTCGGGTTGGAAGCGCCACATTGTGCGG

CAGCTACAGAAGCGGGACCGCACGCAGAAGGCACTTTTCCTGGAGCTGGTGCCGGCCTAT

AATCATCTCCTAGAAAAAGCTGAGCTGTTGGCCAAGTTCTCAGAGAAGCTTCATAAGAAG

CCAAATGATGTTACTTCTGCCACCCATCAGAGCTTCTGGGAGGAGTCAGGGCCTGACTCA

GACCAAAGCTCACCACCAGGCACTCTGAGGGTGAAGTGGCAGGAGGAGGAGGAGGAGGGG

CTCCGCTTGGACTGTGGTGAGATGGCCTACCAGGTGGTGAAGAAGAGTGCAGCCTTGGAT

GCCCTGCAGTCGGAGCTGGAGGAGCGGCAAAGCAGGCTGGCGGCTGTGGAGGCCCGCGTG

GCGCAGCTGCGGGAGGCGAGGGCGCAGCAGGTCAGCCTCGTAGAGGAGCTACAGAAGCAG

AATGCGGCGCAGCGGAAGGCCTACGAGGTGCTGCGCGTGCACACTGGGCACCAAGAGGCG

GCACTGCGCAGGCTCCAGGAGGAGGCGCTCAATCTGCTGGAGCAGCTCTTGCAACGCAAG

GAGCGCGCCGCAGCGGAGTGCAACTTGCGCAACGAGCGCCGAGAGCGGGCCAATCAGGCG

CGGGTGTCCCAGGAACTAAAGAAGGCTGCCAAACGGACTGTGAGCATCAGCGAGATTCCA

CACACTCTACATGATGGGATCAGAGAGCAGACAGAGACTCTGGCTCTGGCTATTAAGCCT

GAGTCCCTGGAGAGTGAGGCTTGTGGGAAGTGGAAAAGGCCTTTCAGGTCTGCCTCAGCC

ACCTCTCTGACTCTGTCCCACTGTGTGGATGTGGTGAAGGGGCTGCTAGATTTCAAGAAG

AGGAGAGGTCACTCAATTGGGGGTGCCCCTGAGCAGCGATACCAGAGCATCCCTGTGTGT

GTGGCTGCCCGACTTCCTACTTGGGCTCAAGATGTCCTGGATGCCCACCTTTCTGAGGTC

AATGCTGTATGTTTTGGCCCTAACAGCAGTCTCCTGGCCACTGGTGGGGCTGACTGTCTT

ATCCACCTCTGGAATGTGGTGGGAGGTCGTCTGGAGGCCAACCAGACCCTTGAGGGAGCT

GGTGGCAGCATCACCAGTGTGGACTTCGACCCCTCGGGCTCCCAGGTTTTGGCAGCTACT

TACAATCAGGCTGCCCAGCTCTGGAAGGTGGGAGAGGCACGGTCCAAGGAAACACTGTCT

GGACACAAGGACAAGGTGACAGCTGCCAAATTCAAGCTAACAAGACACCAGGCGGTGACT

GGGAGCAGAGACCGGACAGTGAAGGAGTGGGACCTTGGCCGTGCCTACTGTGAGGCTCAG

CTCCAACTC

>CL338.Contig16_A-W 31 387 minus strand PREDICTED: autophagy-related protein 16-2 isoform 2 [Pan paniscus]

TTCAAGAAGAGGAGAGGTCACTCAATTGGGGGTGCCCCTGAGCAGCGATACCAGAGCATC

CCTGTGTGTGTGGCTGCCCGACTTCCTACTTGGGCTCAAGATGTCCTGGATGCCCACCTT

TCTGAGGTCAATGCTGTATGTTTTGGCCCTAACAGCAGTCTCCTGGCCACTGGTGGGGCT

GACTGTCTTATCCACCTCTGGAATGTGGTGGGAGGTCGTCTGGAGGCCAACCAGACCCTT

GAGGGAGCTGGTGGCAGCATCACCAGTGTGGACTTCGACCCCTCGGGCTCCCAGGTTTTG

GCAGCTACTTACAATCAGGCTGCCCAGCTCTGGAAGGTGGGAGAGGCACGGTCCAAG

>CL338.Contig17_A-W 25 408 minus strand Autophagy-related protein 16-2 [Cricetulus griseus]

TGCAGTTTCAAGAAGAGGAGAGGTCACTCAATTGGGGGTGCCCCTGAGCAGCGATACCAG

AGCATCCCTGTGTGTGTGGCTGCCCGACTTCCTACTTGGGCTCAAGATGTCCTGGATGCC

CACCTTTCTGAGGTCAATGCTGTATGTTTTGGCCCTAACAGCAGTCTCCTGGCCACTGGT

GGGGCTGACTGTCTTATCCACCTCTGGAATGTGGTGGGAGGTCGTCTGGAGGCCAACCAG

ACCCTTGAGGGAGCTGGTGGCAGCATCACCAGTGTGGACTTCGACCCCTCGGGCTCCCAG

GTTTTGGCAGCTACTTACAATCAGGCTGCCCAGCTCTGGAAGGTGGGAGAGGCACGGTCC

AAGACTTGGGATGGGGGCAGGAAA

>CL338.Contig18_A-W 25 996 minus strand Autophagy-related protein 16-2 [Cricetulus griseus]

TGCAGTTTCAAGAAGAGGAGAGGTCACTCAATTGGGGGTGCCCCTGAGCAGCGATACCAG

AGCATCCCTGTGTGTGTGGCTGCCCGACTTCCTACTTGGGCTCAAGATGTCCTGGATGCC

CACCTTTCTGAGGTCAATGCTGTATGTTTTGGCCCTAACAGCAGTCTCCTGGCCACTGGT

GGGGCTGACTGTCTTATCCACCTCTGGAATGTGGTGGGAGGTCGTCTGGAGGCCAACCAG

ACCCTTGAGGGAGCTGGTGGCAGCATCACCAGTGTGGACTTCGACCCCTCGGGCTCCCAG

GTTTTGGCAGCTACTTACAATCAGGCTGCCCAGCTCTGGAAGGTGGGAGAGGCACGGTCC

AAGGAAACACTGTCTGGACACAAGGACAAGGTGACAGCTGCCAAATTCAAGCTAACAAGA

CACCAGGCGGTGACTGGGAGCAGAGACCGGACAGTGAAGGAGTGGGACCTTGGCCGTGCC

TACTGTTCCAGAACTATCAATGTCCTTTCCTATTGTAACGACGTGGTGTGCGGGGACCAC

ATCATCATTAGTGGCCACAATGACCAGAAGATCCGGTTCTGGGATAGCAGGGGTCCCCGC

TGCACCCAGGTCATTCCTGTGCAGGGCCGGGTCACCTCCCTGAGCCTCAGCCATGACCAG

CTGCACCTGCTCATCTGCTCCAGAGATAATACACTCAAGGTCATTGACCTCCGGGTCAGC

AATATCCGCCAGGTGTTCAGGGCTGATGGCTTCAAATGTGGTTCCGACTGGACCAAAGCT

GTGTTCAGCCCTGACAGAAGCTATGCACTGGCAGGCTCCTCGAATGGAGCCCTTTTCATC

TGGGATGTTGACACTGGGAAACTGGAGAGCAGCCTTCAGGGCCCCCACTGTGCTGCTATC

AACGCTGTGGCCTGGTGCTGCTCCGGGAGCCACGTGGTGAGCGTGGACCAGGGCAGGAAG

GTTGTGCTCTGG

>CL338.Contig19_A-W 25 408 minus strand Autophagy-related protein 16-2 [Cricetulus griseus]

TGCAGTTTCAAGAAGAGGAGAGGTCACTCAATTGGGGGTGCCCCTGAGCAGCGATACCAG

AGCATCCCTGTGTGTGTGGCTGCCCGACTTCCTACTTGGGCTCAAGATGTCCTGGATGCC

CACCTTTCTGAGGTCAATGCTGTATGTTTTGGCCCTAACAGCAGTCTCCTGGCCACTGGT

GGGGCTGACTGTCTTATCCACCTCTGGAATGTGGTGGGAGGTCGTCTGGAGGCCAACCAG

ACCCTTGAGGGAGCTGGTGGCAGCATCACCAGTGTGGACTTCGACCCCTCGGGCTCCCAG

GTTTTGGCAGCTACTTACAATCAGGCTGCCCAGCTCTGGAAGGTGGGAGAGGCACGGTCC

AAGACTTGGGATGGGGGCAGGAAA

>CL338.Contig20_A-W 25 912 minus strand Autophagy-related protein 16-2 [Cricetulus griseus]

TGCAGTTTCAAGAAGAGGAGAGGTCACTCAATTGGGGGTGCCCCTGAGCAGCGATACCAG

AGCATCCCTGTGTGTGTGGCTGCCCGACTTCCTACTTGGGCTCAAGATGTCCTGGATGCC

CACCTTTCTGAGGTCAATGCTGTATGTTTTGGCCCTAACAGCAGTCTCCTGGCCACTGGT

GGGGCTGACTGTCTTATCCACCTCTGGAATGTGGTGGGAGGTCGTCTGGAGGCCAACCAG

ACCCTTGAGGGAGCTGGTGGCAGCATCACCAGTGTGGACTTCGACCCCTCGGGCTCCCAG

GTTTTGGCAGCTACTTACAATCAGGCTGCCCAGCTCTGGAAGGTGGGAGAGGCACGGTCC

AAGGAAACACTGTCTGGACACAAGGACAAGGTGACAGCTGCCAAATTCAAGCTAACAAGA

CACCAGGCGGTGACTGGGAGCAGAGACCGGACAGTGAAGGAGTGGGACCTTGGCCGTGCC

TACTGTTCCAGAACTATCAATGTCCTTTCCTATTGTAACGACGTGGTGTGCGGGGACCAC

ATCATCATTAGTGGCCACAATGACCAGAAGATCCGGTTCTGGGATAGCAGGGGTCCCCGC

TGCACCCAGGTCATTCCTGTGCAGGGCCGGGTCACCTCCCTGAGCCTCAGCCATGACCAG

CTGCACCTGCTCATCTGCTCCAGAGATAATACACTCAAGGTCATTGACCTCCGGGTCAGC

AATATCCGCCAGGTGTTCAGGGCTGATGGCTTCAAATGTGGTTCCGACTGGACCAAAGCT

GTGTTCAGCCCTGACAGAAGCTATGCACTGGCAGGCTCCTCGAATGGAGCCCTTTTCATC

TGGGATGTTGACACTGGGAAACTGGAGAGCAGCCTTCAGGGCCCCCAC

>CL338.Contig21_A-W 100 402 minus strand rCG40272, isoform CRA_b [Rattus norvegicus]

GGGTCAAGGGTCCTTTTTCAGGAGAGGGAGGTGCTATTATGGGGTCTTGGCCATGCCTTT

GATCCAGACTTGGGATGGGGGCAGGAAACACTGTCTGGACACAAGGACAAGGTGACAGCT

GCCAAATTCAAGCTAACAAGACACCAGGCGGTGACTGGGAGCAGAGACCGGACAGTGAAG

GAGTGGGACCTTGGCCGTGCCTACTGTGAGGCTCAGCTCCAACTCCACCTCCCATCCCCC

CTTCCCTCAGATACAAGGGAACCTACCCTGCTTTCTGGGAGTGGCTCTGGGATTCAAAGA

CAT

>CL338.Contig22_A-W 1295 1822 minus strand PREDICTED: autophagy-related protein 16-2 isoform 1 [Saimiri boliviensis boliviensis]

CAGGAAACACTGTCTGGACACAAGGACAAGGTGACAGCTGCCAAATTCAAGCTAACAAGA

CACCAGGCGGTGACTGGGAGCAGAGACCGGACAGTGAAGGAGTGGGACCTTGGCCGTGCC

TACTGTTCCAGAACTATCAATGTCCTTTCCTATTGTAACGACGTGGTGTGCGGGGACCAC

ATCATCATTAGTGGCCACAATGACCAGAAGATCCGGTTCTGGGATAGCAGGGGTCCCCGC

TGCACCCAGGTCATTCCTGTGCAGGGCCGGGTCACCTCCCTGAGCCTCAGCCATGACCAG

CTGCACCTGCTCATCTGCTCCAGAGATAATACACTCAAGGTCATTGACCTCCGGGTCAGC

AATATCCGCCAGGTGTTCAGGGCTGATGGCTTCAAATGTGGTTCCGACTGGACCAAAGCT

GTGTTCAGCCCTGACAGAAGCTATGCACTGGCAGGCTCCTCGAATGGAGCCCTTTTCATC

TGGGATGTTGACACTGGGAAACTGGAGAGCAGCCTTCAGGGCCCCCAC

>CL338.Contig23_A-W 86 229 minus strand Autophagy-related protein 16-2 OS=Mus musculus GN=Atg16l2 PE=2 SV=2

CAGGAAACACTGTCTGGACACAAGGACAAGGTGACAGCTGCCAAATTCAAGCTAACAAGA

CACCAGGCGGTGACTGGGAGCAGAGACCGGACAGTGAAGGAGTGGGACCTTGGCCGTGCC

TACTGTGAGGCTCAGCTCCAACTC

>CL338.Contig24_A-W 3 290 minus strand rCG40272, isoform CRA_b [Rattus norvegicus]

TCCCAGGTTTTGGCAGCTACTTACAATCAGGCTGCCCAGCTCTGGAAGGTGGGAGAGGCA

CGGTCCAAGGAAACACTGTCTGGACACAAGGACAAGGTGACAGCTGCCAAATTCAAGCTA

ACAAGACACCAGGCGGTGACTGGGAGCAGAGACCGGACAGTGAAGGAGTGGGACCTTGGC

CGTGCCTACTGTGAGGCTCAGCTCCAACTCCACCTCCCATCCCCCCTTCCCTCAGATACA

AGGGAACCTACCCTGCTTTCTGGGAGTGGCTCTGGGATTCAAAGACAT

>CL338.Contig25_A-W 1748 2161 minus strand PREDICTED: autophagy-related protein 16-2 isoform 1 [Saimiri boliviensis boliviensis]

TGGTCCCTAGGTTCCAGAACTATCAATGTCCTTTCCTATTGTAACGACGTGGTGTGCGGG

GACCACATCATCATTAGTGGCCACAATGACCAGAAGATCCGGTTCTGGGATAGCAGGGGT

CCCCGCTGCACCCAGGTCATTCCTGTGCAGGGCCGGGTCACCTCCCTGAGCCTCAGCCAT

GACCAGCTGCACCTGCTCATCTGCTCCAGAGATAATACACTCAAGGTCATTGACCTCCGG

GTCAGCAATATCCGCCAGGTGTTCAGGGCTGATGGCTTCAAATGTGGTTCCGACTGGACC

AAAGCTGTGTTCAGCCCTGACAGAAGCTATGCACTGGCAGGCTCCTCGAATGGAGCCCTT

TTCATCTGGGATGTTGACACTGGGAAACTGGAGAGCAGCCTTCAGGGCCCCCAC

>CL338.Contig26_A-W 86 316 minus strand Autophagy-related protein 16-2 OS=Mus musculus GN=Atg16l2 PE=2 SV=2

CAGGAAACACTGTCTGGACACAAGGACAAGGTGACAGCTGCCAAATTCAAGCTAACAAGA

CACCAGGCGGTGACTGGGAGCAGAGACCGGACAGTGAAGGAGTGGGACCTTGGCCGTGCC

TACTGTTCCAGAACTATCAATGTCCTTTCCTATTGTAACGACGTGGTGTGCGGGGACCAC

ATCATCATTAGTGGCCACAATGACCAGAAGATCCGGTTCTGGGATAGCAGG

>CL338.Contig27_A-W 3 299 minus strand PREDICTED: autophagy-related protein 16-2-like [Cavia porcellus]

TCCCAGGTTTTGGCAGCTACTTACAATCAGGCTGCCCAGCTCTGGAAGGTGGGAGAGGCA

CGGTCCAAGGAAACACTGTCTGGACACAAGGACAAGGTGACAGCTGCCAAATTCAAGCTA

ACAAGACACCAGGCGGTGACTGGGAGCAGAGACCGGACAGTGAAGGAGTGGGACCTTGGC

CGTGCCTACTGTTCCAGAACTATCAATGTCCTTTCCTATTGTAACGACGTGGTGTGCGGG

GACCACATCATCATTAGTGGCCACAATGACCAGAAGATCCGGTTCTGGGATAGCAGG

>CL338.Contig28_A-W 33 1421 minus strand PREDICTED: autophagy-related protein 16-2 isoform 1 [Saimiri boliviensis boliviensis]

ATGGCAGGGCCCGAAGCATCCTTCGACCCCACCTCGGGTTGGAAGCGCCACATTGTGCGG

CAGCTACAGAAGCGGGACCGCACGCAGAAGGCACTTTTCCTGGAGCTGGTGCCGGCCTAT

AATCATCTCCTAGAAAAAGCTGAGCTGTTGGCCAAGTTCTCAGAGAAGCTTCATAAGAAG

CCAAATGATGTTACTTCTGCCACCCATCAGAGCTTCTGGGAGGAGTCAGGGCCTGACTCA

GACCAAAGCTCACCACCAGGCACTCTGAGGGTGAAGTGGCAGGAGGAGGAGGAGGAGGGG

CTCCGCTTGGACTGTGGTGAGATGGCCTACCAGGTGGTGAAGAAGAGTGCAGCCTTGGAT

GCCCTGCAGTCGGAGCTGGAGGAGCGGCAAAGCAGGCTGGCGGCTGTGGAGGCCCGCGTG

GCGCAGCTGCGGGAGGCGAGGGCGCAGCAGGTCAGCCTCGTAGAGGAGCTACAGAAGCAG

AATGCGGCGCAGCGGAAGGCCTACGAGGTGCTGCGCGTGCACACTGGGCACCAAGAGGCG

GCACTGCGCAGGCTCCAGGAGGAGGCGCTCAATCTGCTGGAGCAGCTCTTGCAACGCAAG

GAGCGCGCCGCAGCGGAGTGCAACTTGCGCAACGAGCGCCGAGAGCGGGCCAATCAGGCG

CGGGTGTCCCAGGAACTAAAGAAGGCTGCCAAACGGACTGTGAGCATCAGCGAGATTCCA

CACACTCTACATGATGGGATCAGAGAGCAGACAGAGACTCTGGCTCTGGCTATTAAGCCT

GAGTCCCTGGAGAGTGAGGCTTGTGGGAAGTGGAAAAGGCCTTTCAGGTCTGCCTCAGCC

ACCTCTCTGACTCTGTCCCACTGTGTGGATGTGGTGAAGGGGCTGCTAGATTTCAAGAAG

AGGAGAGGTCACTCAATTGGGGGTGCCCCTGAGCAGCGATACCAGAGCATCCCTGTGTGT

GTGGCTGCCCGACTTCCTACTTGGGCTCAAGATGTCCTGGATGCCCACCTTTCTGAGGTC

AATGCTGTATGTTTTGGCCCTAACAGCAGTCTCCTGGCCACTGGTGGGGCTGACTGTCTT

ATCCACCTCTGGAATGTGGTGGGAGGTCGTCTGGAGGCCAACCAGACCCTTGAGGGAGCT

GGTGGCAGCATCACCAGTGTGGACTTCGACCCCTCGGGCTCCCAGGTTTTGGCAGCTACT

TACAATCAGGCTGCCCAGCTCTGGAAGGTGGGAGAGGCACGGTCCAAGGAAACACTGTCT

GGACACAAGGACAAGGTGACAGCTGCCAAATTCAAGCTAACAAGACACCAGGCGGTGACT

GGGAGCAGAGACCGGACAGTGAAGGAGTGGGACCTTGGCCGTGCCTACTGTGAGGCTCAG

CTCCAACTC

>CL338.Contig29_A-W 1345 2001 minus strand PREDICTED: autophagy-related protein 16-2 isoform 1 [Saimiri boliviensis boliviensis]

TTATGGGGTCTTGGCCATGCCTTTGATCCAGACTTGGGATGGGGGCAGGAAACACTGTCT

GGACACAAGGACAAGGTGACAGCTGCCAAATTCAAGCTAACAAGACACCAGGCGGTGACT

GGGAGCAGAGACCGGACAGTGAAGGAGTGGGACCTTGGCCGTGCCTACTGTTCCAGAACT

ATCAATGTCCTTTCCTATTGTAACGACGTGGTGTGCGGGGACCACATCATCATTAGTGGC

CACAATGACCAGAAGATCCGGTTCTGGGATAGCAGGGGTCCCCGCTGCACCCAGGTCATT

CCTGTGCAGGGCCGGGTCACCTCCCTGAGCCTCAGCCATGACCAGCTGCACCTGCTCATC

TGCTCCAGAGATAATACACTCAAGGTCATTGACCTCCGGGTCAGCAATATCCGCCAGGTG

TTCAGGGCTGATGGCTTCAAATGTGGTTCCGACTGGACCAAAGCTGTGTTCAGCCCTGAC

AGAAGCTATGCACTGGCAGGCTCCTCGAATGGAGCCCTTTTCATCTGGGATGTTGACACT

GGGAAACTGGAGAGCAGCCTTCAGGGCCCCCACTGTGCTGCTATCAACGCTGTGGCCTGG

TGCTGCTCCGGGAGCCACGTGGTGAGCGTGGACCAGGGCAGGAAGGTTGTGCTCTGG

>CL338.Contig30_A-W 1345 1917 minus strand PREDICTED: autophagy-related protein 16-2 isoform 1 [Saimiri boliviensis boliviensis]

TTATGGGGTCTTGGCCATGCCTTTGATCCAGACTTGGGATGGGGGCAGGAAACACTGTCT

GGACACAAGGACAAGGTGACAGCTGCCAAATTCAAGCTAACAAGACACCAGGCGGTGACT

GGGAGCAGAGACCGGACAGTGAAGGAGTGGGACCTTGGCCGTGCCTACTGTTCCAGAACT

ATCAATGTCCTTTCCTATTGTAACGACGTGGTGTGCGGGGACCACATCATCATTAGTGGC

CACAATGACCAGAAGATCCGGTTCTGGGATAGCAGGGGTCCCCGCTGCACCCAGGTCATT

CCTGTGCAGGGCCGGGTCACCTCCCTGAGCCTCAGCCATGACCAGCTGCACCTGCTCATC

TGCTCCAGAGATAATACACTCAAGGTCATTGACCTCCGGGTCAGCAATATCCGCCAGGTG

TTCAGGGCTGATGGCTTCAAATGTGGTTCCGACTGGACCAAAGCTGTGTTCAGCCCTGAC

AGAAGCTATGCACTGGCAGGCTCCTCGAATGGAGCCCTTTTCATCTGGGATGTTGACACT

GGGAAACTGGAGAGCAGCCTTCAGGGCCCCCAC

>CL338.Contig31_A-W 1295 1906 minus strand PREDICTED: autophagy-related protein 16-2 isoform 1 [Saimiri boliviensis boliviensis]

CAGGAAACACTGTCTGGACACAAGGACAAGGTGACAGCTGCCAAATTCAAGCTAACAAGA

CACCAGGCGGTGACTGGGAGCAGAGACCGGACAGTGAAGGAGTGGGACCTTGGCCGTGCC

TACTGTTCCAGAACTATCAATGTCCTTTCCTATTGTAACGACGTGGTGTGCGGGGACCAC

ATCATCATTAGTGGCCACAATGACCAGAAGATCCGGTTCTGGGATAGCAGGGGTCCCCGC

TGCACCCAGGTCATTCCTGTGCAGGGCCGGGTCACCTCCCTGAGCCTCAGCCATGACCAG

CTGCACCTGCTCATCTGCTCCAGAGATAATACACTCAAGGTCATTGACCTCCGGGTCAGC

AATATCCGCCAGGTGTTCAGGGCTGATGGCTTCAAATGTGGTTCCGACTGGACCAAAGCT

GTGTTCAGCCCTGACAGAAGCTATGCACTGGCAGGCTCCTCGAATGGAGCCCTTTTCATC

TGGGATGTTGACACTGGGAAACTGGAGAGCAGCCTTCAGGGCCCCCACTGTGCTGCTATC

AACGCTGTGGCCTGGTGCTGCTCCGGGAGCCACGTGGTGAGCGTGGACCAGGGCAGGAAG

GTTGTGCTCTGG

>CL338.Contig32_A-W 33 1805 minus strand PREDICTED: autophagy-related protein 16-2 isoform 1 [Saimiri boliviensis boliviensis]

ATGGCAGGGCCCGAAGCATCCTTCGACCCCACCTCGGGTTGGAAGCGCCACATTGTGCGG

CAGCTACAGAAGCGGGACCGCACGCAGAAGGCACTTTTCCTGGAGCTGGTGCCGGCCTAT

AATCATCTCCTAGAAAAAGCTGAGCTGTTGGCCAAGTTCTCAGAGAAGCTTCATAAGAAG

CCAAATGATGTTACTTCTGCCACCCATCAGAGCTTCTGGGAGGAGTCAGGGCCTGACTCA

GACCAAAGCTCACCACCAGGCACTCTGAGGGTGAAGTGGCAGGAGGAGGAGGAGGAGGGG

CTCCGCTTGGACTGTGGTGAGATGGCCTACCAGGTGGTGAAGAAGAGTGCAGCCTTGGAT

GCCCTGCAGTCGGAGCTGGAGGAGCGGCAAAGCAGGCTGGCGGCTGTGGAGGCCCGCGTG

GCGCAGCTGCGGGAGGCGAGGGCGCAGCAGGTCAGCCTCGTAGAGGAGCTACAGAAGCAG

AATGCGGCGCAGCGGAAGGCCTACGAGGTGCTGCGCGTGCACACTGGGCACCAAGAGGCG

GCACTGCGCAGGCTCCAGGAGGAGGCGCTCAATCTGCTGGAGCAGCTCTTGCAACGCAAG

GAGCGCGCCGCAGCGGAGTGCAACTTGCGCAACGAGCGCCGAGAGCGGGCCAATCAGGCG

CGGGTGTCCCAGGAACTAAAGAAGGCTGCCAAACGGACTGTGAGCATCAGCGAGATTCCA

CACACTCTACATGATGGGATCAGAGAGCAGACAGAGACTCTGGCTCTGGCTATTAAGCCT

GAGTCCCTGGAGAGTGAGGCTTGTGGGAAGTGGAAAAGGCCTTTCAGGTCTGCCTCAGCC

ACCTCTCTGACTCTGTCCCACTGTGTGGATGTGGTGAAGGGGCTGCTAGATTTCAAGAAG

AGGAGAGGTCACTCAATTGGGGGTGCCCCTGAGCAGCGATACCAGAGCATCCCTGTGTGT

GTGGCTGCCCGACTTCCTACTTGGGCTCAAGATGTCCTGGATGCCCACCTTTCTGAGGTC

AATGCTGTATGTTTTGGCCCTAACAGCAGTCTCCTGGCCACTGGTGGGGCTGACTGTCTT

ATCCACCTCTGGAATGTGGTGGGAGGTCGTCTGGAGGCCAACCAGACCCTTGAGGGAGCT

GGTGGCAGCATCACCAGTGTGGACTTCGACCCCTCGGGCTCCCAGGTTTTGGCAGCTACT

TACAATCAGGCTGCCCAGCTCTGGAAGGTGGGAGAGGCACGGTCCAAGGAAACACTGTCT

GGACACAAGGACAAGGTGACAGCTGCCAAATTCAAGCTAACAAGACACCAGGCGGTGACT

GGGAGCAGAGACCGGACAGTGAAGGAGTGGGACCTTGGCCGTGCCTACTGTTCCAGAACT

ATCAATGTCCTTTCCTATTGTAACGACGTGGTGTGCGGGGACCACATCATCATTAGTGGC

CACAATGACCAGAAGATCCGGTTCTGGGATAGCAGGGGTCCCCGCTGCACCCAGGTCATT

CCTGTGCAGGGCCGGGTCACCTCCCTGAGCCTCAGCCATGACCAGCTGCACCTGCTCATC

TGCTCCAGAGATAATACACTCAAGGTCATTGACCTCCGGGTCAGCAATATCCGCCAGGTG

TTCAGGGCTGATGGCTTCAAATGTGGTTCCGACTGGACCAAAGCTGTGTTCAGCCCTGAC

AGAAGCTATGCACTGGCAGGCTCCTCGAATGGAGCCCTTTTCATCTGGGATGTTGACACT

GGGAAACTGGAGAGCAGCCTTCAGGGCCCCCAC

>CL338.Contig33_A-W 33 1889 minus strand PREDICTED: autophagy-related protein 16-2 isoform 1 [Saimiri boliviensis boliviensis]

ATGGCAGGGCCCGAAGCATCCTTCGACCCCACCTCGGGTTGGAAGCGCCACATTGTGCGG

CAGCTACAGAAGCGGGACCGCACGCAGAAGGCACTTTTCCTGGAGCTGGTGCCGGCCTAT

AATCATCTCCTAGAAAAAGCTGAGCTGTTGGCCAAGTTCTCAGAGAAGCTTCATAAGAAG

CCAAATGATGTTACTTCTGCCACCCATCAGAGCTTCTGGGAGGAGTCAGGGCCTGACTCA

GACCAAAGCTCACCACCAGGCACTCTGAGGGTGAAGTGGCAGGAGGAGGAGGAGGAGGGG

CTCCGCTTGGACTGTGGTGAGATGGCCTACCAGGTGGTGAAGAAGAGTGCAGCCTTGGAT

GCCCTGCAGTCGGAGCTGGAGGAGCGGCAAAGCAGGCTGGCGGCTGTGGAGGCCCGCGTG

GCGCAGCTGCGGGAGGCGAGGGCGCAGCAGGTCAGCCTCGTAGAGGAGCTACAGAAGCAG

AATGCGGCGCAGCGGAAGGCCTACGAGGTGCTGCGCGTGCACACTGGGCACCAAGAGGCG

GCACTGCGCAGGCTCCAGGAGGAGGCGCTCAATCTGCTGGAGCAGCTCTTGCAACGCAAG

GAGCGCGCCGCAGCGGAGTGCAACTTGCGCAACGAGCGCCGAGAGCGGGCCAATCAGGCG

CGGGTGTCCCAGGAACTAAAGAAGGCTGCCAAACGGACTGTGAGCATCAGCGAGATTCCA

CACACTCTACATGATGGGATCAGAGAGCAGACAGAGACTCTGGCTCTGGCTATTAAGCCT

GAGTCCCTGGAGAGTGAGGCTTGTGGGAAGTGGAAAAGGCCTTTCAGGTCTGCCTCAGCC

ACCTCTCTGACTCTGTCCCACTGTGTGGATGTGGTGAAGGGGCTGCTAGATTTCAAGAAG

AGGAGAGGTCACTCAATTGGGGGTGCCCCTGAGCAGCGATACCAGAGCATCCCTGTGTGT

GTGGCTGCCCGACTTCCTACTTGGGCTCAAGATGTCCTGGATGCCCACCTTTCTGAGGTC

AATGCTGTATGTTTTGGCCCTAACAGCAGTCTCCTGGCCACTGGTGGGGCTGACTGTCTT

ATCCACCTCTGGAATGTGGTGGGAGGTCGTCTGGAGGCCAACCAGACCCTTGAGGGAGCT

GGTGGCAGCATCACCAGTGTGGACTTCGACCCCTCGGGCTCCCAGGTTTTGGCAGCTACT

TACAATCAGGCTGCCCAGCTCTGGAAGGTGGGAGAGGCACGGTCCAAGGAAACACTGTCT

GGACACAAGGACAAGGTGACAGCTGCCAAATTCAAGCTAACAAGACACCAGGCGGTGACT

GGGAGCAGAGACCGGACAGTGAAGGAGTGGGACCTTGGCCGTGCCTACTGTTCCAGAACT

ATCAATGTCCTTTCCTATTGTAACGACGTGGTGTGCGGGGACCACATCATCATTAGTGGC

CACAATGACCAGAAGATCCGGTTCTGGGATAGCAGGGGTCCCCGCTGCACCCAGGTCATT

CCTGTGCAGGGCCGGGTCACCTCCCTGAGCCTCAGCCATGACCAGCTGCACCTGCTCATC

TGCTCCAGAGATAATACACTCAAGGTCATTGACCTCCGGGTCAGCAATATCCGCCAGGTG

TTCAGGGCTGATGGCTTCAAATGTGGTTCCGACTGGACCAAAGCTGTGTTCAGCCCTGAC

AGAAGCTATGCACTGGCAGGCTCCTCGAATGGAGCCCTTTTCATCTGGGATGTTGACACT

GGGAAACTGGAGAGCAGCCTTCAGGGCCCCCACTGTGCTGCTATCAACGCTGTGGCCTGG

TGCTGCTCCGGGAGCCACGTGGTGAGCGTGGACCAGGGCAGGAAGGTTGTGCTCTGG

>CL338.Contig34_A-W 682 870 minus strand PREDICTED: autophagy-related protein 16-2 isoform 2 [Nomascus leucogenys]

TTATGGGGTCTTGGCCATGCCTTTGATCCAGACTTGGGATGGGGGCAGGAAACACTGTCT

GGACACAAGGACAAGGTGACAGCTGCCAAATTCAAGCTAACAAGACACCAGGCGGTGACT

GGGAGCAGAGACCGGACAGTGAAGGAGTGGGACCTTGGCCGTGCCTACTGTGAGGCTCAG

CTCCAACTC

>CL338.Contig35_A-W 1085 1585 minus strand autophagy-related protein 16-2 [Homo sapiens] >gi|189027648|sp|Q8NAA4.2|A16L2_HUMAN RecName: Full=Autophagy-related protein 16-2; AltName: Full=APG16-like 2; AltName: Full=WD repeat-containing protein 80 >gi|119595280|gb|EAW74874.1| ATG16 autophagy related 16-like 2 (S. cerevisiae), isoform CRA_a [Homo sapiens] >gi|187950669|gb|AAI37490.1| ATG16 autophagy related 16-like 2 (S. cerevisiae) [Homo sapiens] >gi|187952629|gb|AAI37491.1| ATG16 autophagy related 16-like 2 (S. cerevisiae) [Homo sapiens]

TGGTCCCTAGGTTCCAGAACTATCAATGTCCTTTCCTATTGTAACGACGTGGTGTGCGGG

GACCACATCATCATTAGTGGCCACAATGACCAGAAGATCCGGTTCTGGGATAGCAGGGGT

CCCCGCTGCACCCAGGTCATTCCTGTGCAGGGCCGGGTCACCTCCCTGAGCCTCAGCCAT

GACCAGCTGCACCTGCTCATCTGCTCCAGAGATAATACACTCAAGGTCATTGACCTCCGG

GTCAGCAATATCCGCCAGGTGTTCAGGGCTGATGGCTTCAAATGTGGTTCCGACTGGACC

AAAGCTGTGTTCAGCCCTGACAGAAGCTATGCACTGGCAGGCTCCTCGAATGGAGCCCTT

TTCATCTGGGATGTTGACACTGGGAAACTGGAGAGCAGCCTTCAGGGCCCCCACTGTGCT

GCTATCAACGCTGTGGCCTGGTGCTGCTCCGGGAGCCACGTGGTGAGCGTGGACCAGGGC

AGGAAGGTTGTGCTCTGGCGC

>CL338.Contig36_A-W 1068 1568 minus strand PREDICTED: autophagy-related protein 16-2 [Pan troglodytes]

TGGTCCCTAGGTTCCAGAACTATCAATGTCCTTTCCTATTGTAACGACGTGGTGTGCGGG

GACCACATCATCATTAGTGGCCACAATGACCAGAAGATCCGGTTCTGGGATAGCAGGGGT

CCCCGCTGCACCCAGGTCATTCCTGTGCAGGGCCGGGTCACCTCCCTGAGCCTCAGCCAT

GACCAGCTGCACCTGCTCATCTGCTCCAGAGATAATACACTCAAGGTCATTGACCTCCGG

GTCAGCAATATCCGCCAGGTGTTCAGGGCTGATGGCTTCAAATGTGGTTCCGACTGGACC

AAAGCTGTGTTCAGCCCTGACAGAAGCTATGCACTGGCAGGCTCCTCGAATGGAGCCCTT

TTCATCTGGGATGTTGACACTGGGAAACTGGAGAGCAGCCTTCAGGGCCCCCACTGTGCT

GCTATCAACGCTGTGGCCTGGTGCTGCTCCGGGAGCCACGTGGTGAGCGTGGACCAGGGC

AGGAAGGTTGTGCTCTGGCGC

>CL338.Contig37_A-W 1085 1498 minus strand autophagy-related protein 16-2 [Homo sapiens] >gi|189027648|sp|Q8NAA4.2|A16L2_HUMAN RecName: Full=Autophagy-related protein 16-2; AltName: Full=APG16-like 2; AltName: Full=WD repeat-containing protein 80 >gi|119595280|gb|EAW74874.1| ATG16 autophagy related 16-like 2 (S. cerevisiae), isoform CRA_a [Homo sapiens] >gi|187950669|gb|AAI37490.1| ATG16 autophagy related 16-like 2 (S. cerevisiae) [Homo sapiens] >gi|187952629|gb|AAI37491.1| ATG16 autophagy related 16-like 2 (S. cerevisiae) [Homo sapiens]

TGGTCCCTAGGTTCCAGAACTATCAATGTCCTTTCCTATTGTAACGACGTGGTGTGCGGG

GACCACATCATCATTAGTGGCCACAATGACCAGAAGATCCGGTTCTGGGATAGCAGGGGT

CCCCGCTGCACCCAGGTCATTCCTGTGCAGGGCCGGGTCACCTCCCTGAGCCTCAGCCAT

GACCAGCTGCACCTGCTCATCTGCTCCAGAGATAATACACTCAAGGTCATTGACCTCCGG

GTCAGCAATATCCGCCAGGTGTTCAGGGCTGATGGCTTCAAATGTGGTTCCGACTGGACC

AAAGCTGTGTTCAGCCCTGACAGAAGCTATGCACTGGCAGGCTCCTCGAATGGAGCCCTT

TTCATCTGGGATGTTGACACTGGGAAACTGGAGAGCAGCCTTCAGGGCCCCCAC

>CL338.Contig38_A-W 1068 1481 minus strand PREDICTED: autophagy-related protein 16-2 [Pan troglodytes]

TGGTCCCTAGGTTCCAGAACTATCAATGTCCTTTCCTATTGTAACGACGTGGTGTGCGGG

GACCACATCATCATTAGTGGCCACAATGACCAGAAGATCCGGTTCTGGGATAGCAGGGGT

CCCCGCTGCACCCAGGTCATTCCTGTGCAGGGCCGGGTCACCTCCCTGAGCCTCAGCCAT

GACCAGCTGCACCTGCTCATCTGCTCCAGAGATAATACACTCAAGGTCATTGACCTCCGG

GTCAGCAATATCCGCCAGGTGTTCAGGGCTGATGGCTTCAAATGTGGTTCCGACTGGACC

AAAGCTGTGTTCAGCCCTGACAGAAGCTATGCACTGGCAGGCTCCTCGAATGGAGCCCTT

TTCATCTGGGATGTTGACACTGGGAAACTGGAGAGCAGCCTTCAGGGCCCCCAC

>CL338.Contig39_A-W 452 640 minus strand PREDICTED: autophagy-related protein 16-2 isoform 2 [Nomascus leucogenys]

TTATGGGGTCTTGGCCATGCCTTTGATCCAGACTTGGGATGGGGGCAGGAAACACTGTCT

GGACACAAGGACAAGGTGACAGCTGCCAAATTCAAGCTAACAAGACACCAGGCGGTGACT

GGGAGCAGAGACCGGACAGTGAAGGAGTGGGACCTTGGCCGTGCCTACTGTGAGGCTCAG

CTCCAACTC

>CL338.Contig40_A-W 196 582 minus strand PREDICTED: autophagy-related protein 16-2 isoform 2 [Nomascus leucogenys]

AGGGGTCCCCGCTGCACCCAGGTCATTCCTGTGCAGGGCCGGGTCACCTCCCTGAGCCTC

AGCCATGACCAGCTGCACCTGCTCATCTGCTCCAGAGATAATACACTCAAGGTCATTGAC

CTCCGGGTCAGCAATATCCGCCAGGTGTTCAGGGCTGATGGCTTCAAATGTGGTTCCGAC

TGGACCAAAGCTGTGTTCAGCCCTGACAGAAGCTATGCACTGGCAGGCTCCTCGAATGGA

GCCCTTTTCATCTGGGATGTTGACACTGGGAAACTGGAGAGCAGCCTTCAGGGCCCCCAC

TGTGCTGCTATCAACGCTGTGGCCTGGTGCTGCTCCGGGAGCCACGTGGTGAGCGTGGAC

CAGGGCAGGAAGGTTGTGCTCTGGCGC

>CL338.Contig41_A-W 29 340 minus strand Autophagy-related protein 16-2 [Heterocephalus glaber]

GGGTCAAGGGTCCTTTTTCAGGAGAGGGAGGTGCTATTATGGGGTCTTGGCCATGCCTTT

GATCCAGACTTGGGATGGGGGCAGGAAACACTGTCTGGACACAAGGACAAGGTGACAGCT

GCCAAATTCAAGCTAACAAGACACCAGGCGGTGACTGGGAGCAGAGACCGGACAGTGAAG

GAGTGGGACCTTGGCCGTGCCTACTGTTCCAGAACTATCAATGTCCTTTCCTATTGTAAC

GACGTGGTGTGCGGGGACCACATCATCATTAGTGGCCACAATGACCAGAAGATCCGGTTC

TGGGATAGCAGG

>CL339.Contig1_A-W 137 1321 minus strand PREDICTED: coiled-coil domain-containing protein 130 [Canis lupus familiaris]

ATGGGTGAGAGGAAAGGTGTCAACAAGTACTACCCTCCGGACTTCAACCCCGAGAAGCAT

GGCTCCCTCAATCGATACCACAACAGCCATCCGCTTCGGGAGCGGGCCCGGAAGCTGTCC

CAAGGCATCCTCGTCATCCGATTCGAGATGCCGTATAACATCTGGTGCGACGGCTGCAAG

AACCACATCGGCATGGGTGTGCGGTACAATGCAGAGAAAAAGAAAGTGGGCAACTACTAC

ACGACCCCCATCTACAGGTTTCGGATGAAATGCCACCTGTGCGTCAACTACATCGAGATG

CAGACAGACCCCGCCAACTGTGACTATGTGATCGTGAGCGGCGCCCAGCGCAAGGAGGAA

CGCTGGGACATGGCGGACAACGAGCAGGTGCTGACCACAGAGCACGAGAAGAAGCAGAGG

CTGGAGGCGGATGCCATGTTCCGCCTGGAGCACGGAGAGGCGGACCGCAGCGCGCTCAAG

AAGGCCCTGCCCACGCTGAGCCACATCCAGGAGGCCCAGAGCGCGTGGAAGGACGACTTC

GCCCTCAACAGCATGCTGCGGCGGAGGTTCCGGGAAAAGAAAAAAGCCATCCAGGAGGAG

GAGGAGAGGGACCAGGCCCTGCAGGCCAAGGCCAGCCTGGCCATCCCCCTGGTGCCCGAG

TCGGAGGACGACCGCAGGCTGGCCTCCCTGCTCAGGTTCCACACCCTGGACTCCTACGAG

GACAAGCAGAAACTCAAGCGCACGGAGATCATCAGCCGCTCCTGGTTCCCCTCCGCCCCG

GGCCCCGGCAGCGCCAGCAGCAAGGCCAGCAGCGTCCTGAAGAAGCTGGCCCAGGGCCGC

AGGAGCGCACCCAGCAGCTGCCCCATCACCGTGGAGGACCTGGGCATCGTGCGGCGGCCT

CGAGACGTCCCCGGGAGCCCTCAGCCCACGGCCGAGAAGGCCAAGTCCAGGGAGCCGCAG

GCACCTCAGGGGACCACCCAGGACAGACCCGCGTCCTCCCAGGACGGCTCTGAGGAGACA

GCGAACCCCAAGACCTGCGGACAGTGGGGGCAGGAGGGGATCTGTCAGGACAAGTCCCGG

CCCCCATCAGGCTCCTCCCAGGAGGCACCGGACCCTCCAGGCACACCGCAGCCCGGCAGC

CTGAGCTCCTCTCTGGTGGCCGACTACTCTGACTCCGAGAGTGAA

>CL339.Contig2_A-W 137 547 minus strand unnamed protein product [Homo sapiens]

ATGGGTGAGAGGAAAGGTGTCAACAAGTACTACCCTCCGGACTTCAACCCCGAGAAGCAT

GGCTCCCTCAATCGATACCACAACAGCCATCCGCTTCGGGAGCGGGCCCGGAAGCTGTCC

CAAGGCATCCTCGTCATCCGATTCGAGATGCCGTATAACATCTGGTGCGACGGCTGCAAG

AACCACATCGGCATGGGTGTGCGGTACAATGCAGAGAAAAAGAAAGTGGGCAACTACTAC

ACGACCCCCATCTACAGGTTTCGGATGAAATGCCACCTGTGCGTCAACTACATCGAGATG

CAGACAGACCCCGCCAACTGTGACTATGTGATCGTGAGCGGCGCCCAGCGCAAGGAGGAA

CGCTGGGACATGGCGGACAACGAGCAGGTGCTGACCACAGGTGAGCGCCAC

>CL339.Contig3_A-W 3 227 minus strand PREDICTED: coiled-coil domain-containing protein 130 [Papio anubis]

CTGCCCACGCTGAGCCACATCCAGGAGGCCCAGAGCGCGTGGAAGGACGACTTCGCCCTC

AACAGCATGCTGCGGCGGAGGTTCCGGGAAAAGAAAAAAGCCATCCAGGAGGAGGAGGAG

AGGGACCAGGCCCTGCAGGCCAAGGCCAGCCTGGCCATCCCCCTGGTGCCCGAGTCGGAG

GACGACCGCAGGCTGGCCTCCCTGCTCAGGTTCCACACCCTGGAC

>CL340.Contig1_A-W 1 342 PREDICTED: dermatan-sulfate epimerase [Saimiri boliviensis boliviensis]

GCATTTATGTACAGAACCATCTTGCCAGGGTTTCAAAGAACTGTAGCCATTGCAGATTCA

AATTACAACTGGTTTTATGGTCCAGAAAGCCAATTAGTGTTCCTGGATAAATTTGTCATG

CGTAATGGCAGTGGAAACTGGCTGGCTGACCAGATCAGAAGGAATCGTGTGGTAGAAGGT

CCAGGGACACCATCCAAAGGGCAGCGCTGGTGCACTTTGCACACGGAATTTCTCTGGTAT

GATGCCAGCTTGAAATCGGTTCCTCCTCCAGATTTTGGCACCCCTACCCTGCATTATTTT

GAAGACTGGGGTGTTGTGACTTATGGAAGTGCACTGCCTGCA

>CL341.Contig1_A-W 1247 1666 transcription factor 7-like 2 isoform 6 [Mus musculus]

AGGAGAAAAAAAAAGTGCGTTCGCTACATACAAGGTGGAGGCAGCTGCCTCAGCCCACCC

TCTTCAGATGGAAGCTTACTAGACTCGCCGCCCCCCTCCCCGCACCCGCTGGGCTCCCCT

CCCCCCGACGCCAAGGCACAGACTGAGCAGACCCAGCCGCTCTCGCTGTCCCTGAAGCCC

GACCCCCTGGCCCACCTGGCCATGATGCCCCCGCCGCCCGCGCTCCTGCTGGCCGAGGCC

GCCCACGGCAAGGCCTCCGCCCTCTGTCCCAACGGGGCCCTGGACCTGCCACCTGCCGCC

CTGCAGCCACCCATCGCCCCCTCCTCGCTCGCACAGCCGTCGACGTCTTCCTTACATTCC

CACAGCGCGCTGGCCGGGACGCAGCCCCAGCCGCTGTCCCTCGTCACCAAGTCTTTAGAA

>CL341.Contig2_A-W 1321 1740 RecName: Full=Transcription factor 7-like 2; AltName: Full=HMG box transcription factor 4; AltName: Full=T-cell-specific transcription factor 4; Short=T-cell factor 4; Short=TCF-4; Short=hTCF-4 >gi|119569900|gb|EAW49515.1| transcription factor 7-like 2 (T-cell specific, HMG-box), isoform CRA_j [Homo sapiens] >gi|300808106|gb|ADK35185.1| T-cell factor-4 variant M [Homo sapiens]

AGGAGAAAAAAAAAGTGCGTTCGCTACATACAAGGTGGAGGCAGCTGCCTCAGCCCACCC

TCTTCAGATGGAAGCTTACTAGACTCGCCGCCCCCCTCCCCGCACCCGCTGGGCTCCCCT

CCCCCCGACGCCAAGGCACAGACTGAGCAGACCCAGCCGCTCTCGCTGTCCCTGAAGCCC

GACCCCCTGGCCCACCTGGCCATGATGCCCCCGCCGCCCGCGCTCCTGCTGGCCGAGGCC

GCCCACGGCAAGGCCTCCGCCCTCTGTCCCAACGGGGCCCTGGACCTGCCACCTGCCGCC

CTGCAGCCACCCATCGCCCCCTCCTCGCTCGCACAGCCGTCGACGTCTTCCTTACATTCC

CACAGCGCGCTGGCCGGGACGCAGCCCCAGCCGCTGTCCCTCGTCACCAAGTCTTTAGAA

>CL342.Contig1_A-W 999 1205 PREDICTED: L-serine dehydratase/L-threonine deaminase [Callithrix jacchus]

GATGACGAGAAGATCCTGGTGGAACCGGCCTGTGGGGCGGCCTTGGCCGCCGTCTACAGC

CACGTGGTCAAGAAACTGCAGGGGGAGGGGAAGCTGCGCGCCCCGCTGCCCAACCTGGTG

GTCATCGTCTGTGGGGGCAGCAACATCAGCCTGGCCCTGCTGTGGTCCCTCAAGGAACAG

CTGGGCATGAAGAATGGCCTACCAAAG

>CL342.Contig2_A-W 831 1037 PREDICTED: L-serine dehydratase/L-threonine deaminase [Callithrix jacchus]

GATGACGAGAAGATCCTGGTGGAACCGGCCTGTGGGGCGGCCTTGGCCGCCGTCTACAGC

CACGTGGTCAAGAAACTGCAGGGGGAGGGGAAGCTGCGCGCCCCGCTGCCCAACCTGGTG

GTCATCGTCTGTGGGGGCAGCAACATCAGCCTGGCCCTGCTGTGGTCCCTCAAGGAACAG

CTGGGCATGAAGAATGGCCTACCAAAG

>CL342.Contig3_A-W 310 432 PREDICTED: L-serine dehydratase/L-threonine deaminase [Sarcophilus harrisii]

GTTGCCAAGGCTCTGGGTGTGAACACGGTGAGTGCGCAGGCCCTGAAGCTGTTTCGGGAG

CACCCCATCTTCTCTGAAGTGGTCTCGGACCAGGAGGCTGTGGCCGCCATCGAGAAGTTT

GTG

>CL342.Contig4_A-W 1 867 PREDICTED: L-serine dehydratase/L-threonine deaminase [Papio anubis]

TTCAAGATCCGGGGCATCGGGCACCTCTGCAAGACGTGGGCTGAGCAAGGCTGTGAGCAT

TTTGTGTGCTCCTCTGCGGGCAATGCAGGCATGGCGGCCGCGTATGCCGCCAGGAAGCTG

GGCATCCCGGCCACCATCGTCGTGCCCAGCACCACACCGGCTCTCACTGTGGAGCGGCTC

AAGGGGGAAGGCGCCATGGTGGAGGTGGTGGGCGAGACGCTGGGTGAGGCCTTCGCGATG

GCCAAGGCACTGGCCAAGAACAACCCCGGCTGGGTCTACGTCCCCCCCTTCGATGACCCC

CTCATCTGGGAAGGCCACACCTCCCTCATCAGAGAGCTGAAGGAAAGCCTGGGCACCAAG

CCGGGCGCCATCGCACTGTCCGTGGGCGGCGGGGGCCTGCTGTGCGGAGTGGTCCAGGGG

CTGCAGGAGGTGGGCTGGGGGGACGTGCCCGTCATCGCCATGGAGACCATCGGTGCCCAC

AGCTTCCATGCTGCCACCACCGTGGGCAAGCTGGTCTCCCTGCCCAGGATCACCAGCGTT

GCCAAGGCTCTGGGTGTGAACACGGTGAGTGCGCAGGCCCTGAAGCTGTTTCGGGAGCAC

CCCATCTTCTCTGAAGTGGTCTCGGACCAGGAGGCTGTGGCCGCCATCGAGAAGTTTGTG

GATGACGAGAAGATCCTGGTGGAACCGGCCTGTGGGGCGGCCTTGGCCGCCGTCTACAGC

CACGTGGTCAAGAAACTGCAGGGGGAGGGGAAGCTGCGCGCCCCGCTGCCCAACCTGGTG

GTCATCGTCTGTGGGGGCAGCAACATCAGCCTGGCCCTGCTGTGGTCCCTCAAGGAACAG

CTGGGCATGAAGAATGGCCTACCAAAG

>CL342.Contig5_A-W 125 262 PREDICTED: L-serine dehydratase/L-threonine deaminase-like [Loxodonta africana]

CCGGCTCCGGCCAGCGTTGCCAAGGCTCTGGGTGTGAACACGGTGAGTGCGCAGGCCCTG

AAGCTGTTTCGGGAGCACCCCATCTTCTCTGAAGTGGTCTCGGACCAGGAGGCTGTGGCC

GCCATCGAGAAGTTTGTG

>CL342.Contig6_A-W 46 699 PREDICTED: L-serine dehydratase/L-threonine deaminase [Papio anubis]

CAGACGCTGGGTGAGGCCTTCGCGATGGCCAAGGCACTGGCCAAGAACAACCCCGGCTGG

GTCTACGTCCCCCCCTTCGATGACCCCCTCATCTGGGAAGGCCACACCTCCCTCATCAGA

GAGCTGAAGGAAAGCCTGGGCACCAAGCCGGGCGCCATCGCACTGTCCGTGGGCGGCGGG

GGCCTGCTGTGCGGAGTGGTCCAGGGGCTGCAGGAGGTGGGCTGGGGGGACGTGCCCGTC

ATCGCCATGGAGACCATCGGTGCCCACAGCTTCCATGCTGCCACCACCGTGGGCAAGCTG

GTCTCCCTGCCCAGGATCACCAGCGTTGCCAAGGCTCTGGGTGTGAACACGGTGAGTGCG

CAGGCCCTGAAGCTGTTTCGGGAGCACCCCATCTTCTCTGAAGTGGTCTCGGACCAGGAG

GCTGTGGCCGCCATCGAGAAGTTTGTGGATGACGAGAAGATCCTGGTGGAACCGGCCTGT

GGGGCGGCCTTGGCCGCCGTCTACAGCCACGTGGTCAAGAAACTGCAGGGGGAGGGGAAG

CTGCGCGCCCCGCTGCCCAACCTGGTGGTCATCGTCTGTGGGGGCAGCAACATCAGCCTG

GCCCTGCTGTGGTCCCTCAAGGAACAGCTGGGCATGAAGAATGGCCTACCAAAG

>CL342.Contig7_A-W 310 624 PREDICTED: L-serine dehydratase/L-threonine deaminase [Sarcophilus harrisii]

GTTGCCAAGGCTCTGGGTGTGAACACGGTGAGTGCGCAGGCCCTGAAGCTGTTTCGGGAG

CACCCCATCTTCTCTGAAGTGGTCTCGGACCAGGAGGCTGTGGCCGCCATCGAGAAGTTT

GTGGATGACGAGAAGATCCTGGTGGAACCGGCCTGTGGGGCGGCCTTGGCCGCCGTCTAC

AGCCACGTGGTCAAGAAACTGCAGGGGGAGGGGAAGCTGCGCGCCCCGCTGCCCAACCTG

GTGGTCATCGTCTGTGGGGGCAGCAACATCAGCCTGGCCCTGCTGTGGTCCCTCAAGGAA

CAGCTGGGCATGAAG

>CL342.Contig8_A-W 125 469 PREDICTED: L-serine dehydratase/L-threonine deaminase [Papio anubis]

CCGGCTCCGGCCAGCGTTGCCAAGGCTCTGGGTGTGAACACGGTGAGTGCGCAGGCCCTG

AAGCTGTTTCGGGAGCACCCCATCTTCTCTGAAGTGGTCTCGGACCAGGAGGCTGTGGCC

GCCATCGAGAAGTTTGTGGATGACGAGAAGATCCTGGTGGAACCGGCCTGTGGGGCGGCC

TTGGCCGCCGTCTACAGCCACGTGGTCAAGAAACTGCAGGGGGAGGGGAAGCTGCGCGCC

CCGCTGCCCAACCTGGTGGTCATCGTCTGTGGGGGCAGCAACATCAGCCTGGCCCTGCTG

TGGTCCCTCAAGGAACAGCTGGGCATGAAGAATGGCCTACCAAAG

>CL344.Contig1_A-W 121 879 PREDICTED: PAXIP1-associated protein 1-like [Nomascus leucogenys]

ATGTCCCTTGCCCGGGGTCATGGAGACATTGCGTCCACCACGGCGGCGCCTCTGTCTGAA

GAAGGGGAAGTGACTTCCGGCCTCCAGGCTCTGGCCGTGGAGGATACCGGAGGTCCCTCT

GTCTTGGCCAATAAGGCCGAGGAAGAGGGGGAAGGAGGACAGGAGGAGGCCGAGCATGCC

GGGCCCGGGACTGAGGAGGCAACTGAAGTACCCAGCGCCGAGGGGGAGGAGCGTGTCGAG

GGAGAATCCGAGGACTGGTGCGTGCCCTGCAGCGATGAGGAGGTGGAGCTGCCCGAGGAC

GGACAGCCCTGGATGCCCCCGCCCTCTGAAATCCAGCGGCTCTATGAGTTGCTGGCTGTC

CAAGGTACCTTGGAGCTTCAAGCGGAGATCCTGCCCCGACGGCCCCCTACACCCGAAGCC

CAGAGTGAAGAGGAGAGATCTGATGAGGAGCCGGAGGCCAAGGAAGAAGAAGAGGAAAAA

CCTCACATGCCTACAGAATTTGACTTCGATGATGAGCCAATGACACCAAAGGACTCCTTG

ATTGACCGGAGACGCACCCCAGGAAGTTCAGCCCGGAGCCAAAAAAGGGAGGCCCGCCTG

GACAAGGTCCTCTCAGACATGAAGCGACACAAGAAGCTGGAGGAGCAGATTCTTCGTACT

GGGAGGGACCTCTTCAGCTTGGACTCAGAGGACCCCAGCCCAGCCAGCCCCCCACTCCGG

TCATCAGGGAGTAGTCTCTTCCCTCGACAGCGGAAATAC

>CL345.Contig1_A-W 196 747 PREDICTED: prolactin regulatory element-binding protein isoform 1 [Papio anubis]

ATGGGCCGGCGCCGGGCTCCAGAGCTTTACCGGGCCCCGTTCCCGTTGTACGCGCTTCAG

GTAGACCCCAGCGCGGGCCTGCTCATCGCTGCGGGCGGAGGAGGCGCTGCCAAGACCGGC

ATAAAGAATGGAGTGCACTTTCTGCAGCTAGAACAAATTAATGGGCGCCTAAGCGCTTCC

CTGCTGCACTCCCATGACACGGAGACACGGGCTACCATGAACTTGGCACTGGCGGGTGAT

ATCCTTGCAGCAGGGCAGGATGCCCACTGTCAGCTCCTGCGTTTCCGGGTCCATAAGCAG

AAAGACAACAAGGCAGAGAAGGCAGGTTCCAAGGAGCAAGGGCTTCGACAAAGGAAGGGG

GCAACCCCAGCAGAAAAGAAATCAGGAGCTGAGACCCATCCTGAGGGGGGAGAGCTCAAG

GTTGAGAATTTGCAGGCAGTGCAGACAGACTTCGGCTCTGATCCACTGCAGAAAGTTGTG

TGTTTCAACCATGATAACACCTTGCTTGCCACTGGAGGAACAGATGGCTTCGTTCGTGTC

TGGAAGGTACCC

>CL345.Contig2_A-W 699 929 PREDICTED: prolactin regulatory element-binding protein isoform 1 [Papio anubis]

GCAGGTTCCAAGGAGCAAGGGCTTCGACAAAGGAAGGGGGCAACCCCAGCAGAAAAGAAA

TCAGGAGCTGAGACCCATCCTGAGGGGGGAGAGCTCAAGGTTGAGAATTTGCAGGCAGTG

CAGACAGACTTCGGCTCTGATCCACTGCAGAAAGTTGTGTGTTTCAACCATGATAACACC

TTGCTTGCCACTGGAGGAACAGATGGCTTCGTTCGTGTCTGGAAGGTACCC

>CL346.Contig1_A-W 102 203 minus strand UPF0608 protein C19orf42 homolog OS=Bos taurus PE=3 SV=1

GACAACATCCGGGAATTCTTACTGAGCCTCAGATACTTTCGGATCTTCATCGCCCTCTGG

AACGTCTTCATGATGTTCTGCATGATTGTACTCTTTGGCTCT

>CL346.Contig2_A-W 2 151 minus strand hypothetical protein MGC2747, isoform CRA_c [Homo sapiens]

CCGGTTCTGACGGGTACTTCTGCTGTAACGATGATCGGGGACATCCTGCTGTTTGGGACG

CTGCTGATGAACGCTGGGGCCGTGCTCAACTTTAAACTGAAAAAGAAGGACACGCAGGGC

TTTGGGGAGGAGTCGAGAGAGCCCAGTACA

>CL346.Contig3_A-W 2 256 minus strand hypothetical protein MGC2747, isoform CRA_c [Homo sapiens]

CCGGTTCTGACGGGTACTTCTGCTGTAACGATGATCGGGGACATCCTGCTGTTTGGGACG

CTGCTGATGAACGCTGGGGCCGTGCTCAACTTTAAACTGAAAAAGAAGGACACGCAGGGC

TTTGGGGAGGAGTCGAGAGAGCCCAGTACAGGGGACAACATCCGGGAATTCTTACTGAGC

CTCAGATACTTTCGGATCTTCATCGCCCTCTGGAACGTCTTCATGATGTTCTGCATGATT

GTACTCTTTGGCTCT

>CL347.Contig1_A-W 224 325 minus strand cullin 1 [Xenopus (Silurana) tropicalis] >gi|92919088|gb|ABE96895.1| cullin-1 [Xenopus (Silurana) tropicalis]

ACAGCAGAGCTCATTCTCCTTAGCTCACTTTCTTTCTCTCTCCAATTGCAGGACATTCTG

GCACAGGTGCTGCAGATTTTACTGAAGTCGAAGTTACTGGTA

>CL347.Contig2_A-W 130 276 minus strand cullin 1 [Xenopus (Silurana) tropicalis] >gi|92919088|gb|ABE96895.1| cullin-1 [Xenopus (Silurana) tropicalis]

AAGGCATCCACGTTTCAGATGGCGATCCTGCTCCAGTACAACTCAGAGGACGCCTATACT

GTGCAGCAGCTGACCGACAGCACGCAGATCAAAATGGACATTCTGGCACAGGTGCTGCAG

ATTTTACTGAAGTCGAAGTTACTGGTA

>CL347.Contig3_A-W 224 703 minus strand PREDICTED: cullin-1 [Callithrix jacchus]

ACAGCAGAGCTCATTCTCCTTAGCTCACTTTCTTTCTCTCTCCAATTGCAGGACATTCTG

GCACAGGTGCTGCAGATTTTACTGAAGTCGAAGTTACTGGTCCTGGAGGATGAAAACGCA

AATGTTGACGAGGTGGAGTTGAAGCCGGACACCTTAATAAAGTTGTATCTTGGTTATAAA

AATAAGAAATTGAGGGTTAACATCAATGTGCCAATGAAAACTGAACAGAAGCAAGAACAA

GAAACCACACACAAAAACATTGAGGAAGACCGCAAACTCCTGATTCAGGCGGCCATCGTG

AGGATCATGAAGATGAGGAAGGTCCTGAAGCACCAGCAGCTCCTCGGGGAGGTCCTCACC

CAGCTGTCCTCCAGGTTCAAGCCTCGGGTCCCAGTGATCAAGAAATGCATTGACATTCTG

ATTGAGAAGGAATATTTGGAGCGAGTCGATGGTGAAAAGGACACCTACAGTTACTTGGCT

>CL347.Contig5_A-W 130 654 minus strand Cullin-1 [Heterocephalus glaber]

AAGGCATCCACGTTTCAGATGGCGATCCTGCTCCAGTACAACTCAGAGGACGCCTATACT

GTGCAGCAGCTGACCGACAGCACGCAGATCAAAATGGACATTCTGGCACAGGTGCTGCAG

ATTTTACTGAAGTCGAAGTTACTGGTCCTGGAGGATGAAAACGCAAATGTTGACGAGGTG

GAGTTGAAGCCGGACACCTTAATAAAGTTGTATCTTGGTTATAAAAATAAGAAATTGAGG

GTTAACATCAATGTGCCAATGAAAACTGAACAGAAGCAAGAACAAGAAACCACACACAAA

AACATTGAGGAAGACCGCAAACTCCTGATTCAGGCGGCCATCGTGAGGATCATGAAGATG

AGGAAGGTCCTGAAGCACCAGCAGCTCCTCGGGGAGGTCCTCACCCAGCTGTCCTCCAGG

TTCAAGCCTCGGGTCCCAGTGATCAAGAAATGCATTGACATTCTGATTGAGAAGGAATAT

TTGGAGCGAGTCGATGGTGAAAAGGACACCTACAGTTACTTGGCT

>CL347.Contig6_A-W 221 2548 minus strand cullin-1 [Mus musculus] >gi|157820743|ref|NP_001102097.1| cullin-1 [Rattus norvegicus] >gi|13124104|sp|Q9WTX6.1|CUL1_MOUSE RecName: Full=Cullin-1; Short=CUL-1 >gi|5815403|gb|AAD52657.1|AF176910_1 cullin 1 [Mus musculus] >gi|4322381|gb|AAD16038.1| SCF complex protein cul-1 [Mus musculus] >gi|22137706|gb|AAH29260.1| Cullin 1 [Mus musculus] >gi|148681489|gb|EDL13436.1| cullin 1 [Mus musculus] >gi|171847072|gb|AAI61932.1| Cul1 protein [Rattus norvegicus]

ATGTCATCAAACAGGAGTCAGAATCCCCATGGACTGAAGCAGATTGGCCTTGACCAGATC

TGGGATGACCTCAGAGCTGGAATCCAGCAAGTGTACACCAGACAAAGCATGGCCAAGTCC

AGATACATGGAACTCTATACTCACGTTTATAACTACTGTACTAGCGTACACCAGTCAAAC

CAAGCACGGGGAGCTGGAGTCCCTCCTTCTAAATCGAAAAAGGGGCAGACACCTGGAGGG

GCTCAGTTTGTTGGCCTGGAATTGTACAAACGACTTAAAGAATTTTTGAAGAATTACTTG

ACAAATCTTCTTAAGGATGGAGAGGATCTGATGGACGAGAGCGTGCTGAAGTTCTACACT

CAGCAATGGGAAGATTACCGATTCTCAAGCAAAGTGCTAAATGGAATCTGTGCCTACCTC

AATAGACATTGGGTTCGCCGTGAATGTGATGAAGGACGAAAAGGAATATATGAAATCTAT

TCACTTGCATTGGTGACTTGGAGAGATTGTCTGTTCAGGCCATTGAATAAACAGGTAACA

AATGCTGTTTTAAAACTGATTGAAAAGGAAAGAAATGGTGAGACCATCAATACAAGACTG

ATTAGTGGAGTTGTACAGTCCTATGTGGAATTGGGGCTGAATGAAGATGATGCATTTGCA

AAGGGCCCTACGTTAACAGTGTATAAAGAATCCTTTGAATCTCAATTTTTGGCTGACACA

GAGAGATTTTATACCAGAGAGAGTACTGAATTCTTGCAGCAGAACCCAGTTACTGAATAT

ATGAAAAAGGCAGAGGCTCGTCTGCTTGAGGAACAGCGAAGAGTTCAGGTTTATCTTCAT

GAAAGCACGCAAGATGAGCTGGCCAGGAAATGTGAGCAGGTCCTTATCGAAAAACACTTG

GAAATTTTCCACACAGAGTTTCAGAATTTATTGGATGCCGACAAGAATGAAGATTTGGGA

CGCATGTATAATCTTGTATCTAGAATCCAGGATGGCCTAGGAGAGTTGAAAAAGCTGCTG

GAGACACACATTCATAATCAGGGTCTTGCAGCAATTGAGAAGTGTGGTGAAGCTGCCTTA

AATGACCCCAAAATGTATGTACAGACAGTGCTAGATGTTCATAAAAAGTACAATGCCCTG

GTAATGTCGGCCTTCAACAACGACGCTGGCTTCGTGGCTGCACTTGACAAGGCGTGTGGT

CGGTTCATAAACAACAACGCAGTTACCAAAATGGCCCAGTCATCCAGTAAATCCCCTGAG

CTGCTGGCGCGATACTGTGACTCCTTGTTGAAGAAGAGTTCCAAGAACCCAGAAGAGGCA

GAACTAGAAGACACACTCAATCAAGTGATGGTTGTCTTCAAGTACATAGAGGACAAAGAT

GTGTTCCAGAAGTTCTACGCGAAGATGTTGGCCAAGAGACTCGTTCATCAGAACAGTGCG

AGCGATGATGCCGAAGCGAGCATGATCTCCAAGCTCAAGCAAGCGTGTGGTTTTGAGTAT

ACTTCCAAGCTCCAGCGGATGTTCCAGGACATTGGTGTGAGCAAAGATCTGAATGAGCAG

TTCAAAAAGCACCTGACGAATTCAGAACCGCTAGACTTGGACTTCAGTATCCAAGTCCTG

AGCTCCGGGTCGTGGCCCTTCCAGCAGTCCTGCACATTCGCCTTGCCGTCCGAGTTGGAA

CGAAGCTATCAGCGATTCACAGCTTTCTATGCCAGCCGTCACAGTGGCAGAAAACTGACA

TGGTTGTATCAGTTGTCCAAAGGAGAATTGGTAACTAACTGCTTCAAGAACAGGTACACT

TTGCAGGCATCCACGTTTCAGATGGCGATCCTGCTCCAGTACAACTCAGAGGACGCCTAT

ACTGTGCAGCAGCTGACCGACAGCACGCAGATCAAAATGGACATTCTGGCACAGGTGCTG

CAGATTTTACTGAAGTCGAAGTTACTGGTCCTGGAGGATGAAAACGCAAATGTTGACGAG

GTGGAGTTGAAGCCGGACACCTTAATAAAGTTGTATCTTGGTTATAAAAATAAGAAATTG

AGGGTTAACATCAATGTGCCAATGAAAACTGAACAGAAGCAAGAACAAGAAACCACACAC

AAAAACATTGAGGAAGACCGCAAACTCCTGATTCAGGCGGCCATCGTGAGGATCATGAAG

ATGAGGAAGGTCCTGAAGCACCAGCAGCTCCTCGGGGAGGTCCTCACCCAGCTGTCCTCC

AGGTTCAAGCCTCGGGTCCCAGTGATCAAGAAATGCATTGACATTCTGATTGAGAAGGAA

TATTTGGAGCGAGTCGATGGTGAAAAGGACACCTACAGTTACTTGGCT

>CL348.Contig1_A-W 127 1500 PREDICTED: paraspeckle component 1 isoform 2 [Callithrix jacchus]

CCTGTATCCGGTGTTTGAGGCGAACTCCTTAAGATGATGTTAAGAGGAAACCTGAAGCAA

GTGCGCATTGAGAAAAACCCGGCCCGTCTACGCGCGCTTGAATCCGCAGCCGGCGAGAAC

GAGCCGGTGGCCGCGGCGGCCATGGCGCTGGCGCTGGCGGGAGAGCAGGCACCGCCTGCC

CCAGGGCCCCCGGAGGACCACCCGGAGGAAGAGATGGGGTTCACCATTGACATCAAGAGT

TTCCTAAAGCCGGGTGAGAAGACGTACACTCAGCGTTGCCGCCTCTTTGTAGGGAATCTC

CCCACCGACATCACCGAGGAGGATTTCAAGAGACTCTTCGAGCGCTACGGCGAGCCCAGC

GAGGTTTTCATCAACCGGGATCGTGGCTTCGGCTTCATCCGCTTGGAATCCAGAACATTG

GCTGAAATTGCAAAAGCAGAGCTGGATGGCACCATTTTGAAGAGTAGACCTCTCCGAATT

CGTTTTGCTACACATGGAGCAGCCCTAACGGTCAAGAACCTTTCTCCAGTTGTTTCCAAT

GAGCTTCTAGAACAAGCATTTTCCCAGTTTGGTCCAGTAGAGAAAGCCGTTGTGGTTGTG

GATGATCGTGGTAGAGCTACAGGAAAAGGTTTTGTAGAATTTGCAGCAAAACCTCCTGCA

CGAAAGGCTCTGGAAAGATGTGGTGATGGGGCATTCTTGCTAACAACGACTCCTCGTCCA

GTCATTGTGGAGCCCATGGAGCAGTTTGATGATGAAGATGGCTTGCCAGAGAAGCTAATG

CAGAAAACTCAGCAATATCATAAGGAAAGAGAACAGCCACCACGTTTTGCTCAACCTGGA

ACATTTGAGTTTGAGTATGCATCTCGATGGAAGGCTCTTGATGAAATGGAAAAACAGCAG

CGTGAGCAGGTTGATAGAAACATCAGAGAAGCCAAAGAAAAACTGGAGGCAGAAATGGAA

GCAGCTAGGCATGAACACCAATTAATGCTAATGAGGCAAGATCTGATGAGACGTCAAGAA

GAACTCAGACGCTTAGAAGAACTCAGAAACCAAGAGCTGCAGAAACGAAAGCAAATACAG

CTGAGACATGAAGAGGAACATCGTCGGCGTGAGGAAGAAATGATCCGACATAGAGAACAA

GAGGAATTGAGGCGACAGCAGGAAGGCTTTAAACCAAACTATATGGAAAATCACAATGAT

AGATTTCCCCAAGGACCGCCATCCCAAATGGGCTCACCTATGGGGAATAGAACAGGTTCT

GAAACACCTCAAGCACCAATGAGTGGTGTAGGTCCTGTGAGTGGTGGTCCTGGTGGCTTT

GGTAGAGGAAATCAAGGGGGCAACTTTGAAGGTCCTAATAAGCGTCGTAGATAT

>CL348.Contig2_A-W 127 1728 PREDICTED: paraspeckle component 1 isoform 2 [Callithrix jacchus]

CCTGTATCCGGTGTTTGAGGCGAACTCCTTAAGATGATGTTAAGAGGAAACCTGAAGCAA

GTGCGCATTGAGAAAAACCCGGCCCGTCTACGCGCGCTTGAATCCGCAGCCGGCGAGAAC

GAGCCGGTGGCCGCGGCGGCCATGGCGCTGGCGCTGGCGGGAGAGCAGGCACCGCCTGCC

CCAGGGCCCCCGGAGGACCACCCGGAGGAAGAGATGGGGTTCACCATTGACATCAAGAGT

TTCCTAAAGCCGGGTGAGAAGACGTACACTCAGCGTTGCCGCCTCTTTGTAGGGAATCTC

CCCACCGACATCACCGAGGAGGATTTCAAGAGACTCTTCGAGCGCTACGGCGAGCCCAGC

GAGGTTTTCATCAACCGGGATCGTGGCTTCGGCTTCATCCGCTTGGAATCCAGAACATTG

GCTGAAATTGCAAAAGCAGAGCTGGATGGCACCATTTTGAAGAGTAGACCTCTCCGAATT

CGTTTTGCTACACATGGAGCAGCCCTAACGGTCAAGAACCTTTCTCCAGTTGTTTCCAAT

GAGCTTCTAGAACAAGCATTTTCCCAGTTTGGTCCAGTAGAGAAAGCCGTTGTGGTTGTG

GATGATCGTGGTAGAGCTACAGGAAAAGGTTTTGTAGAATTTGCAGCAAAACCTCCTGCA

CGAAAGGCTCTGGAAAGATGTGGTGATGGGGCATTCTTGCTAACAACGACTCCTCGTCCA

GTCATTGTGGAGCCCATGGAGCAGTTTGATGATGAAGATGGCTTGCCAGAGAAGCTAATG

CAGAAAACTCAGCAATATCATAAGGAAAGAGAACAGCCACCACGTTTTGCTCAACCTGGA

ACATTTGAGTTTGAGTATGCATCTCGATGGAAGGCTCTTGATGAAATGGAAAAACAGCAG

CGTGAGCAGGTTGATAGAAACATCAGAGAAGCCAAAGAAAAACTGGAGGCAGAAATGGAA

GCAGCTAGGCATGAACACCAATTAATGCTAATGAGGCAAGATCTGATGAGACGTCAAGAA

GAACTCAGACGCTTAGAAGAACTCAGAAACCAAGAGCTGCAGAAACGAAAGCAAATACAG

CTGAGACATGAAGAGGAACATCGTCGGCGTGAGGAAGAAATGATCCGACATAGAGAACAA

GAGGAATTGAGGCGACAGCAGGAAGGCTTTAAACCAAACTATATGGAAAATAGAGAACAG

GAAATGAGAATGGGTGATATGGGTCCCCGTGGAGCAATAAACATGGGAGATGCGTTTAGC

CCAGCACCTGCTGGTAACCAAGGTCCTCCTCCAATGATGGGTATGAATATGAACAACAGA

GGAACTATACCTGGCCCACCAATGGGTCCTGGTCCTGCCATGGGACCAGAAGGAGCTGCA

AATATGGGAACTCCAATGATGCCAGATAATGGAGCAGTGCACAATGATAGATTTCCCCAA

GGACCGCCATCCCAAATGGGCTCACCTATGGGGAATAGAACAGGTTCTGAAACACCTCAA

GCACCAATGAGTGGTGTAGGTCCTGTGAGTGGTGGTCCTGGTGGCTTTGGTAGAGGAAAT

CAAGGGGGCAACTTTGAAGGTCCTAATAAGCGTCGTAGATAT

>CL349.Contig1_A-W 2 748 minus strand hypothetical protein GW7_14075, partial [Heterocephalus glaber]

CAGGAGAAGAACCATGGACAAGCTAAGAGGATGATCGAGAAGGCCCAGTGCCTGGGCGGA

TGTGAGGAGTTTTGGTACAATTTGACCCTGACTCTGGCCGACGCCGTCTTGTCCTCGGAA

GACAATGGGAGAGAAGCTGCGGCTTGCCAACTATATAAGAAGCTCATAGATGCCTTCAGC

AACCTCCAGAAAGACAGGCCCAACCGAGTGCACACACTGAAGTTCTTCACCATGGACCTG

GAAGCCAGGATCATGAACCTTCAGATCCAAATCACCCAGAACTCAGAGCACAGCAAACTT

CCTGACTGCTGGGTTTTGTTCAAGGAGATGGATGATCAACTGCTGAAAATTGAAGAAGAC

TTTATTAAGTGTGGCTATGAGGAGAATTGTGTCAATATAAAGCTACAGCGTGCAAAGATA

AAGAGGTTTTGTGCCCAAAATGAGACAGATAGAGAACAAAAAGTCACACATTACTTGGAA

GCACACGACCTGATCCAGAAAGCCGTGGCTGCAGAGGAAGAGCTGTTTCATAGGATCCGA

GGTTTGCTGGCCCTTCAAGAGCTGCAGAACACCAACACCCCTCTGATGAGGAAGCTGGCC

CGCCACAAGCTCAGCCTGGCGGAGGGGTCCCTGGACCTGCTGCAGCTTATGTGGGAGGAG

GGGCTGGAGCAGCTGGAGCCACGGTCCCTGGAGAAGCTGCTGGAGACCTACCTGCAGAGC

ACCAGCGACTACACGTCCAGCGGCCTG

>CL349.Contig2_A-W 54 263 minus strand PREDICTED: uncharacterized protein C10orf92 [Canis lupus familiaris]

CTGACGCCCGCCTGGCTCTGTCAGCTGCAGAACACCAACACCCCTCTGATGAGGAAGCTG

GCCCGCCACAAGCTCAGCCTGGCGGAGGGGTCCCTGGACCTGCTGCAGCTTATGTGGGAG

GAGGGGCTGGAGCAGCTGGAGCCACGGTCCCTGGAGAAGCTGCTGGAGACCTACCTGCAG

AGCACCAGCGACTACACGTCCAGCGGCCTG

>CL350.Contig1_A-W 60 254 PREDICTED: dnaJ homolog subfamily C member 4 [Equus caballus]

AGGAAGTTGGAGCAGATACACCGGAGCTTCATGGATGAGAAGGATCGGATCATCACAGCC

ATCTACAACGACACGCGGGCCAGGGCCAGGGCCAACAGAGCCAGGCTCCAGCAGGAGCGC

CGGCAAAGGCAGCAGCAGCCGCTACCCTCTGGGCATCCCCAAGGTCCAGGGATTGTGCCT

CCCAGCACCGGCCCC

>CL350.Contig2_A-W 56 412 PREDICTED: dnaJ homolog subfamily C member 4 [Equus caballus]

AGCTCCTGGGAACCCCCCAATGCACAATACTGGGCCCAGTTTCATGATGTAAGGCCACAG

GGGCCCCAGACGAGGCAGCAGCAGTACAAACACAATCAGCGGGTCCTGGGGTACTGCCTC

CTGCTCATGCTGGCTGGCATGGGCCTGCACTATGCTGCTTTCAGGAAGTTGGAGCAGATA

CACCGGAGCTTCATGGATGAGAAGGATCGGATCATCACAGCCATCTACAACGACACGCGG

GCCAGGGCCAGGGCCAACAGAGCCAGGCTCCAGCAGGAGCGCCGGCAAAGGCAGCAGCAG

CCGCTACCCTCTGGGCATCCCCAAGGTCCAGGGATTGTGCCTCCCAGCACCGGCCCC

>CL350.Contig3_A-W 251 988 PREDICTED: hypothetical protein LOC100664404 [Loxodonta africana]

TGTCCACCCGCCGCCATGCTGCCCCTGTTGCCCCTGCGCCTGTGCCGGCTGTGGCCCCGC

AGCCCTCCCACCCGGCTCCTCGCAGCGGCCTCTGGGCAGCGGTCTGGCCCCAGGAATTAT

TATGAACTATTGGGCGTGCATCCTGGTGCCAGCGCTGAAGAAGTTAAACGAGCTTTCTTT

ACCAAGTCCAAAGAGCTGCACCCTGACCGAGATCCTGGGAACCCGGCCCTGCACAGCCGT

TTTGTGGAGCTGAATGAGGCATACCGTGTGCTCAGCCGTGAGCAGACCCGCCGCAGCTAT

GACCACCAGCTCCGTTCAGCTAGCCCCCGCAAATCTCCAGGGACCACAGCCCATCCCAAG

TCTGCCCAACAAACACAGAGCAGCTCCTGGGAACCCCCCAATGCACAATACTGGGCCCAG

TTTCATGATGTAAGGCCACAGGGGCCCCAGACGAGGCAGCAGCAGTACAAACACAATCAG

CGGGTCCTGGGGTACTGCCTCCTGCTCATGCTGGCTGGCATGGGCCTGCACTATGCTGCT

TTCAGGAAGTTGGAGCAGATACACCGGAGCTTCATGGATGAGAAGGATCGGATCATCACA

GCCATCTACAACGACACGCGGGCCAGGGCCAGGGCCAACAGAGCCAGGCTCCAGCAGGAG

CGCCGGCAAAGGCAGCAGCAGCCGCTACCCTCTGGGCATCCCCAAGGTCCAGGGATTGTG

CCTCCCAGCACCGGCCCC

>CL351.Contig1_A-W 78 713 PREDICTED: LOW QUALITY PROTEIN: cation-independent mannose-6-phosphate receptor [Callithrix jacchus]

GTGGGCTTCGACAGCGAGAGTGCCGGACCAGACGGGCAGGGGCACAAGGGGCTCTCGGAG

CGGAGCCAGGCCGTCGGGGCGGTCCTCAGTCTGCTCCTGGTGGCGCTCATCGGCTGCCTA

CTGGCCCTGCTGCTTCACAAGAAGGAGAGGCGGGAAACCGTGATTAACAAGCTAAGCAAT

TGCTGTCGAAGAAGCTCAAATGTGTCCTACAAATACTCAAAGGTGAACAAAGAGGAGGAG

ACAGACGAGAACGAGACGGAGTGGCTGATGGAAGAGATCCAGCTGCCAGCCCCAAGGCTG

GGCAGAGAGGGGCAGGAAAACGGCCACATCACCACCAAGTCCGTGAAAGCGGAGGCCCTC

ACTTCCCTGCACGGGGATGACCAGGACAGCGAGGATGAGGTCCTGACCATCCCAGAGGTG

AAAGTTCACTCAGGCAGAGGAGCCGGGGCCCAGAGCTCTCAGCCCGTGAGGAACGCACAG

CGGAGGGCCCTTCGGGAGAGGGAGGACGATCGGGTGGGGCTGGTCCAAGGCGAGAAGGCC

AGAAAAGGGAAGCCCAGGCCCGGACAGCAGAAGACGGGGACCTCAGCCATGCTGGCGTCC

TTCCACGACGACAGTGACGAGGACCTTTTACACATC

>CL351.Contig2_A-W 1629 2660 PREDICTED: LOW QUALITY PROTEIN: cation-independent mannose-6-phosphate receptor [Callithrix jacchus]

ATCATATTTAAGTGTGACGAAGACGAGGATATCGGGAGGCCGCAGGTCTTCAGTGAAGTC

CGTGGGTGTGAGGTGACATTTGAGTGGAAGACGAAGGTCGTGTGCCCGCCCCGGAAGATG

GAGTGCAAGTTCGTTCAGAAGCACAAGACCTATGACCTACGGCTGCTCTCCTCCCTCACT

GGGTCCTGGTTCTTTGTCCACGAAGGAACCTCGTACTTCATAAATCTGTGCCAGAGAATA

TACAAGGGGCCACTGGACTGCTCGGAAAGAGCCAGCGTCTGCAAGAAGAGTGCGTCTGGT

CAAGTGCAGGTCTTGGGACTCGTCCACACGCAAAGGCTGGATGTCATAGATGACAAAGTT

GTTGTCACTTACACAAAAGGCTATCGCTGTGGTGAAAATAAGACTGCGTCCTCTGTGATA

GAGCTGACCTGTGCAAAGACAGTGGGCAGGCCTGCATTCAAGAGGTTTGATATCGATAGC

TGCACCTACTACTTCCGCTGGGACTCGCGGGCTGCCTGCGCTGTGAGGCCGCAGGAGGTG

CAGATGGTGAACGGGACCCTCACCAACCCTGTGAATGGCAAGAGCTTCAGCCTTGGGGAT

ATTTATTTTAAGTTGTTCAGTGCCTCTGGGGACATGAGAACCAACGGGGACAACTACCTG

TATGAGATCCAGCTCTCTTCCATCACCGGCTCCACTAACCCCGCGTGCTCCGGTGCCAAC

ATCTGCCAGGTCAAGCCCAATGACGAGCACTTCAGTAGGAAAGTGGGCACCTCCGACAGA

ACTAAGTACTACGTGCAAGATGGTGATCTGGATGTGGTGTTTGCCTCTTCCTCCAAGTGT

GGGAAGGATAAGACGAAGTCCGTCTCTTCCACCATCTTCTTCCACTGCGACCCTCTGGTG

AAGGATGGGATCCCAGAATTCAGTCACGAGACCGCCGACTGCCAGTACCTCTTCTCCTGG

TACACCTCGGCCGTGTGTCCTTTGGGGGTGGGCTTCGACAGCGAGAGTGCCGGACCAGAC

GGGCAGGGGCAC

>CL352.Contig1_A-W 151 1170 Natural killer cell receptor 2B4 OS=Homo sapiens GN=CD244 PE=1 SV=2

ATGTTGGGGCTAGCTGTCACCCTCCTGCATCTCTTGCTCCTCAAGGGGCATCACGGCCAA

GGATCCCCAGATTCTACTTACGATGTGGTTGGTCTCTCGGGAAGGCCCCTCCCTCTACGG

CCTTCTAATACACAGATGGAGATATCTTCTGTTCAATGGAAGATGCAGCTACTCTCATGT

CCAGAAACTTATGTGATACTGACTTGGAGGAATAATTCCACTCCGACGTATGAAGATTTA

GCTCTTAAACATTTCAACAATACACTTGATTTTAACTTTGAGAACTTAACCCTTCTCATC

AAGGCCGCTAAGCCACAGGACAGTGGTCATTATAAACTGGAGGTGACCAATGAGTCTGGA

AAGGTGAACGTGGTCCACTTCAAGGTTTTCGTATTTGATCATGTTGAGCAGCCTCTCCTG

CAGGAACAGTGGAAGGCCCTGGAAGAAAGGAAATGCCAAGTGGTGCTGTCCTGCTTGGTC

TCCAGGGACAGCAATGTGACCTACACTTGGTACAGAGACAATGAATTGATCTCGACGTCT

GGGAACTTTACATATCTGGAGGTGTCGGTTGATCCCAATGACCAGAACACATATACCTGC

AATGTTAGCAACCCTGTCAGTTGGGCAAGCCAAACCCTCAACCTCACCCAGGGATGTCTA

CGTGTCCCCCTGAGATCCAGAATTCTGCCCTTTATGGTGATTGTCATTATTCTGGCCATA

CTGTTTATTGTCACTTTCACTTGCTTCTGTGTGAGGAAGAGAAAGAGAAAGCAGTCACAG

ACCAGCCCAGAGACACCTCTGACAATTTATGAAGATGTCAAAAACCCACAGATGAGGAGG

AATGAACAGCAGGAAGTCCTTGAAGAAGGAATCACCATTTACTCTGTGATCCAGTCCCAG

CCTTCTTCTTCCACATCAAAAGAAGCTACAAATACGTTGTATTCAGTTATACAGCCTTCC

AGGAAGTCCAGATGCAAGGAGAGGAACCATGACCCTTCCGTCAATAGCTCCATCTATGAA

>CL352.Contig2_A-W 151 1251 natural killer cell receptor 2B4 isoform 1 precursor [Homo sapiens] >gi|4894520|gb|AAD32538.1|AF105261_1 natural killer cell receptor 2B4 [Homo sapiens] >gi|5019742|gb|AAD37838.1|AF107761_1 NK cell receptor [Homo sapiens] >gi|5059180|gb|AAD38951.1|AF145782_1 2B4 type I transmembrane protein [Homo sapiens] >gi|6840916|gb|AAF28833.1|AF117711_1 NK cell activation inducing ligand NAIL [Homo sapiens] >gi|9621658|emb|CAC00647.1| PP35 act [Homo sapiens] >gi|32450700|gb|AAH53985.1| CD244 molecule, natural killer cell receptor 2B4 [Homo sapiens] >gi|45710099|gb|AAH28073.1| CD244 molecule, natural killer cell receptor 2B4 [Homo sapiens] >gi|55663398|emb|CAH72353.1| CD244 molecule, natural killer cell receptor 2B4 [Homo sapiens] >gi|119573076|gb|EAW52691.1| CD244 natural killer cell receptor 2B4, isoform CRA_a [Homo sapiens]

ATGTTGGGGCTAGCTGTCACCCTCCTGCATCTCTTGCTCCTCAAGGGGCATCACGGCCAA

GGATCCCCAGATTCTACTTACGATGTGGTTGGTCTCTCGGGAAGGCCCCTCCCTCTACGG

CCTTCTAATACACAGATGGAGATATCTTCTGTTCAATGGAAGATGCAGCTACTCTCATGT

CCAGAAACTTATGTGATACTGACTTGGAGGAATAATTCCACTCCGACGTATGAAGATTTA

GCTCTTAAACATTTCAACAATACACTTGATTTTAACTTTGAGAACTTAACCCTTCTCATC

AAGGCCGCTAAGCCACAGGACAGTGGTCATTATAAACTGGAGGTGACCAATGAGTCTGGA

AAGGTGAACGTGGTCCACTTCAAGGTTTTCGTATTTGATCATGTTGAGCAGCCTCTCCTG

CAGGAACAGTGGAAGGCCCTGGAAGAAAGGAAATGCCAAGTGGTGCTGTCCTGCTTGGTC

TCCAGGGACAGCAATGTGACCTACACTTGGTACAGAGACAATGAATTGATCTCGACGTCT

GGGAACTTTACATATCTGGAGGTGTCGGTTGATCCCAATGACCAGAACACATATACCTGC

AATGTTAGCAACCCTGTCAGTTGGGCAAGCCAAACCCTCAACCTCACCCAGGGATGTCTA

CGTGTCCCCCTGAGATCCAGAATTCTGCCCTTTATGGTGATTGTCATTATTCTGGCCATA

CTGTTTATTGTCACTTTCACTTGCTTCTGTGTGAGGAAGAGAAAGAGAAAGCAGTCACAG

ACCAGCCCAGAGACACCTCTGACAATTTATGAAGATGTCAAAAACCCACAGATGAGGAGG

AATGAACAGCAGGAAGTCCTTGAAGAAGGAATCACCATTTACTCTGTGATCCAGTCCCAG

CCTTCTTCTTCCACATCAAAAGAAGCTACAAATACGTTGTATTCAGTTATACAGCCTTCC

AGGAAGTCCAGATGCAAGGAGAGGAACCATGACCCTTCCGTCAATAGCTCCATCTATGAA

GAGGTTGGAGCAACAGAGTCCAGAGCCCGTAACCCTGCCCGACTGAGCCGCAAAGAGCTG

GACAACTTTGATATTTATTCC

>CL352.Contig3_A-W 151 1251 natural killer cell receptor 2B4 isoform 1 precursor [Homo sapiens] >gi|4894520|gb|AAD32538.1|AF105261_1 natural killer cell receptor 2B4 [Homo sapiens] >gi|5019742|gb|AAD37838.1|AF107761_1 NK cell receptor [Homo sapiens] >gi|5059180|gb|AAD38951.1|AF145782_1 2B4 type I transmembrane protein [Homo sapiens] >gi|6840916|gb|AAF28833.1|AF117711_1 NK cell activation inducing ligand NAIL [Homo sapiens] >gi|9621658|emb|CAC00647.1| PP35 act [Homo sapiens] >gi|32450700|gb|AAH53985.1| CD244 molecule, natural killer cell receptor 2B4 [Homo sapiens] >gi|45710099|gb|AAH28073.1| CD244 molecule, natural killer cell receptor 2B4 [Homo sapiens] >gi|55663398|emb|CAH72353.1| CD244 molecule, natural killer cell receptor 2B4 [Homo sapiens] >gi|119573076|gb|EAW52691.1| CD244 natural killer cell receptor 2B4, isoform CRA_a [Homo sapiens]

ATGTTGGGGCTAGCTGTCACCCTCCTGCATCTCTTGCTCCTCAAGGGGCATCACGGCCAA

GGATCCCCAGATTCTACTTACGATGTGGTTGGTCTCTCGGGAAGGCCCCTCCCTCTACGG

CCTTCTAATACACAGATGGAGATATCTTCTGTTCAATGGAAGATGCAGCTACTCTCATGT

CCAGAAACTTATGTGATACTGACTTGGAGGAATAATTCCACTCCGACGTATGAAGATTTA

GCTCTTAAACATTTCAACAATACACTTGATTTTAACTTTGAGAACTTAACCCTTCTCATC

AAGGCCGCTAAGCCACAGGACAGTGGTCATTATAAACTGGAGGTGACCAATGAGTCTGGA

AAGGTGAACGTGGTCCACTTCAAGGTTTTCGTATTTGATCATGTTGAGCAGCCTCTCCTG

CAGGAACAGTGGAAGGCCCTGGAAGAAAGGAAATGCCAAGTGGTGCTGTCCTGCTTGGTC

TCCAGGGACAGCAATGTGACCTACACTTGGTACAGAGACAATGAATTGATCTCGACGTCT

GGGAACTTTACATATCTGGAGGTGTCGGTTGATCCCAATGACCAGAACACATATACCTGC

AATGTTAGCAACCCTGTCAGTTGGGCAAGCCAAACCCTCAACCTCACCCAGGGATGTCTA

CGTGTCCCCCTGAGATCCAGAATTCTGCCCTTTATGGTGATTGTCATTATTCTGGCCATA

CTGTTTATTGTCACTTTCACTTGCTTCTGTGTGAGGAAGAGAAAGAGAAAGCAGTCACAG

ACCAGCCCAGAGACACCTCTGACAATTTATGAAGATGTCAAAAACCCACAGATGAGGAGG

AATGAACAGCAGGAAGTCCTTGAAGAAGGAATCACCATTTACTCTGTGATCCAGTCCCAG

CCTTCTTCTTCCACATCAAAAGAAGCTACAAATACGTTGTATTCAGTTATACAGCCTTCC

AGGAAGTCCAGATGCAAGGAGAGGAACCATGACCCTTCCGTCAATAGCTCCATCTATGAA

GAGGTTGGAGCAACAGAGTCCAGAGCCCGTAACCCTGCCCGACTGAGCCGCAAAGAGCTG

GACAACTTTGATATTTATTCC

>CL352.Contig4_A-W 151 1251 natural killer cell receptor 2B4 isoform 1 precursor [Homo sapiens] >gi|4894520|gb|AAD32538.1|AF105261_1 natural killer cell receptor 2B4 [Homo sapiens] >gi|5019742|gb|AAD37838.1|AF107761_1 NK cell receptor [Homo sapiens] >gi|5059180|gb|AAD38951.1|AF145782_1 2B4 type I transmembrane protein [Homo sapiens] >gi|6840916|gb|AAF28833.1|AF117711_1 NK cell activation inducing ligand NAIL [Homo sapiens] >gi|9621658|emb|CAC00647.1| PP35 act [Homo sapiens] >gi|32450700|gb|AAH53985.1| CD244 molecule, natural killer cell receptor 2B4 [Homo sapiens] >gi|45710099|gb|AAH28073.1| CD244 molecule, natural killer cell receptor 2B4 [Homo sapiens] >gi|55663398|emb|CAH72353.1| CD244 molecule, natural killer cell receptor 2B4 [Homo sapiens] >gi|119573076|gb|EAW52691.1| CD244 natural killer cell receptor 2B4, isoform CRA_a [Homo sapiens]

ATGTTGGGGCTAGCTGTCACCCTCCTGCATCTCTTGCTCCTCAAGGGGCATCACGGCCAA

GGATCCCCAGATTCTACTTACGATGTGGTTGGTCTCTCGGGAAGGCCCCTCCCTCTACGG

CCTTCTAATACACAGATGGAGATATCTTCTGTTCAATGGAAGATGCAGCTACTCTCATGT

CCAGAAACTTATGTGATACTGACTTGGAGGAATAATTCCACTCCGACGTATGAAGATTTA

GCTCTTAAACATTTCAACAATACACTTGATTTTAACTTTGAGAACTTAACCCTTCTCATC

AAGGCCGCTAAGCCACAGGACAGTGGTCATTATAAACTGGAGGTGACCAATGAGTCTGGA

AAGGTGAACGTGGTCCACTTCAAGGTTTTCGTATTTGATCATGTTGAGCAGCCTCTCCTG

CAGGAACAGTGGAAGGCCCTGGAAGAAAGGAAATGCCAAGTGGTGCTGTCCTGCTTGGTC

TCCAGGGACAGCAATGTGACCTACACTTGGTACAGAGACAATGAATTGATCTCGACGTCT

GGGAACTTTACATATCTGGAGGTGTCGGTTGATCCCAATGACCAGAACACATATACCTGC

AATGTTAGCAACCCTGTCAGTTGGGCAAGCCAAACCCTCAACCTCACCCAGGGATGTCTA

CGTGTCCCCCTGAGATCCAGAATTCTGCCCTTTATGGTGATTGTCATTATTCTGGCCATA

CTGTTTATTGTCACTTTCACTTGCTTCTGTGTGAGGAAGAGAAAGAGAAAGCAGTCACAG

ACCAGCCCAGAGACACCTCTGACAATTTATGAAGATGTCAAAAACCCACAGATGAGGAGG

AATGAACAGCAGGAAGTCCTTGAAGAAGGAATCACCATTTACTCTGTGATCCAGTCCCAG

CCTTCTTCTTCCACATCAAAAGAAGCTACAAATACGTTGTATTCAGTTATACAGCCTTCC

AGGAAGTCCAGATGCAAGGAGAGGAACCATGACCCTTCCGTCAATAGCTCCATCTATGAA

GAGGTTGGAGCAACAGAGTCCAGAGCCCGTAACCCTGCCCGACTGAGCCGCAAAGAGCTG

GACAACTTTGATATTTATTCC

>CL352.Contig5_A-W 139 315 Natural killer cell receptor 2B4 OS=Homo sapiens GN=CD244 PE=1 SV=2

CAGCAGCAGGAAGTCCTTGAAGAAGGAATCACCATTTACTCTGTGATCCAGTCCCAGCCT

TCTTCTTCCACATCAAAAGAAGCTACAAATACGTTGTATTCAGTTATACAGCCTTCCAGG

AAGTCCAGATGCAAGGAGAGGAACCATGACCCTTCCGTCAATAGCTCCATCTATGAA

>CL352.Contig6_A-W 139 315 Natural killer cell receptor 2B4 OS=Homo sapiens GN=CD244 PE=1 SV=2

CAGCAGCAGGAAGTCCTTGAAGAAGGAATCACCATTTACTCTGTGATCCAGTCCCAGCCT

TCTTCTTCCACATCAAAAGAAGCTACAAATACGTTGTATTCAGTTATACAGCCTTCCAGG

AAGTCCAGATGCAAGGAGAGGAACCATGACCCTTCCGTCAATAGCTCCATCTATGAA

>CL352.Contig7_A-W 151 1170 Natural killer cell receptor 2B4 OS=Homo sapiens GN=CD244 PE=1 SV=2

ATGTTGGGGCTAGCTGTCACCCTCCTGCATCTCTTGCTCCTCAAGGGGCATCACGGCCAA

GGATCCCCAGATTCTACTTACGATGTGGTTGGTCTCTCGGGAAGGCCCCTCCCTCTACGG

CCTTCTAATACACAGATGGAGATATCTTCTGTTCAATGGAAGATGCAGCTACTCTCATGT

CCAGAAACTTATGTGATACTGACTTGGAGGAATAATTCCACTCCGACGTATGAAGATTTA

GCTCTTAAACATTTCAACAATACACTTGATTTTAACTTTGAGAACTTAACCCTTCTCATC

AAGGCCGCTAAGCCACAGGACAGTGGTCATTATAAACTGGAGGTGACCAATGAGTCTGGA

AAGGTGAACGTGGTCCACTTCAAGGTTTTCGTATTTGATCATGTTGAGCAGCCTCTCCTG

CAGGAACAGTGGAAGGCCCTGGAAGAAAGGAAATGCCAAGTGGTGCTGTCCTGCTTGGTC

TCCAGGGACAGCAATGTGACCTACACTTGGTACAGAGACAATGAATTGATCTCGACGTCT

GGGAACTTTACATATCTGGAGGTGTCGGTTGATCCCAATGACCAGAACACATATACCTGC

AATGTTAGCAACCCTGTCAGTTGGGCAAGCCAAACCCTCAACCTCACCCAGGGATGTCTA

CGTGTCCCCCTGAGATCCAGAATTCTGCCCTTTATGGTGATTGTCATTATTCTGGCCATA

CTGTTTATTGTCACTTTCACTTGCTTCTGTGTGAGGAAGAGAAAGAGAAAGCAGTCACAG

ACCAGCCCAGAGACACCTCTGACAATTTATGAAGATGTCAAAAACCCACAGATGAGGAGG

AATGAACAGCAGGAAGTCCTTGAAGAAGGAATCACCATTTACTCTGTGATCCAGTCCCAG

CCTTCTTCTTCCACATCAAAAGAAGCTACAAATACGTTGTATTCAGTTATACAGCCTTCC

AGGAAGTCCAGATGCAAGGAGAGGAACCATGACCCTTCCGTCAATAGCTCCATCTATGAA

>CL352.Contig8_A-W 139 315 Natural killer cell receptor 2B4 OS=Homo sapiens GN=CD244 PE=1 SV=2

CAGCAGCAGGAAGTCCTTGAAGAAGGAATCACCATTTACTCTGTGATCCAGTCCCAGCCT

TCTTCTTCCACATCAAAAGAAGCTACAAATACGTTGTATTCAGTTATACAGCCTTCCAGG

AAGTCCAGATGCAAGGAGAGGAACCATGACCCTTCCGTCAATAGCTCCATCTATGAA

>CL352.Contig9_A-W 136 396 PREDICTED: natural killer cell receptor 2B4 [Callithrix jacchus]

CTACAGCAGCAGGAAGTCCTTGAAGAAGGAATCACCATTTACTCTGTGATCCAGTCCCAG

CCTTCTTCTTCCACATCAAAAGAAGCTACAAATACGTTGTATTCAGTTATACAGCCTTCC

AGGAAGTCCAGATGCAAGGAGAGGAACCATGACCCTTCCGTCAATAGCTCCATCTATGAA

GAGGTTGGAGCAACAGAGTCCAGAGCCCGTAACCCTGCCCGACTGAGCCGCAAAGAGCTG

GACAACTTTGATATTTATTCC

>CL352.Contig10_A-W 136 396 PREDICTED: natural killer cell receptor 2B4 [Callithrix jacchus]

CTACAGCAGCAGGAAGTCCTTGAAGAAGGAATCACCATTTACTCTGTGATCCAGTCCCAG

CCTTCTTCTTCCACATCAAAAGAAGCTACAAATACGTTGTATTCAGTTATACAGCCTTCC

AGGAAGTCCAGATGCAAGGAGAGGAACCATGACCCTTCCGTCAATAGCTCCATCTATGAA

GAGGTTGGAGCAACAGAGTCCAGAGCCCGTAACCCTGCCCGACTGAGCCGCAAAGAGCTG

GACAACTTTGATATTTATTCC

>CL352.Contig11_A-W 136 396 PREDICTED: natural killer cell receptor 2B4 [Callithrix jacchus]

CTACAGCAGCAGGAAGTCCTTGAAGAAGGAATCACCATTTACTCTGTGATCCAGTCCCAG

CCTTCTTCTTCCACATCAAAAGAAGCTACAAATACGTTGTATTCAGTTATACAGCCTTCC

AGGAAGTCCAGATGCAAGGAGAGGAACCATGACCCTTCCGTCAATAGCTCCATCTATGAA

GAGGTTGGAGCAACAGAGTCCAGAGCCCGTAACCCTGCCCGACTGAGCCGCAAAGAGCTG

GACAACTTTGATATTTATTCC

>CL352.Contig15_A-W 151 1170 Natural killer cell receptor 2B4 OS=Homo sapiens GN=CD244 PE=1 SV=2

ATGTTGGGGCTAGCTGTCACCCTCCTGCATCTCTTGCTCCTCAAGGGGCATCACGGCCAA

GGATCCCCAGATTCTACTTACGATGTGGTTGGTCTCTCGGGAAGGCCCCTCCCTCTACGG

CCTTCTAATACACAGATGGAGATATCTTCTGTTCAATGGAAGATGCAGCTACTCTCATGT

CCAGAAACTTATGTGATACTGACTTGGAGGAATAATTCCACTCCGACGTATGAAGATTTA

GCTCTTAAACATTTCAACAATACACTTGATTTTAACTTTGAGAACTTAACCCTTCTCATC

AAGGCCGCTAAGCCACAGGACAGTGGTCATTATAAACTGGAGGTGACCAATGAGTCTGGA

AAGGTGAACGTGGTCCACTTCAAGGTTTTCGTATTTGATCATGTTGAGCAGCCTCTCCTG

CAGGAACAGTGGAAGGCCCTGGAAGAAAGGAAATGCCAAGTGGTGCTGTCCTGCTTGGTC

TCCAGGGACAGCAATGTGACCTACACTTGGTACAGAGACAATGAATTGATCTCGACGTCT

GGGAACTTTACATATCTGGAGGTGTCGGTTGATCCCAATGACCAGAACACATATACCTGC

AATGTTAGCAACCCTGTCAGTTGGGCAAGCCAAACCCTCAACCTCACCCAGGGATGTCTA

CGTGTCCCCCTGAGATCCAGAATTCTGCCCTTTATGGTGATTGTCATTATTCTGGCCATA

CTGTTTATTGTCACTTTCACTTGCTTCTGTGTGAGGAAGAGAAAGAGAAAGCAGTCACAG

ACCAGCCCAGAGACACCTCTGACAATTTATGAAGATGTCAAAAACCCACAGATGAGGAGG

AATGAACAGCAGGAAGTCCTTGAAGAAGGAATCACCATTTACTCTGTGATCCAGTCCCAG

CCTTCTTCTTCCACATCAAAAGAAGCTACAAATACGTTGTATTCAGTTATACAGCCTTCC

AGGAAGTCCAGATGCAAGGAGAGGAACCATGACCCTTCCGTCAATAGCTCCATCTATGAA

>CL353.Contig1_A-W 11 1006 PREDICTED: ATP-binding cassette, sub-family A (ABC1), member 6-like [Oryctolagus cuniculus]

TTGGTGTTTATTCTAAGATGCATGGAACTAAAATGTGGACAGAAAGTAATACGAAAAGAT

CCTGTTTTCAGAATCTCCCCACAAATTCAAAATGTTCCACTTAATCCAGAAATACATGTA

GATGAAGATGAAGATGAAGATGTTCAAGCAGAAAGAATGAGAGCATCAAGTGCCCTGACC

ACTTCAAACGTAAATGAGAATCCAGTCATAATTGCCAGCTGTCTACACAAAGAGTATGCA

GGCCAGAGGAAAAGTTGTTTTTCAAAGAGGAAGAAGAAAATAGCAGCAAGAAATATATCT

TTCTGTGTGAAAAAAGGTGAAGTTTTGGGATTGCTAGGACCCAATGGTGCTGGTAAAAGT

TCTTCTATTAGGATGATAGCTGGGATCACAAAGCCAACAGCAGGACAGGTGGAACTGAGA

GTTGGGGAATGCAAGTCATTTCTGGGTCAGCAGGGAGATGGCATGGTCAAGTTCCTGGGG

TACTGTCCTCAGGAGAATGTGCTGTGGCCCACCCTGACAATGAAGGAACACCTGGAGGTG

TATGCCACCGTGAAAGGGCTGCAGAAAGAGGACGCTGCCATTGTGATTTCACGACTAGTG

AATGCTTTCAACCTGTATGAGCAGCTAAATGTTCCAGTGCAGAAATTAGCATCAGGAACC

ACCAAAAAGCTATGTTTTGTGCTGAGCATCCTGGGAAATTCACCCATCTTGCTTCTGGAT

GAACCCTCTACAGGCATAGATCCAACAGGGCAGCAACAGATTTGGCAGACAATCCAGGCA

GCTGTTAAGAGCACTGAGAAGAGTGTCCTCTTGACCACCCACTACCTGGCTGAGGCTGAG

GCTCTGTGTGACCGTGTGGCCATCATGGTGTCTGGAAGGCTGAGATGCATTGGTTCCATC

CAACACCTGAAAAGAAAATTTGGCAAAGATTACATTCTAGAACTCAAAGTGAAGGAGCCT

TCTCAAGTACCTTTGATCCACACCGAGATTCTGAAT

>CL353.Contig2_A-W 11 1006 PREDICTED: ATP-binding cassette, sub-family A (ABC1), member 6-like [Oryctolagus cuniculus]

TTGGTGTTTATTCTAAGATGCATGGAACTAAAATGTGGACAGAAAGTAATACGAAAAGAT

CCTGTTTTCAGAATCTCCCCACAAATTCAAAATGTTCCACTTAATCCAGAAATACATGTA

GATGAAGATGAAGATGAAGATGTTCAAGCAGAAAGAATGAGAGCATCAAGTGCCCTGACC

ACTTCAAACGTAAATGAGAATCCAGTCATAATTGCCAGCTGTCTACACAAAGAGTATGCA

GGCCAGAGGAAAAGTTGTTTTTCAAAGAGGAAGAAGAAAATAGCAGCAAGAAATATATCT

TTCTGTGTGAAAAAAGGTGAAGTTTTGGGATTGCTAGGACCCAATGGTGCTGGTAAAAGT

TCTTCTATTAGGATGATAGCTGGGATCACAAAGCCAACAGCAGGACAGGTGGAACTGAGA

GTTGGGGAATGCAAGTCATTTCTGGGTCAGCAGGGAGATGGCATGGTCAAGTTCCTGGGG

TACTGTCCTCAGGAGAATGTGCTGTGGCCCACCCTGACAATGAAGGAACACCTGGAGGTG

TATGCCACCGTGAAAGGGCTGCAGAAAGAGGACGCTGCCATTGTGATTTCACGACTAGTG

AATGCTTTCAACCTGTATGAGCAGCTAAATGTTCCAGTGCAGAAATTAGCATCAGGAACC

ACCAAAAAGCTATGTTTTGTGCTGAGCATCCTGGGAAATTCACCCATCTTGCTTCTGGAT

GAACCCTCTACAGGCATAGATCCAACAGGGCAGCAACAGATTTGGCAGACAATCCAGGCA

GCTGTTAAGAGCACTGAGAAGAGTGTCCTCTTGACCACCCACTACCTGGCTGAGGCTGAG

GCTCTGTGTGACCGTGTGGCCATCATGGTGTCTGGAAGGCTGAGATGCATTGGTTCCATC

CAACACCTGAAAAGAAAATTTGGCAAAGATTACATTCTAGAACTCAAAGTGAAGGAGCCT

TCTCAAGTACCTTTGATCCACACCGAGATTCTGAAT

>CL353.Contig3_A-W 46 1797 minus strand ATP-binding cassette sub-family A member 6 [Heterocephalus glaber]

CAAAGTGAGATGTTCATTTTATACTGTTTGCTTTATTTTTCCCCATTTATATATTTTGTA

TCACACAATGTTACAAAAGAGAGGAAAAAGAGTAAGGAACTGATGAAAATGATGGGTCTA

CATGATTCAGCTTTCTGGTTGTCCTGGGGTTTAATGTATACTGCATTCATCTTGGTCCTT

TCCATAGTCATTACAATTATCATAACATCCACCCAAGTTATACTCATGACTGGCTTTATG

GTCATATTTACACTCTTATTCTTATATGGCTTGTCTTTGCTAGCTTTATCATTCCTGATG

AGCGTGCTGTTACAGAAAGCTGTCCTCACAAATTTGTTTGTGTTTCTCCTCACCCTCTTT

TGGGGGTTTGTGGGATTCACTGCCTTCTATAAACAACTTCCTCCATCTGTACAGTGGATT

TTGAGTATTTGTAGCCCTTTCGCCTTTAGAGTTGGAATGACTCAGATTCTCCACCTGGAT

TATAACTTGAATGGAGTAGTTTTCCCTGACCCCTCAGAAAAATCATATACAATGCTAGCA

ACATTTTCTGTGTTGGCTTTTGATGGTCTTATCTACTTGATGCTGGCACTGTACTTTGAC

AAAATCTTACCCCATGGAAATGAGCACCGCTATTCTCCATTATTTTTCCTGAGTTCATCA

TCTTGTTTCCGACACCGAAGGACTGATAATACGGTCATTGAGAAAGACCTGGATCCCGAG

CATCCCTCTGATGGTTATTTTGAACCCATAGCTTCTGAATTCCAAGGAAAAGAAGCCATC

AGGATTAGAAACGTTAAAAAGGAATATAAAACAAAGTCTGGACAAGTGAAAGCTTTGAAA

GGCATGCTCTTTGACATATATGAAGGTCAGATCACAGCAATTTTGGGTCACAGTGGAGCT

GGCAAATCTTCACTCCTAAACATCCTTAATGGATTGTCTGTTCCAACAGAAGGATCAGTT

ACCATCTATAATAAAAACCTCTCTGAAATGCAAGACTTGGAGGAAATCAGAAAGATAACT

GGTGTTTGTCCTCAAAATAATGTTCAAATCGACATGCTCACTGTGAAGGAAAACCTCAGC

CTGTTTGCTAAGATCAAAGGGATTCGACCACAGGAAGTGGAACAAGAGGTACAACAAATT

TTATTGGAATTGGACATGCAAAATATTCAGGATAATCTTGCTATACATTTAAGTGACGGA

CAGAAAAGAATGCTGACCTTTGGGATTGCCATTTTAGGAGATCCTCAAGTTTTGCTTTTA

GATGAACCAACTGCTGGATTAGATCCCTTTTCAAGGCACCAAGTGTGGACTTTCCTGAAG

GAACGTAGAGCGAATCGTGTGATCCTCTTCAGTACCCAGTCCATGGAGGAGGCTGACATT

CTAGCTGATAGAAAAGTGATCATGTCCAATGGGGAACTGAAGTGTGCAGGATCTTCTGTC

TTTTTAAAGAGAAAATGGGGTCTTGGATATCACTTAAGTTTGTATAAGAACGAGATAAGT

GATCCAGAACAAATCACATCTTTCATTAATCATCATATCTCTGATGCTAAATTAAAACTA

GAAAACAAAGAAAAACTTGTATTTACTTTGCCTTTGGAAAGCACAAGTAAATTTCCAGAC

CTTTTCAGTGATATTGATAAGTGTTCTGGCCAGGGTATGATGAATTATGACATCTCCATG

TCAACTCTGAATGAGGTCTTCATGAACCTGGAACGAAAACCAGCGATCAGACAAGATTTT

GAACAAACAGAC

>CL353.Contig4_A-W 3921 4625 minus strand ATP-binding cassette sub-family A member 6 [Heterocephalus glaber]

CTGGGGTACTGTCCTCAGGAGAATGTGCTGTGGCCCACCCTGACAATGAAGGAACACCTG

GAGGTGTATGCCACCGTGAAAGGGCTGCAGAAAGAGGACGCTGCCATTGTCATTTCACGA

CTAGTGAATGCTTTCAACCTGTATGAGCAGCTAAATGTTCCAGTGCAGAAATTAGCATCA

GGAACCACCAAAAAGCTATGTTTTGTGCTGAGCATCCTGGGAAATTCACCCATCTTGCTT

CTGGATGAACCCTCTACAGGCATAGATCCAACAGGGCAGCAACAGATTTGGCAGACAATC

CAGGCAGCTGTTAAGAGCACTGAGAAGAGTGTCCTCTTGACCACCCACTACCTGGCTGAG

GCTGAGGCTCTGTGTGACCGTGTGGCCATCATGGTGTCTGGAAGGCTGAGATGCATTGGT

TCTATCCAACACCTGAAAAGAAAATTTGGCAAAGATTACATTCTAGAACTCAAAGTGAAG

GAGCCTTCTCAAGTACCTTTGATCCACACCGAGATTCTGAATCTTTTCCCCCAGGCTGCG

CAGCAGGAAAGGTTTTCTTCCTTGTTGACCTTTAAGCTGCCCATAGCAGATGTTTACCCT

CTATCTCAGGCCTTTTACAAATTAGAAACAGTTAAACATAACTTTAACCTGGAGGAATAT

AGCCTCTCTCAGTGCACGCTGGAAAAGGTGAGTCTACCACTTGAA

>CL353.Contig5_A-W 2 208 PREDICTED: ATP-binding cassette sub-family A member 6 [Equus caballus]

GCTTTGCTATTGGTGTTTATTCTAAGATGCATGGAACTAAAATGTGGACAGAAAGTAATA

CGAAAAGATCCTGTTTTCAGAATCTCCCCACAAATTCAAAATGTTCCACTTAATCCAGAA

ATACATGTAGATGAAGATGAAGATGAAGATGTTCAAGCAGAAAGAATGAGAGCATCAAGT

GCCCTGACCACTTCAAACGTAAATGAG

>CL354.Contig1_A-W 202 1659 minus strand PREDICTED: protein kinase C and casein kinase substrate in neurons protein 2 isoform 2 [Callithrix jacchus]

ATGTCTGTTCCCTATGATGACTCCGTGGGCCTGGAGGTGTCCAGTGACAGCTTCTGGGAG

GTTGGGAACTACAAGCGGACGGTGAAGCGGATTGACGATGGCCACCGCCTGTGCAGCGAC

CTCATGAGCTGCCTGCATGAGCGTGCGCGCATTGAGAAGGCCTACGCGCAGCAGCTCACC

GAGTGGGCACGGCGGTGGAAGCAGCTCGTGGAGAAGGGGCCCCAGTATGGAACGGTGGAG

AGGGCCTGGATGGCGGTCATGTCTGAGGCGGAGCGGGTGAGCGAGCTGCACCTGGAAGTG

AAGGCCTCGCTGATGAACGAGGACTTCGAGAAGATCAAGAACTGGCAGAAGGAGGCCTTT

CACAAGCAGATGATGGGAGGCTTCAAGGAGACCAAGGAAGCGGAGGACGGCTTCCGGAAG

GCCCAGAAGCCCTGGGCCAAGAAGCTGAAAGAGGTAGAAGCAGCAAAGAAAGCCCACCAT

GCAGCATGCAAGGAAGAGAAGCTGGCTATCTCCCGAGAGGCCAACAGCAAAGCAGATCCA

TCCCTCAACCCTGAGCAGCTCAAGAAATTGCAAGACAAGGTAGAAAAATGCAAGCAAGAT

GTCCTGAAGACCAAAGAGAAGTATGAGAAGGCCCTGAAGGAGCTGGACCAGGGCACTCCC

CAGTACATGGAGAACATGGAGCAGGTGTTCGAGCAGTGCCAGCAGTTTGAGGAGAAGCGG

CTGCGCTTCTTCCGGGAGGTTATGCTAGAGGTTCAGAAGCACCTGGACCTGTCAAACGTG

GCTGGTTACAAAACCATTTACCGGGACTTGGAACAGAGCATCAAAGCTGCTGATGCAGTG

GAGGACCTGAGGTGGTTCCGAGCCAATCACGGGCCTGGCATGGCCATGAACTGGCCACAG

TTTGAGGAGTGGTCTGCAGATCTGAATCGAACTCTCAGCCGGAGGGAGAAGAAGAAGGCC

GCTGATGGCATCACCCTGACAGGCATCAACCAGACAGGCGACCCGTCTATGCAGAGCAAG

CCCAGCAGCAACCTTAGTGTCCTGAACAACCCCGCCCAGTCTGCGCAGTCACAGTCCAGC

TACAACCCCTTTGAGGACGAGGACGACACGGGCAGCACCGTCAGTGAGAAGGAGGACACT

AAGGCCAAAAACGTGAGCAGCTATGAGAAGACCCAGAGCTACCCCACTGACTGGTCCGAT

GACGAGTCCAACAACCCCTTCTCTTCCACGGATGCCAACGGGGACTCCAACCCGTTCGAC

GAGGACACCACCTCAGGGACAGAAGTGCGAGTCCGGGCGCTCTATGACTACGAGGGTCAG

GAGCACGACGAGCTGAGCTTCAAGGCCGGAGATGAGCTGACGAAGATAGAGGACGAGGAT

GAGCAGGGCTGGTGCAAAGGACGCTTGGACAGCGGGCAAGTTGGCCTGTACCCAGCCAAC

TACGTCGAGGCCATCCAG

>CL354.Contig2_A-W 202 1677 minus strand PREDICTED: protein kinase C and casein kinase substrate in neurons protein 2 isoform 2 [Callithrix jacchus]

ATGTCTGTTCCCTATGATGACTCCGTGGGCCTGGAGGTGTCCAGTGACAGCTTCTGGGAG

GTTGGGAACTACAAGCGGACGGTGAAGCGGATTGACGATGGCCACCGCCTGTGCAGCGAC

CTCATGAGCTGCCTGCATGAGCGTGCGCGCATTGAGAAGGCCTACGCGCAGCAGCTCACC

GAGTGGGCACGGCGGTGGAAGCAGCTCGTGGAGAAGGGGCCCCAGTATGGAACGGTGGAG

AGGGCCTGGATGGCGGTCATGTCTGAGGCGGAGCGGGTGAGCGAGCTGCACCTGGAAGTG

AAGGCCTCGCTGATGAACGAGGACTTCGAGAAGATCAAGAACTGGCAGAAGGAGGCCTTT

CACAAGCAGATGATGGGAGGCTTCAAGGAGACCAAGGAAGCGGAGGACGGCTTCCGGAAG

GCCCAGAAGCCCTGGGCCAAGAAGCTGAAAGAGGTAGAAGCAGCAAAGAAAGCCCACCAT

GCAGCATGCAAGGAAGAGAAGCTGGCTATCTCCCGAGAGGCCAACAGCAAAGCAGATCCA

TCCCTCAACCCTGAGCAGCTCAAGAAATTGCAAGACAAGGTAGAAAAATGCAAGCAAGAT

GTCCTGAAGACCAAAGAGAAGTATGAGAAGGCCCTGAAGGAGCTGGACCAGGGCACTCCC

CAGTACATGGAGAACATGGAGCAGGTGTTCGAGCAGTGCCAGCAGTTTGAGGAGAAGCGG

CTGCGCTTCTTCCGGGAGGTTATGCTAGAGGTTCAGAAGCACCTGGACCTGTCAAACGTG

GCTGGTTACAAAACCATTTACCGGGACTTGGAACAGAGCATCAAAGCTGCTGATGCAGTG

GAGGACCTGAGGTGGTTCCGAGCCAATCACGGGCCTGGCATGGCCATGAACTGGCCACAG

TTTGAGGAGTGGTCTGCAGATCTGAATCGAACTCTCAGCCGGAGGGAGAAGAAGAAGGCC

GCTGATGGCATCACCCTGACAGGCATCAACCAGACAGGCGACCCGTCTATGCAGAGCAAG

CCCAGCAGCTCTGTCTCTCCCCATAGCAACCTTAGTGTCCTGAACAACCCCGCCCAGTCT

GCGCAGTCACAGTCCAGCTACAACCCCTTTGAGGACGAGGACGACACGGGCAGCACCGTC

AGTGAGAAGGAGGACACTAAGGCCAAAAACGTGAGCAGCTATGAGAAGACCCAGAGCTAC

CCCACTGACTGGTCCGATGACGAGTCCAACAACCCCTTCTCTTCCACGGATGCCAACGGG

GACTCCAACCCGTTCGACGAGGACACCACCTCAGGGACAGAAGTGCGAGTCCGGGCGCTC

TATGACTACGAGGGTCAGGAGCACGACGAGCTGAGCTTCAAGGCCGGAGATGAGCTGACG

AAGATAGAGGACGAGGATGAGCAGGGCTGGTGCAAAGGACGCTTGGACAGCGGGCAAGTT

GGCCTGTACCCAGCCAACTACGTCGAGGCCATCCAG

>CL354.Contig3_A-W 202 1227 minus strand PREDICTED: protein kinase C and casein kinase substrate in neurons protein 2 isoform 2 [Callithrix jacchus]

ATGTCTGTTCCCTATGATGACTCCGTGGGCCTGGAGGTGTCCAGTGACAGCTTCTGGGAG

GTTGGGAACTACAAGCGGACGGTGAAGCGGATTGACGATGGCCACCGCCTGTGCAGCGAC

CTCATGAGCTGCCTGCATGAGCGTGCGCGCATTGAGAAGGCCTACGCGCAGCAGCTCACC

GAGTGGGCACGGCGGTGGAAGCAGCTCGTGGAGAAGGGGCCCCAGTATGGAACGGTGGAG

AGGGCCTGGATGGCGGTCATGTCTGAGGCGGAGCGGGTGAGCGAGCTGCACCTGGAAGTG

AAGGCCTCGCTGATGAACGAGGACTTCGAGAAGATCAAGAACTGGCAGAAGGAGGCCTTT

CACAAGCAGATGATGGGAGGCTTCAAGGAGACCAAGGAAGCGGAGGACGGCTTCCGGAAG

GCCCAGAAGCCCTGGGCCAAGAAGCTGAAAGAGGTAGAAGCAGCAAAGAAAGCCCACCAT

GCAGCATGCAAGGAAGAGAAGCTGGCTATCTCCCGAGAGGCCAACAGCAAAGCAGATCCA

TCCCTCAACCCTGAGCAGCTCAAGAAATTGCAAGACAAGGTAGAAAAATGCAAGCAAGAT

GTCCTGAAGACCAAAGAGAAGTATGAGAAGGCCCTGAAGGAGCTGGACCAGGGCACTCCC

CAGTACATGGAGAACATGGAGCAGGTGTTCGAGCAGTGCCAGCAGTTTGAGGAGAAGCGG

CTGCGCTTCTTCCGGGAGGTTATGCTAGAGGTTCAGAAGCACCTGGACCTGTCAAACGTG

GCTGGTTACAAAACCATTTACCGGGACTTGGAACAGAGCATCAAAGCTGCTGATGCAGTG

GAGGACCTGAGGTGGTTCCGAGCCAATCACGGGCCTGGCATGGCCATGAACTGGCCACAG

TTTGAGGAGTGGTCTGCAGATCTGAATCGAACTCTCAGCCGGAGGGAGAAGAAGAAGGCC

GCTGATGGCATCACCCTGACAGGCATCAACCAGACAGGCGACCCGTCTATGCAGAGCAAG

CCCAGC

>CL354.Contig4_A-W 224 496 minus strand PREDICTED: protein kinase C and casein kinase substrate in neurons protein 2 [Cricetulus griseus]

ATGTCTGTTCCCTATGATGACTCCGTGGGCCTGGAGGTGTCCAGTGACAGCTTCTGGGAG

GTTGGGAACTACAAGCGGACGGTGAAGCGGATTGACGATGGCCACCGCCTGTGCAGCGAC

CTCATGAGCTGCCTGCATGAGCGTGCGCGCATTGAGAAGGCCTACGCGCAGCAGCTCACC

GAGTGGGCACGGCGGTGGAAGCAGCTCGTGGAGAAGGGGCCCCAGTATGGAACGGTGGAG

AGGGCCTGGATGGCGGTCATGTCTGAGGCGGAG

>CL356.Contig1_A-W 191 2254 minus strand PREDICTED: nuclear autoantigen Sp-100 [Pan paniscus]

TCAGTGTTCCAAGAAAAATATGTTTCCCTAAGAAGTGACCCGGAAGGAAGACAACAAACA

TCCATCACTCAGCCAAGCCGTGAGCAAGGAACAGTTGAGAACACGTTTCTGCGAAGACTG

ACCTGGCCACAGCACTCAGATCCCTCGTCTTACAATGGTACAGACCTACCCGAAAATGAA

CTCTTGGCGCATCCTTGCGAAGCAGAAGAGAGAAATGTAAATAAAAGAGAGCCGGCCCGA

GACCCAAATGGTGCAGTAGGAAGCCACCAAGGAAATGAGCAATGCCCTCGAGAGTCAGAG

CCAGTAGAGTCCCACCAACACATGGCTGCCAACGTGGACCGTGGAGAGGCGAGCGTGGGG

ACACCCAGCCCGTTGCCCCACTGTGTAGAAAGAGCAGAGCTCTCCAGCCATGGAATCCAC

ATGACTTCCTGTTCTGTGCATCTGGTGGATATAAAGAAGGAAAAGCCATTTTTTCCCACG

CAAATGAAGCAAGGATCTCAAACCAGGACCAACCATTACCAGCCATCCGAAGTAATAGTC

ATCAGCAGTGAGGACGACGCCAGCGACACAGAAGAGCCTGCAGCAATCTCCAGCTCAGCA

CGGAGGAGCCATCCTGTGATCAAAAACCATGTCCAGTTTGACTTGAGAGATGAAGAAGAG

GCCCAAGAAGCCACTTGCTCATGGCACCAGGCTGCTCCAGCGAGAGGACGTGACTATGGC

TCTTCAGAATCCAGTGAGGAGGAGATACCCTCAGCGCTCCGGATCTCAACACCAACAGAT

TCTGTGAGGATGTCTACTTGGAGGATACACAGTCAGAAAAGAAGGCACAGCAGTGACAGC

TCTTCAGAGCTGAGTTATGGAGAGGAGCCTCGGGAAGCCCTGAGAAGTGGATCAGGTGTG

CCAAGAGGCCAAGAAGCAACGGTGGAAAGTGGCCGAGCATCTCACCCAATAGATACCACG

GATGTTGGAAACAATTCTACTTTGGGAAAAGGCAGTGGGGAAAGAAATATTTCCAAGCTG

CACAACAGAGATAAAGAAATGAACAAAATCTCATCAACCTTACATCCTACTGTGGGGAAA

AAAAGACTTCATAAAGTAAACCATCTCCAAAAAGAAATAAAGAAAGGCAGACATAGATTC

CACTCAATTCAGAATAATGCCCTTCAAAGGAAAAGAGGTTGGCCAAAAGGACCAAGAATT

CCCAGAGAAAAAAATGTGAATTTCAACCTTCCTAAACTTCCTGTGACCTGTGGTGAGGCT

AAGGGGACTCTCCATAAGAAGCAATTTAAGCAAGGGGCCCAGATGAAGAGCATAAGGACT

GAGGATGGAAAATGGCTCACCCCCAGGGACTTCGAGATCAGAGGAAACCACGCAGCAGCC

AAGAACTGGAAGCTAAGCCTGCGCTGCCACGGATGGACCTTGAAAGAACTGATAGAGGCA

GGGCATCTACCAAACCCACCAATAACAAGAAAAAAGAGGAAAACACGGGAGTCTCTCAAC

AATGTCTTAGTTGACCCCCATCAAAAAAACTCAAATAAGTGCGAAGTATGCCGCCAAGAG

AAACAGCTGGTCTTCTGCAATACTTGTTCAAGATCATACCATCAGAACTGCCACATCCCA

CCAGTAGATGCTCAGAGGAGCCAGTGGAGTTGCACCTTATGTAGGACAAAAGCCTGGGAA

AAGCACCCAGAAAGCCAACCATGTCATCTGGAATCTGAGGTCTTGAAGAGGCCTATGCTG

CCTGAGGAACAGCTGAAATGTGAGCTGATCCTATTGAAGGTCTATTGTTATTCACAAAGT

GCCTTTTTTGCCCAAGAACCATATTATACTCAAGACCCCCAGGAGCACATGTGGTTAAAC

AAGGTCAAGGAAAGGCTGAATAAGAAGAAGTACCACCGAGTGGAGGGGTTTGTGCGGGAC

ATGCGCCTCATCTTTCTGAACAACAAGGCCTTCTACAGAAACCACAAATTCATCCAGCTG

CCTCTTAAGATAGAAGCCATATTTGAGAAGTATTTCAAAAACATTTTTTCAATCCAAGAA

ACAAGCGAGAGCATCTGTCAGTTT

>CL356.Contig2_A-W 136 2241 minus strand PREDICTED: nuclear autoantigen Sp-100 [Pan paniscus]

GTCGGGATCCAGGAGGATCCCCGGACATCAGGTTTTTCCTTGGAAGTGTTCCAAGAAAAA

TATGTTTCCCTAAGAAGTGACCCGGAAGGAAGACAACAAACATCCATCACTCAGCCAAGC

CGTGAGCAAGGAACAGTTGAGAACACGTTTCTGCGAAGACTGACCTGGCCACAGCACTCA

GATCCCTCGTCTTACAATGGTACAGACCTACCCGAAAATGAACTCTTGGCGCATCCTTGC

GAAGCAGAAGAGAGAAATGTAAATAAAAGAGAGCCGGCCCGAGACCCAAATGGTGCAGTA

GGAAGCCACCAAGGAAATGAGCAATGCCCTCGAGAGTCAGAGCCAGTAGAGTCCCACCAA

CACATGGCTGCCAACGTGGACCGTGGAGAGGCGAGCGTGGGGACACCCAGCCCGTTGCCC

CACTGTGTAGAAAGAGCAGAGCTCTCCAGCCATGGAATCCACATGACTTCCTGTTCTGTG

CATCTGGTGGATATAAAGAAGGAAAAGCCATTTTTTCCCACGCAAATGAAGCAAGGATCT

CAAACCAGGACCAACCATTACCAGCCATCCGAAGTAATAGTCATCAGCAGTGAGGACGAC

GCCAGCGACACAGAAGAGCCTGCAGCAATCTCCAGCTCAGCACGGAGGAGCCATCCTGTG

ATCAAAAACCATGTCCAGTTTGACTTGAGAGATGAAGAAGAGGCCCAAGAAGCCACTTGC

TCATGGCACCAGGCTGCTCCAGCGAGAGGACGTGACTATGGCTCTTCAGAATCCAGTGAG

GAGGAGATACCCTCAGCGCTCCGGATCTCAACACCAACAGATTCTGTGAGGATGTCTACT

TGGAGGATACACAGTCAGAAAAGAAGGCACAGCAGTGACAGCTCTTCAGAGCTGAGTTAT

GGAGAGGAGCCTCGGGAAGCCCTGAGAAGTGGATCAGGTGTGCCAAGAGGCCAAGAAGCA

ACGGTGGAAAGTGGCCGAGCATCTCACCCAATAGATACCACGGATGTTGGAAACAATTCT

ACTTTGGGAAAAGGCAGTGGGGAAAGAAATATTTCCAAGCTGCACAACAGAGATAAAGAA

ATGAACAAAATCTCATCAACCTTACATCCTACTGTGGGGAAAAAAAGACTTCATAAAGTA

AACCATCTCCAAAAAGAAATAAAGAAAGGCAGACATAGATTCCACTCAATTCAGAATAAT

GCCCTTCAAAGGAAAAGAGGTTGGCCAAAAGGACCAAGAATTCCCAGAGAAAAAAATGTG

AATTTCAACCTTCCTAAACTTCCTGTGACCTGTGGTGAGGCTAAGGGGACTCTCCATAAG

AAGCAATTTAAGCAAGGGGCCCAGATGAAGAGCATAAGGACTGAGGATGGAAAATGGCTC

ACCCCCAGGGACTTCGAGATCAGAGGAAACCACGCAGCAGCCAAGAACTGGAAGCTAAGC

CTGCGCTGCCACGGATGGACCTTGAAAGAACTGATAGAGGCAGGGCATCTACCAAACCCA

CCAATAACAAGAAAAAAGAGGAAAACACGGGAGTCTCTCAACAATGTCTTAGTTGACCCC

CATCAAAAAAACTCAAATAAGTGCGAAGTATGCCGCCAAGAGAAACAGCTGGTCTTCTGC

AATACTTGTTCAAGATCATACCATCAGAACTGCCACATCCCACCAGTAGATGCTCAGAGG

AGCCAGTGGAGTTGCACCTTATGTAGGACAAAAGCCTGGGAAAAGCACCCAGAAAGCCAA

CCATGTCATCTGGAATCTGAGGTCTTGAAGAGGCCTATGCTGCCTGAGGAACAGCTGAAA

TGTGAGCTGATCCTATTGAAGGTCTATTGTTATTCACAAAGTGCCTTTTTTGCCCAAGAA

CCATATTATACTCAAGACCCCCAGGAGCACATGTGGTTAAACAAGGTCAAGGAAAGGCTG

AATAAGAAGAAGTACCACCGAGTGGAGGGGTTTGTGCGGGACATGCGCCTCATCTTTCTG

AACAACAAGGCCTTCTACAGAAACCACAAATTCATCCAGCTGCCTCTTAAGATAGAAGCC

ATATTTGAGAAGTATTTCAAAAACATTTTTTCAATCCAAGAAACAAGCGAGAGCATCTGT

CAGTTT

>CL356.Contig3_A-W 174 2567 minus strand PREDICTED: nuclear autoantigen Sp-100 [Pan paniscus]

CTTGGAAGGATGTTTGCAATGGTCAAGAATAAGGAGATATTTTGTGATATATTTTTCAAC

CACTTCAAGAAAAATAAAGTGGAGATAGCAACTGCAATAACAAAGCTGTTTCCTTTCCTA

GAGGGCCTCAGAGACTGATCCTTTATCACCGAACAAATCTATGCTGATTCTCAAGAAGCC

TTTAGAAACTTGGTTCCTGTAGCCAGAGTGGTGTACCATGTTCTTGGCCACCTTGAGAAG

GTGTTTGATTGGTCACTTCTACAAACCCTGTTCAGCAAAGTGAACCTGAAAGAGTACCCT

GGCTTAATACAGATTTATAAAACCTTTGAAAATGTGTTCCAAGAAAAATATGTTTCCCTA

AGAAGTGACCCGGAAGGAAGACAACAAACATCCATCACTCAGCCAAGCCGTGAGCAAGGA

ACAGTTGAGAACACGTTTCTGCGAAGACTGACCTGGCCACAGCACTCAGATCCCTCGTCT

TACAATGGTACAGACCTACCCGAAAATGAACTCTTGGCGCATCCTTGCGAAGCAGAAGAG

AGAAATGTAAATAAAAGAGAGCCGGCCCGAGACCCAAATGGTGCAGTAGGAAGCCACCAA

GGAAATGAGCAATGCCCTCGAGAGTCAGAGCCAGTAGAGTCCCACCAACACATGGCTGCC

AACGTGGACCGTGGAGAGGCGAGCGTGGGGACACCCAGCCCGTTGCCCCACTGTGTAGAA

AGAGCAGAGCTCTCCAGCCATGGAATCCACATGACTTCCTGTTCTGTGCATCTGGTGGAT

ATAAAGAAGGAAAAGCCATTTTTTCCCACGCAAATGAAGCAAGGATCTCAAACCAGGACC

AACCATTACCAGCCATCCGAAGTAATAGTCATCAGCAGTGAGGACGACGCCAGCGACACA

GAAGAGCCTGCAGCAATCTCCAGCTCAGCACGGAGGAGCCATCCTGTGATCAAAAACCAT

GTCCAGTTTGACTTGAGAGATGAAGAAGAGGCCCAAGAAGCCACTTGCTCATGGCACCAG

GCTGCTCCAGCGAGAGGACGTGACTATGGCTCTTCAGAATCCAGTGAGGAGGAGATACCC

TCAGCGCTCCGGATCTCAACACCAACAGATTCTGTGAGGATGTCTACTTGGAGGATACAC

AGTCAGAAAAGAAGGCACAGCAGTGACAGCTCTTCAGAGCTGAGTTATGGAGAGGAGCCT

CGGGAAGCCCTGAGAAGTGGATCAGGTGTGCCAAGAGGCCAAGAAGCAACGGTGGAAAGT

GGCCGAGCATCTCACCCAATAGATACCACGGATGTTGGAAACAATTCTACTTTGGGAAAA

GGCAGTGGGGAAAGAAATATTTCCAAGCTGCACAACAGAGATAAAGAAATGAACAAAATC

TCATCAACCTTACATCCTACTGTGGGGAAAAAAAGACTTCATAAAGTAAACCATCTCCAA

AAAGAAATAAAGAAAGGCAGACATAGATTCCACTCAATTCAGAATAATGCCCTTCAAAGG

AAAAGAGGTTGGCCAAAAGGACCAAGAATTCCCAGAGAAAAAAATGTGAATTTCAACCTT

CCTAAACTTCCTGTGACCTGTGGTGAGGCTAAGGGGACTCTCCATAAGAAGCAATTTAAG

CAAGGGGCCCAGATGAAGAGCATAAGGACTGAGGATGGAAAATGGCTCACCCCCAGGGAC

TTCGAGATCAGAGGAAACCACGCAGCAGCCAAGAACTGGAAGCTAAGCCTGCGCTGCCAC

GGATGGACCTTGAAAGAACTGATAGAGGCAGGGCATCTACCAAACCCACCAATAACAAGA

AAAAAGAGGAAAACACGGGAGTCTCTCAACAATGTCTTAGTTGACCCCCATCAAAAAAAC

TCAAATAAGTGCGAAGTATGCCGCCAAGAGAAACAGCTGGTCTTCTGCAATACTTGTTCA

AGATCATACCATCAGAACTGCCACATCCCACCAGTAGATGCTCAGAGGAGCCAGTGGAGT

TGCACCTTATGTAGGACAAAAGCCTGGGAAAAGCACCCAGAAAGCCAACCATGTCATCTG

GAATCTGAGGTCTTGAAGAGGCCTATGCTGCCTGAGGAACAGCTGAAATGTGAGCTGATC

CTATTGAAGGTCTATTGTTATTCACAAAGTGCCTTTTTTGCCCAAGAACCATATTATACT

CAAGACCCCCAGGAGCACATGTGGTTAAACAAGGTCAAGGAAAGGCTGAATAAGAAGAAG

TACCACCGAGTGGAGGGGTTTGTGCGGGACATGCGCCTCATCTTTCTGAACAACAAGGCC

TTCTACAGAAACCACAAATTCATCCAGCTGCCTCTTAAGATAGAAGCCATATTTGAGAAG

TATTTCAAAAACATTTTTTCAATCCAAGAAACAAGCGAGAGCATCTGTCAGTTT

>CL356.Contig4_A-W 163 2226 minus strand PREDICTED: nuclear autoantigen Sp-100 [Pan paniscus]

TCAGTGTTCCAAGAAAAATATGTTTCCCTAAGAAGTGACCCGGAAGGAAGACAACAAACA

TCCATCACTCAGCCAAGCCGTGAGCAAGGAACAGTTGAGAACACGTTTCTGCGAAGACTG

ACCTGGCCACAGCACTCAGATCCCTCGTCTTACAATGGTACAGACCTACCCGAAAATGAA

CTCTTGGCGCATCCTTGCGAAGCAGAAGAGAGAAATGTAAATAAAAGAGAGCCGGCCCGA

GACCCAAATGGTGCAGTAGGAAGCCACCAAGGAAATGAGCAATGCCCTCGAGAGTCAGAG

CCAGTAGAGTCCCACCAACACATGGCTGCCAACGTGGACCGTGGAGAGGCGAGCGTGGGG

ACACCCAGCCCGTTGCCCCACTGTGTAGAAAGAGCAGAGCTCTCCAGCCATGGAATCCAC

ATGACTTCCTGTTCTGTGCATCTGGTGGATATAAAGAAGGAAAAGCCATTTTTTCCCACG

CAAATGAAGCAAGGATCTCAAACCAGGACCAACCATTACCAGCCATCCGAAGTAATAGTC

ATCAGCAGTGAGGACGACGCCAGCGACACAGAAGAGCCTGCAGCAATCTCCAGCTCAGCA

CGGAGGAGCCATCCTGTGATCAAAAACCATGTCCAGTTTGACTTGAGAGATGAAGAAGAG

GCCCAAGAAGCCACTTGCTCATGGCACCAGGCTGCTCCAGCGAGAGGACGTGACTATGGC

TCTTCAGAATCCAGTGAGGAGGAGATACCCTCAGCGCTCCGGATCTCAACACCAACAGAT

TCTGTGAGGATGTCTACTTGGAGGATACACAGTCAGAAAAGAAGGCACAGCAGTGACAGC

TCTTCAGAGCTGAGTTATGGAGAGGAGCCTCGGGAAGCCCTGAGAAGTGGATCAGGTGTG

CCAAGAGGCCAAGAAGCAACGGTGGAAAGTGGCCGAGCATCTCACCCAATAGATACCACG

GATGTTGGAAACAATTCTACTTTGGGAAAAGGCAGTGGGGAAAGAAATATTTCCAAGCTG

CACAACAGAGATAAAGAAATGAACAAAATCTCATCAACCTTACATCCTACTGTGGGGAAA

AAAAGACTTCATAAAGTAAACCATCTCCAAAAAGAAATAAAGAAAGGCAGACATAGATTC

CACTCAATTCAGAATAATGCCCTTCAAAGGAAAAGAGGTTGGCCAAAAGGACCAAGAATT

CCCAGAGAAAAAAATGTGAATTTCAACCTTCCTAAACTTCCTGTGACCTGTGGTGAGGCT

AAGGGGACTCTCCATAAGAAGCAATTTAAGCAAGGGGCCCAGATGAAGAGCATAAGGACT

GAGGATGGAAAATGGCTCACCCCCAGGGACTTCGAGATCAGAGGAAACCACGCAGCAGCC

AAGAACTGGAAGCTAAGCCTGCGCTGCCACGGATGGACCTTGAAAGAACTGATAGAGGCA

GGGCATCTACCAAACCCACCAATAACAAGAAAAAAGAGGAAAACACGGGAGTCTCTCAAC

AATGTCTTAGTTGACCCCCATCAAAAAAACTCAAATAAGTGCGAAGTATGCCGCCAAGAG

AAACAGCTGGTCTTCTGCAATACTTGTTCAAGATCATACCATCAGAACTGCCACATCCCA

CCAGTAGATGCTCAGAGGAGCCAGTGGAGTTGCACCTTATGTAGGACAAAAGCCTGGGAA

AAGCACCCAGAAAGCCAACCATGTCATCTGGAATCTGAGGTCTTGAAGAGGCCTATGCTG

CCTGAGGAACAGCTGAAATGTGAGCTGATCCTATTGAAGGTCTATTGTTATTCACAAAGT

GCCTTTTTTGCCCAAGAACCATATTATACTCAAGACCCCCAGGAGCACATGTGGTTAAAC

AAGGTCAAGGAAAGGCTGAATAAGAAGAAGTACCACCGAGTGGAGGGGTTTGTGCGGGAC

ATGCGCCTCATCTTTCTGAACAACAAGGCCTTCTACAGAAACCACAAATTCATCCAGCTG

CCTCTTAAGATAGAAGCCATATTTGAGAAGTATTTCAAAAACATTTTTTCAATCCAAGAA

ACAAGCGAGAGCATCTGTCAGTTT

>CL356.Contig5_A-W 37 2523 minus strand PREDICTED: nuclear autoantigen Sp-100 [Pan paniscus]

GATATATTTTTCAACCACTTCAAGAAAAATAAAGTGGAGATAGCAACTGCAATAACAAAG

CTGTTTCCTTTCCTAGAGGGCCTCAGAGACTGATCCTTTATCACCGAACAAATCTATGCT

GATTCTCAAGAAGCCTTTAGAAACTTGGTTCCTGTAGCCAGAGTGGTGTACCATGTTCTT

GGCCACCTTGAGAAGGTGTTTGATTGGTCACTTCTACAAACCCTGTTCAGCAAAGTGAAC

CTGAAAGAGTACCCTGGCTTAATACAGATTTATAAAACCTTTGAAAATGTGTTCCAAGAA

AAATATGTTTCCCTAAGAAGTGACCCGGAAGGAAGACAACAAACATCCATCACTCAGCCA

AGCCGTGAGCAAGGAACAGTTGAGAACACGTTTCTGCGAAGACTGACCTGGCCACAGCAC

TCAGATCCCTCGTCTTACAATGGTACAGACCTACCCGAAAATGAACTCTTGGCGCATCCT

TGCGAAGCAGAAGAGAGAAATGTAAATAAAAGAGAGCCGGCCCGAGACCCAAATGGTGCA

GTAGGAAGCCACCAAGGAAATGAGCAATGCCCTCGAGAGTCAGAGCCAGTAGAGTCCCAC

CAACACATGGCTGCCAACGTGGACCGTGGAGAGGCGAGCGTGGGGACACCCAGCCCGTTG

CCCCACTGTGTAGAAAGAGCAGAGCTCTCCAGCCATGGAATCCACATGACTTCCTGTTCT

GTGCATCTGGTGGATATAAAGAAGGAAAAGCCATTTTTTCCCACGCAAATGAAGCAAGGA

TCTCAAACCAGGACCAACCATTACCAGCCATCCGAAGTAATAGTCATCAGCAGTGAGGAC

GACGCCAGCGACACAGAAGAGCCTGCAGCAATCTCCAGCTCAGCACGGAGGAGCCATCCT

GTGATCAAAAACCATGTCCAGTTTGACTTGAGAGATGAAGAAGAGGCCCAAGAAGCCACT

TGCTCATGGCACCAGGCTGCTCCAGCGAGAGGACGTGACTATGGCTCTTCAGAATCCAGT

GAGGAGGAGATACCCTCAGCGCTCCGGATCTCAACACCAACAGATTCTGTGAGGATGTCT

ACTTGGAGGATACACAGTCAGAAAAGAAGGCACAGCAGTGACAGCTCTTCAGAGCTGAGT

TATGGAGAGGAGCCTCGGGAAGCCCTGAGAAGTGGATCAGGTGTGCCAAGAGGCCAAGAA

GCAACGGTGGAAAGTGGCCGAGCATCTCACCCAATAGGCAAGTCTGCTCAATGGCATCAG

GGTGGAGGTGGCTCAGAAATACCACAGCTGCTGGGCAGGACTGTGTCAGGAGAGGAAGCT

GTAAAAGATACCACGGATGTTGGAAACAATTCTACTTTGGGAAAAGGCAGTGGGGAAAGA

AATATTTCCAAGCTGCACAACAGAGATAAAGAAATGAACAAAATCTCATCAACCTTACAT

CCTACTGTGGGGAAAAAAAGACTTCATAAAGTAAACCATCTCCAAAAAGAAATAAAGAAA

GGCAGACATAGATTCCACTCAATTCAGAATAATGCCCTTCAAAGGAAAAGAGGTTGGCCA

AAAGGGAGAAAAAGAGTCAACGTTAGACCCTTGAAAAGAGGGAGAAAAAGAGGACCAAGA

ATTCCCAGAGAAAAAAATGTGAATTTCAACCTTCCTAAACTTCCTGTGACCTGTGGTGAG

GCTAAGGGGACTCTCCATAAGAAGCAATTTAAGCAAGGGGCCCAGATGAAGAGCATAAGG

ACTGAGGATGGAAAATGGCTCACCCCCAGGGACTTCGAGATCAGAGGAAACCACGCAGCA

GCCAAGAACTGGAAGCTAAGCCTGCGCTGCCACGGATGGACCTTGAAAGAACTGATAGAG

GCAGGGCATCTACCAAACCCACCAATAACAAGAAAAAAGAGGAAAACACGGGAGTCTCTC

AACAATGTCTTAGTTGACCCCCATCAAAAAAACTCAAATAAGTGCGAAGTATGCCGCCAA

GAGAAACAGCTGGTCTTCTGCAATACTTGTTCAAGATCATACCATCAGAACTGCCACATC

CCACCAGTAGATGCTCAGAGGAGCCAGTGGAGTTGCACCTTATGTAGGACAAAAGCCTGG

GAAAAGCACCCAGAAAGCCAACCATGTCATCTGGAATCTGAGGTCTTGAAGAGGCCTATG

CTGCCTGAGGAACAGCTGAAATGTGAGCTGATCCTATTGAAGGTCTATTGTTATTCACAA

AGTGCCTTTTTTGCCCAAGAACCATATTATACTCAAGACCCCCAGGAGCACATGTGGTTA

AACAAGGTCAAGGAAAGGCTGAATAAGAAGAAGTACCACCGAGTGGAGGGGTTTGTGCGG

GACATGCGCCTCATCTTTCTGAACAACAAGGCCTTCTACAGAAACCACAAATTCATCCAG

CTGCCTCTTAAGATAGAAGCCATATTTGAGAAGTATTTCAAAAACATTTTTTCAATCCAA

GAAACAAGCGAGAGCATCTGTCAGTTT

>CL356.Contig6_A-W 73 291 minus strand PREDICTED: nuclear autoantigen Sp-100 [Pan paniscus]

GAATGTTTAAGTGTACATTTCCCCACACATTCTTGCCGGATGTTTGCAATGGTCAAGAAT

AAGGAGATATTTTGTGATATATTTTTCAACCACTTCAAGAAAAATAAAGTGGAGATAGCA

ACTGCAATAACAAAGCTGTTTCCTTTCCTAGAGGGCCTCAGAGACTGATCCTTTATCACC

GAACAAATCTATGCTTGTTCCAAGAAAAATATGTTTCCC

>CL356.Contig7_A-W 37 2493 minus strand PREDICTED: nuclear autoantigen Sp-100 isoform 14 [Pan troglodytes]

GATATATTTTTCAACCACTTCAAGAAAAATAAAGTGGAGATAGCAACTGCAATAACAAAG

CTGTTTCCTTTCCTAGAGGGCCTCAGAGACTGATCCTTTATCACCGAACAAATCTATGCT

GATTCTCAAGAAGCCTTTAGAAACTTGGTTCCTGTAGCCAGAGTGGTGTACCATGTTCTT

GGCCACCTTGAGAAGGTGTTTGATTGGTCACTTCTACAAACCCTGTTCAGCAAAGTGAAC

CTGAAAGAGTACCCTGGCTTAATACAGATTTATAAAACCTTTGAAAATGTGTTCCAAGAA

AAATATGTTTCCCTAAGAAGTGACCCGGAAGGAAGACAACAAACATCCATCACTCAGCCA

AGCCGTGAGCAAGGAACAGTTGAGAACACGTTTCTGCGAAGACTGACCTGGCCACAGCAC

TCAGATCCCTCGTCTTACAATGGTACAGACCTACCCGAAAATGAACTCTTGGCGCATCCT

TGCGAAGCAGAAGAGAGAAATGTAAATAAAAGAGAGCCGGCCCGAGACCCAAATGGTGCA

GTAGGAAGCCACCAAGGAAATGAGCAATGCCCTCGAGAGTCAGAGCCAGTAGAGTCCCAC

CAACACATGGCTGCCAACGTGGACCGTGGAGAGGCGAGCGTGGGGACACCCAGCCCGTTG

CCCCACTGTGTAGAAAGAGCAGAGCTCTCCAGCCATGGAATCCACATGACTTCCTGTTCT

GTGCATCTGGTGGATATAAAGAAGGAAAAGCCATTTTTTCCCACGCAAATGAAGCAAGGA

TCTCAAACCAGGACCAACCATTACCAGCCATCCGAAGTAATAGTCATCAGCAGTGAGGAC

GACGCCAGCGACACAGAAGAGCCTGCAGCAATCTCCAGCTCAGCACGGAGGAGCCATCCT

GTGATCAAAAACCATGTCCAGTTTGACTTGAGAGATGAAGAAGAGGCCCAAGAAGCCACT

TGCTCATGGCACCAGGCTGCTCCAGCGAGAGGACGTGACTATGGCTCTTCAGAATCCAGT

GAGGAGGAGATACCCTCAGCGCTCCGGATCTCAACACCAACAGATTCTGTGAGGATGTCT

ACTTGGAGGATACACAGTCAGAAAAGAAGGCACAGCAGTGACAGCTCTTCAGAGCTGAGT

TATGGAGAGGAGCCTCGGGAAGCCCTGAGAAGTGGATCAGGAACAGGGCAACAAGAGACT

GGAAATGAGAAGTGCTCCTGTGTCCTGTGTTCCCCAAAAGGTGTGCCAAGAGGCCAAGAA

GCAACGGTGGAAAGTGGCCGAGCATCTCACCCAATAGATACCACGGATGTTGGAAACAAT

TCTACTTTGGGAAAAGGCAGTGGGGAAAGAAATATTTCCAAGCTGCACAACAGAGATAAA

GAAATGAACAAAATCTCATCAACCTTACATCCTACTGTGGGGAAAAAAAGACTTCATAAA

GTAAACCATCTCCAAAAAGAAATAAAGAAAGGCAGACATAGATTCCACTCAATTCAGAAT

AATGCCCTTCAAAGGAAAAGAGGTTGGCCAAAAGGGAGAAAAAGAGTCAACGTTAGACCC

TTGAAAAGAGGGAGAAAAAGAGGACCAAGAATTCCCAGAGAAAAAAATGTGAATTTCAAC

CTTCCTAAACTTCCTGTGACCTGTGGTGAGGCTAAGGGGACTCTCCATAAGAAGCAATTT

AAGCAAGGGGCCCAGATGAAGAGCATAAGGACTGAGGATGGAAAATGGCTCACCCCCAGG

GACTTCGAGATCAGAGGAAACCACGCAGCAGCCAAGAACTGGAAGCTAAGCCTGCGCTGC

CACGGATGGACCTTGAAAGAACTGATAGAGGCAGGGCATCTACCAAACCCACCAATAACA

AGAAAAAAGAGGAAAACACGGGAGTCTCTCAACAATGTCTTAGTTGACCCCCATCAAAAA

AACTCAAATAAGTGCGAAGTATGCCGCCAAGAGAAACAGCTGGTCTTCTGCAATACTTGT

TCAAGATCATACCATCAGAACTGCCACATCCCACCAGTAGATGCTCAGAGGAGCCAGTGG

AGTTGCACCTTATGTAGGACAAAAGCCTGGGAAAAGCACCCAGAAAGCCAACCATGTCAT

CTGGAATCTGAGGTCTTGAAGAGGCCTATGCTGCCTGAGGAACAGCTGAAATGTGAGCTG

ATCCTATTGAAGGTCTATTGTTATTCACAAAGTGCCTTTTTTGCCCAAGAACCATATTAT

ACTCAAGACCCCCAGGAGCACATGTGGTTAAACAAGGTCAAGGAAAGGCTGAATAAGAAG

AAGTACCACCGAGTGGAGGGGTTTGTGCGGGACATGCGCCTCATCTTTCTGAACAACAAG

GCCTTCTACAGAAACCACAAATTCATCCAGCTGCCTCTTAAGATAGAAGCCATATTTGAG

AAGTATTTCAAAAACATTTTTTCAATCCAAGAAACAAGCGAGAGCATCTGTCAGTTT

>CL356.Contig8_A-W 38 2476 minus strand PREDICTED: nuclear autoantigen Sp-100 [Pan paniscus]

GATATATTTTTCAACCACTTCAAGAAAAATAAAGTGGAGATAGCAACTGCAATAACAAAG

CTGTTTCCTTTCCTAGAGGGCCTCAGAGACTGATCCTTTATCACCGAACAAATCTATGCT

GATTCTCAAGAAGCCTTTAGAAACTTGGTTCCTGTAGCCAGAGTGGTGTACCATGTTCTT

GGCCACCTTGAGAAGGTGTTTGATTGGTCACTTCTACAAACCCTGTTCAGCAAAGTGAAC

CTGAAAGAGTACCCTGGCTTAATACAGATTTATAAAACCTTTGAAAATGTGTTCCAAGAA

AAATATGTTTCCCTAAGAAGTGACCCGGAAGGAAGACAACAAACATCCATCACTCAGCCA

AGCCGTGAGCAAGGAACAGTTGAGAACACGTTTCTGCGAAGACTGACCTGGCCACAGCAC

TCAGATCCCTCGTCTTACAATGGTACAGACCTACCCGAAAATGAACTCTTGGCGCATCCT

TGCGAAGCAGAAGAGAGAAATGTAAATAAAAGAGAGCCGGCCCGAGACCCAAATGGTGCA

GTAGGAAGCCACCAAGGAAATGAGCAATGCCCTCGAGAGTCAGAGCCAGTAGAGTCCCAC

CAACACATGGCTGCCAACGTGGACCGTGGAGAGGCGAGCGTGGGGACACCCAGCCCGTTG

CCCCACTGTGTAGAAAGAGCAGAGCTCTCCAGCCATGGAATCCACATGACTTCCTGTTCT

GTGCATCTGGTGGATATAAAGAAGGAAAAGCCATTTTTTCCCACGCAAATGAAGCAAGGA

TCTCAAACCAGGACCAACCATTACCAGCCATCCGAAGTAATAGTCATCAGCAGTGAGGAC

GACGCCAGCGACACAGAAGAGCCTGCAGCAATCTCCAGCTCAGCACGGAGGAGCCATCCT

GTGATCAAAAACCATGTCCAGTTTGACTTGAGAGATGAAGAAGAGGCCCAAGAAGCCACT

TGCTCATGGCACCAGGCTGCTCCAGCGAGAGGACGTGACTATGGCTCTTCAGAATCCAGT

GAGGAGGAGATACCCTCAGCGCTCCGGATCTCAACACCAACAGATTCTGTGAGGATGTCT

ACTTGGAGGATACACAGTCAGAAAAGAAGGCACAGCAGTGACAGCTCTTCAGAGCTGAGT

TATGGAGAGGAGCCTCGGGAAGCCCTGAGAAGTGGATCAGGTGTGCCAAGAGGCCAAGAA

GCAACGGTGGAAAGTGGCCGAGCATCTCACCCAATAGGCAAGTCTGCTCAATGGCATCAG

GGTGGAGGTGGCTCAGAAATACCACAGCTGCTGGGCAGGACTGTGTCAGGAGAGGAAGCT

GTAAAAGATACCACGGATGTTGGAAACAATTCTACTTTGGGAAAAGGCAGTGGGGAAAGA

AATATTTCCAAGCTGCACAACAGAGATAAAGAAATGAACAAAATCTCATCAACCTTACAT

CCTACTGTGGGGAAAAAAAGACTTCATAAAGTAAACCATCTCCAAAAAGAAATAAAGAAA

GGCAGACATAGATTCCACTCAATTCAGAATAATGCCCTTCAAAGGAAAAGAGGTTGGCCA

AAAGGACCAAGAATTCCCAGAGAAAAAAATGTGAATTTCAACCTTCCTAAACTTCCTGTG

ACCTGTGGTGAGGCTAAGGGGACTCTCCATAAGAAGCAATTTAAGCAAGGGGCCCAGATG

AAGAGCATAAGGACTGAGGATGGAAAATGGCTCACCCCCAGGGACTTCGAGATCAGAGGA

AACCACGCAGCAGCCAAGAACTGGAAGCTAAGCCTGCGCTGCCACGGATGGACCTTGAAA

GAACTGATAGAGGCAGGGCATCTACCAAACCCACCAATAACAAGAAAAAAGAGGAAAACA

CGGGAGTCTCTCAACAATGTCTTAGTTGACCCCCATCAAAAAAACTCAAATAAGTGCGAA

GTATGCCGCCAAGAGAAACAGCTGGTCTTCTGCAATACTTGTTCAAGATCATACCATCAG

AACTGCCACATCCCACCAGTAGATGCTCAGAGGAGCCAGTGGAGTTGCACCTTATGTAGG

ACAAAAGCCTGGGAAAAGCACCCAGAAAGCCAACCATGTCATCTGGAATCTGAGGTCTTG

AAGAGGCCTATGCTGCCTGAGGAACAGCTGAAATGTGAGCTGATCCTATTGAAGGTCTAT

TGTTATTCACAAAGTGCCTTTTTTGCCCAAGAACCATATTATACTCAAGACCCCCAGGAG

CACATGTGGTTAAACAAGGTCAAGGAAAGGCTGAATAAGAAGAAGTACCACCGAGTGGAG

GGGTTTGTGCGGGACATGCGCCTCATCTTTCTGAACAACAAGGCCTTCTACAGAAACCAC

AAATTCATCCAGCTGCCTCTTAAGATAGAAGCCATATTTGAGAAGTATTTCAAAAACATT

TTTTCAATCCAAGAAACAAGCGAGAGCATCTGTCAGTTT

>CL356.Contig9_A-W 37 2454 minus strand PREDICTED: nuclear autoantigen Sp-100 [Pan paniscus]

GATATATTTTTCAACCACTTCAAGAAAAATAAAGTGGAGATAGCAACTGCAATAACAAAG

CTGTTTCCTTTCCTAGAGGGCCTCAGAGACTGATCCTTTATCACCGAACAAATCTATGCT

GATTCTCAAGAAGCCTTTAGAAACTTGGTTCCTGTAGCCAGAGTGGTGTACCATGTTCTT

GGCCACCTTGAGAAGGTGTTTGATTGGTCACTTCTACAAACCCTGTTCAGCAAAGTGAAC

CTGAAAGAGTACCCTGGCTTAATACAGATTTATAAAACCTTTGAAAATGTGTTCCAAGAA

AAATATGTTTCCCTAAGAAGTGACCCGGAAGGAAGACAACAAACATCCATCACTCAGCCA

AGCCGTGAGCAAGGAACAGTTGAGAACACGTTTCTGCGAAGACTGACCTGGCCACAGCAC

TCAGATCCCTCGTCTTACAATGGTACAGACCTACCCGAAAATGAACTCTTGGCGCATCCT

TGCGAAGCAGAAGAGAGAAATGTAAATAAAAGAGAGCCGGCCCGAGACCCAAATGGTGCA

GTAGGAAGCCACCAAGGAAATGAGCAATGCCCTCGAGAGTCAGAGCCAGTAGAGTCCCAC

CAACACATGGCTGCCAACGTGGACCGTGGAGAGGCGAGCGTGGGGACACCCAGCCCGTTG

CCCCACTGTGTAGAAAGAGCAGAGCTCTCCAGCCATGGAATCCACATGACTTCCTGTTCT

GTGCATCTGGTGGATATAAAGAAGGAAAAGCCATTTTTTCCCACGCAAATGAAGCAAGGA

TCTCAAACCAGGACCAACCATTACCAGCCATCCGAAGTAATAGTCATCAGCAGTGAGGAC

GACGCCAGCGACACAGAAGAGCCTGCAGCAATCTCCAGCTCAGCACGGAGGAGCCATCCT

GTGATCAAAAACCATGTCCAGTTTGACTTGAGAGATGAAGAAGAGGCCCAAGAAGCCACT

TGCTCATGGCACCAGGCTGCTCCAGCGAGAGGACGTGACTATGGCTCTTCAGAATCCAGT

GAGGAGGAGATACCCTCAGCGCTCCGGATCTCAACACCAACAGATTCTGTGAGGATGTCT

ACTTGGAGGATACACAGTCAGAAAAGAAGGCACAGCAGTGACAGCTCTTCAGAGCTGAGT

TATGGAGAGGAGCCTCGGGAAGCCCTGAGAAGTGGATCAGGTGTGCCAAGAGGCCAAGAA

GCAACGGTGGAAAGTGGCCGAGCATCTCACCCAATAGGCAAGTCTGCTCAATGGCATCAG

GGTGGAGGTGGCTCAGAAATACCACAGCTGCTGGGCAGGACTGTGTCAGGAGAGGAAGCT

GTAAAAGATACCACGGATGTTGGAAACAATTCTACTTTGGGAAAAGGCAGTGGGGAAAGA

AGGAAAAAAAGACTTCATAAAGTAAACCATCTCCAAAAAGAAATAAAGAAAGGCAGACAT

AGATTCCACTCAATTCAGAATAATGCCCTTCAAAGGAAAAGAGGTTGGCCAAAAGGGAGA

AAAAGAGTCAACGTTAGACCCTTGAAAAGAGGGAGAAAAAGAGGACCAAGAATTCCCAGA

GAAAAAAATGTGAATTTCAACCTTCCTAAACTTCCTGTGACCTGTGGTGAGGCTAAGGGG

ACTCTCCATAAGAAGCAATTTAAGCAAGGGGCCCAGATGAAGAGCATAAGGACTGAGGAT

GGAAAATGGCTCACCCCCAGGGACTTCGAGATCAGAGGAAACCACGCAGCAGCCAAGAAC

TGGAAGCTAAGCCTGCGCTGCCACGGATGGACCTTGAAAGAACTGATAGAGGCAGGGCAT

CTACCAAACCCACCAATAACAAGAAAAAAGAGGAAAACACGGGAGTCTCTCAACAATGTC

TTAGTTGACCCCCATCAAAAAAACTCAAATAAGTGCGAAGTATGCCGCCAAGAGAAACAG

CTGGTCTTCTGCAATACTTGTTCAAGATCATACCATCAGAACTGCCACATCCCACCAGTA

GATGCTCAGAGGAGCCAGTGGAGTTGCACCTTATGTAGGACAAAAGCCTGGGAAAAGCAC

CCAGAAAGCCAACCATGTCATCTGGAATCTGAGGTCTTGAAGAGGCCTATGCTGCCTGAG

GAACAGCTGAAATGTGAGCTGATCCTATTGAAGGTCTATTGTTATTCACAAAGTGCCTTT

TTTGCCCAAGAACCATATTATACTCAAGACCCCCAGGAGCACATGTGGTTAAACAAGGTC

AAGGAAAGGCTGAATAAGAAGAAGTACCACCGAGTGGAGGGGTTTGTGCGGGACATGCGC

CTCATCTTTCTGAACAACAAGGCCTTCTACAGAAACCACAAATTCATCCAGCTGCCTCTT

AAGATAGAAGCCATATTTGAGAAGTATTTCAAAAACATTTTTTCAATCCAAGAAACAAGC

GAGAGCATCTGTCAGTTT

>CL356.Contig10_A-W 32 250 minus strand PREDICTED: nuclear autoantigen Sp-100 [Pan paniscus]

GAATGTTTAAGTGTACATTTCCCCACACATTCTTGCCGGATGTTTGCAATGGTCAAGAAT

AAGGAGATATTTTGTGATATATTTTTCAACCACTTCAAGAAAAATAAAGTGGAGATAGCA

ACTGCAATAACAAAGCTGTTTCCTTTCCTAGAGGGCCTCAGAGACTGATCCTTTATCACC

GAACAAATCTATGCTTGTTCCAAGAAAAATATGTTTCCC

>CL356.Contig11_A-W 28 2445 minus strand PREDICTED: nuclear autoantigen Sp-100 [Callithrix jacchus]

ATATTTTGTGATATATTTTTCAACCACTTCAAGAAAAATAAAGTGGAGATAGCAACTGCA

ATAACAAAGCTGTTTCCTTTCCTAGAGGGCCTCAGAGACTGATCCTTTATCACCGAACAA

ATCTATGCTGATTCTCAAGAAGCCTTTAGAAACTTGGTTCCTGTAGCCAGAGTGGTGTAC

CATGTTCTTGGCCACCTTGAGAAGGTGTTTGATTGGTCACTTCTACAAACCCTGTTCAGC

AAAGTGAACCTGAAAGAGTACCCTGGCTTAATACAGATTTATAAAACCTTTGAAAATGTG

TTCCAAGAAAAATATGTTTCCCTAAGAAGTGACCCGGAAGGAAGACAACAAACATCCATC

ACTCAGCCAAGCCGTGAGCAAGGAACAGTTGAGAACACGTTTCTGCGAAGACTGACCTGG

CCACAGCACTCAGATCCCTCGTCTTACAATGGTACAGACCTACCCGAAAATGAACTCTTG

GCGCATCCTTGCGAAGCAGAAGAGAGAAATGTAAATAAAAGAGAGCCGGCCCGAGACCCA

AATGGTGCAGTAGGAAGCCACCAAGGAAATGAGCAATGCCCTCGAGAGTCAGAGCCAGTA

GAGTCCCACCAACACATGGCTGCCAACGTGGACCGTGGAGAGGCGAGCGTGGGGACACCC

AGCCCGTTGCCCCACTGTGTAGAAAGAGCAGAGCTCTCCAGCCATGGAATCCACATGACT

TCCTGTTCTGTGCATCTGGTGGATATAAAGAAGGAAAAGCCATTTTTTCCCACGCAAATG

AAGCAAGGATCTCAAACCAGGACCAACCATTACCAGCCATCCGAAGTAATAGTCATCAGC

AGTGAGGACGACGCCAGCGACACAGAAGAGCCTGCAGCAATCTCCAGCTCAGCACGGAGG

AGCCATCCTGTGATCAAAAACCATGTCCAGTTTGACTTGAGAGATGAAGAAGAGGCCCAA

GAAGCCACTTGCTCATGGCACCAGGCTGCTCCAGCGAGAGGACGTGACTATGGCTCTTCA

GAATCCAGTGAGGAGGAGATACCCTCAGCGCTCCGGATCTCAACACCAACAGATTCTGTG

AGGATGTCTACTTGGAGGATACACAGTCAGAAAAGAAGGCACAGCAGTGACAGCTCTTCA

GAGCTGAGTTATGGAGAGGAGCCTCGGGAAGCCCTGAGAAGTGGATCAGGAACAGGGCAA

CAAGAGACTGGAAATGAGAAGTGCTCCTGTGTCCTGTGTTCCCCAAAAGGTGTGCCAAGA

GGCCAAGAAGCAACGGTGGAAAGTGGCCGAGCATCTCACCCAATAGATACCACGGATGTT

GGAAACAATTCTACTTTGGGAAAAGGCAGTGGGGAAAGAAATATTTCCAAGCTGCACAAC

AGAGATAAAGAAATGAACAAAATCTCATCAACCTTACATCCTACTGTGGGGAAAAAAAGA

CTTCATAAAGTAAACCATCTCCAAAAAGAAATAAAGAAAGGCAGACATAGATTCCACTCA

ATTCAGAATAATGCCCTTCAAAGGAAAAGAGGTTGGCCAAAAGGACCAAGAATTCCCAGA

GAAAAAAATGTGAATTTCAACCTTCCTAAACTTCCTGTGACCTGTGGTGAGGCTAAGGGG

ACTCTCCATAAGAAGCAATTTAAGCAAGGGGCCCAGATGAAGAGCATAAGGACTGAGGAT

GGAAAATGGCTCACCCCCAGGGACTTCGAGATCAGAGGAAACCACGCAGCAGCCAAGAAC

TGGAAGCTAAGCCTGCGCTGCCACGGATGGACCTTGAAAGAACTGATAGAGGCAGGGCAT

CTACCAAACCCACCAATAACAAGAAAAAAGAGGAAAACACGGGAGTCTCTCAACAATGTC

TTAGTTGACCCCCATCAAAAAAACTCAAATAAGTGCGAAGTATGCCGCCAAGAGAAACAG

CTGGTCTTCTGCAATACTTGTTCAAGATCATACCATCAGAACTGCCACATCCCACCAGTA

GATGCTCAGAGGAGCCAGTGGAGTTGCACCTTATGTAGGACAAAAGCCTGGGAAAAGCAC

CCAGAAAGCCAACCATGTCATCTGGAATCTGAGGTCTTGAAGAGGCCTATGCTGCCTGAG

GAACAGCTGAAATGTGAGCTGATCCTATTGAAGGTCTATTGTTATTCACAAAGTGCCTTT

TTTGCCCAAGAACCATATTATACTCAAGACCCCCAGGAGCACATGTGGTTAAACAAGGTC

AAGGAAAGGCTGAATAAGAAGAAGTACCACCGAGTGGAGGGGTTTGTGCGGGACATGCGC

CTCATCTTTCTGAACAACAAGGCCTTCTACAGAAACCACAAATTCATCCAGCTGCCTCTT

AAGATAGAAGCCATATTTGAGAAGTATTTCAAAAACATTTTTTCAATCCAAGAAACAAGC

GAGAGCATCTGTCAGTTT

>CL356.Contig12_A-W 32 250 minus strand PREDICTED: nuclear autoantigen Sp-100 isoform 14 [Pan troglodytes]

GAATGTTTAAGTGTACATTTCCCCACACATTCTTGCCGGATGTTTGCAATGGTCAAGAAT

AAGGAGATATTTTGTGATATATTTTTCAACCACTTCAAGAAAAATAAAGTGGAGATAGCA

ACTGCAATAACAAAGCTGTTTCCTTTCCTAGAGGGCCTCAGAGACTGATCCTTTATCACC

GAACAAATCTATGCTTGTTCCAAGAAAAATATGTTTCCC

>CL356.Contig13_A-W 32 250 minus strand PREDICTED: nuclear autoantigen Sp-100 [Pan paniscus]

GAATGTTTAAGTGTACATTTCCCCACACATTCTTGCCGGATGTTTGCAATGGTCAAGAAT

AAGGAGATATTTTGTGATATATTTTTCAACCACTTCAAGAAAAATAAAGTGGAGATAGCA

ACTGCAATAACAAAGCTGTTTCCTTTCCTAGAGGGCCTCAGAGACTGATCCTTTATCACC

GAACAAATCTATGCTTGTTCCAAGAAAAATATGTTTCCC

>CL356.Contig14_A-W 144 2600 minus strand PREDICTED: nuclear autoantigen Sp-100 [Pan paniscus]

CCAGGAGGATCCCCGGACATCAGGATGTTTGCAATGGTCAAGAATAAGGAGATATTTTGT

GATATATTTTTCAACCACTTCAAGAAAAATAAAGTGGAGATAGCAACTGCAATAACAAAG

CTGTTTCCTTTCCTAGAGGGCCTCAGAGACTGATCCTTTATCACCGAACAAATCTATGCT

GATTCTCAAGAAGCCTTTAGAAACTTGGTTCCTGTAGCCAGAGTGGTGTACCATGTTCTT

GGCCACCTTGAGAAGGTGTTTGATTGGTCACTTCTACAAACCCTGTTCAGCAAAGTGAAC

CTGAAAGAGTACCCTGGCTTAATACAGATTTATAAAACCTTTGAAAATGTGTTCCAAGAA

AAATATGTTTCCCTAAGAAGTGACCCGGAAGGAAGACAACAAACATCCATCACTCAGCCA

AGCCGTGAGCAAGGAACAGTTGAGAACACGTTTCTGCGAAGACTGACCTGGCCACAGCAC

TCAGATCCCTCGTCTTACAATGGTACAGACCTACCCGAAAATGAACTCTTGGCGCATCCT

TGCGAAGCAGAAGAGAGAAATGTAAATAAAAGAGAGCCGGCCCGAGACCCAAATGGTGCA

GTAGGAAGCCACCAAGGAAATGAGCAATGCCCTCGAGAGTCAGAGCCAGTAGAGTCCCAC

CAACACATGGCTGCCAACGTGGACCGTGGAGAGGCGAGCGTGGGGACACCCAGCCCGTTG

CCCCACTGTGTAGAAAGAGCAGAGCTCTCCAGCCATGGAATCCACATGACTTCCTGTTCT

GTGCATCTGGTGGATATAAAGAAGGAAAAGCCATTTTTTCCCACGCAAATGAAGCAAGGA

TCTCAAACCAGGACCAACCATTACCAGCCATCCGAAGTAATAGTCATCAGCAGTGAGGAC

GACGCCAGCGACACAGAAGAGCCTGCAGCAATCTCCAGCTCAGCACGGAGGAGCCATCCT

GTGATCAAAAACCATGTCCAGTTTGACTTGAGAGATGAAGAAGAGGCCCAAGAAGCCACT

TGCTCATGGCACCAGGCTGCTCCAGCGAGAGGACGTGACTATGGCTCTTCAGAATCCAGT

GAGGAGGAGATACCCTCAGCGCTCCGGATCTCAACACCAACAGATTCTGTGAGGATGTCT

ACTTGGAGGATACACAGTCAGAAAAGAAGGCACAGCAGTGACAGCTCTTCAGAGCTGAGT

TATGGAGAGGAGCCTCGGGAAGCCCTGAGAAGTGGATCAGGTGTGCCAAGAGGCCAAGAA

GCAACGGTGGAAAGTGGCCGAGCATCTCACCCAATAGATACCACGGATGTTGGAAACAAT

TCTACTTTGGGAAAAGGCAGTGGGGAAAGAAATATTTCCAAGCTGCACAACAGAGATAAA

GAAATGAACAAAATCTCATCAACCTTACATCCTACTGTGGGGAAAAAAAGACTTCATAAA

GTAAACCATCTCCAAAAAGAAATAAAGAAAGGCAGACATAGATTCCACTCAATTCAGAAT

AATGCCCTTCAAAGGAAAAGAGGTTGGCCAAAAGGGAGAAAAAGAGTCAACGTTAGACCC

TTGAAAAGAGGGAGAAAAAGAGGACCAAGAATTCCCAGAGAAAAAAATGTGAATTTCAAC

CTTCCTAAACTTCCTGTGACCTGTGGTGAGGCTAAGGGGACTCTCCATAAGAAGCAATTT

AAGCAAGGGGCCCAGATGAAGAGCATAAGGACTGAGGATGGAAAATGGCTCACCCCCAGG

GACTTCGAGATCAGAGGAAACCACGCAGCAGCCAAGAACTGGAAGCTAAGCCTGCGCTGC

CACGGATGGACCTTGAAAGAACTGATAGAGGCAGGGCATCTACCAAACCCACCAATAACA

AGAAAAAAGAGGAAAACACGGGAGTCTCTCAACAATGTCTTAGTTGACCCCCATCAAAAA

AACTCAAATAAGTGCGAAGTATGCCGCCAAGAGAAACAGCTGGTCTTCTGCAATACTTGT

TCAAGATCATACCATCAGAACTGCCACATCCCACCAGTAGATGCTCAGAGGAGCCAGTGG

AGTTGCACCTTATGTAGGACAAAAGCCTGGGAAAAGCACCCAGAAAGCCAACCATGTCAT

CTGGAATCTGAGGTCTTGAAGAGGCCTATGCTGCCTGAGGAACAGCTGAAATGTGAGCTG

ATCCTATTGAAGGTCTATTGTTATTCACAAAGTGCCTTTTTTGCCCAAGAACCATATTAT

ACTCAAGACCCCCAGGAGCACATGTGGTTAAACAAGGTCAAGGAAAGGCTGAATAAGAAG

AAGTACCACCGAGTGGAGGGGTTTGTGCGGGACATGCGCCTCATCTTTCTGAACAACAAG

GCCTTCTACAGAAACCACAAATTCATCCAGCTGCCTCTTAAGATAGAAGCCATATTTGAG

AAGTATTTCAAAAACATTTTTTCAATCCAAGAAACAAGCGAGAGCATCTGTCAGTTT

>CL356.Contig15_A-W 37 2514 minus strand PREDICTED: nuclear autoantigen Sp-100 isoform 14 [Pan troglodytes]

GATATATTTTTCAACCACTTCAAGAAAAATAAAGTGGAGATAGCAACTGCAATAACAAAG

CTGTTTCCTTTCCTAGAGGGCCTCAGAGACTGATCCTTTATCACCGAACAAATCTATGCT

GATTCTCAAGAAGCCTTTAGAAACTTGGTTCCTGTAGCCAGAGTGGTGTACCATGTTCTT

GGCCACCTTGAGAAGGTGTTTGATTGGTCACTTCTACAAACCCTGTTCAGCAAAGTGAAC

CTGAAAGAGTACCCTGGCTTAATACAGATTTATAAAACCTTTGAAAATGTGTTCCAAGAA

AAATATGTTTCCCTAAGAAGTGACCCGGAAGGAAGACAACAAACATCCATCACTCAGCCA

AGCCGTGAGCAAGGAACAGTTGAGAACACGTTTCTGCGAAGACTGACCTGGCCACAGCAC

TCAGATCCCTCGTCTTACAATGGTACAGACCTACCCGAAAATGAACTCTTGGCGCATCCT

TGCGAAGCAGAAGAGAGAAATGTAAATAAAAGAGAGCCGGCCCGAGACCCAAATGGTGCA

GTAGGAAGCCACCAAGGAAATGAGCAATGCCCTCGAGAGTCAGAGCCAGTAGAGTCCCAC

CAACACATGGCTGCCAACGTGGACCGTGGAGAGGCGAGCGTGGGGACACCCAGCCCGTTG

CCCCACTGTGTAGAAAGAGCAGAGCTCTCCAGCCATGGAATCCACATGACTTCCTGTTCT

GTGCATCTGGTGGATATAAAGAAGGAAAAGCCATTTTTTCCCACGCAAATGAAGCAAGGA

TCTCAAACCAGGACCAACCATTACCAGCCATCCGAAGTAATAGTCATCAGCAGTGAGGAC

GACGCCAGCGACACAGAAGAGCCTGCAGCAATCTCCAGCTCAGCACGGAGGAGCCATCCT

GTGATCAAAAACCATGTCCAGTTTGACTTGAGAGATGAAGAAGAGGCCCAAGAAGCCACT

TGCTCATGGCACCAGGCTGCTCCAGCGAGAGGACGTGACTATGGCTCTTCAGAATCCAGT

GAGGAGGAGATACCCTCAGCGCTCCGGATCTCAACACCAACAGATTCTGTGAGGATGTCT

ACTTGGAGGATACACAGTCAGAAAAGAAGGCACAGCAGTGACAGCTCTTCAGAGCTGAGT

TATGGAGAGGAGCCTCGGGAAGCCCTGAGAAGTGGATCAGGAACAGGGCAACAAGAGACT

GGAAATGAGAAGTGCTCCTGTGTCCTGTGTTCCCCAAAAGGTGTGCCAAGAGGCCAAGAA

GCAACGGTGGAAAGTGGCCGAGCATCTCACCCAATAGGCAAGTCTGCTCAATGGCATCAG

GGTGGAGGTGGCTCAGAAATACCACAGCTGCTGGGCAGGACTGTGTCAGGAGAGGAAGCT

GTAAAAGATACCACGGATGTTGGAAACAATTCTACTTTGGGAAAAGGCAGTGGGGAAAGA

AGGAAAAAAAGACTTCATAAAGTAAACCATCTCCAAAAAGAAATAAAGAAAGGCAGACAT

AGATTCCACTCAATTCAGAATAATGCCCTTCAAAGGAAAAGAGGTTGGCCAAAAGGGAGA

AAAAGAGTCAACGTTAGACCCTTGAAAAGAGGGAGAAAAAGAGGACCAAGAATTCCCAGA

GAAAAAAATGTGAATTTCAACCTTCCTAAACTTCCTGTGACCTGTGGTGAGGCTAAGGGG

ACTCTCCATAAGAAGCAATTTAAGCAAGGGGCCCAGATGAAGAGCATAAGGACTGAGGAT

GGAAAATGGCTCACCCCCAGGGACTTCGAGATCAGAGGAAACCACGCAGCAGCCAAGAAC

TGGAAGCTAAGCCTGCGCTGCCACGGATGGACCTTGAAAGAACTGATAGAGGCAGGGCAT

CTACCAAACCCACCAATAACAAGAAAAAAGAGGAAAACACGGGAGTCTCTCAACAATGTC

TTAGTTGACCCCCATCAAAAAAACTCAAATAAGTGCGAAGTATGCCGCCAAGAGAAACAG

CTGGTCTTCTGCAATACTTGTTCAAGATCATACCATCAGAACTGCCACATCCCACCAGTA

GATGCTCAGAGGAGCCAGTGGAGTTGCACCTTATGTAGGACAAAAGCCTGGGAAAAGCAC

CCAGAAAGCCAACCATGTCATCTGGAATCTGAGGTCTTGAAGAGGCCTATGCTGCCTGAG

GAACAGCTGAAATGTGAGCTGATCCTATTGAAGGTCTATTGTTATTCACAAAGTGCCTTT

TTTGCCCAAGAACCATATTATACTCAAGACCCCCAGGAGCACATGTGGTTAAACAAGGTC

AAGGAAAGGCTGAATAAGAAGAAGTACCACCGAGTGGAGGGGTTTGTGCGGGACATGCGC

CTCATCTTTCTGAACAACAAGGCCTTCTACAGAAACCACAAATTCATCCAGCTGCCTCTT

AAGATAGAAGCCATATTTGAGAAGTATTTCAAAAACATTTTTTCAATCCAAGAAACAAGC

GAGAGCATCTGTCAGTTT

>CL356.Contig16_A-W 144 347 minus strand PREDICTED: nuclear autoantigen Sp-100 [Pan paniscus]

CCAGGAGGATCCCCGGACATCAGGATGTTTGCAATGGTCAAGAATAAGGAGATATTTTGT

GATATATTTTTCAACCACTTCAAGAAAAATAAAGTGGAGATAGCAACTGCAATAACAAAG

CTGTTTCCTTTCCTAGAGGGCCTCAGAGACTGATCCTTTATCACCGAACAAATCTATGCT

TGTTCCAAGAAAAATATGTTTCCC

>CL356.Contig17_A-W 144 347 minus strand PREDICTED: nuclear autoantigen Sp-100 [Pan paniscus]

CCAGGAGGATCCCCGGACATCAGGATGTTTGCAATGGTCAAGAATAAGGAGATATTTTGT

GATATATTTTTCAACCACTTCAAGAAAAATAAAGTGGAGATAGCAACTGCAATAACAAAG

CTGTTTCCTTTCCTAGAGGGCCTCAGAGACTGATCCTTTATCACCGAACAAATCTATGCT

TGTTCCAAGAAAAATATGTTTCCC

>CL356.Contig18_A-W 144 347 minus strand PREDICTED: nuclear autoantigen Sp-100 [Pan paniscus]

CCAGGAGGATCCCCGGACATCAGGATGTTTGCAATGGTCAAGAATAAGGAGATATTTTGT

GATATATTTTTCAACCACTTCAAGAAAAATAAAGTGGAGATAGCAACTGCAATAACAAAG

CTGTTTCCTTTCCTAGAGGGCCTCAGAGACTGATCCTTTATCACCGAACAAATCTATGCT

TGTTCCAAGAAAAATATGTTTCCC

>CL356.Contig19_A-W 144 347 minus strand PREDICTED: nuclear autoantigen Sp-100 isoform 14 [Pan troglodytes]

CCAGGAGGATCCCCGGACATCAGGATGTTTGCAATGGTCAAGAATAAGGAGATATTTTGT

GATATATTTTTCAACCACTTCAAGAAAAATAAAGTGGAGATAGCAACTGCAATAACAAAG

CTGTTTCCTTTCCTAGAGGGCCTCAGAGACTGATCCTTTATCACCGAACAAATCTATGCT

TGTTCCAAGAAAAATATGTTTCCC

>CL356.Contig20_A-W 32 2476 minus strand PREDICTED: nuclear autoantigen Sp-100 [Pan paniscus]

GAATGTTTAAGTGTACATTTCCCCACACATTCTTGCCGGATGTTTGCAATGGTCAAGAAT

AAGGAGATATTTTGTGATATATTTTTCAACCACTTCAAGAAAAATAAAGTGGAGATAGCA

ACTGCAATAACAAAGCTGTTTCCTTTCCTAGAGGGCCTCAGAGACTGATCCTTTATCACC

GAACAAATCTATGCTGATTCTCAAGAAGCCTTTAGAAACTTGGTTCCTGTAGCCAGAGTG

GTGTACCATGTTCTTGGCCACCTTGAGAAGGTGTTTGATTGGTCACTTCTACAAACCCTG

TTCAGCAAAGTGAACCTGAAAGAGTACCCTGGCTTAATACAGATTTATAAAACCTTTGAA

AATGTGTTCCAAGAAAAATATGTTTCCCTAAGAAGTGACCCGGAAGGAAGACAACAAACA

TCCATCACTCAGCCAAGCCGTGAGCAAGGAACAGTTGAGAACACGTTTCTGCGAAGACTG

ACCTGGCCACAGCACTCAGATCCCTCGTCTTACAATGGTACAGACCTACCCGAAAATGAA

CTCTTGGCGCATCCTTGCGAAGCAGAAGAGAGAAATGTAAATAAAAGAGAGCCGGCCCGA

GACCCAAATGGTGCAGTAGGAAGCCACCAAGGAAATGAGCAATGCCCTCGAGAGTCAGAG

CCAGTAGAGTCCCACCAACACATGGCTGCCAACGTGGACCGTGGAGAGGCGAGCGTGGGG

ACACCCAGCCCGTTGCCCCACTGTGTAGAAAGAGCAGAGCTCTCCAGCCATGGAATCCAC

ATGACTTCCTGTTCTGTGCATCTGGTGGATATAAAGAAGGAAAAGCCATTTTTTCCCACG

CAAATGAAGCAAGGATCTCAAACCAGGACCAACCATTACCAGCCATCCGAAGTAATAGTC

ATCAGCAGTGAGGACGACGCCAGCGACACAGAAGAGCCTGCAGCAATCTCCAGCTCAGCA

CGGAGGAGCCATCCTGTGATCAAAAACCATGTCCAGTTTGACTTGAGAGATGAAGAAGAG

GCCCAAGAAGCCACTTGCTCATGGCACCAGGCTGCTCCAGCGAGAGGACGTGACTATGGC

TCTTCAGAATCCAGTGAGGAGGAGATACCCTCAGCGCTCCGGATCTCAACACCAACAGAT

TCTGTGAGGATGTCTACTTGGAGGATACACAGTCAGAAAAGAAGGCACAGCAGTGACAGC

TCTTCAGAGCTGAGTTATGGAGAGGAGCCTCGGGAAGCCCTGAGAAGTGGATCAGGTGTG

CCAAGAGGCCAAGAAGCAACGGTGGAAAGTGGCCGAGCATCTCACCCAATAGGCAAGTCT

GCTCAATGGCATCAGGGTGGAGGTGGCTCAGAAATACCACAGCTGCTGGGCAGGACTGTG

TCAGGAGAGGAAGCTGTAAAAGATACCACGGATGTTGGAAACAATTCTACTTTGGGAAAA

GGCAGTGGGGAAAGAAGGAAAAAAAGACTTCATAAAGTAAACCATCTCCAAAAAGAAATA

AAGAAAGGCAGACATAGATTCCACTCAATTCAGAATAATGCCCTTCAAAGGAAAAGAGGT

TGGCCAAAAGGACCAAGAATTCCCAGAGAAAAAAATGTGAATTTCAACCTTCCTAAACTT

CCTGTGACCTGTGGTGAGGCTAAGGGGACTCTCCATAAGAAGCAATTTAAGCAAGGGGCC

CAGATGAAGAGCATAAGGACTGAGGATGGAAAATGGCTCACCCCCAGGGACTTCGAGATC

AGAGGAAACCACGCAGCAGCCAAGAACTGGAAGCTAAGCCTGCGCTGCCACGGATGGACC

TTGAAAGAACTGATAGAGGCAGGGCATCTACCAAACCCACCAATAACAAGAAAAAAGAGG

AAAACACGGGAGTCTCTCAACAATGTCTTAGTTGACCCCCATCAAAAAAACTCAAATAAG

TGCGAAGTATGCCGCCAAGAGAAACAGCTGGTCTTCTGCAATACTTGTTCAAGATCATAC

CATCAGAACTGCCACATCCCACCAGTAGATGCTCAGAGGAGCCAGTGGAGTTGCACCTTA

TGTAGGACAAAAGCCTGGGAAAAGCACCCAGAAAGCCAACCATGTCATCTGGAATCTGAG

GTCTTGAAGAGGCCTATGCTGCCTGAGGAACAGCTGAAATGTGAGCTGATCCTATTGAAG

GTCTATTGTTATTCACAAAGTGCCTTTTTTGCCCAAGAACCATATTATACTCAAGACCCC

CAGGAGCACATGTGGTTAAACAAGGTCAAGGAAAGGCTGAATAAGAAGAAGTACCACCGA

GTGGAGGGGTTTGTGCGGGACATGCGCCTCATCTTTCTGAACAACAAGGCCTTCTACAGA

AACCACAAATTCATCCAGCTGCCTCTTAAGATAGAAGCCATATTTGAGAAGTATTTCAAA

AACATTTTTTCAATCCAAGAAACAAGCGAGAGCATCTGTCAGTTT

>CL356.Contig21_A-W 191 2392 minus strand PREDICTED: nuclear autoantigen Sp-100 [Pan paniscus]

TCAGTGTTCCAAGAAAAATATGTTTCCCTAAGAAGTGACCCGGAAGGAAGACAACAAACA

TCCATCACTCAGCCAAGCCGTGAGCAAGGAACAGTTGAGAACACGTTTCTGCGAAGACTG

ACCTGGCCACAGCACTCAGATCCCTCGTCTTACAATGGTACAGACCTACCCGAAAATGAA

CTCTTGGCGCATCCTTGCGAAGCAGAAGAGAGAAATGTAAATAAAAGAGAGCCGGCCCGA

GACCCAAATGGTGCAGTAGGAAGCCACCAAGGAAATGAGCAATGCCCTCGAGAGTCAGAG

CCAGTAGAGTCCCACCAACACATGGCTGCCAACGTGGACCGTGGAGAGGCGAGCGTGGGG

ACACCCAGCCCGTTGCCCCACTGTGTAGAAAGAGCAGAGCTCTCCAGCCATGGAATCCAC

ATGACTTCCTGTTCTGTGCATCTGGTGGATATAAAGAAGGAAAAGCCATTTTTTCCCACG

CAAATGAAGCAAGGATCTCAAACCAGGACCAACCATTACCAGCCATCCGAAGTAATAGTC

ATCAGCAGTGAGGACGACGCCAGCGACACAGAAGAGCCTGCAGCAATCTCCAGCTCAGCA

CGGAGGAGCCATCCTGTGATCAAAAACCATGTCCAGTTTGACTTGAGAGATGAAGAAGAG

GCCCAAGAAGCCACTTGCTCATGGCACCAGGCTGCTCCAGCGAGAGGACGTGACTATGGC

TCTTCAGAATCCAGTGAGGAGGAGATACCCTCAGCGCTCCGGATCTCAACACCAACAGAT

TCTGTGAGGATGTCTACTTGGAGGATACACAGTCAGAAAAGAAGGCACAGCAGTGACAGC

TCTTCAGAGCTGAGTTATGGAGAGGAGCCTCGGGAAGCCCTGAGAAGTGGATCAGGTGTG

CCAAGAGGCCAAGAAGCAACGGTGGAAAGTGGCCGAGCATCTCACCCAATAGGCAAGTCT

GCTCAATGGCATCAGGGTGGAGGTGGCTCAGAAATACCACAGCTGCTGGGCAGGACTGTG

TCAGGAGAGGAAGCTGTAAAAGATACCACGGATGTTGGAAACAATTCTACTTTGGGAAAA

GGCAGTGGGGAAAGAAATATTTCCAAGCTGCACAACAGAGATAAAGAAATGAACAAAATC

TCATCAACCTTACATCCTACTGTGGGGAAAAAAAGACTTCATAAAGTAAACCATCTCCAA

AAAGAAATAAAGAAAGGCAGACATAGATTCCACTCAATTCAGAATAATGCCCTTCAAAGG

AAAAGAGGTTGGCCAAAAGGGAGAAAAAGAGTCAACGTTAGACCCTTGAAAAGAGGGAGA

AAAAGAGGACCAAGAATTCCCAGAGAAAAAAATGTGAATTTCAACCTTCCTAAACTTCCT

GTGACCTGTGGTGAGGCTAAGGGGACTCTCCATAAGAAGCAATTTAAGCAAGGGGCCCAG

ATGAAGAGCATAAGGACTGAGGATGGAAAATGGCTCACCCCCAGGGACTTCGAGATCAGA

GGAAACCACGCAGCAGCCAAGAACTGGAAGCTAAGCCTGCGCTGCCACGGATGGACCTTG

AAAGAACTGATAGAGGCAGGGCATCTACCAAACCCACCAATAACAAGAAAAAAGAGGAAA

ACACGGGAGTCTCTCAACAATGTCTTAGTTGACCCCCATCAAAAAAACTCAAATAAGTGC

GAAGTATGCCGCCAAGAGAAACAGCTGGTCTTCTGCAATACTTGTTCAAGATCATACCAT

CAGAACTGCCACATCCCACCAGTAGATGCTCAGAGGAGCCAGTGGAGTTGCACCTTATGT

AGGACAAAAGCCTGGGAAAAGCACCCAGAAAGCCAACCATGTCATCTGGAATCTGAGGTC

TTGAAGAGGCCTATGCTGCCTGAGGAACAGCTGAAATGTGAGCTGATCCTATTGAAGGTC

TATTGTTATTCACAAAGTGCCTTTTTTGCCCAAGAACCATATTATACTCAAGACCCCCAG

GAGCACATGTGGTTAAACAAGGTCAAGGAAAGGCTGAATAAGAAGAAGTACCACCGAGTG

GAGGGGTTTGTGCGGGACATGCGCCTCATCTTTCTGAACAACAAGGCCTTCTACAGAAAC

CACAAATTCATCCAGCTGCCTCTTAAGATAGAAGCCATATTTGAGAAGTATTTCAAAAAC

ATTTTTTCAATCCAAGAAACAAGCGAGAGCATCTGTCAGTTT

>CL356.Contig22_A-W 163 2364 minus strand PREDICTED: nuclear autoantigen Sp-100 [Pan paniscus]

TCAGTGTTCCAAGAAAAATATGTTTCCCTAAGAAGTGACCCGGAAGGAAGACAACAAACA

TCCATCACTCAGCCAAGCCGTGAGCAAGGAACAGTTGAGAACACGTTTCTGCGAAGACTG

ACCTGGCCACAGCACTCAGATCCCTCGTCTTACAATGGTACAGACCTACCCGAAAATGAA

CTCTTGGCGCATCCTTGCGAAGCAGAAGAGAGAAATGTAAATAAAAGAGAGCCGGCCCGA

GACCCAAATGGTGCAGTAGGAAGCCACCAAGGAAATGAGCAATGCCCTCGAGAGTCAGAG

CCAGTAGAGTCCCACCAACACATGGCTGCCAACGTGGACCGTGGAGAGGCGAGCGTGGGG

ACACCCAGCCCGTTGCCCCACTGTGTAGAAAGAGCAGAGCTCTCCAGCCATGGAATCCAC

ATGACTTCCTGTTCTGTGCATCTGGTGGATATAAAGAAGGAAAAGCCATTTTTTCCCACG

CAAATGAAGCAAGGATCTCAAACCAGGACCAACCATTACCAGCCATCCGAAGTAATAGTC

ATCAGCAGTGAGGACGACGCCAGCGACACAGAAGAGCCTGCAGCAATCTCCAGCTCAGCA

CGGAGGAGCCATCCTGTGATCAAAAACCATGTCCAGTTTGACTTGAGAGATGAAGAAGAG

GCCCAAGAAGCCACTTGCTCATGGCACCAGGCTGCTCCAGCGAGAGGACGTGACTATGGC

TCTTCAGAATCCAGTGAGGAGGAGATACCCTCAGCGCTCCGGATCTCAACACCAACAGAT

TCTGTGAGGATGTCTACTTGGAGGATACACAGTCAGAAAAGAAGGCACAGCAGTGACAGC

TCTTCAGAGCTGAGTTATGGAGAGGAGCCTCGGGAAGCCCTGAGAAGTGGATCAGGTGTG

CCAAGAGGCCAAGAAGCAACGGTGGAAAGTGGCCGAGCATCTCACCCAATAGGCAAGTCT

GCTCAATGGCATCAGGGTGGAGGTGGCTCAGAAATACCACAGCTGCTGGGCAGGACTGTG

TCAGGAGAGGAAGCTGTAAAAGATACCACGGATGTTGGAAACAATTCTACTTTGGGAAAA

GGCAGTGGGGAAAGAAATATTTCCAAGCTGCACAACAGAGATAAAGAAATGAACAAAATC

TCATCAACCTTACATCCTACTGTGGGGAAAAAAAGACTTCATAAAGTAAACCATCTCCAA

AAAGAAATAAAGAAAGGCAGACATAGATTCCACTCAATTCAGAATAATGCCCTTCAAAGG

AAAAGAGGTTGGCCAAAAGGGAGAAAAAGAGTCAACGTTAGACCCTTGAAAAGAGGGAGA

AAAAGAGGACCAAGAATTCCCAGAGAAAAAAATGTGAATTTCAACCTTCCTAAACTTCCT

GTGACCTGTGGTGAGGCTAAGGGGACTCTCCATAAGAAGCAATTTAAGCAAGGGGCCCAG

ATGAAGAGCATAAGGACTGAGGATGGAAAATGGCTCACCCCCAGGGACTTCGAGATCAGA

GGAAACCACGCAGCAGCCAAGAACTGGAAGCTAAGCCTGCGCTGCCACGGATGGACCTTG

AAAGAACTGATAGAGGCAGGGCATCTACCAAACCCACCAATAACAAGAAAAAAGAGGAAA

ACACGGGAGTCTCTCAACAATGTCTTAGTTGACCCCCATCAAAAAAACTCAAATAAGTGC

GAAGTATGCCGCCAAGAGAAACAGCTGGTCTTCTGCAATACTTGTTCAAGATCATACCAT

CAGAACTGCCACATCCCACCAGTAGATGCTCAGAGGAGCCAGTGGAGTTGCACCTTATGT

AGGACAAAAGCCTGGGAAAAGCACCCAGAAAGCCAACCATGTCATCTGGAATCTGAGGTC

TTGAAGAGGCCTATGCTGCCTGAGGAACAGCTGAAATGTGAGCTGATCCTATTGAAGGTC

TATTGTTATTCACAAAGTGCCTTTTTTGCCCAAGAACCATATTATACTCAAGACCCCCAG

GAGCACATGTGGTTAAACAAGGTCAAGGAAAGGCTGAATAAGAAGAAGTACCACCGAGTG

GAGGGGTTTGTGCGGGACATGCGCCTCATCTTTCTGAACAACAAGGCCTTCTACAGAAAC

CACAAATTCATCCAGCTGCCTCTTAAGATAGAAGCCATATTTGAGAAGTATTTCAAAAAC

ATTTTTTCAATCCAAGAAACAAGCGAGAGCATCTGTCAGTTT

>CL356.Contig23_A-W 191 2362 minus strand PREDICTED: nuclear autoantigen Sp-100 isoform 14 [Pan troglodytes]

TCAGTGTTCCAAGAAAAATATGTTTCCCTAAGAAGTGACCCGGAAGGAAGACAACAAACA

TCCATCACTCAGCCAAGCCGTGAGCAAGGAACAGTTGAGAACACGTTTCTGCGAAGACTG

ACCTGGCCACAGCACTCAGATCCCTCGTCTTACAATGGTACAGACCTACCCGAAAATGAA

CTCTTGGCGCATCCTTGCGAAGCAGAAGAGAGAAATGTAAATAAAAGAGAGCCGGCCCGA

GACCCAAATGGTGCAGTAGGAAGCCACCAAGGAAATGAGCAATGCCCTCGAGAGTCAGAG

CCAGTAGAGTCCCACCAACACATGGCTGCCAACGTGGACCGTGGAGAGGCGAGCGTGGGG

ACACCCAGCCCGTTGCCCCACTGTGTAGAAAGAGCAGAGCTCTCCAGCCATGGAATCCAC

ATGACTTCCTGTTCTGTGCATCTGGTGGATATAAAGAAGGAAAAGCCATTTTTTCCCACG

CAAATGAAGCAAGGATCTCAAACCAGGACCAACCATTACCAGCCATCCGAAGTAATAGTC

ATCAGCAGTGAGGACGACGCCAGCGACACAGAAGAGCCTGCAGCAATCTCCAGCTCAGCA

CGGAGGAGCCATCCTGTGATCAAAAACCATGTCCAGTTTGACTTGAGAGATGAAGAAGAG

GCCCAAGAAGCCACTTGCTCATGGCACCAGGCTGCTCCAGCGAGAGGACGTGACTATGGC

TCTTCAGAATCCAGTGAGGAGGAGATACCCTCAGCGCTCCGGATCTCAACACCAACAGAT

TCTGTGAGGATGTCTACTTGGAGGATACACAGTCAGAAAAGAAGGCACAGCAGTGACAGC

TCTTCAGAGCTGAGTTATGGAGAGGAGCCTCGGGAAGCCCTGAGAAGTGGATCAGGAACA

GGGCAACAAGAGACTGGAAATGAGAAGTGCTCCTGTGTCCTGTGTTCCCCAAAAGGTGTG

CCAAGAGGCCAAGAAGCAACGGTGGAAAGTGGCCGAGCATCTCACCCAATAGATACCACG

GATGTTGGAAACAATTCTACTTTGGGAAAAGGCAGTGGGGAAAGAAATATTTCCAAGCTG

CACAACAGAGATAAAGAAATGAACAAAATCTCATCAACCTTACATCCTACTGTGGGGAAA

AAAAGACTTCATAAAGTAAACCATCTCCAAAAAGAAATAAAGAAAGGCAGACATAGATTC

CACTCAATTCAGAATAATGCCCTTCAAAGGAAAAGAGGTTGGCCAAAAGGGAGAAAAAGA

GTCAACGTTAGACCCTTGAAAAGAGGGAGAAAAAGAGGACCAAGAATTCCCAGAGAAAAA

AATGTGAATTTCAACCTTCCTAAACTTCCTGTGACCTGTGGTGAGGCTAAGGGGACTCTC

CATAAGAAGCAATTTAAGCAAGGGGCCCAGATGAAGAGCATAAGGACTGAGGATGGAAAA

TGGCTCACCCCCAGGGACTTCGAGATCAGAGGAAACCACGCAGCAGCCAAGAACTGGAAG

CTAAGCCTGCGCTGCCACGGATGGACCTTGAAAGAACTGATAGAGGCAGGGCATCTACCA

AACCCACCAATAACAAGAAAAAAGAGGAAAACACGGGAGTCTCTCAACAATGTCTTAGTT

GACCCCCATCAAAAAAACTCAAATAAGTGCGAAGTATGCCGCCAAGAGAAACAGCTGGTC

TTCTGCAATACTTGTTCAAGATCATACCATCAGAACTGCCACATCCCACCAGTAGATGCT

CAGAGGAGCCAGTGGAGTTGCACCTTATGTAGGACAAAAGCCTGGGAAAAGCACCCAGAA

AGCCAACCATGTCATCTGGAATCTGAGGTCTTGAAGAGGCCTATGCTGCCTGAGGAACAG

CTGAAATGTGAGCTGATCCTATTGAAGGTCTATTGTTATTCACAAAGTGCCTTTTTTGCC

CAAGAACCATATTATACTCAAGACCCCCAGGAGCACATGTGGTTAAACAAGGTCAAGGAA

AGGCTGAATAAGAAGAAGTACCACCGAGTGGAGGGGTTTGTGCGGGACATGCGCCTCATC

TTTCTGAACAACAAGGCCTTCTACAGAAACCACAAATTCATCCAGCTGCCTCTTAAGATA

GAAGCCATATTTGAGAAGTATTTCAAAAACATTTTTTCAATCCAAGAAACAAGCGAGAGC

ATCTGTCAGTTT

>CL356.Contig24_A-W 191 2344 minus strand PREDICTED: nuclear autoantigen Sp-100 [Pan paniscus]

TCAGTGTTCCAAGAAAAATATGTTTCCCTAAGAAGTGACCCGGAAGGAAGACAACAAACA

TCCATCACTCAGCCAAGCCGTGAGCAAGGAACAGTTGAGAACACGTTTCTGCGAAGACTG

ACCTGGCCACAGCACTCAGATCCCTCGTCTTACAATGGTACAGACCTACCCGAAAATGAA

CTCTTGGCGCATCCTTGCGAAGCAGAAGAGAGAAATGTAAATAAAAGAGAGCCGGCCCGA

GACCCAAATGGTGCAGTAGGAAGCCACCAAGGAAATGAGCAATGCCCTCGAGAGTCAGAG

CCAGTAGAGTCCCACCAACACATGGCTGCCAACGTGGACCGTGGAGAGGCGAGCGTGGGG

ACACCCAGCCCGTTGCCCCACTGTGTAGAAAGAGCAGAGCTCTCCAGCCATGGAATCCAC

ATGACTTCCTGTTCTGTGCATCTGGTGGATATAAAGAAGGAAAAGCCATTTTTTCCCACG

CAAATGAAGCAAGGATCTCAAACCAGGACCAACCATTACCAGCCATCCGAAGTAATAGTC

ATCAGCAGTGAGGACGACGCCAGCGACACAGAAGAGCCTGCAGCAATCTCCAGCTCAGCA

CGGAGGAGCCATCCTGTGATCAAAAACCATGTCCAGTTTGACTTGAGAGATGAAGAAGAG

GCCCAAGAAGCCACTTGCTCATGGCACCAGGCTGCTCCAGCGAGAGGACGTGACTATGGC

TCTTCAGAATCCAGTGAGGAGGAGATACCCTCAGCGCTCCGGATCTCAACACCAACAGAT

TCTGTGAGGATGTCTACTTGGAGGATACACAGTCAGAAAAGAAGGCACAGCAGTGACAGC

TCTTCAGAGCTGAGTTATGGAGAGGAGCCTCGGGAAGCCCTGAGAAGTGGATCAGGTGTG

CCAAGAGGCCAAGAAGCAACGGTGGAAAGTGGCCGAGCATCTCACCCAATAGGCAAGTCT

GCTCAATGGCATCAGGGTGGAGGTGGCTCAGAAATACCACAGCTGCTGGGCAGGACTGTG

TCAGGAGAGGAAGCTGTAAAAGATACCACGGATGTTGGAAACAATTCTACTTTGGGAAAA

GGCAGTGGGGAAAGAAATATTTCCAAGCTGCACAACAGAGATAAAGAAATGAACAAAATC

TCATCAACCTTACATCCTACTGTGGGGAAAAAAAGACTTCATAAAGTAAACCATCTCCAA

AAAGAAATAAAGAAAGGCAGACATAGATTCCACTCAATTCAGAATAATGCCCTTCAAAGG

AAAAGAGGTTGGCCAAAAGGACCAAGAATTCCCAGAGAAAAAAATGTGAATTTCAACCTT

CCTAAACTTCCTGTGACCTGTGGTGAGGCTAAGGGGACTCTCCATAAGAAGCAATTTAAG

CAAGGGGCCCAGATGAAGAGCATAAGGACTGAGGATGGAAAATGGCTCACCCCCAGGGAC

TTCGAGATCAGAGGAAACCACGCAGCAGCCAAGAACTGGAAGCTAAGCCTGCGCTGCCAC

GGATGGACCTTGAAAGAACTGATAGAGGCAGGGCATCTACCAAACCCACCAATAACAAGA

AAAAAGAGGAAAACACGGGAGTCTCTCAACAATGTCTTAGTTGACCCCCATCAAAAAAAC

TCAAATAAGTGCGAAGTATGCCGCCAAGAGAAACAGCTGGTCTTCTGCAATACTTGTTCA

AGATCATACCATCAGAACTGCCACATCCCACCAGTAGATGCTCAGAGGAGCCAGTGGAGT

TGCACCTTATGTAGGACAAAAGCCTGGGAAAAGCACCCAGAAAGCCAACCATGTCATCTG

GAATCTGAGGTCTTGAAGAGGCCTATGCTGCCTGAGGAACAGCTGAAATGTGAGCTGATC

CTATTGAAGGTCTATTGTTATTCACAAAGTGCCTTTTTTGCCCAAGAACCATATTATACT

CAAGACCCCCAGGAGCACATGTGGTTAAACAAGGTCAAGGAAAGGCTGAATAAGAAGAAG

TACCACCGAGTGGAGGGGTTTGTGCGGGACATGCGCCTCATCTTTCTGAACAACAAGGCC

TTCTACAGAAACCACAAATTCATCCAGCTGCCTCTTAAGATAGAAGCCATATTTGAGAAG

TATTTCAAAAACATTTTTTCAATCCAAGAAACAAGCGAGAGCATCTGTCAGTTT

>CL356.Contig25_A-W 163 2334 minus strand PREDICTED: nuclear autoantigen Sp-100 isoform 14 [Pan troglodytes]

TCAGTGTTCCAAGAAAAATATGTTTCCCTAAGAAGTGACCCGGAAGGAAGACAACAAACA

TCCATCACTCAGCCAAGCCGTGAGCAAGGAACAGTTGAGAACACGTTTCTGCGAAGACTG

ACCTGGCCACAGCACTCAGATCCCTCGTCTTACAATGGTACAGACCTACCCGAAAATGAA

CTCTTGGCGCATCCTTGCGAAGCAGAAGAGAGAAATGTAAATAAAAGAGAGCCGGCCCGA

GACCCAAATGGTGCAGTAGGAAGCCACCAAGGAAATGAGCAATGCCCTCGAGAGTCAGAG

CCAGTAGAGTCCCACCAACACATGGCTGCCAACGTGGACCGTGGAGAGGCGAGCGTGGGG

ACACCCAGCCCGTTGCCCCACTGTGTAGAAAGAGCAGAGCTCTCCAGCCATGGAATCCAC

ATGACTTCCTGTTCTGTGCATCTGGTGGATATAAAGAAGGAAAAGCCATTTTTTCCCACG

CAAATGAAGCAAGGATCTCAAACCAGGACCAACCATTACCAGCCATCCGAAGTAATAGTC

ATCAGCAGTGAGGACGACGCCAGCGACACAGAAGAGCCTGCAGCAATCTCCAGCTCAGCA

CGGAGGAGCCATCCTGTGATCAAAAACCATGTCCAGTTTGACTTGAGAGATGAAGAAGAG

GCCCAAGAAGCCACTTGCTCATGGCACCAGGCTGCTCCAGCGAGAGGACGTGACTATGGC

TCTTCAGAATCCAGTGAGGAGGAGATACCCTCAGCGCTCCGGATCTCAACACCAACAGAT

TCTGTGAGGATGTCTACTTGGAGGATACACAGTCAGAAAAGAAGGCACAGCAGTGACAGC

TCTTCAGAGCTGAGTTATGGAGAGGAGCCTCGGGAAGCCCTGAGAAGTGGATCAGGAACA

GGGCAACAAGAGACTGGAAATGAGAAGTGCTCCTGTGTCCTGTGTTCCCCAAAAGGTGTG

CCAAGAGGCCAAGAAGCAACGGTGGAAAGTGGCCGAGCATCTCACCCAATAGATACCACG

GATGTTGGAAACAATTCTACTTTGGGAAAAGGCAGTGGGGAAAGAAATATTTCCAAGCTG

CACAACAGAGATAAAGAAATGAACAAAATCTCATCAACCTTACATCCTACTGTGGGGAAA

AAAAGACTTCATAAAGTAAACCATCTCCAAAAAGAAATAAAGAAAGGCAGACATAGATTC

CACTCAATTCAGAATAATGCCCTTCAAAGGAAAAGAGGTTGGCCAAAAGGGAGAAAAAGA

GTCAACGTTAGACCCTTGAAAAGAGGGAGAAAAAGAGGACCAAGAATTCCCAGAGAAAAA

AATGTGAATTTCAACCTTCCTAAACTTCCTGTGACCTGTGGTGAGGCTAAGGGGACTCTC

CATAAGAAGCAATTTAAGCAAGGGGCCCAGATGAAGAGCATAAGGACTGAGGATGGAAAA

TGGCTCACCCCCAGGGACTTCGAGATCAGAGGAAACCACGCAGCAGCCAAGAACTGGAAG

CTAAGCCTGCGCTGCCACGGATGGACCTTGAAAGAACTGATAGAGGCAGGGCATCTACCA

AACCCACCAATAACAAGAAAAAAGAGGAAAACACGGGAGTCTCTCAACAATGTCTTAGTT

GACCCCCATCAAAAAAACTCAAATAAGTGCGAAGTATGCCGCCAAGAGAAACAGCTGGTC

TTCTGCAATACTTGTTCAAGATCATACCATCAGAACTGCCACATCCCACCAGTAGATGCT

CAGAGGAGCCAGTGGAGTTGCACCTTATGTAGGACAAAAGCCTGGGAAAAGCACCCAGAA

AGCCAACCATGTCATCTGGAATCTGAGGTCTTGAAGAGGCCTATGCTGCCTGAGGAACAG

CTGAAATGTGAGCTGATCCTATTGAAGGTCTATTGTTATTCACAAAGTGCCTTTTTTGCC

CAAGAACCATATTATACTCAAGACCCCCAGGAGCACATGTGGTTAAACAAGGTCAAGGAA

AGGCTGAATAAGAAGAAGTACCACCGAGTGGAGGGGTTTGTGCGGGACATGCGCCTCATC

TTTCTGAACAACAAGGCCTTCTACAGAAACCACAAATTCATCCAGCTGCCTCTTAAGATA

GAAGCCATATTTGAGAAGTATTTCAAAAACATTTTTTCAATCCAAGAAACAAGCGAGAGC

ATCTGTCAGTTT

>CL356.Contig26_A-W 28 2535 minus strand PREDICTED: nuclear autoantigen Sp-100 [Callithrix jacchus]

ATATTTTGTGATATATTTTTCAACCACTTCAAGAAAAATAAAGTGGAGATAGCAACTGCA

ATAACAAAGCTGTTTCCTTTCCTAGAGGGCCTCAGAGACTGATCCTTTATCACCGAACAA

ATCTATGCTGATTCTCAAGAAGCCTTTAGAAACTTGGTTCCTGTAGCCAGAGTGGTGTAC

CATGTTCTTGGCCACCTTGAGAAGGTGTTTGATTGGTCACTTCTACAAACCCTGTTCAGC

AAAGTGAACCTGAAAGAGTACCCTGGCTTAATACAGATTTATAAAACCTTTGAAAATGTG

TTCCAAGAAAAATATGTTTCCCTAAGAAGTGACCCGGAAGGAAGACAACAAACATCCATC

ACTCAGCCAAGCCGTGAGCAAGGAACAGTTGAGAACACGTTTCTGCGAAGACTGACCTGG

CCACAGCACTCAGATCCCTCGTCTTACAATGGTACAGACCTACCCGAAAATGAACTCTTG

GCGCATCCTTGCGAAGCAGAAGAGAGAAATGTAAATAAAAGAGAGCCGGCCCGAGACCCA

AATGGTGCAGTAGGAAGCCACCAAGGAAATGAGCAATGCCCTCGAGAGTCAGAGCCAGTA

GAGTCCCACCAACACATGGCTGCCAACGTGGACCGTGGAGAGGCGAGCGTGGGGACACCC

AGCCCGTTGCCCCACTGTGTAGAAAGAGCAGAGCTCTCCAGCCATGGAATCCACATGACT

TCCTGTTCTGTGCATCTGGTGGATATAAAGAAGGAAAAGCCATTTTTTCCCACGCAAATG

AAGCAAGGATCTCAAACCAGGACCAACCATTACCAGCCATCCGAAGTAATAGTCATCAGC

AGTGAGGACGACGCCAGCGACACAGAAGAGCCTGCAGCAATCTCCAGCTCAGCACGGAGG

AGCCATCCTGTGATCAAAAACCATGTCCAGTTTGACTTGAGAGATGAAGAAGAGGCCCAA

GAAGCCACTTGCTCATGGCACCAGGCTGCTCCAGCGAGAGGACGTGACTATGGCTCTTCA

GAATCCAGTGAGGAGGAGATACCCTCAGCGCTCCGGATCTCAACACCAACAGATTCTGTG

AGGATGTCTACTTGGAGGATACACAGTCAGAAAAGAAGGCACAGCAGTGACAGCTCTTCA

GAGCTGAGTTATGGAGAGGAGCCTCGGGAAGCCCTGAGAAGTGGATCAGGAACAGGGCAA

CAAGAGACTGGAAATGAGAAGTGCTCCTGTGTCCTGTGTTCCCCAAAAGGTGTGCCAAGA

GGCCAAGAAGCAACGGTGGAAAGTGGCCGAGCATCTCACCCAATAGGCAAGTCTGCTCAA

TGGCATCAGGGTGGAGGTGGCTCAGAAATACCACAGCTGCTGGGCAGGACTGTGTCAGGA

GAGGAAGCTGTAAAAGATACCACGGATGTTGGAAACAATTCTACTTTGGGAAAAGGCAGT

GGGGAAAGAAATATTTCCAAGCTGCACAACAGAGATAAAGAAATGAACAAAATCTCATCA

ACCTTACATCCTACTGTGGGGAAAAAAAGACTTCATAAAGTAAACCATCTCCAAAAAGAA

ATAAAGAAAGGCAGACATAGATTCCACTCAATTCAGAATAATGCCCTTCAAAGGAAAAGA

GGTTGGCCAAAAGGACCAAGAATTCCCAGAGAAAAAAATGTGAATTTCAACCTTCCTAAA

CTTCCTGTGACCTGTGGTGAGGCTAAGGGGACTCTCCATAAGAAGCAATTTAAGCAAGGG

GCCCAGATGAAGAGCATAAGGACTGAGGATGGAAAATGGCTCACCCCCAGGGACTTCGAG

ATCAGAGGAAACCACGCAGCAGCCAAGAACTGGAAGCTAAGCCTGCGCTGCCACGGATGG

ACCTTGAAAGAACTGATAGAGGCAGGGCATCTACCAAACCCACCAATAACAAGAAAAAAG

AGGAAAACACGGGAGTCTCTCAACAATGTCTTAGTTGACCCCCATCAAAAAAACTCAAAT

AAGTGCGAAGTATGCCGCCAAGAGAAACAGCTGGTCTTCTGCAATACTTGTTCAAGATCA

TACCATCAGAACTGCCACATCCCACCAGTAGATGCTCAGAGGAGCCAGTGGAGTTGCACC

TTATGTAGGACAAAAGCCTGGGAAAAGCACCCAGAAAGCCAACCATGTCATCTGGAATCT

GAGGTCTTGAAGAGGCCTATGCTGCCTGAGGAACAGCTGAAATGTGAGCTGATCCTATTG

AAGGTCTATTGTTATTCACAAAGTGCCTTTTTTGCCCAAGAACCATATTATACTCAAGAC

CCCCAGGAGCACATGTGGTTAAACAAGGTCAAGGAAAGGCTGAATAAGAAGAAGTACCAC

CGAGTGGAGGGGTTTGTGCGGGACATGCGCCTCATCTTTCTGAACAACAAGGCCTTCTAC

AGAAACCACAAATTCATCCAGCTGCCTCTTAAGATAGAAGCCATATTTGAGAAGTATTTC

AAAAACATTTTTTCAATCCAAGAAACAAGCGAGAGCATCTGTCAGTTT

>CL356.Contig27_A-W 191 2323 minus strand PREDICTED: nuclear autoantigen Sp-100 [Pan paniscus]

TCAGTGTTCCAAGAAAAATATGTTTCCCTAAGAAGTGACCCGGAAGGAAGACAACAAACA

TCCATCACTCAGCCAAGCCGTGAGCAAGGAACAGTTGAGAACACGTTTCTGCGAAGACTG

ACCTGGCCACAGCACTCAGATCCCTCGTCTTACAATGGTACAGACCTACCCGAAAATGAA

CTCTTGGCGCATCCTTGCGAAGCAGAAGAGAGAAATGTAAATAAAAGAGAGCCGGCCCGA

GACCCAAATGGTGCAGTAGGAAGCCACCAAGGAAATGAGCAATGCCCTCGAGAGTCAGAG

CCAGTAGAGTCCCACCAACACATGGCTGCCAACGTGGACCGTGGAGAGGCGAGCGTGGGG

ACACCCAGCCCGTTGCCCCACTGTGTAGAAAGAGCAGAGCTCTCCAGCCATGGAATCCAC

ATGACTTCCTGTTCTGTGCATCTGGTGGATATAAAGAAGGAAAAGCCATTTTTTCCCACG

CAAATGAAGCAAGGATCTCAAACCAGGACCAACCATTACCAGCCATCCGAAGTAATAGTC

ATCAGCAGTGAGGACGACGCCAGCGACACAGAAGAGCCTGCAGCAATCTCCAGCTCAGCA

CGGAGGAGCCATCCTGTGATCAAAAACCATGTCCAGTTTGACTTGAGAGATGAAGAAGAG

GCCCAAGAAGCCACTTGCTCATGGCACCAGGCTGCTCCAGCGAGAGGACGTGACTATGGC

TCTTCAGAATCCAGTGAGGAGGAGATACCCTCAGCGCTCCGGATCTCAACACCAACAGAT

TCTGTGAGGATGTCTACTTGGAGGATACACAGTCAGAAAAGAAGGCACAGCAGTGACAGC

TCTTCAGAGCTGAGTTATGGAGAGGAGCCTCGGGAAGCCCTGAGAAGTGGATCAGGTGTG

CCAAGAGGCCAAGAAGCAACGGTGGAAAGTGGCCGAGCATCTCACCCAATAGGCAAGTCT

GCTCAATGGCATCAGGGTGGAGGTGGCTCAGAAATACCACAGCTGCTGGGCAGGACTGTG

TCAGGAGAGGAAGCTGTAAAAGATACCACGGATGTTGGAAACAATTCTACTTTGGGAAAA

GGCAGTGGGGAAAGAAGGAAAAAAAGACTTCATAAAGTAAACCATCTCCAAAAAGAAATA

AAGAAAGGCAGACATAGATTCCACTCAATTCAGAATAATGCCCTTCAAAGGAAAAGAGGT

TGGCCAAAAGGGAGAAAAAGAGTCAACGTTAGACCCTTGAAAAGAGGGAGAAAAAGAGGA

CCAAGAATTCCCAGAGAAAAAAATGTGAATTTCAACCTTCCTAAACTTCCTGTGACCTGT

GGTGAGGCTAAGGGGACTCTCCATAAGAAGCAATTTAAGCAAGGGGCCCAGATGAAGAGC

ATAAGGACTGAGGATGGAAAATGGCTCACCCCCAGGGACTTCGAGATCAGAGGAAACCAC

GCAGCAGCCAAGAACTGGAAGCTAAGCCTGCGCTGCCACGGATGGACCTTGAAAGAACTG

ATAGAGGCAGGGCATCTACCAAACCCACCAATAACAAGAAAAAAGAGGAAAACACGGGAG

TCTCTCAACAATGTCTTAGTTGACCCCCATCAAAAAAACTCAAATAAGTGCGAAGTATGC

CGCCAAGAGAAACAGCTGGTCTTCTGCAATACTTGTTCAAGATCATACCATCAGAACTGC

CACATCCCACCAGTAGATGCTCAGAGGAGCCAGTGGAGTTGCACCTTATGTAGGACAAAA

GCCTGGGAAAAGCACCCAGAAAGCCAACCATGTCATCTGGAATCTGAGGTCTTGAAGAGG

CCTATGCTGCCTGAGGAACAGCTGAAATGTGAGCTGATCCTATTGAAGGTCTATTGTTAT

TCACAAAGTGCCTTTTTTGCCCAAGAACCATATTATACTCAAGACCCCCAGGAGCACATG

TGGTTAAACAAGGTCAAGGAAAGGCTGAATAAGAAGAAGTACCACCGAGTGGAGGGGTTT

GTGCGGGACATGCGCCTCATCTTTCTGAACAACAAGGCCTTCTACAGAAACCACAAATTC

ATCCAGCTGCCTCTTAAGATAGAAGCCATATTTGAGAAGTATTTCAAAAACATTTTTTCA

ATCCAAGAAACAAGCGAGAGCATCTGTCAGTTT

>CL356.Contig28_A-W 163 2316 minus strand PREDICTED: nuclear autoantigen Sp-100 [Pan paniscus]

TCAGTGTTCCAAGAAAAATATGTTTCCCTAAGAAGTGACCCGGAAGGAAGACAACAAACA

TCCATCACTCAGCCAAGCCGTGAGCAAGGAACAGTTGAGAACACGTTTCTGCGAAGACTG

ACCTGGCCACAGCACTCAGATCCCTCGTCTTACAATGGTACAGACCTACCCGAAAATGAA

CTCTTGGCGCATCCTTGCGAAGCAGAAGAGAGAAATGTAAATAAAAGAGAGCCGGCCCGA

GACCCAAATGGTGCAGTAGGAAGCCACCAAGGAAATGAGCAATGCCCTCGAGAGTCAGAG

CCAGTAGAGTCCCACCAACACATGGCTGCCAACGTGGACCGTGGAGAGGCGAGCGTGGGG

ACACCCAGCCCGTTGCCCCACTGTGTAGAAAGAGCAGAGCTCTCCAGCCATGGAATCCAC

ATGACTTCCTGTTCTGTGCATCTGGTGGATATAAAGAAGGAAAAGCCATTTTTTCCCACG

CAAATGAAGCAAGGATCTCAAACCAGGACCAACCATTACCAGCCATCCGAAGTAATAGTC

ATCAGCAGTGAGGACGACGCCAGCGACACAGAAGAGCCTGCAGCAATCTCCAGCTCAGCA

CGGAGGAGCCATCCTGTGATCAAAAACCATGTCCAGTTTGACTTGAGAGATGAAGAAGAG

GCCCAAGAAGCCACTTGCTCATGGCACCAGGCTGCTCCAGCGAGAGGACGTGACTATGGC

TCTTCAGAATCCAGTGAGGAGGAGATACCCTCAGCGCTCCGGATCTCAACACCAACAGAT

TCTGTGAGGATGTCTACTTGGAGGATACACAGTCAGAAAAGAAGGCACAGCAGTGACAGC

TCTTCAGAGCTGAGTTATGGAGAGGAGCCTCGGGAAGCCCTGAGAAGTGGATCAGGTGTG

CCAAGAGGCCAAGAAGCAACGGTGGAAAGTGGCCGAGCATCTCACCCAATAGGCAAGTCT

GCTCAATGGCATCAGGGTGGAGGTGGCTCAGAAATACCACAGCTGCTGGGCAGGACTGTG

TCAGGAGAGGAAGCTGTAAAAGATACCACGGATGTTGGAAACAATTCTACTTTGGGAAAA

GGCAGTGGGGAAAGAAATATTTCCAAGCTGCACAACAGAGATAAAGAAATGAACAAAATC

TCATCAACCTTACATCCTACTGTGGGGAAAAAAAGACTTCATAAAGTAAACCATCTCCAA

AAAGAAATAAAGAAAGGCAGACATAGATTCCACTCAATTCAGAATAATGCCCTTCAAAGG

AAAAGAGGTTGGCCAAAAGGACCAAGAATTCCCAGAGAAAAAAATGTGAATTTCAACCTT

CCTAAACTTCCTGTGACCTGTGGTGAGGCTAAGGGGACTCTCCATAAGAAGCAATTTAAG

CAAGGGGCCCAGATGAAGAGCATAAGGACTGAGGATGGAAAATGGCTCACCCCCAGGGAC

TTCGAGATCAGAGGAAACCACGCAGCAGCCAAGAACTGGAAGCTAAGCCTGCGCTGCCAC

GGATGGACCTTGAAAGAACTGATAGAGGCAGGGCATCTACCAAACCCACCAATAACAAGA

AAAAAGAGGAAAACACGGGAGTCTCTCAACAATGTCTTAGTTGACCCCCATCAAAAAAAC

TCAAATAAGTGCGAAGTATGCCGCCAAGAGAAACAGCTGGTCTTCTGCAATACTTGTTCA

AGATCATACCATCAGAACTGCCACATCCCACCAGTAGATGCTCAGAGGAGCCAGTGGAGT

TGCACCTTATGTAGGACAAAAGCCTGGGAAAAGCACCCAGAAAGCCAACCATGTCATCTG

GAATCTGAGGTCTTGAAGAGGCCTATGCTGCCTGAGGAACAGCTGAAATGTGAGCTGATC

CTATTGAAGGTCTATTGTTATTCACAAAGTGCCTTTTTTGCCCAAGAACCATATTATACT

CAAGACCCCCAGGAGCACATGTGGTTAAACAAGGTCAAGGAAAGGCTGAATAAGAAGAAG

TACCACCGAGTGGAGGGGTTTGTGCGGGACATGCGCCTCATCTTTCTGAACAACAAGGCC

TTCTACAGAAACCACAAATTCATCCAGCTGCCTCTTAAGATAGAAGCCATATTTGAGAAG

TATTTCAAAAACATTTTTTCAATCCAAGAAACAAGCGAGAGCATCTGTCAGTTT

>CL356.Contig29_A-W 132 2654 minus strand PREDICTED: nuclear autoantigen Sp-100 isoform 14 [Pan troglodytes]

AGGATGTCCACAGAAGAGCAGAGTGTAGACGACAGTACCATCTATGAGGTTATATTCCAT

CACTTCAAAAGACACAAAGTGGAGATTTCAAATGCCATAAAAAAGACATTTCCTTTCCTC

GAGGGCCTCCGTGACCGTGAACTCATTACCAATAAAATGTTTGAAGATTCTCAAGAGTCT

TGTAGAAACCTGGTCCCTGTACAAAGAGTGGTGTACAATGTTCTCTGTGAACTGGAGAAG

AAATTCAACCTGGATTTGTGCGAAGCATTGTTCAGTGAGGTCAACTTGCAGGAATACCCT

GATTTAATTCGTGTTCGTAGAAGCTTCGAACATGAGGTGAGAATGCTATATCCCCAAGAA

GATAATGGAGAAGAGAGGCAGGAAAGGCTTGAACAAGGAACGGGGGAGAACTCTTTTCTG

CGAAGACTGACCTGGCCACAGCACTCAGATCCCTCGTCTTACAATGGTACAGACCTACCC

GAAAATGAACTCTTGGCGCATCCTTGCGAAGCAGAAGAGAGAAATGTAAATAAAAGAGAG

CCGGCCCGAGACCCAAATGGTGCAGTAGGAAGCCACCAAGGAAATGAGCAATGCCCTCGA

GAGTCAGAGCCAGTAGAGTCCCACCAACACATGGCTGCCAACGTGGACCGTGGAGAGGCG

AGCGTGGGGACACCCAGCCCGTTGCCCCACTGTGTAGAAAGAGCAGAGCTCTCCAGCCAT

GGAATCCACATGACTTCCTGTTCTGTGCATCTGGTGGATATAAAGAAGGAAAAGCCATTT

TTTCCCACGCAAATGAAGCAAGGATCTCAAACCAGGACCAACCATTACCAGCCATCCGAA

GTAATAGTCATCAGCAGTGAGGACGACGCCAGCGACACAGAAGAGCCTGCAGCAATCTCC

AGCTCAGCACGGAGGAGCCATCCTGTGATCAAAAACCATGTCCAGTTTGACTTGAGAGAT

GAAGAAGAGGCCCAAGAAGCCACTTGCTCATGGCACCAGGCTGCTCCAGCGAGAGGACGT

GACTATGGCTCTTCAGAATCCAGTGAGGAGGAGATACCCTCAGCGCTCCGGATCTCAACA

CCAACAGATTCTGTGAGGATGTCTACTTGGAGGATACACAGTCAGAAAAGAAGGCACAGC

AGTGACAGCTCTTCAGAGCTGAGTTATGGAGAGGAGCCTCGGGAAGCCCTGAGAAGTGGA

TCAGGAACAGGGCAACAAGAGACTGGAAATGAGAAGTGCTCCTGTGTCCTGTGTTCCCCA

AAAGGTGTGCCAAGAGGCCAAGAAGCAACGGTGGAAAGTGGCCGAGCATCTCACCCAATA

GGCAAGTCTGCTCAATGGCATCAGGGTGGAGGTGGCTCAGAAATACCACAGCTGCTGGGC

AGGACTGTGTCAGGAGAGGAAGCTGTAAAAGATACCACGGATGTTGGAAACAATTCTACT

TTGGGAAAAGGCAGTGGGGAAAGAAATATTTCCAAGCTGCACAACAGAGATAAAGAAATG

AACAAAATCTCATCAACCTTACATCCTACTGTGGGGAAAAAAAGACTTCATAAAGTAAAC

CATCTCCAAAAAGAAATAAAGAAAGGCAGACATAGATTCCACTCAATTCAGAATAATGCC

CTTCAAAGGAAAAGAGGTTGGCCAAAAGGACCAAGAATTCCCAGAGAAAAAAATGTGAAT

TTCAACCTTCCTAAACTTCCTGTGACCTGTGGTGAGGCTAAGGGGACTCTCCATAAGAAG

CAATTTAAGCAAGGGGCCCAGATGAAGAGCATAAGGACTGAGGATGGAAAATGGCTCACC

CCCAGGGACTTCGAGATCAGAGGAAACCACGCAGCAGCCAAGAACTGGAAGCTAAGCCTG

CGCTGCCACGGATGGACCTTGAAAGAACTGATAGAGGCAGGGCATCTACCAAACCCACCA

ATAACAAGAAAAAAGAGGAAAACACGGGAGTCTCTCAACAATGTCTTAGTTGACCCCCAT

CAAAAAAACTCAAATAAGTGCGAAGTATGCCGCCAAGAGAAACAGCTGGTCTTCTGCAAT

ACTTGTTCAAGATCATACCATCAGAACTGCCACATCCCACCAGTAGATGCTCAGAGGAGC

CAGTGGAGTTGCACCTTATGTAGGACAAAAGCCTGGGAAAAGCACCCAGAAAGCCAACCA

TGTCATCTGGAATCTGAGGTCTTGAAGAGGCCTATGCTGCCTGAGGAACAGCTGAAATGT

GAGCTGATCCTATTGAAGGTCTATTGTTATTCACAAAGTGCCTTTTTTGCCCAAGAACCA

TATTATACTCAAGACCCCCAGGAGCACATGTGGTTAAACAAGGTCAAGGAAAGGCTGAAT

AAGAAGAAGTACCACCGAGTGGAGGGGTTTGTGCGGGACATGCGCCTCATCTTTCTGAAC

AACAAGGCCTTCTACAGAAACCACAAATTCATCCAGCTGCCTCTTAAGATAGAAGCCATA

TTTGAGAAGTATTTCAAAAACATTTTTTCAATCCAAGAAACAAGCGAGAGCATCTGTCAG

TTT

>CL356.Contig30_A-W 132 2564 minus strand PREDICTED: nuclear autoantigen Sp-100 isoform 14 [Pan troglodytes]

AGGATGTCCACAGAAGAGCAGAGTGTAGACGACAGTACCATCTATGAGGTTATATTCCAT

CACTTCAAAAGACACAAAGTGGAGATTTCAAATGCCATAAAAAAGACATTTCCTTTCCTC

GAGGGCCTCCGTGACCGTGAACTCATTACCAATAAAATGTTTGAAGATTCTCAAGAGTCT

TGTAGAAACCTGGTCCCTGTACAAAGAGTGGTGTACAATGTTCTCTGTGAACTGGAGAAG

AAATTCAACCTGGATTTGTGCGAAGCATTGTTCAGTGAGGTCAACTTGCAGGAATACCCT

GATTTAATTCGTGTTCGTAGAAGCTTCGAACATGAGGTGAGAATGCTATATCCCCAAGAA

GATAATGGAGAAGAGAGGCAGGAAAGGCTTGAACAAGGAACGGGGGAGAACTCTTTTCTG

CGAAGACTGACCTGGCCACAGCACTCAGATCCCTCGTCTTACAATGGTACAGACCTACCC

GAAAATGAACTCTTGGCGCATCCTTGCGAAGCAGAAGAGAGAAATGTAAATAAAAGAGAG

CCGGCCCGAGACCCAAATGGTGCAGTAGGAAGCCACCAAGGAAATGAGCAATGCCCTCGA

GAGTCAGAGCCAGTAGAGTCCCACCAACACATGGCTGCCAACGTGGACCGTGGAGAGGCG

AGCGTGGGGACACCCAGCCCGTTGCCCCACTGTGTAGAAAGAGCAGAGCTCTCCAGCCAT

GGAATCCACATGACTTCCTGTTCTGTGCATCTGGTGGATATAAAGAAGGAAAAGCCATTT

TTTCCCACGCAAATGAAGCAAGGATCTCAAACCAGGACCAACCATTACCAGCCATCCGAA

GTAATAGTCATCAGCAGTGAGGACGACGCCAGCGACACAGAAGAGCCTGCAGCAATCTCC

AGCTCAGCACGGAGGAGCCATCCTGTGATCAAAAACCATGTCCAGTTTGACTTGAGAGAT

GAAGAAGAGGCCCAAGAAGCCACTTGCTCATGGCACCAGGCTGCTCCAGCGAGAGGACGT

GACTATGGCTCTTCAGAATCCAGTGAGGAGGAGATACCCTCAGCGCTCCGGATCTCAACA

CCAACAGATTCTGTGAGGATGTCTACTTGGAGGATACACAGTCAGAAAAGAAGGCACAGC

AGTGACAGCTCTTCAGAGCTGAGTTATGGAGAGGAGCCTCGGGAAGCCCTGAGAAGTGGA

TCAGGAACAGGGCAACAAGAGACTGGAAATGAGAAGTGCTCCTGTGTCCTGTGTTCCCCA

AAAGGTGTGCCAAGAGGCCAAGAAGCAACGGTGGAAAGTGGCCGAGCATCTCACCCAATA

GATACCACGGATGTTGGAAACAATTCTACTTTGGGAAAAGGCAGTGGGGAAAGAAATATT

TCCAAGCTGCACAACAGAGATAAAGAAATGAACAAAATCTCATCAACCTTACATCCTACT

GTGGGGAAAAAAAGACTTCATAAAGTAAACCATCTCCAAAAAGAAATAAAGAAAGGCAGA

CATAGATTCCACTCAATTCAGAATAATGCCCTTCAAAGGAAAAGAGGTTGGCCAAAAGGA

CCAAGAATTCCCAGAGAAAAAAATGTGAATTTCAACCTTCCTAAACTTCCTGTGACCTGT

GGTGAGGCTAAGGGGACTCTCCATAAGAAGCAATTTAAGCAAGGGGCCCAGATGAAGAGC

ATAAGGACTGAGGATGGAAAATGGCTCACCCCCAGGGACTTCGAGATCAGAGGAAACCAC

GCAGCAGCCAAGAACTGGAAGCTAAGCCTGCGCTGCCACGGATGGACCTTGAAAGAACTG

ATAGAGGCAGGGCATCTACCAAACCCACCAATAACAAGAAAAAAGAGGAAAACACGGGAG

TCTCTCAACAATGTCTTAGTTGACCCCCATCAAAAAAACTCAAATAAGTGCGAAGTATGC

CGCCAAGAGAAACAGCTGGTCTTCTGCAATACTTGTTCAAGATCATACCATCAGAACTGC

CACATCCCACCAGTAGATGCTCAGAGGAGCCAGTGGAGTTGCACCTTATGTAGGACAAAA

GCCTGGGAAAAGCACCCAGAAAGCCAACCATGTCATCTGGAATCTGAGGTCTTGAAGAGG

CCTATGCTGCCTGAGGAACAGCTGAAATGTGAGCTGATCCTATTGAAGGTCTATTGTTAT

TCACAAAGTGCCTTTTTTGCCCAAGAACCATATTATACTCAAGACCCCCAGGAGCACATG

TGGTTAAACAAGGTCAAGGAAAGGCTGAATAAGAAGAAGTACCACCGAGTGGAGGGGTTT

GTGCGGGACATGCGCCTCATCTTTCTGAACAACAAGGCCTTCTACAGAAACCACAAATTC

ATCCAGCTGCCTCTTAAGATAGAAGCCATATTTGAGAAGTATTTCAAAAACATTTTTTCA

ATCCAAGAAACAAGCGAGAGCATCTGTCAGTTT

>CL356.Contig31_A-W 134 1642 minus strand PREDICTED: nuclear autoantigen Sp-100 [Callithrix jacchus]

GGACGTGACTATGGCTCTTCAGAATCCAGTGAGGAGGAGATACCCTCAGCGCTCCGGATC

TCAACACCAACAGATTCTGTGAGGATGTCTACTTGGAGGATACACAGTCAGAAAAGAAGG

CACAGCAGTGACAGCTCTTCAGAGCTGAGTTATGGAGAGGAGCCTCGGGAAGCCCTGAGA

AGTGGATCAGGAACAGGGCAACAAGAGACTGGAAATGAGAAGTGCTCCTGTGTCCTGTGT

TCCCCAAAAGGTGTGCCAAGAGGCCAAGAAGCAACGGTGGAAAGTGGCCGAGCATCTCAC

CCAATAGGCAAGTCTGCTCAATGGCATCAGGGTGGAGGTGGCTCAGAAATACCACAGCTG

CTGGGCAGGACTGTGTCAGGAGAGGAAGCTGTAAAAGATACCACGGATGTTGGAAACAAT

TCTACTTTGGGAAAAGGCAGTGGGGAAAGAAATATTTCCAAGCTGCACAACAGAGATAAA

GAAATGAACAAAATCTCATCAACCTTACATCCTACTGTGGGGAAAAAAAGACTTCATAAA

GTAAACCATCTCCAAAAAGAAATAAAGAAAGGCAGACATAGATTCCACTCAATTCAGAAT

AATGCCCTTCAAAGGAAAAGAGGTTGGCCAAAAGGACCAAGAATTCCCAGAGAAAAAAAT

GTGAATTTCAACCTTCCTAAACTTCCTGTGACCTGTGGTGAGGCTAAGGGGACTCTCCAT

AAGAAGCAATTTAAGCAAGGGGCCCAGATGAAGAGCATAAGGACTGAGGATGGAAAATGG

CTCACCCCCAGGGACTTCGAGATCAGAGGAAACCACGCAGCAGCCAAGAACTGGAAGCTA

AGCCTGCGCTGCCACGGATGGACCTTGAAAGAACTGATAGAGGCAGGGCATCTACCAAAC

CCACCAATAACAAGAAAAAAGAGGAAAACACGGGAGTCTCTCAACAATGTCTTAGTTGAC

CCCCATCAAAAAAACTCAAATAAGTGCGAAGTATGCCGCCAAGAGAAACAGCTGGTCTTC

TGCAATACTTGTTCAAGATCATACCATCAGAACTGCCACATCCCACCAGTAGATGCTCAG

AGGAGCCAGTGGAGTTGCACCTTATGTAGGACAAAAGCCTGGGAAAAGCACCCAGAAAGC

CAACCATGTCATCTGGAATCTGAGGTCTTGAAGAGGCCTATGCTGCCTGAGGAACAGCTG

AAATGTGAGCTGATCCTATTGAAGGTCTATTGTTATTCACAAAGTGCCTTTTTTGCCCAA

GAACCATATTATACTCAAGACCCCCAGGAGCACATGTGGTTAAACAAGGTCAAGGAAAGG

CTGAATAAGAAGAAGTACCACCGAGTGGAGGGGTTTGTGCGGGACATGCGCCTCATCTTT

CTGAACAACAAGGCCTTCTACAGAAACCACAAATTCATCCAGCTGCCTCTTAAGATAGAA

GCCATATTTGAGAAGTATTTCAAAAACATTTTTTCAATCCAAGAAACAAGCGAGAGCATC

TGTCAGTTT

>CL356.Contig32_A-W 134 1630 minus strand PREDICTED: nuclear autoantigen Sp-100 [Pan paniscus]

GGACGTGACTATGGCTCTTCAGAATCCAGTGAGGAGGAGATACCCTCAGCGCTCCGGATC

TCAACACCAACAGATTCTGTGAGGATGTCTACTTGGAGGATACACAGTCAGAAAAGAAGG

CACAGCAGTGACAGCTCTTCAGAGCTGAGTTATGGAGAGGAGCCTCGGGAAGCCCTGAGA

AGTGGATCAGGTGTGCCAAGAGGCCAAGAAGCAACGGTGGAAAGTGGCCGAGCATCTCAC

CCAATAGGCAAGTCTGCTCAATGGCATCAGGGTGGAGGTGGCTCAGAAATACCACAGCTG

CTGGGCAGGACTGTGTCAGGAGAGGAAGCTGTAAAAGATACCACGGATGTTGGAAACAAT

TCTACTTTGGGAAAAGGCAGTGGGGAAAGAAATATTTCCAAGCTGCACAACAGAGATAAA

GAAATGAACAAAATCTCATCAACCTTACATCCTACTGTGGGGAAAAAAAGACTTCATAAA

GTAAACCATCTCCAAAAAGAAATAAAGAAAGGCAGACATAGATTCCACTCAATTCAGAAT

AATGCCCTTCAAAGGAAAAGAGGTTGGCCAAAAGGGAGAAAAAGAGTCAACGTTAGACCC

TTGAAAAGAGGGAGAAAAAGAGGACCAAGAATTCCCAGAGAAAAAAATGTGAATTTCAAC

CTTCCTAAACTTCCTGTGACCTGTGGTGAGGCTAAGGGGACTCTCCATAAGAAGCAATTT

AAGCAAGGGGCCCAGATGAAGAGCATAAGGACTGAGGATGGAAAATGGCTCACCCCCAGG

GACTTCGAGATCAGAGGAAACCACGCAGCAGCCAAGAACTGGAAGCTAAGCCTGCGCTGC

CACGGATGGACCTTGAAAGAACTGATAGAGGCAGGGCATCTACCAAACCCACCAATAACA

AGAAAAAAGAGGAAAACACGGGAGTCTCTCAACAATGTCTTAGTTGACCCCCATCAAAAA

AACTCAAATAAGTGCGAAGTATGCCGCCAAGAGAAACAGCTGGTCTTCTGCAATACTTGT

TCAAGATCATACCATCAGAACTGCCACATCCCACCAGTAGATGCTCAGAGGAGCCAGTGG

AGTTGCACCTTATGTAGGACAAAAGCCTGGGAAAAGCACCCAGAAAGCCAACCATGTCAT

CTGGAATCTGAGGTCTTGAAGAGGCCTATGCTGCCTGAGGAACAGCTGAAATGTGAGCTG

ATCCTATTGAAGGTCTATTGTTATTCACAAAGTGCCTTTTTTGCCCAAGAACCATATTAT

ACTCAAGACCCCCAGGAGCACATGTGGTTAAACAAGGTCAAGGAAAGGCTGAATAAGAAG

AAGTACCACCGAGTGGAGGGGTTTGTGCGGGACATGCGCCTCATCTTTCTGAACAACAAG

GCCTTCTACAGAAACCACAAATTCATCCAGCTGCCTCTTAAGATAGAAGCCATATTTGAG

AAGTATTTCAAAAACATTTTTTCAATCCAAGAAACAAGCGAGAGCATCTGTCAGTTT

>CL356.Contig33_A-W 134 1621 minus strand PREDICTED: nuclear autoantigen Sp-100 isoform 14 [Pan troglodytes]

GGACGTGACTATGGCTCTTCAGAATCCAGTGAGGAGGAGATACCCTCAGCGCTCCGGATC

TCAACACCAACAGATTCTGTGAGGATGTCTACTTGGAGGATACACAGTCAGAAAAGAAGG

CACAGCAGTGACAGCTCTTCAGAGCTGAGTTATGGAGAGGAGCCTCGGGAAGCCCTGAGA

AGTGGATCAGGAACAGGGCAACAAGAGACTGGAAATGAGAAGTGCTCCTGTGTCCTGTGT

TCCCCAAAAGGTGTGCCAAGAGGCCAAGAAGCAACGGTGGAAAGTGGCCGAGCATCTCAC

CCAATAGGCAAGTCTGCTCAATGGCATCAGGGTGGAGGTGGCTCAGAAATACCACAGCTG

CTGGGCAGGACTGTGTCAGGAGAGGAAGCTGTAAAAGATACCACGGATGTTGGAAACAAT

TCTACTTTGGGAAAAGGCAGTGGGGAAAGAAGGAAAAAAAGACTTCATAAAGTAAACCAT

CTCCAAAAAGAAATAAAGAAAGGCAGACATAGATTCCACTCAATTCAGAATAATGCCCTT

CAAAGGAAAAGAGGTTGGCCAAAAGGGAGAAAAAGAGTCAACGTTAGACCCTTGAAAAGA

GGGAGAAAAAGAGGACCAAGAATTCCCAGAGAAAAAAATGTGAATTTCAACCTTCCTAAA

CTTCCTGTGACCTGTGGTGAGGCTAAGGGGACTCTCCATAAGAAGCAATTTAAGCAAGGG

GCCCAGATGAAGAGCATAAGGACTGAGGATGGAAAATGGCTCACCCCCAGGGACTTCGAG

ATCAGAGGAAACCACGCAGCAGCCAAGAACTGGAAGCTAAGCCTGCGCTGCCACGGATGG

ACCTTGAAAGAACTGATAGAGGCAGGGCATCTACCAAACCCACCAATAACAAGAAAAAAG

AGGAAAACACGGGAGTCTCTCAACAATGTCTTAGTTGACCCCCATCAAAAAAACTCAAAT

AAGTGCGAAGTATGCCGCCAAGAGAAACAGCTGGTCTTCTGCAATACTTGTTCAAGATCA

TACCATCAGAACTGCCACATCCCACCAGTAGATGCTCAGAGGAGCCAGTGGAGTTGCACC

TTATGTAGGACAAAAGCCTGGGAAAAGCACCCAGAAAGCCAACCATGTCATCTGGAATCT

GAGGTCTTGAAGAGGCCTATGCTGCCTGAGGAACAGCTGAAATGTGAGCTGATCCTATTG

AAGGTCTATTGTTATTCACAAAGTGCCTTTTTTGCCCAAGAACCATATTATACTCAAGAC

CCCCAGGAGCACATGTGGTTAAACAAGGTCAAGGAAAGGCTGAATAAGAAGAAGTACCAC

CGAGTGGAGGGGTTTGTGCGGGACATGCGCCTCATCTTTCTGAACAACAAGGCCTTCTAC

AGAAACCACAAATTCATCCAGCTGCCTCTTAAGATAGAAGCCATATTTGAGAAGTATTTC

AAAAACATTTTTTCAATCCAAGAAACAAGCGAGAGCATCTGTCAGTTT

>CL356.Contig34_A-W 134 1600 minus strand PREDICTED: nuclear autoantigen Sp-100 isoform 14 [Pan troglodytes]

GGACGTGACTATGGCTCTTCAGAATCCAGTGAGGAGGAGATACCCTCAGCGCTCCGGATC

TCAACACCAACAGATTCTGTGAGGATGTCTACTTGGAGGATACACAGTCAGAAAAGAAGG

CACAGCAGTGACAGCTCTTCAGAGCTGAGTTATGGAGAGGAGCCTCGGGAAGCCCTGAGA

AGTGGATCAGGAACAGGGCAACAAGAGACTGGAAATGAGAAGTGCTCCTGTGTCCTGTGT

TCCCCAAAAGGTGTGCCAAGAGGCCAAGAAGCAACGGTGGAAAGTGGCCGAGCATCTCAC

CCAATAGATACCACGGATGTTGGAAACAATTCTACTTTGGGAAAAGGCAGTGGGGAAAGA

AATATTTCCAAGCTGCACAACAGAGATAAAGAAATGAACAAAATCTCATCAACCTTACAT

CCTACTGTGGGGAAAAAAAGACTTCATAAAGTAAACCATCTCCAAAAAGAAATAAAGAAA

GGCAGACATAGATTCCACTCAATTCAGAATAATGCCCTTCAAAGGAAAAGAGGTTGGCCA

AAAGGGAGAAAAAGAGTCAACGTTAGACCCTTGAAAAGAGGGAGAAAAAGAGGACCAAGA

ATTCCCAGAGAAAAAAATGTGAATTTCAACCTTCCTAAACTTCCTGTGACCTGTGGTGAG

GCTAAGGGGACTCTCCATAAGAAGCAATTTAAGCAAGGGGCCCAGATGAAGAGCATAAGG

ACTGAGGATGGAAAATGGCTCACCCCCAGGGACTTCGAGATCAGAGGAAACCACGCAGCA

GCCAAGAACTGGAAGCTAAGCCTGCGCTGCCACGGATGGACCTTGAAAGAACTGATAGAG

GCAGGGCATCTACCAAACCCACCAATAACAAGAAAAAAGAGGAAAACACGGGAGTCTCTC

AACAATGTCTTAGTTGACCCCCATCAAAAAAACTCAAATAAGTGCGAAGTATGCCGCCAA

GAGAAACAGCTGGTCTTCTGCAATACTTGTTCAAGATCATACCATCAGAACTGCCACATC

CCACCAGTAGATGCTCAGAGGAGCCAGTGGAGTTGCACCTTATGTAGGACAAAAGCCTGG

GAAAAGCACCCAGAAAGCCAACCATGTCATCTGGAATCTGAGGTCTTGAAGAGGCCTATG

CTGCCTGAGGAACAGCTGAAATGTGAGCTGATCCTATTGAAGGTCTATTGTTATTCACAA

AGTGCCTTTTTTGCCCAAGAACCATATTATACTCAAGACCCCCAGGAGCACATGTGGTTA

AACAAGGTCAAGGAAAGGCTGAATAAGAAGAAGTACCACCGAGTGGAGGGGTTTGTGCGG

GACATGCGCCTCATCTTTCTGAACAACAAGGCCTTCTACAGAAACCACAAATTCATCCAG

CTGCCTCTTAAGATAGAAGCCATATTTGAGAAGTATTTCAAAAACATTTTTTCAATCCAA

GAAACAAGCGAGAGCATCTGTCAGTTT

>CL356.Contig35_A-W 134 1582 minus strand PREDICTED: nuclear autoantigen Sp-100 [Pan paniscus]

GGACGTGACTATGGCTCTTCAGAATCCAGTGAGGAGGAGATACCCTCAGCGCTCCGGATC

TCAACACCAACAGATTCTGTGAGGATGTCTACTTGGAGGATACACAGTCAGAAAAGAAGG

CACAGCAGTGACAGCTCTTCAGAGCTGAGTTATGGAGAGGAGCCTCGGGAAGCCCTGAGA

AGTGGATCAGGTGTGCCAAGAGGCCAAGAAGCAACGGTGGAAAGTGGCCGAGCATCTCAC

CCAATAGGCAAGTCTGCTCAATGGCATCAGGGTGGAGGTGGCTCAGAAATACCACAGCTG

CTGGGCAGGACTGTGTCAGGAGAGGAAGCTGTAAAAGATACCACGGATGTTGGAAACAAT

TCTACTTTGGGAAAAGGCAGTGGGGAAAGAAATATTTCCAAGCTGCACAACAGAGATAAA

GAAATGAACAAAATCTCATCAACCTTACATCCTACTGTGGGGAAAAAAAGACTTCATAAA

GTAAACCATCTCCAAAAAGAAATAAAGAAAGGCAGACATAGATTCCACTCAATTCAGAAT

AATGCCCTTCAAAGGAAAAGAGGTTGGCCAAAAGGACCAAGAATTCCCAGAGAAAAAAAT

GTGAATTTCAACCTTCCTAAACTTCCTGTGACCTGTGGTGAGGCTAAGGGGACTCTCCAT

AAGAAGCAATTTAAGCAAGGGGCCCAGATGAAGAGCATAAGGACTGAGGATGGAAAATGG

CTCACCCCCAGGGACTTCGAGATCAGAGGAAACCACGCAGCAGCCAAGAACTGGAAGCTA

AGCCTGCGCTGCCACGGATGGACCTTGAAAGAACTGATAGAGGCAGGGCATCTACCAAAC

CCACCAATAACAAGAAAAAAGAGGAAAACACGGGAGTCTCTCAACAATGTCTTAGTTGAC

CCCCATCAAAAAAACTCAAATAAGTGCGAAGTATGCCGCCAAGAGAAACAGCTGGTCTTC

TGCAATACTTGTTCAAGATCATACCATCAGAACTGCCACATCCCACCAGTAGATGCTCAG

AGGAGCCAGTGGAGTTGCACCTTATGTAGGACAAAAGCCTGGGAAAAGCACCCAGAAAGC

CAACCATGTCATCTGGAATCTGAGGTCTTGAAGAGGCCTATGCTGCCTGAGGAACAGCTG

AAATGTGAGCTGATCCTATTGAAGGTCTATTGTTATTCACAAAGTGCCTTTTTTGCCCAA

GAACCATATTATACTCAAGACCCCCAGGAGCACATGTGGTTAAACAAGGTCAAGGAAAGG

CTGAATAAGAAGAAGTACCACCGAGTGGAGGGGTTTGTGCGGGACATGCGCCTCATCTTT

CTGAACAACAAGGCCTTCTACAGAAACCACAAATTCATCCAGCTGCCTCTTAAGATAGAA

GCCATATTTGAGAAGTATTTCAAAAACATTTTTTCAATCCAAGAAACAAGCGAGAGCATC

TGTCAGTTT

>CL356.Contig36_A-W 134 1573 minus strand PREDICTED: nuclear autoantigen Sp-100 [Callithrix jacchus]

GGACGTGACTATGGCTCTTCAGAATCCAGTGAGGAGGAGATACCCTCAGCGCTCCGGATC

TCAACACCAACAGATTCTGTGAGGATGTCTACTTGGAGGATACACAGTCAGAAAAGAAGG

CACAGCAGTGACAGCTCTTCAGAGCTGAGTTATGGAGAGGAGCCTCGGGAAGCCCTGAGA

AGTGGATCAGGAACAGGGCAACAAGAGACTGGAAATGAGAAGTGCTCCTGTGTCCTGTGT

TCCCCAAAAGGTGTGCCAAGAGGCCAAGAAGCAACGGTGGAAAGTGGCCGAGCATCTCAC

CCAATAGGCAAGTCTGCTCAATGGCATCAGGGTGGAGGTGGCTCAGAAATACCACAGCTG

CTGGGCAGGACTGTGTCAGGAGAGGAAGCTGTAAAAGATACCACGGATGTTGGAAACAAT

TCTACTTTGGGAAAAGGCAGTGGGGAAAGAAGGAAAAAAAGACTTCATAAAGTAAACCAT

CTCCAAAAAGAAATAAAGAAAGGCAGACATAGATTCCACTCAATTCAGAATAATGCCCTT

CAAAGGAAAAGAGGTTGGCCAAAAGGACCAAGAATTCCCAGAGAAAAAAATGTGAATTTC

AACCTTCCTAAACTTCCTGTGACCTGTGGTGAGGCTAAGGGGACTCTCCATAAGAAGCAA

TTTAAGCAAGGGGCCCAGATGAAGAGCATAAGGACTGAGGATGGAAAATGGCTCACCCCC

AGGGACTTCGAGATCAGAGGAAACCACGCAGCAGCCAAGAACTGGAAGCTAAGCCTGCGC

TGCCACGGATGGACCTTGAAAGAACTGATAGAGGCAGGGCATCTACCAAACCCACCAATA

ACAAGAAAAAAGAGGAAAACACGGGAGTCTCTCAACAATGTCTTAGTTGACCCCCATCAA

AAAAACTCAAATAAGTGCGAAGTATGCCGCCAAGAGAAACAGCTGGTCTTCTGCAATACT

TGTTCAAGATCATACCATCAGAACTGCCACATCCCACCAGTAGATGCTCAGAGGAGCCAG

TGGAGTTGCACCTTATGTAGGACAAAAGCCTGGGAAAAGCACCCAGAAAGCCAACCATGT

CATCTGGAATCTGAGGTCTTGAAGAGGCCTATGCTGCCTGAGGAACAGCTGAAATGTGAG

CTGATCCTATTGAAGGTCTATTGTTATTCACAAAGTGCCTTTTTTGCCCAAGAACCATAT

TATACTCAAGACCCCCAGGAGCACATGTGGTTAAACAAGGTCAAGGAAAGGCTGAATAAG

AAGAAGTACCACCGAGTGGAGGGGTTTGTGCGGGACATGCGCCTCATCTTTCTGAACAAC

AAGGCCTTCTACAGAAACCACAAATTCATCCAGCTGCCTCTTAAGATAGAAGCCATATTT

GAGAAGTATTTCAAAAACATTTTTTCAATCCAAGAAACAAGCGAGAGCATCTGTCAGTTT

>CL356.Contig37_A-W 28 2466 minus strand PREDICTED: nuclear autoantigen Sp-100 [Callithrix jacchus]

ATATTTTGTGATATATTTTTCAACCACTTCAAGAAAAATAAAGTGGAGATAGCAACTGCA

ATAACAAAGCTGTTTCCTTTCCTAGAGGGCCTCAGAGACTGATCCTTTATCACCGAACAA

ATCTATGCTGATTCTCAAGAAGCCTTTAGAAACTTGGTTCCTGTAGCCAGAGTGGTGTAC

CATGTTCTTGGCCACCTTGAGAAGGTGTTTGATTGGTCACTTCTACAAACCCTGTTCAGC

AAAGTGAACCTGAAAGAGTACCCTGGCTTAATACAGATTTATAAAACCTTTGAAAATGTG

TTCCAAGAAAAATATGTTTCCCTAAGAAGTGACCCGGAAGGAAGACAACAAACATCCATC

ACTCAGCCAAGCCGTGAGCAAGGAACAGTTGAGAACACGTTTCTGCGAAGACTGACCTGG

CCACAGCACTCAGATCCCTCGTCTTACAATGGTACAGACCTACCCGAAAATGAACTCTTG

GCGCATCCTTGCGAAGCAGAAGAGAGAAATGTAAATAAAAGAGAGCCGGCCCGAGACCCA

AATGGTGCAGTAGGAAGCCACCAAGGAAATGAGCAATGCCCTCGAGAGTCAGAGCCAGTA

GAGTCCCACCAACACATGGCTGCCAACGTGGACCGTGGAGAGGCGAGCGTGGGGACACCC

AGCCCGTTGCCCCACTGTGTAGAAAGAGCAGAGCTCTCCAGCCATGGAATCCACATGACT

TCCTGTTCTGTGCATCTGGTGGATATAAAGAAGGAAAAGCCATTTTTTCCCACGCAAATG

AAGCAAGGATCTCAAACCAGGACCAACCATTACCAGCCATCCGAAGTAATAGTCATCAGC

AGTGAGGACGACGCCAGCGACACAGAAGAGCCTGCAGCAATCTCCAGCTCAGCACGGAGG

AGCCATCCTGTGATCAAAAACCATGTCCAGTTTGACTTGAGAGATGAAGAAGAGGCCCAA

GAAGCCACTTGCTCATGGCACCAGGCTGCTCCAGCGAGAGGACGTGACTATGGCTCTTCA

GAATCCAGTGAGGAGGAGATACCCTCAGCGCTCCGGATCTCAACACCAACAGATTCTGTG

AGGATGTCTACTTGGAGGATACACAGTCAGAAAAGAAGGCACAGCAGTGACAGCTCTTCA

GAGCTGAGTTATGGAGAGGAGCCTCGGGAAGCCCTGAGAAGTGGATCAGGAACAGGGCAA

CAAGAGACTGGAAATGAGAAGTGCTCCTGTGTCCTGTGTTCCCCAAAAGGTGTGCCAAGA

GGCCAAGAAGCAACGGTGGAAAGTGGCCGAGCATCTCACCCAATAGGCAAGTCTGCTCAA

TGGCATCAGGGTGGAGGTGGCTCAGAAATACCACAGCTGCTGGGCAGGACTGTGTCAGGA

GAGGAAGCTGTAAAAGATACCACGGATGTTGGAAACAATTCTACTTTGGGAAAAGGCAGT

GGGGAAAGAAGGAAAAAAAGACTTCATAAAGTAAACCATCTCCAAAAAGAAATAAAGAAA

GGCAGACATAGATTCCACTCAATTCAGAATAATGCCCTTCAAAGGAAAAGAGGTTGGCCA

AAAGGACCAAGAATTCCCAGAGAAAAAAATGTGAATTTCAACCTTCCTAAACTTCCTGTG

ACCTGTGGTGAGGCTAAGGGGACTCTCCATAAGAAGCAATTTAAGCAAGGGGCCCAGATG

AAGAGCATAAGGACTGAGGATGGAAAATGGCTCACCCCCAGGGACTTCGAGATCAGAGGA

AACCACGCAGCAGCCAAGAACTGGAAGCTAAGCCTGCGCTGCCACGGATGGACCTTGAAA

GAACTGATAGAGGCAGGGCATCTACCAAACCCACCAATAACAAGAAAAAAGAGGAAAACA

CGGGAGTCTCTCAACAATGTCTTAGTTGACCCCCATCAAAAAAACTCAAATAAGTGCGAA

GTATGCCGCCAAGAGAAACAGCTGGTCTTCTGCAATACTTGTTCAAGATCATACCATCAG

AACTGCCACATCCCACCAGTAGATGCTCAGAGGAGCCAGTGGAGTTGCACCTTATGTAGG

ACAAAAGCCTGGGAAAAGCACCCAGAAAGCCAACCATGTCATCTGGAATCTGAGGTCTTG

AAGAGGCCTATGCTGCCTGAGGAACAGCTGAAATGTGAGCTGATCCTATTGAAGGTCTAT

TGTTATTCACAAAGTGCCTTTTTTGCCCAAGAACCATATTATACTCAAGACCCCCAGGAG

CACATGTGGTTAAACAAGGTCAAGGAAAGGCTGAATAAGAAGAAGTACCACCGAGTGGAG

GGGTTTGTGCGGGACATGCGCCTCATCTTTCTGAACAACAAGGCCTTCTACAGAAACCAC

AAATTCATCCAGCTGCCTCTTAAGATAGAAGCCATATTTGAGAAGTATTTCAAAAACATT

TTTTCAATCCAAGAAACAAGCGAGAGCATCTGTCAGTTT

>CL356.Contig38_A-W 134 1561 minus strand PREDICTED: nuclear autoantigen Sp-100 [Pan paniscus]

GGACGTGACTATGGCTCTTCAGAATCCAGTGAGGAGGAGATACCCTCAGCGCTCCGGATC

TCAACACCAACAGATTCTGTGAGGATGTCTACTTGGAGGATACACAGTCAGAAAAGAAGG

CACAGCAGTGACAGCTCTTCAGAGCTGAGTTATGGAGAGGAGCCTCGGGAAGCCCTGAGA

AGTGGATCAGGTGTGCCAAGAGGCCAAGAAGCAACGGTGGAAAGTGGCCGAGCATCTCAC

CCAATAGGCAAGTCTGCTCAATGGCATCAGGGTGGAGGTGGCTCAGAAATACCACAGCTG

CTGGGCAGGACTGTGTCAGGAGAGGAAGCTGTAAAAGATACCACGGATGTTGGAAACAAT

TCTACTTTGGGAAAAGGCAGTGGGGAAAGAAGGAAAAAAAGACTTCATAAAGTAAACCAT

CTCCAAAAAGAAATAAAGAAAGGCAGACATAGATTCCACTCAATTCAGAATAATGCCCTT

CAAAGGAAAAGAGGTTGGCCAAAAGGGAGAAAAAGAGTCAACGTTAGACCCTTGAAAAGA

GGGAGAAAAAGAGGACCAAGAATTCCCAGAGAAAAAAATGTGAATTTCAACCTTCCTAAA

CTTCCTGTGACCTGTGGTGAGGCTAAGGGGACTCTCCATAAGAAGCAATTTAAGCAAGGG

GCCCAGATGAAGAGCATAAGGACTGAGGATGGAAAATGGCTCACCCCCAGGGACTTCGAG

ATCAGAGGAAACCACGCAGCAGCCAAGAACTGGAAGCTAAGCCTGCGCTGCCACGGATGG

ACCTTGAAAGAACTGATAGAGGCAGGGCATCTACCAAACCCACCAATAACAAGAAAAAAG

AGGAAAACACGGGAGTCTCTCAACAATGTCTTAGTTGACCCCCATCAAAAAAACTCAAAT

AAGTGCGAAGTATGCCGCCAAGAGAAACAGCTGGTCTTCTGCAATACTTGTTCAAGATCA

TACCATCAGAACTGCCACATCCCACCAGTAGATGCTCAGAGGAGCCAGTGGAGTTGCACC

TTATGTAGGACAAAAGCCTGGGAAAAGCACCCAGAAAGCCAACCATGTCATCTGGAATCT

GAGGTCTTGAAGAGGCCTATGCTGCCTGAGGAACAGCTGAAATGTGAGCTGATCCTATTG

AAGGTCTATTGTTATTCACAAAGTGCCTTTTTTGCCCAAGAACCATATTATACTCAAGAC

CCCCAGGAGCACATGTGGTTAAACAAGGTCAAGGAAAGGCTGAATAAGAAGAAGTACCAC

CGAGTGGAGGGGTTTGTGCGGGACATGCGCCTCATCTTTCTGAACAACAAGGCCTTCTAC

AGAAACCACAAATTCATCCAGCTGCCTCTTAAGATAGAAGCCATATTTGAGAAGTATTTC

AAAAACATTTTTTCAATCCAAGAAACAAGCGAGAGCATCTGTCAGTTT

>CL356.Contig39_A-W 134 1552 minus strand PREDICTED: nuclear autoantigen Sp-100 [Callithrix jacchus]

GGACGTGACTATGGCTCTTCAGAATCCAGTGAGGAGGAGATACCCTCAGCGCTCCGGATC

TCAACACCAACAGATTCTGTGAGGATGTCTACTTGGAGGATACACAGTCAGAAAAGAAGG

CACAGCAGTGACAGCTCTTCAGAGCTGAGTTATGGAGAGGAGCCTCGGGAAGCCCTGAGA

AGTGGATCAGGAACAGGGCAACAAGAGACTGGAAATGAGAAGTGCTCCTGTGTCCTGTGT

TCCCCAAAAGGTGTGCCAAGAGGCCAAGAAGCAACGGTGGAAAGTGGCCGAGCATCTCAC

CCAATAGATACCACGGATGTTGGAAACAATTCTACTTTGGGAAAAGGCAGTGGGGAAAGA

AATATTTCCAAGCTGCACAACAGAGATAAAGAAATGAACAAAATCTCATCAACCTTACAT

CCTACTGTGGGGAAAAAAAGACTTCATAAAGTAAACCATCTCCAAAAAGAAATAAAGAAA

GGCAGACATAGATTCCACTCAATTCAGAATAATGCCCTTCAAAGGAAAAGAGGTTGGCCA

AAAGGACCAAGAATTCCCAGAGAAAAAAATGTGAATTTCAACCTTCCTAAACTTCCTGTG

ACCTGTGGTGAGGCTAAGGGGACTCTCCATAAGAAGCAATTTAAGCAAGGGGCCCAGATG

AAGAGCATAAGGACTGAGGATGGAAAATGGCTCACCCCCAGGGACTTCGAGATCAGAGGA

AACCACGCAGCAGCCAAGAACTGGAAGCTAAGCCTGCGCTGCCACGGATGGACCTTGAAA

GAACTGATAGAGGCAGGGCATCTACCAAACCCACCAATAACAAGAAAAAAGAGGAAAACA

CGGGAGTCTCTCAACAATGTCTTAGTTGACCCCCATCAAAAAAACTCAAATAAGTGCGAA

GTATGCCGCCAAGAGAAACAGCTGGTCTTCTGCAATACTTGTTCAAGATCATACCATCAG

AACTGCCACATCCCACCAGTAGATGCTCAGAGGAGCCAGTGGAGTTGCACCTTATGTAGG

ACAAAAGCCTGGGAAAAGCACCCAGAAAGCCAACCATGTCATCTGGAATCTGAGGTCTTG

AAGAGGCCTATGCTGCCTGAGGAACAGCTGAAATGTGAGCTGATCCTATTGAAGGTCTAT

TGTTATTCACAAAGTGCCTTTTTTGCCCAAGAACCATATTATACTCAAGACCCCCAGGAG

CACATGTGGTTAAACAAGGTCAAGGAAAGGCTGAATAAGAAGAAGTACCACCGAGTGGAG

GGGTTTGTGCGGGACATGCGCCTCATCTTTCTGAACAACAAGGCCTTCTACAGAAACCAC

AAATTCATCCAGCTGCCTCTTAAGATAGAAGCCATATTTGAGAAGTATTTCAAAAACATT

TTTTCAATCCAAGAAACAAGCGAGAGCATCTGTCAGTTT

>CL356.Contig40_A-W 134 1540 minus strand PREDICTED: nuclear autoantigen Sp-100 [Pan paniscus]

GGACGTGACTATGGCTCTTCAGAATCCAGTGAGGAGGAGATACCCTCAGCGCTCCGGATC

TCAACACCAACAGATTCTGTGAGGATGTCTACTTGGAGGATACACAGTCAGAAAAGAAGG

CACAGCAGTGACAGCTCTTCAGAGCTGAGTTATGGAGAGGAGCCTCGGGAAGCCCTGAGA

AGTGGATCAGGTGTGCCAAGAGGCCAAGAAGCAACGGTGGAAAGTGGCCGAGCATCTCAC

CCAATAGATACCACGGATGTTGGAAACAATTCTACTTTGGGAAAAGGCAGTGGGGAAAGA

AATATTTCCAAGCTGCACAACAGAGATAAAGAAATGAACAAAATCTCATCAACCTTACAT

CCTACTGTGGGGAAAAAAAGACTTCATAAAGTAAACCATCTCCAAAAAGAAATAAAGAAA

GGCAGACATAGATTCCACTCAATTCAGAATAATGCCCTTCAAAGGAAAAGAGGTTGGCCA

AAAGGGAGAAAAAGAGTCAACGTTAGACCCTTGAAAAGAGGGAGAAAAAGAGGACCAAGA

ATTCCCAGAGAAAAAAATGTGAATTTCAACCTTCCTAAACTTCCTGTGACCTGTGGTGAG

GCTAAGGGGACTCTCCATAAGAAGCAATTTAAGCAAGGGGCCCAGATGAAGAGCATAAGG

ACTGAGGATGGAAAATGGCTCACCCCCAGGGACTTCGAGATCAGAGGAAACCACGCAGCA

GCCAAGAACTGGAAGCTAAGCCTGCGCTGCCACGGATGGACCTTGAAAGAACTGATAGAG

GCAGGGCATCTACCAAACCCACCAATAACAAGAAAAAAGAGGAAAACACGGGAGTCTCTC

AACAATGTCTTAGTTGACCCCCATCAAAAAAACTCAAATAAGTGCGAAGTATGCCGCCAA

GAGAAACAGCTGGTCTTCTGCAATACTTGTTCAAGATCATACCATCAGAACTGCCACATC

CCACCAGTAGATGCTCAGAGGAGCCAGTGGAGTTGCACCTTATGTAGGACAAAAGCCTGG

GAAAAGCACCCAGAAAGCCAACCATGTCATCTGGAATCTGAGGTCTTGAAGAGGCCTATG

CTGCCTGAGGAACAGCTGAAATGTGAGCTGATCCTATTGAAGGTCTATTGTTATTCACAA

AGTGCCTTTTTTGCCCAAGAACCATATTATACTCAAGACCCCCAGGAGCACATGTGGTTA

AACAAGGTCAAGGAAAGGCTGAATAAGAAGAAGTACCACCGAGTGGAGGGGTTTGTGCGG

GACATGCGCCTCATCTTTCTGAACAACAAGGCCTTCTACAGAAACCACAAATTCATCCAG

CTGCCTCTTAAGATAGAAGCCATATTTGAGAAGTATTTCAAAAACATTTTTTCAATCCAA

GAAACAAGCGAGAGCATCTGTCAGTTT

>CL356.Contig41_A-W 144 2552 minus strand PREDICTED: nuclear autoantigen Sp-100 [Pan paniscus]

CCAGGAGGATCCCCGGACATCAGGATGTTTGCAATGGTCAAGAATAAGGAGATATTTTGT

GATATATTTTTCAACCACTTCAAGAAAAATAAAGTGGAGATAGCAACTGCAATAACAAAG

CTGTTTCCTTTCCTAGAGGGCCTCAGAGACTGATCCTTTATCACCGAACAAATCTATGCT

GATTCTCAAGAAGCCTTTAGAAACTTGGTTCCTGTAGCCAGAGTGGTGTACCATGTTCTT

GGCCACCTTGAGAAGGTGTTTGATTGGTCACTTCTACAAACCCTGTTCAGCAAAGTGAAC

CTGAAAGAGTACCCTGGCTTAATACAGATTTATAAAACCTTTGAAAATGTGTTCCAAGAA

AAATATGTTTCCCTAAGAAGTGACCCGGAAGGAAGACAACAAACATCCATCACTCAGCCA

AGCCGTGAGCAAGGAACAGTTGAGAACACGTTTCTGCGAAGACTGACCTGGCCACAGCAC

TCAGATCCCTCGTCTTACAATGGTACAGACCTACCCGAAAATGAACTCTTGGCGCATCCT

TGCGAAGCAGAAGAGAGAAATGTAAATAAAAGAGAGCCGGCCCGAGACCCAAATGGTGCA

GTAGGAAGCCACCAAGGAAATGAGCAATGCCCTCGAGAGTCAGAGCCAGTAGAGTCCCAC

CAACACATGGCTGCCAACGTGGACCGTGGAGAGGCGAGCGTGGGGACACCCAGCCCGTTG

CCCCACTGTGTAGAAAGAGCAGAGCTCTCCAGCCATGGAATCCACATGACTTCCTGTTCT

GTGCATCTGGTGGATATAAAGAAGGAAAAGCCATTTTTTCCCACGCAAATGAAGCAAGGA

TCTCAAACCAGGACCAACCATTACCAGCCATCCGAAGTAATAGTCATCAGCAGTGAGGAC

GACGCCAGCGACACAGAAGAGCCTGCAGCAATCTCCAGCTCAGCACGGAGGAGCCATCCT

GTGATCAAAAACCATGTCCAGTTTGACTTGAGAGATGAAGAAGAGGCCCAAGAAGCCACT

TGCTCATGGCACCAGGCTGCTCCAGCGAGAGGACGTGACTATGGCTCTTCAGAATCCAGT

GAGGAGGAGATACCCTCAGCGCTCCGGATCTCAACACCAACAGATTCTGTGAGGATGTCT

ACTTGGAGGATACACAGTCAGAAAAGAAGGCACAGCAGTGACAGCTCTTCAGAGCTGAGT

TATGGAGAGGAGCCTCGGGAAGCCCTGAGAAGTGGATCAGGTGTGCCAAGAGGCCAAGAA

GCAACGGTGGAAAGTGGCCGAGCATCTCACCCAATAGATACCACGGATGTTGGAAACAAT

TCTACTTTGGGAAAAGGCAGTGGGGAAAGAAATATTTCCAAGCTGCACAACAGAGATAAA

GAAATGAACAAAATCTCATCAACCTTACATCCTACTGTGGGGAAAAAAAGACTTCATAAA

GTAAACCATCTCCAAAAAGAAATAAAGAAAGGCAGACATAGATTCCACTCAATTCAGAAT

AATGCCCTTCAAAGGAAAAGAGGTTGGCCAAAAGGACCAAGAATTCCCAGAGAAAAAAAT

GTGAATTTCAACCTTCCTAAACTTCCTGTGACCTGTGGTGAGGCTAAGGGGACTCTCCAT

AAGAAGCAATTTAAGCAAGGGGCCCAGATGAAGAGCATAAGGACTGAGGATGGAAAATGG

CTCACCCCCAGGGACTTCGAGATCAGAGGAAACCACGCAGCAGCCAAGAACTGGAAGCTA

AGCCTGCGCTGCCACGGATGGACCTTGAAAGAACTGATAGAGGCAGGGCATCTACCAAAC

CCACCAATAACAAGAAAAAAGAGGAAAACACGGGAGTCTCTCAACAATGTCTTAGTTGAC

CCCCATCAAAAAAACTCAAATAAGTGCGAAGTATGCCGCCAAGAGAAACAGCTGGTCTTC

TGCAATACTTGTTCAAGATCATACCATCAGAACTGCCACATCCCACCAGTAGATGCTCAG

AGGAGCCAGTGGAGTTGCACCTTATGTAGGACAAAAGCCTGGGAAAAGCACCCAGAAAGC

CAACCATGTCATCTGGAATCTGAGGTCTTGAAGAGGCCTATGCTGCCTGAGGAACAGCTG

AAATGTGAGCTGATCCTATTGAAGGTCTATTGTTATTCACAAAGTGCCTTTTTTGCCCAA

GAACCATATTATACTCAAGACCCCCAGGAGCACATGTGGTTAAACAAGGTCAAGGAAAGG

CTGAATAAGAAGAAGTACCACCGAGTGGAGGGGTTTGTGCGGGACATGCGCCTCATCTTT

CTGAACAACAAGGCCTTCTACAGAAACCACAAATTCATCCAGCTGCCTCTTAAGATAGAA

GCCATATTTGAGAAGTATTTCAAAAACATTTTTTCAATCCAAGAAACAAGCGAGAGCATC

TGTCAGTTT

>CL356.Contig42_A-W 134 1513 minus strand PREDICTED: nuclear autoantigen Sp-100 [Pan paniscus]

GGACGTGACTATGGCTCTTCAGAATCCAGTGAGGAGGAGATACCCTCAGCGCTCCGGATC

TCAACACCAACAGATTCTGTGAGGATGTCTACTTGGAGGATACACAGTCAGAAAAGAAGG

CACAGCAGTGACAGCTCTTCAGAGCTGAGTTATGGAGAGGAGCCTCGGGAAGCCCTGAGA

AGTGGATCAGGTGTGCCAAGAGGCCAAGAAGCAACGGTGGAAAGTGGCCGAGCATCTCAC

CCAATAGGCAAGTCTGCTCAATGGCATCAGGGTGGAGGTGGCTCAGAAATACCACAGCTG

CTGGGCAGGACTGTGTCAGGAGAGGAAGCTGTAAAAGATACCACGGATGTTGGAAACAAT

TCTACTTTGGGAAAAGGCAGTGGGGAAAGAAGGAAAAAAAGACTTCATAAAGTAAACCAT

CTCCAAAAAGAAATAAAGAAAGGCAGACATAGATTCCACTCAATTCAGAATAATGCCCTT

CAAAGGAAAAGAGGTTGGCCAAAAGGACCAAGAATTCCCAGAGAAAAAAATGTGAATTTC

AACCTTCCTAAACTTCCTGTGACCTGTGGTGAGGCTAAGGGGACTCTCCATAAGAAGCAA

TTTAAGCAAGGGGCCCAGATGAAGAGCATAAGGACTGAGGATGGAAAATGGCTCACCCCC

AGGGACTTCGAGATCAGAGGAAACCACGCAGCAGCCAAGAACTGGAAGCTAAGCCTGCGC

TGCCACGGATGGACCTTGAAAGAACTGATAGAGGCAGGGCATCTACCAAACCCACCAATA

ACAAGAAAAAAGAGGAAAACACGGGAGTCTCTCAACAATGTCTTAGTTGACCCCCATCAA

AAAAACTCAAATAAGTGCGAAGTATGCCGCCAAGAGAAACAGCTGGTCTTCTGCAATACT

TGTTCAAGATCATACCATCAGAACTGCCACATCCCACCAGTAGATGCTCAGAGGAGCCAG

TGGAGTTGCACCTTATGTAGGACAAAAGCCTGGGAAAAGCACCCAGAAAGCCAACCATGT

CATCTGGAATCTGAGGTCTTGAAGAGGCCTATGCTGCCTGAGGAACAGCTGAAATGTGAG

CTGATCCTATTGAAGGTCTATTGTTATTCACAAAGTGCCTTTTTTGCCCAAGAACCATAT

TATACTCAAGACCCCCAGGAGCACATGTGGTTAAACAAGGTCAAGGAAAGGCTGAATAAG

AAGAAGTACCACCGAGTGGAGGGGTTTGTGCGGGACATGCGCCTCATCTTTCTGAACAAC

AAGGCCTTCTACAGAAACCACAAATTCATCCAGCTGCCTCTTAAGATAGAAGCCATATTT

GAGAAGTATTTCAAAAACATTTTTTCAATCCAAGAAACAAGCGAGAGCATCTGTCAGTTT

>CL356.Contig43_A-W 134 1492 minus strand PREDICTED: nuclear autoantigen Sp-100 [Pan paniscus]

GGACGTGACTATGGCTCTTCAGAATCCAGTGAGGAGGAGATACCCTCAGCGCTCCGGATC

TCAACACCAACAGATTCTGTGAGGATGTCTACTTGGAGGATACACAGTCAGAAAAGAAGG

CACAGCAGTGACAGCTCTTCAGAGCTGAGTTATGGAGAGGAGCCTCGGGAAGCCCTGAGA

AGTGGATCAGGTGTGCCAAGAGGCCAAGAAGCAACGGTGGAAAGTGGCCGAGCATCTCAC

CCAATAGATACCACGGATGTTGGAAACAATTCTACTTTGGGAAAAGGCAGTGGGGAAAGA

AATATTTCCAAGCTGCACAACAGAGATAAAGAAATGAACAAAATCTCATCAACCTTACAT

CCTACTGTGGGGAAAAAAAGACTTCATAAAGTAAACCATCTCCAAAAAGAAATAAAGAAA

GGCAGACATAGATTCCACTCAATTCAGAATAATGCCCTTCAAAGGAAAAGAGGTTGGCCA

AAAGGACCAAGAATTCCCAGAGAAAAAAATGTGAATTTCAACCTTCCTAAACTTCCTGTG

ACCTGTGGTGAGGCTAAGGGGACTCTCCATAAGAAGCAATTTAAGCAAGGGGCCCAGATG

AAGAGCATAAGGACTGAGGATGGAAAATGGCTCACCCCCAGGGACTTCGAGATCAGAGGA

AACCACGCAGCAGCCAAGAACTGGAAGCTAAGCCTGCGCTGCCACGGATGGACCTTGAAA

GAACTGATAGAGGCAGGGCATCTACCAAACCCACCAATAACAAGAAAAAAGAGGAAAACA

CGGGAGTCTCTCAACAATGTCTTAGTTGACCCCCATCAAAAAAACTCAAATAAGTGCGAA

GTATGCCGCCAAGAGAAACAGCTGGTCTTCTGCAATACTTGTTCAAGATCATACCATCAG

AACTGCCACATCCCACCAGTAGATGCTCAGAGGAGCCAGTGGAGTTGCACCTTATGTAGG

ACAAAAGCCTGGGAAAAGCACCCAGAAAGCCAACCATGTCATCTGGAATCTGAGGTCTTG

AAGAGGCCTATGCTGCCTGAGGAACAGCTGAAATGTGAGCTGATCCTATTGAAGGTCTAT

TGTTATTCACAAAGTGCCTTTTTTGCCCAAGAACCATATTATACTCAAGACCCCCAGGAG

CACATGTGGTTAAACAAGGTCAAGGAAAGGCTGAATAAGAAGAAGTACCACCGAGTGGAG

GGGTTTGTGCGGGACATGCGCCTCATCTTTCTGAACAACAAGGCCTTCTACAGAAACCAC

AAATTCATCCAGCTGCCTCTTAAGATAGAAGCCATATTTGAGAAGTATTTCAAAAACATT

TTTTCAATCCAAGAAACAAGCGAGAGCATCTGTCAGTTT

>CL356.Contig44_A-W 15 1484 minus strand PREDICTED: nuclear autoantigen Sp-100 isoform 14 [Pan troglodytes]

TCTACTTGGAGGATACACAGTCAGAAAAGAAGGCACAGCAGTGACAGCTCTTCAGAGCTG

AGTTATGGAGAGGAGCCTCGGGAAGCCCTGAGAAGTGGATCAGGAACAGGGCAACAAGAG

ACTGGAAATGAGAAGTGCTCCTGTGTCCTGTGTTCCCCAAAAGGTGTGCCAAGAGGCCAA

GAAGCAACGGTGGAAAGTGGCCGAGCATCTCACCCAATAGGCAAGTCTGCTCAATGGCAT

CAGGGTGGAGGTGGCTCAGAAATACCACAGCTGCTGGGCAGGACTGTGTCAGGAGAGGAA

GCTGTAAAAGATACCACGGATGTTGGAAACAATTCTACTTTGGGAAAAGGCAGTGGGGAA

AGAAATATTTCCAAGCTGCACAACAGAGATAAAGAAATGAACAAAATCTCATCAACCTTA

CATCCTACTGTGGGGAAAAAAAGACTTCATAAAGTAAACCATCTCCAAAAAGAAATAAAG

AAAGGCAGACATAGATTCCACTCAATTCAGAATAATGCCCTTCAAAGGAAAAGAGGTTGG

CCAAAAGGGAGAAAAAGAGTCAACGTTAGACCCTTGAAAAGAGGGAGAAAAAGAGGACCA

AGAATTCCCAGAGAAAAAAATGTGAATTTCAACCTTCCTAAACTTCCTGTGACCTGTGGT

GAGGCTAAGGGGACTCTCCATAAGAAGCAATTTAAGCAAGGGGCCCAGATGAAGAGCATA

AGGACTGAGGATGGAAAATGGCTCACCCCCAGGGACTTCGAGATCAGAGGAAACCACGCA

GCAGCCAAGAACTGGAAGCTAAGCCTGCGCTGCCACGGATGGACCTTGAAAGAACTGATA

GAGGCAGGGCATCTACCAAACCCACCAATAACAAGAAAAAAGAGGAAAACACGGGAGTCT

CTCAACAATGTCTTAGTTGACCCCCATCAAAAAAACTCAAATAAGTGCGAAGTATGCCGC

CAAGAGAAACAGCTGGTCTTCTGCAATACTTGTTCAAGATCATACCATCAGAACTGCCAC

ATCCCACCAGTAGATGCTCAGAGGAGCCAGTGGAGTTGCACCTTATGTAGGACAAAAGCC

TGGGAAAAGCACCCAGAAAGCCAACCATGTCATCTGGAATCTGAGGTCTTGAAGAGGCCT

ATGCTGCCTGAGGAACAGCTGAAATGTGAGCTGATCCTATTGAAGGTCTATTGTTATTCA

CAAAGTGCCTTTTTTGCCCAAGAACCATATTATACTCAAGACCCCCAGGAGCACATGTGG

TTAAACAAGGTCAAGGAAAGGCTGAATAAGAAGAAGTACCACCGAGTGGAGGGGTTTGTG

CGGGACATGCGCCTCATCTTTCTGAACAACAAGGCCTTCTACAGAAACCACAAATTCATC

CAGCTGCCTCTTAAGATAGAAGCCATATTTGAGAAGTATTTCAAAAACATTTTTTCAATC

CAAGAAACAAGCGAGAGCATCTGTCAGTTT

>CL356.Contig45_A-W 1010 1318 minus strand PREDICTED: nuclear autoantigen Sp-100 isoform 14 [Pan troglodytes]

AAATGTGAGCTGATCCTATTGAAGGTCTATTGTTATTCACAAAGTGCCTTTTTTGCCCAA

GAACCATATTATACTCAAGACCCCCAGGAGCACATGTGGTTAAACAAGGTCAAGGAAAGG

CTGAATAAGAAGAAGTACCACCGAGTGGAGGGGTTTGTGCGGGACATGCGCCTCATCTTT

CTGAACAACAAGGCCTTCTACAGAAACCACAAATTCATCCAGCTGCCTCTTAAGATAGAA

GCCATATTTGAGAAGTATTTCAAAAACATTTTTTCAATCCAAGAAACAAGCGAGAGCATC

TGTCAGTTT

>CL356.Contig46_A-W 989 1297 minus strand PREDICTED: nuclear autoantigen Sp-100 isoform 14 [Pan troglodytes]

AAATGTGAGCTGATCCTATTGAAGGTCTATTGTTATTCACAAAGTGCCTTTTTTGCCCAA

GAACCATATTATACTCAAGACCCCCAGGAGCACATGTGGTTAAACAAGGTCAAGGAAAGG

CTGAATAAGAAGAAGTACCACCGAGTGGAGGGGTTTGTGCGGGACATGCGCCTCATCTTT

CTGAACAACAAGGCCTTCTACAGAAACCACAAATTCATCCAGCTGCCTCTTAAGATAGAA

GCCATATTTGAGAAGTATTTCAAAAACATTTTTTCAATCCAAGAAACAAGCGAGAGCATC

TGTCAGTTT

>CL356.Contig47_A-W 968 1276 minus strand PREDICTED: nuclear autoantigen Sp-100 isoform 14 [Pan troglodytes]

AAATGTGAGCTGATCCTATTGAAGGTCTATTGTTATTCACAAAGTGCCTTTTTTGCCCAA

GAACCATATTATACTCAAGACCCCCAGGAGCACATGTGGTTAAACAAGGTCAAGGAAAGG

CTGAATAAGAAGAAGTACCACCGAGTGGAGGGGTTTGTGCGGGACATGCGCCTCATCTTT

CTGAACAACAAGGCCTTCTACAGAAACCACAAATTCATCCAGCTGCCTCTTAAGATAGAA

GCCATATTTGAGAAGTATTTCAAAAACATTTTTTCAATCCAAGAAACAAGCGAGAGCATC

TGTCAGTTT

>CL356.Contig48_A-W 32 2455 minus strand PREDICTED: nuclear autoantigen Sp-100 [Pan paniscus]

GAATGTTTAAGTGTACATTTCCCCACACATTCTTGCCGGATGTTTGCAATGGTCAAGAAT

AAGGAGATATTTTGTGATATATTTTTCAACCACTTCAAGAAAAATAAAGTGGAGATAGCA

ACTGCAATAACAAAGCTGTTTCCTTTCCTAGAGGGCCTCAGAGACTGATCCTTTATCACC

GAACAAATCTATGCTGATTCTCAAGAAGCCTTTAGAAACTTGGTTCCTGTAGCCAGAGTG

GTGTACCATGTTCTTGGCCACCTTGAGAAGGTGTTTGATTGGTCACTTCTACAAACCCTG

TTCAGCAAAGTGAACCTGAAAGAGTACCCTGGCTTAATACAGATTTATAAAACCTTTGAA

AATGTGTTCCAAGAAAAATATGTTTCCCTAAGAAGTGACCCGGAAGGAAGACAACAAACA

TCCATCACTCAGCCAAGCCGTGAGCAAGGAACAGTTGAGAACACGTTTCTGCGAAGACTG

ACCTGGCCACAGCACTCAGATCCCTCGTCTTACAATGGTACAGACCTACCCGAAAATGAA

CTCTTGGCGCATCCTTGCGAAGCAGAAGAGAGAAATGTAAATAAAAGAGAGCCGGCCCGA

GACCCAAATGGTGCAGTAGGAAGCCACCAAGGAAATGAGCAATGCCCTCGAGAGTCAGAG

CCAGTAGAGTCCCACCAACACATGGCTGCCAACGTGGACCGTGGAGAGGCGAGCGTGGGG

ACACCCAGCCCGTTGCCCCACTGTGTAGAAAGAGCAGAGCTCTCCAGCCATGGAATCCAC

ATGACTTCCTGTTCTGTGCATCTGGTGGATATAAAGAAGGAAAAGCCATTTTTTCCCACG

CAAATGAAGCAAGGATCTCAAACCAGGACCAACCATTACCAGCCATCCGAAGTAATAGTC

ATCAGCAGTGAGGACGACGCCAGCGACACAGAAGAGCCTGCAGCAATCTCCAGCTCAGCA

CGGAGGAGCCATCCTGTGATCAAAAACCATGTCCAGTTTGACTTGAGAGATGAAGAAGAG

GCCCAAGAAGCCACTTGCTCATGGCACCAGGCTGCTCCAGCGAGAGGACGTGACTATGGC

TCTTCAGAATCCAGTGAGGAGGAGATACCCTCAGCGCTCCGGATCTCAACACCAACAGAT

TCTGTGAGGATGTCTACTTGGAGGATACACAGTCAGAAAAGAAGGCACAGCAGTGACAGC

TCTTCAGAGCTGAGTTATGGAGAGGAGCCTCGGGAAGCCCTGAGAAGTGGATCAGGTGTG

CCAAGAGGCCAAGAAGCAACGGTGGAAAGTGGCCGAGCATCTCACCCAATAGATACCACG

GATGTTGGAAACAATTCTACTTTGGGAAAAGGCAGTGGGGAAAGAAATATTTCCAAGCTG

CACAACAGAGATAAAGAAATGAACAAAATCTCATCAACCTTACATCCTACTGTGGGGAAA

AAAAGACTTCATAAAGTAAACCATCTCCAAAAAGAAATAAAGAAAGGCAGACATAGATTC

CACTCAATTCAGAATAATGCCCTTCAAAGGAAAAGAGGTTGGCCAAAAGGACCAAGAATT

CCCAGAGAAAAAAATGTGAATTTCAACCTTCCTAAACTTCCTGTGACCTGTGGTGAGGCT

AAGGGGACTCTCCATAAGAAGCAATTTAAGCAAGGGGCCCAGATGAAGAGCATAAGGACT

GAGGATGGAAAATGGCTCACCCCCAGGGACTTCGAGATCAGAGGAAACCACGCAGCAGCC

AAGAACTGGAAGCTAAGCCTGCGCTGCCACGGATGGACCTTGAAAGAACTGATAGAGGCA

GGGCATCTACCAAACCCACCAATAACAAGAAAAAAGAGGAAAACACGGGAGTCTCTCAAC

AATGTCTTAGTTGACCCCCATCAAAAAAACTCAAATAAGTGCGAAGTATGCCGCCAAGAG

AAACAGCTGGTCTTCTGCAATACTTGTTCAAGATCATACCATCAGAACTGCCACATCCCA

CCAGTAGATGCTCAGAGGAGCCAGTGGAGTTGCACCTTATGTAGGACAAAAGCCTGGGAA

AAGCACCCAGAAAGCCAACCATGTCATCTGGAATCTGAGGTCTTGAAGAGGCCTATGCTG

CCTGAGGAACAGCTGAAATGTGAGCTGATCCTATTGAAGGTCTATTGTTATTCACAAAGT

GCCTTTTTTGCCCAAGAACCATATTATACTCAAGACCCCCAGGAGCACATGTGGTTAAAC

AAGGTCAAGGAAAGGCTGAATAAGAAGAAGTACCACCGAGTGGAGGGGTTTGTGCGGGAC

ATGCGCCTCATCTTTCTGAACAACAAGGCCTTCTACAGAAACCACAAATTCATCCAGCTG

CCTCTTAAGATAGAAGCCATATTTGAGAAGTATTTCAAAAACATTTTTTCAATCCAAGAA

ACAAGCGAGAGCATCTGTCAGTTT

>CL356.Contig49_A-W 950 1258 minus strand PREDICTED: nuclear autoantigen Sp-100 [Pan paniscus]

AAATGTGAGCTGATCCTATTGAAGGTCTATTGTTATTCACAAAGTGCCTTTTTTGCCCAA

GAACCATATTATACTCAAGACCCCCAGGAGCACATGTGGTTAAACAAGGTCAAGGAAAGG

CTGAATAAGAAGAAGTACCACCGAGTGGAGGGGTTTGTGCGGGACATGCGCCTCATCTTT

CTGAACAACAAGGCCTTCTACAGAAACCACAAATTCATCCAGCTGCCTCTTAAGATAGAA

GCCATATTTGAGAAGTATTTCAAAAACATTTTTTCAATCCAAGAAACAAGCGAGAGCATC

TGTCAGTTT

>CL356.Contig50_A-W 941 1249 minus strand PREDICTED: nuclear autoantigen Sp-100 isoform 14 [Pan troglodytes]

AAATGTGAGCTGATCCTATTGAAGGTCTATTGTTATTCACAAAGTGCCTTTTTTGCCCAA

GAACCATATTATACTCAAGACCCCCAGGAGCACATGTGGTTAAACAAGGTCAAGGAAAGG

CTGAATAAGAAGAAGTACCACCGAGTGGAGGGGTTTGTGCGGGACATGCGCCTCATCTTT

CTGAACAACAAGGCCTTCTACAGAAACCACAAATTCATCCAGCTGCCTCTTAAGATAGAA

GCCATATTTGAGAAGTATTTCAAAAACATTTTTTCAATCCAAGAAACAAGCGAGAGCATC

TGTCAGTTT

>CL356.Contig51_A-W 929 1237 minus strand PREDICTED: nuclear autoantigen Sp-100 [Pan paniscus]

AAATGTGAGCTGATCCTATTGAAGGTCTATTGTTATTCACAAAGTGCCTTTTTTGCCCAA

GAACCATATTATACTCAAGACCCCCAGGAGCACATGTGGTTAAACAAGGTCAAGGAAAGG

CTGAATAAGAAGAAGTACCACCGAGTGGAGGGGTTTGTGCGGGACATGCGCCTCATCTTT

CTGAACAACAAGGCCTTCTACAGAAACCACAAATTCATCCAGCTGCCTCTTAAGATAGAA

GCCATATTTGAGAAGTATTTCAAAAACATTTTTTCAATCCAAGAAACAAGCGAGAGCATC

TGTCAGTTT

>CL356.Contig52_A-W 132 2552 minus strand PREDICTED: nuclear autoantigen Sp-100 [Pan paniscus]

AGGATGTCCACAGAAGAGCAGAGTGTAGACGACAGTACCATCTATGAGGTTATATTCCAT

CACTTCAAAAGACACAAAGTGGAGATTTCAAATGCCATAAAAAAGACATTTCCTTTCCTC

GAGGGCCTCCGTGACCGTGAACTCATTACCAATAAAATGTTTGAAGATTCTCAAGAGTCT

TGTAGAAACCTGGTCCCTGTACAAAGAGTGGTGTACAATGTTCTCTGTGAACTGGAGAAG

AAATTCAACCTGGATTTGTGCGAAGCATTGTTCAGTGAGGTCAACTTGCAGGAATACCCT

GATTTAATTCGTGTTCGTAGAAGCTTCGAACATGAGGTGAGAATGCTATATCCCCAAGAA

GATAATGGAGAAGAGAGGCAGGAAAGGCTTGAACAAGGAACGGGGGAGAACTCTTTTCTG

CGAAGACTGACCTGGCCACAGCACTCAGATCCCTCGTCTTACAATGGTACAGACCTACCC

GAAAATGAACTCTTGGCGCATCCTTGCGAAGCAGAAGAGAGAAATGTAAATAAAAGAGAG

CCGGCCCGAGACCCAAATGGTGCAGTAGGAAGCCACCAAGGAAATGAGCAATGCCCTCGA

GAGTCAGAGCCAGTAGAGTCCCACCAACACATGGCTGCCAACGTGGACCGTGGAGAGGCG

AGCGTGGGGACACCCAGCCCGTTGCCCCACTGTGTAGAAAGAGCAGAGCTCTCCAGCCAT

GGAATCCACATGACTTCCTGTTCTGTGCATCTGGTGGATATAAAGAAGGAAAAGCCATTT

TTTCCCACGCAAATGAAGCAAGGATCTCAAACCAGGACCAACCATTACCAGCCATCCGAA

GTAATAGTCATCAGCAGTGAGGACGACGCCAGCGACACAGAAGAGCCTGCAGCAATCTCC

AGCTCAGCACGGAGGAGCCATCCTGTGATCAAAAACCATGTCCAGTTTGACTTGAGAGAT

GAAGAAGAGGCCCAAGAAGCCACTTGCTCATGGCACCAGGCTGCTCCAGCGAGAGGACGT

GACTATGGCTCTTCAGAATCCAGTGAGGAGGAGATACCCTCAGCGCTCCGGATCTCAACA

CCAACAGATTCTGTGAGGATGTCTACTTGGAGGATACACAGTCAGAAAAGAAGGCACAGC

AGTGACAGCTCTTCAGAGCTGAGTTATGGAGAGGAGCCTCGGGAAGCCCTGAGAAGTGGA

TCAGGTGTGCCAAGAGGCCAAGAAGCAACGGTGGAAAGTGGCCGAGCATCTCACCCAATA

GATACCACGGATGTTGGAAACAATTCTACTTTGGGAAAAGGCAGTGGGGAAAGAAATATT

TCCAAGCTGCACAACAGAGATAAAGAAATGAACAAAATCTCATCAACCTTACATCCTACT

GTGGGGAAAAAAAGACTTCATAAAGTAAACCATCTCCAAAAAGAAATAAAGAAAGGCAGA

CATAGATTCCACTCAATTCAGAATAATGCCCTTCAAAGGAAAAGAGGTTGGCCAAAAGGG

AGAAAAAGAGTCAACGTTAGACCCTTGAAAAGAGGGAGAAAAAGAGGACCAAGAATTCCC

AGAGAAAAAAATGTGAATTTCAACCTTCCTAAACTTCCTGTGACCTGTGGTGAGGCTAAG

GGGACTCTCCATAAGAAGCAATTTAAGCAAGGGGCCCAGATGAAGAGCATAAGGACTGAG

GATGGAAAATGGCTCACCCCCAGGGACTTCGAGATCAGAGGAAACCACGCAGCAGCCAAG

AACTGGAAGCTAAGCCTGCGCTGCCACGGATGGACCTTGAAAGAACTGATAGAGGCAGGG

CATCTACCAAACCCACCAATAACAAGAAAAAAGAGGAAAACACGGGAGTCTCTCAACAAT

GTCTTAGTTGACCCCCATCAAAAAAACTCAAATAAGTGCGAAGTATGCCGCCAAGAGAAA

CAGCTGGTCTTCTGCAATACTTGTTCAAGATCATACCATCAGAACTGCCACATCCCACCA

GTAGATGCTCAGAGGAGCCAGTGGAGTTGCACCTTATGTAGGACAAAAGCCTGGGAAAAG

CACCCAGAAAGCCAACCATGTCATCTGGAATCTGAGGTCTTGAAGAGGCCTATGCTGCCT

GAGGAACAGCTGAAATGTGAGCTGATCCTATTGAAGGTCTATTGTTATTCACAAAGTGCC

TTTTTTGCCCAAGAACCATATTATACTCAAGACCCCCAGGAGCACATGTGGTTAAACAAG

GTCAAGGAAAGGCTGAATAAGAAGAAGTACCACCGAGTGGAGGGGTTTGTGCGGGACATG

CGCCTCATCTTTCTGAACAACAAGGCCTTCTACAGAAACCACAAATTCATCCAGCTGCCT

CTTAAGATAGAAGCCATATTTGAGAAGTATTTCAAAAACATTTTTTCAATCCAAGAAACA

AGCGAGAGCATCTGTCAGTTT

>CL356.Contig53_A-W 920 1228 minus strand PREDICTED: nuclear autoantigen Sp-100 isoform 14 [Pan troglodytes]

AAATGTGAGCTGATCCTATTGAAGGTCTATTGTTATTCACAAAGTGCCTTTTTTGCCCAA

GAACCATATTATACTCAAGACCCCCAGGAGCACATGTGGTTAAACAAGGTCAAGGAAAGG

CTGAATAAGAAGAAGTACCACCGAGTGGAGGGGTTTGTGCGGGACATGCGCCTCATCTTT

CTGAACAACAAGGCCTTCTACAGAAACCACAAATTCATCCAGCTGCCTCTTAAGATAGAA

GCCATATTTGAGAAGTATTTCAAAAACATTTTTTCAATCCAAGAAACAAGCGAGAGCATC

TGTCAGTTT

>CL356.Contig54_A-W 908 1216 minus strand PREDICTED: nuclear autoantigen Sp-100 [Pan paniscus]

AAATGTGAGCTGATCCTATTGAAGGTCTATTGTTATTCACAAAGTGCCTTTTTTGCCCAA

GAACCATATTATACTCAAGACCCCCAGGAGCACATGTGGTTAAACAAGGTCAAGGAAAGG

CTGAATAAGAAGAAGTACCACCGAGTGGAGGGGTTTGTGCGGGACATGCGCCTCATCTTT

CTGAACAACAAGGCCTTCTACAGAAACCACAAATTCATCCAGCTGCCTCTTAAGATAGAA

GCCATATTTGAGAAGTATTTCAAAAACATTTTTTCAATCCAAGAAACAAGCGAGAGCATC

TGTCAGTTT

>CL356.Contig55_A-W 26 1189 minus strand nuclear autoantigen Sp-100 isoform 1 [Macaca mulatta]

CTAGATACCACGGATGTTGGAAACAATTCTACTTTGGGAAAAGGCAGTGGGGAAAGAAAT

ATTTCCAAGCTGCACAACAGAGATAAAGAAATGAACAAAATCTCATCAACCTTACATCCT

ACTGTGGGGAAAAAAAGACTTCATAAAGTAAACCATCTCCAAAAAGAAATAAAGAAAGGC

AGACATAGATTCCACTCAATTCAGAATAATGCCCTTCAAAGGAAAAGAGGTTGGCCAAAA

GGGAGAAAAAGAGTCAACGTTAGACCCTTGAAAAGAGGGAGAAAAAGAGGACCAAGAATT

CCCAGAGAAAAAAATGTGAATTTCAACCTTCCTAAACTTCCTGTGACCTGTGGTGAGGCT

AAGGGGACTCTCCATAAGAAGCAATTTAAGCAAGGGGCCCAGATGAAGAGCATAAGGACT

GAGGATGGAAAATGGCTCACCCCCAGGGACTTCGAGATCAGAGGAAACCACGCAGCAGCC

AAGAACTGGAAGCTAAGCCTGCGCTGCCACGGATGGACCTTGAAAGAACTGATAGAGGCA

GGGCATCTACCAAACCCACCAATAACAAGAAAAAAGAGGAAAACACGGGAGTCTCTCAAC

AATGTCTTAGTTGACCCCCATCAAAAAAACTCAAATAAGTGCGAAGTATGCCGCCAAGAG

AAACAGCTGGTCTTCTGCAATACTTGTTCAAGATCATACCATCAGAACTGCCACATCCCA

CCAGTAGATGCTCAGAGGAGCCAGTGGAGTTGCACCTTATGTAGGACAAAAGCCTGGGAA

AAGCACCCAGAAAGCCAACCATGTCATCTGGAATCTGAGGTCTTGAAGAGGCCTATGCTG

CCTGAGGAACAGCTGAAATGTGAGCTGATCCTATTGAAGGTCTATTGTTATTCACAAAGT

GCCTTTTTTGCCCAAGAACCATATTATACTCAAGACCCCCAGGAGCACATGTGGTTAAAC

AAGGTCAAGGAAAGGCTGAATAAGAAGAAGTACCACCGAGTGGAGGGGTTTGTGCGGGAC

ATGCGCCTCATCTTTCTGAACAACAAGGCCTTCTACAGAAACCACAAATTCATCCAGCTG

CCTCTTAAGATAGAAGCCATATTTGAGAAGTATTTCAAAAACATTTTTTCAATCCAAGAA

ACAAGCGAGAGCATCTGTCAGTTT

>CL356.Contig56_A-W 881 1189 minus strand PREDICTED: nuclear autoantigen Sp-100 [Pan paniscus]

AAATGTGAGCTGATCCTATTGAAGGTCTATTGTTATTCACAAAGTGCCTTTTTTGCCCAA

GAACCATATTATACTCAAGACCCCCAGGAGCACATGTGGTTAAACAAGGTCAAGGAAAGG

CTGAATAAGAAGAAGTACCACCGAGTGGAGGGGTTTGTGCGGGACATGCGCCTCATCTTT

CTGAACAACAAGGCCTTCTACAGAAACCACAAATTCATCCAGCTGCCTCTTAAGATAGAA

GCCATATTTGAGAAGTATTTCAAAAACATTTTTTCAATCCAAGAAACAAGCGAGAGCATC

TGTCAGTTT

>CL356.Contig57_A-W 879 1187 minus strand PREDICTED: nuclear autoantigen Sp-100 isoform 14 [Pan troglodytes]

AAATGTGAGCTGATCCTATTGAAGGTCTATTGTTATTCACAAAGTGCCTTTTTTGCCCAA

GAACCATATTATACTCAAGACCCCCAGGAGCACATGTGGTTAAACAAGGTCAAGGAAAGG

CTGAATAAGAAGAAGTACCACCGAGTGGAGGGGTTTGTGCGGGACATGCGCCTCATCTTT

CTGAACAACAAGGCCTTCTACAGAAACCACAAATTCATCCAGCTGCCTCTTAAGATAGAA

GCCATATTTGAGAAGTATTTCAAAAACATTTTTTCAATCCAAGAAACAAGCGAGAGCATC

TGTCAGTTT

>CL356.Contig58_A-W 860 1168 minus strand PREDICTED: nuclear autoantigen Sp-100 [Pan paniscus]

AAATGTGAGCTGATCCTATTGAAGGTCTATTGTTATTCACAAAGTGCCTTTTTTGCCCAA

GAACCATATTATACTCAAGACCCCCAGGAGCACATGTGGTTAAACAAGGTCAAGGAAAGG

CTGAATAAGAAGAAGTACCACCGAGTGGAGGGGTTTGTGCGGGACATGCGCCTCATCTTT

CTGAACAACAAGGCCTTCTACAGAAACCACAAATTCATCCAGCTGCCTCTTAAGATAGAA

GCCATATTTGAGAAGTATTTCAAAAACATTTTTTCAATCCAAGAAACAAGCGAGAGCATC

TGTCAGTTT

>CL356.Contig59_A-W 191 2314 minus strand PREDICTED: nuclear autoantigen Sp-100 isoform 14 [Pan troglodytes]

TCAGTGTTCCAAGAAAAATATGTTTCCCTAAGAAGTGACCCGGAAGGAAGACAACAAACA

TCCATCACTCAGCCAAGCCGTGAGCAAGGAACAGTTGAGAACACGTTTCTGCGAAGACTG

ACCTGGCCACAGCACTCAGATCCCTCGTCTTACAATGGTACAGACCTACCCGAAAATGAA

CTCTTGGCGCATCCTTGCGAAGCAGAAGAGAGAAATGTAAATAAAAGAGAGCCGGCCCGA

GACCCAAATGGTGCAGTAGGAAGCCACCAAGGAAATGAGCAATGCCCTCGAGAGTCAGAG

CCAGTAGAGTCCCACCAACACATGGCTGCCAACGTGGACCGTGGAGAGGCGAGCGTGGGG

ACACCCAGCCCGTTGCCCCACTGTGTAGAAAGAGCAGAGCTCTCCAGCCATGGAATCCAC

ATGACTTCCTGTTCTGTGCATCTGGTGGATATAAAGAAGGAAAAGCCATTTTTTCCCACG

CAAATGAAGCAAGGATCTCAAACCAGGACCAACCATTACCAGCCATCCGAAGTAATAGTC

ATCAGCAGTGAGGACGACGCCAGCGACACAGAAGAGCCTGCAGCAATCTCCAGCTCAGCA

CGGAGGAGCCATCCTGTGATCAAAAACCATGTCCAGTTTGACTTGAGAGATGAAGAAGAG

GCCCAAGAAGCCACTTGCTCATGGCACCAGGCTGCTCCAGCGAGAGGACGTGACTATGGC

TCTTCAGAATCCAGTGAGGAGGAGATACCCTCAGCGCTCCGGATCTCAACACCAACAGAT

TCTGTGAGGATGTCTACTTGGAGGATACACAGTCAGAAAAGAAGGCACAGCAGTGACAGC

TCTTCAGAGCTGAGTTATGGAGAGGAGCCTCGGGAAGCCCTGAGAAGTGGATCAGGAACA

GGGCAACAAGAGACTGGAAATGAGAAGTGCTCCTGTGTCCTGTGTTCCCCAAAAGGTGTG

CCAAGAGGCCAAGAAGCAACGGTGGAAAGTGGCCGAGCATCTCACCCAATAGATACCACG

GATGTTGGAAACAATTCTACTTTGGGAAAAGGCAGTGGGGAAAGAAATATTTCCAAGCTG

CACAACAGAGATAAAGAAATGAACAAAATCTCATCAACCTTACATCCTACTGTGGGGAAA

AAAAGACTTCATAAAGTAAACCATCTCCAAAAAGAAATAAAGAAAGGCAGACATAGATTC

CACTCAATTCAGAATAATGCCCTTCAAAGGAAAAGAGGTTGGCCAAAAGGACCAAGAATT

CCCAGAGAAAAAAATGTGAATTTCAACCTTCCTAAACTTCCTGTGACCTGTGGTGAGGCT

AAGGGGACTCTCCATAAGAAGCAATTTAAGCAAGGGGCCCAGATGAAGAGCATAAGGACT

GAGGATGGAAAATGGCTCACCCCCAGGGACTTCGAGATCAGAGGAAACCACGCAGCAGCC

AAGAACTGGAAGCTAAGCCTGCGCTGCCACGGATGGACCTTGAAAGAACTGATAGAGGCA

GGGCATCTACCAAACCCACCAATAACAAGAAAAAAGAGGAAAACACGGGAGTCTCTCAAC

AATGTCTTAGTTGACCCCCATCAAAAAAACTCAAATAAGTGCGAAGTATGCCGCCAAGAG

AAACAGCTGGTCTTCTGCAATACTTGTTCAAGATCATACCATCAGAACTGCCACATCCCA

CCAGTAGATGCTCAGAGGAGCCAGTGGAGTTGCACCTTATGTAGGACAAAAGCCTGGGAA

AAGCACCCAGAAAGCCAACCATGTCATCTGGAATCTGAGGTCTTGAAGAGGCCTATGCTG

CCTGAGGAACAGCTGAAATGTGAGCTGATCCTATTGAAGGTCTATTGTTATTCACAAAGT

GCCTTTTTTGCCCAAGAACCATATTATACTCAAGACCCCCAGGAGCACATGTGGTTAAAC

AAGGTCAAGGAAAGGCTGAATAAGAAGAAGTACCACCGAGTGGAGGGGTTTGTGCGGGAC

ATGCGCCTCATCTTTCTGAACAACAAGGCCTTCTACAGAAACCACAAATTCATCCAGCTG

CCTCTTAAGATAGAAGCCATATTTGAGAAGTATTTCAAAAACATTTTTTCAATCCAAGAA

ACAAGCGAGAGCATCTGTCAGTTT

>CL356.Contig60_A-W 26 1141 minus strand PREDICTED: nuclear autoantigen Sp-100 [Callithrix jacchus]

CTAGATACCACGGATGTTGGAAACAATTCTACTTTGGGAAAAGGCAGTGGGGAAAGAAAT

ATTTCCAAGCTGCACAACAGAGATAAAGAAATGAACAAAATCTCATCAACCTTACATCCT

ACTGTGGGGAAAAAAAGACTTCATAAAGTAAACCATCTCCAAAAAGAAATAAAGAAAGGC

AGACATAGATTCCACTCAATTCAGAATAATGCCCTTCAAAGGAAAAGAGGTTGGCCAAAA

GGACCAAGAATTCCCAGAGAAAAAAATGTGAATTTCAACCTTCCTAAACTTCCTGTGACC

TGTGGTGAGGCTAAGGGGACTCTCCATAAGAAGCAATTTAAGCAAGGGGCCCAGATGAAG

AGCATAAGGACTGAGGATGGAAAATGGCTCACCCCCAGGGACTTCGAGATCAGAGGAAAC

CACGCAGCAGCCAAGAACTGGAAGCTAAGCCTGCGCTGCCACGGATGGACCTTGAAAGAA

CTGATAGAGGCAGGGCATCTACCAAACCCACCAATAACAAGAAAAAAGAGGAAAACACGG

GAGTCTCTCAACAATGTCTTAGTTGACCCCCATCAAAAAAACTCAAATAAGTGCGAAGTA

TGCCGCCAAGAGAAACAGCTGGTCTTCTGCAATACTTGTTCAAGATCATACCATCAGAAC

TGCCACATCCCACCAGTAGATGCTCAGAGGAGCCAGTGGAGTTGCACCTTATGTAGGACA

AAAGCCTGGGAAAAGCACCCAGAAAGCCAACCATGTCATCTGGAATCTGAGGTCTTGAAG

AGGCCTATGCTGCCTGAGGAACAGCTGAAATGTGAGCTGATCCTATTGAAGGTCTATTGT

TATTCACAAAGTGCCTTTTTTGCCCAAGAACCATATTATACTCAAGACCCCCAGGAGCAC

ATGTGGTTAAACAAGGTCAAGGAAAGGCTGAATAAGAAGAAGTACCACCGAGTGGAGGGG

TTTGTGCGGGACATGCGCCTCATCTTTCTGAACAACAAGGCCTTCTACAGAAACCACAAA

TTCATCCAGCTGCCTCTTAAGATAGAAGCCATATTTGAGAAGTATTTCAAAAACATTTTT

TCAATCCAAGAAACAAGCGAGAGCATCTGTCAGTTT

>CL356.Contig61_A-W 763 1071 minus strand PREDICTED: nuclear autoantigen Sp-100 isoform 14 [Pan troglodytes]

AAATGTGAGCTGATCCTATTGAAGGTCTATTGTTATTCACAAAGTGCCTTTTTTGCCCAA

GAACCATATTATACTCAAGACCCCCAGGAGCACATGTGGTTAAACAAGGTCAAGGAAAGG

CTGAATAAGAAGAAGTACCACCGAGTGGAGGGGTTTGTGCGGGACATGCGCCTCATCTTT

CTGAACAACAAGGCCTTCTACAGAAACCACAAATTCATCCAGCTGCCTCTTAAGATAGAA

GCCATATTTGAGAAGTATTTCAAAAACATTTTTTCAATCCAAGAAACAAGCGAGAGCATC

TGTCAGTTT

>CL356.Contig62_A-W 715 1023 minus strand PREDICTED: nuclear autoantigen Sp-100 isoform 14 [Pan troglodytes]

AAATGTGAGCTGATCCTATTGAAGGTCTATTGTTATTCACAAAGTGCCTTTTTTGCCCAA

GAACCATATTATACTCAAGACCCCCAGGAGCACATGTGGTTAAACAAGGTCAAGGAAAGG

CTGAATAAGAAGAAGTACCACCGAGTGGAGGGGTTTGTGCGGGACATGCGCCTCATCTTT

CTGAACAACAAGGCCTTCTACAGAAACCACAAATTCATCCAGCTGCCTCTTAAGATAGAA

GCCATATTTGAGAAGTATTTCAAAAACATTTTTTCAATCCAAGAAACAAGCGAGAGCATC

TGTCAGTTT

>CL356.Contig63_A-W 124 981 minus strand PREDICTED: nuclear autoantigen Sp-100 [Callithrix jacchus]

TCAGGAGAGGAAGCTGTAAAAGATACCACGGATGTTGGAAACAATTCTACTTTGGGAAAA

GGCAGTGGGGAAAGAAATATTTCCAAGCTGCACAACAGAGATAAAGAAATGAACAAAATC

TCATCAACCTTACATCCTACTGTGGGGAAAAAAAGACTTCATAAAGTAAACCATCTCCAA

AAAGAAATAAAGAAAGGCAGACATAGATTCCACTCAATTCAGAATAATGCCCTTCAAAGG

AAAAGAGGTTGGCCAAAAGGACCAAGAATTCCCAGAGAAAAAAATGTGAATTTCAACCTT

CCTAAACTTCCTGTGACCTGTGGTGAGGCTAAGGGGACTCTCCATAAGAAGCAATTTAAG

CAAGGGGCCCAGATGAAGAGCATAAGGACTGAGGATGGAAAATGGCTCACCCCCAGGGAC

TTCGAGATCAGAGGAAACCACGCAGCAGCCAAGAACTGGAAGCTAAGCCTGCGCTGCCAC

GGATGGACCTTGAAAGAACTGATAGAGGCAGGGCATCTACCAAACCCACCAATAACAAGA

AAAAAGAGGAAAACACGGGAGTCTCTCAACAATGTCTTAGTTGACCCCCATCAAAAAAAC

TCAAATAAGTGCGAAGTATGCCGCCAAGAGAAACAGCTGGTCTTCTGCAATACTTGTTCA

AGATCATACCATCAGAACTGCCACATCCCACCAGTAGATGCTCAGAGGAGCCAGTGGAGT

TGCACCTTATGTAGGACAAAAGCCTGGGAAAAGCACCCAGAAAGCCAACCATGTCATCTG

GAATCTGAGGTCTTGAAGAGGCCTATGCTGCCTGAGGAACAGCTGGTAAGTCAGTGTGAC

CCTGAAGCCCCCTCCTTT

>CL356.Contig64_A-W 1 960 minus strand nuclear autoantigen Sp-100 isoform 1 [Macaca mulatta]

GTGCCAAGAGGCCAAGAAGCAACGGTGGAAAGTGGCCGAGCATCTCACCCAATAGGCAAG

TCTGCTCAATGGCATCAGGGTGGAGGTGGCTCAGAAATACCACAGCTGCTGGGCAGGACT

GTGTCAGGAGAGGAAGCTGTAAAAGATACCACGGATGTTGGAAACAATTCTACTTTGGGA

AAAGGCAGTGGGGAAAGAAGGAAAAAAAGACTTCATAAAGTAAACCATCTCCAAAAAGAA

ATAAAGAAAGGCAGACATAGATTCCACTCAATTCAGAATAATGCCCTTCAAAGGAAAAGA

GGTTGGCCAAAAGGGAGAAAAAGAGTCAACGTTAGACCCTTGAAAAGAGGGAGAAAAAGA

GGACCAAGAATTCCCAGAGAAAAAAATGTGAATTTCAACCTTCCTAAACTTCCTGTGACC

TGTGGTGAGGCTAAGGGGACTCTCCATAAGAAGCAATTTAAGCAAGGGGCCCAGATGAAG

AGCATAAGGACTGAGGATGGAAAATGGCTCACCCCCAGGGACTTCGAGATCAGAGGAAAC

CACGCAGCAGCCAAGAACTGGAAGCTAAGCCTGCGCTGCCACGGATGGACCTTGAAAGAA

CTGATAGAGGCAGGGCATCTACCAAACCCACCAATAACAAGAAAAAAGAGGAAAACACGG

GAGTCTCTCAACAATGTCTTAGTTGACCCCCATCAAAAAAACTCAAATAAGTGCGAAGTA

TGCCGCCAAGAGAAACAGCTGGTCTTCTGCAATACTTGTTCAAGATCATACCATCAGAAC

TGCCACATCCCACCAGTAGATGCTCAGAGGAGCCAGTGGAGTTGCACCTTATGTAGGACA

AAAGCCTGGGAAAAGCACCCAGAAAGCCAACCATGTCATCTGGAATCTGAGGTCTTGAAG

AGGCCTATGCTGCCTGAGGAACAGCTGGTAAGTCAGTGTGACCCTGAAGCCCCCTCCTTT

>CL356.Contig65_A-W 1 939 minus strand nuclear autoantigen Sp-100 isoform 1 [Macaca mulatta]

GTGCCAAGAGGCCAAGAAGCAACGGTGGAAAGTGGCCGAGCATCTCACCCAATAGATACC

ACGGATGTTGGAAACAATTCTACTTTGGGAAAAGGCAGTGGGGAAAGAAATATTTCCAAG

CTGCACAACAGAGATAAAGAAATGAACAAAATCTCATCAACCTTACATCCTACTGTGGGG

AAAAAAAGACTTCATAAAGTAAACCATCTCCAAAAAGAAATAAAGAAAGGCAGACATAGA

TTCCACTCAATTCAGAATAATGCCCTTCAAAGGAAAAGAGGTTGGCCAAAAGGGAGAAAA

AGAGTCAACGTTAGACCCTTGAAAAGAGGGAGAAAAAGAGGACCAAGAATTCCCAGAGAA

AAAAATGTGAATTTCAACCTTCCTAAACTTCCTGTGACCTGTGGTGAGGCTAAGGGGACT

CTCCATAAGAAGCAATTTAAGCAAGGGGCCCAGATGAAGAGCATAAGGACTGAGGATGGA

AAATGGCTCACCCCCAGGGACTTCGAGATCAGAGGAAACCACGCAGCAGCCAAGAACTGG

AAGCTAAGCCTGCGCTGCCACGGATGGACCTTGAAAGAACTGATAGAGGCAGGGCATCTA

CCAAACCCACCAATAACAAGAAAAAAGAGGAAAACACGGGAGTCTCTCAACAATGTCTTA

GTTGACCCCCATCAAAAAAACTCAAATAAGTGCGAAGTATGCCGCCAAGAGAAACAGCTG

GTCTTCTGCAATACTTGTTCAAGATCATACCATCAGAACTGCCACATCCCACCAGTAGAT

GCTCAGAGGAGCCAGTGGAGTTGCACCTTATGTAGGACAAAAGCCTGGGAAAAGCACCCA

GAAAGCCAACCATGTCATCTGGAATCTGAGGTCTTGAAGAGGCCTATGCTGCCTGAGGAA

CAGCTGGTAAGTCAGTGTGACCCTGAAGCCCCCTCCTTT

>CL356.Contig66_A-W 163 2295 minus strand PREDICTED: nuclear autoantigen Sp-100 [Pan paniscus]

TCAGTGTTCCAAGAAAAATATGTTTCCCTAAGAAGTGACCCGGAAGGAAGACAACAAACA

TCCATCACTCAGCCAAGCCGTGAGCAAGGAACAGTTGAGAACACGTTTCTGCGAAGACTG

ACCTGGCCACAGCACTCAGATCCCTCGTCTTACAATGGTACAGACCTACCCGAAAATGAA

CTCTTGGCGCATCCTTGCGAAGCAGAAGAGAGAAATGTAAATAAAAGAGAGCCGGCCCGA

GACCCAAATGGTGCAGTAGGAAGCCACCAAGGAAATGAGCAATGCCCTCGAGAGTCAGAG

CCAGTAGAGTCCCACCAACACATGGCTGCCAACGTGGACCGTGGAGAGGCGAGCGTGGGG

ACACCCAGCCCGTTGCCCCACTGTGTAGAAAGAGCAGAGCTCTCCAGCCATGGAATCCAC

ATGACTTCCTGTTCTGTGCATCTGGTGGATATAAAGAAGGAAAAGCCATTTTTTCCCACG

CAAATGAAGCAAGGATCTCAAACCAGGACCAACCATTACCAGCCATCCGAAGTAATAGTC

ATCAGCAGTGAGGACGACGCCAGCGACACAGAAGAGCCTGCAGCAATCTCCAGCTCAGCA

CGGAGGAGCCATCCTGTGATCAAAAACCATGTCCAGTTTGACTTGAGAGATGAAGAAGAG

GCCCAAGAAGCCACTTGCTCATGGCACCAGGCTGCTCCAGCGAGAGGACGTGACTATGGC

TCTTCAGAATCCAGTGAGGAGGAGATACCCTCAGCGCTCCGGATCTCAACACCAACAGAT

TCTGTGAGGATGTCTACTTGGAGGATACACAGTCAGAAAAGAAGGCACAGCAGTGACAGC

TCTTCAGAGCTGAGTTATGGAGAGGAGCCTCGGGAAGCCCTGAGAAGTGGATCAGGTGTG

CCAAGAGGCCAAGAAGCAACGGTGGAAAGTGGCCGAGCATCTCACCCAATAGGCAAGTCT

GCTCAATGGCATCAGGGTGGAGGTGGCTCAGAAATACCACAGCTGCTGGGCAGGACTGTG

TCAGGAGAGGAAGCTGTAAAAGATACCACGGATGTTGGAAACAATTCTACTTTGGGAAAA

GGCAGTGGGGAAAGAAGGAAAAAAAGACTTCATAAAGTAAACCATCTCCAAAAAGAAATA

AAGAAAGGCAGACATAGATTCCACTCAATTCAGAATAATGCCCTTCAAAGGAAAAGAGGT

TGGCCAAAAGGGAGAAAAAGAGTCAACGTTAGACCCTTGAAAAGAGGGAGAAAAAGAGGA

CCAAGAATTCCCAGAGAAAAAAATGTGAATTTCAACCTTCCTAAACTTCCTGTGACCTGT

GGTGAGGCTAAGGGGACTCTCCATAAGAAGCAATTTAAGCAAGGGGCCCAGATGAAGAGC

ATAAGGACTGAGGATGGAAAATGGCTCACCCCCAGGGACTTCGAGATCAGAGGAAACCAC

GCAGCAGCCAAGAACTGGAAGCTAAGCCTGCGCTGCCACGGATGGACCTTGAAAGAACTG

ATAGAGGCAGGGCATCTACCAAACCCACCAATAACAAGAAAAAAGAGGAAAACACGGGAG

TCTCTCAACAATGTCTTAGTTGACCCCCATCAAAAAAACTCAAATAAGTGCGAAGTATGC

CGCCAAGAGAAACAGCTGGTCTTCTGCAATACTTGTTCAAGATCATACCATCAGAACTGC

CACATCCCACCAGTAGATGCTCAGAGGAGCCAGTGGAGTTGCACCTTATGTAGGACAAAA

GCCTGGGAAAAGCACCCAGAAAGCCAACCATGTCATCTGGAATCTGAGGTCTTGAAGAGG

CCTATGCTGCCTGAGGAACAGCTGAAATGTGAGCTGATCCTATTGAAGGTCTATTGTTAT

TCACAAAGTGCCTTTTTTGCCCAAGAACCATATTATACTCAAGACCCCCAGGAGCACATG

TGGTTAAACAAGGTCAAGGAAAGGCTGAATAAGAAGAAGTACCACCGAGTGGAGGGGTTT

GTGCGGGACATGCGCCTCATCTTTCTGAACAACAAGGCCTTCTACAGAAACCACAAATTC

ATCCAGCTGCCTCTTAAGATAGAAGCCATATTTGAGAAGTATTTCAAAAACATTTTTTCA

ATCCAAGAAACAAGCGAGAGCATCTGTCAGTTT

>CL356.Contig67_A-W 124 912 minus strand PREDICTED: nuclear autoantigen Sp-100 [Callithrix jacchus]

TCAGGAGAGGAAGCTGTAAAAGATACCACGGATGTTGGAAACAATTCTACTTTGGGAAAA

GGCAGTGGGGAAAGAAGGAAAAAAAGACTTCATAAAGTAAACCATCTCCAAAAAGAAATA

AAGAAAGGCAGACATAGATTCCACTCAATTCAGAATAATGCCCTTCAAAGGAAAAGAGGT

TGGCCAAAAGGACCAAGAATTCCCAGAGAAAAAAATGTGAATTTCAACCTTCCTAAACTT

CCTGTGACCTGTGGTGAGGCTAAGGGGACTCTCCATAAGAAGCAATTTAAGCAAGGGGCC

CAGATGAAGAGCATAAGGACTGAGGATGGAAAATGGCTCACCCCCAGGGACTTCGAGATC

AGAGGAAACCACGCAGCAGCCAAGAACTGGAAGCTAAGCCTGCGCTGCCACGGATGGACC

TTGAAAGAACTGATAGAGGCAGGGCATCTACCAAACCCACCAATAACAAGAAAAAAGAGG

AAAACACGGGAGTCTCTCAACAATGTCTTAGTTGACCCCCATCAAAAAAACTCAAATAAG

TGCGAAGTATGCCGCCAAGAGAAACAGCTGGTCTTCTGCAATACTTGTTCAAGATCATAC

CATCAGAACTGCCACATCCCACCAGTAGATGCTCAGAGGAGCCAGTGGAGTTGCACCTTA

TGTAGGACAAAAGCCTGGGAAAAGCACCCAGAAAGCCAACCATGTCATCTGGAATCTGAG

GTCTTGAAGAGGCCTATGCTGCCTGAGGAACAGCTGGTAAGTCAGTGTGACCCTGAAGCC

CCCTCCTTT

>CL356.Contig68_A-W 1 891 minus strand nuclear autoantigen Sp-100 isoform 1 [Macaca mulatta]

GTGCCAAGAGGCCAAGAAGCAACGGTGGAAAGTGGCCGAGCATCTCACCCAATAGATACC

ACGGATGTTGGAAACAATTCTACTTTGGGAAAAGGCAGTGGGGAAAGAAATATTTCCAAG

CTGCACAACAGAGATAAAGAAATGAACAAAATCTCATCAACCTTACATCCTACTGTGGGG

AAAAAAAGACTTCATAAAGTAAACCATCTCCAAAAAGAAATAAAGAAAGGCAGACATAGA

TTCCACTCAATTCAGAATAATGCCCTTCAAAGGAAAAGAGGTTGGCCAAAAGGACCAAGA

ATTCCCAGAGAAAAAAATGTGAATTTCAACCTTCCTAAACTTCCTGTGACCTGTGGTGAG

GCTAAGGGGACTCTCCATAAGAAGCAATTTAAGCAAGGGGCCCAGATGAAGAGCATAAGG

ACTGAGGATGGAAAATGGCTCACCCCCAGGGACTTCGAGATCAGAGGAAACCACGCAGCA

GCCAAGAACTGGAAGCTAAGCCTGCGCTGCCACGGATGGACCTTGAAAGAACTGATAGAG

GCAGGGCATCTACCAAACCCACCAATAACAAGAAAAAAGAGGAAAACACGGGAGTCTCTC

AACAATGTCTTAGTTGACCCCCATCAAAAAAACTCAAATAAGTGCGAAGTATGCCGCCAA

GAGAAACAGCTGGTCTTCTGCAATACTTGTTCAAGATCATACCATCAGAACTGCCACATC

CCACCAGTAGATGCTCAGAGGAGCCAGTGGAGTTGCACCTTATGTAGGACAAAAGCCTGG

GAAAAGCACCCAGAAAGCCAACCATGTCATCTGGAATCTGAGGTCTTGAAGAGGCCTATG

CTGCCTGAGGAACAGCTGGTAAGTCAGTGTGACCCTGAAGCCCCCTCCTTT

>CL356.Contig69_A-W 132 2525 minus strand PREDICTED: nuclear autoantigen Sp-100 [Pan paniscus]

AGGATGTCCACAGAAGAGCAGAGTGTAGACGACAGTACCATCTATGAGGTTATATTCCAT

CACTTCAAAAGACACAAAGTGGAGATTTCAAATGCCATAAAAAAGACATTTCCTTTCCTC

GAGGGCCTCCGTGACCGTGAACTCATTACCAATAAAATGTTTGAAGATTCTCAAGAGTCT

TGTAGAAACCTGGTCCCTGTACAAAGAGTGGTGTACAATGTTCTCTGTGAACTGGAGAAG

AAATTCAACCTGGATTTGTGCGAAGCATTGTTCAGTGAGGTCAACTTGCAGGAATACCCT

GATTTAATTCGTGTTCGTAGAAGCTTCGAACATGAGGTGAGAATGCTATATCCCCAAGAA

GATAATGGAGAAGAGAGGCAGGAAAGGCTTGAACAAGGAACGGGGGAGAACTCTTTTCTG

CGAAGACTGACCTGGCCACAGCACTCAGATCCCTCGTCTTACAATGGTACAGACCTACCC

GAAAATGAACTCTTGGCGCATCCTTGCGAAGCAGAAGAGAGAAATGTAAATAAAAGAGAG

CCGGCCCGAGACCCAAATGGTGCAGTAGGAAGCCACCAAGGAAATGAGCAATGCCCTCGA

GAGTCAGAGCCAGTAGAGTCCCACCAACACATGGCTGCCAACGTGGACCGTGGAGAGGCG

AGCGTGGGGACACCCAGCCCGTTGCCCCACTGTGTAGAAAGAGCAGAGCTCTCCAGCCAT

GGAATCCACATGACTTCCTGTTCTGTGCATCTGGTGGATATAAAGAAGGAAAAGCCATTT

TTTCCCACGCAAATGAAGCAAGGATCTCAAACCAGGACCAACCATTACCAGCCATCCGAA

GTAATAGTCATCAGCAGTGAGGACGACGCCAGCGACACAGAAGAGCCTGCAGCAATCTCC

AGCTCAGCACGGAGGAGCCATCCTGTGATCAAAAACCATGTCCAGTTTGACTTGAGAGAT

GAAGAAGAGGCCCAAGAAGCCACTTGCTCATGGCACCAGGCTGCTCCAGCGAGAGGACGT

GACTATGGCTCTTCAGAATCCAGTGAGGAGGAGATACCCTCAGCGCTCCGGATCTCAACA

CCAACAGATTCTGTGAGGATGTCTACTTGGAGGATACACAGTCAGAAAAGAAGGCACAGC

AGTGACAGCTCTTCAGAGCTGAGTTATGGAGAGGAGCCTCGGGAAGCCCTGAGAAGTGGA

TCAGGTGTGCCAAGAGGCCAAGAAGCAACGGTGGAAAGTGGCCGAGCATCTCACCCAATA

GGCAAGTCTGCTCAATGGCATCAGGGTGGAGGTGGCTCAGAAATACCACAGCTGCTGGGC

AGGACTGTGTCAGGAGAGGAAGCTGTAAAAGATACCACGGATGTTGGAAACAATTCTACT

TTGGGAAAAGGCAGTGGGGAAAGAAGGAAAAAAAGACTTCATAAAGTAAACCATCTCCAA

AAAGAAATAAAGAAAGGCAGACATAGATTCCACTCAATTCAGAATAATGCCCTTCAAAGG

AAAAGAGGTTGGCCAAAAGGACCAAGAATTCCCAGAGAAAAAAATGTGAATTTCAACCTT

CCTAAACTTCCTGTGACCTGTGGTGAGGCTAAGGGGACTCTCCATAAGAAGCAATTTAAG

CAAGGGGCCCAGATGAAGAGCATAAGGACTGAGGATGGAAAATGGCTCACCCCCAGGGAC

TTCGAGATCAGAGGAAACCACGCAGCAGCCAAGAACTGGAAGCTAAGCCTGCGCTGCCAC

GGATGGACCTTGAAAGAACTGATAGAGGCAGGGCATCTACCAAACCCACCAATAACAAGA

AAAAAGAGGAAAACACGGGAGTCTCTCAACAATGTCTTAGTTGACCCCCATCAAAAAAAC

TCAAATAAGTGCGAAGTATGCCGCCAAGAGAAACAGCTGGTCTTCTGCAATACTTGTTCA

AGATCATACCATCAGAACTGCCACATCCCACCAGTAGATGCTCAGAGGAGCCAGTGGAGT

TGCACCTTATGTAGGACAAAAGCCTGGGAAAAGCACCCAGAAAGCCAACCATGTCATCTG

GAATCTGAGGTCTTGAAGAGGCCTATGCTGCCTGAGGAACAGCTGAAATGTGAGCTGATC

CTATTGAAGGTCTATTGTTATTCACAAAGTGCCTTTTTTGCCCAAGAACCATATTATACT

CAAGACCCCCAGGAGCACATGTGGTTAAACAAGGTCAAGGAAAGGCTGAATAAGAAGAAG

TACCACCGAGTGGAGGGGTTTGTGCGGGACATGCGCCTCATCTTTCTGAACAACAAGGCC

TTCTACAGAAACCACAAATTCATCCAGCTGCCTCTTAAGATAGAAGCCATATTTGAGAAG

TATTTCAAAAACATTTTTTCAATCCAAGAAACAAGCGAGAGCATCTGTCAGTTT

>CL356.Contig70_A-W 26 865 minus strand PREDICTED: nuclear autoantigen Sp-100 [Callithrix jacchus]

CTAGATACCACGGATGTTGGAAACAATTCTACTTTGGGAAAAGGCAGTGGGGAAAGAAAT

ATTTCCAAGCTGCACAACAGAGATAAAGAAATGAACAAAATCTCATCAACCTTACATCCT

ACTGTGGGGAAAAAAAGACTTCATAAAGTAAACCATCTCCAAAAAGAAATAAAGAAAGGC

AGACATAGATTCCACTCAATTCAGAATAATGCCCTTCAAAGGAAAAGAGGTTGGCCAAAA

GGACCAAGAATTCCCAGAGAAAAAAATGTGAATTTCAACCTTCCTAAACTTCCTGTGACC

TGTGGTGAGGCTAAGGGGACTCTCCATAAGAAGCAATTTAAGCAAGGGGCCCAGATGAAG

AGCATAAGGACTGAGGATGGAAAATGGCTCACCCCCAGGGACTTCGAGATCAGAGGAAAC

CACGCAGCAGCCAAGAACTGGAAGCTAAGCCTGCGCTGCCACGGATGGACCTTGAAAGAA

CTGATAGAGGCAGGGCATCTACCAAACCCACCAATAACAAGAAAAAAGAGGAAAACACGG

GAGTCTCTCAACAATGTCTTAGTTGACCCCCATCAAAAAAACTCAAATAAGTGCGAAGTA

TGCCGCCAAGAGAAACAGCTGGTCTTCTGCAATACTTGTTCAAGATCATACCATCAGAAC

TGCCACATCCCACCAGTAGATGCTCAGAGGAGCCAGTGGAGTTGCACCTTATGTAGGACA

AAAGCCTGGGAAAAGCACCCAGAAAGCCAACCATGTCATCTGGAATCTGAGGTCTTGAAG

AGGCCTATGCTGCCTGAGGAACAGCTGGTAAGTCAGTGTGACCCTGAAGCCCCCTCCTTT

>CL356.Contig71_A-W 132 2504 minus strand PREDICTED: nuclear autoantigen Sp-100 [Pan paniscus]

AGGATGTCCACAGAAGAGCAGAGTGTAGACGACAGTACCATCTATGAGGTTATATTCCAT

CACTTCAAAAGACACAAAGTGGAGATTTCAAATGCCATAAAAAAGACATTTCCTTTCCTC

GAGGGCCTCCGTGACCGTGAACTCATTACCAATAAAATGTTTGAAGATTCTCAAGAGTCT

TGTAGAAACCTGGTCCCTGTACAAAGAGTGGTGTACAATGTTCTCTGTGAACTGGAGAAG

AAATTCAACCTGGATTTGTGCGAAGCATTGTTCAGTGAGGTCAACTTGCAGGAATACCCT

GATTTAATTCGTGTTCGTAGAAGCTTCGAACATGAGGTGAGAATGCTATATCCCCAAGAA

GATAATGGAGAAGAGAGGCAGGAAAGGCTTGAACAAGGAACGGGGGAGAACTCTTTTCTG

CGAAGACTGACCTGGCCACAGCACTCAGATCCCTCGTCTTACAATGGTACAGACCTACCC

GAAAATGAACTCTTGGCGCATCCTTGCGAAGCAGAAGAGAGAAATGTAAATAAAAGAGAG

CCGGCCCGAGACCCAAATGGTGCAGTAGGAAGCCACCAAGGAAATGAGCAATGCCCTCGA

GAGTCAGAGCCAGTAGAGTCCCACCAACACATGGCTGCCAACGTGGACCGTGGAGAGGCG

AGCGTGGGGACACCCAGCCCGTTGCCCCACTGTGTAGAAAGAGCAGAGCTCTCCAGCCAT

GGAATCCACATGACTTCCTGTTCTGTGCATCTGGTGGATATAAAGAAGGAAAAGCCATTT

TTTCCCACGCAAATGAAGCAAGGATCTCAAACCAGGACCAACCATTACCAGCCATCCGAA

GTAATAGTCATCAGCAGTGAGGACGACGCCAGCGACACAGAAGAGCCTGCAGCAATCTCC

AGCTCAGCACGGAGGAGCCATCCTGTGATCAAAAACCATGTCCAGTTTGACTTGAGAGAT

GAAGAAGAGGCCCAAGAAGCCACTTGCTCATGGCACCAGGCTGCTCCAGCGAGAGGACGT

GACTATGGCTCTTCAGAATCCAGTGAGGAGGAGATACCCTCAGCGCTCCGGATCTCAACA

CCAACAGATTCTGTGAGGATGTCTACTTGGAGGATACACAGTCAGAAAAGAAGGCACAGC

AGTGACAGCTCTTCAGAGCTGAGTTATGGAGAGGAGCCTCGGGAAGCCCTGAGAAGTGGA

TCAGGTGTGCCAAGAGGCCAAGAAGCAACGGTGGAAAGTGGCCGAGCATCTCACCCAATA

GATACCACGGATGTTGGAAACAATTCTACTTTGGGAAAAGGCAGTGGGGAAAGAAATATT

TCCAAGCTGCACAACAGAGATAAAGAAATGAACAAAATCTCATCAACCTTACATCCTACT

GTGGGGAAAAAAAGACTTCATAAAGTAAACCATCTCCAAAAAGAAATAAAGAAAGGCAGA

CATAGATTCCACTCAATTCAGAATAATGCCCTTCAAAGGAAAAGAGGTTGGCCAAAAGGA

CCAAGAATTCCCAGAGAAAAAAATGTGAATTTCAACCTTCCTAAACTTCCTGTGACCTGT

GGTGAGGCTAAGGGGACTCTCCATAAGAAGCAATTTAAGCAAGGGGCCCAGATGAAGAGC

ATAAGGACTGAGGATGGAAAATGGCTCACCCCCAGGGACTTCGAGATCAGAGGAAACCAC

GCAGCAGCCAAGAACTGGAAGCTAAGCCTGCGCTGCCACGGATGGACCTTGAAAGAACTG

ATAGAGGCAGGGCATCTACCAAACCCACCAATAACAAGAAAAAAGAGGAAAACACGGGAG

TCTCTCAACAATGTCTTAGTTGACCCCCATCAAAAAAACTCAAATAAGTGCGAAGTATGC

CGCCAAGAGAAACAGCTGGTCTTCTGCAATACTTGTTCAAGATCATACCATCAGAACTGC

CACATCCCACCAGTAGATGCTCAGAGGAGCCAGTGGAGTTGCACCTTATGTAGGACAAAA

GCCTGGGAAAAGCACCCAGAAAGCCAACCATGTCATCTGGAATCTGAGGTCTTGAAGAGG

CCTATGCTGCCTGAGGAACAGCTGAAATGTGAGCTGATCCTATTGAAGGTCTATTGTTAT

TCACAAAGTGCCTTTTTTGCCCAAGAACCATATTATACTCAAGACCCCCAGGAGCACATG

TGGTTAAACAAGGTCAAGGAAAGGCTGAATAAGAAGAAGTACCACCGAGTGGAGGGGTTT

GTGCGGGACATGCGCCTCATCTTTCTGAACAACAAGGCCTTCTACAGAAACCACAAATTC

ATCCAGCTGCCTCTTAAGATAGAAGCCATATTTGAGAAGTATTTCAAAAACATTTTTTCA

ATCCAAGAAACAAGCGAGAGCATCTGTCAGTTT

>CL356.Contig72_A-W 132 2642 minus strand PREDICTED: nuclear autoantigen Sp-100 [Pan paniscus]

AGGATGTCCACAGAAGAGCAGAGTGTAGACGACAGTACCATCTATGAGGTTATATTCCAT

CACTTCAAAAGACACAAAGTGGAGATTTCAAATGCCATAAAAAAGACATTTCCTTTCCTC

GAGGGCCTCCGTGACCGTGAACTCATTACCAATAAAATGTTTGAAGATTCTCAAGAGTCT

TGTAGAAACCTGGTCCCTGTACAAAGAGTGGTGTACAATGTTCTCTGTGAACTGGAGAAG

AAATTCAACCTGGATTTGTGCGAAGCATTGTTCAGTGAGGTCAACTTGCAGGAATACCCT

GATTTAATTCGTGTTCGTAGAAGCTTCGAACATGAGGTGAGAATGCTATATCCCCAAGAA

GATAATGGAGAAGAGAGGCAGGAAAGGCTTGAACAAGGAACGGGGGAGAACTCTTTTCTG

CGAAGACTGACCTGGCCACAGCACTCAGATCCCTCGTCTTACAATGGTACAGACCTACCC

GAAAATGAACTCTTGGCGCATCCTTGCGAAGCAGAAGAGAGAAATGTAAATAAAAGAGAG

CCGGCCCGAGACCCAAATGGTGCAGTAGGAAGCCACCAAGGAAATGAGCAATGCCCTCGA

GAGTCAGAGCCAGTAGAGTCCCACCAACACATGGCTGCCAACGTGGACCGTGGAGAGGCG

AGCGTGGGGACACCCAGCCCGTTGCCCCACTGTGTAGAAAGAGCAGAGCTCTCCAGCCAT

GGAATCCACATGACTTCCTGTTCTGTGCATCTGGTGGATATAAAGAAGGAAAAGCCATTT

TTTCCCACGCAAATGAAGCAAGGATCTCAAACCAGGACCAACCATTACCAGCCATCCGAA

GTAATAGTCATCAGCAGTGAGGACGACGCCAGCGACACAGAAGAGCCTGCAGCAATCTCC

AGCTCAGCACGGAGGAGCCATCCTGTGATCAAAAACCATGTCCAGTTTGACTTGAGAGAT

GAAGAAGAGGCCCAAGAAGCCACTTGCTCATGGCACCAGGCTGCTCCAGCGAGAGGACGT

GACTATGGCTCTTCAGAATCCAGTGAGGAGGAGATACCCTCAGCGCTCCGGATCTCAACA

CCAACAGATTCTGTGAGGATGTCTACTTGGAGGATACACAGTCAGAAAAGAAGGCACAGC

AGTGACAGCTCTTCAGAGCTGAGTTATGGAGAGGAGCCTCGGGAAGCCCTGAGAAGTGGA

TCAGGTGTGCCAAGAGGCCAAGAAGCAACGGTGGAAAGTGGCCGAGCATCTCACCCAATA

GGCAAGTCTGCTCAATGGCATCAGGGTGGAGGTGGCTCAGAAATACCACAGCTGCTGGGC

AGGACTGTGTCAGGAGAGGAAGCTGTAAAAGATACCACGGATGTTGGAAACAATTCTACT

TTGGGAAAAGGCAGTGGGGAAAGAAATATTTCCAAGCTGCACAACAGAGATAAAGAAATG

AACAAAATCTCATCAACCTTACATCCTACTGTGGGGAAAAAAAGACTTCATAAAGTAAAC

CATCTCCAAAAAGAAATAAAGAAAGGCAGACATAGATTCCACTCAATTCAGAATAATGCC

CTTCAAAGGAAAAGAGGTTGGCCAAAAGGGAGAAAAAGAGTCAACGTTAGACCCTTGAAA

AGAGGGAGAAAAAGAGGACCAAGAATTCCCAGAGAAAAAAATGTGAATTTCAACCTTCCT

AAACTTCCTGTGACCTGTGGTGAGGCTAAGGGGACTCTCCATAAGAAGCAATTTAAGCAA

GGGGCCCAGATGAAGAGCATAAGGACTGAGGATGGAAAATGGCTCACCCCCAGGGACTTC

GAGATCAGAGGAAACCACGCAGCAGCCAAGAACTGGAAGCTAAGCCTGCGCTGCCACGGA

TGGACCTTGAAAGAACTGATAGAGGCAGGGCATCTACCAAACCCACCAATAACAAGAAAA

AAGAGGAAAACACGGGAGTCTCTCAACAATGTCTTAGTTGACCCCCATCAAAAAAACTCA

AATAAGTGCGAAGTATGCCGCCAAGAGAAACAGCTGGTCTTCTGCAATACTTGTTCAAGA

TCATACCATCAGAACTGCCACATCCCACCAGTAGATGCTCAGAGGAGCCAGTGGAGTTGC

ACCTTATGTAGGACAAAAGCCTGGGAAAAGCACCCAGAAAGCCAACCATGTCATCTGGAA

TCTGAGGTCTTGAAGAGGCCTATGCTGCCTGAGGAACAGCTGAAATGTGAGCTGATCCTA

TTGAAGGTCTATTGTTATTCACAAAGTGCCTTTTTTGCCCAAGAACCATATTATACTCAA

GACCCCCAGGAGCACATGTGGTTAAACAAGGTCAAGGAAAGGCTGAATAAGAAGAAGTAC

CACCGAGTGGAGGGGTTTGTGCGGGACATGCGCCTCATCTTTCTGAACAACAAGGCCTTC

TACAGAAACCACAAATTCATCCAGCTGCCTCTTAAGATAGAAGCCATATTTGAGAAGTAT

TTCAAAAACATTTTTTCAATCCAAGAAACAAGCGAGAGCATCTGTCAGTTT

>CL356.Contig73_A-W 132 2633 minus strand PREDICTED: nuclear autoantigen Sp-100 isoform 14 [Pan troglodytes]

AGGATGTCCACAGAAGAGCAGAGTGTAGACGACAGTACCATCTATGAGGTTATATTCCAT

CACTTCAAAAGACACAAAGTGGAGATTTCAAATGCCATAAAAAAGACATTTCCTTTCCTC

GAGGGCCTCCGTGACCGTGAACTCATTACCAATAAAATGTTTGAAGATTCTCAAGAGTCT

TGTAGAAACCTGGTCCCTGTACAAAGAGTGGTGTACAATGTTCTCTGTGAACTGGAGAAG

AAATTCAACCTGGATTTGTGCGAAGCATTGTTCAGTGAGGTCAACTTGCAGGAATACCCT

GATTTAATTCGTGTTCGTAGAAGCTTCGAACATGAGGTGAGAATGCTATATCCCCAAGAA

GATAATGGAGAAGAGAGGCAGGAAAGGCTTGAACAAGGAACGGGGGAGAACTCTTTTCTG

CGAAGACTGACCTGGCCACAGCACTCAGATCCCTCGTCTTACAATGGTACAGACCTACCC

GAAAATGAACTCTTGGCGCATCCTTGCGAAGCAGAAGAGAGAAATGTAAATAAAAGAGAG

CCGGCCCGAGACCCAAATGGTGCAGTAGGAAGCCACCAAGGAAATGAGCAATGCCCTCGA

GAGTCAGAGCCAGTAGAGTCCCACCAACACATGGCTGCCAACGTGGACCGTGGAGAGGCG

AGCGTGGGGACACCCAGCCCGTTGCCCCACTGTGTAGAAAGAGCAGAGCTCTCCAGCCAT

GGAATCCACATGACTTCCTGTTCTGTGCATCTGGTGGATATAAAGAAGGAAAAGCCATTT

TTTCCCACGCAAATGAAGCAAGGATCTCAAACCAGGACCAACCATTACCAGCCATCCGAA

GTAATAGTCATCAGCAGTGAGGACGACGCCAGCGACACAGAAGAGCCTGCAGCAATCTCC

AGCTCAGCACGGAGGAGCCATCCTGTGATCAAAAACCATGTCCAGTTTGACTTGAGAGAT

GAAGAAGAGGCCCAAGAAGCCACTTGCTCATGGCACCAGGCTGCTCCAGCGAGAGGACGT

GACTATGGCTCTTCAGAATCCAGTGAGGAGGAGATACCCTCAGCGCTCCGGATCTCAACA

CCAACAGATTCTGTGAGGATGTCTACTTGGAGGATACACAGTCAGAAAAGAAGGCACAGC

AGTGACAGCTCTTCAGAGCTGAGTTATGGAGAGGAGCCTCGGGAAGCCCTGAGAAGTGGA

TCAGGAACAGGGCAACAAGAGACTGGAAATGAGAAGTGCTCCTGTGTCCTGTGTTCCCCA

AAAGGTGTGCCAAGAGGCCAAGAAGCAACGGTGGAAAGTGGCCGAGCATCTCACCCAATA

GGCAAGTCTGCTCAATGGCATCAGGGTGGAGGTGGCTCAGAAATACCACAGCTGCTGGGC

AGGACTGTGTCAGGAGAGGAAGCTGTAAAAGATACCACGGATGTTGGAAACAATTCTACT

TTGGGAAAAGGCAGTGGGGAAAGAAGGAAAAAAAGACTTCATAAAGTAAACCATCTCCAA

AAAGAAATAAAGAAAGGCAGACATAGATTCCACTCAATTCAGAATAATGCCCTTCAAAGG

AAAAGAGGTTGGCCAAAAGGGAGAAAAAGAGTCAACGTTAGACCCTTGAAAAGAGGGAGA

AAAAGAGGACCAAGAATTCCCAGAGAAAAAAATGTGAATTTCAACCTTCCTAAACTTCCT

GTGACCTGTGGTGAGGCTAAGGGGACTCTCCATAAGAAGCAATTTAAGCAAGGGGCCCAG

ATGAAGAGCATAAGGACTGAGGATGGAAAATGGCTCACCCCCAGGGACTTCGAGATCAGA

GGAAACCACGCAGCAGCCAAGAACTGGAAGCTAAGCCTGCGCTGCCACGGATGGACCTTG

AAAGAACTGATAGAGGCAGGGCATCTACCAAACCCACCAATAACAAGAAAAAAGAGGAAA

ACACGGGAGTCTCTCAACAATGTCTTAGTTGACCCCCATCAAAAAAACTCAAATAAGTGC

GAAGTATGCCGCCAAGAGAAACAGCTGGTCTTCTGCAATACTTGTTCAAGATCATACCAT

CAGAACTGCCACATCCCACCAGTAGATGCTCAGAGGAGCCAGTGGAGTTGCACCTTATGT

AGGACAAAAGCCTGGGAAAAGCACCCAGAAAGCCAACCATGTCATCTGGAATCTGAGGTC

TTGAAGAGGCCTATGCTGCCTGAGGAACAGCTGAAATGTGAGCTGATCCTATTGAAGGTC

TATTGTTATTCACAAAGTGCCTTTTTTGCCCAAGAACCATATTATACTCAAGACCCCCAG

GAGCACATGTGGTTAAACAAGGTCAAGGAAAGGCTGAATAAGAAGAAGTACCACCGAGTG

GAGGGGTTTGTGCGGGACATGCGCCTCATCTTTCTGAACAACAAGGCCTTCTACAGAAAC

CACAAATTCATCCAGCTGCCTCTTAAGATAGAAGCCATATTTGAGAAGTATTTCAAAAAC

ATTTTTTCAATCCAAGAAACAAGCGAGAGCATCTGTCAGTTT

>CL356.Contig74_A-W 163 2286 minus strand PREDICTED: nuclear autoantigen Sp-100 isoform 14 [Pan troglodytes]

TCAGTGTTCCAAGAAAAATATGTTTCCCTAAGAAGTGACCCGGAAGGAAGACAACAAACA

TCCATCACTCAGCCAAGCCGTGAGCAAGGAACAGTTGAGAACACGTTTCTGCGAAGACTG

ACCTGGCCACAGCACTCAGATCCCTCGTCTTACAATGGTACAGACCTACCCGAAAATGAA

CTCTTGGCGCATCCTTGCGAAGCAGAAGAGAGAAATGTAAATAAAAGAGAGCCGGCCCGA

GACCCAAATGGTGCAGTAGGAAGCCACCAAGGAAATGAGCAATGCCCTCGAGAGTCAGAG

CCAGTAGAGTCCCACCAACACATGGCTGCCAACGTGGACCGTGGAGAGGCGAGCGTGGGG

ACACCCAGCCCGTTGCCCCACTGTGTAGAAAGAGCAGAGCTCTCCAGCCATGGAATCCAC

ATGACTTCCTGTTCTGTGCATCTGGTGGATATAAAGAAGGAAAAGCCATTTTTTCCCACG

CAAATGAAGCAAGGATCTCAAACCAGGACCAACCATTACCAGCCATCCGAAGTAATAGTC

ATCAGCAGTGAGGACGACGCCAGCGACACAGAAGAGCCTGCAGCAATCTCCAGCTCAGCA

CGGAGGAGCCATCCTGTGATCAAAAACCATGTCCAGTTTGACTTGAGAGATGAAGAAGAG

GCCCAAGAAGCCACTTGCTCATGGCACCAGGCTGCTCCAGCGAGAGGACGTGACTATGGC

TCTTCAGAATCCAGTGAGGAGGAGATACCCTCAGCGCTCCGGATCTCAACACCAACAGAT

TCTGTGAGGATGTCTACTTGGAGGATACACAGTCAGAAAAGAAGGCACAGCAGTGACAGC

TCTTCAGAGCTGAGTTATGGAGAGGAGCCTCGGGAAGCCCTGAGAAGTGGATCAGGAACA

GGGCAACAAGAGACTGGAAATGAGAAGTGCTCCTGTGTCCTGTGTTCCCCAAAAGGTGTG

CCAAGAGGCCAAGAAGCAACGGTGGAAAGTGGCCGAGCATCTCACCCAATAGATACCACG

GATGTTGGAAACAATTCTACTTTGGGAAAAGGCAGTGGGGAAAGAAATATTTCCAAGCTG

CACAACAGAGATAAAGAAATGAACAAAATCTCATCAACCTTACATCCTACTGTGGGGAAA

AAAAGACTTCATAAAGTAAACCATCTCCAAAAAGAAATAAAGAAAGGCAGACATAGATTC

CACTCAATTCAGAATAATGCCCTTCAAAGGAAAAGAGGTTGGCCAAAAGGACCAAGAATT

CCCAGAGAAAAAAATGTGAATTTCAACCTTCCTAAACTTCCTGTGACCTGTGGTGAGGCT

AAGGGGACTCTCCATAAGAAGCAATTTAAGCAAGGGGCCCAGATGAAGAGCATAAGGACT

GAGGATGGAAAATGGCTCACCCCCAGGGACTTCGAGATCAGAGGAAACCACGCAGCAGCC

AAGAACTGGAAGCTAAGCCTGCGCTGCCACGGATGGACCTTGAAAGAACTGATAGAGGCA

GGGCATCTACCAAACCCACCAATAACAAGAAAAAAGAGGAAAACACGGGAGTCTCTCAAC

AATGTCTTAGTTGACCCCCATCAAAAAAACTCAAATAAGTGCGAAGTATGCCGCCAAGAG

AAACAGCTGGTCTTCTGCAATACTTGTTCAAGATCATACCATCAGAACTGCCACATCCCA

CCAGTAGATGCTCAGAGGAGCCAGTGGAGTTGCACCTTATGTAGGACAAAAGCCTGGGAA

AAGCACCCAGAAAGCCAACCATGTCATCTGGAATCTGAGGTCTTGAAGAGGCCTATGCTG

CCTGAGGAACAGCTGAAATGTGAGCTGATCCTATTGAAGGTCTATTGTTATTCACAAAGT

GCCTTTTTTGCCCAAGAACCATATTATACTCAAGACCCCCAGGAGCACATGTGGTTAAAC

AAGGTCAAGGAAAGGCTGAATAAGAAGAAGTACCACCGAGTGGAGGGGTTTGTGCGGGAC

ATGCGCCTCATCTTTCTGAACAACAAGGCCTTCTACAGAAACCACAAATTCATCCAGCTG

CCTCTTAAGATAGAAGCCATATTTGAGAAGTATTTCAAAAACATTTTTTCAATCCAAGAA

ACAAGCGAGAGCATCTGTCAGTTT

>CL356.Contig75_A-W 589 2415 minus strand PREDICTED: nuclear autoantigen Sp-100 [Saimiri boliviensis boliviensis]

CCAGGAGCAGAGCTCTCCAGCCATGGAATCCACATGACTTCCTGTTCTGTGCATCTGGTG

GATATAAAGAAGGAAAAGCCATTTTTTCCCACGCAAATGAAGCAAGGATCTCAAACCAGG

ACCAACCATTACCAGCCATCCGAAGTAATAGTCATCAGCAGTGAGGACGACGCCAGCGAC

ACAGAAGAGCCTGCAGCAATCTCCAGCTCAGCACGGAGGAGCCATCCTGTGATCAAAAAC

CATGTCCAGTTTGACTTGAGAGATGAAGAAGAGGCCCAAGAAGCCACTTGCTCATGGCAC

CAGGCTGCTCCAGCGAGAGGACGTGACTATGGCTCTTCAGAATCCAGTGAGGAGGAGATA

CCCTCAGCGCTCCGGATCTCAACACCAACAGATTCTGTGAGGATGTCTACTTGGAGGATA

CACAGTCAGAAAAGAAGGCACAGCAGTGACAGCTCTTCAGAGCTGAGTTATGGAGAGGAG

CCTCGGGAAGCCCTGAGAAGTGGATCAGGAACAGGGCAACAAGAGACTGGAAATGAGAAG

TGCTCCTGTGTCCTGTGTTCCCCAAAAGGTGTGCCAAGAGGCCAAGAAGCAACGGTGGAA

AGTGGCCGAGCATCTCACCCAATAGGCAAGTCTGCTCAATGGCATCAGGGTGGAGGTGGC

TCAGAAATACCACAGCTGCTGGGCAGGACTGTGTCAGGAGAGGAAGCTGTAAAAGATACC

ACGGATGTTGGAAACAATTCTACTTTGGGAAAAGGCAGTGGGGAAAGAAATATTTCCAAG

CTGCACAACAGAGATAAAGAAATGAACAAAATCTCATCAACCTTACATCCTACTGTGGGG

AAAAAAAGACTTCATAAAGTAAACCATCTCCAAAAAGAAATAAAGAAAGGCAGACATAGA

TTCCACTCAATTCAGAATAATGCCCTTCAAAGGAAAAGAGGTTGGCCAAAAGGACCAAGA

ATTCCCAGAGAAAAAAATGTGAATTTCAACCTTCCTAAACTTCCTGTGACCTGTGGTGAG

GCTAAGGGGACTCTCCATAAGAAGCAATTTAAGCAAGGGGCCCAGATGAAGAGCATAAGG

ACTGAGGATGGAAAATGGCTCACCCCCAGGGACTTCGAGATCAGAGGAAACCACGCAGCA

GCCAAGAACTGGAAGCTAAGCCTGCGCTGCCACGGATGGACCTTGAAAGAACTGATAGAG

GCAGGGCATCTACCAAACCCACCAATAACAAGAAAAAAGAGGAAAACACGGGAGTCTCTC

AACAATGTCTTAGTTGACCCCCATCAAAAAAACTCAAATAAGTGCGAAGTATGCCGCCAA

GAGAAACAGCTGGTCTTCTGCAATACTTGTTCAAGATCATACCATCAGAACTGCCACATC

CCACCAGTAGATGCTCAGAGGAGCCAGTGGAGTTGCACCTTATGTAGGACAAAAGCCTGG

GAAAAGCACCCAGAAAGCCAACCATGTCATCTGGAATCTGAGGTCTTGAAGAGGCCTATG

CTGCCTGAGGAACAGCTGAAATGTGAGCTGATCCTATTGAAGGTCTATTGTTATTCACAA

AGTGCCTTTTTTGCCCAAGAACCATATTATACTCAAGACCCCCAGGAGCACATGTGGTTA

AACAAGGTCAAGGAAAGGCTGAATAAGAAGAAGTACCACCGAGTGGAGGGGTTTGTGCGG

GACATGCGCCTCATCTTTCTGAACAACAAGGCCTTCTACAGAAACCACAAATTCATCCAG

CTGCCTCTTAAGATAGAAGCCATATTTGAGAAGTATTTCAAAAACATTTTTTCAATCCAA

GAAACAAGCGAGAGCATCTGTCAGTTT

>CL356.Contig76_A-W 589 2403 minus strand PREDICTED: nuclear autoantigen Sp-100 [Pan paniscus]

CCAGGAGCAGAGCTCTCCAGCCATGGAATCCACATGACTTCCTGTTCTGTGCATCTGGTG

GATATAAAGAAGGAAAAGCCATTTTTTCCCACGCAAATGAAGCAAGGATCTCAAACCAGG

ACCAACCATTACCAGCCATCCGAAGTAATAGTCATCAGCAGTGAGGACGACGCCAGCGAC

ACAGAAGAGCCTGCAGCAATCTCCAGCTCAGCACGGAGGAGCCATCCTGTGATCAAAAAC

CATGTCCAGTTTGACTTGAGAGATGAAGAAGAGGCCCAAGAAGCCACTTGCTCATGGCAC

CAGGCTGCTCCAGCGAGAGGACGTGACTATGGCTCTTCAGAATCCAGTGAGGAGGAGATA

CCCTCAGCGCTCCGGATCTCAACACCAACAGATTCTGTGAGGATGTCTACTTGGAGGATA

CACAGTCAGAAAAGAAGGCACAGCAGTGACAGCTCTTCAGAGCTGAGTTATGGAGAGGAG

CCTCGGGAAGCCCTGAGAAGTGGATCAGGTGTGCCAAGAGGCCAAGAAGCAACGGTGGAA

AGTGGCCGAGCATCTCACCCAATAGGCAAGTCTGCTCAATGGCATCAGGGTGGAGGTGGC

TCAGAAATACCACAGCTGCTGGGCAGGACTGTGTCAGGAGAGGAAGCTGTAAAAGATACC

ACGGATGTTGGAAACAATTCTACTTTGGGAAAAGGCAGTGGGGAAAGAAATATTTCCAAG

CTGCACAACAGAGATAAAGAAATGAACAAAATCTCATCAACCTTACATCCTACTGTGGGG

AAAAAAAGACTTCATAAAGTAAACCATCTCCAAAAAGAAATAAAGAAAGGCAGACATAGA

TTCCACTCAATTCAGAATAATGCCCTTCAAAGGAAAAGAGGTTGGCCAAAAGGGAGAAAA

AGAGTCAACGTTAGACCCTTGAAAAGAGGGAGAAAAAGAGGACCAAGAATTCCCAGAGAA

AAAAATGTGAATTTCAACCTTCCTAAACTTCCTGTGACCTGTGGTGAGGCTAAGGGGACT

CTCCATAAGAAGCAATTTAAGCAAGGGGCCCAGATGAAGAGCATAAGGACTGAGGATGGA

AAATGGCTCACCCCCAGGGACTTCGAGATCAGAGGAAACCACGCAGCAGCCAAGAACTGG

AAGCTAAGCCTGCGCTGCCACGGATGGACCTTGAAAGAACTGATAGAGGCAGGGCATCTA

CCAAACCCACCAATAACAAGAAAAAAGAGGAAAACACGGGAGTCTCTCAACAATGTCTTA

GTTGACCCCCATCAAAAAAACTCAAATAAGTGCGAAGTATGCCGCCAAGAGAAACAGCTG

GTCTTCTGCAATACTTGTTCAAGATCATACCATCAGAACTGCCACATCCCACCAGTAGAT

GCTCAGAGGAGCCAGTGGAGTTGCACCTTATGTAGGACAAAAGCCTGGGAAAAGCACCCA

GAAAGCCAACCATGTCATCTGGAATCTGAGGTCTTGAAGAGGCCTATGCTGCCTGAGGAA

CAGCTGAAATGTGAGCTGATCCTATTGAAGGTCTATTGTTATTCACAAAGTGCCTTTTTT

GCCCAAGAACCATATTATACTCAAGACCCCCAGGAGCACATGTGGTTAAACAAGGTCAAG

GAAAGGCTGAATAAGAAGAAGTACCACCGAGTGGAGGGGTTTGTGCGGGACATGCGCCTC

ATCTTTCTGAACAACAAGGCCTTCTACAGAAACCACAAATTCATCCAGCTGCCTCTTAAG

ATAGAAGCCATATTTGAGAAGTATTTCAAAAACATTTTTTCAATCCAAGAAACAAGCGAG

AGCATCTGTCAGTTT

>CL356.Contig77_A-W 174 362 minus strand PREDICTED: nuclear autoantigen Sp-100 [Pan paniscus]

CTTGGAAGGATGTTTGCAATGGTCAAGAATAAGGAGATATTTTGTGATATATTTTTCAAC

CACTTCAAGAAAAATAAAGTGGAGATAGCAACTGCAATAACAAAGCTGTTTCCTTTCCTA

GAGGGCCTCAGAGACTGATCCTTTATCACCGAACAAATCTATGCTTGTTCCAAGAAAAAT

ATGTTTCCC

>CL356.Contig78_A-W 132 2612 minus strand PREDICTED: nuclear autoantigen Sp-100 isoform 14 [Pan troglodytes]

AGGATGTCCACAGAAGAGCAGAGTGTAGACGACAGTACCATCTATGAGGTTATATTCCAT

CACTTCAAAAGACACAAAGTGGAGATTTCAAATGCCATAAAAAAGACATTTCCTTTCCTC

GAGGGCCTCCGTGACCGTGAACTCATTACCAATAAAATGTTTGAAGATTCTCAAGAGTCT

TGTAGAAACCTGGTCCCTGTACAAAGAGTGGTGTACAATGTTCTCTGTGAACTGGAGAAG

AAATTCAACCTGGATTTGTGCGAAGCATTGTTCAGTGAGGTCAACTTGCAGGAATACCCT

GATTTAATTCGTGTTCGTAGAAGCTTCGAACATGAGGTGAGAATGCTATATCCCCAAGAA

GATAATGGAGAAGAGAGGCAGGAAAGGCTTGAACAAGGAACGGGGGAGAACTCTTTTCTG

CGAAGACTGACCTGGCCACAGCACTCAGATCCCTCGTCTTACAATGGTACAGACCTACCC

GAAAATGAACTCTTGGCGCATCCTTGCGAAGCAGAAGAGAGAAATGTAAATAAAAGAGAG

CCGGCCCGAGACCCAAATGGTGCAGTAGGAAGCCACCAAGGAAATGAGCAATGCCCTCGA

GAGTCAGAGCCAGTAGAGTCCCACCAACACATGGCTGCCAACGTGGACCGTGGAGAGGCG

AGCGTGGGGACACCCAGCCCGTTGCCCCACTGTGTAGAAAGAGCAGAGCTCTCCAGCCAT

GGAATCCACATGACTTCCTGTTCTGTGCATCTGGTGGATATAAAGAAGGAAAAGCCATTT

TTTCCCACGCAAATGAAGCAAGGATCTCAAACCAGGACCAACCATTACCAGCCATCCGAA

GTAATAGTCATCAGCAGTGAGGACGACGCCAGCGACACAGAAGAGCCTGCAGCAATCTCC

AGCTCAGCACGGAGGAGCCATCCTGTGATCAAAAACCATGTCCAGTTTGACTTGAGAGAT

GAAGAAGAGGCCCAAGAAGCCACTTGCTCATGGCACCAGGCTGCTCCAGCGAGAGGACGT

GACTATGGCTCTTCAGAATCCAGTGAGGAGGAGATACCCTCAGCGCTCCGGATCTCAACA

CCAACAGATTCTGTGAGGATGTCTACTTGGAGGATACACAGTCAGAAAAGAAGGCACAGC

AGTGACAGCTCTTCAGAGCTGAGTTATGGAGAGGAGCCTCGGGAAGCCCTGAGAAGTGGA

TCAGGAACAGGGCAACAAGAGACTGGAAATGAGAAGTGCTCCTGTGTCCTGTGTTCCCCA

AAAGGTGTGCCAAGAGGCCAAGAAGCAACGGTGGAAAGTGGCCGAGCATCTCACCCAATA

GATACCACGGATGTTGGAAACAATTCTACTTTGGGAAAAGGCAGTGGGGAAAGAAATATT

TCCAAGCTGCACAACAGAGATAAAGAAATGAACAAAATCTCATCAACCTTACATCCTACT

GTGGGGAAAAAAAGACTTCATAAAGTAAACCATCTCCAAAAAGAAATAAAGAAAGGCAGA

CATAGATTCCACTCAATTCAGAATAATGCCCTTCAAAGGAAAAGAGGTTGGCCAAAAGGG

AGAAAAAGAGTCAACGTTAGACCCTTGAAAAGAGGGAGAAAAAGAGGACCAAGAATTCCC

AGAGAAAAAAATGTGAATTTCAACCTTCCTAAACTTCCTGTGACCTGTGGTGAGGCTAAG

GGGACTCTCCATAAGAAGCAATTTAAGCAAGGGGCCCAGATGAAGAGCATAAGGACTGAG

GATGGAAAATGGCTCACCCCCAGGGACTTCGAGATCAGAGGAAACCACGCAGCAGCCAAG

AACTGGAAGCTAAGCCTGCGCTGCCACGGATGGACCTTGAAAGAACTGATAGAGGCAGGG

CATCTACCAAACCCACCAATAACAAGAAAAAAGAGGAAAACACGGGAGTCTCTCAACAAT

GTCTTAGTTGACCCCCATCAAAAAAACTCAAATAAGTGCGAAGTATGCCGCCAAGAGAAA

CAGCTGGTCTTCTGCAATACTTGTTCAAGATCATACCATCAGAACTGCCACATCCCACCA

GTAGATGCTCAGAGGAGCCAGTGGAGTTGCACCTTATGTAGGACAAAAGCCTGGGAAAAG

CACCCAGAAAGCCAACCATGTCATCTGGAATCTGAGGTCTTGAAGAGGCCTATGCTGCCT

GAGGAACAGCTGAAATGTGAGCTGATCCTATTGAAGGTCTATTGTTATTCACAAAGTGCC

TTTTTTGCCCAAGAACCATATTATACTCAAGACCCCCAGGAGCACATGTGGTTAAACAAG

GTCAAGGAAAGGCTGAATAAGAAGAAGTACCACCGAGTGGAGGGGTTTGTGCGGGACATG

CGCCTCATCTTTCTGAACAACAAGGCCTTCTACAGAAACCACAAATTCATCCAGCTGCCT

CTTAAGATAGAAGCCATATTTGAGAAGTATTTCAAAAACATTTTTTCAATCCAAGAAACA

AGCGAGAGCATCTGTCAGTTT

>CL356.Contig79_A-W 589 2394 minus strand PREDICTED: nuclear autoantigen Sp-100 [Saimiri boliviensis boliviensis]

CCAGGAGCAGAGCTCTCCAGCCATGGAATCCACATGACTTCCTGTTCTGTGCATCTGGTG

GATATAAAGAAGGAAAAGCCATTTTTTCCCACGCAAATGAAGCAAGGATCTCAAACCAGG

ACCAACCATTACCAGCCATCCGAAGTAATAGTCATCAGCAGTGAGGACGACGCCAGCGAC

ACAGAAGAGCCTGCAGCAATCTCCAGCTCAGCACGGAGGAGCCATCCTGTGATCAAAAAC

CATGTCCAGTTTGACTTGAGAGATGAAGAAGAGGCCCAAGAAGCCACTTGCTCATGGCAC

CAGGCTGCTCCAGCGAGAGGACGTGACTATGGCTCTTCAGAATCCAGTGAGGAGGAGATA

CCCTCAGCGCTCCGGATCTCAACACCAACAGATTCTGTGAGGATGTCTACTTGGAGGATA

CACAGTCAGAAAAGAAGGCACAGCAGTGACAGCTCTTCAGAGCTGAGTTATGGAGAGGAG

CCTCGGGAAGCCCTGAGAAGTGGATCAGGAACAGGGCAACAAGAGACTGGAAATGAGAAG

TGCTCCTGTGTCCTGTGTTCCCCAAAAGGTGTGCCAAGAGGCCAAGAAGCAACGGTGGAA

AGTGGCCGAGCATCTCACCCAATAGGCAAGTCTGCTCAATGGCATCAGGGTGGAGGTGGC

TCAGAAATACCACAGCTGCTGGGCAGGACTGTGTCAGGAGAGGAAGCTGTAAAAGATACC

ACGGATGTTGGAAACAATTCTACTTTGGGAAAAGGCAGTGGGGAAAGAAGGAAAAAAAGA

CTTCATAAAGTAAACCATCTCCAAAAAGAAATAAAGAAAGGCAGACATAGATTCCACTCA

ATTCAGAATAATGCCCTTCAAAGGAAAAGAGGTTGGCCAAAAGGGAGAAAAAGAGTCAAC

GTTAGACCCTTGAAAAGAGGGAGAAAAAGAGGACCAAGAATTCCCAGAGAAAAAAATGTG

AATTTCAACCTTCCTAAACTTCCTGTGACCTGTGGTGAGGCTAAGGGGACTCTCCATAAG

AAGCAATTTAAGCAAGGGGCCCAGATGAAGAGCATAAGGACTGAGGATGGAAAATGGCTC

ACCCCCAGGGACTTCGAGATCAGAGGAAACCACGCAGCAGCCAAGAACTGGAAGCTAAGC

CTGCGCTGCCACGGATGGACCTTGAAAGAACTGATAGAGGCAGGGCATCTACCAAACCCA

CCAATAACAAGAAAAAAGAGGAAAACACGGGAGTCTCTCAACAATGTCTTAGTTGACCCC

CATCAAAAAAACTCAAATAAGTGCGAAGTATGCCGCCAAGAGAAACAGCTGGTCTTCTGC

AATACTTGTTCAAGATCATACCATCAGAACTGCCACATCCCACCAGTAGATGCTCAGAGG

AGCCAGTGGAGTTGCACCTTATGTAGGACAAAAGCCTGGGAAAAGCACCCAGAAAGCCAA

CCATGTCATCTGGAATCTGAGGTCTTGAAGAGGCCTATGCTGCCTGAGGAACAGCTGAAA

TGTGAGCTGATCCTATTGAAGGTCTATTGTTATTCACAAAGTGCCTTTTTTGCCCAAGAA

CCATATTATACTCAAGACCCCCAGGAGCACATGTGGTTAAACAAGGTCAAGGAAAGGCTG

AATAAGAAGAAGTACCACCGAGTGGAGGGGTTTGTGCGGGACATGCGCCTCATCTTTCTG

AACAACAAGGCCTTCTACAGAAACCACAAATTCATCCAGCTGCCTCTTAAGATAGAAGCC

ATATTTGAGAAGTATTTCAAAAACATTTTTTCAATCCAAGAAACAAGCGAGAGCATCTGT

CAGTTT

>CL356.Contig80_A-W 144 347 minus strand PREDICTED: nuclear autoantigen Sp-100 [Pan paniscus]

CCAGGAGGATCCCCGGACATCAGGATGTTTGCAATGGTCAAGAATAAGGAGATATTTTGT

GATATATTTTTCAACCACTTCAAGAAAAATAAAGTGGAGATAGCAACTGCAATAACAAAG

CTGTTTCCTTTCCTAGAGGGCCTCAGAGACTGATCCTTTATCACCGAACAAATCTATGCT

TGTTCCAAGAAAAATATGTTTCCC

>CL356.Contig81_A-W 589 2373 minus strand PREDICTED: nuclear autoantigen Sp-100 [Saimiri boliviensis boliviensis]

CCAGGAGCAGAGCTCTCCAGCCATGGAATCCACATGACTTCCTGTTCTGTGCATCTGGTG

GATATAAAGAAGGAAAAGCCATTTTTTCCCACGCAAATGAAGCAAGGATCTCAAACCAGG

ACCAACCATTACCAGCCATCCGAAGTAATAGTCATCAGCAGTGAGGACGACGCCAGCGAC

ACAGAAGAGCCTGCAGCAATCTCCAGCTCAGCACGGAGGAGCCATCCTGTGATCAAAAAC

CATGTCCAGTTTGACTTGAGAGATGAAGAAGAGGCCCAAGAAGCCACTTGCTCATGGCAC

CAGGCTGCTCCAGCGAGAGGACGTGACTATGGCTCTTCAGAATCCAGTGAGGAGGAGATA

CCCTCAGCGCTCCGGATCTCAACACCAACAGATTCTGTGAGGATGTCTACTTGGAGGATA

CACAGTCAGAAAAGAAGGCACAGCAGTGACAGCTCTTCAGAGCTGAGTTATGGAGAGGAG

CCTCGGGAAGCCCTGAGAAGTGGATCAGGAACAGGGCAACAAGAGACTGGAAATGAGAAG

TGCTCCTGTGTCCTGTGTTCCCCAAAAGGTGTGCCAAGAGGCCAAGAAGCAACGGTGGAA

AGTGGCCGAGCATCTCACCCAATAGATACCACGGATGTTGGAAACAATTCTACTTTGGGA

AAAGGCAGTGGGGAAAGAAATATTTCCAAGCTGCACAACAGAGATAAAGAAATGAACAAA

ATCTCATCAACCTTACATCCTACTGTGGGGAAAAAAAGACTTCATAAAGTAAACCATCTC

CAAAAAGAAATAAAGAAAGGCAGACATAGATTCCACTCAATTCAGAATAATGCCCTTCAA

AGGAAAAGAGGTTGGCCAAAAGGGAGAAAAAGAGTCAACGTTAGACCCTTGAAAAGAGGG

AGAAAAAGAGGACCAAGAATTCCCAGAGAAAAAAATGTGAATTTCAACCTTCCTAAACTT

CCTGTGACCTGTGGTGAGGCTAAGGGGACTCTCCATAAGAAGCAATTTAAGCAAGGGGCC

CAGATGAAGAGCATAAGGACTGAGGATGGAAAATGGCTCACCCCCAGGGACTTCGAGATC

AGAGGAAACCACGCAGCAGCCAAGAACTGGAAGCTAAGCCTGCGCTGCCACGGATGGACC

TTGAAAGAACTGATAGAGGCAGGGCATCTACCAAACCCACCAATAACAAGAAAAAAGAGG

AAAACACGGGAGTCTCTCAACAATGTCTTAGTTGACCCCCATCAAAAAAACTCAAATAAG

TGCGAAGTATGCCGCCAAGAGAAACAGCTGGTCTTCTGCAATACTTGTTCAAGATCATAC

CATCAGAACTGCCACATCCCACCAGTAGATGCTCAGAGGAGCCAGTGGAGTTGCACCTTA

TGTAGGACAAAAGCCTGGGAAAAGCACCCAGAAAGCCAACCATGTCATCTGGAATCTGAG

GTCTTGAAGAGGCCTATGCTGCCTGAGGAACAGCTGAAATGTGAGCTGATCCTATTGAAG

GTCTATTGTTATTCACAAAGTGCCTTTTTTGCCCAAGAACCATATTATACTCAAGACCCC

CAGGAGCACATGTGGTTAAACAAGGTCAAGGAAAGGCTGAATAAGAAGAAGTACCACCGA

GTGGAGGGGTTTGTGCGGGACATGCGCCTCATCTTTCTGAACAACAAGGCCTTCTACAGA

AACCACAAATTCATCCAGCTGCCTCTTAAGATAGAAGCCATATTTGAGAAGTATTTCAAA

AACATTTTTTCAATCCAAGAAACAAGCGAGAGCATCTGTCAGTTT

>CL356.Contig82_A-W 589 2355 minus strand PREDICTED: nuclear autoantigen Sp-100 [Pan paniscus]

CCAGGAGCAGAGCTCTCCAGCCATGGAATCCACATGACTTCCTGTTCTGTGCATCTGGTG

GATATAAAGAAGGAAAAGCCATTTTTTCCCACGCAAATGAAGCAAGGATCTCAAACCAGG

ACCAACCATTACCAGCCATCCGAAGTAATAGTCATCAGCAGTGAGGACGACGCCAGCGAC

ACAGAAGAGCCTGCAGCAATCTCCAGCTCAGCACGGAGGAGCCATCCTGTGATCAAAAAC

CATGTCCAGTTTGACTTGAGAGATGAAGAAGAGGCCCAAGAAGCCACTTGCTCATGGCAC

CAGGCTGCTCCAGCGAGAGGACGTGACTATGGCTCTTCAGAATCCAGTGAGGAGGAGATA

CCCTCAGCGCTCCGGATCTCAACACCAACAGATTCTGTGAGGATGTCTACTTGGAGGATA

CACAGTCAGAAAAGAAGGCACAGCAGTGACAGCTCTTCAGAGCTGAGTTATGGAGAGGAG

CCTCGGGAAGCCCTGAGAAGTGGATCAGGTGTGCCAAGAGGCCAAGAAGCAACGGTGGAA

AGTGGCCGAGCATCTCACCCAATAGGCAAGTCTGCTCAATGGCATCAGGGTGGAGGTGGC

TCAGAAATACCACAGCTGCTGGGCAGGACTGTGTCAGGAGAGGAAGCTGTAAAAGATACC

ACGGATGTTGGAAACAATTCTACTTTGGGAAAAGGCAGTGGGGAAAGAAATATTTCCAAG

CTGCACAACAGAGATAAAGAAATGAACAAAATCTCATCAACCTTACATCCTACTGTGGGG

AAAAAAAGACTTCATAAAGTAAACCATCTCCAAAAAGAAATAAAGAAAGGCAGACATAGA

TTCCACTCAATTCAGAATAATGCCCTTCAAAGGAAAAGAGGTTGGCCAAAAGGACCAAGA

ATTCCCAGAGAAAAAAATGTGAATTTCAACCTTCCTAAACTTCCTGTGACCTGTGGTGAG

GCTAAGGGGACTCTCCATAAGAAGCAATTTAAGCAAGGGGCCCAGATGAAGAGCATAAGG

ACTGAGGATGGAAAATGGCTCACCCCCAGGGACTTCGAGATCAGAGGAAACCACGCAGCA

GCCAAGAACTGGAAGCTAAGCCTGCGCTGCCACGGATGGACCTTGAAAGAACTGATAGAG

GCAGGGCATCTACCAAACCCACCAATAACAAGAAAAAAGAGGAAAACACGGGAGTCTCTC

AACAATGTCTTAGTTGACCCCCATCAAAAAAACTCAAATAAGTGCGAAGTATGCCGCCAA

GAGAAACAGCTGGTCTTCTGCAATACTTGTTCAAGATCATACCATCAGAACTGCCACATC

CCACCAGTAGATGCTCAGAGGAGCCAGTGGAGTTGCACCTTATGTAGGACAAAAGCCTGG

GAAAAGCACCCAGAAAGCCAACCATGTCATCTGGAATCTGAGGTCTTGAAGAGGCCTATG

CTGCCTGAGGAACAGCTGAAATGTGAGCTGATCCTATTGAAGGTCTATTGTTATTCACAA

AGTGCCTTTTTTGCCCAAGAACCATATTATACTCAAGACCCCCAGGAGCACATGTGGTTA

AACAAGGTCAAGGAAAGGCTGAATAAGAAGAAGTACCACCGAGTGGAGGGGTTTGTGCGG

GACATGCGCCTCATCTTTCTGAACAACAAGGCCTTCTACAGAAACCACAAATTCATCCAG

CTGCCTCTTAAGATAGAAGCCATATTTGAGAAGTATTTCAAAAACATTTTTTCAATCCAA

GAAACAAGCGAGAGCATCTGTCAGTTT

>CL356.Contig83_A-W 589 2346 minus strand PREDICTED: nuclear autoantigen Sp-100 [Saimiri boliviensis boliviensis]

CCAGGAGCAGAGCTCTCCAGCCATGGAATCCACATGACTTCCTGTTCTGTGCATCTGGTG

GATATAAAGAAGGAAAAGCCATTTTTTCCCACGCAAATGAAGCAAGGATCTCAAACCAGG

ACCAACCATTACCAGCCATCCGAAGTAATAGTCATCAGCAGTGAGGACGACGCCAGCGAC

ACAGAAGAGCCTGCAGCAATCTCCAGCTCAGCACGGAGGAGCCATCCTGTGATCAAAAAC

CATGTCCAGTTTGACTTGAGAGATGAAGAAGAGGCCCAAGAAGCCACTTGCTCATGGCAC

CAGGCTGCTCCAGCGAGAGGACGTGACTATGGCTCTTCAGAATCCAGTGAGGAGGAGATA

CCCTCAGCGCTCCGGATCTCAACACCAACAGATTCTGTGAGGATGTCTACTTGGAGGATA

CACAGTCAGAAAAGAAGGCACAGCAGTGACAGCTCTTCAGAGCTGAGTTATGGAGAGGAG

CCTCGGGAAGCCCTGAGAAGTGGATCAGGAACAGGGCAACAAGAGACTGGAAATGAGAAG

TGCTCCTGTGTCCTGTGTTCCCCAAAAGGTGTGCCAAGAGGCCAAGAAGCAACGGTGGAA

AGTGGCCGAGCATCTCACCCAATAGGCAAGTCTGCTCAATGGCATCAGGGTGGAGGTGGC

TCAGAAATACCACAGCTGCTGGGCAGGACTGTGTCAGGAGAGGAAGCTGTAAAAGATACC

ACGGATGTTGGAAACAATTCTACTTTGGGAAAAGGCAGTGGGGAAAGAAGGAAAAAAAGA

CTTCATAAAGTAAACCATCTCCAAAAAGAAATAAAGAAAGGCAGACATAGATTCCACTCA

ATTCAGAATAATGCCCTTCAAAGGAAAAGAGGTTGGCCAAAAGGACCAAGAATTCCCAGA

GAAAAAAATGTGAATTTCAACCTTCCTAAACTTCCTGTGACCTGTGGTGAGGCTAAGGGG

ACTCTCCATAAGAAGCAATTTAAGCAAGGGGCCCAGATGAAGAGCATAAGGACTGAGGAT

GGAAAATGGCTCACCCCCAGGGACTTCGAGATCAGAGGAAACCACGCAGCAGCCAAGAAC

TGGAAGCTAAGCCTGCGCTGCCACGGATGGACCTTGAAAGAACTGATAGAGGCAGGGCAT

CTACCAAACCCACCAATAACAAGAAAAAAGAGGAAAACACGGGAGTCTCTCAACAATGTC

TTAGTTGACCCCCATCAAAAAAACTCAAATAAGTGCGAAGTATGCCGCCAAGAGAAACAG

CTGGTCTTCTGCAATACTTGTTCAAGATCATACCATCAGAACTGCCACATCCCACCAGTA

GATGCTCAGAGGAGCCAGTGGAGTTGCACCTTATGTAGGACAAAAGCCTGGGAAAAGCAC

CCAGAAAGCCAACCATGTCATCTGGAATCTGAGGTCTTGAAGAGGCCTATGCTGCCTGAG

GAACAGCTGAAATGTGAGCTGATCCTATTGAAGGTCTATTGTTATTCACAAAGTGCCTTT

TTTGCCCAAGAACCATATTATACTCAAGACCCCCAGGAGCACATGTGGTTAAACAAGGTC

AAGGAAAGGCTGAATAAGAAGAAGTACCACCGAGTGGAGGGGTTTGTGCGGGACATGCGC

CTCATCTTTCTGAACAACAAGGCCTTCTACAGAAACCACAAATTCATCCAGCTGCCTCTT

AAGATAGAAGCCATATTTGAGAAGTATTTCAAAAACATTTTTTCAATCCAAGAAACAAGC

GAGAGCATCTGTCAGTTT

>CL356.Contig84_A-W 589 2334 minus strand PREDICTED: nuclear autoantigen Sp-100 [Pan paniscus]

CCAGGAGCAGAGCTCTCCAGCCATGGAATCCACATGACTTCCTGTTCTGTGCATCTGGTG

GATATAAAGAAGGAAAAGCCATTTTTTCCCACGCAAATGAAGCAAGGATCTCAAACCAGG

ACCAACCATTACCAGCCATCCGAAGTAATAGTCATCAGCAGTGAGGACGACGCCAGCGAC

ACAGAAGAGCCTGCAGCAATCTCCAGCTCAGCACGGAGGAGCCATCCTGTGATCAAAAAC

CATGTCCAGTTTGACTTGAGAGATGAAGAAGAGGCCCAAGAAGCCACTTGCTCATGGCAC

CAGGCTGCTCCAGCGAGAGGACGTGACTATGGCTCTTCAGAATCCAGTGAGGAGGAGATA

CCCTCAGCGCTCCGGATCTCAACACCAACAGATTCTGTGAGGATGTCTACTTGGAGGATA

CACAGTCAGAAAAGAAGGCACAGCAGTGACAGCTCTTCAGAGCTGAGTTATGGAGAGGAG

CCTCGGGAAGCCCTGAGAAGTGGATCAGGTGTGCCAAGAGGCCAAGAAGCAACGGTGGAA

AGTGGCCGAGCATCTCACCCAATAGGCAAGTCTGCTCAATGGCATCAGGGTGGAGGTGGC

TCAGAAATACCACAGCTGCTGGGCAGGACTGTGTCAGGAGAGGAAGCTGTAAAAGATACC

ACGGATGTTGGAAACAATTCTACTTTGGGAAAAGGCAGTGGGGAAAGAAGGAAAAAAAGA

CTTCATAAAGTAAACCATCTCCAAAAAGAAATAAAGAAAGGCAGACATAGATTCCACTCA

ATTCAGAATAATGCCCTTCAAAGGAAAAGAGGTTGGCCAAAAGGGAGAAAAAGAGTCAAC

GTTAGACCCTTGAAAAGAGGGAGAAAAAGAGGACCAAGAATTCCCAGAGAAAAAAATGTG

AATTTCAACCTTCCTAAACTTCCTGTGACCTGTGGTGAGGCTAAGGGGACTCTCCATAAG

AAGCAATTTAAGCAAGGGGCCCAGATGAAGAGCATAAGGACTGAGGATGGAAAATGGCTC

ACCCCCAGGGACTTCGAGATCAGAGGAAACCACGCAGCAGCCAAGAACTGGAAGCTAAGC

CTGCGCTGCCACGGATGGACCTTGAAAGAACTGATAGAGGCAGGGCATCTACCAAACCCA

CCAATAACAAGAAAAAAGAGGAAAACACGGGAGTCTCTCAACAATGTCTTAGTTGACCCC

CATCAAAAAAACTCAAATAAGTGCGAAGTATGCCGCCAAGAGAAACAGCTGGTCTTCTGC

AATACTTGTTCAAGATCATACCATCAGAACTGCCACATCCCACCAGTAGATGCTCAGAGG

AGCCAGTGGAGTTGCACCTTATGTAGGACAAAAGCCTGGGAAAAGCACCCAGAAAGCCAA

CCATGTCATCTGGAATCTGAGGTCTTGAAGAGGCCTATGCTGCCTGAGGAACAGCTGAAA

TGTGAGCTGATCCTATTGAAGGTCTATTGTTATTCACAAAGTGCCTTTTTTGCCCAAGAA

CCATATTATACTCAAGACCCCCAGGAGCACATGTGGTTAAACAAGGTCAAGGAAAGGCTG

AATAAGAAGAAGTACCACCGAGTGGAGGGGTTTGTGCGGGACATGCGCCTCATCTTTCTG

AACAACAAGGCCTTCTACAGAAACCACAAATTCATCCAGCTGCCTCTTAAGATAGAAGCC

ATATTTGAGAAGTATTTCAAAAACATTTTTTCAATCCAAGAAACAAGCGAGAGCATCTGT

CAGTTT

>CL356.Contig85_A-W 191 2275 minus strand PREDICTED: nuclear autoantigen Sp-100 [Pan paniscus]

TCAGTGTTCCAAGAAAAATATGTTTCCCTAAGAAGTGACCCGGAAGGAAGACAACAAACA

TCCATCACTCAGCCAAGCCGTGAGCAAGGAACAGTTGAGAACACGTTTCTGCGAAGACTG

ACCTGGCCACAGCACTCAGATCCCTCGTCTTACAATGGTACAGACCTACCCGAAAATGAA

CTCTTGGCGCATCCTTGCGAAGCAGAAGAGAGAAATGTAAATAAAAGAGAGCCGGCCCGA

GACCCAAATGGTGCAGTAGGAAGCCACCAAGGAAATGAGCAATGCCCTCGAGAGTCAGAG

CCAGTAGAGTCCCACCAACACATGGCTGCCAACGTGGACCGTGGAGAGGCGAGCGTGGGG

ACACCCAGCCCGTTGCCCCACTGTGTAGAAAGAGCAGAGCTCTCCAGCCATGGAATCCAC

ATGACTTCCTGTTCTGTGCATCTGGTGGATATAAAGAAGGAAAAGCCATTTTTTCCCACG

CAAATGAAGCAAGGATCTCAAACCAGGACCAACCATTACCAGCCATCCGAAGTAATAGTC

ATCAGCAGTGAGGACGACGCCAGCGACACAGAAGAGCCTGCAGCAATCTCCAGCTCAGCA

CGGAGGAGCCATCCTGTGATCAAAAACCATGTCCAGTTTGACTTGAGAGATGAAGAAGAG

GCCCAAGAAGCCACTTGCTCATGGCACCAGGCTGCTCCAGCGAGAGGACGTGACTATGGC

TCTTCAGAATCCAGTGAGGAGGAGATACCCTCAGCGCTCCGGATCTCAACACCAACAGAT

TCTGTGAGGATGTCTACTTGGAGGATACACAGTCAGAAAAGAAGGCACAGCAGTGACAGC

TCTTCAGAGCTGAGTTATGGAGAGGAGCCTCGGGAAGCCCTGAGAAGTGGATCAGGTGTG

CCAAGAGGCCAAGAAGCAACGGTGGAAAGTGGCCGAGCATCTCACCCAATAGGCAAGTCT

GCTCAATGGCATCAGGGTGGAGGTGGCTCAGAAATACCACAGCTGCTGGGCAGGACTGTG

TCAGGAGAGGAAGCTGTAAAAGATACCACGGATGTTGGAAACAATTCTACTTTGGGAAAA

GGCAGTGGGGAAAGAAGGAAAAAAAGACTTCATAAAGTAAACCATCTCCAAAAAGAAATA

AAGAAAGGCAGACATAGATTCCACTCAATTCAGAATAATGCCCTTCAAAGGAAAAGAGGT

TGGCCAAAAGGACCAAGAATTCCCAGAGAAAAAAATGTGAATTTCAACCTTCCTAAACTT

CCTGTGACCTGTGGTGAGGCTAAGGGGACTCTCCATAAGAAGCAATTTAAGCAAGGGGCC

CAGATGAAGAGCATAAGGACTGAGGATGGAAAATGGCTCACCCCCAGGGACTTCGAGATC

AGAGGAAACCACGCAGCAGCCAAGAACTGGAAGCTAAGCCTGCGCTGCCACGGATGGACC

TTGAAAGAACTGATAGAGGCAGGGCATCTACCAAACCCACCAATAACAAGAAAAAAGAGG

AAAACACGGGAGTCTCTCAACAATGTCTTAGTTGACCCCCATCAAAAAAACTCAAATAAG

TGCGAAGTATGCCGCCAAGAGAAACAGCTGGTCTTCTGCAATACTTGTTCAAGATCATAC

CATCAGAACTGCCACATCCCACCAGTAGATGCTCAGAGGAGCCAGTGGAGTTGCACCTTA

TGTAGGACAAAAGCCTGGGAAAAGCACCCAGAAAGCCAACCATGTCATCTGGAATCTGAG

GTCTTGAAGAGGCCTATGCTGCCTGAGGAACAGCTGAAATGTGAGCTGATCCTATTGAAG

GTCTATTGTTATTCACAAAGTGCCTTTTTTGCCCAAGAACCATATTATACTCAAGACCCC

CAGGAGCACATGTGGTTAAACAAGGTCAAGGAAAGGCTGAATAAGAAGAAGTACCACCGA

GTGGAGGGGTTTGTGCGGGACATGCGCCTCATCTTTCTGAACAACAAGGCCTTCTACAGA

AACCACAAATTCATCCAGCTGCCTCTTAAGATAGAAGCCATATTTGAGAAGTATTTCAAA

AACATTTTTTCAATCCAAGAAACAAGCGAGAGCATCTGTCAGTTT

>CL356.Contig86_A-W 132 2594 minus strand PREDICTED: nuclear autoantigen Sp-100 [Pan paniscus]

AGGATGTCCACAGAAGAGCAGAGTGTAGACGACAGTACCATCTATGAGGTTATATTCCAT

CACTTCAAAAGACACAAAGTGGAGATTTCAAATGCCATAAAAAAGACATTTCCTTTCCTC

GAGGGCCTCCGTGACCGTGAACTCATTACCAATAAAATGTTTGAAGATTCTCAAGAGTCT

TGTAGAAACCTGGTCCCTGTACAAAGAGTGGTGTACAATGTTCTCTGTGAACTGGAGAAG

AAATTCAACCTGGATTTGTGCGAAGCATTGTTCAGTGAGGTCAACTTGCAGGAATACCCT

GATTTAATTCGTGTTCGTAGAAGCTTCGAACATGAGGTGAGAATGCTATATCCCCAAGAA

GATAATGGAGAAGAGAGGCAGGAAAGGCTTGAACAAGGAACGGGGGAGAACTCTTTTCTG

CGAAGACTGACCTGGCCACAGCACTCAGATCCCTCGTCTTACAATGGTACAGACCTACCC

GAAAATGAACTCTTGGCGCATCCTTGCGAAGCAGAAGAGAGAAATGTAAATAAAAGAGAG

CCGGCCCGAGACCCAAATGGTGCAGTAGGAAGCCACCAAGGAAATGAGCAATGCCCTCGA

GAGTCAGAGCCAGTAGAGTCCCACCAACACATGGCTGCCAACGTGGACCGTGGAGAGGCG

AGCGTGGGGACACCCAGCCCGTTGCCCCACTGTGTAGAAAGAGCAGAGCTCTCCAGCCAT

GGAATCCACATGACTTCCTGTTCTGTGCATCTGGTGGATATAAAGAAGGAAAAGCCATTT

TTTCCCACGCAAATGAAGCAAGGATCTCAAACCAGGACCAACCATTACCAGCCATCCGAA

GTAATAGTCATCAGCAGTGAGGACGACGCCAGCGACACAGAAGAGCCTGCAGCAATCTCC

AGCTCAGCACGGAGGAGCCATCCTGTGATCAAAAACCATGTCCAGTTTGACTTGAGAGAT

GAAGAAGAGGCCCAAGAAGCCACTTGCTCATGGCACCAGGCTGCTCCAGCGAGAGGACGT

GACTATGGCTCTTCAGAATCCAGTGAGGAGGAGATACCCTCAGCGCTCCGGATCTCAACA

CCAACAGATTCTGTGAGGATGTCTACTTGGAGGATACACAGTCAGAAAAGAAGGCACAGC

AGTGACAGCTCTTCAGAGCTGAGTTATGGAGAGGAGCCTCGGGAAGCCCTGAGAAGTGGA

TCAGGTGTGCCAAGAGGCCAAGAAGCAACGGTGGAAAGTGGCCGAGCATCTCACCCAATA

GGCAAGTCTGCTCAATGGCATCAGGGTGGAGGTGGCTCAGAAATACCACAGCTGCTGGGC

AGGACTGTGTCAGGAGAGGAAGCTGTAAAAGATACCACGGATGTTGGAAACAATTCTACT

TTGGGAAAAGGCAGTGGGGAAAGAAATATTTCCAAGCTGCACAACAGAGATAAAGAAATG

AACAAAATCTCATCAACCTTACATCCTACTGTGGGGAAAAAAAGACTTCATAAAGTAAAC

CATCTCCAAAAAGAAATAAAGAAAGGCAGACATAGATTCCACTCAATTCAGAATAATGCC

CTTCAAAGGAAAAGAGGTTGGCCAAAAGGACCAAGAATTCCCAGAGAAAAAAATGTGAAT

TTCAACCTTCCTAAACTTCCTGTGACCTGTGGTGAGGCTAAGGGGACTCTCCATAAGAAG

CAATTTAAGCAAGGGGCCCAGATGAAGAGCATAAGGACTGAGGATGGAAAATGGCTCACC

CCCAGGGACTTCGAGATCAGAGGAAACCACGCAGCAGCCAAGAACTGGAAGCTAAGCCTG

CGCTGCCACGGATGGACCTTGAAAGAACTGATAGAGGCAGGGCATCTACCAAACCCACCA

ATAACAAGAAAAAAGAGGAAAACACGGGAGTCTCTCAACAATGTCTTAGTTGACCCCCAT

CAAAAAAACTCAAATAAGTGCGAAGTATGCCGCCAAGAGAAACAGCTGGTCTTCTGCAAT

ACTTGTTCAAGATCATACCATCAGAACTGCCACATCCCACCAGTAGATGCTCAGAGGAGC

CAGTGGAGTTGCACCTTATGTAGGACAAAAGCCTGGGAAAAGCACCCAGAAAGCCAACCA

TGTCATCTGGAATCTGAGGTCTTGAAGAGGCCTATGCTGCCTGAGGAACAGCTGAAATGT

GAGCTGATCCTATTGAAGGTCTATTGTTATTCACAAAGTGCCTTTTTTGCCCAAGAACCA

TATTATACTCAAGACCCCCAGGAGCACATGTGGTTAAACAAGGTCAAGGAAAGGCTGAAT

AAGAAGAAGTACCACCGAGTGGAGGGGTTTGTGCGGGACATGCGCCTCATCTTTCTGAAC

AACAAGGCCTTCTACAGAAACCACAAATTCATCCAGCTGCCTCTTAAGATAGAAGCCATA

TTTGAGAAGTATTTCAAAAACATTTTTTCAATCCAAGAAACAAGCGAGAGCATCTGTCAG

TTT

>CL356.Contig87_A-W 589 2325 minus strand PREDICTED: nuclear autoantigen Sp-100 [Saimiri boliviensis boliviensis]

CCAGGAGCAGAGCTCTCCAGCCATGGAATCCACATGACTTCCTGTTCTGTGCATCTGGTG

GATATAAAGAAGGAAAAGCCATTTTTTCCCACGCAAATGAAGCAAGGATCTCAAACCAGG

ACCAACCATTACCAGCCATCCGAAGTAATAGTCATCAGCAGTGAGGACGACGCCAGCGAC

ACAGAAGAGCCTGCAGCAATCTCCAGCTCAGCACGGAGGAGCCATCCTGTGATCAAAAAC

CATGTCCAGTTTGACTTGAGAGATGAAGAAGAGGCCCAAGAAGCCACTTGCTCATGGCAC

CAGGCTGCTCCAGCGAGAGGACGTGACTATGGCTCTTCAGAATCCAGTGAGGAGGAGATA

CCCTCAGCGCTCCGGATCTCAACACCAACAGATTCTGTGAGGATGTCTACTTGGAGGATA

CACAGTCAGAAAAGAAGGCACAGCAGTGACAGCTCTTCAGAGCTGAGTTATGGAGAGGAG

CCTCGGGAAGCCCTGAGAAGTGGATCAGGAACAGGGCAACAAGAGACTGGAAATGAGAAG

TGCTCCTGTGTCCTGTGTTCCCCAAAAGGTGTGCCAAGAGGCCAAGAAGCAACGGTGGAA

AGTGGCCGAGCATCTCACCCAATAGATACCACGGATGTTGGAAACAATTCTACTTTGGGA

AAAGGCAGTGGGGAAAGAAATATTTCCAAGCTGCACAACAGAGATAAAGAAATGAACAAA

ATCTCATCAACCTTACATCCTACTGTGGGGAAAAAAAGACTTCATAAAGTAAACCATCTC

CAAAAAGAAATAAAGAAAGGCAGACATAGATTCCACTCAATTCAGAATAATGCCCTTCAA

AGGAAAAGAGGTTGGCCAAAAGGACCAAGAATTCCCAGAGAAAAAAATGTGAATTTCAAC

CTTCCTAAACTTCCTGTGACCTGTGGTGAGGCTAAGGGGACTCTCCATAAGAAGCAATTT

AAGCAAGGGGCCCAGATGAAGAGCATAAGGACTGAGGATGGAAAATGGCTCACCCCCAGG

GACTTCGAGATCAGAGGAAACCACGCAGCAGCCAAGAACTGGAAGCTAAGCCTGCGCTGC

CACGGATGGACCTTGAAAGAACTGATAGAGGCAGGGCATCTACCAAACCCACCAATAACA

AGAAAAAAGAGGAAAACACGGGAGTCTCTCAACAATGTCTTAGTTGACCCCCATCAAAAA

AACTCAAATAAGTGCGAAGTATGCCGCCAAGAGAAACAGCTGGTCTTCTGCAATACTTGT

TCAAGATCATACCATCAGAACTGCCACATCCCACCAGTAGATGCTCAGAGGAGCCAGTGG

AGTTGCACCTTATGTAGGACAAAAGCCTGGGAAAAGCACCCAGAAAGCCAACCATGTCAT

CTGGAATCTGAGGTCTTGAAGAGGCCTATGCTGCCTGAGGAACAGCTGAAATGTGAGCTG

ATCCTATTGAAGGTCTATTGTTATTCACAAAGTGCCTTTTTTGCCCAAGAACCATATTAT

ACTCAAGACCCCCAGGAGCACATGTGGTTAAACAAGGTCAAGGAAAGGCTGAATAAGAAG

AAGTACCACCGAGTGGAGGGGTTTGTGCGGGACATGCGCCTCATCTTTCTGAACAACAAG

GCCTTCTACAGAAACCACAAATTCATCCAGCTGCCTCTTAAGATAGAAGCCATATTTGAG

AAGTATTTCAAAAACATTTTTTCAATCCAAGAAACAAGCGAGAGCATCTGTCAGTTT

>CL356.Contig88_A-W 589 2313 minus strand PREDICTED: nuclear autoantigen Sp-100 [Pan paniscus]

CCAGGAGCAGAGCTCTCCAGCCATGGAATCCACATGACTTCCTGTTCTGTGCATCTGGTG

GATATAAAGAAGGAAAAGCCATTTTTTCCCACGCAAATGAAGCAAGGATCTCAAACCAGG

ACCAACCATTACCAGCCATCCGAAGTAATAGTCATCAGCAGTGAGGACGACGCCAGCGAC

ACAGAAGAGCCTGCAGCAATCTCCAGCTCAGCACGGAGGAGCCATCCTGTGATCAAAAAC

CATGTCCAGTTTGACTTGAGAGATGAAGAAGAGGCCCAAGAAGCCACTTGCTCATGGCAC

CAGGCTGCTCCAGCGAGAGGACGTGACTATGGCTCTTCAGAATCCAGTGAGGAGGAGATA

CCCTCAGCGCTCCGGATCTCAACACCAACAGATTCTGTGAGGATGTCTACTTGGAGGATA

CACAGTCAGAAAAGAAGGCACAGCAGTGACAGCTCTTCAGAGCTGAGTTATGGAGAGGAG

CCTCGGGAAGCCCTGAGAAGTGGATCAGGTGTGCCAAGAGGCCAAGAAGCAACGGTGGAA

AGTGGCCGAGCATCTCACCCAATAGATACCACGGATGTTGGAAACAATTCTACTTTGGGA

AAAGGCAGTGGGGAAAGAAATATTTCCAAGCTGCACAACAGAGATAAAGAAATGAACAAA

ATCTCATCAACCTTACATCCTACTGTGGGGAAAAAAAGACTTCATAAAGTAAACCATCTC

CAAAAAGAAATAAAGAAAGGCAGACATAGATTCCACTCAATTCAGAATAATGCCCTTCAA

AGGAAAAGAGGTTGGCCAAAAGGGAGAAAAAGAGTCAACGTTAGACCCTTGAAAAGAGGG

AGAAAAAGAGGACCAAGAATTCCCAGAGAAAAAAATGTGAATTTCAACCTTCCTAAACTT

CCTGTGACCTGTGGTGAGGCTAAGGGGACTCTCCATAAGAAGCAATTTAAGCAAGGGGCC

CAGATGAAGAGCATAAGGACTGAGGATGGAAAATGGCTCACCCCCAGGGACTTCGAGATC

AGAGGAAACCACGCAGCAGCCAAGAACTGGAAGCTAAGCCTGCGCTGCCACGGATGGACC

TTGAAAGAACTGATAGAGGCAGGGCATCTACCAAACCCACCAATAACAAGAAAAAAGAGG

AAAACACGGGAGTCTCTCAACAATGTCTTAGTTGACCCCCATCAAAAAAACTCAAATAAG

TGCGAAGTATGCCGCCAAGAGAAACAGCTGGTCTTCTGCAATACTTGTTCAAGATCATAC

CATCAGAACTGCCACATCCCACCAGTAGATGCTCAGAGGAGCCAGTGGAGTTGCACCTTA

TGTAGGACAAAAGCCTGGGAAAAGCACCCAGAAAGCCAACCATGTCATCTGGAATCTGAG

GTCTTGAAGAGGCCTATGCTGCCTGAGGAACAGCTGAAATGTGAGCTGATCCTATTGAAG

GTCTATTGTTATTCACAAAGTGCCTTTTTTGCCCAAGAACCATATTATACTCAAGACCCC

CAGGAGCACATGTGGTTAAACAAGGTCAAGGAAAGGCTGAATAAGAAGAAGTACCACCGA

GTGGAGGGGTTTGTGCGGGACATGCGCCTCATCTTTCTGAACAACAAGGCCTTCTACAGA

AACCACAAATTCATCCAGCTGCCTCTTAAGATAGAAGCCATATTTGAGAAGTATTTCAAA

AACATTTTTTCAATCCAAGAAACAAGCGAGAGCATCTGTCAGTTT

>CL356.Contig89_A-W 132 2585 minus strand PREDICTED: nuclear autoantigen Sp-100 isoform 14 [Pan troglodytes]

AGGATGTCCACAGAAGAGCAGAGTGTAGACGACAGTACCATCTATGAGGTTATATTCCAT

CACTTCAAAAGACACAAAGTGGAGATTTCAAATGCCATAAAAAAGACATTTCCTTTCCTC

GAGGGCCTCCGTGACCGTGAACTCATTACCAATAAAATGTTTGAAGATTCTCAAGAGTCT

TGTAGAAACCTGGTCCCTGTACAAAGAGTGGTGTACAATGTTCTCTGTGAACTGGAGAAG

AAATTCAACCTGGATTTGTGCGAAGCATTGTTCAGTGAGGTCAACTTGCAGGAATACCCT

GATTTAATTCGTGTTCGTAGAAGCTTCGAACATGAGGTGAGAATGCTATATCCCCAAGAA

GATAATGGAGAAGAGAGGCAGGAAAGGCTTGAACAAGGAACGGGGGAGAACTCTTTTCTG

CGAAGACTGACCTGGCCACAGCACTCAGATCCCTCGTCTTACAATGGTACAGACCTACCC

GAAAATGAACTCTTGGCGCATCCTTGCGAAGCAGAAGAGAGAAATGTAAATAAAAGAGAG

CCGGCCCGAGACCCAAATGGTGCAGTAGGAAGCCACCAAGGAAATGAGCAATGCCCTCGA

GAGTCAGAGCCAGTAGAGTCCCACCAACACATGGCTGCCAACGTGGACCGTGGAGAGGCG

AGCGTGGGGACACCCAGCCCGTTGCCCCACTGTGTAGAAAGAGCAGAGCTCTCCAGCCAT

GGAATCCACATGACTTCCTGTTCTGTGCATCTGGTGGATATAAAGAAGGAAAAGCCATTT

TTTCCCACGCAAATGAAGCAAGGATCTCAAACCAGGACCAACCATTACCAGCCATCCGAA

GTAATAGTCATCAGCAGTGAGGACGACGCCAGCGACACAGAAGAGCCTGCAGCAATCTCC

AGCTCAGCACGGAGGAGCCATCCTGTGATCAAAAACCATGTCCAGTTTGACTTGAGAGAT

GAAGAAGAGGCCCAAGAAGCCACTTGCTCATGGCACCAGGCTGCTCCAGCGAGAGGACGT

GACTATGGCTCTTCAGAATCCAGTGAGGAGGAGATACCCTCAGCGCTCCGGATCTCAACA

CCAACAGATTCTGTGAGGATGTCTACTTGGAGGATACACAGTCAGAAAAGAAGGCACAGC

AGTGACAGCTCTTCAGAGCTGAGTTATGGAGAGGAGCCTCGGGAAGCCCTGAGAAGTGGA

TCAGGAACAGGGCAACAAGAGACTGGAAATGAGAAGTGCTCCTGTGTCCTGTGTTCCCCA

AAAGGTGTGCCAAGAGGCCAAGAAGCAACGGTGGAAAGTGGCCGAGCATCTCACCCAATA

GGCAAGTCTGCTCAATGGCATCAGGGTGGAGGTGGCTCAGAAATACCACAGCTGCTGGGC

AGGACTGTGTCAGGAGAGGAAGCTGTAAAAGATACCACGGATGTTGGAAACAATTCTACT

TTGGGAAAAGGCAGTGGGGAAAGAAGGAAAAAAAGACTTCATAAAGTAAACCATCTCCAA

AAAGAAATAAAGAAAGGCAGACATAGATTCCACTCAATTCAGAATAATGCCCTTCAAAGG

AAAAGAGGTTGGCCAAAAGGACCAAGAATTCCCAGAGAAAAAAATGTGAATTTCAACCTT

CCTAAACTTCCTGTGACCTGTGGTGAGGCTAAGGGGACTCTCCATAAGAAGCAATTTAAG

CAAGGGGCCCAGATGAAGAGCATAAGGACTGAGGATGGAAAATGGCTCACCCCCAGGGAC

TTCGAGATCAGAGGAAACCACGCAGCAGCCAAGAACTGGAAGCTAAGCCTGCGCTGCCAC

GGATGGACCTTGAAAGAACTGATAGAGGCAGGGCATCTACCAAACCCACCAATAACAAGA

AAAAAGAGGAAAACACGGGAGTCTCTCAACAATGTCTTAGTTGACCCCCATCAAAAAAAC

TCAAATAAGTGCGAAGTATGCCGCCAAGAGAAACAGCTGGTCTTCTGCAATACTTGTTCA

AGATCATACCATCAGAACTGCCACATCCCACCAGTAGATGCTCAGAGGAGCCAGTGGAGT

TGCACCTTATGTAGGACAAAAGCCTGGGAAAAGCACCCAGAAAGCCAACCATGTCATCTG

GAATCTGAGGTCTTGAAGAGGCCTATGCTGCCTGAGGAACAGCTGAAATGTGAGCTGATC

CTATTGAAGGTCTATTGTTATTCACAAAGTGCCTTTTTTGCCCAAGAACCATATTATACT

CAAGACCCCCAGGAGCACATGTGGTTAAACAAGGTCAAGGAAAGGCTGAATAAGAAGAAG

TACCACCGAGTGGAGGGGTTTGTGCGGGACATGCGCCTCATCTTTCTGAACAACAAGGCC

TTCTACAGAAACCACAAATTCATCCAGCTGCCTCTTAAGATAGAAGCCATATTTGAGAAG

TATTTCAAAAACATTTTTTCAATCCAAGAAACAAGCGAGAGCATCTGTCAGTTT

>CL356.Contig90_A-W 191 2302 minus strand PREDICTED: nuclear autoantigen Sp-100 [Pan paniscus]

TCAGTGTTCCAAGAAAAATATGTTTCCCTAAGAAGTGACCCGGAAGGAAGACAACAAACA

TCCATCACTCAGCCAAGCCGTGAGCAAGGAACAGTTGAGAACACGTTTCTGCGAAGACTG

ACCTGGCCACAGCACTCAGATCCCTCGTCTTACAATGGTACAGACCTACCCGAAAATGAA

CTCTTGGCGCATCCTTGCGAAGCAGAAGAGAGAAATGTAAATAAAAGAGAGCCGGCCCGA

GACCCAAATGGTGCAGTAGGAAGCCACCAAGGAAATGAGCAATGCCCTCGAGAGTCAGAG

CCAGTAGAGTCCCACCAACACATGGCTGCCAACGTGGACCGTGGAGAGGCGAGCGTGGGG

ACACCCAGCCCGTTGCCCCACTGTGTAGAAAGAGCAGAGCTCTCCAGCCATGGAATCCAC

ATGACTTCCTGTTCTGTGCATCTGGTGGATATAAAGAAGGAAAAGCCATTTTTTCCCACG

CAAATGAAGCAAGGATCTCAAACCAGGACCAACCATTACCAGCCATCCGAAGTAATAGTC

ATCAGCAGTGAGGACGACGCCAGCGACACAGAAGAGCCTGCAGCAATCTCCAGCTCAGCA

CGGAGGAGCCATCCTGTGATCAAAAACCATGTCCAGTTTGACTTGAGAGATGAAGAAGAG

GCCCAAGAAGCCACTTGCTCATGGCACCAGGCTGCTCCAGCGAGAGGACGTGACTATGGC

TCTTCAGAATCCAGTGAGGAGGAGATACCCTCAGCGCTCCGGATCTCAACACCAACAGAT

TCTGTGAGGATGTCTACTTGGAGGATACACAGTCAGAAAAGAAGGCACAGCAGTGACAGC

TCTTCAGAGCTGAGTTATGGAGAGGAGCCTCGGGAAGCCCTGAGAAGTGGATCAGGTGTG

CCAAGAGGCCAAGAAGCAACGGTGGAAAGTGGCCGAGCATCTCACCCAATAGATACCACG

GATGTTGGAAACAATTCTACTTTGGGAAAAGGCAGTGGGGAAAGAAATATTTCCAAGCTG

CACAACAGAGATAAAGAAATGAACAAAATCTCATCAACCTTACATCCTACTGTGGGGAAA

AAAAGACTTCATAAAGTAAACCATCTCCAAAAAGAAATAAAGAAAGGCAGACATAGATTC

CACTCAATTCAGAATAATGCCCTTCAAAGGAAAAGAGGTTGGCCAAAAGGGAGAAAAAGA

GTCAACGTTAGACCCTTGAAAAGAGGGAGAAAAAGAGGACCAAGAATTCCCAGAGAAAAA

AATGTGAATTTCAACCTTCCTAAACTTCCTGTGACCTGTGGTGAGGCTAAGGGGACTCTC

CATAAGAAGCAATTTAAGCAAGGGGCCCAGATGAAGAGCATAAGGACTGAGGATGGAAAA

TGGCTCACCCCCAGGGACTTCGAGATCAGAGGAAACCACGCAGCAGCCAAGAACTGGAAG

CTAAGCCTGCGCTGCCACGGATGGACCTTGAAAGAACTGATAGAGGCAGGGCATCTACCA

AACCCACCAATAACAAGAAAAAAGAGGAAAACACGGGAGTCTCTCAACAATGTCTTAGTT

GACCCCCATCAAAAAAACTCAAATAAGTGCGAAGTATGCCGCCAAGAGAAACAGCTGGTC

TTCTGCAATACTTGTTCAAGATCATACCATCAGAACTGCCACATCCCACCAGTAGATGCT

CAGAGGAGCCAGTGGAGTTGCACCTTATGTAGGACAAAAGCCTGGGAAAAGCACCCAGAA

AGCCAACCATGTCATCTGGAATCTGAGGTCTTGAAGAGGCCTATGCTGCCTGAGGAACAG

CTGAAATGTGAGCTGATCCTATTGAAGGTCTATTGTTATTCACAAAGTGCCTTTTTTGCC

CAAGAACCATATTATACTCAAGACCCCCAGGAGCACATGTGGTTAAACAAGGTCAAGGAA

AGGCTGAATAAGAAGAAGTACCACCGAGTGGAGGGGTTTGTGCGGGACATGCGCCTCATC

TTTCTGAACAACAAGGCCTTCTACAGAAACCACAAATTCATCCAGCTGCCTCTTAAGATA

GAAGCCATATTTGAGAAGTATTTCAAAAACATTTTTTCAATCCAAGAAACAAGCGAGAGC

ATCTGTCAGTTT

>CL356.Contig91_A-W 136 2289 minus strand PREDICTED: nuclear autoantigen Sp-100 [Pan paniscus]

GTCGGGATCCAGGAGGATCCCCGGACATCAGGTTTTTCCTTGGAAGTGTTCCAAGAAAAA

TATGTTTCCCTAAGAAGTGACCCGGAAGGAAGACAACAAACATCCATCACTCAGCCAAGC

CGTGAGCAAGGAACAGTTGAGAACACGTTTCTGCGAAGACTGACCTGGCCACAGCACTCA

GATCCCTCGTCTTACAATGGTACAGACCTACCCGAAAATGAACTCTTGGCGCATCCTTGC

GAAGCAGAAGAGAGAAATGTAAATAAAAGAGAGCCGGCCCGAGACCCAAATGGTGCAGTA

GGAAGCCACCAAGGAAATGAGCAATGCCCTCGAGAGTCAGAGCCAGTAGAGTCCCACCAA

CACATGGCTGCCAACGTGGACCGTGGAGAGGCGAGCGTGGGGACACCCAGCCCGTTGCCC

CACTGTGTAGAAAGAGCAGAGCTCTCCAGCCATGGAATCCACATGACTTCCTGTTCTGTG

CATCTGGTGGATATAAAGAAGGAAAAGCCATTTTTTCCCACGCAAATGAAGCAAGGATCT

CAAACCAGGACCAACCATTACCAGCCATCCGAAGTAATAGTCATCAGCAGTGAGGACGAC

GCCAGCGACACAGAAGAGCCTGCAGCAATCTCCAGCTCAGCACGGAGGAGCCATCCTGTG

ATCAAAAACCATGTCCAGTTTGACTTGAGAGATGAAGAAGAGGCCCAAGAAGCCACTTGC

TCATGGCACCAGGCTGCTCCAGCGAGAGGACGTGACTATGGCTCTTCAGAATCCAGTGAG

GAGGAGATACCCTCAGCGCTCCGGATCTCAACACCAACAGATTCTGTGAGGATGTCTACT

TGGAGGATACACAGTCAGAAAAGAAGGCACAGCAGTGACAGCTCTTCAGAGCTGAGTTAT

GGAGAGGAGCCTCGGGAAGCCCTGAGAAGTGGATCAGGTGTGCCAAGAGGCCAAGAAGCA

ACGGTGGAAAGTGGCCGAGCATCTCACCCAATAGATACCACGGATGTTGGAAACAATTCT

ACTTTGGGAAAAGGCAGTGGGGAAAGAAATATTTCCAAGCTGCACAACAGAGATAAAGAA

ATGAACAAAATCTCATCAACCTTACATCCTACTGTGGGGAAAAAAAGACTTCATAAAGTA

AACCATCTCCAAAAAGAAATAAAGAAAGGCAGACATAGATTCCACTCAATTCAGAATAAT

GCCCTTCAAAGGAAAAGAGGTTGGCCAAAAGGGAGAAAAAGAGTCAACGTTAGACCCTTG

AAAAGAGGGAGAAAAAGAGGACCAAGAATTCCCAGAGAAAAAAATGTGAATTTCAACCTT

CCTAAACTTCCTGTGACCTGTGGTGAGGCTAAGGGGACTCTCCATAAGAAGCAATTTAAG

CAAGGGGCCCAGATGAAGAGCATAAGGACTGAGGATGGAAAATGGCTCACCCCCAGGGAC

TTCGAGATCAGAGGAAACCACGCAGCAGCCAAGAACTGGAAGCTAAGCCTGCGCTGCCAC

GGATGGACCTTGAAAGAACTGATAGAGGCAGGGCATCTACCAAACCCACCAATAACAAGA

AAAAAGAGGAAAACACGGGAGTCTCTCAACAATGTCTTAGTTGACCCCCATCAAAAAAAC

TCAAATAAGTGCGAAGTATGCCGCCAAGAGAAACAGCTGGTCTTCTGCAATACTTGTTCA

AGATCATACCATCAGAACTGCCACATCCCACCAGTAGATGCTCAGAGGAGCCAGTGGAGT

TGCACCTTATGTAGGACAAAAGCCTGGGAAAAGCACCCAGAAAGCCAACCATGTCATCTG

GAATCTGAGGTCTTGAAGAGGCCTATGCTGCCTGAGGAACAGCTGAAATGTGAGCTGATC

CTATTGAAGGTCTATTGTTATTCACAAAGTGCCTTTTTTGCCCAAGAACCATATTATACT

CAAGACCCCCAGGAGCACATGTGGTTAAACAAGGTCAAGGAAAGGCTGAATAAGAAGAAG

TACCACCGAGTGGAGGGGTTTGTGCGGGACATGCGCCTCATCTTTCTGAACAACAAGGCC

TTCTACAGAAACCACAAATTCATCCAGCTGCCTCTTAAGATAGAAGCCATATTTGAGAAG

TATTTCAAAAACATTTTTTCAATCCAAGAAACAAGCGAGAGCATCTGTCAGTTT

>CL356.Contig92_A-W 132 2573 minus strand PREDICTED: nuclear autoantigen Sp-100 [Pan paniscus]

AGGATGTCCACAGAAGAGCAGAGTGTAGACGACAGTACCATCTATGAGGTTATATTCCAT

CACTTCAAAAGACACAAAGTGGAGATTTCAAATGCCATAAAAAAGACATTTCCTTTCCTC

GAGGGCCTCCGTGACCGTGAACTCATTACCAATAAAATGTTTGAAGATTCTCAAGAGTCT

TGTAGAAACCTGGTCCCTGTACAAAGAGTGGTGTACAATGTTCTCTGTGAACTGGAGAAG

AAATTCAACCTGGATTTGTGCGAAGCATTGTTCAGTGAGGTCAACTTGCAGGAATACCCT

GATTTAATTCGTGTTCGTAGAAGCTTCGAACATGAGGTGAGAATGCTATATCCCCAAGAA

GATAATGGAGAAGAGAGGCAGGAAAGGCTTGAACAAGGAACGGGGGAGAACTCTTTTCTG

CGAAGACTGACCTGGCCACAGCACTCAGATCCCTCGTCTTACAATGGTACAGACCTACCC

GAAAATGAACTCTTGGCGCATCCTTGCGAAGCAGAAGAGAGAAATGTAAATAAAAGAGAG

CCGGCCCGAGACCCAAATGGTGCAGTAGGAAGCCACCAAGGAAATGAGCAATGCCCTCGA

GAGTCAGAGCCAGTAGAGTCCCACCAACACATGGCTGCCAACGTGGACCGTGGAGAGGCG

AGCGTGGGGACACCCAGCCCGTTGCCCCACTGTGTAGAAAGAGCAGAGCTCTCCAGCCAT

GGAATCCACATGACTTCCTGTTCTGTGCATCTGGTGGATATAAAGAAGGAAAAGCCATTT

TTTCCCACGCAAATGAAGCAAGGATCTCAAACCAGGACCAACCATTACCAGCCATCCGAA

GTAATAGTCATCAGCAGTGAGGACGACGCCAGCGACACAGAAGAGCCTGCAGCAATCTCC

AGCTCAGCACGGAGGAGCCATCCTGTGATCAAAAACCATGTCCAGTTTGACTTGAGAGAT

GAAGAAGAGGCCCAAGAAGCCACTTGCTCATGGCACCAGGCTGCTCCAGCGAGAGGACGT

GACTATGGCTCTTCAGAATCCAGTGAGGAGGAGATACCCTCAGCGCTCCGGATCTCAACA

CCAACAGATTCTGTGAGGATGTCTACTTGGAGGATACACAGTCAGAAAAGAAGGCACAGC

AGTGACAGCTCTTCAGAGCTGAGTTATGGAGAGGAGCCTCGGGAAGCCCTGAGAAGTGGA

TCAGGTGTGCCAAGAGGCCAAGAAGCAACGGTGGAAAGTGGCCGAGCATCTCACCCAATA

GGCAAGTCTGCTCAATGGCATCAGGGTGGAGGTGGCTCAGAAATACCACAGCTGCTGGGC

AGGACTGTGTCAGGAGAGGAAGCTGTAAAAGATACCACGGATGTTGGAAACAATTCTACT

TTGGGAAAAGGCAGTGGGGAAAGAAGGAAAAAAAGACTTCATAAAGTAAACCATCTCCAA

AAAGAAATAAAGAAAGGCAGACATAGATTCCACTCAATTCAGAATAATGCCCTTCAAAGG

AAAAGAGGTTGGCCAAAAGGGAGAAAAAGAGTCAACGTTAGACCCTTGAAAAGAGGGAGA

AAAAGAGGACCAAGAATTCCCAGAGAAAAAAATGTGAATTTCAACCTTCCTAAACTTCCT

GTGACCTGTGGTGAGGCTAAGGGGACTCTCCATAAGAAGCAATTTAAGCAAGGGGCCCAG

ATGAAGAGCATAAGGACTGAGGATGGAAAATGGCTCACCCCCAGGGACTTCGAGATCAGA

GGAAACCACGCAGCAGCCAAGAACTGGAAGCTAAGCCTGCGCTGCCACGGATGGACCTTG

AAAGAACTGATAGAGGCAGGGCATCTACCAAACCCACCAATAACAAGAAAAAAGAGGAAA

ACACGGGAGTCTCTCAACAATGTCTTAGTTGACCCCCATCAAAAAAACTCAAATAAGTGC

GAAGTATGCCGCCAAGAGAAACAGCTGGTCTTCTGCAATACTTGTTCAAGATCATACCAT

CAGAACTGCCACATCCCACCAGTAGATGCTCAGAGGAGCCAGTGGAGTTGCACCTTATGT

AGGACAAAAGCCTGGGAAAAGCACCCAGAAAGCCAACCATGTCATCTGGAATCTGAGGTC

TTGAAGAGGCCTATGCTGCCTGAGGAACAGCTGAAATGTGAGCTGATCCTATTGAAGGTC

TATTGTTATTCACAAAGTGCCTTTTTTGCCCAAGAACCATATTATACTCAAGACCCCCAG

GAGCACATGTGGTTAAACAAGGTCAAGGAAAGGCTGAATAAGAAGAAGTACCACCGAGTG

GAGGGGTTTGTGCGGGACATGCGCCTCATCTTTCTGAACAACAAGGCCTTCTACAGAAAC

CACAAATTCATCCAGCTGCCTCTTAAGATAGAAGCCATATTTGAGAAGTATTTCAAAAAC

ATTTTTTCAATCCAAGAAACAAGCGAGAGCATCTGTCAGTTT

>CL356.Contig93_A-W 589 2286 minus strand PREDICTED: nuclear autoantigen Sp-100 [Pan paniscus]

CCAGGAGCAGAGCTCTCCAGCCATGGAATCCACATGACTTCCTGTTCTGTGCATCTGGTG

GATATAAAGAAGGAAAAGCCATTTTTTCCCACGCAAATGAAGCAAGGATCTCAAACCAGG

ACCAACCATTACCAGCCATCCGAAGTAATAGTCATCAGCAGTGAGGACGACGCCAGCGAC

ACAGAAGAGCCTGCAGCAATCTCCAGCTCAGCACGGAGGAGCCATCCTGTGATCAAAAAC

CATGTCCAGTTTGACTTGAGAGATGAAGAAGAGGCCCAAGAAGCCACTTGCTCATGGCAC

CAGGCTGCTCCAGCGAGAGGACGTGACTATGGCTCTTCAGAATCCAGTGAGGAGGAGATA

CCCTCAGCGCTCCGGATCTCAACACCAACAGATTCTGTGAGGATGTCTACTTGGAGGATA

CACAGTCAGAAAAGAAGGCACAGCAGTGACAGCTCTTCAGAGCTGAGTTATGGAGAGGAG

CCTCGGGAAGCCCTGAGAAGTGGATCAGGTGTGCCAAGAGGCCAAGAAGCAACGGTGGAA

AGTGGCCGAGCATCTCACCCAATAGGCAAGTCTGCTCAATGGCATCAGGGTGGAGGTGGC

TCAGAAATACCACAGCTGCTGGGCAGGACTGTGTCAGGAGAGGAAGCTGTAAAAGATACC

ACGGATGTTGGAAACAATTCTACTTTGGGAAAAGGCAGTGGGGAAAGAAGGAAAAAAAGA

CTTCATAAAGTAAACCATCTCCAAAAAGAAATAAAGAAAGGCAGACATAGATTCCACTCA

ATTCAGAATAATGCCCTTCAAAGGAAAAGAGGTTGGCCAAAAGGACCAAGAATTCCCAGA

GAAAAAAATGTGAATTTCAACCTTCCTAAACTTCCTGTGACCTGTGGTGAGGCTAAGGGG

ACTCTCCATAAGAAGCAATTTAAGCAAGGGGCCCAGATGAAGAGCATAAGGACTGAGGAT

GGAAAATGGCTCACCCCCAGGGACTTCGAGATCAGAGGAAACCACGCAGCAGCCAAGAAC

TGGAAGCTAAGCCTGCGCTGCCACGGATGGACCTTGAAAGAACTGATAGAGGCAGGGCAT

CTACCAAACCCACCAATAACAAGAAAAAAGAGGAAAACACGGGAGTCTCTCAACAATGTC

TTAGTTGACCCCCATCAAAAAAACTCAAATAAGTGCGAAGTATGCCGCCAAGAGAAACAG

CTGGTCTTCTGCAATACTTGTTCAAGATCATACCATCAGAACTGCCACATCCCACCAGTA

GATGCTCAGAGGAGCCAGTGGAGTTGCACCTTATGTAGGACAAAAGCCTGGGAAAAGCAC

CCAGAAAGCCAACCATGTCATCTGGAATCTGAGGTCTTGAAGAGGCCTATGCTGCCTGAG

GAACAGCTGAAATGTGAGCTGATCCTATTGAAGGTCTATTGTTATTCACAAAGTGCCTTT

TTTGCCCAAGAACCATATTATACTCAAGACCCCCAGGAGCACATGTGGTTAAACAAGGTC

AAGGAAAGGCTGAATAAGAAGAAGTACCACCGAGTGGAGGGGTTTGTGCGGGACATGCGC

CTCATCTTTCTGAACAACAAGGCCTTCTACAGAAACCACAAATTCATCCAGCTGCCTCTT

AAGATAGAAGCCATATTTGAGAAGTATTTCAAAAACATTTTTTCAATCCAAGAAACAAGC

GAGAGCATCTGTCAGTTT

>CL356.Contig94_A-W 163 2274 minus strand PREDICTED: nuclear autoantigen Sp-100 [Pan paniscus]

TCAGTGTTCCAAGAAAAATATGTTTCCCTAAGAAGTGACCCGGAAGGAAGACAACAAACA

TCCATCACTCAGCCAAGCCGTGAGCAAGGAACAGTTGAGAACACGTTTCTGCGAAGACTG

ACCTGGCCACAGCACTCAGATCCCTCGTCTTACAATGGTACAGACCTACCCGAAAATGAA

CTCTTGGCGCATCCTTGCGAAGCAGAAGAGAGAAATGTAAATAAAAGAGAGCCGGCCCGA

GACCCAAATGGTGCAGTAGGAAGCCACCAAGGAAATGAGCAATGCCCTCGAGAGTCAGAG

CCAGTAGAGTCCCACCAACACATGGCTGCCAACGTGGACCGTGGAGAGGCGAGCGTGGGG

ACACCCAGCCCGTTGCCCCACTGTGTAGAAAGAGCAGAGCTCTCCAGCCATGGAATCCAC

ATGACTTCCTGTTCTGTGCATCTGGTGGATATAAAGAAGGAAAAGCCATTTTTTCCCACG

CAAATGAAGCAAGGATCTCAAACCAGGACCAACCATTACCAGCCATCCGAAGTAATAGTC

ATCAGCAGTGAGGACGACGCCAGCGACACAGAAGAGCCTGCAGCAATCTCCAGCTCAGCA

CGGAGGAGCCATCCTGTGATCAAAAACCATGTCCAGTTTGACTTGAGAGATGAAGAAGAG

GCCCAAGAAGCCACTTGCTCATGGCACCAGGCTGCTCCAGCGAGAGGACGTGACTATGGC

TCTTCAGAATCCAGTGAGGAGGAGATACCCTCAGCGCTCCGGATCTCAACACCAACAGAT

TCTGTGAGGATGTCTACTTGGAGGATACACAGTCAGAAAAGAAGGCACAGCAGTGACAGC

TCTTCAGAGCTGAGTTATGGAGAGGAGCCTCGGGAAGCCCTGAGAAGTGGATCAGGTGTG

CCAAGAGGCCAAGAAGCAACGGTGGAAAGTGGCCGAGCATCTCACCCAATAGATACCACG

GATGTTGGAAACAATTCTACTTTGGGAAAAGGCAGTGGGGAAAGAAATATTTCCAAGCTG

CACAACAGAGATAAAGAAATGAACAAAATCTCATCAACCTTACATCCTACTGTGGGGAAA

AAAAGACTTCATAAAGTAAACCATCTCCAAAAAGAAATAAAGAAAGGCAGACATAGATTC

CACTCAATTCAGAATAATGCCCTTCAAAGGAAAAGAGGTTGGCCAAAAGGGAGAAAAAGA

GTCAACGTTAGACCCTTGAAAAGAGGGAGAAAAAGAGGACCAAGAATTCCCAGAGAAAAA

AATGTGAATTTCAACCTTCCTAAACTTCCTGTGACCTGTGGTGAGGCTAAGGGGACTCTC

CATAAGAAGCAATTTAAGCAAGGGGCCCAGATGAAGAGCATAAGGACTGAGGATGGAAAA

TGGCTCACCCCCAGGGACTTCGAGATCAGAGGAAACCACGCAGCAGCCAAGAACTGGAAG

CTAAGCCTGCGCTGCCACGGATGGACCTTGAAAGAACTGATAGAGGCAGGGCATCTACCA

AACCCACCAATAACAAGAAAAAAGAGGAAAACACGGGAGTCTCTCAACAATGTCTTAGTT

GACCCCCATCAAAAAAACTCAAATAAGTGCGAAGTATGCCGCCAAGAGAAACAGCTGGTC

TTCTGCAATACTTGTTCAAGATCATACCATCAGAACTGCCACATCCCACCAGTAGATGCT

CAGAGGAGCCAGTGGAGTTGCACCTTATGTAGGACAAAAGCCTGGGAAAAGCACCCAGAA

AGCCAACCATGTCATCTGGAATCTGAGGTCTTGAAGAGGCCTATGCTGCCTGAGGAACAG

CTGAAATGTGAGCTGATCCTATTGAAGGTCTATTGTTATTCACAAAGTGCCTTTTTTGCC

CAAGAACCATATTATACTCAAGACCCCCAGGAGCACATGTGGTTAAACAAGGTCAAGGAA

AGGCTGAATAAGAAGAAGTACCACCGAGTGGAGGGGTTTGTGCGGGACATGCGCCTCATC

TTTCTGAACAACAAGGCCTTCTACAGAAACCACAAATTCATCCAGCTGCCTCTTAAGATA

GAAGCCATATTTGAGAAGTATTTCAAAAACATTTTTTCAATCCAAGAAACAAGCGAGAGC

ATCTGTCAGTTT

>CL356.Contig95_A-W 589 2265 minus strand PREDICTED: nuclear autoantigen Sp-100 [Pan paniscus]

CCAGGAGCAGAGCTCTCCAGCCATGGAATCCACATGACTTCCTGTTCTGTGCATCTGGTG

GATATAAAGAAGGAAAAGCCATTTTTTCCCACGCAAATGAAGCAAGGATCTCAAACCAGG

ACCAACCATTACCAGCCATCCGAAGTAATAGTCATCAGCAGTGAGGACGACGCCAGCGAC

ACAGAAGAGCCTGCAGCAATCTCCAGCTCAGCACGGAGGAGCCATCCTGTGATCAAAAAC

CATGTCCAGTTTGACTTGAGAGATGAAGAAGAGGCCCAAGAAGCCACTTGCTCATGGCAC

CAGGCTGCTCCAGCGAGAGGACGTGACTATGGCTCTTCAGAATCCAGTGAGGAGGAGATA

CCCTCAGCGCTCCGGATCTCAACACCAACAGATTCTGTGAGGATGTCTACTTGGAGGATA

CACAGTCAGAAAAGAAGGCACAGCAGTGACAGCTCTTCAGAGCTGAGTTATGGAGAGGAG

CCTCGGGAAGCCCTGAGAAGTGGATCAGGTGTGCCAAGAGGCCAAGAAGCAACGGTGGAA

AGTGGCCGAGCATCTCACCCAATAGATACCACGGATGTTGGAAACAATTCTACTTTGGGA

AAAGGCAGTGGGGAAAGAAATATTTCCAAGCTGCACAACAGAGATAAAGAAATGAACAAA

ATCTCATCAACCTTACATCCTACTGTGGGGAAAAAAAGACTTCATAAAGTAAACCATCTC

CAAAAAGAAATAAAGAAAGGCAGACATAGATTCCACTCAATTCAGAATAATGCCCTTCAA

AGGAAAAGAGGTTGGCCAAAAGGACCAAGAATTCCCAGAGAAAAAAATGTGAATTTCAAC

CTTCCTAAACTTCCTGTGACCTGTGGTGAGGCTAAGGGGACTCTCCATAAGAAGCAATTT

AAGCAAGGGGCCCAGATGAAGAGCATAAGGACTGAGGATGGAAAATGGCTCACCCCCAGG

GACTTCGAGATCAGAGGAAACCACGCAGCAGCCAAGAACTGGAAGCTAAGCCTGCGCTGC

CACGGATGGACCTTGAAAGAACTGATAGAGGCAGGGCATCTACCAAACCCACCAATAACA

AGAAAAAAGAGGAAAACACGGGAGTCTCTCAACAATGTCTTAGTTGACCCCCATCAAAAA

AACTCAAATAAGTGCGAAGTATGCCGCCAAGAGAAACAGCTGGTCTTCTGCAATACTTGT

TCAAGATCATACCATCAGAACTGCCACATCCCACCAGTAGATGCTCAGAGGAGCCAGTGG

AGTTGCACCTTATGTAGGACAAAAGCCTGGGAAAAGCACCCAGAAAGCCAACCATGTCAT

CTGGAATCTGAGGTCTTGAAGAGGCCTATGCTGCCTGAGGAACAGCTGAAATGTGAGCTG

ATCCTATTGAAGGTCTATTGTTATTCACAAAGTGCCTTTTTTGCCCAAGAACCATATTAT

ACTCAAGACCCCCAGGAGCACATGTGGTTAAACAAGGTCAAGGAAAGGCTGAATAAGAAG

AAGTACCACCGAGTGGAGGGGTTTGTGCGGGACATGCGCCTCATCTTTCTGAACAACAAG

GCCTTCTACAGAAACCACAAATTCATCCAGCTGCCTCTTAAGATAGAAGCCATATTTGAG

AAGTATTTCAAAAACATTTTTTCAATCCAAGAAACAAGCGAGAGCATCTGTCAGTTT

>CL356.Contig96_A-W 163 2247 minus strand PREDICTED: nuclear autoantigen Sp-100 [Pan paniscus]

TCAGTGTTCCAAGAAAAATATGTTTCCCTAAGAAGTGACCCGGAAGGAAGACAACAAACA

TCCATCACTCAGCCAAGCCGTGAGCAAGGAACAGTTGAGAACACGTTTCTGCGAAGACTG

ACCTGGCCACAGCACTCAGATCCCTCGTCTTACAATGGTACAGACCTACCCGAAAATGAA

CTCTTGGCGCATCCTTGCGAAGCAGAAGAGAGAAATGTAAATAAAAGAGAGCCGGCCCGA

GACCCAAATGGTGCAGTAGGAAGCCACCAAGGAAATGAGCAATGCCCTCGAGAGTCAGAG

CCAGTAGAGTCCCACCAACACATGGCTGCCAACGTGGACCGTGGAGAGGCGAGCGTGGGG

ACACCCAGCCCGTTGCCCCACTGTGTAGAAAGAGCAGAGCTCTCCAGCCATGGAATCCAC

ATGACTTCCTGTTCTGTGCATCTGGTGGATATAAAGAAGGAAAAGCCATTTTTTCCCACG

CAAATGAAGCAAGGATCTCAAACCAGGACCAACCATTACCAGCCATCCGAAGTAATAGTC

ATCAGCAGTGAGGACGACGCCAGCGACACAGAAGAGCCTGCAGCAATCTCCAGCTCAGCA

CGGAGGAGCCATCCTGTGATCAAAAACCATGTCCAGTTTGACTTGAGAGATGAAGAAGAG

GCCCAAGAAGCCACTTGCTCATGGCACCAGGCTGCTCCAGCGAGAGGACGTGACTATGGC

TCTTCAGAATCCAGTGAGGAGGAGATACCCTCAGCGCTCCGGATCTCAACACCAACAGAT

TCTGTGAGGATGTCTACTTGGAGGATACACAGTCAGAAAAGAAGGCACAGCAGTGACAGC

TCTTCAGAGCTGAGTTATGGAGAGGAGCCTCGGGAAGCCCTGAGAAGTGGATCAGGTGTG

CCAAGAGGCCAAGAAGCAACGGTGGAAAGTGGCCGAGCATCTCACCCAATAGGCAAGTCT

GCTCAATGGCATCAGGGTGGAGGTGGCTCAGAAATACCACAGCTGCTGGGCAGGACTGTG

TCAGGAGAGGAAGCTGTAAAAGATACCACGGATGTTGGAAACAATTCTACTTTGGGAAAA

GGCAGTGGGGAAAGAAGGAAAAAAAGACTTCATAAAGTAAACCATCTCCAAAAAGAAATA

AAGAAAGGCAGACATAGATTCCACTCAATTCAGAATAATGCCCTTCAAAGGAAAAGAGGT

TGGCCAAAAGGACCAAGAATTCCCAGAGAAAAAAATGTGAATTTCAACCTTCCTAAACTT

CCTGTGACCTGTGGTGAGGCTAAGGGGACTCTCCATAAGAAGCAATTTAAGCAAGGGGCC

CAGATGAAGAGCATAAGGACTGAGGATGGAAAATGGCTCACCCCCAGGGACTTCGAGATC

AGAGGAAACCACGCAGCAGCCAAGAACTGGAAGCTAAGCCTGCGCTGCCACGGATGGACC

TTGAAAGAACTGATAGAGGCAGGGCATCTACCAAACCCACCAATAACAAGAAAAAAGAGG

AAAACACGGGAGTCTCTCAACAATGTCTTAGTTGACCCCCATCAAAAAAACTCAAATAAG

TGCGAAGTATGCCGCCAAGAGAAACAGCTGGTCTTCTGCAATACTTGTTCAAGATCATAC

CATCAGAACTGCCACATCCCACCAGTAGATGCTCAGAGGAGCCAGTGGAGTTGCACCTTA

TGTAGGACAAAAGCCTGGGAAAAGCACCCAGAAAGCCAACCATGTCATCTGGAATCTGAG

GTCTTGAAGAGGCCTATGCTGCCTGAGGAACAGCTGAAATGTGAGCTGATCCTATTGAAG

GTCTATTGTTATTCACAAAGTGCCTTTTTTGCCCAAGAACCATATTATACTCAAGACCCC

CAGGAGCACATGTGGTTAAACAAGGTCAAGGAAAGGCTGAATAAGAAGAAGTACCACCGA

GTGGAGGGGTTTGTGCGGGACATGCGCCTCATCTTTCTGAACAACAAGGCCTTCTACAGA

AACCACAAATTCATCCAGCTGCCTCTTAAGATAGAAGCCATATTTGAGAAGTATTTCAAA

AACATTTTTTCAATCCAAGAAACAAGCGAGAGCATCTGTCAGTTT

>CL356.Contig97_A-W 3 686 nuclear autoantigen Sp-100 isoform 3 [Homo sapiens]

GAGACTGGAAATGAGAAGTGCTCCTGTGTCCTGTGTTCCCCAAAAGGTGTGCCAAGAGGC

CAAGAAGCAACGGTGGAAAGTGGCCGAGCATCTCACCCAATAGATACCACGGATGTTGGA

AACAATTCTACTTTGGGAAAAGGCAGTGGGGAAAGAAATATTTCCAAGCTGCACAACAGA

GATAAAGAAATGAACAAAATCTCATCAACCTTACATCCTACTGTGGGGAAAAAAAGACTT

CATAAAGTAAACCATCTCCAAAAAGAAATAAAGAAAGGCAGACATAGATTCCACTCAATT

CAGAATAATGCCCTTCAAAGGAAAAGAGGTTGGCCAAAAGGGAGAAAAAGAGTCAACGTT

AGACCCTTGAAAAGAGGGAGAAAAAGAGGACCAAGAATTCCCAGAGAAAAAAATGTGAAT

TTCAACCTTCCTAAACTTCCTGTGACCTGTGGTGAAGCTAAGGGGACTCTCCATAAGAAG

CAATTTAAGCAAGGGGCCCAGATGAAGAGCATAAGGACTGAGGATGGAAAATGGCTCACC

CCCAGGGACTTCGAGATCAGAGGAAACCACGCAGCAGCCAAGAACTGGAAGCTAAGCCTG

CGCTGCCACGGATGGACCTTGAAAGAACTGATAGAGGCAGGGCATCTACCAAACCCACCA

ATAACAAGAAAAAAGGTGATTATC

>CL356.Contig98_A-W 232 804 minus strand Nuclear autoantigen Sp-100 [Heterocephalus glaber]

AAAAGAGGACCAAGAATTCCCAGAGAAAAAAATGTGAATTTCAACCTTCCTAAACTTCCT

GTGACCTGTGGTGAGGCTAAGGGGACTCTCCATAAGAAGCAATTTAAGCAAGGGGCCCAG

ATGAAGAGCATAAGGACTGAGGATGGAAAATGGCTCACCCCCAGGGACTTCGAGATCAGA

GGAAACCACGCAGCAGCCAAGAACTGGAAGCTAAGCCTGCGCTGCCACGGATGGACCTTG

AAAGAACTGATAGAGGCAGGGCATCTACCAAACCCACCAATAACAAGAAAAAAGAGGAAA

ACACGGGAGTCTCTCAACAATGTCTTAGTTGACCCCCATCAAAAAAACTCAAATAAGTGC

GAAGTATGCCGCCAAGAGAAACAGCTGGTCTTCTGCAATACTTGTTCAAGATCATACCAT

CAGAACTGCCACATCCCACCAGTAGATGCTCAGAGGAGCCAGTGGAGTTGCACCTTATGT

AGGACAAAAGCCTGGGAAAAGCACCCAGAAAGCCAACCATGTCATCTGGAATCTGAGGTC

TTGAAGAGGCCTATGCTGCCTGAGGAACAGCTG

>CL356.Contig99_A-W 163 726 minus strand Nuclear autoantigen Sp-100 [Heterocephalus glaber]

CCAAGAATTCCCAGAGAAAAAAATGTGAATTTCAACCTTCCTAAACTTCCTGTGACCTGT

GGTGAGGCTAAGGGGACTCTCCATAAGAAGCAATTTAAGCAAGGGGCCCAGATGAAGAGC

ATAAGGACTGAGGATGGAAAATGGCTCACCCCCAGGGACTTCGAGATCAGAGGAAACCAC

GCAGCAGCCAAGAACTGGAAGCTAAGCCTGCGCTGCCACGGATGGACCTTGAAAGAACTG

ATAGAGGCAGGGCATCTACCAAACCCACCAATAACAAGAAAAAAGAGGAAAACACGGGAG

TCTCTCAACAATGTCTTAGTTGACCCCCATCAAAAAAACTCAAATAAGTGCGAAGTATGC

CGCCAAGAGAAACAGCTGGTCTTCTGCAATACTTGTTCAAGATCATACCATCAGAACTGC

CACATCCCACCAGTAGATGCTCAGAGGAGCCAGTGGAGTTGCACCTTATGTAGGACAAAA

GCCTGGGAAAAGCACCCAGAAAGCCAACCATGTCATCTGGAATCTGAGGTCTTGAAGAGG

CCTATGCTGCCTGAGGAACAGCTG

>CL357.Contig1_A-W 3 668 PREDICTED: CDK5 regulatory subunit-associated protein 2-like [Macaca mulatta]

AGGGAGCTGCAGGAGAGAGAGCAGCTGCTCATCAAAGCCTCCAAAGCAGTTGAGAGCTTA

GCTGAAGGTGGTGGCTCTGAAATTCAGCGGGTGAAAGAAGATGCTCAGAAGAAGGTGCAG

CAGGTGGAAGATCTCCTAACCAAGAGGATACTCCTTCTGGAAGGGGATGTGAAAGCCGCC

CAAGCAGAACTGGAAAAGGCCTTTGCAGGAACAGAAACGGAGAAGGCTCTTCGGCTGAGT

TTGGAAAGCAAGCTTTCAGAGATGAAGAAGATGCACAAGGGGGACTTGGAGATGGTCCTG

GTTCTGGAAGAGAAGGACAGACTGATTGAGGAGTTGAAGCTGTCTTTGAAGAGCAGAGAA

GCTTTAATTCAGTGCCTTGAAGAGGAGAAATCTCAGACGGCATGTCTTGATGAGAATGTG

TCATCTGGAGAGCTCCGAAGACACTCTGCTGCTCTGAGGGAGGAAAAGGAGAGAGATGCT

GAGGCTGCACAAATGGAGCATCAGAAGGAGAGAAGTCATTTTGAAGAAAGGATCCAGGCA

CTTCAGAAGGACCTGAGAGAAAAGGAAAGAGAAATTGCTACAGAGAAGAAAAATAGTTTA

AAGAGGGATAAAGCCATTCAGGGCCTAACCAGGGCATTAAAATCAAAGGAAAAAGAGGGC

TCTGAA

>CL357.Contig2_A-W 2 1033 PREDICTED: CDK5 regulatory subunit-associated protein 2-like [Macaca mulatta]

TCAAAGGAAAAAGAGGTTGAAGAACTTAACTCTGAAATCAAAGAGCTCAGTGCTACCTTT

GCTGAGGCCAAAGAGGTCCTGCAGAAAACACAGGAAGCACAGATCCTGAAATGCCAGGGC

TCTGAAGGCTCCCAGGCTGCTCTGACAGAAAAGGAAGCCCTCTTGGCTGAGCTGCGCTCA

GAAAACCTTACTAAGAGTTCGGAGAACCACAGGCTGCGGAGGAGCATTAAGAAGGTCACT

CAGGAGCTCAGTGACCTGCAGCAGGAGAAGGCGAGACTGGAGAGGGACCTGGAGGAAGCC

CGCTGCGAGGGGAGCAGGGGAGCCCGCACCATCCAGGATGTGAAAGCCGCCCAAGCAGAA

CTGGAAAAGGCCTTTGCAGGAACAGAAACGGAGAAGGCTCTTCGGCTGAGTTTGGAAAGC

AAGCTTTCAGAGATGAAGAAGATGCACAAGGGGGACTTGGAGATGGTCCTGGTTCTGGAA

GAGAAGGACAGACTGATTGAGGAGTTGAAGCTGTCTTTGAAGAGCAGAGAAGCTTTAATT

CAGTGCCTTGAAGAGGAGAAATCTCAGACGGCATGTCTTGATGAGAATGTGTCATCTGGA

GAGCTCCGAAGACACTCTGCTGCTCTGAGGGAGGAAAAGGAGAGAGATGCTGAGGCTGCA

CAAATGGAGCATCAGAAGGAGAGAAGTCATTTTGAAGAAAGGATCCAGGCACTTCAGAAG

GACCTGAGAGAAAAGGAAAGAGAAATTGCTACAGAGAAGAAAAATAGTTTAAAGAGGGAT

AAAGCCATTCAGGGCCTAACCAGGGCATTAAAATCAAAGGAAAAAGAGGGCTCTGAAGGC

TCCCAGGCTGCTCTGACAGAAAAGGAAGCCCTCTTGGCTGAGCTGCGCTCAGAAAACCTT

ACTAAGAGTTCGGAGAACCACAGGCTGCGGAGGAGCATTAAGAAGGTCACTCAGGAGCTC

AGTGACCTGCAGCAGGAGAAGGCGAGACTGGAGAGGGACCTGGAGGAAGCCCGCTGCGAG

GGGAGCAGGGGA

>CL358.Contig1_A-W 135 1100 minus strand guanylate-binding protein 6 [Pongo abelii] >gi|75041787|sp|Q5R9T9.1|GBP6_PONAB RecName: Full=Guanylate-binding protein 6; AltName: Full=GTP-binding protein 6; Short=GBP-6; AltName: Full=Guanine nucleotide-binding protein 6

ATGGCATCTGAATCTACCATGAAGTCCCCCATTTGTTTAGTGGAAAACTGGAATGAGCAA

CTGACAGTGAATTCAGAAGCCTTGAGGATTCTTGAAAAGATATGTCAACCTGTGGTGGTG

GTGGCCATTGTAGGGACATATCGTACGGGGAAATCCTACCTCATGAATCAACTTGCAGGA

CAGAACCATGGCTTCCCCCTGGGATCCACAGTGAGGGCTGAGACCAAGGGCATCTGGATG

TGGTGTGTGCCCCACCCCAGCAAGCCAAACCACACCCTGGTCCTCCTGGACACTGAGGGC

CTGGGTGACGTGGAAAAGGGTGACCCTAAGAATGACTCCTGGATCTTTGCCCTGGCTGTG

CTTCTCAGCAGCACCTTTGTCTACAACAGCATGGGCACCATCAATCAGCAGGCCCTGGAC

CAGCTGCATTATGTGACAGAGCTGACAGAATTAATCAGGGCAAAGTCCTCCTCAGAGCCT

GATGAAATAGAAGATTGCACAGAGTTTGTGAGTTTCTTTCCAGACTTTGTCTGGACTGTC

CGGGATTTCACCCTGGAGCTGAAGTTTAATGGTGAGGACATCACAGAAGACCAGTACTTG

GAGAATGCCTTGAAGCTGATTAAAGGGAATGGCTCCAGAGTTGCAATGTTCAATCAGCCC

AGGGAATGCATCAGGAATTTCTTTCCGAAACGGAAGTGTTTTGTCTTTGACCGGCCAACA

AATAACAAAGAACACCTAGCCCATATTGAGAGCATCTCAGAAGACCAACTGGATCCTAAA

TTCCAGGAACAAACAAATAGTTTCAGTTCTTACATCTTTACCCAGGCAAAGATCAAGACA

CTCAGAGAGGGGATTGTGGTTACTGGCAATCGCCTGGGGACTCTGGTGGTGACCTATGTA

GACACCATCGCTAGTGGATCAGTGCCTTGTTTGGAGAATGCGGTGACCACTCTGGCCCAG

CGTGAG

>CL358.Contig2_A-W 70 1035 minus strand guanylate-binding protein 6 [Pongo abelii] >gi|75041787|sp|Q5R9T9.1|GBP6_PONAB RecName: Full=Guanylate-binding protein 6; AltName: Full=GTP-binding protein 6; Short=GBP-6; AltName: Full=Guanine nucleotide-binding protein 6

ATGGCATCTGGACCAAAAATGTCAGCCCCCATTTGTCTGGTGGAAAATAACAATGAGCAG

CTGATGGTGAACCAGCAGGCTCTAAGAATTCTTGAGAAGATTTCTCAGCCAGTCATAGTG

GTGGCCATTGTTGGACTGTACCGTACAGGAAAATCCTACTTGATGAACCGTCTGGCAGGA

CAGAACTGCGGCTTTCCTTTGGGCTCCACAGTGCAATCTGAAACCAAGGGCATCTGGATG

TGGTGTGTGCCCCACCCCAGCAAGCCAAACCACACCCTGGTCCTCCTGGACACTGAGGGC

CTGGGTGACGTGGAAAAGGGTGACCCTAAGAATGACTCCTGGATCTTTGCCCTGGCTGTG

CTTCTCAGCAGCACCTTTGTCTACAACAGCATGGGCACCATCAATCAGCAGGCCCTGGAC

CAGCTGCATTATGTGACAGAGCTGACAGAATTAATCAGGGCAAAGTCCTCCTCAGAGCCT

GATGAAATAGAAGATTGCACAGAGTTTGTGAGTTTCTTTCCAGACTTTGTCTGGACTGTC

CGGGATTTCACCCTGGAGCTGAAGTTTAATGGTGAGGACATCACAGAAGACCAGTACTTG

GAGAATGCCTTGAAGCTGATTAAAGGGAATGGCTCCAGAGTTGCAATGTTCAATCAGCCC

AGGGAATGCATCAGGAATTTCTTTCCGAAACGGAAGTGTTTTGTCTTTGACCGGCCAACA

AATAACAAAGAACACCTAGCCCATATTGAGAGCATCTCAGAAGACCAACTGGATCCTAAA

TTCCAGGAACAAACAAATAGTTTCAGTTCTTACATCTTTACCCAGGCAAAGATCAAGACA

CTCAGAGAGGGGATTGTGGTTACTGGCAATCGCCTGGGGACTCTGGTGGTGACCTATGTA

GACACCATCGCTAGTGGATCAGTGCCTTGTTTGGAGAATGCGGTGACCACTCTGGCCCAG

CGTGAG

>CL359.Contig1_A-W 136 525 dystrobrevin B (mDTN-B) [Mus musculus]

CCCCCAGCTCTCCCCGGGAAGCGAGCCAGGATGATTGAGGAAGGTGGGAACAAGCGGAAG

ACCGTGGCAGAGAAGAGGCAGCTGTTCATAGAAATGCGTGCACAGAATTTTGATGTCATA

CGATTATCAACTTACAGAACAGCTTGCAAATTACGCTTTGTACAGAAACGATGCAACCTT

CATCTGGTTGATATCTGGAACATGATTGAAGCCTTTCGAGACAATGGCCTTAATACACTG

GACCATAGCACCGAGATGAGTGTGTCCCGCCTCGAAACCGTCATCTCATCCATCTACTAT

CAGTTGAATAAGCGCCTTCCTTCTACTCACCAAATCAGTGTGGAACAGTCCATCAGCCTC

CTCCTCAACTTTATGATTGCCGCTTATGAC

>CL359.Contig2_A-W 166 525 minus strand PREDICTED: hypothetical protein LOC100669665 [Loxodonta africana]

ATGATTGAGGAAGGTGGGAACAAGCGGAAGACCGTGGCAGAGAAGAGGCAGCTGTTCATA

GAAATGCGTGCACAGAATTTTGATGTCATACGATTATCAACTTACAGAACAGCTTGCAAA

TTACGCTTTGTACAGAAACGATGCAACCTTCATCTGGTTGATATCTGGAACATGATTGAA

GCCTTTCGAGACAATGGCCTTAATACACTGGACCATAGCACCGAGATGAGTGTGTCCCGC

CTCGAAACCGTCATCTCATCCATCTACTATCAGTTGAATAAGCGCCTTCCTTCTACTCAC

CAAATCAGTGTGGAACAGTCCATCAGCCTCCTCCTCAACTTTATGATTGCCGCTTATGAC

>CL360.Contig1_A-W 54 884 deoxyguanosine kinase, mitochondrial isoform a precursor [Macaca mulatta] >gi|384941442|gb|AFI34326.1| deoxyguanosine kinase, mitochondrial isoform a precursor [Macaca mulatta]

ATGGCTGTGGGCCGTCTCTTTTTAAGTGGGCTTCGCACATTCTTCGGTACCATGGCCCAG

AACCTTCTCGAGGGTGCGCCCTCCTCCAGGGGCCTGCACGCGGGGCACGGACCCCGAAGG

CTCTCCATCGAAGGCAACATTGCTGTGGGAAAGTCCACCTTTGTGAAGTTACTCACGAAA

ACATACCCAGAGTGGCACGTAGCTACAGAACCTGTAGCAACATGGCAGGATATCCAGGCT

GCTGGCACCCAAAAAGACGGTACTACTCAAAGTCTGGGAAACTTGCTAGACATGATGTAC

CAGGAGCCAGCACGATGGTCCTATACATTTCAAACATTTTCATTTCTGAGCCGCCTGAAA

GTACAGCTGGAGCCCTTCCCTGAGAAACACGTACAGGCCAAGAAGCCCATGCAGATCTTT

GAGAGGTCTGTGTACAGTGACAGGTATATCTTTGCCAAGAATCTTTTTGAAAATGGTTCC

CTCAGTGACATCGAATGGCACATCTATCAGGACTGGCATACTTTTCTCCTGCAGGAGTTT

GCCAGTCGACTGTCATTACATGGCTTCATCTACCTCCAGGCTACTCCCCAGGTTTGTTTG

AAGAGACTACACCGGAGGGCCAGGGAGGAAGAGAAGGGAATTGAGCTGGCTTATCTCGAG

CAGCTTCATGGTCAGCACGAAGCCTGGCTTATTCACAAGACAACCAAGCTCCACTTTGAA

GCTCTGTCAAACATTCCTGTTCTGGTATTGGATGTCAATGATGATTTCTCTGAAGAAGTA

ACCAGACAAGAAGAACTCATGAAGAAGGTAAACACCTTTGTAAAGAATCTG

>CL360.Contig2_A-W 54 620 PREDICTED: deoxyguanosine kinase, mitochondrial isoform 3 [Macaca mulatta]

ATGGCTGTGGGCCGTCTCTTTTTAAGTGGGCTTCGCACATTCTTCGGTACCATGGCCCAG

AACCTTCTCGAGGGTGCGCCCTCCTCCAGGGGCCTGCACGCGGGGCACGGACCCCGAAGG

CTCTCCATCGAAGGCAACATTGCTGTGGGAAAGTCCACCTTTGTGAAGTTACTCACGAAA

ACATACCCAGAGTGGCACGTAGCTACAGAACCTGTAGCAACATGGCAGGATATCCAGGCT

GCTGGCACCCAAAAAGACGGTACTACTCAAAGTCTGGGAAACTTGCTAGACATGATGTAC

CAGGAGCCAGCACGATGGTCCTATACATTTCAAACATTTTCATTTCTGAGCCGCCTGAAA

GTACAGCTGGAGCCCTTCCCTGAGAAACACGTACAGGCCAAGAAGCCCATGCAGATCTTT

GAGAGGTCTGTGTACAGTGACAGGCTCCACTTTGAAGCTCTGTCAAACATTCCTGTTCTG

GTATTGGATGTCAATGATGATTTCTCTGAAGAAGTAACCAGACAAGAAGAACTCATGAAG

AAGGTAAACACCTTTGTAAAGAATCTG

>CL360.Contig4_A-W 54 194 minus strand PREDICTED: deoxyguanosine kinase [Oryctolagus cuniculus]

ATGGCTGTGGGCCGTCTCTTTTTAAGTGGGCTTCGCACATTCTTCGGTACCATGGCCCAG

AACCTTCTCGAGGGTGCGCCCTCCTCCAGGGGCCTGCACGCGGGGCACGGACCCCGAAGG

CTCTCCATCGAAGGCAACATT

>CL360.Contig5_A-W 160 507 minus strand PREDICTED: deoxyguanosine kinase, mitochondrial [Canis lupus familiaris]

ACGGACCCCGAAGGCTCTCCATCGAAGGCAACATTGGACGGTACTACTCAAAGTCTGGGA

AACTTGCTAGACATGATGTACCAGGAGCCAGCACGATGGTCCTATACATTTCAAACATTT

TCATTTCTGAGCCGCCTGAAAGTACAGCTGGAGCCCTTCCCTGAGAAACACGTACAGGCC

AAGAAGCCCATGCAGATCTTTGAGAGGTCTGTGTACAGTGACAGGCTCCACTTTGAAGCT

CTGTCAAACATTCCTGTTCTGGTATTGGATGTCAATGATGATTTCTCTGAAGAAGTAACC

AGACAAGAAGAACTCATGAAGAAGGTAAACACCTTTGTAAAGAATCTG

>CL360.Contig6_A-W 116 505 minus strand deoxyguanosine kinase, mitochondrial isoform a, partial [Macaca mulatta]

AGGTATATCTTTGCCAAGAATCTTTTTGAAAATGGTTCCCTCAGTGACATCGAATGGCAC

ATCTATCAGGACTGGCATACTTTTCTCCTGCAGGAGTTTGCCAGTCGACTGTCATTACAT

GGCTTCATCTACCTCCAGGCTACTCCCCAGGTTTGTTTGAAGAGACTACACCGGAGGGCC

AGGGAGGAAGAGAAGGGAATTGAGCTGGCTTATCTCGAGCAGCTTCATGGTCAGCACGAA

GCCTGGCTTATTCACAAGACAACCAAGCTCCACTTTGAAGCTCTGTCAAACATTCCTGTT

CTGGTATTGGATGTCAATGATGATTTCTCTGAAGAAGTAACCAGACAAGAAGAACTCATG

AAGAAGGTAAACACCTTTGTAAAGAATCTG

>CL362.Contig1_A-W 800 1099 minus strand PREDICTED: DIS3 mitotic control homolog (S. cerevisiae)-like [Canis lupus familiaris]

GTAAGAATATCTGTACAAGCCTTGCGTTGCCATTCTGATACAATCAGGCTTGAAATAATA

AGCAACAAACCATACATGATACCAAACACAGAACTTTTTCATCAGAGTTCTCTCTTGCTG

AAGAGTGAATTAGTGAAAGAAGTGACCAGATCTATGGAAGAAGCTCAGCTTGCCCAAGAA

GTCAAAGTAAACATCATTCAGGAAGAATATCAAGAATACTGCCAAACAAAGGGGCAAAGT

CTATACACACTTCTAGAGGAGATAAGGGACCTAGCTCTCCTAGATGTTTCAAGCAATTAC

>CL362.Contig2_A-W 790 1089 minus strand PREDICTED: DIS3 mitotic control homolog (S. cerevisiae)-like [Canis lupus familiaris]

GTAAGAATATCTGTACAAGCCTTGCGTTGCCATTCTGATACAATCAGGCTTGAAATAATA

AGCAACAAACCATACATGATACCAAACACAGAACTTTTTCATCAGAGTTCTCTCTTGCTG

AAGAGTGAATTAGTGAAAGAAGTGACCAGATCTATGGAAGAAGCTCAGCTTGCCCAAGAA

GTCAAAGTAAACATCATTCAGGAAGAATATCAAGAATACTGCCAAACAAAGGGGCAAAGT

CTATACACACTTCTAGAGGAGATAAGGGACCTAGCTCTCCTAGATGTTTCAAGCAATTAC

>CL362.Contig3_A-W 55 1011 minus strand PREDICTED: DIS3 mitotic control homolog (S. cerevisiae)-like [Canis lupus familiaris]

AGGTCCAATAAAACACTGGCTGATTCTCTGGATAATGCTAATGACCCCAATGATCCTATT

GTAAACAGACTGCTACGCTCTATGGCTACGCAGGCTATGTCTAATGCACTGTATTTCTCT

ACTGGATCATGTGCAGAGGAAGAGTTCCATCATTATGGTCTCGCGTTAGATAAATATACT

CATTTTACTTCTCCAATAAGAAGATATTCAGATATTATAGTACACAGACTGTTGATGGCA

GCCATTTCAAAAGATAAGAAAATGGAAATGAAAGAAAACCTACTCAGCAACAAGGATCTT

GAGGAATTGTGCAGACATATCAACAACAGAAACCGAGCAGCACAGCATTCTCAAAAGCAG

TCTACTGAGCTCTTCCAGTGCATGTACTTTAAAGACAAGGACCCTGAAACTGAGGAGCGA

TGCATATCTGATGGAGTTATTTACTCAATTAGAACAAATGGTGTGCTTGTATTTATACCG

AGGTTTGGGATTAAAGGTGCTGCTTATCTAAAAAATAAAGATGGTTTAGTGATCTCATGT

GGTCCAGATAGTCGTTCCGAATGGAAACCAGGATCTCTTCAACGATTTCAAAACAAAATA

ATCTCTACCACAACAGCAGGGCAATCTGTTACATTCCATTTGTTTGACCATGTGACAGTA

AGAATATCTGTACAAGCCTTGCGTTGCCATTCTGATACAATCAGGCTTGAAATAATAAGC

AACAAACCATACATGATACCAAACACAGAACTTTTTCATCAGAGTTCTCTCTTGCTGAAG

AGTGAATTAGTGAAAGAAGTGACCAGATCTATGGAAGAAGCTCAGCTTGCCCAAGAAGTC

AAAGTAAACATCATTCAGGAAGAATATCAAGAATACTGCCAAACAAAGGGGCAAAGTCTA

TACACACTTCTAGAGGAGATAAGGGACCTAGCTCTCCTAGATGTTTCAAGCAATTAC

>CL362.Contig4_A-W 55 540 minus strand PREDICTED: DIS3 mitotic control homolog (S. cerevisiae)-like [Canis lupus familiaris]

AGGTCCAATAAAACACTGGCTGATTCTCTGGATAATGCTAATGACCCCAATGATCCTATT

GTAAACAGACTGCTACGCTCTATGGCTACGCAGGCTATGTCTAATGCACTGTATTTCTCT

ACTGGATCATGTGCAGAGGAAGAGTTCCATCATTATGGTCTCGCGTTAGATAAATATACT

CATTTTACTTCTCCAATAAGAAGATATTCAGATATTATAGTACACAGACTGTTGATGGCA

GCCATTTCAAAAGATAAGAAAATGGAAATGAAAGAAAACCTACTCAGCAACAAGGATCTT

GAGGAATTGTGCAGACATATCAACAACAGAAACCGAGCAGCACAGCATTCTCAAAAGCAG

TCTACTGAGCTCTTCCAGTGCATGTACTTTAAAGACAAGGACCCTGAAACTGAGGAGCGA

TGCATATCTGATGGAGTTATTTACTCAATTAGAACAAATGGTGTGCTTGTATTTATACCG

AGGTAT

>CL362.Contig5_A-W 24 530 minus strand PREDICTED: DIS3 mitotic control homolog (S. cerevisiae)-like [Equus caballus]

AAGGTTTTTCTGTTCTTTATTAGGTCCAATAAAACACTGGCTGATTCTCTGGATAATGCT

AATGACCCCAATGATCCTATTGTAAACAGACTGCTACGCTCTATGGCTACGCAGGCTATG

TCTAATGCACTGTATTTCTCTACTGGATCATGTGCAGAGGAAGAGTTCCATCATTATGGT

CTCGCGTTAGATAAATATACTCATTTTACTTCTCCAATAAGAAGATATTCAGATATTATA

GTACACAGACTGTTGATGGCAGCCATTTCAAAAGATAAGAAAATGGAAATGAAAGAAAAC

CTACTCAGCAACAAGGATCTTGAGGAATTGTGCAGACATATCAACAACAGAAACCGAGCA

GCACAGCATTCTCAAAAGCAGTCTACTGAGCTCTTCCAGTGCATGTACTTTAAAGACAAG

GACCCTGAAACTGAGGAGCGATGCATATCTGATGGAGTTATTTACTCAATTAGAACAAAT

GGTGTGCTTGTATTTATACCGAGGTAT

>CL363.Contig1_A-W 7 318 minus strand protein tyrosine phosphatase, receptor type, A isoform 1 [Bos taurus]

CGCCTCATCACTCAGTTCCATTTTACCAGCTGGCCAGACTTTGGGGTGCCTTTCACCCCG

ATCGGCATGCTCAAGTTCCTCAAGAAAGTGAAGGCCTGTAACCCTCAGTATGCAGGGGCT

ATTGTGGTCCACTGCAGTGCAGGTGTAGGGCGTACTGGTACCTTTGTCGTCATTGATGCC

ATGCTGGACATGATGCATACAGAGCGGAAGGTGGATGTATATGGCTTTGTGAGCCGAATC

CGGGCCCAGCGCTGCCAGATGGTGCAAACAGATATGCAGTATGTTTTTATATACCAAGCC

CTTCTGGAGCAT

>CL363.Contig2_A-W 367 1188 minus strand PREDICTED: receptor-type tyrosine-protein phosphatase alpha [Saimiri boliviensis boliviensis]

CTGCAAAAAATTTACAACAAAATTCCAGGGACCAGCAACAATGGATTGGAAGAAGAGTTT

AAGAAGTTAACATCAATCAAAATCCAGAATGACAAGATGCGGACTGGAAACCTTCCAGCC

AACATGAAGAAGAACAGAGTTTTACAGATCATTCCATATGAATTCAACAGAGTGATCATT

CCAGTTAAGCGAGGTGAAGAGAACACAGACTATGTGAATGCATCCTTCATTGATGGCTAC

CGGCAGAAGGACTCTTACATTGCCAGCCAGGGCCCTCTTCTCCACACAATTGAAGACTTC

TGGCGTATGATCTGGGAGTGGAAATCATGTTCCATCGTGATGCTAACAGAACTGGAGGAG

AGAGGCCAGGAGAAGTGTGCCCAGTACTGGCCATCTGATGGCCTTGTATCCTATGGAGAC

ATAACAGTGGAGCTGAAGAAGGAGGAAGAATGTGAGAGCTACACCGTCCGAGACCTCCTG

GTTACCAACACCAGGGAGAACAAGAGCCGGCAGATCCGACAGTTCCACTTCCATGGCTGG

CCTGAAGTGGGCATCCCCAGTGATGGGAAGGGCATGATCAGCATCATTGCAGCTGTACAG

AAGCAGCAGCAGCAGTCGGGGAACCACCCCATCACTGTGCACTGCAGCGCCGGGGCAGGA

CGGACAGGGACTTTCTGTGCCCTGAGCACAGTCCTGGAACGAGTGAAAGCAGAGGGGATT

TTGGATGTCTTCCAGACTGTCAAGAGCCTGCGGCTTCAGAGGCCACATATGGTCCAGACA

CTGGAGCAGTATGAGTTCTGCTACAAGGTGGTGCAGGAATAC

>CL363.Contig3_A-W 7 318 minus strand PREDICTED: receptor-type tyrosine-protein phosphatase alpha [Saimiri boliviensis boliviensis]

CGCCTCATCACTCAGTTCCATTTTACCAGCTGGCCAGACTTTGGGGTGCCTTTCACCCCG

ATCGGCATGCTCAAGTTCCTCAAGAAAGTGAAGGCCTGTAACCCTCAGTATGCAGGGGCT

ATTGTGGTCCACTGCAGTGCAGGTGTAGGGCGTACTGGTACCTTTGTCGTCATTGATGCC

ATGCTGGACATGATGCATACAGAGCGGAAGGTGGATGTATATGGCTTTGTGAGCCGAATC

CGGGCCCAGCGCTGCCAGATGGTGCAAACAGATATGCAGTATGTTTTTATATACCAAGCC

CTTCTGGAGCAT

>CL363.Contig4_A-W 7 318 minus strand PREDICTED: receptor-type tyrosine-protein phosphatase alpha [Saimiri boliviensis boliviensis]

CGCCTCATCACTCAGTTCCATTTTACCAGCTGGCCAGACTTTGGGGTGCCTTTCACCCCG

ATCGGCATGCTCAAGTTCCTCAAGAAAGTGAAGGCCTGTAACCCTCAGTATGCAGGGGCT

ATTGTGGTCCACTGCAGTGCAGGTGTAGGGCGTACTGGTACCTTTGTCGTCATTGATGCC

ATGCTGGACATGATGCATACAGAGCGGAAGGTGGATGTATATGGCTTTGTGAGCCGAATC

CGGGCCCAGCGCTGCCAGATGGTGCAAACAGATATGCAGTATGTTTTTATATACCAAGCC

CTTCTGGAGCAT

>CL363.Contig5_A-W 2 175 PREDICTED: receptor-type tyrosine-protein phosphatase alpha [Saimiri boliviensis boliviensis]

GTAGGGCGTACTGGTACCTTTGTCGTCATTGATGCCATGCTGGACATGATGCATACAGAG

CGGAAGGTGGATGTATATGGCTTTGTGAGCCGAATCCGGGCCCAGCGCTGCCAGATGGTG

CAAACAGATATGCAGTATGTTTTTATATACCAAGCCCTTCTGGAGCATTATCTC

>CL364.Contig1_A-W 26 475 minus strand PREDICTED: heterogeneous nuclear ribonucleoprotein H2 [Otolemur garnettii]

GGTCCGAATAGTCCTGATACTGCCAACGATGGCTTCGTCCGGCTTAGAGGACTCCCATTT

GGCTGTAGCAAGGAAGAGATTGTTCAGTTCTTTTCAGGGTTGGAAATTGTGCCAAATGGG

ATGACACTGCCGGTGGACTTTCAGGGGCGGAGCACAGGGGAGGCTTTTGTGCAGTTTGCT

TCACAGGAGATAGCTGAAAAGGCCTTAAAGAAACACAAGGAAAGGATAGGGCACAGGTAC

ATTGAAATCTTCAAGAGTAGCAGAGCTGAAGTCCGAACCCATTATGACCCCCCTCGAAAG

CTCATGGCTATGCAGCGACCAGGTCCTTATGATAGGCCAGGAGCTGGCAGAGGGTATAAT

AGCATTGGCAGAGGAGCTGGTTTTGAGAGGATGAGGCGGGGGGCATATGGTGGAGGGTAT

GGAGGCTATGATGACTATGGTGGCTATAAC

>CL364.Contig3_A-W 14 145 PREDICTED: heterogeneous nuclear ribonucleoprotein H2 [Otolemur garnettii]

GAAGGCAGACCAAGTGGTGAAGCATTTGTTGAACTTGAATCTGAAGATGAAGTGAAATTG

GCTTTGAAGAAGGACAGAGAAACCATGGGACATAGATATGTTGAAGTATTCAAGTCCAAC

AGTGTTGAAATG

>CL364.Contig4_A-W 340 1602 minus strand unnamed protein product [Mus musculus]

ATGATGCTGGGCCCTGAGGGAGGTGAAGGTTTTGTGGTCAAACTCCGTGGCCTGCCCTGG

TCCTGCTCAATTGAGGACGTGCAGAACTTTCTCTCCGACTGCACAATTCACGATGGGGTT

GCAGGTGTCCATTTCATTTACACTAGAGAAGGCAGGCAGAGTGGTGAGGCTTTTGTTGAA

CTTGAATCAGAAGATGATGTAAAAATGGCCCTGAAAAAAGACAGGGAAAGCATGGGACAC

CGATACATCGAGGTGTTCAAGTCACACAGAACCGAGATGGATTGGGTGTTGAAGCACAGT

GGTCCAAACAGCGCCGACACTGCCAATGACGGCTTCGTGCGGCTTCGGGGACTCCCGTTT

GGATGCACAAAGGAAGAAATCGTTCAGTTCTTCTCAGGGTTGGAAATTGTGCCAAACGGG

ATCACATTGCCTGTGGACCCTGAGGGCAAGATTACAGGGGAGGCCTTCGTGCAGTTTGCC

TCACAGGAATTAGCTGAAAAGGCTCTAGGGAAGCACAAGGAGAGAATAGGGCATAGGTAT

ATCGAAGTGTTTAAGAGCAGTCAGGAGGAAGTTAGGTCATATTCTGATCCGCCTCTGAAG

TTCATGTCTGTGCAGCGGCCGGGGCCCTATGACCGGCCAGGCACAGCCCGGAGGTACATT

GGCATCGTGAAGCAGGCGGGCCTGGAGAGGATGAGGTCTGGTGCCTACAGTGCAGGCTAT

GGGGGCTATGAGGAGTACAGCGGCCTGAGTGATGGCTACGGCTTTACCACCGACCTGTTT

GGGAGAGACCTCAGTTACTGTCTCTCAGGAATGTATGACCATAGATATGGAGATGGTGAA

TTCACAGTGCAGAGCACCACTGGCCACTGTGTCCACATGAGGGGGCTGCCCTACAAAGCA

ACAGAGAACGACATTTACAACTTCTTCTCTCCACTCAACCCTGTGAGAGTCCATATTGAG

ATTGGCCCTGATGGTAGAGTGACGGGCGAAGCAGATGTTGAGTTTGCCACCCATGAAGAA

GCTGTGGCAGCTATGTCAAAAGACAGGGCCAACATGCAGCACAGATACATAGAACTCTTC

CTGAATTCAACAACAGGGGCCAGCAATGGGGCTTATAGCAGCCAGGTGATGCAGGGCATG

GGGGTGTCAGCTGCCCAGGCCACCTATAGTGGCCTGGAGAGCCAGTCAGTGAGTGGCTGT

TATGGGGCCGGCTATAGCGGTCAGAACAGCATGGGTGGATATGACTAGTTTTGTAGTAAC

ATT

>CL364.Contig5_A-W 320 1582 minus strand unnamed protein product [Mus musculus]

ATGATGCTGGGCCCTGAGGGAGGTGAAGGTTTTGTGGTCAAACTCCGTGGCCTGCCCTGG

TCCTGCTCAATTGAGGACGTGCAGAACTTTCTCTCCGACTGCACAATTCACGATGGGGTT

GCAGGTGTCCATTTCATTTACACTAGAGAAGGCAGGCAGAGTGGTGAGGCTTTTGTTGAA

CTTGAATCAGAAGATGATGTAAAAATGGCCCTGAAAAAAGACAGGGAAAGCATGGGACAC

CGATACATCGAGGTGTTCAAGTCACACAGAACCGAGATGGATTGGGTGTTGAAGCACAGT

GGTCCAAACAGCGCCGACACTGCCAATGACGGCTTCGTGCGGCTTCGGGGACTCCCGTTT

GGATGCACAAAGGAAGAAATCGTTCAGTTCTTCTCAGGGTTGGAAATTGTGCCAAACGGG

ATCACATTGCCTGTGGACCCTGAGGGCAAGATTACAGGGGAGGCCTTCGTGCAGTTTGCC

TCACAGGAATTAGCTGAAAAGGCTCTAGGGAAGCACAAGGAGAGAATAGGGCATAGGTAT

ATCGAAGTGTTTAAGAGCAGTCAGGAGGAAGTTAGGTCATATTCTGATCCGCCTCTGAAG

TTCATGTCTGTGCAGCGGCCGGGGCCCTATGACCGGCCAGGCACAGCCCGGAGGTACATT

GGCATCGTGAAGCAGGCGGGCCTGGAGAGGATGAGGTCTGGTGCCTACAGTGCAGGCTAT

GGGGGCTATGAGGAGTACAGCGGCCTGAGTGATGGCTACGGCTTTACCACCGACCTGTTT

GGGAGAGACCTCAGTTACTGTCTCTCAGGAATGTATGACCATAGATATGGAGATGGTGAA

TTCACAGTGCAGAGCACCACTGGCCACTGTGTCCACATGAGGGGGCTGCCCTACAAAGCA

ACAGAGAACGACATTTACAACTTCTTCTCTCCACTCAACCCTGTGAGAGTCCATATTGAG

ATTGGCCCTGATGGTAGAGTGACGGGCGAAGCAGATGTTGAGTTTGCCACCCATGAAGAA

GCTGTGGCAGCTATGTCAAAAGACAGGGCCAACATGCAGCACAGATACATAGAACTCTTC

CTGAATTCAACAACAGGGGCCAGCAATGGGGCTTATAGCAGCCAGGTGATGCAGGGCATG

GGGGTGTCAGCTGCCCAGGCCACCTATAGTGGCCTGGAGAGCCAGTCAGTGAGTGGCTGT

TATGGGGCCGGCTATAGCGGTCAGAACAGCATGGGTGGATATGACTAGTTTTGTAGTAAC

ATT

>CL364.Contig6_A-W 62 322 minus strand heterogeneous nuclear ribonucleoprotein H2 [Macaca mulatta]

GGTGGGTCCAGTTTCCAGAGCACCACAGGGCATTGTGTACACATGAGGGGGTTACCTTAC

AGAGCCACTGAGAATGATATTTACAATTTTTTCTCCCCTCTTAACCCCATGAGAGTACAC

ATTGAAATTGGACCAGATGGCAGAGTTACTGGTGAGGCAGATGTTGAATTTGCTACTCAT

GAAGATGCCGTGGCAGCAATGGCAAAAGATAAAGCTAATATGCAACACAGATATGTGGAG

CTCTTTTTGAACTCTACTGCA

>CL364.Contig7_A-W 94 324 PREDICTED: heterogeneous nuclear ribonucleoprotein H isoform 1 [Otolemur garnettii] >gi|395853400|ref|XP_003799199.1| PREDICTED: heterogeneous nuclear ribonucleoprotein H isoform 2 [Otolemur garnettii] >gi|395853402|ref|XP_003799200.1| PREDICTED: heterogeneous nuclear ribonucleoprotein H isoform 3 [Otolemur garnettii]

GAACACAGATATGTAGAACTCTTCTTGAATTCTACAGCAGGAGCAAGCGGTGGTGCTTAT

GGTAGCCAAATGATGGGAGGCATGGGCTTGTCAAACCAGTCCAGTTATGGTGGCCCAGCT

AGCCAGCAGCTGAGTGGTGGTTATGGAGGAGGCTATGGAGGCCAGAGCAGCATGAGTGGA

TATGACCAAGTTTTACAGGAAAACTCCAGTGATTTTCAATCAAACATTGCA

>CL364.Contig8_A-W 69 362 PREDICTED: heterogeneous nuclear ribonucleoprotein H2-like [Sus scrofa]

ATCCTCAAAGAGACGATGATGTTGGGCACCGAAGGCGGAGAAGGATTCGTGGTTAAGGTC

CGGGGCTTGCCCTGGTCTTGCTCGGCCGATGAAGTGCAACGTTTTTTTTCCGACTGCAAA

ATTCAAAATGGGGCTCAAGGTATTCGTTTCATCTACACCAGAGAAGGCAGACCGAGTGGC

GAGGCTTTTGTTGAACTTGAATCAGAAGATGAAGTCAAATTGGCCCTGAAAAAAGACAGA

GAAACTATGGGACACAGATATGTTGAAGTATTCAAGTCAAACAACGTTGAAATG

>CL364.Contig9_A-W 84 335 PREDICTED: heterogeneous nuclear ribonucleoprotein H2-like [Sus scrofa]

ATGATGTTGGGCACCGAAGGCGGAGAAGGATTCGTGGTTAAGGTCCGGGGCTTGCCCTGG

TCTTGCTCGGCCGATGAAGTGCAACGTTTTTTTTCCGACTGCAAAATTCAAAATGGGGCT

CAAGGTATTCGTTTCATCTACACCAGAGAAGGCAGACCGAGTGGCGAGGCTTTTGTTGAA

CTTGAATCAGAAGATGAAGTCAAATTGGCCCTGAAAAAAGACAGAGAAACTATGGGACAC

AGATATGTTGAA

>CL364.Contig10_A-W 1986 2288 heterogeneous nuclear ribonucleoprotein H1, isoform CRA_c [Mus musculus]

ACTAATGTTTTCTCTCCCCTCCCCATTCCCCTCCCTCTTGAACACTGCTTTAGGTATATC

GAAATATTTAAGAGCAGTCGAGCTGAAGTTAGAACTCATTATGATCCACCAAGAAAGCTT

ATGGCCATGCAGCGGCCAGGTCCCTATGACAGACCTGGGGCTGGCAGAGGGTATAACAGC

ATTGGCAGAGGAGCTGGCTTTGAGAGGATGAGGCGTGGTGCTTATGGTGGAGGCTATGGA

GGCTATGATGATTATAATGGCTATAATGATGGCTATGGATTTGGGTCAGATAGATTTGGA

AGA

>CL364.Contig11_A-W 300 1562 minus strand unnamed protein product [Mus musculus]

ATGATGCTGGGCCCTGAGGGAGGTGAAGGTTTTGTGGTCAAACTCCGTGGCCTGCCCTGG

TCCTGCTCAATTGAGGACGTGCAGAACTTTCTCTCCGACTGCACAATTCACGATGGGGTT

GCAGGTGTCCATTTCATTTACACTAGAGAAGGCAGGCAGAGTGGTGAGGCTTTTGTTGAA

CTTGAATCAGAAGATGATGTAAAAATGGCCCTGAAAAAAGACAGGGAAAGCATGGGACAC

CGATACATCGAGGTGTTCAAGTCACACAGAACCGAGATGGATTGGGTGTTGAAGCACAGT

GGTCCAAACAGCGCCGACACTGCCAATGACGGCTTCGTGCGGCTTCGGGGACTCCCGTTT

GGATGCACAAAGGAAGAAATCGTTCAGTTCTTCTCAGGGTTGGAAATTGTGCCAAACGGG

ATCACATTGCCTGTGGACCCTGAGGGCAAGATTACAGGGGAGGCCTTCGTGCAGTTTGCC

TCACAGGAATTAGCTGAAAAGGCTCTAGGGAAGCACAAGGAGAGAATAGGGCATAGGTAT

ATCGAAGTGTTTAAGAGCAGTCAGGAGGAAGTTAGGTCATATTCTGATCCGCCTCTGAAG

TTCATGTCTGTGCAGCGGCCGGGGCCCTATGACCGGCCAGGCACAGCCCGGAGGTACATT

GGCATCGTGAAGCAGGCGGGCCTGGAGAGGATGAGGTCTGGTGCCTACAGTGCAGGCTAT

GGGGGCTATGAGGAGTACAGCGGCCTGAGTGATGGCTACGGCTTTACCACCGACCTGTTT

GGGAGAGACCTCAGTTACTGTCTCTCAGGAATGTATGACCATAGATATGGAGATGGTGAA

TTCACAGTGCAGAGCACCACTGGCCACTGTGTCCACATGAGGGGGCTGCCCTACAAAGCA

ACAGAGAACGACATTTACAACTTCTTCTCTCCACTCAACCCTGTGAGAGTCCATATTGAG

ATTGGCCCTGATGGTAGAGTGACGGGCGAAGCAGATGTTGAGTTTGCCACCCATGAAGAA

GCTGTGGCAGCTATGTCAAAAGACAGGGCCAACATGCAGCACAGATACATAGAACTCTTC

CTGAATTCAACAACAGGGGCCAGCAATGGGGCTTATAGCAGCCAGGTGATGCAGGGCATG

GGGGTGTCAGCTGCCCAGGCCACCTATAGTGGCCTGGAGAGCCAGTCAGTGAGTGGCTGT

TATGGGGCCGGCTATAGCGGTCAGAACAGCATGGGTGGATATGACTAGTTTTGTAGTAAC

ATT

>CL364.Contig12_A-W 621 761 PREDICTED: heterogeneous nuclear ribonucleoprotein H isoform 1 [Otolemur garnettii] >gi|395853400|ref|XP_003799199.1| PREDICTED: heterogeneous nuclear ribonucleoprotein H isoform 2 [Otolemur garnettii] >gi|395853402|ref|XP_003799200.1| PREDICTED: heterogeneous nuclear ribonucleoprotein H isoform 3 [Otolemur garnettii]

GCAAACCAGTCCAGTTATGGTGGCCCAGCTAGCCAGCAGCTGAGTGGTGGTTATGGAGGA

GGCTATGGAGGCCAGAGCAGCATGAGTGGATATGACCAAGTTTTACAGGAAAACTCCAGT

GATTTTCAATCAAACATTGCA

>CL364.Contig13_A-W 558 1820 unnamed protein product [Mus musculus]

ATGATGCTGGGCCCTGAGGGAGGTGAAGGTTTTGTGGTCAAACTCCGTGGCCTGCCCTGG

TCCTGCTCAATTGAGGACGTGCAGAACTTTCTCTCCGACTGCACAATTCACGATGGGGTT

GCAGGTGTCCATTTCATTTACACTAGAGAAGGCAGGCAGAGTGGTGAGGCTTTTGTTGAA

CTTGAATCAGAAGATGATGTAAAAATGGCCCTGAAAAAAGACAGGGAAAGCATGGGACAC

CGATACATCGAGGTGTTCAAGTCACACAGAACCGAGATGGATTGGGTGTTGAAGCACAGT

GGTCCAAACAGCGCCGACACTGCCAATGACGGCTTCGTGCGGCTTCGGGGACTCCCGTTT

GGATGCACAAAGGAAGAAATCGTTCAGTTCTTCTCAGGGTTGGAAATTGTGCCAAACGGG

ATCACATTGCCTGTGGACCCTGAGGGCAAGATTACAGGGGAGGCCTTCGTGCAGTTTGCC

TCACAGGAATTAGCTGAAAAGGCTCTAGGGAAGCACAAGGAGAGAATAGGGCATAGGTAT

ATCGAAGTGTTTAAGAGCAGTCAGGAGGAAGTTAGGTCATATTCTGATCCGCCTCTGAAG

TTCATGTCTGTGCAGCGGCCGGGGCCCTATGACCGGCCAGGCACAGCCCGGAGGTACATT

GGCATCGTGAAGCAGGCGGGCCTGGAGAGGATGAGGTCTGGTGCCTACAGTGCAGGCTAT

GGGGGCTATGAGGAGTACAGCGGCCTGAGTGATGGCTACGGCTTTACCACCGACCTGTTT

GGGAGAGACCTCAGTTACTGTCTCTCAGGAATGTATGACCATAGATATGGAGATGGTGAA

TTCACAGTGCAGAGCACCACTGGCCACTGTGTCCACATGAGGGGGCTGCCCTACAAAGCA

ACAGAGAACGACATTTACAACTTCTTCTCTCCACTCAACCCTGTGAGAGTCCATATTGAG

ATTGGCCCTGATGGTAGAGTGACGGGCGAAGCAGATGTTGAGTTTGCCACCCATGAAGAA

GCTGTGGCAGCTATGTCAAAAGACAGGGCCAACATGCAGCACAGATACATAGAACTCTTC

CTGAATTCAACAACAGGGGCCAGCAATGGGGCTTATAGCAGCCAGGTGATGCAGGGCATG

GGGGTGTCAGCTGCCCAGGCCACCTATAGTGGCCTGGAGAGCCAGTCAGTGAGTGGCTGT

TATGGGGCCGGCTATAGCGGTCAGAACAGCATGGGTGGATATGACTAGTTTTGTAGTAAC

ATT

>CL364.Contig14_A-W 328 582 PREDICTED: heterogeneous nuclear ribonucleoprotein H2-like [Sus scrofa]

GAAGGTTTTGTGGTCAAACTCCGTGGCCTGCCCTGGTCCTGCTCAATTGAGGACGTGCAG

AACTTTCTCTCCGACTGCACAATTCACGATGGGGTTGCAGGTGTCCATTTCATTTACACT

AGAGAAGGCAGGCAGAGTGGTGAGGCTTTTGTTGAACTTGAATCAGAAGATGATGTAAAA

ATGGCCCTGAAAAAAGACAGGGAAAGCATGGGACACCGATACATCGAGGTGTTCAAGTCA

CACAGAACCGAGATG

>CL364.Contig15_A-W 582 836 PREDICTED: heterogeneous nuclear ribonucleoprotein H2-like [Sus scrofa]

GAAGGTTTTGTGGTCAAACTCCGTGGCCTGCCCTGGTCCTGCTCAATTGAGGACGTGCAG

AACTTTCTCTCCGACTGCACAATTCACGATGGGGTTGCAGGTGTCCATTTCATTTACACT

AGAGAAGGCAGGCAGAGTGGTGAGGCTTTTGTTGAACTTGAATCAGAAGATGATGTAAAA

ATGGCCCTGAAAAAAGACAGGGAAAGCATGGGACACCGATACATCGAGGTGTTCAAGTCA

CACAGAACCGAGATG

>CL364.Contig16_A-W 328 582 PREDICTED: heterogeneous nuclear ribonucleoprotein H2-like [Sus scrofa]

GAAGGTTTTGTGGTCAAACTCCGTGGCCTGCCCTGGTCCTGCTCAATTGAGGACGTGCAG

AACTTTCTCTCCGACTGCACAATTCACGATGGGGTTGCAGGTGTCCATTTCATTTACACT

AGAGAAGGCAGGCAGAGTGGTGAGGCTTTTGTTGAACTTGAATCAGAAGATGATGTAAAA

ATGGCCCTGAAAAAAGACAGGGAAAGCATGGGACACCGATACATCGAGGTGTTCAAGTCA

CACAGAACCGAGATG

>CL364.Contig17_A-W 360 590 PREDICTED: heterogeneous nuclear ribonucleoprotein H2-like [Sus scrofa]

GGTTTTGTGGTCAAACTCCGTGGCCTGCCCTGGTCCTGCTCAATTGAGGACGTGCAGAAC

TTTCTCTCCGACTGCACAATTCACGATGGGGTTGCAGGTGTCCATTTCATTTACACTAGA

GAAGGCAGGCAGAGTGGTGAGGCTTTTGTTGAACTTGAATCAGAAGATGATGTAAAAATG

GCCCTGAAAAAAGACAGGGAAAGCATGGGACACCGATACATCGAGGTGTTC

>CL365.Contig1_A-W 203 1588 PREDICTED: microspherule protein 1-like isoform 1 [Ailuropoda melanoleuca]

ATGGACAAAGATTCTCAGGGGCTACTAGATTCATCCCTGATGGCATCAGGCACCGCCAGT

CGTTCAGAGGATGAGGAGTCGCTGGCAGGGCAGAAGCGGGCCTCCTCCCAAGCCTTGGGC

ACCATCCCTAAACGGAGAAGCTCCTCCAGGTTTATCAAGAGGAAGAAGTTCGATGATGAG

CTGGTGGAAAGCAGCCTAGCAAAATCCTCTACCCGGGTGAAGGGGACCAGTGGAGTAGAA

CCAGGGCGCTGTTCAGGGAGTGAACCCTCCTCCAGTGAGAAGAAGAAGGTGTCCAAAGTC

CCCAGTACTCCTATACCACCCAGCCCTGCCCCAGTCCCTGGACTCACCAAGCGTGTGAAA

AAGAGCAAACAGCCACTTCAGGTGACCAAGGATCTGGGCCGCTGGAAGCCTGCGGATGAC

CTCTTGCTCATCAATGCTGTATTGCAGACCAATGACCTGACATCTGTCCACCTGGGTGTA

AAGTTCAGCTGCCGTTTTACTCTTCGGGAAGTCCAGGAGCGCTGGTACGCCCTGCTCTAT

GATCCTGTCATCTCCAAATTGGCCTGCCAGGCCATGAGGCAGCTGCACCCAGAGGCCATT

GCAGCCATCCAGAGCAAGGCCCTGTTTAGCAAGGCTGAAGAGCAGCTGCTAAGCAAAGTG

GGATCGACCAGCCAGCCCACCTTGGAGACCTTTCAGGACTTACTGCACAGACACCCTGAT

GCCTTCTACCTGGCCCGAACTGCCAAGGCCTTGCAGGCACACTGGCAACTCATGAAGCAG

TATTACCTGCTGGAGGACCAGACAGTGCAGCCGCTTCCAAAAGGGGACCAAGTGCTGAAC

TTCTCTGATGCAGAAGACCTGATTGATGACAGTAAGCTCAAGGACATGCGAGATGAAGTC

CTGGAACATGAGCTCACAGTGGCTGACCGGCGCCAGAAGCGAGAGATAAGACAGCTGGAA

CAGGAATTGCATAAGTGGCAGGTGCTAGTGGACAGCATCACAGGGATGAGCTCTCCAGAC

TTTGACAACCAGACATTGGCAGTGCTACGGGGCCGCATGGTGCGGTATCTTATGCGCTCA

CGTGAGATCACCCTGGGCAGAGCAACCAAGGATAACCAGATTGATGTGGACCTGTCTCTG

GAGGGTCCAGCTTGGAAGATCTCCCGAAAGCAAGGTGTTATCAAGTTGAAGAACAATGGT

GATTTCTTCATTGCCAATGAGGGCCGACGGCCCATTTACATTGATGGACGGCCTGTACTG

TGTGGCTCCAAATGGCGCCTCAGCAACAACTCTGTGGTGGAGATTGCCAGCCTGCGATTT

GTCTTCCTCATCAACCAGGACCTCATTGCCCTTATCAGGGCAGAGGCTGCCAAGATCACA

CCACAG

>CL365.Contig2_A-W 92 1468 minus strand PREDICTED: microspherule protein 1 isoform 2 [Equus caballus]

GATTCTCAGGGGCTACTAGATTCATCCCTGATGGCATCAGGCACCGCCAGTCGTTCAGAG

GATGAGGAGTCGCTGGCAGGGCAGAAGCGGGCCTCCTCCCAAGCCTTGGGCACCATCCCT

AAACGGAGAAGCTCCTCCAGGTTTATCAAGAGGAAGAAGTTCGATGATGAGCTGGTGGAA

AGCAGCCTAGCAAAATCCTCTACCCGGGTGAAGGGGACCAGTGGAGTAGAACCAGGGCGC

TGTTCAGGGAGTGAACCCTCCTCCAGTGAGAAGAAGAAGGTGTCCAAAGTCCCCAGTACT

CCTATACCACCCAGCCCTGCCCCAGTCCCTGGACTCACCAAGCGTGTGAAAAAGAGCAAA

CAGCCACTTCAGGTGACCAAGGATCTGGGCCGCTGGAAGCCTGCGGATGACCTCTTGCTC

ATCAATGCTGTATTGCAGACCAATGACCTGACATCTGTCCACCTGGGTGTAAAGTTCAGC

TGCCGTTTTACTCTTCGGGAAGTCCAGGAGCGCTGGTACGCCCTGCTCTATGATCCTGTC

ATCTCCAAATTGGCCTGCCAGGCCATGAGGCAGCTGCACCCAGAGGCCATTGCAGCCATC

CAGAGCAAGGCCCTGTTTAGCAAGGCTGAAGAGCAGCTGCTAAGCAAAGTGGGATCGACC

AGCCAGCCCACCTTGGAGACCTTTCAGGACTTACTGCACAGACACCCTGATGCCTTCTAC

CTGGCCCGAACTGCCAAGGCCTTGCAGGCACACTGGCAACTCATGAAGCAGTATTACCTG

CTGGAGGACCAGACAGTGCAGCCGCTTCCAAAAGGGGACCAAGTGCTGAACTTCTCTGAT

GCAGAAGACCTGATTGATGACAGTAAGCTCAAGGACATGCGAGATGAAGTCCTGGAACAT

GAGCTCACAGTGGCTGACCGGCGCCAGAAGCGAGAGATAAGACAGCTGGAACAGGAATTG

CATAAGTGGCAGGTGCTAGTGGACAGCATCACAGGGATGAGCTCTCCAGACTTTGACAAC

CAGACATTGGCAGTGCTACGGGGCCGCATGGTGCGGTATCTTATGCGCTCACGTGAGATC

ACCCTGGGCAGAGCAACCAAGGATAACCAGATTGATGTGGACCTGTCTCTGGAGGGTCCA

GCTTGGAAGATCTCCCGAAAGCAAGGTGTTATCAAGTTGAAGAACAATGGTGATTTCTTC

ATTGCCAATGAGGGCCGACGGCCCATTTACATTGATGGACGGCCTGTACTGTGTGGCTCC

AAATGGCGCCTCAGCAACAACTCTGTGGTGGAGATTGCCAGCCTGCGATTTGTCTTCCTC

ATCAACCAGGACCTCATTGCCCTTATCAGGGCAGAGGCTGCCAAGATCACACCACAG

>CL365.Contig3_A-W 66 296 Microspherule protein 1 [Heterocephalus glaber]

TGCTTCACTACTTCCACAGGTGTTATCAAGTTGAAGAACAATGGTGATTTCTTCATTGCC

AATGAGGGCCGACGGCCCATTTACATTGATGGACGGCCTGTACTGTGTGGCTCCAAATGG

CGCCTCAGCAACAACTCTGTGGTGGAGATTGCCAGCCTGCGATTTGTCTTCCTCATCAAC

CAGGACCTCATTGCCCTTATCAGGGCAGAGGCTGCCAAGATCACACCACAG

>CL366.Contig1_A-W 513 620 PREDICTED: hypothetical protein LOC100427265 [Macaca mulatta] >gi|90082565|dbj|BAE90464.1| unnamed protein product [Macaca fascicularis]

CCTTCCGGAGGGAGAGAGGAACTGCTGTTTGTCTCACTCAGTTACTTGATATCACCGCCT

CTCACCTTCTCCATCGTGCATGTCCCCAGCCACATGGGAGGTGAAAGC

>CL366.Contig2_A-W 172 615 Smg-7 homolog, nonsense mediated mRNA decay factor (C. elegans), isoform CRA_e [Homo sapiens]

TCTAAAGTCTATCTGGTCCAGTTCCATGATGCATCCTGGACCTTCCGCTCTGGAGCAGTT

GTTAATGCAGCAGAAGCAGAAACAGCAGCGGGGACAAGGCACCATGAACCCTCCACACTG

AGGCCAAAGTGGCAACCCTGGGAATGAAGGCTCCATAAACCATGGCATGTTGGGTTTGCA

GGACTGGCCCACACAGTCCCCTGCAGGTGGCAGCCCTCTTGTCTGTTTCTTGCTATCAAG

AGGGTGTAAGTGTTCCACCAGCCCGCTGAGTGTGCACGAAATGTTCGCAGTGCAACAAAA

AGAAAAATCCATCAGGAACTCTCCGTCCCCCCAGGTCCTTCCGGAGGGAGAGAGGAACTG

CTGTTTGTCTCACTCAGTTACTTGATATCACCGCCTCTCACCTTCTCCATCGTGCATGTC

CCCAGCCACATGGGAGGTGAAAGC

>CL366.Contig3_A-W 1720 2163 Smg-7 homolog, nonsense mediated mRNA decay factor (C. elegans), isoform CRA_e [Homo sapiens]

TCTAAAGTCTATCTGGTCCAGTTCCATGATGCATCCTGGACCTTCCGCTCTGGAGCAGTT

GTTAATGCAGCAGAAGCAGAAACAGCAGCGGGGACAAGGCACCATGAACCCTCCACACTG

AGGCCAAAGTGGCAACCCTGGGAATGAAGGCTCCATAAACCATGGCATGTTGGGTTTGCA

GGACTGGCCCACACAGTCCCCTGCAGGTGGCAGCCCTCTTGTCTGTTTCTTGCTATCAAG

AGGGTGTAAGTGTTCCACCAGCCCGCTGAGTGTGCACGAAATGTTCGCAGTGCAACAAAA

AGAAAAATCCATCAGGAACTCTCCGTCCCCCCAGGTCCTTCCGGAGGGAGAGAGGAACTG

CTGTTTGTCTCACTCAGTTACTTGATATCACCGCCTCTCACCTTCTCCATCGTGCATGTC

CCCAGCCACATGGGAGGTGAAAGC

>CL367.Contig1_A-W 71 961 minus strand PREDICTED: lysine-specific demethylase 3A isoform 3 [Callithrix jacchus]

ATGGTGCTCACGCTCGGGGAGAGCTGGCCGGTGCTGGTGGGGAAGCGGTTCCTCAGCCTG

TGCGCGGCCGACGGCGTCGACGACGACTGGGACGTGCAGCGCGTGGCCGAGTGGCCCTGG

CTGGCGGGCACGGTCCGCGCCGTGTCCCACCCCGAAGTTTCCAGGAAGGACCTGAAGGTG

TACAAACCTTTGTTGGACAAAGTTGGTTTGGGATCCATGACTTCTGTTCGCTTCCTAGGA

GATCAGCAAAGCGTATTTCTTTCCAAAGATCTTTTAAAGCCTATACAGGATGTAAACAAT

CTTCGGCTTTCCCTTACTGATAATCAGAGCATCAGTAAAGAATTTCAGGCTTTGATTGTG

AAACATTTAGATGAAAGCCATCTTTTAAAAGGTGACAAAAACTTAGTTGGTTCAGAAGTA

AAAATTTATAGTTTGGACCCATCTACTCAGTGGTTTTCAGCAACTGTTGTGAATGGAAAC

CCAGCATCTAAAACTCTTCAAGTCAACTGTGAGGAGATTCCAGCATTGAAAATTGTCGAT

CCCTCACTGATTCATGTTGAAGTTGTACATGATAACTTCGTGACATATGATAATTCTGCA

AGAATTGGAGGTGTAAAACGGAAGTCTTCTGAGAATAATGGAAGCTTGGTTTCCAAACAA

GCAAAATCTTGCTCTGAGGCTTCTCCCAGTATGTGTCCTGTACAGTCTGTTCCCACAACA

GCTTTTAAGGAGATCCTGCTTGGCTGTACCGCAGCAACCCCTCCTAGTAAAGACCCAAGA

CAGCAAAGTACTCCTCAGACTGCCAACTCTCCACCCAACCTTGGAGCAAAGATTCCTCAA

GGCCTCACCTTGTGCCCTGAGGAAGGAGCCAGCTGTCTGTGGACTGAGACC

>CL367.Contig2_A-W 777 1010 minus strand mKIAA0742 protein [Mus musculus]

AAGACCTTCAGGGAAGCTCTTCAGGCTTCTCCCAGTATGTGTCCTGTACAGTCTGTTCCC

ACAACAGCTTTTAAGGAGATCCTGCTTGGCTGTACCGCAGCAACCCCTCCTAGTAAAGAC

CCAAGACAGCAAAGTACTCCTCAGACTGCCAACTCTCCACCCAACCTTGGAGCAAAGATT

CCTCAAGGCCTCACCTTGTGCCCTGAGGAAGGAGCCAGCTGTCTGTGGACTGAG

>CL367.Contig3_A-W 71 358 minus strand PREDICTED: lysine-specific demethylase 3A isoform 2 [Pan paniscus]

ATGGTGCTCACGCTCGGGGAGAGCTGGCCGGTGCTGGTGGGGAAGCGGTTCCTCAGCCTG

TGCGCGGCCGACGGCGTCGACGACGACTGGGACGTGCAGCGCGTGGCCGAGTGGCCCTGG

CTGGCGGGCACGGTCCGCGCCGTGTCCCACCCCGAAGTTTCCAGGAAGGACCTGAAGGTG

TACAAACCTTTGTTGGACAAAGTTGGTTTGGGATCCATGACTTCTGTTCGCTTCCTAGGA

GATCAGCAAAGCGTATTTCTTTCCAAAGATCTTTTAAAGCCTATACAG

>CL367.Contig4_A-W 71 748 minus strand mKIAA0742 protein [Mus musculus]

ATGGTGCTCACGCTCGGGGAGAGCTGGCCGGTGCTGGTGGGGAAGCGGTTCCTCAGCCTG

TGCGCGGCCGACGGCGTCGACGACGACTGGGACGTGCAGCGCGTGGCCGAGTGGCCCTGG

CTGGCGGGCACGGTCCGCGCCGTGTCCCACCCCGAAGTTTCCAGGAAGGACCTGAAGGTG

TACAAACCTTTGTTGGACAAAGTTGGTTTGGGATCCATGACTTCTGTTCGCTTCCTAGGA

GATCAGCAAAGCGTATTTCTTTCCAAAGATCTTTTAAAGCCTATACAGGATGTAAACAAT

CTTCGGCTTTCCCTTACTGATAATCAGAGCATCAGTAAAGAATTTCAGGCTTTGATTGTG

AAACATTTAGATGAAAGCCATCTTTTAAAAGGTGACAAAAACTTAGTTGGTTCAGAAGTA

AAAATTTATAGTTTGGACCCATCTACTCAGTGGTTTTCAGCAACTGTTGTGAATGGAAAC

CCAGCATCTAAAACTCTTCAAGTCAACTGTGAGGAGATTCCAGCATTGAAAATTGTCGAT

CCCTCACTGATTCATGTTGAAGTTGTACATGATAACTTCGTGACATATGATAATTCTGCA

AGAATTGGAGGTGTAAAACGGAAGTCTTCTGAGAATAATGGAAGCTTGGTTTCCAAACAA

GCAAAATCTTGCTCTGAG

>CL367.Contig5_A-W 71 913 minus strand PREDICTED: lysine-specific demethylase 3A isoform 3 [Callithrix jacchus]

ATGGTGCTCACGCTCGGGGAGAGCTGGCCGGTGCTGGTGGGGAAGCGGTTCCTCAGCCTG

TGCGCGGCCGACGGCGTCGACGACGACTGGGACGTGCAGCGCGTGGCCGAGTGGCCCTGG

CTGGCGGGCACGGTCCGCGCCGTGTCCCACCCCGAAGTTTCCAGGAAGGACCTGAAGGTG

TACAAACCTTTGTTGGACAAAGTTGGTTTGGGATCCATGACTTCTGTTCGCTTCCTAGGA

GATCAGCAAAGCGTATTTCTTTCCAAAGATCTTTTAAAGCCTATACAGGATGTAAACAAT

CTTCGGCTTTCCCTTACTGATAATCAGAGCATCAGTAAAGAATTTCAGGCTTTGATTGTG

AAACATTTAGATGAAAGCCATCTTTTAAAAGGTGACAAAAACTTAGTTGGTTCAGAAGTA

AAAATTTATAGTTTGGACCCATCTACTCAGTGGTTTTCAGCAACTGTTGTGAATGGAAAC

CCAGCATCTAAAACTCTTCAAGTCAACTGTGAGGAGATTCCAGCATTGAAAATTGTCGAT

CCCTCACTGATTCATGTTGAAGTTGTACATGATAACTTCGTGACATATGATAATTCTGCA

AGAATTGGAGGTGTAAAACGGAAGTCTTCTGAGAATAATGGAAGCTTGGTTTCCAAACAA

GCAAAATCTTGCTCTGAGGCTTCTCCCAGTATGTGTCCTGTACAGTCTGTTCCCACAACA

GCTTTTAAGGAGATCCTGCTTGGCTGTACCGCAGCAACCCCTCCTAGTAAAGACCCAAGA

CAGCAAAGTACTCCTCAGACTGCCAACTCTCCACCCAACCTTGGAGCAAAGATTCCTCAA

GGG

>CL367.Contig6_A-W 777 965 minus strand mKIAA0742 protein [Mus musculus]

AAGACCTTCAGGGAAGCTCTTCAGGCTTCTCCCAGTATGTGTCCTGTACAGTCTGTTCCC

ACAACAGCTTTTAAGGAGATCCTGCTTGGCTGTACCGCAGCAACCCCTCCTAGTAAAGAC

CCAAGACAGCAAAGTACTCCTCAGACTGCCAACTCTCCACCCAACCTTGGAGCAAAGATT

CCTCAAGGG

>CL368.Contig1_A-W 318 578 minus strand PREDICTED: beta-hexosaminidase subunit beta [Loxodonta africana]

TATCAATTCAATGATTTTAATGCAGGTACTGAAAAACAGAAAAAACTTATCCTTGGTGGA

GAAGCATGTCTTTGGGGAGAATATGTGGATGCAACTAACCTTACTCCAAGATTATGGCCT

CGGGCAAGTGCTGTTGGTGAAAGACTCTGGAGTCGTGGAGATATCAGGGACCTTGGTGAT

GCTTATGCTAGACTAACAAGGCACCGCTGCAGAATGGTCAAACGTGGAATAGAAGCAGAA

CCGCTGTTTACTGGATATTGT

>CL369.Contig1_A-W 1 636 PREDICTED: zinc finger protein 213 isoform 1 [Papio anubis] >gi|402907432|ref|XP_003916479.1| PREDICTED: zinc finger protein 213 isoform 2 [Papio anubis]

TATGGGGATGTGGGTGGGCCGCATGAAGCCTTCAGCCAGCTCTGGGAGCTCTGCTGCCGT

TGGCTTAGGCCTGAACTGCACACCAAGGAGCAGATTCTGGAGCGGTTGGTGCTGGAGCAG

TTTCTGACAGTGCTGCCAGGGGAGATCCAGGCCCAGGTGCGGGGACAGCTCCCAGGGAGT

GGCGAGGAAGCTGTGGCCTTGGTGGAAGACCTACAGAAGCAGCCAGTGAAAGCCTGGCTG

CAGGATGTGCCCTCAGAGGAAGTGGAACCTGAGTCTGCAGTCCAGAGATCCCAGGCCAGG

GGGCCTCTGCTGATGGTGGGGGCATGGAGCCAGCCGCCTGTACCCCAGGAGCAGCATAGC

CACAATGCCCAGCTTCCTGCTCTTCTTAAAGAGGATAATACCAGAGAGACCACAGATGCC

TGCTTTGCCTCTGGGGTCCATGGACCTGTAGCATTGGGAGACATTCCCTTCTATTTCTCA

CGGGAAGAATGGGGTTCTCTGGACCCTGCCCAAAGGAATCTCTTCTGGGACATAAAGCGG

GAGAATTCCCAGAATGCTGCCCTGGGTTTGGGGCTCAAAAGCCAGAGCTCGAAGACCCAA

CTGGAGGAGGCAGTGCTGGTGCTTCCGCGCCAGACA

>CL369.Contig2_A-W 71 466 PREDICTED: zinc finger protein 213 isoform 1 [Papio anubis] >gi|402907432|ref|XP_003916479.1| PREDICTED: zinc finger protein 213 isoform 2 [Papio anubis]

CAGGATGTGCCCTCAGAGGAAGTGGAACCTGAGTCTGCAGTCCAGAGATCCCAGGCCAGG

GGGCCTCTGCTGATGGTGGGGGCATGGAGCCAGCCGCCTGTACCCCAGGAGCAGCATAGC

CACAATGCCCAGCTTCCTGCTCTTCTTAAAGAGGATAATACCAGAGAGACCACAGATGCC

TGCTTTGCCTCTGGGGTCCATGGACCTGTAGCATTGGGAGACATTCCCTTCTATTTCTCA

CGGGAAGAATGGGGTTCTCTGGACCCTGCCCAAAGGAATCTCTTCTGGGACATAAAGCGG

GAGAATTCCCAGAATGCTGCCCTGGGTTTGGGGCTCAAAAGCCAGAGCTCGAAGACCCAA

CTGGAGGAGGCAGTGCTGGTGCTTCCGCGCCAGACA

>CL370.Contig1_A-W 1238 2260 PREDICTED: B-cell receptor CD22 [Pan paniscus]

CTGGGGGAGAAGAATCTCTCGCTAACTCTGTCCTCAGTGACCAAGGACAGTGGTGGGAAG

TACCGCTGTCAGGCCTCCAATCACATAGGCTTAGGAATGTCGGAAGAAGTGGCCCTCAAA

GTGCTGTGTGAGCCTCCTCGGAGCTGAGGGTGGAGGTGACAGTGGCTGCTTGCTCATGTC

ACAGGGTTCCCCTCACCTCTCGCCTCACCTCTGCCCCTTCTTCCAGATCCCCCGACAGTT

TCCGAGGTTTACATCCACCACACACCTGCTAAGGAGGGACAGTCAGTAGAGCTGGTTTGC

ACGTCCCCGGCCAACCCTCCTCCAACAAATTATACCTGGTATCACAATGGGAAAGAAGTG

CCAGGAGTGACAGAAGAGAAATACTGGATCGCCCAGGTCCTTACTTGGCATGCAGGGCGT

TACGCCTGCGTGGCAGAGAACAGGCTTGGTTCTGGACAGGTTGGCCAGGAAGCCGAGCTG

GACGTCCAATATCCCCCCAAGGGGGTAACCACTGTGATTGAAAGCCCCACACCGATTCGA

GAAGGAGACAGCGTGACCCTGCGCTGTAGGTACAACTCCAGTAACCCAACAGTTACCCGG

TATGAGTGGAGCCCCCAACACTTGTGGAATGAGCCATCCCATGAGGTGATGAAGATTCAG

AATGTTGCCTGGGACACCCCACCCATCTCCTGTTCCGCCTGTAACCAGTGGTGTTCTCCA

GCTTCTCCCATCGACCTGGATGTCCAGTATACTCCCAAGGAGGTGAAGGTCCTGCAGATC

AGTTCCCTGTCAGAGATTCATGCTGGGAACCAGGTCCGACTTCAATGCCACTTCTCAAAG

AGCCACCCGGAAGAAGTCCACTTCTTCTGGAAGAAAAACGGAAGTCTTCTGGAAGCAAAA

GGAAGAGAACTGAACTTTGACTCCGTTTCCCCGGAAGATGCTGGAAATTACAGCTGCTTG

GTCAAAAACTCCATAGGACAGTCAGCATCTGAGGCCTGGAGGCTGCAAGTGCTATATGCA

CCT

>CL370.Contig2_A-W 386 1309 B-cell receptor CD22 isoform 4 precursor [Homo sapiens] >gi|1498646|gb|AAB06449.1| B-cell receptor CD22-A isoform [Homo sapiens]

AGGATGATGACAAAGGACGACAAATGGATGGAAACCGTAAACCTAAGTATCTCTAAATCT

CCTTTTCCACCTCGCATCCAGCTCCCTCTAGAACTTCAGGAGTCCCAGACAGTCACTGTG

ACCTGCTTGTTGAACTTCACCTGCTTTGGATACGACATTGAACTGCGGTGGGTCCTGGAG

GGGCTCCCCTTCACCCCTGCTTCCTCCACCATCTCCTCCTCACCTACCACCCAGACTGTC

TCTACCAAGAGCCAGATCGACTTTAAACCGGAGTGGACTCACCACGGGAAGAACTTGACC

TGCCAGGTGTGGCATTCTGCCGAAGTGCTCTCTGAAGAGACGGTGCAGCTGAACGTGAAG

CATCCCCCCAAGGGGGTAACCACTGTGATTGAAAGCCCCACACCGATTCGAGAAGGAGAC

AGCGTGACCCTGCGCTGTAGGTACAACTCCAGTAACCCAACAGTTACCCGGTATGAGTGG

AGCCCCCAACACTTGTGGAATGAGCCATCCCATGAGGTGATGAAGATTCAGAATGTTGCC

TGGGACACCCCACCCATCTCCTGTTCCGCCTGTAACCAGTGGTGTTCTCCAGCTTCTCCC

ATCGACCTGGATGTCCAGTATACTCCCAAGGAGGTGAAGGTCCTGCAGATCAGTTCCCTG

TCAGAGATTCATGCTGGGAACCAGGTCCGACTTCAATGCCACTTCTCAAAGAGCCACCCG

GAAGAAGTCCACTTCTTCTGGAAGAAAAACGGAAGTCTTCTGGAAGCAAAAGGAAGAGAA

CTGAACTTTGACTCCGTTTCCCCGGAAGATGCTGGAAATTACAGCTGCTTGGTCAAAAAC

TCCATAGGACAGTCAGCATCTGAGGCCTGGAGGCTGCAAGTGCTATATGCACCTCGGAGG

CTGTGGGTGTCCATTACCCCAGGA

>CL370.Contig3_A-W 386 1309 B-cell receptor CD22 isoform 4 precursor [Homo sapiens] >gi|1498646|gb|AAB06449.1| B-cell receptor CD22-A isoform [Homo sapiens]

AGGATGATGACAAAGGACGACAAATGGATGGAAACCGTAAACCTAAGTATCTCTAAATCT

CCTTTTCCACCTCGCATCCAGCTCCCTCTAGAACTTCAGGAGTCCCAGACAGTCACTGTG

ACCTGCTTGTTGAACTTCACCTGCTTTGGATACGACATTGAACTGCGGTGGGTCCTGGAG

GGGCTCCCCTTCACCCCTGCTTCCTCCACCATCTCCTCCTCACCTACCACCCAGACTGTC

TCTACCAAGAGCCAGATCGACTTTAAACCGGAGTGGACTCACCACGGGAAGAACTTGACC

TGCCAGGTGTGGCATTCTGCCGAAGTGCTCTCTGAAGAGACGGTGCAGCTGAACGTGAAG

CATCCCCCCAAGGGGGTAACCACTGTGATTGAAAGCCCCACACCGATTCGAGAAGGAGAC

AGCGTGACCCTGCGCTGTAGGTACAACTCCAGTAACCCAACAGTTACCCGGTATGAGTGG

AGCCCCCAACACTTGTGGAATGAGCCATCCCATGAGGTGATGAAGATTCAGAATGTTGCC

TGGGACACCCCACCCATCTCCTGTTCCGCCTGTAACCAGTGGTGTTCTCCAGCTTCTCCC

ATCGACCTGGATGTCCAGTATACTCCCAAGGAGGTGAAGGTCCTGCAGATCAGTTCCCTG

TCAGAGATTCATGCTGGGAACCAGGTCCGACTTCAATGCCACTTCTCAAAGAGCCACCCG

GAAGAAGTCCACTTCTTCTGGAAGAAAAACGGAAGTCTTCTGGAAGCAAAAGGAAGAGAA

CTGAACTTTGACTCCGTTTCCCCGGAAGATGCTGGAAATTACAGCTGCTTGGTCAAAAAC

TCCATAGGACAGTCAGCATCTGAGGCCTGGAGGCTGCAAGTGCTATATGCACCTCGGAGG

CTGTGGGTGTCCATTACCCCAGGA

>CL370.Contig4_A-W 416 1891 PREDICTED: b-cell receptor CD22-like isoform 1 [Nomascus leucogenys]

GCAGAATCTCCTTTTCCACCTCGCATCCAGCTCCCTCTAGAACTTCAGGAGTCCCAGACA

GTCACTGTGACCTGCTTGTTGAACTTCACCTGCTTTGGATACGACATTGAACTGCGGTGG

GTCCTGGAGGGGCTCCCCTTCACCCCTGCTTCCTCCACCATCTCCTCCTCACCTACCACC

CAGACTGTCTCTACCAAGAGCCAGATCGACTTTAAACCGGAGTGGACTCACCACGGGAAG

AACTTGACCTGCCAGGTGTGGCATTCTGCCGAAGTGCTCTCTGAAGAGACGGTGCAGCTG

AACGTGAAGCATCCCCCCAAGGGGGTAACCACTGTGATTGAAAGCCCCACACCGATTCGA

GAAGGAGACAGCGTGACCCTGCGCTGTAGGTACAACTCCAGTAACCCAACAGTTACCCGG

TATGAGTGGAGCCCCCAACACTTGTGGAATGAGCCATCCCATGAGGTGATGAAGATTCAG

AATGTTGCCTGGGACACCCCACCCATCTCCTGTTCCGCCTGTAACCAGTGGTGTTCTCCA

GCTTCTCCCATCGACCTGGATGTCCAGTATACTCCCAAGGAGGTGAAGGTCCTGCAGATC

AGTTCCCTGTCAGAGATTCATGCTGGGAACCAGGTCCGACTTCAATGCCACTTCTCAAAG

AGCCACCCGGAAGAAGTCCACTTCTTCTGGAAGAAAAACGGAAGTCTTCTGGAAGCAAAA

GGAAGAGAACTGAACTTTGACTCCGTTTCCCCGGAAGATGCTGGAAATTACAGCTGCTTG

GTCAAAAACTCCATAGGACAGTCAGCATCTGAGGCCTGGAGGCTGCAAGTGCTATATGCA

CCTCGGAGGCTGTGGGTGTCCATTACCCCAGGAGACAGAGTGATGGAAGGGAGAAAGGCA

GCCCTGACCTGTGAGAGCGATGCCAACCCTCCCATCTCACAGTACACCTGGTTTGACTGG

AACAACCAAGACCTCCAGCATTCTGGCCAGACGCTGAGCTTGGATCCTGTGAAGGTCGAG

CACTCGGGCTCCTACTGGTGCAAGGGGGTCAACCAGCTGGGCGAGGGCGAGTCACCCCCC

AGCACCCTCACCATCTACTATAGCCCGGAGACCATCGGCAGGCGAACGACCATGGGAATT

GGCATCTGCCTGGCCATCCTCATCCTGGTCATCTGGGGGGTGAAGCTTCAGCAAAACTGG

AAGAGGACTCGGAGCCAACAGGGGCCTCAGGAAAATTCCAATGGTCGGAGCTTCTTTGTG

AGGAATAAAAAGGTTAGAAGGACCCTTCTCTCTGAAGGGCCCCACTCCCTGGGATGCTAC

AATCCAATGATGGAAGACGGCACTAATTATGCTACTCTGCGCTTTCCAGAGACTGACACA

CCAAGAACTGGAGGTACAGGCCCCTCAGAAACACAAGGATCTCTCCCAAATAATGAAGAC

ACGGTCACTTACTCAGTGGTGCAGAAGTGTCATGTG

>CL370.Contig5_A-W 416 2029 PREDICTED: b-cell receptor CD22-like isoform 1 [Nomascus leucogenys]

GCAGAATCTCCTTTTCCACCTCGCATCCAGCTCCCTCTAGAACTTCAGGAGTCCCAGACA

GTCACTGTGACCTGCTTGTTGAACTTCACCTGCTTTGGATACGACATTGAACTGCGGTGG

GTCCTGGAGGGGCTCCCCTTCACCCCTGCTTCCTCCACCATCTCCTCCTCACCTACCACC

CAGACTGTCTCTACCAAGAGCCAGATCGACTTTAAACCGGAGTGGACTCACCACGGGAAG

AACTTGACCTGCCAGGTGTGGCATTCTGCCGAAGTGCTCTCTGAAGAGACGGTGCAGCTG

AACGTGAAGCATCCCCCCAAGGGGGTAACCACTGTGATTGAAAGCCCCACACCGATTCGA

GAAGGAGACAGCGTGACCCTGCGCTGTAGGTACAACTCCAGTAACCCAACAGTTACCCGG

TATGAGTGGAGCCCCCAACACTTGTGGAATGAGCCATCCCATGAGGTGATGAAGATTCAG

AATGTTGCCTGGGACACCCCACCCATCTCCTGTTCCGCCTGTAACCAGTGGTGTTCTCCA

GCTTCTCCCATCGACCTGGATGTCCAGTATACTCCCAAGGAGGTGAAGGTCCTGCAGATC

AGTTCCCTGTCAGAGATTCATGCTGGGAACCAGGTCCGACTTCAATGCCACTTCTCAAAG

AGCCACCCGGAAGAAGTCCACTTCTTCTGGAAGAAAAACGGAAGTCTTCTGGAAGCAAAA

GGAAGAGAACTGAACTTTGACTCCGTTTCCCCGGAAGATGCTGGAAATTACAGCTGCTTG

GTCAAAAACTCCATAGGACAGTCAGCATCTGAGGCCTGGAGGCTGCAAGTGCTATATGCA

CCTCGGAGGCTGTGGGTGTCCATTACCCCAGGAGACAGAGTGATGGAAGGGAGAAAGGCA

GCCCTGACCTGTGAGAGCGATGCCAACCCTCCCATCTCACAGTACACCTGGTTTGACTGG

AACAACCAAGACCTCCAGCATTCTGGCCAGACGCTGAGCTTGGATCCTGTGAAGGTCGAG

CACTCGGGCTCCTACTGGTGCAAGGGGGTCAACCAGCTGGGCGAGGGCGAGTCACCCCCC

AGCACCCTCACCATCTACTATAGCCCGGAGACCATCGGCAGGCGAACGACCATGGGAATT

GGCATCTGCCTGGCCATCCTCATCCTGGTCATCTGGGGGGTGAAGCTTCAGCAAAACTGG

AAGAGGACTCGGAGCCAACAGGGGCCTCAGGAAAATTCCAATGGTCGGAGCTTCTTTGTG

AGGAATAAAAAGGTTAGAAGGACCCTTCTCTCTGAAGGGCCCCACTCCCTGGGATGCTAC

AATCCAATGATGGAAGACGGCACTAATTATGCTACTCTGCGCTTTCCAGAGACTGACACA

CCAAGAACTGGAGGTACAGGCCCCTCAGAAACACAAGGATCTCTCCCAAATAATGAAGAC

ACGGTCACTTACTCAGTGGTGCAGAAGTGTCATGTGCTTTCCCAGGGTGACTACGAGAAT

GTGGCTCCAAATCTTCCAGAAGATGACGGGATTCATTACTCGGAGCTCGTTCATTTTGGG

ACTGGGGAGCGGCCTCGGGCACCAGAAGATGTGGACTATGTGACTCTCAAGCAC

>CL370.Contig6_A-W 377 1300 B-cell receptor CD22 isoform 4 precursor [Homo sapiens] >gi|1498646|gb|AAB06449.1| B-cell receptor CD22-A isoform [Homo sapiens]

AGGATGATGACAAAGGACGACAAATGGATGGAAACCGTAAACCTAAGTATCTCTAAATCT

CCTTTTCCACCTCGCATCCAGCTCCCTCTAGAACTTCAGGAGTCCCAGACAGTCACTGTG

ACCTGCTTGTTGAACTTCACCTGCTTTGGATACGACATTGAACTGCGGTGGGTCCTGGAG

GGGCTCCCCTTCACCCCTGCTTCCTCCACCATCTCCTCCTCACCTACCACCCAGACTGTC

TCTACCAAGAGCCAGATCGACTTTAAACCGGAGTGGACTCACCACGGGAAGAACTTGACC

TGCCAGGTGTGGCATTCTGCCGAAGTGCTCTCTGAAGAGACGGTGCAGCTGAACGTGAAG

CATCCCCCCAAGGGGGTAACCACTGTGATTGAAAGCCCCACACCGATTCGAGAAGGAGAC

AGCGTGACCCTGCGCTGTAGGTACAACTCCAGTAACCCAACAGTTACCCGGTATGAGTGG

AGCCCCCAACACTTGTGGAATGAGCCATCCCATGAGGTGATGAAGATTCAGAATGTTGCC

TGGGACACCCCACCCATCTCCTGTTCCGCCTGTAACCAGTGGTGTTCTCCAGCTTCTCCC

ATCGACCTGGATGTCCAGTATACTCCCAAGGAGGTGAAGGTCCTGCAGATCAGTTCCCTG

TCAGAGATTCATGCTGGGAACCAGGTCCGACTTCAATGCCACTTCTCAAAGAGCCACCCG

GAAGAAGTCCACTTCTTCTGGAAGAAAAACGGAAGTCTTCTGGAAGCAAAAGGAAGAGAA

CTGAACTTTGACTCCGTTTCCCCGGAAGATGCTGGAAATTACAGCTGCTTGGTCAAAAAC

TCCATAGGACAGTCAGCATCTGAGGCCTGGAGGCTGCAAGTGCTATATGCACCTCGGAGG

CTGTGGGTGTCCATTACCCCAGGA

>CL370.Contig7_A-W 440 1288 B-cell receptor CD22 isoform 4 precursor [Homo sapiens] >gi|1498646|gb|AAB06449.1| B-cell receptor CD22-A isoform [Homo sapiens]

ATCCAGCTCCCTCTAGAACTTCAGGAGTCCCAGACAGTCACTGTGACCTGCTTGTTGAAC

TTCACCTGCTTTGGATACGACATTGAACTGCGGTGGGTCCTGGAGGGGCTCCCCTTCACC

CCTGCTTCCTCCACCATCTCCTCCTCACCTACCACCCAGACTGTCTCTACCAAGAGCCAG

ATCGACTTTAAACCGGAGTGGACTCACCACGGGAAGAACTTGACCTGCCAGGTGTGGCAT

TCTGCCGAAGTGCTCTCTGAAGAGACGGTGCAGCTGAACGTGAAGCATCCCCCCAAGGGG

GTAACCACTGTGATTGAAAGCCCCACACCGATTCGAGAAGGAGACAGCGTGACCCTGCGC

TGTAGGTACAACTCCAGTAACCCAACAGTTACCCGGTATGAGTGGAGCCCCCAACACTTG

TGGAATGAGCCATCCCATGAGGTGATGAAGATTCAGAATGTTGCCTGGGACACCCCACCC

ATCTCCTGTTCCGCCTGTAACCAGTGGTGTTCTCCAGCTTCTCCCATCGACCTGGATGTC

CAGTATACTCCCAAGGAGGTGAAGGTCCTGCAGATCAGTTCCCTGTCAGAGATTCATGCT

GGGAACCAGGTCCGACTTCAATGCCACTTCTCAAAGAGCCACCCGGAAGAAGTCCACTTC

TTCTGGAAGAAAAACGGAAGTCTTCTGGAAGCAAAAGGAAGAGAACTGAACTTTGACTCC

GTTTCCCCGGAAGATGCTGGAAATTACAGCTGCTTGGTCAAAAACTCCATAGGACAGTCA

GCATCTGAGGCCTGGAGGCTGCAAGTGCTATATGCACCTCGGAGGCTGTGGGTGTCCATT

ACCCCAGGA

>CL370.Contig8_A-W 377 1300 B-cell receptor CD22 isoform 4 precursor [Homo sapiens] >gi|1498646|gb|AAB06449.1| B-cell receptor CD22-A isoform [Homo sapiens]

AGGATGATGACAAAGGACGACAAATGGATGGAAACCGTAAACCTAAGTATCTCTAAATCT

CCTTTTCCACCTCGCATCCAGCTCCCTCTAGAACTTCAGGAGTCCCAGACAGTCACTGTG

ACCTGCTTGTTGAACTTCACCTGCTTTGGATACGACATTGAACTGCGGTGGGTCCTGGAG

GGGCTCCCCTTCACCCCTGCTTCCTCCACCATCTCCTCCTCACCTACCACCCAGACTGTC

TCTACCAAGAGCCAGATCGACTTTAAACCGGAGTGGACTCACCACGGGAAGAACTTGACC

TGCCAGGTGTGGCATTCTGCCGAAGTGCTCTCTGAAGAGACGGTGCAGCTGAACGTGAAG

CATCCCCCCAAGGGGGTAACCACTGTGATTGAAAGCCCCACACCGATTCGAGAAGGAGAC

AGCGTGACCCTGCGCTGTAGGTACAACTCCAGTAACCCAACAGTTACCCGGTATGAGTGG

AGCCCCCAACACTTGTGGAATGAGCCATCCCATGAGGTGATGAAGATTCAGAATGTTGCC

TGGGACACCCCACCCATCTCCTGTTCCGCCTGTAACCAGTGGTGTTCTCCAGCTTCTCCC

ATCGACCTGGATGTCCAGTATACTCCCAAGGAGGTGAAGGTCCTGCAGATCAGTTCCCTG

TCAGAGATTCATGCTGGGAACCAGGTCCGACTTCAATGCCACTTCTCAAAGAGCCACCCG

GAAGAAGTCCACTTCTTCTGGAAGAAAAACGGAAGTCTTCTGGAAGCAAAAGGAAGAGAA

CTGAACTTTGACTCCGTTTCCCCGGAAGATGCTGGAAATTACAGCTGCTTGGTCAAAAAC

TCCATAGGACAGTCAGCATCTGAGGCCTGGAGGCTGCAAGTGCTATATGCACCTCGGAGG

CTGTGGGTGTCCATTACCCCAGGA

>CL370.Contig9_A-W 440 1288 B-cell receptor CD22 isoform 4 precursor [Homo sapiens] >gi|1498646|gb|AAB06449.1| B-cell receptor CD22-A isoform [Homo sapiens]

ATCCAGCTCCCTCTAGAACTTCAGGAGTCCCAGACAGTCACTGTGACCTGCTTGTTGAAC

TTCACCTGCTTTGGATACGACATTGAACTGCGGTGGGTCCTGGAGGGGCTCCCCTTCACC

CCTGCTTCCTCCACCATCTCCTCCTCACCTACCACCCAGACTGTCTCTACCAAGAGCCAG

ATCGACTTTAAACCGGAGTGGACTCACCACGGGAAGAACTTGACCTGCCAGGTGTGGCAT

TCTGCCGAAGTGCTCTCTGAAGAGACGGTGCAGCTGAACGTGAAGCATCCCCCCAAGGGG

GTAACCACTGTGATTGAAAGCCCCACACCGATTCGAGAAGGAGACAGCGTGACCCTGCGC

TGTAGGTACAACTCCAGTAACCCAACAGTTACCCGGTATGAGTGGAGCCCCCAACACTTG

TGGAATGAGCCATCCCATGAGGTGATGAAGATTCAGAATGTTGCCTGGGACACCCCACCC

ATCTCCTGTTCCGCCTGTAACCAGTGGTGTTCTCCAGCTTCTCCCATCGACCTGGATGTC

CAGTATACTCCCAAGGAGGTGAAGGTCCTGCAGATCAGTTCCCTGTCAGAGATTCATGCT

GGGAACCAGGTCCGACTTCAATGCCACTTCTCAAAGAGCCACCCGGAAGAAGTCCACTTC

TTCTGGAAGAAAAACGGAAGTCTTCTGGAAGCAAAAGGAAGAGAACTGAACTTTGACTCC

GTTTCCCCGGAAGATGCTGGAAATTACAGCTGCTTGGTCAAAAACTCCATAGGACAGTCA

GCATCTGAGGCCTGGAGGCTGCAAGTGCTATATGCACCTCGGAGGCTGTGGGTGTCCATT

ACCCCAGGA

>CL370.Contig10_A-W 440 1333 B-cell receptor CD22 isoform 3 precursor [Homo sapiens]

TTTCCACCTCGCATCCAGCTCCCTCTAGAACTTCAGGAGTCCCAGACAGTCACTGTGACC

TGCTTGTTGAACTTCACCTGCTTTGGATACGACATTGAACTGCGGTGGGTCCTGGAGGGG

CTCCCCTTCACCCCTGCTTCCTCCACCATCTCCTCCTCACCTACCACCCAGACTGTCTCT

ACCAAGAGCCAGATCGACTTTAAACCGGAGTGGACTCACCACGGGAAGAACTTGACCTGC

CAGGTGTGGCATTCTGCCGAAGTGCTCTCTGAAGAGACGGTGCAGCTGAACGTGAAGCAC

ACCCCGAAGTTGGAGATCAAGGTCTTTCCCAATGAAACCACTGTAATGGAAGGGGCGTCC

GTGACCATGACGTGCCAGGTCACCAGCAGCAACCCAGAGCATGGCACTGTATCCTGGCTC

AAGGACGGGACCCTCCTGGGGGAGAAGAATCTCTCGCTAACTCTGTCCTCAGTGACCAAG

GACAGTGGTGGGAAGTACCGCTGTCAGGCCTCCAATCACATAGGCTTAGGAATGTCGGAA

GAAGTGGCCCTCAAAGTGCTGTGTGAGCCTCCTCGGAGCTGAGGGTGGAGGTGACAGTGG

CTGCTTGCTCATGTCACAGGGTTCCCCTCACCTCTCGCCTCACCTCTGCCCCTTCTTCCA

GATCCCCCGACAGTTTCCGAGGTTTACATCCACCACACACCTGCTAAGGAGGGACAGTCA

GTAGAGCTGGTTTGCACGTCCCCGGCCAACCCTCCTCCAACAAATTATACCTGGTATCAC

AATGGGAAAGAAGTGCCAGGAGTGACAGAAGAGAAATACTGGATCGCCCAGGTCCTTACT

TGGCATGCAGGGCGTTACGCCTGCGTGGCAGAGAACAGGCTTGGTTCTGGACAG

>CL370.Contig11_A-W 440 1234 B-cell receptor CD22 isoform 3 precursor [Homo sapiens]

TTTCCACCTCGCATCCAGCTCCCTCTAGAACTTCAGGAGTCCCAGACAGTCACTGTGACC

TGCTTGTTGAACTTCACCTGCTTTGGATACGACATTGAACTGCGGTGGGTCCTGGAGGGG

CTCCCCTTCACCCCTGCTTCCTCCACCATCTCCTCCTCACCTACCACCCAGACTGTCTCT

ACCAAGAGCCAGATCGACTTTAAACCGGAGTGGACTCACCACGGGAAGAACTTGACCTGC

CAGGTGTGGCATTCTGCCGAAGTGCTCTCTGAAGAGACGGTGCAGCTGAACGTGAAGCAC

ACCCCGAAGTTGGAGATCAAGGTCTTTCCCAATGAAACCACTGTAATGGAAGGGGCGTCC

GTGACCATGACGTGCCAGGTCACCAGCAGCAACCCAGAGCATGGCACTGTATCCTGGCTC

AAGGACGGGACCCTCCTGGGGGAGAAGAATCTCTCGCTAACTCTGTCCTCAGTGACCAAG

GACAGTGGTGGGAAGTACCGCTGTCAGGCCTCCAATCACATAGGCTTAGGAATGTCGGAA

GAAGTGGCCCTCAAAGTGCTGTATCCCCCGACAGTTTCCGAGGTTTACATCCACCACACA

CCTGCTAAGGAGGGACAGTCAGTAGAGCTGGTTTGCACGTCCCCGGCCAACCCTCCTCCA

ACAAATTATACCTGGTATCACAATGGGAAAGAAGTGCCAGGAGTGACAGAAGAGAAATAC

TGGATCGCCCAGGTCCTTACTTGGCATGCAGGGCGTTACGCCTGCGTGGCAGAGAACAGG

CTTGGTTCTGGACAG

>CL370.Contig12_A-W 295 747 B-cell receptor CD22 isoform 3 precursor [Homo sapiens]

GTAATGGAAGGGGCGTCCGTGACCATGACGTGCCAGGTCACCAGCAGCAACCCAGAGCAT

GGCACTGTATCCTGGCTCAAGGACGGGACCCTCCTGGGGGAGAAGAATCTCTCGCTAACT

CTGTCCTCAGTGACCAAGGACAGTGGTGGGAAGTACCGCTGTCAGGCCTCCAATCACATA

GGCTTAGGAATGTCGGAAGAAGTGGCCCTCAAAGTGCTGTATCCCCCGACAGTTTCCGAG

GTTTACATCCACCACACACCTGCTAAGGAGGGACAGTCAGTAGAGCTGGTTTGCACGTCC

CCGGCCAACCCTCCTCCAACAAATTATACCTGGTATCACAATGGGAAAGAAGTGCCAGGA

GTGACAGAAGAGAAATACTGGATCGCCCAGGTCCTTACTTGGCATGCAGGGCGTTACGCC

TGCGTGGCAGAGAACAGGCTTGGTTCTGGACAG

>CL370.Contig13_A-W 707 1318 PREDICTED: b-cell receptor CD22-like isoform 2 [Nomascus leucogenys]

GTGCAGCTGAACGTGAAGCACACCCCGAAGTTGGAGATCAAGGTCTTTCCCAATGAAACC

ACTGTAATGGAAGGGGCGTCCGTGACCATGACGTGCCAGGTCACCAGCAGCAACCCAGAG

CATGGCACTGTATCCTGGCTCAAGGACGGGACCCTCCTGGGGGAGAAGAATCTCTCGCTA

ACTCTGTCCTCAGTGACCAAGGACAGTGGTGGGAAGTACCGCTGTCAGGCCTCCAATCAC

ATAGGCTTAGGAATGTCGGAAGAAGTGGCCCTCAAAGTGCTGTGTGAGCCTCCTCGGAGC

TGAGGGTGGAGGTGACAGTGGCTGCTTGCTCATGTCACAGGGTTCCCCTCACCTCTCGCC

TCACCTCTGCCCCTTCTTCCAGATCCCCCGACAGTTTCCGAGGTTTACATCCACCACACA

CCTGCTAAGGAGGGACAGTCAGTAGAGCTGGTTTGCACGTCCCCGGCCAACCCTCCTCCA

ACAAATTATACCTGGTATCACAATGGGAAAGAAGTGCCAGGAGTGACAGAAGAGAAATAC

TGGATCGCCCAGGTCCTTACTTGGCATGCAGGGCGTTACGCCTGCGTGGCAGAGAACAGG

CTTGGTTCTGGA

>CL370.Contig14_A-W 253 876 B-cell receptor CD22 isoform 3 precursor [Homo sapiens]

ACCCCGAAGTTGGAGATCAAGGTCTTTCCCAATGAAACCACTGTAATGGAAGGGGCGTCC

GTGACCATGACGTGCCAGGTCACCAGCAGCAACCCAGAGCATGGCACTGTATCCTGGCTC

AAGGACGGGACCCTCCTGGGGGAGAAGAATCTCTCGCTAACTCTGTCCTCAGTGACCAAG

GACAGTGGTGGGAAGTACCGCTGTCAGGCCTCCAATCACATAGGCTTAGGAATGTCGGAA

GAAGTGGCCCTCAAAGTGCTGTGTGAGCCTCCTCGGAGCTGAGGGTGGAGGTGACAGTGG

CTGCTTGCTCATGTCACAGGGTTCCCCTCACCTCTCGCCTCACCTCTGCCCCTTCTTCCA

GATCCCCCGACAGTTTCCGAGGTTTACATCCACCACACACCTGCTAAGGAGGGACAGTCA

GTAGAGCTGGTTTGCACGTCCCCGGCCAACCCTCCTCCAACAAATTATACCTGGTATCAC

AATGGGAAAGAAGTGCCAGGAGTGACAGAAGAGAAATACTGGATCGCCCAGGTCCTTACT

TGGCATGCAGGGCGTTACGCCTGCGTGGCAGAGAACAGGCTTGGTTCTGGACAGGTTGGC

CAGGAAGCCGAGCTGGACGTCCAA

>CL371.Contig1_A-W 2 496 PREDICTED: tyrosyl-DNA phosphodiesterase 1 [Canis lupus familiaris]

GCTCAGTTCAACTACTGTTTTGATGTAGTCTGGCTCGTAAAGCAGTATCCGCCAGAGTTC

AGGAAGAAGCCGATCCTGCTTGTGCATGGTGATAAACGGGAAGCCAAGGCTCACCTACAT

GCCCAGGCAAAACCTTATGAAAACGTTTCCTTCTGCCAGGCAAAGTTGGATATTGCATTT

GGAACACACCACACGAAGATGATGCTACTGCTGTACGAAGAAGGCCTCCGGGTTGTCATA

CACACCTCCAACCTCATCCATGAAGACTGGCACCAGAAAACCCAAGGAATATGGTTGAGC

CCCCTATACCCACGAATTGGCCAGCAAACCCACAGATCTGGAGAATCAATAACGCATTTT

AAAGCTGATCTCATCAGTTACTTGAAGGCTTACAATGCCCCTCCTCTTCAGGAGTGGATT

AACACCATCCAGGAGCACGATCTCTCTGAAACAAATGTTTACCTTATTGGTTCAACCCCC

GGACGCTTTCAAGGA

>CL371.Contig2_A-W 53 259 PREDICTED: tyrosyl-DNA phosphodiesterase 1 [Canis lupus familiaris]

ATATGGTTGAGCCCCCTATACCCACGAATTGGCCAGCAAACCCACAGATCTGGAGAATCA

ATAACGCATTTTAAAGCTGATCTCATCAGTTACTTGAAGGCTTACAATGCCCCTCCTCTT

CAGGAGTGGATTAACACCATCCAGGAGCACGATCTCTCTGAAACAAATGTTTACCTTATT

GGTTCAACCCCCGGACGCTTTCAAGGA

>CL372.Contig1_A-W 201 884 PREDICTED: pre-B-cell leukemia transcription factor 4 [Pan troglodytes]

CAGGCCTGCCGTGAATTCACTGCACATGTCACCAACCTTCTCCGGGAGCAGAGCAGGATG

AGGCCTGTGTCCCCCAAGGAGATCGAGCACATGGTTGGCATCATACATGGCAAATTCAGC

GCCATCCAGATGCAACTGAAGCAGAGCACCTGTGAGGCTTTGATGACCCTACGCTCAAGG

TTCCTTGATGCCAGGCATAAGCGGCGGAATTTCAGCAAGCAGGCAACAGAAGTGCTGAAT

GAATATTTTTATTCCCACTTGAGCAACCCTTACCCCAGTGAAGAAGCCAAAGAAGACCTA

GCCAGGAAGGGTGGCATCACAGTATCCCAGGTCTCTAACTGGTTTGGCAACAAAAGAATC

CAATATAAAAAGAACATGGGGAAATTTCAAGAAGAGGCTACCATTTACACGGGTAAATCA

GCTATGGATGCCACCAAGGCCAGGGCCCTGGAGAGCCAAGCCAGCTGCCTGTCAATGCCT

AGCCCCGGTTCCTCTGGTCCCTTCCCACTGACCAGTGCTGGGGATACATTCCTCACCCTG

CGGACCCTGGCCTTTCTCCAGCCCCCTCCTGGGGGCAACTGCCTACAGTCCCAGGCCAGG

AGCAGCTGGCAGGGGGCCATCCCCCCACAGTCGATTGTCTCACCCACTGGAGACCCCAGC

AGCATCAACTCAGATGCCTCTAAT

>CL372.Contig2_A-W 201 395 PREDICTED: pre-B-cell leukemia transcription factor 4 [Canis lupus familiaris]

CAGGCCTGCCGTGAATTCACTGCACATGTCACCAACCTTCTCCGGGAGCAGAGCAGGATG

AGGCCTGTGTCCCCCAAGGAGATCGAGCACATGGTTGGCATCATACATGGCAAATTCAGC

GCCATCCAGATGCAACTGAAGCAGAGCACCTGTGAGGCTTTGATGACCCTACGCTCAAGG

TTCCTTGATGCCAGG

>CL373.Contig1_A-W 1 270 WD repeat-containing protein 42A [Cricetulus griseus]

TCAGGCTTTAAAGTAGAGGCCTTAGACCAGAGTTTGGGCTTGACTAGGTGTGATGGTGGC

CCCAATTTCACCAGTAGAGAAAGTCAAGGCATAGAGAGCGCAGGTAAAGAAACGGATAGC

ATGTGGACACTGACCATCACTGTATCAGTGATGATAACTGACCATCACTGTATCAGTGAT

GATAACTGAGTCCGTAACTGCTCAGAGGAAGAAGAAAAAGAATAAAAGCAATCATGGCAC

TGTGTTTAGCACAAGCAAGCTAACCATGAC

>CL373.Contig2_A-W 141 302 WD repeat-containing protein 42A [Cricetulus griseus]

GACACTGACCATCACTGTATCAGTGATGATAACTGAGTCCGTAACTGCTCAGAGGAAGAA

GAAAAAGAATAAAAGCAATCATGGCACTGTGTTTAGCACAAGCAAGCTAACCATGACCAT

GGGGTCCATGAAGAACAGGAGACAACAGCCCTACTCCAGCCT

>CL374.Contig2_A-W 1036 1668 minus strand Retrovirus-related Gag polyprotein [Cricetulus griseus]

TTGCATGAGGAAAAATCTGAAGGATCATGTAGTGAAGTGGCAGAGAGTATTAAAAGTAAC

AGTAGTGAGAAGGCAAAAGAGCCTGAAGATAAAAATAGGAGAAGGCTCTACCCAGACTTG

ACCGAATTAAAAACGCCTGAGAACACAAGCGACTCAGAGAGTTCTGAGGACCTTGATATA

TTGTTACAACAACTACATAAGATAAAAATGAAAAAGAAGAAAAAGGAGACACAGGGCACG

CAGGCCTACTGTAAGAAGGGGAAGGGAGTTAACATCGGTCAACAAAACTCTGAGGAGGAG

GAGGTAGAATATCTAGAAGAAGATATGGTGCCGATTGCCCCACCTCCGTATGTGGGGGGG

AGCCAAGGTTCCGGGAGATCTTTTCATGCACAAGTTTGGAGGACAGTTAATACAGATATG

GGACTTGCATACCCTGTATTTCAGGACAATAACAGGGGAAGATATCATGAGCCTTTAGAC

TTTAAGATAATTAAAACCTTGGCAGAATCTGTCCGCACTTATGGCATAGATGCCGCTTTC

ACTCTCACTCAGGTGGAGGGACTTACTAGATTTTGCATGACTCCCTCAGATTGGGCTAGT

CTAGTCCGCGCTTGTGTTTCCCCAGGGAAATAT

>CL374.Contig4_A-W 163 438 minus strand putative retrovirus-related gag protein [Rattus norvegicus]

ATGGGTGCCGCATCTTCAAGTCCGCTTCTCCTGGCCCTAGATGGCCTGTTACGTTCCAAG

GGACTGGAAGTGAAACATAGTACTTTACAGAGATTTTTACAGAAGATAGATATCGCTGCA

CCGTGGTTTGCATTTTCGGGCAGCCTTACTGTGCCTAGTTGGGATAAATTAGGCAAAGAT

CTTGACTTTGCTTCTGAGCAGGGCATATTAGAGGGTGGGGTGATACCCCTTTGGAAAATG

GTCCGTGGTTGCCTTACAGATGGTAAATGCCAGGAA

>CL375.Contig1_A-W 82 414 minus strand protein canopy homolog 4 precursor [Bos taurus] >gi|122140076|sp|Q3SWX1.1|CNPY4_BOVIN RecName: Full=Protein canopy homolog 4; Flags: Precursor >gi|74356321|gb|AAI04627.1| Canopy 4 homolog (zebrafish) [Bos taurus] >gi|296472918|gb|DAA15033.1| protein canopy homolog 4 precursor [Bos taurus]

CAGGGTCAGAGTCAGACCATGGCAACATTGAAAGGCCTAGTGCAGAAGGGAGTGAAGGTG

GATCTGGGGATCCCATTGGAACTGTGGGATGAGCCCAGCGTGGAGGTTACATTCCTAAAG

AAGCAGTGTGAGACGATGCTGGAAGAGTTTGAAGATGTTGTAGGAGACTGGTACTTCCAC

CATCAGGAGCAGCCCCTACAGCAGTTTCTCTGTGAAGGCCATGTGCTCCCAGCTGCTGAA

ACAGCATGTCTTCAGGAAACTTGGACTGGAAAGGAGAAGATCACAGATGGACAAGAGAAT

GCAGAAGAAAAGGAGGAGGAGGAAGAAGAAGAG

>CL375.Contig2_A-W 272 412 minus strand protein canopy homolog 4 precursor [Bos taurus] >gi|122140076|sp|Q3SWX1.1|CNPY4_BOVIN RecName: Full=Protein canopy homolog 4; Flags: Precursor >gi|74356321|gb|AAI04627.1| Canopy 4 homolog (zebrafish) [Bos taurus] >gi|296472918|gb|DAA15033.1| protein canopy homolog 4 precursor [Bos taurus]

TCCTCACTACCAGTGTGTAAGCTACTAAGCATGGAGCTACAGGAGGAACTGAGTCGCACT

GGCCGATCTCGAGAGGTCCTGGAGCTGGGGCAGGTCCTGGACACAGGCAAGAGGAAGAGA

CATGTGCCTTACAGCATTTCG

>CL375.Contig3_A-W 1202 1501 minus strand canopy 4 homolog [Rattus norvegicus] >gi|171847441|gb|AAI62041.1| Canopy 4 homolog (zebrafish) [Rattus norvegicus]

CAGTGTGAGACGATGCTGGAAGAGTTTGAAGATGTTGTAGGAGACTGGTACTTCCACCAT

CAGGAGCAGCCCCTACAGCAGTTTCTCTGTGAAGGCCATGTGCTCCCAGCTGCTGAAACA

GGTAAATGTAGGGATTGGGCTCCTCTCCTGCCAGGCAACAACCTCCTTATTCCCAGAACC

AATATTTAAATTATTTTCTCTTAACCCCACCTAGCATGTCTTCAGGAAACTTGGACTGGA

AAGGAGAAGATCACAGATGGACAAGAGAATGCAGAAGAAAAGGAGGAGGAGGAAGAAGAA

>CL375.Contig4_A-W 1232 1531 minus strand PREDICTED: canopy 4 homolog [Oryctolagus cuniculus]

CAGTGTGAGACGATGCTGGAAGAGTTTGAAGATGTTGTAGGAGACTGGTACTTCCACCAT

CAGGAGCAGCCCCTACAGCAGTTTCTCTGTGAAGGCCATGTGCTCCCAGCTGCTGAAACA

GGTAAATGTAGGGATTGGGCTCCTCTCCTGCCAGGCAACAACCTCCTTATTCCCAGAACC

AATATTTAAATTATTTTCTCTTAACCCCACCTAGCATGTCTTCAGGAAACTTGGACTGGA

AAGGAGAAGATCACAGATGGACAAGAGAATGCAGAAGAAAAGGAGGAGGAGGAAGAAGAA

>CL375.Contig5_A-W 1173 1475 minus strand RIKEN cDNA 2610019P18 [Mus musculus]

CAGTGTGAGACGATGCTGGAAGAGTTTGAAGATGTTGTAGGAGACTGGTACTTCCACCAT

CAGGAGCAGCCCCTACAGCAGTTTCTCTGTGAAGGCCATGTGCTCCCAGCTGCTGAAACA

GGTAAATGTAGGGATTGGGCTCCTCTCCTGCCAGGCAACAACCTCCTTATTCCCAGAACC

AATATTTAAATTATTTTCTCTTAACCCCACCTAGCATGTCTTCAGGAAACTTGGACTGGA

AAGGAGAAGATCACAGATGGACAAGAGAATGCAGAAGAAAAGGAGGAGGAGGAAGAAGAA

GAG

>CL375.Contig6_A-W 82 249 minus strand protein canopy homolog 4 precursor [Bos taurus] >gi|122140076|sp|Q3SWX1.1|CNPY4_BOVIN RecName: Full=Protein canopy homolog 4; Flags: Precursor >gi|74356321|gb|AAI04627.1| Canopy 4 homolog (zebrafish) [Bos taurus] >gi|296472918|gb|DAA15033.1| protein canopy homolog 4 precursor [Bos taurus]

CAGGGTCAGAGTCAGACCATGGCAACATTGAAAGGCCTAGTGCAGAAGGGAGTGAAGGTG

GATCTGGGGATCCCATTGGAACTGTGGGATGAGCCCAGCGTGGAGGTTACATTCCTAAAG

AAGCAGGTAAGACAAATTACCAAACTGTCCAGGGAGGTGAGAGGAGAG

>CL375.Contig7_A-W 82 249 minus strand protein canopy homolog 4 precursor [Bos taurus] >gi|122140076|sp|Q3SWX1.1|CNPY4_BOVIN RecName: Full=Protein canopy homolog 4; Flags: Precursor >gi|74356321|gb|AAI04627.1| Canopy 4 homolog (zebrafish) [Bos taurus] >gi|296472918|gb|DAA15033.1| protein canopy homolog 4 precursor [Bos taurus]

CAGGGTCAGAGTCAGACCATGGCAACATTGAAAGGCCTAGTGCAGAAGGGAGTGAAGGTG

GATCTGGGGATCCCATTGGAACTGTGGGATGAGCCCAGCGTGGAGGTTACATTCCTAAAG

AAGCAGGTAAGACAAATTACCAAACTGTCCAGGGAGGTGAGAGGAGAG

>CL376.Contig1_A-W 38 316 minus strand MHC class I-like sequence (mapped) [Rattus norvegicus]

AAAGGACTTTGCTGGGGGCTGATGGTGCTCCTGGTACCTCTAATCACTGTATTCATGGTG

AAGCTCAGTCATGCCCGAACACACTCTCTGAGATACTTTCGCCTGGGCGTTTCGGATCCT

GGCCACGGGGTTCCTGAATTTATCTCAGTTGGGTATGTGGACTCCCACCCTATCACCACA

TATGACAGTGTCACTCGGCAGAAGGAGCCGCGGGCCCCGTGGATGGCGGAGAACCTGGCG

CCCGATCATTGGGAGAGGTACACTCAGCTGCTCAGGGGC

>CL376.Contig2_A-W 36 236 minus strand PREDICTED: major histocompatibility complex class I-related gene protein-like [Saimiri boliviensis boliviensis]

ACACACTCTCTGAGATACTTTCGCCTGGGCGTTTCGGATCCTGGCCACGGGGTTCCTGAA

TTTATCTCAGTTGGGTATGTGGACTCCCACCCTATCACCACATATGACAGTGTCACTCGG

CAGAAGGAGCCGCGGGCCCCGTGGATGGCGGAGAACCTGGCGCCCGATCATTGGGAGAGG

TACACTCAGCTGCTCAGGGGC

>CL378.Contig1_A-W 3 683 minus strand PREDICTED: nuclear pore membrane glycoprotein 210 [Equus caballus]

AGAAGGACGCCCCTGGTGGTCACGGCCTCGGTGGCCGGGGGCCAGGCCTCCATGGAGCAG

GTGGGGGCCGAGGTGCCCTTCAGCCCCGGGCTCTACGCTGACCAGGCCGAGATCCTCCTG

AGCAACCACCTGTCCAGCCGCGAGGTCAAGGTCTTTGGGGCCGCGGAGATCCTGGACAGC

TTGGAGGTCAGGTCCGGGTCCCCCGCTGTGTTGGCCTTCGCGAAGGAGAAGAACCTGGGG

CTGCCCAGTTTCATCACCTACACGGTCGGCTTCTCGGACCCCAGGGCGGGCAGCCAGGGC

CCTCTGTCCACCGCCCTGACCTTCTCCAGCCCTGCCACTAACCAGGCCCTCACCATCCCG

GTGACGGTGGCCCTGGTGCTGGATCGCCGTGGGGCTGGTCCTTACGGGGCCGGCCTCTTC

CAGCACCTCCTGGACTCCTACCAGGCCCTGTTCTTCTCGCTCTTCACCCTGCTGGCCGGG

ACGGCCGTCACCGTCATAGCCTACCACGCGGTCTACGCGCCCCGGGAGCTCGCCGGCCCC

GTGGTCCTCACGCCCCGAGCCAGCCCTGGGCACAGTCCCCACTATCCAGCGGCCTCCTCT

CCGGCCTCCTTCAATGCCCTGCCTCCTGGTCGCAGAGGCAGCCCCCCCTCGGGGCTCTGG

AGCCCAGCCTACGCCTCCCAC

>CL378.Contig2_A-W 172 1713 minus strand PREDICTED: nuclear pore membrane glycoprotein 210-like [Cricetulus griseus]

ATATTCTATATATGTCTTTGGTTGAGCCTGGTCCTGCACACCCAGGACAAGGAGGCCCTG

GCGGCCCTGCCTCTGGGGATGACCGTGACCTTCACCGTCCACTTCCATGACCACTTTGGA

GATGTCTTCCACGCGCACAGTTCGGTCCTCAGCTTTGCCACCAACAGAGAGGACTTCGTG

CAGGTCACCAAGGGCACCACCAACAACACCTGCGTCCTGCGCACGGTCAGCGTGGGCCTG

ACGCTGCTGCGCGTGTGGGACCCCGAGCACCCCGGCCTCTCCGACTACGTGCCGCTGCCC

GTCCTGCCGGCCATCGCTCCAGAGCTGCCCGGCACCGTGGTGGTCGGGGATGTCCTCTGC

CTGGCCACCGCGCTCACCGGCCCCGAGGGTGCCCCAGGAACCTGGAGCTCTTCAGCCGAC

AGCATCCTCCAGGTCGACCCCAAGACGGGGGTGGCCGTGGCCTGGGACGCGGGGTCCGTG

ACGGTGCACTACGAGGTCCCTGGGCACCTGAGGACCTACAAGGAGATCGTGGTCAGTGTC

CCCCAGAGGATTGTGGCCCGGCGCGTCCACACGGTCCAGGCCAGCTTCCAGGGGGCCACA

GCCTCCAGAGTGACCGTCACCGTGGGAGACAGAAGCTCTAACCTGAGAGGCGAGTGCTCC

GCTGCCCAGAGGGCGGCCATCCCAGCTCTGCGCCCCGAGGCCCTCCTCGGCTGCCATCTT

CGGTTCCAGCAGGACGTCTTCGACCTCCCGGTGACAGACGTGTTCTCGGCCGAGCCGGAC

TTCGACGTCACACTGGGCCAGTACCTCTGCTCGGTCACCATGCACAGGCTGACCGACGGG

CAGCTGAGGCACCTGAGCTCGAGAAGGACGCCCCTGGTGGTCACGGCCTCGGTGGCCGGG

GGCCAGGCCTCCATGGAGCAGGTGGGGGCCGAGGTGCCCTTCAGCCCCGGGCTCTACGCT

GACCAGGCCGAGATCCTCCTGAGCAACCACCTGTCCAGCCGCGAGGTCAAGGTCTTTGGG

GCCGCGGAGATCCTGGACAGCTTGGAGGTCAGGTCCGGGTCCCCCGCTGTGGTGGCCTTC

GTGAAGGAGAAGTCCCTGGGGCTGCCCAGTTTCGTCACCTACACGGTCGGCGTCTCGGAC

CCCAGGGCGGGCAGCCAGGGCCCTCTGTCCACCGCCCTGACCTTCTCCAGCCCTGCCACT

AACCAGGCCCTCACCATCCCGGTGACGGTGGCCCTGGTGCTGGATCGCCGTGGGGCTGGT

CCTTACGGGGCCGGCCTCTTCCAGCACCTCCTGGACTCCTACCAGGCCCTGTTCTTCTCG

CTCTTCACCCTGCTGGCCGGGACGGCCGTCACCGTCATAGCCTACCACGCGGTCTACGCG

CCCCGGGAGCTCGCCGGCCCCGTGGTCCTCACGCCCCGAGCCAGCCCTGGGCACAGTCCC

CACTATCCAGCGGCCTCCTCTCCGGCCTCCTTCAATGCCCTGCCTCCTGGTCGCAGAGGC

AGCCCCCCCTCGGGGCTCTGGAGCCCAGCCTACGCCTCCCAC

>CL378.Contig3_A-W 167 1687 minus strand PREDICTED: nuclear pore membrane glycoprotein 210 [Papio anubis]

TTGAGCCTGGTCCTGCACACCCAGGACAAGGAGGCCCTGGCGGCCCTGCCTCTGGGGATG

ACCGTGACCTTCACCGTCCACTTCCATGACCACTTTGGAGATGTCTTCCACGCGCACAGT

TCGGTCCTCAGCTTTGCCACCAACAGAGAGGACTTCGTGCAGGTCACCAAGGGCACCACC

AACAACACCTGCGTCCTGCGCACGGTCAGCGTGGGCCTGACGCTGCTGCGCGTGTGGGAC

CCCGAGCACCCCGGCCTCTCCGACTACGTGCCGCTGCCCGTCCTGCCGGCCATCGCTCCA

GAGCTGCCCGGCACCGTGGTGGTCGGGGATGTCCTCTGCCTGGCCACCGCGCTCACCGGC

CCCGAGGGTGCCCCAGGAACCTGGAGCTCTTCAGCCGACAGCATCCTCCAGGTCGACCCC

AAGACGGGGGTGGCCGTGGCCTGGGACGCGGGGTCCGTGACGGTGCACTACGAGGTCCCT

GGGCACCTGAGGACCTACAAGGAGATCGTGGTCAGTGTCCCCCAGAGGATTGTGGCCCGG

CGCGTCCACACGGTCCAGGCCAGCTTCCAGGGGGCCACAGCCTCCAGAGTGACCGTCACC

GTGGGAGACAGAAGCTCTAACCTGAGAGGCGAGTGCTCCGCTGCCCAGAGGGCGGCCATC

CCAGCTCTGCGCCCCGAGGCCCTCCTCGGCTGCCATCTTCGGTTCCAGCAGGACGTCTTC

GACCTCCCGGTGACAGACGTGTTCTCGGCCGAGCCGGACTTCGACGTCACACTGGGCCAG

TACCTCTGCTCGGTCACCATGCACAGGCTGACCGACGGGCAGCTGAGGCACCTGAGCTCG

AGAAGGACGCCCCTGGTGGTCACGGCCTCGGTGGCCGGGGGCCAGGCCTCCATGGAGCAG

GTGGGGGCCGAGGTGCCCTTCAGCCCCGGGCTCTACGCTGACCAGGCCGAGATCCTCCTG

AGCAACCACCTGTCCAGCCGCGAGGTCAAGGTCTTTGGGGCCGCGGAGATCCTGGACAGC

TTGGAGGTCAGGTCCGGGTCCCCCGCTGTGTTGGCCTTCGCGAAGGAGAAGAACCTGGGG

CTGCCCAGTTTCATCACCTACACGGTCGGCTTCTCGGACCCCAGGGCGGGCAGCCAGGGC

CCTCTGTCCACCGCCCTGACCTTCTCCAGCCCTGCCACTAACCAGGCCCTCACCATCCCG

GTGACGGTGGCCCTGGTGCTGGATCGCCGTGGGGCTGGTCCTTACGGGGCCGGCCTCTTC

CAGCACCTCCTGGACTCCTACCAGGCCCTGTTCTTCTCGCTCTTCACCCTGCTGGCCGGG

ACGGCCGTCACCGTCATAGCCTACCACGCGGTCTACGCGCCCCGGGAGCTCGCCGGCCCC

GTGGTCCTCACGCCCCGAGCCAGCCCTGGGCACAGTCCCCACTATCCAGCGGCCTCCTCT

CCGGCCTCCTTCAATGCCCTGCCTCCTGGTCGCAGAGGCAGCCCCCCCTCGGGGCTCTGG

AGCCCAGCCTACGCCTCCCAC

>CL378.Contig4_A-W 112 405 minus strand PREDICTED: LOW QUALITY PROTEIN: nuclear pore membrane glycoprotein 210 [Callithrix jacchus]

CGGTGTCCCCGCGCAGACGGGGCCGGCCTCTTCCAGCACCTCCTGGACTCCTACCAGGCC

CTGTTCTTCTCGCTCTTCACCCTGCTGGCCGGGACGGCCGTCACCGTCATAGCCTACCAC

GCGGTCTACGCGCCCCGGGAGCTCGCCGGCCCCGTGGTCCTCACGCCCCGAGCCAGCCCT

GGGCACAGTCCCCACTATCCAGCGGCCTCCTCTCCGGCCTCCTTCAATGCCCTGCCTCCT

GGTCGCAGAGGCAGCCCCCCCTCGGGGCTCTGGAGCCCAGCCTACGCCTCCCAC

>CL378.Contig5_A-W 59 628 minus strand PREDICTED: nuclear pore membrane glycoprotein 210 [Papio anubis]

GTTTGTCTGTCCATGTGCTGGGTCCCCCGGCCAGTGGCTCTGTGGAGCGTCCCTGCAGCC

TGACGTCTCTCCCAGGTCAGGTCCGGGTCCCCCGCTGTGTTGGCCTTCGCGAAGGAGAAG

AACCTGGGGCTGCCCAGTTTCATCACCTACACGGTCGGCTTCTCGGACCCCAGGGCGGGC

AGCCAGGGCCCTCTGTCCACCGCCCTGACCTTCTCCAGCCCTGCCACTAACCAGGCCCTC

ACCATCCCGGTGACGGTGGCCCTGGTGCTGGATCGCCGTGGGGCTGGTCCTTACGGGGCC

GGCCTCTTCCAGCACCTCCTGGACTCCTACCAGGCCCTGTTCTTCTCGCTCTTCACCCTG

CTGGCCGGGACGGCCGTCACCGTCATAGCCTACCACGCGGTCTACGCGCCCCGGGAGCTC

GCCGGCCCCGTGGTCCTCACGCCCCGAGCCAGCCCTGGGCACAGTCCCCACTATCCAGCG

GCCTCCTCTCCGGCCTCCTTCAATGCCCTGCCTCCTGGTCGCAGAGGCAGCCCCCCCTCG

GGGCTCTGGAGCCCAGCCTACGCCTCCCAC

>CL378.Contig6_A-W 61 630 minus strand PREDICTED: nuclear pore membrane glycoprotein 210 [Papio anubis]

GTTTGTCTGTCCATGTGCTGGGTCCCCCGGCCAGTGGCTCTGTGGAGCGTCCCTGCAGCC

TGACGTCTCTCCCAGGTCAGGTCCGGGTCCCCCGCTGTGTTGGCCTTCGCGAAGGAGAAG

AACCTGGGGCTGCCCAGTTTCATCACCTACACGGTCGGCTTCTCGGACCCCAGGGCGGGC

AGCCAGGGCCCTCTGTCCACCGCCCTGACCTTCTCCAGCCCTGCCACTAACCAGGCCCTC

ACCATCCCGGTGACGGTGGCCCTGGTGCTGGATCGCCGTGGGGCTGGTCCTTACGGGGCC

GGCCTCTTCCAGCACCTCCTGGACTCCTACCAGGCCCTGTTCTTCTCGCTCTTCACCCTG

CTGGCCGGGACGGCCGTCACCGTCATAGCCTACCACGCGGTCTACGCGCCCCGGGAGCTC

GCCGGCCCCGTGGTCCTCACGCCCCGAGCCAGCCCTGGGCACAGTCCCCACTATCCAGCG

GCCTCCTCTCCGGCCTCCTTCAATGCCCTGCCTCCTGGTCGCAGAGGCAGCCCCCCCTCG

GGGCTCTGGAGCCCAGCCTACGCCTCCCAC

>CL379.Contig1_A-W 1 3015 minus strand PREDICTED: HEAT repeat-containing protein 7A-like [Cavia porcellus]

CCAAGTTACAACAGGGTGCCCCAGGAGAAGAACTTCCTGTACAAGTGCCTGGGGACCACG

CTGGCGGCGGCTGCGAGCAGGGAGGTGGTGAGGAAGAGCCTGCGGGAGCTGCTGGGAGCC

GCCAGCTACCAGCAGCAGGCGGAGCACGAGGGCCTGGCCTGCTGCTTTGGGATCTGTGCC

ACTGCCCACCTTGAGGACACTCTGACCCAGCTGGAGGACTTCATGAGGTCAGCTGTGCTC

AGGAAACCCAGCGGCATCTTCAGCATCTTCAAGGATCGGGGCGAGCAGGAGCTGGAGAAG

GTGAAGAGCGCGCTGATCCTCTGTTACGGGCATGTGGCCGCGCGGGCCCCTCACGAGCTG

GTGCTGGCCAAGGTGGAGTCTGAGGTCCTCCGCAACATGTTCCAGTGCTTCAGCACCAAG

GTTCTGGGAATAAAGGTAGAAACCAAGGACCCGGCCCTGAAGCTATGCCTTGTCCAGAGC

CTGTGCATGGTCAGCCAGGCCATCTGCAGCAGTGCGCAGGCCAGCTCCTTCTACTTCTCA

AGGAAAGCAGAGCTGGTGGCACAGATGATGGAATTCATCAGGGCGGAGCCCCTGGACTCC

CTGAAAACACCCCTGCGCAAGAAGGCCATGCTCGCCTGCACCCACCTGGTCTCCTTGGAG

CCAGCACTAGAGGAGCAGGCGCGGGCAGACGTGATCCATGGCTGCCTACACAGTGTCGTG

GCCCTGGTTCCCGAGCCGGACCGGGAGGATGGCGGCAGCCAGGAGTCCCTGTATCAGGAC

ACCGTCCGTGCCCTGGAGGACTTGCTGGCGGGCCTCCTGCAGCGGAACATGACTCCCCAG

GGCCTTCAGATCATGGTCGAGCACCTGAGCCCATGGATCAAGTCCCCAAAGGGCCACGAG

CGGGCACGGGCCTTGGGCTTGAGTACCCGCCTGCTGCAGCACTTCCGGCAGCACCTGCAC

GTCAGCGCTCTGGTGCCCTTCCACAACCTGGGCCTCCTCGTTGGCCTCTTCTCCCCGAGG

TGTGCCGACCTGTGTTCCGCCACCCGCCGGCAGGCCGTGGACTGCGTCTACACCCTGCTC

TACCTGCAGCTGGGCCACGAGGGGTTCTCCCGAGACTACCGCGATGATGTGGCCGAGCGG

CTGCTCAGCCTCAAGGACGGCCTGGTGCACCCTGACCCTGCCACCCTCTTCCACACCTGC

CACAGCGTAGCCCAGATCATTGCCAAGCGCCTTCCGCCAGACCAGCTCATCAGCCTCCTG

CTGACCATGTTTGAGGGTCTGGGAGACCCCGACAAGAACTGCTCCCGTGCAGCTACGGTC

ATGATCAACTGCCTGCTGAAGGAGCGGGGCCCCGCGCTGCTGGAGAAGGTGCCGGAGATC

GTGAGTGTGCTGCGCTCCAAGCTGCAGGAGACCCAGGGAGAGCACATCCTGCCGGCCGCC

CAGCACGGCCTGTACCTGCTGGCCTCCCAGCACTGCGCGGCCGTAGTGTCCAGCCTGCTG

GGCAGCCCCCTGCCCTTCGACAGCCACACCTGTGCCCTGTGGCGGGCCCTGGCCGTGCAG

CCCAGCCTCACCACGCAGGTCCTGGAACTGCTCCTGGAGAAGATGAGCAGGGACGTCCCC

TTCAAAGAGAGCCGGGCCTTTCTGCTGGGCAGCAGCCCTCACCGGGTGGCCACACTGCTG

CCCCTTGTGGCCACCTGTGCACTGAATGAGGTCCTGTCTGCTCCCGCCTCGGGGACTGCG

GTGCTGGAGCTGTACCCCCAGCTGTTGGTGGCGCTGCTGCTGCGCGTCAGCTGCACTGTG

GGCGTCCAGCTGCCCCGGAACCTGCAGGCCAAGGAGAGGAGGGGCACTGGCACAGCCCCG

GCCAGGAGCCTGGAGCCCTGCAGCTCTGCAGTGGACGCGCTCCAGGCCCTGCTGCTCCGA

GGTGGCAGCCAGGACGTGGCACAGTGTATGCAGCTGGAAGGGGGCTGGGAGCTGCTCAGG

ACCTCAGCAGGGCACGAGGAGGGGGTCACCCGGCTGGCCAGTGCCATGGCCCGGTGTGCG

GGGCCCCGGCTCCCCCTAGTGGTGAAGGCGCTTGTGTGCACACAGAACAGCCTGTACGAG

ATCCAGAGAGTCACCTCCACTGCCTTCCTGGCTGAGCTGCTCAGTAGCAATGTGGTCAAT

GACCTGATGCTTCTGGAGTCCTTGCTGGACAACCTGGCAGCTCGACAGAAGGACTCAAGT

ACCAGTGTGCGGAGACTGGTGCTGCGTGGCCTGGCCAACGTGGCCTCTGGCTCCCCGGAC

AAGGTGCGGGCCCACGGCCCCCAGCTCCTGACAGCCATGATCAGCGGGCTGGACGACGGG

GATGACCCACACGGCCTGGTGGCCCTGGAGGCCATGGTGGGCCTGGCTCGACTGCTGGAC

CTGGTGGCACCCTGGGACCTGCGCCTGGTGCTGCTGCACACAGTGATCCGCATCCGGCCC

TTCTTCGACAGTGAGAAGGTGGAGTTCCGAGAAGCATCCATCCGCCTCTTTGGGCACCTC

AACAAGGCCTGCCATGGGGACTGTGAAGAAGTCTTCCTGGAGCAGGTTGTGGGCGGGCTG

GTGCCTCTGCTGCTACACCTGCAGGACCCTCAAGCCCCTGTGGCCAATGCCTGCAGGTTT

GCCCTGTGCATGTGTGTCCCCAACCTGGAGTGCGAGGAGCTGGCAGCCACCTTCCACAAG

CACCTGCAGGAGGGACGCAGCCTGCACTTCGGGGAGTTCCTCAACTCAACCTGCAAGCAC

CTGATGCATCACTTCCCTGACCTGCTGGGCCGCCTGGTGAGCACCAGCCTGTTCTACTTT

AAGAGCACCTTGGAAGAGGTCCGCGCTGCAGCCCCCATGCTCACAGGGTTCCTGGTGCTG

CATGCAGAACCCGAGCAGCAGCCGCAGGTGGACCTGGAGCAACTGATGGCAGCACTGCAG

CTCCTGCTGCGGGACCCAGCACCCGGGGTCCGGGAGAAGTCTGCCGAGACGCTGGGCCGC

CTGGTGAAGTTCGCC

>CL379.Contig2_A-W 1 2955 minus strand PREDICTED: HEAT repeat-containing protein 7A-like [Cavia porcellus]

CCAAGTTACAACAGGGTGCCCCAGGAGAAGAACTTCCTGTACAAGTGCCTGGGGACCACG

CTGGCGGCGGCTGCGAGCAGGGAGGTGGTGAGGAAGAGCCTGCGGGAGCTGCTGGGAGCC

GCCAGCTACCAGCAGCAGGCGGAGCACGAGGGCCTGGCCTGCTGCTTTGGGATCTGTGCC

ACTGCCCACCTTGAGGACACTCTGACCCAGCTGGAGGACTTCATGAGGTCAGCTGTGCTC

AGGAAACCCAGCGGCATCTTCAGCATCTTCAAGGATCGGGGCGAGCAGGAGCTGGAGAAG

GTGAAGAGCGCGCTGATCCTCTGTTACGGGCATGTGGCCGCGCGGGCCCCTCACGAGCTG

GTGCTGGCCAAGGTGGAGTCTGAGGTCCTCCGCAACATGTTCCAGTGCTTCAGCACCAAG

GTTCTGGGAATAAAGGTAGAAACCAAGGACCCGGCCCTGAAGCTATGCCTTGTCCAGAGC

CTGTGCATGGTCAGCCAGGCCATCTGCAGCAGTGCGCAGGCCAGCTCCTTCTACTTCTCA

AGGAAAGCAGAGCTGGTGGCACAGATGATGGAATTCATCAGGGCGGAGCCCCTGGACTCC

CTGAAAACACCCCTGCGCAAGAAGGCCATGCTCGCCTGCACCCACCTGGTCTCCTTGGAG

CCAGCACTAGAGGAGCAGGCGCGGGCAGACGTGATCCATGGCTGCCTACACAGTGTCGTG

GCCCTGGTTCCCGAGCCGGACCGGGAGGATGGCGGCAGCCAGGAGTCCCTGTATCAGGAC

ACCGTCCGTGCCCTGGAGGACTTGCTGGCGGGCCTCCTGCAGCGGAACATGACTCCCCAG

GGCCTTCAGATCATGGTCGAGCACCTGAGCCCATGGATCAAGTCCCCAAAGGGCCACGAG

CGGGCACGGGCCTTGGGCTTGAGTACCCGCCTGCTGCAGCACTTCCGGCAGCACCTGCAC

GTCAGCGCTCTGGTGCCCTTCCACAACCTGGGCCTCCTCGTTGGCCTCTTCTCCCCGAGG

TGTGCCGACCTGTGTTCCGCCACCCGCCGGCAGGCCGTGGACTGCGTCTACACCCTGCTC

TACCTGCAGCTGGGCCACGAGGGGTTCTCCCGAGACTACCGCGATGATGTGGCCGAGCGG

CTGCTCAGCCTCAAGGACGGCCTGGTGCACCCTGACCCTGCCACCCTCTTCCACACCTGC

CACAGCGTAGCCCAGATCATTGCCAAGCGCCTTCCGCCAGACCAGCTCATCAGCCTCCTG

CTGACCATGTTTGAGGGTCTGGGAGACCCCGACAAGAACTGCTCCCGTGCAGCTACGGTC

ATGATCAACTGCCTGCTGAAGGAGCGGGGCCCCGCGCTGCTGGAGAAGGTGCCGGAGATC

GTGAGTGTGCTGCGCTCCAAGCTGCAGGAGACCCAGGGAGAGCACATCCTGCCGGCCGCC

CAGCACGGCCTGTACCTGCTGGCCTCCCAGCACTGCGCGGCCGTAGTGTCCAGCCTGCTG

GGCAGCCCCCTGCCCTTCGACAGCCACACCTGTGCCCTGTGGCGGGCCCTGGCCGTGCAG

CCCAGCCTCACCACGCAGGTCCTGGAACTGCTCCTGGAGAAGATGAGCAGGGACGTCCCC

TTCAAAGAGAGCCGGGCCTTTCTGCTGGGCAGCAGCCCTCACCGGGTGGCCACACTGCTG

CCCCTTGTGGCCACCTGTGCACTGAATGAGGTCCTGTCTGCTCCCGCCTCGGGGACTGCG

GTGCTGGAGCTGTACCCCCAGCTGTTGGTGGCGCTGCTGCTGCGCGTCAGCTGCACTGTG

GGCGTCCAGCTGCCCCGGAACCTGCAGGCCAAGGAGAGGAGGGGCACTGGCACAGCCCCG

GCCAGGAGCCTGGAGCCCTGCAGCTCTGCAGTGGACGCGCTCCAGGCCCTGCTGCTCCGA

GGTGGCAGCCAGGACGTGGCACAGTGTATGCAGCTGGAAGGGGGCTGGGAGCTGCTCAGG

ACCTCAGCAGGGCACGAGGAGGGGGTCACCCGGCTGGCCAGTGCCATGGCCCGGTGTGCG

GGGCCCCGGCTCCCCCTAGTGGTGAAGGCGCTTGTGTGCACACAGAACAGCCTGTACGAG

ATCCAGAGAGTCACCTCCACTGCCTTCCTGGCTGAGCTGCTCAGTAGCAATGTGGTCAAT

GACCTGATGCTTCTGGAGTCCTTGCTGGACAACCTGGCAGCTCGACAGAAGGACTCAAGT

ACCAGTGTGCGGAGACTGGTGCTGCGTGGCCTGGCCAACGTGGCCTCTGGCTCCCCGGAC

AAGGTGCGGGCCCACGGCCCCCAGCTCCTGACAGCCATGATCAGCGGGCTGGACGACGGG

GATGACCCACACGGCCTGGTGGCCCTGGAGGCCATGGTGGGCCTGGCTCGACTGCTGGAC

CTGGTGGCACCCTGGGACCTGCGCCTGGTGCTGCTGCACACAGTGATCCGCATCCGGCCC

TTCTTCGACAGTGAGAAGGTGGAGTTCCGAGAAGCATCCATCCGCCTCTTTGGGCACCTC

AACAAGGCCTGCCATGGGGACTGTGAAGAAGTCTTCCTGGAGCAGGTTGTGGGCGGGCTG

GTGCCTCTGCTGCTACACCTGCAGGACCCTCAAGCCCCTGTGGCCAATGCCTGCAGGTTT

GCCCTGTGCATGTGTGTCCCCAACCTGGAGTGCGAGGAGCTGGCAGCCACCTTCCACAAG

CACCTGCAGGAGGGACGCAGCCTGCACTTCGGGGAGTTCCTCAACTCAACCTGCAAGCAC

CTGATGCATCACTTCCCTGACCTGCTGGGCCGCCTGGTGAGCACCAGCCTGTTCTACTTT

AAGAGCACCTTGGAAGAGGTCCGCGCTGCAGCCCCCATGCTCACAGGGTTCCTGGTGCTG

CATGCAGAACCCGAGCAGCAGCCGCAGGTGGACCTGGAGCAACTGATGGCAGCCCTTCCA

GCAGAGCTCCGTGAG

>CL379.Contig3_A-W 2850 3041 PREDICTED: HEAT repeat-containing protein 7A-like [Cavia porcellus]

ATGCATCACTTCCCTGACCTGCTGGGCCGCCTGGTGAGCACCAGCCTGTTCTACTTTAAG

AGCACCTTGGAAGAGGTCCGCGCTGCAGCCCCCATGCTCACAGGGTTCCTGGTGCTGCAT

GCAGAACCCGAGCAGCAGCCGCAGGTGGACCTGGAGCAACTGATGGCAGCCCTTCCAGCA

GAGCTCCGTGAG

>CL379.Contig4_A-W 3 254 PREDICTED: HEAT repeat-containing protein 7A-like [Cricetulus griseus]

ATGCGTCACTTCCCTGACCTGCTGGGCCGCCTGGTGAGCACCAGCCTGTTCTACTTTAAG

AGCACCTTGGAAGAGGTCCGCGCTGCAGCCCCCATGCTCACAGGGTTCCTGGTGCTGCAT

GCAGAACCCGAGCAGCAGCCGCAGGTGGACCTGGAGCAACTGATGGCAGCACTGCAGCTC

CTGCTGCGGGACCCAGCACCCGGGGTCCGGGAGAAGTCTGCCGAGACGCTGGGCCGCCTG

GTGAAGTTCGCC

>CL380.Contig1_A-W 1 1563 ER degradation-enhancing alpha-mannosidase-like 2 precursor [Bos taurus] >gi|154426006|gb|AAI51592.1| EDEM2 protein [Bos taurus] >gi|296481099|gb|DAA23214.1| ER degradation enhancer, mannosidase alpha-like 2 [Bos taurus]

GACGAGCTGCGACCTCTCACCTGTGACGGACACGACACCTGGGGCAGTTTTTCTCTGACA

TTAATTGATGCTCTGGACACCTTGCTGATTTTGGGGAATGTCTCCGAATTTCAAAGAGTG

GTTGAAGTGCTCCAGGACAATGTGGACTTTGATATAGACGTGAATGCCTCCGTGTTTGAA

ACCAACATTCGAGTGGTAGGAGGACTCCTGTCTGCTCATCTATTGTCCAAGAAGGCTGGG

GTGGAAGTGGAGGCCGGATGGCCCTGTTCTGGACCTCTCTTGAGAATGGCCGAGGAGGCA

GCCCGAAAACTCCTCCCAGCCTTTCAGACCCCCACTGGCATGCCCTATGGAACGGTGAAC

CTGCTGCATGGCGTGAACCCTGGAGAGACCCCTGTCACCTGTACAGCAGGGATCGGGACC

TTCATTGTGGAATTTGCCACCCTGAGCAGCCTCACTGGTGACCCTGTGTTTGAAGATGTG

GCCAGAGTAGCCCTGATGCGCCTCTGGGAGAGCCGGTCAGATATCGGGCTGGTTGGCAAC

CACATTGATGTGCTCACTGGCAAATGGGTGGCCCAGGACGCAGGCATCGGGGCTGGTGTG

GATTCCTACTTTGAGTACCTGGTGAAAGGAGCCATCCTGCTTCAGGATAAGAAGCTCATG

GCCATGTTCCTAGAGTATAACAAAGCCATTCGGAATTACACCCGCTTCGATGACTGGTAC

CTGTGGGTGCAGATGTACAAGGGAACTGTGTCCATGCCGGTCTTCCAGTCCTTGGAGGCC

TACTGGCCTGGTCTTCAGAGCCTCATTGGGGACATTGACAATGCTATGAGGACCTTCCTC

AACTACTACACTGTATGGAAGCAGTTTGGGGGACTCCCAGAATTCTACAACATCCCTCAG

GGATACACAGTGGATAAACGAGAGGGCTACCCACTTCGGCCAGAGCTCATTGAGAGTGCA

ATGTACCTGTATCGTGCCACGGGAGATCCTACCCTCTTAGAACTTGGGAGAGATGCTGTG

GAGTCCATTGAAAAAATCAGCAAGGTGGAGTGCGGATTTGCAACAATCAAAGATCTTCGA

GACCACAAACTAGACAACCGCATGGAGTCCTTCTTCCTGGCTGAGACTGTGAAATACCTC

TACCTTCTGTTTGACCCCACCAACTTCATCCACAACAATGGCTCCACCTTTGATGCTGTG

ATAACCCCCTATGGGGAGTGCATCCTGGGGGCTGGAGGCTACATCTTCAACACAGAAGCC

CATCCCATTGACCCCGCTGCCCTGCACTGCTGCCGGAGGCTGAAGGAGGAGCAGTGGGAA

GTGGAAGACTTGATGAGAGAATTCTACTCTCTCAAACGGAATAGGTCAAGATTTCGGAAA

AAGAGGATGAATTCAGGGCGCTGGGAACCCCCAGAAAGGCCAGGAACCTTCTCCTCACCT

GAAAACCATGACCAGACAAGGGAGAAGAAAGTTGGCAAGCAGAAAATCTCACTTCTCAGC

TGCCCTAGTCAGCCTTTTACCTCCAAGTTGGCCTTGCTGGGACAGGTTTTCCTAGATTCA

TCA

>CL380.Contig2_A-W 380 877 ER degradation-enhancing alpha-mannosidase-like 2 precursor [Bos taurus] >gi|154426006|gb|AAI51592.1| EDEM2 protein [Bos taurus] >gi|296481099|gb|DAA23214.1| ER degradation enhancer, mannosidase alpha-like 2 [Bos taurus]

ATCAAAGATCTTCGAGACCACAAACTAGACAACCGCATGGAGTCCTTCTTCCTGGCTGAG

ACTGTGAAATACCTCTACCTTCTGTTTGACCCCACCAACTTCATCCACAACAATGGCTCC

ACCTTTGATGCTGTGATAACCCCCTATGGGGAGTGCATCCTGGGGGCTGGAGGCTACATC

TTCAACACAGAAGCCCATCCCATTGACCCCGCTGCCCTGCACTGCTGCCGGAGGCTGAAG

GAGGAGCAGTGGGAAGTGGAAGACTTGATGAGAGAATTCTACTCTCTCAAACGGAATAGG

TCAAGATTTCGGAAAAAGAGGATGAATTCAGGGCGCTGGGAACCCCCAGAAAGGCCAGGA

ACCTTCTCCTCACCTGAAAACCATGACCAGACAAGGGAGAAGAAAGTTGGCAAGCAGAAA

ATCTCACTTCTCAGCTGCCCTAGTCAGCCTTTTACCTCCAAGTTGGCCTTGCTGGGACAG

GTTTTCCTAGATTCATCA

>CL380.Contig3_A-W 57 677 ER degradation-enhancing alpha-mannosidase-like 2 precursor [Bos taurus] >gi|154426006|gb|AAI51592.1| EDEM2 protein [Bos taurus] >gi|296481099|gb|DAA23214.1| ER degradation enhancer, mannosidase alpha-like 2 [Bos taurus]

GAGCTCATTGAGAGTGCAATGTACCTGTATCGTGCCACGGGAGATCCTACCCTCTTAGAA

CTTGGGAGAGATGCTGTGGAGTCCATTGAAAAAATCAGCAAGGTGGAGTGCGGATTTGCA

ACAATCAAAGATCTTCGAGACCACAAACTAGACAACCGCATGGAGTCCTTCTTCCTGGCT

GAGACTGTGAAATACCTCTACCTTCTGTTTGACCCCACCAACTTCATCCACAACAATGGC

TCCACCTTTGATGCTGTGATAACCCCCTATGGGGAGTGCATCCTGGGGGCTGGAGGCTAC

ATCTTCAACACAGAAGCCCATCCCATTGACCCCGCTGCCCTGCACTGCTGCCGGAGGCTG

AAGGAGGAGCAGTGGGAAGTGGAAGACTTGATGAGAGAATTCTACTCTCTCAAACGGAAT

AGGTCAAGATTTCGGAAAAAGAGGATGAATTCAGGGCGCTGGGAACCCCCAGAAAGGCCA

GGAACCTTCTCCTCACCTGAAAACCATGACCAGACAAGGGAGAAGAAAGTTGGCAAGCAG

AAAATCTCACTTCTCAGCTGCCCTAGTCAGCCTTTTACCTCCAAGTTGGCCTTGCTGGGA

CAGGTTTTCCTAGATTCATCA

>CL381.Contig1_A-W 5 1936 interleukin 27 receptor, alpha, isoform CRA_a [Homo sapiens] >gi|119604796|gb|EAW84390.1| interleukin 27 receptor, alpha, isoform CRA_a [Homo sapiens]

GGGCCCCGCAGGGCTGGGCGGCACCGGGGGTCCCGGGGAGTCATGCGGGGGCGCGGGGCT

GCCCGCTTCTGGCCTGGGCCAAAGCTCCAGCTGCTGCTTCCGTTGTTCCTGCTGGTCCCC

CGAGCGCGTCCCCTGGGCGGCCCTGGGCCACTGCAGTGCCACGGAGTTGAACCTTTGGGG

AACTTGAACTGCTCATGGGAACCTCTTGGGGACCTGGGAGCCCCCTCTATGCTGCACCTC

CGGAGCCAGAAATACCATTCCAACAGAACCCGGACCGTGGCGGTGCCCTCGGGGCAGAGC

TGGGTGACGGTTCCTCGGGAGCAATTCACCGTGTCTGACACGCTGCTCGTCTGGGGGACC

AAGGCAGGCCAGGTCCTCTGGCCCCCCGTCCTCATCAACCTGGAAACCCGAATGAAGCCC

AGAGCCCCCCGGCTGCGTCCCGAGGTGGACTTTTCAGAGGACGAGCCCCTGGAGGCCACG

GTGCAGTGGGAGCCACCTACCTGGCCACCTCATAAGGTCCTGATCTGCCAGTTCTACTAT

CGAAGATGCCCGGAGGTGGCCTGGACTCCGCTGGAGCCCGAGCTGAAGACGGCACCGCTG

ACCCCCATCGAGATCCCAGACCTGGAGCTGGCCACCCGCTACGAGGTGTCCGGCCGCTGC

CGGGTGGACACAGAGGCCGAGCTGTGGGGCGAGAGCAGCCCGGTGCTGTCCTTCCAGACG

CTGCCCTCTGCTCCAAAAGACGTGTGGGTGTCTGGGAACCGGTGTGGGCCCCCGAGCAGA

CAGGAGCTGCTGCTTCTGTGGAAGGCCCCCGGGCCCTGCGTGCAGGTGAGCTACAGAGTG

TGGTTCTGGGTTGGAGAGAAGAGTCTGACTCAGGAAGGGGTCCCCTGTTGCAAGTGCTCC

ATCCCCCCCTGGGCAGAGTGGGCAGGGGTGTCTGCTGTCAACACCACCAGCTGGGAGCCA

CGCACGAACTTCTCTTTGGTCTGCTTGGCTCCAGACTCTGCCCCCCATGATGTGGTGGTC

AGCGGCATCCCCGGGAGCACGCAGCTGCTGGTGACCTGGCAACCAGGGGACGGGGAACCA

CTGGAACACGTGGTGGACTGGGCTCAAGATGGGGACACTCTAGAAGACCTCAACTGGGTC

CGGCTTCCCCCTGGGAACCTTAGTGCTCTGTTGCCAGGGACTTTCAAAGGAGGGGTGCCC

TATCGGATCACAGTGACAGCTGTCTTTCCTGGGGGCTTGGCCCCCGCCTCCTCGGTCTGG

GCTTTCCGGGAGGAACGAGCACCCCTGGCAGGGCCGACCCTTTGGAGACTCCAGGATGAC

CCCCCAGGGACTCCCACGGTGGCGTGGGAGGAGGTCCCAAGACACCAGCTCCAGGGCCAC

CTTACTCACTACACGTTGTGTTCAAGAAGTGGGACGAACCCCTGCGACTGCACCAATGTG

AGCAGCAGCACCCGGATCGCCACTCTGCCCAACCTTCACTGGGGTCCCTGTGAGCTGTGG

GTGACTGCCTCCACCATTGCAGGACAGGGCCCACCTGGCCCCAGCCTCTGGCTTCACCTA

CCAGATAACACGGTGCCATGGAGAGTTCTGCCGGGTGTCCTGGCCCTGTGGGGGCTGCTC

CTGGTGGGCTGCGGCCTGAGCCTTGTCATGGCTCGAAGGTGCCTCCACCTGCGACAGAAG

GTGCTGCCCCACTGGGTGTGGGAGAAGGTTCCTGACCCCGCCAACAGCAGCGCCGGGCAG

ACCCACGTGGAGGAGGTGTGCCAGGCCCCCCCTCTGACTGACTTGCCTGTCCTGAAGGTA

GAGGAGATGGAGCCACCAGCACCTAAGGAGCCCCCCCAGTCCCCCACCCCACTTCACTCT

GGGTATGAGAAACATTTCCTGCCCACACCAGAGGAACTGGGCCTCCTGAGGCCCCCCAAG

TTTCAAGATCTG

>CL381.Contig2_A-W 1357 2010 PREDICTED: interleukin-27 receptor subunit alpha-like [Nomascus leucogenys]

GCACCCCTGGCAGGGCCCACCCTTTGGAGACTCCAGGATGACCCCCCAGGGACTCCCACG

GTGGCGTGGGAGGAGGTCCCAAGACACCAGCTCCAGGGCCACCTTACTCACTACACGTTG

TGTTCAAGAAGTGGGACGAACCCCTGCGACTGCACCAATGTGAGCAGCAGCACCCGGATC

GCCACTCTGCCCAACCTTCACTGGGGTCCCTGTGAGCTGTGGGTGACTGCCTCCACCATT

GCAGGACAGGGCCCACCTGGCCCCAGCCTCTGGCTTCACCTACCAGATAACACGGTGCCA

TGGAGAGTTCTGCCGGGTGTCCTGGCCCTGTGGGGGCTGCTCCTGGTGGGCTGCGGCCTG

AGCCTTGTCATGGCTCGAAGGTGCCTCCACCTGCGACAGAAGGTGCTGCCCCACTGGGTG

TGGGAGAAGGTTCCTGACCCCGCCAACAGCAGCGCCGGGCAGACCCACGTGGAGGAGGTG

TGCCAGGCCCCCCCTCTGACTGACTTGCCTGTCCTGAAGGTAGAGGAGATGGAGCCACCA

GCACCTAAGGAGCCCCCCCAGTCCCCCACCCCACTTCACTCTGGGTATGAGAAACATTTC

CTGCCCACACCAGAGGAACTGGGCCTCCTGAGGCCCCCCAAGTTTCAAGATCTG

>CL381.Contig3_A-W 116 1912 interleukin-27 receptor subunit alpha precursor [Homo sapiens] >gi|116242518|sp|Q6UWB1.2|I27RA_HUMAN RecName: Full=Interleukin-27 receptor subunit alpha; Short=IL-27 receptor subunit alpha; Short=IL-27R subunit alpha; Short=IL-27R-alpha; Short=IL-27RA; AltName: Full=Cytokine receptor-like 1; AltName: Full=Type I T-cell cytokine receptor; Short=TCCR; AltName: Full=WSX-1; AltName: Full=ZcytoR1; Flags: Precursor >gi|11022747|gb|AAG26090.1|AF106912_1 CRL1 protein [Homo sapiens] >gi|3153241|gb|AAC39755.1| class I cytokine receptor [Homo sapiens] >gi|20380121|gb|AAH28003.1| Interleukin 27 receptor, alpha [Homo sapiens] >gi|117606490|gb|ABK41902.1| interleukin 27 receptor, alpha [Homo sapiens]

GGCGGCCCTGGGCCACTGCAGTGCCACGGAGTTGAACCTTTGGGGAACTTGAACTGCTCA

TGGGAACCTCTTGGGGACCTGGGAGCCCCCTCTATGCTGCACCTCCGGAGCCAGAAATAC

CATTCCAACAGAACCCGGACCGTGGCGGTGCCCTCGGGGCAGAGCTGGGTGACGGTTCCT

CGGGAGCAATTCACCGTGTCTGACACGCTGCTCGTCTGGGGGACCAAGGCAGGCCAGGTC

CTCTGGCCCCCCGTCCTCATCAACCTGGAAACCCGAATGAAGCCCAGAGCCCCCCGGCTG

CGTCCCGAGGTGGACTTTTCAGAGGACGAGCCCCTGGAGGCCACGGTGCAGTGGGAGCCA

CCTACCTGGCCACCTCATAAGGTCCTGATCTGCCAGTTCTACTATCGAAGATGCCCGGAG

GTGGCCTGGACTCCGCTGGAGCCCGAGCTGAAGACGGCACCGCTGACCCCCATCGAGATC

CCAGACCTGGAGCTGGCCACCCGCTACGAGGTGTCCGGCCGCTGCCGGGTGGACACAGAG

GCCGAGCTGTGGGGCGAGAGCAGCCCGGTGCTGTCCTTCCAGACGCTGCCCTCTGCTCCA

AAAGACGTGTGGGTGTCTGGGAACCGGTGTGGGCCCCCGAGCAGACAGGAGCTGCTGCTT

CTGTGGAAGGCCCCCGGGCCCTGCGTGCAGGTGAGCTACAGAGTGTGGTTCTGGGTTGGA

GAGAAGAGTCTGACTCAGGAAGGGGTCCCCTGTTGCAAGTGCTCCATCCCCCCCTGGGCA

GAGTGGGCAGGGGTGTCTGCTGTCAACACCACCAGCTGGGAGCCACGCACGAACTTCTCT

TTGGTCTGCTTGGCTCCAGACTCTGCCCCCCATGATGTGGTGGTCAGCGGCATCCCCGGG

AGCACGCAGCTGCTGGTGACCTGGCAACCAGGGGACGGGGAACCACTGGAACACGTGGTG

GACTGGGCTCAAGATGGGGACACTCTAGAAGACCTCAACTGGGTCCGGCTTCCCCCTGGG

AACCTTAGTGCTCTGTTGCCAGGGACTTTCAAAGGAGGGGTGCCCTATCGGATCACAGTG

ACAGCTGTCTTTCCTGGGGGCTTGGCCCCCGCCTCCTCGGTCTGGGCTTTCCGGGAGGAA

CGAGCACCCCTGGCAGGGCCGACCCTTTGGAGACTCCAGGATGACCCCCCAGGGACTCCC

ACGGTGGCGTGGGAGGAGGTCCCAAGACACCAGCTCCAGGGCCACCTTACTCACTACACG

TTGTGTTCAAGAAGTGGGACGAACCCCTGCGACTGCACCAATGTGAGCAGCAGCACCCGG

ATCGCCACTCTGCCCAACCTTCACTGGGGTCCCTGTGAGCTGTGGGTGACTGCCTCCACC

ATTGCAGGACAGGGCCCACCTGGCCCCAGCCTCTGGCTTCACCTACCAGATAACACGGTG

CCATGGAGAGTTCTGCCGGGTGTCCTGGCCCTGTGGGGGCTGCTCCTGGTGGGCTGCGGC

CTGAGCCTTGTCATGGCTCGAAGGTGCCTCCACCTGCGACAGAAGGTGCTGCCCCACTGG

GTGTGGGAGAAGGTTCCTGACCCCGCCAACAGCAGCGCCGGGCAGACCCACGTGGAGGAG

GTGTGCCAGGCCCCCCCTCTGACTGACTTGCCTGTCCTGAAGGTAGAGGAGATGGAGCCA

CCAGCACCTAAGGAGCCCCCCCAGTCCCCCACCCCACTTCACTCTGGGTATGAGAAACAT

TTCCTGCCCACACCAGAGGAACTGGGCCTCCTGAGGCCCCCCAAGTTTCAAGATCTG

>CL381.Contig4_A-W 1333 1986 PREDICTED: interleukin-27 receptor subunit alpha-like [Nomascus leucogenys]

GCACCCCTGGCAGGGCCCACCCTTTGGAGACTCCAGGATGACCCCCCAGGGACTCCCACG

GTGGCGTGGGAGGAGGTCCCAAGACACCAGCTCCAGGGCCACCTTACTCACTACACGTTG

TGTTCAAGAAGTGGGACGAACCCCTGCGACTGCACCAATGTGAGCAGCAGCACCCGGATC

GCCACTCTGCCCAACCTTCACTGGGGTCCCTGTGAGCTGTGGGTGACTGCCTCCACCATT

GCAGGACAGGGCCCACCTGGCCCCAGCCTCTGGCTTCACCTACCAGATAACACGGTGCCA

TGGAGAGTTCTGCCGGGTGTCCTGGCCCTGTGGGGGCTGCTCCTGGTGGGCTGCGGCCTG

AGCCTTGTCATGGCTCGAAGGTGCCTCCACCTGCGACAGAAGGTGCTGCCCCACTGGGTG

TGGGAGAAGGTTCCTGACCCCGCCAACAGCAGCGCCGGGCAGACCCACGTGGAGGAGGTG

TGCCAGGCCCCCCCTCTGACTGACTTGCCTGTCCTGAAGGTAGAGGAGATGGAGCCACCA

GCACCTAAGGAGCCCCCCCAGTCCCCCACCCCACTTCACTCTGGGTATGAGAAACATTTC

CTGCCCACACCAGAGGAACTGGGCCTCCTGAGGCCCCCCAAGTTTCAAGATCTG

>CL381.Contig5_A-W 353 844 PREDICTED: interleukin-27 receptor subunit alpha-like [Ailuropoda melanoleuca]

TTGTGTTCTTCAGTGAGCAGCAGCACCCGGATCGCCACTCTGCCCAACCTTCACTGGGGT

CCCTGTGAGCTGTGGGTGACTGCCTCCACCATTGCAGGACAGGGCCCACCTGGCCCCAGC

CTCTGGCTTCACCTACCAGATAACACGGTGCCATGGAGAGTTCTGCCGGGTGTCCTGGCC

CTGTGGGGGCTGCTCCTGGTGGGCTGCGGCCTGAGCCTTGTCATGGCTCGAAGGTGCCTC

CACCTGCGACAGAAGGTGCTGCCCCACTGGGTGTGGGAGAAGGTTCCTGACCCCGCCAAC

AGCAGCGCCGGGCAGACCCACGTGGAGGAGGTGTGCCAGGCCCCCCCTCTGACTGACTTG

CCTGTCCTGAAGGTAGAGGAGATGGAGCCACCAGCACCTAAGGAGCCCCCCCAGTCCCCC

ACCCCACTTCACTCTGGGTATGAGAAACATTTCCTGCCCACACCAGAGGAACTGGGCCTC

CTGAGGCCCCCC

>CL381.Contig6_A-W 353 712 PREDICTED: interleukin-27 receptor subunit alpha-like [Ailuropoda melanoleuca]

TTGTGTTCTTCAGTGAGCAGCAGCACCCGGATCGCCACTCTGCCCAACCTTCACTGGGGT

CCCTGTGAGCTGTGGGTGACTGCCTCCACCATTGCAGGACAGGGCCCACCTGGCCCCAGC

CTCTGGCTTCACCTACCAGATAACACGGTGCCATGGAGAGTTCTGCCGGGTGTCCTGGCC

CTGTGGGGGCTGCTCCTGGTGGGCTGCGGCCTGAGCCTTGTCATGGCTCGAAGGTGCCTC

CACCTGCGACAGAAGGTGCTGCCCCACTGGGTGTGGGAGAAGGTTCCTGACCCCGCCAAC

AGCAGCGCCGGGCAGACCCACGTGGAGGTAAGCGCCCGCCCACTGTGCCAGGCGCCAGGG

>CL382.Contig1_A-W 817 1761 minus strand sorting nexin-20 [Macaca mulatta] >gi|383415737|gb|AFH31082.1| sorting nexin-20 isoform 1 [Macaca mulatta] >gi|383415739|gb|AFH31083.1| sorting nexin-20 isoform 1 [Macaca mulatta] >gi|383415741|gb|AFH31084.1| sorting nexin-20 isoform 1 [Macaca mulatta] >gi|383415743|gb|AFH31085.1| sorting nexin-20 isoform 1 [Macaca mulatta]

ATGGCATGTCCTGAGCACCCTGGGAGCCCTGGCTGGACAGGACCTGTAACCCAGTGTGCT

GCTAAGACCAAGCAGGAAGCACCCACCTCGGACCCAGATCTCCCAAGCCCAGGACCTAGA

GAACACTCAGGTGCCCACCCCAGTCCGAGCTCCAACTCCAGCATGACCACCCGGGAGCTG

CAGGAGTACTGGAAGAAGGAGAAGTGCCGCTGGAAGCACGTCAAGCTGCTCTTTGAGATC

GCCTCAGCCCGCATCGAGGAGCGAAAGGTCTCCAAGTTTGTGATGTACCAGATCGTGGTC

ATCCAGACGGGGAGCTTCGACAGCAACAAGGCTGTCCTGGATCGGCGCTACTCGGACTTC

GAGAGGCTGCACAAGAGCCTGCTGAAGACGTTCCGGGAGGAGATGGAGGACGTGGTGTTC

CCCCGGAAGCGCCTGACGGGGAACTTCTCGGAGGAGATGATCTGCGAGCGCAAGCTGGAG

CTCAAGGAGTACCTGGGGCTGCTCTACGCGCTGCGCTGCGTGCGCCGCTCGCGCGAGTTC

GCCGACTTCCTCACGCGGCCCGAGCTGCGCGAGGCCTTCGGCTGCCTGCGCGCCGGCCAG

TACGCGCGAGCGCTCGAGCTGCTGGTGCGCGTGGTGCCGCTGCAGGAGAAGCTCACGGCC

CACTGCCCCACGGCCGCCGTCCCCGCGCTCTGCGCCATGCTCGTGTGCCACCGCGACCTG

GAGCGCCCAGCCGAGGCCTTCGCCGCCGGGGAGCGGGCCCTGCGGTGCCTACAGGCCCGG

GAGGGCCATCGCTACTATGCGCCCCTGCTGGACGCCATGATCCGCCTGGCCTACGTGCTG

GGCAAGGACTTCGTGTCCCTGCAGGGCAGGCTGGAGGAGAGCCAGCTCCGGAAGCCCACC

CACCGGGGCTTCACCCTCAAGGAGCTCACGGTGCGGGAGTATCTG

>CL382.Contig2_A-W 817 1761 minus strand sorting nexin-20 [Macaca mulatta] >gi|383415737|gb|AFH31082.1| sorting nexin-20 isoform 1 [Macaca mulatta] >gi|383415739|gb|AFH31083.1| sorting nexin-20 isoform 1 [Macaca mulatta] >gi|383415741|gb|AFH31084.1| sorting nexin-20 isoform 1 [Macaca mulatta] >gi|383415743|gb|AFH31085.1| sorting nexin-20 isoform 1 [Macaca mulatta]

ATGGCATGTCCTGAGCACCCTGGGAGCCCTGGCTGGACAGGACCTGTAACCCAGTGTGCT

GCTAAGACCAAGCAGGAAGCACCCACCTCGGACCCAGATCTCCCAAGCCCAGGACCTAGA

GAACACTCAGGTGCCCACCCCAGTCCGAGCTCCAACTCCAGCATGACCACCCGGGAGCTG

CAGGAGTACTGGAAGAAGGAGAAGTGCCGCTGGAAGCACGTCAAGCTGCTCTTTGAGATC

GCCTCAGCCCGCATCGAGGAGCGAAAGGTCTCCAAGTTTGTGATGTACCAGATCGTGGTC

ATCCAGACGGGGAGCTTCGACAGCAACAAGGCTGTCCTGGATCGGCGCTACTCGGACTTC

GAGAGGCTGCACAAGAGCCTGCTGAAGACGTTCCGGGAGGAGATGGAGGACGTGGTGTTC

CCCCGGAAGCGCCTGACGGGGAACTTCTCGGAGGAGATGATCTGCGAGCGCAAGCTGGAG

CTCAAGGAGTACCTGGGGCTGCTCTACGCGCTGCGCTGCGTGCGCCGCTCGCGCGAGTTC

GCCGACTTCCTCACGCGGCCCGAGCTGCGCGAGGCCTTCGGCTGCCTGCGCGCCGGCCAG

TACGCGCGAGCGCTCGAGCTGCTGGTGCGCGTGGTGCCGCTGCAGGAGAAGCTCACGGCC

CACTGCCCCACGGCCGCCGTCCCCGCGCTCTGCGCCATGCTCGTGTGCCACCGCGACCTG

GAGCGCCCAGCCGAGGCCTTCGCCGCCGGGGAGCGGGCCCTGCGGTGCCTACAGGCCCGG

GAGGGCCATCGCTACTATGCGCCCCTGCTGGACGCCATGATCCGCCTGGCCTACGTGCTG

GGCAAGGACTTCGTGTCCCTGCAGGGCAGGCTGGAGGAGAGCCAGCTCCGGAAGCCCACC

CACCGGGGCTTCACCCTCAAGGAGCTCACGGTGCGGGAGTATCTG

>CL382.Contig3_A-W 232 540 conserved hypothetical protein [Culex quinquefasciatus] >gi|167875108|gb|EDS38491.1| conserved hypothetical protein [Culex quinquefasciatus]

ATCTACTGTCTATTATCTATCTACCAATCTGTCTATCATCTATCATTACCTATCATCTAC

CATTCATCTATTGTCTATCATCTGTTATCTATCATTATCTATCATCTGTCTATCTATCAT

CTATCATCTACCTATCAAGTTACCTATCTATCACCTATCTATCATCATCATGTATCCTTT

ATCCTATTTTTTCTATTTCTGTACCATCTATCTATAATCTATACATCACCTATCATCTAT

CTATTTATAAGGAAACTTTTACTGACAGCTTATTTGGCCCACTTCTTAGTCACCTAGTTC

TTCATATTC

>CL383.Contig1_A-W 319 930 Mndal [Mus musculus]

GAATACAAGAAAATTGTCCTGCTGGTGGGGCTAGAGGCCATTAAGGATTATGAATTTAGA

TTGATTAAGTCCTTATTGGCCAAAGACTTAAAACTGACTAGAAATATGCAAGAAGATTAT

GACAGAATTAGGATTGCTGACTTGATGGAAGAGAAGTTCCAAAGGGATGCTGGGCTGGGA

AAACTGATAACACTCTGCAAAGACATTCCGGCACTTGCAGACCTCGCGGAAACTCTTAAA

ACTCGAAGGTTGAAAGTTAAAAGCAAATCCAAAGGGGGAAGAAAATCCACAGGGAAAAAA

AAATCTACAGTGAAAAAAGTGGAGCAGGATGAATCTCCTACTGCCCAGTCCAGGTCCACA

ACAAACACAGATTCGGAACCAGAGTCAGAGGAGGAGATACCTTCGTTGAAGGAAAACGAA

ATCAAGAACTCTGAAGACCCTGAGAGAACACAGCTTCCCCCAGAACAGGGTCGGTTTATG

GAACCTTTAGCCACCAGCGAAGAACAAACTGCGGTCTGTCCCCAGACTCTTCATGGGCCT

CCACCAACATCATCCAGCAGTCCTCCTATCAAGCGACCCAACATTGAGAAGTCTGTATCT

TTGGTGAATGAG

>CL383.Contig2_A-W 310 879 interferon-activable protein 203 isoform 2 [Mus musculus]

ATGGTGAACGAATACAAGAAAATTGTCCTGCTGGTGGGGCTAGAGGCCATTAAGGATTAT

GAATTTAGATTGATTAAGTCCTTATTGGCCAAAGACTTAAAACTGACTAGAAATATGCAA

GAAGATTATGACAGAATTAGGATTGCTGACTTGATGGAAGAGAAGTTCCAAAGGGATGCT

GGGCTGGGAAAACTGATAACACTCTGCAAAGACATTCCGGCACTTGCAGACCTCGCGGAA

ACTCTTAAAACTCGGAGGTTGAAAGTTAAAAGGAAATCCAAAGGGGAAAAAAAATCTACA

GGGAAAAAAGTGGAGCAGGATGAATCTCCTACTGCCCAGTCCAGGTCCACAACAAACACA

GATTCGGAACCAGAGTCAGAGGAGGAGATACCTTCGTTGAAGGAAAACGAAATCAAGAAC

TCTGAAGACCCTGAGAGAACACAGCTTCCCCCAGAACAGGGTCGGTTTATGGAACCTTTA

GCCACCAGCGAAGAACAAACTGCGGTCTGTCCCCAGACTCTTCATGGGCCTCCACCAACA

TCATCCAGCAGTCCTCCTATCAAGCGACCC

>CL383.Contig3_A-W 294 905 Mndal [Mus musculus]

GAATACAAGAAAATTGTCCTGCTGGTGGGGCTAGAGGCCATTAAGGATTATGAATTTAGA

TTGATTAAGTCCTTATTGGCCAAAGACTTAAAACTGACTAGAAATATGCAAGAAGATTAT

GACAGAATTAGGATTGCTGACTTGATGGAAGAGAAGTTCCAAAGGGATGCTGGGCTGGGA

AAACTGATAACACTCTGCAAAGACATTCCGGCACTTGCAGACCTCGCGGAAACTCTTAAA

ACTCGAAGGTTGAAAGTTAAAAGCAAATCCAAAGGGGGAAGAAAATCCACAGGGAAAAAA

AAATCTACAGTGAAAAAAGTGGAGCAGGATGAATCTCCTACTGCCCAGTCCAGGTCCACA

ACAAACACAGATTCGGAACCAGAGTCAGAGGAGGAGATACCTTCGTTGAAGGAAAACGAA

ATCAAGAACTCTGAAGACCCTGAGAGAACACAGCTTCCCCCAGAACAGGGTCGGTTTATG

GAACCTTTAGCCACCAGCGAAGAACAAACTGCGGTCTGTCCCCAGACTCTTCATGGGCCT

CCACCAACATCATCCAGCAGTCCTCCTATCAAGCGACCCAACATTGAGAAGTCTGTATCT

TTGGTGAATGAG

>CL383.Contig4_A-W 285 854 interferon-activable protein 203 isoform 2 [Mus musculus]

ATGGTGAACGAATACAAGAAAATTGTCCTGCTGGTGGGGCTAGAGGCCATTAAGGATTAT

GAATTTAGATTGATTAAGTCCTTATTGGCCAAAGACTTAAAACTGACTAGAAATATGCAA

GAAGATTATGACAGAATTAGGATTGCTGACTTGATGGAAGAGAAGTTCCAAAGGGATGCT

GGGCTGGGAAAACTGATAACACTCTGCAAAGACATTCCGGCACTTGCAGACCTCGCGGAA

ACTCTTAAAACTCGGAGGTTGAAAGTTAAAAGGAAATCCAAAGGGGAAAAAAAATCTACA

GGGAAAAAAGTGGAGCAGGATGAATCTCCTACTGCCCAGTCCAGGTCCACAACAAACACA

GATTCGGAACCAGAGTCAGAGGAGGAGATACCTTCGTTGAAGGAAAACGAAATCAAGAAC

TCTGAAGACCCTGAGAGAACACAGCTTCCCCCAGAACAGGGTCGGTTTATGGAACCTTTA

GCCACCAGCGAAGAACAAACTGCGGTCTGTCCCCAGACTCTTCATGGGCCTCCACCAACA

TCATCCAGCAGTCCTCCTATCAAGCGACCC

>CL383.Contig5_A-W 233 844 Mndal [Mus musculus]

GAATACAAGAAAATTGTCCTGCTGGTGGGGCTAGAGGCCATTAAGGATTATGAATTTAGA

TTGATTAAGTCCTTATTGGCCAAAGACTTAAAACTGACTAGAAATATGCAAGAAGATTAT

GACAGAATTAGGATTGCTGACTTGATGGAAGAGAAGTTCCAAAGGGATGCTGGGCTGGGA

AAACTGATAACACTCTGCAAAGACATTCCGGCACTTGCAGACCTCGCGGAAACTCTTAAA

ACTCGAAGGTTGAAAGTTAAAAGCAAATCCAAAGGGGGAAGAAAATCCACAGGGAAAAAA

AAATCTACAGTGAAAAAAGTGGAGCAGGATGAATCTCCTACTGCCCAGTCCAGGTCCACA

ACAAACACAGATTCGGAACCAGAGTCAGAGGAGGAGATACCTTCGTTGAAGGAAAACGAA

ATCAAGAACTCTGAAGACCCTGAGAGAACACAGCTTCCCCCAGAACAGGGTCGGTTTATG

GAACCTTTAGCCACCAGCGAAGAACAAACTGCGGTCTGTCCCCAGACTCTTCATGGGCCT

CCACCAACATCATCCAGCAGTCCTCCTATCAAGCGACCCAACATTGAGAAGTCTGTATCT

TTGGTGAATGAG

>CL383.Contig6_A-W 224 793 interferon-activable protein 203 isoform 2 [Mus musculus]

ATGGTGAACGAATACAAGAAAATTGTCCTGCTGGTGGGGCTAGAGGCCATTAAGGATTAT

GAATTTAGATTGATTAAGTCCTTATTGGCCAAAGACTTAAAACTGACTAGAAATATGCAA

GAAGATTATGACAGAATTAGGATTGCTGACTTGATGGAAGAGAAGTTCCAAAGGGATGCT

GGGCTGGGAAAACTGATAACACTCTGCAAAGACATTCCGGCACTTGCAGACCTCGCGGAA

ACTCTTAAAACTCGGAGGTTGAAAGTTAAAAGGAAATCCAAAGGGGAAAAAAAATCTACA

GGGAAAAAAGTGGAGCAGGATGAATCTCCTACTGCCCAGTCCAGGTCCACAACAAACACA

GATTCGGAACCAGAGTCAGAGGAGGAGATACCTTCGTTGAAGGAAAACGAAATCAAGAAC

TCTGAAGACCCTGAGAGAACACAGCTTCCCCCAGAACAGGGTCGGTTTATGGAACCTTTA

GCCACCAGCGAAGAACAAACTGCGGTCTGTCCCCAGACTCTTCATGGGCCTCCACCAACA

TCATCCAGCAGTCCTCCTATCAAGCGACCC

>CL383.Contig7_A-W 298 618 mCG62472, isoform CRA_b [Mus musculus]

GTAACTTGAGAGATGGTGAACGAATACAAGAAAATTGTCCTGCTGGTGGGGCTAGAGGCC

ATTAAGGATTATGAATTTAGATTGATTAAGTCCTTATTGGCCAAAGACTTAAAACTGACT

AGAAATATGCAAGAAGATTATGACAGAATTAGGATTGCTGACTTGATGGAAGAGAAGTTC

CAAAGGGATGCTGGGCTGGGAAAACTGATAACACTCTGCAAAGACATTCCGGCACTTGCA

GACCTCGCGGAAACTCTTAAAACTCGGAGGTTGAAAGGTAACCAGGAGGCACCCCTGACT

CCCATCCTTTCTGCCCCCACC

>CL383.Contig8_A-W 273 593 mCG62472, isoform CRA_b [Mus musculus]

GTAACTTGAGAGATGGTGAACGAATACAAGAAAATTGTCCTGCTGGTGGGGCTAGAGGCC

ATTAAGGATTATGAATTTAGATTGATTAAGTCCTTATTGGCCAAAGACTTAAAACTGACT

AGAAATATGCAAGAAGATTATGACAGAATTAGGATTGCTGACTTGATGGAAGAGAAGTTC

CAAAGGGATGCTGGGCTGGGAAAACTGATAACACTCTGCAAAGACATTCCGGCACTTGCA

GACCTCGCGGAAACTCTTAAAACTCGGAGGTTGAAAGGTAACCAGGAGGCACCCCTGACT

CCCATCCTTTCTGCCCCCACC

>CL383.Contig9_A-W 212 532 mCG62472, isoform CRA_b [Mus musculus]

GTAACTTGAGAGATGGTGAACGAATACAAGAAAATTGTCCTGCTGGTGGGGCTAGAGGCC

ATTAAGGATTATGAATTTAGATTGATTAAGTCCTTATTGGCCAAAGACTTAAAACTGACT

AGAAATATGCAAGAAGATTATGACAGAATTAGGATTGCTGACTTGATGGAAGAGAAGTTC

CAAAGGGATGCTGGGCTGGGAAAACTGATAACACTCTGCAAAGACATTCCGGCACTTGCA

GACCTCGCGGAAACTCTTAAAACTCGGAGGTTGAAAGGTAACCAGGAGGCACCCCTGACT

CCCATCCTTTCTGCCCCCACC

>CL383.Contig10_A-W 310 1740 PREDICTED: gamma-interferon-inducible protein 16-like [Cavia porcellus]

ATGGTGAACGAATACAAGAAAATTGTCCTGCTGGTGGGGCTAGAGGCCATTAAGGATTAT

GAATTTAGATTGATTAAGTCCTTATTGGCCAAAGACTTAAAACTGACTAGAAATATGCAA

GAAGATTATGACAGAATTAGGATTGCTGACTTGATGGAAGAGAAGTTCCAAAGGGATGCT

GGGCTGGGAAAACTGATAACACTCTGCAAAGACATTCCGGCACTTGCAGACCTCGCGGAA

ACTCTTAAAACTCGGAGGTTGAAAGTTAAAAGGAAATCCAAAGGGGGAAGAAAATCCACA

GGGAAAAAAAAATCTACAGTGAAAAAAGTGGAGCAGGATGAATCTCCTACTGCCCAGTCC

AGGTCCACAACAAACACAGATTCGGAACCAGAGTCAGAGGAGGAGATACCTTCGTTGAAG

CACCAAAAAAGAAAACCTACCTCTGTCAAGAAGAAAGAAAGACTAAAATTGTCTCAAGAA

AACAAAATGAAAAAATCTGAAGACCCTGAGAGAACACAGCTTCCCCCAGAACAGGGTCGG

TTTATGGAACCTTCAGCAACCAGCGAAGAACAAACTGCGGTCTGTCCCCAGACTCCTCAT

GGGGCTCCACCAGAACCATCCAATAGTCCTCCAGTCAAGCGACTCAAGATCAAGAAGTCT

GTACCGTTGGTGAATAAGCAAGAAAAAATTTCTTCAAGTTTTCAGGAGGGGCAGGAAATG

GAAGCCACGAATAGCAAAACAAATAACGTGAAGGCCCAGGAATCTCAGGGACAGCGTCAA

CTTCCACAACTGTCAGAAATTGGCGTAAAACCACCTCAGTGTATTATCCACACTCATCAG

ATGCCTCCAACAGCACCAACCAGCAGTGTCCACACTCTTCAGAAGCCTCCAACAGCACCA

ACCAGCATTGTCCACACTCCTCAGATGCCTCCGACAGCCCCATCTGGCAAAACCAACACC

TTTCACTTACTTTTAACATTACCCAGCGGAGTCCAGACCTCTCATATGTCTCCCCCAGAT

CCATCAGGCAGAATCCAGACTCCTCATTTTTCTCCAACGTCATCCAGAGGAATCCAGGGT

CCTCACACATCTCCAACATCATGCAGCAAAATCCAGACTCCTCAGGTGCCTCCGGCAGCA

CCATCCACCATAATCCAGAATCCACAAGTGGGGCCAGCAACACCATCTGCCAGTCTGCAG

ACTCCTCAGAAGACTGTGACATCTTTCAGCAGTTGCTTACCCACGAATCCAAGATTGAAA

ACTGTACCTAAAGAAGCCTCTGAAGAATGTGGTCACCAGAAGGGCCCCAAAGAAGTAGTG

GTGTTGAAAGTAACAGAACCATTTATTTATGAGTTCAAAGAAGGCGGGAAGAAGATGTTT

CATGCTACAGTGGCCACTGAGAGCGAATTCTTCCGAGTGAAAGTTTTCGAC

>CL383.Contig11_A-W 310 1716 PREDICTED: gamma-interferon-inducible protein 16-like [Cavia porcellus]

ATGGTGAACGAATACAAGAAAATTGTCCTGCTGGTGGGGCTAGAGGCCATTAAGGATTAT

GAATTTAGATTGATTAAGTCCTTATTGGCCAAAGACTTAAAACTGACTAGAAATATGCAA

GAAGATTATGACAGAATTAGGATTGCTGACTTGATGGAAGAGAAGTTCCAAAGGGATGCT

GGGCTGGGAAAACTGATAACACTCTGCAAAGACATTCCGGCACTTGCAGACCTCGCGGAA

ACTCTTAAAACTCGGAGGTTGAAAGTTAAAAGGAAATCCAAAGGGGGAAGAAAATCCACA

GGGAAAAAAAAATCTACAGGGAAAAAAGTGGAGCAGGATGAATCTCCTACTGCCCAGTCC

AGGTCCACAACAAACACAGATTCGGAACCAGAGTCAGAGGAGGAGATACCTTCGTTGAAG

CACCAAAAAAGAAAACCTACCTCTGTCAAGAAGAAAGAAAGACTAAAATTGTCTCAAGAA

AACAAAATGAAAAAATCTGAAGACCCTGAGAGAACACAGCTTCCCCCAGAACAGGGTCGG

TTTATGGAACCTTCAGCAACCAGCGAAGAACAAACTGCGGTCTGTCCCCAGACTCCTCAT

GGGGCTCCACCAGAACCATCCAATAGTCCTCCAGTCAAGCGACTCAAGATCAAGAAGTCT

GTACCGTTGGTGAATAAGCAAGAAAAAATTTCTTCAAGTTTTCAGGAGGGGCAGGAAATG

GAAGCCACGAATAGCAAAACAAATAACGTGAAGGCCCAGGAATCTCAGGGACAGCGTCAA

CTTCCACAACTGTCAGAAATTGGCGTAAAACCACCTCAGTGTATTATCCACACTCATCAG

ATGCCTCCAACAGCACCAACCAGCAGTGTCCACACTCCTCAGATGCCTCCGACAGCCCCA

TCTGGCAAAACCAACACCTTTCACTTACTTTTAACATTACCCAGCGGAGTCCAGACCTCT

CATATGTCTCCCCCAGATCCATCAGGCAGAATCCAGACTCCTCATTTTTCTCCAACGTCA

TCCAGAGGAATCCAGGGTCCTCACACATCTCCAACATCATGCAGCAAAATCCAGACTCCT

CAGGTGCCTCCGGCAGCACCAGCTGGCAGAACCCCCACTCCACAGTTACCTCGAACATCA

CCCAGTGAGTTCCAGACCACTCACGTATCTCTAACATCATCCAGCAGAGTCCAGACCCCT

CACATGTGTCCCGAAGATCCATCAGGCAAAATCCAGACTCCTCATTTTTCTCCAACGTCA

TCCAGAGGAATCCAGGGTCCTCATTCATCTCCAGCATCATCCAGTAGAATCCAAACTCCT

CAGATGCATCCCACAGAACCATCCACCATAATCCAGAATCCACAAGTGGGGCCAGCAACA

CCATCTGCCAGTCTGCAGACTCCTCAG

>CL383.Contig12_A-W 2 286 PREDICTED: gamma-interferon-inducible protein 16-like [Loxodonta africana]

CTGATTAAAAATGCTAATGCAACTCCTAAAATTGAGTATCTTTGCTCAAGAAGCACAACA

AAATACGTGAATGGGGTGTACATGGTGTATAAGAAAAATGTGAGGGAAGATTGCACTTAT

TATGAAATACAAGATGATACCGGGAAGATGGAAGTGGTGGTGTATGGACGGCTGACCTAT

ATCAACTGTGAAGAAGGAGATAAACTTAATCTCTTCTGTTTTGAATTGACCTTGAATGCA

GATAAGTGGCAGCTGAGATCTGTTTTCCACAGTTACATCAAGGTA

>CL383.Contig13_A-W 310 1815 PREDICTED: interferon-activable protein 203 isoform 1 [Sus scrofa]

ATGGTGAACGAATACAAGAAAATTGTCCTGCTGGTGGGGCTAGAGGCCATTAAGGATTAT

GAATTTAGATTGATTAAGTCCTTATTGGCCAAAGACTTAAAACTGACTAGAAATATGCAA

GAAGATTATGACAGAATTAGGATTGCTGACTTGATGGAAGAGAAGTTCCAAAGGGATGCT

GGGCTGGGAAAACTGATAACACTCTGCAAAGACATTCCGGCACTTGCAGACCTCGCGGAA

ACTCTTAAAACTCGGAGGTTGAAAGTTAAAAGGAAATCCAAAGGGGGAAGAAAATCCACA

GGGAAAAAAAAATCTACAGTGAAAAAAGTGGAGCAGGATGAATCTCCTACTGCCCAGTCC

AGGTCCACAACAAACACAGATTCGGAACCAGAGTCAGAGGAGGAGATACCTTCGTTGAAG

GAAAACGAAATCAAGAACTCTGAAGACCCTGAGAGAACACAGCTTCCCCCAGAACAGGGT

CGGTTTATGGAACCTTTAGCCACCAGCGAAGAACAAACTGCGGTCTGTCCCCAGACTCTT

CATGGGCCTCCACCAACATCATCCAGCAGTCCTCCTATCAAGCGACCCAACATTGAGAAG

TCTGTATCTTTGGTGAATGAGCAAGAAAAAATTGCTTCAGATCTTCAAGGCGGGCACGTA

AGAGCACAAGAAATGGACAACACAAACACCCAAACACATGATAACATGAAGGTACAGGAA

TCTCAGGGACAGCATCAACTTCCAGAACTGTCATCATTTGCCACACAAACAACTGTGAGT

ATGTTCCACACTTTTAAGATGGCTCCCAGAACACCATCCTGCAATTTCCAGACTCTTCAG

GTGACTCTAACAGAACCATCCACCAGAATCCAAACTCTGCAGAATCCAGGATTGACAACT

ATACCTACGGAAGCTTCCGAAGAACGTGGTCACCAGAAGGGCCCCAAAGAAGTAGTGGTG

TTGAAAGTAACAGAACCATTTATTTATGAGTTCAAAGAAGGCGGGAAGAAGATGTTTCAT

GCTACAGTGGCCACTGAGAGCGAATTCTTCCGAGTGAAAGTTTTCGACTTCCACTTGAAG

GAAAAATTCATCCCAAAGACAGTTATTGCCATATCCGATTATATTGGCCGCAACGGGTTC

CTGGAGATATACAGTGCTTCAAGTGTGTCTCACGTGAGCGCAGACAGAAAGATGGAGATC

TCGAGTAGACTGATTAAAAATGCTAATGCAACTCCTAAAATTGAGTATCTTTGCTCACAA

TGTACAGTAAAATATGTGAATGGGGTGTACACAGTGTATAAGAAAGATATGAGGGAAGAT

TGCACTTATTATGGAATACGAGATGATACAGGGAACATGGAAGTGGTGGTGTATGGATGG

ATGACCTATGTCAACTGTGAGGAAGGAGATAAAATTAATCTCTTCTGTTTTGAATTGGCT

TTCAATGAAGATAAGTGGCAGCTTAGATCTGTAAGGCACAGCTATATCAAGGTAATCAAG

GCCAGG

>CL384.Contig1_A-W 230 2152 unnamed protein product [Homo sapiens]

ATGGCTCCAGGCCAAGTACCCCATCACAGCACACCCTGGAGGGAAGCCCACTACTCCTAC

CTCCTGTCCCCACTGATGGGCTTCCTCAGCCGGGCCTGGAGCCGGCTGAGAGGCCCAGGA

CCTTCAGAGCCCTGGCTGGTAGAAGCAGTAACTGGAGAAAATCTGGTAGAAGGTGGCCTG

CAGGGAGAAGTGAAGGCTTCTTTGGCCATCTCCCATGGTCCCTGGGCCAGGCATCCTCAA

GGGGAAGCTGAAGACAGTGGAGTACCTCAAGAGAATGGAGAAGCAGACCTGGAGTCCTGC

CTTGATCTGAAGCCCAGTAATTCCCCTCCTGAAGTTTGGGGACTTTTAGGCAGTAAAGAA

AATAGTGAGAAAGATGCAAACAGTGTCCCCAGAGAGCAGGGAAGGGAAATTACAGATGGC

CAGCCGCTGTCCCCCAGCCTGCAGATAAGAGCCCTGCAAGGTTCTGATAAGAGTCCTGGG

GAGGAGAAAGCTGAAGAAGAGGGAGTGACCAAGTTCTCTTATCCTTCATCACATTGGGAG

TGGAGCGATGTCAAGAAAGAAGCTCCTACTGCCTCCACTTTCCCTGAGTCTTCAGGATCC

CAACTTGGCACTTGTGTGTATTGCCCAGAGGAGGAAGAGGATCAAGCCACGGAGGAAAAA

GGAACAGAAAACGAAGCCAGGAAGACCTCCGTTTCCCCCTCATTGGCAGGCTCCAGCTCC

AGGGCTTGGGGATATTGTCCAGGCGAGAGGCCTAGGGAGAAGGGCAAGAAGGCCTGTGAA

GCCCCTGGGAAAGAAGCTGACCCAGAGCCATGGTCCTCGCTTCCAGCCCAGAGGCACCTG

CTTGGGGCTTTGGAGAATCAACCGAGTAAGAACGGAAAGGAAGAGGAGGATGACAGTGCT

TTAGGGGCAACTGAGAAGGGAGGAGCTAAGGGTCCTTCTTCCATTTCAAGTGCCTCCGGG

AGGGCCTGGGTGTGTCAGCCAGGGGAGAATGTGGAGGAAGACGAGAAGAGTGATTCAGGA

TCAGCTGAGGAGGAAGGAGAAACTGAGGCTTCCTCTCCCACCCTCCCTACAAGTGCCTTC

TTGAAAGCCTGGGTGTATCATCCTGGAGAGGACACAGATGAAGATGAGGACAGTGACTGG

GAATCAACTGAGGAGGAGGAAGACGCTGAGGCTTCCTCTCCCACCCCCGCTACCAGTGCC

CTCCTGAAGGCCTGGGTGTATCGTCCTGGGGAGGACACTGAGGAGGAAGATGAGTGTGAG

CAGAATGAGGACAAAGGAGAGGACTGTGGAGCAGCTGCCTGGGGCCAGCACCCCTCCCTT

CAGGCCCAGAGTGCCCACCTCAGGGGCTGGCTATATCAACCTGGAAATGAGACAGAGGAA

GGGGAAGCTGAGGAGGAATGGGGAGACTCCGAGACCAGCCCCTTCAGAGTGGCCTTCTAC

TTACCTGGAGAGAAACCACCACCTCCCTGGACTGCTCCTAAGCTGCCCCTCAGACTACAA

AGACGACTCAGGTTGTCACAGACCCTCACCCAGGATCCAGACCCTGAAACTCCCCCAAAA

TCCAGAAAGGTGCGCTTCTCTGAGAAGGTCACTGTGCATTTCCTGGCTGTCTGGGCAGGC

CCAGCCCAGGCTGCCCGCCGGGGCCCCTGGGAACAGTTCGCTCGGGATCGGAGTCGCTTT

GCCCGCCGCATCGCCCAGGCCCAGGAAAATCTAGGTCCCTGCCTTACCCCTGCCTCCCGG

GCAAGAGCCTGGGCACGCCTTGGGAATGCACCCAGTTCTCTTACCCCCATACCTGCCTCT

ACCCAGACCTTATCTTTCCCCTTGATCTCCTTCGAGGCTCCAGCTCAGGCCACGCCCTTG

AGTCAGGCTATGGCCACACCCCCTCATACCCCTTCTTTGGAAGCACCAGCTCTTAGCCTG

AAT

>CL384.Contig2_A-W 70 426 unnamed protein product [Homo sapiens]

CAGGTGCGCTTCTCTGAGAAGGTCACTGTGCATTTCCTGGCTGTCTGGGCAGGCCCAGCC

CAGGCTGCCCGCCGGGGCCCCTGGGAACAGTTCGCTCGGGATCGGAGTCGCTTTGCCCGC

CGCATCGCCCAGGCCCAGGAAAATCTAGGTCCCTGCCTTACCCCTGCCTCCCGGGCAAGA

GCCTGGGCACGCCTTGGGAATGCACCCAGTTCTCTTACCCCCATACCTGCCTCTACCCAG

ACCTTATCTTTCCCCTTGATCTCCTTCGAGGCTCCAGCTCAGGCCACGCCCTTGAGTCAG

GCTATGGCCACACCCCCTCATACCCCTTCTTTGGAAGCACCAGCTCTTAGCCTGAAT

>CL385.Contig1_A-W 42 1592 minus strand PREDICTED: nucleosome-remodeling factor subunit BPTF [Canis lupus familiaris]

CTGTCCTGCAGGTATAGACTTCAGACTGTTAAGTCCTTAGCAGGAGTGAGCCTGATGTTA

CGGTTACTGTGGGCCAGTTTGAGATGGGATGACATGGCAGCCAAGGCACCTCCAGGAGGA

GGGACTACACGGACAGAAACATCTGAAACGGAAATCACAACAACAGAAATAATTAAGAGG

CGAGATGTTGGTCCTTATGGTATTCGTTCTGAGTATTGTATCAGGAAAATCATTTGCCCC

ATTGGAGTTCCAGAAGCACCAAAAGAAACACCTACCCCTCAGCGGAAAGGCCTTCGATCA

AGTGCATTGCGACCAAAGAGACCAGAAACACCCAAACAAACTGGCCCTGTTATTATTGAA

ACCTGGGTTGCAGAAGAAGAGTTGGAACTATGGGAAATCAGGGCATTTGCTGAGAGAGTG

GAGAAAGAAAAGGCACAAGCAGTCGAGCAGCAGGCTAAGAAACGATTGGAGCAGCAGAAG

CCTACAGTCATTGCAGCTTCTACTACTTCCCCAACAAATAGTACTACCAGCACCATCTCT

CCTGCTCAGAAAGTGATGGTGGCCCCCATAAGTGGCTCAGTTACCACTGGAACCAAAATG

GTATTAACTACTAAAGTTGGATCTCCAGCTACAGTTACATTCCAGCAAAACAAGAATTTC

CATCAAACCTTTGCTACATGGGTTAAGCAAGGCCAGTCAAATTCAGGTGTTGTTCAAGTA

CAGCAGAAAGTACTGGGTATCATTCCATCAAGTACAGGTACAAGTCAGCAAACCTTTACT

TCATTCCAGCCCAGGACAGCAACAGTCACAATTAGGCCTAATACCTCAGGCTCTGGAGGA

ACCACCAGCACGTCACAAGTAATCACAGGGCCTCAGATCCGCCCTGGTATGACGGTGATT

AGAACACCACTCCAACAGTCAACACTAGGAAAGGCAATTATTCGAACACCTGTGATGGTA

CAGCCAGGCACTCCCCAGCAAGTGGTGACTCAGATCATCAGAGGTCAGCCTGTTTCCACT

GCCATCTCTGCCCCAAGCCCAGCTTCCTCAACACCTGGACAGAAAGGAGCATCAACTGCA

AGTCTACAGTCTCCAACTCCACAGTCCCCTCGCCCCCAACAAGGACAGGTGAAGCTTACC

ATGGCTCAGCTTACTCAGTTAACTCAGGGCCACGGTGGCAATCAAGGTTTGACAGTAGTA

ATTCAAGGACAAGGTCAAACTACTGGACAGTTGCAGTTAATACCTCAAGGGGTGACTATA

CTCCCAGGTCCAGGACAGCAGCTCATGCAAGCTGCAATGCCAAATGGCACCGTTCAGAGG

TTCCTCTTTACCCCATTGGCAACAACAGCCACGGCAGCCAGTACTACCACTACCACGGTG

TCCAGTGCGGCAGCAGGTTCAGGTGAACAAAAACAGAATAAATCATCACCCCAGACACAG

GTGCAACAAGCCAAAACCCTGCCACCAGCTCAGTCATCCAGTGTGAGTCCTGCAGAAGCC

CAGCCACAGACTGCTCAGCCTTCAGCGCAGCCCCAGCCCCAGCCCCAGCCC

>CL385.Contig2_A-W 88 672 minus strand PREDICTED: LOW QUALITY PROTEIN: nucleosome-remodeling factor subunit BPTF-like [Callithrix jacchus]

GGCACTCCCCAGCAAGTGGTGACTCAGATCATCAGAGGTCAGCCTGTTTCCACTGCCATC

TCTGCCCCAAGCCCAGCTTCCTCAACACCTGGACAGAAAGGAGCATCAACTGCAAGTCTA

CAGTCTCCAACTCCACAGTCCCCTCGCCCCCAACAAGGACAGGTGAAGCTTACCATGGCT

CAGCTTACTCAGTTAACTCAGGGCCACGGTGGCAATCAAGGTTTGACAGTAGTAATTCAA

GGACAAGGTCAAACTACTGGACAGTTGCAGTTAATACCTCAAGGGGTGACTATACTCCCA

GGTCCAGGACAGCAGCTCATGCAAGCTGCAATGCCAAATGGCACCGTTCAGAGGTTCCTC

TTTACCCCATTGGCAACAACAGCCACGGCAGCCAGTACTACCACTACCACGGTGTCCAGT

GCGGCAGCAGGTTCAGGTGAACAAAAACAGAATAAATCATCACCCCAGACACAGGTGCAA

CAAGCCAAAACCCTGCCACCAGCTCAGTCATCCAGTGTGAGTCCTGCAGAAGCCCAGCCA

CAGACTGCTCAGCCTTCAGCGCAGCCCCAGCCCCAGCCCCAGCCC

>CL385.Contig3_A-W 2 2170 minus strand PREDICTED: nucleosome-remodeling factor subunit BPTF [Canis lupus familiaris]

AGTAGTGATTTTATTGATGAAAATGGTCTGCCCTCCAACAAAGATGAAAACGTTAATGGA

GAATCTAAAAGAAAAACAGTCATCACAGAAGTTACCACGATGACATCCACAGTGGCCACA

GAGTCAAAAACAGTAATCAAGGTAGCAAAAGGTGATAAGCAAACTGTAGTCTCTTCCACA

GAAAATTGTGCCAAGTCCACTGTCACAACCACCACGACAGTAACAAAGCTATCCACCCCC

TCCACTGGCAGCAGTGTGGACATCATCTCTGTAAAGGAGCAGAGCAAAACTGTGGTCACC

ACGACAGTGACAGACTCACTGACTACTGCAGGAGGCACACTGGTAACATCCATGACTGTG

AGCAAAGAGTACTCCACAAGAGACAAGGTGAAACTGATGAAATTTTCAAGACCGAAGAAG

ACTCGTTCAGGTACAGCTCTACCATCCTATAGAAAGTTTATTACCAAGAGCAGCAAGAAG

AGCATATTTGTTTTGCCTAATGATGACTTAAAAAAGTTGGCAAGAAAAGGAGGAATCCGA

GAAGTCCCTTATTTTAATTATAATGCCAAACCTGCCTTGGACATATGGCCGTATCCTTCT

CCTAGACCAACCTTTGGTATCACATGGAGGTATAGACTTCAGACTGTTAAGTCCTTAGCA

GGAGTGAGCCTGATGTTACGGTTACTGTGGGCCAGTTTGAGATGGGATGACATGGCAGCC

AAGGCACCTCCAGGAGGAGGGACTACACGGACAGAAACATCTGAAACGGAAATCACAACA

ACAGAAATAATTAAGAGGCGAGATGTTGGTCCTTATGGTATTCGTTCTGAGTATTGTATC

AGGAAAATCATTTGCCCCATTGGAGTTCCAGAAGCACCAAAAGAAACACCTACCCCTCAG

CGGAAAGGCCTTCGATCAAGTGCATTGCGACCAAAGAGACCAGAAACACCCAAACAAACT

GGCCCTGTTATTATTGAAACCTGGGTTGCAGAAGAAGAGTTGGAACTATGGGAAATCAGG

GCATTTGCTGAGAGAGTGGAGAAAGAAAAGGCACAAGCAGTCGAGCAGCAGGCTAAGAAA

CGATTGGAGCAGCAGAAGCCTACAGTCATTGCAGCTTCTACTACTTCCCCAACAAATAGT

ACTACCAGCACCATCTCTCCTGCTCAGAAAGTGATGGTGGCCCCCATAAGTGGCTCAGTT

ACCACTGGAACCAAAATGGTATTAACTACTAAAGTTGGATCTCCAGCTACAGTTACATTC

CAGCAAAACAAGAATTTCCATCAAACCTTTGCTACATGGGTTAAGCAAGGCCAGTCAAAT

TCAGGTGTTGTTCAAGTACAGCAGAAAGTACTGGGTATCATTCCATCAAGTACAGGTACA

AGTCAGCAAACCTTTACTTCATTCCAGCCCAGGACAGCAACAGTCACAATTAGGCCTAAT

ACCTCAGGCTCTGGAGGAACCACCAGCACGTCACAAGTAATCACAGGGCCTCAGATCCGC

CCTGGTATGACGGTGATTAGAACACCACTCCAACAGTCAACACTAGGAAAGGCAATTATT

CGAACACCTGTGATGGTACAGCCAGGCACTCCCCAGCAAGTGGTGACTCAGATCATCAGA

GGTCAGCCTGTTTCCACTGCCATCTCTGCCCCAAGCCCAGCTTCCTCAACACCTGGACAG

AAAGGAGCATCAACTGCAAGTCTACAGTCTCCAACTCCACAGTCCCCTCGCCCCCAACAA

GGACAGGTGAAGCTTACCATGGCTCAGCTTACTCAGTTAACTCAGGGCCACGGTGGCAAT

CAAGGTTTGACAGTAGTAATTCAAGGACAAGGTCAAACTACTGGACAGTTGCAGTTAATA

CCTCAAGGGGTGACTATACTCCCAGGTCCAGGACAGCAGCTCATGCAAGCTGCAATGCCA

AATGGCACCGTTCAGAGGTTCCTCTTTACCCCATTGGCAACAACAGCCACGGCAGCCAGT

ACTACCACTACCACGGTGTCCAGTGCGGCAGCAGGTTCAGGTGAACAAAAACAGAATAAA

TCATCACCCCAGACACAGGTGCAACAAGCCAAAACCCTGCCACCAGCTCAGTCATCCAGT

GTGAGTCCTGCAGAAGCCCAGCCACAGACTGCTCAGCCTTCAGCGCAGCCCCAGCCCCAG

CCCCAGCCC

>CL386.Contig1_A-W 1 360 minus strand PREDICTED: pregnancy zone protein [Papio anubis]

GAAGGTATTGAACAAGAGGAGACCTTCAACTCTATGGCCTGTGCTTCAGGTACTAAAGTG

TCTGAGCTGTTATCCCTGAAGCTTTCACCAAATGTGGTCAAAGAATCTGCCAGAGCCTCT

GTCTCAGTTGTAGGTGACATACTGGGTTCCACTATGCAAAATATACAAAATCTCCTCCAG

ATGCCCTATGGCTGTGGAGAACAAAACATGGTCCTATTTGCTCCTAACATCTATATCTTG

AATTATCTGAATGAAACCCAACAGCTAACTCAGGAGATCAAGTCCAAGGCAATTGGTTAT

CTCATTGGTGGTTACCAGAGACAGCTGAACTATAAACACCAAGATGGCTCTTATAGCACC

>CL386.Contig2_A-W 161 4342 PREDICTED: alpha-2-macroglobulin isoform 3 [Macaca mulatta]

TTTCAGGTTCCACAGTCTTCATCCAGTGAGGAGGTAATGTTCCTCACTGTCCAAGTGAAA

GGATCAACTCAAGAATTCAGGAGGCGGAACACAGTGGTGGTTAAAAACCAAGAGAGTCTG

GTCTTTGTCCAAACAGATAAACCTATCTACAAACCAGGACAGACAGTGAAGTTTCGCATT

GTCTCATTGGATGAAAACTTTCACCCCCTGAATGAGTTGATACCACTACTATATATTAAG

GATCCCAAAGGAAATCGCATTATACAATGGCAGAATCTCAAGCTAGAGAGTGGTCTCAAA

CAACTGTCCTTTCCCCTCTCATCAGAGCCCCTTCAGGGCATCTACAATGTTGTGGTACAC

AAGGAATCAAGTGGAACAACAGAACACCCCTTTGCTGTGGAGGAATTTGTGCTTCCCAAG

TTTGAAGTGCAAGTAACAATGCCAAAGATAATCACTATCATGGAAGAAGAGGTGAACGTG

TCAGTGTGTGGCCTATACACATATGGGAAGCCTGTCCCAGGACATGTGACTATGAGCATT

TGCAGAAAGTATAATAATCCTTCTAACTGTTTTGGTGAAGAGTCAGAAGCTTTCTGTGAG

AAATTCAGCCAGAAGCTCAACAGCCATGGCTGCTTCTTCCAACAAGTAAAAGCCAGTATC

TTCCACCTGAAGAAGACAGGGTATGAAATGCAACTGCAGGTGGAGGCCAAGATCCAAGAA

GAAGGAACTGGTGTGGAAGTAACAGGAAGAGGGTCCAGTGAAATCACAAACATCATAACC

AAGCTCTCATTTGTGACAGTGGACTCACATGTTAGACAAGGGATCCCCTTCTTTGGACAG

GTGCGCCTTGTGGATGGGAAAGGCATCCCTATGCCAAATAAAATCATCTTCATCACAGCA

AATGAAGCTAACTATCACTCAAATGCTACCACTAATGAGGATGGCCTGGTGCAGTTCTCC

ATCAACACCACCAACATTATAGATACCTCTCTTAGTATCATGGTCAAACACAAGGATCAC

AGCCCCTGCTATAACCACCAGTGGCTCTCAGAGGAAAATGAGAATGCATATCACACTGCT

AATGTTGTATTCTCCCTGAGCAAAAGCTTTATCCACCTTGAACCTATTCCTGGTAAACTG

CCATGTGGCCAATCTCAGACAGTCCAGGCACATTATCTTCTAAATGAACAGGTCCTACAG

GAGCTGAAGCAGCTCGTCTTCTATTATCTGATAATGGCAAAGGGAGGCATTGTCCGAACT

GGGACTCATGTCCTGCCTGTGGAGCAGGGAGACATGAAAGGCCATTTTTCCGTGACCTTC

CTTGTGGAGTCAGACTTGGCTCCAATTGCTCGATTGTTAATCTATGCCATTTTACCTGAT

GGGGAAATGGTTGGAGATTCTGCAAAATATGAGATTGAAAATTGTCTGGCAAACAAGGTG

GATTTGAGCTTCAGACCAGTACAAACTCTTCCAGCCTCACATGCCCAGCTGCGAGTCACA

GCTTCTCCTCAGTCCCTCTGTGCTCTCCGAGCTGTGGACCAAAGTGTGCTGCTCATGAGG

CCTGAAGCTGAGCTCTCTGCATCCTCGGTCTACAATCTTCTAACTGTGAGGGATCTCACG

GATTTTCCCAACAATTTAGACCAGCAGGAGGAAGAGCAAACAAGCTGTGTGCATTCTTTC

ATCATTCGTGGAGGATCGGTCTATGTTCCCTTATCAAGTTATGATGAAGAAGATGTTTAT

AATTTCCTCAAGGGTATGGGATTAAAGGTGTTCACCAACTCAAAAATTCGAAAAATATCG

TCTTGCTCAGTTACCCACACACCTACAAGAGAAGCTGGAAGAGGATTTCCTGGAGCAGGA

TCATTAGCCATGCCATATATTCCTCAAGCAGCTTCATTTAATATGAGAACTTCAAATAAT

ATGCAAAATCCAGAGCCAGTCCTTGAAACTGTGCGGAGCTATTTCCCTGAGACCTGGATC

TGGGAGTTAGCAGTCGTAGACTCATCCGGTATGGCTGAAGTAGAAAAAACTGTCCCTGAC

ACCATCACTGAGTGGAAGGCAGGGGCCCTCTGCCTGTCCAATGACACTGGGCTTGGTCTC

TCTCCCACTGCCTCTCTCCGAGTCTTCCAGCCCTTCTTTGTGGAGCTCACAATGCCCTAC

TCTGTGGTCCGTGGAGAGGCCTTCACGCTCAAGGCCACTGTGTTGAACTACCTTCCCAAA

TGCATCCGGGTCCGCATACAGCTGGAAACCTCCCCCACATTCCTAGCTGTCCCAGTGGAA

AAGGAACAAGGGTCTCATTGCATCTGTGGGGATGGGCGGCACACTGTGTCCTGGGAAGTA

ACTCCAAAGTCATTAGGAAATGTGAATTTCACTGTGAGTGCAGAAGCACTGGAGTCTCAG

GAGCTCTGTGGGAGTGAAGTGGCTATGGTCCCTAAATATGGAAAGAAAGACACAATCATC

AAGCTACTGTTGGTTGAACCTGAAGGACTAGAGAAGGAAGTAACATTCAACTCACTCCTT

TGTCCCTCAGGCGCCGAGGTATCTGAACAATTATCCCTGAAGTTGCCACCAAATGTAGTA

GAGGAATCTGCCCGAGCCTCTGTCTCAGTATTGGGAGATATATTGGGCTCTGCAATGCAA

AACACACAGAATCTCCTCCAGATGCCTTATGGTTGTGGAGAACAGAATATGGTTCTCTTT

GCTCCTAACATCTATGTTCTGGATTATCTAAATGAAACACAGCAGCTGACTCCAGAGATC

AAATCCAAAGCCATTGGCTATCTCAACACTGGTTACCAAAGACAGTTAAACTACAAGCAC

CAAGATGGCTCTTATAGCACCTTTGGGGAGAAATATGGCAGGAACCAGGGCAACACCTGG

CTCACAGCCTTTGTACTGAAGACTTTTGCCCAGGCTCGAAAATATATCTTCATTGATGAA

ACACACATTACCCAAGCCCTCACCTGGCTCTCCCAGAAGCAGAAGGACAATGGCTGCTTC

AGGAGCTCTGGGTCACTGCTCAACAATGCCATAAAGGGAGGAGTAGAAGATGAAGTCACC

CTCTCTGCCTATATCACCATAGCCCTTCTGGAGATTCCACTCCCAGTCACTCATCCTGTT

GTCCGCAATGCCTTGTTTTGCCTGGATTCAGCCTGGAAGTCGGCAAAGGAAGGAGCCCAT

GGCACCCATGTCTACACCAAGGCACTTTTGGCCTATGCATTTGCCCTGGCAGATAACCAG

GACAAGAGGAAAGAAGTACTGAACTCACTTGATGAGGAAGCTGTAAAAGAAGATAATACT

ATCCACTGGACACGGCCTCAGAAACCCAAGGCACCACAGGGACTTTTGTATCAACCCCAG

GCTCCCTCTGCTGAGGTAGAGATGACGGCCTATGTACTCCTCACTCTCCTCACAGTCCAG

CCGGCCCCGACCTCAAAGGACCTGACTTCTGCAGTGCCCATTGTAAAGTGGATCACAAAG

CAACAGAATTCCCATGGTGGTTTCTCCTCCACCCAGGACACAGTGGTGACTCTCCATGCT

TTGTCCAAATATGGAGCAGCTACTTTTACTAGGACTGGGAAAGCTGCACAGGTCACCATC

CAGTCTTCAGGGACATTTACCTCAAAATTCCAAGTGGACAACAACAACCGCCTGTTACTA

CAGCGGGTCTCCTTACCAACAGTGCCTGGGGAATACAGTGCAAGAGTGACGGGAGAAGGA

TGTGTCTACCTCCAGACATCCTTGAAATACAATATTCTCCCAGACAAGGAAGACTTCCCA

TTTGCTTTAGAGGTACTGACTCTTCCCCAAACTTGTGATGGACCCAAAAGCCACACCACC

TTCCAGATCTCAATGAATATCAGATACACTGGGAGTCGTCCTGCCTCCAACATGGTCATT

GCTGATGTAAAGATGGTTTCTGGCTTCATCCCCCTGAAACCAACAGTGAAAATGCTTGAA

AGACTGAACCATGTGAACCGGACTGAAGTCAGCAACAATCATGTCTTGATTTACCTGGAT

AAGGTGTCAAATCAGACACTGAACTTGTCCTTCACAGTTCTGCAAGATATTCCAGTAAGA

GATCTGAAACCAGCCATAGTGAAAGTCTATGATTACTATGAGACAGATGAGTTTGCAATT

GCTGAGTACAGTGCTCCTTGCAGCAAAGATCATGGAAATGCC

>CL386.Contig3_A-W 161 4330 PREDICTED: alpha-2-macroglobulin isoform 3 [Macaca mulatta]

TTTCAGGTTCCACAGTCTTCATCCAGTGAGGAGGTAATGTTCCTCACTGTCCAAGTGAAA

GGATCAACTCAAGAATTCAGGAGGCGGAACACAGTGGTGGTTAAAAACCAAGAGAGTCTG

GTCTTTGTCCAAACAGATAAACCTATCTACAAACCAGGACAGACAGTGAAGTTTCGCATT

GTCTCATTGGATGAAAACTTTCACCCCCTGAATGAGTTGATACCACTACTATATATTAAG

GATCCCAAAGGAAATCGCATTATACAATGGCAGAATCTCAAGCTAGAGAGTGGTCTCAAA

CAACTGTCCTTTCCCCTCTCATCAGAGCCCCTTCAGGGCATCTACAATGTTGTGGTACAC

AAGGAATCAAGTGGAACAACAGAACACCCCTTTGCTGTGGAGGAATTTGTGCTTCCCAAG

TTTGAAGTGCAAGTAACAATGCCAAAGATAATCACTATCATGGAAGAAGAGGTGAACGTG

TCAGTGTGTGGCCTATACACATATGGGAAGCCTGTCCCAGGACATGTGACTATGAGCATT

TGCAGAAAGTATAATAATCCTTCTAACTGTTTTGGTGAAGAGTCAGAAGCTTTCTGTGAG

AAATTCAGCCAGAAGCTCAACAGCCATGGCTGCTTCTTCCAACAAGTAAAAGCCAGTATC

TTCCACCTGAAGAAGACAGGGTATGAAATGCAACTGCAGGTGGAGGCCAAGATCCAAGAA

GAAGGAACTGGTGTGGAAGTAACAGGAAGAGGGTCCAGTGAAATCACAAACATCATAACC

AAGCTCTCATTTGTGACAGTGGACTCACATGTTAGACAAGGGATCCCCTTCTTTGGACAG

GTGCGCCTTGTGGATGGGAAAGGCATCCCTATGCCAAATAAAATCATCTTCATCACAGCA

AATGAAGCTAACTATCACTCAAATGCTACCACTAATGAGGATGGCCTGGTGCAGTTCTCC

ATCAACACCACCAACATTATAGATACCTCTCTTAGTATCATGGTCAAACACAAGGATCAC

AGCCCCTGCTATAACCACCAGTGGCTCTCAGAGGAAAATGAGAATGCATATCACACTGCT

AATGTTGTATTCTCCCTGAGCAAAAGCTTTATCCACCTTGAACCTATTCCTGGTAAACTG

CCATGTGGCCAATCTCAGACAGTCCAGGCACATTATCTTCTAAATGAACAGGTCCTACAG

GAGCTGAAGCAGCTCGTCTTCTATTATCTGATAATGGCAAAGGGAGGCATTGTCCGAACT

GGGACTCATGTCCTGCCTGTGGAGCAGGGAGACATGAAAGGCCATTTTTCCGTGACCTTC

CTTGTGGAGTCAGACTTGGCTCCAATTGCTCGATTGTTAATCTATGCCATTTTACCTGAT

GGGGAAATGGTTGGAGATTCTGCAAAATATGAGATTGAAAATTGTCTGGCAAACAAGGTG

GATTTGAGCTTCAGACCAGTACAAACTCTTCCAGCCTCACATGCCCAGCTGCGAGTCACA

GCTTCTCCTCAGTCCCTCTGTGCTCTCCGAGCTGTGGACCAAAGTGTGCTGCTCATGAGG

CCTGAAGCTGAGCTCTCTGCATCCTCGGTCTACAATCTTCTAACTGTGAGGGATCTCACG

GATTTTCCCAACAATTTAGACCAGCAGGAGGAAGAGCAAACAAGCTGTGTGCATTCTTTC

ATCATTCGTGGAGGATCGGTCTATGTTCCCTTATCAAGTTATGATGAAGAAGATGTTTAT

AATTTCCTCAAGGGTATGGGATTAAAGGTGTTCACCAACTCAAAAATTCGAAAAATATCG

TCTTGCTCAGTTACCCACACACCTACAAGAGAAGCTGGAAGAGGATTTCCTGGAGCAGGA

TCATTAGCCATGCCATATATTCCTCAAGCAGCTTCATTTAATATGAGAACTTCAAATAAT

ATGCAAAATCCAGAGCCAGTCCTTGAAACTGTGCGGAGCTATTTCCCTGAGACCTGGATC

TGGGAGTTAGCAGTCGTAGACTCATCCGGTATGGCTGAAGTAGAAAAAACTGTCCCTGAC

ACCATCACTGAGTGGAAGGCAGGGGCCCTCTGCCTGTCCAATGACACTGGGCTTGGTCTC

TCTCCCACTGCCTCTCTCCGAGTCTTCCAGCCCTTCTTTGTGGAGCTCACAATGCCCTAC

TCTGTGGTCCGTGGAGAGGCCTTCACGCTCAAGGCCACTGTGTTGAACTACCTTCCCAAA

TGCATCCGGGTCCGCATACAGCTGGAAACCTCCCCCACATTCCTAGCTGTCCCAGTGGAA

AAGGAACAAGGGTCTCATTGCATCTGTGGGGATGGGCGGCACACTGTGTCCTGGGAAGTA

ACTCCAAAGTCATTAGGAAATGTGAATTTCACTGTGAGTGCAGAAGCACTGGAGTCTCAG

GAGCTCTGTGGGAGTGAAGTGGCTATGGTCCCTAAATATGGAAAGAAAGACACAATCATC

AAGCTACTGTTGGTTGAACCTGAAGGACTAGAGAAGGAAGTAACATTCAACTCACTCCTT

TGTCCCTCAGGCGCCGAGGTATCTGAACAATTATCCCTGAAGTTGCCACCAAATGTAGTA

GAGGAATCTGCCCGAGCCTCTGTCTCAGTATTGGGAGATATATTGGGCTCTGCAATGCAA

AACACACAGAATCTCCTCCAGATGCCTTATGGTTGTGGAGAACAGAATATGGTTCTCTTT

GCTCCTAACATCTATGTTCTGGATTATCTAAATGAAACACAGCAGCTGACTCCAGAGATC

AAATCCAAAGCCATTGGCTATCTCAACACTGGTTACCAAAGACAGTTAAACTACAAGCAC

CAAGATGGCTCTTATAGCACCTTTGGGGAGAAATATGGCAGGAACCAGGGCAACACCTGG

CTCACAGCCTTTGTACTGAAGACTTTTGCCCAGGCTCGAAAATATATCTTCATTGATGAA

ACACACATTACCCAAGCCCTCACCTGGCTCTCCCAGAAGCAGAAGGACAATGGCTGCTTC

AGGAGCTCTGGGTCACTGCTCAACAATGCCATAAAGGGAGGAGTAGAAGATGAAGTCACC

CTCTCTGCCTATATCACCATAGCCCTTCTGGAGATTCCACTCCCAGTCACTCATCCTGTT

GTCCGCAATGCCTTGTTTTGCCTGGATTCAGCCTGGAAGTCGGCAAAGGAAGGAGCCCAT

GGCACCCATGTCTACACCAAGGCACTTTTGGCCTATGCATTTGCCCTGGCAGATAACCAG

GACAAGAGGAAAGAAGTACTGAACTCACTTGATGAGGAAGCTGTAAAAGAAGATAATACT

ATCCACTGGACACGGCCTCAGAAACCCAAGGCACCACAGGGACTTTTGTATCAACCCCAG

GCTCCCTCTGCTGAGGTAGAGATGACGGCCTATGTACTCCTCACTCTCCTCACAGTCCAG

CCGGCCCCGACCTCAAAGGACCTGACTTCTGCAGTGCCCATTGTAAAGTGGATCACAAAG

CAACAGAATTCCCATGGTGGTTTCTCCTCCACCCAGGACACAGTGGTGGCTCTGCATGCC

CTATCCAGATATGGAGCAGCTACTTTCACCAAAACTGAGAAAACTGCACAGGTTACCATT

CAGAACTCCAGGGCCTTTTCCACAAATTTCCAGGTGGACAACAACAACCCCCTGCTACTA

CAGCAGATCTCATTGCCAGAGCTGCCTGGGGACTATACCATAACAATAACTGGGGAAAGA

TGTGTATATCTTCAGACATCCATGAAATACAATATTCATCCAAAGAAGGAGGATTCCCCA

TTTTCTCTGAAGGTCCAGACTCTACCTGAAATTTGTGATGGACCCAAAGCCCACACCAGC

TTCCAGATCTCACTGAATATTAGTTACACAGGGAGCCGTCCTGTTTCCAATATGGTGATT

GCTGATATAAAAATGATATCTGGTTTTATTCCCCTGAAACCAACAGTAAAAATGCTTGAA

AGATCTAGCTACGTGAGCAGGACAGAAGTGAGCAACAATCATGTCCTTATTTATGTGGAT

CAGGTGACAAATCAGACACTAAGTTTTTCCTTCACGGTTTTGCAAGACATCCCAGTAAGA

GACCTGAAGCCAGCAATTGTTAAAGTCTATGACTACTATGAAACAGATGAGTTTTCTATT

GCTGAGTATATCAGTCCCTGTAGTACAGAT

>CL386.Contig4_A-W 161 4306 PREDICTED: alpha-2-macroglobulin isoform 3 [Macaca mulatta]

TTTCAGGTTCCACAGTCTTCATCCAGTGAGGAGGTAATGTTCCTCACTGTCCAAGTGAAA

GGATCAACTCAAGAATTCAGGAGGCGGAACACAGTGGTGGTTAAAAACCAAGAGAGTCTG

GTCTTTGTCCAAACAGATAAACCTATCTACAAACCAGGACAGACAGTGAAGTTTCGCATT

GTCTCATTGGATGAAAACTTTCACCCCCTGAATGAGTTGATACCACTACTATATATTAAG

GATCCCAAAGGAAATCGCATTATACAATGGCAGAATCTCAAGCTAGAGAGTGGTCTCAAA

CAACTGTCCTTTCCCCTCTCATCAGAGCCCCTTCAGGGCATCTACAATGTTGTGGTACAC

AAGGAATCAAGTGGAACAACAGAACACCCCTTTGCTGTGGAGGAATTTGTGCTTCCCAAG

TTTGAAGTGCAAGTAACAATGCCAAAGATAATCACTATCATGGAAGAAGAGGTGAACGTG

TCAGTGTGTGGCCTATACACATATGGGAAGCCTGTCCCAGGACATGTGACTATGAGCATT

TGCAGAAAGTATAATAATCCTTCTAACTGTTTTGGTGAAGAGTCAGAAGCTTTCTGTGAG

AAATTCAGCCAGAAGCTCAACAGCCATGGCTGCTTCTTCCAACAAGTAAAAGCCAGTATC

TTCCACCTGAAGAAGACAGGGTATGAAATGCAACTGCAGGTGGAGGCCAAGATCCAAGAA

GAAGGAACTGGTGTGGAAGTAACAGGAAGAGGGTCCAGTGAAATCACAAACATCATAACC

AAGCTCTCATTTGTGACAGTGGACTCACATGTTAGACAAGGGATCCCCTTCTTTGGACAG

GTGCGCCTTGTGGATGGGAAAGGCATCCCTATGCCAAATAAAATCATCTTCATCACAGCA

AATGAAGCTAACTATCACTCAAATGCTACCACTAATGAGGATGGCCTGGTGCAGTTCTCC

ATCAACACCACCAACATTATAGATACCTCTCTTAGTATCATGGTCAAACACAAGGATCAC

AGCCCCTGCTATAACCACCAGTGGCTCTCAGAGGAAAATGAGAATGCATATCACACTGCT

AATGTTGTATTCTCCCTGAGCAAAAGCTTTATCCACCTTGAACCTATTCCTGGTAAACTG

CCATGTGGCCAATCTCAGACAGTCCAGGCACATTATCTTCTAAATGAACAGGTCCTACAG

GAGCTGAAGCAGCTCGTCTTCTATTATCTGATAATGGCAAAGGGAGGCATTGTCCGAACT

GGGACTCATGTCCTGCCTGTGGAGCAGGGAGACATGAAAGGCCATTTTTCCGTGACCTTC

CTTGTGGAGTCAGACTTGGCTCCAATTGCTCGATTGTTAATCTATGCCATTTTACCTGAT

GGGGAAATGGTTGGAGATTCTGCAAAATATGAGATTGAAAATTGTCTGGCAAACAAGGTG

GATTTGAGCTTCAGACCAGTACAAACTCTTCCAGCCTCACATGCCCAGCTGCGAGTCACA

GCTTCTCCTCAGTCCCTCTGTGCTCTCCGAGCTGTGGACCAAAGTGTGCTGCTCATGAGG

CCCGAGGCTGAGCTCTCTGCATCATCGGTTTATAAGCTTTTACCAGTGAAGGACCTCACA

GGCATCCCTGAAAGTTTGAATGAGGCTGAAGATCATGGAGACTGTGTCAATCGTGATAAT

GTCTATATTAATGGAATCCTGTATTCCCCAGTGTCAAATACAAATGAAAAAGATATGTAT

AGCTTACTAAAGGATATGGGCTTAAGAGTATTCACAAACTCAAAGATTCATAAACCAAAA

GTATGTACACAGACTATAGAGTATCAAGCGAGATTTTCAACATATGACGCACCAGTTGCA

CTTTACAGAAGCCAAATTCAGGAGATAGATGTGCCAGTATCTCACAAAGAGACTGTAAGA

AAGTACTTCCCTGAGACATGGATCTGGGACTTAGTGGTGGTAGACTCATCGGGTGTAGCT

GAAGTAGGAGGAACTGTCCCTGACACCATCACTGAGTGGAAGGCAGGGGCCCTCTGCCTG

TCCAATGACACTGGGCTTGGTCTCTCTCCCACTGCCTCTCTCCGAGTCTTCCAGCCCTTC

TTTGTGGAGCTCACAATGCCCTACTCTGTGGTCCGTGGAGAGGCCTTCACGCTCAAGGCC

ACTGTGTTGAACTACCTTCCCAAATGCATCCGGGTCCGCATACAGCTGGAAACCTCCCCC

ACATTCCTAGCTGTCCCAGTGGAAAAGGAACAAGGGTCTCATTGCATCTGTGGGGATGGG

CGGCACACTGTGTCCTGGGAAGTAACTCCAAAGTCATTAGGAAATGTGAATTTCACTGTG

AGTGCAGAAGCACTGGAGTCTCAGGAGCTCTGTGGGAGTGAAGTGGCTATGGTCCCTAAA

TATGGAAAGAAAGACACAATCATCAAGCTACTGTTGGTTGAACCTGAAGGACTAGAGAAG

GAAGTAACATTCAACTCACTCCTTTGTCCCTCAGGCGCCGAGGTATCTGAACAATTATCC

CTGAAGTTGCCACCAAATGTAGTAGAGGAATCTGCCCGAGCCTCTGTCTCAGTATTGGGA

GATATATTGGGCTCTGCAATGCAAAACACACAGAATCTCCTCCAGATGCCTTATGGTTGT

GGAGAACAGAATATGGTTCTCTTTGCTCCTAACATCTATGTTCTGGATTATCTAAATGAA

ACACAGCAGCTGACTCCAGAGATCAAATCCAAAGCCATTGGCTATCTCAACACTGGTTAC

CAAAGACAGTTAAACTACAAGCACCAAGATGGCTCTTATAGCACCTTTGGGGAGAAATAT

GGCAGGAACCAGGGCAACACCTGGCTCACAGCCTTTGTACTGAAGACTTTTGCCCAGGCT

CGAAAATATATCTTCATTGATGAAACACACATTACCCAAGCCCTCACCTGGCTCTCCCAG

AAGCAGAAGGACAATGGCTGCTTCAGGAGCTCTGGGTCACTGCTCAACAATGCCATAAAG

GGAGGAGTAGAAGATGAAGTCACCCTCTCTGCCTATATCACCATAGCCCTTCTGGAGATT

CCACTCCCAGTCACTCATCCTGTTGTCCGCAATGCCTTGTTTTGCCTGGATTCAGCCTGG

AAGTCGGCAAAGGAAGGAGCCCATGGCACCCATGTCTACACCAAGGCACTTTTGGCCTAT

GCATTTGCCCTGGCAGATAACCAGGACAAGAGGAAAGAAGTACTGAACTCACTTGATGAG

GAAGCTGTAAAAGAAGATAATACTATCCACTGGACACGGCCTCAGAAACCCAAGGCACCA

CAGGGACTTTTGTATCAACCCCAGGCTCCCTCTGCTGAGGTAGAGATGACGGCCTATGTA

CTCCTCACTCTCCTCACAGTCCAGCCGGCCCCGACCTCAAAGGACCTGACTTCTGCAGTG

CCCATTGTAAAGTGGATCACAAAGCAACAGAATTCCCATGGTGGTTTCTCCTCCACCCAG

GACACAGTGGTGACTCTCCATGCTTTGTCCAAATATGGAGCAGCTACTTTTACTAGGACT

GGGAAAGCTGCACAGGTCACCATCCAGTCTTCAGGGACATTTACCTCAAAATTCCAAGTG

GACAACAACAACCGCCTGTTACTACAGCGGGTCTCCTTACCAACAGTGCCTGGGGAATAC

AGTGCAAGAGTGACGGGAGAAGGATGTGTCTACCTCCAGACATCCTTGAAATACAATATT

CTCCCAGACAAGGAAGACTTCCCATTTGCTTTAGAGGTACTGACTCTTCCCCAAACTTGT

GATGGACCCAAAAGCCACACCACCTTCCAGATCTCAATGAATATCAGATACACTGGGAGT

CGTCCTGCCTCCAACATGGTCATTGCTGATGTAAAGATGGTTTCTGGCTTCATCCCCCTG

AAACCAACAGTGAAAATGCTTGAAAGACTGAACCATGTGAACCGGACTGAAGTCAGCAAC

AATCATGTCTTGATTTACCTGGATAAGGTGTCAAATCAGACACTGAACTTGTCCTTCACA

GTTCTGCAAGATATTCCAGTAAGAGATCTGAAACCAGCCATAGTGAAAGTCTATGATTAC

TATGAGACAGATGAGTTTGCAATTGCTGAGTACAGTGCTCCTTGCAGCAAAGATCATGGA

AATGCC

>CL386.Contig5_A-W 161 4294 PREDICTED: alpha-2-macroglobulin isoform 3 [Macaca mulatta]

TTTCAGGTTCCACAGTCTTCATCCAGTGAGGAGGTAATGTTCCTCACTGTCCAAGTGAAA

GGATCAACTCAAGAATTCAGGAGGCGGAACACAGTGGTGGTTAAAAACCAAGAGAGTCTG

GTCTTTGTCCAAACAGATAAACCTATCTACAAACCAGGACAGACAGTGAAGTTTCGCATT

GTCTCATTGGATGAAAACTTTCACCCCCTGAATGAGTTGATACCACTACTATATATTAAG

GATCCCAAAGGAAATCGCATTATACAATGGCAGAATCTCAAGCTAGAGAGTGGTCTCAAA

CAACTGTCCTTTCCCCTCTCATCAGAGCCCCTTCAGGGCATCTACAATGTTGTGGTACAC

AAGGAATCAAGTGGAACAACAGAACACCCCTTTGCTGTGGAGGAATTTGTGCTTCCCAAG

TTTGAAGTGCAAGTAACAATGCCAAAGATAATCACTATCATGGAAGAAGAGGTGAACGTG

TCAGTGTGTGGCCTATACACATATGGGAAGCCTGTCCCAGGACATGTGACTATGAGCATT

TGCAGAAAGTATAATAATCCTTCTAACTGTTTTGGTGAAGAGTCAGAAGCTTTCTGTGAG

AAATTCAGCCAGAAGCTCAACAGCCATGGCTGCTTCTTCCAACAAGTAAAAGCCAGTATC

TTCCACCTGAAGAAGACAGGGTATGAAATGCAACTGCAGGTGGAGGCCAAGATCCAAGAA

GAAGGAACTGGTGTGGAAGTAACAGGAAGAGGGTCCAGTGAAATCACAAACATCATAACC

AAGCTCTCATTTGTGACAGTGGACTCACATGTTAGACAAGGGATCCCCTTCTTTGGACAG

GTGCGCCTTGTGGATGGGAAAGGCATCCCTATGCCAAATAAAATCATCTTCATCACAGCA

AATGAAGCTAACTATCACTCAAATGCTACCACTAATGAGGATGGCCTGGTGCAGTTCTCC

ATCAACACCACCAACATTATAGATACCTCTCTTAGTATCATGGTCAAACACAAGGATCAC

AGCCCCTGCTATAACCACCAGTGGCTCTCAGAGGAAAATGAGAATGCATATCACACTGCT

AATGTTGTATTCTCCCTGAGCAAAAGCTTTATCCACCTTGAACCTATTCCTGGTAAACTG

CCATGTGGCCAATCTCAGACAGTCCAGGCACATTATCTTCTAAATGAACAGGTCCTACAG

GAGCTGAAGCAGCTCGTCTTCTATTATCTGATAATGGCAAAGGGAGGCATTGTCCGAACT

GGGACTCATGTCCTGCCTGTGGAGCAGGGAGACATGAAAGGCCATTTTTCCGTGACCTTC

CTTGTGGAGTCAGACTTGGCTCCAATTGCTCGATTGTTAATCTATGCCATTTTACCTGAT

GGGGAAATGGTTGGAGATTCTGCAAAATATGAGATTGAAAATTGTCTGGCAAACAAGGTG

GATTTGAGCTTCAGACCAGTACAAACTCTTCCAGCCTCACATGCCCAGCTGCGAGTCACA

GCTTCTCCTCAGTCCCTCTGTGCTCTCCGAGCTGTGGACCAAAGTGTGCTGCTCATGAGG

CCCGAGGCTGAGCTCTCTGCATCATCGGTTTATAAGCTTTTACCAGTGAAGGACCTCACA

GGCATCCCTGAAAGTTTGAATGAGGCTGAAGATCATGGAGACTGTGTCAATCGTGATAAT

GTCTATATTAATGGAATCCTGTATTCCCCAGTGTCAAATACAAATGAAAAAGATATGTAT

AGCTTACTAAAGGATATGGGCTTAAGAGTATTCACAAACTCAAAGATTCATAAACCAAAA

GTATGTACACAGACTATAGAGTATCAAGCGAGATTTTCAACATATGACGCACCAGTTGCA

CTTTACAGAAGCCAAATTCAGGAGATAGATGTGCCAGTATCTCACAAAGAGACTGTAAGA

AAGTACTTCCCTGAGACATGGATCTGGGACTTAGTGGTGGTAGACTCATCGGGTGTAGCT

GAAGTAGGAGGAACTGTCCCTGACACCATCACTGAGTGGAAGGCAGGGGCCCTCTGCCTG

TCCAATGACACTGGGCTTGGTCTCTCTCCCACTGCCTCTCTCCGAGTCTTCCAGCCCTTC

TTTGTGGAGCTCACAATGCCCTACTCTGTGGTCCGTGGAGAGGCCTTCACGCTCAAGGCC

ACTGTGTTGAACTACCTTCCCAAATGCATCCGGGTCCGCATACAGCTGGAAACCTCCCCC

ACATTCCTAGCTGTCCCAGTGGAAAAGGAACAAGGGTCTCATTGCATCTGTGGGGATGGG

CGGCACACTGTGTCCTGGGAAGTAACTCCAAAGTCATTAGGAAATGTGAATTTCACTGTG

AGTGCAGAAGCACTGGAGTCTCAGGAGCTCTGTGGGAGTGAAGTGGCTATGGTCCCTAAA

TATGGAAAGAAAGACACAATCATCAAGCTACTGTTGGTTGAACCTGAAGGACTAGAGAAG

GAAGTAACATTCAACTCACTCCTTTGTCCCTCAGGCGCCGAGGTATCTGAACAATTATCC

CTGAAGTTGCCACCAAATGTAGTAGAGGAATCTGCCCGAGCCTCTGTCTCAGTATTGGGA

GATATATTGGGCTCTGCAATGCAAAACACACAGAATCTCCTCCAGATGCCTTATGGTTGT

GGAGAACAGAATATGGTTCTCTTTGCTCCTAACATCTATGTTCTGGATTATCTAAATGAA

ACACAGCAGCTGACTCCAGAGATCAAATCCAAAGCCATTGGCTATCTCAACACTGGTTAC

CAAAGACAGTTAAACTACAAGCACCAAGATGGCTCTTATAGCACCTTTGGGGAGAAATAT

GGCAGGAACCAGGGCAACACCTGGCTCACAGCCTTTGTACTGAAGACTTTTGCCCAGGCT

CGAAAATATATCTTCATTGATGAAACACACATTACCCAAGCCCTCACCTGGCTCTCCCAG

AAGCAGAAGGACAATGGCTGCTTCAGGAGCTCTGGGTCACTGCTCAACAATGCCATAAAG

GGAGGAGTAGAAGATGAAGTCACCCTCTCTGCCTATATCACCATAGCCCTTCTGGAGATT

CCACTCCCAGTCACTCATCCTGTTGTCCGCAATGCCTTGTTTTGCCTGGATTCAGCCTGG

AAGTCGGCAAAGGAAGGAGCCCATGGCACCCATGTCTACACCAAGGCACTTTTGGCCTAT

GCATTTGCCCTGGCAGATAACCAGGACAAGAGGAAAGAAGTACTGAACTCACTTGATGAG

GAAGCTGTAAAAGAAGATAATACTATCCACTGGACACGGCCTCAGAAACCCAAGGCACCA

CAGGGACTTTTGTATCAACCCCAGGCTCCCTCTGCTGAGGTAGAGATGACGGCCTATGTA

CTCCTCACTCTCCTCACAGTCCAGCCGGCCCCGACCTCAAAGGACCTGACTTCTGCAGTG

CCCATTGTAAAGTGGATCACAAAGCAACAGAATTCCCATGGTGGTTTCTCCTCCACCCAG

GACACAGTGGTGGCTCTGCATGCCCTATCCAGATATGGAGCAGCTACTTTCACCAAAACT

GAGAAAACTGCACAGGTTACCATTCAGAACTCCAGGGCCTTTTCCACAAATTTCCAGGTG

GACAACAACAACCCCCTGCTACTACAGCAGATCTCATTGCCAGAGCTGCCTGGGGACTAT

ACCATAACAATAACTGGGGAAAGATGTGTATATCTTCAGACATCCATGAAATACAATATT

CATCCAAAGAAGGAGGATTCCCCATTTTCTCTGAAGGTCCAGACTCTACCTGAAATTTGT

GATGGACCCAAAGCCCACACCAGCTTCCAGATCTCACTGAATATTAGTTACACAGGGAGC

CGTCCTGTTTCCAATATGGTGATTGCTGATATAAAAATGATATCTGGTTTTATTCCCCTG

AAACCAACAGTAAAAATGCTTGAAAGATCTAGCTACGTGAGCAGGACAGAAGTGAGCAAC

AATCATGTCCTTATTTATGTGGATCAGGTGACAAATCAGACACTAAGTTTTTCCTTCACG

GTTTTGCAAGACATCCCAGTAAGAGACCTGAAGCCAGCAATTGTTAAAGTCTATGACTAC

TATGAAACAGATGAGTTTTCTATTGCTGAGTATATCAGTCCCTGTAGTACAGAT

>CL386.Contig6_A-W 1 267 PREDICTED: pregnancy zone protein [Saimiri boliviensis boliviensis]

ACTCATCCTGTTGTCCGCAATGCCCTGTTCTGCCTAGAATCAGCCTGGAACAGAGCAAAA

GAGGGAGCCCATGGCAGCCATGTCTACACCCGGGCTTTGTTGGCCTATGCTTTTGCCCTA

GTGGGAAACCAAAATAGGAGTAGAGAAATATTGCGCTCACTCAATGAGGAAGCTGTTAAA

GAAGACAATTCAGTCCATTGGGAACGGCCTCAGAAACCCAGAGCACCACAAGGACTTTTG

TACCAACCCCAGGCTCCCTCTGCTGAG

>CL387.Contig1_A-W 46 228 minus strand PREDICTED: mediator of RNA polymerase II transcription subunit 13-like [Saimiri boliviensis boliviensis]

CTGCCTGGCCTTCCTTCTGGCTCCTGCCCTCACCCATGTCTTCTCTTTGGCAGGTTTGTT

TTGGAGCAGTACAACGCACTGTCCTGGCTCACGTGCAATCCAGCCACCCAGGACCGTACT

TCCTGCCTTCCCGTCCACTTTGTGGTGCTCACTCAGTTGTACAATGCCATCATGAATATA

CTT

>CL387.Contig2_A-W 2 1534 PREDICTED: mediator of RNA polymerase II transcription subunit 13-like [Saimiri boliviensis boliviensis]

AGCTTGATGCGCTGCTACACAGAAATGCTGGATAATTTACCTGAGCATATGAGAAATTCT

TTCATTCTCCAGATTGTGCCTTGCCAGTACATGCTGCAGACAGTGAAGGATGAACAAGTT

TTCTACATTCAATACTTGAAGTCCATGGCATTTTCAGTGTACTGCCAGTGCAGGCGACCT

CTGCCTACACAGATCCACATTAAATCCCTCACAGGATTTGGGCCTGCAGCCAGCATTGAG

ATGACCCTCAAGAACCCTGAGCGGCCCAGCCCAATCCAGCTTTACTCCCCTCCCTTTATA

TTGGCCCCAATCAAAGACAAATCAAAGCAGACAGAGCTGGGAGAGAACTTTGGTGAGGCC

AGCCAGAAGTACAATGTGCTCTTTGTGGGCTATTGTCTATCCCATGACCAGCGCTGGCTC

TTGGCTTCCTGCACTGACCTCCATGGGGAGTTATTAGAGACCTGCGTTGTGAATATTGCT

TTACCAAACAGGTCCCGGAAGAGTAAAGTATCTGCACGTAAGATTGGACTGCAGAAACTG

TGGGAATGGTGCATAGGCATTGTGCAAATGACATCGCTGCCCTGGAGAGTTGTGATTGGA

AGACTTGGGCGCCTTGGCCATGGGGAGCTTAAAGATTGGAGTATCCTCCTTGGAGAATGT

TCACTACAGACAATCAGCAAACAGCTCAAGGATGTGTGCCGGATGTGTGGAATCTCTGCC

GCAGACTCTCCTTCTATCCTTAGTGCCTGCCTGGTTGCCATGGAGCCCCAGGGGTCCTTT

GTAGTGATGCCAGATGCTGTCACAATGGGCTCTGTTTTTGGCCGAAGTACTGCACTGAAC

ATGCAGTCTTCTCAGCTGAATACCCCTCAAGACGCGTCTTGTACACACATCTTGGTGTTC

CCAACATCATCAACCATCCAGGTGGCTCCAGCAAACTATCCCAACGAGGATGGGTTCAGC

CCTAACAATGATGATATGTTTGTTGACCTTCCATTCCCAGATGATATGGACAATGACATT

GGCATATTAATGACTGGGAACCTCCATTCCTCTCCCAACTCCTCCCCAGTTCCCTCCCCA

GGCTCTCCTTCTGGAATTGGTGTGGGCTCTCACTTCCAGCACAGTCGGAGCCAGGGTGAG

CGTCTTCTCTCGAGAGAGGCACCAGAGGAGCTCAAGCAGCAGCCCTTGGCCCTTGGGTAT

TTTGTGTCAACTGCCAAAGCTGAGAATCTCCCCCAGTGGTTTTGGTCATCATGTCCCCAG

GCTCAGAACCAGTGCCCTCTCTTCCTAAAGGCTTCACTACATCACCATATCTCTGTAGCA

CAGTCGGATGAACTTCTCCCTGCCAGGAATTCTCAACGGGTTCCACACCCTCTGGACTCC

AAGACCACATCTGATGTTTTAAGGTTTGTTTTGGAGCAGTACAACGCACTGTCCTGGCTC

ACGTGCAATCCAGCCACCCAGGACCGTACTTCCTGCCTTCCCGTCCACTTTGTGGTGCTC

ACTCAGTTGTACAATGCCATCATGAATATACTT

>CL388.Contig1_A-W 370 1785 minus strand lysophospholipid acyltransferase 7 isoform 1 [Homo sapiens] >gi|167008974|sp|Q96N66.2|MBOA7_HUMAN RecName: Full=Lysophospholipid acyltransferase 7; Short=LPLAT 7; AltName: Full=1-acylglycerophosphatidylinositol O-acyltransferase; AltName: Full=Bladder and breast carcinoma-overexpressed gene 1 protein; AltName: Full=Leukocyte receptor cluster member 4; AltName: Full=Lysophosphatidylinositol acyltransferase; Short=LPIAT; Short=Lyso-PI acyltransferase; AltName: Full=Membrane-bound O-acyltransferase domain-containing protein 7; Short=O-acyltransferase domain-containing protein 7; Short=h-mboa-7 >gi|37589905|gb|AAH03164.2| Membrane bound O-acyltransferase domain containing 7 [Homo sapiens] >gi|119592605|gb|EAW72199.1| leukocyte receptor cluster (LRC) member 4, isoform CRA_a [Homo sapiens] >gi|119592608|gb|EAW72202.1| leukocyte receptor cluster (LRC) member 4, isoform CRA_d [Homo sapiens] >gi|157696155|gb|ABV66273.1| lysophosphatidylinositol acyltransferase [Homo sapiens] >gi|159571305|emb|CAP19119.1| leukocyte receptor cluster (LRC) member 4 [Homo sapiens] >gi|169158354|emb|CAQ09590.1| leukocyte receptor cluster (LRC) member 4 [Homo sapiens]

ATGTCACCAGAAGAATGGACTTATCTGGTAGTTCTTCTTATCTCCATCCCTATCGGCTTC

CTCTTTAAGAAAGCTGGGCCTGGGCTGAAGAGATGGGGGGCAGCAGCTGTGGGCCTGGGA

CTCACCTTGTTCACCTGTGGTCCCCATACTTTGCATTCTTTGATCACCATCCTTGGGACC

TGGGCCCTCATTCAGGCCCAGCCCTGCTCCTGCCATGCCCTGGCTCTCGCCTGGACCTTC

TCCTATCTCCTGTTCTTCCGAGCCCTCAGCCTGCTGGGCCTGCCCACTCCCACGCCCTTC

ACCAATGCCGTCCAGCTGCTGTTGACACTGAAGCTGGTGAGTCTGGCCAGTGAGGTCCAG

GACCTGCACCTGGCCCAGAGGAAGGAAATGGCCTCCGGCTTCAGCAAGGGGCCCACCCTG

GGGCTGCTACCCGATGTGCCCTCCTTGATGGAGACACTCAGCTACAGCTACTGCTATGTG

GGAATCATGACAGGCCCGTTCTTCCGCTACCGCACCTACCTGGACTGGCTGGAGCAGCCC

TTCCCGGAGGCCGTGCCCAGCCTGCGGCCACTGCTGCGCCGCGCCTGGCCGGCCCCGCTG

TTTGGCCTGCTCTTCCTGCTGTCCTCCCACCTCTTCCCGCTGGAGGCCGTGCGCGAGGAC

GCCTTCTACGCCCGCCCGCTGCCCGCCCGCCTCTTCTACATGATCCCCGTCTTCTTCGCC

TTCCGCATGCGCTTCTACGTGGCCTGGATTGCCGCCGAGTGCGGCTGCATTGCCGCGGGC

TTCGGGGCTTACCCCGTGGCCGCCAAAGCCCGGGCCGGGGGCGGCCCCACCCTCCAATGC

CCACCCCCCAGCAGTCCGGAGAAGGCGGCTTCCCTGGAGTATGATTTTGAGACCATCCGC

AACATCGACTGCTACAGCACAGATTTCTGCGTGCGGGTGCGGGATGGCATGCGGTACTGG

AACATGACAGTGCAGTGGTGGCTGGCGCAGTACATCTACAAGAGCGCACCTGCCCGCTCC

TACGTCCTGAGGAGCGCCTGGACCATGCTGCTGAGCGCCTACTGGCATGGCCTGCATCCT

GGCTACTACCTGAGCTTCCTGACCATCCCGCTGTGCCTGGCTGCAGAGGGTCGCTTGGAG

TCGGCCCTGAGGTGGCGGCTCAGTCCCAGGGGCCAGAAGGCCTGGGACTGGGTGCACTGG

TTCCTGAAGATGCGCGCCTATGACTACATGTGCATGGGCTTTGTGCTCCTCTCCACGGGC

GACACGCTCCGGTACTGGTCCTCCATCTACTTCTGCGTCCACTTCGTGGCCCTGGCCTGC

CTAGGGCTGGGGCTAGCTCTGGGTGGGGGCAGCCCGAGCCGGCGAAAGACAGCACTCCAA

TCCTCTGGCCTTGCCCCAGAAAAGCTCCGGGAGGAG

>CL388.Contig2_A-W 370 1290 minus strand leukocyte receptor cluster (LRC) member 4, isoform CRA_c [Homo sapiens]

ATGTCACCAGAAGAATGGACTTATCTGGTAGTTCTTCTTATCTCCATCCCTATCGGCTTC

CTCTTTAAGAAAGCTGGGCCTGGGCTGAAGAGATGGGGGGCAGCAGCTGTGGGCCTGGGA

CTCACCTTGTTCACCTGTGGTCCCCATACTTTGCATTCTTTGATCACCATCCTTGGGACC

TGGGCCCTCATTCAGGCCCAGCCCTGCTCCTGCCATGCCCTGGCTCTCGCCTGGACCTTC

TCCTATCTCCTGTTCTTCCGAGCCCTCAGCCTGCTGGGCCTGCCCACTCCCACGCCCTTC

ACCAATGCCGTCCAGCTGCTGTTGACACTGAAGCTGGTGAGTCTGGCCAGTGAGGTCCAG

GACCTGCACCTGGCCCAGAGGAAGGAAATGGCCTCCGGCTTCAGCAAGGGGCCCACCCTG

GGGCTGCTACCCGATGTGCCCTCCTTGATGGAGACACTCAGCTACAGCTACTGCTATGTG

GGAATCATGACAGGCCCGTTCTTCCGCTACCGCACCTACCTGGACTGGCTGGAGCAGCCC

TTCCCGGAGGCCGTGCCCAGCCTGCGGCCACTGCTGCGCCGCGCCTGGCCGGCCCCGCTG

TTTGGCCTGCTCTTCCTGCTGTCCTCCCACCTCTTCCCGCTGGAGGCCGTGCGCGAGGAC

GCCTTCTACGCCCGCCCGCTGCCCGCCCGCCTCTTCTACATGATCCCCGTCTTCTTCGCC

TTCCGCATGCGCTTCTACGTGGCCTGGATTGCCGCCGAGTGCGGCTGCATTGCCGCGGGC

TTCGGGGCTTACCCCGTGGCCGCCAAAGCCCGGGCCGGGGGCGGCCCCACCCTCCAATGC

CCACCCCCCAGCAGGTCAGGCGGCGGGAGGGAGGCTTCCCAAGACCCAGCAGGCCCCACC

ACCAAGGGCTGGCCCTGCCCC

>CL388.Contig3_A-W 168 1088 minus strand leukocyte receptor cluster (LRC) member 4, isoform CRA_c [Homo sapiens]

ATGTCACCAGAAGAATGGACTTATCTGGTAGTTCTTCTTATCTCCATCCCTATCGGCTTC

CTCTTTAAGAAAGCTGGGCCTGGGCTGAAGAGATGGGGGGCAGCAGCTGTGGGCCTGGGA

CTCACCTTGTTCACCTGTGGTCCCCATACTTTGCATTCTTTGATCACCATCCTTGGGACC

TGGGCCCTCATTCAGGCCCAGCCCTGCTCCTGCCATGCCCTGGCTCTCGCCTGGACCTTC

TCCTATCTCCTGTTCTTCCGAGCCCTCAGCCTGCTGGGCCTGCCCACTCCCACGCCCTTC

ACCAATGCCGTCCAGCTGCTGTTGACACTGAAGCTGGTGAGTCTGGCCAGTGAGGTCCAG

GACCTGCACCTGGCCCAGAGGAAGGAAATGGCCTCCGGCTTCAGCAAGGGGCCCACCCTG

GGGCTGCTACCCGATGTGCCCTCCTTGATGGAGACACTCAGCTACAGCTACTGCTATGTG

GGAATCATGACAGGCCCGTTCTTCCGCTACCGCACCTACCTGGACTGGCTGGAGCAGCCC

TTCCCGGAGGCCGTGCCCAGCCTGCGGCCACTGCTGCGCCGCGCCTGGCCGGCCCCGCTG

TTTGGCCTGCTCTTCCTGCTGTCCTCCCACCTCTTCCCGCTGGAGGCCGTGCGCGAGGAC

GCCTTCTACGCCCGCCCGCTGCCCGCCCGCCTCTTCTACATGATCCCCGTCTTCTTCGCC

TTCCGCATGCGCTTCTACGTGGCCTGGATTGCCGCCGAGTGCGGCTGCATTGCCGCGGGC

TTCGGGGCTTACCCCGTGGCCGCCAAAGCCCGGGCCGGGGGCGGCCCCACCCTCCAATGC

CCACCCCCCAGCAGGTCAGGCGGCGGGAGGGAGGCTTCCCAAGACCCAGCAGGCCCCACC

ACCAAGGGCTGGCCCTGCCCC

>CL389.Contig1_A-W 385 1326 minus strand WAS protein family homolog 1 [Sus scrofa]

ATGACTCCTTTGAGGACCCAGCATTCCTTGGCAGGTCAGACGTACTCAGTGCCTCTCATC

CAACCTGACCTGCGGCGAGAGGAAGCCATTCAACAGGTGGCAGATGCCCTGCAATACCTA

CAGAAAGTCTCTGGAGACATCTTTAGCAGGATCTCCCAGCGGGTAGAGCTGAGCCGGAGC

CAGCTGCAGGCCATTGGGGAGAGGGTCTCCTTGGCCCAGGCCAAGATCGAGAAGATCAAG

GGCAGCAAGAAGGCCATCAAGGTATTCTCCAGTGCCAAGTACCCAGCCCCAGAGCGTCTG

CAGGAATACGGCTCTATCTTTACTGGGGCACAGGACCCTGGTCTGCAGAGACGGCCTCGC

CACAGGATCCAGAGCAAGCACCGCCCCTTGGATGAGCGGGCCCTGCAGGAGAAGCTGAAA

TACTTTCCTGTCTGTGTGAACACCAAGCCAGAGCCCGAGGATGAAGCTGAGGAAGGTCTT

GGGGGTCTTCCAAGCAACATCAGCTCTGTCAGCTCCTTGCTGCTCTTCAACACTACTGAG

AACTTGTACAAGAAGTATGTCTTCCTGGACCCCCTGGCTGGTGCTGTAACAAAGACCCAT

GTGATGCTGGGGGCAGAGACGGAGGAGAAGCTATTTGATGCCCCTCTGTCCATCAGCAAG

AGAGAGCAGCTGGAACAGCAGGTTCCAGAGAATTACTTCTATGTGCCAGACCTGGGCCAA

GTACCCGAGATTGATGTGCCATCCTACCTGCCTGACCTGCCTGGCATTGCTGATGACCTT

ATGTACAATGCTGACTTGGAGCCAGGCATTGCTCCCTCTGCCCCCGGTGCCATTCCAGAA

CTGCCTACCTTTCCTACAGAGGTTGCAGAGCCTTTCAAGGCGGACCTAGAAGATGGGGTG

CTGACAGCACCCCCACCACCACCACCGCCCCCACCGCCACCC

>CL389.Contig2_A-W 118 558 minus strand WAS protein family homolog 1 [Sus scrofa]

TCAGGAACCCAGGCCTGCCTGATGCCACCCACCCCACCCTGTAGGTACAAGAAGTATGTC

TTCCTGGACCCCCTGGCTGGTGCTGTAACAAAGACCCATGTGATGCTGGGGGCAGAGACG

GAGGAGAAGCTATTTGATGCCCCTCTGTCCATCAGCAAGAGAGAGCAGCTGGAACAGCAG

GTTCCAGAGAATTACTTCTATGTGCCAGACCTGGGCCAAGTACCCGAGATTGATGTGCCA

TCCTACCTGCCTGACCTGCCTGGCATTGCTGATGACCTTATGTACAATGCTGACTTGGAG

CCAGGCATTGCTCCCTCTGCCCCCGGTGCCATTCCAGAACTGCCTACCTTTCCTACAGAG

GTTGCAGAGCCTTTCAAGGCGGACCTAGAAGATGGGGTGCTGACAGCACCCCCACCACCA

CCACCGCCCCCACCGCCACCC

>CL389.Contig3_A-W 118 495 minus strand PREDICTED: WAS protein family homolog 1 [Equus caballus]

TCAGGAACCCAGGCCTGCCTGATGCCACCCACCCCACCCTGTAGGTACAAGAAGTATGTC

TTCCTGGACCCCCTGGCTGGTGCTGTAACAAAGACCCATGTGATGCTGGGGGCAGAGACG

GAGGAGAAGCTATTTGATGCCCCTCTGTCCATCAGCAAGAGAGAGCAGCTGGAACAGCAG

GTTCCAGAGAATTACTTCTATGTGCCAGACCTGGGCCAAGTACCCGAGATTGATGTGCCA

TCCTACCTGCCTGACCTGCCTGGCATTGCTGATGACCTTATGTACAATGCTGACTTGGAG

CCAGGCATTGCTCCCTCTGCCCCCGGTGCCATTCCAGAACTGCCTACCTTTCCTACAGAG

GTTGCAGAGCCTTTCAAG

>CL390.Contig1_A-W 20 796 PREDICTED: estradiol 17-beta-dehydrogenase 8 [Otolemur garnettii]

ATGGCGTCTCAGCTTCGGCTCGGTTCTGCGCTGGCCTTGGTCACAGGTGCGGGTAGCGGC

ATCGGCCGGGCGGTTAGTGCGCGCCTGGCCAGAGAGGGGGCTACCGTAGCCGCCTGCGAC

GTGGACGGGACAGCAGCTCAGGAGACGGTGCGGCTGCTAGACGGGTCGAGGAGTGAGGAA

GGGGCGCCGGGCGGGAACCACGCTGCCTTCCAGGCTGATGTGTCTGAGGCCAGAGCTACC

AGGCGCCTGCTGGAAAAAGTGCAGGCCAGCTTTTCTCGCCCGCCATCTGTCGTTGTGTCT

TGTGCGGGCATCACTCGGGATGAGTTTCTGCTCCACATGTCTGAGGATGACTGGGACCAA

GTCATAGATGTCAACCTCAAGGGCATCTTCCTAGTTACTCAGGCTGCAGCTCAAGCCTTG

GTGTCCAGTGGTTATCACGGCTCTATTATCAATATCAGTAGCATCGTTGGAAAGGTGGGG

AACATCGGGCAGATAAATTATGCTGCATCCAAGGCTGGAGTGATTGGGCTCACCCAGACT

GCAGCCCGCGAGCTTGGACGACATGGGATCCGCTGTAACTCTGTCCTCCCAGGGTTCATT

GCAACACCCATGACAGAGAAAATGCCACAGAAAGTGAAGGATAAGGTGACCAGAATGATC

CCAATGGGACACTGGGGGAACCCTGAGGATGTGGCAGATGTGGTTGCATTCTTGGCATCT

GAAGACAGTGGATACATCACAGGGGCCTCAGTGGAAGTCACTGGAGGTCTTTTCATG

>CL390.Contig2_A-W 1050 1184 minus strand PREDICTED: estradiol 17-beta-dehydrogenase 8 [Otolemur garnettii]

CAGGTGACCAGAATGATCCCAATGGGACACTGGGGGAACCCTGAGGATGTGGCAGATGTG

GTTGCATTCTTGGCATCTGAAGACAGTGGATACATCACAGGGGCCTCAGTGGAAGTCACT

GGAGGTCTTTTCATG

>CL390.Contig3_A-W 102 593 minus strand Estradiol 17-beta-dehydrogenase 8 [Cricetulus griseus]

GCCAGCTTTTCTCGCCCGCCATCTGTCGTTGTGTCTTGTGCGGGCATCACTCGGGATGAG

TTTCTGCTCCACATGTCTGAGGATGACTGGGACCAAGTCATAGATGTCAACCTCAAGGTG

GTGACCTCTGAACCTGTGACCTCTGATCCCCCAAGCCAGGAGAGGGAATTGGAGGAGGGC

TGTCATCCCAGCTGATCTCACCTTACTACCCTCTTCTGCCAGGGCATCTTCCTAGTTACT

CAGGCTGCAGCTCAAGCCTTGGTGTCCAGTGGTTATCACGGCTCTATTATCAATATCAGT

AGCATCGTTGGAAAGGTGGGGAACATCGGGCAGATAAATTATGCTGCATCCAAGGCTGGA

GTGATTGGGCTCACCCAGACTGCAGCCCGCGAGCTTGGACGACATGGGATCCGCTGTAAC

TCTGTCCTCCCAGGGTTCATTGCAACACCCATGACAGAGAAAATGCCACAGAAAGTGAAG

GATAAGGTAGGA

>CL390.Contig4_A-W 585 944 minus strand PREDICTED: estradiol 17-beta-dehydrogenase 8-like [Equus caballus]

TCAGCCACCTCTGGTGCAGGGGACTTCACTGAGGCATGTAGTGGCGTGAGGGAAGGGTCA

GTAATCGGTCTAGAGTTCAGCAGCCACTCTCCCTTCCACAGACATGGGATCCGCTGTAAC

TCTGTCCTCCCAGGGTTCATTGCAACACCCATGACAGAGAAAATGCCACAGAAAGTGAAG

GATAAGGTGACCAGAATGATCCCAATGGGACACTGGGGGAACCCTGAGGATGTGGCAGAT

GTGGTTGCATTCTTGGCATCTGAAGACAGTGGATACATCACAGGGGCCTCAGTGGAAGTC

ACTGGAGGTCTTTTCATGTAACTGCCTCAAGGACCCTGGACTCTTCTCACTCCCCCACCA

>CL390.Contig5_A-W 20 901 minus strand PREDICTED: estradiol 17-beta-dehydrogenase 8 [Otolemur garnettii]

ATGGCGTCTCAGCTTCGGCTCGGTTCTGCGCTGGCCTTGGTCACAGGTGCGGGTAGCGGC

ATCGGCCGGGCGGTTAGTGCGCGCCTGGCCAGAGAGGGGGCTACCGTAGCCGCCTGCGAC

GTGGACGGGACAGCAGCTCAGGAGACGGTGCGGCTGCTAGACGGGTCGAGGAGTGAGGAA

GGGGCGCCGGGCGGGAACCACGCTGCCTTCCAGGCTGATGTGTCTGAGGCCAGAGCTACC

AGGCGCCTGCTGGAAAAAGTGCAGGCCAGCTTTTCTCGCCCGCCATCTGTCGTTGTGTCT

TGTGCGGGCATCACTCGGGATGAGTTTCTGCTCCACATGTCTGAGGATGACTGGGACCAA

GTCATAGATGTCAACCTCAAGGTGGTGACCTCTGAACCTGTGACCTCTGATCCCCCAAGC

CAGGAGAGGGAATTGGAGGAGGGCTGTCATCCCAGCTGATCTCACCTTACTACCCTCTTC

TGCCAGGGCATCTTCCTAGTTACTCAGGCTGCAGCTCAAGCCTTGGTGTCCAGTGGTTAT

CACGGCTCTATTATCAATATCAGTAGCATCGTTGGAAAGGTGGGGAACATCGGGCAGATA

AATTATGCTGCATCCAAGGCTGGAGTGATTGGGCTCACCCAGACTGCAGCCCGCGAGCTT

GGACGACATGGGATCCGCTGTAACTCTGTCCTCCCAGGGTTCATTGCAACACCCATGACA

GAGAAAATGCCACAGAAAGTGAAGGATAAGGTGACCAGAATGATCCCAATGGGACACTGG

GGGAACCCTGAGGATGTGGCAGATGTGGTTGCATTCTTGGCATCTGAAGACAGTGGATAC

ATCACAGGGGCCTCAGTGGAAGTCACTGGAGGTCTTTTCATG

>CL390.Contig6_A-W 585 779 minus strand PREDICTED: estradiol 17-beta-dehydrogenase 8-like [Equus caballus]

TCAGCCACCTCTGGTGCAGGGGACTTCACTGAGGCATGTAGTGGCGTGAGGGAAGGGTCA

GTAATCGGTCTAGAGTTCAGCAGCCACTCTCCCTTCCACAGACATGGGATCCGCTGTAAC

TCTGTCCTCCCAGGGTTCATTGCAACACCCATGACAGAGAAAATGCCACAGAAAGTGAAG

GATAAGGTAGGAGGC

>CL390.Contig7_A-W 20 775 minus strand Estradiol 17-beta-dehydrogenase 8 [Cricetulus griseus]

ATGGCGTCTCAGCTTCGGCTCGGTTCTGCGCTGGCCTTGGTCACAGGTGCGGGTAGCGGC

ATCGGCCGGGCGGTTAGTGCGCGCCTGGCCAGAGAGGGGGCTACCGTAGCCGCCTGCGAC

GTGGACGGGACAGCAGCTCAGGAGACGGTGCGGCTGCTAGACGGGTCGAGGAGTGAGGAA

GGGGCGCCGGGCGGGAACCACGCTGCCTTCCAGGCTGATGTGTCTGAGGCCAGAGCTACC

AGGCGCCTGCTGGAAAAAGTGCAGGCCAGCTTTTCTCGCCCGCCATCTGTCGTTGTGTCT

TGTGCGGGCATCACTCGGGATGAGTTTCTGCTCCACATGTCTGAGGATGACTGGGACCAA

GTCATAGATGTCAACCTCAAGGTGGTGACCTCTGAACCTGTGACCTCTGATCCCCCAAGC

CAGGAGAGGGAATTGGAGGAGGGCTGTCATCCCAGCTGATCTCACCTTACTACCCTCTTC

TGCCAGGGCATCTTCCTAGTTACTCAGGCTGCAGCTCAAGCCTTGGTGTCCAGTGGTTAT

CACGGCTCTATTATCAATATCAGTAGCATCGTTGGAAAGGTGGGGAACATCGGGCAGATA

AATTATGCTGCATCCAAGGCTGGAGTGATTGGGCTCACCCAGACTGCAGCCCGCGAGCTT

GGACGACATGGGATCCGCTGTAACTCTGTCCTCCCAGGGTTCATTGCAACACCCATGACA

GAGAAAATGCCACAGAAAGTGAAGGATAAGGTAGGA

>CL390.Contig8_A-W 404 511 minus strand H2-K region expressed gene 6, isoform CRA_c [Mus musculus]

CATCGTTGGAAAGGTGGGGAACATCGGGCAGATAAATTATGCTGCATCCAAGGCTGGAGT

GATTGGGCTCACCCAGACTGCAGCCCGCGAGCTTGGACGGTTGGTCAG

>CL390.Contig9_A-W 496 711 minus strand predicted protein [Pediculus humanus corporis] >gi|212507348|gb|EEB11304.1| predicted protein [Pediculus humanus corporis]

TTCCACAGACATGGGATCCGCTGTAACTCTGTCCTCCCAGGGTTCATTGCAACACCCATG

ACAGAGAAAATGCCACAGAAAGTGAAGGATAAGGTGACCAGAATGATCCCAATGGGACAC

TGGGGGAACCCTGAGGATGTGGCAGATGTGGTTGCATTCTTGGCATCTGAAGACAGTGGA

TACATCACAGGGGCCTCAGTGGAAGTCACTGGAGGT

>CL390.Contig10_A-W 102 719 minus strand PREDICTED: estradiol 17-beta-dehydrogenase 8 [Otolemur garnettii]

GCCAGCTTTTCTCGCCCGCCATCTGTCGTTGTGTCTTGTGCGGGCATCACTCGGGATGAG

TTTCTGCTCCACATGTCTGAGGATGACTGGGACCAAGTCATAGATGTCAACCTCAAGGTG

GTGACCTCTGAACCTGTGACCTCTGATCCCCCAAGCCAGGAGAGGGAATTGGAGGAGGGC

TGTCATCCCAGCTGATCTCACCTTACTACCCTCTTCTGCCAGGGCATCTTCCTAGTTACT

CAGGCTGCAGCTCAAGCCTTGGTGTCCAGTGGTTATCACGGCTCTATTATCAATATCAGT

AGCATCGTTGGAAAGGTGGGGAACATCGGGCAGATAAATTATGCTGCATCCAAGGCTGGA

GTGATTGGGCTCACCCAGACTGCAGCCCGCGAGCTTGGACGACATGGGATCCGCTGTAAC

TCTGTCCTCCCAGGGTTCATTGCAACACCCATGACAGAGAAAATGCCACAGAAAGTGAAG

GATAAGGTGACCAGAATGATCCCAATGGGACACTGGGGGAACCCTGAGGATGTGGCAGAT

GTGGTTGCATTCTTGGCATCTGAAGACAGTGGATACATCACAGGGGCCTCAGTGGAAGTC

ACTGGAGGTCTTTTCATG

>CL390.Contig11_A-W 460 600 minus strand Estradiol 17-beta-dehydrogenase 8 [Cricetulus griseus]

GAAGGGTCAGTAATCGGTCTAGAGTTCAGCAGCCACTCTCCCTTCCACAGACATGGGATC

CGCTGTAACTCTGTCCTCCCAGGGTTCATTGCAACACCCATGACAGAGAAAATGCCACAG

AAAGTGAAGGATAAGGTAGGA

>CL390.Contig12_A-W 403 597 minus strand PREDICTED: estradiol 17-beta-dehydrogenase 8-like [Equus caballus]

TCAGCCACCTCTGGTGCAGGGGACTTCACTGAGGCATGTAGTGGCGTGAGGGAAGGGTCA

GTAATCGGTCTAGAGTTCAGCAGCCACTCTCCCTTCCACAGACATGGGATCCGCTGTAAC

TCTGTCCTCCCAGGGTTCATTGCAACACCCATGACAGAGAAAATGCCACAGAAAGTGAAG

GATAAGGTAGGAGGC

>CL390.Contig13_A-W 102 593 minus strand Estradiol 17-beta-dehydrogenase 8 [Cricetulus griseus]

GCCAGCTTTTCTCGCCCGCCATCTGTCGTTGTGTCTTGTGCGGGCATCACTCGGGATGAG

TTTCTGCTCCACATGTCTGAGGATGACTGGGACCAAGTCATAGATGTCAACCTCAAGGTG

GTGACCTCTGAACCTGTGACCTCTGATCCCCCAAGCCAGGAGAGGGAATTGGAGGAGGGC

TGTCATCCCAGCTGATCTCACCTTACTACCCTCTTCTGCCAGGGCATCTTCCTAGTTACT

CAGGCTGCAGCTCAAGCCTTGGTGTCCAGTGGTTATCACGGCTCTATTATCAATATCAGT

AGCATCGTTGGAAAGGTGGGGAACATCGGGCAGATAAATTATGCTGCATCCAAGGCTGGA

GTGATTGGGCTCACCCAGACTGCAGCCCGCGAGCTTGGACGACATGGGATCCGCTGTAAC

TCTGTCCTCCCAGGGTTCATTGCAACACCCATGACAGAGAAAATGCCACAGAAAGTGAAG

GATAAGGTAGGA

>CL390.Contig14_A-W 102 614 minus strand PREDICTED: estradiol 17-beta-dehydrogenase 8 [Otolemur garnettii]

GCCAGCTTTTCTCGCCCGCCATCTGTCGTTGTGTCTTGTGCGGGCATCACTCGGGATGAG

TTTCTGCTCCACATGTCTGAGGATGACTGGGACCAAGTCATAGATGTCAACCTCAAGGGC

ATCTTCCTAGTTACTCAGGCTGCAGCTCAAGCCTTGGTGTCCAGTGGTTATCACGGCTCT

ATTATCAATATCAGTAGCATCGTTGGAAAGGTGGGGAACATCGGGCAGATAAATTATGCT

GCATCCAAGGCTGGAGTGATTGGGCTCACCCAGACTGCAGCCCGCGAGCTTGGACGACAT

GGGATCCGCTGTAACTCTGTCCTCCCAGGGTTCATTGCAACACCCATGACAGAGAAAATG

CCACAGAAAGTGAAGGATAAGGTGACCAGAATGATCCCAATGGGACACTGGGGGAACCCT

GAGGATGTGGCAGATGTGGTTGCATTCTTGGCATCTGAAGACAGTGGATACATCACAGGG

GCCTCAGTGGAAGTCACTGGAGGTCTTTTCATG

>CL390.Contig15_A-W 108 491 minus strand PREDICTED: estradiol 17-beta-dehydrogenase 8-like [Equus caballus]

TTTTCTCGCCCGCCATCTGTCGTTGTGTCTTGTGCGGGCATCACTCGGGATGAGTTTCTG

CTCCACATGTCTGAGGATGACTGGGACCAAGTCATAGATGTCAACCTCAAGGGCATCTTC

CTAGTTACTCAGGCTGCAGCTCAAGCCTTGGTGTCCAGTGGTTATCACGGCTCTATTATC

AATATCAGTAGCATCGTTGGAAAGGTGGGGAACATCGGGCAGATAAATTATGCTGCATCC

AAGGCTGGAGTGATTGGGCTCACCCAGACTGCAGCCCGCGAGCTTGGACGACATGGGATC

CGCTGTAACTCTGTCCTCCCAGGGTTCATTGCAACACCCATGACAGAGAAAATGCCACAG

AAAGTGAAGGATAAGGTAGGAGGC

>CL390.Contig16_A-W 460 597 minus strand Estradiol 17-beta-dehydrogenase 8 [Cricetulus griseus]

GAAGGGTCAGTAATCGGTCTAGAGTTCAGCAGCCACTCTCCCTTCCACAGACATGGGATC

CGCTGTAACTCTGTCCTCCCAGGGTTCATTGCAACACCCATGACAGAGAAAATGCCACAG

AAAGTGAAGGATAAGGTG

>CL390.Contig17_A-W 304 531 minus strand PREDICTED: estradiol 17-beta-dehydrogenase 8-like [Equus caballus]

TCAGCCACCTCTGGTGCAGGGGACTTCACTGAGGCATGTAGTGGCGTGAGGGAAGGGTCA

GTAATCGGTCTAGAGTTCAGCAGCCACTCTCCCTTCCACAGACATGGGATCCGCTGTAAC

TCTGTCCTCCCAGGGTTCATTGCAACACCCATGACAGAGAAAATGCCACAGAAAGTGAAG

GATAAGGTGACCAGAATGATCCCAATGGGACACTGGGGGAACCCTGAG

>CL390.Contig18_A-W 3 491 minus strand Estradiol 17-beta-dehydrogenase 8 [Cricetulus griseus]

GCCAGCTTTTCTCGCCCGCCATCTGTCGTTGTGTCTTGTGCGGGCATCACTCGGGATGAG

TTTCTGCTCCACATGTCTGAGGATGACTGGGACCAAGTCATAGATGTCAACCTCAAGGTG

GTGACCTCTGAACCTGTGACCTCTGATCCCCCAAGCCAGGAGAGGGAATTGGAGGAGGGC

TGTCATCCCAGCTGATCTCACCTTACTACCCTCTTCTGCCAGGGCATCTTCCTAGTTACT

CAGGCTGCAGCTCAAGCCTTGGTGTCCAGTGGTTATCACGGCTCTATTATCAATATCAGT

AGCATCGTTGGAAAGGTGGGGAACATCGGGCAGATAAATTATGCTGCATCCAAGGCTGGA

GTGATTGGGCTCACCCAGACTGCAGCCCGCGAGCTTGGACGACATGGGATCCGCTGTAAC

TCTGTCCTCCCAGGGTTCATTGCAACACCCATGACAGAGAAAATGCCACAGAAAGTGAAG

GATAAGGTG

>CL390.Contig19_A-W 3 503 minus strand Estradiol 17-beta-dehydrogenase 8 [Cricetulus griseus]

GCCAGCTTTTCTCGCCCGCCATCTGTCGTTGTGTCTTGTGCGGGCATCACTCGGGATGAG

TTTCTGCTCCACATGTCTGAGGATGACTGGGACCAAGTCATAGATGTCAACCTCAAGGTG

GTGACCTCTGAACCTGTGACCTCTGATCCCCCAAGCCAGGAGAGGGAATTGGAGGAGGGC

TGTCATCCCAGCTGATCTCACCTTACTACCCTCTTCTGCCAGGGCATCTTCCTAGTTACT

CAGGCTGCAGCTCAAGCCTTGGTGTCCAGTGGTTATCACGGCTCTATTATCAATATCAGT

AGCATCGTTGGAAAGGTCAGGTTGGGTTGGGACAAGGTCAGCCAGCCAAGTGGGCACAGA

GAGAAGGGCCCCTCCTTGAGATTCCTTACTCACCTTAAATCTCTGACTCACCTGTAGGTG

GGGAACATCGGGCAGATAAATTATGCTGCATCCAAGGCTGGAGTGATTGGGCTCACCCAG

ACTGCAGCCCGCGAGCTTGGA

>CL390.Contig20_A-W 741 881 minus strand Estradiol 17-beta-dehydrogenase 8 [Cricetulus griseus]

GAAGGGTCAGTAATCGGTCTAGAGTTCAGCAGCCACTCTCCCTTCCACAGACATGGGATC

CGCTGTAACTCTGTCCTCCCAGGGTTCATTGCAACACCCATGACAGAGAAAATGCCACAG

AAAGTGAAGGATAAGGTAGGA

>CL390.Contig21_A-W 3 431 minus strand H2-K region expressed gene 6, isoform CRA_e [Mus musculus]

GCCAGCTTTTCTCGCCCGCCATCTGTCGTTGTGTCTTGTGCGGGCATCACTCGGGATGAG

TTTCTGCTCCACATGTCTGAGGATGACTGGGACCAAGTCATAGATGTCAACCTCAAGGGC

ATCTTCCTAGTTACTCAGGCTGCAGCTCAAGCCTTGGTGTCCAGTGGTTATCACGGCTCT

ATTATCAATATCAGTAGCATCGTTGGAAAGGTGGGGAACATCGGGCAGATAAATTATGCT

GCATCCAAGGCTGGAGTGATTGGGCTCACCCAGACTGCAGCCCGCGAGCTTGGACGACAT

GGGATCCGCTGTAACTCTGTCCTCCCAGGGTTCATTGCAACACCCATGACAGAGAAAATG

CCACAGAAAGTGAAGGATAAGGTGACCAGAATGATCCCAATGGGACACTGGGGGAACCCT

GAGGGTGAG

>CL390.Contig22_A-W 9 398 minus strand PREDICTED: estradiol 17-beta-dehydrogenase 8-like [Equus caballus]

TTTTCTCGCCCGCCATCTGTCGTTGTGTCTTGTGCGGGCATCACTCGGGATGAGTTTCTG

CTCCACATGTCTGAGGATGACTGGGACCAAGTCATAGATGTCAACCTCAAGGGCATCTTC

CTAGTTACTCAGGCTGCAGCTCAAGCCTTGGTGTCCAGTGGTTATCACGGCTCTATTATC

AATATCAGTAGCATCGTTGGAAAGGTCAGGTTGGGTTGGGACAAGGTCAGCCAGCCAAGT

GGGCACAGAGAGAAGGGCCCCTCCTTGAGATTCCTTACTCACCTTAAATCTCTGACTCAC

CTGTAGGTGGGGAACATCGGGCAGATAAATTATGCTGCATCCAAGGCTGGAGTGATTGGG

CTCACCCAGACTGCAGCCCGCGAGCTTGGA

>CL390.Contig23_A-W 585 779 minus strand PREDICTED: estradiol 17-beta-dehydrogenase 8-like [Equus caballus]

TCAGCCACCTCTGGTGCAGGGGACTTCACTGAGGCATGTAGTGGCGTGAGGGAAGGGTCA

GTAATCGGTCTAGAGTTCAGCAGCCACTCTCCCTTCCACAGACATGGGATCCGCTGTAAC

TCTGTCCTCCCAGGGTTCATTGCAACACCCATGACAGAGAAAATGCCACAGAAAGTGAAG

GATAAGGTAGGAGGC

>CL390.Contig24_A-W 20 775 minus strand Estradiol 17-beta-dehydrogenase 8 [Cricetulus griseus]

ATGGCGTCTCAGCTTCGGCTCGGTTCTGCGCTGGCCTTGGTCACAGGTGCGGGTAGCGGC

ATCGGCCGGGCGGTTAGTGCGCGCCTGGCCAGAGAGGGGGCTACCGTAGCCGCCTGCGAC

GTGGACGGGACAGCAGCTCAGGAGACGGTGCGGCTGCTAGACGGGTCGAGGAGTGAGGAA

GGGGCGCCGGGCGGGAACCACGCTGCCTTCCAGGCTGATGTGTCTGAGGCCAGAGCTACC

AGGCGCCTGCTGGAAAAAGTGCAGGCCAGCTTTTCTCGCCCGCCATCTGTCGTTGTGTCT

TGTGCGGGCATCACTCGGGATGAGTTTCTGCTCCACATGTCTGAGGATGACTGGGACCAA

GTCATAGATGTCAACCTCAAGGTGGTGACCTCTGAACCTGTGACCTCTGATCCCCCAAGC

CAGGAGAGGGAATTGGAGGAGGGCTGTCATCCCAGCTGATCTCACCTTACTACCCTCTTC

TGCCAGGGCATCTTCCTAGTTACTCAGGCTGCAGCTCAAGCCTTGGTGTCCAGTGGTTAT

CACGGCTCTATTATCAATATCAGTAGCATCGTTGGAAAGGTGGGGAACATCGGGCAGATA

AATTATGCTGCATCCAAGGCTGGAGTGATTGGGCTCACCCAGACTGCAGCCCGCGAGCTT

GGACGACATGGGATCCGCTGTAACTCTGTCCTCCCAGGGTTCATTGCAACACCCATGACA

GAGAAAATGCCACAGAAAGTGAAGGATAAGGTAGGA

>CL390.Contig25_A-W 559 699 minus strand Estradiol 17-beta-dehydrogenase 8 [Cricetulus griseus]

GAAGGGTCAGTAATCGGTCTAGAGTTCAGCAGCCACTCTCCCTTCCACAGACATGGGATC

CGCTGTAACTCTGTCCTCCCAGGGTTCATTGCAACACCCATGACAGAGAAAATGCCACAG

AAAGTGAAGGATAAGGTAGGA

>CL390.Contig26_A-W 741 878 minus strand Estradiol 17-beta-dehydrogenase 8 [Cricetulus griseus]

GAAGGGTCAGTAATCGGTCTAGAGTTCAGCAGCCACTCTCCCTTCCACAGACATGGGATC

CGCTGTAACTCTGTCCTCCCAGGGTTCATTGCAACACCCATGACAGAGAAAATGCCACAG

AAAGTGAAGGATAAGGTG

>CL390.Contig27_A-W 403 597 minus strand PREDICTED: estradiol 17-beta-dehydrogenase 8-like [Equus caballus]

TCAGCCACCTCTGGTGCAGGGGACTTCACTGAGGCATGTAGTGGCGTGAGGGAAGGGTCA

GTAATCGGTCTAGAGTTCAGCAGCCACTCTCCCTTCCACAGACATGGGATCCGCTGTAAC

TCTGTCCTCCCAGGGTTCATTGCAACACCCATGACAGAGAAAATGCCACAGAAAGTGAAG

GATAAGGTAGGAGGC

>CL390.Contig28_A-W 870 1046 PREDICTED: estradiol 17-beta-dehydrogenase 8-like [Equus caballus]

CAGGTGACCAGAATGATCCCAATGGGACACTGGGGGAACCCTGAGGATGTGGCAGATGTG

GTTGCATTCTTGGCATCTGAAGACAGTGGATACATCACAGGGGCCTCAGTGGAAGTCACT

GGAGGTCTTTTCATGTAACTGCCTCAAGGACCCTGGACTCTTCTCACTCCCCCACCA

>CL390.Contig29_A-W 20 673 minus strand PREDICTED: estradiol 17-beta-dehydrogenase 8-like [Equus caballus]

ATGGCGTCTCAGCTTCGGCTCGGTTCTGCGCTGGCCTTGGTCACAGGTGCGGGTAGCGGC

ATCGGCCGGGCGGTTAGTGCGCGCCTGGCCAGAGAGGGGGCTACCGTAGCCGCCTGCGAC

GTGGACGGGACAGCAGCTCAGGAGACGGTGCGGCTGCTAGACGGGTCGAGGAGTGAGGAA

GGGGCGCCGGGCGGGAACCACGCTGCCTTCCAGGCTGATGTGTCTGAGGCCAGAGCTACC

AGGCGCCTGCTGGAAAAAGTGCAGGCCAGCTTTTCTCGCCCGCCATCTGTCGTTGTGTCT

TGTGCGGGCATCACTCGGGATGAGTTTCTGCTCCACATGTCTGAGGATGACTGGGACCAA

GTCATAGATGTCAACCTCAAGGGCATCTTCCTAGTTACTCAGGCTGCAGCTCAAGCCTTG

GTGTCCAGTGGTTATCACGGCTCTATTATCAATATCAGTAGCATCGTTGGAAAGGTGGGG

AACATCGGGCAGATAAATTATGCTGCATCCAAGGCTGGAGTGATTGGGCTCACCCAGACT

GCAGCCCGCGAGCTTGGACGACATGGGATCCGCTGTAACTCTGTCCTCCCAGGGTTCATT

GCAACACCCATGACAGAGAAAATGCCACAGAAAGTGAAGGATAAGGTAGGAGGC

>CL391.Contig1_A-W 225 353 hCG2007893 [Homo sapiens]

AAATATACAGCTTGCTGGGTGTGGTGGCACACACCTGTAATCCCATCGGTTCAGGAGGCT

GACACAGGAGGATCAGTTGAGCCCAGGAGTTCAGGGCCAGCCTCACCAACATACTGAGAC

CCCATCTTT

>CL392.Contig1_A-W 2771 3019 minus strand transmembrane channel-like protein 6 isoform 1 [Mus musculus] >gi|123261068|emb|CAM15524.1| transmembrane channel-like gene family 6 [Mus musculus] >gi|148702677|gb|EDL34624.1| transmembrane channel-like gene family 6, isoform CRA_b [Mus musculus] >gi|148702680|gb|EDL34627.1| transmembrane channel-like gene family 6, isoform CRA_b [Mus musculus]

GCCATCATCTACTTCAACATCCAGGTGGTGAAGGGCCAGCGGAAGGTCATCTCCCTCCTT

AAGGAGCAGATCCGCAATGAGGGAGAAGACAAGATCTTCTTGATCAACAGGCTTCACTCT

GTCTACGAGAGGAAGGAGAGGAGCAGAGCTCACAGGAACGAGGAGGCCGTGATGCCCTCT

GCCCTGCTTGCAGACGGAGGGGACAGCTGGTGGGACAGTGAGGGGCCTTGCAGGCTCCCC

CAGCACCCC

>CL392.Contig2_A-W 400 2865 minus strand RecName: Full=Transmembrane channel-like protein 6 >gi|32264653|gb|AAP78773.1| Tmc6 protein [Mus musculus] >gi|34849670|gb|AAH58195.1| Transmembrane channel-like gene family 6 [Mus musculus] >gi|37514839|gb|AAH04840.2| Transmembrane channel-like gene family 6 [Mus musculus]

ATGGCCCAGCCACTGGCCTTGGTCCTCAATGTCCCTGAGGCTCTAGGGGACCAGGACCAG

GAGCCCAGTCCCTATGAGGAGAGCGAGGTGCATGACTCCTTCCACCAGCTCATCCAAGAG

CAGAGCCAGCTGGCGGAGGAGGAGCTGGAACTGCAGCAGAGGCAGCTGGTGGCCGGAACC

CTGGGGGTCCCAGGCAGTGGTCACCAGACCCTCCTGGGGCCAGAGGGTGCCCCTGTCCAC

AGCGCAGCCACACTCCGCATCTTGGCCAGCATGCCTAGCCGCACCATCGGCCGCAGCCGT

GGGGCCATCATCTCCCAGTACTACAACCGGACGGTGAAGCTGCGGCGCCGGGGCAGCCGG

CCCCTGCTCGGCGACATGGTGCGCTCTGCCCGGCCCAGCCTCCGCCTGTATGACCTGGAG

CTGGGCCCCAGGGCCCTCGAGGAGGATGAGAAGCAAGGCCTCCTGGTGAAGGAGCTTCAG

GGCCTGTCGGTGGTCCAGCGGGACCACATGCTCCGGGGGATGCCCTTGAACTTGGCTGAG

AAACGCTGCCTGCGAGAGAAGAGCCAGATTCAGAGGGGGAAGCGGAGGGCCCGGCAGGAC

CGTGGGGGCGTCTTTTCCTGCTGCAGCCGGCTCAGATACGCCTGCATCCTGGCCCTGCAT

AGCCTAGGGCTGGCGCTGCTCTCGGGCCTGCACGCCGCGAGGCCGTGGCGCCACACACTG

AAGCAGATCGGAGGCCAGTTCGGCTCCAGCGTGCTCTCCTACTTCCTGTTCCTCAAGACC

CTGCTGGCCTTCAACGCGCTGCTGCTGCTGCTGCTGCTGGGCCTCCTCGCGGGGGTGCAA

GCTGCCCTCCCGCCCGGCCCGCCGGACCCTGCCCCCGCCTTCACAGGGCTGGAGCTCCTC

ACAGGCGGGGGCTGCTTCACCCACACTGTCATGTACTATGGGTACTACAGCAACGCCACG

CTGAACCAGCCCTGCGTCCCTCCGCGGGATGGCCACCAGTGCAGCCCCGGGGCCAGCAGC

CTTCCCTACAACATGCCCCTGGCCTACCTCTTCACCATGGGGGCAGCCTTCTTCATCACC

TGCATCTCCTTGGTTTACAGCATGTCGCATGCTTTTGGGGAGAGCTACCGGGTGGGCAGC

ACCAAGGGCGTCCACGCCATCACCGTTTTCTGCTCCTGGGACTACAAGGTGACTGAGAAG

CGGGCCTCTCGCCTCCAGCAGGACAACATCCGCACCCAACTGAAGGAGCTGCTGGCTGAG

TGGCAGCTGCACAGGAGCCCCCAGAGCCTGCGTGGGCGGCTGCGGCGGGCGGTCCTGCTG

GGGCTGGCATGGCTGCTGTGTCTGGGCACCATGCTGGGCTGCTCCGTGGCCGTCTTCGTC

TTCTCAGAGGTCACGATCCAGAGCCCGGTGTCTGCCGGCCAGGAGGCGAGGCTGCTGGCT

CTGCCCCTGGTGGTCTCCCTCCTCAACCTGGGGGCCTCCTACCTGTTCCGTGGCTTGGCT

GCCCTGGAGCGGCATGAGTCCCCGGGGCTGGAGGTGTACGTGTCCATCTGCAGGAACCTC

ATCCTCAAGATGGCCATCCTAGGCATTCTTTGCTACCACTGGCTGGGCCGCAGGGTGGCT

GCCCTGCAGGGCCAGTGTTGGGAGGACTTCGTGGGCCAGGAGCTGTACCGGTTCATGGTG

ATGGACTTCCTGTTCGCACTGCTCGACACGCTGTTTGGGGAGCTGGTGTGGAGGCTCATC

TCGGAGAGGAAGCTCAAGAGGAGGCGGAAACCTGAGTTCGATATTGCAAGGAATGTTCTG

GACCTGATTTATGGGCAGACTCTGACCTGGCTGGGGGTCCTCTTCTCGCCTCTCCTGCCG

GCTGTGCAGGTCCTCAGGCTGTTGCTCCTCTTCCTGGTCAAGAAGGCTAGCCTGATGGCC

AACTGCCAAGCTCCCCGCAGGCCCTGGCTGGCCTCGCACATGAGCACGGTCTTCCTCACA

CTGCTCTGCTTCCCTTCCTTCCTGGGTGCCGCCATCTTCCTCTGCTACGCCGTGTGGCAG

GTGAAGCCCTCGAGCACCTGCGGCCCCTTTCGGACCCTGGACACCATGTATGAGGCAGGC

AGGGTGTGGGTGCGCCACCTGGAGAGTGCGGGCCCCGGGGCCTCCTGGGTGCCCTGGCTG

CACCGGTACCTGGTGGAGAGCACCTTCTTCCTCTTCTTGGCATCTGCCCTGTTGCTGGCC

ATCATCTACTTCAACATCCAGGTGGTGAAGGGCCAGCGGAAGGTCATCTCCCTCCTTAAG

GAGCAGATCCGCAATGAGGGAGAAGACAAGATCTTCTTGATCAACAGGCTTCACTCTGTC

TACGAGAGGAAGGAGAGGAGCAGAGCTCACAGGAACGAGGAGGCCGTGATGCCCTCTGCC

CTGCTTGCAGACGGAGGGGACAGCTGGTGGGACAGTGAGGGGCCTTGCAGGCTCCCCCAG

CACCCC

>CL392.Contig3_A-W 370 2235 minus strand PREDICTED: LOW QUALITY PROTEIN: transmembrane channel-like protein 6 [Callithrix jacchus]

CTGCAGACGGGCTGGGCGTGGACCGCAGACATGGCCCAGCCACTGGCCTTGGTCCTCAAT

GTCCCTGAGGCTCTAGGGGACCAGGACCAGGAGCCCAGTCCCTATGAGGAGAGCGAGGTG

CATGACTCCTTCCACCAGCTCATCCAAGAGCAGAGCCAGCTGGCGGAGGAGGAGCTGGAA

CTGCAGCAGAGGCAGCTGGTGGCCGGAACCCTGGGGGTCCCAGGCAGTGGTCACCAGACC

CTCCTGGGGCCAGAGGGTGCCCCTGTCCACAGCGCAGCCACACTCCGCATCTTGGCCAGC

ATGCCTAGCCGCACCATCGGCCGCAGCCGTGGGGCCATCATCTCCCAGTACTACAACCGG

ACGGTGAAGCTGCGGCGCCGGGGCAGCCGGCCCCTGCTCGGCGACATGGTGCGCTCTGCC

CGGCCCAGCCTCCGCCTGTATGACCTGGAGCTGGGCCCCAGGGCCCTCGAGGAGGATGAG

AAGCAAGGCCTCCTGGTGAAGGAGCTTCAGGGCCTGTCGGTGGTCCAGCGGGACCACATG

CTCCGGGGGATGCCCTTGAACTTGGCTGAGAAACGCTGCCTGCGAGAGAAGAGCCAGATT

CAGAGGGGGAAGCGGAGGGCCCGGCAGGACCGTGGGGGCGTCTTTTCCTGCTGCAGCCGG

CTCAGATACGCCTGCATCCTGGCCCTGCATAGCCTAGGGCTGGCGCTGCTCTCGGGCCTG

CACGCCGCGAGGCCGTGGCGCCACACACTGAAGCAGATCGGAGGCCAGTTCGGCTCCAGC

GTGCTCTCCTACTTCCTGTTCCTCAAGACCCTGCTGGCCTTCAACGCGCTGCTGCTGCTG

CTGCTGCTGGGCCTCCTCGCGGGGGTGCAAGCTGCCCTCCCGCCCGGCCCGCCGGACCCT

GCCCCCGCCTTCACAGGGCTGGAGCTCCTCACAGGCGGGGGCTGCTTCACCCACACTGTC

ATGTACTATGGGTACTACAGCAACGCCACGCTGAACCAGCCCTGCGTCCCTCCGCGGGAT

GGCCACCAGTGCAGCCCCGGGGCCAGCAGCCTTCCCTACAACATGCCCCTGGCCTACCTC

TTCACCATGGGGGCAGCCTTCTTCATCACCTGCATCTCCTTGGTTTACAGCATGTCGCAT

GCTTTTGGGGAGAGCTACCGGGTGGGCAGCACCAAGGGCGTCCACGCCATCACCGTTTTC

TGCTCCTGGGACTACAAGGTGACTGAGAAGCGGGCCTCTCGCCTCCAGCAGGACAACATC

CGCACCCAACTGAAGGAGCTGCTGGCTGAGTGGCAGCTGCACAGGAGCCCCCAGAGCCTG

CGTGGGCGGCTGCGGCGGGCGGTCCTGCTGGGGCTGGCATGGCTGCTGTGTCTGGGCACC

ATGCTGGGCTGCTCCGTGGCCGTCTTCGTCTTCTCAGAGGTCACGATCCAGAGCCCGGTG

TCTGCCGGCCAGGAGGCGAGGCTGCTGGCTCTGCCCCTGGTGGTCTCCCTCCTCAACCTG

GGGGCCTCCTACCTGTTCCGTGGCTTGGCTGCCCTGGAGCGGCATGAGTCCCCGGGGCTG

GAGGTGTACGTGTCCATCTGCAGGAACCTCATCCTCAAGATGGCCATCCTAGGCATTCTT

TGCTACCACTGGCTGGGCCGCAGGGTGGCTGCCCTGCAGGGCCAGTGTTGGGAGGACTTC

GTGGGCCAGGAGCTGTACCGGTTCATGGTGATGGACTTCCTGTTCGCACTGCTCGACACG

CTGTTTGGGGAGCTGGTGTGGAGGCTCATCTCGGAGAGGAAGCTCAAGAGGAGGCGGAAA

CCTGAGTTCGATATTGCAAGGAATGTTCTGGACCTGATTTATGGGCAGACTCTGACCTGG

TGAGGT

>CL392.Contig4_A-W 370 2133 minus strand PREDICTED: LOW QUALITY PROTEIN: transmembrane channel-like protein 6 [Callithrix jacchus]

CTGCAGACGGGCTGGGCGTGGACCGCAGACATGGCCCAGCCACTGGCCTTGGTCCTCAAT

GTCCCTGAGGCTCTAGGGGACCAGGACCAGGAGCCCAGTCCCTATGAGGAGAGCGAGGTG

CATGACTCCTTCCACCAGCTCATCCAAGAGCAGAGCCAGCTGGCGGAGGAGGAGCTGGAA

CTGCAGCAGAGGCAGCTGGTGGCCGGAACCCTGGGGGTCCCAGGCAGTGGTCACCAGACC

CTCCTGGGGCCAGAGGGTGCCCCTGTCCACAGCGCAGCCACACTCCGCATCTTGGCCAGC

ATGCCTAGCCGCACCATCGGCCGCAGCCGTGGGGCCATCATCTCCCAGTACTACAACCGG

ACGGTGAAGCTGCGGCGCCGGGGCAGCCGGCCCCTGCTCGGCGACATGGTGCGCTCTGCC

CGGCCCAGCCTCCGCCTGTATGACCTGGAGCTGGGCCCCAGGGCCCTCGAGGAGGATGAG

AAGCAAGGCCTCCTGGTGAAGGAGCTTCAGGGCCTGTCGGTGGTCCAGCGGGACCACATG

CTCCGGGGGATGCCCTTGAACTTGGCTGAGAAACGCTGCCTGCGAGAGAAGAGCCAGATT

CAGAGGGGGAAGCGGAGGGCCCGGCAGGACCGTGGGGGCGTCTTTTCCTGCTGCAGCCGG

CTCAGATACGCCTGCATCCTGGCCCTGCATAGCCTAGGGCTGGCGCTGCTCTCGGGCCTG

CACGCCGCGAGGCCGTGGCGCCACACACTGAAGCAGATCGGAGGCCAGTTCGGCTCCAGC

GTGCTCTCCTACTTCCTGTTCCTCAAGACCCTGCTGGCCTTCAACGCGCTGCTGCTGCTG

CTGCTGCTGGGCCTCCTCGCGGGGGTGCAAGCTGCCCTCCCGCCCGGCCCGCCGGACCCT

GCCCCCGCCTTCACAGGGCTGGAGCTCCTCACAGGCGGGGGCTGCTTCACCCACACTGTC

ATGTACTATGGGTACTACAGCAACGCCACGCTGAACCAGCCCTGCGTCCCTCCGCGGGAT

GGCCACCAGTGCAGCCCCGGGGCCAGCAGCCTTCCCTACAACATGCCCCTGGCCTACCTC

TTCACCATGGGGGCAGCCTTCTTCATCACCTGCATCTCCTTGGTTTACAGCATGTCGCAT

GCTTTTGGGGAGAGCTACCGGGTGGGCAGCACCAAGGGCGTCCACGCCATCACCGTTTTC

TGCTCCTGGGACTACAAGGTGACTGAGAAGCGGGCCTCTCGCCTCCAGCAGGACAACATC

CGCACCCAACTGAAGGAGCTGCTGGCTGAGTGGCAGCTGCACAGGAGCCCCCAGAGCCTG

CGTGGGCGGCTGCGGCGGGCGGTCCTGCTGGGGCTGGCATGGCTGCTGTGTCTGGGCACC

ATGCTGGGCTGCTCCGTGGCCGTCTTCGTCTTCTCAGAGGTCACGATCCAGAGCCCGGTG

TCTGCCGGCCAGGAGGCGAGGCTGCTGGCTCTGCCCCTGGTGGTCTCCCTCCTCAACCTG

GGGGCCTCCTACCTGTTCCGTGGCTTGGCTGCCCTGGAGCGGCATGAGTCCCCGGGGCTG

GAGGTGTACGTGTCCATCTGCAGGAACCTCATCCTCAAGATGGCCATCCTAGGCATTCTT

TGCTACCACTGGCTGGGCCGCAGGGTGGCTGCCCTGCAGGGCCAGTGTTGGGAGGACTTC

GTGGGCCAGGAGCTGTACCGGTTCATGGTGATGGACTTCCTGTTCGCACTGCTCGACACG

CTGTTTGGGGAGCTGGTGTGGAGG

>CL392.Contig5_A-W 33 1688 minus strand transmembrane channel-like protein 6 isoform 1 [Mus musculus] >gi|123261068|emb|CAM15524.1| transmembrane channel-like gene family 6 [Mus musculus] >gi|148702677|gb|EDL34624.1| transmembrane channel-like gene family 6, isoform CRA_b [Mus musculus] >gi|148702680|gb|EDL34627.1| transmembrane channel-like gene family 6, isoform CRA_b [Mus musculus]

CCTTCTTTTATAGGCAGTGGTCACCAGACCCTCCTGGGGCCAGAGGGTGCCCCTGTCCAC

AGCGCAGCCACACTCCGCATCTTGGCCAGCATGCCTAGCCGCACCATCGGCCGCAGCCGT

GGGGCCATCATCTCCCAGTACTACAACCGGACGGTGAAGCTGCGGCGCCGGGGCAGCCGG

CCCCTGCTCGGCGACATGGTGCGCTCTGCCCGGCCCAGCCTCCGCCTGTATGACCTGGAG

CTGGGCCCCAGGGCCCTCGAGGAGGATGAGAAGCAAGGCCTCCTGGTGAAGGAGCTTCAG

GGCCTGTCGGTGGTCCAGCGGGACCACATGCTCCGGGGGATGCCCTTGAACTTGGCTGAG

AAACGCTGCCTGCGAGAGAAGAGCCAGATTCAGAGGGGGAAGCGGAGGGCCCGGCAGGAC

CGTGGGGGCGTCTTTTCCTGCTGCAGCCGGCTCAGATACGCCTGCATCCTGGCCCTGCAT

AGCCTAGGGCTGGCGCTGCTCTCGGGCCTGCACGCCGCGAGGCCGTGGCGCCACACACTG

AAGCAGATCGGAGGCCAGTTCGGCTCCAGCGTGCTCTCCTACTTCCTGTTCCTCAAGACC

CTGCTGGCCTTCAACGCGCTGCTGCTGCTGCTGCTGCTGGGCCTCCTCGCGGGGGTGCAA

GCTGCCCTCCCGCCCGGCCCGCCGGACCCTGCCCCCGCCTTCACAGGGCTGGAGCTCCTC

ACAGGCGGGGGCTGCTTCACCCACACTGTCATGTACTATGGGTACTACAGCAACGCCACG

CTGAACCAGCCCTGCGTCCCTCCGCGGGATGGCCACCAGTGCAGCCCCGGGGCCAGCAGC

CTTCCCTACAACATGCCCCTGGCCTACCTCTTCACCATGGGGGCAGCCTTCTTCATCACC

TGCATCTCCTTGGTTTACAGCATGTCGCATGCTTTTGGGGAGAGCTACCGGGTGGGCAGC

ACCAAGGGCGTCCACGCCATCACCGTTTTCTGCTCCTGGGACTACAAGGTGACTGAGAAG

CGGGCCTCTCGCCTCCAGCAGGACAACATCCGCACCCAACTGAAGGAGCTGCTGGCTGAG

TGGCAGCTGCACAGGAGCCCCCAGAGCCTGCGTGGGCGGCTGCGGCGGGCGGTCCTGCTG

GGGCTGGCATGGCTGCTGTGTCTGGGCACCATGCTGGGCTGCTCCGTGGCCGTCTTCGTC

TTCTCAGAGGTCACGATCCAGAGCCCGGTGTCTGCCGGCCAGGAGGCGAGGCTGCTGGCT

CTGCCCCTGGTGGTCTCCCTCCTCAACCTGGGGGCCTCCTACCTGTTCCGTGGCTTGGCT

GCCCTGGAGCGGCATGAGTCCCCGGGGCTGGAGGTGTACGTGTCCATCTGCAGGAACCTC

ATCCTCAAGATGGCCATCCTAGGCATTCTTTGCTACCACTGGCTGGGCCGCAGGGTGGCT

GCCCTGCAGGGCCAGTGTTGGGAGGACTTCGTGGGCCAGGAGCTGTACCGGTTCATGGTG

ATGGACTTCCTGTTCGCACTGCTCGACACGCTGTTTGGGGAGCTGGTGTGGAGGCTCATC

TCGGAGAGGAAGCTCAAGAGGAGGCGGAAACCTGAGTTCGATATTGCAAGGAATGTTCTG

GACCTGATTTATGGGCAGACTCTGACCTGGTGAGGT

>CL392.Contig6_A-W 33 1586 minus strand transmembrane channel-like protein 6 isoform 1 [Mus musculus] >gi|123261068|emb|CAM15524.1| transmembrane channel-like gene family 6 [Mus musculus] >gi|148702677|gb|EDL34624.1| transmembrane channel-like gene family 6, isoform CRA_b [Mus musculus] >gi|148702680|gb|EDL34627.1| transmembrane channel-like gene family 6, isoform CRA_b [Mus musculus]

CCTTCTTTTATAGGCAGTGGTCACCAGACCCTCCTGGGGCCAGAGGGTGCCCCTGTCCAC

AGCGCAGCCACACTCCGCATCTTGGCCAGCATGCCTAGCCGCACCATCGGCCGCAGCCGT

GGGGCCATCATCTCCCAGTACTACAACCGGACGGTGAAGCTGCGGCGCCGGGGCAGCCGG

CCCCTGCTCGGCGACATGGTGCGCTCTGCCCGGCCCAGCCTCCGCCTGTATGACCTGGAG

CTGGGCCCCAGGGCCCTCGAGGAGGATGAGAAGCAAGGCCTCCTGGTGAAGGAGCTTCAG

GGCCTGTCGGTGGTCCAGCGGGACCACATGCTCCGGGGGATGCCCTTGAACTTGGCTGAG

AAACGCTGCCTGCGAGAGAAGAGCCAGATTCAGAGGGGGAAGCGGAGGGCCCGGCAGGAC

CGTGGGGGCGTCTTTTCCTGCTGCAGCCGGCTCAGATACGCCTGCATCCTGGCCCTGCAT

AGCCTAGGGCTGGCGCTGCTCTCGGGCCTGCACGCCGCGAGGCCGTGGCGCCACACACTG

AAGCAGATCGGAGGCCAGTTCGGCTCCAGCGTGCTCTCCTACTTCCTGTTCCTCAAGACC

CTGCTGGCCTTCAACGCGCTGCTGCTGCTGCTGCTGCTGGGCCTCCTCGCGGGGGTGCAA

GCTGCCCTCCCGCCCGGCCCGCCGGACCCTGCCCCCGCCTTCACAGGGCTGGAGCTCCTC

ACAGGCGGGGGCTGCTTCACCCACACTGTCATGTACTATGGGTACTACAGCAACGCCACG

CTGAACCAGCCCTGCGTCCCTCCGCGGGATGGCCACCAGTGCAGCCCCGGGGCCAGCAGC

CTTCCCTACAACATGCCCCTGGCCTACCTCTTCACCATGGGGGCAGCCTTCTTCATCACC

TGCATCTCCTTGGTTTACAGCATGTCGCATGCTTTTGGGGAGAGCTACCGGGTGGGCAGC

ACCAAGGGCGTCCACGCCATCACCGTTTTCTGCTCCTGGGACTACAAGGTGACTGAGAAG

CGGGCCTCTCGCCTCCAGCAGGACAACATCCGCACCCAACTGAAGGAGCTGCTGGCTGAG

TGGCAGCTGCACAGGAGCCCCCAGAGCCTGCGTGGGCGGCTGCGGCGGGCGGTCCTGCTG

GGGCTGGCATGGCTGCTGTGTCTGGGCACCATGCTGGGCTGCTCCGTGGCCGTCTTCGTC

TTCTCAGAGGTCACGATCCAGAGCCCGGTGTCTGCCGGCCAGGAGGCGAGGCTGCTGGCT

CTGCCCCTGGTGGTCTCCCTCCTCAACCTGGGGGCCTCCTACCTGTTCCGTGGCTTGGCT

GCCCTGGAGCGGCATGAGTCCCCGGGGCTGGAGGTGTACGTGTCCATCTGCAGGAACCTC

ATCCTCAAGATGGCCATCCTAGGCATTCTTTGCTACCACTGGCTGGGCCGCAGGGTGGCT

GCCCTGCAGGGCCAGTGTTGGGAGGACTTCGTGGGCCAGGAGCTGTACCGGTTCATGGTG

ATGGACTTCCTGTTCGCACTGCTCGACACGCTGTTTGGGGAGCTGGTGTGGAGG

>CL392.Contig7_A-W 2224 2472 minus strand transmembrane channel-like protein 6 isoform 1 [Mus musculus] >gi|123261068|emb|CAM15524.1| transmembrane channel-like gene family 6 [Mus musculus] >gi|148702677|gb|EDL34624.1| transmembrane channel-like gene family 6, isoform CRA_b [Mus musculus] >gi|148702680|gb|EDL34627.1| transmembrane channel-like gene family 6, isoform CRA_b [Mus musculus]

GCCATCATCTACTTCAACATCCAGGTGGTGAAGGGCCAGCGGAAGGTCATCTCCCTCCTT

AAGGAGCAGATCCGCAATGAGGGAGAAGACAAGATCTTCTTGATCAACAGGCTTCACTCT

GTCTACGAGAGGAAGGAGAGGAGCAGAGCTCACAGGAACGAGGAGGCCGTGATGCCCTCT

GCCCTGCTTGCAGACGGAGGGGACAGCTGGTGGGACAGTGAGGGGCCTTGCAGGCTCCCC

CAGCACCCC

>CL392.Contig8_A-W 33 2318 minus strand RecName: Full=Transmembrane channel-like protein 6 >gi|32264653|gb|AAP78773.1| Tmc6 protein [Mus musculus] >gi|34849670|gb|AAH58195.1| Transmembrane channel-like gene family 6 [Mus musculus] >gi|37514839|gb|AAH04840.2| Transmembrane channel-like gene family 6 [Mus musculus]

CCTTCTTTTATAGGCAGTGGTCACCAGACCCTCCTGGGGCCAGAGGGTGCCCCTGTCCAC

AGCGCAGCCACACTCCGCATCTTGGCCAGCATGCCTAGCCGCACCATCGGCCGCAGCCGT

GGGGCCATCATCTCCCAGTACTACAACCGGACGGTGAAGCTGCGGCGCCGGGGCAGCCGG

CCCCTGCTCGGCGACATGGTGCGCTCTGCCCGGCCCAGCCTCCGCCTGTATGACCTGGAG

CTGGGCCCCAGGGCCCTCGAGGAGGATGAGAAGCAAGGCCTCCTGGTGAAGGAGCTTCAG

GGCCTGTCGGTGGTCCAGCGGGACCACATGCTCCGGGGGATGCCCTTGAACTTGGCTGAG

AAACGCTGCCTGCGAGAGAAGAGCCAGATTCAGAGGGGGAAGCGGAGGGCCCGGCAGGAC

CGTGGGGGCGTCTTTTCCTGCTGCAGCCGGCTCAGATACGCCTGCATCCTGGCCCTGCAT

AGCCTAGGGCTGGCGCTGCTCTCGGGCCTGCACGCCGCGAGGCCGTGGCGCCACACACTG

AAGCAGATCGGAGGCCAGTTCGGCTCCAGCGTGCTCTCCTACTTCCTGTTCCTCAAGACC

CTGCTGGCCTTCAACGCGCTGCTGCTGCTGCTGCTGCTGGGCCTCCTCGCGGGGGTGCAA

GCTGCCCTCCCGCCCGGCCCGCCGGACCCTGCCCCCGCCTTCACAGGGCTGGAGCTCCTC

ACAGGCGGGGGCTGCTTCACCCACACTGTCATGTACTATGGGTACTACAGCAACGCCACG

CTGAACCAGCCCTGCGTCCCTCCGCGGGATGGCCACCAGTGCAGCCCCGGGGCCAGCAGC

CTTCCCTACAACATGCCCCTGGCCTACCTCTTCACCATGGGGGCAGCCTTCTTCATCACC

TGCATCTCCTTGGTTTACAGCATGTCGCATGCTTTTGGGGAGAGCTACCGGGTGGGCAGC

ACCAAGGGCGTCCACGCCATCACCGTTTTCTGCTCCTGGGACTACAAGGTGACTGAGAAG

CGGGCCTCTCGCCTCCAGCAGGACAACATCCGCACCCAACTGAAGGAGCTGCTGGCTGAG

TGGCAGCTGCACAGGAGCCCCCAGAGCCTGCGTGGGCGGCTGCGGCGGGCGGTCCTGCTG

GGGCTGGCATGGCTGCTGTGTCTGGGCACCATGCTGGGCTGCTCCGTGGCCGTCTTCGTC

TTCTCAGAGGTCACGATCCAGAGCCCGGTGTCTGCCGGCCAGGAGGCGAGGCTGCTGGCT

CTGCCCCTGGTGGTCTCCCTCCTCAACCTGGGGGCCTCCTACCTGTTCCGTGGCTTGGCT

GCCCTGGAGCGGCATGAGTCCCCGGGGCTGGAGGTGTACGTGTCCATCTGCAGGAACCTC

ATCCTCAAGATGGCCATCCTAGGCATTCTTTGCTACCACTGGCTGGGCCGCAGGGTGGCT

GCCCTGCAGGGCCAGTGTTGGGAGGACTTCGTGGGCCAGGAGCTGTACCGGTTCATGGTG

ATGGACTTCCTGTTCGCACTGCTCGACACGCTGTTTGGGGAGCTGGTGTGGAGGCTCATC

TCGGAGAGGAAGCTCAAGAGGAGGCGGAAACCTGAGTTCGATATTGCAAGGAATGTTCTG

GACCTGATTTATGGGCAGACTCTGACCTGGCTGGGGGTCCTCTTCTCGCCTCTCCTGCCG

GCTGTGCAGGTCCTCAGGCTGTTGCTCCTCTTCCTGGTCAAGAAGGCTAGCCTGATGGCC

AACTGCCAAGCTCCCCGCAGGCCCTGGCTGGCCTCGCACATGAGCACGGTCTTCCTCACA

CTGCTCTGCTTCCCTTCCTTCCTGGGTGCCGCCATCTTCCTCTGCTACGCCGTGTGGCAG

GTGAAGCCCTCGAGCACCTGCGGCCCCTTTCGGACCCTGGACACCATGTATGAGGCAGGC

AGGGTGTGGGTGCGCCACCTGGAGAGTGCGGGCCCCGGGGCCTCCTGGGTGCCCTGGCTG

CACCGGTACCTGGTGGAGAGCACCTTCTTCCTCTTCTTGGCATCTGCCCTGTTGCTGGCC

ATCATCTACTTCAACATCCAGGTGGTGAAGGGCCAGCGGAAGGTCATCTCCCTCCTTAAG

GAGCAGATCCGCAATGAGGGAGAAGACAAGATCTTCTTGATCAACAGGCTTCACTCTGTC

TACGAGAGGAAGGAGAGGAGCAGAGCTCACAGGAACGAGGAGGCCGTGATGCCCTCTGCC

CTGCTTGCAGACGGAGGGGACAGCTGGTGGGACAGTGAGGGGCCTTGCAGGCTCCCCCAG

CACCCC

>CL393.Contig1_A-W 252 1139 minus strand PREDICTED: nuclear pore glycoprotein p62-like [Ailuropoda melanoleuca] >gi|281348339|gb|EFB23923.1| hypothetical protein PANDA_006250 [Ailuropoda melanoleuca]

ATGAGCGGGTTTAATTTTGGAGGCACTGGGGCTCCTACAGGCGGGTTCACATTTGGCACT

ACAAAGACAGTAACCACCACCCCTGCAACGGGGTTTTCTTTCTCTGCTTCGGGCACTGGA

GGGTTTAACTTTGGGACTTCCTCACAGCCAACTACAACCACCCCTTCAACTGGTCTGTTC

TCACTCACTACCCAAGCTCCAGCAACACAGTCCTCAGGGTTCACATTTGGAACAACTCCT

GCCTCTGGAGGAACTGGCTTTTCCTTAGGAATCAATGCTCCAAAGCTAAATTTGAGCAAT

ACAACTACCAGCCCAGCCACAGGGAACCCCAGTGGCTTTTGGCTTGGCAGCAGCACCCTT

ACCAATGCCATTTCAAGCACTGTTACCTCCAGCCAAGGGGCAGCACCCACTGGTTTTGTG

TTTGGCTCCTCTACCACCTCTGCAGCCCCATCCACCACATCTGGAGGGTTCTCCTTCACT

GGTGGAAGCACGTCCCAGACTGGAACCTCTGGTTTCAACATTGGCTCCGTTGGCAGTTCT

GCCCAGCCCACCGCACTTTCTGGATTACCCTTCACTCCAGCAGCACCAGCAACCACTGGA

GCAGGGACCACACAGCCAGCCGCTCCCACACCCACTGCTGCCACCACCAGTGCAGGGCCT

ACTCTCTTTGCCTCAATAGCAACTGTTCCCACTTCATCCAGCACCACAGGGCTCTCCCTC

TGTGCCCCAGCAACCACTGCCGGAGCTCCTGGAGCTGGTACACTGGGGTTTAGCTTAAAG

GCTCCTGGAGCAGCTTCTGGTGCCTCCACAGCAACATCCTCTTCCAGTACCACCACCACC

ACCACCACCACTCCCCAAAAAACACAATTTTCCAACAAAGAAAAGCCC

>CL393.Contig2_A-W 318 764 minus strand cyclic AMP-dependent transcription factor ATF-5 [Rattus norvegicus] >gi|24781899|gb|AAM92263.1| activating transcription factor 5 [Rattus norvegicus] >gi|149056027|gb|EDM07458.1| activating transcription factor 5, isoform CRA_a [Rattus norvegicus] >gi|149056028|gb|EDM07459.1| activating transcription factor 5, isoform CRA_a [Rattus norvegicus]

ATGTCACTCCTGGCGACCCTGGGGCTGGAGCTAGACAGGGCCCTGGTCCCAGCTAGCGGA

CTGGGCTGGCTGGTAGACTATGGGAAACTCCCCCTGGCCCCTGCCCCCCTGGGCCCCTAT

GAGGTCCTTGGGGGAGCCCTGGAGGGTGGGCTTCCAGGGGGAGGAGAGCCTCTGGCAGGT

GACGGCTTCTCTGACTGGATGACTGAGCGAGTTGATTTTACAGCCCTCCTCCCCCTGGAG

GCTCCCTTGCCCCCAGGTGCCCTGCCCCCACCTTCCCCACCTCCCCCTGACCTGGAAGCC

ATGGCTTCCCTGCTCAAGAAGGAGCTGGAGCAGATGGAAGACTTCTTCCTTGAGGGCCCA

CTCCTCCCTCCACCTTCACCACCACCACCACCCCCACCCCCACCATCACCACCATCATCA

TCCAGGCTTGAGAGCCACTGGCTACCC

>CL393.Contig3_A-W 177 479 minus strand unknown [Homo sapiens]

CACAATTTTCCAACAAAGAAAAGCCCAGGACCAGTTGGATTCTCAGCTGAATTCGACCTG

ACTTTAAAAGAAGAATTAATGCCAATAATACTCCTTAAATTACTCCATGAAATAGAATAG

TGTAGAACACTTCCAAATTCATTTTAAGAAGCCAGTATCATCCCGATACTAAAACCAGAG

GATACTTCAAAGAAAGAAAACTATAGACCAATATCCCTGATGAACATTGGTGCAAAAAAA

AAAAATCCTAATAAAATATTAGCAAATAAATATTTGACAACAAAAACTATATATCATGAT

CAG

>CL394.Contig1_A-W 32 220 minus strand hypothetical protein GW7_19349 [Heterocephalus glaber]

AGGTGTGTTCATCCTAGAGGGGGTGTGATTCAGAGTGTTTCTTCATGGAAACACGGCTCT

GGCACACAGTATGTTAGCACCCGGCAAAACCAGTCATGGACTACTGTGACCCCCCAGCAG

ACTTGGGCTTCACCAGCAGAAGTTGTTGATCTTACCTTGGATGAGGATAGCAGGCGTAAA

TACCTACTG

>CL394.Contig2_A-W 3 251 minus strand hypothetical protein GW7_19349 [Heterocephalus glaber]

GAAATTAATATTGCCTCTTCAGATAGTGAAGTAGAGATTGTGGGTGTTCAGGAACATGCG

AGGTGTGTTCATCCTAGAGGGGGTGTGATTCAGAGTGTTTCTTCATGGAAACACGGCTCT

GGCACACAGTATGTTAGCACCCGGCAAAACCAGTCATGGACTACTGTGACCCCCCAGCAG

ACTTGGGCTTCACCAGCAGAAGTTGTTGATCTTACCTTGGATGAGGATAGCAGGCGTAAA

TACCTACTG

>CL395.Contig1_A-W 1402 1713 minus strand c-fos [Ictidomys tridecemlineatus]

TTGTCTCCTGAAGAAGAAGAGAAAAGGAGAATTCGAAGGGAAAGGAATAAGATGGCTGCA

GCCAAGTGCCGGAACCGGAGGAGGGAGCTGACTGATACCCTCCAAGCGGTAGGTAGATCC

TCTGGGTCACTCCTTTTAAAAAGTAAGGGACAAAGTTGGAGATTGGGCATAGGGAGCAAG

GCCCTGGAGGAAGACTGTGCCTTATGCTGTCCTTCATCCCCCTGTGTACAGGAGACAGAC

CAACTTGAAGATGAGAAGTCTGCTTTGCAGACTGAGATTGCCAACCTGTTGAAGGAGAAG

GAAAAACTGGAG

>CL395.Contig2_A-W 1402 1590 minus strand c-fos [Ictidomys tridecemlineatus]

TTGTCTCCTGAAGAAGAAGAGAAAAGGAGAATTCGAAGGGAAAGGAATAAGATGGCTGCA

GCCAAGTGCCGGAACCGGAGGAGGGAGCTGACTGATACCCTCCAAGCGGAGACAGACCAA

CTTGAAGATGAGAAGTCTGCTTTGCAGACTGAGATTGCCAACCTGTTGAAGGAGAAGGAA

AAACTGGAG

>CL395.Contig3_A-W 763 1326 minus strand c-fos [Ictidomys tridecemlineatus]

GACTTCTGCACGGATCTGAATGTCTCCAGTGCCAACTTCATCCCCACAGTGACTGCTATC

TCGACCAGCCCGGACCTGCAGTGGCTGGTGCAACCCACCTTGGTCTCCTCCGTGGCCCCA

TCGCAGAACAGAGCTCCCCACCCCTACGGAGTCCCCACTCCCTCTACTGCGGCTTACTCC

AGGGCTGGAGCGGCAAAGACCATGACAGGCGGCAGAGCGCAGAGCATCGGCAGGAGGGGC

AAGGTAGAACAGTTGTCTCCTGAAGAAGAAGAGAAAAGGAGAATTCGAAGGGAAAGGAAT

AAGATGGCTGCAGCCAAGTGCCGGAACCGGAGGAGGGAGCTGACTGATACCCTCCAAGCG

GTAGGTAGATCCTCTGGGTCACTCCTTTTAAAAAGTAAGGGACAAAGTTGGAGATTGGGC

ATAGGGAGCAAGGCCCTGGAGGAAGACTGTGCCTTATGCTGTCCTTCATCCCCCTGTGTA

CAGGAGACAGACCAACTTGAAGATGAGAAGTCTGCTTTGCAGACTGAGATTGCCAACCTG

TTGAAGGAGAAGGAAAAACTGGAG

>CL395.Contig4_A-W 992 1303 minus strand RecName: Full=Proto-oncogene c-Fos; AltName: Full=Cellular oncogene fos >gi|3201684|gb|AAC19354.1| c-fos proto-oncogene protein [Mesocricetus auratus]

TTGTCTCCTGAAGAAGAAGAGAAAAGGAGAATTCGAAGGGAAAGGAATAAGATGGCTGCA

GCCAAGTGCCGGAACCGGAGGAGGGAGCTGACTGATACCCTCCAAGCGGTAGGTAGATCC

TCTGGGTCACTCCTTTTAAAAAGTAAGGGACAAAGTTGGAGATTGGGCATAGGGAGCAAG

GCCCTGGAGGAAGACTGTGCCTTATGCTGTCCTTCATCCCCCTGTGTACAGGAGACAGAC

CAACTTGAAGATGAGAAGTCTGCTTTGCAGACTGAGATTGCCAACCTGTTGAAGGAGAAG

GAAAAACTGGAG

>CL395.Contig5_A-W 992 1180 minus strand RecName: Full=Proto-oncogene c-Fos; AltName: Full=Cellular oncogene fos >gi|3201684|gb|AAC19354.1| c-fos proto-oncogene protein [Mesocricetus auratus]

TTGTCTCCTGAAGAAGAAGAGAAAAGGAGAATTCGAAGGGAAAGGAATAAGATGGCTGCA

GCCAAGTGCCGGAACCGGAGGAGGGAGCTGACTGATACCCTCCAAGCGGAGACAGACCAA

CTTGAAGATGAGAAGTCTGCTTTGCAGACTGAGATTGCCAACCTGTTGAAGGAGAAGGAA

AAACTGGAG

>CL395.Contig6_A-W 212 916 minus strand RecName: Full=Proto-oncogene c-Fos; AltName: Full=Cellular oncogene fos >gi|3201684|gb|AAC19354.1| c-fos proto-oncogene protein [Mesocricetus auratus]

ATGATGTTCTCGGGCTTCAACGCCGACTACGAGGCGTCATCCTCCCGCTGCAGCAGCGCC

TCCCCGGCCGGGGACAGTCTCTCCTACTACCACTCACCGGCCGACTCCTTCTCCAGCATG

GGCTCTCCGGTCAATGCACAGGACTTCTGCACGGATCTGAATGTCTCCAGTGCCAACTTC

ATCCCCACAGTGACTGCTATCTCGACCAGCCCGGACCTGCAGTGGCTGGTGCAACCCACC

TTGGTCTCCTCCGTGGCCCCATCGCAGAACAGAGCTCCCCACCCCTACGGAGTCCCCACT

CCCTCTACTGCGGCTTACTCCAGGGCTGGAGCGGCAAAGACCATGACAGGCGGCAGAGCG

CAGAGCATCGGCAGGAGGGGCAAGGTAGAACAGTTGTCTCCTGAAGAAGAAGAGAAAAGG

AGAATTCGAAGGGAAAGGAATAAGATGGCTGCAGCCAAGTGCCGGAACCGGAGGAGGGAG

CTGACTGATACCCTCCAAGCGGTAGGTAGATCCTCTGGGTCACTCCTTTTAAAAAGTAAG

GGACAAAGTTGGAGATTGGGCATAGGGAGCAAGGCCCTGGAGGAAGACTGTGCCTTATGC

TGTCCTTCATCCCCCTGTGTACAGGAGACAGACCAACTTGAAGATGAGAAGTCTGCTTTG

CAGACTGAGATTGCCAACCTGTTGAAGGAGAAGGAAAAACTGGAG

>CL395.Contig7_A-W 763 1203 minus strand c-fos [Ictidomys tridecemlineatus]

GACTTCTGCACGGATCTGAATGTCTCCAGTGCCAACTTCATCCCCACAGTGACTGCTATC

TCGACCAGCCCGGACCTGCAGTGGCTGGTGCAACCCACCTTGGTCTCCTCCGTGGCCCCA

TCGCAGAACAGAGCTCCCCACCCCTACGGAGTCCCCACTCCCTCTACTGCGGCTTACTCC

AGGGCTGGAGCGGCAAAGACCATGACAGGCGGCAGAGCGCAGAGCATCGGCAGGAGGGGC

AAGGTAGAACAGTTGTCTCCTGAAGAAGAAGAGAAAAGGAGAATTCGAAGGGAAAGGAAT

AAGATGGCTGCAGCCAAGTGCCGGAACCGGAGGAGGGAGCTGACTGATACCCTCCAAGCG

GAGACAGACCAACTTGAAGATGAGAAGTCTGCTTTGCAGACTGAGATTGCCAACCTGTTG

AAGGAGAAGGAAAAACTGGAG

>CL396.Contig1_A-W 13 633 minus strand Ras-related protein Rab-8A [Heterocephalus glaber]

ATGGCGAAGACCTACGATTACCTGTTCAAGCTGCTGCTGATCGGGGACTCGGGGGTGGGG

AAGACCTGTGTCCTGTTCCGCTTCTCCGAGGACGCCTTCAACTCCACTTTCATCTCCACC

ATAGGAATTGACTTTAAAATCAGGACCATAGAGCTCGATGGCAAGAGGATCAAACTGCAG

ATATGGGACACAGCCGGCCAGGAACGGTTCCGGACGATCACCACGGCCTACTACCGGGGA

GCCATGGGTATCATGCTGGTCTACGACATCACCAATGAGAAGTCCTTTGACAACATCCGG

AACTGGATTCGGAACATTGAGGAGCACGCCTCTGCAGACGTGGAGAAGATGATACTTGGG

AACAAGTGTGATGTGAACGACAAGAGACAGGTGTCCAGGGAACGGGGAGAGAAGCTGGCC

CTTGACTATGGAATCAAGTTCATGGAGACCAGCGCCAAGGCCAACATCAACGTGGAGAAC

GCATTTTTCACCCTCGCCAGAGACATCAAAGCAAAAATGGACAAAAAATTGGAAGGCAAC

AGCCCCCAGGGCAGCAGCCAAGGCGTCAAGATCACCCCCGACCAGCAGAAGAGGAGCAGC

TTCTTCCGCTGCGCATTGGTG

>CL396.Contig2_A-W 13 633 minus strand Ras-related protein Rab-8A [Heterocephalus glaber]

ATGGCGAAGACCTACGATTACCTGTTCAAGCTGCTGCTGATCGGGGACTCGGGGGTGGGG

AAGACCTGTGTCCTGTTCCGCTTCTCCGAGGACGCCTTCAACTCCACTTTCATCTCCACC

ATAGGAATTGACTTTAAAATCAGGACCATAGAGCTCGATGGCAAGAGGATCAAACTGCAG

ATATGGGACACAGCCGGCCAGGAACGGTTCCGGACGATCACCACGGCCTACTACCGGGGA

GCCATGGGTATCATGCTGGTCTACGACATCACCAATGAGAAGTCCTTTGACAACATCCGG

AACTGGATTCGGAACATTGAGGAGCACGCCTCTGCAGACGTGGAGAAGATGATACTTGGG

AACAAGTGTGATGTGAACGACAAGAGACAGGTGTCCAGGGAACGGGGAGAGAAGCTGGCC

CTTGACTATGGAATCAAGTTCATGGAGACCAGCGCCAAGGCCAACATCAACGTGGAGAAC

GCATTTTTCACCCTCGCCAGAGACATCAAAGCAAAAATGGACAAAAAATTGGAAGGCAAC

AGCCCCCAGGGCAGCAGCCAAGGCGTCAAGATCACCCCCGACCAGCAGAAGAGGAGCAGC

TTCTTCCGCTGCGCATTGGTG

>CL397.Contig1_A-W 66 290 minus strand PREDICTED: cysteine-rich protein 2 [Otolemur garnettii]

TGCCCCAAGTGCGACAAGACCGTGTACTTCGCTGAGAAAGTGAGCTCCCTGGGCAAGGAT

TGGCACAAGTTCTGCCTCAAGTGTGAGCGCTGCAACAAGACGCTGACCCCGGGGGGCCAT

GCTGAGCATGATGGGAAGCCCTTCTGCCACAAACCCTGCTACGCCACACTGTTTGGACCC

AAAGGCGTGAATATTGGGGGTGCTGGCTCCTACATCTATGAGAAG

>CL397.Contig2_A-W 66 191 minus strand PREDICTED: cysteine-rich protein 2-like [Cricetulus griseus]

TGCCCCAAGTGCGACAAGACCGTGTACTTCGCTGAGAAAGTGAGCTCCCTGGGCAAGGAT

TGGCACAAGTTCTGCCTCAAGTGTGAGCGCTGCAACAAGACGCTGACCCCGGGGGGCCAT

GCTGAG

>CL397.Contig3_A-W 54 677 minus strand PREDICTED: cysteine-rich protein 2-like [Cricetulus griseus]

ATGGCCTCCAAGTGCCCCAAGTGCGACAAGACCGTGTACTTCGCTGAGAAAGTGAGCTCC

CTGGGCAAGGATTGGCACAAGTTCTGCCTCAAGTGTGAGCGCTGCAACAAGACGCTGACC

CCGGGGGGCCATGCTGAGCATGATGGGAAGCCCTTCTGCCACAAACCCTGCTACGCCACA

CTGTTTGGACCCAAAGGCGTGAATATTGGGGGTGCTGGCTCCTACATCTATGAGAAGCCC

CTGGCTGAGGGCCCTCAGGTCACCGGCCCCATCGAGGTGCCAGTGGTCCGAGCTGAGGAG

CGGAAGGCCAGCGGTCCCACTAAGGGGCCCAGCAGAGCCTCCAGCGTGACCACGTTCACT

GGGGAGCCCAACATGTGTCCTCGCTGCAACAAAAGGGTCTACTTTGCCGAGAAGGTGACA

TCTCTGGGCAAGGACTGGCACCGGCCCTGTCTGCGCTGCGAGCGCTGCTCAAAGACGCTG

ACCCCAGGCGGGCACGCGGAGCATGACGGCCAGCCTTACTGCCACAAGCCCTGCTATGGC

ATACTGTTTGGACCCAAGGGAGTGAACACCGGGGCTGTGGGCAGCTACATCTATGACAGG

GACCCGGAAGGCACGGTTCAGCCC

>CL398.Contig1_A-W 30 347 PREDICTED: uncharacterized protein LOC100991126 [Pan paniscus]

GAAACGATAGATAATTCCCAAGGAGCTTACCAAGAGGCATTTGATATAAGCAAGAAAGAG

ATGCAACCCACACATCCAATCCGCCTGGGGCTGGCTCTTAACTTTTCTGTATTTTACTAT

GAGATCCTTAATAACCCAGAGCTTGCCTGCACACTGGCTAAAACGGCTTTTGATGAGGCC

ATTGCAGAACTTGATACACTGAATGAAGACTCATATAAGGACAGCACCCTCATCATGCAG

TTGCTTAGAGACAACTTAACATTATGGACATCAGACAGTGCAGGAGAAGAATGTGATGCA

GCAGAAGGGGCTGAAAAC

>CL398.Contig2_A-W 147 881 14-3-3 protein theta OS=Pongo abelii GN=YWHAQ PE=2 SV=2

ATGGAGAAGACCGAGCTGATCCAGAAGGCCAAGCTGGCCGAGCAGGCCGAGCGCTACGAC

GACATGGCCACCTGCATGAAGGCCGTGACCGAGCAGGGCGCCGAGCTGTCCAACGAGGAG

CGCAACCTGCTCTCGGTGGCCTACAAGAACGTGGTCGGGGGCCGCAGGTCCGCCTGGAGG

GTCATCTCGAGCATCGAGCAGAAGACCGACACCTCCGACAAGAAGTTGCAGCTGATTAAG

GACTATCGGGAGAAAGTGGAGTCGGAGCTGAGGTCCATCTGCACCACGGTCCTGGAATTG

TTGGATAAGTATTTAATAGCCAATGCAACTAATCCAGAGAGTAAGGTCTTCTATCTGAAA

ATGAAAGGAGATTACTTCCGGTACCTTGCTGAAGTTGCCTGTGGTGATGATCGGAAACAA

ACGATAGATAATTCCCAAGGAGCTTACCAAGAGGCATTTGATATAAGCAAGAAAGAGATG

CAACCCACACATCCAATCCGCCTGGGGCTGGCTCTTAACTTTTCTGTATTTTACTATGAG

ATCCTTAATAACCCAGAGCTTGCCTGCACACTGGCTAAAACGGCTTTTGATGAGGCCATT

GCAGAACTTGATACACTGAATGAAGACTCATATAAGGACAGCACCCTCATCATGCAGTTG

CTTAGAGACAACTTAACATTATGGACATCAGACAGTGCAGGAGAAGAATGTGATGCAGCA

GAAGGGGCTGAAAAC

>CL399.Contig1_A-W 321 551 minus strand PREDICTED: coiled-coil domain-containing protein 15-like [Macaca mulatta]

CAGCATGTGTTCCCTGAAGACCAGAATATTCTACACAAATATCAGGACCAAGATTTTCTA

CATGATGACCAGGATTTTCTATGTAGAGACCAGTGTGTTCTTCCCAAAGACCAGAATATT

CTTCTCAAATGCCAGGACCAGGATTTTCTCCCTAAAGATCAGTACATTCTCCTCTCAGAT

GAGAATCTTCTATACAAATGTCAGGAGCAGGATTTTCCACTCAAAGATCAG

>CL399.Contig2_A-W 215 445 minus strand PREDICTED: coiled-coil domain-containing protein 15-like [Macaca mulatta]

CAGCATGTGTTCCCTGAAGACCAGAATATTCTACACAAATATCAGGACCAAGATTTTCTA

CATGATGACCAGGATTTTCTATGTAGAGACCAGTGTGTTCTTCCCAAAGACCAGAATATT

CTTCTCAAATGCCAGGACCAGGATTTTCTCCCTAAAGATCAGTACATTCTCCTCTCAGAT

GAGAATCTTCTATACAAATGTCAGGAGCAGGATTTTCCACTCAAAGATCAG

>CL399.Contig3_A-W 215 445 minus strand PREDICTED: coiled-coil domain-containing protein 15-like [Macaca mulatta]

CAGCATGTGTTCCCTGAAGACCAGAATATTCTACACAAATATCAGGACCAAGATTTTCTA

CATGATGACCAGGATTTTCTATGTAGAGACCAGTGTGTTCTTCCCAAAGACCAGAATATT

CTTCTCAAATGCCAGGACCAGGATTTTCTCCCTAAAGATCAGTACATTCTCCTCTCAGAT

GAGAATCTTCTATACAAATGTCAGGAGCAGGATTTTCCACTCAAAGATCAG

>CL399.Contig4_A-W 21 239 PREDICTED: LOW QUALITY PROTEIN: coiled-coil domain-containing protein 15-like [Ailuropoda melanoleuca]

TCTATTATAACAAATGAGAAAGGGAGAGACGATTTATTTTGGGGAGACAAGCAGAGTCTC

TTTTCTGAAGACAATGATAAGCCTTTTAGCAGAGTTCAGAAAGTACATTTTCTGGAGCCA

TTTTCTGATGAGGAAGGGAGAGAAGACTTTTCTCTGGTGGGTTATCAGTGTCTGCCTCCC

CAAACCCAATTCCAGGACCTCATCAGAGAGCAGAGCTTA

>CL399.Contig5_A-W 88 366 minus strand PREDICTED: coiled-coil domain-containing protein 15 [Saimiri boliviensis boliviensis]

GAATCTGACAGTGAGGAAGTATTATCTATTATAACAAATGAGAAAGGGAGAGACGATTTA

TTTTGGGGAGACAAGCAGAGTCTCTTTTCTGAAGACAATGATAAGCCTTTTAGCAGAGTT

CAGAAAGTAAAATTCAAAAATCCATTATTTGTTGTCATAGAAGAGAAAGAACAAAAGCAG

TCACATTTTCAGGGCCTTGAAGCCCAGGATTATCCTCTGGAAACCAAAGGTGATTTGATA

GAAGTTCAGAGTATTGAGCCTGAAAAGAGTATTGAGCCA

>CL400.Contig1_A-W 167 1339 minus strand PREDICTED: CDK5 regulatory subunit-associated protein 3 isoform 2 [Otolemur garnettii]

GTGGAACTTTCTAGCCTCCTGGTTCGGAATGTCAACTACGAGATCCCCTCGCTGAAGAAG

CAGATTGCTAAGTGCCAGCAATTGCAACAAGAATACAGCCGCAAAGAGGAGGAGGGCCAG

GCAGGGGCTGCTGAGATGCGTGAGCAGTTCTATCATTCCTGCAAACAGTATGGCATCACG

GGAGACAATGTCCGAAGAGAATTGCTGGCCCTGGTGAAGGACCTGCCAAGTCAGCTGACT

GAGATTGGGGCAGGGGCTCAGTCCCTGGGGGAAGCCATCGACCTGTACCAGGCCTGTGTA

GAGTTTGTGTGTGAAAGCCCTACAGAGCAGGTGTTGCCGATGCTGCGGTACGTGCAGAAA

CGGGGAAACTCAACTGTGTATGAATGGAGGACGGGAACAGAGCCGTCTGTGGTAGAGCGG

CCATGCCTGGAGGAGCCTCCTGAGCAATTGGAAGAAGATGCGATTGACTGGGGCGACTTT

GGGATGGAGACAGTTTCCAATGCCGGCATCTCTGCTGAAGTCTCTGGAATCGACTGGGGC

ATCTCCCTGGAATCTGAGTCAAAGGATGCTGGAGTTGAGGGGATAGACTGGGGAGATGAT

GCTACTGCTTTGCAGATCACAGTGCTTGAAGCAGGAACCCAGGCTCCAGAAGGTGTTGCT

AGAGGCCCAGATGCTCTGACACTTCTTGAATACCCTGAGACCCGGAATCAGTTCATTGAT

GAGCTCATGGAGCTCGAGATCTTCTTGTCCCAGAGAGCAGTGGAGATGAGTGAGGAGGCC

GACATCCTGTCCGTGAGCCAGTTTCAGTTGGCTCCCGCCATTCTACAGGGCCAGACCAAA

GAAAAGATGGTCACCATGGTGTCCACACTGCAGGATCTGATTGGTCGGCTCACCAGTCTT

CAAATGCAACACCTGTTTATGATTCTGGCCTCACCAAGGTATGTGGACCGAGTGACAGAA

TTCCTCCAGCAAAAACTAAAACAGTCCCAGCTGTTGGCTTTGAAGAAAGAGCTGATGGTG

CAGAAGCAGCAGGAGGCACTTCAGGAGCAGGCAGCGCTGGAGCCCAAGCTGGACCTGCTA

TTGGAGAAGACCAAGGAGCTGCAGAAGCTGATTGAAGCTGACATCTCCAAGAGGTACAGC

GGGCGCCCTGTGAACCTGATGGGAACCTCTGTC

>CL400.Contig2_A-W 69 1574 minus strand PREDICTED: CDK5 regulatory subunit-associated protein 3 isoform 1 [Otolemur garnettii]

ATGCAGGATCATCAGCACTTGCCCATCGACATCCAGACCAGCAAACTGCTCGACTGGCTG

GTGGATAGAAGGCACTGCAACCTGAAATGGCAGAGTCTGGTGTTGACGATTCGAGAGAAG

ATCAATGCTGCCATCCAGGACATGCCAGAGAGCGAAGAGATCGCCCAGCTGCTCTCTGGG

TCCTACATTCACTATTTCCACTGCCTGAGAATTGTGGACCTTCTCAAAGGCACTGAGGCT

TCCACAAAAAACATTTTTGGCCGGTACTCTTCACAGCGGATGAAGGATTGGCAAGAAATC

GTAGCCCTGTATGAGAAGGACAATACCTACTTAGTGGAACTTTCTAGCCTCCTGGTTCGG

AATGTCAACTACGAGATCCCCTCGCTGAAGAAGCAGATTGCTAAGTGCCAGCAATTGCAA

CAAGAATACAGCCGCAAAGAGGAGGAGGGCCAGGCAGGGGCTGCTGAGATGCGTGAGCAG

TTCTATCATTCCTGCAAACAGTATGGCATCACGGGAGACAATGTCCGAAGAGAATTGCTG

GCCCTGGTGAAGGACCTGCCAAGTCAGCTGACTGAGATTGGGGCAGGGGCTCAGTCCCTG

GGGGAAGCCATCGACCTGTACCAGGCCTGTGTAGAGTTTGTGTGTGAAAGCCCTACAGAG

CAGGTGTTGCCGATGCTGCGGTACGTGCAGAAACGGGGAAACTCAACTGTGTATGAATGG

AGGACGGGAACAGAGCCGTCTGTGGTAGAGCGGCCATGCCTGGAGGAGCCTCCTGAGCAA

TTGGAAGAAGATGCGATTGACTGGGGCGACTTTGGGATGGAGACAGTTTCCAATGCCGGC

ATCTCTGCTGAAGTCTCTGGAATCGACTGGGGCATCTCCCTGGAATCTGAGTCAAAGGAT

GCTGGAGTTGAGGGGATAGACTGGGGAGATGATGCTACTGCTTTGCAGATCACAGTGCTT

GAAGCAGGAACCCAGGCTCCAGAAGGTGTTGCTAGAGGCCCAGATGCTCTGACACTTCTT

GAATACCCTGAGACCCGGAATCAGTTCATTGATGAGCTCATGGAGCTCGAGATCTTCTTG

TCCCAGAGAGCAGTGGAGATGAGTGAGGAGGCCGACATCCTGTCCGTGAGCCAGTTTCAG

TTGGCTCCCGCCATTCTACAGGGCCAGACCAAAGAAAAGATGGTCACCATGGTGTCCACA

CTGCAGGATCTGATTGGTCGGCTCACCAGTCTTCAAATGCAACACCTGTTTATGATTCTG

GCCTCACCAAGGTATGTGGACCGAGTGACAGAATTCCTCCAGCAAAAACTAAAACAGTCC

CAGCTGTTGGCTTTGAAGAAAGAGCTGATGGTGCAGAAGCAGCAGGAGGCACTTCAGGAG

CAGGCAGCGCTGGAGCCCAAGCTGGACCTGCTATTGGAGAAGACCAAGGAGCTGCAGAAG

CTGATTGAAGCTGACATCTCCAAGAGGTACAGCGGGCGCCCTGTGAACCTGATGGGAACC

TCTGTC

>CL400.Contig3_A-W 69 1550 minus strand PREDICTED: CDK5 regulatory subunit-associated protein 3 isoform 1 [Otolemur garnettii]

ATGCAGGATCATCAGCACTTGCCCATCGACATCCAGACCAGCAAACTGCTCGACTGGCTG

GTGGATAGAAGGCACTGCAACCTGAAATGGCAGAGTCTGGTGTTGACGATTCGAGAGAAG

ATCAATGCTGCCATCCAGGACATGCCAGAGAGCGAAGAGATCGCCCAGCTGCTCTCTGGG

TCCTACATTCACTATTTCCACTGCCTGAGAATTGTGGACCTTCTCAAAGGCACTGAGGCT

TCCACAAAAAACATTTTTGGCCGGTACTCTTCACAGCGGATGAAGGATTGGCAAGAAATC

GTAGCCCTGTATGAGAAGGACAATACCTACTTAGTGGAACTTTCTAGCCTCCTGGTTCGG

AATGTCAACTACGAGATCCCCTCGCTGAAGAAGCAGATTGCTAAGTGCCAGCAATTGCAA

CAAGAATACAGCCGCAAAGAGGAGGAGGGCCAGGCAGGGGCTGCTGAGATGCGTGAGCAG

TTCTATCATTCCTGCAAACAGTATGGCATCACGGGAGACAATGTCCGAAGAGAATTGCTG

GCCCTGGTGAAGGACCTGCCAAGTCAGCTGACTGAGATTGGGGCAGGGGCTCAGTCCCTG

GGGGAAGCCATCGACCTGTACCAGGCCTGTGTAGAGTTTGTGTGTGAAAGCCCTACAGAG

CAGGTGTTGCCGATGCTGCGGTACGTGCAGAAACGGGGAAACTCAACTGTGTATGAATGG

AGGACGGGAACAGAGCCGTCTGTGGTAGAGCGGCCATGCCTGGAGGAGCCTCCTGAGCAA

TTGGAAGAAGATGCGATTGACTGGGGCGACTTTGGGATGGAGACAGTTTCCAATGCCGGC

ATCTCTGCTGAAGTCTCTGGAATCGACTGGGGCATCTCCCTGGAATCTGAGTCAAAGGAT

GCTGGAGTTGAGGGGATAGACTGGGGAGATGATGCTACTGCTTTGCAGATCACAGTGCTT

GAAGCAGGAACCCAGGCTCCAGAAGGTGTTGCTAGAGGCCCAGATGCTCTGACACTTCTT

GAATACCCTGAGACCCGGAATCAGTTCATTGATGAGCTCATGGAGCTCGAGATCTTCTTG

TCCCAGAGAGCAGTGGAGATGAGTGAGGAGGCCGACATCCTGTCCGTGAGCCAGTTTCAG

TTGGCTCCCGCCATTCTACAGGGCCAGACCAAAGAAAAGATGGTCACCATGGTGTCCACA

CTGCAGGATCTGATTGGTCGGCTCACCAGTCTTCAAATGCAACACCTGTTTATGATTCTG

GCCTCACCAAGGTATGTGGACCGAGTGACAGAATTCCTCCAGCAAAAACTAAAACAGTCC

CAGCTGTTGGCTTTGAAGAAAGAGCTGATGGTGCAGAAGCAGCAGGAGGCACTTCAGGAG

CAGGCAGCGCTGGAGCCCAAGCTGGACCTGCTATTGGAGAAGACCAAGGAGCTGCAGAAG

CTGGTAAGACAGGACAGGCAGACCCCTCACATGGGAACCCCC

>CL400.Contig4_A-W 425 1705 CDK5 regulatory subunit-associated protein 3 OS=Mus musculus GN=Cdk5rap3 PE=2 SV=1

AAGGCACTGAGGCTTCCACAAAAAAACATTTTTGGCCGGTACTCTTCACAGCGGATGAAG

GATTGGCAAGAAATCGTAGCCCTGTATGAGAAGGACAATACCTACTTAGTGGAACTTTCT

AGCCTCCTGGTTCGGAATGTCAACTACGAGATCCCCTCGCTGAAGAAGCAGATTGCTAAG

TGCCAGCAATTGCAACAAGAATACAGCCGCAAAGAGGAGGAGGGCCAGGCAGGGGCTGCT

GAGATGCGTGAGCAGTTCTATCATTCCTGCAAACAGTATGGCATCACGGGAGACAATGTC

CGAAGAGAATTGCTGGCCCTGGTGAAGGACCTGCCAAGTCAGCTGACTGAGATTGGGGCA

GGGGCTCAGTCCCTGGGGGAAGCCATCGACCTGTACCAGGCCTGTGTAGAGTTTGTGTGT

GAAAGCCCCACAGAGCAGGTGTTGCCGATGCTGCGGTACGTGCAGAAACGGGGAAACTCA

ACTGTGTATGAATGGAGGACGGGAACAGAGCCGTCTGTGGTAGAGCGGCCATGCCTGGAG

GAGCCTCCTGAGCAATTGGAAGAAGATGTGATTGACTGGGGCGACTTTGGGATGGAGACA

GTTTCCAATGCCGGCATCTCTGCTGAAGTCTCTGGAATCGACTGGGGCATCTCCCTGGAA

TCTGAGTCAAAGGATGCTGGAGTTGAGGGGATAGACTGGGGAGATGATGCTACTGCTTTG

CAGATCACAGTGCTTGAAGCAGGAACCCAGGCTCCAGAAGGTGTTGCTAGAGGCCCAGAT

GCTCTGACACTTCTTGAATACCCTGAGACCCGGAATCAGTTCATTGATGAGCTCATGGAG

CTCGAGATCTTCTTGTCCCAGAGAGCAGTGGAGATGAGTGAGGAGGCCGACATCCTGTCC

GTGAGCCAGTTTCAGTTGGCTCCCGCCATTCTACAGGGCCAGACCAAAGAAAAGATGGTC

ACCATGGTGTCCACACTGCAGGATCTGATTGGTCGGCTCACCAGTCTTCAAATGCAACAC

CTGTTTATGATTCTGGCCTCACCAAGGTATGTGGACCGAGTGACAGAATTCCTCCAGCAA

AAACTAAAACAGTCCCAGCTGTTGGCTTTGAAGAAAGAGCTGATGGTGCAGAAGCAGCAG

GAGGCACTTCAGGAGCAGGCAGCGCTGGAGCCCAAGCTGGACCTGCTATTGGAGAAGACC

AAGGAGCTGCAGAAGCTGATTGAAGCTGACATCTCCAAGAGGTACAGCGGGCGCCCTGTG

AACCTGATGGGAACCTCTGTC

>CL400.Contig5_A-W 425 1657 CDK5 regulatory subunit-associated protein 3 OS=Mus musculus GN=Cdk5rap3 PE=2 SV=1

AAGGCACTGAGGCTTCCACAAAAAAACATTTTTGGCCGGTACTCTTCACAGCGGATGAAG

GATTGGCAAGAAATCGTAGCCCTGTATGAGAAGGACAATACCTACTTAGTGGAACTTTCT

AGCCTCCTGGTTCGGAATGTCAACTACGAGATCCCCTCGCTGAAGAAGCAGATTGCTAAG

TGCCAGCAATTGCAACAAGAATACAGCCGCAAAGAGGAGGAGGGCCAGGCAGGGGCTGCT

GAGATGCGTGAGCAGTTCTATCATTCCTGCAAACAGTATGGCATCACGGGAGACAATGTC

CGAAGAGAATTGCTGGCCCTGGTGAAGGACCTGCCAAGTCAGCTGACTGAGATTGGGGCA

GGGGCTCAGTCCCTGGGGGAAGCCATCGACCTGTACCAGGCCTGTGTAGAGTTTGTGTGT

GAAAGCCCCACAGAGCAGGTGTTGCCGATGCTGCGGTACGTGCAGAAACGGGGAAACTCA

ACTGTGTATGAATGGAGGACGGGAACAGAGCCGTCTGTGGTAGAGCGGCCATGCCTGGAG

GAGCCTCCTGAGCAATTGGAAGAAGATGTGATTGACTGGGGCGACTTTGGGATGGAGACA

GTTTCCAATGCCGGCATCTCTGCTGAAGTCTCTGGAATCGACTGGGGCATCTCCCTGGAA

TCTGAGTCAAAGGATGCTGGAGTTGAGGGGATAGACTGGGGAGATGATGCTACTGCTTTG

CAGATCACAGTGCTTGAAGCAGGAACCCAGGCTCCAGAAGGTGTTGCTAGAGGCCCAGAT

GCTCTGACACTTCTTGAATACCCTGAGACCCGGAATCAGTTCATTGATGAGCTCATGGAG

CTCGAGATCTTCTTGTCCCAGAGAGCAGTGGAGATGAGTGAGGAGGCCGACATCCTGTCC

GTGAGCCAGTTTCAGTTGGCTCCCGCCATTCTACAGGGCCAGACCAAAGAAAAGATGGTC

ACCATGGTGTCCACACTGCAGGATCTGATTGGTCGGCTCACCAGTCTTCAAATGCAACAC

CTGTTTATGATTCTGGCCTCACCAAGGTATGTGGACCGAGTGACAGAATTCCTCCAGCAA

AAACTAAAACAGTCCCAGCTGTTGGCTTTGAAGAAAGAGCTGATGGTGCAGAAGCAGCAG

GAGGCACTTCAGGAGCAGGCAGCGCTGGAGCCCAAGCTGGACCTGCTATTGGAGAAGACC

AAGGAGCTGCAGAAGCTGCCTCAGGTGGAGGTG

>CL400.Contig6_A-W 425 1681 CDK5 regulatory subunit-associated protein 3 OS=Mus musculus GN=Cdk5rap3 PE=2 SV=1

AAGGCACTGAGGCTTCCACAAAAAAACATTTTTGGCCGGTACTCTTCACAGCGGATGAAG

GATTGGCAAGAAATCGTAGCCCTGTATGAGAAGGACAATACCTACTTAGTGGAACTTTCT

AGCCTCCTGGTTCGGAATGTCAACTACGAGATCCCCTCGCTGAAGAAGCAGATTGCTAAG

TGCCAGCAATTGCAACAAGAATACAGCCGCAAAGAGGAGGAGGGCCAGGCAGGGGCTGCT

GAGATGCGTGAGCAGTTCTATCATTCCTGCAAACAGTATGGCATCACGGGAGACAATGTC

CGAAGAGAATTGCTGGCCCTGGTGAAGGACCTGCCAAGTCAGCTGACTGAGATTGGGGCA

GGGGCTCAGTCCCTGGGGGAAGCCATCGACCTGTACCAGGCCTGTGTAGAGTTTGTGTGT

GAAAGCCCCACAGAGCAGGTGTTGCCGATGCTGCGGTACGTGCAGAAACGGGGAAACTCA

ACTGTGTATGAATGGAGGACGGGAACAGAGCCGTCTGTGGTAGAGCGGCCATGCCTGGAG

GAGCCTCCTGAGCAATTGGAAGAAGATGTGATTGACTGGGGCGACTTTGGGATGGAGACA

GTTTCCAATGCCGGCATCTCTGCTGAAGTCTCTGGAATCGACTGGGGCATCTCCCTGGAA

TCTGAGTCAAAGGATGCTGGAGTTGAGGGGATAGACTGGGGAGATGATGCTACTGCTTTG

CAGATCACAGTGCTTGAAGCAGGAACCCAGGCTCCAGAAGGTGTTGCTAGAGGCCCAGAT

GCTCTGACACTTCTTGAATACCCTGAGACCCGGAATCAGTTCATTGATGAGCTCATGGAG

CTCGAGATCTTCTTGTCCCAGAGAGCAGTGGAGATGAGTGAGGAGGCCGACATCCTGTCC

GTGAGCCAGTTTCAGTTGGCTCCCGCCATTCTACAGGGCCAGACCAAAGAAAAGATGGTC

ACCATGGTGTCCACACTGCAGGATCTGATTGGTCGGCTCACCAGTCTTCAAATGCAACAC

CTGTTTATGATTCTGGCCTCACCAAGGTATGTGGACCGAGTGACAGAATTCCTCCAGCAA

AAACTAAAACAGTCCCAGCTGTTGGCTTTGAAGAAAGAGCTGATGGTGCAGAAGCAGCAG

GAGGCACTTCAGGAGCAGGCAGCGCTGGAGCCCAAGCTGGACCTGCTATTGGAGAAGACC

AAGGAGCTGCAGAAGCTGGTAAGACAGGACAGGCAGACCCCTCACATGGGAACCCCC

>CL400.Contig7_A-W 86 943 PREDICTED: CDK5 regulatory subunit-associated protein 3 isoform 2 [Otolemur garnettii]

AGCCCCACAGAGCAGGTGTTGCCGATGCTGCGGTACGTGCAGAAACGGGGAAACTCAACT

GTGTATGAATGGAGGACGGGAACAGAGCCGTCTGTGGTAGAGCGGCCATGCCTGGAGGAG

CCTCCTGAGCAATTGGAAGAAGATGTGATTGACTGGGGCGACTTTGGGATGGAGACAGTT

TCCAATGCCGGCATCTCTGCTGAAGTCTCTGGAATCGACTGGGGCATCTCCCTGGAATCT

GAGTCAAAGGATGCTGGAGTTGAGGGGATAGACTGGGGAGATGATGCTACTGCTTTGCAG

ATCACAGTGCTTGAAGCAGGAACCCAGGCTCCAGAAGGTGTTGCTAGAGGCCCAGATGCT

CTGACACTTCTTGAATACCCTGAGACCCGGAATCAGTTCATTGATGAGCTCATGGAGCTC

GAGATCTTCTTGTCCCAGAGAGCAGTGGAGATGAGTGAGGAGGCCGACATCCTGTCCGTG

AGCCAGTTTCAGTTGGCTCCCGCCATTCTACAGGGCCAGACCAAAGAAAAGATGGTCACC

ATGGTGTCCACACTGCAGGATCTGATTGGTCGGCTCACCAGTCTTCAAATGCAACACCTG

TTTATGATTCTGGCCTCACCAAGGTATGTGGACCGAGTGACAGAATTCCTCCAGCAAAAA

CTAAAACAGTCCCAGCTGTTGGCTTTGAAGAAAGAGCTGATGGTGCAGAAGCAGCAGGAG

GCACTTCAGGAGCAGGCAGCGCTGGAGCCCAAGCTGGACCTGCTATTGGAGAAGACCAAG

GAGCTGCAGAAGCTGATTGAAGCTGACATCTCCAAGAGGTACAGCGGGCGCCCTGTGAAC

CTGATGGGAACCTCTGTC

>CL400.Contig8_A-W 86 895 PREDICTED: CDK5 regulatory subunit-associated protein 3 isoform 2 [Otolemur garnettii]

AGCCCCACAGAGCAGGTGTTGCCGATGCTGCGGTACGTGCAGAAACGGGGAAACTCAACT

GTGTATGAATGGAGGACGGGAACAGAGCCGTCTGTGGTAGAGCGGCCATGCCTGGAGGAG

CCTCCTGAGCAATTGGAAGAAGATGTGATTGACTGGGGCGACTTTGGGATGGAGACAGTT

TCCAATGCCGGCATCTCTGCTGAAGTCTCTGGAATCGACTGGGGCATCTCCCTGGAATCT

GAGTCAAAGGATGCTGGAGTTGAGGGGATAGACTGGGGAGATGATGCTACTGCTTTGCAG

ATCACAGTGCTTGAAGCAGGAACCCAGGCTCCAGAAGGTGTTGCTAGAGGCCCAGATGCT

CTGACACTTCTTGAATACCCTGAGACCCGGAATCAGTTCATTGATGAGCTCATGGAGCTC

GAGATCTTCTTGTCCCAGAGAGCAGTGGAGATGAGTGAGGAGGCCGACATCCTGTCCGTG

AGCCAGTTTCAGTTGGCTCCCGCCATTCTACAGGGCCAGACCAAAGAAAAGATGGTCACC

ATGGTGTCCACACTGCAGGATCTGATTGGTCGGCTCACCAGTCTTCAAATGCAACACCTG

TTTATGATTCTGGCCTCACCAAGGTATGTGGACCGAGTGACAGAATTCCTCCAGCAAAAA

CTAAAACAGTCCCAGCTGTTGGCTTTGAAGAAAGAGCTGATGGTGCAGAAGCAGCAGGAG

GCACTTCAGGAGCAGGCAGCGCTGGAGCCCAAGCTGGACCTGCTATTGGAGAAGACCAAG

GAGCTGCAGAAGCTGCCTCAGGTGGAGGTG

>CL400.Contig9_A-W 86 919 PREDICTED: CDK5 regulatory subunit-associated protein 3 isoform 2 [Otolemur garnettii]

AGCCCCACAGAGCAGGTGTTGCCGATGCTGCGGTACGTGCAGAAACGGGGAAACTCAACT

GTGTATGAATGGAGGACGGGAACAGAGCCGTCTGTGGTAGAGCGGCCATGCCTGGAGGAG

CCTCCTGAGCAATTGGAAGAAGATGTGATTGACTGGGGCGACTTTGGGATGGAGACAGTT

TCCAATGCCGGCATCTCTGCTGAAGTCTCTGGAATCGACTGGGGCATCTCCCTGGAATCT

GAGTCAAAGGATGCTGGAGTTGAGGGGATAGACTGGGGAGATGATGCTACTGCTTTGCAG

ATCACAGTGCTTGAAGCAGGAACCCAGGCTCCAGAAGGTGTTGCTAGAGGCCCAGATGCT

CTGACACTTCTTGAATACCCTGAGACCCGGAATCAGTTCATTGATGAGCTCATGGAGCTC

GAGATCTTCTTGTCCCAGAGAGCAGTGGAGATGAGTGAGGAGGCCGACATCCTGTCCGTG

AGCCAGTTTCAGTTGGCTCCCGCCATTCTACAGGGCCAGACCAAAGAAAAGATGGTCACC

ATGGTGTCCACACTGCAGGATCTGATTGGTCGGCTCACCAGTCTTCAAATGCAACACCTG

TTTATGATTCTGGCCTCACCAAGGTATGTGGACCGAGTGACAGAATTCCTCCAGCAAAAA

CTAAAACAGTCCCAGCTGTTGGCTTTGAAGAAAGAGCTGATGGTGCAGAAGCAGCAGGAG

GCACTTCAGGAGCAGGCAGCGCTGGAGCCCAAGCTGGACCTGCTATTGGAGAAGACCAAG

GAGCTGCAGAAGCTGGTAAGACAGGACAGGCAGACCCCTCACATGGGAACCCCC

>CL400.Contig10_A-W 147 1271 PREDICTED: CDK5 regulatory subunit-associated protein 3 isoform 2 [Otolemur garnettii]

GTGGAACTTTCTAGCCTCCTGGTTCGGAATGTCAACTACGAGATCCCCTCGCTGAAGAAG

CAGATTGCTAAGTGCCAGCAATTGCAACAAGAATACAGCCGCAAAGAGGAGGAGGGCCAG

GCAGGGGCTGCTGAGATGCGTGAGCAGTTCTATCATTCCTGCAAACAGTATGGCATCACG

GGAGACAATGTCCGAAGAGAATTGCTGGCCCTGGTGAAGGACCTGCCAAGTCAGCTGACT

GAGATTGGGGCAGGGGCTCAGTCCCTGGGGGAAGCCATCGACCTGTACCAGGCCTGTGTA

GAGTTTGTGTGTGAAAGCCCCACAGAGCAGGTGTTGCCGATGCTGCGGTACGTGCAGAAA

CGGGGAAACTCAACTGTGTATGAATGGAGGACGGGAACAGAGCCGTCTGTGGTAGAGCGG

CCATGCCTGGAGGAGCCTCCTGAGCAATTGGAAGAAGATGTGATTGACTGGGGCGACTTT

GGGATGGAGACAGTTTCCAATGCCGGCATCTCTGCTGAAGTCTCTGGAATCGACTGGGGC

ATCTCCCTGGAATCTGAGTCAAAGGATGCTGGAGTTGAGGGGATAGACTGGGGAGATGAT

GCTACTGCTTTGCAGATCACAGTGCTTGAAGCAGGAACCCAGGCTCCAGAAGGTGTTGCT

AGAGGCCCAGATGCTCTGACACTTCTTGAATACCCTGAGACCCGGAATCAGTTCATTGAT

GAGCTCATGGAGCTCGAGATCTTCTTGTCCCAGAGAGCAGTGGAGATGAGTGAGGAGGCC

GACATCCTGTCCGTGAGCCAGTTTCAGTTGGCTCCCGCCATTCTACAGGGCCAGACCAAA

GAAAAGATGGTCACCATGGTGTCCACACTGCAGGATCTGATTGGTCGGCTCACCAGTCTT

CAAATGCAACACCTGTTTATGATTCTGGCCTCACCAAGGTATGTGGACCGAGTGACAGAA

TTCCTCCAGCAAAAACTAAAACAGTCCCAGCTGTTGGCTTTGAAGAAAGAGCTGATGGTG

CAGAAGCAGCAGGAGGCACTTCAGGAGCAGGCAGCGCTGGAGCCCAAGCTGGACCTGCTA

TTGGAGAAGACCAAGGAGCTGCAGAAGCTGCCTCAGGTGGAGGTG

>CL400.Contig11_A-W 167 1315 minus strand PREDICTED: CDK5 regulatory subunit-associated protein 3 isoform 2 [Otolemur garnettii]

GTGGAACTTTCTAGCCTCCTGGTTCGGAATGTCAACTACGAGATCCCCTCGCTGAAGAAG

CAGATTGCTAAGTGCCAGCAATTGCAACAAGAATACAGCCGCAAAGAGGAGGAGGGCCAG

GCAGGGGCTGCTGAGATGCGTGAGCAGTTCTATCATTCCTGCAAACAGTATGGCATCACG

GGAGACAATGTCCGAAGAGAATTGCTGGCCCTGGTGAAGGACCTGCCAAGTCAGCTGACT

GAGATTGGGGCAGGGGCTCAGTCCCTGGGGGAAGCCATCGACCTGTACCAGGCCTGTGTA

GAGTTTGTGTGTGAAAGCCCCACAGAGCAGGTGTTGCCGATGCTGCGGTACGTGCAGAAA

CGGGGAAACTCAACTGTGTATGAATGGAGGACGGGAACAGAGCCGTCTGTGGTAGAGCGG

CCATGCCTGGAGGAGCCTCCTGAGCAATTGGAAGAAGATGTGATTGACTGGGGCGACTTT

GGGATGGAGACAGTTTCCAATGCCGGCATCTCTGCTGAAGTCTCTGGAATCGACTGGGGC

ATCTCCCTGGAATCTGAGTCAAAGGATGCTGGAGTTGAGGGGATAGACTGGGGAGATGAT

GCTACTGCTTTGCAGATCACAGTGCTTGAAGCAGGAACCCAGGCTCCAGAAGGTGTTGCT

AGAGGCCCAGATGCTCTGACACTTCTTGAATACCCTGAGACCCGGAATCAGTTCATTGAT

GAGCTCATGGAGCTCGAGATCTTCTTGTCCCAGAGAGCAGTGGAGATGAGTGAGGAGGCC

GACATCCTGTCCGTGAGCCAGTTTCAGTTGGCTCCCGCCATTCTACAGGGCCAGACCAAA

GAAAAGATGGTCACCATGGTGTCCACACTGCAGGATCTGATTGGTCGGCTCACCAGTCTT

CAAATGCAACACCTGTTTATGATTCTGGCCTCACCAAGGTATGTGGACCGAGTGACAGAA

TTCCTCCAGCAAAAACTAAAACAGTCCCAGCTGTTGGCTTTGAAGAAAGAGCTGATGGTG

CAGAAGCAGCAGGAGGCACTTCAGGAGCAGGCAGCGCTGGAGCCCAAGCTGGACCTGCTA

TTGGAGAAGACCAAGGAGCTGCAGAAGCTGGTAAGACAGGACAGGCAGACCCCTCACATG

GGAACCCCC

>CL401.Contig1_A-W 182 553 aurora kinase C [Bos taurus] >gi|296477144|gb|DAA19259.1| aurora kinase C-like [Bos taurus]

GCTGCAGTGGGCCAGACGCAACCCGGAACCCCCGCCGTGAAGCCCTTCACAATCTATGAC

CTTGAAATCGGGCGTCCTCTGGGCAAGGGCAGATTTGGGAACCTCTACCTGGCTCGACTG

AAGGAGAACCATTTCATCGTGGCCCTGAAGGTCCTCTTCAAGTCCCAGATTGAGAAGGCG

GGACTGGAGCACCAGCTGCGCAGGGAAGTCGAGATCCAGGCGCACCTACGACACCCGAAC

ATCCTACACCTCTACAACTATTTCTATGACTCTCGCCGGATATACCTGATTCTGGAGTTT

GCTCCCAGGGGTGAGCTCTACAACTTGCTGCAGAGAAGCGGCCCCTTGGATGAGCAGCGC

ACAGCCACGGTG

>CL401.Contig2_A-W 191 1042 PREDICTED: serine/threonine-protein kinase 13-like isoform 1 [Equus caballus]

GGCCAGACGCAACCCGGAACCCCCGCCGTGAAGCCCTTCACAATCTATGACCTTGAAATC

GGGCGTCCTCTGGGCAAGGGCAGATTTGGGAACCTCTACCTGGCTCGACTGAAGGAGAAC

CATTTCATCGTGGCCCTGAAGGTCCTCTTCAAGTCCCAGATTGAGAAGGCGGGACTGGAG

CACCAGCTGCGCAGGGAAGTCGAGATCCAGGCGCACCTACGACACCCGAACATCCTACAC

CTCTACAACTATTTCTATGACTCTCGCCGGATATACCTGATTCTGGAGTTTGCTCCCAGG

GGTGAGCTCTACAACTTGCTGCAGAGAAGCGGCCCCTTGGATGAGCAGCGCACAGCCACG

ATAATGGAGGAGTTGGCAGACGCCCTGACATACTGCCACAACAAGAAGGTGATTCACAGG

GACATTAAGCCAGAGAATCTGCTGCTGGGGCTGATGGGTGAGGTGAAGATTGCAGACTTT

GGCTGGTCTGTGCACACCCCCTCCCTCAGGAGACAGACTATGTGTGGGACTCTGGACTAC

TTGCCCCCAGAAATAGTGGAAGGCAGGACCTACGATGAGAAGGTGGACCTGTGGTGCCTC

GAGGTGCTCTGCTACGAGCTGCTGGTGGGACGGCCACCCTTTGAGAGCCCCTCTCAGAGC

GAGACCCAGAGGCGCATCCTCAAGGTGGATGTGACGTTTCCCCCCTCCATGCCCGAGGGG

GCCCAGGACTTGGTCTCCAAGCTTCTGAGATACCAGCCCTCAGAGCGGCTTCCCCTGGCT

CAGGTCCTGGAGCACCCCTGGGTGAAAGCGCACTCATGGGGGGTGTTGCCTCCCGGTGCT

CAGAAGGCTTCC

>CL402.Contig1_A-W 502 681 PREDICTED: spatacsin-like [Sus scrofa]

TCTCTTCTAGCCCCCACCATCACTGTGTGTGGGCAAGAACAGCCTACCTTAGGAGCAGTT

AGACTCAGCAAGTGTCTTTTATCCTCAGCAGAGGACAACCTAAAGGATGGACTCCAGCTG

AAGCAGCTATTGCTGAAGGCCCTGACCCTGATGCTAGATGCAGCAGAGAGTTATGCCAAG

>CL402.Contig2_A-W 1 456 PREDICTED: spatacsin-like [Sus scrofa]

GAGACCGTGGTATTCCCTTTTTCCTCCCTTTGGCAGAGTGGTACCCTGAAGACAGCCCTG

CTGGATTATATCAAACGGTGCCGCCCTGGAGACAGTGAAAAGCACAACATGATAGCCCTG

TGCTTTAGTATGTGCCGAGAGATCGGAGAGAACCATGAGGCAGCTGCCTGCATCCAACTG

AAATTGATTGATTCTCAGCCTTGGGAGGACAACCTAAAGGATGGACTCCAGCTGAAGCAG

CTATTGCTGAAGGCCCTGACCCTGATGCTAGATGCAGCAGAGAGTTATGCCAAGGACTCC

TGTGTACGACAGGCCCTGCACTGTCACCGCCTTACCAGGTTGATAACCCTACAGATTCAC

TTTCTGAACACTGGCCAGAACACAATGCTTATCAACTTAGGCCGCCAAAAGCTGATGGAC

TGTATCATGGCCCTACCTCGGTTCTACCAGGTGAAC

>CL402.Contig3_A-W 1 780 PREDICTED: spatacsin-like [Sus scrofa]

GAGACCGTGGTATTCCCTTTTTCCTCCCTTTGGCAGAGTGGTACCCTGAAGACAGCCCTG

CTGGATTATATCAAACGGTGCCGCCCTGGAGACAGTGAAAAGCACAACATGATAGCCCTG

TGCTTTAGTATGTGCCGAGAGATCGGAGAGAACCATGAGGCAGCTGCCTGCATCCAACTG

AAATTGATTGATTCTCAGCCTTGGGAGGACAACCTAAAGGATGGACTCCAGCTGAAGCAG

CTATTGCTGAAGGCCCTGACCCTGATGCTAGATGCAGCAGAGAGTTATGCCAAGGACTCC

TGTGTACGACAGGCCCTGCACTGTCACCGCCTTACCAGGTTGATAACCCTACAGATTCAC

TTTCTGAACACTGGCCAGAACACAATGCTTATCAACTTAGGCCGCCAAAAGCTGATGGAC

TGTATCATGGCCCTACCTCGGTTCTACCAGGCTTCTATCGTGGCCGAGGCCTATGACTTT

GTTCCAGATTGGGCAGAAATTCTATACCAACAAGTGATTCTTAAAGGAGATTTTACTTAT

TTGGAAGAATTTAAACAACAAAGGTTGTTAAGACCTAGTATAATTGAAGAAATTTCAAAA

AAATATAAACAACATCAGCCTACCAACAAGGTCACAGAAAACTTGAAGAAGTTACTTTCA

TACTGTGAAGATATCTACCTGTATTACAAGTTGGCATATGAACACAAATTTTATGAAATT

ATAAATGTACTTCTGAAGGACCCTCAAACAGGTTGCTGTCTAAAGGATATGCTTTCAGGT

>CL402.Contig4_A-W 1164 1829 PREDICTED: spatacsin isoform 1 [Pan paniscus]

TCTCTTCTAGCCCCCACCATCACTGTGTGTGGGCAAGAACAGCCTACCTTAGGAGCAGTT

AGACTCAGCAAGTGTCTTTTATCCTCAGCAGAGGACAACCTAAAGGATGGACTCCAGCTG

AAGCAGCTATTGCTGAAGGCCCTGACCCTGATGCTAGATGCAGCAGAGAGTTATGCCAAG

GACTCCTGTGTACGACAGGCCCTGCACTGTCACCGCCTTACCAGGTTGATAACCCTACAG

ATTCACTTTCTGAACACTGGCCAGAACACAATGCTTATCAACTTAGGCCGCCAAAAGCTG

ATGGACTGTATCATGGCCCTACCTCGGTTCTACCAGGCTTCTATCGTGGCCGAGGCCTAT

GACTTTGTTCCAGATTGGGCAGAAATTCTATACCAACAAGTGATTCTTAAAGGAGATTTT

ACTTATTTGGAAGAATTTAAACAACAAAGGTTGTTAAGACCTAGTATAATTGAAGAAATT

TCAAAAAAATATAAACAACATCAGCCTACCAACAAGGTCACAGAAAACTTGAAGAAGTTA

CTTTCATACTGTGAAGATATCTACCTGTATTACAAGTTGGCATATGAACACAAATTTTAT

GAAATTATAAATGTACTTCTGAAGGACCCTCAAACAGGTTGCTGTCTAAAGGATATGCTT

TCAGGT

>CL402.Contig5_A-W 365 1168 PREDICTED: LOW QUALITY PROTEIN: spatacsin-like [Equus caballus]

TCAGGACATAAGCAGATGTTCAGCCCAGCAGAGGAAAGTCAGACATTTCTTCAGCTGACC

ATGCTGTGTCAAGACCGGACCTTGTTAGGCATGAAGTTGTTGGATAAGATTTCCTCTGTT

CCCCATGGGGAACTGTCTTGCACCACAGAGCTCCTGATCCTGGCCCATCACTGCTTCAGC

CTGACATGCCACATGGAAGGCATCATCCGAGTCCTACAGGCTGCCCGGATGCTCACAGAT

AACCACTTGGCTCCCAATGAAGAGTATGGACTGGTGGTACGTCTCCTCACTGGCATTGGA

AGGTACAATGAGATGACATACATATTTGATTTGCTGCATAAAAAGCACTACTTCGAAGTG

CTGATGAGGAAGAAGCTAGATCCGAGTGGTACCCTGAAGACAGCCCTGCTGGATTATATC

AAACGGTGCCGCCCTGGAGACAGTGAAAAGCACAACATGATAGCCCTGTGCTTTAGTATG

TGCCGAGAGATCGGAGAGAACCATGAGGCAGCTGCCTGCATCCAACTGAAATTGATTGAT

TCTCAGCCTTGGGAGGACAACCTAAAGGATGGACTCCAGCTGAAGCAGCTATTGCTGAAG

GCCCTGACCCTGATGCTAGATGCAGCAGAGAGTTATGCCAAGGACTCCTGTGTACGACAG

GCCCTGCACTGTCACCGCCTTACCAGGTTGATAACCCTACAGATTCACTTTCTGAACACT

GGCCAGAACACAATGCTTATCAACTTAGGCCGCCAAAAGCTGATGGACTGTATCATGGCC

CTACCTCGGTTCTACCAGGTGAAC

>CL402.Contig6_A-W 362 1492 PREDICTED: spatacsin isoform 2 [Pan paniscus]

CATTCAGGACATAAGCAGATGTTCAGCCCAGCAGAGGAAAGTCAGACATTTCTTCAGCTG

ACCATGCTGTGTCAAGACCGGACCTTGTTAGGCATGAAGTTGTTGGATAAGATTTCCTCT

GTTCCCCATGGGGAACTGTCTTGCACCACAGAGCTCCTGATCCTGGCCCATCACTGCTTC

AGCCTGACATGCCACATGGAAGGCATCATCCGAGTCCTACAGGCTGCCCGGATGCTCACA

GATAACCACTTGGCTCCCAATGAAGAGTATGGACTGGTGGTACGTCTCCTCACTGGCATT

GGAAGGTACAATGAGATGACATACATATTTGATTTGCTGCATAAAAAGCACTACTTCGAA

GTGCTGATGAGGAAGAAGCTAGATCCGAGTGGTACCCTGAAGACAGCCCTGCTGGATTAT

ATCAAACGGTGCCGCCCTGGAGACAGTGAAAAGCACAACATGATAGCCCTGTGCTTTAGT

ATGTGCCGAGAGATCGGAGAGAACCATGAGGCAGCTGCCTGCATCCAACTGAAATTGATT

GATTCTCAGCCTTGGGAGGACAACCTAAAGGATGGACTCCAGCTGAAGCAGCTATTGCTG

AAGGCCCTGACCCTGATGCTAGATGCAGCAGAGAGTTATGCCAAGGACTCCTGTGTACGA

CAGGCCCTGCACTGTCACCGCCTTACCAGGTTGATAACCCTACAGATTCACTTTCTGAAC

ACTGGCCAGAACACAATGCTTATCAACTTAGGCCGCCAAAAGCTGATGGACTGTATCATG

GCCCTACCTCGGTTCTACCAGGCTTCTATCGTGGCCGAGGCCTATGACTTTGTTCCAGAT

TGGGCAGAAATTCTATACCAACAAGTGATTCTTAAAGGAGATTTTACTTATTTGGAAGAA

TTTAAACAACAAAGGTTGTTAAGACCTAGTATAATTGAAGAAATTTCAAAAAAATATAAA

CAACATCAGCCTACCAACAAGGTCACAGAAAACTTGAAGAAGTTACTTTCATACTGTGAA

GATATCTACCTGTATTACAAGTTGGCATATGAACACAAATTTTATGAAATTATAAATGTA

CTTCTGAAGGACCCTCAAACAGGTTGCTGTCTAAAGGATATGCTTTCAGGT

>CL403.Contig1_A-W 1 1440 LAS1-like protein [Heterocephalus glaber]

GAAAGAGCCCGAGACTTGCTGGTATCATATGAAGAGGAGCAGTTTAAGGTGCTGGAGAAA

TTTAGGCATTTACCTCAGGCTATTAAGGCGTGGAATAAACTATCCCCATGTGTAGAAGGC

ATCCTGGCAGAGCTCAAGGGCATTTCATGGGAAAACAGGAATGCTGTGCTGGATGCTTTC

TTGGATGACGGCTTCCTCATTCCCACATTTGAGCAGTTGGCAGCTTTGCAGATAGAATAT

GAAGAAAACGTGGACTTGAATGACGTCTTGGTGCCAAAGCCGTTCTCTCAGTTCTGGCAG

CCCCTGCTCAGGGGCCTGCACTCCCAGACCTTCACACAGGCCCTGCTGGAGAGGATGTTC

TCTGAGCTGCCAACCTTGGGGGACAGTGGGATCCGGCCCACCTACATCCTTAGATGGACC

ATTGAACTGATCGTGGCCAACACCAAAACTGGAAAGAATGCTCGCCGATTTTCTACAAGA

CAGTGGGAAGCAAGAAGGAGCTGGAGGCTGTTCAACTGCTCTGCCTCCCTTGACTGGCCC

CAGGTGGTTGAGTCCTGCTTGGGCTCACCTTGCTGGGCCAGTCCCCAACTCCTTCAGCTT

GTCTTCAAAGCCATGGGACAGGTCCTGCCAGATGGGGAGCAGGAGAAGCTGCTGCGCATC

TGTTCCATTTATACTCAGAATGGAGAAAACAGCCAGGTGAAAGAGGGATCAGAGGCTCCC

CCCTGCGTGAAGTCTCCATACACACTGGATAGCCTATACTGGGGTCTTAAACCAGCTGGT

TCTAACTGTTGTTCTGAAGGAAAGGCCCAGCAGCAGGGGGAACAGGGCAGCCATAATGAT

ATCAATCAAGAAGAGAAGGAAGAGAAAAAAGTGTTGCAAGACCAGATGGAAGAAGACGAT

GAAGAAGATGATGACGATAACCTGGAAGATGAAGAGGGAATTGAAGATGACGATGAAGAT

GGTGATGAAGATGATGATGATGACCACGATGATGATGATGACGATGACGATGATGATGAT

GATGAAGACGAGGAAGAAGATGAAGATGAAGACAGAATGGAGGTGGGACCTTTCTCTACT

GTGCAGGAATCCCCTACAGTGGAGAATGCCAGGCTCCTGGTCCAGAAACGAGGAGCTTTG

CAGGGTTCTGCATGGCAAGTTAGTTCAGAGGATGTGCGATGGGACACGTTCCCCTTAGGC

CGAATGCCAGGTCAGACTGAGGACCCAGCAGAACTTATGCTGGACAATTATGACACCATG

TATCTTTTGGACCAGCCTGTGCTAGACCAGCGGCTGGAACCCCCAACAGGCAAAGCTGGC

ACCCTGGGCCTAAGCTGTGGTGTTGGCAGTGGCAACTGCAGCAATAGCAGCACCAATTTT

GAGGGTCTTCTCTGGAGCCAGGGGCAGCTGCATGGGCTCAAAACTGGCCTGCAGCTCTTC

>CL403.Contig2_A-W 1 294 PREDICTED: LOW QUALITY PROTEIN: protein LAS1 homolog [Equus caballus]

GAAAGAGCCCGAGACTTGCTGGTATCATATGAAGAGGAGCAGTTTAAGGTGCTGGAGAAA

TTTAGGCATTTACCTCAGGCTATTAAGGCGTGGAATAAACTATCCCCATGTGTAGAAGGC

ATCCTGGCAGAGCTCAAGGGCATTTCATGGGAAAACAGGAATGCTGTGCTGGATGCTTTC

TTGGATGACGGCTTCCTCATTCCCACATTTGAGCAGTTGGCAGCTTTGCAGATAGAATAT

GAAGATGGTCAGACTGAGGTCCAGAGAGGGGAAGGTACTGACCCAGAGTCACAC

>CL404.Contig1_A-W 6 2201 PREDICTED: leucine-rich repeat-containing protein 33 [Papio anubis]

ATGCCCCAGAGAGGAGGCAGCCCAGCGCCGCGTTTGGCTGCTCTCGAGGAGGAGGCCGAG

CCTGGGAGACGATGCGCCCCGCGGAGCCACCTGGGCCTCCGGGAGCCTGCTGCCCTTGAG

ATGGAGTTGCTGCCCCTTTGGCTCTGCCTGGGTTTTCATTTCTTGACCATGAAATGGAAG

AATGGAAGTGGGATGGCCATGGTGGCTTCCCAAGGGGGTTGCAAGTTGGGGGATGGCGTT

GCCGACTGCAGAGGGCAGAACCTTGCTTCAGTGCCCAGCAGCCTCCCGTCCCACTCACAG

ACACTCATCCTGGACGCCAACCCTCTCAAGACCCTGTGGAATCATTCCCTCCAGCCGTAC

CCTCTTCTGGAGAACCTCAGCCTGCACAGCTGCCACCTGGGACTCATCGGCCTTGATGCC

TTCCAGGGGCAAGGCCACCTGCACAGTCTGGCTCTGGCTGACAACTGCCTCTCTGAGAAC

TACAAAGAGACAGCAGCTGCTCTCCACACTCTGCCAGATCTGCGGAGGCTCGACTTGTCA

GGAAACTCCCTAACGGAAGACATGGCAGCCCTCATGCTGCAGAACCTCTCTTCACTGGAG

GCTGTGTCCCTGGCAAGGAACACCCTGATGAGGCTTGATGACTGTGTCTTTGAGGGCCTG

GAGCACCTCAGGGAACTGGATTTGCAGAGGAACTACATCTTTGAGATTGAGGGTGGTGCT

TTTGATGGCTTGACTGAGCTTAGACACCTCAACTTGGCCTACAACAACCTCCCCTGCATT

GTAGACTTTGGTCTCACACAGCTACAGTTTCTAAACATCAGCTACAATGCCCTGGAGTGG

TTCCTGGCAGCCAAGGAGGAGGCTGCCTTTGAGCTGGAGATGCTGGACCTGTCTCACAAC

CAACTGCTGTTCTTCCCTCTCCTACCCCAGTGCAGTAAGTTGCACACTCTCCTGCTACGG

GACAACAACATGGGCTTCTACAGGGACTTGTACAATACCTCGTCACCGCAGGAGATGGTG

GCCCAGTTCCTCCTTGTGGACGGCAACGTGACCAACATCACCACTGTCAGCCTCTGGGAA

GAGTTTGCCTCCAGTGACCTGGCAGCTCTTCACTTCCTGGACATGAGCCAGAACCAGTTC

CAGTACCTGCCAGATGGCTTCCTAAAGAAAATGCCTTCCCTCTCCCACCTGAACCTCAAC

CAGAATTGCCTGATGATGCTCCACATCCGGGAGCATGAGCCCCTAGCCGCACTTACAGAA

CTAGACCTGAGCCACAACCAGCTCTCAGAGCTGCACTTGGCTCCAGGGCTTACGGGCTGC

CTAAAGAGCCTCCGCTTGTTTAACCTGAGCTCCAACCAGCTCCAGGGTGTCCCCACTGGC

CTTTTTGCCAATGCCAGTAACATCACTACAATTGACATGAGCCATAATCAGGTCTTACTT

TGTCCCCAGCCAGCTCCCTTAGATAGGGCAGGACCCCCCAGCTGTGTGGATTTCAGAAAT

ATGACATCTTTAAGGAGCCTCTCTCTGGAGGGCTGTGGGTTAGGGGCATTACAAGACTGC

CCATTCCAAGGGACGGCCCTCACTCACTTAGACCTATCCAGCAACTGGGGGATTCTGAAT

GGGAGCATTGCTCCTCTCCAGGATATTGCCCGCACCTTACAGGTCCTATCTCTCAGGAAC

GTGGGCCTCAATTCTGGCTTCACAGAATTGGACTTTTCTGGATTTGGGAATCTGAGGGAC

TTGGATCTGTCAGGAAATTTCTTGACTAGCTTCCCGAGGTTCAGGAGCAGCCTGGCTCTG

CAGACCCTGGATCTCCGCAGAAACTCTCTCACTGCCCTTCCTCAGAGGGCTGTGTCTGAG

CAGCTCCTGAGGAGTCTGCAGACCATCTACCTCAGTCAGAATCCATATGACTGCTGTGGA

GTGGAGGGATGGGGGGCCCTGCAGCACCTAAAGAGTGTCGCCGACTTGGCCATGGTCACT

TGCAACCTCTCCTCCAAGGTCATTCGTGTGCTGGAGCTGCCAGGCGGCGTGCCTCAGGAC

TGCAAGTGGGAGCGGGTAGACATGGGCCTGCTCTACCTTGTGCTTATTCTCCCCAGCTGC

CTCACCCTATTGGTGGCCTGCACTGTGATCTTCCTCACTTTTAAGAAGCCTTTGCTTCAG

GTAATCAAGAGCCGCTGCCACTGGTCCTCCGTATAC

>CL404.Contig2_A-W 6 236 PREDICTED: leucine-rich repeat-containing protein 33 [Papio anubis]

ATGCCCCAGAGAGGAGGCAGCCCAGCGCCGCGTTTGGCTGCTCTCGAGGAGGAGGCCGAG

CCTGGGAGACGATGCGCCCCGCGGAGCCACCTGGGCCTCCGGGAGCCTGCTGCCCTTGAG

ATGGAGTTGCTGCCCCTTTGGCTCTGCCTGGGTTTTCATTTCTTGACCATGAAATGGAAG

AATGGAAGTGGGATGGCCATGGTGGCTTCCCAAGGGGGTTGCAAGTTGGTG

>CL404.Contig3_A-W 42 164 hCG40298, isoform CRA_g [Homo sapiens]

GCTGCCCTTGAGATGGAGTTGCTGCCCCTTTGGCTCTGCCTGGGTTTTCATTTCTTGACC

ATGAAATGGAAGAATGGAAGTGGGATGGCCATGGTGGCTTCCCAAGGGGGTTGCAAGTTG

GTG

>CL405.Contig1_A-W 145 306 PREDICTED: thyroid transcription factor 1-associated protein 26-like [Loxodonta africana]

CAGGAGTTCGAGAGGAGAAAAAAAGAGAGAGAAGAAGCTCAAAGACTCTACAAAAAGAAG

AAAATGGAAGTGTTCAAAATATTGAGCAAAAAGACTAGAAAGGACCAACCAAACTTGAAT

TTACAAATGGAATATCTTCTTCAAAAAATACAAGAAAAAAGT

>CL405.Contig2_A-W 185 904 coiled-coil domain containing 59 [Sus scrofa]

ATGGCACCCGTCAGGCCGGCAGCGAGGTTGAGAACCGGTGGTTTGGGGGCGCGTGTTGAG

GGGGTTTCACCTGTCGGATGCAGGAATAAGAATGGGAAAAAGAGGACCTGGCGGCCTAAC

TATCCTCAAGCTTTTTCGGGCAGCGTTCGCGAGGGGCAAGGCTTTGCATTTCGGAGAAAA

CTGAAGATTCAACAACATTACAAGAAATTGCTGTGGAAGAAAAAGAAGGCTCAAATATCA

CAGGAGTCCCAATTCACAGATCAGTATCCAGATCATCTGAAACATCTTTATTTAGCTGAA

GAAGAAAGACTCAGGAAGCAACTGAGAAAAGTTACCCAGCCTTTGTCAGAAGAACAAGTT

GACCAGCCTTTGCCTGAAAAAGAGCATAGCATTGATCAGACTTTGTCTGAAGACGAATGT

AGTGTTCATCAGCCTCAACCAGAACAGTGTGGCAAAACGAAAAATTCTGTGACTATTCCA

AAAAAGAATAAAAAGAAAACATCAAATCAAAAAGCACTAGAAGAATATGAACGGATACAA

GCTAAACGTGCTGCCAAGAAACAAGAGTTCGAGAGGAGAAAAAAAGAGAGAGAAGAAGCT

CAAAGACTCTACAAAAAGAAGAAAATGGAAGTGTTCAAAATATTGAGCAAAAAGACTAGA

AAGGACCAACCAAACTTGAATTTACAAATGGAATATCTTCTTCAAAAAATACAAGAAAAA

>CL406.Contig1_A-W 2 433 PREDICTED: thioredoxin domain-containing protein 17-like [Saimiri boliviensis boliviensis]

CCACCCCGAGGGCGACCCGCCCAGCGGCAGCTGCAACGTAGACTCCTCCAGTACGCAGTA

CTCATGGCTATCTACGAGGAGGTGAGCGTGTCGGGCTTCGAGGAGTTCAATCAGGCTGTG

AAGCAGCACCTGGGCAAGACTATTTTCGCTTACTTTACAGGTTCTAAGGACGCCGAAGGG

AAAAGCTGGTGTCCCGACTGCGTGCAGGCGGAACCAGTCGTTCGAGAGGGGCTGAAGCAT

ATTAGTAAAGAATGTGTGTTCATCCACTGCCAAGTAGGAGAAAAACCTTATTGGAAAGAT

CCAAATAATGACTTCAGAAAACAACTGAAAGTAACTGCAGTGCCTACACTAATTAAATAT

GGAACGCCTCAAAAACTGGTAGAATCAGAGTGCCTTCACGCCAACCTTGTGGAGATGCTG

TTCTCTGAAGAT

>CL406.Contig2_A-W 2 214 PREDICTED: thioredoxin domain-containing protein 17-like [Saimiri boliviensis boliviensis]

CCACCCCGAGGGCGACCCGCCCAGCGGCAGCTGCAACGTAGACTCCTCCAGTACGCAGTA

CTCATGGCTATCTACGAGGAGGTGAGCGTGTCGGGCTTCGAGGAGTTCAATCAGGCTGTG

AAGCAGCACCTGGGCAAGACTATTTTCGCTTACTTTACAGGTTCTAAGGACGCCGAAGGG

AAAAGCTGGTGTCCCGACTGCGTGCAGGGTGAG

>CL407.Contig1_A-W 18 1802 minus strand PREDICTED: tyrosine-protein kinase HCK [Otolemur garnettii]

ATGGCCTGTGGGCGCCACCGGGTTCTTGGTTCCAGCCCGGGGGCGCAGAGTAGGTTTAGA

GGCGAGTGGGACCGGACAGTCCAGCGCTGGGAGAGCCCAAGAGGACCCGCGAGGCAGAAG

CCGCCGCCCCCGGAGTGGCCCCAGCCTCCTGATCTCCACAGTCAGCCCATCTTCTTCTGG

GCTCGGGTGCCCCGGGCCCCGGGAGCTGCTGAGCTGGGGGGGCGCTGGAGCTGCGAGGAT

CCGGGCTGCCCGCGAGAAGAAGAGCGGGCGCCCAGGATGGGGTGCATGAAGTCCAAGTTC

CTCCGGAATAAAGGCAAGGCCTCCAAGTCTGAGTCCAGCACCAACCTGCACAGCCCTGTG

TACATGCCAGATCCCACGACCCCGATCAAGTTGGGGCCTAACTACAATGGCAACCCCCCA

GGGTTTGTGGAGGCAGGCTCTGAGGACACTGTTGTGGTCGCCCTGTACGATTACGAGGCC

ATTCACCGTGAAGACCTCAGCTTCCAGAAGGGGGACCAGATGGTGGTCCTGGAGGAGGCT

GGGGAGTGGTGGAGGGCGCGATCTCTGGCCACCAGGAAAGAGGGCTACATCCCAAGCAAC

TATGTGGCCCGCGTGAATTCCCTGGAGACGGAGGAGTGGTTCTTCAAGGGCATCAGCAGG

AAGGACGCAGAGCGCCACCTCCTGGCTCCCGGCAACGTGCTGGGCTCCTTCATGATCCGG

GACAGCGAGACCACAAAAGGGAGCTACTCTCTGTCCGTGCGAGACTACGAGCCCCACCAA

GGAGACACGGTGAAGCACTATAAGATCCGGACCCTGGACAACGGGGGCTTCTACATTTCC

CCGAGGAGCACCTTCAGCAACCTGCAGGATCTGGTGGCCCACTACAAGAAGGGGAAGGAC

GGGCTCTGCCAGAAGCTGTCAGTGCCCTGTGTGTCCCCCAAGCCCCAGAAGCCGTGGGAG

AAAGACGCCTGGGAGATCCCTCGGGAGTCCCTCGAGCTGGAGAAGAAGCTGGGCGCTGGG

CAGTTCGGGGAAGTCTGGATGGCCACCTACAACAAGCACACCAAGGTGGCGGTGAAGACG

ATGAAGCCTGGGAGTATGTCGGTGGAGGCCTTCTTGGCCGAGGCCAACCTGATGAAGACG

CTGCAGCATGACAAGCTGGTGAAGCTGCACGCGGTGGTCACCCAGGAGCCCATCTTCATC

GTCACGGAGTTCATGGCCAAAGGAAGCCTGCTGGACTTTTTGAAAAGTGGAGAAGGCAGC

AAGCAGCCCTTGCCAAAACTCATCGACTTCTCAGCCCAGATTGCAGAAGGCATGGCCTTC

ATCGAGCAGAGAAACTACATCCATCGGGACCTCCGAGCCGCCAACATCCTGGTCTCTGCT

TCCCTGGTGTGTAAGATTGCTGACTTTGGCCTGGCACGGATCATCGAGGACAACGAGTAC

ACGGCTCGGGAAGGGGCCAAGTTCCCCATCAAGTGGACAGCTCCTGAGGCCATCAACTTC

GGCTCCTTCACCATCAAGTCAGACGTCTGGTCCTTTGGAATCCTGCTGATGGAAATTGTC

ACCTATGGCCGGATCCCTTACCCAGGAATGTCAAACCCAGAGGTGATCAGGGCGCTGGAG

CACGGGTACCGAATGCCTCGACCCGAGCACTGCCCTGAGGAGCTCTACAACATCATGACC

CGCTGCTGGAAAAACCGCCCCGAAGAGCGGCCCACCTTTGAATATATCCAGAGTGTGCTG

GATGATTTCTACACGGCCACCGAGAGCCAGTACCAACAACAGCCT

>CL407.Contig2_A-W 18 1604 minus strand PREDICTED: tyrosine-protein kinase HCK [Otolemur garnettii]

ATGGCCTGTGGGCGCCACCGGGTTCTTGGTTCCAGCCCGGGGGCGCAGAGTAGGTTTAGA

GGCGAGTGGGACCGGACAGTCCAGCGCTGGGAGAGCCCAAGAGGACCCGCGAGGCAGAAG

CCGCCGCCCCCGGAGTGGCCCCAGCCTCCTGATCTCCACAGTCAGCCCATCTTCTTCTGG

GCTCGGGTGCCCCGGGCCCCGGGAGCTGCTGAGCTGGGGGGGCGCTGGAGCTGCGAGGAT

CCGGGCTGCCCGCGAGAAGAAGAGCGGGCGCCCAGGATGGGGTGCATGAAGTCCAAGTTC

CTCCGGAATAAAGGCAAGGCCTCCAAGTCTGAGTCCAGCACCAACCTGCACAGCCCTGTG

TACATGCCAGATCCCACGACCCCGATCAAGTTGGGGCCTAACTACAATGGCAACCCCCCA

GGGTTTGTGGAGGCAGGCTCTGAGGACACTGTTGTGGTCGCCCTGTACGATTACGAGGCC

ATTCACCGTGAAGACCTCAGCTTCCAGAAGGGGGACCAGATGGTGGTCCTGGAGGAGGCT

GGGGAGTGGTGGAGGGCGCGATCTCTGGCCACCAGGAAAGAGGGCTACATCCCAAGCAAC

TATGTGGCCCGCGTGAATTCCCTGGAGACGGAGGAGTGGTTCTTCAAGGGCATCAGCAGG

AAGGACGCAGAGCGCCACCTCCTGGCTCCCGGCAACGTGCTGGGCTCCTTCATGATCCGG

GACAGCGAGACCACAAAAGGGAGCTACTCTCTGTCCGTGCGAGACTACGAGCCCCACCAA

GGAGACACGGTGAAGCACTATAAGATCCGGACCCTGGACAACGGGGGCTTCTACATTTCC

CCGAGGAGCACCTTCAGCAACCTGCAGGATCTGGTGGCCCACTACAAGAAGGGGAAGGAC

GGGCTCTGCCAGAAGCTGTCAGTGCCCTGTGTGTCCCCCAAGCCCCAGAAGCCGTGGGAG

AAAGACGCCTGGGAGATCCCTCGGGAGTCCCTCGAGCTGGAGAAGAAGCTGGGCGCTGGG

CAGTTCGGGGAAGTCTGGATGGCCACCTACAACAAGCACACCAAGGTGGCGGTGAAGACG

ATGAAGCCTGGGAGTATGTCGGTGGAGGCCTTCTTGGCCGAGGCCAACCTGATGAAGACG

CTGCAGCATGACAAGCTGGTGAAGCTGCACGCGGTGGTCACCCAGGAGCCCATCTTCATC

GTCACGGAGTTCATGGCCAAAGGAAGCCTGCTGGACTTTTTGAAAAGTGGAGAAGGCAGC

AAGCAGCCCTTGCCAAAACTCATCGACTTCTCAGCCCAGATTGCAGAAGGCATGGCCTTC

ATCGAGCAGAGAAACTACATCCATCGGGACCTCCGAGCCGCCAACATCCTGGTCTCTGCT

TCCCTGGTGTGTAAGATTGCTGACTTTGGCCTGGCACGGATCATCGAGGACAACGAGTAC

ACGGCTCGGGAAGGGGCCAAGTTCCCCATCAAGTGGACAGCTCCTGAGGCCATCAACTTC

GGCTCCTTCACCATCAAGTCAGACGTCTGGTCCTTTGGAATCCTGCTGATGGAAATTGTC

ACCTATGGCCGGATCCCTTACCCAGGT

>CL407.Contig3_A-W 18 1472 minus strand PREDICTED: tyrosine-protein kinase HCK [Otolemur garnettii]

ATGGCCTGTGGGCGCCACCGGGTTCTTGGTTCCAGCCCGGGGGCGCAGAGTAGGTTTAGA

GGCGAGTGGGACCGGACAGTCCAGCGCTGGGAGAGCCCAAGAGGACCCGCGAGGCAGAAG

CCGCCGCCCCCGGAGTGGCCCCAGCCTCCTGATCTCCACAGTCAGCCCATCTTCTTCTGG

GCTCGGGTGCCCCGGGCCCCGGGAGCTGCTGAGCTGGGGGGGCGCTGGAGCTGCGAGGAT

CCGGGCTGCCCGCGAGAAGAAGAGCGGGCGCCCAGGATGGGGTGCATGAAGTCCAAGTTC

CTCCGGAATAAAGGCAAGGCCTCCAAGTCTGAGTCCAGCACCAACCTGCACAGCCCTGTG

TACATGCCAGATCCCACGACCCCGATCAAGTTGGGGCCTAACTACAATGGCAACCCCCCA

GGGTTTGTGGAGGCAGGCTCTGAGGACACTGTTGTGGTCGCCCTGTACGATTACGAGGCC

ATTCACCGTGAAGACCTCAGCTTCCAGAAGGGGGACCAGATGGTGGTCCTGGAGGAGGCT

GGGGAGTGGTGGAGGGCGCGATCTCTGGCCACCAGGAAAGAGGGCTACATCCCAAGCAAC

TATGTGGCCCGCGTGAATTCCCTGGAGACGGAGGAGTGGTTCTTCAAGGGCATCAGCAGG

AAGGACGCAGAGCGCCACCTCCTGGCTCCCGGCAACGTGCTGGGCTCCTTCATGATCCGG

GACAGCGAGACCACAAAAGGGAGCTACTCTCTGTCCGTGCGAGACTACGAGCCCCACCAA

GGAGACACGGTGAAGCACTATAAGATCCGGACCCTGGACAACGGGGGCTTCTACATTTCC

CCGAGGAGCACCTTCAGCAACCTGCAGGATCTGGTGGCCCACTACAAGAAGGGGAAGGAC

GGGCTCTGCCAGAAGCTGTCAGTGCCCTGTGTGTCCCCCAAGCCCCAGAAGCCGTGGGAG

AAAGACGCCTGGGAGATCCCTCGGGAGTCCCTCGAGCTGGAGAAGAAGCTGGGCGCTGGG

CAGTTCGGGGAAGTCTGGATGGCCACCTACAACAAGCACACCAAGGTGGCGGTGAAGACG

ATGAAGCCTGGGAGTATGTCGGTGGAGGCCTTCTTGGCCGAGGCCAACCTGATGAAGACG

CTGCAGCATGACAAGCTGGTGAAGCTGCACGCGGTGGTCACCCAGGAGCCCATCTTCATC

GTCACGGAGTTCATGGCCAAAGGAAGCCTGCTGGACTTTTTGAAAAGTGGAGAAGGCAGC

AAGCAGCCCTTGCCAAAACTCATCGACTTCTCAGCCCAGATTGCAGAAGGCATGGCCTTC

ATCGAGCAGAGAAACTACATCCATCGGGACCTCCGAGCCGCCAACATCCTGGTCTCTGCT

TCCCTGGTGTGTAAGATTGCTGACTTTGGCCTGGCACGGATCATCGAGGACAACGAGTAC

ACGGCTCGGGAAGGT

>CL407.Contig4_A-W 46 390 minus strand PREDICTED: tyrosine-protein kinase HCK [Canis lupus familiaris]

AGAACCTCTTGGGCTGGGCCAGATCCAAGGGTGAGAGTGTCTCCGTTTCGGATGCAGATT

GCAGAAGGCATGGCCTTCATCGAGCAGAGAAACTACATCCATCGGGACCTCCGAGCCGCC

AACATCCTGGTCTCTGCTTCCCTGGTGTGTAAGATTGCTGACTTTGGCCTGGCACGGATC

ATCGAGGACAACGAGTACACGGCTCGGGAAGGGGCCAAGTTCCCCATCAAGTGGACAGCT

CCTGAGGCCATCAACTTCGGCTCCTTCACCATCAAGTCAGACGTCTGGTCCTTTGGAATC

CTGCTGATGGAAATTGTCACCTATGGCCGGATCCCTTACCCAGGT

>CL407.Contig5_A-W 70 270 minus strand PREDICTED: LOW QUALITY PROTEIN: tyrosine-protein kinase HCK [Pan troglodytes]

CCAAGGGTGAGAGTGTCTCCGTTTCGGATGCAGATTGCAGAAGGCATGGCCTTCATCGAG

CAGAGAAACTACATCCATCGGGACCTCCGAGCCGCCAACATCCTGGTCTCTGCTTCCCTG

GTGTGTAAGATTGCTGACTTTGGCCTGGCACGGATCATCGAGGACAACGAGTACACGGCT

CGGGAAGGTAGGGAATGCCAC

>CL408.Contig1_A-W 4 912 Crossover junction endonuclease MUS81 [Heterocephalus glaber]

GGTGCCAGTGAAGGAGGGGTCCATCAGCCACTGCTGGAGCTGAGGCCTAGAGAGTACAGG

GTGCTGTTGTGTGTGGACATCAGCGAGACCAGGGGGGCAGGACATAGGCAAGAGCTGCTC

CGAGAGCTACAGCGGATGCATGTGGCCCATACAGTGCGCAAGCTCCATGTTGGGGACTTT

GTGTGGGTGGCACAGGAGACCAGGCCCAGAGACCCAGCCAGACCTGGAGAGTTAGTCCTG

GATCACATCGTGGAGCGCAAGCGCCTGGATGACCTATGCAGTAGCATCATCGACGGCCGC

TTCCGGGAGCAGAAGTTCCGACTAAAGCGCTGTGGTCTGGGGCATCGGGTGTACCTGGTG

GAGGAGTATGGCTCAGTCCACAACCTTAGCCTTCCTGAGAGCACACTGCTGCAGGCTGTC

ACCAACACCCAGGTCATTGATGGCTTCTTTGTGAAGCGCACAGTAGACATTAAGGAGTCA

GCTGCCTACCTTGCCCTTTTGACAAGGGGCCTGGAGAGACTCTACCAGGGACACACCCTA

CGCAGCCGTCCCTGGGGAACCCCAGGGGACCCTGAGTCGGGGGCCAGGCCCACCCCAAAC

CCACTCTGCTCACTCCTCACCTTCAGTGACTTCAACACAGGAGCCATCAAGAACAAGGCC

CAGTCTGTGCGTGAGGTGTTCGCCAGGCAGCTGATGCAGGTTCGTGGAATCAGTGGGGAG

AAGGCAGCAGCCCTGGTGGACCGATACAGCACCCCTGCCAGCCTCCTGGCTGCCTATGAT

GCCTGTGCCACCCCCAAGGAGCAGGAGATGCTGCTGAGCACCATCAAGTGTGGGCGTCTG

CAGAGGAATCTGGGACCTGTTCTGAGCAGGACTTTATCTCAGCTCTACTGCACCTACAGC

CCCTTGACT

>CL408.Contig2_A-W 530 814 Crossover junction endonuclease MUS81 [Heterocephalus glaber]

AGTGACTTCAACACAGGAGCCATCAAGAACAAGGCCCAGTCTGTGCGTGAGGTGTTCGCC

AGGCAGCTGATGCAGGTTCGTGGAATCAGTGGGGAGAAGGCAGCAGCCCTGGTGGACCGA

TACAGCACCCCTGCCAGCCTCCTGGCTGCCTATGATGCCTGTGCCACCCCCAAGGAGCAG

GAGATGCTGCTGAGCACCATCAAGTGTGGGCGTCTGCAGAGGAATCTGGGACCTGTTCTG

AGCAGGACTTTATCTCAGCTCTACTGCACCTACAGCCCCTTGACT

>CL408.Contig3_A-W 4 984 Crossover junction endonuclease MUS81 [Heterocephalus glaber]

GGTGCCAGTGAAGGAGGGGTCCATCAGCCACTGCTGGAGCTGAGGCCTAGAGAGTACAGG

GTGCTGTTGTGTGTGGACATCAGCGAGACCAGGGGGGCAGGACATAGGCAAGAGCTGCTC

CGAGAGCTACAGCGGATGCATGTGGCCCATACAGTGCGCAAGCTCCATGTTGGGGACTTT

GTGTGGGTGGCACAGGAGACCAGGCCCAGAGACCCAGCCAGACCTGGAGAGTTAGTCCTG

GATCACATCGTGGAGCGCAAGCGCCTGGATGACCTATGCAGTAGCATCATCGACGGCCGC

TTCCGGGAGCAGAAGTTCCGACTAAAGCGCTGTGGTCTGGGGCATCGGGTGTACCTGGTG

GAGGAGTATGGCTCAGTCCACAACCTTAGCCTTCCTGAGAGCACACTGCTGCAGGCTGTC

ACCAACACCCAGGTCATTGATGGCTTCTTTGTGAAGCGCACAGTAGACATTAAGGAGTCA

GCTGCCTACCTTGCCCTTTTGACAAGGGGCCTGGAGAGACTCTACCAGGGACACACCCTA

CGCAGCCGTCCCTGGGGAACCCCAGGGGACCCTGAGTCGGGGGCCAGGCCCACCCCAAAC

CCACTCTGCTCACTCCTCACCTTCAGTGACTTCAACACAGGAGCCATCAAGAACAAGGCC

CAGTCTGTGCGTGAGGTGTTCGCCAGGCAGCTGATGCAGGTTCGTGGAATCAGTGGGGAG

AAGGCAGCAGCCCTGGTGGACCGATACAGCACCCCTGCCAGGTTGACCCTATTCCTGCTT

CAGTTCTCATTGTGCTGGCTCAGATAGTCAGCCATCAAATACCCCATCCCCAGCCTCCTG

GCTGCCTATGATGCCTGTGCCACCCCCAAGGAGCAGGAGATGCTGCTGAGCACCATCAAG

TGTGGGCGTCTGCAGAGGAATCTGGGACCTGTTCTGAGCAGGACTTTATCTCAGCTCTAC

TGCACCTACAGCCCCTTGACT

>CL408.Contig4_A-W 541 672 minus strand PREDICTED: crossover junction endonuclease MUS81-like, partial [Cricetulus griseus]

CAGGGACACACCCTACGCAGCCGTCCCTGGGGAACCCCAGGGGACCCTGAGTCGGGGGCC

AGGCCCACCCCAAACCCACTCTGCTCACTCCTCACCTTCAGTGACTTCAACACAGGAGCC

ATCAAGAACAAG

>CL408.Contig5_A-W 3 656 minus strand Crossover junction endonuclease MUS81 [Macaca mulatta]

GCCAGTGAAGGAGGGGTCCATCAGCCACTGCTGGAGCTGAGGCCTAGAGAGTACAGGGTG

CTGTTGTGTGTGGACATCAGCGAGACCAGGGGGGCAGGACATAGGCAAGAGCTGCTCCGA

GAGCTACAGCGGATGCATGTGGCCCATACAGTGCGCAAGCTCCATGTTGGGGACTTTGTG

TGGGTGGCACAGGAGACCAGGCCCAGAGACCCAGCCAGACCTGGAGAGTTAGTCCTGGAT

CACATCGTGGAGCGCAAGCGCCTGGATGACCTATGCAGTAGCATCATCGACGGCCGCTTC

CGGGAGCAGAAGTTCCGACTAAAGCGCTGTGGTCTGGGGCATCGGGTGTACCTGGTGGAG

GAGTATGGCTCAGTCCACAACCTTAGCCTTCCTGAGAGCACACTGCTGCAGGCTGTCACC

AACACCCAGGTCATTGATGGCTTCTTTGTGAAGCGCACAGTAGACATTAAGGAGTCAGCT

GCCTACCTTGCCCTTTTGACAAGGGGCCTGGAGAGACTCTACCAGGGACACACCCTACGC

AGCCGTCCCTGGGGAACCCCAGGGGACCCTGAGTCGGGGGCCAGGCCCACCCCAAACCCA

CTCTGCTCACTCCTCACCTTCAGTGACTTCAACACAGGAGCCATCAAGAACAAG

>CL408.Contig6_A-W 113 577 minus strand PREDICTED: crossover junction endonuclease MUS81-like [Cavia porcellus]

TTCCGACTAAAGCGCTGTGGTCTGGGGCATCGGGTGTACCTGGTGGAGGAGTATGGCTCA

GTCCACAACCTTAGCCTTCCTGAGAGCACACTGCTGCAGGCTGTCACCAACACCCAGGTC

ATTGATGGCTTCTTTGTGAAGCGCACAGTAGACATTAAGGAGTCAGCTGCCTACCTTGCC

CTTTTGACAAGGGGCCTGGAGAGACTCTACCAGGCCCAGTCTGTGCGTGAGGTGTTCGCC

AGGCAGCTGATGCAGGTTCGTGGAATCAGTGGGGAGAAGGCAGCAGCCCTGGTGGACCGA

TACAGCACCCCTGCCAGCCTCCTGGCTGCCTATGATGCCTGTGCCACCCCCAAGGAGCAG

GAGATGCTGCTGAGCACCATCAAGTGTGGGCGTCTGCAGAGGAATCTGGGACCTGTTCTG

AGCAGGACTTTATCTCAGCTCTACTGCACCTACAGCCCCTTGACT

>CL408.Contig7_A-W 541 924 minus strand PREDICTED: crossover junction endonuclease MUS81-like, partial [Cricetulus griseus]

CAGGGACACACCCTACGCAGCCGTCCCTGGGGAACCCCAGGGGACCCTGAGTCGGGGGCC

AGGCCCACCCCAAACCCACTCTGCTCACTCCTCACCTTCAGTGACTTCAACACAGGAGCC

ATCAAGAACAAGGCCCAGTCTGTGCGTGAGGTGTTCGCCAGGCAGCTGATGCAGGTTCGT

GGAATCAGTGGGGAGAAGGCAGCAGCCCTGGTGGACCGATACAGCACCCCTGCCAGCCTC

CTGGCTGCCTATGATGCCTGTGCCACCCCCAAGGAGCAGGAGATGCTGCTGAGCACCATC

AAGTGTGGGCGTCTGCAGAGGAATCTGGGACCTGTTCTGAGCAGGACTTTATCTCAGCTC

TACTGCACCTACAGCCCCTTGACT

>CL408.Contig8_A-W 114 518 minus strand PREDICTED: crossover junction endonuclease MUS81 [Equus caballus]

GGCCTTAGTCCCCTCTTCCCTCAGGGACACACCCTACGCAGCCGTCCCTGGGGAACCCCA

GGGGACCCTGAGTCGGGGGCCAGGCCCACCCCAAACCCACTCTGCTCACTCCTCACCTTC

AGTGACTTCAACACAGGAGCCATCAAGAACAAGGCCCAGTCTGTGCGTGAGGTGTTCGCC

AGGCAGCTGATGCAGGTTCGTGGAATCAGTGGGGAGAAGGCAGCAGCCCTGGTGGACCGA

TACAGCACCCCTGCCAGCCTCCTGGCTGCCTATGATGCCTGTGCCACCCCCAAGGAGCAG

GAGATGCTGCTGAGCACCATCAAGTGTGGGCGTCTGCAGAGGAATCTGGGACCTGTTCTG

AGCAGGACTTTATCTCAGCTCTACTGCACCTACAGCCCCTTGACT

>CL408.Contig9_A-W 113 229 minus strand PREDICTED: crossover junction endonuclease MUS81 [Equus caballus]

TTCCGACTAAAGCGCTGTGGTCTGGGGCATCGGGTGTACCTGGTGGAGGAGTATGGCTCA

GTCCACAACCTTAGCCTTCCTGAGAGCACACTGCTGCAGGCTGTCACCAACACCCAG

>CL408.Contig10_A-W 3 779 minus strand Crossover junction endonuclease MUS81 [Macaca mulatta]

GCCAGTGAAGGAGGGGTCCATCAGCCACTGCTGGAGCTGAGGCCTAGAGAGTACAGGGTG

CTGTTGTGTGTGGACATCAGCGAGACCAGGGGGGCAGGACATAGGCAAGAGCTGCTCCGA

GAGCTACAGCGGATGCATGTGGCCCATACAGTGCGCAAGCTCCATGTTGGGGACTTTGTG

TGGGTGGCACAGGAGACCAGGCCCAGAGACCCAGCCAGACCTGGAGAGTTAGTCCTGGAT

CACATCGTGGAGCGCAAGCGCCTGGATGACCTATGCAGTAGCATCATCGACGGCCGCTTC

CGGGAGCAGAAGTTCCGACTAAAGCGCTGTGGTCTGGGGCATCGGGTGTACCTGGTGGAG

GAGTATGGCTCAGTCCACAACCTTAGCCTTCCTGAGAGCACACTGCTGCAGGCTGTCACC

AACACCCAGGTCATTGATGGCTTCTTTGTGAAGCGCACAGTAGACATTAAGGAGTCAGCT

GCCTACCTTGCCCTTTTGACAAGGGGCCTGGAGAGACTCTACCAGGCCCAGTCTGTGCGT

GAGGTGTTCGCCAGGCAGCTGATGCAGGTTCGTGGAATCAGTGGGGAGAAGGCAGCAGCC

CTGGTGGACCGATACAGCACCCCTGCCAGCCTCCTGGCTGCCTATGATGCCTGTGCCACC

CCCAAGGAGCAGGAGATGCTGCTGAGCACCATCAAGTGTGGGCGTCTGCAGAGGAATCTG

GGACCTGTTCTGAGCAGGACTTTATCTCAGCTCTACTGCACCTACAGCCCCTTGACT

>CL408.Contig11_A-W 113 325 minus strand PREDICTED: crossover junction endonuclease MUS81-like, partial [Cricetulus griseus]

TTCCGACTAAAGCGCTGTGGTCTGGGGCATCGGGTGTACCTGGTGGAGGAGTATGGCTCA

GTCCACAACCTTAGCCTTCCTGAGAGCACACTGCTGCAGGCTGTCACCAACACCCAGGTC

ATTGATGGCTTCTTTGTGAAGCGCACAGTAGACATTAAGGAGTCAGCTGCCTACCTTGCC

CTTTTGACAAGGGGCCTGGAGAGACTCTACCAG

>CL408.Contig12_A-W 113 706 minus strand Crossover junction endonuclease MUS81 [Heterocephalus glaber]

TTCCGACTAAAGCGCTGTGGTCTGGGGCATCGGGTGTACCTGGTGGAGGAGTATGGCTCA

GTCCACAACCTTAGCCTTCCTGAGAGCACACTGCTGCAGGCTGTCACCAACACCCAGGTC

ATTGATGGCTTCTTTGTGAAGCGCACAGTAGACATTAAGGAGTCAGCTGCCTACCTTGCC

CTTTTGACAAGGGGCCTGGAGAGACTCTACCAGGGACACACCCTACGCAGCCGTCCCTGG

GGAACCCCAGGGGACCCTGAGTCGGGGGCCAGGCCCACCCCAAACCCACTCTGCTCACTC

CTCACCTTCAGTGACTTCAACACAGGAGCCATCAAGAACAAGGCCCAGTCTGTGCGTGAG

GTGTTCGCCAGGCAGCTGATGCAGGTTCGTGGAATCAGTGGGGAGAAGGCAGCAGCCCTG

GTGGACCGATACAGCACCCCTGCCAGCCTCCTGGCTGCCTATGATGCCTGTGCCACCCCC

AAGGAGCAGGAGATGCTGCTGAGCACCATCAAGTGTGGGCGTCTGCAGAGGAATCTGGGA

CCTGTTCTGAGCAGGACTTTATCTCAGCTCTACTGCACCTACAGCCCCTTGACT

>CL408.Contig13_A-W 411 683 minus strand PREDICTED: crossover junction endonuclease MUS81-like, partial [Cricetulus griseus]

CAGGCTGTCACCAACACCCAGGCCCAGTCTGTGCGTGAGGTGTTCGCCAGGCAGCTGATG

CAGGTTCGTGGAATCAGTGGGGAGAAGGCAGCAGCCCTGGTGGACCGATACAGCACCCCT

GCCAGCCTCCTGGCTGCCTATGATGCCTGTGCCACCCCCAAGGAGCAGGAGATGCTGCTG

AGCACCATCAAGTGTGGGCGTCTGCAGAGGAATCTGGGACCTGTTCTGAGCAGGACTTTA

TCTCAGCTCTACTGCACCTACAGCCCCTTGACT

>CL409.Contig1_A-W 2 631 PREDICTED: telomere-associated protein RIF1 isoform 1 [Nomascus leucogenys]

GACAGTCCTAGTGGTATGCAAACACGATGTGTCTGGTCCCCATTGGCTTCTCCATCTACC

AGCATTTTAAAGAGAGGACTGAAAAGACCCCAAGAAGATGAGATCTCCTCACCTGTTAAT

AAGGTTCGACGTGTCTCATTTGCAGATCCAATATACCAAGCAGGATTGGCAGATGACATT

GATAGGCGATGTTCCATTGTTAGGTCCCATTCTTCAAGTAGTTCTCCCATAATGAAAAAT

GTTAAAACATCACCTACTGCACAATCTAAGCATAATACCACTTCAGCTAAAGGATTTCTG

TCCCCAGGATCACGTAGCTCTAAATTTAAGAGCTCAAAGAAGTGTTTAATAACAGAAATG

GCCAAAGAATCCATTCCATGCCCAACAGAAAGTGTTTACCCAGCTTTGGTGAACTGTGCA

GCACCAGTTGACATTATTTTGCCTCAGATAACATCAAATATGTGGGCAAGAGGCCTAGGA

CAACTTATCAGAGCCAAGAATATAAAAACAATTGGCGATCTGAGTTCTCTTACAGCATCT

GAAATAAAAACTCTTCCTATCCGGTCTCCCAAAGTTTCTAATGTAAAAAAGGCTCTTAGA

GTGTATCATGAACAGCAGGTAAAAACTCAG

>CL409.Contig2_A-W 2 553 PREDICTED: telomere-associated protein RIF1 isoform 2 [Nomascus leucogenys] >gi|332251744|ref|XP_003275009.1| PREDICTED: telomere-associated protein RIF1 isoform 3 [Nomascus leucogenys]

GACAGTCCTAGTGGTATGCAAACACGATGTGTCTGGTCCCCATTGGCTTCTCCATCTACC

AGCATTTTAAAGAGAGGACTGAAAAGACCCCAAGAAGATGAGATCTCCTCACCTGTTAAT

AAGGTTCGACGTGTCTCATTTGCAGATCCAATATACCAAGCAGGATTGGCAGATGACATT

GATAGGCGATGTTCCATTGTTAGGTCCCATTCTTCAAGTAGTTCTCCCATAATGAAAAAT

GTTAAAACATCACCTACTGCACAATCTAAGATAACAGAAATGGCCAAAGAATCCATTCCA

TGCCCAACAGAAAGTGTTTACCCAGCTTTGGTGAACTGTGCAGCACCAGTTGACATTATT

TTGCCTCAGATAACATCAAATATGTGGGCAAGAGGCCTAGGACAACTTATCAGAGCCAAG

AATATAAAAACAATTGGCGATCTGAGTTCTCTTACAGCATCTGAAATAAAAACTCTTCCT

ATCCGGTCTCCCAAAGTTTCTAATGTAAAAAAGGCTCTTAGAGTGTATCATGAACAGCAG

GTAAAAACTCAG

>CL409.Contig3_A-W 2 355 RAP1 interacting factor homolog (yeast), isoform CRA_c [Homo sapiens]

GACAGTCCTAGTGGTATGCAAACACGATGTGTCTGGTCCCCATTGGCTTCTCCATCTACC

AGCATTTTAAAGAGAGGACTGAAAAGACCCCAAGAAGATGAGATCTCCTCACCTGTTAAT

AAGGTTCGACGTGTCTCATTTGCAGATCCAATATACCAAGCAGGATTGGCAGATGACATT

GATAGGCGATGTTCCATTGTTAGGTCCCATTCTTCAAGTAGTTCTCCCATAATGAAAAAT

GTTAAAACATCACCTACTGCACAATCTAAGCATAATACCACTTCAGCTAAAGGATTTCTG

TCCCCAGGATCACGTAGCTCTAAATTTAAGAGCTCAAAGAAGTGTTTAGTAAGA

>CL410.Contig1_A-W 3 1781 Integrator complex subunit 11 [Heterocephalus glaber]

GTTGCGCGTCCCTGGCCCTCCCTGCAAGCGGAGCACCGAGGATCTGGGCTGAGGGCCCGC

CGCGAGCGCAGGATGCCTGAGATCAGAGTGACTCCCTTGGGGGCTGGGCAGGATGTGGGC

CGCAGCTGCATCTTGGTCTCCATCGCGGGCAAGAATGTCATGCTGGACTGCGGGATGCAC

ATGGGCTTCAATGATGATAGGCGCTTTCCTGACTTCTCCTACATCACCCAGAATGGCCGG

CTGACTGACTTCCTGGACTGTGTGATCATCAGCCACTTCCACCTGGACCACTGTGGAGCA

CTCCCCTACTTCAGTGAGATGGTGGGCTATGATGGACCCATCTACATGACCCATCCCACC

CAGGCCATCTGCCCAATCCTGCTGGAGGACTACCGCAAGATTGCCGTGGACAAGAAAGGC

GAGGCCAACTTCTTTACCTCCCAGATGATCAAAGACTGCATGAAGAAGGTGGTAGCTGTC

CACTTGCACCAGACAGTTCAGGTGGATGATGAGCTAGAGATCAAGGCCTACTATGCGGGC

CATGTGCTGGGGGCGGCCATGTTCCAGATCAAAGTGGGCTCAGAGTCTGTGGTCTACACG

GGTGATTATAACATGACCCCGGACCGCCACTTGGGTGCTGCTTGGATTGACAAGTGCCGT

CCCAACCTGCTCATCACGGAGTCCACCTATGCCACAACCATCCGGGACTCCAAGCGCTGC

CGCGAGCGAGACTTCCTGAAGAAAGTCCACGAGACCGTGGAGCGTGGCGGGAAGGTGCTG

ATCCCTGTGTTTGCACTGGGCCGTGCTCAGGAGCTCTGCATCTTGCTGGAGACCTTTTGG

GAACGCATGAACCTGAAAGTGCCCATCTACTTCTCCACGGGCTTGACGGAGAAGGCCAAC

CACTACTACAAGCTCTTCATCACCTGGACCAACCAGAAGATTCGGAAGACCTTTGTTCAG

AGGAATATGTTTGAATTCAAGCACATCAAAGCCTTCGACCGGGCATTTGCTGACAACCCA

GGCCCCATGGTTGTGTTTGCCACGCCAGGGATGCTGCATGCTGGCCAGTCCCTGCAGATC

TTCCGGAAGTGGGCAGGAAATGAGAAGAACATGGTCATTATGCCCGGTTACTGTGTGCAG

GGCACTGTGGGCCACAAGATTCTCAGTGGGCAGCGCAAGTTGGAGATGGAAGGGCGACAG

GTGCTGGAGGTGAAGATGCAGGTGGAGTACATGTCGTTCAGCGCCCATGCTGACGCCAAG

GGCATCATGCAGCTGGTGGGGCAGGCAGAGCCAGAGCGTGTGCTGCTGGTACATGGGGAG

GCCAAGAAGATGGAGTTCCTCCGGCAGAAGATTGAGCAGGAGTTCCGAGTCAGCTGCTAC

ATGCCAGCCAACGGTGAGACGGTGACGCTGCCTACGAGCCCCAGCATCCCTGTGGGCATC

TCTCTGGGACTGCTGAAGCGGGAGATGGCGCAGGGGCTACTTCCTGAGGCCAAGAAGCCC

CGGCTACTGCATGGCACCCTGATCATGAAGGACAGTGACGCCCGCAAGGAACAGGAGACA

GCCCTGCGAGTCTACAGCCACCTCAAAAGCACCCTGAAGGACCACTGTGTGCAGCACCTC

CCTGATGGCTCTGTGACGGTGGAGTCTATCCTTATCCAGGCTGCTGCCCACTCTGAGGAC

CCAGGCACCAAGGTGCTGTTAGTTTCTTGGACATACCAGGATGAGGAGCTGGGGAGCTTC

CTCACGGCACTGCTCAAGAAGGGTCTCCCCCAGGCCCCC

>CL410.Contig2_A-W 183 1337 Integrator complex subunit 11 [Cricetulus griseus]

CATTATAGTGCTGCTTGGATTGACAAGTGCCGTCCCAACCTGCTCATCACGGAGTCCACC

TATGCCACAACCATCCGGGACTCCAAGCGCTGCCGCGAGCGAGACTTCCTGAAGAAAGTC

CACGAGACCGTGGAGCGTGGCGGGAAGGTGCTGATCCCTGTGTTTGCACTGGGCCGTGCT

CAGGAGCTCTGCATCTTGCTGGAGACCTTTTGGGAACGCATGAACCTGAAAGTGCCCATC

TACTTCTCCACGGGCTTGACGGAGAAGGCCAACCACTACTACAAGCTCTTCATCACCTGG

ACCAACCAGAAGATTCGGAAGACCTTTGTTCAGAGGAATATGTTTGAATTCAAGCACATC

AAAGCCTTCGACCGGGCATTTGCTGACAACCCAGGCCCCATGGTTGTGTTTGCCACGCCA

GGGATGCTGCATGCTGGCCAGTCCCTGCAGATCTTCCGGAAGTGGGCAGGAAATGAGAAG

AACATGGTCATTATGCCCGGTTACTGTGTGCAGGGCACTGTGGGCCACAAGATTCTCAGT

GGGCAGCGCAAGTTGGAGATGGAAGGGCGACAGGTGCTGGAGGTGAAGATGCAGGTGGAG

TACATGTCGTTCAGCGCCCATGCTGACGCCAAGGGCATCATGCAGCTGGTGGGGCAGGCA

GAGCCAGAGCGTGTGCTGCTGGTACATGGGGAGGCCAAGAAGATGGAGTTCCTCCGGCAG

AAGATTGAGCAGGAGTTCCGAGTCAGCTGCTACATGCCAGCCAACGGTGAGACGGTGACG

CTGCCTACGAGCCCCAGCATCCCTGTGGGCATCTCTCTGGGACTGCTGAAGCGGGAGATG

GCGCAGGGGCTACTTCCTGAGGCCAAGAAGCCCCGGCTACTGCATGGCACCCTGATCATG

AAGGACAGTGACGCCCGCAAGGAACAGGAGACAGCCCTGCGAGTCTACAGCCACCTCAAA

AGCACCCTGAAGGACCACTGTGTGCAGCACCTCCCTGATGGCTCTGTGACGGTGGAGTCT

ATCCTTATCCAGGCTGCTGCCCACTCTGAGGACCCAGGCACCAAGGTGCTGTTAGTTTCT

TGGACATACCAGGATGAGGAGCTGGGGAGCTTCCTCACGGCACTGCTCAAGAAGGGTCTC

CCCCAGGCCCCCAGC

>CL410.Contig3_A-W 80 1090 Integrator complex subunit 11 [Cricetulus griseus]

CAGGTGCTGATCCCTGTGTTTGCACTGGGCCGTGCTCAGGAGCTCTGCATCTTGCTGGAG

ACCTTTTGGGAACGCATGAACCTGAAAGTGCCCATCTACTTCTCCACGGGCTTGACGGAG

AAGGCCAACCACTACTACAAGCTCTTCATCACCTGGACCAACCAGAAGATTCGGAAGACC

TTTGTTCAGAGGAATATGTTTGAATTCAAGCACATCAAAGCCTTCGACCGGGCATTTGCT

GACAACCCAGGCCCCATGGTTGTGTTTGCCACGCCAGGGATGCTGCATGCTGGCCAGTCC

CTGCAGATCTTCCGGAAGTGGGCAGGAAATGAGAAGAACATGGTCATTATGCCCGGTTAC

TGTGTGCAGGGCACTGTGGGCCACAAGATTCTCAGTGGGCAGCGCAAGTTGGAGATGGAA

GGGCGACAGGTGCTGGAGGTGAAGATGCAGGTGGAGTACATGTCGTTCAGCGCCCATGCT

GACGCCAAGGGCATCATGCAGCTGGTGGGGCAGGCAGAGCCAGAGCGTGTGCTGCTGGTA

CATGGGGAGGCCAAGAAGATGGAGTTCCTCCGGCAGAAGATTGAGCAGGAGTTCCGAGTC

AGCTGCTACATGCCAGCCAACGGTGAGACGGTGACGCTGCCTACGAGCCCCAGCATCCCT

GTGGGCATCTCTCTGGGACTGCTGAAGCGGGAGATGGCGCAGGGGCTACTTCCTGAGGCC

AAGAAGCCCCGGCTACTGCATGGCACCCTGATCATGAAGGACAGTGACGCCCGCAAGGAA

CAGGAGACAGCCCTGCGAGTCTACAGCCACCTCAAAAGCACCCTGAAGGACCACTGTGTG

CAGCACCTCCCTGATGGCTCTGTGACGGTGGAGTCTATCCTTATCCAGGCTGCTGCCCAC

TCTGAGGACCCAGGCACCAAGGTGCTGTTAGTTTCTTGGACATACCAGGATGAGGAGCTG

GGGAGCTTCCTCACGGCACTGCTCAAGAAGGGTCTCCCCCAGGCCCCCAGC

>CL410.Contig4_A-W 10 1908 Integrator complex subunit 11 [Heterocephalus glaber]

CCTGCGCAGTGCGTCGCCGCGGCTAGTTGGGTTGCGCGTCCCTGGCCCTCCCTGCAAGCG

GAGCACCGAGGATCTGGGCTGAGGGCCCGCCGCGAGCGCAGGATGCCTGAGATCAGAGTG

ACTCCCTTGGGGGCTGGGCAGGATGTGGGCCGCAGCTGCATCTTGGTCTCCATCGCGGGC

AAGAATGTCATGCTGGACTGCGGGATGCACATGGGCTTCAATGATGATAGGCGCTTTCCT

GACTTCTCCTACATCACCCAGAATGGCCGGCTGACTGACTTCCTGGACTGTGTGATCATC

AGCCACTTCCACCTGGACCACTGTGGAGCACTCCCCTACTTCAGTGAGATGGTGGGCTAT

GATGGACCCATCTACATGACCCATCCCACCCAGGCCATCTGCCCAATCCTGCTGGAGGAC

TACCGCAAGATTGCCGTGGACAAGAAAGGCGAGGCCAACTTCTTTACCTCCCAGATGATC

AAAGACTGCATGAAGAAGGTGGTAGCTGTCCACTTGCACCAGACAGTTCAGGTGGATGAT

GAGCTAGAGATCAAGGCCTACTATGCGGGCCATGTGCTGGGGGCGGCCATGTTCCAGATC

AAAGTGGGCTCAGAGTCTGTGGTCTACACGGGTGATTATAACATGACCCCGGACCGCCAC

TTGGGTGCTGCTTGGATTGACAAGTGCCGTCCCAACCTGCTCATCACGGAGTCCACCTAT

GCCACAACCATCCGGGACTCCAAGCGCTGCCGCGAGCGAGACTTCCTGAAGAAAGTCCAC

GAGACCGTGGAGCGTGGCGGGAAGGTGCTGATCCCTGTGTTTGCACTGGGCCGTGCTCAG

GAGCTCTGCATCTTGCTGGAGACCTTTTGGGAACGCATGAACCTGAAAGTGCCCATCTAC

TTCTCCACGGGCTTGACGGAGAAGGCCAACCACTACTACAAGCTCTTCATCACCTGGACC

AACCAGAAGATTCGGAAGACCTTTGTTCAGAGGAATATGTTTGAATTCAAGCACATCAAA

GCCTTCGACCGGGCATTTGCTGACAACCCAGGCCCCATGGTTGTGTTTGCCACGCCAGGG

ATGCTGCATGCTGGCCAGTCCCTGCAGATCTTCCGGAAGTGGGCAGGAAATGAGAAGAAC

ATGGTCATTATGCCCGGTTACTGTGTGCAGGGCACTGTGGGCCACAAGATTCTCAGTGGG

CAGCGCAAGTTGGAGATGGAAGGGCGACAGGTGCTGGAGGTGAAGATGCAGGTGGAGTAC

ATGTCGTTCAGCGCCCATGCTGACGCCAAGGGCATCATGCAGCTGGTGGGGCAGGCAGAG

CCAGAGCGTGTGCTGCTGGTACATGGGGAGGCCAAGAAGATGGAGTTCCTCCGGCAGAAG

ATTGAGCAGGAGTTCCGAGTCAGCTGCTACATGCCAGCCAACGGTGAGACGGTGACGCTG

CCTACGAGCCCCAGCATCCCTGTGGGCATCTCTCTGGGACTGCTGAAGCGGGAGATGGCG

CAGGGGCTACTTCCTGAGGCCAAGAAGCCCCGGCTACTGCATGGCACCCTGATCATGAAG

GACAGTAGCTTCCGGCTGGTATCCTCAGAGCAAGCCCTCAAGGAGCTAGGCCTGGCCGAG

CACCAGCTGCGCTTCACCTGCCGTGTGCACCTGCAGGACACCCGCAAGGAACAGGAGACA

GCCCTGCGAGTCTACAGCCACCTCAAAAGCACCCTGAAGGACCACTGTGTGCAGCACCTC

CCTGATGGCTCTGTGACGGTGGAGTCTATCCTTATCCAGGCTGCTGCCCACTCTGAGGAC

CCAGGCACCAAGGTGCTGTTAGTTTCTTGGACATACCAGGATGAGGAGCTGGGGAGCTTC

CTCACGGCACTGCTCAAGAAGGGTCTCCCCCAGGCCCCC

>CL410.Contig5_A-W 1641 1973 Integrator complex subunit 11 [Heterocephalus glaber]

AGCTTCCGGCTGGTATCCTCAGAGCAAGCCCTCAAGGAGCTAGGCCTGGCCGAGCACCAG

CTGCGCTTCACCTGCCGTGTGCACCTGCAGGACACCCGCAAGGAACAGGAGACAGCCCTG

CGAGTCTACAGCCACCTCAAAAGCACCCTGAAGGACCACTGTGTGCAGCACCTCCCTGAT

GGCTCTGTGACGGTGGAGTCTATCCTTATCCAGGCTGCTGCCCACTCTGAGGACCCAGGC

ACCAAGGTGCTGTTAGTTTCTTGGACATACCAGGATGAGGAGCTGGGGAGCTTCCTCACG

GCACTGCTCAAGAAGGGTCTCCCCCAGGCCCCC

>CL410.Contig6_A-W 112 675 Integrator complex subunit 11 [Cricetulus griseus]

ATGCCTGAGATCAGAGTGACTCCCTTGGGGGCTGGGCAGGATGTGGGCCGCAGCTGCATC

TTGGTCTCCATCGCGGGCAAGAATGTCATGCTGGACTGCGGGATGCACATGGGCTTCAAT

GATGATAGGCGCTTTCCTGACTTCTCCTACATCACCCAGAATGGCCGGCTGACTGACTTC

CTGGACTGTGTGATCATCAGCCACTTCCACCTGGACCACTGTGGAGCACTCCCCTACTTC

AGTGAGATGGTGGGCTATGATGGACCCATCTACATGACCCATCCCACCCAGGCCATCTGC

CCAATCCTGCTGGAGGACTACCGCAAGATTGCCGTGGACAAGAAAGGCGAGGCCAACTTC

TTTACCTCCCAGATGATCAAAGACTGCATGAAGAAGGTGGTAGCTGTCCACTTGCACCAG

ACAGTTCAGGTGGATGATGAGCTAGAGATCAAGGCCTACTATGCGGGCCATGTGCTGGGG

GCGGCCATGTTCCAGATCAAAGTGGGCTCAGAGTCTGTGGTCTACACGGGTGATTATAAC

ATGACCCCGGACCGCCACTTGGGG

>CL410.Contig7_A-W 112 675 Integrator complex subunit 11 [Cricetulus griseus]

ATGCCTGAGATCAGAGTGACTCCCTTGGGGGCTGGGCAGGATGTGGGCCGCAGCTGCATC

TTGGTCTCCATCGCGGGCAAGAATGTCATGCTGGACTGCGGGATGCACATGGGCTTCAAT

GATGATAGGCGCTTTCCTGACTTCTCCTACATCACCCAGAATGGCCGGCTGACTGACTTC

CTGGACTGTGTGATCATCAGCCACTTCCACCTGGACCACTGTGGAGCACTCCCCTACTTC

AGTGAGATGGTGGGCTATGATGGACCCATCTACATGACCCATCCCACCCAGGCCATCTGC

CCAATCCTGCTGGAGGACTACCGCAAGATTGCCGTGGACAAGAAAGGCGAGGCCAACTTC

TTTACCTCCCAGATGATCAAAGACTGCATGAAGAAGGTGGTAGCTGTCCACTTGCACCAG

ACAGTTCAGGTGGATGATGAGCTAGAGATCAAGGCCTACTATGCGGGCCATGTGCTGGGG

GCGGCCATGTTCCAGATCAAAGTGGGCTCAGAGTCTGTGGTCTACACGGGTGATTATAAC

ATGACCCCGGACCGCCACTTGGGG

>CL410.Contig8_A-W 986 1141 Integrator complex subunit 11 [Cricetulus griseus]

CATTATAGTGCTGCTTGGATTGACAAGTGCCGTCCCAACCTGCTCATCACGGAGTCCACC

TATGCCACAACCATCCGGGACTCCAAGCGCTGCCGCGAGCGAGACTTCCTGAAGAAAGTC

CACGAGACCGTGGAGCGTGGCGGGAAGGTAGCTGTG

>CL410.Contig9_A-W 112 822 Integrator complex subunit 11 [Cricetulus griseus]

ATGCCTGAGATCAGAGTGACTCCCTTGGGGGCTGGGCAGGATGTGGGCCGCAGCTGCATC

TTGGTCTCCATCGCGGGCAAGAATGTCATGCTGGACTGCGGGATGCACATGGGCTTCAAT

GATGATAGGCGCTTTCCTGACTTCTCCTACATCACCCAGAATGGCCGGCTGACTGACTTC

CTGGACTGTGTGATCATCAGCCACTTCCACCTGGACCACTGTGGAGCACTCCCCTACTTC

AGTGAGATGGTGGGCTATGATGGACCCATCTACATGACCCATCCCACCCAGGCCATCTGC

CCAATCCTGCTGGAGGACTACCGCAAGATTGCCGTGGACAAGAAAGGCGAGGCCAACTTC

TTTACCTCCCAGATGATCAAAGACTGCATGAAGAAGGTGGTAGCTGTCCACTTGCACCAG

ACAGTTCAGGTGGATGATGAGCTAGAGATCAAGGCCTACTATGCGGGCCATGTGCTGGGG

GCGGCCATGTTCCAGATCAAAGTGGGCTCAGAGTCTGTGGTCTACACGGGTGATTATAAC

ATGACCCCGGACCGCCACTTGGGTGCTGCTTGGATTGACAAGTGCCGTCCCAACCTGCTC

ATCACGGAGTCCACCTATGCCACAACCATCCGGGACTCCAAGCGCTGCCGCGAGCGAGAC

TTCCTGAAGAAAGTCCACGAGACCGTGGAGCGTGGCGGGAAGGTAGCTGTG

>CL410.Contig10_A-W 112 675 Integrator complex subunit 11 [Cricetulus griseus]

ATGCCTGAGATCAGAGTGACTCCCTTGGGGGCTGGGCAGGATGTGGGCCGCAGCTGCATC

TTGGTCTCCATCGCGGGCAAGAATGTCATGCTGGACTGCGGGATGCACATGGGCTTCAAT

GATGATAGGCGCTTTCCTGACTTCTCCTACATCACCCAGAATGGCCGGCTGACTGACTTC

CTGGACTGTGTGATCATCAGCCACTTCCACCTGGACCACTGTGGAGCACTCCCCTACTTC

AGTGAGATGGTGGGCTATGATGGACCCATCTACATGACCCATCCCACCCAGGCCATCTGC

CCAATCCTGCTGGAGGACTACCGCAAGATTGCCGTGGACAAGAAAGGCGAGGCCAACTTC

TTTACCTCCCAGATGATCAAAGACTGCATGAAGAAGGTGGTAGCTGTCCACTTGCACCAG

ACAGTTCAGGTGGATGATGAGCTAGAGATCAAGGCCTACTATGCGGGCCATGTGCTGGGG

GCGGCCATGTTCCAGATCAAAGTGGGCTCAGAGTCTGTGGTCTACACGGGTGATTATAAC

ATGACCCCGGACCGCCACTTGGGG

>CL410.Contig11_A-W 112 675 Integrator complex subunit 11 [Cricetulus griseus]

ATGCCTGAGATCAGAGTGACTCCCTTGGGGGCTGGGCAGGATGTGGGCCGCAGCTGCATC

TTGGTCTCCATCGCGGGCAAGAATGTCATGCTGGACTGCGGGATGCACATGGGCTTCAAT

GATGATAGGCGCTTTCCTGACTTCTCCTACATCACCCAGAATGGCCGGCTGACTGACTTC

CTGGACTGTGTGATCATCAGCCACTTCCACCTGGACCACTGTGGAGCACTCCCCTACTTC

AGTGAGATGGTGGGCTATGATGGACCCATCTACATGACCCATCCCACCCAGGCCATCTGC

CCAATCCTGCTGGAGGACTACCGCAAGATTGCCGTGGACAAGAAAGGCGAGGCCAACTTC

TTTACCTCCCAGATGATCAAAGACTGCATGAAGAAGGTGGTAGCTGTCCACTTGCACCAG

ACAGTTCAGGTGGATGATGAGCTAGAGATCAAGGCCTACTATGCGGGCCATGTGCTGGGG

GCGGCCATGTTCCAGATCAAAGTGGGCTCAGAGTCTGTGGTCTACACGGGTGATTATAAC

ATGACCCCGGACCGCCACTTGGGG

>CL410.Contig12_A-W 1641 1781 Integrator complex subunit 11 [Heterocephalus glaber]

AGCTTCCGGCTGGTATCCTCAGAGCAAGCCCTCAAGGAGCTAGGCCTGGCCGAGCACCAG

CTGCGCTTCACCTGCCGTGTGCACCTGCAGGACACCCGCAAGGAACAGGAGACAGCCCTG

CGAGTCTACAGCCACCTCAAA

>CL410.Contig13_A-W 10 1716 Integrator complex subunit 11 [Heterocephalus glaber]

CCTGCGCAGTGCGTCGCCGCGGCTAGTTGGGTTGCGCGTCCCTGGCCCTCCCTGCAAGCG

GAGCACCGAGGATCTGGGCTGAGGGCCCGCCGCGAGCGCAGGATGCCTGAGATCAGAGTG

ACTCCCTTGGGGGCTGGGCAGGATGTGGGCCGCAGCTGCATCTTGGTCTCCATCGCGGGC

AAGAATGTCATGCTGGACTGCGGGATGCACATGGGCTTCAATGATGATAGGCGCTTTCCT

GACTTCTCCTACATCACCCAGAATGGCCGGCTGACTGACTTCCTGGACTGTGTGATCATC

AGCCACTTCCACCTGGACCACTGTGGAGCACTCCCCTACTTCAGTGAGATGGTGGGCTAT

GATGGACCCATCTACATGACCCATCCCACCCAGGCCATCTGCCCAATCCTGCTGGAGGAC

TACCGCAAGATTGCCGTGGACAAGAAAGGCGAGGCCAACTTCTTTACCTCCCAGATGATC

AAAGACTGCATGAAGAAGGTGGTAGCTGTCCACTTGCACCAGACAGTTCAGGTGGATGAT

GAGCTAGAGATCAAGGCCTACTATGCGGGCCATGTGCTGGGGGCGGCCATGTTCCAGATC

AAAGTGGGCTCAGAGTCTGTGGTCTACACGGGTGATTATAACATGACCCCGGACCGCCAC

TTGGGTGCTGCTTGGATTGACAAGTGCCGTCCCAACCTGCTCATCACGGAGTCCACCTAT

GCCACAACCATCCGGGACTCCAAGCGCTGCCGCGAGCGAGACTTCCTGAAGAAAGTCCAC

GAGACCGTGGAGCGTGGCGGGAAGGTGCTGATCCCTGTGTTTGCACTGGGCCGTGCTCAG

GAGCTCTGCATCTTGCTGGAGACCTTTTGGGAACGCATGAACCTGAAAGTGCCCATCTAC

TTCTCCACGGGCTTGACGGAGAAGGCCAACCACTACTACAAGCTCTTCATCACCTGGACC

AACCAGAAGATTCGGAAGACCTTTGTTCAGAGGAATATGTTTGAATTCAAGCACATCAAA

GCCTTCGACCGGGCATTTGCTGACAACCCAGGCCCCATGGTTGTGTTTGCCACGCCAGGG

ATGCTGCATGCTGGCCAGTCCCTGCAGATCTTCCGGAAGTGGGCAGGAAATGAGAAGAAC

ATGGTCATTATGCCCGGTTACTGTGTGCAGGGCACTGTGGGCCACAAGATTCTCAGTGGG

CAGCGCAAGTTGGAGATGGAAGGGCGACAGGTGCTGGAGGTGAAGATGCAGGTGGAGTAC

ATGTCGTTCAGCGCCCATGCTGACGCCAAGGGCATCATGCAGCTGGTGGGGCAGGCAGAG

CCAGAGCGTGTGCTGCTGGTACATGGGGAGGCCAAGAAGATGGAGTTCCTCCGGCAGAAG

ATTGAGCAGGAGTTCCGAGTCAGCTGCTACATGCCAGCCAACGGTGAGACGGTGACGCTG

CCTACGAGCCCCAGCATCCCTGTGGGCATCTCTCTGGGACTGCTGAAGCGGGAGATGGCG

CAGGGGCTACTTCCTGAGGCCAAGAAGCCCCGGCTACTGCATGGCACCCTGATCATGAAG

GACAGTAGCTTCCGGCTGGTATCCTCAGAGCAAGCCCTCAAGGAGCTAGGCCTGGCCGAG

CACCAGCTGCGCTTCACCTGCCGTGTGCACCTGCAGGACACCCGCAAGGAACAGGAGACA

GCCCTGCGAGTCTACAGCCACCTCAAA

>CL410.Contig14_A-W 112 675 Integrator complex subunit 11 [Cricetulus griseus]

ATGCCTGAGATCAGAGTGACTCCCTTGGGGGCTGGGCAGGATGTGGGCCGCAGCTGCATC

TTGGTCTCCATCGCGGGCAAGAATGTCATGCTGGACTGCGGGATGCACATGGGCTTCAAT

GATGATAGGCGCTTTCCTGACTTCTCCTACATCACCCAGAATGGCCGGCTGACTGACTTC

CTGGACTGTGTGATCATCAGCCACTTCCACCTGGACCACTGTGGAGCACTCCCCTACTTC

AGTGAGATGGTGGGCTATGATGGACCCATCTACATGACCCATCCCACCCAGGCCATCTGC

CCAATCCTGCTGGAGGACTACCGCAAGATTGCCGTGGACAAGAAAGGCGAGGCCAACTTC

TTTACCTCCCAGATGATCAAAGACTGCATGAAGAAGGTGGTAGCTGTCCACTTGCACCAG

ACAGTTCAGGTGGATGATGAGCTAGAGATCAAGGCCTACTATGCGGGCCATGTGCTGGGG

GCGGCCATGTTCCAGATCAAAGTGGGCTCAGAGTCTGTGGTCTACACGGGTGATTATAAC

ATGACCCCGGACCGCCACTTGGGG

>CL410.Contig15_A-W 112 675 Integrator complex subunit 11 [Cricetulus griseus]

ATGCCTGAGATCAGAGTGACTCCCTTGGGGGCTGGGCAGGATGTGGGCCGCAGCTGCATC

TTGGTCTCCATCGCGGGCAAGAATGTCATGCTGGACTGCGGGATGCACATGGGCTTCAAT

GATGATAGGCGCTTTCCTGACTTCTCCTACATCACCCAGAATGGCCGGCTGACTGACTTC

CTGGACTGTGTGATCATCAGCCACTTCCACCTGGACCACTGTGGAGCACTCCCCTACTTC

AGTGAGATGGTGGGCTATGATGGACCCATCTACATGACCCATCCCACCCAGGCCATCTGC

CCAATCCTGCTGGAGGACTACCGCAAGATTGCCGTGGACAAGAAAGGCGAGGCCAACTTC

TTTACCTCCCAGATGATCAAAGACTGCATGAAGAAGGTGGTAGCTGTCCACTTGCACCAG

ACAGTTCAGGTGGATGATGAGCTAGAGATCAAGGCCTACTATGCGGGCCATGTGCTGGGG

GCGGCCATGTTCCAGATCAAAGTGGGCTCAGAGTCTGTGGTCTACACGGGTGATTATAAC

ATGACCCCGGACCGCCACTTGGGG

>CL410.Contig16_A-W 1641 1913 Integrator complex subunit 11 [Heterocephalus glaber]

AGCTTCCGGCTGGTATCCTCAGAGCAAGCCCTCAAGGAGCTAGGCCTGGCCGAGCACCAG

CTGCGCTTCACCTGCCGTGTGCACCTGCAGGACACCCGCAAGGAACAGGAGACAGCCCTG

CGAGTCTACAGCCACCTCAAAAGCACCCTGAAGGACCACTGTGTGCAGCACCTCCCTGAT

GGCTCTGTGACGGTGGAGTCTATCCTTATCCAGGCTGCTGCCCACTCTGAGGACCCAGGC

ACCAAGGTGCTGTTAGTTTCTTGGACATACCAG

>CL410.Contig17_A-W 10 1848 Integrator complex subunit 11 [Heterocephalus glaber]

CCTGCGCAGTGCGTCGCCGCGGCTAGTTGGGTTGCGCGTCCCTGGCCCTCCCTGCAAGCG

GAGCACCGAGGATCTGGGCTGAGGGCCCGCCGCGAGCGCAGGATGCCTGAGATCAGAGTG

ACTCCCTTGGGGGCTGGGCAGGATGTGGGCCGCAGCTGCATCTTGGTCTCCATCGCGGGC

AAGAATGTCATGCTGGACTGCGGGATGCACATGGGCTTCAATGATGATAGGCGCTTTCCT

GACTTCTCCTACATCACCCAGAATGGCCGGCTGACTGACTTCCTGGACTGTGTGATCATC

AGCCACTTCCACCTGGACCACTGTGGAGCACTCCCCTACTTCAGTGAGATGGTGGGCTAT

GATGGACCCATCTACATGACCCATCCCACCCAGGCCATCTGCCCAATCCTGCTGGAGGAC

TACCGCAAGATTGCCGTGGACAAGAAAGGCGAGGCCAACTTCTTTACCTCCCAGATGATC

AAAGACTGCATGAAGAAGGTGGTAGCTGTCCACTTGCACCAGACAGTTCAGGTGGATGAT

GAGCTAGAGATCAAGGCCTACTATGCGGGCCATGTGCTGGGGGCGGCCATGTTCCAGATC

AAAGTGGGCTCAGAGTCTGTGGTCTACACGGGTGATTATAACATGACCCCGGACCGCCAC

TTGGGTGCTGCTTGGATTGACAAGTGCCGTCCCAACCTGCTCATCACGGAGTCCACCTAT

GCCACAACCATCCGGGACTCCAAGCGCTGCCGCGAGCGAGACTTCCTGAAGAAAGTCCAC

GAGACCGTGGAGCGTGGCGGGAAGGTGCTGATCCCTGTGTTTGCACTGGGCCGTGCTCAG

GAGCTCTGCATCTTGCTGGAGACCTTTTGGGAACGCATGAACCTGAAAGTGCCCATCTAC

TTCTCCACGGGCTTGACGGAGAAGGCCAACCACTACTACAAGCTCTTCATCACCTGGACC

AACCAGAAGATTCGGAAGACCTTTGTTCAGAGGAATATGTTTGAATTCAAGCACATCAAA

GCCTTCGACCGGGCATTTGCTGACAACCCAGGCCCCATGGTTGTGTTTGCCACGCCAGGG

ATGCTGCATGCTGGCCAGTCCCTGCAGATCTTCCGGAAGTGGGCAGGAAATGAGAAGAAC

ATGGTCATTATGCCCGGTTACTGTGTGCAGGGCACTGTGGGCCACAAGATTCTCAGTGGG

CAGCGCAAGTTGGAGATGGAAGGGCGACAGGTGCTGGAGGTGAAGATGCAGGTGGAGTAC

ATGTCGTTCAGCGCCCATGCTGACGCCAAGGGCATCATGCAGCTGGTGGGGCAGGCAGAG

CCAGAGCGTGTGCTGCTGGTACATGGGGAGGCCAAGAAGATGGAGTTCCTCCGGCAGAAG

ATTGAGCAGGAGTTCCGAGTCAGCTGCTACATGCCAGCCAACGGTGAGACGGTGACGCTG

CCTACGAGCCCCAGCATCCCTGTGGGCATCTCTCTGGGACTGCTGAAGCGGGAGATGGCG

CAGGGGCTACTTCCTGAGGCCAAGAAGCCCCGGCTACTGCATGGCACCCTGATCATGAAG

GACAGTAGCTTCCGGCTGGTATCCTCAGAGCAAGCCCTCAAGGAGCTAGGCCTGGCCGAG

CACCAGCTGCGCTTCACCTGCCGTGTGCACCTGCAGGACACCCGCAAGGAACAGGAGACA

GCCCTGCGAGTCTACAGCCACCTCAAAAGCACCCTGAAGGACCACTGTGTGCAGCACCTC

CCTGATGGCTCTGTGACGGTGGAGTCTATCCTTATCCAGGCTGCTGCCCACTCTGAGGAC

CCAGGCACCAAGGTGCTGTTAGTTTCTTGGACATACCAG

>CL411.Contig1_A-W 94 615 PREDICTED: RAS guanyl-releasing protein 2 isoform 4 [Pan troglodytes]

TGGGGGGACAGCTCTGGTGACTGCAAGGAAAAGTCAGCTCAGTAACTCCGTAGAGGAGCC

CCCCTACACCAGAATGGGGGAGATCGGTCTGAGCCCCTTGGGGAAGCTTCCGGAGCGTAG

CCTGGGTCCAGCCCACCAGCCCCGGCAGCCATGGCGGGCACCCTGGACCTCGACAAGGGC

TGCACAGTGGAGGAGCTGCTCCGCGGCTGCATCGAAGCCTTTGATGACTCCGGGAAGGTG

CGGGACCCGCAGCTGGTACGCATGTTCCTCATGATGCACCCCTGGTACATCCCCTCCTCT

CAGCTGGCTGCAAAACTACTCCACATCTACCAACAATCCCGGAAGGACAACTCCAATTCA

CTGCAGGTGAAAACTTGCCATCTGGTCAGGTACTGGATCTCAGCATTCCCAGCAGAGTTC

GACTTGAACCCAGAGTTGGCTGAGCAGATCAAGGAGCTGAAGGCTCTGCTAGACCAAGAA

GGAAACAGGCGGCACAGCAGCCTCATCGACATCGAGAGCGTG

>CL411.Contig2_A-W 55 489 minus strand RAS guanyl releasing protein 2 [Mustela putorius furo]

TCTGAGCCCCTTGGGGAAGCTTCCGGAGCGTAGCCTGGGTCCAGCCCACCAGCCCCGGCA

GCCATGGCGGGCACCCTGGACCTCGACAAGGGCTGCACAGTGGAGGAGCTGCTCCGCGGC

TGCATCGAAGCCTTTGATGACTCCGGGAAGGTGCGGGACCCGCAGCTGGTACGCATGTTC

CTCATGATGCACCCCTGGTACATCCCCTCCTCTCAGCTGGCTGCAAAACTACTCCACATC

TACCAACAATCCCGGAAGGACAACTCCAATTCACTGCAGGTGAAAACTTGCCATCTGGTC

AGGTACTGGATCTCAGCATTCCCAGCAGAGTTCGACTTGAACCCAGAGTTGGCTGAGCAG

ATCAAGGAGCTGAAGGCTCTGCTAGACCAAGAAGGAAACAGGCGGCACAGCAGCCTCATC

GACATCGAGAGCGTG

>CL411.Contig3_A-W 302 1885 RAS guanyl-releasing protein 2 OS=Homo sapiens GN=RASGRP2 PE=1 SV=1

TACTGGATCTCAGCATTCCCAGCAGAGTTCGACTTGAACCCAGAGTTGGCTGAGCAGATC

AAGGAGCTGAAGGCTCTGCTAGACCAAGAAGGAAACAGGCGGCACAGCAGCCTCATCGAC

ATCGAGAGCGTCCCCACCTACAAGTGGAAGCGGCAGGTGACCCAGCGGAACCCCGTGGGA

CAGAAAAAGCGCAAGATGTCCCTGTTGTTCGACCACCTGGAGCCCCTGGAGCTGGCGGAG

CACCTCACCTACTTGGAGTATCGCTCCTTCTGCAAGATCCTGTTCCAGGACTACCACAGT

TTCGTGACTCATGGCTGCACCGTGGACAACCCCGTCCTAGAGCGATTCATCTCCCTCTTC

AACAGTGTCTCGCAGTGGGTGCAGCTTATGGTCCTCAGCAAGCCCACAGCTCCTCAGCGG

GCGCTGGTCATCACACATTTCGTCCACGTGGCTGAGAAGCTGCTCCAGCTGCAGAACTTC

AACACTCTCATGGCAGTGGTTGGGGGCCTGAGCCACAGTTCCATCTCCCGCCTCAAGGAG

ACCCACAATCACGTTGGCCCTGAAACCATCAAGCTCTGGGAAGGTCTGACGGAACTAGTG

ACAGCTACAGGCAACTATGGTAACTACCGGCGCCGGCTGGCAGCCTGCGTGGGCTTCCGC

TTCCCCATCCTGGGCGTCCACCTTAAGGACCTGGTGGCCCTGCAGCTGGCACTGCCTGAC

TGGCTGGACTCTGCTCGGACCCGGCTCAATGGGGCCAAGATGAAACAGCTTTTCAGCATC

CTGGAGGAGCTGGCCATGGTGACCAGCCTGCGGCCTCCCGTACAGGCCAACCCTGACTTG

CTGAGCTTGCTCACGGTATCTCTGGATCAGTATCAGACGGAGGATGAGCTGTACCAGCTG

TCTCTGCAGCGGGAACCACGCTCCAAGTCCTCGCCAACTAGCCCTACCAGCTGCACCCCA

CCTCCCCGGCCCCCAGTGCTAGAGGAGTGGACTTCGGCCGCCAAGCCCAAACTGGACCAG

GCCCTTGTGGTGGAGCACATAGAGAAGATGGTGGAGTCTGTGTTCCGGAACTTTGATGTC

GACGGGGACGGCCACATCTCACAAGAAGAGTTCCAGATCATCCGTGGCAACTTCCCTTAT

CTCAGCGCCTTTGGGGACCTGGACCAGAACCAGGATGGCTGCATTAGCCGGGAGGAGATG

GTCTCCTACTTCCTGCGCTCCAGCTCGGTGCTAGGAGGCCGCATGGGCTTCGTACACAAC

TTCCAGGAAAGCAACTCGTTGCGCCCTGTCGCCTGCCGCCACTGCAAAGCCCTGATCCTG

GGCATCTACAAGCAGGGCCTCAAATGTCGAGCTTGTGGGGTGAACTGCCACAAGCAGTGC

AAGGACCGCCTGTCAGTGGAGTGCCGTCGCAGGGCCCAGAGTGTGAGCCTGGAGGGCTCT

GCACTCTCCCCCTCCCCCACGCACACCCACCACCGGGCCTTCAGCTTCTCCCTGCCTCGC

CCTGGAAGGCGTGGTTCCCGGCCCCCAGAGATCCGAGAGGAGGAGGTGCAGGCCGTGGAG

GATGGCGTGTTTGACATCCACCTG

>CL411.Contig4_A-W 69 2051 RASGRP2 protein [Homo sapiens]

GGGCGGCGTGGCAGGGGAGGGGTTAAGCTGCCGCAGGGACCGCCGCGTGCGGGGCGAGGG

GGAGCCCCGGTGGAGAAGGCGCAGCCGGCGGATCGGTCTGAGCCCCTTGGGGAAGCTTCC

GGAGCGTAGCCTGGGTCCAGCCCACCAGCCCCGGCAGCCATGGCGGGCACCCTGGACCTC

GACAAGGGCTGCACAGTGGAGGAGCTGCTCCGCGGCTGCATCGAAGCCTTTGATGACTCC

GGGAAGGTGCGGGACCCGCAGCTGGTACGCATGTTCCTCATGATGCACCCCTGGTACATC

CCCTCCTCTCAGCTGGCTGCAAAACTACTCCACATCTACCAACAATCCCGGAAGGACAAC

TCCAATTCACTGCAGGTGAAAACTTGCCATCTGGTCAGGTACTGGATCTCAGCATTCCCA

GCAGAGTTCGACTTGAACCCAGAGTTGGCTGAGCAGATCAAGGAGCTGAAGGCTCTGCTA

GACCAAGAAGGAAACAGGCGGCACAGCAGCCTCATCGACATCGAGAGCGTCCCCACCTAC

AAGTGGAAGCGGCAGGTGACCCAGCGGAACCCCGTGGGACAGAAAAAGCGCAAGATGTCC

CTGTTGTTCGACCACCTGGAGCCCCTGGAGCTGGCGGAGCACCTCACCTACTTGGAGTAT

CGCTCCTTCTGCAAGATCCTGTTCCAGGACTACCACAGTTTCGTGACTCATGGCTGCACC

GTGGACAACCCCGTCCTAGAGCGATTCATCTCCCTCTTCAACAGTGTCTCGCAGTGGGTG

CAGCTTATGGTCCTCAGCAAGCCCACAGCTCCTCAGCGGGCGCTGGTCATCACACATTTC

GTCCACGTGGCTGAGAAGCTGCTCCAGCTGCAGAACTTCAACACTCTCATGGCAGTGGTT
[truncated: 26,017,830 more chars]
